# Transcriptomic analysis of seed germination improvement of *Andrographis paniculata* responding to air plasma treatment

**DOI:** 10.1371/journal.pone.0240939

**Published:** 2020-10-22

**Authors:** Jiayun Tong, Rui He, Xiaoting Tang, Mingzhi Li, Jinglin Wan

**Affiliations:** 1 School of Pharmaceutical Sciences, Guangzhou University of Chinese Medicine, Guangzhou, Guangdong, P. R. China; 2 Research Center of Chinese Herbal Resource Science and Engineering, Guangzhou University of Chinese Medicine, Guangzhou, Guangdong, P. R. China; 3 Genepioneer Biotechnologies Co. Ltd, Nanjing, Jiangsu, P. R. China; 4 Nanjing Suman Plasma Technology Co., Ltd (Corona Lab.), Nanjing, Jiangsu, P. R. China; Universite Toulouse III Paul Sabatier, FRANCE

## Abstract

The plasma seed treatment is effective for promoting seed germination in many crops. However, the biological mechanism remains unclear. Therefore, mRNA sequencing was used to screen differentially expressed genes in the germination process of *Andrographis paniculata* seeds treated with air plasma (power density = 8.99 J/cm^3^). Following plasma treatment, the germination percentages were significantly higher than those of the control, they were 3.5±0.6% *vs*. 0 at 28 hours after sowing (HAS) and 50.3±2.6% *vs*. 37.3±1.7% at 48 HAS. After unigenes were assembled and annotated, 125 differentially expressed genes (DEGs) were detected at 28 HAS, compared with nine DEGs at 48 HAS, but no DEGs were detected at 0 HAS, indicating that air plasma treatment mainly changed the gene expression of *A*. *paniculata* seeds at 28 HAS. The *NCED5* expression level of the treated group was less than one-fifth of the control, and the expressions of three ethylene response factors were significantly higher than the control at 28 HAS, indicating that lower abscisic acid levels play an important role and ethylene signal transduction also participates in radicle protrusion. *ACO*, *NRT1* and *PRP3* expressions were significantly higher than in the control at 48 HAS, suggesting that higher ethylene levels cause the endosperm cap to weaken and start to grow root hairs and lateral roots earlier. These findings reveal that plasma promotes seed germination mainly by regulating the expression of hormone-related genes. And the possible signal transduction of related hormones was discussed.

## Introduction

Seed germination is a crucial event in the life cycle of seed plants. Under natural conditions, there is heterogeneity in the germination duration of seed populations within the same species [1, 2]. This is an important strategy for plants to ensure the reproduction of their species under different conditions [2]. However, early and uniform germination within a seed lot is necessary in agricultural practice, which is beneficial for obtaining stronger seedlings. A previous investigation found that germination percentages of *Andrographis paniculata* (Burm.f.) Nees seeds were diverse in different seed lots and the germination duration of the same lot was not uniform [3]. A study found that there are unknown proteins in *A*. *paniculata* seeds that may inhibit (delay) their germination [4]. However, the germination index or germination energy (germination percentage in the early two days) of *A*. *paniculata* seeds can be increased by air plasma treatment generated by dielectric barrier discharge (DBD) [5, 6].

Plasma seed treatment is a promising agricultural technology, which has been proved effective for promoting seed germination in many crops [7]. However, the biological mechanism of its regulation on seed germination is not clear, and the mechanism of plasma interaction with living tissues and cells can be quite complex, owing to the complexity of both the plasma and the tissue [8]. Plasmas excited by different gases have different characteristics. A previous study proved that air plasma is more effective in improving seed germination than helium plasma [9]. And the air plasma needs no additional gas source, so it is more convenient in practical application [5]. At present, research on the physical mechanism through which the plasma affects seeds is still in its infancy, and it is only limited to investigation from a biological point of view [10, 11]. From what we know so far, when the air plasma is being generated, ultraviolet light, charged particles and reactive species (including reactive oxygen species (ROS) and reactive nitrogen species (RNS)) are produced [8]. The energy of generated UV is too low, and the processing time is too short to affect the organism [12]. As for the charged particles, a previous investigation showed that tiny holes on the seed coat of *A*. *paniculata* produced by etching effect of charged particles [5]. However, the limit of the bombarded seed coat was less than 0.1 mm, and particles remained in the seed coat, that is, did not enter the interior tissue [13]. Therefore, reactive species may play a major role in the biochemical effects of air plasma treatment on seeds [12]. A previous study showed that after air plasma seed treatment, not only germination and seedling emergence, but also light sensitivity of *Paulownia tomentosa* seeds was improved [14]. Another studies showed that the catalase activity and its isoenzyme expression in *A*. *paniculata* seedlings, and the peroxidase activity of tomato seedlings were promoted by air plasma seed treatment [5, 15]. In addition, the secondary metabolism of seedlings of oat (*Avena sativa*) and wheat (*Triticum aestivum*) were promoted by air plasma seed treatment [16]. Thus, we presume that the expression of germination-related genes may be changed by reactive species, which can be produced by air plasma.

Seed germination is the integrated process of a series of complex physiological and biochemical metabolic reactions involving a series of gene expressions and the signal transductions of many molecules. It begins when the dry seed comes into water uptake under favourable conditions, follows by expansion of the embryo, and ends with the radicle rupture the seed coat [17]. During the process, stored energy reactivation, translation of stored mRNA, transcription and translation of new mRNA occur; and hormone contents, signalling, and interactions play important roles in determining the physiological state of the seed and in regulating the germination process [17, 18]. Most of the previous studies on the germination mechanism at the molecular level focused on the expression of single or several genes. Only Nakano et al. [19] used DNA microarray analysis to study the changes of polygene expressions responding to plasma seed treatment. In this investigation, RNA-seq was used to screen differentially expressed genes in the germination process of *A*. *paniculata* seeds treated with air plasma. The biological mechanism of seed germination promotion by plasma may be revealed so as to provide a biological basis for the application of plasma technology in seed processing.

## Materials and methods

### Plasma seed treatment and germination

**Fig 1:**
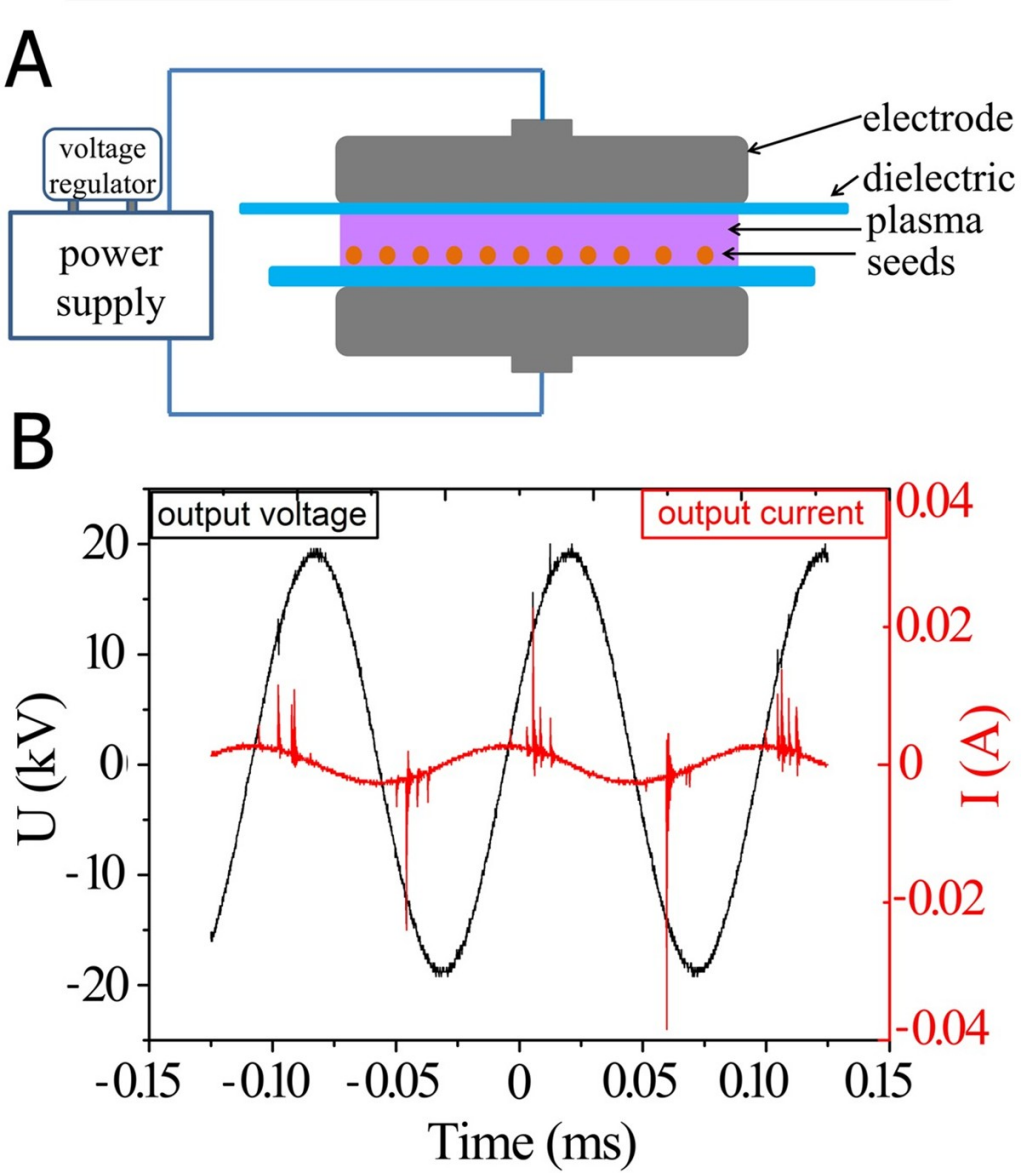
Characteristics of atmospheric pressure DBD air plasma treatment. (A) Schematic of the plasma device. (B) Electrical characteristics of plasma excitation.

An atmospheric pressure dielectric barrier discharge-generating device DBD-50 (Corona Lab., Nanjing, China) was used to generate low temperature air plasma. The DBD generator consisted of two parallel metal electrodes covered by quartz plates acting as dielectric barriers. The diameter of the metal electrodes was 50 mm, while that of the upper dielectric disc was 90 mm, avoiding arching formation due to border effects. The inter-electrode distance was fixed at 8 mm, and the thicknesses of upper and lower dielectric discs were 1 mm and 2 mm, respectively. The active electrode was connected to a power supply (Corona Lab, Model CTP-2000K) as a plasma generator, as shown in Fig 1A.

As plasma with different power may have different effects on seeds, power density was measured at the same time. The voltage/current wave-form was recorded by means of an oscilloscope (Tektronix TBS1102- bandwidth 100 MHz, sample rate on each channel 1.0 Gs/s) using a high voltage probe (Tektronix, attenuation factor: 1:1000) and a current probe (coil with a conversion factor of 0.05 V mA^−1^). The frequency and the voltage amplitude of the input signal were maintained constant at 9.7 kHz and 30 V, respectively [6]. Under these conditions, the input current reached a value of 2.4 A. The V(t), I(t) curves are shown in the Fig 1B and the Lissajous figure is shown in the S1 Fig.

Seeds of *A*. *paniculata* were collected from Guigang, Guangxi autonomous region, P. R. China on October 2nd, 2012. After air drying, all seeds were sealed in a glass bottle and stored at 4°C in a refrigerator before plasma treatment. The initial moisture content of the seeds was 11.6%; the thousand seed weight was 1.1080 g, which was calculated from measured weight according to the standard moisture content (13.0%). Seeds were spread evenly on the lower dielectric disc. An automatic timing outlet was used to control seed treatment time to 3 seconds. After air plasma treatment, seeds were stored for 4 days. Then, treated and control seeds were soaked in distilled water for 20 h at the room temperature about 25°C, surface-sterilized by washing with 75% (v/v) ethanol for about 45 s, followed by 0.1% (w/v) HgCl_2_ for 5 min. Germination tests were conducted according to the TP (top of paper) method [20]. After washing with distilled water three times, one hundred seeds were sown on filter paper (filtration rate less than 70 s) in an 11 cm diameter Petri dish for one replicate, with four replicates for each group [21]. All samples were cultured in an incubator under laboratory conditions of 27.5 ±1°C and a light/dark (12 h/12 h) cycle [21]. The number of germinated seeds of which the radicle protruded by more than 1 mm was counted to calculate the germination percentage (GP) [22]. Independent sample t-tests were conducted using statistical software SPSS 17.0 for Windows (SPSS Inc.).

### RNA extraction, library construction and RNA sequencing

Seeds at three germination phases of plasma-treated and control groups were harvested for RNA isolation. The first phase was at 0 h after sowing (HAS), representing accomplishment of imbibition (seeds were soaked for 20 h and surface-sterilized); the second phase was at 28 HAS, seeds with the radicles had just protruded were selected; and the third phase was at 48 HAS, seeds with the radicles had grown to a length of 1 mm were selected, which was considered as germination accomplishment [22]. There were three replicates for each group. Seeds were frozen in liquid nitrogen and stored at -80°C until use for RNA extraction.

Total RNA was isolated using a plant RNA extraction kit (TIANDZ, Inc., Beijing, China), following the manufacturer’s protocol [22]. The concentration and quality of the RNA samples were detected using a NanoDrop 2000 Spectrophotometer (Thermo Fisher Scientific, USA) and agarose gel electrophoresis (1.2%). Accurate quantification was determined by an Agilent 2100 Bioanalyzer (Agilent Technologies, Inc., USA). High-quality RNA samples (RNA integrity number ≥7.0) were delivered to Genepioneer Biotech Corporation (Nanjing, China) for cDNA library construction and sequencing. Nine libraries for plasma-treated groups and nine for control groups were constructed and sequenced with PE125 using the Illumina HiSeq 2500 platform according to the standard procedure [23]. All sequencing data have been deposited into the NCBI Sequence Read Archive under BioProject ID: PRJNA419905.

### Sequence data processing and *de novo* assembly

Raw sequencing image data were transformed by base calling into raw reads. Raw reads were cleaned by discarding adaptor sequences, ambiguous reads (‘*N*’ > 10%), and low-quality reads (more than 50% bases in a read had a quality value *Q* ≤ 5) using Perl Script. Then, clean reads were *de novo* assembled using the Trinity program with default parameters [23]. Firstly, short clean reads with overlapping sequences were assembled to form contigs. Then, contigs from the same transcript were clustered using TGICL 2.1 to yield unigenes that could not be extended on either end [24]. Finally, redundancies were removed to acquire non-redundant unigenes.

### Gene annotation and classification

For functional annotations, all sequences of the generated unigenes were aligned with public databases using BLASTX with an *E*-value cut-off < 10^−5^ [25]. These databases include the NCBI NR database, Swiss-Prot, KOG/COG, and Pfam databases [22]. A website tool (http://bioinformatics.psb.ugent.be/webtools/Venn/) was used to draw Venn diagrams. For all annotated unigenes, the WEGO tool was used to obtain the Gene Ontology (GO) functional classifications (http://geneontology.org/). Furthermore, KOBAS 2.0 was used to retrieve Kyoto Encyclopedia of Genes and Genomes (KEGG) annotation from the BLAST results (http://www.genome.jp/kegg/).

### Differential gene expression analysis

The relative expression levels of unigenes were normalized and expressed as FPKM (fragments per kb of per million mapped reads) values [26, 27]. The DESeq2 package was used to obtain the “base mean” value for identifying differentially expressed genes (DEGs) [28]. The Benjamin-Hochberg false discovery rate (FDR) method was used to determine the threshold of the *P* value in multiple tests [29]. FDR ≤ 0.01 and |log2 Fold Change|≥ 1 were considered to indicate significant differential expression between two samples. Gene set enrichment analyses of GO terms and KEGG pathways were conducted on each set of differentially expressed unigenes. GO enrichment analysis of DEGs was performed using the Singular Enrichment Analysis (SEA) method with *P* < 0.01 and FDR < 0.05 by agriGO [30]. With the KEGG annotation information, associations between unigenes and pathways were established and an enrichment map of KEGG pathway was drawn using bubble plot in R-3.2.1 for Windows. The hypergeometric Fisher exact test (*P* < 0.01) and Benjamini (FDR < 0.05) were used to detect statistically significant enrichment of the KEGG pathway. Expression changes of GA signal-related genes were drawn in the heatmap by Pheatmap with a scaled algorithm (https://cran.r-project.org/web/packages/pheatmap/index.html).

### QPCR validation

To verify the differential expression data detected by RNA-seq, quantitative real-time PCR (qPCR) was performed on sixteen selected unigenes of plasma-treated samples. Total RNA was isolated as described above. The first-strand cDNA was synthesized from 2 μg of total RNA using a Prime Script RT Reagent Kit (Takara, Japan). The qPCR analysis was performed using an ABI 7500 Real-Time PCR system (Applied Biosystems, USA) with the SYBR Premix Ex Taq Kit (Takara, Dalian, China). Three biological replicates and three technical repeats were performed in parallel for each gene and sample. The relative mRNA expression levels were calculated from cycle threshold values using the 2^−ΔΔ*Ct*^ method [31], and the *GAPDH* gene was used as an internal control [22, 32]. Each unigene primer was designed using NCBI primer-BLAST (https://www.ncbi.nlm.nih.gov/tools/primer-blast/). The gene names with function descriptions and corresponding primer sequences are listed in S1 Table.

## Results

### Discharge and seed germination traits

Results of the discharge under atmospheric pressure showed that the highest DBD voltage (output voltage) was close to 20 kV with the frequency was 9.709 kHz (Fig 1B). Based on the data of the discharge frequency, sampling capacitance (C = 0.47 μF) and the integral area (25.8) of the corresponding Lissajous figure in 2.5 discharge periods (Fig 1B and S1 Fig), the output discharged power (47.09 W) was calculated according to a formula in literature [33, 34]. Thus, the power-supply efficiency was 65.4% and the discharge power density was 8.99 J/cm^3^.

**Fig 2:**
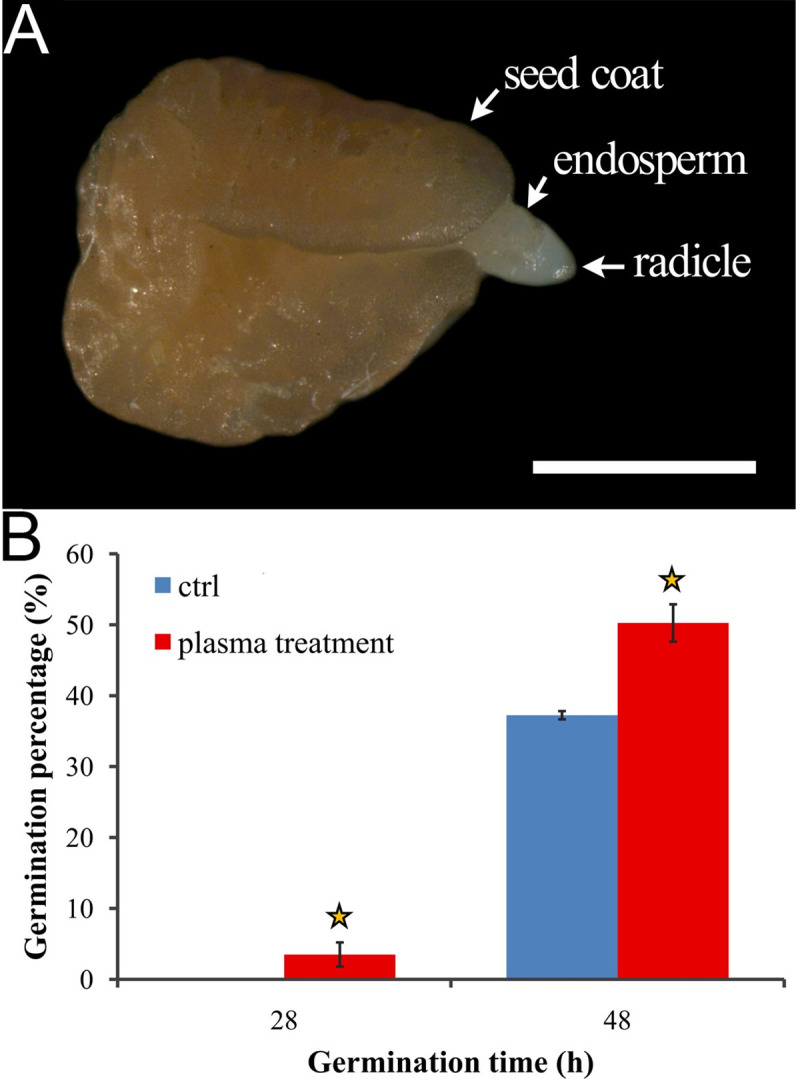
Seed germination characteristics of *A*. *paniculata* before and after air plasma treated. (A) The radicle protruding the endosperm and seed coat at 28 HAS. The scale bar is 1 mm. (B) Seed germination characteristics of control and treated groups at 28 and 48 HAS. Replications = 4. Data are shown as the mean ± SD. The asterisk indicates a significant difference (*P* < 0.05).

Germination assay results showed that air plasma-treated seeds initiated germination after 24 hours, and the GP was 3.5±0.6% at 28 HAS with most of the micropylar endosperms just being ruptured by seed radicles (Fig 2A). However, the non-treated seeds still had not germinated at 28 HAS. Then, the germinated seed number per day achieved a peak at 48 HAS, and the GP of the plasma-treated group was significantly higher than that of the control group (50.3±2.6% *vs*. 37.3±1.7%, *P*<0.01; Fig 2B). After 72 HAS, there was no significant difference between treated and control groups, as their GP values were 67.0±3.6% *vs*. 63.0±4.6% at 72 HAS and 78.0±2.2% *vs*. 73±2.6% after 7 days of a germination test. Thereafter, the germination count of the treated group was not obviously higher than the control. That is to say, air plasma treatment mainly stimulated the seeds of *A*. *paniculata* to initiate germination earlier.

### Library construction and functional annotation of the transcriptome of *A*. *paniculata* seeds

**Fig 3:**
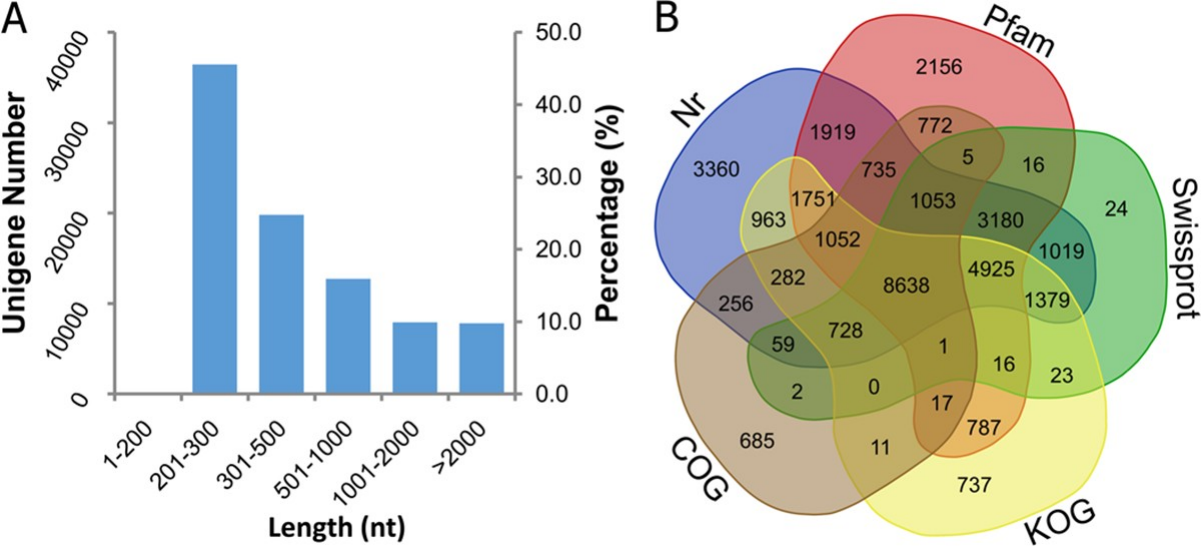
Sequence and annotation characteristics of assembled unigenes. (A) Length distribution of unigenes. (B) Unigene numbers annotated in five databases. A website tool (http://bioinformatics.psb.ugent.be/webtools/Venn/) was used to draw this Venn diagram.

RNA-seq library was prepared from air plasma-treated and untreated seeds in parallel at three germination time points and sequenced using the Illumina Hiseq 2500 platform. The transcriptome data of control groups were reported previously [22]; in this paper, assembly and annotation of transcriptome data from the treated groups at three germination time points are reported. After removing adaptors, primers, poly-A tails, short and low-quality sequences, a total of 227.9 million clean reads from nine samples was obtained, ranging from 16.3 to 36 million reads for each sample (S2 Table). The percentage of Q30 bases in all samples exceeded 85.02% and the GC contents were between 49.96% and 53.74%. Subsequently, 579,209 transcripts (longer than 200 bp) were assembled, with 64.57% transcripts longer than 2000 bp. Finally, 84,749 unigenes were identified, with an N50 length of 1620 bp and average length of 758 bp (S3 Table). There were 36,434 (42.99%) unigenes in the size range 201–300 bp, 19,811 (23.38%) in the 301–500 bp range, 12,735 (15.03%) in the 501–1000 bp, 7936 (9.36%) in the 1001–2000 bp, and 7833 (9.24%) > 2000 bp (Fig 3A).

All assembled unigenes were annotated by BLASTX searches against the public protein databases. In total, 43.1% (36,567) of these unigenes could be assigned at least one putative function from one of these databases, and the remaining 56.9% of the unigenes had no significant protein matches. The highest numbers of annotation hits were 31,299 in NR, 27,023 in Pfam, 21,068 in Swiss-Prot, 21,310 in KOG, and 14,296 in COG (Fig 3B). In the NR database, most of the unigenes (13,111, 41.9%) were homologous to the plant *Sesamum indicum*, followed by *Erythranthe guttata* (2694, 8.6%), *Vitis vinifera* (591), *Coffea canephora* (418), etc.

### Transcriptome changes during the germination process of *A*. *paniculata* seeds treated with plasma

The transcriptome changes of plasma-treated seeds at the three germination stages were further analyzed based on the RNA-seq data. A cut-off was performed using a fold change value of |log2 Fold change | ≥ 1 with a statistically significant *P*< 0.01 [22].

**Fig 4:**
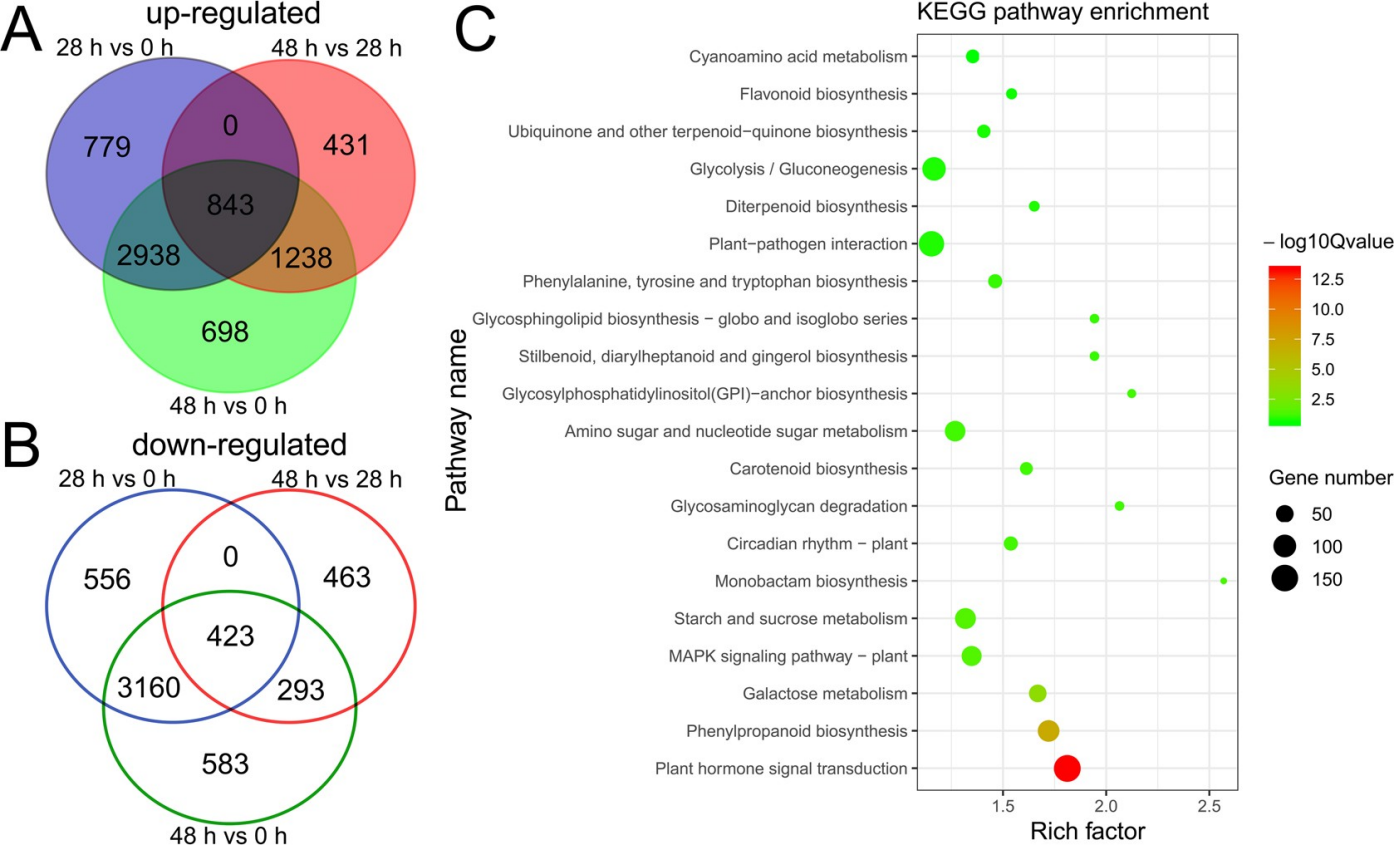
Differential gene expressions during the germination process of *A*. *paniculata* seeds treated with plasma. (A) Venn diagram of up-regulated DEG numbers in three germination stages. (B) Venn diagram of down-regulated DEG numbers in three germination stages. (C) KEGG pathway enrichment map of differentially expressed genes. The bubble plot in R-3.2.1 for Windows was used to draw this map.

A total of 8699 differentially expressed genes (DEGs) were identified by the comparative analysis of 28 HAS and 0 HAS samples. Among these DEGs, 4560 genes were up-regulated while 4139 genes were down-regulated at 28 HAS (Fig 4A and 4B, and S4 Table). In contrast, fewer DEGs (3691) were scored between 48 HAS and 28 HAS samples, with only 2512 genes of the 48 HAS sample being activated and 1179 genes being repressed (Fig 4A and 4B, and S5 Table). This suggests that most of the mRNA transcription occurred at the early germination stage of *A*. *paniculata* seeds. Furthermore, results showed that 843 genes were continuously activated, while 423 genes were continuously repressed during the germination process (Fig 4A and 4B, and S6 Table).

Gene ontology (GO) classification of the unigenes and differentially expressed genes (DEGs) during seed germination was performed. Overall, the DEGs tested at three time points showed a similar classification pattern to the unigenes (S2 Fig). The majority of DEGs within the cellular component term were involved in “intracellular part”, “intracellular”, and “membrane” categories. Furthermore, the DEGs within the molecular function term were mainly clustered in “binding”, “organic cyclic compound binding” and “heterocyclic compound binding” categories. In addition, the DEGs were enriched in the “metabolic process”, “biological regulation”, “regulation of biological process” and “response to stimulus” with respect to the biological process terms.

To gain insight into the role of the DEGs during seed germination, KEGG pathway enrichment of all the DEGs in three germination stages was analyzed. Results predicted that the DEGs were predominantly associated with plant hormone signal transduction, followed by phenylpropanoid biosynthesis and galactose metabolism (Fig 4C). This may confirm that plant hormones play crucial roles in controlling seed germination in *A*. *paniculata*.

### Expression profiles of transcripts between plasma-treated and control groups

RNA-seq analysis demonstrated that there were no DEGs at 0 HAS between plasma and control groups. However, there were 125 DEGs at 28 HAS (S7 Table), and only 9 DEGs at 48 HAS between plasma and control groups (S8 Table). That means the plasma seed treatment did not significantly affect the transcriptional events at the early germination stage, but mainly changed gene expression at the radicle protrusion stage, as well as the expression of some genes after radicle protrusion.

**Fig 5:**
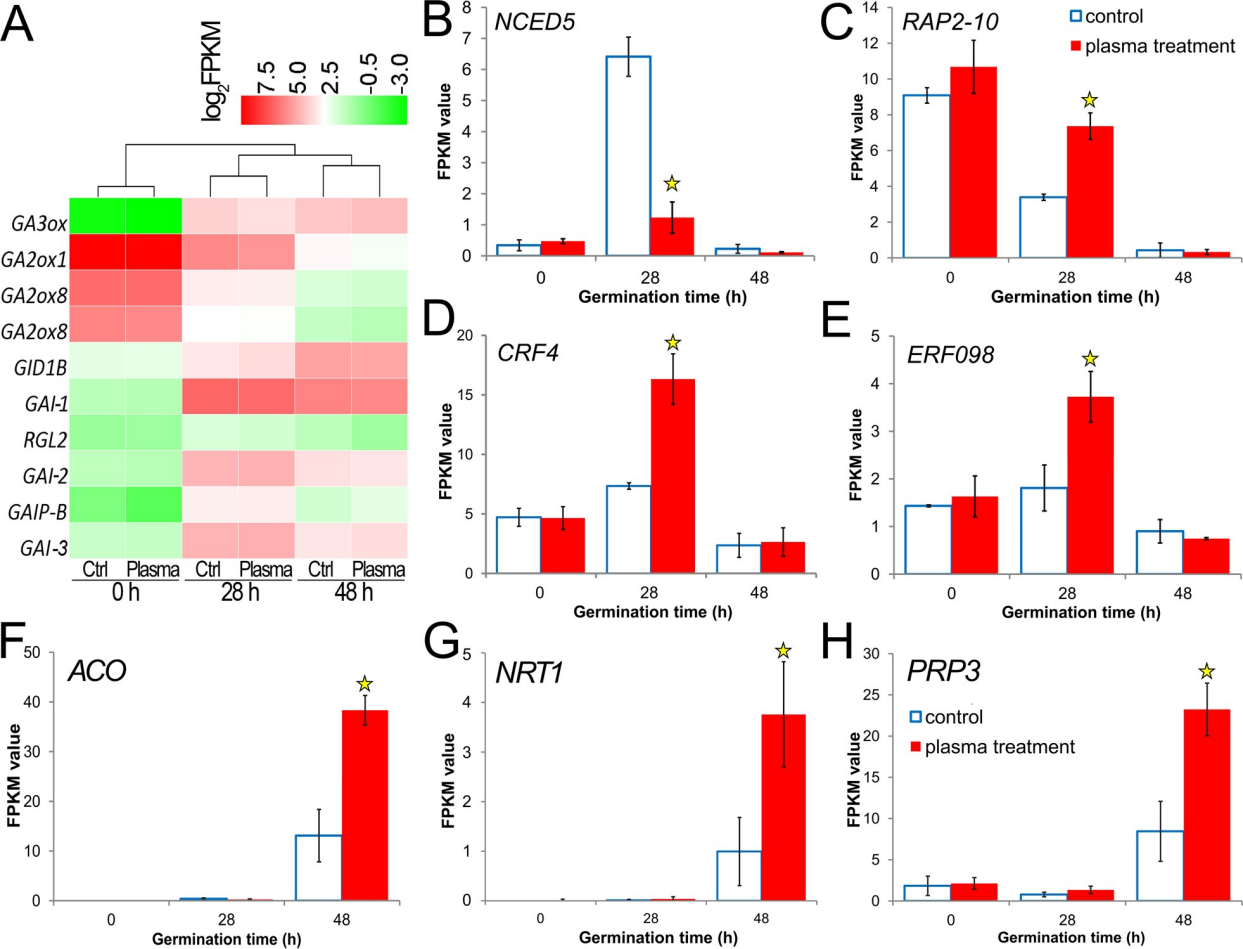
Differential expressions of genes related to GA, ABA, and ethylene signaling between control and plasma-treated groups. (A) Heatmap of expression changes of GA synthesis or signal-related genes. Pheatmap in R-3.2.1 for Windows was used to draw this map. (B-H) Bar charts of genes expression changes related to ABA and ethylene syntheses, *ERF*s signal or other hormones during seed germination. Replications = 3. Data are shown as the mean ± SD. Asterisk represents a significant difference, FDR≤0.01.

As small molecular signaling substances, plant hormones still have significant physiological activity when the concentration is very low or even close to zero [35]; we therefore focused on the expression changes of the plant hormone-related genes. Genes related to gibberellic acid (GA), abscisic acid (ABA), and ethylene signaling were proved to play crucial roles in seed germination of the control group [22]. In the plasma treatment group, the expression of the GA biosynthesis-related gene *GA3ox* (GA3-oxidase, c21977.graph_c0) [17], was continuously increased at 28 and 48 HAS compared to 0 HAS (Fig 5A). On the other hand, the GA catabolic genes, *GA2ox1* (c37960.graph_c0) and *GA2ox8* (c41543.graph_c0, c41543.graph_c1) [36], were down-regulated gradually in the three germination stages (Fig 5A). In the GA signal transduction pathway, most genes encoding DELLA family proteins (repressors of GA signal) [37], including RGL2 (c28002.graph_c0) and GAI (c21104.graph_c0, c26940.graph_c0, c26940.graph_c1, c26940.graph_c2), were up-regulated at 28 HAS and then down-regulated at 48 HAS; however, a GA receptor, *GID1B* (c41362.graph_c0), which is necessary for DELLA protein degradation [38], was activated continuously (Fig 5A). Thus, the expression of these genes means that the GA level was increasing in the germination process, but there was no significant change between the plasma-treated and control groups.

As for the ABA signal transduction, the expression of *NCED5*, a key ABA biosynthetic gene [39], was strikingly increased at 28 HAS in the control group [22]. Interestingly, this expression in the plasma-treated group was decreased by more than a factor of five compared to the control (Fig 5B). This means that treated seeds had a lower ABA level. Ethylene has important roles during the late phase of germination and counteracts ABA inhibition by interfering with ABA signaling, even without affecting ABA contents [40]. In the treated group, the expressions of genes encoding ethylene-responsive transcription factors, *RAP2-10* (c26161.graph_c0), *CRF4* (c43253.graph_c0) and *ERF098* (c43253.graph_c1) [40, 41], were significantly higher than in the control (Fig 5C–5E). Therefore, signal transduction of ethylene may also help treated seeds to germinate at 28 HAS.

At 48 HAS, with respect to treated seeds, expression of *ACO* (c50472.graph_c0), a gene encoding the enzyme that catalyzes the final step of ethylene formation [42], was 2.9 fold of the control group (Fig 5F). That means more ethylene was synthesized at this stage. Additionally, expressions of *NRT1* (c27911.graph_c0), *PRP3* (c51145.graph_c0) in treated seeds were up-regulated significantly compared to the control group (Fig 5G and 5H), indicating earlier growth of root hairs and lateral roots at this post-germination stage [43, 44].

### Validation of RNA-seq data by qPCR analysis

**Fig 6:**
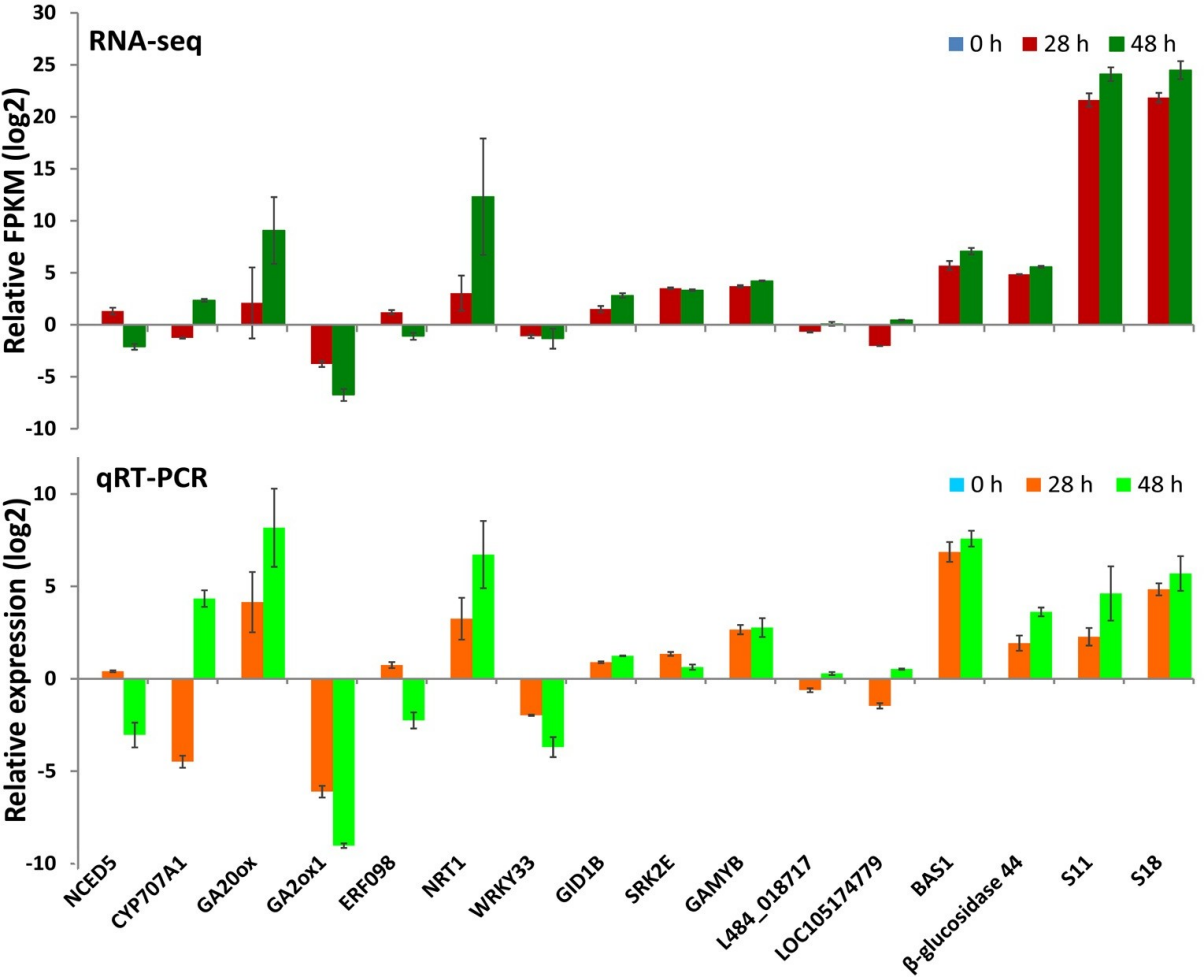
Validation of RNA-seq data on relative gene expression based on qPCR (relative FPKM value of 16 genes with the corresponding relative expression analyzed by qPCR at 0, 28, and 48 HAS). Replications = 3. Data are shown as the mean ± SD.

RNA-seq data of sixteen significant DEGs were validated by qPCR analysis (Fig 6). Ten genes are involved in the metabolism or signal transduction of plant hormones, which include *NCED5* (c21161.graph_c0), *CYP707A1* (c41899.graph_c0), *GA20ox* (c41142.graph_c0), *GA2ox1* (c37960.graph_c0), *ERF098* (c43253.graph_c1), *NRT1* (c27911.graph_c0), *WRKY33* (c42797.graph_c0), *GID1B* (c41362.graph_c0), *SRK2E* (c42159.graph_c0) and *GAMYB* (c40439. graph_c0). Also, six genes that were not directly related to seed germination but showed significant differences in expression during seed germination were selected, such as genes encoding wound-induced protein L484_018717 and LOC105174779 (c21137.graph_c0 and c40715.graph_c2), 2-Cys peroxiredoxin BAS1 (c27825. graph_c0), beta-glucosidase 44 (c46740.graph_c0) and 40S ribosomal proteins (c47954.graph_c0 and c46166.graph_c0). Trends of gene expression changes at the three time points detected by qPCR were generally consistent with those detected by RNA-seq.

## Discussion

### Air plasma activated expressions of different genes at different germination stages due to appropriate dose of ROS, the generation products

Short-term (3 s) air plasma treatment of *A*. *paniculata* seeds altered the gene expression during germination, with the major changes occurring in the late germination stage (125 DEGs at 28 HAS), followed by the post-germination stage (9 DEGs at 48 HAS). Among these DEGs, those related to the metabolism and signaling of ABA and ethylene may play the important roles. These findings will facilitate the application of air plasma technology in seed processing before sowing.

**Fig 7:**
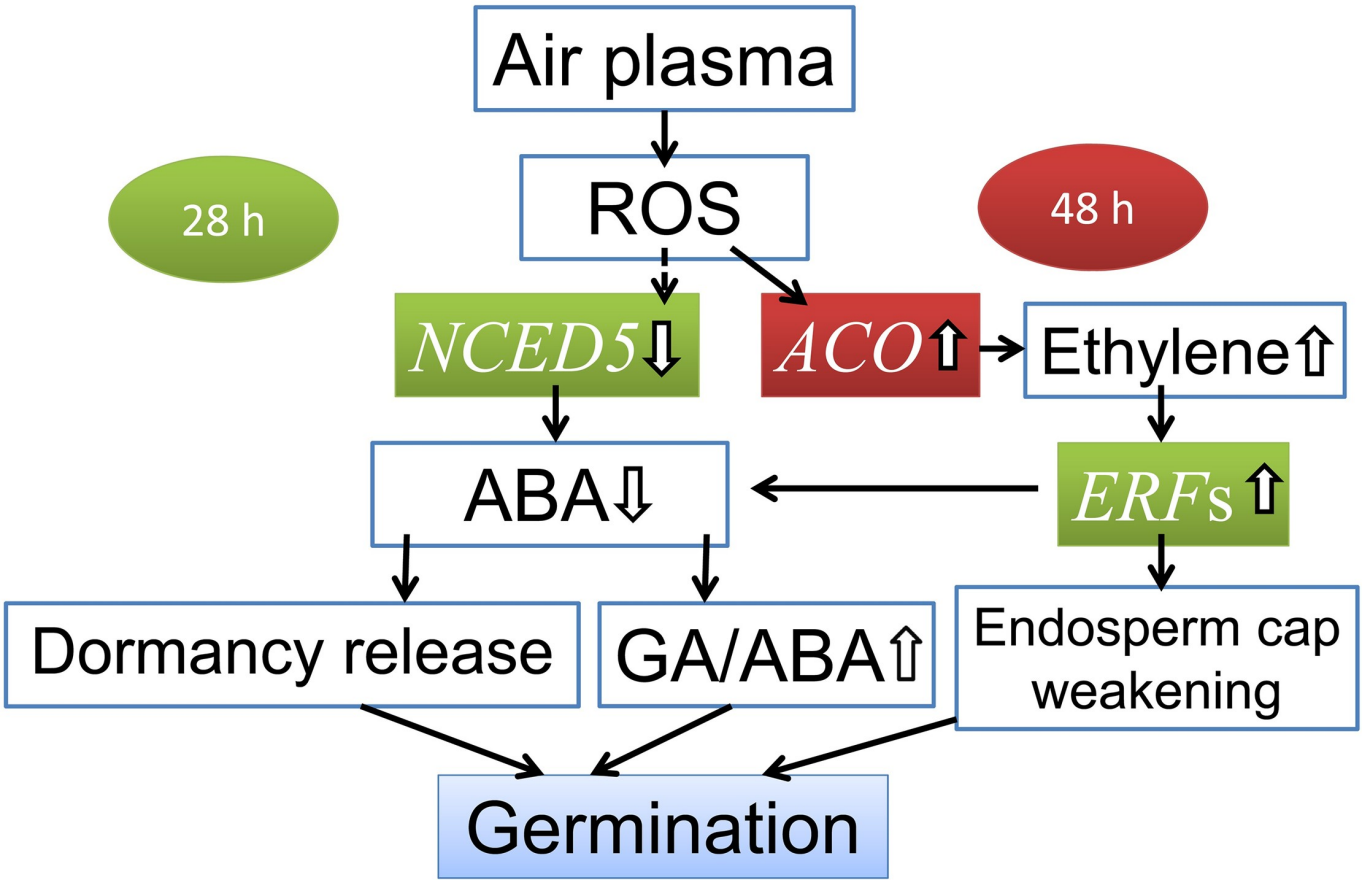
Proposed molecular mechanism of earlier seed germination improved by air plasma treatment. Air plasma treatment decreases ABA synthesis by down-regulates *NCED5* and up-regulated *ERF*s signal at late germination stage (28 HAS), and increases ethylene synthesis by up-regulated *ACO* at post germination stage (48 HAS).

Recent studies on the mechanism of plasma effects focused on the role of active species. While air plasma is generated, ROS and RNS are produced [8]. Studies showed that RNS promoted ABA degradation by activating the *CYP707A* gene and promoted GA synthesis by activating *GA3ox*, thus promoting seed germination [45, 46]. But there were no gene expression changes of the *CYP707A* or *GA3ox* in our results, RNS may not the main factor. As for the ROS, a study proved that an appropriate dose of ROS can decrease the production of ABA [47]. Results of the control group indicated that the primary factor of germination inhibition at the late germination stage (28 HAS) may be the high ABA level increased by high expression of *NCED5* [22]. However, in the plasma-treated group, the expression of *NCED5* was significantly decreased at this stage. Also, Results showed that the expressions of ethylene response factors (*ERFs*), including *CRF*, *ERF098*, and *PAP2-10*, were significantly increased at 28 HAS. And this increased ethylene signal conduction can relieve the germination inhibition of ABA [40, 41]. In addition, studies proved that ROS can activate signal transduction associated with ethylene synthesis [40, 48]. In our results, the *ACO*, a gene encoding the enzyme related to ethylene synthesis, was up-regulated at the post-germination stage (48 HAS). And more ethylene synthesis by up-regulated *ACO* is favorable for endosperm cap weakening and seed germination [42]. Thus, the mechanism of *A*. *paniculata* seed germination promotion by air plasma was proposed (Fig 7). Due to the effect of air plasma generated ROS, germination inhibition was released earlier by decreasing the ABA level at the late germination stage (28 HAS), which resulted from down-regulated *NCED5* gene expression. At the same time, ethylene signal conduction played a synergistic role, and germination inhibition by ABA was resisted. And the endosperm cap was weakened by activated ethylene synthesis via up-regulated *ACO* at the post-germination stage (48 HAS).

In this investigation, results showed that there was no significant difference in gene expression between the treatment group and control group at the early germination stage (0 HAS), when the air plasma power density was 8.99 J/cm^3^ which was excited by about 20 kV electric output. This may indicate that the treatment with this power for 3 s did not result in gene mutation of *A*. *paniculata* seeds. In a previous study, germination was inhibited significantly by a treatment of 50 V input for 9 s [6]. The reason may be that this treatment exceeded the high dose for living tissue [8], and excessive ROS produced by plasma may cause damage to seeds [49].

### Investigation limitations and perspectives

Differential gene analysis in this investigation may be incomplete due to several technical limitations. Firstly, gene expression during seed germination is time specific [50, 51]. Three sampling time points were set according to the observed phenotypes, including the imbibition accomplishment, the radicle protrusion and the germination accomplishment. However, the phenotypic change occurs after the translation of mRNA, and mRNA transcription occurs prior to translation. These time intervals make it hard to decide the accurate stage of gene expression change according to the phenotype. That is to say, these time intervals may lead to systematic errors in the analysis of differential gene expression, and expression changes may be hidden. Secondly, gene expression during seed germination is also tissue specific [51]. A study showed that there were significant differences between the gene expression of the endosperm, scutellum, and embryo in barley seeds at the same germination time [52]. Also, an article proved that ROS may promote germination by inhibiting the transport of ABA from cotyledons to radicles [53]. Gene expression in different tissues of *A*. *paniculata* seeds has not been studied because we could not separate the small embryo and endosperm to obtain enough samples for RNA-Seq in a short time period, which limited by our experimental conditions. This may cause the equalization of gene expressions and differences being obscured. In addition, the genes annotated in all current databases are incomplete because of the incomplete reference data, and the functions of these un-annotated genes remain to be uncovered. However, the data provide us with useful resources to further elucidate the biological processes that occur during seed germination. In the future, as more genes function are discovered, and if there is a more accurate identification method for seed germination stages, the technology of single cell transcriptome sequencing could be used to detect gene expression in different tissues of seeds. Then, on these bases, the establishment of signal transmission network could explain the whole biological mechanism more comprehensively.

## Supporting information

S1 Fig Lissajous figure of the air plasma generating power. (TIF)

S2 Fig GO analysis plot of differentially expressed genes of the plasma treatment groups in the three germination stages. (TIF)

S1 Table Gene names with the corresponding primers for qPCR analysis. (PDF)

S2 Table A summary of the high-throughput RNA-seq quality data for plasma-treated groups. (PDF)

S3 Table Assembly results of the RNA sequencing data. (PDF)

S4 Table Differentially expressed genes of plasma-treated groups (28 HAS *vs*. 0 HAS). (PDF)

S5 Table Differentially expressed genes of plasma-treated groups (48 HAS *vs*. 28 HAS). (PDF)

S6 Table Differentially expressed genes of plasma-treated groups (48 HAS *vs*. 0 HAS). (PDF)

S7 Table Differentially expressed genes at 28 HAS (plasma *vs*. control). (PDF)

S8 Table Differentially expressed genes at 48 HAS (plasma *vs*. control). (PDF)
