# Supplementary figures and images for: Transcriptomic analysis of seed germination improvement of Andrographis paniculata responding to air plasma treatment
Source: PLoS One. 2020 Oct 22;15(10):e0240939. doi: 10.1371/journal.pone.0240939 (PMC7580921; doi:10.1371/journal.pone.0240939)

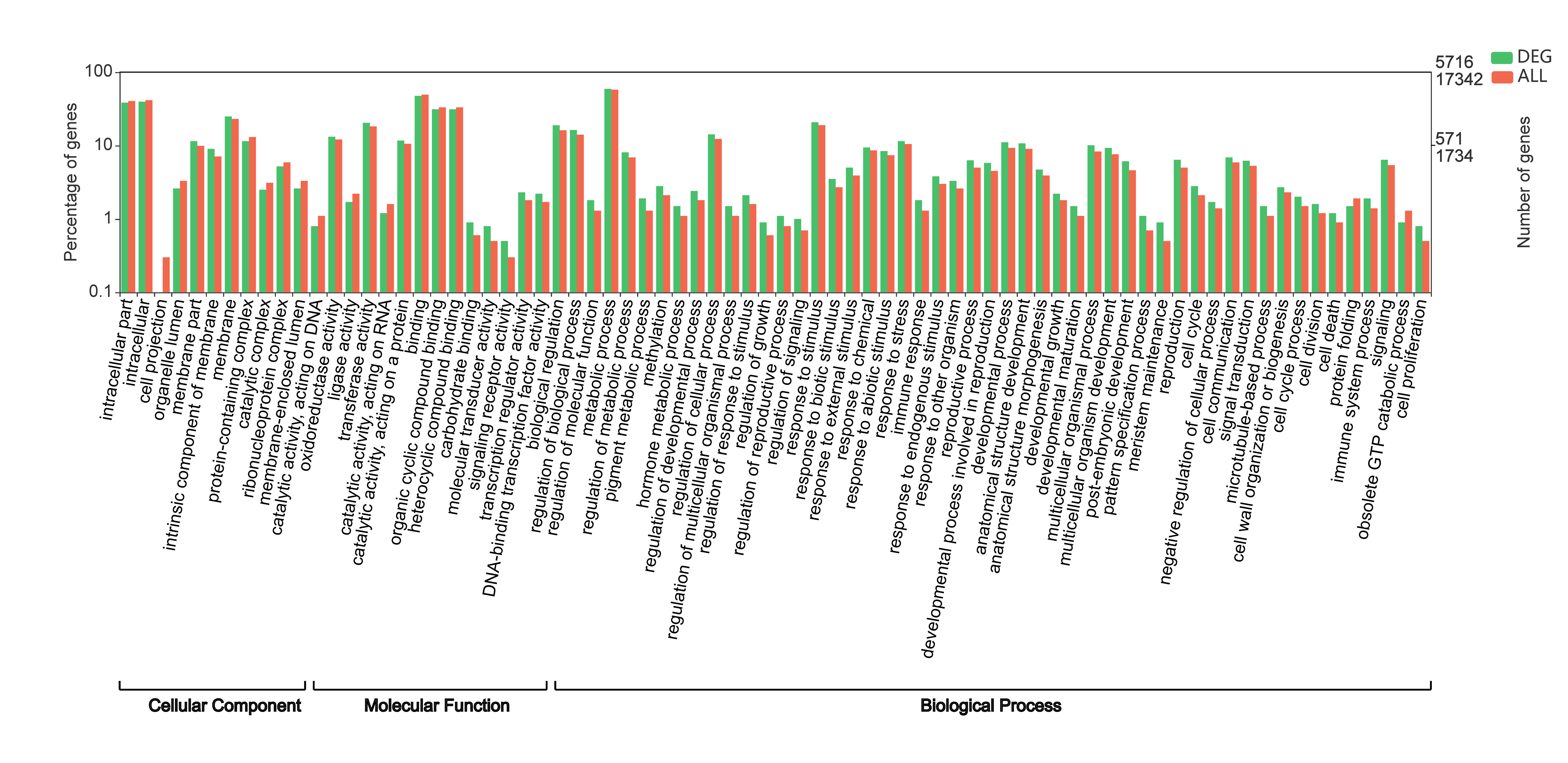

Supplement: S2 Fig — (TIF) [file pone.0240939.s002.tif]
