# Supplementary material for: Transcriptomic analysis of seed germination improvement of Andrographis paniculata responding to air plasma treatment
Source: PLoS One. 2020 Oct 22;15(10):e0240939. doi: 10.1371/journal.pone.0240939 (PMC7580921; doi:10.1371/journal.pone.0240939)
Supplement: S1 Table — (PDF) [file pone.0240939.s003.pdf]

S1 Table. Gene names with the corresponding primers for qPCR analysis.

ID	Gene name	Gene fuction description	Forward primer	Reverse primer
c21161.graph_c0	*NCED5*	9-cis-epoxycarotenoid dioxygenase, NCED5	TCATGCCGTCACCGTCAAA	TTAACGGCGTCTACGTCAG
c41899.graph_c0	*CYP707A1*	CYP707A1	AACAGCCTCTCTGAAAGTGA	GGTTTGAGTTGGGCAGATAC
c41142.graph_c0	*GA20OX*	gibberelin 20-oxidase, GA20OX	CCAAGGTCCTGGTGAAGT	AAACGCTGTCGTTTCAGT
c37960.graph_c0	*GA2OX1*	Gibberellin 2-beta-dioxygenase1, GA2OX1	ATGGTGGTCCAGTCGAAGA	AATGGCGGGATGAATGTC
c43253.graph_c1	*ERF098*	Ethylene-responsive transcription factor, ERF	AACAACCGCAGAGAGAATC	AGGCTTTCAATTTCTTCCCA
c27911.graph_c0	*NRT1*	NRT1/PTR FAMILY 6.3-like	GAATGTATCGCCTACGAAGC	GCCAATACTGTCACCAACT
c42797.graph_c0	*WRKY33*	WRKY transcription factor 33	TTCTACTTCGGCCATGTTCTC	GTGTGTGTGTGTTCGTTAGTT
c41362.graph_c0	*GID1B*	Gibberellin receptor GID1B	AAATTCCCGAAGAGTTTGGTC	TCGTGGCCTTCTCCAGGTA
c42159.graph_c0	*SRK2E*	SRK2E isoform X1	CAAACTCCATACACCCTG	CAAACTCCATACACCCTG
c40439.graph_c0	*GAMYB*	transcription factor GAMYB-like isoform X1	CCTTGGGTCTTTACTCCT	CTCAAACTTCTCGGCACT
c21137.graph_c0	*L484_018717*	Wound-induced protein L484_018717	GATAGCCGCAGTGAGCAT	TTAGCGAGTCTTCCGATT
c40715.graph_c2	*LOC105174779*	Wound-induced protein LOC105174779	TATGTCTGGGTCGTCGTT	GATTGCTGGTAAGATTAGGG
c27825.graph_c0	*BAS1*	2-Cys peroxiredoxin BAS1, chloroplastic-like	GTCTACGGATACACCCAATA	TGCTTCTGAACTCCCACT
c46740.graph_c0	*β-glucosidase 44*	Beta-glucosidase 44	GACAACTATGCTGCTCAACG	AGGCAAATCCACAGTCCC
c47954.graph_c0	*S11*	40S ribosomal protein S11	TACAGTTCCGTGTGCAGAT	CGCTGCTTCTGGAAAGAC
c46166.graph_c0	*S18*	40S ribosomal protein S18	CGCTGAAGCTCATCATCG	CGTGTACTTTCCGTCCTTG
	GAPDH		GATGCTCCCATGTTTGTGG	CCACCTCTCCAGTCCTTC

Transcriptomic analysis of seed germination improvement of *Andrographis paniculata* responding to air plasma treatment

Jia-Yun Tong 1*, Rui He 2*, Xiao-Ting Tang 2, Ming-Zhi Li 3 and Jing-Lin Wan 4
