# Supplementary material for: Transcriptomic analysis of seed germination improvement of Andrographis paniculata responding to air plasma treatment
Source: PLoS One. 2020 Oct 22;15(10):e0240939. doi: 10.1371/journal.pone.0240939 (PMC7580921; doi:10.1371/journal.pone.0240939)
Supplement: S2 Table — (PDF) [file pone.0240939.s004.pdf]

S2 Table. A summary of the high-throughput RNA-seq quality data for plasma treated groups.

Samples	Read number	Base number	GC content	%⩾Q30
0 h-1	21,307,771	5,312,610,744	53.44%	85.11%
0 h-2	21,377,437	5,332,029,262	53.74%	85.02%
0 h-3	16,310,466	4,068,149,700	53.36%	85.06%
28 h-1	29,289,465	7,305,114,988	50.05%	86.28%
28 h-2	32,398,245	8,078,339,204	50.00%	86.25%
28 h-3	36,051,611	8,985,250,144	49.99%	86.40%
48 h-1	23,207,149	5,787,809,202	50.63%	86.55%
48 h-2	21,948,975	5,474,104,454	49.96%	85.88%
48 h-3	26,037,677	6,495,254,468	50.14%	86.04%
Total	227,928,796	56,838,662,166		

Transcriptomic analysis of seed germination improvement of *Andrographis paniculata* responding to air plasma treatment
Jia-Yun Tong 1*, Rui He 2*, Xiao-Ting Tang 2, Ming-Zhi Li 3 and Jing-Lin Wan 4
