# Supplementary material for: Transcriptomic analysis of seed germination improvement of Andrographis paniculata responding to air plasma treatment
Source: PLoS One. 2020 Oct 22;15(10):e0240939. doi: 10.1371/journal.pone.0240939 (PMC7580921; doi:10.1371/journal.pone.0240939)
Supplement: S3 Table — (PDF) [file pone.0240939.s005.pdf]

S3 Table. Assembly results of the RNA sequencing data.

**Length range**	**Contig**	**Transcript**	**Unigene**
201-300	1,782,060(95.85%)*	41,460(7.16%)	36,434(42.99%)
301-500	32,250(1.73%)	28,789(4.97%)	19,811(23.38%)
501-1000	23,514(1.26%)	38,952(6.73%)	12,735(15.03%)
1001-2000	13,238(0.71%)	96,026(16.58%)	7,936(9.36%)
2001+	8,222(0.44%)	373,982(64.57%)	7,833(9.24%)
Total Number	1,859,284	579,209	84,749
Total Length	165,679,144	1,790,544,098	64,241,916
N50 Length	216	4,309	1,620
Mean Length	89.11	3091.36	758.03

Transcriptomic analysis of seed germination improvement of *Andrographis paniculata* responding to air plasma treatment

Jia-Yun Tong 1*, Rui He 2*, Xiao-Ting Tang 2, Ming-Zhi Li 3 and Jing-Lin Wan 4
