# Supplementary material for: Transcriptomic analysis of seed germination improvement of Andrographis paniculata responding to air plasma treatment
Source: PLoS One. 2020 Oct 22;15(10):e0240939. doi: 10.1371/journal.pone.0240939 (PMC7580921; doi:10.1371/journal.pone.0240939)
Supplement: S4 Table — (PDF) [file pone.0240939.s006.pdf]

S4 Table. Differentially expressed genes of plasma-treated groups (28 HAS vs. 0 HAS).

#ID	0 h-1 FPKM	0 h-2 FPKM	0 h-3 FPKM	28 h-1 FPKM	28 h-2 FPKM	28 h-3 FPKM	FDR	log2FC	regulated	nr_annotation
c13791.graph_c0	0	0	0	35.2465444	32.5315321	29.7195888	5.414E-43	Inf	up	--
c13810.graph_c0	2.08421284	1.25177992	1.60697761	23.0248669	23.3423675	24.5563808	7.216E-19	2.75492	up	PREDICTED: SNF2 domain-containing protein CLASSY 3-like [Sesamum indicum]
c13899.graph_c0	1.02487953	0.92331521	0.92190754	0.37243827	0.23539378	0.12156263	1.445E-12	-3.06885	down	-
c13905.graph_c0	33.5266557	27.6762916	27.9760259	23.101163	16.7865524	22.530054	2.852E-23	-1.6025	down	PREDICTED: monothiol glutaredoxin-S4-like [Sesamum indicum]
c13919.graph_c0	18.3674562	18.7499246	16.8258357	19.1649246	17.5428077	19.7367671	1.188E-06	-1.02352	down	-
c13925.graph_c0	0	0	0	5.90772173	8.97101801	6.5736173	1.159E-14	Inf	up	PREDICTED: homeobox-leucine zipper protein HOX21-like [Sesamum indicum]
c13954.graph_c0	30.8512077	29.2567268	34.4285729	0	0.79916041	1.20372109	1.261E-58	-6.64825	down	flavin-containing monooxygenase family protein FMO1 [Gossypium hirsutum]
c14063.graph_c0	0.68622231	0.68690946	1.17576379	6.42823566	5.55391762	5.42626324	5.255E-06	1.67327	up	PREDICTED: zinc finger protein ZAT5 [Sesamum indicum]
c14220.graph_c0	75.8344644	85.6198711	65.9489167	61.0121875	66.8929173	53.0277762	4.45E-16	-1.41918	down	PREDICTED: transcription factor GTE7-like [Sesamum indicum]
c14260.graph_c0	44.0279329	43.5981275	43.8020415	22.7091437	25.7856195	23.0929035	1.259E-56	-1.96735	down	-
c14261.graph_c0	0.13025651	0	0	24.2354383	29.2334232	21.4754128	7.233E-39	8.10824	up	PREDICTED: probable mitochondrial chaperone BCS1-B [Sesamum indicum]
c14340.graph_c0	3.26653414	3.38255698	4.92135426	22.1036727	22.4707163	21.7105013	1.477E-09	1.4219	up	-
c14382.graph_c0	61.137143	69.9492103	62.8952639	12.7492075	15.4365169	14.87153	1.96E-114	-3.26201	down	PREDICTED: protein SHORT INTERNODES [Sesamum indicum]
c14398.graph_c0	3.61799512	5.94980099	6.64180996	27.7979732	26.116489	26.5144933	6.985E-05	1.21409	up	--
c14444.graph_c0	1.00984902	0.44927122	1.00931909	9.54138186	9.20403123	10.1147421	5.595E-10	2.45526	up	hypothetical protein PHAVU_001G014100g [Phaseolus vulgaris]
c14524.graph_c0	2.37243356	1.65684362	1.91428182	0.16039731	0.07241186	0.13088289	4.888E-28	-5.11782	down	hypothetical protein MIMGU_mgv1a007850mg [Erythranthe guttata]
c14644.graph_c0	0.63711619	0.3826525	0.81871952	24.0324181	35.7051317	36.6889393	1.081E-21	4.61779	up	-
c14914.graph_c0	38.4457824	40.0916187	43.1551146	39.4124685	37.2115093	37.2210028	4.835E-23	-1.18824	down	PREDICTED: uncharacterized protein LOC105172581 [Sesamum indicum]
c14919.graph_c0	65.4938286	66.8890088	73.0018135	1.55945597	0.93869502	1.39369414	1.54E-213	-6.81272	down	hypothetical protein MIMGU_mgv1a025595mg [Erythranthe guttata]
c14921.graph_c0	2.11867605	1.749658	1.70161412	0	0.03475831	0	6.326E-36	-8.42225	down	-
c14990.graph_c0	27.6138836	31.9065371	27.3760719	15.8753762	14.6171165	18.7435751	4.561E-39	-1.90849	down	PREDICTED: F-box protein SKIP1 [Sesamum indicum]
c15003.graph_c0	9.23689238	13.4993669	10.6827952	5.77345533	6.18272863	8.32658192	1.792E-08	-1.80996	down	-
c15072.graph_c0	3.61535952	2.37310148	3.35112664	1.46457645	1.71130911	1.79262364	6.155E-09	-2.00068	down	hypothetical protein MIMGU_mgv1a012722mg [Erythranthe guttata]
c15088.graph_c0	35.864785	35.6411751	38.2027904	13.3786168	12.5333318	11.1218531	2.98E-101	-2.65884	down	PREDICTED: uncharacterized protein LOC105156392 [Sesamum indicum]
c15089.graph_c0	17.7664581	18.5101362	11.6482663	8.82797423	7.97083303	7.41858637	2.407E-09	-2.07049	down	hypothetical protein MIMGU_mgv1a022353mg [Erythranthe guttata]

#ID	0 h-1 FPKM	0 h-2 FPKM	0 h-3 FPKM	28 h-1 FPKM	28 h-2 FPKM	28 h-3 FPKM	FDR	log2FC	regulated	nr_annotation
c15094.graph_c0	13.2998175	15.4867083	14.4748544	17.4583962	12.9134337	14.3263941	3.521E-07	-1.04406	down	-
c15121.graph_c0	4.43412995	1.47952335	1.50364747	0	0	0	2.188E-06	-Inf	down	PREDICTED: oxygen-evolving enhancer protein 1, chloroplastic [Elaeis guineensis]
c15133.graph_c0	0.68325502	0.78916061	1.41832344	6.11182535	5.69085542	5.01836958	2.582E-06	1.43966	up	PREDICTED: uncharacterized protein LOC105171932 [Sesamum indicum]
c15145.graph_c0	1.4974279	1.32691929	1.45107689	0.03568269	0	0.02911678	3.083E-48	-7.13041	down	PREDICTED: cucumisin-like [Sesamum indicum]
c15147.graph_c0	2.4447761	1.34094475	1.63537116	0	0	0	1.033E-21	-Inf	down	UPF0012 hydrolase C26A3.11 [Taphrina deformans PYCC 5710]
c15152.graph_c0	0.14374472	0	0	16.6111811	22.2616857	16.6235515	3.181E-28	7.53284	up	cytochrome P450 98A3 [Sesamum indicum]
c15159.graph_c0	0	0	0	1.31330477	2.96447768	2.80519091	1.496E-07	Inf	up	PREDICTED: probable xyloglucan endotransglucosylase/hydrolase protein 23 [Sesamum indicum]
c15161.graph_c0	2.07852285	2.68465055	2.0678587	10.7695327	10.2518717	10.418176	2.127E-05	1.11271	up	PREDICTED: transmembrane protein 184A-like [Sesamum indicum]
c15169.graph_c0	6.98435129	7.34091231	7.2549274	7.36047008	6.64581437	6.6964379	2.519E-09	-1.15091	down	PREDICTED: uncharacterized protein LOC105156896 isoform X1 [Sesamum indicum]
c15170.graph_c0	1.59297725	1.01472787	0.18609444	12.840685	15.6803215	13.6556161	5.79E-12	2.84372	up	hypothetical protein MIMGU_mgv1a006023mg [Erythranthe guttata]
c15173.graph_c0	1.22328875	1.34696506	0.94318469	12.0914773	15.0115217	15.4527534	2.459E-12	2.51182	up	BnaC09g03800D [Brassica napus]
c15187.graph_c1	4.64768984	3.48925785	1.82492	14.82529	15.3344196	12.9395338	0.0013104	1.03227	up	hypothetical protein MIMGU_mgv1a005900mg [Erythranthe guttata]
c15220.graph_c0	2.54410705	1.99737615	2.05131187	0	0	0	1.046E-43	-Inf	down	malate dehydrogenase [Symphyocladia latiuscula]
c15238.graph_c0	25.807402	24.5196894	24.7321265	17.3487853	16.8535032	17.2694076	1.907E-44	-1.6343	down	PREDICTED: uncharacterized protein LOC105180187 [Sesamum indicum]
c15278.graph_c0	29.8150822	27.094252	25.9543478	19.8220361	18.2130121	17.1116882	2.212E-47	-1.67685	down	hypothetical protein MIMGU_mgv1a025332mg [Erythranthe guttata]
c15296.graph_c0	7.67964238	3.52336068	6.44203183	0	0	0	1.262E-16	-Inf	down	unnamed protein product [Chondrus crispus]
c15303.graph_c0	0.7314863	1.00680081	0.70499164	18.7403109	17.8807859	18.5455451	4.409E-27	3.40789	up	hypothetical protein MIMGU_mgv1a012143mg [Erythranthe guttata]
c15307.graph_c0	130.942733	103.676379	112.1313	82.8784619	84.0075841	86.3030312	3.759E-21	-1.5426	down	PREDICTED: zinc finger A20 and AN1 domain-containing stress-associated protein 8-like isoform X2 [Sesamum indicum]
c15329.graph_c0	0.5199872	0.20820316	0	79.2128082	79.0970376	69.1393914	4.75E-153	7.22094	up	PREDICTED: dof zinc finger protein DOF3.1-like isoform X1 [Sesamum indicum]
c15339.graph_c0	252.184041	235.786193	251.24662	25.5104477	29.2313229	30.848277	0	-4.20071	down	hypothetical protein ZEAMMB73_313798 [Zea mays]
c15356.graph_c0	0.08034583	0.08042628	0.10324757	4.0292469	4.16528321	2.85898484	5.325E-12	4.29204	up	PREDICTED: putative late blight resistance protein homolog R1B-19 isoform X1 [Sesamum indicum]
c15361.graph_c0	1.23328096	1.23451591	1.30514166	7.69799168	7.04577795	6.19545043	3.169E-05	1.38041	up	hypothetical protein MIMGU_mgv1a014572mg [Erythranthe guttata]
c15363.graph_c0	0.38282205	0	0.24597075	16.9722045	14.4450457	12.7140016	1.714E-17	5.04505	up	PREDICTED: GTP-binding protein SAR1A-like [Elaeis guineensis]
c15363.graph_c1	1.43603069	0.47915622	0.92267815	23.6571735	25.7578998	24.4139587	1.188E-16	3.61491	up	PREDICTED: GTP-binding protein SAR1A-like [Sesamum indicum]
c15371.graph_c0	33.9535964	34.6334408	21.0903899	12.2214074	3.7576486	19.3233463	1.325E-07	-2.42442	down	PREDICTED: protein notum homolog isoform X1 [Sesamum indicum]

#ID	0 h-1 FPKM	0 h-2 FPKM	0 h-3 FPKM	28 h-1 FPKM	28 h-2 FPKM	28 h-3 FPKM	FDR	log2FC	regulated	nr_annotation
c15380.graph_c0	22.647299	23.528688	24.8034169	20.2008187	19.4216316	16.9415837	6.275E-26	-1.41987	down	PREDICTED: uncharacterized protein LOC105168435 [Sesamum indicum]
c15395.graph_c0	4.04650337	1.53641754	2.779266	0	0	0	1.519E-11	-Inf	down	--
c15399.graph_c0	3.69184075	4.83262605	4.92662928	0.10320033	0.09318022	0	8.004E-32	-7.1985	down	-
c15416.graph_c0	3.14448248	1.41643404	1.91937263	0	0	0	2.524E-12	-Inf	down	predicted protein [Physcomitrella patens]
c15439.graph_c0	23.5591092	24.6669622	30.6223695	21.7477016	28.2547108	21.0381814	3.746E-06	-1.24551	down	-
c15446.graph_c0	4.90674413	3.91113467	2.80238243	28.0012219	30.6490418	33.3031577	3.726E-21	1.90079	up	PREDICTED: UPF0329 protein ECU05_1680/ECU11_0050-like isoform X2 [Sesamum indicum]
c15446.graph_c1	0.46345301	1.15979273	2.08444416	13.3049817	12.3172814	13.1930205	1.522E-07	2.28188	up	-
c15451.graph_c0	2.29466803	1.62468312	2.22953457	0.04067687	0.11018219	0.09957597	2.38E-30	-5.70827	down	-
c15452.graph_c0	0.58381897	0.19480119	0	12.022294	7.91776786	7.15559781	1.448E-07	4.05529	up	-
c15456.graph_c0	1.56347764	1.02068036	1.04824209	5.08876666	5.57606697	5.76497148	0.0018607	1.09145	up	hypothetical protein MIMGU_mgv1a022502mg, partial [Erythranthe guttata]
c15476.graph_c0	1.60801872	1.463299	1.7845903	0.10624534	0.19185916	0.13004299	5.108E-20	-4.59702	down	-
c15480.graph_c1	2.10475236	0.52671499	2.70469011	13.767497	15.5384487	14.3547665	3.739E-06	1.93459	up	-
c15485.graph_c0	1.7698855	0.61622879	0.69220046	5.87243353	5.35275456	6.25223351	0.0002168	1.42612	up	hypothetical protein MIMGU_mgv1a013787mg [Erythranthe guttata]
c15487.graph_c0	4.77082613	4.77560339	5.50191067	4.88993568	4.49542896	5.58620661	0.0007425	-1.09894	down	hypothetical protein MIMGU_mgv1a022391mg, partial [Erythranthe guttata]
c15495.graph_c0	15.5429054	10.3723128	16.3114865	57.9886695	63.5779741	59.7623741	6.973E-07	1.00985	up	hypothetical protein M569_09676, partial [Genlisea aurea]
c15497.graph_c0	3.29711056	4.28560976	3.66777942	3.32623654	3.45538636	3.29787688	4.164E-07	-1.24983	down	-
c15500.graph_c0	1.76409036	1.47154736	2.26692596	8.97491495	10.611735	8.54403353	0.0007412	1.25848	up	-
c15510.graph_c0	0.17067871	0.85424809	0.4386578	4.58978716	4.48015943	4.65623045	1.371E-05	2.13574	up	-
c15525.graph_c0	13.7003403	7.67642305	9.63318703	0	0	0	7.964E-29	-Inf	down	--
c15553.graph_c0	8.10423045	4.5333696	5.20712849	0	0	0	8.707E-18	-Inf	down	hypothetical protein OIDMADRAFT_111959 [Oidiodendron maius Zn]
c15569.graph_c0	0	0	0	12.4446714	13.764555	10.4932419	6.856E-23	Inf	up	PREDICTED: CDGSH iron-sulfur domain-containing protein NEET [Sesamum indicum]
c15575.graph_c0	0	0	0.09336734	5.59752189	8.58232774	9.04888445	9.936E-17	6.82947	up	PREDICTED: probable LRR receptor-like serine/threonine-protein kinase At4g30520 [Sesamum indicum]
c15576.graph_c0	13.9419651	13.4223169	13.0681245	9.59649012	8.77236723	9.2654675	1.344E-38	-1.63872	down	PREDICTED: uncharacterized protein LOC105180173 [Sesamum indicum]
c15577.graph_c0	0.19460632	0.58440357	1.00030732	15.9825791	17.240301	18.6968846	5.161E-16	3.76069	up	PREDICTED: uncharacterized protein LOC103494735 isoform X1 [Cucumis melo]
c15590.graph_c1	4.87900989	7.32584322	5.82188367	0	0	0.10334059	1.915E-31	-8.52369	down	PREDICTED: cirhin-like [Phoenix dactylifera]

#ID	0 h-1 FPKM	0 h-2 FPKM	0 h-3 FPKM	28 h-1 FPKM	28 h-2 FPKM	28 h-3 FPKM	FDR	log2FC	regulated	nr_annotation
c15640.graph_c0	2.48649798	1.42227876	1.27809934	0	0	0	9.703E-14	-Inf	down	-
c15678.graph_c0	3.42647893	3.0215874	4.08864957	21.4051094	19.0055857	19.4501124	3.248E-11	1.4127	up	PREDICTED: uncharacterized protein LOC105165199 [Sesamum indicum]
c15749.graph_c0	26.6632874	22.8771314	15.9895773	7.56693018	9.16518455	10.9937167	1.291E-09	-2.32422	down	hypothetical protein MIMGU_mgv1a004686mg [Erythranthe guttata]
c15817.graph_c0	14.6969744	6.85593374	8.61797199	0	0	0	1.117E-15	-Inf	down	hypothetical protein MIMGU_mgv1a016137mg [Erythranthe guttata]
c15957.graph_c0	0.10804568	0	0.13884298	6.20363146	6.52303007	5.25434474	1.21E-14	5.08612	up	-
c15999.graph_c0	12.694969	11.1579639	11.3398982	2.36291752	1.32073374	0.91815305	5.705E-38	-4.02668	down	hypothetical protein EUTSA_v10020227mg [Eutrema salsugineum]
c16017.graph_c0	8.69616319	9.2939225	9.07436625	2.89879397	4.03327675	3.10215372	1.343E-33	-2.52322	down	PREDICTED: uncharacterized protein LOC105174057 [Sesamum indicum]
c16072.graph_c0	10.3328465	9.14398248	9.04451438	4.35353814	4.86441049	3.77447979	3.256E-26	-2.22451	down	hypothetical protein MIMGU_mgv1a016741mg [Erythranthe guttata]
c16076.graph_c0	16.9084791	16.2891168	15.0299398	12.8433755	13.2649055	13.9483206	2.362E-12	-1.35662	down	PREDICTED: dof zinc finger protein DOF3.1-like [Solanum lycopersicum]
c16083.graph_c0	9.01720259	10.7025322	5.62819599	39.6963996	43.7882588	41.7122596	7.3E-10	1.21959	up	PREDICTED: phosphoglycerate kinase, chloroplastic-like [Fragaria vesca subsp. vesca]
c16084.graph_c0	0	0	0	5.21243168	4.377988	5.63809759	2.557E-14	Inf	up	acetylajmalan acetylesterase [Striga asiatica]
c16100.graph_c0	44.676479	46.7279369	38.8260588	39.1312449	41.0638733	40.5928485	4.827E-22	-1.19718	down	hypothetical protein M569_09334 [Genlisea aurea]
c16107.graph_c0	3.23517637	3.65627602	5.90072605	26.3194747	23.2161444	21.7859357	7.154E-09	1.37999	up	Fructose-bisphosphate aldolase, chloroplast precursor, putative, expressed [Oryza sativa Japonica Group]
c16116.graph_c0	1.48219569	1.48367989	1.90468014	30.163036	28.2444602	28.4331831	9.75E-59	3.06212	up	PREDICTED: transcription repressor OFP6-like [Sesamum indicum]
c16134.graph_c0	0	0	0	3.85468682	3.80876237	3.32343408	2.221E-14	Inf	up	PREDICTED: uncharacterized protein LOC104223573 [Nicotiana sylvestris]
c16163.graph_c0	14.1685582	17.6940082	16.9697786	10.2976265	12.6377745	11.4620433	3.636E-15	-1.59794	down	PREDICTED: xyloglucan galactosyltransferase KATAMARI1 homolog [Sesamum indicum]
c16187.graph_c0	3.87316065	3.55395245	2.40562964	18.532023	15.8007325	15.9642163	2.37E-09	1.26978	up	PREDICTED: uncharacterized protein LOC105171815 [Sesamum indicum]
c16231.graph_c0	27.5459711	15.1503045	15.9483978	0	0	0	2.287E-19	-Inf	down	-
c16250.graph_c0	138.859848	149.508724	147.58971	101.57395	98.1395586	102.702659	4.748E-74	-1.61863	down	hypothetical protein MIMGU_mgv1a014113mg [Erythranthe guttata]
c16273.graph_c0	5.76379997	7.89881818	7.23036089	62.0385258	67.9023939	69.0571648	3.898E-67	2.15942	up	40S ribosomal protein S9-2 [Morus notabilis]
c16382.graph_c0	11.9521371	9.62192599	9.91425535	8.77866757	9.58627473	6.26321788	1.976E-15	-1.44491	down	PREDICTED: peroxidase 9 [Sesamum indicum]
c16384.graph_c0	1.79710656	1.19927073	1.15467654	0	0	0	1.433E-28	-Inf	down	PREDICTED: L-lactate dehydrogenase A-like [Solanum tuberosum]
c16509.graph_c0	11.8766476	13.4251543	14.3275565	55.0796089	60.9717282	65.2605801	9.279E-15	1.10163	up	Peptidylprolyl cis/trans isomerase [Theobroma cacao]
c16681.graph_c0	0.78693784	1.05030112	1.10755545	0	0	0	3.577E-31	-Inf	down	--
c16725.graph_c0	31.6667139	33.0968832	32.7139652	3.49740559	3.15782933	2.8538544	5.23E-106	-4.44916	down	-

#ID	0 h-1 FPKM	0 h-2 FPKM	0 h-3 FPKM	28 h-1 FPKM	28 h-2 FPKM	28 h-3 FPKM	FDR	log2FC	regulated	nr_annotation
c16744.graph_c0	1.77680778	1.7172564	2.83440209	9.79662774	8.64440368	10.1741414	1.065E-05	1.08011	up	PREDICTED: uncharacterized protein LOC105171253 isoform X1 [Sesamum indicum]
c16837.graph_c0	0.26345674	0.5274411	0.50782851	5.45714688	6.56972263	4.29674212	2.768E-07	2.55379	up	PREDICTED: uncharacterized protein LOC105156659 [Sesamum indicum]
c16854.graph_c0	1.70989262	1.53605561	1.29583163	0.12746052	0	0.02600169	2.804E-31	-5.97581	down	-
c16937.graph_c0	0.68840394	0.92762556	0.61243361	0	0	0	7.677E-31	-Inf	down	PREDICTED: subtilisin-like protease [Sesamum indicum]
c16945.graph_c0	1.10561168	0.40437802	0.87431064	0	0	0	4.046E-11	-Inf	down	polyunsaturated fatty acids delta-5-elongase [Thraustochytrium sp. FJN-10]
c17004.graph_c0	1.26627964	0.89954993	1.20729186	0	0	0	3.591E-32	-Inf	down	PREDICTED: prostaglandin reductase 1-like [Pyrus x bretschneideri]
c17007.graph_c0	18.7777388	13.4261013	10.7245057	9.53162385	8.01937884	6.89389459	4.197E-06	-1.89744	down	-
c17223.graph_c0	2.33728246	2.67385474	3.8616435	16.0165351	11.8320787	16.4357127	0.0027672	1.22331	up	-
c17325.graph_c0	1.40953713	0.68826759	1.19281457	0	0	0	4.079E-16	-Inf	down	unnamed protein product [Chondrus crispus]
c17409.graph_c0	0.3694757	0.18492283	0.71218634	4.6993241	4.48550901	6.35449672	4.317E-07	2.51592	up	-
c17428.graph_c0	0.60200763	1.08469881	0.81228382	0.02187678	0	0.01785128	1.006E-16	-7.06326	down	gag-pol polymerase [Arabidopsis lyrata subsp. lyrata]
c17559.graph_c0	0	0	0	31.7446849	37.5068947	28.8637926	3.276E-32	Inf	up	PREDICTED: 3'-hydroxy-N-methyl-(S)-coclaurine 4'-O-methyltransferase-like [Sesamum indicum]
c17663.graph_c0	1.29924554	0.43351551	0.55652732	40.6042193	39.5038032	43.663263	2.992E-19	4.67624	up	-
c17703.graph_c0	8.90752367	6.17292222	4.40250792	2.20540707	2.31244932	2.43815982	6.669E-06	-2.56803	down	-
c17748.graph_c0	11.2922392	11.5971453	13.9456118	11.7244777	11.6447145	10.6977327	5.567E-07	-1.20648	down	PREDICTED: ABC transporter B family member 20 [Nelumbo nucifera]
c17782.graph_c0	1.51149176	1.56181191	1.56639255	0	0	0	4.809E-35	-Inf	down	--
c17811.graph_c0	0.19867237	0.19887131	0	2.21404028	2.21636109	2.00301257	1.223E-07	2.94559	up	PREDICTED: uncharacterized protein LOC105158387 [Sesamum indicum]
c17821.graph_c0	1.76219832	0.5039894	0.97049769	12.4416257	12.2248234	15.3776955	7.156E-08	2.54596	up	-
c17845.graph_c0	0.10381222	0.20783235	0	4.1497568	3.06559744	4.00183451	1.46E-10	4.09632	up	hypothetical protein MIMGU_mgv1a009966mg [Erythranthe guttata]
c17960.graph_c0	76.7099437	74.2008113	73.5080526	42.8775767	41.5168167	38.6147223	3.54E-116	-1.95789	down	PREDICTED: probable ADP-ribosylation factor GTPase-activating protein AGD6 [Sesamum indicum]
c18032.graph_c0	1.21310507	1.05241051	0.88336989	0	0	0	9.685E-32	-Inf	down	hypothetical protein GUITHDRAFT_137181 [Guillardia theta CCMP2712]
c18118.graph_c0	63.8274276	66.1772567	60.4518029	60.2481379	59.1938727	61.3509114	1.757E-31	-1.16471	down	PREDICTED: E3 ubiquitin-protein ligase ATL42 [Sesamum indicum]
c18221.graph_c0	1.93596322	0.81448046	1.04559264	0	0	0	1.517E-11	-Inf	down	hypothetical protein PHAVU_001G265900g [Phaseolus vulgaris]
c18282.graph_c0	5.85528607	5.86114926	6.84024948	2.45018713	2.09585297	2.20978876	9.962E-12	-2.5504	down	-
c18320.graph_c0	2.09189538	0.87249587	1.45609121	0	0	0	1.415E-12	-Inf	down	predicted protein [Physcomitrella patens]
c18333.graph_c0	7.39263791	6.66003646	6.3332216	1.43278043	0.8085415	1.60756356	1.901E-16	-3.49158	down	-

#ID	0 h-1 FPKM	0 h-2 FPKM	0 h-3 FPKM	28 h-1 FPKM	28 h-2 FPKM	28 h-3 FPKM	FDR	log2FC	regulated	nr_annotation
c18333.graph_c1	3.97975493	5.80962091	3.83560699	3.25402082	2.50280523	1.5734841	2.803E-07	-1.98553	down	-
c18358.graph_c0	14.726961	13.777297	12.5575297	91.5296764	119.222279	90.9309725	1.264E-13	1.78759	up	PREDICTED: protein P21-like [Sesamum indicum]
c18381.graph_c0	1.52956927	2.04146786	1.31037138	8.89345702	8.36453942	6.34986476	0.0059942	1.18475	up	--
c18400.graph_c0	3.1384297	0.39269655	2.01650321	15.1115761	32.437479	16.9841011	0.0037218	2.45104	up	-
c18422.graph_c1	0.83602348	0.74387611	0.67642576	0	0	0	1.5E-30	-Inf	down	--
c18557.graph_c0	1.32208413	1.10284	0.28315507	17.936485	16.6287579	18.1643528	4.16E-12	3.2078	up	mitochondrial ATP synthase, beta chain [Plantago major]
c18610.graph_c0	1.10290418	1.38001071	3.18886995	73.7457647	82.1462556	74.4023193	5.18E-46	4.23839	up	betaine aldehyde dehydrogenase 1 [Jatropha curcas]
c18652.graph_c0	4.00552561	3.06498226	2.99431893	13.898147	14.2041948	15.4179713	6.216E-15	1.02546	up	unnamed protein product [Coffea canephora]
c18653.graph_c1	2.80292996	3.32531753	2.13444502	2.33895383	2.52060234	2.77050082	0.0007906	-1.20107	down	unnamed protein product [Coffea canephora]
c18663.graph_c0	13.4998478	10.5211206	12.3913135	0.28033176	0.82261821	0.45749685	1.137E-77	-5.63626	down	-
c18669.graph_c0	311.128593	341.87719	284.800189	32.2291542	36.9952273	36.6958976	3.79E-156	-4.23528	down	PREDICTED: uncharacterized protein LOC105174162 [Sesamum indicum]
c18688.graph_c0	13.7462915	14.5845603	13.1484309	9.05949933	8.93660734	8.77110397	1.061E-73	-1.72183	down	PREDICTED: topless-related protein 4-like isoform X5 [Sesamum indicum]
c18696.graph_c0	10.3862138	11.0746541	12.2828289	11.596462	10.7174641	13.3458626	1.427E-08	-1.01199	down	BnaC04g40780D [Brassica napus]
c18699.graph_c0	18.5435267	18.4034449	16.2934468	13.5925192	10.1926398	10.7154055	4.939E-16	-1.715	down	-
c18707.graph_c0	0.15319321	0.30669323	0.19685934	2.82061193	4.02118123	2.99813202	1.277E-09	2.81314	up	PREDICTED: protein kinase PVPK-1 [Sesamum indicum]
c18710.graph_c0	88.4938063	51.544474	57.3873292	0	0	0	1.242E-51	-Inf	down	hypothetical protein CHLNCDRAFT_59390 [Chlorella variabilis]
c18715.graph_c0	0.19385313	0.33958268	0.24910895	6.37533298	7.55319274	7.17101549	3.369E-41	3.66115	up	PREDICTED: homeobox-leucine zipper protein MERISTEM L1-like [Sesamum indicum]
c18725.graph_c0	0.26750845	0	0.13750358	2.33308318	2.03633685	1.74512875	7.8E-08	2.83182	up	PREDICTED: basic 7S globulin-like [Nelumbo nucifera]
c18726.graph_c0	0.21384123	0.07135179	0.1831963	0.82889905	1.61377659	1.05683572	0.0040785	1.80947	up	PREDICTED: sulfite reductase 1 [ferredoxin], chloroplastic-like [Sesamum indicum]
c18732.graph_c0	8.95554731	8.0307113	10.9088457	6.16983808	5.99930725	6.97089806	1.008E-08	-1.63638	down	PREDICTED: uncharacterized protein LOC105163289 [Sesamum indicum]
c18736.graph_c0	34.5668746	32.7331572	33.0030498	12.8870707	13.1301004	12.7960007	1.53E-146	-2.45993	down	PREDICTED: cation/calcium exchanger 3-like [Sesamum indicum]
c18737.graph_c0	1.91235189	1.38252604	1.91134836	0.92659089	1.88240557	1.260151	0.0004921	-1.4481	down	-
c18755.graph_c0	5.97281527	8.56717739	6.64569013	6.69679848	6.1660788	6.45808431	5.424E-06	-1.22357	down	PREDICTED: uncharacterized protein LOC105174054 [Sesamum indicum]
c18784.graph_c0	16.284431	14.8157841	16.9835328	8.18433504	8.71717591	8.8978024	1.682E-46	-1.98943	down	PREDICTED: probable carboxylesterase 16 [Sesamum indicum]
c18793.graph_c0	13.2742561	8.70948543	8.02726808	0	0	0	5.878E-30	-Inf	down	Ubiquitin-conjugating enzyme [Pestalotiopsis fici W106-1]
c18797.graph_c0	0	0	0	2.60171562	2.74062282	3.11370209	5.951E-12	Inf	up	-

#ID	0 h-1 FPKM	0 h-2 FPKM	0 h-3 FPKM	28 h-1 FPKM	28 h-2 FPKM	28 h-3 FPKM	FDR	log2FC	regulated	nr_annotation
c18808.graph_c0	0.32186867	0.83769654	1.24084197	10.8076721	10.4342152	9.8115838	1.732E-17	2.5891	up	PREDICTED: uncharacterized protein LOC105161274 [Sesamum indicum]
c18817.graph_c0	0.34194817	0.59900852	0.43941694	4.59773029	4.09522047	3.44751764	8.075E-06	2.044	up	hypothetical protein MIMGU_mgv1a015124mg [Erythranthe guttata]
c18827.graph_c0	3.78286749	4.13089687	2.82829643	1.54964282	0.496484	1.26449588	7.799E-18	-2.78287	down	-
c18840.graph_c0	12.3547473	12.3334208	10.7716853	8.0496162	8.77025938	7.76630943	2.181E-40	-1.61756	down	hypothetical protein MIMGU_mgv1a005412mg [Erythranthe guttata]
c18846.graph_c0	5.94156695	5.54720292	6.75417006	25.4115147	31.4545695	28.9349552	1.674E-12	1.14102	up	PREDICTED: 50S ribosomal protein L17, chloroplastic-like [Sesamum indicum]
c18858.graph_c0	1.9472711	1.79533513	1.18530969	0.93109567	1.0088305	0.63820383	1.133E-08	-2.02119	down	PREDICTED: cytochrome P450 71A8-like [Sesamum indicum]
c18864.graph_c0	13.9296556	17.5087301	13.3234138	0.34513614	0.46743836	1.36120331	2.177E-57	-5.44712	down	hypothetical protein MIMGU_mgv1a017080mg [Erythranthe guttata]
c18867.graph_c0	31.4213272	21.8243855	32.4669263	0.9321159	1.17825857	1.67331771	9.952E-30	-5.5915	down	PREDICTED: uncharacterized protein LOC105160499 [Sesamum indicum]
c18869.graph_c0	6.22804306	5.0797833	5.33552127	22.2972323	25.8541247	23.9673066	3.673E-13	1.02627	up	PREDICTED: DNA-directed RNA polymerases II, IV and V subunit 3 [Sesamum indicum]
c18874.graph_c0	0.22724936	0.22747691	0.33374219	4.05830367	3.40862113	3.15751742	7.104E-13	2.65561	up	hypothetical protein MIMGU_mgv1a002578mg [Erythranthe guttata]
c18878.graph_c0	8.90314232	9.10579785	8.45631027	86.1594766	95.7659144	88.4987526	6.079E-69	2.26302	up	40S ribosomal protein S17-like protein [Solanum tuberosum]
c18882.graph_c0	48.1873733	49.5632117	52.6200777	46.3081627	48.9558581	46.2097021	3.238E-32	-1.17984	down	PREDICTED: 30S ribosomal protein S5, chloroplastic [Sesamum indicum]
c18889.graph_c0	10.7276735	10.4096887	12.5194713	9.78579376	10.6315189	8.30972649	1.585E-09	-1.32229	down	histone h4, partial [Cysticapnos vesicaria]
c18892.graph_c0	1.76071547	0.88123928	0.67877673	5.56659868	7.85691804	6.63071243	0.0001261	1.5142	up	hypothetical protein CICLE_v10006109mg [Citrus clementina]
c18903.graph_c0	31.8662863	27.7924872	35.6787195	12.8030992	14.8036992	12.9108982	1.514E-20	-2.32748	down	PREDICTED: transmembrane ascorbate ferrireductase 1 [Sesamum indicum]
c18924.graph_c0	2.62904921	2.30272158	1.37248813	1.43308336	1.24002575	2.29004429	0.0014186	-1.42518	down	-
c18926.graph_c0	0.05423749	0	0	1.28113354	0.90759872	1.15797545	2.653E-12	4.889	up	PREDICTED: BTB/POZ domain-containing protein At5g66560 [Sesamum indicum]
c18936.graph_c0	2.13649196	1.9247682	1.83031783	0	0	0	1.501E-32	-Inf	down	-
c18949.graph_c0	135.04453	137.152955	138.637056	17.8970631	17.9457298	19.4099552	0	-3.98491	down	hypothetical protein MIMGU_mgv1a005690mg [Erythranthe guttata]
c18956.graph_c0	0.05843633	0.05849484	0.075093	1.44402038	2.64597752	1.69815097	3.117E-06	3.81829	up	unnamed protein product [Coffea canephora]
c18990.graph_c0	2.005296	3.19343818	1.75696688	85.7900754	100.48916	96.2217156	7.72E-107	4.25633	up	-
c19012.graph_c0	11.1347011	6.74617284	5.91706198	0	0	0	3.943E-25	-Inf	down	uncharacterized protein LOC100191867 precursor [Zea mays]
c19020.graph_c0	51.9644699	49.6064734	45.3770139	50.3080295	54.2447963	42.7167766	9.255E-16	-1.08674	down	-
c19035.graph_c0	8.95781246	8.45439479	8.16743051	4.99137296	4.28280432	5.41369434	1.172E-30	-1.88896	down	PREDICTED: putative pectinesterase 11 [Sesamum indicum]
c19039.graph_c0	0.1035807	0.10368442	0	2.74778776	2.85484323	3.50147466	2.034E-13	4.38772	up	PREDICTED: probable WRKY transcription factor 65 [Sesamum indicum]

#ID	0 h-1 FPKM	0 h-2 FPKM	0 h-3 FPKM	28 h-1 FPKM	28 h-2 FPKM	28 h-3 FPKM	FDR	log2FC	regulated	nr_annotation
c19040.graph_c0	3.43721516	3.56808875	4.49875308	16.0066531	17.9612081	17.1004858	6.982E-15	1.05606	up	PREDICTED: UDP-sulfoquinovose synthase, chloroplastic isoform X2 [Sesamum indicum]
c19065.graph_c0	1.63163204	0.94226877	1.33060554	16.1231454	14.5988701	13.9193119	3.847E-41	2.42578	up	PREDICTED: protein SCARECROW [Sesamum indicum]
c19067.graph_c0	0.55034134	0.55089242	0	2.59990194	3.19014839	3.04625461	0.0001285	1.93406	up	-
c19070.graph_c0	37.3547876	42.2776049	44.1428931	41.7470793	40.1573388	40.1881194	4.231E-14	-1.1122	down	-
c19074.graph_c0	3.32657844	2.61635747	3.47326282	17.2479705	18.2630567	19.6404711	2.021E-26	1.45878	up	PREDICTED: probable galacturonosyltransferase 9 [Sesamum indicum]
c19080.graph_c0	5.28747724	5.85915702	4.73868164	4.96316529	4.95500748	5.58886524	6.391E-18	-1.12303	down	PREDICTED: protein INVOLVED IN DE NOVO 2-like isoform X2 [Sesamum indicum]
c19095.graph_c0	0	0.12329361	0.07913934	3.13318064	4.0817972	3.72540402	2.165E-14	4.65517	up	PREDICTED: probable glutathione S-transferase [Sesamum indicum]
c19100.graph_c0	0.95226465	0.32996015	0.32945709	15.4391641	15.0697479	15.8563953	1.932E-59	3.76822	up	PREDICTED: protein notum homolog [Sesamum indicum]
c19110.graph_c0	0.0576275	0.11537041	0.14810723	2.42923335	2.45808702	2.73411666	3.442E-10	3.46924	up	-
c19118.graph_c0	172.324738	179.907123	170.886095	51.1950384	48.8635237	41.4290591	0	-2.9776	down	hypothetical protein M569_08491 [Genlisea aurea]
c19122.graph_c0	320.795999	336.387866	305.974354	94.1178354	99.0290833	105.586303	0	-2.77831	down	-
c19130.graph_c0	13.0659878	12.9776832	13.1459029	11.6311083	9.83712937	11.1728192	2.362E-12	-1.35409	down	hypothetical protein MIMGU_mgv1a000569mg [Erythranthe guttata]
c19155.graph_c0	0.44680628	0.48452483	0.33492886	6.27823377	6.49940783	4.88008722	3.853E-17	2.71366	up	PREDICTED: transcription factor bHLH143-like [Sesamum indicum]
c19173.graph_c0	0.495028	1.02644193	0.63613057	9.71415434	10.0239664	8.76547174	1.421E-26	2.63206	up	PREDICTED: protein ECERIFERUM 26-like [Sesamum indicum]
c19181.graph_c0	0.34341309	0.44197325	0.31521387	1.67582299	2.57550814	2.50215696	0.0004294	1.52906	up	PREDICTED: myb-related protein 308-like [Sesamum indicum]
c19184.graph_c0	0.51117079	0.63960331	0.78003871	0.41795548	0.35640936	0.60630802	0.0001357	-1.57698	down	PREDICTED: F-box protein At5g51370-like [Sesamum indicum]
c19192.graph_c0	2.62112622	2.68338158	1.68412548	11.9496673	13.9558921	11.7999186	1.505E-08	1.34603	up	PREDICTED: probable carbohydrate esterase At4g34215 [Sesamum indicum]
c19206.graph_c0	0.27214243	0.61293361	0.21857112	16.639214	20.0687392	16.1598033	7.558E-56	4.4954	up	PREDICTED: lysine histidine transporter-like 8 [Sesamum indicum]
c19214.graph_c0	0	0	0	14.342881	12.150877	15.0991841	1.223E-28	Inf	up	hypothetical protein MIMGU_mgv1a015553mg [Erythranthe guttata]
c19228.graph_c0	9.13013374	9.48199904	10.4126677	1.57598554	1.5727532	1.42135884	1.29E-70	-3.75969	down	PREDICTED: zinc finger CCCH domain-containing protein 48-like [Sesamum indicum]
c19232.graph_c0	3.24809785	3.40804192	2.96701357	3.21396343	2.85054644	3.08673922	6.748E-07	-1.16158	down	-
c19237.graph_c0	1.87028547	1.94149747	2.3143764	2.01379659	1.77281277	1.43783638	0.0002963	-1.32419	down	hypothetical protein MIMGU_mgv1a013302mg [Erythranthe guttata]
c19281.graph_c0	561.128627	554.807207	558.743233	363.863427	377.712377	385.797435	6.614E-82	-1.66129	down	PREDICTED: putative DNA-binding protein ESCAROLA [Sesamum indicum]
c19282.graph_c0	0	0	0	12.3319158	11.5799463	12.3229894	8.932E-54	Inf	up	PREDICTED: cytosolic sulfotransferase 12-like [Sesamum indicum]
c19300.graph_c0	1.07845777	0.53976884	1.24727484	3.99746616	5.37862005	4.02940556	0.0060032	1.1304	up	BnaC04g47180D [Brassica napus]
c19303.graph_c0	0.15964309	0.15980295	0.30772154	3.48083032	3.79762663	2.88766793	4.262E-12	2.91762	up	PREDICTED: aspartic proteinase PCS1 [Sesamum indicum]

#ID	0 h-1 FPKM	0 h-2 FPKM	0 h-3 FPKM	28 h-1 FPKM	28 h-2 FPKM	28 h-3 FPKM	FDR	log2FC	regulated	nr_annotation
c19305.graph_c0	21.3995982	22.6834076	17.2693773	18.0736814	17.068834	18.9199476	6.206E-14	-1.26974	down	PREDICTED: uncharacterized protein LOC105174914 [Sesamum indicum]
c19327.graph_c0	0	0	0.14040454	2.46171515	3.65669667	3.88788283	1.091E-11	5.02587	up	predicted protein [Hordeum vulgare subsp. vulgare]
c19336.graph_c0	101.882514	120.656094	89.2795173	31.1892368	36.5666341	34.3419662	3.212E-27	-2.69922	down	PREDICTED: probable WRKY transcription factor 7 [Sesamum indicum]
c19337.graph_c0	0	0.151852	0.09747031	2.20509508	1.84166955	1.48445515	1.104E-08	3.36946	up	unnamed protein product [Coffea canephora]
c19349.graph_c0	1.00335107	1.86523214	1.84192263	31.7735829	34.3635723	31.1973875	9.727E-84	3.27258	up	PREDICTED: LRR receptor-like serine/threonine-protein kinase HSL2 [Sesamum indicum]
c19358.graph_c0	0.16094826	0.53703142	0.13788326	1.32573104	1.65469144	1.74994746	0.0095291	1.41577	up	hypothetical protein MIMGU_mgv1a000332mg [Erythranthe guttata]
c19362.graph_c0	0	0.03629732	0	3.58418386	3.73588654	3.35476252	7.596E-24	7.11802	up	PREDICTED: lactosylceramide 4-alpha-galactosyltransferase-like [Sesamum indicum]
c19374.graph_c0	1.26716495	1.5993296	1.20356831	7.86827163	8.27932492	7.92345042	2.834E-13	1.47484	up	PREDICTED: pentatricopeptide repeat-containing protein At1g77360, mitochondrial-like [Sesamum indicum]
c19380.graph_c0	0	0	0	4.16164644	5.60340361	4.08100108	3.98E-24	Inf	up	PREDICTED: uncharacterized protein LOC105170375 [Sesamum indicum]
c19383.graph_c0	5.93652485	2.97123469	4.0773923	1.33902374	0.67167383	1.33543964	1.416E-06	-3.04115	down	-
c19389.graph_c0	0.0529669	0	0	3.15667515	2.78066507	2.35593398	3.508E-15	6.23088	up	PREDICTED: probable receptor-like serine/threonine-protein kinase At4g34500 [Sesamum indicum]
c19395.graph_c0	1.06117983	0.69045759	0.81819486	9.87212012	8.91359887	10.0694617	9.767E-17	2.40149	up	PREDICTED: uncharacterized protein LOC105159543 [Sesamum indicum]
c19414.graph_c0	2.22766283	1.95960338	1.99516981	1.66811471	1.59474831	1.24106492	1.508E-05	-1.54762	down	-
c19429.graph_c0	1.14471221	1.32815413	1.2369777	6.22086837	6.5046322	6.78880921	5.118E-10	1.30427	up	PREDICTED: uncharacterized protein LOC105163794 isoform X2 [Sesamum indicum]
c19430.graph_c0	8.9739926	6.41641335	7.00153244	0	0	0	1.281E-60	-Inf	down	40S ribosomal protein S6-B [Mucor circinelloides f. circinelloides 1006PhL]
c19433.graph_c0	0.23146885	0.1853605	0.41642529	175.363187	160.888371	135.489922	4.83E-126	8.04418	up	PREDICTED: GDSL esterase/lipase At1g71250 [Sesamum indicum]
c19445.graph_c0	0	0	0	2.64546592	3.61703451	2.46706125	5.574E-14	Inf	up	PREDICTED: T-complex protein 1 subunit epsilon [Nicotiana tomentosiformis]
c19453.graph_c0	41.5376555	46.7950207	35.8365055	10.6142693	10.0171232	11.6207484	4.446E-44	-3.03254	down	PREDICTED: protein ECERIFERUM 1-like [Sesamum indicum]
c19467.graph_c0	0.84134946	0.90381575	0.76472814	4.34007295	5.03637776	4.38119268	1.696E-09	1.36497	up	hypothetical protein MIMGU_mgv1a023991mg [Erythranthe guttata]
c19468.graph_c0	0.65296846	0.20111456	0.12909082	5.54886192	5.40563643	4.79592097	3.881E-13	2.92809	up	PREDICTED: probable sugar phosphate/phosphate translocator At1g12500 [Sesamum indicum]
c19492.graph_c0	135.002675	136.635693	133.050524	20.9953265	20.4024387	19.3998501	0	-3.82533	down	l-asparaginase, putative [Ricinus communis]
c19498.graph_c0	0	0	0	0.92276334	1.13072906	2.07066022	1.782E-05	Inf	up	predicted protein [Physcomitrella patens]
c19507.graph_c0	347.873114	190.615216	181.239426	0	0	0	9.609E-31	-Inf	down	-

#ID	0 h-1 FPKM	0 h-2 FPKM	0 h-3 FPKM	28 h-1 FPKM	28 h-2 FPKM	28 h-3 FPKM	FDR	log2FC	regulated	nr_annotation
c19511.graph_c0	4.36867519	4.5533817	4.2249093	29.099884	29.1708648	29.354386	2.168E-32	1.64666	up	PREDICTED: histone-lysine N-methyltransferase family member SUVH9 [Sesamum indicum]
c19514.graph_c0	3.74253578	2.59822878	2.28829913	22.1991458	23.6088407	20.5486574	3.675E-35	1.85745	up	PREDICTED: omega-6 fatty acid desaturase, chloroplastic [Sesamum indicum]
c19516.graph_c0	5.54320052	5.25975374	4.37781975	22.4519782	24.6674623	23.6284772	4.916E-12	1.13274	up	PREDICTED: myosin heavy chain 1B isoform X1 [Sesamum indicum]
c19520.graph_c0	16.5809509	17.5435288	20.0927796	12.987541	14.0380117	12.9414556	1.13E-15	-1.53321	down	PREDICTED: calcium-binding protein PBP1-like [Sesamum indicum]
c19522.graph_c0	0.54875865	0.5053635	0.42310596	8.18408654	8.62824216	7.87578832	6.866E-42	2.9751	up	PREDICTED: putative leucine-rich repeat receptor-like serine/threonine-protein kinase At2g14440 [Sesamum indicum]
c19529.graph_c0	1.00161765	0.91543621	1.06327166	0.2848578	0.60013307	0.36157588	8.602E-09	-2.35077	down	-
c19541.graph_c0	0.73820271	0.7389419	0.63241314	7.04631534	6.9434764	8.17221328	1.018E-13	2.30532	up	PREDICTED: origin recognition complex subunit 1-like isoform X1 [Sesamum indicum]
c19554.graph_c0	2.92788845	1.85756215	1.5367764	18.2825802	21.2702627	20.2988557	4.837E-31	2.15969	up	PREDICTED: uncharacterized protein LOC105168795 [Sesamum indicum]
c19560.graph_c0	26.3138584	25.35245	26.1457384	14.7875382	15.2327244	15.9679225	4.255E-68	-1.84903	down	PREDICTED: glutathione S-transferase omega-like 2 [Sesamum indicum]
c19565.graph_c0	27.6032281	28.6247847	23.9877457	3.17525954	2.21537946	1.76658126	7.024E-73	-4.57655	down	-
c19570.graph_c0	0.85576057	0.32946826	0.84591247	7.79844476	6.30689944	7.65177656	3.427E-11	2.32906	up	PREDICTED: probable glucan 1,3-alpha-glucosidase [Sesamum indicum]
c19577.graph_c0	2406.73122	2236.25457	2518.98785	239.328416	250.905393	287.38666	6.72E-160	-4.29387	down	PREDICTED: reticulon-like protein B13 [Sesamum indicum]
c19580.graph_c0	1.13834323	1.82317297	1.1702529	17.3741673	19.6215053	16.7875798	2.713E-23	2.61236	up	expansin 5 [Cucumis sativus]
c19592.graph_c0	0	0.12837874	0	4.66057949	6.39626079	5.32419273	9.775E-14	5.91334	up	PREDICTED: protein WALLS ARE THIN 1-like [Nicotiana sylvestris]
c19593.graph_c0	1.57099471	1.50704416	0.7570464	12.4169651	10.2233815	10.947372	1.224E-12	2.04847	up	PREDICTED: transcription factor bHLH95-like isoform X1 [Sesamum indicum]
c19602.graph_c0	0	0	0	7.24874476	5.98394229	5.06992846	1.136E-10	Inf	up	-
c19602.graph_c1	0.11008628	0.66117911	0.4243957	75.6895326	70.9412489	71.7510386	1.51E-134	6.41584	up	PREDICTED: NAC domain-containing protein 100-like isoform X2 [Nicotiana tomentosiformis]
c19608.graph_c0	129.339462	132.063344	124.510637	20.1409501	22.9606897	24.0315354	0	-3.61275	down	hypothetical protein MIMGU_mgv1a012664mg [Erythranthe guttata]
c19622.graph_c0	5.11909416	5.32438501	4.00861419	22.4975686	24.4073274	21.986706	8.993E-17	1.16533	up	PREDICTED: cyclin-D3-3-like [Sesamum indicum]
c19632.graph_c0	0	0	0	3.57255279	2.86050887	6.1603675	7.294E-07	Inf	up	NADH dehydrogenase subunit 1 (mitochondrion) [Cyanoptyche gloeocystis]
c19652.graph_c0	0	0.1268796	0.16288221	3.17824745	2.91124873	3.04445422	1.416E-11	3.86925	up	PREDICTED: probable nucleoredoxin 2 [Sesamum indicum]
c19656.graph_c0	0.03091457	0	0.03972644	3.66237006	4.46313431	3.11680301	8.226E-26	6.2134	up	hypothetical protein M569_04675 [Genlisea aurea]
c19657.graph_c0	15.6245196	15.3621178	16.5979178	5.75361081	4.83041173	6.79524918	9.477E-54	-2.54316	down	PREDICTED: auxin-induced protein 15A [Sesamum indicum]
c19668.graph_c0	2.41526061	1.53316237	1.89250336	15.5845045	14.7285247	15.5466721	6.129E-17	1.88484	up	PREDICTED: uncharacterized protein LOC105169149 [Sesamum indicum]
c19685.graph_c0	0.73197928	0.87925469	0.56437341	3.29838958	4.00287169	3.6754332	0.000732	1.24796	up	hypothetical protein MIMGU_mgv1a026794mg [Erythranthe guttata]

#ID	0 h-1 FPKM	0 h-2 FPKM	0 h-3 FPKM	28 h-1 FPKM	28 h-2 FPKM	28 h-3 FPKM	FDR	log2FC	regulated	nr_annotation
c19686.graph_c0	5.6757177	4.99963295	6.12655845	2.31004419	2.60719165	2.28890088	1.545E-12	-2.31376	down	-
c19697.graph_c0	0	0	0	7.178556	6.85193804	8.36806153	2.443E-13	Inf	up	PREDICTED: non-specific lipid-transfer protein 2-like [Sesamum indicum]
c19719.graph_c0	36.3377323	36.5027631	33.0294781	4.43670233	5.94563787	5.06843802	0	-3.8662	down	SPla/RYanodine receptor domain-containing protein [Theobroma cacao]
c19727.graph_c0	17.4585684	9.5244475	11.8905243	0	0	0	2.554E-32	-Inf	down	--
c19728.graph_c0	0.14285693	0.07149999	0.27536522	2.90717267	2.01555155	1.90625557	2.828E-07	2.69816	up	PREDICTED: uncharacterized protein LOC105160245 [Sesamum indicum]
c19729.graph_c0	15.076573	11.8159586	15.3940644	7.72975614	10.3921726	8.31747413	1.037E-12	-1.76972	down	hypothetical protein MIMGU_mgv1a012036mg [Erythranthe guttata]
c19742.graph_c0	2.04525583	1.78594591	1.73351652	0.28464514	0.31412073	0.49034378	5.392E-20	-3.43942	down	-
c19746.graph_c0	2976.86331	2819.2395	3061.80676	213.463914	193.085385	247.669028	0	-4.84915	down	PREDICTED: 17.3 kDa class I heat shock protein-like [Sesamum indicum]
c19755.graph_c0	0.06001965	0.06007975	0	33.748839	31.2728966	31.6203536	0	8.58189	up	PREDICTED: probable beta-D-xylosidase 2 [Sesamum indicum]
c19769.graph_c0	3.95417972	2.63875949	2.29155651	31.4999243	32.9697279	27.910795	1.251E-31	2.29318	up	-
c19778.graph_c0	0.7245456	0.72527112	1.70696126	6.7579764	8.87537319	6.94677916	8.011E-06	1.73472	up	PREDICTED: uncharacterized protein LOC102622885 [Citrus sinensis]
c19787.graph_c0	493.291072	478.612443	506.13531	68.2793471	69.9715933	72.1362992	0	-3.90337	down	hypothetical protein MIMGU_mgv1a013874mg [Erythranthe guttata]
c19789.graph_c0	16.0787182	19.2998474	18.4256651	78.7155458	76.5082705	78.4314659	1.462E-23	1.0269	up	PREDICTED: plasminogen activator inhibitor 1 RNA-binding protein-like [Sesamum indicum]
c19792.graph_c0	12.6417582	11.3223731	12.458694	1.03623575	1.09156102	0.76100392	1.53E-137	-4.74853	down	hypothetical protein MIMGU_mgv1a004859mg [Erythranthe guttata]
c19801.graph_c0	11.7672543	5.68643188	7.99521649	124.353464	130.386255	129.225647	1.984E-93	2.82862	up	unnamed protein product [Coffea canephora]
c19806.graph_c0	3.35690653	3.2861444	2.66437981	24.4157427	24.5557764	22.6750913	1.511E-52	1.85645	up	PREDICTED: probable dolichyl-diphosphooligosaccharide--protein glycosyltransferase subunit 3B [Sesamum indicum]
c19810.graph_c0	11.2872278	10.9715105	11.0620691	5.60354308	6.29218485	6.28711242	5.008E-86	-1.96404	down	PREDICTED: probable receptor protein kinase TMK1 [Sesamum indicum]
c19815.graph_c0	0.18894528	0.41609586	0.63128564	4.17465999	3.91811576	2.93585518	3.071E-08	2.05249	up	PREDICTED: radical S-adenosyl methionine domain-containing protein 1, mitochondrial [Sesamum indicum]
c19818.graph_c0	4.86687728	5.16075288	5.03510465	29.0021929	27.7696163	27.689308	9.391E-44	1.3964	up	PREDICTED: nuclear pore complex protein NUP93A-like [Sesamum indicum]
c19819.graph_c0	15.0248126	8.39114011	10.1835179	0	0	0	1.779E-32	-Inf	down	unnamed protein product [Chondrus crispus]
c19832.graph_c0	0	0	0	5.88857755	6.43927603	11.8524103	8.111E-09	Inf	up	ATPase subunit 6 [Cycas taitungensis]
c19836.graph_c0	5.16103918	3.92922799	3.45618546	26.9439	20.1300747	21.8135631	6.683E-08	1.37092	up	PREDICTED: gibberellin 2-beta-dioxygenase [Sesamum indicum]
c19846.graph_c0	4.48013357	4.09465282	4.6307503	3.8932052	3.45128656	3.63890709	1.515E-05	-1.35702	down	hypothetical protein MIMGU_mgv1a0026501mg, partial [Erythranthe guttata]
c19852.graph_c0	2.85872384	2.95101099	1.83678666	11.2325873	13.1904275	11.7087838	5.85E-05	1.15498	up	PREDICTED: ubiquitin domain-containing protein 1-like [Sesamum indicum]

#ID	0 h-1 FPKM	0 h-2 FPKM	0 h-3 FPKM	28 h-1 FPKM	28 h-2 FPKM	28 h-3 FPKM	FDR	log2FC	regulated	nr_annotation
c19859.graph_c0	15.988221	19.2431822	17.1212475	4.35755508	7.55666833	5.07961337	5.371E-45	-2.71538	down	PREDICTED: uncharacterized protein DDB_G0287625-like [Sesamum indicum]
c19875.graph_c0	0.15775389	0.42109829	0.06757333	17.0835383	15.321311	16.4348873	7.876E-57	5.15829	up	PREDICTED: vestitone reductase-like [Sesamum indicum]
c19884.graph_c0	65.2938074	65.2196819	53.1011106	25.930676	26.4310473	26.4490248	4.642E-50	-2.30807	down	PREDICTED: uncharacterized protein LOC105166661 [Sesamum indicum]
c19885.graph_c0	16.9539225	15.4690498	16.0024453	10.1411259	9.15648583	8.80895175	2.524E-17	-1.87522	down	atypical dual-speci###city phosphatase 1 [Arabidopsis thaliana]
c19888.graph_c0	6.66948108	5.00711967	5.53514509	0	0	0	1.119E-41	-Inf	down	60S ribosomal protein L26B [Populus trichocarpa]
c19916.graph_c0	1344.43037	1348.4148	1364.68462	47.7791925	38.4736873	50.5774917	0	-5.97991	down	hypothetical protein MIMGU_mgv1a010720mg [Erythranthe guttata]
c19917.graph_c0	0.95666917	0	0.52686761	10.9261686	12.9145821	13.454548	3.804E-15	3.56772	up	-
c19925.graph_c0	5.91768344	8.57181082	7.24660069	36.4314767	33.853622	32.4528346	6.405E-15	1.14857	up	PREDICTED: uncharacterized protein At1g27050 [Sesamum indicum]
c19932.graph_c0	0.12207268	0.16292656	0	8.75387666	6.96934356	7.02243153	1.257E-35	5.24622	up	PREDICTED: uncharacterized protein LOC105155205 [Sesamum indicum]
c19933.graph_c0	3.79883537	2.53509288	3.3629171	1.59522806	2.38210304	1.45188843	2.569E-08	-1.92849	down	-
c19944.graph_c0	174.505401	168.680333	188.870882	8.05908869	8.40040093	9.29293863	5.13E-203	-5.45997	down	PREDICTED: F-box/kelch-repeat protein At1g23390-like [Sesamum indicum]
c19947.graph_c0	0	0	0	5.28585035	5.10340342	5.5089523	1.322E-20	Inf	up	PREDICTED: uncharacterized protein At1g08160-like [Sesamum indicum]
c19952.graph_c0	26.371973	26.2305942	23.5500035	24.3648279	23.9424139	25.4317529	3.376E-38	-1.13528	down	PREDICTED: DEAD-box ATP-dependent RNA helicase 7 [Sesamum indicum]
c19957.graph_c0	0.12222524	0.04893905	0.09423857	3.8731015	3.384757	3.49385792	1.799E-25	4.25142	up	PREDICTED: probable beta-D-xylosidase 7 [Sesamum indicum]
c19964.graph_c0	8.62530099	9.09812814	8.34268525	2.69626626	1.70413294	1.98011812	2.919E-32	-3.12046	down	PREDICTED: probable E3 ubiquitin-protein ligase XERICO [Sesamum indicum]
c19983.graph_c0	1.65724115	1.45153805	0.8518482	8.43131321	9.21678686	8.62442442	3.152E-11	1.64674	up	PREDICTED: glucuronokinase 1-like [Sesamum indicum]
c19987.graph_c0	39.3644392	33.1474258	39.2107517	33.0113625	30.5194752	34.5576413	9.652E-17	-1.27827	down	PREDICTED: uncharacterized protein LOC105163283 [Sesamum indicum]
c19990.graph_c0	26.4654423	24.9156474	31.5939046	4.91026067	3.56680478	4.82012946	4.397E-35	-3.73385	down	PREDICTED: benzoate carboxyl methyltransferase-like [Sesamum indicum]
c20007.graph_c0	45.7618919	52.6208884	53.2230097	50.310225	47.8962546	50.1564768	2.604E-15	-1.12317	down	unnamed protein product [Coffea canephora]
c20019.graph_c0	0.58928846	0.19662618	0.50483933	2.99804019	2.38469334	1.86390576	0.0025347	1.39707	up	PREDICTED: uncharacterized protein LOC105167731 [Sesamum indicum]
c20028.graph_c0	5.3707566	6.68656741	6.08206572	1.87852807	1.98249512	2.18980509	6.984E-37	-2.67483	down	PREDICTED: probable L-type lectin-domain containing receptor kinase S.7 [Sesamum indicum]
c20029.graph_c0	8.20482025	6.85923897	6.4883193	0.19658954	0.23666923	0.16041546	3.109E-59	-6.27148	down	hypothetical protein MIMGU_mgv1a021012mg [Erythranthe guttata]
c20032.graph_c0	32.646766	36.0153002	28.9914869	22.3557029	23.5525131	23.1917413	6.949E-30	-1.58772	down	PREDICTED: DNA topoisomerase 6 subunit A [Sesamum indicum]

#ID	0 h-1 FPKM	0 h-2 FPKM	0 h-3 FPKM	28 h-1 FPKM	28 h-2 FPKM	28 h-3 FPKM	FDR	log2FC	regulated	nr_annotation
c20045.graph_c0	15.3763512	15.2984649	12.4542963	3.92833769	4.28076626	5.52670944	1.11E-42	-2.73714	down	PREDICTED: uncharacterized protein LOC105176125 [Sesamum indicum]
c20049.graph_c0	21.4425848	18.1618938	22.1042158	103.867104	115.971463	111.65542	4E-32	1.33396	up	V-type proton ATPase 16 kDa proteolipid subunit [Morus notabilis]
c20061.graph_c0	2.35129873	2.22756464	2.05031169	9.12436072	9.6712148	9.99775799	9.679E-12	1.03019	up	PREDICTED: polygalacturonate 4-alpha-galacturonosyltransferase-like [Sesamum indicum]
c20070.graph_c0	0.92940246	1.05168092	1.03853823	0.32305773	0.31820819	0.28757724	1.09E-11	-2.793	down	-
c20073.graph_c0	27.71263	30.3129516	26.4203944	22.774387	27.8374472	24.4783503	1.748E-34	-1.25941	down	PREDICTED: ocs element-binding factor 1 [Sesamum indicum]
c20074.graph_c0	9.16627718	7.23697924	9.12459999	2.34577374	2.11801375	2.06726279	2.958E-23	-3.05811	down	-
c20085.graph_c0	26.8514898	27.2175369	26.994626	16.59126	19.5105886	18.9134778	2.673E-61	-1.64972	down	PREDICTED: UDP-glycosyltransferase 86A1-like [Sesamum indicum]
c20086.graph_c0	14.8492964	12.2915216	12.2931689	8.6650383	6.69935062	8.89117427	2.539E-26	-1.7887	down	-
c20092.graph_c0	0.20772227	0.13862018	0.3855675	2.39876703	1.74177691	1.82049499	4.153E-07	1.92441	up	PREDICTED: pentatricopeptide repeat-containing protein At4g20770 [Sesamum indicum]
c20097.graph_c0	2.14236831	1.60838517	2.06477107	0.51902031	0.62483557	0.31763725	5.585E-15	-3.086	down	hypothetical protein MIMGU_mgv1a023283mg [Erythranthe guttata]
c20112.graph_c0	0.60693055	0.36002269	0.54883948	11.4362791	12.1402938	12.0115018	3.474E-69	3.46283	up	PREDICTED: pentatricopeptide repeat-containing protein At2g15820 isoform X1 [Sesamum indicum]
c20115.graph_c0	4.93631992	4.41559663	3.91399211	0	0	0	1.78E-42	-Inf	down	-
c20120.graph_c0	0.56860528	0.15177991	0.09742404	3.49892656	3.38308313	3.32719791	3.994E-10	2.57004	up	PREDICTED: probable protein phosphatase 2C 38 [Sesamum indicum]
c20121.graph_c0	27.8863766	24.0969603	29.0968744	178.944363	188.472245	179.093626	8.473E-63	1.66109	up	hypothetical protein CICLE_v10026787mg [Citrus clementina]
c20128.graph_c0	1.80586613	1.20511629	2.02592891	7.0207964	7.63704178	8.14287567	4.824E-08	1.08555	up	PREDICTED: uncharacterized protein LOC105158144 [Sesamum indicum]
c20131.graph_c0	0.83122105	1.01035055	0.68666912	14.4343122	12.9549063	12.8170232	3.196E-48	2.90338	up	PREDICTED: protein ASPARTIC PROTEASE IN GUARD CELL 2 [Sesamum indicum]
c20135.graph_c0	0.09579202	0.03196265	0	4.01481604	5.23844286	3.69266552	2.376E-23	5.59706	up	PREDICTED: membrane protein of ER body-like protein [Sesamum indicum]
c20139.graph_c0	0.63491358	0.34221888	0.56484629	5.57291467	7.05095717	6.57497961	4.613E-14	2.54775	up	PREDICTED: protein IQ-DOMAIN 14-like [Sesamum indicum]
c20147.graph_c0	36.6726467	45.3606213	29.1159419	37.1790417	34.4888926	37.1257028	4.992E-05	-1.11779	down	PREDICTED: uncharacterized protein LOC105166783 [Sesamum indicum]
c20172.graph_c0	30.4805458	30.6905444	29.3995715	19.8856516	20.0962865	21.0328146	5.227E-72	-1.6597	down	PREDICTED: ethylene-responsive transcription factor ERF008-like [Nelumbo nucifera]
c20173.graph_c0	7.63749349	7.40472804	7.90096625	6.0047323	6.46192212	5.58350729	4.191E-16	-1.43774	down	PREDICTED: uncharacterized protein At3g17611 [Sesamum indicum]
c20177.graph_c0	1.42254605	2.10205171	2.1762238	0.93543177	1.11132524	1.48643544	4.36E-06	-1.78324	down	PREDICTED: LOW QUALITY PROTEIN: dof zinc finger protein DOF5.1-like [Sesamum indicum]
c20191.graph_c0	6.54293365	6.09637635	7.13880746	6.72929217	7.2370957	7.00413609	4.196E-09	-1.00755	down	hypothetical protein MIMGU_mgv1a014744mg [Erythranthe guttata]
c20195.graph_c0	7.69703814	6.34159827	4.71724556	30.0793839	30.616859	28.5123887	1.333E-14	1.16473	up	monodehydroascorbate reductase [Acanthus ebracteatus]

#ID	0 h-1 FPKM	0 h-2 FPKM	0 h-3 FPKM	28 h-1 FPKM	28 h-2 FPKM	28 h-3 FPKM	FDR	log2FC	regulated	nr_annotation
c20199.graph_c0	81.4503773	87.8036251	75.2413583	35.019864	27.2633857	29.5048617	4.42E-103	-2.50275	down	PREDICTED: NAC domain-containing protein 18 [Sesamum indicum]
c20225.graph_c0	0.64099947	2.08533434	2.26520167	7.27929252	7.88702238	6.55758695	0.0057578	1.01995	up	-
c20239.graph_c0	1.84894046	1.2125878	1.26991056	41.8431291	42.8010765	40.3069021	1.56E-167	3.76365	up	PREDICTED: trihelix transcription factor GTL1 isoform X1 [Sesamum indicum]
c20244.graph_c0	1.10666135	0.94951672	1.21894599	0.25278474	0.22824092	0.15001475	7.374E-18	-3.47006	down	PREDICTED: G2/mitotic-specific cyclin S13-7-like [Sesamum indicum]
c20255.graph_c0	10.6277847	6.17538818	7.43658472	0	0	0	1.541E-41	-Inf	down	--
c20259.graph_c0	4.28935444	1.66619238	2.7971294	0	0	0	5.656E-12	-Inf	down	uncharacterized protein LOC100273024 [Zea mays]
c20263.graph_c0	6.41187082	4.81371851	5.29682648	0	0	0	2.938E-30	-Inf	down	putative 40s ribosomal protein s21 protein [Neofusicoccum parvum UCRNP2]
c20279.graph_c0	0.60035519	0.41604671	0.29672315	5.23601633	5.93984511	6.29926737	7.153E-14	2.65222	up	PREDICTED: haloacid dehalogenase-like hydrolase domain-containing protein At2g33255 [Sesamum indicum]
c20280.graph_c0	5.58849168	7.14800096	6.71596981	45.6187235	48.727809	41.7663252	9.246E-42	1.71398	up	PREDICTED: 50S ribosomal protein L3, chloroplastic-like [Sesamum indicum]
c20281.graph_c0	0.08475428	0.04241957	0.10891261	1.41677523	2.00224988	1.80951186	9.845E-10	3.37275	up	PREDICTED: BTB/POZ domain-containing protein At5g60050 [Sesamum indicum]
c20284.graph_c0	3.6578188	3.20379636	2.93777624	1.82770612	1.50022478	2.03371809	1.535E-07	-1.95686	down	PREDICTED: uncharacterized protein At4g08330, chloroplastic-like [Sesamum indicum]
c20285.graph_c0	7.66766925	8.67029968	6.75130679	6.50162079	8.4794002	8.50527328	0.0001303	-1.06358	down	PREDICTED: dentin sialophosphoprotein [Sesamum indicum]
c20293.graph_c0	24.3837383	23.9031587	22.906285	153.478289	165.282071	162.361844	6.141E-90	1.66684	up	PREDICTED: 40S ribosomal protein S16-like [Sesamum indicum]
c20297.graph_c0	35.682738	39.3392451	36.4945448	25.2590136	22.197064	25.5682605	1.101E-71	-1.70088	down	PREDICTED: uncharacterized protein At4g18255 [Sesamum indicum]
c20315.graph_c0	6.05323205	5.95571578	5.85060316	28.352038	27.602355	28.9034478	1.498E-16	1.15839	up	BnaA09g38920D [Brassica napus]
c20317.graph_c0	0.64283071	0.34066292	0.43732743	2.3085456	2.60550028	2.55652377	0.0006575	1.30796	up	hypothetical protein PRUPE_ppa024913mg [Prunus persica]
c20335.graph_c0	0.89392663	0.85008067	0.516929	6.33458202	9.20991571	7.50162932	7.476E-11	2.26502	up	PREDICTED: perakine reductase-like [Sesamum indicum]
c20336.graph_c0	5.72828534	3.71649532	2.86263967	0	0	0	4.665E-17	-Inf	down	probable 40S ribosomal protein S27 [Piriformospora indica DSM 11827]
c20338.graph_c0	3.08081037	2.63695398	2.98356654	14.4406706	15.6755861	13.5311299	1.352E-11	1.23545	up	PREDICTED: BAG family molecular chaperone regulator 8, chloroplastic [Sesamum indicum]
c20366.graph_c0	2.27842677	2.91423834	3.09052797	0.55198248	0.99677685	0.75068866	8.398E-12	-2.94338	down	-
c20368.graph_c0	0.25634208	0.1140439	0	9.00488398	10.4669355	8.12493235	6.524E-49	5.1524	up	PREDICTED: protein NRT1/ PTR FAMILY 2.13-like [Sesamum indicum]
c20375.graph_c0	1.35356305	1.16522986	1.11320455	5.03686054	6.0400639	4.54351754	1.401E-05	1.01516	up	PREDICTED: exopolygalacturonase [Sesamum indicum]
c20377.graph_c0	1.32632866	1.96859453	2.23333186	9.77264135	9.93425444	8.43550004	2.769E-08	1.25101	up	PREDICTED: diphthamide biosynthesis protein 7 homolog isoform X1 [Sesamum indicum]
c20380.graph_c0	0	0	0	1.71826917	2.03410492	1.99408869	1.89E-14	Inf	up	chlorophyllase [Picrorhiza kurrooa]
c20382.graph_c0	1.76004365	1.66570756	1.80938062	25.3046958	29.8406966	27.4047112	3.926E-74	2.88771	up	PREDICTED: fasciclin-like arabinogalactan protein 1 [Sesamum indicum]

#ID	0 h-1 FPKM	0 h-2 FPKM	0 h-3 FPKM	28 h-1 FPKM	28 h-2 FPKM	28 h-3 FPKM	FDR	log2FC	regulated	nr_annotation
c20385.graph_c0	40.9855089	41.8933078	38.5207617	20.3781303	19.130111	18.0466916	5.01E-124	-2.16679	down	hypothetical protein MIMGU_mgv1a003020mg [Erythranthe guttata]
c20387.graph_c0	94.224968	102.419745	95.4753915	32.7523391	29.5391014	27.8054652	4.35E-205	-2.78792	down	PREDICTED: uncharacterized protein LOC105158345 [Sesamum indicum]
c20394.graph_c0	2.08641542	1.79014684	0.76603612	16.8969696	10.9532979	12.0201239	2.631E-05	2.02238	up	-
c20398.graph_c0	15.505617	8.50788608	9.88886834	0	0	0	5.916E-24	-Inf	down	--
c20399.graph_c0	0.83630069	1.17199337	1.1463248	8.75258674	8.01252582	6.97671432	7.279E-10	1.81757	up	myb family transcription factor family protein [Populus trichocarpa]
c20408.graph_c0	0.21806392	0.13096937	0.11208831	2.18712888	2.11787176	2.48303208	1.489E-08	2.79753	up	PREDICTED: aspartic proteinase nepenthesin-1 [Sesamum indicum]
c20413.graph_c0	2.67950017	2.42673726	2.24085422	2.13291626	1.36761372	1.58909945	1.014E-08	-1.61811	down	PREDICTED: probable methyltransferase PMT26 [Sesamum indicum]
c20415.graph_c0	27.789405	27.1489501	26.9169126	224.898322	220.641099	205.588489	7.26E-100	1.90124	up	PREDICTED: probable E3 ubiquitin-protein ligase RHA2B [Sesamum indicum]
c20444.graph_c0	0.41049386	0.24654294	0.66816775	7.65751061	8.24294151	6.86519785	4.443E-33	3.00239	up	PREDICTED: transcription factor DIVARICATA-like [Sesamum indicum]
c20447.graph_c0	1.1749342	0.48428088	0.97695333	17.0787081	17.8242516	18.9776705	3.02E-33	3.26377	up	PREDICTED: uncharacterized protein At5g01610-like [Sesamum indicum]
c20460.graph_c0	4.7822646	5.08013821	4.13873834	2.30532605	2.75397572	2.69145896	1.316E-16	-1.9415	down	PREDICTED: lysM domain receptor-like kinase 3 [Sesamum indicum]
c20472.graph_c0	118.075129	112.735985	114.014348	68.6639145	66.211898	76.0491109	1.82E-100	-1.79873	down	PREDICTED: uncharacterized protein LOC105175062 [Sesamum indicum]
c20473.graph_c0	0.43637762	0.39313313	0.47664814	4.02789056	4.3097601	4.06311773	2.08E-15	2.15476	up	PREDICTED: uncharacterized protein LOC105174198 [Sesamum indicum]
c20480.graph_c0	1.1853104	1.08479754	0.82686415	22.7922139	21.8459884	22.173304	2.022E-81	3.34499	up	PREDICTED: protein notum homolog [Sesamum indicum]
c20483.graph_c0	2.52553295	2.85426342	3.35010046	0.35526635	0.3207722	0.57978887	1.638E-20	-3.88849	down	-
c20506.graph_c0	18.1048829	18.0305478	19.7044467	13.6620395	13.3660282	11.9424499	5.001E-43	-1.61119	down	PREDICTED: probable 2-oxoglutarate-dependent dioxygenase At3g49630 isoform X1 [Sesamum indicum]
c20508.graph_c0	11.7511843	12.3604346	10.4986077	3.00957755	3.40282814	3.82749362	1.46E-86	-2.84517	down	PREDICTED: cellulose synthase-like protein D3 isoform X1 [Sesamum indicum]
c20512.graph_c0	10.7333417	15.471489	7.17223993	8.42500539	9.57917253	10.1847935	0.009312	-1.32823	down	-
c20514.graph_c0	0	0.06217232	0	3.65644354	3.62749243	4.45702465	3.264E-17	6.4804	up	PREDICTED: uncharacterized protein LOC105156631 [Sesamum indicum]
c20522.graph_c0	44.3085878	46.2779976	42.5554404	26.5844341	30.8685348	29.7219386	9.248E-85	-1.70084	down	PREDICTED: zinc finger protein GIS2-like isoform X2 [Sesamum indicum]
c20541.graph_c0	1.47445508	0.32798478	0.63157772	8.45393411	8.27816141	7.67561908	1.6E-06	2.24476	up	Os06g0708300 [Oryza sativa Japonica Group]
c20547.graph_c0	1.02782737	1.1079994	1.01599913	6.95302311	8.6386364	6.44728418	4.167E-11	1.71453	up	hypothetical protein MIMGU_mgv1a006570mg [Erythranthe guttata]
c20552.graph_c0	165.075907	173.46402	178.445608	20.3262129	18.9652367	19.4204844	1.11E-232	-4.22984	down	hypothetical protein MIMGU_mgv1a010327mg [Erythranthe guttata]
c20557.graph_c0	0.28981944	0.72527412	0.74485891	2.42234963	3.13809138	3.52353557	0.0021519	1.27003	up	PREDICTED: UDP-galactose transporter 2-like [Sesamum indicum]

#ID	0 h-1 FPKM	0 h-2 FPKM	0 h-3 FPKM	28 h-1 FPKM	28 h-2 FPKM	28 h-3 FPKM	FDR	log2FC	regulated	nr_annotation
c20560.graph_c1	0.71046499	0.38791441	0.82997786	3.28593912	4.32318977	4.51990486	5.492E-05	1.55912	up	poly(A)-binding protein [Nicotiana tabacum]
c20573.graph_c0	2.41971637	3.41583755	3.26888936	0.36074583	0.32571966	0.36795706	1.79E-24	-4.20301	down	-
c20574.graph_c0	35.9055724	37.7164166	28.2536799	33.0548866	34.4997267	32.8612543	7.807E-11	-1.10836	down	PREDICTED: LOW QUALITY PROTEIN: serrate RNA effector molecule [Pyrus x bretschneideri]
c20588.graph_c0	54.9067961	56.770743	52.3745457	4.3874181	5.45011638	4.1045702	0	-4.64751	down	PREDICTED: protein BPS1, chloroplastic-like [Sesamum indicum]
c20601.graph_c0	2182.4846	2192.99073	2245.65722	180.48652	161.371419	229.601929	0	-4.6237	down	PREDICTED: chaperone protein dnaJ 20, chloroplastic-like [Nelumbo nucifera]
c20602.graph_c0	0	0.04873447	0	5.23690559	5.33546352	5.13947602	9.075E-26	7.25088	up	PREDICTED: anthocyanidin 3-O-glucosyltransferase 2-like [Sesamum indicum]
c20607.graph_c0	0	0	0	2.3705486	2.44615215	2.43175215	5.101E-13	Inf	up	unnamed protein product [Coffea canephora]
c20619.graph_c0	2.43403027	2.50919795	2.56761775	0.71289638	0.90591802	0.73253331	5.134E-21	-2.76736	down	PREDICTED: G2/mitotic-specific cyclin-1-like [Sesamum indicum]
c20625.graph_c0	3.98220196	2.75711443	2.04691487	1.76066354	2.59145175	1.81061932	0.0007457	-1.59581	down	PREDICTED: probable anion transporter 3, chloroplastic [Sesamum indicum]
c20635.graph_c0	1.31827676	0.92371777	1.41169819	18.3319518	20.0123892	19.4671883	7.252E-48	2.89139	up	PREDICTED: uncharacterized protein LOC105169209 [Sesamum indicum]
c20639.graph_c0	11.4129153	12.8556998	11.1951967	70.4772804	74.373329	73.9983254	1.236E-70	1.53563	up	PREDICTED: T-complex protein 1 subunit alpha [Sesamum indicum]
c20647.graph_c0	13.8998822	15.255741	15.4138214	3.28198311	4.44498455	4.14264173	2.127E-63	-3.00035	down	PREDICTED: uncharacterized protein LOC105174183 [Sesamum indicum]
c20653.graph_c0	0	0	0	2.6670525	3.89000502	5.85925055	2.655E-07	Inf	up	predicted protein [Hordeum vulgare subsp. vulgare]
c20673.graph_c0	20.6115627	11.1616831	11.7236099	0	0	0	8.956E-20	-Inf	down	-
c20674.graph_c0	0.04193356	0.1679022	0.10777257	2.49911966	2.00880931	1.54188044	8.502E-09	3.15474	up	PREDICTED: probable transcription factor GLK1 [Sesamum indicum]
c20676.graph_c0	3.89879309	2.6017981	3.24183167	0	0	0	1.578E-39	-Inf	down	-
c20678.graph_c0	8.43904527	6.99935358	7.74607663	1.48955201	1.50315237	1.42995562	3.165E-27	-3.4803	down	PREDICTED: homeobox-leucine zipper protein HAT22-like [Sesamum indicum]
c20681.graph_c0	0.63210801	0.2711747	0.46416218	3.15025686	3.61474128	3.26678367	0.0001164	1.78678	up	PREDICTED: ras-related protein RABC2a-like [Sesamum indicum]
c20682.graph_c0	15.0215957	11.5907415	10.2549022	7.16468104	5.64757043	6.49591247	8.983E-13	-2.01902	down	PREDICTED: protein MKS1 [Sesamum indicum]
c20683.graph_c0	1.18632972	0.86780136	0.70360646	4.34425023	4.0721625	4.41079582	0.0007469	1.13341	up	PREDICTED: GDSL esterase/lipase At4g10955-like [Sesamum indicum]
c20685.graph_c0	164.27113	165.182303	166.309129	25.3601118	23.3601104	26.6412236	0	-3.80794	down	PREDICTED: F-box/kelch-repeat protein At5g43190 [Sesamum indicum]
c20709.graph_c0	23.8910808	23.6049948	22.6277628	24.2129937	22.3265957	23.9032912	7.72E-27	-1.08311	down	PREDICTED: ABSCISIC ACID-INSENSITIVE 5-like protein 2 [Sesamum indicum]
c20713.graph_c0	234.818836	248.452814	226.559352	16.3067513	19.3663792	21.2823211	0	-4.72876	down	PREDICTED: uncharacterized protein At1g76070 [Sesamum indicum]
c20718.graph_c0	0.09706982	0.09716702	0	1.83429443	2.29319431	2.70569831	1.685E-09	4.06751	up	-
c20743.graph_c0	13.3143983	12.5536453	13.7826761	57.3523111	55.093331	57.6263999	1.151E-28	1.00969	up	PREDICTED: COP9 signalosome complex subunit 1-like [Sesamum indicum]

#ID	0 h-1 FPKM	0 h-2 FPKM	0 h-3 FPKM	28 h-1 FPKM	28 h-2 FPKM	28 h-3 FPKM	FDR	log2FC	regulated	nr_annotation
c20752.graph_c0	0	0.19241464	0.37051962	2.16544231	2.90125172	1.88098216	2.048E-06	2.51007	up	-
c20754.graph_c0	0.06319426	0.06325754	0	1.83716883	1.24409341	2.09876067	1.293E-08	4.28783	up	PREDICTED: ATP-dependent 6-phosphofructokinase 6-like [Sesamum indicum]
c20761.graph_c0	0.33520481	0.16777024	0.28716764	3.32954066	3.50119659	3.97591901	9.905E-15	2.68438	up	PREDICTED: inorganic phosphate transporter 2-1, chloroplastic-like [Sesamum indicum]
c20762.graph_c0	21.3045264	10.9775737	12.7460876	0	0	0	1.041E-23	-Inf	down	cytochrome c oxidase subunit I [Lingulodinium polyedrum]
c20763.graph_c0	0.68363213	1.12710982	0.98184662	13.7659416	14.5141083	14.4286668	2.94E-38	2.84148	up	PREDICTED: uncharacterized protein LOC105157658 [Sesamum indicum]
c20765.graph_c0	1.05501725	0.56865506	1.0428761	0.91423824	0.63907464	0.67381633	0.0049132	-1.3521	down	PREDICTED: uncharacterized protein LOC105172642 [Sesamum indicum]
c20780.graph_c0	18.9011012	16.6289306	16.6035784	76.8425262	89.5371441	81.7501854	2.915E-26	1.16142	up	PREDICTED: NADH dehydrogenase [ubiquinone] 1 beta subcomplex subunit 3-B [Sesamum indicum]
c20793.graph_c0	27.3477849	27.0229156	24.5484096	188.824019	200.576923	201.602406	8.33E-105	1.81567	up	PREDICTED: aquaporin AQPcic-like [Sesamum indicum]
c20797.graph_c0	106.189933	109.450259	111.044639	545.60722	593.947745	559.31674	3.335E-60	1.28738	up	PREDICTED: 40S ribosomal protein S3-3-like [Sesamum indicum]
c20802.graph_c0	0.31497287	0.39411034	0.2023763	1.23044751	1.31767245	1.82127301	0.0089709	1.17813	up	PREDICTED: pentatricopeptide repeat-containing protein At1g09220, mitochondrial [Sesamum indicum]
c20804.graph_c0	20.7643723	21.2505042	15.9301735	18.6953488	16.7784595	16.4193073	1.159E-11	-1.24653	down	PREDICTED: LOW QUALITY PROTEIN: probable LRR receptor-like serine/threonine-protein kinase At1g56140 [Sesamum indicum]
c20809.graph_c0	2.52116588	2.00154759	1.89919191	0.44229592	0.62755275	0.51558552	1.186E-13	-3.10599	down	-
c20810.graph_c0	27.3130531	30.9896517	30.8597821	171.719298	182.00772	172.449367	1.516E-68	1.46908	up	PREDICTED: adenylate kinase [Sesamum indicum]
c20811.graph_c0	14.046012	12.8267369	11.3997989	14.6859994	14.3920386	11.2529449	0.0002706	-1.01363	down	-
c20815.graph_c0	39.8855724	40.3745011	40.1700804	315.163302	304.736811	293.061624	3.19E-117	1.83151	up	PREDICTED: tubulin alpha-3 chain [Sesamum indicum]
c20826.graph_c0	3.86244748	2.28653046	2.56175377	0	0	0	8.637E-27	-Inf	down	Fructose-bisphosphate aldolase A [Rozella allomycis CSF55]
c20831.graph_c0	6.59956081	4.39115957	5.9863743	27.0862462	25.2460767	24.6577174	1.119E-14	1.09061	up	PREDICTED: isocitrate dehydrogenase [NADP] isoform X1 [Sesamum indicum]
c20841.graph_c0	0.13293002	0.13306313	0.17082034	2.99499601	3.66375563	4.80894933	1.616E-09	3.62209	up	Chain O, Localization Of The Small Subunit Ribosomal Proteins Into A 5.5 A Cryo-em Map Of Triticum Aestivum Translating 80s Ribosome
c20850.graph_c0	1.45604889	1.80604116	1.66770215	7.95986493	10.6974307	8.95434433	7.034E-10	1.39357	up	PREDICTED: uncharacterized protein LOC105161748 [Sesamum indicum]
c20853.graph_c0	0.02622689	0.05250631	0.10110779	1.35337045	1.32523141	1.11989331	3.841E-10	3.29392	up	PREDICTED: B3 domain-containing protein Os01g0234100-like [Sesamum indicum]
c20865.graph_c0	0.97699654	1.32725158	1.07612505	0.8749144	0.68692667	1.03467102	5.195E-08	-1.46981	down	PREDICTED: 125 kDa kinesin-related protein [Sesamum indicum]
c20868.graph_c0	2.49157738	2.36280535	4.04434809	0.09530864	0.25816429	0.46662641	9.759E-09	-4.53285	down	-
c20871.graph_c0	196.431259	208.112584	192.338467	136.735304	131.271493	134.694673	4.852E-87	-1.65778	down	PREDICTED: branchpoint-bridging protein [Sesamum indicum]

#ID	0 h-1 FPKM	0 h-2 FPKM	0 h-3 FPKM	28 h-1 FPKM	28 h-2 FPKM	28 h-3 FPKM	FDR	log2FC	regulated	nr_annotation
c20872.graph_c0	1.89138441	1.19575475	2.04674008	18.0874643	21.1653446	22.1978717	5.607E-20	2.48881	up	hypothetical protein MIMGU_mgv1a013356mg [Erythranthe guttata]
c20880.graph_c0	0	0	0	14.5181669	14.4584748	27.9473095	2.172E-15	Inf	up	cytochrome c oxidase subunit I [Lingulodinium polyedrum]
c20899.graph_c0	0.44279184	0.18995796	0.08128644	3.63195232	4.35857979	5.17699739	6.048E-11	3.13274	up	PREDICTED: polyadenylate-binding protein 8-like [Sesamum indicum]
c20900.graph_c0	0	0	0	1.26154096	1.03159535	1.35959431	1.697E-14	Inf	up	PREDICTED: raucaffricine-O-beta-D-glucosidase-like [Sesamum indicum]
c20914.graph_c0	59.4792406	60.1244276	57.9388557	14.967214	15.7663228	15.5439765	0	-3.02994	down	PREDICTED: uncharacterized protein LOC105168301 [Sesamum indicum]
c20944.graph_c0	1673.56929	1658.2327	1660.80325	822.875729	952.815926	928.70749	1.907E-94	-1.97487	down	PREDICTED: 18 kDa seed maturation protein-like [Nicotiana sylvestris]
c20994.graph_c0	1.7279142	1.94585	2.15104947	1.80526724	1.16933854	1.15284782	4.366E-06	-1.59084	down	hypothetical protein MIMGU_mgv1a011912mg [Erythranthe guttata]
c20998.graph_c0	0.29132832	0.19441336	0.43676317	2.08206818	1.78432359	1.18062671	0.0040175	1.35202	up	hypothetical protein MIMGU_mgv1a024355mg [Erythranthe guttata]
c21005.graph_c0	0.70997679	0.95435208	0.86021409	5.24852872	6.08340086	5.13690185	2.143E-14	1.61296	up	PREDICTED: probable LRR receptor-like serine/threonine-protein kinase At2g24230 [Sesamum indicum]
c21008.graph_c0	0.1666016	0.12507632	0.05352241	1.05949448	0.76529934	0.71633219	0.000417	1.80076	up	hypothetical protein MIMGU_mgv1a020944mg, partial [Erythranthe guttata]
c21009.graph_c0	480.2178	457.952132	475.338859	67.3038134	66.3002858	76.8398209	0	-3.83762	down	PREDICTED: histone H1-like [Sesamum indicum]
c21011.graph_c0	0.56152507	0.71822272	0.52114251	2.76610046	2.8250742	2.99715322	0.0004876	1.16511	up	PREDICTED: la-related protein 1B-like [Sesamum indicum]
c21017.graph_c0	1.98691854	1.98890814	2.13640875	0.64836287	0.50558213	0.76954009	2.257E-16	-2.75786	down	PREDICTED: uncharacterized protein LOC105175392 [Sesamum indicum]
c21036.graph_c0	1.39662388	0.73784515	1.29618449	5.7519964	6.7210174	5.86697761	3.984E-07	1.32693	up	PREDICTED: uncharacterized mitochondrial carrier YMR166C isoform X1 [Sesamum indicum]
c21047.graph_c0	0.04437748	0	0.05702682	0.93534556	1.16486806	1.31592127	1.712E-10	3.9757	up	PREDICTED: PAN domain-containing protein At5g03700 [Sesamum indicum]
c21056.graph_c0	320.714039	178.609573	216.018971	0	0	0	1.166E-57	-Inf	down	--
c21058.graph_c1	5.73341899	4.97393879	7.04021838	4.44481694	4.51490957	4.15586225	1.792E-06	-1.53059	down	-
c21062.graph_c0	28.2441686	28.6066406	25.2262251	7.37638356	5.56475795	5.90027134	2.24E-133	-3.21228	down	-
c21068.graph_c0	23.4138299	21.8259626	18.1466431	146.825119	140.363746	142.039783	4.83E-95	1.67206	up	PREDICTED: LOW QUALITY PROTEIN: protein SENSITIVE TO PROTON RHIZOTOXICITY 1 [Sesamum indicum]
c21082.graph_c0	10.1731722	9.86710574	10.7993762	46.6131056	50.546182	49.4654244	3.284E-22	1.15783	up	BnaC01g37340D [Brassica napus]
c21098.graph_c0	46.04187	48.0986108	38.6839276	39.4730282	40.0741009	39.8366459	2.195E-22	-1.2421	down	PREDICTED: receptor-like protein kinase HAIKU2 [Sesamum indicum]
c21098.graph_c1	67.1025111	66.9482656	50.6952969	49.9487665	51.5832516	55.407848	1.82E-12	-1.32192	down	unnamed protein product [Coffea canephora]
c21099.graph_c0	12.0112033	7.30823829	6.80950228	0	0	0	1.121E-22	-Inf	down	--
c21103.graph_c0	4.69730471	5.11689144	5.5627933	47.5278732	46.1770352	47.4674193	5.4E-68	2.10613	up	hypothetical protein MIMGU_mgv1a010545mg [Erythranthe guttata]
c21104.graph_c0	3.28913351	4.11553386	2.4201076	172.040563	159.653334	149.64927	7.03E-233	4.52784	up	PREDICTED: DELLA protein GAI-like [Sesamum indicum]
c21106.graph_c0	2.05893627	2.18223315	1.75090673	34.6818039	36.9176314	33.9385343	1.54E-120	3.04995	up	PREDICTED: GDP-mannose 4,6 dehydratase 1 [Sesamum indicum]

#ID	0 h-1 FPKM	0 h-2 FPKM	0 h-3 FPKM	28 h-1 FPKM	28 h-2 FPKM	28 h-3 FPKM	FDR	log2FC	regulated	nr_annotation
c21107.graph_c0	1.32177034	1.74648393	1.1549985	1.03750747	1.4918961	1.91268351	0.005117	-1.01336	down	PREDICTED: probable CCR4-associated factor 1 homolog 9 [Sesamum indicum]
c21108.graph_c0	0.43017333	0.28706939	0.3685265	25.8455262	23.5242811	23.7259556	2.124E-32	4.98346	up	-
c21110.graph_c0	0	0	0	2.93276119	3.74175098	4.00585668	5.437E-15	Inf	up	PREDICTED: uncharacterized protein At1g08160-like [Sesamum indicum]
c21116.graph_c0	6.39291694	5.11945478	7.11979786	0.46463352	0.13984017	0.37913716	1.685E-25	-5.33426	down	-
c21117.graph_c0	1.00561267	1.16555958	0.47609287	8.34734459	10.7669565	8.3180264	2.446E-13	2.29046	up	PREDICTED: BURP domain-containing protein 17-like [Sesamum indicum]
c21119.graph_c0	1.40734402	0.37072454	0.95183831	20.0263169	18.8596021	16.6488089	1.013E-31	3.25859	up	Signal peptidase complex catalytic subunit SEC11C [Gossypium arboreum]
c21130.graph_c0	0.17812424	0.0891513	0.11444835	2.13608457	2.3377989	2.85222693	7.73E-09	3.17768	up	PREDICTED: uncharacterized protein LOC101301302 [Fragaria vesca subsp. vesca]
c21136.graph_c0	15.9021166	18.8751465	20.4348495	17.9530582	16.2924215	18.0044074	4.978E-08	-1.1726	down	Arabinoxylan arabinofuranohydrolase [Gossypium arboreum]
c21137.graph_c0	353.410059	329.253381	340.108395	228.51831	215.28593	195.903522	9.13E-103	-1.76777	down	hypothetical protein L484_018717 [Morus notabilis]
c21144.graph_c0	30.1080582	33.1599796	26.6440692	27.4828624	21.6865764	22.0017743	9.709E-21	-1.42622	down	hypothetical protein MIMGU_mgv1a000612mg [Erythranthe guttata]
c21148.graph_c0	158.91922	149.175399	177.046655	151.628858	169.748961	165.775842	7.521E-12	-1.08621	down	PREDICTED: CBS domain-containing protein CBSX3, mitochondrial-like [Sesamum indicum]
c21154.graph_c0	1.35307603	0.84651933	1.04325376	6.66258825	7.52516517	5.75759871	5.709E-10	1.53154	up	PREDICTED: uncharacterized protein At5g39865-like [Sesamum indicum]
c21157.graph_c0	0	0	0.13961939	6.87005105	6.41690893	7.21679795	3.536E-17	6.06854	up	PREDICTED: transcription factor PRE6-like [Solanum lycopersicum]
c21162.graph_c0	0	0.1213478	0.05192691	1.23349317	1.59104097	1.43788622	1.411E-09	3.52402	up	PREDICTED: vegetative incompatibility protein HET-E-1-like [Sesamum indicum]
c21165.graph_c0	15.341046	14.6294772	13.1522885	8.16441269	8.53330025	8.45211128	2.702E-77	-1.86654	down	PREDICTED: protein PHOTOPERIOD-INDEPENDENT EARLY FLOWERING 1 isoform X2 [Sesamum indicum]
c21182.graph_c0	4.92426671	4.39470631	4.42189043	5.08811545	5.13915286	4.01111962	1.255E-05	-1.03924	down	-
c21184.graph_c0	62.6680933	76.622035	75.1528136	24.6945353	24.7542014	24.9513969	5.653E-32	-2.61949	down	PREDICTED: uncharacterized protein LOC105158345 [Sesamum indicum]
c21193.graph_c0	1.41290041	1.64522383	1.18571949	1.04784477	1.2867036	1.12864327	2.901E-06	-1.38153	down	hypothetical protein MIMGU_mgv1a002822mg [Erythranthe guttata]
c21202.graph_c0	5.17184689	6.2762572	4.09686659	0.79811592	1.32501803	1.05041302	5.518E-19	-3.37992	down	hypothetical protein MIMGU_mgv1a027064mg [Erythranthe guttata]
c21208.graph_c0	3.53909575	3.5848139	2.05737345	0.88801949	0.52531616	0.87453734	3.861E-10	-3.08721	down	-
c21212.graph_c0	0.33760743	0.50691824	0.54229864	3.49653868	3.91399929	3.82088108	3.069E-10	1.92234	up	PREDICTED: protein LUTEIN DEFICIENT 5, chloroplastic [Sesamum indicum]
c21220.graph_c0	1.18139245	1.65560561	1.03233253	0.89297439	0.93031402	1.26114198	9.882E-05	-1.41339	down	hypothetical protein MIMGU_mgv1a017885mg [Erythranthe guttata]
c21224.graph_c0	9.21333427	6.31781671	9.78856241	60.687592	68.8391548	64.1917503	4.977E-39	1.843	up	PREDICTED: elongation factor 1-gamma 2-like [Sesamum indicum]

#ID	0 h-1 FPKM	0 h-2 FPKM	0 h-3 FPKM	28 h-1 FPKM	28 h-2 FPKM	28 h-3 FPKM	FDR	log2FC	regulated	nr_annotation
c21231.graph_c0	0	0.07137492	0.18325568	1.24375164	1.59090408	1.39547537	6.852E-07	2.93719	up	PREDICTED: probable xyloglucan glycosyltransferase 5 [Sesamum indicum]
c21237.graph_c0	3.12424179	2.38667729	2.64129894	15.1777093	15.8082134	14.1889846	7.215E-15	1.38083	up	unnamed protein product [Coffea canephora]
c21242.graph_c0	0.02327536	0	0	9.2025205	10.7833884	10.0904627	9.298E-97	9.27663	up	PREDICTED: probable inactive receptor kinase At4g23740 [Sesamum indicum]
c21243.graph_c0	8.39005168	8.15849724	6.1608849	2.26491245	1.41577171	1.99031545	1.234E-32	-3.08752	down	PREDICTED: uncharacterized protein DDB_G0284459-like isoform X1 [Sesamum indicum]
c21247.graph_c0	0.43096847	0.21570001	0.46150962	5.89907838	5.89194102	5.49517144	4.282E-19	2.86965	up	PREDICTED: uncharacterized protein LOC105161548 [Sesamum indicum]
c21248.graph_c0	0.19188993	0.12805472	0.08219539	2.23143194	2.51846729	2.57950951	1.35E-11	3.10726	up	PREDICTED: phosphatidylinositol/phosphatidylcholine transfer protein SFH13-like isoform X1 [Sesamum indicum]
c21250.graph_c0	0.06446166	0	0.08283578	1.42893599	1.43825041	1.37626241	6.893E-11	3.74882	up	hypothetical protein MIMGU_mgv1a007352mg [Erythranthe guttata]
c21251.graph_c0	8.07500501	8.16473829	8.28039788	8.0030006	6.04839547	7.01411522	1.767E-09	-1.30969	down	PREDICTED: metal tolerance protein 1-like isoform X1 [Sesamum indicum]
c21257.graph_c0	8.55112898	10.3286946	9.66991458	63.5163931	63.8212572	66.1299716	1.409E-51	1.66915	up	PREDICTED: tubulin beta chain-like [Sesamum indicum]
c21269.graph_c0	0	0.4811318	0.61765492	4.36667513	4.73123787	6.69876195	4.467E-07	2.7384	up	hypothetical protein MIMGU_mgv1a022779mg, partial [Erythranthe guttata]
c21278.graph_c0	3.01726265	3.37561151	3.68723815	18.4034051	18.2277334	16.297686	1.199E-21	1.2991	up	PREDICTED: cationic amino acid transporter 5-like [Nelumbo nucifera]
c21285.graph_c0	0.54944316	0.3882306	0.08306544	2.25505199	2.20577555	1.6484265	0.0022555	1.50654	up	PREDICTED: pentatricopeptide repeat-containing protein At3g49740 [Sesamum indicum]
c21292.graph_c0	0.28082885	0.52708136	0.45109524	31.8148344	35.7748091	32.7891067	1.14E-170	5.22253	up	PREDICTED: cytochrome P450 71D95-like [Sesamum indicum]
c21295.graph_c0	11.4378769	11.8433355	10.9789635	11.8973259	12.0336612	12.0973895	4.186E-23	-1.01726	down	PREDICTED: uncharacterized protein LOC105161140 [Sesamum indicum]
c21305.graph_c0	5.58324641	2.55142567	3.04144581	0	0	0	8.498E-14	-Inf	down	hypothetical protein M569_16047 [Genlisea aurea]
c21328.graph_c0	34.8213697	28.3206934	23.9715207	23.0483306	27.33926	25.9982567	2.336E-08	-1.27579	down	F-box/WD repeat-containing protein 4 [Theobroma cacao]
c21331.graph_c0	83.3967348	82.8380883	87.3082822	82.0172973	86.7215527	80.6218149	1.877E-40	-1.11545	down	PREDICTED: serine acetyltransferase 5-like [Sesamum indicum]
c21343.graph_c0	2.92448309	2.92741152	2.69732914	2.74257736	2.60010473	2.60158285	7.477E-10	-1.19544	down	PREDICTED: putative pentatricopeptide repeat-containing protein At5g09950 [Sesamum indicum]
c21346.graph_c0	0	0	0	2.56632007	2.87774634	2.26297494	1.628E-15	Inf	up	PREDICTED: transcription factor MYB28-like [Sesamum indicum]
c21350.graph_c0	108.562925	118.51188	111.813428	52.1927992	54.1385725	51.7973083	2.25E-133	-2.19054	down	PREDICTED: ethylene-responsive transcription factor 3-like [Sesamum indicum]
c21351.graph_c0	0	0	0.05402426	2.71940428	2.56572068	2.36860798	9.905E-17	6.01578	up	PREDICTED: uncharacterized protein LOC105175340 [Sesamum indicum]
c21361.graph_c0	2.59261096	2.68318018	2.31518503	29.1905993	28.5479327	28.6404818	1.68E-55	2.41933	up	PREDICTED: cyclin-D3-3-like [Sesamum indicum]
c21364.graph_c0	1.90877171	1.3699237	1.61980476	1.59668567	1.58345983	1.1747301	3.794E-05	-1.26023	down	conserved hypothetical protein [Ricinus communis]

#ID	0 h-1 FPKM	0 h-2 FPKM	0 h-3 FPKM	28 h-1 FPKM	28 h-2 FPKM	28 h-3 FPKM	FDR	log2FC	regulated	nr_annotation
c21372.graph_c0	2.71048774	2.65783042	2.41683384	0.20101703	0.36299906	0.29525085	4.112E-48	-4.26893	down	PREDICTED: galactose oxidase-like [Sesamum indicum]
c21382.graph_c0	1.58291541	2.21830065	1.52558173	0.63274959	0.31162557	0.61019459	5.778E-12	-2.86397	down	-
c21391.graph_c0	19.9278375	9.84208249	10.7170296	0	0	0	2.179E-18	-Inf	down	-
c21397.graph_c0	0.34141048	0.57835013	0.33748152	5.000878	5.75617634	5.24880794	5.606E-20	2.58127	up	hypothetical protein MIMGU_mgv1a003249mg [Erythranthe guttata]
c21401.graph_c0	11.997393	4.52710613	3.14799933	5118.79158	4443.51251	4010.85951	7.8E-187	8.35391	up	hypothetical protein MIMGU_mgv1a011768mg [Erythranthe guttata]
c21405.graph_c0	0.36351466	0.7641452	0.14013922	2.21928304	2.76715861	2.06961924	0.0083314	1.39482	up	PREDICTED: uncharacterized protein LOC105157791 isoform X1 [Sesamum indicum]
c21408.graph_c0	384.755952	388.145761	389.462542	94.8222604	100.112487	109.249508	0	-3.02422	down	PREDICTED: zinc finger CCCH domain-containing protein 20-like [Sesamum indicum]
c21417.graph_c0	10.1092089	11.3741289	9.45725416	3.76182518	5.25407762	4.38859538	1.973E-52	-2.29656	down	PREDICTED: uncharacterized protein LOC105168262 [Sesamum indicum]
c21437.graph_c0	287.072556	269.564162	293.561768	125.461781	119.187659	129.847699	2.92E-106	-2.27355	down	PREDICTED: 7-deoxyloganetic acid glucosyltransferase-like [Sesamum indicum]
c21448.graph_c0	6.03100089	4.78406945	5.26420183	0.41351856	0.56005273	0.40491332	9.843E-53	-4.63451	down	PREDICTED: ethylene-responsive transcription factor ERF053-like [Nelumbo nucifera]
c21449.graph_c1	0	0	0	1.73847488	1.71917314	1.35102982	5.574E-14	Inf	up	PREDICTED: uncharacterized protein LOC105176193 [Sesamum indicum]
c21453.graph_c0	2.29001996	2.36248592	2.28214531	11.7015634	11.34747	10.9480701	1E-15	1.20289	up	PREDICTED: serine/threonine protein phosphatase 2A 57 kDa regulatory subunit B' theta isoform-like [Sesamum indicum]
c21454.graph_c1	2.29956846	2.79014682	1.52229154	18.8909026	14.6788342	16.4893139	2.75E-13	1.83545	up	PREDICTED: calcium-dependent protein kinase 10-like [Sesamum indicum]
c21469.graph_c0	30.2881285	28.2812489	32.7678562	261.896617	282.033369	276.823172	6.36E-140	2.07586	up	PREDICTED: 40S ribosomal protein S3a [Sesamum indicum]
c21482.graph_c0	2.11140665	1.74054663	1.75562662	8.84627661	10.0249498	11.7852889	0.0001144	1.36316	up	PREDICTED: uncharacterized protein LOC105165376 isoform X2 [Sesamum indicum]
c21484.graph_c0	0.11535308	0.28867146	0.29646654	2.80857133	2.23308535	1.94971586	3.234E-06	2.21946	up	PREDICTED: uncharacterized protein LOC105160233 [Sesamum indicum]
c21504.graph_c0	4.3992891	2.83094635	4.71105056	1.52256094	2.4057771	1.18027743	1.047E-06	-2.3204	down	PREDICTED: EPIDERMAL PATTERNING FACTOR-like protein 6 [Vitis vinifera]
c21505.graph_c0	0	0.08396257	0.05389365	2.28609266	1.32104149	1.71619831	1.967E-08	4.17053	up	PREDICTED: uncharacterized protein LOC105160466 [Sesamum indicum]
c21513.graph_c0	3.98588123	5.98480872	5.44502515	3.54937395	4.65395955	4.19531802	1.845E-05	-1.40755	down	PREDICTED: cyclic nucleotide-gated ion channel 2-like isoform X1 [Sesamum indicum]
c21536.graph_c0	0.38474834	0.26663096	0.22819239	4.83979978	5.49634478	4.72153235	1.611E-20	3.01243	up	PREDICTED: protein TRANSPORT INHIBITOR RESPONSE 1-like [Sesamum indicum]
c21538.graph_c0	1.48819132	1.34071337	1.72114629	0.64896533	1.17190969	0.70606721	2.373E-07	-1.94311	down	hypothetical protein MIMGU_mgv1a023150mg, partial [Erythranthe guttata]

#ID	0 h-1 FPKM	0 h-2 FPKM	0 h-3 FPKM	28 h-1 FPKM	28 h-2 FPKM	28 h-3 FPKM	FDR	log2FC	regulated	nr_annotation
c21541.graph_c0	3.16582966	3.68289162	3.07865029	17.5366407	16.8446195	15.8066981	1.678E-13	1.24776	up	PREDICTED: uncharacterized protein LOC105166059 [Sesamum indicum]
c21544.graph_c0	0.50208441	0.40206974	0.32259917	7.59017079	8.59946386	7.44413147	2.072E-20	3.18203	up	hypothetical protein JCGZ_21387 [Jatropha curcas]
c21554.graph_c0	0	0.04716574	0.03027461	6.8662223	6.10679355	5.99399798	3.262E-57	6.8351	up	PREDICTED: LRR receptor-like serine/threonine-protein kinase RCH1 [Sesamum indicum]
c21556.graph_c0	3.77911651	3.58024533	4.33599494	0.83380146	0.68641708	0.66036415	8.311E-45	-3.51624	down	PREDICTED: ribulose bisphosphate carboxylase/oxygenase activase, chloroplastic isoform X2 [Sesamum indicum]
c21558.graph_c0	0.05364942	0.05370314	0	1.55968358	1.79551625	1.14542016	4.231E-11	4.31752	up	hypothetical protein CICLE_v10024014mg, partial [Citrus clementina]
c21564.graph_c0	0.54592569	0.34775513	0.5739841	7.03375004	5.60174611	6.03381504	9.117E-14	2.57655	up	PREDICTED: methylsterol monooxygenase 1-1-like [Sesamum indicum]
c21575.graph_c0	0.07506663	0.0751418	0.03215453	23.5508667	24.7453176	21.7400512	4.57E-196	7.50354	up	PREDICTED: uncharacterized protein LOC105169481 [Sesamum indicum]
c21576.graph_c0	0	0.06896954	0.08853993	3.15482084	3.02936504	3.26896259	4.84E-13	4.79846	up	hypothetical protein MIMGU_mgv1a000209mg [Erythranthe guttata]
c21581.graph_c0	5.12324476	4.68457324	5.15924179	4.8871525	4.57427568	5.3025611	3.575E-13	-1.11	down	PREDICTED: uncharacterized protein LOC105164695 [Sesamum indicum]
c21589.graph_c0	39.7640546	37.7191384	37.7791953	207.476594	214.188964	220.456636	1.127E-66	1.3881	up	PREDICTED: 40S ribosomal protein S9-2-like [Sesamum indicum]
c21609.graph_c0	21.2824483	22.9760682	21.7483542	8.18270963	6.1012394	7.10779999	4.462E-79	-2.71593	down	PREDICTED: pathogenesis-related genes transcriptional activator PTI6 [Sesamum indicum]
c21617.graph_c0	22.5448093	19.8799707	18.2229628	4.75302765	4.63034399	4.96031008	2.748E-90	-3.16782	down	PREDICTED: probable galacturonosyltransferase-like 9 [Sesamum indicum]
c21618.graph_c0	1.21918692	1.38873985	0.81036386	0.45832656	0.60694468	0.52358703	2.323E-08	-2.19092	down	PREDICTED: protein ODORANT1-like [Sesamum indicum]
c21625.graph_c0	0.22637184	0.3776642	0	2.74209584	2.42633834	2.10327614	2.442E-06	2.51558	up	PREDICTED: transcription factor bHLH68-like [Sesamum indicum]
c21625.graph_c1	0.37881644	0.37919577	0.24339706	1.92725124	1.67797977	1.79728145	0.005128	1.34661	up	PREDICTED: transcription factor bHLH68-like [Sesamum indicum]
c21628.graph_c0	0	0.04455761	0	2.62049728	3.65133233	3.00946542	2.21E-18	6.62035	up	PREDICTED: F-box/kelch-repeat protein At5g26960 [Sesamum indicum]
c21629.graph_c0	0	0	0	38.5808792	41.766253	77.7645877	2.531E-18	Inf	up	-
c21636.graph_c0	33.8016237	34.1979938	33.5080991	12.6343673	15.5270779	14.2472123	1.135E-58	-2.34969	down	PREDICTED: probable histone H2A.3-like [Cicer arietinum]
c21641.graph_c0	1.32509301	1.06113591	1.36223758	8.51781877	8.98547274	7.63154681	1.176E-15	1.65305	up	PREDICTED: uncharacterized protein LOC105157824 isoform X1 [Sesamum indicum]
c21652.graph_c0	7.82113138	7.85633706	7.52027687	37.2265509	35.8193924	37.1070864	1.463E-31	1.15755	up	PREDICTED: probable phenylalanine--tRNA ligase beta subunit [Sesamum indicum]
c21657.graph_c0	2.12078345	1.74827643	1.68326779	0.45334506	0.65492493	0.66586655	2.99E-12	-2.73335	down	PREDICTED: uncharacterized protein LOC105157568 [Sesamum indicum]
c21659.graph_c0	5.7155553	6.348268	4.82940154	38.5812896	29.9542366	38.354198	5.298E-12	1.57427	up	PREDICTED: probable polygalacturonase [Sesamum indicum]
c21672.graph_c0	0.1123666	0	0.14439552	3.10336096	3.42881697	2.79887691	8.219E-13	4.08331	up	PREDICTED: wall-associated receptor kinase-like 20 [Sesamum indicum]
c21676.graph_c1	7.24258934	8.09530428	7.29908682	7.30496549	6.77583356	6.76224323	6.444E-25	-1.20956	down	PREDICTED: exocyst complex component SEC15B [Sesamum indicum]

#ID	0 h-1 FPKM	0 h-2 FPKM	0 h-3 FPKM	28 h-1 FPKM	28 h-2 FPKM	28 h-3 FPKM	FDR	log2FC	regulated	nr_annotation
c21704.graph_c0	15.3339836	16.2165325	15.2517193	16.6854943	15.6149938	14.6085393	1.891E-27	-1.08744	down	PREDICTED: protein DGCR14 [Sesamum indicum]
c21705.graph_c0	104.474391	102.906771	98.8325417	80.6012119	79.9432442	78.1160822	1.192E-68	-1.44956	down	PREDICTED: tetraspanin-3-like [Sesamum indicum]
c21709.graph_c0	1.89049311	2.75256168	2.87106004	0.99927236	0.97743669	0.84937301	1.032E-09	-2.50626	down	PREDICTED: uncharacterized protein LOC105173813 [Sesamum indicum]
c21716.graph_c0	7.29466678	5.414945	6.74080763	4.05075371	3.17337644	3.35399051	2.345E-14	-1.96911	down	unnamed protein product [Vitis vinifera]
c21718.graph_c0	0	0.0165736	0	0.84234818	0.97786469	0.80518053	2.38E-15	6.22512	up	PREDICTED: uncharacterized protein LOC105157238 isoform X1 [Sesamum indicum]
c21719.graph_c0	0.24338589	0.16241973	0.3127605	3.47886536	3.67347725	2.93495371	7.833E-08	2.71489	up	hypothetical protein MIMGU_mgv1a015222mg [Erythranthe guttata]
c21723.graph_c0	0.16046604	0.05354224	0	0.89413106	0.94771939	0.6502989	1.427E-05	2.47541	up	PREDICTED: scarecrow-like protein 28 [Sesamum indicum]
c21733.graph_c0	14.7732486	15.9710852	14.4279992	8.6510445	8.14346855	7.81015844	1.535E-29	-1.9668	down	-
c21735.graph_c0	1.70382433	2.88628231	2.02106005	13.05009	16.1263791	13.3692547	1.841E-11	1.59453	up	PREDICTED: RING-H2 finger protein ATL66 [Sesamum indicum]
c21738.graph_c0	0	0	0	1.78473192	1.73230401	1.78400018	2.091E-14	Inf	up	PREDICTED: uncharacterized protein LOC105171956 [Sesamum indicum]
c21743.graph_c0	0.54860612	0.45762955	0.50915264	4.76253386	5.00014133	4.98878182	8.86E-14	2.19312	up	hypothetical protein MIMGU_mgv1a004026mg [Erythranthe guttata]
c21746.graph_c0	3.86869203	3.4679695	3.33901506	2.76977675	2.31139048	2.26011521	3.61E-09	-1.62949	down	unnamed protein product [Coffea canephora]
c21766.graph_c0	11.0619096	4.23887762	4.66429418	0	0	0	1.554E-09	-Inf	down	60S ribosomal protein L7a [Zea mays]
c21771.graph_c0	1.00339128	0.89279646	0.5253101	6.05014871	6.24310517	7.00859498	1.1E-11	1.91238	up	PREDICTED: replication factor C subunit 3 [Sesamum indicum]
c21772.graph_c0	29.819347	26.1671498	25.5250194	13.5809766	15.2233355	15.793983	5.818E-75	-1.95849	down	PREDICTED: activating signal cointegrator 1 [Sesamum indicum]
c21774.graph_c0	39.6863671	37.3484846	44.1309174	22.9455848	20.295564	23.1254654	6.36E-28	-1.96021	down	PREDICTED: F-box protein At5g39250 [Sesamum indicum]
c21785.graph_c0	0	0.20678885	0	5.90560892	6.41672867	6.45246379	3.475E-20	5.42298	up	PREDICTED: protein TORNADO 2 [Sesamum indicum]
c21789.graph_c0	3.56497991	4.84683616	3.41876144	0.27070221	0.24441871	0.31555827	1.58E-29	-4.92009	down	PREDICTED: COBRA-like protein 10 [Sesamum indicum]
c21813.graph_c0	0.37704398	0.12580718	0	10.9613357	9.40220546	9.68972376	3.999E-17	4.83653	up	PREDICTED: probable receptor-like protein kinase At1g80640 [Nicotiana tomentosiformis]
c21818.graph_c0	6.46476606	7.33407148	4.9844882	2.08824654	2.59254989	1.87439133	7.872E-18	-2.60695	down	-
c21827.graph_c0	0	0	0	0.72218722	0.86241166	0.77939529	2.231E-12	Inf	up	PREDICTED: L-ascorbate oxidase-like [Sesamum indicum]
c21839.graph_c0	0.15460317	0.15475798	0.06622373	0.93637242	1.25127562	1.10026403	5.066E-05	2.04867	up	PREDICTED: uncharacterized protein LOC105177291 [Sesamum indicum]
c21856.graph_c0	0	0	0	1.10461481	0.97469625	0.77844439	1.168E-13	Inf	up	PREDICTED: cytochrome P450 71A8-like [Sesamum indicum]
c21857.graph_c0	15.1495907	11.0685323	15.7756504	120.547472	115.356508	121.395215	3.445E-78	1.99706	up	PREDICTED: DNA-binding protein S1FA-like [Nicotiana sylvestris]
c21859.graph_c0	0.09844465	0.03941729	0.20240843	4.90826161	4.79347148	4.27366505	1.525E-39	4.2552	up	PREDICTED: oligopeptide transporter 4-like [Sesamum indicum]
c21881.graph_c0	0.35376525	0.56659119	0.27276141	2.51972256	3.8072654	2.01411236	0.0040504	1.71686	up	PREDICTED: LOW QUALITY PROTEIN: alkaline/neutral invertase CINV2 [Sesamum indicum]

#ID	0 h-1 FPKM	0 h-2 FPKM	0 h-3 FPKM	28 h-1 FPKM	28 h-2 FPKM	28 h-3 FPKM	FDR	log2FC	regulated	nr_annotation
c21889.graph_c0	11.0064193	11.5284536	9.86646644	6.81210683	7.34331195	7.38727747	1.913E-57	-1.67771	down	hypothetical protein MIMGU_mgv1a009265mg [Erythranthe guttata]
c21894.graph_c0	0.04729404	0.0946828	0	1.99363608	2.14145837	2.49628237	2.928E-12	4.47233	up	PREDICTED: probable membrane-associated kinase regulator 2 [Sesamum indicum]
c21898.graph_c0	0	0	0	4.31457212	5.87649516	4.65442607	1.859E-33	Inf	up	PREDICTED: uncharacterized protein LOC105164396 [Sesamum indicum]
c21901.graph_c0	0.35569413	0.32637944	0.30472069	3.31762766	2.74263927	2.72473602	1.118E-08	2.06516	up	vacuolar invertase CvINV [Elsholtzia haichowensis]
c21905.graph_c0	27.7995146	28.5442154	30.7874461	15.8986442	13.2441826	15.7145169	2.944E-52	-2.04917	down	PREDICTED: auxin-induced protein 22D [Sesamum indicum]
c21908.graph_c0	5.94914524	5.18590168	5.25585993	4.46793129	3.23705841	4.63074825	2.002E-23	-1.49813	down	PREDICTED: lysine-specific demethylase JMJ18 [Sesamum indicum]
c21911.graph_c0	0.70849569	0.70920514	1.05051348	4.87203555	5.82955758	4.75125658	3.348E-06	1.5495	up	PREDICTED: inositol monophosphatase 3-like [Sesamum indicum]
c21920.graph_c0	3.48093045	3.42328597	3.21751704	1.42030607	0.52097631	0.97787094	3.597E-19	-2.88252	down	PREDICTED: WW domain-containing protein C11B10.08 [Sesamum indicum]
c21925.graph_c0	0.24587332	0.28127946	0.31595695	7.65854197	8.11353586	6.49928001	2.161E-30	3.62886	up	PREDICTED: inactive rhomboid protein 1-like [Sesamum indicum]
c21938.graph_c0	23.5215631	23.6241269	27.0818099	22.02889	23.205026	22.469243	8.731E-16	-1.22527	down	hypothetical protein MIMGU_mgv1a014940mg [Erythranthe guttata]
c21940.graph_c0	0.16519651	0	0	1.48078771	1.8067736	1.40425295	6.464E-10	3.76808	up	PREDICTED: uncharacterized protein LOC105161910 [Sesamum indicum]
c21944.graph_c0	0.43855056	0.36582475	1.03318386	3.08111485	3.74125363	2.9476412	0.0010143	1.30586	up	PREDICTED: transcription repressor KAN1-like isoform X4 [Sesamum indicum]
c21947.graph_c0	9.43796014	9.51871204	9.65675534	52.7789767	54.5724284	55.1699671	7.781E-48	1.41514	up	PREDICTED: diaminopimelate decarboxylase 1, chloroplastic-like [Sesamum indicum]
c21950.graph_c0	1.04646697	0.68915451	0.81392853	5.46406744	6.18047942	5.42222206	1.412E-11	1.6543	up	PREDICTED: uncharacterized protein LOC105173886 [Sesamum indicum]
c21965.graph_c0	23.9577838	26.1619352	25.3364236	17.0791279	18.4297978	14.4802896	6.212E-28	-1.68606	down	PREDICTED: probable methyltransferase PMT16 [Sesamum indicum]
c21969.graph_c0	37.4194181	35.5840436	35.3179221	36.8555636	37.0028009	35.6218118	2.503E-27	-1.07474	down	hypothetical protein MIMGU_mgv1a013699mg [Erythranthe guttata]
c21976.graph_c0	1.50828221	4.75032283	4.11277085	53.6608563	85.0241371	62.5713012	2.299E-13	3.17959	up	PREDICTED: epidermis-specific secreted glycoprotein EP1-like [Sesamum indicum]
c21977.graph_c0	0.05250836	0.21024378	0.13495066	19.4248535	23.7756126	19.5251025	6.436E-67	6.2052	up	gibberellin 3-oxidase [Torenia fournieri]
c21979.graph_c0	11.4215614	10.7564896	9.11894471	5.10838462	4.70109221	5.09025786	2.414E-30	-2.15826	down	PREDICTED: uncharacterized protein LOC105170847 [Sesamum indicum]
c21982.graph_c0	4.62797861	5.25374528	3.85400976	0.46977489	0.42416268	0.72066504	2.32E-47	-4.17405	down	PREDICTED: exocyst complex component EXO70B1 [Sesamum indicum]
c21992.graph_c0	9.58099868	8.23022486	9.34312058	38.5210171	39.1507705	40.4597009	9.393E-15	1.03068	up	hypothetical protein MIMGU_mgv1a007464mg [Erythranthe guttata]
c21997.graph_c0	0	0	0	1.35653183	1.05470702	1.01467566	5.437E-11	Inf	up	PREDICTED: uncharacterized protein LOC105158717 [Sesamum indicum]
c22004.graph_c0	0.35812537	0.35848398	0.69030792	28.1106134	26.6738118	24.9557281	4.459E-28	4.7243	up	H(+)-transporting atpase plant/fungi plasma membrane type [Theobroma cacao]

#ID	0 h-1 FPKM	0 h-2 FPKM	0 h-3 FPKM	28 h-1 FPKM	28 h-2 FPKM	28 h-3 FPKM	FDR	log2FC	regulated	nr_annotation
c22008.graph_c0	4.48035217	3.91675901	3.68475515	2.14915413	2.0188881	2.07254535	1.7E-24	-2.04138	down	hypothetical protein MIMGU_mgv1a027091mg [Erythranthe guttata]
c22023.graph_c0	1.63980724	1.69274455	1.11945915	0.63314505	0.47078757	0.79015712	7.428E-10	-2.31665	down	CLAVATA3/ESR (CLE)-related protein 26 [Arabidopsis thaliana]
c22042.graph_c0	5.72738724	6.29976816	7.35991936	5.61471298	9.46172076	5.5887132	0.0011801	-1.00343	down	PREDICTED: putative methyltransferase DDB_G0268948 isoform X2 [Sesamum indicum]
c22045.graph_c0	0.07749917	0.21333614	0.14938426	0.95754121	0.66114172	0.66644201	0.0035797	1.28106	up	PREDICTED: receptor-like protein kinase HERK 1 [Sesamum indicum]
c22047.graph_c0	56.7538854	51.8116829	58.802476	55.3730184	56.7797251	54.0462262	3.296E-23	-1.10161	down	PREDICTED: probable serine incorporator [Sesamum indicum]
c22049.graph_c0	70.8123996	78.4844891	59.6063898	403.616133	389.223006	332.385929	9.358E-20	1.34045	up	hypothetical protein MIMGU_mgv1a015271mg [Erythranthe guttata]
c22059.graph_c0	3.31047319	1.88079867	2.06955687	0	0.23485644	0.10612449	1.23E-13	-5.50014	down	-
c22064.graph_c0	1.14636132	1.89588481	0.89668154	6.5566762	7.39190294	7.38977105	2.276E-06	1.35144	up	PREDICTED: homeobox-leucine zipper protein HOX11-like [Nicotiana tomentosiformis]
c22065.graph_c0	2.26529571	1.53870418	2.183246	1.58760373	2.01745844	1.72729557	0.0010475	-1.25862	down	-
c22068.graph_c0	3.93736921	4.33544308	3.66826461	29.4512225	29.8645163	26.8535229	2.473E-45	1.76141	up	PREDICTED: glycerol-3-phosphate 2-O-acyltransferase 4 [Sesamum indicum]
c22080.graph_c0	6.17516755	6.87150189	5.89371953	2.15689098	3.08841254	2.77334189	2.334E-44	-2.32971	down	PREDICTED: vacuolar protein sorting-associated protein 4-like [Sesamum indicum]
c22084.graph_c0	0.30095808	0.25822238	0.19337152	2.39045666	2.53924499	2.20557261	8.186E-10	2.15923	up	PREDICTED: uncharacterized protein LOC105169955 isoform X1 [Sesamum indicum]
c22085.graph_c0	0.05843516	0.01949789	0.0250305	0.33976269	0.35790283	0.40431355	3.348E-05	2.33974	up	PREDICTED: WD repeat and HMG-box DNA-binding protein 1 [Sesamum indicum]
c22088.graph_c0	9.17962269	9.04744831	11.2517396	0.30792447	0.55605385	0.20938654	2.176E-55	-5.8765	down	PREDICTED: nuclear transcription factor Y subunit C-2 [Sesamum indicum]
c22090.graph_c0	21.4134281	20.7251065	21.4426872	111.55313	110.710465	107.454375	4.172E-49	1.28374	up	PREDICTED: uncharacterized protein LOC105172629 [Sesamum indicum]
c22094.graph_c0	244.890801	271.317847	244.597046	27.7576198	26.7333634	31.5269573	0	-4.2343	down	PREDICTED: ethylene-responsive transcription factor ERF008-like [Sesamum indicum]
c22096.graph_c0	1.20964319	0.64766634	0.57839605	4.74338405	4.24591011	4.8048364	1.907E-07	1.41991	up	PREDICTED: probable inactive purple acid phosphatase 29 isoform X2 [Nicotiana sylvestris]
c22097.graph_c0	0.15539659	0.28517903	0.26625437	12.4988427	12.0161071	11.1205457	3.392E-77	4.56017	up	PREDICTED: MATE efflux family protein 5-like [Sesamum indicum]
c22111.graph_c0	0.10675351	0.21372082	0.41154747	3.84059798	3.43267262	4.55839504	2.76E-11	2.90994	up	PREDICTED: (DL)-glycerol-3-phosphatase 2 [Sesamum indicum]
c22122.graph_c0	1.39248111	0.85181279	2.28644707	9.72689853	9.69626116	9.58871434	5.588E-08	1.57926	up	PREDICTED: plastidial pyruvate kinase 2-like [Sesamum indicum]
c22136.graph_c0	3.47190995	3.96411277	3.69471426	1.41939233	1.56637335	1.86600911	7.584E-16	-2.2879	down	hypothetical protein MIMGU_mgv1a003925mg [Erythranthe guttata]
c22145.graph_c0	0.1851713	0.10591813	0.10197962	2.07639924	1.70120181	2.44735808	4.628E-11	2.90285	up	PREDICTED: putative pentatricopeptide repeat-containing protein At3g25060, mitochondrial [Sesamum indicum]
c22147.graph_c0	0.70050943	0.88216853	0.69691537	0.39416223	0.53383731	0.42884422	2.227E-07	-1.83896	down	PREDICTED: glutathione transferase GST 23-like [Sesamum indicum]

#ID	0 h-1 FPKM	0 h-2 FPKM	0 h-3 FPKM	28 h-1 FPKM	28 h-2 FPKM	28 h-3 FPKM	FDR	log2FC	regulated	nr_annotation
c22152.graph_c0	0.18850776	0.02695665	0.03460571	1.25262954	0.90127123	0.95825201	1.416E-06	2.56584	up	PREDICTED: pyruvate kinase isozyme A, chloroplastic-like [Sesamum indicum]
c22156.graph_c0	31.3519044	30.5419233	26.2468915	5.13151799	5.35033479	5.2839445	4.17E-134	-3.57131	down	PREDICTED: serine/threonine-protein kinase OXI1 [Sesamum indicum]
c22160.graph_c0	1.44161863	1.50719829	1.35852715	0.51223828	0.48352601	0.60797418	1.64E-13	-2.51396	down	-
c22176.graph_c0	0.26967307	0.22495259	0.4620541	2.09063288	1.82865656	1.41273103	0.0012151	1.37821	up	hypothetical protein MIMGU_mgv1a009135mg [Erythranthe guttata]
c22178.graph_c0	8.85643996	11.2758151	10.4181433	6.22289742	7.69058593	7.45806792	2.132E-14	-1.60725	down	PREDICTED: uncharacterized protein LOC105169534 [Sesamum indicum]
c22183.graph_c0	1.26984785	0.88272182	1.17852567	4.89659326	5.1618833	4.58131961	9.732E-05	1.04486	up	PREDICTED: malonyl-coenzyme:anthocyanin 5-O-glucoside-6'''-O-malonyltransferase-like [Sesamum indicum]
c22206.graph_c0	1.05529926	0.94856456	1.19004811	5.29063414	5.22920188	5.6454571	1.243E-09	1.24732	up	hypothetical protein MIMGU_mgv1a010455mg [Erythranthe guttata]
c22209.graph_c0	1.75618537	1.1796729	1.30654974	0.26871272	0.0909834	0.13704211	1.942E-23	-4.18209	down	PREDICTED: receptor-like protein kinase HSL1 [Sesamum indicum]
c22212.graph_c0	2.23207803	1.7874505	2.29464691	8.76021182	9.86264991	8.38376326	0.0074208	1.00447	up	hypothetical protein CISIN_1g035370mg [Citrus sinensis]
c22220.graph_c0	2.67243728	2.93823922	3.09639875	12.9912017	12.7360731	12.7832145	1.411E-08	1.05274	up	PREDICTED: ankyrin repeat domain-containing protein EMB506, chloroplastic [Sesamum indicum]
c22221.graph_c0	14.0137811	14.7087756	13.8121633	9.29517205	9.82120823	9.48098061	4.289E-26	-1.66306	down	PREDICTED: probable calcium-binding protein CML15 [Sesamum indicum]
c22228.graph_c0	1.88009308	2.16226996	2.00476101	1.74439253	2.33628397	2.08766808	0.0002863	-1.06248	down	PREDICTED: uncharacterized protein LOC105161595 [Sesamum indicum]
c22236.graph_c0	3.72716238	4.94560443	4.23262666	0.18899164	0.34128346	0.56545732	4.756E-31	-4.64573	down	-
c22271.graph_c0	7.66546434	7.45179956	5.49350634	4.28555023	4.15965881	6.60053842	4.958E-09	-1.53815	down	PREDICTED: calcium uniporter protein 2, mitochondrial-like [Sesamum indicum]
c22278.graph_c0	0.34187654	0.34221888	0.4393249	27.4031218	29.133273	27.6612578	1.51E-104	5.13362	up	PREDICTED: probable serine/threonine-protein kinase At4g35230 [Sesamum indicum]
c22283.graph_c0	0.11865703	0.05938792	0.07623949	1.7247849	1.94664881	2.14630085	2.802E-09	3.4307	up	PREDICTED: ethylene-responsive transcription factor 1-like [Sesamum indicum]
c22291.graph_c0	1.02004313	0.69862311	1.0348388	5.03345418	4.33335407	5.09427288	9.487E-05	1.30158	up	PREDICTED: ras-related protein Rab7 [Sesamum indicum]
c22292.graph_c0	0.85849438	1.0868301	1.00585815	0.82581454	0.49708874	0.88350261	6.75E-06	-1.50961	down	-
c22303.graph_c0	47.456204	50.3718192	46.5884248	46.3157475	44.0864021	42.4811403	4.824E-51	-1.21045	down	PREDICTED: receptor-like protein kinase FERONIA [Sesamum indicum]
c22326.graph_c0	70.5696857	74.2135978	70.6502731	32.5521802	29.2714376	30.597324	3.27E-171	-2.31132	down	PREDICTED: EIN3-binding F-box protein 2, partial [Sesamum indicum]
c22331.graph_c0	3.88724561	3.20446667	3.62401543	17.5053324	18.4566255	19.1661529	3.426E-17	1.27319	up	PREDICTED: actin-related protein 6 [Sesamum indicum]
c22337.graph_c0	0.31795031	0.59981407	0.17286024	5.38604537	5.86620743	5.24350267	1.011E-45	2.83556	up	PREDICTED: myosin-9-like [Sesamum indicum]
c22345.graph_c0	31.6982939	29.4759887	33.8333417	156.986652	163.341183	174.943066	6.828E-31	1.29084	up	ribosomal protein L32 [Medicago sativa]
c22345.graph_c1	59.1561086	55.9854166	59.8929237	286.107649	261.622186	271.733883	4.233E-21	1.13621	up	PREDICTED: 60S ribosomal protein L32-1 [Sesamum indicum]

#ID	0 h-1 FPKM	0 h-2 FPKM	0 h-3 FPKM	28 h-1 FPKM	28 h-2 FPKM	28 h-3 FPKM	FDR	log2FC	regulated	nr_annotation
c22346.graph_c0	2.67204732	3.04621228	2.25733101	18.9527606	20.5870336	19.8378394	1.327E-43	1.80815	up	PREDICTED: uncharacterized protein LOC105163060 [Sesamum indicum]
c22348.graph_c0	0.07990306	0.07998307	0.0684524	1.80026566	1.78277476	1.64275501	8.227E-12	3.42747	up	PREDICTED: LOW QUALITY PROTEIN: recQ-mediated genome instability protein 1 [Sesamum indicum]
c22359.graph_c0	0	0	0	2.19273993	2.79506621	4.68364562	1.6E-06	Inf	up	calmodulin [Chara corallina]
c22362.graph_c0	6685.5471	6693.30449	6723.05173	192.765877	195.974085	211.784147	0	-6.15518	down	hypothetical protein MIMGU_mgv1a011867mg [Erythranthe guttata]
c22366.graph_c0	0.08612068	0.02873564	0.03688949	5.67500926	7.12085471	6.02679952	4.168E-44	5.87334	up	PREDICTED: probable LRR receptor-like serine/threonine-protein kinase At4g37250 [Sesamum indicum]
c22379.graph_c0	0.4735244	0.35549892	0.3042488	3.09739323	2.56360103	2.94868884	0.0001157	1.84037	up	hypothetical protein MIMGU_mgv1a016703mg [Erythranthe guttata]
c22396.graph_c0	288.044775	309.121092	258.335753	178.923662	181.691295	178.1546	9.013E-54	-1.75611	down	PREDICTED: ethylene-responsive transcription factor 4-like [Sesamum indicum]
c22400.graph_c0	4.6239143	7.98423918	5.12489991	1.38626163	1.55509805	1.1654563	3.041E-08	-3.20154	down	PREDICTED: uncharacterized protein LOC105158187 [Sesamum indicum]
c22403.graph_c0	786.901076	826.627547	722.444604	177.686982	161.718163	181.722212	7.45E-243	-3.25319	down	-
c22409.graph_c0	0.81724096	0.53173855	0.68262154	27.4709284	30.3007029	28.5471468	5.09E-112	4.31971	up	GDP-mannose 3,5-epimerase 1 [Theobroma cacao]
c22410.graph_c0	7.12881067	5.93950254	7.02145453	6.36012931	6.27484146	6.65815108	4.167E-11	-1.14897	down	PREDICTED: uncharacterized protein LOC105168803 isoform X1 [Sesamum indicum]
c22444.graph_c0	0.55421266	0.63798277	0.56974817	8.09625127	8.14664129	8.06910245	7.946E-30	2.69591	up	asparagine synthetase [Striga hermonthica]
c22447.graph_c0	0.4365081	0.71783854	0.40066442	24.8136051	28.7062242	24.4821494	3.42E-102	4.55994	up	hypothetical protein SBB1_14t00005 [Solanum bulbocastanum]
c22453.graph_c0	3.71997801	3.79892932	4.29745851	17.0139322	17.5935421	17.8052979	3.184E-22	1.05679	up	PREDICTED: probable receptor protein kinase TMK1 [Sesamum indicum]
c22457.graph_c0	0.11409707	0.14276415	0.10996444	2.05239608	1.72209245	1.69165499	1.6E-09	2.8076	up	PREDICTED: putative pentatricopeptide repeat-containing protein At1g68930 [Sesamum indicum]
c22469.graph_c0	4.00702817	4.0110406	2.7355071	0	0	0	1.183E-33	-Inf	down	-
c22477.graph_c0	0.34554891	0.17294746	0.0555055	1.22432191	1.13379271	0.89657138	0.0059373	1.42941	up	PREDICTED: kinetochore protein Spc25 [Sesamum indicum]
c22480.graph_c0	0.20337371	0.67859118	0.37749582	4.56571251	5.48666122	4.86470158	1.438E-20	2.473	up	PREDICTED: protein LONGIFOLIA 1-like [Sesamum indicum]
c22481.graph_c0	11.6759149	11.1488162	9.94946954	8.30543471	9.45529723	9.84653597	3.623E-23	-1.3357	down	PREDICTED: uncharacterized protein LOC105156396 [Sesamum indicum]
c22502.graph_c0	4.65501698	4.96690981	5.45600146	31.5645093	31.8902271	31.1867615	3.9E-29	1.55744	up	PREDICTED: NAP1-related protein 2-like [Sesamum indicum]
c22518.graph_c0	0.09031828	0.22602181	0.11606257	1.4769632	1.48173255	2.31664283	7.799E-06	2.52094	up	PREDICTED: DNA (cytosine-5)-methyltransferase CMT3 [Sesamum indicum]
c22532.graph_c0	0.35416865	0.23634886	0.60682765	2.31666958	2.40162216	2.38048288	0.0005233	1.46787	up	unnamed protein product [Coffea canephora]
c22543.graph_c0	0.84718558	0.93889468	0.77761929	0.83563302	0.97290566	0.75364548	0.0021351	-1.09099	down	PREDICTED: uncharacterized protein LOC105155579 [Sesamum indicum]

#ID	0 h-1 FPKM	0 h-2 FPKM	0 h-3 FPKM	28 h-1 FPKM	28 h-2 FPKM	28 h-3 FPKM	FDR	log2FC	regulated	nr_annotation
c22552.graph_c0	5.78221411	5.13213963	5.26230457	1.85718658	1.84885154	2.06917086	1.6E-78	-2.57504	down	PREDICTED: pentatricopeptide repeat-containing protein At3g26540 [Sesamum indicum]
c22556.graph_c0	15.6975032	15.9025378	14.6226117	14.7307101	15.1207295	13.2913203	2.332E-30	-1.18963	down	PREDICTED: E3 ubiquitin-protein ligase RING1-like [Sesamum indicum]
c22557.graph_c0	62.6485339	60.626933	64.6838882	61.9164312	66.5391073	61.0467531	4.258E-30	-1.07959	down	PREDICTED: uncharacterized protein LOC105167256 isoform X2 [Sesamum indicum]
c22559.graph_c0	0.64735733	0.43200371	0.55458653	7.99842482	6.42884268	5.98916057	3.672E-16	2.55321	up	PREDICTED: formin-like protein 6 [Sesamum indicum]
c22564.graph_c0	0	0.0816331	0	3.4080893	2.32795742	2.78097304	3.61E-17	5.62358	up	PREDICTED: polygalacturonase-like [Sesamum indicum]
c22577.graph_c0	5.37840698	4.1724393	4.14687955	4.98396888	3.97063818	3.26945086	0.000182	-1.25313	down	PREDICTED: uncharacterized protein LOC105177681 [Sesamum indicum]
c22594.graph_c0	0.26892655	0.40379376	0.11519376	3.87650966	2.64715318	2.68473297	4.425E-07	2.46513	up	hypothetical protein MIMGU_mgv1a000346mg [Erythranthe guttata]
c22603.graph_c0	6.39350022	6.92833465	5.95464378	1.30023993	1.27022383	0.99141237	3.249E-84	-3.52669	down	PREDICTED: uncharacterized protein LOC105179085 [Sesamum indicum]
c22607.graph_c0	10.8315808	11.722795	9.48753044	11.8757806	9.64436961	10.0199635	4.041E-16	-1.11154	down	PREDICTED: uncharacterized protein LOC105179586 [Sesamum indicum]
c22632.graph_c0	0.41524888	0.51958086	1.46743095	8.9031146	9.74178742	8.61933587	2.552E-10	2.39643	up	PREDICTED: zinc finger HIT domain-containing protein 3 [Sesamum indicum]
c22634.graph_c0	0.06592552	0.04399435	0.05647793	1.16591429	2.1631054	2.51528319	7.423E-07	4.04652	up	PREDICTED: DNA replication licensing factor MCM3 homolog 2 [Sesamum indicum]
c22637.graph_c0	0.19764727	0.16487099	0.29631533	4.74041358	6.65800957	5.50916734	4.256E-21	3.58415	up	sucrose transporter 1 [Verbascum phoeniceum]
c22642.graph_c0	0.22355023	0.11188704	0.28727089	2.43712523	3.22739351	2.51898743	2.327E-07	2.61904	up	-
c22646.graph_c1	0.07766931	0.23324125	0.09980814	2.82248028	2.19165415	2.76374503	1.701E-08	3.15449	up	hypothetical protein MIMGU_mgv1a025026mg, partial [Erythranthe guttata]
c22658.graph_c0	0	0	0.11456155	1.38226895	1.69658055	1.26891019	1.494E-12	4.11481	up	-
c22663.graph_c0	1.68171604	1.37165928	1.62747375	0.9959168	0.53135697	0.7387818	1.328E-15	-2.13686	down	PREDICTED: putative calcium-transporting ATPase 13, plasma membrane-type [Sesamum indicum]
c22683.graph_c0	2.20462883	2.24488533	2.53996271	1.68518971	1.57145579	1.98375232	9.354E-08	-1.5069	down	PREDICTED: glycerate dehydrogenase HPR, peroxisomal [Sesamum indicum]
c22686.graph_c0	0	0	0.07880629	2.3622818	1.52926266	1.81849307	2.347E-12	5.04895	up	PREDICTED: uncharacterized protein LOC105161798 [Sesamum indicum]
c22691.graph_c0	0.86634041	0.74106859	0.78941796	0	0	0	2.208E-48	-Inf	down	PREDICTED: geraniol 8-hydroxylase [Vitis vinifera]
c22722.graph_c0	4.87144316	2.73875573	4.54492957	0.38800403	0.61307971	0.59364023	1.787E-11	-4.02075	down	PREDICTED: protein UPSTREAM OF FLC [Sesamum indicum]
c22728.graph_c0	2.53850363	2.79515011	1.9572466	1.89109405	1.91570997	2.25822044	2.976E-05	-1.35178	down	hypothetical protein MIMGU_mgv1a020957mg, partial [Erythranthe guttata]
c22730.graph_c0	16.6542668	17.4440888	15.7563873	15.0162561	14.2868701	12.8257191	8.708E-29	-1.33319	down	PREDICTED: ABC transporter B family member 20 isoform X2 [Nicotiana tomentosiformis]

#ID	0 h-1 FPKM	0 h-2 FPKM	0 h-3 FPKM	28 h-1 FPKM	28 h-2 FPKM	28 h-3 FPKM	FDR	log2FC	regulated	nr_annotation
c22740.graph_c0	0.16513605	0.08265071	0.15915478	8.85146999	10.8908579	7.93275804	4.649E-32	4.99466	up	PREDICTED: probable WRKY transcription factor 29 [Sesamum indicum]
c22750.graph_c0	4.66532669	4.56386199	6.7445207	26.0084599	25.3270669	25.0899368	9.352E-14	1.16244	up	hypothetical protein MIMGU_mgv1a013190mg [Erythranthe guttata]
c22752.graph_c0	4.12406373	4.12819335	3.31224073	26.8824447	25.2026253	24.8402543	5.708E-11	1.64587	up	PREDICTED: transcription termination factor 4, mitochondrial [Sesamum indicum]
c22756.graph_c0	0.03756984	0	0	0.98299966	0.88755652	1.15861733	3.97E-12	5.27546	up	PREDICTED: uncharacterized protein LOC105158433 [Sesamum indicum]
c22766.graph_c0	88.5579153	79.2467087	81.9792512	16.0301254	14.2982615	16.9253114	0	-3.49137	down	PREDICTED: uncharacterized protein LOC104211375 [Nicotiana sylvestris]
c22771.graph_c0	1.47319632	2.06454011	2.27173872	8.99396901	7.73401024	12.7558901	0.0040687	1.25055	up	PREDICTED: CBL-interacting serine/threonine-protein kinase 23-like [Tarenaya hassleriana]
c22778.graph_c0	0.14139626	0	0	3.13436252	3.66512791	4.06702589	5.412E-14	5.20586	up	PREDICTED: LOW QUALITY PROTEIN: 4-coumarate--CoA ligase 1-like [Sesamum indicum]
c22778.graph_c1	0	0	0	3.21648381	3.66006663	3.5594223	1.283E-17	Inf	up	4-coumarate coenzyme A ligase [Paulownia fortunei]
c22780.graph_c0	1.29267178	2.10868565	2.70703387	37.510663	32.8003994	34.4699361	1.386E-72	3.00182	up	PREDICTED: uncharacterized protein At4g06744-like [Sesamum indicum]
c22800.graph_c0	1.43368979	1.15372827	1.11985868	0.10215662	0.07379028	0.01667179	2.213E-33	-5.3593	down	-
c22803.graph_c0	3.70717423	2.83199225	3.25948703	0.99265632	1.02431495	2.14071283	5.285E-10	-2.32241	down	-
c22837.graph_c0	106.098355	108.183843	122.423567	23.5156308	23.5915664	27.8767175	1.651E-58	-3.25845	down	PREDICTED: cytochrome P450 86B1-like [Sesamum indicum]
c22838.graph_c0	8.5955291	7.56645145	6.82717693	5.3367878	6.77441143	5.17449766	1.278E-19	-1.50019	down	PREDICTED: uncharacterized protein LOC105171100 [Sesamum indicum]
c22841.graph_c0	1.60960748	1.99484289	2.06840934	0.61278037	0.65388017	0.36365366	3.337E-12	-2.89458	down	-
c22844.graph_c0	0.54395153	0.27224811	0.34949962	4.34874918	3.56955707	4.03243616	6.214E-06	2.27219	up	PREDICTED: solute carrier family 35 member F1-like isoform X1 [Sesamum indicum]
c22846.graph_c0	0.51988252	0.59474641	0.76350814	4.15632383	5.4585756	3.3915263	3.179E-05	1.69461	up	PREDICTED: protein MIZU-KUSSEI 1-like [Sesamum indicum]
c22847.graph_c0	3.02674162	2.7267952	2.59298854	12.0623374	11.7518592	12.1763128	1.062E-06	1.01972	up	PREDICTED: scarecrow-like protein 9 [Sesamum indicum]
c22852.graph_c0	0.11113576	0.11124705	0.14281386	5.73487114	5.39684183	5.33870774	1.211E-12	4.40089	up	-
c22852.graph_c1	5.48943407	4.93612437	4.06509769	75.4659826	81.5710682	82.271704	1.628E-90	2.9589	up	PREDICTED: HMG-Y-related protein A-like [Sesamum indicum]
c22864.graph_c0	0.09697454	0.48535822	0	17.4086923	16.35479	15.1830469	7.402E-33	5.31488	up	PREDICTED: NADPH--cytochrome P450 reductase-like [Sesamum indicum]
c22871.graph_c0	26.7655364	27.4205487	24.019671	25.1607412	26.3130114	24.5747838	1.041E-43	-1.12968	down	PREDICTED: calcium-transporting ATPase 4, endoplasmic reticulum-type-like [Sesamum indicum]
c22886.graph_c0	0	0.10780318	0.13839277	1.80026405	1.1307615	1.56480516	1.206E-08	3.0823	up	PREDICTED: alcohol dehydrogenase-like 4 [Sesamum indicum]
c22889.graph_c0	29.676431	30.4608071	27.3905449	21.4690347	20.9586749	20.6889687	1.107E-43	-1.56124	down	PREDICTED: zinc finger protein 598 [Sesamum indicum]

#ID	0 h-1 FPKM	0 h-2 FPKM	0 h-3 FPKM	28 h-1 FPKM	28 h-2 FPKM	28 h-3 FPKM	FDR	log2FC	regulated	nr_annotation
c22923.graph_c0	0.44268615	0.88625887	1.13773846	7.85049856	7.6692694	8.29622089	4.459E-09	2.17139	up	PREDICTED: uncharacterized protein LOC102582737 [Solanum tuberosum]
c22943.graph_c0	41.2114345	46.4975438	41.5638688	39.1795714	32.6644615	35.8544415	2.576E-34	-1.35356	down	PREDICTED: dof zinc finger protein DOF3.1-like [Sesamum indicum]
c22951.graph_c0	441.109685	421.822618	432.806823	323.592201	379.262146	388.269323	8.217E-55	-1.33804	down	PREDICTED: 1-Cys peroxiredoxin [Phoenix dactylifera]
c22986.graph_c0	34.8029518	32.7689609	32.1551537	29.076702	30.443445	30.7085525	1.305E-43	-1.23395	down	PREDICTED: ninja-family protein mc410 [Sesamum indicum]
c22991.graph_c0	5.41089771	4.92392355	5.82946542	44.8873584	49.6391515	47.1946524	5.904E-57	2.04022	up	PREDICTED: uncharacterized protein LOC105174944 [Sesamum indicum]
c22996.graph_c0	26.1039194	27.9591626	25.1584274	24.3223324	24.4275198	22.8811285	5.506E-33	-1.23548	down	PREDICTED: putative F-box/LRR-repeat protein 23 [Sesamum indicum]
c22998.graph_c0	0.19127284	0.09573219	0.49158635	8.68849992	8.66077049	8.39425806	8.936E-17	3.94001	up	PREDICTED: probable ADP-ribosylation factor GTPase-activating protein AGD11 [Sesamum indicum]
c23011.graph_c0	0.33222277	0.16627772	0.42691942	6.27789753	8.17550893	7.68406968	2.946E-10	3.48407	up	-
c23025.graph_c0	16.4244684	11.6121148	16.2354555	6.92858981	7.46181724	7.22035309	2.972E-11	-2.12604	down	PREDICTED: MATH domain-containing protein At5g43560-like [Sesamum indicum]
c23026.graph_c0	8.11233381	6.11315309	6.44221188	1.98741487	1.79444929	1.45954288	1.075E-26	-3.0686	down	-
c23028.graph_c0	0.90521784	0.69469528	0.34897223	3.07023319	3.16815167	2.95265757	0.0005767	1.15729	up	PREDICTED: sodium-coupled neutral amino acid transporter 1 [Sesamum indicum]
c23036.graph_c0	1.90976021	1.99856675	1.8963635	7.57092388	9.45623761	10.0904264	0.0002441	1.13427	up	PREDICTED: anthocyanidin 3-O-glucoside 2'''-O-xylosyltransferase-like isoform X3 [Sesamum indicum]
c23056.graph_c0	7.20146288	9.84410328	8.75662917	76.5962271	71.2426263	77.5188832	9.477E-62	2.03488	up	PREDICTED: uncharacterized protein At1g66480-like [Sesamum indicum]
c23062.graph_c0	0.61304676	0.20455354	0.39389468	2.89613252	3.75478009	2.90857906	7.786E-05	1.89301	up	-
c23070.graph_c0	0.27430736	0	0.11749861	196.374535	311.653697	208.664495	5.521E-39	9.75501	up	PREDICTED: EG45-like domain containing protein [Sesamum indicum]
c23071.graph_c0	1.41954971	1.06572838	1.29212579	0.17195343	0.11644337	0.10523444	4.763E-19	-4.35355	down	-
c23072.graph_c0	2.74060658	2.46198156	2.4381609	18.0799334	21.0281498	18.3371591	6.706E-17	1.82067	up	PREDICTED: protein RDM1 [Sesamum indicum]
c23075.graph_c1	23.6754363	25.3668612	23.1559478	9.52767411	10.9906081	10.8947067	1.637E-97	-2.29049	down	PREDICTED: E3 ubiquitin-protein ligase CIP8-like [Sesamum indicum]
c23078.graph_c0	0.09888119	0.29694062	0.12706625	4.02451175	3.89331083	3.16668412	4.675E-09	3.31554	up	PREDICTED: probable sugar phosphate/phosphate translocator At3g17430 [Nicotiana tomentosiformis]
c23083.graph_c0	17.9512557	17.2099679	16.2451227	18.3758721	17.0894403	16.6439633	2.222E-05	-1.06998	down	-
c23084.graph_c1	0.81292692	0.48824456	1.25357193	26.5873769	18.2444905	20.8272791	4.95E-16	3.58516	up	PREDICTED: elongation of fatty acids protein 3-like [Populus euphratica]
c23085.graph_c0	291.313747	326.521369	313.623422	18.1876681	27.2737749	23.4143388	2.4E-222	-4.84882	down	PREDICTED: zinc finger protein ZAT10-like [Sesamum indicum]
c23086.graph_c0	0	0.14711949	0	3.41819623	4.58124246	3.13787274	8.519E-14	5.1592	up	myb family transcription factor family protein [Populus trichocarpa]
c23103.graph_c0	0.55078956	0.39381507	0.20222468	8.40652427	6.66087834	6.48634059	4.766E-13	3.15223	up	PREDICTED: uncharacterized protein LOC105172657 [Sesamum indicum]

#ID	0 h-1 FPKM	0 h-2 FPKM	0 h-3 FPKM	28 h-1 FPKM	28 h-2 FPKM	28 h-3 FPKM	FDR	log2FC	regulated	nr_annotation
c23112.graph_c0	1160.41187	1150.0643	1254.61612	200.819277	215.119334	210.580553	3.34E-140	-3.60015	down	hypothetical protein MIMGU_mgv1a018508mg [Erythranthe guttata]
c23113.graph_c0	8.81079134	7.42704338	9.53449743	38.2195742	43.3945281	41.5824806	2.739E-14	1.16489	up	-
c23115.graph_c0	2.11809863	2.43825252	1.53103534	23.610883	27.5558305	23.0347548	2.324E-44	2.52071	up	PREDICTED: probable glucan endo-1,3-beta-glucosidase A6 [Sesamum indicum]
c23120.graph_c0	0.54008683	0.84955773	0.39659026	11.6077319	11.493321	10.5699958	7.813E-19	3.15032	up	hypothetical protein MIMGU_mgv1a016344mg [Erythranthe guttata]
c23122.graph_c0	12.6156819	13.8613823	12.2815536	10.3730156	9.68641828	11.2731324	2.654E-45	-1.39614	down	PREDICTED: exocyst complex component SEC15B [Sesamum indicum]
c23128.graph_c0	52.9299247	51.6512187	50.8601451	19.2345821	21.1709326	21.6972203	8.5E-154	-2.41347	down	PREDICTED: omega-6 fatty acid desaturase, endoplasmic reticulum [Sesamum indicum]
c23134.graph_c0	13.6457391	12.2359178	12.5974325	12.1386692	13.1044436	12.7760853	5.518E-24	-1.10714	down	PREDICTED: U-box domain-containing protein 8 [Sesamum indicum]
c23146.graph_c0	0.44216564	0.4426084	0.56820036	3.33414569	4.60630676	3.441768	2.259E-06	1.8747	up	PREDICTED: uncharacterized protein LOC105156632 [Sesamum indicum]
c23151.graph_c0	0.41287815	0.4959499	0.74279068	6.84176608	5.41883622	8.52115433	2.109E-08	2.5571	up	PREDICTED: copper transporter 6-like [Sesamum indicum]
c23152.graph_c0	132.359843	104.083664	138.333754	41.6978151	36.7661868	39.1034463	1.334E-21	-2.7643	down	-
c23170.graph_c0	1.27508361	1.81635905	1.32343044	40.9540466	41.8694005	38.5079668	5.84E-112	3.69017	up	PREDICTED: BES1/BZR1 homolog protein 2 [Sesamum indicum]
c23171.graph_c0	1.05479129	0.9838579	1.47867116	7.09120011	6.65439159	7.20807179	3.326E-14	1.47938	up	PREDICTED: probable arabinosyltransferase ARAD1 [Sesamum indicum]
c23186.graph_c0	1.62755949	1.3033514	1.43415661	15.1073338	17.0582614	15.5541119	2.227E-29	2.36081	up	PREDICTED: probable aquaporin SIP2-1 [Sesamum indicum]
c23196.graph_c0	0.24161531	0.4837145	0.15524262	3.51209231	2.85398106	2.36431688	7.9E-06	2.22422	up	BnaC01g07200D [Brassica napus]
c23201.graph_c0	0	0	0.23369216	6.21207902	4.77355312	5.71611287	2.032E-12	5.0295	up	PREDICTED: cytochrome P450 71D11-like [Beta vulgaris subsp. vulgaris]
c23225.graph_c0	3.03923356	3.61629141	3.53708872	14.920477	18.5143061	19.2827315	4.609E-10	1.27926	up	PREDICTED: GATA transcription factor 15-like [Sesamum indicum]
c23258.graph_c1	10.1705138	12.3440963	12.4160372	2.77195154	2.75309358	2.1864935	3.458E-33	-3.27305	down	-
c23259.graph_c0	12.7371725	14.3436677	13.412479	5.5715253	7.85542067	5.77032814	7.935E-43	-2.16868	down	PREDICTED: uncharacterized protein LOC105165738 [Sesamum indicum]
c23266.graph_c0	1078.60554	1065.59275	1153.72731	167.729416	154.211522	193.326208	5.93E-172	-3.76869	down	PREDICTED: stem-specific protein TSJT1-like [Sesamum indicum]
c23267.graph_c0	18.0768003	22.4951505	18.9639552	11.9318854	12.9221615	12.0506925	8.61E-22	-1.78066	down	PREDICTED: U-box domain-containing protein 38-like [Sesamum indicum]
c23284.graph_c0	28.1138564	24.8633276	29.4631374	28.7648871	28.3005199	27.842545	1.022E-06	-1.04911	down	Zinc finger A20 and AN1 domain-containing stress-associated protein 8 [Morus notabilis]
c23287.graph_c0	0.04441129	0.08891152	0.11414053	1.61389348	1.36976282	1.5539702	1.431E-08	3.09675	up	PREDICTED: GDSL esterase/lipase At5g33370-like isoform X1 [Sesamum indicum]
c23298.graph_c0	2.52925199	2.95374876	2.76867947	1.49782989	2.21301811	1.49999354	1.256E-09	-1.75579	down	hypothetical protein MIMGU_mgv1a008021mg [Erythranthe guttata]
c23299.graph_c0	5.85058812	4.93174451	5.38147517	1.16375281	1.17200118	1.53398989	1.233E-30	-3.15099	down	PREDICTED: B-box zinc finger protein 21 [Vitis vinifera]

#ID	0 h-1 FPKM	0 h-2 FPKM	0 h-3 FPKM	28 h-1 FPKM	28 h-2 FPKM	28 h-3 FPKM	FDR	log2FC	regulated	nr_annotation
c23300.graph_c0	11.854125	11.8242134	13.4093485	4.823472	4.05384377	4.13394716	6.98E-50	-2.6036	down	PREDICTED: uncharacterized protein LOC105160924 [Sesamum indicum]
c23305.graph_c0	0.20244915	0.33775313	0.26015518	4.61035153	3.67558893	3.52188095	4.563E-09	2.79046	up	PREDICTED: uncharacterized protein LOC105163284 [Sesamum indicum]
c23307.graph_c0	0.59926318	0.59986325	1.30320708	6.13107752	7.19954962	5.79571993	2.3E-10	1.8316	up	PREDICTED: thermospermine synthase ACAULIS5-like [Sesamum indicum]
c23310.graph_c0	8.59442473	6.22346905	4.22968444	28.3080551	28.9194565	27.6539022	8.026E-05	1.07323	up	-
c23312.graph_c0	0.20642916	0.16071679	0.29474405	10.2021438	11.5897322	9.79396006	1.204E-76	4.47941	up	PREDICTED: probable inactive receptor kinase At5g67200 [Sesamum indicum]
c23318.graph_c0	0.8798262	0.73392268	0.94217625	7.35370502	6.15856803	8.60950379	5.106E-06	2.02287	up	-
c23327.graph_c0	8.5912281	5.29989579	5.26328835	0	0	0	2.5E-25	-Inf	down	--
c23334.graph_c0	2.84465406	3.25428863	2.93744981	15.4322478	13.300515	11.9298204	5.725E-08	1.0786	up	hypothetical protein MIMGU_mgv1a011374mg [Erythranthe guttata]
c23343.graph_c0	0.16433457	0.5263972	0.33788228	7.93064454	8.64883135	7.62136868	3.08E-33	3.4616	up	PREDICTED: probable dolichyl-diphosphooligosaccharide--protein glycosyltransferase subunit 3B [Sesamum indicum]
c23352.graph_c0	2.81004587	1.92458822	1.8055101	14.83373	14.4610626	16.972197	2.696E-12	1.73776	up	PREDICTED: phosphatidyl-N-methylethanolamine N-methyltransferase [Nicotiana sylvestris]
c23358.graph_c1	3.30421376	3.09181445	2.03071741	25.5288139	23.8985963	23.9836891	2.094E-23	2.03818	up	PREDICTED: polyadenylate-binding protein-interacting protein 12 [Sesamum indicum]
c23362.graph_c0	25.4398285	23.3779827	23.8484887	12.2758293	9.57869881	12.490304	5.029E-23	-2.17025	down	-
c23376.graph_c0	1.81058011	1.44251698	1.56693908	1.71875159	1.86709544	1.00803772	0.0001505	-1.16108	down	4-coumarate coenzyme A ligase [Paulownia fortunei]
c23392.graph_c0	0.32761525	0.24595748	0.31574892	1.48818046	3.4935873	3.15729206	0.0037152	2.10377	up	PREDICTED: uncharacterized protein LOC105168788 [Sesamum indicum]
c23395.graph_c0	0.97939151	1.30716296	0.83903791	14.7504115	15.8533457	14.1336791	2.264E-28	2.75102	up	PREDICTED: uncharacterized protein LOC105176156 [Sesamum indicum]
c23407.graph_c0	0.50227045	0.33518227	0.43029161	13.963058	14.9969573	15.9115211	2.306E-55	4.05643	up	PREDICTED: uncharacterized protein LOC105166016 [Sesamum indicum]
c23417.graph_c0	3.62999824	3.04211146	4.23076667	0.30677463	0.33238647	0.45058606	1.976E-26	-4.41421	down	-
c23432.graph_c0	0	0	0	3.54462612	2.15415917	2.78114036	1.173E-11	Inf	up	PREDICTED: beta-galactosidase-like [Sesamum indicum]
c23438.graph_c0	18.4933751	20.1188286	19.1438016	20.2546571	19.5943461	19.079136	1.204E-27	-1.06194	down	PREDICTED: metal tolerance protein 1-like isoform X1 [Sesamum indicum]
c23443.graph_c0	11.1443802	12.7330907	14.1763136	11.4540807	10.5635744	12.4841685	4.927E-09	-1.23406	down	PREDICTED: LOW QUALITY PROTEIN: expansin-A11-like [Sesamum indicum]
c23452.graph_c0	1.46810616	1.53637516	1.62931437	0.29100303	0.04379141	0.03957602	2.467E-21	-4.7246	down	PREDICTED: uncharacterized protein LOC105168585 [Sesamum indicum]
c23454.graph_c0	27.7416549	27.769434	28.5408402	28.0203579	27.1147385	26.3934511	5.825E-21	-1.13508	down	hypothetical protein CICLE_v100253982mg, partial [Citrus clementina]

#ID	0 h-1 FPKM	0 h-2 FPKM	0 h-3 FPKM	28 h-1 FPKM	28 h-2 FPKM	28 h-3 FPKM	FDR	log2FC	regulated	nr_annotation
c23455.graph_c0	10.5904054	11.5802199	9.23669179	3.5239486	4.77269278	5.34744575	6.398E-39	-2.29034	down	anthocyanidin synthase [Ipomoea nil]
c23458.graph_c0	1.12058316	1.19906424	0.94344417	5.19552332	6.41535578	4.05617467	0.0002932	1.17345	up	PREDICTED: uncharacterized protein LOC105155953 [Sesamum indicum]
c23463.graph_c0	1.2423378	0.96277301	1.80244722	7.07775871	7.02171819	5.98929677	5.255E-07	1.22807	up	PREDICTED: uncharacterized protein LOC101217532 [Cucumis sativus]
c23471.graph_c0	0.33532534	0.67132223	0.43090633	2.35588772	4.1809416	3.11558971	0.0008975	1.65538	up	-
c23501.graph_c0	7.08762116	5.76445865	5.76357639	36.823333	36.9906901	35.8600138	1.563E-27	1.47017	up	PREDICTED: protein transport protein Sec24-like At4g32640 [Sesamum indicum]
c23505.graph_c0	26.2081291	13.6700179	19.6135229	0	0	0	1.365E-28	-Inf	down	cytochrome c oxidase subunit 2 (mitochondrion) [Gelidium vagum]
c23509.graph_c0	7.52124197	4.56289294	8.64000801	33.7094378	34.2503673	34.1974269	2.337E-10	1.20744	up	-
c23513.graph_c0	532.834615	566.225916	524.298132	35.1363829	37.8273483	42.1116749	0	-4.90785	down	PREDICTED: zinc finger protein ZAT10-like [Sesamum indicum]
c23524.graph_c0	2.69133504	2.2001245	1.67159484	16.4307931	19.4568309	14.5246757	9.614E-14	1.85524	up	PREDICTED: uncharacterized protein LOC105161815 [Sesamum indicum]
c23535.graph_c0	1.10634827	0.9756161	1.08320102	6.08809145	6.93172101	5.88953775	1.532E-11	1.48753	up	PREDICTED: HIPL1 protein-like [Sesamum indicum]
c23540.graph_c0	0.20905168	0.62778303	0.71637249	3.03875109	3.56681953	2.68622908	0.0004212	1.47835	up	-
c23541.graph_c0	0	0	0	10.0056518	8.85874477	7.37185733	5.417E-19	Inf	up	PREDICTED: non-specific lipid transfer protein GPI-anchored 2-like [Nicotiana tomentosiformis]
c23552.graph_c0	0.57952362	0.29005196	0.49647386	3.43975449	4.43682256	4.4107039	7.574E-06	2.07943	up	PREDICTED: phosphoglycolate phosphatase 2 [Sesamum indicum]
c23561.graph_c0	3.04000074	4.23852675	3.69724339	19.8850952	21.4098815	20.6528319	2.262E-29	1.40468	up	PREDICTED: leukotriene A-4 hydrolase homolog [Sesamum indicum]
c23565.graph_c0	6.30206352	7.00930453	8.818259	4.68208796	2.94086013	4.48498825	1.248E-08	-1.96366	down	PREDICTED: protein CUP-SHAPED COTYLEDON 3 [Sesamum indicum]
c23578.graph_c0	0.45666827	0.71834016	0.8383383	3.17679416	4.45235994	4.68150461	7.792E-05	1.51597	up	-
c23583.graph_c0	0.68228463	0.63743665	0.64295936	9.98372815	12.2977504	9.63030036	1.338E-26	2.93236	up	PREDICTED: glyoxylate/succinic semialdehyde reductase 2, chloroplastic [Sesamum indicum]
c23586.graph_c0	1.16998165	0.557692	0.7159394	4.98053895	4.27759581	4.19624429	0.0001525	1.37537	up	PREDICTED: glucan endo-1,3-beta-glucosidase 14-like [Sesamum indicum]
c23589.graph_c0	0.06590589	0	0	2.49080103	2.55170438	3.20505405	1.196E-13	5.9097	up	PREDICTED: calcium-dependent protein kinase 8-like [Sesamum indicum]
c23596.graph_c0	17.9611653	18.232775	16.6775121	12.2970171	12.617946	12.0878673	6.126E-59	-1.60455	down	PREDICTED: probable 1-acyl-sn-glycerol-3-phosphate acyltransferase 4 [Sesamum indicum]
c23601.graph_c0	6.45407707	5.56097101	6.61399883	31.4698303	34.4188388	34.497239	4.167E-16	1.33876	up	PREDICTED: uncharacterized protein LOC105163419 [Sesamum indicum]
c23609.graph_c0	2.20108011	1.81446931	2.49571332	15.7150177	15.7185611	11.8251031	3.004E-07	1.63779	up	PREDICTED: uncharacterized protein LOC105170698 [Sesamum indicum]
c23610.graph_c1	0	0	0	3.32254569	5.19221638	4.69240988	1.067E-12	Inf	up	hypothetical protein MIMGU_mgv1a005845mg [Erythranthe guttata]

#ID	0 h-1 FPKM	0 h-2 FPKM	0 h-3 FPKM	28 h-1 FPKM	28 h-2 FPKM	28 h-3 FPKM	FDR	log2FC	regulated	nr_annotation
c23625.graph_c0	0.20257345	0.4055526	0.26031491	2.72374055	3.4562887	2.82323925	5.276E-06	2.28215	up	-
c23632.graph_c0	6.01974268	5.40455708	5.10390609	28.956942	25.3309051	27.7875612	1.331E-14	1.2236	up	-
c23633.graph_c0	132.335231	131.755553	135.313385	20.3146182	22.2107314	23.3277444	0	-3.69101	down	conserved hypothetical protein [Ricinus communis]
c23650.graph_c0	0.46338178	0.49476884	0.27788317	43.8267217	47.3356901	43.0356139	1.41E-253	5.67787	up	PREDICTED: glucan endo-1,3-beta-glucosidase 3 [Sesamum indicum]
c23652.graph_c0	25.0259724	23.9043299	26.384268	19.7258085	21.5114502	20.1723833	2.108E-25	-1.38605	down	PREDICTED: uncharacterized protein LOC105169025 [Sesamum indicum]
c23673.graph_c0	0	0	0	4.39526919	7.33574166	5.86882434	1.033E-11	Inf	up	PREDICTED: EPIDERMAL PATTERNING FACTOR-like protein 4-like [Fragaria vesca subsp. vesca]
c23690.graph_c0	3.50055966	3.17034447	4.391254	20.3534927	19.9087369	21.3535078	4.371E-11	1.38393	up	PREDICTED: uncharacterized protein LOC105170479 [Sesamum indicum]
c23691.graph_c0	1.30835682	2.73839451	1.68129031	10.1141841	8.81994933	10.087112	0.0004088	1.25047	up	-
c23713.graph_c1	10.2787059	11.1869475	9.94243137	7.19884954	8.75645044	7.47021216	1.176E-29	-1.51341	down	PREDICTED: uncharacterized protein LOC105162910 isoform X1 [Sesamum indicum]
c23716.graph_c0	0.29654297	0.29683991	0	20.9059948	14.4003643	15.6521832	6.425E-15	5.35367	up	hypothetical protein [Arachis diogoi]
c23716.graph_c1	0.12336449	0.24697603	0	42.6784442	41.4490176	34.8252619	5.881E-69	7.25235	up	PREDICTED: abscisic acid receptor PYL4-like [Sesamum indicum]
c23720.graph_c0	1.72191326	0.86181875	0.66381801	11.138142	7.90976243	12.0500976	1.764E-06	2.18108	up	unnamed protein product [Coffea canephora]
c23737.graph_c0	9.56463597	6.58227178	7.16971261	0	0	0	3.178E-40	-Inf	down	hypothetical protein BOTBODRAFT_366584 [Botryobasidium botryosum FD-172 SS1]
c23783.graph_c0	0.05184724	0	0	0.64059872	0.83357731	0.52272332	2.706E-10	4.20665	up	PREDICTED: putative late blight resistance protein homolog R1A-10 [Sesamum indicum]
c23800.graph_c0	2.46207885	2.83422588	3.63844912	0.89471236	0.6462731	1.02210917	8.922E-12	-2.89533	down	-
c23802.graph_c0	0.32865828	0.14099459	0	68.1112036	75.1471429	69.3613533	0	7.75646	up	PREDICTED: nicotianamine synthase-like [Sesamum indicum]
c23803.graph_c1	5.49089093	4.08877735	5.85132722	28.1299058	27.859438	30.935968	2.464E-18	1.40215	up	hypothetical protein MIMGU_mgv1a015927mg [Erythranthe guttata]
c23816.graph_c0	5.29489973	4.18436982	3.10365001	0.40508449	0.36575331	0.11018188	5.264E-20	-4.92486	down	PREDICTED: elongation of fatty acids protein 3-like [Sesamum indicum]
c23817.graph_c0	0.82907181	0.47422971	0.91319151	10.587921	11.7361374	8.00748707	6.542E-11	2.67938	up	hypothetical protein MIMGU_mgv1a012373mg [Erythranthe guttata]
c23825.graph_c0	92.7354826	104.654642	96.7769624	30.7787651	33.5962189	33.3696503	8.63E-105	-2.68019	down	PREDICTED: zinc finger A20 and AN1 domain-containing stress-associated protein 5-like [Sesamum indicum]
c23833.graph_c0	79.4240499	80.931372	75.1502342	71.0752567	68.9228709	67.8383401	4.121E-56	-1.2702	down	PREDICTED: U3 small nucleolar RNA-associated protein 15 homolog [Sesamum indicum]
c23834.graph_c0	0.14383872	0	0	4.39072856	5.19149743	4.81971723	1.061E-16	5.58666	up	hypothetical protein MIMGU_mgv1a026155mg [Erythranthe guttata]
c23837.graph_c0	0.46977167	0.70536311	1.60980051	5.23521923	5.65174275	5.89707402	4.259E-05	1.48554	up	-
c23837.graph_c1	8.75419606	8.05879547	8.78866291	41.8107093	39.2899201	36.2726996	1.625E-24	1.10548	up	PREDICTED: protein FAR1-RELATED SEQUENCE 7-like [Sesamum indicum]
c23840.graph_c0	0	0.04599782	0.05904989	6.91328391	7.4783947	6.458746	1.34E-33	6.52317	up	Integrase, catalytic region, putative [Theobroma cacao]

#ID	0 h-1 FPKM	0 h-2 FPKM	0 h-3 FPKM	28 h-1 FPKM	28 h-2 FPKM	28 h-3 FPKM	FDR	log2FC	regulated	nr_annotation
c23846.graph_c0	18.973588	16.6566005	13.038402	1.84356877	1.79773533	2.16624537	5.061E-36	-4.15218	down	PREDICTED: galactinol--sucrose galactosyltransferase [Sesamum indicum]
c23859.graph_c0	3.88167234	4.27411517	2.32778123	0	0.25472584	0	1.24E-17	-6.45725	down	putative histone h2a protein [Eutypa lata UCREL1]
c23876.graph_c0	5.41358174	7.90271215	5.79722239	2.78698	3.70056123	3.47811624	7.487E-08	-2.02967	down	hypothetical protein, partial [Oryza eichingeri]
c23879.graph_c0	2.26868941	2.4907316	1.50469972	1.38292355	1.00852525	1.08505224	9.844E-08	-1.93497	down	-
c23891.graph_c0	56.3928194	51.347141	61.1788759	22.621106	21.2075675	18.0727429	1.353E-40	-2.54107	down	-
c23894.graph_c0	0.90172026	0.77367702	0.6621407	7.02176879	6.84719877	7.33402367	2.454E-06	2.09459	up	PREDICTED: uncharacterized protein LOC105169824 isoform X1 [Sesamum indicum]
c23905.graph_c0	3.30542899	6.47961363	4.24760712	44.2435874	46.455147	40.0230293	1.01E-23	2.12776	up	PREDICTED: macrophage migration inhibitory factor homolog [Sesamum indicum]
c23936.graph_c0	0.36167337	0.24135702	0	43.021767	43.5914189	45.6155843	2.51E-76	6.70878	up	actin [Ipomoea nil]
c23939.graph_c0	3.83367221	2.16902798	3.85545957	24.3497593	23.845024	24.6140895	5.117E-10	1.79271	up	hypothetical protein PHAVU_002G125300g [Phaseolus vulgaris]
c23951.graph_c0	14.6767768	12.9106888	14.7167019	5.09106667	5.58177498	5.37417316	8.317E-58	-2.4895	down	PREDICTED: uncharacterized protein LOC105166244 [Sesamum indicum]
c23961.graph_c0	0.26908484	0.20201572	0.08644617	2.00458545	2.91358241	3.15176349	1.059E-07	2.77792	up	PREDICTED: N-alpha-acetyltransferase 10 [Sesamum indicum]
c23962.graph_c0	37.7891168	37.0900681	40.0466553	16.0509228	11.4329551	13.0974219	2.565E-39	-2.59313	down	PREDICTED: NAC transcription factor 25-like [Sesamum indicum]
c23973.graph_c0	0.15981051	0.07998527	0	9.23387382	11.7980976	8.00865027	1.431E-21	5.85211	up	PREDICTED: transcription factor MYB1R1-like [Malus domestica]
c23976.graph_c0	1.9675891	1.0743051	1.89632242	0	0	0	6.255E-19	-Inf	down	predicted protein [Physcomitrella patens]
c23978.graph_c0	28.5261757	31.8144139	33.811724	31.2409947	29.2334232	28.3506349	1.438E-14	-1.17677	down	PREDICTED: ran-binding protein 6 isoform X2 [Elaeis guineensis]
c23979.graph_c0	0.04907925	0.1965136	0.06306879	5.49325996	4.09030607	4.71531009	9.221E-19	4.44578	up	PREDICTED: ferredoxin--NADP reductase, leaf isozyme, chloroplastic isoform 1 [Glycine max]
c23985.graph_c0	0.35236662	1.32269797	0.45280505	4.86586249	4.68245825	5.79902567	4.939E-05	1.7631	up	hypothetical protein MIMGU_mgv1a016798mg [Erythranthe guttata]
c23986.graph_c0	0.78002256	0.39040182	1.33647983	9.16514038	10.663998	7.70997908	1.076E-08	2.35539	up	PREDICTED: uncharacterized protein LOC105178874 isoform X1 [Sesamum indicum]
c23987.graph_c0	0.16019151	0.04008798	0.20585244	6.14148569	7.27969884	7.0539642	5.215E-31	4.5582	up	PREDICTED: lysophospholipid acyltransferase 1-like [Sesamum indicum]
c23990.graph_c0	0.50516519	1.01134208	0.32457864	9.36235433	8.12182509	6.89062383	6.682E-07	2.64164	up	PREDICTED: phosphoribosylformylglycinamidine cyclo-ligase, chloroplastic [Sesamum indicum]
c23994.graph_c0	31.4707959	29.7994816	31.2872942	30.5614515	32.0594199	32.8196568	4.289E-26	-1.04619	down	PREDICTED: transmembrane and coiled-coil domain-containing protein 1 [Sesamum indicum]
c23996.graph_c0	0.14539625	0.08732511	0.22420793	0.67630822	1.10679028	0.7933016	0.0023757	1.39573	up	Quinohemoprotein ethanol dehydrogenase type-1 [Theobroma cacao]
c24007.graph_c0	13.3183623	14.3071888	12.9701411	6.50935134	6.89749296	6.30233812	6.534E-91	-2.13279	down	PREDICTED: uncharacterized protein LOC105164548 [Sesamum indicum]

#ID	0 h-1 FPKM	0 h-2 FPKM	0 h-3 FPKM	28 h-1 FPKM	28 h-2 FPKM	28 h-3 FPKM	FDR	log2FC	regulated	nr_annotation
c24031.graph_c0	8.38389819	9.41297773	12.0839488	2.71731315	4.38652307	3.02359819	6.342E-11	-2.65736	down	hypothetical protein MIMGU_mgv1a025777mg, partial [Erythranthe guttata]
c24032.graph_c0	0.69570898	0.19897304	0.38314868	1.9503152	2.21749481	1.94509489	0.0092746	1.17356	up	PREDICTED: coatomer subunit beta-1-like [Camelina sativa]
c24033.graph_c0	2.20737218	1.38741228	2.17689537	0	0	0	6.679E-26	-Inf	down	CK1/CK1/CK1-D protein kinase [Coniosporium apollinis CBS 100218]
c24064.graph_c0	30.9563711	29.2147867	30.2565219	7.43608112	8.90907306	7.46803873	4.97E-131	-3.01601	down	PREDICTED: 21 kDa protein [Sesamum indicum]
c24065.graph_c0	3.69052017	3.36584095	3.26703113	29.6239478	30.1381942	27.3100263	1.298E-45	1.98667	up	PREDICTED: zinc finger CCCH domain-containing protein 53 [Sesamum indicum]
c24089.graph_c0	5.80369921	6.52878349	7.60004008	1.00430837	1.1969711	1.31121188	1.651E-28	-3.59769	down	PREDICTED: ubiquitin-conjugating enzyme E2 5B-like isoform X1 [Citrus sinensis]
c24100.graph_c0	2.07410805	1.64364642	2.55425634	0.12562081	0.17013573	0.15375834	5.83E-18	-4.8968	down	hypothetical protein MIMGU_mgv1a023310mg [Erythranthe guttata]
c24101.graph_c0	0.970612	1.7272603	0.41575828	17.4791167	22.0806507	24.5601863	6.724E-17	3.28377	up	PREDICTED: methionine gamma-lyase [Sesamum indicum]
c24101.graph_c1	0.44213769	1.32774128	0.75755258	6.85532002	6.18971123	9.35227675	1.671E-05	2.05702	up	PREDICTED: methionine gamma-lyase [Sesamum indicum]
c24103.graph_c0	4.20368666	2.08311684	2.78117819	0	0	0	3.261E-19	-Inf	down	isocitrate dehydrogenase [Lichtheimia corymbifera JMRC:FSU:9682]
c24107.graph_c0	0.13476328	0.40469466	0	1.46917758	1.45918256	1.19883691	0.0023714	1.85865	up	-
c24109.graph_c0	3.2107792	2.99972803	3.64461425	42.4905264	44.5981703	44.4943116	1.65E-131	2.64651	up	PREDICTED: dihydroxy-acid dehydratase, chloroplastic [Sesamum indicum]
c24123.graph_c0	0.08503434	0.08511948	0	2.90471584	3.29230803	3.02581839	1.241E-11	4.68974	up	PREDICTED: fasciclin-like arabinogalactan protein 1 [Sesamum indicum]
c24123.graph_c1	0	0.17250389	0	2.19186474	2.4879465	1.99294873	2.155E-10	4.19105	up	PREDICTED: fasciclin-like arabinogalactan protein 1 [Sesamum indicum]
c24136.graph_c0	1.70937318	2.50959112	2.92881628	2.23625263	1.71999661	2.56818621	0.0040936	-1.22652	down	PREDICTED: uncharacterized protein LOC104116785 [Nicotiana tomentosiformis]
c24143.graph_c0	10.6053634	12.1088557	10.3809407	8.40042515	9.46060613	11.031364	1.487E-20	-1.28486	down	PREDICTED: phosphoglycerate mutase-like protein 1 isoform X1 [Sesamum indicum]
c24146.graph_c0	0.07800556	0.23425102	0.10024023	1.98428974	7.9855401	3.46963762	0.0079044	3.93375	up	PREDICTED: glutamate decarboxylase [Sesamum indicum]
c24157.graph_c0	19.1514645	21.5201521	21.0696413	17.5040412	17.3671007	17.6522039	1.673E-32	-1.32467	down	PREDICTED: plant intracellular Ras-group-related LRR protein 1-like [Sesamum indicum]
c24158.graph_c0	0.26580064	0.15964008	0.20493861	1.27500367	1.08143854	1.60787944	0.000715	1.56632	up	-
c24171.graph_c0	2.21557016	1.26730784	1.76248761	6.59441292	9.20814145	10.1988476	0.0006386	1.22179	up	exocyst subunit Exo70-interacting protein Roh1 [Nicotiana tabacum]
c24187.graph_c0	4.73589486	5.64980043	4.33509924	25.8858846	27.8001093	27.4710141	2.045E-16	1.374	up	PREDICTED: probable methyltransferase PMT11 [Sesamum indicum]
c24197.graph_c0	0.61769397	0.53587083	0.3704219	21.848239	21.1050928	20.0747991	1.836E-80	4.28529	up	PREDICTED: very-long-chain enoyl-CoA reductase [Sesamum indicum]
c24237.graph_c1	0.18130731	0.36297771	0	1.97659657	1.62604304	1.61288666	2.503E-05	2.18592	up	hypothetical protein MIMGU_mgv1a021532mg [Erythranthe guttata]
c24238.graph_c0	1080.13099	1042.58625	1033.19528	91.5596853	81.6058497	95.192839	0	-4.64532	down	-
c24240.graph_c0	10.0380567	4.77285144	8.70702768	34.1069382	40.6762339	38.993142	5.897E-06	1.18415	up	PREDICTED: nudix hydrolase 26, chloroplastic isoform X2 [Vitis vinifera]

#ID	0 h-1 FPKM	0 h-2 FPKM	0 h-3 FPKM	28 h-1 FPKM	28 h-2 FPKM	28 h-3 FPKM	FDR	log2FC	regulated	nr_annotation
c24241.graph_c0	5.79789672	6.33942882	7.22127985	72.9973314	78.9043852	76.4931769	2.72E-73	2.46719	up	PREDICTED: 14 kDa zinc-binding protein-like [Sesamum indicum]
c24247.graph_c0	0.14473161	0.36219135	0.27897878	6.83735659	5.50865434	6.09423272	4.895E-14	3.45754	up	PREDICTED: cytochrome P450 71D11-like [Sesamum indicum]
c24250.graph_c0	0.5241299	0.07495068	0.19243648	1.74141526	2.11282476	2.35349917	9.389E-05	1.89351	up	hypothetical protein MIMGU_mgv1a016982mg [Erythranthe guttata]
c24255.graph_c0	50.4433572	51.7964418	48.0342978	16.8752486	18.3008645	18.8695287	8.83E-202	-2.5649	down	PREDICTED: uncharacterized protein LOC105164653 isoform X2 [Sesamum indicum]
c24261.graph_c0	0.4936893	0.14119533	0.54378026	3.79314001	4.07279434	3.97353069	3.446E-07	2.23346	up	-
c24269.graph_c0	6.36450519	7.77156782	5.16240469	30.6082606	30.5096262	29.5804718	2.313E-18	1.14504	up	unknown [Glycine max]
c24270.graph_c0	7.26712524	6.96703306	9.60160122	112.998725	105.116954	113.451668	2.38E-100	2.70377	up	PREDICTED: peptidyl-prolyl cis-trans isomerase FKBP12 [Sesamum indicum]
c24272.graph_c0	2.34945165	1.50515473	2.17377964	0.06830272	0.03083547	0.05573446	2.352E-26	-6.37201	down	PREDICTED: acyl-[acyl-carrier-protein] desaturase 6, chloroplastic [Sesamum indicum]
c24284.graph_c0	0	0.07344123	0	1.27975839	1.68510685	2.13205615	9.007E-10	5.03685	up	-
c24286.graph_c0	1.26591511	0	0.29577291	4.76758262	7.55207035	6.8251026	4.083E-05	2.54321	up	PREDICTED: topless-related protein 3-like [Sesamum indicum]
c24289.graph_c0	6.27219362	6.27847428	5.09053714	43.4266077	42.2429848	41.3090872	1.555E-08	1.75956	up	hypothetical protein MIMGU_mgv1a018579mg [Erythranthe guttata]
c24292.graph_c0	53.7044584	57.0568334	57.5675202	29.1931558	33.6058592	25.9869565	6.277E-80	-2.01519	down	hypothetical protein MIMGU_mgv1a016421mg [Erythranthe guttata]
c24300.graph_c0	5.5979338	2.66168116	2.69758746	0	0	0	1.796E-10	-Inf	down	--
c24304.graph_c0	0	0	0	3.44398214	3.3583604	3.82195537	1.688E-09	Inf	up	-
c24309.graph_c0	1.10159605	1.55898842	0.97627209	8.94501052	16.6764889	10.4529684	9.818E-05	2.22064	up	PREDICTED: chitotriosidase-1 [Vitis vinifera]
c24314.graph_c0	5.92516869	4.48449164	6.03554833	2.94094138	3.12957189	3.12829006	3.45E-13	-1.92965	down	hypothetical protein MIMGU_mgv1a013918mg [Erythranthe guttata]
c24323.graph_c0	4.45600816	3.95866728	3.57884181	182.049861	160.682213	185.647708	1.04E-134	4.37377	up	PREDICTED: alpha-xylosidase 1 [Sesamum indicum]
c24336.graph_c0	4.4781711	1.73193501	3.13888754	35.8017984	54.966992	38.7507687	3.171E-10	2.70368	up	PREDICTED: hydroxymethylglutaryl-CoA synthase isoform X4 [Sesamum indicum]
c24341.graph_c0	0.78328947	0.52271588	0.33551933	3.13109649	3.25543301	2.47752646	0.0044535	1.35076	up	PREDICTED: methylsterol monooxygenase 1-1-like [Sesamum indicum]
c24348.graph_c0	0.42416922	0.08491879	0.2180297	3.69940492	3.06186448	3.42117543	1.296E-07	2.7247	up	hypothetical protein MIMGU_mgv1a011727mg [Erythranthe guttata]
c24351.graph_c0	35.2569645	30.0166206	30.5936288	164.710436	160.047846	164.149808	1.137E-27	1.26145	up	40S ribosomal protein S13, putative [Ricinus communis]
c24361.graph_c0	1.44073375	1.44217643	1.38854993	8.46419564	5.12117917	7.40513722	0.0022115	1.20773	up	unnamed protein product [Coffea canephora]
c24366.graph_c0	1.04162414	0.80205167	0.72074599	8.44397933	7.99218268	8.22074307	1.798E-09	2.17899	up	PREDICTED: chaperone protein dnaJ 20, chloroplastic-like [Sesamum indicum]
c24372.graph_c0	0	0	0	1.80014994	3.02654473	2.88716331	1.405E-10	Inf	up	early dehydration-responsive 1 [Lindernia subracemosa]
c24393.graph_c0	1.01263412	0.25341203	1.4639343	4.78384817	5.98066113	5.63016496	0.0003268	1.48666	up	PREDICTED: serine/threonine-protein kinase STE20 [Sesamum indicum]
c24395.graph_c0	0.68860663	0.34464808	0.70790943	8.25784298	8.76651664	8.29019082	9.956E-15	2.76892	up	PREDICTED: uncharacterized protein LOC104250007 [Nicotiana sylvestris]

#ID	0 h-1 FPKM	0 h-2 FPKM	0 h-3 FPKM	28 h-1 FPKM	28 h-2 FPKM	28 h-3 FPKM	FDR	log2FC	regulated	nr_annotation
c24403.graph_c0	4.7488648	6.90041623	7.67731265	3.34011055	3.31738733	3.54315381	2.485E-06	-2.01726	down	-
c24417.graph_c0	10.0718919	11.2126664	12.0355756	2.2234231	1.82223097	1.73055839	3.043E-58	-3.62135	down	PREDICTED: transcription factor TGA3 [Sesamum indicum]
c24436.graph_c0	1.6114729	1.36104178	1.16482865	5.45344293	7.30330491	5.82377956	0.0002221	1.07957	up	PREDICTED: myb family transcription factor APL isoform X4 [Sesamum indicum]
c24438.graph_c0	0.02838536	0.22731027	0.10942894	1.21718866	1.24802517	1.29623106	1.831E-06	2.27041	up	PREDICTED: ornithine decarboxylase-like [Sesamum indicum]
c24441.graph_c0	0.61013615	0.34899835	0.50403157	15.5838694	16.9878844	16.2830801	1.131E-56	3.97204	up	PREDICTED: cysteine-rich repeat secretory protein 3-like [Sesamum indicum]
c24454.graph_c0	2.74780725	3.83411221	3.31703861	1.39191154	1.6392595	0.98764213	1.058E-11	-2.39446	down	hypothetical protein TRIUR3_08605 [Triticum urartu]
c24465.graph_c0	2528.85708	2899.14667	2538.28124	86.4538622	118.468905	118.242721	4.53E-244	-5.71331	down	legumin-like protein [Perilla frutescens]
c24469.graph_c0	19.9370719	21.8370465	21.7212422	20.3702094	21.1417669	18.0784849	3.911E-11	-1.18367	down	PREDICTED: uncharacterized protein LOC105159964 [Sesamum indicum]
c24473.graph_c0	31.584226	33.5155178	27.8708881	25.0734202	26.4195184	26.5292849	6.995E-36	-1.34155	down	PREDICTED: uncharacterized protein LOC105159991 [Sesamum indicum]
c24478.graph_c0	0.39788268	0	0.17043166	2.31343068	3.56838546	2.51698739	1.049E-06	2.80365	up	PREDICTED: indole-3-acetic acid-amido synthetase GH3.6 [Sesamum indicum]
c24482.graph_c0	5.9714425	5.51365649	4.5975089	37.1332751	40.1996578	40.2683049	6.648E-68	1.78309	up	PREDICTED: probable methylenetetrahydrofolate reductase [Sesamum indicum]
c24492.graph_c0	1.5829336	1.70640472	2.03413235	79.2052292	73.8321382	72.5020598	9.75E-99	4.31093	up	hypothetical protein MIMGU_mgv1a017195mg [Erythranthe guttata]
c24494.graph_c0	23.9298511	24.1255251	20.2801015	25.059553	20.6001797	21.9235233	1.428E-17	-1.10436	down	-
c24500.graph_c0	4.54786624	5.31115695	5.45457563	5.50893829	4.37716819	5.84382326	0.0024521	-1.05277	down	-
c24502.graph_c0	23.3114681	20.8785151	23.0189436	9.27386648	7.4072667	9.89582987	1.122E-21	-2.42814	down	-
c24503.graph_c0	57.9925772	59.6072111	49.0745289	23.9994798	32.533933	27.1237943	7.454E-47	-2.08326	down	PREDICTED: uncharacterized protein LOC105178379 [Sesamum indicum]
c24520.graph_c0	2.36183844	1.35097341	1.30073824	83.621509	83.694723	94.247609	6.707E-87	4.62195	up	-
c24523.graph_c0	66.3616252	66.1381381	67.5766896	43.9975631	47.8144214	43.8416242	1.247E-89	-1.65172	down	PREDICTED: anthocyanidin 3-O-glucoside 6''-O-acyltransferase-like [Sesamum indicum]
c24539.graph_c0	0.13595458	0.20413607	0.08735351	2.37146408	2.05199264	2.13666775	1.371E-07	2.85533	up	PREDICTED: BTB/POZ domain-containing protein At1g30440-like [Camelina sativa]
c24542.graph_c0	21.4777708	20.8615871	22.1032183	2.38117424	1.79164757	2.21288245	9.41E-118	-4.42558	down	-
c24558.graph_c0	3.52549709	2.48636017	3.50076622	1.39762416	1.31450373	1.90074984	1.351E-09	-2.13384	down	PREDICTED: uncharacterized protein LOC105160970 [Sesamum indicum]
c24559.graph_c0	153.220404	156.896774	146.418925	65.2719527	71.1420099	71.0772935	2.15E-160	-2.22744	down	PREDICTED: uncharacterized protein LOC105168445 [Sesamum indicum]
c24566.graph_c0	0.54732374	0.44825875	0.51151467	5.49675859	6.95481103	6.2853359	1.681E-13	2.5457	up	PREDICTED: 2-C-methyl-D-erythritol 4-phosphate cytidylyltransferase, chloroplastic isoform X2 [Sesamum indicum]

#ID	0 h-1 FPKM	0 h-2 FPKM	0 h-3 FPKM	28 h-1 FPKM	28 h-2 FPKM	28 h-3 FPKM	FDR	log2FC	regulated	nr_annotation
c24579.graph_c0	1.22482873	0.11145956	0.71543337	5.50304503	3.72655027	5.34890624	0.0011079	1.74462	up	unnamed protein product [Vitis vinifera]
c24593.graph_c0	198.631914	203.43666	202.034914	104.440484	113.763849	105.961476	5.26E-130	-1.98899	down	hypothetical protein MIMGU_mgv1a023553mg [Erythranthe guttata]
c24594.graph_c0	0.85792605	0.99090592	0.63603977	15.8282141	18.2106997	17.671017	4.9E-64	3.29277	up	PREDICTED: uncharacterized protein LOC105165456 [Sesamum indicum]
c24616.graph_c0	1.40760658	1.6103041	1.35662264	0.94996604	2.07834641	1.01367669	0.0007458	-1.20628	down	PREDICTED: uncharacterized protein LOC105170552 [Sesamum indicum]
c24619.graph_c0	8.17252621	7.95080691	6.960355	4.42351029	4.9987987	5.17595601	1.29E-50	-1.74925	down	PREDICTED: ribonucleoside-diphosphate reductase large subunit isoform X2 [Sesamum indicum]
c24628.graph_c0	0.15081109	0	0	4.49395383	6.8286758	5.45582456	5.433E-13	5.73828	up	hypothetical protein MIMGU_mgv1a009447mg [Erythranthe guttata]
c24648.graph_c0	1.54106536	1.69686936	1.2872145	56.7298955	69.7283613	60.5943142	1.57E-72	4.28095	up	PREDICTED: protein YLS3-like [Sesamum indicum]
c24651.graph_c0	12.8744382	8.25928335	8.22637932	0	0	0	2.613E-35	-Inf	down	uncharacterized protein LOC100274375 precursor [Zea mays]
c24658.graph_c0	0	0.10074915	0	4.4621896	3.03821617	3.10389728	1.498E-12	5.63556	up	PREDICTED: protein LURP-one-related 11-like [Sesamum indicum]
c24676.graph_c0	0.59102025	0.50287026	0.49366487	3.75856348	3.87843477	3.36489008	4.835E-08	1.70367	up	PREDICTED: LOW QUALITY PROTEIN: uncharacterized protein LOC105175967 [Sesamum indicum]
c24677.graph_c0	19.7965123	19.5670735	17.1728622	16.3486188	18.2745611	16.6385045	8.083E-31	-1.23031	down	PREDICTED: probable inactive receptor kinase At1g27190 [Sesamum indicum]
c24678.graph_c0	9.63993462	11.4847852	10.6397403	9.62821938	11.7205383	11.4691577	9.065E-11	-1.04405	down	PREDICTED: vacuolar protein sorting-associated protein 2 homolog 3 [Beta vulgaris subsp. vulgaris]
c24685.graph_c0	11.3973257	11.1434189	9.48017232	6.13236638	8.5228474	6.07810956	3.564E-32	-1.71646	down	PREDICTED: LOW QUALITY PROTEIN: U-box domain-containing protein 28-like [Sesamum indicum]
c24693.graph_c1	4.53925679	5.76287104	7.96719469	3.86236761	4.64980777	5.05578861	0.0026655	-1.526	down	PREDICTED: mitoferrin-like [Sesamum indicum]
c24702.graph_c0	1.24265803	1.1978319	0.65057453	16.457522	15.3730407	13.3746133	1.516E-33	2.7866	up	PREDICTED: mitogen-activated protein kinase kinase kinase 2 [Sesamum indicum]
c24737.graph_c0	3.63095419	3.98074147	3.61053325	18.5355284	18.6363554	16.2271586	8.887E-13	1.15961	up	hypothetical protein MIMGU_mgv1a010118mg [Erythranthe guttata]
c24739.graph_c0	0.9279034	0.82562893	1.14822991	21.5306797	20.6805681	19.1382349	1.917E-74	3.30772	up	PREDICTED: homeobox-leucine zipper protein ATHB-6-like [Sesamum indicum]
c24741.graph_c0	3.03223027	2.49737124	2.71277819	13.9481486	13.6517556	13.9424298	3.859E-23	1.24395	up	PREDICTED: uncharacterized protein LOC105162811 [Sesamum indicum]
c24752.graph_c0	5.66846869	6.35504219	5.19606744	87.0927914	96.4668782	83.6622944	4.4E-113	2.86629	up	hypothetical protein MIMGU_mgv1a006173mg [Erythranthe guttata]
c24769.graph_c0	4.51302474	4.35903354	4.78197416	49.4307838	65.7780045	50.7123693	6.277E-20	2.51079	up	PREDICTED: inorganic phosphate transporter 1-4-like [Sesamum indicum]
c24790.graph_c0	89.7149166	82.5351042	77.5364442	66.0376927	68.4048905	66.677718	1.078E-64	-1.40155	down	PREDICTED: cell number regulator 8-like [Sesamum indicum]
c24791.graph_c0	12.9341724	13.8588934	9.71504693	166.494452	152.958888	141.206007	1.355E-69	2.57013	up	unnamed protein product [Coffea canephora]
c24796.graph_c0	0.15451505	0.30933955	0.39711592	3.6497711	4.05587829	2.52000135	3.02E-07	2.46785	up	hypothetical protein L484_021121 [Morus notabilis]

#ID	0 h-1 FPKM	0 h-2 FPKM	0 h-3 FPKM	28 h-1 FPKM	28 h-2 FPKM	28 h-3 FPKM	FDR	log2FC	regulated	nr_annotation
c24800.graph_c0	0	0	0	2.8675005	2.36502874	2.49734736	1.71E-19	Inf	up	PREDICTED: uncharacterized protein LOC105163689 [Sesamum indicum]
c24813.graph_c0	4.26672518	4.14538009	3.22524164	24.8082502	21.6583632	22.4016257	5.626E-16	1.47798	up	PREDICTED: polyadenylate-binding protein-interacting protein 6-like [Sesamum indicum]
c24821.graph_c0	0.32283847	0.21544116	0.41486022	2.42458377	3.46030384	2.36136454	2.238E-05	2.0162	up	PREDICTED: protein RADIALIS-like 4 [Sesamum indicum]
c24843.graph_c0	630.067796	663.918723	606.604714	77.3075587	82.1836523	78.5512226	0	-4.08718	down	PREDICTED: zinc finger CCCH domain-containing protein 29 [Sesamum indicum]
c24844.graph_c0	3.40973176	2.99090122	2.93615072	14.8434922	14.2557833	15.2600814	5.135E-13	1.1596	up	PREDICTED: protein trichome birefringence-like 35 [Sesamum indicum]
c24858.graph_c0	2.87378081	2.20886275	4.08859235	17.0821514	15.0531457	13.9084618	1.972E-07	1.23076	up	PREDICTED: uncharacterized protein LOC105174534 [Sesamum indicum]
c24859.graph_c0	4.36843102	2.55553559	4.15552712	29.0693519	30.565538	27.6232772	3.079E-32	1.88576	up	PREDICTED: O-acetyl-ADP-ribose deacetylase MACROD2 isoform X1 [Sesamum indicum]
c24860.graph_c0	13.7655464	8.03794282	6.63347706	0	0	0	1.986E-16	-Inf	down	40S ribosomal protein S26E [Uncinocarpus reesii 1704]
c24873.graph_c0	3.37351285	3.14400189	3.88663921	17.0784013	16.1072336	16.2469772	1.339E-09	1.15553	up	PREDICTED: plastid division protein PDV2-like [Sesamum indicum]
c24876.graph_c0	2.36562097	2.03564034	3.25324409	0.0301635	0.02723482	0.04922634	1.137E-21	-7.25843	down	retrotransposon protein, putative, unclassified [Oryza sativa Japonica Group]
c24896.graph_c0	145.115897	143.656112	147.741354	144.627096	165.414311	149.301218	2.799E-30	-1.01758	down	PREDICTED: thiamine thiazole synthase 2, chloroplastic-like [Sesamum indicum]
c24896.graph_c1	145.213955	147.057819	142.906883	136.113546	166.137001	141.38156	2.733E-39	-1.06304	down	PREDICTED: thiamine thiazole synthase 2, chloroplastic [Vitis vinifera]
c24901.graph_c0	22.4095756	20.8578389	29.0497989	119.439103	118.936138	130.686787	1.279E-17	1.25748	up	PREDICTED: 40S ribosomal protein S24-1-like [Elaeis guineensis]
c24909.graph_c0	5.19017097	5.70534292	5.68755184	33.8339659	33.9548295	33.4811281	1.463E-42	1.51888	up	PREDICTED: DEAD-box ATP-dependent RNA helicase 31-like [Sesamum indicum]
c24911.graph_c0	0	0	0.50715	2.75361125	4.8689117	3.18314275	7.653E-06	3.28169	up	PREDICTED: auxin-induced protein 10A5-like [Sesamum indicum]
c24920.graph_c0	873.207101	776.991163	836.622975	33.1507516	31.3405862	36.8367465	0	-5.7068	down	-
c24925.graph_c0	6.92784282	7.23305011	6.22221398	4.71027002	5.47503915	3.9760778	3.261E-12	-1.61572	down	PREDICTED: protein MKS1 [Sesamum indicum]
c24930.graph_c0	1.87441894	1.96782251	1.76246528	10.4998082	10.80039	11.4146392	4.559E-11	1.45595	up	PREDICTED: potassium transporter 8-like [Sesamum indicum]
c24941.graph_c0	1.32199744	1.62039333	1.38679106	0.84316867	1.15080574	0.9760266	3.668E-08	-1.63455	down	PREDICTED: scarecrow-like protein 14 [Sesamum indicum]
c24946.graph_c0	2.01775041	1.6831424	1.23470913	13.1285715	15.3848089	13.0206193	1.69E-19	1.98765	up	hypothetical protein MIMGU_mgv1a010693mg [Erythranthe guttata]
c24966.graph_c0	14.1134348	12.9624071	11.779261	2.53795375	3.62826261	3.53787212	1.3E-30	-3.08947	down	hypothetical protein MIMGU_mgv1a019945mg [Erythranthe guttata]
c24971.graph_c0	5.89281907	7.23657382	7.3383046	0.97137188	1.51491791	1.33306211	1.366E-37	-3.5148	down	PREDICTED: zinc transporter 8-like [Sesamum indicum]
c24986.graph_c0	0.12156351	0	0	3.97582451	5.26503526	4.54193647	4.649E-13	5.76579	up	-
c24998.graph_c0	20.2942048	20.4249314	17.4331488	4.48904251	5.28361551	4.18631089	6.256E-59	-3.14807	down	PREDICTED: uncharacterized protein LOC105177785 [Sesamum indicum]

#ID	0 h-1 FPKM	0 h-2 FPKM	0 h-3 FPKM	28 h-1 FPKM	28 h-2 FPKM	28 h-3 FPKM	FDR	log2FC	regulated	nr_annotation
c24998.graph_c1	14.3980554	14.0898056	16.2928687	3.27989399	3.73705114	2.86753576	1.5E-48	-3.27301	down	PREDICTED: uncharacterized protein LOC105177785 [Sesamum indicum]
c24999.graph_c0	22.369013	21.9608081	22.9407748	2.97015119	3.81093354	4.27322318	1.372E-83	-3.69537	down	hypothetical protein MIMGU_mgv1a011964mg [Erythranthe guttata]
c25013.graph_c0	12.5422114	15.5491554	10.7448254	6.46998538	7.99402867	7.7375012	2.055E-11	-1.8946	down	PREDICTED: probable LRR receptor-like serine/threonine-protein kinase RLK [Sesamum indicum]
c25019.graph_c0	1.00467236	1.08017308	1.91265742	14.6038016	15.9939642	13.9247478	1.668E-35	2.37735	up	PREDICTED: 4-diphosphocytidyl-2-C-methyl-D-erythritol kinase, chloroplastic [Sesamum indicum]
c25027.graph_c0	1.76575889	3.41721892	2.77330788	12.5767652	12.3083805	12.2405778	2.294E-08	1.1284	up	PREDICTED: putative methyltransferase C9orf114 [Nelumbo nucifera]
c25041.graph_c0	3.58343813	4.52481108	3.03980946	3.04700373	3.12002912	3.37529737	1.115E-06	-1.3118	down	PREDICTED: TATA box-binding protein-associated factor RNA polymerase I subunit B isoform X1 [Sesamum indicum]
c25044.graph_c0	28.3636885	27.3958768	27.3541094	20.2729985	23.0939318	22.6415582	4.411E-58	-1.42251	down	PREDICTED: zinc finger Ran-binding domain-containing protein 2-like isoform X1 [Sesamum indicum]
c25045.graph_c0	1.86562029	1.7806285	2.28588914	0.40992993	0.22777126	0.61753749	1.819E-19	-3.33029	down	-
c25047.graph_c0	148.446321	148.429078	136.401223	39.3257742	39.0691109	38.7727778	0	-2.97619	down	hypothetical protein MIMGU_mgv1a010693mg [Erythranthe guttata]
c25052.graph_c0	0.73141491	1.46429462	1.35762916	0.59065349	0.26665236	0.45787	6.959E-05	-2.52872	down	-
c25059.graph_c0	7.0898624	8.15240743	7.42876855	6.88370733	6.51358331	6.25314473	4.012E-31	-1.2973	down	PREDICTED: lysine-specific demethylase JMJ706 [Sesamum indicum]
c25062.graph_c0	1.14013102	1.14127269	0.45784797	6.4737551	7.57537127	7.26876306	1.859E-07	1.87973	up	hypothetical protein MIMGU_mgv1a015838mg [Erythranthe guttata]
c25075.graph_c0	4.25003957	4.2004435	2.41963821	1.60310221	2.18882833	1.56336091	1.522E-06	-2.10633	down	-
c25081.graph_c0	0.32712571	0.53211157	0.63055423	2.55584562	2.01251944	1.91579515	0.007672	1.02372	up	PREDICTED: putative RING-H2 finger protein ATL21A [Sesamum indicum]
c25082.graph_c0	2.10830795	1.48688618	1.72407403	11.2485195	12.8290844	12.4466583	2.102E-14	1.69092	up	PREDICTED: delta(7)-sterol-C5(6)-desaturase-like [Solanum tuberosum]
c25091.graph_c0	62.6735645	34.4773403	37.7854548	0	0	0	3.872E-33	-Inf	down	-
c25102.graph_c0	0	0.07307332	0	1.91002101	1.96409349	2.25125557	1.209E-12	5.30836	up	PREDICTED: ubiquitin-conjugating enzyme E2 20-like [Sesamum indicum]
c25105.graph_c0	0	0	0	8.53987796	9.58809915	8.11978322	6.747E-23	Inf	up	PREDICTED: protein IWS1 homolog [Sesamum indicum]
c25108.graph_c0	28.4828901	28.7615115	25.0432284	16.7062319	17.6419101	16.2400381	5.329E-66	-1.79103	down	PREDICTED: LOW QUALITY PROTEIN: probable carboxylesterase 13 [Sesamum indicum]
c25114.graph_c0	0.20725532	0.10373143	0.53266258	2.93732343	2.99214402	2.08954579	6.402E-06	2.14008	up	-
c25122.graph_c0	7.11268892	10.0122345	8.56883143	184.727469	180.794201	194.763968	2.25E-179	3.35517	up	PREDICTED: pEARLI1-like lipid transfer protein 1 [Sesamum indicum]
c25137.graph_c0	6.01441083	9.73893631	7.04680698	5.14263427	2.20557537	3.35707805	9.156E-07	-2.18132	down	PREDICTED: PHD finger protein ALFIN-LIKE 4-like [Sesamum indicum]
c25141.graph_c0	29.3142446	27.9363566	29.4401147	7.26579293	5.91113048	6.57729322	7.44E-167	-3.22429	down	hypothetical protein MIMGU_mgv1a024254mg, partial [Erythranthe guttata]

#ID	0 h-1 FPKM	0 h-2 FPKM	0 h-3 FPKM	28 h-1 FPKM	28 h-2 FPKM	28 h-3 FPKM	FDR	log2FC	regulated	nr_annotation
c25145.graph_c0	14.5828492	14.7371403	12.9562839	93.7658048	94.98689	86.0089045	8.24E-75	1.61084	up	PREDICTED: protein STRICTOSIDINE SYNTHASE-LIKE 11 [Sesamum indicum]
c25166.graph_c0	0	0	0	46.8989485	40.113294	38.2115247	3.864E-85	Inf	up	hypothetical protein MIMGU_mgv1a015417mg [Erythranthe guttata]
c25182.graph_c0	63.8314566	57.7060787	53.7491973	38.0655055	36.756359	43.0326233	5.882E-39	-1.66054	down	-
c25187.graph_c1	3.43660287	4.49851922	2.24205592	3.1509486	2.98379219	3.32367955	0.0057466	-1.19013	down	hypothetical protein MIMGU_mgv1a002261mg [Erythranthe guttata]
c25203.graph_c0	2.31816883	2.17078108	0.67266358	80.5458272	72.9216198	70.824835	1.29E-137	4.36319	up	hypothetical protein MIMGU_mgv1a012619mg [Erythranthe guttata]
c25206.graph_c0	1.41362179	1.50347715	1.19211748	0.06421326	0.17393566	0.23578873	2.236E-21	-4.20137	down	-
c25210.graph_c0	1.86423018	1.73280429	4.19231679	23.0818895	25.7779066	28.0742648	4.7E-17	2.20198	up	PREDICTED: 60S acidic ribosomal protein P1-like [Tarenaya hassleriana]
c25213.graph_c0	4.97758299	4.85057874	4.40546149	4.71975291	5.32145996	4.7310138	3.393E-09	-1.03582	down	PREDICTED: hydroxymethylglutaryl-CoA lyase, mitochondrial [Sesamum indicum]
c25220.graph_c0	3.4938305	3.49732904	2.87927088	16.3122159	17.3451226	17.4096799	8.324E-16	1.28327	up	PREDICTED: probable RNA 3'-terminal phosphate cyclase-like protein [Sesamum indicum]
c25222.graph_c0	0	0	0	2.00196177	1.60674113	1.40021525	1.762E-14	Inf	up	PREDICTED: fructose-1,6-bisphosphatase, chloroplastic [Sesamum indicum]
c25224.graph_c0	0.30007715	0.15018882	0.38561102	23.0453021	25.0743199	24.2919771	1.225E-90	5.34052	up	PREDICTED: alpha carbonic anhydrase 1, chloroplastic [Sesamum indicum]
c25226.graph_c0	1.53320517	1.98947836	1.82428643	15.2703753	15.3900735	14.7168626	7.982E-19	1.993	up	PREDICTED: uncharacterized protein LOC105161424 [Sesamum indicum]
c25229.graph_c0	5.18272245	3.2709317	3.78604852	0	0	0	7.24E-50	-Inf	down	--
c25236.graph_c0	2.34768629	2.64379179	2.01124656	2.32244329	1.43362808	2.82330444	3.502E-06	-1.17577	down	PREDICTED: uncharacterized protein LOC105167293 [Sesamum indicum]
c25243.graph_c0	3.0344506	2.87180792	2.22147019	0.05346476	0.10861574	0.07634691	1.619E-71	-6.17885	down	hypothetical protein MIMGU_mgv1a019304mg, partial [Erythranthe guttata]
c25252.graph_c0	103.496319	104.193991	106.68728	39.7236847	38.0475866	38.6084473	3.98E-203	-2.52459	down	hypothetical protein MIMGU_mgv1a004537mg [Erythranthe guttata]
c25257.graph_c0	22.0544113	11.7446956	14.5104853	0	0	0	5.241E-31	-Inf	down	secreted protein [Achlya hypogyna]
c25258.graph_c0	0	0.15909452	0.13615882	2.27176172	2.22501731	1.97941623	1.345E-09	3.3503	up	PREDICTED: uncharacterized protein At4g06744-like [Nicotiana tomentosiformis]
c25260.graph_c0	0.83073167	0.95035832	0.76251649	3.45011996	4.33522932	3.40178423	0.0013036	1.04699	up	PREDICTED: probable leucine-rich repeat receptor-like protein kinase At1g35710-like [Solanum tuberosum]
c25262.graph_c0	4.35303401	2.31486498	2.85517949	0	0	0	1.915E-21	-Inf	down	--
c25264.graph_c0	0.15727397	0.19678932	0.15157746	1.08590524	1.23848921	1.02599873	0.00054	1.63803	up	PREDICTED: F-box protein At3g07870-like [Sesamum indicum]
c25282.graph_c0	0.8141608	0.67914672	0.87185739	9.91143359	13.9356558	9.25452358	1.25E-10	2.71336	up	unnamed protein product [Coffea canephora]
c25287.graph_c0	0	0	0	4.89856468	6.10651709	5.20923957	7.938E-31	Inf	up	PREDICTED: GDSL esterase/lipase At3g48460 [Sesamum indicum]

#ID	0 h-1 FPKM	0 h-2 FPKM	0 h-3 FPKM	28 h-1 FPKM	28 h-2 FPKM	28 h-3 FPKM	FDR	log2FC	regulated	nr_annotation
c25288.graph_c0	12.0281435	10.3785908	10.2387938	7.33138902	6.803899	6.92135591	1.76E-51	-1.72144	down	PREDICTED: uncharacterized aarF domain-containing protein kinase At5g05200, chloroplastic [Sesamum indicum]
c25291.graph_c0	0.09626664	0.03212101	0.08247096	1.81911695	1.41085855	1.25601772	8.165E-10	3.31956	up	PREDICTED: purple acid phosphatase 22-like [Sesamum indicum]
c25311.graph_c0	23.2037019	12.4851816	14.5117501	0	0	0	9.505E-29	-Inf	down	putative Trypsin [Glarea lozoyensis 74030]
c25312.graph_c0	0.40427861	0.32374675	0.41561122	26.9439692	34.1439247	34.2138561	1.914E-58	5.28958	up	PREDICTED: uncharacterized protein LOC105158282 [Sesamum indicum]
c25316.graph_c0	5.95631368	4.25877002	4.81114788	3.46321156	2.23353952	2.62409862	1.47E-07	-1.94115	down	-
c25324.graph_c0	0.85452749	0.85538317	1.2478428	7.81978124	7.74873972	7.04891176	4.107E-13	1.83852	up	PREDICTED: protein BASIC PENTACYSTEINE4 isoform X2 [Sesamum indicum]
c25329.graph_c0	0.94418337	1.03514109	0.80887534	8.43078921	7.5532028	7.7327209	7.928E-13	2.00013	up	PREDICTED: probable plastid-lipid-associated protein 13, chloroplastic [Sesamum indicum]
c25337.graph_c0	4.66134813	3.70602209	3.98379115	28.1401527	27.6764837	27.6709596	1.609E-54	1.66786	up	PREDICTED: peroxisomal fatty acid beta-oxidation multifunctional protein AIM1 [Sesamum indicum]
c25352.graph_c0	0.67709229	0.79737682	0.61418146	4.77633047	5.01827327	4.67693599	7.144E-08	1.70348	up	PREDICTED: transcription factor bHLH62 isoform X2 [Sesamum indicum]
c25353.graph_c0	3544.28683	3972.98757	3769.0428	515.558583	510.278448	575.037981	8.51E-173	-3.90809	down	hypothetical protein MIMGU_mgv1a005193mg [Erythranthe guttata]
c25360.graph_c0	0.20923553	0.38398259	0.26887594	3.24418514	3.54707208	2.97813216	4.168E-10	2.41012	up	PREDICTED: subtilisin-like protease [Sesamum indicum]
c25363.graph_c0	14.5785624	16.6689119	14.5350232	5.02378303	6.84524399	5.62731141	8.36E-56	-2.47839	down	PREDICTED: probable WRKY transcription factor 7 [Sesamum indicum]
c25365.graph_c0	11.9350735	14.7216069	10.518026	9.31425313	8.73088572	9.14750044	1.282E-10	-1.53933	down	PREDICTED: uncharacterized protein LOC105168447 isoform X2 [Sesamum indicum]
c25367.graph_c0	0.49928036	0.58307703	0.85546004	5.50359034	5.07843983	6.070097	6.409E-07	2.00641	up	hypothetical protein MIMGU_mgv1a014003mg [Erythranthe guttata]
c25379.graph_c0	3.26135044	4.64579996	4.02977349	18.6435626	20.2082983	21.0155063	1.99E-11	1.23454	up	PREDICTED: CBL-interacting protein kinase 23-like [Malus domestica]
c25389.graph_c0	105.964818	101.507201	100.604851	60.2396698	63.6234148	68.9269538	4.87E-104	-1.76576	down	PREDICTED: vacuolar cation/proton exchanger 3-like [Sesamum indicum]
c25391.graph_c0	6.69734689	3.86335273	4.23024274	0	0	0	3.077E-21	-Inf	down	PREDICTED: 60S ribosomal protein L9-like [Phoenix dactylifera]
c25397.graph_c0	26.4134521	24.0362738	18.5139991	22.5130152	23.7937863	21.716989	4.151E-08	-1.1064	down	PREDICTED: U3 small nucleolar ribonucleoprotein protein MPP10 [Sesamum indicum]
c25411.graph_c0	114.718066	124.119637	134.439611	10.0519479	8.8962444	12.3440666	2.02E-99	-4.6674	down	PREDICTED: basic 7S globulin [Sesamum indicum]
c25414.graph_c0	8.31181414	8.19407448	6.79700652	7.32239832	8.76015686	7.54345925	4.131E-05	-1.06858	down	PREDICTED: protein SOMBRERO-like [Sesamum indicum]
c25418.graph_c0	0.37335694	0.2802981	0.9595569	6.44464974	6.55393524	5.70162632	7.492E-09	2.42884	up	-
c25429.graph_c0	2.31751076	2.3198314	3.37517216	20.6614025	15.6136828	16.7679078	1.751E-07	1.63088	up	PREDICTED: alpha-soluble NSF attachment protein-like [Sesamum indicum]
c25448.graph_c0	3.72238792	2.78248872	3.35460332	25.7716662	27.6790047	26.1184	2.524E-62	1.92255	up	PREDICTED: putative invertase inhibitor [Sesamum indicum]
c25449.graph_c0	0.02876356	0.37430067	0.18481155	2.17413951	2.64255941	2.42230165	5.195E-10	2.52606	up	PREDICTED: F-box/LRR-repeat protein 17-like [Sesamum indicum]
c25457.graph_c0	3.1410753	3.98267945	3.5366606	37.3963289	34.8843477	35.9262067	7.065E-84	2.25215	up	PREDICTED: biotin carboxylase 2, chloroplastic [Sesamum indicum]

#ID	0 h-1 FPKM	0 h-2 FPKM	0 h-3 FPKM	28 h-1 FPKM	28 h-2 FPKM	28 h-3 FPKM	FDR	log2FC	regulated	nr_annotation
c25460.graph_c0	0.2311598	0.23139128	0.198033	7.33624512	7.83748804	7.54001647	1.265E-17	4.01506	up	-
c25462.graph_c0	13.5460463	13.3702305	11.8155056	4.37258365	4.51913219	4.39828013	7.764E-85	-2.63205	down	PREDICTED: uncharacterized protein LOC105160924 [Sesamum indicum]
c25464.graph_c0	0.13398266	0.10058762	0.30130279	5.55053597	5.09953661	5.78067929	2.563E-26	3.83525	up	PREDICTED: alanine--glyoxylate aminotransferase 2 homolog 3, mitochondrial-like [Sesamum indicum]
c25466.graph_c0	0.07882468	0.23671083	0	3.55193801	2.58634435	3.45932384	2.281E-10	3.85165	up	PREDICTED: uncharacterized protein LOC105177131 [Sesamum indicum]
c25483.graph_c0	1.05602619	1.31334634	0.74020116	0.06977398	0.04199957	0	7.675E-19	-5.89034	down	hypothetical protein VITISV_009673 [Vitis vinifera]
c25484.graph_c0	23.2951542	26.6633448	22.5740818	5.89801896	6.57838357	8.71954396	1.328E-71	-2.86298	down	hypothetical protein MIMGU_mgv1a012871mg [Erythranthe guttata]
c25495.graph_c0	29.027103	33.7022065	30.579227	31.3773717	28.9593243	28.8369082	2.56E-23	-1.15631	down	PREDICTED: uncharacterized protein LOC105164353 [Sesamum indicum]
c25502.graph_c0	7.84809105	6.90371339	8.12920804	5.80765548	5.39983291	6.54433256	2.821E-16	-1.45677	down	PREDICTED: chaperone protein dnaJ 10 [Sesamum indicum]
c25506.graph_c0	13.3334905	15.1599978	12.672741	4.24196856	4.68857055	4.17756585	5.287E-73	-2.74092	down	PREDICTED: serine acetyltransferase 1, chloroplastic-like [Sesamum indicum]
c25512.graph_c0	1.26174657	0.84200668	0.54046477	10.0873315	10.4879016	9.97718802	1.236E-06	2.44923	up	-
c25519.graph_c0	0	0	0	3.37850101	4.49542896	4.20779199	2.777E-14	Inf	up	PREDICTED: ethylene-responsive transcription factor CRF4-like [Sesamum indicum]
c25523.graph_c0	3.1296977	4.20084239	4.47880652	3.6704519	3.64081361	3.24816233	1.512E-05	-1.25628	down	PREDICTED: uncharacterized protein LOC105168847 isoform X1 [Sesamum indicum]
c25529.graph_c0	4.67367251	4.8516248	5.00487851	0	0	0	3.741E-52	-Inf	down	-
c25539.graph_c0	1.18988269	1.48884272	0.99388013	21.317317	22.4879964	19.8469586	1.301E-78	3.02714	up	PREDICTED: E3 ubiquitin-protein ligase ATL6-like [Sesamum indicum]
c25564.graph_c0	0	0	0	41.3792518	40.8034396	37.8899774	5.13E-233	Inf	up	PREDICTED: low-temperature-induced cysteine proteinase [Sesamum indicum]
c25568.graph_c0	4.28856398	4.68760393	6.01773079	23.681302	21.6731269	22.3348627	1.883E-13	1.07962	up	PREDICTED: uncharacterized membrane protein At4g09580 [Sesamum indicum]
c25571.graph_c0	50.8473951	48.2284602	45.1278551	23.1127789	23.135055	22.9562769	7.27E-138	-2.14833	down	PREDICTED: leucine-rich repeat extensin-like protein 5 [Nicotiana tomentosiformis]
c25584.graph_c0	5.09613289	5.14637958	4.34650389	4.4904963	3.78814331	3.79793854	4.725E-11	-1.36137	down	PREDICTED: uncharacterized protein At5g19025-like [Sesamum indicum]
c25587.graph_c0	17.5449738	18.6118767	21.3406974	20.5709545	19.732233	18.781695	3.843E-09	-1.05404	down	hypothetical protein MIMGU_mgv1a012710mg [Erythranthe guttata]
c25589.graph_c0	0	0	0	4.78819576	3.7593842	3.05775285	1.391E-14	Inf	up	putative protein, contains domain DUF1677 [Arachis hypogaea]
c25603.graph_c0	9.74293119	6.38969165	6.23604138	0	0	0	2.075E-40	-Inf	down	-
c25609.graph_c0	2.64255414	2.36675813	2.94897266	17.2853381	15.561405	17.2803131	1.433E-12	1.563	up	PREDICTED: histidine-containing phosphotransfer protein 1 [Sesamum indicum]

#ID	0 h-1 FPKM	0 h-2 FPKM	0 h-3 FPKM	28 h-1 FPKM	28 h-2 FPKM	28 h-3 FPKM	FDR	log2FC	regulated	nr_annotation
c25625.graph_c0	10.4362608	9.55972623	10.9438889	2.7549344	2.64897003	2.27719883	1.531E-66	-3.1023	down	PREDICTED: LOW QUALITY PROTEIN: benzyl alcohol O-benzoyltransferase-like [Sesamum indicum]
c25626.graph_c0	0.30293634	0.30323968	0.06488086	4.07318911	4.40662294	3.8926088	6.171E-12	3.12785	up	PREDICTED: serine/threonine-protein kinase WAG1 [Sesamum indicum]
c25628.graph_c0	0.51863669	0.43718402	0.66646868	4.78121367	5.05141295	4.41946369	1.45E-13	2.03987	up	PREDICTED: uncharacterized protein At5g08430 [Sesamum indicum]
c25636.graph_c0	0.92690603	1.85566837	2.08444416	8.08403952	11.7090206	11.2690383	0.0002026	1.57597	up	-
c25645.graph_c0	0	0.0623051	0	2.26188428	2.40987793	2.14098776	7.282E-13	5.69061	up	-
c25649.graph_c0	10.8224193	6.1270056	12.0833373	53.9636674	56.6410759	56.7127324	4.743E-15	1.43319	up	PREDICTED: V-type proton ATPase subunit F [Sesamum indicum]
c25664.graph_c0	8.61478446	8.62341086	8.01645245	6.89538961	7.11530321	6.84879406	2.692E-36	-1.36558	down	PREDICTED: putative leucine-rich repeat-containing protein DDB_G0290503 [Sesamum indicum]
c25683.graph_c0	0.15225949	0.30482391	0.13043965	9.51687353	10.624486	8.48808096	7.77E-33	4.51905	up	PREDICTED: DNA-damage-repair/toleration protein DRT100-like [Sesamum indicum]
c25690.graph_c0	16.7306371	16.3517826	17.7752028	143.810357	168.057983	172.19385	6.438E-39	2.15956	up	lipid transfer protein 2 [Tamarix hispida]
c25692.graph_c0	0.17288249	0.46148163	0.22216085	1.80098335	1.92865289	1.26452895	0.0021658	1.452	up	PREDICTED: uncharacterized protein LOC105177785 [Sesamum indicum]
c25695.graph_c0	16.1641509	19.5279928	12.8927011	10.937804	9.87581153	9.58628165	6.675E-10	-1.76396	down	PREDICTED: plasma membrane ATPase 4-like [Cicer arietinum]
c25697.graph_c0	0.62249915	1.63569652	0.19998401	10.9148446	9.24233014	10.7061678	5.468E-13	2.57072	up	hypothetical protein F383_00208 [Gossypium arboreum]
c25703.graph_c0	5.61586181	4.26816472	5.34563316	4.38391496	3.34405077	2.83712043	1.375E-07	-1.61933	down	hypothetical protein MIMGU_mgv1a015966mg [Erythranthe guttata]
c25707.graph_c0	16.1342975	18.9433891	17.7713238	3.96754999	4.90911293	5.53970236	4.571E-65	-2.96457	down	PREDICTED: heterogeneous nuclear ribonucleoprotein 1-like [Sesamum indicum]
c25715.graph_c0	54.9727546	67.2245384	41.6316857	41.4643076	49.2826607	44.3867246	1.539E-05	-1.36468	down	PREDICTED: L-lactate dehydrogenase B [Sesamum indicum]
c25716.graph_c0	1.2244194	1.13809937	1.01148905	30.1930685	30.7624618	24.1186174	9.803E-39	3.56685	up	hypothetical protein MIMGU_mgv1a000915mg [Erythranthe guttata]
c25721.graph_c0	16.3504774	15.2447728	17.2359829	78.9981393	86.0145636	74.2732398	5.156E-32	1.20081	up	PREDICTED: phospho-2-dehydro-3-deoxyheptonate aldolase 2, chloroplastic [Sesamum indicum]
c25731.graph_c0	1.6763807	1.38622293	2.4352002	140.537926	139.04135	133.524208	0	5.13475	up	PREDICTED: major pollen allergen Lol p 11-like [Sesamum indicum]
c25751.graph_c0	0.29458342	0.0737196	0.18927568	10.5445006	10.8738907	10.1765723	5.63E-27	4.73764	up	-
c25755.graph_c0	75.0770244	66.9483368	74.5678409	23.3479678	24.7319298	26.1473753	5.395E-94	-2.6354	down	PREDICTED: zinc finger protein CONSTANS-LIKE 1-like [Sesamum indicum]
c25776.graph_c0	0	0	0	7.03293736	6.41056002	13.6638558	3.546E-08	Inf	up	--
c25782.graph_c0	5.23884172	5.57479585	3.63898326	59.5831681	59.4039099	57.3244027	2.433E-98	2.52266	up	PREDICTED: uncharacterized protein LOC105165646 [Sesamum indicum]
c25784.graph_c0	0	0.04984908	0	22.2591756	20.4574241	21.6778313	9.376E-97	9.25363	up	PREDICTED: uncharacterized protein LOC105164223 [Sesamum indicum]
c25786.graph_c0	0.84317208	0.62190681	1.19756313	4.77348767	3.49460417	3.89512696	0.0004782	1.09498	up	PREDICTED: glutathione S-transferase U17-like [Sesamum indicum]

#ID	0 h-1 FPKM	0 h-2 FPKM	0 h-3 FPKM	28 h-1 FPKM	28 h-2 FPKM	28 h-3 FPKM	FDR	log2FC	regulated	nr_annotation
c25786.graph_c1	1.69570615	2.73470667	2.54222446	12.6324019	11.869528	12.0398937	2.233E-10	1.29564	up	PREDICTED: pentatricopeptide repeat-containing protein At1g59720, mitochondrial [Sesamum indicum]
c25787.graph_c0	0.2619825	0.61190461	0.44887717	2.66570316	3.20917336	3.05562687	0.000309	1.66133	up	PREDICTED: uncharacterized protein LOC105177716 [Sesamum indicum]
c25799.graph_c0	19.0708916	18.6908944	22.2867179	205.59106	212.316137	218.991261	2.02E-151	2.31431	up	PREDICTED: 60S ribosomal protein L11 [Sesamum indicum]
c25801.graph_c0	2.22893739	1.673377	2.27839881	18.4272594	18.5495561	18.0708305	4.968E-50	2.06305	up	hypothetical protein MIMGU_mgv1a003796mg [Erythranthe guttata]
c25802.graph_c0	45.4154571	42.6499905	42.1889659	32.8817089	34.512157	32.6292136	8.982E-67	-1.47094	down	hypothetical protein MIMGU_mgv1a008591mg [Erythranthe guttata]
c25803.graph_c0	17.365853	16.4683348	11.6864367	12.8206872	10.8561347	11.6811898	2.368E-08	-1.4501	down	PREDICTED: uncharacterized protein LOC105171564 [Sesamum indicum]
c25804.graph_c0	8.46040903	7.84616902	8.63361574	103.62834	101.731306	84.7812398	1.312E-42	2.44829	up	PREDICTED: photosystem II 10 kDa polypeptide, chloroplastic [Sesamum indicum]
c25813.graph_c0	0	0	0	7.4811127	6.98766553	6.42027826	1.036E-14	Inf	up	PREDICTED: root phototropism protein 2-like [Malus domestica]
c25815.graph_c0	3.17671004	2.62686651	3.99345473	37.0916076	41.3303147	37.0241811	4.642E-50	2.46397	up	hypothetical protein MIMGU_mgv1a012869mg [Erythranthe guttata]
c25818.graph_c0	0.43468377	0.34809523	0.44686869	90.7338496	99.5526863	101.570347	4.28E-208	6.79954	up	PREDICTED: kirola-like isoform X2 [Sesamum indicum]
c25832.graph_c0	0.25351165	0.03625222	0	1.02654022	1.33088964	1.54642788	3.643E-05	2.69134	up	unnamed protein product [Coffea canephora]
c25837.graph_c0	1.12303395	1.51329029	1.38763759	0.03139287	0.17006891	0.1024653	3.228E-24	-4.82031	down	PREDICTED: uncharacterized protein LOC104248035 [Nicotiana sylvestris]
c25838.graph_c0	24.6119354	26.6700761	21.2856826	11.4709149	14.151368	12.4416159	5.532E-37	-2.01969	down	PREDICTED: myb-related protein 306-like [Sesamum indicum]
c25840.graph_c0	0.40941551	0.51228184	0.7891727	2.97560831	5.10472109	4.06702073	0.0001732	1.72848	up	-
c25843.graph_c0	0.27103202	0.13565171	0.08707172	1.60049916	1.55626227	1.26580981	1.442E-05	2.08407	up	PREDICTED: serine/threonine-protein kinase D6PKL1-like [Sesamum indicum]
c25847.graph_c0	18.592017	11.9040993	16.7886061	74.2582719	70.8952883	78.4743825	3.485E-13	1.15181	up	PREDICTED: probable small nuclear ribonucleoprotein G [Sesamum indicum]
c25849.graph_c0	0.43318055	0.28907621	0.33399249	4.34469713	3.37327555	3.92202589	5.466E-13	2.37439	up	PREDICTED: cell division cycle 20.2, cofactor of APC complex-like [Sesamum indicum]
c25850.graph_c0	30.1776056	31.3102992	29.579921	2.28134281	1.9514261	2.841324	0	-4.7749	down	PREDICTED: vacuolar iron transporter 1 [Nicotiana sylvestris]
c25855.graph_c0	9.58700699	12.079826	6.77416562	153.629251	154.279846	141.574286	1.41E-193	2.89603	up	PREDICTED: luminal-binding protein 5 [Sesamum indicum]
c25861.graph_c0	0.64957853	0.68635281	0.46374118	3.04248622	2.84180668	2.88928386	0.0008279	1.19919	up	PREDICTED: uncharacterized protein LOC104225770 [Nicotiana sylvestris]
c25862.graph_c0	1.24426506	1.09723589	1.25630204	11.1318061	11.8395457	13.3704357	9.711E-33	2.24609	up	PREDICTED: cell division protein FtsZ homolog 2-2, chloroplastic [Sesamum indicum]
c25866.graph_c0	2.3414135	2.61161614	2.40704724	18.0382939	17.2965874	15.9489984	1.171E-14	1.70969	up	PREDICTED: protein DA1-related 1-like [Sesamum indicum]
c25870.graph_c0	0.73219586	0.97723873	0.41817808	30.3328769	30.9647006	23.7381654	4.918E-39	4.23599	up	PREDICTED: transcription factor DIVARICATA-like [Sesamum indicum]

#ID	0 h-1 FPKM	0 h-2 FPKM	0 h-3 FPKM	28 h-1 FPKM	28 h-2 FPKM	28 h-3 FPKM	FDR	log2FC	regulated	nr_annotation
c25871.graph_c0	33.5445046	30.3021827	29.8062333	28.6613101	29.0193282	29.7437202	2.377E-38	-1.1884	down	hypothetical protein MIMGU_mgv1a005352mg [Erythranthe guttata]
c25878.graph_c0	1.558299	1.49746502	1.52188169	8.99254808	8.77389048	9.1492032	2.918E-13	1.46558	up	hypothetical protein MIMGU_mgv1a004635mg [Erythranthe guttata]
c25880.graph_c0	7.72696086	9.0117668	9.06333032	5.12604925	4.83369492	4.48260649	1.811E-28	-1.92981	down	PREDICTED: uncharacterized protein LOC105170968 [Sesamum indicum]
c25881.graph_c0	0.92666394	0.49692421	0.72298562	3.10288312	2.88848424	3.51329446	0.0008831	1.05894	up	PREDICTED: pentatricopeptide repeat-containing protein At5g44230 [Sesamum indicum]
c25882.graph_c0	37.6074378	40.7464205	36.596318	30.4893681	30.7965826	28.3734658	1.754E-72	-1.44881	down	PREDICTED: putative nuclear matrix constituent protein 1-like protein [Sesamum indicum]
c25883.graph_c0	11.3283925	12.1217869	12.5495111	9.65296388	11.2791688	8.34007733	4.484E-07	-1.39137	down	hypothetical protein CICLE_v10005060mg [Citrus clementina]
c25884.graph_c0	0.77774645	1.02876551	1.0708232	0.10093958	0.07291118	0.0658927	5.954E-25	-4.67998	down	-
c25886.graph_c0	0	0	0	8.19529096	8.50951626	7.72753569	5.194E-32	Inf	up	hypothetical protein MIMGU_mgv1a010584mg [Erythranthe guttata]
c25892.graph_c0	1.11969429	0.74721033	0.83933004	9.76544831	7.83758598	9.01992882	3.292E-09	2.21102	up	PREDICTED: 3-phosphoinositide-dependent protein kinase 2 [Nicotiana sylvestris]
c25894.graph_c0	75.0972479	76.0151868	76.0195032	51.0313071	53.5901572	47.6992319	5.314E-85	-1.66758	down	PREDICTED: uncharacterized protein LOC105164821 isoform X1 [Sesamum indicum]
c25905.graph_c0	1.26373636	1.58125225	1.50795474	11.3825456	13.3280034	11.7506058	7.826E-19	1.97376	up	PREDICTED: uncharacterized protein LOC102616798 [Citrus sinensis]
c25908.graph_c0	0	0.018544	0.04761188	1.17138475	1.65333887	1.20853383	8.622E-17	4.81214	up	PREDICTED: putative receptor-like protein kinase At3g47110 [Sesamum indicum]
c25914.graph_c0	5.57398691	5.69828264	3.04799739	26.3755174	24.5150493	29.1182798	1.987E-09	1.39946	up	PREDICTED: uncharacterized protein LOC105157918 [Sesamum indicum]
c25915.graph_c0	339.022346	362.994381	337.218742	47.0650705	43.3743705	45.1075397	0	-4.02887	down	MYB-related transcription factor [Salvia miltiorrhiza]
c25916.graph_c0	0.35866772	0.37897281	0.20484543	3.21502411	2.82440951	3.07248959	1.145E-12	2.18963	up	PREDICTED: putative pentatricopeptide repeat-containing protein At3g25970 [Sesamum indicum]
c25918.graph_c0	173.437929	173.357225	168.502999	65.3816929	74.4589688	67.5929124	1.62E-185	-2.40321	down	PREDICTED: probable calcium-binding protein CML49 [Sesamum indicum]
c25922.graph_c0	0	0	0	1.81062133	0.9008199	1.26638762	5.451E-07	Inf	up	hypothetical protein MIMGU_mgv1a027056mg [Erythranthe guttata]
c25924.graph_c0	27.2664905	32.9951641	30.9896242	162.06007	148.313161	141.223369	1.891E-27	1.21512	up	hypothetical protein MIMGU_mgv1a012865mg [Erythranthe guttata]
c25927.graph_c0	2.18595484	3.71984436	2.52813466	11.1211756	12.3365512	14.7789413	0.0050273	1.09117	up	PREDICTED: V-type proton ATPase subunit H [Beta vulgaris subsp. vulgaris]
c25928.graph_c0	0	0.12266246	0	5.78897724	4.98566197	3.92435293	2.988E-13	5.82193	up	BnaA07g08260D [Brassica napus]
c25929.graph_c0	0.18163122	0.44443201	0.18153591	4.16560231	3.9068266	3.65043918	1.326E-20	2.77147	up	PREDICTED: leucine-rich repeat receptor-like protein kinase TDR [Sesamum indicum]
c25931.graph_c0	0.37946311	0.25322873	0.43344456	8.94790538	8.38347001	8.50165077	1.165E-28	3.50509	up	PREDICTED: putative GATA transcription factor 22 [Nicotiana tomentosiformis]
c25936.graph_c0	90.9913325	99.3036651	89.1537202	2.35624618	2.41113196	3.33264124	0	-6.19687	down	PREDICTED: heat shock 70 kDa protein-like [Sesamum indicum]

#ID	0 h-1 FPKM	0 h-2 FPKM	0 h-3 FPKM	28 h-1 FPKM	28 h-2 FPKM	28 h-3 FPKM	FDR	log2FC	regulated	nr_annotation
c25937.graph_c0	25.4693758	31.0309105	25.43523	18.8283269	20.8204866	17.4352793	1.024E-20	-1.61203	down	PREDICTED: DEAD-box ATP-dependent RNA helicase 37-like [Vitis vinifera]
c25938.graph_c0	1.93217941	1.02078249	2.20704626	13.2628037	12.6090424	12.0318951	5.793E-15	1.78235	up	PREDICTED: ataxin-3 homolog [Sesamum indicum]
c25949.graph_c0	3.9174485	4.09643244	4.26997553	0.68637351	0.96402588	0.89197158	8.864E-43	-3.36401	down	PREDICTED: abscisic acid 8'-hydroxylase 4-like [Sesamum indicum]
c25952.graph_c0	0.25653754	0.237041	0.40573648	5.20625778	5.68494435	5.59413254	4.031E-34	3.09887	up	PREDICTED: probably inactive leucine-rich repeat receptor-like protein kinase At2g25790 isoform X1 [Sesamum indicum]
c25953.graph_c0	0.20442775	0.13642164	0.26269776	16.145358	17.3054819	16.1245939	3.034E-46	5.26436	up	PREDICTED: probable pectate lyase 5 [Sesamum indicum]
c25953.graph_c1	0	0.09716702	0	5.36178373	6.05148499	4.77814808	8.22E-16	6.29794	up	PREDICTED: probable pectate lyase 5 [Sesamum indicum]
c25955.graph_c0	1.07686384	1.02404504	0.48433443	41.7939601	41.8346971	40.4580269	2.67E-124	4.50325	up	PREDICTED: probable xyloglucan endotransglucosylase/hydrolase protein 7 [Sesamum indicum]
c25958.graph_c0	2.64049313	2.40285198	2.36491451	18.8420255	19.7955048	22.3506056	1.784E-17	1.95266	up	-
c25968.graph_c0	0.52086309	0.78207698	0.7529959	8.23368627	8.88691562	7.91561641	1.411E-13	2.51175	up	PREDICTED: probable lipid phosphate phosphatase beta [Sesamum indicum]
c25975.graph_c0	0.77442366	0.65593772	1.76067664	6.36449673	6.37201797	7.13647773	1.786E-06	1.53603	up	hypothetical protein MIMGU_mgv1a014840mg [Erythranthe guttata]
c25976.graph_c0	19.3563291	10.6376456	13.8186941	0	0	0	2.223E-32	-Inf	down	cytochrome c oxidase subunit I [Lingulodinium polyedrum]
c25980.graph_c0	0.85810301	1.18618599	1.10269634	8.10763618	9.33154391	9.91153058	1.672E-16	2.0273	up	PREDICTED: thioredoxin-like protein CITRX1, chloroplastic [Sesamum indicum]
c25983.graph_c0	12.9533551	7.0480859	8.0810464	0	0	0	3.613E-26	-Inf	down	ats1, partial [Pyramimonas parkeae]
c25990.graph_c0	109.405698	118.806256	113.26768	29.9976985	29.2882042	35.213788	4.5E-193	-2.94331	down	PREDICTED: espin-like [Sesamum indicum]
c25994.graph_c0	1.67740909	1.4815489	1.33136114	0.25099761	0.3237533	0.29258857	4.969E-17	-3.46013	down	PREDICTED: pathogenesis-related genes transcriptional activator PTI6-like [Nicotiana tomentosiformis]
c25999.graph_c0	5.2469955	5.07599959	4.57048519	20.0399974	29.6255607	26.4396326	2.711E-06	1.26445	up	PREDICTED: glutamate decarboxylase [Sesamum indicum]
c26000.graph_c0	2.08330899	1.25123707	0.96376843	5.75372049	7.21841324	5.97993327	0.0042463	1.05814	up	PREDICTED: uncharacterized protein LOC105169481 [Sesamum indicum]
c26003.graph_c0	0.27318352	0.41018561	0.35105164	6.40317981	5.96074143	6.11601789	5.826E-13	3.06668	up	PREDICTED: uncharacterized protein LOC105170399 [Sesamum indicum]
c26004.graph_c0	3.00873073	1.28369396	2.47192109	10.1449847	10.1309916	9.97482196	1.829E-05	1.07125	up	unnamed protein product [Vitis vinifera]
c26005.graph_c0	5.20278063	2.25036624	3.30161931	0	0	0	3.212E-14	-Inf	down	uncharacterized protein LOC100273290 [Zea mays]
c26009.graph_c0	177.484823	173.577147	170.383878	862.653706	913.495574	909.96749	1.81E-58	1.27493	up	PREDICTED: 40S ribosomal protein S20-2-like [Glycine max]
c26010.graph_c0	24.0990123	22.6988992	22.8547491	19.3893247	18.498791	20.0406054	3.761E-23	-1.3556	down	PREDICTED: uncharacterized protein LOC105158897 [Sesamum indicum]
c26011.graph_c0	38.663364	40.9215536	34.1911078	12.1616934	12.2085436	12.6564947	1.077E-97	-2.70817	down	PREDICTED: LOW QUALITY PROTEIN: benzyl alcohol O-benzoyltransferase-like [Sesamum indicum]

#ID	0 h-1 FPKM	0 h-2 FPKM	0 h-3 FPKM	28 h-1 FPKM	28 h-2 FPKM	28 h-3 FPKM	FDR	log2FC	regulated	nr_annotation
c26016.graph_c0	0.94593453	0.69623657	1.21556332	5.90440974	7.68631869	6.53393237	3.354E-12	1.72049	up	PREDICTED: probable aminotransferase ACS10 [Sesamum indicum]
c26017.graph_c0	17.4103225	14.6648193	16.6432948	121.90781	135.568856	127.303811	2.264E-37	1.89093	up	PREDICTED: 60S ribosomal protein L34-like [Sesamum indicum]
c26029.graph_c0	42.8225006	37.366942	36.8778111	31.5712548	31.9265889	29.5846176	1E-50	-1.42002	down	PREDICTED: abscisic acid receptor PYL9-like [Sesamum indicum]
c26034.graph_c0	1.68880236	1.37352592	1.28854312	0	0	0.06259738	9.631E-30	-7.19221	down	-
c26035.graph_c0	0.09157801	0	0.11768136	40.6893488	38.1809701	40.7333134	4.77E-269	8.05956	up	PREDICTED: polyphenol oxidase I, chloroplastic-like [Sesamum indicum]
c26038.graph_c0	0.58814789	0.5352153	0.27483394	3.39379315	3.68414985	3.78401559	6.313E-13	1.87604	up	PREDICTED: DNA mismatch repair protein MSH2-like isoform X1 [Sesamum indicum]
c26039.graph_c0	0.398227	0	0.12793436	10.6365189	9.27712018	8.44314083	4.115E-19	4.67486	up	PREDICTED: uncharacterized protein LOC105177716 [Sesamum indicum]
c26045.graph_c0	0.11283371	0.02823667	0.07249789	2.56271538	2.38793625	2.32536417	4.969E-16	4.00368	up	PREDICTED: uncharacterized protein LOC105165819 [Sesamum indicum]
c26046.graph_c0	2.1382737	2.48288123	1.53874916	1.20182573	1.49673925	1.11594619	1.137E-07	-1.77864	down	PREDICTED: uncharacterized protein LOC104242602 [Nicotiana sylvestris]
c26048.graph_c0	1.43467578	2.20203899	1.78216134	15.3974113	13.6199769	14.1240466	1.16E-19	1.90112	up	PREDICTED: uncharacterized protein LOC105164113 [Sesamum indicum]
c26049.graph_c0	1.23404064	1.28278697	0.97587143	31.0808137	32.392434	30.2032115	1.529E-89	3.65754	up	PREDICTED: LOW QUALITY PROTEIN: plasma membrane ATPase 3 [Sesamum indicum]
c26053.graph_c0	7.23232055	8.49084506	7.74484854	4.60746952	4.45307869	4.34213966	2.6E-27	-1.8993	down	-
c26054.graph_c0	1.75640471	2.05119073	2.91535572	0.85102988	0.72037518	0.30381463	2.589E-07	-2.94263	down	PREDICTED: solute carrier family 40 member 2 isoform X2 [Sesamum indicum]
c26056.graph_c0	10.7537969	12.8011046	9.33719918	6.75960875	4.38674179	5.17104894	1.082E-09	-2.09956	down	PREDICTED: ubiquitin-activating enzyme E1 1-like isoform X1 [Sesamum indicum]
c26057.graph_c0	120.065047	116.530154	120.726648	36.254455	32.3688534	33.4005443	0	-2.89905	down	hypothetical protein MIMGU_mgv1a015990mg [Erythranthe guttata]
c26058.graph_c0	6.13329594	5.28936155	7.33588386	28.5632121	31.7961943	30.3862902	6.973E-19	1.1807	up	hypothetical protein MIMGU_mgv1a007272mg [Erythranthe guttata]
c26065.graph_c0	143.336503	154.010305	146.326241	141.356608	142.571223	138.62698	1.66E-42	-1.16109	down	PREDICTED: DEAD-box ATP-dependent RNA helicase 3, chloroplastic [Sesamum indicum]
c26066.graph_c0	1.8247941	2.61538967	1.9718752	0.48227267	1.03418663	0.95923074	4.361E-12	-2.46277	down	PREDICTED: origin of replication complex subunit 6 [Sesamum indicum]
c26067.graph_c0	1.53308309	0.86739292	1.3704852	7.1214211	6.91113019	7.6689671	4.994E-06	1.43487	up	PREDICTED: probable glutathione peroxidase 2 [Sesamum indicum]
c26072.graph_c0	0	0	0	3.61177692	2.68134554	4.51901006	1.165E-10	Inf	up	PREDICTED: uncharacterized protein LOC105161043 [Sesamum indicum]
c26073.graph_c0	7.93912324	11.6185279	9.54581304	132.341331	131.648171	118.810417	7.58E-129	2.62519	up	-
c26074.graph_c0	2292.59361	2294.71831	2444.78119	1242.65302	1420.56001	1543.22814	1.308E-49	-1.83216	down	PREDICTED: embryonic protein DC-8 [Nicotiana sylvestris]
c26075.graph_c0	0.7340472	1.10217335	1.13193565	5.17496233	4.33531465	5.83345792	0.0004598	1.27634	up	-
c26076.graph_c0	8.36624157	9.43246572	8.31784154	9.31282117	7.33946909	6.94633499	5.987E-16	-1.23699	down	-

#ID	0 h-1 FPKM	0 h-2 FPKM	0 h-3 FPKM	28 h-1 FPKM	28 h-2 FPKM	28 h-3 FPKM	FDR	log2FC	regulated	nr_annotation
c26079.graph_c0	0	0	0	2.80666391	2.75841581	2.5131561	1.459E-21	Inf	up	PREDICTED: putative E3 ubiquitin-protein ligase XBAT31 [Sesamum indicum]
c26082.graph_c0	1.18204939	1.38723873	1.414223	12.6792296	12.2238484	12.6184327	1.085E-23	2.14327	up	PREDICTED: uncharacterized protein LOC105156132 [Sesamum indicum]
c26084.graph_c0	7.69898482	3.51334588	4.75275995	0	0	0	1.758E-18	-Inf	down	NADH dehydrogenase subunit 4 (mitochondrion) [Saccharina latissima]
c26086.graph_c0	0.39647589	0.46690929	0.29969836	17.8656962	17.6615086	17.5105123	9.61E-122	4.42427	up	PREDICTED: protein FAR1-RELATED SEQUENCE 6 isoform X1 [Sesamum indicum]
c26089.graph_c1	0	0.04730302	0	17.3786555	19.0714374	16.9553533	1.143E-84	9.05874	up	Phosphoenolpyruvate carboxylase family protein [Theobroma cacao]
c26091.graph_c0	1.50137122	1.42773089	1.35052502	0	0.29557212	0.08904005	3.302E-19	-4.56959	down	PREDICTED: uncharacterized protein LOC105162488 [Sesamum indicum]
c26092.graph_c0	1.28159516	0.85525232	0.90030562	6.39599769	7.52430245	6.94188561	1.105E-21	1.69319	up	PREDICTED: receptor-like protein kinase THESEUS 1 [Sesamum indicum]
c26096.graph_c0	0.13697373	0	0	4.03184454	2.92129067	2.96501867	2.271E-13	5.11877	up	PREDICTED: thylakoid lumenal 16.5 kDa protein, chloroplastic [Sesamum indicum]
c26098.graph_c0	12.8006086	18.9150582	14.2952153	13.5120934	14.7001849	16.4482617	0.0001128	-1.13297	down	PREDICTED: UDP-glycosyltransferase 73C3 isoform X2 [Vitis vinifera]
c26103.graph_c0	2.67710326	1.67486499	2.04260906	11.8262274	12.5720111	12.8998833	2.021E-13	1.45645	up	hypothetical protein MIMGU_mgv1a009994mg [Erythranthe guttata]
c26109.graph_c0	1.58095082	1.2308597	1.42963346	0.97879988	0.88376452	0.86814425	7.721E-06	-1.72537	down	PREDICTED: uncharacterized protein LOC105156347 [Sesamum indicum]
c26113.graph_c0	7.98137312	8.09660506	8.09955772	49.3264017	48.5678362	47.2600596	1.341E-67	1.49499	up	PREDICTED: uncharacterized protein LOC105163882 [Sesamum indicum]
c26115.graph_c0	1.88605357	1.61823615	1.21182676	19.8762066	23.9579365	23.4493809	4.506E-34	2.74945	up	PREDICTED: macrophage migration inhibitory factor homolog isoform X2 [Sesamum indicum]
c26117.graph_c0	12.9357711	14.4314026	15.1637089	13.6718561	14.515205	15.1969234	3.118E-13	-1.06338	down	PREDICTED: uncharacterized protein LOC105176303 [Sesamum indicum]
c26118.graph_c0	3.40203987	2.12840406	1.40520726	0	0	0	1.573E-12	-Inf	down	hypothetical protein AALP_AAs67754U000100, partial [Arabis alpina]
c26120.graph_c0	5.39038939	6.62210229	6.8219113	3.02731668	2.81377675	3.2694693	8.391E-19	-2.14016	down	PREDICTED: cytochrome P450 76C1-like [Sesamum indicum]
c26125.graph_c0	0	0	0.05845085	1.95046345	1.94017907	1.64551351	2.253E-13	5.43457	up	PREDICTED: cytochrome P450 90B1 [Sesamum indicum]
c26131.graph_c0	0.73867046	1.30484139	1.11673033	0.85266227	0.94095722	0.79884181	0.0007565	-1.38012	down	hypothetical protein L484_013769 [Morus notabilis]
c26134.graph_c0	0.12996492	0.28620913	0.46762818	10.1258711	9.22799872	9.41877947	1.78E-56	3.92052	up	PREDICTED: probable methyltransferase PMT2 [Sesamum indicum]
c26136.graph_c0	1.19146389	0.62225581	0.73225483	43.5610042	49.1558796	41.4747813	3.02E-109	4.63402	up	PREDICTED: uncharacterized protein LOC105162013 [Sesamum indicum]
c26138.graph_c0	0	0.09086078	0.07776193	1.91315981	2.89886178	2.51215161	2.543E-15	4.3364	up	PREDICTED: glycerol-3-phosphate 2-O-acyltransferase 6-like [Sesamum indicum]
c26142.graph_c0	0.07748707	0.19391165	0	6.39199715	6.68665812	5.23879464	8.73E-30	5.00039	up	PREDICTED: uncharacterized protein LOC105156886 [Sesamum indicum]

#ID	0 h-1 FPKM	0 h-2 FPKM	0 h-3 FPKM	28 h-1 FPKM	28 h-2 FPKM	28 h-3 FPKM	FDR	log2FC	regulated	nr_annotation
c26151.graph_c0	0.2931574	0.16302831	0.37671886	1.37308625	1.73140034	1.27496885	0.0015795	1.29744	up	PREDICTED: pentatricopeptide repeat-containing protein At2g13600-like [Sesamum indicum]
c26155.graph_c0	60.6819133	63.1452779	53.3829576	8.9458955	10.1774042	8.43124322	1.18E-176	-3.77458	down	hypothetical protein MIMGU_mgv1a001787mg [Erythranthe guttata]
c26158.graph_c0	3.04919887	2.84876871	3.2652844	0.36935669	0.13339779	0.48222727	2.445E-21	-4.30536	down	PREDICTED: shikimate O-hydroxycinnamoyltransferase-like [Sesamum indicum]
c26159.graph_c0	6.53295605	2.61579913	4.4773905	0	0	0	1.279E-11	-Inf	down	metallothionein type 2a-FL [Elaeis guineensis]
c26161.graph_c0	9.63539728	10.0232828	12.381867	6.59101291	7.43883336	8.06731906	4.547E-08	-1.62934	down	hypothetical protein MIMGU_mgv1a020481mg, partial [Erythranthe guttata]
c26161.graph_c1	22.6554834	22.6515206	24.1526609	15.5758275	21.9775129	20.4619049	1.014E-23	-1.35104	down	PREDICTED: beta-galactosidase 15-like [Sesamum indicum]
c26163.graph_c0	0.1018095	0.10191145	0.13082924	4.4396736	4.60990035	3.47179001	5.546E-16	4.13075	up	PREDICTED: hyoscyamine 6-dioxygenase-like [Sesamum indicum]
c26164.graph_c0	0.82453564	1.78828279	1.94252839	8.83919932	8.02605845	8.23145944	2.49E-06	1.36333	up	PREDICTED: uncharacterized protein LOC104422511 isoform X2 [Eucalyptus grandis]
c26166.graph_c0	5.00892175	4.23445976	4.38125785	25.4678922	24.2242873	23.4775824	2.835E-36	1.3356	up	PREDICTED: kinesin-13A [Sesamum indicum]
c26171.graph_c0	1.54884731	2.08707456	1.07171622	1.42876457	0.97730337	1.20118932	0.0011283	-1.46922	down	PREDICTED: peroxidase 11 [Sesamum indicum]
c26174.graph_c0	0.70239315	0.31248733	0.75216917	10.181545	9.32101646	9.25688866	2.556E-28	2.93082	up	hypothetical protein L484_002404 [Morus notabilis]
c26175.graph_c0	4.80588221	2.61151991	2.8231995	0	0	0	2.935E-15	-Inf	down	PREDICTED: protein translation factor SUI1 homolog 2-like [Tarenaya hassleriana]
c26185.graph_c0	0	0	0	3.42676344	3.36309358	2.73542379	3.583E-14	Inf	up	PREDICTED: probable receptor-like serine/threonine-protein kinase At4g34500 [Sesamum indicum]
c26189.graph_c0	0.22273528	0.22295831	0.28622365	5.08311728	5.32040289	4.70258108	2.079E-16	3.27276	up	PREDICTED: calcium-dependent protein kinase 13 [Sesamum indicum]
c26189.graph_c1	0.74588258	0.44797768	0.28754659	7.31839263	9.78936648	7.21033352	7.228E-14	2.95605	up	PREDICTED: calcium-dependent protein kinase 13 [Sesamum indicum]
c26190.graph_c0	1.44332529	1.6098872	1.11283809	12.8277776	13.0706576	12.7418119	3.699E-23	2.12625	up	PREDICTED: RING-H2 finger protein ATL65 [Sesamum indicum]
c26191.graph_c0	1.52214548	1.93921596	1.24473823	1.55885834	0.9080664	1.18995015	0.0001016	-1.451	down	hypothetical protein MIMGU_mgv1a008195mg [Erythranthe guttata]
c26193.graph_c0	0.1512023	0.1513537	0.19430094	2.96710988	5.25882101	3.04883973	3.664E-05	3.40818	up	PREDICTED: GATA transcription factor 2-like [Sesamum indicum]
c26199.graph_c0	3.26428577	2.33396747	3.14605234	23.6963972	25.9858663	27.6789225	2.397E-38	2.05469	up	hypothetical protein MIMGU_mgv1a007643mg [Erythranthe guttata]
c26203.graph_c0	2.11657411	1.78575599	1.94277252	1.79103686	2.00406103	1.72148784	5.757E-10	-1.17387	down	PREDICTED: receptor-like protein kinase isoform X1 [Sesamum indicum]
c26206.graph_c0	1.59242128	1.34232914	0.96931155	6.39595126	7.75492522	6.31255798	0.0003216	1.30459	up	hypothetical protein MIMGU_mgv1a006425mg [Erythranthe guttata]
c26207.graph_c0	5.82380999	4.84711778	6.39068323	37.0956741	38.0885939	37.5655572	2.137E-29	1.63188	up	PREDICTED: transport and Golgi organization 2 homolog [Sesamum indicum]
c26208.graph_c0	2.09529116	2.85244942	2.69253209	0.24365529	0.71499311	0.34793627	9.661E-18	-3.64307	down	-
c26211.graph_c0	4.0661284	4.92708423	4.05635557	1.39984991	1.72035354	1.90377682	1.626E-20	-2.46622	down	hypothetical protein MIMGU_mgv1a013710mg [Erythranthe guttata]
c26213.graph_c0	17.5979997	17.4408633	14.8517377	11.4451315	11.7315896	12.5281064	3.881E-48	-1.57066	down	PREDICTED: myb-related protein 3R-1-like isoform X1 [Sesamum indicum]

#ID	0 h-1 FPKM	0 h-2 FPKM	0 h-3 FPKM	28 h-1 FPKM	28 h-2 FPKM	28 h-3 FPKM	FDR	log2FC	regulated	nr_annotation
c26214.graph_c0	0	0	0	5.74066323	4.89926528	3.6576309	2.807E-15	Inf	up	hypothetical protein MIMGU_mgv1a024317mg, partial [Erythranthe guttata]
c26221.graph_c0	29.8270016	31.4896663	27.8483253	28.791217	30.430342	28.606667	3.009E-09	-1.1115	down	putative phosphoprotein phosphatase [Arabidopsis thaliana]
c26223.graph_c0	8.71151389	6.51901224	7.93406542	47.0865263	50.7845528	46.5480693	1.294E-26	1.54992	up	PREDICTED: UPF0587 protein C1orf123 homolog [Sesamum indicum]
c26228.graph_c0	8.39223796	7.03591636	6.50176098	70.4186921	72.2697522	72.3204621	1.26E-120	2.20583	up	hypothetical protein MIMGU_mgv1a005139mg [Erythranthe guttata]
c26229.graph_c0	4.18597011	3.60672148	4.0173304	24.7751653	23.3084813	25.1080565	6.24E-52	1.54152	up	PREDICTED: uncharacterized protein LOC105163825 [Sesamum indicum]
c26231.graph_c0	0.67543264	0.72118291	0.6365023	5.53081284	6.17577121	6.03526729	1.34E-10	2.03586	up	PREDICTED: alpha-ketoglutarate-dependent dioxygenase alkB isoform X1 [Sesamum indicum]
c26233.graph_c0	0	0.07126464	0	3.25980364	2.87321855	3.48329817	4.449E-21	5.99522	up	PREDICTED: uncharacterized protein LOC105176469 [Sesamum indicum]
c26234.graph_c0	0	0	0.04671307	2.35138322	3.17269047	2.69481672	1.663E-19	6.32841	up	PREDICTED: glucan endo-1,3-beta-glucosidase 12 [Sesamum indicum]
c26239.graph_c0	2.37674512	2.73599383	3.35963274	0	0.38992111	0.21143221	1.897E-21	-4.91268	down	-
c26246.graph_c0	2.71421837	3.23444791	2.6574309	0.28181118	0.25444907	0.3066075	9.124E-20	-4.44098	down	hypothetical protein MIMGU_mgv1a027137mg [Erythranthe guttata]
c26252.graph_c0	2.32820571	2.19543346	2.55823702	1.61855577	1.3064067	1.46080554	4.196E-11	-1.78313	down	-
c26261.graph_c0	91.2087954	97.6281411	86.1395797	84.4406212	88.2014876	82.4132215	1.505E-50	-1.19836	down	PREDICTED: beta-amylase 1, chloroplastic [Sesamum indicum]
c26268.graph_c0	8.50817845	10.4591029	7.09708435	5.64126567	5.68124944	7.43598162	2.464E-08	-1.56089	down	hypothetical protein MIMGU_mgv1a005615mg [Erythranthe guttata]
c26268.graph_c1	9.97669274	12.1755449	9.30799444	9.3369077	9.14782902	10.8609002	8.924E-07	-1.18865	down	PREDICTED: patatin-like protein 6 [Sesamum indicum]
c26270.graph_c0	11.7840835	11.4689767	10.3518062	49.2118583	48.7377616	47.7261636	3.732E-29	1.02698	up	hypothetical protein MIMGU_mgv1a027170mg [Erythranthe guttata]
c26274.graph_c0	14.6624301	13.7049104	13.2858868	11.6129285	12.130152	12.1702273	6.739E-33	-1.30325	down	PREDICTED: protein FIZZY-RELATED 2-like [Sesamum indicum]
c26278.graph_c0	119.257931	119.064571	113.767239	630.501497	689.647365	656.308473	7.272E-70	1.39886	up	PREDICTED: 60S acidic ribosomal protein P2A-like [Nicotiana tomentosiformis]
c26279.graph_c0	93.3152559	90.7774661	100.535586	13.8759031	16.4324909	13.4148475	8.85E-135	-3.79499	down	hypothetical protein MIMGU_mgv1a015010mg [Erythranthe guttata]
c26282.graph_c0	0.33015285	0.39658014	0.67881517	5.4709327	4.20311149	5.52155556	5.71E-09	2.33526	up	PREDICTED: inorganic pyrophosphatase 3 [Sesamum indicum]
c26284.graph_c0	288.192432	294.360382	271.715883	38.9885245	43.9394886	47.3731445	0	-3.802	down	PREDICTED: 18.2 kDa class I heat shock protein-like [Sesamum indicum]
c26286.graph_c0	0.1795195	0.14974938	0.07689656	1.06553553	1.82598606	2.2357756	0.0002322	2.57886	up	PREDICTED: mini-chromosome maintenance complex-binding protein isoform X1 [Sesamum indicum]
c26288.graph_c0	1.54517531	1.66883225	1.30632332	0	0.08005151	0.04823046	6.492E-33	-6.228	down	hypothetical protein MIMGU_mgv1a008148mg [Erythranthe guttata]
c26291.graph_c0	16.7912953	15.6724262	15.5999343	12.3687331	12.2845869	10.0927852	7.946E-17	-1.55878	down	PREDICTED: uncharacterized protein LOC105169169 isoform X2 [Sesamum indicum]
c26292.graph_c0	0.41129652	0.09149075	0.11745162	2.09249558	2.09925257	1.73456183	6.968E-06	2.17934	up	PREDICTED: putative kinase-like protein TMKL1 [Sesamum indicum]
c26293.graph_c0	0.73942009	0.41120028	0.52788005	3.82155562	4.69053217	5.35967813	5.273E-06	1.96067	up	-
c26293.graph_c1	3.13299168	2.75099026	4.16728215	29.8811604	28.3144527	29.0115999	4.467E-37	2.02209	up	-

#ID	0 h-1 FPKM	0 h-2 FPKM	0 h-3 FPKM	28 h-1 FPKM	28 h-2 FPKM	28 h-3 FPKM	FDR	log2FC	regulated	nr_annotation
c26296.graph_c0	3.88331017	2.8120161	2.65436559	0.18015111	0.10843971	0.09800123	3.54E-31	-5.68354	down	-
c26297.graph_c0	0.0200172	0	0	2.86603199	3.42845119	3.33585328	5.887E-37	7.85144	up	O-Glycosyl hydrolases family 17 protein [Theobroma cacao]
c26298.graph_c0	2.37951036	2.54068595	1.90260934	13.3357067	15.7537906	13.6728441	2.648E-13	1.55993	up	hypothetical protein MIMGU_mgv1a013638mg [Erythranthe guttata]
c26299.graph_c0	0.80123465	0.26734566	0.51480913	10.5790446	12.9695324	7.99884231	4.045E-10	3.22824	up	PREDICTED: cytochrome b561 and DOMON domain-containing protein At3g25290-like [Sesamum indicum]
c26299.graph_c1	0.53625235	0.53678933	0	7.32720824	7.17882733	8.20514393	1.388E-11	3.33392	up	PREDICTED: cytochrome b561 and DOMON domain-containing protein At3g25290-like [Sesamum indicum]
c26301.graph_c0	0	0	0.04240265	0.71946428	1.42913933	0.88061546	3.549E-06	5.02692	up	PREDICTED: adenine/guanine permease AZG2 [Sesamum indicum]
c26302.graph_c0	8.16585954	6.55582616	7.13767856	7.98349445	7.20834696	7.9156911	2.779E-10	-1.00891	down	PREDICTED: upstream activation factor subunit spp27-like isoform X2 [Sesamum indicum]
c26306.graph_c0	96.3485699	94.7159539	90.9473592	9.03218456	9.32024664	10.3296469	0	-4.38681	down	PREDICTED: uncharacterized protein LOC105172717 [Sesamum indicum]
c26310.graph_c0	0.35862941	0.40386208	0.34563974	2.47617296	3.17712129	2.57884317	1.332E-05	1.80276	up	PREDICTED: uncharacterized protein LOC105176355 [Sesamum indicum]
c26313.graph_c0	2.30470081	2.15816936	1.76742543	8.24013221	8.65974988	9.67797895	1.694E-06	1.00619	up	PREDICTED: uncharacterized protein LOC105171071 [Sesamum indicum]
c26314.graph_c0	2.42770198	2.36618209	2.70920722	23.3556111	23.7291585	26.4841622	3.438E-30	2.20197	up	PREDICTED: galactokinase [Sesamum indicum]
c26316.graph_c0	0.47401167	0.91247369	0.98396915	72.4793828	79.0329848	74.3228749	0	5.47626	up	PREDICTED: uncharacterized protein LOC105167296 [Sesamum indicum]
c26318.graph_c0	1.0357385	0.69118376	0.51759749	3.9729463	4.30463761	3.99264561	0.0003976	1.36782	up	PREDICTED: alpha-ketoglutarate-dependent dioxygenase alkB homolog 2 [Sesamum indicum]
c26329.graph_c0	11.5352725	9.4579508	11.9182166	9.35274799	9.16739524	8.73184036	1.041E-12	-1.36406	down	PREDICTED: septum-promoting GTP-binding protein 1 [Sesamum indicum]
c26333.graph_c0	0.06482693	0.03244592	0	23.6050353	23.1636753	25.6051291	5.11E-163	8.47315	up	4-coumarate coenzyme A ligase [Paulownia fortunei]
c26334.graph_c0	5.24746078	4.54205384	4.20458032	27.9082198	27.6292299	29.4363265	3.041E-37	1.51449	up	PREDICTED: aspartate aminotransferase, chloroplastic [Nicotiana sylvestris]
c26336.graph_c0	26.376128	26.8809132	27.3989322	1.64314438	1.94195493	2.34366151	0	-4.85578	down	PREDICTED: uncharacterized protein LOC104091376 [Nicotiana tomentosiformis]
c26336.graph_c1	13.9967733	13.7413507	15.2192682	14.6722704	11.3046908	13.0898818	2.349E-05	-1.22844	down	-
c26338.graph_c0	58.6710932	52.3582094	59.3021816	7.59305857	8.08169507	8.53471356	9.04E-132	-3.90537	down	PREDICTED: uncharacterized protein LOC105158260 [Sesamum indicum]
c26341.graph_c0	0.05422336	0	0	3.58623955	5.80000226	3.11928782	7.039E-10	6.78825	up	PREDICTED: transcription factor JUNGBRUNNEN 1-like [Sesamum indicum]
c26342.graph_c0	1.49965636	1.47482194	1.58902696	0.7648713	1.15676684	0.85817701	7.913E-10	-1.80745	down	PREDICTED: uncharacterized protein LOC105158927 [Sesamum indicum]
c26348.graph_c0	0.34373531	0.17203975	0.7729986	6.68281455	6.14673917	7.10944423	4.489E-19	2.84706	up	hypothetical protein MIMGU_mgv1a024370mg [Erythranthe guttata]

#ID	0 h-1 FPKM	0 h-2 FPKM	0 h-3 FPKM	28 h-1 FPKM	28 h-2 FPKM	28 h-3 FPKM	FDR	log2FC	regulated	nr_annotation
c26349.graph_c0	2.01499525	1.85472457	1.36908091	10.8910695	10.1223916	11.2770426	5.406E-21	1.53835	up	PREDICTED: FACT complex subunit SPT16-like [Sesamum indicum]
c26358.graph_c0	60.6539279	63.2800719	57.594479	21.7310195	22.203847	21.2968955	2.61E-203	-2.56646	down	PREDICTED: amidophosphoribosyltransferase, chloroplastic [Sesamum indicum]
c26361.graph_c0	2.01996468	0.86656602	1.3596705	11.1855138	11.4250257	10.2681989	1.704E-08	1.86573	up	-
c26363.graph_c0	37.6848377	41.6316483	42.4045592	24.6927843	22.9359377	20.2649098	1.157E-32	-1.93473	down	PREDICTED: protein phosphatase 2C 37-like [Sesamum indicum]
c26363.graph_c1	45.0172776	42.4791632	40.0152845	26.9665462	29.993146	27.9222245	1.008E-69	-1.67644	down	PREDICTED: protein phosphatase 2C 37-like [Sesamum indicum]
c26364.graph_c0	0.77478315	1.24089436	0.73012648	146.595763	157.919495	144.556016	0	6.26486	up	PREDICTED: protein YLS3-like [Sesamum indicum]
c26365.graph_c0	1.43056541	1.56931277	1.43541085	9.71361105	8.65474057	9.19302902	5.862E-22	1.54534	up	PREDICTED: mechanosensitive ion channel protein 10-like [Sesamum indicum]
c26367.graph_c0	11.021348	12.1570448	10.3815202	0.93323327	0.98305917	0.76151073	3.78E-114	-4.73821	down	PREDICTED: dehydration-responsive protein RD22 [Sesamum indicum]
c26371.graph_c0	22.0280349	21.665498	20.2975812	8.93572958	8.3762836	8.43077649	2.73E-99	-2.40328	down	PREDICTED: uncharacterized protein LOC105176235 [Sesamum indicum]
c26381.graph_c0	77.2222938	80.5114303	71.957321	49.8238608	51.100521	50.2434749	4.099E-97	-1.69309	down	PREDICTED: protein SDE2 homolog [Sesamum indicum]
c26382.graph_c0	5.93040678	6.67838834	5.71560563	0	0	0	4.563E-36	-Inf	down	PREDICTED: myb-related protein Myb4-like [Sesamum indicum]
c26384.graph_c0	17.7179454	18.4199344	15.7761563	14.3677289	13.4751131	13.5076762	1.895E-48	-1.41723	down	PREDICTED: uncharacterized protein LOC105161020 isoform X2 [Sesamum indicum]
c26387.graph_c0	3.18311774	3.71735601	3.18144736	26.819134	26.2653291	26.3938766	1.2E-51	1.88871	up	PREDICTED: serine carboxypeptidase-like 45 [Sesamum indicum]
c26388.graph_c0	1.31984395	1.72253233	1.48136164	0.20642196	0.26312425	0.3071528	5.458E-38	-3.63214	down	PREDICTED: kinesin-like protein KCA1 [Sesamum indicum]
c26390.graph_c0	5.64961927	8.58041953	7.059709	4.5308808	4.34664558	4.78320299	2.207E-07	-1.73171	down	PREDICTED: uncharacterized protein LOC105171007 isoform X3 [Sesamum indicum]
c26393.graph_c0	34.7435714	37.8765216	33.7678471	22.0977557	24.8705587	23.2323158	2.01E-84	-1.68986	down	PREDICTED: uncharacterized protein LOC105156254 [Sesamum indicum]
c26394.graph_c0	7.63524136	8.10409559	7.52785588	47.5513269	49.1110676	47.3503146	2.393E-32	1.53951	up	hypothetical protein M569_01696, partial [Genlisea aurea]
c26398.graph_c0	0.07263453	0	0.15556375	14.4293649	14.3470882	11.5014261	3.544E-73	6.35467	up	PREDICTED: glycerophosphodiester phosphodiesterase GDPDL3-like [Sesamum indicum]
c26401.graph_c0	8.38353329	9.46781635	8.19497714	6.1972593	6.20682202	6.11928965	5.22E-15	-1.58169	down	PREDICTED: LOW QUALITY PROTEIN: uncharacterized protein LOC105173575 [Sesamum indicum]
c26404.graph_c0	0	0	0	1.44397798	1.86253819	2.31446726	3.823E-13	Inf	up	--
c26407.graph_c0	0	0	0	2.27008375	1.53725459	1.66713282	1.244E-11	Inf	up	PREDICTED: EPIDERMAL PATTERNING FACTOR-like protein 4 [Sesamum indicum]
c26409.graph_c0	0.36403001	0.36439453	0.24948959	1.85202441	1.64035288	1.64077138	0.0006434	1.30605	up	PREDICTED: zinc finger CCCH domain-containing protein 31 isoform X1 [Sesamum indicum]
c26413.graph_c0	0.1375434	0.51630423	0.22093589	11.1211897	12.027105	10.3187613	4.069E-51	4.16651	up	PREDICTED: protein WVD2-like 1 isoform X2 [Sesamum indicum]
c26418.graph_c0	31.8411698	33.9911999	33.8818287	35.2988409	33.1058497	35.5164302	6.537E-32	-1.03132	down	PREDICTED: GATA transcription factor 15-like [Sesamum indicum]

#ID	0 h-1 FPKM	0 h-2 FPKM	0 h-3 FPKM	28 h-1 FPKM	28 h-2 FPKM	28 h-3 FPKM	FDR	log2FC	regulated	nr_annotation
c26420.graph_c0	0.05548801	0.44434855	0.07130428	3.5892243	3.71409852	3.68565286	7.908E-11	3.18046	up	PREDICTED: 3-ketoacyl-CoA synthase 11 [Sesamum indicum]
c26422.graph_c0	285.842613	280.100125	294.371219	37.6878361	37.7565902	43.3936009	0	-3.94619	down	hypothetical protein MIMGU_mgv1a006265mg [Erythranthe guttata]
c26423.graph_c0	0.23799961	0.19059035	0.24467114	15.7754795	13.8377062	12.4774491	8.051E-53	4.87395	up	hypothetical protein MIMGU_mgv1a008823mg [Erythranthe guttata]
c26429.graph_c0	7.54124191	7.95409766	6.79656388	4.81880525	4.36753593	4.02215395	4.106E-43	-1.84463	down	PREDICTED: mitogen-activated protein kinase homolog NTF6 [Sesamum indicum]
c26438.graph_c0	7.36137478	9.13083753	8.2257896	3.43110957	4.72571733	3.98609527	1.014E-19	-2.11717	down	PREDICTED: probable trehalose-phosphate phosphatase J [Nicotiana tomentosiformis]
c26442.graph_c0	90.8368424	90.4089556	79.0126698	50.762646	54.167348	54.8705749	1.231E-96	-1.79225	down	PREDICTED: uncharacterized protein At4g13200, chloroplastic-like [Sesamum indicum]
c26444.graph_c0	34.0354039	39.1989703	32.0796162	3.01974389	3.15824866	2.48462041	5.58E-118	-4.69417	down	PREDICTED: receptor-like protein kinase FERONIA [Sesamum indicum]
c26444.graph_c1	19.6387413	20.0094495	17.0604297	1.23799239	1.3150483	1.30730688	2.54E-211	-4.96537	down	PREDICTED: receptor-like protein kinase FERONIA [Sesamum indicum]
c26447.graph_c0	122.9693	111.146755	119.45988	14.5813106	17.9323958	15.5907847	0	-3.96861	down	PREDICTED: uncharacterized protein LOC105156412 [Sesamum indicum]
c26450.graph_c0	1.74992952	2.00192207	2.64136389	32.9264905	31.8804136	30.4588994	7.34E-110	2.80129	up	PREDICTED: coatomer subunit alpha-1-like [Sesamum indicum]
c26452.graph_c0	0	0.07097769	0	9.17315343	7.84045408	7.94778796	3.994E-51	7.37627	up	PREDICTED: subtilisin-like protease [Sesamum indicum]
c26453.graph_c0	6.83268368	5.57294677	5.04052482	30.1635792	31.2204767	28.5528526	2.082E-16	1.27857	up	PREDICTED: DAG protein, chloroplastic [Sesamum indicum]
c26454.graph_c0	9.03314413	4.63702024	5.45672762	0	0	0	2.483E-19	-Inf	down	hypothetical protein SNOG_05978 [Phaeosphaeria nodorum SN15]
c26459.graph_c0	0.70506285	0.84692263	0.96643592	9.36049055	12.6157772	9.92393376	1.396E-17	2.568	up	hypothetical protein MIMGU_mgv1a021565mg [Erythranthe guttata]
c26464.graph_c0	19.0647235	22.1372242	18.1292041	181.584599	189.788644	197.467846	1.24E-108	2.17197	up	PREDICTED: 60S ribosomal protein L12 [Sesamum indicum]
c26468.graph_c0	2.01543682	1.21420901	1.82253356	0	0	0	1.678E-32	-Inf	down	PREDICTED: uncharacterized protein LOC104812256 [Tarenaya hassleriana]
c26470.graph_c0	0	0	0	5.31014859	5.36341315	4.75532548	8.673E-40	Inf	up	PREDICTED: tyrosine decarboxylase 1-like [Setaria italica]
c26474.graph_c0	0.18175733	0.07277573	0.04671307	0.89828123	0.73949928	0.97013402	2.859E-05	2.03921	up	PREDICTED: acetylornithine deacetylase [Sesamum indicum]
c26475.graph_c0	6.21392652	3.52784561	4.05216141	0	0	0	3.877E-22	-Inf	down	predicted protein [Hordeum vulgare subsp. vulgare]
c26481.graph_c0	1.11494777	0.98476255	0.80065555	19.7897157	19.5611156	19.4608734	1.25E-178	3.25517	up	PREDICTED: LOW QUALITY PROTEIN: UDP-glucose:glycoprotein glucosyltransferase [Sesamum indicum]
c26482.graph_c0	2.62904921	2.6316818	1.68921616	1.13452433	1.18611158	0.68214085	7.658E-10	-2.29815	down	-
c26483.graph_c0	4.86044051	6.25539537	5.71049919	3.83532931	5.19441415	4.61203827	1.47E-08	-1.3947	down	-
c26487.graph_c0	189.426176	201.646716	189.169362	132.39502	134.236355	137.134416	1.45E-77	-1.61336	down	hypothetical protein H632_c16p1 [Helicosporidium sp. ATCC 50920]
c26488.graph_c0	11.5626991	11.3112256	9.79311945	11.6505883	10.0020419	9.74055831	2.736E-12	-1.14618	down	PREDICTED: mechanosensitive ion channel protein 2, chloroplastic-like [Sesamum indicum]
c26490.graph_c0	2.19642595	2.5983754	3.25014496	39.279878	42.8475273	41.2097911	1.269E-65	2.84296	up	PREDICTED: 50S ribosomal protein L21, chloroplastic [Sesamum indicum]

#ID	0 h-1 FPKM	0 h-2 FPKM	0 h-3 FPKM	28 h-1 FPKM	28 h-2 FPKM	28 h-3 FPKM	FDR	log2FC	regulated	nr_annotation
c26491.graph_c0	3.08618573	2.87172143	1.95500676	28.6222245	29.2090745	26.6293962	2.553E-51	2.33055	up	hypothetical protein MIMGU_mgv1a006583mg [Erythranthe guttata]
c26496.graph_c0	317.258495	348.859207	361.694843	15.2394521	16.5643409	17.5440252	6.93E-171	-5.47205	down	PREDICTED: methionine gamma-lyase [Sesamum indicum]
c26498.graph_c0	1.85233419	1.66237636	0.82080095	14.9017698	15.047693	15.5689905	1.359E-19	2.31101	up	PREDICTED: uncharacterized protein LOC105161253 [Sesamum indicum]
c26502.graph_c0	34.216199	34.344556	31.5676095	17.9223656	17.8271678	16.5942593	8.12E-114	-2.02558	down	PREDICTED: uncharacterized protein LOC105166690 [Sesamum indicum]
c26504.graph_c0	0.2132239	0	0.06850029	1.70466895	1.15436718	1.13808759	1.277E-05	2.74938	up	clathrin heavy chain 1, partial [Nicotiana tabacum]
c26507.graph_c0	62.246062	63.0164419	60.9004526	24.7406683	25.7037849	24.9074988	4.23E-123	-2.39517	down	hypothetical protein MIMGU_mgv1a002702mg [Erythranthe guttata]
c26512.graph_c0	78.9197461	79.4784248	69.0262374	46.1792717	47.8901407	43.9906444	3.867E-96	-1.80922	down	PREDICTED: PRA1 family protein F2-like [Nicotiana tomentosiformis]
c26516.graph_c0	16.5811142	14.2475983	16.4048033	15.1971309	14.7326485	16.4908016	6.082E-14	-1.11538	down	PREDICTED: zinc finger A20 and AN1 domain-containing stress-associated protein 8-like [Sesamum indicum]
c26519.graph_c0	0.28693327	0.31594265	0.25810443	2.39822666	2.59844858	2.1441183	2.872E-07	1.96234	up	PREDICTED: uncharacterized protein LOC105156252 [Sesamum indicum]
c26520.graph_c0	5.38868501	4.34157736	3.37788955	3.15227687	3.88119608	3.58553577	4.051E-05	-1.38899	down	-
c26523.graph_c0	1.0424481	0.52174598	0.37210752	4.50378024	3.61043619	3.19420051	0.0004998	1.46616	up	PREDICTED: epidermal growth factor receptor substrate 15-like [Sesamum indicum]
c26525.graph_c0	0.34799442	0.5515429	0.52171771	1.83367247	2.47393633	2.52816642	0.0006478	1.17229	up	PREDICTED: probable receptor-like protein kinase At5g15080 [Sesamum indicum]
c26527.graph_c0	17.0549249	18.6817699	18.8164362	4.67519606	3.91577751	4.26668886	2.36E-111	-3.17649	down	PREDICTED: F-box/kelch-repeat protein SKIP25-like [Sesamum indicum]
c26529.graph_c0	0	0	0.03575775	3.53918453	3.72510038	3.45728354	3.856E-56	7.09759	up	PREDICTED: protein STICHEL [Sesamum indicum]
c26530.graph_c0	0	0.1307766	0.05596167	2.05730633	1.77182143	2.89260705	8.776E-09	4.07558	up	hypothetical protein MIMGU_mgv1a009832mg [Erythranthe guttata]
c26533.graph_c0	2.65958304	3.03496068	3.34931611	19.4456744	19.0236647	18.1072597	1.663E-17	1.55168	up	PREDICTED: 54S ribosomal protein L12, mitochondrial [Sesamum indicum]
c26539.graph_c1	24.134704	23.6555614	25.1989157	17.2716946	17.6612818	17.3893835	1.009E-66	-1.57149	down	PREDICTED: grpE protein homolog, mitochondrial isoform X2 [Sesamum indicum]
c26540.graph_c0	0.73732778	0.54552712	0.6385295	5.62679858	5.78521605	5.75115967	9.581E-26	2.06974	up	PREDICTED: chromosome-associated kinesin KIF4A [Sesamum indicum]
c26542.graph_c0	2.87400532	2.40612049	2.75312064	17.0902677	18.0027276	18.1291746	5.711E-18	1.63762	up	PREDICTED: 50S ribosomal protein L21, mitochondrial [Sesamum indicum]
c26546.graph_c0	11.765041	6.03440461	5.49765217	0	0.1914183	0	3.944E-12	-8.01913	down	heat shock protein 70-1 [Nicotiana tabacum]
c26547.graph_c0	0	0	0.33361398	2.35857248	2.38511819	3.38725355	2.574E-08	3.47879	up	-
c26548.graph_c0	0.12173276	0.12185466	0.26071903	4.98407297	5.99132474	4.93329812	7.736E-22	3.87712	up	PREDICTED: amino acid permease 6 [Sesamum indicum]
c26549.graph_c0	2.76018101	2.32669045	2.24017382	0.47512508	0.61965713	0.38769818	1.725E-17	-3.39526	down	PREDICTED: uncharacterized protein LOC105160363 [Sesamum indicum]

#ID	0 h-1 FPKM	0 h-2 FPKM	0 h-3 FPKM	28 h-1 FPKM	28 h-2 FPKM	28 h-3 FPKM	FDR	log2FC	regulated	nr_annotation
c26550.graph_c0	3.8962461	4.0290781	4.01374015	22.0690774	20.4123152	23.011522	1.234E-25	1.36526	up	PREDICTED: glycoprotein 3-alpha-L-fucosyltransferase A [Sesamum indicum]
c26554.graph_c0	16.216429	14.0597906	16.4084672	77.4906769	98.7076755	93.1437895	7.42E-15	1.43735	up	hypothetical protein MIMGU_mgv1a016711mg [Erythranthe guttata]
c26555.graph_c0	3.04088308	1.88933466	2.56018786	19.9670769	19.7486653	19.3401305	8.238E-13	1.88952	up	PREDICTED: cytochrome c oxidase subunit 6b-2-like [Sesamum indicum]
c26558.graph_c0	1.42630536	1.00781195	1.83285886	0.03048913	0	0	8.126E-17	-8.22851	down	-
c26561.graph_c0	2.72465804	2.35547005	2.54639524	15.3470597	14.0601371	14.6898245	6.272E-11	1.44162	up	PREDICTED: vesicle-associated membrane protein 724 [Sesamum indicum]
c26569.graph_c0	0.3729222	0.07465913	0.57506375	9.26948953	9.15258918	9.55430875	3.166E-20	3.67299	up	PREDICTED: uncharacterized protein LOC105155725 [Sesamum indicum]
c26574.graph_c0	6.91418328	7.11335976	5.75879582	0.93059047	1.00828314	1.29090219	6.881E-44	-3.70174	down	PREDICTED: zinc finger CCCH domain-containing protein 48-like [Sesamum indicum]
c26576.graph_c0	1.62758065	2.50415677	2.40135826	17.3056265	16.258284	17.0884998	1.801E-34	1.86082	up	PREDICTED: ATP-dependent zinc metalloprotease FTSH 7, chloroplastic [Sesamum indicum]
c26578.graph_c0	0.63278934	0.63342298	0.5227453	9.85516376	10.8262515	10.4810589	4.657E-27	3.03453	up	PREDICTED: transcription repressor KAN1 [Sesamum indicum]
c26579.graph_c0	44.8314832	44.2409039	41.6829249	21.5976724	19.064239	22.9123744	9.05E-136	-2.12928	down	PREDICTED: uncharacterized protein LOC105161451 [Sesamum indicum]
c26585.graph_c0	0.03186086	0	0	1.62094021	2.00716393	2.43749918	2.091E-16	6.51514	up	PREDICTED: probable isoprenylcysteine alpha-carbonyl methylesterase ICMEL2 [Sesamum indicum]
c26586.graph_c0	1.90805198	1.90996261	2.29114055	13.4811374	14.5943278	12.2990391	2.141E-22	1.63064	up	hypothetical protein NitaMp096 [Nicotiana tabacum]
c26587.graph_c0	0.13548058	0.3390406	0.43524476	2.8062953	4.93428406	5.18243707	8.085E-06	2.72756	up	Selenoprotein H [Gossypium arboreum]
c26588.graph_c0	18.3667864	17.6398329	17.861009	80.3638321	91.1288049	85.006225	3.752E-20	1.16101	up	-
c26591.graph_c0	20784.3207	19032.3289	20528.9155	9887.92024	10697.4993	11635.0432	6.747E-20	-1.99538	down	-
c26595.graph_c0	0.47198792	0.53995491	1.039754	8.23291445	8.71672213	8.23753654	9.703E-14	2.51628	up	PREDICTED: homeobox-leucine zipper protein HAT22-like [Sesamum indicum]
c26599.graph_c1	6.26271054	5.1676741	7.12341797	1.26094445	1.24958921	1.53083275	1.204E-21	-3.29074	down	PREDICTED: uncharacterized protein LOC103430095 [Malus domestica]
c26604.graph_c0	0.93702604	0.39081847	0.50171482	13.3934941	12.2467949	13.1518256	2.22E-22	3.32278	up	-
c26607.graph_c0	14.4314151	14.445866	13.5996225	4.07892696	4.20901556	5.32539375	4.126E-25	-2.73019	down	PREDICTED: heat stress transcription factor A-8 [Sesamum indicum]
c26607.graph_c1	41.5186745	43.9383561	42.3772219	9.61999291	9.39122122	11.8418461	1.32E-196	-3.14033	down	PREDICTED: heat stress transcription factor A-8 [Sesamum indicum]
c26609.graph_c1	20.3570047	22.8790233	21.357922	10.2985537	10.5405685	11.0656147	2.111E-79	-2.10818	down	PREDICTED: homeobox-leucine zipper protein HDG5 isoform X2 [Sesamum indicum]
c26612.graph_c0	0	0	0	5.7478571	7.20463027	5.7386031	2.43E-19	Inf	up	PREDICTED: gibberellin-regulated protein 9-like [Solanum tuberosum]
c26614.graph_c0	79.6598938	76.4765612	78.0505533	363.690394	367.891914	371.077152	2.666E-47	1.14492	up	PREDICTED: dnaJ protein homolog [Sesamum indicum]
c26616.graph_c0	62.770186	64.0623829	57.3402847	20.8269826	20.6554449	23.1450916	7.25E-190	-2.5996	down	PREDICTED: uncharacterized protein LOC105165909 [Sesamum indicum]

#ID	0 h-1 FPKM	0 h-2 FPKM	0 h-3 FPKM	28 h-1 FPKM	28 h-2 FPKM	28 h-3 FPKM	FDR	log2FC	regulated	nr_annotation
c26621.graph_c0	2.73607994	2.36870894	2.37565611	12.5763435	11.9133166	11.709426	9.317E-11	1.18555	up	PREDICTED: protein ROOT PRIMORDIUM DEFECTIVE 1 isoform X2 [Sesamum indicum]
c26624.graph_c0	0.04105661	0.08219545	0	2.267818	2.20928179	2.84882833	1.934E-14	4.82043	up	PREDICTED: vinorine synthase-like [Sesamum indicum]
c26625.graph_c0	0.22899539	0.45844938	0.58853628	6.32443603	4.80873496	5.43230332	3.495E-07	2.59839	up	PREDICTED: V-type proton ATPase subunit c''2 [Sesamum indicum]
c26630.graph_c0	32.3789992	30.748145	30.4127211	10.8035783	11.3754763	10.6000647	2.44E-140	-2.60293	down	PREDICTED: annexin D5 [Sesamum indicum]
c26634.graph_c0	1.45007138	1.6203052	2.16674318	14.2154429	12.4147483	13.1796424	1.426E-24	1.8306	up	PREDICTED: pentatricopeptide repeat-containing protein At3g59040 [Sesamum indicum]
c26636.graph_c0	56.2039248	60.248626	50.3582056	45.277654	43.6499736	47.4267948	3.132E-37	-1.37943	down	PREDICTED: ethylene-responsive transcription factor ERF008-like [Sesamum indicum]
c26638.graph_c0	0.47739881	0.47787686	0.24539055	12.1439747	11.8420602	10.2491345	4.895E-19	3.7501	up	-
c26639.graph_c0	3.9333911	3.82795953	4.49294515	0.39705083	0.64529941	0.97197071	8.694E-20	-3.69449	down	PREDICTED: UDP-glucuronate 4-epimerase 3-like [Sesamum indicum]
c26640.graph_c0	1.22171486	1.22293822	1.50956919	0.71717852	0.64754497	0.69668074	1.374E-07	-2.03304	down	-
c26644.graph_c0	0.04765713	0	0	3.56760372	4.75363274	4.0134101	1.47E-21	6.95649	up	PREDICTED: protein polybromo-1-like isoform X1 [Cucumis melo]
c26648.graph_c0	0.16870275	0	0	7.23411882	3.54263378	6.60333566	7.288E-07	5.6295	up	-
c26649.graph_c0	26.3751529	21.7601928	23.4466074	25.7779065	23.8677755	24.3915201	1.492E-18	-1.04084	down	PREDICTED: ganglioside-induced differentiation-associated protein 2 [Sesamum indicum]
c26650.graph_c0	4.71048956	5.52987557	6.05316558	5.34146537	4.96849874	4.62186279	1.618E-08	-1.21977	down	PREDICTED: uncharacterized protein LOC105161159 [Sesamum indicum]
c26651.graph_c0	5.6199084	6.56312521	6.10414453	37.7331574	37.1866872	37.178069	1.737E-25	1.52468	up	hypothetical protein AMTR_s00062p00211520 [Amborella trichopoda]
c26653.graph_c0	0	0.04438812	0	2.96504573	2.99725331	2.05127546	1.391E-16	6.41278	up	PREDICTED: basic leucine zipper 61-like [Sesamum indicum]
c26655.graph_c0	0	0.44280995	0	7.71623459	5.22527728	6.68990844	4.377E-11	4.38917	up	BnaA05g08010D [Brassica napus]
c26655.graph_c1	0.9964087	0.66493764	0.42680821	16.173445	15.4437948	15.1952995	2.333E-42	3.40548	up	PREDICTED: uncharacterized protein LOC105174078 [Sesamum indicum]
c26658.graph_c0	1.16106525	1.93704647	1.65779424	0.12501563	0.08465805	0.0765088	1.852E-17	-5.14905	down	-
c26666.graph_c0	22.505543	21.3951387	20.0821362	13.1930887	14.1402888	14.8702655	1.027E-54	-1.68915	down	PREDICTED: potassium/sodium hyperpolarization-activated cyclic nucleotide-gated channel 4 [Sesamum indicum]
c26667.graph_c0	70.096248	73.1312179	67.1248351	3.26155884	3.00377947	4.57761725	0	-5.36585	down	hypothetical protein CARUB_v10021660mg, partial [Capsella rubella]
c26671.graph_c0	18.012511	10.3855956	10.9252848	0	0	0	3.747E-24	-Inf	down	predicted protein [Hordeum vulgare subsp. vulgare]
c26672.graph_c0	1.08462259	0.82513859	0.50176187	8.32442104	7.40229032	8.10487792	7.712E-15	2.22334	up	PREDICTED: alpha/beta hydrolase domain-containing protein 17B-like [Sesamum indicum]
c26673.graph_c0	25.9940895	25.4979758	26.2535353	6.76080912	10.953648	9.64144917	9.592E-72	-2.59765	down	PREDICTED: uncharacterized protein LOC105177028 [Sesamum indicum]

#ID	0 h-1 FPKM	0 h-2 FPKM	0 h-3 FPKM	28 h-1 FPKM	28 h-2 FPKM	28 h-3 FPKM	FDR	log2FC	regulated	nr_annotation
c26676.graph_c0	1.04607464	0.4430132	0.93063276	10.4977263	8.71279294	8.20814564	7.635E-19	2.41085	up	PREDICTED: probable receptor-like protein kinase At2g42960 [Sesamum indicum]
c26684.graph_c0	3.21498733	1.68572729	2.16405933	0	0	0	2.206E-17	-Inf	down	predicted protein [Nectria haematococca mpVI 77-13-4]
c26685.graph_c0	0.08453855	0	0.07242359	4.28046852	5.14082087	4.09446269	3.691E-32	5.33503	up	PREDICTED: kinesin-like protein NACK1 [Sesamum indicum]
c26693.graph_c0	1.77994775	1.83829296	1.23441767	14.9283768	16.2229148	16.0687687	2.432E-41	2.19671	up	PREDICTED: ATP-dependent 6-phosphofructokinase 4, chloroplastic [Sesamum indicum]
c26694.graph_c0	0.09804912	0.07361048	0.0629985	5.11301513	4.29486085	4.41931113	2.249E-34	4.79521	up	PREDICTED: putative inactive cadmium/zinc-transporting ATPase HMA3 [Sesamum indicum]
c26695.graph_c0	4.80948768	5.11519763	4.63528656	1.23799239	1.05203864	2.34721008	4.714E-27	-2.73671	down	-
c26696.graph_c0	0.23335796	0.13348094	0.12851752	1.59911611	1.15945684	1.40371915	2.597E-05	1.98739	up	PREDICTED: glutamate synthase 1 [NADH], chloroplastic isoform X1 [Sesamum indicum]
c26697.graph_c0	2.15414048	1.64463371	1.73596212	0	0	0	2.12E-48	-Inf	down	hypothetical protein VHEMI06393 [Torrubiella hemipterigena]
c26698.graph_c0	1.19765436	1.04416284	1.16668651	23.4740248	21.2835779	20.8042921	1E-124	3.17481	up	PREDICTED: auxin response factor 9 [Sesamum indicum]
c26701.graph_c0	1.0291294	1.07698537	1.38258438	11.8314513	11.4194286	11.3189138	2.305E-20	2.21416	up	PREDICTED: 3-deoxy-manno-octulosonate cytidylyltransferase, mitochondrial [Sesamum indicum]
c26703.graph_c0	1.41739228	1.0286384	1.13837825	8.88506564	9.09203205	8.93133313	9.041E-16	1.81982	up	PREDICTED: probable arabinosyltransferase ARAD1 [Sesamum indicum]
c26708.graph_c0	0	0	0	11.1558966	8.51945744	9.86880358	1.458E-33	Inf	up	PREDICTED: intracellular ribonuclease LX-like [Sesamum indicum]
c26709.graph_c0	0	0	0	1.08919371	1.39320638	2.11083628	9.532E-07	Inf	up	RecName: Full=60S ribosomal protein L5 [Helianthus annuus]
c26715.graph_c1	0.51613562	0.53510432	0.54481636	3.85842232	4.23377694	4.24164982	1.182E-14	1.85902	up	hypothetical protein MIMGU_mgv1a001258mg [Erythranthe guttata]
c26721.graph_c0	0	0	0	16.8236646	28.4076314	21.4299006	4.783E-26	Inf	up	hypothetical protein MIMGU_mgv1a020088mg [Erythranthe guttata]
c26721.graph_c1	0	0	0	21.9607705	34.0902038	24.867329	1.635E-29	Inf	up	hypothetical protein MIMGU_mgv1a026045mg [Erythranthe guttata]
c26722.graph_c0	4.91935208	4.67389105	4.98224817	66.6288939	70.5555808	68.8075973	1.39E-136	2.73006	up	PREDICTED: aldose 1-epimerase-like [Sesamum indicum]
c26727.graph_c0	1.23801183	1.40448505	0.58332788	9.53766655	9.50527719	6.92607616	7.472E-09	1.9257	up	PREDICTED: uncharacterized protein LOC105174183 [Sesamum indicum]
c26737.graph_c0	15.3864814	8.15842617	10.8160014	0	0	0	2.664E-31	-Inf	down	cytochrome c oxidase subunit III (mitochondrion) [Chlorella sorokiniana]
c26738.graph_c0	0.20906542	0.13951651	0.13432867	3.36817059	3.10973944	3.53365676	2.251E-14	3.28866	up	PREDICTED: UPF0496 protein At4g34320-like [Sesamum indicum]
c26739.graph_c0	0.13113565	0.13126696	0	24.3037021	17.8993905	22.1647545	1.134E-33	6.86859	up	PREDICTED: NAC domain-containing protein 100-like [Sesamum indicum]
c26741.graph_c0	2.21161621	1.64360166	1.55018967	11.349147	9.82941072	10.8109041	2.996E-14	1.47879	up	PREDICTED: calcium uptake protein 1, mitochondrial [Sesamum indicum]
c26743.graph_c0	26.9953053	28.7808405	24.7194726	16.89621	16.3124481	15.6277739	2.619E-82	-1.81043	down	PREDICTED: endo-1,3(4)-beta-glucanase 2 [Sesamum indicum]
c26744.graph_c0	1.74652054	1.20193522	1.4728534	0	0	0	5.027E-32	-Inf	down	-

#ID	0 h-1 FPKM	0 h-2 FPKM	0 h-3 FPKM	28 h-1 FPKM	28 h-2 FPKM	28 h-3 FPKM	FDR	log2FC	regulated	nr_annotation
c26746.graph_c0	0	0.0352468	0	6.11637979	5.869119	6.03504064	4.122E-39	7.91634	up	PREDICTED: uncharacterized protein LOC105157258 [Sesamum indicum]
c26750.graph_c1	3.65393183	4.07165756	4.16388702	0.15032018	0.85959187	0.28620677	7.818E-32	-4.2933	down	hypothetical protein CICLE_v10032119mg [Citrus clementina]
c26752.graph_c0	2.20550616	2.89286746	2.29664911	1.520038	1.09796144	1.17268376	2.382E-13	-2.05453	down	PREDICTED: cytochrome P450 84A1-like [Sesamum indicum]
c26755.graph_c0	4.74696891	2.40113626	2.95268038	0	0	0	1.168E-21	-Inf	down	NADH dehydrogenase subunit 5 [Pneumocystis carinii]
c26756.graph_c0	3.06699925	1.94767906	2.7122343	2.9481346	2.64024775	2.32742112	0.0005743	-1.05589	down	PREDICTED: uncharacterized protein LOC105171097 [Sesamum indicum]
c26757.graph_c0	56.2531835	55.7077798	53.1382741	35.1128321	31.4959751	27.7511388	1.3E-115	-1.89765	down	PREDICTED: protein SPA1-RELATED 4-like [Solanum lycopersicum]
c26758.graph_c0	0.75856059	0.89737839	0.443082	2.85682688	3.71078314	3.14909386	0.009312	1.1264	up	hypothetical protein MIMGU_mgv1a001946mg [Erythranthe guttata]
c26760.graph_c0	3.27785664	2.91656793	2.63260972	14.8895457	12.6073567	12.1227483	2.367E-08	1.07781	up	PREDICTED: NAC domain-containing protein 18 [Sesamum indicum]
c26762.graph_c0	1.09647608	1.20733144	0.98631062	8.75275558	7.86694077	7.60820684	2.608E-25	1.79123	up	PREDICTED: uncharacterized protein LOC105177385 [Sesamum indicum]
c26763.graph_c0	0	0	0	2.02205018	1.61093094	1.60144772	1.136E-10	Inf	up	unnamed protein product [Coffea canephora]
c26765.graph_c0	0.46446595	0.19925616	0.42632652	9.54844175	8.6648898	9.44418241	4.496E-21	3.57417	up	PREDICTED: uncharacterized protein LOC105158032 [Sesamum indicum]
c26766.graph_c0	1.81079752	2.24763734	1.58232362	8.52818026	8.05663548	8.69865681	5.818E-07	1.07628	up	hypothetical protein MIMGU_mgv1a004061mg [Erythranthe guttata]
c26767.graph_c0	54.6529913	54.2397096	49.0132595	15.5952858	15.9058081	16.4615894	0	-2.80802	down	unnamed protein product [Coffea canephora]
c26775.graph_c0	0.28165492	0.56387391	0.12064591	3.00234253	2.77244367	2.61692474	5.216E-05	2.03698	up	PREDICTED: transcription factor bHLH35-like isoform X2 [Sesamum indicum]
c26777.graph_c0	14.1023944	13.2036696	14.6483772	13.9422136	13.8281274	14.003618	2.523E-23	-1.09743	down	PREDICTED: pentatricopeptide repeat-containing protein At5g47360 [Sesamum indicum]
c26779.graph_c0	0.05748938	0.28773475	0.18469033	1.83844991	0.9808783	1.29559282	0.0027194	1.86087	up	PREDICTED: probable leucine-rich repeat receptor-like protein kinase At1g35710 [Sesamum indicum]
c26780.graph_c0	31.5938168	31.4146168	29.3668309	7.34790526	8.01665067	8.55655005	7.966E-90	-3.03837	down	PREDICTED: desiccation-related protein PCC3-06 isoform X2 [Sesamum indicum]
c26781.graph_c0	1.36078361	1.05566333	1.04919649	0.37087863	0.1785966	0.38333632	8.9E-15	-2.97894	down	PREDICTED: rop guanine nucleotide exchange factor 5-like [Sesamum indicum]
c26785.graph_c0	1.50732417	1.19666108	0.80150534	0.5099809	0.61395325	0.63191667	4.184E-06	-2.08026	down	1-deoxy-D-xylulose-5-phosphate synthase [Andrographis paniculata]
c26786.graph_c0	9.49467719	8.73058825	10.2148209	1.28384682	1.12296852	1.11308405	9.777E-80	-4.10637	down	hypothetical protein MIMGU_mgv1a013028mg [Erythranthe guttata]
c26787.graph_c0	15.3789918	18.2412996	17.7998881	16.3832575	15.1381657	17.9913928	6.313E-13	-1.14576	down	PREDICTED: CASP-like protein 4A3 [Sesamum indicum]
c26789.graph_c0	1.84425579	2.50318987	1.56657164	8.63304918	9.47687711	8.19386304	7.178E-08	1.06483	up	PREDICTED: uncharacterized protein LOC105174843 [Sesamum indicum]
c26790.graph_c0	4.95812546	3.69050302	3.1040093	0.73918708	1.08455202	0.75396327	2.201E-15	-3.27523	down	-
c26796.graph_c0	5.56169541	4.86335758	4.92896468	47.1590953	47.9499665	45.4194691	4.346E-51	2.10464	up	PREDICTED: acylpyruvase FAHD1, mitochondrial [Sesamum indicum]

#ID	0 h-1 FPKM	0 h-2 FPKM	0 h-3 FPKM	28 h-1 FPKM	28 h-2 FPKM	28 h-3 FPKM	FDR	log2FC	regulated	nr_annotation
c26804.graph_c0	0	0	0	2.43854532	2.31766083	1.78037695	1.326E-20	Inf	up	PREDICTED: NAC domain-containing protein 45 [Sesamum indicum]
c26806.graph_c0	0	0	0	4.27010543	4.5068915	4.27988969	5.519E-39	Inf	up	PREDICTED: uncharacterized protein LOC105179467 isoform X2 [Sesamum indicum]
c26810.graph_c0	18.5002897	11.3270422	13.3870784	0	0	0	4.78E-33	-Inf	down	-
c26813.graph_c0	2.84650355	2.21616414	1.4225049	113.095623	149.436211	166.000646	1.161E-30	4.96449	up	PREDICTED: non-specific lipid-transfer protein 2-like [Sesamum indicum]
c26813.graph_c1	2.35516353	3.17358713	2.79367235	0.921695	1.84273779	0.69837448	4.911E-10	-2.36043	down	PREDICTED: PRA1 family protein F2-like [Sesamum indicum]
c26814.graph_c0	6.87094134	6.14418725	9.18260892	53.0004455	55.7299741	57.7544602	1.995E-36	1.81214	up	hypothetical protein MIMGU_mgv1a016555mg [Erythranthe guttata]
c26817.graph_c0	0.3319957	0.66465629	1.27988287	20.5098679	16.9934358	15.5545282	5.731E-11	3.43607	up	unknown [Medicago truncatula]
c26817.graph_c1	1.3492705	1.75080577	1.92651779	46.8528967	40.7624726	43.2105777	1.64E-107	3.60753	up	PREDICTED: protein BRASSINAZOLE-RESISTANT 1 [Sesamum indicum]
c26818.graph_c0	64.4910871	62.5058671	62.554912	29.0687745	31.7576069	31.9288374	1.06E-145	-2.12117	down	PREDICTED: uncharacterized protein LOC105165319 [Sesamum indicum]
c26819.graph_c0	1.19267902	1.42029756	1.13626741	19.4289984	18.4466807	16.6222086	2.205E-82	2.77156	up	PREDICTED: BTB/POZ domain-containing protein At1g63850-like [Sesamum indicum]
c26821.graph_c0	4.8033793	6.01023645	4.56230668	84.5066229	82.3316758	81.2184379	5.24E-144	2.9226	up	hypothetical protein MIMGU_mgv1a008879mg [Erythranthe guttata]
c26822.graph_c0	1.39291113	1.81865989	1.08953231	0.96834414	0.079484	0.50282967	1.081E-07	-2.5573	down	-
c26824.graph_c0	0	0.2833105	0.36370102	65.344814	57.1429401	59.195638	1.53E-135	7.02425	up	PREDICTED: arabinogalactan peptide 22-like [Sesamum indicum]
c26829.graph_c0	78.0159633	93.9857191	79.7650926	5.74443676	8.49543691	9.29400949	8.671E-95	-4.50853	down	-
c26830.graph_c1	8.35110496	8.1365482	7.44050702	2.99430391	5.91863867	5.480978	8.556E-12	-1.82188	down	PREDICTED: type 1 phosphatases regulator ypi1 [Sesamum indicum]
c26833.graph_c0	1.62011843	0.94601542	0.86746513	17.6133288	18.3396055	18.1755959	2.673E-29	2.89515	up	unnamed protein product [Coffea canephora]
c26837.graph_c0	7.1977646	8.40580075	6.43831653	4.82099627	3.88983234	5.69159073	9.637E-15	-1.7011	down	-
c26839.graph_c0	0	0	0	4.54193622	4.1952171	5.70837361	2.034E-19	Inf	up	PREDICTED: 36.4 kDa proline-rich protein-like, partial [Cucumis sativus]
c26840.graph_c0	0.08246542	0.082548	0	20.3980127	16.4873541	16.905448	2.269E-79	7.27809	up	PREDICTED: uncharacterized protein LOC105157872 [Sesamum indicum]
c26843.graph_c0	5.75859373	7.30152277	4.44001256	3.34825018	2.01543733	3.64285974	1.334E-07	-2.04312	down	-
c26844.graph_c0	17.7153225	15.8396879	17.9036381	158.125936	167.90044	159.316925	3.65E-137	2.14634	up	PREDICTED: alcohol dehydrogenase class-3 [Sesamum indicum]
c26847.graph_c0	24.4006139	27.7004369	23.9072614	2.2422584	1.10429951	1.55244265	3.38E-156	-5.04523	down	PREDICTED: WRKY transcription factor 22 [Sesamum indicum]
c26848.graph_c0	56.9290091	50.0111641	52.961918	376.152302	390.608851	412.707891	3.885E-89	1.79327	up	TPA: hypothetical protein ZEAMMB73_262778, partial [Zea mays]
c26851.graph_c0	1.21368323	1.95733655	1.73292334	1.61717962	1.28317229	1.39958145	0.0007915	-1.28304	down	-
c26853.graph_c0	49.7629291	54.8733634	47.231831	9.0170259	10.1141461	8.47607841	7.7E-161	-3.54996	down	PREDICTED: scarecrow-like protein 21 [Sesamum indicum]

#ID	0 h-1 FPKM	0 h-2 FPKM	0 h-3 FPKM	28 h-1 FPKM	28 h-2 FPKM	28 h-3 FPKM	FDR	log2FC	regulated	nr_annotation
c26854.graph_c0	1.58550047	2.01438107	1.56725452	50.0821479	50.1416047	48.461306	1.47E-112	3.75692	up	PREDICTED: lipoxygenase homology domain-containing protein 1-like [Sesamum indicum]
c26856.graph_c0	0.17672281	0.35379955	0.22709581	61.3948566	55.0858883	50.83135	2.255E-66	6.69517	up	PREDICTED: dof zinc finger protein DOF1.4-like isoform X2 [Sesamum indicum]
c26859.graph_c0	2.232391	2.00737626	1.7017776	11.0549244	10.2298578	9.3573224	5.196E-10	1.27878	up	hypothetical protein MIMGU_mgv1a011616mg [Erythranthe guttata]
c26860.graph_c0	2.32599749	2.12586344	2.46917297	23.7408029	24.2230262	21.7713479	2.49E-36	2.24071	up	hypothetical protein MIMGU_mgv1a011282mg [Erythranthe guttata]
c26862.graph_c0	73.3254192	76.640103	74.0922918	7.17398932	8.29386463	7.96008779	0	-4.34812	down	PREDICTED: probable serine/threonine-protein kinase WNK11 [Sesamum indicum]
c26866.graph_c0	0.03798013	0.03801816	0	0.93852715	1.59510969	0.65320836	0.0003804	4.31635	up	PREDICTED: methylthioribose kinase [Sesamum indicum]
c26868.graph_c0	0.03221302	0	0	0.77260373	0.8455621	0.64954253	2.258E-10	5.07746	up	-
c26870.graph_c0	0	0.10318111	0	2.99665668	3.17919752	3.60673988	1.341E-12	5.48601	up	PREDICTED: uncharacterized protein LOC104095178 [Nicotiana tomentosiformis]
c26871.graph_c0	1.74445425	1.30965079	2.03153408	0.67355025	0.6797001	0.79208717	9.302E-09	-2.33803	down	PREDICTED: LOW QUALITY PROTEIN: anthranilate synthase alpha subunit 1, chloroplastic [Sesamum indicum]
c26878.graph_c0	0	0.06439695	0	7.71482407	7.05019629	8.88963268	6.351E-29	7.44024	up	PREDICTED: probable receptor-like protein kinase At1g80640 isoform X1 [Sesamum indicum]
c26881.graph_c0	9.10731444	6.7715245	7.21854597	4.98607409	5.4745057	5.54293008	2.55E-13	-1.6173	down	hypothetical protein MIMGU_mgv1a005840mg [Erythranthe guttata]
c26885.graph_c0	3.42620939	4.15236134	4.20038645	112.667327	120.817653	107.28028	0	3.76139	up	PREDICTED: LOW QUALITY PROTEIN: SNF2 domain-containing protein CLASSY 1-like [Sesamum indicum]
c26889.graph_c0	0.89078453	0.44583826	0.57234669	8.82105747	8.76835359	8.32052081	4.486E-10	2.67648	up	PREDICTED: premnaspirodiene oxygenase-like [Sesamum indicum]
c26891.graph_c0	14.3484255	4.78759775	4.43884875	0	0	0	2.444E-07	-Inf	down	PREDICTED: chlorophyll a-b binding protein of LHCII type 1 [Elaeis guineensis]
c26893.graph_c0	1.40606647	1.11770029	1.85999377	1.14214701	1.19408087	1.10366365	0.000733	-1.4454	down	hypothetical protein MIMGU_mgv1a018417mg [Erythranthe guttata]
c26895.graph_c0	3.57527569	2.72674727	2.84413431	26.5417169	28.0146572	28.5489501	1.228E-45	2.09572	up	hypothetical protein MIMGU_mgv1a000048mg [Erythranthe guttata]
c26897.graph_c0	1.82912893	1.09857631	1.5983421	14.67658	16.4204307	14.1021379	3.383E-17	2.22948	up	PREDICTED: hypoxanthine-guanine phosphoribosyltransferase [Nicotiana sylvestris]
c26898.graph_c0	0.6932653	0.56778504	0.48593092	7.12276546	6.57594285	6.22326509	4.552E-19	2.42463	up	PREDICTED: probable receptor-like protein kinase At2g42960 [Nelumbo nucifera]
c26899.graph_c0	10.3937641	12.5877635	8.90426558	55.117155	60.2913473	55.2486594	2.151E-16	1.33176	up	hypothetical protein MIMGU_mgv1a011204mg [Erythranthe guttata]
c26901.graph_c0	3.23386552	3.39895893	3.15829037	26.7705414	28.5216965	26.1597515	5.597E-52	1.96599	up	PREDICTED: LL-diaminopimelate aminotransferase, chloroplastic [Sesamum indicum]
c26913.graph_c0	0.44704711	0.84526788	0.63830359	4.47655517	5.11757909	4.09470736	9.979E-07	1.7323	up	-
c26916.graph_c0	14.7571685	17.4153464	13.4740984	7.33844872	6.72786855	7.87667333	7.329E-27	-2.14502	down	hypothetical protein MIMGU_mgv1a026902mg, partial [Erythranthe guttata]

#ID	0 h-1 FPKM	0 h-2 FPKM	0 h-3 FPKM	28 h-1 FPKM	28 h-2 FPKM	28 h-3 FPKM	FDR	log2FC	regulated	nr_annotation
c26918.graph_c0	0	0.09688252	0	2.50890819	2.56170441	2.20031391	3.478E-17	5.14741	up	PREDICTED: probable beta-1,4-xylosyltransferase IRX14 [Sesamum indicum]
c26922.graph_c0	5.49329034	4.56282661	3.67974131	36.8243655	47.3231875	42.5945339	1.205E-22	2.11987	up	PREDICTED: uncharacterized protein LOC105157862 isoform X2 [Sesamum indicum]
c26924.graph_c0	1.51205362	1.18247477	1.21440548	7.36641165	7.17831213	7.72033426	1.54E-13	1.42201	up	PREDICTED: cell wall protein RBR3 isoform X2 [Sesamum indicum]
c26928.graph_c0	21.2644127	16.8132229	15.7361502	2.94664505	3.04062195	3.63119253	4.704E-38	-3.57032	down	PREDICTED: probable protein phosphatase 2C 51 [Sesamum indicum]
c26932.graph_c0	5.84509046	5.65176238	4.76240309	29.0683843	28.4331944	26.6550078	9.207E-33	1.2835	up	PREDICTED: uncharacterized protein LOC105170351 [Sesamum indicum]
c26933.graph_c0	2.64060791	2.10481184	2.10509392	12.4568908	12.5149436	12.0932631	4.738E-19	1.34752	up	PREDICTED: ABC transporter G family member 7 isoform X2 [Sesamum indicum]
c26934.graph_c0	0.39252387	0.7421764	0.78463577	20.9208328	20.8643671	22.2960593	1.036E-73	3.96441	up	PREDICTED: BAG family molecular chaperone regulator 7 [Sesamum indicum]
c26939.graph_c0	0.37174691	0.42936826	0.33072196	18.9128458	19.365907	16.8064101	1.3E-105	4.51565	up	PREDICTED: protein NRT1/ PTR FAMILY 5.10-like [Sesamum indicum]
c26940.graph_c0	3.92851751	3.26853097	2.88474316	44.3857531	49.0154703	44.9931318	4.83E-84	2.69173	up	PREDICTED: DELLA protein GAI-like [Sesamum indicum]
c26940.graph_c1	0.97629113	0.97726874	0	16.3199436	16.7855187	16.7909253	4.949E-14	3.60447	up	DELLA protein GAIP-B, putative [Ricinus communis]
c26940.graph_c2	4.88713012	3.94518052	4.05171335	47.3782699	51.3647988	47.4020862	1.578E-52	2.41489	up	PREDICTED: LOW QUALITY PROTEIN: DELLA protein GAI [Sesamum indicum]
c26945.graph_c0	32.0972311	32.8175509	33.3503514	32.165897	33.6106841	33.1019368	1.273E-31	-1.08203	down	PREDICTED: uncharacterized protein LOC105161789, partial [Sesamum indicum]
c26946.graph_c1	0	0	0	1.60460352	1.78970219	1.41203697	1.999E-14	Inf	up	PREDICTED: protein NRT1/ PTR FAMILY 2.10-like [Sesamum indicum]
c26949.graph_c0	9.56422759	9.78193091	8.93848081	41.5150265	46.2784456	42.215991	2.734E-37	1.11065	up	PREDICTED: uncharacterized protein LOC105168910 isoform X1 [Sesamum indicum]
c26950.graph_c0	0.1146561	0.09564243	0.1718939	36.2769328	38.5858027	37.4100876	0	7.10144	up	PREDICTED: alpha-xylosidase 1 [Sesamum indicum]
c26952.graph_c0	0.46903035	0.26083334	0.73666091	4.99969877	5.57442616	4.91420019	1.881E-10	2.30115	up	hypothetical protein MIMGU_mgv1a006305mg [Erythranthe guttata]
c26954.graph_c0	0	0	0.13123956	2.59793204	2.74780717	1.48392393	2.917E-08	4.5688	up	PREDICTED: protein NRT1/ PTR FAMILY 4.4 [Sesamum indicum]
c26959.graph_c0	0.30637837	0.30668517	0	8.01626532	8.54478554	9.53926026	4.345E-13	4.3422	up	-
c26965.graph_c0	1.62333324	1.57079347	1.59930307	25.1500268	24.9984578	24.9668247	3.093E-94	2.87952	up	PREDICTED: ethylene-responsive transcription factor ERF118-like [Sesamum indicum]
c26968.graph_c0	4.9854943	4.18394324	4.14161296	54.6076298	59.7483044	55.7888216	8.727E-96	2.58733	up	PREDICTED: V-type proton ATPase subunit E [Sesamum indicum]
c26969.graph_c0	0.10425848	0.27830102	0.13397628	5.68308489	5.5189905	5.13200162	7.612E-25	3.89261	up	PREDICTED: protein trichome birefringence-like 5 [Sesamum indicum]
c26972.graph_c0	4.54346189	2.99755302	3.71542717	2.55167057	2.91378027	3.42941093	2.908E-05	-1.4275	down	PREDICTED: telomere repeat-binding factor 4-like isoform X2 [Sesamum indicum]

#ID	0 h-1 FPKM	0 h-2 FPKM	0 h-3 FPKM	28 h-1 FPKM	28 h-2 FPKM	28 h-3 FPKM	FDR	log2FC	regulated	nr_annotation
c26974.graph_c0	299.484742	322.388712	290.318029	67.2390976	69.8890068	70.6964896	0	-3.22378	down	PREDICTED: WW domain-containing protein C11B10.08 [Sesamum indicum]
c26976.graph_c0	0.15704198	0.39299808	0.45406161	3.02463592	3.4781128	2.70091577	6.081E-08	2.09499	up	PREDICTED: 12-oxophytodienoate reductase 3 [Sesamum indicum]
c26982.graph_c0	0.14325226	0.14339571	0.18408483	1.35349393	1.39128894	1.17240554	1.365E-05	1.96205	up	PREDICTED: pentatricopeptide repeat-containing protein At2g22410, mitochondrial [Sesamum indicum]
c26985.graph_c0	2.9476693	2.74944225	2.84090382	56.2853526	54.1615295	52.2057976	2.34E-162	3.16127	up	hypothetical protein MIMGU_mgv1a004588mg [Erythranthe guttata]
c26992.graph_c0	2.53283036	2.30487874	2.36711803	9.78995644	11.3325805	12.0852037	0.0004907	1.11511	up	hypothetical protein L484_015058 [Morus notabilis]
c26994.graph_c0	1.21261836	0.58957584	0.97947935	4.60806566	4.81998942	4.17108864	2.079E-05	1.19971	up	PREDICTED: plastidal glycolate/glycerate translocator 1, chloroplastic [Sesamum indicum]
c26995.graph_c0	36.6078112	32.13438	37.9441597	168.000072	171.434721	172.730179	4.306E-41	1.17188	up	hypothetical protein PHAVU_003G132300g [Phaseolus vulgaris]
c27002.graph_c0	4.82077705	4.53136017	3.24853955	22.688678	23.4563754	22.9417412	4.692E-15	1.36912	up	PREDICTED: ER membrane protein complex subunit 4-like [Fragaria vesca subsp. vesca]
c27004.graph_c1	4.7158969	4.50105548	3.94612089	43.919655	38.3598751	41.1715146	7.707E-28	2.14115	up	PREDICTED: membrin-11 [Sesamum indicum]
c27007.graph_c0	1.44852102	1.20830958	1.08582067	10.9664242	8.55502809	9.16327692	1.556E-06	1.85086	up	-
c27014.graph_c0	1.38727706	1.41589495	1.36324572	19.1372645	20.7599772	21.084626	3.325E-75	2.78144	up	PREDICTED: lysosomal beta glucosidase-like [Sesamum indicum]
c27015.graph_c0	3.40258416	5.03494371	2.70902125	3.06434299	3.858978	3.70685024	0.0069311	-1.15505	down	PREDICTED: uncharacterized protein LOC105163552 [Sesamum indicum]
c27017.graph_c0	26.4437438	25.7548118	24.9051826	14.5136745	13.7390204	13.7379263	8.126E-82	-1.96664	down	PREDICTED: monoglyceride lipase [Sesamum indicum]
c27018.graph_c0	3.2162122	3.01172741	3.11083011	2.31238982	2.22403671	2.35861401	7.714E-11	-1.52774	down	PREDICTED: vesicle-associated protein 2-2-like [Sesamum indicum]
c27022.graph_c1	11.7363788	12.7271419	10.6444051	9.4293471	8.99844897	8.93719081	9.452E-42	-1.44862	down	PREDICTED: uncharacterized protein LOC105173472 isoform X1 [Sesamum indicum]
c27023.graph_c0	0	0.06195051	0.07952923	12.4145451	13.3210429	12.7361163	1.318E-45	6.97781	up	PREDICTED: UPF0483 protein C25G4.2-like [Sesamum indicum]
c27028.graph_c0	3.09944386	4.48145748	4.42545342	0.12514766	0.56498303	0.10211947	5.216E-22	-5.0216	down	PREDICTED: protein UPSTREAM OF FLC-like [Sesamum indicum]
c27031.graph_c0	17.3178179	21.69316	18.0270494	17.1570035	13.7134908	15.3196445	6.316E-14	-1.39454	down	PREDICTED: uncharacterized protein LOC104093193 [Nicotiana tomentosiformis]
c27035.graph_c0	8.47105347	7.64548323	6.87044434	4.23904818	5.42223934	6.25919479	3.072E-14	-1.61691	down	hypothetical protein MIMGU_mgv1a013760mg [Erythranthe guttata]
c27037.graph_c0	1.9051786	2.20979847	1.90417884	11.3191509	12.2840033	10.097197	3.267E-15	1.39423	up	PREDICTED: metal transporter Nramp5-like isoform X2 [Sesamum indicum]
c27038.graph_c0	0.51375486	0.42855776	0.22006511	2.24036442	1.6576042	1.95507179	0.0049947	1.25138	up	PREDICTED: uncharacterized protein LOC105163339 [Sesamum indicum]
c27043.graph_c0	1.68280096	2.52672905	2.04232887	0.67947224	0.49079978	0.60988818	5.677E-12	-2.90345	down	PREDICTED: lysine histidine transporter 1-like [Musa acuminata subsp. malaccensis]
c27050.graph_c0	14.2419932	14.3755536	15.5448101	8.14220809	8.79851789	10.5314077	5.405E-33	-1.77554	down	hypothetical protein MIMGU_mgv1a016029mg [Erythranthe guttata]
c27052.graph_c0	282.016234	255.957385	280.989177	28.6521853	27.1735234	45.1992721	3.08E-205	-4.10736	down	PREDICTED: L-idonate 5-dehydrogenase-like [Cucumis sativus]

#ID	0 h-1 FPKM	0 h-2 FPKM	0 h-3 FPKM	28 h-1 FPKM	28 h-2 FPKM	28 h-3 FPKM	FDR	log2FC	regulated	nr_annotation
c27054.graph_c0	0.29370099	0.17639705	0.07548348	1.66498945	1.50332937	1.49796321	5.32E-05	2.01992	up	PREDICTED: uncharacterized protein LOC105156067 [Sesamum indicum]
c27056.graph_c0	0	0	0	3.95788071	4.31162029	4.51090638	4.549E-35	Inf	up	PREDICTED: probable polygalacturonase non-catalytic subunit JP650 [Sesamum indicum]
c27058.graph_c0	0.25070779	0.32266135	0.32216943	1.40564294	1.69221838	1.5080836	0.0027481	1.26933	up	PREDICTED: pentatricopeptide repeat-containing protein At5g42450, mitochondrial [Sesamum indicum]
c27063.graph_c0	0.33207294	0.16620273	0.24892401	7.6427087	7.40911829	6.48256109	8.248E-38	3.76029	up	PREDICTED: uncharacterized protein LOC105165907 [Sesamum indicum]
c27067.graph_c0	28.0643843	29.9903109	25.566529	29.4812826	28.2162713	28.9982683	1.559E-33	-1.03699	down	PREDICTED: probable beta-1,3-galactosyltransferase 19 [Sesamum indicum]
c27068.graph_c0	0	0	0	6.84984501	8.21558709	12.6804698	3.366E-10	Inf	up	--
c27069.graph_c0	2.4004483	2.24266185	1.64515792	2.67510238	1.57523911	2.37267576	0.0040544	-1.01012	down	hypothetical protein MIMGU_mgv1a001232mg [Erythranthe guttata]
c27070.graph_c0	0.27931492	0.59413856	0.35893074	2.66443376	2.88688064	3.4786499	1.15E-06	1.78344	up	PREDICTED: uncharacterized protein LOC105177060 [Sesamum indicum]
c27071.graph_c0	2.01053102	3.45007588	2.46058282	22.8696027	21.5496258	21.0650591	2.621E-43	1.95594	up	PREDICTED: uncharacterized protein LOC105167501 [Sesamum indicum]
c27072.graph_c0	110.208019	110.008119	100.540574	53.2595845	48.1465241	49.3940212	1.91E-155	-2.17822	down	PREDICTED: growth-regulating factor 8 isoform X2 [Sesamum indicum]
c27073.graph_c0	2.75900836	1.87405895	2.91232159	13.0340039	13.4496954	12.5640811	1.797E-10	1.27889	up	PREDICTED: uncharacterized protein LOC105170966 [Sesamum indicum]
c27077.graph_c0	0.89516456	0.83426362	0.79332544	9.40005463	10.2901741	8.54907343	9.841E-27	2.39474	up	PREDICTED: LOW QUALITY PROTEIN: 4-coumarate--CoA ligase-like 7 [Sesamum indicum]
c27079.graph_c0	6.95490107	6.42113795	7.07176547	213.184205	204.360596	212.119694	0	3.85367	up	hypothetical protein MIMGU_mgv1a008423mg [Erythranthe guttata]
c27082.graph_c0	32.4560732	31.4931015	34.4463121	16.9853127	18.5101633	18.1223917	7.12E-70	-1.9673	down	PREDICTED: probable BOI-related E3 ubiquitin-protein ligase 2 [Sesamum indicum]
c27085.graph_c0	31.8839211	24.8918969	25.3109504	0.35788494	0.32313655	0.29203119	7.24E-99	-7.48719	down	-
c27086.graph_c0	0	0	0	3.23981534	3.16902029	2.20305239	1.194E-15	Inf	up	PREDICTED: flavonoid 3',5'-methyltransferase-like [Sesamum indicum]
c27087.graph_c0	0.41980472	0.14007503	0.26973284	6.63617837	6.84126577	6.14122582	3.565E-26	3.47692	up	PREDICTED: 3-ketoacyl-CoA thiolase 2, peroxisomal [Sesamum indicum]
c27090.graph_c0	0.21709742	0.21731481	0.09299293	3.05051294	2.89679237	2.44627651	6.146E-08	2.91038	up	PREDICTED: uncharacterized protein LOC103992241 isoform X1 [Musa acuminata subsp. malaccensis]
c27097.graph_c0	9.88172009	5.50240971	5.83881486	0	0	0	8.555E-27	-Inf	down	--
c27099.graph_c0	0.32293863	0.25860961	0.74698007	7.65154396	6.9086268	6.70324873	1.382E-14	2.89644	up	PREDICTED: GDSL esterase/lipase CPRD49-like [Sesamum indicum]
c27099.graph_c1	0.5003733	0	0	6.18236332	6.23881141	6.13284303	1.034E-10	4.15363	up	PREDICTED: GDSL esterase/lipase CPRD49-like [Sesamum indicum]
c27102.graph_c0	11.3355401	8.54902738	10.5757636	46.798287	49.9648863	49.3914109	4.889E-26	1.17217	up	PREDICTED: ATP-dependent Clp protease proteolytic subunit 3, chloroplastic [Sesamum indicum]

#ID	0 h-1 FPKM	0 h-2 FPKM	0 h-3 FPKM	28 h-1 FPKM	28 h-2 FPKM	28 h-3 FPKM	FDR	log2FC	regulated	nr_annotation
c27104.graph_c0	0.30035216	0.60130584	0.77192884	5.08313682	5.51877797	5.92906114	5.053E-12	2.20439	up	PREDICTED: protein GLUTAMINE DUMPER 5-like [Sesamum indicum]
c27105.graph_c0	0.04911234	0.14748455	0.0631113	4.53320953	4.80209775	5.18451204	6.848E-20	4.71609	up	PREDICTED: squamosa promoter-binding-like protein 9 [Sesamum indicum]
c27114.graph_c0	5.58817179	5.91524839	4.95242896	39.4941102	44.0474647	44.0357723	2.652E-39	1.86604	up	PREDICTED: 14-3-3-like protein 16R [Sesamum indicum]
c27115.graph_c0	10.8440727	11.3338255	13.7301354	7.76550649	8.16267005	8.98471699	6.453E-10	-1.62052	down	-
c27116.graph_c0	2.22823056	1.21661552	2.16921552	20.169702	20.4711524	19.4616538	1.142E-25	2.32837	up	PREDICTED: growth-regulating factor 6-like [Sesamum indicum]
c27117.graph_c0	5.15371226	5.6610641	6.0366425	61.8535796	51.4483831	56.8283523	7.58E-44	2.24349	up	PREDICTED: very-long-chain 3-oxoacyl-CoA reductase 1-like [Sesamum indicum]
c27118.graph_c0	26.4244873	27.8541252	23.3635861	21.8053893	23.6011533	22.7189811	9.867E-37	-1.27752	down	PREDICTED: LOW QUALITY PROTEIN: putative SWI/SNF-related matrix-associated actin-dependent regulator of chromatin subfamily A member 3-like 1 [Sesamum indicum]
c27119.graph_c0	4.41421521	2.70027718	3.62405973	18.6255353	16.8975767	15.9981944	1.848E-05	1.17296	up	-
c27120.graph_c0	0.65255488	0.6205479	0.37735148	4.90872684	4.90316668	5.7082942	7.276E-13	2.14995	up	PREDICTED: aureusidin synthase-like [Sesamum indicum]
c27121.graph_c0	172.753427	189.200933	170.704446	23.8007952	25.3833484	26.4586033	0	-3.90576	down	PREDICTED: uncharacterized protein LOC105159027 [Sesamum indicum]
c27129.graph_c0	0	0	0	2.50583191	3.28226367	3.11030626	2.635E-18	Inf	up	PREDICTED: kinesin-like protein NACK1 [Sesamum indicum]
c27130.graph_c0	0.02561602	0	0	0.59576243	0.75644665	1.33687751	3.663E-05	5.65841	up	gelsolin with villin headpeace [Emiliania huxleyi CCMP1516]
c27132.graph_c0	1.13231781	1.29537332	1.03933801	13.402525	14.9673027	17.4597883	2.034E-19	2.63639	up	hypothetical protein MIMGU_mgv1a012883mg [Erythranthe guttata]
c27143.graph_c0	138.692907	145.008625	139.333365	106.033471	120.130817	112.052232	1.228E-66	-1.41347	down	hypothetical protein MIMGU_mgv1a010222mg [Erythranthe guttata]
c27150.graph_c0	1.68049299	1.17361099	1.65729072	0	0	0	7.328E-37	-Inf	down	mRNA cleavage factor complex subunit [Schizosaccharomyces japonicus yFS275]
c27151.graph_c0	138.37461	137.403401	140.775385	26.4392496	29.5105411	27.1218662	0	-3.41712	down	PREDICTED: probable protein phosphatase 2C 63 [Sesamum indicum]
c27152.graph_c0	1.44769156	1.16736375	1.55028414	0	0	0	1.235E-36	-Inf	down	hypothetical protein KNAG_0E02970 [Kazachstania naganishii CBS 8797]
c27155.graph_c1	3.96400649	4.21597433	3.37471225	29.4764059	28.223978	25.6393548	3.224E-54	1.7617	up	PREDICTED: plant intracellular Ras-group-related LRR protein 5-like [Sesamum indicum]
c27157.graph_c0	1.54452424	1.11660672	0.90049968	13.9383455	13.7675042	12.518567	5.752E-74	2.41266	up	PREDICTED: serine/threonine-protein kinase D6PKL1-like [Sesamum indicum]
c27158.graph_c0	562.953248	538.606693	581.511915	143.561816	171.265479	175.764025	1.99E-151	-2.86924	down	hypothetical protein MIMGU_mgv1a015139mg [Erythranthe guttata]
c27160.graph_c0	0.41872408	0.7335009	0.58291677	7.50671414	7.85407315	7.11872837	1.666E-21	2.60234	up	PREDICTED: NAC domain-containing protein 8 [Sesamum indicum]
c27165.graph_c0	13.2215243	13.8147732	12.6805875	6.84010573	7.086239	6.95601165	1.838E-96	-2.01738	down	PREDICTED: uncharacterized protein LOC105161407 [Sesamum indicum]
c27166.graph_c0	19.0175871	20.5880738	16.9022966	7.00227032	7.00961027	7.85025803	5.871E-67	-2.45826	down	PREDICTED: probable LRR receptor-like serine/threonine-protein kinase RLK [Sesamum indicum]

#ID	0 h-1 FPKM	0 h-2 FPKM	0 h-3 FPKM	28 h-1 FPKM	28 h-2 FPKM	28 h-3 FPKM	FDR	log2FC	regulated	nr_annotation
c27168.graph_c0	28.9551007	29.3347089	31.2070877	33.4334424	29.3444721	29.9818607	1.335E-14	-1.03996	down	PREDICTED: NAC domain-containing protein 78 isoform X2 [Sesamum indicum]
c27171.graph_c0	0	0.10623295	0.06818849	2.50679837	2.40269029	2.29728417	5.87E-12	4.2676	up	PREDICTED: short-chain dehydrogenase TIC 32, chloroplastic [Sesamum indicum]
c27176.graph_c0	3.62223083	3.77693536	2.61827439	1.64538531	3.11982019	1.74540726	5.476E-08	-1.71006	down	PREDICTED: phospholipase A1-Ibeta2, chloroplastic [Sesamum indicum]
c27178.graph_c0	83.9686391	74.4664161	82.4040443	58.5778968	64.8811739	61.4072852	1.441E-43	-1.47234	down	PREDICTED: multiprotein-bridging factor 1b-like [Sesamum indicum]
c27180.graph_c0	0.1196463	0	0	7.79725549	8.76752366	9.12982813	1.264E-45	6.68803	up	PREDICTED: uncharacterized protein LOC105169888 [Sesamum indicum]
c27182.graph_c0	0	0	0	2.31477376	2.56898743	2.59715016	1.513E-14	Inf	up	PREDICTED: heme oxygenase 1, chloroplastic-like [Sesamum indicum]
c27183.graph_c0	1.22659341	1.49640765	1.42845037	8.85909268	9.30693121	8.04731867	7.301E-12	1.56607	up	PREDICTED: putative MO25-like protein At5g47540 [Sesamum indicum]
c27185.graph_c0	49.7589501	46.0797769	48.726024	18.6624385	20.6046736	20.9094576	7.606E-74	-2.35471	down	hypothetical protein MIMGU_mgv1a015987mg [Erythranthe guttata]
c27186.graph_c0	43.0350575	40.4286972	41.5707937	30.3514149	31.0069491	31.9755368	1.41E-40	-1.51194	down	PREDICTED: homeobox-leucine zipper protein HAT4-like [Sesamum indicum]
c27187.graph_c0	5.13882144	4.53879458	5.16078805	28.7490511	27.9130264	31.0396255	6.653E-29	1.47277	up	biotin carboxyl carrier protein subunit [Camellia chekiangoleosa]
c27197.graph_c0	0.03628889	0.01816261	0.09326532	0.51430385	0.35720627	0.40890703	1.227E-05	2.0087	up	PREDICTED: uncharacterized protein LOC105158016 [Sesamum indicum]
c27200.graph_c0	0.43515993	0.47519528	0.47447081	5.2424435	5.20072103	5.5525259	7.634E-36	2.43878	up	hypothetical protein MIMGU_mgv1a000304mg [Erythranthe guttata]
c27204.graph_c0	0	0.11023399	0.09434223	16.5410391	14.0678135	13.9545212	8.569E-85	6.66255	up	PREDICTED: serine/threonine-protein kinase-like protein ACR4 [Sesamum indicum]
c27212.graph_c0	48.8195597	54.169429	46.8386391	17.5905408	13.6291791	16.1448878	1.04E-105	-2.75073	down	PREDICTED: uncharacterized protein LOC105171942 [Sesamum indicum]
c27216.graph_c0	0.52483189	0.85370582	0.33721488	7.24745106	6.1563088	5.36916317	1.035E-09	2.36643	up	PREDICTED: GDSL esterase/lipase CPRD49-like isoform X1 [Sesamum indicum]
c27217.graph_c0	0.4430605	0.49894218	0.35584393	3.22013819	2.76210872	2.95605727	7.744E-05	1.6965	up	-
c27218.graph_c0	0.89082739	1.48619903	1.14474846	46.0407617	46.875059	45.2979011	1.47E-197	4.20237	up	PREDICTED: glucan endo-1,3-beta-glucosidase 13-like [Sesamum indicum]
c27220.graph_c0	1.11348969	0.97950108	0.82383904	0.22071215	0.11071243	0.14007724	2.066E-18	-3.71618	down	-
c27222.graph_c0	1.88874381	1.96477765	1.80843533	19.8910996	20.0255392	18.4712447	1.056E-41	2.27606	up	hypothetical protein MIMGU_mgv1a006093mg [Erythranthe guttata]
c27224.graph_c0	19.5930506	17.7634754	15.1426758	13.1009069	12.5819722	14.1263794	6.16E-22	-1.48614	down	PREDICTED: uncharacterized protein LOC105158173 [Sesamum indicum]
c27230.graph_c0	5.36102275	5.9030301	4.97001268	71.9991746	82.0710377	72.9217709	3.39E-101	2.71588	up	PREDICTED: BAG family molecular chaperone regulator 7 [Sesamum indicum]
c27232.graph_c0	0.97101237	1.20340962	1.30720789	6.11629371	5.18866593	4.85373458	0.0001188	1.12009	up	1-aminocyclopropane-1-carboxylate oxidase-like protein [Medicago truncatula]

#ID	0 h-1 FPKM	0 h-2 FPKM	0 h-3 FPKM	28 h-1 FPKM	28 h-2 FPKM	28 h-3 FPKM	FDR	log2FC	regulated	nr_annotation
c27233.graph_c1	15.7967045	15.1800216	15.3695387	4.59238152	3.58375202	3.55997853	4.35E-106	-3.07221	down	hypothetical protein MIMGU_mgv1a022117mg [Erythranthe guttata]
c27235.graph_c1	0.38645154	0.25789234	0.33107050	5.43016786	7.26986536	5.11853735	3.599E-09	3.10051	up	PREDICTED: enolase-like [Sesamum indicum]
c27237.graph_c0	6.85949209	6.67294221	6.60483163	6.12296209	6.1244186	6.02763072	6.467E-25	-1.2302	down	PREDICTED: U-box domain-containing protein 45 [Sesamum indicum]
c27239.graph_c0	0.11329592	0.34022811	0.14558974	3.95245549	2.00739191	2.88921605	0.0001888	2.79571	up	hypothetical protein MIMGU_mgv1a019829mg [Erythranthe guttata]
c27244.graph_c0	18.9355193	17.0427624	17.9624782	120.58191	124.393326	125.432282	7.496E-87	1.68962	up	PREDICTED: ATP synthase subunit gamma, mitochondrial [Sesamum indicum]
c27255.graph_c0	0.4255344	0.73021801	0.54682855	5.30187336	3.15150391	4.65075677	4.075E-05	1.85331	up	PREDICTED: uncharacterized protein LOC105159742 [Sesamum indicum]
c27256.graph_c0	2.70946149	1.59539683	1.63847778	8.45606663	9.41305726	8.97955788	0.0066662	1.09047	up	hypothetical protein M569_10374, partial [Genlisea aurea]
c27258.graph_c0	3.07647529	2.5662966	1.67719684	0.3387825	0.45883325	0.46995438	7.785E-12	-3.61269	down	-
c27260.graph_c0	0	0	0.11933787	2.26109111	1.43213438	2.5610144	1.172E-08	4.58354	up	PREDICTED: WAT1-related protein At1g43650-like [Sesamum indicum]
c27262.graph_c0	7.37727859	7.68005245	7.58407605	48.9623633	47.9104944	47.7355177	1.953E-75	1.58427	up	PREDICTED: ER membrane protein complex subunit 1 [Sesamum indicum]
c27264.graph_c0	0.75378519	0.51739885	1.27307472	0.26609698	0.14132978	0.22990549	0.0014645	-3.09555	down	PREDICTED: probable leucine-rich repeat receptor-like protein kinase At5g63930 [Sesamum indicum]
c27265.graph_c0	5.21926027	5.5443531	3.55879303	28.1015953	26.6312763	27.2262252	4.471E-11	1.43097	up	PREDICTED: probable methyltransferase PMT11 [Sesamum indicum]
c27266.graph_c0	0.04928634	0.09867138	0	8.8119722	11.837548	11.4580283	9.581E-37	6.68772	up	PREDICTED: palmitoyl-protein thioesterase 1 [Sesamum indicum]
c27273.graph_c0	2.51463262	2.30738809	2.87235776	35.486298	36.349577	32.5191353	5.417E-49	2.6684	up	unnamed protein product [Coffea canephora]
c27276.graph_c0	0.29370078	0.04199927	0.10783346	4.29969494	4.06118905	3.12282803	9.928E-21	3.61444	up	hypothetical protein PHAVU_002G220200g [Phaseolus vulgaris]
c27279.graph_c0	2.68775679	2.12993814	2.15867118	0	0	0	2.335E-28	-Inf	down	PREDICTED: inorganic phosphate transporter 1-11-like [Sesamum indicum]
c27280.graph_c0	4.0199322	2.12720223	2.00259004	0	0	0	2.392E-18	-Inf	down	predicted protein [Hordeum vulgare subsp. vulgare]
c27282.graph_c0	806.354253	753.647759	837.92116	198.994044	247.777846	231.646503	3.03E-105	-2.91283	down	hypothetical protein MIMGU_mgv1a005561mg [Erythranthe guttata]
c27283.graph_c0	45.0235688	47.2002786	43.4709866	345.889583	336.54131	314.645822	1.46E-108	1.78703	up	PREDICTED: blue copper protein-like [Nicotiana tomentosiformis]
c27284.graph_c0	4.86722414	5.10410259	6.15529674	0.4492026	0.35488935	0.50400027	9.955E-41	-4.71621	down	-
c27285.graph_c0	0.16678156	0.38954664	0.2857612	5.17187725	5.03454214	5.275262	2.04E-13	3.10675	up	PREDICTED: protein XRI1-like [Sesamum indicum]
c27290.graph_c0	1.76571973	1.61153302	1.93534039	13.5502958	13.1207214	15.030034	1.019E-17	1.88112	up	-
c27300.graph_c0	3.3095266	2.98155653	2.4099612	2.04454313	2.46137434	2.06087885	2.366E-07	-1.49332	down	PREDICTED: uncharacterized protein LOC105168317 [Sesamum indicum]
c27312.graph_c0	2.18560687	2.22907458	1.90772243	9.74071896	10.3915787	10.5407311	1.42E-08	1.18987	up	PREDICTED: uncharacterized protein LOC105166961 [Sesamum indicum]
c27313.graph_c0	10.1805561	7.69113235	11.3545511	41.4632705	48.0258142	45.4533413	6.673E-08	1.11404	up	unnamed protein product [Coffea canephora]

#ID	0 h-1 FPKM	0 h-2 FPKM	0 h-3 FPKM	28 h-1 FPKM	28 h-2 FPKM	28 h-3 FPKM	FDR	log2FC	regulated	nr_annotation
c27314.graph_c0	6.78563859	7.7220821	6.64091813	40.292395	40.5284493	40.4559903	9.911E-62	1.42978	up	PREDICTED: carbamoyl-phosphate synthase large chain, chloroplastic [Sesamum indicum]
c27317.graph_c0	0.08865708	0.17749172	0	5.02596563	5.58520091	6.99298128	1.168E-15	4.97471	up	PREDICTED: transcription factor bHLH79-like [Sesamum indicum]
c27318.graph_c0	28.8449762	32.9883852	29.8388838	2.83018893	4.02238141	3.37858284	1.58E-158	-4.25449	down	hypothetical protein MIMGU_mgv1a022206mg [Erythranthe guttata]
c27319.graph_c0	0.40234334	0.33562186	0.34468475	2.7048926	4.24645974	3.28092878	8.342E-08	2.15085	up	hypothetical protein MIMGU_mgv1a003421mg [Erythranthe guttata]
c27323.graph_c0	3.3804496	4.59854447	4.12124175	27.0888014	34.8109127	28.3242712	1.245E-15	1.80571	up	1-deoxy-D-xylulose 5-phosphate reductoisomerase [Lavandula angustifolia]
c27324.graph_c0	42.3037815	43.5208323	39.1715604	28.1458279	25.8450466	26.0731353	1.859E-94	-1.7322	down	PREDICTED: lysM domain receptor-like kinase 3 [Sesamum indicum]
c27328.graph_c0	0.37027189	0.48468656	0.47581404	2.09078915	1.88778658	1.72295863	0.0057921	1.00554	up	PREDICTED: protein ROOT PRIMORDIUM DEFECTIVE 1 [Sesamum indicum]
c27330.graph_c0	8.78339159	8.31693348	7.62635536	4.83092202	4.67343176	3.51963653	1.216E-08	-2.01506	down	PREDICTED: T-complex protein 1 subunit alpha [Sesamum indicum]
c27332.graph_c0	0	0	0	1.90356985	2.3680488	3.00304174	2.391E-13	Inf	up	predicted protein [Hordeum vulgare subsp. vulgare]
c27334.graph_c0	0.65362323	0.73606245	0.20998293	3.08782238	3.91394287	3.5371839	0.0004119	1.64082	up	PREDICTED: uncharacterized protein LOC105170524 [Sesamum indicum]
c27335.graph_c0	8.7574214	8.31014604	8.97688662	38.8880895	45.5097911	42.7501282	9.916E-25	1.19634	up	PREDICTED: L-ascorbate oxidase homolog [Sesamum indicum]
c27337.graph_c0	0.04464943	0.04469414	0	0.97352836	0.87900483	1.16510708	5.257E-09	4.00881	up	PREDICTED: uncharacterized protein LOC105180005 [Sesamum indicum]
c27340.graph_c0	100.811686	100.912633	98.9307675	478.992997	505.929895	498.561832	2.635E-53	1.21252	up	PREDICTED: 60S ribosomal protein L22-2-like [Sesamum indicum]
c27341.graph_c0	1.52639033	0.9822335	0.84063086	8.08256522	10.0165863	8.27646395	9.898E-07	1.89292	up	hypothetical protein MIMGU_mgv1a004267mg [Erythranthe guttata]
c27342.graph_c0	4.64636634	3.89387635	4.95249476	35.2616645	34.745231	36.8690389	4.736E-57	1.89399	up	PREDICTED: pyruvate dehydrogenase E1 component subunit beta-like [Sesamum indicum]
c27344.graph_c0	4.76048247	6.31913503	4.78753704	4.249649	7.02890409	4.35761417	0.0001252	-1.11257	down	PREDICTED: UDP-glycosyltransferase 86A1-like [Sesamum indicum]
c27346.graph_c0	4.71467144	5.16784153	5.42801273	25.7692425	24.4991681	22.4698104	5.814E-29	1.15555	up	PREDICTED: filament-like plant protein 4 [Sesamum indicum]
c27349.graph_c0	4.4570799	2.43563826	3.0683165	0	0	0	1.215E-32	-Inf	down	alpha-2 macroglobulin family-like protein [Micromonas sp. RCC299]
c27350.graph_c0	0.07439785	0	0	6.0019843	4.93101024	6.17711553	1.028E-19	6.78725	up	-
c27353.graph_c0	64.6405875	67.1728908	61.3753555	48.0255214	50.1477699	50.0109384	1.311E-72	-1.47232	down	PREDICTED: xyloglucan 6-xylosyltransferase 2-like [Sesamum indicum]
c27356.graph_c0	6.4844187	5.75435449	5.20057205	42.2484213	43.9407476	40.3928301	3.063E-47	1.77135	up	PREDICTED: protein TRIGALACTOSYLDIACYLGLYCEROL 1, chloroplastic-like [Sesamum indicum]
c27361.graph_c0	11.4866023	11.9153744	10.7729585	1.95244754	2.18839912	1.81293039	1.417E-96	-3.61139	down	unnamed protein product [Coffea canephora]
c27363.graph_c0	43.6070316	38.3520579	51.9878631	11.541505	14.2253514	10.5389374	3.708E-19	-2.97793	down	-
c27369.graph_c0	4.71124535	3.75678404	5.13062441	2.90178472	3.30124981	3.93060217	2.635E-07	-1.51565	down	-
c27373.graph_c0	0.15111292	0.09075854	0.34953496	1.82314483	2.24111517	2.16877381	1.708E-08	2.29299	up	PREDICTED: probable glycerol-3-phosphate dehydrogenase [NAD(+)] 1, cytosolic [Sesamum indicum]

#ID	0 h-1 FPKM	0 h-2 FPKM	0 h-3 FPKM	28 h-1 FPKM	28 h-2 FPKM	28 h-3 FPKM	FDR	log2FC	regulated	nr_annotation
c27376.graph_c0	15.7918563	20.2768535	14.5558272	14.9627231	14.4865579	15.0534304	7.621E-08	-1.27459	down	-
c27382.graph_c0	0.38009145	0.35120497	0.41328909	24.6923458	24.7315761	21.9347435	7.07E-141	4.86969	up	PREDICTED: protein NRT1/ PTR FAMILY 5.2-like [Sesamum indicum]
c27384.graph_c0	54.5700025	54.3273114	49.4034993	12.6755351	13.8117533	15.4266195	2.57E-235	-3.00574	down	unnamed protein product [Coffea canephora]
c27389.graph_c0	2.8235241	2.42258694	2.07333729	0.87948045	0.49157852	0.95686515	5.929E-15	-2.73747	down	PREDICTED: expansin-A18-like [Sesamum indicum]
c27391.graph_c0	4.0257316	3.45408237	4.2230404	0.47769445	1.4556824	1.26683301	4.031E-17	-2.96176	down	unnamed protein product [Coffea canephora]
c27391.graph_c1	4.07624457	3.62695672	3.37568742	0.79002428	1.3077494	1.02070116	9.246E-16	-2.91785	down	serine glyoxylate aminotransferase 2 [Glycine max]
c27395.graph_c0	0	0.1379293	0	4.75693214	4.38548611	4.33106826	2.218E-16	5.5282	up	PREDICTED: uncharacterized protein LOC100247103 isoform X1 [Vitis vinifera]
c27396.graph_c0	1665.78492	1427.69534	1516.48269	1220.08575	1249.27861	1362.20893	3.002E-36	-1.35607	down	PREDICTED: abscisic acid 8'-hydroxylase 2 isoform X2 [Sesamum indicum]
c27400.graph_c0	9.40437917	11.4977664	8.25654551	60.6545342	59.170141	53.3679426	5.149E-34	1.48153	up	PREDICTED: ferredoxin--NADP reductase, root-type isozyme, chloroplastic-like [Sesamum indicum]
c27405.graph_c0	259.280677	273.745016	369.65664	291.284753	262.337665	273.633429	0.000318	-1.22116	down	ATP synthase subunit alpha [Medicago truncatula]
c27411.graph_c0	18.4209297	19.7246235	18.1830397	17.6732195	15.5273771	18.3373052	1.063E-38	-1.21781	down	PREDICTED: ankyrin repeat and zinc finger domain-containing protein 1 [Sesamum indicum]
c27412.graph_c0	0.63426819	0.81267624	0.45643345	3.13468077	2.91356762	3.05440237	0.000117	1.17188	up	PREDICTED: uncharacterized protein LOC105164594 [Sesamum indicum]
c27414.graph_c0	18.8857477	16.6935877	16.0846733	9.17745815	9.56678763	7.16123538	5.581E-74	-2.08592	down	PREDICTED: LOW QUALITY PROTEIN: BAG family molecular chaperone regulator 2 [Sesamum indicum]
c27419.graph_c1	59.3911928	52.2657175	56.158485	30.7465322	27.8446029	27.1231559	2.781E-90	-2.05969	down	PREDICTED: uncharacterized protein LOC105161274 [Sesamum indicum]
c27420.graph_c1	1.15536398	1.67882067	1.10154305	7.47614452	6.89700228	7.89082905	6.302E-09	1.41171	up	PREDICTED: uncharacterized protein LOC105174515 [Sesamum indicum]
c27427.graph_c0	1.58099053	1.0550491	1.40279588	0	0	0	1.43E-36	-Inf	down	DEAD-domain-containing protein [Punctularia strigosozonata HHB-11173 SS5]
c27428.graph_c0	0	0.14587534	0.2809021	4.71323069	5.40318439	3.32740165	3.714E-13	3.86154	up	PREDICTED: U-box domain-containing protein 26-like [Sesamum indicum]
c27434.graph_c0	1.41243565	1.18580967	0.93679264	8.11305718	9.86677097	8.48464964	3.558E-12	1.8181	up	PREDICTED: putative thiol protease ulp-4 [Sesamum indicum]
c27437.graph_c0	0.22450844	0.0642095	0.08242921	7.15623327	6.86129936	6.60026542	8.21E-38	4.71656	up	hypothetical protein PHAVU_010G112100g [Phaseolus vulgaris]
c27438.graph_c0	0.13861709	0.13875589	0	5.33954335	4.54821417	4.68585575	1.838E-11	4.64495	up	unknown [Lotus japonicus]
c27438.graph_c1	0	0	0	9.62996924	12.2519881	11.889016	2.899E-32	Inf	up	PREDICTED: auxin-induced protein IAA6 [Sesamum indicum]
c27438.graph_c2	0.250355	0.50121139	0.32171609	7.15695847	8.87164509	7.07731197	2.95E-14	3.33622	up	PREDICTED: PGR5-like protein 1A, chloroplastic [Sesamum indicum]
c27439.graph_c1	0.40540756	0.20290676	0.39072358	10.1162439	11.7500739	10.939577	2.424E-49	3.94539	up	PREDICTED: uncharacterized protein LOC105174869 [Sesamum indicum]

#ID	0 h-1 FPKM	0 h-2 FPKM	0 h-3 FPKM	28 h-1 FPKM	28 h-2 FPKM	28 h-3 FPKM	FDR	log2FC	regulated	nr_annotation
c27440.graph_c0	0.04680039	0.09369451	0.12028072	0.8503564	0.82921547	1.02694814	2.277E-05	2.27633	up	hypothetical protein MIMGU_mgv1a018556mg, partial [Erythranthe guttata]
c27451.graph_c0	2.80558051	2.55308171	1.63876456	3.05861962	1.92478409	2.26891694	0.0056976	-1.03256	down	PREDICTED: cyclic dof factor 1 [Sesamum indicum]
c27455.graph_c0	22.7763983	21.2974614	20.1550058	15.0668908	18.3474862	13.9930365	6.343E-18	-1.52824	down	PREDICTED: pectinesterase 1 [Sesamum indicum]
c27456.graph_c0	716.622161	716.657582	798.109808	67.7173105	70.4622129	79.1345526	1.38E-130	-4.45147	down	PREDICTED: CBL-interacting protein kinase 2 [Sesamum indicum]
c27459.graph_c0	0.01683437	0	0	2.19008645	2.11000872	1.97678406	3.373E-29	7.48324	up	PREDICTED: filament-like plant protein 7 isoform X2 [Sesamum indicum]
c27464.graph_c0	0.26442882	0.1764624	0.11326717	2.85074925	3.61511137	3.50235064	1.925E-11	3.08818	up	PREDICTED: fasciclin-like arabinogalactan protein 2 [Sesamum indicum]
c27465.graph_c0	12.7363586	12.353177	11.9954879	9.96581833	7.2850808	8.14766488	1.395E-50	-1.63591	down	PREDICTED: uncharacterized protein LOC105174651 [Sesamum indicum]
c27466.graph_c0	29.8422622	31.4036274	28.7930768	26.6391965	23.5992797	23.1618082	6.561E-65	-1.38496	down	PREDICTED: flowering time control protein FPA [Sesamum indicum]
c27470.graph_c1	7.12226207	7.45102073	6.19334577	3.50283994	3.76014174	4.0016043	2.668E-22	-1.97067	down	PREDICTED: RING-H2 finger protein ATL43-like [Sesamum indicum]
c27470.graph_c2	0.07236581	0.07243827	0	2.68234758	2.51688517	2.87544783	9.102E-12	4.73145	up	-
c27474.graph_c0	19.1257073	23.0283355	19.6793256	1.08829176	1.6525973	1.29169046	2.92E-104	-5.02962	down	PREDICTED: uncharacterized protein LOC105176742 [Sesamum indicum]
c27475.graph_c0	38.3030693	36.6442182	41.4961418	177.393101	178.52787	181.453346	5.325E-39	1.11461	up	PREDICTED: 60S ribosomal protein L22-2 [Sesamum indicum]
c27476.graph_c0	0	0.08282376	0.31897602	2.10474646	2.44335692	2.50257864	9.85E-09	3.01324	up	hypothetical protein PRUPE_ppa003008mg [Prunus persica]
c27478.graph_c0	0.07219573	0.07226802	0.04638719	0.9182502	0.91200322	0.53520333	5.371E-06	2.54419	up	PREDICTED: probably inactive leucine-rich repeat receptor-like protein kinase At3g28040 [Sesamum indicum]
c27480.graph_c0	1.32360445	0.98520424	0.9594731	6.93124751	6.48098075	6.54145354	9.055E-10	1.52301	up	PREDICTED: pentatricopeptide repeat-containing protein At1g09190 [Sesamum indicum]
c27481.graph_c0	1.04726577	0.58239692	0.29906177	7.01946427	9.16325508	10.4895118	2.659E-09	2.71366	up	PREDICTED: 60S ribosomal protein L32-1-like [Brachypodium distachyon]
c27481.graph_c1	4.41970067	2.39640176	3.0763906	19.0055934	20.7856779	18.457191	6.265E-06	1.47047	up	PREDICTED: 60S ribosomal protein L32-1-like [Sesamum indicum]
c27482.graph_c0	4.83637779	8.06870114	5.14951917	3.95024835	3.71783541	3.93360455	0.000144	-1.72751	down	PREDICTED: uncharacterized protein LOC105173594 [Sesamum indicum]
c27486.graph_c0	28.0810388	28.8087069	25.2270163	15.8840344	18.3441548	15.071212	2.323E-57	-1.82591	down	PREDICTED: probable membrane-associated kinase regulator 1 [Solanum lycopersicum]
c27490.graph_c0	0	0.03700263	0	10.5584927	11.8620811	10.0406231	1.178E-65	8.69458	up	PREDICTED: cytochrome P450 76A1 [Sesamum indicum]
c27492.graph_c0	3.22592452	1.63941705	3.69900865	0.68533975	0.58622924	0.64753137	2.836E-05	-3.25209	down	PREDICTED: heat stress transcription factor A-6b-like [Sesamum indicum]
c27498.graph_c0	1.8214495	2.31415471	1.98053707	8.85940407	12.2286792	8.72489821	3.453E-05	1.19318	up	unnamed protein product [Coffea canephora]
c27501.graph_c0	37.1437689	32.9831121	34.2936872	27.4708779	29.2353991	30.1356473	8.038E-44	-1.35551	down	unknown [Picea sitchensis]

#ID	0 h-1 FPKM	0 h-2 FPKM	0 h-3 FPKM	28 h-1 FPKM	28 h-2 FPKM	28 h-3 FPKM	FDR	log2FC	regulated	nr_annotation
c27502.graph_c0	31.3811051	35.0796924	31.9688173	20.5165255	24.2272118	19.9582102	8.071E-56	-1.69643	down	PREDICTED: calcium-dependent protein kinase 32 isoform X1 [Sesamum indicum]
c27503.graph_c0	3.44282208	3.37080379	4.32728321	1.33330144	1.15437306	0.80479461	2.169E-19	-2.85419	down	hypothetical protein MIMGU_mgv1a026576mg, partial [Erythranthe guttata]
c27505.graph_c0	0	0	0	2.65120636	3.63987364	3.31913188	7.345E-19	Inf	up	plasma membrane intrinsic protein [Olea europaea]
c27507.graph_c0	1.36373328	0.85950668	0.71396162	15.4546525	15.2467625	14.3482348	8.761E-32	2.85553	up	PREDICTED: uncharacterized protein LOC105172180 [Sesamum indicum]
c27508.graph_c0	0.25709585	0.20016367	0.25696094	3.52949812	3.11182221	4.04899972	4.128E-15	2.81363	up	-
c27510.graph_c0	0.47363158	0.60956467	0.78253115	2.80303774	4.12933096	2.80891076	0.0015165	1.28649	up	PREDICTED: uncharacterized protein LOC105170878 [Sesamum indicum]
c27511.graph_c0	227.333194	220.181959	209.626456	56.5850766	60.7060439	62.7963056	0	-2.9568	down	PREDICTED: glutathione S-transferase-like [Sesamum indicum]
c27512.graph_c0	28.5727871	29.0564207	28.5392492	24.1175009	26.3355585	27.9597856	4.24E-29	-1.22613	down	PREDICTED: multiple myeloma tumor-associated protein 2 homolog [Sesamum indicum]
c27515.graph_c0	128.590267	134.819816	148.612866	48.9988085	46.5620247	43.1508938	5.527E-49	-2.66344	down	hypothetical protein MIMGU_mgv1a022248mg, partial [Erythranthe guttata]
c27518.graph_c0	0	0	0	3.31288887	3.11331898	2.26193675	4.126E-14	Inf	up	hypothetical protein MIMGU_mgv1a013970mg [Erythranthe guttata]
c27519.graph_c0	15.6308104	18.75908	15.4481865	9.74611889	10.6764002	11.3610763	2.535E-24	-1.73837	down	PREDICTED: F-box protein At5g51370-like [Sesamum indicum]
c27524.graph_c0	0.43806578	1.15107416	0.84439781	4.89514919	4.13239014	4.15677582	0.0001493	1.34281	up	PREDICTED: zinc finger protein ZAT5-like [Sesamum indicum]
c27527.graph_c0	10.8459486	13.2641886	12.7255192	3.8385677	3.49681197	3.7475013	2.379E-39	-2.8246	down	hypothetical protein MIMGU_mgv1a009219mg [Erythranthe guttata]
c27529.graph_c0	0.84356256	0.50664436	0.57813935	3.2698475	3.17379314	2.73487308	0.004199	1.1636	up	PREDICTED: alpha/beta hydrolase domain-containing protein 11 isoform X1 [Sesamum indicum]
c27531.graph_c0	1.7063004	1.39171104	0.81209762	171.505289	158.875895	160.897726	0	5.89108	up	PREDICTED: RING-H2 finger protein ATL79-like [Sesamum indicum]
c27535.graph_c0	22.21318	20.8571944	25.5959711	24.8837412	19.9378118	22.1557811	7.37E-09	-1.12849	down	-
c27537.graph_c0	2.72745383	3.08020867	2.6061974	2.84637622	2.5241178	2.61294722	0.0002437	-1.16538	down	BnaC03g60650D [Brassica napus]
c27538.graph_c0	38.5841192	40.574808	31.1454477	16.0967212	17.9159162	19.0000151	2.807E-29	-2.14431	down	-
c27542.graph_c0	0.4914054	0.60763805	0.26001928	4.11773651	3.43339454	3.82290194	8.332E-10	1.98235	up	PREDICTED: pentatricopeptide repeat-containing protein At5g15010, mitochondrial [Sesamum indicum]
c27545.graph_c0	0.16552945	0.2711376	0.25138678	5.12940385	5.96449452	4.39081325	1.015E-28	3.39676	up	PREDICTED: probably inactive leucine-rich repeat receptor-like protein kinase At3g28040 [Sesamum indicum]
c27546.graph_c0	0	0.06114516	0.07849535	1.3984581	1.62344128	1.59395991	5.273E-12	3.9378	up	PREDICTED: peptide-N4-(N-acetyl-beta-glucosaminyl)asparagine amidase A-like [Sesamum indicum]
c27548.graph_c0	16.9430342	16.5147495	18.9145083	14.6358511	17.619735	15.9955908	3.781E-16	-1.21058	down	PREDICTED: mitochondrial substrate carrier family protein B-like [Sesamum indicum]
c27555.graph_c0	4.9016927	5.15679596	4.47879934	27.8946482	29.8152737	27.3240483	4.285E-57	1.45874	up	PREDICTED: primary amine oxidase-like [Sesamum indicum]

#ID	0 h-1 FPKM	0 h-2 FPKM	0 h-3 FPKM	28 h-1 FPKM	28 h-2 FPKM	28 h-3 FPKM	FDR	log2FC	regulated	nr_annotation
c27557.graph_c0	5.87585541	6.26120624	5.84570852	1.51535214	1.3682209	1.57374633	6.331E-15	-3.10214	down	PREDICTED: uncharacterized protein At5g65660-like [Sesamum indicum]
c27557.graph_c1	8.11961278	7.83218904	8.34721911	3.43347652	2.80947231	2.88924158	2.306E-15	-2.50308	down	PREDICTED: uncharacterized protein At5g65660 [Sesamum indicum]
c27561.graph_c0	59.9359416	60.3875769	56.9606564	27.2683368	26.5205801	27.4711953	2.04E-147	-2.21517	down	PREDICTED: cytosolic Fe-S cluster assembly factor NBP35-like [Sesamum indicum]
c27569.graph_c0	6.23966465	6.47512054	5.48034037	2.89155216	1.87827374	3.3949395	1.866E-42	-2.24271	down	hypothetical protein MIMGU_mgv1a002973mg [Erythranthe guttata]
c27570.graph_c0	2.39134585	1.70981459	2.92664199	9.60047143	10.835407	10.1300512	0.0011292	1.02616	up	-
c27572.graph_c0	2.77776659	1.60092163	1.83885396	0.06117772	0.27618872	0.14976156	2.024E-13	-4.76189	down	PREDICTED: protein IAL1-like [Sesamum indicum]
c27575.graph_c0	11.1081151	10.6874231	15.7988147	3.6839394	3.04316661	4.73295256	4.997E-09	-2.80768	down	-
c27576.graph_c0	8.84781085	8.11241256	8.50345433	44.8516511	48.8401597	48.9009957	3.253E-25	1.39526	up	PREDICTED: 60S ribosomal protein L14-2 [Sesamum indicum]
c27577.graph_c0	0.62653174	0.49276788	0.69010122	10.9936832	13.6265889	12.9518603	5.16E-36	3.28352	up	PREDICTED: protein INVOLVED IN DE NOVO 2-like [Sesamum indicum]
c27582.graph_c0	1.73758231	2.48014467	2.06746487	16.4640153	16.2802149	15.1901472	9.614E-30	1.83908	up	PREDICTED: glycerol-3-phosphate acyltransferase 1 [Sesamum indicum]
c27584.graph_c0	0.45873328	0.09183853	0.35369426	3.53408987	3.07053853	3.37348841	1.637E-06	2.3747	up	PREDICTED: uncharacterized protein LOC105159891 [Sesamum indicum]
c27587.graph_c0	0.72846948	0.36459947	0.73551667	4.34903239	5.63405914	4.44368338	1.124E-07	1.88669	up	Tubulin beta-7 chain [Gossypium arboreum]
c27588.graph_c0	31.0915017	31.8990834	32.1457894	31.2413464	32.4073941	32.0862798	4.317E-25	-1.08186	down	PREDICTED: E3 ubiquitin-protein ligase CIP8-like [Sesamum indicum]
c27593.graph_c0	0	0.04725455	0	0.51464973	0.89838221	0.67191996	1.328E-07	4.38101	up	PREDICTED: wall-associated receptor kinase 2-like [Sesamum indicum]
c27595.graph_c0	4.73583626	2.93167353	3.04286854	2.87586361	2.92377035	3.03036	0.0002976	-1.36401	down	hypothetical protein MIMGU_mgv1a009836mg [Erythranthe guttata]
c27597.graph_c0	5.5536178	3.27010524	6.29701655	2.96789516	3.96600197	3.58423175	0.0021548	-1.61761	down	PREDICTED: uncharacterized protein LOC105165182 [Sesamum indicum]
c27600.graph_c0	3.29282473	2.19741466	3.12318368	15.2709337	16.9265852	18.1799786	2.387E-10	1.45774	up	PREDICTED: 50S ribosomal protein 5, chloroplastic [Sesamum indicum]
c27601.graph_c0	0.83325537	1.13739511	0.68139664	44.8698399	48.8147482	46.6764987	1.01E-103	4.63857	up	PREDICTED: protein SPIRAL1-like 1-like [Oryza brachyantha]
c27604.graph_c0	1.83039173	1.32327332	1.43741024	12.04677	13.5018446	11.9006115	4.74E-24	1.9395	up	PREDICTED: deoxyribodipyrimidine photo-lyase [Sesamum indicum]
c27609.graph_c0	41.1686019	39.923359	39.7462753	166.418619	173.706094	178.1484	1.383E-36	1.01065	up	PREDICTED: protein disulfide-isomerase like 2-1-like [Sesamum indicum]
c27612.graph_c0	0.07281783	0.07289074	0	1.85232519	1.62469094	1.51148222	5.257E-09	4.02659	up	PREDICTED: uncharacterized protein LOC105160557 [Sesamum indicum]
c27614.graph_c0	30.9455574	27.0083076	33.1645464	19.4040456	18.9800367	21.6631653	9.133E-19	-1.69294	down	PREDICTED: uncharacterized protein LOC105163129 [Sesamum indicum]
c27616.graph_c0	0	0	0.14474571	1.55544364	2.62404746	1.33603085	1.585E-05	4.11923	up	PREDICTED: myb-related protein Zm38-like [Sesamum indicum]
c27622.graph_c0	23.4439774	24.3941047	21.7100697	22.5560008	20.8215681	21.6583883	8.956E-43	-1.18614	down	PREDICTED: 1-phosphatidylinositol-3-phosphate 5-kinase FAB1A isoform X2 [Sesamum indicum]
c27630.graph_c0	0.72589485	0.7556866	0.52237003	0.10551524	0.17148666	0.10331949	1.175E-15	-3.48569	down	-

#ID	0 h-1 FPKM	0 h-2 FPKM	0 h-3 FPKM	28 h-1 FPKM	28 h-2 FPKM	28 h-3 FPKM	FDR	log2FC	regulated	nr_annotation
c27631.graph_c0	3.67344539	4.33245278	3.78576578	26.5662191	30.6458286	24.4172038	1.532E-22	1.70001	up	PREDICTED: uncharacterized protein LOC100264843 [Vitis vinifera]
c27632.graph_c0	54.1287191	50.2338521	56.3110863	29.4128376	30.5934812	29.3089519	7.217E-58	-1.93861	down	PREDICTED: monothiol glutaredoxin-S16, chloroplastic [Sesamum indicum]
c27637.graph_c0	1.66504701	1.37040954	1.14114738	9.33157467	9.00828308	8.07530793	5.82E-12	1.57427	up	-
c27639.graph_c0	4.73420112	5.96924388	5.0891954	4.23481857	4.98866069	3.80653445	1.472E-11	-1.36922	down	PREDICTED: uncharacterized protein LOC105162275 [Sesamum indicum]
c27641.graph_c0	1.37360702	1.85093796	1.15412961	1.19031614	0.90139809	0.90862449	6.105E-06	-1.63333	down	PREDICTED: heat shock factor protein HSF30-like [Sesamum indicum]
c27644.graph_c0	2.28789938	2.02845432	2.52003539	11.3547677	11.4533958	8.02484235	4.33E-05	1.07933	up	PREDICTED: uncharacterized protein LOC105159746 [Sesamum indicum]
c27652.graph_c0	3.15962805	4.99388201	3.84654992	0.12086313	0.32738422	0.49311663	2.648E-18	-4.76012	down	-
c27656.graph_c0	0.17104361	0.26905196	0.21979781	1.01226715	1.28278222	1.02887935	0.0032382	1.23965	up	PREDICTED: LOW QUALITY PROTEIN: uncharacterized protein ycf45 [Sesamum indicum]
c27659.graph_c0	3.23727108	2.27404401	1.60562234	11.2688172	10.9946234	12.091939	7.551E-07	1.18912	up	PREDICTED: methyltransferase-like protein 13 [Sesamum indicum]
c27662.graph_c0	0.19317134	0.12890985	0.12411641	1.05296862	1.18313308	1.08833747	0.0001356	1.81152	up	PREDICTED: MATE efflux family protein 5-like [Sesamum indicum]
c27667.graph_c0	75.2251956	73.1638146	71.5073582	52.1589116	56.2720173	58.3634726	3.892E-63	-1.48841	down	PREDICTED: uncharacterized protein LOC105180257 [Sesamum indicum]
c27670.graph_c1	53.4949168	53.789802	60.9363003	45.7656739	49.3270854	51.8561246	2.979E-17	-1.287	down	hypothetical protein MIMGU_mgv1a001800mg [Erythranthe guttata]
c27672.graph_c0	1.19885073	1.16368601	1.21378294	10.5350243	12.5398126	9.82456435	1.064E-22	2.10979	up	PREDICTED: uncharacterized protein LOC105164990 isoform X1 [Sesamum indicum]
c27675.graph_c0	41.2079142	43.5771737	47.2568315	26.09372	27.7094456	28.2316435	4.776E-32	-1.77874	down	PREDICTED: DNL-type zinc finger protein-like [Sesamum indicum]
c27677.graph_c0	0	0	0	1.41975113	1.30655412	1.40357376	2.017E-14	Inf	up	PREDICTED: formin-like protein 6 [Sesamum indicum]
c27682.graph_c0	1.21841638	1.55226456	1.20986936	0.04025173	0.18171768	0.1642254	3.047E-20	-4.4543	down	PREDICTED: ammonium transporter 3 member 1-like [Solanum tuberosum]
c27685.graph_c0	20.9534597	22.9118396	18.4913668	18.4703367	19.1067413	16.6187332	4.36E-21	-1.29166	down	PREDICTED: probable WRKY transcription factor 65 [Sesamum indicum]
c27686.graph_c0	1.20948591	1.41247987	1.46789076	0.04883597	0.30866009	0.0796995	2.229E-18	-4.32324	down	-
c27688.graph_c0	0.10753606	0.03588125	0.18425079	9.40484254	8.25646412	6.93023146	2.614E-36	5.12695	up	PREDICTED: allene oxide cyclase 4, chloroplastic-like [Pyrus x bretschneideri]
c27692.graph_c0	1.12071689	0.85105037	0.74491323	9.52158161	9.43398085	9.67180784	8.585E-21	2.31172	up	PREDICTED: uncharacterized protein LOC105172843 [Sesamum indicum]
c27693.graph_c0	3.25245818	3.47769559	3.46711636	15.0696576	16.3229347	16.4175427	3.134E-13	1.13808	up	PREDICTED: uncharacterized protein LOC105170243 [Sesamum indicum]
c27694.graph_c0	1.73179554	0.95344132	1.66906938	0	0	0	7.043E-21	-Inf	down	hypothetical protein V501_06377 [Pseudogymnoascus pannorum VKM F-4519 (FW-2642)]
c27696.graph_c0	10.579298	10.1759544	9.96362789	4.25773737	5.15593519	5.57928321	6.155E-53	-2.12403	down	hypothetical protein MIMGU_mgv1a008679mg [Erythranthe guttata]

#ID	0 h-1 FPKM	0 h-2 FPKM	0 h-3 FPKM	28 h-1 FPKM	28 h-2 FPKM	28 h-3 FPKM	FDR	log2FC	regulated	nr_annotation
c27697.graph_c0	3.99936244	4.25754924	4.97618545	20.3931333	22.1623585	22.0996605	8.76E-12	1.19538	up	PREDICTED: glycine-rich RNA-binding protein 3, mitochondrial-like [Solanum tuberosum]
c27698.graph_c0	0.03000741	0.09011236	0	3.29319066	2.04793384	2.66942055	8.878E-14	4.98377	up	hypothetical protein MIMGU_mgv1a004055mg [Erythranthe guttata]
c27703.graph_c0	0.19891022	0.1991094	0.04260125	1.48180973	2.01234164	1.4450753	1.419E-08	2.40928	up	PREDICTED: probable LRR receptor-like serine/threonine-protein kinase At1g74360 [Sesamum indicum]
c27705.graph_c0	0	0	0.14040454	3.25581681	3.36989693	5.37823791	4.041E-08	5.29006	up	hypothetical protein MIMGU_mgv1a024655mg [Erythranthe guttata]
c27710.graph_c0	0.28420376	0.22759068	0.29217047	23.8367533	23.9468818	23.5632004	4.688E-79	5.37993	up	PREDICTED: glucan endo-1,3-beta-glucosidase 4-like [Nicotiana sylvestris]
c27714.graph_c0	1.04243035	0.72049408	0.82925407	9.10962729	9.38154506	9.71491441	4.521E-32	2.35564	up	PREDICTED: pentatricopeptide repeat-containing protein At3g62470, mitochondrial-like [Sesamum indicum]
c27725.graph_c0	5.76024151	5.59388984	7.18117905	53.0499593	51.6227473	55.3213686	1.413E-42	2.01626	up	PREDICTED: 40S ribosomal protein S13-like [Sesamum indicum]
c27727.graph_c0	27.3597056	31.0483826	25.2925727	18.1740663	16.4094779	16.9453772	5.763E-34	-1.78903	down	PREDICTED: autophagy-related protein 9 [Sesamum indicum]
c27733.graph_c0	0.79409193	0.67259677	0.74570584	0.19977973	0.16033988	0.21735817	6.677E-13	-3.02681	down	-
c27737.graph_c0	5.23289862	6.26859207	4.96068337	6.48421341	5.51686925	5.39281645	7.33E-05	-1.01025	down	PREDICTED: uncharacterized protein LOC105168421 isoform X1 [Sesamum indicum]
c27740.graph_c0	3.33067998	3.6515404	3.26099477	0.23054467	0.31224033	0.18812257	2.498E-21	-4.90078	down	PREDICTED: uncharacterized protein LOC103423918 [Malus domestica]
c27741.graph_c0	0.78114804	0.54735117	0.90342554	11.0140375	11.3799526	10.42349	1.782E-16	2.78406	up	PREDICTED: transcription factor TCP4-like [Sesamum indicum]
c27744.graph_c0	1.59169862	1.49672928	1.30161547	0.3155011	0.28486792	0.28605147	1.063E-16	-3.39639	down	-
c27745.graph_c1	6.9930733	7.36635884	6.47855018	40.7193021	42.2085315	37.229244	2.939E-36	1.43769	up	PREDICTED: equilibrative nucleotide transporter 1 [Sesamum indicum]
c27749.graph_c0	1.38113785	1.6671575	1.40941336	8.85708556	9.0634002	8.26322385	3.8E-10	1.46365	up	PREDICTED: tRNA (adenine(58)-N(1))-methyltransferase catalytic subunit trmt61a [Sesamum indicum]
c27751.graph_c0	26.0567852	30.0224784	17.9627712	20.8119865	18.3459816	20.2823108	2.357E-05	-1.4023	down	PREDICTED: probable glutathione S-transferase [Sesamum indicum]
c27752.graph_c0	0.72781003	1.11304542	0.88330555	4.59908406	4.1525417	4.46021467	9.164E-08	1.18654	up	PREDICTED: CSC1-like protein At1g32090 [Sesamum indicum]
c27754.graph_c0	104.926041	103.51742	102.236858	84.1452544	95.2140757	90.4430578	1.42E-54	-1.29384	down	PREDICTED: topless-related protein 4 [Sesamum indicum]
c27755.graph_c0	0.13297161	0.15211973	0.29292649	1.10449144	0.99725221	1.16036709	0.0002504	1.39586	up	PREDICTED: glutamate receptor 3.2-like [Sesamum indicum]
c27758.graph_c0	0	0	0	1.78981028	1.93923705	1.31442352	3.653E-14	Inf	up	PREDICTED: myosin heavy chain kinase B-like [Sesamum indicum]
c27760.graph_c0	11.6993865	11.7935742	9.31695343	8.38328546	8.38031941	9.62583155	8.519E-14	-1.4011	down	PREDICTED: uncharacterized protein LOC105179312 [Sesamum indicum]
c27765.graph_c0	40.4608418	41.8292706	40.9131412	16.2701041	17.410819	20.553636	2.34E-49	-2.27343	down	PREDICTED: polyubiquitin 4-like [Cicer arietinum]
c27768.graph_c0	140.34812	133.942591	133.163783	80.7990294	77.9881004	82.5633512	7.172E-96	-1.84527	down	PREDICTED: uncharacterized protein LOC105170296 [Sesamum indicum]
c27771.graph_c0	1.18331957	1.42957438	0.99626336	0.56346783	0.54892377	0.59288085	1.445E-13	-2.16919	down	PREDICTED: uncharacterized protein LOC105176570 isoform X1 [Sesamum indicum]

#ID	0 h-1 FPKM	0 h-2 FPKM	0 h-3 FPKM	28 h-1 FPKM	28 h-2 FPKM	28 h-3 FPKM	FDR	log2FC	regulated	nr_annotation
c27772.graph_c0	1.51363599	1.59705176	1.73480302	12.4579109	12.1879231	12.7130073	1.793E-19	1.85468	up	PREDICTED: uncharacterized protein At2g39795, mitochondrial isoform X1 [Sesamum indicum]
c27778.graph_c0	0.29460773	0.15612498	0.26723477	1.17135797	0.93253082	0.97637378	0.0076572	1.01019	up	PREDICTED: phosphoenolpyruvate carboxylase 4 [Sesamum indicum]
c27779.graph_c0	1.39791127	2.13579058	1.41818789	18.5552562	13.4705193	15.795445	3.352E-13	2.18243	up	PREDICTED: transcription factor BEE 1-like [Sesamum indicum]
c27780.graph_c0	2.24519132	2.14972478	2.73462967	12.9266314	12.2865019	11.9142903	9.58E-23	1.28696	up	PREDICTED: NAD kinase 2, chloroplastic [Sesamum indicum]
c27785.graph_c0	5.04004086	4.71426228	6.05195356	48.4005993	50.3160255	46.6975838	9.741E-43	2.108	up	hypothetical protein MIMGU_mgv1a013628mg [Erythranthe guttata]
c27785.graph_c1	12.4363384	14.1367632	15.1685845	134.04791	124.767418	137.008768	1.739E-46	2.15282	up	PREDICTED: polyadenylate-binding protein 2 isoform X1 [Sesamum indicum]
c27788.graph_c0	2.1744026	2.53934326	2.9494264	0	0.23781697	0.42984907	2.349E-19	-4.6092	down	-
c27790.graph_c0	3.45263063	4.63262849	3.87037126	39.0284329	45.4588122	41.5621421	3.081E-46	2.30693	up	PREDICTED: nucleoside diphosphate kinase 2, chloroplastic [Sesamum indicum]
c27793.graph_c0	1.47155985	1.09113585	0.98052509	0.67340225	0.32189246	0.80807461	1.882E-07	-2.05728	down	-
c27795.graph_c1	0.60917852	0.99090635	0.39140926	3.76335325	2.84828592	3.4773036	0.0048307	1.2569	up	PREDICTED: exonuclease DPD1, chloroplastic/mitochondrial [Sesamum indicum]
c27796.graph_c0	2.21699923	3.00668411	2.29752564	19.5435372	15.674888	15.8625403	3.225E-13	1.67341	up	unnamed protein product [Coffea canephora]
c27798.graph_c0	23.9436946	22.5806526	21.6163628	11.1985988	10.1476563	10.1733821	1.6E-117	-2.20176	down	PREDICTED: uncharacterized protein LOC105164093 [Sesamum indicum]
c27801.graph_c0	0.77136419	0.66684524	0.49561676	6.80392517	7.04064341	6.36290599	2.286E-16	2.29964	up	PREDICTED: chorismate mutase 1, chloroplastic-like [Sesamum indicum]
c27803.graph_c0	2.39313581	1.75998282	2.44766727	1.49084341	1.98708793	2.51992611	0.0001213	-1.22827	down	hypothetical protein MIMGU_mgv1a020303mg [Erythranthe guttata]
c27807.graph_c0	0.53456058	1.60528759	0.68693151	39.4990808	37.5348632	39.0998135	1.29E-35	4.27124	up	hypothetical protein MIMGU_mgv1a0223741mg, partial [Erythranthe guttata]
c27818.graph_c0	0	0	0	17.3087547	13.3595755	14.3971664	1.087E-60	Inf	up	hypothetical protein MIMGU_mgv1a023677mg [Erythranthe guttata]
c27824.graph_c0	3.13417841	3.64009194	2.94320475	32.1680761	32.3276184	30.5621408	1.281E-96	2.20059	up	PREDICTED: LOW QUALITY PROTEIN: uncharacterized protein LOC105170443 [Sesamum indicum]
c27825.graph_c0	1.17831881	0.8256491	1.58989564	59.7335735	61.1249918	58.9796979	1.79E-164	4.54887	up	PREDICTED: 2-Cys peroxiredoxin BAS1, chloroplastic-like [Sesamum indicum]
c27827.graph_c0	0	0	0	2.33657903	2.67230159	2.54217254	7.501E-16	Inf	up	PREDICTED: cyclin-D5-3-like [Sesamum indicum]
c27833.graph_c0	1.49385644	1.07665366	1.07501222	6.81836086	6.03870349	5.35110028	0.000146	1.23271	up	PREDICTED: uncharacterized protein LOC105175923 [Sesamum indicum]
c27839.graph_c0	14.6785035	14.7674099	13.4323566	12.2307735	10.8486471	13.7612605	9.53E-17	-1.30734	down	-
c27841.graph_c0	1.95382384	2.1335785	2.47269945	10.0262576	9.84925897	10.5163632	8.318E-11	1.1187	up	PREDICTED: uncharacterized protein LOC105159050 [Sesamum indicum]
c27843.graph_c0	131.848771	125.004989	108.372557	35.4272527	46.3671838	41.99225	3.745E-83	-2.64918	down	PREDICTED: NAC domain-containing protein 2 [Glycine max]
c27846.graph_c0	0.047995	0.14412917	0.18502642	4.11613361	4.12592574	5.63583745	4.349E-16	4.10024	up	PREDICTED: lignin-forming anionic peroxidase-like [Sesamum indicum]

#ID	0 h-1 FPKM	0 h-2 FPKM	0 h-3 FPKM	28 h-1 FPKM	28 h-2 FPKM	28 h-3 FPKM	FDR	log2FC	regulated	nr_annotation
c27847.graph_c0	1.46725879	1.75431403	1.20461582	8.08683051	7.27490376	6.55044489	1.333E-06	1.21936	up	PREDICTED: cytochrome P450 CYP736A12-like [Sesamum indicum]
c27850.graph_c0	0.04795551	0.33602472	0.18487419	4.49613884	4.75192531	4.72110766	1.207E-15	3.52237	up	PREDICTED: protein COBRA-like [Sesamum indicum]
c27854.graph_c0	0.1551319	0.15528724	0.19935064	4.45358444	4.6319797	4.27810423	1.04E-11	3.61798	up	PREDICTED: hexose carrier protein HEX6-like [Sesamum indicum]
c27858.graph_c0	0.29546901	0.39435318	0.50625251	10.1407445	7.10947455	8.39158488	1.512E-19	3.32543	up	hypothetical protein MIMGU_mgv1a026536mg, partial [Erythranthe guttata]
c27862.graph_c0	2.91723347	2.27123139	3.60991753	0.15705396	0.28361	0.2563095	2.397E-16	-4.75217	down	PREDICTED: ethylene-responsive transcription factor ERF109-like [Sesamum indicum]
c27868.graph_c0	1.43253145	1.28141635	1.33168569	7.177328	8.48059444	6.65198735	2E-10	1.37245	up	PREDICTED: F-box/kelch-repeat protein At3g27150-like [Sesamum indicum]
c27870.graph_c0	8.45255533	10.0579666	9.53110955	77.279063	75.3960112	91.3103508	6.984E-28	2.03047	up	PREDICTED: asparagine synthetase [glutamine-hydrolyzing] [Solanum lycopersicum]
c27876.graph_c0	0.27004538	0.16218948	0.1735095	1.00096388	1.4531309	1.29723621	0.0003958	1.54485	up	hypothetical protein MIMGU_mgv1a018375mg [Erythranthe guttata]
c27877.graph_c0	0.7519986	0.37637581	0.48317399	2.5765846	3.77161108	3.24927489	0.000229	1.48808	up	PREDICTED: RING-H2 finger protein ATL3 [Sesamum indicum]
c27879.graph_c0	3.57069196	3.06365783	3.47413527	35.6278166	35.6498809	33.3072634	6.806E-54	2.2801	up	PREDICTED: hsp70 nucleotide exchange factor FES1 [Sesamum indicum]
c27879.graph_c1	2.20511295	2.0601663	2.83365745	27.8863429	25.7575749	23.5396842	3.041E-16	2.3478	up	-
c27881.graph_c0	29.1757675	31.6799812	31.2963018	20.3062721	24.0946635	29.6935824	7.713E-23	-1.40456	down	PREDICTED: serine carboxypeptidase-like 50 [Sesamum indicum]
c27884.graph_c0	29.0378283	28.6356455	30.2282765	4.88472714	4.69317181	5.57003618	5.7E-131	-3.62713	down	hypothetical protein CICLE_v10017323mg [Citrus clementina]
c27885.graph_c0	0.58582963	0.39094416	0.15056285	6.7556712	8.38073203	8.06040017	3.816E-25	3.28655	up	PREDICTED: cytochrome P450 81E8-like isoform X1 [Sesamum indicum]
c27888.graph_c1	7.2733907	7.48535148	8.07035312	39.0843445	43.1869855	39.5321557	4.395E-41	1.32346	up	PREDICTED: aspartokinase 2, chloroplastic [Sesamum indicum]
c27896.graph_c0	8.03810346	9.25392581	7.59955572	8.34931068	7.49403736	8.12315575	6.153E-18	-1.14383	down	PREDICTED: U-box domain-containing protein 44 [Sesamum indicum]
c27905.graph_c0	13.3845651	12.7640854	13.0939622	7.82409992	6.74331825	7.42570779	5.202E-64	-1.92553	down	PREDICTED: transcription factor RF2b-like [Sesamum indicum]
c27907.graph_c0	0.49605815	0.16551829	0.84993914	6.60975703	6.61904442	6.27608176	1.618E-07	2.58767	up	PREDICTED: protein RER1A-like [Sesamum indicum]
c27908.graph_c0	8.67355296	8.84814085	7.09927321	39.9112418	42.2354803	36.5316709	1.541E-20	1.18058	up	PREDICTED: vacuolar protein sorting-associated protein 29 [Sesamum indicum]
c27913.graph_c0	1.4030446	1.40444954	1.54540102	14.7131711	20.4530443	16.5228714	2.38E-15	2.47735	up	hypothetical protein OsI_11409 [Oryza sativa Indica Group]
c27915.graph_c0	6.10573662	7.95961938	7.4811778	42.518536	41.558379	44.1263683	4.011E-14	1.48081	up	PREDICTED: mitochondrial import inner membrane translocase subunit TIM8 [Sesamum indicum]
c27923.graph_c0	0.97241116	1.36994909	1.57355379	52.3251447	52.0424356	50.3434575	3.38E-203	4.20811	up	PREDICTED: protein disulfide isomerase-like 2-3 [Sesamum indicum]
c27931.graph_c0	0.40804837	0.24507418	0.89140913	3.61811989	3.56137265	4.01714052	1.854E-07	1.75439	up	PREDICTED: pentatricopeptide repeat-containing protein At2g29760, chloroplastic-like [Sesamum indicum]
c27934.graph_c0	1.70236905	1.57784603	1.45840775	0.13747467	0.37238021	0.26174915	5.88E-17	-3.70795	down	PREDICTED: ADP,ATP carrier protein 1, mitochondrial-like [Malus domestica]
c27935.graph_c0	28.4094746	31.453245	28.1395948	11.9823035	12.4523843	11.7350936	1.52E-118	-2.3731	down	PREDICTED: DNA ligase 1-like [Sesamum indicum]

#ID	0 h-1 FPKM	0 h-2 FPKM	0 h-3 FPKM	28 h-1 FPKM	28 h-2 FPKM	28 h-3 FPKM	FDR	log2FC	regulated	nr_annotation
c27941.graph_c0	9.59838261	9.97753216	9.07282767	6.00342692	5.42053163	5.28188959	2.12E-31	-1.86809	down	hypothetical protein JCGZ_04255 [Jatropha curcas]
c27942.graph_c0	0.23949333	0.29966643	0	6.26624542	6.40435161	5.75235573	1.097E-17	4.02258	up	PREDICTED: 1-aminocyclopropane-1-carboxylate oxidase [Sesamum indicum]
c27943.graph_c0	8.94174521	7.78022299	10.4298354	9.64823165	8.44062633	10.7691184	5.549E-05	-1.00423	down	PREDICTED: LOW QUALITY PROTEIN: auxilin-related protein 2-like [Sesamum indicum]
c27946.graph_c0	1.59187668	1.53852343	2.11616333	1.91497952	1.40485065	2.14858552	0.0046599	-1.03336	down	PREDICTED: putative germin-like protein 2-1 [Sesamum indicum]
c27947.graph_c0	1.77093816	1.64300089	1.22112108	0	0	0	2.277E-38	-Inf	down	-
c27949.graph_c0	5.61574579	6.50895371	6.4025697	2.82328748	3.07562164	2.55419034	2.276E-29	-2.22468	down	PREDICTED: uncharacterized protein LOC105156723 [Sesamum indicum]
c27952.graph_c0	0	0.06881418	0	1.51556606	2.09021488	1.79387932	7.07E-18	5.21204	up	PREDICTED: uncharacterized protein LOC105176065 [Sesamum indicum]
c27954.graph_c0	2.38106559	3.85018825	2.11829785	1.79710416	1.2019382	1.46642232	0.0001687	-1.99036	down	PREDICTED: probable polyamine transporter At3g13620 [Sesamum indicum]
c27954.graph_c1	3.6205255	2.83342707	4.22956237	2.00941521	1.64152177	1.71774576	6.318E-09	-2.08634	down	PREDICTED: probable polyamine transporter At3g13620 [Sesamum indicum]
c27955.graph_c0	0.0774356	0.07751314	0	6.8098494	5.56427917	5.8782482	1.667E-32	5.80976	up	hypothetical protein MIMGU_mgv1a005882mg [Erythranthe guttata]
c27956.graph_c0	0.80611286	1.07589342	1.43051054	8.78817275	8.28755806	8.30934342	8.919E-15	1.84052	up	PREDICTED: surfeit locus protein 1 [Sesamum indicum]
c27958.graph_c0	110.868602	106.039489	112.652421	97.0656654	101.67515	98.2806928	1.702E-52	-1.24097	down	PREDICTED: peptide methionine sulfoxide reductase B5-like [Sesamum indicum]
c27959.graph_c0	0.20719884	0.24197403	0.1775058	2.33415925	3.01399016	2.17089697	6.181E-09	2.49539	up	PREDICTED: uncharacterized protein LOC105161116 [Sesamum indicum]
c27961.graph_c0	1.65015259	1.82184372	1.30972764	8.37761269	7.37310323	8.04496741	4.782E-11	1.22795	up	hypothetical protein MIMGU_mgv1a003065mg [Erythranthe guttata]
c27962.graph_c0	4.91038074	4.05827147	4.62735802	34.6451659	37.841659	36.4391023	7.511E-75	1.91177	up	PREDICTED: ent-kaurene oxidase, chloroplastic-like [Sesamum indicum]
c27963.graph_c0	1.29986727	0.7319075	1.14838687	5.07795622	7.51713359	5.54082143	0.000105	1.42049	up	-
c27967.graph_c0	1.50655594	1.37692848	0.9048621	9.47375127	8.7795776	9.15812181	3.06E-30	1.77076	up	PREDICTED: LOW QUALITY PROTEIN: alpha,alpha-trehalose-phosphate synthase [UDP-forming] 1-like [Sesamum indicum]
c27970.graph_c0	2.57657718	2.26334206	2.36500263	15.095783	16.1490529	16.933401	1.679E-16	1.6519	up	Os05g0129800 [Oryza sativa Japonica Group]
c27976.graph_c0	0	0	0	4.89014477	3.67257438	3.9903182	3.192E-19	Inf	up	PREDICTED: homeobox-leucine zipper protein ATHB-40-like [Sesamum indicum]
c27983.graph_c0	8.96596812	8.00468172	4.67092865	4.05074381	2.86234581	3.59279768	6.047E-06	-2.12506	down	-
c27987.graph_c0	0.75989639	0.82404542	1.22062118	9.06673317	8.7681853	9.23858648	1.062E-12	2.1743	up	PREDICTED: uncharacterized protein LOC105171687 [Sesamum indicum]
c27988.graph_c0	6.72300873	9.51680518	6.02135288	134.4951	132.176351	140.073295	5.82E-180	3.10361	up	-
c27993.graph_c0	10.5415026	10.5018104	10.9660104	3.64833492	3.3929275	2.7686207	1.392E-58	-2.79831	down	PREDICTED: protein ECERIFERUM 1 [Sesamum indicum]

#ID	0 h-1 FPKM	0 h-2 FPKM	0 h-3 FPKM	28 h-1 FPKM	28 h-2 FPKM	28 h-3 FPKM	FDR	log2FC	regulated	nr_annotation
c27997.graph_c0	2.78044175	2.27718486	2.31431512	12.3085641	10.5329245	12.0861542	1.696E-11	1.15605	up	PREDICTED: probable lysine--tRNA ligase, cytoplasmic [Sesamum indicum]
c27998.graph_c0	1.24868964	1.11105779	0.93602578	1.08400663	0.59180617	1.25481335	0.0002771	-1.25401	down	PREDICTED: pectinesterase 2.1-like [Sesamum indicum]
c28000.graph_c0	435.520406	440.309382	438.873921	223.66438	176.704469	184.690473	1.27E-165	-2.25887	down	PREDICTED: uncharacterized protein LOC105170296 [Sesamum indicum]
c28000.graph_c1	26.4632623	32.5866111	19.4321942	12.9748766	12.4042205	13.9504478	6.86E-08	-2.0825	down	PREDICTED: probable membrane-associated kinase regulator 1 [Sesamum indicum]
c28009.graph_c0	2.28776084	1.55723514	2.23429607	20.1523045	19.696932	20.4601706	3.136E-18	2.2193	up	hypothetical protein MIMGU_mgv1a006302mg [Erythranthe guttata]
c28010.graph_c0	26.4577206	25.4793929	27.9794833	9.9012753	11.3866391	11.5237184	2.558E-84	-2.37545	down	PREDICTED: uncharacterized protein LOC105174992 [Sesamum indicum]
c28013.graph_c0	171.941286	160.79534	163.834681	713.170108	734.818607	716.630812	2.538E-39	1.03387	up	PREDICTED: 60S ribosomal protein L21-1 [Sesamum indicum]
c28022.graph_c0	3.58059824	3.80362348	3.94389471	27.8824089	30.9774854	27.7788863	2.765E-24	1.84307	up	PREDICTED: uncharacterized protein LOC105178683 [Sesamum indicum]
c28025.graph_c0	81.2593957	89.3854557	79.5592691	23.5081667	26.6297521	27.9499209	4.31E-149	-2.7695	down	PREDICTED: probable mitochondrial chaperone BCS1-B [Sesamum indicum]
c28031.graph_c0	10.3213707	10.5436385	9.38638495	50.452482	49.6003914	50.6016981	4.739E-41	1.227	up	PREDICTED: serine acetyltransferase 1, chloroplastic-like [Sesamum indicum]
c28033.graph_c0	1.44047719	1.04609854	1.81477472	8.25227584	9.61961511	8.91138338	3.637E-16	1.54347	up	PREDICTED: ATP sulfurylase 2 [Sesamum indicum]
c28034.graph_c0	0.45793189	0.36115611	0.39230708	6.2025967	6.19227158	5.01189003	7.512E-37	2.75485	up	PREDICTED: phytosulfokine receptor 2-like [Sesamum indicum]
c28035.graph_c0	0.03140662	0.22006647	0.12107625	5.40979741	4.55478188	4.56335812	3.031E-26	4.18927	up	PREDICTED: ADP-ribosylation factor GTPase-activating protein AGD3-like [Sesamum indicum]
c28036.graph_c0	0.15564349	0	0	8.1446968	8.7838221	7.19983992	3.945E-15	6.21662	up	PREDICTED: MADS-box protein SVP-like isoform X2 [Nicotiana sylvestris]
c28041.graph_c0	0.25444783	0.08490087	0.16348777	2.58903707	2.97773068	2.89229532	1.85E-10	2.98613	up	PREDICTED: uncharacterized protein LOC105158497 [Sesamum indicum]
c28043.graph_c1	1.64646237	2.07343003	2.25227058	10.2679377	9.30583839	10.9614157	7.776E-08	1.2608	up	PREDICTED: uncharacterized protein LOC105176055 isoform X2 [Sesamum indicum]
c28049.graph_c0	0.27174964	0.18134784	0	12.2453764	12.6613919	11.9798083	3.649E-27	5.27887	up	-
c28056.graph_c0	12.4253844	12.8860365	12.5147626	67.4454815	69.2711945	70.768721	1.206E-23	1.36516	up	hypothetical protein MIMGU_mgv1a017013mg [Erythranthe guttata]
c28063.graph_c0	0.67928371	0.42497744	0.32733992	4.56672021	4.84768724	4.17961799	2.138E-06	2.16443	up	hypothetical protein MIMGU_mgv1a014814mg [Erythranthe guttata]
c28065.graph_c0	0.5242006	0.32795344	0.67361852	3.52412027	2.88095489	2.48705155	0.0005996	1.44641	up	hypothetical protein MIMGU_mgv1a017254mg [Erythranthe guttata]
c28072.graph_c0	198.493523	183.280961	193.992839	83.7346706	93.5055459	102.76597	7.11E-135	-2.12986	down	PREDICTED: uncharacterized protein LOC104888288 [Beta vulgaris subsp. vulgaris]
c28078.graph_c0	4.19414264	2.87664204	2.69481967	28.1683532	32.5180313	28.8350731	1.089E-27	2.11051	up	hypothetical protein MIMGU_mgv1a007042mg [Erythranthe guttata]
c28080.graph_c0	0.8954879	1.43893317	1.12045484	1.02763491	0.86600078	1.27178824	0.0007448	-1.21639	down	PREDICTED: receptor-like protein kinase HSL1 [Sesamum indicum]

#ID	0 h-1 FPKM	0 h-2 FPKM	0 h-3 FPKM	28 h-1 FPKM	28 h-2 FPKM	28 h-3 FPKM	FDR	log2FC	regulated	nr_annotation
c28081.graph_c0	0.80136317	0.87856234	0.83363418	0.13867289	0.02504172	0.2036807	7.971E-17	-3.85951	down	PREDICTED: ent-kaurenoic acid oxidase 1-like [Sesamum indicum]
c28093.graph_c0	4386.13677	4135.01396	4879.89869	571.850921	623.612114	643.248635	4.007E-75	-3.95749	down	hypothetical protein MIMGU_mgv1a010438mg [Erythranthe guttata]
c28095.graph_c0	2.71875862	3.06166618	1.52849921	12.5351111	13.6039376	12.7982821	2.155E-06	1.32967	up	PREDICTED: transcription factor TCP2-like [Sesamum indicum]
c28098.graph_c0	25.4807645	15.972379	16.2924204	0	0	0	1.605E-40	-Inf	down	hypothetical protein GYMLUDRAFT_153379 [Gymnopus luxurians FD-317 M1]
c28103.graph_c0	6.44589236	5.37695579	5.13560067	5.4344114	5.52715942	5.83612465	2.003E-09	-1.10145	down	hypothetical protein MIMGU_mgv1a006429mg [Erythranthe guttata]
c28105.graph_c0	0.58816115	0.58875011	1.07073135	7.96166853	9.48836179	8.02271676	5.38E-28	2.40276	up	PREDICTED: probable receptor-like serine/threonine-protein kinase At5g57670 [Sesamum indicum]
c28106.graph_c0	1.21782295	0.87074458	0.96877901	12.1386061	11.302524	11.6246222	1.33E-18	2.43146	up	PREDICTED: protein GLUTAMINE DUMPER 5-like [Sesamum indicum]
c28107.graph_c1	5.37034887	6.11550534	7.59755331	53.4264478	52.0865595	50.0530622	3.03E-62	1.93202	up	PREDICTED: isoaspartyl peptidase/L-asparaginase 1 [Sesamum indicum]
c28115.graph_c0	2.26394451	1.51080767	1.31609305	11.9097199	14.644379	11.2207243	4.728E-12	1.8061	up	PREDICTED: CASP-like protein 5A1 [Sesamum indicum]
c28116.graph_c0	4.94836881	5.70811606	4.5420356	43.9451441	37.8227715	38.9053462	1.043E-41	1.89997	up	PREDICTED: coatomer subunit alpha-1-like [Sesamum indicum]
c28117.graph_c0	0.70203915	0.95572931	0.59541775	3.87780889	3.78693004	3.57228343	2.673E-09	1.23026	up	PREDICTED: protein STICHEL [Sesamum indicum]
c28118.graph_c0	0.08732436	0.20978832	0.22443052	9.43117255	8.90513588	9.41513545	2.565E-90	4.63481	up	PREDICTED: phosphoenolpyruvate carboxylase [Sesamum indicum]
c28121.graph_c1	0	0	0	0.79112094	0.85716965	0.81769443	2.03E-11	Inf	up	PREDICTED: truncated FRIGIDA-like protein 1 [Sesamum indicum]
c28123.graph_c0	7.96623088	8.27856694	7.34557767	7.20411559	7.26285061	6.56372336	2.748E-11	-1.25554	down	PREDICTED: uncharacterized protein LOC105171936 [Sesamum indicum]
c28130.graph_c0	62.9381511	58.4921438	65.9244265	56.3831272	60.351416	60.6268968	4.11E-26	-1.17036	down	unnamed protein product [Coffea canephora]
c28134.graph_c0	0.2340272	0.13386374	0.08592406	1.09343327	1.11890342	1.13016131	0.0001994	1.80148	up	PREDICTED: pentatricopeptide repeat-containing protein At3g24000, mitochondrial [Sesamum indicum]
c28142.graph_c1	19.6552174	17.9340021	19.6882606	8.46684839	6.97449418	6.89250739	2.63E-102	-2.44993	down	PREDICTED: trihelix transcription factor PTL [Sesamum indicum]
c28145.graph_c0	0.04488097	0.02246296	0.02883691	1.54941342	1.8849348	1.18445762	1.754E-13	4.49786	up	PREDICTED: G-type lectin S-receptor-like serine/threonine-protein kinase At2g19130 isoform X2 [Vitis vinifera]
c28146.graph_c0	0.88948183	1.26136106	1.04776776	5.01014459	4.23183979	4.61575153	0.0067487	1.02381	up	PREDICTED: T-complex protein 1 subunit gamma-like [Malus domestica]
c28147.graph_c0	13.7864041	15.2664813	13.3977818	12.1177912	13.4291574	12.1875579	4.1E-25	-1.26009	down	PREDICTED: uncharacterized protein LOC105164004 [Sesamum indicum]
c28148.graph_c0	0.219217	0.13166191	0.16902152	8.92223663	9.37941951	9.38660884	3.771E-37	4.64681	up	PREDICTED: uncharacterized protein LOC105177785 [Sesamum indicum]
c28150.graph_c0	9.49960364	7.88414053	11.5892723	1.31093491	0.78910087	0.96274096	1.117E-21	-4.33568	down	-
c28151.graph_c0	0.45751377	0.68695785	0.54269843	2.92871948	3.00232911	3.42296099	4.597E-08	1.38029	up	PREDICTED: uncharacterized protein LOC105163554 [Sesamum indicum]
c28152.graph_c0	9.27489285	8.2624762	8.2688883	36.6397432	38.9357148	36.7142034	3.379E-19	1.03191	up	PREDICTED: probable prolyl 4-hydroxylase 4 [Sesamum indicum]

#ID	0 h-1 FPKM	0 h-2 FPKM	0 h-3 FPKM	28 h-1 FPKM	28 h-2 FPKM	28 h-3 FPKM	FDR	log2FC	regulated	nr_annotation
c28153.graph_c0	6.55382365	7.17194775	6.56624495	3.22934323	2.40553018	2.99744639	9.871E-27	-2.32284	down	PREDICTED: probable alpha,alpha-trehalose-phosphate synthase [UDP-forming] 7 [Sesamum indicum]
c28156.graph_c0	46.5717805	42.5833036	46.7310325	206.57939	225.13195	218.872725	3.486E-43	1.16847	up	PREDICTED: 60S ribosomal protein L13a-4 [Sesamum indicum]
c28158.graph_c0	46.0224572	49.6505449	47.9001184	51.0763318	48.0740188	49.4593107	2.215E-23	-1.04107	down	hypothetical protein MIMGU_mgv1a015858mg [Erythranthe guttata]
c28160.graph_c0	7.31249736	8.24027609	8.32774608	8.0515825	7.38476201	7.79054415	2.373E-12	-1.13183	down	unnamed protein product [Coffea canephora]
c28161.graph_c0	1.86998308	1.3370397	1.65921587	9.67544725	9.40802338	9.13611952	2.999E-18	1.44586	up	PREDICTED: uncharacterized protein LOC105175750 [Sesamum indicum]
c28172.graph_c0	0.77184763	1.01406443	0.92986384	48.629469	47.2001484	43.8868326	4.41E-157	4.59251	up	PREDICTED: 1,4-dihydroxy-2-naphthoyl-CoA synthase, peroxisomal [Sesamum indicum]
c28176.graph_c0	1.23246868	1.96270901	1.43979186	21.620175	21.1753122	18.8379464	8.209E-36	2.64155	up	PREDICTED: PRA1 family protein B1 [Sesamum indicum]
c28177.graph_c0	0.63623994	0.38212622	1.14463091	4.7166383	6.17926431	7.32014939	1.075E-05	1.97438	up	-
c28178.graph_c0	10.5705561	5.18882872	5.74690054	0	0	0	1.345E-15	-Inf	down	60S ribosomal protein L13a [Rozella allomycis CSF55]
c28179.graph_c0	7.23674932	6.44892312	6.12406716	33.0330276	32.4897723	35.8000032	1.351E-13	1.26648	up	PREDICTED: heavy metal-associated isoprenylated plant protein 26-like [Sesamum indicum]
c28185.graph_c0	0	0.03425619	0.13192957	1.84054979	1.79658799	1.35980447	1.227E-11	3.78857	up	PREDICTED: lysosomal Pro-X carboxypeptidase [Sesamum indicum]
c28187.graph_c0	0.18789201	0.68962724	0.72434602	2.59461777	2.54819628	2.56291047	0.004473	1.16499	up	PREDICTED: pentatricopeptide repeat-containing protein At1g74600, chloroplastic [Sesamum indicum]
c28192.graph_c0	12.8697776	11.857519	11.668844	12.4302222	12.0969972	12.2889752	2.599E-20	-1.07288	down	PREDICTED: probable WRKY transcription factor 21 isoform X1 [Sesamum indicum]
c28196.graph_c0	0.75146804	0.81008364	0.96566618	23.022856	25.9843504	24.8886346	1.539E-61	3.77609	up	PREDICTED: uncharacterized protein LOC105157188 [Sesamum indicum]
c28198.graph_c0	10.2774906	9.98520013	10.9626585	4.73564203	5.33378072	4.86018472	7.961E-56	-2.15633	down	PREDICTED: flavonoid 3'-monooxygenase-like [Sesamum indicum]
c28203.graph_c0	8.12679349	7.49468202	7.90496112	4.15643062	4.59232427	3.84903499	2.072E-63	-1.99222	down	PREDICTED: disease resistance protein RGA2-like [Sesamum indicum]
c28206.graph_c0	0	0	0	1.36455869	1.20925248	1.50524479	1.035E-14	Inf	up	PREDICTED: cytokinin dehydrogenase 3-like [Sesamum indicum]
c28207.graph_c0	0.72439552	0.60426741	0.6723	6.02534539	8.02843629	6.53959875	4.431E-15	2.27277	up	PREDICTED: piriformospora indica-insensitive protein 2-like [Sesamum indicum]
c28208.graph_c0	4.26456225	3.59480637	3.51882117	17.3569719	17.6159539	15.6806274	2.595E-10	1.06543	up	PREDICTED: biotin carboxyl carrier protein of acetyl-CoA carboxylase 2, chloroplastic-like isoform X2 [Sesamum indicum]
c28209.graph_c0	11.0782775	9.92885514	13.5738844	4.40030844	3.719466	4.50736871	1.371E-14	-2.54653	down	PREDICTED: zinc finger CCCH domain-containing protein 23 [Sesamum indicum]
c28209.graph_c1	14.116436	14.4752195	15.6624961	5.50522866	6.10041106	7.55510001	4.788E-43	-2.29831	down	PREDICTED: zinc finger CCCH domain-containing protein 20-like [Sesamum indicum]
c28210.graph_c0	0.03074336	0	0	11.9987795	13.9204903	12.434633	1.988E-93	9.22566	up	PREDICTED: LRR receptor-like serine/threonine-protein kinase GSO1 [Sesamum indicum]

#ID	0 h-1 FPKM	0 h-2 FPKM	0 h-3 FPKM	28 h-1 FPKM	28 h-2 FPKM	28 h-3 FPKM	FDR	log2FC	regulated	nr_annotation
c28213.graph_c0	3.08007553	3.11309335	2.03665055	18.2129223	17.0725158	16.5109138	4.23E-26	1.56825	up	PREDICTED: probable mitochondrial chaperone BCS1-A [Sesamum indicum]
c28214.graph_c0	1.66114017	1.24710266	2.25322149	8.14932293	9.08404264	9.33158563	3.556E-08	1.26811	up	PREDICTED: myb family transcription factor APL-like [Sesamum indicum]
c28215.graph_c0	8.34731783	6.18320054	6.65051302	50.9003051	50.6142785	53.0687701	1.447E-34	1.77953	up	Uncharacterized protein isoform 1 [Theobroma cacao]
c28218.graph_c0	0.21827051	0.37143143	0.16829175	2.04642699	1.73314369	1.46275233	9.027E-06	1.70283	up	PREDICTED: pentatricopeptide repeat-containing protein At4g35130, chloroplastic [Sesamum indicum]
c28219.graph_c0	67.100205	65.0535516	76.6607825	32.9213063	39.6331372	39.982181	8.367E-25	-1.98399	down	PREDICTED: light-induced protein, chloroplastic-like [Sesamum indicum]
c28222.graph_c0	236.478816	231.936076	229.20001	90.0030211	90.6642633	88.0874484	4.72E-193	-2.46646	down	PREDICTED: uncharacterized protein LOC105155796 [Sesamum indicum]
c28223.graph_c0	4.92755412	4.70828431	5.46863366	22.2204499	24.9868652	22.1831125	6.306E-07	1.10724	up	PREDICTED: 50S ribosomal protein L29, chloroplastic-like [Solanum lycopersicum]
c28224.graph_c1	3.53225771	4.36774644	2.93705889	3.32229059	3.95417229	3.57354081	0.0054802	-1.08618	down	probable WRKY transcription factor 75-like [Solanum tuberosum]
c28226.graph_c0	0.04976265	0.04981248	0.06394698	4.23156331	2.7104146	3.74804256	2.087E-14	4.93737	up	hypothetical protein MIMGU_mgv1a020800mg [Erythranthe guttata]
c28228.graph_c1	3.09350378	2.54609453	2.73852266	1.79867258	2.43604904	2.2423227	8.367E-06	-1.46071	down	PREDICTED: LOW QUALITY PROTEIN: omega-hydroxypalmitate O-feruloyl transferase [Sesamum indicum]
c28230.graph_c0	20.5152463	17.7396215	19.1248744	1.76394917	1.20202318	2.14547323	1.2E-199	-4.57658	down	PREDICTED: inorganic pyrophosphatase 2 [Sesamum indicum]
c28233.graph_c0	0.36569308	0.41597644	0.7262556	3.32229533	3.56694119	3.71648774	2.122E-13	1.71468	up	hypothetical protein MIMGU_mgv1a025694mg [Erythranthe guttata]
c28237.graph_c0	1.43895253	1.46362558	0.83508237	12.0606939	11.9253372	11.2178512	3.887E-36	2.15123	up	hypothetical protein MIMGU_mgv1a003416mg [Erythranthe guttata]
c28239.graph_c0	25.9110514	21.2928728	25.197523	158.863213	173.252213	163.219915	1.634E-71	1.68343	up	PREDICTED: cytochrome c oxidase subunit 6a, mitochondrial [Sesamum indicum]
c28240.graph_c0	1.77790439	1.73574186	1.55132425	53.7606807	50.6582148	51.6265829	1.07E-259	3.85644	up	PREDICTED: LOW QUALITY PROTEIN: pyrophosphate--fructose 6-phosphate 1-phosphotransferase subunit beta [Sesamum indicum]
c28244.graph_c0	66.8375991	60.0360013	52.9049874	37.494935	41.0255422	39.3371496	6.706E-31	-1.69702	down	-
c28246.graph_c0	4.15667685	4.53909724	4.78653387	30.4851286	25.8956636	27.0526326	3.955E-24	1.53734	up	PREDICTED: thebaine 6-O-demethylase-like isoform X2 [Sesamum indicum]
c28248.graph_c0	1.8767961	2.75247795	2.74828156	2.28398163	1.97629539	2.07078929	5.134E-05	-1.31494	down	-
c28250.graph_c0	0.21478813	0.14333547	0.1840075	1.53504993	1.90282201	1.78334592	2.036E-06	2.17822	up	PREDICTED: probable glycosyltransferase At5g11130 [Sesamum indicum]
c28254.graph_c0	2.06750906	2.00194605	1.73649085	10.2828653	10.463865	10.2580131	2.5E-26	1.32846	up	PREDICTED: protein SCAR1-like isoform X2 [Sesamum indicum]
c28256.graph_c0	5.64154181	4.09326593	3.30854437	2.97198609	3.80151845	3.09875982	5.339E-05	-1.48691	down	PREDICTED: malate dehydrogenase [NADP], chloroplastic [Nicotiana sylvestris]
c28257.graph_c0	40.8207442	39.6693712	39.0987071	18.6771145	17.455807	20.3989823	1.41E-128	-2.17035	down	PREDICTED: lanC-like protein GCL1 [Sesamum indicum]
c28260.graph_c0	10.598748	9.68277929	11.1218542	44.267648	43.0674784	46.9641407	6.485E-21	1.00536	up	hypothetical protein M569_06331, partial [Genlisea aurea]

#ID	0 h-1 FPKM	0 h-2 FPKM	0 h-3 FPKM	28 h-1 FPKM	28 h-2 FPKM	28 h-3 FPKM	FDR	log2FC	regulated	nr_annotation
c28264.graph_c0	0.63848138	0.69998937	0.66419312	11.0707781	10.6343337	11.4859167	6.111E-41	2.96032	up	hypothetical protein MIMGU_mgv1a005920mg [Erythranthe guttata]
c28267.graph_c1	0.09485823	0.37981287	0	1.86144522	2.6766871	3.82543847	0.0005911	3.06439	up	PREDICTED: protein YLS9 [Sesamum indicum]
c28269.graph_c1	4.0257316	4.60544316	2.74497626	18.2718128	19.6786694	19.4897386	2.095E-08	1.25054	up	PREDICTED: vesicle-fusing ATPase [Sesamum indicum]
c28271.graph_c0	0	0	0	6.22164339	4.90259838	3.32300304	6.969E-09	Inf	up	PREDICTED: transcription factor bHLH79-like [Sesamum indicum]
c28275.graph_c0	0.03595314	0.05398371	0.04620121	3.01807662	3.12612838	2.96379997	3.287E-32	4.97139	up	PREDICTED: protein argonaute 7 isoform X1 [Sesamum indicum]
c28276.graph_c0	0.28330451	0.28358819	0.242705	4.97601533	4.77174342	4.9284708	2.537E-14	3.09169	up	PREDICTED: uncharacterized protein LOC105160853 [Sesamum indicum]
c28279.graph_c0	2.2700451	2.18262144	2.07267477	1.0203055	0.88203849	0.86798908	2.112E-17	-2.32574	down	PREDICTED: QWRF motif-containing protein 2-like [Sesamum indicum]
c28280.graph_c0	26.4104309	27.949284	31.2202668	13.6165542	13.4842593	17.5267017	7.356E-25	-2.03069	down	-
c28281.graph_c0	1.96001472	1.80501919	1.41047005	0	0	0	2.413E-29	-Inf	down	PREDICTED: extracellular ribonuclease LE-like isoform X1 [Phoenix dactylifera]
c28293.graph_c0	0.15330805	0.46038468	0	4.23408937	5.78478101	4.22780734	3.33E-11	3.45987	up	PREDICTED: nudix hydrolase 17, mitochondrial [Sesamum indicum]
c28296.graph_c1	0.25151993	0.37765768	0.32321307	4.57008085	2.80592107	2.75957	0.0001027	2.31848	up	-
c28302.graph_c0	3.36936384	2.56730783	3.48966209	65.2413295	71.8431997	71.9660583	1.93E-149	3.37935	up	PREDICTED: reticulon-like protein B2 isoform X1 [Nicotiana tomentosiformis]
c28305.graph_c0	0.74081264	1.01121062	0.77888747	4.1605055	3.53557307	4.15380713	0.003714	1.13745	up	PREDICTED: uncharacterized protein LOC105178720 [Sesamum indicum]
c28307.graph_c0	0.14860603	0.29750967	0.28644695	5.88632728	6.77759108	3.78967679	1.89E-08	3.39063	up	-
c28308.graph_c0	0	0	0	2.76868532	2.35281247	3.62140415	1.383E-14	Inf	up	PREDICTED: uncharacterized oxidoreductase At4g09670-like [Sesamum indicum]
c28311.graph_c1	0.0633247	0.25355244	0.32549898	1.19662468	1.57910446	1.53976441	0.0001966	1.64404	up	PREDICTED: uncharacterized protein LOC105155826 [Sesamum indicum]
c28312.graph_c0	0.40101606	0.6221973	0.77298226	10.1863927	9.52630454	10.0243594	1.914E-55	2.95163	up	PREDICTED: uncharacterized protein LOC105156537 [Sesamum indicum]
c28317.graph_c0	0.4857728	0.91173606	1.24847457	10.3269135	9.12499888	8.46268854	1.775E-14	2.29794	up	PREDICTED: cyclin-P3-1-like [Sesamum indicum]
c28318.graph_c0	18.4180081	17.9704427	15.6664097	8.0358907	7.33203119	8.93852783	6.02E-94	-2.18637	down	PREDICTED: pectinesterase 1-like [Sesamum indicum]
c28321.graph_c0	151.958408	144.991047	155.320504	85.8962794	88.036866	81.9113581	8.57E-111	-1.91321	down	PREDICTED: AP2/ERF and B3 domain-containing transcription factor RAV1-like [Sesamum indicum]
c28326.graph_c0	2.18156737	1.84120256	1.81396499	9.17132203	8.39312652	8.80288877	1.755E-06	1.08708	up	PREDICTED: WEB family protein At2g38370-like [Sesamum indicum]
c28328.graph_c0	3.09425412	2.49684542	2.2721366	12.8966731	13.820058	13.763045	5.576E-15	1.27707	up	PREDICTED: protein ASPARTIC PROTEASE IN GUARD CELL 2 [Sesamum indicum]
c28329.graph_c0	2.4695985	2.89829064	3.28296398	0.12378551	0.11176671	0.75755977	1.539E-21	-4.20741	down	PREDICTED: uncharacterized protein LOC105163289 [Sesamum indicum]
c28336.graph_c0	6.02666034	5.57451576	6.37205461	2.43957276	2.25276696	2.94076464	5.845E-18	-2.32586	down	PREDICTED: mavicyanin [Sesamum indicum]

#ID	0 h-1 FPKM	0 h-2 FPKM	0 h-3 FPKM	28 h-1 FPKM	28 h-2 FPKM	28 h-3 FPKM	FDR	log2FC	regulated	nr_annotation
c28337.graph_c0	1.85000861	0.79365476	1.35847675	8.25953582	6.0701285	10.8148896	0.0025569	1.56578	up	-
c28341.graph_c0	0.23098545	0.1849734	0.29682545	1.49412216	1.45515746	1.50686588	5.918E-05	1.54956	up	PREDICTED: pentatricopeptide repeat-containing protein At1g53600, mitochondrial isoform X1 [Sesamum indicum]
c28346.graph_c1	0.26645469	0.2667215	0.24457487	18.1208306	19.7585895	16.6601526	8.927E-86	5.04175	up	PREDICTED: lysM domain-containing GPI-anchored protein 1 [Sesamum indicum]
c28347.graph_c0	5.34714701	5.40347757	5.36615467	3.22005695	2.70689852	3.47318529	9.305E-17	-1.86745	down	PREDICTED: 60S ribosomal protein L3-like [Nicotiana tomentosiformis]
c28348.graph_c0	22.4992334	25.0919129	22.0808716	16.6394057	16.8491475	16.0569256	3.43E-58	-1.58202	down	PREDICTED: scarecrow-like protein 4 [Sesamum indicum]
c28350.graph_c0	9.52936894	9.28248882	8.42709084	62.4074858	61.626541	65.4172848	5.552E-56	1.70935	up	PREDICTED: gamma carbonic anhydrase 1, mitochondrial [Sesamum indicum]
c28351.graph_c0	3.35829492	2.49723147	1.84951797	0	0	0	3.418E-21	-Inf	down	-
c28352.graph_c0	5.9863192	6.70326606	7.04073708	1.62234052	1.93090091	1.50433706	6.761E-23	-3.05721	down	-
c28353.graph_c0	15.5665914	14.0127509	12.9520271	0.65114872	0.14698156	0.26566602	4.571E-91	-6.41015	down	hypothetical protein MIMGU_mgv1a016475mg [Erythranthe guttata]
c28357.graph_c0	2.24564729	1.70530039	2.18918638	17.8970785	20.0721906	18.0941029	1.844E-18	2.09942	up	hypothetical protein MIMGU_mgv1a013733mg [Erythranthe guttata]
c28360.graph_c0	0.15079841	0.03773735	0.04844548	3.01398388	2.49868937	2.14637344	2.073E-13	3.93599	up	-
c28362.graph_c0	8.88730854	6.44208154	7.87623679	2.00459316	3.21770609	2.54447145	5.477E-11	-2.66937	down	MAP kinase 20 [Theobroma cacao]
c28364.graph_c0	0.37376394	0.93534551	0.72045218	11.8167521	8.70722719	10.8615239	3.381E-09	2.85645	up	PREDICTED: uncharacterized protein At3g50808-like [Malus domestica]
c28364.graph_c1	0.74604487	0.18669798	0.7190229	12.199975	9.79149651	11.61426	6.179E-11	3.2542	up	hypothetical protein glysoja_041042 [Glycine soja]
c28372.graph_c0	0.11173369	0.33553672	0.23930369	6.41538466	7.087858	6.11842917	9.85E-27	3.74121	up	PREDICTED: endoplasmic reticulum-Golgi intermediate compartment protein 3 [Sesamum indicum]
c28382.graph_c0	2.3141341	1.55695911	1.75500233	22.8021434	23.9490279	24.9959723	2.049E-58	2.58525	up	hypothetical protein JCGZ_02248 [Jatropha curcas]
c28384.graph_c0	30.2889641	30.1068996	27.9478676	10.2936891	10.99992	10.1612731	1E-119	-2.57963	down	PREDICTED: uncharacterized protein LOC102593344 [Solanum tuberosum]
c28385.graph_c0	7.20413563	6.50780319	9.0318044	74.3245834	73.911188	88.8882472	1.993E-26	2.28896	up	hypothetical protein M569_02037, partial [Genlisea aurea]
c28386.graph_c0	25.0636073	26.0149755	27.0096451	14.7381197	14.4001648	16.6359617	4.473E-69	-1.86134	down	hypothetical protein MIMGU_mgv1a002759mg [Erythranthe guttata]
c28387.graph_c0	0.20487204	0.30761578	0.13163434	1.53863146	1.05313351	1.21501036	0.0015752	1.47865	up	PREDICTED: transcription factor HBP-1b(c38)-like [Sesamum indicum]
c28389.graph_c0	7.81179128	6.50639606	7.81620634	3.77059652	4.69585536	5.1633252	8.742E-18	-1.78981	down	PREDICTED: endo-1,3;1,4-beta-D-glucanase-like [Sesamum indicum]
c28393.graph_c0	0.39687429	0.6621195	0.6799989	2.6601126	2.3439568	2.64136923	0.0056677	1.04099	up	PREDICTED: uncharacterized protein LOC105167417 [Sesamum indicum]
c28395.graph_c0	1.08222216	1.44440779	0.92713222	12.322661	12.3098466	12.6224735	3.062E-06	2.3435	up	PREDICTED: protein transport protein SEC31 homolog B [Sesamum indicum]
c28395.graph_c1	7.1100379	8.56694887	6.26031791	43.6369586	44.3682943	45.1729868	4.98E-29	1.51361	up	PREDICTED: protein transport protein SEC31 homolog B [Sesamum indicum]
c28396.graph_c0	0	0	0	2.22344052	1.71232916	1.60086116	3.034E-10	Inf	up	PREDICTED: leucine-rich repeat extensin-like protein 3 [Vitis vinifera]

#ID	0 h-1 FPKM	0 h-2 FPKM	0 h-3 FPKM	28 h-1 FPKM	28 h-2 FPKM	28 h-3 FPKM	FDR	log2FC	regulated	nr_annotation
c28399.graph_c0	3.89165192	3.72617714	3.7688144	0.61487613	0.2590819	0.56863175	2.327E-34	-4.06887	down	hypothetical protein MIMGU_mgv1a013527mg [Erythranthe guttata]
c28400.graph_c0	9.01366711	10.3792517	12.595412	0.18324703	0.1034093	0.39251121	1.155E-43	-6.6467	down	-
c28401.graph_c0	13.2583821	12.8174952	10.6889547	9.59947648	8.99824506	10.3444682	2.045E-24	-1.43242	down	-
c28409.graph_c0	37.3504938	36.3941964	35.9577829	5.50137389	6.14795858	5.96090442	1.55E-214	-3.72922	down	PREDICTED: uncharacterized protein LOC105172478 [Sesamum indicum]
c28414.graph_c0	0.1132684	0.22676365	0.65499471	10.125707	9.32838854	8.39684374	8.922E-26	3.69498	up	PREDICTED: vacuole membrane protein KMS1-like [Sesamum indicum]
c28414.graph_c1	0.14955928	0.07485452	0.28828439	7.60891755	8.39138424	7.18448545	5.19E-20	4.39567	up	PREDICTED: vacuole membrane protein KMS1-like [Sesamum indicum]
c28415.graph_c0	19.8742326	20.4280158	18.4329362	15.0973355	15.7867257	15.4157758	2.282E-51	-1.43275	down	PREDICTED: DEAD-box ATP-dependent RNA helicase 18 [Sesamum indicum]
c28416.graph_c0	0.53584134	1.0727558	0.64554125	10.8801587	11.120412	10.6855233	8.81E-34	2.76747	up	PREDICTED: serine carboxypeptidase-like 51 [Sesamum indicum]
c28425.graph_c0	2.75118341	1.74815214	1.47563671	0	0	0	1.36E-22	-Inf	down	ATP dependent RNA helicase dbp2 [Aspergillus fumigatus var. RP-2014]
c28426.graph_c0	22.1569478	22.3221233	21.170854	19.3334787	19.8622909	19.5881709	1.375E-50	-1.24932	down	PREDICTED: methyltransferase-like protein 1 [Sesamum indicum]
c28428.graph_c0	64.5639069	70.791766	63.864618	46.7268897	45.0696353	44.786163	7.694E-86	-1.63481	down	PREDICTED: uncharacterized protein LOC105164194 [Sesamum indicum]
c28433.graph_c0	99.3998819	100.29014	110.983717	108.604108	107.735666	107.749507	8.47E-18	-1.03104	down	PREDICTED: iron-sulfur cluster assembly protein 1-like [Sesamum indicum]
c28434.graph_c0	131.03845	116.822983	145.114592	64.1024936	56.8449961	64.7300502	2.404E-23	-2.17351	down	Uncharacterized protein TCM_029816 [Theobroma cacao]
c28436.graph_c0	0.30932448	0	0	5.48549202	7.75410681	6.86093296	3.095E-21	4.96295	up	-
c28439.graph_c0	33.684611	38.1317364	28.555201	13.7149245	11.4574374	11.4004489	2.372E-30	-2.54491	down	PREDICTED: probable protein phosphatase 2C 23 [Sesamum indicum]
c28441.graph_c0	0.0333178	0	0	0.62959475	0.63405714	0.75085687	6.12E-09	4.85995	up	PREDICTED: LOW QUALITY PROTEIN: protein NRT1/ PTR FAMILY 2.7-like [Sesamum indicum]
c28442.graph_c0	0.6238483	0.07805912	0.10020872	4.25071262	3.94034148	3.46854678	3.787E-08	2.79065	up	--
c28444.graph_c0	1.27017838	0.9535877	1.22417213	43.6192308	48.9696179	41.0542861	3.006E-24	4.18501	up	enolase [Solanum lycopersicum]
c28444.graph_c1	3.93914738	4.02698741	2.69253209	110.436758	118.798854	115.862777	1.96E-181	3.93112	up	PREDICTED: enolase [Sesamum indicum]
c28447.graph_c0	0	0	0	17.0785352	11.3011905	12.4723528	6.851E-28	Inf	up	PREDICTED: putative expansin-B2 [Sesamum indicum]
c28450.graph_c0	0.29853022	0.22986858	0.32460421	6.60924143	5.95245583	6.59155082	5.802E-37	3.39673	up	PREDICTED: beta-galactosidase-like [Sesamum indicum]
c28463.graph_c0	31.6080866	30.7130504	32.8000211	27.6824337	25.5153638	24.3141609	4.415E-22	-1.38683	down	Vacuolar protein sorting-associated protein 32-2-like protein [Morus notabilis]
c28547.graph_c1	0.09233161	0.09242407	0.05932489	8.75734399	9.39152785	7.72088036	1.1E-37	5.64184	up	PREDICTED: uncharacterized protein LOC105173291 [Sesamum indicum]
c28558.graph_c0	0.45161367	0.7749701	0.33162368	3.70431237	3.51399596	3.13747429	0.0001499	1.64626	up	PREDICTED: thylakoid lumenal 17.9 kDa protein, chloroplastic [Nicotiana tomentosiformis]
c28609.graph_c0	0.62932319	0.22907395	0.51463059	11.1436462	11.2067456	10.8574619	3.484E-52	3.50617	up	PREDICTED: allantoinase [Sesamum indicum]

#ID	0 h-1 FPKM	0 h-2 FPKM	0 h-3 FPKM	28 h-1 FPKM	28 h-2 FPKM	28 h-3 FPKM	FDR	log2FC	regulated	nr_annotation
c28614.graph_c0	38.2836168	42.6919992	25.8926123	3.17294787	7.0519986	3.98323007	8.05E-18	-3.99845	down	-
c28687.graph_c0	3.66268429	3.86453309	3.68903068	3.11767854	3.50788745	3.15064644	2.789E-10	-1.28907	down	PREDICTED: pentatricopeptide repeat-containing protein At5g50390, chloroplastic [Sesamum indicum]
c28711.graph_c0	14.3117709	15.6349063	12.3970259	86.8350377	81.998279	80.9371501	6.756E-61	1.47178	up	hypothetical protein MIMGU_mgv1a018814mg [Erythranthe guttata]
c28741.graph_c0	1.65159489	1.53923156	1.2807374	18.2952892	17.733558	18.4583547	9.053E-57	2.51977	up	PREDICTED: LOW QUALITY PROTEIN: replication factor C subunit 2 [Sesamum indicum]
c28754.graph_c0	2.97872363	2.57043653	1.97988507	12.0936864	8.56032125	11.6958214	0.0012587	1.01843	up	-
c28816.graph_c0	6.05164464	5.1012248	6.54871828	59.2613113	55.284021	56.5263917	2.071E-53	2.1805	up	hypothetical protein MIMGU_mgv1a005703mg [Erythranthe guttata]
c28825.graph_c0	2.64666955	2.14468745	2.75325132	1.92358417	2.39846041	2.31707135	0.0015445	-1.27602	down	PREDICTED: transcription factor GAMYB-like isoform X1 [Sesamum indicum]
c28850.graph_c0	31.6129873	30.3441781	27.6105811	21.3297963	22.9313141	20.585638	5.136E-67	-1.55523	down	PREDICTED: cyclic dof factor 2-like [Sesamum indicum]
c28909.graph_c0	1.53850122	1.89543606	0.45623884	15.6974402	13.1637106	16.9148762	4.163E-15	2.47911	up	PREDICTED: uncharacterized protein LOC105163227 [Sesamum indicum]
c28943.graph_c0	1.37994784	0.92088643	0.72245051	6.61196253	5.39981539	6.94115395	5.471E-07	1.56598	up	PREDICTED: uncharacterized protein LOC105157329 isoform X2 [Sesamum indicum]
c28984.graph_c0	0.26828908	0.98471168	0.68952418	16.2492465	11.795923	12.0394012	2.005E-15	3.27065	up	PREDICTED: cyclin-dependent kinase inhibitor 7-like isoform X1 [Sesamum indicum]
c29041.graph_c0	2.14227092	2.09927048	2.05743273	10.0302945	9.24879033	8.93355955	1.46E-11	1.0728	up	PREDICTED: protein NRT1/ PTR FAMILY 1.2 isoform X1 [Sesamum indicum]
c29044.graph_c0	0.15069022	0	0	162.96693	165.24018	157.287799	9.81E-235	10.5946	up	Gip1-like protein [Populus tomentosa]
c29248.graph_c0	0.30337157	0.49347244	0.31674864	3.45891659	4.429544	5.25132697	2.268E-10	2.47236	up	hypothetical protein MIMGU_mgv1a000889mg [Erythranthe guttata]
c29280.graph_c1	0.10651454	0.1599318	0.0684377	2.16759636	2.55126694	2.11617218	2.973E-09	3.26599	up	PREDICTED: proteasome subunit beta type-3-A [Sesamum indicum]
c29287.graph_c0	9.62217067	10.6168769	9.97620215	67.4698195	66.3004513	70.9422556	3.878E-32	1.66982	up	hypothetical protein MIMGU_mgv1a016558mg [Erythranthe guttata]
c29351.graph_c0	2.9997934	3.3781469	2.08804553	13.1722384	15.6663419	13.935907	6.068E-05	1.25086	up	Rho GDP-dissociation inhibitor 1 [Arabidopsis thaliana]
c29353.graph_c0	37.7367091	30.4723635	33.7108621	216.86562	241.470546	231.537979	4.974E-65	1.66926	up	hypothetical protein PHAVU_003G132300g [Phaseolus vulgaris]
c29363.graph_c0	47.3257733	50.7745232	42.7181486	18.1501908	19.262523	20.3703801	6.873E-85	-2.37377	down	PREDICTED: cell division cycle protein 48 homolog [Nicotiana sylvestris]
c29442.graph_c0	0.04482006	0.40378445	0.05759555	2.37797208	3.35298335	2.57834743	1.341E-08	2.95086	up	PREDICTED: uncharacterized protein LOC105176814 [Sesamum indicum]
c29513.graph_c0	7.96650322	7.97448047	6.94672032	38.6689862	39.0220536	34.6751875	1.157E-16	1.20626	up	PREDICTED: ribosomal RNA-processing protein 17-like [Sesamum indicum]
c29525.graph_c0	0.85691168	0.81262397	1.33298974	5.70352285	7.8134083	6.25886534	3.137E-08	1.6218	up	PREDICTED: F-box/FBD/LRR-repeat protein At1g13570-like [Sesamum indicum]
c29552.graph_c0	16.5943951	20.4719498	16.8290292	17.0805741	16.7171473	14.2567909	1.755E-13	-1.25635	down	PREDICTED: succinate dehydrogenase assembly factor 2, mitochondrial-like isoform X1 [Sesamum indicum]

#ID	0 h-1 FPKM	0 h-2 FPKM	0 h-3 FPKM	28 h-1 FPKM	28 h-2 FPKM	28 h-3 FPKM	FDR	log2FC	regulated	nr_annotation
c29644.graph_c0	0.16175295	0.08095746	0	13.9897726	14.8074596	12.2789004	3.789E-36	6.3356	up	PREDICTED: homeobox-leucine zipper protein HOX21-like [Sesamum indicum]
c29668.graph_c0	13.3825095	12.329569	15.8281348	157.605271	169.740961	158.969044	4.22E-148	2.45597	up	PREDICTED: 20 kDa chaperonin, chloroplastic-like [Sesamum indicum]
c29678.graph_c1	25.479594	24.2227282	25.5170338	12.10422	11.5361411	10.4256638	7.771E-67	-2.23403	down	PREDICTED: myelin transcription factor 1 [Sesamum indicum]
c29682.graph_c0	0.66166739	0.27597081	0.14171146	8.53590477	10.9274683	10.6930974	7.24E-27	3.73021	up	PREDICTED: topless-related protein 3-like [Sesamum indicum]
c29739.graph_c0	84.5375655	82.962958	77.4497364	56.7188596	60.43603	57.8821494	1.231E-75	-1.57439	down	PREDICTED: mitochondrial fission 1 protein A [Sesamum indicum]
c29787.graph_c0	1.01790939	1.49842452	2.3852747	8.96476471	11.9450484	9.23274489	2.815E-06	1.51877	up	PREDICTED: cell division topological specificity factor homolog, chloroplastic-like [Sesamum indicum]
c29798.graph_c0	2.82753416	2.66054358	2.10742614	12.5768174	12.4318795	14.6560379	4.177E-09	1.2985	up	PREDICTED: chorismate mutase 1, chloroplastic-like isoform X1 [Sesamum indicum]
c29807.graph_c0	1.59721223	0.9326401	2.22352103	69.9731355	74.0972419	73.332144	5.94E-236	4.41824	up	PREDICTED: annexin D1-like [Sesamum indicum]
c29876.graph_c0	138.1294	140.868528	135.528206	31.9480534	34.2743129	30.5347883	0	-3.18971	down	PREDICTED: heat stress transcription factor B-2a-like [Sesamum indicum]
c29887.graph_c0	0.30527121	1.22230759	0.72853039	9.98412117	8.07032552	8.89699905	6.429E-18	2.4841	up	PREDICTED: ribokinase [Sesamum indicum]
c29900.graph_c0	15.3985536	16.9251467	14.0232521	15.7998777	15.5961514	17.0366119	3.436E-19	-1.02515	down	PREDICTED: remorin-like [Sesamum indicum]
c30136.graph_c0	71.4869259	70.6695836	61.4800954	6.53487445	6.41028821	8.55817955	0	-4.33056	down	PREDICTED: peroxisomal membrane protein 13 [Glycine max]
c30145.graph_c0	0.39657595	0.5954596	0.54146679	11.7273222	10.9465077	12.2888136	4.638E-61	3.41817	up	PREDICTED: oligopeptide transporter 3 [Sesamum indicum]
c30224.graph_c0	0.47901618	0.2996849	0.23083304	5.30922959	5.10807991	5.53964693	7.266E-12	2.90053	up	PREDICTED: glucosidase 2 subunit beta [Sesamum indicum]
c30362.graph_c0	1.10057778	1.00725014	1.2930615	4.7536533	5.55084504	5.25895058	7.599E-06	1.10107	up	PREDICTED: putative E3 ubiquitin-protein ligase XBAT34 isoform X2 [Sesamum indicum]
c30397.graph_c0	0.13062285	0.06537682	0.16785553	1.70884677	1.84294227	2.13034099	5.529E-08	2.86959	up	PREDICTED: peptidyl-prolyl cis-trans isomerase CYP20-2, chloroplastic [Sesamum indicum]
c30461.graph_c0	1.40598168	2.5802142	2.10786582	19.2450184	21.9127764	17.1629785	6.826E-14	2.1641	up	PREDICTED: trafficking protein particle complex subunit 2-like protein [Vitis vinifera]
c30490.graph_c0	0.95191813	0.97404625	1.16888571	6.68786992	6.26062595	5.41961105	1.125E-13	1.4752	up	PREDICTED: pentatricopeptide repeat-containing protein At3g61520, mitochondrial [Sesamum indicum]
c30494.graph_c0	1.7030138	1.42059926	0.36474003	10.9333754	10.803116	9.76320027	2.593E-05	2.09845	up	-
c30514.graph_c1	1.92862843	2.46454425	2.3729016	11.6312607	11.1482109	9.73437304	4.594E-09	1.17148	up	PREDICTED: uncharacterized protein LOC105170005 [Sesamum indicum]
c30648.graph_c0	1.00648748	1.00749532	1.50893897	1.01598514	0.99072647	0.99484269	0.0012115	-1.32735	down	PREDICTED: WEB family protein At1g75720 [Sesamum indicum]
c30774.graph_c0	0.36960455	0.09249366	0.23747824	6.44703498	7.73110609	6.52111062	1.615E-23	3.79992	up	PREDICTED: probable 6-phosphogluconolactonase 4, chloroplastic [Sesamum indicum]
c30802.graph_c0	1.93148443	1.41288276	1.43194267	10.5284381	12.723673	11.0583128	7.428E-10	1.75696	up	-
c30813.graph_c0	0.36799448	0.49115063	0.55170206	2.31795432	2.01239944	2.69165294	0.0042217	1.22152	up	--

#ID	0 h-1 FPKM	0 h-2 FPKM	0 h-3 FPKM	28 h-1 FPKM	28 h-2 FPKM	28 h-3 FPKM	FDR	log2FC	regulated	nr_annotation
c30953.graph_c0	4.2618401	2.80807088	2.07973415	19.094597	20.2497758	19.9322072	9.075E-18	1.61333	up	PREDICTED: protein sym-1-like [Sesamum indicum]
c30987.graph_c0	0.55284466	0.69174781	0.17760682	12.6568359	11.3825871	11.1885309	5.449E-24	3.55124	up	-
c30990.graph_c0	0	0	0	0.67959747	0.58507267	0.5674424	5.574E-14	Inf	up	PREDICTED: sucrose synthase 5-like [Sesamum indicum]
c31059.graph_c0	0.7824693	0.7205926	0.98539395	4.53817955	4.40563715	4.0557953	4.259E-11	1.29067	up	hypothetical protein MIMGU_mgv1a000521mg [Erythranthe guttata]
c31079.graph_c0	2.53451683	2.37069052	3.04338369	11.1128292	12.0242518	11.5321023	3.61E-08	1.03149	up	hypothetical protein MIMGU_mgv1a018718mg [Erythranthe guttata]
c31084.graph_c0	0.71505966	0.63624506	0.61258665	4.21535452	3.85820785	3.53393308	0.0005764	1.47392	up	PREDICTED: uncharacterized protein LOC105169539 [Sesamum indicum]
c31162.graph_c0	0.12962359	0.19463008	0.33314288	6.3120482	6.71993775	5.91932304	7.151E-17	3.74681	up	-
c31209.graph_c0	3.6357687	3.2438214	2.84389105	2.87223449	2.69709279	2.2499711	9.569E-06	-1.40299	down	hypothetical protein MIMGU_mgv1a021709mg [Erythranthe guttata]
c31272.graph_c0	7.02627349	9.43944135	8.3162207	7.59278979	8.12962262	8.60812169	9.326E-07	-1.11792	down	PREDICTED: choline-phosphate cytidylyltransferase 2-like isoform X1 [Sesamum indicum]
c31392.graph_c0	0.25057653	0.18812058	0.16100038	1.5935288	1.72656852	1.74612585	4.335E-05	1.99331	up	PREDICTED: transcription repressor MYB5-like [Sesamum indicum]
c31417.graph_c0	1.83118465	0.6110061	1.08606682	5.90371129	6.10080092	6.37676339	3.684E-05	1.29636	up	PREDICTED: probable polygalacturonase [Sesamum indicum]
c31482.graph_c0	1.98169641	1.7569744	2.54655834	32.797359	40.0907935	33.7467867	6.713E-45	2.98955	up	hypothetical protein MIMGU_mgv1a010578mg [Erythranthe guttata]
c31565.graph_c0	0.74818645	0.27234023	0.87404474	7.11854313	7.09689546	6.33306716	1.181E-10	2.34312	up	PREDICTED: dual specificity tyrosine-phosphorylation-regulated kinase 3-like isoform X3 [Cucumis melo]
c31599.graph_c0	57.9215481	56.7653164	58.3983734	36.8703384	43.1269221	38.4873363	1.827E-81	-1.63783	down	PREDICTED: uncharacterized protein LOC105172751 isoform X2 [Sesamum indicum]
c31611.graph_c0	3.809044	5.73615832	4.93808891	23.0045961	24.5756908	22.0101069	4.349E-16	1.17157	up	PREDICTED: alpha/beta hydrolase domain-containing protein 17B-like [Sesamum indicum]
c31665.graph_c0	6.23893187	6.83593942	7.04220055	4.71824756	6.36253953	5.5500742	3.748E-08	-1.36671	down	PREDICTED: synaptotagmin-3 [Sesamum indicum]
c31685.graph_c0	0.6702548	0.31309878	0.8038837	10.944352	11.7290489	13.0645027	3.775E-34	3.22697	up	PREDICTED: uncharacterized protein At1g04910 [Sesamum indicum]
c31685.graph_c1	0.23303277	0.93306445	0.49909388	12.4202372	11.0613873	11.655034	1.078E-20	3.30604	up	PREDICTED: uncharacterized protein At1g04910 [Sesamum indicum]
c31695.graph_c0	1.26413638	0.67488118	0.32489306	4.34883063	6.13874762	5.54782734	5.333E-05	1.74823	up	hypothetical protein MIMGU_mgv1a022283mg [Erythranthe guttata]
c31982.graph_c0	9.82888783	10.3481788	10.4232364	53.657757	55.2945066	53.3674109	5.966E-45	1.31591	up	PREDICTED: LOW QUALITY PROTEIN: probable phenylalanine--tRNA ligase alpha subunit [Sesamum indicum]
c32009.graph_c0	3.39750251	2.93411377	4.02349973	23.0466623	24.5248636	22.5986724	2.503E-19	1.66684	up	hypothetical protein MIMGU_mgv1a016742mg [Erythranthe guttata]
c32020.graph_c0	2.30050561	1.66953668	1.77374447	13.2087543	13.7001102	13.1999278	1.919E-13	1.71588	up	PREDICTED: uncharacterized protein LOC105165412 [Sesamum indicum]
c32140.graph_c0	6.28590933	5.4714815	5.70702993	26.7409891	25.870822	25.4875575	5.511E-19	1.07114	up	PREDICTED: cytochrome P450 CYP72A219-like [Sesamum indicum]
c32190.graph_c0	90.6177498	86.4050063	87.891596	85.7443921	91.7818091	90.3971395	1.022E-38	-1.07396	down	glutathione peroxidase, partial [Eleutherococcus senticosus]
c32191.graph_c0	0.73024081	0.73097204	1.02369639	4.39062438	4.22570604	5.07879187	0.0001952	1.36796	up	PREDICTED: structure-specific endonuclease subunit SLX1 homolog [Sesamum indicum]

#ID	0 h-1 FPKM	0 h-2 FPKM	0 h-3 FPKM	28 h-1 FPKM	28 h-2 FPKM	28 h-3 FPKM	FDR	log2FC	regulated	nr_annotation
c32206.graph_c0	1.65948116	1.42383676	2.38636793	29.6930665	33.1366057	32.8056283	4.544E-91	3.03187	up	PREDICTED: magnesium-chelatase subunit ChlI, chloroplastic-like [Sesamum indicum]
c32223.graph_c0	3.31717611	3.73555998	3.06381752	15.2565049	16.4281937	13.7402099	1.804E-08	1.07685	up	PREDICTED: cell division topological specificity factor homolog, chloroplastic-like [Sesamum indicum]
c32244.graph_c0	5.81760121	5.5128439	5.88100033	31.6269287	31.5084807	33.9037283	2.101E-17	1.40469	up	PREDICTED: calcineurin subunit B-like isoform X1 [Sesamum indicum]
c32295.graph_c0	1.02712074	0.69914149	0.52795633	0	0	0	1.719E-18	-Inf	down	--
c32333.graph_c0	1.64870335	1.2519929	0.94973924	8.51183542	7.49885075	8.36167899	7.063E-08	1.57832	up	Alpha-soluble NSF attachment protein 2 [Theobroma cacao]
c32358.graph_c0	2.28533813	3.1126722	1.97388113	18.6791118	23.6018472	19.3968926	2.432E-18	1.97617	up	PREDICTED: uncharacterized protein LOC105174506 [Sesamum indicum]
c32363.graph_c1	0.15991793	0.37351548	0.41100174	1.51095657	2.20379188	2.33940227	0.0002969	1.58204	up	PREDICTED: disease resistance response protein 206-like [Sesamum indicum]
c32364.graph_c0	1.01538057	1.01639732	0.86986949	5.82178499	5.70073947	5.01816445	0.0008633	1.42192	up	CTP synthase 1a [Malus domestica]
c32392.graph_c0	1.47489552	1.29182586	1.73735755	0.71463038	1.45179951	0.80180726	1.638E-05	-1.69692	down	hypothetical protein MIMGU_mgv1a018953mg, partial [Erythranthe guttata]
c32414.graph_c0	1.24662515	1.31532607	0.82262948	25.099756	28.0354507	26.5756005	2.92E-100	3.4723	up	hypothetical protein MIMGU_mgv1a007303mg [Erythranthe guttata]
c32453.graph_c1	6.1624421	6.42563839	5.54328746	37.8460915	34.6095681	35.9986629	1.243E-29	1.49153	up	PREDICTED: mitochondrial import inner membrane translocase subunit TIM17-2-like [Sesamum indicum]
c32471.graph_c0	16.6823588	17.4124218	15.5265716	10.7826916	11.2450148	10.5852012	6.091E-57	-1.69523	down	PREDICTED: double-stranded RNA-binding protein 3-like isoform X1 [Sesamum indicum]
c32488.graph_c0	2.38068354	3.40438205	2.07593498	26.2011871	30.0736771	24.9600887	7.743E-26	2.28079	up	PREDICTED: 30S ribosomal protein S31, chloroplastic [Sesamum indicum]
c32525.graph_c0	0	0.84821266	0.2177793	8.49885953	7.34003534	6.53297099	2.102E-09	3.29895	up	hypothetical protein MIMGU_mgv1a012373mg [Erythranthe guttata]
c32529.graph_c0	2.62442529	3.15246391	3.56520414	80.2205166	77.3522305	76.3098747	9.2E-133	3.55169	up	PREDICTED: FK506-binding protein 2-like [Sesamum indicum]
c32549.graph_c0	14.6502265	10.7778155	9.52647094	67.2216255	64.1697197	72.4385302	1.998E-16	1.4587	up	vacuolar ATPase subunit c [Cucumis sativus]
c32550.graph_c0	3.6642444	3.95748571	3.46956778	44.5026668	52.3311737	44.0340721	2.357E-45	2.57663	up	hypothetical protein MIMGU_mgv1a022013mg [Erythranthe guttata]
c32563.graph_c0	22.282251	22.0951308	17.7447506	4.10567619	4.73677417	3.53632037	1.192E-68	-3.41605	down	-
c32594.graph_c0	24.8678592	17.1878585	15.5976611	0	0	0	1.688E-32	-Inf	down	-
c32609.graph_c0	7.67890211	8.43650272	8.18296511	44.6478543	49.2848883	51.0940576	2.284E-12	1.4866	up	hypothetical protein MIMGU_mgv1a016321mg [Erythranthe guttata]
c32611.graph_c0	39.6636704	37.8349929	36.1782706	19.7834597	22.9662119	19.740756	4.649E-39	-1.95321	down	-
c32641.graph_c0	7.90086449	7.16910631	6.86600594	31.9751248	36.1814258	33.7772915	6.009E-17	1.12716	up	hypothetical protein MIMGU_mgv1a011069mg [Erythranthe guttata]
c32655.graph_c0	3.88549272	3.05049683	2.44755489	1.43966268	2.54976543	1.53621545	7.176E-08	-1.85091	down	hypothetical protein AMTR_s00033p00078670, partial [Amborella trichopoda]
c32658.graph_c0	1.90706085	0.82998717	1.1187744	0	0	0	9.039E-12	-Inf	down	Glucose-6-phosphate isomerase 2 [Neocallimastix frontalis]

#ID	0 h-1 FPKM	0 h-2 FPKM	0 h-3 FPKM	28 h-1 FPKM	28 h-2 FPKM	28 h-3 FPKM	FDR	log2FC	regulated	nr_annotation
c32667.graph_c0	0.24301298	0.3040704	0.3122813	1.32465325	1.55484899	1.22502706	0.0098459	1.16181	up	PREDICTED: S-adenosylmethionine decarboxylase proenzyme 4 [Sesamum indicum]
c32674.graph_c0	25.3558486	24.1121767	18.6613269	18.3447188	18.2652968	21.8385409	3.556E-11	-1.30699	down	unnamed protein product [Coffea canephora]
c32693.graph_c0	1.19626477	0.61491325	0.66475571	7.5194667	9.75972428	8.53262945	1.049E-17	2.2977	up	PREDICTED: BTB/POZ domain-containing protein At3g05675 isoform X2 [Sesamum indicum]
c32721.graph_c0	1.13821912	1.22481079	1.42609022	8.41727991	7.73072734	7.81347386	1.654E-14	1.56731	up	PREDICTED: histone-lysine N-methyltransferase setd3 [Sesamum indicum]
c32725.graph_c0	0.3001718	0.26291333	0.48216581	28.7975337	31.7631629	25.2787457	1.436E-82	5.26094	up	PREDICTED: putative E3 ubiquitin-protein ligase XBAT31 [Sesamum indicum]
c32747.graph_c1	6.03968112	4.45474765	4.79970763	1.44394915	2.81610152	2.87493175	3.804E-14	-2.18722	down	PREDICTED: protein ODORANT1-like [Sesamum indicum]
c32765.graph_c0	2.98241767	1.83717176	2.21107199	13.0889932	11.9686832	12.4152444	6.166E-09	1.32657	up	hypothetical protein MIMGU_mgv1a014004mg [Erythranthe guttata]
c32792.graph_c1	9.29295874	9.2530459	8.52987136	4.68139164	5.51750131	5.56958529	7.388E-28	-1.86911	down	PREDICTED: alpha/beta hydrolase domain-containing protein 17C-like [Sesamum indicum]
c32815.graph_c0	1.06229048	0.54686788	0.62403901	0	0	0	5.173E-14	-Inf	down	PREDICTED: aldehyde dehydrogenase family 2 member B7, mitochondrial-like [Solanum tuberosum]
c32819.graph_c0	5.50723908	2.78958623	2.72848974	0	0	0	7.519E-14	-Inf	down	--
c32832.graph_c0	4.05438797	3.70296334	3.72689641	59.1061866	53.4838673	49.1603689	7.191E-68	2.72582	up	Cytochrome P450 77A3 [Morus notabilis]
c32844.graph_c0	36.8034373	33.7666071	31.1722436	35.994143	36.7884428	33.8455055	5.176E-30	-1.02129	down	PREDICTED: uncharacterized protein LOC105156000 [Sesamum indicum]
c32856.graph_c0	1.478013	0.87029	1.00551453	6.9507792	7.18876038	7.06394302	1.857E-05	1.5739	up	-
c32883.graph_c0	1.50947781	0.94436833	0.60616836	17.2104389	17.5824431	16.8411031	1.825E-21	2.99625	up	PREDICTED: uncharacterized protein LOC105167888 isoform X3 [Sesamum indicum]
c32889.graph_c0	1.38257699	0.5766506	0.61689797	0	0	0	1.168E-09	-Inf	down	phosphoenolpyruvate carboxylase [Dendrobium catenatum]
c32893.graph_c0	0.67508453	0.59129046	0.54219396	4.10917317	4.54083947	4.05368943	7.583E-05	1.72392	up	-
c32893.graph_c1	0.59268189	1.18655074	0.30464788	5.38447226	6.80634305	4.53428332	2.88E-05	1.92064	up	PREDICTED: RNA polymerase sigma factor sigE, chloroplastic/mitochondrial-like [Sesamum indicum]
c32947.graph_c0	110.849502	116.44729	113.390789	54.0121932	55.0144828	51.6924062	2.47E-151	-2.17488	down	PREDICTED: uncharacterized protein LOC105174506 [Sesamum indicum]
c32955.graph_c0	2.34736609	4.06681723	2.55238785	0.08202174	0	0.02677163	1.625E-20	-7.4545	down	Uncharacterized protein TCM_033752 [Theobroma cacao]
c32968.graph_c0	7.2876849	5.37227387	6.24330909	6.19995159	5.19017548	7.30388047	8.162E-07	-1.1039	down	PREDICTED: transcription factor DIVARICATA [Sesamum indicum]
c32986.graph_c0	0.68964474	0.86291914	0.92314666	0.0835383	0.11314088	0.15337479	1.25E-18	-3.91387	down	PREDICTED: nucleoprotein TPR-like [Phoenix dactylifera]
c32989.graph_c1	3.35152678	1.54143265	2.59962693	0	0	0	1.449E-18	-Inf	down	hypothetical protein CHLNCDRAFT_59390 [Chlorella variabilis]
c32997.graph_c0	7.35476974	6.58717291	5.47173018	228.164361	205.248962	211.203249	5.905E-82	3.96627	up	-
c32998.graph_c0	203.362964	201.806297	195.028611	41.03611	45.3564646	45.2738335	0	-3.27843	down	PREDICTED: cytochrome P450 CYP82D47-like [Sesamum indicum]

#ID	0 h-1 FPKM	0 h-2 FPKM	0 h-3 FPKM	28 h-1 FPKM	28 h-2 FPKM	28 h-3 FPKM	FDR	log2FC	regulated	nr_annotation
c33046.graph_c0	1.31151944	1.14159368	1.90518324	6.71387707	7.59621177	6.62827097	2.819E-05	1.16748	up	PREDICTED: E3 ubiquitin-protein ligase RING1-like [Sesamum indicum]
c33052.graph_c0	0.68591139	0.21126099	0.40681075	4.67838507	4.34532751	3.95833432	4.62E-14	2.22981	up	PREDICTED: uncharacterized protein LOC105165576 [Sesamum indicum]
c33085.graph_c0	12.9225782	14.7560727	11.9317362	13.009736	12.2490989	15.2141426	3.702E-09	-1.05708	down	PREDICTED: uncharacterized protein LOC105171255 [Sesamum indicum]
c33101.graph_c1	3.10871924	4.38485439	2.90532894	2.15669926	4.82187883	2.76547697	0.0026493	-1.18433	down	PREDICTED: E3 ubiquitin-protein ligase RHA1B-like [Sesamum indicum]
c33104.graph_c0	1.56109463	1.11618416	1.0030342	0.36469111	0.80491127	0.69436493	1.767E-07	-2.06677	down	hypothetical protein MIMGU_mgv1a001430mg [Erythranthe guttata]
c33108.graph_c0	1.11620127	1.02610926	1.02454487	19.4368553	21.3764999	19.9267163	5.42E-102	3.17169	up	unnamed protein product [Coffea canephora]
c33114.graph_c0	3.43614575	4.28702089	5.24750482	4.16228487	3.79083258	3.86893175	0.0004106	-1.22939	down	PREDICTED: uncharacterized protein LOC105172002 isoform X2 [Sesamum indicum]
c33115.graph_c0	2.63172072	1.39465905	1.88986591	0	0	0	2.653E-23	-Inf	down	--
c33125.graph_c0	9.02732222	8.25736501	8.60034582	0.22624158	1.2256496	1.29227896	3.976E-31	-4.32584	down	-
c33126.graph_c0	6.63961022	4.99429851	6.51008893	26.5549143	28.9633273	27.1312638	2.376E-19	1.09626	up	hypothetical protein MIMGU_mgv1a010921mg [Erythranthe guttata]
c33148.graph_c0	1.38654401	0.76701529	0.70332787	0	0	0	3.379E-13	-Inf	down	PREDICTED: serpin-ZX-like isoform X2 [Pyrus x bretschneideri]
c33167.graph_c0	6.35116868	6.61182955	9.14088359	8.30878703	8.00219271	5.1225929	0.0021373	-1.14194	down	hypothetical protein EUGRSUZ_A02667 [Eucalyptus grandis]
c33253.graph_c0	3.90726761	3.64601539	4.93589198	3.46549372	2.30330338	2.08158573	4.292E-08	-1.76495	down	PREDICTED: uncharacterized protein LOC105179909 [Sesamum indicum]
c33309.graph_c0	6.61061767	6.61723721	6.04445194	2.95665158	3.79580788	3.27963295	1.752E-18	-2.03164	down	PREDICTED: transcription factor DIVARICATA-like [Sesamum indicum]
c33310.graph_c0	0.06813807	0	0.02918671	15.5335127	16.2610079	15.6655476	1.06E-150	7.84894	up	PREDICTED: beta-xylosidase/alpha-L-arabinofuranosidase 2-like [Sesamum indicum]
c33328.graph_c0	0.30731526	0.30762299	0.23694737	1.83151068	1.53268127	2.40577688	0.0003651	1.67455	up	PREDICTED: uncharacterized protein LOC105177453 isoform X1 [Sesamum indicum]
c33366.graph_c0	2.11798459	2.48207465	1.92510067	22.2640981	21.7295683	22.8853115	3.047E-34	2.26881	up	PREDICTED: lamin-like protein-like [Solanum tuberosum]
c33380.graph_c0	0	0	0	1.48050406	1.01593483	1.47385665	3.334E-14	Inf	up	PREDICTED: protein PHYTOCHROME KINASE SUBSTRATE 4-like [Sesamum indicum]
c33390.graph_c0	30.1209212	23.5398819	32.0197259	22.8372012	20.948193	24.03555	2.83E-08	-1.42882	down	Uncharacterized protein isoform 2, partial [Theobroma cacao]
c33412.graph_c0	10.3104198	12.384893	8.89595703	59.4131759	65.0017021	59.3997354	5.006E-32	1.45218	up	PREDICTED: plastidic ATP/ADP-transporter-like [Nicotiana sylvestris]
c33412.graph_c1	6.9414468	8.6468948	7.58202887	42.4340852	44.1597957	39.565885	8.455E-36	1.35343	up	PREDICTED: plastidic ATP/ADP-transporter [Sesamum indicum]
c33416.graph_c0	0	0	0	2.46857924	2.7905071	2.91841459	3.333E-26	Inf	up	PREDICTED: uncharacterized protein LOC105176193 [Sesamum indicum]
c33420.graph_c0	2.97469568	2.90999996	2.60631935	14.0201884	13.9750879	12.6699306	7.07E-18	1.17044	up	PREDICTED: ceramide kinase isoform X1 [Sesamum indicum]
c33422.graph_c0	2.06308793	1.00128669	2.89216327	15.9940311	14.9744469	14.6823736	1.676E-15	1.8402	up	hypothetical protein MIMGU_mgv1a008498mg [Erythranthe guttata]

#ID	0 h-1 FPKM	0 h-2 FPKM	0 h-3 FPKM	28 h-1 FPKM	28 h-2 FPKM	28 h-3 FPKM	FDR	log2FC	regulated	nr_annotation
c33426.graph_c1	26.7865892	29.9615453	23.1336979	11.3499626	13.5216034	13.1847413	1.26E-30	-2.15796	down	PREDICTED: dnaJ homolog subfamily C member 21 [Sesamum indicum]
c33429.graph_c0	268.414368	245.710036	279.17083	95.0409578	109.726458	100.917253	3.82E-66	-2.46736	down	hypothetical protein MIMGU_mgv1a013716mg [Erythranthe guttata]
c33432.graph_c0	0.44987949	0.39028598	0.30832691	2.98632434	2.79477858	2.86370379	3.145E-07	1.82606	up	PREDICTED: WEB family protein At2g38370 [Sesamum indicum]
c33440.graph_c0	8.38115755	7.40254412	5.38505963	5.73306071	4.36760125	3.65478962	1.69E-06	-1.70839	down	-
c33444.graph_c0	1.09699236	0.93541071	1.30525797	11.1029698	11.0914237	10.1924242	2.022E-21	2.18461	up	PREDICTED: uncharacterized protein LOC105178046 isoform X1 [Sesamum indicum]
c33448.graph_c0	0.71702904	1.67474308	2.14995831	12.4203263	11.0575455	12.1902112	3.236E-08	1.87229	up	hypothetical protein MIMGU_mgv1a007817mg [Erythranthe guttata]
c33454.graph_c0	2.74468706	3.32584291	3.52703154	0.73493469	0.56878039	0.25701459	3.071E-16	-3.71709	down	-
c33454.graph_c1	3.1991485	2.73141785	3.14373072	2.39350865	2.77857502	2.17629306	8.223E-05	-1.39607	down	-
c33455.graph_c0	1.48042556	1.55083393	1.37150161	12.7113977	12.7649964	12.802151	8.704E-26	2.03066	up	hypothetical protein MIMGU_mgv1a006888mg [Erythranthe guttata]
c33456.graph_c0	3.85122789	3.85508431	2.41603763	132.56919	117.389167	117.832929	2.14E-175	4.09826	up	PREDICTED: hexose carrier protein HEX6-like [Sesamum indicum]
c33458.graph_c0	1.94462046	2.50890948	2.08242856	17.3212539	18.3051256	17.2862814	2.488E-49	1.92632	up	PREDICTED: protein argonaute 10 [Sesamum indicum]
c33459.graph_c0	0.57983108	0.90286263	0.49673726	21.445488	17.6721501	17.5757594	4.402E-42	3.75229	up	-
c33460.graph_c0	6.61440114	7.62857166	7.04794373	6.55406363	5.99858475	6.12773436	1.818E-26	-1.27969	down	PREDICTED: protein PHLOEM PROTEIN 2-LIKE A10 [Sesamum indicum]
c33463.graph_c0	0.54027516	0.59232246	0.95876067	0.33657332	0.21947911	0.33567243	0.0003231	-2.32683	down	PREDICTED: 187-kDa microtubule-associated protein AIR9-like [Nicotiana sylvestris]
c33471.graph_c0	0.62858041	0.96628653	1.06738487	7.7664265	8.24982936	7.4157536	2.577E-25	2.0413	up	hypothetical protein MIMGU_mgv1a002735mg [Erythranthe guttata]
c33473.graph_c0	26.1000759	28.8792958	20.9171892	27.6585868	21.9527639	20.1391659	2.172E-08	-1.21005	down	-
c33474.graph_c0	0	0	0	1.99185707	1.98291763	2.75057387	2.494E-14	Inf	up	60s ribosomal protein l6 [Lichtheimia corymbifera JMRC:FSU:9682]
c33475.graph_c0	6.1476594	6.90888471	5.96133892	5.89348426	4.50451162	6.5537082	6.81E-14	-1.25435	down	PREDICTED: uncharacterized protein LOC102591997 [Solanum tuberosum]
c33480.graph_c0	0.13709011	0.20584107	0.15414544	1.05863568	0.94460348	0.96546608	0.0002636	1.48752	up	PREDICTED: uncharacterized protein LOC105159750 isoform X2 [Sesamum indicum]
c33489.graph_c0	7.22543633	6.93543844	5.34203854	2.80554539	2.79295413	2.99370279	9.77E-16	-2.26906	down	hypothetical protein 2_207_02, partial [Pinus radiata]
c33494.graph_c0	0	0	0	5.55233471	5.48322869	7.07915591	4.298E-21	Inf	up	putative receptor protein kinase [Arabidopsis thaliana]
c33494.graph_c1	0	0.04276892	0	4.08348357	3.42064138	3.58548007	3.428E-38	6.93671	up	PREDICTED: probable LRR receptor-like serine/threonine-protein kinase At4g36180 [Sesamum indicum]
c33496.graph_c0	4.2681701	5.40194072	4.16085815	34.4438007	34.8340408	36.9798635	6.424E-42	1.85239	up	PREDICTED: myosin heavy chain, muscle [Sesamum indicum]
c33503.graph_c0	80.1748543	86.0690748	84.2955235	23.0516594	22.5697419	22.0856691	3.78E-204	-2.9788	down	hypothetical protein MIMGU_mgv1a015119mg [Erythranthe guttata]
c33510.graph_c0	8.33610726	9.37372655	6.84261252	2.21527172	2.67494308	3.02725862	3.024E-27	-2.71958	down	PREDICTED: non-structural maintenance of chromosomes element 4 homolog A-like [Sesamum indicum]

#ID	0 h-1 FPKM	0 h-2 FPKM	0 h-3 FPKM	28 h-1 FPKM	28 h-2 FPKM	28 h-3 FPKM	FDR	log2FC	regulated	nr_annotation
c33512.graph_c0	0.53952087	0.74258403	0.38998444	7.18070953	5.70902703	6.13937168	4.695E-17	2.42259	up	PREDICTED: probable Histone-lysine N-methyltransferase ATXR5 [Sesamum indicum]
c33518.graph_c0	1.44939576	1.16585928	1.79601197	6.94123583	6.72586699	6.44681988	1.879E-08	1.09441	up	unnamed protein product [Coffea canephora]
c33518.graph_c1	1.24683606	0.91770925	1.03673945	6.23674187	6.83443956	5.39360832	4.613E-08	1.43825	up	PREDICTED: protein STICHEL isoform X2 [Sesamum indicum]
c33519.graph_c0	1.02502371	1.33386515	0.92203723	0.2234944	0.16816207	0.45592401	1.378E-12	-3.03654	down	PREDICTED: protein IQ-DOMAIN 1 [Sesamum indicum]
c33520.graph_c0	16.1483398	13.5875591	14.4356563	8.70883825	11.2420108	8.60533468	1.719E-36	-1.71975	down	-
c33525.graph_c0	19.6987782	18.7226195	21.9896874	2.53077548	3.2643614	1.88808423	2.841E-74	-4.0694	down	PREDICTED: mitogen-activated protein kinase 9-like [Sesamum indicum]
c33528.graph_c0	32.9971994	34.6324543	32.3821673	19.7166881	21.1419392	20.3481344	9.565E-82	-1.79865	down	-
c33538.graph_c0	0.77284396	0.89263597	0.53476504	4.40717311	4.29136325	4.47764307	9.322E-05	1.49665	up	-
c33540.graph_c0	0.92892036	1.77516919	1.62777196	11.2010862	12.9674858	11.2434457	3.252E-19	1.93541	up	PREDICTED: uncharacterized protein LOC105174130 [Sesamum indicum]
c33544.graph_c0	0.12569352	0.18872908	0.16152115	1.18759326	1.44346107	1.26724068	8.664E-05	1.94122	up	-
c33557.graph_c0	0.0239816	0.02400562	0.03081731	15.965597	15.8790132	14.8767199	9.17E-142	8.11724	up	PREDICTED: prolyl endopeptidase-like [Sesamum indicum]
c33558.graph_c0	71.811315	73.8799793	77.1104292	26.4287611	27.8755743	27.480693	7.97E-122	-2.53726	down	PREDICTED: uncharacterized protein LOC102628603 [Citrus sinensis]
c33565.graph_c0	3.91661588	3.25977298	3.95854441	17.3672989	20.705907	19.2608133	8.286E-16	1.27297	up	PREDICTED: protein FLX-like 3 [Nicotiana sylvestris]
c33566.graph_c0	18.9745102	18.0292589	18.6830755	18.9355687	18.3319113	17.9356954	3.263E-34	-1.10323	down	PREDICTED: exocyst complex component EXO70A1 [Sesamum indicum]
c33567.graph_c0	11.5390552	11.8696875	11.5921669	10.4947322	9.85227939	9.96252868	2.721E-28	-1.29819	down	PREDICTED: WPP domain-interacting protein 1 [Sesamum indicum]
c33572.graph_c0	2.34996888	2.17411581	1.83018367	15.190349	14.8837284	14.9080268	4.41E-23	1.73602	up	hypothetical protein MIMGU_mgv1a022739mg, partial [Erythranthe guttata]
c33576.graph_c0	0	0.12788772	0.16417638	2.04281167	1.55102941	2.38672298	4.63E-09	3.24844	up	PREDICTED: pathogenesis-related protein 5-like [Sesamum indicum]
c33583.graph_c0	56.9118346	53.0603592	51.4674546	19.1766988	22.9439995	22.9106801	9.17E-142	-2.40094	down	PREDICTED: nifU-like protein 2, chloroplastic [Sesamum indicum]
c33588.graph_c0	6.41683312	5.69883094	6.57190804	4.66371733	5.00242194	5.00730955	2.388E-13	-1.43999	down	PREDICTED: light-induced protein, chloroplastic-like [Sesamum indicum]
c33589.graph_c0	4.73223879	4.73697741	4.67207555	62.4118714	58.8136841	55.2057677	1.27E-138	2.55036	up	PREDICTED: protein TRANSPARENT TESTA 12 [Sesamum indicum]
c33596.graph_c0	206.761828	196.890444	210.455799	143.405238	137.013149	153.842761	1.223E-77	-1.59037	down	PREDICTED: uncharacterized protein LOC105160439 [Sesamum indicum]
c33599.graph_c0	157.350601	150.934988	142.178082	44.6640227	41.912878	50.4064471	0	-2.80593	down	hypothetical protein MIMGU_mgv1a014857mg [Erythranthe guttata]
c33599.graph_c1	0.04893134	0	0.18863615	14.1540817	10.9174245	12.188036	5.112E-47	6.17745	up	PREDICTED: probable plastid-lipid-associated protein 6, chloroplastic [Nicotiana sylvestris]
c33602.graph_c0	1.13821794	1.82987751	1.37400935	12.5090264	11.6113571	11.6800549	5.007E-23	1.95211	up	PREDICTED: protein ABIL2-like [Sesamum indicum]
c33606.graph_c0	2.4955156	1.9775948	2.93959998	14.283095	13.5445222	14.5223616	1.755E-12	1.42097	up	PREDICTED: uncharacterized protein LOC105160133 isoform X1 [Sesamum indicum]
c33607.graph_c0	0.18237379	0.45639101	0.64448322	4.37409114	5.26585892	5.13751745	7.425E-12	2.4226	up	PREDICTED: neurofilament medium polypeptide [Sesamum indicum]

#ID	0 h-1 FPKM	0 h-2 FPKM	0 h-3 FPKM	28 h-1 FPKM	28 h-2 FPKM	28 h-3 FPKM	FDR	log2FC	regulated	nr_annotation
c33613.graph_c0	0.28145908	0.22539273	0.21701163	1.7183278	1.77313017	1.8027533	0.0001749	1.7831	up	PREDICTED: uncharacterized protein LOC105174187 [Sesamum indicum]
c33615.graph_c0	0.2635421	0.35174134	0.11288735	2.87311324	2.65179969	4.32418369	1.78E-05	2.67885	up	PREDICTED: putative axial regulator YABBY 2 [Sesamum indicum]
c33617.graph_c0	17.430542	14.2707375	15.6930821	96.1470327	108.064573	99.7998557	1.81E-59	1.59146	up	PREDICTED: 20 kDa chaperonin, chloroplastic-like [Sesamum indicum]
c33622.graph_c0	0	0.03280733	0	0.90517239	1.01085349	1.20510588	1.238E-12	5.49129	up	PREDICTED: aldehyde dehydrogenase family 3 member F1-like [Sesamum indicum]
c33624.graph_c0	0.97174121	0.94133638	0.80562963	8.54341825	7.61105358	8.25408967	4.17E-19	2.07841	up	PREDICTED: uncharacterized protein LOC105177218 isoform X2 [Sesamum indicum]
c33625.graph_c0	0	0	0	5.96737993	4.96704828	5.51604162	7.185E-23	Inf	up	PREDICTED: growth-regulating factor 1-like [Sesamum indicum]
c33625.graph_c1	0	0	0	12.7020075	14.2867507	11.4900484	2.138E-59	Inf	up	PREDICTED: growth-regulating factor 1-like [Sesamum indicum]
c33637.graph_c0	0.6341679	0.95220439	1.08657448	14.2113786	13.4557796	13.8529596	4.162E-16	2.86108	up	hypothetical protein MIMGU_mgv1a025918mg [Erythranthe guttata]
c33642.graph_c0	2.72129482	1.65092108	1.58953254	28.4087072	32.6853186	30.1258739	4.145E-39	2.85085	up	PREDICTED: 50S ribosomal protein L18, chloroplastic [Nicotiana tomentosiformis]
c33647.graph_c0	0.21202422	0.09095851	0.15569115	3.87446344	3.45852407	3.43097915	7.893E-18	3.46444	up	PREDICTED: U-box domain-containing protein 45-like [Sesamum indicum]
c33649.graph_c0	22.3165817	25.4955161	19.2015844	7.86296204	9.3280656	7.42312604	4.367E-30	-2.53408	down	hypothetical protein MIMGU_mgv1a010621mg [Erythranthe guttata]
c33657.graph_c0	0.82369816	0.41226149	0.92617416	4.11578116	4.39183077	4.27438304	2.354E-05	1.46942	up	PREDICTED: uncharacterized protein LOC105180227 [Sesamum indicum]
c33659.graph_c0	11.8054162	9.39318881	14.7814419	2.42003059	3.17827014	5.02657323	1.849E-09	-2.85139	down	-
c33663.graph_c0	0.29355603	0.58769997	1.50892449	6.18729013	8.47613401	5.04877822	0.0007109	1.93215	up	hypothetical protein MIMGU_mgv1a005080mg [Erythranthe guttata]
c33667.graph_c0	8.30689252	7.30347396	8.52351335	8.26412388	8.31153705	8.05468546	6.173E-15	-1.06204	down	PREDICTED: rop guanine nucleotide exchange factor 9-like [Sesamum indicum]
c33672.graph_c0	12.9979819	13.7580403	13.8258631	59.0315643	62.6254216	59.898087	1.542E-33	1.07017	up	PREDICTED: carbamoyl-phosphate synthase small chain, chloroplastic [Sesamum indicum]
c33678.graph_c0	8.20972946	8.17834568	7.37216854	8.81358705	8.00976995	7.93094124	5.568E-21	-1.03021	down	PREDICTED: U-box domain-containing protein 5 [Sesamum indicum]
c33687.graph_c0	97.6515586	102.595896	97.1648796	26.3462319	25.8925953	27.8191488	0	-2.98336	down	PREDICTED: probable WRKY transcription factor 4 isoform X1 [Sesamum indicum]
c33688.graph_c0	30.5721737	27.6491669	36.2671194	3.23664681	3.31682143	3.14336816	1.573E-38	-4.37802	down	PREDICTED: peptidyl-prolyl cis-trans isomerase FKBP62 [Sesamum indicum]
c33691.graph_c0	1.31387749	1.57823177	1.01303076	0.49110099	0.51732117	0.46752346	7.013E-12	-2.49099	down	PREDICTED: probable LRR receptor-like serine/threonine-protein kinase At2g16250 [Nicotiana tomentosiformis]
c33693.graph_c0	7.48936881	7.16681748	7.80826083	1.50630267	1.85461374	1.70402207	1.436E-54	-3.24039	down	PREDICTED: probable calcium-binding protein CML45 [Sesamum indicum]
c33699.graph_c0	44.1213133	44.5058104	43.4455332	28.0861814	29.3006459	28.5636077	1.479E-85	-1.71013	down	PREDICTED: trihelix transcription factor ASIL1-like [Sesamum indicum]
c33701.graph_c0	93.8671965	91.4860221	88.1758263	63.2454465	65.9668598	70.0511997	2.818E-79	-1.54645	down	PREDICTED: protein slowmo homolog 1-like [Sesamum indicum]

#ID	0 h-1 FPKM	0 h-2 FPKM	0 h-3 FPKM	28 h-1 FPKM	28 h-2 FPKM	28 h-3 FPKM	FDR	log2FC	regulated	nr_annotation
c33707.graph_c0	8.68004208	6.1489501	4.63328214	1.84405364	3.15475113	4.27660876	2.107E-05	-2.15015	down	40S ribosomal protein S12 [Pseudozyma brasiliensis GHG001]
c33713.graph_c0	38.0141503	36.917967	35.9327034	6.98681384	7.33985541	7.37035069	0	-3.44302	down	PREDICTED: uncharacterized protein LOC105174809 [Sesamum indicum]
c33724.graph_c0	3.97449361	3.72452835	3.74903546	193.139324	170.75191	188.493243	1.12E-202	4.50283	up	PREDICTED: alpha-xylosidase 1-like [Sesamum indicum]
c33735.graph_c0	10.6203626	13.2123256	9.98497532	98.6923668	100.710725	84.5165154	9.878E-42	1.97973	up	unnamed protein product [Coffea canephora]
c33740.graph_c0	4.5505993	3.94467121	2.89370735	1.39795178	1.41614858	1.02942775	1.795E-14	-2.65373	down	-
c33743.graph_c0	30.3353515	29.7161935	23.0348936	12.1438425	13.5728716	13.8537455	4.101E-26	-2.15701	down	PREDICTED: uncharacterized protein LOC105160397 [Sesamum indicum]
c33751.graph_c0	2.46029839	1.96015752	2.25827203	9.87121126	9.65413689	10.0052574	8.04E-11	1.05471	up	PREDICTED: two-component response regulator-like APRR1 isoform X2 [Sesamum indicum]
c33759.graph_c0	18.7282955	18.166645	19.6705347	15.2551252	15.9427737	12.7231528	4.192E-31	-1.4573	down	PREDICTED: uncharacterized protein LOC105159346 [Sesamum indicum]
c33768.graph_c0	43.7196028	42.9529483	36.5526032	32.4812699	35.1009582	33.2586045	4.419E-40	-1.37763	down	PREDICTED: pollen-specific leucine-rich repeat extensin-like protein 1 [Sesamum indicum]
c33769.graph_c0	31.0238986	31.1485034	32.7821508	418.903635	426.490688	362.548503	6.705E-82	2.57715	up	hypothetical protein MIMGU_mgv1a013324mg [Erythranthe guttata]
c33774.graph_c1	4021.99952	4070.62006	4025.80509	557.12096	621.810496	659.395538	1.36E-193	-3.81081	down	hypothetical protein MIMGU_mgv1a012797mg [Erythranthe guttata]
c33780.graph_c0	14.7557296	14.0431698	15.5141156	13.4460967	12.0305293	13.5574296	7.418E-33	-1.27398	down	PREDICTED: homocysteine S-methyltransferase 2 [Sesamum indicum]
c33784.graph_c0	1.65127198	2.50300145	2.0006956	1.71447852	1.05264908	1.37102046	8.353E-07	-1.66398	down	PREDICTED: probable methyltransferase PMT26 [Sesamum indicum]
c33788.graph_c0	0	0.10286187	0	1.19495406	1.31494774	1.21884095	1.068E-09	4.09802	up	PREDICTED: WUSCHEL-related homeobox 1 [Sesamum indicum]
c33796.graph_c0	3.38657107	3.36194599	2.84130871	21.9689881	20.184902	21.4288247	3.468E-36	1.64093	up	hypothetical protein MIMGU_mgv1a003584mg [Erythranthe guttata]
c33799.graph_c0	0.49523629	0.2478661	0.15909956	7.0787193	5.76852722	5.84960312	3.614E-20	3.29499	up	PREDICTED: beta-galactosidase 8-like isoform X1 [Sesamum indicum]
c33801.graph_c0	0	0	0	2.23804256	1.77203581	1.71384141	8.382E-15	Inf	up	PREDICTED: sulfate transporter 3.1-like [Sesamum indicum]
c33802.graph_c0	0.04103234	0.08214685	0.05272818	1.61039155	2.01948969	1.89809584	4.329E-11	3.88273	up	PREDICTED: dof zinc finger protein DOF1.1-like [Sesamum indicum]
c33805.graph_c0	0.20154904	0.10087543	0.12949925	2.70996657	1.917798	3.37673103	5.222E-07	3.12824	up	PREDICTED: 7-methylguanosine phosphate-specific 5'-nucleotidase A [Sesamum indicum]
c33806.graph_c0	14.5045286	15.1770158	14.3592684	14.7776399	13.1415307	14.4493298	1.391E-21	-1.14586	down	PREDICTED: protein Mpv17-like [Sesamum indicum]
c33810.graph_c0	1.9885819	1.97086452	1.84697695	11.8198953	12.6620001	12.4006346	2.14E-29	1.57762	up	PREDICTED: conserved oligomeric Golgi complex subunit 3 [Sesamum indicum]
c33812.graph_c0	32.5236056	27.8026402	35.6917535	23.6379691	19.8934389	20.4345589	1.233E-13	-1.67845	down	-
c33813.graph_c0	42.3387302	44.4961151	45.4261438	43.4699755	35.5696891	39.1339263	1.484E-34	-1.25369	down	PREDICTED: uncharacterized protein C9orf85 homolog [Sesamum indicum]
c33815.graph_c0	57.4628091	58.5116176	62.0451516	6.53590243	8.0440436	7.17448167	1.42E-203	-4.12445	down	PREDICTED: heat shock 70 kDa protein 8 [Sesamum indicum]
c33818.graph_c0	0.29695994	0.77286899	0.45792634	4.27340976	3.58566594	2.67694068	0.000142	1.69228	up	unnamed protein product [Coffea canephora]

#ID	0 h-1 FPKM	0 h-2 FPKM	0 h-3 FPKM	28 h-1 FPKM	28 h-2 FPKM	28 h-3 FPKM	FDR	log2FC	regulated	nr_annotation
c33821.graph_c0	0.10709349	0.10720072	0.04587312	3.32095695	2.34258809	2.75221553	1.123E-14	3.9347	up	PREDICTED: ras GTPase-activating protein-binding protein 1-like [Sesamum indicum]
c33827.graph_c0	0.55581659	0.8559587	0.4395362	3.85319788	4.29273138	3.37238447	1.534E-05	1.54974	up	hypothetical protein MIMGU_mgv1a007488mg [Erythranthe guttata]
c33832.graph_c0	1.4508947	1.633891	1.16528571	23.5944802	24.6756822	22.085267	6.852E-43	2.96118	up	PREDICTED: G-type lectin S-receptor-like serine/threonine-protein kinase At1g11330 [Sesamum indicum]
c33838.graph_c0	3.03543869	2.18390622	2.37696401	0.03447093	0	0.028128	1.964E-47	-8.00707	down	-
c33842.graph_c0	2.36571081	2.63119967	3.04003205	0.19104278	0.68997486	0.62355738	2.109E-14	-3.51006	down	PREDICTED: ethylene-responsive transcription factor ERF109-like [Solanum tuberosum]
c33845.graph_c0	48.5565885	51.0549577	48.8170053	25.8717241	28.3601857	27.0156307	1.412E-98	-1.95994	down	PREDICTED: transcription factor TGA1 [Sesamum indicum]
c33851.graph_c0	34.3975658	34.5226203	33.0353538	34.0788827	31.1264456	35.5922079	4.596E-22	-1.10597	down	PREDICTED: selenoprotein O-like [Malus domestica]
c33853.graph_c0	14.3768218	13.5242772	13.5778542	8.93829371	10.3438049	10.169914	2.343E-10	-1.58384	down	-
c33856.graph_c0	1.53541534	1.19957294	1.01059657	1.46976281	1.25333257	1.06605729	0.0010736	-1.06999	down	-
c33857.graph_c0	0.30573958	0.68860289	0.98221865	3.61090862	3.36062922	4.03440005	0.0005774	1.37546	up	PREDICTED: vesicle transport protein SFT2B isoform X1 [Nicotiana sylvestris]
c33864.graph_c0	14.3408748	13.2785924	12.4392995	10.6834368	10.5872288	8.9302215	7.334E-06	-1.49721	down	-
c33869.graph_c0	0.66265423	0.47379841	0.30412033	7.29300098	7.3924777	6.76508513	4.569E-17	2.81421	up	hypothetical protein MIMGU_mgv1a014159mg [Erythranthe guttata]
c33871.graph_c0	13.7154942	11.9933488	10.9396261	11.8588898	8.94875145	10.2844763	1.775E-14	-1.32493	down	PREDICTED: uncharacterized protein LOC105163723 [Sesamum indicum]
c33873.graph_c0	45.96523	36.703923	45.5289133	4.4415857	4.52700444	4.98062992	2.079E-58	-4.29067	down	PREDICTED: RING finger and CHY zinc finger domain-containing protein 1-like [Sesamum indicum]
c33874.graph_c0	0.15929443	0.21260525	0.27293286	1.81379692	2.23004399	1.95239777	7.431E-06	2.11982	up	-
c33875.graph_c0	0.4266468	0.65317203	0.32250472	3.17380539	3.50794926	2.88747736	7.988E-08	1.68368	up	PREDICTED: probable serine/threonine-protein kinase At1g54610 isoform X2 [Sesamum indicum]
c33877.graph_c0	6.20736275	7.65440828	7.91890467	50.7377532	54.8733607	55.9401668	1.968E-58	1.79797	up	hypothetical protein MIMGU_mgv1a007222mg [Erythranthe guttata]
c33887.graph_c0	5.58803901	4.1078254	5.49783933	73.4851339	64.1155942	70.5267466	8.656E-73	2.68499	up	metallothionein class I type 3 [Avicennia marina]
c33911.graph_c0	6.59394844	6.1964359	5.53370437	2.88524673	3.35573141	2.87309016	8.526E-18	-2.09652	down	PREDICTED: uncharacterized protein LOC105171510 [Sesamum indicum]
c33912.graph_c0	3.19434873	2.41126524	1.68232155	18.4208357	15.6357248	15.1554767	8.502E-14	1.67189	up	-
c33919.graph_c0	20.5139896	18.4276646	15.8091544	164.887307	166.716655	171.906128	6.13E-146	2.1138	up	PREDICTED: malate dehydrogenase, mitochondrial [Sesamum indicum]
c33924.graph_c0	0.59225031	0.71141203	0.50737673	3.58703426	3.31648575	2.36500845	0.0005338	1.26583	up	PREDICTED: F-box protein At2g32560-like [Sesamum indicum]
c33931.graph_c0	180.579699	179.955865	188.371675	10.3075797	12.5095155	18.4563089	0	-4.82209	down	hypothetical protein MIMGU_mgv1a010609mg [Erythranthe guttata]
c33932.graph_c0	8.41775773	8.16955171	7.57749731	7.7328755	7.17834441	5.92984499	1.265E-16	-1.3037	down	PREDICTED: uncharacterized protein LOC105175305 isoform X1 [Sesamum indicum]

#ID	0 h-1 FPKM	0 h-2 FPKM	0 h-3 FPKM	28 h-1 FPKM	28 h-2 FPKM	28 h-3 FPKM	FDR	log2FC	regulated	nr_annotation
c33943.graph_c0	2.33306931	2.45026153	1.76936248	13.0927293	15.0843433	12.9291492	5.536E-17	1.56173	up	PREDICTED: uncharacterized protein LOC105159044 [Sesamum indicum]
c33952.graph_c0	2.49342949	2.53752506	2.45651931	20.8404171	20.0986765	21.4418563	6.144E-33	1.96851	up	PREDICTED: Golgi to ER traffic protein 4 homolog [Sesamum indicum]
c33967.graph_c0	3.20188913	3.70112199	2.98795014	71.1432167	74.1411524	69.626571	9.25E-192	3.35201	up	PREDICTED: auxin-responsive protein IAA9-like [Sesamum indicum]
c33968.graph_c0	11.7109947	10.6646136	11.6174699	54.4451898	56.0772914	55.9418423	2.163E-41	1.20122	up	PREDICTED: glucose-6-phosphate isomerase, cytosolic [Sesamum indicum]
c33982.graph_c1	21.7316012	21.0058239	26.0066574	21.4934137	22.7879795	23.6946232	6.269E-08	-1.10907	down	hypothetical protein MIMGU_mgv1a015522mg [Erythranthe guttata]
c33985.graph_c0	0.03504283	0.07015583	0.09006284	0.86594343	0.80486178	1.01833928	1.143E-07	2.68447	up	PREDICTED: PI-PLC X domain-containing protein At5g67130-like isoform X2 [Nicotiana tomentosiformis]
c33989.graph_c0	1.78708374	1.2384507	1.58986618	7.64318659	6.22450432	6.44059411	0.0008888	1.04749	up	PREDICTED: hydroxyacyl-thioester dehydratase type 2, mitochondrial-like [Sesamum indicum]
c33996.graph_c0	611.235202	582.437609	654.740645	100.2461	117.339124	118.709297	1.2E-111	-3.54984	down	PREDICTED: glutaredoxin [Sesamum indicum]
c34001.graph_c0	0.06286269	0	0.04039052	0.75385596	0.74253955	0.57785908	9.818E-08	3.23997	up	PREDICTED: 5'-3' exoribonuclease 3-like [Sesamum indicum]
c34003.graph_c1	5.44525343	5.97607528	5.47986513	40.6247849	48.4766416	40.0361831	5.56E-28	1.84269	up	PREDICTED: 3-oxoacyl-[acyl-carrier-protein] reductase, chloroplastic-like isoform X2 [Sesamum indicum]
c34004.graph_c0	1.26733778	1.08737728	1.16327088	7.23716555	6.17805566	6.69286372	5.794E-06	1.42527	up	PREDICTED: SEC14 cytosolic factor [Sesamum indicum]
c34006.graph_c0	50.929112	57.2257588	61.0360927	29.0224692	25.3856758	27.3824256	2.433E-28	-2.14111	down	PREDICTED: uncharacterized protein LOC105156884 [Sesamum indicum]
c34007.graph_c0	249.198178	261.628215	242.405968	100.298376	119.483306	118.889677	3.82E-161	-2.24302	down	-
c34008.graph_c0	0.19626193	0.19645846	0.08406812	5.04001881	5.45221117	6.55690376	2.99E-16	4.08019	up	PREDICTED: acyl-protein thioesterase 1 homolog 1 [Sesamum indicum]
c34010.graph_c0	0.51714305	0.29118425	0.5399463	8.12789867	7.82656512	7.80157708	2.409E-29	3.04633	up	PREDICTED: retinol dehydrogenase 11-like [Sesamum indicum]
c34011.graph_c0	10.1814765	11.3136906	10.3228406	0.2715537	0.49037507	0.38777481	1.639E-76	-5.88135	down	PREDICTED: uncharacterized protein LOC105163738 [Sesamum indicum]
c34014.graph_c0	3.4001042	3.64151651	2.9026609	15.0171473	15.4938579	17.3866757	6.596E-18	1.18031	up	PREDICTED: NEDD8-activating enzyme E1 regulatory subunit [Sesamum indicum]
c34025.graph_c0	3.65065378	2.79447186	2.20763983	13.4848756	15.8677097	14.2893301	1.505E-11	1.24949	up	hypothetical protein MIMGU_mgv1a009463mg [Erythranthe guttata]
c34026.graph_c0	34.4049956	31.0973565	30.4824298	25.9759854	26.1456502	27.2495529	1.286E-54	-1.36319	down	PREDICTED: probable receptor-like protein kinase At5g24010 [Sesamum indicum]
c34030.graph_c0	18.9270343	17.9279935	17.2068992	14.6183796	13.1619502	14.2404587	4.742E-31	-1.45273	down	PREDICTED: dnaJ homolog subfamily B member 4 [Sesamum indicum]
c34031.graph_c0	0.30020852	0.50084855	0.77155966	3.05465748	3.80876237	3.14539297	0.0004064	1.568	up	PREDICTED: maltose excess protein 1-like, chloroplastic [Sesamum indicum]
c34032.graph_c0	2.40924644	2.94104748	3.77558178	0.64061942	0.30849028	0.55758957	1.181E-12	-3.69322	down	PREDICTED: uncharacterized protein LOC105176742 [Sesamum indicum]
c34034.graph_c0	25.6378604	25.5681293	21.9229522	27.2228458	25.8305515	23.4006072	4.381E-15	-1.02491	down	hypothetical protein MIMGU_mgv1a010704mg [Erythranthe guttata]

#ID	0 h-1 FPKM	0 h-2 FPKM	0 h-3 FPKM	28 h-1 FPKM	28 h-2 FPKM	28 h-3 FPKM	FDR	log2FC	regulated	nr_annotation
c34036.graph_c0	0.26304655	0.23937269	0.3687548	4.48405686	4.40961202	4.53823724	1.033E-21	2.85102	up	PREDICTED: uncharacterized protein LOC105158907 [Sesamum indicum]
c34037.graph_c0	19.9600514	18.1636713	18.3901762	14.5566165	14.2666155	14.9440462	8.451E-46	-1.45827	down	PREDICTED: cyclin-C1-2-like [Sesamum indicum]
c34044.graph_c0	0	0	0	3.70814788	3.96193	3.68141237	1.885E-15	Inf	up	PREDICTED: receptor-like serine/threonine-protein kinase At3g01300 [Sesamum indicum]
c34044.graph_c1	0	0.0749921	0.09627142	4.46484282	4.81793629	4.75402994	6.15E-16	5.24777	up	PREDICTED: receptor-like serine/threonine-protein kinase At3g01300 [Sesamum indicum]
c34050.graph_c0	1.24536008	1.4840561	1.21930439	7.86592843	9.59282894	8.12428058	6.886E-15	1.60546	up	PREDICTED: polygalacturonase-1 non-catalytic subunit beta-like [Sesamum indicum]
c34051.graph_c0	155.160122	128.920345	163.32293	4.56007742	3.17145101	4.32438906	1.313E-92	-6.3047	down	PREDICTED: HVA22-like protein e [Sesamum indicum]
c34063.graph_c0	2.0533487	1.6747743	1.73465779	13.3068945	14.0111195	12.8879093	3.439E-39	1.79056	up	PREDICTED: homeobox-leucine zipper protein MERISTEM L1-like [Sesamum indicum]
c34065.graph_c0	0.41454667	0.27664118	0.2367595	2.74508784	2.9319515	2.86825332	9.937E-07	2.11885	up	PREDICTED: WUSCHEL-related homeobox 9 [Sesamum indicum]
c34069.graph_c0	94.6321948	97.3658158	93.841366	83.987543	87.8712568	86.3595046	1.953E-52	-1.23698	down	PREDICTED: uncharacterized protein LOC105174506 [Sesamum indicum]
c34071.graph_c0	0.82150735	1.19611631	1.03647548	5.55814919	5.47916907	5.35921803	2.647E-14	1.33216	up	PREDICTED: conserved oligomeric Golgi complex subunit 4 [Sesamum indicum]
c34074.graph_c0	3.09795119	1.31883877	2.47095926	23.3438955	22.6429613	20.4422155	9.094E-34	2.18001	up	Phosphoribulokinase family protein [Populus trichocarpa]
c34076.graph_c0	0.80230215	0.49421879	0.71376233	6.45904889	5.386422	5.74634299	1.919E-08	2.03932	up	PREDICTED: uncharacterized protein ycf49 [Sesamum indicum]
c34081.graph_c0	1.00355862	1.00456353	0.62827278	9.03309843	10.1317279	8.50022887	6.133E-29	2.30594	up	PREDICTED: probable glycosyltransferase At3g07620 [Sesamum indicum]
c34084.graph_c0	0.61081395	0.44738458	0.61262058	6.23676026	6.64795597	6.5204676	1.173E-38	2.44636	up	PREDICTED: conserved oligomeric Golgi complex subunit 1 [Sesamum indicum]
c34085.graph_c1	13.5662347	16.4503502	12.0472979	10.9821219	7.59972093	7.71851071	4.688E-13	-1.7665	down	-
c34103.graph_c0	5.10096457	3.07716947	3.16894519	0	0	0	1.693E-28	-Inf	down	carboxylesterase [Cryptococcus neoformans var. grubii H99]
c34109.graph_c0	1.90663785	1.8351414	1.88469625	0.15989232	0.09624518	0.06523541	6.432E-36	-5.2227	down	PREDICTED: ATP-dependent 6-phosphofructokinase 6 [Sesamum indicum]
c34111.graph_c0	0	0.2155088	0.41499046	3.52066207	5.36868687	4.72421179	6.574E-10	3.31856	up	PREDICTED: uncharacterized protein At5g01610 [Sesamum indicum]
c34112.graph_c0	5.0873499	6.41787477	7.6120947	34.7459619	32.9272434	31.9068063	7.685E-18	1.28552	up	hypothetical protein M569_08891, partial [Genlisea aurea]
c34113.graph_c0	1.85048995	3.41221068	3.50435158	0	0	0.17328129	3.975E-13	-6.7431	down	-
c34114.graph_c0	28.9536618	34.1188211	27.3162392	11.9867209	11.5444108	14.345564	2.303E-19	-2.34313	down	hypothetical protein PRUPE_ppb013116mg [Prunus persica]
c34116.graph_c0	7.11396317	6.33711388	6.0385702	3.91336664	4.34715661	4.50929167	5.158E-26	-1.69832	down	PREDICTED: GATA transcription factor 26 [Sesamum indicum]
c34122.graph_c0	39.3733105	21.1584167	23.9666508	0	0	0	2.259E-30	-Inf	down	-
c34134.graph_c0	5.07503065	4.72802552	3.61594512	31.4800951	32.4134265	31.4388783	1.413E-33	1.74218	up	PREDICTED: F-box only protein 6-like [Sesamum indicum]

#ID	0 h-1 FPKM	0 h-2 FPKM	0 h-3 FPKM	28 h-1 FPKM	28 h-2 FPKM	28 h-3 FPKM	FDR	log2FC	regulated	nr_annotation
c34139.graph_c0	8.29340796	8.1440851	7.62203422	0.8774356	0.51667962	0.65372113	5.389E-83	-4.64375	down	PREDICTED: hyoscyamine 6-dioxygenase [Sesamum indicum]
c34142.graph_c0	0.47892337	0.38352235	0.45131936	3.73604516	3.47805932	3.99534705	1.344E-09	2.00315	up	PREDICTED: probable BOI-related E3 ubiquitin-protein ligase 2 [Sesamum indicum]
c34152.graph_c1	1.15109959	2.17647646	1.97227806	0.69717712	0.54555416	0.64474295	7.258E-06	-2.58415	down	PREDICTED: UDP-glucuronic acid decarboxylase 1-like [Nicotiana tomentosiformis]
c34153.graph_c0	0.32571588	0	0	4.14274745	3.09928181	4.24970617	2.117E-09	4.08298	up	-
c34155.graph_c0	24.3538805	25.5281855	26.8178567	22.3758004	23.4196876	22.0963827	4.128E-34	-1.2679	down	PREDICTED: acyl-CoA-binding domain-containing protein 1 [Sesamum indicum]
c34158.graph_c0	0.37637809	0.4362426	0.63639571	6.66596754	5.23877691	5.19266476	7.573E-19	2.46284	up	hypothetical protein MIMGU_mgv1a022010mg [Erythranthe guttata]
c34160.graph_c0	2.80760472	2.41826502	3.60788322	30.9404466	29.1360417	31.7525529	1.306E-38	2.2836	up	PREDICTED: uncharacterized protein LOC105157271 [Sesamum indicum]
c34161.graph_c0	8.29288931	8.2015221	7.27506527	6.76124287	7.19690585	6.82469304	1.119E-37	-1.28274	down	PREDICTED: uncharacterized protein LOC105166332 [Sesamum indicum]
c34162.graph_c1	0.17981579	0.05999861	0.38511734	1.26332774	1.21933316	1.17305341	0.0009072	1.44278	up	PREDICTED: serine/threonine-protein kinase Aurora-2 [Sesamum indicum]
c34163.graph_c0	1.04513087	0.7692481	0.82952134	6.56826346	7.03997823	6.61752623	4.066E-14	1.84706	up	hypothetical protein MIMGU_mgv1a004090mg [Erythranthe guttata]
c34177.graph_c0	0.03781357	0.07570287	0	15.5552001	18.2633163	17.9405177	5.53E-99	7.75879	up	PREDICTED: cytochrome P450 71D95-like [Sesamum indicum]
c34185.graph_c0	2.9523746	3.94044127	5.05855767	0.08940704	0	0	2.198E-18	-8.16395	down	hypothetical protein SORBIDRAFT_07g005790 [Sorghum bicolor]
c34187.graph_c0	2.73917496	3.63014473	3.27206459	18.7024599	18.5321853	17.434668	1.451E-19	1.41116	up	hypothetical protein MIMGU_mgv1a013909mg [Erythranthe guttata]
c34191.graph_c0	23.0175205	23.3794009	25.0111683	7.99547853	7.66342392	8.43133224	1.084E-36	-2.65887	down	Mitogen-activated protein kinase 10 [Morus notabilis]
c34193.graph_c1	3.03555952	2.21735615	1.37055633	10.3156027	9.96008137	8.61207061	0.0007868	1.04255	up	PREDICTED: abscisic acid receptor PYL2 [Sesamum indicum]
c34201.graph_c0	45.2777152	46.2227672	43.2840205	27.3222756	25.7606304	28.2515709	4.05E-108	-1.81825	down	PREDICTED: uncharacterized protein LOC105177120 [Sesamum indicum]
c34203.graph_c0	0.61121199	0.35689735	0.42544212	7.88499994	9.05803588	7.53665184	3.202E-37	3.04726	up	PREDICTED: zinc finger CCCH domain-containing protein 46-like [Sesamum indicum]
c34204.graph_c0	0.48672048	0.34800561	0.35740291	2.08878377	1.52094831	1.66319428	0.0032885	1.05775	up	PREDICTED: receptor protein kinase CLAVATA1 [Sesamum indicum]
c34210.graph_c0	1.23512998	1.64204963	0.94239461	7.25161449	7.38338261	7.06179557	3.482E-15	1.41929	up	PREDICTED: protein DENND6A [Sesamum indicum]
c34215.graph_c0	1.84418838	2.23467402	2.30749048	0.141089	0.0955426	0.20147307	2.937E-30	-4.95555	down	PREDICTED: vinorine synthase-like [Sesamum indicum]
c34218.graph_c0	12.5829769	13.7842005	11.0504733	9.86905884	10.1173794	9.81361595	9.484E-26	-1.41713	down	PREDICTED: uncharacterized protein LOC105168895 [Sesamum indicum]
c34223.graph_c0	0.20827172	0.1563602	0.40145611	2.72467235	2.87014428	1.82187934	1.764E-06	2.172	up	PREDICTED: putative receptor-like protein kinase At1g80870 [Sesamum indicum]
c34225.graph_c0	2.1745378	2.94259657	2.38038684	10.4777421	11.970865	12.7291	2.214E-09	1.13993	up	PREDICTED: CAP-Gly domain-containing linker protein 1 isoform X1 [Sesamum indicum]

#ID	0 h-1 FPKM	0 h-2 FPKM	0 h-3 FPKM	28 h-1 FPKM	28 h-2 FPKM	28 h-3 FPKM	FDR	log2FC	regulated	nr_annotation
c34228.graph_c0	0	0	0	1.12940704	1.54419084	1.60619435	2.506E-14	Inf	up	PREDICTED: transcription factor SPEECHLESS-like [Sesamum indicum]
c34229.graph_c0	0.93262725	1.72349748	1.50576113	6.46543736	6.76355293	6.31103795	4.691E-09	1.13646	up	PREDICTED: gamma-tubulin complex component 4 isoform X1 [Sesamum indicum]
c34232.graph_c0	9.51475593	9.09963393	9.81262164	43.6939338	48.2406255	43.596948	1.382E-21	1.16185	up	PREDICTED: probable signal peptidase complex subunit 2 [Sesamum indicum]
c34236.graph_c0	0.4230115	0.61590558	0.51887805	2.30581743	2.61188453	2.15520502	0.0001443	1.09014	up	PREDICTED: receptor protein kinase CLAVATA1-like [Sesamum indicum]
c34242.graph_c0	1.74887411	1.86236739	1.67357525	19.2012947	22.7822422	18.6251777	5.904E-37	2.42909	up	hypothetical protein MIMGU_mgv1a006918mg [Erythranthe guttata]
c34246.graph_c0	1.0846981	1.40513258	0.86092589	6.65462113	6.76217826	6.65993382	5.765E-10	1.4959	up	PREDICTED: F-box/kelch-repeat protein At1g55270-like [Sesamum indicum]
c34255.graph_c0	0.04321715	0.02163021	0	4.74290654	4.934686	4.22899699	1.9E-45	6.67731	up	PREDICTED: subtilisin-like protease [Sesamum indicum]
c34257.graph_c0	2.4359044	2.77700242	2.05783875	13.4911134	13.2616824	13.5634986	1.069E-22	1.38323	up	PREDICTED: ABC transporter B family member 25, mitochondrial [Sesamum indicum]
c34260.graph_c0	0.05366103	0.05371476	0.13791309	0.9360126	1.03880762	0.79560275	1.03E-06	2.39217	up	hypothetical protein MIMGU_mgv1a026332mg [Erythranthe guttata]
c34261.graph_c0	39.3081028	38.5708692	40.9306076	13.5952892	15.3299473	15.5413275	2.28E-151	-2.50877	down	PREDICTED: kinesin KP1-like isoform X2 [Sesamum indicum]
c34266.graph_c0	9.29976489	10.4463853	9.13866818	8.3799559	10.1068288	6.88788781	4.418E-19	-1.27842	down	PREDICTED: aspartic proteinase nepenthesin-2-like [Sesamum indicum]
c34267.graph_c0	52.2993027	51.1691912	54.7865499	9.75637366	10.0599815	10.7952804	1.19E-206	-3.46095	down	hypothetical protein MIMGU_mgv1a010327mg [Erythranthe guttata]
c34268.graph_c0	0.06412093	0	0	3.7282175	5.00726839	3.95485355	1.032E-17	6.56909	up	PREDICTED: auxin transporter-like protein 2 [Sesamum indicum]
c34279.graph_c0	0.15336048	0.15351405	0.49268573	3.62249925	3.17013788	3.72901914	4.556E-08	2.61109	up	-
c34284.graph_c0	1.6180142	1.19090764	2.20151891	13.6273405	14.7400698	12.7002776	3.902E-20	1.93843	up	PREDICTED: putative lipoyltransferase-like protein, chloroplastic isoform X1 [Sesamum indicum]
c34292.graph_c0	0.54698231	0.37642689	0.57110131	8.05025764	8.34546163	9.2046321	1.943E-29	3.00675	up	PREDICTED: probable fructokinase-6, chloroplastic [Sesamum indicum]
c34294.graph_c0	0.57971382	0.23211773	0.07449552	9.26931863	9.2062582	7.87311192	3.292E-23	3.82202	up	PREDICTED: ABC transporter B family member 19 [Sesamum indicum]
c34294.graph_c1	0.38241826	0.44169369	0.34021565	10.1340977	11.0805494	10.8338834	2.656E-51	3.69407	up	PREDICTED: ABC transporter B family member 19 [Sesamum indicum]
c34294.graph_c2	0.33295022	0.49992543	0.07130904	10.1230937	11.3979442	11.4855229	1.531E-31	4.11297	up	PREDICTED: ABC transporter B family member 19 [Sesamum indicum]
c34296.graph_c0	18.781885	18.7497418	17.2676503	11.467955	12.2917794	13.8253322	5.459E-41	-1.63276	down	serine/threonine-protein phosphatase, partial [Genlisea aurea]
c34297.graph_c1	0.03189573	0.03192767	0	0.60272241	0.71164854	0.73772485	1.636E-08	3.93696	up	PREDICTED: zinc finger protein 91-like [Sesamum indicum]
c34299.graph_c0	1.8366291	1.92601432	2.0229781	0.50851484	0.45914122	0.36307596	1.429E-13	-3.21317	down	PREDICTED: receptor-like cytosolic serine/threonine-protein kinase RBK2 [Sesamum indicum]
c34304.graph_c0	19.3439892	19.4475478	20.9670071	16.2596546	17.5509025	14.5645927	5.946E-22	-1.39727	down	PREDICTED: mitogen-activated protein kinase 9-like [Sesamum indicum]
c34305.graph_c0	6.91294675	7.2081969	6.13378328	4.9345704	4.64447369	4.51463814	2.076E-20	-1.61232	down	PREDICTED: monoacylglycerol lipase abhd6-B [Sesamum indicum]
c34311.graph_c1	14.0691597	16.7965341	18.1900048	6.09770617	6.126808	6.78733572	5.325E-20	-2.46044	down	PREDICTED: vesicle transport v-SNARE 13-like isoform X2 [Sesamum indicum]

#ID	0 h-1 FPKM	0 h-2 FPKM	0 h-3 FPKM	28 h-1 FPKM	28 h-2 FPKM	28 h-3 FPKM	FDR	log2FC	regulated	nr_annotation
c34324.graph_c0	17.1834914	18.2431646	18.11445	104.317144	106.197098	100.20963	1.767E-64	1.44559	up	PREDICTED: chaperone protein dnaJ 1, mitochondrial-like isoform X1 [Sesamum indicum]
c34335.graph_c0	275.155832	250.574913	259.617161	91.4979188	105.379333	104.872684	8.63E-195	-2.46983	down	PREDICTED: uncharacterized protein LOC105176201 [Sesamum indicum]
c34337.graph_c0	0	0.06893287	0	2.05204583	3.20851596	2.9405022	3.252E-13	5.81297	up	PREDICTED: MLO-like protein 4 [Sesamum indicum]
c34342.graph_c0	0.28931237	0.28960207	0.18588892	3.36432896	2.80035537	3.17421261	4.16E-07	2.52529	up	-
c34344.graph_c0	1.63603508	1.12051333	2.2130209	11.8280265	10.8491164	11.9495666	5.084E-09	1.7049	up	hypothetical protein MIMGU_mgv1a014272mg [Erythranthe guttata]
c34351.graph_c0	26.5837143	36.219621	31.3143649	22.0714337	22.6542091	18.6122714	3.747E-11	-1.66394	down	hypothetical protein MIMGU_mgv11b020149mg [Erythranthe guttata]
c34359.graph_c0	0.88264154	0.48826402	0.56711466	0.45580177	0.73163779	0.3030545	0.0032661	-1.46791	down	-
c34360.graph_c0	0	0.09571884	0.12287945	3.30116958	4.45528231	3.17576285	1.511E-16	4.53381	up	PREDICTED: uncharacterized oxidoreductase At1g06690, chloroplastic [Sesamum indicum]
c34362.graph_c0	1.15759876	1.18702031	1.66897002	1.27226436	1.27843136	1.33955784	0.0011126	-1.14039	down	PREDICTED: uncharacterized protein LOC105166158 [Sesamum indicum]
c34363.graph_c0	9.0094781	7.59826355	8.02222971	37.4320841	39.6633724	34.5831874	1.71E-14	1.09085	up	PREDICTED: threonine dehydratase biosynthetic, chloroplastic-like [Sesamum indicum]
c34364.graph_c0	0.1427817	0.07146234	0.18348013	1.50470725	1.31176096	1.56654023	8.575E-06	2.36648	up	unnamed protein product [Coffea canephora]
c34370.graph_c1	4.15431181	4.15847172	3.99262612	55.5658188	57.2725166	59.6889664	1.93E-135	2.71969	up	PREDICTED: uridine 5'-monophosphate synthase [Sesamum indicum]
c34376.graph_c0	8.16160689	7.1708545	8.56442687	0	0.02338807	0.04227343	3.94E-140	-9.59342	down	hypothetical protein MIMGU_mgv1a019938mg [Erythranthe guttata]
c34389.graph_c0	15.4784927	14.8601469	13.6520994	3.4771736	3.50892195	3.67185857	4.666E-68	-3.13404	down	PREDICTED: serine/threonine-protein kinase OXI1 [Sesamum indicum]
c34389.graph_c1	11.4592633	12.4145329	13.4208064	4.16426327	4.09309847	3.44101754	8.75E-47	-2.76592	down	PREDICTED: serine/threonine-protein kinase OXI1 [Sesamum indicum]
c34390.graph_c0	6.49634188	6.29307772	5.95434855	6.29533845	5.16458582	5.88263029	8.467E-20	-1.20127	down	PREDICTED: ubiquitin carboxyl-terminal hydrolase 20-like [Sesamum indicum]
c34399.graph_c0	1.876392	2.04159882	2.30640157	11.0285941	10.8411385	9.4588815	1.207E-09	1.23773	up	PREDICTED: protein SENSITIVITY TO RED LIGHT REDUCED 1-like [Sesamum indicum]
c34400.graph_c0	2.25400177	1.62450634	2.20132595	0	0	0.05347017	1.873E-27	-7.90593	down	PREDICTED: serine/threonine-protein kinase CDL1-like [Sesamum indicum]
c34401.graph_c0	0.01743838	0.01745584	0.02240901	11.6221632	8.12491148	8.66657431	8.531E-39	7.85945	up	PREDICTED: ABC transporter G family member 28 [Sesamum indicum]
c34402.graph_c1	0.96339031	0.91613725	1.08324536	8.99739685	8.7876187	8.22738941	1.005E-25	2.04152	up	PREDICTED: uncharacterized protein LOC105169037 [Sesamum indicum]
c34407.graph_c0	1.49303801	1.7483217	1.26700856	8.55820631	8.57762598	7.80205779	4.68E-12	1.37919	up	hypothetical protein MIMGU_mgv1a004065mg [Erythranthe guttata]
c34409.graph_c0	10.9000268	12.3341078	11.3425975	11.9692245	10.8459619	10.6099655	1.353E-13	-1.13982	down	hypothetical protein MIMGU_mgv1a015267mg [Erythranthe guttata]
c34410.graph_c0	7.08433936	7.47381446	6.47073628	3.1296974	2.64351213	3.08928329	3.582E-43	-2.33532	down	PREDICTED: pentatricopeptide repeat-containing protein At5g18390, mitochondrial [Sesamum indicum]

#ID	0 h-1 FPKM	0 h-2 FPKM	0 h-3 FPKM	28 h-1 FPKM	28 h-2 FPKM	28 h-3 FPKM	FDR	log2FC	regulated	nr_annotation
c34411.graph_c0	82.9953131	73.9079305	79.0164569	48.7529702	48.7066078	52.4901726	3.06E-83	-1.74367	down	PREDICTED: polyadenylate-binding protein 1 isoform X1 [Sesamum indicum]
c34413.graph_c0	46.5133807	29.6182932	34.2203449	0	0	0	8.551E-52	-Inf	down	-
c34414.graph_c0	0.12401871	0.2482858	0.15936896	4.21386206	3.94714511	4.02687951	4.346E-19	3.42761	up	PREDICTED: ABC transporter G family member 8-like [Sesamum indicum]
c34416.graph_c1	2.81888424	2.01550495	2.24242439	10.4876428	10.6144898	11.1052782	1.915E-05	1.09818	up	PREDICTED: outer envelope pore protein 21B, chloroplastic [Sesamum indicum]
c34420.graph_c0	0.85186696	1.05737277	0.87574622	12.6055275	12.2983992	12.448292	5.202E-36	2.65523	up	PREDICTED: dnaJ protein P58IPK homolog [Sesamum indicum]
c34428.graph_c0	22.0660752	21.6567614	21.9314238	22.0828806	22.3992998	21.8533758	1.476E-25	-1.07573	down	hypothetical protein PGTG_21261 [Puccinia graminis f. sp. tritici CRL 75-36-700-3]
c34430.graph_c0	159.04022	165.53313	147.142251	49.9028658	53.7905344	50.3072817	1.47E-233	-2.70461	down	hypothetical protein PRUPE_ppa024708mg, partial [Prunus persica]
c34441.graph_c0	3.13104731	3.89245256	2.5958192	17.9113878	19.4863005	18.3892323	1.281E-15	1.44815	up	PREDICTED: 1-aminocyclopropane-1-carboxylate oxidase 5-like [Sesamum indicum]
c34445.graph_c0	16.8109404	18.1777633	16.2020429	14.5659747	16.5682725	16.5544407	3.574E-32	-1.19189	down	PREDICTED: glycerophosphodiester phosphodiesterase GDPDL3-like [Sesamum indicum]
c34445.graph_c1	11.2498504	10.7059901	13.3366333	9.50066104	9.20207568	9.58486189	1.674E-11	-1.4121	down	hypothetical protein MIMGU_mgv1a001772mg [Erythranthe guttata]
c34451.graph_c0	2.95018253	2.44397519	3.07209937	1.18299347	1.40192349	1.0558111	1.214E-13	-2.31026	down	PREDICTED: putative nuclease HARBI1 isoform X2 [Sesamum indicum]
c34453.graph_c0	0.2034014	0.05090127	0.03267236	0.99785903	0.85091908	0.79916621	2.435E-05	2.13412	up	PREDICTED: uncharacterized protein DDB_G0290685 [Sesamum indicum]
c34455.graph_c0	9.49446596	8.52340456	8.1338431	52.192052	47.1739781	49.7831617	3.315E-26	1.42309	up	hypothetical protein MIMGU_mgv1a015463mg [Erythranthe guttata]
c34456.graph_c1	0.33938971	0.28310796	0.58150562	8.01666076	8.09204042	7.38018737	4.722E-19	3.18663	up	hypothetical protein MIMGU_mgv1a023921mg, partial [Erythranthe guttata]
c34459.graph_c0	10.0183833	7.21790395	8.7740131	57.0909829	57.5358926	57.5604915	4.489E-42	1.63726	up	PREDICTED: uncharacterized protein LOC105175711 [Sesamum indicum]
c34467.graph_c0	0.74174273	0.78156365	0.40133422	15.3500019	13.9877051	13.8683184	2.806E-47	3.40515	up	hypothetical protein MIMGU_mgv1a014915mg [Erythranthe guttata]
c34469.graph_c0	0.08690824	0.26098579	0.11168053	2.71606946	2.39532453	2.78324853	4.121E-08	3.01367	up	-
c34477.graph_c0	7.66906458	7.29290679	6.1902043	40.0444988	41.0789864	37.8211368	8.54E-48	1.40316	up	hypothetical protein MIMGU_mgv1a003188mg [Erythranthe guttata]
c34486.graph_c0	0.52757718	0.28165625	0.40677454	9.38150002	9.94777937	9.80369722	1.631E-37	3.4941	up	hypothetical protein MIMGU_mgv1a001741mg [Erythranthe guttata]
c34487.graph_c0	11.5426736	12.0083283	11.6589636	10.6238127	11.1469221	11.8077002	3.865E-16	-1.15866	down	PREDICTED: SNAP25 homologous protein SNAP33 [Sesamum indicum]
c34489.graph_c0	0	0	0	4.08451343	5.57918019	7.34953708	8.643E-10	Inf	up	hypothetical protein PAXINDRAFT_10917 [Paxillus involutus ATCC 200175]
c34493.graph_c0	59.8991401	62.6598233	54.425757	40.7776412	38.6693657	39.8771163	1.206E-87	-1.65784	down	hypothetical protein MIMGU_mgv1a000078mg [Erythranthe guttata]
c34498.graph_c0	1.67900073	1.4829547	1.77683258	23.400818	24.1749133	24.8936272	1.334E-50	2.78364	up	PREDICTED: cytochrome P450 71D95-like [Sesamum indicum]

#ID	0 h-1 FPKM	0 h-2 FPKM	0 h-3 FPKM	28 h-1 FPKM	28 h-2 FPKM	28 h-3 FPKM	FDR	log2FC	regulated	nr_annotation
c34501.graph_c0	0	0	0	5.63647358	6.48873915	5.05924806	7.097E-18	Inf	up	PREDICTED: probable galactinol--sucrose galactosyltransferase 1 [Sesamum indicum]
c34508.graph_c0	0.35199211	0.16442747	0.21108444	4.2125988	3.98837114	3.61836444	7.798E-20	2.93647	up	PREDICTED: cellulose synthase-like protein G2 [Sesamum indicum]
c34509.graph_c0	0.42647199	0.25613942	0.21921336	4.15342895	4.03001938	4.35027034	4.516E-08	2.71418	up	hypothetical protein CARUB_v10021088mg [Capsella rubella]
c34512.graph_c0	3.3126713	2.90876179	2.76325982	19.2293639	18.0487962	18.3535631	1.906E-48	1.54186	up	PREDICTED: uncharacterized protein LOC105177031 isoform X1 [Sesamum indicum]
c34518.graph_c0	0.04734052	0	0	3.09661338	2.05036447	2.97602212	4.186E-15	6.36497	up	PREDICTED: serine/threonine-protein kinase At5g01020 [Sesamum indicum]
c34523.graph_c0	0.03950503	0	0	2.64150655	2.77389678	2.85831117	1.963E-18	6.65168	up	Serine carboxypeptidase-like 40 [Theobroma cacao]
c34527.graph_c1	18.7416056	17.4139342	15.9789814	12.7305845	14.1033496	14.6425457	1.093E-45	-1.41825	down	PREDICTED: uncharacterized protein LOC105168318 [Sesamum indicum]
c34529.graph_c0	0.2314741	0.23170589	0.08498668	4.20585108	5.40328462	4.3536612	8.729E-21	3.59036	up	PREDICTED: acetolactate synthase small subunit 1, chloroplastic-like isoform X2 [Sesamum indicum]
c34533.graph_c0	0	0	0	5.12608014	4.9550782	6.39728399	2.934E-19	Inf	up	PREDICTED: GDSL esterase/lipase At2g40250 [Sesamum indicum]
c34535.graph_c0	1.24343366	1.75177012	1.1836011	18.4760619	18.8580844	17.3705375	7.794E-38	2.62125	up	PREDICTED: poly(U)-specific endoribonuclease-B [Sesamum indicum]
c34540.graph_c0	83.7779649	83.9287314	82.160054	86.7211841	74.7060871	81.0653359	3.32E-41	-1.13335	down	PREDICTED: zinc finger A20 and AN1 domain-containing stress-associated protein 5-like [Sesamum indicum]
c34545.graph_c0	0.40782283	0.57152368	0.52406848	22.9712543	22.641022	22.7109035	5.249E-91	4.41336	up	PREDICTED: LOW QUALITY PROTEIN: signal peptide peptidase-like [Sesamum indicum]
c34552.graph_c0	576.771256	604.116531	584.298523	73.2339637	75.7240123	74.4680557	0	-4.07257	down	PREDICTED: zinc finger CCCH domain-containing protein 29 [Sesamum indicum]
c34555.graph_c0	30.1506338	27.1497523	28.6277625	17.386024	16.4076218	16.367473	4.011E-78	-1.86681	down	PREDICTED: RNA-directed DNA methylation 4 [Sesamum indicum]
c34558.graph_c0	0.26038098	0.14034554	0.18016916	5.45894411	4.5477453	5.70170089	2.313E-36	3.67108	up	PREDICTED: histidine kinase 5-like [Sesamum indicum]
c34564.graph_c0	6.7417725	7.26199797	9.04011608	7.32319231	8.70400177	8.17036395	0.0001197	-1.02394	down	PREDICTED: putative nuclease HARBI1 isoform X1 [Sesamum indicum]
c34566.graph_c0	8.41170875	8.94833195	8.77514144	7.42202322	7.27172224	6.71900691	2.614E-25	-1.37885	down	PREDICTED: chlorophyll a-b binding protein, chloroplastic [Sesamum indicum]
c34574.graph_c0	131.093961	130.672372	136.877674	57.058046	59.4658278	58.7718371	3.7E-153	-2.27645	down	PREDICTED: heat shock 70 kDa protein 14-like [Sesamum indicum]
c34580.graph_c0	109.402773	104.024982	126.257267	13.0422252	14.7583163	14.6714371	6.284E-57	-4.09214	down	hypothetical protein MIMGU_mgv1a010728mg [Erythranthe guttata]
c34593.graph_c0	0.53802107	0.17951994	0.23045946	114.832657	109.967662	110.890167	0	7.38717	up	PREDICTED: uncharacterized protein LOC105177617 [Sesamum indicum]
c34601.graph_c0	3.0180559	4.00468483	4.14890188	3.26475257	3.13200082	2.99701287	5.775E-06	-1.34417	down	PREDICTED: uncharacterized protein LOC105178335 [Sesamum indicum]
c34605.graph_c0	9.96441786	9.18989268	10.6465808	54.3562969	55.1032631	59.6923812	4.339E-21	1.4136	up	40S ribosomal protein s13, partial [Camellia sinensis]
c34608.graph_c0	21.5194268	22.7128384	20.5143289	16.1971162	14.4149576	16.094853	5.047E-70	-1.56058	down	PREDICTED: uncharacterized protein LOC105172586 [Sesamum indicum]

#ID	0 h-1 FPKM	0 h-2 FPKM	0 h-3 FPKM	28 h-1 FPKM	28 h-2 FPKM	28 h-3 FPKM	FDR	log2FC	regulated	nr_annotation
c34610.graph_c0	1.8238076	1.4605071	1.61452476	12.1949023	10.6119094	12.5949104	2.801E-17	1.76442	up	PREDICTED: F-actin-capping protein subunit alpha [Sesamum indicum]
c34613.graph_c0	8338.39758	8509.41383	8986.97563	6137.99925	6406.62925	6827.4128	5.296E-08	-1.50646	down	hypothetical protein MIMGU_mgv1a024110mg [Erythranthe guttata]
c34619.graph_c1	0.34967357	0.70004743	0.53921325	2.54140734	2.98304831	2.2811444	0.0061061	1.20202	up	PREDICTED: uncharacterized protein LOC105177084 [Sesamum indicum]
c34623.graph_c0	1.28444308	1.19389145	1.41476589	21.471094	25.6478259	24.0767237	1.794E-60	3.10064	up	hypothetical protein MIMGU_mgv1a008681mg [Erythranthe guttata]
c34627.graph_c0	65.4916	61.7546709	67.7846923	61.7877283	65.3180135	62.7376809	1.721E-35	-1.1302	down	PREDICTED: quinone oxidoreductase-like protein 2 homolog [Sesamum indicum]
c34632.graph_c2	9.63446044	13.5935997	10.3761748	5.26837481	4.63642196	4.29895083	2.305E-13	-2.33331	down	PREDICTED: cyclin-D3-1 [Sesamum indicum]
c34639.graph_c0	90.6784481	92.1497697	91.8246703	406.26536	439.674356	429.965153	5.385E-46	1.12518	up	40S ribosomal protein S15D [Hevea brasiliensis]
c34641.graph_c0	32.55315	34.5534854	33.0412858	10.7248516	9.92433387	9.2021735	1.83E-215	-2.8373	down	PREDICTED: BTB/POZ domain-containing protein POB1-like [Sesamum indicum]
c34644.graph_c0	0.45846422	0.4589233	0.58914467	6.14056864	5.32946016	6.87508321	7.914E-11	2.51308	up	PREDICTED: cytokinin riboside 5'-monophosphate phosphoribohydrolase LOG3-like isoform X1 [Nicotiana sylvestris]
c34647.graph_c0	0.05552871	0	0.14271318	21.6722168	18.9485093	25.2915871	2.834E-48	7.26779	up	PREDICTED: probable pectinesterase 15 [Sesamum indicum]
c34648.graph_c0	97.3794263	110.931612	93.1389953	23.7741103	25.4987596	23.6791227	1.508E-87	-3.13674	down	PREDICTED: uncharacterized protein LOC105163464 [Sesamum indicum]
c34652.graph_c0	3.1243141	2.16515259	2.05890632	0.40756755	0.21028302	0.4275923	3.88E-18	-3.89704	down	PREDICTED: protein YLS9-like [Sesamum indicum]
c34656.graph_c0	0.13392417	0	0.08604893	1.89803863	2.02134724	2.50188188	3.688E-10	3.78143	up	hypothetical protein MIMGU_mgv1a014357mg [Erythranthe guttata]
c34662.graph_c0	0	0	0	3.22790734	3.57190317	2.79237887	7.149E-25	Inf	up	PREDICTED: probable GABA transporter 2 [Sesamum indicum]
c34664.graph_c0	24.9426898	24.1067121	24.3155712	18.4407584	19.8486339	19.6382735	4.592E-17	-1.43109	down	PREDICTED: cyclin-dependent kinase F-4-like isoform X1 [Sesamum indicum]
c34673.graph_c0	1.0042957	0.50265068	0.43018658	5.53519883	7.6888701	5.26118425	5.094E-07	2.1721	up	hypothetical protein MIMGU_mgv1a009336mg [Erythranthe guttata]
c34674.graph_c0	0.34296255	0.3719148	0.55090057	2.90807071	2.98206225	1.9831182	1.135E-05	1.53822	up	hypothetical protein MIMGU_mgv1a023462mg, partial [Erythranthe guttata]
c34676.graph_c0	0.9102361	0.79725412	0.77979295	8.76559515	9.03448848	7.60250652	1.319E-18	2.26272	up	PREDICTED: uncharacterized protein LOC105168235 isoform X2 [Sesamum indicum]
c34678.graph_c0	4.37685739	5.38266647	4.33884988	25.4485978	22.0750025	25.5123275	8.587E-08	1.28374	up	-
c34681.graph_c0	3.538411	3.58220366	3.51358954	3.36074004	3.95795629	3.2431109	1.239E-06	-1.101	down	hypothetical protein POPTR_0005s12710g [Populus trichocarpa]
c34682.graph_c0	27.0744031	25.633923	25.7902224	17.0964736	18.301461	19.4187476	2.846E-66	-1.60771	down	PREDICTED: E3 ubiquitin-protein ligase At1g63170-like [Sesamum indicum]
c34684.graph_c0	1.13317547	1.18586972	1.25760597	25.1193546	26.5675287	23.2769858	1.944E-60	3.29715	up	hypothetical protein CISIN_1g0023491mg, partial [Citrus sinensis]
c34685.graph_c0	3.08252285	1.92218298	2.27279896	0.14690884	0.23212859	0.26972193	2.841E-19	-4.57375	down	PREDICTED: uncharacterized protein LOC105169662 [Sesamum indicum]
c34687.graph_c0	70.415942	62.1374789	71.1455417	66.9249472	72.160036	77.4663318	2.672E-15	-1.00232	down	PREDICTED: probable fructokinase-7 [Sesamum indicum]

#ID	0 h-1 FPKM	0 h-2 FPKM	0 h-3 FPKM	28 h-1 FPKM	28 h-2 FPKM	28 h-3 FPKM	FDR	log2FC	regulated	nr_annotation
c34693.graph_c0	9.34410696	11.6851294	8.29174476	53.3180689	56.2233194	53.7487458	1.502E-43	1.38889	up	PREDICTED: sterol 14-demethylase-like [Sesamum indicum]
c34696.graph_c0	0.08094611	0.32410867	0.72813271	2.64740254	2.07164211	2.25627036	0.0026267	1.51044	up	hypothetical protein EUGRSUZ_K01314 [Eucalyptus grandis]
c34696.graph_c1	0.46945007	0.36549345	0.13405821	2.84328005	2.19069039	2.38196246	6.344E-05	1.8585	up	PREDICTED: 2,3-bisphosphoglycerate-independent phosphoglycerate mutase [Sesamum indicum]
c34700.graph_c0	0.53247906	0.79951838	0.25659625	5.24872426	5.39428182	5.90134527	2.845E-14	2.29894	up	PREDICTED: tRNA (guanine(37)-N1)-methyltransferase 2 [Sesamum indicum]
c34704.graph_c0	0.44075183	0.66178977	0.64729549	2.42540034	3.20222557	2.68859801	0.0004443	1.15407	up	PREDICTED: putative cyclin-A3-1 [Sesamum indicum]
c34710.graph_c0	0.08067945	0.08076024	0.10367629	2.58004396	3.97080306	3.6364183	1.532E-11	4.17011	up	hypothetical protein MIMGU_mgv1a014352mg [Erythranthe guttata]
c34722.graph_c0	4.77646478	3.92307503	5.19364691	2.62588356	2.79287122	3.19589084	1.555E-12	-1.78105	down	PREDICTED: probable protein phosphatase 2C 50 [Sesamum indicum]
c34723.graph_c0	1.16332272	1.00386863	0.67013465	4.69396195	5.50177252	5.54313457	3.262E-06	1.38795	up	hypothetical protein MIMGU_mgv1a001992mg [Erythranthe guttata]
c34724.graph_c0	4.19810803	4.65161557	4.81793946	21.3773147	25.1407383	19.7455329	3.223E-12	1.18445	up	PREDICTED: protein TRANSPORT INHIBITOR RESPONSE 1-like [Sesamum indicum]
c34730.graph_c0	0.9281043	1.15023215	1.07906498	13.6193011	17.6913696	14.6254412	1.354E-26	2.77041	up	PREDICTED: methylsterol monooxygenase 2-2 [Sesamum indicum]
c34731.graph_c0	64.4846162	61.3109342	67.6225067	1.93320039	1.46244486	1.83328261	0	-6.29979	down	hypothetical protein MIMGU_mgv1a016001mg [Erythranthe guttata]
c34735.graph_c0	93.5654991	90.0888493	86.5524122	7.53577853	6.69523584	8.16604961	0	-4.68152	down	PREDICTED: peroxisomal membrane protein 13 [Glycine max]
c34754.graph_c0	1.77904446	1.97162868	2.12284667	11.267565	10.4237241	10.2869992	1.34E-07	1.35201	up	PREDICTED: uncharacterized protein LOC105178220 [Sesamum indicum]
c34755.graph_c0	0.26437485	0.26463958	0.39635417	8.45442916	5.95648754	6.29771812	6.015E-16	3.38749	up	PREDICTED: beta-galactosidase 8-like isoform X1 [Sesamum indicum]
c34762.graph_c0	1.18013965	1.45927935	0.89207422	7.21492785	6.33218231	5.72264187	8.65E-05	1.36076	up	TPA: hypothetical protein ZEAMMB73_114394 [Zea mays]
c34763.graph_c0	0.11420885	0.17148482	0.25683507	8.79867146	9.04984271	7.75537024	9.348E-51	4.45969	up	PREDICTED: transmembrane 9 superfamily member 5 isoform X1 [Sesamum indicum]
c34764.graph_c0	758.64986	773.011633	758.835563	9.0493111	9.13905629	9.77262193	0	-7.44626	down	PREDICTED: proline dehydrogenase 2, mitochondrial [Sesamum indicum]
c34777.graph_c0	23.4607797	23.8180587	22.0371392	14.6276444	15.2705579	15.6227911	1.105E-79	-1.69616	down	PREDICTED: zinc finger CCCH domain-containing protein 1 [Sesamum indicum]
c34783.graph_c1	10.5904577	10.8242427	8.8817659	10.9379609	10.3331732	10.0492118	3.102E-20	-1.04048	down	hypothetical protein MIMGU_mgv1a012299mg [Erythranthe guttata]
c34786.graph_c0	0.93215901	0.69981932	0.74866327	8.12985358	8.29628922	7.20399441	1.045E-21	2.22225	up	PREDICTED: uncharacterized protein LOC103931436 [Pyrus x bretschneideri]
c34789.graph_c0	3.96076528	5.83048733	4.19155075	2.96332789	3.44006645	3.10892317	6.044E-07	-1.64609	down	-
c34792.graph_c0	3.93654626	4.30283185	3.0816867	2.40065881	2.25664804	3.94467047	8.342E-08	-1.48074	down	unnamed protein product [Coffea canephora]
c34794.graph_c0	0.93477376	1.06938263	0	54.4481101	57.5742081	51.7152874	5.369E-75	5.28073	up	hypothetical protein MIMGU_mgv1a016621mg [Erythranthe guttata]
c34794.graph_c1	51.78846	52.3829817	49.6258117	52.1483972	53.6770291	48.0183198	1.341E-40	-1.09	down	PREDICTED: nephrocystin-3 [Sesamum indicum]
c34805.graph_c0	0.13341277	0.22257728	0.34288139	7.75709082	7.29575595	7.35830271	4.25E-27	3.90157	up	PREDICTED: 1-aminocyclopropane-1-carboxylate oxidase homolog 11-like [Sesamum indicum]

#ID	0 h-1 FPKM	0 h-2 FPKM	0 h-3 FPKM	28 h-1 FPKM	28 h-2 FPKM	28 h-3 FPKM	FDR	log2FC	regulated	nr_annotation
c34808.graph_c0	12.6441363	12.8863249	13.7646802	69.0892928	65.9924679	65.0988894	2.357E-45	1.25711	up	PREDICTED: probable purple acid phosphatase 20 [Sesamum indicum]
c34821.graph_c0	133.452253	123.586002	147.946617	99.4993472	106.242354	107.970818	1.608E-17	-1.46045	down	PREDICTED: protein lin-12 [Sesamum indicum]
c34824.graph_c0	5.58922581	3.53249747	3.69604044	0	0	0	5.339E-40	-Inf	down	VTT-domain-containing protein [Auricularia delicata TFB-10046 SS5]
c34827.graph_c0	1.60551838	1.60712607	1.26684937	7.73826683	8.22535342	8.41915006	1.266E-11	1.35715	up	quercetin 3-O-glucoside-6''-O-malonyltransferase [Glandularia x hybrida]
c34832.graph_c0	173.156058	179.205022	177.717436	27.5552771	26.0589706	27.2447137	0	-3.80339	down	-
c34836.graph_c0	2.67308576	2.67576246	2.95940274	20.4141694	18.1082377	18.5357608	6.334E-25	1.68781	up	hypothetical protein MIMGU_mgv1a008311mg [Erythranthe guttata]
c34840.graph_c0	42.3422532	42.3145953	40.4713426	19.4309229	17.6591202	18.9062005	1.92E-141	-2.24978	down	PREDICTED: tubby-like F-box protein 6 [Sesamum indicum]
c34841.graph_c0	43.8769888	45.7336544	40.4333438	29.3943161	32.7792653	28.5499309	1.476E-77	-1.60931	down	PREDICTED: uncharacterized protein LOC105169164 isoform X2 [Sesamum indicum]
c34847.graph_c0	56.8883055	56.8357077	57.4562224	57.4151848	57.1017947	61.2608124	3.451E-38	-1.05212	down	PREDICTED: DNA-directed RNA polymerase III subunit RPC5 [Sesamum indicum]
c34848.graph_c0	6.71469516	7.63279775	5.65493389	139.706114	135.10362	115.978902	1.144E-81	3.19937	up	hypothetical protein MIMGU_mgv1a0036351mg, partial [Erythranthe guttata]
c34849.graph_c0	0.02193226	0.02195422	0	3.15616731	2.27402151	2.66645649	1.257E-27	6.45729	up	PREDICTED: uncharacterized protein LOC105171410 [Sesamum indicum]
c34854.graph_c0	2.32926988	1.97289425	2.07221817	60.1196512	64.0414839	58.6206311	3.87E-137	3.75194	up	chalcone isomerase [Camellia nitidissima]
c34856.graph_c0	86.1756905	90.9279293	88.2098911	76.0483214	80.6260067	77.2934459	9.737E-55	-1.2722	down	PREDICTED: DEAD-box ATP-dependent RNA helicase 21 [Sesamum indicum]
c34858.graph_c0	2.21805564	2.15299557	2.85028931	17.68392	17.3783623	19.8511251	1.281E-17	1.8333	up	PREDICTED: rac-like GTP-binding protein 5 isoform X2 [Sesamum indicum]
c34867.graph_c0	0.04178775	0.209148	0.21479568	1.42744167	1.39853502	1.13999818	2.763E-05	1.98689	up	PREDICTED: pentatricopeptide repeat-containing protein At3g03580 [Sesamum indicum]
c34868.graph_c0	13.103545	11.7644326	11.891158	87.8231256	77.6117275	85.7231527	2.356E-52	1.68316	up	hypothetical protein VITISV_041718 [Vitis vinifera]
c34871.graph_c0	15.4722335	13.294186	15.6158162	69.9321614	73.5569415	64.1527773	1.71E-26	1.13435	up	PREDICTED: uncharacterized protein LOC105170170 isoform X1 [Sesamum indicum]
c34875.graph_c0	15.0346524	13.2284052	15.2592059	73.705717	75.1586637	73.375917	2.632E-25	1.26108	up	hypothetical protein PRUPE_ppa013245mg [Prunus persica]
c34877.graph_c0	83.1645899	91.8660379	79.3284569	13.7129628	14.1846536	15.5351669	1.09E-166	-3.63915	down	hypothetical protein MIMGU_mgv1a003677mg [Erythranthe guttata]
c34877.graph_c1	109.843143	110.3038	98.8369625	17.2283906	19.5403126	18.3518714	0	-3.62209	down	PREDICTED: uncharacterized protein LOC105162623 [Sesamum indicum]
c34887.graph_c0	10.127689	11.0736301	9.61069389	53.9221828	53.6115788	49.3287346	6.368E-44	1.25793	up	PREDICTED: serine/threonine-protein kinase D6PK [Sesamum indicum]
c34889.graph_c0	0.69569118	0.69638781	1.78798142	11.6293599	10.7284873	8.87058467	5.838E-06	2.19047	up	-
c34898.graph_c0	6.39533017	6.66847304	5.33208047	3.37539804	3.27249592	3.31870241	1.542E-27	-1.97228	down	PREDICTED: probable trehalose-phosphate phosphatase F [Sesamum indicum]

#ID	0 h-1 FPKM	0 h-2 FPKM	0 h-3 FPKM	28 h-1 FPKM	28 h-2 FPKM	28 h-3 FPKM	FDR	log2FC	regulated	nr_annotation
c34900.graph_c0	0.2563308	0.59870412	0.47579311	9.91528677	9.79363034	9.05358051	1.772E-43	3.33851	up	PREDICTED: sulfate transporter 3.1 [Sesamum indicum]
c34902.graph_c0	1487.49793	1517.3602	1443.9736	457.868111	550.111836	547.011603	7.2E-172	-2.60638	down	PREDICTED: desiccation-related protein PCC3-06 isoform X2 [Sesamum indicum]
c34906.graph_c0	0.08028496	0.24109606	0.05158468	2.91753226	2.76597099	2.61875075	8.057E-12	3.39243	up	PREDICTED: manganese-dependent ADP-ribose/CDP-alcohol diphosphatase-like [Sesamum indicum]
c34910.graph_c0	1.54666663	2.22135251	1.98752786	10.0681013	10.2820315	9.01310866	3.262E-06	1.25781	up	PREDICTED: uncharacterized protein LOC105173417 [Sesamum indicum]
c34912.graph_c0	13.7709284	13.806392	13.2164927	62.3178935	63.4000791	62.2281372	1.518E-40	1.11384	up	PREDICTED: probable cytosolic oligopeptidase A [Sesamum indicum]
c34914.graph_c0	5.08203703	3.8463635	6.84918556	28.1974465	26.191717	24.4791024	2.037E-08	1.22541	up	PREDICTED: uncharacterized protein LOC105169952 [Sesamum indicum]
c34917.graph_c0	0.6557594	0.82052005	0.80054295	11.6171774	10.6183201	9.52813486	8.772E-51	2.70918	up	PREDICTED: 125 kDa kinesin-related protein-like [Sesamum indicum]
c34925.graph_c0	23.363432	23.2810042	20.812279	14.3834042	14.6795979	14.1944339	2.942E-86	-1.73019	down	PREDICTED: probable serine/threonine-protein kinase At1g54610 [Sesamum indicum]
c34926.graph_c0	85.0498513	89.1475268	81.4467465	32.7646609	33.0144832	33.4540729	1.17E-194	-2.45501	down	PREDICTED: uncharacterized protein LOC105172852 [Sesamum indicum]
c34927.graph_c0	0.94727966	1.65939938	1.21729187	9.38050454	8.54741991	6.24993527	6.248E-05	1.56728	up	-
c34929.graph_c0	0.59966604	0.60026651	0.32446088	7.38623632	8.74022137	7.22504313	1.713E-26	2.85326	up	hypothetical protein CISIN_1g018827mg [Citrus sinensis]
c34938.graph_c0	81.6703747	76.036959	88.8084703	24.705253	25.60798	27.0099082	4.219E-53	-2.76431	down	unnamed protein product [Coffea canephora]
c34944.graph_c0	0.09559642	0	0	2.987594	2.60341782	3.17487742	1.105E-15	5.46065	up	PREDICTED: AP2-like ethylene-responsive transcription factor ANT isoform X2 [Sesamum indicum]
c34945.graph_c0	33.1832292	35.9700245	33.9796575	24.6757358	24.3940246	24.9852833	1.571E-76	-1.56836	down	chloroplast glyceraldehyde-3-phosphate dehydrogenase, partial [Chlorokybus atmophyticus]
c34946.graph_c0	55.2857926	61.0010436	56.0877097	54.3362052	56.3729216	56.4960146	9.391E-44	-1.13431	down	PREDICTED: zinc finger CCCH domain-containing protein 49-like [Sesamum indicum]
c34949.graph_c0	33.3360881	36.594438	36.7430945	15.0249077	15.2398204	16.6388456	2.022E-70	-2.27655	down	PREDICTED: fructose-1,6-bisphosphatase, chloroplastic [Sesamum indicum]
c34953.graph_c0	0	0.84859333	0.15562645	28.0781874	41.6439894	27.6518968	1.384E-19	5.50865	up	-
c34956.graph_c0	19.5573359	19.1571416	15.1869489	4.43326597	4.85342351	4.38622946	4.803E-50	-3.06671	down	PREDICTED: receptor-like serine/threonine-protein kinase At4g25390 [Sesamum indicum]
c34956.graph_c1	15.6560784	16.5783861	20.9500311	3.85560868	5.43415124	4.37390918	9.771E-18	-3.05565	down	PREDICTED: receptor-like serine/threonine-protein kinase At4g25390 [Sesamum indicum]
c34957.graph_c0	2.76577343	2.16173901	3.01857477	18.9724946	19.5172007	21.4807243	6.87E-32	1.82395	up	PREDICTED: uncharacterized protein LOC105178545 [Sesamum indicum]
c34964.graph_c0	21.4608283	23.542867	16.2091185	17.5393492	18.6817642	18.987381	9.156E-08	-1.23557	down	PREDICTED: uncharacterized protein LOC105159829 [Sesamum indicum]
c34966.graph_c0	0	0.36110195	0	17.5663466	39.0600903	20.538262	9.834E-07	6.65551	up	-

#ID	0 h-1 FPKM	0 h-2 FPKM	0 h-3 FPKM	28 h-1 FPKM	28 h-2 FPKM	28 h-3 FPKM	FDR	log2FC	regulated	nr_annotation
c34969.graph_c0	6.39545902	5.03976457	4.89607884	4.45038775	4.82193945	3.79287917	1.164E-05	-1.41051	down	-
c34970.graph_c0	4.15548272	4.51282111	4.886567	5.21408588	4.29621707	4.51039558	1.881E-07	-1.04381	down	PREDICTED: uncharacterized protein LOC105172443 [Sesamum indicum]
c34973.graph_c0	2.30438953	1.99214743	2.82663033	1.1419207	0.6186284	0.62119864	1.45E-10	-2.67535	down	-
c34978.graph_c0	20.9348545	20.5897771	21.8506277	12.9562867	12.4781996	13.8252162	1.527E-31	-1.78161	down	PREDICTED: uncharacterized protein LOC104117218 isoform X2 [Nicotiana tomentosiformis]
c34985.graph_c0	16.7360592	19.8950562	16.2691648	17.9618981	16.6845305	16.0658684	7.253E-15	-1.15089	down	PREDICTED: uncharacterized protein LOC105167921 [Sesamum indicum]
c34990.graph_c1	0.4372602	0.21884903	0.09364945	5.31429206	4.38383573	4.23557197	3.331E-23	3.14092	up	PREDICTED: nuclear valosin-containing protein-like [Sesamum indicum]
c34991.graph_c0	0	0	0	0.49872937	0.6671198	1.00986146	9.202E-07	Inf	up	--
c34993.graph_c0	4.21012724	3.77837652	3.17148493	0.10551366	0	0.17219657	1.556E-27	-6.40855	down	-
c34994.graph_c0	253.151977	245.025957	266.913895	218.538337	227.287884	223.36122	6.353E-42	-1.28461	down	PREDICTED: nucleoside diphosphate kinase 1-like [Sesamum indicum]
c34999.graph_c1	0	0	0	73.2947179	73.8293799	71.9975807	2.56E-264	Inf	up	PREDICTED: probable chalcone--flavonone isomerase 3 isoform X2 [Pyrus x bretschneideri]
c35002.graph_c0	4.25697529	3.98376205	2.74791271	18.3909853	16.3974439	17.4023605	1.939E-14	1.16264	up	PREDICTED: auxin response factor 18-like [Sesamum indicum]
c35003.graph_c0	0.92955856	1.24065249	0.72998417	4.42891892	3.79556471	4.13461717	0.0048171	1.00469	up	PREDICTED: uncharacterized protein LOC105158948 [Sesamum indicum]
c35005.graph_c0	11.5890668	10.9424774	11.3013322	58.0042826	49.7295359	56.2025408	6.288E-24	1.1866	up	PREDICTED: GDSL esterase/lipase At4g01130-like [Sesamum indicum]
c35007.graph_c0	0.45799077	2.29224691	1.76560883	11.983142	13.975386	11.2720294	5.948E-06	1.94405	up	PREDICTED: LOW QUALITY PROTEIN: piriformospora indica-insensitive protein 2 [Sesamum indicum]
c35010.graph_c0	2.94554705	2.39217646	3.49947328	2.30237693	2.18824263	3.09824127	6.949E-05	-1.31166	down	PREDICTED: uncharacterized aarF domain-containing protein kinase 1-like [Sesamum indicum]
c35017.graph_c0	27.1286185	25.4474251	21.7483644	14.9104389	14.9793269	13.6639239	1.163E-42	-1.85931	down	PREDICTED: beta-carotene hydroxylase 2, chloroplastic isoform X1 [Sesamum indicum]
c35018.graph_c0	1.29171394	1.12343266	1.22451902	12.0352396	12.7704486	11.7420901	1.04E-44	2.23737	up	PREDICTED: uncharacterized protein At5g41620 [Sesamum indicum]
c35026.graph_c0	1.62000661	1.49189849	1.08252177	17.9437507	16.0314298	16.7171916	3.971E-24	2.50958	up	hypothetical protein MIMGU_mgv1a011760mg [Erythranthe guttata]
c35027.graph_c0	9.7547834	10.7485759	11.401601	1.52053434	1.07516261	1.74900002	1.23E-78	-3.96703	down	PREDICTED: uncharacterized protein LOC104748542 [Camelina sativa]
c35030.graph_c0	6.9167932	8.48713982	6.88130563	0.89190225	0.14641893	0.72778496	1.605E-40	-4.74446	down	-
c35039.graph_c0	4.87328522	4.1584358	2.87452794	27.5801789	28.1002973	25.1110661	1.427E-17	1.67766	up	PREDICTED: acyl carrier protein 1, chloroplastic-like [Sesamum indicum]
c35040.graph_c0	16.60546	16.7051983	14.7236847	3.9223446	4.35878083	5.07172185	1.494E-57	-2.93496	down	PREDICTED: serine/threonine-protein kinase tricorner [Sesamum indicum]
c35042.graph_c0	0.82717687	0.50748703	0.61719969	0	0	0	2.589E-25	-Inf	down	-
c35053.graph_c0	8.56749731	7.83773866	8.60350757	0.90721701	1.45209746	1.81705501	2.47E-59	-3.67113	down	PREDICTED: uncharacterized protein LOC105175543 [Sesamum indicum]

#ID	0 h-1 FPKM	0 h-2 FPKM	0 h-3 FPKM	28 h-1 FPKM	28 h-2 FPKM	28 h-3 FPKM	FDR	log2FC	regulated	nr_annotation
c35055.graph_c0	13.9821822	13.3631901	13.2274465	86.5571721	81.5880547	79.8180889	1.06E-65	1.52141	up	hypothetical protein MIMGU_mgv1a005945mg [Erythranthe guttata]
c35065.graph_c0	0.57527114	0.69101663	0.59139696	2.98944182	2.7180611	3.00228937	0.0003211	1.13912	up	PREDICTED: cytokinin dehydrogenase 7 [Sesamum indicum]
c35070.graph_c0	6.84740432	7.77862694	5.79895441	2.3680262	2.60688815	2.58327546	1.026E-28	-2.52221	down	PREDICTED: LOW QUALITY PROTEIN: uncharacterized protein LOC105172331 [Sesamum indicum]
c35074.graph_c0	0.64938514	0.61753363	1.33517699	4.43651216	4.64497066	3.54313306	0.0005066	1.1761	up	PREDICTED: protease Do-like 1, chloroplastic isoform X2 [Cucumis melo]
c35077.graph_c0	72.9451706	30.0316848	42.7110074	0	0	0	7.591E-16	-Inf	down	hypothetical protein ASPNIDRAFT_138440 [Aspergillus niger ATCC 1015]
c35089.graph_c0	10.1193128	10.5030728	12.2576026	3.22519847	3.53800689	3.44339239	1.275E-31	-2.78076	down	PREDICTED: uncharacterized protein LOC105179874 [Sesamum indicum]
c35092.graph_c0	2.72584703	1.50269433	1.3199037	0	0	0	2.683E-14	-Inf	down	Chain G, Crystal Structure Of The Fab Complexed With Antigen Asf1p, Northeast Structural Genomics Consortium (nesg) Target Pdr16
c35093.graph_c0	18.5941435	19.473137	15.7944782	12.722843	12.1267752	12.2507883	2.122E-35	-1.62617	down	PREDICTED: U-box domain-containing protein 17 isoform X1 [Sesamum indicum]
c35093.graph_c1	6.2623213	6.52620544	7.60638117	4.80081185	4.84133424	4.1718014	9.376E-11	-1.65585	down	PREDICTED: U-box domain-containing protein 17 isoform X1 [Sesamum indicum]
c35096.graph_c0	3.29030414	2.84447176	3.55550751	0	0	0	1.377E-42	-Inf	down	--
c35097.graph_c0	2.01905027	1.19767232	1.44142196	5.27189287	8.04787728	8.29320234	0.0027156	1.12768	up	PREDICTED: B3 domain-containing transcription factor FUS3-like isoform X2 [Sesamum indicum]
c35103.graph_c0	1.17678102	1.09381943	1.26017517	10.0596944	10.0942217	11.0168435	1.336E-26	2.05086	up	hypothetical protein MIMGU_mgv1a002945mg [Erythranthe guttata]
c35106.graph_c0	6.50055684	6.26783579	6.69506285	35.1563386	37.8279747	36.2276191	3.287E-29	1.39713	up	hypothetical protein MIMGU_mgv1a011325mg [Erythranthe guttata]
c35108.graph_c0	7.24279257	7.67296443	7.91118392	1.93014063	1.84175511	2.25508356	6.634E-76	-3.01187	down	PREDICTED: ethylene-responsive transcription factor RAP2-11-like [Sesamum indicum]
c35113.graph_c0	249.308429	270.460022	251.555118	39.5394393	37.9017721	41.4182612	0	-3.78814	down	hypothetical protein MIMGU_mgv1a002687mg [Erythranthe guttata]
c35115.graph_c0	71.904909	78.0509118	149.322577	32.1939693	24.4889089	34.0070755	0.0017778	-2.82207	down	hypothetical protein EUGRSUZ_J02069 [Eucalyptus grandis]
c35120.graph_c0	9.50994623	9.60761223	10.9759608	42.5586091	46.5739934	47.1823698	4.759E-25	1.08714	up	PREDICTED: uncharacterized protein LOC105156523 [Sesamum indicum]
c35121.graph_c0	1.86752454	1.38172643	1.90422323	9.64867576	9.12479656	8.72798082	2.349E-17	1.32393	up	PREDICTED: LETM1 and EF-hand domain-containing protein 1, mitochondrial [Sesamum indicum]
c35122.graph_c0	2.77493643	2.90397489	2.30973274	19.2513472	18.3754279	17.691261	1.73E-30	1.70341	up	PREDICTED: cytochrome P450 82C2 [Sesamum indicum]
c35127.graph_c0	0.67464013	0.8441446	1.1378578	5.76131357	5.28493544	4.55114701	6.917E-07	1.45584	up	PREDICTED: uncharacterized protein LOC105162824 isoform X1 [Sesamum indicum]
c35128.graph_c0	0	0	0.1012367	15.5168031	10.8049286	12.0542024	3.504E-27	7.43607	up	PREDICTED: anthocyanidin 3-O-glucosyltransferase 2-like [Sesamum indicum]
c35134.graph_c0	1.17905054	0.56651097	1.09089117	4.28463568	4.3947572	4.39126203	0.0005058	1.11299	up	PREDICTED: protein-ribulosamine 3-kinase, chloroplastic [Sesamum indicum]

#ID	0 h-1 FPKM	0 h-2 FPKM	0 h-3 FPKM	28 h-1 FPKM	28 h-2 FPKM	28 h-3 FPKM	FDR	log2FC	regulated	nr_annotation
c35135.graph_c0	2.91442428	2.56852993	2.40178136	29.0099493	28.0018468	27.0347919	3.508E-68	2.32551	up	PREDICTED: cytochrome b561 and DOMON domain-containing protein At3g61750 [Sesamum indicum]
c35136.graph_c0	62.3449402	64.945192	60.1238094	44.3068724	42.8030295	42.851771	1.742E-54	-1.61814	down	hypothetical protein MIMGU_mgv1a007434mg [Erythranthe guttata]
c35138.graph_c0	253.440277	272.417552	239.292681	187.025514	187.825248	182.540459	2.648E-82	-1.54677	down	hypothetical protein JCGZ_08206 [Jatropha curcas]
c35139.graph_c0	0.81062676	0.44260281	0.37879545	3.85631252	5.65806905	4.41414823	2.466E-06	2.01071	up	PREDICTED: thaumatin-like protein 1 [Sesamum indicum]
c35141.graph_c1	0.19627074	0	0	11.839821	11.0337101	12.5323964	1.385E-38	6.43662	up	hypothetical protein MIMGU_mgv1a011475mg [Erythranthe guttata]
c35143.graph_c0	15.1794938	14.7319112	16.3377225	143.700801	148.962837	144.539831	1.39E-105	2.14907	up	PREDICTED: CBL-interacting serine/threonine-protein kinase 6-like [Sesamum indicum]
c35152.graph_c0	1.1013109	0.91867808	0.7665819	7.80415757	7.66373738	7.27980542	1.443E-21	1.94259	up	PREDICTED: AP-3 complex subunit mu [Sesamum indicum]
c35154.graph_c0	0.39700398	0.50578376	0.41740841	10.7022186	13.6183394	11.4726635	9.204E-40	3.66992	up	PREDICTED: transcription factor bHLH66-like [Sesamum indicum]
c35155.graph_c0	0.42829578	0	0.55037703	2.55251981	2.47332163	2.64164479	5.058E-05	1.87072	up	PREDICTED: SUN domain-containing protein 2-like [Sesamum indicum]
c35156.graph_c0	245.809332	239.388072	248.401916	39.3458472	37.4785896	47.1082496	0	-3.65515	down	hypothetical protein MIMGU_mgv1a014857mg [Erythranthe guttata]
c35156.graph_c1	276.095132	240.603564	258.441988	46.5473175	47.6657476	56.2785496	1.91E-163	-3.45396	down	Lactoylglutathione lyase / glyoxalase I family protein [Theobroma cacao]
c35159.graph_c0	41.1443734	40.6863542	42.8584842	189.207558	223.568603	184.56034	1.679E-17	1.16839	up	PREDICTED: stromal cell-derived factor 2-like protein [Sesamum indicum]
c35168.graph_c0	10.2281164	6.51531893	7.72680515	36.3126743	33.7632263	34.1526745	1.199E-10	1.00281	up	PREDICTED: E3 ubiquitin-protein ligase RMA1H1 [Sesamum indicum]
c35189.graph_c0	0.19264081	0.23140045	0.14853064	5.3483908	6.16910621	5.25537141	1.073E-22	3.78511	up	PREDICTED: BTB/POZ and TAZ domain-containing protein 3 [Sesamum indicum]
c35192.graph_c0	8.13406141	6.40454043	6.88341356	62.3982115	65.1601089	58.6524341	1.028E-73	2.03041	up	Triosephosphate isomerase, chloroplastic [Gossypium arboreum]
c35198.graph_c0	18.1620311	16.7568554	17.6495459	8.26765293	8.33440842	10.5794835	5.697E-79	-2.04076	down	PREDICTED: uncharacterized protein LOC105165319 [Sesamum indicum]
c35200.graph_c0	0.07197531	0.14409477	0.09249112	11.0376719	12.941608	10.0311032	1.278E-32	5.69214	up	PREDICTED: probable flavin-containing monooxygenase 1 [Sesamum indicum]
c35204.graph_c0	7.45130019	7.97179805	7.9540152	5.04306306	5.56240806	4.62948556	9.768E-26	-1.70966	down	hypothetical protein MIMGU_mgv1a009915mg [Erythranthe guttata]
c35206.graph_c0	1.29459644	1.00791661	0.61615099	16.5181141	18.1551812	19.2795826	7.292E-44	3.1267	up	hypothetical protein MIMGU_mgv1a006135mg [Erythranthe guttata]
c35207.graph_c0	0.65666315	1.18317726	1.18137341	10.5633324	10.9740991	9.89176181	1.445E-20	2.28249	up	PREDICTED: nitrilase-like protein 2 [Sesamum indicum]
c35209.graph_c0	7.45048521	7.14934109	6.40478856	40.8176682	39.9903655	39.3405189	5.707E-30	1.42726	up	PREDICTED: protein bem46-like isoform X2 [Sesamum indicum]
c35210.graph_c0	1.1140593	0.84391611	0.96730426	4.39859641	5.78933036	3.96421175	4.398E-05	1.1836	up	PREDICTED: uncharacterized protein LOC105169721 [Sesamum indicum]
c35213.graph_c0	21.6541825	21.6291506	20.3301235	20.1475263	21.2538402	20.5641097	3.62E-26	-1.12766	down	PREDICTED: tubby-like F-box protein 8 [Sesamum indicum]
c35214.graph_c0	70.4413124	66.5048305	74.8131162	10.3946224	12.4330641	12.158197	6.3E-122	-3.68898	down	hypothetical protein MIMGU_mgv1a007518mg [Erythranthe guttata]
c35218.graph_c0	0.08753076	0.05257105	0.1124805	4.74581731	4.75603652	4.33974567	1.991E-46	4.67995	up	PREDICTED: probable disease resistance protein At5g63020 [Vitis vinifera]

#ID	0 h-1 FPKM	0 h-2 FPKM	0 h-3 FPKM	28 h-1 FPKM	28 h-2 FPKM	28 h-3 FPKM	FDR	log2FC	regulated	nr_annotation
c35223.graph_c0	5.76352858	4.9039049	5.18445369	23.8767263	26.6013564	25.2370283	7.007E-09	1.16627	up	PREDICTED: uncharacterized protein LOC105165107 isoform X2 [Sesamum indicum]
c35227.graph_c0	9.04413256	9.11649791	8.12732057	41.2320378	42.3335982	41.7093053	3.215E-19	1.16347	up	PREDICTED: V-type proton ATPase subunit C-like [Sesamum indicum]
c35231.graph_c0	0.09021589	0.04515311	0	0.91795743	1.09523897	1.20382356	1.104E-08	3.50496	up	PREDICTED: transcription factor TGA4 isoform X1 [Sesamum indicum]
c35234.graph_c0	84.8779191	86.3763098	78.860794	47.9117493	45.8310837	47.511355	1.03E-119	-1.91372	down	PREDICTED: uncharacterized protein LOC105155881 [Sesamum indicum]
c35235.graph_c0	10.3705674	12.5182067	9.82509356	4.71448762	4.12330016	3.83492389	1.075E-30	-2.45801	down	PREDICTED: uncharacterized protein LOC105164201 isoform X1 [Sesamum indicum]
c35239.graph_c0	3.06959217	2.9702437	2.80501153	42.5370068	42.2789562	40.5555068	4.55E-110	2.73557	up	PREDICTED: ran-binding protein 1 homolog c-like [Sesamum indicum]
c35240.graph_c0	17.5115622	19.7327554	17.616674	12.4363927	10.2439057	10.8601349	2.379E-26	-1.80018	down	PREDICTED: disease resistance protein RPM1-like [Sesamum indicum]
c35243.graph_c0	0	0	0	3.1911553	3.20402121	2.47896675	1.418E-22	Inf	up	PREDICTED: lycopene epsilon cyclase, chloroplastic [Sesamum indicum]
c35244.graph_c0	6.79262366	6.71750466	6.62546736	6.54279964	6.98163183	6.86772961	1.487E-10	-1.07197	down	PREDICTED: zinc finger CCCH domain-containing protein 39-like isoform X2 [Sesamum indicum]
c35245.graph_c0	6.36424758	5.67467028	7.42233187	34.166497	32.8145041	34.5085163	8.191E-23	1.28979	up	PREDICTED: alpha-soluble NSF attachment protein 2-like isoform X1 [Sesamum indicum]
c35253.graph_c0	3.74979124	4.56200217	4.78156382	34.6116486	38.2167883	35.3249114	6.311E-71	1.95277	up	PREDICTED: argininosuccinate synthase, chloroplastic-like isoform X2 [Sesamum indicum]
c35256.graph_c0	3.88442926	3.78322923	2.02364002	0.76302184	0.68893718	0.62261959	1.079E-08	-3.3074	down	PREDICTED: uncharacterized protein LOC105170847 [Sesamum indicum]
c35258.graph_c0	143.807394	142.294968	135.535553	24.5117089	25.8807322	26.2634606	0	-3.54918	down	PREDICTED: zinc finger protein 622 [Sesamum indicum]
c35265.graph_c0	0.14203193	0.3317397	0.06083889	2.92479309	3.72820791	4.09934898	5.465E-12	3.24958	up	PREDICTED: auxin response factor 4 isoform X2 [Sesamum indicum]
c35265.graph_c1	0.23165513	0.40580243	0.14884301	6.81880746	6.99284482	7.2814033	6.841E-19	3.66143	up	PREDICTED: auxin response factor 4 isoform X2 [Sesamum indicum]
c35270.graph_c0	5.59192407	5.13106324	5.94543031	4.88669044	5.41698702	5.13242509	1.778E-12	-1.20257	down	PREDICTED: kinesin-13A-like [Sesamum indicum]
c35272.graph_c0	0.23412227	0.14061403	0.15042819	3.70946006	4.02529906	3.58228161	4.816E-22	3.34343	up	PREDICTED: interactor of constitutive active ROPs 2, chloroplastic-like [Sesamum indicum]
c35275.graph_c0	0.14554898	0.43708418	0.35329061	2.06866803	2.31354144	2.11002028	9.22E-09	1.69727	up	PREDICTED: serine/threonine-protein kinase Nek5 [Sesamum indicum]
c35277.graph_c0	3.37189249	3.28644606	3.99093519	2.19270655	2.21272707	2.10497722	1.368E-07	-1.80311	down	-
c35281.graph_c0	7.87877539	7.18666496	6.47011915	3.76102715	3.64060118	3.04131909	8.907E-30	-2.13328	down	PREDICTED: uncharacterized protein LOC105158079 isoform X1 [Sesamum indicum]
c35282.graph_c0	0	0	0	2.43730793	3.05091575	2.89283399	1.794E-14	Inf	up	lavandulyl diphsophate synthase [Lavandula x intermedia]
c35283.graph_c0	67.2061862	68.4781565	65.4847995	53.6284415	52.6169934	53.7525446	4.763E-67	-1.42043	down	PREDICTED: DNA-damage-repair/toleration protein DRT111, chloroplastic-like [Sesamum indicum]
c35285.graph_c0	8.86805911	8.59751974	9.93339114	7.3816108	7.49625393	8.31551203	3.584E-18	-1.33208	down	-
c35288.graph_c0	4.17401583	4.35156458	4.13967743	48.6517002	49.3038378	48.7177768	1.31E-154	2.4433	up	PREDICTED: valine--tRNA ligase [Sesamum indicum]

#ID	0 h-1 FPKM	0 h-2 FPKM	0 h-3 FPKM	28 h-1 FPKM	28 h-2 FPKM	28 h-3 FPKM	FDR	log2FC	regulated	nr_annotation
c35288.graph_c1	148.275141	158.617944	134.860455	63.3549168	62.6605526	58.7044897	1.647E-97	-2.34735	down	PREDICTED: protein TIME FOR COFFEE [Sesamum indicum]
c35290.graph_c0	14.9940452	14.9224687	12.8946995	14.2506889	12.7345812	12.6715858	1.515E-31	-1.19921	down	PREDICTED: protein FRIGIDA [Sesamum indicum]
c35292.graph_c0	586.521041	592.817883	629.515466	257.821975	252.780092	283.877385	8.488E-92	-2.27793	down	hypothetical protein MIMGU_mgv1a0189502mg, partial [Erythranthe guttata]
c35295.graph_c1	0.04021615	0.12076927	0.25839673	2.33830937	3.61555694	2.55200531	1.819E-09	3.23137	up	PREDICTED: ras-related protein RABA6a-like [Sesamum indicum]
c35299.graph_c0	3.58774883	4.33805597	3.97133404	46.0840196	43.8473734	40.6588653	8.531E-91	2.36465	up	PREDICTED: ammonium transporter 2 [Sesamum indicum]
c35306.graph_c0	7.0319596	7.12717225	7.20643917	6.40182177	6.20412945	6.50367368	4.303E-32	-1.25165	down	PREDICTED: uncharacterized protein LOC105157223 [Sesamum indicum]
c35309.graph_c0	2.80381689	2.71094411	2.57943171	21.3042514	22.3720037	23.733069	3.865E-45	1.96881	up	PREDICTED: WD-40 repeat-containing protein MSI4-like [Sesamum indicum]
c35310.graph_c0	1.25281019	1.07491259	1.30326104	11.2732933	10.1395779	12.2416334	5.169E-15	2.12157	up	PREDICTED: uncharacterized protein LOC105164773 [Sesamum indicum]
c35323.graph_c0	0.7032665	0.4399817	0.53658689	6.34120705	5.79762518	6.0476301	8.794E-24	2.34859	up	PREDICTED: probable thimet oligopeptidase isoform X2 [Sesamum indicum]
c35324.graph_c0	5.71594504	2.83358831	3.56767619	3593.64153	3214.21357	3176.74463	0	8.60075	up	PREDICTED: vignain-like [Sesamum indicum]
c35328.graph_c0	1.31134471	0.99761995	1.01107792	6.36655957	6.2303087	5.95721023	3.342E-10	1.39447	up	PREDICTED: uncharacterized protein LOC105175059 [Sesamum indicum]
c35329.graph_c0	16.4931436	17.5116904	20.0304914	7.06755155	7.38233035	5.25820573	2.108E-28	-2.54961	down	PREDICTED: chromo domain protein LHP1 isoform X3 [Sesamum indicum]
c35338.graph_c0	0	0	0	5.07408212	5.32253254	5.6017306	1.375E-26	Inf	up	PREDICTED: dehydrodolichyl diphosphate synthase 2-like [Nicotiana sylvestris]
c35341.graph_c0	0.26674821	0.13350766	0.25708651	5.76766414	6.08289692	5.93239518	1.231E-26	3.66591	up	PREDICTED: transcription factor bHLH79-like [Sesamum indicum]
c35344.graph_c0	30.7258265	29.9497374	29.1998387	30.0727035	30.2131926	30.5486512	4.511E-39	-1.07457	down	PREDICTED: microfibrillar-associated protein 1-like [Sesamum indicum]
c35358.graph_c0	11.7751118	7.24052602	6.26875802	3.05645836	1.76620501	2.29452146	1.13E-06	-2.91198	down	ubiquitin-like protein [Vanilla planifolia]
c35359.graph_c0	1.1090211	0.44405265	0.78382481	3.18381908	4.148287	4.83419803	0.0012662	1.29392	up	PREDICTED: sphinganine C(4)-monooxygenase 1-like [Sesamum indicum]
c35363.graph_c0	4.96527998	4.18921236	4.14737788	59.7245953	59.5812762	54.8555509	1.65E-127	2.62173	up	PREDICTED: F-box protein At4g00755-like [Sesamum indicum]
c35365.graph_c0	2.55642063	2.76873299	3.01583108	12.7622714	14.7683132	14.1668932	6.526E-12	1.22914	up	PREDICTED: probable 6-phosphogluconolactonase 1 [Solanum lycopersicum]
c35372.graph_c0	0.912464	1.3986096	0.4763494	5.07742271	5.72586782	6.47681994	4.009E-06	1.5502	up	PREDICTED: vacuolar amino acid transporter 1 isoform X2 [Sesamum indicum]
c35373.graph_c0	40.5765862	44.2626839	41.4219381	28.8338086	29.4918964	30.2986233	3.471E-82	-1.60103	down	PREDICTED: E3 ubiquitin-protein ligase KEG [Sesamum indicum]
c35374.graph_c0	0.05080182	0	0	2.87995273	2.266952	2.28976104	1.266E-14	6.13442	up	PREDICTED: mitogen-activated protein kinase 19 isoform X2 [Sesamum indicum]
c35375.graph_c0	0.5971671	0.52304444	0.38369169	19.693549	20.0510505	18.8292315	3.77E-120	4.19792	up	PREDICTED: uncharacterized protein At1g04910 [Sesamum indicum]

#ID	0 h-1 FPKM	0 h-2 FPKM	0 h-3 FPKM	28 h-1 FPKM	28 h-2 FPKM	28 h-3 FPKM	FDR	log2FC	regulated	nr_annotation
c35382.graph_c0	0.0278963	0.02792424	0	0.75017024	0.6956397	0.69485344	2.706E-10	4.19067	up	PREDICTED: bidirectional sugar transporter SWEET3b [Sesamum indicum]
c35387.graph_c0	0.90459799	0.9531619	0.79535658	8.5123329	8.68562237	7.90600958	3.823E-14	2.15301	up	PREDICTED: transcription factor bHLH121 [Sesamum indicum]
c35389.graph_c0	9.09735913	9.86534115	7.79364444	9.6423661	10.1986358	7.58708871	7.126E-07	-1.05385	down	PREDICTED: protein ETHYLENE INSENSITIVE 3 [Sesamum indicum]
c35396.graph_c0	0.52541626	0.5976618	0.55242057	4.79072657	4.85843784	4.61738103	1.843E-14	1.99894	up	PREDICTED: ycf20-like protein [Sesamum indicum]
c35397.graph_c0	0.27248524	0.27275809	0.30013228	4.13056323	4.24040328	4.17850384	2.28E-13	2.8	up	PREDICTED: 2-amino-3-ketobutyrate coenzyme A ligase, mitochondrial [Sesamum indicum]
c35400.graph_c0	0.35725835	0.22351005	0.05738639	1.72020317	2.10998333	1.82742169	2.07E-05	2.07438	up	PREDICTED: transcription factor bHLH68-like [Sesamum indicum]
c35403.graph_c1	0.4753781	0.95170824	1.5271992	4.83702888	3.9774386	5.07468352	0.0050308	1.13057	up	PREDICTED: deSI-like protein At4g17486 [Sesamum indicum]
c35407.graph_c0	0	0.09979194	0.25621664	14.4911313	14.0327325	13.8939576	3.73E-57	5.77978	up	PREDICTED: rac-like GTP-binding protein ARAC8 [Sesamum indicum]
c35415.graph_c0	8.12513095	6.38612078	7.81150082	1.74971803	2.13277229	1.85608231	6.382E-38	-3.05082	down	hypothetical protein MIMGU_mgv1a019839mg, partial [Erythranthe guttata]
c35430.graph_c0	4.42442609	4.19369597	4.07549185	0.28457057	0.3340227	0.37153161	8.807E-61	-4.76918	down	unnamed protein product [Coffea canephora]
c35431.graph_c0	1.88937126	2.30641851	2.25026441	1.30619955	1.57250096	1.2298248	4.592E-08	-1.74293	down	PREDICTED: cysteine-rich repeat secretory protein 3-like isoform X1 [Sesamum indicum]
c35440.graph_c0	1.77447552	1.42567626	1.23014636	0	0	0	1.207E-55	-Inf	down	predicted protein [Thalassiosira pseudonana CCMP1335]
c35441.graph_c0	5.69298877	4.73280987	4.3398315	1.68310431	2.40616853	1.48784932	7.894E-15	-2.49388	down	-
c35451.graph_c0	9.57140232	9.08385997	8.99265469	44.4293388	44.4411306	41.2074384	6.772E-25	1.14391	up	hypothetical protein MIMGU_mgv1a005627mg [Erythranthe guttata]
c35463.graph_c0	131.951969	132.147419	134.691624	37.376927	40.7630986	39.4652264	0	-2.85253	down	PREDICTED: BTB/POZ and TAZ domain-containing protein 1 [Sesamum indicum]
c35467.graph_c0	18.3740197	20.9125335	19.6382803	10.3045102	11.1300266	11.6826938	2.499E-56	-1.92197	down	PREDICTED: common plant regulatory factor 1-like isoform X2 [Sesamum indicum]
c35468.graph_c0	1.35796447	1.50829131	1.60161032	18.9820945	19.665963	17.2102601	1.385E-86	2.55109	up	PREDICTED: alpha,alpha-trehalose-phosphate synthase [UDP-forming] 5 isoform X1 [Sesamum indicum]
c35474.graph_c1	4.96277249	5.30535549	4.76754033	4.83256499	4.45820859	5.94357523	2.819E-07	-1.06959	down	hypothetical protein MIMGU_mgv1a015318mg [Erythranthe guttata]
c35476.graph_c0	2.30957079	2.03445746	2.07752293	1.91358432	1.3943546	1.72583436	2.953E-06	-1.44027	down	hypothetical protein MIMGU_mgv1a012316mg [Erythranthe guttata]
c35479.graph_c0	1.70482551	1.80691691	1.54642467	13.5203023	15.5638232	15.9390743	8.962E-22	2.06519	up	PREDICTED: glycine-rich RNA-binding protein 8-like [Sesamum indicum]
c35484.graph_c0	15.6273844	16.5143954	14.3821965	119.242812	125.999153	126.112924	3.4E-105	1.90753	up	PREDICTED: 60S ribosomal protein L4-1 [Sesamum indicum]
c35495.graph_c0	32.7029237	34.5576378	37.8834462	10.2576681	9.45752041	13.1303777	5.345E-48	-2.76947	down	PREDICTED: CBL-interacting protein kinase 18-like [Sesamum indicum]
c35497.graph_c0	1.18330517	1.63572438	1.7378217	1.43336563	1.55303373	1.16961465	0.0011104	-1.22859	down	PREDICTED: probable protein phosphatase 2C 4 [Sesamum indicum]
c35500.graph_c0	7.25863693	5.95967518	6.39309872	143.50642	147.930333	151.586787	9.53E-192	3.40842	up	PREDICTED: nascent polypeptide-associated complex subunit alpha-like protein 2 [Vitis vinifera]
c35504.graph_c0	58.6007934	60.1087073	63.167052	356.910225	397.176687	379.55106	2.285E-78	1.54838	up	PREDICTED: 40S ribosomal protein S8 [Sesamum indicum]

#ID	0 h-1 FPKM	0 h-2 FPKM	0 h-3 FPKM	28 h-1 FPKM	28 h-2 FPKM	28 h-3 FPKM	FDR	log2FC	regulated	nr_annotation
c35508.graph_c0	3.01749558	2.41641372	2.4898282	28.025489	28.8478371	26.8244184	1.78E-67	2.31209	up	PREDICTED: acyl-CoA-binding domain-containing protein 4 [Sesamum indicum]
c35510.graph_c0	0	0.32190479	0.2066233	10.9850518	14.5611608	10.8708855	2.675E-17	5.00465	up	PREDICTED: beta-glucosidase 24-like [Sesamum indicum]
c35510.graph_c1	0.24092456	0.48233162	0.4127968	26.090285	26.5610042	21.2418321	3.545E-55	4.92759	up	PREDICTED: NADPH--cytochrome P450 reductase-like [Sesamum indicum]
c35517.graph_c0	10.8808782	10.0247669	10.6432884	7.06226191	6.85613453	7.28770923	1.989E-44	-1.66335	down	PREDICTED: putative lysine-specific demethylase JMJ16 isoform X1 [Sesamum indicum]
c35521.graph_c0	2.59215621	2.41943079	3.15096805	12.3985211	11.6084693	12.7969788	1.544E-10	1.07965	up	PREDICTED: rhodanese-like domain-containing protein 8, chloroplastic [Sesamum indicum]
c35522.graph_c0	2.78382228	1.42728798	1.48327979	0	0	0	6.009E-13	-Inf	down	hypothetical protein SNOG_05978 [Phaeosphaeria nodorum SN15]
c35526.graph_c1	0	0.0675518	0	2.86925796	2.05925124	2.08114761	4.525E-17	5.61507	up	PREDICTED: L-ascorbate oxidase homolog [Sesamum indicum]
c35528.graph_c0	16.2079293	11.9416399	13.6385288	12.3777905	12.0398285	13.1741426	8.209E-10	-1.24126	down	PREDICTED: protein N-terminal glutamine amidohydrolase [Sesamum indicum]
c35533.graph_c0	55.4728921	48.070331	53.5441039	32.9683302	35.9303154	38.5797955	9.966E-40	-1.63751	down	hypothetical protein MIMGU_mgv1a026579mg [Erythranthe guttata]
c35535.graph_c0	1.17143599	1.60690864	0.72479412	0.81985986	0.59789955	0.90057551	0.0025713	-1.67842	down	PREDICTED: peregrin [Sesamum indicum]
c35537.graph_c0	2.37791456	1.45139981	3.57742169	11.6762567	11.5321199	12.2106288	2.078E-05	1.15973	up	hypothetical protein MIMGU_mgv1a013138mg [Erythranthe guttata]
c35540.graph_c0	41.3697896	37.3264354	44.5727484	9.66449721	11.4963364	12.2673457	1.805E-49	-2.97398	down	PREDICTED: ras-related protein RABC2a-like [Sesamum indicum]
c35552.graph_c0	0	0	0	2.04682507	1.80958923	1.53100983	3.62E-14	Inf	up	PREDICTED: probable glycosyltransferase At3g07620 [Sesamum indicum]
c35552.graph_c1	0.43638088	0.10920446	0.42057504	4.51951734	3.50797053	5.30538453	8.707E-08	2.69529	up	hypothetical protein MIMGU_mgv1a009597mg [Erythranthe guttata]
c35557.graph_c0	1.41596184	1.0838786	0.21406671	9.44362431	9.18260789	9.237225	4.194E-10	2.28617	up	--
c35559.graph_c0	4.54759744	3.37962739	2.74483523	18.028187	18.6742109	18.5111571	6.961E-10	1.28647	up	PREDICTED: AP-2 complex subunit sigma-like [Camelina sativa]
c35583.graph_c0	628.040481	704.20716	655.342701	76.9606386	90.0551628	109.898692	1.87E-186	-3.93303	down	PREDICTED: probable xyloglucan endotransglucosylase/hydrolase protein 23 [Nicotiana tomentosiformis]
c35583.graph_c1	355.409371	376.017552	381.101463	46.6868657	53.9923487	64.3934098	5.56E-219	-3.84288	down	PREDICTED: probable xyloglucan endotransglucosylase/hydrolase protein 25 [Prunus mume]
c35586.graph_c0	1.21413932	1.51386337	1.39598372	6.71884887	5.99660014	5.76044187	1.504E-08	1.0715	up	PREDICTED: probable galactinol--sucrose galactosyltransferase 6 [Sesamum indicum]
c35595.graph_c0	1584.50283	1516.13061	1578.39165	628.63883	666.759956	702.638324	1.77E-100	-2.31791	down	PREDICTED: cysteine proteinase 15A [Nicotiana tomentosiformis]
c35599.graph_c0	3.14639204	2.04720274	3.23459067	0.91471184	2.16798495	2.79899022	0.0003267	-1.60862	down	-
c35599.graph_c1	18.4584802	18.9017213	20.7208128	18.5041614	18.6567338	17.8359827	2.608E-22	-1.17097	down	PREDICTED: uncharacterized protein LOC105161992 [Sesamum indicum]
c35601.graph_c0	7.37851039	7.21108468	7.91074908	44.2974658	44.1078421	42.5418958	2.182E-57	1.44922	up	PREDICTED: protein transport protein SEC24 [Sesamum indicum]

#ID	0 h-1 FPKM	0 h-2 FPKM	0 h-3 FPKM	28 h-1 FPKM	28 h-2 FPKM	28 h-3 FPKM	FDR	log2FC	regulated	nr_annotation
c35616.graph_c0	32.1422369	29.4444715	29.6638156	134.855484	135.759678	126.965911	3.363E-26	1.03332	up	PREDICTED: uncharacterized protein LOC105173072 [Sesamum indicum]
c35618.graph_c1	9.14005645	8.25857789	10.2901602	54.3192645	53.5038577	53.726153	3.305E-28	1.4521	up	PREDICTED: uncharacterized protein LOC105168327 [Sesamum indicum]
c35623.graph_c0	1.08079519	1.08187745	1.14195535	6.17939031	5.59517112	5.38418505	2.13E-09	1.28476	up	PREDICTED: pentatricopeptide repeat-containing protein At1g51965, mitochondrial [Sesamum indicum]
c35626.graph_c0	1.88259169	1.51288984	1.9592152	0.87684062	1.07010658	1.16366185	7.438E-15	-1.87463	down	PREDICTED: phosphatidylinositol/phosphatidylcholine transfer protein SFH2 [Sesamum indicum]
c35628.graph_c0	2.23381846	1.63404041	1.43527258	22.0737145	20.9453429	19.336755	3.886E-42	2.46966	up	PREDICTED: uncharacterized protein At4g15545 [Sesamum indicum]
c35629.graph_c0	7.59210431	6.84393472	6.73767584	33.6561271	33.388624	32.5378328	1.462E-21	1.14424	up	PREDICTED: KH domain-containing protein At1g09660/At1g09670 [Sesamum indicum]
c35636.graph_c0	5.90986294	5.98620673	5.67320349	28.4049259	29.4444007	27.3402474	2.847E-36	1.18735	up	PREDICTED: extra-large guanine nucleotide-binding protein 3 [Sesamum indicum]
c35639.graph_c0	1.91413673	1.91605345	2.00663121	1.79389457	1.78499601	1.64304454	0.0001473	-1.25218	down	hypothetical protein EUTSA_v10027555mg, partial [Eutrema salsugineum]
c35645.graph_c0	0.03630618	0	0	1.02909782	0.92917885	0.99045716	3.97E-12	5.28499	up	hypothetical protein MIMGU_mgv1a021537mg [Erythranthe guttata]
c35649.graph_c0	0.21863511	0.25532972	0.18730318	2.51596006	2.70209887	1.88011841	9.365E-08	2.33444	up	PREDICTED: pto-interacting protein 1-like [Sesamum indicum]
c35651.graph_c0	2.72863175	2.84061862	1.89345591	1.42787076	1.03854914	1.52114309	3.965E-10	-1.9888	down	PREDICTED: uncharacterized protein LOC105171337 [Sesamum indicum]
c35653.graph_c0	16.875585	14.2025975	14.0888604	114.760506	121.745833	117.867313	7.515E-60	1.88327	up	PREDICTED: macrophage migration inhibitory factor homolog isoform X1 [Eucalyptus grandis]
c35660.graph_c0	1.6693277	1.0152907	1.00469153	0	0	0	1.796E-23	-Inf	down	hypothetical protein THAOC_20916 [Thalassiosira oceanica]
c35679.graph_c0	1.44421421	1.22325109	1.4989739	19.2569774	20.8136624	20.3584181	5.958E-38	2.76682	up	PREDICTED: serine/threonine-protein kinase At5g01020 [Sesamum indicum]
c35683.graph_c0	3.46573409	2.36201157	2.17942398	2.78684105	2.37108755	2.18657648	0.0001791	-1.21055	down	-
c35685.graph_c0	6.61998551	6.77278975	5.75469706	30.5309558	39.3215214	45.3226266	3.459E-07	1.50103	up	hypothetical protein POPTR_0014s02030g [Populus trichocarpa]
c35685.graph_c2	0.38816723	0.51807457	0.49881026	3.10329479	3.82088759	3.76002719	0.0001061	1.83411	up	-
c35687.graph_c0	246.167261	242.351856	259.752647	63.7470108	65.6123263	66.1274097	6.38E-160	-3.02773	down	PREDICTED: ubiquitin carboxyl-terminal hydrolase 19-like [Sesamum indicum]
c35689.graph_c0	33.5426055	31.0305894	30.5282525	25.9227046	25.7505738	25.6581672	5.891E-56	-1.38795	down	hypothetical protein MIMGU_mgv1a010259mg [Erythranthe guttata]
c35690.graph_c0	4.75461583	4.66124538	4.93408712	1.62687612	1.30808632	1.67635438	3.117E-76	-2.72806	down	PREDICTED: UDP-glycosyltransferase 73C3-like [Sesamum indicum]
c35693.graph_c0	130.313951	138.415702	144.453854	64.3373701	60.3388898	62.6763541	1.771E-66	-2.23269	down	hypothetical protein MIMGU_mgv1a013946mg [Erythranthe guttata]
c35694.graph_c0	39.109906	40.0161576	42.2989047	178.387208	184.685525	182.524645	4.803E-42	1.07623	up	PREDICTED: splicing factor U2af small subunit B-like [Sesamum indicum]

#ID	0 h-1 FPKM	0 h-2 FPKM	0 h-3 FPKM	28 h-1 FPKM	28 h-2 FPKM	28 h-3 FPKM	FDR	log2FC	regulated	nr_annotation
c35699.graph_c0	11.9143396	14.201954	12.1184776	11.1071245	11.2995455	12.9083689	1.481E-16	-1.20416	down	PREDICTED: trihelix transcription factor GT-4-like isoform X2 [Sesamum indicum]
c35713.graph_c0	28.3624123	28.9570848	28.05381	26.986485	27.3192411	27.3124416	3.574E-41	-1.15522	down	PREDICTED: uncharacterized protein LOC105178130 [Sesamum indicum]
c35716.graph_c0	4.26491438	3.57420144	3.44129683	1.2975552	1.30174521	0.99997253	5.475E-14	-2.7374	down	PREDICTED: wound-induced protein 1 [Sesamum indicum]
c35717.graph_c0	16.466383	17.9401986	17.0311826	17.5136667	18.0863463	17.5064861	1.271E-18	-1.04479	down	PREDICTED: vacuolar protein-sorting-associated protein 37 homolog 1 isoform X2 [Sesamum indicum]
c35719.graph_c1	9.51224092	8.90185935	8.84938156	6.19329168	5.60821742	6.56683989	1.015E-42	-1.65891	down	PREDICTED: solute carrier family 25 member 44-like [Sesamum indicum]
c35725.graph_c0	1.12722524	1.16154087	0.85207592	5.66253794	6.41812149	5.56436373	5.676E-08	1.4026	up	PREDICTED: LOW QUALITY PROTEIN: ankyrin repeat-containing protein At3g12360-like [Sesamum indicum]
c35730.graph_c0	17.6672111	17.7454669	17.3383002	15.0831046	13.9362649	13.4918119	5.606E-27	-1.40191	down	PREDICTED: 26S proteasome non-ATPase regulatory subunit 9 [Sesamum indicum]
c35732.graph_c0	1.71253766	1.61341412	1.52105733	0.58572353	0.61148678	0.56756042	6.49E-18	-2.54705	down	PREDICTED: zinc finger BED domain-containing protein DAYSLEEPER-like isoform X1 [Sesamum indicum]
c35735.graph_c0	14.4521098	15.1856804	16.0736077	12.438604	12.839268	12.6809838	1.384E-29	-1.35996	down	PREDICTED: uncharacterized protein LOC105166332 [Sesamum indicum]
c35736.graph_c1	0.60692411	0.70878716	0.12998692	7.27829759	6.3392903	8.24865472	2.642E-16	2.84333	up	PREDICTED: RING-H2 finger protein ATL57 [Solanum lycopersicum]
c35742.graph_c0	0.04162066	0	0	0.78649105	1.17444193	1.01202236	1.107E-10	5.09936	up	PREDICTED: thiol-disulfide oxidoreductase LTO1-like [Sesamum indicum]
c35746.graph_c0	0.53269439	0.5332278	0.47917337	6.23326334	7.0612831	6.28678326	2.606E-13	2.57418	up	PREDICTED: probable pectinesterase/pectinesterase inhibitor 61 [Sesamum indicum]
c35749.graph_c0	1.55297444	1.34011164	1.10103925	0.15568178	0.14056605	0.06351753	1.508E-21	-4.5625	down	PREDICTED: oligopeptide transporter 7 isoform X1 [Malus domestica]
c35752.graph_c0	0.08353479	0	0	8.10513229	7.07149449	8.3724268	1.894E-41	7.08079	up	PREDICTED: probable galactinol--sucrose galactosyltransferase 6 [Sesamum indicum]
c35755.graph_c0	1.11070489	1.76582714	1.8470938	7.45522728	11.2761187	7.36208198	0.0005092	1.36864	up	PREDICTED: dynein light chain 1, cytoplasmic-like [Nicotiana tomentosiformis]
c35760.graph_c0	32.9544458	37.5759238	31.8404267	7.9236726	7.56082966	10.5066847	1.824E-84	-3.06585	down	Ubiquitin-like protein [Medicago truncatula]
c35764.graph_c0	1.83027332	3.09167898	1.76398026	410.626022	412.193881	379.706963	0	6.40186	up	PREDICTED: pistil-specific extensin-like protein [Sesamum indicum]
c35766.graph_c0	19.9220317	20.9637428	20.8287563	118.366326	120.085658	118.420602	1.026E-74	1.44069	up	PREDICTED: staphylococcal nuclease domain-containing protein 1 [Sesamum indicum]
c35772.graph_c0	176.962155	175.50605	158.591457	13.7455339	14.4064109	15.1749176	0	-4.64896	down	-
c35783.graph_c0	14.938512	8.19708525	8.41843347	0	0	0	2.782E-24	-Inf	down	uncharacterized protein LOC100273336 [Zea mays]
c35784.graph_c0	2.61913173	1.74783626	2.46817118	0	0	0	2.584E-34	-Inf	down	NAD(P)-binding protein [Auricularia delicata TFB-10046 SS5]
c35787.graph_c0	4.00547614	3.35873258	3.66501873	0.36579983	0.38533011	0.47260865	3.361E-85	-4.26069	down	PREDICTED: probable LRR receptor-like serine/threonine-protein kinase At4g36180 [Sesamum indicum]

#ID	0 h-1 FPKM	0 h-2 FPKM	0 h-3 FPKM	28 h-1 FPKM	28 h-2 FPKM	28 h-3 FPKM	FDR	log2FC	regulated	nr_annotation
c35789.graph_c0	2.0794413	2.04974456	1.87663454	17.2129643	17.2083623	15.8719478	2.469E-62	1.97631	up	PREDICTED: pentatricopeptide repeat-containing protein At4g01570 [Sesamum indicum]
c35793.graph_c0	1.47073022	1.63322513	1.62417283	9.8874938	10.9180666	9.67628561	5.078E-22	1.59662	up	PREDICTED: serine/threonine-protein kinase D6PKL1 [Sesamum indicum]
c35799.graph_c0	66.5904956	72.6654824	69.2457515	28.3460411	28.0313238	29.1002358	2.16E-140	-2.37708	down	PREDICTED: GTP cyclohydrolase 1 [Sesamum indicum]
c35804.graph_c0	2.2056856	2.48929255	3.33458038	16.439424	15.5811643	15.5946021	3.357E-27	1.47208	up	hypothetical protein MIMGU_mgv1a001397mg [Erythranthe guttata]
c35812.graph_c0	1294.50295	1071.59934	1135.28011	926.428892	865.54204	945.94862	1.204E-28	-1.44381	down	-
c35813.graph_c0	6.53720099	6.71371446	7.74597284	816.311694	699.279622	688.307971	2.48E-173	5.62064	up	PREDICTED: cysteine proteinase COT44 [Sesamum indicum]
c35814.graph_c0	300.204405	299.751082	328.509247	260.200227	253.512202	248.986135	8.756E-33	-1.37564	down	PREDICTED: thioredoxin H-type-like [Nelumbo nucifera]
c35816.graph_c0	4.31965006	4.22003382	4.93711755	28.9648764	32.3670392	31.4559938	1.21E-59	1.69103	up	PREDICTED: oligopeptide transporter 7-like isoform X2 [Sesamum indicum]
c35818.graph_c0	10.8106673	8.85591209	9.78827114	3.12089623	3.46924516	4.9396863	2.94E-41	-2.4409	down	PREDICTED: probable galactinol--sucrose galactosyltransferase 1 [Sesamum indicum]
c35827.graph_c1	11.8323388	12.3950795	12.3761822	10.3329436	12.8809765	12.2394159	1.39E-09	-1.13701	down	PREDICTED: ethylene-responsive transcription factor ERF060 [Sesamum indicum]
c35828.graph_c0	0.04272613	0.06415337	0.08235716	0.85396044	0.60281795	0.98822419	3.366E-07	2.59544	up	PREDICTED: K(+) efflux antiporter 3, chloroplastic [Sesamum indicum]
c35844.graph_c0	0.31564105	0.34755283	0.24336675	1.97289242	2.60986566	1.85321566	1.523E-05	1.7394	up	PREDICTED: uncharacterized protein LOC105173814 [Sesamum indicum]
c35849.graph_c0	0.25361014	0.2855971	0.04073738	48.6841501	46.2663813	46.2309094	0	6.8521	up	PREDICTED: cytochrome P450 78A5 [Sesamum indicum]
c35854.graph_c0	1.52062746	1.28995775	1.39103038	6.81841885	6.10565343	6.5420201	4.072E-08	1.12231	up	PREDICTED: putative pentatricopeptide repeat-containing protein At1g16830 [Sesamum indicum]
c35855.graph_c0	4.04441503	4.18969042	2.77991556	244.863649	221.829583	216.791984	1.13E-260	4.86943	up	PREDICTED: cysteine proteinase 3-like [Sesamum indicum]
c35856.graph_c0	12.7984981	13.56216	13.6753587	3.85022649	3.96862624	3.3641784	7.909E-83	-2.93198	down	PREDICTED: probable methyltransferase PMT19 [Sesamum indicum]
c35864.graph_c0	0.28652612	0.38241737	0.42956364	3.36663609	3.32179602	2.97371642	5.753E-07	2.04126	up	PREDICTED: ras-related protein RABA5a [Beta vulgaris subsp. vulgaris]
c35878.graph_c0	58.6301038	57.9062954	62.1822678	275.728187	272.297995	276.870373	1.859E-43	1.1152	up	PREDICTED: 60S ribosomal protein L11-like [Sesamum indicum]
c35883.graph_c0	0.03389161	0	0	9.7050974	10.3196344	9.48705652	4.011E-65	8.70695	up	PREDICTED: uncharacterized protein LOC105165376 isoform X1 [Sesamum indicum]
c35884.graph_c0	8.8338195	8.07630092	6.50836981	0.72764183	0.73428557	0.94301438	2.055E-57	-4.36915	down	-
c35887.graph_c0	0	0.10880836	0.20952478	3.35759277	2.46093895	2.15958205	1.366E-09	3.53261	up	PREDICTED: putative UDP-rhamnose:rhamnosyltransferase 1 [Sesamum indicum]
c35890.graph_c0	7.98813074	8.59961113	10.168224	7.39408676	8.65429066	7.82122239	1.622E-08	-1.25857	down	PREDICTED: thioredoxin X, chloroplastic [Sesamum indicum]
c35890.graph_c1	134.996533	137.792093	139.724935	134.360898	135.626907	136.942438	1.534E-41	-1.11055	down	PREDICTED: probable prefoldin subunit 5 [Sesamum indicum]
c35891.graph_c0	34.6483865	33.3618214	36.8917687	362.144704	360.763709	347.563343	5.012E-78	2.25943	up	40S ribosomal protein S6 [Morus notabilis]

#ID	0 h-1 FPKM	0 h-2 FPKM	0 h-3 FPKM	28 h-1 FPKM	28 h-2 FPKM	28 h-3 FPKM	FDR	log2FC	regulated	nr_annotation
c35901.graph_c0	8.9690827	10.2983674	9.15270329	3.06761299	2.6832117	2.7378172	9.823E-23	-2.83381	down	PREDICTED: uncharacterized protein LOC104435912 [Eucalyptus grandis]
c35910.graph_c0	2.5865758	2.07133269	2.06078843	19.5886454	18.773034	18.5919575	7.391E-25	1.99534	up	PREDICTED: exosome complex component RRP42 isoform X1 [Sesamum indicum]
c35914.graph_c0	0.94110453	0.82429104	0.30233915	5.81390949	6.56176895	5.16269996	1.544E-05	2.00484	up	PREDICTED: GTP-binding protein OBGC, chloroplastic [Sesamum indicum]
c35914.graph_c1	3.48304068	3.5666785	3.54980825	26.4784258	26.5478737	26.2716894	6.293E-65	1.81251	up	PREDICTED: uncharacterized protein LOC105163283 [Sesamum indicum]
c35921.graph_c0	1.12285783	1.31131257	0.84170145	5.50858455	8.16675066	6.82568001	4.99E-05	1.5588	up	PREDICTED: rho-N domain-containing protein 1, chloroplastic [Sesamum indicum]
c35922.graph_c0	38.4544225	39.4585438	39.6112487	33.6528688	35.4783902	35.9898534	7.096E-44	-1.25163	down	PREDICTED: protein DEHYDRATION-INDUCED 19 homolog 7-like [Sesamum indicum]
c35923.graph_c0	14.1166392	15.9854391	18.1404403	71.3062496	72.0254273	74.4060444	2.571E-21	1.08083	up	PREDICTED: uncharacterized protein At5g64816 [Sesamum indicum]
c35931.graph_c0	40.3877794	40.9242735	45.3195407	2.16100162	2.6015758	2.17970848	4.65E-153	-5.28175	down	PREDICTED: 22.0 kDa heat shock protein-like [Sesamum indicum]
c35933.graph_c0	4.42916949	3.81782621	3.95254039	0.73770924	0.86792539	1.00327452	1.827E-47	-3.31376	down	hypothetical protein VITISV_009861 [Vitis vinifera]
c35935.graph_c0	28.8407443	28.9535473	35.2838392	140.544209	138.149356	144.093268	8.755E-30	1.09028	up	hypothetical protein MIMGU_mgv1a007974mg [Erythranthe guttata]
c35936.graph_c0	3.78240134	4.96411426	2.91632128	4.15408599	3.25432735	3.38970024	0.0005191	-1.198	down	-
c35937.graph_c0	5.09852834	3.60774109	3.5018299	16.8667825	20.0170673	19.9148708	4.764E-07	1.13187	up	hypothetical protein MIMGU_mgv1a016751mg [Erythranthe guttata]
c35939.graph_c0	1.89737421	1.89927414	2.14266147	18.88813	19.1671215	20.0499672	9.739E-27	2.19834	up	PREDICTED: uncharacterized protein LOC105156528 [Sesamum indicum]
c35942.graph_c0	11.5174834	12.7009219	11.1409771	10.5555379	10.4927249	10.4772439	4.652E-48	-1.25541	down	PREDICTED: putative uncharacterized protein At4g01020, chloroplastic [Sesamum indicum]
c35944.graph_c0	0.68271918	0.77254232	0.83917678	0.51777012	0.58437234	0.56332825	5.146E-05	-1.55515	down	PREDICTED: DNA-directed RNA polymerase III subunit RPC5 [Sesamum indicum]
c35957.graph_c0	0.01980486	0	0	0.90682582	0.8057822	0.76345333	1.548E-13	5.90673	up	PREDICTED: glutamate receptor 3.2-like [Sesamum indicum]
c35961.graph_c0	1.61313137	0	1.81382047	9.96552396	10.5858044	13.15436	7.659E-07	2.20212	up	-
c35962.graph_c0	10.1818575	13.019906	10.1344975	663.401092	821.06563	651.487658	1.39E-80	4.91109	up	PREDICTED: (-)-isopiperitenol/(-)-carveol dehydrogenase, mitochondrial-like [Sesamum indicum]
c35963.graph_c0	1.65706473	1.90576803	2.03878128	311.489765	341.030505	328.150107	0	6.35884	up	tubulin alpha chain-like [Sesamum indicum]
c35966.graph_c0	2.35110521	2.29605804	2.06330175	9.33571748	10.4613356	10.4065535	1.036E-05	1.08148	up	-
c35974.graph_c1	0	0.19879677	0	0.90212384	1.17292787	1.06002099	1.049E-06	2.89733	up	PREDICTED: pentatricopeptide repeat-containing protein At2g03380, mitochondrial [Sesamum indicum]
c35979.graph_c0	1.77228675	2.17956118	1.43154543	15.0522259	14.0559577	14.7149677	5.41E-19	1.93789	up	PREDICTED: uncharacterized protein LOC105171592 [Sesamum indicum]

#ID	0 h-1 FPKM	0 h-2 FPKM	0 h-3 FPKM	28 h-1 FPKM	28 h-2 FPKM	28 h-3 FPKM	FDR	log2FC	regulated	nr_annotation
c35983.graph_c0	0.16718133	0.35860443	0.49105057	2.4648446	2.36657788	2.08211324	5.436E-07	1.66304	up	PREDICTED: uncharacterized mitochondrial protein ymf11 [Sesamum indicum]
c35991.graph_c2	4.9122176	4.19074129	4.23217332	29.6973352	29.487974	30.7213424	1.12E-27	1.6644	up	PREDICTED: ribonuclease 2 [Sesamum indicum]
c35992.graph_c0	0.06156216	0.04108253	0.07910981	1.05891814	0.67331254	0.68151886	3.099E-06	2.63513	up	PREDICTED: putative leucine-rich repeat-containing protein DDB_G0290503 [Sesamum indicum]
c35993.graph_c0	2.74660134	2.69324243	2.23294358	13.3624122	12.6167587	11.4687459	8.55E-08	1.19861	up	PREDICTED: tetraspanin-19 [Sesamum indicum]
c35995.graph_c0	0	0.0522414	0	14.792999	16.3533847	14.9184764	7E-130	8.70229	up	PREDICTED: LOW QUALITY PROTEIN: interactor of constitutive active ROPs 3 [Sesamum indicum]
c36002.graph_c0	17.2559374	14.8549662	16.4978746	17.7086203	16.3968811	17.561157	6.977E-13	-1.00213	down	PREDICTED: uncharacterized protein LOC105166164 [Sesamum indicum]
c36012.graph_c0	4.00026863	3.66105078	4.40614591	0.33227111	0.5250169	0.54226102	2.125E-22	-4.19944	down	-
c36015.graph_c0	21.7776219	20.9819504	22.3880851	18.74611	19.6948735	17.3147034	7.423E-26	-1.31617	down	PREDICTED: uncharacterized protein LOC105158384 [Sesamum indicum]
c36019.graph_c0	2.69984528	2.73192429	3.58254094	1.79160312	1.94503111	1.68818513	5.187E-09	-1.82764	down	PREDICTED: F-box protein SKIP14 [Sesamum indicum]
c36022.graph_c0	34.8079776	35.6322684	37.4512756	15.5280634	14.6320129	15.4983112	1.16E-103	-2.33191	down	PREDICTED: protein trichome birefringence-like 39 [Sesamum indicum]
c36025.graph_c0	9.97672382	10.7549228	7.53091433	4.36075652	5.72289866	4.4272391	5.422E-15	-2.04945	down	-
c36034.graph_c0	13.969574	15.3585152	13.9330335	6.83947255	6.59732436	6.51689009	1.747E-85	-2.20676	down	hypothetical protein MIMGU_mgv1a022687mg [Erythranthe guttata]
c36037.graph_c0	74.9731039	74.7315191	79.5513232	18.3401982	18.3958729	17.5775511	1.41E-171	-3.16915	down	PREDICTED: ethylene-responsive transcription factor ERF107-like [Sesamum indicum]
c36039.graph_c0	2.89349168	4.16355929	4.76400956	14.8522474	19.5812463	19.0905985	2.453E-05	1.08315	up	floricaula, partial [Salvia coccinea]
c36040.graph_c0	7.34374668	8.10291741	7.7211861	7.39956723	7.17398461	7.62172058	2.529E-07	-1.15254	down	CYP72A52v1 [Nicotiana tabacum]
c36046.graph_c0	7.99675015	5.56852709	9.82935183	5.18025367	6.3884846	5.51577783	0.0009701	-1.54852	down	PREDICTED: uncharacterized protein LOC105174651 [Sesamum indicum]
c36056.graph_c0	3.89290927	3.89680743	5.40504595	25.7893081	29.68659	26.8554744	1.014E-23	1.54605	up	PREDICTED: uncharacterized protein At2g34160-like [Sesamum indicum]
c36057.graph_c0	22.4777427	21.6458109	21.9378147	21.3824575	20.4266457	20.6877549	1.049E-24	-1.17036	down	hypothetical protein MIMGU_mgv1a011414mg [Erythranthe guttata]
c36070.graph_c1	2.16449255	2.25332636	2.44768359	14.9762996	14.0903538	13.4015145	5.836E-16	1.53653	up	PREDICTED: uncharacterized protein LOC105157576 isoform X2 [Sesamum indicum]
c36076.graph_c0	0.08161367	0.49017238	0.83901441	15.8374648	17.084715	15.730538	1.964E-32	3.99822	up	PREDICTED: uncharacterized protein LOC105165595 [Sesamum indicum]
c36079.graph_c0	16.9912507	19.3043807	16.4304085	83.3258432	80.7268571	70.8399036	2.235E-20	1.06502	up	PREDICTED: abscisic acid receptor PYL4 [Sesamum indicum]
c36079.graph_c1	3.62148874	5.3335027	4.17233334	24.0214857	24.0383519	21.9465842	1.619E-20	1.3234	up	PREDICTED: probable E3 ubiquitin-protein ligase XBOS32 [Sesamum indicum]
c36085.graph_c0	0.53327678	0.86744252	0.770942	10.4647273	11.5046277	10.0018537	3.033E-18	2.78579	up	PREDICTED: putative receptor-like protein kinase At1g72540 [Sesamum indicum]

#ID	0 h-1 FPKM	0 h-2 FPKM	0 h-3 FPKM	28 h-1 FPKM	28 h-2 FPKM	28 h-3 FPKM	FDR	log2FC	regulated	nr_annotation
c36088.graph_c0	34.1609475	35.2392814	31.5664703	17.6259704	20.4322912	20.1047766	9.332E-79	-1.88499	down	PREDICTED: uncharacterized protein LOC105178899 isoform X2 [Sesamum indicum]
c36099.graph_c0	0.84127951	0.52269636	0.41006387	2.90959722	3.23627458	2.87313524	0.0001234	1.26316	up	PREDICTED: phytosulfokine receptor 2 [Sesamum indicum]
c36106.graph_c0	7.29621738	4.97967507	4.04869627	0	0	0	8.249E-21	-Inf	down	-
c36117.graph_c0	0.90537184	0.90627843	0.49861657	3.05508837	4.4559712	3.1449235	0.0069174	1.12089	up	-
c36119.graph_c0	0.36667573	0.3670429	0.34554141	9.13199161	8.90303381	8.42295207	9.032E-49	3.52553	up	PREDICTED: uncharacterized protein LOC105159055 [Sesamum indicum]
c36125.graph_c0	5.31800136	4.47159429	5.74042753	31.384553	31.9318196	30.2772875	2.004E-26	1.499	up	PREDICTED: protein OS-9 homolog [Sesamum indicum]
c36129.graph_c0	0.42000941	0.63064498	0.53972872	2.4115573	3.27989628	2.41617307	0.0009498	1.25645	up	PREDICTED: glycerol-3-phosphate dehydrogenase [NAD(+)]-like [Cicer arietinum]
c36141.graph_c0	9.28059381	7.1264886	7.02486316	1.66317093	1.00112494	1.50792655	1.212E-27	-3.57604	down	ubiquitin extension protein [Capsicum annuum]
c36144.graph_c0	11.3751387	11.1967537	11.9863529	1.92905656	1.26899453	1.50663321	1.09E-132	-3.96807	down	PREDICTED: type I inositol 1,4,5-trisphosphate 5-phosphatase CVP2 isoform X1 [Cucumis melo]
c36150.graph_c0	1.48202465	0.52493384	0.70318536	0	0	0	1.684E-08	-Inf	down	hypothetical protein EMIHUDRAFT_239943 [Emiliania huxleyi CCMP1516]
c36156.graph_c0	11.6191778	11.3840984	9.45636555	11.3364391	10.975118	10.5659673	8.433E-19	-1.06971	down	PREDICTED: UDP-glycosyltransferase 90A1-like [Solanum tuberosum]
c36157.graph_c0	1.30711437	1.49099394	1.87500695	8.28491158	10.1734778	7.93222636	2.461E-10	1.40159	up	PREDICTED: katanin p60 ATPase-containing subunit A-like 2 isoform X1 [Sesamum indicum]
c36162.graph_c0	560.709512	561.688486	606.77246	571.367304	579.801569	619.77934	1.378E-24	-1.05683	down	hypothetical protein MIMGU_mgv1a009201mg [Erythranthe guttata]
c36172.graph_c0	17.8985072	18.2125692	20.3390998	363.271316	345.18135	312.655752	1.87E-120	3.08424	up	-
c36188.graph_c0	1.74423702	2.13793912	1.23506465	1.42293016	1.61180553	1.56220645	0.0002732	-1.24053	down	PREDICTED: WEB family protein At1g12150-like [Sesamum indicum]
c36190.graph_c0	0.0931565	0.09324978	0.17956468	99.1210054	79.501923	87.5944683	7.8E-127	8.40677	up	unnamed protein product [Coffea canephora]
c36193.graph_c0	1.73641286	2.15199724	0.95629683	33.5335377	36.0021764	34.5716496	5.94E-101	3.34212	up	PREDICTED: GDSL esterase/lipase At1g54790 [Sesamum indicum]
c36202.graph_c0	0	0.20102967	0.12903634	3.86796785	2.89936117	4.34726044	9.552E-11	3.97422	up	PREDICTED: NAC domain-containing protein 8-like [Sesamum indicum]
c36202.graph_c1	0.21770556	0	0	4.11390574	3.2144462	2.77590923	4.723E-11	4.47653	up	PREDICTED: NAC domain-containing protein 8-like [Sesamum indicum]
c36206.graph_c0	80.255123	77.7708505	80.5293913	54.3028031	55.6646583	55.1521731	8.35E-77	-1.62151	down	PREDICTED: early nodulin-93-like [Solanum tuberosum]
c36211.graph_c0	210.783866	241.942607	201.227282	68.933664	75.4796657	69.4829146	1.561E-63	-2.70208	down	PREDICTED: probable WRKY transcription factor 17 [Sesamum indicum]
c36215.graph_c0	14.2416674	14.7663707	11.6546688	1.64129726	1.57754642	1.90092088	1.526E-83	-4.07658	down	PREDICTED: phosphoenolpyruvate carboxylase kinase 2 [Sesamum indicum]
c36225.graph_c0	0.86939937	0.68566723	0.3724042	2.22113444	2.80075117	3.14049949	0.0076794	1.0022	up	PREDICTED: pentatricopeptide repeat-containing protein At3g07290, mitochondrial [Sesamum indicum]
c36234.graph_c0	12.293973	11.5528376	12.574111	0.41028928	0.493937	0.6695854	9.18E-123	-5.62163	down	PREDICTED: high mobility group B protein 6 [Sesamum indicum]

#ID	0 h-1 FPKM	0 h-2 FPKM	0 h-3 FPKM	28 h-1 FPKM	28 h-2 FPKM	28 h-3 FPKM	FDR	log2FC	regulated	nr_annotation
c36238.graph_c0	3.63785479	3.9289842	2.89098696	27.1007119	25.945733	28.1605237	9.407E-34	1.86992	up	PREDICTED: probable UDP-arabinopyranose mutase 5 isoform X1 [Sesamum indicum]
c36244.graph_c0	0.11539944	0.13861799	0.17795141	4.00906461	4.60427647	4.83176354	2.429E-30	3.86457	up	PREDICTED: uncharacterized protein LOC105158603 isoform X1 [Sesamum indicum]
c36245.graph_c0	0	0	0	3.27745674	2.75747005	3.40375374	2.481E-18	Inf	up	-
c36254.graph_c1	0.55944952	0.33600583	0	4.18802825	3.92824721	3.31786102	1.617E-08	2.60623	up	PREDICTED: pentatricopeptide repeat-containing protein DOT4, chloroplastic isoform X1 [Sesamum indicum]
c36264.graph_c0	4.88198056	6.14799665	7.08302712	31.7048422	28.8848618	29.0463809	2.633E-13	1.21213	up	-
c36266.graph_c0	413.501845	410.015453	403.882408	209.000847	187.480799	198.828163	1.1E-144	-2.13404	down	PREDICTED: uncharacterized protein LOC105176605 [Sesamum indicum]
c36267.graph_c0	0.03976429	0.19902054	0.15329606	12.571699	12.5775024	12.1214241	2.829E-60	5.47201	up	PREDICTED: transcription factor bHLH30-like isoform X2 [Sesamum indicum]
c36268.graph_c0	0	0	0	5.16509788	4.03580692	9.15881882	2.206E-06	Inf	up	-
c36275.graph_c0	17.0330058	17.3390459	17.3744418	122.710825	121.500265	117.509144	4.087E-83	1.7144	up	PREDICTED: gamma carbonic anhydrase 2, mitochondrial [Sesamum indicum]
c36277.graph_c0	17.281138	20.282027	14.3020885	5.13916041	7.88622521	5.43464231	2.456E-19	-2.57934	down	PREDICTED: ethylene-responsive transcription factor ABR1-like [Sesamum indicum]
c36280.graph_c0	7.91456347	7.64929945	10.0536248	44.3651249	44.2961263	41.9204581	3E-17	1.2555	up	-
c36282.graph_c0	32.0829521	32.858483	32.4478557	171.752168	180.808405	173.357627	1.896E-37	1.34225	up	hypothetical protein M569_11398, partial [Genlisea aurea]
c36287.graph_c0	2.86635903	2.15192194	3.03879249	0.88538262	0.70536838	0.93495469	6.741E-16	-2.76477	down	PREDICTED: glutamate decarboxylase-like [Sesamum indicum]
c36289.graph_c0	5.40734069	4.31506368	4.61623394	3.51778376	3.57325753	2.96018541	6.213E-16	-1.6024	down	PREDICTED: transcription factor GAMYB-like [Sesamum indicum]
c36295.graph_c0	0.44881411	0.39934536	0.83307452	4.31302949	4.51603405	3.6376959	6.051E-07	1.78959	up	PREDICTED: uncharacterized protein LOC105171863 [Sesamum indicum]
c36304.graph_c0	0.34367535	0.11467316	0.36803036	1.79009908	2.25529065	1.69849562	0.0001944	1.70193	up	PREDICTED: E3 ubiquitin-protein ligase RNF5-like [Sesamum indicum]
c36310.graph_c1	0.07390919	0.14796641	0.18995248	2.63212203	2.03705092	2.93677419	1.211E-08	3.10812	up	PREDICTED: histone H3-like centromeric protein HTR12 [Sesamum indicum]
c36317.graph_c0	22.8430936	25.3102606	21.2565474	22.5561315	19.4873328	21.3953644	4.724E-22	-1.21888	down	PREDICTED: zinc finger CCCH domain-containing protein 44-like isoform X3 [Sesamum indicum]
c36326.graph_c0	0.69600685	0.48769266	0.26831891	2.78219572	3.8366036	3.38473485	0.0001525	1.70425	up	PREDICTED: glycosyltransferase 6-like [Sesamum indicum]
c36336.graph_c0	6.33676463	3.41552074	4.38468877	0	0	0	5.572E-22	-Inf	down	hypothetical protein EMIHUDRAFT_109093 [Emiliania huxleyi CCMP1516]
c36337.graph_c0	0.64222967	0.61492178	0.68176176	0.22323726	0.23821001	0.36431957	1.263E-08	-2.32065	down	-
c36340.graph_c0	2.91235034	3.44531509	3.74248549	2.88638106	2.95361566	3.92543895	0.0007586	-1.14054	down	PREDICTED: putative DNA-binding protein ESCAROLA [Sesamum indicum]

#ID	0 h-1 FPKM	0 h-2 FPKM	0 h-3 FPKM	28 h-1 FPKM	28 h-2 FPKM	28 h-3 FPKM	FDR	log2FC	regulated	nr_annotation
c36343.graph_c0	1.93851606	2.04826037	1.52232049	0.50877028	0.88340742	0.76643518	3.049E-11	-2.43905	down	PREDICTED: brefeldin A-inhibited guanine nucleotide-exchange protein 1-like [Sesamum indicum]
c36346.graph_c0	3.5450544	3.9775564	2.50304135	23.7549952	25.5388378	22.6876904	6.508E-33	1.75749	up	PREDICTED: xylosyltransferase 1 [Sesamum indicum]
c36365.graph_c0	42.4170837	38.7494995	35.1036205	21.3504766	22.790664	25.7256701	2.085E-40	-1.8224	down	unnamed protein product [Vitis vinifera]
c36372.graph_c0	0.55630159	0.87506358	0.20424845	13.6312517	13.8201343	14.5164447	3.66E-25	3.6015	up	PREDICTED: uncharacterized protein LOC105168457 [Sesamum indicum]
c36376.graph_c0	0.23981899	0.28807096	0.18490614	2.09158874	2.04588423	1.45071143	7.281E-05	1.88239	up	hypothetical protein CICLE_v10011295mg [Citrus clementina]
c36380.graph_c0	0.35686282	0.17861008	0.34393714	9.09941819	10.2455078	9.96473581	2.641E-50	3.96698	up	PREDICTED: uncharacterized protein LOC105162690 isoform X1 [Sesamum indicum]
c36386.graph_c0	44.502157	45.4816503	45.0436141	38.6929012	37.3877154	40.8918779	1.954E-45	-1.29735	down	PREDICTED: uncharacterized protein LOC105179031 [Sesamum indicum]
c36389.graph_c1	0.30790509	0.13698374	0.30774351	2.56107947	2.67162467	2.7796634	7.871E-09	2.31992	up	PREDICTED: transcription factor MYB3-like [Sesamum indicum]
c36392.graph_c0	20.5208776	19.7855017	22.4040711	231.830297	252.090166	223.500974	1.63E-140	2.40348	up	PREDICTED: aspartic proteinase-like isoform X1 [Sesamum indicum]
c36394.graph_c0	0.15836895	0.07926377	0.10175519	6.38814122	5.71592958	5.77620308	2.419E-16	4.63326	up	PREDICTED: auxin-induced protein 15A-like [Sesamum indicum]
c36395.graph_c0	5.75674353	5.06954821	5.80574542	2.80696589	2.48660764	1.68543374	2.557E-33	-2.34577	down	PREDICTED: uncharacterized protein LOC105174263 [Sesamum indicum]
c36397.graph_c0	0.3102294	0.31054005	0.33221422	3.00630591	2.54476159	2.63710515	5.061E-06	2.01145	up	PREDICTED: RING-H2 finger protein ATL38-like [Sesamum indicum]
c36398.graph_c0	3.81032755	4.10753863	4.1431261	0.79884349	1.34639078	1.04295962	9.824E-20	-3.01158	down	hypothetical protein MIMGU_mgv1a020815mg [Erythranthe guttata]
c36403.graph_c0	0.43806562	0.24556239	0.29272447	2.48339183	2.50674285	2.50445649	6.1E-10	1.85408	up	hypothetical protein MIMGU_mgv1a000577mg [Erythranthe guttata]
c36404.graph_c0	0.68405378	0.65840265	0.81141802	0.439801	0.91505437	0.73335126	0.002802	-1.13793	down	PREDICTED: putative E3 ubiquitin-protein ligase RF4 [Sesamum indicum]
c36405.graph_c0	14.2342176	13.5487693	14.8618707	160.583806	167.885614	156.850153	3.66E-146	2.41695	up	cyclophilin [Ricinus communis]
c36418.graph_c0	14.9485316	11.8782425	11.0899946	12.7125928	12.2873211	11.9727905	1.076E-09	-1.12372	down	PREDICTED: acyl-coenzyme A thioesterase 13 [Sesamum indicum]
c36426.graph_c0	20.3023279	21.0407728	25.167412	129.515716	132.476128	135.168005	1.246E-58	1.48481	up	-
c36433.graph_c0	36.2507744	34.6861738	32.761706	11.9882744	11.1741221	11.883862	2.16E-183	-2.65421	down	PREDICTED: uncharacterized protein At5g19025-like [Sesamum indicum]
c36438.graph_c0	3.44900567	3.10431217	3.87343966	3.28611699	2.52960504	3.33461703	6.122E-10	-1.27997	down	PREDICTED: uncharacterized protein LOC105171186 [Sesamum indicum]
c36443.graph_c0	0.58104785	1.2974816	0.68923338	13.1888235	14.4307127	14.5525191	3.651E-35	2.94811	up	PREDICTED: (S)-ureidoglycine aminohydrolase [Sesamum indicum]
c36444.graph_c0	2.20677469	2.65078133	3.48397407	0.50407378	0.41375579	0.44871283	3.88E-12	-3.70593	down	M protein repeat-containing [Populus trichocarpa]
c36448.graph_c0	6.74594766	6.15246247	4.2380848	2.17907372	2.29541581	3.46730741	7.628E-10	-2.19231	down	PREDICTED: LOW QUALITY PROTEIN: protein LOW PSII ACCUMULATION 2, chloroplastic [Sesamum indicum]
c36451.graph_c0	780.803198	806.213247	747.287511	69.0433179	64.4168439	75.3459234	0	-4.57198	down	hypothetical protein MIMGU_mgv1a024078mg [Erythranthe guttata]

#ID	0 h-1 FPKM	0 h-2 FPKM	0 h-3 FPKM	28 h-1 FPKM	28 h-2 FPKM	28 h-3 FPKM	FDR	log2FC	regulated	nr_annotation
c36459.graph_c0	28.1867476	34.7984659	27.3304562	169.474807	176.701506	163.23799	6.378E-63	1.40595	up	PREDICTED: shikimate O-hydroxycinnamoyltransferase [Sesamum indicum]
c36463.graph_c1	0.56335408	0.77538751	0.40721191	3.60821199	4.29762514	3.23661053	2.435E-06	1.58701	up	PREDICTED: leucine-rich repeat receptor-like serine/threonine/tyrosine-protein kinase SOBIR1 [Sesamum indicum]
c36469.graph_c0	1.7759811	2.07667479	2.10043741	10.3718737	10.4993353	11.1850629	3.456E-25	1.33693	up	PREDICTED: uncharacterized protein LOC105171114 [Sesamum indicum]
c36477.graph_c0	18.8265878	18.5641646	16.9034423	10.8749658	10.0726186	10.4361866	9.53E-99	-1.8799	down	PREDICTED: uncharacterized protein LOC105159352 [Sesamum indicum]
c36479.graph_c0	6.2517644	7.10045098	4.37737183	5.65055634	6.75873249	4.94354654	0.0002204	-1.11808	down	PREDICTED: UDP-glycosyltransferase 74B1 [Vitis vinifera]
c36480.graph_c0	2.50489207	2.10127212	1.81345661	13.1284092	14.2730859	12.3446837	2.079E-32	1.54291	up	PREDICTED: methionine S-methyltransferase-like isoform X1 [Sesamum indicum]
c36483.graph_c0	2.3160295	1.86963601	2.35214985	9.39377105	10.3692392	10.1907821	1.508E-08	1.10454	up	PREDICTED: triose phosphate/phosphate translocator, chloroplastic isoform X2 [Sesamum indicum]
c36491.graph_c0	0.25380118	0.10162213	0.26091565	76.957226	81.4102254	78.2397029	0	7.49125	up	acetylajmalan acetylesterase [Striga asiatica]
c36492.graph_c0	3.93749026	5.30577528	5.64365752	2.97181236	4.07459203	5.20920472	0.0001617	-1.3732	down	-
c36493.graph_c0	4.94490717	5.2052086	3.60587068	2.99494169	3.10333582	3.31665081	8.712E-11	-1.63313	down	PREDICTED: plastidial pyruvate kinase 4, chloroplastic [Sesamum indicum]
c36504.graph_c0	4.08400932	4.84116968	5.66243844	49.5226483	58.9608228	57.8743683	1.444E-39	2.4172	up	hypothetical protein MIMGU_mgv1a014610mg [Erythranthe guttata]
c36508.graph_c0	0.18668804	0.22424997	0.16793106	8.99583524	6.90954935	6.97516244	9.536E-34	4.2157	up	PREDICTED: uncharacterized protein LOC105176006 [Sesamum indicum]
c36519.graph_c1	5.93133978	6.01540121	5.44070855	4.38176593	4.6477202	4.38546568	1.027E-29	-1.46377	down	PREDICTED: uncharacterized protein LOC105168551 [Sesamum indicum]
c36532.graph_c0	1.25095327	1.14608677	1.30781653	14.2079517	15.1520392	14.1838935	6.326E-60	2.46334	up	PREDICTED: uncharacterized protein LOC105171185 [Sesamum indicum]
c36534.graph_c0	56.5780661	60.2174521	57.9930426	38.8694839	37.5496251	40.6703798	5.89E-93	-1.66836	down	hypothetical protein MIMGU_mgv1a004539mg [Erythranthe guttata]
c36536.graph_c0	7.92304631	7.7668908	9.54947748	35.0668189	38.0088025	37.2989669	3.191E-17	1.03549	up	unnamed protein product [Coffea canephora]
c36541.graph_c0	0.49983457	0.44474229	0.2141024	2.48239203	3.44405212	3.14546195	4.883E-08	1.88789	up	PREDICTED: organic cation/carnitine transporter 7-like [Sesamum indicum]
c36548.graph_c0	1.94952592	2.10759633	2.30480056	1.58693271	1.17698512	1.75739704	2.934E-05	-1.58385	down	hypothetical protein MIMGU_mgv1a019201mg [Erythranthe guttata]
c36549.graph_c0	2.89047464	2.36193389	2.50151742	18.7784036	16.9164274	16.302578	7.659E-16	1.65597	up	-
c36558.graph_c0	1.01066323	1.46692912	1.10393092	7.67598699	8.15766198	6.74304836	2.924E-08	1.56491	up	PREDICTED: uncharacterized protein LOC105159405 [Sesamum indicum]
c36561.graph_c0	1032.83313	1046.67282	1077.48139	206.181746	200.142557	235.285718	0	-3.38911	down	hypothetical protein MIMGU_mgv1a011962mg [Erythranthe guttata]
c36563.graph_c0	0.47436479	1.38799324	0.84403043	4.90627962	8.28512988	6.27573183	0.0001942	1.75285	up	PREDICTED: cytochrome P450 CYP736A12-like [Sesamum indicum]

#ID	0 h-1 FPKM	0 h-2 FPKM	0 h-3 FPKM	28 h-1 FPKM	28 h-2 FPKM	28 h-3 FPKM	FDR	log2FC	regulated	nr_annotation
c36567.graph_c0	0.15150885	0.25276761	0.19469487	10.9381656	11.5000667	10.7524762	7.96E-112	4.69995	up	PREDICTED: uncharacterized protein LOC105164077 [Sesamum indicum]
c36568.graph_c0	1.49904116	0.69255795	0.59271607	6.6207741	7.34000014	7.7960092	8.17E-06	1.88652	up	hypothetical protein MIMGU_mgv1a007969mg [Erythranthe guttata]
c36568.graph_c1	0.74314382	0.83687396	0.71622693	4.45592934	6.0958885	4.79291166	8.365E-05	1.64993	up	PREDICTED: probable purine permease 9 [Sesamum indicum]
c36569.graph_c0	0.21343015	0	0.45711032	7.13551097	7.09630437	6.24444033	3.067E-17	3.82401	up	PREDICTED: uncharacterized protein LOC105167095 [Sesamum indicum]
c36570.graph_c0	6.17352697	5.38430076	4.79135466	30.4752703	32.2694939	30.5950882	2.641E-36	1.42552	up	PREDICTED: protein trichome birefringence-like 25 [Sesamum indicum]
c36574.graph_c0	29.5570487	29.8924507	25.6157524	27.449085	30.9736728	31.8195268	2.42E-25	-1.00311	down	PREDICTED: uncharacterized protein LOC105176146 [Sesamum indicum]
c36575.graph_c0	32.6154049	31.9629003	31.2691433	28.9530506	34.1820933	32.3936621	2.637E-35	-1.09487	down	PREDICTED: PCI domain-containing protein 2 [Sesamum indicum]
c36586.graph_c0	1.40038005	1.86904309	1.39964519	51.3983016	50.747415	43.417051	2.138E-84	3.87196	up	PREDICTED: GDP-mannose 3,5-epimerase 2 [Sesamum indicum]
c36586.graph_c1	1.44949874	1.10548586	1.41917201	36.7717244	39.9051167	31.765636	2.227E-55	3.67818	up	GDP-mannose 3,5-epimerase 1 [Gossypium arboreum]
c36593.graph_c0	5.5431604	5.14621386	4.94562796	4.71758844	5.10767855	4.32644454	1.354E-14	-1.23371	down	PREDICTED: protein YIPF5 homolog [Nicotiana tomentosiformis]
c36599.graph_c0	3.99975509	4.05860627	4.92861719	33.5300426	31.3171927	32.591831	3.513E-35	1.81414	up	PREDICTED: trafficking protein particle complex subunit 3-like isoform X1 [Sesamum indicum]
c36600.graph_c0	0.08138728	0.10862504	0.06972392	6.99963049	5.60789625	5.84035436	6.225E-42	5.06229	up	hypothetical protein MIMGU_mgv1a021873mg [Erythranthe guttata]
c36602.graph_c0	19.1787674	17.6649216	19.5016829	19.4539102	19.0497497	19.3215618	3.153E-25	-1.05358	down	PREDICTED: TOM1-like protein 2 [Sesamum indicum]
c36605.graph_c0	9.60312159	10.0071064	9.80902909	6.7289475	5.33231614	6.16250889	8.593E-28	-1.78134	down	PREDICTED: protein FAR-RED IMPAIRED RESPONSE 1 isoform X1 [Sesamum indicum]
c36606.graph_c0	170.246824	155.806892	158.850346	52.931889	70.3134747	78.5934397	2.86E-142	-2.35339	down	Gibberellin 20 oxidase 1-D GN=GA20ox1D OS=Triticum aestivum (Wheat)
c36607.graph_c0	54.7426035	48.1577325	54.3772636	48.2376329	51.4729797	49.2196663	7.747E-26	-1.16962	down	PREDICTED: nuclear pore complex protein NUP50A-like [Sesamum indicum]
c36608.graph_c0	1.95914512	2.54943898	4.02812648	10.8216049	17.870452	18.5901921	0.0024075	1.37079	up	-
c36617.graph_c0	2.60037857	1.60183535	1.54227204	13.0842011	13.126452	10.3207145	0.0001205	1.58311	up	predicted Rac-like GTPase ortholog, partial [Nicotiana alata]
c36625.graph_c0	3.05897799	3.33422252	4.28032185	78.2583145	74.94234	66.1963858	3.749E-88	3.26588	up	PREDICTED: uncharacterized protein LOC105164615 [Sesamum indicum]
c36627.graph_c0	14.155991	14.0109507	11.446028	58.0895231	61.2170526	57.1165155	6.276E-18	1.06691	up	PREDICTED: uncharacterized protein LOC105159910 [Sesamum indicum]
c36632.graph_c0	0.67359575	0.41205405	0.76941974	18.0867258	18.3197605	15.9348715	3.357E-66	3.7239	up	PREDICTED: protein trichome birefringence-like 23 [Sesamum indicum]
c36639.graph_c0	0.09696325	0.19412068	0.31150402	2.74842081	2.89516068	2.70272769	5.379E-09	2.68827	up	PREDICTED: B-box zinc finger protein 24 [Sesamum indicum]
c36654.graph_c0	20.0131601	18.9028003	23.2991103	149.923002	146.27642	148.269082	4.338E-85	1.74395	up	PREDICTED: uncharacterized protein LOC105177676 [Sesamum indicum]
c36664.graph_c0	5.84940272	3.00895306	3.34076181	0	0	0	3.297E-16	-Inf	down	predicted protein [Hordeum vulgare subsp. vulgare]

#ID	0 h-1 FPKM	0 h-2 FPKM	0 h-3 FPKM	28 h-1 FPKM	28 h-2 FPKM	28 h-3 FPKM	FDR	log2FC	regulated	nr_annotation
c36665.graph_c0	0.57681259	0.73486023	0.33692137	7.4316994	7.88009876	7.65022913	6.06E-16	2.71605	up	PREDICTED: LOW QUALITY PROTEIN: single-stranded DNA-binding protein WHY1, chloroplastic, partial [Sesamum indicum]
c36666.graph_c0	12.7048608	11.8970936	13.16633	3.41190989	3.9363666	4.02146421	4.154E-91	-2.82289	down	PREDICTED: uncharacterized protein LOC105167713 [Sesamum indicum]
c36669.graph_c0	1.30437649	1.45490349	1.67617543	24.7566905	29.4941819	25.7709694	8.083E-64	3.07922	up	PREDICTED: enoyl-[acyl-carrier-protein] reductase [NADH], chloroplastic-like [Sesamum indicum]
c36674.graph_c0	9.79846317	10.4599875	10.4579843	88.2257435	91.4087742	93.3823596	3.05E-118	2.06084	up	PREDICTED: peroxidase 17 [Sesamum indicum]
c36675.graph_c0	3.2911882	2.83119703	2.51115111	2.16776505	2.12602037	1.98236393	4.958E-08	-1.54782	down	PREDICTED: nuclear transcription factor Y subunit B-3-like [Sesamum indicum]
c36679.graph_c0	23.9546694	28.701725	18.656181	22.0703991	23.9765153	23.5340202	2.098E-05	-1.1224	down	hypothetical protein MIMGU_mgv1a014406mg [Erythranthe guttata]
c36684.graph_c0	18.2798318	18.9581893	22.1251394	12.4070645	12.2601548	12.1228055	8.057E-18	-1.78337	down	PREDICTED: uncharacterized protein LOC105176815 isoform X1 [Sesamum indicum]
c36690.graph_c0	2.90119621	2.52380234	2.08598981	11.9987827	11.6270438	11.4295545	3.887E-10	1.1358	up	unnamed protein product [Coffea canephora]
c36691.graph_c0	0.63963765	0.57436716	0.54394392	4.23865695	4.22216631	4.26760119	1.689E-25	1.76744	up	PREDICTED: uncharacterized protein LOC105169716 isoform X1 [Sesamum indicum]
c36695.graph_c0	7.15790988	6.79397782	6.55967079	4.97433719	5.05278011	4.32961977	2.021E-28	-1.60495	down	PREDICTED: two-pore potassium channel 3-like [Sesamum indicum]
c36697.graph_c0	0.15812729	0.11871422	0.30479974	1.81008453	1.89375513	2.36791164	3.072E-07	2.28386	up	PREDICTED: rho GTPase-activating protein REN1 [Sesamum indicum]
c36705.graph_c0	6.8441927	4.89360438	5.70631778	29.4017832	30.0224986	28.2922267	7.149E-23	1.24094	up	PREDICTED: putative tRNA (cytidine(32)/guanosine(34)-2'-O)-methyltransferase [Sesamum indicum]
c36707.graph_c0	0.13521344	0	0.08687731	3.04644281	4.79145872	3.92928227	4.329E-13	4.63989	up	PREDICTED: uncharacterized protein LOC105163665 [Sesamum indicum]
c36710.graph_c0	6.73422813	6.74097144	6.34608316	6.52586426	5.89224356	4.52629415	2.007E-05	-1.31733	down	hypothetical protein MIMGU_mgv1a009490mg [Erythranthe guttata]
c36710.graph_c1	11.2816424	9.61991121	10.3808236	9.31293587	13.5270519	9.25129693	1.683E-05	-1.05436	down	PREDICTED: zinc finger CCCH domain-containing protein 20-like [Sesamum indicum]
c36719.graph_c0	0.27511764	0.55078625	0.61868984	8.74798365	9.2526563	9.2185829	6.214E-18	3.13756	up	PREDICTED: uncharacterized protein LOC105157890 [Sesamum indicum]
c36719.graph_c1	0.30257204	0.70670837	0.64802841	12.6446877	12.4097472	13.9666325	3.311E-35	3.46114	up	PREDICTED: uncharacterized protein LOC105157890 [Sesamum indicum]
c36723.graph_c0	0.91393027	0.77562982	0.56168706	23.7682132	22.7642632	22.3639609	1.31E-154	3.85069	up	PREDICTED: dolichyl-diphosphooligosaccharide--protein glycosyltransferase subunit STT3A [Sesamum indicum]
c36725.graph_c0	1.43323798	2.08679731	1.50690072	21.6856568	26.1638216	23.5680011	7.69E-20	2.7383	up	PREDICTED: uncharacterized protein LOC104897046 [Beta vulgaris subsp. vulgaris]
c36729.graph_c1	0.17854026	0.40211785	0.34414696	30.2994348	30.901728	27.9006342	1.42E-125	5.49411	up	PREDICTED: probable inactive receptor kinase At5g58300-like isoform X1 [Glycine max]
c36731.graph_c0	6.63703525	8.00648764	7.21672182	141.620616	148.195347	145.536546	1.77E-100	3.22472	up	hypothetical protein 0_2321_02, partial [Pinus lambertiana]
c36731.graph_c2	5.3185548	5.89943518	6.46511816	78.250155	75.840658	80.134043	1.63E-52	2.63481	up	PREDICTED: tubulin beta-1 chain [Sesamum indicum]

#ID	0 h-1 FPKM	0 h-2 FPKM	0 h-3 FPKM	28 h-1 FPKM	28 h-2 FPKM	28 h-3 FPKM	FDR	log2FC	regulated	nr_annotation
c36731.graph_c3	2.76625666	2.42289832	1.333031	30.6601158	40.1633456	34.8617107	3.453E-13	2.93579	up	PREDICTED: tubulin beta-6 chain-like [Citrus sinensis]
c36732.graph_c0	1.26794341	1.60766988	1.62935748	11.345107	12.2589992	12.4324746	1.615E-27	1.90695	up	PREDICTED: LOW QUALITY PROTEIN: probable galacturonosyltransferase-like 7 [Sesamum indicum]
c36736.graph_c0	86.5260092	84.9418416	88.079676	32.4411919	27.9133536	32.6027194	4.57E-193	-2.57169	down	PREDICTED: uncharacterized protein LOC105162600 [Sesamum indicum]
c36738.graph_c0	3.09743751	3.62989947	2.62119282	13.7817349	15.7156406	13.485979	3.647E-06	1.11242	up	PREDICTED: uncharacterized protein LOC105156818 [Sesamum indicum]
c36759.graph_c0	1418.62285	1555.23514	1461.46938	45.2661566	46.7775056	49.9817323	0	-6.05511	down	PREDICTED: GEM-like protein 5 [Sesamum indicum]
c36760.graph_c0	16.3360711	16.2620843	16.701214	4.78854282	4.95536486	4.74598881	2.79E-137	-2.85754	down	PREDICTED: sodium/hydrogen exchanger 1-like [Sesamum indicum]
c36762.graph_c0	0	0.42407859	0.09073544	16.3705244	16.2174515	13.4838401	8.684E-42	5.39248	up	PREDICTED: glucomannan 4-beta-mannosyltransferase 2-like [Sesamum indicum]
c36762.graph_c1	0.10188959	0	0.06546608	14.3662364	13.3391094	13.11254	6E-57	6.84275	up	PREDICTED: glucomannan 4-beta-mannosyltransferase 2-like [Sesamum indicum]
c36764.graph_c1	0.32280271	0	0	13.8420579	13.3454079	17.4211113	2.7E-14	6.0529	up	PREDICTED: probable small nuclear ribonucleoprotein F isoform X2 [Nicotiana tomentosiformis]
c36764.graph_c2	2.20539106	0.36793324	0	10.1514816	13.0251355	8.28352606	0.0002241	2.54789	up	hypothetical protein MIMGU_mgv1a017211mg [Erythranthe guttata]
c36767.graph_c0	77.8982265	71.4447157	75.248726	18.3612828	18.1155979	16.7091352	0	-3.16892	down	PREDICTED: uncharacterized protein LOC105169073 [Sesamum indicum]
c36770.graph_c0	99.3520527	106.805938	103.460261	8.85919403	8.41906153	8.74745638	0	-4.66324	down	PREDICTED: probable protein phosphatase 2C 49 [Sesamum indicum]
c36772.graph_c0	5.19648461	5.89882156	4.95663473	26.8267837	27.8430766	24.1144285	1.098E-15	1.20497	up	PREDICTED: protein CREG1 isoform X1 [Sesamum indicum]
c36783.graph_c0	0.60219266	0.42698026	0.4191641	47.3048491	46.5977321	48.1835004	0	5.52982	up	PREDICTED: probable methyltransferase PMT13 [Sesamum indicum]
c36792.graph_c0	6.55267324	6.55923475	5.47328895	44.7372191	47.6498342	39.2254869	5.42E-33	1.73504	up	PREDICTED: hexose carrier protein HEX6-like [Sesamum indicum]
c36799.graph_c0	105.804709	112.891859	112.811027	22.640738	23.9512448	25.006276	1.11E-196	-3.30196	down	PREDICTED: uncharacterized protein LOC105174622 [Sesamum indicum]
c36800.graph_c0	8.61204457	9.34408094	7.35208204	1.26934508	1.462265	2.07155044	4.178E-54	-3.48383	down	hypothetical protein MIMGU_mgv1a002177mg [Erythranthe guttata]
c36801.graph_c0	0.0635073	0.0211903	0.02720313	1.58471483	2.08376055	2.19703897	9.336E-19	4.63179	up	PREDICTED: BTB/POZ domain-containing protein At3g08570 [Sesamum indicum]
c36813.graph_c0	0.90059375	0.95300959	0.82664187	4.37610984	4.47466869	4.05919398	3.919E-06	1.17835	up	PREDICTED: WPP domain-interacting tail-anchored protein 2-like [Sesamum indicum]
c36814.graph_c0	32.9132465	32.54531	36.2593907	20.3753225	18.8987424	19.4762848	6.327E-43	-1.88378	down	PREDICTED: uncharacterized protein At5g19025-like isoform X2 [Sesamum indicum]
c36816.graph_c0	1.27391533	0.7847329	0.62962755	3.27617066	5.2730901	4.7073828	0.0049456	1.21951	up	-
c36816.graph_c1	1.39785011	1.2126832	0.71851709	6.70526605	5.13691951	6.24518166	0.0006967	1.35947	up	PREDICTED: uncharacterized protein LOC105168668 [Sesamum indicum]
c36825.graph_c0	0	0.13483276	0.1730921	2.59428747	3.44730323	2.87581231	5.9E-11	3.74635	up	PREDICTED: transcription factor bHLH104 [Sesamum indicum]

#ID	0 h-1 FPKM	0 h-2 FPKM	0 h-3 FPKM	28 h-1 FPKM	28 h-2 FPKM	28 h-3 FPKM	FDR	log2FC	regulated	nr_annotation
c36829.graph_c0	77.2286372	76.2254546	76.291389	46.0731209	45.2702709	46.9650145	1.203E-81	-1.82242	down	PREDICTED: transcription factor ILR3-like [Sesamum indicum]
c36831.graph_c0	0.41742301	0.64997489	0.65560623	3.23606016	3.77406776	3.63082316	2.67E-05	1.53176	up	PREDICTED: 1-aminocyclopropane-1-carboxylate oxidase 5-like [Sesamum indicum]
c36843.graph_c0	2.24591195	1.54040654	1.33615082	0.75570179	0.90067278	0.51798305	9.625E-08	-2.32311	down	--
c36851.graph_c0	1.51207359	2.09949261	1.5669955	8.72082989	11.010925	8.36000165	3.35E-08	1.34856	up	PREDICTED: serine/threonine-protein kinase D6PKL2 [Sesamum indicum]
c36851.graph_c1	0	1.18320614	1.32907752	5.36929473	6.88411701	6.30907295	0.0001075	1.77771	up	PREDICTED: serine/threonine-protein kinase D6PKL2 [Sesamum indicum]
c36852.graph_c0	1.28556562	0.7218931	0.68497674	11.2576778	9.62964715	9.61386948	3.997E-28	2.41821	up	PREDICTED: probable polygalacturonase isoform X2 [Sesamum indicum]
c36853.graph_c0	171.511192	167.237817	150.169434	40.4281122	35.4803076	43.4540103	0	-3.12234	down	PREDICTED: uncharacterized protein LOC105157533 [Sesamum indicum]
c36854.graph_c0	0.294792	0.20656103	0.35987842	7.670262	8.23131821	7.25539533	7.195E-73	3.65392	up	PREDICTED: aldehyde dehydrogenase 22A1 [Sesamum indicum]
c36865.graph_c0	0	0.09257952	0	10.5533753	9.80182425	9.76332046	2.424E-47	7.26395	up	PREDICTED: cinnamoyl-CoA reductase 1-like [Sesamum indicum]
c36866.graph_c0	0.87074821	0.87162013	1.35045198	7.92159605	9.06372152	7.35430998	2.921E-18	1.87875	up	PREDICTED: adenine/guanine permease AZG1 [Sesamum indicum]
c36868.graph_c0	5.85884804	1.90060202	2.43990564	0	0	0.06434514	8.829E-07	-8.37457	down	PREDICTED: ribulose bisphosphate carboxylase/oxygenase activase 2, chloroplastic-like isoform X2 [Phoenix dactylifera]
c36878.graph_c0	6.97047104	8.00166389	6.20439	34.6725618	35.3976838	33.48834	1.343E-28	1.20101	up	PREDICTED: transcription factor bHLH143-like [Sesamum indicum]
c36881.graph_c0	3.71753715	3.63794792	2.95900105	51.8802165	56.8378377	52.5512097	2.48E-160	2.87882	up	PREDICTED: 65-kDa microtubule-associated protein 1 [Sesamum indicum]
c36885.graph_c0	20.9165267	22.1464593	16.2259053	11.6708353	17.8149669	11.8512256	2.196E-11	-1.60892	down	PREDICTED: uncharacterized protein LOC105173633 [Sesamum indicum]
c36890.graph_c0	25.8125898	27.6692356	24.5228884	13.0921798	13.8591161	13.3231911	7.24E-99	-2.04348	down	PREDICTED: uncharacterized protein LOC105180144 isoform X2 [Sesamum indicum]
c36891.graph_c0	0	0.17453754	0	0.6843203	0.77806742	0.70317009	3.058E-05	2.55116	up	PREDICTED: guanosine nucleotide diphosphate dissociation inhibitor At5g09550 [Nicotiana sylvestris]
c36892.graph_c0	2.12702127	2.23053931	2.21267672	19.6551007	18.6107853	18.8616409	4.662E-14	2.02922	up	PREDICTED: uncharacterized protein At1g76660-like [Sesamum indicum]
c36892.graph_c1	4.15314489	3.34391814	2.78449858	32.9408228	30.1572102	29.4495883	2.097E-24	2.084	up	PREDICTED: uncharacterized protein At1g76660-like [Sesamum indicum]
c36897.graph_c0	0.54051123	0.59023906	0.50514788	3.05344167	3.07942435	2.30216484	3.362E-05	1.27558	up	PREDICTED: probable LRR receptor-like serine/threonine-protein kinase At2g24230 [Sesamum indicum]
c36900.graph_c1	0.09710944	0	0	1.27041448	1.52942022	1.06544362	1.079E-10	4.2545	up	PREDICTED: probable LRR receptor-like serine/threonine-protein kinase At1g06840 [Sesamum indicum]
c36904.graph_c0	15.496594	15.6374849	15.348648	8.30023786	7.86046069	8.25851334	8.27E-121	-2.01892	down	PREDICTED: LOW QUALITY PROTEIN: U-box domain-containing protein 17-like [Sesamum indicum]

#ID	0 h-1 FPKM	0 h-2 FPKM	0 h-3 FPKM	28 h-1 FPKM	28 h-2 FPKM	28 h-3 FPKM	FDR	log2FC	regulated	nr_annotation
c36905.graph_c0	3.06531597	3.42937193	3.01221652	15.5949778	16.5656504	14.9353836	2.578E-09	1.21816	up	PREDICTED: outer envelope pore protein 24A, chloroplastic-like [Sesamum indicum]
c36911.graph_c0	0.97667465	0.81471053	0.69725866	5.48155385	5.19857598	5.69571125	1.687E-06	1.63189	up	-
c36918.graph_c0	9.2002345	9.91016595	9.76654983	47.0247857	49.7432784	52.2497558	9.04E-18	1.2767	up	PREDICTED: probable small nuclear ribonucleoprotein F isoform X1 [Sesamum indicum]
c36920.graph_c0	0.25863176	0.17259383	0.08308801	1.1591611	1.15976123	1.31654321	6.335E-05	1.74377	up	hypothetical protein MIMGU_mgv1a002741mg [Erythranthe guttata]
c36922.graph_c0	3.13179653	3.71073649	2.62823349	27.406965	29.9048149	26.1164394	6.548E-30	2.05059	up	PREDICTED: probable inactive receptor kinase At1g27190 [Sesamum indicum]
c36925.graph_c0	25.5276409	29.5790905	24.1902797	7.42945652	9.53063766	6.98995249	5.197E-55	-2.81779	down	PREDICTED: protein ECERIFERUM 1-like [Sesamum indicum]
c36926.graph_c0	4.22277186	4.93941611	5.36545791	126.28083	129.21408	124.710968	2.97E-260	3.6165	up	hypothetical protein CICLE_v10008112mg [Citrus clementina]
c36926.graph_c1	3.65784063	3.50230761	3.47426046	87.2680698	89.6487498	82.1509115	8.062E-72	3.51616	up	PREDICTED: serine carboxypeptidase II-2 [Sesamum indicum]
c36930.graph_c0	19.5663794	22.4875976	16.451967	136.062557	143.142082	134.520456	1.439E-30	1.73383	up	PREDICTED: protein transport protein Sec61 subunit beta-like [Vitis vinifera]
c36938.graph_c0	71.2579824	67.6811949	61.30252	49.8922461	52.4727337	49.9056627	1.523E-68	-1.48398	down	PREDICTED: uncharacterized protein LOC105174627 [Sesamum indicum]
c36942.graph_c0	2.08721367	1.8716679	1.89985781	1.51697487	1.48382658	1.46993394	2.408E-06	-1.47975	down	-
c36943.graph_c0	23.1671247	22.1223476	23.6598502	17.9011684	23.5044742	20.7357262	1.087E-30	-1.24119	down	PREDICTED: probable protein phosphatase 2C 24 [Sesamum indicum]
c36948.graph_c0	17.9822936	18.579709	17.9012302	18.3981638	17.0423061	17.7589865	1.878E-26	-1.12418	down	PREDICTED: mitochondrial ribosome-associated GTPase 1 [Sesamum indicum]
c36949.graph_c0	64.2194328	65.8768922	56.4479945	30.5956777	26.5806038	26.0512937	2.29E-104	-2.25349	down	PREDICTED: probable receptor-like protein kinase At5g47070 [Sesamum indicum]
c36951.graph_c0	13.2609844	13.3270437	13.9918242	113.088504	116.087225	104.490433	3.24E-122	1.94785	up	PREDICTED: mechanosensitive ion channel protein 8-like [Sesamum indicum]
c36954.graph_c0	34.8819259	33.943078	34.1691267	1.27938293	0.75997554	0.74176533	0	-6.30204	down	PREDICTED: heat stress transcription factor B-2b [Sesamum indicum]
c36959.graph_c0	3.43120575	3.00193083	2.96842214	20.8436856	19.4226977	19.6840966	1.044E-55	1.58378	up	PREDICTED: protein PIR [Sesamum indicum]
c36972.graph_c0	0.03841162	0	0	153.740826	162.180205	151.261369	0	12.511	up	chalcone synthase [Misopates orontium]
c36976.graph_c0	12.0267499	10.9337829	13.7376442	2.36470753	3.12640988	2.30860708	1.393E-35	-3.32797	down	hypothetical protein MIMGU_mgv1a008959mg [Erythranthe guttata]
c36993.graph_c0	3.63666985	1.96694246	2.78888379	0	0	0	2.166E-26	-Inf	down	--
c37003.graph_c0	49.5921483	54.221855	49.1235695	37.1160044	32.9311344	34.5316984	1.968E-64	-1.63847	down	PREDICTED: BTB/POZ and MATH domain-containing protein 2-like [Sesamum indicum]
c37003.graph_c1	29.1925526	28.1356651	28.483767	21.1794268	21.1913084	22.2156445	6.229E-48	-1.50005	down	PREDICTED: BTB/POZ and MATH domain-containing protein 2-like [Sesamum indicum]
c37013.graph_c0	3.75058623	1.62688148	2.4098259	0	0	0	2.056E-13	-Inf	down	-

#ID	0 h-1 FPKM	0 h-2 FPKM	0 h-3 FPKM	28 h-1 FPKM	28 h-2 FPKM	28 h-3 FPKM	FDR	log2FC	regulated	nr_annotation
c37020.graph_c0	0.28393242	0.21316255	0.2736483	6.86148475	6.98715496	5.5147213	4.774E-15	3.55904	up	PREDICTED: 7-deoxyloganetin glucosyltransferase-like [Sesamum indicum]
c37021.graph_c0	1.83691161	1.99198025	1.34885951	16.3226584	15.971962	14.8365282	5.513E-49	2.09941	up	PREDICTED: putative late blight resistance protein homolog R1B-16 [Sesamum indicum]
c37022.graph_c0	18.4447141	16.2284467	14.2759575	15.0667136	15.1037392	17.0859459	9.618E-15	-1.13769	down	PREDICTED: protein OSB4, chloroplastic-like [Sesamum indicum]
c37023.graph_c0	0.26600159	0.17751197	0.11394086	5.21986746	4.74214382	6.25759182	1.372E-19	3.78299	up	PREDICTED: bifunctional riboflavin kinase/FMN phosphatase-like isoform X2 [Sesamum indicum]
c37025.graph_c0	9.5134281	7.9357953	8.61316101	7.1762102	6.2902641	6.58235221	2.936E-13	-1.46814	down	PREDICTED: syntaxin-51-like [Sesamum indicum]
c37038.graph_c0	0.25122182	0.12573669	0.08070749	1.91715896	1.85465871	1.22916047	4.161E-06	2.37003	up	-
c37038.graph_c1	0.72828745	0.24300557	0.10398647	4.58739769	3.71717051	3.95923746	5.236E-10	2.4405	up	unnamed protein product [Coffea canephora]
c37039.graph_c0	2.75929989	1.49611741	1.9945179	0	0	0	2.569E-20	-Inf	down	hypothetical protein POPTR_0005s04730g [Populus trichocarpa]
c37041.graph_c0	67.1090667	63.5866948	62.1199475	57.9810473	66.1651419	63.8560919	1.234E-41	-1.12613	down	hypothetical protein JCGZ_06000 [Jatropha curcas]
c37048.graph_c0	1.00423355	1.03990256	1.06798334	5.71312941	6.24918747	5.70924674	4.315E-08	1.41389	up	PREDICTED: mannose-6-phosphate isomerase 2-like [Sesamum indicum]
c37049.graph_c0	30.3509647	31.4121526	31.4106933	14.1807254	15.8270004	17.2606062	6.284E-84	-2.06936	down	PREDICTED: uncharacterized protein LOC105155538 isoform X3 [Sesamum indicum]
c37051.graph_c0	1.56493984	1.38221196	0.94635748	10.6379348	10.5111952	10.5912628	8.399E-09	1.94262	up	PREDICTED: transcription repressor OFP1 [Sesamum indicum]
c37063.graph_c0	0.17172866	0.34380124	0.1765425	28.1324899	33.1768172	29.4535984	5.2E-129	5.94607	up	PREDICTED: fasciclin-like arabinogalactan protein 8 [Sesamum indicum]
c37064.graph_c0	1.53315439	0.98902219	0.83184706	30.0857699	35.1239776	27.9038638	1.068E-54	3.71058	up	hypothetical protein MIMGU_mgv1a007836mg [Erythranthe guttata]
c37065.graph_c0	1.91116503	1.70810605	1.66651899	0	0.04479136	0.08095943	1.94E-27	-6.47678	down	--
c37067.graph_c0	8.36272045	6.4243283	6.78940923	32.9341546	31.5231934	29.8728168	3.383E-21	1.03953	up	PREDICTED: polyadenylate-binding protein-interacting protein 8-like [Sesamum indicum]
c37069.graph_c0	13.0820354	13.3908317	12.6353145	11.3481959	10.5786712	11.5124762	1.504E-25	-1.31565	down	PREDICTED: peptide chain release factor APG3, chloroplastic isoform X2 [Sesamum indicum]
c37074.graph_c0	10.3307979	11.0275997	11.0013115	7.1064177	6.63418101	6.5072269	3.696E-58	-1.76782	down	PREDICTED: uncharacterized protein LOC105165632 [Sesamum indicum]
c37077.graph_c0	0.05061038	0.10132213	0.06503635	4.78183363	4.18469981	3.87192229	5.053E-18	4.79513	up	hypothetical protein MIMGU_mgv1a0032972mg, partial [Erythranthe guttata]
c37078.graph_c0	45.1292829	50.4367324	43.3387915	1114.34737	1380.26684	1033.4789	4.792E-40	3.57595	up	3-hydroxy-3-methylglutaryl-coenzyme A reductase [Andrographis paniculata]
c37079.graph_c0	19.780804	20.3681449	23.3953013	146.467074	147.748462	140.064159	8.525E-79	1.6798	up	PREDICTED: translocon-associated protein subunit beta [Sesamum indicum]
c37096.graph_c0	9.22197731	9.32445629	8.25951324	9.27514904	8.55797513	9.28101391	5.693E-07	-1.07231	down	-
c37097.graph_c0	11.8023746	12.0603219	9.98462475	5.36118614	5.29244228	5.30795005	4.847E-44	-2.17277	down	PREDICTED: LOW QUALITY PROTEIN: transcription factor bHLH96-like [Sesamum indicum]

#ID	0 h-1 FPKM	0 h-2 FPKM	0 h-3 FPKM	28 h-1 FPKM	28 h-2 FPKM	28 h-3 FPKM	FDR	log2FC	regulated	nr_annotation
c37104.graph_c0	13.75557	13.6207541	12.6532876	90.0611326	88.6442505	86.2541347	5.16E-92	1.63701	up	PREDICTED: calcium-transporting ATPase 4, plasma membrane-type-like [Sesamum indicum]
c37113.graph_c0	0.10534813	0.08436289	0	3.55268144	5.06048361	3.93608663	3.74E-24	4.97784	up	PREDICTED: LOW QUALITY PROTEIN: respiratory burst oxidase homolog protein C-like [Sesamum indicum]
c37117.graph_c0	0.38044831	0.598446	0.41904955	4.1081135	3.78057288	4.31916411	3.4E-07	2.03667	up	PREDICTED: psbP-like protein 1, chloroplastic [Sesamum indicum]
c37119.graph_c0	2.57804928	3.10482143	1.91526765	12.266954	11.6310265	13.5931714	2.35E-10	1.21708	up	PREDICTED: uncharacterized protein LOC105157824 isoform X3 [Sesamum indicum]
c37122.graph_c0	15.3210229	14.9801652	16.1823653	0.18678452	0.23351388	0.30482929	0	-7.09199	down	PREDICTED: uncharacterized protein LOC105166759 [Sesamum indicum]
c37123.graph_c0	0.41352759	0.11826905	0	4.03594999	3.56655072	2.83784554	4.453E-10	3.22996	up	PREDICTED: phospholipase D alpha 1-like [Sesamum indicum]
c37127.graph_c0	0.46082408	0.49011587	0.55516611	3.72603267	3.98796893	3.689489	1.905E-09	1.82741	up	PREDICTED: uncharacterized protein LOC105170631 [Sesamum indicum]
c37133.graph_c0	6.90883624	6.47346778	6.29727691	6.36420881	6.74792966	7.05123925	2.556E-10	-1.05416	down	PREDICTED: uncharacterized protein LOC105173649 [Sesamum indicum]
c37135.graph_c0	0.14802243	0.06350171	0	0.95286783	1.01299311	0.91548166	1.829E-07	2.70169	up	hypothetical protein MIMGU_mgv1a001054mg [Erythranthe guttata]
c37136.graph_c0	361.428755	351.449907	357.726262	60.1964355	65.6799714	62.4208397	0	-3.59795	down	PREDICTED: uncharacterized protein LOC105171465 [Sesamum indicum]
c37142.graph_c0	0.88301914	0.49105742	0.50431758	0	0	0	2.928E-16	-Inf	down	predicted protein [Hordeum vulgare subsp. vulgare]
c37145.graph_c0	0.84300135	0.25315365	0.37915154	8.11812297	8.98950478	8.54912506	5.486E-24	3.03873	up	PREDICTED: ACT domain-containing protein DS12, chloroplastic-like isoform X1 [Sesamum indicum]
c37146.graph_c0	4.97232749	4.26087604	3.43685057	17.5491181	22.7913916	19.9942959	1.636E-08	1.16512	up	PREDICTED: arogenate dehydratase/prephenate dehydratase 6, chloroplastic-like [Sesamum indicum]
c37147.graph_c0	7.1172503	7.96253917	8.37738132	8.25905491	5.84797727	7.16496271	1.127E-10	-1.23308	down	conserved hypothetical protein [Ricinus communis]
c37156.graph_c0	1.58653351	0.68062379	1.45625598	32.5059705	28.0112528	33.9622495	4.099E-48	3.5747	up	hypothetical protein MIMGU_mgv1a016103mg [Erythranthe guttata]
c37157.graph_c0	28.7792422	27.8442218	28.0461821	16.4326101	16.9667224	14.9527998	9.979E-89	-1.89912	down	PREDICTED: probable protein phosphatase 2C 8 isoform X1 [Sesamum indicum]
c37160.graph_c0	0.23686964	0.23710683	0.25365572	1.72155488	1.83937646	1.73255549	6.959E-05	1.77127	up	hypothetical protein MIMGU_mgv1a007966mg [Erythranthe guttata]
c37161.graph_c0	0.06087761	0	0	1.2388739	1.23843558	1.66078233	3.615E-10	5.02949	up	hypothetical protein MIMGU_mgv1a014011mg [Erythranthe guttata]
c37167.graph_c0	0.56353449	0.28204939	0.99572568	3.73735929	3.7442916	4.09405694	7.71E-05	1.55144	up	-
c37173.graph_c0	1.89470668	1.97788697	1.80868859	49.1414374	43.7484316	63.1021676	5.118E-22	3.69093	up	PREDICTED: vignain-like [Sesamum indicum]
c37180.graph_c1	3.05762296	1.70038039	2.61944437	14.3212488	18.9502296	17.7305162	7.191E-05	1.69963	up	PREDICTED: LOW QUALITY PROTEIN: protein transport protein SEC31 homolog B-like [Sesamum indicum]
c37182.graph_c0	326.859368	311.578992	348.117387	124.445863	123.159833	124.692672	6.507E-76	-2.49745	down	hypothetical protein CISIN_1g033114mg [Citrus sinensis]
c37183.graph_c0	0.03642495	0	0.02340375	5.12261187	4.69694735	4.27721898	6.849E-55	6.79255	up	hypothetical protein MIMGU_mgv1a024814mg, partial [Erythranthe guttata]

#ID	0 h-1 FPKM	0 h-2 FPKM	0 h-3 FPKM	28 h-1 FPKM	28 h-2 FPKM	28 h-3 FPKM	FDR	log2FC	regulated	nr_annotation
c37190.graph_c0	25.8032818	26.6355598	32.1821264	17.6829938	17.8391172	16.6816047	1.065E-13	-1.79066	down	PREDICTED: zinc finger CCCH domain-containing protein 30-like [Sesamum indicum]
c37191.graph_c0	13.4873583	11.6429468	12.9590903	1.96350458	1.39392852	1.54105089	1.19E-143	-4.04949	down	PREDICTED: kinesin-3-like [Sesamum indicum]
c37195.graph_c0	10.7141459	10.3123793	10.061305	6.24669782	6.25828463	6.59850057	4.317E-25	-1.79208	down	PREDICTED: ethylene-responsive transcription factor ERF069-like [Sesamum indicum]
c37196.graph_c0	2.91575648	3.82447223	1.67962803	13.5187475	12.6350266	11.9554222	4.45E-05	1.09423	up	PREDICTED: protein IWS1 homolog A-like isoform X2 [Sesamum indicum]
c37196.graph_c1	2.9323532	2.88906448	2.46267533	12.0489299	12.7730397	11.1874705	6.432E-13	1.0311	up	hypothetical protein MIMGU_mgv1a000837mg [Erythranthe guttata]
c37209.graph_c0	2.32529435	2.58888656	2.896623	12.0715038	13.1883177	11.5951497	5.663E-16	1.14464	up	hypothetical protein MIMGU_mgv1a002141mg [Erythranthe guttata]
c37212.graph_c1	5.43275581	6.3861383	4.67555377	46.621228	58.7754407	47.3240938	1.709E-22	2.12193	up	Pollen Ole e 1 allergen and extensin family protein [Theobroma cacao]
c37217.graph_c0	12.9364216	13.1049235	11.8812845	12.7337977	14.3526618	13.1553778	3.78E-17	-1.004	down	PREDICTED: IAA-amino acid hydrolase ILR1-like 4 isoform X2 [Sesamum indicum]
c37229.graph_c1	27.085447	30.0051519	25.7718636	18.4003439	18.4534719	17.8281561	2.573E-59	-1.68941	down	PREDICTED: NAC domain-containing protein 100-like [Sesamum indicum]
c37234.graph_c0	22.8842419	19.0063006	21.309534	105.939469	115.023495	113.933324	1.617E-39	1.3157	up	PREDICTED: 60S ribosomal protein L21-1-like [Sesamum indicum]
c37236.graph_c0	0	0	0	9.15873645	11.4394478	22.54492	2.273E-06	Inf	up	-
c37239.graph_c0	4.89723339	4.87428418	4.7198541	25.8250105	26.9877512	24.2578751	1.667E-30	1.32047	up	BnaC09g00400D [Brassica napus]
c37240.graph_c0	1.34908544	1.10035554	1.6052113	10.6766375	10.6236764	11.2901043	3.807E-15	1.91346	up	PREDICTED: uncharacterized protein LOC105173599 [Sesamum indicum]
c37244.graph_c0	0.32655398	0.14009185	0.05994783	1.22059311	0.94901431	1.68765652	0.0081468	1.80201	up	PREDICTED: F-box/kelch-repeat protein At1g57790-like [Sesamum indicum]
c37245.graph_c0	0.27362275	0.06847418	0.08790402	2.03839121	1.88536581	2.3124074	2.083E-07	2.78176	up	PREDICTED: secretory carrier-associated membrane protein 2 [Sesamum indicum]
c37251.graph_c0	19.3012341	22.3646369	19.4594707	12.1786718	12.2179987	12.4773304	1.093E-45	-1.81927	down	PREDICTED: uncharacterized protein LOC105176792 [Sesamum indicum]
c37255.graph_c0	0.07245576	0.07252831	0	1.84311502	1.37887547	1.54693729	1.305E-08	3.96944	up	PREDICTED: probable WRKY transcription factor 57 isoform X1 [Nicotiana tomentosiformis]
c37256.graph_c0	6.79662951	3.25381689	5.69604653	0	0	0	4.895E-16	-Inf	down	predicted protein [Hordeum vulgare subsp. vulgare]
c37260.graph_c0	2.69893719	2.2403842	1.35345996	0.33490254	0.25918765	0.11711903	3.633E-11	-4.2313	down	PREDICTED: uncharacterized protein LOC105156178 [Sesamum indicum]
c37266.graph_c0	2.61480383	2.7076781	2.08559573	12.2544605	12.8988721	12.2454256	1.003E-05	1.24808	up	PREDICTED: protein TRANSPORT INHIBITOR RESPONSE 1-like [Sesamum indicum]
c37269.graph_c0	1.99189318	1.46489713	1.72385373	13.4133511	13.6848947	13.524777	2.769E-21	1.8817	up	PREDICTED: GPN-loop GTPase 2 isoform X1 [Sesamum indicum]
c37276.graph_c1	0.06086077	0.09138256	0.03910425	1.34911424	1.39784684	1.28133608	1.822E-09	3.31098	up	PREDICTED: AT-rich interactive domain-containing protein 2 isoform X1 [Nicotiana tomentosiformis]

#ID	0 h-1 FPKM	0 h-2 FPKM	0 h-3 FPKM	28 h-1 FPKM	28 h-2 FPKM	28 h-3 FPKM	FDR	log2FC	regulated	nr_annotation
c37277.graph_c0	7.38132822	5.73881126	6.53840999	67.9182299	61.6529858	62.5608138	5.97E-105	2.19922	up	hypothetical protein MIMGU_mgv1a004994mg [Erythranthe guttata]
c37279.graph_c0	7.88963908	7.81352299	9.49136038	9.51623934	8.20672122	7.01852228	2.568E-07	-1.12024	down	-
c37282.graph_c0	33.0854992	31.7422707	30.20305	17.3321095	20.4427399	18.5258723	9.696E-82	-1.84494	down	hypothetical protein MIMGU_mgv1a011134mg [Erythranthe guttata]
c37288.graph_c0	6.72958949	5.9675081	7.00282908	123.445265	114.699602	111.814135	4.75E-222	3.05924	up	monodehydroascorbate reductase [Acanthus ebracteatus]
c37289.graph_c0	6.93863421	6.50274307	7.77942656	5.26295423	5.82152591	5.37161178	1.746E-17	-1.45942	down	PREDICTED: uncharacterized protein LOC105168518 isoform X1 [Sesamum indicum]
c37290.graph_c0	85.4188277	79.5799022	85.7165954	59.6017354	63.7519682	68.2085045	2.7E-62	-1.47861	down	hypothetical protein MIMGU_mgv1a012378mg [Erythranthe guttata]
c37293.graph_c0	3.2043211	1.98112131	2.66438084	20.0009875	16.0181002	16.2088573	1.8E-10	1.64463	up	PREDICTED: cytochrome P450 734A1 [Sesamum indicum]
c37293.graph_c1	4.99997804	5.30329511	4.04232533	26.0634007	22.8591996	24.6451895	1.22E-23	1.27094	up	PREDICTED: BTB/POZ domain-containing protein At3g50780-like [Sesamum indicum]
c37295.graph_c0	2.73084264	1.98259443	1.85102817	15.7472222	16.1284753	16.1776879	6.068E-30	1.78577	up	PREDICTED: ATP-dependent 6-phosphofructokinase 5, chloroplastic-like [Sesamum indicum]
c37299.graph_c0	1.65082269	2.06559468	1.25956493	11.39818	11.6794845	11.7484064	7.62E-13	1.72053	up	PREDICTED: peptide methionine sulfoxide reductase A5 isoform X1 [Sesamum indicum]
c37303.graph_c0	21.0586751	20.390881	23.9659924	21.507481	21.6772898	20.3252664	2.687E-13	-1.13537	down	PREDICTED: uncharacterized protein LOC105163935 [Sesamum indicum]
c37304.graph_c0	0	0.22251673	0.19043784	9.42445554	6.85614492	7.95394546	7.191E-22	4.77088	up	PREDICTED: uncharacterized protein LOC105169888 [Sesamum indicum]
c37307.graph_c0	0	0.03710522	0.04763398	0.72740393	0.70542774	0.79140756	7.936E-08	3.60552	up	PREDICTED: ferric reduction oxidase 7, chloroplastic [Sesamum indicum]
c37312.graph_c0	0.97957256	0.58833207	0.75527371	5.98036061	4.75688291	3.36947232	0.001366	1.51224	up	hypothetical protein JCGZ_14002 [Jatropha curcas]
c37319.graph_c0	0.31052036	0.36411666	0.39903095	3.85597926	4.33160748	3.66208615	4.499E-38	2.37018	up	PREDICTED: sporulation-specific protein 15 [Sesamum indicum]
c37329.graph_c0	0.47381605	0.62022604	0.23418168	3.0728112	2.39177622	2.01023416	0.0011341	1.40909	up	PREDICTED: E3 ubiquitin-protein ligase RGLG2-like [Sesamum indicum]
c37333.graph_c0	0.39916062	0.44656741	0.39224609	2.0136883	2.44990936	2.26977943	4.662E-05	1.35327	up	PREDICTED: uncharacterized protein LOC105172792, partial [Sesamum indicum]
c37339.graph_c0	0.44937542	0.2249127	0.3849768	2.66726163	2.45743589	2.4873871	0.0001681	1.75518	up	PREDICTED: uncharacterized protein LOC105170136 [Sesamum indicum]
c37340.graph_c0	2.01715435	1.72126327	1.97596277	34.7767747	34.709445	34.4178473	9.11E-187	3.09375	up	PREDICTED: probable glucan 1,3-alpha-glucosidase [Sesamum indicum]
c37349.graph_c0	0.17787149	0.1780496	0	9.37250784	9.86318479	9.93346614	9.415E-61	5.28652	up	PREDICTED: uncharacterized protein LOC105169807 [Sesamum indicum]
c37350.graph_c0	0.07641185	0.03824418	0.14728837	2.27696331	3.18411362	2.76431687	2.854E-14	3.86985	up	PREDICTED: OTU domain-containing protein DDB_G0284757-like [Sesamum indicum]
c37352.graph_c0	14.5202732	14.5348131	14.3065191	8.69684729	8.67229778	7.72223883	4.701E-68	-1.88013	down	hypothetical protein MIMGU_mgv1a003689mg [Erythranthe guttata]
c37358.graph_c0	1381.26568	1564.69499	1419.82621	121.604139	125.879831	130.62801	3.97E-222	-4.61966	down	hypothetical protein MIMGU_mgv1a011603mg [Erythranthe guttata]

#ID	0 h-1 FPKM	0 h-2 FPKM	0 h-3 FPKM	28 h-1 FPKM	28 h-2 FPKM	28 h-3 FPKM	FDR	log2FC	regulated	nr_annotation
c37359.graph_c0	0.45962675	0.5981131	0.53157472	0.4676757	0.57307707	0.49065371	0.0028439	-1.14561	down	PREDICTED: oxysterol-binding protein-related protein 1C-like isoform X1 [Sesamum indicum]
c37361.graph_c0	19.3089128	21.3015505	17.2130108	13.3968286	11.841775	13.1799962	8.372E-32	-1.67832	down	PREDICTED: uncharacterized protein LOC105159352 [Sesamum indicum]
c37362.graph_c0	0.30367901	0.87817338	0.30351965	2.72211653	3.23280303	3.24178763	0.0001281	1.54344	up	PREDICTED: uncharacterized protein LOC105174401 isoform X2 [Sesamum indicum]
c37364.graph_c0	8.08760645	7.47295843	7.46156527	3.59212685	3.22067296	3.30010164	3.498E-45	-2.27657	down	PREDICTED: uncharacterized protein LOC105156132 [Sesamum indicum]
c37366.graph_c0	0.23917368	0.50542781	0.71714451	5.77502059	5.47588919	6.20961083	1.65E-21	2.47683	up	hypothetical protein MIMGU_mgv1a005273mg [Erythranthe guttata]
c37371.graph_c0	0	0	0	0.65932618	0.75558541	1.40708941	2.111E-05	Inf	up	-
c37372.graph_c0	6.05579039	6.27681372	6.07100954	36.365463	34.1592614	31.4059609	4.626E-29	1.37856	up	PREDICTED: interactor of constitutive active ROPs 1-like [Sesamum indicum]
c37374.graph_c0	1.58615318	2.64623578	1.92503243	1.02471732	0.86739719	0.41808048	7.864E-08	-2.50866	down	-
c37384.graph_c0	9.63052192	9.59047385	8.10155385	2.59771837	1.88942744	1.44258516	5.676E-66	-3.29399	down	hypothetical protein MIMGU_mgv1a022389mg [Erythranthe guttata]
c37384.graph_c1	14.7307393	15.3613949	11.3019379	6.839352	8.81166895	7.55561959	1.173E-17	-1.92237	down	PREDICTED: ankyrin repeat-containing protein At5g02620-like [Sesamum indicum]
c37387.graph_c0	0.04412318	0	0	15.1363192	12.1031166	11.9324311	9.022E-53	8.73477	up	hypothetical protein MIMGU_mgv1a011856mg [Erythranthe guttata]
c37388.graph_c0	4.53429769	4.52103874	4.93560174	4.18722534	4.23575277	4.68220157	3.498E-18	-1.18556	down	PREDICTED: uncharacterized protein LOC105155378 [Sesamum indicum]
c37395.graph_c0	3.85326308	2.62985559	3.03847912	14.5118341	18.3899307	16.3080832	1.406E-07	1.28117	up	hypothetical protein MIMGU_mgv1a013530mg [Erythranthe guttata]
c37400.graph_c0	1.36499	1.58373179	1.47501021	7.55323639	6.53485502	6.01619403	7.196E-07	1.09246	up	PREDICTED: aspartic proteinase-like protein 2 [Sesamum indicum]
c37416.graph_c0	12.3354427	12.3477947	13.6618311	8.42687045	9.60200064	9.49055345	6.721E-33	-1.57039	down	PREDICTED: rho GTPase-activating protein 2 [Sesamum indicum]
c37417.graph_c0	1.66005351	1.89091798	2.05967467	11.1914883	12.4338668	12.4930706	1.7E-11	1.59363	up	PREDICTED: uncharacterized protein LOC105162643 [Sesamum indicum]
c37423.graph_c0	0.86393071	0.95679536	1.0865642	11.7697824	12.351939	10.2363218	1.283E-49	2.46875	up	PREDICTED: vacuolar protein sorting-associated protein 18 homolog [Sesamum indicum]
c37427.graph_c0	21.3741468	19.7314514	25.0251471	166.047685	158.497115	170.916246	1.566E-68	1.81269	up	hypothetical protein MIMGU_mgv1a021808mg [Erythranthe guttata]
c37428.graph_c0	0.9061252	0.73696395	0.83691736	0.10290103	0.13007397	0.25189919	3.471E-16	-3.44083	down	-
c37430.graph_c0	6.22482527	6.54261141	4.89947749	28.9546696	27.1645766	29.025861	1.67E-11	1.18172	up	-
c37432.graph_c0	2.55203528	2.69919023	2.75351421	20.6828203	21.5816993	20.2895387	3.739E-50	1.87455	up	PREDICTED: uncharacterized protein LOC105168937 [Sesamum indicum]
c37434.graph_c0	8.20562597	7.76743817	5.38689184	5.96380166	5.56034354	6.50618321	6.023E-07	-1.32925	down	PREDICTED: uncharacterized protein LOC105168847 isoform X2 [Sesamum indicum]
c37435.graph_c1	1.38635072	1.51389702	0.97173576	12.3658983	13.3982961	12.6317761	1.398E-09	2.22369	up	PREDICTED: auxin efflux carrier component 2 [Sesamum indicum]

#ID	0 h-1 FPKM	0 h-2 FPKM	0 h-3 FPKM	28 h-1 FPKM	28 h-2 FPKM	28 h-3 FPKM	FDR	log2FC	regulated	nr_annotation
c37435.graph_c3	1.33209216	1.03232985	0.66262884	16.7709761	17.7086866	16.641139	6.987E-44	2.99519	up	PREDICTED: auxin efflux carrier component 2 [Sesamum indicum]
c37436.graph_c0	3.64172577	1.6004074	1.99745931	0	0	0	7.316E-13	-Inf	down	--
c37439.graph_c1	0.83341551	1.48973224	1.22396792	9.1290897	9.53185661	9.63814551	2.046E-11	1.90282	up	hypothetical protein MIMGU_mgv1a015229mg [Erythranthe guttata]
c37444.graph_c1	11.7362554	16.7731668	12.2919015	8.18470465	7.52357551	8.40866447	6.271E-09	-1.84817	down	PREDICTED: E3 ubiquitin-protein ligase RNF170-like [Sesamum indicum]
c37447.graph_c0	18.5128669	21.2036997	16.6371415	18.261324	17.2325253	16.8465115	3.867E-15	-1.19557	down	PREDICTED: uncharacterized protein LOC105157015 isoform X1 [Sesamum indicum]
c37456.graph_c0	8.64740309	3.82864287	6.62461375	0	0.21825615	0	9.91E-13	-7.54995	down	hypothetical protein TSTA_040370 [Talaromyces stipitatus ATCC 10500]
c37460.graph_c0	1.10707358	1.07023071	0.69182856	14.2350669	13.2858401	13.2795808	1.24E-141	2.74495	up	PREDICTED: acetyl-CoA carboxylase 1-like isoform X1 [Sesamum indicum]
c37470.graph_c1	2.20763437	2.20984498	1.37792168	12.3316973	9.10617334	8.75330875	6.704E-05	1.29572	up	-
c37473.graph_c0	1.60810108	2.53734163	2.06647356	11.4498724	13.2178221	12.834505	3.149E-19	1.50174	up	PREDICTED: uncharacterized protein LOC105162786 [Sesamum indicum]
c37475.graph_c0	0.35434448	0.1182331	0.75891114	4.80732981	5.58073039	4.34303563	2.302E-07	2.47405	up	PREDICTED: reticulon-like protein B8 [Sesamum indicum]
c37477.graph_c0	0.66450709	0.95618546	0.96065766	4.46737932	5.34182726	5.09855691	1.605E-06	1.43574	up	hypothetical protein MIMGU_mgv1a010755mg [Erythranthe guttata]
c37479.graph_c0	1.39530165	1.54118492	1.73118966	0.97912833	0.88406108	0.9987009	9.069E-07	-1.79847	down	hypothetical protein MIMGU_mgv1a022974mg [Erythranthe guttata]
c37480.graph_c0	6.91765299	6.92457997	5.15808055	27.8386183	27.2092842	29.3764312	3.164E-16	1.06515	up	PREDICTED: 60S ribosomal protein L11-like [Sesamum indicum]
c37481.graph_c0	0.97053277	0.85720995	0.917039	3.98330895	5.11385094	4.2491523	5.19E-06	1.19099	up	PREDICTED: uncharacterized protein LOC105162739 [Sesamum indicum]
c37483.graph_c0	59.6199521	57.8878846	61.5742993	360.961883	367.433401	373.015698	1.377E-82	1.52969	up	hypothetical protein MIMGU_mgv1a016379mg [Erythranthe guttata]
c37485.graph_c0	44.8184972	50.0323316	45.5885568	27.9899978	28.7902953	30.2502097	9.05E-72	-1.78051	down	PREDICTED: replication protein A 70 kDa DNA-binding subunit A [Sesamum indicum]
c37486.graph_c0	0.08022014	0.08030046	0.03436202	2.13779619	2.36891805	2.2201763	1.178E-15	4.02804	up	PREDICTED: nucleobase-ascorbate transporter 1 [Sesamum indicum]
c37489.graph_c0	0.14161268	0.14175448	0.36395579	2.6760052	2.50911187	2.68750572	3.621E-07	2.50024	up	PREDICTED: CASP-like protein 3A1 [Sesamum indicum]
c37490.graph_c0	0.76319402	0.51949161	0.54921134	3.21717311	3.78626395	3.15022438	1.197E-05	1.38291	up	PREDICTED: beta-hexosaminidase 3 [Sesamum indicum]
c37493.graph_c0	0	0.06917437	0.08880288	3.89245621	3.87731247	4.13932207	5.502E-24	5.12738	up	PREDICTED: serine/threonine-protein kinase-like protein CCR1 [Sesamum indicum]
c37495.graph_c0	0.73902028	0.69624499	0.81001292	8.89400376	8.47262556	8.98478118	2.304E-36	2.46113	up	PREDICTED: uncharacterized protein LOC105159731 [Sesamum indicum]
c37499.graph_c0	0.67590827	0.9533699	0.59220606	3.39406489	3.68952364	3.15216156	0.0003151	1.11547	up	PREDICTED: uncharacterized protein LOC105178641 [Sesamum indicum]
c37501.graph_c0	8.60699543	8.85493664	8.75609449	37.8806166	42.4787439	41.2964094	5.437E-19	1.12351	up	PREDICTED: DNA-directed RNA polymerases II and V subunit 8A-like [Sesamum indicum]

#ID	0 h-1 FPKM	0 h-2 FPKM	0 h-3 FPKM	28 h-1 FPKM	28 h-2 FPKM	28 h-3 FPKM	FDR	log2FC	regulated	nr_annotation
c37503.graph_c0	0.0873203	0	0.05610502	306.245018	241.871512	298.986579	2.92E-149	11.4415	up	PREDICTED: WAT1-related protein At2g39510-like [Sesamum indicum]
c37506.graph_c0	1.62265211	0.84744884	1.6318739	35.9440554	34.9541434	33.3885655	4.823E-67	3.57552	up	PREDICTED: cold-regulated 413 plasma membrane protein 2-like [Sesamum indicum]
c37508.graph_c0	0.1540825	0.27762622	0	1.92616391	1.63803231	1.55345804	2.513E-07	2.49328	up	PREDICTED: kinesin-4-like [Sesamum indicum]
c37508.graph_c1	0.2172957	0.29001772	0.23269465	2.10572352	2.78060871	2.1478165	3.717E-07	2.15738	up	PREDICTED: kinesin-4-like [Sesamum indicum]
c37510.graph_c0	1.72032766	2.30754741	1.5474824	8.52721121	8.263735	8.5701366	1.836E-07	1.09724	up	PREDICTED: laccase-4-like [Sesamum indicum]
c37512.graph_c1	6.17331981	6.14860395	6.3067035	54.7381458	55.2772522	52.0430724	4.396E-92	2.0298	up	PREDICTED: dolichyl-diphosphooligosaccharide--protein glycosyltransferase 48 kDa subunit-like [Sesamum indicum]
c37513.graph_c0	12.4690316	12.1179781	9.17833745	10.5581596	9.29470247	9.90767779	6.078E-09	-1.26876	down	-
c37514.graph_c0	1.46084451	2.05513462	1.57282525	14.0034023	14.2501386	12.3866889	5.001E-22	1.90648	up	PREDICTED: uncharacterized protein LOC105161074 [Sesamum indicum]
c37515.graph_c0	4.88266602	4.18374731	4.54267946	21.3205507	20.9806925	20.177272	1.41E-25	1.10858	up	PREDICTED: uncharacterized protein LOC105164119 [Sesamum indicum]
c37518.graph_c0	36.3781275	36.9126261	38.0088507	23.5061654	20.752222	20.8530147	3.067E-78	-1.86473	down	hypothetical protein MIMGU_mgv1a009711mg [Erythranthe guttata]
c37520.graph_c0	102.078254	107.261005	101.367731	63.1464418	61.725954	58.2597147	1.28E-107	-1.85329	down	PREDICTED: probable E3 ubiquitin ligase SUD1 [Sesamum indicum]
c37521.graph_c0	1.79678597	2.13581991	2.54945581	14.2832122	12.7981432	12.5651833	4.435E-17	1.51767	up	PREDICTED: inactive beta-amylase 9 [Sesamum indicum]
c37522.graph_c0	3.41678101	2.16012783	3.00416239	28.231163	29.3844131	26.1292482	2.174E-13	2.19636	up	PREDICTED: elongation factor Tu, mitochondrial [Sesamum indicum]
c37522.graph_c1	1.66852606	1.27720935	1.51349792	13.1967587	13.203591	14.2027089	1.652E-11	2.09715	up	hypothetical protein MIMGU_mgv1a006302mg [Erythranthe guttata]
c37524.graph_c0	24.907545	20.8541537	25.9619803	13.7372632	13.2027948	14.6221629	5.804E-20	-1.87838	down	PREDICTED: uncharacterized protein LOC105159531 [Sesamum indicum]
c37525.graph_c0	11.7932581	12.5444242	11.1050857	10.0027997	9.74248465	10.177201	2.519E-36	-1.33367	down	PREDICTED: probable protein phosphatase 2C 33 [Sesamum indicum]
c37526.graph_c0	12.0341671	11.0155251	12.3218866	11.973631	12.7114489	11.983988	6.999E-11	-1.03935	down	-
c37531.graph_c0	59.2718691	60.1059929	60.9333588	38.5781609	36.0354252	38.9688226	4.121E-95	-1.75736	down	PREDICTED: F-box/LRR-repeat protein 14 [Sesamum indicum]
c37532.graph_c0	22.8716531	19.0787963	20.393741	4.74942447	4.33933601	4.52140685	7.689E-92	-3.285	down	hypothetical protein JCGZ_04972 [Jatropha curcas]
c37536.graph_c0	3.09622239	2.86293377	2.89978033	18.212308	20.1288415	18.1756633	2.306E-33	1.58324	up	PREDICTED: lysM domain receptor-like kinase 3 [Sesamum indicum]
c37539.graph_c0	0.58002341	0.77413895	0.49690203	11.2883776	10.5729736	10.5489536	1.375E-20	3.0421	up	PREDICTED: growth-regulating factor 3 [Sesamum indicum]
c37539.graph_c1	15.4605972	13.0324873	14.116367	6.65329318	7.07526437	6.63548466	6.888E-15	-2.15515	down	-
c37540.graph_c0	1.722227	1.72395155	2.28690087	8.65762387	8.60814298	7.30287256	4.514E-08	1.00416	up	hypothetical protein MIMGU_mgv1a025916mg, partial [Erythranthe guttata]
c37544.graph_c0	379.406992	354.534787	356.753969	163.370728	188.030034	195.869964	5.16E-136	-2.08443	down	PREDICTED: uncharacterized GPI-anchored protein At3g06035 [Sesamum indicum]
c37545.graph_c0	3.09313898	3.61227567	3.09151582	15.4242964	14.2086103	16.5606577	2.578E-06	1.14783	up	Uncharacterized protein TCM_037774 [Theobroma cacao]

#ID	0 h-1 FPKM	0 h-2 FPKM	0 h-3 FPKM	28 h-1 FPKM	28 h-2 FPKM	28 h-3 FPKM	FDR	log2FC	regulated	nr_annotation
c37546.graph_c0	7.00358441	7.70395323	7.34331133	34.5264199	34.0655228	33.2168524	2.741E-34	1.11593	up	PREDICTED: calcium-transporting ATPase 4, endoplasmic reticulum-type-like [Sesamum indicum]
c37546.graph_c1	6.74675816	6.66580604	5.29198719	32.8598513	31.0493353	32.7372545	4.15E-13	1.28203	up	PREDICTED: calcium-transporting ATPase 4, endoplasmic reticulum-type-like [Sesamum indicum]
c37547.graph_c0	105.359496	113.329673	98.6421047	59.1594058	61.5936416	53.6472426	2.331E-94	-1.95366	down	PREDICTED: F-box protein SKIP2 [Sesamum indicum]
c37548.graph_c0	1.61628554	1.9985873	1.5271992	10.9524154	10.9808501	10.0929817	2.745E-11	1.54924	up	PREDICTED: protein-S-isoprenylcysteine O-methyltransferase A isoform X2 [Sesamum indicum]
c37552.graph_c0	1.18961132	1.16833456	1.70175776	8.71127954	9.8392005	7.93364504	2.271E-16	1.60918	up	PREDICTED: uncharacterized protein LOC105164201 isoform X1 [Sesamum indicum]
c37555.graph_c0	24.6451084	25.2972646	26.4976071	20.9811679	21.8018693	20.5641202	6.024E-48	-1.36272	down	hypothetical protein M569_08441 [Genlisea aurea]
c37557.graph_c0	0.37618014	0.25103788	0.32227092	2.41508242	2.30402279	2.9746227	2.859E-05	1.93022	up	PREDICTED: cancer-related nucleoside-triphosphatase homolog isoform X1 [Sesamum indicum]
c37559.graph_c0	4.8194351	4.69503976	3.76013584	101.610666	108.970018	103.201628	1.22E-232	3.47544	up	PREDICTED: betaine aldehyde dehydrogenase 1, chloroplastic [Sesamum indicum]
c37565.graph_c0	0.19413641	0.16194234	0.2910518	6.74915046	6.20001471	6.38994705	2.085E-30	3.80434	up	PREDICTED: uncharacterized protein LOC105172319 [Sesamum indicum]
c37571.graph_c0	12.9196786	11.1763345	11.3244073	59.8754087	58.3271689	57.1111784	6.822E-36	1.21797	up	hypothetical protein MIMGU_mgv1a014386mg [Erythranthe guttata]
c37573.graph_c0	0.61506506	0.41045397	0.50057587	14.9455606	15.7950872	15.247359	2.59E-106	3.82453	up	PREDICTED: putative potassium transporter 12 [Sesamum indicum]
c37575.graph_c0	23.6741513	22.1975614	23.4185448	14.557219	15.0885504	14.2017619	1.469E-70	-1.75098	down	hypothetical protein MIMGU_mgv1a005560mg [Erythranthe guttata]
c37577.graph_c0	0.9835976	1.8707068	1.39035805	0	0	0.02916653	7.572E-16	-8.2634	down	PREDICTED: glycerol-3-phosphate acyltransferase 5-like [Sesamum indicum]
c37578.graph_c0	18.8518484	20.0154901	16.5788799	10.766325	11.4095482	10.8517236	1.649E-52	-1.83629	down	PREDICTED: lysine-specific demethylase JMJ25-like [Sesamum indicum]
c37579.graph_c0	0	0	0	1.27083966	1.45432497	1.56755025	4.935E-20	Inf	up	PREDICTED: protein STICHEL-like 2 [Sesamum indicum]
c37580.graph_c0	50.9575476	48.1747642	44.4817762	2.89925558	3.03997466	2.48024156	0	-5.18212	down	PREDICTED: protein PLANT CADMIUM RESISTANCE 2-like [Sesamum indicum]
c37584.graph_c0	32.6052918	35.8628143	28.2658262	10.1329334	10.2790559	12.6496498	2.566E-46	-2.63626	down	PREDICTED: LOW QUALITY PROTEIN: ethylene-responsive transcription factor ERF061 [Sesamum indicum]
c37587.graph_c0	0.38234673	0.51941873	0.42114059	5.91503177	6.0934379	6.08996049	8.353E-23	2.68331	up	PREDICTED: G-type lectin S-receptor-like serine/threonine-protein kinase At1g11300 [Sesamum indicum]
c37588.graph_c0	12.5258256	13.3281868	11.0265183	5.37619953	6.73116303	7.19457326	2.706E-22	-2.02218	down	PREDICTED: CBL-interacting serine/threonine-protein kinase 1-like [Sesamum indicum]
c37590.graph_c0	0.03021021	0.03024046	0.03882132	1.339353	1.28860921	1.79164117	1.277E-12	4.38395	up	PREDICTED: LOW QUALITY PROTEIN: interactor of constitutive active ROPs 3 [Sesamum indicum]
c37595.graph_c0	1488.66418	1562.82425	1488.39846	87.6148833	101.769178	98.0701905	0	-5.07162	down	hypothetical protein CISIN_1g036208mg, partial [Citrus sinensis]
c37599.graph_c0	2.03544939	2.95635453	2.7182069	0	0.02619053	0.04733882	1.838E-37	-7.79958	down	-

#ID	0 h-1 FPKM	0 h-2 FPKM	0 h-3 FPKM	28 h-1 FPKM	28 h-2 FPKM	28 h-3 FPKM	FDR	log2FC	regulated	nr_annotation
c37600.graph_c0	7.88081605	7.91111859	7.13504499	115.563495	104.754379	112.19733	1.43E-177	2.76925	up	PREDICTED: alpha-L-arabinofuranosidase 1-like [Sesamum indicum]
c37601.graph_c0	10.6611181	13.0718757	11.7192419	10.0823064	10.255348	10.4615952	5.71E-17	-1.29401	down	PREDICTED: UDP-glycosyltransferase 74F2-like [Sesamum indicum]
c37602.graph_c0	1.18989297	0.9966217	1.09218541	7.81856445	8.0155612	7.50320531	1.794E-18	1.74129	up	PREDICTED: probable serine/threonine-protein kinase At1g54610 isoform X1 [Sesamum indicum]
c37606.graph_c0	0.13301875	0.16643994	0.21366796	1.54683623	2.20408526	2.22858199	3.342E-08	2.44692	up	PREDICTED: aspartic proteinase-like protein 1 [Sesamum indicum]
c37607.graph_c0	4.21568496	3.49731963	4.41548854	21.258676	20.5588662	19.1449565	5.685E-23	1.2374	up	PREDICTED: protein IQ-DOMAIN 1 [Sesamum indicum]
c37612.graph_c0	25.1190175	27.3367703	21.4284893	26.5500327	24.8068119	25.8550567	1.29E-13	-1.02463	down	hypothetical protein MIMGU_mgv1a010621mg [Erythranthe guttata]
c37614.graph_c0	0.61995837	0.84221458	0.62595582	6.27596591	6.94522821	6.43424897	2.26E-12	2.1447	up	PREDICTED: 3-phosphoinositide-dependent protein kinase 2-like [Sesamum indicum]
c37617.graph_c0	7.18120744	6.93349059	6.47932945	0.33314427	0.36764205	0.33225256	1.115E-84	-5.40689	down	PREDICTED: uncharacterized protein LOC104880778 [Vitis vinifera]
c37620.graph_c0	0.67753259	0.64251572	0.59571214	9.6671153	8.65829633	10.1934434	1.354E-28	2.80755	up	-
c37621.graph_c0	2.97544777	2.52227171	2.41125922	1.40272754	0.8443543	1.2082039	1.061E-21	-2.28208	down	PREDICTED: polyadenylate-binding protein-interacting protein 4-like [Sesamum indicum]
c37623.graph_c0	14.4927563	11.0531571	11.9090746	58.5419503	59.7158318	59.6975743	8.005E-18	1.15972	up	ATP binding protein, putative [Ricinus communis]
c37626.graph_c0	4.24527231	2.66343362	3.41919385	14.3625033	14.4197814	15.2480022	1.805E-10	1.00353	up	PREDICTED: uncharacterized protein LOC105173972 [Sesamum indicum]
c37627.graph_c0	19.1109789	16.4950192	14.4977823	201.289552	202.251144	186.148011	1.16E-191	2.46927	up	PREDICTED: low-temperature-induced cysteine proteinase [Sesamum indicum]
c37630.graph_c0	0.03845902	0.11549258	0.09884272	5.19903325	6.73850519	5.49683253	1.776E-29	5.00977	up	hypothetical protein MIMGU_mgv1a004581mg [Erythranthe guttata]
c37632.graph_c0	17.6676907	18.5985369	16.1246383	10.0128283	8.47865914	8.64515184	4.61E-103	-2.03845	down	PREDICTED: uncharacterized protein LOC105164604 [Sesamum indicum]
c37634.graph_c0	0.88851321	0.64683849	0.72658387	13.942681	12.7479518	11.880102	1.868E-36	3.00279	up	PREDICTED: ureidoglycolate hydrolase [Sesamum indicum]
c37635.graph_c0	138.873848	133.309605	138.847793	124.259251	125.428611	122.981287	8.818E-52	-1.2321	down	PREDICTED: uncharacterized protein LOC105176574 [Sesamum indicum]
c37636.graph_c0	2.26648961	2.11229301	1.22527077	34.5131799	30.4441433	29.5715236	2.567E-74	2.99257	up	PREDICTED: LRR receptor-like serine/threonine-protein kinase RCH1 [Sesamum indicum]
c37639.graph_c0	1.59698029	1.51216972	2.1631117	1.4743723	0.79306721	0.89590748	3.388E-06	-1.83247	down	PREDICTED: LOW QUALITY PROTEIN: transcription factor GAMYB [Sesamum indicum]
c37640.graph_c0	3.72685691	4.61882422	3.02173107	22.572151	21.8654036	21.8129925	1.226E-20	1.45561	up	hypothetical protein MIMGU_mgv1a011727mg [Erythranthe guttata]
c37641.graph_c0	64.0167555	57.8258215	59.7119228	36.8640787	38.2578971	38.2022055	2.947E-83	-1.7698	down	PREDICTED: probable prefoldin subunit 2 [Sesamum indicum]
c37644.graph_c0	28.9225562	32.2920775	24.5054702	27.8351652	29.4082055	27.3313177	3.062E-12	-1.10781	down	PREDICTED: uncharacterized protein LOC102585852 isoform X1 [Solanum tuberosum]
c37646.graph_c0	0.90462727	0.70430354	0.60276837	5.94165203	6.66196997	6.29886735	1.341E-13	2.00938	up	PREDICTED: trehalose-phosphate phosphatase A-like isoform X2 [Sesamum indicum]

#ID	0 h-1 FPKM	0 h-2 FPKM	0 h-3 FPKM	28 h-1 FPKM	28 h-2 FPKM	28 h-3 FPKM	FDR	log2FC	regulated	nr_annotation
c37647.graph_c0	3.54267988	3.2608987	2.61637006	20.8055623	19.5604062	18.8125385	1.867E-22	1.56415	up	PREDICTED: acetyl-coenzyme A carboxylase carboxyl transferase subunit alpha, chloroplastic-like [Sesamum indicum]
c37648.graph_c0	0.12406632	0.28386413	0.09110294	3.76140588	4.48949309	3.69994973	2.66E-30	3.4961	up	PREDICTED: probable LRR receptor-like serine/threonine-protein kinase At2g24230 [Sesamum indicum]
c37651.graph_c0	3.97891729	3.7173748	3.54505927	31.0006765	29.6966054	29.134792	4.011E-35	1.90906	up	PREDICTED: ras-related protein RABA1d [Sesamum indicum]
c37654.graph_c0	0.37448132	0.56228446	0.42107041	14.3910345	16.4341817	14.5468428	4.032E-49	3.97132	up	Alpha/beta-Hydrolases superfamily protein isoform 1 [Theobroma cacao]
c37656.graph_c0	55.7857726	72.2102272	37.5517346	225.41597	310.837173	242.891946	0.0001568	1.14864	up	PREDICTED: cytochrome P450 CYP736A12-like [Sesamum indicum]
c37664.graph_c0	17.9047204	9.02770482	10.0554682	0	0	0	7.515E-18	-Inf	down	metallothionein-like protein type 2 [Narcissus pseudonarcissus]
c37666.graph_c0	0.56297104	0.81968693	0.52613823	7.66252777	8.22836561	7.37559272	2.962E-15	2.51852	up	PREDICTED: beta-galactosidase 9-like [Sesamum indicum]
c37666.graph_c1	0.33227813	0.20468361	0.3612997	4.82995664	5.63575125	4.72944621	1.145E-23	2.98661	up	PREDICTED: beta-galactosidase 9-like [Sesamum indicum]
c37669.graph_c0	1.57992616	1.42721474	1.93123022	12.8271415	12.5932089	13.5520475	3.98E-22	1.887	up	PREDICTED: alkylated DNA repair protein alkB homolog 8 [Sesamum indicum]
c37670.graph_c0	22.5102761	22.7994773	20.0261049	19.4028365	19.8165083	18.484481	1.268E-50	-1.26849	down	PREDICTED: cationic amino acid transporter 1-like isoform X1 [Sesamum indicum]
c37671.graph_c0	81.6068808	78.2675227	84.3015049	396.035436	436.790453	427.471173	7.519E-59	1.2767	up	BnaC02g12080D [Brassica napus]
c37673.graph_c0	106.354669	106.088201	105.920549	45.4941675	39.3110928	44.4148613	7.21E-183	-2.39099	down	PREDICTED: ethylene receptor 1 [Sesamum indicum]
c37673.graph_c1	0.2788139	0.31397973	0.31350104	7.87764308	8.78233078	7.77158441	1.489E-33	3.66045	up	PREDICTED: ethylene receptor 1 [Sesamum indicum]
c37674.graph_c0	6820.08107	6352.57917	6186.29338	6416.47393	7280.45621	6194.2988	3.131E-14	-1.05091	down	2S albumin precursor isoform 3 [Sesamum indicum]
c37675.graph_c0	1.92646068	1.24412241	2.23600615	9.89131526	9.33873532	8.95575111	8.089E-07	1.28793	up	hypothetical protein EUGRSUZ_B02864 [Eucalyptus grandis]
c37682.graph_c0	1.43952262	1.274699	1.42295659	7.12245785	6.46724448	5.45067794	0.0001864	1.11068	up	PREDICTED: calcineurin B-like protein 7 [Sesamum indicum]
c37684.graph_c0	0.70571057	0.57797774	0.61831775	8.57952487	8.58851813	7.95202091	5.132E-26	2.63358	up	PREDICTED: uncharacterized protein LOC105162014 [Sesamum indicum]
c37685.graph_c0	240.100628	228.699912	242.721351	161.895813	154.635085	158.112975	1.8E-90	-1.67487	down	PREDICTED: protein DEHYDRATION-INDUCED 19 homolog 3-like isoform X2 [Sesamum indicum]
c37693.graph_c0	1.55631132	1.3052422	1.24319454	5.74730276	6.23817164	6.31120896	7.309E-05	1.06837	up	PREDICTED: uncharacterized protein LOC105162119 isoform X1 [Sesamum indicum]
c37694.graph_c0	8.1426415	8.06705408	8.09855266	82.6091423	74.771304	79.3818082	7.88E-130	2.19355	up	PREDICTED: transcription factor DIVARICATA [Sesamum indicum]
c37697.graph_c0	0.90584938	0.90675645	0.6008012	112.155853	101.803135	94.2736856	6.73E-189	5.91084	up	PREDICTED: granule-bound starch synthase 1, chloroplastic/amyloplastic [Sesamum indicum]
c37698.graph_c0	67.4279551	57.5471615	61.5964174	344.099434	371.693639	382.114236	1.011E-56	1.46744	up	PREDICTED: 40S ribosomal protein S8 [Sesamum indicum]
c37704.graph_c1	0	0	0	0.97285601	0.79706463	1.55828354	5.403E-08	Inf	up	--
c37707.graph_c0	1.07914519	1.02878647	0.79242531	17.8338633	18.0413277	18.8646399	3.47E-82	3.1518	up	Dihydrolipoyllysine-residue acetyltransferase component of pyruvate dehydrogenase, putative [Ricinus communis]

#ID	0 h-1 FPKM	0 h-2 FPKM	0 h-3 FPKM	28 h-1 FPKM	28 h-2 FPKM	28 h-3 FPKM	FDR	log2FC	regulated	nr_annotation
c37710.graph_c0	502.178741	501.604659	521.849556	151.874762	149.782524	164.886342	1.33E-208	-2.79995	down	PREDICTED: uncharacterized protein LOC105175543 [Sesamum indicum]
c37714.graph_c0	10.2166985	9.04119804	11.4164017	10.7615037	10.0081277	11.5913149	9.958E-05	-1.01463	down	hypothetical protein glysoja_022734 [Glycine soja]
c37716.graph_c0	41.9987933	60.4758733	39.2509907	210.733181	245.393793	195.931907	1.779E-07	1.11243	up	PREDICTED: cytochrome P450 71D95-like [Sesamum indicum]
c37716.graph_c1	95.8591727	124.788746	82.9068767	484.752196	569.972101	473.165319	3.533E-12	1.24286	up	PREDICTED: cytochrome P450 71D95-like [Sesamum indicum]
c37717.graph_c0	18.8792366	18.2802185	17.5839529	6.87935409	7.02159588	8.60329394	9.347E-74	-2.37114	down	PREDICTED: uncharacterized protein LOC105174387 [Sesamum indicum]
c37721.graph_c0	47.8418295	55.7522298	57.6247598	39.6495086	36.2683724	31.2526339	1.044E-18	-1.68248	down	-
c37727.graph_c0	0.60325684	0.84540528	0.15504178	5.48054493	4.79006884	5.08028278	6.089E-08	2.18098	up	PREDICTED: mitochondrial inner membrane protease subunit 2 [Sesamum indicum]
c37732.graph_c0	0	0.16106347	0	2.06599126	1.79500408	1.04966898	1.442E-05	3.84611	up	PREDICTED: expansin-A2-like [Sesamum indicum]
c37733.graph_c0	0.2075223	0.15107644	0.24243124	1.19289662	1.29991666	0.99577078	0.0002046	1.4423	up	PREDICTED: protein argonaute 16 isoform X1 [Sesamum indicum]
c37734.graph_c0	3.19433115	2.1604931	2.36675574	0.64838077	0.83092871	1.33121698	2.881E-12	-2.54263	down	Adenosylhomocysteinase [Rozella allomycis CSF55]
c37737.graph_c0	0.19271342	0.11574383	0.34670212	3.69766323	3.21217945	3.22298548	3.829E-12	2.84985	up	PREDICTED: glucose-6-phosphate 1-dehydrogenase, chloroplastic [Sesamum indicum]
c37742.graph_c0	1.47448983	0.95223633	0.97794984	0	0	0	1.029E-24	-Inf	down	hypothetical protein WALSEDRAFT_61591 [Wallemia sebi CBS 633.66]
c37744.graph_c0	10.9755882	12.8017524	10.3020999	2.94801961	3.69518431	3.48098672	1.662E-50	-2.84035	down	PREDICTED: uncharacterized protein LOC105167616 isoform X1 [Sesamum indicum]
c37745.graph_c0	37.2538958	40.8591036	34.67465	13.7202555	14.8830512	14.1237007	2.459E-91	-2.48987	down	PREDICTED: U-box domain-containing protein 14 [Sesamum indicum]
c37751.graph_c0	0.15614381	0.10420011	0.10032549	2.00489149	2.22009222	1.9292159	1.631E-11	3.0067	up	PREDICTED: proline-rich receptor-like protein kinase PERK10 [Sesamum indicum]
c37753.graph_c0	0.32946337	0.17357541	0.1559797	1.62575358	1.66134764	1.70709979	3.24E-07	1.83998	up	PREDICTED: BTB/POZ domain-containing protein At5g67385 [Sesamum indicum]
c37755.graph_c0	0.59490187	0.92302125	0.80269609	10.0310131	10.2672755	11.5722143	3.762E-36	2.68737	up	PREDICTED: uncharacterized protein LOC105157935 [Sesamum indicum]
c37758.graph_c0	0	0	0	22.8939848	20.9992348	18.1306086	1.046E-66	Inf	up	PREDICTED: non-specific phospholipase C6-like [Eucalyptus grandis]
c37758.graph_c1	0.11457534	0	0	14.572722	13.3833639	13.0123945	6.118E-52	7.42292	up	PREDICTED: non-specific phospholipase C6 [Sesamum indicum]
c37760.graph_c0	0.15844967	0.23791251	0.35632468	2.64868714	3.43130578	2.96005099	1.467E-09	2.4857	up	-
c37761.graph_c0	10.7545561	8.95094452	11.1026087	68.8102219	67.1645366	68.6539104	1.586E-50	1.64017	up	unnamed protein product [Triticum aestivum]
c37763.graph_c0	0	0	0.03116116	1.6390474	1.84590441	1.25116224	1.651E-17	6.11602	up	PREDICTED: probable membrane-associated kinase regulator 4 [Sesamum indicum]
c37764.graph_c0	1.16043173	2.01750489	1.49120071	62.8551289	59.0368083	71.0660675	1.896E-74	4.27803	up	PREDICTED: acid phosphatase 1-like [Camelina sativa]
c37766.graph_c0	0.29313528	0.44014322	0	2.91167015	2.98163121	3.07128416	6.976E-08	2.53938	up	PREDICTED: indole-3-acetate O-methyltransferase 1 [Sesamum indicum]
c37767.graph_c0	47.2597556	45.3227218	47.7897285	27.3747585	28.9837474	29.4150651	2.27E-93	-1.80132	down	PREDICTED: U-box domain-containing protein 4-like [Sesamum indicum]

#ID	0 h-1 FPKM	0 h-2 FPKM	0 h-3 FPKM	28 h-1 FPKM	28 h-2 FPKM	28 h-3 FPKM	FDR	log2FC	regulated	nr_annotation
c37769.graph_c0	2.49950625	3.12751141	0.80299112	14.5330139	11.8917662	18.1588191	0.0003927	1.71634	up	-
c37772.graph_c0	21.0443411	18.507643	18.8649166	11.0550808	11.8008705	11.0080172	4.866E-88	-1.87631	down	PREDICTED: histone-lysine N-methyltransferase setd3 [Sesamum indicum]
c37775.graph_c0	191.417992	192.68639	195.677962	114.223743	116.636876	120.6778	6.55E-98	-1.81246	down	PREDICTED: acetate/butyrate--CoA ligase AAE7, peroxisomal [Sesamum indicum]
c37776.graph_c0	1.28799006	1.38845516	0.9548759	14.5456122	16.6117044	14.2194177	1.064E-28	2.55622	up	PREDICTED: GDSL esterase/lipase At2g04570-like [Sesamum indicum]
c37780.graph_c0	12.3177879	12.3113263	11.1236244	11.5181519	10.7941169	12.2495139	1.083E-32	-1.13757	down	PREDICTED: AP-3 complex subunit delta isoform X1 [Sesamum indicum]
c37786.graph_c0	3.80051131	3.62940583	3.36814311	20.5418017	18.6046542	20.0262465	1.339E-17	1.36528	up	PREDICTED: THO complex subunit 7A [Sesamum indicum]
c37787.graph_c0	0	0	0	0.88948176	0.94726813	1.09802003	2.567E-14	Inf	up	--
c37793.graph_c0	35.8461888	33.6303314	34.9560058	214.084106	211.996184	204.448366	5.403E-80	1.50341	up	PREDICTED: DEAD-box ATP-dependent RNA helicase 7-like [Sesamum indicum]
c37795.graph_c0	0	0	0	0.77541738	0.80784137	0.82742171	6.334E-10	Inf	up	PREDICTED: putative late blight resistance protein homolog R1B-16 [Sesamum indicum]
c37801.graph_c0	3.46379579	2.88188198	3.52620777	12.8815835	14.7599773	14.9932284	9.191E-09	1.01958	up	PREDICTED: telomere repeat-binding factor 1 isoform X2 [Sesamum indicum]
c37803.graph_c0	0.46527115	0.36985001	0.29894593	10.0054509	10.596522	10.6396462	7.5E-104	3.6986	up	PREDICTED: LOW QUALITY PROTEIN: LRR receptor-like serine/threonine-protein kinase RCH1 [Sesamum indicum]
c37804.graph_c0	147.625928	129.726671	142.60595	22.6303323	23.9128143	23.1204454	1.07E-163	-3.6823	down	PREDICTED: SPX domain-containing protein 1-like [Sesamum indicum]
c37805.graph_c0	140.224353	147.734754	138.478325	64.6186621	66.8364037	65.2653307	3.79E-158	-2.20648	down	hypothetical protein MIMGU_mgv1a026895mg [Erythranthe guttata]
c37807.graph_c0	28.2497598	28.5007095	29.5847495	25.6647033	24.924467	27.2413347	1.095E-32	-1.24037	down	PREDICTED: pollen-specific leucine-rich repeat extensin-like protein 1 [Nicotiana sylvestris]
c37812.graph_c0	0.22038068	0.11030068	0.2831979	2.162314	3.7239589	3.75758361	1.241E-06	2.87812	up	PREDICTED: AMSH-like ubiquitin thioesterase 2 isoform X2 [Sesamum indicum]
c37826.graph_c0	1.23976729	2.1510818	1.27452004	16.9999179	18.6036073	17.401014	1.195E-36	2.41674	up	PREDICTED: UDP-N-acetylglucosamine--dolichyl-phosphate N-acetylglucosaminephosphotransferase-like [Sesamum indicum]
c37828.graph_c0	47.1714843	44.257978	50.2413035	14.5042784	14.5350021	14.4867381	1.068E-75	-2.79421	down	PREDICTED: uncharacterized protein LOC105157890 [Sesamum indicum]
c37829.graph_c0	0.31868284	0.27912671	0.23035504	6.19575078	6.84895185	5.75261017	3.418E-41	3.4171	up	PREDICTED: uncharacterized aarF domain-containing protein kinase At1g79600, chloroplastic [Sesamum indicum]
c37835.graph_c0	105.53838	105.748638	106.434692	76.643942	76.1129248	76.4071256	4.447E-79	-1.56203	down	PREDICTED: DEAD-box ATP-dependent RNA helicase 38 [Sesamum indicum]
c37837.graph_c1	0.89538416	0.30729626	0.49311604	12.3644409	12.27193	12.7140135	1.368E-61	3.37717	up	PREDICTED: serine/threonine protein phosphatase 2A 57 kDa regulatory subunit B' iota isoform [Sesamum indicum]
c37839.graph_c0	1.66893868	1.5099743	1.79408412	1.14299568	1.18997987	1.03736317	1.574E-13	-1.65366	down	PREDICTED: histidine kinase 1 [Sesamum indicum]

#ID	0 h-1 FPKM	0 h-2 FPKM	0 h-3 FPKM	28 h-1 FPKM	28 h-2 FPKM	28 h-3 FPKM	FDR	log2FC	regulated	nr_annotation
c37843.graph_c0	29.2098478	30.6463264	32.5176544	23.0459811	22.9609491	22.5108166	3.642E-21	-1.52296	down	PREDICTED: coiled-coil domain-containing protein 150 [Sesamum indicum]
c37845.graph_c0	22.0777098	21.0817161	18.3654852	15.6253673	14.7411563	15.3189262	1.165E-49	-1.51761	down	PREDICTED: retinoblastoma-related protein [Sesamum indicum]
c37847.graph_c0	0.77583503	1.1036064	0.94450616	0.92000238	0.58951192	0.65384805	0.0001172	-1.47662	down	PREDICTED: probable LRR receptor-like serine/threonine-protein kinase At1g56140 isoform X1 [Sesamum indicum]
c37849.graph_c0	905.230238	914.777562	981.434342	290.771407	293.751266	317.859847	8.16E-102	-2.72557	down	hypothetical protein MIMGU_mgv1a008920mg [Erythranthe guttata]
c37850.graph_c0	7.21689876	7.37006732	7.02575806	31.4712026	35.6868632	31.8625253	4.733E-13	1.10535	up	PREDICTED: GRF1-interacting factor 2-like [Sesamum indicum]
c37851.graph_c0	4.12253059	3.58839886	3.12592191	2.43792747	2.04999108	2.03488606	8.111E-22	-1.81997	down	PREDICTED: clathrin interactor EPSIN 1-like isoform X3 [Sesamum indicum]
c37854.graph_c0	0.50872867	0.44932773	0.36532333	6.8184603	5.84222658	6.30919846	1.356E-42	2.75495	up	PREDICTED: uncharacterized protein LOC105166467 isoform X1 [Sesamum indicum]
c37856.graph_c0	40.3831501	40.113489	40.3208857	36.2495576	35.1694513	34.8810531	1.365E-54	-1.27532	down	PREDICTED: VHS domain-containing protein At3g16270 [Sesamum indicum]
c37858.graph_c0	8.86561839	9.212572	8.46312675	6.99577866	5.43000138	6.15916918	1.696E-13	-1.60374	down	PREDICTED: kxDL motif-containing protein 1 [Sesamum indicum]
c37865.graph_c0	11.1452614	12.7590016	10.7613585	49.3179202	49.863295	49.7377436	6.188E-18	1.01326	up	hypothetical protein MIMGU_mgv1a009799mg [Erythranthe guttata]
c37867.graph_c0	5.67617404	5.09910322	3.86525401	1.0930575	0.98692844	1.00701322	3.565E-26	-3.33167	down	PREDICTED: uncharacterized protein LOC105158642 [Sesamum indicum]
c37868.graph_c0	0.06329101	0	0	8.44091541	8.65968697	9.36504443	4.929E-61	7.64944	up	Nucleobase-ascorbate transporter 7 [Theobroma cacao]
c37870.graph_c0	4.93389022	4.29678277	4.69178076	4.5720518	4.53285313	4.79877754	3.792E-12	-1.09194	down	PREDICTED: putative pentatricopeptide repeat-containing protein At5g43820 [Sesamum indicum]
c37874.graph_c0	4.31565832	3.51626263	3.67569922	18.6372134	17.2228263	18.4815116	2.9E-11	1.15075	up	PREDICTED: aspartic proteinase-like protein 1 isoform X1 [Sesamum indicum]
c37877.graph_c0	43.0877936	24.3011224	26.8181863	0	0	0	9.466E-34	-Inf	down	hypothetical protein SELMODRAFT_140819 [Selaginella moellendorffii]
c37878.graph_c0	21.499304	24.4615671	21.5642882	24.9750315	22.4916918	23.9959772	6.818E-23	-1.00829	down	hypothetical protein MIMGU_mgv1a013275mg [Erythranthe guttata]
c37880.graph_c0	64.6268924	64.6916065	71.5000047	305.354266	315.188482	329.601433	7.168E-48	1.1506	up	elongation factor-like protein [Solanum tuberosum]
c37881.graph_c1	157.250435	162.222933	179.707888	69.4758397	73.9791291	68.7250925	5.303E-44	-2.3268	down	heat shock protein 70 [Saussurea medusa]
c37881.graph_c2	21.9345614	24.5703976	30.8712351	5.69354029	8.56788844	9.91121575	4.727E-13	-2.77206	down	70 kD heatshockprotein [Medicago sativa]
c37887.graph_c0	18.7813448	21.029819	18.0197621	17.3110686	15.9126466	16.2272812	1.511E-35	-1.31555	down	PREDICTED: zinc finger Ran-binding domain-containing protein 2 isoform X1 [Sesamum indicum]
c37888.graph_c0	17.7056823	19.0277322	12.410456	5.0693649	5.5831307	4.81841097	2.406E-15	-2.75399	down	PREDICTED: myb-related protein 306-like [Sesamum indicum]
c37890.graph_c0	39.8098354	39.8496989	36.5630472	22.6551083	26.8774951	22.7138897	4.013E-71	-1.77588	down	PREDICTED: probable acetyltransferase NATA1-like [Sesamum indicum]
c37891.graph_c0	0.02624142	0.07880308	0.10116378	0.74381232	0.56827082	0.96488657	2.224E-05	2.36427	up	PREDICTED: receptor-like serine/threonine-protein kinase NCRK isoform X1 [Sesamum indicum]

#ID	0 h-1 FPKM	0 h-2 FPKM	0 h-3 FPKM	28 h-1 FPKM	28 h-2 FPKM	28 h-3 FPKM	FDR	log2FC	regulated	nr_annotation
c37892.graph_c0	2.56655709	2.47031452	2.9175743	1.14791317	1.03645796	0.58542986	5.566E-11	-2.61694	down	PREDICTED: serine/threonine-protein kinase BLUS1-like isoform X2 [Sesamum indicum]
c37893.graph_c0	0.28549307	0.50805146	0.7337398	4.51877386	5.32901762	4.87248053	1.003E-13	2.16755	up	PREDICTED: riboflavin biosynthesis protein PYRD, chloroplastic [Sesamum indicum]
c37897.graph_c0	5.40577053	7.24894405	5.63594441	5.63386977	5.68924704	7.19823441	6.879E-05	-1.07089	down	hypothetical protein MIMGU_mgv11b019635mg [Erythranthe guttata]
c37897.graph_c1	8.00774592	8.6694105	6.7129662	5.64513429	6.20213461	6.40001953	1.979E-19	-1.446	down	PREDICTED: CBL-interacting serine/threonine-protein kinase 11-like [Sesamum indicum]
c37899.graph_c0	0.11160091	0.13405519	0.02868232	1.08688604	1.27429833	1.20458212	7.427E-08	2.62251	up	PREDICTED: G-type lectin S-receptor-like serine/threonine-protein kinase At1g34300 [Sesamum indicum]
c37902.graph_c0	18.9312674	21.0384032	18.831976	20.3923283	20.8080208	19.4545414	2.943E-20	-1.04569	down	PREDICTED: small nuclear ribonucleoprotein-associated protein N-like [Sesamum indicum]
c37904.graph_c0	13.9875966	14.6051205	12.3962865	7.62457847	6.80514983	10.2262978	2.098E-17	-1.8203	down	PREDICTED: uncharacterized protein LOC105164967 [Sesamum indicum]
c37905.graph_c0	2.95536001	2.23872816	3.38718656	11.1460599	12.7894723	13.5005299	3.083E-05	1.03233	up	PREDICTED: uncharacterized protein LOC104093968 [Nicotiana tomentosiformis]
c37906.graph_c0	7.41778373	6.69800008	6.43665513	35.5708204	29.9843385	32.4496008	2.459E-20	1.16471	up	PREDICTED: protein TRANSPORT INHIBITOR RESPONSE 1-like [Sesamum indicum]
c37908.graph_c0	0.64828552	0.58404121	0.54149716	3.9107125	3.47782891	3.35450964	9.616E-11	1.50967	up	PREDICTED: pentatricopeptide repeat-containing protein ELI1, chloroplastic [Sesamum indicum]
c37916.graph_c0	92.6675273	94.2689492	97.8823277	61.9622068	64.4194995	65.4655396	1.495E-87	-1.66119	down	PREDICTED: protein DJ-1 homolog B [Sesamum indicum]
c37916.graph_c1	0	0	0	4.10915413	3.56177368	3.15185404	4.025E-14	Inf	up	PREDICTED: probable calcium-binding protein CML47 [Sesamum indicum]
c37918.graph_c0	32.7384782	34.5541533	33.4600101	20.4346024	19.0905936	19.3152216	6.081E-86	-1.86666	down	hypothetical protein MIMGU_mgv1a008971mg [Erythranthe guttata]
c37919.graph_c0	0.03302333	0.09916919	0.29705399	10.2004949	10.4886678	9.96863991	6.58E-58	5.04439	up	PREDICTED: uncharacterized protein LOC104231484 isoform X1 [Nicotiana sylvestris]
c37920.graph_c0	0.46892763	0.19558216	0.35151116	3.06732508	3.97475535	3.70801809	1.282E-09	2.31508	up	PREDICTED: squamosa promoter-binding-like protein 16 isoform X1 [Nicotiana tomentosiformis]
c37921.graph_c0	13.9914072	15.0682232	14.1592449	14.6959952	13.362005	14.3705846	1.403E-30	-1.11691	down	PREDICTED: uncharacterized protein LOC105178567 [Sesamum indicum]
c37925.graph_c0	19.3854976	17.7188587	19.4015609	18.1847265	19.2929485	18.8524537	4.337E-29	-1.09534	down	PREDICTED: transcription factor RF2b-like [Sesamum indicum]
c37926.graph_c0	1.5109383	1.47727799	1.62553858	9.75558037	10.8144701	9.94017293	1.255E-15	1.63341	up	PREDICTED: uncharacterized protein C630.12 isoform X1 [Sesamum indicum]
c37927.graph_c0	7.06482747	7.18460145	7.19776103	38.4384701	35.1681006	37.3080589	4.253E-37	1.27999	up	PREDICTED: sphingosine-1-phosphate lyase [Sesamum indicum]
c37931.graph_c0	0.58076952	0.89438626	0.74631182	0.7467922	0.60099167	0.86107525	0.0015524	-1.0991	down	hypothetical protein MIMGU_mgv1a001348mg [Erythranthe guttata]
c37932.graph_c0	43.620691	45.3524261	41.5206447	33.2720883	33.1106567	32.4703571	1.037E-74	-1.49051	down	PREDICTED: serine/threonine-protein kinase WNK8-like isoform X1 [Sesamum indicum]

#ID	0 h-1 FPKM	0 h-2 FPKM	0 h-3 FPKM	28 h-1 FPKM	28 h-2 FPKM	28 h-3 FPKM	FDR	log2FC	regulated	nr_annotation
c37933.graph_c0	119.012437	121.993927	117.457654	51.7050412	52.9612092	54.1201248	7.04E-165	-2.2649	down	PREDICTED: protein PPLZ12 [Sesamum indicum]
c37935.graph_c0	1.1858099	1.02659227	1.11197141	1.05983003	1.00950556	0.95034357	1.598E-06	-1.22913	down	PREDICTED: uncharacterized protein LOC105178399 [Sesamum indicum]
c37936.graph_c0	0.11044925	0.38695946	0.21289748	2.64903792	2.06567384	1.83407917	1.133E-05	2.11091	up	-
c37937.graph_c0	1.72103201	0.77934171	1.05314013	0	0	0	1.355E-12	-Inf	down	Mitochondrial uncoupling protein 3 [Aegilops tauschii]
c37938.graph_c1	0	0	0	2.01864819	2.08302863	1.83545192	8.377E-21	Inf	up	PREDICTED: chromosome-associated kinesin KIF4A [Sesamum indicum]
c37943.graph_c1	4.81558472	5.28390745	3.86763564	2.82687398	4.34516083	3.15799048	2.079E-11	-1.52371	down	hypothetical protein MIMGU_mgv1a008064mg [Erythranthe guttata]
c37947.graph_c0	0.46530971	0.98330414	0.5979414	5.29822251	4.93081797	5.63154083	1.177E-17	1.8638	up	PREDICTED: pentatricopeptide repeat-containing protein At5g57250, mitochondrial [Sesamum indicum]
c37953.graph_c0	127.815646	127.108393	128.669186	57.1156788	61.0528293	64.1731232	7.96E-152	-2.16325	down	PREDICTED: uncharacterized protein LOC105167724 [Sesamum indicum]
c37954.graph_c0	1.97482734	2.56012428	1.66408611	12.611756	15.8061595	12.4990663	1.398E-12	1.63407	up	PREDICTED: O-acyltransferase WSD1-like isoform X1 [Sesamum indicum]
c37955.graph_c0	1.8305107	1.79641537	1.93717071	11.0997484	11.0937183	10.7069893	2.129E-26	1.47234	up	hypothetical protein MIMGU_mgv1a024346mg [Erythranthe guttata]
c37960.graph_c0	1043.70309	1022.36532	1039.80801	81.7585336	60.2133103	89.6728932	0	-4.83416	down	Gibberellin 2-beta-dioxygenase 1 GN=GA2OX1 OS=Pisum sativum (Garden pea) PE=1 SV=1
c37966.graph_c0	4.23292668	2.93342214	3.48684511	2.28762909	2.27918851	2.28508194	2.964E-10	-1.72552	down	-
c37969.graph_c0	0.21115021	0.04696926	0.09044546	1.31295915	1.5241873	1.32181266	5.63E-08	2.49547	up	PREDICTED: uncharacterized protein At5g41620 [Sesamum indicum]
c37971.graph_c0	46.3680145	48.7252638	44.4978457	36.6241883	39.8203554	39.2730088	7.652E-47	-1.36041	down	PREDICTED: protein vip1-like [Sesamum indicum]
c37976.graph_c0	2.67979769	2.10471594	1.85427099	18.9072987	17.8561044	16.7974237	3.134E-27	1.9255	up	PREDICTED: ninja-family protein AFP1-like [Sesamum indicum]
c37977.graph_c0	11.6354543	10.7115949	11.6493645	94.4148412	96.1337029	107.110188	2.537E-57	2.03996	up	PREDICTED: glutathione S-transferase L3-like [Malus domestica]
c37980.graph_c0	0.95162421	1.25339095	1.22287479	17.6912951	17.5512184	17.8221634	1.88E-38	2.85947	up	PREDICTED: methionine adenosyltransferase 2 subunit beta [Sesamum indicum]
c37983.graph_c0	0.83883291	0.77249904	0.51740808	5.90149839	5.85694598	6.42739901	4.836E-13	2.01044	up	PREDICTED: cytochrome P450 71D11-like [Sesamum indicum]
c37985.graph_c0	4.30828312	3.27757388	4.5397778	2.31711415	1.69632716	2.14625247	2.871E-10	-2.0686	down	PREDICTED: uncharacterized protein LOC100267301 [Vitis vinifera]
c37985.graph_c1	2.69168717	2.31716894	2.69796117	1.7215464	1.87233941	2.1071517	1.05E-06	-1.52521	down	hypothetical protein MIMGU_mgv1a007684mg [Erythranthe guttata]
c37986.graph_c0	1.58161942	1.93861613	1.74209449	1.26680901	0.72017652	0.4211394	3.03E-13	-2.22178	down	PREDICTED: tonoplast dicarboxylate transporter [Sesamum indicum]
c37987.graph_c0	5.0978445	5.50581363	4.82700253	0.68251547	1.58463638	1.35253713	6.161E-17	-3.18128	down	-
c37991.graph_c0	107.092761	106.998747	119.877889	64.8782372	66.0112815	72.613802	2.077E-33	-1.80626	down	hypothetical protein EUTSA_v10023737mg [Eutrema salsugineum]
c37996.graph_c0	0.07062959	0	0.09076183	2.15599366	2.17840539	2.76457221	2.223E-11	4.36077	up	unnamed protein product [Coffea canephora]
c37997.graph_c0	4.02702729	4.03105975	4.15268911	23.9565656	23.881674	24.5607624	1.786E-21	1.47699	up	PREDICTED: mitochondrial inner membrane protease subunit 2-like isoform X1 [Sesamum indicum]
c38000.graph_c0	0.06897801	0.20714124	0.17727894	1.17812123	0.97320254	0.87952136	0.000432	1.64269	up	hypothetical protein MIMGU_mgv1a004734mg [Erythranthe guttata]

#ID	0 h-1 FPKM	0 h-2 FPKM	0 h-3 FPKM	28 h-1 FPKM	28 h-2 FPKM	28 h-3 FPKM	FDR	log2FC	regulated	nr_annotation
c38007.graph_c0	1.53917311	1.40064942	1.43847155	19.118953	16.6198648	18.0904164	1.462E-13	2.53023	up	-
c38008.graph_c0	1.04353948	0.81750086	1.04947008	5.40799612	5.44861683	4.92412904	8.444E-06	1.34703	up	PREDICTED: uncharacterized protein LOC105167885 [Sesamum indicum]
c38010.graph_c0	17.3164442	19.0637367	14.7322648	10.9687784	9.92623605	9.97536991	3.505E-25	-1.81579	down	PREDICTED: tyrosine-sulfated glycopeptide receptor 1 [Sesamum indicum]
c38012.graph_c0	0.07426168	0.07433604	0.06361946	3.36431445	3.6711833	3.42055603	8.923E-26	4.53391	up	PREDICTED: uncharacterized protein LOC105173003 [Sesamum indicum]
c38014.graph_c0	0.12532598	0.06272574	0.08052442	3.37018934	3.74202451	4.60818683	4.126E-14	4.36285	up	acyl-CoA thioesterase, putative [Ricinus communis]
c38015.graph_c0	13.0393057	9.841621	13.9782875	55.2903178	59.851458	58.4322097	4.842E-19	1.14311	up	PREDICTED: protein yippee-like [Sesamum indicum]
c38018.graph_c0	5.59850988	6.80499792	6.42348825	5.9581269	5.77326142	6.8776441	0.0017803	-1.10773	down	-
c38021.graph_c0	36.3865117	37.6805789	37.6610791	28.5351586	27.2390991	26.7090626	1.872E-74	-1.52889	down	hypothetical protein MIMGU_mgv1a011020mg [Erythranthe guttata]
c38024.graph_c0	2.48134812	2.61882373	2.01022363	14.3294236	19.7346245	16.6352946	3.235E-12	1.74585	up	PREDICTED: lysine histidine transporter 1-like [Solanum tuberosum]
c38028.graph_c0	0.23139901	0.32428301	0.05947138	3.83449189	4.58587882	5.24230339	7.783E-15	3.39616	up	hypothetical protein MIMGU_mgv1a013394mg [Erythranthe guttata]
c38030.graph_c0	7.49861061	7.22017193	8.25943845	116.213631	127.612517	119.690849	7.87E-151	2.89152	up	PREDICTED: serine/threonine-protein kinase Nek6-like [Sesamum indicum]
c38031.graph_c0	20.1300671	17.3326082	18.304851	13.0805844	12.9020368	12.8741419	2.826E-46	-1.61097	down	PREDICTED: uncharacterized protein LOC105175857 [Sesamum indicum]
c38035.graph_c0	14.3065347	16.2534334	14.5040058	2.19471393	2.2739912	1.70279314	3.38E-133	-3.9599	down	hypothetical protein MIMGU_mgv1a008930mg [Erythranthe guttata]
c38036.graph_c0	7.77892878	6.96149534	6.68226342	3.50282385	2.99626249	3.4600179	1.965E-63	-2.19344	down	PREDICTED: pentatricopeptide repeat-containing protein At1g74900, mitochondrial [Sesamum indicum]
c38041.graph_c0	85.7662091	92.3659067	84.0673572	71.9825009	69.17831	68.7967996	1.361E-67	-1.41072	down	hypothetical protein MIMGU_mgv1a027042mg [Erythranthe guttata]
c38045.graph_c0	20.2820253	18.6030203	21.1835305	11.1692853	10.6418277	11.8429604	3.041E-47	-1.92679	down	PREDICTED: cyclin-dependent kinase inhibitor 3 isoform X2 [Sesamum indicum]
c38047.graph_c0	297.951936	281.049148	303.908687	54.6842802	61.2247245	62.8161566	2.14E-209	-3.39522	down	PREDICTED: uncharacterized protein C6C3.02c-like [Malus domestica]
c38050.graph_c0	8.38721558	12.3935256	7.18526939	1.54813459	1.6599117	1.5001274	7.465E-11	-3.65837	down	Ethylene-responsive transcription factor 1B [Theobroma cacao]
c38051.graph_c0	4.08551253	3.13167839	3.216244	16.8529368	16.3035234	15.650925	4.267E-13	1.13763	up	PREDICTED: cysteine desulfurase 2, chloroplastic isoform X1 [Sesamum indicum]
c38054.graph_c0	23.770779	21.510302	22.2377735	20.9621111	20.8403017	20.4883006	1.722E-24	-1.2063	down	PREDICTED: proline synthase co-transcribed bacterial homolog protein, partial [Sesamum indicum]
c38063.graph_c1	798.182712	762.336116	876.599721	259.154691	305.141838	323.965661	1.84E-49	-2.54755	down	PREDICTED: peroxygenase-like [Nicotiana tomentosiformis]
c38064.graph_c0	96.6655172	98.582478	91.1291209	89.0988569	88.1847708	81.6093026	1.326E-51	-1.23566	down	PREDICTED: ubiquitin-conjugating enzyme E2 32-like [Sesamum indicum]
c38065.graph_c1	0.45008328	0.09010679	0.23134994	2.5515199	1.94935505	2.0820194	3.282E-05	2.00951	up	PREDICTED: nudix hydrolase 16, mitochondrial-like [Sesamum indicum]
c38066.graph_c0	103.788976	96.7965648	98.691183	509.863547	511.491839	515.5839	3.148E-58	1.27048	up	RecName: Full=40S ribosomal protein S7 [Avicennia marina]

#ID	0 h-1 FPKM	0 h-2 FPKM	0 h-3 FPKM	28 h-1 FPKM	28 h-2 FPKM	28 h-3 FPKM	FDR	log2FC	regulated	nr_annotation
c38067.graph_c0	0.56454144	0.79533542	0.69858929	7.65903537	7.76609368	6.37371251	4.917E-29	2.31154	up	PREDICTED: LOW QUALITY PROTEIN: auxin response factor 19-like [Sesamum indicum]
c38068.graph_c0	4.17200056	3.57420656	2.60814449	2.29462722	2.244486	2.74172428	2.2E-07	-1.59209	down	hypothetical protein MIMGU_mgv1a007990mg [Erythranthe guttata]
c38071.graph_c0	1.1213697	1.06013189	1.32092076	22.1409702	22.7711853	19.6001317	4.503E-80	3.10949	up	PREDICTED: calcium-dependent protein kinase 26-like isoform X1 [Sesamum indicum]
c38072.graph_c0	0.08360128	0.20084399	0.21486191	7.42498562	7.65865461	7.81387459	1.567E-75	4.42026	up	Lysosomal alpha-mannosidase [Glycine soja]
c38075.graph_c0	18.112022	18.0124302	16.9270294	5.06033652	4.58444526	4.33844293	7.34E-187	-3.01377	down	PREDICTED: MAR-binding filament-like protein 1 [Sesamum indicum]
c38078.graph_c0	26.2758557	30.3710106	25.8837856	195.851199	224.515299	170.467178	6.571E-19	1.74873	up	PREDICTED: polyol transporter 5-like [Sesamum indicum]
c38081.graph_c0	0.17729124	0.22183597	0.39869597	9.18086565	8.75483413	7.91208747	7.006E-31	3.91612	up	hypothetical protein VITISV_034764 [Vitis vinifera]
c38083.graph_c0	2.24485664	2.56065399	2.91827302	26.2755253	22.6965668	22.6945417	1.484E-43	2.12001	up	PREDICTED: lysosomal beta glucosidase-like [Sesamum indicum]
c38085.graph_c0	0.07725753	0.07733489	0	2.97596721	2.99121024	2.52000135	1.23E-11	4.70762	up	PREDICTED: uncharacterized protein LOC105176978 [Sesamum indicum]
c38086.graph_c0	0.32431482	0.23188541	0.31752944	5.331539	5.9084041	5.33049792	2.188E-43	3.15376	up	PREDICTED: calcium-transporting ATPase, endoplasmic reticulum-type [Sesamum indicum]
c38087.graph_c0	0.34216005	0.30444682	0.26869896	4.57294805	4.32852972	4.74609232	9.097E-26	2.81092	up	hypothetical protein MIMGU_mgv1a023205mg [Erythranthe guttata]
c38089.graph_c0	46.9909455	41.5770028	43.1828002	21.2246836	18.2011916	19.439883	3.59E-130	-2.25199	down	RING/U-box superfamily protein isoform 1 [Theobroma cacao]
c38090.graph_c0	0.14321456	0.19114396	0.10224243	1.27218077	1.46193086	1.24570695	8.406E-08	2.10195	up	PREDICTED: DNA (cytosine-5)-methyltransferase CMT2 [Sesamum indicum]
c38091.graph_c0	2.70131736	1.73057428	2.29105784	18.4943038	18.932196	19.2565005	5.366E-24	1.98649	up	unnamed protein product [Coffea canephora]
c38093.graph_c0	10.6724823	10.4568309	11.15761	6.44200395	6.884867	7.99221194	1.295E-29	-1.68918	down	PREDICTED: transcription intermediary factor 1-alpha isoform X1 [Sesamum indicum]
c38094.graph_c0	23.2188981	23.9118373	22.9594751	18.5628975	22.5712264	17.5744547	8.253E-42	-1.34688	down	PREDICTED: beta-D-glucosyl crocetin beta-1,6-glucosyltransferase-like [Sesamum indicum]
c38097.graph_c0	0.99420141	0.89224554	0.68284885	16.6940862	18.6615149	16.3771832	1.11E-112	3.24514	up	PREDICTED: uncharacterized protein LOC105172430 [Sesamum indicum]
c38098.graph_c0	0.07345591	0.02450982	0.15732291	2.36683826	2.76368182	2.3088721	1.337E-17	3.75897	up	PREDICTED: pentatricopeptide repeat-containing protein At2g37310 [Sesamum indicum]
c38099.graph_c0	0.71092767	0.75908219	0.79176084	3.5135473	3.39011777	3.34486356	0.0023446	1.08798	up	-
c38100.graph_c0	0.48094641	0.44439509	0.42787051	2.90394622	2.96187891	1.98563402	3.21E-06	1.4456	up	PREDICTED: DNA-binding protein SMUBP-2 [Sesamum indicum]
c38101.graph_c0	2.70077739	3.28409535	3.21438664	15.4859063	14.9874309	15.0228255	3.664E-39	1.21389	up	PREDICTED: uncharacterized protein At3g06530 [Sesamum indicum]
c38102.graph_c0	0.38793186	0.20135127	0.24002227	8.91786774	8.77799829	7.69443471	2.011E-84	3.85022	up	PREDICTED: histidine kinase 1 [Sesamum indicum]
c38104.graph_c0	2.40196804	1.72434849	2.21363946	12.7491668	12.355146	12.6047264	5.763E-23	1.48211	up	PREDICTED: probable sulfate transporter 3.3 [Sesamum indicum]
c38107.graph_c0	21.9477474	24.4785408	22.3944194	21.1608431	21.0430138	21.2678134	4.474E-43	-1.20717	down	PREDICTED: uncharacterized aarF domain-containing protein kinase At1g79600, chloroplastic-like [Citrus sinensis]

#ID	0 h-1 FPKM	0 h-2 FPKM	0 h-3 FPKM	28 h-1 FPKM	28 h-2 FPKM	28 h-3 FPKM	FDR	log2FC	regulated	nr_annotation
c38114.graph_c0	33.3256603	33.6683882	35.7424856	14.225566	13.5879727	12.5549097	2.2E-109	-2.43955	down	PREDICTED: iron-sulfur assembly protein IscA-like 2, mitochondrial isoform X1 [Sesamum indicum]
c38115.graph_c0	25.058219	22.2678373	22.3434147	14.123723	10.0676823	13.4961973	3.677E-16	-1.97495	down	PREDICTED: pyrophosphate--fructose 6-phosphate 1-phosphotransferase subunit alpha 2 [Tarenaya hassleriana]
c38119.graph_c0	1.17797579	1.23530561	1.0812468	6.1316362	5.41849914	5.80177439	8.411E-17	1.22277	up	PREDICTED: uncharacterized protein LOC105168860 isoform X1 [Sesamum indicum]
c38120.graph_c0	0.39415893	0.49976792	0.77664841	4.6599517	4.89725348	4.92452656	2.635E-14	2.01676	up	PREDICTED: pentatricopeptide repeat-containing protein At1g73400, mitochondrial [Sesamum indicum]
c38123.graph_c0	1.30693137	1.04659205	1.59548563	9.49870188	10.6776643	11.4325232	7.389E-12	1.90721	up	-
c38138.graph_c0	6.80330923	5.78860345	6.19261954	29.13197	29.3169855	24.8473851	4.854E-13	1.05822	up	PREDICTED: xanthoxin dehydrogenase-like [Malus domestica]
c38142.graph_c0	2.88268274	1.79546535	1.72870201	16.295356	17.4456261	16.2221905	1.56E-16	1.87812	up	PREDICTED: uncharacterized protein LOC105179724 [Sesamum indicum]
c38148.graph_c0	11.0669437	7.9761784	10.4527737	63.2210524	60.3870874	63.8941274	1.872E-47	1.57788	up	PREDICTED: eukaryotic translation initiation factor 3 subunit K [Sesamum indicum]
c38149.graph_c0	43.1585196	42.2047732	39.7288489	14.748693	17.782815	17.5723037	5.06E-149	-2.4093	down	PREDICTED: homeobox-leucine zipper protein HAT4-like [Sesamum indicum]
c38152.graph_c0	0.23631917	0.2183592	0.30367948	2.86699411	3.03000565	2.7814585	1.29E-15	2.42184	up	PREDICTED: serine/threonine-protein kinase-like protein ACR4 [Sesamum indicum]
c38154.graph_c0	24.6763553	25.8760345	25.8480114	18.7295733	18.9242687	19.0644208	4.135E-64	-1.52074	down	squalene epoxidase [Withania somnifera]
c38155.graph_c0	1.89042535	2.28191328	2.2863731	13.0121171	12.1135889	11.3761942	6.199E-10	1.40578	up	PREDICTED: glucuronoxylan 4-O-methyltransferase 1-like [Sesamum indicum]
c38156.graph_c0	18.1328602	17.5141397	17.7145475	12.3824751	12.177619	12.1583385	5.208E-52	-1.62967	down	PREDICTED: mitogen-activated protein kinase 9-like [Sesamum indicum]
c38161.graph_c0	7.51549101	7.21734102	7.12882402	2.47834159	2.25997586	2.48512291	8.06E-105	-2.68729	down	PREDICTED: uncharacterized protein LOC105168894 [Sesamum indicum]
c38166.graph_c0	21.7951588	22.3415785	20.6392949	12.6366281	12.1278535	11.3260695	5.91E-114	-1.93398	down	hypothetical protein MIMGU_mgv1a001457mg [Erythranthe guttata]
c38167.graph_c0	3.88114464	3.30227637	4.05228243	0	0	0	1.799E-68	-Inf	down	-
c38168.graph_c0	17.9023545	16.7508193	18.3688627	17.881211	16.7764279	17.831085	1.21E-26	-1.10548	down	hypothetical protein MIMGU_mgv1a012288mg [Erythranthe guttata]
c38169.graph_c0	0.25023633	0.15655431	0.04019545	2.43251552	2.21685992	2.20751984	4.194E-10	2.86567	up	PREDICTED: nudix hydrolase 8-like isoform X3 [Sesamum indicum]
c38175.graph_c0	3.72770066	3.01951518	3.27753464	15.310948	14.7255887	15.5770137	7.066E-17	1.09674	up	PREDICTED: protein CASP isoform X1 [Sesamum indicum]
c38176.graph_c0	44.8262197	46.266063	38.3824138	30.3512071	31.2756462	29.3669804	4.746E-44	-1.59754	down	PREDICTED: probable protein phosphatase 2C 9 [Sesamum indicum]
c38180.graph_c0	1.50999059	1.42594586	1.09833831	1.40805221	0.95350434	1.53757762	0.0001019	-1.13415	down	PREDICTED: polyadenylate-binding protein 7-like [Sesamum indicum]
c38182.graph_c0	4.25202691	4.93729022	3.9340965	95.5535803	103.0735	91.6385512	5.92E-201	3.37759	up	PREDICTED: homeobox-leucine zipper protein ATHB-6 [Sesamum indicum]
c38184.graph_c0	1.26098834	1.61287632	1.44037329	6.36443793	8.20598875	7.58226089	2.285E-08	1.26877	up	hypothetical protein CICLE_v10008173mg [Citrus clementina]

#ID	0 h-1 FPKM	0 h-2 FPKM	0 h-3 FPKM	28 h-1 FPKM	28 h-2 FPKM	28 h-3 FPKM	FDR	log2FC	regulated	nr_annotation
c38185.graph_c0	3.73059573	4.04552563	4.26130063	2.25947956	1.97209492	1.96663133	4.121E-09	-2.04991	down	-
c38187.graph_c0	5.23422477	5.12832587	5.07521312	24.358436	23.5859006	24.7301245	2.306E-33	1.14497	up	PREDICTED: RRP12-like protein [Sesamum indicum]
c38190.graph_c0	311.498325	330.084368	288.486018	18.0466655	16.5457752	24.8334478	0	-5.05566	down	PREDICTED: early nodulin-like protein 2 [Sesamum indicum]
c38193.graph_c0	7.60540689	7.02989742	7.07000989	37.7991421	36.6563748	37.3119666	1.169E-33	1.27463	up	PREDICTED: dolichyl-diphosphooligosaccharide--protein glycosyltransferase subunit 1B [Sesamum indicum]
c38200.graph_c0	6.98927563	6.19585312	6.85029349	2.86274906	3.69256285	2.63456375	1.478E-43	-2.21631	down	hypothetical protein MIMGU_mgv1a005237mg [Erythranthe guttata]
c38202.graph_c0	0	0	0	1.37731133	1.9897328	3.66062077	2.089E-05	Inf	up	--
c38204.graph_c0	0	0.15686325	0.30206075	2.448705	2.67370822	2.09105925	4.656E-08	2.85957	up	PREDICTED: uncharacterized protein LOC105164617 isoform X2 [Sesamum indicum]
c38206.graph_c0	0.21455029	0	0.0689264	3.58647998	4.57579533	4.19894637	7.366E-16	4.36982	up	PREDICTED: putative UDP-glucuronate:xylan alpha-glucuronosyltransferase 4 [Sesamum indicum]
c38207.graph_c1	0.24440056	0.3844426	0.44866342	1.70016249	2.49738087	2.17415619	0.0001457	1.46681	up	PREDICTED: uncharacterized permease C29B12.14c [Sesamum indicum]
c38208.graph_c0	0.93758288	0.7647214	1.16020774	4.8204851	4.36383992	4.91169707	5.082E-10	1.20615	up	PREDICTED: pentatricopeptide repeat-containing protein At2g26790, mitochondrial isoform X1 [Sesamum indicum]
c38209.graph_c0	0.14603641	0.19491019	0.18766257	2.37042473	2.90693562	3.40658466	7.579E-10	2.94629	up	PREDICTED: T-complex protein 1 subunit epsilon [Sesamum indicum]
c38212.graph_c0	39.3199978	41.4080447	39.609296	16.2269987	14.203775	14.4916466	9.12E-127	-2.51218	down	hypothetical protein MIMGU_mgv1a000768mg [Erythranthe guttata]
c38212.graph_c1	17.5635205	20.3617931	19.6910158	3.25639426	4.0575018	2.81662259	1.577E-83	-3.60078	down	PREDICTED: uncharacterized protein At4g14450, chloroplastic-like [Sesamum indicum]
c38217.graph_c0	112.276987	111.548597	112.617847	19.5102677	20.7624667	20.6942092	0	-3.5548	down	PREDICTED: dnaJ protein homolog [Sesamum indicum]
c38218.graph_c0	3.28902837	2.64198666	2.33068002	18.7891091	16.7693978	18.0047837	4.46E-40	1.61032	up	PREDICTED: histidine kinase 4 [Sesamum indicum]
c38222.graph_c0	11.6019744	12.0298498	11.5424489	10.2154117	12.8529476	10.4812702	3.692E-19	-1.15926	down	PREDICTED: protein kinase APK1A, chloroplastic [Sesamum indicum]
c38225.graph_c0	10.3170679	11.0158921	8.41767662	81.6966949	76.1501942	72.2385798	2.211E-71	1.86305	up	PREDICTED: LOW QUALITY PROTEIN: transcription factor BIM2 [Sesamum indicum]
c38231.graph_c0	18.4526055	20.9119047	15.5823874	90.9569953	94.8834394	86.8833216	4.283E-34	1.22292	up	PREDICTED: protein BRASSINAZOLE-RESISTANT 1 [Sesamum indicum]
c38235.graph_c0	1.32644661	1.3769517	1.13635761	0.53558542	0.51582231	0.40789777	2.399E-10	-2.4853	down	PREDICTED: uncharacterized protein LOC105167746 [Sesamum indicum]
c38238.graph_c0	3.18338658	2.37298084	3.04632389	14.1773639	13.8231189	14.5611884	2.857E-14	1.21616	up	PREDICTED: probable glucuronosyltransferase Os03g0107900 [Sesamum indicum]
c38241.graph_c0	0	0	0	2.05867811	1.74261873	1.36488993	2.79E-14	Inf	up	PREDICTED: lipid phosphate phosphatase epsilon 2, chloroplastic isoform X1 [Sesamum indicum]
c38243.graph_c0	5.53297976	6.31585637	4.98954249	0.987697	1.210297	0.3454083	7.299E-29	-3.82028	down	-
c38246.graph_c0	1.68565245	1.30854968	1.19358191	26.0276022	27.5639688	24.3393899	2.663E-86	3.13081	up	PREDICTED: uncharacterized GPI-anchored protein At4g28100 [Nicotiana sylvestris]

#ID	0 h-1 FPKM	0 h-2 FPKM	0 h-3 FPKM	28 h-1 FPKM	28 h-2 FPKM	28 h-3 FPKM	FDR	log2FC	regulated	nr_annotation
c38247.graph_c0	42.1607284	40.9595316	41.9716771	42.2723931	42.6752848	40.4740306	6.784E-28	-1.08701	down	hypothetical protein EUGRSUZ_J00392, partial [Eucalyptus grandis]
c38248.graph_c0	8.74166794	8.40860806	9.30265059	8.58700891	6.81211629	6.88542171	4.271E-12	-1.33921	down	hypothetical protein MIMGU_mgv1a027006mg [Erythranthe guttata]
c38250.graph_c0	5.1895992	4.26520076	4.00130426	20.7248879	20.9710516	19.9566763	7.384E-11	1.10807	up	PREDICTED: exosome complex exonuclease RRP46 homolog [Sesamum indicum]
c38251.graph_c0	0.08556296	0.12847296	0.0549759	0.77733336	1.12297463	0.86264484	1.248E-05	2.27512	up	PREDICTED: transcription initiation factor IIB-2 [Sesamum indicum]
c38255.graph_c0	0.5989865	0.666207	0.14254099	15.930217	17.7027598	15.4855638	5.1E-108	4.04729	up	PREDICTED: probable protein S-acyltransferase 22 isoform X1 [Sesamum indicum]
c38256.graph_c0	4.09997867	4.97050195	5.09301375	33.010015	33.3325152	29.4214624	1.081E-35	1.66391	up	PREDICTED: cyclin-dependent kinase inhibitor 4-like [Musa acuminata subsp. malaccensis]
c38257.graph_c2	31.1715947	31.2581652	32.2396298	141.023099	171.266117	159.010728	7.258E-21	1.22459	up	PREDICTED: chaperonin CPN60-2, mitochondrial [Sesamum indicum]
c38259.graph_c0	6.63049026	6.39016673	7.68821323	41.7572136	51.5049652	41.5905754	3.523E-18	1.60963	up	PREDICTED: lysine histidine transporter 1-like [Sesamum indicum]
c38268.graph_c0	0	0	0.10204909	2.30868189	2.29297561	2.77870171	1.619E-12	5.04712	up	hypothetical protein MIMGU_mgv1a003097mg [Erythranthe guttata]
c38269.graph_c0	0.23295128	0.26233261	0.22451371	6.85695959	6.40138638	6.2859913	3.619E-32	3.67311	up	PREDICTED: uncharacterized protein LOC105168472 [Sesamum indicum]
c38278.graph_c0	44.7620074	45.1987262	44.0490864	36.8957642	36.482044	39.1360552	8.222E-53	-1.34222	down	PREDICTED: probable BOI-related E3 ubiquitin-protein ligase 2 [Sesamum indicum]
c38278.graph_c1	3.11786497	3.64115155	3.56140438	0.37767438	0.45467274	0.71908483	4.666E-16	-3.82298	down	zinc ion binding protein, partial [Genlisea aurea]
c38283.graph_c0	3.79713416	3.74585038	2.82867694	2.67974289	2.18482348	2.39878584	2.102E-15	-1.60066	down	hypothetical protein SORBIDRAFT_03g008610 [Sorghum bicolor]
c38284.graph_c0	3.53468035	2.68046955	2.89049358	15.9977721	14.4444883	16.6113599	4.933E-11	1.28145	up	PREDICTED: E3 ubiquitin ligase BIG BROTHER-like [Sesamum indicum]
c38288.graph_c0	4.53247844	4.92357902	4.20507037	3.14645816	3.18774016	3.07374865	2.243E-27	-1.62761	down	PREDICTED: G-type lectin S-receptor-like serine/threonine-protein kinase At4g27290 [Sesamum indicum]
c38291.graph_c0	0.26880006	0.18834845	0.06908374	1.26985515	1.09364198	1.37096098	0.0001489	1.7514	up	PREDICTED: two-component response regulator ARR9 [Sesamum indicum]
c38296.graph_c2	5.19854445	6.95127799	6.68033544	34.3709981	37.8057229	35.2018657	6.983E-35	1.4185	up	PREDICTED: LOW QUALITY PROTEIN: succinate dehydrogenase [ubiquinone] flavoprotein subunit 1, mitochondrial-like [Sesamum indicum]
c38298.graph_c0	1309.473	1284.57546	1277.70993	326.293903	321.72818	337.283245	5.69E-191	-3.06442	down	hypothetical protein ZEAMMB73_433257, partial [Zea mays]
c38303.graph_c0	2.5330782	2.06387242	2.34670416	1.88384121	1.66227444	1.81668978	1.03E-05	-1.46255	down	PREDICTED: uncharacterized protein LOC104427989 [Eucalyptus grandis]
c38304.graph_c1	4.56562355	4.32783648	4.26691419	21.7698549	23.6055235	23.9178427	1.561E-23	1.3069	up	PREDICTED: delta(8)-fatty-acid desaturase [Sesamum indicum]
c38304.graph_c2	0.7076121	0.81456877	1.18210244	15.2229366	15.6023013	15.1495503	1.323E-49	2.99019	up	PREDICTED: peroxidase 31 [Sesamum indicum]
c38305.graph_c0	0.18935694	0.25272874	0.16222078	2.79834522	2.73374528	2.32086008	2.602E-07	2.61127	up	PREDICTED: probable xyloglucan endotransglucosylase/hydrolase protein 26 [Sesamum indicum]
c38308.graph_c0	55.8993162	52.9116976	55.2271269	254.813071	262.994601	264.76156	1.817E-48	1.16372	up	PREDICTED: 60S ribosomal protein L7a [Sesamum indicum]

#ID	0 h-1 FPKM	0 h-2 FPKM	0 h-3 FPKM	28 h-1 FPKM	28 h-2 FPKM	28 h-3 FPKM	FDR	log2FC	regulated	nr_annotation
c38310.graph_c0	31.655055	33.9297018	29.7362978	27.418552	24.7029122	25.4901153	2.111E-51	-1.38606	down	PREDICTED: histone acetyltransferase HAC1-like isoform X1 [Sesamum indicum]
c38315.graph_c0	10.4488424	10.2221926	8.99654625	63.5460814	58.7406075	58.7834776	3.85E-64	1.52082	up	PREDICTED: transcription factor PIF3 isoform X2 [Sesamum indicum]
c38316.graph_c1	13.0848142	12.2247222	17.3749878	14.2649513	15.169676	14.7441061	0.0038987	-1.04517	down	-
c38317.graph_c0	0.46087873	0.92268047	0.64160137	3.04258974	3.32685202	3.37104404	0.0005884	1.17404	up	PREDICTED: uncharacterized protein LOC105171729 [Sesamum indicum]
c38319.graph_c0	37.2585785	36.9423718	38.3483815	36.9613469	39.0216438	39.9517361	4.855E-31	-1.04801	down	PREDICTED: NAC domain-containing protein 78-like [Sesamum indicum]
c38321.graph_c0	2.12384068	2.25481389	1.53001691	19.4118786	20.3567837	22.5003406	2.779E-44	2.31186	up	hypothetical protein MIMGU_mgv1a017270mg [Erythranthe guttata]
c38322.graph_c0	107.160284	109.211482	110.300283	35.7725095	37.9759146	34.7600613	4.26E-227	-2.68125	down	PREDICTED: protein phosphatase 2C 37-like [Sesamum indicum]
c38324.graph_c0	37.469595	33.3396579	38.4989501	10.3828322	10.4461211	9.63422262	2.696E-73	-2.9349	down	Mitogen-activated protein kinase 10 [Morus notabilis]
c38327.graph_c0	27.6189555	24.8904747	30.0200045	140.100477	161.330537	153.712802	2.756E-33	1.3713	up	PREDICTED: ent-kaurenoic acid oxidase 1-like [Sesamum indicum]
c38330.graph_c0	25.0624638	21.9308472	21.9685073	22.6182792	22.7094703	22.9350675	1.194E-17	-1.10379	down	PREDICTED: U-box domain-containing protein 4 [Sesamum indicum]
c38331.graph_c0	0.09796823	0.09806633	0.04196435	22.7374104	21.2797928	27.2880371	2.474E-64	7.14697	up	PREDICTED: raucaffricine-O-beta-D-glucosidase-like [Sesamum indicum]
c38333.graph_c0	5.46421833	5.69895836	4.87737413	26.9196541	29.4161797	28.5638452	1.33E-28	1.31507	up	PREDICTED: uncharacterized protein At2g39795, mitochondrial-like [Sesamum indicum]
c38335.graph_c0	3.33747891	3.7613438	3.86891075	3.91837455	3.67576606	4.02784518	5.118E-09	-1.00822	down	PREDICTED: uncharacterized protein LOC105173958 [Sesamum indicum]
c38338.graph_c0	0.17699739	0.35434925	0.34117298	3.68769959	4.04590824	3.32404199	1.041E-13	2.56662	up	PREDICTED: uncharacterized protein LOC105156552 [Sesamum indicum]
c38345.graph_c0	0.09343087	0.07014332	0	1.00159905	1.65542041	1.75926567	9.749E-08	3.68712	up	PREDICTED: uncharacterized protein LOC105160841 isoform X1 [Sesamum indicum]
c38347.graph_c0	1.40535855	1.44814126	1.75282545	29.1401264	28.128146	25.7636995	5.611E-77	3.07802	up	cyclin dependent kinase A [Camellia sinensis]
c38350.graph_c0	0	0	0	1.33844241	0.99432558	1.39630337	6.712E-17	Inf	up	PREDICTED: uncharacterized protein LOC105176095 isoform X2 [Sesamum indicum]
c38351.graph_c0	2.71687605	2.126864	2.95417124	19.0878952	16.5259962	19.1286137	2.709E-26	1.71954	up	PREDICTED: uncharacterized protein LOC105170284 [Sesamum indicum]
c38352.graph_c0	12.5143996	13.8736829	13.4469872	12.680779	13.6077325	13.0324199	4.168E-30	-1.11003	down	PREDICTED: U-box domain-containing protein 33-like [Sesamum indicum]
c38353.graph_c0	3.34141503	3.89783166	2.87383702	16.5024707	16.5404126	17.0547026	9.376E-20	1.22047	up	PREDICTED: uncharacterized protein LOC105156425 isoform X2 [Sesamum indicum]
c38354.graph_c0	1.36312996	1.15457263	1.61693166	16.4611108	20.6428328	19.4641542	1.759E-19	2.68073	up	PREDICTED: auxin-responsive protein IAA26 [Sesamum indicum]
c38355.graph_c0	110.727094	100.198137	112.907262	14.7835403	13.1976078	14.2854255	5.03E-149	-4.02856	down	hypothetical protein MIMGU_mgv1a006265mg [Erythranthe guttata]
c38359.graph_c0	1.62838226	1.30401027	1.30202219	6.10159402	7.12392326	5.98749741	8.572E-06	1.09311	up	PREDICTED: probable beta-1,3-galactosyltransferase 2-like isoformX1 [Glycine max]

#ID	0 h-1 FPKM	0 h-2 FPKM	0 h-3 FPKM	28 h-1 FPKM	28 h-2 FPKM	28 h-3 FPKM	FDR	log2FC	regulated	nr_annotation
c38361.graph_c0	1.40040248	1.15259504	1.95953464	7.05677754	6.9434195	7.23475306	3.619E-08	1.1386	up	PREDICTED: uncharacterized protein LOC105173063 [Sesamum indicum]
c38362.graph_c0	1.17260636	1.45833341	0.95890175	8.44495879	7.08869059	7.860396	1.421E-11	1.61691	up	PREDICTED: phospholipase D delta [Sesamum indicum]
c38362.graph_c1	0.74452019	0	0	9.7400337	13.435793	13.0255414	2.418E-11	4.545	up	-
c38367.graph_c0	0.14939764	0.04984908	0.38396377	1.80968908	2.32025098	2.21503726	4.28E-07	2.33554	up	-
c38369.graph_c0	5.18625011	3.20323101	4.46665759	1.84456902	2.35338559	2.19228844	5.15E-08	-2.09819	down	-
c38371.graph_c0	7.67124357	3.02830852	2.77685964	0	0	0	1.266E-07	-Inf	down	PREDICTED: plastocyanin, chloroplastic-like [Solanum tuberosum]
c38372.graph_c0	45.264079	48.5567486	50.3537522	20.0319344	21.1545336	22.7691196	2.228E-63	-2.264	down	PREDICTED: myosin-14 [Sesamum indicum]
c38374.graph_c0	0	0	0	0.78896459	0.94133425	1.44852449	2.05E-07	Inf	up	Fructose-bisphosphate aldolase A [Rozella allomycis CSF55]
c38375.graph_c0	22.603314	26.5740998	21.2485283	4.52044492	5.17658469	5.48798572	1.403E-56	-3.30225	down	PREDICTED: WRKY transcription factor 22 [Sesamum indicum]
c38376.graph_c0	0.13795438	0.36824673	0.05909228	1.4037023	1.90111735	1.19995293	0.0015207	1.91133	up	-
c38376.graph_c1	0.247906	0.16543616	0.26547419	2.40235539	2.30467052	2.54839309	1.441E-07	2.32573	up	PREDICTED: receptor-like serine/threonine-protein kinase ALE2 [Sesamum indicum]
c38383.graph_c0	0.02887026	0.08669752	0.1112983	0.92324063	1.23145414	1.1985222	4.849E-08	2.78307	up	PREDICTED: transcription factor LHW-like [Sesamum indicum]
c38383.graph_c1	0.21250368	0.04254329	0.05461513	1.01934659	0.94826453	1.05862717	6.735E-06	2.21434	up	PREDICTED: transcription factor LHW-like [Sesamum indicum]
c38384.graph_c0	0.10878371	0.21778527	0.06989568	2.13471102	1.67759006	1.45158896	6.757E-07	2.6457	up	-
c38387.graph_c0	4.13896733	4.52608861	4.78239368	22.4728562	22.3450772	21.658662	1.453E-20	1.21314	up	hypothetical protein MIMGU_mgv1a005705mg [Erythranthe guttata]
c38388.graph_c0	10.8320539	14.4572008	10.0530575	11.8090115	10.0042555	9.51709331	2.673E-06	-1.26307	down	-
c38389.graph_c0	10.4665468	9.61721066	9.5253283	7.07522776	8.22541606	7.82984036	7.238E-30	-1.44579	down	PREDICTED: glucan endo-1,3-beta-glucosidase 4-like [Sesamum indicum]
c38390.graph_c0	23.5147851	21.6576917	21.5338128	11.2541233	11.931215	10.9264771	2.736E-98	-2.05749	down	PREDICTED: F-box protein At3g07870-like [Sesamum indicum]
c38393.graph_c1	46.040942	48.2769969	46.2825319	12.8390777	12.8567284	12.3550087	0	-2.97627	down	PREDICTED: light-inducible protein CPRF2 [Sesamum indicum]
c38399.graph_c0	0.86999904	0.54429388	0.65215687	3.16155144	3.33034861	3.54722522	0.0003201	1.19309	up	PREDICTED: probable flavin-containing monooxygenase 1 [Sesamum indicum]
c38402.graph_c0	0.22160395	0.35492136	0.39867778	1.38512004	1.89049255	1.57708834	0.0044305	1.21793	up	hypothetical protein MIMGU_mgv1a004574mg [Erythranthe guttata]
c38405.graph_c0	1.13613035	1.63766593	1.10957908	0.03302932	0.23857903	0.24256489	6.486E-17	-4.00421	down	PREDICTED: BEACH domain-containing protein lvsA-like [Sesamum indicum]
c38406.graph_c0	0	0	0	0.90970657	0.46935985	0.75746238	5.839E-08	Inf	up	PREDICTED: potassium channel SKOR-like [Sesamum indicum]
c38408.graph_c0	2.98673649	2.92328887	3.41162191	19.1990147	19.1206673	18.6577824	4.374E-14	1.51917	up	PREDICTED: N-alpha-acetyltransferase MAK3-like isoform X2 [Sesamum indicum]
c38414.graph_c0	34.0496939	37.4197837	32.4830883	161.850286	162.022184	152.80269	9.854E-44	1.10715	up	PREDICTED: phosphoenolpyruvate carboxylase 1-like [Sesamum indicum]
c38415.graph_c0	7.09079872	6.97999379	6.72044676	6.52326637	5.96719316	6.60825952	1.798E-21	-1.21203	down	PREDICTED: autophagy-related protein 13 [Sesamum indicum]

#ID	0 h-1 FPKM	0 h-2 FPKM	0 h-3 FPKM	28 h-1 FPKM	28 h-2 FPKM	28 h-3 FPKM	FDR	log2FC	regulated	nr_annotation
c38416.graph_c0	3.67987069	2.9835133	3.14538729	22.5578481	19.023621	21.0633977	1.749E-29	1.58628	up	PREDICTED: probable sucrose-phosphate synthase 2 [Sesamum indicum]
c38418.graph_c0	58.8608432	62.1081538	53.2936188	36.7560722	48.5012151	51.8703303	3.88E-28	-1.43411	down	hypothetical protein MIMGU_mgv1a006647mg [Erythranthe guttata]
c38422.graph_c0	0.58956661	0.13114599	0.0841796	2.33290968	1.97743553	2.25328254	0.0001369	1.95776	up	hypothetical protein CICLE_v10010274mg [Citrus clementina]
c38423.graph_c0	15.3066269	14.6835394	15.0254054	11.8586939	10.6026573	9.92875628	1.983E-31	-1.56577	down	PREDICTED: coiled-coil domain-containing protein 97 [Sesamum indicum]
c38428.graph_c0	1.66741002	1.65238888	1.60701592	9.37982729	8.81924846	9.3547208	6.054E-22	1.39363	up	PREDICTED: structural maintenance of chromosomes protein 5 [Sesamum indicum]
c38433.graph_c0	7.73187122	8.5326067	8.10334576	37.079352	38.3035072	38.901148	5.129E-29	1.13883	up	PREDICTED: uncharacterized protein LOC105163069 [Sesamum indicum]
c38435.graph_c0	0.09024382	0.31616966	0.23193377	1.04941905	1.5397314	1.632355	0.0003647	1.62986	up	PREDICTED: BEL1-like homeodomain protein 4 [Sesamum indicum]
c38436.graph_c0	3.04965361	2.93252205	2.25261079	13.2813715	12.433057	12.1476735	2.091E-14	1.11396	up	PREDICTED: putative tRNA pseudouridine synthase Pus10 [Sesamum indicum]
c38437.graph_c0	1.39223764	1.27066425	1.15764032	0.89282673	1.04798047	0.72853932	6.006E-06	-1.60712	down	PREDICTED: probably inactive leucine-rich repeat receptor-like protein kinase At2g25790 [Sesamum indicum]
c38437.graph_c1	1.71318311	2.22936819	2.75188516	2.17898032	2.02362679	1.5748267	0.000669	-1.30986	down	PREDICTED: probable leucine-rich repeat receptor-like protein kinase At1g35710 [Sesamum indicum]
c38439.graph_c0	2.77462121	2.01992696	2.64711223	2.32212093	2.56564666	1.96962771	1.257E-05	-1.20982	down	PREDICTED: telomere repeat-binding protein 5-like isoform X2 [Nicotiana sylvestris]
c38441.graph_c0	56.0023774	55.3104843	52.6599815	23.7810637	25.1883908	24.7229122	1.89E-147	-2.24364	down	PREDICTED: UBA and UBX domain-containing protein At4g15410-like [Sesamum indicum]
c38442.graph_c0	1438.04255	1446.16627	1541.26606	1177.83144	1132.30583	1260.07167	1.097E-43	-1.40084	down	--
c38448.graph_c0	817.785776	823.214906	818.062214	127.601353	128.040782	136.605464	4.01E-231	-3.73849	down	PREDICTED: probable alpha,alpha-trehalose-phosphate synthase [UDP-forming] 11 [Sesamum indicum]
c38450.graph_c1	225.332758	232.468593	226.179785	118.303721	114.56524	112.120494	1.39E-138	-2.07806	down	PREDICTED: uncharacterized protein LOC104242030 [Nicotiana sylvestris]
c38451.graph_c0	5.35131419	5.02775422	4.48389791	62.7406195	61.7521237	65.7277663	8.5E-192	2.58976	up	PREDICTED: delta(24)-sterol reductase [Sesamum indicum]
c38454.graph_c0	357.327064	350.468193	373.693893	350.399323	372.333069	363.661699	4.223E-38	-1.08471	down	PREDICTED: endo-1,3;1,4-beta-D-glucanase-like isoform X1 [Nicotiana tomentosiformis]
c38459.graph_c0	1.47218704	0.85963571	0.86708354	0	0	0	1.148E-18	-Inf	down	PREDICTED: metalloendoproteinase 1-like [Glycine max]
c38460.graph_c0	29.9799067	24.2994157	27.9872092	10.9701938	10.801971	10.4493937	5.177E-42	-2.44265	down	PREDICTED: uncharacterized protein LOC105164190 [Sesamum indicum]
c38462.graph_c0	35.8331971	38.8422571	36.5925441	20.7950793	20.6714894	21.1703671	3.17E-110	-1.91943	down	PREDICTED: NPL4-like protein 2 [Sesamum indicum]
c38464.graph_c0	0	0.05540722	0.07112925	0.70401391	1.10786205	0.91915137	2.792E-09	3.32235	up	PREDICTED: cation/H(+) antiporter 14 isoform X1 [Nicotiana tomentosiformis]
c38465.graph_c0	0.97865154	0.76193562	2.23574395	11.3014219	10.2041243	11.7369228	4.611E-09	1.96036	up	PREDICTED: F-box protein PP2-A15-like [Nicotiana tomentosiformis]

#ID	0 h-1 FPKM	0 h-2 FPKM	0 h-3 FPKM	28 h-1 FPKM	28 h-2 FPKM	28 h-3 FPKM	FDR	log2FC	regulated	nr_annotation
c38467.graph_c0	1.28403239	1.15349065	1.48079836	9.88263824	9.938557	9.76289601	6.964E-19	1.82358	up	PREDICTED: uncharacterized protein LOC105163544 [Sesamum indicum]
c38474.graph_c0	50.1529913	51.4234289	49.1756088	54.8345646	51.4246088	51.173521	7.238E-34	-1.02778	down	PREDICTED: transcription elongation factor S-II [Sesamum indicum]
c38476.graph_c1	17.9179692	15.5727339	16.413305	246.066997	249.267085	250.725551	1.9E-220	2.81236	up	hypothetical protein CISIN_1g024108mg [Citrus sinensis]
c38477.graph_c0	1.12669131	1.30589629	1.14303391	6.2061935	5.01990138	5.80273286	0.000356	1.16177	up	-
c38478.graph_c0	0.47106089	0.38312023	0.49183216	19.4291763	21.4840415	21.3540773	4.5E-115	4.44008	up	hypothetical protein MIMGU_mgv1a002464mg [Erythranthe guttata]
c38479.graph_c1	545.50221	518.186758	554.788777	148.693906	147.222355	154.437527	7.48E-206	-2.93622	down	PREDICTED: putative G3BP-like protein isoform X1 [Sesamum indicum]
c38482.graph_c0	3.85380163	5.65437928	3.52765697	47.0017534	44.9325019	47.6203954	8.74E-68	2.33217	up	PREDICTED: 40S ribosomal protein S7 [Sesamum indicum]
c38484.graph_c0	567.450345	568.018561	576.68671	267.451295	274.925686	278.582276	1.32E-141	-2.15109	down	-
c38489.graph_c0	4.28451968	4.72644365	3.90460746	3.78124226	3.91618182	3.69477689	1.392E-16	-1.27036	down	PREDICTED: uncharacterized protein LOC105171501 [Sesamum indicum]
c38492.graph_c1	7.48986328	6.56548758	6.19900495	6.18170329	6.19240876	7.20244263	1.721E-11	-1.13675	down	PREDICTED: uncharacterized protein LOC105168847 isoform X2 [Sesamum indicum]
c38493.graph_c0	4.26806306	4.21401146	3.40683503	0.70933189	0.61178287	0.76022678	1.24E-76	-3.60089	down	PREDICTED: structural maintenance of chromosomes protein 2-1 [Sesamum indicum]
c38494.graph_c0	0.12021529	0.06016783	0	1.94402206	2.6230434	2.13883971	1.211E-14	4.14918	up	Putative gag-pol polyprotein, identical [Solanum demissum]
c38495.graph_c0	0.26403554	0.13214997	0.8482403	4.02988967	3.55197891	4.38447609	4.555E-06	2.15604	up	PREDICTED: cytochrome c oxidase subunit 5b-1, mitochondrial-like [Sesamum indicum]
c38500.graph_c0	19.7577445	20.0498889	19.1496955	18.3604836	19.0363083	18.0355035	8.443E-41	-1.17924	down	PREDICTED: uncharacterized protein LOC105159907 [Sesamum indicum]
c38502.graph_c0	2.29561308	2.81494195	2.87620486	2.29410274	1.46878232	1.8038975	3.37E-07	-1.61346	down	PREDICTED: protein ROOT HAIR DEFECTIVE 3 homolog 1-like [Sesamum indicum]
c38504.graph_c0	3.85063158	3.78244091	4.48576412	93.8190523	94.0616573	89.6171962	1.19E-238	3.42422	up	PREDICTED: probable pectinesterase/pectinesterase inhibitor 51 [Sesamum indicum]
c38505.graph_c0	2.48048904	2.01271286	2.41479289	16.1159853	15.8830306	15.1788118	1.418E-41	1.68077	up	PREDICTED: nuclear pore complex protein NUP96 [Sesamum indicum]
c38507.graph_c0	0	0.31884833	0.51165356	1.85204248	2.50833107	2.97527647	0.0003778	2.03245	up	hypothetical protein EUGRSUZ_J00099 [Eucalyptus grandis]
c38509.graph_c0	0.02719466	0.08166567	0.10483864	6.9177222	7.33465263	6.35443743	2.637E-49	5.48797	up	PREDICTED: sulfate transporter 3.1-like [Sesamum indicum]
c38512.graph_c0	0.09191375	0.09200579	0	1.16904176	1.26664213	1.09020404	1.562E-08	3.18991	up	PREDICTED: microtubule-associated protein RP/EB family member 1B [Sesamum indicum]
c38514.graph_c0	3.35755108	3.20086967	2.72229835	3.12293742	2.97709939	3.23573749	1.556E-10	-1.07908	down	PREDICTED: serine/threonine-protein kinase D6PK [Sesamum indicum]
c38514.graph_c1	5.15052449	6.70238655	7.05986975	2.62036006	2.47860324	3.36001665	6.148E-10	-2.25325	down	-
c38515.graph_c0	9.20949729	7.23236535	7.7807385	45.8923826	47.1127541	48.5825731	8.516E-36	1.45856	up	hypothetical protein MIMGU_mgv1a012548mg [Erythranthe guttata]
c38516.graph_c0	0.02504371	0.02506879	0	5.93372965	5.75202619	6.59445544	3.805E-53	7.44119	up	PREDICTED: uncharacterized protein LOC105155823 [Sesamum indicum]

#ID	0 h-1 FPKM	0 h-2 FPKM	0 h-3 FPKM	28 h-1 FPKM	28 h-2 FPKM	28 h-3 FPKM	FDR	log2FC	regulated	nr_annotation
c38518.graph_c0	0.96603278	1.48273351	1.32414972	1.2637927	1.22561123	0.84027331	0.0012953	-1.27517	down	PREDICTED: LOW QUALITY PROTEIN: ethylene-responsive transcription factor ERF025 [Sesamum indicum]
c38524.graph_c0	222.006809	224.180776	212.295353	44.2115516	39.4071053	39.6993088	0	-3.50683	down	Histone H3.3 [Arabidopsis thaliana]
c38525.graph_c1	29.1873033	30.4911892	27.5164994	31.5274863	31.4570367	29.1245301	5.59E-36	-1.01092	down	PREDICTED: pectinesterase 3-like [Sesamum indicum]
c38527.graph_c0	0.15792058	0.07903936	0.1014671	12.7018515	13.5066722	12.5967439	2.425E-95	5.75511	up	PREDICTED: auxin transporter-like protein 2 [Sesamum indicum]
c38531.graph_c0	35.6877425	37.389506	38.7857693	26.868144	28.6912215	27.2581223	2.727E-52	-1.5254	down	PREDICTED: deSI-like protein At4g17486 [Sesamum indicum]
c38532.graph_c0	15.411083	15.9814255	14.5893124	8.44483401	10.8843897	9.66569267	2.879E-72	-1.75521	down	PREDICTED: autophagy-related protein 13 isoform X2 [Sesamum indicum]
c38536.graph_c0	0.22331707	0.29805426	0.0318857	1.00990128	1.12352474	1.29496902	0.0015515	1.55701	up	PREDICTED: uncharacterized protein LOC105175444 [Sesamum indicum]
c38540.graph_c0	3.11451672	1.8339032	2.04037666	0	0	0	2.268E-22	-Inf	down	hypothetical protein PAXRUDRAFT_143168 [Paxillus rubicundulus Ve08.2h10]
c38540.graph_c1	1.45384243	0.81398037	0.82329444	0.28654761	0.42042914	0.74530287	0.0002646	-2.17035	down	PREDICTED: heat shock protein 83-like [Nelumbo nucifera]
c38542.graph_c0	1.36876631	0.77070202	0.76952702	14.0827998	14.8487239	14.0789284	5.307E-35	2.80184	up	PREDICTED: homoserine dehydrogenase [Sesamum indicum]
c38545.graph_c1	32.4407005	33.8787945	33.3593212	20.1758242	17.956974	17.3814949	3.082E-98	-1.93555	down	PREDICTED: protein SSUH2 homolog [Sesamum indicum]
c38547.graph_c0	35.5152993	35.436551	38.8881669	220.525146	220.396272	241.576946	5.264E-53	1.54408	up	-
c38549.graph_c0	28.3750862	31.9202942	32.5933667	30.7583997	30.9479048	29.2232358	2.48E-17	-1.12305	down	PREDICTED: 3-ketoacyl-CoA synthase 10 [Sesamum indicum]
c38552.graph_c0	4.53231428	6.00035358	6.26258418	1.84183994	1.85489441	2.16768224	9.47E-15	-2.61113	down	PREDICTED: uncharacterized protein LOC105175383 [Sesamum indicum]
c38553.graph_c0	1.59876792	2.29242023	1.88790061	8.57349349	9.61252602	9.04598146	3.301E-07	1.14476	up	PREDICTED: uncharacterized protein LOC105174166 [Sesamum indicum]
c38556.graph_c0	2.95573304	3.12571573	2.84867548	47.7630181	56.1867586	52.7007398	3.967E-91	3.04275	up	PREDICTED: thaumatin-like protein [Sesamum indicum]
c38559.graph_c0	13.8974043	14.3373014	12.7964019	10.1624115	10.2608316	9.46780869	8.131E-72	-1.54675	down	PREDICTED: filaggrin-like [Sesamum indicum]
c38566.graph_c0	0	0	0	1.24631602	1.08779629	1.54242527	1.285E-15	Inf	up	PREDICTED: homeobox protein ATH1 isoform X2 [Sesamum indicum]
c38569.graph_c1	6.64626937	6.61772394	7.23023854	7.1818103	6.64603696	6.52766352	1.492E-12	-1.10171	down	PREDICTED: transcription factor HBP-1b(c38)-like isoform X1 [Sesamum indicum]
c38572.graph_c0	22.5052068	22.4802155	21.9036062	18.5996558	17.6661476	17.7395461	1.815E-38	-1.39892	down	PREDICTED: cyclin-dependent kinase F-4-like [Sesamum indicum]
c38576.graph_c0	108.547577	111.653137	111.229456	54.6677295	58.1573249	58.2313518	4.49E-135	-2.04493	down	PREDICTED: HIPL1 protein-like [Solanum tuberosum]
c38580.graph_c0	3.26708642	3.66769111	2.98199523	24.4329349	24.945966	26.1662797	3.014E-46	1.84062	up	PREDICTED: NADP-dependent glyceraldehyde-3-phosphate dehydrogenase [Sesamum indicum]
c38581.graph_c1	13.6162419	11.0460136	9.53650303	85.829655	81.1803735	67.8931103	1.615E-19	1.69273	up	-
c38583.graph_c0	11.0792944	11.435305	10.626908	10.8071245	9.65345846	10.3275778	2.515E-27	-1.19602	down	PREDICTED: internal alternative NAD(P)H-ubiquinone oxidoreductase A1, mitochondrial-like [Sesamum indicum]
c38584.graph_c0	24.3240699	23.9261997	23.8495714	195.026568	209.214396	205.96071	1.02E-112	1.99091	up	hypothetical protein M569_17345, partial [Genlisea aurea]

#ID	0 h-1 FPKM	0 h-2 FPKM	0 h-3 FPKM	28 h-1 FPKM	28 h-2 FPKM	28 h-3 FPKM	FDR	log2FC	regulated	nr_annotation
c38588.graph_c0	1.06743738	0.94639126	1.29331648	5.12032843	5.82400155	6.05921632	8.903E-08	1.26895	up	hypothetical protein MIMGU_mgv1a003578mg [Erythranthe guttata]
c38591.graph_c0	66.1167518	62.4407873	63.2072657	13.8570427	14.8246812	15.9631535	0	-3.19242	down	PREDICTED: E3 ubiquitin-protein ligase RNF170-like [Sesamum indicum]
c38596.graph_c0	0	0	0	3.06435481	4.48295709	3.5133446	9.205E-20	Inf	up	PREDICTED: uncharacterized protein LOC105168232 isoform X2 [Sesamum indicum]
c38600.graph_c0	0.23745343	0.29051147	0.54246589	3.24066488	3.25497698	2.9729447	1.716E-10	2.04302	up	PREDICTED: phosphatidylinositol 4-kinase gamma 8 [Sesamum indicum]
c38601.graph_c0	1.32814492	1.35660699	1.35840884	0.53189343	0.48024989	0.69121814	7.14E-13	-2.33624	down	-
c38602.graph_c0	0.81465389	1.25456868	1.12739038	10.293183	9.7461313	8.91945484	1.674E-12	2.08563	up	hypothetical protein MIMGU_mgv1a015466mg [Erythranthe guttata]
c38604.graph_c1	3.29310271	4.14891756	3.35622928	19.807515	16.9901125	16.5331673	1.091E-10	1.21373	up	PREDICTED: pentatricopeptide repeat-containing protein At4g21190 isoform X1 [Sesamum indicum]
c38608.graph_c0	16.7925579	16.8093731	15.6409274	15.6012751	15.0591544	15.2021562	5.291E-42	-1.19221	down	PREDICTED: DNA ligase 1-like [Sesamum indicum]
c38610.graph_c0	1.12794784	0.74281402	1.37317039	7.48594049	8.5121837	8.06247138	3.511E-17	1.79615	up	hypothetical protein MIMGU_mgv1a005256mg [Erythranthe guttata]
c38614.graph_c0	13.8997873	13.7090926	12.7834287	11.0431845	11.5806219	12.7691609	1.711E-16	-1.27955	down	PREDICTED: sphingoid long-chain bases kinase 1-like [Sesamum indicum]
c38619.graph_c0	3.02348843	2.80773171	2.01286748	1.85327443	2.7251424	2.76526958	1.524E-05	-1.1801	down	PREDICTED: uncharacterized protein LOC105177754 isoform X2 [Sesamum indicum]
c38629.graph_c0	21.7974091	18.6656745	18.7101612	82.8622957	88.3945559	84.2283403	3.638E-21	1.02116	up	hypothetical protein M569_15947, partial [Genlisea aurea]
c38630.graph_c0	43.1488629	43.0498036	45.5491782	14.5645871	13.7100504	14.5817947	2.04E-154	-2.71128	down	PREDICTED: WPP domain-interacting protein 1-like [Sesamum indicum]
c38632.graph_c1	1.04988829	1.00715045	0.89943202	7.94846626	7.66473674	6.95286717	2.361E-11	1.84306	up	PREDICTED: protein PHR1-LIKE 1-like isoform X1 [Sesamum indicum]
c38638.graph_c0	0.16199196	0	0.20816608	5.57278149	5.59865121	5.1237681	1.76E-19	4.36034	up	PREDICTED: ABC transporter I family member 17-like [Sesamum indicum]
c38642.graph_c0	0	0.09304754	0.29862539	2.56723298	2.68396605	1.81920415	2.228E-09	3.05359	up	hypothetical protein MIMGU_mgv1a025521mg [Erythranthe guttata]
c38644.graph_c0	241.157655	235.6063	248.322257	55.5190611	60.9744928	57.7408608	0	-3.14826	down	PREDICTED: pheophytinase, chloroplastic [Sesamum indicum]
c38645.graph_c0	43.2521494	42.3623681	35.2170969	14.6788925	14.4362983	15.9581902	2.67E-63	-2.51013	down	PREDICTED: cell number regulator 6-like [Sesamum indicum]
c38646.graph_c0	2.07624441	2.80573667	2.80145909	0.07545012	0.34062194	0	1.192E-23	-5.30931	down	PREDICTED: uncharacterized protein LOC105168686 [Sesamum indicum]
c38650.graph_c0	31.4532265	33.7951118	29.1363155	20.9522807	22.9272426	20.9886467	1.484E-64	-1.63068	down	PREDICTED: probable histone-lysine N-methyltransferase ATXR3 isoform X1 [Sesamum indicum]
c38660.graph_c0	1.14980496	1.541111	1.4024155	7.46438889	7.55811992	8.00944991	1.717E-16	1.40041	up	PREDICTED: anaphase-promoting complex subunit 5 [Sesamum indicum]
c38664.graph_c0	0.71242512	1.1410216	1.92253841	11.5466382	12.9968512	13.6470578	4.573E-16	2.23634	up	PREDICTED: uncharacterized protein LOC105179255 [Sesamum indicum]
c38669.graph_c0	217.033901	218.697641	226.070098	61.8038581	67.181799	58.4867633	0	-2.91134	down	PREDICTED: BTB/POZ and TAZ domain-containing protein 1 [Sesamum indicum]
c38681.graph_c1	0.39206183	0.32704535	0.25190749	4.63040952	4.35234671	3.352098	2.161E-13	2.5805	up	PREDICTED: 3-ketoacyl-CoA synthase 12 [Sesamum indicum]

#ID	0 h-1 FPKM	0 h-2 FPKM	0 h-3 FPKM	28 h-1 FPKM	28 h-2 FPKM	28 h-3 FPKM	FDR	log2FC	regulated	nr_annotation
c38697.graph_c0	1.61620645	1.01114052	2.07688928	13.6553008	16.5718499	16.4143883	2.171E-08	2.2142	up	hypothetical protein AMTR_s00007p00249170 [Amborella trichopoda]
c38697.graph_c1	1.9088292	2.66707543	2.50402362	21.6191645	22.4167747	20.0230767	2.424E-38	2.08414	up	PREDICTED: uncharacterized membrane protein At1g16860-like [Sesamum indicum]
c38703.graph_c0	0.08523162	0.08531697	0.10952601	3.69610248	4.36264109	3.97638831	3.623E-25	4.33106	up	PREDICTED: BTB/POZ domain-containing protein NPY2 [Sesamum indicum]
c38706.graph_c0	1.33702047	1.59739658	1.27473734	0.81500671	0.45284594	0.38367624	3.631E-11	-2.44072	down	-
c38710.graph_c0	6.98902706	6.70229925	7.438608	108.164131	103.876451	96.1395211	2.57E-178	2.77446	up	PREDICTED: protein NRT1/ PTR FAMILY 8.3-like [Sesamum indicum]
c38711.graph_c0	22.1611926	21.4027367	21.4628989	149.35236	155.577829	147.153962	9.776E-80	1.70706	up	unnamed protein product [Coffea canephora]
c38714.graph_c1	32.2512875	31.2928692	29.1644239	23.6136799	20.9626038	22.7903464	1.551E-66	-1.54976	down	PREDICTED: putative E3 ubiquitin-protein ligase XBAT34 isoform X2 [Sesamum indicum]
c38715.graph_c0	0.45691732	0.3201624	0.41100979	2.25817715	3.08836752	1.68006699	0.0019796	1.47181	up	PREDICTED: F-box/kelch-repeat protein At3g24760 [Sesamum indicum]
c38757.graph_c0	26.1340665	24.5895918	27.69962	20.0036173	21.9775055	21.1128403	6.172E-32	-1.40536	down	PREDICTED: uncharacterized protein At5g02240 [Sesamum indicum]
c38759.graph_c0	1.188999	1.56603895	1.36707807	12.5530265	11.4163379	11.5421194	5.567E-14	2.01526	up	-
c38760.graph_c0	12.4644723	9.45761962	8.60718292	62.7367314	57.8097313	52.9288966	5.739E-21	1.41959	up	PREDICTED: NADPH-dependent 1-acyldihydroxyacetone phosphate reductase-like [Sesamum indicum]
c38761.graph_c0	0.16190234	0.08103223	0.15603819	2.47106346	2.39050566	2.16039386	5.412E-10	3.04523	up	PREDICTED: putative glucose-6-phosphate 1-epimerase [Sesamum indicum]
c38762.graph_c0	6.79808054	6.42102233	7.12303908	3.29387051	3.0426883	3.99030682	1.329E-33	-2.06821	down	PREDICTED: exocyst complex component EXO70A1-like [Sesamum indicum]
c38763.graph_c0	2.79422498	2.89237603	3.2234601	17.6312674	17.5029848	18.5674773	1.701E-25	1.49935	up	PREDICTED: 54S ribosomal protein L10, mitochondrial [Sesamum indicum]
c38770.graph_c0	3.89767001	1.91177074	2.85493636	0	0	0	4.563E-20	-Inf	down	cytochrome P450 [Aureobasidium pullulans var. namibiae CBS 147.97]
c38775.graph_c0	16.4387003	15.5180791	15.3500625	105.296312	99.200951	99.1344108	7.572E-75	1.59231	up	PREDICTED: uncharacterized protein LOC105168156 [Sesamum indicum]
c38777.graph_c0	62.5234583	59.4662169	67.6015581	368.132596	370.815718	367.327719	4.56E-72	1.45294	up	hypothetical protein [Solanum tuberosum]
c38791.graph_c0	0.26355243	0.14070205	0.22578354	35.4875314	37.6570312	35.7827197	0	6.34368	up	PREDICTED: probable methyltransferase PMT26 [Sesamum indicum]
c38793.graph_c0	44.4013752	45.1631675	41.1079392	36.3956419	44.9569701	39.490397	2.624E-48	-1.20264	down	PREDICTED: uncharacterized protein LOC105159283 [Sesamum indicum]
c38793.graph_c1	0.92475413	0.92568013	0.7130074	6.67625264	6.63487885	4.24121507	1.315E-05	1.68631	up	PREDICTED: uncharacterized protein LOC105166230 isoform X2 [Sesamum indicum]
c38793.graph_c2	1.48580459	2.08220936	1.81385184	12.7425157	12.6753272	12.2923012	4.404E-19	1.7166	up	PREDICTED: uncharacterized protein LOC105166230 isoform X2 [Sesamum indicum]
c38806.graph_c0	1.45459062	1.30392284	1.6739163	12.9387198	12.2380796	11.0600332	3.802E-36	1.93789	up	PREDICTED: uncharacterized protein LOC105171651 isoform X2 [Sesamum indicum]
c38814.graph_c0	0.99765733	0.99865633	0.82416161	6.21507082	5.82206222	4.52204028	1.116E-07	1.46431	up	PREDICTED: probable amino acid permease 7 [Sesamum indicum]

#ID	0 h-1 FPKM	0 h-2 FPKM	0 h-3 FPKM	28 h-1 FPKM	28 h-2 FPKM	28 h-3 FPKM	FDR	log2FC	regulated	nr_annotation
c38822.graph_c0	36.9342021	35.2806547	31.043676	167.145221	162.962797	158.554547	7.757E-39	1.15401	up	PREDICTED: protein ETHYLENE INSENSITIVE 3 [Sesamum indicum]
c38823.graph_c0	0.57097993	0.42866376	0.67258749	2.0749246	2.52917419	2.53968229	0.0098697	1.00148	up	PREDICTED: protein cornichon homolog 4 [Sesamum indicum]
c38825.graph_c0	0.52418989	0.38161075	0.48989437	2.45904103	2.34536989	2.93918267	0.0006291	1.38259	up	PREDICTED: tRNA (guanine(9)-N1)-methyltransferase [Sesamum indicum]
c38829.graph_c0	1.75342253	1.48515088	1.64658193	14.9004126	12.5464375	12.5985649	6.166E-26	1.94476	up	PREDICTED: heparan-alpha-glucosaminide N-acetyltransferase isoform X1 [Sesamum indicum]
c38844.graph_c0	0.89775221	1.02702991	1.81287406	4.28773313	5.72297018	6.54115107	0.0072037	1.04784	up	PREDICTED: 14 kDa zinc-binding protein [Sesamum indicum]
c38846.graph_c0	3.9000548	2.92797009	4.87251025	1.41726868	0.42655358	0.96373305	3.32E-09	-3.15263	down	-
c38847.graph_c0	85.8989643	87.0406362	89.5746276	6.62632623	6.63036022	7.12194372	0	-4.77799	down	hypothetical protein EUGRSUZ_J00099 [Eucalyptus grandis]
c38848.graph_c0	5.98185102	4.25802023	4.66908935	1.61021291	1.71556811	2.54900593	3.969E-16	-2.42948	down	predicted protein [Hordeum vulgare subsp. vulgare]
c38855.graph_c0	0	0.07096172	0.36438958	2.93680956	3.16338825	3.44747078	2.415E-10	3.33306	up	PREDICTED: UDP-N-acetylglucosamine--peptide N-acetylglucosaminyltransferase 110 kDa subunit [Sesamum indicum]
c38857.graph_c0	2.01215942	1.63651662	1.29285226	0.30467206	0.08252709	0.19888793	7.994E-24	-4.15998	down	-
c38868.graph_c0	0.94802055	0.72568282	1.14658253	0.28371242	0.23786816	0.39686917	1.948E-09	-2.71037	down	-
c38873.graph_c1	0.5585085	0.35577039	0.78295149	7.52798498	6.36391834	7.28701653	5.171E-15	2.54485	up	PREDICTED: raucaffricine-O-beta-D-glucosidase-like [Sesamum indicum]
c38877.graph_c0	0.75729994	0.89183325	0.28622365	4.20895061	3.71258883	3.27595536	0.0001141	1.45162	up	PREDICTED: probable dimethyladenosine transferase [Sesamum indicum]
c38893.graph_c0	0.07256716	0.29055931	0.2331292	1.34490561	1.73814956	1.14046854	0.0001362	1.72471	up	-
c38898.graph_c0	6.23957423	5.81594781	6.07038334	32.2399225	34.0330664	30.6371669	1.095E-38	1.32779	up	PREDICTED: CDPK-related kinase 1 [Sesamum indicum]
c38900.graph_c0	2.09331965	2.77334443	2.53176342	1.83464468	1.41409572	1.20494677	5.658E-08	-1.82544	down	hypothetical protein AMTR_s00334p00011530 [Amborella trichopoda]
c38902.graph_c0	1.79614463	0.8989716	1.61568188	8.09365093	8.36861876	6.17826577	0.002472	1.30083	up	PREDICTED: copper transporter 6-like [Nicotiana sylvestris]
c38904.graph_c0	28.1343657	29.5194352	27.1953166	21.8754273	23.2007038	23.3627226	2.455E-57	-1.39992	down	PREDICTED: 20S-pre-rRNA D-site endonuclease nob1 [Sesamum indicum]
c38906.graph_c0	0.77160191	0.80595605	0.47421431	0.73147281	0.22015046	0.27854206	8.464E-05	-1.82377	down	gag-pol polyprotein [Oryza sativa Japonica Group]
c38908.graph_c0	23.2929401	22.8641486	20.1483687	23.588296	24.2619588	22.538743	4.893E-18	-1.00279	down	PREDICTED: uncharacterized protein LOC105158092 [Sesamum indicum]
c38923.graph_c0	57.3795797	56.9894027	51.0003629	50.1331558	46.8061816	50.4888641	9.455E-55	-1.25444	down	PREDICTED: large subunit GTPase 1 homolog isoform X1 [Cicer arietinum]
c38925.graph_c0	27.4062434	29.4951775	28.8259195	29.3138784	30.9586654	30.7031365	1.384E-29	-1.00523	down	PREDICTED: probable nucleoredoxin 1 [Sesamum indicum]
c38926.graph_c0	3.65483242	3.94642906	3.00060834	15.3106823	15.6895327	14.7813381	3.42E-19	1.02237	up	PREDICTED: pleiotropic drug resistance protein 2-like [Sesamum indicum]
c38930.graph_c0	1.30892097	1.94936905	2.01021337	12.5759364	12.9261402	12.5906602	2.828E-23	1.75915	up	PREDICTED: uncharacterized protein LOC105159306 [Sesamum indicum]
c38931.graph_c0	1.76168665	2.29248592	1.47149411	66.5799525	69.6530027	67.3362415	1.16E-207	4.11554	up	PREDICTED: uncharacterized protein LOC105171035 [Sesamum indicum]

#ID	0 h-1 FPKM	0 h-2 FPKM	0 h-3 FPKM	28 h-1 FPKM	28 h-2 FPKM	28 h-3 FPKM	FDR	log2FC	regulated	nr_annotation
c38934.graph_c0	27.9185733	28.9288329	26.3556508	20.3267191	20.4782198	22.2316451	1.689E-48	-1.48972	down	PREDICTED: formin-like protein 14 isoform X2 [Sesamum indicum]
c38934.graph_c1	23.6241239	23.3396655	18.4916409	17.4495168	18.3811572	17.6614188	3.444E-19	-1.37874	down	PREDICTED: formin-like protein 5 [Sesamum indicum]
c38938.graph_c0	2.47164023	3.45345248	2.24977685	15.9802426	12.9418667	15.788172	8.825E-10	1.36356	up	PREDICTED: EPIDERMAL PATTERNING FACTOR-like protein 2 [Sesamum indicum]
c38944.graph_c0	4.21522924	4.81165368	3.80121857	1.45118099	1.40733818	1.44729669	9.467E-18	-2.6637	down	hypothetical protein MIMGU_mgv1a003797mg [Erythranthe guttata]
c38944.graph_c1	14.2897216	15.1454442	14.6238145	2.16172677	2.50344258	2.3774997	1.297E-92	-3.73596	down	PREDICTED: probable protein S-acyltransferase 22 isoform X2 [Sesamum indicum]
c38952.graph_c0	7.6726155	7.21198758	7.75542863	8.56863419	7.36826027	8.07402078	2.274E-09	-1.00586	down	hypothetical protein MIMGU_mgv1a006823mg [Erythranthe guttata]
c38956.graph_c0	42.9873913	36.8091687	36.3833011	176.63581	183.16342	168.270792	9.78E-42	1.09528	up	PREDICTED: REF/SRPP-like protein At1g67360 [Sesamum indicum]
c38964.graph_c0	1.83947561	1.39121772	1.83851033	10.7102169	11.2932189	11.1394648	2.618E-27	1.61756	up	PREDICTED: DNA gyrase subunit B, chloroplastic/mitochondrial-like isoform X1 [Sesamum indicum]
c38967.graph_c1	0.90296948	1.02439015	0.69621092	11.3535231	11.1004928	11.5313889	8.614E-36	2.60849	up	PREDICTED: phosphomevalonate kinase [Sesamum indicum]
c38969.graph_c0	14.3940413	13.4971508	12.5842245	12.7326367	13.14333	12.2429514	4.53E-32	-1.17578	down	PREDICTED: chaperone protein ClpB4, mitochondrial [Sesamum indicum]
c38969.graph_c1	19.2277736	19.1335871	18.2522148	15.704305	15.2454575	17.1383868	1.793E-36	-1.32481	down	PREDICTED: chaperone protein ClpB4, mitochondrial [Sesamum indicum]
c38972.graph_c0	103.586863	96.6875213	110.202666	57.4898919	51.4636913	59.2915779	6.593E-44	-1.97501	down	PREDICTED: zinc finger A20 and AN1 domain-containing stress-associated protein 8-like isoform X1 [Sesamum indicum]
c38986.graph_c0	17.807236	15.1012368	14.6039841	16.3740599	15.1781371	16.3750593	2.047E-19	-1.07556	down	PREDICTED: ABC transporter I family member 1 [Sesamum indicum]
c38987.graph_c0	1.40863495	1.16121393	1.17127462	24.0891239	29.3627974	25.6517695	1.278E-40	3.31324	up	PREDICTED: NADPH:quinone oxidoreductase [Sesamum indicum]
c38988.graph_c1	0.75812376	0.67456258	0.61339718	25.6112953	26.9203492	24.4788474	5.38E-137	4.14567	up	PREDICTED: probable protein S-acyltransferase 7 [Sesamum indicum]
c38995.graph_c0	2.20585906	2.2080679	2.6226823	31.2550186	28.9779447	29.7218768	2.55E-117	2.58333	up	PREDICTED: SNF2 domain-containing protein CLASSY 3-like [Sesamum indicum]
c38996.graph_c0	11.6308942	13.628801	10.5133091	11.7813247	13.3418633	12.8903708	9.025E-10	-1.00114	down	PREDICTED: uncharacterized protein LOC105173283 [Sesamum indicum]
c39015.graph_c0	0.6633793	0.88539143	0.87141241	0.01071423	0	0	1.189E-47	-8.91713	down	PREDICTED: uncharacterized protein LOC103452707 [Malus domestica]
c39021.graph_c0	9.53150499	9.37424778	10.363998	52.7309202	62.0890434	52.5348758	3.072E-25	1.42312	up	PREDICTED: uncharacterized protein LOC105158512 [Sesamum indicum]
c39024.graph_c0	71.5366187	73.8872967	66.4862285	29.7133701	30.574401	31.564636	3.9E-168	-2.29536	down	PREDICTED: uncharacterized protein LOC105169073 [Sesamum indicum]
c39028.graph_c0	8.6009005	10.4413243	10.5821786	2.52703433	3.12229219	2.49615293	5.534E-19	-2.95618	down	-
c39028.graph_c1	9.99733907	6.60485093	8.22206497	2.03448236	0.78726292	0.94864056	3.397E-21	-3.80872	down	-
c39029.graph_c0	19.4573194	18.1234299	18.2804503	7.775671	7.63790644	7.53019205	3.648E-67	-2.37375	down	PREDICTED: CBL-interacting serine/threonine-protein kinase 1-like [Sesamum indicum]

#ID	0 h-1 FPKM	0 h-2 FPKM	0 h-3 FPKM	28 h-1 FPKM	28 h-2 FPKM	28 h-3 FPKM	FDR	log2FC	regulated	nr_annotation
c39034.graph_c0	14.0081415	15.6479272	13.6703371	14.6075741	14.9478391	15.0259971	1.366E-19	-1.04865	down	PREDICTED: histone-lysine N-methyltransferase ATXR6 [Sesamum indicum]
c39040.graph_c0	0.5066508	0.84526356	0.65106634	10.862811	9.14314392	9.26459626	6.91E-14	2.77708	up	PREDICTED: uncharacterized protein LOC105178874 isoform X1 [Sesamum indicum]
c39047.graph_c0	2.45067276	2.1720393	2.29630007	20.7984599	20.3537529	18.7881128	2.833E-51	2.02447	up	PREDICTED: transcription factor LHW-like [Nicotiana sylvestris]
c39052.graph_c0	0.62843976	0.41937936	0.71783997	9.64242385	9.07278172	9.64882324	7.458E-17	2.91236	up	unnamed protein product [Vitis vinifera]
c39052.graph_c1	1.18180146	1.26457002	0.68077937	12.4988614	13.344466	11.1415275	5.043E-27	2.47939	up	hypothetical protein MIMGU_mgv1a002912mg [Erythranthe guttata]
c39060.graph_c0	16.2703123	17.1321845	15.1972337	10.6506603	11.7133889	11.1957339	1.101E-47	-1.6238	down	hypothetical protein MIMGU_mgv1a009934mg [Erythranthe guttata]
c39068.graph_c0	1.41249524	0.99805387	1.17448444	12.1379573	14.3535915	13.2429224	3.661E-50	2.38127	up	PREDICTED: protein NRT1/ PTR FAMILY 5.8 [Nicotiana sylvestris]
c39070.graph_c0	14.0818408	17.0703147	11.4550907	6.57267938	9.49522117	7.0488464	7.804E-11	-1.97057	down	-
c39073.graph_c0	1.75695129	2.08439776	2.92671551	19.5797784	22.3759443	19.2958204	7.319E-24	2.08036	up	LEC14B protein [Morus notabilis]
c39074.graph_c0	0.34321666	0.34356034	0.441047	2.92210387	3.12101738	3.51846294	1.12E-06	1.99037	up	-
c39081.graph_c0	3.96931897	3.35608298	3.71412527	17.7854139	17.1207022	17.323884	6.348E-14	1.15209	up	PREDICTED: UDP-galactose/UDP-glucose transporter 2-like [Sesamum indicum]
c39087.graph_c0	81.4900172	86.8394515	79.3069015	15.1833037	12.5579539	15.4158828	7.47E-187	-3.60987	down	ubiquitin [Galdieria sulphuraria]
c39094.graph_c0	3.98382019	3.29427732	5.56452899	24.2963299	24.2106041	26.1944546	1.028E-07	1.44461	up	-
c39102.graph_c1	12419.2522	12680.8182	12985.2777	370.04556	377.492734	381.132713	0	-6.16755	down	15 kDa oleosin [Sesamum indicum]
c39103.graph_c0	1.88858879	1.73719777	2.23013477	17.0649488	16.2454428	16.104402	1.386E-22	1.98374	up	hypothetical protein MIMGU_mgv1a011278mg [Erythranthe guttata]
c39104.graph_c0	0	0.06766919	0.02895686	0.52407867	0.42883197	0.60137425	1.604E-07	2.91264	up	PREDICTED: probable RNA helicase SDE3 [Sesamum indicum]
c39110.graph_c0	1.54254487	1.22955274	2.16577115	14.8028402	14.3028542	13.7561645	2.155E-35	2.02133	up	PREDICTED: tubulin gamma-1 chain [Sesamum indicum]
c39112.graph_c0	10.6251316	9.24459426	6.3371672	86.606886	83.4052071	77.9289954	1.251E-74	2.15717	up	PREDICTED: probable voltage-gated potassium channel subunit beta [Sesamum indicum]
c39115.graph_c0	3.65323793	2.58133842	3.3138038	0.31237086	0	0.31861508	1.281E-24	-5.00464	down	PREDICTED: stem-specific protein TSJT1-like [Sesamum indicum]
c39130.graph_c0	1.13022838	0.95730473	1.00549953	6.98226951	5.67675388	6.47088778	4.791E-08	1.5396	up	PREDICTED: xylosyltransferase 1 [Sesamum indicum]
c39132.graph_c0	84.8566816	87.7806117	84.7716573	52.0265803	50.6974001	55.909133	3.46E-99	-1.78837	down	PREDICTED: histidine kinase 3 isoform X2 [Sesamum indicum]
c39133.graph_c0	0.95594446	0.8657682	0.93594398	0	0	0	8.146E-28	-Inf	down	-
c39134.graph_c0	150.985195	143.330085	159.722013	80.636067	83.3585195	80.340766	1.514E-59	-1.98555	down	PREDICTED: high mobility group B protein 1 [Sesamum indicum]
c39144.graph_c0	27.0993377	27.5085366	24.4721188	27.2293352	28.368941	27.1749841	3.043E-35	-1.02346	down	PREDICTED: ankyrin repeat domain-containing protein 50 [Sesamum indicum]
c39146.graph_c0	1.80691612	1.28255079	1.09765323	7.92729209	7.24383766	7.20898818	4.841E-09	1.33319	up	PREDICTED: phytoene synthase 2, chloroplastic [Sesamum indicum]
c39157.graph_c0	0.5576278	0.41863964	0.3582869	4.58473761	5.10015563	5.37396794	6.853E-14	2.41114	up	PREDICTED: probable beta-1,3-galactosyltransferase 11 [Sesamum indicum]

#ID	0 h-1 FPKM	0 h-2 FPKM	0 h-3 FPKM	28 h-1 FPKM	28 h-2 FPKM	28 h-3 FPKM	FDR	log2FC	regulated	nr_annotation
c39167.graph_c0	14.0149597	14.9351726	13.2703628	12.7203057	12.4753506	12.6067203	1.153E-29	-1.24909	down	PREDICTED: uncharacterized protein LOC105176476 isoform X2 [Sesamum indicum]
c39170.graph_c0	65.9160176	62.6829214	66.9937112	5.60734126	4.96459462	5.99707313	0	-4.65149	down	PREDICTED: protein REVEILLE 6 isoform X2 [Nicotiana sylvestris]
c39171.graph_c0	0	0	0	3.3434172	4.21042078	6.461529	6.254E-08	Inf	up	-
c39178.graph_c0	15.6781098	18.6506137	17.2271379	3.1376875	3.72768108	4.58163846	2.407E-28	-3.26111	down	hypothetical protein SERLADRAFT_461711 [Serpula lacrymans var. lacrymans S7.9]
c39191.graph_c1	3.09783151	3.52480213	2.89255	25.3332679	22.7858155	23.2755331	2.696E-55	1.81817	up	PREDICTED: protein FAR1-RELATED SEQUENCE 5-like [Prunus mume]
c39191.graph_c2	1.24203038	0.88805292	0.22800832	11.6061496	11.7600639	11.7855401	1.966E-09	2.82342	up	-
c39195.graph_c0	1.89823259	1.9544229	2.26506803	17.324142	17.6707411	17.2241636	3.738E-43	2.00054	up	PREDICTED: armadillo repeat-containing protein LFR [Sesamum indicum]
c39213.graph_c0	2.3393633	1.36599506	1.25257269	0	0	0	4.458E-15	-Inf	down	PREDICTED: uncharacterized protein LOC104436401 [Eucalyptus grandis]
c39216.graph_c0	0.41945304	0.74285234	0.82925195	20.542608	21.3006133	19.4415548	4.484E-92	3.84582	up	PREDICTED: cell division protein FtsZ homolog 2-2, chloroplastic [Sesamum indicum]
c39218.graph_c0	28.9893274	25.5058176	27.2603777	25.9091235	27.2924276	26.3679206	8.501E-33	-1.12927	down	PREDICTED: F-box protein At4g00755-like [Sesamum indicum]
c39224.graph_c0	0.91197553	1.46406684	1.37093073	0.06253005	0.03387526	0.08163842	1.888E-20	-5.48644	down	hypothetical protein VITISV_033646 [Vitis vinifera]
c39225.graph_c0	4.94504722	4.80179538	5.32719404	29.6561428	30.7407586	29.6782278	4.154E-40	1.4873	up	PREDICTED: serine hydroxymethyltransferase, mitochondrial [Sesamum indicum]
c39227.graph_c1	1.81839612	2.18426037	1.76551499	0	0	0.02396477	1.28E-44	-8.98633	down	PREDICTED: phospholipase D delta-like isoform X2 [Sesamum indicum]
c39231.graph_c0	3.20044132	2.51165853	3.15854885	19.0737109	19.9436528	22.4880709	1.764E-26	1.70321	up	PREDICTED: glucose-1-phosphate adenylyltransferase large subunit 1 [Sesamum indicum]
c39235.graph_c0	1.58915372	1.08840449	2.14960573	7.3554484	6.86082672	6.94444641	0.0012264	1.03597	up	-
c39242.graph_c0	5.34134277	3.80530283	4.88507313	25.0410172	25.5148465	25.2562012	3.792E-20	1.3435	up	PREDICTED: sn1-specific diacylglycerol lipase beta isoform X1 [Sesamum indicum]
c39247.graph_c0	104.644516	94.5967513	100.746163	103.584367	101.570607	100.202986	2.667E-39	-1.06482	down	PREDICTED: protein CWC15 homolog A [Sesamum indicum]
c39252.graph_c0	1.05350156	0.35151883	0.67689561	8.47351277	9.12563818	9.10107418	7.045E-25	2.5947	up	hypothetical protein MIMGU_mgv1a009282mg [Erythranthe guttata]
c39255.graph_c0	14.9621014	14.1074465	16.1257809	165.991981	164.127909	160.923336	3.19E-113	2.34974	up	hypothetical protein MIMGU_mgv1a016658mg [Erythranthe guttata]
c39260.graph_c0	0.11986528	0.0399951	0	2.03273913	2.01891013	2.55913488	8.079E-13	4.30648	up	PREDICTED: probable galactinol--sucrose galactosyltransferase 2 [Sesamum indicum]
c39266.graph_c0	1.75138826	1.06712992	1.85919399	1.43892918	1.29921814	1.12899468	0.0010107	-1.3681	down	-
c39271.graph_c0	31.8571455	27.6564121	25.7481419	15.0515794	13.7478235	14.3337096	1.162E-48	-2.07115	down	PREDICTED: glycerophosphodiester phosphodiesterase GDPD1, chloroplastic-like [Sesamum indicum]
c39272.graph_c0	5.02700417	4.80839182	4.47407846	20.4601469	21.5769627	20.9464341	7.144E-24	1.04881	up	PREDICTED: ATP-dependent zinc metalloprotease FtsH [Sesamum indicum]

#ID	0 h-1 FPKM	0 h-2 FPKM	0 h-3 FPKM	28 h-1 FPKM	28 h-2 FPKM	28 h-3 FPKM	FDR	log2FC	regulated	nr_annotation
c39285.graph_c0	128.443647	127.704265	124.870853	30.0144734	34.6043943	39.372923	0	-2.96272	down	hypothetical protein MIMGU_mgv11b009377mg [Erythranthe guttata]
c39287.graph_c0	6.82663674	6.19645395	6.46785905	34.142262	34.4338991	38.9419715	5.312E-24	1.37422	up	-
c39293.graph_c0	11.0793087	10.564216	10.6520322	10.5797663	9.97709425	9.40038097	2.031E-19	-1.19896	down	hypothetical protein MIMGU_mgv1a0098172mg, partial [Erythranthe guttata]
c39296.graph_c0	1.54794011	1.0151832	0.96028623	0	0	0	3.884E-22	-Inf	down	glycosyltransferase family 35 protein [Micromonas sp. RCC299]
c39314.graph_c1	8.04413931	10.3372765	7.40355388	72.4483792	76.899011	71.8819778	2.226E-44	2.01194	up	PREDICTED: mitochondrial import inner membrane translocase subunit Tim9 [Elaeis guineensis]
c39316.graph_c0	21.5655409	23.4283912	23.3926727	11.4786882	12.2372237	10.4950118	6.699E-61	-2.09127	down	PREDICTED: splicing factor 3B subunit 2 [Solanum lycopersicum]
c39319.graph_c0	0	0	0	0.97818611	1.40407798	0.90052417	2.821E-12	Inf	up	PREDICTED: auxilin-like protein 1 [Sesamum indicum]
c39327.graph_c0	0.95615242	0.90925437	1.16725903	8.99929257	9.38042359	9.58321253	1.609E-15	2.11154	up	unknown [Glycine max]
c39328.graph_c0	11.1172394	9.09180693	9.99092767	74.0925726	69.3304573	64.3803619	5.868E-46	1.69251	up	PREDICTED: DNA-directed RNA polymerases II, IV and V subunit 9A [Sesamum indicum]
c39339.graph_c0	1.88498907	1.95807949	2.23947158	12.8986717	13.069986	12.0860636	1.393E-17	1.55219	up	PREDICTED: WD repeat domain-containing protein 83 [Sesamum indicum]
c39340.graph_c0	23.7726243	25.4663538	24.3235768	23.2935608	23.8740502	21.9944973	1.656E-37	-1.17999	down	PREDICTED: protein BASIC PENTACYSTEINE1-like [Sesamum indicum]
c39360.graph_c0	4.51259873	2.55969987	2.80278621	14.04818	14.3128986	14.0502259	2.078E-05	1.01719	up	-
c39362.graph_c0	0.82811828	0.4736843	0.60809416	9.11405561	7.63394848	8.84020154	4.755E-22	2.65677	up	PREDICTED: U-box domain-containing protein 4 [Sesamum indicum]
c39383.graph_c0	0.06582625	0.08785622	0.02819645	19.0092765	21.1377152	20.2481214	1.01E-191	7.29412	up	PREDICTED: probable auxin efflux carrier component 1c [Sesamum indicum]
c39389.graph_c0	53.9672251	50.7178065	61.9374802	35.8167483	34.1954373	42.6633065	3.536E-15	-1.65603	down	hypothetical protein MIMGU_mgv1a004314mg [Erythranthe guttata]
c39396.graph_c0	6.74864984	3.8413102	3.91102925	0.38469646	0.95519828	1.64802312	1.804E-07	-3.3592	down	hypothetical protein AALP_AA2G024800 [Arabis alpina]
c39398.graph_c0	0.16250198	0.13013176	0.0417643	1.01570733	0.57584626	0.73243576	0.0035893	1.72025	up	-
c39416.graph_c1	18.7431915	14.07147	15.3272911	3.40561089	2.79540666	4.04211007	1.372E-36	-3.31971	down	MYB-related transcription factor [Salvia miltiorrhiza]
c39418.graph_c0	1.13938771	0.985002	1.53071097	11.2922785	8.59851593	10.4737093	3.401E-14	1.95968	up	PREDICTED: metal transporter Nramp5-like isoform X1 [Sesamum indicum]
c39418.graph_c2	0.56164659	0.64252457	0.61863266	6.00639359	5.73912542	7.04245516	1.623E-08	2.2751	up	PREDICTED: metal transporter Nramp5-like isoform X2 [Sesamum indicum]
c39426.graph_c0	0.03494053	0.27980412	0.17959984	21.5599929	22.2639803	20.2866076	3.26E-120	5.92061	up	PREDICTED: serine/threonine-protein kinase HT1-like [Sesamum indicum]
c39433.graph_c0	1.1490699	0.69013231	0.88596019	5.84596165	7.13834712	6.5420667	1.735E-06	1.75271	up	PREDICTED: peroxisome biogenesis factor 10-like [Sesamum indicum]
c39444.graph_c0	5.22940674	5.48793238	6.71999469	36.4744854	39.0768514	35.2652638	8.111E-22	1.57368	up	PREDICTED: uncharacterized protein LOC104232221 [Nicotiana sylvestris]
c39445.graph_c0	0.12673338	0.25372058	0	3.13170919	2.57814214	2.17964778	3.581E-07	3.29898	up	-

#ID	0 h-1 FPKM	0 h-2 FPKM	0 h-3 FPKM	28 h-1 FPKM	28 h-2 FPKM	28 h-3 FPKM	FDR	log2FC	regulated	nr_annotation
c39446.graph_c0	2.88144344	1.71500629	1.90142187	0.11320089	0	0.0461855	2.561E-18	-6.43445	down	PREDICTED: polyubiquitin [Sesamum indicum]
c39449.graph_c0	56.9708962	62.4224792	53.9887364	38.5284737	35.2567267	35.5807693	1.715E-68	-1.75446	down	-
c39464.graph_c0	2.32825901	1.84360137	2.27742023	10.8349087	9.25841521	10.2013812	1.736E-09	1.14117	up	PREDICTED: ribulose-1,5 bisphosphate carboxylase/oxygenase large subunit N-methyltransferase, chloroplastic isoform X1 [Sesamum indicum]
c39470.graph_c0	4.32948521	3.73417241	3.39412145	3.58202973	3.50226769	3.50425868	1.929E-11	-1.20158	down	hypothetical protein MIMGU_mgv1a003704mg [Erythranthe guttata]
c39481.graph_c0	10.0800191	5.35552135	6.37697162	0	0	0	7.319E-26	-Inf	down	-
c39483.graph_c0	0.25629874	0.15393323	0.19761242	2.34708328	2.28738631	2.46240101	3.489E-07	2.45757	up	PREDICTED: protein DYAD-like [Sesamum indicum]
c39502.graph_c1	1.86295396	1.6735559	1.35044439	12.4809569	11.2848073	12.139076	2.973E-28	1.79069	up	PREDICTED: guanylate-binding protein 4 isoform X1 [Sesamum indicum]
c39522.graph_c0	62.5869409	58.4308949	58.7635145	39.3134462	38.3273536	36.7831251	1.992E-98	-1.74199	down	hypothetical protein MIMGU_mgv1a011861mg [Erythranthe guttata]
c39525.graph_c0	2.92912773	2.70058233	2.57539928	20.5884443	16.6925538	18.2171455	1.231E-22	1.66886	up	PREDICTED: WAT1-related protein At2g37460-like [Sesamum indicum]
c39536.graph_c0	96.1937466	99.5586813	85.9855126	59.5642158	63.6869861	57.3342052	8.86E-86	-1.73106	down	PREDICTED: ubiquitin-activating enzyme E1 1-like isoform X1 [Sesamum indicum]
c39539.graph_c0	0.36494821	0.18265683	0.30148258	3.35341996	3.4041635	3.46296825	1.157E-13	2.50007	up	PREDICTED: probable inactive leucine-rich repeat receptor-like protein kinase At5g20690 [Sesamum indicum]
c39541.graph_c0	3.516826	2.57138431	2.98664182	23.715317	21.6535255	23.142006	1.408E-42	1.82744	up	PREDICTED: UPF0160 protein-like [Sesamum indicum]
c39547.graph_c2	0.19264372	0.16528853	0.14145989	4.84042681	4.89418337	4.73316999	1.212E-26	3.76947	up	PREDICTED: homeobox-leucine zipper protein ATHB-14-like [Sesamum indicum]
c39550.graph_c0	26.0202306	29.5692131	22.0555771	12.5954464	11.1317034	11.0493569	1.212E-24	-2.2471	down	PREDICTED: uncharacterized protein LOC105155212 [Sesamum indicum]
c39552.graph_c0	0.77605837	0.83982214	0.37734379	7.88124232	7.11602288	8.14762909	7.771E-32	2.45581	up	PREDICTED: anaphase-promoting complex subunit 2 [Sesamum indicum]
c39554.graph_c0	1.60274	1.79089664	1.5566626	15.4818313	13.8196549	14.4346112	4.752E-52	2.05343	up	PREDICTED: cleavage and polyadenylation specificity factor subunit 6 [Nicotiana sylvestris]
c39567.graph_c1	2.05905749	1.63171946	1.54348273	8.23081113	8.78285996	6.30922772	0.0014618	1.06695	up	PREDICTED: DCN1-like protein 1 [Sesamum indicum]
c39579.graph_c0	2.31561616	2.14749851	2.01294542	21.6559474	22.2348841	22.8815192	2.795E-51	2.27734	up	PREDICTED: parafibromin [Sesamum indicum]
c39585.graph_c0	4.73915814	3.95325308	4.19533736	18.9441605	18.3833414	18.8622248	1.396E-09	1.03499	up	PREDICTED: E3 ubiquitin ligase BIG BROTHER-like isoform X2 [Sesamum indicum]
c39586.graph_c0	1.5916474	1.41621439	1.5150592	11.9964461	11.1024577	10.9427058	1.915E-12	1.82157	up	PREDICTED: uncharacterized protein LOC105155506 isoform X4 [Sesamum indicum]
c39590.graph_c0	0.09643865	0.09653522	0.04130916	0.93454742	0.67504696	0.99135798	2.82E-06	2.39296	up	PREDICTED: pentatricopeptide repeat-containing protein At3g09040, mitochondrial [Sesamum indicum]
c39594.graph_c0	1.82864045	1.54446037	1.3952385	10.840026	11.887533	8.98093399	2.718E-10	1.64471	up	unnamed protein product [Coffea canephora]
c39612.graph_c0	2.11962849	2.81456762	3.05733386	16.3799611	16.8050382	15.5978426	5.693E-18	1.51502	up	PREDICTED: MOB kinase activator-like 1 [Sesamum indicum]
c39649.graph_c0	4.9320028	9.35420486	7.00495769	3.01856944	3.40685648	1.23156401	7.132E-06	-2.5719	down	-

#ID	0 h-1 FPKM	0 h-2 FPKM	0 h-3 FPKM	28 h-1 FPKM	28 h-2 FPKM	28 h-3 FPKM	FDR	log2FC	regulated	nr_annotation
c39654.graph_c0	4.34557966	4.26070175	4.15238501	25.0561819	29.7599123	27.873209	1.436E-35	1.60615	up	PREDICTED: glucan endo-1,3-beta-glucosidase 3 [Sesamum indicum]
c39655.graph_c0	0.76196945	0.65377067	1.95832182	6.40819932	5.42884247	6.39104683	0.0008466	1.32912	up	PREDICTED: UDP-arabinose 4-epimerase 1-like [Sesamum indicum]
c39655.graph_c1	0.11906544	0.357554	0.45901141	6.66327777	4.06296603	5.5784057	3.844E-08	3.02099	up	PREDICTED: probable UDP-arabinose 4-epimerase 3 [Sesamum indicum]
c39662.graph_c0	0.46078635	0.6259791	0.33835926	12.8935292	12.5271908	10.8528462	8.106E-50	3.58298	up	PREDICTED: folic acid synthesis protein fol1 isoform X2 [Sesamum indicum]
c39674.graph_c0	0.5809124	0.74008339	0.67863221	3.93417159	4.53986965	4.91716631	6.18E-10	1.65228	up	PREDICTED: auxin response factor 18-like [Sesamum indicum]
c39675.graph_c0	49.7075032	52.2378459	50.2017518	33.937604	34.1818238	33.2256059	1.164E-79	-1.67685	down	LIM domain and RING finger protein [Medicago truncatula]
c39682.graph_c0	25.042137	26.16026	23.8544624	12.7509183	13.4237357	12.4769396	1.7E-57	-2.04751	down	PREDICTED: uncharacterized protein LOC105170284 [Sesamum indicum]
c39688.graph_c1	65.0624152	69.5398775	66.6738114	48.7886923	49.2040266	51.2480372	4.802E-73	-1.522	down	PREDICTED: delta(3,5)-Delta(2,4)-dienoyl-CoA isomerase, mitochondrial [Sesamum indicum]
c39689.graph_c0	0.45674427	0.3657613	0.23477379	2.12453826	2.31789611	2.09477376	8.144E-05	1.5451	up	PREDICTED: uncharacterized protein LOC105163339 [Sesamum indicum]
c39695.graph_c0	28.1681222	25.4109025	25.5952134	542.891914	572.224302	543.169473	0	3.29879	up	hypothetical protein MIMGU_mgv1a004702mg [Erythranthe guttata]
c39709.graph_c0	31.446007	35.061271	31.2079908	12.7934708	13.7440947	12.9165058	4.34E-105	-2.39873	down	PREDICTED: uncharacterized protein LOC105156369 [Sesamum indicum]
c39709.graph_c1	15.2156393	16.5279087	14.5099104	15.8211185	16.2285222	15.7641339	9.887E-22	-1.04192	down	PREDICTED: F-box/kelch-repeat protein At5g15710-like [Sesamum indicum]
c39711.graph_c0	0.097301	0.06493229	0	0.68360543	0.68108315	1.15410299	0.0003418	2.89124	up	PREDICTED: LOW QUALITY PROTEIN: BTB/POZ domain-containing protein At3g22104-like [Sesamum indicum]
c39713.graph_c0	27.0728429	27.4641989	25.5778927	26.7905634	24.4281563	27.5149292	1.431E-33	-1.11442	down	PREDICTED: eukaryotic translation initiation factor 4G-like [Sesamum indicum]
c39715.graph_c0	19.5371557	20.5964435	19.5763385	9.23865691	8.99079593	8.80000225	1.444E-74	-2.234	down	PREDICTED: serine carboxypeptidase-like 50 [Sesamum indicum]
c39715.graph_c1	26.3388863	24.9054392	21.9775168	23.4790218	22.0983704	21.4388625	1.306E-34	-1.21633	down	PREDICTED: zinc finger CCHC domain-containing protein 10 [Sesamum indicum]
c39718.graph_c0	0.35238792	0.61729637	0.56604053	51.094645	59.2569119	51.4890567	4.64E-146	5.62444	up	PREDICTED: GDSL esterase/lipase At1g71691 [Sesamum indicum]
c39721.graph_c0	76.9641607	73.1921743	69.1001439	19.3880376	20.9357276	19.8468639	1.5E-227	-2.95489	down	PREDICTED: UDP-glucuronic acid decarboxylase 2-like [Sesamum indicum]
c39727.graph_c0	4.19624405	3.85483964	3.78828931	3.01120085	3.57282416	3.59116852	3.03E-13	-1.30768	down	-
c39735.graph_c0	1.33993719	1.82901672	0.78266924	11.0665753	11.2710661	7.22418804	2.659E-05	1.8175	up	-
c39737.graph_c0	0.33116918	0.57259229	0.38687774	14.944776	14.1852111	14.5159366	3.183E-72	3.98909	up	PREDICTED: LRR receptor-like serine/threonine-protein kinase FEI 1 [Sesamum indicum]
c39750.graph_c0	1.53840651	1.11770346	0.98845664	10.1711486	11.2840963	11.1249611	1.555E-30	2.07476	up	PREDICTED: uncharacterized membrane protein At3g27390 [Sesamum indicum]

#ID	0 h-1 FPKM	0 h-2 FPKM	0 h-3 FPKM	28 h-1 FPKM	28 h-2 FPKM	28 h-3 FPKM	FDR	log2FC	regulated	nr_annotation
c39753.graph_c0	30.1535127	28.5156599	27.756132	17.9936962	17.7775521	18.3251505	4.17E-101	-1.7654	down	PREDICTED: RNA polymerase-associated protein RTF1 homolog [Sesamum indicum]
c39760.graph_c0	75.0796781	73.5094568	75.9283297	52.4364397	49.8232312	52.5888315	1.239E-84	-1.62655	down	PREDICTED: uncharacterized protein LOC105175444 [Sesamum indicum]
c39762.graph_c0	2.35388898	2.7282849	2.8656384	17.8882912	13.8750709	17.5601234	2.403E-12	1.54164	up	PREDICTED: flavonol synthase/flavanone 3-hydroxylase-like [Sesamum indicum]
c39764.graph_c0	59.6739735	53.9118827	65.3191202	22.6407793	23.1611967	22.4909282	1.832E-34	-2.48159	down	PREDICTED: putative cytochrome c oxidase subunit 6b-like isoform X2 [Sesamum indicum]
c39775.graph_c0	1.27748844	1.0893206	1.45922065	13.5831903	12.6990331	12.7393232	6.93E-44	2.25737	up	PREDICTED: LOW QUALITY PROTEIN: ARF guanine-nucleotide exchange factor GNOM [Sesamum indicum]
c39776.graph_c0	0.26292315	0.31582372	0.13514666	1.22298261	1.13874609	1.21624403	0.0086458	1.24289	up	-
c39778.graph_c0	1.32659878	0.90687709	1.24736485	6.18476157	6.00891836	6.06373353	6.689E-08	1.30027	up	PREDICTED: uncharacterized protein LOC105176769 [Sesamum indicum]
c39783.graph_c0	22.6527503	23.1350708	21.8322608	16.6492925	15.3675552	14.1605822	1.29E-48	-1.64076	down	PREDICTED: histone H2AX [Beta vulgaris subsp. vulgaris]
c39791.graph_c0	10.9044414	9.86122918	10.782311	9.06099583	9.18437774	10.2947684	1.596E-22	-1.23466	down	hypothetical protein M569_06867, partial [Genlisea aurea]
c39798.graph_c1	44.6331686	44.1613549	43.8572172	46.4219751	43.5351666	41.6176744	8.696E-39	-1.10242	down	PREDICTED: LOW QUALITY PROTEIN: mitogen-activated protein kinase homolog MMK1-like [Sesamum indicum]
c39800.graph_c0	5.72728228	6.36588284	4.97024407	5.75730114	5.4667601	5.4919238	4.65E-11	-1.11817	down	PREDICTED: cyclin-dependent kinase inhibitor 3 isoform X2 [Sesamum indicum]
c39801.graph_c0	19.2034721	23.0502305	19.6544245	11.9190078	12.2115154	9.97777707	1.023E-32	-1.9512	down	hypothetical protein VITISV_001313 [Vitis vinifera]
c39804.graph_c0	0.8507405	1.36254783	2.07714694	11.067808	9.32325819	9.0816948	5.129E-08	1.67886	up	-
c39812.graph_c0	105.887973	109.314788	96.136581	42.7603388	44.4731707	44.9702391	2.99E-162	-2.32481	down	hypothetical protein M569_12244, partial [Genlisea aurea]
c39821.graph_c0	8.01847922	8.83282444	7.81038585	8.54209507	9.27447484	8.25142159	7.309E-13	-1.01035	down	PREDICTED: tubulin beta chain [Vitis vinifera]
c39822.graph_c0	3.20402339	2.15168711	2.60588412	12.4981553	15.9954767	11.954248	4.352E-07	1.25518	up	PREDICTED: uric acid degradation bifunctional protein TTL isoform X1 [Sesamum indicum]
c39823.graph_c0	18.4329858	19.8707854	15.8110033	12.299964	12.0662085	11.8541213	8.944E-31	-1.6677	down	hypothetical protein MIMGU_mgv1a001998mg [Erythranthe guttata]
c39824.graph_c0	0.41940471	0.64881996	0.19598242	8.83980691	10.0081716	8.75082247	5.662E-31	3.36602	up	PREDICTED: apurinic endonuclease-redox protein [Sesamum indicum]
c39835.graph_c0	1.29639201	0.97326761	0.83295753	7.3806469	6.2386684	6.85545312	1.324E-07	1.63701	up	PREDICTED: methionine aminopeptidase 2B [Sesamum indicum]
c39843.graph_c0	0	0	0.5181663	1.52393863	2.11688261	1.67397122	0.0018674	2.22765	up	PREDICTED: uncharacterized protein LOC105177798 [Sesamum indicum]
c39865.graph_c0	0.7211291	0.56988253	0.56088493	8.48234662	7.3723384	6.88776204	2.323E-36	2.52999	up	PREDICTED: uncharacterized protein LOC105169651 [Sesamum indicum]
c39874.graph_c0	0.59010848	0.72968747	0.75831276	6.23148224	6.4009345	5.72301674	1.351E-13	2.04917	up	PREDICTED: uncharacterized protein LOC105156620 [Sesamum indicum]

#ID	0 h-1 FPKM	0 h-2 FPKM	0 h-3 FPKM	28 h-1 FPKM	28 h-2 FPKM	28 h-3 FPKM	FDR	log2FC	regulated	nr_annotation
c39877.graph_c0	12.6592941	14.1862311	14.3749121	13.1355934	12.9051616	14.2362947	1.495E-18	-1.12484	down	hypothetical protein MIMGU_mgv1a019094mg, partial [Erythranthe guttata]
c39878.graph_c0	71.4927016	67.0544349	76.025879	38.5610553	41.9360278	40.4656957	4.508E-46	-1.91854	down	PREDICTED: uncharacterized protein LOC105157942 isoform X1 [Sesamum indicum]
c39881.graph_c0	652.275533	554.684526	688.800879	59.2828218	58.1148438	58.6773958	1.174E-68	-4.52028	down	-
c39883.graph_c0	0	0.1269072	0	2.53393667	2.53749712	3.00752238	1.445E-12	4.9115	up	PREDICTED: adenine phosphoribosyltransferase 5-like isoform X1 [Sesamum indicum]
c39885.graph_c0	0.05817756	0.01941194	0.04984031	0.4510199	0.50903585	0.62104819	4.951E-07	2.54373	up	--
c39886.graph_c0	85.567761	80.6418064	81.518036	50.612201	47.3408133	51.252776	6.51E-102	-1.82124	down	PREDICTED: membrane-anchored ubiquitin-fold protein 3 [Sesamum indicum]
c39894.graph_c0	392.557405	381.642285	443.490563	41.6168886	31.6913415	52.7919198	7.003E-88	-4.36176	down	PREDICTED: beta-amyrin 28-oxidase-like [Sesamum indicum]
c39900.graph_c0	18.3967698	19.5719245	16.9879527	17.0772748	17.1885918	16.6487989	4.951E-46	-1.19969	down	PREDICTED: septin and tuftelin-interacting protein 1 homolog 1 [Sesamum indicum]
c39908.graph_c0	5.05182786	6.08067333	5.45629715	1.91465857	2.03383196	1.80129275	2.626E-45	-2.6201	down	-
c39912.graph_c0	11.8171362	13.1227627	13.0203694	62.0494712	62.9162603	61.3769311	2.282E-46	1.20378	up	hypothetical protein MIMGU_mgv1a002912mg [Erythranthe guttata]
c39913.graph_c0	0.4939992	1.29329165	0.1953257	98.4310378	103.686331	99.9704665	0	6.17042	up	PREDICTED: uncharacterized protein LOC105168623 [Sesamum indicum]
c39917.graph_c0	11.3335328	10.501396	12.9397962	6.15489373	7.32677796	6.49656311	4.805E-19	-1.89256	down	PREDICTED: protein OPI10 homolog [Sesamum indicum]
c39921.graph_c0	17.4650339	19.7850986	18.2283063	10.0309499	8.80542373	9.92831194	5.446E-68	-2.03799	down	PREDICTED: autophagy-related protein 18a-like [Sesamum indicum]
c39926.graph_c0	1055.18742	1151.14368	1166.76017	52.418733	46.4805396	56.401703	2.01E-245	-5.53181	down	-
c39935.graph_c0	5.86637835	6.32396438	4.73574222	2.24114934	2.81322489	2.85464908	2.079E-16	-2.1849	down	MYB5b [Vitis vinifera]
c39936.graph_c0	4.58045339	5.42316561	5.94935496	33.433656	39.6573976	33.5031949	3.101E-24	1.64614	up	PREDICTED: ABC transporter I family member 19-like [Nicotiana sylvestris]
c39938.graph_c0	1.43519285	1.40539889	1.08251196	0.70295188	0.81896728	0.61060964	2.137E-09	-1.9676	down	PREDICTED: thioredoxin reductase NTRC [Sesamum indicum]
c39942.graph_c0	0.84248656	1.05416273	0.99240953	5.23018529	5.41344503	5.64180819	1.572E-07	1.40318	up	PREDICTED: uncharacterized protein LOC105158797 [Sesamum indicum]
c39944.graph_c1	1.46626252	1.01222811	1.23447939	8.89284201	10.252419	8.36594926	1.251E-11	1.79948	up	Reticulon-4-interacting 1, mitochondrial [Gossypium arboreum]
c39945.graph_c0	0.07594888	0.15204986	0.19519463	23.4044638	28.209275	24.0975247	5.482E-69	6.38231	up	PREDICTED: protodermal factor 1-like [Sesamum indicum]
c39951.graph_c0	10.0060133	10.246817	9.48064385	47.5548933	48.6868319	45.5315952	1.111E-26	1.16337	up	PREDICTED: peptide chain release factor PrfB2, chloroplastic isoform X1 [Sesamum indicum]
c39953.graph_c0	0.30958405	0.2213529	0.28416269	5.59295424	6.53006029	6.34736096	4.009E-20	3.41192	up	hypothetical protein MIMGU_mgv1a010093mg [Erythranthe guttata]
c39981.graph_c0	4.54478588	3.90599624	4.01147097	43.4744527	45.9411864	44.9764903	5.069E-75	2.34151	up	PREDICTED: 2-aminoethanethiol dioxygenase-like isoform X1 [Sesamum indicum]
c39982.graph_c0	11.1973922	13.5376654	10.4647944	5.98915161	10.0518529	6.98567653	3.119E-13	-1.70252	down	hypothetical protein MIMGU_mgv1a011446mg [Erythranthe guttata]

#ID	0 h-1 FPKM	0 h-2 FPKM	0 h-3 FPKM	28 h-1 FPKM	28 h-2 FPKM	28 h-3 FPKM	FDR	log2FC	regulated	nr_annotation
c39988.graph_c0	86.2966659	90.8810335	99.1667717	12.8198542	12.0513182	13.6719942	3.633E-91	-3.93368	down	hypothetical protein JCGZ_17893 [Jatropha curcas]
c39996.graph_c0	1.90481188	1.82381842	1.27709148	8.87825775	8.42383947	9.16010343	1.956E-08	1.31696	up	PREDICTED: exosome complex component MTR3 [Sesamum indicum]
c39997.graph_c0	14.6520722	12.7245725	12.5523286	64.1373787	65.81285	70.0320651	7.076E-29	1.23584	up	Pollen-specific protein SF3, putative [Solanum lycopersicum]
c40001.graph_c0	2.34215324	1.8399862	2.43828629	12.1959636	10.6810682	11.6045803	1.446E-14	1.28922	up	PREDICTED: protein IQ-DOMAIN 1-like [Sesamum indicum]
c40010.graph_c0	4.97607701	4.71889876	3.77684241	36.9483766	37.4474893	37.8293448	6.584E-71	1.97131	up	PREDICTED: protein RRP45A-like isoform X1 [Sesamum indicum]
c40013.graph_c0	2.67608149	2.47270263	2.99798981	0.39896628	0.13508593	0.12208246	1.659E-27	-4.72861	down	PREDICTED: transcription repressor OFP7 [Sesamum indicum]
c40024.graph_c0	16.1854622	15.7848169	16.3394915	7.99030232	7.99788584	9.00619124	7.776E-83	-2.04088	down	PREDICTED: pheophytinase, chloroplastic [Sesamum indicum]
c40027.graph_c0	74.765173	72.4832512	77.1820432	30.5219768	28.2837065	31.0038718	8.46E-153	-2.41208	down	hypothetical protein MIMGU_mgv1a025482mg [Erythranthe guttata]
c40032.graph_c0	92.7411866	103.667521	88.1293072	64.3436867	62.4169169	66.2792899	5.666E-46	-1.64904	down	PREDICTED: ubiquitin-activating enzyme E1 1-like isoform X2 [Sesamum indicum]
c40037.graph_c0	61.9454695	58.230392	60.2068769	29.2526198	42.0947195	30.3968244	7.34E-30	-1.91755	down	elicitor and UV light related transcription factor [Oryza sativa]
c40039.graph_c0	1.7391013	1.56380789	1.32573714	0.2356552	0.25146083	0.33214197	1.7E-24	-3.58399	down	hypothetical protein [Vitis hybrid cultivar]
c40040.graph_c0	0.86367174	1.40487193	1.47980317	20.7929481	22.0093647	20.6376967	5.88E-66	2.98498	up	PREDICTED: G-type lectin S-receptor-like serine/threonine-protein kinase At1g11330 [Sesamum indicum]
c40045.graph_c0	7.77920686	8.26375145	8.10947765	5.30772663	6.09465653	5.13136644	1.077E-24	-1.6383	down	unnamed protein product [Coffea canephora]
c40053.graph_c0	18.8819513	18.0194715	17.0979869	85.1128608	84.1678792	81.1136644	4.238E-22	1.12363	up	PREDICTED: serine/threonine-protein phosphatase PP2A-2 catalytic subunit [Sesamum indicum]
c40056.graph_c0	0.69253372	0.9358567	1.02342324	7.12211267	7.58947135	7.18747357	4.699E-15	1.95091	up	PREDICTED: uncharacterized protein LOC105176075 isoform X2 [Sesamum indicum]
c40061.graph_c0	1.74351121	0.96958726	1.12024047	6.12467385	6.35632902	7.69758202	3.402E-06	1.31136	up	PREDICTED: uncharacterized protein LOC105157824 isoform X1 [Sesamum indicum]
c40063.graph_c0	2.69619634	2.6624246	2.43466743	15.0411002	16.5455047	16.4221749	2.104E-20	1.53386	up	PREDICTED: zinc finger protein VAR3, chloroplastic [Sesamum indicum]
c40070.graph_c0	6.73457168	7.90512548	5.29963503	3.38001743	3.72842574	3.53865152	7.719E-12	-1.9924	down	PREDICTED: protein TRM32 isoform X2 [Sesamum indicum]
c40072.graph_c0	0.90089815	0.75150022	0.72355612	3.81947757	3.05450098	3.25023376	0.0025282	1.00306	up	PREDICTED: putative zinc finger protein At1g68190 isoform X1 [Sesamum indicum]
c40081.graph_c0	0.56837703	0.42670963	0.41084268	4.44075138	4.12613976	3.93962996	8.632E-10	2.06582	up	PREDICTED: RING-H2 finger protein ATL52-like [Sesamum indicum]
c40085.graph_c0	0.29534307	0.25340469	0.16265466	2.69850288	2.29805787	2.30204524	5.084E-07	2.27508	up	PREDICTED: uncharacterized protein LOC105172351 [Sesamum indicum]
c40091.graph_c0	2.26961146	2.59643901	1.24994596	25.4500538	27.181193	25.2857893	4.524E-30	2.58767	up	PREDICTED: universal stress protein A-like protein [Sesamum indicum]
c40095.graph_c0	0.03654176	0.18289173	0.23478802	7.94094294	7.69748009	7.95339888	3.771E-38	4.5937	up	PREDICTED: alcohol-forming fatty acyl-CoA reductase-like isoform X1 [Sesamum indicum]
c40096.graph_c0	51.9092037	47.4031844	47.1640833	45.4451113	46.0391345	45.5612466	2.847E-49	-1.18557	down	PREDICTED: uncharacterized protein LOC105168199 [Sesamum indicum]
c40099.graph_c0	0	0.09194924	0	4.1725831	4.67163963	4.05851413	9.687E-22	6.05045	up	PREDICTED: glucan 1,3-beta-glucosidase A-like [Sesamum indicum]

#ID	0 h-1 FPKM	0 h-2 FPKM	0 h-3 FPKM	28 h-1 FPKM	28 h-2 FPKM	28 h-3 FPKM	FDR	log2FC	regulated	nr_annotation
c40100.graph_c0	0.40807616	0.22280988	0.19068874	4.28703607	4.28464984	4.42223566	1.882E-14	2.90156	up	PREDICTED: uncharacterized protein LOC105169180 [Sesamum indicum]
c40100.graph_c1	0.36630627	0.22917067	0.11767952	2.92852217	3.42541622	3.04137295	5.13E-09	2.6411	up	-
c40102.graph_c0	1.28530288	1.85420312	1.06872447	19.232515	18.456682	19.3030974	2.728E-48	2.67235	up	PREDICTED: protein Brevis radix-like 2 [Sesamum indicum]
c40109.graph_c0	0.43065006	0.64662194	0.33204143	2.09705968	2.76952135	2.37522496	0.0019396	1.27461	up	PREDICTED: uncharacterized protein LOC101260668 [Solanum lycopersicum]
c40115.graph_c0	31.1628105	34.853573	29.5746882	33.1204142	31.4361564	30.4502393	1.028E-25	-1.09861	down	PREDICTED: LOW QUALITY PROTEIN: DEAD-box ATP-dependent RNA helicase 42 [Sesamum indicum]
c40116.graph_c0	34.6005774	36.3686526	35.5191365	16.555236	14.8385581	14.2989337	4.81E-142	-2.31175	down	PREDICTED: LOW QUALITY PROTEIN: uncharacterized protein LOC105178147 [Sesamum indicum]
c40121.graph_c0	2.04138988	2.64889595	2.67184583	16.512702	16.6955754	15.6937787	1.649E-22	1.63816	up	hypothetical protein MIMGU_mgv1a007621mg [Erythranthe guttata]
c40126.graph_c0	19.5486758	20.5900916	19.5129103	7.4192506	7.50275419	7.7340462	6.3E-139	-2.48687	down	PREDICTED: cryptochrome-1 isoform X1 [Sesamum indicum]
c40127.graph_c0	312.704077	328.723827	320.467489	349.072322	338.545394	333.928702	2.453E-32	-1.00403	down	PREDICTED: protein YLS9-like [Sesamum indicum]
c40129.graph_c0	0.02007546	0.04019112	0	0.80248993	0.60600665	0.86913167	7.09E-11	4.16719	up	PREDICTED: probable linoleate 9S-lipoxygenase 5 isoform X1 [Sesamum indicum]
c40130.graph_c0	0	0.03273848	0	17.1384143	15.409994	15.9632391	1.25E-110	9.45147	up	PREDICTED: bifunctional 3-dehydroquinate dehydratase/shikimate dehydrogenase, chloroplastic-like isoform X1 [Sesamum indicum]
c40132.graph_c0	0.32051135	0.14583286	0.37442707	1.33414414	1.31933156	1.43425413	0.0030158	1.18694	up	PREDICTED: putative pentatricopeptide repeat-containing protein At3g05240 isoform X1 [Sesamum indicum]
c40136.graph_c1	1.12724178	1.60347392	1.44855031	0.30183736	0.23359786	0.24629682	1.923E-15	-3.51129	down	-
c40148.graph_c0	3.6894133	3.13750742	4.61517378	36.7571439	36.0378594	34.7762289	2.476E-74	2.13845	up	PREDICTED: signal peptide peptidase-like 2 [Sesamum indicum]
c40152.graph_c0	43.6727573	45.0671868	44.0680585	43.987866	45.591308	45.6332588	5.611E-37	-1.06463	down	PREDICTED: peptide methionine sulfoxide reductase A1-like [Nicotiana tomentosiformis]
c40153.graph_c0	0.13820625	0.27668928	0.88800251	7.13177289	8.43460521	7.45875533	3.193E-10	3.0305	up	Lysine decarboxylase family protein isoform 3 [Theobroma cacao]
c40154.graph_c0	8.15228533	6.81220058	6.14896205	37.4307582	35.8433262	35.1887854	3.696E-30	1.27352	up	PREDICTED: serine/threonine-protein kinase At5g01020 [Sesamum indicum]
c40159.graph_c0	51.0736622	52.3218119	48.485756	49.6992324	46.0921323	44.4920092	3.786E-49	-1.20467	down	PREDICTED: pentatricopeptide repeat-containing protein At5g01110 [Sesamum indicum]
c40161.graph_c0	73.0220927	73.9855503	65.692891	39.2105698	38.4749583	38.5329646	9.92E-126	-1.96113	down	PREDICTED: DNA-directed RNA polymerase II subunit RPB2 [Sesamum indicum]
c40167.graph_c0	5.18621546	4.80968744	5.09637638	23.8907832	21.9715296	21.6205544	1.128E-15	1.06998	up	PREDICTED: probable methyltransferase PMT23 [Sesamum indicum]
c40167.graph_c1	1.54231144	1.54385584	1.5155945	7.45097452	7.73963788	6.18754382	0.0013764	1.12458	up	PREDICTED: probable methyltransferase PMT23 [Sesamum indicum]
c40171.graph_c0	0.033963	0.0169985	0.0218219	3.72730078	3.25396595	3.52485579	6.921E-43	6.08767	up	PREDICTED: 125 kDa kinesin-related protein-like [Sesamum indicum]
c40173.graph_c0	55.1260178	51.5620071	53.7629061	305.88163	312.326803	310.053778	1.243E-74	1.44199	up	PREDICTED: 40S ribosomal protein S5-like, partial [Nicotiana sylvestris]

#ID	0 h-1 FPKM	0 h-2 FPKM	0 h-3 FPKM	28 h-1 FPKM	28 h-2 FPKM	28 h-3 FPKM	FDR	log2FC	regulated	nr_annotation
c40175.graph_c0	379.855921	371.686769	378.37508	336.521564	321.706828	339.288619	7.997E-53	-1.27018	down	hypothetical protein EUTSA_v10026962mg [Eutrema salsugineum]
c40179.graph_c0	0	0	0	0.98127046	1.29945966	1.5213465	2.671E-14	Inf	up	PREDICTED: RING-H2 finger protein ATL46-like [Sesamum indicum]
c40181.graph_c0	4.19194185	4.52250585	5.98534583	89.8431838	91.7260867	89.1392536	1.59E-181	3.10752	up	PREDICTED: histone deacetylase HDT1 [Sesamum indicum]
c40182.graph_c0	10.017294	9.83705296	7.98737481	9.07646313	9.09329879	10.1005518	6.184E-15	-1.06512	down	PREDICTED: WPP domain-associated protein [Sesamum indicum]
c40184.graph_c0	54.8806914	54.255137	58.6110469	5.56980215	4.86678302	5.86440396	5.67E-217	-4.45404	down	PREDICTED: ADP-ribosylation factor-like protein 8B isoform X1 [Nelumbo nucifera]
c40186.graph_c0	0	0.04339346	0.05570654	1.16574313	1.05255675	1.0797824	3.4E-10	3.94743	up	PREDICTED: tetratricopeptide repeat protein 7A-like [Sesamum indicum]
c40190.graph_c0	50.8635568	53.8755901	42.9760993	22.9328894	19.681721	20.0775437	2.622E-46	-2.32483	down	PREDICTED: SNF1-related protein kinase regulatory subunit beta-3-like [Sesamum indicum]
c40192.graph_c0	0.3642044	0.18228455	0.07800284	2.64701657	4.38168092	4.24788967	1.7E-07	3.10039	up	PREDICTED: endo-1,3;1,4-beta-D-glucanase-like [Sesamum indicum]
c40195.graph_c0	1.9488645	1.43744336	2.10894151	22.2900327	20.9335345	22.7508136	1.219E-20	2.49341	up	PREDICTED: growth-regulating factor 6-like [Sesamum indicum]
c40198.graph_c0	40.2043098	38.1351979	37.0556518	20.8773725	21.6159924	22.0017897	3.459E-85	-1.92879	down	hypothetical protein MIMGU_mgv1a012947mg [Erythranthe guttata]
c40199.graph_c0	2.57280587	2.41834665	1.93531165	18.3569498	15.7716113	14.5883614	1.917E-19	1.7266	up	hypothetical protein MIMGU_mgv1a006303mg [Erythranthe guttata]
c40200.graph_c0	0.88965153	1.00186018	0.85742808	10.2916149	9.82752561	9.87079922	2.171E-23	2.35777	up	PREDICTED: patatin-like protein 2 [Sesamum indicum]
c40209.graph_c0	0	0	0.04902479	3.82639476	5.50777286	4.68346031	2.941E-26	7.02903	up	PREDICTED: leucine-rich repeat receptor-like tyrosine-protein kinase At2g41820 [Sesamum indicum]
c40211.graph_c1	0.93400196	1.32180779	0.53803391	5.96899815	6.38279449	5.80658329	8.323E-10	1.61635	up	PREDICTED: histone deacetylase 15 isoform X8 [Sesamum indicum]
c40212.graph_c0	5.23069748	5.28227094	4.40179069	21.901488	21.324183	22.2912528	3.412E-12	1.04702	up	PREDICTED: protein TIFY 7 [Sesamum indicum]
c40215.graph_c1	0.02279387	0	0.05858205	29.9355819	38.7111861	36.6069903	2.281E-98	9.22651	up	PREDICTED: subtilisin-like protease [Sesamum indicum]
c40216.graph_c0	7.74045047	8.61715852	9.8538247	58.2024886	58.7227368	59.2050609	2.647E-40	1.65494	up	PREDICTED: ubiquitin-conjugating enzyme E2 36-like [Brassica rapa]
c40228.graph_c0	0.22705082	0.51137589	0.58353858	2.14524993	2.16045488	3.02972265	0.0009169	1.37446	up	PREDICTED: membrane protein of ER body-like protein [Sesamum indicum]
c40230.graph_c0	0.41668932	0.39724435	0.58645867	3.01405263	2.27869001	2.49474533	1.078E-06	1.37977	up	-
c40233.graph_c0	122.60205	137.885592	176.158814	80.0270849	50.0575287	71.3333084	1.042E-08	-2.21058	down	PREDICTED: endoglucanase 6 [Sesamum indicum]
c40236.graph_c0	2.29268373	2.24201844	1.69972463	1.30741193	1.34249574	1.38061318	5.394E-17	-1.71591	down	PREDICTED: uveal autoantigen with coiled-coil domains and ankyrin repeats [Sesamum indicum]
c40242.graph_c0	49.9966987	46.8033141	44.3689949	48.1627654	43.7379604	48.0200755	2.146E-40	-1.10166	down	hypothetical protein MIMGU_mgv1a007434mg [Erythranthe guttata]
c40244.graph_c0	0.21722552	0.54360759	0.1860956	3.2101912	3.23111661	2.87714454	2.243E-08	2.21206	up	PREDICTED: sulfhydryl oxidase 2-like [Sesamum indicum]
c40250.graph_c0	1.19326248	0.75648966	1.58450252	8.93273983	9.39661345	9.78949108	2.042E-16	1.89625	up	PREDICTED: Werner Syndrome-like exonuclease isoform X2 [Sesamum indicum]
c40251.graph_c0	1.16045182	0.68181682	0.97253904	9.82755642	10.0156418	10.009046	8.838E-33	2.31738	up	PREDICTED: APO protein 2, chloroplastic [Sesamum indicum]

#ID	0 h-1 FPKM	0 h-2 FPKM	0 h-3 FPKM	28 h-1 FPKM	28 h-2 FPKM	28 h-3 FPKM	FDR	log2FC	regulated	nr_annotation
c40255.graph_c0	43.0542947	46.2197702	43.8659895	30.81266	31.799482	27.565975	4.919E-77	-1.65331	down	PREDICTED: DEAD-box ATP-dependent RNA helicase 37-like [Sesamum indicum]
c40260.graph_c0	0.21187402	0.26510773	0.38571088	9.12383748	9.47772053	8.7433976	2.016E-76	3.88788	up	PREDICTED: proteoglycan 4-like [Sesamum indicum]
c40269.graph_c0	37.3000991	33.4931819	31.6462927	34.610838	36.3222216	35.9859795	5.327E-29	-1.02689	down	PREDICTED: COP9 signalosome complex subunit 5b-like [Sesamum indicum]
c40273.graph_c0	121.146377	122.925756	121.547661	90.9128669	93.3679064	94.3730873	1.567E-73	-1.48233	down	hypothetical protein 29 [Hevea brasiliensis]
c40274.graph_c0	4.19741006	4.15643449	5.16184507	3.90352429	5.00540571	3.39937799	5.539E-07	-1.22992	down	-
c40281.graph_c1	0.32566811	0.08149855	0.15693616	2.8994979	3.15225532	2.41425165	5.951E-10	2.82418	up	hypothetical protein MIMGU_mgv1a006975mg [Erythranthe guttata]
c40283.graph_c0	0.96493648	1.13243768	1.15446561	7.42421568	8.18815636	8.26822031	6.994E-15	1.78427	up	unnamed protein product [Coffea canephora]
c40289.graph_c0	4.1767579	3.66213748	4.03526879	18.0698345	17.5057579	17.6287142	8.133E-26	1.07325	up	PREDICTED: RNA polymerase-associated protein CTR9 homolog [Sesamum indicum]
c40295.graph_c0	5.10845251	5.73971902	6.49757838	49.0998043	44.2641053	44.2075666	4.326E-52	1.89392	up	hypothetical protein MIMGU_mgv1a011840mg [Erythranthe guttata]
c40300.graph_c0	4.67852065	4.52263843	3.53854102	48.2262789	51.4034481	47.9456903	3.11E-123	2.44675	up	PREDICTED: protein INVOLVED IN DE NOVO 2 [Sesamum indicum]
c40302.graph_c0	10.5792869	10.4204424	11.7459094	3.59843552	2.97135331	4.09073437	7.157E-59	-2.7098	down	hypothetical protein MIMGU_mgv1a018285mg [Erythranthe guttata]
c40303.graph_c0	51.5860484	51.1423423	42.0865146	12.795386	12.3107711	12.1573696	3.7E-78	-3.04643	down	PREDICTED: leucine-rich repeat receptor-like serine/threonine/tyrosine-protein kinase SOBIR1 [Sesamum indicum]
c40304.graph_c0	66.2415585	60.8887404	65.8932286	31.9970033	29.9963029	31.6219285	3.17E-115	-2.13453	down	PREDICTED: probable serine/threonine-protein kinase Cx32, chloroplastic [Sesamum indicum]
c40310.graph_c0	77.7729053	81.0945657	76.5792865	81.0749086	71.1125553	77.1011072	3.265E-42	-1.12828	down	hypothetical protein MIMGU_mgv1a008821mg [Erythranthe guttata]
c40310.graph_c1	40.0487648	37.2253771	41.3848449	40.2403845	38.6344029	40.2238858	6.856E-23	-1.08574	down	protein phosphatase 2C, partial [Nicotiana attenuata]
c40312.graph_c0	2.45464406	2.11819139	2.0122353	15.9639134	14.8860492	15.2602412	4.993E-20	1.71953	up	hypothetical protein MIMGU_mgv1a010279mg [Erythranthe guttata]
c40316.graph_c0	63.1802962	64.4881269	57.2234473	34.5047012	37.4670021	36.8292504	4.75E-112	-1.85415	down	PREDICTED: glucose-6-phosphate/phosphate translocator 2, chloroplastic-like [Solanum tuberosum]
c40320.graph_c0	5.78919409	4.83416355	4.58695279	35.5570921	38.620148	39.0118656	9.665E-62	1.80786	up	PREDICTED: sugar transport protein 14-like [Sesamum indicum]
c40322.graph_c0	305.692846	299.893572	311.850951	1572.50451	1650.22933	1511.0752	8.387E-40	1.27609	up	RecName: Full=Catalase [Avicennia marina]
c40323.graph_c0	14.7054921	13.3299746	13.7529182	11.1628832	13.3493627	11.143771	4.237E-15	-1.3195	down	PREDICTED: RPA-interacting protein B [Sesamum indicum]
c40323.graph_c1	19.3780981	16.6860235	19.5464355	14.5382091	15.5878846	14.8288248	2.771E-09	-1.39812	down	PREDICTED: RPA-interacting protein B [Sesamum indicum]
c40329.graph_c0	222.876342	228.095854	236.780308	71.2169905	76.5191209	73.1832981	7.56E-160	-2.72977	down	PREDICTED: transmembrane 9 superfamily member 8 [Sesamum indicum]
c40333.graph_c0	0.05491298	0.05496797	0.07056536	1.91570115	1.47745082	1.79116265	5.18E-10	3.75098	up	PREDICTED: transcription factor TCP5 [Sesamum indicum]
c40336.graph_c0	25.1821953	26.2332945	24.8031524	24.7222583	26.8855371	25.4118153	1.464E-35	-1.0753	down	YSL transporter [Medicago truncatula]
c40338.graph_c0	38.2156083	37.0261328	39.8177592	30.9652422	29.6531777	32.5030449	1.297E-43	-1.39602	down	PREDICTED: vesicle transport v-SNARE 12 [Sesamum indicum]
c40343.graph_c0	1.77867698	2.07030007	2.28567034	16.955836	19.3269246	17.2947797	2.204E-30	2.03278	up	hypothetical protein MIMGU_mgv1a009694mg [Erythranthe guttata]

#ID	0 h-1 FPKM	0 h-2 FPKM	0 h-3 FPKM	28 h-1 FPKM	28 h-2 FPKM	28 h-3 FPKM	FDR	log2FC	regulated	nr_annotation
c40349.graph_c0	2.3494512	1.6518622	2.65971664	11.505753	10.7006418	10.8814875	6.288E-14	1.21911	up	hypothetical protein MIMGU_mgv1a009342mg [Erythranthe guttata]
c40351.graph_c0	13.0413442	9.573229	12.1881045	1.26378318	0.8298747	1.40623194	2.004E-39	-4.40245	down	PREDICTED: calcium-dependent protein kinase 11 [Sesamum indicum]
c40354.graph_c0	4.79289792	5.48308261	5.5724861	1.07821089	1.19818258	0.81213349	3.884E-21	-3.45312	down	PREDICTED: UDP-glucuronic acid decarboxylase 2-like [Sesamum indicum]
c40359.graph_c0	6.08160605	7.57249973	5.33714457	50.453553	54.5587202	46.0959746	4.154E-38	1.90321	up	chloroplast 1-deoxy-D-xylulose 5-phosphate reductoisomerase [Mitragyna speciosa]
c40360.graph_c0	5.91991072	4.12484845	5.70548974	2.8894623	2.55178405	2.77081898	1.334E-10	-2.03037	down	PREDICTED: type I inositol 1,4,5-trisphosphate 5-phosphatase CVP2-like isoform X1 [Sesamum indicum]
c40362.graph_c0	5.28292275	6.78892184	5.32092384	0.51886457	0.60903187	0.93145635	3.116E-41	-4.16564	down	PREDICTED: uncharacterized protein LOC105160931 [Sesamum indicum]
c40362.graph_c1	0.44862987	0.79836286	0.6405635	3.15192887	3.36927946	2.95626232	0.0012136	1.23447	up	PREDICTED: protein SHI RELATED SEQUENCE 5-like [Sesamum indicum]
c40365.graph_c1	2.85125968	2.9001489	2.48205204	0.80217256	0.3319647	0.76366063	2.101E-23	-3.2037	down	-
c40366.graph_c0	21.9938837	22.3515156	22.6621097	129.951454	122.240313	119.957857	2.98E-64	1.3824	up	PREDICTED: F-box/kelch-repeat protein At1g67480-like [Sesamum indicum]
c40367.graph_c0	21.1762944	22.6695478	23.0549303	21.4830039	20.1450379	19.841119	9.63E-35	-1.21381	down	PREDICTED: probable protein phosphatase 2C 55 [Sesamum indicum]
c40373.graph_c0	2.01848688	3.00612179	2.0877213	10.6269833	11.145904	10.9489035	4.476E-07	1.11213	up	PREDICTED: probable glycosyltransferase At5g03795 isoform X1 [Sesamum indicum]
c40374.graph_c1	3.08691282	3.09000389	3.21835138	1.9895636	2.1021578	2.00342817	1.044E-08	-1.71553	down	Gibberellin 2-beta-dioxygenase 8 GN=GA2OX8 OS=Arabidopsis thaliana
c40376.graph_c0	0.35852187	0.71776175	0.9214296	7.55657724	8.70505475	9.78071879	2.327E-07	2.60499	up	-
c40376.graph_c1	316.99601	287.237436	317.935424	101.060508	94.7635867	114.442008	2.01E-101	-2.66158	down	PREDICTED: protein SRG1 [Sesamum indicum]
c40377.graph_c0	9.57542268	4.56429096	7.03131271	0	0	0	5.062E-17	-Inf	down	predicted protein [Hordeum vulgare subsp. vulgare]
c40382.graph_c0	0.11301803	0.1319864	0.14523265	1.23211283	1.27317426	1.36286743	9.987E-08	2.21617	up	PREDICTED: uncharacterized protein LOC105160401 [Sesamum indicum]
c40383.graph_c0	7.55999292	8.86647319	8.2649066	6.10962189	6.77519516	6.26799929	1.576E-27	-1.4579	down	PREDICTED: uncharacterized protein LOC105169210 [Sesamum indicum]
c40384.graph_c0	18.2566152	16.8821998	16.2217399	16.2283869	16.5010022	17.0659758	2.394E-28	-1.13366	down	PREDICTED: uncharacterized protein LOC105179476 isoform X3 [Sesamum indicum]
c40386.graph_c0	12.9190282	12.8332474	11.2788564	7.66926604	8.86611271	9.59179054	1.405E-15	-1.59137	down	PREDICTED: uncharacterized protein LOC105178241 [Sesamum indicum]
c40386.graph_c1	25.1870859	26.5214887	24.2341406	18.5511331	17.248279	16.989111	4.398E-58	-1.61476	down	PREDICTED: uncharacterized protein LOC105178241 [Sesamum indicum]
c40388.graph_c0	14.1305795	13.4018987	14.9001811	7.84297216	7.75106295	8.10519239	3.436E-62	-1.93157	down	PREDICTED: uncharacterized protein LOC105162418 [Sesamum indicum]
c40392.graph_c0	1.58224697	1.64359857	1.07416981	1.12827047	1.15585816	1.02688943	7.19E-07	-1.46326	down	hypothetical protein [Lotus japonicus]

#ID	0 h-1 FPKM	0 h-2 FPKM	0 h-3 FPKM	28 h-1 FPKM	28 h-2 FPKM	28 h-3 FPKM	FDR	log2FC	regulated	nr_annotation
c40395.graph_c0	49.7588082	51.0113994	46.6849543	396.524317	400.000181	354.621417	2.509E-96	1.87453	up	PREDICTED: bidirectional sugar transporter SWEET7-like [Sesamum indicum]
c40402.graph_c0	0.4549434	0.5692487	0.14615507	1.98390144	2.1644597	2.0910112	0.0078634	1.33538	up	-
c40408.graph_c0	31.5894447	32.1750848	29.2963874	20.5919569	19.8358385	20.7163843	1.849E-90	-1.69531	down	PREDICTED: nucleolin-like isoform X1 [Sesamum indicum]
c40413.graph_c1	3.09140736	4.54505118	4.22086715	1.96596492	3.42337242	2.69278328	1.188E-06	-1.64675	down	PREDICTED: uncharacterized protein LOC100784245 [Glycine max]
c40414.graph_c0	1.70245234	1.54923371	1.29274284	0.11248478	0.17773561	0.06883999	6.617E-28	-4.75258	down	hypothetical protein MIMGU_mgv1a005040mg [Erythranthe guttata]
c40418.graph_c0	0	0.2933403	0.75315363	3.30126392	3.46149499	4.51863964	8.047E-06	2.31498	up	PREDICTED: organ-specific protein S2-like [Nicotiana tomentosiformis]
c40422.graph_c0	2.87166403	2.20512624	2.02202848	2.23006153	2.55564244	2.4496123	5.424E-05	-1.0591	down	PREDICTED: enoyl-CoA hydratase 2, peroxisomal [Sesamum indicum]
c40426.graph_c0	0.80322063	1.05141723	0.63518173	6.82569011	7.0549653	6.96213423	6.379E-09	1.97847	up	hypothetical protein JCGZ_05663 [Jatropha curcas]
c40427.graph_c0	3.83103857	4.44845474	3.93842899	0.89100056	0.90505121	1.09057377	3.041E-14	-3.17105	down	-
c40428.graph_c0	0.50569709	0.78231445	0.53168791	17.0069732	17.0451312	16.1677562	2.085E-51	3.69646	up	PREDICTED: cytokinin hydroxylase [Sesamum indicum]
c40435.graph_c0	139.760249	132.304054	124.560655	39.8372074	37.903865	40.0831303	0	-2.84012	down	PREDICTED: probable polyamine oxidase 4 [Sesamum indicum]
c40439.graph_c0	15.9636002	16.5513596	15.1825731	234.055236	197.14526	198.208016	6.091E-57	2.63199	up	PREDICTED: transcription factor GAMYB-like isoform X1 [Sesamum indicum]
c40441.graph_c0	3.36191983	1.762769	2.88013339	58.9912355	61.3003632	61.3809879	2.287E-96	3.41449	up	PREDICTED: 60S acidic ribosomal protein P2B-like [Sesamum indicum]
c40442.graph_c0	142.320902	142.543857	140.857834	119.616627	131.074444	120.172857	1.063E-56	-1.28972	down	PREDICTED: la-related protein 6A [Sesamum indicum]
c40444.graph_c0	90.0435533	88.2219808	89.9605393	16.7931273	13.4984295	19.6261908	0	-3.51495	down	PREDICTED: UDP-glycosyltransferase 76C4-like [Sesamum indicum]
c40445.graph_c0	14.5409622	15.4876499	12.0122435	12.4684969	11.5869241	11.4486206	7.139E-18	-1.33191	down	PREDICTED: zinc transporter 4, chloroplastic-like [Sesamum indicum]
c40449.graph_c0	8.09037747	7.2157062	6.15903754	7.41275149	7.74981215	7.5268214	2.186E-12	-1.00734	down	hypothetical protein MIMGU_mgv1a001653mg [Erythranthe guttata]
c40451.graph_c0	0.18339036	0.30595666	0.23566387	4.13189871	4.03158142	4.3685728	1.935E-17	3.01988	up	PREDICTED: rop guanine nucleotide exchange factor 1-like [Sesamum indicum]
c40458.graph_c0	8.07987442	8.35982957	6.54388143	5.82305821	5.61412818	6.04012875	4.649E-13	-1.48258	down	PREDICTED: protein tesmin/TSO1-like CXC 5 [Sesamum indicum]
c40461.graph_c0	3.034377	4.13847858	3.65558867	3.28048448	2.9370794	3.03676095	1.057E-08	-1.31859	down	PREDICTED: protein phosphatase 2C 16-like [Sesamum indicum]
c40463.graph_c0	68.4468411	69.4612898	63.7515502	48.57922	50.2909595	49.8772191	4.1E-79	-1.52862	down	PREDICTED: lipase 3-like [Sesamum indicum]
c40465.graph_c0	1.99538377	2.09725094	1.88037458	12.5445191	12.3085932	11.3209889	1.171E-16	1.50838	up	PREDICTED: uncharacterized protein LOC105160301 [Sesamum indicum]
c40469.graph_c0	1.74211814	1.22070383	1.56708355	11.9525639	14.8619257	12.8113996	1.426E-24	2.03846	up	PREDICTED: pentatricopeptide repeat-containing protein At3g58590 [Sesamum indicum]
c40470.graph_c0	10.989404	12.3754593	11.5181088	10.7325789	9.37500745	9.24649846	2.706E-28	-1.34004	down	PREDICTED: mechanosensitive ion channel protein 2, chloroplastic-like [Sesamum indicum]
c40477.graph_c0	8.3372749	8.41403017	8.29874859	51.6794461	49.1281943	49.2827749	9.551E-51	1.49242	up	PREDICTED: dolichyl-diphosphooligosaccharide--protein glycosyltransferase 48 kDa subunit-like [Sesamum indicum]
c40478.graph_c0	4.12455477	4.46612548	5.01991629	1.85915212	1.92588161	1.90513626	1.023E-27	-2.35115	down	PREDICTED: disease resistance protein RPM1-like [Solanum tuberosum]

#ID	0 h-1 FPKM	0 h-2 FPKM	0 h-3 FPKM	28 h-1 FPKM	28 h-2 FPKM	28 h-3 FPKM	FDR	log2FC	regulated	nr_annotation
c40483.graph_c0	2.18100831	1.09159613	1.33461049	0	0	0	1.179E-14	-Inf	down	-
c40490.graph_c0	1.36277783	0.62006475	0.63680853	7.65354739	5.52834857	4.92271242	0.0002832	1.706	up	-
c40492.graph_c0	7.27085128	7.62470965	8.2628104	4.99682571	5.25819964	5.07471103	4.242E-20	-1.68743	down	PREDICTED: zinc finger CCCH domain-containing protein 32 [Sesamum indicum]
c40493.graph_c0	1.35554618	1.61536137	1.74193051	14.965705	17.1555314	19.370589	5.29E-21	2.35713	up	PREDICTED: clp protease-related protein At4g12060, chloroplastic isoform X1 [Sesamum indicum]
c40496.graph_c0	0.03726599	0.03730331	0.04788828	0.94796482	1.46729718	1.3923568	2.136E-10	3.86484	up	PREDICTED: uncharacterized protein LOC105173890 [Sesamum indicum]
c40498.graph_c0	19.9657721	20.5087568	21.2447959	21.5630868	20.0816924	22.0881914	3.764E-25	-1.04464	down	hypothetical protein M569_11555, partial [Genlisea aurea]
c40499.graph_c0	1114.41988	1191.90246	1168.8041	214.990573	237.905045	255.426993	3.75E-176	-3.38507	down	PREDICTED: legumin B-like [Sesamum indicum]
c40500.graph_c0	6.92641418	6.08928995	6.03700768	0.74416804	0.98810877	0.67867446	3.211E-50	-4.07278	down	hypothetical protein M569_14517 [Genlisea aurea]
c40504.graph_c0	1.34690873	0.70783517	1.29812329	8.3208668	7.62344731	7.60852446	6.116E-14	1.72064	up	PREDICTED: isochorismate synthase, chloroplastic-like [Sesamum indicum]
c40512.graph_c0	1.30129141	1.6673209	1.5384341	0.68095519	0.85394262	0.71000217	3.464E-08	-2.09805	down	hypothetical protein MIMGU_mgv1a023619mg [Erythranthe guttata]
c40514.graph_c0	1.82619674	1.76605844	1.35235583	1.0573162	1.66557247	1.28496376	1.102E-06	-1.39052	down	PREDICTED: rho guanine nucleotide exchange factor 8-like [Sesamum indicum]
c40517.graph_c0	59.4167606	58.8321103	63.6733485	42.560167	44.5509578	39.2447582	9.993E-54	-1.61788	down	hypothetical protein MIMGU_mgv1a015340mg [Erythranthe guttata]
c40522.graph_c0	1.59900543	1.65988832	1.90257897	12.181032	10.687423	12.7845323	5.813E-12	1.69602	up	PREDICTED: branched-chain-amino-acid aminotransferase-like protein 1 [Sesamum indicum]
c40522.graph_c1	1.08163354	1.79208271	1.62958709	12.849078	13.0211071	13.5815022	2.818E-25	2.03722	up	hypothetical protein MIMGU_mgv1a0045111mg, partial [Erythranthe guttata]
c40525.graph_c0	25.8431431	24.2547942	27.5240022	16.4085208	16.8775666	15.9150241	1.935E-41	-1.74961	down	PREDICTED: U-box domain-containing protein 4 [Sesamum indicum]
c40526.graph_c0	0.25772169	0	0.11039419	1.34239293	1.09930565	1.42654356	4.506E-06	2.31374	up	PREDICTED: SNARE-interacting protein KEULE-like isoform X1 [Sesamum indicum]
c40528.graph_c0	1.38491192	1.27450042	1.34910214	14.4812748	13.6175917	12.161032	1.923E-46	2.23715	up	PREDICTED: magnesium-chelatase subunit ChlH, chloroplastic [Sesamum indicum]
c40528.graph_c1	0.85256645	0.85342017	0.76214375	6.92380778	9.34083624	7.05671737	3.741E-13	2.15014	up	PREDICTED: magnesium-chelatase subunit ChlH, chloroplastic [Sesamum indicum]
c40537.graph_c2	2.44661951	2.72737278	2.88200269	21.3652098	18.9258863	18.9617707	2.015E-50	1.78616	up	PREDICTED: transcription factor bHLH143-like [Sesamum indicum]
c40538.graph_c0	2.03557613	2.51147827	2.61579591	18.6134824	16.681969	17.6028797	2.838E-22	1.79149	up	PREDICTED: DNA-repair protein XRCC1 [Sesamum indicum]
c40553.graph_c0	0.47623041	0.39725607	0.4419819	4.0188554	4.21895626	4.33062909	2.257E-13	2.16632	up	PREDICTED: uncharacterized protein LOC105177782 [Sesamum indicum]
c40558.graph_c0	74.463538	64.9781049	76.5125234	45.1901054	51.0193562	47.3176882	3.048E-27	-1.68091	down	unnamed protein product [Coffea canephora]
c40560.graph_c1	1.6603941	1.42796424	1.3222757	7.17260242	6.75242298	5.86665416	3.594E-08	1.07716	up	PREDICTED: formin-like protein 14 [Sesamum indicum]

#ID	0 h-1 FPKM	0 h-2 FPKM	0 h-3 FPKM	28 h-1 FPKM	28 h-2 FPKM	28 h-3 FPKM	FDR	log2FC	regulated	nr_annotation
c40564.graph_c0	2.72879673	2.91671763	3.80378233	1.78158488	1.69965717	1.23432344	4.098E-09	-2.09921	down	hypothetical protein MIMGU_mgv1a001712mg [Erythranthe guttata]
c40566.graph_c0	37.5048816	34.4490528	32.6115227	3.09676429	3.34916011	4.6373411	0	-4.3251	down	hypothetical protein F775_27779 [Aegilops tauschii]
c40570.graph_c0	0.92824794	0.25341203	0.27109894	23.7965781	23.9503327	23.2463256	5.463E-92	4.53426	up	PREDICTED: transmembrane emp24 domain-containing protein p24delta3-like [Sesamum indicum]
c40576.graph_c0	67.6700635	69.7781208	69.1478905	443.639849	463.764805	453.936017	4.669E-94	1.62936	up	PREDICTED: 40S ribosomal protein SA-like [Sesamum indicum]
c40579.graph_c0	68.5207675	69.2775685	58.8975914	24.816971	24.5127911	24.4636257	3.845E-67	-2.50298	down	-
c40586.graph_c0	0.49447727	0.44547517	0.38125372	8.26581423	7.52815313	7.4193206	1.67E-19	3.04738	up	PREDICTED: uncharacterized protein LOC105171062 [Sesamum indicum]
c40588.graph_c0	0.20644942	0.28046191	0.32214475	6.01256741	6.18358892	5.52713393	8.585E-52	3.3573	up	PREDICTED: protein phosphatase 2C and cyclic nucleotide-binding/kinase domain-containing protein isoform X1 [Sesamum indicum]
c40590.graph_c0	4.55473685	3.98938551	4.04833688	29.9311399	28.9927329	30.3437481	5.256E-41	1.73642	up	PREDICTED: urease accessory protein G isoform X1 [Sesamum indicum]
c40591.graph_c0	50.1982016	51.0302069	60.5481856	6.96880878	4.92555341	8.33675612	1.105E-50	-4.09045	down	PREDICTED: cytokinin hydroxylase [Sesamum indicum]
c40592.graph_c0	2.85022471	4.10564995	3.9306505	0.30315028	0.36495504	0.41228025	2.245E-23	-4.42555	down	-
c40593.graph_c0	0.24220263	0.09697806	0.18674398	1.83072737	2.16158291	1.66622688	1.859E-06	2.33774	up	Phototropic-responsive NPH3 family protein [Theobroma cacao]
c40601.graph_c0	4.71563378	4.77461274	4.66672413	21.3122625	21.1992847	21.0552016	4.724E-11	1.07635	up	PREDICTED: probable sphingolipid transporter spinster homolog 2 [Populus euphratica]
c40608.graph_c0	88.1698792	88.6187733	90.1553533	57.4703106	51.0037935	52.1296319	1.558E-90	-1.82394	down	cellular nucleic acid binding protein, putative [Ricinus communis]
c40610.graph_c0	1.36968901	0.82263633	1.12646695	8.16295307	6.9748974	8.77289669	1.312E-09	1.76072	up	PREDICTED: vesicle transport protein SFT2B [Sesamum indicum]
c40618.graph_c0	5.17681386	4.4309835	5.88111676	2.97181271	2.17861688	3.05902797	3.607E-13	-2.00757	down	PREDICTED: uncharacterized protein LOC105170597 [Sesamum indicum]
c40622.graph_c0	12.2280945	11.4572871	13.0682008	57.3336138	57.0084092	57.063491	1.235E-31	1.1298	up	PREDICTED: NADH dehydrogenase [ubiquinone] 1 alpha subcomplex subunit 9, mitochondrial [Sesamum indicum]
c40624.graph_c0	37.5408725	34.771925	36.0290599	8.41558463	9.22331692	8.57301317	1.77E-228	-3.13773	down	hypothetical protein MIMGU_mgv1a008212mg [Erythranthe guttata]
c40628.graph_c0	3.25749988	3.03382231	2.83667772	13.2252253	13.0452044	12.6315681	1.61E-21	1.00239	up	PREDICTED: uncharacterized protein LOC105164793 [Sesamum indicum]
c40632.graph_c1	61.9054208	61.6134941	67.3663266	13.6893078	12.8031024	17.460841	1.42E-103	-3.20894	down	PREDICTED: cytochrome P450 71A1-like [Vitis vinifera]
c40645.graph_c0	0.52365902	0.10483668	0	4.11039747	4.19239844	3.10560257	3.367E-08	3.11799	up	PREDICTED: auxin response factor 17-like isoform X3 [Zea mays]
c40646.graph_c0	389.052741	403.702315	394.744712	46.7227809	45.2038231	49.7822644	0	-4.15714	down	PREDICTED: transcription factor PIF3 isoform X2 [Sesamum indicum]
c40646.graph_c1	308.731804	313.052762	320.830782	30.1789745	30.1806157	34.7176976	0	-4.39983	down	unnamed protein product [Vitis vinifera]
c40647.graph_c0	35.1366365	36.1097357	32.0168507	19.0367509	17.9150653	17.6302693	3.53E-100	-2.00925	down	PREDICTED: protein FAM63A-like [Sesamum indicum]
c40647.graph_c1	17.5658582	15.8073419	13.680499	7.09264695	7.45192168	8.10255879	5.355E-18	-2.14206	down	-
c40651.graph_c0	203.782242	200.262068	196.890152	140.733099	153.148414	131.485925	1.351E-75	-1.58924	down	PREDICTED: type I inositol 1,4,5-trisphosphate 5-phosphatase CVP2 [Sesamum indicum]

#ID	0 h-1 FPKM	0 h-2 FPKM	0 h-3 FPKM	28 h-1 FPKM	28 h-2 FPKM	28 h-3 FPKM	FDR	log2FC	regulated	nr_annotation
c40652.graph_c0	49.848473	49.6224529	49.3565627	23.5742724	20.7728207	23.1872353	2.61E-158	-2.23005	down	PREDICTED: uncharacterized protein LOC105173727 [Sesamum indicum]
c40653.graph_c1	0.76706531	0.92948255	0.5706738	4.13722681	4.07993828	4.28577198	2.964E-05	1.37706	up	PREDICTED: protein ROOT PRIMORDIUM DEFECTIVE 1 [Sesamum indicum]
c40656.graph_c0	11.4675273	10.3231377	10.5404954	9.49210675	9.04081378	8.92619525	3.409E-23	-1.32549	down	PREDICTED: putative GTP diphosphokinase RSH1, chloroplastic [Sesamum indicum]
c40658.graph_c0	17.5678315	16.7950669	14.9149563	9.46855182	8.78237492	9.99731342	3.529E-59	-1.89079	down	PREDICTED: protein DOS2-like [Sesamum indicum]
c40661.graph_c0	0.59885284	1.02763286	0.9344531	5.96904401	4.5894851	5.47952757	4.132E-10	1.55284	up	unnamed protein product [Coffea canephora]
c40662.graph_c0	2.06203679	2.06410161	1.71878849	8.9110492	10.2401625	9.40316905	2.9E-11	1.20006	up	PREDICTED: pentatricopeptide repeat-containing protein At2g40720 [Sesamum indicum]
c40668.graph_c0	3.17720903	3.92001622	2.94344235	0	0.04848769	0.21910113	1.601E-37	-6.30868	down	hypothetical protein MIMGU_mgv1a010491mg [Erythranthe guttata]
c40670.graph_c0	5.85216245	7.04385702	5.96749411	27.6723965	28.8725707	25.7841474	8.829E-24	1.03509	up	PREDICTED: probable methyltransferase PMT9 [Sesamum indicum]
c40673.graph_c0	0.13113713	0.06563422	0	1.62026503	1.63505887	1.90541261	9.699E-10	3.64723	up	PREDICTED: GDSL esterase/lipase At4g10955-like [Sesamum indicum]
c40682.graph_c0	149.821317	165.358277	144.258667	61.0733252	61.0344248	62.2901166	5.5E-106	-2.40672	down	PREDICTED: splicing factor 3B subunit 1-like [Sesamum indicum]
c40685.graph_c0	8.03374693	8.59009548	5.27915602	40.7394649	42.7148745	42.5554618	1.218E-18	1.43912	up	PREDICTED: uncharacterized protein LOC105159330 [Sesamum indicum]
c40689.graph_c0	97.5574953	99.0684287	91.0304068	8.38845213	8.26884686	8.56136865	0	-4.60123	down	PREDICTED: zinc finger AN1 domain-containing stress-associated protein 12 [Sesamum indicum]
c40691.graph_c1	11.2227473	9.58998731	10.6403818	45.5577516	58.0641388	48.4976312	2.398E-11	1.18313	up	PREDICTED: thaumatin-like protein 1b [Sesamum indicum]
c40696.graph_c0	0.25298123	0.11510662	0	0.93604153	0.78478933	0.90019551	0.0025658	1.76633	up	PREDICTED: pentatricopeptide repeat-containing protein At2g27610 [Sesamum indicum]
c40701.graph_c1	161.828015	154.559325	174.613496	126.813224	140.481105	142.932899	1.585E-25	-1.35079	down	PREDICTED: glutathione S-transferase F9-like [Sesamum indicum]
c40702.graph_c0	8.19098373	9.28437211	9.44220649	40.5340143	42.6454527	37.4688126	1.468E-17	1.07159	up	PREDICTED: 60S ribosome subunit biogenesis protein NIP7 homolog [Sesamum indicum]
c40705.graph_c0	0.34490636	0.34525174	0.531862	2.05554285	2.12756673	1.84094581	0.0062997	1.20415	up	PREDICTED: putative transcription factor bHLH041 [Sesamum indicum]
c40707.graph_c0	57.3609618	56.7819892	55.1017757	17.9696622	16.7812032	16.2969857	1.53E-173	-2.81947	down	PREDICTED: uncharacterized protein LOC104230730, partial [Nicotiana sylvestris]
c40708.graph_c0	5.82118911	5.82701815	6.10247928	3.34011055	4.82529066	5.81440625	2.312E-05	-1.43422	down	PREDICTED: nuclear transcription factor Y subunit B-3-like [Sesamum indicum]
c40708.graph_c1	5.65590466	4.83185561	5.45104606	4.85485354	4.67144324	4.82900716	2.573E-09	-1.24135	down	PREDICTED: nuclear transcription factor Y subunit B-3-like [Solanum tuberosum]
c40710.graph_c0	2.66927956	3.02185097	3.47096479	15.0120363	16.890944	14.6619474	2.259E-17	1.25126	up	PREDICTED: probable tRNA modification GTPase MnmE [Sesamum indicum]
c40711.graph_c1	5.62647142	6.16011537	7.23023854	3.32254569	2.76918207	3.64965213	1.259E-08	-2.05772	down	-

#ID	0 h-1 FPKM	0 h-2 FPKM	0 h-3 FPKM	28 h-1 FPKM	28 h-2 FPKM	28 h-3 FPKM	FDR	log2FC	regulated	nr_annotation
c40712.graph_c0	7.75888091	6.39354082	5.3432901	37.6356066	39.0448813	34.599986	9.095E-32	1.42627	up	PREDICTED: uncharacterized protein LOC105166784 [Sesamum indicum]
c40713.graph_c0	8.09539644	8.96080028	7.02261593	5.51317819	5.31241814	5.10337349	3.722E-26	-1.6848	down	PREDICTED: protein tipD-like [Sesamum indicum]
c40715.graph_c1	84.0139855	94.3883788	88.1125125	77.8621041	82.801106	75.6042946	2.385E-38	-1.26496	down	PREDICTED: nematode resistance protein-like HSPRO2 [Sesamum indicum]
c40715.graph_c2	405.898417	380.961208	396.335706	100.621086	96.8175419	92.9578975	0	-3.11725	down	PREDICTED: uncharacterized protein LOC105174779 [Sesamum indicum]
c40718.graph_c0	6.9313805	6.05257809	7.51733833	34.0490503	32.4528422	32.2443066	1.606E-20	1.17583	up	PREDICTED: uncharacterized protein DDB_G0290685 [Sesamum indicum]
c40719.graph_c0	1.80873568	2.20660398	2.28797982	9.65393711	9.56971411	9.11782641	6.916E-10	1.07545	up	PREDICTED: CBL-interacting serine/threonine-protein kinase 8 [Sesamum indicum]
c40728.graph_c0	0.73221957	0.99681578	0.63983318	24.4586696	24.3326361	23.2583655	2.12E-115	3.83851	up	PREDICTED: shaggy-related protein kinase kappa isoform X1 [Sesamum indicum]
c40735.graph_c0	3.24786543	2.75388792	2.74968938	17.1068989	15.2202548	14.8202025	1.113E-15	1.34052	up	-
c40736.graph_c0	131.097979	129.870194	126.182798	44.5497191	45.6747976	48.5011856	4.34E-211	-2.5704	down	hypothetical protein MIMGU_mgv1a008470mg [Erythranthe guttata]
c40740.graph_c0	31.4146665	26.4433311	26.2136949	3.03932448	2.54342809	2.54055332	1.61E-128	-4.46035	down	-
c40743.graph_c0	0.3403906	0.2433796	0.06248791	2.36791327	2.80812422	2.68200603	1.507E-07	2.52949	up	PREDICTED: protein N-terminal asparagine amidohydrolase [Sesamum indicum]
c40748.graph_c0	83.5682079	89.8793576	79.3639643	33.771948	34.3379181	32.5754308	2.88E-159	-2.4181	down	PREDICTED: uncharacterized protein LOC105170953 [Sesamum indicum]
c40751.graph_c0	0.62681183	0.75292738	0.72493022	9.20237715	8.02095215	8.81014016	1.589E-14	2.53704	up	PREDICTED: 15-cis-zeta-carotene isomerase, chloroplastic [Sesamum indicum]
c40754.graph_c0	0.94341927	0.80945482	1.15460106	0.91422892	0.85494382	0.6660744	0.000699	-1.3508	down	hypothetical chloroplast RF21 (chloroplast) [Penthorum chinense]
c40759.graph_c0	328.124177	320.817172	313.354095	79.0458284	70.0388213	79.7868447	0	-3.16151	down	hypothetical protein MIMGU_mgv1a008881mg [Erythranthe guttata]
c40760.graph_c0	1.23405132	1.38969791	0.5451204	9.58563354	10.3251777	8.96533651	5.427E-18	2.10592	up	PREDICTED: UDP-glycosyltransferase 86A1-like [Sesamum indicum]
c40767.graph_c0	0.25073821	0.37648394	0.08055213	3.50802546	3.78444757	2.78816895	2.752E-08	2.75158	up	conserved hypothetical protein [Ricinus communis]
c40770.graph_c0	34.2059952	37.4502706	32.7094886	15.5865062	18.6765218	16.9183277	3.492E-83	-2.11788	down	PREDICTED: O-glucosyltransferase rumi homolog isoform X1 [Sesamum indicum]
c40770.graph_c1	21.0263151	24.5373673	18.8172544	13.1376739	15.2763979	13.5832053	2.665E-19	-1.70538	down	PREDICTED: O-glucosyltransferase rumi homolog isoform X1 [Sesamum indicum]
c40774.graph_c0	27.3662502	23.9728404	28.9977274	5.57855909	5.25049529	5.30805427	3.931E-56	-3.40746	down	PREDICTED: uncharacterized protein LOC105165423 [Sesamum indicum]
c40777.graph_c0	16.3683458	19.1213732	17.0703973	2.13982705	2.36907749	2.1202417	1.12E-119	-4.07808	down	PREDICTED: formin-like protein 6 [Sesamum indicum]
c40778.graph_c0	53.9724016	58.0442363	53.7002087	39.1233841	39.0047981	40.3486119	3.973E-82	-1.57422	down	hypothetical protein MIMGU_mgv1a004204mg [Erythranthe guttata]
c40780.graph_c0	0.32711161	0.28066214	0.18015059	2.88687729	2.2999231	2.93765693	4.267E-07	2.28339	up	hypothetical protein MIMGU_mgv1a009114mg [Erythranthe guttata]

#ID	0 h-1 FPKM	0 h-2 FPKM	0 h-3 FPKM	28 h-1 FPKM	28 h-2 FPKM	28 h-3 FPKM	FDR	log2FC	regulated	nr_annotation
c40785.graph_c0	3.5182278	3.71923212	3.78867814	27.6891846	28.7335865	28.2621857	6.609E-96	1.84978	up	PREDICTED: alpha-mannosidase-like [Sesamum indicum]
c40788.graph_c0	2.49911446	2.15192855	2.4862924	30.6043859	27.9150392	29.5149103	9.174E-92	2.53383	up	PREDICTED: phosphoribosylaminoimidazole carboxylase, chloroplastic-like isoform X1 [Sesamum indicum]
c40792.graph_c0	15.4836094	15.1335687	15.2669197	14.1198296	14.1707501	15.7953662	3.574E-32	-1.14757	down	unnamed protein product [Vitis vinifera]
c40796.graph_c0	126.990996	124.539503	135.663433	106.165043	111.415855	107.90532	2.648E-40	-1.34205	down	PREDICTED: 2-alkenal reductase (NADP(+)-dependent)-like [Sesamum indicum]
c40799.graph_c0	0.15345267	0.05120211	0.19719276	3.234327	3.86015877	2.82119713	2.712E-12	3.5266	up	hypothetical protein MIMGU_mgv1a026254mg [Erythranthe guttata]
c40806.graph_c1	5.57899117	5.58457769	5.29230309	35.1878214	40.3819239	35.9835205	3.608E-53	1.67085	up	PREDICTED: phenylalanine ammonia-lyase [Sesamum indicum]
c40807.graph_c0	9.53061026	10.0157626	9.44579657	8.32840167	10.1241726	10.4756432	3.886E-20	-1.09302	down	PREDICTED: probable protein phosphatase 2C 38 isoform X2 [Sesamum indicum]
c40809.graph_c0	3.27471402	2.99295026	3.75073095	1.65568228	1.63507516	1.98431535	8.248E-10	-2.01716	down	PREDICTED: protein tesmin/TSO1-like CXC 2 isoform X4 [Sesamum indicum]
c40809.graph_c1	0.23852944	0.28652195	0.2758678	1.69894587	1.69051831	1.57022634	5.98E-05	1.53827	up	PREDICTED: LOW QUALITY PROTEIN: protein tesmin/TSO1-like CXC 2 [Sesamum indicum]
c40812.graph_c0	8.49866481	8.50717493	8.41936622	2.122418	1.99005002	2.30916768	5.052E-71	-3.07504	down	hypothetical protein MIMGU_mgv1a008008mg [Erythranthe guttata]
c40815.graph_c0	0.24014702	0.32051666	0.30859843	4.33434725	4.88532018	4.08273971	6.846E-14	2.84218	up	PREDICTED: myosin-11 [Sesamum indicum]
c40816.graph_c0	4.2552552	3.62058876	2.05056427	0	0	0.25236138	5.75E-14	-6.36553	down	PREDICTED: uncharacterized protein LOC105168692 [Sesamum indicum]
c40818.graph_c0	1.01271476	0.84477403	1.44597617	5.19313759	6.49802579	5.87252113	1.809E-05	1.31411	up	PREDICTED: protein SAWADEE HOMEODOMAIN HOMOLOG 1-like isoform X1 [Sesamum indicum]
c40827.graph_c0	16.3515963	18.5999658	17.1920034	18.0514493	18.6561938	15.2808266	1.497E-17	-1.096	down	PREDICTED: random slug protein 5 [Sesamum indicum]
c40830.graph_c0	0.30639798	0.40004973	0.23966389	1.94610198	2.25544322	1.95142697	1.21E-08	1.61388	up	PREDICTED: auxilin-like protein 1 [Sesamum indicum]
c40838.graph_c0	0.15097515	0.22668949	0.19400904	8.50390981	6.68749346	6.55858852	2.155E-29	4.15682	up	PREDICTED: indole-3-acetic acid-amido synthetase GH3.6 [Sesamum indicum]
c40843.graph_c0	2.92246105	3.05257821	3.18399235	0.32322163	0.2084563	0.3767803	6.27E-29	-4.42317	down	-
c40848.graph_c0	313.954084	307.037415	302.055882	171.135014	173.278996	177.898205	4.36E-115	-1.91144	down	Histone [Morus notabilis]
c40850.graph_c0	22.2386991	21.9184914	21.103465	4.66239183	4.93938448	4.66682102	1.67E-87	-3.28345	down	unnamed protein product [Coffea canephora]
c40855.graph_c0	0.6828788	0.54685008	0.96527892	7.54394811	5.51191956	7.97824086	2.922E-09	2.16543	up	PREDICTED: transcription factor BEE 1-like [Sesamum indicum]
c40857.graph_c0	35.6586579	31.5272806	30.4781365	23.9259919	24.8379748	22.3171138	5.139E-54	-1.54761	down	PREDICTED: vacuolar protein sorting-associated protein 32 homolog 2 [Sesamum indicum]
c40858.graph_c0	0.66286219	0.60320541	0.77436742	8.10240164	8.0670521	6.9331338	6.809E-13	2.40739	up	PREDICTED: GATA transcription factor 5-like [Sesamum indicum]
c40862.graph_c0	1.06726148	0.62319261	0.57144719	4.13695729	5.01928862	3.63945175	0.0011416	1.41534	up	-
c40879.graph_c0	221.066044	222.780212	215.274793	99.6714079	96.191978	100.14984	1.73E-142	-2.24492	down	PREDICTED: triacylglycerol lipase SDP1-like [Sesamum indicum]

#ID	0 h-1 FPKM	0 h-2 FPKM	0 h-3 FPKM	28 h-1 FPKM	28 h-2 FPKM	28 h-3 FPKM	FDR	log2FC	regulated	nr_annotation
c40880.graph_c0	1.14507785	0.86741311	0.87492833	8.9858993	8.80392877	9.01181086	1.746E-41	2.12691	up	PREDICTED: chlorophyllide a oxygenase, chloroplastic-like [Sesamum indicum]
c40884.graph_c0	3.15467743	2.7187789	2.68813791	44.850628	42.9646348	42.6806958	1.34E-184	2.84108	up	PREDICTED: bifunctional aspartokinase/homoserine dehydrogenase 1, chloroplastic-like [Sesamum indicum]
c40885.graph_c0	1.10372681	0.80841367	0.72646306	7.83595132	7.72876177	7.55953365	1.318E-38	2.04564	up	PREDICTED: putative ATP-dependent RNA helicase C550.03c isoform X1 [Sesamum indicum]
c40889.graph_c0	5.84827626	6.45382403	4.61913943	32.407421	31.3014475	30.3186117	3.766E-36	1.38673	up	PREDICTED: putative E3 ubiquitin-protein ligase RF298 [Sesamum indicum]
c40890.graph_c0	23.7750733	24.3378795	25.5752491	13.7273368	15.1805421	14.0017434	5.393E-75	-1.87184	down	PREDICTED: low molecular weight phosphotyrosine protein phosphatase [Sesamum indicum]
c40893.graph_c0	60.2639037	49.9360852	59.1924473	262.002799	277.902918	279.765377	1.43E-35	1.18396	up	PREDICTED: 40S ribosomal protein S25-2 [Eucalyptus grandis]
c40896.graph_c0	7.67777897	3.53935985	5.58222037	0	0	0	9.536E-17	-Inf	down	-
c40897.graph_c0	10.1755677	10.8046385	10.990467	2.56503677	1.84264716	2.4138814	2.88E-138	-3.31924	down	PREDICTED: protein root UVB sensitive 6 [Sesamum indicum]
c40898.graph_c0	2.06255478	2.47754414	1.32523228	0.19987372	0.13535042	0.36696448	5.622E-12	-4.14281	down	PREDICTED: probable 2-oxoglutarate-dependent dioxygenase AOP1 [Sesamum indicum]
c40899.graph_c1	11.314966	9.25124955	11.3768578	66.0089322	65.8631069	62.2892093	3.18E-53	1.51221	up	hypothetical protein ARALYDRAFT_496862 [Arabidopsis lyrata subsp. lyrata]
c40901.graph_c0	4.90022557	5.26847555	5.13087575	23.9189124	22.5493236	25.7245328	1.998E-12	1.14797	up	hypothetical protein MIMGU_mgv1a003594mg [Erythranthe guttata]
c40903.graph_c0	23.852298	23.4216042	20.8138797	12.1491441	11.7642264	12.5555197	5.47E-110	-1.98928	down	PREDICTED: serine/threonine-protein kinase/endoribonuclease IRE1a-like isoform X1 [Sesamum indicum]
c40904.graph_c0	4.47052244	6.29094061	4.49592913	22.2260692	23.9371099	22.3245446	7.116E-10	1.07655	up	hypothetical protein MIMGU_mgv1a017323mg [Erythranthe guttata]
c40909.graph_c0	0.10059509	0.06713055	0.17235824	1.04793772	1.25425121	1.29260606	1.772E-06	2.30249	up	PREDICTED: pentatricopeptide repeat-containing protein At2g17140-like [Sesamum indicum]
c40909.graph_c1	0.04141366	0	0.05321819	1.20396841	1.0055403	0.98242839	2.706E-10	3.97617	up	hypothetical protein MIMGU_mgv1a026472mg [Erythranthe guttata]
c40910.graph_c0	9.82537833	10.9788468	11.4514885	234.159301	207.000487	211.365017	1.4E-145	3.24606	up	PREDICTED: hippocampus abundant transcript-like protein 1 isoform X1 [Sesamum indicum]
c40910.graph_c1	9.94780145	11.038086	12.72302	222.936515	207.295314	203.286601	1.72E-248	3.13842	up	PREDICTED: hippocampus abundant transcript-like protein 1 isoform X1 [Sesamum indicum]
c40929.graph_c0	20132.0579	19213.0311	21254.6743	3669.99954	3972.13879	4094.39391	4.786E-53	-3.45947	down	--
c40931.graph_c0	4.36942192	5.84498358	4.3898154	3.031322	3.96213269	5.0177404	1.432E-06	-1.3701	down	PREDICTED: cyclin-dependent kinase inhibitor 1 [Sesamum indicum]
c40932.graph_c0	6.7489863	5.8212322	6.34832711	4.45277858	3.99491496	4.41779066	4.459E-38	-1.64611	down	PREDICTED: uncharacterized protein LOC105163461 [Sesamum indicum]
c40935.graph_c0	117.450405	118.36284	110.369065	55.8341433	56.0361516	56.2733282	1.06E-149	-2.13152	down	hypothetical protein M569_02358 [Genlisea aurea]
c40938.graph_c0	1.73324258	1.80437728	1.3363712	16.0067242	15.8174519	15.528349	4.528E-50	2.19301	up	PREDICTED: endoglucanase 10-like [Sesamum indicum]

#ID	0 h-1 FPKM	0 h-2 FPKM	0 h-3 FPKM	28 h-1 FPKM	28 h-2 FPKM	28 h-3 FPKM	FDR	log2FC	regulated	nr_annotation
c40939.graph_c0	0	0	0.05648198	3.89730777	3.40352962	3.25837225	2.451E-20	6.41592	up	PREDICTED: uncharacterized protein LOC105156631 [Sesamum indicum]
c40940.graph_c0	0.08042897	0.04025476	0.05167721	1.66597646	1.58339005	1.59791848	2.669E-10	3.72773	up	PREDICTED: squamosa promoter-binding-like protein 12 [Sesamum indicum]
c40943.graph_c0	7.04410164	7.0123059	4.8127445	58.5864193	59.5580525	56.725077	1.33E-123	2.12616	up	PREDICTED: uncharacterized protein LOC105157708 [Sesamum indicum]
c40949.graph_c0	65.7595936	65.0380562	77.2258523	59.6849475	78.3571703	82.1970127	1.226E-06	-1.00984	down	PREDICTED: malate synthase [Tarenaya hassleriana]
c40949.graph_c1	121.071073	130.770536	139.118586	104.057935	114.660369	122.03089	1.114E-18	-1.29004	down	PREDICTED: malate synthase [Tarenaya hassleriana]
c40952.graph_c0	8.95382337	6.24679252	8.13556794	34.772865	38.9342037	37.1709662	5.741E-13	1.1581	up	putative nitrilase-associated protein [Plantago major]
c40953.graph_c0	3.67825963	3.13462702	2.29948018	1.62567845	1.36997954	1.44445504	1.162E-10	-2.12224	down	-
c40954.graph_c0	28.0425268	30.1028683	29.7580348	154.933605	156.544152	140.421561	1.392E-48	1.27039	up	PREDICTED: putative 4-hydroxy-4-methyl-2-oxoglutarate aldolase 3 [Sesamum indicum]
c40955.graph_c0	5.9564231	5.48003711	5.72777923	50.7330902	48.3369419	43.5172905	7.793E-69	1.96354	up	PREDICTED: probable alanine--tRNA ligase, chloroplastic isoform X2 [Sesamum indicum]
c40956.graph_c0	1.21421697	0.83161193	1.02652426	10.7756173	11.6375032	12.0332715	1.959E-31	2.39802	up	PREDICTED: uncharacterized protein LOC105174334 [Sesamum indicum]
c40959.graph_c0	52.1536428	52.0088636	50.2266864	51.321895	52.7446771	53.9001521	1.547E-36	-1.05686	down	hypothetical protein MIMGU_mgv1a008968mg [Erythranthe guttata]
c40960.graph_c0	13.5099371	12.088322	12.0462722	4.20811903	5.60884024	3.98973784	1.797E-54	-2.53763	down	PREDICTED: transcription factor bHLH113-like [Sesamum indicum]
c40961.graph_c0	0.39790078	0.49787403	0.12782956	3.47030395	2.61113258	2.59576138	7.186E-05	2.00406	up	Peptidyl-prolyl cis-trans isomerase CYP38 [Morus notabilis]
c40966.graph_c0	0.88233765	1.00939563	1.78174671	5.40505675	5.70742209	5.15802152	0.0080611	1.04659	up	-
c40970.graph_c0	0.13931366	0.13945316	0.04475589	0.8100189	1.53130829	1.32193762	0.0001511	2.42249	up	PREDICTED: glycerophosphodiester phosphodiesterase GDPDL3-like [Sesamum indicum]
c40971.graph_c0	1.43337841	1.34322987	2.11628069	34.7774248	41.4473941	39.3024854	8.136E-79	3.46509	up	PREDICTED: uncharacterized protein LOC105164832 [Sesamum indicum]
c40977.graph_c0	0.11641632	0.11653289	0.14959958	10.9147813	8.55630008	8.69924751	3.258E-18	5.10806	up	class I KNOX homeobox transcription factor STM-like 2 [Prunus persica]
c40979.graph_c0	0.88026104	0.68088284	1.07975346	24.8637363	29.6702616	27.5023323	1.597E-81	3.8628	up	PREDICTED: uncharacterized protein LOC105165458 [Sesamum indicum]
c40984.graph_c1	21.3471099	20.4042601	20.5239986	7.45259918	8.66613419	8.01620646	3.04E-124	-2.45785	down	PREDICTED: uncharacterized protein LOC105159405 [Sesamum indicum]
c40985.graph_c0	1.61098934	1.40585859	1.11471504	11.5584677	11.3849592	10.4605187	1.058E-17	1.92868	up	PREDICTED: zinc transporter 4, chloroplastic-like [Sesamum indicum]
c40989.graph_c0	2.48932088	1.86886017	2.13258393	22.2132511	19.9884181	21.160473	4.596E-46	2.19808	up	PREDICTED: GDSL esterase/lipase At4g10955-like [Sesamum indicum]
c40992.graph_c0	0	0	0	4.62579805	5.3787256	3.92191542	3.688E-36	Inf	up	PREDICTED: hippocampus abundant transcript 1 protein-like [Sesamum indicum]
c40996.graph_c0	69.4210047	75.6683288	65.3658601	30.7985614	36.4757083	32.0320118	1.177E-88	-2.17346	down	PREDICTED: probable methionine--tRNA ligase [Sesamum indicum]

#ID	0 h-1 FPKM	0 h-2 FPKM	0 h-3 FPKM	28 h-1 FPKM	28 h-2 FPKM	28 h-3 FPKM	FDR	log2FC	regulated	nr_annotation
c40998.graph_c0	15.0762415	15.0650006	14.3695743	15.3555538	13.726497	14.8706054	4.945E-29	-1.10791	down	PREDICTED: protein FAM63A-like [Sesamum indicum]
c40999.graph_c0	0.15939825	0.10637191	0.17069422	1.56397133	1.42955305	1.32345405	7.213E-07	2.21293	up	PREDICTED: tyrosine-sulfated glycopeptide receptor 1 [Sesamum indicum]
c41000.graph_c0	3.27286716	3.53209323	4.20576388	22.3189054	22.5848702	19.8800919	9.353E-33	1.4624	up	PREDICTED: ATP-dependent zinc metalloprotease FTSH 2, chloroplastic [Sesamum indicum]
c41001.graph_c0	40.1248656	38.1541695	38.6097999	261.383537	296.77137	258.963033	1.881E-59	1.71476	up	PREDICTED: uncharacterized protein LOC105155456 [Sesamum indicum]
c41003.graph_c0	2001.21231	1906.87187	2046.74872	118.90689	127.933423	144.284401	0	-5.01865	down	hypothetical protein M569_01497, partial [Genlisea aurea]
c41005.graph_c0	0.52003141	0.67365572	0.58964202	0.5780501	0.4014808	0.58053435	0.0012173	-1.28318	down	PREDICTED: putative nuclease HARBI1 [Camelina sativa]
c41011.graph_c0	15.9990072	14.413525	13.3372244	12.2839239	11.7911637	12.0672245	9.072E-31	-1.36396	down	PREDICTED: xyloglucan galactosyltransferase KATAMARI1 homolog [Sesamum indicum]
c41012.graph_c0	3.32648265	3.48468867	2.38585781	13.9437621	15.2804953	14.8648012	1.413E-06	1.1749	up	PREDICTED: UMP-CMP kinase isoform X2 [Sesamum indicum]
c41013.graph_c0	27.2127615	30.0006168	27.7641192	13.6431088	13.3583171	14.5212608	4.16E-110	-2.12333	down	PREDICTED: uncharacterized protein LOC105172362 [Sesamum indicum]
c41014.graph_c0	16.5728759	15.0507375	16.9139816	150.999799	154.212423	140.820759	1.46E-127	2.10847	up	PREDICTED: short-chain dehydrogenase TIC 32, chloroplastic-like [Sesamum indicum]
c41017.graph_c0	0.10299957	0.13747028	0.22059754	4.81596069	5.42981861	4.84605562	3.134E-24	3.93217	up	PREDICTED: protein PNS1 isoform X1 [Sesamum indicum]
c41021.graph_c0	165.918449	174.937366	170.78556	130.368155	123.585051	127.133942	1.003E-72	-1.51573	down	PREDICTED: glycosyltransferase 6 [Sesamum indicum]
c41024.graph_c0	6.13656721	5.25154139	5.43420897	56.5937466	56.8785043	50.328507	4.184E-95	2.19359	up	PREDICTED: glycerol-3-phosphate acyltransferase, chloroplastic [Sesamum indicum]
c41028.graph_c0	1.64702555	1.56057767	1.26020196	15.3971528	14.5704804	14.15961	9.479E-79	2.21657	up	PREDICTED: DNA-directed RNA polymerase V subunit 1 [Sesamum indicum]
c41039.graph_c0	58.8712785	61.5639824	63.5645213	48.093888	45.3235439	46.1880281	1.226E-56	-1.48979	down	hypothetical protein MIMGU_mgv1a0073611mg, partial [Erythranthe guttata]
c41040.graph_c0	0.01929667	0.01931599	0.07439095	5.18914211	4.97655772	4.45173449	2.309E-51	5.90459	up	PREDICTED: glucomannan 4-beta-mannosyltransferase 9-like [Nicotiana tomentosiformis]
c41044.graph_c0	2.03609404	2.44575945	1.15124304	17.2843141	20.2558822	14.5385671	7.186E-12	2.12379	up	PREDICTED: phytochrome B [Sesamum indicum]
c41044.graph_c1	1.18362858	1.51586472	0.87234373	11.1706568	11.171192	10.8184514	1.545E-44	2.12835	up	PREDICTED: phytochrome B [Sesamum indicum]
c41045.graph_c0	25.6669068	28.094442	26.3998969	24.2999351	23.7967186	24.2273911	5.763E-53	-1.23902	down	PREDICTED: probable pre-mRNA-splicing factor ATP-dependent RNA helicase [Sesamum indicum]
c41058.graph_c0	0.55793016	0.40617371	0.0651784	10.1743743	12.1488215	11.7313786	9.093E-34	3.97571	up	PREDICTED: axial regulator YABBY 5 [Beta vulgaris subsp. vulgaris]
c41070.graph_c1	41.580037	39.7417026	43.6099361	21.0302909	21.8115591	24.0118126	1.919E-68	-1.99287	down	probable tocopherol cyclase, chloroplastic [Sesamum indicum]
c41074.graph_c0	37.9708001	35.4263411	35.6179063	25.289199	26.8275159	28.2074634	1.88E-49	-1.53018	down	PREDICTED: uncharacterized protein LOC105177909 [Sesamum indicum]

#ID	0 h-1 FPKM	0 h-2 FPKM	0 h-3 FPKM	28 h-1 FPKM	28 h-2 FPKM	28 h-3 FPKM	FDR	log2FC	regulated	nr_annotation
c41077.graph_c0	2.53352231	2.47775904	2.13302941	10.9846017	10.7971232	10.4140601	2.473E-10	1.08344	up	PREDICTED: ubiquitin carboxyl-terminal hydrolase 24-like [Sesamum indicum]
c41079.graph_c0	0.33164247	0.96272622	0.59664319	7.47211092	8.40062457	7.1789395	4.895E-22	2.51384	up	PREDICTED: HIPL1 protein-like [Sesamum indicum]
c41083.graph_c0	0.03722506	0	0	3.24659496	2.17410005	2.25181573	8.307E-15	6.62791	up	hypothetical protein MIMGU_mgv1a013647mg [Erythranthe guttata]
c41084.graph_c0	3.67889597	3.71157652	2.9035205	14.1269296	15.625709	15.4787492	3.41E-13	1.04813	up	PREDICTED: WD repeat-containing protein 43 [Sesamum indicum]
c41088.graph_c0	6.69602699	7.99171898	6.50865291	112.806076	98.1352138	97.2673137	3.587E-76	2.77211	up	PREDICTED: transcription factor ILR3-like [Sesamum indicum]
c41093.graph_c0	2.29088888	3.16374301	2.53501028	13.3662888	12.6252994	12.5044312	2.452E-18	1.17757	up	PREDICTED: uncharacterized protein LOC105174509 [Sesamum indicum]
c41098.graph_c0	6.36257991	5.58061185	7.16413337	1.05439755	1.1832275	1.04474689	7.989E-37	-3.63434	down	-
c41099.graph_c0	4.14188601	0.99504804	1.9160955	0	0	0	2.238E-06	-Inf	down	PREDICTED: LOW QUALITY PROTEIN: oryzain gamma chain-like [Phoenix dactylifera]
c41100.graph_c0	501.726751	516.389476	501.810512	252.864563	247.134379	254.020033	1.68E-125	-2.1017	down	PREDICTED: SNF1-related protein kinase catalytic subunit alpha KIN10-like isoform X2 [Sesamum indicum]
c41100.graph_c1	708.821262	714.438608	702.827095	290.721589	286.369774	295.744951	3.49E-148	-2.3747	down	PREDICTED: uncharacterized protein LOC105162997 [Sesamum indicum]
c41101.graph_c0	32.9508027	34.2318335	32.5774734	505.549696	537.541265	474.072109	2.65E-190	2.83598	up	-
c41104.graph_c0	16.8621193	18.4314762	18.4457799	5.6599779	4.89388575	6.26236712	1.25E-97	-2.76663	down	PREDICTED: UPF0496 protein At4g34320 [Sesamum indicum]
c41105.graph_c0	41.6796703	39.1225199	34.6740104	22.2392426	22.9702469	20.4566637	1.068E-70	-1.90318	down	PREDICTED: probable BOI-related E3 ubiquitin-protein ligase 2 [Sesamum indicum]
c41105.graph_c1	0.28385933	0.5276952	0.10422014	4.42087124	3.88518871	3.65549343	4.492E-11	2.62652	up	PREDICTED: B3 domain-containing protein At3g19184-like isoform X1 [Sesamum indicum]
c41107.graph_c0	129.647597	121.413229	135.118396	110.412538	122.965254	131.174718	8.294E-24	-1.17397	down	PREDICTED: isopentenyl-diphosphate Delta-isomerase I [Sesamum indicum]
c41118.graph_c0	25.5906067	24.277726	25.4784241	26.3235988	26.1127448	26.0738753	5.654E-33	-1.03133	down	hypothetical protein MIMGU_mgv1a002787mg [Erythranthe guttata]
c41122.graph_c0	111.385994	118.892775	112.359212	507.323979	589.550851	551.337048	3.463E-31	1.17556	up	PREDICTED: uncharacterized protein LOC105165162 [Sesamum indicum]
c41124.graph_c0	1.83300727	1.49080974	1.71754254	10.2414345	10.0489678	8.90046688	1.203E-11	1.4428	up	hypothetical protein MIMGU_mgv1a016809mg [Erythranthe guttata]
c41125.graph_c0	15.7388567	14.1114973	14.43465	154.015922	153.846772	142.167811	1.65E-159	2.25506	up	PREDICTED: isocitrate dehydrogenase [NADP] [Sesamum indicum]
c41127.graph_c2	0.23381423	0.46809673	0.66101321	1.93725715	2.57771179	2.05224851	0.0029211	1.16628	up	PREDICTED: leucine-rich repeat receptor-like serine/threonine-protein kinase BAM3 [Sesamum indicum]
c41129.graph_c0	1.86144977	1.77664797	1.39071898	14.9132632	13.1243853	14.1972845	6.574E-22	1.98339	up	hypothetical protein MIMGU_mgv1a010701mg [Erythranthe guttata]
c41131.graph_c0	13.9506951	15.3581019	13.2995681	10.3592259	10.64422	9.6375467	3.987E-47	-1.56575	down	PREDICTED: lysM domain receptor-like kinase 4 [Sesamum indicum]
c41135.graph_c0	4.06981597	5.49247841	4.24927315	5.01789795	4.69761158	4.82727515	3.605E-06	-1.0157	down	hypothetical protein MIMGU_mgv1a008665mg [Erythranthe guttata]

#ID	0 h-1 FPKM	0 h-2 FPKM	0 h-3 FPKM	28 h-1 FPKM	28 h-2 FPKM	28 h-3 FPKM	FDR	log2FC	regulated	nr_annotation
c41136.graph_c1	146.777213	135.821772	148.843321	24.907351	27.8061699	33.2434507	2.8E-167	-3.41739	down	PREDICTED: protein TRANSPARENT TESTA 12-like [Nicotiana tomentosiformis]
c41136.graph_c2	69.5671798	64.7003918	77.7361568	12.2340477	14.713881	14.6233638	6.815E-53	-3.44249	down	PREDICTED: protein TRANSPARENT TESTA 12-like [Sesamum indicum]
c41137.graph_c0	2.12897569	2.04042211	2.34775876	15.5281424	14.9815763	13.4051191	7.329E-46	1.6598	up	PREDICTED: ABC transporter G family member 29-like [Vitis vinifera]
c41138.graph_c1	87.0492902	85.1440967	85.2316083	86.4974131	90.507315	85.995863	2.06E-37	-1.0596	down	PREDICTED: syntaxin-51-like [Sesamum indicum]
c41143.graph_c0	17.8342171	19.3024231	15.9629684	8.70314823	8.96273899	8.40325461	2.04E-60	-2.11551	down	PREDICTED: formin-like protein 5 [Sesamum indicum]
c41144.graph_c0	48.3231579	52.4413695	46.3143668	249.35597	274.782029	253.731458	1.754E-61	1.31294	up	hypothetical protein MIMGU_mgv1a008949mg [Erythranthe guttata]
c41149.graph_c0	0.08841046	0.28319677	0.29538845	1.10520686	1.42722645	1.38421918	4.895E-05	1.45369	up	PREDICTED: uncharacterized protein LOC105158269 isoform X1 [Sesamum indicum]
c41152.graph_c0	16.428353	15.0418203	14.7629958	10.670882	10.9918209	9.97461983	1.918E-63	-1.63709	down	PREDICTED: AP2-associated protein kinase 1 isoform X4 [Sesamum indicum]
c41160.graph_c0	2.99498584	3.85455197	3.71122269	16.3514519	16.7058212	18.2483487	1.165E-15	1.18832	up	hypothetical protein POPTR_0019s10450g, partial [Populus trichocarpa]
c41163.graph_c0	0.33364585	0.61229656	0.28583208	24.8150023	37.1480808	30.670066	4.634E-29	5.14533	up	-
c41164.graph_c1	47.8637264	48.9719402	46.4491061	629.439262	668.50478	622.489089	3.37E-230	2.65414	up	PREDICTED: CASP-like protein 2A1 [Sesamum indicum]
c41167.graph_c0	2.66078802	2.43351406	2.58286248	13.8847317	13.8430313	13.5208665	7.423E-26	1.33518	up	PREDICTED: uncharacterized protein LOC105165931 isoform X2 [Sesamum indicum]
c41168.graph_c0	6.02115991	5.01326952	5.53190008	32.1462564	26.7540111	28.2501419	1.451E-19	1.30532	up	hypothetical protein MIMGU_mgv1a003607mg [Erythranthe guttata]
c41178.graph_c0	15.3907975	16.7594571	13.7642686	15.116139	13.9214254	12.9513783	3.614E-12	-1.21826	down	PREDICTED: probable E3 ubiquitin-protein ligase ARI2 isoform X1 [Sesamum indicum]
c41185.graph_c0	0.65225977	0.88001306	0.80173704	4.39019527	3.77783449	3.80100539	4.271E-06	1.26597	up	-
c41190.graph_c0	0.39633158	0.34005296	0.36378698	9.5056998	11.1464359	10.0231046	1.78E-51	3.71117	up	PREDICTED: pectinesterase-like [Sesamum indicum]
c41192.graph_c1	66.3198153	65.3430125	71.1008223	32.4322926	35.1897214	34.4431632	2.347E-73	-2.08183	down	hypothetical protein MIMGU_mgv1a014126mg [Erythranthe guttata]
c41194.graph_c0	12.9402526	14.1307749	11.2117999	2.99246851	3.69905508	2.7034545	2.034E-63	-3.11639	down	unnamed protein product [Coffea canephora]
c41196.graph_c0	362.369009	351.300466	367.666676	105.936113	110.638558	113.752292	0	-2.80149	down	-
c41198.graph_c0	67.9110422	67.3090005	62.6361745	7.47436635	9.46408597	8.15608846	0	-4.06891	down	-
c41199.graph_c0	0.02624224	0.05253704	0.13488929	2.42223441	2.11816708	2.22553433	4.416E-16	3.87511	up	PREDICTED: U-box domain-containing protein 13-like [Sesamum indicum]
c41200.graph_c0	0.14849897	0.2147133	0.10601503	0.75549737	0.96366274	0.58712351	0.0055724	1.20961	up	PREDICTED: uncharacterized protein LOC105159953 isoform X1 [Sesamum indicum]
c41201.graph_c0	1.7541774	1.47271879	1.81789307	47.2956428	71.4448642	52.1842563	2.962E-20	3.98988	up	cytochrome P450 98A3 [Sesamum indicum]
c41202.graph_c0	162.028601	148.498406	157.668906	78.2200158	72.0480091	81.5208724	3.69E-139	-2.10426	down	PREDICTED: GATA transcription factor 15-like [Sesamum indicum]
c41204.graph_c0	9.37608627	9.56666178	8.69921262	1.23660547	1.23531952	1.8678344	2.48E-104	-3.75858	down	PREDICTED: uncharacterized protein LOC100265977 [Vitis vinifera]

#ID	0 h-1 FPKM	0 h-2 FPKM	0 h-3 FPKM	28 h-1 FPKM	28 h-2 FPKM	28 h-3 FPKM	FDR	log2FC	regulated	nr_annotation
c41210.graph_c0	1.22673056	0.95934292	0.83746108	25.7443926	23.3202428	25.1904689	7.614E-85	3.53224	up	PREDICTED: probable beta-1,3-galactosyltransferase 6 [Sesamum indicum]
c41214.graph_c0	0.50876506	0.145507	0.46698803	2.6411965	3.62482427	2.75864932	2.55E-05	1.91594	up	-
c41219.graph_c0	5.21990891	4.70262227	3.73719707	29.3206521	34.6459338	24.8983499	2.077E-10	1.61385	up	hypothetical protein MIMGU_mgv1a008823mg [Erythranthe guttata]
c41223.graph_c0	15.4207032	16.2877941	15.7163078	99.7612186	110.220939	98.8751575	8.045E-79	1.61221	up	PREDICTED: 6-phosphogluconate dehydrogenase, decarboxylating 3 [Sesamum indicum]
c41223.graph_c1	1.3510688	1.35242169	0.43404422	14.483748	14.6290303	12.8201981	2.658E-07	2.66102	up	-
c41230.graph_c0	2.33233694	1.92045634	1.98198318	0.38277107	0.19748937	0.51312683	2.512E-25	-3.59701	down	hypothetical protein MIMGU_mgv1a002463mg [Erythranthe guttata]
c41232.graph_c0	55.8667625	56.227385	51.5942266	577.661706	506.557579	487.793868	1.125E-56	2.17373	up	PREDICTED: uncharacterized protein LOC105163662 isoform X2 [Sesamum indicum]
c41239.graph_c0	0	0	0	4.53097772	4.50015286	8.42970906	1.887E-07	Inf	up	--
c41241.graph_c0	18.8661384	18.7323209	16.6634835	10.9768324	10.2614411	10.9323721	7.332E-81	-1.84293	down	PREDICTED: uncharacterized protein LOC105172605 [Sesamum indicum]
c41241.graph_c1	10.7234493	9.94875889	9.18670461	6.71655742	7.03701753	5.79088182	4.915E-16	-1.70096	down	PREDICTED: uncharacterized protein LOC105172605 [Sesamum indicum]
c41242.graph_c0	2.01551522	1.73851287	1.40522191	11.0799883	10.5107319	10.1220524	4.246E-22	1.53331	up	PREDICTED: pentatricopeptide repeat-containing protein At3g02490, mitochondrial [Sesamum indicum]
c41245.graph_c0	0.16575239	0.08295918	0.26624798	0.99386003	1.22367603	1.11817169	6.546E-05	1.59553	up	PREDICTED: COP9 signalosome complex subunit 1-like [Sesamum indicum]
c41246.graph_c0	0.85830699	0.91644421	0.77207092	7.38178208	8.39233952	7.32997387	5.493E-20	2.09193	up	PREDICTED: phosphoglycolate phosphatase 2 [Sesamum indicum]
c41251.graph_c0	0.15472012	0.54206268	0.29823223	2.24899388	2.84288285	2.89037801	5.547E-05	1.91153	up	-
c41253.graph_c0	0.14028516	0.28085128	0.18027199	34.2580388	38.2965106	39.0195157	2.306E-55	6.44455	up	-
c41257.graph_c0	5.532751	3.81951118	3.67748487	0	0	0	1.028E-28	-Inf	down	metallothionein-like protein [Oryza officinalis]
c41258.graph_c0	3.28521468	3.76546297	3.89936903	1.95023844	2.40269942	2.64734016	1.395E-19	-1.73707	down	PREDICTED: phosphoenolpyruvate carboxykinase [ATP]-like [Sesamum indicum]
c41275.graph_c0	0.08226679	0.74114247	0.31714823	3.34829744	2.69928451	2.92733932	7.281E-05	1.8825	up	-
c41277.graph_c0	1.36006256	1.32976342	1.30063943	12.160672	10.8969225	11.8550883	7.112E-26	2.03934	up	PREDICTED: preprotein translocase subunit SCY1, chloroplastic [Sesamum indicum]
c41278.graph_c2	0.62091596	0.62153771	0.3989508	21.6613508	20.9842853	22.5546558	3.32E-24	4.22707	up	PREDICTED: uncharacterized protein LOC101261197 [Solanum lycopersicum]
c41279.graph_c1	53.6534929	55.5784361	57.1888097	57.5868212	56.9238817	62.3361566	1.1E-31	-1.00321	down	PREDICTED: nucleolar GTP-binding protein 2 [Sesamum indicum]
c41280.graph_c0	3.53934305	3.71159608	3.97064726	2.49071146	2.54381386	2.06571825	1.703E-10	-1.75323	down	hypothetical protein MIMGU_mgv1a019699mg [Erythranthe guttata]
c41281.graph_c0	2.88664037	1.90446355	1.09597303	1.43045017	1.07630199	1.05051209	0.0069163	-1.8071	down	-
c41283.graph_c0	1.33806722	1.42490116	1.2804559	10.1595165	9.84565965	10.4343605	1.573E-21	1.82274	up	PREDICTED: armadillo repeat-containing protein 6 [Sesamum indicum]

#ID	0 h-1 FPKM	0 h-2 FPKM	0 h-3 FPKM	28 h-1 FPKM	28 h-2 FPKM	28 h-3 FPKM	FDR	log2FC	regulated	nr_annotation
c41284.graph_c0	0.06186708	0.08257205	0.0530011	0.4496464	0.4736533	0.67266428	5.486E-05	1.92899	up	PREDICTED: putative late blight resistance protein homolog R1A-3 [Sesamum indicum]
c41289.graph_c0	7.09874359	6.98872248	7.31778197	88.0770641	77.7848344	75.2011286	8.374E-67	2.40202	up	PREDICTED: uncharacterized protein LOC105166112 [Sesamum indicum]
c41291.graph_c0	5.90919927	5.50634823	5.49452354	3.50914295	3.23148035	2.93466164	4.579E-36	-1.89585	down	hypothetical protein MIMGU_mgv11b004345mg [Erythranthe guttata]
c41293.graph_c0	10.3931163	10.5630867	10.3239344	8.71219338	8.05458273	8.37585597	1.072E-29	-1.40546	down	PREDICTED: cell wall / vacuolar inhibitor of fructosidase 1-like [Sesamum indicum]
c41296.graph_c0	8.3318276	7.75145274	7.93557512	54.9271673	58.0848978	58.1905838	4.292E-32	1.74346	up	-
c41298.graph_c0	35.1650395	38.2370615	34.4981956	30.7996519	36.4236635	30.5864523	8.242E-42	-1.2323	down	unnamed protein product [Coffea canephora]
c41304.graph_c0	2.61759397	2.08646758	1.86872883	1.72629923	2.95832307	2.09859517	0.0004547	-1.04223	down	PREDICTED: anthocyanidin 3-O-glucosyltransferase 2-like [Sesamum indicum]
c41305.graph_c0	0.44239496	0.55354744	0.52112046	7.28801581	6.24169816	7.80538682	1.131E-20	2.72273	up	PREDICTED: dehydrogenase/reductase SDR family member on chromosome X-like isoform X1 [Sesamum indicum]
c41313.graph_c0	0	0	0	8.29505438	8.24533919	6.00987414	2.203E-42	Inf	up	PREDICTED: uncharacterized protein LOC105168700 [Sesamum indicum]
c41315.graph_c0	11.3013936	10.6928357	9.94708098	2.30661163	2.99698995	2.29533689	4.415E-90	-3.16149	down	-
c41319.graph_c0	6.81877715	7.48402362	7.635402	3.71290215	4.0718022	3.6278356	1.017E-53	-2.03502	down	hypothetical protein MIMGU_mgv1a0011691mg, partial [Erythranthe guttata]
c41321.graph_c0	7.2384167	8.84987113	8.16897855	6.48383914	5.50374201	5.87686192	1.756E-19	-1.53277	down	PREDICTED: probable amino acid permease 7 [Sesamum indicum]
c41326.graph_c0	6.11389799	6.25939947	5.87211791	71.3451301	66.1623568	59.5758166	2.08E-65	2.34265	up	PREDICTED: ABC transporter C family member 4 [Sesamum indicum]
c41330.graph_c0	24.176733	21.7297193	25.543633	7.1770957	6.45231241	6.73996787	2.357E-58	-2.90211	down	PREDICTED: E3 ubiquitin-protein ligase RNF5-like [Sesamum indicum]
c41334.graph_c1	49.1611456	54.8161931	49.1688643	47.7944087	47.4670562	48.2476437	4.484E-42	-1.18392	down	PREDICTED: dnaJ homolog subfamily B member 4-like [Malus domestica]
c41336.graph_c0	0	0.06135122	0	7.21630773	4.38398625	5.59766062	1.493E-15	7.04954	up	-
c41339.graph_c0	4.67610746	4.08896587	4.83481408	0.85940799	0.4937958	0.54189038	8.894E-40	-3.93515	down	PREDICTED: C2 and GRAM domain-containing protein At1g03370-like [Sesamum indicum]
c41341.graph_c0	17.8974575	18.1076042	19.0012714	16.106186	15.5253094	14.6458197	1.147E-35	-1.34094	down	PREDICTED: E3 ubiquitin-protein ligase PRT1 [Nicotiana tomentosiformis]
c41344.graph_c0	0.16754379	0.06708463	0.25836049	2.60587508	2.70469053	2.60331621	1.428E-11	2.9049	up	PREDICTED: O-glucosyltransferase rumi homolog isoform X2 [Sesamum indicum]
c41345.graph_c0	43.3034388	43.3468007	40.9385707	25.0133131	22.2385512	23.9297384	4.94E-117	-1.93156	down	PREDICTED: F-box/kelch-repeat protein At1g51550 [Sesamum indicum]
c41349.graph_c0	5.42297203	4.36154868	5.23662074	28.0909103	31.7608917	29.354229	8.918E-38	1.47927	up	PREDICTED: peptidyl-prolyl cis-trans isomerase CYP23 isoform X2 [Sesamum indicum]
c41356.graph_c0	0.99640199	0.96300664	0.83889325	0.0249717	0	0.0203767	1.717E-29	-7.03175	down	hypothetical protein MIMGU_mgv1a004102mg [Erythranthe guttata]

#ID	0 h-1 FPKM	0 h-2 FPKM	0 h-3 FPKM	28 h-1 FPKM	28 h-2 FPKM	28 h-3 FPKM	FDR	log2FC	regulated	nr_annotation
c41358.graph_c0	12.2850868	11.2995097	12.1697943	63.4600451	64.8023	64.2330266	1.03E-51	1.33796	up	PREDICTED: glucose-6-phosphate isomerase 1, chloroplastic [Sesamum indicum]
c41361.graph_c0	0.39625075	0.57694187	0.55548862	7.40926836	6.92626597	7.64817345	5.082E-22	2.75277	up	hypothetical protein MIMGU_mgv1a007271mg [Erythranthe guttata]
c41376.graph_c0	6.53293407	4.12399375	3.93282977	29.6007603	28.9668112	31.4490387	5.547E-20	1.54076	up	hypothetical protein MIMGU_mgv1a014628mg [Erythranthe guttata]
c41377.graph_c0	1.03828247	0.62359329	1.51213196	11.8726521	10.8561614	9.35958078	4.075E-14	2.23917	up	hypothetical protein MIMGU_mgv1a013935mg [Erythranthe guttata]
c41378.graph_c0	33.4580047	34.2013471	33.0538524	33.6878306	32.8917671	33.1091368	2.313E-37	-1.10508	down	PREDICTED: uncharacterized protein At1g47420, mitochondrial [Sesamum indicum]
c41379.graph_c0	20.4963394	20.7757512	21.1456645	12.6409815	12.7501604	12.63484	4.398E-72	-1.80593	down	PREDICTED: uncharacterized protein LOC105169116 [Sesamum indicum]
c41381.graph_c0	10.1966006	9.42319765	8.23874046	9.93872316	9.6890329	9.41455575	8.301E-22	-1.02811	down	PREDICTED: uncharacterized protein LOC105157976 isoform X1 [Sesamum indicum]
c41384.graph_c0	14.7876585	14.0111393	13.3258115	7.90860027	6.77453354	6.97734346	1.11E-51	-2.04902	down	hypothetical protein MIMGU_mgv1a000172mg [Erythranthe guttata]
c41385.graph_c0	8.48109528	9.81132005	9.07124256	6.75457512	6.39868645	6.05381041	1.915E-21	-1.60195	down	PREDICTED: uncharacterized protein LOC105163010 [Sesamum indicum]
c41389.graph_c0	45.3886524	47.1378811	46.7824852	206.107593	214.515936	215.849601	4.208E-37	1.10106	up	unnamed protein product [Vitis vinifera]
c41394.graph_c1	0.21726735	0.36247486	0.09306572	2.42126613	2.23370187	2.01868411	1.302E-05	2.22843	up	PREDICTED: squamosa promoter-binding-like protein 6, partial [Sesamum indicum]
c41407.graph_c0	2.27192133	1.98540948	2.87316744	14.0484692	13.4417311	13.0460747	2.164E-16	1.41264	up	PREDICTED: paramyosin [Sesamum indicum]
c41408.graph_c0	3.45172411	2.4149111	2.52781628	0.16185117	0.07306822	0.33017309	1.519E-31	-4.97425	down	PREDICTED: probable glycosyltransferase At3g07620 isoform X1 [Sesamum indicum]
c41415.graph_c0	33.5689255	33.128597	32.946264	31.6929241	33.7112668	33.639173	1.374E-39	-1.09898	down	PREDICTED: nuclear pore complex protein NUP50A-like [Sesamum indicum]
c41418.graph_c0	0.91054134	0.76562061	0.79565553	4.07654136	3.65683376	4.49283304	6.446E-05	1.21796	up	PREDICTED: kirola-like [Sesamum indicum]
c41421.graph_c0	28.9822446	28.9454807	30.8249798	10.3648885	12.7655423	13.1347139	2.416E-88	-2.38215	down	hypothetical protein MIMGU_mgv1a015977mg [Erythranthe guttata]
c41425.graph_c0	0.37406076	0.65526182	0.90128041	7.13646164	7.60953251	6.98795331	2.457E-15	2.39231	up	PREDICTED: uncharacterized protein LOC105166018 [Sesamum indicum]
c41426.graph_c0	7.68078262	9.09622248	7.61110577	3.83295058	2.99935567	2.83894963	1.846E-43	-2.42495	down	PREDICTED: uncharacterized protein LOC105177245 [Sesamum indicum]
c41428.graph_c0	1.54016858	1.71201609	1.85260229	14.1354978	13.9793952	13.8445272	1.309E-81	1.9463	up	PREDICTED: uncharacterized protein LOC105169752 [Sesamum indicum]
c41431.graph_c0	61.0710242	61.4519623	64.2471529	28.6748707	27.3931502	28.3876057	3.54E-137	-2.23611	down	PREDICTED: uncharacterized protein LOC105163802 [Sesamum indicum]
c41432.graph_c1	0.33516741	0.047929	0.06152906	4.14116163	3.36203053	4.17424915	5.946E-14	3.64427	up	-
c41433.graph_c0	2.81908084	2.55568639	2.39230316	13.5304169	13.368558	12.6494989	2.909E-09	1.25926	up	PREDICTED: palmitoyl-monogalactosyldiacylglycerol delta-7 desaturase, chloroplastic [Sesamum indicum]

#ID	0 h-1 FPKM	0 h-2 FPKM	0 h-3 FPKM	28 h-1 FPKM	28 h-2 FPKM	28 h-3 FPKM	FDR	log2FC	regulated	nr_annotation
c41436.graph_c0	2.70928434	1.94556327	2.49762483	14.297322	14.416496	13.7972057	1.484E-11	1.48082	up	PREDICTED: protein N-lysine methyltransferase METTL21A [Sesamum indicum]
c41437.graph_c0	0.83189641	1.13806355	1.38972577	5.11909496	5.40453799	4.35803944	6.009E-06	1.04981	up	PREDICTED: pentatricopeptide repeat-containing protein At3g57430, chloroplastic-like [Sesamum indicum]
c41438.graph_c0	1.53207402	1.60663712	1.82814893	10.2866428	10.2932624	10.1028668	4.812E-14	1.53412	up	-
c41439.graph_c0	23.4714234	24.4408261	20.499516	20.6331399	21.0635323	20.3074146	3.257E-36	-1.2307	down	PREDICTED: mediator of RNA polymerase II transcription subunit 14 [Sesamum indicum]
c41444.graph_c0	0.10022092	0.20064255	0.25757569	2.95001941	2.79512629	3.09071533	1.076E-09	2.88406	up	PREDICTED: squamosa promoter-binding-like protein 6 [Sesamum indicum]
c41445.graph_c0	0.76752863	0.7387473	0.53108713	0.10727604	0.11623224	0.0700291	8.181E-18	-3.88323	down	unnamed protein product [Coffea canephora]
c41446.graph_c0	3.13854744	2.29188877	3.10751497	32.2716472	31.7043304	30.6053312	2.542E-92	2.37812	up	PREDICTED: ADP-ribosylation factor GTPase-activating protein AGD3-like [Sesamum indicum]
c41448.graph_c0	0.72569242	0.90075967	0.59682783	8.81861398	9.06720966	9.41666649	3.639E-29	2.53098	up	PREDICTED: phosphoacetylglucosamine mutase [Sesamum indicum]
c41449.graph_c0	15.8612738	17.3102489	13.7496237	5.06569652	6.89785877	7.26167175	1.039E-40	-2.375	down	PREDICTED: sodium-coupled neutral amino acid transporter 2-like [Sesamum indicum]
c41451.graph_c0	1.86128018	0.95429325	1.34175197	0	0	0	2.327E-17	-Inf	down	hypothetical protein GUITHDRAFT_154386 [Guillardia theta CCMP2712]
c41452.graph_c0	0.0862945	0	0.05544593	1.19164862	1.44403409	0.92119788	9.294E-10	3.56029	up	PREDICTED: uncharacterized protein LOC105161758 [Sesamum indicum]
c41454.graph_c0	1.52325973	1.28653738	1.16223538	8.96056144	10.0897553	11.0099635	1.948E-13	1.8331	up	PREDICTED: BURP domain-containing protein 3-like [Sesamum indicum]
c41459.graph_c0	1.96138289	2.39964623	1.40025259	1.82150005	1.68039689	1.93869022	0.0003346	-1.16802	down	PREDICTED: heptahelical transmembrane protein 4-like [Sesamum indicum]
c41459.graph_c1	1.80680798	1.29186944	1.76900555	1.12558082	1.1292155	1.58180042	0.0004503	-1.43288	down	hypothetical protein MIMGU_mgv1a007578mg [Erythranthe guttata]
c41464.graph_c0	0.42753476	0.53495359	0.85843608	8.99174354	10.7840202	9.79348377	5.289E-42	2.92164	up	PREDICTED: nucleobase-ascorbate transporter 12 [Sesamum indicum]
c41482.graph_c0	22.3336105	23.8194916	20.8776458	10.0731274	9.34963463	10.1130151	4.6E-142	-2.27158	down	PREDICTED: autophagy-related protein 18g-like [Sesamum indicum]
c41483.graph_c0	0	0	0	1.72125606	2.94467276	1.84806696	4.902E-09	Inf	up	PREDICTED: uncharacterized protein LOC105112702 isoform X1 [Populus euphratica]
c41486.graph_c0	1250.05621	1227.48123	1331.97371	58.4288292	55.0684394	61.399918	0	-5.53606	down	glucose and ribitol dehydrogenase homolog 1-like precursor [Sesamum indicum]
c41487.graph_c0	6.01185273	7.28036346	6.15876532	0.94720823	0.57935628	0.77291418	2.556E-74	-4.17024	down	hypothetical protein JCGZ_16285 [Jatropha curcas]
c41489.graph_c0	397.669757	375.472013	386.714498	124.686956	129.569242	133.011794	6E-192	-2.6727	down	PREDICTED: protein DJ-1 homolog D [Sesamum indicum]
c41491.graph_c0	33.9097872	36.7992544	33.6127894	35.6305483	31.2652431	31.8984349	8.118E-46	-1.16884	down	PREDICTED: CTD small phosphatase-like protein 2 isoform X2 [Sesamum indicum]
c41493.graph_c0	28.24164	27.478362	26.7106364	21.4563669	23.4503318	22.2649178	4.933E-49	-1.38534	down	PREDICTED: ethylene-responsive transcription factor 3-like [Sesamum indicum]

#ID	0 h-1 FPKM	0 h-2 FPKM	0 h-3 FPKM	28 h-1 FPKM	28 h-2 FPKM	28 h-3 FPKM	FDR	log2FC	regulated	nr_annotation
c41493.graph_c1	10.7867313	10.9230853	9.50954919	11.212643	8.3131748	9.81889527	3.661E-07	-1.17791	down	PREDICTED: ethylene-responsive transcription factor 3-like [Sesamum indicum]
c41495.graph_c0	3.38945407	3.21659625	2.09294232	1.08775082	1.21322807	1.22697065	1.493E-10	-2.38653	down	ATP binding protein, putative [Ricinus communis]
c41499.graph_c0	2.76138325	2.33225017	1.7742434	0.06271742	0	0.1023538	2.734E-28	-6.45681	down	-
c41499.graph_c1	99.2115304	106.476872	99.9499896	18.7891932	17.8438892	18.994873	0	-3.54816	down	PREDICTED: UV-stimulated scaffold protein A homolog [Sesamum indicum]
c41502.graph_c0	15.8935714	16.3117953	15.7756777	16.7295502	15.2970276	14.1712212	3.25E-26	-1.1455	down	PREDICTED: MORN repeat-containing protein 1-like [Sesamum indicum]
c41503.graph_c0	0.73076411	0.27431095	0.3521478	2.7883545	2.27784909	2.54614051	0.0030126	1.40559	up	-
c41504.graph_c0	0.48058256	1.30574458	0.44111973	4.39100684	6.66784986	4.88594417	0.0004092	1.7521	up	PREDICTED: uncharacterized protein LOC105130684 isoform X1 [Populus euphratica]
c41507.graph_c0	0.722804	0.28311956	0.60575984	3.95139356	4.53700774	4.43574891	2.501E-10	1.91347	up	PREDICTED: endoglucanase 6 [Sesamum indicum]
c41510.graph_c0	1.12753426	1.12866332	0.94250537	7.22420141	6.58024594	6.49866106	1.138E-28	1.57772	up	PREDICTED: 1-phosphatidylinositol-3-phosphate 5-kinase FAB1B-like [Sesamum indicum]
c41514.graph_c0	0.25111178	0.4608326	0.26890715	2.0684081	1.86757858	1.38995633	0.0023751	1.35008	up	PREDICTED: shugoshin-1-like isoform X2 [Sesamum indicum]
c41516.graph_c0	16.7274208	17.5464099	13.9429609	6.48195158	6.45943037	7.04416798	3.773E-45	-2.35824	down	PREDICTED: LOW QUALITY PROTEIN: ubiquitin carboxyl-terminal hydrolase 22 [Sesamum indicum]
c41519.graph_c0	0	0	0.06244676	1.20083621	0.92479508	1.35452969	1.107E-10	4.67163	up	PREDICTED: solute carrier family 35 member F1-like isoform X1 [Sesamum indicum]
c41521.graph_c0	77.8841589	79.4215844	77.1180402	57.4944955	59.8748807	56.3426745	2.411E-75	-1.52301	down	PREDICTED: uncharacterized protein LOC105162845 [Sesamum indicum]
c41523.graph_c0	4.16878059	4.62005731	3.63513978	1.94775795	1.46553568	1.32446215	1.26E-10	-2.48024	down	-
c41525.graph_c0	0.30398167	0.30428606	0	4.19771042	5.38598691	3.0647398	1.419E-06	3.30555	up	PREDICTED: LOW QUALITY PROTEIN: LOB domain-containing protein 6 [Sesamum indicum]
c41525.graph_c1	18.0441911	18.0622596	18.057522	6.80092623	5.97293734	6.11770848	8.55E-123	-2.61023	down	PREDICTED: LOB domain-containing protein 36 [Sesamum indicum]
c41527.graph_c0	54.6246814	55.2877734	60.5296586	50.4129694	48.8086559	48.9763999	2.029E-27	-1.29369	down	PREDICTED: phosphoribosylaminoimidazole-succinocarboxamide synthase, chloroplastic [Sesamum indicum]
c41530.graph_c0	1.55962074	1.40506422	1.5031308	0.68011402	0.51173266	0.32373103	2.831E-10	-2.65234	down	retrotransposon protein, putative, unclassified [Oryza sativa Japonica Group]
c41535.graph_c0	260.403141	293.444909	249.876169	110.865898	106.41278	108.046186	9.046E-73	-2.39447	down	PREDICTED: adagio protein 1-like [Sesamum indicum]
c41538.graph_c0	3.03051511	3.981534	2.79905105	19.8459916	19.7213345	18.7028588	6.526E-26	1.48127	up	PREDICTED: allantoate deiminase [Sesamum indicum]
c41539.graph_c0	0.80211589	0.94890438	1.26501219	14.6273277	14.3076976	12.811503	4.97E-75	2.69411	up	PREDICTED: poly [ADP-ribose] polymerase 1 [Sesamum indicum]
c41543.graph_c0	155.144725	159.08041	169.199722	14.465702	13.9988978	18.1598379	3.52E-196	-4.46454	down	Gibberellin 2-beta-dioxygenase 8 GN=GA2OX8 OS=Arabidopsis thaliana
c41543.graph_c1	99.0368586	94.355885	93.6487752	12.3738203	10.3549069	11.8823702	0	-4.14152	down	PREDICTED: gibberellin 2-beta-dioxygenase 8 [Sesamum indicum]

#ID	0 h-1 FPKM	0 h-2 FPKM	0 h-3 FPKM	28 h-1 FPKM	28 h-2 FPKM	28 h-3 FPKM	FDR	log2FC	regulated	nr_annotation
c41545.graph_c0	2.96274525	2.04775352	2.49283895	55.1912879	61.4979598	56.2474857	3.84E-176	3.43718	up	PREDICTED: 60S ribosomal protein L5-like [Nicotiana sylvestris]
c41549.graph_c0	1.58246896	1.32004463	1.52515145	0.7028568	1.413458	1.14705083	1.721E-05	-1.53094	down	PREDICTED: interferon-activable protein 203 isoform X1 [Sesamum indicum]
c41551.graph_c0	34.8039768	34.1373748	29.7162706	14.599989	16.2481005	15.3766903	3.846E-28	-2.18241	down	-
c41551.graph_c1	41.1516742	39.9078984	38.7656497	18.3931121	19.194618	17.1620413	2.9E-158	-2.22032	down	PREDICTED: uncharacterized protein LOC105172898 isoform X1 [Sesamum indicum]
c41556.graph_c0	4.00111236	4.14564936	4.41996144	4.84664234	4.46819127	3.20548615	5.048E-05	-1.09871	down	transcription factor, putative [Ricinus communis]
c41558.graph_c0	0.21568756	0.17991962	0.27716706	1.51535129	1.65130017	1.53498324	9.7E-05	1.71006	up	PREDICTED: NAC domain-containing protein 8-like [Sesamum indicum]
c41561.graph_c0	15.4320969	17.6043775	16.3885183	7.57606291	10.5855387	9.04852197	3.164E-44	-1.95235	down	PREDICTED: probable transcription factor GLK1 [Sesamum indicum]
c41564.graph_c0	0.70928964	0.75018856	0.46433136	0.02917968	0.02634651	0.03174717	7.675E-25	-5.54677	down	Strong similarity to gi|3600044 T12H20.12 protease homolog from Arabidopsis thaliana BAC gb|AF080119 and is a member of the reverse transcriptase family PF|00078 [Arabidopsis thaliana]
c41567.graph_c0	3.7865713	6.06458077	4.91711398	31.4024325	31.7537741	30.139081	2.994E-35	1.56681	up	chloroplast omega-3 desaturase [Portulaca oleracea]
c41570.graph_c0	31.9958752	32.5131857	28.8746219	26.7778143	25.9593819	28.7218781	4.664E-53	-1.28581	down	PREDICTED: RNA polymerase-associated protein LEO1 [Sesamum indicum]
c41572.graph_c0	4.56175876	4.76272782	4.22319055	33.0119993	33.4762525	30.6028866	9.467E-36	1.75147	up	PREDICTED: ER lumen protein-retaining receptor-like [Sesamum indicum]
c41575.graph_c0	3.22592004	2.95432901	2.55782158	29.4319629	30.0725021	30.5562573	1.452E-89	2.27787	up	PREDICTED: glycosylphosphatidylinositol anchor attachment 1 protein [Sesamum indicum]
c41577.graph_c0	7.76203014	5.06346689	6.94853927	35.8133742	36.6285243	34.5508764	4.523E-16	1.34548	up	PREDICTED: ergosterol biosynthetic protein 28 [Sesamum indicum]
c41581.graph_c0	15.1052992	15.7949419	14.0350242	3.14252869	3.22432814	2.7640916	4.85E-176	-3.38915	down	PREDICTED: trehalose-phosphate phosphatase A-like isoform X2 [Sesamum indicum]
c41582.graph_c0	5.75070916	7.40431141	5.35907977	26.7365645	37.651287	26.2426645	7.775E-05	1.20119	up	PREDICTED: lysine histidine transporter-like 8 [Sesamum indicum]
c41586.graph_c0	0.03510107	0.21081731	0.09021253	6.68394889	5.78160218	7.76473205	2.081E-29	4.82006	up	PREDICTED: protein Brevis radix-like 4 [Sesamum indicum]
c41597.graph_c0	31.5110882	27.7158806	34.7495647	29.7870015	30.9161875	32.0911891	3.137E-09	-1.11032	down	PREDICTED: ninja-family protein AFP1-like [Sesamum indicum]
c41598.graph_c0	4.86810013	4.00575047	3.55196605	24.4968942	24.0405606	22.7539988	3.459E-23	1.43319	up	hypothetical protein MIMGU_mgv1a006127mg [Erythranthe guttata]
c41602.graph_c0	3.19864573	3.18681654	3.35778044	15.7821551	15.728002	14.8641529	4.108E-27	1.15936	up	PREDICTED: pentatricopeptide repeat-containing protein At1g63080, mitochondrial-like [Sesamum indicum]
c41604.graph_c0	13.4411416	13.8476272	13.1519144	9.24577782	9.99707208	8.69322706	2.746E-75	-1.6244	down	Calcium-transporting ATPase 12, plasma membrane-type [Morus notabilis]
c41605.graph_c1	25.130711	24.4762786	23.2095517	21.6953906	22.4600569	22.183401	3.372E-52	-1.22399	down	PREDICTED: WD repeat-containing protein LWD1 [Sesamum indicum]
c41609.graph_c1	1.5862564	1.26567339	1.15214042	6.06516187	5.76290936	5.35814095	9.658E-07	1.0144	up	PREDICTED: protein transport protein SEC23 [Sesamum indicum]
c41610.graph_c0	1.06087295	1.00293885	0.83310562	6.12545543	5.41468302	4.85850842	4.15E-05	1.41274	up	PREDICTED: uncharacterized protein LOC105179150 [Sesamum indicum]

#ID	0 h-1 FPKM	0 h-2 FPKM	0 h-3 FPKM	28 h-1 FPKM	28 h-2 FPKM	28 h-3 FPKM	FDR	log2FC	regulated	nr_annotation
c41614.graph_c0	12.8006014	12.1658096	13.0050261	10.1430606	11.5842547	10.5239582	5.289E-23	-1.32675	down	PREDICTED: zinc finger CCCH domain-containing protein 44-like [Sesamum indicum]
c41615.graph_c0	2.68720295	2.07855428	1.80506205	16.512096	14.968991	16.3713131	4.509E-29	1.77875	up	hypothetical protein MIMGU_mgv1a003382mg [Erythranthe guttata]
c41620.graph_c0	0.30594418	0.91875162	1.17945116	6.67075406	6.62537202	6.7133797	3.529E-05	1.95431	up	-
c41627.graph_c0	0.67614824	0.48881827	0.28962575	2.9212227	3.17989848	2.71785656	6.955E-05	1.51839	up	hypothetical protein MIMGU_mgv1a021778mg, partial [Erythranthe guttata]
c41630.graph_c0	70.7283071	67.3570377	70.6141754	374.33959	386.619669	374.874879	8.214E-66	1.35348	up	unknown [Lotus japonicus]
c41639.graph_c0	6.0705277	5.55016774	5.52239696	40.1668967	41.6606386	43.0862647	3.215E-91	1.77592	up	PREDICTED: alpha-1,4 glucan phosphorylase L-1 isozyme, chloroplastic/amyloplastic [Sesamum indicum]
c41640.graph_c0	1186.59981	1029.49954	1151.16288	447.995136	605.119625	547.610334	6.095E-53	-2.16347	down	PREDICTED: ubiquitin carboxyl-terminal hydrolase isozyme L3 [Sesamum indicum]
c41642.graph_c0	0.19054544	0.25431499	0.24485844	3.92380764	4.50147864	4.10583123	7.965E-11	3.09022	up	PREDICTED: protein polybromo-1-like [Sesamum indicum]
c41645.graph_c0	0	0.06579297	0	5.44579352	5.95220748	5.22332358	2.909E-37	6.89872	up	PREDICTED: MATE efflux family protein 5-like isoform X3 [Sesamum indicum]
c41646.graph_c0	7.4233126	7.43074592	6.88662476	6.63583309	6.59068862	6.38003	1.003E-14	-1.23878	down	PREDICTED: protein PHR1-LIKE 1-like isoform X1 [Sesamum indicum]
c41648.graph_c0	3.74827025	2.98237771	3.21111711	1.32718469	1.89208949	1.25396743	6.652E-10	-2.24273	down	hypothetical protein VITISV_011460 [Vitis vinifera]
c41653.graph_c0	18.8351338	19.0782242	18.9025445	5.11482017	5.48794421	5.77890206	5.63E-201	-2.88441	down	hypothetical protein MIMGU_mgv1a026893mg [Erythranthe guttata]
c41653.graph_c1	9.52065653	9.3408485	10.0468089	2.1537698	2.48253472	2.76706243	2.524E-48	-3.05581	down	PREDICTED: uncharacterized protein LOC105160086 [Sesamum indicum]
c41654.graph_c0	7.4687153	7.35998382	6.98685015	48.9946006	47.132524	47.9888029	9.598E-76	1.63412	up	PREDICTED: coatomer subunit beta-1 [Sesamum indicum]
c41654.graph_c1	0.19815456	0.06611766	0	1.44017594	1.43037823	1.56689574	3.002E-07	3.00545	up	PREDICTED: coatomer subunit beta-1 [Sesamum indicum]
c41657.graph_c0	16.1589179	15.4092761	14.6575406	8.74497009	8.58050264	8.45574542	8.082E-84	-1.93186	down	PREDICTED: cysteine synthase 2 [Sesamum indicum]
c41660.graph_c0	27.2929906	29.6002339	27.929079	18.7702174	18.4930567	16.2321196	1.083E-74	-1.75627	down	hypothetical protein MIMGU_mgv1a007698mg [Erythranthe guttata]
c41662.graph_c0	287.467525	307.739525	280.128695	115.782539	107.834748	120.428744	1.8E-143	-2.43682	down	PREDICTED: uncharacterized protein LOC105179373 [Sesamum indicum]
c41665.graph_c0	1.03419893	1.03523452	1.52833455	20.3697101	23.7194925	23.0309213	5.836E-52	3.12559	up	NAD(P)-binding Rossmann-fold superfamily protein [Theobroma cacao]
c41667.graph_c0	2.87274291	2.95549785	2.51233098	1.37259632	1.40515106	1.46707813	4.985E-37	-2.06292	down	PREDICTED: phospholipase A I-like [Sesamum indicum]
c41668.graph_c0	51.5986078	57.7922768	57.4070139	52.6273781	58.7494462	56.6905415	4.014E-20	-1.08069	down	PREDICTED: NASP-related protein sim3 isoform X1 [Sesamum indicum]
c41676.graph_c0	5.00707781	6.00433313	5.61775855	3.82384196	3.60268254	3.4518418	6.68E-28	-1.70404	down	PREDICTED: uncharacterized protein LOC105161819 isoform X2 [Sesamum indicum]
c41679.graph_c0	2.24829945	1.45623874	1.78447735	1.4898701	0.9546673	1.2941555	9.07E-06	-1.64178	down	hypothetical protein MIMGU_mgv1a009883mg [Erythranthe guttata]
c41683.graph_c0	39.564922	44.783998	40.9695108	170.975909	187.987238	184.05682	1.466E-36	1.02481	up	PREDICTED: histone deacetylase HDT1-like isoform X2 [Sesamum indicum]

#ID	0 h-1 FPKM	0 h-2 FPKM	0 h-3 FPKM	28 h-1 FPKM	28 h-2 FPKM	28 h-3 FPKM	FDR	log2FC	regulated	nr_annotation
c41686.graph_c0	2.0055034	2.14134572	1.34011866	44.3689883	48.8523545	45.1964461	1.95E-193	3.57119	up	PREDICTED: BTB/POZ domain-containing protein NPY4-like [Sesamum indicum]
c41688.graph_c0	19.395995	17.6853305	17.8503065	166.185206	178.026444	168.064599	1.24E-121	2.13134	up	PREDICTED: 60S ribosomal protein L18a-2-like [Sesamum indicum]
c41690.graph_c0	14.8190637	15.8464558	13.8436068	73.1873257	65.6830131	66.9490812	1.289E-29	1.11967	up	PREDICTED: uncharacterized protein LOC105167598 [Sesamum indicum]
c41690.graph_c1	35.2247556	35.4659258	32.7759736	162.576642	155.700188	159.559416	2.961E-41	1.11782	up	PREDICTED: uncharacterized protein LOC105167598 [Sesamum indicum]
c41692.graph_c0	34.778187	33.7123665	31.5037365	28.3547649	26.0827281	24.5380509	1.496E-55	-1.43018	down	PREDICTED: protein kinase 2B, chloroplastic-like isoform X2 [Sesamum indicum]
c41697.graph_c0	1.40906561	1.07859974	1.27814478	0.39156794	0.4351374	0.46698528	1.42E-11	-2.63089	down	-
c41702.graph_c1	1.8885449	1.30876338	1.96015209	1.84770863	1.66830765	1.68002545	0.0053438	-1.08147	down	-
c41705.graph_c0	2.503111	2.21329545	1.60829837	15.5090777	15.070939	13.5707165	2.003E-35	1.71757	up	PREDICTED: putative phospholipid:diacylglycerol acyltransferase 2 [Sesamum indicum]
c41709.graph_c0	33.9643868	32.2742311	32.3710495	24.0200698	22.358994	20.7812992	2.578E-60	-1.64462	down	PREDICTED: B-box zinc finger protein 20 [Sesamum indicum]
c41712.graph_c0	18.4695199	16.9033274	18.4786644	14.6380515	16.448841	15.2827398	1.69E-30	-1.30681	down	PREDICTED: syntaxin-61 [Vitis vinifera]
c41713.graph_c0	3.26564156	3.6899078	2.35257139	15.12175	14.9036071	14.4079887	3.939E-17	1.1687	up	PREDICTED: poly(A)-specific ribonuclease PARN [Sesamum indicum]
c41715.graph_c0	17.3394242	21.0503112	19.6436759	8.87194426	10.1952249	10.217474	5.801E-30	-2.0781	down	Vesicle-associated protein 4-2 [Triticum urartu]
c41722.graph_c0	1.39038517	1.06281186	1.03953454	9.42543231	9.42269002	8.75553268	6.977E-23	1.89474	up	PREDICTED: uncharacterized protein LOC105159385 [Sesamum indicum]
c41723.graph_c0	0.62676782	0.84882912	0.94755467	4.86346589	5.20176059	5.4444549	6.805E-15	1.58349	up	PREDICTED: katanin p80 WD40 repeat-containing subunit B1 homolog [Sesamum indicum]
c41725.graph_c0	0	0	0	1.36025778	1.22818523	1.86798018	9.453E-13	Inf	up	similar to AHNAK nucleoprotein [Ectocarpus siliculosus]
c41730.graph_c0	2.08370984	1.92738144	2.03365795	1.66778813	2.00780841	2.31509679	7.949E-06	-1.10241	down	PREDICTED: vacuolar amino acid transporter 1-like isoform X1 [Sesamum indicum]
c41730.graph_c1	26.2679998	26.5593074	28.1988165	18.3004085	18.3187522	19.2419174	2.105E-61	-1.62778	down	PREDICTED: glucan endo-1,3-beta-glucosidase 5 [Sesamum indicum]
c41733.graph_c0	230.359054	237.078221	228.966949	50.5194543	52.295091	47.9734366	0	-3.29815	down	PREDICTED: protein NLP5-like [Sesamum indicum]
c41734.graph_c0	13.216879	12.8711572	14.4168318	7.96772722	9.61455907	7.5649462	1.703E-35	-1.78039	down	hypothetical protein M569_06103, partial [Genlisea aurea]
c41738.graph_c0	8.03273279	7.20404582	7.15230875	3.89703514	2.92998463	3.27667733	4.776E-70	-2.23714	down	hypothetical protein MIMGU_mgv1a0006002mg, partial [Erythranthe guttata]
c41741.graph_c0	0.62758698	0.80770553	0.63365824	3.35578856	3.67713888	3.32317483	0.0006095	1.23326	up	hypothetical protein MIMGU_mgv1a006910mg [Erythranthe guttata]
c41744.graph_c0	38.6832684	41.8368653	33.7146664	6.51872578	4.68461489	7.12848789	8.435E-93	-3.72666	down	hypothetical protein MIMGU_mgv1a021012mg [Erythranthe guttata]
c41745.graph_c1	1.69524092	1.08604061	1.13279488	9.80744699	9.1221962	9.37010864	3.995E-09	1.76791	up	hypothetical protein MIMGU_mgv1a015492mg [Erythranthe guttata]
c41751.graph_c0	0.69692502	0.41857373	0.53734575	2.93782231	3.20138737	3.00343739	0.0003475	1.37932	up	PREDICTED: protein DA1-related 2 isoform X2 [Sesamum indicum]
c41751.graph_c1	1.30680877	0.96387594	0.26515279	4.9988478	6.27375232	6.15931867	1.144E-05	1.70627	up	hypothetical protein MIMGU_mgv1a005034mg [Erythranthe guttata]

#ID	0 h-1 FPKM	0 h-2 FPKM	0 h-3 FPKM	28 h-1 FPKM	28 h-2 FPKM	28 h-3 FPKM	FDR	log2FC	regulated	nr_annotation
c41753.graph_c2	5.67388346	3.27142944	4.60801621	30.8126286	29.697486	28.5885549	1.577E-31	1.62756	up	PREDICTED: omega-amidase,chloroplastic-like isoform X2 [Nicotiana sylvestris]
c41755.graph_c0	84.0368231	91.9837531	102.920662	18.1955101	17.2055613	20.1014059	3.961E-43	-3.42168	down	PREDICTED: protein PHLOEM PROTEIN 2-LIKE A10 [Sesamum indicum]
c41756.graph_c0	90.7807759	91.4953694	98.8821281	63.8505916	65.1743615	62.3371163	2.133E-48	-1.64694	down	PREDICTED: protein DEHYDRATION-INDUCED 19 homolog 4-like [Sesamum indicum]
c41758.graph_c0	3.04789808	2.71866839	2.75330246	0.52638291	0.29704651	0.42952416	1.727E-42	-3.8543	down	PREDICTED: tetratricopeptide repeat protein 28-like [Sesamum indicum]
c41762.graph_c1	1.66857317	1.33282097	1.12623732	32.4124139	34.6295828	30.746358	5E-195	3.47982	up	PREDICTED: lanC-like protein GCR2 [Sesamum indicum]
c41766.graph_c0	0.13032058	0.04348369	0.19537829	1.24709724	1.18302514	1.23660294	3.03E-07	2.21254	up	PREDICTED: uncharacterized protein LOC102666339 [Glycine max]
c41767.graph_c0	12.6033443	12.4176004	11.3064994	9.3904792	10.3253152	9.82499299	6.96E-28	-1.38761	down	PREDICTED: uncharacterized protein LOC105162543 [Sesamum indicum]
c41770.graph_c0	17.0092243	17.6703419	15.4584482	18.7466351	16.6877945	17.5700977	5.953E-26	-1.00891	down	PREDICTED: nuclear pore complex protein NUP1 [Sesamum indicum]
c41771.graph_c0	0.44417966	0.36378363	0.41511887	11.5043882	11.6063116	10.536975	9.128E-40	3.69129	up	PREDICTED: uncharacterized protein LOC105163485 [Sesamum indicum]
c41771.graph_c1	0.22919949	0.34414349	0.34361882	6.08020163	6.49256469	6.11678821	5.285E-22	3.25459	up	PREDICTED: uncharacterized protein LOC105163485 [Sesamum indicum]
c41776.graph_c0	0.22895205	0.08333866	0.24071927	1.67913766	1.52976246	1.55531973	2.048E-07	2.01307	up	unnamed protein product [Coffea canephora]
c41779.graph_c0	2.00547986	2.0271693	2.22339853	12.2035864	12.2960371	11.2873184	4.866E-25	1.4238	up	gtpase-activating protein [Gossypium arboreum]
c41780.graph_c0	45.7214835	52.4199517	48.87585	11.5810719	11.6389034	11.5395206	1.18E-103	-3.17155	down	PREDICTED: E3 ubiquitin-protein ligase PUB23-like [Sesamum indicum]
c41780.graph_c2	0	0	0	0.42454032	0.67818172	0.38639314	1.421E-07	Inf	up	PREDICTED: leucine-rich repeat receptor-like protein kinase TDR [Sesamum indicum]
c41782.graph_c0	61.3542228	65.5060849	60.933056	40.61765	36.9589266	36.6441855	8.85E-110	-1.80777	down	PREDICTED: protein NLP7 isoform X1 [Sesamum indicum]
c41785.graph_c0	4.02981264	2.72792591	3.53924126	19.3851585	18.7205793	20.0133745	1.441E-26	1.40731	up	PREDICTED: heterogeneous nuclear ribonucleoprotein 1 [Sesamum indicum]
c41786.graph_c0	19.7581168	21.6576195	16.524459	17.6534108	18.9397277	16.3660612	1.45E-13	-1.21829	down	PREDICTED: transmembrane protein 45B-like [Sesamum indicum]
c41791.graph_c0	3.84125074	4.14471513	4.64768244	4.45962681	3.60104664	4.1123874	3.243E-10	-1.14619	down	PREDICTED: probable beta-1,3-galactosyltransferase 16 [Sesamum indicum]
c41797.graph_c0	0.90058274	0.73967962	0.77152298	22.3046527	22.4726502	21.9391001	8.55E-129	3.70077	up	PREDICTED: probable acyl-activating enzyme 17, peroxisomal isoform X1 [Sesamum indicum]
c41799.graph_c1	0	0	0	0.72477534	1.14172644	0.90599095	1.359E-14	Inf	up	PREDICTED: respiratory burst oxidase homolog protein A [Sesamum indicum]
c41800.graph_c0	0.42404402	0.12127675	0.31137906	48.2541795	40.1502644	44.9076373	6.08E-104	6.19365	up	PREDICTED: acyl-CoA-binding domain-containing protein 3-like [Sesamum indicum]
c41802.graph_c0	156.84102	162.890867	151.74885	143.799424	151.63473	149.384884	3.314E-47	-1.17398	down	PREDICTED: uncharacterized protein LOC105170559 [Sesamum indicum]

#ID	0 h-1 FPKM	0 h-2 FPKM	0 h-3 FPKM	28 h-1 FPKM	28 h-2 FPKM	28 h-3 FPKM	FDR	log2FC	regulated	nr_annotation
c41805.graph_c0	2.68317814	2.24735637	3.02578726	16.4765106	14.4455343	16.6597346	6.031E-14	1.48795	up	hypothetical protein MIMGU_mgv1a014951mg [Erythranthe guttata]
c41811.graph_c0	3.47575943	3.17669728	3.49551272	25.7009517	21.66843	22.0024305	5.942E-17	1.68208	up	PREDICTED: zinc finger SWIM domain-containing protein 7 isoform X2 [Vitis vinifera]
c41812.graph_c0	2.14754823	2.23926946	2.52971079	2.43879012	2.0551989	2.25537053	0.0001299	-1.12778	down	PREDICTED: probable 1-acyl-sn-glycerol-3-phosphate acyltransferase 5 [Sesamum indicum]
c41815.graph_c0	2.04901019	2.58824486	2.38229168	11.2399807	11.3331913	11.4574324	2.82E-08	1.18567	up	PREDICTED: calcineurin B-like protein 10 isoform X3 [Sesamum indicum]
c41816.graph_c0	0	0.02992607	0.03841771	2.62912858	1.72644117	1.71982399	2.939E-12	5.36421	up	--
c41819.graph_c0	13.6183594	13.1737778	13.4314731	15.3318013	12.4413382	14.5467119	3.42E-18	-1.01676	down	PREDICTED: deoxyhypusine synthase [Sesamum indicum]
c41820.graph_c0	1.08798645	1.01128477	0.99864691	6.18473429	6.06871222	6.5445695	2.117E-08	1.51194	up	PREDICTED: uncharacterized protein LOC105162931 [Sesamum indicum]
c41824.graph_c0	1.03872036	0.86646707	0.62290517	7.80100506	7.38439436	7.26905476	9.281E-16	2.06619	up	PREDICTED: probable protein phosphatase 2C 53 [Sesamum indicum]
c41826.graph_c0	0.53388599	0.41566046	0.22868821	9.26949229	16.8557484	11.2929741	2.411E-09	3.90681	up	hypothetical protein MIMGU_mgv1a008654mg [Erythranthe guttata]
c41827.graph_c0	0.43734405	1.24038228	0.65567182	4.45002786	4.01795793	3.37181594	0.0023537	1.25093	up	PREDICTED: agamous-like MADS-box protein AGL15 [Sesamum indicum]
c41829.graph_c0	0.6164017	0.41134595	0.85810897	7.09328904	5.96636718	6.42779953	3.362E-12	2.27269	up	PREDICTED: uncharacterized protein LOC105164835 isoform X1 [Sesamum indicum]
c41839.graph_c0	13.4470995	13.961423	12.7390175	11.7052008	10.4866119	10.6641267	1.401E-35	-1.37897	down	PREDICTED: putative F-box protein At5g62060 [Sesamum indicum]
c41840.graph_c0	0.98608859	0.82914385	0.55755167	5.41812731	5.97918563	5.89486352	2.053E-09	1.78168	up	PREDICTED: probable glycosyltransferase At3g07620 [Sesamum indicum]
c41843.graph_c0	13.0686798	12.9281641	11.3711247	10.2882123	10.1619977	10.3213435	3.573E-55	-1.36891	down	PREDICTED: uncharacterized protein LOC105155405 [Sesamum indicum]
c41846.graph_c2	3.29210692	3.12196118	2.95020855	16.3395154	14.2982358	14.1549789	5.461E-11	1.16853	up	PREDICTED: BRCA1-A complex subunit BRE [Sesamum indicum]
c41847.graph_c0	19.6602845	17.4562457	20.8870115	140.65632	173.406393	174.478181	2.058E-25	1.98286	up	hypothetical protein MIMGU_mgv1a012525mg [Erythranthe guttata]
c41848.graph_c0	35.7417674	36.5305289	33.8320612	17.0142137	16.1400747	17.4252962	9.72E-142	-2.15833	down	PREDICTED: LOW QUALITY PROTEIN: F-box protein SKIP14-like [Sesamum indicum]
c41849.graph_c0	3.63086695	2.95606221	3.48380315	0.95000154	2.16029029	1.78007396	3.016E-14	-2.13272	down	PREDICTED: uncharacterized protein LOC105177931 [Sesamum indicum]
c41851.graph_c0	1.36077476	1.2054313	0.71183959	9.00605615	7.91825807	8.58439229	1.413E-27	1.87727	up	hypothetical protein MIMGU_mgv1a000152mg [Erythranthe guttata]
c41852.graph_c0	67.6675866	76.6758049	67.6320772	65.9773013	68.5450953	67.2438033	1.348E-31	-1.16142	down	PREDICTED: PHD finger protein ALFIN-LIKE 4-like [Sesamum indicum]
c41853.graph_c0	1.08130329	1.04075582	1.22918859	6.77070154	7.75080207	7.64598001	2.827E-10	1.63338	up	PREDICTED: homogentisate solanesyltransferase, chloroplastic [Sesamum indicum]
c41856.graph_c0	40.1971167	40.5042826	44.1165282	33.1878896	32.8527446	36.6088313	1.194E-30	-1.37338	down	PREDICTED: uncharacterized protein LOC105160829 [Sesamum indicum]

#ID	0 h-1 FPKM	0 h-2 FPKM	0 h-3 FPKM	28 h-1 FPKM	28 h-2 FPKM	28 h-3 FPKM	FDR	log2FC	regulated	nr_annotation
c41857.graph_c0	21.0082901	17.8420694	19.5724513	6.39376987	7.0438908	7.82589601	2.51E-74	-2.54752	down	PREDICTED: monoglyceride lipase-like [Sesamum indicum]
c41859.graph_c0	10.1745242	11.2696818	10.8281602	1.82963767	2.43209825	1.72106194	2.54E-115	-3.52373	down	PREDICTED: probable microtubule-binding protein TANGLED [Sesamum indicum]
c41861.graph_c0	0.81779185	0.70698201	0.71651925	0.5943664	0.78059219	0.52908887	0.000699	-1.3259	down	PREDICTED: calcium-transporting ATPase 2, plasma membrane-type-like [Sesamum indicum]
c41867.graph_c0	1.27438305	1.02412073	0.9918057	0.96535007	1.26031627	1.26673534	0.0001415	-1.00137	down	PREDICTED: regulator of telomere elongation helicase 1 [Sesamum indicum]
c41869.graph_c0	555.445129	554.398658	554.344749	209.903143	200.460496	205.997689	1.51E-167	-2.52344	down	PREDICTED: probable 2-aminoethanethiol dioxygenase [Sesamum indicum]
c41875.graph_c0	370.321664	426.445212	351.088867	288.405234	366.888327	326.547177	1.713E-18	-1.31506	down	PREDICTED: basic 7S globulin 2-like [Sesamum indicum]
c41879.graph_c0	3.7606396	1.5724731	1.65163811	0.24218327	0.40609917	0.31054504	0.0001826	-3.9467	down	PREDICTED: heat shock cognate 70 kDa protein 2-like [Elaeis guineensis]
c41882.graph_c0	0.5052771	0.39338682	0.64930108	4.57000426	4.47628279	3.8622678	8.819E-12	1.96424	up	hypothetical protein MIMGU_mgv1a004014mg [Erythranthe guttata]
c41887.graph_c0	0.88975544	0.68822676	0.46774267	0.14697026	0.19905054	0.29981632	1.42E-07	-2.74388	down	hypothetical protein VITISV_000584 [Vitis vinifera]
c41892.graph_c0	0.09631117	0.04820381	0	4.72489242	6.92061877	7.42535663	6.519E-18	5.97838	up	PREDICTED: tetraspanin-10 [Nicotiana sylvestris]
c41899.graph_c0	40.9931655	47.5692925	42.5324788	18.9520377	17.1368213	18.2785195	5.889E-63	-2.36022	down	hypothetical protein AALP_AA7G017200 [Arabis alpina]
c41900.graph_c2	14.246858	15.5889754	14.6769047	10.9040766	10.8812542	10.5733198	8.646E-71	-1.55088	down	PREDICTED: LRR receptor-like serine/threonine-protein kinase RPK2 [Sesamum indicum]
c41909.graph_c0	1.24568401	0.73348904	0.28248587	496.135064	487.681974	469.375288	0	8.25205	up	hypothetical protein MIMGU_mgv1a015091mg [Erythranthe guttata]
c41910.graph_c0	101.022111	101.512477	95.7505618	64.5848959	71.0485702	64.54479	5.88E-94	-1.66556	down	hypothetical protein MIMGU_mgv1a007193mg [Erythranthe guttata]
c41914.graph_c0	0.91836278	0.78795633	0.84295181	5.62574083	5.4238905	5.05739504	0.0004471	1.56934	up	-
c41915.graph_c0	13.7134237	13.37176	13.0598866	13.3858818	13.8918448	14.8049596	6.924E-26	-1.0216	down	PREDICTED: zinc finger BED domain-containing protein RICESLEEPER 1-like [Sesamum indicum]
c41927.graph_c0	1.11476237	1.56920433	1.611578	9.31733294	7.9554673	7.80946725	2.343E-11	1.45117	up	hypothetical protein MIMGU_mgv1a006603mg [Erythranthe guttata]
c41931.graph_c0	1.47187514	1.56264288	1.23228737	7.84483604	7.12705525	7.20810035	4.129E-12	1.28983	up	PREDICTED: zeaxanthin epoxidase, chloroplastic-like [Sesamum indicum]
c41932.graph_c0	3.35247379	3.29919229	3.07199325	17.3848972	16.8108481	18.581812	8.386E-39	1.35135	up	PREDICTED: kinesin-like calmodulin-binding protein homolog [Sesamum indicum]
c41933.graph_c0	0.94121077	1.04683694	0.76153269	4.71245854	4.98693544	3.86600149	3.109E-05	1.21397	up	PREDICTED: uncharacterized protein LOC105167458 [Sesamum indicum]
c41936.graph_c0	2.1474924	3.15512087	1.86941509	1.00695813	1.25013457	1.25304616	2.987E-06	-2.11828	down	PREDICTED: cyclic nucleotide-gated ion channel 4 [Sesamum indicum]
c41941.graph_c0	0	0	0	3.84676527	4.98725714	7.00222633	3.399E-09	Inf	up	60S ribosomal protein L35 [Galdieria sulphuraria]
c41944.graph_c0	24.0428783	9.7337457	15.2420516	0	0	0	3.714E-16	-Inf	down	--
c41954.graph_c0	49.0201818	50.2668909	48.1735337	24.0335238	20.2606204	22.3508086	5.63E-149	-2.23581	down	PREDICTED: pheophorbide a oxygenase, chloroplastic-like [Citrus sinensis]
c41957.graph_c0	0.18666452	0.18685144	0.3198284	2.39677834	1.79658319	1.95575155	1.71E-05	2.05043	up	PREDICTED: RING-H2 finger protein ATL39-like [Sesamum indicum]

#ID	0 h-1 FPKM	0 h-2 FPKM	0 h-3 FPKM	28 h-1 FPKM	28 h-2 FPKM	28 h-3 FPKM	FDR	log2FC	regulated	nr_annotation
c41963.graph_c0	6.52687248	5.02569858	4.70978567	4.99912294	5.56804337	4.61520249	3.876E-08	-1.18675	down	PREDICTED: lachrymatory-factor synthase-like [Sesamum indicum]
c41964.graph_c0	1.26295542	1.03201639	1.25861252	0.74931324	0.45667773	0.50443263	6.465E-11	-2.14636	down	hypothetical protein M569_02915, partial [Genlisea aurea]
c41968.graph_c0	5.08219914	5.45952879	6.05296171	1.17117731	1.22014996	1.32323693	3.365E-32	-3.25169	down	-
c41974.graph_c0	0.41854669	0.37706922	0.32270944	2.06854177	2.14236093	1.7872022	0.0018015	1.33494	up	unnamed protein product [Coffea canephora]
c41980.graph_c0	0.63563064	0.40489727	0.59404403	2.22586841	3.29902437	2.09044749	0.0068953	1.12756	up	PREDICTED: probable serine/threonine-protein kinase At5g41260, partial [Sesamum indicum]
c41981.graph_c1	63.2914967	67.4521272	57.6906595	42.0281119	39.8619617	38.7751024	9.002E-71	-1.73242	down	PREDICTED: homeobox-leucine zipper protein ANTHOCYANINLESS 2-like [Sesamum indicum]
c41985.graph_c0	0.06753857	0.1014093	0.08678973	1.93892236	1.92794764	1.18160182	2.243E-08	3.20857	up	PREDICTED: probable GABA transporter 2 [Sesamum indicum]
c41988.graph_c0	13.3213072	16.58585	17.6023197	8.62129334	9.29781781	9.43570587	1.487E-12	-1.88979	down	hypothetical protein MIMGU_mgv1a002241mg [Erythranthe guttata]
c41989.graph_c0	0.38546974	0.16077322	0.04127866	2.80157186	3.56245905	3.06713018	1.778E-12	2.93407	up	PREDICTED: transcription factor bHLH18-like [Sesamum indicum]
c41992.graph_c0	1.92634212	1.22708158	1.68779038	1.14549901	1.26411781	1.5059343	0.0005884	-1.39498	down	-
c41994.graph_c0	66.2339926	66.5577929	61.4385831	50.9426676	50.2796084	51.369885	3.452E-70	-1.43755	down	PREDICTED: neurofilament medium polypeptide [Sesamum indicum]
c41997.graph_c0	2.00293685	2.0806007	2.71954295	12.3873784	12.5734216	12.8423144	2.351E-14	1.3797	up	unnamed protein product [Coffea canephora]
c41999.graph_c0	4.73631583	6.77294076	5.73856174	25.6207485	28.7721125	29.7343268	1.078E-13	1.19465	up	-
c41999.graph_c1	5.80139966	7.43322737	6.73082551	32.3580412	34.2415641	33.6389192	1.283E-29	1.23616	up	PREDICTED: uncharacterized protein LOC105156173 isoform X1 [Sesamum indicum]
c42001.graph_c0	47.2488427	49.0984337	45.555003	31.6635554	31.3819072	31.8960833	5.544E-94	-1.66968	down	PREDICTED: ubiquitin-activating enzyme E1 1 [Sesamum indicum]
c42002.graph_c0	3.24942901	2.11139061	1.75816629	0	0	0	6.833E-20	-Inf	down	predicted protein [Hordeum vulgare subsp. vulgare]
c42004.graph_c0	95.13054	94.1304626	93.8600093	43.4177402	43.8022863	45.162746	1.91E-155	-2.18694	down	PREDICTED: uncharacterized protein At1g04910-like [Sesamum indicum]
c42005.graph_c0	4.10026854	3.22753073	3.28115461	2.73622914	3.48568879	3.61438707	1.607E-10	-1.19674	down	PREDICTED: chromatin assembly factor 1 subunit FAS1-like [Sesamum indicum]
c42006.graph_c0	116.269162	112.846983	116.559007	77.4852765	80.9668708	83.6810052	5.814E-86	-1.60406	down	PREDICTED: 50S ribosomal protein L1, chloroplastic [Sesamum indicum]
c42007.graph_c0	9.85294818	8.82462342	9.66266912	8.40483003	9.80074783	8.5497749	3.457E-13	-1.17407	down	PREDICTED: phytochromobilin:ferredoxin oxidoreductase, chloroplastic isoform X1 [Sesamum indicum]
c42013.graph_c0	44.9178524	45.2853762	40.2475068	35.2221223	34.0225126	33.9196884	2.132E-65	-1.42771	down	PREDICTED: CBL-interacting protein kinase 18 [Sesamum indicum]
c42014.graph_c0	0	0	0	2.41552374	1.92812289	2.68519535	3.965E-16	Inf	up	hypothetical protein MIMGU_mgv1a013619mg [Erythranthe guttata]
c42016.graph_c1	2.02107492	1.9545191	2.2890235	1.56849242	1.19140769	1.84871123	6.459E-08	-1.53357	down	PREDICTED: uncharacterized protein LOC105163887 isoform X1 [Sesamum indicum]
c42018.graph_c0	1.83423134	1.29937123	1.23292364	12.8183535	13.2589125	14.0912698	5.449E-34	2.11576	up	PREDICTED: GPI transamidase component PIG-S [Sesamum indicum]
c42019.graph_c0	2.11082769	2.65241576	2.53935904	12.1098931	11.7003691	12.3053561	1.081E-09	1.21408	up	PREDICTED: uncharacterized protein LOC105165798 [Sesamum indicum]

#ID	0 h-1 FPKM	0 h-2 FPKM	0 h-3 FPKM	28 h-1 FPKM	28 h-2 FPKM	28 h-3 FPKM	FDR	log2FC	regulated	nr_annotation
c42029.graph_c1	6.9755975	5.4582159	6.56512374	27.2056756	28.3036024	29.0168119	2.367E-15	1.06327	up	PREDICTED: vesicle-associated membrane protein 724 [Nicotiana sylvestris]
c42030.graph_c0	2.16296909	0.88372856	1.07776566	0	0	0	1.373E-09	-Inf	down	--
c42033.graph_c0	1.86908674	1.48456477	1.7491737	11.3990958	10.1456626	10.4100462	8.634E-25	1.55633	up	PREDICTED: protein ROOT PRIMORDIUM DEFECTIVE 1 [Sesamum indicum]
c42041.graph_c0	5.26157556	6.01925055	4.24998068	3.60556377	3.6500904	3.7445044	1.005E-09	-1.58496	down	PREDICTED: probable LRR receptor-like serine/threonine-protein kinase RLK [Sesamum indicum]
c42043.graph_c0	39.3487022	40.7362088	39.7293602	31.3859038	32.872693	33.16361	1.059E-60	-1.38894	down	PREDICTED: probable zinc metalloprotease EGY1, chloroplastic [Sesamum indicum]
c42045.graph_c0	1.32328592	1.64768685	1.24424986	7.71751014	6.92582827	7.44589186	5.463E-09	1.30073	up	PREDICTED: membrane-bound transcription factor site-2 protease [Sesamum indicum]
c42048.graph_c0	0.49804745	0.565019	0.34133904	2.50971426	2.68002414	2.4811173	0.0002537	1.36352	up	PREDICTED: probable glucan 1,3-beta-glucosidase A [Beta vulgaris subsp. vulgaris]
c42052.graph_c0	14.4849852	12.5634783	13.8545745	1.9739257	1.91729028	3.09939123	3.47E-127	-3.63667	down	PREDICTED: homeobox-leucine zipper protein HOX11-like [Sesamum indicum]
c42061.graph_c0	0.56910493	0.55068564	0.51192566	3.41927671	4.35705725	3.73513638	4.475E-12	1.72896	up	PREDICTED: putative ion channel POLLUX-like 2 isoform X4 [Sesamum indicum]
c42063.graph_c0	0.9466137	0.54858829	0.60821804	3.13218038	3.89063307	3.64908036	5.977E-06	1.25725	up	PREDICTED: uncharacterized CRM domain-containing protein At3g25440, chloroplastic [Sesamum indicum]
c42066.graph_c1	4.25717172	3.92998972	3.55591224	24.8042711	24.8481537	25.6682961	6.071E-45	1.593	up	PREDICTED: uncharacterized protein LOC105165741 [Sesamum indicum]
c42067.graph_c0	0	0	0	1.15828787	1.12329389	1.71527848	2.559E-11	Inf	up	-
c42073.graph_c0	0.33216219	0.53199168	0.2987891	3.09009365	3.46578367	2.83667758	1.311E-07	1.92508	up	PREDICTED: protein OBERON 2 isoform X2 [Sesamum indicum]
c42075.graph_c0	16.8105268	15.0266462	17.1382708	86.4713243	97.81826	90.0208873	8.865E-46	1.39431	up	PREDICTED: ATP synthase subunit d, mitochondrial [Sesamum indicum]
c42078.graph_c0	2.16740261	1.17274213	1.4302369	13.6238333	16.6448503	15.6679327	1.572E-20	2.18127	up	PREDICTED: uncharacterized protein LOC105156356 isoform X1 [Sesamum indicum]
c42079.graph_c0	0.82334184	1.06766997	0.72138195	10.5264019	9.18508622	10.5204214	2.205E-46	2.44491	up	PREDICTED: uncharacterized protein LOC105156622 [Sesamum indicum]
c42083.graph_c0	3.31687134	2.95128239	2.55010059	23.4268935	22.7894048	23.1848514	2.747E-47	1.88902	up	PREDICTED: imidazole glycerol phosphate synthase hisHF, chloroplastic [Sesamum indicum]
c42089.graph_c0	5.28745057	4.53003515	4.62862445	3.37301576	3.33340167	3.92997712	6.885E-23	-1.53006	down	PREDICTED: U-box domain-containing protein 9-like [Sesamum indicum]
c42091.graph_c0	15.2817122	14.8003582	13.3892763	232.398453	233.124786	206.897991	3.15E-165	2.86177	up	PREDICTED: calnexin homolog 1 [Sesamum indicum]
c42092.graph_c0	1.93549567	2.2572044	2.36645126	22.3980548	22.8375805	21.1852886	6.735E-83	2.24697	up	PREDICTED: protein PAT1 homolog 1-like [Sesamum indicum]
c42096.graph_c0	27.0197639	26.7402737	26.9114068	16.0595157	16.6180754	15.647085	6.991E-96	-1.83006	down	PREDICTED: uncharacterized protein LOC105170420 [Sesamum indicum]
c42097.graph_c0	4.29819141	2.34921528	2.91415808	0	0	0	1.512E-26	-Inf	down	predicted protein [Hordeum vulgare subsp. vulgare]

#ID	0 h-1 FPKM	0 h-2 FPKM	0 h-3 FPKM	28 h-1 FPKM	28 h-2 FPKM	28 h-3 FPKM	FDR	log2FC	regulated	nr_annotation
c42099.graph_c0	11.5852891	12.1767345	11.3517608	128.301599	122.686636	76.5229809	2.136E-07	2.12919	up	--
c42100.graph_c0	1.99919642	2.08635569	2.15909231	20.9603618	24.9266087	22.1865992	1.609E-54	2.35463	up	PREDICTED: 4-hydroxy-3-methylbut-2-en-1-yl diphosphate synthase, chloroplastic [Sesamum indicum]
c42109.graph_c1	2.43700469	1.14186787	1.46587756	10.2503545	8.37042953	10.7320138	9.021E-07	1.45644	up	hypothetical protein MIMGU_mgv1a002479mg [Erythranthe guttata]
c42117.graph_c0	28.7682554	30.5436298	27.6168865	190.589951	178.158932	168.83317	6.423E-77	1.53839	up	PREDICTED: monosaccharide-sensing protein 2-like [Sesamum indicum]
c42120.graph_c0	9.24910311	8.30060283	7.45915307	107.833277	112.097602	101.080029	3.15E-127	2.59367	up	PREDICTED: chaperone protein dnaJ 11, chloroplastic-like [Sesamum indicum]
c42122.graph_c0	69.1541318	62.415697	65.9593961	68.9395366	72.7690247	64.9575632	3.243E-31	-1.02543	down	PREDICTED: cyclic dof factor 1 [Sesamum indicum]
c42123.graph_c1	42.254896	39.1095922	41.3933141	191.073434	213.551992	197.68038	9.71E-49	1.20397	up	PREDICTED: elongation factor 1-delta-like [Sesamum indicum]
c42128.graph_c0	156.365536	164.873214	150.780296	127.616352	128.76837	125.226875	3.152E-67	-1.39677	down	PREDICTED: glycine-rich RNA-binding protein 2, mitochondrial-like [Sesamum indicum]
c42129.graph_c1	0.19090324	0.34396992	0.34344551	1.44297268	1.57847595	2.10583106	0.0005633	1.451	up	PREDICTED: cytochrome P450 90A1 [Sesamum indicum]
c42134.graph_c1	47.2791889	46.4541533	48.4365157	24.5444585	28.3093308	25.1966139	1.258E-34	-1.95642	down	70 kDa heat shock protein-like protein, partial [Arachis diogoi]
c42138.graph_c0	0.49482988	0.2251479	0.28903454	11.7046312	12.7379095	11.4850707	1.546E-80	4.06899	up	PREDICTED: LOW QUALITY PROTEIN: putative kinase-like protein TMKL1 [Sesamum indicum]
c42141.graph_c0	35.205188	36.7590665	35.9463526	23.5176189	23.98906	23.1711932	7.863E-93	-1.70134	down	PREDICTED: uncharacterized protein LOC105177867 [Sesamum indicum]
c42144.graph_c1	81.6563307	82.959765	87.2424157	35.1298359	34.2519939	36.1224338	1.7E-112	-2.34649	down	hypothetical protein MIMGU_mgv1a005576mg [Erythranthe guttata]
c42147.graph_c0	38.616984	36.8917054	38.0431809	20.9310248	19.6586678	20.7223854	2.209E-92	-1.97943	down	PREDICTED: F-box protein CPR30-like [Sesamum indicum]
c42152.graph_c2	53.1071726	51.3777763	50.1919797	29.9407634	29.0592821	29.665046	1.04E-106	-1.89266	down	PREDICTED: shaggy-related protein kinase alpha [Sesamum indicum]
c42153.graph_c0	9.68564675	12.003761	9.82168018	46.6424895	48.5994922	48.4540701	5.463E-19	1.09906	up	PREDICTED: mediator-associated protein 2-like isoform X1 [Sesamum indicum]
c42159.graph_c0	13.3463733	13.6369107	11.6353779	144.031911	158.084618	140.683215	5.61E-139	2.43016	up	PREDICTED: serine/threonine-protein kinase SRK2E isoform X1 [Sesamum indicum]
c42163.graph_c0	109.164041	99.6404399	111.103397	8.30554339	9.96594504	9.54166246	6.86E-197	-4.61483	down	-
c42163.graph_c1	117.671901	119.655907	113.345388	12.7337869	13.3127971	13.2677155	0	-4.24696	down	PREDICTED: BAG family molecular chaperone regulator 6 [Sesamum indicum]
c42164.graph_c0	1.44810974	2.07079972	1.68365184	1.85436567	0.99554063	1.18598027	9.193E-05	-1.45792	down	-
c42173.graph_c0	1.67635	2.30728934	1.11555549	1.24011798	1.11971026	1.59777812	0.0036816	-1.44906	down	PREDICTED: rop guanine nucleotide exchange factor 12-like [Sesamum indicum]
c42176.graph_c0	3.35393568	3.28430949	3.65407968	19.3949855	24.0668368	20.6691245	1.038E-14	1.54723	up	ribosomal protein L7Ae/L30e/S12e/Gadd45 family protein [Medicago truncatula]
c42181.graph_c1	7.0167447	6.37741163	5.4211404	29.0340434	27.5427193	27.4188755	2.49E-16	1.07087	up	PREDICTED: ethanolamine-phosphate cytidylyltransferase [Sesamum indicum]

#ID	0 h-1 FPKM	0 h-2 FPKM	0 h-3 FPKM	28 h-1 FPKM	28 h-2 FPKM	28 h-3 FPKM	FDR	log2FC	regulated	nr_annotation
c42188.graph_c0	5.13143019	6.62046612	4.39606064	3.72949389	3.66670627	4.39578614	4.102E-07	-1.54058	down	-
c42189.graph_c0	0.02244671	0.06740755	0.11537963	12.1214038	13.4486143	12.9261488	1.64E-117	6.44455	up	PREDICTED: uncharacterized protein At1g04910 [Sesamum indicum]
c42192.graph_c2	3.42697869	3.04310591	3.12527976	38.6059796	44.4334396	39.4350712	9.013E-68	2.58398	up	PREDICTED: rac-like GTP-binding protein ARAC7 isoform X3 [Sesamum indicum]
c42206.graph_c0	0.16767392	0.83920911	0.53866907	2.74194991	2.36569152	2.63517029	0.0062069	1.22869	up	F15O4.13 [Arabidopsis thaliana]
c42211.graph_c0	1.28695203	1.53130501	1.84100518	10.2711944	9.70415919	8.10759735	5.057E-14	1.49575	up	PREDICTED: uncharacterized protein LOC105176468 [Sesamum indicum]
c42219.graph_c0	191.547767	182.752306	196.438422	41.0792763	48.543931	45.9810081	7.91E-201	-3.16448	down	PREDICTED: uncharacterized protein LOC105161471 [Sesamum indicum]
c42226.graph_c0	2.82484639	3.33261702	2.56695647	19.2843066	17.5443308	17.2436051	2.573E-37	1.54269	up	PREDICTED: filament-like plant protein 3 [Sesamum indicum]
c42229.graph_c0	58.4229465	54.4847262	54.6657454	50.1429311	48.9155804	49.778502	4.215E-54	-1.26087	down	PREDICTED: zinc finger CCCH domain-containing protein 17 isoform X1 [Sesamum indicum]
c42230.graph_c1	1.60876864	1.54036307	1.67783418	8.72696441	8.55284179	8.75276964	1.069E-14	1.33996	up	hypothetical protein MIMGU_mgv1a006437mg [Erythranthe guttata]
c42233.graph_c2	2.87116539	2.10762965	1.47582448	10.4337323	11.5560366	10.3301263	0.002687	1.24073	up	PREDICTED: probable serine/threonine protein kinase IREH1 [Sesamum indicum]
c42237.graph_c1	132.721667	131.735007	135.459963	26.7571843	40.1838311	36.6473567	0	-3.03959	down	PREDICTED: E3 ubiquitin-protein ligase RNF5-like [Sesamum indicum]
c42239.graph_c0	0	0	0	7.24571773	10.8243735	21.2848074	1.154E-05	Inf	up	--
c42243.graph_c0	87.3297975	79.3778156	83.5142306	392.297812	402.368819	428.348693	4.117E-51	1.19929	up	unnamed protein product [Coffea canephora]
c42246.graph_c0	1.17864354	1.02758845	1.12373817	0.38686576	0.14970151	0.38332487	2.204E-13	-2.94376	down	hypothetical protein VITISV_034155 [Vitis vinifera]
c42248.graph_c0	1.76387973	1.40531007	1.54965209	0.40552353	0.53150767	0.54439027	3.378E-25	-2.76034	down	PREDICTED: 65-kDa microtubule-associated protein 3 [Sesamum indicum]
c42251.graph_c0	5.1352324	4.91118588	5.00177234	28.1702224	30.0284071	28.9030721	2.839E-34	1.4427	up	PREDICTED: ATPase family AAA domain-containing protein At1g05910 isoform X1 [Sesamum indicum]
c42253.graph_c0	14.2631371	14.9428925	12.8533863	4.41449055	4.24366373	4.17567095	7.17E-120	-2.80167	down	PREDICTED: lysM domain receptor-like kinase 3 [Sesamum indicum]
c42256.graph_c0	77.1738581	79.1782509	78.9217926	37.6060594	32.9097443	34.196055	7.97E-166	-2.25871	down	PREDICTED: mpv17-like protein isoform X2 [Sesamum indicum]
c42268.graph_c0	64.7965441	62.6401462	56.4612762	52.3837382	50.5595079	58.2738741	5.861E-42	-1.27809	down	-
c42279.graph_c0	134.012253	130.081402	107.59526	62.2632781	62.2813513	64.8372614	3.012E-43	-2.06039	down	PREDICTED: transcription factor bHLH130-like [Sesamum indicum]
c42284.graph_c0	26.2859333	27.5477867	23.6155529	6.27955453	5.72984738	6.39830365	3.77E-166	-3.16157	down	PREDICTED: DNA repair protein RAD51 homolog [Sesamum indicum]
c42290.graph_c0	78.541665	77.2889526	71.468992	81.8860086	76.8983162	76.9894774	1.657E-38	-1.03663	down	unknown [Lotus japonicus]
c42290.graph_c1	2.90700641	2.50854942	2.5762885	0.14571003	0.06578125	0.35669459	6.136E-23	-4.89669	down	-
c42293.graph_c1	10.0265538	7.91119751	11.2171103	4.11514814	3.79300112	5.80641592	6.542E-11	-2.17885	down	hypothetical protein CISIN_1g015935mg [Citrus sinensis]
c42299.graph_c0	6.71034372	7.32770522	5.48740013	6.57661339	5.33758676	5.90913883	2.276E-06	-1.21919	down	hypothetical protein MIMGU_mgv1a012462mg [Erythranthe guttata]

#ID	0 h-1 FPKM	0 h-2 FPKM	0 h-3 FPKM	28 h-1 FPKM	28 h-2 FPKM	28 h-3 FPKM	FDR	log2FC	regulated	nr_annotation
c42300.graph_c0	0.3715127	0	0.11935213	5.19775587	4.38834036	3.24984746	1.755E-09	3.63061	up	PREDICTED: putative B3 domain-containing protein At5g66980 [Sesamum indicum]
c42303.graph_c0	29.961936	31.3316359	29.4037539	13.2100351	14.222248	14.1590078	6.67E-108	-2.21486	down	PREDICTED: high mobility group B protein 10 [Sesamum indicum]
c42308.graph_c0	0.43596474	0.5727767	0.28011598	27.4477082	26.0343589	25.9037233	1.85E-163	4.85987	up	PREDICTED: zinc finger CCCH domain-containing protein 53 [Sesamum indicum]
c42309.graph_c1	1.84959068	1.51757603	1.0909889	10.7541747	11.5803826	11.3647571	8.173E-22	1.83386	up	PREDICTED: serine/threonine-protein kinase-like protein At3g51990 [Sesamum indicum]
c42316.graph_c0	0	0.12859377	0	4.57501826	5.3110447	6.01879645	1.372E-13	5.86969	up	-
c42329.graph_c0	8.13081282	9.18914229	10.6506438	5.68067585	7.88300636	7.52861205	1.294E-08	-1.5008	down	PREDICTED: AP2-like ethylene-responsive transcription factor BBM2 [Sesamum indicum]
c42333.graph_c0	2.00739047	1.50705043	2.07799197	0	0.18295888	0	6.536E-30	-6.03569	down	PREDICTED: uncharacterized protein LOC105169088 [Sesamum indicum]
c42334.graph_c1	689.972084	727.482483	684.610474	180.467254	188.342673	195.26202	1.8E-192	-2.98791	down	PREDICTED: protein EXORDIUM-like 2 [Sesamum indicum]
c42337.graph_c0	50.0922986	47.3485971	48.2307477	13.8260405	15.0863804	14.8538233	3.59E-154	-2.82498	down	unnamed protein product [Coffea canephora]
c42339.graph_c0	40.4468872	38.1878028	40.6385207	10.5507911	10.7292003	11.4066765	0	-2.95795	down	PREDICTED: uncharacterized protein LOC105156535 [Sesamum indicum]
c42340.graph_c0	189.989555	195.368149	196.193148	161.234315	178.706586	178.569684	5.925E-46	-1.25628	down	phosphoglycerate kinase [Gossypium hirsutum]
c42346.graph_c0	9.83535735	10.2454176	10.069957	50.1541995	48.0918663	44.9800211	1.69E-29	1.15691	up	PREDICTED: uncharacterized protein LOC105168920 [Sesamum indicum]
c42352.graph_c0	2.5301706	2.58386992	2.52884286	14.4512589	12.5114478	13.0652933	8.107E-19	1.29832	up	PREDICTED: putative ABC transporter C family member 15 [Sesamum indicum]
c42353.graph_c0	2.75948121	1.18381903	1.74999505	0	0	0	6.975E-14	-Inf	down	predicted protein [Physcomitrella patens]
c42358.graph_c0	248.889005	279.994799	241.911836	23.8790996	21.7270791	31.4470765	1.06E-182	-4.41056	down	hypothetical protein MIMGU_mgv1a016283mg [Erythranthe guttata]
c42361.graph_c1	2.58740504	2.73388459	2.77076493	2.71629377	2.8298753	1.8754776	0.0021479	-1.21785	down	copalyl diphosphate synthase [Isodon eriocalyx]
c42363.graph_c0	4.31296147	3.2294616	2.9239044	0.8145116	1.06971288	0.96674154	5.764E-18	-2.96248	down	PREDICTED: uncharacterized protein LOC105168320 [Sesamum indicum]
c42364.graph_c0	27.6071161	25.7869272	26.6956915	26.913121	26.9940009	27.4511087	4.776E-34	-1.06754	down	PREDICTED: probable mediator of RNA polymerase II transcription subunit 26b [Sesamum indicum]
c42367.graph_c0	872.339612	928.883009	773.152763	767.07005	782.174312	804.683357	6.471E-31	-1.21779	down	PREDICTED: fructose-bisphosphate aldolase cytoplasmic isozyme-like [Solanum lycopersicum]
c42368.graph_c0	13.5472044	15.0006903	12.7757351	15.5498459	13.1677773	13.0773028	2.862E-21	-1.07341	down	PREDICTED: pentatricopeptide repeat-containing protein At4g19191, mitochondrial-like [Sesamum indicum]
c42372.graph_c1	0.50863441	0.71280122	1.04578456	57.2253115	51.2685553	59.0630281	3.84E-100	5.10885	up	-
c42373.graph_c0	9.71320584	9.59946634	8.95523498	56.9914719	55.6668643	54.4961209	3.621E-55	1.4743	up	PREDICTED: ethylene-responsive transcription factor ERF118-like [Sesamum indicum]

#ID	0 h-1 FPKM	0 h-2 FPKM	0 h-3 FPKM	28 h-1 FPKM	28 h-2 FPKM	28 h-3 FPKM	FDR	log2FC	regulated	nr_annotation
c42379.graph_c0	2.1364789	2.24045723	2.18983117	1.22004186	1.15165539	1.29722426	9.689E-15	-1.93007	down	-
c42385.graph_c0	69.4182426	60.1528676	70.25138	35.3065709	36.2184985	40.0403629	9.789E-35	-1.93143	down	PREDICTED: uncharacterized protein LOC105170258 [Sesamum indicum]
c42387.graph_c0	88.5108245	85.3409809	88.8007943	75.9557228	75.1724744	81.0639309	7.682E-55	-1.26819	down	PREDICTED: homogentisate 1,2-dioxygenase isoform X1 [Sesamum indicum]
c42388.graph_c0	0	0	0	1.70899358	2.00026422	1.34287574	1.418E-14	Inf	up	PREDICTED: ATP-citrate synthase beta chain protein 2-like [Citrus sinensis]
c42389.graph_c0	2.31305633	2.36880418	2.76658957	13.0480466	12.8436858	12.1138455	6.011E-24	1.25797	up	PREDICTED: protein FAR1-RELATED SEQUENCE 3-like [Sesamum indicum]
c42390.graph_c0	0.95799343	1.66774386	1.76630275	13.138201	11.397901	11.9804663	1.771E-20	1.9591	up	hypothetical protein JCGZ_15647 [Jatropha curcas]
c42391.graph_c0	14.5398584	12.8320016	12.3824905	2.87635695	2.70999719	3.26550787	4.626E-54	-3.25499	down	-
c42395.graph_c0	0	0.18019828	0.28916286	2.32234009	2.21498806	1.86832026	2.67E-08	2.65798	up	PREDICTED: tetraketide alpha-pyrone reductase 1-like [Sesamum indicum]
c42396.graph_c0	19.2956679	21.0623114	19.8244327	14.1611341	13.683995	13.8216771	7.731E-46	-1.62109	down	BnaA08g23740D [Brassica napus]
c42401.graph_c0	2.28542801	2.94688908	3.03642024	1.57657645	1.39808106	1.03377336	7.156E-14	-2.13963	down	PREDICTED: serine/threonine-protein kinase BLUS1-like isoform X2 [Sesamum indicum]
c42402.graph_c0	0.28747763	0.95921833	0.61570023	10.4468543	11.3504777	11.593385	6.061E-28	3.07001	up	PREDICTED: transcription factor bHLH71-like [Nicotiana sylvestris]
c42403.graph_c0	0.90646604	0.63516161	1.04836027	11.6280723	10.974937	12.1763474	2.757E-26	2.65395	up	PREDICTED: LOW QUALITY PROTEIN: protein YIF1B-like [Sesamum indicum]
c42407.graph_c0	10.3476127	11.0485059	11.0316615	7.24204509	8.09816093	8.40960143	4.526E-16	-1.54017	down	PREDICTED: myb family transcription factor APL-like [Sesamum indicum]
c42407.graph_c1	8.97683436	8.32992379	7.49392184	6.42906605	3.99889244	6.17869924	5.027E-17	-1.66576	down	PREDICTED: protein ENDOSPERM DEFECTIVE 1-like [Sesamum indicum]
c42409.graph_c0	51.6321754	46.8900103	54.6180482	350.133592	356.240199	362.924031	1.34E-104	1.71225	up	PREDICTED: 60S ribosomal protein L5-like [Sesamum indicum]
c42413.graph_c0	560.082674	502.626025	566.234461	428.57195	499.410286	475.07777	6.196E-28	-1.3064	down	PREDICTED: extra-large guanine nucleotide-binding protein 3-like [Sesamum indicum]
c42414.graph_c0	3.66399016	3.00544287	2.8773387	15.5709594	13.3912249	12.4039758	2.2E-07	1.02694	up	PREDICTED: paired amphipathic helix protein Sin3-like 2 isoform X1 [Sesamum indicum]
c42426.graph_c0	14.681265	14.9537901	14.6873518	13.1272263	13.2470845	13.0029842	1.494E-31	-1.26125	down	PREDICTED: uncharacterized membrane protein At3g27390 [Sesamum indicum]
c42427.graph_c0	6.4723034	3.23939222	3.366472	0	0	0	1.738E-14	-Inf	down	PREDICTED: serpin-ZX-like [Malus domestica]
c42432.graph_c0	0.06562578	0.11496011	0.08433172	1.28780376	0.93667265	1.00218747	1.438E-08	2.5145	up	PREDICTED: uncharacterized protein LOC103330039 [Prunus mume]
c42437.graph_c0	2.68665745	3.41619847	3.07922215	2.13735702	2.00130829	2.1962311	3.161E-11	-1.62672	down	PREDICTED: post-GPI attachment to proteins factor 3 [Sesamum indicum]
c42439.graph_c0	4.87239703	5.51279984	4.45874978	97.4911372	140.879326	93.8336742	6.467E-16	3.39362	up	PREDICTED: probable serine/threonine-protein kinase At1g54610 [Nicotiana tomentosiformis]

#ID	0 h-1 FPKM	0 h-2 FPKM	0 h-3 FPKM	28 h-1 FPKM	28 h-2 FPKM	28 h-3 FPKM	FDR	log2FC	regulated	nr_annotation
c42441.graph_c0	26.6614838	29.1544158	22.6522535	22.3938087	22.8756661	22.1869795	1.262E-17	-1.30648	down	PREDICTED: uncharacterized protein LOC105162071 [Sesamum indicum]
c42442.graph_c0	1.80249619	2.49418096	1.97564971	17.0691007	15.4117975	14.5570615	4.636E-18	1.81565	up	PREDICTED: uncharacterized protein LOC101292603 isoform 2 [Fragaria vesca subsp. vesca]
c42445.graph_c0	2.81482136	2.96593682	2.63065956	36.1978631	35.3700575	33.7145363	5.29E-159	2.556	up	PREDICTED: ABC transporter B family member 1-like [Sesamum indicum]
c42449.graph_c0	2.75377124	2.65808127	3.12796251	22.0514154	26.7999263	21.6683546	3.04E-25	1.95259	up	PREDICTED: heme-binding protein 2-like [Sesamum indicum]
c42450.graph_c0	2.47121235	3.21579296	2.06414371	9.87834591	12.4869082	14.2893334	0.0003644	1.15495	up	PREDICTED: protein PLANT CADMIUM RESISTANCE 2-like [Nicotiana tomentosiformis]
c42453.graph_c0	0.0700688	0.03506948	0	1.22221446	1.5633555	1.3713109	4.031E-11	4.23832	up	hypothetical protein MIMGU_mgv1a006178mg [Erythranthe guttata]
c42460.graph_c0	3.42680096	2.52017073	3.14541065	0	0	0	6.964E-43	-Inf	down	hypothetical protein [Dendrobium catenatum]
c42462.graph_c0	0	0	0	1.02934531	1.3708684	1.80586533	3.072E-10	Inf	up	PREDICTED: 65-kDa microtubule-associated protein 8 isoform X1 [Sesamum indicum]
c42463.graph_c0	19.3639746	20.1828797	19.4261326	19.3264571	19.2908556	19.0629168	5.336E-38	-1.12251	down	PREDICTED: TBC1 domain family member 5 homolog A-like [Sesamum indicum]
c42465.graph_c0	12.1460642	14.2561167	11.1705554	7.68528257	8.39254756	7.81066145	1.902E-22	-1.7426	down	PREDICTED: uncharacterized protein LOC105168867 isoform X1 [Sesamum indicum]
c42465.graph_c1	1.43261352	2.02070409	2.00832555	20.0433935	22.8620029	18.9041148	7.911E-42	2.40588	up	PREDICTED: transcription factor bHLH93-like [Nicotiana sylvestris]
c42466.graph_c0	0.79630752	0.76644702	0.94457233	0.6010114	0.50246013	0.81736732	0.0001102	-1.47531	down	PREDICTED: TSL-kinase interacting protein 1 isoform X2 [Sesamum indicum]
c42472.graph_c0	48.0496938	44.8838994	40.2627875	3.70143563	5.30368698	4.39919269	6.12E-196	-4.40163	down	-
c42475.graph_c0	0	0	0	1.88662038	2.70201037	3.8752096	3.869E-07	Inf	up	--
c42478.graph_c0	0.35313514	0.45448553	0.25931008	3.29987034	3.07878931	2.84225988	2.76E-06	2.0248	up	PREDICTED: pentatricopeptide repeat-containing protein At4g21170 [Sesamum indicum]
c42478.graph_c1	0.65361224	0.55612672	0.79792172	4.60790359	5.4043672	4.74846838	2.922E-10	1.78392	up	PREDICTED: pentatricopeptide repeat-containing protein At3g16610 [Sesamum indicum]
c42481.graph_c0	0.14125679	0.25451683	0.2541288	1.86849614	1.9280878	1.87652627	6.435E-07	2.02979	up	hypothetical protein PGTG_21261 [Puccinia graminis f. sp. tritici CRL 75-36-700-3]
c42487.graph_c1	0.18445075	0.2051505	0.1316814	4.02173221	3.2681224	3.69495183	9.704E-24	3.31143	up	PREDICTED: bifunctional dethiobiotin synthetase/7,8-diamino-pelargonic acid aminotransferase, mitochondrial [Sesamum indicum]
c42491.graph_c0	7.94474307	7.4203003	8.24433771	43.4876496	46.4857171	48.6743693	2.439E-48	1.46306	up	PREDICTED: bidirectional sugar transporter SWEET3b [Sesamum indicum]
c42492.graph_c0	13.8314218	14.6004686	11.5415095	9.03428259	10.5382319	9.01246834	9.954E-25	-1.57239	down	PREDICTED: uncharacterized protein At5g01610 [Sesamum indicum]
c42493.graph_c0	0.25370258	0.15872289	0.2445134	1.79779836	1.20702711	1.61744917	6.946E-05	1.72421	up	PREDICTED: MATE efflux family protein 1 [Sesamum indicum]
c42494.graph_c1	1.35584778	1.53423226	1.36355328	0.81404455	0.61895234	0.55937154	2.616E-08	-2.18533	down	Transcription factor jumonji domain-containing protein, putative isoform 9 [Theobroma cacao]

#ID	0 h-1 FPKM	0 h-2 FPKM	0 h-3 FPKM	28 h-1 FPKM	28 h-2 FPKM	28 h-3 FPKM	FDR	log2FC	regulated	nr_annotation
c42496.graph_c0	31.472329	30.1165185	30.3509435	20.6914428	22.5856591	23.4595803	2.245E-49	-1.55208	down	hypothetical protein MIMGU_mgv1a013377mg [Erythranthe guttata]
c42499.graph_c0	0.07723091	0.27057886	0	3.22753142	2.61007218	2.63363928	1.728E-12	3.52928	up	PREDICTED: protein PHR1-LIKE 1-like isoform X1 [Sesamum indicum]
c42500.graph_c0	0	0	0	0.91417803	1.11365792	1.13670371	7.562E-17	Inf	up	PREDICTED: LOW QUALITY PROTEIN: intracellular protein transport protein USO1-like [Sesamum indicum]
c42503.graph_c0	14.305093	13.9216558	13.0720789	6.87347911	8.6051066	8.15382848	5.574E-27	-1.89471	down	-
c42504.graph_c0	2.0602098	2.64228701	2.19242049	1.84828969	1.85895243	1.5654623	8.133E-09	-1.47836	down	PREDICTED: crossover junction endonuclease EME1B-like isoform X1 [Sesamum indicum]
c42506.graph_c0	92.2368761	103.075513	94.2908575	2.43381294	2.84382986	2.07358787	0	-6.39171	down	PREDICTED: mitogen-activated protein kinase kinase kinase 2-like [Sesamum indicum]
c42509.graph_c0	0.94924966	1.4823123	0.97585872	0.35875278	0.52325558	0.4278498	3.903E-08	-2.46923	down	PREDICTED: putative late blight resistance protein homolog R1B-14 [Sesamum indicum]
c42517.graph_c0	0.94358399	1.46401971	1.27316993	1.57732024	1.05264908	1.09122037	0.0035218	-1.07762	down	PREDICTED: beta-carotene isomerase D27, chloroplastic [Sesamum indicum]
c42517.graph_c1	6.91811984	5.26037246	4.9579169	27.6059263	28.8106334	27.9309229	1.065E-13	1.21239	up	hypothetical protein MIMGU_mgv1a017465mg [Erythranthe guttata]
c42520.graph_c1	37.4656734	39.2756967	33.5426202	36.9329526	37.4516246	36.1810063	2.566E-39	-1.08544	down	PREDICTED: uncharacterized protein LOC105178356 [Sesamum indicum]
c42522.graph_c0	0.11349594	0.03786986	0.04861559	0.59116537	0.71996466	0.50482268	2.313E-05	2.10057	up	hypothetical protein MIMGU_mgv1a005054mg [Erythranthe guttata]
c42524.graph_c0	8.12616914	8.90322079	7.58506251	56.4160277	58.8975059	55.9852952	3.801E-61	1.70937	up	T1N15.23 [Arabidopsis thaliana]
c42526.graph_c0	32.915255	30.4137366	34.3666422	23.5009048	23.3998708	23.0556521	1.56E-40	-1.57329	down	PREDICTED: BI1-like protein [Sesamum indicum]
c42531.graph_c0	0.02069566	0.04143276	0.07978421	1.83506094	1.45317224	1.05554055	1.286E-09	3.82826	up	PREDICTED: probable LRR receptor-like serine/threonine-protein kinase MRH1 isoform X1 [Sesamum indicum]
c42536.graph_c0	10.8401826	12.1836209	12.3008165	57.1773129	58.4481729	54.7768841	2.467E-25	1.17805	up	PREDICTED: signal peptidase complex subunit 3B-like [Sesamum indicum]
c42537.graph_c0	18.8283772	17.2151643	16.7613779	10.813034	12.3454026	12.0309922	5.905E-82	-1.67471	down	PREDICTED: uncharacterized protein LOC105173836 [Sesamum indicum]
c42538.graph_c0	8.43460466	7.9057656	8.57250619	38.0352414	42.0158014	42.3596334	1.04E-29	1.20599	up	PREDICTED: triose phosphate/phosphate translocator, non-green plastid, chloroplastic-like [Sesamum indicum]
c42542.graph_c0	18.1468028	17.8972797	17.6981669	12.8280298	12.3722246	12.166848	9.988E-72	-1.61468	down	PREDICTED: mitogen-activated protein kinase kinase kinase 2-like [Sesamum indicum]
c42544.graph_c0	1.22657794	1.07746256	0.93285378	5.47617149	4.74734771	5.18109773	5.376E-07	1.16357	up	PREDICTED: non-lysosomal glucosylceramidase isoform X3 [Sesamum indicum]
c42546.graph_c0	89.9819463	86.4951812	91.7320303	390.147256	409.609605	419.884434	5.098E-44	1.09429	up	PREDICTED: 40S ribosomal protein S9-2-like [Sesamum indicum]
c42549.graph_c0	21.9241144	23.6924395	20.9245077	131.997033	136.468478	127.677512	1.001E-61	1.48372	up	hypothetical protein M569_16894, partial [Genlisea aurea]
c42549.graph_c1	21.9392806	21.7048925	23.3660538	133.705111	124.532542	125.303081	3.727E-48	1.42541	up	hypothetical protein M569_06257, partial [Genlisea aurea]
c42554.graph_c0	26.900506	26.2749199	28.9836893	20.6566087	20.0198602	16.855624	1.163E-38	-1.60653	down	PREDICTED: protein REVEILLE 6 isoform X2 [Sesamum indicum]

#ID	0 h-1 FPKM	0 h-2 FPKM	0 h-3 FPKM	28 h-1 FPKM	28 h-2 FPKM	28 h-3 FPKM	FDR	log2FC	regulated	nr_annotation
c42556.graph_c0	1263.48934	1254.2425	1261.84737	636.66845	652.304681	682.790339	2.61E-63	-2.02903	down	PREDICTED: LOW QUALITY PROTEIN: low-temperature-induced 65 kDa protein [Sesamum indicum]
c42557.graph_c1	0.03129634	0.09398302	0	1.38750577	1.0474126	1.24355666	3.878E-10	3.80023	up	PREDICTED: MLO-like protein 11 [Sesamum indicum]
c42559.graph_c0	0.25652133	0.19971637	0.36626673	4.43308658	3.609877	4.39492634	2.039E-17	2.82268	up	PREDICTED: caffeoylshikimate esterase [Sesamum indicum]
c42560.graph_c0	7.36087219	7.70535215	7.32609768	5.36732262	5.03561668	5.17859279	9.37E-36	-1.61348	down	hypothetical protein PRUPE_ppa004658mg [Prunus persica]
c42560.graph_c1	3.32325057	3.85883083	3.75804753	1.93225549	1.74464554	3.3899151	6.663E-06	-1.71898	down	PREDICTED: probable folate-biopterin transporter 2 [Sesamum indicum]
c42561.graph_c0	19.6541767	20.2197469	20.9956049	135.806204	122.663214	125.228365	2.041E-78	1.56477	up	PREDICTED: probable WRKY transcription factor 4 [Sesamum indicum]
c42562.graph_c0	9.62075456	9.87668979	8.82171635	8.69120757	9.41358531	9.67482668	5.38E-23	-1.11684	down	PREDICTED: NADPH-dependent diflavin oxidoreductase 1 isoform X1 [Sesamum indicum]
c42564.graph_c0	16.0732871	16.3873335	16.9573442	4.18243456	2.53926732	2.82441306	5.935E-63	-3.4636	down	PREDICTED: uncharacterized protein LOC103940260 [Pyrus x bretschneideri]
c42565.graph_c0	1.23026906	0.76552764	0.64092343	6.37989867	6.72052538	6.21163917	8.916E-12	1.78927	up	Mitochondrial transcription termination factor family protein [Theobroma cacao]
c42569.graph_c0	4.37158891	4.37596639	3.32174964	24.920657	22.6506909	22.5444042	6.969E-25	1.45118	up	PREDICTED: probable leucine-rich repeat receptor-like protein kinase At1g35710 [Sesamum indicum]
c42571.graph_c0	14.1956888	12.949547	13.2294317	72.6161641	76.1934666	71.3627269	2.162E-58	1.35701	up	hypothetical protein MIMGU_mgv1a020880mg [Erythranthe guttata]
c42575.graph_c0	2.52917444	1.7322206	2.00992364	1.7656293	1.87809594	1.85519828	1.777E-06	-1.27794	down	PREDICTED: nucleobase-ascorbate transporter 2 [Sesamum indicum]
c42588.graph_c0	2.71037449	1.92177104	2.08008829	17.9751966	18.601412	18.7754385	9.134E-32	1.95605	up	PREDICTED: alpha-galactosidase-like [Sesamum indicum]
c42589.graph_c0	35.5341043	35.5248034	32.0935534	14.8927115	13.490858	14.1865833	1.12E-167	-2.36599	down	hypothetical protein M569_03173, partial [Genlisea aurea]
c42592.graph_c1	233.05321	226.491291	245.03122	156.211574	149.91806	151.78552	8.314E-69	-1.71299	down	hypothetical protein MIMGU_mgv1a008823mg [Erythranthe guttata]
c42595.graph_c0	1.59394858	1.50690331	1.650009	0.28961816	0.3777194	0.23632605	2.237E-18	-3.48734	down	-
c42595.graph_c1	61.3563007	62.5279665	62.7683648	48.9140973	44.7471093	46.5701397	9.327E-71	-1.50329	down	PREDICTED: ABSCISIC ACID-INSENSITIVE 5-like protein 6 isoform X2 [Sesamum indicum]
c42597.graph_c0	5.93219562	5.26589403	6.9998334	172.405191	146.019247	152.983644	3.33E-103	3.60186	up	hypothetical protein MIMGU_mgv1a007920mg [Erythranthe guttata]
c42598.graph_c1	29.7761856	29.6310156	27.2562469	24.6057025	25.4478064	25.1062049	1.096E-49	-1.29488	down	PREDICTED: mechanosensitive ion channel protein 1, mitochondrial-like [Sesamum indicum]
c42599.graph_c0	27.4138918	25.7845069	22.9092366	15.0121143	16.4962498	14.9492096	5.179E-70	-1.80082	down	-
c42602.graph_c0	0.1963911	0.27522286	0.35331847	2.82617121	2.21668702	2.63225239	3.082E-07	2.12093	up	PREDICTED: uncharacterized protein LOC105171438 isoform X1 [Sesamum indicum]
c42604.graph_c0	3.37282137	3.14600337	3.4279653	2.45134713	3.16909562	2.9004049	3.331E-18	-1.31428	down	PREDICTED: uncharacterized protein LOC105176546 [Sesamum indicum]
c42605.graph_c0	3.02388081	2.07104284	2.40306459	11.046531	13.3160489	12.1758157	2.102E-10	1.19632	up	-
c42613.graph_c0	157.675664	174.582388	159.039181	91.984743	97.3004327	87.4831729	2.092E-89	-1.9187	down	hypothetical protein MIMGU_mgv1a008930mg [Erythranthe guttata]
c42615.graph_c0	1.66856052	1.48114854	1.94188648	8.16856528	8.90425304	9.27939729	5.064E-11	1.27903	up	hypothetical protein MIMGU_mgv1a007358mg [Erythranthe guttata]

#ID	0 h-1 FPKM	0 h-2 FPKM	0 h-3 FPKM	28 h-1 FPKM	28 h-2 FPKM	28 h-3 FPKM	FDR	log2FC	regulated	nr_annotation
c42619.graph_c0	1.46118793	1.15651482	1.00434279	16.2014146	16.2785085	16.6461942	1.209E-46	2.67551	up	PREDICTED: uncharacterized protein LOC103434215 [Malus domestica]
c42624.graph_c0	0.1111824	0	0.22859806	5.34940542	5.67635798	4.91894724	1.703E-41	4.44451	up	PREDICTED: uncharacterized protein LOC105179248 isoform X1 [Sesamum indicum]
c42629.graph_c0	0.18594783	0.17450065	0.13440956	0.86155455	1.11346898	1.40604296	4.254E-05	1.68741	up	PREDICTED: DNA polymerase alpha catalytic subunit [Sesamum indicum]
c42630.graph_c0	1.25021141	1.0092446	1.14014728	13.2486514	13.6296004	12.7959588	1.081E-29	2.45463	up	PREDICTED: uncharacterized protein LOC101231346, partial [Cucumis sativus]
c42633.graph_c0	35.0491982	33.1463051	32.5143071	3.88168461	3.85527699	3.84050032	0	-4.21055	down	PREDICTED: probable leucine-rich repeat receptor-like serine/threonine-protein kinase At5g15730 [Sesamum indicum]
c42636.graph_c0	21.2249058	19.1262543	22.9205454	727.628069	694.626637	672.390152	0	3.95686	up	hypothetical protein MIMGU_mgv1a009141mg [Erythranthe guttata]
c42641.graph_c0	4.55662471	4.91753025	4.39158237	21.1640154	19.7865844	19.0853008	7.246E-14	1.02419	up	PREDICTED: uncharacterized GPI-anchored protein At1g61900-like [Sesamum indicum]
c42642.graph_c0	585.482624	572.994012	617.057386	242.555045	249.555431	266.125963	1.95E-103	-2.3185	down	PREDICTED: leucine-rich repeat receptor-like protein kinase PXL1 [Sesamum indicum]
c42644.graph_c0	0.61175993	0.30618626	0.78613572	10.0040307	14.8537763	10.7028719	2.519E-09	3.28591	up	-
c42644.graph_c1	55.9377296	58.82255	50.1199421	19.0892264	19.2811671	19.877485	9.36E-121	-2.58996	down	PREDICTED: scarecrow-like protein 14 [Sesamum indicum]
c42649.graph_c0	2.29513172	1.88829859	2.06049441	1.48528429	1.52675941	1.56625088	1.076E-08	-1.53709	down	hypothetical protein JCGZ_16864 [Jatropha curcas]
c42653.graph_c0	0.13414578	0.1342801	0	2.22291738	2.25357022	1.67068119	1.809E-12	3.44565	up	PREDICTED: protein trichome birefringence-like 14 [Sesamum indicum]
c42659.graph_c0	0.27310139	0.18224991	0.29245509	5.29302718	4.77910727	5.45282203	1.475E-16	3.28359	up	hypothetical protein MIMGU_mgv1a021608mg, partial [Erythranthe guttata]
c42665.graph_c0	2.34147002	2.27487892	2.25666125	24.6756202	23.2288084	21.1357335	3.431E-51	2.23775	up	PREDICTED: uncharacterized protein LOC105168054 [Sesamum indicum]
c42669.graph_c0	19.0756285	15.4865284	16.1506647	337.953774	300.953968	337.383408	4.37E-133	3.1784	up	PREDICTED: zeaxanthin epoxidase, chloroplastic-like [Sesamum indicum]
c42676.graph_c0	18.9072705	21.1725374	17.2809236	10.1389124	10.0776289	10.3291723	2.873E-43	-1.99804	down	PREDICTED: uncharacterized protein At1g04910 [Sesamum indicum]
c42680.graph_c1	99.6258533	92.3567258	86.7151985	36.5604352	34.6209205	36.3817275	1.507E-83	-2.46228	down	PREDICTED: alpha,alpha-trehalose-phosphate synthase [UDP-forming] 6 [Solanum lycopersicum]
c42680.graph_c2	39.1565275	40.7807795	40.8986589	19.4602573	20.5329489	19.363788	3.57E-140	-2.11677	down	PREDICTED: nuclear pore complex protein GP210 [Sesamum indicum]
c42680.graph_c3	154.441695	154.307919	167.731442	64.2071504	64.8178638	64.7302118	1.456E-78	-2.38985	down	PREDICTED: alpha,alpha-trehalose-phosphate synthase [UDP-forming] 6 [Sesamum indicum]
c42683.graph_c1	52.0569167	53.0425934	51.3153725	47.4781937	50.2402683	46.4600132	4.327E-46	-1.20812	down	hypothetical protein PRUPE_ppa012854mg [Prunus persica]
c42686.graph_c0	46.7055174	44.6532037	44.0951797	33.9453008	32.1923202	32.7489901	1.068E-58	-1.54386	down	unnamed protein product [Coffea canephora]
c42690.graph_c0	30.0734074	28.976493	29.7361089	15.6768734	16.2157401	15.2873826	2.77E-107	-2.0029	down	PREDICTED: probable polyamine oxidase 2 isoform X1 [Sesamum indicum]
c42691.graph_c0	0.69246996	0.83179604	0.62289592	4.84410056	4.5441752	4.15808398	4.949E-13	1.56822	up	PREDICTED: pentatricopeptide repeat-containing protein At5g46100 [Sesamum indicum]

#ID	0 h-1 FPKM	0 h-2 FPKM	0 h-3 FPKM	28 h-1 FPKM	28 h-2 FPKM	28 h-3 FPKM	FDR	log2FC	regulated	nr_annotation
c42692.graph_c0	64.9675728	62.6078029	64.7023343	63.887801	63.236817	60.7586746	1.484E-41	-1.12416	down	PREDICTED: multiple C2 and transmembrane domain-containing protein 1 [Sesamum indicum]
c42694.graph_c0	0.85502949	0.76579245	0.4915446	75.7164015	72.2038721	66.1208527	3.7E-274	5.5784	up	hypothetical protein MIMGU_mgv1a002094mg [Erythranthe guttata]
c42695.graph_c0	0	0	0	1.56013175	1.96469981	1.139049	1.077E-10	Inf	up	PREDICTED: dehydrodolichyl diphosphate synthase 2-like [Glycine max]
c42698.graph_c0	3.78177587	4.48087018	2.90922715	0.54223547	0.60776415	0.51874587	1.751E-22	-3.83048	down	hypothetical protein MIMGU_mgv1a010940mg [Erythranthe guttata]
c42706.graph_c0	1.52113436	1.38423413	2.19165337	15.6117298	14.7311525	16.9762797	1.818E-26	2.11924	up	PREDICTED: uncharacterized protein LOC105176645 [Sesamum indicum]
c42707.graph_c0	0.03246437	0.06499377	0	1.95837838	1.34215205	1.46324793	1.673E-12	4.53685	up	PREDICTED: uncharacterized protein LOC105170754 [Sesamum indicum]
c42707.graph_c1	40.6117638	39.1322234	42.732695	10.4858376	9.17562045	10.0160632	5.05E-140	-3.13621	down	PREDICTED: uncharacterized protein LOC105175855 [Sesamum indicum]
c42708.graph_c0	11.8608327	5.03462997	4.05157507	0	0	0	4.67E-09	-Inf	down	PREDICTED: ribulose bisphosphate carboxylase small chain, chloroplastic-like [Phoenix dactylifera]
c42709.graph_c0	1.2109541	0.89594929	0.60891807	4.68756614	4.49156125	3.74695414	1.323E-05	1.16728	up	PREDICTED: two-component response regulator-like APRR5 [Sesamum indicum]
c42710.graph_c0	53.80395	55.4573044	45.4090252	33.5452552	35.066952	33.3070209	1.132E-39	-1.69022	down	PREDICTED: cysteine-rich receptor-like protein kinase 10 [Sesamum indicum]
c42713.graph_c1	100.954797	95.7371566	93.8120854	89.2283204	89.5273659	88.8760655	5.264E-51	-1.20806	down	PREDICTED: probable CCR4-associated factor 1 homolog 7 [Sesamum indicum]
c42715.graph_c0	3.82392509	5.67563549	3.89722717	20.2211247	16.8733035	19.9410878	0.0002036	1.00094	up	-
c42720.graph_c0	74.3202553	73.2753577	69.5371078	8.85262635	7.62619557	7.67366997	0	-4.25824	down	PREDICTED: mitochondrial carrier protein MTM1-like [Sesamum indicum]
c42722.graph_c0	19.5932882	20.6451663	16.6970897	17.2007701	16.7487754	17.1853085	8.097E-25	-1.24328	down	PREDICTED: DNA-directed RNA polymerase II subunit 1 [Sesamum indicum]
c42722.graph_c1	28.2533191	29.3702851	24.8801804	27.2662111	27.8736316	28.0256668	4.963E-34	-1.0772	down	PREDICTED: DNA-directed RNA polymerase II subunit 1 [Sesamum indicum]
c42724.graph_c0	0.45369634	0.51470406	0.4664142	1.84656574	2.18333727	2.24223596	0.0042189	1.03794	up	PREDICTED: U-box domain-containing protein 11-like [Sesamum indicum]
c42727.graph_c0	0.19755014	0.13183197	0.08461992	2.48869058	2.59275491	3.20233842	3.545E-09	3.24273	up	-
c42728.graph_c0	9.35726117	8.38223989	7.95247666	6.97407236	7.47679367	7.12821033	1.705E-50	-1.3405	down	PREDICTED: G-type lectin S-receptor-like serine/threonine-protein kinase SD2-2 [Sesamum indicum]
c42730.graph_c0	2.06079656	2.29206681	2.64820516	0.24962918	0.11269587	0.37344151	3.943E-25	-4.33961	down	PREDICTED: uncharacterized protein LOC104646173 [Solanum lycopersicum]
c42731.graph_c0	33.0929906	33.2099919	29.4450814	22.1337403	22.4037472	24.9424968	9.069E-62	-1.55111	down	PREDICTED: lipase-like [Sesamum indicum]

#ID	0 h-1 FPKM	0 h-2 FPKM	0 h-3 FPKM	28 h-1 FPKM	28 h-2 FPKM	28 h-3 FPKM	FDR	log2FC	regulated	nr_annotation
c42733.graph_c0	20.9317812	20.3779777	24.0809031	22.9144916	23.909548	22.8153213	2.881E-10	-1.0019	down	PREDICTED: uncharacterized protein LOC105161097 [Sesamum indicum]
c42736.graph_c0	0.30251359	0.20187767	0.21596774	7.78078267	8.22745158	7.11652332	2.024E-67	3.91702	up	PREDICTED: probable polyribonucleotide nucleotidyltransferase 1, chloroplastic-like [Solanum tuberosum]
c42737.graph_c0	0	0	0	2.24572525	2.64724804	2.23971424	7.128E-14	Inf	up	hypothetical protein MIMGU_mgv1a011219mg [Erythranthe guttata]
c42739.graph_c0	2.71986182	3.27718607	2.86945868	16.7416174	16.3721142	16.2299327	2.048E-34	1.38568	up	PREDICTED: nardilysin isoform X1 [Sesamum indicum]
c42741.graph_c0	20.6137178	20.8071593	20.3172726	17.3362348	16.5525919	16.9164533	1.364E-63	-1.37148	down	PREDICTED: zinc finger CCCH domain-containing protein 41 [Sesamum indicum]
c42744.graph_c0	21.3280843	21.6784565	20.508644	14.9835397	15.1344445	13.8941809	4.627E-78	-1.61957	down	PREDICTED: MAR-binding filament-like protein 1-1 [Sesamum indicum]
c42747.graph_c0	239.098083	246.669119	227.588765	46.594433	41.5781835	50.3453422	0	-3.45361	down	hypothetical protein MIMGU_mgv1a015419mg [Erythranthe guttata]
c42749.graph_c0	0.05573454	0	0	2.01187472	1.52393812	1.4984401	5.523E-20	5.43766	up	-
c42749.graph_c1	0.11477048	0	0	9.34242694	10.318223	10.5501637	7.516E-22	6.98154	up	-
c42751.graph_c0	3.09280956	2.09722701	3.58976505	13.4145061	13.7488036	14.2595498	1.592E-05	1.14456	up	PREDICTED: uncharacterized protein LOC105166611 isoform X2 [Sesamum indicum]
c42754.graph_c0	0.20685667	0.37271485	0.0531638	3.36766605	2.93209074	3.16509331	3.297E-10	2.82463	up	-
c42755.graph_c0	4.00330412	3.96950799	2.81486273	3.40365771	3.02361631	3.18052161	2.9E-08	-1.25319	down	PREDICTED: uncharacterized protein LOC105156766 [Sesamum indicum]
c42757.graph_c1	2.69903379	2.66368383	2.00285882	11.438308	11.4004056	11.0018845	8.232E-09	1.11292	up	hypothetical protein MIMGU_mgv1a010783mg [Erythranthe guttata]
c42758.graph_c0	0.24055833	0.36119882	0.92738093	3.23447352	3.78866118	3.49529446	0.0001688	1.67417	up	PREDICTED: uncharacterized protein LOC100262706 [Vitis vinifera]
c42760.graph_c0	16.6812682	16.9842973	15.1205468	10.1110827	8.72835039	9.49971123	9.16E-105	-1.87271	down	PREDICTED: protein PHOTOPERIOD-INDEPENDENT EARLY FLOWERING 1 isoform X1 [Sesamum indicum]
c42764.graph_c0	2.26045893	2.0437493	1.40553833	16.1109068	14.9772954	15.4815786	2.444E-14	1.94308	up	PREDICTED: LOW QUALITY PROTEIN: protein IQ-DOMAIN 14 [Sesamum indicum]
c42766.graph_c0	57.2857277	58.8910446	57.496092	55.7963099	51.4387314	52.214034	1.911E-49	-1.21385	down	PREDICTED: LOW QUALITY PROTEIN: neurofilament heavy polypeptide [Sesamum indicum]
c42771.graph_c0	1.0375451	0.99863851	1.07688528	18.3299884	22.3638019	19.8323849	2.61E-54	3.18968	up	PREDICTED: uncharacterized protein LOC105176977 [Sesamum indicum]
c42773.graph_c0	0.08966356	0.26926003	0.23044244	1.62917393	1.97112835	1.86114967	6.265E-06	2.11461	up	PREDICTED: probable beta-1,3-galactosyltransferase 20 [Sesamum indicum]
c42773.graph_c1	0	0	0	1.21566706	1.14868609	1.1534586	2.684E-14	Inf	up	PREDICTED: probable beta-1,3-galactosyltransferase 20 [Sesamum indicum]
c42774.graph_c0	12.4726889	11.6455319	11.5757049	11.3578541	10.5303011	9.7113054	6.347E-40	-1.26602	down	PREDICTED: uncharacterized protein LOC105163756 [Sesamum indicum]
c42776.graph_c0	1.71231077	0.99984815	0.97794984	0	0	0	1.382E-16	-Inf	down	unnamed protein product [Coffea canephora]

#ID	0 h-1 FPKM	0 h-2 FPKM	0 h-3 FPKM	28 h-1 FPKM	28 h-2 FPKM	28 h-3 FPKM	FDR	log2FC	regulated	nr_annotation
c42779.graph_c0	27.2465449	29.8257653	27.8465081	30.4566793	27.0290016	30.3331594	3.838E-19	-1.04175	down	PREDICTED: serine-threonine kinase receptor-associated protein [Solanum lycopersicum]
c42783.graph_c0	7.20083563	4.83577782	5.1537695	0	0	0	3.33E-39	-Inf	down	PREDICTED: glycine-rich RNA-binding protein-like isoform X1 [Eucalyptus grandis]
c42786.graph_c0	6.00304211	4.81427073	4.73675679	33.832203	33.887699	32.5410487	1.092E-41	1.60025	up	PREDICTED: protein TIC 40, chloroplastic-like [Sesamum indicum]
c42792.graph_c0	2.89511177	1.97912932	2.26849588	0.25660373	0.23168911	0.33501847	2.53E-22	-4.20345	down	PREDICTED: external alternative NAD(P)H-ubiquinone oxidoreductase B1, mitochondrial-like isoform X2 [Sesamum indicum]
c42794.graph_c0	5.53287244	5.83162286	5.31155846	59.5382995	55.7224169	56.4965263	4.92E-109	2.27463	up	PREDICTED: LOW QUALITY PROTEIN: cytochrome P450 78A7-like [Sesamum indicum]
c42797.graph_c0	68.4494852	70.999687	61.7742379	27.9946258	37.3394153	28.8321544	2.869E-97	-2.18548	down	PREDICTED: LOW QUALITY PROTEIN: probable WRKY transcription factor 33 [Sesamum indicum]
c42799.graph_c0	38.4660335	44.0537368	36.9758597	18.7201538	18.3873938	18.0040579	3.861E-53	-2.20629	down	PREDICTED: uncharacterized protein LOC105164926 [Sesamum indicum]
c42801.graph_c0	21.5200614	19.3198301	23.5618156	5.36551673	6.23776492	6.72471367	1.222E-39	-2.90458	down	vacuolar H+-ATPase A subunit [Citrus unshiu]
c42803.graph_c0	30.9510395	31.7744116	29.6011088	192.11874	202.883994	188.784286	3.739E-87	1.57048	up	PREDICTED: 2,3-bisphosphoglycerate-independent phosphoglycerate mutase [Sesamum indicum]
c42810.graph_c0	0.80684377	0.56834749	0.7680194	8.77442215	9.60894915	8.84348652	1.71E-29	2.57611	up	hypothetical protein MIMGU_mgv1a004563mg [Erythranthe guttata]
c42811.graph_c1	2.18045566	1.69154526	2.17152818	20.2450868	25.469557	21.8216873	4.432E-29	2.39056	up	hypothetical protein MIMGU_mgv1a016107mg [Erythranthe guttata]
c42812.graph_c0	0.68415176	0.59144999	0.31969531	2.37317923	2.28560889	2.26846542	0.0059796	1.03651	up	PREDICTED: protein TPX2-like isoform X2 [Sesamum indicum]
c42817.graph_c0	1.84452032	1.93777165	2.06519588	10.2335879	10.5942072	10.3975398	2.386E-21	1.32487	up	PREDICTED: probable glutamate carboxypeptidase 2 isoform X2 [Sesamum indicum]
c42818.graph_c0	21.1990576	20.7275065	19.808499	98.8619841	94.3713506	96.4908354	4.324E-43	1.14084	up	V-type proton ATPase subunit B2 -like protein [Gossypium arboreum]
c42820.graph_c0	4.37690861	4.43107883	5.04926781	50.3374818	51.7493838	49.8504173	4.5E-127	2.36216	up	PREDICTED: glucosidase 2 subunit beta [Sesamum indicum]
c42822.graph_c0	16.7051912	17.6386214	17.1653496	8.65263937	8.47565393	9.00820253	1.315E-81	-2.06925	down	PREDICTED: uncharacterized protein LOC105177905 [Sesamum indicum]
c42826.graph_c0	2.21721753	1.65104515	2.60598687	10.9264911	11.0899247	10.6157267	1.457E-14	1.23959	up	PREDICTED: B3 domain-containing protein Os01g0723500-like [Sesamum indicum]
c42828.graph_c0	7.51600979	6.65001679	8.1029023	5.23754197	4.26719201	4.99165489	5.62E-20	-1.71087	down	hypothetical protein ARALYDRAFT_319569 [Arabidopsis lyrata subsp. lyrata]
c42831.graph_c0	35.1806492	32.2477965	31.8210362	19.9194302	20.2466972	22.0523358	1.07E-51	-1.76273	down	PREDICTED: uncharacterized protein LOC105163175 [Sesamum indicum]
c42834.graph_c0	15.5343019	15.1394041	12.7271646	8.11521897	7.61706136	7.5759625	2.673E-52	-1.98503	down	PREDICTED: calmodulin-binding transcription activator 1 [Sesamum indicum]
c42838.graph_c0	0.80017006	0.84004308	0.62698183	10.7801178	12.2052161	9.95392077	1.4E-47	2.77271	up	PREDICTED: WD repeat-containing protein 44 [Sesamum indicum]
c42839.graph_c0	11.3705617	11.4394322	12.8405163	10.6848436	9.7604676	10.5578554	6.052E-17	-1.29346	down	PREDICTED: probable complex I intermediate-associated protein 30-like [Citrus sinensis]

#ID	0 h-1 FPKM	0 h-2 FPKM	0 h-3 FPKM	28 h-1 FPKM	28 h-2 FPKM	28 h-3 FPKM	FDR	log2FC	regulated	nr_annotation
c42843.graph_c0	0.03198604	0.16009036	0.28772332	0.76715996	0.9445548	0.83466169	0.0079531	1.29816	up	Receptor-like protein kinase [Medicago truncatula]
c42847.graph_c0	71.487786	70.375624	70.0994816	66.9007609	65.3616884	67.4179918	4.674E-47	-1.17634	down	PREDICTED: uncharacterized protein LOC105164616 [Sesamum indicum]
c42849.graph_c0	0.30173407	0.20135748	0.4523636	2.19298587	1.38604244	2.29647182	0.0009614	1.52427	up	PREDICTED: monothiol glutaredoxin-S10-like [Sesamum indicum]
c42851.graph_c0	10.3016933	9.7616489	8.99892214	10.1371794	10.6530904	9.71342382	4.1E-18	-1.01944	down	hypothetical protein MIMGU_mgv1a008129mg [Erythranthe guttata]
c42854.graph_c0	9.42569303	10.6269375	13.6423747	1.29799693	0.91153186	0.70610327	6.261E-22	-4.62752	down	-
c42859.graph_c0	44.975602	45.2581446	45.2945422	34.0675726	31.9016396	34.1622674	1.066E-75	-1.5271	down	PREDICTED: soluble starch synthase 1, chloroplastic/amyloplastic-like, partial [Sesamum indicum]
c42863.graph_c1	31.0968348	31.1681906	31.5451752	26.9518389	26.7869374	27.2582856	9.322E-45	-1.30254	down	PREDICTED: uncharacterized protein LOC105166783 [Sesamum indicum]
c42865.graph_c0	11.4783505	11.0424521	10.9385955	9.67127666	9.8521179	8.84350406	4.36E-39	-1.3287	down	PREDICTED: putative amidase C869.01 isoform X1 [Sesamum indicum]
c42869.graph_c0	3.25445027	3.56552415	2.8978313	15.476987	16.8666558	16.2612896	3.205E-20	1.23356	up	PREDICTED: small RNA degrading nuclease 5 isoform X1 [Sesamum indicum]
c42872.graph_c0	6.77736797	5.08811586	6.41729507	5.99515866	6.3201205	5.6324114	3.784E-08	-1.11774	down	PREDICTED: uncharacterized protein LOC105163723 [Sesamum indicum]
c42875.graph_c0	1.02767578	0.85725404	0.88040271	0.68466643	0.36529385	0.73644474	1.252E-05	-1.71716	down	--
c42877.graph_c1	2.38608402	3.00172998	2.48611819	13.5923166	11.256925	11.9326222	1.772E-11	1.13345	up	PREDICTED: protein DEK-like [Sesamum indicum]
c42878.graph_c0	13.7380393	14.3018677	12.2871314	8.69336964	8.90708973	8.73407317	6.746E-72	-1.70383	down	PREDICTED: long chain acyl-CoA synthetase 4 [Sesamum indicum]
c42879.graph_c0	5.01540422	5.88882447	5.2953456	26.7180881	27.8421446	25.0655718	1.415E-27	1.20607	up	PREDICTED: uncharacterized protein LOC105179636 isoform X3 [Sesamum indicum]
c42880.graph_c0	6.87570357	6.64928046	6.28971449	32.9477839	31.7753462	28.6820717	9.177E-16	1.14694	up	PREDICTED: uncharacterized protein LOC105164000 [Sesamum indicum]
c42882.graph_c0	0.3361044	0.37849608	0.10797687	4.03059924	4.41121601	4.06133772	2.553E-12	2.84682	up	PREDICTED: transcription factor bHLH110 [Sesamum indicum]
c42883.graph_c0	0.49300285	0.41757398	0.29239762	5.18173744	4.85282713	5.93755019	3.955E-15	2.64737	up	PREDICTED: uncharacterized protein LOC101301730 isoform 2 [Fragaria vesca subsp. vesca]
c42883.graph_c1	1.48822958	1.42891493	1.24893645	18.6968626	19.6917778	19.6695207	1.858E-62	2.71244	up	PREDICTED: uncharacterized protein At1g01500-like [Sesamum indicum]
c42886.graph_c0	3.60303267	3.66928692	3.03307807	19.6172326	19.2086109	19.0244835	5.747E-54	1.40058	up	PREDICTED: LOW QUALITY PROTEIN: translational activator GCN1 [Sesamum indicum]
c42887.graph_c0	10.0495079	10.9984642	9.03981086	51.1761637	54.5166739	56.0234612	8.457E-28	1.33752	up	-
c42891.graph_c0	0.08844409	0	0	2.48551856	2.37961549	2.44778275	1.3E-18	5.31072	up	PREDICTED: uncharacterized protein LOC104093672 [Nicotiana tomentosiformis]
c42892.graph_c0	11.0482363	11.210108	10.7771332	8.13929163	10.7104511	8.87531596	1.832E-19	-1.34354	down	PREDICTED: UDP-galactose/UDP-glucose transporter 5B-like [Sesamum indicum]
c42898.graph_c0	33.0156385	30.9476645	31.7104555	31.3756583	32.7219909	33.7438736	2.505E-26	-1.05766	down	unknown [Medicago truncatula]

#ID	0 h-1 FPKM	0 h-2 FPKM	0 h-3 FPKM	28 h-1 FPKM	28 h-2 FPKM	28 h-3 FPKM	FDR	log2FC	regulated	nr_annotation
c42901.graph_c0	28.4815192	25.9411133	25.7039791	23.0408631	23.74268	23.7849546	6.109E-49	-1.27264	down	PREDICTED: MACPF domain-containing protein At4g24290-like [Sesamum indicum]
c42911.graph_c1	49.251218	48.519865	47.5584319	6.75030329	7.45965027	7.66661888	0	-3.82171	down	hypothetical protein MIMGU_mgv1a023461mg [Erythranthe guttata]
c42913.graph_c0	1.40381267	0.84313102	0.96210926	4.78399342	4.76987166	4.93975253	3.456E-05	1.0888	up	PREDICTED: uncharacterized protein LOC105176347 isoform X1 [Sesamum indicum]
c42916.graph_c0	40.1535778	39.1804968	38.6630692	29.673866	31.8486451	30.116956	3.083E-52	-1.45499	down	hypothetical protein MIMGU_mgv1a013052mg [Erythranthe guttata]
c42919.graph_c0	18.8064427	18.8478197	16.6709048	12.4079736	11.7796566	12.6092559	5.496E-71	-1.65075	down	PREDICTED: pentatricopeptide repeat-containing protein At4g02820, mitochondrial [Sesamum indicum]
c42920.graph_c0	1.18665484	0.67006534	0.54739944	11.7647557	14.1566395	10.2134749	3.932E-19	2.82679	up	PREDICTED: uncharacterized protein LOC105169238 [Sesamum indicum]
c42921.graph_c0	0.31391227	0.25138128	0.32271175	1.9620868	2.43077313	2.08508421	0.0001344	1.77488	up	PREDICTED: protein notum homolog isoform X1 [Sesamum indicum]
c42925.graph_c0	11.7413575	12.8380176	13.7948428	4.20113279	4.92781091	4.91257548	1.015E-41	-2.54267	down	PREDICTED: formin-like protein 5 [Sesamum indicum]
c42928.graph_c0	15.591991	15.8634664	15.4583538	2.39183079	2.77812486	2.54859792	0	-3.6942	down	PREDICTED: probably inactive leucine-rich repeat receptor-like protein kinase At5g06940 [Sesamum indicum]
c42929.graph_c0	2.4391503	1.05432414	2.49327647	0.20145004	0.4365372	0.13150527	4.991E-06	-4.0571	down	PREDICTED: mitochondrial phosphate carrier protein 3, mitochondrial-like [Sesamum indicum]
c42932.graph_c0	18.6742762	21.0004891	22.1219796	10.0985709	8.97927939	10.2095069	1.631E-31	-2.16928	down	PREDICTED: sulfate transporter 4.1, chloroplastic [Sesamum indicum]
c42937.graph_c0	23.8166415	23.4105015	23.4599954	25.2015393	23.0051942	23.7062073	2.43E-34	-1.06559	down	hypothetical protein MIMGU_mgv1a002036mg [Erythranthe guttata]
c42939.graph_c0	32.9914529	34.189712	30.9396865	25.5094198	23.6707976	25.2284994	1.114E-75	-1.4885	down	PREDICTED: protein transport protein SEC16B homolog [Sesamum indicum]
c42941.graph_c0	6.43423001	7.17105851	7.88466215	30.4204533	32.1461878	30.9597088	1.674E-21	1.02866	up	PREDICTED: wall-associated receptor kinase-like 20 [Sesamum indicum]
c42942.graph_c1	14.6034759	14.7327509	13.0258428	6.70121281	6.57670312	7.3700949	3.731E-45	-2.12527	down	PREDICTED: uncharacterized protein LOC105166066 [Sesamum indicum]
c42944.graph_c0	32.155664	32.6903622	32.2542682	31.3161333	30.3252795	30.7140697	3.967E-42	-1.16281	down	PREDICTED: uncharacterized protein LOC105162695 [Sesamum indicum]
c42945.graph_c0	111.577083	104.961575	115.648847	68.3819418	75.7673936	73.7716664	8.337E-57	-1.69939	down	PREDICTED: cell division cycle protein 48 homolog [Solanum lycopersicum]
c42945.graph_c1	100.088888	104.116071	109.249175	63.8526738	68.2554348	69.032228	1.837E-55	-1.73151	down	PREDICTED: cell division cycle protein 48 homolog [Sesamum indicum]
c42947.graph_c0	2.72746655	2.1234871	2.53131848	13.9495676	13.0260384	13.239916	4.145E-11	1.35538	up	PREDICTED: 39S ribosomal protein L45, mitochondrial [Sesamum indicum]
c42950.graph_c0	0.05386135	0.16174586	0	7.86837014	7.86432395	6.73995493	1.174E-53	5.62689	up	hypothetical protein CISIN_1g0086311mg, partial [Citrus sinensis]
c42952.graph_c0	14.3514206	14.0426346	13.1998984	68.1717245	65.5778661	66.819225	1.996E-41	1.18018	up	hypothetical protein MIMGU_mgv1a007743mg [Erythranthe guttata]
c42953.graph_c0	0.78871099	0.48056568	0.44066298	10.8415353	11.1615848	10.6769362	2.664E-38	3.17227	up	hypothetical protein CICLE_v10018965mg [Citrus clementina]
c42956.graph_c1	3.89591947	4.73637522	3.77454944	21.3660066	20.4044719	19.7449938	4.428E-38	1.22003	up	PREDICTED: protein NAP1 isoform X2 [Sesamum indicum]

#ID	0 h-1 FPKM	0 h-2 FPKM	0 h-3 FPKM	28 h-1 FPKM	28 h-2 FPKM	28 h-3 FPKM	FDR	log2FC	regulated	nr_annotation
c42957.graph_c0	0.1154822	0.23119568	0.2967984	1.51077235	1.46512918	1.34692401	0.000209	1.64733	up	PREDICTED: putative pectate lyase 2 [Sesamum indicum]
c42958.graph_c0	1.40473928	1.32802669	1.50428799	8.13929138	10.0120754	9.76569065	3.689E-13	1.62853	up	hypothetical protein MIMGU_mgv1a008941mg [Erythranthe guttata]
c42960.graph_c0	0.39332024	0.31497127	0.40434566	8.46154127	7.38187104	7.13781035	1.271E-14	3.27647	up	PREDICTED: SEC14 cytosolic factor [Sesamum indicum]
c42963.graph_c0	20.3407552	20.31743	19.8283732	20.2084063	20.1940954	20.1787641	6.411E-35	-1.08796	down	PREDICTED: ubiquitin carboxyl-terminal hydrolase 25 isoform X1 [Sesamum indicum]
c42964.graph_c0	0.77642246	0.24543156	0.57763524	3.14819647	3.4593008	3.36865531	1.677E-05	1.55239	up	PREDICTED: uncharacterized protein LOC105168903 [Sesamum indicum]
c42966.graph_c0	2.16349917	4.84089956	3.10726234	18.0365678	13.5293508	12.6798577	0.0070903	1.03683	up	NAD(P)H-quinone oxidoreductase subunit H [Medicago truncatula]
c42978.graph_c0	24.9943599	26.39646	23.8953906	24.3558197	20.25649	22.6609312	7.274E-53	-1.25191	down	PREDICTED: uncharacterized protein LOC105176008 [Sesamum indicum]
c42979.graph_c0	103.278708	97.5754529	92.6361014	63.1557789	63.9484689	62.408556	3.07E-100	-1.72038	down	PREDICTED: heterogeneous nuclear ribonucleoprotein A2 homolog 1 [Sesamum indicum]
c42981.graph_c0	1.03973855	0.59473125	0.71577064	12.3876885	12.2814821	11.9581289	1.03E-35	2.87521	up	PREDICTED: petal death protein [Sesamum indicum]
c42985.graph_c1	2.03857477	1.90636504	1.65451534	35.8514114	35.3276343	34.6472058	3.62E-138	3.15215	up	PREDICTED: protein NSP-INTERACTING KINASE 3 [Sesamum indicum]
c42986.graph_c0	72.7450531	62.785977	70.1657158	40.6262867	41.1606405	43.8593737	1.172E-47	-1.8013	down	thioredoxin peroxidase, partial [Fargesia spathacea]
c42986.graph_c1	0.1851051	0.92645228	0.23786738	8.15271923	12.1713917	8.78224728	1.356E-12	3.34261	up	PREDICTED: cytochrome P450 CYP82D47-like [Sesamum indicum]
c42996.graph_c0	0.12918691	0.25863253	0.16601029	5.30491484	5.84954	5.55461277	1.885E-15	3.82379	up	-
c42998.graph_c0	3.10135118	3.79433598	2.9386991	29.2115016	31.8024219	30.5618008	3.218E-65	2.12973	up	PREDICTED: exocyst complex component EXO70B1 [Sesamum indicum]
c42999.graph_c0	2.75567881	3.01589244	2.54963259	13.3787807	10.9224399	11.5924773	1.527E-08	1.01955	up	PREDICTED: uncharacterized protein LOC105170455 [Sesamum indicum]
c43005.graph_c0	0.66823858	0.55086518	0.50512531	3.37113469	3.52102739	3.18209075	3.127E-08	1.45888	up	PREDICTED: myosin heavy chain, striated muscle [Sesamum indicum]
c43008.graph_c0	6.22339052	5.33592121	6.20887676	36.3376742	36.6866874	35.4133047	8.867E-51	1.51838	up	PREDICTED: uncharacterized protein LOC105169979 [Sesamum indicum]
c43010.graph_c0	8.68574599	9.40816642	7.37160584	115.702346	110.699891	97.0069662	3.619E-70	2.57784	up	hypothetical protein MIMGU_mgv1a004602mg [Erythranthe guttata]
c43011.graph_c0	11.4300259	12.139122	9.7323164	6.75388369	6.06763312	5.31822495	5.625E-39	-1.96551	down	PREDICTED: uncharacterized protein LOC105158337 [Sesamum indicum]
c43012.graph_c0	0.67954997	0.71511405	0.49260178	7.71232917	7.43231788	7.80191166	2.082E-39	2.51769	up	PREDICTED: heat shock 70 kDa protein 16 [Sesamum indicum]
c43013.graph_c0	13.1658265	11.1298347	14.8037823	58.2299337	54.4727058	57.3943024	1.485E-19	1.02859	up	PREDICTED: uncharacterized protein LOC105176983 [Sesamum indicum]
c43015.graph_c0	3.93982718	3.38437199	3.91382196	18.0132947	16.3926704	16.3889692	1.669E-16	1.08526	up	PREDICTED: protein PHLOEM PROTEIN 2-LIKE A9 [Sesamum indicum]
c43016.graph_c0	2.16973319	1.97836968	1.98762277	11.2572341	11.4611879	12.2180206	2.893E-22	1.42013	up	PREDICTED: protein STRUBBELIG-RECEPTOR FAMILY 1 [Sesamum indicum]

#ID	0 h-1 FPKM	0 h-2 FPKM	0 h-3 FPKM	28 h-1 FPKM	28 h-2 FPKM	28 h-3 FPKM	FDR	log2FC	regulated	nr_annotation
c43017.graph_c0	1.24395948	0.96849287	0.8436724	11.3012822	10.974967	11.721874	2.682E-28	2.38987	up	hypothetical protein MIMGU_mgv1a007209mg [Erythranthe guttata]
c43022.graph_c1	16.1807069	13.1064646	14.0052983	69.2355723	72.3259279	75.9084622	1.684E-39	1.23977	up	PREDICTED: eukaryotic translation initiation factor 3 subunit M [Sesamum indicum]
c43024.graph_c0	2.52998112	2.85193076	2.31386438	14.8592955	14.2092788	14.7609472	1.969E-25	1.42083	up	PREDICTED: uncharacterized protein LOC105166682 isoform X3 [Sesamum indicum]
c43027.graph_c0	0	0.22369319	0.04102386	4.68686991	5.00693653	3.50258813	3.725E-21	4.54872	up	PREDICTED: phototropin-2 isoform X1 [Sesamum indicum]
c43030.graph_c0	6.81193458	6.97372742	6.56520422	77.076119	75.688203	80.9810987	1.025E-43	2.43208	up	Succinyl-CoA ligase [ADP-forming] subunit alpha-1 [Morus notabilis]
c43030.graph_c1	5.46072553	6.41901636	4.18459773	57.6025626	59.7355556	53.3020088	8.437E-82	2.32171	up	hypothetical protein M569_06331, partial [Genlisea aurea]
c43031.graph_c0	2.40877652	2.15051951	2.09146838	14.0527949	14.0127301	14.7873668	1.142E-11	1.59908	up	PREDICTED: serine protease SPPA, chloroplastic-like [Sesamum indicum]
c43032.graph_c0	0	0	0.03968561	0.98759907	0.79037875	1.04397155	1.808E-12	5.02274	up	Protein kinase G11A [Glycine soja]
c43034.graph_c0	5.44012392	5.27697784	6.06011423	5.23915822	4.86309862	4.83447055	2.211E-19	-1.2599	down	hypothetical protein MIMGU_mgv1a004587mg [Erythranthe guttata]
c43040.graph_c0	11.0554949	12.5801538	11.8177214	6.05332627	5.31918644	5.20407899	3.065E-67	-2.18811	down	PREDICTED: mitogen-activated protein kinase kinase kinase YODA-like isoform X5 [Sesamum indicum]
c43041.graph_c0	5.45498038	4.247011	4.15399452	22.7233083	21.8428338	22.0767029	1.099E-13	1.17839	up	PREDICTED: uncharacterized protein LOC105157371 [Sesamum indicum]
c43042.graph_c0	0.40868792	0.88113234	0.82816871	8.19122928	8.2726938	7.8026333	3.064E-45	2.42142	up	hypothetical protein MIMGU_mgv1a000774mg [Erythranthe guttata]
c43044.graph_c1	61.4178725	66.468645	55.7393634	7.23012382	7.5958709	8.57475123	3.46E-166	-4.06053	down	PREDICTED: uncharacterized protein LOC100243914 [Vitis vinifera]
c43045.graph_c0	19.9331374	18.370342	20.5518534	12.3019506	11.7368767	12.467158	7.967E-54	-1.78003	down	PREDICTED: putative MO25-like protein At5g47540 [Sesamum indicum]
c43047.graph_c0	16.9779486	17.8855145	16.767909	85.8734076	93.2179113	89.9020381	3.727E-56	1.29096	up	PREDICTED: putative serine/threonine-protein kinase {ECO:0000250|UniProtKB:Q9FE20, ECO:0000303|Ref.2} isoform X1 [Nicotiana tomentosiformis]
c43050.graph_c0	11.7228524	13.0100901	11.2436662	8.52010157	8.08307006	8.4385205	3.16E-33	-1.6123	down	PREDICTED: putative vesicle-associated membrane protein 726 [Pyrus x bretschneideri]
c43052.graph_c0	56.9464359	55.611655	55.3110811	56.6783019	54.6662134	52.523125	3.136E-37	-1.12528	down	PLAC8 family protein isoform 1 [Theobroma cacao]
c43053.graph_c0	19.8850525	21.5596558	20.7423533	10.9187617	9.47575916	11.1298171	1.484E-68	-2.07046	down	PREDICTED: F-box protein PP2-A15 [Sesamum indicum]
c43056.graph_c0	0.06354077	0	0	2.0088762	1.68873535	1.78996005	5.22E-15	5.37346	up	PREDICTED: flap endonuclease GEN-like 2 isoform X2 [Sesamum indicum]
c43058.graph_c0	0.48554697	0.22872149	0.33032492	2.3456988	2.43657514	1.86325502	2.434E-05	1.58151	up	unnamed protein product [Coffea canephora]
c43062.graph_c0	10.9373974	10.8846964	11.6035372	9.84411379	8.12327712	8.46012235	1.533E-42	-1.43039	down	PREDICTED: chloride channel protein CLC-f isoform X1 [Sesamum indicum]
c43063.graph_c0	2.19149854	2.48342603	1.85972988	20.4655727	20.8663162	22.2908426	1.428E-38	2.1956	up	PREDICTED: uncharacterized protein LOC105177601 [Sesamum indicum]
c43066.graph_c0	0.73629421	1.0318441	1.19847847	4.70918262	3.63992721	4.48309698	0.0014789	1.01754	up	PREDICTED: transcription factor TCP5-like [Sesamum indicum]

#ID	0 h-1 FPKM	0 h-2 FPKM	0 h-3 FPKM	28 h-1 FPKM	28 h-2 FPKM	28 h-3 FPKM	FDR	log2FC	regulated	nr_annotation
c43066.graph_c1	1.61440524	2.50098616	1.87699612	25.6179326	25.0476304	23.662352	2.43E-58	2.5414	up	PREDICTED: zinc finger CCCH domain-containing protein 67 isoform X1 [Sesamum indicum]
c43072.graph_c0	6.42113669	8.38467807	6.82328537	31.6358252	32.0486093	29.6959154	1.08E-22	1.01931	up	PREDICTED: phosphatidylinositol 4-phosphate 5-kinase 9-like [Sesamum indicum]
c43077.graph_c0	1.35488899	0.96874693	0.64668908	6.61172842	5.84275445	6.19864437	2.423E-08	1.56872	up	PREDICTED: microtubule-associated protein TORTIFOLIA1-like [Sesamum indicum]
c43079.graph_c0	0.39530079	0.49462079	0.38098288	4.22334438	4.61744594	4.71217089	1.028E-19	2.32551	up	PREDICTED: ATP-dependent DNA helicase DDM1 [Sesamum indicum]
c43082.graph_c1	0	0	0.14227133	8.12706519	7.0473649	9.91459845	1.434E-19	6.33355	up	PREDICTED: auxin-responsive protein IAA28-like [Sesamum indicum]
c43088.graph_c0	1.5536107	1.43553822	1.68930491	0.1737162	0.1960618	0	2.294E-22	-4.76063	down	PREDICTED: putative clathrin assembly protein At5g57200 [Sesamum indicum]
c43090.graph_c0	0.19142737	0.22994287	0.04919835	2.17040163	2.0099171	2.2932568	2.438E-08	2.70498	up	hypothetical protein MIMGU_mgv1a018481mg, partial [Erythranthe guttata]
c43092.graph_c0	1.5705004	2.37935376	1.36361837	12.0929949	14.6791555	13.8702795	3.859E-18	1.84762	up	PREDICTED: uncharacterized protein LOC105160850 [Sesamum indicum]
c43096.graph_c0	0	0.08865349	0.11380928	9.33341686	10.4322928	8.69273563	2.775E-44	6.02522	up	PREDICTED: uncharacterized protein LOC104227860 [Nicotiana sylvestris]
c43097.graph_c0	30.9847032	34.7956099	31.574075	34.2867903	32.417388	33.5610181	8.072E-28	-1.04786	down	PREDICTED: uncharacterized protein LOC105164803 [Sesamum indicum]
c43099.graph_c0	5.5004032	5.26800128	6.37013851	0.03701538	0.07798331	0.0604085	5.9E-106	-7.7035	down	PREDICTED: putative RING-H2 finger protein ATL21A [Sesamum indicum]
c43100.graph_c0	16.5962635	16.263505	14.0609238	12.1508536	11.3604519	11.1259103	3.792E-57	-1.52654	down	PREDICTED: kinesin-like protein NACK1 isoform X1 [Sesamum indicum]
c43104.graph_c0	1.4032262	1.66001883	0.88520788	6.84231143	5.99379869	7.1871365	5.793E-08	1.25829	up	hypothetical protein MIMGU_mgv1a007414mg [Erythranthe guttata]
c43106.graph_c0	14.5307122	15.6461654	13.9187515	14.9934832	13.3846175	14.5470695	7.581E-26	-1.12836	down	PREDICTED: uncharacterized protein LOC105166690 [Sesamum indicum]
c43106.graph_c1	46.3082371	48.2622048	46.7387293	22.6553563	27.3694979	24.4442714	1.06E-126	-2.01497	down	PREDICTED: bifunctional riboflavin biosynthesis protein RIBA 1, chloroplastic-like [Sesamum indicum]
c43107.graph_c0	5.90546584	6.1268983	6.8773114	5.07446504	5.26802188	4.70619508	1.557E-19	-1.42248	down	PREDICTED: secretory carrier-associated membrane protein 2 [Sesamum indicum]
c43108.graph_c0	26.367832	27.9986795	23.2660393	22.5767534	20.5920939	20.3905421	2.89E-35	-1.3775	down	PREDICTED: U-box domain-containing protein 32 isoform X3 [Sesamum indicum]
c43115.graph_c0	0	0.06908632	0	2.58330753	2.4457119	2.35354517	1.669E-17	5.65765	up	PREDICTED: MLO-like protein 4 [Sesamum indicum]
c43116.graph_c0	1.82084575	1.57249879	2.20222012	11.962319	11.902024	12.9795831	1.929E-18	1.62581	up	PREDICTED: tRNA (adenine(58)-N(1))-methyltransferase non-catalytic subunit trm6 [Sesamum indicum]
c43117.graph_c0	8.8346542	8.37094729	8.44966291	38.9424827	36.6882435	38.7560336	6.444E-25	1.06666	up	PREDICTED: transcription factor BIM2 isoform X2 [Sesamum indicum]
c43123.graph_c0	14.8565587	14.3694459	13.6941571	62.3713006	62.0333662	61.0808016	4.893E-21	1.02193	up	PREDICTED: GDSL esterase/lipase At4g01130-like [Sesamum indicum]

#ID	0 h-1 FPKM	0 h-2 FPKM	0 h-3 FPKM	28 h-1 FPKM	28 h-2 FPKM	28 h-3 FPKM	FDR	log2FC	regulated	nr_annotation
c43124.graph_c0	157.487903	165.811311	167.884458	119.030324	116.919079	115.562076	4.719E-75	-1.57402	down	PREDICTED: putative DNA-binding protein ESCAROLA [Sesamum indicum]
c43128.graph_c0	8.88543989	7.97590031	9.21518529	5.10664395	5.02278405	4.76839952	9.088E-42	-1.89929	down	PREDICTED: transmembrane protein 53-B [Sesamum indicum]
c43139.graph_c0	76.7318865	76.2418678	74.1527519	27.3696788	29.6175433	31.0317194	3.54E-170	-2.45738	down	PREDICTED: transcription factor bHLH112 isoform X1 [Sesamum indicum]
c43140.graph_c0	0.67926502	0.60439573	0.43644122	23.0386752	24.8382964	24.5958435	2.8E-101	4.31168	up	PREDICTED: uncharacterized protein LOC102583254 [Solanum tuberosum]
c43142.graph_c0	1.80162025	1.3917731	1.81185914	0.83972854	0.73249457	0.85941798	1.027E-16	-2.13232	down	PREDICTED: protein WEAK CHLOROPLAST MOVEMENT UNDER BLUE LIGHT 1 isoform X1 [Sesamum indicum]
c43143.graph_c0	12.2106154	11.1086055	13.263768	3.9224773	3.54162923	4.08090454	6.688E-45	-2.75535	down	PREDICTED: vacuolar amino acid transporter 1-like isoform X1 [Eucalyptus grandis]
c43146.graph_c0	81.3470457	80.5463857	78.0199031	83.1514581	83.1142048	83.0970916	6.843E-38	-1.03429	down	hypothetical protein MIMGU_mgv1a000959mg [Erythranthe guttata]
c43147.graph_c0	1.19707318	2.99567966	3.84571607	37.846126	38.2956504	44.1933926	2.432E-16	2.80296	up	hypothetical protein [Asterochloris sp. DA2]
c43150.graph_c0	42.3389847	41.5084378	46.338634	36.5394487	39.9877672	39.9915669	8.86E-24	-1.25172	down	PREDICTED: patellin-3 [Sesamum indicum]
c43151.graph_c0	7.89256139	6.96094989	6.46911328	2.32551411	2.49166916	2.17591526	5.133E-45	-2.69718	down	PREDICTED: F-box protein At1g61340-like [Sesamum indicum]
c43153.graph_c0	0.28949557	0.28978546	0.12400442	2.38457541	2.31136037	2.80423261	6.982E-07	2.33458	up	PREDICTED: uncharacterized protein LOC105157169 [Sesamum indicum]
c43157.graph_c0	36.5862507	32.6939943	33.8650527	34.1297951	31.5059155	31.7568961	2.501E-29	-1.1727	down	hypothetical protein MIMGU_mgv1a012602mg [Erythranthe guttata]
c43159.graph_c1	0.5095856	0.53328205	0.68460302	5.97632621	7.08328169	7.14323763	4.624E-27	2.45362	up	PREDICTED: endonuclease 4-like [Sesamum indicum]
c43163.graph_c0	0.99496473	0.89433236	1.04372985	17.960303	18.748211	18.2440614	1.59E-102	3.13614	up	hypothetical protein SELMODRAFT_91337 [Selaginella moellendorffii]
c43164.graph_c0	59.0424375	54.8653309	56.4947116	52.1987873	52.7388299	53.7295733	5.021E-46	-1.193	down	PREDICTED: F-box protein SKP2A-like [Sesamum indicum]
c43165.graph_c0	3.44385889	2.99680699	3.09281754	17.7910453	17.065216	16.8846849	1.659E-30	1.35063	up	PREDICTED: uncharacterized protein LOC105161979 [Sesamum indicum]
c43166.graph_c1	14.8243822	14.4805746	14.8485767	14.0293437	13.7546199	12.2712237	6.695E-24	-1.23175	down	PREDICTED: syntaxin-81 [Sesamum indicum]
c43168.graph_c0	1.51507641	2.21655977	2.54598953	9.9103392	9.59818327	8.4669025	1.705E-05	1.05909	up	PREDICTED: annexin D5 [Sesamum indicum]
c43170.graph_c0	40.7768342	46.7402294	36.9881269	2.55686313	2.20367097	3.03473372	2.087E-97	-5.08503	down	PREDICTED: hyoscyamine 6-dioxygenase [Sesamum indicum]
c43170.graph_c1	36.9351945	38.3454319	29.1559579	1.34221432	2.49303845	2.31564167	1.507E-82	-5.1727	down	hypothetical protein MIMGU_mgv1a019348mg [Erythranthe guttata]
c43172.graph_c0	102.133094	104.588501	106.484902	23.1262416	19.8340317	22.0076631	0	-3.3601	down	PREDICTED: RING-H2 finger protein ATL47 [Sesamum indicum]
c43177.graph_c0	1.50481999	1.25527237	0.87479314	15.8325298	17.2813127	17.5089362	3.829E-46	2.71582	up	PREDICTED: formin-like protein 1 [Sesamum indicum]
c43181.graph_c1	3.18198331	2.81429368	3.24877429	1.75824542	1.7019474	1.82247442	3.314E-22	-1.89833	down	PREDICTED: nodulation-signaling pathway 2 protein-like [Sesamum indicum]
c43182.graph_c0	0.29576804	0.70315248	0.28505523	3.60062194	3.95460385	3.17926362	6.453E-08	1.97527	up	PREDICTED: rho GTPase-activating protein 5-like [Sesamum indicum]
c43183.graph_c1	18.9229477	20.0018218	19.400718	8.44052415	9.25727588	10.270327	2.582E-89	-2.1504	down	PREDICTED: pentatricopeptide repeat-containing protein At5g42310, mitochondrial-like [Sesamum indicum]

#ID	0 h-1 FPKM	0 h-2 FPKM	0 h-3 FPKM	28 h-1 FPKM	28 h-2 FPKM	28 h-3 FPKM	FDR	log2FC	regulated	nr_annotation
c43183.graph_c2	3.6544276	2.48749913	3.6159848	21.3278159	21.6071896	20.979349	2.211E-29	1.61992	up	PREDICTED: O-glucosyltransferase rumi homolog [Beta vulgaris subsp. vulgaris]
c43188.graph_c0	3.17222643	2.49915971	2.4156661	11.1221724	12.2010797	11.2878886	7.503E-10	1.00978	up	PREDICTED: probable monogalactosyldiacylglycerol synthase, chloroplastic [Sesamum indicum]
c43190.graph_c0	3.33855733	3.52377252	3.47300152	17.2657188	17.7056581	17.806159	3.695E-25	1.26149	up	PREDICTED: LETM1 and EF-hand domain-containing protein 1, mitochondrial-like [Sesamum indicum]
c43191.graph_c0	1.5130821	1.80309193	1.6666032	11.4159266	9.55099675	10.3265972	8.09E-21	1.55966	up	hypothetical protein MIMGU_mgv1a002186mg [Erythranthe guttata]
c43192.graph_c0	1.89631175	2.24333982	2.52544788	11.6522951	11.3807049	10.9804116	2.788E-12	1.25732	up	PREDICTED: heparan-alpha-glucosaminide N-acetyltransferase-like [Sesamum indicum]
c43193.graph_c0	3.62297285	3.98331553	2.97657177	2.89215471	2.68929512	2.91180949	7.988E-15	-1.40486	down	PREDICTED: rho GTPase-activating protein 7-like [Sesamum indicum]
c43196.graph_c0	0.71781438	0.42458778	0.3773536	8.34725372	7.00150428	7.39179861	7.757E-25	2.8201	up	PREDICTED: basic leucine zipper 43-like [Sesamum indicum]
c43198.graph_c0	18.0700348	16.1288185	19.0650414	9.48394142	10.6108101	9.53893529	4.694E-32	-1.93789	down	hypothetical protein MIMGU_mgv1a005420mg [Erythranthe guttata]
c43199.graph_c1	3.12593216	4.14499163	3.3387608	21.2733342	22.1382852	22.1500106	9.552E-25	1.53703	up	PREDICTED: single-stranded DNA-binding protein, mitochondrial [Sesamum indicum]
c43200.graph_c0	3.30326925	3.26472157	2.79406648	20.0572918	19.9757177	20.4582329	1.599E-23	1.60354	up	PREDICTED: uncharacterized aarF domain-containing protein kinase At1g79600, chloroplastic [Sesamum indicum]
c43201.graph_c0	1.6494096	1.81616735	1.37771181	16.5431831	19.0500147	18.5857206	3.331E-37	2.39577	up	PREDICTED: ATP-dependent RNA helicase-like protein DB10 [Sesamum indicum]
c43201.graph_c1	0.5038334	0.60520549	0.77693504	7.83632067	7.50528068	7.55970346	1.166E-15	2.50614	up	PREDICTED: ATP-dependent RNA helicase-like protein DB10 [Sesamum indicum]
c43202.graph_c0	20.1522391	25.2777837	23.8982442	21.9697969	23.4284911	24.0873357	1.562E-09	-1.08856	down	PREDICTED: probable phytol kinase 1, chloroplastic [Nicotiana sylvestris]
c43205.graph_c0	28.2188275	26.440737	26.9530797	12.4880424	11.9963416	12.0358434	3.48E-134	-2.25026	down	PREDICTED: protein TRANSPARENT TESTA 12-like [Sesamum indicum]
c43206.graph_c0	0.12642643	0.21092172	0.16246297	1.65394806	1.88052744	2.02441216	7.267E-07	2.38395	up	hypothetical protein MIMGU_mgv1a010976mg [Erythranthe guttata]
c43207.graph_c1	8.04206005	8.29405577	7.08530366	4.23978308	4.45781845	4.73327746	1.082E-68	-1.89072	down	PREDICTED: BRCT domain-containing protein At4g02110 [Sesamum indicum]
c43211.graph_c0	2.81169693	5.62902484	4.30135937	1.04609024	0.54914035	0.81389872	1.522E-07	-3.49564	down	-
c43214.graph_c0	0.42180977	0.14074405	0.54204225	8.94159314	9.27289983	8.54705561	2.012E-19	3.50107	up	-
c43218.graph_c0	6.65294303	6.16169055	5.75279676	43.2467463	45.6169201	45.4663814	4.389E-39	1.76616	up	-
c43222.graph_c0	40.6669995	40.9720572	41.3997528	13.4347913	14.0798785	12.6853868	3.37E-138	-2.70504	down	--
c43223.graph_c0	1.64077962	2.02144322	1.73002402	0.09173147	0.33129968	0.57386636	2.869E-20	-3.52138	down	PREDICTED: uncharacterized protein LOC105164927 [Sesamum indicum]
c43228.graph_c0	15.5547174	14.6958745	13.108353	69.5712922	64.4990999	61.9836532	1.823E-35	1.08809	up	PREDICTED: 3-oxoacyl-[acyl-carrier-protein] synthase II, chloroplastic-like [Sesamum indicum]

#ID	0 h-1 FPKM	0 h-2 FPKM	0 h-3 FPKM	28 h-1 FPKM	28 h-2 FPKM	28 h-3 FPKM	FDR	log2FC	regulated	nr_annotation
c43229.graph_c0	2.85503296	2.67511969	3.30621343	12.7034746	14.2150149	14.6678424	1.603E-17	1.14212	up	unnamed protein product [Vitis vinifera]
c43231.graph_c0	69.6869052	72.5409009	71.2906911	13.1399256	13.8679272	13.2143262	0	-3.49918	down	hypothetical protein MIMGU_mgv1a010924mg [Erythranthe guttata]
c43245.graph_c0	16.4294765	16.9589967	16.1993257	11.3468268	10.1814865	10.3926631	4.018E-88	-1.72582	down	PREDICTED: uncharacterized protein LOC105163648 [Sesamum indicum]
c43246.graph_c0	20.8235224	19.9166689	19.5017282	84.9758521	84.5935067	89.5214275	1.444E-34	1.01508	up	unnamed protein product [Coffea canephora]
c43248.graph_c0	23.7665796	26.5266518	26.1501178	5.79459545	5.15723387	5.76407475	1.24E-110	-3.2843	down	PREDICTED: cell cycle checkpoint protein RAD17 [Sesamum indicum]
c43250.graph_c0	0.64251763	0.71462335	0.45870033	10.0659726	12.0400917	10.0766642	7.327E-19	3.06085	up	PREDICTED: uncharacterized protein LOC105177156 [Sesamum indicum]
c43250.graph_c1	0.83391374	0.34781199	0.44650507	7.62655323	8.2541544	8.77842847	2.264E-14	2.83755	up	PREDICTED: uncharacterized protein LOC102585981 isoform X1 [Solanum tuberosum]
c43252.graph_c0	14.5610972	16.23096	16.350503	11.1671522	11.0776162	11.7411165	4.932E-34	-1.56359	down	PREDICTED: alkaline/neutral invertase CINV2 [Sesamum indicum]
c43257.graph_c0	4.51445744	3.49193754	4.74648263	35.8433314	33.3955571	31.8035044	3.741E-31	1.89374	up	PREDICTED: transcriptional regulator ATRX homolog [Solanum tuberosum]
c43258.graph_c0	4.87535993	5.64525276	6.29889557	4.11798623	3.44145553	3.89163042	7.202E-12	-1.64789	down	PREDICTED: zinc finger BED domain-containing protein DAYSLEEPER-like isoform X1 [Sesamum indicum]
c43259.graph_c0	0.03502619	0	0.09002008	11.6592299	9.88360768	10.8017356	4.227E-66	6.90452	up	PREDICTED: 1-acyl-sn-glycerol-3-phosphate acyltransferase-like [Sesamum indicum]
c43260.graph_c0	1.47107704	1.3743801	1.44930072	10.5847861	11.4234332	12.2140795	1.072E-15	1.90419	up	PREDICTED: UPF0057 membrane protein At2g24040-like [Sesamum indicum]
c43263.graph_c0	0.64995008	1.1325275	0.86614534	14.8362301	15.6862674	14.3476107	8.151E-69	2.98988	up	PREDICTED: oxysterol-binding protein-related protein 2A-like isoform X3 [Sesamum indicum]
c43264.graph_c0	1.89001568	2.01266834	1.96366615	13.6780717	9.6583461	11.3996819	1.981E-08	1.47524	up	PREDICTED: chlorophyll a-b binding protein CP26, chloroplastic [Sesamum indicum]
c43269.graph_c0	66.6720576	61.9267217	58.9636536	29.0837587	27.2171864	31.4199761	6.95E-151	-2.18491	down	PREDICTED: AP2-like ethylene-responsive transcription factor At2g41710 isoform X1 [Sesamum indicum]
c43272.graph_c0	0.08719686	0.61098923	0	2.21809616	2.05995381	1.96508678	0.0019048	2.08141	up	PREDICTED: histone-lysine N-methyltransferase CLF isoform X3 [Sesamum indicum]
c43272.graph_c1	0.23600488	0.33073769	0.24262049	3.44769246	3.5775614	3.14920398	5.636E-15	2.56163	up	PREDICTED: histone-lysine N-methyltransferase CLF isoform X2 [Sesamum indicum]
c43274.graph_c0	0.08596319	0.05736618	0.14728813	1.20789929	1.27865771	1.35949787	6.566E-08	2.6267	up	PREDICTED: uncharacterized protein LOC105179654 [Sesamum indicum]
c43278.graph_c1	9.20153643	8.7773033	8.06837223	46.7740266	48.0222219	51.1999342	1.42E-32	1.39814	up	hypothetical protein MIMGU_mgv1a009792mg [Erythranthe guttata]
c43281.graph_c0	0.0623444	0.16641822	0.16023006	4.59157082	4.99127437	4.92984775	1.748E-37	4.12322	up	PREDICTED: probable beta-D-xylosidase 6 [Sesamum indicum]
c43283.graph_c0	13.2937308	6.34000454	6.70797269	0	0	0	1.02E-15	-Inf	down	glyceraldehyde 3-phosphate dehydrogenase [Cymbidium faberi]
c43285.graph_c0	17.4310852	17.0272917	16.7356863	10.1575864	9.72366393	10.0486661	8.051E-85	-1.86437	down	PREDICTED: pentatricopeptide repeat-containing protein At2g15980 [Sesamum indicum]

#ID	0 h-1 FPKM	0 h-2 FPKM	0 h-3 FPKM	28 h-1 FPKM	28 h-2 FPKM	28 h-3 FPKM	FDR	log2FC	regulated	nr_annotation
c43287.graph_c0	35.2367142	39.1911094	38.5723399	237.760498	240.22524	239.159373	1.587E-68	1.57451	up	PREDICTED: 60S ribosomal protein L31 [Eucalyptus grandis]
c43288.graph_c0	10.2166752	5.68019914	6.86688603	0	0	0	4.374E-38	-Inf	down	alpha-2 macroglobulin family-like protein [Micromonas sp. RCC299]
c43296.graph_c0	0.58775737	0.05883459	0.1510583	4.57081172	4.78270831	4.46175108	2.045E-11	3.03976	up	hypothetical protein VITISV_034944 [Vitis vinifera]
c43299.graph_c1	63.0447828	61.1304937	63.4629916	34.5397783	34.7048123	38.2929911	3.83E-100	-1.89335	down	unnamed protein product [Coffea canephora]
c43304.graph_c0	0	0	0	4.74949132	3.0408267	4.29833298	3.341E-14	Inf	up	metallothionein-like type 1 protein [Ipomoea batatas]
c43305.graph_c2	2.61758962	2.27084931	2.82551329	38.048981	37.8130318	37.3606815	2.98E-103	2.78345	up	PREDICTED: probable methyltransferase PMT26 [Sesamum indicum]
c43305.graph_c3	1.65111495	1.71179574	1.81864122	31.5434048	30.9960195	27.7325468	7.557E-58	3.03053	up	PREDICTED: probable methyltransferase PMT26 [Sesamum indicum]
c43307.graph_c1	2.98925096	2.80124993	2.69708707	1.10939764	0.79299829	0.82982113	3.224E-15	-2.72497	down	PREDICTED: uncharacterized protein LOC105160017 [Sesamum indicum]
c43309.graph_c0	2.05260369	2.36067211	1.57138205	8.77634844	9.72973821	8.22334032	8.179E-11	1.07158	up	hypothetical protein MIMGU_mgv1a002245mg [Erythranthe guttata]
c43312.graph_c0	7.62454427	7.78636453	6.87828249	57.4858045	63.0735697	54.3986027	6.875E-68	1.88288	up	PREDICTED: IAA-amino acid hydrolase ILR1-like 4 [Sesamum indicum]
c43314.graph_c0	0.44278704	0.32659084	0.44920964	7.79131216	8.01358254	7.06251666	1.246E-39	3.13785	up	PREDICTED: LOW QUALITY PROTEIN: probable serine/threonine-protein kinase WNK3 [Sesamum indicum]
c43319.graph_c0	3.70325272	1.9247682	2.47092907	0	0	0	6.473E-18	-Inf	down	predicted protein [Hordeum vulgare subsp. vulgare]
c43322.graph_c0	2.64912464	3.5085054	2.35677465	26.8662961	33.5115284	29.367204	2.933E-30	2.30925	up	hypothetical protein MIMGU_mgv1a008032mg [Erythranthe guttata]
c43323.graph_c0	22.9477132	24.0933197	20.7307919	24.5784969	23.4092458	24.0465581	9.155E-24	-1.00103	down	Ribosome production factor 1 [Gossypium arboreum]
c43328.graph_c0	0.87506275	0.82032382	0.66041493	0.70665619	0.57423985	0.79080093	3.856E-06	-1.27128	down	PREDICTED: leucine-rich repeat receptor protein kinase EXS-like [Nicotiana tomentosiformis]
c43336.graph_c0	20.0470329	20.8037649	18.3183192	12.4900447	12.0911679	11.3637073	2.23E-102	-1.80866	down	PREDICTED: uncharacterized protein LOC105160884 [Sesamum indicum]
c43338.graph_c1	34.1207419	32.3690311	34.6759935	11.8320874	14.122399	16.2678228	2.943E-62	-2.35059	down	PREDICTED: calcium-dependent protein kinase 26-like [Sesamum indicum]
c43342.graph_c0	1.80407069	1.54603875	1.70120022	11.7818276	11.9495225	11.8306854	4.27E-46	1.7254	up	PREDICTED: tRNA(adenine(34)) deaminase, chloroplastic [Sesamum indicum]
c43345.graph_c0	0	0.04589777	0	0.53319785	0.42124921	0.4486815	6.333E-08	3.85234	up	PREDICTED: uncharacterized protein LOC105155881 [Sesamum indicum]
c43349.graph_c0	0.84691833	0.93700496	1.37472453	6.57650941	8.33656082	7.00537026	2.176E-10	1.69726	up	Transcription factor SPEECHLESS [Glycine soja]
c43349.graph_c1	19.9871317	19.500208	17.6579257	14.9682172	15.8412314	15.8581031	6.63E-43	-1.38111	down	PREDICTED: protein DEFECTIVE IN MERISTEM SILENCING 3-like [Sesamum indicum]
c43350.graph_c0	3.31847026	3.0993517	2.96982609	2.79946302	2.95540923	2.92571105	5.678E-16	-1.2022	down	PREDICTED: pentatricopeptide repeat-containing protein At2g32630 [Sesamum indicum]
c43360.graph_c0	3.59412775	3.66968126	4.71096839	23.0656495	20.3072348	23.1270658	7.082E-20	1.37954	up	PREDICTED: palmitoyl-protein thioesterase 1-like [Sesamum indicum]
c43361.graph_c0	20.3230543	22.0975153	20.5686972	10.0024365	10.1110872	10.5319011	5.19E-119	-2.12968	down	hypothetical protein MIMGU_mgv1a001131mg [Erythranthe guttata]
c43362.graph_c0	59.988471	58.8307072	59.168611	32.4103815	29.1407087	39.3785255	5.51E-106	-1.90753	down	PREDICTED: protein SRG1-like [Citrus sinensis]

#ID	0 h-1 FPKM	0 h-2 FPKM	0 h-3 FPKM	28 h-1 FPKM	28 h-2 FPKM	28 h-3 FPKM	FDR	log2FC	regulated	nr_annotation
c43363.graph_c0	7.46029793	8.54862949	8.45150184	6.27822284	5.79747946	5.35584123	1.239E-10	-1.58102	down	-
c43363.graph_c2	12.8422355	12.2228772	12.038914	8.83637741	9.84350558	10.1133049	1.511E-23	-1.45538	down	hypothetical protein MIMGU_mgv1a021390mg, partial [Erythranthe guttata]
c43367.graph_c0	1.40385711	1.7608113	1.84748214	0.70069843	0.81025516	0.99306413	5.196E-14	-2.09341	down	PREDICTED: centromere-associated protein E isoform X1 [Sesamum indicum]
c43369.graph_c0	2.37230986	2.10388792	2.13930673	11.4189189	11.9352666	12.3907491	1.533E-21	1.34457	up	PREDICTED: B2 protein [Sesamum indicum]
c43372.graph_c0	13.468978	13.423071	11.8946014	10.0910444	8.61481976	9.70334773	4.659E-65	-1.53781	down	PREDICTED: nuclear pore complex protein NUP214 [Sesamum indicum]
c43375.graph_c0	16.5546918	17.9359616	16.3680307	126.971454	149.969308	131.536694	4.436E-47	1.91513	up	hypothetical protein MIMGU_mgv1a007068mg [Erythranthe guttata]
c43376.graph_c0	1.45034717	1.01625963	2.23650424	8.0814637	7.93130871	7.36853459	1.364E-06	1.21523	up	PREDICTED: transmembrane protein 64-like [Tarenaya hassleriana]
c43381.graph_c0	2.1997349	2.1120626	2.48061422	13.8667495	12.8738911	14.5366454	7.016E-15	1.5115	up	PREDICTED: caffeoyl-CoA O-methyltransferase-like [Sesamum indicum]
c43386.graph_c0	18.1090614	17.5927321	15.8379077	14.519757	14.7158786	13.2993175	8.173E-33	-1.36636	down	PREDICTED: vacuolar protein sorting-associated protein VTA1 homolog [Sesamum indicum]
c43387.graph_c0	11.575851	11.5874424	14.8754268	1.18496645	0.32097408	0.38676918	3.503E-37	-5.42452	down	PREDICTED: uncharacterized protein LOC104587006 isoform X2 [Nelumbo nucifera]
c43389.graph_c0	0.02750534	0.01376644	0.03534545	1.37935972	1.57935281	1.29682505	9.601E-21	4.69855	up	PREDICTED: ABC transporter C family member 8-like isoform X3 [Sesamum indicum]
c43390.graph_c0	19.6959177	18.6887839	17.0616747	12.0568776	12.3479853	10.4293078	4.151E-70	-1.76015	down	hypothetical protein MIMGU_mgv1a008049mg [Erythranthe guttata]
c43392.graph_c0	0.8192594	0.72895979	0.70185384	6.66554175	5.94368977	6.07335592	1.08E-18	1.96477	up	PREDICTED: probable receptor-like protein kinase At5g18500 [Sesamum indicum]
c43393.graph_c0	2.1698033	1.3637989	1.49140726	1.50365167	2.15238222	1.61600595	0.0023976	-1.01822	down	hypothetical protein MIMGU_mgv1a0000292mg, partial [Erythranthe guttata]
c43400.graph_c1	21.7139436	20.1424951	20.0143925	16.5527452	15.592141	16.9229607	9.911E-44	-1.42385	down	PREDICTED: putative G3BP-like protein isoform X2 [Sesamum indicum]
c43403.graph_c0	0.4404849	0.94064209	0.67924864	4.82347108	4.91398781	4.85893686	2.572E-10	1.73155	up	-
c43406.graph_c1	17.927768	17.6133918	9.81243534	4.10197359	5.01088492	4.134748	2.414E-08	-2.85954	down	-
c43407.graph_c0	213.601327	203.116589	209.315054	58.2213397	64.2235394	61.4591867	0	-2.85778	down	PREDICTED: uncharacterized protein LOC105176746 isoform X1 [Sesamum indicum]
c43412.graph_c0	0.23895416	0.31892458	0.25588796	38.2943461	40.5087803	37.1526117	4.78E-188	6.0639	up	PREDICTED: uncharacterized protein LOC105177232 [Sesamum indicum]
c43416.graph_c1	0.26646774	0.26673457	0.1712108	3.87334435	3.75956179	2.84455562	1.218E-07	2.80831	up	hypothetical protein POPTR_0004s23860g, partial [Populus trichocarpa]
c43418.graph_c0	24.5804177	24.3306257	22.4278093	22.8679651	21.8069338	22.6702723	5.132E-38	-1.17237	down	PREDICTED: UBX domain-containing protein 4 [Sesamum indicum]
c43421.graph_c0	1.19591357	0.88928253	1.09771163	7.40047316	6.7267786	7.96382278	1.089E-12	1.70555	up	PREDICTED: GDT1-like protein 2, chloroplastic [Sesamum indicum]
c43426.graph_c0	133.800454	122.405109	138.516376	30.2768716	31.5476355	30.4969438	1.06E-101	-3.18749	down	PREDICTED: F-box protein SKIP23-like [Sesamum indicum]
c43433.graph_c0	73.5132952	69.2945357	66.3677534	15.2556006	17.1635578	16.9725984	0	-3.17161	down	PREDICTED: probable 1-acylglycerol-3-phosphate O-acyltransferase [Beta vulgaris subsp. vulgaris]

#ID	0 h-1 FPKM	0 h-2 FPKM	0 h-3 FPKM	28 h-1 FPKM	28 h-2 FPKM	28 h-3 FPKM	FDR	log2FC	regulated	nr_annotation
c43435.graph_c0	0	0	0	1.04249436	0.87635917	1.70133377	2.587E-06	Inf	up	predicted protein [Hordeum vulgare subsp. vulgare]
c43443.graph_c0	0.21466815	0.10744156	0.13792854	0.98812398	1.1504469	1.3155439	0.0001193	1.82348	up	PREDICTED: CDT1-like protein a, chloroplastic [Sesamum indicum]
c43444.graph_c0	82.7826441	75.1905826	76.1535934	17.7044036	18.5535654	17.9043713	0	-3.20173	down	PREDICTED: probable polygalacturonase At1g80170 [Sesamum indicum]
c43453.graph_c0	8.57484837	10.4126914	8.87714932	6.64081597	5.86425322	7.074281	1.836E-21	-1.59879	down	PREDICTED: uncharacterized protein LOC105177800 [Sesamum indicum]
c43454.graph_c0	2.54483334	2.21511444	2.65408478	25.7063453	23.3072306	23.1853368	3.245E-49	2.19194	up	hypothetical protein MIMGU_mgv1a003626mg [Erythranthe guttata]
c43456.graph_c1	2.94332776	2.59272205	4.91698052	0.21391937	0.19314913	0.59349195	2.032E-07	-4.47779	down	PREDICTED: putative Myb family transcription factor At1g14600 [Sesamum indicum]
c43457.graph_c0	0	0	0	1.51584677	1.5102538	3.19892737	4.491E-05	Inf	up	ADP ribosylation factor [Karlodinium veneficum]
c43463.graph_c0	1.62124122	2.20245917	1.87502324	9.0561446	8.70880065	8.2825525	5.43E-11	1.1007	up	F3H9.11 protein isoform 5 [Theobroma cacao]
c43466.graph_c0	32.9210148	31.8206423	27.6436176	26.8068821	26.404474	28.0464909	3.42E-42	-1.27325	down	PREDICTED: 50S ribosomal protein L3-2, chloroplastic-like [Sesamum indicum]
c43467.graph_c0	0.07717569	0.38626486	0.29752146	5.72126659	5.67221508	5.30928226	2.063E-12	3.35723	up	hypothetical protein VITISV_020777 [Vitis vinifera]
c43471.graph_c0	0.01644231	0	0	7.68396372	6.45235378	6.64059902	2.865E-94	9.24433	up	PREDICTED: pentatricopeptide repeat-containing protein MRL1, chloroplastic [Sesamum indicum]
c43477.graph_c0	25.4891655	22.8653083	26.026218	16.9362942	17.0664997	16.4814839	1.513E-41	-1.6504	down	PREDICTED: high mobility group B protein 14-like isoform X2 [Nelumbo nucifera]
c43479.graph_c0	0.38280194	0.07663705	0.29514939	2.22574595	2.76325487	2.86050006	3.061E-06	2.28985	up	PREDICTED: diacylglycerol kinase 4-like [Sesamum indicum]
c43480.graph_c1	1.0337102	1.09561267	1.40649726	1.10484502	0.31922287	0.68517383	6.573E-06	-1.83912	down	-
c43481.graph_c0	2.48324815	1.42041986	1.08268521	0.06445752	0.37829411	0.23668563	4.729E-06	-3.95763	down	-
c43483.graph_c0	0.52873211	0.45365276	0.42707764	0.40623828	0.43618874	0.43003734	0.0003977	-1.23561	down	hypothetical protein MIMGU_mgv1a000607mg [Erythranthe guttata]
c43488.graph_c1	56.2874843	53.5079984	55.470229	38.2814631	37.5978394	39.2401389	2.796E-81	-1.61198	down	hypothetical protein CISIN_1g018310mg [Citrus sinensis]
c43489.graph_c0	0.28202774	0.16132008	0.38830372	1.2005745	1.25586085	1.44559436	0.0014402	1.13398	up	PREDICTED: pentatricopeptide repeat-containing protein At1g43980, mitochondrial [Sesamum indicum]
c43491.graph_c0	1.70550907	1.62961611	1.89278576	12.1701681	12.0822855	11.9307021	1.209E-17	1.69882	up	PREDICTED: cytochrome b561 and DOMON domain-containing protein At4g17280 [Sesamum indicum]
c43493.graph_c0	89.8759357	92.2204127	87.608149	36.2994491	37.029158	41.0998556	4.28E-175	-2.32653	down	PREDICTED: E3 ubiquitin-protein ligase RNF170-like [Sesamum indicum]
c43494.graph_c0	0.83879053	1.02621499	1.25752554	7.28166922	7.15568145	6.2181446	5.257E-09	1.62966	up	PREDICTED: uncharacterized protein LOC105160516 isoform X2 [Sesamum indicum]
c43495.graph_c0	39.2122608	34.8156935	40.388011	33.7227535	35.7757476	36.7488811	9.153E-20	-1.19794	down	PREDICTED: JmjC domain-containing protein 7 [Sesamum indicum]
c43496.graph_c0	0.89059982	0.89149162	1.14445602	8.6920835	7.97337266	7.99811628	2.454E-10	1.98097	up	-
c43498.graph_c0	2.73053086	2.57658109	2.45842311	1.56740272	1.70054385	1.93911057	2.599E-19	-1.66516	down	PREDICTED: chromosome transmission fidelity protein 18 homolog [Sesamum indicum]

#ID	0 h-1 FPKM	0 h-2 FPKM	0 h-3 FPKM	28 h-1 FPKM	28 h-2 FPKM	28 h-3 FPKM	FDR	log2FC	regulated	nr_annotation
c43504.graph_c0	85.2011869	75.5729352	75.8802576	346.482786	346.62311	360.276068	5.168E-41	1.06504	up	PREDICTED: 40S ribosomal protein S12-like [Sesamum indicum]
c43506.graph_c0	1.21393017	1.65971125	1.82628098	0.58101198	0.3691625	0.47410103	1.266E-11	-2.81695	down	PREDICTED: uncharacterized protein LOC103328554 [Prunus mume]
c43510.graph_c2	33.25454	31.5453352	29.0803104	34.4651161	30.8036417	29.867607	1.173E-26	-1.07006	down	PREDICTED: dynamin-related protein 3A-like [Nicotiana sylvestris]
c43511.graph_c0	22.2370477	23.6881221	21.2868068	16.35286	15.0608943	15.6605869	3.451E-57	-1.60351	down	PREDICTED: uncharacterized protein LOC105169670 [Sesamum indicum]
c43512.graph_c0	3.3210317	3.14170022	3.6579919	15.3575404	13.9143169	15.1072125	3.959E-11	1.04077	up	PREDICTED: malonyl-CoA-acyl carrier protein transacylase, mitochondrial [Sesamum indicum]
c43522.graph_c0	63.0121231	63.8412191	60.1075259	39.0707075	38.1803348	38.4618762	1.44E-106	-1.78207	down	PREDICTED: TOM1-like protein 2 isoform X2 [Sesamum indicum]
c43525.graph_c0	1.21988665	1.17502863	1.24225115	1.35500252	1.41979483	1.07837047	0.0003841	-1.00888	down	PREDICTED: putative U-box domain-containing protein 50 [Sesamum indicum]
c43530.graph_c0	6.447602	6.97384823	5.8387215	52.2387841	45.0085839	46.1679301	2.873E-42	1.80736	up	hypothetical protein MIMGU_mgv1a026892mg [Erythranthe guttata]
c43539.graph_c0	30.3404579	29.7981048	28.6339145	23.1948403	22.6010827	21.8487664	1.943E-73	-1.48217	down	PREDICTED: autophagy-related protein 18f [Sesamum indicum]
c43541.graph_c0	0.94771995	0.94866895	0.635404	8.08588253	8.30127295	6.74463665	3.13E-14	2.10513	up	PREDICTED: DNA-3-methyladenine glycosylase isoform X1 [Sesamum indicum]
c43545.graph_c0	14.1602913	13.4182404	13.6660707	9.62991354	10.1440605	9.67306688	1.871E-60	-1.57646	down	PREDICTED: probable beta-1,4-xylosyltransferase IRX10L [Sesamum indicum]
c43547.graph_c0	2.7057567	2.22613652	2.02428343	20.3791605	18.4612791	16.03572	1.043E-23	1.8918	up	PREDICTED: protein LHY-like [Sesamum indicum]
c43548.graph_c0	29.7826751	31.9806796	28.1588526	16.6607656	14.9009697	15.072306	9.28E-104	-2.03702	down	unnamed protein product [Coffea canephora]
c43550.graph_c0	2.08605274	1.87932745	1.80944574	0.41693606	0.20533864	0.58764654	9.406E-18	-3.34004	down	PREDICTED: subtilisin-like protease SDD1, partial [Sesamum indicum]
c43552.graph_c1	2.18504381	2.08430324	1.81685564	0.28024893	0.50607701	0.42687089	1.151E-31	-3.4151	down	hypothetical protein MIMGU_mgv1a005717mg [Erythranthe guttata]
c43557.graph_c0	0.2422373	0.40413311	0.4496332	1.64319564	1.9428771	1.58026891	0.0018529	1.13931	up	PREDICTED: pentatricopeptide repeat-containing protein At4g02750-like [Sesamum indicum]
c43560.graph_c0	11.7519198	10.9725245	12.0214053	12.1196912	11.5296295	11.2035677	6.81E-34	-1.08683	down	PREDICTED: protein HASTY 1 [Sesamum indicum]
c43561.graph_c0	10.9970005	12.3241878	11.9811231	3.27231484	3.73899885	3.54448963	2.619E-81	-2.83313	down	PREDICTED: uncharacterized protein LOC105156369 [Sesamum indicum]
c43567.graph_c0	0.53306695	0.50551648	0.43263924	6.18868421	5.34845589	5.98169595	3.565E-37	2.48615	up	PREDICTED: uncharacterized protein LOC105158074 [Sesamum indicum]
c43568.graph_c0	89.4368897	94.3236194	84.0180281	62.9779647	57.7106826	59.0301064	5.412E-90	-1.6649	down	PREDICTED: nucleolin [Sesamum indicum]
c43573.graph_c0	1.22333915	1.36062682	1.68848659	0.09879065	0	0.2149663	4.226E-24	-4.85354	down	PREDICTED: phospholipase D delta-like [Vitis vinifera]
c43574.graph_c0	76.5668623	74.5711822	80.2836467	42.5068	43.5290806	46.3294487	2.387E-81	-1.89693	down	PREDICTED: uncharacterized Rho GTPase-activating protein At5g61530 [Sesamum indicum]
c43575.graph_c1	23.4937614	25.1418363	23.0400524	15.8955983	16.5329632	17.0956292	5.515E-36	-1.62322	down	PREDICTED: ELMO domain-containing protein A [Sesamum indicum]
c43575.graph_c2	23.3680183	20.2602832	25.2998793	16.0476293	17.5081515	16.2047339	1.086E-15	-1.56241	down	PREDICTED: ELMO domain-containing protein A [Sesamum indicum]

#ID	0 h-1 FPKM	0 h-2 FPKM	0 h-3 FPKM	28 h-1 FPKM	28 h-2 FPKM	28 h-3 FPKM	FDR	log2FC	regulated	nr_annotation
c43582.graph_c0	1.52428048	1.55211383	1.6210434	7.62116327	7.01916472	6.54611263	3.446E-08	1.08142	up	PREDICTED: uncharacterized protein LOC105156687 isoform X2 [Sesamum indicum]
c43583.graph_c0	15.6639298	13.8110597	13.5060917	9.23143541	10.2258349	10.3004092	4.17E-40	-1.61917	down	PREDICTED: solute carrier family 25 member 44-like [Sesamum indicum]
c43584.graph_c0	2.92859049	3.715535	3.06319571	31.5064938	31.0619793	33.1006435	4.984E-67	2.21081	up	PREDICTED: nodulation-signaling pathway 1 protein [Sesamum indicum]
c43587.graph_c0	0	0.07643826	0	1.83147732	2.5055338	2.2643493	4.385E-15	5.35059	up	PREDICTED: vacuolar cation/proton exchanger 3-like [Sesamum indicum]
c43590.graph_c0	4.33573558	3.72006614	3.18376679	2.77027484	3.06409059	2.34529121	2.627E-10	-1.54649	down	PREDICTED: uncharacterized protein LOC101267356 [Solanum lycopersicum]
c43592.graph_c0	0	0	0	2.95901246	3.55008149	2.71221178	3.566E-17	Inf	up	PREDICTED: suppressor of disruption of TFIIS-like [Sesamum indicum]
c43602.graph_c0	6.79684083	6.32526542	6.96007325	2.95236495	3.03159033	3.60578563	5.838E-46	-2.15654	down	PREDICTED: DDT domain-containing protein DDB_G0282237 [Sesamum indicum]
c43605.graph_c0	154.036641	143.834088	152.571266	95.5646507	98.8299558	104.477757	6.075E-94	-1.68211	down	PREDICTED: probable proteasome inhibitor [Sesamum indicum]
c43607.graph_c0	1.63943401	1.39613899	1.88663069	11.5418469	10.8065814	13.6699626	6.764E-16	1.77941	up	-
c43609.graph_c0	4.54861651	4.852722	4.92223352	24.3854233	23.7929959	24.732679	3.73E-45	1.25649	up	PREDICTED: 116 kDa U5 small nuclear ribonucleoprotein component-like [Sesamum indicum]
c43611.graph_c0	0	0	0	0.42889202	0.42730955	0.83269292	1.069E-05	Inf	up	--
c43613.graph_c0	10.485471	8.67777099	10.2913528	0.78007983	0.48761927	0.29378713	4.909E-74	-5.33096	down	PREDICTED: benzoate carboxyl methyltransferase-like [Sesamum indicum]
c43614.graph_c0	0.05233699	0.23575227	0.10088265	1.65466254	1.42531524	1.84681314	1.422E-08	2.57255	up	PREDICTED: taxadiene 5-alpha hydroxylase [Sesamum indicum]
c43620.graph_c0	0.53285022	0.33336487	0.42795852	1.98477141	2.62253047	2.291081	0.0031113	1.32529	up	PREDICTED: uncharacterized protein LOC105161412 [Sesamum indicum]
c43623.graph_c0	17.8156993	16.3125549	16.5968419	14.632461	15.0065428	13.449361	6.399E-34	-1.32573	down	PREDICTED: uncharacterized protein LOC105169540 isoform X1 [Sesamum indicum]
c43628.graph_c0	35.8837256	37.9151942	36.6382622	37.589221	34.5813081	36.160086	2.534E-37	-1.11836	down	PREDICTED: uncharacterized protein LOC105165517 [Sesamum indicum]
c43629.graph_c0	1.70328902	1.47249534	1.54210474	6.78053851	7.29074142	6.5430135	1.721E-05	1.03741	up	PREDICTED: uncharacterized protein LOC105176133 [Sesamum indicum]
c43633.graph_c0	0	0	0	3.01758632	5.5925949	5.57263205	2.023E-09	Inf	up	hypothetical protein SELMODRAFT_227399 [Selaginella moellendorffii]
c43638.graph_c0	0.41890706	0.35169322	0.45148762	2.51424405	2.50068638	2.68471436	2.948E-10	1.56367	up	PREDICTED: protein ROS1-like isoform X1 [Sesamum indicum]
c43640.graph_c0	18.2771894	18.2954912	17.668696	12.106157	11.3480796	11.8632108	1.915E-86	-1.70899	down	PREDICTED: LOW QUALITY PROTEIN: ubiquitin carboxyl-terminal hydrolase 2-like [Sesamum indicum]
c43647.graph_c0	1.84630568	1.23210298	0.86994449	7.17908114	7.06764177	7.08078435	3.741E-09	1.35141	up	PREDICTED: uncharacterized protein LOC105166821 isoform X5 [Sesamum indicum]
c43650.graph_c0	0.63211513	0.80531576	0.29537926	5.84713354	4.78918212	4.46449237	1.445E-07	2.04098	up	PREDICTED: solute carrier family 35 member F1-like isoform X2 [Sesamum indicum]
c43652.graph_c0	116.078884	121.311759	105.998491	49.7433397	49.9825577	48.735309	1.66E-157	-2.29911	down	PREDICTED: protein SMG7-like [Sesamum indicum]

#ID	0 h-1 FPKM	0 h-2 FPKM	0 h-3 FPKM	28 h-1 FPKM	28 h-2 FPKM	28 h-3 FPKM	FDR	log2FC	regulated	nr_annotation
c43655.graph_c0	1.13897041	1.49779277	1.23403429	8.21304603	8.19234443	8.31762232	2.852E-18	1.58451	up	PREDICTED: pentatricopeptide repeat-containing protein At1g79490, mitochondrial [Sesamum indicum]
c43656.graph_c0	0.22641141	0.05665953	0.14547381	1.89237685	2.48866965	1.64487041	1.843E-07	2.72562	up	-
c43658.graph_c0	1.05304781	1.51240762	1.23553786	7.25417825	5.7686689	6.89685759	2.119E-06	1.29882	up	PREDICTED: potassium transporter 8-like [Sesamum indicum]
c43663.graph_c0	43.7727795	43.181588	39.4020021	23.2071709	21.7270329	23.1112128	3.86E-119	-1.98193	down	PREDICTED: VIN3-like protein 2 isoform X2 [Sesamum indicum]
c43666.graph_c0	3.08869046	2.41309917	2.75900126	14.20821	15.3252071	13.8499916	2.321E-14	1.30275	up	PREDICTED: uncharacterized protein LOC105173066 [Sesamum indicum]
c43670.graph_c0	1.26944227	1.31378845	1.02300834	11.5875234	12.6933064	11.7649247	1.046E-42	2.23334	up	PREDICTED: ABC transporter B family member 15-like [Sesamum indicum]
c43680.graph_c0	2.45376842	4.86019088	3.48863527	1.66955168	2.22691275	2.38409593	0.0007735	-1.87408	down	PREDICTED: probable receptor protein kinase TMK1 [Sesamum indicum]
c43684.graph_c0	1.42779806	1.60246751	1.64944602	14.2030744	13.4676128	13.4884043	2.204E-61	2.04453	up	PREDICTED: villin-4-like [Sesamum indicum]
c43685.graph_c0	5.92846234	5.23164104	6.0812816	5.15918784	6.07451132	5.72108519	3.257E-14	-1.1155	down	PREDICTED: uncharacterized protein LOC105159738 isoform X2 [Sesamum indicum]
c43685.graph_c1	0.34445747	0.30423741	0.15622641	42.10293	39.4244385	36.9151968	9.27E-260	6.11915	up	PREDICTED: LOW QUALITY PROTEIN: LRR receptor-like serine/threonine-protein kinase ERECTA [Sesamum indicum]
c43693.graph_c0	0.28960345	0.09663115	0	1.89433904	2.59729009	2.11827081	1.975E-07	3.03189	up	PREDICTED: uncharacterized protein LOC105171344 [Sesamum indicum]
c43694.graph_c0	0.17641527	0.13244394	0	7.24429429	6.59880276	7.96454104	2.008E-59	5.07381	up	PREDICTED: probable LRR receptor-like serine/threonine-protein kinase At1g67720 [Sesamum indicum]
c43698.graph_c0	1.74698648	1.35385999	0.86901166	8.76502377	9.31928344	8.48904536	5.826E-09	1.65994	up	PREDICTED: coiled-coil domain-containing protein 93 [Sesamum indicum]
c43703.graph_c0	2.77519777	2.0896285	2.7141288	2.49893526	2.30465362	2.65084399	2.861E-07	-1.11438	down	PREDICTED: protein hunchback-like [Sesamum indicum]
c43708.graph_c0	43.6127045	46.8695278	41.6580484	18.1431338	18.6446745	19.1626594	1.75E-164	-2.32941	down	PREDICTED: uncharacterized protein LOC105161926 [Sesamum indicum]
c43712.graph_c0	3.25390864	3.053594	2.926981	14.1304092	12.5182131	14.3766902	1.906E-09	1.06257	up	PREDICTED: mitochondrial ribosome-associated GTPase 1 [Sesamum indicum]
c43713.graph_c0	1.47369333	1.19857482	1.42031571	35.9311124	36.3258459	35.287179	1.99E-185	3.62503	up	PREDICTED: fatty-acid-binding protein 1-like [Nicotiana sylvestris]
c43715.graph_c0	0.08498505	0.04253508	0.10920916	0.74119976	0.73894575	0.70561511	9.784E-06	2.11036	up	hypothetical protein MIMGU_mgv1a021755mg, partial [Erythranthe guttata]
c43717.graph_c0	2.026549	1.50843	1.20193648	0	0	0	4.613E-31	-Inf	down	glutathione peroxidase [Micromonas pusilla CCMP1545]
c43730.graph_c0	0.62776199	0.86403708	0.40334951	4.33441108	3.39862381	4.9329667	0.0001237	1.65732	up	PREDICTED: basic leucine zipper 43-like [Sesamum indicum]
c43731.graph_c2	20.2268938	20.1556699	22.3910084	4.58292695	4.57773583	5.07650388	8.695E-87	-3.23194	down	hypothetical protein MIMGU_mgv1a004451mg [Erythranthe guttata]
c43738.graph_c0	4.8886825	4.19881056	4.45955132	1.16242291	1.14857389	1.14539183	4.11E-48	-3.0604	down	Wee1-like protein kinase [Morus notabilis]
c43741.graph_c0	0.07235098	0.10863515	0.04648694	1.34089911	1.28192418	0.79380433	7.563E-07	2.82211	up	unnamed protein product [Coffea canephora]

#ID	0 h-1 FPKM	0 h-2 FPKM	0 h-3 FPKM	28 h-1 FPKM	28 h-2 FPKM	28 h-3 FPKM	FDR	log2FC	regulated	nr_annotation
c43743.graph_c0	0.12629723	0.06321185	0.10819796	3.04443787	2.55545609	2.94612965	1.736E-21	3.75371	up	hypothetical protein MIMGU_mgv1a003562mg [Erythranthe guttata]
c43744.graph_c0	5.9561807	4.83484861	4.82390421	24.5365971	26.506267	24.3851684	7.396E-27	1.18362	up	hypothetical protein MIMGU_mgv1a003216mg [Erythranthe guttata]
c43744.graph_c1	7.61706708	9.36267624	6.69342388	54.8312242	58.5856589	58.161059	5.096E-51	1.76927	up	PREDICTED: D-3-phosphoglycerate dehydrogenase 3, chloroplastic [Sesamum indicum]
c43748.graph_c0	15.0342719	16.2010606	15.2782825	6.52264383	7.75177536	6.50518501	9.965E-76	-2.25362	down	PREDICTED: phosphoglucan phosphatase LSF1, chloroplastic [Sesamum indicum]
c43749.graph_c0	0.134143	0.13427732	0.08618953	1.34054741	1.25440275	1.31265089	1.888E-06	2.37699	up	PREDICTED: cytochrome c oxidase copper chaperone 1 [Sesamum indicum]
c43754.graph_c1	7.96811394	7.65598205	6.78056754	4.76464918	4.28454355	3.76147857	2.706E-43	-1.89575	down	PREDICTED: folylpolyglutamate synthase [Sesamum indicum]
c43757.graph_c0	1.25042338	1.51989166	1.56858537	6.66453702	7.67811255	6.72713366	1.689E-08	1.1864	up	hypothetical protein M569_12213, partial [Genlisea aurea]
c43758.graph_c0	8.37706927	9.43363984	7.42775731	8.18890562	8.82310145	9.34000789	3.951E-12	-1.02599	down	PREDICTED: feruloyl CoA ortho-hydroxylase 1-like [Sesamum indicum]
c43759.graph_c0	0.84949411	1.20838465	1.35017836	7.76633551	9.00739644	7.72934369	1.63E-21	1.74991	up	PREDICTED: BTB/POZ domain-containing protein At5g66560 [Sesamum indicum]
c43761.graph_c0	0.04954292	0.17357386	0.03183231	1.24225909	1.08913208	1.23403703	6.835E-08	2.72275	up	PREDICTED: probable inactive leucine-rich repeat receptor-like protein kinase At5g20690 [Sesamum indicum]
c43763.graph_c0	0.69913324	0.75815275	0.82354606	15.79425	20.2632313	15.6176046	6.476E-28	3.40842	up	PREDICTED: squamosa promoter-binding-like protein 16-like isoform X1 [Solanum tuberosum]
c43763.graph_c1	0.43552266	0.43595877	0.47971188	5.5619784	6.45095375	5.23960253	5.843E-11	2.58186	up	-
c43768.graph_c0	8.77071002	7.53841246	8.99886501	1.48515873	1.08482083	0.95316199	1.164E-74	-3.93705	down	PREDICTED: QWRF motif-containing protein 2 [Sesamum indicum]
c43769.graph_c0	1.70178386	1.3448589	0.95914896	17.3158538	15.2231558	15.8293963	1.744E-47	2.51002	up	PREDICTED: telomere repeat-binding protein 4 [Sesamum indicum]
c43777.graph_c0	4.08575449	3.60584034	3.47952606	30.8722867	30.4131781	29.0627428	1.098E-67	1.92673	up	PREDICTED: probable sugar phosphate/phosphate translocator At3g14410 [Sesamum indicum]
c43779.graph_c0	1.9627234	0.71135283	0.65228723	0	0	0	2.116E-06	-Inf	down	PREDICTED: 2,3-bisphosphoglycerate-independent phosphoglycerate mutase [Elaeis guineensis]
c43780.graph_c0	19.9620858	23.3258495	23.9970983	2.39855438	2.64106063	3.03127439	3.951E-65	-4.15117	down	PREDICTED: mitogen-activated protein kinase 9-like [Sesamum indicum]
c43781.graph_c0	12.1876941	12.3957682	10.8841419	10.4019914	10.73112	10.7425492	9.668E-30	-1.24302	down	PREDICTED: probable serine/threonine-protein kinase abkC isoform X1 [Sesamum indicum]
c43782.graph_c0	3.64355587	3.47887183	4.0338209	16.4590558	18.0980302	16.2894133	2.437E-09	1.09584	up	PREDICTED: pentatricopeptide repeat-containing protein At1g06140, mitochondrial-like [Sesamum indicum]
c43783.graph_c0	106.392227	101.219448	109.85987	51.0138479	49.0944056	50.2766253	2.05E-104	-2.16904	down	PREDICTED: uncharacterized protein LOC105163767 [Sesamum indicum]
c43784.graph_c0	8.94943769	9.95694687	10.2917429	7.45727358	7.50005767	8.29936528	2.702E-23	-1.41982	down	PREDICTED: histone-lysine N-methyltransferase ATXR4 [Sesamum indicum]
c43789.graph_c2	5.64326574	5.16472377	5.38706622	2.57808133	3.49164858	2.77305027	5.509E-08	-1.96387	down	PREDICTED: coiled-coil domain-containing protein 94-like [Sesamum indicum]

#ID	0 h-1 FPKM	0 h-2 FPKM	0 h-3 FPKM	28 h-1 FPKM	28 h-2 FPKM	28 h-3 FPKM	FDR	log2FC	regulated	nr_annotation
c43789.graph_c4	3.07860923	3.03637299	3.49070713	0.95423453	0.95071372	1.18139633	9.593E-21	-2.72923	down	PREDICTED: uncharacterized protein LOC105175802 [Sesamum indicum]
c43791.graph_c1	2.29487247	1.08477493	1.8840846	8.61748375	9.16124081	8.92206421	4.912E-06	1.25324	up	PREDICTED: uncharacterized protein LOC105165414 [Sesamum indicum]
c43796.graph_c0	0	0	0.25520617	3.64458031	3.68169026	3.9750787	1.44E-16	4.3389	up	geranyl diphosphate synthase large subunit [Antirrhinum majus]
c43798.graph_c0	4.99631328	4.80441411	4.19604891	32.3099182	29.8956933	30.7976202	1.77E-37	1.64384	up	PREDICTED: methyltransferase-like protein 13 isoform X1 [Sesamum indicum]
c43799.graph_c0	6.27907187	2.86538444	4.47940165	0	0	0	1.035E-21	-Inf	down	hypothetical protein THAPSDRAFT_261727 [Thalassiosira pseudonana CCMP1335]
c43802.graph_c0	23.0745188	20.4920997	24.2275842	173.021725	154.88361	175.562529	9.411E-50	1.80153	up	PREDICTED: ethylene-responsive transcription factor RAP2-3 [Sesamum indicum]
c43805.graph_c0	15.7888083	15.8724494	13.1488233	12.8542107	14.8078428	14.5076784	2.618E-16	-1.17567	down	PREDICTED: NEP1-interacting protein-like 1 isoform X1 [Sesamum indicum]
c43808.graph_c0	38.483976	32.7868766	38.1609167	36.7993808	37.7276807	39.6343384	9.491E-15	-1.02957	down	PREDICTED: (S)-coclaurine N-methyltransferase isoform X1 [Sesamum indicum]
c43813.graph_c0	2.30854631	2.00274357	1.87882982	1.00670289	1.86841433	1.14091852	4.533E-06	-1.71404	down	-
c43813.graph_c1	74.0821308	75.3966033	70.979846	60.4305843	60.2120427	61.3968592	5.473E-64	-1.3661	down	PREDICTED: FACT complex subunit SSRP1 [Sesamum indicum]
c43816.graph_c0	0	0.27364625	0.87823623	4.76845348	5.47153023	6.07971734	3.208E-08	2.70648	up	hypothetical protein VITISV_012452 [Vitis vinifera]
c43820.graph_c0	14.2792034	14.7357513	13.1715571	6.43490024	6.10003732	6.57139308	2.43E-118	-2.23185	down	PREDICTED: metal tolerance protein 9-like [Sesamum indicum]
c43826.graph_c0	24.632141	24.5708941	23.4918044	16.0623459	15.601062	16.0843915	1.957E-60	-1.69624	down	PREDICTED: protein REVEILLE 6-like isoform X1 [Sesamum indicum]
c43829.graph_c0	6.39743516	3.75497963	3.84890281	0	0	0	4.506E-28	-Inf	down	predicted protein, partial [Micromonas sp. RCC299]
c43831.graph_c1	1.34132746	1.50298947	1.56930137	18.304258	18.4056974	15.7078644	2.869E-62	2.47699	up	ABC transporter-like [Arabidopsis thaliana]
c43836.graph_c0	9.87198853	10.3000378	8.81514384	9.77960239	9.13305642	9.20577359	2.53E-26	-1.13292	down	hypothetical protein MIMGU_mgv1a020553mg, partial [Erythranthe guttata]
c43838.graph_c0	19.6826702	19.8437841	18.7882291	20.0033957	19.5753026	20.4556202	1.794E-31	-1.0478	down	PREDICTED: uncharacterized protein LOC105159284 [Sesamum indicum]
c43840.graph_c0	10.5318327	10.1948277	10.9063766	8.63581826	8.65807888	7.25266891	5.828E-27	-1.45785	down	hypothetical protein M569_07122 [Genlisea aurea]
c43843.graph_c0	0.27720661	0.27748419	0.25907016	2.61913816	2.59635861	2.76490307	6.227E-10	2.20442	up	PREDICTED: interactor of constitutive active ROPs 2, chloroplastic-like isoform X2 [Sesamum indicum]
c43844.graph_c0	51.616544	52.0817138	46.4681109	20.4522781	18.6133179	17.8729356	1.61E-201	-2.48856	down	PREDICTED: uncharacterized protein LOC105180214 [Sesamum indicum]
c43851.graph_c0	0.41669978	0.48663655	0.3792953	5.27472452	5.82219914	5.28234398	3.521E-30	2.58547	up	PREDICTED: serine/threonine-protein kinase D6PKL1-like [Sesamum indicum]
c43851.graph_c1	0.68565278	0.11438989	0	4.9832849	6.8241492	6.09947967	3.336E-10	3.42201	up	PREDICTED: serine/threonine-protein kinase D6PKL1-like [Sesamum indicum]

#ID	0 h-1 FPKM	0 h-2 FPKM	0 h-3 FPKM	28 h-1 FPKM	28 h-2 FPKM	28 h-3 FPKM	FDR	log2FC	regulated	nr_annotation
c43852.graph_c0	4.85052311	5.35687484	4.76994188	29.2784635	28.3036913	28.1451765	1.345E-42	1.42686	up	PREDICTED: putative 3,4-dihydroxy-2-butanone kinase [Sesamum indicum]
c43853.graph_c0	88.2481205	80.8890157	85.1622751	3.96956853	3.92280787	4.18282065	0	-5.48644	down	PREDICTED: BTB/POZ and TAZ domain-containing protein 1 [Sesamum indicum]
c43854.graph_c0	0.73228832	0.73302159	0.6843778	3.53202488	4.21571131	3.71120172	1.52E-05	1.32426	up	PREDICTED: uncharacterized protein LOC105167611 [Sesamum indicum]
c43855.graph_c1	4.79136477	4.8345319	5.22121523	33.0543362	30.4612342	29.9159225	1.501E-58	1.56183	up	hypothetical protein MIMGU_mgv1a001401mg [Erythranthe guttata]
c43856.graph_c0	50.840675	47.2664303	54.5330632	5.97285443	6.00229788	6.69114975	3.5E-107	-4.12282	down	PREDICTED: uncharacterized protein LOC105161639 [Sesamum indicum]
c43864.graph_c0	2.5538267	2.41894397	2.18784561	2.11556228	2.2885812	2.31256584	2.246E-07	-1.18082	down	PREDICTED: uncharacterized protein LOC105178718 [Sesamum indicum]
c43865.graph_c0	31.1317159	31.8157435	31.7920704	35.9935958	30.4444818	33.6509892	9.988E-28	-1.01131	down	PREDICTED: chavicol O-methyltransferase-like [Sesamum indicum]
c43866.graph_c0	8.53029009	7.73567443	8.57405029	36.8302911	38.7689711	35.9386384	1.412E-21	1.0756	up	hypothetical protein MIMGU_mgv1a010682mg [Erythranthe guttata]
c43867.graph_c0	0.40732267	0.32618444	0.23554159	2.7235659	2.69969119	2.83840093	2.157E-10	2.00786	up	unnamed protein product [Coffea canephora]
c43870.graph_c0	1.36595579	1.8406279	1.8903309	23.0627828	22.6852369	20.8442705	7.524E-41	2.61338	up	PREDICTED: nitrogen regulatory protein P-II homolog [Musa acuminata subsp. malaccensis]
c43873.graph_c0	0	0	0	3.49779475	4.28108941	3.48843239	2.634E-14	Inf	up	hypothetical protein MIMGU_mgv1a024870mg, partial [Erythranthe guttata]
c43875.graph_c0	38.9510379	41.6269995	36.0781266	30.8050861	27.5584972	29.0637379	2.58E-63	-1.50538	down	PREDICTED: uncharacterized protein LOC105157181 [Sesamum indicum]
c43879.graph_c0	66.2147348	66.3561874	64.1058337	64.9844272	73.5036679	65.4486482	3.516E-37	-1.0382	down	PREDICTED: probable 3-hydroxyacyl-CoA dehydrogenase B0272.3 [Sesamum indicum]
c43881.graph_c0	0	0	0	5.2022902	6.24053952	6.00368008	1.548E-17	Inf	up	hypothetical protein MIMGU_mgv1a004314mg [Erythranthe guttata]
c43889.graph_c0	18.800262	14.1623236	18.3042016	4.56625966	5.06708092	5.31883757	5.291E-24	-2.86824	down	PREDICTED: uncharacterized protein LOC102584927 isoform X1 [Solanum tuberosum]
c43890.graph_c0	28.9198883	32.6976188	27.3153987	161.900268	166.637877	151.975047	3.447E-62	1.34394	up	PREDICTED: plasma membrane ATPase 3 [Sesamum indicum]
c43900.graph_c0	0.33211551	0.49867211	0.42678158	2.61494607	3.12385225	2.85597528	7.684E-05	1.68024	up	PREDICTED: 5'-nucleotidase domain-containing protein 4 [Sesamum indicum]
c43900.graph_c1	0.85613143	0.73456176	1.02157973	4.71118054	4.53466262	4.06188588	0.000484	1.25481	up	PREDICTED: 5'-nucleotidase domain-containing protein 4 [Sesamum indicum]
c43902.graph_c1	0	0	0	8.51151567	8.82547039	9.45460682	1.451E-29	Inf	up	PREDICTED: uncharacterized protein LOC105173250 [Sesamum indicum]
c43902.graph_c4	0.79336444	0.73533229	0.75518868	8.11528993	7.80940815	8.17295483	3.256E-23	2.30939	up	PREDICTED: la-related protein 1A-like [Sesamum indicum]
c43903.graph_c0	0.66584973	0.08331456	0.32086623	3.38755024	4.69719753	3.35654476	1.483E-06	2.33495	up	PREDICTED: uncharacterized protein LOC105156092 [Sesamum indicum]

#ID	0 h-1 FPKM	0 h-2 FPKM	0 h-3 FPKM	28 h-1 FPKM	28 h-2 FPKM	28 h-3 FPKM	FDR	log2FC	regulated	nr_annotation
c43907.graph_c0	0.5841318	0.45345378	0.45957091	10.4663	10.3184317	9.7635046	2.44E-101	3.26253	up	PREDICTED: uncharacterized protein LOC105157864 isoform X2 [Sesamum indicum]
c43908.graph_c0	0.17455214	0.17472692	0.22430641	80.2411552	83.6375495	73.1883352	0	7.59647	up	hypothetical protein MIMGU_mgv1a002930mg [Erythranthe guttata]
c43909.graph_c0	23.8778326	27.2802181	23.3307773	12.2931961	12.9071078	12.9875754	2.759E-54	-2.05366	down	PREDICTED: heterogeneous nuclear ribonucleoprotein 1-like [Sesamum indicum]
c43909.graph_c3	3.64963511	2.94444241	5.10991894	1.46483208	1.57282879	1.42142715	9.309E-06	-2.48865	down	PREDICTED: AP2-like ethylene-responsive transcription factor ANT [Sesamum indicum]
c43910.graph_c0	0.80762656	0.73914082	0.74130865	5.46728387	6.49611899	5.55604648	3.908E-16	1.84659	up	PREDICTED: tetratricopeptide repeat protein 7A [Sesamum indicum]
c43913.graph_c0	5.84772385	4.99475313	4.17797745	4.44700745	4.26710872	4.48568926	1.019E-10	-1.27282	down	hypothetical protein MIMGU_mgv1a003864mg [Erythranthe guttata]
c43917.graph_c1	17.828969	18.6893759	15.585333	12.763344	12.3777402	12.9560826	4.066E-45	-1.5402	down	PREDICTED: nicotinate phosphoribosyltransferase-like [Sesamum indicum]
c43920.graph_c0	73.1107296	68.1635414	66.642173	52.7982475	54.220387	51.7301905	7.22E-69	-1.47895	down	PREDICTED: serine/arginine-rich splicing factor RS31-like [Eucalyptus grandis]
c43922.graph_c0	3.4797001	3.39537312	3.30669421	15.9604917	16.9629448	17.2203269	2.23E-18	1.21031	up	PREDICTED: zinc finger CCCH domain-containing protein 33 isoform X4 [Sesamum indicum]
c43923.graph_c0	0.42317762	0.21180069	1.3595	3.69075668	4.58205996	4.01550308	0.0026684	1.51265	up	-
c43924.graph_c0	11.7778451	10.3944153	10.5676312	6.60999483	6.15113909	6.01366753	7.648E-47	-1.89191	down	PREDICTED: uncharacterized vacuolar membrane protein YML018C isoform X2 [Sesamum indicum]
c43925.graph_c0	82.8736261	86.0661668	79.0938349	74.1994861	71.7978247	70.3821959	3.515E-55	-1.28694	down	-
c43929.graph_c0	185.05218	189.018643	198.966735	174.04544	169.101846	179.916458	7.749E-40	-1.22285	down	PREDICTED: sucrose synthase 2, partial [Sesamum indicum]
c43936.graph_c0	16.1762239	17.4801845	16.3480193	17.4533735	18.0569123	17.1099526	9.835E-28	-1.0171	down	PREDICTED: LMBR1 domain-containing protein 2 homolog A [Sesamum indicum]
c43937.graph_c1	87.1924424	88.2269745	83.0064956	96.0061926	86.5958803	88.0943876	9.186E-37	-1.02301	down	PREDICTED: protein decapping 5-like isoform X2 [Sesamum indicum]
c43939.graph_c0	6.74310063	6.32236215	5.48792914	5.89735216	5.47225624	5.15877562	3.212E-22	-1.25516	down	PREDICTED: pentatricopeptide repeat-containing protein At5g02830, chloroplastic [Sesamum indicum]
c43949.graph_c0	0.35461754	0.26622948	0.45569758	3.15724134	4.30512807	2.9443236	4.589E-06	2.1766	up	PREDICTED: probable nucleoside diphosphate kinase 5 [Sesamum indicum]
c43951.graph_c0	8.55668398	8.33718336	8.36061883	5.37258281	6.0968988	6.0655117	1.256E-37	-1.61646	down	PREDICTED: switch 2 isoform X1 [Sesamum indicum]
c43953.graph_c0	1.48204522	1.83675052	1.99517663	0.25646236	0.25471762	0.25112543	1.203E-25	-3.89526	down	PREDICTED: G-type lectin S-receptor-like serine/threonine-protein kinase At5g24080 isoform X1 [Sesamum indicum]
c43955.graph_c0	1.02749211	1.10470773	1.36927056	6.85926982	7.26711194	8.57621411	3.421E-11	1.60347	up	PREDICTED: alkylated DNA repair protein alkB homolog 8 isoform X2 [Sesamum indicum]
c43963.graph_c0	1.88259878	1.72295673	1.58976918	91.4389912	91.4205066	84.2471888	1.04E-242	4.59511	up	PREDICTED: uncharacterized protein LOC105158952 [Sesamum indicum]
c43965.graph_c0	2.02896774	1.09072192	1.49678555	6.28088283	6.85457143	7.10835897	1.335E-05	1.04521	up	PREDICTED: calmodulin-lysine N-methyltransferase isoform X2 [Sesamum indicum]

#ID	0 h-1 FPKM	0 h-2 FPKM	0 h-3 FPKM	28 h-1 FPKM	28 h-2 FPKM	28 h-3 FPKM	FDR	log2FC	regulated	nr_annotation
c43967.graph_c1	67.5156205	71.504195	74.5881856	55.7472058	63.7476862	60.990249	1.725E-31	-1.33482	down	phytosulfokine precursor [Avicennia marina]
c43968.graph_c0	0.9393793	1.27334993	0.70416476	8.86133736	10.2741018	7.72985355	9.271E-17	2.11582	up	PREDICTED: probable serine/threonine-protein kinase At1g54610 isoform X2 [Sesamum indicum]
c43972.graph_c0	105.992448	103.077721	106.598932	447.817691	475.100054	467.082391	8.086E-41	1.0479	up	hypothetical protein JCGZ_19923 [Jatropha curcas]
c43977.graph_c0	68.4922666	72.6386535	66.0114557	67.9022291	67.4637825	62.947567	6.607E-47	-1.15307	down	PREDICTED: LOW QUALITY PROTEIN: protein EARLY FLOWERING 3 [Sesamum indicum]
c43979.graph_c1	3.72760029	3.73133291	3.23332692	3.38649801	2.62961356	1.65801283	1.707E-06	-1.56945	down	PREDICTED: LOW QUALITY PROTEIN: uncharacterized protein LOC105178099 [Sesamum indicum]
c43983.graph_c0	6.17394724	5.53711026	5.96178996	3.76093898	4.14518776	2.98423631	1.137E-23	-1.79025	down	PREDICTED: cryptochrome DASH, chloroplastic/mitochondrial [Sesamum indicum]
c43985.graph_c0	21.9929503	21.524073	21.4993371	9.91139338	8.98619196	9.97808233	3.97E-140	-2.26091	down	PREDICTED: uncharacterized protein LOC105162976 [Sesamum indicum]
c43986.graph_c0	0	0.33387802	0	8.12099876	7.55138107	6.23104666	1.16E-12	4.95304	up	hypothetical protein MIMGU_mgv1a005877mg [Erythranthe guttata]
c43987.graph_c0	2.58477753	2.82856091	2.61782505	2.16516773	2.02681638	2.02657671	2.578E-13	-1.45957	down	unnamed protein product [Coffea canephora]
c43988.graph_c0	12.5089188	13.2398881	5.66558692	4.6202248	1.9512462	2.06745539	2.64E-05	-2.94526	down	uncharacterized protein UHOR_12387 [Ustilago hordei]
c43990.graph_c0	29.2198758	32.0074033	27.9955415	22.154736	20.2296793	20.5804173	6.507E-66	-1.59264	down	PREDICTED: ethanolamine-phosphate cytidylyltransferase-like [Sesamum indicum]
c43993.graph_c0	8.70042327	11.3218761	14.5345049	85.682307	82.2161248	90.8134838	3.923E-13	1.80808	up	hypothetical protein M569_00228 [Genlisea aurea]
c43994.graph_c0	3.11305743	3.16643557	2.70994978	16.3487643	15.0908969	15.2164601	1.694E-11	1.28699	up	hypothetical protein MIMGU_mgv1a014320mg [Erythranthe guttata]
c43994.graph_c1	8.25180505	7.66620689	8.17816831	48.3708994	47.4613881	51.0170689	7.541E-38	1.51714	up	PREDICTED: heat stress transcription factor A-6b-like [Sesamum indicum]
c43996.graph_c0	3.49028305	2.43045429	4.192642	2.97788588	2.98750194	3.14990999	0.0029665	-1.2439	down	PREDICTED: probable prolyl 4-hydroxylase 9 [Sesamum indicum]
c43997.graph_c0	3.44819432	3.09602291	2.39009026	13.2141282	12.8773799	14.2157079	5.85E-16	1.08792	up	PREDICTED: histone-lysine N-methyltransferase family member SUVH9 [Sesamum indicum]
c43998.graph_c0	7.78864019	4.847786	5.26098912	0	0	0	5.731E-35	-Inf	down	--
c43999.graph_c0	3.89604689	4.39877877	4.51173865	62.378052	64.065929	60.3438149	9.09E-186	2.77407	up	hypothetical protein MIMGU_mgv1a001702mg [Erythranthe guttata]
c44005.graph_c1	3.77934887	4.42859682	3.65972254	4.19181275	4.33725568	3.97286143	6.814E-13	-1.01491	down	PREDICTED: putative late blight resistance protein homolog R1B-17 [Sesamum indicum]
c44006.graph_c0	0.29301634	0.37711254	0.26895543	3.346555	3.57101183	3.07831302	1.134E-08	2.32267	up	PREDICTED: uncharacterized protein LOC105178295 isoform X2 [Sesamum indicum]
c44010.graph_c0	76.7650805	69.464068	78.9000209	23.8905382	22.628949	20.3531	5.869E-83	-2.84306	down	photosystem II protein I (chloroplast) [Salvia miltiorrhiza]
c44013.graph_c0	2.99082791	1.78941131	2.95980761	0.7995279	0.56398338	0.93783684	2.615E-08	-2.83974	down	PREDICTED: peroxidase 46 [Sesamum indicum]
c44014.graph_c0	1.54717674	1.8472756	1.07793074	11.2447914	11.7554526	12.5363809	9.667E-36	1.90484	up	PREDICTED: uncharacterized protein At1g04910-like isoform X1 [Sesamum indicum]

#ID	0 h-1 FPKM	0 h-2 FPKM	0 h-3 FPKM	28 h-1 FPKM	28 h-2 FPKM	28 h-3 FPKM	FDR	log2FC	regulated	nr_annotation
c44015.graph_c0	11.1278792	9.89407254	11.2715797	9.22946835	10.395203	9.4721942	4.633E-13	-1.24173	down	PREDICTED: uncharacterized protein LOC105159125 isoform X1 [Sesamum indicum]
c44025.graph_c0	7.9640684	9.30071708	6.54985165	0.07717652	0.55746529	0.40934017	1.422E-49	-5.59932	down	PREDICTED: WAT1-related protein At1g25270-like [Sesamum indicum]
c44029.graph_c0	4.84019714	3.88921889	4.06193957	3.326381	4.01895173	4.17884995	7.109E-11	-1.2388	down	PREDICTED: vesicle-associated protein 1-4-like [Sesamum indicum]
c44030.graph_c0	120.681183	113.648583	119.742543	17.0074134	16.9422836	19.5911221	0	-3.81524	down	PREDICTED: RING-H2 finger protein ATL3 [Sesamum indicum]
c44032.graph_c1	50.561844	56.6022477	49.2521569	11.5272171	13.9551815	11.9258457	8.51E-127	-3.15432	down	PREDICTED: glycosyltransferase family protein 64 protein C5-like [Sesamum indicum]
c44036.graph_c0	23.1416118	24.3781781	20.4270964	19.4021371	18.7476658	17.7109105	7.743E-41	-1.37168	down	PREDICTED: glucose-6-phosphate 1-dehydrogenase, chloroplastic-like [Sesamum indicum]
c44039.graph_c1	49.4157286	52.6289757	46.9973109	41.7854482	48.9875854	45.4026073	2.142E-44	-1.2201	down	PREDICTED: probable serine/threonine-protein kinase NAK [Sesamum indicum]
c44041.graph_c0	1.96161953	1.89428083	1.95729503	1.77792399	1.13582453	1.38233884	2.586E-10	-1.52677	down	PREDICTED: kinesin-like protein NACK1 [Sesamum indicum]
c44043.graph_c0	2.31655134	2.95656055	2.38148815	0	0.03800459	0.03434624	1.102E-40	-7.8137	down	PREDICTED: uncharacterized protein LOC105165513 [Sesamum indicum]
c44044.graph_c0	0.11205158	0.44865514	0.14399072	2.22598413	2.6961469	2.32585867	1.965E-07	2.27392	up	PREDICTED: scarecrow-like protein 3 isoform X1 [Sesamum indicum]
c44050.graph_c2	4.75962045	4.35172309	4.67150527	21.8723588	22.25724	22.4484966	2.106E-18	1.18169	up	PREDICTED: elongation factor Ts, mitochondrial [Sesamum indicum]
c44052.graph_c0	13.0450051	13.3677452	8.21602826	2.4358389	1.92864687	1.95704581	1.87E-15	-3.53877	down	PREDICTED: ferredoxin--nitrite reductase, chloroplastic [Sesamum indicum]
c44052.graph_c1	5.90335295	9.88273507	7.19366077	1.55346519	1.33584135	1.32797752	1.518E-11	-3.53751	down	PREDICTED: ferredoxin--nitrite reductase, chloroplastic [Sesamum indicum]
c44054.graph_c0	195.776595	182.911959	221.898007	63.5673822	65.7527442	71.2709812	1.457E-33	-2.67415	down	PREDICTED: uncharacterized protein LOC105157696 [Sesamum indicum]
c44056.graph_c0	71.3816532	66.5944965	68.839105	329.208398	350.603354	337.558602	3.749E-53	1.20808	up	hypothetical protein Csa_5G600925 [Cucumis sativus]
c44062.graph_c0	47.6143051	50.2536293	46.7344556	13.583586	15.1052314	15.1626585	1.52E-206	-2.81136	down	PREDICTED: transcription factor E2FB [Sesamum indicum]
c44077.graph_c0	8.9808397	9.55761155	9.71851333	1.13367549	1.24073052	1.42965355	3.163E-88	-3.98357	down	PREDICTED: PXMP2/4 family protein 4-like [Sesamum indicum]
c44077.graph_c1	10.5885087	10.8000425	9.41502994	2.51659255	2.93086947	2.55945843	4.703E-64	-3.03317	down	PREDICTED: uncharacterized protein LOC105169472 [Sesamum indicum]
c44080.graph_c0	0.33077737	0.35870098	0.24795284	7.41255184	7.01843691	9.18730528	7.111E-26	3.56956	up	PREDICTED: 6-phosphogluconate dehydrogenase, decarboxylating 3-like [Citrus sinensis]
c44081.graph_c0	1.10936792	1.53169487	1.27810773	18.6557022	19.6559255	14.156617	1.444E-19	2.65007	up	PREDICTED: pheophytinase, chloroplastic isoform X1 [Nicotiana sylvestris]
c44083.graph_c0	11.8688864	12.0491223	11.2383077	60.8202183	58.5885649	56.2972709	9.093E-46	1.23128	up	PREDICTED: translation factor GUF1 homolog, organellar chromatophore isoform X2 [Sesamum indicum]
c44084.graph_c1	131.224501	128.075532	145.127529	7.17091813	5.75782863	6.4365517	8.49E-164	-5.47616	down	PREDICTED: chaperone protein dnaJ 6 [Sesamum indicum]
c44085.graph_c1	4.71437121	4.63329027	4.95667118	3.42637783	4.27492804	4.01592381	1.859E-06	-1.37909	down	-

#ID	0 h-1 FPKM	0 h-2 FPKM	0 h-3 FPKM	28 h-1 FPKM	28 h-2 FPKM	28 h-3 FPKM	FDR	log2FC	regulated	nr_annotation
c44085.graph_c2	8.98922812	9.03988794	8.98451092	7.68269942	6.74558678	7.4290355	1.96E-20	-1.39584	down	PREDICTED: CTD small phosphatase-like protein 2 isoform X3 [Sesamum indicum]
c44086.graph_c0	3.57736257	3.20958752	3.74574651	21.3393126	23.1452233	23.1016932	5E-39	1.59046	up	PREDICTED: probable apyrase 7 [Sesamum indicum]
c44088.graph_c0	0.17429008	0.41435344	0.16797724	2.4542899	2.98801727	2.7908322	4.952E-12	2.35634	up	hypothetical protein MIMGU_mgv1a001438mg [Erythranthe guttata]
c44089.graph_c1	3.62370135	3.75241028	3.85373768	2.76991035	2.54196884	3.33475706	1.927E-07	-1.4672	down	PREDICTED: COBRA-like protein 1 [Sesamum indicum]
c44095.graph_c0	2.92365619	2.1356152	1.69234863	9.74389462	9.47191974	9.49738815	1.944E-08	1.00414	up	hypothetical protein MIMGU_mgv1a003860mg [Erythranthe guttata]
c44100.graph_c0	0.49611288	0.49660966	0.56668861	4.84768995	5.22106093	5.01270163	7.649E-22	2.18125	up	PREDICTED: probable LRR receptor-like serine/threonine-protein kinase At4g36180 [Sesamum indicum]
c44101.graph_c0	27.3294547	27.4973527	23.3778749	21.3083916	20.4755357	18.9208461	9.64E-47	-1.45438	down	PREDICTED: uncharacterized protein LOC105175040 isoform X2 [Sesamum indicum]
c44102.graph_c0	1.44589862	1.8790112	1.56466271	13.2004119	12.2683061	12.2908617	4.847E-30	1.85822	up	hypothetical protein MIMGU_mgv1a001743mg [Erythranthe guttata]
c44103.graph_c0	1.15224998	0.57670189	0.74034342	7.81618735	6.1751238	6.94740443	3.188E-05	1.99828	up	hypothetical protein M569_03256, partial [Genlisea aurea]
c44103.graph_c1	0.35422066	0.38412331	0.34139068	3.79732164	4.24219754	4.35902417	6.829E-14	2.43189	up	ABC transporter family protein [Hevea brasiliensis]
c44105.graph_c0	0.19193416	0.48031589	0.49328599	2.78993286	2.3301192	3.4717577	0.0001204	1.78492	up	PREDICTED: UDP-galactose transporter 2-like [Sesamum indicum]
c44106.graph_c0	0.20994016	0.73552634	0.13489071	2.16159672	2.08948758	2.46938338	0.0042542	1.55439	up	PREDICTED: phospholipase D alpha 1 [Sesamum indicum]
c44106.graph_c1	0.91672586	0.30588127	0.39267634	3.99762641	3.2084283	2.71835876	0.0010401	1.53729	up	phospholipase D [Plantago major]
c44106.graph_c2	0.35492139	0.3806537	0.32577717	6.48572113	5.1739074	5.15698111	9.479E-25	2.89632	up	PREDICTED: phospholipase D alpha 1 [Sesamum indicum]
c44110.graph_c0	0.98344868	0.80309045	1.06422778	4.3638274	4.8062756	4.54315029	1.21E-06	1.17377	up	PREDICTED: glucan endo-1,3-beta-glucosidase 9 [Sesamum indicum]
c44112.graph_c1	0.21381639	0.21403049	0.27476252	7.10957374	7.89255553	7.38642354	6.756E-23	3.8997	up	PREDICTED: 2-hydroxyisoflavanone dehydratase-like [Sesamum indicum]
c44123.graph_c0	3.78015682	4.14188254	4.39814363	21.1623761	17.3980103	20.7825819	1.031E-12	1.17629	up	PREDICTED: ribonuclease H2 subunit B [Sesamum indicum]
c44127.graph_c0	32.4685377	33.6063247	33.2099509	21.3639493	20.4842302	22.2998358	1.112E-84	-1.72057	down	PREDICTED: ran-binding protein 9 [Sesamum indicum]
c44130.graph_c1	8.01032785	6.91490646	6.73647683	7.29957996	7.58749936	8.07745631	5.985E-16	-1.00401	down	PREDICTED: rab3 GTPase-activating protein catalytic subunit [Sesamum indicum]
c44133.graph_c1	119.040684	124.472746	112.049548	15.0993342	19.0069859	17.1174039	0	-3.88503	down	PREDICTED: syntaxin-121 [Sesamum indicum]
c44134.graph_c0	2.58891767	2.49183662	3.58277415	11.4344019	13.3299705	13.5821967	9.105E-05	1.05114	up	PREDICTED: non-specific lipid-transfer protein 3 [Sesamum indicum]
c44139.graph_c0	41.9746165	44.0066107	42.3337558	32.9425083	33.5085874	37.8622045	1.911E-49	-1.38866	down	hypothetical protein F383_14596 [Gossypium arboreum]
c44142.graph_c0	1.29787847	1.85960787	0.75215647	15.9249647	18.4368642	15.5603659	7.917E-36	2.59031	up	PREDICTED: probable LRR receptor-like serine/threonine-protein kinase At1g56130 [Sesamum indicum]
c44144.graph_c0	0.98932602	1.01637765	0.86985265	6.64162616	6.09927453	5.8209581	2.025E-12	1.60128	up	hypothetical protein MIMGU_mgv1a003454mg [Erythranthe guttata]
c44152.graph_c0	78.1455376	76.3379313	70.460519	33.0904282	31.8350491	32.7277034	1.59E-165	-2.293	down	PREDICTED: WD repeat-containing protein 55 homolog [Sesamum indicum]

#ID	0 h-1 FPKM	0 h-2 FPKM	0 h-3 FPKM	28 h-1 FPKM	28 h-2 FPKM	28 h-3 FPKM	FDR	log2FC	regulated	nr_annotation
c44162.graph_c1	0	0	0	1.28415396	1.6368997	1.64370062	1.998E-15	Inf	up	hypothetical protein MIMGU_mgv1a0019072mg, partial [Erythranthe guttata]
c44163.graph_c0	0.24736507	0.30951596	0.27813967	17.05696	16.7197477	16.0455172	3.155E-93	4.80724	up	PREDICTED: probable LRR receptor-like serine/threonine-protein kinase At4g30520 [Sesamum indicum]
c44166.graph_c0	90.6443452	96.4799792	80.9080568	34.0177355	36.2058327	36.2100712	3.412E-86	-2.42126	down	PREDICTED: probable ADP-ribosylation factor GTPase-activating protein AGD14 [Sesamum indicum]
c44169.graph_c0	2.06064815	0.68757053	1.0788207	0	0	0	2.508E-08	-Inf	down	PREDICTED: vacuolar-processing enzyme-like [Musa acuminata subsp. malaccensis]
c44170.graph_c0	0.87792774	0.74507538	0.68671334	39.5743862	41.217624	38.7666974	0	4.6059	up	PREDICTED: pyruvate, phosphate dikinase, chloroplastic [Populus euphratica]
c44173.graph_c0	2.84483524	3.09411809	3.26906259	1.2723753	0.96933003	1.20047408	2.589E-27	-2.51116	down	hypothetical protein MIMGU_mgv1a025700mg, partial [Erythranthe guttata]
c44178.graph_c0	3.00183179	2.81703532	2.7859525	27.9229096	29.3156927	26.3206621	2.255E-86	2.18941	up	PREDICTED: LOW QUALITY PROTEIN: homeobox-leucine zipper protein ATHB-15 [Sesamum indicum]
c44178.graph_c1	1.11294206	0.92838042	1.43017461	11.5939449	15.8849535	12.3757383	4.581E-14	2.42565	up	PREDICTED: homeobox-leucine zipper protein ATHB-15-like [Sesamum indicum]
c44188.graph_c0	54.9997636	56.3748336	57.1204704	48.49933	45.7914711	47.6725473	4.121E-60	-1.33807	down	PREDICTED: uncharacterized protein LOC105157865 [Sesamum indicum]
c44190.graph_c0	0.14990815	0.18006991	0.07705516	1.96114281	2.34129603	2.11592118	2.619E-10	2.89606	up	PREDICTED: DNA replication complex GINS protein PSF1 isoform X2 [Sesamum indicum]
c44197.graph_c0	1.67694022	2.01434331	1.58028536	20.1506694	19.5513912	20.0396402	1.092E-59	2.41326	up	PREDICTED: 5'-methylthioadenosine/S-adenosylhomocysteine nucleosidase 1-like isoform X3 [Populus euphratica]
c44204.graph_c0	14.0562408	14.1952004	16.0855343	10.4577935	10.0427912	8.8294327	1.647E-25	-1.68915	down	PREDICTED: uncharacterized protein At2g39910 [Sesamum indicum]
c44207.graph_c0	35.3215556	37.9991985	35.5103109	27.4460862	27.336233	27.591134	3.986E-75	-1.4922	down	PREDICTED: stress response protein NST1-like [Sesamum indicum]
c44208.graph_c1	11.0236324	11.9504113	12.1085297	2.49333377	3.00166216	2.7398472	2.453E-83	-3.18268	down	PREDICTED: uncharacterized protein LOC105160931 [Sesamum indicum]
c44209.graph_c0	43.2573315	43.8281678	43.0590596	16.086646	15.562214	14.3506767	2.09E-167	-2.5913	down	PREDICTED: probable RNA methyltransferase At5g51130 [Sesamum indicum]
c44211.graph_c1	0.99313384	0.76021577	0.90085857	5.49844776	5.38628481	5.40481391	1.324E-09	1.52765	up	PREDICTED: glucose-6-phosphate 1-dehydrogenase 4, chloroplastic [Sesamum indicum]
c44213.graph_c0	4.06784255	1.9434144	1.90085045	0	0	0	1.993E-10	-Inf	down	PREDICTED: small heat shock protein, chloroplastic-like isoform X2 [Phoenix dactylifera]
c44215.graph_c0	157.672713	160.102614	147.915422	23.635769	21.499597	24.5606174	0	-3.82939	down	PREDICTED: PAX3- and PAX7-binding protein 1 [Sesamum indicum]
c44221.graph_c0	4.67099423	4.72173726	3.81434096	29.8178353	28.7195702	26.6100194	9.908E-50	1.60033	up	PREDICTED: kinesin-like protein KCA2 [Sesamum indicum]
c44223.graph_c0	11.8722385	12.1755631	9.43645589	104.602106	106.015464	106.515618	8.18E-132	2.15603	up	PREDICTED: 14-3-3 protein 7 isoform X1 [Sesamum indicum]
c44225.graph_c0	20.3383769	18.553534	20.8569969	13.2890702	12.8863658	12.9234004	1.039E-46	-1.70298	down	unnamed protein product [Coffea canephora]

#ID	0 h-1 FPKM	0 h-2 FPKM	0 h-3 FPKM	28 h-1 FPKM	28 h-2 FPKM	28 h-3 FPKM	FDR	log2FC	regulated	nr_annotation
c44229.graph_c0	1.1655477	1.27783052	1.2838071	10.649425	10.4167183	11.3560459	1.447E-14	2.02965	up	PREDICTED: uncharacterized protein LOC105172855 [Sesamum indicum]
c44231.graph_c0	121.952411	118.257214	112.915522	120.969953	126.220801	125.284584	6.927E-35	-1.01262	down	unnamed protein product [Coffea canephora]
c44232.graph_c0	68.9403254	69.4893841	64.7633131	25.9406389	24.6657416	27.2209677	2.31E-197	-2.4738	down	PREDICTED: WEB family protein At5g55860-like [Sesamum indicum]
c44236.graph_c0	3.04913507	2.93010079	3.13460743	25.9135235	24.571358	27.1247166	2.033E-42	1.99946	up	PREDICTED: actin-depolymerizing factor 5-like [Sesamum indicum]
c44238.graph_c0	3.02738353	2.37612085	1.85673776	58.9576341	57.3419712	52.2098379	1.72E-163	3.45133	up	hypothetical protein MIMGU_mgv1a008109mg [Erythranthe guttata]
c44241.graph_c0	4.34738849	1.90076076	2.37589603	0	0	0	6.602E-13	-Inf	down	DNAJ [Theobroma cacao]
c44242.graph_c0	5.65740275	7.69823276	6.9292059	38.1944672	37.0093904	37.8767111	1.788E-31	1.38676	up	PREDICTED: 50S ribosomal protein L4, chloroplastic [Sesamum indicum]
c44243.graph_c0	18.8366518	22.9335668	17.9010655	86.8224159	101.706149	87.6292071	1.163E-15	1.12058	up	PREDICTED: alcohol dehydrogenase-like 4 [Sesamum indicum]
c44249.graph_c0	0.59214869	0.52688146	0.33819312	4.44715933	3.88583992	4.60434186	1.112E-06	2.06692	up	PREDICTED: protein RADIALIS-like 4 [Solanum lycopersicum]
c44256.graph_c0	68.1445947	61.3118152	62.0497487	20.5614315	19.9529171	18.960724	4.46E-239	-2.77691	down	hypothetical protein MIMGU_mgv1a026885mg [Erythranthe guttata]
c44260.graph_c0	11.134091	11.3516334	10.3612637	49.8624223	50.4404297	50.2895906	1.53E-43	1.10727	up	PREDICTED: tripeptidyl-peptidase 2 isoform X4 [Sesamum indicum]
c44261.graph_c0	29.4386291	31.2518912	29.3799264	16.254093	16.8743868	17.1629071	4.71E-117	-1.93095	down	PREDICTED: phosphatidylinositol:ceramide inositolphosphotransferase 1 [Sesamum indicum]
c44264.graph_c0	1.39285728	1.32940308	1.1238761	0.42376213	0.51015663	0.51867966	1.661E-12	-2.49231	down	-
c44266.graph_c0	81.9136492	82.7533336	85.7732698	35.4720517	33.186903	36.6757377	4.92E-159	-2.34024	down	PREDICTED: interferon-related developmental regulator 1 [Sesamum indicum]
c44271.graph_c0	7.30424283	7.47915709	6.13198567	44.2187318	45.2267767	43.0081045	1.873E-65	1.57442	up	PREDICTED: DNA-directed RNA polymerases IV and V subunit 2-like [Sesamum indicum]
c44271.graph_c2	1.7037012	2.84234533	2.18932349	0.23585528	0.1597164	0.19245598	1.947E-15	-4.60953	down	-
c44274.graph_c0	0.62806356	0.05239104	0.60531491	2.01608898	2.3355297	2.82462652	0.006172	1.38798	up	PREDICTED: uncharacterized protein LOC105159603 [Sesamum indicum]
c44287.graph_c0	0.93233776	0.93327136	0.98025622	0.49281288	0.41715359	0.4775309	1.174E-07	-2.12722	down	-
c44292.graph_c0	10.4866312	13.452207	13.4191156	11.496482	11.6813903	11.0534205	3.624E-08	-1.21934	down	hypothetical protein MIMGU_mgv1a008797mg [Erythranthe guttata]
c44293.graph_c0	1.66438254	1.41251995	0.92991194	0.23667332	0.35615636	0.47207966	3.944E-10	-2.99338	down	-
c44295.graph_c0	0	0.11657946	0	13.8817012	23.5392279	14.3664012	3.422E-14	7.71176	up	hypothetical protein MIMGU_mgv1a006801mg [Erythranthe guttata]
c44295.graph_c2	0.0675832	0.06765088	0	14.5392485	25.2794581	15.9922275	7.703E-17	7.61708	up	PREDICTED: spermidine hydroxycinnamoyl transferase-like [Sesamum indicum]
c44297.graph_c0	19.1119704	17.7689248	18.3031484	11.516782	12.0258322	11.2269039	6.743E-67	-1.75687	down	PREDICTED: BTB/POZ domain-containing protein At5g48130 [Sesamum indicum]
c44299.graph_c0	4.37925802	4.64591243	5.14653533	2.32590655	2.25973041	2.51946205	2.827E-31	-2.08816	down	PREDICTED: DNA polymerase alpha subunit B isoform X1 [Sesamum indicum]
c44303.graph_c0	5251.31481	5319.33153	4740.25765	2581.7455	2630.93946	3104.19336	3.72E-24	-1.96877	down	hypothetical protein MIMGU_mgv1a024110mg [Erythranthe guttata]

#ID	0 h-1 FPKM	0 h-2 FPKM	0 h-3 FPKM	28 h-1 FPKM	28 h-2 FPKM	28 h-3 FPKM	FDR	log2FC	regulated	nr_annotation
c44309.graph_c0	3.24948701	1.87032601	1.77468077	0.05904271	0.10662006	0.09635674	4.511E-12	-5.80116	down	PREDICTED: probable rRNA-processing protein EBP2 homolog [Phoenix dactylifera]
c44315.graph_c0	46.691315	50.6506636	45.0560884	42.3308271	42.1698241	41.5302604	5.033E-56	-1.26607	down	PREDICTED: exocyst complex component EXO70B1 [Sesamum indicum]
c44315.graph_c1	32.2840125	30.3414526	27.2733577	30.3943556	26.7566817	28.4180688	8.138E-39	-1.15962	down	PREDICTED: translation initiation factor eIF-2B subunit beta [Sesamum indicum]
c44316.graph_c0	0.38917012	0.35414529	0.18185419	1.69707961	1.78768773	1.55265802	0.0020262	1.36245	up	PREDICTED: uncharacterized protein LOC105169064 [Sesamum indicum]
c44319.graph_c0	53.2209163	51.8539499	50.493235	20.9818353	18.2985744	20.0746804	3.35E-187	-2.4798	down	PREDICTED: F-box-like/WD repeat-containing protein TBL1XR1 [Sesamum indicum]
c44321.graph_c0	8.45361575	4.47281412	5.27642645	0	0	0	3.96E-24	-Inf	down	--
c44328.graph_c0	5.2166481	4.46543241	4.60480889	21.7209949	22.4434475	21.396206	1.595E-22	1.10866	up	PREDICTED: actin-related protein 5 [Sesamum indicum]
c44329.graph_c0	0.67141641	1.23805819	1.40772088	0.02568324	0.18551647	0.12574389	9.142E-08	-4.39775	down	hypothetical protein MIMGU_mgv1a000906mg [Erythranthe guttata]
c44346.graph_c0	30.9508983	32.4070009	32.3453971	33.1733027	34.436831	30.5140009	8.849E-35	-1.05548	down	hypothetical protein MIMGU_mgv1a012735mg [Erythranthe guttata]
c44347.graph_c0	16.3022131	17.386762	16.2575705	9.46031407	9.28814545	8.93534561	1.14E-108	-1.94188	down	hypothetical protein MIMGU_mgv1a000491mg [Erythranthe guttata]
c44349.graph_c0	13.9799296	14.1407179	13.7562347	65.8303019	66.8804431	69.2552003	3.078E-33	1.17978	up	PREDICTED: proteasome subunit alpha type-6-like [Sesamum indicum]
c44353.graph_c0	19.6075407	18.6032991	18.2902729	11.8314395	11.5814364	11.1657431	3.228E-92	-1.79838	down	PREDICTED: uncharacterized TPR repeat-containing protein At1g05150-like [Sesamum indicum]
c44355.graph_c0	0.31372567	0.45361306	0.5375332	5.95369903	6.77100883	5.27163105	7.688E-19	2.68825	up	PREDICTED: potassium transporter 8-like [Sesamum indicum]
c44355.graph_c1	0.09680832	0.13566737	0.14928304	4.62967038	6.41635237	5.40830148	5.618E-30	4.3341	up	PREDICTED: potassium transporter 6-like [Sesamum indicum]
c44358.graph_c0	16.7876969	15.2704858	16.1125059	16.5045296	17.004785	17.164944	9.042E-22	-1.01708	down	PREDICTED: protein HEADING DATE 3A-like [Sesamum indicum]
c44360.graph_c0	0.45240108	0.37737841	0.43601497	2.74002079	2.99351807	2.07932617	6.825E-05	1.53371	up	PREDICTED: probable WRKY transcription factor 30 [Sesamum indicum]
c44363.graph_c0	93.8099031	89.480965	90.4238108	36.3593931	36.6871439	34.4549838	1.09E-189	-2.43879	down	PREDICTED: pentatricopeptide repeat-containing protein At2g17210 isoform X1 [Sesamum indicum]
c44363.graph_c1	19.1002982	20.8752897	19.4639706	12.263096	10.907984	11.3275877	2.537E-82	-1.87541	down	PREDICTED: uncharacterized protein LOC105157994 [Sesamum indicum]
c44364.graph_c0	52.6839602	53.3589774	51.5531653	45.5031286	46.5630666	48.0059012	1.129E-54	-1.2601	down	PREDICTED: probable pre-mRNA-splicing factor ATP-dependent RNA helicase [Sesamum indicum]
c44366.graph_c0	53.6055241	58.5797295	50.5884348	37.7282433	37.6245182	38.6771912	1.141E-62	-1.60286	down	PREDICTED: rac-like GTP-binding protein RAC1 [Sesamum indicum]
c44371.graph_c0	2.16573758	1.933538	1.88044496	10.7843394	9.21869571	9.94548628	7.877E-12	1.23547	up	-
c44373.graph_c0	0.43515432	0.91473912	1.00654295	12.9986004	13.3356536	13.6778064	7.598E-36	2.98745	up	PREDICTED: inositol polyphosphate multikinase alpha [Nicotiana sylvestris]
c44376.graph_c0	8.47384316	6.79550175	6.99137674	3.81421989	4.13897858	3.85477301	1.018E-26	-2.0035	down	hypothetical protein F383_25407 [Gossypium arboreum]

#ID	0 h-1 FPKM	0 h-2 FPKM	0 h-3 FPKM	28 h-1 FPKM	28 h-2 FPKM	28 h-3 FPKM	FDR	log2FC	regulated	nr_annotation
c44380.graph_c0	4.22351861	4.35064747	4.19675981	22.2369635	21.6731671	20.7956002	5.052E-27	1.25042	up	PREDICTED: peptidyl-prolyl cis-trans isomerase FKBP43-like isoform X3 [Sesamum indicum]
c44384.graph_c0	0.29916589	0.19964364	0.89702671	2.53670723	2.55216979	2.72048256	0.0027739	1.37612	up	-
c44385.graph_c0	17.8906258	15.8392764	15.1657794	15.4340002	14.6260617	16.1381754	2.909E-27	-1.17016	down	PREDICTED: uncharacterized protein KIAA0930 homolog [Sesamum indicum]
c44385.graph_c1	3.22203769	2.89056686	3.47641221	0.53020946	0.25931177	0.3965927	4.987E-50	-4.10642	down	hypothetical protein MIMGU_mgv1a001279mg [Erythranthe guttata]
c44391.graph_c0	0.34419685	0.68908303	0.32167752	89.6938349	83.7982475	91.0970957	0	6.52204	up	PREDICTED: raucaffricine-O-beta-D-glucosidase-like [Sesamum indicum]
c44391.graph_c1	10.1422808	7.18655639	7.76136313	8.696538	7.85215844	7.97489401	4.658E-06	-1.12081	down	-
c44394.graph_c0	0.63478391	0.37248732	0.50631049	10.2612225	8.92018055	10.1255776	1.161E-53	3.18692	up	PREDICTED: protein ENHANCED DISEASE RESISTANCE 2 [Sesamum indicum]
c44395.graph_c0	4.97552992	5.53050737	5.9230472	3.63837167	3.52548228	3.74730783	1.545E-22	-1.68277	down	unnamed protein product [Coffea canephora]
c44396.graph_c0	46.7776308	48.8594674	50.3395987	9.67506987	8.00450062	8.24256558	2.45E-193	-3.58504	down	PREDICTED: uncharacterized protein LOC105156238 [Sesamum indicum]
c44409.graph_c0	31.0580677	30.7784702	30.3880838	29.6647239	27.5482799	27.3009731	2.595E-49	-1.2164	down	PREDICTED: nuclear poly(A) polymerase 1 isoform X1 [Sesamum indicum]
c44411.graph_c0	0.59465044	0.51021077	0.21832839	3.08706081	2.00687506	2.16635411	0.0047794	1.37601	up	PREDICTED: uncharacterized protein LOC105170709 [Sesamum indicum]
c44415.graph_c0	6.97970619	5.46352951	5.31350639	31.7351161	34.3411864	30.8393002	9.454E-33	1.36043	up	PREDICTED: serine/threonine-protein kinase CDL1 [Sesamum indicum]
c44423.graph_c0	8.665834	7.59822953	6.57845331	64.2191107	58.3629528	60.9583253	3.412E-95	1.91908	up	PREDICTED: cytochrome P450 734A1 [Sesamum indicum]
c44427.graph_c1	5.54168326	6.25538975	4.24246611	1.24257702	0.92849425	1.0488959	2.428E-24	-3.40331	down	hypothetical protein MIMGU_mgv1a004986mg [Erythranthe guttata]
c44434.graph_c0	20.6673665	19.0927137	19.6435147	20.6132897	19.8376646	19.1070205	2.317E-37	-1.08658	down	PREDICTED: COP9 signalosome complex subunit 6a [Sesamum indicum]
c44435.graph_c0	7.31099625	8.37849525	7.63750147	44.5788523	40.4259663	42.1632056	6.989E-60	1.35601	up	hypothetical protein MIMGU_mgv1a001619mg [Erythranthe guttata]
c44436.graph_c0	23.0952057	24.0459195	22.7624878	18.9484039	18.9914003	18.8225318	9.474E-61	-1.39071	down	PREDICTED: LOW QUALITY PROTEIN: U-box domain-containing protein 4-like [Sesamum indicum]
c44438.graph_c0	4.91872847	5.26053541	6.2542265	33.3218637	33.0764833	31.458535	6.31E-47	1.48016	up	PREDICTED: prolyl-tRNA synthetase associated domain-containing protein 1 [Sesamum indicum]
c44443.graph_c0	46.5279596	49.3392497	45.4569863	42.1802424	40.8731973	41.3696795	4.037E-52	-1.27381	down	PREDICTED: F-box/FBD/LRR-repeat protein At1g13570-like [Sesamum indicum]
c44445.graph_c0	0.50015202	1.03981745	0.7415944	3.41137906	2.97916674	3.60506457	0.0017492	1.03928	up	PREDICTED: RING-H2 zinc finger protein RHA4a [Eucalyptus grandis]
c44446.graph_c0	1751.22491	1783.3599	1734.69118	419.413553	411.576959	448.032465	5.72E-162	-3.13255	down	PREDICTED: REF/SRPP-like protein At3g05500 [Sesamum indicum]
c44457.graph_c0	0.35205536	0.48456085	0.39585444	12.9215262	13.6595453	11.9531877	1.461E-43	3.87494	up	hypothetical protein MIMGU_mgv1a011349mg [Erythranthe guttata]
c44460.graph_c0	8.76199046	11.8956773	8.32663966	47.4371031	52.6438836	42.4733233	1.895E-11	1.20831	up	PREDICTED: ammonium transporter 1 member 1-like [Sesamum indicum]
c44463.graph_c1	9.39043954	8.11804592	8.36468502	1.31844994	1.22544952	1.61376658	3.039E-67	-3.72576	down	PREDICTED: metalloendoproteinase 1-like [Sesamum indicum]
c44465.graph_c0	4.59300877	4.27684462	3.84329179	19.1750828	21.7993396	19.7325929	7.101E-12	1.16685	up	PREDICTED: tubulin-folding cofactor B isoform X2 [Sesamum indicum]

#ID	0 h-1 FPKM	0 h-2 FPKM	0 h-3 FPKM	28 h-1 FPKM	28 h-2 FPKM	28 h-3 FPKM	FDR	log2FC	regulated	nr_annotation
c44467.graph_c0	0.91189746	0.58891006	0.79381649	8.78693895	8.35846135	8.35635945	1.658E-25	2.38446	up	PREDICTED: probable LRR receptor-like serine/threonine-protein kinase At1g51880 isoform X1 [Sesamum indicum]
c44468.graph_c0	3.54054424	3.44282986	3.76978418	19.6301997	17.1931668	20.2775728	4.856E-08	1.31785	up	PREDICTED: transcription factor TCP2-like [Sesamum indicum]
c44471.graph_c0	7.38942699	6.86303479	6.460996	1.63332466	2.17461518	2.57520123	3.857E-50	-2.78592	down	PREDICTED: probable WRKY transcription factor 31 [Sesamum indicum]
c44480.graph_c0	0.55805642	0.71096484	0.45635201	20.7589457	15.9135638	16.6081702	4.579E-32	3.86102	up	hypothetical protein M569_00618, partial [Genlisea aurea]
c44483.graph_c0	48.5004093	49.8281828	47.936251	21.8575408	22.3949046	21.617785	1.32E-162	-2.24133	down	PREDICTED: uncharacterized protein LOC105160446 [Sesamum indicum]
c44495.graph_c0	29.1235878	29.4973458	31.4971323	24.719685	24.8045289	25.846724	1.549E-33	-1.34917	down	PREDICTED: structural maintenance of chromosomes protein 1A [Sesamum indicum]
c44496.graph_c0	2.49586733	2.58921626	3.20728834	17.7770235	16.5274526	16.9818677	2.347E-18	1.53484	up	PREDICTED: uncharacterized protein LOC105165753 [Sesamum indicum]
c44497.graph_c0	14.3968365	14.5867619	14.3948448	6.20166382	5.7529325	5.01429236	4.4E-132	-2.44534	down	PREDICTED: uncharacterized protein LOC105166424 [Sesamum indicum]
c44501.graph_c0	3.76538453	3.50231216	2.6548445	20.5370574	19.5817098	19.7322335	9.574E-44	1.50628	up	PREDICTED: calcium-transporting ATPase 3, endoplasmic reticulum-type [Sesamum indicum]
c44504.graph_c0	0.05332012	0.10674702	0.06851846	1.10445348	0.85725753	0.85379213	8.052E-07	2.53102	up	-
c44505.graph_c0	4.36260543	3.87721983	2.56728892	19.1428303	22.8761219	20.3355274	1.935E-14	1.44393	up	hypothetical protein CICLE_v10012334mg [Citrus clementina]
c44506.graph_c0	1.47243791	1.2452018	1.4027942	11.587243	12.0115317	9.78632515	3.324E-28	1.92707	up	PREDICTED: uncharacterized protein LOC105166535 [Sesamum indicum]
c44511.graph_c0	0.36177906	0.18107066	0.17433766	1.90630729	1.39477909	1.26051665	0.0008772	1.58593	up	PREDICTED: uncharacterized protein LOC105162704 [Sesamum indicum]
c44513.graph_c0	4.57034652	6.6336384	4.84528789	39.071514	38.5395276	37.4385231	3.223E-36	1.75142	up	PREDICTED: uncharacterized protein LOC105176019 [Sesamum indicum]
c44514.graph_c0	609.893772	607.120252	654.001086	265.163901	262.847381	289.97041	2.627E-85	-2.28473	down	PREDICTED: alcohol dehydrogenase 1 [Sesamum indicum]
c44519.graph_c0	37.7609227	39.1440583	33.0639288	14.0517318	13.5693511	14.8848011	1.449E-97	-2.45969	down	PREDICTED: calmodulin-binding transcription activator 4 [Sesamum indicum]
c44531.graph_c0	1.72449521	1.50701924	2.04016807	8.59442182	9.55624347	8.11687271	1.392E-11	1.223	up	Alg9-like mannosyltransferase family isoform 3 [Theobroma cacao]
c44533.graph_c0	4.79128194	3.6333937	5.3640417	24.8507886	23.366891	21.2252101	3.162E-15	1.23884	up	unnamed protein product [Coffea canephora]
c44536.graph_c1	5.3411715	3.82698265	3.46793272	23.1281462	24.0186192	27.708386	9.357E-14	1.48167	up	uncharacterized protein LOC100499956 [Glycine max]
c44537.graph_c0	45.0239036	45.130078	44.9567077	47.837178	46.9270247	45.8662797	1.992E-37	-1.03289	down	PREDICTED: probable UDP-N-acetylglucosamine--peptide N-acetylglucosaminyltransferase SEC [Sesamum indicum]
c44546.graph_c0	329.500278	332.973957	392.704109	14.646446	17.9759801	15.779961	1.213E-87	-5.53954	down	--
c44547.graph_c0	0	0	0	2.46352871	3.37020522	4.93417466	1.471E-07	Inf	up	-

#ID	0 h-1 FPKM	0 h-2 FPKM	0 h-3 FPKM	28 h-1 FPKM	28 h-2 FPKM	28 h-3 FPKM	FDR	log2FC	regulated	nr_annotation
c44548.graph_c0	127.798123	118.148303	121.648628	16.9156945	16.1635684	17.0214006	0	-3.96515	down	PREDICTED: uncharacterized protein LOC102619543 isoform X1 [Citrus sinensis]
c44550.graph_c0	0.04855391	0.12960675	0.0831916	0.62343376	0.94525092	1.09422116	3.159E-05	2.25716	up	hypothetical protein MIMGU_mgv1a020534mg, partial [Erythranthe guttata]
c44555.graph_c0	7.06763217	7.67468468	6.57896949	5.79014795	5.67045269	5.80591746	1.5E-26	-1.39361	down	PREDICTED: protein tesmin/TSO1-like CXC 5 [Sesamum indicum]
c44559.graph_c0	0.15133577	0.23805149	0.08334534	0.81706832	0.99310627	1.33344216	0.0012468	1.65201	up	PREDICTED: probable receptor-like protein kinase At5g24010 [Sesamum indicum]
c44560.graph_c1	0.60215378	0.5241363	0.40371728	5.70837255	5.77262022	6.11748747	2.635E-20	2.43836	up	PREDICTED: uncharacterized protein LOC102583109 [Solanum tuberosum]
c44561.graph_c0	2.36033552	4.4300607	6.82453034	92.6358257	57.1162783	97.6371831	2.271E-09	3.08291	up	BnaCnng13060D [Brassica napus]
c44563.graph_c0	71.2716066	72.9695942	61.399815	13.1157076	12.7402451	12.7649988	2.84E-147	-3.50125	down	PREDICTED: uncharacterized protein LOC105165633 [Sesamum indicum]
c44566.graph_c0	82.9759821	81.9871424	73.9550815	56.8597465	54.1699944	54.1813481	3.1E-90	-1.62135	down	hypothetical protein MIMGU_mgv1a005930mg [Erythranthe guttata]
c44568.graph_c0	103.587954	104.441589	109.834879	108.451084	116.535566	101.359517	2.506E-33	-1.05388	down	PREDICTED: probable cyclic nucleotide-gated ion channel 17 [Sesamum indicum]
c44568.graph_c1	15.5801375	10.1466252	10.1311558	5.59356291	6.52864711	5.78887039	1.224E-07	-2.08758	down	-
c44571.graph_c1	0.57240976	0.4456534	0.57210938	3.0970684	3.08851965	3.20612708	0.0004527	1.47113	up	PREDICTED: probable serine/threonine-protein kinase At1g09600 isoform X2 [Sesamum indicum]
c44571.graph_c2	32.2304616	31.0452738	26.2766173	29.4468845	27.9845069	34.036501	1.745E-16	-1.05658	down	PREDICTED: probable serine/threonine-protein kinase At1g09600 isoform X1 [Sesamum indicum]
c44572.graph_c0	5.6724729	3.15452946	3.41326812	0	0	0	3.124E-26	-Inf	down	--
c44575.graph_c0	7.21038567	7.62614951	6.55587103	2.66967174	2.83452626	2.64235532	5.012E-49	-2.48223	down	unknown protein [Arabidopsis thaliana]
c44583.graph_c0	2.41395468	1.89519364	2.00719416	0	0.03106083	0.02807089	6.221E-41	-7.82607	down	actin [Dendrobium catenatum]
c44585.graph_c0	0.95387859	0.65102302	1.50435625	7.68903295	7.48307847	7.30274353	1.741E-11	1.75501	up	PREDICTED: methyltransferase-like protein 6 isoform X3 [Sesamum indicum]
c44590.graph_c0	3.80051416	1.9021599	2.80819142	0.96676713	0.93525008	0.95791853	4.941E-06	-2.6611	down	hypothetical protein MIMGU_mgv1a013990mg [Erythranthe guttata]
c44591.graph_c0	56.0964009	60.1213003	52.3803175	29.4586209	29.5656932	30.3924194	5.87E-109	-2.00428	down	PREDICTED: uncharacterized protein LOC105165989 [Sesamum indicum]
c44592.graph_c0	38.7344985	39.1203607	40.2913114	30.276011	27.6614457	28.0831852	1.382E-61	-1.54893	down	PREDICTED: E3 ubiquitin-protein ligase RNF185-like [Sesamum indicum]
c44594.graph_c0	154.948309	150.189297	159.134055	85.78851	80.881915	85.451726	2.15E-123	-1.97168	down	PREDICTED: uncharacterized protein LOC105169586 [Sesamum indicum]
c44595.graph_c0	0.15148391	0.1010904	0.12977522	4.44060466	6.13015031	4.94113246	4.317E-19	4.25229	up	PREDICTED: putative 4-hydroxy-tetrahydrodipicolinate reductase 3, chloroplastic isoform X2 [Sesamum indicum]
c44602.graph_c0	30.5111821	30.7493927	28.2782103	23.1379842	25.5828393	25.2306581	4.955E-63	-1.36538	down	PREDICTED: uncharacterized protein At1g51745 [Sesamum indicum]

#ID	0 h-1 FPKM	0 h-2 FPKM	0 h-3 FPKM	28 h-1 FPKM	28 h-2 FPKM	28 h-3 FPKM	FDR	log2FC	regulated	nr_annotation
c44606.graph_c0	0	0	0.06420405	4.28488718	4.16393326	5.33354179	9.839E-23	6.61695	up	--
c44609.graph_c0	21.8897191	20.2400288	20.8213751	18.6975533	15.8751329	17.3983913	1.133E-36	-1.36644	down	PREDICTED: extra-large guanine nucleotide-binding protein 3-like [Sesamum indicum]
c44609.graph_c1	38.9641467	34.7739047	39.2118148	37.3946185	32.7164162	32.7966628	1.132E-26	-1.22549	down	Extra-large GTP-binding protein 3 isoform 2 [Theobroma cacao]
c44609.graph_c2	47.058659	50.3437491	47.0909062	43.7025072	37.3947065	38.7618358	1.094E-55	-1.36009	down	PREDICTED: extra-large guanine nucleotide-binding protein 3 [Sesamum indicum]
c44611.graph_c0	8.04535954	7.93795459	9.07870379	5.09282792	5.58235433	5.45543297	1.824E-28	-1.72792	down	PREDICTED: MACPF domain-containing protein NSL1 [Sesamum indicum]
c44615.graph_c0	10.7782511	12.0866952	10.2569701	9.19300489	10.0747429	10.3899567	2.435E-28	-1.24866	down	PREDICTED: pentatricopeptide repeat-containing protein At3g22470, mitochondrial-like [Sesamum indicum]
c44618.graph_c1	0.89725626	0.73485387	0.8385526	8.73446486	9.3316486	7.8367522	1.532E-26	2.29924	up	hypothetical protein MIMGU_mgv1a005181mg [Erythranthe guttata]
c44622.graph_c0	446.771074	481.905289	406.651674	39.0185529	38.4278615	44.5348281	1.82E-215	-4.54087	down	PREDICTED: telomere repeat-binding protein 2-like [Sesamum indicum]
c44627.graph_c0	7.23527912	6.6917173	7.34181007	4.6288935	5.10907867	4.63810487	1.354E-59	-1.65637	down	PREDICTED: uncharacterized protein LOC105176363 [Sesamum indicum]
c44631.graph_c0	4.01527079	5.80103925	5.37255645	3.42972605	4.21045406	2.77407907	2.933E-07	-1.6392	down	PREDICTED: WD repeat-containing protein 48-like [Sesamum indicum]
c44633.graph_c0	3.33840862	3.23853141	3.04771295	14.0989803	14.4471348	13.3775033	1.018E-22	1.0332	up	PREDICTED: proline-rich receptor-like protein kinase PERK1 isoform X1 [Sesamum indicum]
c44635.graph_c0	17.0762981	18.2978102	15.9671777	4.17061903	4.6463607	3.93837031	2.68E-193	-3.09912	down	PREDICTED: uncharacterized protein LOC105175356 [Sesamum indicum]
c44641.graph_c0	20.7809077	21.1100979	22.0616297	13.5768155	11.615337	12.5402111	1.715E-82	-1.85239	down	PREDICTED: chloroplast envelope membrane protein [Sesamum indicum]
c44642.graph_c0	23.2092946	10.7227086	13.4375754	0	0	0	7.635E-15	-Inf	down	-
c44646.graph_c1	3.70834971	3.87345711	3.35648237	3.18737547	3.21647781	3.65269573	1.823E-09	-1.20974	down	PREDICTED: non-specific lipid-transfer protein-like protein At2g13820 [Sesamum indicum]
c44646.graph_c3	82.7252259	84.897333	78.015323	65.2168373	68.6027829	67.8049915	6.771E-63	-1.37454	down	PREDICTED: heat shock 70 kDa protein, mitochondrial [Sesamum indicum]
c44656.graph_c0	73.4399385	72.7223295	71.4604	28.4226305	31.7208422	29.9423097	3.48E-177	-2.36279	down	PREDICTED: MLO-like protein 10 isoform X1 [Sesamum indicum]
c44657.graph_c0	140.474524	138.727925	131.191827	142.631816	140.564581	146.042065	6.991E-36	-1.02475	down	PREDICTED: very-long-chain 3-oxoacyl-CoA reductase 1-like [Sesamum indicum]
c44658.graph_c0	62.4680854	67.0787825	61.4205088	44.4236807	43.0587342	44.4084776	3.237E-89	-1.624	down	PREDICTED: serine/threonine-protein kinase CDL1 [Sesamum indicum]
c44658.graph_c1	0.04040068	0.08088227	0.05191647	3.55292078	4.34797118	3.80963257	2.502E-36	4.98772	up	PREDICTED: protein LONGIFOLIA 1 [Sesamum indicum]
c44661.graph_c0	4.29673155	2.88213624	2.56150677	1.44874008	0.98832444	0.76183649	9.714E-09	-2.69274	down	hypothetical protein MIMGU_mgv1a0244072mg, partial [Erythranthe guttata]
c44662.graph_c0	0.32044688	0.48115163	0.51473365	2.73656258	2.20800204	2.80314362	0.0011748	1.46266	up	PREDICTED: transcription factor JUNGBRUNNEN 1 [Sesamum indicum]
c44663.graph_c0	46.4128499	47.6857362	43.0184339	39.4419902	40.5073532	39.1725755	2.437E-53	-1.29256	down	hypothetical protein MIMGU_mgv1a008429mg [Erythranthe guttata]

#ID	0 h-1 FPKM	0 h-2 FPKM	0 h-3 FPKM	28 h-1 FPKM	28 h-2 FPKM	28 h-3 FPKM	FDR	log2FC	regulated	nr_annotation
c44666.graph_c0	14.6297915	14.0954958	12.7302567	65.1856589	66.5763801	66.6303395	2.076E-46	1.16993	up	PREDICTED: coatomer subunit beta'-2-like isoform X1 [Sesamum indicum]
c44670.graph_c0	24.0734273	21.8559022	26.402925	23.4777653	20.5736632	22.477884	4.917E-13	-1.21255	down	PREDICTED: peroxisome biogenesis factor 10 isoform X1 [Sesamum indicum]
c44676.graph_c1	2.62481372	2.35183626	2.57102001	13.0070419	13.105255	12.3444798	4.044E-23	1.25828	up	PREDICTED: zinc-metallopeptidase, peroxisomal isoform X1 [Sesamum indicum]
c44677.graph_c2	116.144763	116.617148	120.451878	49.1549377	50.8018306	49.524413	7.75E-174	-2.33168	down	PREDICTED: putative peptidyl-tRNA hydrolase PTRHD1 [Sesamum indicum]
c44677.graph_c3	42.1865402	43.3901115	40.5513753	37.0587298	36.753321	35.4945721	1.808E-58	-1.29663	down	PREDICTED: DEAD-box ATP-dependent RNA helicase 37-like [Sesamum indicum]
c44677.graph_c4	14.6817979	11.8388469	14.3746222	8.89220511	9.51989545	9.12178678	1.089E-19	-1.6617	down	PREDICTED: purple acid phosphatase 17-like [Sesamum indicum]
c44680.graph_c0	0.25574099	0.44799488	0.24647797	3.39214712	2.7271425	2.73004703	5.631E-06	2.13055	up	PREDICTED: uncharacterized protein LOC102579233 [Solanum tuberosum]
c44681.graph_c0	66.765262	68.2191021	64.4953827	30.062132	30.8813819	26.5695863	4.87E-149	-2.27937	down	PREDICTED: tubby-like F-box protein 8 [Sesamum indicum]
c44681.graph_c1	39.3070642	51.1637803	41.8914863	23.0105217	23.9456164	21.5610331	1.331E-18	-2.04094	down	unknown [Lotus japonicus]
c44683.graph_c0	640.030326	738.274837	640.229928	54.7660068	52.753344	58.4621759	7.6E-166	-4.6939	down	PREDICTED: protein FAR1-RELATED SEQUENCE 5 [Sesamum indicum]
c44684.graph_c0	0	0	0	2.7830699	2.72225545	3.59569033	2.091E-14	Inf	up	-
c44686.graph_c0	2.00148416	1.9331905	2.1207607	11.0248458	11.2217449	10.5371971	1.151E-12	1.34497	up	PREDICTED: cytochrome P450 734A1-like [Sesamum indicum]
c44689.graph_c0	3.59108013	3.85770113	4.61468014	27.7548703	31.7273972	31.6341244	1.206E-21	1.82349	up	PREDICTED: uncharacterized protein LOC105180344 [Sesamum indicum]
c44691.graph_c0	1.36869887	1.41900047	1.75883224	0.07105446	0.09623327	0.11595972	3.536E-25	-5.09712	down	PREDICTED: uncharacterized protein LOC103492548 [Cucumis melo]
c44694.graph_c0	3.84319686	2.42221367	1.64622004	10.6555916	12.516641	13.4221868	0.0014381	1.12921	up	PREDICTED: crocetin glucosyltransferase, chloroplastic-like [Sesamum indicum]
c44702.graph_c0	8.86931675	9.59035295	9.99560107	38.1599375	58.5763501	45.2305523	3.398E-05	1.22616	up	PREDICTED: hydroxymethylglutaryl-CoA synthase isoform X1 [Sesamum indicum]
c44705.graph_c0	2.01307151	1.88366856	1.68709343	8.04698826	8.64414408	8.1754038	6.691E-06	1.06631	up	PREDICTED: pyridoxal kinase isoform X1 [Sesamum indicum]
c44710.graph_c0	14.711464	14.7817658	15.908579	3.14714024	2.73228131	3.9179083	5.955E-93	-3.30233	down	PREDICTED: uncharacterized protein LOC100244117 [Vitis vinifera]
c44710.graph_c1	13.4005448	13.3491617	12.7279965	4.98734544	5.86253334	4.99105952	4.065E-45	-2.4078	down	PREDICTED: dehydration-responsive element-binding protein 1D-like [Sesamum indicum]
c44711.graph_c0	32.1643932	32.7140464	31.3315253	12.3146147	13.2485026	12.0413184	1.65E-159	-2.44579	down	PREDICTED: gamma-glutamyltranspeptidase 3 [Sesamum indicum]
c44714.graph_c0	4.70995938	5.04360655	4.081908	25.9524152	23.7919933	23.9052787	4.759E-09	1.32369	up	hypothetical protein MIMGU_mgv1a014633mg [Erythranthe guttata]
c44715.graph_c0	6.48985561	6.67075299	7.16432442	0.53815721	0.4859055	0.6199509	1.473E-84	-4.71873	down	PREDICTED: uncharacterized membrane protein At1g06890 isoform X2 [Sesamum indicum]

#ID	0 h-1 FPKM	0 h-2 FPKM	0 h-3 FPKM	28 h-1 FPKM	28 h-2 FPKM	28 h-3 FPKM	FDR	log2FC	regulated	nr_annotation
c44722.graph_c0	7.45885883	8.15711004	7.33964044	5.7732108	6.64831971	6.30441115	1.793E-40	-1.38394	down	PREDICTED: protein NRT1/ PTR FAMILY 5.5-like isoform X2 [Sesamum indicum]
c44724.graph_c0	73.364664	73.0149469	72.4625209	57.315364	57.9604479	56.7976574	7.143E-70	-1.43734	down	PREDICTED: cytochrome P450 78A3-like [Sesamum indicum]
c44725.graph_c0	1.78859779	1.15096423	1.64172784	16.0171495	15.3422789	16.0247888	5.959E-21	2.28023	up	hypothetical protein MIMGU_mgv1a006677mg [Erythranthe guttata]
c44728.graph_c0	67.008013	63.5359012	65.2806575	321.398068	316.69831	327.462089	3.793E-52	1.21159	up	PREDICTED: ruBisCO large subunit-binding protein subunit beta, chloroplastic [Sesamum indicum]
c44735.graph_c0	2.99180356	1.68754569	2.13588113	0	0	0	5.15E-36	-Inf	down	hypothetical protein SELMODRAFT_444263 [Selaginella moellendorffii]
c44740.graph_c0	5.70418914	5.00137315	6.74155999	39.4907459	33.7711578	33.6069239	2.172E-21	1.52123	up	PREDICTED: transcription factor TGA1-like [Sesamum indicum]
c44741.graph_c0	6.68674714	6.65646256	5.83926178	28.1121773	31.7101429	30.7610192	3.588E-22	1.1507	up	PREDICTED: aspartate-semialdehyde dehydrogenase [Sesamum indicum]
c44742.graph_c0	10.7137706	11.3754077	11.579125	9.45207174	9.87544428	10.8163025	8.404E-26	-1.25042	down	PREDICTED: probable zinc metallopeptidase EGY3, chloroplastic [Sesamum indicum]
c44743.graph_c0	28.4500503	27.1378741	26.1604722	11.3633111	11.1389048	11.7388694	9.56E-172	-2.34481	down	PREDICTED: COBRA-like protein 7 [Sesamum indicum]
c44744.graph_c0	3.37918669	3.28308307	4.34238868	0.65011061	0.71743091	0.67784188	1.559E-21	-3.52194	down	PREDICTED: serine/threonine-protein kinase Nek2 [Sesamum indicum]
c44747.graph_c0	2.83974747	2.43650661	2.22243776	131.004104	136.261078	115.964753	1.06E-189	4.58701	up	PREDICTED: endoplasmin homolog [Sesamum indicum]
c44753.graph_c0	0	0	0	5.76830795	7.13721949	13.5279568	1.207E-08	Inf	up	apocytochrome b [Chara vulgaris]
c44762.graph_c1	2.42039588	2.4810604	3.05049099	26.8013571	26.4899721	26.1483906	8.12E-124	2.22674	up	PREDICTED: LOW QUALITY PROTEIN: cullin-associated NEDD8-dissociated protein 1 [Sesamum indicum]
c44762.graph_c2	63.0526965	55.3339584	60.2053126	50.0014582	46.681614	41.4331937	2.301E-42	-1.46173	down	PREDICTED: zeatin O-glucosyltransferase-like [Sesamum indicum]
c44762.graph_c4	165.397321	146.537528	173.860517	75.3327334	73.1888906	78.2356849	1.866E-36	-2.19051	down	PREDICTED: uncharacterized protein LOC105162036 [Sesamum indicum]
c44762.graph_c5	0	0	0	22.7424718	20.5343197	20.6519539	1.267E-50	Inf	up	unnamed protein product [Coffea canephora]
c44762.graph_c6	5.31181574	4.9787716	5.58482198	25.8660003	23.8932811	22.9679361	1.803E-14	1.10397	up	PREDICTED: UPF0496 protein At1g20180-like isoform X2 [Nicotiana sylvestris]
c44767.graph_c0	0.9973398	0.51996796	0.77431277	32.6942432	37.4008825	29.8820882	6.28E-80	4.3578	up	PREDICTED: putative E3 ubiquitin-protein ligase XBAT31 [Sesamum indicum]
c44770.graph_c0	2.96117197	3.02892701	2.76554474	25.9200362	24.8050158	24.4805001	8.015E-84	2.01284	up	PREDICTED: SNF2 domain-containing protein CLASSY 3-like [Sesamum indicum]
c44773.graph_c0	12.3859457	12.6237729	13.0225341	13.3230103	11.7486439	12.0066945	2.138E-29	-1.1279	down	PREDICTED: zinc finger CCCH domain-containing protein 5 [Sesamum indicum]
c44775.graph_c0	3.92970027	2.89846809	2.74639356	16.3347934	17.825221	18.3172331	1.541E-10	1.36814	up	hypothetical protein MIMGU_mgv1a015597mg [Erythranthe guttata]
c44781.graph_c0	1.06605236	1.3042576	1.48407945	13.6451014	13.6416618	14.0671409	4.383E-39	2.32924	up	PREDICTED: protein S-acyltransferase 21 [Sesamum indicum]
c44781.graph_c1	1.50655171	0.94253768	1.20998662	14.7818669	12.4815798	15.7474579	3.58E-09	2.46735	up	-
c44785.graph_c0	59.8466496	56.3426245	61.1143862	26.7033064	27.6720038	29.4879182	1.35E-104	-2.17076	down	PREDICTED: phytosulfokine receptor 2-like [Sesamum indicum]
c44796.graph_c0	23.0028605	22.5559782	20.4730299	13.7542519	14.2864333	14.2858547	2.495E-74	-1.73061	down	PREDICTED: AMP deaminase-like [Sesamum indicum]

#ID	0 h-1 FPKM	0 h-2 FPKM	0 h-3 FPKM	28 h-1 FPKM	28 h-2 FPKM	28 h-3 FPKM	FDR	log2FC	regulated	nr_annotation
c44797.graph_c0	0	0	0.05621366	0.85842147	1.03343214	0.95989636	1.177E-09	4.53451	up	hypothetical protein MIMGU_mgv1a000411mg [Erythranthe guttata]
c44797.graph_c1	84.3931498	82.4960087	75.5298408	77.0050918	70.2298975	71.5118067	4.477E-53	-1.23745	down	PREDICTED: uncharacterized protein LOC105172555 [Sesamum indicum]
c44802.graph_c0	77.6366872	74.9863793	74.1287236	5.67560213	5.42260806	6.29932204	0	-4.79353	down	PREDICTED: adagio protein 3 isoform X1 [Sesamum indicum]
c44803.graph_c0	15.1091255	16.79711	15.3847965	15.8496017	14.1399107	14.6147795	1.036E-38	-1.17487	down	PREDICTED: ribosome biogenesis protein BMS1 homolog [Sesamum indicum]
c44812.graph_c0	5.76585752	5.14920034	4.06788128	28.6904911	28.625139	28.6298381	7.778E-47	1.43374	up	PREDICTED: pyrophosphate-energized membrane proton pump 3 [Sesamum indicum]
c44815.graph_c0	16.7146686	17.0005437	16.929837	3.76711287	4.53840035	4.23440962	4.72E-237	-3.10464	down	PREDICTED: LOW QUALITY PROTEIN: leucine-rich repeat receptor-like protein kinase PXL2 [Sesamum indicum]
c44820.graph_c0	71.7394847	67.3604614	60.3653355	54.8627856	52.77801	52.8982673	5.646E-56	-1.40171	down	PREDICTED: protein TIME FOR COFFEE [Sesamum indicum]
c44826.graph_c0	2.38916166	1.73931203	2.58173162	0.55250011	0.71265114	1.03048133	1.15E-11	-2.63772	down	PREDICTED: uncharacterized protein LOC105177426 isoform X1 [Sesamum indicum]
c44832.graph_c0	1.17168327	1.33843628	1.09824567	1.1621462	0.63320374	0.9973519	3.808E-10	-1.45798	down	Ty3/gypsy retrotransposon protein [Beta vulgaris subsp. vulgaris]
c44833.graph_c0	1.36357558	1.81992133	1.66238971	8.23324603	7.81242488	7.95201634	2.364E-15	1.21588	up	PREDICTED: putative receptor-like protein kinase At5g39000 isoform X1 [Sesamum indicum]
c44846.graph_c0	1.96276064	2.10848649	2.24539554	10.6989234	11.2779993	10.4904765	2.747E-16	1.26935	up	PREDICTED: uncharacterized protein LOC105173505 [Sesamum indicum]
c44848.graph_c0	2.93735929	3.09781672	3.16798715	70.945833	67.6027959	61.8885454	2.07E-161	3.35299	up	PREDICTED: L-type lectin-domain containing receptor kinase S.4-like [Sesamum indicum]
c44849.graph_c0	111.012853	111.252483	134.409905	7.13559294	7.57973106	8.18206469	2.968E-64	-5.05407	down	hypothetical protein MIMGU_mgv1a011926mg [Erythranthe guttata]
c44852.graph_c0	6.84805344	3.05580358	4.02892472	0	0	0	4.751E-14	-Inf	down	Ribosomal protein L7 eukaryotic [Macrophomina phaseolina MS6]
c44854.graph_c0	0.12921769	0.19402063	0.16604985	1.62002812	1.54752965	1.49435534	2.626E-06	2.15953	up	PREDICTED: GDSL esterase/lipase At4g18970-like [Sesamum indicum]
c44858.graph_c0	60.6791425	64.8343482	61.9891189	29.5810063	29.7701914	27.2252602	5.07E-152	-2.20591	down	PREDICTED: F-box protein SKIP22-like [Sesamum indicum]
c44859.graph_c0	0.43534477	0.34862456	0.11188705	3.2779669	3.4853572	3.50098549	1.972E-17	2.4415	up	PREDICTED: DNA topoisomerase 2-binding protein 1-A isoform X1 [Sesamum indicum]
c44861.graph_c0	80.2069051	78.9718656	71.2455868	31.3141548	33.2320156	31.9663448	4.41E-180	-2.34464	down	PREDICTED: scopoletin glucosyltransferase-like [Sesamum indicum]
c44864.graph_c0	38.759963	38.7213326	37.5797543	29.0139464	27.4153325	25.5792488	9.91E-71	-1.5791	down	PREDICTED: UPF0183 protein At3g51130 isoform X1 [Sesamum indicum]
c44866.graph_c0	8.02596963	7.93892942	7.75052348	5.48806702	4.3942428	3.83042535	4.583E-26	-1.88138	down	PREDICTED: transcription repressor OFP13-like [Sesamum indicum]
c44868.graph_c1	5.71177044	4.84800988	5.41187181	30.7615723	31.5575797	31.0798961	6.584E-43	1.45758	up	PREDICTED: mitochondrial inner membrane protein OXA1-like [Sesamum indicum]
c44868.graph_c2	0.14868058	0.22324419	0.12737362	1.96309339	2.61808049	2.2631899	2.939E-11	2.68911	up	PREDICTED: uncharacterized protein LOC105178110 [Sesamum indicum]
c44869.graph_c0	3.84943796	3.42350226	3.08216227	19.3475126	18.7028952	17.5435205	1.448E-37	1.3363	up	PREDICTED: kinesin-like protein KIF3A [Sesamum indicum]

#ID	0 h-1 FPKM	0 h-2 FPKM	0 h-3 FPKM	28 h-1 FPKM	28 h-2 FPKM	28 h-3 FPKM	FDR	log2FC	regulated	nr_annotation
c44871.graph_c0	0.91417554	0.84972731	0.75519757	13.6561529	11.6231927	11.1917163	2.144E-44	2.76711	up	PREDICTED: synaptotagmin-2-like [Sesamum indicum]
c44877.graph_c0	13.1432057	11.7605445	14.2207637	11.9458985	11.9736573	12.0879208	4.373E-14	-1.2117	down	PREDICTED: vinorine synthase-like [Sesamum indicum]
c44878.graph_c1	6.7836841	6.72641583	8.01827366	35.0472932	32.9673119	36.7394284	2.086E-25	1.19025	up	Taxane 13-alpha-hydroxylase [Medicago truncatula]
c44881.graph_c0	5.48615953	4.24949346	3.44103834	0	0.04285912	0.07746694	5.166E-35	-7.8543	down	PREDICTED: xylosyltransferase 2-like [Sesamum indicum]
c44881.graph_c1	32.9856868	30.6118085	26.6338841	18.6572339	19.668171	16.6702439	2.941E-43	-1.80297	down	PREDICTED: protein SPA1-RELATED 3-like isoform X1 [Solanum tuberosum]
c44882.graph_c0	0.51901797	0.17317923	0.62249473	10.6878792	10.9898211	10.0140151	4.603E-42	3.49488	up	PREDICTED: uncharacterized protein LOC105178220 [Sesamum indicum]
c44892.graph_c0	0.04949118	0	0.25439251	3.09341183	2.63067359	2.75900798	1.893E-19	3.68417	up	PREDICTED: uncharacterized protein LOC105176998 [Sesamum indicum]
c44894.graph_c0	6.37475982	4.44746343	4.22002784	0	0	0	3.366E-35	-Inf	down	unknown [Lotus japonicus]
c44899.graph_c0	2.51658082	2.04327064	1.97627588	16.2174564	15.5419645	15.0740358	1.303E-30	1.75295	up	PREDICTED: uncharacterized protein LOC105166230 isoform X2 [Sesamum indicum]
c44900.graph_c1	31.8178694	33.2519196	30.4886242	32.169627	32.09112	32.4436759	7.564E-41	-1.07261	down	PREDICTED: cell division cycle and apoptosis regulator protein 1 isoform X2 [Sesamum indicum]
c44901.graph_c0	1.75540613	3.5593833	2.42928687	17.3380539	19.7898242	15.4557063	1.966E-11	1.67049	up	unnamed protein product [Coffea canephora]
c44904.graph_c0	0	0.08000995	0	5.22833368	5.97954713	5.38025006	7.126E-31	6.61377	up	PREDICTED: uncharacterized protein LOC105161004 [Sesamum indicum]
c44905.graph_c0	1.7191845	2.20927121	1.46286199	9.0841546	9.01015738	8.69395549	1.197E-12	1.22488	up	PREDICTED: uncharacterized protein LOC105163513 isoform X1 [Sesamum indicum]
c44906.graph_c0	16.1707019	17.6508254	15.5178752	13.0890055	12.2842897	12.662985	4.915E-37	-1.46507	down	PREDICTED: pentatricopeptide repeat-containing protein At1g66345, mitochondrial [Sesamum indicum]
c44907.graph_c0	6.86643477	6.4578906	6.10867422	6.85506211	5.8923834	6.42153856	8.216E-12	-1.10873	down	hypothetical protein MIMGU_mgv1a005181mg [Erythranthe guttata]
c44908.graph_c0	38.1384558	37.1558797	36.3311852	22.1139034	21.673201	21.0534853	2.69E-117	-1.87384	down	hypothetical protein MIMGU_mgv1a001607mg [Erythranthe guttata]
c44911.graph_c0	5.97639451	6.93008256	4.67637692	27.6958837	31.3361436	26.8458158	7.42E-15	1.20046	up	PREDICTED: amino-acid permease BAT1 homolog [Sesamum indicum]
c44912.graph_c0	1.84846455	2.20614541	1.73583278	9.81754392	14.4161863	10.1613663	1.243E-05	1.48005	up	PREDICTED: uncharacterized protein LOC105178220 [Sesamum indicum]
c44917.graph_c0	10.4436366	10.5841608	9.05830657	6.26825669	6.62956895	6.11662408	1.27E-68	-1.75096	down	PREDICTED: serine/threonine-protein kinase CTR1-like [Sesamum indicum]
c44919.graph_c0	84.2917399	83.6764122	80.546239	19.1648964	19.7506349	21.2350573	0	-3.13625	down	PREDICTED: tubby-like F-box protein 6 [Sesamum indicum]
c44920.graph_c0	5.49603322	5.87362127	5.2201976	46.9303777	45.8061425	45.128664	3.037E-86	1.9651	up	PREDICTED: molybdate-anion transporter-like [Sesamum indicum]
c44922.graph_c0	24.7942644	24.8613734	24.5339836	16.1783869	15.6331452	15.3557449	1.344E-64	-1.74397	down	PREDICTED: transcription factor IIIA-like isoform X2 [Sesamum indicum]
c44927.graph_c0	19.9533199	19.9733001	18.8580677	128.07641	130.361796	125.804824	2.505E-81	1.61856	up	PREDICTED: S-adenosylmethionine synthase 1 [Sesamum indicum]

#ID	0 h-1 FPKM	0 h-2 FPKM	0 h-3 FPKM	28 h-1 FPKM	28 h-2 FPKM	28 h-3 FPKM	FDR	log2FC	regulated	nr_annotation
c44928.graph_c0	3.06764635	3.20821298	2.05927829	13.2441788	12.4389854	11.9204403	8.004E-08	1.08777	up	hypothetical protein MIMGU_mgv1a008719mg [Erythranthe guttata]
c44935.graph_c0	2.50390849	3.20821219	3.31415019	19.6723218	19.7011585	19.334223	2.601E-37	1.6078	up	PREDICTED: ABC transporter B family member 19 [Sesamum indicum]
c44940.graph_c0	0.4173852	0.35352574	0.41258193	1.98346297	2.31761083	2.11355699	0.0003744	1.34723	up	PREDICTED: trimethylguanosine synthase [Sesamum indicum]
c44943.graph_c0	0.81198698	0.78377149	0.63351436	5.24809706	4.357934	5.53788835	4.356E-10	1.67751	up	PREDICTED: GDSL esterase/lipase At4g10955-like [Sesamum indicum]
c44946.graph_c0	1.19838373	1.55945884	1.65546832	1.08872298	1.45486187	1.45695806	6.025E-05	-1.23523	down	PREDICTED: protein TONSOKU [Sesamum indicum]
c44948.graph_c0	2.11903549	1.85601271	2.26920375	2.16042195	2.25967367	2.05961017	4.897E-05	-1.03887	down	PREDICTED: protein LURP-one-related 15-like isoform X1 [Sesamum indicum]
c44949.graph_c1	56.5040731	59.8192919	58.2672607	55.3303773	54.2306781	58.1946713	1.043E-41	-1.14809	down	-
c44950.graph_c0	1.01188815	1.01290141	1.23187864	0.11612123	0.06989772	0.06316932	2.706E-20	-4.80257	down	hypothetical protein MIMGU_mgv1a010141mg [Erythranthe guttata]
c44950.graph_c1	7.54125966	10.7320447	10.5081151	42.4608076	41.8559346	43.3769458	5.284E-13	1.05592	up	PREDICTED: U6 snRNA-associated Sm-like protein LSm7 [Sesamum indicum]
c44951.graph_c0	3.85662586	3.34885679	3.07505689	15.1799586	16.0539506	14.6601474	1.265E-16	1.07011	up	PREDICTED: protein arginine N-methyltransferase 1.5 [Sesamum indicum]
c44953.graph_c0	2.8128905	2.66142186	3.0204824	15.4869741	17.2199231	16.019364	4.117E-18	1.42816	up	PREDICTED: sucrose nonfermenting 4-like protein [Sesamum indicum]
c44953.graph_c1	2.34556591	2.70010184	3.46626682	15.76888	16.9314618	12.9368302	2.672E-06	1.32604	up	PREDICTED: sucrose nonfermenting 4-like protein [Sesamum indicum]
c44955.graph_c0	9.85043514	10.0468451	9.47654369	0.9287662	0.698824	0.85259884	3.01E-192	-4.65551	down	PREDICTED: LOW QUALITY PROTEIN: serine carboxypeptidase 24-like [Sesamum indicum]
c44957.graph_c1	1.39953572	1.24527746	1.59863005	7.14282534	8.02080861	7.10116314	9.188E-10	1.29828	up	PREDICTED: digalactosyldiacylglycerol synthase 2, chloroplastic isoform X1 [Sesamum indicum]
c44963.graph_c1	9.59943051	9.40603494	8.51333472	9.38430181	9.515652	9.7623276	1.215E-15	-1.03015	down	PREDICTED: glutathione gamma-glutamylcysteinyltransferase 1 isoform X2 [Sesamum indicum]
c44975.graph_c0	9.42345285	9.75538094	6.83100564	2.69273432	1.37420548	1.38522233	1.553E-25	-3.34164	down	-
c44976.graph_c1	12.0529966	12.841891	12.7772976	12.5848584	12.0631848	12.8436184	1.408E-26	-1.09783	down	PREDICTED: N-alpha-acetyltransferase 35, NatC auxiliary subunit isoform X1 [Sesamum indicum]
c44978.graph_c0	2.5745445	2.65703329	2.51335176	16.1007144	17.9426005	15.3277267	4.372E-37	1.58161	up	PREDICTED: uncharacterized protein LOC105173611 isoform X1 [Sesamum indicum]
c44980.graph_c0	8.39767196	7.47569918	9.01024584	8.11808741	7.36197341	7.16584107	3.178E-14	-1.22809	down	PREDICTED: probable inactive leucine-rich repeat receptor-like protein kinase At3g03770 [Sesamum indicum]
c44981.graph_c2	2.13231431	2.08152926	2.19661575	29.1635478	30.3678959	27.340149	4.08E-132	2.66892	up	hypothetical protein MIMGU_mgv1a006718mg [Erythranthe guttata]
c44987.graph_c0	2.65136654	2.48814514	3.46518673	0.75547078	0.90949237	0.8487465	1.94E-13	-2.86989	down	unnamed protein product [Coffea canephora]
c44990.graph_c0	17.7974046	18.1043169	17.2107649	7.7138033	6.82270153	8.58521973	8.552E-90	-2.28893	down	PREDICTED: protein WEAK CHLOROPLAST MOVEMENT UNDER BLUE LIGHT 1-like isoform X2 [Sesamum indicum]
c44996.graph_c0	69.7346194	65.9890045	71.4421605	18.4156888	17.9973958	20.2047106	3.43E-166	-2.96197	down	PREDICTED: protein FAR1-RELATED SEQUENCE 5-like [Sesamum indicum]

#ID	0 h-1 FPKM	0 h-2 FPKM	0 h-3 FPKM	28 h-1 FPKM	28 h-2 FPKM	28 h-3 FPKM	FDR	log2FC	regulated	nr_annotation
c45000.graph_c0	67.5039454	68.850497	64.6347832	66.4264561	64.3647309	67.7164203	1.587E-41	-1.10764	down	PREDICTED: filaggrin-like [Sesamum indicum]
c45006.graph_c0	49.9460539	43.7946321	47.8284221	25.3754486	24.7710558	26.4594519	2.127E-73	-1.97603	down	PREDICTED: uncharacterized protein LOC103488200 [Cucumis melo]
c45009.graph_c0	0	0	0	0.40895065	0.46641359	0.60592965	2.388E-14	Inf	up	--
c45012.graph_c0	16.6244461	17.0790165	16.7472278	6.09148033	6.39152489	6.06303737	5.62E-138	-2.53451	down	PREDICTED: peptide-N(4)-(N-acetyl-beta-glucosaminyl)asparagine amidase isoform X2 [Sesamum indicum]
c45014.graph_c1	32.0951012	33.760828	29.0601729	22.4793238	22.7955644	22.9517248	1.218E-73	-1.56537	down	PREDICTED: TBC1 domain family member 15 [Sesamum indicum]
c45015.graph_c0	13.7244357	16.8696752	14.1350905	5.79420897	4.63561925	4.90757254	9.39E-39	-2.63454	down	PREDICTED: protein HEADING DATE 3A-like [Sesamum indicum]
c45017.graph_c0	0.24815557	0.34155558	0.2590979	1.70212896	2.11700315	1.78444455	1.804E-07	1.63222	up	PREDICTED: calcium-transporting ATPase 12, plasma membrane-type-like [Sesamum indicum]
c45018.graph_c0	0	0	0	2.60459486	5.43831771	6.44240092	7.33E-07	Inf	up	hypothetical protein DI09_20p90 [Microsporidia sp. UGP3]
c45019.graph_c0	55.6912742	51.0043415	45.9243801	17.2963001	19.2235793	15.4784399	1.171E-93	-2.64253	down	BnaAnng27240D [Brassica napus]
c45024.graph_c0	0.05279945	0	0.06784939	0.716321	0.50817698	0.8558927	8.521E-07	3.01052	up	PREDICTED: uncharacterized protein LOC105156561 isoform X1 [Sesamum indicum]
c45027.graph_c0	0.84021256	0.8831066	0.70180916	11.9995013	12.5161037	8.39626929	3.154E-13	2.67264	up	PREDICTED: uncharacterized protein LOC101264598 [Solanum lycopersicum]
c45030.graph_c0	0.96081742	1.37633968	1.04309884	5.89957096	4.81582537	5.64908366	1.381E-09	1.18536	up	PREDICTED: protein FAM91A1 isoform X1 [Sesamum indicum]
c45031.graph_c0	28.4434448	30.0712008	28.2764391	19.2474466	19.094264	18.9359199	1.13E-84	-1.68993	down	PREDICTED: uncharacterized protein LOC105156468 [Sesamum indicum]
c45033.graph_c1	1.22881852	1.02156611	0.66910188	4.2081577	4.40094293	4.2613982	6.628E-06	1.05673	up	hypothetical protein MIMGU_mgv1a021825mg [Erythranthe guttata]
c45035.graph_c0	4.04935496	4.48008449	4.30371398	0.95151171	0.39959342	0.55974882	1.661E-61	-3.83872	down	PREDICTED: protein NRT1/ PTR FAMILY 4.5-like [Sesamum indicum]
c45041.graph_c0	97.5218459	95.8197111	88.4167488	51.074351	48.3754179	49.020285	7.98E-135	-2.01361	down	PREDICTED: NF-kappa-B-activating protein [Sesamum indicum]
c45042.graph_c0	1.79073689	2.30468148	2.08200896	11.5895231	9.90466705	10.8729711	7.883E-06	1.29794	up	putative retroelement pol polyprotein [Arabidopsis thaliana]
c45045.graph_c0	0.43575289	0.43618923	0	15.5184319	11.1521606	12.6629137	1.341E-13	4.42511	up	-
c45047.graph_c0	0.44647374	0.37988269	0.45898914	4.57537299	5.44957947	4.84556355	1.216E-20	2.44051	up	PREDICTED: subtilisin-like protease SBT3.5 [Sesamum indicum]
c45055.graph_c0	56.1000721	60.1566661	52.4367033	49.770832	45.7271337	47.0813819	2.335E-58	-1.33209	down	PREDICTED: transcription factor GTE9-like isoform X2 [Sesamum indicum]
c45056.graph_c0	2.14655714	1.32928459	2.22075454	8.4748096	8.66664876	9.10542412	3.047E-10	1.11191	up	PREDICTED: uncharacterized protein LOC105167041 isoform X1 [Sesamum indicum]
c45058.graph_c0	0.60482662	0.53816201	1.29537693	9.62201635	9.6579874	9.52540781	4.889E-15	2.46017	up	-
c45063.graph_c0	89.4575033	81.6406427	82.1117382	85.2060697	86.6589259	89.5277854	8.868E-33	-1.04359	down	PREDICTED: serine/arginine-rich splicing factor RSZ22 isoform X2 [Vitis vinifera]
c45063.graph_c2	0	0	0	2.10876535	2.76389622	3.44147062	1.012E-11	Inf	up	PREDICTED: KH domain-containing protein At4g18375 [Sesamum indicum]

#ID	0 h-1 FPKM	0 h-2 FPKM	0 h-3 FPKM	28 h-1 FPKM	28 h-2 FPKM	28 h-3 FPKM	FDR	log2FC	regulated	nr_annotation
c45070.graph_c0	20.2945197	21.5979651	17.9122309	20.484788	18.5059169	18.3541864	1.097E-25	-1.14954	down	PREDICTED: CCR4-NOT transcription complex subunit 1 isoform X1 [Sesamum indicum]
c45072.graph_c0	51.0523768	51.2982707	46.9138125	30.8821769	31.1636774	29.5269546	1.78E-107	-1.79454	down	PREDICTED: extensin [Sesamum indicum]
c45073.graph_c0	46.9195677	48.8014571	49.6028436	40.9415421	38.9095374	40.808727	5.091E-58	-1.35935	down	hypothetical protein MIMGU_mgv1a005118mg [Erythranthe guttata]
c45073.graph_c1	12.6562556	11.7237866	12.3914587	60.578651	61.7102718	61.8581118	5.291E-50	1.23375	up	PREDICTED: acyl-CoA dehydrogenase family member 10 [Sesamum indicum]
c45073.graph_c2	2.90514396	2.90805303	2.28141529	2.18964344	2.29478174	2.83962632	2.217E-05	-1.23063	down	PREDICTED: phosphatidylcholine:diacylglycerol cholinephosphotransferase 1-like [Sesamum indicum]
c45075.graph_c0	152.483753	147.835513	148.12963	71.0141041	64.6834299	67.7551432	1.46E-159	-2.23042	down	PREDICTED: uncharacterized protein LOC105179151 [Sesamum indicum]
c45078.graph_c0	273.655042	248.88511	259.444151	28.1741218	31.2761305	37.213546	0	-4.10516	down	PREDICTED: uncharacterized protein LOC105174597 [Sesamum indicum]
c45079.graph_c0	1.24903221	1.25620844	1.19428326	7.58498928	7.11268635	6.90897411	3.813E-43	1.45589	up	PREDICTED: probable LRR receptor-like serine/threonine-protein kinase At4g20940 [Sesamum indicum]
c45081.graph_c0	4.7870349	5.10893468	3.79947402	31.1080462	33.4926736	30.456519	1.185E-43	1.70731	up	PREDICTED: uncharacterized protein LOC105177718 [Sesamum indicum]
c45092.graph_c0	1.01112717	0.82470639	0.67373105	0	0	0	1.406E-29	-Inf	down	-
c45094.graph_c0	27.2853971	27.3875487	26.8975024	23.7209796	23.2525125	23.8870374	3.751E-56	-1.29331	down	PREDICTED: pentatricopeptide repeat-containing protein At1g63330-like [Sesamum indicum]
c45095.graph_c1	0.3721209	0.10642672	0.06831287	1.66136841	1.4651748	1.00886553	0.0038444	1.84456	up	PREDICTED: putative phospholipid-transporting ATPase 5 [Sesamum indicum]
c45095.graph_c2	0.54124028	0.61917972	0.39743725	4.1865841	4.21137868	4.1269753	2.477E-08	1.92069	up	PREDICTED: putative phospholipid-transporting ATPase 5 [Sesamum indicum]
c45097.graph_c0	1.17966417	1.01012078	1.1506344	6.12556939	5.5494685	6.32177226	1.976E-16	1.33946	up	PREDICTED: RNA polymerase II C-terminal domain phosphatase-like 3 [Sesamum indicum]
c45104.graph_c0	64.4749031	62.2293275	58.7939802	66.50554	61.3544873	60.1190647	7.861E-41	-1.07048	down	PREDICTED: mRNA-decapping enzyme-like protein [Sesamum indicum]
c45105.graph_c0	0.63454337	0.40116554	0.47208141	3.83509626	3.2654881	4.11972597	4.34E-08	1.80889	up	hypothetical protein VITISV_036763 [Vitis vinifera]
c45106.graph_c0	16.4335965	15.3204349	11.3293037	58.3095351	80.1638775	63.4749965	4.835E-06	1.14207	up	PREDICTED: UDP-glycosyltransferase 73C3-like [Sesamum indicum]
c45111.graph_c0	3.98126084	4.48045574	3.87489917	22.2581341	21.0091032	21.9765714	4.42E-30	1.31318	up	PREDICTED: uncharacterized protein LOC105175953 [Sesamum indicum]
c45113.graph_c0	5.26336289	2.4085181	3.60726917	0	0	0	2.428E-17	-Inf	down	--
c45114.graph_c0	161.019614	176.007028	192.62196	30.5077141	27.5859358	30.72574	8.871E-59	-3.66842	down	PREDICTED: serine/arginine repetitive matrix protein 1-like [Sesamum indicum]
c45124.graph_c0	24.886053	22.1069053	21.1803644	23.1046501	22.2963648	22.7224538	7.852E-38	-1.08974	down	PREDICTED: elongation factor Tu GTP-binding domain-containing protein 1 isoform X1 [Sesamum indicum]
c45132.graph_c0	2.04244581	2.68173496	2.82913987	1.23381847	1.0269894	0.94386168	6.405E-13	-2.33158	down	ribosomal protein S3 (mitochondrion) [Rhazya stricta]

#ID	0 h-1 FPKM	0 h-2 FPKM	0 h-3 FPKM	28 h-1 FPKM	28 h-2 FPKM	28 h-3 FPKM	FDR	log2FC	regulated	nr_annotation
c45134.graph_c0	1.35216416	1.43638661	1.89715857	6.93938392	6.98090702	6.50530648	5.324E-13	1.02866	up	PREDICTED: filament-like plant protein [Sesamum indicum]
c45137.graph_c0	4.82192231	7.00657365	5.09700669	1.97837237	2.90909232	2.49068936	1.488E-10	-2.28818	down	PREDICTED: receptor-like protein kinase HAIKU2 [Sesamum indicum]
c45137.graph_c2	9.29012208	10.7320388	10.3571732	6.02206046	6.37653564	6.73062538	7.976E-36	-1.7585	down	PREDICTED: receptor-like protein kinase HAIKU2 [Sesamum indicum]
c45141.graph_c0	2.39613337	2.47121555	1.95944398	16.0780533	15.1999396	15.1004128	3.267E-32	1.67618	up	PREDICTED: uncharacterized protein LOC105159160 [Sesamum indicum]
c45142.graph_c0	155.033464	162.348159	144.768678	148.577037	151.647192	144.895718	6.071E-45	-1.14387	down	PREDICTED: suppressor of mec-8 and unc-52 protein homolog 1 [Sesamum indicum]
c45147.graph_c0	108.327671	112.504683	107.060343	36.3612184	34.5020725	38.6295284	4.38E-217	-2.6722	down	PREDICTED: phytochrome C [Sesamum indicum]
c45150.graph_c0	5.02032182	3.96531437	3.85566712	20.9945134	20.757741	20.7250974	3.872E-28	1.19489	up	hypothetical protein MIMGU_mgv1a000966mg [Erythranthe guttata]
c45151.graph_c0	0	0	0	2.61383076	2.20094001	3.83436324	6.295E-12	Inf	up	mitochondrial carrier, adenine nucleotidetranslocator [Galdieria sulphuraria]
c45153.graph_c1	18.9850355	20.4318113	20.3515031	19.4819349	21.8185023	20.5349692	1.236E-20	-1.04233	down	PREDICTED: vesicle-associated protein 4-2 [Sesamum indicum]
c45156.graph_c1	58.1948419	55.7806183	55.4940473	30.5775641	27.6086734	29.8607639	1.57E-133	-2.0345	down	PREDICTED: casein kinase I isoform alpha-like isoform X2 [Sesamum indicum]
c45158.graph_c0	0.01463921	0	0.03762392	0.75541801	0.82617127	0.59905112	1.159E-12	4.2709	up	PREDICTED: uncharacterized protein LOC105177021 [Sesamum indicum]
c45162.graph_c0	0	0	0	0.65873689	0.66086406	1.22436005	1.155E-06	Inf	up	-
c45163.graph_c0	1.31455666	0.83547492	0.64352661	0	0	0	9.404E-17	-Inf	down	-
c45166.graph_c0	0.43845343	0.34136081	0.31301667	3.29287604	3.00512777	2.97588039	2.207E-06	1.9982	up	hypothetical protein MIMGU_mgv1a006017mg [Erythranthe guttata]
c45169.graph_c0	21.094801	20.0008919	22.0081947	18.6964314	18.6628942	18.7037329	7.32E-37	-1.26196	down	hypothetical protein MIMGU_mgv1a012191mg [Erythranthe guttata]
c45170.graph_c0	0.48349414	0.31393186	0.50376415	6.25873238	5.84827784	5.86654842	6.147E-46	2.69556	up	PREDICTED: probable LRR receptor-like serine/threonine-protein kinase At4g26540 [Sesamum indicum]
c45175.graph_c0	0	0	0	8.65041327	16.1467318	17.9181536	2.5E-12	Inf	up	predicted protein [Hordeum vulgare subsp. vulgare]
c45180.graph_c0	6.30938785	6.25944111	5.86868437	45.5498779	36.8024509	37.776689	1.48E-20	1.61405	up	PREDICTED: pentatricopeptide repeat-containing protein At5g24830 isoform X1 [Sesamum indicum]
c45183.graph_c0	2.35360189	2.35595866	1.84098272	11.0568202	11.6620733	10.4180978	2.661E-11	1.2509	up	hypothetical protein OsJ_01978 [Oryza sativa Japonica Group]
c45187.graph_c0	1.02135072	0.4868445	0.62498863	5.40827182	6.98962458	5.59570257	1.027E-09	1.99039	up	PREDICTED: dicer-like protein 4 isoform X1 [Sesamum indicum]
c45187.graph_c1	0.39843388	0.15953314	0.20480132	2.83787857	2.71921612	3.96974698	3.727E-07	2.56141	up	PREDICTED: dicer-like protein 4 isoform X1 [Sesamum indicum]
c45187.graph_c2	0.19175547	0.28792123	0.12320669	2.64796865	2.65512118	3.28657005	1.531E-16	2.74916	up	PREDICTED: dicer-like protein 4 isoform X2 [Sesamum indicum]
c45194.graph_c0	1.76913513	1.46947573	2.0799271	12.0919653	12.671693	12.076962	1.256E-18	1.69864	up	hypothetical protein OsI_30754 [Oryza sativa Indica Group]
c45195.graph_c0	0.05059501	0.05064567	0.06501659	1.50765859	2.25772331	1.2302371	1.74E-06	3.81285	up	PREDICTED: glutamine-dependent NAD(+) synthetase-like [Sesamum indicum]
c45195.graph_c1	0.19245222	0.04816123	0.24730873	1.71344628	1.95753149	2.22563954	2.623E-07	2.49834	up	PREDICTED: glutamine-dependent NAD(+) synthetase-like [Sesamum indicum]

#ID	0 h-1 FPKM	0 h-2 FPKM	0 h-3 FPKM	28 h-1 FPKM	28 h-2 FPKM	28 h-3 FPKM	FDR	log2FC	regulated	nr_annotation
c45196.graph_c0	13.9914558	14.8162022	14.362844	11.2726261	11.8789076	11.3478595	7.503E-50	-1.41434	down	PREDICTED: eukaryotic translation initiation factor 2D [Sesamum indicum]
c45198.graph_c0	36.1787492	37.5813431	35.6287127	7.44510426	6.37707987	7.06291224	0	-3.47891	down	hypothetical protein MIMGU_mgv1a000338mg [Erythranthe guttata]
c45198.graph_c1	34.7965878	35.8175002	35.6305148	9.09680474	9.31630849	10.1205994	2.42E-229	-2.98699	down	cryptochrome 1 family protein [Populus trichocarpa]
c45201.graph_c0	22.0402413	20.0152927	25.4027288	21.4683881	22.8134082	22.7734382	5.983E-07	-1.10138	down	-
c45206.graph_c0	15.4898248	17.1338545	14.781552	13.7060124	12.4654646	13.0864962	3.518E-43	-1.36162	down	PREDICTED: uncharacterized protein LOC105171856 [Sesamum indicum]
c45208.graph_c0	18.2590242	18.9000901	15.6076185	14.3518579	14.7335029	14.1654958	7.554E-35	-1.37547	down	PREDICTED: uncharacterized protein At2g33490 [Sesamum indicum]
c45213.graph_c1	0.04501424	0.3604745	0.17353523	80.2525644	71.5152753	71.2250902	1.88E-234	7.49433	up	PREDICTED: nudix hydrolase 18, mitochondrial-like [Sesamum indicum]
c45215.graph_c1	2.21491176	3.56569307	2.64084987	26.9182623	27.526309	25.9057967	3.712E-77	2.16275	up	PREDICTED: U-box domain-containing protein 13 [Sesamum indicum]
c45216.graph_c1	2.836692	2.14696361	2.93399099	0.10057031	0.27241669	0.57445187	8.436E-23	-4.14938	down	-
c45216.graph_c2	31.437625	31.3199624	27.139813	27.694555	28.7209816	31.7439772	1.527E-33	-1.11626	down	PREDICTED: calpain-15 [Sesamum indicum]
c45218.graph_c0	98.9272002	98.191523	96.7822241	49.1240316	54.6941529	54.7391899	3.18E-127	-1.98044	down	PREDICTED: 4-coumarate--CoA ligase 1-like [Sesamum indicum]
c45223.graph_c0	3.90105376	3.37465685	2.78500474	17.1865966	16.4028156	15.8806764	3.053E-11	1.21105	up	PREDICTED: uncharacterized protein LOC105166056 isoform X1 [Sesamum indicum]
c45225.graph_c0	5.29028877	3.32024849	3.56097789	21.6965114	22.8411252	23.4312655	2.333E-19	1.3953	up	hypothetical protein MIMGU_mgv1a011604mg [Erythranthe guttata]
c45227.graph_c0	123.706499	131.599807	115.83258	49.9560008	49.4376113	47.9530936	1.96E-171	-2.42241	down	PREDICTED: uncharacterized protein LOC105178162 [Sesamum indicum]
c45234.graph_c0	9.91884346	10.1702864	9.95574268	8.76765194	9.02465912	9.205241	1.29E-26	-1.24468	down	-
c45235.graph_c0	48.9696439	50.1154831	42.9989478	16.9684455	15.9108904	17.2784827	3.28E-132	-2.59069	down	PREDICTED: outer envelope protein 61 [Sesamum indicum]
c45238.graph_c0	403.374093	396.509747	391.610168	345.722559	385.272968	369.973591	1.961E-43	-1.20424	down	PREDICTED: 8-hydroxygeraniol dehydrogenase [Sesamum indicum]
c45240.graph_c0	71.0528903	74.2869097	66.4231309	69.0997895	72.1466438	69.1657534	6.12E-42	-1.09891	down	PREDICTED: crt homolog 1 [Sesamum indicum]
c45242.graph_c0	4.05869351	4.31005597	4.80740588	1.43644142	1.57489447	1.4023632	1.004E-32	-2.67095	down	PREDICTED: serine/threonine-protein kinase TIO isoform X2 [Sesamum indicum]
c45246.graph_c0	0.13556302	0.09046585	0.05806795	7.48800831	5.96032832	6.27094715	3.083E-28	5.03599	up	PREDICTED: WAT1-related protein At5g40240-like [Sesamum indicum]
c45248.graph_c0	194.914687	191.294407	202.196762	179.410406	178.690113	196.226543	7.087E-43	-1.17673	down	PREDICTED: aldehyde dehydrogenase [Nicotiana sylvestris]
c45251.graph_c0	0.60149642	0.88730339	0.44749547	2.87607982	3.03309793	2.44073196	0.0032199	1.02148	up	PREDICTED: uncharacterized protein LOC105179935 isoform X1 [Sesamum indicum]
c45253.graph_c0	0	0	0	12.367585	14.3163714	17.8548087	2.328E-14	Inf	up	-
c45255.graph_c1	78.5206363	81.9231123	67.3684844	68.2832696	66.8061269	68.3682163	1.171E-28	-1.25149	down	PREDICTED: anamorsin homolog isoform X1 [Sesamum indicum]
c45257.graph_c0	2.92607756	3.2014734	2.66706577	20.1043207	20.586022	20.9854366	3.308E-32	1.72105	up	PREDICTED: acyl-coenzyme A thioesterase 8-like [Sesamum indicum]
c45258.graph_c0	29.6324645	25.4331977	23.9331176	411.87866	440.611306	405.961258	1.22E-260	2.90526	up	ascorbate peroxidase [Acanthus ebracteatus]

#ID	0 h-1 FPKM	0 h-2 FPKM	0 h-3 FPKM	28 h-1 FPKM	28 h-2 FPKM	28 h-3 FPKM	FDR	log2FC	regulated	nr_annotation
c45259.graph_c0	0.08349071	0.16714864	0.21457775	9.28412722	8.30964459	7.97189079	9.631E-54	4.67955	up	PREDICTED: LRR receptor-like serine/threonine-protein kinase FEI 2 isoform X1 [Sesamum indicum]
c45266.graph_c0	2.253657	1.73531823	2.3231957	100.364625	93.6589266	86.8757292	3.44E-205	4.3836	up	PREDICTED: phosphomethylpyrimidine synthase, chloroplastic isoform X1 [Sesamum indicum]
c45267.graph_c2	0.54284464	1.19545407	0.13951537	25.1714193	26.2184791	25.7550754	4.285E-38	4.28162	up	-
c45271.graph_c0	2.66471019	2.87256146	1.77798017	15.0466382	18.4953885	15.6817176	6.811E-16	1.66445	up	PREDICTED: dof zinc finger protein DOF5.4-like [Sesamum indicum]
c45275.graph_c0	4.47874616	4.58749213	4.73813863	22.4528055	21.571439	20.7797954	1.853E-26	1.13949	up	hypothetical protein MIMGU_mgv1a001439mg [Erythranthe guttata]
c45282.graph_c0	144.030981	136.330166	148.096257	78.2481755	74.3954225	89.0385504	4.285E-73	-1.91633	down	branched-chain amino acid aminotransferase, putative [Ricinus communis]
c45285.graph_c0	11.6343034	11.0198269	11.7353705	3.77326241	4.82302417	3.7652236	2.208E-85	-2.56779	down	PREDICTED: U-box domain-containing protein 19-like [Sesamum indicum]
c45287.graph_c0	1.45650738	2.1634332	1.99242253	1.6732412	1.60327666	1.28175823	2.387E-05	-1.39447	down	hypothetical protein MIMGU_mgv1a006543mg [Erythranthe guttata]
c45290.graph_c0	0.80401608	0.98367033	0.688795	7.66153353	6.97627056	6.51665341	3.404E-07	2.0061	up	-
c45290.graph_c1	18.9139961	18.949558	17.1139575	17.2345224	17.0213786	16.3677084	1.145E-45	-1.20846	down	PREDICTED: WD repeat-containing protein 26-like [Sesamum indicum]
c45292.graph_c0	0.62928407	0.71390276	0.43128264	47.3215182	41.4054438	40.9775854	7.56E-130	5.10581	up	PREDICTED: pentatricopeptide repeat-containing protein At5g59600 [Sesamum indicum]
c45297.graph_c0	5.17558453	2.10743068	3.15632659	0	0	0	8.168E-12	-Inf	down	hypothetical protein SORBIDRAFT_05g002010 [Sorghum bicolor]
c45300.graph_c0	68.3283747	74.0017582	60.5873056	21.4095283	19.8100726	21.8976587	4.589E-76	-2.77319	down	PREDICTED: uncharacterized protein LOC105166619 [Sesamum indicum]
c45303.graph_c0	2.46053958	3.39724612	3.05286006	39.7743917	38.8078216	36.1288459	9.811E-43	2.59367	up	PREDICTED: S-type anion channel SLAH2 [Sesamum indicum]
c45304.graph_c0	62.763009	60.6965632	58.2143091	46.0744995	45.3284722	46.5380407	1.006E-75	-1.48684	down	PREDICTED: cytochrome b-c1 complex subunit Rieske-4, mitochondrial-like [Sesamum indicum]
c45320.graph_c1	7.15344582	10.0037919	7.29989557	1.03217093	1.20808796	1.06060236	4.5E-24	-3.9795	down	PREDICTED: laccase-17-like [Sesamum indicum]
c45321.graph_c1	66.4375836	71.807053	60.1158516	66.5490416	66.1855854	68.1926654	4.468E-27	-1.07034	down	PREDICTED: UDP-glucuronate 4-epimerase 1 [Sesamum indicum]
c45321.graph_c2	19.9779263	20.3534499	21.1845058	21.7474962	18.5288787	19.4483912	4.724E-33	-1.13393	down	PREDICTED: protein FAR1-RELATED SEQUENCE 4 [Sesamum indicum]
c45326.graph_c0	1.7671061	1.96541733	2.46003475	0.21405376	0.19327047	0.08733306	4.034E-25	-4.74407	down	conserved hypothetical protein [Ricinus communis]
c45328.graph_c0	5.61503585	5.2279487	7.02648984	30.8835409	27.8205796	28.7634268	3.49E-20	1.19734	up	PREDICTED: RNA polymerase sigma factor sigA [Sesamum indicum]
c45330.graph_c0	3.27671551	3.70875438	2.58697164	14.4446412	12.7470216	14.2126375	3.65E-12	1.0259	up	PREDICTED: uncharacterized protein LOC105167904 [Sesamum indicum]
c45331.graph_c0	138.570724	153.051674	120.498966	95.1523948	98.6236118	84.4717205	4.338E-25	-1.65585	down	PREDICTED: ferredoxin--nitrite reductase, chloroplastic [Sesamum indicum]
c45334.graph_c0	549.462852	508.971473	599.222047	347.162078	344.07339	373.833173	6.522E-26	-1.72978	down	hypothetical protein MIMGU_mgv1a010052mg [Erythranthe guttata]
c45339.graph_c0	0.7027507	0.4689696	0.66893517	0	0	0	1.159E-29	-Inf	down	-

#ID	0 h-1 FPKM	0 h-2 FPKM	0 h-3 FPKM	28 h-1 FPKM	28 h-2 FPKM	28 h-3 FPKM	FDR	log2FC	regulated	nr_annotation
c45340.graph_c0	12.746407	11.6195955	10.9720061	12.6475231	11.7530417	12.8521188	1.589E-25	-1.01237	down	PREDICTED: B3 domain-containing transcription repressor VAL1 [Populus euphratica]
c45342.graph_c0	62.5889661	61.4687891	59.2969945	35.5841408	37.1953632	35.355224	1.05E-114	-1.85187	down	PREDICTED: uncharacterized protein At4g26450 [Sesamum indicum]
c45349.graph_c0	7.64421738	6.17467656	6.56139036	55.150165	50.8219384	50.9008406	8.042E-80	1.85518	up	PREDICTED: uncharacterized protein LOC105168326 [Sesamum indicum]
c45351.graph_c1	45.7970811	52.5144262	43.708727	16.5362338	18.4268308	17.3840998	1.106E-58	-2.52958	down	PREDICTED: cytochrome P450 710A1-like [Sesamum indicum]
c45351.graph_c2	0.08565664	0.08574241	0.03669072	2.36568054	1.57388569	1.82877726	1.967E-12	3.71184	up	PREDICTED: potassium channel KAT3-like [Sesamum indicum]
c45352.graph_c0	1.72126275	1.66357301	1.37289773	39.8595709	37.1189945	34.2498994	2.072E-80	3.45891	up	PREDICTED: uncharacterized protein LOC105164615 [Sesamum indicum]
c45353.graph_c0	1.12739325	0.75234811	2.99407291	11.908365	12.5277194	13.1938994	4.618E-08	1.84256	up	PREDICTED: protein ZINC INDUCED FACILITATOR-LIKE 1-like isoform X2 [Pyrus x bretschneideri]
c45353.graph_c1	1.32552492	1.99027835	2.64965868	13.0592036	16.2371125	14.9798271	2.884E-13	1.79328	up	PREDICTED: protein ZINC INDUCED FACILITATOR 1-like [Sesamum indicum]
c45356.graph_c1	19.9560017	19.3257717	18.904714	13.2449784	13.0640933	13.5257939	7.984E-86	-1.63643	down	PREDICTED: uncharacterized protein LOC105174921 [Sesamum indicum]
c45357.graph_c0	4.949178	4.6789042	4.71102939	0.93256438	1.44345977	1.03273811	1.584E-20	-3.16375	down	PREDICTED: two-component response regulator-like APRR9 [Sesamum indicum]
c45368.graph_c1	4.92600197	3.67578762	3.65419846	19.3330251	18.7636329	19.5601389	1.248E-22	1.14688	up	PREDICTED: E3 ubiquitin-protein ligase HOS1 isoform X1 [Sesamum indicum]
c45371.graph_c0	0.07905292	0.07913208	0.05079307	2.35566351	2.46414161	2.20350007	1.061E-12	3.98516	up	-
c45372.graph_c0	72.9417579	74.0937109	66.8371382	35.5974813	33.1610211	35.0083333	1.23E-150	-2.13261	down	PREDICTED: uncharacterized RING finger protein C4G3.12c [Sesamum indicum]
c45377.graph_c0	15.2341062	17.0401115	15.4097033	91.0346855	91.7342388	89.4685055	4.754E-65	1.42282	up	PREDICTED: transmembrane 9 superfamily member 1-like [Sesamum indicum]
c45382.graph_c0	1684.32576	1714.14098	1965.92845	313.682817	322.034612	324.870773	2.951E-59	-3.57395	down	Polyubiquitin [Triticum urartu]
c45383.graph_c1	3.59887452	3.34515837	2.1471804	18.1537959	18.8934305	18.8279447	2.029E-19	1.53534	up	PREDICTED: GTP-binding protein OBGC, chloroplastic [Sesamum indicum]
c45387.graph_c0	0.67738532	1.0431748	0.80350796	10.1493609	10.497477	10.6303645	1.466E-19	2.54013	up	PREDICTED: nudix hydrolase 9 isoform X1 [Sesamum indicum]
c45390.graph_c0	16.8134457	17.7510875	17.9940076	16.9741683	17.3382596	20.3063991	5.754E-18	-1.03501	down	General transcription factor IIE subunit 1 isoform 2 [Theobroma cacao]
c45393.graph_c1	13.3086455	12.0405483	13.4706482	73.2909265	73.9731628	71.8230551	2.869E-63	1.4055	up	PREDICTED: aldehyde dehydrogenase family 7 member B4 isoform X1 [Sesamum indicum]
c45395.graph_c0	45.9040321	48.8606238	45.3026363	17.9818914	19.6118678	17.5097353	5.72E-191	-2.43651	down	PREDICTED: probable protein phosphatase 2C 4 [Sesamum indicum]
c45397.graph_c0	0.47487361	0.50036749	0.32117441	14.5501818	14.2855418	13.9776258	7.398E-84	3.95972	up	PREDICTED: choline transporter-like protein 2 [Sesamum indicum]
c45401.graph_c1	5.49494379	5.15231664	4.06690537	28.6130444	27.7291511	26.6687082	2.647E-29	1.40901	up	PREDICTED: mitogen-activated protein kinase kinase 2 [Sesamum indicum]

#ID	0 h-1 FPKM	0 h-2 FPKM	0 h-3 FPKM	28 h-1 FPKM	28 h-2 FPKM	28 h-3 FPKM	FDR	log2FC	regulated	nr_annotation
c45402.graph_c0	1.50636294	1.23371291	1.26116134	1.29387334	0.88367349	0.97457546	2.3E-07	-1.43298	down	PREDICTED: cellulose synthase-like protein E1 [Sesamum indicum]
c45405.graph_c0	0.82191259	0.68931902	0.91346181	10.3165916	10.2187095	10.1704108	1.529E-43	2.57005	up	PREDICTED: beta-amylase 7 [Sesamum indicum]
c45406.graph_c0	931.010988	935.738944	932.214349	296.21273	323.017887	336.277759	5.82E-139	-2.64078	down	15.5 kDa oleosin [Sesamum indicum]
c45407.graph_c0	0.29075334	0.40746228	0.44835547	3.63466709	3.43440377	3.89700034	3.177E-07	2.16301	up	PREDICTED: root phototropism protein 3 [Populus euphratica]
c45407.graph_c1	0.48980468	0.57681782	0.62941841	8.52275283	9.2456404	7.84303191	3.947E-32	2.82248	up	hypothetical protein MIMGU_mgv1a005061mg [Erythranthe guttata]
c45420.graph_c0	8.04712808	5.76150596	5.74882719	30.3334497	27.0660462	27.4211641	3.132E-12	1.02972	up	PREDICTED: L-aminoadipate-semialdehyde dehydrogenase-phosphopantetheinyl transferase [Sesamum indicum]
c45423.graph_c0	19.8316666	20.0199366	20.7010702	9.64469178	10.4747442	10.5016345	2.21E-111	-2.07463	down	PREDICTED: aspartic proteinase nepenthesin-1 [Sesamum indicum]
c45424.graph_c0	139.42402	138.631966	132.480324	39.504822	39.0894798	37.7002108	0	-2.9098	down	hypothetical protein F383_22559 [Gossypium arboreum]
c45426.graph_c0	0.33770523	0.28170283	0.28930973	2.0453518	2.28998352	2.63700477	5.207E-05	1.85166	up	hypothetical protein MIMGU_mgv1a010549mg [Erythranthe guttata]
c45427.graph_c0	1.10820511	0.82828839	1.13926995	6.01393024	5.23608603	5.50320657	2.97E-15	1.35368	up	PREDICTED: alpha-N-acetylglucosaminidase [Sesamum indicum]
c45430.graph_c0	0	0	0	3.74681775	4.15189447	8.06034616	2.752E-06	Inf	up	predicted protein [Bathycoccus prasinos]
c45432.graph_c1	0.48075456	0.54139046	0.23167074	3.53777264	3.43089011	3.5639427	6.002E-06	1.98849	up	PREDICTED: charged multivesicular body protein 5-like [Nelumbo nucifera]
c45434.graph_c1	6.8795423	6.88643112	5.82668279	3.18182562	3.7450176	3.70906247	4.212E-15	-1.96957	down	-
c45437.graph_c1	42.9840523	37.8407927	41.0880952	37.9872675	39.0054041	39.2480729	4.898E-38	-1.15896	down	PREDICTED: rop guanine nucleotide exchange factor 1 [Sesamum indicum]
c45443.graph_c2	1.59609211	1.71751713	1.48700512	0.66701756	0.81173408	0.85191791	5.754E-10	-2.13136	down	PREDICTED: protein IQ-DOMAIN 1-like isoform X1 [Sesamum indicum]
c45444.graph_c0	2.29272264	0.8980507	1.47311968	0	0	0	5.715E-11	-Inf	down	PREDICTED: malate dehydrogenase-like [Musa acuminata subsp. malaccensis]
c45449.graph_c0	447.950348	445.741815	402.582816	132.15183	146.016468	137.15944	1.46E-195	-2.73137	down	PREDICTED: putative DNA-binding protein ESCAROLA [Sesamum indicum]
c45449.graph_c1	13.869955	16.3972981	14.7504341	0.43450876	0.2353924	0.70911121	2.179E-86	-6.11533	down	-
c45449.graph_c2	1.92429955	1.85353865	1.58632524	10.1594223	9.8163075	9.92647428	1.421E-11	1.39069	up	PREDICTED: protein IQ-DOMAIN 1-like [Sesamum indicum]
c45449.graph_c4	23.8629523	24.3710403	19.890706	0.35155654	0.10580753	1.91244846	1.668E-94	-5.92299	down	-
c45455.graph_c0	141.868009	146.577644	140.608988	151.547902	149.567173	154.768656	2.112E-35	-1.00269	down	PREDICTED: 3-ketoacyl-CoA synthase 11 [Sesamum indicum]
c45461.graph_c0	0.16278119	0.10184012	0.20918028	4.36262343	4.37967829	3.55986509	3.929E-29	3.60192	up	PREDICTED: putative pentatricopeptide repeat-containing protein At4g17915 [Sesamum indicum]
c45462.graph_c0	2.43419505	0.82470639	0.86622563	0	0	0	1.51E-06	-Inf	down	PREDICTED: serine hydroxymethyltransferase, mitochondrial [Musa acuminata subsp. malaccensis]
c45464.graph_c1	7.50119927	9.49017588	7.22950354	7.42054421	7.99904607	8.52657429	3.722E-06	-1.1054	down	f-box family protein, partial [Galium verum var. asiaticum]
c45467.graph_c1	0.19285497	0	0.24782627	6.5177134	5.82160578	7.14838832	5.576E-15	4.3681	up	hypothetical protein MIMGU_mgv1a008026mg [Erythranthe guttata]

#ID	0 h-1 FPKM	0 h-2 FPKM	0 h-3 FPKM	28 h-1 FPKM	28 h-2 FPKM	28 h-3 FPKM	FDR	log2FC	regulated	nr_annotation
c45471.graph_c0	0.27334174	0.43778472	0.84301189	3.93353136	4.69960517	4.92806949	3.784E-08	2.02152	up	PREDICTED: mitochondrial thiamine pyrophosphate carrier-like isoform X2 [Sesamum indicum]
c45473.graph_c0	5.55256967	4.16859729	4.21179242	115.686501	117.687949	114.843262	6.75E-268	3.55576	up	PREDICTED: peroxisomal (S)-2-hydroxy-acid oxidase GLO5 [Musa acuminata subsp. malaccensis]
c45475.graph_c0	4.18825973	3.67379958	4.21688709	2.98124009	3.31513992	3.25209243	5.625E-10	-1.43011	down	PREDICTED: probable carboxylesterase 18 [Sesamum indicum]
c45476.graph_c1	2.13410973	2.00391285	1.71097604	13.2114509	12.9511616	12.0236863	8.505E-54	1.61857	up	PREDICTED: nuclear pore complex protein NUP205 isoform X2 [Sesamum indicum]
c45479.graph_c0	3.84863039	3.91778055	4.06548572	16.5696314	17.8716562	16.0546032	8.461E-13	1.00185	up	PREDICTED: reticulon-like protein B17 [Sesamum indicum]
c45485.graph_c0	7.92041863	7.92834973	8.56445799	7.61685165	6.94068817	8.82170016	8.942E-12	-1.15301	down	PREDICTED: phospholipase A1-II 1-like [Sesamum indicum]
c45487.graph_c2	2.27426744	2.07858436	2.28719246	0.28746512	0.06488851	0.35185379	4.436E-19	-4.32356	down	unnamed protein product [Coffea canephora]
c45501.graph_c0	12.3552923	12.8230627	12.4923995	12.112228	13.355999	12.8087617	1.103E-25	-1.06761	down	PREDICTED: probable serine/threonine-protein kinase At1g18390 [Sesamum indicum]
c45502.graph_c0	14.1513631	13.5775303	13.4272092	13.1054539	13.5968484	13.3331199	6.719E-35	-1.12984	down	PREDICTED: uncharacterized protein LOC105163310 [Sesamum indicum]
c45503.graph_c0	0	0	0.11497538	3.1213399	5.34298399	9.12670652	2.411E-05	6.13086	up	40s ribosomal protein s22 [Lichtheimia corymbifera JMRC:FSU:9682]
c45506.graph_c0	14.9035417	13.3850899	14.4765082	12.5018086	11.7277517	12.8889342	3.911E-32	-1.2944	down	hypothetical protein MIMGU_mgv1a011631mg [Erythranthe guttata]
c45510.graph_c0	28.3430575	30.8218336	33.9205475	7.22378289	7.49011359	6.97900473	6.477E-48	-3.19434	down	PREDICTED: 26.5 kDa heat shock protein, mitochondrial [Sesamum indicum]
c45512.graph_c0	3.51025659	2.83804627	2.77588843	2.84560744	1.94913682	1.96168326	8.497E-07	-1.52197	down	hypothetical protein MIMGU_mgv1a000874mg [Erythranthe guttata]
c45512.graph_c1	1.29201339	1.27583002	0.83014418	1.29433573	0.84785414	0.91120324	6.705E-05	-1.23975	down	PREDICTED: LOW QUALITY PROTEIN: putative E3 ubiquitin-protein ligase LIN [Sesamum indicum]
c45515.graph_c0	20.157587	20.672009	19.7516091	6.00682742	6.3533623	6.65843135	1.76E-181	-2.76135	down	PREDICTED: V-type proton ATPase subunit a3-like [Sesamum indicum]
c45519.graph_c0	1.17599104	1.37336338	0.37779876	4.84331987	4.69461255	6.65465491	0.0030213	1.39051	up	predicted protein [Arabidopsis lyrata subsp. lyrata]
c45522.graph_c1	41.8510652	41.5023623	49.743621	4.73627773	5.45434932	5.87814398	4.77E-54	-4.14252	down	PREDICTED: G-type lectin S-receptor-like serine/threonine-protein kinase At4g27290 [Sesamum indicum]
c45525.graph_c0	31.5596968	24.7218889	26.0970026	20.0376073	22.7787412	20.160125	3.264E-17	-1.47648	down	PREDICTED: MATE efflux family protein 5 [Sesamum indicum]
c45528.graph_c0	0.47557095	0.38348244	0.54322444	7.46971255	7.27325658	7.14504459	3.122E-59	2.8711	up	PREDICTED: uncharacterized protein LOC105168379 [Sesamum indicum]
c45530.graph_c0	1.90536193	1.21371718	2.00328997	46.3280576	38.1925104	42.4260048	4.963E-34	3.53895	up	hypothetical protein AMTR_s00028p00129850 [Amborella trichopoda]
c45532.graph_c0	9.32956503	8.22229871	8.81789372	3.02182215	3.28297941	3.0872395	6.106E-63	-2.57969	down	PREDICTED: uncharacterized protein LOC105175116 [Sesamum indicum]
c45533.graph_c0	39.4281412	40.4786158	33.0831234	21.1956664	20.5715931	20.7453738	5.625E-46	-1.94219	down	PREDICTED: uncharacterized protein LOC105157181 [Sesamum indicum]
c45537.graph_c0	7.27823661	8.4512086	7.29520131	36.8697695	36.5377445	36.6463856	1.815E-15	1.16683	up	PREDICTED: DNA polymerase epsilon subunit 3 [Sesamum indicum]

#ID	0 h-1 FPKM	0 h-2 FPKM	0 h-3 FPKM	28 h-1 FPKM	28 h-2 FPKM	28 h-3 FPKM	FDR	log2FC	regulated	nr_annotation
c45541.graph_c0	0.11651871	0.19439231	0.14973116	49.1173744	41.4937753	48.7171731	2.21E-124	7.14918	up	PREDICTED: WAT1-related protein At1g09380 [Sesamum indicum]
c45543.graph_c0	1.50325583	0.77832471	0.46628286	11.7167392	13.2664167	12.542738	5.371E-24	2.69374	up	PREDICTED: protein SCARECROW [Sesamum indicum]
c45543.graph_c1	69.7189926	72.7736629	75.1767135	37.1521188	35.4084844	44.1263683	6.711E-71	-1.98992	down	-
c45543.graph_c2	146.990011	142.961081	135.276899	135.710438	129.710402	133.222631	7.983E-49	-1.1826	down	PREDICTED: E3 ubiquitin-protein ligase UPL5 [Sesamum indicum]
c45544.graph_c0	25.022116	23.3852742	25.6524122	13.7615487	13.1257755	14.9802296	5.453E-74	-1.91338	down	PREDICTED: F-box protein FBW2 [Sesamum indicum]
c45550.graph_c0	55.5144229	56.6075405	62.3876542	56.9204972	55.3932895	55.1999755	4.323E-20	-1.15116	down	PREDICTED: uncharacterized protein LOC104114664 [Nicotiana tomentosiformis]
c45551.graph_c0	0.20950179	0.29958796	0.23075837	2.37097879	2.02293091	2.04119654	5.528E-07	2.02984	up	hypothetical protein CICLE_v10013877mg [Citrus clementina]
c45552.graph_c0	2.02343353	1.56060009	1.87554847	10.4630984	9.88255156	9.81650607	1.034E-12	1.37555	up	PREDICTED: probable anion transporter 6, chloroplastic isoform X2 [Sesamum indicum]
c45554.graph_c0	48.8193415	51.1306446	46.7025634	31.9334545	33.2231123	32.8130702	1.881E-57	-1.67183	down	PREDICTED: protein polybromo-1-like [Sesamum indicum]
c45554.graph_c1	58.8663967	59.2507585	57.8107582	40.3846686	38.9251064	37.9811281	5.735E-94	-1.67535	down	PREDICTED: vacuolar protein 8 [Sesamum indicum]
c45555.graph_c0	17.85357	16.570461	14.635761	12.4289959	12.0751066	10.6693411	1.176E-43	-1.56882	down	PREDICTED: abscisic acid 8'-hydroxylase 3-like [Solanum lycopersicum]
c45556.graph_c0	39.062434	40.5698522	39.8705807	31.5422603	30.6141121	31.8843868	3.385E-63	-1.43625	down	PREDICTED: granule-bound starch synthase 1, chloroplastic/amyloplastic [Sesamum indicum]
c45559.graph_c0	6.60479391	6.89475365	6.09275627	6.99476096	6.34657221	6.47708383	4.792E-17	-1.07287	down	PREDICTED: uncharacterized protein LOC105170176 [Sesamum indicum]
c45560.graph_c0	7.42109233	6.20120216	7.46326757	2.90785776	2.49848149	3.06166217	6.426E-25	-2.40656	down	PREDICTED: uncharacterized protein LOC105171076 [Sesamum indicum]
c45565.graph_c0	48.0017798	43.8730288	46.695901	41.1437504	41.5473156	41.2890955	3.243E-50	-1.25096	down	hypothetical protein MIMGU_mgv1a003857mg [Erythranthe guttata]
c45566.graph_c0	74.0480001	73.2147613	76.4440263	26.3392219	28.3300178	25.9277466	5.87E-208	-2.56414	down	hypothetical protein MIMGU_mgv1a020815mg [Erythranthe guttata]
c45569.graph_c0	20.3805894	19.7634663	29.4635973	3.00878803	2.2987068	1.51085936	3.959E-15	-4.44975	down	-
c45569.graph_c1	0	0.0523539	0.06720954	2.31875947	1.75040561	1.55089245	1.885E-11	4.44464	up	-
c45569.graph_c3	74.1422938	70.1946862	68.5655201	67.1241291	65.036294	65.5112485	1.836E-46	-1.19676	down	PREDICTED: putative glycerol-3-phosphate transporter 1 [Eucalyptus grandis]
c45574.graph_c0	1.04175747	0.82704878	0.73859288	4.12512569	4.57324517	4.72953982	1.637E-05	1.27827	up	PREDICTED: probable lipid-A-disaccharide synthase, mitochondrial isoform X1 [Sesamum indicum]
c45580.graph_c1	1.45148261	0.72646802	1.17802902	19.5994739	18.6991212	17.0123952	3.723E-53	2.95306	up	PREDICTED: ABC transporter G family member 15-like isoform X2 [Sesamum indicum]
c45581.graph_c0	1.04783714	1.16875912	0.8463791	3.82956889	4.81333354	5.06020243	4.959E-05	1.07452	up	hypothetical protein MIMGU_mgv1a001682mg [Erythranthe guttata]
c45588.graph_c0	9.91802783	11.5826191	10.0898379	46.8972824	44.8232693	43.5545678	3.58E-17	1.00783	up	PREDICTED: probable receptor-like protein kinase At5g56460 [Sesamum indicum]
c45590.graph_c0	1.87274859	1.79311848	2.5635054	15.327202	13.6252962	15.9353964	7.562E-22	1.75463	up	hypothetical protein MIMGU_mgv1a014871mg [Erythranthe guttata]

#ID	0 h-1 FPKM	0 h-2 FPKM	0 h-3 FPKM	28 h-1 FPKM	28 h-2 FPKM	28 h-3 FPKM	FDR	log2FC	regulated	nr_annotation
c45594.graph_c0	0.13529975	0.47402332	0.43466384	3.44172777	2.79680174	4.53359512	8.759E-06	2.27001	up	hypothetical protein MIMGU_mgv1a011617mg [Erythranthe guttata]
c45596.graph_c0	46.0805474	47.9573992	43.8239507	36.4753005	33.5456223	34.7826236	7.274E-76	-1.48526	down	PREDICTED: scarecrow-like protein 13 [Sesamum indicum]
c45602.graph_c0	1.74774985	0.78899018	2.28996556	6.97394639	6.99396761	7.43853603	0.0005814	1.05243	up	PREDICTED: probable alpha-mannosidase I MNS5 isoform X1 [Sesamum indicum]
c45604.graph_c0	6.03702796	6.08670543	5.09719577	516.367822	456.263217	440.222867	4.49E-209	5.26962	up	PREDICTED: glucose-6-phosphate 1-dehydrogenase, chloroplastic-like [Sesamum indicum]
c45606.graph_c0	1.02930215	1.03033284	1.20767714	0.71556518	0.73419121	0.55735465	4.211E-06	-1.79695	down	hypothetical protein VITISV_002159 [Vitis vinifera]
c45611.graph_c0	12.4630261	12.1824571	14.2419554	59.059738	57.3323588	58.5847284	1.388E-29	1.0774	up	PREDICTED: AP-4 complex subunit mu [Sesamum indicum]
c45616.graph_c0	2.02221232	2.14331003	3.21006357	0.30259194	0.35127276	0.59964476	2.288E-09	-3.65062	down	PREDICTED: la-related protein 6C-like isoform X1 [Sesamum indicum]
c45617.graph_c0	20.7012453	19.3619831	17.5508348	14.0061298	14.7343737	14.863915	1.423E-47	-1.49039	down	PREDICTED: uncharacterized protein LOC105158248 [Sesamum indicum]
c45623.graph_c0	2.73168274	2.11783363	1.27335155	0	0	0	4.707E-20	-Inf	down	--
c45625.graph_c0	19.6003543	20.1151225	20.7377519	16.2526662	14.985709	15.7922207	1.916E-59	-1.45332	down	PREDICTED: protein VACUOLELESS1 [Sesamum indicum]
c45627.graph_c0	2.71028197	1.84096151	1.74140982	1.96981732	1.96912039	1.60735485	0.000727	-1.26867	down	-
c45628.graph_c0	26.5138553	27.8998425	21.3986359	23.9130158	22.1602128	23.394326	5.105E-14	-1.21362	down	PREDICTED: E3 ubiquitin-protein ligase UPL2-like [Sesamum indicum]
c45629.graph_c0	2.20137795	1.40227965	1.80018225	1.59994881	1.57593138	1.7209459	0.0021729	-1.23061	down	-
c45632.graph_c0	0	0.04848218	0.1867176	2.28808596	2.06592709	1.3787516	2.821E-08	3.48506	up	PREDICTED: probable protein phosphatase 2C 14 [Sesamum indicum]
c45632.graph_c1	0.34123575	0.38176303	0.23214783	5.28111528	5.7035812	5.21407172	1.214E-30	2.99812	up	PREDICTED: protein-tyrosine-phosphatase MKP1-like isoform X1 [Sesamum indicum]
c45637.graph_c0	56.9376088	54.8948213	57.735979	46.5157739	44.2047013	44.3672034	3.706E-68	-1.41888	down	PREDICTED: activator of 90 kDa heat shock protein ATPase homolog [Sesamum indicum]
c45639.graph_c0	15.0454639	15.0247564	14.6956897	68.6486694	65.8763237	67.2921653	2.573E-33	1.08238	up	PREDICTED: dolichyl-diphosphooligosaccharide--protein glycosyltransferase subunit 1A [Sesamum indicum]
c45641.graph_c0	26.0770213	29.771682	27.8948148	17.8257157	17.6674451	17.7222722	1.113E-26	-1.74516	down	PREDICTED: F-box protein PP2-A15-like [Nicotiana sylvestris]
c45642.graph_c0	95.6197768	98.4392499	91.0526769	48.6011875	46.263107	47.4822258	1.3E-144	-2.09189	down	PREDICTED: uncharacterized protein LOC105169167 [Sesamum indicum]
c45653.graph_c0	3.32709187	2.65350812	2.50269939	3.1455105	2.59159222	2.63087866	5.15E-08	-1.10732	down	PREDICTED: integrator complex subunit 9 homolog isoform X2 [Sesamum indicum]
c45660.graph_c0	30.8114009	29.6885536	28.3871262	27.0844665	26.9758404	28.2980141	4.423E-39	-1.1994	down	PREDICTED: uncharacterized protein LOC105168018 [Sesamum indicum]
c45661.graph_c0	0.12640267	0.06326462	0.08121622	3.53694625	3.38707817	3.47333833	1.276E-27	4.17655	up	PREDICTED: chaperonin 60 subunit beta 4, chloroplastic isoform X1 [Sesamum indicum]
c45662.graph_c1	399.967544	413.856287	388.466041	259.760579	254.038665	256.61738	7.079E-74	-1.73212	down	PREDICTED: glucan endo-1,3-beta-glucosidase-like [Sesamum indicum]

#ID	0 h-1 FPKM	0 h-2 FPKM	0 h-3 FPKM	28 h-1 FPKM	28 h-2 FPKM	28 h-3 FPKM	FDR	log2FC	regulated	nr_annotation
c45663.graph_c0	31.8507435	34.345873	32.695842	27.2995479	25.8219655	27.8521441	1.487E-60	-1.3788	down	PREDICTED: uncharacterized protein LOC105167456 isoform X2 [Sesamum indicum]
c45665.graph_c0	3.44833649	3.29488996	2.25590854	2.36952758	2.69489829	2.34252797	5.436E-07	-1.36676	down	hypothetical protein MIMGU_mgv1a015231mg [Erythranthe guttata]
c45667.graph_c0	21.9553657	21.9371727	19.3935674	20.5370227	19.5307371	21.1736919	2.702E-40	-1.13599	down	hypothetical protein MIMGU_mgv1a002103mg [Erythranthe guttata]
c45668.graph_c0	128.196915	135.435528	121.037185	684.811085	690.027084	683.676976	2.563E-60	1.33024	up	triosephosphate isomerase, putative [Ricinus communis]
c45669.graph_c0	1.03052518	1.22993345	1.27333236	8.06593586	7.93303238	7.54549225	2.846E-11	1.64303	up	PREDICTED: uncharacterized protein LOC105168837 isoform X1 [Sesamum indicum]
c45670.graph_c0	5.29637586	6.05015177	4.62411226	26.2210398	25.3413961	24.2508227	6.38E-34	1.15833	up	PREDICTED: RRP12-like protein isoform X1 [Sesamum indicum]
c45672.graph_c0	1.36223194	1.16473826	1.45876831	8.43615604	7.98953021	7.5234083	1.586E-13	1.49473	up	PREDICTED: alpha-1,3-mannosyl-glycoprotein 2-beta-N-acetylglucosaminyltransferase isoform X1 [Sesamum indicum]
c45673.graph_c0	66.7131895	59.4534997	68.8247622	3.70210263	3.42378389	7.28825291	1.53E-136	-4.8454	down	PREDICTED: probable galactinol--sucrose galactosyltransferase 2-like isoform X1 [Citrus sinensis]
c45676.graph_c0	4.53732006	4.07694834	4.49923087	23.7330397	24.6406716	24.9808256	9.914E-27	1.39321	up	PREDICTED: GABA transporter 1 [Sesamum indicum]
c45681.graph_c1	2.10616574	2.55506145	2.04332269	30.5136084	30.223642	29.4153918	4.65E-153	2.65943	up	PREDICTED: uncharacterized protein LOC105164103 [Sesamum indicum]
c45682.graph_c0	21.3627598	23.4535854	21.6121612	17.4349729	17.0621395	18.0044074	3.254E-62	-1.42964	down	PREDICTED: GTPase-activating protein gyp7 isoform X3 [Sesamum indicum]
c45685.graph_c0	1.67522936	1.36304728	1.43899469	8.34703373	7.2250138	7.5230372	1.152E-25	1.27782	up	PREDICTED: probable receptor-like serine/threonine-protein kinase At5g57670 [Sesamum indicum]
c45687.graph_c1	2.19069896	1.5049263	1.8767566	10.2399502	8.00543611	9.29828124	6.573E-08	1.21629	up	PREDICTED: uncharacterized protein LOC105177833 [Sesamum indicum]
c45690.graph_c0	0.63286126	0.72087359	0.4767338	3.87000754	4.23893104	4.72389964	1.714E-11	1.72354	up	PREDICTED: U-box domain-containing protein 35 [Sesamum indicum]
c45693.graph_c1	20.7942628	21.3227701	17.4785544	13.3371039	12.435491	13.2892522	1.268E-40	-1.69747	down	PREDICTED: abl interactor 2-like isoform X1 [Sesamum indicum]
c45697.graph_c0	13.8478987	13.023352	12.0547438	8.72533541	9.14232962	8.75901277	2.071E-50	-1.63688	down	PREDICTED: uncharacterized protein LOC105177954 [Sesamum indicum]
c45699.graph_c0	0	0	0	3.19760091	6.23620905	8.61041357	3.178E-07	Inf	up	60s ribosomal protein l9 [Pfiesteria piscicida]
c45702.graph_c0	43.5171089	44.6480028	47.3716316	30.7398346	27.8888411	29.8344132	2.862E-55	-1.70698	down	PREDICTED: uncharacterized protein LOC105178309 [Sesamum indicum]
c45702.graph_c1	12.4147356	12.8182317	14.0568655	0.82026893	0.48425542	0.51487135	7.66E-140	-5.52535	down	PREDICTED: uncharacterized protein LOC105162982 [Sesamum indicum]
c45705.graph_c0	3.49376546	3.72208805	3.27101392	19.5884892	20.6179543	17.8932529	2.012E-28	1.37985	up	PREDICTED: tyrosine-sulfated glycopeptide receptor 1 [Sesamum indicum]
c45711.graph_c0	0.19728714	0.19748469	0.38028268	2.15080735	2.71876833	1.93054536	1.415E-05	2.03189	up	hypothetical protein VITISV_029424 [Vitis vinifera]
c45712.graph_c0	98.326615	99.7086369	95.5472928	60.4013432	59.4392035	62.2149219	5.69E-102	-1.77932	down	PREDICTED: plastidial pyruvate kinase 2-like [Sesamum indicum]

#ID	0 h-1 FPKM	0 h-2 FPKM	0 h-3 FPKM	28 h-1 FPKM	28 h-2 FPKM	28 h-3 FPKM	FDR	log2FC	regulated	nr_annotation
c45713.graph_c0	1.42197674	1.49457067	2.13184582	10.8860368	10.6066869	10.8365966	3.232E-23	1.58295	up	PREDICTED: probable sodium/metabolite cotransporter BASS2, chloroplastic isoform X2 [Sesamum indicum]
c45720.graph_c0	1.18509867	1.54217098	0.91373923	11.0033999	11.8209459	10.876332	2.105E-27	2.12339	up	PREDICTED: serine/threonine-protein kinase BLUS1-like isoform X4 [Sesamum indicum]
c45721.graph_c0	9.089999	7.71155503	9.00910634	8.52459211	9.16631702	8.69499694	9.957E-16	-1.05924	down	hypothetical protein MIMGU_mgv1a008794mg [Erythranthe guttata]
c45722.graph_c0	1.97190877	2.20163911	1.31572082	17.6941612	17.9918533	17.137037	3.613E-34	2.18091	up	hypothetical protein MIMGU_mgv1a007079mg [Erythranthe guttata]
c45729.graph_c0	9.08714367	3.88254277	4.12979076	0	0	0	2.295E-10	-Inf	down	chaperone [Agave tequilana]
c45742.graph_c0	0.60578347	0.92081455	0.86495083	7.89242552	9.14322455	8.22317283	2.227E-31	2.30673	up	PREDICTED: DNA replication licensing factor MCM5 [Sesamum indicum]
c45743.graph_c0	14.1836219	14.3529922	17.0313344	16.1106686	13.783499	15.9960439	1.376E-08	-1.08239	down	PREDICTED: WAT1-related protein At3g28050-like [Sesamum indicum]
c45745.graph_c0	67.853071	65.2664207	65.8360819	376.011944	340.073621	393.405513	1.116E-34	1.38967	up	PREDICTED: alpha-galactosidase [Sesamum indicum]
c45745.graph_c1	6.0229221	4.93277985	5.11041954	3.92711579	4.75612302	4.04193563	7.949E-23	-1.42572	down	PREDICTED: CSC1-like protein ERD4 [Sesamum indicum]
c45746.graph_c0	1.89722416	1.76347223	1.69789854	18.9597593	21.2985608	18.6254872	3.323E-48	2.36819	up	PREDICTED: LOW QUALITY PROTEIN: uncharacterized protein LOC105161466 [Sesamum indicum]
c45747.graph_c0	7.71565169	6.6859091	6.75792911	38.026284	36.7776133	36.8115296	4.503E-31	1.30995	up	hypothetical protein CISIN_1g034493mg [Citrus sinensis]
c45752.graph_c0	0	0	0	3.09563284	4.28276275	7.94471239	1.368E-06	Inf	up	PREDICTED: 60S ribosomal protein L6-3-like isoform X2 [Cicer arietinum]
c45753.graph_c0	3.12808951	3.46585621	3.22191207	2.62057322	2.36613192	2.77562784	3.857E-14	-1.42864	down	PREDICTED: phosphatidylinositol/phosphatidylcholine transfer protein SFH9 isoform X1 [Sesamum indicum]
c45754.graph_c1	5.05019401	4.27204311	3.19914752	1.60259167	1.9604381	2.40448381	7.568E-11	-2.153	down	PREDICTED: uncharacterized protein LOC105170987 isoform X2 [Sesamum indicum]
c45762.graph_c0	15.9583686	15.4761232	15.9499942	15.1254645	16.2902649	15.9766715	5.392E-26	-1.09038	down	PREDICTED: caffeoylshikimate esterase-like [Sesamum indicum]
c45764.graph_c0	1.22255414	0.99834549	1.36431593	5.30788903	4.70807633	5.62864432	2.583E-05	1.03352	up	PREDICTED: tRNA pseudouridine(38/39) synthase isoform X3 [Sesamum indicum]
c45766.graph_c0	60.1959704	53.9878771	53.1341736	40.7382224	39.6029438	41.488854	2.094E-79	-1.54672	down	PREDICTED: uncharacterized protein DDB_G0286299-like [Sesamum indicum]
c45772.graph_c0	11.7065894	10.960068	11.5922908	10.7104252	10.5742957	10.5773899	1.536E-12	-1.19527	down	PREDICTED: diphthamide biosynthesis protein 3-like [Sesamum indicum]
c45779.graph_c0	119.360801	115.23231	119.872952	39.2893376	39.9512052	41.7741503	3.3E-212	-2.64093	down	hypothetical protein MIMGU_mgv1a025324mg, partial [Erythranthe guttata]
c45783.graph_c0	9.67954925	10.0095474	9.73158	6.18230842	5.72203344	6.61031382	7.475E-47	-1.7581	down	PREDICTED: uncharacterized protein LOC105166430 [Sesamum indicum]
c45785.graph_c0	0.30562976	0.24474864	0.47129561	16.2154863	16.646689	14.500498	2.494E-85	4.43656	up	hypothetical protein EUTSA_v10017458mg [Eutrema salsugineum]
c45789.graph_c0	0.04423468	0	0	0.93233589	0.92889588	1.60025817	3.89E-06	5.23397	up	--
c45791.graph_c1	3.03545884	2.47969409	2.15210196	11.9436613	11.0129661	11.1529858	5.805E-09	1.06678	up	PREDICTED: cyclic nucleotide-gated ion channel 1 [Sesamum indicum]

#ID	0 h-1 FPKM	0 h-2 FPKM	0 h-3 FPKM	28 h-1 FPKM	28 h-2 FPKM	28 h-3 FPKM	FDR	log2FC	regulated	nr_annotation
c45791.graph_c2	31.9947119	34.0797465	30.2208936	27.3776032	32.5704007	31.8678074	5.965E-28	-1.1582	down	PREDICTED: uncharacterized protein LOC105178732 [Sesamum indicum]
c45792.graph_c0	42.7163756	42.7318448	41.1867016	37.390029	36.2657121	34.9424761	9.984E-57	-1.31196	down	PREDICTED: probable protein phosphatase 2C 60 [Sesamum indicum]
c45800.graph_c0	332.182346	330.019441	328.140203	306.414521	325.105148	317.755064	4.255E-38	-1.15153	down	PREDICTED: uncharacterized protein LOC105158790 isoform X1 [Sesamum indicum]
c45801.graph_c0	60.3363111	58.3350719	63.5450575	60.6173789	58.7736226	62.7830093	2.354E-31	-1.09142	down	PREDICTED: protein CPR-5 [Sesamum indicum]
c45803.graph_c0	0.03215615	0	0	2.7343957	2.30008906	2.66986528	2.612E-20	6.84598	up	unnamed protein product [Vitis vinifera]
c45804.graph_c2	38.3731817	40.2275865	35.2861276	24.830433	24.0005409	24.9179445	3.87E-100	-1.7161	down	PREDICTED: putative SWI/SNF-related matrix-associated actin-dependent regulator of chromatin subfamily A member 3-like 2 isoform X2 [Sesamum indicum]
c45806.graph_c0	0.59181134	0.52658129	0.63375084	7.79004301	9.47173264	8.73546609	1.675E-28	2.79921	up	unnamed protein product [Coffea canephora]
c45813.graph_c1	5.60474374	6.2478965	4.71970055	1.85158884	1.81112871	1.81305765	2.18E-35	-2.68573	down	hypothetical protein MIMGU_mgv1a004624mg [Erythranthe guttata]
c45815.graph_c0	2.9933396	3.90431788	3.72999748	20.2391329	16.5478272	15.9232257	2.365E-07	1.2172	up	-
c45815.graph_c1	65.932923	64.2302848	66.1638823	466.377553	414.504467	389.035145	7.54E-36	1.60226	up	hypothetical protein MIMGU_mgv1a010438mg [Erythranthe guttata]
c45817.graph_c0	2.58666228	2.58925243	2.86862599	12.8250093	12.6261467	12.6085576	1.744E-11	1.1502	up	PREDICTED: LOW QUALITY PROTEIN: actin-related protein 2 [Tarenaya hassleriana]
c45818.graph_c0	105.119902	102.011447	102.794686	28.1541854	29.4828378	27.4200857	0	-2.95604	down	PREDICTED: uncharacterized protein LOC101291143 [Fragaria vesca subsp. vesca]
c45819.graph_c2	1.674902	1.67657916	1.78682786	8.93458958	8.68923782	8.37757533	3.209E-10	1.24759	up	PREDICTED: uncharacterized protein LOC105166519 [Sesamum indicum]
c45820.graph_c0	4.25093441	7.36475379	6.72322341	4.27784488	4.61353311	6.10870135	0.0015978	-1.38286	down	PREDICTED: probable polyamine transporter At3g13620 [Sesamum indicum]
c45820.graph_c2	44.53843	46.8490689	50.6713957	19.093105	18.3448089	17.7029192	6.789E-54	-2.45766	down	PREDICTED: uncharacterized protein LOC105161926 [Sesamum indicum]
c45822.graph_c0	40.7677868	36.5746038	39.4935479	28.7467854	27.3139682	26.6594824	4.376E-67	-1.58879	down	hypothetical protein EUGRSUZ_K00825 [Eucalyptus grandis]
c45825.graph_c1	31.9956587	30.4483204	29.4158319	31.6197784	33.6248194	31.4004944	1.462E-33	-1.01655	down	hypothetical protein POPTR_0010s08110g [Populus trichocarpa]
c45829.graph_c0	48.0823459	50.3227045	47.5484003	39.7685635	39.6395825	37.5685156	2.998E-64	-1.40981	down	PREDICTED: nucleobase-ascorbate transporter 3 [Sesamum indicum]
c45829.graph_c3	0.39198713	0.50448811	0.21587956	2.44194844	2.97654815	3.12175564	3.322E-05	1.85791	up	PREDICTED: uncharacterized protein LOC105167094 [Sesamum indicum]
c45831.graph_c0	128.125325	127.479651	134.797021	32.9680697	33.5513846	31.1814877	1.61E-193	-3.09009	down	PREDICTED: uncharacterized protein LOC105166052 [Sesamum indicum]
c45832.graph_c1	52.6036305	56.1343713	57.7678654	43.0085704	43.8348258	41.6119904	2.345E-43	-1.46613	down	PREDICTED: probable mitochondrial chaperone BCS1-B [Sesamum indicum]
c45833.graph_c0	0	0	0	2.25583801	6.5992651	5.1540871	1.139E-05	Inf	up	60S ribosomal protein L35a-4 [Auxenochlorella protothecoides]

#ID	0 h-1 FPKM	0 h-2 FPKM	0 h-3 FPKM	28 h-1 FPKM	28 h-2 FPKM	28 h-3 FPKM	FDR	log2FC	regulated	nr_annotation
c45837.graph_c0	1.50607729	0.89512883	1.02816475	0	0	0	2.119E-21	-Inf	down	hypothetical protein EMIHUDRAFT_61288, partial [Emiliania huxleyi CCMP1516]
c45838.graph_c0	33.8427139	36.1916321	35.2916565	31.0820352	29.9612454	31.7405054	3.429E-54	-1.2736	down	PREDICTED: uncharacterized protein LOC105173340 isoform X4 [Sesamum indicum]
c45839.graph_c0	2.71880769	2.93212476	2.16281342	2.77582885	2.6868527	2.35143284	1.567E-09	-1.08805	down	-
c45840.graph_c0	1.72307764	1.14986869	1.19937078	8.37490937	10.7890503	10.410456	1.241E-13	1.77398	up	PREDICTED: uncharacterized protein LOC101257656 isoform X1 [Solanum lycopersicum]
c45842.graph_c0	11.2074837	11.7985495	11.9747467	52.7720812	55.4656602	52.2026527	9.071E-37	1.1059	up	PREDICTED: CTP synthase isoform X2 [Elaeis guineensis]
c45844.graph_c0	113.01763	106.896536	109.187362	32.8255945	32.9725659	34.7280083	0	-2.80095	down	PREDICTED: LOW QUALITY PROTEIN: uncharacterized protein LOC105169583 [Sesamum indicum]
c45845.graph_c0	60.6352427	58.4281516	57.5669965	28.3491556	26.5643925	28.1394786	3.79E-155	-2.17841	down	hypothetical protein MIMGU_mgv1a000962mg [Erythranthe guttata]
c45846.graph_c0	0	0	0	3.92531617	2.50686785	6.56229705	2.984E-05	Inf	up	--
c45850.graph_c0	126.036413	122.193321	116.101354	74.0889844	88.5133115	86.1434775	5.366E-89	-1.64	down	PREDICTED: uncharacterized protein LOC105163078 [Sesamum indicum]
c45851.graph_c0	2.19023645	2.01602725	2.26457286	2.04927974	1.93291048	2.46350235	2.881E-06	-1.09576	down	-
c45854.graph_c0	10.8414317	11.2759507	11.4632952	10.6716217	10.5968848	10.7353074	4.413E-22	-1.16051	down	PREDICTED: uncharacterized protein LOC105159449 [Sesamum indicum]
c45857.graph_c0	0.51056181	0.70763963	0.45421764	4.99521861	6.03080052	5.03101966	1.087E-11	2.17444	up	PREDICTED: prolyl 3-hydroxylase 1 isoform X1 [Sesamum indicum]
c45858.graph_c0	1.14398087	0.87881793	0.95724883	0.09667892	0.0698336	0.03155568	2.765E-28	-5.00348	down	PREDICTED: putative ABC transporter B family member 8 [Sesamum indicum]
c45860.graph_c0	12.606571	12.7884175	11.7930619	11.8654617	11.0145157	11.5297433	9.953E-32	-1.20155	down	PREDICTED: serine/threonine-protein kinase svkA-like isoform X2 [Sesamum indicum]
c45863.graph_c0	39.592594	38.1032184	37.1378365	33.4161898	32.5774022	31.9779745	8.696E-55	-1.31904	down	hypothetical protein MIMGU_mgv1a004300mg [Erythranthe guttata]
c45865.graph_c0	0.05673674	0.02839677	0	1.36078578	1.21004588	1.02626962	1.944E-11	4.33351	up	PREDICTED: type I inositol 1,4,5-trisphosphate 5-phosphatase CVP2-like [Sesamum indicum]
c45867.graph_c0	50.7797155	52.3265368	48.8047738	51.8767567	51.0398702	50.0573344	1.1E-39	-1.07999	down	PREDICTED: uncharacterized protein LOC105177822 isoform X2 [Sesamum indicum]
c45870.graph_c0	0.07286516	0.40115969	0.09363462	1.74761331	1.88873555	1.36121827	8.549E-05	2.04984	up	PREDICTED: kinesin-4-like isoform X2 [Sesamum indicum]
c45871.graph_c0	0.24791215	0.1240802	0.31857693	2.16217349	1.34216496	1.3967501	0.0006792	1.73042	up	PREDICTED: pentatricopeptide repeat-containing protein At2g03880, mitochondrial [Sesamum indicum]
c45871.graph_c1	1.94439037	1.9014219	1.94123445	17.6755169	18.0106881	17.7405627	4.247E-76	2.11603	up	PREDICTED: pentatricopeptide repeat-containing protein At2g03880, mitochondrial [Sesamum indicum]
c45873.graph_c0	18.2166288	20.3047742	18.0692862	13.0536688	12.0964032	13.0576603	3.895E-63	-1.65657	down	hypothetical protein PHAVU_004G031700g [Phaseolus vulgaris]
c45879.graph_c0	26.6741422	25.1693099	26.1531567	24.9466262	25.4797349	27.1873123	1.077E-34	-1.0972	down	PREDICTED: oral cancer-overexpressed protein 1 homolog [Sesamum indicum]

#ID	0 h-1 FPKM	0 h-2 FPKM	0 h-3 FPKM	28 h-1 FPKM	28 h-2 FPKM	28 h-3 FPKM	FDR	log2FC	regulated	nr_annotation
c45882.graph_c0	7.03864343	6.50716737	5.87632751	5.99540616	6.59009174	5.74302029	3.163E-11	-1.17252	down	PREDICTED: auxin-binding protein T92-like [Sesamum indicum]
c45884.graph_c1	0.28737606	0.14383191	0.46161201	8.77225701	7.73194166	7.62677455	2.523E-18	3.65633	up	hypothetical protein MIMGU_mgv1a007716mg [Erythranthe guttata]
c45885.graph_c0	2.70944439	2.32967374	2.36579995	2.52462637	1.73242069	2.20427972	3.388E-07	-1.28529	down	PREDICTED: ADP,ATP carrier protein ER-ANT1 [Sesamum indicum]
c45897.graph_c0	0.28665998	0.43042055	0.36836943	2.36121868	2.32007312	1.75672912	0.0006792	1.47463	up	PREDICTED: uncharacterized protein At1g04910 isoform X2 [Sesamum indicum]
c45898.graph_c0	0.90639556	1.20973757	1.39770502	10.9354669	11.8563723	11.39596	8.167E-16	2.18689	up	PREDICTED: uncharacterized protein LOC105158788 [Sesamum indicum]
c45900.graph_c0	2.3085226	2.05407488	1.75795137	23.2409268	18.6278435	23.173085	1.56E-21	2.32302	up	-
c45902.graph_c1	1.16714335	0.89870159	1.09602627	0	0	0	1.018E-29	-Inf	down	--
c45903.graph_c0	24.8382072	24.242364	21.5590912	21.1319173	24.3475246	25.1705657	2.083E-31	-1.08818	down	PREDICTED: probable xyloglucan glycosyltransferase 12 [Sesamum indicum]
c45904.graph_c0	16.3871601	15.1170149	19.4065299	6.07181236	4.74427841	7.24126437	1.984E-20	-2.58687	down	-
c45908.graph_c0	0	0	0.08585955	3.20499609	2.54304601	2.53600062	1.839E-13	5.46167	up	PREDICTED: chlorophyll a-b binding protein CP24 10A, chloroplastic [Cucumis melo]
c45916.graph_c0	749.525642	742.087625	782.110347	324.800612	373.731201	344.780032	3.74E-117	-2.21523	down	PREDICTED: translocator protein homolog [Sesamum indicum]
c45917.graph_c1	104.635673	113.471287	106.383371	70.6764223	70.2557482	69.4371172	3.23E-98	-1.71584	down	PREDICTED: signal recognition particle receptor subunit alpha homolog [Sesamum indicum]
c45918.graph_c1	15.5233174	16.0421446	17.4444726	14.7993987	12.9912897	13.790709	6.981E-22	-1.32908	down	-
c45918.graph_c4	33.7718362	36.3636482	33.8041943	26.6209549	22.5236868	22.5545331	1.104E-63	-1.62631	down	PREDICTED: branchpoint-bridging protein [Sesamum indicum]
c45921.graph_c0	1.02637137	0.68493275	1.09910654	0.2486536	0.43031253	0.49033999	1.228E-07	-2.35636	down	PREDICTED: serine/threonine-protein kinase CTR1-like [Sesamum indicum]
c45923.graph_c0	4.52820123	2.98442488	3.6008155	24.6667367	24.4636203	23.4514779	5.93E-47	1.61866	up	PREDICTED: alpha-glucosidase 2 [Sesamum indicum]
c45925.graph_c0	0.5693625	0.9973821	0.18291334	4.24154616	5.13742726	3.5454833	0.0002876	1.80403	up	-
c45925.graph_c2	0.91159577	1.3687629	1.03627089	5.37678766	6.21222031	5.59343406	8.577E-07	1.28201	up	PREDICTED: protein LATERAL ROOT PRIMORDIUM 1-like [Sesamum indicum]
c45931.graph_c0	85.8311486	90.9976071	75.8016161	16.8045003	19.6137336	19.3979374	1.79E-118	-3.26688	down	PREDICTED: uncharacterized protein LOC105168927 [Sesamum indicum]
c45931.graph_c1	18.3572667	18.1581854	20.3328382	19.1313644	18.2004849	20.1418883	1.902E-17	-1.07578	down	PREDICTED: uncharacterized protein LOC105168924 isoform X1 [Sesamum indicum]
c45933.graph_c0	26.5326757	25.6508661	27.2097947	21.7963424	19.8218407	23.0905754	6.451E-44	-1.38546	down	PREDICTED: N-acylphosphatidylethanolamine synthase isoform X3 [Sesamum indicum]
c45935.graph_c0	7.12576487	6.12941238	6.16262177	33.4958905	34.1907914	33.3258917	7.553E-36	1.28998	up	PREDICTED: 4-hydroxy-tetrahydrodipicolinate reductase 2, chloroplastic-like [Sesamum indicum]
c45937.graph_c0	21.0026522	19.9286997	21.0291159	21.1478404	20.2143459	22.2906819	3.011E-23	-1.05144	down	PREDICTED: uncharacterized protein LOC105161374 [Sesamum indicum]

#ID	0 h-1 FPKM	0 h-2 FPKM	0 h-3 FPKM	28 h-1 FPKM	28 h-2 FPKM	28 h-3 FPKM	FDR	log2FC	regulated	nr_annotation
c45939.graph_c0	66.0537789	60.5230487	66.2147865	44.9995666	42.8966264	45.1066839	4.514E-65	-1.62625	down	PREDICTED: pentatricopeptide repeat-containing protein At5g65560-like isoform X1 [Sesamum indicum]
c45940.graph_c0	51.6757703	50.202837	47.9302893	43.1737495	40.3226869	42.7203621	3.212E-57	-1.33668	down	hypothetical protein MIMGU_mgv1a013847mg [Erythranthe guttata]
c45941.graph_c0	6.12962639	4.95454763	5.77071296	5.55085386	4.19450908	4.76272789	1.033E-14	-1.30648	down	PREDICTED: exonuclease V, chloroplastic-like [Sesamum indicum]
c45944.graph_c0	33.8870908	35.6901473	32.6350039	13.0684184	13.6652983	14.8107259	6.11E-139	-2.38828	down	PREDICTED: probable E3 ubiquitin-protein ligase LUL4 isoform X2 [Sesamum indicum]
c45947.graph_c0	28.3022102	36.5447339	26.6852847	280.018186	307.295364	270.226262	5.663E-80	2.13833	up	hypothetical protein JCGZ_17777 [Jatropha curcas]
c45950.graph_c0	2.59269447	3.89293599	2.57450708	2.26979173	1.70139625	2.13169854	9.713E-06	-1.65864	down	Gag-pol polyprotein, putative [Solanum demissum]
c45951.graph_c0	0.80840209	0.35964959	0.46170164	16.8428702	18.1842008	15.7679022	1.99E-55	3.87696	up	PREDICTED: probable ribose-5-phosphate isomerase 4, chloroplastic [Sesamum indicum]
c45957.graph_c1	53.6807718	50.5769009	52.5207238	22.9464087	25.1105522	22.2378977	1.46E-157	-2.24811	down	hypothetical protein MIMGU_mgv1a008606mg [Erythranthe guttata]
c45959.graph_c0	33.8891143	33.9626326	36.9936586	36.3851841	35.6809348	35.81094	2.141E-24	-1.05062	down	PREDICTED: methionine aminopeptidase 1A [Sesamum indicum]
c45960.graph_c0	24.532705	23.6030113	24.6538193	14.2252097	14.3102442	14.390312	5.49E-107	-1.85258	down	PREDICTED: F-box/LRR-repeat protein 3 isoform X1 [Sesamum indicum]
c45961.graph_c1	28.8907065	29.7745868	28.4169092	15.1544368	14.1216725	14.5131361	1.09E-132	-2.08202	down	PREDICTED: digalactosyldiacylglycerol synthase 1, chloroplastic [Sesamum indicum]
c45963.graph_c0	65.9154636	64.7431545	62.5543002	30.494433	27.1818331	27.4876915	5.29E-169	-2.2719	down	PREDICTED: protein MEI2-like 2 [Sesamum indicum]
c45964.graph_c2	824.165106	820.355132	822.794958	499.134779	516.308566	514.442549	1.025E-91	-1.78003	down	hypothetical protein MIMGU_mgv1a027154mg [Erythranthe guttata]
c45964.graph_c5	179.862678	185.174559	169.028982	78.9758604	76.4835529	80.5386253	2.61E-164	-2.26769	down	PREDICTED: uncharacterized protein LOC105172390 isoform X1 [Sesamum indicum]
c45964.graph_c6	32.6480749	30.9616051	31.9690862	11.2582809	11.1937255	10.43265	2.2E-209	-2.63004	down	hypothetical protein MIMGU_mgv1a000896mg [Erythranthe guttata]
c45969.graph_c0	14.497262	7.60140798	9.31477785	0	0	0	2.583E-23	-Inf	down	--
c45973.graph_c0	9.00342193	10.1389922	9.50711314	6.6240897	6.51364652	6.16018086	7.8E-55	-1.66126	down	PREDICTED: F-box/kelch-repeat protein At1g55270-like [Sesamum indicum]
c45974.graph_c1	5.67069907	2.73307061	4.04837379	4.42672262	3.44561679	2.74026652	0.0087449	-1.31939	down	hypothetical protein POPTR_0012s15190g, partial [Populus trichocarpa]
c45974.graph_c2	45.2739311	51.5421536	41.9586627	47.2148636	48.7685079	44.4662333	7.207E-17	-1.07227	down	PREDICTED: EIN3-binding F-box protein 1-like [Sesamum indicum]
c45974.graph_c3	3.96752017	2.69761791	3.55927469	3.78128623	3.6106446	3.15209218	5.063E-05	-1.04634	down	hypothetical protein MIMGU_mgv1a004105mg [Erythranthe guttata]
c45979.graph_c0	1.15627696	1.3268155	0.83353216	6.60002168	5.94069393	6.37235965	8.514E-10	1.42559	up	PREDICTED: uncharacterized protein LOC105161942 [Sesamum indicum]
c45981.graph_c1	44.5813044	46.6543979	41.9212747	45.2416965	44.426342	43.3828056	9.814E-41	-1.0909	down	PREDICTED: DEAD-box ATP-dependent RNA helicase 51-like [Sesamum indicum]
c45985.graph_c0	12.0303205	11.738667	10.8826308	10.4500972	9.79902612	10.6550809	7.065E-45	-1.25411	down	PREDICTED: uncharacterized protein LOC105175876 isoform X1 [Sesamum indicum]
c45987.graph_c0	2.70987778	2.53997187	2.05772287	12.1215222	12.3834005	11.2351958	3.724E-15	1.20248	up	hypothetical protein MIMGU_mgv1a004935mg [Erythranthe guttata]

#ID	0 h-1 FPKM	0 h-2 FPKM	0 h-3 FPKM	28 h-1 FPKM	28 h-2 FPKM	28 h-3 FPKM	FDR	log2FC	regulated	nr_annotation
c45989.graph_c0	0.40063175	0.47264594	0.47805426	2.46460403	2.89195932	2.48629262	7.132E-09	1.44412	up	PREDICTED: pentatricopeptide repeat-containing protein At4g21300 [Sesamum indicum]
c45991.graph_c0	0.54265933	0.50698921	0.46489248	3.8651364	3.34741268	2.98227785	2.245E-06	1.66145	up	PREDICTED: uncharacterized protein LOC105179859 isoform X3 [Sesamum indicum]
c45992.graph_c0	67.9098448	72.7093439	67.0455364	73.0345503	69.3789233	69.1230856	4.75E-40	-1.06362	down	PREDICTED: putative clathrin assembly protein At2g01600 [Sesamum indicum]
c45999.graph_c0	0.12562038	0.25149235	0	1.18690221	0.96861705	0.94987746	0.0002628	1.96719	up	PREDICTED: heparanase-like protein 1 isoform X1 [Sesamum indicum]
c46005.graph_c0	0.31399005	0.12572179	0.48418758	3.62847797	3.62645831	3.03529414	1.163E-11	2.37684	up	MYB-related transcription factor [Salvia miltiorrhiza]
c46007.graph_c1	39.7646948	34.6807407	37.4546317	449.068478	415.807438	425.016832	3.88E-188	2.43683	up	PREDICTED: glyoxysomal fatty acid beta-oxidation multifunctional protein MFP-a [Sesamum indicum]
c46010.graph_c0	59.179845	60.9146952	53.910594	51.3502896	46.2271885	49.7605179	1.224E-59	-1.32906	down	PREDICTED: phosphoinositide phosphatase SAC1 isoform X4 [Sesamum indicum]
c46015.graph_c0	0.08872793	0	0	10.2211818	11.4413443	10.1821298	3.549E-52	7.42816	up	PREDICTED: cytochrome P450 724B1 isoform X1 [Vitis vinifera]
c46019.graph_c1	56.9482476	63.0751092	52.6676104	60.3720863	61.821526	56.6504325	2.242E-22	-1.03911	down	PREDICTED: malate dehydrogenase, chloroplastic-like [Solanum tuberosum]
c46019.graph_c2	90.191711	82.2390824	82.1280425	85.5518442	80.4308935	84.1447984	7.232E-44	-1.11477	down	PREDICTED: protein ACCUMULATION AND REPLICATION OF CHLOROPLASTS 3 [Sesamum indicum]
c46020.graph_c0	99.4260407	99.697493	90.9464743	82.7280899	80.0725118	77.6749084	1.44E-63	-1.36007	down	PREDICTED: flowering time control protein FCA isoform X4 [Sesamum indicum]
c46021.graph_c1	1.42281992	1.24621407	0.45709498	7.04478796	7.11940851	6.11765849	4.171E-05	1.61845	up	PREDICTED: probable cyclic nucleotide-gated ion channel 5 [Sesamum indicum]
c46024.graph_c0	8.94807643	7.45225443	8.03371122	43.7202601	44.7835565	43.7274532	3.692E-48	1.34643	up	PREDICTED: KH domain-containing protein At4g18375-like [Sesamum indicum]
c46028.graph_c0	126.903244	126.947635	116.654494	563.761186	535.963505	524.802221	5.234E-35	1.04284	up	PREDICTED: mannose-6-phosphate isomerase 2-like [Sesamum indicum]
c46030.graph_c0	0.67837597	1.01858288	0.87173997	6.76168789	5.45861571	5.94853539	5.739E-18	1.73002	up	PREDICTED: uncharacterized protein LOC105166222 isoform X2 [Sesamum indicum]
c46032.graph_c1	5.85874743	5.36901289	5.00147522	35.6241366	32.8240834	34.1993087	2.06E-70	1.57241	up	PREDICTED: CAP-Gly domain-containing linker protein 1 [Sesamum indicum]
c46041.graph_c1	19.4887847	20.9781032	18.6971283	19.0636446	20.497547	17.7723777	2.222E-31	-1.1357	down	PREDICTED: pentatricopeptide repeat-containing protein At2g03880, mitochondrial-like isoform X1 [Sesamum indicum]
c46042.graph_c0	3.70115574	3.16002925	3.82355682	29.5370206	25.7166902	25.7159386	4.522E-40	1.83016	up	PREDICTED: methylmalonate-semialdehyde dehydrogenase [acylating], mitochondrial-like [Sesamum indicum]
c46047.graph_c0	3.00559397	2.48711232	2.42037921	16.6600596	15.0161738	17.1119235	6.635E-19	1.53653	up	PREDICTED: histone-lysine N-methyltransferase ATXR2 [Sesamum indicum]
c46051.graph_c0	194.468858	201.839392	190.278287	197.231802	192.246831	191.124561	3.14E-41	-1.10501	down	PREDICTED: uncharacterized protein LOC105174291 isoform X1 [Sesamum indicum]

#ID	0 h-1 FPKM	0 h-2 FPKM	0 h-3 FPKM	28 h-1 FPKM	28 h-2 FPKM	28 h-3 FPKM	FDR	log2FC	regulated	nr_annotation
c46053.graph_c0	3.00966655	2.68402425	3.51594607	28.3169948	25.8907771	24.8588909	3.835E-33	2.00846	up	PREDICTED: (RS)-norcoclaurine 6-O-methyltransferase-like [Cucumis melo]
c46054.graph_c0	6.91518015	6.48947312	6.14898858	0.53849069	0.70905129	0.73234001	2.03E-105	-4.39256	down	PREDICTED: exocyst complex component EXO70A1-like [Sesamum indicum]
c46055.graph_c0	1.55264924	1.46169184	1.97146318	11.0024511	9.99482997	10.7756847	3.005E-28	1.57803	up	PREDICTED: importin-13 [Sesamum indicum]
c46056.graph_c2	8.33841775	8.82307751	8.35668891	47.494417	56.1166654	46.3743758	4.913E-24	1.46414	up	PREDICTED: cytochrome P450 93A3-like [Sesamum indicum]
c46064.graph_c0	59.719391	52.4351838	58.8749237	33.2985501	36.5633808	38.0337975	3.857E-45	-1.755	down	hypothetical protein M569_12324 [Genlisea aurea]
c46067.graph_c1	2.32595713	1.57501715	2.32962046	12.2063484	13.9167752	11.0151372	1.291E-13	1.4829	up	PREDICTED: diacylglycerol kinase 5 [Sesamum indicum]
c46068.graph_c0	23.8187642	22.487921	22.8864775	19.0031306	18.6382079	19.8939934	2.697E-57	-1.35607	down	PREDICTED: cation/H(+) antiporter 18-like [Sesamum indicum]
c46070.graph_c0	66.8278468	60.9005303	73.2896106	18.7517451	18.8739771	20.1464533	9.016E-44	-2.89065	down	PREDICTED: glucan endo-1,3-beta-glucosidase 14-like [Sesamum indicum]
c46073.graph_c0	200.953026	197.225619	206.108563	146.127364	146.063432	162.397934	3.541E-65	-1.5011	down	PREDICTED: RING finger and CHY zinc finger domain-containing protein 1-like isoform X1 [Sesamum indicum]
c46074.graph_c0	0.26143258	0.57572761	0.47033171	3.42013849	3.2253117	2.54273352	3.171E-05	1.71698	up	PREDICTED: violaxanthin de-epoxidase, chloroplastic [Sesamum indicum]
c46077.graph_c0	9.29998946	11.3156171	10.3024609	48.6544332	50.7698535	51.4932391	1.187E-19	1.1961	up	PREDICTED: 40S ribosomal protein S20-2-like [Sesamum indicum]
c46079.graph_c0	0.38714259	0.44715029	0.30614988	10.692174	11.9795968	10.9147391	3.548E-54	3.7927	up	PREDICTED: probable beta-D-xylosidase 7 [Sesamum indicum]
c46082.graph_c0	22.2352414	23.1487972	22.31186	7.83754632	8.3750226	8.28758301	2.53E-157	-2.55671	down	PREDICTED: uncharacterized protein LOC105165640 isoform X3 [Sesamum indicum]
c46087.graph_c1	1107.63223	1134.59727	1185.00262	108.898599	110.281759	123.256788	0	-4.41396	down	PREDICTED: SUMO-activating enzyme subunit 2 [Sesamum indicum]
c46087.graph_c2	102.103117	108.075582	96.7042365	57.0670942	57.8292025	54.0296717	1.69E-127	-1.95127	down	PREDICTED: zinc finger protein JACKDAW-like [Sesamum indicum]
c46089.graph_c0	2.08686183	1.88005636	1.60902005	8.4177913	8.53627068	8.1064794	3.434E-07	1.08047	up	PREDICTED: 1-(5-phosphoribosyl)-5-[(5-phosphoribosylamino)methylideneamino] imidazole-4-carboxamide isomerase, chloroplastic [Sesamum indicum]
c46092.graph_c0	0.27137535	0.29428435	0.20342471	1.36420212	1.17238533	1.15341313	0.0042793	1.17537	up	hypothetical protein M569_17037, partial [Genlisea aurea]
c46093.graph_c0	0.53558359	0.31427718	0.71197874	3.54359979	4.29029008	4.06350185	1.63E-14	1.833	up	PREDICTED: uncharacterized protein LOC105169441 [Sesamum indicum]
c46094.graph_c0	11.6622083	13.5975969	11.8149497	7.17484921	6.68397915	7.04233808	2.018E-43	-1.91696	down	PREDICTED: probable protein phosphatase 2C 23 [Sesamum indicum]
c46097.graph_c0	5.39513255	5.26881459	6.73004305	3.67249287	3.78221752	3.69908142	4.219E-11	-1.73481	down	PREDICTED: uncharacterized protein LOC105179152 isoform X1 [Sesamum indicum]
c46098.graph_c0	66.511901	67.4782121	61.6595381	44.7224688	50.1803337	46.8260667	1.082E-67	-1.55482	down	-
c46100.graph_c0	1.43916961	1.31349801	1.19666407	0.83063132	0.86109068	1.07944074	8.151E-06	-1.5983	down	hypothetical protein M569_02521, partial [Genlisea aurea]
c46103.graph_c1	11.3382725	11.0237995	10.0852249	9.50230841	9.64770521	9.2766836	4.792E-39	-1.2801	down	PREDICTED: uncharacterized protein LOC105171484 [Sesamum indicum]

#ID	0 h-1 FPKM	0 h-2 FPKM	0 h-3 FPKM	28 h-1 FPKM	28 h-2 FPKM	28 h-3 FPKM	FDR	log2FC	regulated	nr_annotation
c46112.graph_c0	1.91746757	2.69979798	2.3557131	11.011006	11.5044031	11.3467052	4.697E-17	1.18774	up	CAI-1 autoinducer sensor kinase/phosphatase cqsS isoform 2 [Theobroma cacao]
c46114.graph_c0	0	0	0	3.29689138	4.04842597	5.16525397	7.097E-14	Inf	up	40S ribosomal protein S17 [Auxenochlorella protothecoides]
c46117.graph_c1	6.37633378	6.60281246	5.65092507	5.67300116	5.9157666	5.41150955	9.761E-06	-1.22132	down	hypothetical protein MIMGU_mgv1a015893mg [Erythranthe guttata]
c46120.graph_c0	10.1528231	10.2850676	9.83405916	9.43981886	11.0842543	8.37183895	6.761E-22	-1.1583	down	PREDICTED: PHD finger protein ALFIN-LIKE 3-like isoform X2 [Elaeis guineensis]
c46130.graph_c0	110.017843	121.043923	115.133348	16.4375936	16.3024772	16.2065079	2.76E-241	-3.91321	down	PREDICTED: GATA transcription factor 5-like [Sesamum indicum]
c46132.graph_c1	4.73239017	4.82822758	3.04065437	3.77019254	4.24023258	4.64165586	0.0001907	-1.07865	down	-
c46142.graph_c0	29.4889497	27.600508	26.8262817	22.8514993	22.1655492	22.9105805	1.379E-63	-1.3943	down	PREDICTED: homeobox protein LUMINIDEPENDENS [Sesamum indicum]
c46143.graph_c0	0.32799317	0.2872814	0.52685526	3.78433875	2.9864271	3.1123044	1.043E-07	2.01521	up	hypothetical protein MIMGU_mgv1a018015mg [Erythranthe guttata]
c46144.graph_c1	0.7761794	1.06831536	0.78962516	34.0118987	33.9778842	30.7646885	7.18E-150	4.13821	up	hypothetical protein MIMGU_mgv1a007954mg [Erythranthe guttata]
c46148.graph_c0	2.97624609	2.78492898	2.41115713	16.5055347	18.7666781	18.0729564	2.231E-14	1.61856	up	PREDICTED: proline synthase co-transcribed bacterial homolog protein-like [Sesamum indicum]
c46149.graph_c1	2.07611406	0.95103746	2.21570423	0.15344824	0	0.14608127	2.7E-08	-5.21838	down	PREDICTED: geraniol 8-hydroxylase-like [Vitis vinifera]
c46151.graph_c0	1.46209687	0.75395564	0.91095873	10.1112147	10.1761707	10.5366819	1.124E-18	2.21591	up	PREDICTED: uncharacterized protein LOC105155656 [Sesamum indicum]
c46153.graph_c0	23.1959549	23.9859135	21.9333639	25.1071025	23.548991	24.1364241	2.913E-36	-1.01491	down	PREDICTED: uncharacterized protein LOC105171827 [Sesamum indicum]
c46154.graph_c1	2.11936722	2.3572105	1.96695112	0.55623529	0.34769653	0.48879757	1.452E-17	-3.29842	down	hypothetical protein MIMGU_mgv1a012700mg [Erythranthe guttata]
c46155.graph_c0	41.1855307	44.2292818	39.9976894	45.9649674	42.3196813	43.8152265	3.283E-36	-1.01497	down	PREDICTED: uncharacterized protein LOC105178641 [Sesamum indicum]
c46159.graph_c0	2.85587877	1.53932073	2.54071197	37.8404451	30.2740023	43.1242641	4.361E-15	2.91438	up	dehydration responsive protein, partial [Corchorus olitorius]
c46164.graph_c0	31.6762686	30.7937336	30.6756541	25.4058099	21.5768871	22.577513	4.991E-58	-1.51147	down	PREDICTED: protein Brevis radix-like 1 [Sesamum indicum]
c46166.graph_c0	0	0	0	2.68582259	4.09948248	4.95721151	4.029E-10	Inf	up	ribosomal protein S18 [Micromonas pusilla CCMP1545]
c46170.graph_c0	3.99701356	3.4453193	3.40043452	20.7923144	18.8585134	19.5233844	4.116E-35	1.35946	up	PREDICTED: E3 ubiquitin-protein ligase UPL7 [Sesamum indicum]
c46179.graph_c0	82.1261691	76.7874023	83.9799927	22.3335569	23.4128217	22.9995301	6.79E-144	-2.91205	down	PREDICTED: peptidyl-prolyl cis-trans isomerase FKBP53 [Sesamum indicum]
c46180.graph_c1	4.35480189	3.86135696	3.93799126	0.55681346	0.28224581	0.38261493	3.1E-150	-4.40387	down	PREDICTED: replication protein A 70 kDa DNA-binding subunit A isoform X1 [Sesamum indicum]
c46182.graph_c0	25.0297438	22.9480968	19.0280772	15.6785373	15.5373466	16.1368228	6.864E-22	-1.5879	down	PREDICTED: uncharacterized protein LOC105163032 [Sesamum indicum]
c46184.graph_c0	3.41155127	3.45765452	4.05518007	24.7019769	24.934103	23.7225866	7.362E-30	1.65431	up	PREDICTED: DNA repair protein RAD50 [Sesamum indicum]
c46186.graph_c0	0	0	0	2.29555054	3.40230228	5.15999947	5.578E-08	Inf	up	ATP synthase F0 subunit 9 (mitochondrion) [Hildenbrandia rubra]

#ID	0 h-1 FPKM	0 h-2 FPKM	0 h-3 FPKM	28 h-1 FPKM	28 h-2 FPKM	28 h-3 FPKM	FDR	log2FC	regulated	nr_annotation
c46190.graph_c1	38.0099736	40.559052	37.6405934	18.7430481	19.0511639	19.5758173	3.29E-145	-2.10843	down	PREDICTED: protein ASPARTIC PROTEASE IN GUARD CELL 1-like [Sesamum indicum]
c46190.graph_c3	1.82599866	1.82782712	4.35774963	33.0833378	33.8939148	28.1560086	7.022E-23	2.46577	up	-
c46194.graph_c0	1.53447072	1.45516478	1.2453825	6.54472418	6.06826618	6.84917607	1.75E-05	1.11273	up	PREDICTED: F-box protein At5g07610-like [Sesamum indicum]
c46194.graph_c1	0.33937193	0.2717694	0.08722127	2.61452773	2.85062449	3.05926281	5.977E-07	2.53319	up	-
c46199.graph_c0	1.2621641	1.34117738	1.47221414	8.70761272	8.90704757	8.29148367	6.763E-22	1.57547	up	PREDICTED: IAA-amino acid hydrolase ILR1-like 3 [Sesamum indicum]
c46204.graph_c0	6.73143247	5.54908363	5.99291939	32.7756567	31.7298402	31.4934776	5.37E-25	1.30369	up	PREDICTED: probable cinnamyl alcohol dehydrogenase 6 [Sesamum indicum]
c46205.graph_c0	10.9438953	8.8039009	12.136854	109.539468	101.953624	113.566594	8.089E-78	2.25754	up	PREDICTED: uncharacterized protein LOC104222596 [Nicotiana sylvestris]
c46205.graph_c1	24.9804604	21.471606	26.5306026	183.050572	179.20703	202.346156	2.061E-50	1.86034	up	PREDICTED: uncharacterized protein LOC102603928 [Solanum tuberosum]
c46206.graph_c0	3.60387609	3.48948299	4.32815277	2.32552575	2.27655123	2.41695593	7.752E-15	-1.79534	down	hypothetical protein MIMGU_mgv1a005772mg [Erythranthe guttata]
c46208.graph_c0	17.8031739	17.5971895	17.8496991	5.3625753	5.63054595	4.59129352	7.49E-154	-2.86418	down	PREDICTED: uncharacterized protein LOC105158293 isoform X2 [Sesamum indicum]
c46211.graph_c0	128.458572	128.041881	123.665751	133.66977	131.237456	133.240187	9.555E-36	-1.02334	down	PREDICTED: interferon-induced guanylate-binding protein 1 [Sesamum indicum]
c46212.graph_c0	9.1786692	8.27444724	8.09322333	8.4135273	9.0408072	9.06186339	2.465E-23	-1.03528	down	PREDICTED: protein root UVB sensitive 1, chloroplastic [Sesamum indicum]
c46212.graph_c1	3.87999963	3.157076	3.32396876	36.4121328	36.003606	35.6194166	1.21E-103	2.29318	up	PREDICTED: heterogeneous nuclear ribonucleoprotein 1-like [Sesamum indicum]
c46212.graph_c2	130.254003	133.060098	121.799553	29.6132403	32.9958045	31.233465	0	-3.12668	down	PREDICTED: ubiquinol oxidase 2, mitochondrial-like [Sesamum indicum]
c46213.graph_c2	64.445769	67.0081753	59.7274448	54.2037043	55.2346835	50.4711078	1.487E-63	-1.34753	down	PREDICTED: protein ETHYLENE INSENSITIVE 3-like [Sesamum indicum]
c46214.graph_c0	5.22047418	6.81613264	8.26411392	1.70464532	2.08527954	3.00630439	1.485E-08	-2.67239	down	-
c46218.graph_c0	0.96392878	0.69126735	0.7025387	0.18821495	0.16049932	0.08532326	3.835E-24	-3.53141	down	hypothetical protein MIMGU_mgv1a020311mg [Erythranthe guttata]
c46221.graph_c0	1.26585852	1.50620648	1.65737036	0.26038264	0.26644793	0.16997609	6.417E-29	-3.76387	down	hypothetical protein MIMGU_mgv1a023003mg, partial [Erythranthe guttata]
c46226.graph_c0	3.84435133	3.5828077	3.81157636	3.70934541	3.67541104	3.95055785	6.031E-14	-1.07796	down	PREDICTED: CSC1-like protein At3g54510 isoform X2 [Sesamum indicum]
c46227.graph_c0	1.44304694	1.06212642	0.54540366	7.58836343	7.04654438	6.14170078	3.761E-09	1.68727	up	-
c46231.graph_c0	0.04575881	0.04580463	0	0.79817372	0.87081688	1.51970756	0.0001283	4.05476	up	PREDICTED: vestitone reductase-like [Sesamum indicum]
c46232.graph_c0	0.68352591	0.45614024	0.29278595	4.51835686	5.08354619	4.96096425	5.779E-13	2.26482	up	PREDICTED: uncharacterized protein LOC103980779 [Musa acuminata subsp. malaccensis]

#ID	0 h-1 FPKM	0 h-2 FPKM	0 h-3 FPKM	28 h-1 FPKM	28 h-2 FPKM	28 h-3 FPKM	FDR	log2FC	regulated	nr_annotation
c46234.graph_c2	64.9349124	75.9587399	51.0114136	29.8836622	38.5537561	48.6705233	3.167E-09	-1.7981	down	PREDICTED: uncharacterized protein LOC105177376 [Sesamum indicum]
c46239.graph_c0	0.22884524	0.13744464	0.02940752	4.15808512	4.17484944	3.75940356	1.105E-26	3.86083	up	PREDICTED: subtilisin-like protease SBT3.5 [Sesamum indicum]
c46241.graph_c0	2.36650158	2.21353545	4.28737946	1.21243061	1.06925277	0.73624817	0.0008931	-2.65614	down	hypothetical protein (mitochondrion) [Vicia faba]
c46242.graph_c0	0	0	0	1.83429443	2.73909321	3.68435515	2.693E-07	Inf	up	unknown [Picea sitchensis]
c46243.graph_c1	2.68067734	3.04927459	2.66187276	11.9850022	13.4600327	13.0796983	1.384E-09	1.10869	up	hypothetical protein MIMGU_mgv1a008659mg [Erythranthe guttata]
c46243.graph_c2	170.124301	154.721201	168.292911	83.6629513	90.2072142	87.6222886	5.661E-93	-2.00593	down	PREDICTED: probable phospholipid hydroperoxide glutathione peroxidase [Sesamum indicum]
c46246.graph_c0	24.3230999	23.9463445	23.0172939	225.939146	250.80817	213.689812	2.481E-74	2.18533	up	PREDICTED: serine carboxypeptidase-like 25 [Sesamum indicum]
c46247.graph_c0	37.8996991	40.8348492	34.403446	37.6793318	38.5510355	39.0501218	4.743E-29	-1.06192	down	PREDICTED: UDP-glycosyltransferase 91C1-like [Sesamum indicum]
c46249.graph_c2	6.22398768	3.36768652	3.45862512	0	0	0.1995236	1.605E-12	-7.101	down	-
c46249.graph_c6	11.7563845	14.7516331	11.9157112	0.60172342	0.76061976	1.17840335	6.275E-48	-5.00605	down	-
c46250.graph_c0	0.86089941	1.16337798	1.2169188	14.5474489	13.2197242	13.6830997	2.635E-31	2.58267	up	PREDICTED: uncharacterized protein LOC105168273 [Sesamum indicum]
c46259.graph_c0	1.42853113	2.33993715	1.60208209	26.6924172	26.5721849	25.4776761	2.147E-96	2.78344	up	PREDICTED: phosphoglycerate kinase, cytosolic-like [Sesamum indicum]
c46268.graph_c0	1.60683248	1.68503393	1.44211281	12.6052203	12.6868378	11.1933236	1.167E-28	1.85661	up	PREDICTED: trimethylguanosine synthase isoform X2 [Sesamum indicum]
c46269.graph_c0	0	0.07531056	0.2900407	1.91381832	2.17234097	1.78475442	1.679E-08	2.88517	up	hypothetical protein MIMGU_mgv1a009279mg [Erythranthe guttata]
c46272.graph_c0	6.12378538	4.98055791	5.016683	0	0	0	1.349E-63	-Inf	down	hypothetical protein S7711_01318 [Stachybotrys chartarum IBT 7711]
c46273.graph_c1	36.0555233	38.9338431	34.2862534	32.3467309	31.838308	32.4754652	5.865E-54	-1.26657	down	hypothetical protein MIMGU_mgv1a002387mg [Erythranthe guttata]
c46275.graph_c0	35.1155901	33.8258052	33.4657133	20.8598029	19.9183363	22.1406051	2.61E-107	-1.79233	down	PREDICTED: DIS3-like exonuclease 2 [Sesamum indicum]
c46280.graph_c0	27.4148293	30.5477586	26.8083187	17.6471466	16.5361671	18.1735721	2.782E-65	-1.78477	down	PREDICTED: uncharacterized protein LOC105162504 [Sesamum indicum]
c46282.graph_c0	20.1072363	19.2435909	18.0796966	18.2155307	18.3719531	18.1911716	1.08E-34	-1.1575	down	PREDICTED: uncharacterized PKHD-type hydroxylase At1g22950 [Sesamum indicum]
c46288.graph_c0	24.90261	25.2628943	22.6732407	25.0789851	22.6683993	22.760128	2.295E-33	-1.13634	down	PREDICTED: magnesium transporter MRS2-1-like [Sesamum indicum]
c46291.graph_c0	6.06142864	6.17662591	5.23948155	39.4110005	32.0503184	35.0943604	9.307E-22	1.51953	up	hypothetical protein MIMGU_mgv11b005497mg [Erythranthe guttata]
c46293.graph_c0	38.7463702	39.5814826	39.3273735	208.517965	214.865501	206.615394	5.826E-62	1.32996	up	hypothetical protein POPTR_0016s08070g [Populus trichocarpa]
c46303.graph_c0	3.51617589	3.21789653	2.74728305	1.94653327	1.74212005	2.00169917	2.041E-31	-1.82365	down	PREDICTED: sacsin [Sesamum indicum]
c46307.graph_c0	27.9436035	28.797924	26.6618534	28.4489493	28.9099902	28.6892134	1.805E-38	-1.04482	down	PREDICTED: LOW QUALITY PROTEIN: translocase of chloroplast 120, chloroplastic-like [Sesamum indicum]
c46308.graph_c0	3.0007704	2.83454844	2.87850372	21.8094263	19.7196021	20.0271193	1.055E-26	1.73029	up	PREDICTED: glycine--tRNA ligase 2, chloroplastic/mitochondrial isoform X1 [Cucumis melo]

#ID	0 h-1 FPKM	0 h-2 FPKM	0 h-3 FPKM	28 h-1 FPKM	28 h-2 FPKM	28 h-3 FPKM	FDR	log2FC	regulated	nr_annotation
c46309.graph_c0	4.48997595	4.11196373	5.15598869	21.0725128	19.1205388	18.5264068	1.126E-10	1.00054	up	PREDICTED: persulfide dioxygenase ETHE1 homolog, mitochondrial [Sesamum indicum]
c46314.graph_c0	30.6954798	32.321003	29.0377299	23.2163367	23.349389	24.2827715	2.815E-65	-1.46722	down	PREDICTED: uncharacterized protein LOC105166052 [Sesamum indicum]
c46315.graph_c0	0.53551212	0.75940183	0.83151974	6.74625674	5.93017094	5.30639677	4.105E-19	1.98421	up	hypothetical protein MIMGU_mgv1a001332mg [Erythranthe guttata]
c46320.graph_c0	1.05642002	2.04673135	1.22616656	8.29719334	8.45319442	8.44789481	3.62E-11	1.45104	up	PREDICTED: MORC family CW-type zinc finger protein 3-like isoform X1 [Sesamum indicum]
c46324.graph_c1	5.10169226	4.81288424	4.90511626	1.58718627	1.52942193	1.3277814	5.742E-75	-2.82836	down	PREDICTED: kinesin-4-like [Sesamum indicum]
c46325.graph_c0	1.20794422	1.17076796	1.03483743	5.22575536	5.48588818	5.27620425	7.584E-09	1.13891	up	PREDICTED: probable leucine-rich repeat receptor-like serine/threonine-protein kinase At5g15730 [Sesamum indicum]
c46327.graph_c0	14.571728	20.0680031	15.2066804	5.21526508	5.70024178	5.35684709	3.295E-17	-2.70532	down	PREDICTED: high affinity nitrate transporter 2.4-like [Sesamum indicum]
c46328.graph_c0	1.56935355	1.90415154	1.99631092	13.8369408	14.6051774	13.6787332	1.297E-50	1.85159	up	PREDICTED: kinesin-13A [Sesamum indicum]
c46332.graph_c0	0.93237986	1.28059293	0.89164281	8.3366501	8.22444111	8.07572196	8.88E-24	1.89919	up	PREDICTED: uncharacterized protein LOC105162624 [Sesamum indicum]
c46334.graph_c0	25.8960103	25.3758781	25.0302118	25.0014723	24.995635	24.6448614	7.485E-35	-1.12196	down	PREDICTED: glycine-rich RNA-binding protein RZ1A [Populus euphratica]
c46335.graph_c1	19.7252948	21.1200054	17.1379759	18.1669642	20.2077381	17.1732161	9.977E-20	-1.15107	down	PREDICTED: phospholipid-transporting ATPase 1-like [Sesamum indicum]
c46342.graph_c0	2.41318285	2.37663801	2.70090189	1.75388739	1.45588653	1.26957546	6.312E-11	-1.83485	down	PREDICTED: protein GAMETE EXPRESSED 3-like [Solanum tuberosum]
c46342.graph_c1	18.536616	15.897535	18.1133421	18.9103458	16.6941371	17.8068419	1.75E-18	-1.06703	down	PREDICTED: zinc finger CCHC domain-containing protein 8 [Sesamum indicum]
c46345.graph_c0	1.21612964	1.58255164	1.40649726	0.44193801	0.23941715	0.28849424	7.66E-14	-3.20814	down	-
c46348.graph_c0	8.25514268	6.34725615	7.84083252	54.345925	56.1352504	51.8668839	7.015E-26	1.76385	up	PREDICTED: transmembrane protein 33 homolog [Sesamum indicum]
c46350.graph_c0	4.14487504	4.35910274	4.51726596	32.9465214	31.0559642	30.7113417	5.63E-34	1.77101	up	PREDICTED: uncharacterized protein LOC105177167 [Sesamum indicum]
c46351.graph_c0	21.1344624	22.2418556	19.7547426	20.505578	19.9388154	18.1727176	2.058E-40	-1.19702	down	PREDICTED: chromodomain-helicase-DNA-binding protein 1-like isoform X2 [Sesamum indicum]
c46352.graph_c0	17.3037824	15.1401669	18.1377962	17.0055299	16.6598338	18.72655	1.356E-11	-1.04032	down	PREDICTED: CDP-diacylglycerol--serine O-phosphatidyltransferase 1-like [Sesamum indicum]
c46354.graph_c0	83.8131888	79.9199658	97.8790633	7.13023705	7.80954943	8.57656291	7.312E-61	-4.56793	down	PREDICTED: uncharacterized protein LOC105172385 [Sesamum indicum]
c46354.graph_c1	6.14009796	5.77499656	7.20185625	2.45592081	3.73183341	3.1282128	6.194E-18	-2.13034	down	PREDICTED: pentatricopeptide repeat-containing protein At4g22760 [Sesamum indicum]
c46355.graph_c0	64.4795694	65.6111541	65.9915949	24.5482078	24.7158685	26.2288899	1.66E-194	-2.46755	down	PREDICTED: fimbrin-2 [Sesamum indicum]
c46360.graph_c0	14.0195403	14.0785582	12.1836705	10.9077989	9.11154008	10.0998675	1.013E-31	-1.50794	down	unnamed protein product [Coffea canephora]

#ID	0 h-1 FPKM	0 h-2 FPKM	0 h-3 FPKM	28 h-1 FPKM	28 h-2 FPKM	28 h-3 FPKM	FDR	log2FC	regulated	nr_annotation
c46360.graph_c1	19.5923747	19.1431809	19.0582696	13.1049936	14.035182	13.2396553	6.958E-29	-1.60771	down	PREDICTED: mitochondrial ubiquitin ligase activator of NFKB 1 [Sesamum indicum]
c46363.graph_c0	1.71102938	1.80012755	1.43591216	9.63129406	9.47525772	9.25691334	5.314E-22	1.4311	up	PREDICTED: transcription termination factor 2 [Sesamum indicum]
c46364.graph_c0	0	0.05099425	0.03273204	3.27673461	2.75800215	2.62847023	1.462E-24	5.59177	up	PREDICTED: uncharacterized protein LOC105172001 isoform X1 [Sesamum indicum]
c46365.graph_c0	14.5925212	14.5475124	14.5423421	12.6403347	11.3544085	13.2992306	2.325E-35	-1.31802	down	PREDICTED: CDK5RAP3-like protein [Sesamum indicum]
c46368.graph_c2	1.65880447	1.71490701	1.15317657	0	0	0	1.087E-44	-Inf	down	PREDICTED: uncharacterized protein LOC104097920 [Nicotiana tomentosiformis]
c46368.graph_c3	63.8863421	64.8428042	59.6003692	52.0025969	50.1827158	50.6092732	1.736E-66	-1.39127	down	PREDICTED: LOW QUALITY PROTEIN: nuclear pore complex protein NUP1-like [Sesamum indicum]
c46369.graph_c0	20.9958161	20.5922576	19.2587759	23.8913526	21.0149636	19.4532236	6.504E-16	-1.00904	down	PREDICTED: retrovirus-related Pol polyprotein from transposon TNT 1-94 [Sesamum indicum]
c46372.graph_c1	69.8299143	77.8195245	81.8511504	46.4184769	48.5331032	44.7792982	1.001E-27	-1.80851	down	hypothetical protein MIMGU_mgv1a026333mg, partial [Erythranthe guttata]
c46373.graph_c0	0	0	0.0963358	4.249886	3.29609815	2.57867425	1.013E-11	5.58397	up	PREDICTED: uncharacterized protein LOC104110773 [Nicotiana tomentosiformis]
c46375.graph_c0	17.5890931	17.022043	15.740387	11.9467711	11.6190963	11.0152736	3.95E-76	-1.63152	down	PREDICTED: ubiquitin carboxyl-terminal hydrolase 8-like [Sesamum indicum]
c46376.graph_c0	22.4577305	23.0586945	23.165646	173.616798	155.706269	159.377436	2.494E-88	1.74	up	hypothetical protein MIMGU_mgv1a025345mg [Erythranthe guttata]
c46380.graph_c0	5.10847992	4.67528712	3.65741942	4.24321573	4.35801954	3.54899012	2.388E-06	-1.23309	down	-
c46382.graph_c0	6.84045399	3.22801459	4.77185228	0	0	0	9.465E-17	-Inf	down	-
c46385.graph_c0	0.6459963	0.43109545	0.79938524	9.07712354	10.6451085	9.70553896	5.04E-45	2.87602	up	PREDICTED: uncharacterized protein LOC105177282 [Sesamum indicum]
c46386.graph_c0	2.77812002	1.80979329	1.41666437	0	0	0	2.008E-18	-Inf	down	--
c46388.graph_c0	13.0316093	12.525181	13.7451675	70.4480215	73.4840304	71.522843	1.753E-56	1.36325	up	hypothetical protein MIMGU_mgv1a008721mg [Erythranthe guttata]
c46390.graph_c0	2.3626663	1.91215365	2.77772657	13.1162731	12.3045997	11.8356557	3.209E-10	1.30752	up	hypothetical protein PHAVU_011G040900g [Phaseolus vulgaris]
c46391.graph_c0	70.1259689	67.290978	59.8748322	49.0478054	52.8304025	52.3813918	2.662E-66	-1.44349	down	hypothetical protein MIMGU_mgv1a003239mg [Erythranthe guttata]
c46391.graph_c1	5.65013405	4.88102581	4.87358427	26.5796452	24.6084241	25.8658705	3.347E-21	1.23365	up	unnamed protein product [Coffea canephora]
c46392.graph_c0	89.0341303	90.9149978	91.0031045	64.6465458	64.7353142	63.5717824	6.046E-84	-1.58065	down	PREDICTED: tobamovirus multiplication protein 1 [Sesamum indicum]
c46394.graph_c0	2.28902889	3.27331572	3.99202588	2.49547951	2.41412574	2.76353765	0.0018307	-1.41252	down	-
c46398.graph_c0	0.1280409	0.08544608	0.10969175	5.24234553	4.7333465	4.10052784	1.937E-20	4.35454	up	PREDICTED: squalene synthase-like [Sesamum indicum]
c46400.graph_c0	0.32591014	0.23561524	0.37227328	2.36869613	2.67338815	2.17981027	9.043E-10	1.857	up	PREDICTED: flavin-containing monooxygenase FMO GS-OX-like 2 isoform X1 [Sesamum indicum]
c46401.graph_c0	7.2657167	4.94563471	5.60204018	5.06945378	3.05149431	4.99843141	9.659E-05	-1.52776	down	-

#ID	0 h-1 FPKM	0 h-2 FPKM	0 h-3 FPKM	28 h-1 FPKM	28 h-2 FPKM	28 h-3 FPKM	FDR	log2FC	regulated	nr_annotation
c46401.graph_c5	29.3382453	29.3676231	27.239682	18.8255948	17.9690469	18.7473386	1.706E-60	-1.71928	down	-
c46403.graph_c0	18.3709167	18.5904106	15.4609532	12.9949611	11.5574527	12.4438892	2.199E-40	-1.59091	down	PREDICTED: LOW QUALITY PROTEIN: uncharacterized protein LOC105173575 [Sesamum indicum]
c46404.graph_c0	23.3969713	24.9736908	23.9803169	25.5437925	26.1185253	25.3851201	3.737E-32	-1.00005	down	PREDICTED: kanadaptin [Sesamum indicum]
c46405.graph_c1	60.5520054	59.3129529	55.7675971	37.2316575	36.7666474	36.3308711	2.802E-97	-1.76034	down	PREDICTED: protein RTF2 homolog [Sesamum indicum]
c46407.graph_c0	142.081968	140.840865	136.576304	102.403421	104.639069	102.450225	1.55E-78	-1.52888	down	PREDICTED: ras-related protein Rab11D [Sesamum indicum]
c46411.graph_c0	15.918535	17.1278057	15.2293179	11.6714334	13.0001936	11.7279913	8.543E-43	-1.49747	down	PREDICTED: pentatricopeptide repeat-containing protein At1g80150, mitochondrial [Sesamum indicum]
c46412.graph_c0	1.83850493	1.86955776	1.65003606	12.9379527	11.8541121	10.5399568	2.124E-20	1.63142	up	PREDICTED: uncharacterized protein LOC105174617 [Sesamum indicum]
c46416.graph_c0	616.78973	611.26002	629.19279	547.986485	536.901984	586.868361	7.481E-37	-1.24224	down	PREDICTED: xylose isomerase-like [Sesamum indicum]
c46430.graph_c0	0.62881709	0.68666918	0.36729766	25.9670815	22.5455197	21.7991675	2.396E-65	4.30049	up	PREDICTED: baicalein 7-O-glucuronosyltransferase-like [Sesamum indicum]
c46434.graph_c0	0.30644173	0.10224953	0.42004241	2.96960111	2.86896037	2.58067632	5.284E-13	2.24594	up	PREDICTED: putative ion channel POLLUX-like 2 isoform X4 [Sesamum indicum]
c46435.graph_c0	5.03191463	4.57255338	4.08150814	1.45249168	1.12411183	1.67200869	1.936E-36	-2.77418	down	PREDICTED: putative beta-glucosidase 41 isoform X1 [Sesamum indicum]
c46436.graph_c0	10.6985563	10.2197027	10.9984547	44.8321708	47.4196925	44.7766266	4.767E-27	1.01084	up	PREDICTED: cyprosin-like [Sesamum indicum]
c46439.graph_c0	219.066072	233.960244	228.421032	52.2230213	52.173652	51.3460019	0	-3.22042	down	PREDICTED: uncharacterized protein LOC105159071 [Sesamum indicum]
c46440.graph_c0	24.9885251	27.6370659	24.3469561	18.9758034	19.6541431	20.7641195	1.824E-53	-1.46361	down	PREDICTED: uncharacterized protein DDB_G0271670 isoform X1 [Sesamum indicum]
c46442.graph_c0	0	0	0.04533257	14.7425697	14.8158978	13.2641802	7.015E-91	8.75292	up	PREDICTED: heat stress transcription factor C-1-like [Sesamum indicum]
c46445.graph_c0	53.0146281	54.763369	53.855738	21.9784734	23.0970227	20.4643986	5.25E-137	-2.39345	down	PREDICTED: DNA-directed RNA polymerase III subunit RPC9-like [Sesamum indicum]
c46448.graph_c1	80.2274773	88.015929	81.8090286	36.5731963	37.5661427	36.2333928	9.02E-136	-2.27051	down	hypothetical protein MIMGU_mgv1a021536mg, partial [Erythranthe guttata]
c46448.graph_c2	194.303433	192.913263	192.615114	193.674767	190.49085	197.906756	1.537E-37	-1.08473	down	PREDICTED: pentatricopeptide repeat-containing protein At4g21065-like [Sesamum indicum]
c46449.graph_c0	1.93984296	1.51965816	1.73410435	8.73504359	7.74855826	9.20351762	1.281E-07	1.21721	up	hypothetical protein POPTR_0001s04230g [Populus trichocarpa]
c46450.graph_c0	3.20957617	3.23125439	3.31850887	39.8838325	42.3905167	39.3820416	7.68E-147	2.54864	up	PREDICTED: probable metal-nicotianamine transporter YSL7 [Sesamum indicum]
c46452.graph_c0	27.9603367	30.4089475	29.3915016	21.6395572	20.001332	22.7966609	2.883E-51	-1.53612	down	PREDICTED: TATA-box-binding protein-like isoform X4 [Sesamum indicum]
c46453.graph_c0	1.32540352	1.71369384	1.987062	0.72247173	0.97848635	0.91704827	9.957E-08	-2.03603	down	acidic class III chitinase [Rehmannia glutinosa]

#ID	0 h-1 FPKM	0 h-2 FPKM	0 h-3 FPKM	28 h-1 FPKM	28 h-2 FPKM	28 h-3 FPKM	FDR	log2FC	regulated	nr_annotation
c46454.graph_c0	18.3829866	20.823987	18.5939083	8.86967438	7.29887181	8.91718946	1.343E-73	-2.2939	down	PREDICTED: kinesin-like protein KIF3A isoform X1 [Sesamum indicum]
c46459.graph_c0	20.2472482	15.3482211	21.7241974	10.4294911	10.3198384	14.8057288	2.281E-08	-1.77951	down	-
c46459.graph_c1	12.7457258	13.2310253	14.8116585	9.43506671	9.26761831	8.70213226	1.19E-24	-1.66653	down	PREDICTED: putative lipid phosphate phosphatase 3, chloroplastic [Sesamum indicum]
c46462.graph_c0	4.48746923	5.98928366	4.08465773	2.5140473	2.20859903	1.55244265	5.491E-12	-2.30469	down	-
c46462.graph_c2	3.59510361	2.29704483	2.01504116	1.52882481	1.1545042	0.90727895	8.363E-06	-2.22436	down	PREDICTED: restin homolog [Sesamum indicum]
c46468.graph_c0	4.39359714	3.17471282	3.62686615	1.92440356	2.2721886	1.94992998	2.128E-15	-1.9543	down	hypothetical protein MIMGU_mgv1a003714mg [Erythranthe guttata]
c46470.graph_c0	1.60120634	1.14486408	1.69018255	13.4662215	13.0969095	14.1762982	1.952E-18	2.10709	up	hypothetical protein MIMGU_mgv1a002711mg [Erythranthe guttata]
c46471.graph_c0	52.0607448	49.9500884	48.1918836	6.0195942	6.65043756	6.98654779	0	-4.02308	down	hypothetical protein AMTR_s00109p00039350 [Amborella trichopoda]
c46474.graph_c0	0.56489301	0	1.08886483	5.85049407	5.28244761	5.69524502	1.296E-06	2.24104	up	PREDICTED: non-specific lipid-transfer protein 1-like [Pyrus x bretschneideri]
c46476.graph_c0	0.59241617	0.59300939	0.56390981	0.4465115	0.35996252	0.32531227	4.34E-06	-1.71888	down	PREDICTED: uncharacterized protein LOC105176110 [Sesamum indicum]
c46477.graph_c0	1.17490262	1.17607911	0.75489819	8.79529829	7.45302789	6.57301074	8.93E-12	1.79139	up	PREDICTED: UPF0554 protein isoform X2 [Sesamum indicum]
c46479.graph_c0	4.27347372	3.97219917	3.35596734	27.0906646	29.0007372	27.2550557	1.368E-42	1.75699	up	hypothetical protein MIMGU_mgv1a004800mg [Erythranthe guttata]
c46480.graph_c0	4.44555629	4.50000793	4.36477165	24.559266	23.2891865	23.5357095	9.627E-32	1.33276	up	PREDICTED: gamma-tubulin complex component 2 [Sesamum indicum]
c46484.graph_c0	1.407838	2.18748903	1.21508568	35.2472437	39.8363512	31.7522564	2.91E-57	3.38469	up	PREDICTED: formin-like protein 1 [Nelumbo nucifera]
c46485.graph_c0	2.54937237	1.6769794	1.26361714	0.31763471	0.43019154	0.21598944	5.261E-08	-3.59518	down	PREDICTED: heat shock protein 81-1 [Musa acuminata subsp. malaccensis]
c46487.graph_c0	2.13343651	2.80293934	2.91289728	11.9684573	12.8626723	12.8897541	1.613E-12	1.17107	up	PREDICTED: putative bifunctional dihydrofolate reductase-thymidylate synthase isoform X1 [Sesamum indicum]
c46488.graph_c0	32.5373723	33.8236084	31.8336271	26.651759	24.6886525	23.8570177	4.081E-68	-1.47538	down	PREDICTED: pentatricopeptide repeat-containing protein At5g67570, chloroplastic [Sesamum indicum]
c46488.graph_c1	19.0863946	21.3799718	18.6870649	12.7158914	14.3142954	16.0357346	9.552E-11	-1.54711	down	-
c46488.graph_c2	34.2171521	37.567284	34.876408	28.1244842	28.1357069	28.0169587	8.963E-58	-1.43036	down	PREDICTED: F-box/kelch-repeat protein SKIP11-like isoform X1 [Sesamum indicum]
c46489.graph_c0	12.7370054	11.5805676	10.8997918	9.11569679	10.5300831	9.28554794	4.676E-35	-1.3728	down	PREDICTED: dr1-associated corepressor [Sesamum indicum]
c46493.graph_c0	21.072462	22.2695106	19.7666885	111.796674	102.556022	101.545157	3.977E-48	1.23382	up	PREDICTED: probable receptor-like protein kinase At1g67000 [Sesamum indicum]
c46493.graph_c1	0.70953726	1.04993146	1.38749658	10.5155426	9.67674719	9.75150988	1.13E-25	2.15191	up	PREDICTED: uncharacterized protein LOC105170658 [Sesamum indicum]
c46496.graph_c0	0.17937402	0.29925607	0.30733696	1.89033609	1.64794121	2.05666508	1.459E-05	1.73671	up	PREDICTED: pentatricopeptide repeat-containing protein At3g12770 [Sesamum indicum]

#ID	0 h-1 FPKM	0 h-2 FPKM	0 h-3 FPKM	28 h-1 FPKM	28 h-2 FPKM	28 h-3 FPKM	FDR	log2FC	regulated	nr_annotation
c46499.graph_c0	0.5436114	0.49468704	0.57155087	2.80157305	2.72413916	2.3153692	0.0030126	1.19182	up	PREDICTED: uncharacterized protein LOC105177245 [Sesamum indicum]
c46499.graph_c1	0.24637613	0.57545329	0.36937026	5.55100725	5.14677133	5.69849958	5.49E-15	2.69101	up	PREDICTED: probable serine incorporator [Sesamum indicum]
c46504.graph_c0	19.0700761	17.1340034	17.1648575	15.0811687	15.3121003	13.8381465	3.72E-51	-1.36082	down	PREDICTED: uncharacterized protein LOC105157171 isoform X1 [Sesamum indicum]
c46506.graph_c0	2.8602521	2.12265512	1.90113992	0	0	0	3.746E-39	-Inf	down	putative Trypsin [Glarea lozoyensis 74030]
c46507.graph_c0	50.8021785	56.2191993	54.3379608	24.3784695	26.1869121	25.4637575	1.391E-85	-2.17682	down	PREDICTED: mitogen-activated protein kinase kinase kinase YODA-like [Sesamum indicum]
c46508.graph_c0	154.12016	149.047117	154.001047	64.0356021	64.1447502	62.3377428	2.19E-149	-2.35357	down	hypothetical protein VITISV_004365 [Vitis vinifera]
c46509.graph_c0	0.11284093	0.22590785	0.09667004	1.25751948	1.28352073	1.09304686	6.831E-08	1.97364	up	PREDICTED: probable LRR receptor-like serine/threonine-protein kinase At3g47570 [Solanum lycopersicum]
c46518.graph_c0	68.9973951	67.0993531	70.4304216	37.0675516	40.0078833	40.0843771	3.97E-117	-1.90864	down	PREDICTED: uncharacterized protein LOC105175391 [Sesamum indicum]
c46523.graph_c1	1.05373545	0.86048707	0.78394787	12.5558904	13.6842174	13.4359178	8.619E-49	2.79109	up	PREDICTED: glutathione synthetase, chloroplastic isoform X2 [Sesamum indicum]
c46525.graph_c1	31.4372141	33.0560381	29.5160067	16.8481598	15.1908526	15.0083522	3.92E-140	-2.08848	down	PREDICTED: lon protease homolog 2, peroxisomal [Sesamum indicum]
c46529.graph_c0	0.63351753	0.34146641	1.12720861	4.46269598	5.0207554	4.73976072	3.679E-07	1.657	up	PREDICTED: uncharacterized protein LOC105164965 [Sesamum indicum]
c46530.graph_c0	5.36334203	6.15822916	5.6758527	2.17831987	3.51957013	3.27432594	1.181E-08	-2.02925	down	PREDICTED: diacylglycerol kinase 3-like [Sesamum indicum]
c46530.graph_c1	7.97107497	6.12677575	6.95070684	2.2759516	3.64290297	3.96756465	1.569E-11	-2.17849	down	PREDICTED: diacylglycerol kinase 3-like [Sesamum indicum]
c46530.graph_c2	2.96929877	2.76490425	2.9874598	2.49265361	3.23245205	2.86669025	7.169E-09	-1.11271	down	PREDICTED: glutamate receptor 2.8-like [Sesamum indicum]
c46532.graph_c0	48.1967446	48.4643018	46.873326	31.2873697	31.0529529	33.3906932	2.74E-49	-1.67416	down	PREDICTED: uncharacterized protein At1g04910 [Eucalyptus grandis]
c46533.graph_c0	0.23714751	0.71215492	1.21897574	23.6129694	25.2108627	26.1594612	4.983E-19	4.00552	up	Cell wall-associated hydrolase, partial [Medicago truncatula]
c46533.graph_c1	0	0.92731807	1.19044838	8.41619029	10.638643	12.3615766	1.975E-07	2.78288	up	-
c46537.graph_c0	0.23128401	0.08681835	0.03715114	1.42881504	1.29008601	1.33735749	8.095E-07	2.44116	up	hypothetical protein MIMGU_mgv1a001125mg [Erythranthe guttata]
c46540.graph_c0	11.6547932	11.5366921	12.3446731	9.89340192	10.0643042	10.4333076	4.461E-48	-1.3168	down	PREDICTED: L-ascorbate oxidase homolog [Sesamum indicum]
c46546.graph_c0	0.65574649	0.41025195	0.4213301	4.88507588	4.35697567	4.08340598	3.348E-06	2.07724	up	PREDICTED: vacuolar cation/proton exchanger 3-like [Sesamum indicum]
c46546.graph_c1	0.26276986	0.4383883	0.11255656	2.16443567	2.29915585	2.90897243	2.595E-05	2.09965	up	PREDICTED: vacuolar cation/proton exchanger 3-like [Sesamum indicum]
c46547.graph_c0	2.21648948	1.69943665	1.81804898	1.30245548	1.39262587	1.65012603	5.534E-06	-1.48772	down	PREDICTED: NAC domain-containing protein 7 [Sesamum indicum]
c46550.graph_c1	7.03776048	7.31294921	7.16619514	4.92031503	5.92877104	5.38694769	5.592E-30	-1.49715	down	PREDICTED: G-type lectin S-receptor-like serine/threonine-protein kinase At4g27290 [Sesamum indicum]
c46550.graph_c2	0	0	0	0.60805462	0.65195687	0.99233521	4.69E-09	Inf	up	PREDICTED: uncharacterized protein LOC105175473 [Sesamum indicum]

#ID	0 h-1 FPKM	0 h-2 FPKM	0 h-3 FPKM	28 h-1 FPKM	28 h-2 FPKM	28 h-3 FPKM	FDR	log2FC	regulated	nr_annotation
c46552.graph_c1	141.748757	144.262824	143.286029	95.9515855	94.2470771	88.7622601	1.91E-97	-1.71287	down	PREDICTED: uncharacterized protein LOC105165908 isoform X2 [Sesamum indicum]
c46558.graph_c0	31.4553008	30.3819986	32.3167843	19.7961381	19.9951458	21.5301903	7.585E-73	-1.70927	down	-
c46559.graph_c0	0	0.95831244	1.53779691	25.3966715	19.6325455	17.0329944	1.032E-12	3.5232	up	-
c46560.graph_c0	1.82739429	1.52935134	2.27128143	15.5022201	15.7073651	15.5811429	5.794E-35	1.96132	up	hypothetical protein L484_026123 [Morus notabilis]
c46562.graph_c0	2.91953347	2.45277636	2.44531495	12.8829649	12.0255484	13.9289222	2.192E-16	1.22461	up	hypothetical protein MIMGU_mgv1a0057401mg, partial [Erythranthe guttata]
c46564.graph_c0	4.22969034	3.38197726	2.75080197	15.0519146	14.4028492	14.6530328	4.973E-13	1.00415	up	PREDICTED: probable methyltransferase PMT28 isoform X2 [Sesamum indicum]
c46568.graph_c0	31.5271648	32.5024765	31.6694659	23.0919189	25.1682907	25.5524257	8.326E-70	-1.46496	down	PREDICTED: uncharacterized protein LOC105175670 isoform X1 [Sesamum indicum]
c46569.graph_c0	3.14319745	2.81762229	3.37599191	0.06819277	0.33864423	0.25040135	8.323E-41	-4.92039	down	PREDICTED: U-box domain-containing protein 33 [Sesamum indicum]
c46569.graph_c1	1.85324107	2.66670167	1.04190107	0.21045702	0.03800459	0.24042369	7.89E-07	-4.58699	down	PREDICTED: U-box domain-containing protein 33 [Sesamum indicum]
c46571.graph_c1	0.99602202	1.20153618	1.41120237	6.57081244	6.87135395	6.63400404	1.252E-11	1.38114	up	PREDICTED: uncharacterized protein LOC105165701 isoform X1 [Sesamum indicum]
c46572.graph_c0	722.003728	756.032668	673.132973	74.0642428	80.9865635	75.7792371	0	-4.30991	down	PREDICTED: short-chain type dehydrogenase/reductase [Sesamum indicum]
c46574.graph_c0	196.454845	189.888292	187.093228	90.3685122	86.7670639	88.4044823	3.61E-157	-2.20067	down	hypothetical protein MIMGU_mgv1a002799mg [Erythranthe guttata]
c46576.graph_c0	120.374546	128.847253	117.145289	47.5508399	44.6187	39.4764326	8.75E-205	-2.56728	down	PREDICTED: EIN3-binding F-box protein 1-like [Sesamum indicum]
c46579.graph_c0	0	0	0	4.1175438	7.435512	7.97972004	3.512E-11	Inf	up	60S ribosomal protein L11 [Candida albicans WO-1]
c46581.graph_c0	5.91682064	2.01763856	1.5039591	0	0	0	1.244E-05	-Inf	down	PREDICTED: photosystem II 22 kDa protein, chloroplastic [Phoenix dactylifera]
c46582.graph_c0	0.66518186	0.52566942	0.53986422	9.26189071	9.35051073	9.63389839	2.254E-30	2.94043	up	PREDICTED: uncharacterized protein LOC105162786 [Sesamum indicum]
c46584.graph_c0	86.9018845	85.9442331	86.5525282	30.8068069	31.2399343	32.2558043	2.23E-201	-2.55015	down	PREDICTED: phosphoglucan phosphatase DSP4, amyloplastic [Sesamum indicum]
c46585.graph_c0	2.69711072	1.52171192	1.63842264	23.1308854	19.3403659	21.375718	7.046E-32	2.36146	up	PREDICTED: uncharacterized protein LOC105160704 isoform X2 [Sesamum indicum]
c46587.graph_c0	32.6414798	31.2751334	31.748875	6.65179393	6.56807915	6.60428056	3.38E-210	-3.36103	down	PREDICTED: inositol-tetrakisphosphate 1-kinase 2-like isoform X1 [Sesamum indicum]
c46589.graph_c0	0.22660799	0.34025236	0.33973362	1.92146947	2.18102567	1.63509926	0.0001926	1.5663	up	PREDICTED: uncharacterized protein LOC105173906 [Sesamum indicum]
c46589.graph_c1	70.948567	73.5744107	66.2065761	57.060663	55.9450361	55.8348365	3.153E-68	-1.40931	down	PREDICTED: transcription factor GTE7-like [Sesamum indicum]
c46590.graph_c0	8.74249731	7.52795836	7.85203856	3.34780469	3.51626456	2.84328257	1.41E-38	-2.40366	down	PREDICTED: uncharacterized protein LOC104210866 [Nicotiana sylvestris]

#ID	0 h-1 FPKM	0 h-2 FPKM	0 h-3 FPKM	28 h-1 FPKM	28 h-2 FPKM	28 h-3 FPKM	FDR	log2FC	regulated	nr_annotation
c46592.graph_c0	0.16761703	0.06711395	0.0430789	6.16424829	4.75177653	5.84511076	6.825E-31	4.83977	up	hypothetical protein MIMGU_mgv1a019962mg [Erythranthe guttata]
c46601.graph_c0	0.09612178	0	0.12352029	0.85579423	0.52039111	0.52730369	0.0024054	2.01527	up	PREDICTED: zinc finger MYM-type protein 1-like [Setaria italica]
c46603.graph_c3	130.042218	130.879217	125.265333	130.61699	134.751588	132.963203	1.658E-38	-1.04538	down	PREDICTED: DEAD-box ATP-dependent RNA helicase 53-like [Sesamum indicum]
c46604.graph_c0	0.25677747	0.42060206	0.44995802	6.68454616	7.95034029	6.83893485	8.791E-38	3.15497	up	PREDICTED: carboxy-terminal kinesin 2-like [Sesamum indicum]
c46615.graph_c0	0.74333743	0.59526541	0.68229859	4.52269239	4.36230897	4.69811966	4.537E-12	1.65906	up	hypothetical protein MIMGU_mgv1a001336mg [Erythranthe guttata]
c46617.graph_c0	150.514395	151.506556	164.671464	12.5073902	13.5761175	14.0418063	2.79E-185	-4.63135	down	BnaC09g43060D [Brassica napus]
c46618.graph_c0	12.0894006	5.22443884	4.92751455	0	0	0	1.372E-11	-Inf	down	PREDICTED: mitochondrial uncoupling protein 5-like [Musa acuminata subsp. malaccensis]
c46620.graph_c0	8.96307387	5.17731354	5.13585263	0	0	0	3.747E-27	-Inf	down	PREDICTED: alpha carbonic anhydrase 1, chloroplastic-like [Camelina sativa]
c46622.graph_c0	2.18972226	2.23036958	2.12275142	15.1609174	16.1594411	14.9912331	2.049E-22	1.73303	up	PREDICTED: uncharacterized protein LOC105163866 [Sesamum indicum]
c46624.graph_c0	1.4546899	1.12733926	1.20602201	0.13642068	0.15396885	0.1391477	3.015E-20	-4.22959	down	PREDICTED: folylpolyglutamate synthase-like [Solanum tuberosum]
c46629.graph_c0	6.26704428	6.63843363	6.73247198	4.28975448	4.96123657	4.77864227	2.019E-22	-1.57647	down	Uncharacterized protein TCM_005954 [Theobroma cacao]
c46635.graph_c0	0.34570825	0.27684354	0.53309855	2.91460168	3.13071089	2.87035141	3.08E-05	1.84975	up	hypothetical protein MIMGU_mgv1a014966mg [Erythranthe guttata]
c46636.graph_c0	78.3586473	83.2555768	63.7277783	46.1407856	41.1784648	41.5736783	1.874E-24	-1.89382	down	PREDICTED: ABC transporter B family member 4-like [Sesamum indicum]
c46638.graph_c0	16.0591194	14.1359697	14.2818404	136.354811	116.759098	139.473569	5.068E-37	2.05317	up	unknown [Populus trichocarpa x Populus deltoides]
c46643.graph_c0	0.73854566	0.42564906	0.4313911	0	0	0	2.033E-16	-Inf	down	alpha-2 macroglobulin family-like protein [Micromonas sp. RCC299]
c46644.graph_c0	4.35236168	2.59328566	3.72863805	0.30126394	0.47602291	0.12291446	4.741E-17	-4.6622	down	PREDICTED: protein ROOT HAIR DEFECTIVE 3-like [Sesamum indicum]
c46648.graph_c0	10.2794665	11.6319024	11.1993769	8.08011056	9.05532069	10.7016948	2.772E-17	-1.34058	down	PREDICTED: ABC transporter G family member 12-like [Sesamum indicum]
c46653.graph_c0	0.42861882	0.58227945	0.29506723	3.60470468	3.8875707	3.94911472	1.15E-17	2.04587	up	PREDICTED: DNA mismatch repair protein MSH1, mitochondrial-like [Sesamum indicum]
c46657.graph_c2	106.950267	103.157921	108.30996	32.1304621	33.9298481	33.1490392	7.26E-226	-2.77323	down	PREDICTED: putative clathrin assembly protein At5g35200 [Sesamum indicum]
c46659.graph_c0	2.77244759	3.11784399	2.90294474	0.39802448	0.29199526	0.50747613	1.472E-40	-3.96498	down	PREDICTED: probable serine/threonine-protein kinase At5g41260 [Sesamum indicum]
c46660.graph_c0	9.9894918	9.84014027	9.6148836	7.20245403	6.79042804	6.8920721	3.071E-49	-1.58564	down	PREDICTED: GDP-mannose transporter GONST1-like isoform X2 [Sesamum indicum]
c46667.graph_c1	10.6262266	10.5232254	11.7293995	8.87824181	9.05922253	9.26443512	6.94E-28	-1.36524	down	PREDICTED: 3-ketoacyl-CoA synthase 4 [Sesamum indicum]
c46670.graph_c1	48.443091	45.170257	45.6225207	47.9590596	46.6502474	50.1537622	1.089E-33	-1.03347	down	PREDICTED: UDP-galactose transporter 1 [Sesamum indicum]

#ID	0 h-1 FPKM	0 h-2 FPKM	0 h-3 FPKM	28 h-1 FPKM	28 h-2 FPKM	28 h-3 FPKM	FDR	log2FC	regulated	nr_annotation
c46679.graph_c0	0	0	0	5.21987107	5.23672685	5.20589932	2.759E-20	Inf	up	RecName: Full=Tubulin beta-1 chain; AltName: Full=Beta-1-tubulin [Cyanophora paradoxa]
c46684.graph_c0	0.18360544	0.22054716	0	2.96244299	4.21704006	3.57154958	1.138E-15	3.66173	up	hypothetical protein MIMGU_mgv1a002647mg [Erythranthe guttata]
c46687.graph_c0	112.375138	124.833601	107.757127	23.4962482	22.8132927	25.1084189	7.71E-144	-3.36137	down	PREDICTED: uncharacterized protein LOC105168969 [Sesamum indicum]
c46690.graph_c0	4.89292819	5.03238342	4.31841102	40.4072657	41.7413339	37.1811902	8.683E-79	1.97721	up	tyrosine aminotransferase 2 [Salvia miltiorrhiza]
c46691.graph_c0	20.2031272	23.0500745	19.7270852	13.1270086	12.3499378	12.5099002	2.844E-47	-1.81928	down	PREDICTED: fimbrin-1 [Sesamum indicum]
c46692.graph_c0	38.2432148	41.4168223	37.1535737	26.7124416	25.4986918	28.1232393	6.478E-87	-1.62965	down	PREDICTED: E3 ubiquitin-protein ligase MBR2-like isoform X2 [Sesamum indicum]
c46692.graph_c1	36.5070894	39.8571062	36.8923939	38.7934432	34.9432956	36.6572857	2.256E-40	-1.12729	down	PREDICTED: transcription factor SPATULA-like [Sesamum indicum]
c46698.graph_c0	20.651992	23.8739517	19.7820739	623.121249	651.439834	662.572372	0	3.82337	up	hypothetical protein MIMGU_mgv1a006007mg [Erythranthe guttata]
c46699.graph_c0	0.03100056	0.0310316	0	3.46977793	3.62112584	5.05591153	3.603E-19	6.54478	up	PREDICTED: gamma-cadinene synthase-like [Sesamum indicum]
c46710.graph_c0	5.29739268	5.23557447	4.8254699	108.933872	87.3825495	89.3902249	2.874E-46	3.12784	up	PREDICTED: uncharacterized protein LOC105177873 [Sesamum indicum]
c46712.graph_c0	2.11783371	3.34300502	2.61682709	20.8979983	18.762027	16.9076731	1.901E-13	1.71564	up	PREDICTED: sucrose transport protein SUC3 isoform X2 [Nicotiana tomentosiformis]
c46713.graph_c0	2.71437042	2.71708845	3.1821021	14.6401694	11.4062071	12.5958188	2.771E-08	1.07284	up	PREDICTED: probable plastid-lipid-associated protein 10, chloroplastic [Sesamum indicum]
c46714.graph_c0	0.45386591	0.63100054	0.61563764	2.14413632	3.06112395	2.63187351	0.0002684	1.11065	up	PREDICTED: boron transporter 4-like [Sesamum indicum]
c46718.graph_c0	0.18582979	0.14881269	0.04775973	4.32192056	3.99984627	4.5405643	1.715E-19	3.99557	up	PREDICTED: probable receptor-like protein kinase At1g80640 isoform X1 [Sesamum indicum]
c46718.graph_c1	0.44147464	0.47137783	0.60513322	2.41715457	2.83913451	2.86256686	5.194E-05	1.32469	up	PREDICTED: uncharacterized protein LOC105166978 [Sesamum indicum]
c46719.graph_c2	4.02228851	4.22541978	4.31679999	25.4911972	24.7130046	24.7588846	5.431E-43	1.48544	up	PREDICTED: 1,4-alpha-glucan-branching enzyme-like isoform X1 [Sesamum indicum]
c46725.graph_c0	1.28400595	1.28529169	1.59677273	0.93320813	0.84259944	1.03169641	4.42E-06	-1.66213	down	PREDICTED: GDSL esterase/lipase At1g33811 [Sesamum indicum]
c46728.graph_c0	2.05177401	2.02966586	2.66762978	12.5262272	12.1020261	13.0271459	1.512E-21	1.38616	up	hypothetical protein MIMGU_mgv1a007859mg [Erythranthe guttata]
c46730.graph_c0	17.9809927	14.5244439	16.493377	16.1925971	17.8252304	17.8327991	1.477E-12	-1.0082	down	PREDICTED: protein-lysine N-methyltransferase EFM1 isoform X1 [Sesamum indicum]
c46732.graph_c0	2.16230317	1.39487963	2.03767313	8.38159722	9.1444207	8.26417216	3.283E-06	1.11342	up	hypothetical protein MIMGU_mgv1a009658mg [Erythranthe guttata]
c46734.graph_c0	49.2876437	48.4121098	49.0515233	44.7407449	45.266281	47.5529761	9.354E-47	-1.18359	down	PREDICTED: protein RCC2 homolog isoform X1 [Sesamum indicum]
c46737.graph_c0	0	0	0	3.48596601	4.75336805	5.68903959	3.584E-13	Inf	up	60S ribosomal protein L12 [Triticum urartu]
c46738.graph_c0	1.45296223	1.89074229	2.98738238	40.1281738	33.9436431	39.4654527	9.365E-32	3.06538	up	cell wall-associated hydrolase [Phaseolus vulgaris]
c46740.graph_c0	267.531722	273.896711	257.507924	8005.65746	7682.10383	7556.60064	8.6E-201	3.77254	up	hypothetical protein MIMGU_mgv1a004735mg [Erythranthe guttata]

#ID	0 h-1 FPKM	0 h-2 FPKM	0 h-3 FPKM	28 h-1 FPKM	28 h-2 FPKM	28 h-3 FPKM	FDR	log2FC	regulated	nr_annotation
c46745.graph_c0	6.56906508	7.54414856	6.34749104	58.6987039	68.1373454	55.3873354	4.509E-34	2.06431	up	PREDICTED: uncharacterized protein LOC105166530 [Sesamum indicum]
c46745.graph_c2	3.83353249	3.98785634	4.53986918	20.5959485	22.3943754	22.7350773	7.999E-12	1.31809	up	PREDICTED: BTB/POZ domain-containing protein At5g48800-like isoform X2 [Sesamum indicum]
c46746.graph_c0	0.87470229	0.78243156	0.90878785	6.87127916	6.57050592	6.22499199	6.204E-22	1.84649	up	PREDICTED: putative leucine--tRNA ligase, mitochondrial isoform X3 [Sesamum indicum]
c46749.graph_c1	0.10474247	0	0.04486607	15.9865068	17.8710588	15.3225328	3.18E-100	7.27952	up	PREDICTED: BTB/POZ domain-containing protein NPY1 [Sesamum indicum]
c46751.graph_c1	2.20664946	2.04879684	1.19178733	11.8307837	13.7251196	13.9401961	2.148E-18	1.7751	up	transposase [Pisum sativum]
c46753.graph_c0	1.32854418	1.14431063	0.43673374	9.31895293	10.3808194	10.0228714	4.706E-25	2.27391	up	PREDICTED: uncharacterized protein LOC105158178 [Sesamum indicum]
c46756.graph_c1	100.530254	95.6048031	97.4615591	19.8458754	17.9393041	22.9860242	0	-3.3616	down	PREDICTED: uncharacterized protein LOC105173509 [Sesamum indicum]
c46756.graph_c2	144.928747	140.16661	140.734433	140.513688	148.203202	142.066427	2.677E-40	-1.07369	down	PREDICTED: trihelix transcription factor ASIL2 [Sesamum indicum]
c46761.graph_c1	41.007158	37.9467994	39.0534589	26.7571589	26.4615063	25.2383713	2.561E-74	-1.67926	down	PREDICTED: uncharacterized protein LOC105178437 [Sesamum indicum]
c46762.graph_c0	1.07059126	0.98477167	0.96674476	0.02102972	0.01898786	0.06864031	2.465E-30	-5.88062	down	PREDICTED: phospholipase D beta 1-like [Sesamum indicum]
c46762.graph_c2	4.18122222	3.10530351	3.23537657	0.22873352	0.23602848	0.10665411	1.981E-49	-5.29376	down	hypothetical protein JCGZ_20700 [Jatropha curcas]
c46767.graph_c0	15.7976799	15.0567473	14.8737392	13.0921269	13.138205	12.4146225	7.861E-41	-1.33295	down	PREDICTED: MYST-like histone acetyltransferase 2 isoform X2 [Sesamum indicum]
c46769.graph_c0	1.39768571	1.25917675	1.05199057	1.42216188	1.08753616	1.07758159	1.093E-05	-1.13567	down	PREDICTED: endoglucanase 11-like [Sesamum indicum]
c46770.graph_c0	29.2311509	27.517102	27.1630984	10.9948004	11.2494165	11.6044366	1.09E-134	-2.39932	down	PREDICTED: diphthamide biosynthesis protein 2 [Sesamum indicum]
c46771.graph_c0	5.29875796	5.3757404	4.69276759	0.28623091	0.79881353	0.33972667	1.404E-75	-4.52301	down	PREDICTED: LOW QUALITY PROTEIN: uncharacterized protein LOC105163603 [Sesamum indicum]
c46775.graph_c0	12.6482602	13.7921422	11.7851948	87.2199199	88.6773393	91.71514	1.082E-75	1.71834	up	PREDICTED: gamma-tubulin complex component 3 [Sesamum indicum]
c46778.graph_c0	0.22975133	0.17248604	0.07380989	2.4629836	1.95999746	2.00977451	3.635E-07	2.67762	up	PREDICTED: probable ubiquitin-like-specific protease 2B isoform X1 [Sesamum indicum]
c46780.graph_c0	3.6595009	2.97073774	3.09683648	14.4499781	14.3648517	13.7231577	1.116E-15	1.0396	up	PREDICTED: subtilisin-like protease SBT3.5 [Sesamum indicum]
c46787.graph_c1	0.10627276	0.21275836	0.13656471	6.4107899	6.76468909	6.61772267	5.285E-14	4.34993	up	hypothetical protein MIMGU_mgv1a019568mg [Erythranthe guttata]
c46792.graph_c1	24.4781464	26.6396106	22.8953966	17.8139076	18.2752065	19.9239388	2.047E-49	-1.49099	down	PREDICTED: LOW QUALITY PROTEIN: protein ALWAYS EARLY 3-like [Sesamum indicum]
c46797.graph_c0	0	0	0	0.57613714	0.55375897	0.87958534	2.958E-09	Inf	up	hypothetical protein GUITHDRAFT_89280 [Guillardia theta CCMP2712]
c46802.graph_c0	0.11272624	0.1253768	0.0482859	4.06002639	4.21651857	3.95176802	1.388E-52	4.33476	up	PREDICTED: callose synthase 7 [Sesamum indicum]
c46804.graph_c0	1.04418354	1.47773774	0.97166073	0.94209093	1.20504468	1.13175391	0.0005373	-1.18008	down	hypothetical protein MIMGU_mgv1a013877mg [Erythranthe guttata]

#ID	0 h-1 FPKM	0 h-2 FPKM	0 h-3 FPKM	28 h-1 FPKM	28 h-2 FPKM	28 h-3 FPKM	FDR	log2FC	regulated	nr_annotation
c46806.graph_c1	3.35063814	3.30092378	3.35190777	15.9599681	15.8506944	15.8592562	5.084E-34	1.16197	up	PREDICTED: uncharacterized protein LOC105173395 isoform X2 [Sesamum indicum]
c46807.graph_c1	63.8047329	61.1200834	62.5552312	20.8356775	23.424209	21.3073789	1.4E-188	-2.60639	down	hypothetical protein MIMGU_mgv1a012384mg [Erythranthe guttata]
c46809.graph_c1	515.97447	510.28739	517.220676	332.313963	316.518424	320.95601	9.988E-83	-1.76107	down	PREDICTED: pentatricopeptide repeat-containing protein At5g14080-like [Sesamum indicum]
c46812.graph_c0	0.58256445	0.74976145	0.6416729	3.65942445	3.00374289	3.23284233	0.0004933	1.23476	up	PREDICTED: LOW QUALITY PROTEIN: phosphatidylinositol 4-phosphate 5-kinase 4 [Sesamum indicum]
c46816.graph_c0	3.3554227	5.27808701	11.0876169	41.1095459	36.4889458	34.9664543	0.0001026	1.4072	up	cell wall-associated hydrolase [Phaseolus vulgaris]
c46818.graph_c0	11.6693665	12.8491568	10.6079971	9.64347072	9.5863735	8.48415896	7.57E-27	-1.43157	down	PREDICTED: geranylgeranyl transferase type-1 subunit beta [Sesamum indicum]
c46820.graph_c0	1.19184463	0.9941984	1.27630628	5.72671504	4.21476147	5.65466546	0.0016261	1.08023	up	putative retrotransposon protein [Phyllostachys edulis]
c46821.graph_c0	7.15802122	7.05567727	6.90879735	41.414807	39.4859292	40.9960232	4.702E-62	1.4388	up	PREDICTED: nodal modulator 1 [Sesamum indicum]
c46822.graph_c0	5.57503254	5.70744725	7.13157328	7.20133407	6.01987434	6.05657444	2.348E-05	-1.02799	down	PREDICTED: uncharacterized protein LOC105160395 [Sesamum indicum]
c46832.graph_c0	1.56198078	1.53911448	1.16041663	7.21940817	5.20514945	6.45546987	1.989E-05	1.06109	up	PREDICTED: cytochrome c-type biogenesis ccda-like chloroplastic protein [Sesamum indicum]
c46833.graph_c0	61.0235244	57.9088477	62.939766	47.7490763	50.2615801	49.5111338	2.1E-54	-1.39312	down	PREDICTED: F-box protein SKIP17-like [Sesamum indicum]
c46836.graph_c0	2.22752375	2.90837515	2.57206211	52.1583916	48.9584126	47.1174908	6.77E-157	3.17355	up	PREDICTED: probable pectate lyase 12 [Sesamum indicum]
c46837.graph_c0	4.60673214	4.98025269	4.69943681	4.83163276	4.62295982	5.00177156	2.936E-19	-1.07321	down	PREDICTED: protein ROOT PRIMORDIUM DEFECTIVE 1-like [Sesamum indicum]
c46838.graph_c0	69.644263	73.0703166	71.0439443	33.4656638	32.1815116	31.8008054	9.29E-161	-2.22406	down	PREDICTED: regulator of nonsense transcripts UPF3-like [Sesamum indicum]
c46841.graph_c0	241.047589	221.643557	249.413677	194.618883	198.162002	211.290987	3.997E-31	-1.32829	down	PREDICTED: uncharacterized protein LOC104591752 [Nelumbo nucifera]
c46842.graph_c0	12.5275414	13.1638074	11.1255866	13.1092138	12.2452845	12.7586173	2.245E-20	-1.03886	down	PREDICTED: uncharacterized protein LOC105174093 isoform X2 [Sesamum indicum]
c46843.graph_c1	4.82520577	5.5687491	4.44982832	5.21913959	4.71239371	4.91526634	8.746E-12	-1.08875	down	hypothetical protein F383_09329 [Gossypium arboreum]
c46844.graph_c1	22.3812256	23.4670243	23.1140959	14.669722	13.2295397	13.7458832	8.399E-89	-1.81847	down	PREDICTED: uncharacterized protein LOC105173956 [Sesamum indicum]
c46846.graph_c0	7.16144652	6.56564979	6.23550297	37.3585206	34.0826085	32.8062901	8.516E-32	1.29537	up	nucleotide binding protein, putative [Ricinus communis]
c46849.graph_c0	3.4500809	3.13766347	3.67655314	14.8462283	13.8465154	15.1086918	1.989E-16	1.00195	up	PREDICTED: patellin-3 [Sesamum indicum]
c46852.graph_c0	476.026893	447.305302	455.919056	418.53408	438.0514	433.157238	3.05E-42	-1.18694	down	PREDICTED: uncharacterized protein LOC105178178 [Sesamum indicum]
c46855.graph_c0	2.49235888	3.11856826	2.0017374	15.2839581	16.9688657	17.8297475	9.122E-07	1.63101	up	-

#ID	0 h-1 FPKM	0 h-2 FPKM	0 h-3 FPKM	28 h-1 FPKM	28 h-2 FPKM	28 h-3 FPKM	FDR	log2FC	regulated	nr_annotation
c46855.graph_c1	0.50419743	0.6234558	0.26678798	2.65135753	3.48384536	2.97259471	2.525E-06	1.62408	up	PREDICTED: peptide-N4-(N-acetyl-beta-glucosaminyl)asparagine amidase A-like [Sesamum indicum]
c46858.graph_c0	0	0	0	1.35757144	1.51417378	2.74986919	1E-08	Inf	up	predicted protein [Hordeum vulgare subsp. vulgare]
c46860.graph_c1	11.3484286	9.76082998	9.92636627	50.1636143	48.4220933	46.1080615	1.062E-36	1.13156	up	hypothetical protein CISIN_1g014750mg [Citrus sinensis]
c46861.graph_c0	6.85524604	5.64972351	5.35403944	4.61263805	4.92779276	4.53963547	3.219E-19	-1.43106	down	PREDICTED: pentatricopeptide repeat-containing protein At3g54980, mitochondrial-like [Sesamum indicum]
c46863.graph_c0	2.52902963	3.68674088	2.90282616	16.1323115	15.0010101	14.6200108	1.702E-19	1.23569	up	PREDICTED: myosin-2 heavy chain [Sesamum indicum]
c46875.graph_c0	39.2994449	39.2313143	34.1229017	36.0700502	34.1110072	34.5272613	1.173E-50	-1.19425	down	PREDICTED: uncharacterized protein LOC105163168 [Sesamum indicum]
c46878.graph_c0	3.52640345	2.99599492	2.43714584	14.064027	13.7291569	13.8135362	2.659E-13	1.12913	up	PREDICTED: LOW QUALITY PROTEIN: ATP-dependent Clp protease ATP-binding subunit clpX-like, mitochondrial [Sesamum indicum]
c46883.graph_c1	6.73632821	7.01830112	5.21153529	31.773551	30.5379558	27.965241	4.15E-13	1.16327	up	PREDICTED: probable serine/threonine-protein kinase WNK5 isoform X1 [Sesamum indicum]
c46888.graph_c1	6.11006754	4.64954945	4.80715023	31.0400192	30.7879356	31.299989	8.762E-39	1.4928	up	PREDICTED: probable sphingolipid transporter spinster homolog 2 [Sesamum indicum]
c46891.graph_c0	125.717258	134.820094	117.264677	89.805086	111.964959	88.9778563	1.741E-42	-1.4681	down	PREDICTED: primary amine oxidase-like [Sesamum indicum]
c46896.graph_c0	0.22856138	0.4575805	0	3.48845901	1.79985795	1.94288611	0.008731	2.32277	up	-
c46899.graph_c0	19.4485729	20.029369	16.8368412	12.6213277	12.5816073	13.0385873	1.836E-53	-1.6468	down	PREDICTED: nuclear-pore anchor [Sesamum indicum]
c46900.graph_c2	87.7322834	89.5017231	85.6229526	58.5306892	57.4710568	61.1900463	2.668E-89	-1.65886	down	PREDICTED: 1,4-alpha-glucan-branching enzyme 3, chloroplastic/amyloplastic [Sesamum indicum]
c46907.graph_c0	6.59517583	5.79668479	4.7543031	3.97496138	4.96128881	5.15149839	4.256E-05	-1.36958	down	-
c46910.graph_c0	1.78511556	1.1562314	1.07950314	20.5868709	21.5855253	19.1651738	6.159E-79	2.84583	up	PREDICTED: cytosolic endo-beta-N-acetylglucosaminidase [Sesamum indicum]
c46912.graph_c0	48.2427553	44.1536762	49.1718293	39.1696164	38.8628787	39.7754673	7.8E-40	-1.35603	down	PREDICTED: uncharacterized protein LOC105158577 [Sesamum indicum]
c46913.graph_c2	220.391984	217.392697	199.780805	129.765228	125.958113	130.547746	2.1E-102	-1.81212	down	PREDICTED: serine/threonine-protein kinase HT1-like [Sesamum indicum]
c46915.graph_c1	158.037291	149.981082	155.610949	81.0704103	70.8490924	83.9917481	4.97E-135	-2.06461	down	PREDICTED: uncharacterized protein At1g04910-like [Sesamum indicum]
c46925.graph_c0	148.998171	142.405006	153.303666	693.303834	625.309925	653.53746	1.623E-36	1.05783	up	PREDICTED: aquaporin PIP2-7 [Sesamum indicum]
c46926.graph_c0	1.4750977	1.52271775	1.48090543	1.13909829	1.45199852	1.94100406	0.002348	-1.07155	down	PREDICTED: kinesin-4-like isoform X2 [Sesamum indicum]
c46929.graph_c1	0.12891214	0.30109621	0.27609535	3.49785721	3.04544292	2.98164359	6.739E-10	2.6567	up	-
c46933.graph_c1	0.16645887	0.45822027	0.40107419	4.58216848	4.83364044	3.96113159	9.169E-22	2.60693	up	PREDICTED: probable LRR receptor-like serine/threonine-protein kinase At1g07650 [Sesamum indicum]

#ID	0 h-1 FPKM	0 h-2 FPKM	0 h-3 FPKM	28 h-1 FPKM	28 h-2 FPKM	28 h-3 FPKM	FDR	log2FC	regulated	nr_annotation
c46938.graph_c0	22.2719481	21.0287552	23.0898356	22.599414	23.1940914	22.764029	2.265E-30	-1.04492	down	PREDICTED: EH domain-containing protein 1-like [Sesamum indicum]
c46941.graph_c0	19.2166944	19.9168552	18.0508142	398.949875	369.186769	358.6808	1.36E-251	3.21061	up	Expansin A1, ALPHA 1.2,EXPA1 [Theobroma cacao]
c46945.graph_c0	1.96555781	2.17739546	1.48181417	17.8664748	17.54199	14.6566102	5.514E-30	2.06632	up	PREDICTED: mitogen-activated protein kinase 9-like [Sesamum indicum]
c46945.graph_c1	2.59052261	2.24736774	2.21928274	19.3298428	15.8663961	14.8511956	6.961E-07	1.73666	up	PREDICTED: mitogen-activated protein kinase 9-like [Sesamum indicum]
c46948.graph_c1	0.06146046	0.15380501	0.11846869	3.03749935	3.12573128	3.04354389	1.076E-16	3.69088	up	PREDICTED: long-chain-alcohol oxidase FAO4A-like isoform X1 [Sesamum indicum]
c46955.graph_c0	8.5615848	10.1155962	7.99597121	8.38168762	9.51741257	8.39316457	2.798E-12	-1.11035	down	PREDICTED: probable LRR receptor-like serine/threonine-protein kinase At2g16250 [Sesamum indicum]
c46956.graph_c0	0.73203614	0.51724882	0.40117893	4.03724206	3.94902197	4.42439746	1.976E-22	1.82752	up	PREDICTED: uncharacterized protein LOC105155208 [Sesamum indicum]
c46957.graph_c0	4.26585206	2.92474224	3.90483585	0.16988475	0.5752125	0.34656142	5.219E-27	-4.43704	down	BRI1 kinase inhibitor 1 [Glycine soja]
c46957.graph_c2	16.1265408	15.694281	10.2177103	6.34869998	7.27558721	7.77073101	2.903E-09	-2.05889	down	PREDICTED: CBL-interacting serine/threonine-protein kinase 6-like [Sesamum indicum]
c46957.graph_c6	3.64265611	3.87940072	3.82598795	30.9474948	30.9770872	28.4095159	1.283E-84	1.9014	up	PREDICTED: alpha-glucan phosphorylase, H isozyme [Sesamum indicum]
c46959.graph_c0	54.6267508	55.5915447	55.4959728	268.720873	261.970526	266.094437	3.261E-47	1.17482	up	PREDICTED: 60S ribosomal protein L27a-3-like [Sesamum indicum]
c46964.graph_c0	5.20673258	4.92830299	4.18747544	23.73506	23.8013737	25.7324604	1.568E-22	1.26744	up	hypothetical protein MIMGU_mgv1a000577mg [Erythranthe guttata]
c46965.graph_c0	0.38176897	0.70970946	0.49058824	6.14392286	4.68843616	5.82200231	3.486E-10	2.30513	up	-
c46971.graph_c1	45.4522506	51.2434819	48.1615199	28.2585985	27.5090127	27.2484915	3.921E-64	-1.8942	down	PREDICTED: uncharacterized protein LOC105159071 [Sesamum indicum]
c46972.graph_c0	127.101446	120.24303	134.709411	104.463256	119.06992	115.797656	1.231E-26	-1.26254	down	unnamed protein product [Coffea canephora]
c46976.graph_c0	23.5194074	26.5507881	22.4805265	20.5468256	20.8747106	19.6583474	4.292E-32	-1.33816	down	PREDICTED: serine/threonine-protein kinase EDR1 [Sesamum indicum]
c46979.graph_c0	3.98613871	4.69427085	4.59504573	3.64978127	4.14170242	3.56919473	4.547E-18	-1.31716	down	PREDICTED: probable boron transporter 2 [Sesamum indicum]
c46980.graph_c0	5.36422422	5.7826415	6.18624148	27.2908107	24.6410433	25.7766625	3.891E-06	1.07236	up	-
c46981.graph_c0	5.13779598	4.68579042	3.81464561	109.893784	122.400141	109.083024	5.48E-183	3.5583	up	PREDICTED: heterodimeric geranylgeranyl pyrophosphate synthase small subunit, chloroplastic [Sesamum indicum]
c46981.graph_c1	0.68697652	0.9578183	0.4729243	9.59351178	8.45273582	9.09413019	1.602E-34	2.59466	up	PREDICTED: putative membrane-bound O-acyltransferase C24H6.01c [Sesamum indicum]
c46982.graph_c0	0.22131996	0.16615619	0.28440492	2.01067593	2.50532355	2.26415929	2.291E-06	2.23981	up	PREDICTED: LOW QUALITY PROTEIN: ABC transporter B family member 13-like [Sesamum indicum]
c46982.graph_c1	0.16741026	0.12568342	0.18823771	1.59695566	1.78521104	1.87398589	2.291E-09	2.35626	up	PREDICTED: LOW QUALITY PROTEIN: ABC transporter B family member 13-like [Sesamum indicum]
c46985.graph_c0	3.9280582	3.84194595	3.9688104	3.57406084	3.42381269	3.27206354	1.681E-11	-1.28389	down	PREDICTED: sucrose nonfermenting 4-like protein [Sesamum indicum]

#ID	0 h-1 FPKM	0 h-2 FPKM	0 h-3 FPKM	28 h-1 FPKM	28 h-2 FPKM	28 h-3 FPKM	FDR	log2FC	regulated	nr_annotation
c46992.graph_c0	6.99707137	7.04527834	6.82297219	4.45723083	5.18588332	4.34494889	1.247E-21	-1.66774	down	PREDICTED: pentatricopeptide repeat-containing protein At4g16835, mitochondrial [Sesamum indicum]
c47001.graph_c0	2.41325432	3.07210312	3.10112734	1.62048742	1.25658611	1.29119146	2.466E-16	-2.13598	down	PREDICTED: ATP-dependent zinc metalloprotease FTSH 10, mitochondrial-like [Sesamum indicum]
c47003.graph_c0	0.19581067	0.50090613	0.25162447	3.33647426	3.02680079	2.5806026	1.723E-11	2.14648	up	PREDICTED: transcription factor HBP-1b(c38)-like [Sesamum indicum]
c47004.graph_c1	42.0591468	41.5198265	42.2067169	464.531392	473.260237	350.869053	7.625E-21	2.26486	up	PREDICTED: glucan endo-1,3-beta-glucosidase 5 [Sesamum indicum]
c47005.graph_c0	0.36377804	0.23172692	0.29748039	8.07596713	9.11480141	6.6095361	7.456E-27	3.646	up	PREDICTED: (6-4)DNA photolyase [Sesamum indicum]
c47005.graph_c1	4.93305483	4.47793294	4.21298927	52.8221685	52.2175085	52.9331621	2.19E-119	2.44678	up	PREDICTED: 3-oxoacyl-[acyl-carrier-protein] reductase 4-like [Sesamum indicum]
c47006.graph_c0	0.18585978	0.08586733	0.16534881	3.19000902	3.17112157	3.22198078	9.349E-30	3.36392	up	PREDICTED: putative ATP-dependent helicase hrq1 isoform X1 [Sesamum indicum]
c47007.graph_c1	38.7305071	40.2961273	37.347542	37.5421331	36.1965053	36.7095468	7.064E-47	-1.16534	down	PREDICTED: uncharacterized protein LOC105162827 [Sesamum indicum]
c47008.graph_c0	2.69800569	2.99006883	2.16690258	0	0.0632323	0.14286378	3.099E-43	-6.33279	down	PREDICTED: pyruvate decarboxylase 1-like [Sesamum indicum]
c47018.graph_c0	66.0199894	64.3955238	65.0755799	26.4278174	24.197687	25.1615597	2.48E-192	-2.45746	down	PREDICTED: 65-kDa microtubule-associated protein 6-like [Sesamum indicum]
c47021.graph_c0	7.36544331	8.18995728	6.79469165	50.6730021	54.5797136	51.7684664	2.04E-86	1.72326	up	PREDICTED: uncharacterized protein LOC105163284 [Sesamum indicum]
c47022.graph_c0	1.89150628	1.24792295	1.10484566	8.1859272	8.51954075	8.71923746	1.795E-09	1.49838	up	PREDICTED: dihydroorotase, mitochondrial isoform X3 [Vitis vinifera]
c47023.graph_c0	113.86264	113.315573	111.71551	67.0022978	64.6730611	64.5540497	1.24E-116	-1.87866	down	unnamed protein product [Coffea canephora]
c47028.graph_c0	2.05268382	2.44186406	1.72029129	1.40539304	1.60081428	1.0762176	6.901E-11	-1.69577	down	PREDICTED: probable LRR receptor-like serine/threonine-protein kinase At4g36180 [Sesamum indicum]
c47029.graph_c0	0.07593404	0	0.19515649	1.54527578	1.94336881	2.34173163	1.511E-09	3.31748	up	PREDICTED: uncharacterized protein LOC105170663 [Sesamum indicum]
c47030.graph_c0	0	0.16196886	0.1039641	2.58720607	2.60145957	2.63892379	4.116E-10	3.77869	up	PREDICTED: uncharacterized protein LOC105173611 isoform X1 [Sesamum indicum]
c47033.graph_c0	27.1938824	27.5824552	24.7994467	12.9901203	12.2572972	13.4158413	3.2E-150	-2.13032	down	PREDICTED: protein CHROMATIN REMODELING 5 isoform X2 [Sesamum indicum]
c47033.graph_c3	2.85110597	1.70444889	1.22126135	42.07651	39.3424017	38.937017	1.46E-116	3.2998	up	PREDICTED: polyadenylate-binding protein-interacting protein 9 [Sesamum indicum]
c47034.graph_c0	0.91002078	0.82812003	1.11625761	10.5222387	10.3149317	10.671247	9.23E-23	2.37091	up	PREDICTED: primase homolog protein isoform X2 [Sesamum indicum]
c47036.graph_c0	4.378654	3.05247328	2.81337187	0	0	0	1.833E-31	-Inf	down	hypothetical protein SNOG_05978 [Phaeosphaeria nodorum SN15]
c47037.graph_c0	32.9645728	33.5582204	33.9811606	28.465211	29.5868197	25.7185695	1.157E-49	-1.35425	down	PREDICTED: L-2-hydroxyglutarate dehydrogenase, mitochondrial isoform X1 [Sesamum indicum]
c47037.graph_c1	27.1154278	23.9113203	17.0073849	8.44600142	10.3797617	8.04051289	2.883E-12	-2.42638	down	glyceraldehyde-3-phosphate dehydrogenase [Leucophyllum frutescens]

#ID	0 h-1 FPKM	0 h-2 FPKM	0 h-3 FPKM	28 h-1 FPKM	28 h-2 FPKM	28 h-3 FPKM	FDR	log2FC	regulated	nr_annotation
c47037.graph_c3	252.046144	242.631421	248.036854	1258.09536	1273.83191	1264.96419	5.191E-47	1.26348	up	glyceraldehyde 3-phosphate dehydrogenase [Salvia miltiorrhiza]
c47037.graph_c4	67.8617337	63.2782961	76.63094	21.3187156	23.3145548	26.5818505	3.28E-33	-2.63639	down	PREDICTED: glyceraldehyde-3-phosphate dehydrogenase, cytosolic [Sesamum indicum]
c47049.graph_c0	16.1878922	15.1297968	14.2511503	9.42521369	10.0360304	9.06995486	4.562E-45	-1.76495	down	PREDICTED: lycopene beta cyclase, chloroplastic/chromoplastic [Sesamum indicum]
c47049.graph_c1	9.83851594	9.92459348	9.08095586	6.60819548	6.18645494	4.59739845	1.904E-74	-1.82074	down	PREDICTED: uncharacterized protein At5g05190 [Sesamum indicum]
c47051.graph_c0	46.7080568	51.5133968	42.9557578	36.6344004	39.7622385	39.2014873	1.232E-33	-1.37752	down	PREDICTED: nuclear transcription factor Y subunit C-9-like isoform X1 [Sesamum indicum]
c47051.graph_c1	36.2044343	35.7173563	35.2710022	36.8574425	33.964976	35.3075567	3.446E-28	-1.10456	down	PREDICTED: probable ribonuclease P/MRP protein subunit POP5 [Sesamum indicum]
c47061.graph_c0	9.55091099	10.07819	9.90282409	9.87353625	10.917331	9.64148684	4.008E-26	-1.04799	down	hypothetical protein MIMGU_mgv1a005188mg [Erythranthe guttata]
c47064.graph_c0	122.479737	121.544928	116.027181	52.3898413	55.7034954	54.4321391	8.4E-163	-2.23741	down	PREDICTED: pentatricopeptide repeat-containing protein At3g46790, chloroplastic-like [Nicotiana tomentosiformis]
c47064.graph_c1	0.89495602	0.73587858	0.28751348	3.71685073	3.08329535	2.67276061	0.0028149	1.2245	up	lipoxygenase [Actinidia arguta]
c47064.graph_c2	61.5252139	62.2719659	59.6143693	29.8199928	33.3376669	34.2780012	1.01E-123	-2.00236	down	PREDICTED: RNA pseudouridine synthase 6, chloroplastic [Sesamum indicum]
c47065.graph_c0	0	0	0	1.93453983	3.00821948	3.55177959	1.136E-09	Inf	up	predicted protein [Physcomitrella patens]
c47067.graph_c0	1.64347234	2.58518548	2.21249498	0.45503475	0.38517536	0.41771772	1.968E-14	-3.44895	down	-
c47068.graph_c0	1.61141186	1.55098601	1.39375926	13.5134406	15.0076849	14.0408648	1.678E-33	2.13476	up	hypothetical protein MIMGU_mgv1a009241mg [Erythranthe guttata]
c47069.graph_c0	28.0906474	24.8591972	26.316961	163.214909	178.519924	167.943381	9.969E-86	1.59462	up	PREDICTED: 60S ribosomal protein L27a-3-like [Sesamum indicum]
c47073.graph_c0	0	0	0	3.56502552	4.67399564	6.81430716	9.728E-11	Inf	up	component of cytosolic 80S ribosome and 40S small subunit [Volvox carteri f. nagariensis]
c47082.graph_c2	6.89630178	7.22771713	7.08204228	44.0815894	40.7298581	40.3922116	1.297E-48	1.47081	up	PREDICTED: uncharacterized protein LOC103719963 isoform X1 [Phoenix dactylifera]
c47083.graph_c0	1.55411614	1.69179368	1.57271686	0.11295227	0.17847428	0.04608406	2.417E-50	-4.93086	down	PREDICTED: pleiotropic drug resistance protein 2-like [Sesamum indicum]
c47085.graph_c1	2.92182624	2.43729334	2.37795286	12.3166856	10.3538597	11.8408882	0.0002872	1.0693	up	PREDICTED: uncharacterized protein LOC105177480 [Sesamum indicum]
c47086.graph_c0	56.3256488	52.4709027	46.2040406	54.4977895	52.2313776	54.1552006	1.174E-25	-1.03429	down	hypothetical protein MIMGU_mgv1a001579mg [Erythranthe guttata]
c47088.graph_c0	7.75824143	8.73952708	7.78257375	35.9041935	36.0007941	32.6795253	2.409E-27	1.01612	up	PREDICTED: serine/threonine-protein kinase D6PKL2-like [Sesamum indicum]
c47090.graph_c0	7.69394272	6.73894116	6.53249602	30.9552339	30.0684395	28.8851381	6.659E-11	1.01149	up	Geranylgeranyl transferase type-2 subunit beta [Morus notabilis]
c47092.graph_c0	0.11740396	0.18803444	0.15086874	0.97274519	0.67798419	0.91908141	0.0017718	1.4023	up	PREDICTED: uncharacterized protein LOC105177054 [Sesamum indicum]

#ID	0 h-1 FPKM	0 h-2 FPKM	0 h-3 FPKM	28 h-1 FPKM	28 h-2 FPKM	28 h-3 FPKM	FDR	log2FC	regulated	nr_annotation
c47102.graph_c0	4.81657012	3.97340946	4.66543967	59.4407769	61.0233859	60.1588823	3.6E-166	2.65604	up	PREDICTED: dolichyl-diphosphooligosaccharide--protein glycosyltransferase subunit 2 [Sesamum indicum]
c47103.graph_c0	3.49395298	4.18709	3.85749215	19.3851635	20.1187449	19.7872613	1.775E-14	1.27005	up	PREDICTED: uncharacterized protein LOC105164063 [Sesamum indicum]
c47103.graph_c2	398.471809	399.301431	397.543187	309.974195	316.535959	321.600993	3.993E-64	-1.42467	down	PREDICTED: two-pore potassium channel 1 [Sesamum indicum]
c47105.graph_c0	48.8088317	44.366608	52.1512602	12.7963234	12.1784143	14.6748151	1.264E-57	-2.96483	down	R3-MYB anthocyanin repressor [Petunia x hybrida]
c47107.graph_c0	45.672473	42.5626968	43.9318457	11.3134779	11.5620441	11.3186218	0	-3.04084	down	hypothetical protein MIMGU_mgv1a008454mg [Erythranthe guttata]
c47116.graph_c0	19.7034079	19.1324082	18.4944944	16.8850267	15.6644745	17.0350621	6.609E-56	-1.29891	down	hypothetical protein JCGZ_07037 [Jatropha curcas]
c47126.graph_c0	30.6717857	33.7837532	30.2318789	30.8413683	30.4439808	28.9477701	6.724E-32	-1.15985	down	PREDICTED: INO80 complex subunit D-like [Sesamum indicum]
c47129.graph_c0	0.12475197	0.24975378	0.12023341	1.38270242	1.20751784	1.44271087	3.092E-05	1.93979	up	PREDICTED: pentatricopeptide repeat-containing protein At2g41080 [Sesamum indicum]
c47134.graph_c0	33.974959	34.7371813	31.2031666	19.9200054	18.3600658	19.4844854	5.04E-113	-1.87973	down	PREDICTED: dynamin-related protein 4C-like [Sesamum indicum]
c47142.graph_c0	1.01793628	0.81100547	0.85426183	114.749177	95.7826369	104.821237	2.67E-164	5.78823	up	hypothetical protein MIMGU_mgv1a004716mg [Erythranthe guttata]
c47143.graph_c0	3.14062802	4.16549907	3.32956086	18.0324637	17.8788719	17.0891264	2.825E-08	1.22645	up	PREDICTED: 15-cis-phytoene desaturase, chloroplastic/chromoplastic [Sesamum indicum]
c47145.graph_c0	88.0486774	91.6675493	90.4272971	75.5449745	73.7822768	75.3829393	5.883E-62	-1.35638	down	PREDICTED: uncharacterized protein LOC105156360 [Sesamum indicum]
c47147.graph_c0	11.76611	11.9960011	10.0488338	11.6483952	12.1061412	11.3858933	1.551E-23	-1.03304	down	PREDICTED: CDT1-like protein a, chloroplastic [Sesamum indicum]
c47150.graph_c1	0.20756529	0.10388657	0.40009443	3.64571709	3.01932079	2.93384261	9.683E-12	2.65076	up	hypothetical protein MIMGU_mgv1a011470mg [Erythranthe guttata]
c47157.graph_c0	0.66951509	0.72044942	0.51621204	3.02908318	2.87776813	3.49414021	6.938E-07	1.21593	up	PREDICTED: GPI mannosyltransferase 1-like isoform X2 [Sesamum indicum]
c47165.graph_c0	0	0	0	3.23400759	4.19418992	8.73243871	1.173E-05	Inf	up	40S ribosomal protein S8-B [Rozella allomycis CSF55]
c47166.graph_c0	154.787709	159.036635	155.603271	133.776226	131.906238	129.925641	1.424E-55	-1.33746	down	PREDICTED: splicing factor U2af large subunit B isoform X2 [Sesamum indicum]
c47166.graph_c1	2.54027767	2.27037623	2.68143642	0.39562697	0.89303514	0.59185202	1.027E-13	-3.08699	down	-
c47175.graph_c0	5.75987232	4.30643479	5.57409262	4.05703701	3.54646403	3.77440206	5.245E-11	-1.54985	down	PREDICTED: O-acyltransferase WSD1-like isoform X1 [Sesamum indicum]
c47176.graph_c0	463.042836	472.071847	416.757961	184.293414	185.212763	186.683761	6.45E-151	-2.37038	down	PREDICTED: very-long-chain (3R)-3-hydroxyacyl-CoA dehydratase PASTICCINO 2 [Sesamum indicum]
c47178.graph_c2	0.87416788	0.87504323	1.05726149	10.2028158	10.5282126	10.2466638	2.667E-18	2.37115	up	PREDICTED: uncharacterized protein LOC105158099 [Sesamum indicum]
c47184.graph_c2	14.1375266	14.4634706	13.0528692	9.09287352	8.67558694	9.00011914	1.893E-76	-1.72724	down	PREDICTED: myosin-6-like [Sesamum indicum]
c47184.graph_c3	267.060312	265.322948	253.593389	268.610005	271.85165	258.502683	3.385E-36	-1.06646	down	PREDICTED: calcium-dependent protein kinase 33-like [Sesamum indicum]

#ID	0 h-1 FPKM	0 h-2 FPKM	0 h-3 FPKM	28 h-1 FPKM	28 h-2 FPKM	28 h-3 FPKM	FDR	log2FC	regulated	nr_annotation
c47184.graph_c4	12.7942346	15.6758244	10.1277164	8.10853263	7.72424902	8.43755047	1.412E-08	-1.75608	down	PREDICTED: calcium-dependent protein kinase 10-like [Sesamum indicum]
c47185.graph_c0	1.15154259	1.56171674	1.33657352	7.96437687	7.72736993	8.85608246	6.053E-11	1.50879	up	PREDICTED: protein LOW PSII ACCUMULATION 1, chloroplastic [Sesamum indicum]
c47185.graph_c1	0.33072419	0.60693484	0.92081965	3.04466689	2.49584688	3.33435761	0.0020529	1.15474	up	PREDICTED: rapid alkalinization factor-like [Sesamum indicum]
c47186.graph_c0	39.490125	41.8058489	38.6685423	18.840324	17.6576648	17.1041987	5.52E-136	-2.2526	down	PREDICTED: BTB/POZ domain-containing protein At1g04390 [Sesamum indicum]
c47186.graph_c1	42.6558914	41.117175	37.8349306	17.6409006	15.3154608	17.5463618	3.76E-105	-2.35644	down	PREDICTED: BTB/POZ domain-containing protein At1g04390 [Sesamum indicum]
c47189.graph_c1	42.9880869	50.8915353	46.4177896	18.2629768	20.6361624	19.8410392	1.142E-48	-2.34712	down	PREDICTED: inactive rhomboid protein 1-like [Sesamum indicum]
c47192.graph_c2	0.19928369	0.19948324	0	8.11094118	7.32341941	7.24879146	6.27E-22	4.7594	up	-
c47197.graph_c0	0.40936998	0.29026076	0.24110929	1.59921161	1.60064461	1.62865056	0.0001714	1.27532	up	PREDICTED: acyltransferase-like protein At1g54570, chloroplastic isoform X1 [Nelumbo nucifera]
c47199.graph_c0	0	0	0	4.42726398	5.57085064	9.49271255	3.682E-09	Inf	up	RecName: Full=60S ribosomal protein L13a [Cyanophora paradoxa]
c47200.graph_c0	0	0	0	2.44778307	3.29432774	3.80631077	2.918E-15	Inf	up	--
c47201.graph_c0	8.70754544	7.86230636	8.61897049	58.1332657	53.2224588	55.1649452	7.959E-68	1.63418	up	PREDICTED: probable serine/threonine-protein kinase At1g01540 [Sesamum indicum]
c47205.graph_c0	0.77524342	0.51734647	0.79697479	8.78970225	8.34326558	9.22593976	1.39E-17	2.56536	up	unnamed protein product [Vitis vinifera]
c47206.graph_c0	15.7871858	9.25087252	10.2685883	0	0	0	9.172E-44	-Inf	down	alpha-2 macroglobulin family-like protein [Micromonas sp. RCC299]
c47207.graph_c0	39.2797961	37.869003	34.4131004	280.940831	291.716788	231.871964	5.493E-26	1.76038	up	hypothetical protein MIMGU_mgv1a001548mg [Erythranthe guttata]
c47210.graph_c0	0	0	0	1.14190604	1.26669903	2.1830415	5.935E-07	Inf	up	hypothetical protein V492_07146 [Pseudogymnoascus pannorum VKM F-4246]
c47214.graph_c1	3.53836707	3.52044408	3.47221008	1.73002904	1.80128752	1.70420182	5.536E-30	-2.09876	down	PREDICTED: protein LONGIFOLIA 2-like [Sesamum indicum]
c47221.graph_c0	6.51291471	6.30913201	5.77755354	53.5349108	67.6662923	53.5272901	7.697E-23	2.14191	up	hypothetical protein MIMGU_mgv1a010747mg [Erythranthe guttata]
c47222.graph_c0	0.54154259	0.49691113	0.57991973	2.52553606	2.50243158	2.59609389	0.0001171	1.14443	up	hypothetical protein MIMGU_mgv1a000984mg [Erythranthe guttata]
c47224.graph_c0	0.10302809	0.06875417	0	1.02336304	0.9916107	0.79432145	8.512E-08	2.96244	up	-
c47233.graph_c0	9.60038565	8.52548499	9.66839785	7.48058526	7.42574396	7.01435648	7.57E-29	-1.43377	down	PREDICTED: glycerophosphodiester phosphodiesterase GDPD4 isoform X3 [Sesamum indicum]
c47234.graph_c0	179.270387	158.829879	176.282782	120.176792	135.471063	134.581122	1.11E-40	-1.48905	down	PREDICTED: uncharacterized protein LOC105178088 [Sesamum indicum]
c47234.graph_c1	223.560896	244.569721	177.885203	192.413808	192.580942	193.335459	1.263E-10	-1.24707	down	-
c47234.graph_c2	38.7861674	38.0326589	32.3462856	35.6396617	34.023283	32.7902317	1.975E-31	-1.18003	down	PREDICTED: formin-1 isoform X1 [Sesamum indicum]
c47234.graph_c3	71.2458435	69.2673502	78.725243	474.711472	476.605228	476.572557	4.61E-92	1.61132	up	hypothetical protein OsJ_19397 [Oryza sativa Japonica Group]

#ID	0 h-1 FPKM	0 h-2 FPKM	0 h-3 FPKM	28 h-1 FPKM	28 h-2 FPKM	28 h-3 FPKM	FDR	log2FC	regulated	nr_annotation
c47234.graph_c6	65.6647471	70.6830291	59.3397382	39.9214802	40.2859835	41.0388709	4.187E-57	-1.77953	down	PREDICTED: uncharacterized protein LOC105169758 [Sesamum indicum]
c47235.graph_c0	25.0054393	25.482642	26.6185456	8.98137925	8.6598471	7.80710889	8.06E-150	-2.69116	down	PREDICTED: aspartic proteinase-like protein 1 isoform X1 [Sesamum indicum]
c47238.graph_c0	3.17265212	3.22322949	3.04252507	19.6858946	19.2816221	18.2820936	6.908E-34	1.51042	up	PREDICTED: WUSCHEL-related homeobox 8 [Sesamum indicum]
c47239.graph_c0	108.309949	111.207238	107.326854	44.7542759	42.2638818	41.5242216	4.74E-186	-2.43688	down	PREDICTED: transcription factor GTE8 [Sesamum indicum]
c47240.graph_c0	1.36206804	0.43824598	0.71887788	0	0	0	3.607E-08	-Inf	down	sucrose synthase 1 [Oncidium hybrid cultivar]
c47246.graph_c0	0	0	0	3.6459255	4.28817053	7.98564828	9.265E-08	Inf	up	allergen Ale o 13 [Metarhizium acridum CQMa 102]
c47250.graph_c1	2.91526028	1.93114818	2.75457712	2.21227059	2.7570753	2.51710252	0.0001995	-1.11289	down	PREDICTED: uncharacterized protein LOC105163644 [Sesamum indicum]
c47250.graph_c2	0.49216111	0.36949045	0.39527907	1.87792425	1.9378166	2.73637652	0.003544	1.29554	up	-
c47250.graph_c3	74.8056247	74.3808517	79.9999135	354.197274	372.406138	371.321904	3.759E-50	1.16877	up	PREDICTED: 60S ribosomal protein L6-like [Sesamum indicum]
c47253.graph_c0	1171.19964	1109.53842	1166.40438	480.018153	483.024001	484.772938	8.42E-118	-2.34215	down	PREDICTED: ABC transporter G family member 3 [Sesamum indicum]
c47256.graph_c0	3.3152479	3.01380121	3.5646779	3.07334575	3.39653031	3.77176886	2.233E-06	-1.0413	down	PREDICTED: CDPK-related protein kinase-like [Sesamum indicum]
c47259.graph_c0	31.5861763	33.5153306	31.0217555	27.9696	27.9217002	30.5028591	3.892E-50	-1.24366	down	PREDICTED: uncharacterized protein LOC105174955 isoform X1 [Sesamum indicum]
c47262.graph_c0	1.30872502	1.52291628	1.51358712	7.3121486	6.61291986	7.0629655	7.132E-14	1.18022	up	PREDICTED: putative chloride channel-like protein CLC-g [Sesamum indicum]
c47266.graph_c0	0	0	0	1.1476099	1.05670264	1.95632579	2.432E-10	Inf	up	--
c47269.graph_c0	30.0996563	31.892566	29.9059188	29.7131156	28.534919	27.9411545	1.452E-43	-1.18298	down	PREDICTED: uncharacterized protein LOC105174622 [Sesamum indicum]
c47269.graph_c1	59.7904738	62.7437367	62.0786895	57.4801359	53.0583427	54.5856252	1.208E-53	-1.25185	down	PREDICTED: uncharacterized protein LOC105168306 isoform X2 [Sesamum indicum]
c47270.graph_c0	5.09966745	3.14139938	3.94113016	0	0	0	1.66E-38	-Inf	down	-
c47277.graph_c1	62.921997	67.0361067	56.6805659	55.2470361	51.8114987	52.4386908	1.418E-41	-1.31577	down	PREDICTED: zinc finger CCCH domain-containing protein 44-like isoform X4 [Sesamum indicum]
c47283.graph_c0	8.83854777	9.62009677	9.27477848	40.5064421	38.5997532	39.55364	2.399E-21	1.00625	up	PREDICTED: probable trehalose-phosphate phosphatase J [Sesamum indicum]
c47287.graph_c0	36.5231047	35.0221205	31.4718537	28.8395412	28.6433419	27.4549684	3.671E-51	-1.36732	down	PREDICTED: GATA transcription factor 15-like [Sesamum indicum]
c47290.graph_c0	0	0	0	2.80542034	2.58174368	5.39282714	2.222E-08	Inf	up	--
c47293.graph_c0	1.03268034	0.91028582	0.75264678	5.98196458	5.44161123	5.04577023	2.741E-16	1.52383	up	PREDICTED: uncharacterized protein LOC105174459 isoform X2 [Sesamum indicum]
c47294.graph_c0	3.57713678	2.97327538	2.38046624	20.7058877	18.8421886	19.2824667	1.305E-29	1.6339	up	PREDICTED: uncharacterized protein LOC105159979 [Sesamum indicum]

#ID	0 h-1 FPKM	0 h-2 FPKM	0 h-3 FPKM	28 h-1 FPKM	28 h-2 FPKM	28 h-3 FPKM	FDR	log2FC	regulated	nr_annotation
c47295.graph_c0	0	0	0	0.76913499	1.03176436	1.68557542	1.301E-06	Inf	up	--
c47296.graph_c0	166.821295	170.652449	175.434841	163.860243	159.693946	160.804113	3.179E-47	-1.17379	down	PREDICTED: NEDD8-activating enzyme E1 catalytic subunit [Sesamum indicum]
c47297.graph_c0	318.434749	304.932656	307.966573	259.130574	271.872818	260.898694	1.567E-54	-1.32433	down	PREDICTED: pentatricopeptide repeat-containing protein At5g15340, mitochondrial [Sesamum indicum]
c47300.graph_c0	161.61969	170.012016	149.513931	27.1568323	27.8533034	27.0172014	0	-3.64179	down	PREDICTED: chaperone protein ClpD, chloroplastic [Sesamum indicum]
c47302.graph_c1	2.07434105	2.44186779	1.91923947	16.1978598	16.1061765	16.3568042	3.186E-52	1.82964	up	PREDICTED: paladin [Sesamum indicum]
c47303.graph_c0	3.95417446	2.17697368	2.37125967	0.7184678	0.90819269	1.0943592	3.699E-06	-2.72734	down	hypothetical protein M569_00480, partial [Genlisea aurea]
c47312.graph_c1	59.2010528	60.9296388	58.8723506	60.0627069	56.1764994	68.7907727	3.998E-35	-1.04173	down	hypothetical protein MIMGU_mgv1a004264mg [Erythranthe guttata]
c47315.graph_c0	9.77123146	10.2157277	9.46560838	3.89681638	3.79248318	4.12082387	4.63E-111	-2.40801	down	PREDICTED: uncharacterized protein LOC105167449 isoform X2 [Sesamum indicum]
c47316.graph_c0	4.10239494	4.36862007	5.1034929	2.28377642	2.5202662	2.25178102	2.299E-18	-2.03775	down	PREDICTED: putative pentatricopeptide repeat-containing protein At1g53330 [Sesamum indicum]
c47327.graph_c0	5.97779165	3.08805661	3.81341483	0	0	0	7.016E-29	-Inf	down	alpha-2 macroglobulin family-like protein [Micromonas sp. RCC299]
c47328.graph_c0	25.7565265	25.9997372	25.4225474	127.249199	112.138443	121.471004	4.333E-37	1.13499	up	PREDICTED: DNA repair protein RAD16 [Sesamum indicum]
c47331.graph_c0	15.4930184	17.4470989	15.879911	8.89201217	8.89607059	9.17003037	8.447E-73	-1.94712	down	PREDICTED: uncharacterized protein LOC104812881 [Tarenaya hassleriana]
c47334.graph_c0	477.83099	438.829954	522.768726	304.889002	407.318576	341.938915	2.525E-17	-1.54192	down	PREDICTED: em-like protein GEA6 [Sesamum indicum]
c47334.graph_c1	11.3596507	9.82919169	9.15442769	1.46931103	1.89521443	2.16952098	4.702E-42	-3.54199	down	hypothetical protein M569_09902, partial [Genlisea aurea]
c47334.graph_c2	67.917916	64.7581071	65.3499988	70.6745811	64.8264672	68.8652086	5.533E-38	-1.04455	down	PREDICTED: probable E3 ubiquitin-protein ligase ARI8 [Sesamum indicum]
c47342.graph_c1	2.17573899	1.92943042	2.34556271	10.1246583	9.74530889	9.90703822	4.09E-19	1.11474	up	PREDICTED: pentatricopeptide repeat-containing protein At5g50990 [Sesamum indicum]
c47345.graph_c0	0	0	0.27463156	3.18419219	4.69821485	5.06980453	1.528E-10	4.43032	up	PREDICTED: 40S ribosomal protein S24-1-like [Musa acuminata subsp. malaccensis]
c47346.graph_c0	0	2.0899367	1.60977889	71.6229069	76.4516386	87.170275	2.48E-35	4.88838	up	PREDICTED: probable aquaporin PIP-type pTOM75 [Sesamum indicum]
c47348.graph_c0	23.7092651	25.8805406	19.1569613	19.3507436	17.2192143	19.4113203	5.264E-13	-1.38356	down	hypothetical protein MIMGU_mgv1a010183mg [Erythranthe guttata]
c47349.graph_c0	1.34228618	2.39516702	0.82494782	7.38036782	7.85100537	7.8913157	0.0002062	1.25762	up	Ubiquitin carboxyl-terminal hydrolase [Medicago truncatula]
c47350.graph_c3	11.9110197	11.0872262	9.63189605	10.5985862	9.26515523	10.023735	6.794E-25	-1.21446	down	PREDICTED: uncharacterized protein LOC105166536 [Sesamum indicum]
c47351.graph_c0	1.9138469	2.37189745	2.75215106	0.29806562	0.32893093	0.67560865	3.785E-17	-3.52456	down	hypothetical protein MIMGU_mgv1a003544mg [Erythranthe guttata]
c47352.graph_c0	3.93001002	4.30699187	4.09241842	22.6042356	21.6545296	22.4432697	2.68E-24	1.34492	up	RUBISCO SUBUNIT BINDING-protein ALPHA SUBUNIT [Populus trichocarpa]

#ID	0 h-1 FPKM	0 h-2 FPKM	0 h-3 FPKM	28 h-1 FPKM	28 h-2 FPKM	28 h-3 FPKM	FDR	log2FC	regulated	nr_annotation
c47357.graph_c0	33.1769776	31.6526021	33.1595676	32.0965948	30.5240444	29.6840987	1.209E-46	-1.17711	down	hypothetical protein MIMGU_mgv1a004228mg [Erythranthe guttata]
c47358.graph_c0	7.38837045	6.65939354	6.74057646	1.02282382	0.90252506	1.13811257	2.912E-92	-3.85122	down	hypothetical protein MIMGU_mgv1a009769mg [Erythranthe guttata]
c47359.graph_c0	58.4066837	63.282334	52.6404133	41.2469626	39.203419	40.3465626	5.329E-44	-1.61814	down	hypothetical protein VITISV_031499 [Vitis vinifera]
c47360.graph_c1	1.3065725	0.85012254	1.17529817	0.59350585	0.72879699	0.46492409	3.43E-08	-1.99063	down	-
c47362.graph_c2	2.3749482	1.65092108	1.86505151	0.71920778	1.12558713	0.86073925	4.644E-09	-2.21148	down	hypothetical protein MIMGU_mgv1a003741mg [Erythranthe guttata]
c47364.graph_c0	11.817542	11.5490585	13.0268611	2.48305311	1.92955931	2.87314863	2.382E-83	-3.41095	down	PREDICTED: pleiotropic drug resistance protein 1-like [Sesamum indicum]
c47367.graph_c0	13.1885499	10.4195679	12.1505984	56.3040998	50.7657968	52.0361125	4.06E-25	1.06351	up	PREDICTED: inositol transporter 1 [Sesamum indicum]
c47367.graph_c4	37.5855224	36.9899498	36.8056015	29.5263057	27.754923	28.0114995	2.923E-73	-1.47538	down	PREDICTED: ubiquitin carboxyl-terminal hydrolase 5 [Sesamum indicum]
c47370.graph_c0	39.1110723	38.2916783	35.4586122	28.5681866	27.0165723	23.8346057	1.17E-82	-1.59697	down	PREDICTED: hexokinase-3-like [Sesamum indicum]
c47371.graph_c0	0.18052678	0.18070755	0.27838087	3.28014552	3.34075684	2.2911456	8.446E-11	2.70213	up	PREDICTED: DNA repair protein RAD51 homolog 2 isoform X3 [Sesamum indicum]
c47373.graph_c1	3.24609284	3.05359974	3.11595403	20.8352298	22.0973465	21.2227289	1.156E-28	1.67815	up	PREDICTED: acyl-protein thioesterase 2-like [Sesamum indicum]
c47378.graph_c0	29.9811228	31.1516573	30.7756101	24.8913818	25.6705783	24.8689207	1.119E-59	-1.37593	down	PREDICTED: E3 ubiquitin protein ligase RIE1-like isoform X2 [Sesamum indicum]
c47382.graph_c0	0	0	0	5.53161268	8.79471166	13.4431273	3.998E-07	Inf	up	ubiquitin/40s ribosomal protein S27a fusion [Wallemia sebi CBS 633.66]
c47383.graph_c0	6.23280714	3.44126882	4.59732603	0	0	0	3.223E-31	-Inf	down	--
c47388.graph_c0	0.51101114	0.34101523	0.21888985	1.60939979	1.90025623	1.76784608	0.0080753	1.21993	up	-
c47389.graph_c0	4.23416533	2.78842448	4.24785297	2.07857242	2.55921274	2.35691557	1.768E-06	-1.78005	down	PREDICTED: uncharacterized protein LOC104811210 [Tarenaya hassleriana]
c47391.graph_c0	4.97260435	7.27492997	6.38999346	2.91904861	3.13765144	2.49534458	2.239E-09	-2.21735	down	-
c47395.graph_c0	4.86723221	4.33076089	3.99598555	24.6149394	24.4430445	24.9366001	5.634E-15	1.39937	up	hypothetical protein MIMGU_mgv1a009112mg [Erythranthe guttata]
c47396.graph_c0	8.15458655	8.95142384	9.31463695	6.48979135	6.28191426	6.85315108	8.268E-28	-1.52066	down	hypothetical protein MIMGU_mgv1a001351mg [Erythranthe guttata]
c47400.graph_c0	11.8269707	7.08214741	6.78487747	0	0	0	4.895E-27	-Inf	down	glyceraldehyde-3-phosphate dehydrogenase 3 [Pilobolus crystallinus]
c47404.graph_c1	22.297474	22.6812558	22.3598871	24.9318086	22.8369039	21.843195	3.276E-33	-1.04298	down	PREDICTED: uncharacterized protein LOC105169219 [Sesamum indicum]
c47411.graph_c0	23.8290284	24.1512061	22.0204888	22.4629987	20.7057113	21.2826886	1.267E-50	-1.20862	down	PREDICTED: mediator of RNA polymerase II transcription subunit 13 [Sesamum indicum]
c47412.graph_c0	260.870499	263.526052	267.529967	48.6547339	50.8567567	47.6989809	0	-3.51859	down	PREDICTED: L-type lectin-domain containing receptor kinase IV.1 [Sesamum indicum]
c47419.graph_c0	10.5038195	11.8378205	8.19623108	47.9045195	41.4939155	42.1317812	1.964E-11	1.01945	up	unnamed protein product [Coffea canephora]
c47421.graph_c1	96.93358	91.5459834	93.422616	86.9142242	83.1810235	84.0500244	3.828E-53	-1.23978	down	PREDICTED: probable serine/threonine-protein kinase DDB_G0282963 isoform X2 [Sesamum indicum]

#ID	0 h-1 FPKM	0 h-2 FPKM	0 h-3 FPKM	28 h-1 FPKM	28 h-2 FPKM	28 h-3 FPKM	FDR	log2FC	regulated	nr_annotation
c47421.graph_c2	12.0693212	14.9352824	12.1308335	11.3044864	11.0799859	12.5308632	1.435E-12	-1.25401	down	PREDICTED: transcription factor bHLH48-like isoform X2 [Sesamum indicum]
c47423.graph_c0	19.045105	20.6490619	17.1888961	19.0003344	17.7526496	17.344321	2.438E-27	-1.16164	down	PREDICTED: mediator of RNA polymerase II transcription subunit 12 isoform X1 [Sesamum indicum]
c47426.graph_c0	18.8616889	18.1192624	17.8713489	8.51257774	8.21367405	7.80963547	6.83E-123	-2.25089	down	PREDICTED: sucrose transport protein SUC3 isoform X2 [Sesamum indicum]
c47429.graph_c0	31.8594916	29.723375	31.1251361	18.8414646	17.9125877	17.590446	4.29E-111	-1.86116	down	PREDICTED: pentatricopeptide repeat-containing protein At1g03560, mitochondrial [Sesamum indicum]
c47432.graph_c0	0.72185187	0.72257469	0.9469334	4.44849116	5.01331149	3.99560133	3.391E-13	1.39727	up	PREDICTED: ABC transporter C family member 13 [Sesamum indicum]
c47433.graph_c1	3.41132882	2.59817536	2.28714396	1.88644988	1.65462202	1.36340453	4.445E-08	-1.84547	down	hypothetical protein VITISV_042890 [Vitis vinifera]
c47434.graph_c0	18.0655343	20.3932888	19.3064631	13.6253951	13.5929215	13.170804	4.998E-50	-1.60745	down	PREDICTED: type I inositol 1,4,5-trisphosphate 5-phosphatase 2 isoform X1 [Sesamum indicum]
c47435.graph_c0	21.3837584	24.1377461	23.0355673	2.51310856	2.38852779	2.75222313	1.6E-119	-4.2536	down	PREDICTED: protein TIC 62, chloroplastic [Sesamum indicum]
c47437.graph_c1	13.0304915	11.5467399	13.7937876	3.10507741	3.60962698	3.42051904	4.109E-53	-3.01238	down	hypothetical protein MIMGU_mgv1a006304mg [Erythranthe guttata]
c47438.graph_c0	6.17812351	6.47089994	5.38311966	49.3158078	48.3939224	44.8972403	3.88E-105	1.89439	up	PREDICTED: clustered mitochondria protein [Sesamum indicum]
c47439.graph_c1	50.7544499	55.6217636	50.9926915	36.5088385	36.7112708	36.907728	1.952E-84	-1.60527	down	PREDICTED: long chain base biosynthesis protein 2a [Sesamum indicum]
c47448.graph_c0	20.6730004	23.2276239	21.2645471	21.4983811	20.7030398	23.1583286	1.434E-24	-1.08578	down	PREDICTED: ADP-ribosylation factor-related protein 1 [Nicotiana tomentosiformis]
c47449.graph_c0	1.86459419	2.5451745	2.55944627	0.12319785	0	0.07539633	4.517E-35	-6.2241	down	PREDICTED: E3 ubiquitin-protein ligase CHIP-like [Glycine max]
c47449.graph_c1	9.40506853	8.95524305	8.35203464	8.16931538	7.72736993	4.98823408	6.268E-13	-1.44615	down	hypothetical protein MIMGU_mgv1a013929mg [Erythranthe guttata]
c47451.graph_c0	0.57854054	0.71073802	0.60827523	3.23005039	3.48591359	3.41548609	5.029E-06	1.32608	up	PREDICTED: uncharacterized protein LOC105174442 [Sesamum indicum]
c47452.graph_c0	15.2717543	9.33425657	10.1289336	0	0	0	3.832E-41	-Inf	down	lectin [Bryopsis maxima]
c47453.graph_c0	45.5899734	45.7866362	44.5881263	18.4860415	17.2059549	19.77263	1.04E-166	-2.38327	down	PREDICTED: probable plastidic glucose transporter 2-like isoform X2 [Glycine max]
c47456.graph_c0	0.48088662	0.30632519	0.33706819	3.27264356	3.08398745	2.70934048	3.858E-10	1.92399	up	PREDICTED: SWI/SNF-related matrix-associated actin-dependent regulator of chromatin subfamily A-like protein 1 isoform X3 [Sesamum indicum]
c47458.graph_c0	0	0	0	9.66441683	11.6524856	19.2824401	5.564E-12	Inf	up	--
c47458.graph_c1	0	0	0	4.94459416	7.41047076	10.6495401	4.264E-12	Inf	up	--
c47462.graph_c0	0.51065417	0.96852413	0.69074824	3.43793311	2.64556642	3.79356496	0.0010699	1.09574	up	PREDICTED: leucine-rich repeat receptor-like serine/threonine-protein kinase BAM3 [Sesamum indicum]
c47466.graph_c0	0.29556557	0.32544769	0.34183211	4.01704707	4.86834885	4.68017819	3.408E-17	2.72438	up	hypothetical protein MIMGU_mgv1a004441mg [Erythranthe guttata]
c47468.graph_c0	80.0914389	73.4736252	75.0244425	46.8130135	49.7090334	53.7391683	1.655E-89	-1.69473	down	hypothetical protein MIMGU_mgv1a012537mg [Erythranthe guttata]

#ID	0 h-1 FPKM	0 h-2 FPKM	0 h-3 FPKM	28 h-1 FPKM	28 h-2 FPKM	28 h-3 FPKM	FDR	log2FC	regulated	nr_annotation
c47468.graph_c1	17.1266498	2.60112821	2.42851565	13005.4582	11549.5541	11593.6636	0	9.60229	up	PREDICTED: non-specific lipid-transfer protein 1-like [Sesamum indicum]
c47468.graph_c2	16.1569824	19.8251653	18.52985	13.5105353	18.4691304	14.8366921	1.015E-09	-1.31191	down	PREDICTED: zinc finger protein ZAT11-like [Nicotiana tomentosiformis]
c47468.graph_c3	63.8762735	63.5020891	82.5335188	1.56941213	1.26383936	1.22871015	1.436E-57	-6.78606	down	PREDICTED: LOW QUALITY PROTEIN: small heat shock protein, chloroplastic-like [Sesamum indicum]
c47471.graph_c1	0.36412477	0.24299292	0.07798579	1.58786269	1.1150931	1.2237006	0.0026373	1.44351	up	PREDICTED: protein DYAD-like [Sesamum indicum]
c47474.graph_c0	95.7833967	93.7327576	89.6997632	43.0197213	46.1675154	45.0658341	2.36E-151	-2.14618	down	PREDICTED: ankyrin repeat domain-containing protein 6 [Sesamum indicum]
c47474.graph_c2	3.32174435	4.13865167	4.04152125	4.28910124	3.73351893	3.91901788	4.473E-07	-1.03829	down	hypothetical protein MIMGU_mgv1a025582mg, partial [Erythranthe guttata]
c47475.graph_c0	462.409017	271.598512	313.741882	0	0	0	6.814E-65	-Inf	down	--
c47476.graph_c0	30.2165419	29.5019636	26.8563452	11.1687088	10.1904474	10.1496448	4.83E-157	-2.54741	down	PREDICTED: uncharacterized protein LOC105163261 [Sesamum indicum]
c47482.graph_c3	41.7415311	41.750762	41.2643853	13.3598518	12.7885902	13.5642046	1.03E-202	-2.74163	down	PREDICTED: YTH domain-containing family protein 2-like isoform X2 [Sesamum indicum]
c47487.graph_c0	0.28142601	0.42256172	0.18082178	2.99990241	3.32422847	3.11550383	8.819E-08	2.33057	up	PREDICTED: vinorine synthase-like [Sesamum indicum]
c47492.graph_c0	13.5790167	12.7012951	16.5198836	101.482377	104.155485	106.473161	6.889E-79	1.77303	up	PREDICTED: uncharacterized protein LOC105161817 [Sesamum indicum]
c47495.graph_c2	72.8984159	76.5802252	74.4589671	54.2256524	52.6196989	52.7275908	2.307E-81	-1.5796	down	PREDICTED: uncharacterized protein LOC105175572 isoform X2 [Sesamum indicum]
c47495.graph_c3	0	0	0.16423398	44.1216343	44.240813	39.4136937	2.46E-147	8.47294	up	hypothetical protein M569_11087, partial [Genlisea aurea]
c47497.graph_c0	0	0	0	3.30911995	5.22869385	6.69860994	1.209E-09	Inf	up	alpha-tubulin, partial [Syntrichia caninervis]
c47498.graph_c0	93.9221566	89.3994331	82.4562682	65.4661018	68.2732977	66.4029094	3.918E-76	-1.49823	down	hypothetical protein MIMGU_mgv1a005594mg [Erythranthe guttata]
c47499.graph_c0	14.4408307	13.4744945	13.332356	14.0162013	13.4965997	14.2176367	8.489E-39	-1.0727	down	replication factor C / DNA polymerase III gamma-tau subunit, putative [Ricinus communis]
c47500.graph_c0	4.81071851	3.5400695	3.27476998	52.3893388	54.1966996	50.6760859	1.92E-147	2.67132	up	hypothetical protein MIMGU_mgv1a001160mg [Erythranthe guttata]
c47501.graph_c0	147.700461	158.649894	135.016049	121.354522	117.298925	123.993692	4.187E-46	-1.37237	down	PREDICTED: malonate--CoA ligase [Camelina sativa]
c47502.graph_c0	898.215909	944.828922	878.431335	348.351608	357.057643	346.095584	1.95E-118	-2.46215	down	PREDICTED: probable inactive receptor kinase At1g27190 [Sesamum indicum]
c47503.graph_c0	1.68796457	1.72037581	1.18314617	9.21217467	8.27745044	8.70013159	4.185E-12	1.42598	up	PREDICTED: pentatricopeptide repeat-containing protein At1g80270, mitochondrial [Sesamum indicum]
c47506.graph_c0	9.98219732	9.61448122	8.90432574	161.953617	162.143934	147.369934	5.89E-245	2.95848	up	-
c47507.graph_c3	2.1221453	2.26132	1.583443	0.49753644	0.80861177	0.64957708	3.921E-12	-2.69645	down	PREDICTED: uncharacterized protein LOC105173228 [Sesamum indicum]

#ID	0 h-1 FPKM	0 h-2 FPKM	0 h-3 FPKM	28 h-1 FPKM	28 h-2 FPKM	28 h-3 FPKM	FDR	log2FC	regulated	nr_annotation
c47508.graph_c0	5.72549101	6.22959155	6.20587665	31.0465723	33.7496558	31.8591092	1.593E-23	1.32049	up	PREDICTED: probable serine/threonine-protein kinase At5g41260 [Sesamum indicum]
c47511.graph_c1	63.0128752	64.7325457	65.0141737	58.5812595	58.1475576	59.9439054	1.196E-49	-1.21648	down	hypothetical protein MIMGU_mgv1a002091mg [Erythranthe guttata]
c47512.graph_c1	3.81854803	2.31229895	2.48378273	14.9729078	17.1077344	15.4329733	1.757E-13	1.37731	up	PREDICTED: glycosyltransferase family protein 64 protein C5 [Sesamum indicum]
c47513.graph_c0	12.1801989	9.79231767	12.570928	66.2194759	59.0347363	66.4340592	7.866E-26	1.38105	up	-
c47517.graph_c0	113.42782	115.788633	121.05734	55.8429297	56.9215098	55.2034264	6.77E-106	-2.15178	down	PREDICTED: calcium and calcium/calmodulin-dependent serine/threonine-protein kinase-like [Sesamum indicum]
c47522.graph_c1	8.09712044	8.69990557	8.79345768	0.5757007	0.6353157	0.56111067	2.84E-213	-4.94394	down	PREDICTED: ABC transporter C family member 4-like [Sesamum indicum]
c47524.graph_c0	63.113418	67.9381945	60.760191	62.0087973	59.9837147	60.4949781	2.344E-47	-1.16174	down	PREDICTED: histone-lysine N-methyltransferase ASHH2 [Sesamum indicum]
c47525.graph_c0	0	0	0	0.8983183	0.62126594	1.23209812	6.694E-08	Inf	up	glycoside hydrolase family 13 protein [Serpula lacrymans var. lacrymans S7.9]
c47526.graph_c0	1.37936907	1.21830909	0.93840558	11.2046073	10.5959224	8.78196534	1.203E-17	2.0256	up	-
c47533.graph_c1	1.72960211	2.17909285	1.83939905	10.1215393	10.0976902	9.86846713	1.218E-12	1.29728	up	PREDICTED: riboflavin biosynthesis protein PYRR, chloroplastic [Sesamum indicum]
c47533.graph_c2	23.9202255	23.2670932	24.17985	3.62002993	2.7439659	2.77157444	2.65E-171	-4.05686	down	hypothetical protein MIMGU_mgv1a0267602mg, partial [Erythranthe guttata]
c47540.graph_c0	0.68129576	0.86383877	0.93385816	4.3904349	3.48726071	4.92938572	8.35E-05	1.27644	up	hypothetical protein MIMGU_mgv1a008287mg [Erythranthe guttata]
c47543.graph_c0	100.473274	97.2032702	92.0327537	454.744305	526.633364	470.854665	3.3E-35	1.23578	up	PREDICTED: mediator of RNA polymerase II transcription subunit 33A-like [Sesamum indicum]
c47547.graph_c0	4.81950626	3.17027549	5.83935599	0	0	0	5.563E-18	-Inf	down	PREDICTED: uncharacterized protein LOC101507298 [Cicer arietinum]
c47550.graph_c0	15.6573649	18.6497067	20.7442202	0.97036053	1.03544359	1.43964776	2.657E-44	-5.09001	down	PREDICTED: uncharacterized protein LOC105155796 [Sesamum indicum]
c47550.graph_c2	5.16204908	4.92827159	6.32668889	0.91082888	0.80281229	0.86710041	1.295E-30	-3.76269	down	PREDICTED: chloride channel protein CLC-a-like [Sesamum indicum]
c47550.graph_c6	8.5943049	8.39807959	10.6934182	1.33849099	0.80568791	1.01129418	3.152E-34	-4.22669	down	PREDICTED: chloride channel protein CLC-b-like [Sesamum indicum]
c47550.graph_c7	0.92628527	0.52983589	0.680179	6.9245362	8.24943973	8.00468374	1.536E-07	2.35215	up	hypothetical protein MIMGU_mgv1a002946mg [Erythranthe guttata]
c47552.graph_c0	2.20099348	1.62340864	1.83595543	9.65414502	8.5647508	8.79371554	5.754E-08	1.16575	up	PREDICTED: folylpolyglutamate synthase isoform X1 [Vitis vinifera]
c47554.graph_c2	2.22533938	2.57027045	2.6396762	13.9342115	13.367617	14.9233912	5.974E-05	1.41374	up	-
c47554.graph_c4	17.1231238	17.733467	15.9117397	12.2863084	12.3214345	13.1700654	7.53E-49	-1.51555	down	PREDICTED: zinc finger protein JACKDAW [Sesamum indicum]
c47554.graph_c5	394.290269	392.242733	369.627339	280.341585	315.708882	304.515536	3.958E-66	-1.45015	down	hypothetical protein POPTR_0003s102801g, partial [Populus trichocarpa]
c47555.graph_c0	0	0	0	0.42449342	0.42160553	0.51957474	7.62E-12	Inf	up	PREDICTED: kinesin-4 [Sesamum indicum]

#ID	0 h-1 FPKM	0 h-2 FPKM	0 h-3 FPKM	28 h-1 FPKM	28 h-2 FPKM	28 h-3 FPKM	FDR	log2FC	regulated	nr_annotation
c47556.graph_c0	55.1822147	28.1452627	38.8353215	0	0	0	6.185E-31	-Inf	down	mannose-binding lectin precursor [Dendrobium catenatum]
c47557.graph_c0	37.320859	36.4957381	31.3503644	174.932104	169.885822	172.745952	6.225E-52	1.21075	up	PREDICTED: pentatricopeptide repeat-containing protein At5g16860 [Sesamum indicum]
c47558.graph_c0	104.06505	91.3569944	109.651955	459.330883	472.797736	427.450902	1.392E-34	1.06384	up	PREDICTED: NADH dehydrogenase [ubiquinone] 1 beta subcomplex subunit 2-like [Fragaria vesca subsp. vesca]
c47560.graph_c0	35.6117574	33.2993837	33.3399583	7.82671982	8.25625458	8.45215288	1.62E-226	-3.14886	down	PREDICTED: CBL-interacting serine/threonine-protein kinase 24 [Sesamum indicum]
c47563.graph_c2	1.28905493	1.34195955	1.25892983	1.12425323	0.57522065	0.64216702	1.516E-06	-1.82299	down	-
c47564.graph_c3	63.1922398	59.5292584	52.3862557	57.9375999	55.0529519	57.8303842	9.033E-33	-1.12385	down	PREDICTED: uncharacterized protein LOC105179697 [Sesamum indicum]
c47564.graph_c8	0.92208555	0.57688055	1.03680189	9.63364295	8.16881605	10.3218006	2.544E-09	2.37862	up	PREDICTED: probable transcription factor KAN2 isoform X2 [Sesamum indicum]
c47564.graph_c9	3.18029712	1.91008903	1.86825496	2.245381	1.66959761	2.74831884	0.0079981	-1.1452	down	-
c47568.graph_c0	1.42892391	1.04296702	0.68858393	8.11354426	8.94720679	8.35076563	3.135E-18	1.92488	up	PREDICTED: D-2-hydroxyglutarate dehydrogenase, mitochondrial isoform X1 [Sesamum indicum]
c47574.graph_c1	947.355017	933.596707	956.402819	88.9852365	87.2550234	93.9879714	0	-4.48269	down	PREDICTED: NAC domain-containing protein 2 [Sesamum indicum]
c47575.graph_c0	0.01750384	0.07008547	0.11246563	4.6815818	5.01959744	3.42566231	2.306E-26	4.92715	up	PREDICTED: uncharacterized protein At5g05190 [Sesamum indicum]
c47580.graph_c0	40.5618678	39.8265258	38.6409666	16.5612829	15.2608691	16.3577718	1.76E-181	-2.39456	down	PREDICTED: two-component response regulator ARR2-like isoform X1 [Sesamum indicum]
c47580.graph_c1	388.161765	366.175821	396.904401	208.645352	200.332427	213.11291	2.635E-92	-1.97878	down	PREDICTED: probable copper-transporting ATPase HMA5 [Nicotiana tomentosiformis]
c47585.graph_c0	26.8630866	22.8110219	21.9461309	110.522458	97.3898829	98.4358734	1.604E-20	1.0083	up	PREDICTED: uncharacterized protein LOC105167876 [Sesamum indicum]
c47585.graph_c2	217.199107	229.446516	276.854933	201.404187	186.799722	190.356228	3.055E-09	-1.41626	down	unnamed protein product [Triticum aestivum]
c47586.graph_c0	0	0	0	6.05538369	7.87475104	10.9147651	5.375E-15	Inf	up	60S ribosomal protein L8 [Rozella allomycis CSF55]
c47591.graph_c0	3.65889165	2.95934483	3.27246716	16.0406645	16.4616926	16.0401636	9.53E-18	1.20541	up	PREDICTED: uncharacterized protein LOC100250241 isoform X4 [Vitis vinifera]
c47593.graph_c0	1.28051448	1.6022459	1.09700787	10.5863775	11.5892512	9.20796223	5.511E-13	1.89032	up	PREDICTED: beta-galactosidase-like [Sesamum indicum]
c47593.graph_c2	4.35404072	4.03264388	4.29727882	36.5547727	34.434152	32.8365252	1.1E-110	1.94217	up	PREDICTED: pentatricopeptide repeat-containing protein At3g09060 [Sesamum indicum]
c47594.graph_c0	0.02661866	0	0.13682415	1.35424041	0.94326592	1.16819478	4.953E-10	3.28789	up	PREDICTED: uncharacterized protein LOC104815505 isoform X1 [Tarenaya hassleriana]
c47596.graph_c5	0.42355031	0.35613852	0.37010967	4.33430167	4.83624601	4.01903951	1.889E-23	2.43002	up	PREDICTED: probably inactive leucine-rich repeat receptor-like protein kinase At3g28040 [Sesamum indicum]
c47598.graph_c0	211.556645	225.315966	237.99075	37.7924073	46.1725226	37.6912503	4.22E-104	-3.56442	down	PREDICTED: B2 protein [Sesamum indicum]

#ID	0 h-1 FPKM	0 h-2 FPKM	0 h-3 FPKM	28 h-1 FPKM	28 h-2 FPKM	28 h-3 FPKM	FDR	log2FC	regulated	nr_annotation
c47598.graph_c1	162.165233	168.562163	161.354457	101.20407	96.8716	98.6199244	3.005E-91	-1.82026	down	transferase, transferring glycosyl groups, putative [Ricinus communis]
c47601.graph_c0	12.819353	12.8195347	11.5346125	11.834652	11.1999491	11.7788157	2.494E-42	-1.18363	down	PREDICTED: LOW QUALITY PROTEIN: mediator of RNA polymerase II transcription subunit 16 [Sesamum indicum]
c47604.graph_c0	0	0	0.11054	2.18817718	5.08041986	5.15254303	1.763E-06	5.68304	up	large subunit ribosomal protein L19e, cytoplasmic [Guillardia theta CCMP2712]
c47605.graph_c0	8.30276232	7.17540126	8.18427466	35.5692262	37.3103615	37.220705	9.264E-32	1.12744	up	PREDICTED: arginine--tRNA ligase, cytoplasmic-like isoform X1 [Sesamum indicum]
c47606.graph_c1	0.03360826	0.13456765	0.04318794	34.8807416	29.4429569	29.3194614	1.98E-115	7.70288	up	PREDICTED: serine carboxypeptidase-like 27 [Sesamum indicum]
c47610.graph_c0	0.08159668	0.5717487	0.10485496	39.7485034	59.3489903	43.2983631	9.993E-40	6.46606	up	PREDICTED: uncharacterized protein LOC105174809 [Sesamum indicum]
c47615.graph_c0	0.42646644	0.18973044	0.24356723	2.68626239	2.23887053	2.64160267	4.336E-06	2.05373	up	PREDICTED: vinorine synthase-like [Sesamum indicum]
c47616.graph_c0	1.35021542	1.19440845	1.0087676	6.7551935	7.89200791	7.18818424	2.204E-11	1.53209	up	PREDICTED: UPF0505 protein isoform X2 [Sesamum indicum]
c47617.graph_c1	0.2719415	0	0.06989112	12.2935512	11.7423634	12.3215726	5.807E-44	5.65923	up	PREDICTED: transcription factor UNE12-like [Sesamum indicum]
c47617.graph_c3	5.23907807	3.74594586	4.56842972	0.13599042	1.10507939	0.33290122	4.731E-19	-4.20003	down	-
c47618.graph_c0	4.49150342	1.38847089	1.78245521	0.09601173	0.04334479	0.03917239	4.392E-05	-6.50513	down	HSP70 [Dendrobium catenatum]
c47622.graph_c0	11.8354658	14.8091466	11.5588721	2.23650657	2.1746908	3.6499422	9.636E-36	-3.33162	down	-
c47622.graph_c1	2339.59651	2200.16903	2471.13004	710.668904	800.90527	884.331202	9.125E-76	-2.63984	down	PREDICTED: probable glycosyltransferase At5g03795 [Sesamum indicum]
c47626.graph_c0	1.64031481	2.12846322	2.10786948	9.28716203	7.7741369	8.86330792	1.075E-10	1.04859	up	PREDICTED: serine/threonine-protein kinase HT1-like [Sesamum indicum]
c47628.graph_c0	2.56348723	1.65271286	2.40084493	15.0629141	14.1136218	15.4606477	1.212E-18	1.66355	up	-
c47631.graph_c0	2.54274883	1.05536622	0.9563511	0.40567065	0.30523547	0.64365766	0.0070706	-2.8256	down	hypothetical protein EUGRSUZ_H03284 [Eucalyptus grandis]
c47633.graph_c0	205.02863	213.141217	183.744597	114.888861	108.276959	113.435004	4.144E-98	-1.9274	down	-
c47636.graph_c1	2.8935163	2.70954831	3.718283	14.7209368	14.3941568	13.3960541	1.75E-05	1.09458	up	PREDICTED: chlorophyll synthase, chloroplastic [Sesamum indicum]
c47637.graph_c0	3.68997875	3.56175678	2.54023327	1.05358601	1.55665535	1.87574744	1.367E-08	-2.21059	down	hypothetical protein VITISV_006175 [Vitis vinifera]
c47647.graph_c0	18.3206339	19.3931524	17.84955	13.4868864	12.9161399	12.5235176	1.326E-73	-1.60363	down	zeaxanthin epoxidase [Scutellaria baicalensis]
c47647.graph_c1	12.3028679	15.8636311	17.149475	5.0012638	2.59993254	2.5969939	1.507E-18	-3.24691	down	zeaxanthin epoxidase [Scutellaria baicalensis]
c47648.graph_c1	6.96330141	6.86739183	6.04245607	150.351306	169.999141	143.745092	1.42E-114	3.45617	up	PREDICTED: ATP-citrate synthase beta chain protein 2 [Sesamum indicum]
c47650.graph_c0	8.6783183	8.56881773	9.7485018	9.22503823	9.95647237	9.50572752	2.518E-14	-1.00461	down	PREDICTED: uncharacterized protein LOC105179099 isoform X1 [Sesamum indicum]
c47652.graph_c0	18.1838584	17.5773468	16.1126431	13.0715998	15.1160448	13.7282588	9.263E-46	-1.39682	down	PREDICTED: E3 ubiquitin-protein ligase SDIR1 [Sesamum indicum]
c47652.graph_c1	2.82717454	2.83000552	2.69113423	43.416617	41.1938301	38.7499131	9.89E-79	2.79499	up	PREDICTED: auxin-responsive protein IAA9-like [Sesamum indicum]

#ID	0 h-1 FPKM	0 h-2 FPKM	0 h-3 FPKM	28 h-1 FPKM	28 h-2 FPKM	28 h-3 FPKM	FDR	log2FC	regulated	nr_annotation
c47652.graph_c5	0.34374291	2.06452271	0.44172325	19.4867687	24.1363536	22.8322685	1.118E-11	3.45681	up	AUX/IAA8A [Malus domestica]
c47653.graph_c0	20.9158782	19.7252007	19.660563	20.5150409	18.1101206	21.1045932	1.481E-23	-1.10309	down	PREDICTED: protein TIFY 10B-like [Sesamum indicum]
c47657.graph_c0	3.12529107	3.24547033	3.29212785	18.7973542	17.4256788	17.1541408	7.652E-48	1.37437	up	PREDICTED: uncharacterized protein LOC105164082 isoform X2 [Sesamum indicum]
c47660.graph_c0	0.19599722	0.13079566	0.16790946	21.0350654	17.3635219	18.8563467	4.16E-101	5.76535	up	PREDICTED: beta-galactosidase-like [Sesamum indicum]
c47663.graph_c2	43.9531541	43.9558547	45.1322003	8.24868287	9.66858198	8.81130484	0	-3.40655	down	PREDICTED: zinc finger protein ZAT10-like [Sesamum indicum]
c47664.graph_c0	5.37810993	5.99008632	6.18104009	1.34880413	1.36696738	1.05569017	6.138E-55	-3.31146	down	PREDICTED: cytochrome P450 704C1-like [Sesamum indicum]
c47666.graph_c1	83.3886171	80.5056232	75.5573016	65.3494732	65.0869533	65.945299	1.261E-65	-1.37536	down	acetyl-CoA carboxylase beta subunit (chloroplast) [Andrographis paniculata]
c47671.graph_c2	16.5305629	14.6853915	14.8928859	15.6256745	15.5020654	16.0018217	2.608E-35	-1.05775	down	PREDICTED: pentatricopeptide repeat-containing protein At1g77360, mitochondrial-like [Sesamum indicum]
c47676.graph_c1	83.2216796	79.775591	89.024426	28.3688603	29.7071474	30.9981103	1.009E-77	-2.59179	down	PREDICTED: BI1-like protein-like [Solanum tuberosum]
c47677.graph_c0	17.8458459	20.1039538	19.4964608	17.0049799	21.552683	19.7020894	1.076E-16	-1.07118	down	UDP-galactose/UDP-glucose transporter 4 [Morus notabilis]
c47678.graph_c2	38.7139371	41.7304083	38.3629327	17.5123125	15.4633877	17.5536474	1.3E-167	-2.32322	down	hypothetical protein MIMGU_mgv1a024577mg, partial [Erythranthe guttata]
c47679.graph_c0	0.32395783	0.59867487	0.28820675	6.68317702	5.85439849	6.94608781	6.555E-29	2.92145	up	PREDICTED: outer envelope protein 80, chloroplastic isoform X1 [Sesamum indicum]
c47680.graph_c0	2.0719942	1.58024304	1.85958986	10.6370076	11.3452104	9.85000954	3.022E-35	1.43952	up	PREDICTED: pentatricopeptide repeat-containing protein At5g21222-like [Sesamum indicum]
c47682.graph_c0	9.26123854	8.96854118	10.5830257	66.7400946	61.6061988	66.8218643	1.954E-67	1.66772	up	PREDICTED: probable tRNA N6-adenosine threonylcarbamoyltransferase, mitochondrial isoform X1 [Sesamum indicum]
c47683.graph_c0	14.407761	15.9974272	15.8631889	12.1997798	12.7363913	12.9206575	2.491E-35	-1.38085	down	alpha-expansin 3 [Striga asiatica]
c47689.graph_c0	0.98137459	0.80139674	0.49780469	47.7694757	45.8260084	46.3006031	4.72E-269	4.85609	up	PREDICTED: major facilitator superfamily domain-containing protein 12-like [Sesamum indicum]
c47692.graph_c0	167.922715	159.836402	176.772216	119.593459	120.52625	122.299025	1.591E-47	-1.5686	down	hypothetical protein MIMGU_mgv1a020592mg, partial [Erythranthe guttata]
c47694.graph_c0	28.3421579	28.2006548	28.8603904	24.4500785	25.1326328	25.2966971	5.168E-53	-1.28046	down	PREDICTED: truncated FRIGIDA-like protein 1 [Sesamum indicum]
c47695.graph_c0	183.845786	162.403378	185.7542	61.1749455	54.8844091	54.7580794	8.062E-72	-2.73021	down	hypothetical protein VITISV_032357 [Vitis vinifera]
c47702.graph_c1	109.090209	108.07668	101.749072	101.960883	104.437709	113.749164	1.856E-41	-1.08364	down	-
c47705.graph_c0	0.70409477	0	7.23831726	36.3329736	41.1220973	38.6251465	1.32E-10	2.74528	up	-
c47705.graph_c1	0.38095805	0.10895415	0.27974067	2.63034206	2.71423116	2.19475127	4.386E-09	2.20282	up	hypothetical protein MIMGU_mgv1a003510mg [Erythranthe guttata]
c47706.graph_c0	0.5169776	0.66535107	0.28471572	3.27427381	2.32631784	2.73748022	0.0002173	1.42373	up	PREDICTED: pentatricopeptide repeat-containing protein At3g09040, mitochondrial [Sesamum indicum]

#ID	0 h-1 FPKM	0 h-2 FPKM	0 h-3 FPKM	28 h-1 FPKM	28 h-2 FPKM	28 h-3 FPKM	FDR	log2FC	regulated	nr_annotation
c47714.graph_c2	2.32424863	2.57147875	2.82955406	0.44453961	0.64220414	1.01567393	9.607E-12	-2.96745	down	hypothetical protein MIMGU_mgv1a022738mg [Erythranthe guttata]
c47718.graph_c0	0.48949962	0.3392237	0.43547981	4.07762003	5.68317431	5.02445332	2.025E-13	2.45782	up	hypothetical protein MIMGU_mgv1a004721mg [Erythranthe guttata]
c47719.graph_c0	22.4539097	22.0858774	22.5875735	22.5415266	21.7325006	23.3938064	3.157E-35	-1.07881	down	PREDICTED: arginine/serine-rich coiled-coil protein 2 [Sesamum indicum]
c47721.graph_c0	35.3616898	34.0522631	43.6170097	13.2280163	12.1266393	14.822894	2.597E-18	-2.585	down	PREDICTED: 17.4 kDa class III heat shock protein [Sesamum indicum]
c47722.graph_c0	19.8341018	17.4742069	17.8936961	165.2824	170.808437	149.451709	6.338E-91	2.04671	up	Heterogeneous nuclear ribonucleoprotein F [Gossypium arboreum]
c47726.graph_c0	84.864226	74.369074	77.2739779	23.71689	23.4343288	30.5506285	1.62E-127	-2.69421	down	PREDICTED: E3 ubiquitin-protein ligase At4g11680-like [Sesamum indicum]
c47726.graph_c1	37.2192574	37.5429686	37.2868896	17.7334181	16.7752639	18.2038717	2.77E-149	-2.17815	down	PREDICTED: LOW QUALITY PROTEIN: uncharacterized protein LOC105174988 [Sesamum indicum]
c47730.graph_c0	1.108891	0.59200074	0.56998753	3.81476178	3.54139678	3.81429377	0.0040619	1.21559	up	hypothetical protein MIMGU_mgv1a012535mg [Erythranthe guttata]
c47731.graph_c0	10.7384266	8.73211257	9.11212904	6.52548349	6.27688186	6.79207051	8.438E-29	-1.63326	down	hypothetical protein MIMGU_mgv1a003230mg [Erythranthe guttata]
c47747.graph_c3	9.61239059	7.95131221	7.46698671	69.4804169	66.2837695	70.0398723	3.114E-96	1.952	up	PREDICTED: ultraviolet-B receptor UVR8 [Sesamum indicum]
c47749.graph_c0	1.80626656	1.90045867	1.98227382	14.718514	15.8504236	13.6131094	1.808E-59	1.86495	up	PREDICTED: importin-11 [Sesamum indicum]
c47753.graph_c0	173.504354	165.705319	160.335146	46.4082702	44.6928516	46.6154144	0	-2.9484	down	PREDICTED: transcription factor PIF1 [Sesamum indicum]
c47755.graph_c0	2.67213774	1.83415781	1.76595572	0	0.20040332	0.04527809	7.832E-24	-5.7655	down	--
c47757.graph_c0	35.521868	32.9087715	36.1915554	18.2300261	16.1745707	19.0673627	5.514E-77	-2.05876	down	PREDICTED: uncharacterized protein LOC105172510 isoform X1 [Sesamum indicum]
c47763.graph_c2	212.122713	218.481498	219.793375	90.3578115	88.9579885	94.0598842	8.36E-166	-2.34113	down	PREDICTED: uncharacterized protein LOC105161925 [Sesamum indicum]
c47763.graph_c3	0.03476091	0.10438716	0	1.46531372	1.30022985	1.42245169	2.65E-10	3.8358	up	PREDICTED: U-box domain-containing protein 5 [Sesamum indicum]
c47764.graph_c2	54.2831268	54.9711564	43.6229007	278.670382	311.96561	259.010932	5.824E-25	1.38606	up	PREDICTED: arginine decarboxylase [Sesamum indicum]
c47770.graph_c0	11.1935456	11.3458721	8.65947527	8.79115494	10.0653805	9.63156673	2.152E-12	-1.21839	down	PREDICTED: probable serine/threonine-protein kinase At1g18390 isoform X1 [Sesamum indicum]
c47776.graph_c0	9.66083816	10.066574	9.4486238	5.82324329	5.04147058	5.70988452	9.427E-45	-1.90557	down	GTPase-activating protein gyp7 [Glycine soja]
c47778.graph_c0	11.617805	10.8454314	11.5744317	5.57856987	4.91699875	5.18687958	3.359E-77	-2.20862	down	PREDICTED: CBS domain-containing protein CBSCBSPB1-like isoform X3 [Sesamum indicum]
c47782.graph_c0	0	0	0	2.25840422	1.06389248	1.94967067	6.936E-08	Inf	up	squalene epoxidase [Chlorophytum borivilianum]
c47784.graph_c0	5.72459622	5.13899242	4.71745174	23.1542187	22.1982971	23.1645366	7.377E-29	1.04853	up	PREDICTED: uncharacterized protein LOC105163660 [Sesamum indicum]
c47792.graph_c1	4.07483999	3.97379352	3.18498467	16.135978	15.8098001	15.8824063	1.925E-16	1.00259	up	PREDICTED: cleavage stimulation factor subunit 77 [Sesamum indicum]
c47795.graph_c0	0	0	0	6.06291411	8.05301861	10.1398969	2.075E-17	Inf	up	unnamed protein product [Vitis vinifera]
c47796.graph_c0	15.0616242	14.4145535	15.1699654	14.8298222	13.389939	14.9980146	6.151E-19	-1.1373	down	PREDICTED: uncharacterized protein LOC105157941 [Sesamum indicum]

#ID	0 h-1 FPKM	0 h-2 FPKM	0 h-3 FPKM	28 h-1 FPKM	28 h-2 FPKM	28 h-3 FPKM	FDR	log2FC	regulated	nr_annotation
c47801.graph_c0	2.08557046	1.82001027	1.58053677	10.5197764	9.88438932	9.9689171	1.041E-21	1.38161	up	PREDICTED: uncharacterized protein LOC105174380 isoform X3 [Sesamum indicum]
c47804.graph_c0	9.08082723	8.67799038	8.24954099	7.41740228	6.35515656	6.45929639	5.687E-31	-1.45191	down	PREDICTED: MORC family CW-type zinc finger protein 3 isoform X1 [Sesamum indicum]
c47809.graph_c1	2.74325731	2.70501913	2.36766778	11.2187525	12.9506901	11.388378	1.158E-08	1.0966	up	PREDICTED: probable E3 ubiquitin-protein ligase LUL4 [Sesamum indicum]
c47810.graph_c1	3.04350875	3.04655637	2.3806265	0	0.08683621	0.07847729	4.758E-26	-6.76498	down	-
c47810.graph_c2	325.27905	329.012967	313.966124	207.400886	193.328125	203.005588	6.182E-90	-1.7714	down	PREDICTED: E3 ubiquitin-protein ligase RKP [Sesamum indicum]
c47810.graph_c3	23.8975471	23.0594417	26.8360499	0.86060628	0.49448432	0.7022477	8.39E-118	-6.25623	down	-
c47816.graph_c0	2.89179242	3.09998514	2.29289287	14.7866962	15.0602513	13.5253987	1.675E-23	1.30036	up	PREDICTED: malonate--CoA ligase isoform X2 [Sesamum indicum]
c47820.graph_c0	0	0	0	2.47116597	2.77448746	5.34797941	1.274E-08	Inf	up	predicted protein [Hordeum vulgare subsp. vulgare]
c47823.graph_c0	21.4256518	24.1513067	20.0510687	20.4806181	19.6535638	18.811378	6.983E-26	-1.24454	down	hypothetical protein MIMGU_mgv1a017876mg [Erythranthe guttata]
c47828.graph_c0	12.2428195	12.4663733	11.4603181	5.06266122	3.91314584	5.50812029	1.702E-50	-2.40872	down	-
c47831.graph_c0	241.94522	245.600643	229.973898	243.923466	237.11763	243.527353	2.653E-39	-1.07565	down	PREDICTED: uncharacterized protein LOC100254102 [Vitis vinifera]
c47832.graph_c0	0.17811172	0.05348702	0.04577612	3.23626431	2.65321527	2.60907607	1.243E-24	3.86164	up	hypothetical protein MIMGU_mgv1a000209mg [Erythranthe guttata]
c47833.graph_c0	12.0750729	11.6972557	10.8739449	10.4942162	9.97770424	9.78992219	9.997E-47	-1.28462	down	PREDICTED: uncharacterized protein LOC105171928 isoform X1 [Sesamum indicum]
c47836.graph_c0	2.57795022	1.34187646	1.59012869	10.1925981	8.5262722	7.09397731	0.0011843	1.14156	up	-
c47836.graph_c1	50.9098757	50.9400709	53.6283132	28.3845387	29.3481952	28.2469962	3.46E-106	-1.94603	down	PREDICTED: uncharacterized protein LOC105173629 [Sesamum indicum]
c47840.graph_c3	8.34547202	12.8401442	4.76633865	107.605416	141.274049	112.91851	9.719E-21	2.7171	up	-
c47843.graph_c3	0.18382992	0.061338	0.11811436	0.73483591	0.80422779	0.65413102	0.0011919	1.50676	up	PREDICTED: serine/threonine-protein kinase STN7, chloroplastic [Sesamum indicum]
c47846.graph_c0	5.50867929	4.54466653	5.60086495	70.7462811	63.4005628	63.6879103	2.461E-93	2.5682	up	hypothetical protein MIMGU_mgv1a012852mg [Erythranthe guttata]
c47848.graph_c3	4.12744426	3.68249279	3.68968994	3.32586821	2.35525268	4.31028207	4.853E-05	-1.28981	down	-
c47849.graph_c0	0	0	0	2.09799358	2.41091646	4.39658765	9.443E-07	Inf	up	Eukaryotic translation initiation factor 5A [Rozella allomycis CSF55]
c47850.graph_c0	22.9760556	23.569759	21.7592231	11.6021889	9.83966484	10.5830793	6.243E-72	-2.18247	down	unnamed protein product [Coffea canephora]
c47851.graph_c0	122.008247	117.662985	119.289821	93.8647428	93.9815758	90.4839907	2.742E-71	-1.45756	down	hypothetical protein MIMGU_mgv1a004485mg [Erythranthe guttata]
c47851.graph_c1	170.694755	170.459897	163.089613	62.2339535	66.2592258	61.9892926	1.54E-194	-2.49464	down	PREDICTED: NADH dehydrogenase [ubiquinone] iron-sulfur protein 7, mitochondrial-like [Sesamum indicum]
c47853.graph_c3	96.7143345	106.93826	94.7916698	99.0286045	100.681601	102.714571	2.001E-37	-1.07075	down	PREDICTED: probable indole-3-acetic acid-amido synthetase GH3.1 [Sesamum indicum]
c47861.graph_c0	0.77935794	0.78013835	0.70694527	7.95227304	7.11998805	7.92998063	2.319E-39	2.25436	up	PREDICTED: uncharacterized protein LOC105167319 isoform X5 [Sesamum indicum]

#ID	0 h-1 FPKM	0 h-2 FPKM	0 h-3 FPKM	28 h-1 FPKM	28 h-2 FPKM	28 h-3 FPKM	FDR	log2FC	regulated	nr_annotation
c47863.graph_c0	1.06299099	1.50219588	0.7231688	12.7929451	11.407214	14.0916402	1.028E-24	2.45724	up	PREDICTED: LOW QUALITY PROTEIN: uncharacterized protein LOC105171608 [Sesamum indicum]
c47863.graph_c1	2.01928437	1.69671703	1.74253396	21.681978	20.8438683	21.1538572	4.47E-107	2.45522	up	PREDICTED: LOW QUALITY PROTEIN: uncharacterized protein LOC105171608 [Sesamum indicum]
c47865.graph_c0	49.8391422	45.7295142	42.6208742	20.5514216	18.772161	20.3010656	2.62E-134	-2.30115	down	PREDICTED: transcription factor GTE4-like isoform X3 [Sesamum indicum]
c47865.graph_c1	28.0397058	34.1364932	26.7806387	16.6153895	12.4327118	10.7864928	2.948E-24	-2.24952	down	PREDICTED: transcription factor GTE4-like isoform X3 [Sesamum indicum]
c47865.graph_c3	0.60747661	0.55280446	0.3548325	8.46897418	10.2559866	8.90846894	7.455E-21	3.10435	up	hypothetical protein MIMGU_mgv1a012348mg [Erythranthe guttata]
c47865.graph_c4	235.782617	232.609558	223.254425	83.9397757	79.8629008	81.0910755	5.37E-185	-2.58774	down	PREDICTED: patellin-3-like [Sesamum indicum]
c47865.graph_c6	375.978214	345.168882	378.285883	90.3451128	95.9539705	102.194683	4.95E-147	-3.02063	down	hypothetical protein VITISV_027452 [Vitis vinifera]
c47874.graph_c1	22.4028717	23.0570035	22.1996422	20.9834991	19.567317	20.1788151	1.732E-43	-1.2462	down	PREDICTED: dymeclin-like [Sesamum indicum]
c47881.graph_c1	23.9163023	23.692392	21.4310217	18.2820416	16.6694585	17.4057691	7.045E-73	-1.4881	down	PREDICTED: uncharacterized protein LOC105166430 [Sesamum indicum]
c47886.graph_c0	1.35183649	1.46144536	1.70242014	0.98250697	1.08227624	0.91395814	1.376E-08	-1.69436	down	PREDICTED: ferric reduction oxidase 2-like isoform X2 [Sesamum indicum]
c47889.graph_c0	36.6568199	34.6722726	34.8966332	20.5647375	19.2815314	20.2350476	7.24E-110	-1.91206	down	PREDICTED: serine/threonine-protein kinase EDR1 [Sesamum indicum]
c47891.graph_c1	54.680133	51.1395561	58.0040062	12.623677	11.9256818	12.6216806	1.95E-94	-3.23126	down	hypothetical protein MIMGU_mgv1a011954mg [Erythranthe guttata]
c47891.graph_c2	786.407989	791.290727	812.491392	607.566185	641.985668	675.78401	8.701E-39	-1.40265	down	PREDICTED: fumarate hydratase 1, mitochondrial [Sesamum indicum]
c47894.graph_c0	2.97689395	2.88957562	3.12989448	49.2380489	54.0473408	38.144879	3.39E-21	2.88182	up	PREDICTED: uncharacterized protein LOC105169824 isoform X1 [Sesamum indicum]
c47896.graph_c1	886.559198	830.280553	912.005776	266.226231	254.54085	267.909758	5.09E-127	-2.82787	down	PREDICTED: uncharacterized protein LOC105160195 [Sesamum indicum]
c47896.graph_c2	22.0835764	20.8888628	22.7807214	20.7622061	17.3503225	19.5251133	1.206E-17	-1.28091	down	PREDICTED: cytochrome P450 CYP72A219-like [Sesamum indicum]
c47900.graph_c0	109.067233	113.480735	98.9436018	81.5807835	80.2733081	81.6575755	1.185E-76	-1.48989	down	PREDICTED: glycine-rich RNA-binding protein RZ1C-like isoform X1 [Sesamum indicum]
c47902.graph_c0	5.50678276	5.2285758	5.09919499	85.1665465	82.4508127	79.4608287	1.17E-165	2.87392	up	PREDICTED: probable starch synthase 4, chloroplastic/amyloplastic isoform X3 [Sesamum indicum]
c47904.graph_c0	153.121301	143.562816	152.233291	150.239668	158.135757	159.665648	7.387E-36	-1.03035	down	PREDICTED: ATP-dependent RNA helicase DHX36 isoform X1 [Sesamum indicum]
c47910.graph_c0	23.4819139	21.0411488	19.9951185	10.7812691	12.1784498	12.2602448	3.096E-86	-1.96181	down	PREDICTED: cytochrome P450 CYP72A219-like [Sesamum indicum]
c47911.graph_c2	120.574232	119.426922	129.895818	30.4035936	27.7160971	26.7555642	1.44E-125	-3.2156	down	hypothetical protein MIMGU_mgv1a008076mg [Erythranthe guttata]
c47913.graph_c1	29.2355588	30.0596812	25.5097134	28.54084	30.7436341	27.9126622	2.123E-21	-1.04892	down	PREDICTED: lysophospholipid acyltransferase LPEAT2 [Sesamum indicum]
c47914.graph_c0	0	0	0	3.60660162	4.55007064	5.52308456	3.042E-17	Inf	up	hypothetical protein JCGZ_08960 [Jatropha curcas]

#ID	0 h-1 FPKM	0 h-2 FPKM	0 h-3 FPKM	28 h-1 FPKM	28 h-2 FPKM	28 h-3 FPKM	FDR	log2FC	regulated	nr_annotation
c47915.graph_c0	4.24654579	1.92614287	2.13163261	0	0	0	1.441E-11	-Inf	down	PREDICTED: stem-specific protein TSJT1-like isoform X1 [Elaeis guineensis]
c47919.graph_c0	1.3542199	1.06918666	0.90687844	5.33014057	5.87652476	5.667165	3.219E-19	1.25513	up	PREDICTED: pentatricopeptide repeat-containing protein At1g09900 [Sesamum indicum]
c47920.graph_c0	6.58369259	6.11647381	6.58023772	31.0555222	31.344058	29.5262818	9.268E-21	1.16231	up	indole-3-glycerol phosphate lyase IGL1 [Aphelandra squarrosa]
c47923.graph_c0	0	0	0	0.93612895	0.95089125	0.98667004	4.502E-09	Inf	up	--
c47924.graph_c0	22.1390296	22.7450864	20.6812748	15.5702269	17.3120826	15.6456091	6.575E-62	-1.52403	down	PREDICTED: cysteine-rich receptor-like protein kinase 3 [Nicotiana sylvestris]
c47934.graph_c0	14.2326956	13.5532759	11.6450493	5.88885638	5.94674	5.21623479	1.615E-51	-2.29807	down	hypothetical protein MIMGU_mgv1a010597mg [Erythranthe guttata]
c47937.graph_c1	39.199188	36.420621	38.9785371	12.3837103	11.4661521	12.4363653	1.12E-196	-2.74951	down	PREDICTED: ATP-dependent DNA helicase 2 subunit KU80 isoform X2 [Sesamum indicum]
c47944.graph_c1	4.77697983	4.49263338	4.65392157	23.3019761	20.2813195	20.9643989	2.039E-24	1.12241	up	PREDICTED: MATE efflux family protein DTX1 [Sesamum indicum]
c47946.graph_c0	42.6491057	44.1070106	41.161116	25.0032052	24.7306646	24.8130353	8.03E-117	-1.86897	down	PREDICTED: scarecrow-like protein 1 [Sesamum indicum]
c47948.graph_c0	1.8445318	2.20539692	1.48143524	332.616535	275.507696	325.302865	9.24E-156	6.31192	up	PREDICTED: alpha-L-arabinofuranosidase 1-like isoform X2 [Sesamum indicum]
c47950.graph_c0	3.96320485	5.18066174	5.2127075	26.7710966	25.9770252	21.7343886	1.371E-13	1.28103	up	unnamed protein product [Coffea canephora]
c47952.graph_c0	1.9012037	1.93770942	2.13217922	14.3705525	14.0414106	12.259265	3.931E-21	1.67522	up	PREDICTED: ABC transporter I family member 11, chloroplastic isoform X2 [Sesamum indicum]
c47954.graph_c0	0	0	0	1.99105427	3.59547065	4.75370511	2.039E-06	Inf	up	40S ribosomal protein S11 [Auxenochlorella protothecoides]
c47956.graph_c0	20.4064264	16.8019499	19.0541701	12.5905772	11.9140369	12.0151316	7.517E-32	-1.71369	down	hypothetical protein MIMGU_mgv1a0158002mg, partial [Erythranthe guttata]
c47957.graph_c0	56.5884466	58.3645245	53.1592309	53.3348273	54.7629737	51.6703191	4.539E-43	-1.16332	down	PREDICTED: nucleolar complex protein 4 homolog isoform X1 [Sesamum indicum]
c47958.graph_c0	3.44671142	3.58159755	3.24805113	23.6190428	21.6058167	20.9666292	9.794E-32	1.59738	up	PREDICTED: 3-oxoacyl-[acyl-carrier-protein] synthase II, chloroplastic [Vitis vinifera]
c47958.graph_c1	11.1702903	10.6537363	11.009171	10.8725669	11.1575436	10.9238029	2.238E-19	-1.08526	down	hypothetical protein MIMGU_mgv1a024121mg, partial [Erythranthe guttata]
c47961.graph_c0	0.3050715	0.45806547	0.29402171	2.60525942	1.6516184	2.6460302	0.0001788	1.61962	up	PREDICTED: probable polygalacturonase At3g15720-like [Glycine max]
c47963.graph_c0	2.90894205	3.95713616	2.87546592	30.2493562	29.8575678	21.0559433	2.817E-10	1.96755	up	PREDICTED: protein ENHANCED DISEASE RESISTANCE 2-like [Sesamum indicum]
c47963.graph_c2	7.13039425	4.48170756	8.94974964	5.90544519	4.46152236	5.6055371	0.0083723	-1.45899	down	-
c47968.graph_c0	179.286408	160.616204	172.227328	69.4375954	67.5108205	76.6870706	5.14E-123	-2.3511	down	PREDICTED: alpha-galactosidase-like [Sesamum indicum]
c47971.graph_c0	221.818253	214.295197	224.086907	86.4549941	79.9351609	81.761115	1.19E-191	-2.50248	down	PREDICTED: LOW QUALITY PROTEIN: manganese-dependent ADP-ribose/CDP-alcohol diphosphatase-like [Sesamum indicum]
c47972.graph_c1	0.15624427	0.25024115	0.40156013	6.74531144	7.13620728	6.50486797	3.772E-31	3.55499	up	PREDICTED: NF-X1-type zinc finger protein NFXL1 [Prunus mume]

#ID	0 h-1 FPKM	0 h-2 FPKM	0 h-3 FPKM	28 h-1 FPKM	28 h-2 FPKM	28 h-3 FPKM	FDR	log2FC	regulated	nr_annotation
c47972.graph_c3	170.258586	168.897847	173.211786	134.964113	136.076933	140.446904	1.148E-64	-1.40697	down	PREDICTED: putative lactoylglutathione lyase [Sesamum indicum]
c47972.graph_c5	0.19527785	0.0651578	0.50187954	10.3606576	12.6865142	10.8476419	6.61E-31	4.36595	up	hypothetical protein MIMGU_mgv1a026403mg, partial [Erythranthe guttata]
c47975.graph_c0	57.0319485	50.9962193	49.8231944	52.600916	51.4879808	54.5919325	9.569E-30	-1.08124	down	hypothetical protein PRUPE_ppa009199mg [Prunus persica]
c47976.graph_c1	172.240021	170.155899	181.558412	767.29367	797.600994	783.068792	6.015E-39	1.07257	up	PREDICTED: UPF0586 protein C9orf41 homolog isoform X2 [Sesamum indicum]
c47977.graph_c0	0	0	0	1.84969625	1.79857169	4.06359971	0.0001496	Inf	up	hypothetical protein CY34DRAFT_809502 [Suillus luteus UH-Slu-Lm8-n1]
c47978.graph_c0	5.30333039	5.22482023	3.71237587	26.5144209	25.0939934	26.6016299	5.274E-35	1.37144	up	PREDICTED: ABC transporter D family member 1 [Sesamum indicum]
c47978.graph_c1	9.75787293	9.262421	8.51804431	5.6980277	5.74096155	6.48541555	3.151E-35	-1.70781	down	hypothetical protein MIMGU_mgv1a010030mg [Erythranthe guttata]
c47979.graph_c0	8.03468871	7.19364792	6.75205619	55.501559	56.0192306	58.0607968	2.17E-92	1.85933	up	hypothetical protein MIMGU_mgv1a024130mg [Erythranthe guttata]
c47981.graph_c0	0.98467056	0.83963337	0.93728938	0.55661891	0.74189597	0.90839298	0.0002255	-1.41234	down	hypothetical protein PHAVU_007G150900g [Phaseolus vulgaris]
c47981.graph_c1	0.82393781	0.48348167	0.40161102	3.53105123	4.30687773	4.19559025	7.662E-09	1.7336	up	PREDICTED: uncharacterized protein LOC105174256 [Sesamum indicum]
c47982.graph_c0	119.879118	124.380467	116.865072	89.955118	84.0932942	82.77626	2.39E-49	-1.58199	down	flavone synthase II [Verbena x hybrida]
c47982.graph_c2	125.566937	136.501197	132.040924	108.602244	119.543468	114.129943	1.37E-43	-1.29448	down	flavone synthase II [Verbena x hybrida]
c47982.graph_c3	98.4547239	96.138865	89.1826638	42.1528913	41.0531153	40.9896032	7.47E-169	-2.2815	down	PREDICTED: uncharacterized protein LOC105178089 [Sesamum indicum]
c47983.graph_c0	0	0	0	2.75419664	4.10859525	5.42307865	2.387E-09	Inf	up	PREDICTED: 40S ribosomal protein S19-3 [Prunus mume]
c47984.graph_c0	0.78341904	0.54291013	0.54208241	0.28469222	0.27682349	0.42887359	5.631E-07	-2.00006	down	hypothetical protein [Beta vulgaris subsp. vulgaris]
c47984.graph_c1	3.17213568	3.21475696	3.443351	21.4940397	20.16994	20.7406099	5.267E-45	1.57477	up	PREDICTED: putative nuclease HARBI1 [Sesamum indicum]
c47990.graph_c0	0.48098542	0.64195608	0.41205687	4.69987438	4.8748172	5.26132077	7.287E-09	2.18541	up	PREDICTED: kinetochore protein Spc25 [Sesamum indicum]
c47999.graph_c0	30.6202899	15.7295958	16.8407466	0	0	0	2.537E-23	-Inf	down	--
c48000.graph_c0	7.69961942	8.11796583	7.7248654	5.05707624	4.80420518	4.6505371	1.176E-60	-1.7887	down	PREDICTED: uncharacterized protein LOC105159735 isoform X3 [Sesamum indicum]
c48005.graph_c0	6.15667496	6.36164123	5.44452203	61.0571283	63.12233	60.9722217	3.504E-69	2.27665	up	PREDICTED: uncharacterized protein LOC105173858 [Sesamum indicum]
c48007.graph_c0	2.01089875	2.09918003	1.40278873	1.04393544	0.98027886	0.86887965	5.497E-12	-2.01681	down	PREDICTED: uncharacterized protein LOC104226160 [Nicotiana sylvestris]
c48011.graph_c0	1.75428331	1.20414169	1.24524537	0.47357254	0.64686935	0.70350319	2.485E-11	-2.29055	down	PREDICTED: NADP-dependent malic enzyme-like isoform X1 [Sesamum indicum]
c48012.graph_c1	0.99958718	0.86097117	0.77667992	4.17311786	4.34761634	3.36387103	1.782E-05	1.08325	up	PREDICTED: LOW QUALITY PROTEIN: phosphatidylinositol 4-phosphate 5-kinase 4 [Sesamum indicum]
c48013.graph_c0	47.3825275	51.1030022	46.3069789	49.2355517	50.8020746	49.3297897	7.86E-39	-1.04524	down	PREDICTED: OBERON-like protein [Sesamum indicum]

#ID	0 h-1 FPKM	0 h-2 FPKM	0 h-3 FPKM	28 h-1 FPKM	28 h-2 FPKM	28 h-3 FPKM	FDR	log2FC	regulated	nr_annotation
c48014.graph_c0	2.84142316	3.35513216	3.03096774	14.1952624	15.414785	14.9534765	1.31E-28	1.18094	up	PREDICTED: uncharacterized protein LOC105158471, partial [Sesamum indicum]
c48016.graph_c0	65.9167395	62.5008061	61.0645323	52.0933523	54.8218091	58.3579033	5.589E-57	-1.28651	down	PREDICTED: probable F-box protein At1g60180 [Sesamum indicum]
c48019.graph_c0	7.41937765	6.14014529	5.93796295	6.24378633	5.60552209	5.79928422	1.429E-17	-1.23182	down	PREDICTED: protein NRT1/ PTR FAMILY 5.10-like [Sesamum indicum]
c48023.graph_c1	48.7110722	50.9131656	51.7257519	57.3442612	54.0734152	49.1277467	1.438E-31	-1.00666	down	unnamed protein product [Coffea canephora]
c48024.graph_c0	4.30159372	3.12296125	3.94837017	3.53864256	3.38118259	3.44818371	4.463E-07	-1.22366	down	hypothetical protein MIMGU_mgv1a020521mg, partial [Erythranthe guttata]
c48031.graph_c1	1.06918099	1.19723062	0.9547718	5.95318154	5.6724637	5.54557018	3.632E-12	1.32538	up	PREDICTED: cytochrome P450 CYP749A22-like [Sesamum indicum]
c48046.graph_c0	31.8626314	36.6153891	35.1060521	28.0064558	26.8723717	27.355429	1.13E-30	-1.42437	down	PREDICTED: uncharacterized protein LOC105172478 [Sesamum indicum]
c48046.graph_c1	33.6910287	32.8451899	32.0027229	28.5703207	26.1756786	26.796407	6.497E-60	-1.3631	down	PREDICTED: probable NOT transcription complex subunit VIP2 isoform X2 [Sesamum indicum]
c48050.graph_c1	0	0	0	4.29993541	5.17658469	6.92745738	8.131E-15	Inf	up	unknown [Lotus japonicus]
c48052.graph_c1	0	0	0	0.8931728	0.96774155	1.7491721	3.35E-08	Inf	up	--
c48055.graph_c0	0.36873413	0.30199366	0.17230471	4.14172299	3.21164577	3.87661393	1.541E-12	2.65424	up	farnesyl diphosphate synthase [Leibnitzia anandria]
c48059.graph_c1	3.91055298	3.66788809	3.75901939	28.074795	26.268298	27.7206565	2.561E-74	1.7656	up	PREDICTED: protein transport protein Sec24-like At4g32640 [Sesamum indicum]
c48062.graph_c3	76.9083771	74.9822743	73.076858	53.9971674	53.7523158	54.0337029	2.918E-80	-1.56558	down	PREDICTED: probable protein phosphatase 2C 6 [Sesamum indicum]
c48066.graph_c0	0	0	0	1.69073652	1.9740214	3.18741454	4.406E-09	Inf	up	predicted protein [Hordeum vulgare subsp. vulgare]
c48067.graph_c0	0	0	0	7.11479202	7.76718805	12.1390073	1.046E-16	Inf	up	--
c48069.graph_c1	47.843159	50.2609823	44.6417854	50.8208087	50.3781572	48.6427448	3.41E-37	-1.01969	down	PREDICTED: protein FAR-RED IMPAIRED RESPONSE 1 [Sesamum indicum]
c48076.graph_c1	2.8987529	2.9923323	3.37579235	29.0342948	24.4913409	25.1780778	6.072E-37	1.99362	up	PREDICTED: aspartokinase 2, chloroplastic isoform X1 [Sesamum indicum]
c48076.graph_c2	37.8500862	36.1069281	35.3082089	38.5991255	36.7266439	38.0708809	7.958E-38	-1.03612	down	PREDICTED: eukaryotic translation initiation factor 4G-like [Sesamum indicum]
c48076.graph_c3	0	0	0	0.90046655	0.81303687	1.20712766	3.324E-11	Inf	up	-
c48078.graph_c0	27.8483402	29.5806582	26.6249475	11.7392076	11.8107643	11.1551658	2.75E-172	-2.36621	down	PREDICTED: ankyrin repeat protein SKIP35-like [Sesamum indicum]
c48078.graph_c1	38.3818327	36.728808	38.3099909	9.1231203	8.79175741	8.30335993	4.85E-103	-3.20402	down	-
c48082.graph_c0	24.0632653	23.8961915	26.8553769	116.831563	112.506154	114.733633	8.442E-45	1.10928	up	PREDICTED: transmembrane 9 superfamily member 3 [Sesamum indicum]
c48090.graph_c0	0.32247233	0.96838571	0.20719485	11.2498094	10.7923683	9.0841302	4.984E-11	3.29249	up	hypothetical protein [Arabidopsis thaliana]
c48090.graph_c2	51.6566556	53.9833048	47.0427886	41.1597459	41.7987386	44.4049395	3.199E-50	-1.35047	down	hypothetical protein MIMGU_mgv1a003910mg [Erythranthe guttata]

#ID	0 h-1 FPKM	0 h-2 FPKM	0 h-3 FPKM	28 h-1 FPKM	28 h-2 FPKM	28 h-3 FPKM	FDR	log2FC	regulated	nr_annotation
c48090.graph_c3	0.99459919	0.9244812	1.46068496	9.55219698	9.18418075	9.47981522	1.373E-10	1.96536	up	hypothetical protein MIMGU_mgv1a003194mg [Erythranthe guttata]
c48091.graph_c1	21.4541658	22.0810455	17.3024902	200.646948	205.897286	199.631651	1.55E-162	2.22887	up	PREDICTED: pentatricopeptide repeat-containing protein At2g13420, mitochondrial-like [Sesamum indicum]
c48094.graph_c0	31.198067	26.8513669	27.726314	26.616137	29.6189047	30.4336375	4.45E-25	-1.07414	down	PREDICTED: sugar transporter ERD6-like 5 [Sesamum indicum]
c48096.graph_c0	8.72749305	8.21178235	6.18340563	4.86003073	4.77720473	4.75889446	2.681E-14	-1.76999	down	PREDICTED: putative SWI/SNF-related matrix-associated actin-dependent regulator of chromatin subfamily A member 3-like 2 isoform X3 [Sesamum indicum]
c48100.graph_c0	51.3881423	53.4628498	47.4214159	47.7652609	47.0866051	46.1682906	2.889E-49	-1.20025	down	PREDICTED: uncharacterized protein LOC105166426 [Sesamum indicum]
c48103.graph_c0	13.7729291	12.2408412	11.8261732	69.68119	61.9228306	59.924674	2.465E-28	1.25041	up	PREDICTED: LOW QUALITY PROTEIN: probable serine/threonine-protein kinase drkD [Sesamum indicum]
c48105.graph_c0	27.7789966	15.5684852	17.6882389	1.48097473	1.82839079	3.62538985	1.79E-11	-4.21945	down	hypothetical protein AOL_s00169g48 [Arthrobotrys oligospora ATCC 24927]
c48109.graph_c0	116.935893	113.453806	107.41553	98.8871054	97.3666075	103.128321	3.354E-55	-1.26346	down	PREDICTED: cleft lip and palate transmembrane protein 1 homolog [Sesamum indicum]
c48112.graph_c3	7.9967825	6.88784262	7.24908971	1.48679465	1.54583558	2.0955485	7.859E-41	-3.19777	down	PREDICTED: uncharacterized protein LOC105163706 [Sesamum indicum]
c48114.graph_c0	159.373403	156.0553	157.89243	68.2115888	68.4282869	74.9292893	3.65E-164	-2.25173	down	ribosomal protein L20 (chloroplast) [Andrographis paniculata]
c48118.graph_c0	2.55953286	2.19608216	3.21078914	0.53150107	0.27993912	0.61440908	5.419E-16	-3.57375	down	PREDICTED: sodium/hydrogen exchanger 4 isoform X3 [Nelumbo nucifera]
c48119.graph_c1	12.7431571	13.399073	11.1463225	12.8573538	10.2597567	10.8852443	3.312E-23	-1.22185	down	PREDICTED: uncharacterized protein LOC105173907 [Sesamum indicum]
c48122.graph_c1	181.404108	201.097442	159.934232	74.9320283	73.3906212	71.0651393	3.318E-45	-2.39479	down	PREDICTED: NF-X1-type zinc finger protein NFXL1 [Sesamum indicum]
c48126.graph_c0	0.08555543	0.0856411	0.05497106	1.6322563	2.03521227	2.28327053	1.814E-15	3.63341	up	PREDICTED: cysteine-rich receptor-like protein kinase 2 [Sesamum indicum]
c48128.graph_c0	6.89529398	7.06271482	5.93462562	6.182743	5.79815819	6.11494551	8.515E-35	-1.22495	down	PREDICTED: uncharacterized protein LOC105167197 [Sesamum indicum]
c48134.graph_c1	295.021142	296.319902	290.191209	194.669421	185.707728	188.152547	2.435E-90	-1.7231	down	PREDICTED: protein ALUMINUM SENSITIVE 3 [Sesamum indicum]
c48135.graph_c0	3.10375323	2.9938844	2.75565345	1.4355027	1.66644559	2.87819513	4.646E-08	-1.65202	down	PREDICTED: formin-like protein 3 [Nicotiana tomentosiformis]
c48139.graph_c0	0	0	0.18038052	2.09140657	1.56594384	1.54007598	8.487E-10	3.71798	up	hypothetical protein VITISV_002420 [Vitis vinifera]
c48142.graph_c0	11.8967129	11.6379751	10.4234639	113.517571	108.764936	116.895857	1.367E-84	2.23162	up	PREDICTED: pentatricopeptide repeat-containing protein At5g39350 [Vitis vinifera]
c48143.graph_c2	3.07489539	2.25351699	2.75184161	24.6228861	23.1329743	20.4828437	7.119E-28	1.98762	up	PREDICTED: protein transport protein SEC13 homolog B-like [Sesamum indicum]

#ID	0 h-1 FPKM	0 h-2 FPKM	0 h-3 FPKM	28 h-1 FPKM	28 h-2 FPKM	28 h-3 FPKM	FDR	log2FC	regulated	nr_annotation
c48143.graph_c3	642.847733	657.767596	591.781889	397.408776	369.060911	357.624118	8.806E-87	-1.84111	down	PREDICTED: haloacid dehalogenase-like hydrolase domain-containing protein 3 [Sesamum indicum]
c48148.graph_c0	0.14414463	0.14428897	0	2.40956022	1.93912795	2.47909837	3.885E-09	3.49561	up	PREDICTED: SNF2 domain-containing protein CLASSY 3-like [Sesamum indicum]
c48150.graph_c0	4.66837668	5.34250533	4.53298916	2.51611968	2.38369009	2.64418658	1.018E-51	-2.03636	down	PREDICTED: CST complex subunit CTC1 [Sesamum indicum]
c48154.graph_c0	32.8359313	30.4537196	31.3819369	14.2419956	15.0216926	15.7045299	5.184E-87	-2.16388	down	hypothetical protein MIMGU_mgv1a024172mg [Erythranthe guttata]
c48157.graph_c1	269.174604	258.621146	281.562204	284.5087	287.96606	282.904434	4.575E-32	-1.01156	down	Metal tolerance protein 3 [Triticum urartu]
c48161.graph_c2	1.02361066	0.81970852	0.78922814	11.4568967	10.6131919	10.4009726	4.985E-16	2.53676	up	hypothetical protein MIMGU_mgv1a011390mg [Erythranthe guttata]
c48161.graph_c4	0.32271534	0.43071798	0.59901398	27.1814662	24.471724	23.7534971	5.52E-120	4.70272	up	PREDICTED: WD repeat-containing protein 44 [Sesamum indicum]
c48162.graph_c0	3.25757107	2.73334534	3.93986616	0.03481741	0.09431057	0.02841072	1.527E-34	-7.07624	down	PREDICTED: aluminum-activated malate transporter 9-like [Sesamum indicum]
c48163.graph_c0	2.00399786	3.26400743	2.66251208	2.61675211	2.20665565	2.63884466	0.0007659	-1.17969	down	PREDICTED: uncharacterized aarF domain-containing protein kinase 1 [Sesamum indicum]
c48166.graph_c0	68.3290786	69.0716222	64.9055587	68.5994472	68.1259569	68.3270765	1.13E-37	-1.07038	down	PREDICTED: uroporphyrinogen decarboxylase 1, chloroplastic isoform X2 [Sesamum indicum]
c48169.graph_c0	2.37666436	2.60973943	2.79497138	12.6253287	12.127316	12.1558714	7.472E-24	1.15336	up	PREDICTED: GPI inositol-deacylase A isoform X1 [Sesamum indicum]
c48175.graph_c2	35.2834311	32.6548165	35.412006	12.665913	13.1543685	13.4918701	8.43E-140	-2.48514	down	PREDICTED: transcription factor TGA1-like [Sesamum indicum]
c48181.graph_c0	48.6639999	47.3497024	47.9800816	36.2009197	33.7725246	35.4953044	4.482E-80	-1.5395	down	PREDICTED: probable leucine-rich repeat receptor-like protein kinase At1g35710 [Sesamum indicum]
c48182.graph_c0	16.0058479	17.278493	18.6188792	16.5940724	16.5275195	16.4255723	2.183E-16	-1.15941	down	PREDICTED: C2 domain-containing protein At1g53590 isoform X2 [Sesamum indicum]
c48187.graph_c1	2.00113514	2.12381	2.10680212	29.9819455	31.7066364	30.1129969	1.27E-116	2.78959	up	PREDICTED: protein IQ-DOMAIN 31-like isoform X1 [Sesamum indicum]
c48188.graph_c0	31.15958	30.3344349	29.3016016	15.3528186	12.7825486	16.0207211	4.18E-116	-2.12902	down	PREDICTED: nitronate monooxygenase [Sesamum indicum]
c48195.graph_c0	0.30643743	0.15337214	0.06563071	1.29918227	1.40764774	1.87793062	3.985E-05	2.0522	up	hypothetical protein VITISV_040416 [Vitis vinifera]
c48199.graph_c0	5.76441842	5.58028561	5.36340933	27.4070559	26.0292697	25.332521	7.598E-36	1.14719	up	PREDICTED: protein RIK isoform X2 [Sesamum indicum]
c48202.graph_c0	3.2066345	3.30026364	2.90186781	26.8178924	25.1624504	24.0527499	3.199E-35	1.92508	up	hypothetical protein MIMGU_mgv1a010335mg [Erythranthe guttata]
c48203.graph_c1	4.53447169	3.53565167	3.98687808	17.5188641	19.1067603	17.6638337	7.958E-11	1.08109	up	Hypothetical protein [Arabidopsis thaliana]
c48205.graph_c0	8.51564998	7.11107824	7.43184127	5.26241138	4.87099776	4.6451734	4.277E-23	-1.73133	down	PREDICTED: uncharacterized protein LOC105167185 isoform X1 [Sesamum indicum]
c48208.graph_c0	0	0	0	3.53153752	5.24798195	5.46323386	1.542E-14	Inf	up	PREDICTED: 40S ribosomal protein S13-1-like [Camelina sativa]
c48210.graph_c1	9.16181657	9.92822852	11.1252256	8.98015648	8.65982023	9.62076687	8.235E-10	-1.24093	down	-
c48210.graph_c2	60.9603583	62.2634004	58.2383641	59.5883528	57.1452765	57.4924317	1.366E-45	-1.14863	down	PREDICTED: uncharacterized protein LOC105170049 isoform X1 [Sesamum indicum]

#ID	0 h-1 FPKM	0 h-2 FPKM	0 h-3 FPKM	28 h-1 FPKM	28 h-2 FPKM	28 h-3 FPKM	FDR	log2FC	regulated	nr_annotation
c48211.graph_c0	0.50948854	0.35999909	0.23107516	3.68115137	3.6580747	3.50145871	1.117E-10	2.21836	up	PREDICTED: wall-associated receptor kinase-like 2 [Sesamum indicum]
c48213.graph_c0	17.6485428	19.7836098	17.3081469	18.5225403	18.5303219	18.8550164	2.955E-30	-1.05942	down	PREDICTED: probable ATP-dependent DNA helicase CHR12 [Sesamum indicum]
c48214.graph_c0	0.53140258	0.35462313	0.50077379	3.24425076	2.95250231	3.61375021	7.763E-07	1.73261	up	PREDICTED: DNA mismatch repair protein MLH3 isoform X2 [Sesamum indicum]
c48215.graph_c0	2.44028257	1.35129531	1.80145133	0	0	0	1.909E-21	-Inf	down	--
c48219.graph_c0	24.9993548	26.5097389	24.4332208	18.5715264	17.5110396	16.1983034	1.659E-75	-1.62915	down	PREDICTED: transmembrane protein 45B-like [Sesamum indicum]
c48220.graph_c0	1.77918022	3.49831782	1.55142941	0.09236414	0.16679231	0.30147353	4.099E-06	-4.68946	down	hypothetical protein VITISV_010987 [Vitis vinifera]
c48221.graph_c0	36.4775065	39.4000221	37.0087301	18.5627043	16.4724732	18.0649064	1.52E-133	-2.17834	down	hypothetical protein MIMGU_mgv1a025040mg, partial [Erythranthe guttata]
c48222.graph_c0	15.1128631	14.33699	14.8510681	7.41236596	6.85472165	6.98571708	6.458E-93	-2.15018	down	hypothetical protein MIMGU_mgv1a001261mg [Erythranthe guttata]
c48223.graph_c0	6.10230826	4.32679665	5.88128078	3.69593539	5.0056247	3.99600536	7.839E-06	-1.45289	down	-
c48223.graph_c1	0.0411732	0.20607214	0.15872757	2.69319992	3.24227602	2.90575408	2.416E-12	3.34596	up	PREDICTED: uncharacterized protein LOC100255010 isoform X5 [Vitis vinifera]
c48224.graph_c0	573.009602	567.068485	517.664962	537.794633	501.884336	487.896438	5.622E-30	-1.20745	down	-
c48231.graph_c0	68.1693603	69.4663903	74.6127635	54.8386321	48.789153	50.4748275	4.686E-41	-1.5536	down	PREDICTED: putative lysine-specific demethylase JMJD5 [Sesamum indicum]
c48235.graph_c0	1.13225851	0.89574553	0.96217545	11.1890433	11.9242512	10.7872447	1.274E-52	2.41365	up	PREDICTED: pentatricopeptide repeat-containing protein At1g19720 isoform X1 [Sesamum indicum]
c48239.graph_c2	0.38343273	0.47977086	0.49272624	3.48345877	3.58556979	3.32569447	2.309E-06	1.84502	up	-
c48239.graph_c3	5507.69182	5503.88487	5444.07256	230.610798	273.697894	262.579239	0	-5.51378	down	PREDICTED: beta-hexosaminidase 2 [Sesamum indicum]
c48239.graph_c6	79.6979073	80.0794895	73.5143895	60.1326108	58.0925606	59.2731757	8.948E-71	-1.48376	down	PREDICTED: putative transcription elongation factor SPT5 homolog 1 [Sesamum indicum]
c48244.graph_c0	21.9910376	21.0604066	21.9261139	18.0580267	21.590909	18.9682974	4.264E-37	-1.23963	down	hypothetical protein MIMGU_mgv1a007762mg [Erythranthe guttata]
c48244.graph_c1	0	0	0	21.8802953	20.5008386	37.4288653	1.231E-18	Inf	up	PREDICTED: putative mannan endo-1,4-beta-mannosidase 9 [Sesamum indicum]
c48244.graph_c2	2586.72989	2714.42794	2573.21681	523.943497	548.343325	509.210625	3.58E-125	-3.4065	down	PREDICTED: heat shock cognate 70 kDa protein 2 [Sesamum indicum]
c48244.graph_c5	8.25090777	8.36371625	8.3658775	7.16063104	6.56818482	7.09214188	1.715E-36	-1.35323	down	PREDICTED: uncharacterized protein LOC105164739 [Sesamum indicum]
c48244.graph_c6	10.8041679	11.4365376	11.1309643	60.8561538	60.7131091	58.4960555	5.361E-49	1.34087	up	PREDICTED: uncharacterized protein LOC105158481 isoform X1 [Sesamum indicum]
c48245.graph_c0	6.666108	6.59801802	7.10251809	3.93562402	3.14913575	3.5547286	2.453E-58	-2.028	down	PREDICTED: putative pentatricopeptide repeat-containing protein At3g16890, mitochondrial [Sesamum indicum]
c48250.graph_c1	1.997647	2.71380711	1.28352774	8.19274199	11.3300079	9.39314395	0.0022711	1.18486	up	PREDICTED: peptidyl-tRNA hydrolase, mitochondrial [Sesamum indicum]

#ID	0 h-1 FPKM	0 h-2 FPKM	0 h-3 FPKM	28 h-1 FPKM	28 h-2 FPKM	28 h-3 FPKM	FDR	log2FC	regulated	nr_annotation
c48250.graph_c2	46.6439445	42.8995301	42.008751	31.6550008	36.8550892	32.627649	2.233E-59	-1.46907	down	hypothetical protein MIMGU_mgv1a010855mg [Erythranthe guttata]
c48250.graph_c3	0.2846882	0.33246882	0.42680821	3.89680017	3.45617183	3.26417545	2.832E-08	2.2502	up	PREDICTED: probable pectin methyltransferase QUA2 [Sesamum indicum]
c48251.graph_c0	1.45375946	1.9029737	1.50888107	0.93467046	0.58707464	1.16060527	1.904E-07	-1.94681	down	PREDICTED: uncharacterized protein LOC104593668 [Nelumbo nucifera]
c48253.graph_c0	2.99646679	3.15133906	3.16819746	3.28050582	3.53447449	2.81183399	8.549E-06	-1.04476	down	PREDICTED: serine/threonine-protein kinase-like protein At3g51990 [Sesamum indicum]
c48253.graph_c1	5.06731958	6.01172591	4.52201825	56.624567	54.944626	52.2434408	9.1E-128	2.30344	up	PREDICTED: uncharacterized protein LOC105168293 [Sesamum indicum]
c48253.graph_c2	28.369835	24.9372072	26.8865664	1.03095163	1.27992241	1.68249622	9.32E-183	-5.41626	down	-
c48256.graph_c0	87.7635569	89.0117409	89.2813527	65.5913128	67.3572094	63.7072848	6.646E-78	-1.52703	down	PREDICTED: uncharacterized protein LOC105170589 [Sesamum indicum]
c48257.graph_c0	2.59611333	2.41130576	2.38980843	12.8450276	12.0237655	11.2882714	7.221E-27	1.19918	up	PREDICTED: protein SHOOT GRAVITROPISM 6 [Sesamum indicum]
c48258.graph_c1	180.188273	192.747522	186.461088	33.6832503	32.9201039	34.5425164	0	-3.55804	down	PREDICTED: pentatricopeptide repeat-containing protein At1g79540 [Sesamum indicum]
c48258.graph_c2	84.9896801	87.1819714	91.7805206	76.4307196	79.9851513	78.3289684	3.904E-41	-1.26073	down	PREDICTED: LRR receptor-like serine/threonine-protein kinase ERECTA [Sesamum indicum]
c48259.graph_c1	82.9022101	79.572592	86.6579304	90.9533971	77.1022837	80.1102253	2.722E-26	-1.09696	down	PREDICTED: dehydrodolichyl diphosphate synthase 6 [Sesamum indicum]
c48264.graph_c0	50.9217011	50.0028489	48.4607675	36.0075095	38.221381	36.6969048	1.267E-78	-1.51949	down	PREDICTED: phytosulfokine receptor 1 [Sesamum indicum]
c48265.graph_c0	0.17151973	0.26534138	0.28052137	3.5357982	3.65295016	4.25379464	6.94E-28	2.90084	up	PREDICTED: osmotic avoidance abnormal protein 3-like [Sesamum indicum]
c48267.graph_c0	0.36827736	0.12288205	0.15775033	3.07811144	2.0139464	2.4389105	9.839E-07	2.45609	up	hypothetical protein MIMGU_mgv1a013848mg [Erythranthe guttata]
c48268.graph_c0	1.99837233	0.75014002	0.49384376	0	0	0	4.71E-06	-Inf	down	PREDICTED: BAG family molecular chaperone regulator 6-like [Phoenix dactylifera]
c48270.graph_c0	6.5982191	6.36216899	5.72403673	5.40118508	5.21977768	5.4181768	1.249E-47	-1.30889	down	PREDICTED: calcium-transporting ATPase 1, chloroplastic [Sesamum indicum]
c48272.graph_c0	1.12104021	1.2871867	1.31347096	8.36332887	7.98404119	8.56472989	5.122E-14	1.65085	up	PREDICTED: uncharacterized protein LOC105162718 [Sesamum indicum]
c48275.graph_c0	64.1686189	58.9482889	62.3491818	420.924073	453.632625	434.975798	2.75E-105	1.72933	up	PREDICTED: actin-1-like [Tarenaya hassleriana]
c48277.graph_c0	2.61766503	2.47196814	2.22137947	12.3483269	11.4086694	10.6912468	1.152E-07	1.14732	up	PREDICTED: histone deacetylase 5 [Sesamum indicum]
c48278.graph_c0	23.6692639	24.3387699	23.7835043	13.5980335	14.8708946	15.2568409	1.18E-68	-1.8056	down	G patch domain-containing protein 8 [Glycine soja]
c48280.graph_c0	0.89800829	0.91653315	1.24448415	28.2566528	26.1832894	22.931887	3.346E-73	3.5649	up	PREDICTED: LRR receptor-like serine/threonine-protein kinase ERL2 isoform X2 [Sesamum indicum]
c48283.graph_c1	15.1411206	15.9848716	15.2907611	14.8146861	12.8104404	14.4000316	5.306E-30	-1.23358	down	PREDICTED: probable mitochondrial chaperone BCS1-B [Sesamum indicum]

#ID	0 h-1 FPKM	0 h-2 FPKM	0 h-3 FPKM	28 h-1 FPKM	28 h-2 FPKM	28 h-3 FPKM	FDR	log2FC	regulated	nr_annotation
c48286.graph_c0	1.3564722	1.07312411	0.7872157	9.60434213	9.23175588	9.52385021	1.812E-29	2.05603	up	PREDICTED: uncharacterized protein LOC105179489 [Sesamum indicum]
c48289.graph_c0	6.02672607	6.20678288	5.58503482	26.0143963	26.4547363	26.0504126	1.096E-26	1.05016	up	PREDICTED: glutamate receptor 3.3 [Sesamum indicum]
c48291.graph_c1	7.70223242	6.36908503	8.00420561	38.1633347	39.995792	44.1296137	1.281E-22	1.37869	up	PREDICTED: pyridoxal reductase, chloroplastic [Sesamum indicum]
c48293.graph_c0	25.2260864	22.4793291	24.0760773	264.383195	246.734706	237.405494	4.22E-165	2.29183	up	PREDICTED: apyrase 2-like [Sesamum indicum]
c48295.graph_c0	0.27826821	0.37853803	0.33007908	0.36818731	0.29498075	0.36814214	0.0006827	-1.02753	down	PREDICTED: phragmoplast orienting kinesin 2 [Sesamum indicum]
c48300.graph_c0	8.19241125	8.48339453	7.6616274	6.36483158	6.33226342	6.19887329	7.554E-46	-1.45478	down	hypothetical protein MIMGU_mgv1a0002422mg, partial [Erythranthe guttata]
c48302.graph_c0	16.7475409	16.4509594	18.1019886	457.815694	442.534869	439.433144	0	3.61514	up	hypothetical protein MIMGU_mgv1a010747mg [Erythranthe guttata]
c48303.graph_c0	2.40777323	2.29894498	2.23726069	10.7958806	11.2061719	10.5448583	2.42E-09	1.13875	up	PREDICTED: altered inheritance rate of mitochondria protein 25 [Sesamum indicum]
c48303.graph_c3	38.8734828	46.5924893	44.0383634	29.3682741	29.5376991	31.244557	2.96E-24	-1.61393	down	hexose transport protein [Actinidia deliciosa]
c48306.graph_c0	3.87315567	3.73856855	3.17738236	3.06004191	2.66081029	3.20965652	2.277E-28	-1.36014	down	PREDICTED: helicase and polymerase-containing protein TEBICHI [Sesamum indicum]
c48309.graph_c2	0.38122797	0.25440647	0.32659536	3.50960768	5.00344212	2.93917466	5.013E-06	2.48165	up	-
c48309.graph_c3	2.9383954	2.36081057	2.90649124	2.96456228	2.68940767	2.53370594	1.57E-09	-1.09461	down	PREDICTED: aluminum-activated malate transporter 4-like [Sesamum indicum]
c48311.graph_c0	6.94288388	7.82352981	7.03554018	47.5193548	46.3160944	54.069103	3.759E-30	1.67249	up	cytochrome P450 CYP72A219-like [Sesamum indicum]
c48316.graph_c1	0.08012273	0.18714024	0.13728118	1.04818931	0.80620679	0.90283135	0.000304	1.67508	up	PREDICTED: probable inactive receptor kinase At5g10020 [Nicotiana sylvestris]
c48331.graph_c0	62.6073514	62.6990035	62.9979121	15.9700887	16.0712319	17.2952122	0	-3.0226	down	PREDICTED: uncharacterized protein LOC105164372 [Sesamum indicum]
c48334.graph_c1	4.16459769	4.73402457	6.07732348	31.8071097	28.6725171	30.0149404	5.474E-27	1.49991	up	PREDICTED: 3-hydroxyisobutyryl-CoA hydrolase-like protein 1, mitochondrial isoform X2 [Sesamum indicum]
c48336.graph_c0	11.0866325	12.4591662	10.1157304	11.8319396	10.5479026	11.1701977	1.674E-17	-1.09372	down	PREDICTED: pentatricopeptide repeat-containing protein At2g21090-like [Sesamum indicum]
c48336.graph_c2	3.95123763	3.41063847	3.56896513	2.16420739	2.12317913	2.86971029	3.636E-17	-1.69906	down	PREDICTED: ABC transporter G family member 5 [Sesamum indicum]
c48336.graph_c3	2.06430917	1.52259304	1.25655111	1.42136338	1.54478261	1.56790604	0.0001292	-1.17987	down	PREDICTED: pentatricopeptide repeat-containing protein At2g13600 [Sesamum indicum]
c48336.graph_c4	5.65315089	6.75679005	6.18026559	30.5595664	31.0253267	31.3747738	3.896E-31	1.23098	up	hypothetical protein MIMGU_mgv1a004418mg [Erythranthe guttata]
c48340.graph_c0	81.3504491	87.2309412	81.811064	65.4073513	62.3756281	68.132748	4.025E-71	-1.44402	down	PREDICTED: serine/threonine-protein kinase PBS1 [Sesamum indicum]
c48340.graph_c3	140.626259	152.314374	135.124054	124.185764	123.929062	123.761915	4.169E-56	-1.29281	down	hypothetical protein MIMGU_mgv1a000810mg [Erythranthe guttata]
c48342.graph_c0	2.78737316	3.10904021	2.42203643	0.84892626	0.95812602	1.13353673	2.617E-27	-2.58762	down	hypothetical protein MIMGU_mgv11b010420mg [Erythranthe guttata]

#ID	0 h-1 FPKM	0 h-2 FPKM	0 h-3 FPKM	28 h-1 FPKM	28 h-2 FPKM	28 h-3 FPKM	FDR	log2FC	regulated	nr_annotation
c48344.graph_c0	0.96360532	0.88418937	1.13508174	6.59489462	7.43004056	6.52432815	2.422E-06	1.69076	up	PREDICTED: uncharacterized protein LOC105167681 [Sesamum indicum]
c48347.graph_c1	1.67334669	1.82067641	1.68285659	8.56612847	9.16670916	7.55080805	6.675E-05	1.19686	up	PREDICTED: sulfhydryl oxidase 2 isoform X3 [Sesamum indicum]
c48347.graph_c4	3.68510194	3.50435241	3.39377635	16.3823347	17.9757593	18.0666329	4.386E-10	1.21903	up	PREDICTED: sulfhydryl oxidase 2 isoform X1 [Sesamum indicum]
c48348.graph_c0	523.793944	520.219895	501.906429	143.115546	141.020066	155.30762	2.82E-226	-2.90427	down	PREDICTED: uncharacterized protein LOC105164444 [Sesamum indicum]
c48351.graph_c0	0.40058979	0.86881367	0.3431825	6.74489053	7.01007626	7.83991056	2.494E-12	2.65731	up	-
c48354.graph_c0	1.17986169	0.81196716	1.08974645	6.02941411	6.6295769	7.30339642	3.972E-10	1.60573	up	PREDICTED: uncharacterized protein LOC105158848 isoform X1 [Sesamum indicum]
c48359.graph_c0	0	0	0	3.85661726	4.78797522	4.032053	8.341E-13	Inf	up	-
c48360.graph_c0	0.14221097	0.14235338	0	2.41168937	3.20407895	2.81131262	5.057E-12	3.81763	up	hypothetical protein MIMGU_mgv1a011575mg [Erythranthe guttata]
c48364.graph_c0	0	0	0	2.7268429	3.8945684	4.97609143	3.635E-11	Inf	up	predicted protein [Physcomitrella patens]
c48368.graph_c0	50.6210105	48.2156127	48.397202	48.6334513	45.2558341	42.9197252	1.587E-49	-1.19639	down	PREDICTED: protein strawberry notch isoform X1 [Sesamum indicum]
c48372.graph_c0	10.5213438	9.1822564	10.1789313	46.0385991	43.7488723	42.621762	7.588E-32	1.05694	up	PREDICTED: acyl-CoA-binding domain-containing protein 4-like [Sesamum indicum]
c48373.graph_c0	91.0589542	96.7124474	92.5211978	34.1762099	33.4065337	35.6832538	3.87E-187	-2.53086	down	PREDICTED: uncharacterized protein LOC105162592 [Sesamum indicum]
c48374.graph_c0	0	0	0	4.77808944	4.5128455	5.92527456	2.067E-29	Inf	up	PREDICTED: GDSL esterase/lipase At1g29670-like [Sesamum indicum]
c48377.graph_c0	0	0	0	1.49253921	1.32516259	2.88236288	1.06E-06	Inf	up	-
c48384.graph_c0	0.90514359	0.78319572	0.94628774	6.37783048	5.84919002	6.22327306	2.173E-22	1.71655	up	PREDICTED: LOW QUALITY PROTEIN: probable serine/threonine-protein kinase GCN2 [Sesamum indicum]
c48386.graph_c0	1102.79499	1085.33769	1175.1755	487.540828	495.767895	483.341574	1.963E-90	-2.28891	down	PREDICTED: uncharacterized protein LOC105166153 [Sesamum indicum]
c48390.graph_c0	0	0.10518253	0.0450095	1.52739167	2.29848579	1.49560696	1.505E-09	4.04905	up	PREDICTED: 7-hydroxymethyl chlorophyll a reductase, chloroplastic isoform X1 [Sesamum indicum]
c48395.graph_c0	0.95892001	0.93854955	1.01318348	5.12636278	4.29301468	4.17043249	3.534E-07	1.13134	up	PREDICTED: protein FAM135B-like [Sesamum indicum]
c48398.graph_c0	4.69575535	4.625847	6.13001299	44.367023	44.8526779	41.2423918	2.337E-39	1.98311	up	hypothetical protein MIMGU_mgv1a027078mg [Erythranthe guttata]
c48399.graph_c0	37.3037488	37.7549821	35.5393473	156.629426	162.132984	155.244485	3.446E-35	1.00955	up	PREDICTED: uncharacterized protein LOC105163762 isoform X1 [Sesamum indicum]
c48400.graph_c0	3.17388977	1.96335659	1.87889365	0	0	0	1.299E-28	-Inf	down	lectin [Bryopsis plumosa]
c48404.graph_c0	12.1733976	5.50316851	5.92931452	0	0	0	6.812E-15	-Inf	down	PREDICTED: BTB/POZ and TAZ domain-containing protein 1-like [Phoenix dactylifera]
c48409.graph_c2	2.03328549	1.50666658	1.59485793	0.01919195	0.06931413	0.0626419	7.692E-49	-6.17345	down	PREDICTED: subtilisin-like protease SDD1 [Sesamum indicum]
c48410.graph_c0	28.4445447	30.4858485	29.365708	28.9548423	31.2842397	29.7510311	3.397E-37	-1.06348	down	PREDICTED: DNA polymerase eta [Sesamum indicum]

#ID	0 h-1 FPKM	0 h-2 FPKM	0 h-3 FPKM	28 h-1 FPKM	28 h-2 FPKM	28 h-3 FPKM	FDR	log2FC	regulated	nr_annotation
c48412.graph_c0	5.0552222	3.99496124	5.47045109	0.90241209	0.87299304	0.99934693	1.274E-23	-3.47941	down	PREDICTED: auxin-induced protein 15A-like [Sesamum indicum]
c48415.graph_c3	1.20581159	1.11761021	1.31995776	18.8907913	17.3496823	17.0303648	2.071E-41	2.77776	up	hypothetical protein MTR_4g125190 [Medicago truncatula]
c48417.graph_c0	140.228979	140.396089	133.290965	93.390565	90.2722483	92.4465482	3.079E-91	-1.67391	down	PREDICTED: E3 ubiquitin-protein ligase UPL1-like [Sesamum indicum]
c48418.graph_c0	0.503599	0.6398234	0.7218152	0.42232252	0.43215999	0.41353408	6.596E-06	-1.65156	down	PREDICTED: putative multidrug resistance protein [Sesamum indicum]
c48419.graph_c0	135.851885	127.64814	133.021202	116.736192	117.285132	114.839393	2.276E-55	-1.27529	down	PREDICTED: asparagine--tRNA ligase, chloroplastic/mitochondrial [Sesamum indicum]
c48420.graph_c1	0.98505564	1.23567292	0.6569526	7.34963889	7.56884367	7.13605639	6.636E-34	1.85267	up	PREDICTED: protein MON2 homolog isoform X2 [Sesamum indicum]
c48421.graph_c2	26.7137185	26.9526942	26.5362326	15.9329361	16.3615804	17.7036878	4.86E-69	-1.77178	down	PREDICTED: peptide chain release factor PrfB1, chloroplastic [Sesamum indicum]
c48422.graph_c0	3.24039605	2.90114458	3.02603947	13.3992092	13.8416426	13.929655	9.642E-18	1.07714	up	PREDICTED: myosin-1 [Sesamum indicum]
c48423.graph_c0	2.24473163	1.98771253	1.94153684	10.2070706	8.75333785	8.36302105	4.328E-13	1.05648	up	PREDICTED: protein WEAK CHLOROPLAST MOVEMENT UNDER BLUE LIGHT 1 isoform X2 [Sesamum indicum]
c48428.graph_c0	0	0	0.09659047	3.76945304	6.7575998	6.15168579	5.1E-12	6.30202	up	hypothetical protein MGL_1430 [Malassezia globosa CBS 7966]
c48429.graph_c0	28.010976	29.3651948	25.7533319	23.139894	22.9217503	25.5417856	1.148E-54	-1.30413	down	PREDICTED: uncharacterized protein LOC105159568 [Sesamum indicum]
c48429.graph_c6	2.30294875	2.47601441	2.5209536	18.845409	19.3105095	19.7279702	3.107E-15	1.89581	up	PREDICTED: protein GLUTAMINE DUMPER 2-like [Sesamum indicum]
c48431.graph_c0	0.43342642	0.82433483	0.61266713	5.89072115	5.14811321	6.14342516	3.5E-11	2.10757	up	PREDICTED: urease accessory protein D isoform X1 [Sesamum indicum]
c48435.graph_c0	0	0	0	2.56908608	4.32702797	5.64402802	8.116E-09	Inf	up	60s ribosomal protein l10 [Lichtheimia corymbifera JMRC:FSU:9682]
c48437.graph_c0	52.1613896	54.9147417	49.9702234	44.2844807	44.3958612	43.8087327	2.015E-61	-1.33521	down	PREDICTED: uncharacterized protein LOC105158158 [Sesamum indicum]
c48450.graph_c1	28.2379813	29.6384057	26.6675958	20.150683	18.9920304	19.8925274	8.485E-88	-1.60765	down	PREDICTED: uncharacterized protein LOC105178025 [Sesamum indicum]
c48454.graph_c0	22.4617397	21.8653997	21.2823135	5.09222147	4.86825729	5.02999243	0	-3.21968	down	PREDICTED: DNA repair protein UVH3 [Sesamum indicum]
c48457.graph_c0	2.20226858	1.94921896	2.62147583	14.0852458	12.7785064	11.7409121	1.287E-19	1.41701	up	PREDICTED: beta-galactosidase 3-like [Sesamum indicum]
c48458.graph_c0	0	0	0	0.62718406	0.63707445	1.11951235	6.341E-07	Inf	up	Inositol-3-phosphate synthase [Ectocarpus siliculosus]
c48459.graph_c1	17.1861742	16.1318925	13.9081467	13.2687868	15.8438795	12.3437381	4.943E-22	-1.27689	down	PREDICTED: carboxyl-terminal-processing peptidase 1, chloroplastic [Sesamum indicum]
c48463.graph_c0	2.34722391	1.9509858	2.20834468	14.7290402	13.6977731	14.5045628	1.184E-33	1.632	up	PREDICTED: uncharacterized protein LOC105169809 [Sesamum indicum]
c48464.graph_c0	0.8999303	0.40946884	0.10513146	6.30280006	5.20765319	5.87082112	1.289E-08	2.54849	up	PREDICTED: auxin response factor 8-like isoform X2 [Nicotiana tomentosiformis]
c48464.graph_c2	289.038472	267.831424	288.709742	148.213469	147.101586	149.354423	1.27E-114	-2.0179	down	PREDICTED: probable LRR receptor-like serine/threonine-protein kinase At5g10290 [Sesamum indicum]

#ID	0 h-1 FPKM	0 h-2 FPKM	0 h-3 FPKM	28 h-1 FPKM	28 h-2 FPKM	28 h-3 FPKM	FDR	log2FC	regulated	nr_annotation
c48465.graph_c0	1.93038801	2.35239079	1.79755436	19.7639279	16.9453815	13.7877637	3.322E-12	1.96377	up	PREDICTED: elongation factor G-1, chloroplastic [Sesamum indicum]
c48468.graph_c0	1.48522514	1.37658552	1.48444575	0.39979743	0.21658776	0.48934716	1.862E-13	-3.06273	down	PREDICTED: scarecrow-like protein 21 [Sesamum indicum]
c48469.graph_c2	168.329457	173.348289	155.66403	68.7022407	61.2045928	63.884017	1.45E-187	-2.44915	down	PREDICTED: rhomboid protein 2-like [Sesamum indicum]
c48469.graph_c3	4.62646793	5.03658871	3.80821212	20.0587263	19.0135586	19.0103688	2.291E-28	1.0203	up	PREDICTED: probable ubiquitin-conjugating enzyme E2 24 [Sesamum indicum]
c48471.graph_c1	8.16750424	8.58041953	8.52115284	42.1403972	44.2576322	36.4004756	4.195E-16	1.18904	up	PREDICTED: methyltransferase-like protein 22 [Sesamum indicum]
c48474.graph_c2	3.94079196	4.24639451	3.00865555	65.8247886	60.3615496	58.9503857	3.4E-153	2.96035	up	PREDICTED: LOW QUALITY PROTEIN: probable acyl-activating enzyme 16, chloroplastic [Sesamum indicum]
c48475.graph_c2	0.29560054	0.19726436	0.12661947	52.6359888	48.6892493	44.1776953	2.72E-102	6.79423	up	PREDICTED: MADS-box protein SVP-like isoform X1 [Sesamum indicum]
c48479.graph_c1	2.41993983	2.11956765	1.63260221	15.5653651	15.4038908	15.6791784	8.16E-16	1.83221	up	PREDICTED: dystrophia myotonica WD repeat-containing protein-like [Sesamum indicum]
c48480.graph_c0	0	0	0	3.68363669	5.78806657	6.79236671	6.698E-13	Inf	up	hypothetical protein SELMODRAFT_270900 [Selaginella moellendorffii]
c48484.graph_c0	26.3009618	24.5694197	24.4561556	25.9719939	25.1654955	26.9816337	7.213E-35	-1.03696	down	PREDICTED: uncharacterized protein LOC105160250 [Sesamum indicum]
c48489.graph_c0	13.960049	13.6982247	12.451232	6.32461435	7.26247303	7.14891207	1.553E-84	-2.04078	down	PREDICTED: BTB/POZ domain-containing protein At2g04740 isoform X1 [Sesamum indicum]
c48492.graph_c0	26.7311619	26.7579291	29.2324602	5.2327711	4.52365055	5.72348095	1.7E-117	-3.50872	down	hypothetical protein MIMGU_mgv1a013606mg [Erythranthe guttata]
c48492.graph_c2	10.8446446	11.3489358	10.4518482	0.82400899	0.1940877	0.46774582	2.29E-139	-5.54671	down	hypothetical protein VITISV_043907 [Vitis vinifera]
c48492.graph_c3	1.03781954	1.45440226	1.1669343	0.39599766	0.30647037	0.36159882	6E-19	-2.87251	down	OSJNBb0012A12.6 [Oryza sativa Japonica Group]
c48494.graph_c0	40.4070459	40.307389	39.7329529	30.9388542	31.1601032	30.4481398	1.477E-73	-1.47058	down	PREDICTED: chromatin modification-related protein eaf-1 isoform X2 [Sesamum indicum]
c48495.graph_c0	0.92749032	1.04744714	1.1001801	8.48667838	7.74070619	8.12389961	3.811E-22	1.89291	up	PREDICTED: uncharacterized protein LOC105160781 [Sesamum indicum]
c48498.graph_c0	3530.09566	3650.77638	3524.72972	1741.83808	1873.00714	1923.67125	2.765E-59	-2.04099	down	hypothetical protein MIMGU_mgv1a004937mg [Erythranthe guttata]
c48506.graph_c0	41.3151423	40.6495642	35.4952345	27.5466939	28.0390239	28.5976306	4.437E-71	-1.56905	down	hypothetical protein MIMGU_mgv1a010122mg [Erythranthe guttata]
c48506.graph_c1	0.88428517	0.66387798	0.67470273	4.67961104	5.13195602	5.6868153	3.881E-11	1.71463	up	PREDICTED: uncharacterized protein LOC105174972 isoform X1 [Sesamum indicum]
c48506.graph_c2	0.55393766	0.63370553	0.33049335	5.07561929	4.54386095	4.49364666	9.546E-18	2.13229	up	PREDICTED: probable L-type lectin-domain containing receptor kinase S.7 [Sesamum indicum]
c48506.graph_c5	2.19585028	2.97277131	2.24352616	16.7834417	21.8036258	18.5946639	7.99E-18	1.8573	up	PREDICTED: CO(2)-response secreted protease-like [Sesamum indicum]
c48510.graph_c0	150.853964	153.062023	148.623132	152.454072	163.27342	153.728231	1.349E-35	-1.03755	down	hypothetical protein M569_12680 [Genlisea aurea]
c48519.graph_c2	33.0446698	32.2610242	34.3379375	14.0838529	16.5759373	15.9884228	2.65E-119	-2.18604	down	hypothetical protein MIMGU_mgv1a005028mg [Erythranthe guttata]
c48526.graph_c0	3.15509773	3.20152088	3.6101049	0.09423727	0.19853731	0.15379368	9.022E-52	-5.5729	down	hypothetical protein MIMGU_mgv1a026399mg [Erythranthe guttata]

#ID	0 h-1 FPKM	0 h-2 FPKM	0 h-3 FPKM	28 h-1 FPKM	28 h-2 FPKM	28 h-3 FPKM	FDR	log2FC	regulated	nr_annotation
c48529.graph_c0	8.18357843	6.66427142	7.02015265	5.49781258	5.32736905	5.71695806	1.501E-25	-1.49141	down	PREDICTED: LOW QUALITY PROTEIN: uncharacterized protein LOC105155975 [Sesamum indicum]
c48536.graph_c0	0	0	0.29304319	1.35906571	1.73591016	1.677004	1.06E-07	2.89541	up	PREDICTED: glycerophosphodiester phosphodiesterase GDPD6 [Solanum lycopersicum]
c48537.graph_c0	0.87745541	0.61483384	1.80410391	72.0634378	74.6825544	95.7503055	9.566E-41	5.09973	up	hypothetical protein CARUB_v10002736mg [Capsella rubella]
c48546.graph_c0	11.0039397	9.90831546	8.45786808	5.10127287	5.33145374	5.73310064	1.471E-30	-1.94837	down	PREDICTED: cytosolic Fe-S cluster assembly factor narfl [Sesamum indicum]
c48549.graph_c0	1.36240801	1.55859686	1.50064134	27.1877962	27.0002795	27.4253937	8.337E-81	3.1148	up	dimethylaniline monooxygenase, putative [Ricinus communis]
c48551.graph_c0	7.73833146	7.69642587	8.35046257	4.95720527	4.6223753	4.42747907	2.344E-45	-1.85582	down	PREDICTED: probable galactinol--sucrose galactosyltransferase 1 [Sesamum indicum]
c48553.graph_c0	2.35059646	2.43903377	2.24703878	1.29171704	1.48609114	1.41104091	2.956E-13	-1.83824	down	PREDICTED: uncharacterized protein LOC103423938 [Malus domestica]
c48554.graph_c1	57.8001558	60.7583452	54.0284198	15.8320837	15.5855906	14.6622688	0	-2.99494	down	unnamed protein product [Coffea canephora]
c48555.graph_c0	42.8824173	43.8463307	41.8432478	36.2400035	35.0838759	35.7278376	1.342E-62	-1.35439	down	PREDICTED: cytokinesis protein sepH-like [Sesamum indicum]
c48560.graph_c1	20.9709972	19.6728273	20.2482362	18.5730972	20.0033972	20.7623494	1.383E-21	-1.12725	down	PREDICTED: glucose-induced degradation protein 8 homolog isoform X1 [Sesamum indicum]
c48561.graph_c0	0	0	0	1.623625	3.94298377	9.95932805	0.0022523	Inf	up	putative 40S ribosomal protein S21-2 [Wolffia australiana]
c48562.graph_c0	18.0271954	13.9705138	12.8105031	5.9774383	8.06834161	6.70046015	5.393E-16	-2.19808	down	TBC1 domain family member 15 [Morus notabilis]
c48565.graph_c0	0.05165816	0	0.06638278	9.31112367	6.81379599	7.59780088	3.684E-31	6.55143	up	PREDICTED: shikimate O-hydroxycinnamoyltransferase-like [Sesamum indicum]
c48566.graph_c0	0	0	0.09451842	3.52821752	4.05446315	5.36540292	2.761E-14	5.9694	up	predicted protein [Hordeum vulgare subsp. vulgare]
c48568.graph_c1	31.3670313	29.8177411	28.0172029	26.4100054	26.7308012	26.6479375	6.592E-53	-1.25008	down	PREDICTED: DEAD-box ATP-dependent RNA helicase ISE2, chloroplastic [Sesamum indicum]
c48571.graph_c0	4.71332335	3.59082585	4.60973743	1.13062211	1.35604884	1.273709	2.151E-25	-2.87102	down	hypothetical protein VITISV_032357 [Vitis vinifera]
c48572.graph_c0	44.3257829	42.8917044	43.4242122	38.4323791	35.6700725	34.9701423	1.028E-62	-1.3509	down	hypothetical protein MIMGU_mgv1a000350mg [Erythranthe guttata]
c48573.graph_c0	31.5285691	32.7981346	29.2785752	20.6739445	17.729301	18.904886	2.55E-105	-1.79725	down	PREDICTED: transcription initiation factor TFIID subunit 1 isoform X2 [Sesamum indicum]
c48574.graph_c0	1.14820929	0.53876205	0.6455288	4.45942293	4.77992679	4.53260599	9.931E-07	1.47744	up	PREDICTED: glucan endo-1,3-beta-glucosidase 4-like isoform X1 [Sesamum indicum]
c48574.graph_c1	57.9622402	58.3229425	55.3674373	44.0604472	43.3142616	44.7932703	6.171E-72	-1.46691	down	PREDICTED: thylakoid lumenal 19 kDa protein, chloroplastic [Sesamum indicum]
c48575.graph_c1	34.2174392	37.123103	37.5857879	12.7323516	12.9751527	12.5463784	1.078E-93	-2.60136	down	hypothetical protein MIMGU_mgv1a015601mg [Erythranthe guttata]
c48576.graph_c0	4.06726727	4.86165896	6.51787698	5.2339764	4.96129444	4.46952768	0.0037893	-1.17197	down	orf25 gene product (mitochondrion) [Beta vulgaris subsp. vulgaris]
c48580.graph_c0	64.162535	64.944975	62.5629616	50.1341574	53.3377262	54.5859414	5.612E-44	-1.36815	down	PREDICTED: homeobox-leucine zipper protein HOX11 [Sesamum indicum]

#ID	0 h-1 FPKM	0 h-2 FPKM	0 h-3 FPKM	28 h-1 FPKM	28 h-2 FPKM	28 h-3 FPKM	FDR	log2FC	regulated	nr_annotation
c48580.graph_c2	0.69241881	1.07320593	0.6601635	15.0973801	14.8041224	13.8559445	5.934E-74	3.08404	up	PREDICTED: cinnamoyl-CoA reductase 2 [Sesamum indicum]
c48580.graph_c3	2.58146671	2.13614937	2.56536855	1.70108358	1.60368011	2.28623345	2.534E-08	-1.47075	down	-
c48580.graph_c4	40.6532393	39.579848	41.740125	10.6648534	9.07197446	9.5535695	0	-3.14906	down	PREDICTED: uncharacterized protein LOC105176765 [Sesamum indicum]
c48582.graph_c0	55.5944087	53.9995446	52.3078534	44.7163522	44.7512881	46.1210573	5.46E-62	-1.34556	down	PREDICTED: uncharacterized protein LOC105174456 [Sesamum indicum]
c48587.graph_c1	3.66530488	3.17457423	3.03982718	13.6974737	14.1586946	14.2295112	6.291E-13	1.00242	up	PREDICTED: rab3 GTPase-activating protein catalytic subunit isoform X4 [Sesamum indicum]
c48588.graph_c0	446.523713	412.155812	456.124903	429.054818	464.432122	474.877669	3.674E-25	-1.03324	down	vacuolar-processing enzyme precursor [Sesamum indicum]
c48589.graph_c2	1.13414962	1.07336065	1.24543776	6.77419311	6.08939724	6.65279054	9.569E-14	1.40704	up	PREDICTED: GPI ethanolamine phosphate transferase 2 isoform X1 [Sesamum indicum]
c48596.graph_c0	1.11405151	1.37755932	1.17896495	13.5741513	14.7934314	11.9702812	2.681E-18	2.36656	up	PREDICTED: early nodulin-like protein 1 [Nelumbo nucifera]
c48596.graph_c1	18.9954413	19.1119724	17.7754101	18.6200996	18.0427552	18.0824137	1.345E-34	-1.11936	down	hypothetical protein MIMGU_mgv1a011046mg [Erythranthe guttata]
c48601.graph_c0	0.40448621	0.17352482	0.185636	2.89778559	3.03354051	2.60445323	3.224E-10	2.4002	up	PREDICTED: lactosylceramide alpha-2,3-sialyltransferase [Sesamum indicum]
c48601.graph_c2	289.713444	303.527481	314.682415	123.92504	124.036969	141.235623	1.067E-86	-2.31295	down	PREDICTED: B2 protein [Sesamum indicum]
c48601.graph_c3	0.89603625	1.02981253	0.76762804	28.364858	35.2366379	30.1324396	4.954E-64	4.03238	up	PREDICTED: cytochrome P450 CYP736A12-like [Sesamum indicum]
c48601.graph_c8	39.9759573	38.244221	29.4577054	19.0766864	20.3123968	8.01363413	1.142E-17	-2.27407	down	PREDICTED: B2 protein [Sesamum indicum]
c48602.graph_c0	26.7012916	24.2013024	22.655186	26.1713336	25.260826	24.513322	4.148E-28	-1.04275	down	PREDICTED: mediator of RNA polymerase II transcription subunit 15a isoform X2 [Sesamum indicum]
c48604.graph_c0	0.71166757	0.61523745	0.58196791	4.34948363	3.33279253	4.54674011	1.823E-07	1.59231	up	PREDICTED: probable serine/threonine-protein kinase At5g41260 [Sesamum indicum]
c48612.graph_c0	23.4005982	25.7291448	23.5430479	12.1587054	11.1337321	11.9097019	4.59E-103	-2.13591	down	PREDICTED: autophagy-related protein 9 [Sesamum indicum]
c48612.graph_c1	37.4865573	37.6019452	35.079399	2.93929609	3.11323915	3.92052955	5.18E-232	-4.55394	down	hypothetical protein CICLE_v10018809mg [Citrus clementina]
c48616.graph_c0	10.0101968	9.12893406	8.8761612	8.51079072	8.39268993	8.65691711	8.575E-25	-1.22138	down	PREDICTED: deSI-like protein At4g17486 [Sesamum indicum]
c48617.graph_c0	0.6290517	0.53708137	0.66570522	0.30927637	0.25496517	0.34014681	1.194E-08	-2.10899	down	hypothetical protein VITISV_012155 [Vitis vinifera]
c48618.graph_c3	2.68109055	3.70992462	3.95197037	3.83989052	3.46706112	3.22685123	0.0007617	-1.06871	down	PREDICTED: dnaJ homolog subfamily C member 21 [Sesamum indicum]
c48620.graph_c1	236.748792	237.107703	249.172695	218.954988	210.662541	217.406415	6.363E-50	-1.25138	down	hypothetical protein VITISV_027576 [Vitis vinifera]
c48620.graph_c2	7.48928205	7.03237904	8.00582523	4.47977463	4.52323563	3.3803174	4.057E-19	-1.95625	down	-
c48620.graph_c4	0.60275155	0.42234858	0.54219172	7.79775905	6.96153694	6.39865438	2.079E-13	2.66428	up	-
c48622.graph_c1	56.6734261	62.1634886	53.7146262	46.7979366	45.501782	47.8754345	1.24E-48	-1.38917	down	PREDICTED: uncharacterized protein LOC105161451 [Sesamum indicum]
c48623.graph_c1	2.70995311	1.87800004	2.74573613	1.43930722	2.01773708	1.63806673	7.781E-07	-1.61774	down	-

#ID	0 h-1 FPKM	0 h-2 FPKM	0 h-3 FPKM	28 h-1 FPKM	28 h-2 FPKM	28 h-3 FPKM	FDR	log2FC	regulated	nr_annotation
c48623.graph_c3	584.225012	601.156751	572.264226	2656.68077	2742.5137	2722.20038	2.396E-23	1.1179	up	PREDICTED: CBL-interacting serine/threonine-protein kinase 3-like [Sesamum indicum]
c48623.graph_c4	3.074017	1.53854758	0.65837226	12.6603411	10.5905788	8.96343179	0.0018275	1.53592	up	-
c48624.graph_c0	1.86339681	2.08048534	1.93405047	22.4502274	22.2927924	23.5471881	9.49E-29	2.44785	up	PREDICTED: pyridoxal biosynthesis protein PDX2 [Sesamum indicum]
c48625.graph_c0	222.081868	232.184438	219.954492	68.8673372	75.2456568	71.4575456	7.07E-214	-2.73547	down	hypothetical protein MIMGU_mgv1a004857mg [Erythranthe guttata]
c48627.graph_c1	6.95907194	9.27134874	7.5272943	2.51071312	2.62833454	4.81004155	2.813E-15	-2.3432	down	hypothetical protein MIMGU_mgv1a018596mg [Erythranthe guttata]
c48628.graph_c2	26.8079395	26.2365559	25.5626701	212.709102	221.357859	220.505402	4.92E-105	1.96788	up	PREDICTED: non-specific lipid-transfer protein-like protein At2g13820 [Sesamum indicum]
c48633.graph_c0	8.88027629	9.27864242	8.49980776	40.0222822	39.9362442	39.5260495	1.528E-31	1.07425	up	PREDICTED: homeobox-leucine zipper protein MERISTEM L1-like [Sesamum indicum]
c48637.graph_c0	14.8027536	14.0899275	13.5023116	4.99504386	5.68093627	4.70297885	7.678E-52	-2.55312	down	PREDICTED: kinesin-1-like protein PSS1 isoform X2 [Sesamum indicum]
c48639.graph_c1	4.74010102	7.5648984	5.11432411	0.58501326	1.14445961	1.00777276	9.672E-13	-3.75967	down	PREDICTED: multiple C2 and transmembrane domain-containing protein 1 [Sesamum indicum]
c48639.graph_c2	216.740662	209.092159	224.914944	201.50545	201.782612	200.793497	3.387E-46	-1.19853	down	PREDICTED: uncharacterized protein LOC105176680 [Sesamum indicum]
c48646.graph_c1	3.09969039	2.3270957	3.37042105	0.43323791	0.86058106	0.56562975	1.914E-17	-3.33599	down	BnaC03g12490D [Brassica napus]
c48646.graph_c2	152.514859	159.352951	149.523227	130.382397	132.865063	131.909221	1.913E-56	-1.31373	down	PREDICTED: WD-40 repeat-containing protein MSI1 [Sesamum indicum]
c48649.graph_c0	29.2552007	31.6708577	28.9330782	28.599575	27.5609892	27.8578036	1.488E-42	-1.18713	down	PREDICTED: DNA (cytosine-5)-methyltransferase DRM2-like [Sesamum indicum]
c48649.graph_c1	20.6978063	20.4363731	20.5173327	21.0427049	21.3646289	21.2066199	2.505E-33	-1.04528	down	PREDICTED: uncharacterized protein LOC105175481 [Sesamum indicum]
c48652.graph_c0	8.5244646	7.71251974	8.84768326	65.5296627	67.8270593	62.1729686	8.163E-47	1.87079	up	PREDICTED: uncharacterized protein LOC105158144 [Sesamum indicum]
c48653.graph_c0	1.69299816	2.95669919	3.19392142	14.5037354	16.5230392	15.445224	4.23E-14	1.46983	up	PREDICTED: serine carboxypeptidase-like 7 [Sesamum indicum]
c48658.graph_c0	53.7132597	51.0684712	50.0749632	39.0978617	36.5347165	40.0187101	3.221E-70	-1.51057	down	PREDICTED: calmodulin-binding receptor-like cytoplasmic kinase 2 [Sesamum indicum]
c48669.graph_c0	2.2211366	2.56304084	2.21997103	1.47978351	1.76123032	1.48192109	7.26E-11	-1.65922	down	PREDICTED: pentatricopeptide repeat-containing protein At4g25270, chloroplastic [Sesamum indicum]
c48669.graph_c1	3.17070809	2.67513002	2.91034673	0.16460374	0.20807044	0.29549368	2.578E-39	-4.7997	down	PREDICTED: probable serine/threonine protein kinase IRE [Sesamum indicum]
c48671.graph_c0	59.0592952	57.1006374	57.103794	29.9352496	31.5697072	31.564076	7.28E-126	-1.98671	down	PREDICTED: protein DAMAGED DNA-BINDING 2 [Sesamum indicum]
c48672.graph_c0	7.45352615	5.16530058	4.91183395	35.142218	40.0075526	36.4964255	3.246E-10	1.58485	up	-
c48676.graph_c0	2.27398857	2.32368782	2.37425934	15.7697204	14.1453131	14.1603785	1.413E-15	1.56912	up	PREDICTED: probable receptor-like protein kinase At1g80640 isoform X2 [Sesamum indicum]

#ID	0 h-1 FPKM	0 h-2 FPKM	0 h-3 FPKM	28 h-1 FPKM	28 h-2 FPKM	28 h-3 FPKM	FDR	log2FC	regulated	nr_annotation
c48680.graph_c0	29.2128647	26.8476754	34.0815793	15.7119235	13.676233	16.012702	4.46E-17	-2.08189	down	PREDICTED: zinc finger A20 and AN1 domain-containing stress-associated protein 5 [Sesamum indicum]
c48681.graph_c1	12.0052704	12.2244866	9.73512533	7.7324754	9.5353183	10.0414064	2.464E-18	-1.4019	down	PREDICTED: uncharacterized protein LOC105173885 isoform X2 [Sesamum indicum]
c48681.graph_c2	1.96716664	1.8351816	2.13236728	8.76314668	10.0638131	9.96806227	5.427E-22	1.18686	up	PREDICTED: subtilisin-like protease [Sesamum indicum]
c48683.graph_c0	0.51029758	0.178783	0.3278763	5.00689807	4.65470795	5.03889179	8.205E-21	2.76758	up	PREDICTED: probable ATP-dependent RNA helicase DHX35 isoform X3 [Sesamum indicum]
c48684.graph_c0	7.35794011	7.43512132	7.21466513	8.23699172	7.89490699	6.59723211	8.575E-13	-1.04462	down	PREDICTED: ATPase ASNA1 homolog 2-like [Sesamum indicum]
c48684.graph_c1	15.2928437	18.0014422	15.558821	143.643414	133.915892	136.711963	7.65E-136	1.99377	up	Beta-galactosidase 8 [Morus notabilis]
c48695.graph_c0	96.0625346	90.8982787	98.029186	437.756957	435.153362	454.759997	4.17E-47	1.12913	up	PREDICTED: pre-mRNA-splicing factor 18 [Sesamum indicum]
c48696.graph_c1	17.9791517	16.820126	16.9136305	103.048489	106.036305	102.037824	8.282E-68	1.49882	up	hypothetical protein MIMGU_mgv1a0087951mg, partial [Erythranthe guttata]
c48697.graph_c0	1.85551436	1.92880977	1.696599	20.513933	20.2783678	21.2467239	2.432E-49	2.41163	up	PREDICTED: pentatricopeptide repeat-containing protein At1g07740, mitochondrial [Sesamum indicum]
c48702.graph_c1	19.5881959	22.3311175	23.6857832	16.8070829	17.8978331	17.3850726	6.673E-16	-1.42554	down	PREDICTED: phospholipase SGR2-like isoform X2 [Sesamum indicum]
c48703.graph_c0	8.64905604	9.78068421	8.97521018	10.0472139	9.07169213	9.27153932	9.571E-15	-1.03975	down	calcium-dependent protein kinase 34 [Brassica napus]
c48704.graph_c0	1.82642369	2.25484485	1.2517476	0.53097368	0.43946783	0.54158774	2.228E-09	-2.90326	down	PREDICTED: uncharacterized protein LOC104774238, partial [Camelina sativa]
c48706.graph_c0	1.07175988	1.32040996	0.79456955	6.53118698	4.78797137	5.20716141	1.095E-05	1.28815	up	PREDICTED: uncharacterized protein LOC105173691 [Sesamum indicum]
c48706.graph_c3	0.68505914	0.68574513	0.52819687	12.6133916	8.06200789	9.75071801	2.082E-11	2.9149	up	-
c48708.graph_c1	660.78492	638.12528	697.367549	483.254048	502.067416	514.457463	1.496E-48	-1.50377	down	PREDICTED: 2-alkenal reductase (NADP(+)-dependent)-like [Sesamum indicum]
c48710.graph_c0	19.2124042	17.1896139	18.5067435	14.7822415	16.2841125	17.1362526	9.194E-38	-1.27789	down	unnamed protein product [Vitis vinifera]
c48714.graph_c0	39.1761932	44.4406617	39.1071889	34.5432313	36.5331	30.6326538	2.979E-33	-1.36187	down	hypothetical protein MIMGU_mgv1a0225922mg, partial [Erythranthe guttata]
c48714.graph_c2	1.3731092	0.8246905	1.76449969	8.9817107	10.902964	11.9707042	4.025E-07	1.91215	up	-
c48714.graph_c5	4.32498834	3.57639409	4.41860869	3.55338224	3.75485178	2.78801047	8.35E-13	-1.37987	down	PREDICTED: leucine-rich repeat receptor-like protein FASCIATED EAR2 [Sesamum indicum]
c48716.graph_c0	0	0	0	2.29607672	3.69120404	6.35189266	7.501E-06	Inf	up	unknown [Lotus japonicus]
c48717.graph_c0	48.3775243	48.5310695	47.8817715	14.0699257	14.900078	13.660378	0	-2.85475	down	hypothetical protein MIMGU_mgv1a002354mg [Erythranthe guttata]
c48722.graph_c0	194.910592	182.679612	183.06037	1302.17076	1387.49018	1375.44825	4.57E-103	1.76828	up	PREDICTED: 60S ribosomal protein L7-2-like [Sesamum indicum]
c48725.graph_c0	36.3825832	36.15212	36.2562037	33.647878	34.071122	32.3295442	3.779E-48	-1.21161	down	hypothetical protein MIMGU_mgv1a0000431mg, partial [Erythranthe guttata]

#ID	0 h-1 FPKM	0 h-2 FPKM	0 h-3 FPKM	28 h-1 FPKM	28 h-2 FPKM	28 h-3 FPKM	FDR	log2FC	regulated	nr_annotation
c48725.graph_c1	0.25179482	0.20163756	0.19413978	1.20781994	1.37144693	1.49328971	0.0001836	1.56578	up	PREDICTED: DNA polymerase epsilon catalytic subunit A [Sesamum indicum]
c48727.graph_c1	0.72000353	0.60060375	0.38551377	2.61647208	2.51992423	2.84669313	0.0090124	1.14349	up	hypothetical protein MIMGU_mgv1a024288mg, partial [Erythranthe guttata]
c48728.graph_c0	45.9005398	46.6562834	42.187843	30.6804338	31.6567045	32.0156228	1.445E-85	-1.60338	down	PREDICTED: protein cfxQ homolog [Sesamum indicum]
c48730.graph_c1	1.41100183	2.82482947	2.53847051	14.357112	10.5556883	9.53959014	0.0037314	1.25016	up	PREDICTED: 3-oxoacyl-[acyl-carrier-protein] reductase 4 [Sesamum indicum]
c48730.graph_c2	67.0908578	73.4480572	68.4497681	316.456289	314.468336	307.073069	1.041E-41	1.07549	up	PREDICTED: LOW QUALITY PROTEIN: dedicator of cytokinesis protein 7 [Sesamum indicum]
c48735.graph_c0	0	0	0	0.62309522	0.68889375	1.16214997	3.17E-08	Inf	up	predicted protein [Hordeum vulgare subsp. vulgare]
c48736.graph_c1	75.128419	79.189465	73.340915	39.4345052	41.4923833	38.89617	1.06E-134	-2.01628	down	PREDICTED: crossover junction endonuclease MUS81 isoform X1 [Sesamum indicum]
c48736.graph_c4	84.7408446	88.8407055	87.1460658	82.0121558	80.4394045	82.5824992	2.244E-47	-1.18024	down	PREDICTED: vacuolar-sorting receptor 1-like [Sesamum indicum]
c48739.graph_c1	8.28706107	8.59810965	9.45732013	70.6638667	70.1540213	66.7013332	4.48E-110	1.8853	up	PREDICTED: pentatricopeptide repeat-containing protein At1g74850, chloroplastic [Sesamum indicum]
c48739.graph_c3	11.343087	12.3185021	12.5136297	11.2772933	7.79475566	9.32910582	4.839E-12	-1.44043	down	Os01g0916200 [Oryza sativa Japonica Group]
c48743.graph_c0	85.088583	88.4511418	79.1937555	68.5591069	65.2050551	65.9407455	5.807E-69	-1.42926	down	PREDICTED: non-lysosomal glucosylceramidase [Sesamum indicum]
c48745.graph_c1	21.221314	21.4460368	20.2437043	12.5279237	12.04519	11.1388673	7.95E-101	-1.90723	down	PREDICTED: phosphoinositide phosphatase SAC2 [Sesamum indicum]
c48751.graph_c0	680.067546	668.498249	714.345067	179.368139	171.683895	169.389507	7.99E-177	-3.07827	down	PREDICTED: UBP1-associated protein 2C [Sesamum indicum]
c48751.graph_c1	193.601193	208.676203	192.893131	85.1411371	75.9825195	78.8452136	7.13E-184	-2.40073	down	PREDICTED: UBP1-associated protein 2C [Sesamum indicum]
c48756.graph_c1	6.89438335	6.50916845	7.09771213	30.0078476	30.6417204	28.9234037	2.526E-18	1.03599	up	PREDICTED: probable beta-1,3-galactosyltransferase 9 isoform X1 [Sesamum indicum]
c48756.graph_c3	45.550011	34.1967168	32.5925547	11.285961	9.17114813	15.0417782	1.412E-19	-2.74671	down	-
c48757.graph_c1	62.5392369	63.0335974	58.5651272	62.2760495	59.562946	57.8935768	2.116E-41	-1.1248	down	putative movement protein [Nicotiana tabacum]
c48762.graph_c2	4.24403252	4.43709482	2.54508324	65.049487	68.5122041	64.2663307	2.174E-77	3.05477	up	PREDICTED: uncharacterized GPI-anchored protein At3g06035-like [Sesamum indicum]
c48762.graph_c3	0.10589435	0.31800116	0.13607843	5.77225521	6.18467557	5.65213574	4.69E-12	3.88512	up	-
c48765.graph_c0	2.08574268	2.48933724	2.88643591	23.2487469	22.03114	21.3971454	7.125E-71	2.06505	up	PREDICTED: probable galacturonosyltransferase 4 [Sesamum indicum]
c48769.graph_c0	0.06857308	0.16016406	0.08811912	3.28934206	3.05996649	2.73830002	6.894E-21	3.75091	up	PREDICTED: uncharacterized protein At5g41620-like [Sesamum indicum]
c48772.graph_c0	1.18838513	0.97876434	0.96653288	5.81638608	5.77484211	5.39737734	1.303E-14	1.35001	up	hypothetical protein MIMGU_mgv1a002385mg [Erythranthe guttata]
c48773.graph_c0	140.508411	130.60974	137.682426	115.383594	109.349464	107.962975	1.102E-65	-1.38782	down	PREDICTED: uncharacterized protein LOC105180349 [Sesamum indicum]
c48774.graph_c0	996.611934	1006.31246	973.257363	613.360498	632.20189	670.367882	1.081E-80	-1.72529	down	-

#ID	0 h-1 FPKM	0 h-2 FPKM	0 h-3 FPKM	28 h-1 FPKM	28 h-2 FPKM	28 h-3 FPKM	FDR	log2FC	regulated	nr_annotation
c48776.graph_c0	12.2160605	11.8554792	10.3377872	95.0655823	93.4118865	89.279331	3.601E-67	1.92426	up	PREDICTED: uncharacterized protein LOC104109628 isoform X2 [Nicotiana tomentosiformis]
c48781.graph_c0	0.46229822	0.42419771	0.29703576	4.87193738	5.3848636	5.43769968	1.004E-14	2.64407	up	PREDICTED: pullulanase 1, chloroplastic isoform X2 [Sesamum indicum]
c48782.graph_c0	0.53919581	0.28407144	0.36467787	3.14538799	2.56995857	2.53295044	2.623E-11	1.70871	up	PREDICTED: uncharacterized protein LOC105163648 [Sesamum indicum]
c48784.graph_c2	85.6961487	81.1126469	79.9804274	15.6919466	14.4015805	16.7960266	0	-3.48511	down	beta-D-glucosidase [Tropaeolum majus]
c48784.graph_c3	77.8386146	76.3447544	78.6625433	67.0792657	69.8564987	66.546603	2.434E-56	-1.28539	down	PREDICTED: ubiquitin domain-containing protein DSK2a-like isoform X2 [Sesamum indicum]
c48784.graph_c4	64.5642357	62.0357526	59.6649709	54.1664135	53.5712477	52.1174167	1.38E-49	-1.31035	down	PREDICTED: probable LRR receptor-like serine/threonine-protein kinase At3g47570 [Sesamum indicum]
c48784.graph_c5	2.73719116	2.83778676	2.00994229	1.13678558	1.02641079	1.4493871	1.895E-08	-2.15513	down	-
c48784.graph_c6	0.05383449	0.0538884	0	1.34986758	1.16581239	0.71835712	8.084E-07	3.83536	up	PREDICTED: auxin response factor 18-like [Sesamum indicum]
c48787.graph_c0	0.04887022	0	0	1.27866932	1.25072839	1.62304135	8.422E-13	5.35123	up	PREDICTED: phosphate transporter PHO1 homolog 3-like isoform X3 [Sesamum indicum]
c48795.graph_c1	69.509698	65.2057454	64.9619738	19.9190177	23.0321809	19.4017164	1.41E-228	-2.76958	down	hypothetical protein MIMGU_mgv1a013489mg [Erythranthe guttata]
c48796.graph_c6	4.67929302	4.18509333	3.87825663	25.7782922	25.3103869	25.6983457	5.031E-37	1.50288	up	hypothetical protein MIMGU_mgv1a006632mg [Erythranthe guttata]
c48796.graph_c7	2.24395288	3.025124	2.48824038	31.5524996	33.4884684	32.2395717	4.56E-131	2.5575	up	hypothetical protein MIMGU_mgv1a025096mg, partial [Erythranthe guttata]
c48798.graph_c0	3.66660695	1.92845142	2.71523717	1.49051984	2.36534486	2.54307239	0.0022379	-1.46385	down	PREDICTED: uncharacterized protein LOC105168156 [Sesamum indicum]
c48800.graph_c1	165.431296	162.251253	166.810563	157.263416	157.011589	160.541012	4.17E-43	-1.1492	down	photosystem II protein D1 [Phaseolus vulgaris]
c48801.graph_c1	17.2652332	16.1643563	15.6136608	14.5850517	12.6545227	13.2262306	2.007E-51	-1.36676	down	PREDICTED: molybdenum cofactor sulfurase isoform X3 [Sesamum indicum]
c48802.graph_c0	39.0645936	40.8979432	39.1570034	35.0013769	32.6754212	33.4538548	2.435E-57	-1.32663	down	PREDICTED: cyclin-T1-3-like [Sesamum indicum]
c48804.graph_c0	11.634823	14.1539296	14.1888237	9.55805326	10.2738404	10.7509057	5.567E-14	-1.4787	down	PREDICTED: uncharacterized protein LOC105155609 [Sesamum indicum]
c48808.graph_c0	18.1327747	16.6296111	18.5871908	15.8831156	17.6080904	15.540159	1.847E-23	-1.21336	down	hypothetical protein MIMGU_mgv1a005510mg [Erythranthe guttata]
c48808.graph_c2	487.48894	468.605443	478.682683	173.157193	170.96675	179.934152	2.96E-155	-2.54322	down	PREDICTED: 26S proteasome non-ATPase regulatory subunit 14-like [Cucumis sativus]
c48810.graph_c0	2.20864639	2.83781775	2.28750291	80.6928294	83.6526471	77.7897925	0	3.95456	up	hypothetical protein MIMGU_mgv1a006553mg [Erythranthe guttata]
c48810.graph_c1	8.36340862	7.56594321	8.67831011	8.48386388	9.01019551	8.01024759	6.874E-11	-1.04019	down	PREDICTED: alpha,alpha-trehalose-phosphate synthase [UDP-forming] 1-like [Sesamum indicum]
c48810.graph_c2	54.8362296	54.7939873	53.5671838	47.1378294	46.5098387	46.9972682	2.469E-58	-1.30477	down	PREDICTED: uncharacterized protein LOC105170911 [Sesamum indicum]

#ID	0 h-1 FPKM	0 h-2 FPKM	0 h-3 FPKM	28 h-1 FPKM	28 h-2 FPKM	28 h-3 FPKM	FDR	log2FC	regulated	nr_annotation
c48814.graph_c0	2.37542317	2.48788521	2.26112061	1.59855914	1.42891553	1.48702877	9.774E-16	-1.74796	down	PREDICTED: molybdopterin biosynthesis protein CNX1 [Sesamum indicum]
c48816.graph_c0	4.9534952	5.07988694	4.57272241	26.6118819	25.5538343	25.4801443	7.334E-38	1.32084	up	PREDICTED: uncharacterized protein LOC105176358 [Sesamum indicum]
c48816.graph_c1	20.7865124	20.5770937	21.3913603	23.5284434	21.1307778	21.2791954	4.877E-28	-1.01971	down	PREDICTED: serrate RNA effector molecule-like [Sesamum indicum]
c48816.graph_c2	11.1626754	10.7440896	11.2312548	49.4913346	48.8115188	46.4135589	1.09E-36	1.03566	up	PREDICTED: endoglucanase 6 [Sesamum indicum]
c48824.graph_c4	4.30109086	6.25307768	5.85606442	5.91708666	5.14096789	6.43772586	0.0005398	-1.00002	down	PREDICTED: UDP-N-acetylglucosamine diphosphorylase 1 [Vitis vinifera]
c48824.graph_c6	26.8584881	27.3032903	25.6323227	26.8703173	28.0969274	27.730699	4.005E-26	-1.03831	down	PREDICTED: hippocampus abundant transcript 1 protein-like isoform X1 [Sesamum indicum]
c48828.graph_c1	78.5264157	82.2280246	74.9812531	54.8230792	55.2603138	53.6922522	1.823E-86	-1.61538	down	PREDICTED: LOW QUALITY PROTEIN: B3 domain-containing transcription repressor VAL1 [Sesamum indicum]
c48832.graph_c0	34.3337096	34.6522615	32.0600891	281.467703	254.577287	245.719246	3.362E-74	1.86179	up	PREDICTED: aconitate hydratase, cytoplasmic [Sesamum indicum]
c48832.graph_c2	207.492414	196.034086	211.442555	55.6511371	59.5634886	62.7010258	1.05E-172	-2.87994	down	PREDICTED: (+)-neomenthol dehydrogenase-like isoform X1 [Sesamum indicum]
c48837.graph_c0	39.2241732	34.1421307	41.024974	31.2347459	29.947888	32.7693826	1.563E-17	-1.37518	down	-
c48837.graph_c5	4.68802065	4.3493456	4.16312871	38.7814386	39.8682205	38.4490847	7.578E-65	2.05975	up	PREDICTED: 3-ketoacyl-CoA synthase 1 [Sesamum indicum]
c48837.graph_c6	1.3376508	0.77520489	1.1761129	5.93551787	7.02242114	6.22117916	2.002E-05	1.45339	up	-
c48837.graph_c8	0.32542795	0.21716921	0.13939594	2.6805505	3.84398329	3.28096098	2.191E-07	2.7645	up	TPA: hypothetical protein ZEAMMB73_870894 [Zea mays]
c48837.graph_c9	32.7406247	31.6624465	28.1080912	19.023114	18.3292533	19.1154167	4.052E-75	-1.80069	down	PREDICTED: probable WRKY transcription factor 33-like [Cicer arietinum]
c48839.graph_c1	1.27716826	1.30303267	1.19934707	9.44304197	8.12324289	8.34635172	4.949E-19	1.6876	up	hypothetical protein MIMGU_mgv1a005831mg [Erythranthe guttata]
c48847.graph_c0	15.1463903	15.3393309	14.8993569	5.27366237	5.61072321	5.38660497	3.24E-124	-2.57031	down	PREDICTED: uncharacterized protein LOC105164002 isoform X1 [Sesamum indicum]
c48847.graph_c1	1.34141809	1.31693899	1.65747631	0	0	0.0611953	4.482E-46	-7.21927	down	PREDICTED: ABC transporter B family member 19-like [Sesamum indicum]
c48848.graph_c1	1.02487585	1.44386223	1.21945005	1.43457189	1.32019322	1.17059894	0.0035115	-1.00243	down	PREDICTED: O-acyltransferase WSD1-like [Nicotiana tomentosiformis]
c48848.graph_c2	0.40972361	0.31363179	0.46456858	10.947969	11.8461715	11.020308	1.459E-66	3.73777	up	PREDICTED: probable inactive leucine-rich repeat receptor-like protein kinase At1g66830 [Sesamum indicum]
c48848.graph_c3	54.7236139	56.2630125	52.9959838	47.2447543	48.6188066	47.0472201	2.263E-57	-1.28859	down	PREDICTED: myb-related protein 3R-1-like isoform X1 [Sesamum indicum]
c48848.graph_c4	34.794188	34.006121	33.6338626	23.1727899	22.2205111	22.8750449	1.158E-88	-1.6755	down	PREDICTED: protein STRUBBELIG-RECEPTOR FAMILY 3-like [Sesamum indicum]
c48849.graph_c1	244.99828	253.804355	247.589247	119.256714	116.48589	116.259216	1.32E-139	-2.17498	down	PREDICTED: probable inactive receptor kinase At5g10020 [Sesamum indicum]

#ID	0 h-1 FPKM	0 h-2 FPKM	0 h-3 FPKM	28 h-1 FPKM	28 h-2 FPKM	28 h-3 FPKM	FDR	log2FC	regulated	nr_annotation
c48850.graph_c0	0.16351582	0	0	2.07974127	3.48736398	6.64274598	0.0007631	5.16782	up	-
c48861.graph_c0	12.0432512	11.6298291	10.8514453	11.986414	10.822608	11.8478956	1.358E-33	-1.08347	down	PREDICTED: phospholipase A I [Sesamum indicum]
c48861.graph_c1	5.48297191	5.3085127	4.62022032	5.68351373	4.12893795	4.6910077	1.503E-05	-1.17595	down	PREDICTED: phospholipase A I [Sesamum indicum]
c48863.graph_c0	60.8142887	33.5626899	40.4483079	0	0	0	3.414E-36	-Inf	down	predicted protein [Hordeum vulgare subsp. vulgare]
c48864.graph_c0	4.18595266	3.89570169	3.79445672	22.2500943	20.7133775	20.4706712	6.942E-47	1.32759	up	PREDICTED: probable splicing factor 3B subunit 3 isoform X1 [Sesamum indicum]
c48865.graph_c3	73.1994211	78.8623462	71.667927	70.0900975	68.9862211	67.6010145	1.579E-49	-1.20455	down	PREDICTED: enhancer of mRNA-decapping protein 4-like [Sesamum indicum]
c48866.graph_c0	14.3838429	14.1116041	12.6810808	58.6261755	61.4768011	58.315395	9.014E-34	1.02624	up	hypothetical protein MIMGU_mgv1a005571mg [Erythranthe guttata]
c48869.graph_c0	101.644942	102.615364	98.1677696	36.0755344	36.2742825	36.7109037	3.13E-210	-2.56145	down	PREDICTED: uncharacterized protein LOC105165944 [Sesamum indicum]
c48870.graph_c0	226.363051	235.333954	204.303126	150.49657	172.223122	164.849842	6.436E-70	-1.53903	down	PREDICTED: DNA repair helicase XPB1-like [Sesamum indicum]
c48871.graph_c0	6.586903	4.34890346	6.24326836	28.2486018	28.7247248	28.2037737	5.482E-20	1.21885	up	hypothetical protein MIMGU_mgv1a012747mg [Erythranthe guttata]
c48872.graph_c0	5.22314178	4.48146169	4.83990518	0	0.09326713	0.08428917	2.25E-104	-7.44437	down	-
c48872.graph_c1	119.354218	127.462319	112.999087	110.140299	110.036906	114.660253	4.325E-49	-1.19324	down	PREDICTED: FACT complex subunit SPT16-like [Sesamum indicum]
c48872.graph_c2	0.47902338	0.2950788	0.37880862	3.52168561	3.01048756	3.21238787	6.392E-13	1.99091	up	PREDICTED: putative late blight resistance protein homolog R1A-10 [Sesamum indicum]
c48879.graph_c1	1.17368585	1.07487294	1.37987255	11.9060945	11.4055801	11.6553691	8.516E-36	2.17555	up	PREDICTED: multisubstrate pseudouridine synthase 7 isoform X2 [Sesamum indicum]
c48887.graph_c2	37.0780082	37.0570531	35.0453099	173.496733	176.375484	174.229124	6.368E-45	1.17329	up	hypothetical protein MIMGU_mgv1a000942mg [Erythranthe guttata]
c48889.graph_c0	1.44584185	0.692182	0.76741992	0	0	0	4.082E-12	-Inf	down	PREDICTED: granule-bound starch synthase 1b, chloroplastic/amyloplastic-like [Elaeis guineensis]
c48892.graph_c0	0	0	0	1.11777666	2.10259901	2.54626975	1.561E-06	Inf	up	predicted protein [Micromonas sp. RCC299]
c48893.graph_c0	5.81286319	4.52564303	5.60231951	3.87269251	2.43707877	1.72368298	9.429E-09	-2.08145	down	PREDICTED: calcium-dependent protein kinase 20-like [Malus domestica]
c48895.graph_c0	61.2924419	60.7836143	62.2200115	41.9594591	43.1935334	40.7248183	5.368E-89	-1.64102	down	neutral/alkaline invertase 1 [Orobanche ramosa]
c48896.graph_c0	1.52905525	1.1704484	1.04023934	7.51767545	7.02385195	9.28155608	8.79E-06	1.58648	up	PREDICTED: N-acetyl-D-glucosamine kinase-like isoform X1 [Sesamum indicum]
c48898.graph_c0	0	0	0	1.13299039	0.884743	1.9989425	1.122E-05	Inf	up	hypothetical protein EUGRSUZ_K01803 [Eucalyptus grandis]
c48899.graph_c0	1.95226345	1.60815884	2.14287851	0.50252489	0.44038778	0.39799573	6.641E-26	-3.18168	down	PREDICTED: phosphate transporter PHO1 homolog 9-like [Sesamum indicum]
c48900.graph_c0	5.32465352	2.43914584	2.9573044	0	0	0	2.819E-17	-Inf	down	--
c48903.graph_c0	54.4673358	53.2023733	52.5114514	12.2630151	13.3214246	14.1967506	5.14E-226	-3.09924	down	PREDICTED: phosphoenolpyruvate carboxylase kinase 2 [Sesamum indicum]

#ID	0 h-1 FPKM	0 h-2 FPKM	0 h-3 FPKM	28 h-1 FPKM	28 h-2 FPKM	28 h-3 FPKM	FDR	log2FC	regulated	nr_annotation
c48903.graph_c1	50.9896189	55.0331229	45.2233981	37.684189	39.41391	37.1968211	3.887E-36	-1.49313	down	hypothetical protein MIMGU_mgv1a0000982mg, partial [Erythranthe guttata]
c48903.graph_c2	53.4103412	52.3878221	53.5693674	28.8085706	28.1717102	30.0020194	1.74E-118	-1.96394	down	PREDICTED: suppressor of mec-8 and unc-52 protein homolog 2 [Sesamum indicum]
c48904.graph_c0	0	0	0	1.5483212	1.13989862	2.7795184	2.107E-06	Inf	up	PREDICTED: cytochrome P450 6k1-like [Pyrus x bretschneideri]
c48909.graph_c1	17.5864922	16.6317828	16.7524168	14.1565436	13.5536503	12.9766275	1.553E-30	-1.41544	down	hypothetical protein MIMGU_mgv1a025365mg [Erythranthe guttata]
c48914.graph_c1	34.865895	34.5033867	34.6466143	5.69239332	6.19763825	6.28601818	0	-3.60699	down	hypothetical protein MIMGU_mgv1a000556mg [Erythranthe guttata]
c48915.graph_c1	6.56614996	6.36007798	4.74003726	35.3847556	32.8489133	32.5616217	2.171E-42	1.42571	up	PREDICTED: vacuolar protein sorting-associated protein 8 homolog [Sesamum indicum]
c48920.graph_c0	7.31708244	7.25690332	6.71624331	30.3641407	30.8014796	29.6162836	4.696E-19	1.00261	up	PREDICTED: cell division cycle protein 27 homolog B [Sesamum indicum]
c48922.graph_c1	0	0	0	1.72940355	2.80267303	2.74999019	4.924E-12	Inf	up	-
c48927.graph_c0	39.5444731	38.8384806	37.9816391	28.4700208	28.9939638	28.8132428	5.025E-69	-1.52167	down	PREDICTED: coiled-coil domain-containing protein 130-like [Sesamum indicum]
c48936.graph_c0	101.326145	95.5542945	103.122502	93.554821	94.7191441	97.24513	6.127E-46	-1.1622	down	carotenoid cleavage dioxygenase 1 [Ipomoea sp. Kenyan]
c48938.graph_c0	3.07291205	3.54204806	4.54711875	22.9428499	17.6598969	18.168931	1.392E-07	1.30076	up	cytochrome b, partial (mitochondrion) [Erythranthe guttata]
c48942.graph_c0	16.3103917	16.7348922	16.567985	93.2538164	89.2325617	90.0396101	1.636E-65	1.36689	up	PREDICTED: heparanase-like protein 2 isoform X1 [Sesamum indicum]
c48943.graph_c0	389.230273	381.899263	396.053806	322.165551	309.127581	320.775063	3.163E-40	-1.38462	down	PREDICTED: DNA mismatch repair protein msh2 isoform X2 [Sesamum indicum]
c48950.graph_c0	245.361664	237.41112	250.336273	177.169736	173.905001	176.555563	1.287E-72	-1.56537	down	PREDICTED: methyl-CpG-binding domain-containing protein 11 [Sesamum indicum]
c48950.graph_c2	0.5818557	0.47323115	0.37385381	3.93815725	3.91375187	4.24872697	5.922E-09	1.99709	up	hypothetical protein MIMGU_mgv1a004080mg [Erythranthe guttata]
c48950.graph_c3	9.2544081	10.5071213	10.9345132	5.8232249	5.42085947	4.34651991	2.62E-29	-2.07087	down	-
c48952.graph_c2	4.05129797	4.06505654	3.6118725	4.21945347	3.79069021	3.56949512	5.973E-24	-1.10787	down	PREDICTED: protein RST1 [Sesamum indicum]
c48953.graph_c1	0	0	0	1.60828918	1.13645296	1.85440879	7.11E-12	Inf	up	PREDICTED: protein ECERIFERUM 1-like [Sesamum indicum]
c48956.graph_c2	31.8432848	30.8904097	33.3347329	16.8402006	16.0036035	15.6146046	9.881E-97	-2.07877	down	PREDICTED: DEAD-box ATP-dependent RNA helicase 13 [Sesamum indicum]
c48956.graph_c3	64.2968181	58.1402709	58.0125655	20.4819036	22.2637048	20.9550787	1.76E-195	-2.59184	down	PREDICTED: spermatogenesis-associated protein 5 [Sesamum indicum]
c48957.graph_c2	74.5098353	81.0202516	69.7018076	616.140028	617.11856	611.33848	4.09E-118	1.94434	up	PREDICTED: uncharacterized protein LOC105169334 [Sesamum indicum]
c48958.graph_c0	1.30096611	1.16020314	1.55020802	0.80800236	0.80716211	0.9539146	7.116E-09	-1.73538	down	PREDICTED: probable plastidic glucose transporter 2 [Sesamum indicum]
c48960.graph_c0	0.49217669	0.4187691	0.98032313	3.75596743	4.21488456	4.15942402	5.547E-09	1.58007	up	PREDICTED: uncharacterized protein LOC105166139 [Sesamum indicum]
c48965.graph_c0	0	0.14476592	0.05068469	0.65932503	0.53491505	0.56919247	7.454E-05	2.07907	up	hypothetical protein VITISV_029627 [Vitis vinifera]

#ID	0 h-1 FPKM	0 h-2 FPKM	0 h-3 FPKM	28 h-1 FPKM	28 h-2 FPKM	28 h-3 FPKM	FDR	log2FC	regulated	nr_annotation
c48967.graph_c0	0	0	0	1.359078	1.57224751	1.24762112	5.951E-12	Inf	up	PREDICTED: FAS-associated factor 2-like [Sesamum indicum]
c48967.graph_c3	47.4255923	46.8446461	45.8170711	46.3320977	45.369736	45.1290268	2.762E-43	-1.12411	down	PREDICTED: mitochondrial carnitine/acylcarnitine carrier-like protein [Sesamum indicum]
c48969.graph_c0	337.103831	309.66971	324.247717	295.490344	314.278577	295.494583	1.273E-45	-1.19164	down	putative aldo/keto reductase 2 [Salvia miltiorrhiza]
c48971.graph_c0	14.8837985	14.3502839	12.5161687	75.9213072	76.5393118	76.4642022	5.63E-55	1.36664	up	PREDICTED: LOW QUALITY PROTEIN: T-complex protein 1 subunit beta-like [Sesamum indicum]
c48978.graph_c0	26.86928	23.5513447	25.3519696	16.9002494	17.2718915	16.5865638	1.852E-57	-1.66832	down	PREDICTED: LOW QUALITY PROTEIN: ADP-ribosylation factor-binding protein GGA3 [Sesamum indicum]
c48979.graph_c0	8854.65546	8951.01217	8042.78709	4278.56395	4314.16447	4494.00888	4.695E-60	-2.07102	down	hypothetical protein PHAVU_003G123600g, partial [Phaseolus vulgaris]
c48981.graph_c0	55.7266216	55.5421113	59.2322916	35.6817039	32.8080137	35.8432651	8.552E-73	-1.79967	down	hypothetical protein MIMGU_mgv1a010195mg [Erythranthe guttata]
c48983.graph_c0	70.7943042	70.8280431	67.6041484	22.8199983	25.2195889	25.7193373	2.05E-214	-2.59387	down	PREDICTED: protein SPA1-RELATED 4-like isoform X6 [Sesamum indicum]
c48984.graph_c0	204.912448	194.785218	204.100268	150.722601	147.697055	147.289289	1.591E-74	-1.52865	down	PREDICTED: uncharacterized protein LOC105157959 [Sesamum indicum]
c48986.graph_c0	13.0754007	12.2623098	13.7322002	1.54702049	0.99772461	1.05625714	6.66E-153	-4.53145	down	anion exchange family protein [Populus trichocarpa]
c48988.graph_c0	0	0	0	3.01184858	5.3551594	6.95702259	3.966E-09	Inf	up	-
c48990.graph_c0	1.30586357	1.20584009	1.27482532	21.652589	20.6264187	20.6700918	3.39E-175	2.96472	up	PREDICTED: alpha-glucan water dikinase, chloroplastic-like [Sesamum indicum]
c48992.graph_c0	1.75179028	1.98735035	1.50074657	9.29429485	9.50312992	10.215983	1.752E-07	1.38127	up	Papain family cysteine protease [Arabidopsis thaliana]
c48992.graph_c1	1.87943482	1.99069568	1.57265462	68.6475522	69.0668146	60.8371963	4.53E-204	4.10033	up	PREDICTED: subtilisin-like protease [Sesamum indicum]
c48995.graph_c0	16.5619278	18.2268536	13.4288755	16.6402164	17.0195169	16.2451076	3.475E-09	-1.0382	down	PREDICTED: proline-rich receptor-like protein kinase PERK9 isoform X1 [Sesamum indicum]
c48999.graph_c0	1.32657046	0.51073032	0.52452171	6.82317127	5.55800832	4.23625699	0.000459	1.73331	up	PREDICTED: uncharacterized protein LOC100792643 [Glycine max]
c49001.graph_c2	8.36795122	8.6936157	9.28681017	3.54770425	4.28485927	4.21076029	1.478E-27	-2.22114	down	PREDICTED: uncharacterized protein LOC105156426 [Sesamum indicum]
c49003.graph_c1	2.80076841	4.15344142	3.15476517	0.57800486	0.58995605	0.86126889	7.863E-17	-3.40545	down	hypothetical protein MIMGU_mgv1a000068mg [Erythranthe guttata]
c49008.graph_c0	44.787024	41.1119165	50.6368468	1.95585113	1.97618236	1.63395756	5.901E-86	-5.70987	down	PREDICTED: uncharacterized protein LOC105161665 [Sesamum indicum]
c49013.graph_c0	64.9923843	66.5123608	57.117181	53.645581	49.7644988	50.4255421	1.035E-55	-1.38296	down	PREDICTED: probable histone-lysine N-methyltransferase ATXR3 isoform X2 [Sesamum indicum]
c49015.graph_c1	56.4582066	56.6532574	55.6580211	45.5927768	41.6503087	43.0573875	1.885E-65	-1.46354	down	PREDICTED: E3 ubiquitin-protein ligase SINAT5 isoform X1 [Vitis vinifera]
c49015.graph_c3	47.2820439	49.031176	44.4217278	30.7470205	30.7528882	29.0054138	2.317E-98	-1.72681	down	PREDICTED: AP-5 complex subunit mu [Sesamum indicum]
c49018.graph_c1	4.21720788	4.24814869	3.77291582	30.1266507	30.0915952	30.6299423	2.72E-57	1.80307	up	hypothetical protein MIMGU_mgv1a008196mg [Erythranthe guttata]

#ID	0 h-1 FPKM	0 h-2 FPKM	0 h-3 FPKM	28 h-1 FPKM	28 h-2 FPKM	28 h-3 FPKM	FDR	log2FC	regulated	nr_annotation
c49020.graph_c0	1.54733418	1.67116388	1.72675599	0	0	0	7.303E-41	-Inf	down	-
c49020.graph_c1	37.2319069	35.4916125	34.2295677	23.9275956	22.3768955	21.7138582	5.48E-99	-1.74279	down	PREDICTED: uncharacterized protein LOC105161915 [Sesamum indicum]
c49022.graph_c0	8.69725988	8.56832509	9.67436679	72.7553227	65.3077353	65.0354121	1.873E-66	1.82213	up	PREDICTED: uncharacterized protein LOC105176567 [Sesamum indicum]
c49023.graph_c0	17.8032157	16.0876892	14.9505622	6.68594733	6.14331505	7.83050315	1.308E-62	-2.32874	down	PREDICTED: DNA polymerase zeta catalytic subunit [Sesamum indicum]
c49023.graph_c1	36.0681975	35.9791453	30.405413	18.3175973	16.6758349	18.1110957	1.264E-65	-2.03614	down	PREDICTED: DNA polymerase zeta catalytic subunit [Sesamum indicum]
c49028.graph_c0	14.909801	8.67079527	10.043641	0	0	0	1.425E-45	-Inf	down	alpha-2 macroglobulin family-like protein [Micromonas sp. RCC299]
c49030.graph_c2	2432.47793	2372.10496	2596.92043	589.918193	636.893527	681.800808	4.259E-89	-3.04636	down	PREDICTED: probable xyloglucan endotransglucosylase/hydrolase protein 28 [Sesamum indicum]
c49032.graph_c0	17.067669	17.748962	18.142975	109.873881	116.671454	110.993532	6.894E-78	1.58069	up	PREDICTED: tyrosine--tRNA ligase, cytoplasmic-like [Sesamum indicum]
c49034.graph_c1	8.22911111	7.20409471	7.81130146	35.8435446	33.5299464	35.3357131	8.426E-27	1.08136	up	PREDICTED: probable NOT transcription complex subunit VIP2 isoform X1 [Sesamum indicum]
c49035.graph_c1	11.325483	12.1186736	12.2117203	3.21682822	1.70852605	1.46685899	3.944E-38	-3.57271	down	PREDICTED: histone-lysine N-methyltransferase, H3 lysine-9 specific SUVH5-like isoform X1 [Sesamum indicum]
c49038.graph_c0	5.50031078	4.87053175	2.44665592	2.7675652	3.74827783	2.7601574	0.0028718	-1.54899	down	-
c49038.graph_c1	28.0918035	25.9105099	26.0429075	18.9099947	21.4631431	16.3030718	1.174E-42	-1.58895	down	PREDICTED: E3 ubiquitin-protein ligase RING1-like [Sesamum indicum]
c49038.graph_c2	1.16361174	0.98829557	1.08748157	29.779113	30.9139673	28.6282559	5.43E-115	3.69481	up	ATP-citrate synthase [Camellia sinensis]
c49038.graph_c3	68.0801967	67.4513608	61.9436516	71.0344722	67.0244852	66.7481804	1.848E-38	-1.03684	down	PREDICTED: probable monodehydroascorbate reductase, cytoplasmic isoform 2 [Sesamum indicum]
c49043.graph_c0	0.59751195	0.76558114	0.98281793	0.66008773	0.65873389	0.63784681	0.0009035	-1.35841	down	Cytochrome c biogenesis [Medicago truncatula]
c49045.graph_c1	8.4857651	8.25924319	8.49089008	34.4204589	36.7790971	37.495498	1.411E-22	1.01629	up	hypothetical protein MIMGU_mgv1a008892mg [Erythranthe guttata]
c49047.graph_c0	0.25494122	0.55292576	0.49141434	4.63224522	4.76803076	4.96423488	1.439E-11	2.37114	up	PREDICTED: uncharacterized protein LOC105176173 [Sesamum indicum]
c49051.graph_c3	6.38798023	6.80741073	5.57727591	3.77639725	5.04439859	5.20231279	4.675E-24	-1.50888	down	alcohol dehydrogenase class III [Solanum lycopersicum]
c49059.graph_c1	20.3157567	17.4309427	18.6475369	125.622872	142.099516	138.461269	2.138E-44	1.75875	up	PREDICTED: 60S ribosomal protein L35a-3-like [Sesamum indicum]
c49059.graph_c5	23.1102402	21.1041376	19.2773724	104.756429	119.329049	119.263779	4.159E-21	1.34682	up	unknown [Glycine max]
c49060.graph_c2	1.43217298	0.86016425	1.04289276	4.92690334	5.51367318	5.18110745	0.0002059	1.14018	up	PREDICTED: probable serine/threonine-protein kinase DDB_G0291350 [Sesamum indicum]
c49060.graph_c3	21.159166	20.9125305	21.3740917	106.400749	103.210033	102.240147	5.059E-52	1.20645	up	PREDICTED: 1,4-alpha-glucan-branching enzyme 2-2, chloroplastic/amyloplastic-like isoform X1 [Nicotiana sylvestris]

#ID	0 h-1 FPKM	0 h-2 FPKM	0 h-3 FPKM	28 h-1 FPKM	28 h-2 FPKM	28 h-3 FPKM	FDR	log2FC	regulated	nr_annotation
c49060.graph_c5	6.59052495	5.15573585	5.26649014	5.11196937	5.05175148	5.55081865	1.208E-08	-1.20224	down	PREDICTED: uncharacterized protein At1g15400-like [Sesamum indicum]
c49071.graph_c0	15.0950327	15.5104169	16.0577108	72.849263	79.9458956	71.9735765	6.64E-42	1.17642	up	hypothetical protein MIMGU_mgv1a005383mg [Erythranthe guttata]
c49074.graph_c0	4.37574243	5.82016487	4.6216457	0.1306958	0.2360121	0.24884231	1.408E-48	-5.67857	down	PREDICTED: uncharacterized protein LOC105167219 [Sesamum indicum]
c49075.graph_c0	5.39123566	4.22979434	5.49242983	2.8240137	2.42232861	2.33317677	1.273E-17	-2.08756	down	-
c49078.graph_c2	3.37595104	2.93854917	3.96099329	2.84477885	2.69699701	4.41050099	0.0002761	-1.13691	down	WRKY transcription factor [Medicago truncatula]
c49078.graph_c3	4.64072708	4.54992117	5.39167384	31.2104475	32.4978594	32.4988296	2.139E-46	1.62931	up	hypothetical protein EUGRSUZ_H01869 [Eucalyptus grandis]
c49080.graph_c1	211.977807	213.744404	211.812821	91.3441969	98.223046	91.502783	8.71E-165	-2.27232	down	hypothetical protein MIMGU_mgv1a009220mg [Erythranthe guttata]
c49084.graph_c0	0.14016878	0.28061827	0.36024486	1.73185563	2.16158979	1.78725712	0.0001403	1.76176	up	PREDICTED: uncharacterized protein LOC105173906 [Sesamum indicum]
c49085.graph_c0	101.41198	97.0057601	98.2855975	56.0008256	53.7746143	51.9418566	6.95E-128	-1.96603	down	-
c49085.graph_c1	33.6047021	30.5007184	32.6919676	10.8078863	10.1890304	9.81346254	2.49E-110	-2.74227	down	PREDICTED: cellulose synthase A catalytic subunit 2 [UDP-forming]-like [Nicotiana sylvestris]
c49085.graph_c3	7.17812944	7.06071638	6.9847841	5.80506907	6.58923017	5.42589098	1.758E-28	-1.34314	down	PREDICTED: leucine-rich repeat receptor-like protein kinase PXL2 [Sesamum indicum]
c49086.graph_c0	4.32450516	3.39287107	5.05651527	4.16238503	3.57927945	4.04341928	0.000134	-1.20939	down	PREDICTED: uncharacterized protein LOC105159651 [Sesamum indicum]
c49094.graph_c0	88.2778485	84.7222765	79.1623751	47.6237925	45.512261	48.7432849	3.67E-123	-1.91865	down	PREDICTED: uridine-cytidine kinase C-like isoform X1 [Sesamum indicum]
c49097.graph_c1	11.4378988	11.6163219	11.8503857	4.86656814	4.47224077	4.91792097	6.749E-95	-2.38243	down	PREDICTED: probable galacturonosyltransferase 14 [Sesamum indicum]
c49099.graph_c0	131.282506	133.492378	116.369645	70.8467057	71.6890247	66.1707963	8.82E-119	-1.95815	down	PREDICTED: uncharacterized protein LOC105161154 isoform X1 [Sesamum indicum]
c49103.graph_c5	16.4305875	17.1247313	14.1540462	117.069867	118.228232	108.268218	1.1E-100	1.75934	up	PREDICTED: V-type proton ATPase catalytic subunit A [Sesamum indicum]
c49112.graph_c0	358.668528	352.973443	347.767126	134.880036	130.171304	134.763402	2.04E-168	-2.49593	down	PREDICTED: THO complex subunit 4D [Solanum lycopersicum]
c49114.graph_c0	5.4511327	5.00628997	5.44071561	32.5025709	31.6389481	31.0101425	6.894E-45	1.49046	up	PREDICTED: signal recognition particle subunit SRP68 [Sesamum indicum]
c49115.graph_c1	0.46621198	0.46667882	0.68468669	6.29274619	5.33211305	5.45081901	2.841E-09	2.30392	up	ALA-interacting subunit 5 [Arabidopsis thaliana]
c49119.graph_c0	43.201845	43.689934	45.0633665	39.051971	39.2161129	37.4578376	1.186E-55	-1.28063	down	PREDICTED: pentatricopeptide repeat-containing protein At1g62680, mitochondrial-like [Sesamum indicum]
c49119.graph_c2	29.3052442	27.2294973	26.5328515	26.7377398	28.0846327	26.2234458	1.025E-38	-1.12523	down	PREDICTED: cytochrome P450 86A8-like [Sesamum indicum]
c49121.graph_c0	4.1242365	3.91108386	1.67362332	13.7252634	16.5235013	15.7053305	0.0028011	1.16225	up	PREDICTED: uncharacterized protein LOC105160017 [Sesamum indicum]
c49124.graph_c0	0	0	0	2.76816209	3.87160539	7.04213387	2.537E-06	Inf	up	-

#ID	0 h-1 FPKM	0 h-2 FPKM	0 h-3 FPKM	28 h-1 FPKM	28 h-2 FPKM	28 h-3 FPKM	FDR	log2FC	regulated	nr_annotation
c49128.graph_c3	0	0	0	1.45129482	1.38980031	1.11247231	6.947E-11	Inf	up	PREDICTED: calcium-dependent protein kinase 29 [Sesamum indicum]
c49128.graph_c4	12.2497484	12.8202852	11.7228724	8.42532208	8.66602047	8.70596321	6.247E-58	-1.60198	down	PREDICTED: uncharacterized protein LOC105173091 [Sesamum indicum]
c49132.graph_c3	1.79096041	1.51263601	2.01377316	0	0	0	1.081E-34	-Inf	down	-
c49134.graph_c0	1.2336301	2.14759198	1.58526351	0.35084162	0.17598727	0.4453305	7.497E-10	-3.44154	down	retrotransposon protein, putative, unclassified [Oryza sativa Japonica Group]
c49138.graph_c2	29.5557684	30.2277262	28.6972984	16.2972808	14.823203	16.8432511	6.08E-121	-1.97304	down	hypothetical protein MIMGU_mgv1a026550mg, partial [Erythranthe guttata]
c49142.graph_c2	70.958353	73.7247194	66.3121488	73.2139947	70.0758526	71.5964921	2.566E-40	-1.06315	down	PREDICTED: ethylene-responsive transcription factor RAP2-12-like [Sesamum indicum]
c49146.graph_c0	18.8429163	20.460241	17.1002073	15.2810581	12.959042	13.7003567	1.264E-36	-1.51649	down	PREDICTED: beta-galactosidase 3 [Sesamum indicum]
c49149.graph_c0	0	0	0	0.87829464	0.46933701	1.18471799	4.941E-06	Inf	up	--
c49152.graph_c0	143.379752	132.474897	143.607577	137.452186	139.741618	140.514528	1.918E-41	-1.0968	down	PREDICTED: xyloglucan galactosyltransferase KATAMARI1 homolog [Sesamum indicum]
c49154.graph_c0	87.9182565	90.2744968	89.7898008	90.5478022	90.4616944	88.7417071	1.62E-40	-1.08136	down	PREDICTED: uncharacterized protein LOC105158432 [Sesamum indicum]
c49154.graph_c1	102.157883	108.387619	101.910049	96.7890245	97.8355363	95.6784041	1.383E-38	-1.19652	down	hypothetical protein MIMGU_mgv1a005704mg [Erythranthe guttata]
c49157.graph_c4	2.35944133	2.11956765	0.85517258	11.520129	11.5926189	11.337804	1.112E-09	1.61151	up	MYB-related transcription factor [Salvia miltiorrhiza]
c49158.graph_c0	0	0	0	3.59546684	5.88071844	5.00201057	1.883E-19	Inf	up	S-adenosyl-methionine synthetase [Undaria pinnatifida]
c49159.graph_c0	19.1985957	19.0516719	18.6276379	96.9099803	107.687492	107.066683	9.126E-50	1.36401	up	PREDICTED: DNA ligase 1-like isoform X2 [Sesamum indicum]
c49159.graph_c1	0	0.6754867	0.43357941	5.76277262	4.98182875	8.40424539	8.817E-06	3.01141	up	-
c49160.graph_c0	472.266599	439.129256	451.413504	173.467583	176.850494	173.741961	2.27E-149	-2.46899	down	PREDICTED: G-type lectin S-receptor-like serine/threonine-protein kinase At1g11300 [Sesamum indicum]
c49160.graph_c3	3.74589925	4.28531451	4.51891687	3.11142627	1.60532925	1.49613657	4.547E-12	-2.10835	down	PREDICTED: dof zinc finger protein DOF1.4-like [Sesamum indicum]
c49161.graph_c0	20.2874361	20.4244621	18.4289052	6.65210428	7.30693794	9.12744313	2.117E-74	-2.44511	down	hypothetical protein CICLE_v10004556mg [Citrus clementina]
c49163.graph_c0	0.95841634	1.26463206	0.8117383	6.5541043	5.66044856	7.44082864	1.901E-10	1.60865	up	putative retroelement pol polyprotein [Arabidopsis thaliana]
c49165.graph_c0	202.012994	190.75215	186.988449	90.1429249	94.184555	89.0753135	6.23E-154	-2.1743	down	PREDICTED: UDP-D-apiose/UDP-D-xylose synthase 2 isoform X1 [Phoenix dactylifera]
c49168.graph_c0	19.5656038	20.2420905	18.0526815	17.4528274	17.9114712	17.7584628	1.118E-40	-1.21264	down	PREDICTED: kinesin-like protein NACK1 [Sesamum indicum]
c49169.graph_c1	57.2586257	52.1416039	64.3821508	34.9683607	30.5294106	34.42933	1.777E-20	-1.89043	down	hypothetical protein (mitochondrion) [Vicia faba]
c49169.graph_c2	303.595306	327.392522	335.249452	195.540701	156.063708	172.761665	2.42E-52	-1.97333	down	Cell wall-associated hydrolase, partial [Medicago truncatula]
c49169.graph_c4	21.921697	16.4214056	25.3718795	11.7120165	7.33570871	10.7622859	6.872E-08	-2.18832	down	hypothetical protein (mitochondrion) [Capsicum annuum]
c49170.graph_c0	20.5873319	20.7043961	18.4487127	7.18959171	7.81933962	7.90473692	9.53E-121	-2.47142	down	PREDICTED: type I inositol 1,4,5-trisphosphate 5-phosphatase 1-like isoform X2 [Sesamum indicum]

#ID	0 h-1 FPKM	0 h-2 FPKM	0 h-3 FPKM	28 h-1 FPKM	28 h-2 FPKM	28 h-3 FPKM	FDR	log2FC	regulated	nr_annotation
c49172.graph_c1	0	0	0	1.98919683	2.18092785	2.28016509	2.935E-14	Inf	up	predicted protein [Hordeum vulgare subsp. vulgare]
c49175.graph_c0	0.06271153	0	0.0805868	3.91974055	5.72026733	8.62844764	3.828E-08	5.89751	up	60S ribosomal protein L7a, putative [Ricinus communis]
c49177.graph_c0	0	0	0	4.4303876	5.8336609	6.57758297	4.356E-19	Inf	up	40S ribosomal protein S12 [Blumeria graminis f. sp. hordei DH14]
c49179.graph_c2	36.4475922	37.3449011	38.1433725	25.0211324	26.8335655	27.7355539	2.321E-83	-1.58283	down	hypothetical protein MIMGU_mgv1a003978mg [Erythranthe guttata]
c49183.graph_c1	7.72243628	8.58048773	8.15722909	5.47791925	4.9055061	5.36758873	4.34E-59	-1.72568	down	PREDICTED: mitochondrial fission protein ELM1 [Sesamum indicum]
c49185.graph_c0	0	0	0	5.23926295	7.32849548	8.48030639	3.462E-19	Inf	up	hypothetical protein CHLNCDRAFT_59606 [Chlorella variabilis]
c49189.graph_c0	20.1221532	16.9169011	18.3370415	12.0199452	11.5001729	12.791579	6.485E-42	-1.69815	down	PREDICTED: josephin-like protein [Sesamum indicum]
c49192.graph_c0	1.45165346	1.78401264	1.90237142	8.5344416	8.24341458	9.43596861	5.721E-18	1.25815	up	PREDICTED: 2-oxoisovalerate dehydrogenase subunit alpha 1, mitochondrial isoform X1 [Sesamum indicum]
c49193.graph_c0	1.527031	1.22856231	1.44879746	1.19281582	1.22684444	1.22723961	4.643E-09	-1.29561	down	PREDICTED: probable ubiquitin conjugation factor E4 [Sesamum indicum]
c49194.graph_c1	0.17018834	0.13628701	0.13121925	1.08848877	0.9828033	1.09006112	0.0002041	1.76523	up	-
c49197.graph_c2	2.13997294	1.80132465	1.06248066	0.07069636	0.03191609	0.05768766	7.13E-15	-6.04542	down	-
c49199.graph_c0	36.1157866	38.600282	29.3574707	33.3971791	33.0402033	33.6867129	1.255E-12	-1.14346	down	PREDICTED: ABC transporter C family member 3-like [Sesamum indicum]
c49220.graph_c0	60.1722097	59.914893	59.9486564	55.503234	53.3956814	53.2820444	3.644E-52	-1.24126	down	unnamed protein product [Coffea canephora]
c49221.graph_c0	76.4187236	74.4084694	75.0094027	48.2680179	50.7734184	51.8203718	9.472E-84	-1.67225	down	PREDICTED: mannosyl-oligosaccharide 1,2-alpha-mannosidase MNS3 [Sesamum indicum]
c49224.graph_c0	29.1199115	30.5223158	31.2334263	32.7715629	31.7468734	31.8685398	6.675E-32	-1.00643	down	PREDICTED: uncharacterized protein LOC105173178 [Sesamum indicum]
c49230.graph_c0	34.3969378	35.601853	36.1374839	27.634004	25.5903538	25.2073983	2.96E-76	-1.5277	down	PREDICTED: protein NLP7 isoform X1 [Sesamum indicum]
c49230.graph_c1	39.8531658	41.0831385	41.0500256	22.315487	21.8448187	22.7768966	1.97E-92	-1.95652	down	PREDICTED: uncharacterized protein LOC105156041 [Sesamum indicum]
c49231.graph_c0	1.06036903	1.06143083	0.93679863	11.8972197	14.1777979	13.2453745	8.372E-20	2.59533	up	PREDICTED: methyl-CpG-binding domain-containing protein 13-like isoform X1 [Sesamum indicum]
c49233.graph_c2	10.7700719	10.088508	10.3693956	10.3650219	10.5473851	10.9189266	4.715E-18	-1.06243	down	PREDICTED: uncharacterized protein LOC105166728 [Sesamum indicum]
c49236.graph_c1	24.5667215	25.7011435	23.9206127	11.7477948	12.0622907	13.0813991	5.486E-70	-2.09754	down	PREDICTED: kinesin KP1-like isoform X2 [Sesamum indicum]
c49238.graph_c0	19.7238033	20.0019772	18.3458967	13.5283255	13.8750749	12.5547608	7.273E-67	-1.62934	down	PREDICTED: guanine nucleotide-binding protein alpha-1 subunit [Sesamum indicum]
c49238.graph_c1	265.148778	266.512525	279.593719	153.766877	139.273779	145.205931	8.93E-100	-1.97962	down	PREDICTED: uncharacterized protein LOC105157579 [Sesamum indicum]
c49239.graph_c1	57.7586141	58.1576098	53.7923488	55.5003795	56.6239156	56.2620469	5.285E-43	-1.10088	down	hypothetical protein MIMGU_mgv1a000365mg [Erythranthe guttata]

#ID	0 h-1 FPKM	0 h-2 FPKM	0 h-3 FPKM	28 h-1 FPKM	28 h-2 FPKM	28 h-3 FPKM	FDR	log2FC	regulated	nr_annotation
c49244.graph_c1	25.2409747	25.8404827	25.8625178	22.410079	21.8654846	18.6550054	5.055E-41	-1.38163	down	PREDICTED: protein ENHANCED DISEASE RESISTANCE 2 [Sesamum indicum]
c49245.graph_c0	4.99249682	5.64934336	5.29980719	71.9393117	73.7859801	75.1796146	1.724E-34	2.70172	up	-
c49245.graph_c3	85.9601126	92.9550067	71.1150471	41.4982015	35.2455509	42.8673044	2.513E-28	-2.15098	down	hypothetical protein JCGZ_25510 [Jatropha curcas]
c49246.graph_c0	0.96759881	0.70137662	1.02902298	9.67568444	8.76907886	8.91434439	2.023E-43	2.24965	up	PREDICTED: uncharacterized protein LOC105164785 isoform X2 [Sesamum indicum]
c49249.graph_c2	5.21867007	5.51411221	5.29044481	25.0331356	26.4838245	24.0032496	2.793E-13	1.14574	up	PREDICTED: 15 kDa selenoprotein [Sesamum indicum]
c49250.graph_c0	0.02842689	0	0	0.5991545	0.57828933	0.74178717	4.578E-10	5.01942	up	ADP-glucose pyrophosphorylase beta subunit IbAGPb1A [Ipomoea batatas]
c49255.graph_c2	1233.81734	1168.46724	1280.4424	741.024366	771.773379	786.972871	1.5E-65	-1.77034	down	PREDICTED: rop guanine nucleotide exchange factor 7-like isoform X1 [Sesamum indicum]
c49256.graph_c0	2.12491994	0.96683988	1.48942159	14.3206105	14.0076827	13.1748298	1.035E-11	2.09189	up	PREDICTED: uncharacterized protein LOC105160829 [Sesamum indicum]
c49257.graph_c2	2.6646774	2.13387653	2.22574037	19.3667217	16.6994489	17.4624109	3.152E-24	1.84116	up	PREDICTED: uncharacterized protein LOC105174437 [Sesamum indicum]
c49258.graph_c1	42.6192204	38.4618197	42.3361376	26.0057857	27.2285338	27.0958939	2.972E-64	-1.71024	down	PREDICTED: probable serine/threonine-protein kinase NAK [Sesamum indicum]
c49260.graph_c2	37004.0695	37864.8467	39206.8195	5586.08096	4115.49667	5205.14571	8.831E-13	-4.0266	down	hypothetical protein MTR_5g051050, partial [Medicago truncatula]
c49262.graph_c0	38.0841397	39.5639604	36.6981106	33.1972554	30.959641	32.7992779	1.065E-59	-1.32778	down	PREDICTED: probable LRR receptor-like serine/threonine-protein kinase At1g56140 [Nicotiana tomentosiformis]
c49266.graph_c0	35.7557528	36.7780297	36.5911811	23.6950892	23.197603	23.742444	1.919E-98	-1.71822	down	PREDICTED: C2 and GRAM domain-containing protein At1g03370 isoform X1 [Sesamum indicum]
c49268.graph_c1	1.92083326	2.16310127	3.18828127	25.6524288	21.3760258	21.6916163	1.018E-22	2.14281	up	PREDICTED: auxin-responsive protein IAA14 [Sesamum indicum]
c49275.graph_c1	3.37275195	2.5932587	3.70598648	2.13156233	2.37367448	2.89889575	1.047E-06	-1.47696	down	hypothetical protein CICLE_v10022328mg [Citrus clementina]
c49275.graph_c2	31.8384719	27.6126244	30.4642262	15.9797962	17.6710241	18.1030413	5.845E-56	-1.88699	down	hypothetical protein MIMGU_mgv1a010148mg [Erythranthe guttata]
c49277.graph_c0	7.4625693	9.28615305	8.05006675	2.56259859	2.20146654	2.4564874	9.67E-42	-2.87065	down	PREDICTED: cyclic pyranopterin monophosphate synthase, mitochondrial [Sesamum indicum]
c49281.graph_c0	15.6738367	15.3750791	16.9545544	14.6841787	14.0287713	14.6296215	1.298E-29	-1.2389	down	PREDICTED: peroxisome biogenesis protein 1 isoform X2 [Sesamum indicum]
c49284.graph_c2	63.9069131	67.2292996	56.1713899	53.8825869	53.1847018	52.28065	3.901E-38	-1.32206	down	PREDICTED: probable glycerol-3-phosphate dehydrogenase [NAD(+)] 1, cytosolic [Sesamum indicum]
c49287.graph_c0	271.677864	218.103826	265.328776	52.1278311	77.7311106	69.9264162	8.495E-39	-3.00907	down	PREDICTED: peroxygenase-like [Solanum lycopersicum]
c49287.graph_c1	178.244103	178.37497	185.343425	59.2589252	66.4133178	67.9054851	3.54E-193	-2.57608	down	PREDICTED: probable helicase DDB_G0274399 isoform X1 [Sesamum indicum]
c49288.graph_c0	6.55130786	5.84677388	5.17292998	2.40940985	3.10781598	2.57460076	1.823E-16	-2.20697	down	PREDICTED: GPI-anchored protein LORELEI-like [Pyrus x bretschneideri]

#ID	0 h-1 FPKM	0 h-2 FPKM	0 h-3 FPKM	28 h-1 FPKM	28 h-2 FPKM	28 h-3 FPKM	FDR	log2FC	regulated	nr_annotation
c49288.graph_c3	26.2166147	27.550227	23.291934	6.66777196	6.85653582	6.36654721	1.65E-135	-3.04304	down	PREDICTED: probable protein phosphatase 2C 25 [Sesamum indicum]
c49289.graph_c0	146.296335	153.475486	135.25508	132.024491	128.662425	133.428187	5.573E-52	-1.23174	down	PREDICTED: BTB/POZ domain-containing protein At5g17580 [Sesamum indicum]
c49290.graph_c0	189.868841	179.435037	181.311991	160.144069	160.649819	162.980847	2.757E-54	-1.27673	down	PREDICTED: probable acyl-activating enzyme 18, peroxisomal isoform X2 [Vitis vinifera]
c49303.graph_c0	0	0	0	0.46501141	0.41986171	0.94319314	2.916E-05	Inf	up	PREDICTED: delta-1-pyrroline-5-carboxylate synthase [Brachypodium distachyon]
c49306.graph_c0	22.6785808	22.3437894	23.1060099	4.05327723	3.50648941	4.34203477	0	-3.60712	down	unnamed protein product [Coffea canephora]
c49307.graph_c0	54.4230837	56.9109965	51.6257427	322.889592	308.708995	295.226307	2.71E-63	1.41778	up	PREDICTED: pentatricopeptide repeat-containing protein At3g21470 [Sesamum indicum]
c49321.graph_c0	19.0625171	20.768629	18.7013154	20.1776214	18.7565364	19.9495255	6.452E-34	-1.08123	down	PREDICTED: serine hydroxymethyltransferase 6-like [Sesamum indicum]
c49322.graph_c1	2.90130667	4.44900545	3.56964314	34.276827	30.3816371	29.8000828	1.161E-34	2.02069	up	-
c49328.graph_c0	115.048104	123.448797	105.787476	44.5074017	43.3335689	43.6859077	3.86E-115	-2.4775	down	PREDICTED: uncharacterized protein LOC105166619 [Sesamum indicum]
c49330.graph_c0	186.515212	187.99822	181.881244	181.267107	176.073958	182.650664	1.119E-43	-1.13323	down	PREDICTED: membrin-11 [Sesamum indicum]
c49343.graph_c0	59.796504	54.5843116	59.8105951	22.130469	23.2611229	23.2157157	2.75E-111	-2.43499	down	PREDICTED: serine/threonine-protein kinase HT1 [Sesamum indicum]
c49345.graph_c0	0	0	0	1.07329081	1.36811432	2.39556112	4.072E-06	Inf	up	-
c49348.graph_c1	0.7656602	1.44088255	1.29875284	25.8650704	21.2836521	23.3942546	2.743E-54	3.23609	up	PREDICTED: LRR receptor-like serine/threonine-protein kinase GSO2 isoform X2 [Sesamum indicum]
c49350.graph_c0	104.359473	105.154074	104.206274	83.0505922	80.4723649	83.7141591	2.684E-69	-1.43395	down	PREDICTED: autophagy-related protein 18f [Sesamum indicum]
c49353.graph_c4	1.36477884	0.95142273	0.68899083	6.34116063	4.8618546	5.3188698	1.06E-06	1.37613	up	PREDICTED: QWRF motif-containing protein 8 [Sesamum indicum]
c49353.graph_c5	101.752668	99.3385947	95.9004453	95.8591251	93.341796	94.9400464	5.045E-45	-1.1536	down	PREDICTED: nuclear poly(A) polymerase 4-like isoform X2 [Sesamum indicum]
c49353.graph_c7	1.39625006	2.70512554	2.72029422	13.0940193	12.2955786	11.2188435	2.394E-09	1.32682	up	PREDICTED: QWRF motif-containing protein 8 [Sesamum indicum]
c49354.graph_c0	6.67941997	6.86205862	9.7127032	5.62107403	7.38225865	6.98437009	0.0005298	-1.31398	down	-
c49354.graph_c1	22.9590578	23.0316851	23.2585397	17.5334504	18.6783751	18.7478101	2.099E-56	-1.42403	down	fructose-6-phosphate 2-kinase/fructose-2,6-bisphosphatase [Solanum tuberosum]
c49354.graph_c2	1.45919827	1.13606845	1.66678005	7.07024597	6.49016536	7.21157871	0.0043033	1.19222	up	PREDICTED: 6-phosphofructo-2-kinase/fructose-2,6-bisphosphatase [Sesamum indicum]
c49355.graph_c1	0	0	0	191.726728	188.899412	177.551792	0	Inf	up	PREDICTED: (RS)-norcoclaurine 6-O-methyltransferase-like [Sesamum indicum]
c49355.graph_c3	2.63241936	2.24260028	2.66302627	564.605564	571.827602	523.227074	0	6.69091	up	PREDICTED: (RS)-norcoclaurine 6-O-methyltransferase-like [Sesamum indicum]
c49360.graph_c2	2.44384408	2.96763197	3.06321255	2.62058133	2.40557489	2.60168711	3.095E-10	-1.24458	down	PREDICTED: rho GTPase-activating protein 2-like [Sesamum indicum]

#ID	0 h-1 FPKM	0 h-2 FPKM	0 h-3 FPKM	28 h-1 FPKM	28 h-2 FPKM	28 h-3 FPKM	FDR	log2FC	regulated	nr_annotation
c49362.graph_c1	4.52959179	4.64748067	4.87484025	54.772001	51.7204676	46.741818	6.442E-70	2.35488	up	-
c49364.graph_c1	7.75482441	8.62128324	9.78888017	6.08505952	5.6518598	5.53515415	4.401E-14	-1.69278	down	Cationic amino acid transporter 2 isoform 1 [Theobroma cacao]
c49366.graph_c0	23.5762998	22.6509606	27.1450093	2.24673761	2.20440484	2.55442523	1.411E-69	-4.48058	down	PREDICTED: MLO-like protein 3 [Sesamum indicum]
c49366.graph_c1	124.967387	116.459377	122.476653	670.638151	672.688243	683.665674	1.733E-69	1.38731	up	PREDICTED: 40S ribosomal protein S26-like [Musa acuminata subsp. malaccensis]
c49369.graph_c4	98.3436651	100.658719	78.001304	48.4708766	46.499948	42.4100779	2.366E-29	-2.09974	down	hypothetical protein MIMGU_mgv1a000459mg [Erythranthe guttata]
c49372.graph_c0	0	0	0	3.42532447	5.53975532	8.0165341	1.511E-09	Inf	up	small subunit ribosomal protein S6e, cytoplasmic [Guillardia theta CCMP2712]
c49375.graph_c0	2.25532291	2.05641067	2.29558751	2.23963847	2.35921415	2.33075869	2.113E-06	-1.02233	down	PREDICTED: protein-L-isoaspartate O-methyltransferase 1 isoform X1 [Sesamum indicum]
c49376.graph_c0	0.71572306	1.09573137	1.1361401	4.13086811	4.08895124	4.74402506	0.000514	1.04247	up	hypothetical protein MIMGU_mgv1a024598mg, partial [Erythranthe guttata]
c49376.graph_c1	1.55062723	1.02117102	1.31093249	6.88055181	6.77483092	6.46148527	2.798E-12	1.28374	up	PREDICTED: uncharacterized protein LOC105163785 [Sesamum indicum]
c49378.graph_c0	53.6530519	49.6193589	49.9097965	13.030451	12.5504581	13.9442099	0	-3.04375	down	PREDICTED: uncharacterized protein LOC105170029 [Sesamum indicum]
c49378.graph_c2	143.158693	145.333797	137.015717	20.5604444	21.4361863	20.2755301	0	-3.86266	down	PREDICTED: uncharacterized protein LOC105158337 [Sesamum indicum]
c49380.graph_c0	0.29697507	0.17836347	0.53427462	11.5258438	9.78313049	12.6808914	8.115E-31	3.97323	up	PREDICTED: uncharacterized protein LOC105167081 [Sesamum indicum]
c49380.graph_c2	0.38560504	0.17155163	0.36705018	8.3972857	6.3261114	7.77533127	1.888E-31	3.51421	up	PREDICTED: gamma-cadinene synthase-like [Sesamum indicum]
c49386.graph_c0	21.7069217	22.2178101	20.6893399	12.5613565	10.7003795	11.6379803	1.525E-98	-1.97833	down	hypothetical protein MIMGU_mgv1a000652mg [Erythranthe guttata]
c49386.graph_c1	529.836112	515.772176	543.915697	303.040285	311.460407	350.94991	5.693E-92	-1.80968	down	chloroplast chlorophyll a/b binding protein cab-BO3 [Bambusa oldhamii]
c49392.graph_c1	5.53459272	4.68780635	4.10317544	0.20628257	0.04656345	0	9.635E-55	-6.91675	down	-
c49392.graph_c2	11.0139353	9.73256395	9.68386617	77.1569372	79.2792818	76.3045172	3.42E-99	1.84583	up	PREDICTED: auxin response factor 6-like isoform X2 [Nicotiana tomentosiformis]
c49392.graph_c4	16.2406554	17.255807	14.5864789	6.8537118	6.85947245	6.25835534	9.97E-96	-2.35699	down	PREDICTED: uncharacterized protein LOC105175883 [Sesamum indicum]
c49395.graph_c0	1.12070299	1.41132849	1.57951685	0.76197077	1.04384411	1.37216425	2.682E-05	-1.46409	down	-
c49398.graph_c0	18.6131658	18.2039785	16.6414523	12.4407362	12.6491912	12.6102141	1.553E-67	-1.59298	down	PREDICTED: SWI/SNF complex subunit SWI3C [Sesamum indicum]
c49402.graph_c0	3.81323882	3.13340517	3.5837012	3.68146507	2.46500196	3.74661847	1.267E-05	-1.17899	down	Pollen-specific SF21 [Gossypium arboreum]
c49405.graph_c0	8.42025304	9.34609251	8.09686693	135.30151	137.53852	132.689737	1.62E-237	2.88096	up	hypothetical protein MIMGU_mgv1a009036mg [Erythranthe guttata]
c49405.graph_c1	0	0	0	3.50705729	3.18366036	3.63517068	1.817E-32	Inf	up	PREDICTED: subtilisin-like protease SBT5.3 [Sesamum indicum]
c49405.graph_c2	14.4546168	13.4286169	12.8562348	14.707745	12.8107682	13.2920801	1.629E-23	-1.08672	down	unnamed protein product [Coffea canephora]

#ID	0 h-1 FPKM	0 h-2 FPKM	0 h-3 FPKM	28 h-1 FPKM	28 h-2 FPKM	28 h-3 FPKM	FDR	log2FC	regulated	nr_annotation
c49409.graph_c1	21.5742805	20.9018763	18.0283342	20.304051	19.6975673	19.2284849	4.699E-28	-1.11916	down	PREDICTED: bifunctional riboflavin kinase/FMN phosphatase-like [Sesamum indicum]
c49414.graph_c0	34.4797943	33.5515373	35.6560621	23.8522738	24.6061599	24.951347	1.184E-83	-1.58917	down	PREDICTED: E3 ubiquitin-protein ligase RGLG2-like isoform X1 [Sesamum indicum]
c49415.graph_c0	237.305843	232.929864	236.419897	59.3825927	57.566318	61.9193954	0	-3.07236	down	-
c49417.graph_c0	15.59661	15.3923371	12.4205563	15.0874258	14.9919917	14.395655	5.191E-09	-1.05287	down	-
c49417.graph_c1	20.8426626	19.5735837	20.0397356	19.5598022	19.7931839	19.3720732	2.437E-39	-1.1322	down	PREDICTED: exocyst complex component EXO70A1 [Sesamum indicum]
c49417.graph_c4	13.0790362	14.8125735	13.3594734	10.0541779	10.1508321	9.49689857	1.065E-35	-1.56463	down	PREDICTED: uncharacterized RING finger protein C4G3.12c-like [Sesamum indicum]
c49417.graph_c6	143.411344	149.959197	132.326661	107.451258	104.052156	103.924174	6.948E-77	-1.52194	down	PREDICTED: omega-amidase,chloroplastic isoform X2 [Nicotiana sylvestris]
c49418.graph_c0	28.9705904	30.5409174	26.8952884	16.6621547	16.5038912	16.3357114	4.991E-68	-1.89315	down	PREDICTED: putative pentatricopeptide repeat-containing protein At1g13630 [Sesamum indicum]
c49421.graph_c1	81.3259596	74.4969283	72.9869367	36.7947312	37.6838506	35.7933131	4.22E-138	-2.14264	down	hypothetical protein MIMGU_mgv1a006935mg [Erythranthe guttata]
c49428.graph_c1	7.59385121	6.89103892	6.61199963	48.6666761	51.8355367	46.6774518	1.408E-60	1.71309	up	PREDICTED: magnesium-protoporphyrin IX monomethyl ester [oxidative] cyclase, chloroplastic [Sesamum indicum]
c49428.graph_c2	0.06473118	0.58316399	0.33272852	24.793373	25.9542727	23.8013999	6.911E-71	5.15117	up	PREDICTED: GATA transcription factor 12-like [Sesamum indicum]
c49430.graph_c0	0.05039946	0	0	1.09890108	2.18285021	3.07865045	3.843E-05	5.92347	up	hypothetical protein A1O3_04014 [Capronia epimyces CBS 606.96]
c49432.graph_c0	4.54681319	2.79061865	3.02803856	1.83320744	2.02545977	3.16890856	0.0001099	-1.64438	down	mitochondrial carrier, adenine nucleotidetranslocator [Galdieria sulphuraria]
c49434.graph_c0	216.622941	223.61775	201.209839	180.686781	180.294354	180.658012	2.154E-55	-1.33349	down	PREDICTED: protein misato homolog 1 isoform X1 [Sesamum indicum]
c49437.graph_c0	2.22736594	1.6827142	1.94417213	9.54502789	10.5349674	9.234241	9.513E-16	1.23413	up	FIZZY-related 3 isoform 1 [Theobroma cacao]
c49438.graph_c0	0.6765703	0.75249753	0.46369046	5.60569228	4.60756451	4.52961629	6.273E-17	1.87541	up	PREDICTED: ABC transporter B family member 4-like [Sesamum indicum]
c49438.graph_c2	2.14721053	2.17745685	1.24436789	14.1064213	14.5694717	14.2240263	4.245E-50	1.86143	up	PREDICTED: wall-associated receptor kinase-like 14 [Sesamum indicum]
c49439.graph_c0	8.04482393	8.41677542	8.79804339	41.3639835	39.8482548	39.501886	3.587E-50	1.16497	up	PREDICTED: random slug protein 5 [Sesamum indicum]
c49439.graph_c2	2.77931852	3.55490759	1.78576717	44.1031736	46.1032289	35.9878295	1.889E-28	2.87216	up	hypothetical protein EUGRSUZ_H01271 [Eucalyptus grandis]
c49446.graph_c1	4.34577073	3.12316478	4.0093756	17.5538662	16.5624485	15.6785434	6.217E-15	1.02643	up	PREDICTED: serine/threonine-protein kinase STE20 [Sesamum indicum]
c49447.graph_c0	0.39736854	0.53638202	0.61121379	0.46821443	0.52547894	0.39634174	0.0004086	-1.24891	down	PREDICTED: uncharacterized protein LOC105155261 [Sesamum indicum]
c49449.graph_c0	0.26384192	0.10564245	0.13561893	2.14769845	1.97379838	2.47228299	2.111E-07	2.62432	up	PREDICTED: protein DA1-related 2 isoform X2 [Sesamum indicum]
c49451.graph_c1	8.09365087	7.24893908	5.83896804	47.2657511	43.9810648	40.0842582	1.19E-14	1.54517	up	hypothetical protein M569_10128, partial [Genlisea aurea]
c49452.graph_c0	0.57054479	0.97905618	0.68080317	5.80536129	5.08123683	5.12383169	1.102E-08	1.75256	up	Rop guanine nucleotide exchange factor 1 [Morus notabilis]

#ID	0 h-1 FPKM	0 h-2 FPKM	0 h-3 FPKM	28 h-1 FPKM	28 h-2 FPKM	28 h-3 FPKM	FDR	log2FC	regulated	nr_annotation
c49456.graph_c0	0.68837441	0.76562635	0.88458837	5.55896172	7.52883184	5.21647747	6.949E-07	1.87351	up	PREDICTED: uncharacterized protein LOC105161573 [Sesamum indicum]
c49456.graph_c3	1.68681526	1.83896513	2.38224066	10.3296875	9.72128993	8.99351125	3.046E-15	1.20202	up	PREDICTED: pentatricopeptide repeat-containing protein At1g31920 [Sesamum indicum]
c49471.graph_c0	14.0316067	14.6214675	13.4375777	12.3631321	12.0120888	12.8661291	1.178E-40	-1.26616	down	PREDICTED: pleiotropic drug resistance protein 1-like [Sesamum indicum]
c49473.graph_c0	42.590805	43.5301905	41.7717582	42.3767573	40.0930481	41.5768752	1.327E-43	-1.13419	down	PREDICTED: transcription factor GTE4-like isoform X3 [Sesamum indicum]
c49474.graph_c0	247.894053	255.032471	226.648262	215.383563	220.832532	218.138179	8.514E-41	-1.2463	down	PREDICTED: pyruvate decarboxylase 1 [Sesamum indicum]
c49474.graph_c1	10.2082867	9.46929023	10.7936726	6.28602601	6.15321429	5.56090144	3.699E-43	-1.85148	down	PREDICTED: lipoxygenase 6, chloroplastic [Sesamum indicum]
c49474.graph_c6	11.2157525	12.4597502	12.4909945	5.53026337	5.39739215	5.32127485	2.246E-53	-2.24607	down	PREDICTED: uncharacterized protein LOC105170638 isoform X1 [Sesamum indicum]
c49479.graph_c0	0.05179035	0	0	4.29106581	7.57892906	9.61369798	1.724E-10	7.6395	up	hypothetical protein PISMIDRAFT_677145 [Pisolithus microcarpus 441]
c49487.graph_c0	231.275744	219.907485	219.599078	228.577077	225.617175	231.344485	7.164E-37	-1.05851	down	dead box ATP-dependent RNA helicase, putative [Ricinus communis]
c49488.graph_c0	2.3323775	0.94971377	1.26999891	0	0	0	8.68E-11	-Inf	down	Fructose-bisphosphate aldolase A [Rozella allomycis CSF55]
c49494.graph_c0	218.726948	233.092716	219.219013	149.903453	143.965371	150.845614	3.095E-89	-1.68374	down	hypothetical protein MIMGU_mgv1a000743mg [Erythranthe guttata]
c49495.graph_c0	85.7893061	86.2121234	78.7172924	45.0102628	44.0758	42.9158776	1.57E-133	-2.01507	down	PREDICTED: E3 ubiquitin-protein ligase RHF2A [Sesamum indicum]
c49495.graph_c1	10.9415026	10.8015391	11.2648178	4.85273329	5.14480321	5.36487777	1.764E-85	-2.19409	down	PREDICTED: BI1-like protein [Sesamum indicum]
c49495.graph_c2	3.32389025	2.580292	3.05095041	25.0699246	23.8822067	24.7212127	8.764E-46	1.95039	up	PREDICTED: transcription factor bHLH143 [Sesamum indicum]
c49495.graph_c3	1.54483092	1.34467638	1.81254551	12.7410465	11.0632052	11.3127645	1.521E-11	1.80748	up	-
c49495.graph_c4	20.628298	15.5388594	12.9863304	11.6608552	11.4858097	10.4419642	3.263E-07	-1.635	down	-
c49500.graph_c1	117.000092	116.371935	103.367516	49.7483631	52.3314486	48.2989446	2.3E-165	-2.25228	down	PREDICTED: pectinesterase-like isoform X1 [Sesamum indicum]
c49500.graph_c8	0.03320279	0.03323603	0.04266689	9.38718968	14.7508591	10.0818856	1.539E-23	7.19768	up	PREDICTED: tyrosine/DOPA decarboxylase 1-like [Sesamum indicum]
c49500.graph_c9	64.8536178	62.4836653	70.6064096	16.6650226	15.8685494	20.3235327	1.928E-74	-2.9956	down	hypothetical protein MIMGU_mgv1a019874mg [Erythranthe guttata]
c49506.graph_c0	59.4601009	59.1284323	59.440856	55.5103041	54.0225327	55.1630561	1.52E-49	-1.20281	down	PREDICTED: cullin-4 [Sesamum indicum]
c49508.graph_c0	3.61428907	3.54093146	3.09633636	1.75122964	1.5475537	1.67222127	6.435E-26	-2.13264	down	PREDICTED: uncharacterized protein LOC103341621 [Prunus mume]
c49510.graph_c0	48.9470891	48.6587011	50.5954309	16.247856	16.0691692	17.3587337	5.66E-218	-2.6676	down	PREDICTED: nuclear valosin-containing protein-like [Sesamum indicum]
c49511.graph_c0	16.1213842	16.5432193	15.4506612	119.623955	122.666554	124.211821	1.29E-114	1.83944	up	PREDICTED: L-ascorbate oxidase homolog [Sesamum indicum]
c49511.graph_c1	0.56510112	0.50910029	0.36308866	10.0213787	9.60461731	10.1211626	3.279E-23	3.28628	up	hypothetical protein MIMGU_mgv1a004264mg [Erythranthe guttata]
c49512.graph_c0	2.15633271	2.53388185	2.71073436	1.66941954	1.59961479	2.08505007	2.752E-07	-1.55893	down	PREDICTED: lipoxygenase homology domain-containing protein 1 [Sesamum indicum]
c49514.graph_c0	39.179932	37.6256715	38.8145107	7.61015632	6.86225206	6.99927897	0	-3.51949	down	ankyrin repeat BTB/POZ domain-containing protein [Populus trichocarpa]

#ID	0 h-1 FPKM	0 h-2 FPKM	0 h-3 FPKM	28 h-1 FPKM	28 h-2 FPKM	28 h-3 FPKM	FDR	log2FC	regulated	nr_annotation
c49515.graph_c0	35.55673	35.3336382	32.1862933	34.3104778	35.0069986	33.6169194	4.881E-41	-1.09141	down	PREDICTED: LOW QUALITY PROTEIN: cytokinin dehydrogenase 5 [Sesamum indicum]
c49519.graph_c0	1.73371723	2.23782134	1.87612234	14.2253655	11.9160377	13.2583059	1.11E-15	1.66172	up	PREDICTED: synaptotagmin-5 [Sesamum indicum]
c49520.graph_c0	14.0244251	14.0916445	11.2637135	72.1222867	64.4573107	62.6632927	1.354E-25	1.25098	up	-
c49521.graph_c0	81.4697907	83.8754225	77.4490231	80.9684015	78.2645688	80.1096169	1.738E-42	-1.11039	down	PREDICTED: rubisco accumulation factor 1, chloroplastic [Sesamum indicum]
c49523.graph_c2	75.5408435	80.3946368	71.7848138	44.4785102	39.1404121	42.8906008	1.29E-125	-1.93748	down	PREDICTED: pentatricopeptide repeat-containing protein At2g34400 [Sesamum indicum]
c49527.graph_c0	2.00300178	2.38331079	2.47680691	12.4976494	11.5074097	11.9910313	2.415E-12	1.29814	up	PREDICTED: fe-S cluster assembly factor HCF101, chloroplastic [Sesamum indicum]
c49528.graph_c0	156.84621	160.007235	149.925649	119.558906	110.000175	120.441529	2.347E-77	-1.50497	down	hypothetical protein MIMGU_mgv1a021345mg [Erythranthe guttata]
c49528.graph_c2	1107.05383	1085.44464	1217.30885	423.461014	474.398558	459.813344	6.999E-57	-2.42044	down	PREDICTED: LOW QUALITY PROTEIN: pre-mRNA-splicing factor 38B-like [Sesamum indicum]
c49537.graph_c0	24.4895838	17.9551482	19.4714571	94.9472474	98.1443084	97.6831316	2.121E-28	1.14331	up	-
c49537.graph_c4	94.5968071	92.4953245	92.3043667	51.6509733	46.9281132	47.822915	3.79E-135	-2.02272	down	PREDICTED: uncharacterized protein LOC105163060 [Sesamum indicum]
c49541.graph_c1	0.23905379	0.65805621	0.49918958	5.60320907	5.39252877	4.53672965	3.25E-16	2.37938	up	PREDICTED: CBL-interacting serine/threonine-protein kinase 1-like [Sesamum indicum]
c49548.graph_c1	6.92287001	7.01902714	7.48346679	6.43513993	5.61535056	5.86775236	4.582E-21	-1.34945	down	PREDICTED: 4-hydroxy-3-methylbut-2-enyl diphosphate reductase, chloroplastic-like [Sesamum indicum]
c49548.graph_c2	5.73956642	5.68147691	6.84289003	31.3324783	35.0280664	33.6040248	9.056E-36	1.35938	up	hypothetical protein MIMGU_mgv1a010472mg [Erythranthe guttata]
c49549.graph_c0	18.8673114	20.0549468	16.7216065	16.1108302	17.0894427	15.7755671	3.771E-34	-1.27326	down	PREDICTED: uncharacterized protein LOC105168432 [Sesamum indicum]
c49552.graph_c0	0	0	0	3.85566822	5.75172444	6.90794721	2.477E-14	Inf	up	predicted protein [Physcomitrella patens]
c49554.graph_c0	0.70548194	0.82388644	0.65474661	4.38677734	3.34357353	3.7887704	7.483E-05	1.30992	up	PREDICTED: uncharacterized protein At4g17910 isoform X1 [Sesamum indicum]
c49560.graph_c0	0.21581469	0.21603079	0.34182586	1.19645911	1.11981322	1.0685732	6.254E-07	1.0319	up	PREDICTED: anaphase-promoting complex subunit 1 [Sesamum indicum]
c49561.graph_c1	0.37164562	0.31690402	0.35376247	3.39137976	3.13435985	3.29794876	1.243E-18	2.14627	up	PREDICTED: probable inactive leucine-rich repeat receptor-like protein kinase At3g03770 [Sesamum indicum]
c49563.graph_c0	2.39110668	1.43610061	0.73744002	28.5006179	32.13535	28.7016423	1.722E-37	3.21253	up	-
c49567.graph_c0	2.64733068	0.75082811	1.13397491	8.62622332	13.7532292	19.075756	0.0021932	2.11489	up	predicted protein [Hordeum vulgare subsp. vulgare]
c49570.graph_c2	4.23444696	5.16589993	2.89076075	0.96174114	0.95519828	2.04040958	2.597E-08	-2.71655	down	-
c49570.graph_c3	14.2008194	12.9441583	12.5383697	7.04020257	7.25151003	7.20882188	3.831E-78	-1.97329	down	PREDICTED: DNA polymerase kappa isoform X1 [Sesamum indicum]
c49571.graph_c1	7.09935531	7.53758046	6.65921984	7.26002936	6.71011489	6.71510655	9.6E-29	-1.13164	down	PREDICTED: uncharacterized protein LOC105156089 isoform X1 [Sesamum indicum]

#ID	0 h-1 FPKM	0 h-2 FPKM	0 h-3 FPKM	28 h-1 FPKM	28 h-2 FPKM	28 h-3 FPKM	FDR	log2FC	regulated	nr_annotation
c49571.graph_c2	25.2670932	33.5799508	20.450877	167.415292	247.828861	191.729776	1.183E-08	1.84836	up	PREDICTED: cannabidiolic acid synthase-like [Sesamum indicum]
c49571.graph_c4	68.4677408	70.6447687	66.3012319	33.7762522	34.5853375	33.7939624	3.84E-145	-2.09787	down	PREDICTED: scarecrow-like protein 21 [Sesamum indicum]
c49573.graph_c0	0.83758354	0.52401391	0.134541	10.5009529	8.51949717	9.5621633	5.187E-13	3.18261	up	-
c49576.graph_c0	1.35331597	1.5882351	1.25932265	22.3849754	23.8863583	23.0538519	3.05E-102	2.95539	up	PREDICTED: DNA (cytosine-5)-methyltransferase 1B-like [Sesamum indicum]
c49584.graph_c0	11.8956093	11.6240086	11.2827666	9.46905652	11.8951934	12.1779082	1.478E-06	-1.14268	down	-
c49585.graph_c0	0	0	0	3.27883405	5.01837417	7.21080365	8.141E-10	Inf	up	60S ribosomal protein L5 [Rozella allomycis CSF55]
c49586.graph_c0	0.29568552	0.0739954	0	2.20275092	2.13440518	2.32350289	3.928E-08	3.1082	up	PREDICTED: probable polyamine oxidase 4 [Musa acuminata subsp. malaccensis]
c49587.graph_c2	7.65362216	7.45097629	7.79102712	6.43517451	7.03151947	6.74626405	3.954E-20	-1.27073	down	PREDICTED: psbP domain-containing protein 5, chloroplastic [Sesamum indicum]
c49592.graph_c3	45.4048134	48.163729	45.9809059	38.9432325	40.2614798	42.8699625	1.51E-48	-1.2832	down	PREDICTED: DNA annealing helicase and endonuclease ZRANB3 isoform X1 [Sesamum indicum]
c49593.graph_c0	1.15023478	1.08365794	0.95641585	12.6872867	11.189025	11.7972883	2.689E-16	2.39485	up	-
c49594.graph_c0	1.11344498	0.86288511	1.06157679	13.8354858	12.892838	12.9085357	8.688E-36	2.615	up	hypothetical protein MIMGU_mgv1a0042561mg, partial [Erythranthe guttata]
c49595.graph_c0	15.9516749	15.9833334	15.1423354	4.58960208	4.74038448	4.99033942	2.81E-192	-2.80652	down	PREDICTED: uncharacterized protein LOC105169231 [Sesamum indicum]
c49596.graph_c0	37.6199032	36.6981452	37.258031	32.9922071	33.1433934	32.5109115	1.264E-51	-1.26827	down	PREDICTED: probable serine/threonine-protein kinase DDB_G0282963 isoform X2 [Sesamum indicum]
c49599.graph_c0	13.7381142	5.44885449	8.32736824	0	0	0	3.504E-14	-Inf	down	--
c49600.graph_c2	96.4460323	99.0295844	86.5096975	68.1601754	65.5691105	71.1337099	8.714E-83	-1.54968	down	PREDICTED: transcription initiation factor TFIID subunit 15b [Sesamum indicum]
c49602.graph_c0	0	0	0	5.16801612	4.03434815	5.40310678	3.923E-19	Inf	up	sporamin A [Ipomoea trifida]
c49603.graph_c0	730.120419	773.602999	730.564182	156.77878	163.043159	164.727888	4.14E-192	-3.29538	down	PREDICTED: GRIP and coiled-coil domain-containing protein 2-like [Sesamum indicum]
c49607.graph_c1	25.1913399	27.4172837	22.9153595	25.2552142	24.2057411	23.2881814	1.618E-28	-1.14333	down	PREDICTED: uncharacterized protein LOC105164316 isoform X1 [Sesamum indicum]
c49610.graph_c2	112.899898	116.166523	105.436952	99.6670099	91.6932864	89.6705526	1.379E-59	-1.34109	down	PREDICTED: alpha,alpha-trehalose-phosphate synthase [UDP-forming] 1-like [Sesamum indicum]
c49611.graph_c0	38.1760675	36.995986	33.0701077	38.6362378	35.5859705	35.9779877	4.643E-41	-1.06296	down	PREDICTED: nipped-B-like protein isoform X2 [Sesamum indicum]
c49616.graph_c0	23.0603137	19.6015507	20.0315271	519.610012	479.726722	491.805042	0	3.48286	up	hypothetical protein MIMGU_mgv1a012029mg [Erythranthe guttata]
c49618.graph_c3	1.60934337	1.93314586	2.06806995	0.04678646	0.12673136	0.07635474	2.586E-26	-5.58367	down	-
c49624.graph_c0	0	0	0	8.59062201	9.37755305	17.0284887	2.61E-14	Inf	up	-

#ID	0 h-1 FPKM	0 h-2 FPKM	0 h-3 FPKM	28 h-1 FPKM	28 h-2 FPKM	28 h-3 FPKM	FDR	log2FC	regulated	nr_annotation
c49634.graph_c0	5.48318464	5.36449705	4.78242716	22.8289416	22.6638643	21.7770776	2.115E-19	1.01662	up	PREDICTED: uncharacterized protein At1g04910 [Sesamum indicum]
c49635.graph_c0	7.04059481	7.0476449	7.26979297	4.33674903	4.63377784	4.72649893	2.013E-69	-1.7317	down	PREDICTED: uncharacterized protein LOC105155337 [Sesamum indicum]
c49636.graph_c0	19.5286373	16.1401193	16.4273663	2.61455546	1.98130048	2.97159929	9.448E-96	-3.87052	down	-
c49641.graph_c0	26.1821501	28.1659934	21.1416593	13.7570039	11.938194	13.8385157	1.006E-22	-2.02034	down	PREDICTED: LOW QUALITY PROTEIN: auxin transport protein BIG [Sesamum indicum]
c49643.graph_c2	9.02041435	7.85418558	9.53086253	81.7520943	79.7902917	80.8048927	1.12E-123	2.10647	up	PREDICTED: phosphoglucomutase, cytoplasmic [Sesamum indicum]
c49647.graph_c0	5.62087615	3.34073711	3.72438433	0.19149471	0.28816961	0.26043019	1.229E-16	-5.18547	down	PREDICTED: uncharacterized protein LOC105167219 [Sesamum indicum]
c49648.graph_c1	157.417783	145.413425	153.844003	1524.52669	1481.09805	1496.59966	5.32E-131	2.21094	up	PREDICTED: uncharacterized protein LOC105179524 [Sesamum indicum]
c49659.graph_c0	48.105363	49.030048	48.4551956	18.7342798	17.7413106	19.9932143	7.13E-145	-2.45649	down	hypothetical protein MIMGU_mgv1a000745mg [Erythranthe guttata]
c49661.graph_c0	0	0	0	0.80357958	0.92021866	1.45536605	2.046E-08	Inf	up	transferrin-like protein IDI-100 [Dunaliella salina]
c49662.graph_c0	0	0	0	1.37287283	1.9532704	3.11464287	2.909E-10	Inf	up	predicted protein [Hordeum vulgare subsp. vulgare]
c49663.graph_c0	0.15239218	0.15254478	0.32638331	3.35965204	4.53350888	3.34396481	1.13E-11	3.05112	up	PREDICTED: uncharacterized protein LOC105174845 isoform X2 [Sesamum indicum]
c49668.graph_c0	4.20392411	4.96343975	5.84084981	3.52544748	3.24209572	4.74128769	3.268E-06	-1.47572	down	NADH-ubiquinone oxidoreductase chain [Medicago truncatula]
c49671.graph_c1	0.14142313	0.18875299	0.24231243	4.24846797	3.77409779	4.1376923	1.017E-13	3.31216	up	PREDICTED: systemin receptor SR160-like [Sesamum indicum]
c49671.graph_c2	37.6918059	37.214366	33.5741401	20.353111	20.641786	20.1270671	1.6E-107	-1.91683	down	PREDICTED: uncharacterized protein LOC104231501 [Nicotiana sylvestris]
c49671.graph_c3	35.6883239	40.5239555	29.5534999	202.92071	202.038269	185.343123	3.415E-47	1.39235	up	PREDICTED: receptor-like protein kinase BRI1-like 3 [Sesamum indicum]
c49671.graph_c5	4.50009095	5.35161536	3.3609407	0.69885474	0.8329204	0.50182859	2.92E-19	-3.78837	down	-
c49674.graph_c2	1.3440458	1.02064195	1.78670904	47.1243378	38.3821727	51.6189239	3.082E-36	3.95135	up	PREDICTED: NAC domain-containing protein 100-like [Sesamum indicum]
c49679.graph_c2	56.1167668	56.1167863	54.5168739	22.4319385	20.769492	20.434231	6.13E-156	-2.48014	down	hypothetical protein MIMGU_mgv1a010138mg [Erythranthe guttata]
c49680.graph_c0	70.0135872	73.9470045	61.7497552	61.749994	55.9684835	61.5094897	9.187E-33	-1.28728	down	OSJNBa0006B20.7 [Oryza sativa Japonica Group]
c49682.graph_c0	52.9688171	51.8598523	52.818245	50.2466469	52.4779233	53.0104319	8.497E-39	-1.10806	down	hypothetical protein MIMGU_mgv1a027053mg [Erythranthe guttata]
c49684.graph_c2	4.49946205	3.54858052	2.45296413	3.17108663	0.98422291	3.55792359	0.0013469	-1.52509	down	-
c49686.graph_c0	7.96232432	7.16642619	7.24576751	6.80527971	7.51247222	7.6810473	1.017E-24	-1.11376	down	PREDICTED: DNA repair protein REV1 [Sesamum indicum]
c49686.graph_c1	2.64320037	2.96176918	2.71222412	15.1677943	15.1836935	14.8685331	1.597E-27	1.35215	up	PREDICTED: probable mitochondrial import inner membrane translocase subunit TIM21 [Sesamum indicum]
c49687.graph_c2	60.988951	83.4833389	68.2567925	0.50420752	0.51463283	0.44720568	3.897E-89	-8.27256	down	PREDICTED: uncharacterized protein LOC103716664 [Phoenix dactylifera]

#ID	0 h-1 FPKM	0 h-2 FPKM	0 h-3 FPKM	28 h-1 FPKM	28 h-2 FPKM	28 h-3 FPKM	FDR	log2FC	regulated	nr_annotation
c49689.graph_c1	36.0723116	38.3812911	34.6006034	28.1631954	28.563388	26.6014831	2.152E-72	-1.47831	down	PREDICTED: TBC1 domain family member 5 homolog A-like [Sesamum indicum]
c49693.graph_c0	3.33164391	3.50325886	3.41717988	19.6712573	19.4762544	18.8431654	2.592E-42	1.40899	up	PREDICTED: beta-galactosidase 8-like [Sesamum indicum]
c49701.graph_c0	2.64981634	3.46861427	2.92490983	17.0118627	15.9174507	14.9896466	2.32E-17	1.31432	up	hypothetical protein MIMGU_mgv1a007501mg [Erythranthe guttata]
c49702.graph_c0	335.328193	327.490437	321.635988	203.147167	196.258846	198.598085	3.064E-93	-1.80923	down	hypothetical protein MIMGU_mgv1a004852mg [Erythranthe guttata]
c49703.graph_c0	2.4340113	2.90390676	2.50951467	0.87422766	0.63147649	0.74693264	2.275E-54	-2.89076	down	PREDICTED: stromal 70 kDa heat shock-related protein, chloroplastic-like [Sesamum indicum]
c49707.graph_c0	250.126673	228.811187	263.601089	265.883548	252.908002	262.1603	8.095E-17	-1.01867	down	PREDICTED: uncharacterized protein LOC105178836 [Sesamum indicum]
c49707.graph_c1	18.2925959	17.133783	15.2233931	13.3266079	12.6900446	13.5090567	3.257E-36	-1.44585	down	PREDICTED: non-structural maintenance of chromosomes element 1 homolog [Sesamum indicum]
c49708.graph_c0	0	0	0	266.103206	225.323725	238.680366	3.32E-172	Inf	up	-
c49712.graph_c0	18.1389785	15.6828983	15.8816023	78.5470712	75.2627208	73.0019455	1.017E-34	1.10071	up	PREDICTED: uncharacterized protein LOC103874877 [Brassica rapa]
c49716.graph_c0	0.35524791	0.35560364	0.15216921	8.26214837	8.1593152	6.95252828	3.953E-20	3.67754	up	-
c49716.graph_c1	1.92697895	2.36729684	3.03902703	1.27319745	1.26453572	0.5714053	3.247E-07	-2.33728	down	NADH dehydrogenase subunit 5 [Arabidopsis thaliana]
c49722.graph_c0	3.66879225	4.42579235	4.31159088	3.28179331	2.77795468	2.77089976	1.213E-18	-1.58282	down	hypothetical protein VITISV_035665 [Vitis vinifera]
c49726.graph_c0	0.02036919	0.04077918	0.05235044	0.53295166	0.66834081	0.38656374	4.412E-07	2.70391	up	unnamed protein product [Coffea canephora]
c49738.graph_c0	0	0	0	1.01603004	0.982907	1.2139986	6.947E-11	Inf	up	F-type H+-transporting ATPase subunit gamma [Galdieria sulphuraria]
c49740.graph_c0	375.689845	356.186915	392.598077	231.021034	252.948096	247.741283	2.381E-55	-1.71118	down	putative galactinol synthase family protein [Populus trichocarpa]
c49740.graph_c1	0.70167913	1.05357264	1.40888354	5.13163703	5.35285715	4.47346778	6.678E-05	1.14173	up	PREDICTED: RPM1-interacting protein 4-like [Sesamum indicum]
c49740.graph_c2	506.353189	494.623171	508.834806	58.0937301	59.9058168	59.2442868	0	-4.18125	down	galactinol synthase [Boea hygrometrica]
c49746.graph_c0	1.10415771	1.47368448	0.15765404	4.9933051	4.58899502	5.31139681	0.006237	1.37003	up	-
c49750.graph_c0	9.78968098	8.16623655	8.38675176	7.11508371	7.37599474	6.02088169	1.551E-06	-1.45079	down	-
c49751.graph_c1	34.6220885	38.2947594	36.2255521	20.7317813	20.6174141	19.6537405	1.661E-87	-1.93034	down	PREDICTED: polycomb group protein EMBRYONIC FLOWER 2 isoform X5 [Sesamum indicum]
c49754.graph_c1	3.08556282	3.12259378	3.05005415	15.0202854	15.0638436	14.3779288	2.321E-24	1.17319	up	PREDICTED: putative pentatricopeptide repeat-containing protein At1g74580 [Sesamum indicum]
c49759.graph_c0	3.77118598	4.70019809	2.89817177	16.6333567	17.2747552	15.2171926	3.19E-09	1.02401	up	PREDICTED: methyltransferase-like protein 16 homolog isoform X1 [Sesamum indicum]
c49761.graph_c1	73.5638926	70.1463179	70.1180036	74.8788213	72.577558	71.8781462	1.297E-37	-1.05346	down	50S ribosomal protein L2 [Medicago truncatula]
c49762.graph_c2	37.5980002	36.279015	36.8541706	17.1581922	15.6213408	15.6343883	4.27E-159	-2.28409	down	PREDICTED: autophagy-related protein 18a-like [Sesamum indicum]
c49770.graph_c0	0	0	0	12.6216705	17.3733678	25.6638752	1.029E-17	Inf	up	Elongation factor 1-alpha [Auxenochlorella protothecoides]
c49775.graph_c0	451.031763	419.995523	492.212876	365.386363	306.362312	376.237132	3.34E-19	-1.47068	down	-

#ID	0 h-1 FPKM	0 h-2 FPKM	0 h-3 FPKM	28 h-1 FPKM	28 h-2 FPKM	28 h-3 FPKM	FDR	log2FC	regulated	nr_annotation
c49777.graph_c1	0.77990659	0	0.66814057	5.29042843	6.31214826	6.83516513	1.591E-09	2.57892	up	-
c49784.graph_c1	46.7565191	48.4307929	47.2843138	29.3101325	28.8801808	29.5580085	2.55E-106	-1.78974	down	PREDICTED: BTB/POZ domain-containing protein POB1-like isoform X1 [Sesamum indicum]
c49785.graph_c0	863.708163	889.458883	721.286403	371.212294	343.999938	346.894949	1.506E-52	-2.3084	down	-
c49787.graph_c1	100.509869	97.815778	93.5030827	61.2758176	57.0906079	56.1944228	7.34E-113	-1.83124	down	PREDICTED: uncharacterized protein LOC105163549 [Sesamum indicum]
c49790.graph_c0	5.88601469	6.66791612	5.64514914	32.0948456	28.9974782	27.6194876	1.121E-25	1.19524	up	PREDICTED: UDP-glycosyltransferase 76F1-like [Sesamum indicum]
c49795.graph_c1	35.7040434	37.0489456	35.9934222	12.1509531	12.4730918	14.1680913	2.24E-195	-2.57701	down	PREDICTED: putative receptor-like protein kinase At5g39000 [Sesamum indicum]
c49798.graph_c2	16.1605151	17.7541267	14.7744625	13.6386527	12.282768	12.7502245	3.594E-34	-1.42142	down	PREDICTED: leukocyte receptor cluster member 8 homolog isoform X1 [Sesamum indicum]
c49809.graph_c0	3.83310858	2.33046664	2.86887083	0	0	0	7.958E-55	-Inf	down	--
c49812.graph_c0	0	0	0	1.16255272	1.3901116	1.41013092	2.359E-14	Inf	up	Short-chain dehydrogenase/reductase SDR [Macrophomina phaseolina MS6]
c49818.graph_c0	75.8049888	74.9820914	77.9369225	65.041721	60.6728199	65.6278437	1.213E-61	-1.34814	down	PREDICTED: uncharacterized protein LOC105158481 isoform X1 [Sesamum indicum]
c49823.graph_c0	10.8938452	11.1220586	9.82721226	5.30707616	5.16736509	4.81625251	3.37E-127	-2.14784	down	PREDICTED: uncharacterized protein LOC105179488 isoform X2 [Sesamum indicum]
c49823.graph_c2	2.6870455	2.21654184	2.68563544	11.8079885	12.0166444	12.4092218	2.613E-15	1.16441	up	PREDICTED: transmembrane protein 87B [Sesamum indicum]
c49825.graph_c0	1.51217241	0.86496378	0.83280061	9.00164614	8.50567236	7.64420514	1.486E-08	1.88563	up	PREDICTED: protein CHROMATIN REMODELING 4 [Sesamum indicum]
c49837.graph_c0	20.6940312	22.6322339	19.4247506	5.30197718	5.8292973	5.50361364	6.29E-134	-3.00503	down	PREDICTED: leucine-rich repeat receptor protein kinase EXS [Sesamum indicum]
c49840.graph_c0	0	0	0	3.84158186	4.99476706	7.04680431	4.417E-14	Inf	up	predicted protein [Hordeum vulgare subsp. vulgare]
c49842.graph_c0	2.06303875	1.6826778	1.27644904	12.9670738	12.4100335	11.0115177	4.298E-18	1.77157	up	-
c49847.graph_c1	13.4277615	12.5970736	12.170338	12.1636651	12.6215328	12.4260496	1.106E-23	-1.12709	down	hypothetical protein JCGZ_09714 [Jatropha curcas]
c49857.graph_c2	1.87316663	3.303646	2.98021073	1.4262356	0.64387851	1.11089676	2.208E-06	-2.45199	down	-
c49858.graph_c0	0.04385115	0	0	2.03972892	2.96396014	3.47183884	1.216E-15	6.53701	up	protein of unknown function [Taphrina deformans PYCC 5710]
c49861.graph_c1	25.3056211	29.4924759	24.1567672	19.7932733	25.3838135	19.1526904	3.805E-17	-1.38604	down	hypothetical protein POPTR_0001s24240g [Populus trichocarpa]
c49861.graph_c2	6.56471608	6.07534326	7.16257365	27.5829247	29.3427903	28.1636847	1.112E-23	1.0112	up	PREDICTED: putative late blight resistance protein homolog R1A-10 [Sesamum indicum]
c49862.graph_c0	1.96765452	2.70823414	3.0552877	1.13214664	0.96842109	1.60453366	1.882E-07	-2.15425	down	hypothetical protein PHAVU_001G023000g [Phaseolus vulgaris]
c49865.graph_c0	6.60425595	9.71854263	6.7458626	1.92817908	1.77800673	1.60685452	4.274E-14	-3.20852	down	PREDICTED: monosaccharide-sensing protein 2-like [Sesamum indicum]

#ID	0 h-1 FPKM	0 h-2 FPKM	0 h-3 FPKM	28 h-1 FPKM	28 h-2 FPKM	28 h-3 FPKM	FDR	log2FC	regulated	nr_annotation
c49865.graph_c1	4.24873402	4.04552563	3.8618037	83.6007438	92.2804418	74.6705333	2.027E-61	3.27508	up	PREDICTED: uncharacterized protein LOC105161592 [Sesamum indicum]
c49872.graph_c2	145.743505	148.195115	144.080977	118.211066	112.70649	111.419484	2.073E-70	-1.44605	down	PREDICTED: putative RNA polymerase II subunit B1 CTD phosphatase RPAP2 homolog isoform X3 [Sesamum indicum]
c49874.graph_c0	0.28889397	0.49574272	0.21213725	3.50943962	3.1957778	2.64339145	1.868E-07	2.1428	up	hypothetical protein MIMGU_mgv11b019086mg [Erythranthe guttata]
c49876.graph_c0	12.4322794	11.0334706	11.8172777	60.7348689	62.2813791	58.2814167	3.467E-44	1.27083	up	PREDICTED: serine/threonine-protein kinase HT1 [Sesamum indicum]
c49876.graph_c4	4.31846532	5.22337078	6.01184882	3.13863571	3.66044612	3.30808893	5.928E-06	-1.71655	down	-
c49876.graph_c7	0	0	0	4.69398128	3.83776279	3.136583	1.633E-20	Inf	up	PREDICTED: squalene monooxygenase-like [Sesamum indicum]
c49876.graph_c8	0.32663153	0.1089862	0.34977871	4.47091873	1.92910003	2.16311171	0.0092253	2.35152	up	PREDICTED: uncharacterized protein LOC105160027 [Sesamum indicum]
c49876.graph_c9	5.97661245	8.10799347	6.26541295	40.579933	43.4001897	44.259347	1.334E-25	1.56574	up	PREDICTED: uncharacterized protein LOC105160027 [Sesamum indicum]
c49878.graph_c0	0	0	0	0.92579407	1.1507267	2.18783389	2.848E-07	Inf	up	--
c49879.graph_c0	10.3102536	9.60881382	9.9531297	3.21147125	3.49958656	3.28319817	3.287E-96	-2.67009	down	PREDICTED: protein RMD5 homolog A-like isoform X1 [Sesamum indicum]
c49879.graph_c1	53.227144	45.1155428	49.1187359	48.6810169	44.5795232	44.7725866	6.823E-27	-1.18535	down	-
c49880.graph_c0	0.68619741	0.75335723	0.56889732	0.19305458	0.05810339	0.18378612	4.199E-16	-3.29232	down	PREDICTED: probable sulfate transporter 3.4 [Eucalyptus grandis]
c49880.graph_c1	7.40400713	6.72698909	7.30528419	6.10530759	6.70476309	6.4648597	5.868E-26	-1.24401	down	hypothetical protein MIMGU_mgv1a013752mg [Erythranthe guttata]
c49886.graph_c0	18.3498417	16.2323772	24.67703	0	0	0	4.11E-27	-Inf	down	-
c49891.graph_c1	155.030149	154.995171	162.306206	34.5736217	32.9833319	35.1990014	2.33E-244	-3.29151	down	PREDICTED: zinc finger protein AZF2-like [Sesamum indicum]
c49906.graph_c0	0.12087279	0.12099383	0	5.09527955	7.37675952	5.16129083	1.692E-13	5.11639	up	-
c49906.graph_c2	4.29398683	5.73104881	3.88299698	29.5902329	27.7608469	27.4465158	6.145E-09	1.51951	up	PREDICTED: uncharacterized protein LOC105174944 [Sesamum indicum]
c49906.graph_c3	25.4387981	24.9802396	23.9635605	21.5753271	23.5366351	22.5053469	4.131E-47	-1.22747	down	PREDICTED: receptor-like protein kinase FERONIA [Sesamum indicum]
c49907.graph_c0	0.20853313	0.24353227	0.17864889	3.66271638	3.42112705	2.94752256	1.275E-12	2.90245	up	hypothetical protein MIMGU_mgv1a022633mg, partial [Erythranthe guttata]
c49909.graph_c0	30.2317121	30.0268748	27.2071981	134.881717	135.172926	123.414731	2.685E-40	1.07985	up	PREDICTED: putative late blight resistance protein homolog R1A-10 [Sesamum indicum]
c49911.graph_c1	6.96848646	6.68482	5.96985399	2.32130057	2.00064783	2.92734175	1.095E-13	-2.52363	down	-
c49912.graph_c2	34.5771473	29.1693685	33.6465087	176.190601	182.994643	173.638519	6.941E-63	1.36091	up	PREDICTED: translocon-associated protein subunit alpha-like [Sesamum indicum]
c49918.graph_c0	45.5957752	46.4237029	47.8573665	18.4104148	19.9120863	20.5364163	2.25E-158	-2.3397	down	PREDICTED: uncharacterized protein At1g04910-like [Sesamum indicum]
c49920.graph_c0	0.03615507	0.4704866	0.0464607	11.8510617	24.2479076	12.3720693	2.867E-08	5.36672	up	hypothetical protein MIMGU_mgv1a020088mg [Erythranthe guttata]

#ID	0 h-1 FPKM	0 h-2 FPKM	0 h-3 FPKM	28 h-1 FPKM	28 h-2 FPKM	28 h-3 FPKM	FDR	log2FC	regulated	nr_annotation
c49921.graph_c0	4.93395916	4.10583234	5.19448957	24.6265621	22.3525054	22.4218716	4.75E-13	1.19365	up	PREDICTED: transcription factor GTE1-like isoform X2 [Sesamum indicum]
c49926.graph_c0	0	0	0	7.845358	10.2739566	20.7201617	1.693E-07	Inf	up	cysteine protease 1 [Brachiaria hybrid cultivar]
c49930.graph_c0	0	0	0	0.63099372	0.71216021	1.12631252	9.005E-08	Inf	up	hypothetical protein CHLNCDRAFT_58466 [Chlorella variabilis]
c49931.graph_c2	0.40644173	0.40684872	0.52229371	5.61259061	6.84576347	5.78504603	2.735E-11	2.67727	up	PREDICTED: ethylene-responsive transcription factor CRF2-like [Sesamum indicum]
c49931.graph_c4	0.18640286	0.06219651	0.19961255	4.17719288	4.17935553	4.27451257	1.459E-21	3.72258	up	PREDICTED: uncharacterized protein LOC105177468 [Sesamum indicum]
c49932.graph_c0	3.60354619	1.69085372	2.46005912	56.6366665	47.5904829	55.698494	6.024E-49	3.2799	up	Cell wall-associated hydrolase, partial [Medicago truncatula]
c49936.graph_c0	326.525351	327.548735	345.453129	351.01978	357.601457	353.73864	1.812E-31	-1.00337	down	PREDICTED: pentatricopeptide repeat-containing protein At5g52850, chloroplastic [Sesamum indicum]
c49936.graph_c1	138.427737	146.948886	155.535118	7.31832452	7.94266207	6.67542792	5.09E-194	-5.42173	down	PREDICTED: heat shock protein 83-like [Sesamum indicum]
c49944.graph_c0	0	0	0	0.84998403	0.95931986	1.38716006	9.311E-10	Inf	up	predicted protein [Hordeum vulgare subsp. vulgare]
c49945.graph_c0	71.3102584	73.1982641	68.041477	70.5004062	69.6681799	70.8756301	6.709E-42	-1.10005	down	PREDICTED: BEACH domain-containing protein lvsA isoform X2 [Sesamum indicum]
c49949.graph_c0	0	0	0	2.1697509	3.74184592	3.11607827	2.813E-16	Inf	up	ATP synthase subunit alpha [Taphrina deformans PYCC 5710]
c49950.graph_c0	597.243677	597.665692	607.296269	597.034466	584.804159	549.508372	4.792E-30	-1.14897	down	PREDICTED: phosphoenolpyruvate carboxykinase [ATP]-like [Fragaria vesca subsp. vesca]
c49955.graph_c0	2.30265162	1.21534116	1.56019921	0.12171301	0.10989544	0.07448762	8.865E-13	-5.14067	down	predicted protein [Hordeum vulgare subsp. vulgare]
c49963.graph_c0	2.83546742	2.2924785	2.24226948	0.63409273	1.07348679	0.25870725	8.864E-13	-2.99918	down	-
c49968.graph_c0	26.0905411	22.190632	21.8037525	112.473385	110.281436	106.542647	9.217E-30	1.14344	up	PREDICTED: uncharacterized protein LOC105161736 [Sesamum indicum]
c49974.graph_c0	18.2763036	11.0260411	12.2568852	0.04472428	0.08076367	0.1459786	1.897E-31	-8.34047	down	predicted protein [Hordeum vulgare subsp. vulgare]
c49975.graph_c1	48.5475955	48.8760332	50.7706082	318.437423	327.347039	327.869967	2.589E-93	1.62484	up	PREDICTED: 60S ribosomal protein L3-like [Sesamum indicum]
c49983.graph_c0	4.34998247	5.37888851	3.78140274	25.1063644	23.2983791	22.1179278	3.05E-13	1.29549	up	PREDICTED: uncharacterized protein LOC104590989 [Nelumbo nucifera]
c50004.graph_c1	19.910867	20.6706954	17.7896681	14.885203	13.773415	12.7763455	3.569E-66	-1.58385	down	hypothetical protein MIMGU_mgv1a002674mg [Erythranthe guttata]
c50010.graph_c0	49.3063131	50.1481665	45.8621427	289.101009	264.169048	256.468902	1.401E-60	1.38844	up	PREDICTED: putative GTP diphosphokinase RSH1, chloroplastic [Sesamum indicum]
c50014.graph_c2	27.2344229	32.187331	23.2898031	24.1137612	18.6392766	17.4807085	4.801E-11	-1.5463	down	PREDICTED: uncharacterized protein LOC104210641 [Nicotiana sylvestris]
c50017.graph_c2	218.172584	227.042797	205.305941	47.9697207	43.3121602	56.073758	7.79E-219	-3.23082	down	hypothetical protein MIMGU_mgv1a011129mg [Erythranthe guttata]
c50017.graph_c3	111.650546	107.283219	107.866323	86.2798819	98.8111191	86.8375987	4.46E-62	-1.35581	down	PREDICTED: bifunctional epoxide hydrolase 2 [Sesamum indicum]
c50018.graph_c0	327.876509	340.304545	331.371981	58.2020006	55.3272262	60.0375082	0	-3.61606	down	PREDICTED: uncharacterized protein LOC103433973 [Malus domestica]

#ID	0 h-1 FPKM	0 h-2 FPKM	0 h-3 FPKM	28 h-1 FPKM	28 h-2 FPKM	28 h-3 FPKM	FDR	log2FC	regulated	nr_annotation
c50018.graph_c5	236.112247	241.150458	235.173646	163.775877	161.758184	161.439807	8.773E-71	-1.63938	down	PREDICTED: UBP1-associated protein 2A-like [Sesamum indicum]
c50021.graph_c0	17.2431989	16.592825	16.5115092	14.5140366	13.1434335	13.2510402	5.164E-53	-1.38971	down	PREDICTED: pentatricopeptide repeat-containing protein At3g53170-like isoform X1 [Sesamum indicum]
c50022.graph_c0	5.43715381	5.11274386	4.41095828	22.4527958	23.624534	22.5392679	5.974E-21	1.10923	up	PREDICTED: xyloglucan galactosyltransferase KATAMARI1 homolog [Sesamum indicum]
c50023.graph_c0	3.18766239	3.56080848	3.14641211	57.6843312	55.3273954	46.6315735	1.047E-52	2.92113	up	hypothetical protein MIMGU_mgv1a000846mg [Erythranthe guttata]
c50024.graph_c0	5.75482065	5.70821429	4.43710343	2.31942545	2.78085444	2.51316738	1.511E-20	-2.1499	down	hypothetical protein MIMGU_mgv1a022598mg, partial [Erythranthe guttata]
c50024.graph_c1	0.22769295	0.15954467	0.14629723	1.80379661	2.51022705	2.61968221	3.026E-11	2.61511	up	hypothetical protein CICLE_v10007524mg [Citrus clementina]
c50026.graph_c0	0	0	0	0.67417384	0.66958734	1.10024069	8.951E-09	Inf	up	PREDICTED: uncharacterized protein LOC100820133 [Glycine max]
c50030.graph_c0	1.84634767	1.97259435	1.27756992	12.6191607	11.9181791	12.3818276	5.587E-37	1.77104	up	PREDICTED: uncharacterized protein LOC105168379 [Sesamum indicum]
c50031.graph_c0	183.628845	188.776871	193.922113	135.110956	135.162	135.124964	2.28E-81	-1.5735	down	PREDICTED: putative ATP-dependent RNA helicase DHX33 [Sesamum indicum]
c50036.graph_c0	0.3007526	0.07526344	0.09661975	1.3388347	1.01148021	1.36002386	0.0001024	1.89634	up	hypothetical protein VITISV_027174 [Vitis vinifera]
c50039.graph_c2	0.18258516	0.18276799	0.11731458	36.493006	38.7009943	34.9214668	2.852E-84	6.74826	up	hypothetical protein MIMGU_mgv1a001711mg [Erythranthe guttata]
c50042.graph_c1	51.2449039	51.0799607	50.3049475	43.5094906	41.1847339	41.6661931	1.504E-63	-1.36283	down	PREDICTED: uncharacterized calcium-binding protein At1g02270 [Sesamum indicum]
c50042.graph_c2	0.61786393	0.49877631	0.43540817	0.08691483	0.02615865	0.03546089	1.459E-21	-4.47274	down	PREDICTED: uncharacterized protein LOC105170683 [Sesamum indicum]
c50043.graph_c0	4.21949852	3.25115575	3.60292426	3.93426253	3.46118634	3.73714884	4.112E-09	-1.08125	down	hypothetical protein MIMGU_mgv1a025306mg, partial [Erythranthe guttata]
c50044.graph_c0	12.9025087	11.6519628	12.254956	60.2400454	62.6740235	67.6198445	2.23E-16	1.28219	up	Gag-protease-integrase-RT-RNaseH polyprotein [Glycine max]
c50046.graph_c1	43.0975468	43.2489602	39.8514528	35.9998902	34.1191063	33.1437769	6.459E-63	-1.37909	down	hypothetical protein VITISV_006175 [Vitis vinifera]
c50050.graph_c0	3.75610476	4.73464599	4.24574644	28.2596787	28.1404571	25.1635007	6.995E-36	1.58701	up	hypothetical protein MIMGU_mgv1a001814mg [Erythranthe guttata]
c50051.graph_c0	0	0	0	2.28765497	3.06935079	3.73341555	1.165E-17	Inf	up	hypothetical protein GUITHDRAFT_64866 [Guillardia theta CCMP2712]
c50052.graph_c4	1.27677951	1.38112721	0.89974541	6.06164802	5.81094549	5.94771931	5.849E-10	1.23855	up	hypothetical protein MIMGU_mgv1a002839mg [Erythranthe guttata]
c50057.graph_c0	4.76717722	4.65190174	5.50955748	1.35103729	1.45596194	1.61809066	1.711E-39	-2.84628	down	PREDICTED: transcription factor bHLH79-like [Sesamum indicum]
c50057.graph_c2	3.32285308	3.46738619	3.40391332	3.67950397	3.29139032	3.69960739	1.678E-12	-1.02447	down	PREDICTED: LOW QUALITY PROTEIN: probable galacturonosyltransferase 14 [Sesamum indicum]
c50057.graph_c3	372.787274	393.753475	357.477558	251.423811	250.470978	248.152746	2.03E-61	-1.6735	down	hypothetical protein MIMGU_mgv1a006830mg [Erythranthe guttata]
c50059.graph_c0	9.97944634	4.02014019	7.037552	42.8104144	35.0599462	45.6871	1.258E-06	1.46769	up	hypothetical protein AMTR_s00028p00129850 [Amborella trichopoda]
c50061.graph_c0	13.5722587	14.8570398	13.3611495	241.702708	234.693648	225.517099	1.64E-210	2.97974	up	-

#ID	0 h-1 FPKM	0 h-2 FPKM	0 h-3 FPKM	28 h-1 FPKM	28 h-2 FPKM	28 h-3 FPKM	FDR	log2FC	regulated	nr_annotation
c50063.graph_c0	14.6387507	14.3905678	12.6533907	12.1183738	15.1202997	15.3388451	3.951E-13	-1.05765	down	-
c50066.graph_c0	29.1815625	31.9101297	28.5268193	15.9942394	16.4637124	15.9355627	1.657E-83	-1.97908	down	PREDICTED: flowering time control protein FPA [Vitis vinifera]
c50067.graph_c0	1.6672662	1.68614124	1.63448688	1.13680527	1.43248823	1.2232402	4.491E-11	-1.48597	down	PREDICTED: uncharacterized protein LOC105173219 [Sesamum indicum]
c50071.graph_c0	28.4115149	27.669285	27.0506425	25.1531438	24.3128952	23.9548338	7.698E-53	-1.2693	down	PREDICTED: uncharacterized protein LOC105171564 [Sesamum indicum]
c50075.graph_c0	20.6042023	21.2753393	23.136862	9.02946205	7.87681815	7.93473401	4.468E-60	-2.48028	down	PREDICTED: uncharacterized protein LOC104218703 [Nicotiana sylvestris]
c50080.graph_c0	0	0	0	2.2467483	3.003893	5.18267799	4.768E-07	Inf	up	carbonyl reductase (NADPH) [Galdieria sulphuraria]
c50085.graph_c0	5.84936635	5.74262315	6.33134672	69.7701396	69.8018723	67.0053484	4.14E-160	2.43489	up	PREDICTED: subtilisin-like protease [Sesamum indicum]
c50092.graph_c0	28.3427544	28.0747716	27.4807925	25.1760644	24.5026301	24.6494543	2.511E-55	-1.26491	down	PREDICTED: RNA polymerase II C-terminal domain phosphatase-like 2 [Sesamum indicum]
c50101.graph_c0	0	0	0	0.4120535	0.43405329	0.51835812	8.087E-10	Inf	up	predicted protein [Micromonas sp. RCC299]
c50102.graph_c0	0	0	0	0.91318707	1.0027974	1.59100276	2.301E-08	Inf	up	protein disulfide isomerase [Rhizoctonia solani 123E]
c50103.graph_c0	0	0.08453615	0	4.97169309	4.59981155	4.30728448	1.157E-15	6.27701	up	hypothetical protein MIMGU_mgv1a018847mg, partial [Erythranthe guttata]
c50108.graph_c1	0.46589557	0.34977157	0.07483678	24.1259777	21.3440043	24.1765273	1.12E-123	5.21572	up	PREDICTED: cationic amino acid transporter 7, chloroplastic [Sesamum indicum]
c50109.graph_c0	7.02373725	5.91373217	6.41083471	89.2626429	91.0633435	85.7622692	2.63E-116	2.69158	up	PREDICTED: pentatricopeptide repeat-containing protein At5g66520-like [Sesamum indicum]
c50109.graph_c2	4.84451144	4.79297455	6.44255374	3.68472872	3.73359355	3.37419514	1.461E-07	-1.67026	down	ribosomal protein S12 (chloroplast) [Andrographis paniculata]
c50110.graph_c0	2.26471942	2.15903542	1.94016941	9.43175544	9.55395096	8.78351522	2.946E-07	1.03647	up	PREDICTED: rRNA methyltransferase 1, mitochondrial [Sesamum indicum]
c50110.graph_c3	0.24452432	0.12238459	0	3.28779908	4.0115869	3.33539436	6.066E-10	3.79009	up	PREDICTED: uncharacterized protein LOC101248594 [Solanum lycopersicum]
c50110.graph_c4	0	0.1035005	0.06643459	1.01450225	1.45881548	1.19574802	1.375E-08	3.33104	up	PREDICTED: uncharacterized protein LOC105159914 [Sesamum indicum]
c50121.graph_c0	0.41516015	0.3324607	0.4054579	2.3052631	2.24490003	2.45229262	9.911E-08	1.51191	up	PREDICTED: uncharacterized protein LOC104898733 [Beta vulgaris subsp. vulgaris]
c50123.graph_c0	1.52340525	1.9177159	1.750008	2.02987062	1.87822375	1.57422441	3.058E-05	-1.01382	down	unnamed protein product [Coffea canephora]
c50126.graph_c0	8.12288733	3.70678907	3.12123599	0	0	0	2.64E-11	#NAME?	down	-
c50128.graph_c0	90.1259294	87.5077218	88.6948628	69.8055143	69.1401463	68.7477203	4.616E-68	-1.44921	down	PREDICTED: uncharacterized protein LOC105173692 [Sesamum indicum]
c50129.graph_c0	2.8710987	3.75719485	3.02357017	24.0937275	26.6254457	24.0209311	5.036E-67	1.86228	up	PREDICTED: geranylgeranyl diphosphate reductase, chloroplastic [Sesamum indicum]

#ID	0 h-1 FPKM	0 h-2 FPKM	0 h-3 FPKM	28 h-1 FPKM	28 h-2 FPKM	28 h-3 FPKM	FDR	log2FC	regulated	nr_annotation
c50133.graph_c0	1.68122665	1.87387867	1.99189762	18.3112556	17.9339476	16.5753248	1.259E-91	2.15842	up	PREDICTED: purple acid phosphatase 2-like [Sesamum indicum]
c50140.graph_c0	34.3826433	38.7797997	32.4725675	30.5244661	33.9164551	33.3669391	4.228E-24	-1.20045	down	PREDICTED: uncharacterized protein LOC105161920 [Sesamum indicum]
c50140.graph_c1	3.71336722	5.03605145	4.00217488	28.3379659	25.3507057	24.7929908	3.705E-20	1.53086	up	PREDICTED: uncharacterized protein LOC105179028 [Sesamum indicum]
c50141.graph_c0	4.06501043	3.69231417	3.43409864	2.29788701	2.32177373	2.27685497	3.801E-15	-1.78711	down	hypothetical protein VITISV_003658 [Vitis vinifera]
c50144.graph_c0	0	0	0	3.24308376	4.04780667	5.02024342	3.649E-16	Inf	up	molecular chaperone DnaK [Galdieria sulphuraria]
c50145.graph_c0	3.33884596	3.8085413	2.39472516	18.2845557	14.3691529	15.9331378	9.471E-08	1.26216	up	PREDICTED: E3 ubiquitin-protein ligase MARCH11 isoform X1 [Sesamum indicum]
c50150.graph_c0	0	0	0	0.36988159	0.2652102	0.53262416	8.647E-07	Inf	up	Eukaryotic translation initiation factor 3 subunit, putative [Ricinus communis]
c50151.graph_c0	100.433113	107.00824	95.6950621	74.3154562	70.6614911	72.5549033	1.574E-76	-1.56842	down	PREDICTED: ethylene-insensitive protein 2-like [Sesamum indicum]
c50153.graph_c1	0	0	0	0.54552606	0.95775339	0.95211529	5.763E-10	Inf	up	hypothetical protein EMIHUDRAFT_422478 [Emiliania huxleyi CCMP1516]
c50154.graph_c0	54.7983168	55.7814292	51.5380863	52.1480178	52.0480685	50.3719538	1.592E-46	-1.15865	down	PREDICTED: splicing factor U2AF-associated protein 2 isoform X3 [Sesamum indicum]
c50157.graph_c0	12.3520956	12.2686158	11.5663224	94.0890534	97.6463641	108.633354	9.842E-46	1.96407	up	ATP-binding cassette superfamily [Micromonas sp. RCC299]
c50163.graph_c0	0	0	0	0.56193374	0.97415709	1.17384537	3.415E-07	Inf	up	-
c50166.graph_c1	2.60198304	2.4214534	3.39589577	1.12284435	1.17390052	1.01267728	6.459E-11	-2.44228	down	PREDICTED: putative late blight resistance protein homolog R1A-10 [Sesamum indicum]
c50173.graph_c0	9.96485562	8.69094441	8.68473791	52.130936	52.3136626	45.5810559	3.515E-34	1.36637	up	PREDICTED: uncharacterized protein LOC102612426 [Citrus sinensis]
c50178.graph_c0	39.384112	41.0047076	36.5367343	34.4982182	32.73805	32.162809	6.193E-58	-1.3239	down	PREDICTED: DDB1- and CUL4-associated factor homolog 1 [Sesamum indicum]
c50179.graph_c0	0	0	0	2.57702125	3.99060179	5.1214044	2.287E-15	Inf	up	EF2, translation elongation factor 2 [Ectocarpus siliculosus]
c50180.graph_c0	0	0	0	1.99728118	2.53893773	4.34604036	1.079E-11	Inf	up	Myosin tail domain-containing protein [Rozella allomycis CSF55]
c50181.graph_c0	0	0	0	1.26316787	1.33623306	2.4152126	1.916E-11	Inf	up	--
c50182.graph_c0	1.29921872	1.3005197	1.14394921	9.18035106	8.95211597	9.58859712	1.593E-22	1.79987	up	PREDICTED: putative late blight resistance protein homolog R1A-10 [Sesamum indicum]
c50183.graph_c0	270.542582	300.15235	362.054404	301.671805	242.89737	277.977136	2.338E-06	-1.2753	down	Cell wall-associated hydrolase, partial [Medicago truncatula]
c50185.graph_c0	44.9326937	51.1383794	67.076259	278.575142	215.269415	252.261241	4.064E-07	1.09788	up	BnaCnng12640D [Brassica napus]
c50188.graph_c0	16.4507368	15.6830569	13.5899024	7.5438564	7.19694568	6.15572521	2.453E-17	-2.21865	down	PREDICTED: putative ribonuclease H protein At1g65750 [Malus domestica]
c50188.graph_c1	7.27170714	7.16381479	6.41690396	3.01046288	2.20473442	2.01979968	5.888E-76	-2.61697	down	PREDICTED: uncharacterized protein LOC105167219 [Sesamum indicum]

#ID	0 h-1 FPKM	0 h-2 FPKM	0 h-3 FPKM	28 h-1 FPKM	28 h-2 FPKM	28 h-3 FPKM	FDR	log2FC	regulated	nr_annotation
c50189.graph_c0	13.9401126	11.8138765	13.4077226	59.5153423	57.609189	56.7045318	1.12E-27	1.05954	up	PREDICTED: probable LRR receptor-like serine/threonine-protein kinase At2g16250 [Sesamum indicum]
c50190.graph_c1	21.3741212	16.7208718	14.3103252	2.21922687	0.47147148	0.95869641	1.568E-33	-4.93012	down	-
c50191.graph_c0	8.19328842	8.51999734	8.89317379	0.54923234	0.31320335	0.37740556	1.1E-132	-5.45992	down	PREDICTED: uncharacterized protein LOC105157045 [Sesamum indicum]
c50194.graph_c0	7.04809722	6.26054694	5.90410594	40.4031928	34.5544637	38.6037367	5.176E-31	1.4751	up	hypothetical protein M569_07983, partial [Genlisea aurea]
c50195.graph_c0	37.0903537	42.949412	34.4193271	27.3649337	24.473612	24.5685701	2.453E-25	-1.67236	down	hypothetical protein VITISV_039063 [Vitis vinifera]
c50196.graph_c1	4.8048227	4.55732533	4.73707099	1.96932525	1.51966896	1.5135258	1.702E-72	-2.58582	down	hypothetical protein VITISV_021503 [Vitis vinifera]
c50197.graph_c0	6.71677483	6.65212592	5.845842	4.16655779	3.21923332	3.61976968	3.137E-83	-1.8926	down	PREDICTED: uncharacterized protein LOC103415359 [Malus domestica]
c50198.graph_c0	7.81267953	8.4845077	6.91406588	7.71379534	6.23933042	6.49109382	8.049E-18	-1.27201	down	PREDICTED: uncharacterized protein LOC105162629 [Sesamum indicum]
c50199.graph_c0	257.142571	245.816206	258.271166	40.9401081	79.2906531	69.8413939	8.44E-191	-3.09234	down	defensin-like protein 4 [Arabidopsis thaliana]
c50201.graph_c0	2.22889401	3.68620803	2.11702999	2.11297477	2.48016354	2.41383822	0.0042774	-1.28527	down	-
c50202.graph_c0	44.0154056	40.8946444	41.9431692	19.960047	19.7306879	20.0741095	3.02E-138	-2.17584	down	PREDICTED: squalene monooxygenase-like [Sesamum indicum]
c50208.graph_c0	1.57431195	1.74177137	1.70362374	1.62596963	1.1418541	1.32677794	0.0004381	-1.38505	down	hypothetical protein AALP_AA3G181900 [Arabis alpina]
c50212.graph_c0	10560.0812	9984.95233	10690.9067	3900.98327	4367.39198	4538.99836	2.939E-38	-2.37676	down	hypothetical protein MIMGU_mgv1a016953mg [Erythranthe guttata]
c50213.graph_c0	0.60983977	0.61045044	0.65305688	3.54582427	4.93571808	4.34004547	0.0001557	1.68323	up	PREDICTED: LOW QUALITY PROTEIN: uncharacterized protein LOC105161466 [Sesamum indicum]
c50216.graph_c0	239.587447	251.5836	226.064149	12.2197035	15.5763481	18.4760069	0	-5.04265	down	-
c50218.graph_c0	2.02802411	1.47640354	1.97431157	0.17866136	0.04032861	0.07289311	5.294E-26	-5.32195	down	hypothetical protein VITISV_044399 [Vitis vinifera]
c50219.graph_c0	40.1403106	34.9634463	33.7221001	282.035034	293.592904	300.633046	7.12E-109	1.92091	up	60S ribosomal L34-1 -like protein [Gossypium arboreum]
c50224.graph_c0	12.64576	6.25386368	7.83496719	0.01367688	0	0	1.194E-25	-12.0227	down	predicted protein [Hordeum vulgare subsp. vulgare]
c50229.graph_c0	0	0	0	6.7923066	6.24040929	4.57010414	3.044E-14	Inf	up	PREDICTED: homeobox-leucine zipper protein ATHB-13 [Sesamum indicum]
c50230.graph_c0	1.34178033	0.61990335	0.99475433	28.2432405	26.2121813	21.1486869	4.226E-34	3.58745	up	PREDICTED: zinc finger protein CONSTANS-LIKE 4-like [Sesamum indicum]
c50231.graph_c0	29.052712	14.837655	21.6193672	0	0	0	1.148E-29	-Inf	down	predicted protein [Physcomitrella patens]
c50236.graph_c0	14.0486943	16.3309493	8.73538468	12.9278232	11.3752208	10.2802339	0.0011674	-1.26287	down	-
c50238.graph_c0	30.0943133	26.2713211	20.2355498	4.06927879	3.67417721	5.18827853	4.245E-27	-3.65071	down	-
c50239.graph_c0	7223.6407	7097.40063	7562.77428	3283.07678	4019.61288	3655.18711	1.402E-49	-2.0893	down	hypothetical protein MIMGU_mgv1a015957mg [Erythranthe guttata]
c50240.graph_c0	1.71133495	2.29799201	1.39457225	320.321011	343.097615	310.24407	0	6.40567	up	hypothetical protein MIMGU_mgv1a027056mg [Erythranthe guttata]
c50242.graph_c0	2.6309155	2.3679819	2.92626513	10.7497294	12.9413303	11.0400062	9.61E-12	1.03887	up	PREDICTED: pentatricopeptide repeat-containing protein At1g11290 [Sesamum indicum]

#ID	0 h-1 FPKM	0 h-2 FPKM	0 h-3 FPKM	28 h-1 FPKM	28 h-2 FPKM	28 h-3 FPKM	FDR	log2FC	regulated	nr_annotation
c50244.graph_c0	1.63257204	1.9677184	2.0979197	9.49235157	11.1506601	9.26715449	8.38E-12	1.29809	up	PREDICTED: U-box domain-containing protein 28 [Sesamum indicum]
c50245.graph_c0	0.20082858	0.10051484	0	7.29805255	5.66693319	6.07425431	5.225E-15	4.91482	up	PREDICTED: protein RALF-like 24 [Sesamum indicum]
c50247.graph_c0	9.55358467	4.91080734	6.4701709	0	0	0	2.05E-19	-Inf	down	40S ribosomal protein S11 [Lachancea thermotolerans]
c50248.graph_c0	40.850923	46.8697947	42.7797947	26.7812207	28.8920438	29.0874392	8.422E-47	-1.71318	down	PREDICTED: uncharacterized protein LOC105176358 [Sesamum indicum]
c50251.graph_c0	25.3623616	20.8886618	18.5648575	7.93328379	7.79504187	8.56786075	2.465E-18	-2.50213	down	PREDICTED: trihelix transcription factor ASIL2 [Sesamum indicum]
c50253.graph_c0	24.9242288	19.3233897	35.7966938	11.721332	7.05550922	6.66617448	1.825E-05	-2.75119	down	conserved hypothetical protein [Ricinus communis]
c50254.graph_c0	2.0113616	3.09750104	1.39175039	9.67067368	10.9654053	9.95574647	8.746E-05	1.14898	up	PREDICTED: uncharacterized protein LOC105175348 [Sesamum indicum]
c50255.graph_c0	3759.77414	3929.75954	3850.37212	1042.18329	1311.23894	1255.42556	7.77E-119	-2.76764	down	oleosin, partial [Genlisea aurea]
c50256.graph_c0	0.03788731	0.22755147	0.09737338	3.80000617	5.19629928	4.22424285	2.407E-20	4.09432	up	PREDICTED: probable indole-3-pyruvate monooxygenase YUCCA5 [Sesamum indicum]
c50257.graph_c0	1.14820929	0.61059699	0.5533104	44.3595228	44.4085809	43.4534339	2.78E-196	4.75525	up	PREDICTED: 5-methyltetrahydropteroyltriglutamate--homocysteine methyltransferase [Sesamum indicum]
c50258.graph_c0	6.10865909	4.4162271	5.88740159	0	0	0	3.123E-35	-Inf	down	RecName: Full=60S ribosomal protein L31 [Cyanophora paradoxa]
c50260.graph_c0	65.5419441	65.7201088	56.1974842	21.0805504	21.4683112	26.5357351	1.41E-100	-2.5276	down	hypothetical protein POPTR_0001s05450g [Populus trichocarpa]
c50261.graph_c0	2.09356461	2.10918139	2.11753701	13.5077987	13.4814937	12.1036518	3.473E-40	1.53766	up	PREDICTED: U-box domain-containing protein 30-like [Sesamum indicum]
c50267.graph_c0	8.13674358	9.44533612	7.7321959	0.84481976	1.30123518	0.93267168	3.235E-59	-4.13006	down	hypothetical protein MIMGU_mgv1a021404mg, partial [Erythranthe guttata]
c50270.graph_c0	0	0	0	6.69013381	5.32222628	5.57712904	2.084E-29	Inf	up	hypothetical protein MIMGU_mgv1a025449mg [Erythranthe guttata]
c50271.graph_c0	0.06204401	0.12421227	0	13.3926889	9.69015989	12.400153	4.964E-32	6.50021	up	PREDICTED: uncharacterized protein LOC105180227 [Sesamum indicum]
c50272.graph_c0	77.9722749	90.1034846	87.7539312	6.21164655	6.24398415	8.51433805	1.45E-127	-4.69889	down	hypothetical protein MIMGU_mgv1a027115mg, partial [Erythranthe guttata]
c50273.graph_c0	0.28382527	0.21308211	0.18236335	6.13690642	6.19293948	6.77507689	1.791E-15	3.7288	up	-
c50276.graph_c0	0.73507531	0.55185853	0.59037555	12.2376296	12.7528235	13.0237795	3.61E-67	3.25165	up	PREDICTED: glycerophosphodiester phosphodiesterase GDPDL3-like [Sesamum indicum]
c50277.graph_c0	55.1234473	62.7352794	61.1349519	34.3190187	38.8998087	49.3075408	7.628E-24	-1.63695	down	PREDICTED: serine carboxypeptidase-like 50 [Sesamum indicum]
c50278.graph_c0	4.27734925	3.05830883	2.9165437	1.07854688	1.20296241	1.06127951	9.852E-15	-2.70352	down	PREDICTED: probable carboxylesterase 6 [Sesamum indicum]
c50282.graph_c0	32.6504941	27.5795075	33.8933357	0.57009468	0.51474204	0.6977889	1.17E-105	-6.81269	down	hypothetical protein MIMGU_mgv1a026849mg [Erythranthe guttata]
c50283.graph_c0	42.5126105	44.5375646	34.2712369	45.6751865	37.081714	38.2884731	4.879E-11	-1.09113	down	hypothetical protein PHAVU_005G057600g [Phaseolus vulgaris]
c50286.graph_c0	13.480203	7.25231509	8.46380843	0	0	0	3.512E-27	-Inf	down	predicted protein [Physcomitrella patens]

#ID	0 h-1 FPKM	0 h-2 FPKM	0 h-3 FPKM	28 h-1 FPKM	28 h-2 FPKM	28 h-3 FPKM	FDR	log2FC	regulated	nr_annotation
c50287.graph_c0	2.41019564	2.72729723	1.88525024	1.25666604	1.06588485	1.46045981	8.213E-10	-1.97837	down	PREDICTED: uncharacterized protein LOC105168059 [Sesamum indicum]
c50290.graph_c0	0.43567418	0.2907403	0.09330976	280.706222	232.270887	271.686706	6.72E-199	8.82791	up	PREDICTED: GDSL esterase/lipase At5g03810-like [Sesamum indicum]
c50294.graph_c0	7.15865176	7.06536465	7.99552371	5.61617869	5.99286148	5.47550062	3.531E-22	-1.47157	down	PREDICTED: mitoferrin-like [Sesamum indicum]
c50297.graph_c0	2217.19253	2063.96425	2415.48696	358.938876	423.163673	384.386757	9.818E-76	-3.61344	down	PREDICTED: uncharacterized protein LOC100246645 [Vitis vinifera]
c50300.graph_c0	196.129956	231.129658	194.181325	167.977646	173.899063	164.957905	2.528E-23	-1.38423	down	putative heat shock protein 90, partial [Liriodendron tulipifera]
c50301.graph_c0	121.356152	122.957718	118.531762	106.634703	106.282435	92.3417525	7.434E-54	-1.34018	down	-
c50309.graph_c0	39.9011934	43.4474324	39.2387785	6.86258534	6.89585013	6.14173104	5.62E-200	-3.71357	down	PREDICTED: uncharacterized protein LOC105167450 [Sesamum indicum]
c50310.graph_c0	18.7132801	16.7008359	14.4863288	202.208059	247.822519	234.129027	1.181E-45	2.68972	up	PREDICTED: 60S ribosomal protein L15 [Solanum lycopersicum]
c50311.graph_c0	174.21718	181.944648	187.984746	38.7899494	42.5158011	34.1825902	4.54E-166	-3.32858	down	PREDICTED: vegetative incompatibility protein HET-E-1-like [Sesamum indicum]
c50313.graph_c0	0.70889398	1.48371711	0.41407129	6.744699	6.76647869	6.26801088	2.385E-07	1.83998	up	unnamed protein product [Coffea canephora]
c50316.graph_c0	13.0823184	12.2296055	11.2885776	2.7699373	2.71384421	2.96557128	1.51E-142	-3.20334	down	PREDICTED: scarecrow-like protein 8 [Sesamum indicum]
c50321.graph_c0	5.17771059	6.27403113	7.28390065	3.8819659	4.25613365	3.81411217	1.735E-08	-1.74344	down	hypothetical protein MIMGU_mgv1a011362mg [Erythranthe guttata]
c50322.graph_c0	4.884407	3.13653079	2.96068697	31.815669	27.8798884	27.3823671	5.849E-20	1.90202	up	PREDICTED: 40S ribosomal protein S17-like [Sesamum indicum]
c50323.graph_c0	16.9367803	13.488742	20.6523819	91.108672	92.8090742	98.0987983	7.891E-22	1.37124	up	hypothetical protein MIMGU_mgv1a017626mg [Erythranthe guttata]
c50330.graph_c0	0.2403748	0.7218465	0.51481856	15.5485688	13.7234189	11.7371303	9.969E-26	3.69915	up	hypothetical protein MIMGU_mgv1a027157mg [Erythranthe guttata]
c50331.graph_c0	26.9534761	26.160114	26.5622853	5.01322485	4.10818417	4.15825468	0	-3.67562	down	PREDICTED: UPF0392 protein RCOM_0530710-like [Sesamum indicum]
c50341.graph_c0	9.9267909	10.2727558	7.82634084	1.60327431	1.63642461	1.79174568	2.826E-51	-3.56467	down	PREDICTED: U-box domain-containing protein 8 [Sesamum indicum]
c50344.graph_c0	4.62674263	5.47937397	5.86180736	1.84711134	2.39474431	1.89369643	2.98E-20	-2.47387	down	-
c50349.graph_c0	0.8258109	0.68886485	1.0611997	6.30204329	8.30943156	8.40744181	1.576E-06	2.06545	up	PREDICTED: RNA-binding protein 38-like isoform X1 [Nelumbo nucifera]
c50366.graph_c0	0.04692523	0.25051849	0.10050129	3.93344169	4.22898835	3.73841471	2.93E-37	3.81044	up	PREDICTED: BTB/POZ domain-containing protein At1g67900 [Sesamum indicum]
c50372.graph_c0	0	0	0	1.70454592	1.34962421	1.09131805	1.805E-14	Inf	up	PREDICTED: cytochrome P450 CYP82D47-like [Sesamum indicum]
c50373.graph_c0	0.3941234	0.07890361	0.20258567	6.8174294	7.50039862	6.9653953	4.082E-16	3.89388	up	PREDICTED: auxin-induced in root cultures protein 12-like [Sesamum indicum]
c50374.graph_c0	1.17101687	0.67338544	0.92849549	40.381424	38.9621994	29.1682066	2.669E-37	4.20033	up	PREDICTED: succinate dehydrogenase [ubiquinone] flavoprotein subunit 1, mitochondrial isoform X1 [Nicotiana sylvestris]
c50376.graph_c0	5.09929737	4.92838963	6.02556231	0.63898964	0.46155819	1.11234204	8.836E-42	-3.94795	down	-
c50380.graph_c0	13.7275447	11.8834122	6.07515101	461.517249	502.493298	381.599503	1.603E-63	4.32577	up	hypothetical protein MIMGU_mgv1a001548mg [Erythranthe guttata]

#ID	0 h-1 FPKM	0 h-2 FPKM	0 h-3 FPKM	28 h-1 FPKM	28 h-2 FPKM	28 h-3 FPKM	FDR	log2FC	regulated	nr_annotation
c50385.graph_c0	0	0	0	3.01963986	3.05362588	2.56256153	3.146E-14	Inf	up	PREDICTED: uncharacterized protein LOC105157280 [Sesamum indicum]
c50389.graph_c0	0.06993471	0.21001423	0.08986888	3.25300139	4.03858848	3.1521268	4.318E-11	3.72943	up	PREDICTED: uncharacterized protein At1g08160-like [Sesamum indicum]
c50390.graph_c0	2.72507421	1.70487685	2.40750692	0.12378551	0.50295021	0.20201594	4.28E-17	-4.13764	down	-
c50393.graph_c0	73.6508277	72.5687614	72.1225064	3.80637325	3.54504378	4.5489002	0	-5.28671	down	hypothetical protein MIMGU_mgv1a014429mg [Erythranthe guttata]
c50401.graph_c0	2.54984714	2.04192034	2.44656871	0.46948093	0.60238032	0.22179046	1.316E-29	-3.53817	down	PREDICTED: cytochrome P450 71A21-like [Sesamum indicum]
c50411.graph_c0	0	0	0	2.119839	2.42066712	3.408667	1.441E-10	Inf	up	hypothetical protein MIMGU_mgv1a015512mg [Erythranthe guttata]
c50414.graph_c0	0	0	0	1.37982792	1.82984988	2.8851913	8.399E-07	Inf	up	60S ribosomal protein L13a [Rozella allomycis CSF55]
c50416.graph_c0	2.3127972	1.37191889	2.20150813	1.68092768	1.01181339	1.11761895	4.885E-05	-1.71901	down	PREDICTED: cysteine proteinase inhibitor 1-like [Sesamum indicum]
c50419.graph_c0	15.4534843	17.6788099	14.3779534	5.43342207	6.70029991	6.26361545	3.933E-48	-2.458	down	PREDICTED: uncharacterized protein At5g05190 [Sesamum indicum]
c50427.graph_c0	0	0.03751075	0.04815458	4.57553508	3.22141412	3.62248024	2.074E-22	5.9494	up	PREDICTED: geraniol 8-hydroxylase-like [Sesamum indicum]
c50432.graph_c0	0.80679455	0.08076024	0.31102888	2.9905055	4.44729942	4.88045613	9.084E-05	2.28318	up	hypothetical protein M569_10692 [Genlisea aurea]
c50436.graph_c0	2.57430903	3.55855797	1.57528049	1.42551845	2.65466335	1.96291926	0.0099545	-1.43603	down	-
c50437.graph_c0	0.21504522	0.4783568	0.27634163	6.49486858	6.836407	6.22084096	4.086E-34	3.24252	up	PREDICTED: probable leucine-rich repeat receptor-like protein kinase At1g68400 [Sesamum indicum]
c50454.graph_c0	87.9634008	84.0334318	84.5277496	21.4881921	23.0555967	23.2679972	0	-3.00949	down	PREDICTED: LOW QUALITY PROTEIN: putative ETHYLENE INSENSITIVE 3-like 4 protein [Sesamum indicum]
c50458.graph_c0	0.05331743	0.10674165	0.13703003	1.8406632	2.36171315	2.24504413	2.238E-13	3.33961	up	PREDICTED: uncharacterized protein LOC105166786 isoform X1 [Sesamum indicum]
c50460.graph_c0	0.18373335	0.06130578	0	2.71523887	3.57693352	2.1429697	1.473E-10	4.03871	up	unnamed protein product [Coffea canephora]
c50465.graph_c0	0.06713446	0.03360084	0.04313522	1.56137398	1.47585748	2.11017581	3.679E-12	4.0768	up	PREDICTED: cannabidiolic acid synthase-like 1 [Sesamum indicum]
c50466.graph_c0	0.36903826	0.22164468	0.18969151	3.64772235	2.78498821	3.21725892	4.254E-10	2.54556	up	PREDICTED: pentatricopeptide repeat-containing protein At1g09410-like [Sesamum indicum]
c50469.graph_c0	0	0	0	1.45497138	1.30142515	1.43135117	8.712E-21	Inf	up	PREDICTED: uncharacterized protein LOC105170283 [Sesamum indicum]
c50477.graph_c0	0	0	0	4.38682541	6.08803777	2.65156571	3.507E-07	Inf	up	--
c50479.graph_c0	0.85391267	0.44773548	0.41802346	6.14708154	4.90982618	5.1365445	3.905E-11	2.15263	up	PREDICTED: putative receptor-like protein kinase At5g39000 [Sesamum indicum]
c50480.graph_c0	50.399795	48.3273557	55.547837	36.040932	38.2312427	35.9198014	3.446E-27	-1.57771	down	PREDICTED: calcyclin-binding protein-like [Sesamum indicum]
c50481.graph_c0	0.75901224	0.81605171	0.43349368	9.13280141	9.29757483	8.95275272	5.87E-32	2.68513	up	hypothetical protein MIMGU_mgv1a003752mg [Erythranthe guttata]
c50482.graph_c0	0.44260548	0.08860974	0	287.134344	236.658405	283.59505	1.86E-152	9.50759	up	PREDICTED: putative lipid-binding protein AIR1B [Beta vulgaris subsp. vulgaris]
c50489.graph_c0	36.6009765	38.3560896	32.826525	21.0615044	20.9692915	20.5933536	4.028E-86	-1.87244	down	PREDICTED: transcription factor MYC4-like [Sesamum indicum]

#ID	0 h-1 FPKM	0 h-2 FPKM	0 h-3 FPKM	28 h-1 FPKM	28 h-2 FPKM	28 h-3 FPKM	FDR	log2FC	regulated	nr_annotation
c50490.graph_c0	4.79986033	5.55691245	4.79734154	4.19326049	5.55191702	4.19801077	5.838E-16	-1.21121	down	PREDICTED: lysM domain receptor-like kinase 4 [Sesamum indicum]
c50491.graph_c0	0	0.05785681	0.07427391	16.6351396	19.0025395	16.1107194	2.147E-65	7.50367	up	PREDICTED: adenine phosphoribosyltransferase 1-like [Sesamum indicum]
c50495.graph_c0	0.0576275	0.0576852	0.14810723	1.34026668	1.92865289	1.4695877	1.065E-08	3.06388	up	PREDICTED: glycerophosphodiester phosphodiesterase GDPDL3-like [Sesamum indicum]
c50496.graph_c0	0.09932885	0.23199939	0.29783017	1.25132166	1.36882782	1.17815568	0.0005543	1.49196	up	PREDICTED: putative pentatricopeptide repeat-containing protein At1g69350, mitochondrial [Sesamum indicum]
c50497.graph_c0	0	0	0	1.51983368	2.00939143	3.18901339	2.04E-06	Inf	up	60S ribosomal protein L7a [Zea mays]
c50502.graph_c0	41.2494328	42.6694525	35.4893789	19.8667842	21.020186	20.0230539	1.584E-63	-2.05981	down	PREDICTED: myb-like protein X [Sesamum indicum]
c50505.graph_c0	0.91038616	1.06318074	0.90990843	0.18379539	0.16595001	0.17997065	8.593E-15	-3.53436	down	hypothetical protein MIMGU_mgv1a023911mg [Erythranthe guttata]
c50507.graph_c0	2.55333697	2.2757958	1.57314888	13.7274701	17.1229359	13.3173431	1.218E-12	1.70067	up	hypothetical protein MIMGU_mgv1a005093mg [Erythranthe guttata]
c50509.graph_c0	19.5535103	18.8324868	18.7434098	87.9569107	94.0517379	91.0158249	6.495E-24	1.16657	up	PREDICTED: serine/threonine-protein phosphatase PP2A-2 catalytic subunit [Sesamum indicum]
c50510.graph_c0	0.08613714	0.03448936	0.04427585	1.17695476	1.11920532	1.55296375	7.268E-13	3.4633	up	PREDICTED: probable RNA-dependent RNA polymerase 5 [Sesamum indicum]
c50512.graph_c0	3.64857777	3.68734888	3.47134279	2.62626755	2.18709679	2.76719138	4.173E-13	-1.60064	down	-
c50516.graph_c0	25.3328646	24.7730417	29.8617906	22.3065796	25.1919222	26.8695996	1.248E-09	-1.19712	down	ferritin [Avicennia marina]
c50519.graph_c0	4.96080443	4.28862121	3.76694506	3.58207184	2.79035492	2.90383717	7.116E-17	-1.57624	down	PREDICTED: vinorine synthase-like isoform X1 [Sesamum indicum]
c50520.graph_c0	31.6957508	41.3448845	14.5101675	987.896856	1177.32287	941.927153	1.449E-71	4.06685	up	PREDICTED: dirigent protein 22-like [Sesamum indicum]
c50525.graph_c0	2.29575436	2.01079656	2.67356006	0.88641242	0.75326806	0.68075793	3.858E-12	-2.68264	down	PREDICTED: uncharacterized protein LOC105162053 [Sesamum indicum]
c50526.graph_c0	19.7784066	26.8037327	19.1597806	13.9325294	13.3784832	16.0607284	3.637E-09	-1.68845	down	hypothetical protein M569_14808, partial [Genlisea aurea]
c50528.graph_c0	0.44034514	0.22039304	0.14146523	4.56057438	4.55122065	4.76599256	1.603E-08	3.03493	up	hypothetical protein CICLE_v10032302mg [Citrus clementina]
c50530.graph_c0	0.06171555	0.06177735	0.39653463	2.42214333	2.9969566	2.78166922	6.155E-09	2.86285	up	-
c50532.graph_c0	0	0	0.14587044	2.64005051	3.35210355	3.56799255	1.041E-12	4.90476	up	PREDICTED: chromosome-associated kinesin KIF4A [Sesamum indicum]
c50533.graph_c0	0	0	0	0.8741685	0.74982759	0.98080724	1.638E-14	Inf	up	hypothetical protein MIMGU_mgv1a002430mg [Erythranthe guttata]
c50535.graph_c0	9.42879944	3.88114582	7.24718091	0	0	0.15678018	4.132E-13	-8.10908	down	60S ribosomal protein l10 [Lichtheimia corymbifera JMRC:FSU:9682]
c50536.graph_c0	0.04182974	0.04187162	0.16125861	1.4288758	1.31759069	2.45593931	5.446E-05	3.30161	up	PREDICTED: mitogen-activated protein kinase kinase kinase 2-like [Sesamum indicum]
c50538.graph_c0	0	0	0	1.54328705	2.10158693	2.43604158	2.118E-15	Inf	up	PREDICTED: beta-glucosidase 12-like [Sesamum indicum]
c50540.graph_c0	0.14491152	0.07252831	0	3.63356961	3.99398413	3.17981553	1.131E-12	4.56779	up	-
c50544.graph_c0	0	0	0	6.24224579	6.38212539	5.76777739	4.6E-16	Inf	up	PREDICTED: glutaredoxin-C5-like [Sesamum indicum]

#ID	0 h-1 FPKM	0 h-2 FPKM	0 h-3 FPKM	28 h-1 FPKM	28 h-2 FPKM	28 h-3 FPKM	FDR	log2FC	regulated	nr_annotation
c50545.graph_c0	1.11761958	0.88727553	1.18856723	6.89037067	7.38470217	6.33101088	5.739E-10	1.59717	up	PREDICTED: uncharacterized protein LOC105163839 [Sesamum indicum]
c50549.graph_c0	1.53756357	2.19871886	1.62300326	0.03991044	0.32431847	0	3E-25	-4.97858	down	PREDICTED: probable N-acetyltransferase HLS1 [Sesamum indicum]
c50558.graph_c0	16.2148041	16.6269198	13.5523026	16.4476571	16.2348345	15.7665983	2.478E-19	-1.02579	down	PREDICTED: general transcriptional corepressor ssn6 isoform X1 [Sesamum indicum]
c50559.graph_c0	713.74997	751.060965	713.806162	97.3414861	110.529223	107.488933	0	-3.87869	down	PREDICTED: nematode resistance protein-like HSPRO2 [Sesamum indicum]
c50561.graph_c0	0.31962102	0.55989687	0.10268142	3.1941073	2.41205551	2.93808471	4.102E-05	2.04187	up	-
c50562.graph_c0	1.31345813	1.23509013	1.32981782	22.5346712	24.2645442	24.242088	4.332E-69	3.10434	up	PREDICTED: coatomer subunit gamma-2 isoform X1 [Sesamum indicum]
c50563.graph_c0	0	0	0	0.81147457	0.62118984	0.86368255	3.032E-14	Inf	up	PREDICTED: putative transcription factor bHLH086 [Sesamum indicum]
c50566.graph_c0	0.07078398	0.07085486	0.09096022	3.39539575	2.22961747	2.01499288	1.812E-07	3.94254	up	PREDICTED: glycine-rich RNA-binding protein 3, mitochondrial [Sesamum indicum]
c50570.graph_c0	0.42947954	0.19107093	0.49057619	8.01167682	9.45716661	9.22602984	9.764E-27	3.49193	up	PREDICTED: uncharacterized protein LOC105162704 [Sesamum indicum]
c50575.graph_c0	0.61530483	0.57192661	0.5930183	3.7852286	3.98011393	3.93583173	4.898E-10	1.62626	up	PREDICTED: probable beta-D-xylosidase 7 [Sesamum indicum]
c50578.graph_c0	48.7998054	50.9257011	53.0316523	8.82496368	8.42199474	7.79359373	1.2E-171	-3.70087	down	PREDICTED: stachyose synthase [Sesamum indicum]
c50580.graph_c0	0.61297274	0.68176282	0.35008633	7.21058178	8.68560509	7.2705697	4.401E-34	2.73172	up	PREDICTED: centrosome-associated protein CEP250 [Sesamum indicum]
c50581.graph_c0	0.26525512	0	0	7.61504267	5.57016267	7.94424146	2.858E-14	5.25803	up	-
c50582.graph_c0	0.05957105	0	0.07655116	1.55865226	1.13367191	1.5544803	1.685E-09	3.86497	up	PREDICTED: metalloendoproteinase 1-like [Sesamum indicum]
c50588.graph_c0	20.8989179	22.2468501	26.0542672	12.4120808	8.64828195	10.5443822	7.619E-19	-2.22367	down	PREDICTED: 1,2-dihydroxy-3-keto-5-methylthiopentene dioxygenase 2 [Sesamum indicum]
c50589.graph_c0	286.708522	275.459707	283.258028	289.221216	310.818846	286.525491	2.005E-35	-1.02224	down	PREDICTED: coiled-coil domain-containing protein 9-like [Sesamum indicum]
c50590.graph_c0	208.681287	224.602756	197.887598	114.474056	120.879864	103.229092	3.02E-107	-1.98883	down	PREDICTED: uncharacterized protein At1g01500 [Sesamum indicum]
c50592.graph_c0	0.44148119	0.41430306	0.31911796	14.2785363	16.0065825	15.1039734	5.751E-85	4.18505	up	PREDICTED: dihydrolipoyl dehydrogenase 2, chloroplastic-like isoform X1 [Sesamum indicum]
c50596.graph_c0	0.36980387	0	0.23760631	2.08297773	2.79076598	1.86417922	1.461E-06	2.38285	up	-
c50601.graph_c0	0.08169489	0.16355339	0.27994977	1.76146942	2.3588574	2.14794203	1.069E-09	2.47281	up	PREDICTED: protein CYPRO4 [Sesamum indicum]
c50602.graph_c0	0.17728643	0.4056319	0.45564016	4.16003868	3.37386439	3.80010208	1.93E-14	2.34889	up	PREDICTED: putative 1-phosphatidylinositol-3-phosphate 5-kinase FAB1C isoform X2 [Populus euphratica]
c50608.graph_c0	0.54939381	0.43995516	0.84719136	10.0622656	14.7816103	12.7070768	3.747E-15	3.25598	up	PREDICTED: palmitoyl-monogalactosyldiacylglycerol delta-7 desaturase, chloroplastic [Sesamum indicum]
c50612.graph_c0	2.89303925	3.20901037	2.11002891	1.19339268	2.46290675	2.0403401	2.266E-06	-1.61408	down	PREDICTED: uncharacterized protein LOC105161205 [Sesamum indicum]

#ID	0 h-1 FPKM	0 h-2 FPKM	0 h-3 FPKM	28 h-1 FPKM	28 h-2 FPKM	28 h-3 FPKM	FDR	log2FC	regulated	nr_annotation
c50618.graph_c0	14.5959802	8.74138216	10.3400735	0	0	0	2.821E-37	-Inf	down	NADH dehydrogenase subunit 2 [Amorphotheca resinae]
c50623.graph_c0	10.376967	9.93907495	10.1778662	44.2099714	43.5028093	42.2746507	6.616E-35	1.0012	up	PREDICTED: probable phosphoribosylformylglycinamidine synthase, chloroplastic/mitochondrial [Sesamum indicum]
c50625.graph_c0	2.81922329	3.03108678	1.94558505	0.53122099	0.51390294	0.83598169	2.522E-16	-3.13451	down	PREDICTED: salicylate carboxymethyltransferase [Sesamum indicum]
c50630.graph_c0	565.932905	652.502523	574.70912	211.580395	212.362054	202.327983	3.063E-76	-2.60818	down	hypothetical protein AMTR_s00033p00173430 [Amborella trichopoda]
c50631.graph_c0	1.58383495	1.05694728	1.91193967	21.2434353	21.8894533	19.896214	2.663E-44	2.69634	up	PREDICTED: uncharacterized protein LOC105167007 [Sesamum indicum]
c50633.graph_c0	4.53091983	4.751431	4.34370372	1.56811734	2.17098984	2.21792297	6.209E-15	-2.28299	down	PREDICTED: uncharacterized protein LOC105158623 isoform X2 [Sesamum indicum]
c50636.graph_c0	0	0.04654826	0	1.68985837	1.31217396	1.26859778	1.975E-12	5.43737	up	-
c50640.graph_c0	303.97244	300.207546	317.481325	96.7382652	104.384426	107.71293	4.95E-168	-2.66831	down	PREDICTED: uncharacterized protein LOC105162278 [Sesamum indicum]
c50641.graph_c0	0	0.16129723	0	1.63957675	2.06196354	1.5767886	1.305E-08	3.94966	up	PREDICTED: KH domain-containing protein At4g18375-like [Glycine max]
c50642.graph_c0	49.6168481	42.571313	48.8371513	201.789488	234.847068	239.072444	2.368E-17	1.16999	up	PREDICTED: 60S ribosomal protein L6-like [Sesamum indicum]
c50643.graph_c0	0.54553226	1.25598062	1.12164889	5.51120689	5.76368776	6.60006017	1.328E-06	1.51677	up	PREDICTED: nucleolin [Sesamum indicum]
c50648.graph_c0	4.53925679	2.54896219	3.84132601	0	0.07265325	0	1.361E-20	-8.33255	down	60S ribosomal protein L30 [Nectria haematococca mpVI 77-13-4]
c50650.graph_c0	0	0	0	0.78602488	0.75145422	1.05640686	9.128E-14	Inf	up	PREDICTED: asparagine--tRNA ligase, cytoplasmic 2 [Sesamum indicum]
c50651.graph_c0	0.07805779	0	0	1.4750308	1.66476814	1.2499059	9.51E-12	4.75338	up	PREDICTED: scarecrow-like protein 32 [Sesamum indicum]
c50652.graph_c0	4.50628147	5.2537481	5.99512951	0.65502788	1.39159708	1.00611275	7.163E-25	-3.46231	down	PREDICTED: uncharacterized protein LOC105162763 [Sesamum indicum]
c50658.graph_c0	0.20065574	0.26780889	0.08595019	6.56259457	8.07611109	4.83935464	7.547E-12	4.05156	up	hypothetical protein MIMGU_mgv1a000413mg [Erythranthe guttata]
c50662.graph_c0	9.02014408	10.3940519	9.70429585	4.26888568	5.29980485	4.04320412	1.265E-35	-2.18913	down	hypothetical protein MIMGU_mgv1a005797mg [Erythranthe guttata]
c50663.graph_c0	1.23502862	0.78671429	1.0099477	0.46240602	0.54030613	0.68805327	4.781E-07	-1.92986	down	PREDICTED: cell division cycle 20.2, cofactor of APC complex-like [Sesamum indicum]
c50665.graph_c0	2.23813461	4.10735557	1.91739439	15.7244122	15.6663944	14.268948	0.000167	1.37878	up	PREDICTED: pleiotropic drug resistance protein 2-like [Sesamum indicum]
c50666.graph_c0	0.23901213	0.13956335	0.10238002	10.871516	10.4956266	9.30812487	1.02E-89	4.91348	up	PREDICTED: protein LONGIFOLIA 1 [Sesamum indicum]
c50671.graph_c0	5.04412659	5.86457265	5.91825869	2.75522421	2.71386451	2.34114296	1.369E-34	-2.20025	down	PREDICTED: uncharacterized protein LOC105159806 [Sesamum indicum]
c50675.graph_c0	4.38530982	4.81111235	3.69675466	1.37687813	1.611545	2.87122114	2.411E-20	-2.22256	down	PREDICTED: BTB/POZ domain-containing protein NPY4-like [Sesamum indicum]
c50679.graph_c0	2.58355395	2.58614099	2.45923638	13.8394338	14.6515876	14.5085886	1.984E-18	1.40492	up	PREDICTED: uncharacterized protein LOC105164931 [Sesamum indicum]

#ID	0 h-1 FPKM	0 h-2 FPKM	0 h-3 FPKM	28 h-1 FPKM	28 h-2 FPKM	28 h-3 FPKM	FDR	log2FC	regulated	nr_annotation
c50680.graph_c0	345.41294	353.58575	361.081399	135.965866	131.689277	145.972557	2.58E-174	-2.44839	down	PREDICTED: transcription factor HBP-1b(c38)-like [Sesamum indicum]
c50681.graph_c0	1.63616802	1.09187093	0.87605862	10.5042245	8.67905654	9.54170418	2.836E-06	1.91135	up	-
c50683.graph_c0	7.53892895	5.65985854	5.65122961	0.68490626	1.0306769	1.11775584	2.155E-16	-3.82041	down	hypothetical protein MIMGU_mgv1a022729mg [Erythranthe guttata]
c50685.graph_c0	40.9749491	44.192071	38.0988354	31.6152831	27.7844194	28.8733168	1.14E-59	-1.57113	down	hypothetical protein MIMGU_mgv1a008021mg [Erythranthe guttata]
c50687.graph_c0	1.87746579	0.51254885	1.09664449	6.82265658	5.71220327	9.2112385	0.0029139	1.55792	up	-
c50690.graph_c0	0	0.03794991	0	2.61764774	2.5874001	2.42827107	6.706E-18	6.57009	up	PREDICTED: uncharacterized protein LOC105174731 [Sesamum indicum]
c50693.graph_c0	273.781085	280.142221	299.616389	51.7252234	54.2959637	50.73899	3.56E-137	-3.53685	down	PREDICTED: organic cation/carnitine transporter 3-like [Sesamum indicum]
c50694.graph_c0	0	0	0	1.41489929	2.23566245	1.8857589	6.844E-16	Inf	up	PREDICTED: protein kinase PINOID [Sesamum indicum]
c50696.graph_c0	0	0.0494687	0	3.59176032	2.6592787	2.98946403	5.317E-17	6.46371	up	PREDICTED: (RS)-norcoclaurine 6-O-methyltransferase-like [Sesamum indicum]
c50700.graph_c0	0.99395712	2.27417697	1.64220976	29.3088937	38.5766119	27.7052961	8.618E-18	3.18823	up	-
c50702.graph_c0	0.20345731	0.13577403	0.17430047	2.11949297	1.8691987	2.89588849	2.608E-07	2.65739	up	-
c50703.graph_c0	0.7552396	0.54431701	0.23292307	11.021988	12.7867033	11.9320828	6.264E-51	3.46549	up	PREDICTED: fructokinase-like 2, chloroplastic [Sesamum indicum]
c50708.graph_c0	5.56235426	7.37269952	6.40841316	3.01110481	4.17881226	4.52731806	5.005E-13	-1.81382	down	PREDICTED: UPF0496 protein 1-like [Sesamum indicum]
c50711.graph_c0	0	0.05944843	0.15263434	1.94236003	1.71479635	1.16229661	4.478E-08	3.38757	up	-
c50715.graph_c0	0.20363145	0.47561584	0.34889931	4.80994044	4.34292525	4.58908125	6.06E-15	2.6467	up	PREDICTED: aspartic proteinase nepenthesin-2-like [Sesamum indicum]
c50718.graph_c0	11.2470347	12.1916272	10.6711715	5.40010725	4.99051502	4.38916324	4.369E-77	-2.2969	down	PREDICTED: formin BNI1-like [Sesamum indicum]
c50719.graph_c0	6.27084837	5.7064797	6.34895451	28.1743562	26.4987522	30.8224373	1.217E-09	1.13158	up	PREDICTED: transmembrane protein 256 homolog [Sesamum indicum]
c50721.graph_c0	2.07970969	0.80958586	1.33625465	6.54992441	6.975449	6.23546564	0.0040803	1.13885	up	PREDICTED: LOW QUALITY PROTEIN: GDSL esterase/lipase At2g27360-like [Sesamum indicum]
c50723.graph_c0	2.82617691	2.73470667	2.23957869	38.2395472	37.1540953	35.58892	2.364E-76	2.74275	up	hypothetical protein MIMGU_mgv1a005711mg [Erythranthe guttata]
c50724.graph_c0	2.2845635	2.68456439	2.72301872	36.721478	36.9583511	33.4399854	4.05E-99	2.70685	up	PREDICTED: protein ASPARTIC PROTEASE IN GUARD CELL 1-like [Sesamum indicum]
c50725.graph_c0	0.06516539	0.06523064	0	1.46821671	1.32566203	1.15940598	7.936E-08	3.85098	up	-
c50726.graph_c0	5.91185096	6.075578	7.09049837	2.57801952	3.51742832	3.5995667	3.319E-15	-2.06899	down	PREDICTED: magnesium-dependent phosphatase 1-like [Sesamum indicum]
c50728.graph_c0	1.95585023	2.84772177	1.82788726	0.60306089	0.23336033	0.4217938	4.444E-13	-3.48569	down	PREDICTED: UDP-glycosyltransferase 76C4-like [Sesamum indicum]
c50734.graph_c0	7.20201781	7.8138746	7.40390242	5.7071682	6.00679553	7.21973191	2.079E-14	-1.33349	down	PREDICTED: putative chromatin-remodeling complex ATPase chain isoform X1 [Sesamum indicum]
c50735.graph_c0	0.28607846	0.28636492	0.18381107	7.27720609	6.00743753	7.04093831	1.086E-10	3.66363	up	PREDICTED: uncharacterized protein LOC104450130 [Eucalyptus grandis]

#ID	0 h-1 FPKM	0 h-2 FPKM	0 h-3 FPKM	28 h-1 FPKM	28 h-2 FPKM	28 h-3 FPKM	FDR	log2FC	regulated	nr_annotation
c50737.graph_c0	4.20627716	5.62608458	5.12565168	5.40263008	4.64011386	5.39772687	1.651E-06	-1.04589	down	PREDICTED: uncharacterized protein LOC105171448 [Sesamum indicum]
c50738.graph_c0	0	0	0.24172594	4.7508687	5.92518768	8.98048748	2.716E-10	5.21783	up	elongation factor 1-alpha [Lichtheimia corymbifera JMRC:FSU:9682]
c50739.graph_c0	0.31447309	0.44969712	0.34638032	16.1622612	14.5930065	15.7459989	5.529E-56	4.2975	up	PREDICTED: uncharacterized protein LOC105163344 [Sesamum indicum]
c50740.graph_c0	3.76372764	2.47925572	2.87071994	0	0	0	6.599E-48	-Inf	down	hypothetical protein SELMODRAFT_118280 [Selaginella moellendorffii]
c50742.graph_c0	7.37808179	7.71371292	7.37421005	46.9503299	46.0499168	42.2005361	4.612E-13	1.49844	up	hypothetical protein MIMGU_mgv1a016086mg [Erythranthe guttata]
c50746.graph_c0	0.73528336	0.44161178	0.18897365	4.40879761	4.3426155	3.4885264	4.835E-09	2.08636	up	hypothetical protein MIMGU_mgv1a005428mg [Erythranthe guttata]
c50747.graph_c0	10.3226229	11.1191629	11.044537	10.927442	10.9561835	10.8065122	7.909E-25	-1.08226	down	PREDICTED: exocyst complex component EXO70B1 [Sesamum indicum]
c50748.graph_c0	0.12265192	0	0.07880629	5.43770527	4.38656922	3.81883545	5.581E-17	4.99341	up	hypothetical protein MIMGU_mgv1a006779mg [Erythranthe guttata]
c50749.graph_c0	0.23840847	0.2386472	0.20424288	3.46547797	3.65050197	3.1105819	4.523E-08	2.81868	up	PREDICTED: protein IRX15-LIKE-like [Nicotiana tomentosiformis]
c50763.graph_c0	1.80695601	2.08008021	2.28330984	26.0249353	29.0316857	28.0588457	9.489E-86	2.65843	up	PREDICTED: dihydrolipoyllysine-residue acetyltransferase component 5 of pyruvate dehydrogenase complex, chloroplastic [Sesamum indicum]
c50766.graph_c0	1.18041195	0.89903888	1.15414486	20.8884043	22.6154852	19.4949526	4.622E-96	3.19253	up	PREDICTED: uncharacterized protein LOC105178721 [Sesamum indicum]
c50767.graph_c0	6.10538876	6.48498308	5.84066267	3.94432121	3.62812759	3.78177733	7.238E-26	-1.78863	down	PREDICTED: uncharacterized protein LOC102659506 [Glycine max]
c50770.graph_c0	0.47975642	0.38418946	0.1233012	8.78684182	10.7671566	12.9742697	1.68E-15	3.96655	up	cyclin D [Scutellaria baicalensis]
c50771.graph_c0	2.89812941	2.53129215	3.10350919	2.66390533	2.98326419	2.30852965	4.478E-08	-1.19399	down	PREDICTED: pentatricopeptide repeat-containing protein At3g05340 [Sesamum indicum]
c50773.graph_c0	0.19101167	0	0.08181919	2.12866815	1.9637701	1.81249652	5.555E-09	3.35471	up	PREDICTED: probable leucine-rich repeat receptor-like protein kinase At1g68400 [Sesamum indicum]
c50774.graph_c0	2.83675133	2.97481057	2.43022518	0.39271134	0.4432269	0.24033693	1.281E-17	-4.02784	down	-
c50776.graph_c0	17.667833	19.8000983	19.0021478	11.3055567	9.0736513	8.9974582	5.793E-18	-2.03436	down	PREDICTED: RNA-binding protein cabeza [Nicotiana sylvestris]
c50777.graph_c0	0	0	0	4.0922528	4.45285302	3.89578567	1.348E-17	Inf	up	hypothetical protein CICLE_v10023373mg [Citrus clementina]
c50779.graph_c0	2.50631299	2.58484761	1.75675168	2.98075718	2.04342805	1.66655778	0.0008265	-1.12098	down	PREDICTED: uncharacterized protein LOC105126503 [Populus euphratica]
c50786.graph_c0	0.35852555	0.29907046	0.46071952	4.64690836	4.86233268	5.77635328	5.292E-11	2.67901	up	PREDICTED: uncharacterized protein LOC105162253 isoform X1 [Sesamum indicum]
c50787.graph_c0	0.36092687	0.44157456	0.46380531	2.30258285	3.00009966	2.87779172	3.218E-05	1.59848	up	-
c50789.graph_c0	21.9447532	22.9743756	25.0953031	12.7301913	11.0978185	10.865329	1.092E-21	-2.10553	down	PREDICTED: uncharacterized protein LOC102593092 [Solanum tuberosum]
c50794.graph_c0	24.1375075	19.2950702	22.6582854	3.88185562	2.92079305	5.9493321	8.336E-52	-3.46095	down	PREDICTED: cytochrome P450 86B1-like [Sesamum indicum]
c50795.graph_c0	10.965696	9.76266621	8.57169893	8.88798493	8.38979057	8.6011328	2.081E-16	-1.26672	down	PREDICTED: caffeoylshikimate esterase [Sesamum indicum]

#ID	0 h-1 FPKM	0 h-2 FPKM	0 h-3 FPKM	28 h-1 FPKM	28 h-2 FPKM	28 h-3 FPKM	FDR	log2FC	regulated	nr_annotation
c50796.graph_c0	0.72185373	0.12042943	0.15460177	2.5357558	2.44744972	4.06696185	0.005037	2.11205	up	PREDICTED: glucomannan 4-beta-mannosyltransferase 2-like [Sesamum indicum]
c50803.graph_c0	176.448717	201.715778	152.243778	56.093549	49.404702	54.0065382	2.349E-35	-2.82157	down	-
c50811.graph_c0	0.10369449	0.18164706	0.1665644	5.78422963	5.59687623	5.33485276	1.533E-34	4.11418	up	PREDICTED: probable inactive leucine-rich repeat receptor-like protein kinase At1g66830 [Sesamum indicum]
c50814.graph_c0	25.2028735	24.4352269	29.795755	9.31565705	10.4903292	12.3417614	2.3E-23	-2.397	down	-
c50818.graph_c0	1.40460155	1.15788898	1.27409549	38.4322376	37.791239	37.6814711	4.994E-66	3.80207	up	PREDICTED: uncharacterized protein LOC105164615 [Sesamum indicum]
c50820.graph_c0	0.11291623	0.24220563	0.24874598	3.73990419	4.16011177	3.95098752	5.14E-31	3.19687	up	PREDICTED: probable RNA-dependent RNA polymerase 1 [Sesamum indicum]
c50823.graph_c0	3.65353427	4.65460893	2.1340619	1.35182353	1.00261099	0.98489014	1.236E-05	-2.72918	down	PREDICTED: uncharacterized protein LOC105158347 [Sesamum indicum]
c50824.graph_c0	1.25416558	1.65487372	1.025597	9.73669431	11.1855545	10.3454863	2.481E-12	1.90288	up	PREDICTED: tetraspanin-3-like [Sesamum indicum]
c50830.graph_c0	0.02938713	0.02941655	0.03776362	4.18624539	3.72193321	4.54877998	2.161E-30	5.91796	up	PREDICTED: delta(8)-fatty-acid desaturase 2-like [Sesamum indicum]
c50831.graph_c0	19.8766683	22.3290681	21.1384599	2.12844406	2.6168993	2.51280685	3.29E-158	-4.21645	down	PREDICTED: uncharacterized protein LOC105179506 [Sesamum indicum]
c50835.graph_c0	46.864079	46.3310625	44.5048653	23.6739485	23.7410079	25.5406598	2.776E-92	-2.00597	down	PREDICTED: RING-H2 finger protein ATL80-like [Sesamum indicum]
c50836.graph_c0	0.39088309	0.68473037	0.75345036	3.1960302	3.20635069	4.11474117	0.0010974	1.42774	up	hypothetical protein JCGZ_25112 [Jatropha curcas]
c50838.graph_c0	24.7811436	29.5796133	23.7467728	17.2061865	20.1180084	17.9289145	1.702E-20	-1.58921	down	ring finger protein, putative [Ricinus communis]
c50839.graph_c0	5.95162086	6.64189718	5.16761381	0.14613526	0.05277858	0.38158458	1.374E-98	-6.01629	down	PREDICTED: E3 ubiquitin-protein ligase PUB23-like [Sesamum indicum]
c50841.graph_c0	3.75864793	3.69970479	4.66901102	34.7389278	39.2588794	35.6655493	6.088E-42	2.08285	up	hypothetical protein MIMGU_mgv1a012263mg [Erythranthe guttata]
c50843.graph_c0	0.14955928	0.14970904	0.1921896	1.35873528	1.32495541	1.4635063	0.0001675	1.98341	up	PREDICTED: serine/threonine-protein kinase D6PKL2 [Sesamum indicum]
c50844.graph_c0	0	0	0	3.38486775	2.57868412	3.62515708	4.249E-11	Inf	up	-
c50849.graph_c0	29.5396833	28.7478944	34.309695	23.0290049	20.5030896	22.0481707	6.819E-17	-1.59023	down	PREDICTED: polyadenylate-binding protein-interacting protein 6-like [Sesamum indicum]
c50851.graph_c0	2.21116078	2.2896982	1.2737441	14.4635302	14.1099529	14.6056919	1.018E-11	1.81878	up	PREDICTED: fibroin heavy chain [Sesamum indicum]
c50852.graph_c0	5.35562966	5.39874598	5.81594041	4.63255275	4.43026142	3.98143365	5.691E-15	-1.4374	down	PREDICTED: F-box/kelch-repeat protein At3g23880-like [Sesamum indicum]
c50857.graph_c0	0.06191221	0	0	2.02488459	1.747024	1.50541913	1.441E-12	5.35365	up	PREDICTED: uncharacterized protein LOC105165400 [Sesamum indicum]
c50859.graph_c0	0.36126166	0.39778575	0.13927066	9.37349536	9.60132191	9.89831204	1.033E-39	3.92632	up	-
c50868.graph_c0	5.20226469	3.85738813	5.44713163	23.2460005	23.7706282	24.3391592	2.438E-05	1.2064	up	hypothetical protein PHAVU_008G011200g [Phaseolus vulgaris]

#ID	0 h-1 FPKM	0 h-2 FPKM	0 h-3 FPKM	28 h-1 FPKM	28 h-2 FPKM	28 h-3 FPKM	FDR	log2FC	regulated	nr_annotation
c50871.graph_c0	1.21390322	1.57515395	2.13765952	16.7628995	15.7844049	17.624591	7.786E-31	2.25121	up	PREDICTED: protein arginine N-methyltransferase PRMT10 isoform X1 [Sesamum indicum]
c50874.graph_c0	7.45218227	11.7222985	14.1365156	474.820474	449.094718	446.166874	0	4.26455	up	hypothetical protein JCGZ_21814 [Jatropha curcas]
c50879.graph_c0	5.33924024	4.9882809	4.80279457	0.77610581	0.46716718	0.42219733	4.43E-21	-4.27399	down	-
c50880.graph_c0	1.48593803	1.40629364	1.21512949	8.24705275	8.77601457	9.03679433	6.213E-16	1.57771	up	PREDICTED: peptide-N4-(N-acetyl-beta-glucosaminyl)asparagine amidase A-like [Sesamum indicum]
c50883.graph_c0	3.51069822	1.9388765	1.40008537	13.9015768	13.6640071	14.2153638	2.556E-06	1.52874	up	PREDICTED: uncharacterized protein LOC105175099 [Sesamum indicum]
c50885.graph_c0	0.05306334	0	0.06818849	4.70506771	3.76073262	4.24840223	4.007E-19	5.6131	up	hypothetical protein MIMGU_mgv1a010676mg [Erythranthe guttata]
c50890.graph_c0	3.04018261	3.62846283	3.75649521	31.6990779	31.1534742	32.0380139	1.694E-20	2.0935	up	PREDICTED: ADP-ribosylation factor GTPase-activating protein AGD3-like [Sesamum indicum]
c50892.graph_c0	0.84437723	1.2960082	0.86804653	23.5656384	26.7447134	21.6330396	9.868E-53	3.48942	up	PREDICTED: endo-1,3;1,4-beta-D-glucanase-like [Sesamum indicum]
c50894.graph_c0	6.84003352	7.74778841	5.93691201	33.5344504	35.0820702	29.3565229	4.091E-16	1.16586	up	PREDICTED: auxin-induced protein X15-like [Sesamum indicum]
c50896.graph_c0	0.27197032	0.11667543	0.17474632	14.1190332	15.004588	14.4934378	1.36E-135	5.18848	up	PREDICTED: WEB family protein At3g02930, chloroplastic-like [Sesamum indicum]
c50897.graph_c0	1.38358736	1.84663042	1.64626346	0.48416975	0.67255367	0.66859448	5.381E-11	-2.50913	down	PREDICTED: RING-H2 finger protein ATL16-like [Sesamum indicum]
c50901.graph_c0	0	0	0.12787867	1.95279582	1.2734161	1.50493958	1.339E-10	4.07923	up	hypothetical protein MIMGU_mgv1a012192mg [Erythranthe guttata]
c50904.graph_c0	3.83982119	1.38371983	1.77635601	25.6749912	28.8264496	26.3248632	1.691E-16	2.44736	up	hypothetical protein EMIHUDRAFT_447448 [Emiliania huxleyi CCMP1516]
c50906.graph_c0	0.02424172	0	0	10.1836326	9.27440579	8.74106352	4.761E-87	9.12463	up	PREDICTED: probable LRR receptor-like serine/threonine-protein kinase At4g26540 [Sesamum indicum]
c50907.graph_c0	563.298612	588.693763	568.599103	147.141056	145.57855	153.110622	3.1E-225	-3.03865	down	hypothetical protein MIMGU_mgv1a008386mg [Erythranthe guttata]
c50912.graph_c0	0	0	0	3.31876805	2.44615215	2.43175215	2.743E-14	Inf	up	PREDICTED: uncharacterized protein LOC105157276 [Sesamum indicum]
c50915.graph_c0	2308.10462	2107.65866	1983.73098	409.642676	366.048778	387.55628	0	-3.54849	down	hypothetical protein NitaMp027 [Nicotiana tabacum]
c50917.graph_c0	7.37066986	4.01693859	4.84104132	0	0	0	3.98E-26	-Inf	down	--
c50920.graph_c0	0	0.05184221	0	3.1994789	3.8744301	4.33077129	3.709E-19	6.70046	up	PREDICTED: DNA-damage-repair/toleration protein DRT100-like [Sesamum indicum]
c50921.graph_c0	0.58956557	0.46591257	0.43861926	11.8624864	12.0953679	10.2501682	5.379E-48	3.42902	up	PREDICTED: glucan endo-1,3-beta-glucosidase 2-like [Sesamum indicum]
c50926.graph_c0	11.1841858	12.0510833	9.52038623	3.57244281	3.85667283	3.97127408	2.71E-50	-2.61068	down	PREDICTED: putative nuclease HARBI1 [Sesamum indicum]
c50930.graph_c0	0.06750493	0.06757253	0	3.67966479	3.49958656	4.12353829	9.431E-15	5.31714	up	PREDICTED: NADPH:quinone oxidoreductase [Sesamum indicum]
c50940.graph_c0	0.02189264	0.08765823	0.05626581	1.82981576	1.93948287	1.81770488	6.87E-16	3.97883	up	LINE-1 reverse transcriptase like [Glycine soja]
c50942.graph_c0	108.411885	114.415537	109.367658	43.1705366	46.3342493	51.5633302	1.97E-164	-2.32551	down	PREDICTED: uncharacterized protein LOC105163758 [Sesamum indicum]

#ID	0 h-1 FPKM	0 h-2 FPKM	0 h-3 FPKM	28 h-1 FPKM	28 h-2 FPKM	28 h-3 FPKM	FDR	log2FC	regulated	nr_annotation
c50948.graph_c0	2.92707014	2.74687609	2.40964753	12.3321427	12.8455012	12.6396882	9.583E-09	1.13779	up	hypothetical protein MIMGU_mgv1a009797mg [Erythranthe guttata]
c50949.graph_c0	22.3746796	21.4179769	21.2107494	15.2843036	16.7689609	16.5687302	1.819E-35	-1.50879	down	PREDICTED: mitogen-activated protein kinase kinase 9-like [Sesamum indicum]
c50952.graph_c0	5.13309968	4.71005306	4.39749088	41.5041183	45.1937582	41.6044311	3.415E-15	2.08245	up	-
c50955.graph_c0	7.16955544	6.45906119	6.26495056	7.2951082	6.21041022	5.35747384	9.354E-05	-1.16669	down	-
c50957.graph_c0	6.70870104	6.41977142	5.80151742	368.724297	485.042693	334.456822	8.368E-36	4.88184	up	PREDICTED: basic 7S globulin-like [Sesamum indicum]
c50959.graph_c0	0.15850677	0.23799823	0	6.27849833	7.64520745	6.48625747	1.88E-17	4.61267	up	hypothetical protein JCGZ_13997 [Jatropha curcas]
c50961.graph_c0	0	0	0	8.68034174	8.27908599	9.97618025	6.277E-17	Inf	up	-
c50963.graph_c0	93.6727037	96.6244078	85.1696695	57.9526534	59.3739611	55.674305	1.076E-95	-1.76058	down	PREDICTED: zinc finger A20 and AN1 domain-containing stress-associated protein 4 isoform X1 [Sesamum indicum]
c50965.graph_c0	91.1674743	90.4654259	90.8650201	40.8432297	39.33178	42.7429391	3.61E-162	-2.23873	down	PREDICTED: probable ribose-5-phosphate isomerase 1 [Sesamum indicum]
c50969.graph_c0	12.3235742	12.9425987	8.56716827	3.37711741	2.12119679	3.47457855	1.288E-17	-2.99918	down	-
c50970.graph_c0	0.18549034	0.18567608	0.11918122	4.92068592	5.2949845	5.88535109	1.619E-12	3.95273	up	PREDICTED: putative B3 domain-containing protein At5g66980 [Sesamum indicum]
c50977.graph_c0	15.0390245	16.7979375	14.6656339	85.5042182	93.41734	90.2865896	1.896E-61	1.44341	up	hypothetical protein MIMGU_mgv1a020088mg [Erythranthe guttata]
c50978.graph_c0	2.86038043	1.82206479	1.44800386	1.82691916	1.30825325	0.77107816	0.0010902	-1.73556	down	-
c50981.graph_c0	21.0499929	11.1748953	12.5615121	0	0	0	5.33E-26	-Inf	down	cytochrome b (mitochondrion) [Gracilaria salicornia]
c50983.graph_c0	38.5825151	40.7286489	36.8075314	37.614109	40.2596058	35.193643	1.093E-35	-1.12873	down	PREDICTED: ATP synthase delta chain, chloroplastic-like [Sesamum indicum]
c50984.graph_c0	0.03940448	0.09860984	0.15190895	8.76351834	7.49890283	6.4265158	2.909E-41	5.18498	up	PREDICTED: putative late blight resistance protein homolog R1A-10 [Sesamum indicum]
c50987.graph_c0	9.42447382	11.7923888	8.96200827	55.1397087	54.3007412	53.3774437	3.291E-21	1.3423	up	unnamed protein product [Coffea canephora]
c50996.graph_c0	11.5997631	12.7790032	14.239958	0.70647829	0.72293482	1.61414545	7.176E-61	-4.75425	down	SLF-interacting SKP1-like protein 1, partial [Antirrhinum hispanicum]
c50997.graph_c0	29.8601201	24.7167477	23.6131917	20.7283482	18.9669731	18.1628594	5.727E-16	-1.52272	down	hypothetical protein MIMGU_mgv1a007364mg [Erythranthe guttata]
c50999.graph_c0	1.45655389	1.12154801	1.0798439	10.341854	10.2935546	9.83427183	6.333E-13	1.97082	up	PREDICTED: probable F-actin-capping protein subunit beta isoform X2 [Sesamum indicum]
c51000.graph_c0	0	0	0	1.60708811	0.99423788	1.26280149	2.017E-12	Inf	up	PREDICTED: polygalacturonase-like [Sesamum indicum]
c51004.graph_c0	6.4841629	6.62875487	6.91412417	48.530291	52.9622046	51.9549668	5.572E-23	1.84641	up	putative: proteolipid subunit of vacuolar H+ ATPase, partial [Zea mays]
c51010.graph_c0	0.04377754	0	0	1.04996979	0.89056826	0.80484151	3.82E-09	4.91107	up	PREDICTED: uncharacterized protein LOC105169592 [Sesamum indicum]
c51014.graph_c0	0.42433391	0.79288312	0.72704783	2.87843209	2.97023348	2.71787062	0.0006272	1.04399	up	PREDICTED: polyprenol reductase 2 [Sesamum indicum]
c51018.graph_c0	8.81333327	7.81710246	7.1680267	1.94596856	1.39097983	2.51416621	1.126E-25	-3.10989	down	PREDICTED: pathogenesis-related genes transcriptional activator PTI6-like [Sesamum indicum]

#ID	0 h-1 FPKM	0 h-2 FPKM	0 h-3 FPKM	28 h-1 FPKM	28 h-2 FPKM	28 h-3 FPKM	FDR	log2FC	regulated	nr_annotation
c51019.graph_c0	2.0326933	2.03472874	1.52132794	12.4032443	13.3830584	11.6576342	7.247E-28	1.65672	up	PREDICTED: two-component response regulator ARR12-like [Sesamum indicum]
c51021.graph_c0	0.67065068	0.44754815	0.71817722	3.1682628	3.30074337	4.37508342	0.0010307	1.47079	up	-
c51022.graph_c0	5.67195016	5.67762976	6.59921074	33.5357986	42.9548975	33.8197902	1.225E-12	1.52618	up	PREDICTED: uncharacterized protein LOC105178343 [Sesamum indicum]
c51026.graph_c0	0.37198926	0.22341705	0	2.48730881	2.05051911	2.64733523	2.035E-06	2.52652	up	PREDICTED: uncharacterized protein LOC105161197 [Sesamum indicum]
c51029.graph_c0	3.3684447	3.73965234	4.87949559	0.68993774	0.68323449	0.54482281	2.063E-16	-3.74026	down	PREDICTED: uncharacterized protein LOC104606632 [Nelumbo nucifera]
c51031.graph_c0	0	0	0.05980424	0.94707625	1.25214155	0.96600811	4.578E-10	4.59487	up	PREDICTED: uncharacterized protein C594.04c [Sesamum indicum]
c51032.graph_c0	1.47571685	2.33241247	0.99808136	7.95939438	8.56274179	9.16642101	3.577E-05	1.33363	up	hypothetical protein M569_01741 [Genlisea aurea]
c51033.graph_c0	3.87345378	2.90799935	3.24922846	3.59720496	3.38915354	2.99910052	3.132E-05	-1.09608	down	PREDICTED: uncharacterized protein LOC105173717 [Sesamum indicum]
c51036.graph_c0	0.20898547	0.17432895	0.0895182	1.82267322	2.12569974	1.59057025	2.178E-07	2.46804	up	PREDICTED: beta-amyrin 28-oxidase-like [Sesamum indicum]
c51037.graph_c0	13.7724466	16.1810301	17.6150493	102.447051	101.283347	104.341559	4.592E-61	1.60197	up	unnamed protein product [Coffea canephora]
c51039.graph_c0	0.96177684	1.51287702	1.41248151	1.69760511	1.21720625	1.14077922	0.0098293	-1.03355	down	PREDICTED: uncharacterized protein LOC105173120 [Sesamum indicum]
c51043.graph_c0	5.25502725	6.1185471	6.25533463	27.117197	28.2594766	27.0974674	8.116E-26	1.13346	up	PREDICTED: probable acyl-activating enzyme 1, peroxisomal [Sesamum indicum]
c51044.graph_c0	30.9481898	31.6448481	38.0605403	0.07435691	0.03356866	0.1820239	7.88E-109	-9.52597	down	PREDICTED: uncharacterized protein LOC104420277 [Eucalyptus grandis]
c51046.graph_c1	0.24642126	0.49333603	0.94998324	8.05938994	11.8047059	14.7603563	1.122E-06	3.25273	up	-
c51050.graph_c0	0.30227914	0.33620202	0.30212051	23.8979049	23.2526391	20.3570032	5.8E-120	5.07444	up	PREDICTED: protein TRANSPARENT TESTA 12-like [Sesamum indicum]
c51051.graph_c0	0.63924203	0.2399558	0.30804425	2.03261373	2.77910743	2.1798693	0.0017309	1.47495	up	PREDICTED: uncharacterized protein LOC105159339 isoform X2 [Sesamum indicum]
c51053.graph_c0	7.72952234	7.57935897	5.47314368	0.11464841	0.62110061	0.46776085	2.393E-32	-5.19621	down	hypothetical protein MIMGU_mgv1a015250mg [Erythranthe guttata]
c51054.graph_c0	34.2469858	20.1978457	17.9680625	0	0	0	2.499E-31	-Inf	down	uncharacterized protein LOC100274422 [Zea mays]
c51055.graph_c0	0.25744471	0.04295042	0.11027555	0.8108071	1.15443825	0.78884504	0.0022201	1.66342	up	PREDICTED: uncharacterized protein PB18E9.04c [Sesamum indicum]
c51057.graph_c0	0	0	0	7.22644684	8.41605147	9.57149015	7.925E-18	Inf	up	PREDICTED: uncharacterized protein LOC105177738 [Sesamum indicum]
c51059.graph_c0	7.69323132	9.64732507	6.73558927	1.84331742	4.2718132	3.40936579	5.653E-14	-2.42628	down	PREDICTED: E3 ubiquitin-protein ligase RHA1B-like [Sesamum indicum]
c51060.graph_c0	0.33298763	0.26665685	0.21395114	2.39593356	2.11960026	1.73783308	1.021E-05	1.85579	up	PREDICTED: PTI1-like tyrosine-protein kinase At3g15890 [Sesamum indicum]

#ID	0 h-1 FPKM	0 h-2 FPKM	0 h-3 FPKM	28 h-1 FPKM	28 h-2 FPKM	28 h-3 FPKM	FDR	log2FC	regulated	nr_annotation
c51061.graph_c0	9.63089819	10.5843714	9.52006575	1.12582287	1.54686683	1.75744085	1.412E-63	-3.83576	down	-
c51063.graph_c0	6.07591027	4.18137113	4.3918792	1.93197225	2.24278689	1.75664199	3.743E-13	-2.39239	down	-
c51066.graph_c0	0.48968106	0.14004897	0.26968267	2.26249045	2.68550066	2.26104423	2.424E-06	1.91801	up	PREDICTED: guanine nucleotide-binding protein subunit beta-like protein [Sesamum indicum]
c51067.graph_c0	0.4224885	0.53568798	0.61530305	4.29887094	2.86489999	3.89203597	4.098E-07	1.7187	up	PREDICTED: spindle pole body protein pcp1 [Sesamum indicum]
c51070.graph_c0	10.0991523	7.37703126	8.41803926	53.363825	46.749593	46.4582002	2.756E-08	1.4118	up	PREDICTED: ER membrane protein complex subunit 4 [Sesamum indicum]
c51071.graph_c0	0.59232435	0.75462224	0.4843747	2.66125991	2.93291263	2.87413194	0.0063864	1.12184	up	PREDICTED: protein disulfide-isomerase SCO2 [Sesamum indicum]
c51073.graph_c0	3.54989601	1.85748559	2.90293622	2.16958242	2.01187355	1.81820915	0.0006865	-1.55983	down	-
c51074.graph_c0	0	0.27968341	0	6.14283556	8.1133356	7.49804266	9.324E-22	5.19969	up	-
c51076.graph_c0	24.0265811	27.2439183	21.0158298	126.932431	121.391823	116.244408	2.8E-46	1.24564	up	PREDICTED: protein ETHYLENE INSENSITIVE 3 [Sesamum indicum]
c51078.graph_c0	19.5028141	15.5591607	12.6251617	14.3876837	14.4341438	12.0011265	1.346E-06	-1.31055	down	Dof zinc finger DOF5.2 -like protein [Gossypium arboreum]
c51081.graph_c0	0.15947166	0.63852538	0.81970958	4.28841403	6.06967773	4.44507051	1.156E-05	2.08858	up	PREDICTED: uncharacterized protein LOC105170400 [Sesamum indicum]
c51084.graph_c0	20.8119302	20.2289218	15.891454	13.1530356	13.8552842	12.3426851	1.478E-16	-1.62049	down	PREDICTED: 5'-adenylylsulfate reductase-like 5 isoform X2 [Sesamum indicum]
c51087.graph_c0	0.33009987	0.49564563	0.70698568	6.03785294	6.06537309	5.93830889	5.458E-12	2.45761	up	PREDICTED: protein CURVATURE THYLAKOID 1D, chloroplastic [Sesamum indicum]
c51088.graph_c0	1.01111028	0.70848593	0.5197267	33.8774668	34.5029319	37.4179857	3.876E-58	4.47962	up	PREDICTED: indole-3-acetic acid-induced protein ARG7-like [Nicotiana sylvestris]
c51090.graph_c0	13.1168316	15.8465108	15.6931894	8.5470406	8.60761972	7.77904402	1.829E-21	-1.93357	down	hypothetical protein MIMGU_mgv1a017353mg [Erythranthe guttata]
c51097.graph_c0	2.50639066	1.69520299	1.82802799	26.5859555	27.872037	27.5593165	9.986E-40	2.67859	up	PREDICTED: 30S ribosomal protein S20, chloroplastic isoform X1 [Sesamum indicum]
c51098.graph_c0	20.595011	17.8920236	19.687679	20.3837086	17.9650654	18.2465832	2.648E-26	-1.13016	down	PREDICTED: pre-mRNA-splicing factor 38 [Sesamum indicum]
c51100.graph_c0	0.03555325	0	0	1.05943577	1.7498253	1.45487498	1.745E-11	5.84697	up	hypothetical protein MIMGU_mgv1a004964mg [Erythranthe guttata]
c51103.graph_c0	5.4975839	2.82209687	6.15889336	0	0	0	1.376E-13	-Inf	down	40S ribosomal protein S16 [Arabidopsis lyrata subsp. lyrata]
c51104.graph_c0	118.04178	114.260312	116.895156	33.9770636	38.6032793	35.7537062	5.27E-202	-2.77926	down	-
c51106.graph_c0	0.08227375	0	0	2.93000838	2.59153259	2.00051795	2.059E-12	5.45433	up	PREDICTED: transcription factor HEC2-like [Sesamum indicum]
c51110.graph_c0	8.75421562	4.8196399	6.56221631	224.384641	247.12013	237.176858	2.38E-213	4.04932	up	beta-tubulin [Deschampsia antarctica]
c51112.graph_c0	0.09563642	0	0	3.54490797	2.76140508	2.09856452	8.598E-10	5.39708	up	PREDICTED: major allergen Pru ar 1-like [Sesamum indicum]
c51114.graph_c0	2.84344351	2.53003626	1.35331018	1.76043564	1.24396303	1.87369709	0.0013849	-1.54425	down	PREDICTED: lysine-specific demethylase JMJ18 [Sesamum indicum]
c51116.graph_c0	6.77944461	3.98322383	5.68164439	0	0	0	2.118E-25	-Inf	down	ribosomal protein L27

#ID	0 h-1 FPKM	0 h-2 FPKM	0 h-3 FPKM	28 h-1 FPKM	28 h-2 FPKM	28 h-3 FPKM	FDR	log2FC	regulated	nr_annotation
c51117.graph_c0	6.73336377	2.77533785	4.07183013	0	0	0	1.613E-11	-Inf	down	hypothetical protein MVLG_03147 [Microbotryum violaceum p1A1 Lamole]
c51118.graph_c0	0.36927205	0.36964182	0.42707629	5.58240383	5.74313356	5.56259504	2.034E-17	2.76373	up	PREDICTED: beta-1,4-mannosyl-glycoprotein 4-beta-N-acetylglucosaminyltransferase [Sesamum indicum]
c51119.graph_c0	4.64115492	3.98211629	2.98203391	0.40156722	0.50760857	0.39321069	1.662E-23	-4.24176	down	-
c51126.graph_c0	14.5385011	13.2796666	12.610721	12.4156361	12.3430966	10.8316096	5.783E-13	-1.27343	down	PREDICTED: suppressor of glycerol defect protein 1 isoform X2 [Sesamum indicum]
c51132.graph_c0	3.72277151	3.07841247	3.0679415	43.6733477	48.5675717	48.2360681	9.91E-106	2.74293	up	PREDICTED: 24-methylenesterol C-methyltransferase 2-like [Sesamum indicum]
c51133.graph_c0	0	0	0.89156985	13.8670165	13.5450298	15.224319	1.089E-17	4.44995	up	-
c51138.graph_c0	19.295474	16.1705264	15.9152178	1.89158762	3.06248848	2.34189249	8.957E-84	-3.90522	down	hypothetical protein MIMGU_mgv1a008111mg [Erythranthe guttata]
c51148.graph_c0	0.03963936	0.0793581	0.05093814	2.47763045	2.26308042	2.4918951	8.903E-14	4.32063	up	PREDICTED: GDSL esterase/lipase 7 [Sesamum indicum]
c51153.graph_c0	7.0969408	8.28051375	6.33161554	4.99374766	6.5260191	5.06676336	2.003E-14	-1.47779	down	hypothetical protein MIMGU_mgv1a020747mg [Erythranthe guttata]
c51154.graph_c0	55.6563405	54.7886674	54.1343776	13.1855897	15.3357067	22.430475	9.188E-49	-2.77961	down	PREDICTED: heat stress transcription factor A-2-like [Nicotiana sylvestris]
c51156.graph_c0	0.1016834	0.15267784	0.2613344	1.18244658	1.2344569	1.32669179	0.0001101	1.75819	up	PREDICTED: uncharacterized protein At4g26485-like [Sesamum indicum]
c51164.graph_c0	30.5132635	29.6111823	21.2516288	19.552884	20.7878896	19.7538016	3.069E-10	-1.52359	down	PREDICTED: probable WRKY transcription factor 31 [Sesamum indicum]
c51165.graph_c0	0.2236553	0.44775852	0.3592574	2.31635723	1.9813769	1.69116768	0.0014083	1.44385	up	-
c51166.graph_c0	0.50195686	0.25122975	0.80629305	4.28662741	3.87042264	3.05131854	0.0001562	1.74444	up	-
c51170.graph_c0	2.23454477	1.2340868	2.2773795	43.0654389	61.4863578	53.0086006	2.544E-26	3.68456	up	PREDICTED: CASP-like protein 1 [Sesamum indicum]
c51171.graph_c0	0.10844773	0	0.27871925	2.83749065	2.91781969	3.15147475	8.98E-10	3.41374	up	SUPERMAN like protein [Arabidopsis thaliana]
c51172.graph_c0	75.4740889	80.4397044	66.5649364	33.0889541	36.5153839	33.5318215	5.786E-64	-2.19813	down	PREDICTED: ethylene-responsive transcription factor TINY-like [Sesamum indicum]
c51176.graph_c0	88.2408922	86.9624552	72.5978651	8.68338111	8.73631088	9.2112385	3.72E-138	-4.3058	down	-
c51177.graph_c0	0	0	0	26.7548685	25.5744404	24.9912388	2.78E-122	Inf	up	PREDICTED: trans-resveratrol di-O-methyltransferase [Vitis vinifera]
c51184.graph_c0	151.995192	113.692556	130.928683	44.6125733	76.4516386	70.5782056	8.84E-18	-2.13829	down	hypothetical protein CARUB_v10018292mg, partial [Capsella rubella]
c51186.graph_c0	0.13202674	0.26431789	0	5.08568253	5.45828968	5.16776938	4.072E-11	4.23466	up	PREDICTED: nucleobase-ascorbate transporter 12 [Sesamum indicum]
c51193.graph_c0	10.8699855	10.4275006	9.07943514	5.06932248	6.06320177	4.62001577	1.271E-20	-2.03674	down	PREDICTED: cyclin-D3-1 [Sesamum indicum]
c51202.graph_c0	0.35552205	0.2588204	0.49839262	3.4060681	2.82084758	2.85599476	4.593E-08	1.93235	up	PREDICTED: U-box domain-containing protein 9-like [Sesamum indicum]
c51203.graph_c0	0.03031958	0.09104983	0.03896186	18.3340327	17.1906015	18.1251254	1.97E-125	7.29717	up	PREDICTED: aldehyde dehydrogenase family 3 member F1 [Sesamum indicum]
c51204.graph_c0	34.2745661	21.986681	22.0220872	0	0.15839364	0	6.327E-40	-10.0447	down	histone H3 [Neurospora crassa OR74A]

#ID	0 h-1 FPKM	0 h-2 FPKM	0 h-3 FPKM	28 h-1 FPKM	28 h-2 FPKM	28 h-3 FPKM	FDR	log2FC	regulated	nr_annotation
c51207.graph_c0	0.25671103	0.32121011	0.28864836	14.0864954	14.6981981	13.6068586	1.39E-73	4.52064	up	PREDICTED: argininosuccinate lyase, chloroplastic isoform X1 [Sesamum indicum]
c51208.graph_c0	7.23447398	5.26670415	7.18372515	6.45296808	4.85535429	5.85063251	9.14E-05	-1.28904	down	-
c51209.graph_c0	0.75359724	0.60348148	1.54944366	6.62729258	6.23108928	6.03351442	3.398E-06	1.5982	up	PREDICTED: U11/U12 small nuclear ribonucleoprotein 25 kDa protein-like isoform X1 [Sesamum indicum]
c51211.graph_c0	3.60094432	3.69466387	2.89209751	110.836115	126.127104	115.000413	5.91E-158	4.02259	up	PREDICTED: acyl carrier protein 1, chloroplastic-like [Sesamum indicum]
c51213.graph_c0	10.4737729	8.15442505	6.48036173	8.45808641	8.27326392	9.31733474	0.0005199	-1.03139	down	-
c51215.graph_c0	24.476775	25.8742017	22.641187	10.8884492	11.2851658	9.0100249	1.363E-48	-2.31732	down	PREDICTED: uncharacterized protein LOC105162031 [Sesamum indicum]
c51218.graph_c0	0.14491152	0.29011326	0.37243408	2.05375674	1.80680235	2.01961257	0.0001411	1.76449	up	-
c51221.graph_c0	0.32666738	0.28612018	0.20989026	2.78968464	3.88541949	2.80912496	5.517E-09	2.44074	up	PREDICTED: uncharacterized protein LOC105171540 [Sesamum indicum]
c51222.graph_c0	4.34024535	3.33422134	2.98325357	0.07335966	0	0.05986088	6.511E-35	-7.4042	down	PREDICTED: transcription factor PAR2-like [Sesamum indicum]
c51225.graph_c0	11.030075	10.4398708	7.64839133	67.7838817	78.5455841	72.862975	1.033E-40	1.82599	up	PREDICTED: S-formylglutathione hydrolase [Sesamum indicum]
c51231.graph_c0	4.90061722	4.57848948	4.47821308	4.07621395	4.28789012	4.2303554	1.068E-07	-1.23776	down	PREDICTED: uncharacterized protein LOC105173527 isoform X1 [Sesamum indicum]
c51232.graph_c0	28.1604181	25.2025342	24.6870661	137.890928	141.651048	131.624679	8.814E-35	1.30784	up	hypothetical protein MIMGU_mgv1a017391mg [Erythranthe guttata]
c51234.graph_c0	2.86224339	2.75491297	3.53663084	0.80010077	0.50569118	0.32643785	8.726E-17	-3.58353	down	hypothetical protein MIMGU_mgv1a020559mg [Erythranthe guttata]
c51236.graph_c0	0	0.20413607	0.08735351	29.54449	25.1592142	23.7452322	5.22E-70	6.97528	up	hypothetical protein MIMGU_mgv1a014485mg [Erythranthe guttata]
c51241.graph_c0	8.55215087	4.65256226	5.2560164	0	0	0	2.581E-17	-Inf	down	predicted protein [Hordeum vulgare subsp. vulgare]
c51249.graph_c0	4.0475686	3.85154154	3.66016415	15.2535161	17.4123685	17.8996041	3.099E-09	1.04008	up	PREDICTED: NADP-dependent D-sorbitol-6-phosphate dehydrogenase-like [Sesamum indicum]
c51262.graph_c0	0.19147915	0.19167088	0.16403885	2.96887337	3.03663338	3.19855793	4.31E-13	2.98392	up	hypothetical protein MIMGU_mgv1a003907mg [Erythranthe guttata]
c51265.graph_c0	4.01757012	4.09340728	4.24081941	0.99069624	1.03574358	1.10623164	5.159E-21	-3.07009	down	kunitz trypsin inhibitor [Populus tremula]
c51267.graph_c0	20.3306598	21.6700653	20.319991	10.1635282	10.0943843	9.42571807	1.16E-109	-2.16073	down	PREDICTED: exocyst complex component EXO70B1-like [Sesamum indicum]
c51269.graph_c0	0.07245576	0.07252831	0.09310852	2.47504017	3.37586754	2.06258305	1.568E-09	3.95881	up	PREDICTED: uncharacterized protein LOC102579011 [Solanum tuberosum]
c51271.graph_c0	91.1880693	91.236806	93.4053626	56.8159433	53.9788762	58.3174273	3.21E-105	-1.79638	down	PREDICTED: alcohol dehydrogenase-like 7 [Sesamum indicum]
c51275.graph_c0	0.0678191	0	0	5.52053983	5.6075961	4.94714283	4.017E-20	6.82948	up	PREDICTED: homeobox-leucine zipper protein ATHB-52 [Sesamum indicum]
c51276.graph_c0	12.4014537	12.0828352	11.6866632	9.85454684	12.9125621	11.6695895	3.594E-06	-1.16084	down	PREDICTED: uncharacterized protein LOC103341433 [Prunus mume]
c51277.graph_c0	0	0	0	1.13755732	1.45783015	1.04801006	4.657E-12	Inf	up	PREDICTED: formin-like protein 6 [Sesamum indicum]
c51279.graph_c0	0	0.04741835	0	4.3036132	4.22770603	3.51171323	3.389E-21	6.90599	up	hypothetical protein MIMGU_mgv1a008158mg [Erythranthe guttata]

#ID	0 h-1 FPKM	0 h-2 FPKM	0 h-3 FPKM	28 h-1 FPKM	28 h-2 FPKM	28 h-3 FPKM	FDR	log2FC	regulated	nr_annotation
c51281.graph_c0	3.86986257	2.55314527	2.42995278	13.2319754	16.2759409	13.1704779	3.372E-07	1.18322	up	PREDICTED: protein TRANSPARENT TESTA GLABRA 1 [Sesamum indicum]
c51282.graph_c0	6.09062293	6.61754724	6.37147759	6.91799909	5.64376714	6.26217239	9.526E-14	-1.11019	down	PREDICTED: scarecrow-like protein 15 [Sesamum indicum]
c51286.graph_c0	10.8798077	11.261593	8.70024062	62.3777343	63.7482231	60.8878933	3.694E-57	1.51241	up	hypothetical protein JCGZ_13359 [Jatropha curcas]
c51305.graph_c0	0.76571954	0.447117	0.49198996	3.94202055	4.81548965	4.35194714	4.358E-06	1.85695	up	PREDICTED: uncharacterized protein LOC105156993 [Sesamum indicum]
c51306.graph_c0	13.2999157	8.90609417	8.13291971	3.33320761	3.25033987	2.23029356	4.82E-10	-2.87002	down	PREDICTED: uncharacterized protein LOC105166496 [Sesamum indicum]
c51308.graph_c0	2.43760221	2.40131225	1.54134723	63.6945049	65.2035789	64.4571815	3.98E-204	3.83653	up	PREDICTED: adenosine kinase 2-like [Sesamum indicum]
c51311.graph_c0	0.54382398	0.54436854	0.51493131	6.36557526	7.06230924	6.84080309	4.711E-23	2.57047	up	PREDICTED: pentatricopeptide repeat-containing protein At1g02150 [Sesamum indicum]
c51312.graph_c0	9.49344487	5.9551827	6.01839657	0	0	0	9.463E-27	-Inf	down	60s ribosomal protein L21-a, putative [Candida maltosa Xu316]
c51323.graph_c0	0.02973189	0.17856999	0.07641332	1.14527536	1.03407627	1.02269905	1.918E-06	2.39869	up	PREDICTED: uncharacterized protein LOC105175605 isoform X2 [Sesamum indicum]
c51328.graph_c0	2.9678455	3.34216952	3.21789288	25.7493428	24.2230325	29.7017719	6.462E-20	1.97353	up	-
c51331.graph_c0	0.88235673	1.76648056	1.700795	26.6136092	30.2541271	26.5569097	2.082E-31	3.16477	up	--
c51334.graph_c0	23.8753218	23.1104759	22.58021	9.73569593	10.5743589	10.3508883	6.09E-101	-2.27186	down	PREDICTED: GATA transcription factor 8-like [Sesamum indicum]
c51335.graph_c0	0.84250178	0.73792724	0.13533102	41.1023451	49.343867	42.095728	6.718E-74	5.19651	up	PREDICTED: uncharacterized protein LOC105168965 [Sesamum indicum]
c51336.graph_c0	3.1215495	3.29132461	2.6742098	2.51072143	3.46870058	2.54239763	1.627E-06	-1.18212	down	-
c51337.graph_c0	0.04182974	0.08374325	0	1.1856629	0.96074321	1.3892182	5.662E-10	3.74271	up	PREDICTED: cell division control protein 2 homolog D [Sesamum indicum]
c51339.graph_c0	1.84451312	1.92663664	2.09545819	8.7040654	8.73607048	9.2902534	3.979E-10	1.09604	up	PREDICTED: uncharacterized protein LOC105161977 [Sesamum indicum]
c51341.graph_c0	5.90274641	7.72670547	5.39720625	0.7425142	1.04287666	1.14445044	4.565E-24	-3.7868	down	PREDICTED: putative calcium-binding protein CML23 [Sesamum indicum]
c51343.graph_c0	14.9802184	18.2611564	15.9591604	12.3664055	11.2347709	12.129869	5.623E-22	-1.55204	down	PREDICTED: F-box/kelch-repeat protein SKIP30 [Sesamum indicum]
c51348.graph_c0	0.07858398	0.07866267	0.05049176	2.77004748	3.71296304	3.30894567	5.33E-17	4.47349	up	PREDICTED: BTB/POZ domain-containing protein At1g63850-like [Sesamum indicum]
c51349.graph_c0	2.93372511	4.01859119	4.56362721	0.44888726	0.10132578	0.18314417	1.636E-18	-5.06888	down	-
c51352.graph_c0	3.09124341	0.92830165	1.19171105	9.43613115	7.70851868	8.79977824	0.0091593	1.23649	up	-
c51356.graph_c0	1.5981637	1.59976402	0.55505504	13.1536123	13.5771677	13.0899421	8.012E-25	2.32794	up	PREDICTED: A-kinase anchor protein 17B [Sesamum indicum]
c51358.graph_c0	10.4842046	8.3514185	10.1519022	59.1880631	63.1314387	63.4472516	1.935E-38	1.58937	up	PREDICTED: 50S ribosomal protein HLP, mitochondrial [Sesamum indicum]

#ID	0 h-1 FPKM	0 h-2 FPKM	0 h-3 FPKM	28 h-1 FPKM	28 h-2 FPKM	28 h-3 FPKM	FDR	log2FC	regulated	nr_annotation
c51363.graph_c0	6.98448335	7.28898692	10.5030547	0.75604204	0.29255789	0.79318816	1.384E-16	-4.84314	down	-
c51366.graph_c0	0.80679455	0.48456146	0.31102888	147.41433	130.348228	129.714868	2.9E-208	6.91028	up	PREDICTED: putative invertase inhibitor [Sesamum indicum]
c51369.graph_c0	0.87150579	1.96285155	1.3998993	6.9674592	9.00751434	9.17415174	0.0008639	1.47751	up	-
c51370.graph_c0	4.26194463	2.34641678	3.55989893	91.0681697	105.019966	94.152394	1.73E-64	3.74534	up	putative plasma membrane intrinsic protein PIP family member 2 aquaporin [Cedrela odorata]
c51372.graph_c0	0.08081761	0	0	5.13955486	6.12550773	5.77550964	2.304E-31	6.66116	up	PREDICTED: 2-alkenal reductase (NADP(+)-dependent) isoform X1 [Sesamum indicum]
c51373.graph_c0	6.53177224	6.30480165	8.54347139	3.68759941	4.36286717	4.42710953	1.347E-08	-1.87103	down	PREDICTED: F-box protein At5g52880 [Sesamum indicum]
c51375.graph_c0	71.8959022	72.6455589	71.3362017	321.492041	343.854499	342.713581	3.095E-40	1.13296	up	hypothetical protein MIMGU_mgv1a013134mg [Erythranthe guttata]
c51377.graph_c0	9.39448121	6.12677575	5.85322681	0	0	0	4.57E-27	-Inf	down	unknown [Lotus japonicus]
c51379.graph_c0	90.0258061	92.7453312	87.5782263	61.1607839	61.8353579	66.2212628	1.128E-84	-1.60453	down	PREDICTED: glutelin type-B 5 [Sesamum indicum]
c51380.graph_c0	24.7052913	25.7848653	23.3716134	25.0471576	26.1240539	26.7109519	2.001E-26	-1.01306	down	PREDICTED: protein YLS9 [Sesamum indicum]
c51381.graph_c0	4.40598105	2.3816122	3.96330206	0	0	0	2.475E-20	-Inf	down	--
c51384.graph_c0	0.8088742	0.64099996	0.77957653	7.17709731	9.22273912	7.43549638	1.606E-20	2.32696	up	PREDICTED: afadin- and alpha-actinin-binding protein [Sesamum indicum]
c51386.graph_c0	0.20327992	0.5087087	0.13061138	4.80163703	5.00241697	4.94282957	5.638E-09	3.04556	up	-
c51391.graph_c0	5.89714488	4.83312217	5.35200816	4.01813523	3.45869274	4.45912112	2.662E-16	-1.51893	down	hypothetical protein MIMGU_mgv1a026169mg, partial [Erythranthe guttata]
c51392.graph_c0	0	0.07816369	0	5.56170519	4.76548923	5.32556319	7.433E-18	6.56432	up	PREDICTED: 21 kDa protein-like [Sesamum indicum]
c51395.graph_c0	50.7126173	58.1688823	51.3674977	33.4754077	29.5987264	31.4200848	7.628E-52	-1.85223	down	PREDICTED: E3 ubiquitin-protein ligase RING1-like [Sesamum indicum]
c51396.graph_c0	0.03725766	0	0.04787756	1.1914606	1.27137306	1.36994935	1.999E-11	4.39355	up	-
c51398.graph_c0	3.19502962	2.23132253	1.43223473	1.83610209	1.80410698	1.71857436	0.0051533	-1.4377	down	hypothetical protein JCGZ_03974 [Jatropha curcas]
c51401.graph_c0	8.24779118	7.43630044	7.66717233	1.8280938	1.34350943	2.28960079	3.279E-45	-3.18397	down	PREDICTED: uncharacterized protein LOC105157495 [Sesamum indicum]
c51405.graph_c0	4.32583663	4.54667672	3.61326637	25.623544	27.29913	26.7236742	5.558E-18	1.58521	up	PREDICTED: DEAD-box ATP-dependent RNA helicase 31-like [Sesamum indicum]
c51408.graph_c0	2.3589027	2.95158098	3.03128336	18.5730663	19.3496971	17.0207591	2.396E-06	1.62582	up	hypothetical protein POPTR_0013s01180g [Populus trichocarpa]
c51409.graph_c0	5.61440049	4.29766423	5.80007457	0.32004031	0.36120799	0	6.037E-36	-5.62671	down	-
c51417.graph_c0	3.20253016	3.20573701	3.35492776	14.420879	15.0537585	14.8226937	2.968E-12	1.09061	up	PREDICTED: ubiquitin receptor RAD23c-like [Sesamum indicum]
c51420.graph_c0	2.53872621	2.80877029	2.40384693	1.89368767	2.01671362	1.78296193	3.627E-06	-1.5352	down	PREDICTED: embryonic protein DC-8-like isoform X2 [Sesamum indicum]
c51422.graph_c0	2.01278187	1.67899781	1.2932522	10.3620544	12.1077175	10.8427452	3.93E-05	1.65474	up	PREDICTED: GTP-binding protein OBGC, chloroplastic [Sesamum indicum]

#ID	0 h-1 FPKM	0 h-2 FPKM	0 h-3 FPKM	28 h-1 FPKM	28 h-2 FPKM	28 h-3 FPKM	FDR	log2FC	regulated	nr_annotation
c51426.graph_c0	1.28685488	1.23556619	1.28242979	6.9477847	7.85873177	6.97764379	2.369E-12	1.42635	up	hypothetical protein MIMGU_mgv1a026964mg, partial [Erythranthe guttata]
c51429.graph_c0	0.28261084	0.14144692	0.54474918	2.97829898	3.15276693	3.77110497	2.216E-06	2.25178	up	-
c51440.graph_c0	17.8315849	19.7384743	16.6784376	12.5960085	12.7377764	12.1054827	1.231E-49	-1.62464	down	hypothetical protein MIMGU_mgv1a026899mg [Erythranthe guttata]
c51441.graph_c0	9.47979429	10.3422565	10.5394061	5.41899089	9.57598822	6.2221779	4.449E-17	-1.61011	down	PREDICTED: phospholipid hydroperoxide glutathione peroxidase 1, chloroplastic-like [Sesamum indicum]
c51444.graph_c0	1.33021638	1.22502452	1.09400366	0.73476314	0.38408655	0.63111655	4.999E-08	-2.14734	down	-
c51446.graph_c0	1.66639466	1.40117318	1.45614052	8.91388764	7.87343946	8.02474393	4.508E-06	1.36616	up	PREDICTED: phospholipase D delta [Sesamum indicum]
c51448.graph_c0	0	0.10205227	0	1.25964564	1.27114685	1.66271561	2.704E-11	4.28047	up	PREDICTED: uncharacterized protein LOC105162545 [Sesamum indicum]
c51455.graph_c0	0.15920705	0.07968323	0.30688104	1.64887838	1.85444848	1.69954269	2.152E-06	2.15003	up	PREDICTED: uncharacterized protein LOC105171871 [Sesamum indicum]
c51460.graph_c0	0	0	0	20.1402292	26.6071439	24.73789	1.225E-48	Inf	up	PREDICTED: MLP-like protein 423 [Sesamum indicum]
c51462.graph_c0	18.6289537	10.3317827	17.4689584	11.7097654	12.2248234	12.0931391	0.0010799	-1.45706	down	-
c51463.graph_c0	47.6161678	37.0281134	40.8345815	12.1554061	16.2045483	12.602599	7.821E-37	-2.70502	down	PREDICTED: uncharacterized protein LOC105175709 [Sesamum indicum]
c51466.graph_c0	125.48772	128.249015	126.701684	29.926231	31.9835445	41.0315058	0	-2.9751	down	PREDICTED: zinc-finger homeodomain protein 6 [Sesamum indicum]
c51468.graph_c0	9.40967601	14.971409	11.3281132	4.6072578	5.45989739	5.11054957	1.681E-08	-2.32575	down	PREDICTED: oleosin 1-like [Solanum lycopersicum]
c51470.graph_c0	0.73263557	0.1833423	0.11768321	6.32314428	8.11308282	8.0381668	5.541E-13	3.37153	up	PREDICTED: probable stress-associated endoplasmic reticulum protein [Musa acuminata subsp. malaccensis]
c51473.graph_c0	0	0.29656079	0	1.79435883	1.84695708	1.52274944	5.626E-08	3.03958	up	PREDICTED: DNA replication complex GINS protein PSF2 [Sesamum indicum]
c51477.graph_c0	5.36844353	3.72503377	4.15487627	0	0	0	2.549E-50	-Inf	down	RecName: Full=60S ribosomal protein L5 [Helianthus annuus]
c51480.graph_c0	0.92155709	0.46123994	0.78949156	42.6428114	38.7040442	34.1585595	1.146E-43	4.6419	up	PREDICTED: uncharacterized protein LOC105179276 [Sesamum indicum]
c51483.graph_c0	0.29925074	0.25675748	0.32961346	4.90914373	5.0216258	5.14671964	1.896E-15	2.99736	up	PREDICTED: UPF0553 protein-like isoform X1 [Sesamum indicum]
c51484.graph_c0	6.07423294	6.8861403	5.4545365	27.1265774	25.9335064	28.2547602	5.306E-10	1.05418	up	PREDICTED: multiple C2 and transmembrane domain-containing protein 1-like [Sesamum indicum]
c51490.graph_c0	7.48351737	9.56683331	7.18349637	0.26211939	0.05916731	0.2673591	6.638E-55	-6.44922	down	unnamed protein product [Coffea canephora]
c51492.graph_c0	2.56160418	3.05518038	3.43183781	12.1608524	15.7369648	12.444349	1.197E-06	1.06181	up	hypothetical protein MIMGU_mgv1a000973mg [Erythranthe guttata]
c51494.graph_c0	0.57979126	0.49746157	0.69183653	2.16714314	3.12532795	2.79992091	0.002438	1.10001	up	PREDICTED: uncharacterized protein LOC105164785 isoform X2 [Sesamum indicum]
c51496.graph_c0	0.12853312	0.64330914	0.3303403	2.98934824	3.96430487	3.12533231	1.677E-05	2.09838	up	-

#ID	0 h-1 FPKM	0 h-2 FPKM	0 h-3 FPKM	28 h-1 FPKM	28 h-2 FPKM	28 h-3 FPKM	FDR	log2FC	regulated	nr_annotation
c51497.graph_c0	1.75802705	1.83311193	2.02380787	8.33182479	8.868799	7.99336091	3.186E-07	1.073	up	PREDICTED: uncharacterized protein LOC105176842 [Sesamum indicum]
c51499.graph_c0	19.6961322	19.1282963	19.737013	84.6577918	91.8668932	88.8835349	9.345E-39	1.08949	up	hypothetical protein MIMGU_mgv1a004619mg [Erythranthe guttata]
c51500.graph_c0	0.43468377	0.87023808	0.44686869	7.26629018	6.2755275	4.74338676	7.674E-08	2.2937	up	PREDICTED: BRI1 kinase inhibitor 1 [Sesamum indicum]
c51503.graph_c0	3.19921874	2.04955025	2.13778382	9.48670886	14.3599909	11.156258	0.0006842	1.15766	up	PREDICTED: protein ENHANCED DISEASE RESISTANCE 2 [Sesamum indicum]
c51505.graph_c0	115.068372	134.938921	178.601483	83.7525859	55.0966731	79.7481954	3.714E-06	-2.06643	down	hypothetical protein M569_00222, partial [Genlisea aurea]
c51511.graph_c0	4.18386607	4.34316875	4.11529481	19.2209209	19.7274008	20.0033725	1.36E-11	1.13116	up	PREDICTED: protein FLUORESCENT IN BLUE LIGHT, chloroplastic [Sesamum indicum]
c51514.graph_c0	0.40461607	0.25313827	0.25997384	7.13126127	7.00309034	7.10884061	3.342E-20	3.44716	up	PREDICTED: cyclin-D3-3-like [Sesamum indicum]
c51516.graph_c0	12.2523612	6.67875898	8.41799546	0	0	0	7.679E-24	-Inf	down	predicted protein [Physcomitrella patens]
c51518.graph_c0	0	0.07172588	0	5.05154749	4.70213789	6.16178472	2.92E-19	6.71328	up	PREDICTED: uncharacterized protein LOC105177191 [Sesamum indicum]
c51521.graph_c0	6.02716485	5.64604292	5.38432572	29.7968291	31.2607957	31.5775764	1.913E-32	1.35204	up	PREDICTED: methylthioribose kinase [Sesamum indicum]
c51523.graph_c0	0.78529933	0.42877401	0.5504404	5.08487278	4.96595221	5.33470456	4.884E-07	2.03755	up	-
c51525.graph_c0	25.800674	30.1971495	22.494316	19.3864568	31.387655	24.7174851	1.04E-06	-1.14515	down	PREDICTED: E3 ubiquitin-protein ligase ATL31-like [Sesamum indicum]
c51527.graph_c0	0	0	0.27893722	11.0433154	15.6688375	12.7444895	9.087E-17	6.01347	up	-
c51533.graph_c0	16.9552304	16.2521754	15.7138724	1.12026943	1.01149826	0.97507276	2.77E-182	-5.06693	down	PREDICTED: uncharacterized protein LOC105155796 [Sesamum indicum]
c51534.graph_c0	434.593424	445.102392	438.608057	56.7431607	64.2333631	63.6416298	0	-3.92653	down	PREDICTED: zinc finger A20 and AN1 domain-containing stress-associated protein 5 [Sesamum indicum]
c51535.graph_c0	32.2664208	19.5524884	19.3814661	0	0	0	8.071E-31	-Inf	down	-
c51537.graph_c0	134.719924	125.495728	131.122502	124.486464	127.747636	132.035712	1.335E-41	-1.11649	down	PREDICTED: anthocyanidin 3-O-glucosyltransferase 5-like [Sesamum indicum]
c51538.graph_c0	0.4549434	1.02464765	0.73077535	4.13312801	4.40355595	2.83298291	0.0051504	1.26767	up	PREDICTED: uncharacterized protein LOC105159330 [Sesamum indicum]
c51540.graph_c0	857.850664	933.26925	780.863924	154.887004	166.220161	157.959565	2.55E-131	-3.51381	down	Polyubiquitin-A [Triticum urartu]
c51544.graph_c0	0.58553734	0.41028657	0.82768253	3.48963435	5.18731348	4.82688202	1.341E-06	1.79204	up	PREDICTED: polyol transporter 5-like [Sesamum indicum]
c51547.graph_c0	0.47543626	0	1.22190884	12.0940517	10.6078006	12.9702224	3.662E-09	3.28365	up	-
c51554.graph_c0	6.41493548	7.18000064	6.46953995	2.67543454	3.71231109	3.88469208	3.196E-42	-2.05549	down	PREDICTED: 3-ketoacyl-CoA synthase 1 [Sesamum indicum]
c51558.graph_c0	4.28800689	5.74095216	1.99746875	50.7989287	74.4981646	74.9560299	5.36E-14	2.97631	up	PREDICTED: blue copper protein-like [Sesamum indicum]
c51564.graph_c0	0.07018735	0.21077289	0	1.63237629	1.88841226	1.3736309	1.604E-07	3.04541	up	PREDICTED: uncharacterized protein LOC105170763 isoform X2 [Sesamum indicum]

#ID	0 h-1 FPKM	0 h-2 FPKM	0 h-3 FPKM	28 h-1 FPKM	28 h-2 FPKM	28 h-3 FPKM	FDR	log2FC	regulated	nr_annotation
c51565.graph_c1	11.2072158	15.3643826	12.2101505	8.05680354	5.99549852	7.5857145	1.834E-12	-1.93182	down	PREDICTED: floral homeotic protein APETALA 2-like isoform X2 [Sesamum indicum]
c51566.graph_c0	2.87210383	3.13372799	2.17755261	26.8025821	27.1215846	26.7081301	6.112E-66	2.21368	up	PREDICTED: ankyrin repeat-containing protein At2g01680 [Sesamum indicum]
c51569.graph_c0	5.84030455	5.84615273	6.54284019	4.35353814	5.99452606	4.88462091	4.121E-08	-1.35175	down	PREDICTED: uncharacterized protein LOC105171557 [Sesamum indicum]
c51570.graph_c0	2.12078345	1.93559176	2.32451266	1.49603869	1.14611863	1.47970345	1.478E-06	-1.72176	down	PREDICTED: uncharacterized protein LOC105175073 [Sesamum indicum]
c51573.graph_c0	34.7559086	35.4565623	35.9011326	15.7122068	14.0775215	14.1031357	1.37E-45	-2.36463	down	-
c51574.graph_c0	3.72890961	4.08477973	4.88220773	1.38064246	1.15425064	1.37694696	8.829E-19	-2.79183	down	hypothetical protein JCGZ_21459 [Jatropha curcas]
c51578.graph_c0	0.40585215	0.10156464	0.13038402	3.02345278	4.72737808	3.00867412	3.047E-06	2.9974	up	hypothetical protein CICLE_v10033008mg [Citrus clementina]
c51579.graph_c0	6.82941825	4.91730758	5.38881665	0.17416077	0.31450168	0.28422753	8.761E-36	-5.55849	down	-
c51582.graph_c0	13.0688342	12.9828152	11.8321148	0	0.03248531	0.4403738	1.09E-190	-7.4006	down	PREDICTED: LOW QUALITY PROTEIN: EID1-like F-box protein 3 [Sesamum indicum]
c51588.graph_c0	2.25541527	2.33552455	3.39800446	2.82624624	2.14354171	2.67518487	0.0037283	-1.15909	down	-
c51592.graph_c0	8.02379469	6.34447021	6.41181635	2.45027216	4.07075348	3.51894765	3.648E-20	-2.13766	down	PREDICTED: glutaredoxin-C9-like [Sesamum indicum]
c51596.graph_c0	0.62892811	0.45786028	0.44083501	2.86727196	2.25119804	2.61093665	0.0036764	1.25279	up	hypothetical protein MIMGU_mgv1a007994mg [Erythranthe guttata]
c51597.graph_c0	11.9019547	10.1736441	11.3419761	3.86346421	3.35671025	3.80685916	1.362E-80	-2.68951	down	PREDICTED: pentatricopeptide repeat-containing protein At3g26630, chloroplastic [Sesamum indicum]
c51601.graph_c0	0.26610555	0.33901893	0.18652155	4.1493945	3.74651419	3.67281034	1.02E-17	2.78329	up	PREDICTED: uncharacterized protein LOC105171071 [Sesamum indicum]
c51602.graph_c0	8.66082409	10.8368707	9.37221208	5.71486783	8.15128828	5.60945935	1.104E-11	-1.66009	down	-
c51603.graph_c0	0.32182691	0.08053729	0.20678016	1.57883813	2.2703089	1.67004317	1.415E-05	2.09213	up	hypothetical protein MIMGU_mgv1a020884mg [Erythranthe guttata]
c51610.graph_c0	30.121116	13.0104829	18.0278371	0	0	0	1.851E-15	-Inf	down	-
c51619.graph_c0	0	0	0	2.60286824	2.9067595	3.4653419	2.556E-14	Inf	up	hypothetical protein MIMGU_mgv1a023502mg, partial [Erythranthe guttata]
c51620.graph_c0	67.9826049	59.5785063	75.0807732	51.459821	53.2716543	50.1406779	2.85E-13	-1.48042	down	PREDICTED: ethylene-responsive transcription factor ERF069-like [Sesamum indicum]
c51622.graph_c0	25.9222439	26.1828141	25.1791028	182.541513	188.626947	193.988379	1.37E-102	1.78046	up	PREDICTED: 40S ribosomal protein SA-like [Sesamum indicum]
c51629.graph_c0	51.9814082	43.846862	53.7947909	25.6902557	27.7441064	35.0206062	2.763E-19	-1.84855	down	-
c51630.graph_c0	4.10138072	2.86821739	3.3932948	1.14334351	1.32728399	1.49939834	2.138E-16	-2.47223	down	hypothetical protein MIMGU_mgv1a010366mg [Erythranthe guttata]
c51632.graph_c0	0.19285497	0.38609617	0.86739194	170.371627	171.990484	162.297008	0	7.3385	up	PREDICTED: non-specific lipid-transfer protein 2-like [Sesamum indicum]
c51638.graph_c0	0.06877941	0	0	2.64938933	1.85053126	2.48820158	1.27E-12	5.6087	up	-

#ID	0 h-1 FPKM	0 h-2 FPKM	0 h-3 FPKM	28 h-1 FPKM	28 h-2 FPKM	28 h-3 FPKM	FDR	log2FC	regulated	nr_annotation
c51644.graph_c0	1.59097771	1.20815718	1.90347146	8.73216003	9.3963835	8.97991996	2.822E-08	1.43316	up	-
c51645.graph_c0	56.3960131	62.0483583	55.7795129	47.3217659	49.8483041	47.5851444	6.841E-30	-1.35759	down	PREDICTED: uncharacterized protein LOC105177839 [Sesamum indicum]
c51648.graph_c0	0.67447312	0.311607	0.20001338	2.22476826	2.79183196	2.18462515	0.0007513	1.52403	up	hypothetical protein MIMGU_mgv1a011849mg [Erythranthe guttata]
c51653.graph_c0	0	0	0	2.69129297	4.0220445	2.42325306	1.486E-09	Inf	up	-
c51657.graph_c0	3.41874635	2.85180809	4.2467836	0.49694503	0.74782461	0.74342232	4.236E-15	-3.49689	down	-
c51658.graph_c0	49.7268238	55.1871197	51.3985801	6.54731322	5.20221677	6.51791603	3.27E-135	-4.18705	down	hypothetical protein MIMGU_mgv1a005887mg [Erythranthe guttata]
c51660.graph_c0	0.35354746	0.17695074	0.68148372	11.2418362	10.6143389	10.3264547	1.625E-19	3.62797	up	PREDICTED: nudix hydrolase 1 [Sesamum indicum]
c51665.graph_c0	5.18143695	3.30870929	2.64038082	19.8680446	19.4632086	19.5499602	1.455E-08	1.32043	up	PREDICTED: uncharacterized protein LOC104227211 [Nicotiana sylvestris]
c51668.graph_c0	77.04052	83.0838721	72.3068655	53.0117663	54.8739609	54.7014193	3.728E-75	-1.60506	down	hypothetical protein MIMGU_mgv1a003993mg [Erythranthe guttata]
c51669.graph_c0	2.44309736	2.90408321	4.218672	20.7526649	16.8338791	16.9792832	5.864E-09	1.41419	up	PREDICTED: probable calcium-binding protein CML13 [Sesamum indicum]
c51672.graph_c0	29.5589602	16.8348698	21.7755592	0	0	0	6.107E-34	-Inf	down	--
c51676.graph_c0	158.237019	168.590537	155.25803	12.0724272	14.8587889	15.2949768	0	-4.60275	down	hypothetical protein MIMGU_mgv1a009467mg [Erythranthe guttata]
c51680.graph_c0	6.24373048	5.87684934	5.98764244	58.3832256	58.7076515	52.11687	7.292E-44	2.13336	up	PREDICTED: cytochrome b-c1 complex subunit 6 [Sesamum indicum]
c51684.graph_c0	1.81063396	0.55767601	0.35795944	20.650423	26.2315127	26.6800795	8.278E-24	3.68128	up	PREDICTED: 60S ribosomal protein L12-1-like [Cucumis sativus]
c51687.graph_c0	4.11647333	5.75328408	4.39155798	0.84674408	0.30581221	0.69093626	7.261E-26	-4.04021	down	PREDICTED: LOB domain-containing protein 37-like [Sesamum indicum]
c51688.graph_c0	0.86678195	1.7352998	1.33661895	5.98473518	6.54126627	6.27143573	0.0012586	1.16128	up	PREDICTED: uncharacterized protein LOC105159746 [Sesamum indicum]
c51689.graph_c0	0	0	0	1.25634949	1.7349124	1.86942934	2.386E-14	Inf	up	PREDICTED: transcription factor PCF7-like [Sesamum indicum]
c51693.graph_c0	2.52684129	1.95451437	2.50911585	2.67126354	2.03504107	2.1116127	0.0043009	-1.12749	down	PREDICTED: zinc finger protein NUTCRACKER-like isoform X1 [Sesamum indicum]
c51694.graph_c0	2.19521648	1.09870733	0.90673074	7.69244796	7.97453102	9.02024515	1.042E-05	1.47524	up	PREDICTED: uncharacterized protein LOC105176347 isoform X3 [Sesamum indicum]
c51700.graph_c0	0	0	0	61.6514862	61.5329575	71.3817092	2.53E-106	Inf	up	--
c51701.graph_c0	2.23459088	2.07315811	1.68090015	8.99190061	9.79983714	8.82417476	1.159E-05	1.11775	up	PREDICTED: protein SAWADEE HOMEODOMAIN HOMOLOG 1-like [Sesamum indicum]
c51703.graph_c0	2.32665346	1.66355946	1.38814084	9.90441874	11.5601551	11.679367	2.734E-07	1.53917	up	hypothetical protein MIMGU_mgv1a001920mg [Erythranthe guttata]
c51705.graph_c0	0	0	0	1.70028505	2.45631677	3.56104136	4.68E-07	Inf	up	60S ribosomal protein L8 [Rozella allomycis CSF55]
c51715.graph_c0	7.67698065	7.88689609	9.60561291	4.99226052	3.97724398	5.27177435	2.09E-08	-1.91416	down	BnaCnng51820D [Brassica napus]
c51716.graph_c0	22.053418	19.903493	19.8224516	11.7559961	18.0965342	14.8103424	4.415E-36	-1.55788	down	PREDICTED: SNF1-related protein kinase regulatory subunit gamma-1-like [Sesamum indicum]

#ID	0 h-1 FPKM	0 h-2 FPKM	0 h-3 FPKM	28 h-1 FPKM	28 h-2 FPKM	28 h-3 FPKM	FDR	log2FC	regulated	nr_annotation
c51717.graph_c0	11.6189151	6.44395319	8.37333493	0	0	0	3.035E-28	-Inf	down	60S ribosomal protein L8 [Rozella allomycis CSF55]
c51719.graph_c0	2.54892225	1.02954239	1.32167927	0	0	0	3.672E-10	-Inf	down	Bifunctional monodehydroascorbate reductase and carbonic anhydrase nectarin-3 [Gossypium arboreum]
c51720.graph_c0	12.6927661	15.6642855	11.8420202	151.391576	162.992564	140.497053	3.97E-82	2.41061	up	hypothetical protein MIMGU_mgv1a021151mg [Erythranthe guttata]
c51724.graph_c0	13.1862118	12.733554	13.456168	131.127056	157.131004	142.373469	1.15E-55	2.35992	up	PREDICTED: 60S acidic ribosomal protein P3-like [Sesamum indicum]
c51729.graph_c0	14.4472132	13.7921577	13.3222794	2.18753152	1.75567632	2.18167627	1.034E-88	-3.85141	down	PREDICTED: homeobox-leucine zipper protein HOX3 [Sesamum indicum]
c51730.graph_c0	0.16859296	0.25314266	0.21664862	6.92307997	5.64239819	4.44935158	2.517E-12	3.64248	up	PREDICTED: uncharacterized protein LOC105170277 [Sesamum indicum]
c51731.graph_c0	7.3777705	8.67463029	8.57780079	2.8512866	2.19019903	2.0835462	1.764E-41	-2.88213	down	hypothetical protein MIMGU_mgv1a020203mg [Erythranthe guttata]
c51733.graph_c0	3.61757604	2.80350851	4.04889251	0	0	0	1.753E-33	-Inf	down	-
c51740.graph_c0	0.07567624	0.11362802	0.2431174	1.67753209	2.38371808	1.77277617	1.689E-08	2.64776	up	hypothetical protein MIMGU_mgv1a007527mg [Erythranthe guttata]
c51743.graph_c0	1.02070686	0.40869158	1.04931898	10.6825516	11.5208258	8.95901027	6.617E-08	2.55788	up	-
c51745.graph_c0	8.36342989	3.88525739	5.99713366	0	0	0	1.319E-20	-Inf	down	predicted protein [Physcomitrella patens]
c51751.graph_c0	5.70270474	4.1100589	5.56943304	0	0	0	5.234E-29	-Inf	down	30S ribosomal protein S28e [Mucor circinelloides f. circinelloides 1006PhL]
c51753.graph_c0	0	0	0	9.74711242	8.04638046	11.3622345	1.834E-25	Inf	up	Putative non-specific lipid-transfer protein AKCS9 [Aegilops tauschii]
c51754.graph_c0	47.5834595	48.0071422	40.6035074	39.3158472	43.4692572	40.8938964	7.209E-35	-1.22768	down	PREDICTED: uncharacterized protein LOC105174255 [Sesamum indicum]
c51757.graph_c0	3.0903147	4.29640164	4.41241859	2.49558101	1.3519654	2.64728584	1.121E-06	-1.95338	down	-
c51758.graph_c0	21.0961927	18.8902051	15.3976124	8.94238806	8.74258515	8.60050405	1.508E-23	-2.16208	down	PREDICTED: uncharacterized protein LOC105123074 [Populus euphratica]
c51759.graph_c0	0.12874747	0.0644382	0.41361399	4.35114073	7.39266259	5.91749217	1.861E-10	3.75258	up	PREDICTED: uncharacterized protein LOC105158157 [Sesamum indicum]
c51760.graph_c0	4.71381345	4.58746325	4.54307769	2.60120131	2.31999893	2.69202608	5.008E-18	-1.95231	down	PREDICTED: uncharacterized protein LOC105156249 [Sesamum indicum]
c51767.graph_c0	6.03785111	4.80412334	2.98418374	0	0	0	7.056E-18	-Inf	down	predicted protein [Physcomitrella patens]
c51771.graph_c0	0.39182902	0.78444276	0.2517579	7.54664553	5.91396253	5.34468022	7.246E-07	2.63405	up	hypothetical protein MIMGU_mgv1a000217mg [Erythranthe guttata]
c51779.graph_c0	0.05179035	0.15552663	0.06655265	14.0024253	15.5996791	13.1458873	2.91E-56	6.1962	up	PREDICTED: acid phosphatase 1-like [Sesamum indicum]
c51780.graph_c0	0	0.10174174	0	2.6593682	4.66892251	3.43586933	4.921E-09	5.6427	up	hypothetical protein M569_05207, partial [Genlisea aurea]
c51782.graph_c0	44.2094699	43.083568	45.3394835	7.72384325	8.71738284	10.4623044	2.64E-142	-3.39143	down	PREDICTED: uncharacterized protein LOC105172990 [Sesamum indicum]
c51793.graph_c0	2.95948388	2.73009854	3.20649647	1.47613243	2.17057495	1.82397533	8.162E-09	-1.79369	down	PREDICTED: uncharacterized protein LOC105170241 [Sesamum indicum]

#ID	0 h-1 FPKM	0 h-2 FPKM	0 h-3 FPKM	28 h-1 FPKM	28 h-2 FPKM	28 h-3 FPKM	FDR	log2FC	regulated	nr_annotation
c51794.graph_c0	22.7299907	25.0232962	23.0149428	16.8291328	15.3811918	16.2266935	2.054E-52	-1.6373	down	PREDICTED: uncharacterized protein LOC105156609 [Sesamum indicum]
c51795.graph_c0	22.8768532	24.6376892	20.3421322	8.83296892	8.54501011	8.35842866	1.097E-75	-2.48765	down	PREDICTED: E3 ubiquitin-protein ligase ATL4-like [Sesamum indicum]
c51802.graph_c0	1.59199747	1.45699805	1.34436957	11.0746653	13.7603469	11.8423021	1.659E-18	1.97251	up	hypothetical protein MIMGU_mgv1a010707mg [Erythranthe guttata]
c51806.graph_c0	0.74150419	0.90461316	1.13152391	6.39968653	5.8391413	6.1977986	7.823E-15	1.63513	up	PREDICTED: uncharacterized protein LOC105177377 [Sesamum indicum]
c51809.graph_c0	13.9807256	13.1026311	14.3153673	0.85013277	0.73103827	0.7267348	4.75E-147	-5.25625	down	PREDICTED: LOW QUALITY PROTEIN: myb-like protein AA [Sesamum indicum]
c51812.graph_c0	0	0.27372327	0.35139338	3.64359394	4.96464304	3.94617148	8.476E-10	3.21809	up	PREDICTED: calmodulin-like protein 1 [Sesamum indicum]
c51816.graph_c0	9.10935506	9.20615436	9.45475077	0.25463949	0.17243669	0.25972968	7.02E-76	-6.42722	down	hypothetical protein Csa_5G205920 [Cucumis sativus]
c51818.graph_c0	1.42221111	1.63299336	1.39757462	10.36695	10.4858259	10.0718319	4.2E-14	1.70589	up	PREDICTED: FACT complex subunit SPT16-like [Sesamum indicum]
c51820.graph_c0	18.3593667	15.1198768	16.8364752	16.8613089	16.7575518	16.1837833	3.237E-13	-1.10487	down	PREDICTED: uncharacterized protein LOC105171189 [Sesamum indicum]
c51821.graph_c0	1.29892749	1.74348778	1.44155872	7.08039763	7.18720897	8.03604215	3.464E-09	1.22405	up	PREDICTED: bifunctional aspartate aminotransferase and glutamate/aspartate-prephenate aminotransferase [Sesamum indicum]
c51822.graph_c0	4.97993555	2.65862518	3.41302099	20.7511605	15.6862485	17.326563	0.0033331	1.19569	up	-
c51823.graph_c0	0.76433414	0.54649965	0.65479976	3.88859555	5.03965202	4.4897751	5.783E-08	1.68161	up	hypothetical protein MIMGU_mgv1a021162mg [Erythranthe guttata]
c51825.graph_c0	16.9418648	18.7607051	18.5053219	12.6980387	12.0905096	10.1103081	5.537E-23	-1.72763	down	hypothetical protein MIMGU_mgv1a016748mg [Erythranthe guttata]
c51833.graph_c0	2.89418265	2.4731177	2.99345358	14.185632	14.8465055	15.9292023	1.224E-16	1.33592	up	PREDICTED: uncharacterized CRM domain-containing protein At3g25440, chloroplastic [Sesamum indicum]
c51834.graph_c0	53.7006178	49.0866426	49.0977161	13.228418	13.5233959	19.7151746	4.482E-90	-2.79635	down	unnamed protein product [Coffea canephora]
c51835.graph_c0	60.1854436	70.9537485	54.5188385	9.62409021	10.7342749	12.7498671	3.245E-49	-3.57517	down	hypothetical protein CICLE_v10001854mg [Citrus clementina]
c51837.graph_c0	36.9800656	37.5546882	34.5561356	34.2373769	35.1926478	33.6250055	7.851E-41	-1.1719	down	PREDICTED: protein MOS2 [Sesamum indicum]
c51840.graph_c0	1.04933545	1.47054068	1.48328136	17.3884419	19.3497249	19.5407524	1.271E-19	2.71962	up	PREDICTED: putative DNA-binding protein ESCAROLA [Populus euphratica]
c51841.graph_c0	8.05330231	3.9531701	4.37834367	0	0	0	3.905E-18	-Inf	down	uncharacterized protein LOC100191800 [Zea mays]
c51842.graph_c0	4.90721966	5.70869569	4.77208658	5.49439787	5.3090613	4.79800722	0.0008101	-1.07039	down	-
c51844.graph_c0	0	0	0	10.2518055	9.0133622	7.59657914	9.289E-51	Inf	up	hypothetical protein MIMGU_mgv1a004963mg [Erythranthe guttata]
c51845.graph_c0	15.8772504	17.3494934	16.988865	6.48234527	8.84169043	10.1289079	5.128E-45	-2.07034	down	PREDICTED: replication protein A 32 kDa subunit A-like isoform X2 [Sesamum indicum]
c51847.graph_c0	0.31437334	0.47203221	0.80796439	53.0465392	58.3142161	48.1330264	8.15E-120	5.54153	up	PREDICTED: S-antigen protein-like [Sesamum indicum]
c51849.graph_c0	21.8346699	20.3599879	21.3565645	22.6921057	18.5722928	21.0734271	1.891E-30	-1.11798	down	PREDICTED: uncharacterized protein LOC104243797 [Nicotiana sylvestris]
c51856.graph_c0	45.6076529	38.5013656	45.1416546	5.71208102	6.72034439	8.96891601	1.083E-74	-3.68357	down	-

#ID	0 h-1 FPKM	0 h-2 FPKM	0 h-3 FPKM	28 h-1 FPKM	28 h-2 FPKM	28 h-3 FPKM	FDR	log2FC	regulated	nr_annotation
c51857.graph_c0	4.34570843	4.93564499	3.00698859	0	0.10968352	0.0991253	3.005E-28	-6.96308	down	-
c51858.graph_c0	1.25155202	0.8816037	1.25089525	23.6164509	25.156185	23.2569517	4.426E-65	3.31997	up	PREDICTED: LRR receptor-like serine/threonine-protein kinase HSL2 [Sesamum indicum]
c51863.graph_c0	1.62342693	1.40545085	1.74787038	55.1043944	58.2480021	53.5257301	8.76E-177	4.0343	up	-
c51865.graph_c0	92.9783406	98.260481	95.5279859	7.77566292	8.54063876	8.503444	0	-4.62094	down	PREDICTED: ethylene-responsive transcription factor 14-like [Sesamum indicum]
c51866.graph_c0	1426.45982	1423.86615	1367.47214	166.205917	159.372296	170.3113	0	-4.17814	down	hypothetical protein M569_15403, partial [Genlisea aurea]
c51869.graph_c0	9.83560339	5.21229826	5.20435167	0	0	0	1.017E-16	-Inf	down	predicted protein [Hordeum vulgare subsp. vulgare]
c51870.graph_c0	737.950664	768.931021	704.285105	43.7167797	45.2471138	49.7155694	0	-5.08428	down	PREDICTED: zinc finger A20 and AN1 domain-containing stress-associated protein 1-like [Sesamum indicum]
c51872.graph_c0	0.85324734	0.72756814	0.73097107	23.7948191	25.1551034	23.7831889	2E-110	3.88644	up	PREDICTED: amino-acid permease BAT1 homolog isoform X1 [Sesamum indicum]
c51874.graph_c0	0.91383771	0.52271588	1.17431764	4.0799136	5.65417312	4.72278482	0.0014211	1.37284	up	PREDICTED: coatomer subunit alpha-1 [Solanum lycopersicum]
c51875.graph_c0	20.1945219	17.9838373	19.9306514	3.47097416	2.80564417	3.23689894	5.59E-156	-3.70108	down	hypothetical protein MIMGU_mgv1a017259mg [Erythranthe guttata]
c51877.graph_c0	0	0	0	3.21761526	3.04354799	2.33382025	7.412E-15	Inf	up	PREDICTED: myb-related protein 305 [Sesamum indicum]
c51878.graph_c0	1.68342197	1.40425638	0.54081597	8.66646814	8.28530277	7.90373985	8.188E-05	1.6973	up	-
c51882.graph_c0	3.69274782	3.06325812	2.46054	15.0719059	13.8665525	13.7788396	2.904E-17	1.12659	up	PREDICTED: pre-mRNA-splicing factor SYF1 [Sesamum indicum]
c51887.graph_c0	6.61988652	3.15915266	4.3523246	0	0	0	3.252E-20	-Inf	down	predicted protein [Physcomitrella patens]
c51891.graph_c0	0.32319136	1.0167614	0.11866106	4.46297878	7.4836383	3.36793813	0.0055021	2.30857	up	PREDICTED: E3 ubiquitin-protein ligase PUB24-like [Sesamum indicum]
c51894.graph_c0	1.73999691	2.40077573	1.81294453	13.6373857	14.4117846	14.0843049	4.223E-17	1.73302	up	PREDICTED: activating signal cointegrator 1 [Sesamum indicum]
c51895.graph_c0	0.27571083	0.18399127	0	9.38469941	10.6748053	9.81075342	4.59E-39	4.95359	up	PREDICTED: cyclin-D1-1-like [Sesamum indicum]
c51898.graph_c0	0.12030896	0.36128828	0	2.18599638	2.68429969	3.6388606	1.062E-05	3.06899	up	PREDICTED: uncharacterized protein LOC105163238 [Sesamum indicum]
c51905.graph_c0	28.3646374	24.1678855	30.4832163	9.14188391	9.97159414	11.6651725	2.655E-26	-2.52256	down	PREDICTED: cysteine proteinase inhibitor isoform X4 [Sesamum indicum]
c51907.graph_c0	0.07829528	0	0	3.64189164	4.93243004	3.34322307	5.585E-15	6.18962	up	PREDICTED: dof zinc finger protein DOF5.6 isoform X2 [Sesamum indicum]
c51908.graph_c0	1.7674532	1.34460951	0.99934883	9.7113855	8.21174228	7.63091455	9.808E-07	1.55131	up	hypothetical protein MIMGU_mgv1a014525mg [Erythranthe guttata]
c51910.graph_c0	3.46672274	4.16423297	4.32759381	24.3320802	27.1044906	24.6128767	2.094E-13	1.57566	up	PREDICTED: uncharacterized protein LOC105168519 isoform X2 [Sesamum indicum]
c51913.graph_c0	2.28435017	1.82155877	1.79114059	8.77964055	8.35912259	8.61071514	1.905E-06	1.03866	up	PREDICTED: uncharacterized protein LOC105168217 [Sesamum indicum]
c51919.graph_c0	0.45510028	0.075926	0	7.49732328	6.07253204	6.61257296	1.841E-16	4.18607	up	PREDICTED: F-box/kelch-repeat protein At5g26960 [Sesamum indicum]
c51920.graph_c0	0.04566381	0.13712861	0.05867981	5.14417025	4.49487428	3.65597499	1.741E-19	4.6924	up	PREDICTED: protein TIFY 6B [Sesamum indicum]

#ID	0 h-1 FPKM	0 h-2 FPKM	0 h-3 FPKM	28 h-1 FPKM	28 h-2 FPKM	28 h-3 FPKM	FDR	log2FC	regulated	nr_annotation
c51922.graph_c0	0	0	0	8.06905007	6.51288098	6.68404077	4.554E-15	Inf	up	PREDICTED: uncharacterized protein LOC102617723 [Citrus sinensis]
c51924.graph_c0	5.64326574	3.71214521	6.21584563	0.70311309	0.42323013	1.14746908	5.925E-12	-3.86479	down	-
c51926.graph_c0	0	0.15227936	0.13032617	17.5798427	23.46002	14.0442742	2.447E-22	6.50048	up	PREDICTED: WD repeat-containing protein RUP2-like [Sesamum indicum]
c51927.graph_c0	0.31568965	0.13543104	0.28976693	37.1039549	38.2661664	34.3954153	1.18E-160	6.1192	up	geranyl diphosphate synthase large subunit [Antirrhinum majus]
c51931.graph_c0	10.5402208	9.80476082	7.6615925	3.48207806	4.05225379	4.41987352	3.91E-20	-2.31424	down	PREDICTED: histone H3.2-like [Oryza brachyantha]
c51933.graph_c0	0.14304274	0.09545732	0.3063593	4.22781396	3.66087208	3.11053101	1.157E-12	3.23111	up	hypothetical protein MIMGU_mgv1a008682mg [Erythranthe guttata]
c51938.graph_c0	0	0.21471573	0.2756422	4.05334924	2.4633231	2.98947104	3.441E-07	3.16787	up	PREDICTED: ABC transporter B family member 15-like [Sesamum indicum]
c51940.graph_c0	0	0	0	5.18638696	4.3561127	4.13362965	4.102E-17	Inf	up	PREDICTED: kirola-like isoform X2 [Sesamum indicum]
c51942.graph_c0	19.6257528	23.2457581	22.506975	14.0365711	15.8036516	16.3227208	4.589E-23	-1.59358	down	PREDICTED: set1/Ash2 histone methyltransferase complex subunit ASH2 [Sesamum indicum]
c51943.graph_c0	34.3959697	34.9382352	34.4213813	7.07930407	5.06029203	6.43856262	1.06E-129	-3.57144	down	PREDICTED: ethylene-responsive transcription factor ERF008-like [Sesamum indicum]
c51945.graph_c0	22.41656	25.1565336	23.7227259	6.42669147	7.58423049	8.97021854	3.643E-79	-2.72322	down	PREDICTED: chaperone protein dnaJ 11, chloroplastic-like [Sesamum indicum]
c51947.graph_c0	0.14468401	0.25345056	0.09296234	4.60055335	3.67914052	4.82658938	8.773E-19	3.65471	up	PREDICTED: aldehyde dehydrogenase family 2 member C4-like [Sesamum indicum]
c51949.graph_c0	2.52124681	3.07241742	1.90168159	1.55357996	1.87031606	1.4302353	2.284E-07	-1.71437	down	PREDICTED: neural Wiskott-Aldrich syndrome protein [Sesamum indicum]
c51950.graph_c0	16.5715046	15.6070819	13.7387352	6.24867283	6.6651201	6.92177665	1.727E-69	-2.29892	down	PREDICTED: U-box domain-containing protein 26-like [Sesamum indicum]
c51953.graph_c0	3.72458321	3.976867	3.03128336	0.81210168	0.8961965	1.54622613	1.414E-12	-2.80625	down	-
c51958.graph_c0	7.06947151	3.6076532	5.16572495	0	0	0	2.598E-18	-Inf	down	predicted protein [Physcomitrella patens]
c51963.graph_c0	1.22193814	0.78631825	1.53285224	6.82989341	6.83497744	7.21229526	7.561E-12	1.46476	up	PREDICTED: D-3-phosphoglycerate dehydrogenase 2, chloroplastic-like [Sesamum indicum]
c51965.graph_c0	0.07411406	0	0	10.9347315	9.28940951	8.35125029	4.928E-30	7.53119	up	PREDICTED: subtilisin-like protease [Sesamum indicum]
c51971.graph_c0	1.48248172	1.07924814	0.17318615	15.6721333	14.5926691	15.905489	2.397E-14	3.00519	up	PREDICTED: UDP-glucose 6-dehydrogenase 3 [Nicotiana sylvestris]
c51972.graph_c0	47.7576768	49.4687654	46.5616854	37.0863345	33.3447875	37.3190751	2.946E-62	-1.50599	down	PREDICTED: transcription factor GTE7-like [Sesamum indicum]
c51974.graph_c0	14.6783689	12.8137213	15.5723517	6.07836678	6.16021921	5.66845446	3.015E-19	-2.35811	down	hypothetical protein MIMGU_mgv1a0003262mg, partial [Erythranthe guttata]
c51983.graph_c0	2.59302187	3.91991348	3.12812745	15.2305912	15.2103196	16.4137977	4.319E-10	1.18974	up	hypothetical protein MIMGU_mgv1a013022mg [Erythranthe guttata]
c51984.graph_c0	4.90800446	3.94113289	4.22775585	31.0456111	27.0933608	27.9558226	1.397E-40	1.62973	up	PREDICTED: uncharacterized protein LOC105164204 [Sesamum indicum]

#ID	0 h-1 FPKM	0 h-2 FPKM	0 h-3 FPKM	28 h-1 FPKM	28 h-2 FPKM	28 h-3 FPKM	FDR	log2FC	regulated	nr_annotation
c51987.graph_c0	0.78242579	1.34264446	0.57454178	3.81816286	4.54769086	4.64024	0.008681	1.18311	up	PREDICTED: RPM1-interacting protein 4 [Sesamum indicum]
c51991.graph_c0	14.3425789	14.9704853	14.3350524	70.8304479	72.0781322	81.3520982	4.884E-21	1.27139	up	PREDICTED: mitochondrial import inner membrane translocase subunit Tim13 [Sesamum indicum]
c51992.graph_c0	0.4492542	0.14990135	0	1.30606144	1.76887654	1.46538627	0.0043465	1.85667	up	PREDICTED: cucumber peeling cupredoxin-like [Sesamum indicum]
c51993.graph_c0	1.9884685	1.89788013	2.02043873	19.157401	28.2219707	21.1447193	2.348E-12	2.44437	up	PREDICTED: U-box domain-containing protein 26-like [Sesamum indicum]
c51995.graph_c0	5.28104004	2.67158522	3.64857287	0	0	0	3.155E-20	-Inf	down	--
c52000.graph_c0	1.30455013	1.89942755	0.76199935	17.0665113	12.9190418	15.121812	2.348E-12	2.4242	up	PREDICTED: probable serine/threonine-protein kinase DDB_G0282963 isoform X2 [Nicotiana tomentosiformis]
c52001.graph_c0	1.90659104	1.93928246	2.7661798	13.9910937	14.9533396	16.2130569	2.315E-22	1.67657	up	PREDICTED: kynurenine--oxoglutarate transaminase 1-like [Sesamum indicum]
c52003.graph_c0	16.1798278	8.14178779	9.32817679	0	0	0	1.157E-19	-Inf	down	-
c52004.graph_c0	0.116696	0	0	3.47737323	2.29737219	2.62988561	1.404E-10	5.11141	up	hypothetical protein MIMGU_mgv1a000070mg [Erythranthe guttata]
c52011.graph_c0	3.68857871	3.69227227	3.87815701	2.51836274	1.90709617	2.58527657	7.295E-07	-1.77366	down	-
c52012.graph_c0	18.7140174	20.116707	19.2893641	10.6584915	10.2068681	11.0985007	1.635E-58	-1.95299	down	PREDICTED: RING-H2 finger protein ATL52-like [Sesamum indicum]
c52017.graph_c0	10.854695	11.0497265	8.27466016	8.06741519	8.24996963	8.1104791	5.807E-14	-1.39212	down	hypothetical protein MIMGU_mgv1a014249mg [Erythranthe guttata]
c52018.graph_c0	2.24672554	1.12448765	0.36089141	11.2262368	12.3477824	12.9912795	5.921E-06	2.22	up	hypothetical protein JCGZ_17967 [Jatropha curcas]
c52023.graph_c0	11.0624211	9.50093058	9.25278932	1.28451364	0.77319692	1.47517779	6.977E-73	-4.16353	down	-
c52026.graph_c0	1.28557144	1.96415282	1.56506229	16.9657547	12.6988027	12.038189	1.004E-09	2.02228	up	PREDICTED: uncharacterized protein LOC105168444 [Sesamum indicum]
c52029.graph_c0	0.18194257	0.31871832	0.05845085	5.48774461	15.4318858	5.28722373	0.0020587	4.46731	up	PREDICTED: epidermis-specific secreted glycoprotein EP1-like [Vitis vinifera]
c52032.graph_c0	19.9690778	20.7203814	20.698726	12.2883582	11.6659778	11.8015604	1.301E-72	-1.87072	down	PREDICTED: cyclin-dependent kinase F-1 [Sesamum indicum]
c52038.graph_c0	0.54335573	0.54389982	0.34911681	7.89815623	6.77472982	8.37828028	6.544E-08	2.92002	up	PREDICTED: uncharacterized protein LOC104246111 isoform X2 [Nicotiana sylvestris]
c52039.graph_c0	0.10764661	0.1077544	0.27666031	7.43250881	9.53648326	5.55414089	5.309E-11	4.41001	up	hypothetical protein MIMGU_mgv1a019582mg [Erythranthe guttata]
c52040.graph_c0	0.47198428	0.52495212	0.4717366	15.8939762	16.8630154	16.63934	5.822E-48	3.98162	up	PREDICTED: transmembrane protein 87A-like [Sesamum indicum]
c52041.graph_c0	9.28622104	10.302041	7.14469554	5.5025096	6.09386946	4.41984661	1.837E-12	-1.82742	down	PREDICTED: protein MKS1 [Sesamum indicum]
c52042.graph_c0	26.3790729	26.0781468	20.7311019	9.50685527	9.15605222	10.311032	2.478E-39	-2.42382	down	PREDICTED: uncharacterized protein LOC105164219 [Sesamum indicum]
c52044.graph_c0	0.5181383	0.20746285	0	3.0879554	2.7881342	2.70411808	2.414E-06	2.49803	up	PREDICTED: LOW QUALITY PROTEIN: BTB/POZ domain-containing protein At1g63850 [Sesamum indicum]
c52045.graph_c0	0.1002416	0.20068395	0.12881442	5.09985121	4.73624999	2.73465856	4.158E-07	3.77689	up	-
c52048.graph_c0	4.26825674	1.67550226	3.22639935	0	0	0	4.757E-12	-Inf	down	tamavidin1 [Pleurotus cornucopiae]

#ID	0 h-1 FPKM	0 h-2 FPKM	0 h-3 FPKM	28 h-1 FPKM	28 h-2 FPKM	28 h-3 FPKM	FDR	log2FC	regulated	nr_annotation
c52049.graph_c0	0.84173703	0.96294846	1.5452363	9.43873133	9.98212603	8.9855823	2.057E-12	1.98496	up	PREDICTED: serine/threonine-protein kinase BLUS1 [Sesamum indicum]
c52052.graph_c0	12.045374	14.2846053	11.8309166	9.08907564	8.20658312	8.50862791	1.014E-26	-1.65427	down	PREDICTED: 5'-adenylylsulfate reductase-like 5 isoform X2 [Sesamum indicum]
c52053.graph_c0	33.9792454	28.0625141	30.3159903	3.5929607	2.5049431	2.74626794	2.36E-126	-4.47412	down	PREDICTED: uncharacterized protein LOC105180043 [Sesamum indicum]
c52056.graph_c0	2.08753852	1.23611849	1.54908951	0	0	0	3.723E-28	-Inf	down	putative Bax inhibitor 1 [Auxenochlorella protothecoides]
c52059.graph_c0	3.07400349	2.69244643	3.16017295	0.72610461	1.00862212	0.59249543	6.381E-16	-3.03286	down	PREDICTED: shikimate O-hydroxycinnamoyltransferase-like [Sesamum indicum]
c52060.graph_c0	0	0	0	4.03072271	2.50206323	5.68729283	1.137E-06	Inf	up	--
c52068.graph_c0	1.05107943	0.61374362	1.57579179	14.9600701	17.6460212	19.6875098	5.088E-21	2.91486	up	PREDICTED: outer envelope pore protein 16, chloroplastic [Nicotiana tomentosiformis]
c52071.graph_c0	0	0.16320341	0.20951305	7.10979868	7.59638656	6.09161455	1.711E-12	4.69214	up	hypothetical protein MIMGU_mgv1a026849mg [Erythranthe guttata]
c52074.graph_c0	0.83946836	0.31511586	0.26968752	3.88951989	4.8890764	3.60943834	2.583E-05	2.04184	up	-
c52081.graph_c0	61.5450933	66.7713567	76.5621209	44.9984671	48.7713989	42.6195502	1.423E-14	-1.68083	down	PREDICTED: probable cyclic nucleotide-gated ion channel 17 [Sesamum indicum]
c52084.graph_c0	75.9287299	78.5947004	82.9932558	14.4651542	17.9910839	15.580551	5.59E-135	-3.39786	down	PREDICTED: transcription factor TGA5-like [Sesamum indicum]
c52089.graph_c0	17.250026	17.7537021	15.9227505	13.655563	14.7478009	13.664373	1.811E-34	-1.36537	down	PREDICTED: uncharacterized protein LOC105176476 isoform X2 [Sesamum indicum]
c52091.graph_c0	6.74689105	4.890572	4.18552792	0	0	0	1.595E-26	-Inf	down	-
c52093.graph_c0	5.53492119	6.00216888	4.14901251	5.11224329	2.95113408	5.53926969	1.726E-05	-1.2903	down	-
c52094.graph_c0	4.14557806	4.8136859	5.11414265	2.41038579	1.30581142	2.75359717	1.19E-08	-2.21161	down	-
c52102.graph_c0	47.4168469	48.1497331	51.4736859	30.8543256	30.1052214	31.0650184	6.321E-38	-1.76762	down	PREDICTED: transcription factor ILR3-like [Sesamum indicum]
c52103.graph_c0	0.64829814	0.64894731	0.29403133	20.4547271	19.294537	18.7489811	1.845E-77	4.11834	up	PREDICTED: brefeldin A-inhibited guanine nucleotide-exchange protein 2 [Sesamum indicum]
c52106.graph_c0	6.04812392	4.88041057	5.07564323	0.35883605	0.48599296	0.54901366	1.374E-48	-4.609	down	PREDICTED: ethylene-responsive transcription factor ERF054-like [Sesamum indicum]
c52108.graph_c0	35.6084285	35.644085	33.0370699	32.9674697	36.7476105	33.385511	1.977E-15	-1.1064	down	-
c52109.graph_c0	0	0	0	3.53965204	3.12939112	3.73075591	2.875E-14	Inf	up	PREDICTED: serine/threonine-protein kinase D6PK-like [Sesamum indicum]
c52110.graph_c0	181.367503	177.293589	183.896985	43.474757	48.1731228	50.1053963	0	-3.02685	down	PREDICTED: oleosin 1 [Sesamum indicum]
c52113.graph_c0	0	0	0	30.6682257	28.1598634	28.8423979	3.917E-48	Inf	up	-
c52114.graph_c0	12.5223818	12.214426	10.6516456	106.473639	103.676226	108.211711	3.79E-121	2.08098	up	PREDICTED: protein rough sheath 2 homolog [Sesamum indicum]
c52117.graph_c0	0.04752541	0.09514599	0.06107203	2.27971866	2.90043356	2.53667974	1.265E-12	4.15171	up	PREDICTED: serine/threonine protein phosphatase 2A 57 kDa regulatory subunit B' iota isoform [Sesamum indicum]

#ID	0 h-1 FPKM	0 h-2 FPKM	0 h-3 FPKM	28 h-1 FPKM	28 h-2 FPKM	28 h-3 FPKM	FDR	log2FC	regulated	nr_annotation
c52118.graph_c0	0	0.32407226	0.03782083	3.23000301	5.25336454	3.31638844	3.279E-09	3.93864	up	PREDICTED: MLO-like protein 6 [Sesamum indicum]
c52120.graph_c0	0.32428432	0.05410151	0.2083591	8.75973648	9.64712126	8.84669538	1.041E-29	4.4506	up	PREDICTED: probable pectinesterase/pectinesterase inhibitor 12 [Sesamum indicum]
c52127.graph_c0	6.36372991	4.39996751	5.31125389	0	0	0	1.805E-53	-Inf	down	NADH dehydrogenase subunit 1 (mitochondrion) [Micromonas pusilla CCMP1545]
c52131.graph_c0	1.60464789	0.62853445	1.34480613	8.41727421	9.33977061	8.44071754	8.678E-09	1.78184	up	PREDICTED: uncharacterized protein LOC105174462 isoform X1 [Sesamum indicum]
c52132.graph_c0	2.72443107	2.0094857	3.50100178	2.08431663	3.01110799	2.21102095	0.0042387	-1.26869	down	-
c52133.graph_c0	10.6526597	9.22787887	8.95055847	4.91337739	4.70518694	5.34570111	2.5E-10	-2.03416	down	-
c52136.graph_c0	52.1327056	62.5931119	51.5990727	21.8353614	23.9916456	25.5469083	1.099E-35	-2.30995	down	PREDICTED: cell division cycle protein 48 homolog [Nicotiana tomentosiformis]
c52137.graph_c0	6.85728129	4.94857168	4.71333007	0	0	0	2.367E-37	-Inf	down	rpl13 protein isoform 1 [Lichtheimia corymbifera JMRC:FSU:9682]
c52139.graph_c0	32.2669683	40.9124198	30.8678915	8.0777205	8.70505475	8.50497286	2.999E-29	-3.13	down	PREDICTED: ethylene-responsive transcription factor ERF008-like [Sesamum indicum]
c52140.graph_c0	54.1557491	43.2526419	48.1223224	257.794608	267.924759	267.987584	5.861E-42	1.35779	up	PREDICTED: 60S ribosomal protein L17-2 [Sesamum indicum]
c52147.graph_c0	9.27974669	7.25898408	7.97621875	38.6801848	41.7804448	38.5240066	1.014E-19	1.18962	up	PREDICTED: transmembrane emp24 domain-containing protein p24beta3 [Sesamum indicum]
c52153.graph_c0	0.06339357	0.1269141	0	17.4160027	16.0994227	18.7228449	1.051E-58	7.02712	up	PREDICTED: probable 3-beta-hydroxysteroid-Delta(8),Delta(7)-isomerase [Sesamum indicum]
c52155.graph_c0	17.9292118	15.781128	13.5060586	93.2379625	94.7336375	92.7643289	9.383E-14	1.48459	up	hypothetical protein MIMGU_mgv1a013595mg [Erythranthe guttata]
c52157.graph_c0	0.30978149	0.41345559	0.39808148	10.1316332	9.48672647	10.4719512	3.264E-16	3.65351	up	PREDICTED: choline transporter-like protein 2 [Sesamum indicum]
c52158.graph_c0	6.61206508	6.61868606	7.33405014	42.4917165	47.3637849	42.2266312	1.228E-30	1.59083	up	PREDICTED: 50S ribosomal protein L12, chloroplastic-like [Sesamum indicum]
c52160.graph_c0	6.08778224	3.14850375	2.34691236	0	0	0	6.665E-10	-Inf	down	-
c52162.graph_c0	0	0.38492448	0	20.1226346	22.4587203	18.2443395	2.57E-15	6.22147	up	-
c52165.graph_c0	23.8591949	21.9913567	20.4906008	1.54521315	0.85261159	1.54107716	7.61E-112	-5.16107	down	hypothetical protein MIMGU_mgv1a011603mg [Erythranthe guttata]
c52171.graph_c0	0.27723418	0.27751178	0.71251379	6.24625826	6.27653555	8.13859157	2.807E-09	2.92418	up	enolase [Prunus armeniaca]
c52173.graph_c0	12.3555667	11.1271425	9.58295692	9.38681348	8.94772648	9.66338075	4.469E-20	-1.32722	down	PREDICTED: pentatricopeptide repeat-containing protein At3g09650, chloroplastic [Sesamum indicum]
c52175.graph_c0	19.5522181	20.6951228	18.3587173	14.4927661	14.6434195	15.0256483	2.009E-42	-1.49767	down	PREDICTED: mitogen-activated protein kinase kinase kinase 1-like [Sesamum indicum]
c52177.graph_c0	9.61483347	8.85450437	9.94613055	5.031364	4.76368294	5.64513391	1.251E-31	-1.9705	down	PREDICTED: dnaJ protein ERDJ3A [Sesamum indicum]
c52179.graph_c0	16.4029273	16.0772825	12.7348733	8.19606771	15.4733142	10.5385474	1.837E-06	-1.49095	down	hypothetical protein MIMGU_mgv1a0261451mg, partial [Erythranthe guttata]

#ID	0 h-1 FPKM	0 h-2 FPKM	0 h-3 FPKM	28 h-1 FPKM	28 h-2 FPKM	28 h-3 FPKM	FDR	log2FC	regulated	nr_annotation
c52180.graph_c0	0.69492397	1.39123967	0.66975362	6.31333425	7.52446054	7.6244112	5.663E-05	1.87357	up	-
c52181.graph_c0	1.34331797	0.67233155	1.23301198	74.1999976	67.3734359	78.47157	2.29E-107	4.99138	up	Alpha-xylosidase, partial [Medicago truncatula]
c52184.graph_c0	0.95167982	0.85736951	0.978357	5.94840852	6.43253585	5.98265575	3.951E-05	1.62839	up	PREDICTED: probable LRR receptor-like serine/threonine-protein kinase At1g67720 [Sesamum indicum]
c52190.graph_c0	17.4657531	16.9282189	18.7034869	111.62668	134.445211	110.158714	7.81E-23	1.65383	up	PREDICTED: probable E3 ubiquitin-protein ligase RHA2B [Sesamum indicum]
c52194.graph_c0	18.0588731	12.3008874	12.495736	0	0	0	1.712E-46	-Inf	down	--
c52196.graph_c0	1.16834006	1.75426497	1.87670404	19.3179992	16.8673198	18.8813509	3.264E-08	2.42522	up	PREDICTED: aldo-keto reductase family 4 member C9-like [Sesamum indicum]
c52197.graph_c0	19.237124	20.7376476	20.2834592	9.08195508	12.8397147	12.0913072	1.017E-19	-1.91592	down	PREDICTED: splicing factor 3B subunit 2 [Sesamum indicum]
c52199.graph_c0	4.31632389	2.19890022	3.16951262	0	0	0	3.095E-22	-Inf	down	--
c52203.graph_c0	0	0	0	4.4198441	3.39634453	4.37390918	3.196E-14	Inf	up	-
c52213.graph_c0	0	0.1486544	0.1908357	4.31732336	3.31341747	2.73024797	4.189E-09	3.82127	up	hypothetical protein MIMGU_mgv1a0183231mg, partial [Erythranthe guttata]
c52214.graph_c0	3.15333585	2.59946518	3.81379897	0.40443994	0.06086189	0.27501641	2.856E-22	-4.78354	down	PREDICTED: uncharacterized protein LOC105178332 [Sesamum indicum]
c52215.graph_c0	0	0.71665974	0.46000744	4.50964041	4.22838949	4.59978718	1.1E-06	2.40223	up	-
c52217.graph_c0	0.43598431	0.52370505	0.11205142	6.52754013	5.20710545	5.17128101	1.346E-09	2.90149	up	PREDICTED: zinc finger protein JACKDAW-like [Sesamum indicum]
c52218.graph_c0	158.251382	160.732572	149.09039	31.3680525	26.7997002	28.0731142	1.03E-126	-3.53004	down	-
c52220.graph_c0	11.7609519	14.333564	14.7023535	0.38736218	0.34975173	0.31608437	2.443E-79	-6.36912	down	PREDICTED: transcription factor MYB44-like [Sesamum indicum]
c52223.graph_c0	14.5762838	15.0311218	15.7841828	12.5346628	11.7299249	11.4019083	1.898E-40	-1.43962	down	PREDICTED: ectonucleotide pyrophosphatase/phosphodiesterase family member 1-like [Sesamum indicum]
c52226.graph_c0	13.7597318	11.981067	10.6575233	2.9453509	3.64891086	4.02426801	2.477E-38	-2.86404	down	hypothetical protein MIMGU_mgv1a016982mg [Erythranthe guttata]
c52230.graph_c0	14.1895426	10.2482763	13.1562667	10.182335	14.497748	15.0195771	8.906E-05	-1.01108	down	PREDICTED: protein-L-isoaspartate O-methyltransferase 1 isoform X2 [Sesamum indicum]
c52231.graph_c0	25.609214	19.645405	20.2989192	19.8302777	20.5749077	26.4011414	1.697E-06	-1.05948	down	PREDICTED: L-ascorbate oxidase homolog [Nicotiana tomentosiformis]
c52232.graph_c0	14.1648219	14.5200816	15.3875595	78.8568952	85.446848	77.6835593	2.009E-47	1.36505	up	PREDICTED: cytochrome c1-2, heme protein, mitochondrial-like [Sesamum indicum]
c52233.graph_c0	234.458802	248.35368	215.778928	58.375412	67.7180807	55.2645263	1.25E-188	-3.03567	down	PREDICTED: probable calcium-binding protein CML18 [Sesamum indicum]
c52235.graph_c0	0	0	0	11.9582673	17.6875089	6.29124442	4.103E-08	Inf	up	PREDICTED: pathogenesis-related protein STH-2-like [Sesamum indicum]
c52237.graph_c0	22.6625815	26.6021566	18.2276357	11.1386846	14.1228595	14.0203827	8.497E-12	-1.86795	down	-
c52248.graph_c0	9.2696467	7.38250543	7.30362403	0	0	0	2.31E-101	-Inf	down	-

#ID	0 h-1 FPKM	0 h-2 FPKM	0 h-3 FPKM	28 h-1 FPKM	28 h-2 FPKM	28 h-3 FPKM	FDR	log2FC	regulated	nr_annotation
c52251.graph_c0	17.6023506	16.5380483	14.2861374	3.92776355	1.51988667	3.20502298	7.302E-43	-3.57101	down	PREDICTED: dynamin-related protein 4C-like [Sesamum indicum]
c52252.graph_c0	6.68791822	3.01914017	4.54989161	0	0	0	3.358E-14	-Inf	down	--
c52253.graph_c0	12.0430756	7.90500647	9.13327607	0.71744573	1.48990832	0.8196018	1.769E-23	-4.35399	down	hypothetical protein L484_021523 [Morus notabilis]
c52257.graph_c0	9.27455744	7.17387985	6.22995433	0	0	0	2.404E-38	-Inf	down	60s ribosomal protein l43 [Moniliophthora roreri MCA 2997]
c52260.graph_c0	0.13731092	0.54979367	0.70579988	12.6741918	12.7051068	12.4593044	7.096E-16	3.65934	up	PREDICTED: uncharacterized protein LOC105162704 [Sesamum indicum]
c52262.graph_c0	0.56132979	0.56189188	0.24044367	11.2872048	18.5407764	9.54316817	3.287E-06	3.76805	up	hypothetical protein MIMGU_mgv1a010766mg [Erythranthe guttata]
c52266.graph_c0	31.6340027	35.57658	27.3705572	28.4873656	23.9018985	27.8048562	4.873E-15	-1.32598	down	hypothetical protein M569_00407, partial [Genlisea aurea]
c52267.graph_c0	7.57177698	3.15806624	5.6758527	0	0	0	6.722E-13	-Inf	down	-
c52269.graph_c0	5.31268265	4.78022696	6.13663599	35.8356585	33.4922336	34.6580279	1.481E-28	1.58688	up	PREDICTED: probable protein Pop3 [Sesamum indicum]
c52273.graph_c0	28.8567249	28.4654661	26.2945332	25.7776024	23.6879195	23.6480443	6.78E-22	-1.28315	down	hypothetical protein MIMGU_mgv1a000759mg [Erythranthe guttata]
c52275.graph_c0	0	0.07901046	0	1.72100703	2.07187732	2.76184445	3.617E-10	5.29444	up	-
c52276.graph_c0	8.31860792	11.0629316	9.00992415	7.77332095	7.33051688	6.90678565	2.696E-09	-1.4583	down	-
c52278.graph_c0	3.93935063	2.55154401	3.87111057	14.9891566	17.6395635	17.8655486	0.0008155	1.19365	up	-
c52279.graph_c0	9.20084667	9.09774212	7.06523366	0.57085191	0.883587	0.53235485	2.281E-45	-4.7633	down	PREDICTED: 8-hydroxygeraniol dehydrogenase-like [Sesamum indicum]
c52290.graph_c0	0.49356867	0.39525032	1.01480845	4.23292981	2.72070215	3.16132123	0.0047321	1.30766	up	hypothetical protein MIMGU_mgv1a011727mg [Erythranthe guttata]
c52293.graph_c0	6.16745921	6.51661471	6.8246752	0.99610529	1.29287267	1.21922067	6.392E-32	-3.56699	down	PREDICTED: putative calcium-binding protein CML23 [Sesamum indicum]
c52298.graph_c0	1.16670308	1.07803509	0.80729365	11.0233899	11.8965899	10.2191672	7.407E-12	2.35401	up	PREDICTED: LOW QUALITY PROTEIN: uncharacterized protein LOC105174495 [Sesamum indicum]
c52301.graph_c0	0.28142714	0.28170895	0.09041125	7.31229154	8.12592451	6.92646049	2.423E-18	4.01742	up	hypothetical protein MIMGU_mgv1a015992mg [Erythranthe guttata]
c52307.graph_c0	3.54989601	3.95725191	4.25072804	3.16641759	1.80009739	2.67946611	4.62E-08	-1.71263	down	PREDICTED: probable L-type lectin-domain containing receptor kinase S.7 [Sesamum indicum]
c52308.graph_c0	4.35129799	4.20009605	3.16191112	19.1444094	19.5631596	16.2361038	4.64E-13	1.14239	up	PREDICTED: cytochrome P450 703A2 [Sesamum indicum]
c52310.graph_c0	8.72220717	4.91115439	5.90441527	0	0	0	8.23E-26	-Inf	down	unknown [Picea sitchensis]
c52312.graph_c0	5.99810416	4.36662571	3.92397158	0.87187751	1.35974993	0.77612174	4.852E-17	-3.33591	down	-
c52313.graph_c0	6.34669649	6.47464125	5.3851405	28.3606413	29.6323805	24.6908582	8.825E-18	1.094	up	PREDICTED: LRR receptor-like serine/threonine-protein kinase GSO1 [Sesamum indicum]
c52320.graph_c0	1.44784459	1.44929438	0.37210752	14.3110774	15.5058733	13.7041506	4.104E-24	2.65694	up	PREDICTED: coatomer subunit alpha-1-like [Nicotiana sylvestris]
c52321.graph_c0	0.16629621	0.33292547	0.42739444	3.86762014	3.38297029	2.86007647	1.269E-05	2.34656	up	-
c52322.graph_c0	0	0	0	17.7332773	15.2109124	17.9430597	3.834E-80	Inf	up	hypothetical protein MIMGU_mgv1a018712mg, partial [Erythranthe guttata]

#ID	0 h-1 FPKM	0 h-2 FPKM	0 h-3 FPKM	28 h-1 FPKM	28 h-2 FPKM	28 h-3 FPKM	FDR	log2FC	regulated	nr_annotation
c52323.graph_c0	97.8367903	87.5534244	108.864589	10.8068437	7.70765767	12.375256	1.819E-58	-4.3425	down	PREDICTED: putative defensin-like protein 120 [Solanum lycopersicum]
c52324.graph_c0	3.55532983	2.41086094	2.35805914	14.7120905	13.0578541	13.0592044	2.928E-08	1.20804	up	PREDICTED: uncharacterized protein LOC105157576 isoform X2 [Sesamum indicum]
c52327.graph_c0	42.8914015	45.0568116	39.4175844	40.1406619	41.0138471	40.083785	1.547E-47	-1.16051	down	PREDICTED: F-box/kelch-repeat protein At5g60570 [Sesamum indicum]
c52334.graph_c0	2.39493683	1.71238214	1.86853596	0	0	0	7.56E-30	-Inf	down	Serpin-ZX [Aegilops tauschii]
c52338.graph_c0	9.35784155	10.6363182	10.9390501	5.92364785	5.66544668	4.61881946	3.056E-29	-2.0257	down	unnamed protein product [Coffea canephora]
c52343.graph_c0	4.27145965	3.39110166	5.48899476	36.8789894	38.5660656	37.2121872	1.964E-35	2.00367	up	PREDICTED: uncharacterized protein LOC105158873 [Sesamum indicum]
c52347.graph_c0	1.47105848	1.32527837	1.41777629	11.5468937	14.8663649	13.0427274	2.052E-14	2.13631	up	PREDICTED: LIM domain-containing protein WLIM1 [Sesamum indicum]
c52349.graph_c0	7.56385219	8.29251445	9.25699955	7.59159815	7.48480032	7.47634046	1.746E-06	-1.24818	down	-
c52357.graph_c0	50.7529527	54.7072751	44.6514604	8.07959647	7.8680331	6.73095562	1.86E-113	-3.81608	down	hypothetical protein MIMGU_mgv1a001601mg [Erythranthe guttata]
c52362.graph_c0	16.5844453	13.7952405	19.8108471	14.4302217	12.5692853	14.4069891	5.07E-06	-1.37074	down	-
c52365.graph_c0	1.3776527	0.82741933	1.06220296	7.2091443	13.7416051	8.00687614	0.0021685	2.05778	up	zinc finger family protein [Populus trichocarpa]
c52366.graph_c0	0.27867893	0.092986	0.11937115	4.38841526	2.86506759	4.95818448	1.245E-07	3.55736	up	PREDICTED: myosin-9-like [Sesamum indicum]
c52371.graph_c0	1003.62253	984.370931	1005.92888	44.4816353	37.8307157	49.8786238	0	-5.59089	down	hypothetical protein MIMGU_mgv1a015982mg [Erythranthe guttata]
c52372.graph_c0	0.36126166	0.28929873	0.37138843	6.59032867	7.58622966	5.44192913	2.404E-21	3.1699	up	PREDICTED: ATP synthase gamma chain, chloroplastic-like [Sesamum indicum]
c52377.graph_c0	0.08936731	0.0447284	0	8.83342357	7.68252705	8.48000176	7.8E-40	6.47601	up	PREDICTED: uncharacterized protein LOC105168503 isoform X2 [Sesamum indicum]
c52380.graph_c0	4.44109999	3.27065249	3.95412831	18.4674319	19.2556586	17.8159827	1.239E-17	1.16114	up	PREDICTED: LOW QUALITY PROTEIN: ATP-dependent Clp protease ATP-binding subunit clpA homolog CD4B, chloroplastic [Sesamum indicum]
c52381.graph_c0	33.0319057	35.7936458	31.3203428	16.8984117	15.2576814	11.1262691	7.915E-44	-2.30142	down	PREDICTED: importin subunit beta-1-like [Nicotiana sylvestris]
c52383.graph_c0	12.6465586	7.46286027	7.80631501	0	0	0	9.249E-30	-Inf	down	-
c52389.graph_c0	0	0	0	3.67988321	4.8177548	2.47727258	3.834E-08	Inf	up	hypothetical protein MIMGU_mgv1a020102mg [Erythranthe guttata]
c52394.graph_c0	0.37777545	0.07563075	0	15.8149452	18.6425667	19.4021078	3.772E-47	5.82998	up	PREDICTED: protein RALF-like 34 [Phoenix dactylifera]
c52396.graph_c0	1.06671739	0.15254079	0.19582487	4.31943969	6.80008553	4.0668779	0.001143	2.35054	up	protein with unknown function [Ricinus communis]
c52400.graph_c0	2.93891524	2.52159268	2.87742663	2.64455491	3.76535348	2.32393023	0.0095016	-1.02558	down	hypothetical protein MIMGU_mgv1a005057mg [Erythranthe guttata]
c52401.graph_c0	424.357015	410.235511	394.499635	80.3730243	80.1384078	85.2549863	0	-3.41161	down	unnamed protein product [Coffea canephora]
c52403.graph_c0	0	0.09781862	0.12557506	8.87786168	12.1200056	11.3590038	3.587E-25	6.06932	up	PREDICTED: dirigent protein 22-like [Sesamum indicum]
c52404.graph_c0	8.48318608	10.6789318	8.58884786	2.52226258	3.62691722	2.59173899	5.871E-22	-2.75782	down	PREDICTED: cyclic pyranopterin monophosphate synthase, mitochondrial [Sesamum indicum]

#ID	0 h-1 FPKM	0 h-2 FPKM	0 h-3 FPKM	28 h-1 FPKM	28 h-2 FPKM	28 h-3 FPKM	FDR	log2FC	regulated	nr_annotation
c52410.graph_c0	4.0525019	4.59743452	5.3812129	2.74897936	1.50697145	2.96415551	2.141E-08	-2.05041	down	-
c52413.graph_c0	9.06071015	5.62952053	8.02991122	4.54156684	4.92073085	6.67058661	4.897E-05	-1.58236	down	-
c52415.graph_c0	0	0	0	35.3124145	29.7154568	25.2818122	1.416E-58	Inf	up	PREDICTED: GDSL esterase/lipase 1-like [Sesamum indicum]
c52418.graph_c0	0.07882468	0.15780722	0.20258567	1.43223307	1.81044105	2.1503905	7.878E-07	2.51908	up	PREDICTED: auxin response factor 4 isoform X2 [Sesamum indicum]
c52420.graph_c0	0.17839682	0	0.22924698	2.85247121	3.04378934	2.06309398	8.822E-09	3.18619	up	flavonoid glycosyltransferase [Veronica persica]
c52421.graph_c0	2.96697983	2.90321035	2.69886801	14.7554804	15.2917044	15.2629744	9.697E-17	1.31331	up	PREDICTED: uncharacterized protein LOC105173356 [Sesamum indicum]
c52437.graph_c0	0	0	0	9.60245161	10.098523	8.97632608	9.467E-45	Inf	up	PREDICTED: probable LRR receptor-like serine/threonine-protein kinase At4g26540 [Sesamum indicum]
c52440.graph_c0	182.010223	202.337482	169.066375	45.5551751	56.0838727	50.3171526	4.212E-89	-2.95422	down	hypothetical protein MIMGU_mgv1a009891mg [Erythranthe guttata]
c52441.graph_c0	0	0	0	9.16868329	9.27586635	11.988541	2.269E-19	Inf	up	-
c52442.graph_c0	4.62906739	4.75564225	3.28734822	3.2758432	2.07843949	3.1787756	1.131E-06	-1.65549	down	PREDICTED: mitoferrin-like [Sesamum indicum]
c52443.graph_c0	0.26852745	0.57599215	0.19718194	2.98322421	2.8949603	2.63903967	1.636E-06	1.94579	up	PREDICTED: cinnamoyl-CoA reductase 1-like [Sesamum indicum]
c52446.graph_c0	0.21005617	0.77097719	0.26993049	14.8596475	14.7953139	12.9558545	1.624E-31	4.00098	up	PREDICTED: vegetative incompatibility protein HET-E-1-like [Sesamum indicum]
c52447.graph_c0	1.99797913	2.41858023	3.34369787	2.39768605	1.18916257	1.73604241	0.0011288	-1.64045	down	ATPase subunit 1 [Silene latifolia]
c52451.graph_c0	4.83638833	6.0098043	6.64356776	4.12110385	3.72097038	3.26388158	9.08E-07	-1.75003	down	PREDICTED: calcium uniporter protein 2, mitochondrial-like [Sesamum indicum]
c52454.graph_c0	2.04273284	1.61429868	1.42762754	0.07813929	0	0.06376102	4.942E-39	-6.24563	down	PREDICTED: cytochrome P450 86B1-like [Sesamum indicum]
c52455.graph_c0	2.37234374	3.26523901	1.90534727	20.0438919	24.9087382	18.2901916	1.237E-06	1.97953	up	-
c52456.graph_c0	0.24203836	0.40380122	0	7.74013188	9.15931905	7.22498898	8.425E-18	4.14911	up	PREDICTED: uncharacterized protein LOC105159948 [Sesamum indicum]
c52460.graph_c0	0	0	0	4.38126835	4.73292132	3.83044199	8.098E-15	Inf	up	hypothetical protein MIMGU_mgv1a007773mg [Erythranthe guttata]
c52461.graph_c0	63.6210898	64.2637493	82.8704685	56.3280467	46.6839569	55.0529686	1.371E-07	-1.50875	down	PREDICTED: branchpoint-bridging protein [Sesamum indicum]
c52465.graph_c0	12.6074591	9.96322383	11.0849522	7.39472482	7.11218296	9.18222727	1.51E-07	-1.59484	down	-
c52466.graph_c0	3.94576305	4.42846736	2.91935676	0	0	0	1.652E-35	-Inf	down	-
c52471.graph_c0	0.20585242	0.20605855	0.08817617	13.0162748	10.716817	13.4290705	1.373E-34	5.13522	up	PREDICTED: bidirectional sugar transporter SWEET14-like [Solanum tuberosum]
c52472.graph_c0	15.2130213	5.55625516	6.60450498	0	0	0	2.343E-08	-Inf	down	RecName: Full=40S ribosomal protein S29 [Griffithsia japonica]
c52482.graph_c0	50.727242	44.5804408	45.4768609	32.5218001	34.6275192	35.8006211	2.125E-56	-1.54073	down	hypothetical protein MIMGU_mgv1a027140mg [Erythranthe guttata]
c52484.graph_c0	55.6838217	43.5560657	44.5758162	7.2979916	5.79068675	12.9929488	4.487E-45	-3.54759	down	histone H4 [Zea mays]
c52487.graph_c0	0.07458826	0.07466295	0	2.65630547	4.01364026	3.58304917	4.215E-13	5.03182	up	PREDICTED: long chain acyl-CoA synthetase 4 [Sesamum indicum]

#ID	0 h-1 FPKM	0 h-2 FPKM	0 h-3 FPKM	28 h-1 FPKM	28 h-2 FPKM	28 h-3 FPKM	FDR	log2FC	regulated	nr_annotation
c52489.graph_c0	1.45229415	0.79295367	0.67863835	8.82797722	10.6566608	8.61294897	2.031E-07	2.18184	up	hypothetical protein MIMGU_mgv1a012466mg [Erythranthe guttata]
c52491.graph_c0	18.5394591	14.7184325	16.9779795	6.96950278	5.59360672	7.45636409	3.021E-19	-2.41626	down	PREDICTED: cation/calcium exchanger 3-like [Sesamum indicum]
c52493.graph_c0	9.41343717	8.61749037	8.0644263	7.6018132	10.1899911	6.29844853	3.504E-09	-1.20593	down	PREDICTED: E3 ubiquitin-protein ligase RHA2A-like [Sesamum indicum]
c52496.graph_c0	5.86007934	6.00396962	5.13841377	2.25480277	2.08111717	1.92167401	7.492E-22	-2.53132	down	-
c52500.graph_c0	7.01101327	5.48846228	5.54426438	0	0	0	4.698E-61	-Inf	down	-
c52505.graph_c0	8.59357281	10.6667007	11.0430813	10.9925362	8.79736253	9.1736793	0.0014336	-1.15858	down	PREDICTED: UDP-glycosyltransferase 73C3-like [Sesamum indicum]
c52508.graph_c0	0.30598701	0	0	7.63537613	6.2916379	6.65383013	9.524E-16	5.01276	up	hypothetical protein MIMGU_mgv1a018889mg [Erythranthe guttata]
c52510.graph_c0	18.9334696	18.3259021	13.6732029	16.4901389	16.8400282	14.3838062	2.992E-09	-1.18139	down	PREDICTED: zinc-finger homeodomain protein 1-like [Sesamum indicum]
c52513.graph_c0	27.9032811	33.2602052	29.4875173	7.87182033	7.83031391	9.253966	7.4E-50	-2.95084	down	PREDICTED: cyclin-dependent protein kinase inhibitor SMR3-like [Sesamum indicum]
c52518.graph_c0	0	0	0	25.9645351	23.3095777	23.7292704	1.017E-29	Inf	up	PREDICTED: protein YLS9 [Sesamum indicum]
c52520.graph_c0	11.2256034	9.39473856	8.86803986	9.09494857	10.8686724	8.34907924	1.707E-08	-1.14758	down	unnamed protein product [Coffea canephora]
c52522.graph_c0	21.8215658	12.1088506	16.6115858	0	0	0	1.021E-30	-Inf	down	--
c52528.graph_c0	1.78100202	2.30276451	1.90721509	0.862948	0.68176599	1.18826751	1.105E-08	-2.22057	down	-
c52531.graph_c0	53.8185969	32.4843062	33.0999924	0	0	0	1.059E-41	-Inf	down	uncharacterized protein LOC100191904 [Zea mays]
c52533.graph_c0	0	0	0	12.6878378	21.0641256	14.1382027	2.431E-14	Inf	up	-
c52540.graph_c0	0.71427591	0.35749557	0.65562345	26.3644699	21.9966967	23.9035468	1.967E-73	4.29572	up	PREDICTED: flavonol synthase/flavanone 3-hydroxylase-like [Sesamum indicum]
c52546.graph_c0	17.3418837	13.403977	16.3611445	6.38176258	5.61807993	6.11877273	2.222E-18	-2.46871	down	-
c52548.graph_c0	0.17373099	2.26076447	1.56275844	14.6469355	11.1726838	11.9517797	2.212E-07	2.14024	up	-
c52555.graph_c0	0.47528673	0.88355922	0.43625876	4.44131559	5.83691105	5.83879112	1.568E-07	2.07918	up	PREDICTED: regulatory protein NPR5-like [Sesamum indicum]
c52558.graph_c0	2.28589468	2.37971101	2.58496935	67.8505583	74.4632727	77.1104242	6.76E-114	3.8277	up	eukaryotic translation initiation factor 5A [Quercus suber]
c52562.graph_c0	1.23367469	1.67594933	1.35884642	19.7258085	18.5622998	17.5593803	3.18E-21	2.61854	up	hypothetical protein MIMGU_mgv1a012628mg [Erythranthe guttata]
c52564.graph_c0	19.8700416	10.659967	12.572193	0	0	0	1.322E-29	-Inf	down	--
c52571.graph_c0	40.9594764	46.2112559	39.2558675	12.5448429	11.6863985	11.780087	6.251E-89	-2.90149	down	PREDICTED: uncharacterized protein LOC105157856 [Sesamum indicum]
c52573.graph_c0	0.63465124	0.4235245	0.54370132	6.45765212	4.02592804	4.0147744	4.812E-05	2.08785	up	PREDICTED: microtubule-associated protein 70-2 [Sesamum indicum]
c52577.graph_c0	0	0.41234525	0.17644997	3.19349714	3.7844999	2.68730095	1.302E-07	2.93924	up	hypothetical protein MIMGU_mgv1a001002mg [Erythranthe guttata]
c52580.graph_c0	0.08629323	0.25913891	0.11089021	5.95815487	9.79663529	7.21594322	3.423E-12	4.56337	up	hypothetical protein POPTR_0001s10630g [Populus trichocarpa]
c52585.graph_c0	1.3827387	2.42221578	2.33214709	1.50744978	2.04162872	1.89635284	0.0040019	-1.26771	down	hypothetical protein MIMGU_mgv1a017183mg [Erythranthe guttata]

#ID	0 h-1 FPKM	0 h-2 FPKM	0 h-3 FPKM	28 h-1 FPKM	28 h-2 FPKM	28 h-3 FPKM	FDR	log2FC	regulated	nr_annotation
c52590.graph_c0	0.31192415	0.23417737	0.40083488	24.597457	27.6335636	24.7885477	4.909E-62	5.25042	up	PREDICTED: probable serine/threonine-protein kinase At4g35230 [Sesamum indicum]
c52594.graph_c0	7.97435889	6.44030028	6.98684106	32.6009142	31.6655362	29.7459619	5.958E-17	1.04547	up	PREDICTED: GDP-mannose transporter GONST4 [Sesamum indicum]
c52596.graph_c0	6.16315085	3.50677268	4.41846653	0	0	0	8.93E-27	-Inf	down	translationally-controlled tumor protein [Tilletiaria anomala UBC 951]
c52599.graph_c0	2.03440068	0.29091969	0.74693869	16.0532703	13.541006	8.27326578	0.0002228	2.54259	up	-
c52600.graph_c0	0	0	0	3.00969378	3.22411849	2.62238626	4.508E-15	Inf	up	PREDICTED: LOW QUALITY PROTEIN: protein E6-like [Sesamum indicum]
c52601.graph_c0	21.3518917	28.1342055	21.2784693	3.95878364	2.00166985	3.23033512	4.056E-33	-4.03345	down	-
c52604.graph_c0	14.8941634	13.0454429	15.949658	0.06443448	0.11635658	0.26289003	2.88E-103	-7.71481	down	-
c52605.graph_c0	15.6961061	17.2067917	15.804943	16.0263118	14.7200754	14.883506	2.883E-32	-1.18462	down	PREDICTED: inositol-tetrakisphosphate 1-kinase 1-like [Sesamum indicum]
c52610.graph_c0	113.926978	111.547765	102.007316	44.0374743	46.7972945	45.0863665	1.5E-175	-2.35789	down	PREDICTED: cysteine proteinase inhibitor 1-like [Sesamum indicum]
c52621.graph_c0	7.67071966	5.04975904	5.50581139	0	0	0	1.218E-40	-Inf	down	-
c52622.graph_c0	21.3740828	19.0840621	19.097236	0.12909607	0.11656165	0.1755689	2.2E-261	-8.23096	down	PREDICTED: LOW QUALITY PROTEIN: EID1-like F-box protein 3 [Sesamum indicum]
c52631.graph_c0	28.0337786	25.6098439	22.5007491	14.3085086	13.4550628	16.1952234	2.441E-42	-1.87968	down	hypothetical protein MIMGU_mgv1a017584mg [Erythranthe guttata]
c52635.graph_c0	5.01279177	4.54605129	4.95510453	4.67085663	4.97646781	4.90397671	7.407E-08	-1.08694	down	PREDICTED: thioredoxin-like protein CDSP32, chloroplastic [Sesamum indicum]
c52638.graph_c0	0.7246657	0.45336959	0.34920904	23.1082103	21.8750809	20.6826287	1.048E-39	4.34357	up	hypothetical protein MIMGU_mgv1a016092mg [Erythranthe guttata]
c52657.graph_c0	21.5617086	17.7228719	15.5433154	78.8642514	76.383626	90.2391663	1.279E-10	1.07668	up	PREDICTED: 60S ribosomal protein L23A-like isoform X1 [Musa acuminata subsp. malaccensis]
c52662.graph_c0	5.31967927	4.56429096	4.29691333	0	0	0	6.5E-35	-Inf	down	hypothetical protein M569_00609, partial [Genlisea aurea]
c52666.graph_c0	3.21245743	4.56418277	4.12813499	0.5272119	0.20400982	0.4302006	2.672E-25	-4.44865	down	-
c52667.graph_c0	1.04980163	1.40113713	0.44967888	35.8605193	39.497405	40.4685725	7.165E-20	4.23859	up	PIP2;1 [Karelinia caspia]
c52673.graph_c0	1.21000952	0.69878144	0.47843389	16.2017687	19.1791437	15.0697265	2.383E-34	3.32046	up	PREDICTED: uncharacterized protein LOC105176754 [Sesamum indicum]
c52674.graph_c0	2819.40896	2596.66673	2878.14897	122.509356	129.244267	147.319251	0	-5.46773	down	hypothetical protein PHAVU_005G056100g [Phaseolus vulgaris]
c52675.graph_c0	98.5272191	84.2259575	90.8602309	29.5905021	27.8752051	31.5502612	4.059E-96	-2.70938	down	PREDICTED: uncharacterized protein LOC102593371 [Solanum tuberosum]
c52676.graph_c0	25.7252892	24.7461302	22.5762054	26.540608	24.7871748	23.4430571	2.71E-12	-1.05566	down	PREDICTED: zinc finger protein NUTCRACKER-like [Sesamum indicum]
c52679.graph_c0	43.5058974	55.9921654	58.3270085	17.5665753	29.332496	25.9132225	1.228E-12	-2.20964	down	PREDICTED: transcription factor TGA5-like [Sesamum indicum]
c52682.graph_c0	5.57455068	6.07249741	8.0062852	48.737779	45.4043572	42.2005361	3.551E-16	1.69865	up	PREDICTED: uncharacterized protein At2g34160-like [Sesamum indicum]

#ID	0 h-1 FPKM	0 h-2 FPKM	0 h-3 FPKM	28 h-1 FPKM	28 h-2 FPKM	28 h-3 FPKM	FDR	log2FC	regulated	nr_annotation
c52686.graph_c0	1.00870062	0.72122191	0.55552296	7.95956463	6.99761583	5.29850347	1.024E-05	2.06267	up	-
c52687.graph_c0	7.90859801	8.05788365	6.71474781	2.617358	1.39013463	2.68014777	3.735E-32	-2.84835	down	-
c52691.graph_c0	20.2010636	16.9915023	18.387743	1.83525208	0.4602946	0.91516988	1.633E-80	-5.20324	down	RNA recognition motif containing protein [Zea mays]
c52692.graph_c0	49.4771286	46.7136256	53.7311771	33.0507323	36.2124151	29.090295	1.563E-29	-1.70092	down	PREDICTED: universal stress protein A-like protein [Sesamum indicum]
c52694.graph_c0	0.22836289	0.22859156	0.58691071	18.4229843	20.3057462	18.2833842	5.975E-31	4.66662	up	PREDICTED: expansin-A10-like isoform X2 [Camelina sativa]
c52698.graph_c0	0.82270269	0.8235265	0.70480369	4.7834846	6.83847698	5.85492655	0.0001508	1.80527	up	-
c52700.graph_c0	10.0736203	6.09223998	8.22546936	0	0	0	1.828E-33	-Inf	down	PREDICTED: 60S ribosomal protein L18-3 [Nelumbo nucifera]
c52718.graph_c0	0.70641293	0.23570677	0	5.13416847	7.26255398	6.28415932	7.038E-09	3.24464	up	hypothetical protein M569_04419, partial [Genlisea aurea]
c52719.graph_c0	9.83682169	8.11685106	8.37019677	5.60354092	7.24027899	6.5433245	1.159E-08	-1.5303	down	-
c52726.graph_c0	9.46555246	8.79824286	6.95063409	4.00134706	3.86637395	2.46312036	3.909E-21	-2.37608	down	-
c52732.graph_c0	10.5895353	9.6783879	10.0580664	6.69253325	3.92777452	5.18799985	3.219E-07	-2.02962	down	-
c52734.graph_c0	5.56920824	5.95925289	4.68884167	0.5582991	0.25204587	1.02502688	1.173E-20	-4.22623	down	-
c52737.graph_c0	0.87150579	0.87237847	1.67987916	11.5596482	10.2943021	9.43257855	2.822E-06	2.09131	up	PREDICTED: serine/threonine-protein kinase At5g01020 [Sesamum indicum]
c52739.graph_c0	0.81088562	1.6233952	1.56303019	7.8088477	6.38550097	8.41579088	0.0013387	1.40458	up	-
c52742.graph_c0	0	0	0	1.68853995	2.26734382	1.34250571	7.97E-11	Inf	up	PREDICTED: palmitoyl-protein thioesterase 1-like [Sesamum indicum]
c52743.graph_c0	8.30412485	4.64201204	4.85051327	0	0	0	2.535E-19	-Inf	down	-
c52746.graph_c0	4.23704957	2.5447754	3.42243091	0.08798466	0.63553521	0.35897383	4.34E-16	-4.32696	down	-
c52747.graph_c0	25.4571872	29.4920563	25.6362928	14.8352989	16.1496808	15.5544033	1.27E-45	-1.88232	down	hypothetical protein MIMGU_mgv1a022353mg [Erythranthe guttata]
c52748.graph_c0	45.7607402	49.9707956	49.6400466	5.32714183	6.10988517	8.75255041	1.74E-165	-3.93737	down	PREDICTED: pentatricopeptide repeat-containing protein At3g57430, chloroplastic [Sesamum indicum]
c52749.graph_c0	7.20043393	4.67522859	4.00122926	0	0	0	1.051E-18	-Inf	down	-
c52753.graph_c0	8.49020982	11.1488473	8.44664962	1.92417668	2.51616371	2.65294864	1.779E-24	-3.07431	down	-
c52756.graph_c0	0.52466773	0.21007724	0.13484376	4.42337556	5.16451733	5.04076733	1.106E-08	2.99676	up	PREDICTED: WUSCHEL-related homeobox 11-like [Sesamum indicum]
c52758.graph_c0	24.5720259	24.5055324	21.0506841	18.3879566	24.5455717	21.6970425	2.729E-19	-1.20653	down	PREDICTED: auxin-induced protein X15-like [Sesamum indicum]
c52760.graph_c0	50.8722032	51.144549	47.4663339	33.1155068	23.8040409	25.1855377	1.143E-36	-1.95432	down	hypothetical protein MIMGU_mgv1a001181mg [Erythranthe guttata]
c52761.graph_c0	1.29663561	0.353982	0	36.4960895	35.8919761	35.5129098	3.718E-54	4.96668	up	PREDICTED: callose synthase 3-like [Cucumis sativus]
c52765.graph_c0	0	0	0	4.07147866	5.09007253	4.08897642	2.379E-10	Inf	up	PREDICTED: transcription factor HEC2-like [Sesamum indicum]
c52769.graph_c0	0.86678195	1.12794487	1.67077368	10.8985178	12.1155627	11.1035256	1.298E-11	2.11946	up	PREDICTED: uncharacterized protein LOC105168965 [Sesamum indicum]
c52771.graph_c0	0.17157045	0.17174225	0.11023741	2.68097285	1.85772128	2.0859007	1.285E-07	2.78333	up	PREDICTED: CASP-like protein 2C1 isoform X2 [Sesamum indicum]

#ID	0 h-1 FPKM	0 h-2 FPKM	0 h-3 FPKM	28 h-1 FPKM	28 h-2 FPKM	28 h-3 FPKM	FDR	log2FC	regulated	nr_annotation
c52778.graph_c0	0.17018039	0.5110524	0	41.4348526	51.929794	38.1503568	4.945E-38	6.5157	up	PREDICTED: uncharacterized protein LOC104588233 isoform X2 [Nelumbo nucifera]
c52779.graph_c0	4.50510001	5.26121306	5.54801389	0	0	0	6.531E-32	-Inf	down	-
c52787.graph_c0	3.51001911	4.99291654	4.74790897	0.1342664	0.36368992	0.43824115	9.07E-22	-4.91419	down	-
c52791.graph_c0	96.25799	99.4209325	98.4175559	23.7886595	24.44154	30.6947978	6.25E-158	-2.98731	down	hypothetical protein MIMGU_mgv1a018283mg [Erythranthe guttata]
c52792.graph_c0	8.1125241	6.49651806	5.21245733	39.7006475	41.8794412	46.0271336	9.42E-09	1.60173	up	-
c52797.graph_c0	5.96555068	3.44121738	3.11836019	2.71901577	2.85312706	2.87830659	0.0018333	-1.6508	down	uncharacterized protein [Arabidopsis thaliana]
c52799.graph_c0	28.4638294	29.7728858	26.0971784	5.57861047	7.24063317	7.77650517	1.336E-57	-3.12231	down	PREDICTED: uncharacterized protein LOC105178281 [Sesamum indicum]
c52801.graph_c0	85.7546789	77.8276281	75.5081715	75.6948419	70.5247961	72.7762415	1.411E-43	-1.21573	down	PREDICTED: UPF0369 protein C6orf57 homolog [Nicotiana tomentosiformis]
c52803.graph_c0	203.439453	244.526075	194.231766	47.1259352	61.4775996	47.4640629	4.423E-45	-3.13113	down	PREDICTED: probable CCR4-associated factor 1 homolog 11 [Sesamum indicum]
c52805.graph_c0	10.4744979	11.1140857	9.82590545	5.70960351	6.87364885	4.28627065	6.272E-18	-1.98845	down	PREDICTED: dnaJ protein ERDJ3A [Sesamum indicum]
c52817.graph_c0	1.05714778	2.11641271	0.67923838	33.9985544	45.6126798	37.7737447	3.012E-23	3.84452	up	-
c52828.graph_c0	0	0	0	6.93892612	7.18486204	14.0254028	8.321E-09	Inf	up	cytochrome c oxidase subunit 3 (mitochondrion) [Microbotryum lychnidis-dioicae]
c52831.graph_c0	0	0	0	4.37604223	3.73164718	5.65378998	7.128E-14	Inf	up	-
c52832.graph_c0	68.4917306	67.576749	56.2996251	27.515193	30.8743856	29.8905263	5.058E-57	-2.21174	down	PREDICTED: acidic leucine-rich nuclear phosphoprotein 32-related protein [Sesamum indicum]
c52837.graph_c0	0.08503434	0	0	3.21372816	3.57131719	3.47969115	2.112E-13	5.85657	up	hypothetical protein CISIN_1g041913mg [Citrus sinensis]
c52838.graph_c0	1.62089096	1.62251403	1.92268528	9.3338141	10.0639782	8.35576424	0.0002335	1.33187	up	-
c52840.graph_c0	6.19315683	5.36160722	5.05469231	3.04131804	2.91078654	2.38242305	6.776E-14	-2.08405	down	hypothetical protein MIMGU_mgv1a013481mg [Erythranthe guttata]
c52846.graph_c0	0	0	0	5.47780266	4.62684916	4.32565378	1E-14	Inf	up	PREDICTED: ethylene-responsive transcription factor CRF4-like [Sesamum indicum]
c52849.graph_c0	8.79298705	4.28805246	6.08425811	0	0	0	3.049E-15	-Inf	down	unknown [Picea sitchensis]
c52852.graph_c0	1.10882214	1.87540311	1.32661274	0	0.05018201	0	3.15E-20	-7.52562	down	PREDICTED: uncharacterized protein LOC103504028 [Cucumis melo]
c52856.graph_c0	3.62546949	4.42296544	2.6206154	25.3615894	32.2669663	23.9200213	7.608E-11	1.84703	up	PREDICTED: protein transport protein SEC31 homolog B [Sesamum indicum]
c52858.graph_c0	0.46345301	1.62370983	2.97777736	19.3680114	20.0726068	18.8275396	4.463E-10	2.41548	up	PREDICTED: membrin-11 [Sesamum indicum]
c52861.graph_c0	119.662825	122.156934	119.786297	3.70636356	2.87954547	2.3210223	0	-6.43517	down	PREDICTED: heat shock 70 kDa protein-like [Sesamum indicum]
c52869.graph_c0	143.492551	139.341466	131.097117	74.1460652	79.3039745	80.4345096	2.239E-75	-1.91286	down	-
c52870.graph_c0	4.53542719	3.92088209	4.90098932	4.56990143	3.11155502	3.05655922	1.084E-05	-1.40764	down	hypothetical protein M569_00403, partial [Genlisea aurea]

#ID	0 h-1 FPKM	0 h-2 FPKM	0 h-3 FPKM	28 h-1 FPKM	28 h-2 FPKM	28 h-3 FPKM	FDR	log2FC	regulated	nr_annotation
c52872.graph_c0	79.3171032	94.7481216	82.9877765	64.7007245	62.5072098	60.8957473	1.327E-26	-1.54131	down	-
c52876.graph_c0	1.84647139	0.69312013	1.48299303	11.5747911	14.8433799	12.7301298	6.388E-07	2.19289	up	PREDICTED: muscle M-line assembly protein unc-89-like [Populus euphratica]
c52879.graph_c0	0.07585005	0.075926	0	4.68582705	4.4299619	4.2734315	3.077E-15	5.3924	up	-
c52880.graph_c0	3.23132494	3.00352058	1.48299303	52.1704353	65.1291157	52.4262335	2.354E-31	3.37644	up	hypothetical protein MIMGU_mgv1a011350mg [Erythranthe guttata]
c52881.graph_c0	4.86229399	4.05596903	3.27288776	22.7178466	19.90432	18.5375773	1.232E-06	1.24033	up	PREDICTED: uncharacterized protein LOC105170351 [Sesamum indicum]
c52884.graph_c0	1.26417293	1.01235105	0.64980491	6.06403778	7.46626031	8.84679131	0.0001266	1.85317	up	-
c52890.graph_c0	6.41122314	3.14333535	4.37154214	0	0	0	3.798E-16	-Inf	down	60S ribosomal protein L11 [Zea mays]
c52894.graph_c0	23.5685791	21.3828784	20.9676166	13.6921481	15.6215199	19.166524	1.004E-29	-1.53133	down	proliferating cell nuclear antigen [Nicotiana benthamiana]
c52898.graph_c0	0.40444411	0.50606138	0.5197267	16.2406077	14.5973943	14.7513213	2.839E-24	3.90111	up	PREDICTED: vegetative incompatibility protein HET-E-1-like [Sesamum indicum]
c52903.graph_c0	1.4682682	1.11700122	1.43395491	0.76833278	0.57811039	1.25390655	1.658E-05	-1.71578	down	-
c52907.graph_c0	0.44285432	0.55412222	0.426814	39.1064721	34.5829453	32.0418811	1.213E-58	5.12458	up	-
c52908.graph_c0	43.1549937	46.3629107	45.2991217	23.6097675	23.9626321	23.4371989	1.685E-80	-2.01598	down	PREDICTED: transcription factor TCP9 [Sesamum indicum]
c52911.graph_c0	20.7480141	23.906521	18.6058698	19.8529157	23.0188555	20.1833787	5.798E-10	-1.09386	down	-
c52917.graph_c0	0.52372489	1.04849865	0.26920289	3.04512187	3.29935145	3.41659255	0.0056235	1.32405	up	PREDICTED: scarecrow-like protein 8 [Sesamum indicum]
c52924.graph_c0	5.7802941	5.40034338	4.20914717	2.94075911	3.54030633	3.6565873	5.17E-06	-1.68848	down	PREDICTED: probable galacturonosyltransferase-like 1 [Sesamum indicum]
c52927.graph_c0	1.03620904	0.86437221	0.44385655	16.3173998	12.3531227	17.4117465	3.372E-13	3.21708	up	unknown [Lotus japonicus]
c52930.graph_c0	2.16052922	1.73015413	3.88691182	22.1092634	20.3028634	19.7323201	6.064E-17	1.89804	up	PREDICTED: uncharacterized protein LOC105172794 [Sesamum indicum]
c52932.graph_c0	34.0637966	22.0360619	20.5466638	0	0	0	1.296E-30	-Inf	down	-
c52935.graph_c0	0	0.04486597	0.17279063	1.23787703	1.82359442	1.59489087	1.718E-09	3.29863	up	PREDICTED: GDSL esterase/lipase At4g10955-like [Sesamum indicum]
c52939.graph_c0	7.01848112	14.0510181	9.01902611	4.73663508	5.59265657	5.79758332	0.0008213	-1.99048	down	-
c52943.graph_c0	46.0578735	46.9473593	46.1941003	18.1660923	12.9006682	21.1524678	3.021E-39	-2.50321	down	-
c52952.graph_c0	6.42665612	7.07640059	4.78993438	34.5643391	35.1726623	35.8269801	5.717E-12	1.44223	up	unnamed protein product [Coffea canephora]
c52953.graph_c0	0	0	0	11.8204386	16.3325382	12.2759399	3.996E-17	Inf	up	-
c52955.graph_c0	1.04486402	1.34474181	1.34269163	5.31580803	5.77920202	6.99336367	0.0051405	1.18497	up	hypothetical protein POPTR_0005s25720g [Populus trichocarpa]
c52958.graph_c0	3.47695978	1.28226789	1.64611667	13.9651897	14.7708438	11.7210659	4.764E-05	1.57597	up	PREDICTED: phosphatidylinositol/phosphatidylcholine transfer protein SFH6-like isoform X2 [Populus euphratica]
c52960.graph_c0	5.29649341	5.18654059	4.66076994	27.2809619	28.5612055	30.4894335	1.433E-20	1.42262	up	hypothetical protein MIMGU_mgv1a007612mg [Erythranthe guttata]

#ID	0 h-1 FPKM	0 h-2 FPKM	0 h-3 FPKM	28 h-1 FPKM	28 h-2 FPKM	28 h-3 FPKM	FDR	log2FC	regulated	nr_annotation
c52961.graph_c0	0.30799728	0.15415285	0.19789435	9.62557794	13.2386077	11.3249387	3.681E-16	4.60859	up	-
c52974.graph_c0	0	0	0	29.8584455	27.5562634	31.5566456	2.677E-46	Inf	up	-
c52975.graph_c0	0.89836991	2.24817373	0.28861023	10.6101137	7.07441565	7.45899851	0.0040833	1.79143	up	-
c52984.graph_c0	15.6017172	16.9514845	19.4443371	5.81207179	6.12238188	6.50945526	1.521E-25	-2.58821	down	-
c52991.graph_c0	17.3519715	14.9247722	13.5439524	8.40754193	8.51903811	9.52845217	2.77E-19	-1.87957	down	hypothetical protein M569_07000 [Genlisea aurea]
c52992.graph_c0	6.13009916	6.68411587	8.29942476	1.75030457	2.15503762	2.59678953	8.539E-16	-2.79237	down	-
c53000.graph_c0	7.86357134	8.01370055	8.94840406	2.4788812	1.70973405	2.19130905	8.337E-55	-3.05193	down	-
c53001.graph_c0	1.25343881	1.25469393	1.38061585	5.72623605	8.81293341	7.64601031	0.0010355	1.42022	up	hypothetical protein MIMGU_mgv1a027115mg, partial [Erythranthe guttata]
c53003.graph_c0	24.2430563	29.8225044	20.2684037	16.7705708	14.7589048	11.605968	7.562E-11	-1.87415	down	-
c53005.graph_c0	1.09056439	0.43666257	0.14014184	5.07274186	5.009605	4.26866956	2.74E-05	2.03375	up	-
c53010.graph_c0	4.12118102	3.00022382	1.44433112	26.68496	28.7653059	24.8853822	3.454E-07	2.1497	up	-
c53025.graph_c0	1.48396473	1.32040062	0.84753486	8.14894631	6.27571471	9.38749221	0.0002367	1.62258	up	PREDICTED: protein Brevis radix-like 2 [Sesamum indicum]
c53027.graph_c0	1.80885321	2.80652997	2.44067038	2.36639535	1.83987853	2.95007668	0.0045109	-1.07063	down	-
c53031.graph_c0	11.73195	13.0815874	15.2668559	8.31084747	8.08863677	6.60543863	1.883E-13	-1.89572	down	-
c53036.graph_c0	11.6687294	7.32680506	6.40686022	0	0	0	6.45E-23	-Inf	down	hypothetical protein GUITHDRAFT_93413, partial [Guillardia theta CCMP2712]
c53038.graph_c0	1.89370724	1.08067115	1.2736016	0	0	0	4.449E-26	-Inf	down	predicted protein [Physcomitrella patens]
c53039.graph_c0	0.18710993	0	0	6.52754013	9.45456808	7.32382673	1.397E-13	5.90102	up	PREDICTED: premnaspirodiene oxygenase-like [Sesamum indicum]
c53043.graph_c0	2.51894925	1.14612345	2.64841324	21.469796	18.1830291	14.1239886	2.688E-07	1.99582	up	hypothetical protein M569_02184 [Genlisea aurea]
c53045.graph_c0	12.2530294	12.0883956	9.46251678	5.65152362	8.46600237	5.93917812	1.548E-19	-1.84157	down	PREDICTED: uncharacterized protein At1g76070 [Sesamum indicum]
c53051.graph_c0	30.1519528	30.4265353	30.7461734	6.74285094	5.36719562	9.26671377	2.848E-81	-3.18341	down	PREDICTED: histone H2A.1 [Sesamum indicum]
c53054.graph_c0	2.07580077	3.56207892	4.19176399	180.179502	208.610683	212.44761	2.435E-90	4.83712	up	PREDICTED: LOW QUALITY PROTEIN: 36.4 kDa proline-rich protein [Sesamum indicum]
c53073.graph_c0	0.27789504	0	0.35710614	4.84734041	3.64724492	2.80173472	2.399E-06	3.05198	up	-
c53074.graph_c0	5.47554551	7.30803792	7.03629277	4.737613	4.79093479	3.86585352	1.504E-05	-1.65919	down	PREDICTED: probable galacturonosyltransferase-like 1 [Sesamum indicum]
c53076.graph_c0	7.84791442	8.10918495	8.29562771	0.4599854	0.08306474	0.15013773	1.251E-48	-6.221	down	-
c53085.graph_c0	0.37515921	0.84495346	1.44628329	6.33942749	5.90855086	5.28416668	2.441E-05	1.61136	up	unnamed protein product [Coffea canephora]
c53092.graph_c0	6.059923	6.86414782	6.35274922	1.62264315	1.88369309	2.17524727	5.572E-15	-2.85235	down	-
c53100.graph_c0	0.36775843	0	0	3.56379612	3.94177286	4.79824708	2.381E-10	4.00646	up	hypothetical protein MIMGU_mgv1a006779mg [Erythranthe guttata]
c53102.graph_c0	0.29062867	1.45459844	0.74693869	5.49190827	7.62873577	7.06674785	3.689E-05	1.92429	up	-

#ID	0 h-1 FPKM	0 h-2 FPKM	0 h-3 FPKM	28 h-1 FPKM	28 h-2 FPKM	28 h-3 FPKM	FDR	log2FC	regulated	nr_annotation
c53107.graph_c0	57.2474771	57.6249403	58.9755134	33.3554418	31.3760771	31.7698687	2.316E-54	-1.94017	down	PREDICTED: R3H domain-containing protein 1-like, partial [Sesamum indicum]
c53108.graph_c0	39.3821837	38.7074593	42.5397766	17.8373623	22.6600158	21.2403525	2.731E-47	-2.05799	down	-
c53115.graph_c0	1.6316135	2.17766308	1.16482663	14.2301674	12.9674746	12.4718264	2.189E-06	1.9094	up	-
c53119.graph_c0	33.9437957	51.7161879	37.2045447	33.9214633	38.8171792	34.1924928	8.687E-05	-1.29114	down	-
c53120.graph_c0	0.29504194	0	0	10.0784347	10.2615686	8.04894341	1.742E-12	5.52828	up	-
c53141.graph_c0	0.0937898	0.09388372	0	6.20309572	8.00116262	6.73035966	4.166E-18	5.7307	up	PREDICTED: squamosa promoter-binding-like protein 9 [Sesamum indicum]
c53164.graph_c0	23.5806769	24.3419234	20.6433079	3.74900314	3.77185528	3.05915595	7.664E-67	-3.78584	down	PREDICTED: uncharacterized protein LOC103930947 [Pyrus x bretschneideri]
c53168.graph_c0	2.12647036	0.38701813	0.74525416	17.141071	16.6185096	14.5602106	6.207E-11	2.81137	up	hypothetical protein M569_03938, partial [Genlisea aurea]
c53169.graph_c0	1.16791868	1.16908818	0.30016435	5.60232135	4.75180298	4.57144847	0.0028167	1.42303	up	-
c53171.graph_c0	2.59364375	1.46454615	1.965577	21.7988576	19.2895509	18.4187386	9.73E-24	2.21591	up	PREDICTED: uncharacterized protein LOC105166615 isoform X1 [Sesamum indicum]
c53173.graph_c0	1.24885611	0.81256932	0.64193182	14.4318166	15.2023402	14.5166285	1.763E-26	2.94653	up	PREDICTED: 30S ribosomal protein 2, chloroplastic [Sesamum indicum]
c53178.graph_c0	15.4743775	15.4898727	11.2255092	13.9676613	13.1028453	10.9534392	4.71E-06	-1.2374	down	PREDICTED: uncharacterized protein LOC105161374 [Sesamum indicum]
c53184.graph_c0	0	0	0.14672768	3.07049739	3.82137659	2.97951446	1.653E-12	4.94098	up	PREDICTED: MATE efflux family protein LAL5-like [Sesamum indicum]
c53185.graph_c0	6.03893014	4.21316594	5.87898812	65.9688961	65.9284005	67.938771	2.182E-34	2.53989	up	PREDICTED: 60S ribosomal protein L38 [Sesamum indicum]
c53215.graph_c0	1.81856002	0.72815241	2.10322946	21.147503	22.4356997	19.4132135	1.37E-13	2.66431	up	-
c53222.graph_c0	0.59373033	0.19810829	0.76296698	9.63727514	11.9484051	7.5118212	1.258E-09	3.12709	up	PREDICTED: zeatin O-glucosyltransferase-like [Solanum lycopersicum]
c53226.graph_c0	2.54984714	1.1911202	1.26697308	0	0	0	2.696E-12	-Inf	down	carboxypeptidase, partial [Metarhizium majus ARSEF 297]
c53230.graph_c0	4.48999776	4.16156834	2.56436661	19.9424163	25.5359693	23.7682217	1.868E-07	1.54229	up	hypothetical protein MIMGU_mgv1a007612mg [Erythranthe guttata]
c53235.graph_c0	0.24111558	0.36203553	0.9295292	4.03055251	4.03477743	3.28889793	7.893E-05	1.78036	up	PREDICTED: elicitor-responsive protein 3-like [Sesamum indicum]
c53236.graph_c0	1.43918239	1.28055423	1.84940634	10.6923367	9.44430414	7.49199888	0.0001805	1.50049	up	-
c53238.graph_c0	1.99943352	1.50107674	1.28467562	9.44564893	9.51259714	11.2649135	0.0009072	1.57439	up	-
c53246.graph_c0	6.36010843	7.51244299	5.96628353	7.44220914	6.63614357	6.41225416	2.987E-07	-1.04294	down	hypothetical protein MIMGU_mgv1a026702mg [Erythranthe guttata]
c53264.graph_c0	1.09180025	1.63934028	0.70150327	5.35622036	6.44821972	5.50375891	0.0069804	1.249	up	PREDICTED: coatomer subunit beta'-2-like [Glycine max]
c53267.graph_c0	0	0.15790331	0	6.64960801	6.21100611	6.4550997	1.881E-13	5.853	up	hypothetical protein JCGZ_13755 [Jatropha curcas]
c53270.graph_c0	49.0072083	46.0440538	43.6414038	8.12343921	10.7199519	15.0419594	6.49E-36	-3.12016	down	Histone H3.3 [Triticum urartu]
c53274.graph_c0	0.89951367	1.80082879	2.31182135	26.150454	25.3822637	20.0048971	1.077E-08	2.73382	up	PREDICTED: 8-hydroxygeraniol dehydrogenase-like [Sesamum indicum]

#ID	0 h-1 FPKM	0 h-2 FPKM	0 h-3 FPKM	28 h-1 FPKM	28 h-2 FPKM	28 h-3 FPKM	FDR	log2FC	regulated	nr_annotation
c53275.graph_c0	0.27167786	0	0.69823362	10.2676031	12.3014831	9.18388415	5.715E-11	3.92121	up	ATP binding protein, putative [Ricinus communis]
c53277.graph_c0	3.22802712	5.1700152	2.07407133	17.3612251	20.3358597	18.7611967	0.0011453	1.346	up	prohibitin-like protein [Petunia x hybrida]
c53292.graph_c0	15.5221661	15.3173161	14.1465234	64.568132	67.9793434	64.4388309	5.963E-15	1.041	up	PREDICTED: U6 snRNA-associated Sm-like protein LSm6 [Sesamum indicum]
c53293.graph_c0	5.21768387	3.13374515	2.23497678	23.0059075	29.6745393	22.279608	1.37E-05	1.74194	up	-
c53299.graph_c0	1.38647313	1.15655123	1.33625465	9.32104628	11.2213745	8.56520005	9.442E-07	1.81596	up	PREDICTED: probable polygalacturonase [Sesamum indicum]
c53303.graph_c0	13.0587973	5.58277938	10.6629725	0	0	0	6.511E-16	-Inf	down	hypothetical protein CICLE_v10032980mg [Citrus clementina]
c53304.graph_c0	16.9487067	16.7771708	17.4238074	20.1197632	17.5483598	16.7526148	6.419E-06	-1.00194	down	PREDICTED: vacuolar-sorting receptor 3-like [Sesamum indicum]
c53313.graph_c0	10.3982339	12.9651557	11.4867538	5.96634982	6.94331468	6.7077011	1.169E-11	-1.92013	down	PREDICTED: uncharacterized protein LOC100815796 [Glycine max]
c53319.graph_c0	3.57671215	2.68522027	0.38301806	14.9473192	13.6916224	11.3130578	0.0025609	1.51462	up	PREDICTED: succinyl-CoA ligase [ADP-forming] subunit beta, mitochondrial [Prunus mume]
c53339.graph_c0	0	0	0	5.2255944	6.43393881	5.2977496	5.101E-13	Inf	up	hypothetical protein MIMGU_mgv1a021216mg, partial [Erythranthe guttata]
c53342.graph_c0	45.9986597	38.7001638	42.7913599	29.3295288	29.8151898	36.4847483	2.044E-16	-1.50388	down	hypothetical protein MIMGU_mgv1a006294mg [Erythranthe guttata]
c53351.graph_c0	87.9821338	90.7781042	81.4433066	70.9666766	69.8245208	64.8602718	1.682E-50	-1.42926	down	PREDICTED: zinc finger A20 and AN1 domain-containing stress-associated protein 8-like [Sesamum indicum]
c53354.graph_c0	0	0	0	6.93521085	6.36620924	3.96135279	1.283E-10	Inf	up	PREDICTED: uncharacterized protein DDB_G0271670-like [Solanum tuberosum]
c53357.graph_c0	3.66676612	2.09739305	1.68283558	12.5634983	13.2342712	14.1349362	0.0017072	1.34155	up	-
c53362.graph_c0	10.8480194	12.4761624	10.6775498	3.69051311	3.7865765	3.96961037	1.35E-15	-2.66033	down	PREDICTED: uncharacterized protein LOC105161926 [Sesamum indicum]
c53364.graph_c0	12.0072761	8.82429586	8.00788393	9.05821723	5.28624641	9.10408	1.344E-05	-1.38287	down	-
c53370.graph_c0	8.2008455	8.40451115	8.02926564	2.1286883	2.05013968	3.35818506	6.425E-15	-2.79637	down	-
c53377.graph_c0	0	0	0	18.1224201	20.7528782	21.2799284	1.368E-25	Inf	up	-
c53452.graph_c0	7.11333189	5.53813152	5.75538973	2.87218257	3.80352363	4.29674212	9.235E-09	-1.83367	down	PREDICTED: trihelix transcription factor GT-3b [Sesamum indicum]
c53470.graph_c0	0.97742074	0.24459987	0.31400609	16.6940183	15.2334858	15.6510183	5.608E-13	3.87566	up	-
c53487.graph_c0	1.15219476	1.25819838	0.53840578	6.31862788	6.32375694	6.21198645	0.0001515	1.59477	up	PREDICTED: rapid alkalinization factor-like [Sesamum indicum]
c53493.graph_c0	39.7799322	37.4424663	46.0323412	1.72607913	1.42861365	1.52583868	1.005E-73	-5.81157	down	-
c53497.graph_c0	0	0.8801094	1.12984406	12.1413526	11.25099	9.64652765	2.959E-08	2.92898	up	PREDICTED: UDP-arabinopyranose mutase 3-like [Sesamum indicum]
c53522.graph_c0	118.232222	138.137357	123.920424	6.71332015	8.00117823	10.5177877	5.42E-153	-5.00278	down	PREDICTED: uncharacterized protein LOC103335027 [Prunus mume]
c53537.graph_c0	23.7420201	23.0345389	29.570684	2.92017226	2.87633647	3.46594432	1.816E-31	-4.13591	down	--
c53566.graph_c0	4.81448288	4.81930385	3.68261971	1.7495692	2.1062626	2.71930321	1.046E-09	-2.10365	down	-

#ID	0 h-1 FPKM	0 h-2 FPKM	0 h-3 FPKM	28 h-1 FPKM	28 h-2 FPKM	28 h-3 FPKM	FDR	log2FC	regulated	nr_annotation
c53570.graph_c0	39.5187624	31.4438043	24.7405208	25.0396999	19.5053737	22.4353466	2.841E-07	-1.59965	down	-
c53579.graph_c0	0	0.37847426	0.16195596	5.03795855	5.62397612	6.12902744	5.102E-11	3.86165	up	-
c53583.graph_c0	17.4130014	13.7392863	9.47706191	1.63779246	0.26886783	1.57941169	2.755E-15	-4.62268	down	-
c53596.graph_c0	37.1588891	34.3981615	33.5944794	22.3463895	28.0531581	29.2531625	2.697E-22	-1.48956	down	PREDICTED: proliferating cell nuclear antigen-like [Nelumbo nucifera]
c53619.graph_c0	4.40334048	5.58314969	6.03569797	22.6156122	25.428402	24.0252205	0.0013085	1.07522	up	-
c53622.graph_c0	0.24538388	0	0	15.515892	15.7806986	16.8811216	1.25E-17	6.55865	up	-
c53646.graph_c0	0.15199084	0.60857213	0.39062848	7.51169234	5.58546791	5.76892198	2.32E-08	2.93892	up	-
c53652.graph_c0	1.65933192	0.95203286	1.01414612	0	0	0	7.69E-22	-Inf	down	hypothetical protein VHEMI09090 [Torrubiella hemipterigena]
c53655.graph_c0	1.15083192	0.92158744	0.29577291	12.2953447	15.9348684	13.5819542	1.578E-15	3.06481	up	-
c53686.graph_c0	0	0.26217413	0	6.0913931	6.53119876	4.65986909	1.177E-09	4.95953	up	PREDICTED: cytochrome b561 and DOMON domain-containing protein At3g25290-like [Sesamum indicum]
c53698.graph_c0	0.970612	0.64772261	1.52444703	8.15169569	10.2618409	9.59382276	2.176E-08	2.05717	up	hypothetical protein POPTR_0007s07730g [Populus trichocarpa]
c53715.graph_c0	0	1.38558188	0	8.65181964	10.7184915	6.89563245	2.997E-06	3.16122	up	PREDICTED: purple acid phosphatase 2 [Sesamum indicum]
c53744.graph_c0	0	0.19286941	0	6.7217351	9.35652388	9.48427331	4.985E-14	5.96913	up	-
c53761.graph_c0	4.78310974	5.54388339	6.14648538	0.54889525	0.49560095	2.68736425	4.786E-13	-3.22726	down	-
c53764.graph_c0	24.9395847	19.5035609	16.5249324	16.7098569	16.1103109	15.0217278	5.473E-07	-1.43595	down	-
c53797.graph_c0	7.16445354	9.05889809	7.75293175	0	0.7423434	0	1.454E-24	-6.11417	down	-
c53803.graph_c0	5.85961153	5.44651625	4.3027616	4.10663432	3.57057591	4.21975268	6.685E-05	-1.47796	down	-
c53824.graph_c0	1.93995142	0.52960745	1.1331429	20.1237677	15.2765832	18.8264294	6.593E-13	2.82739	up	-
c53867.graph_c0	8.42534016	5.95325426	6.36876217	64.4767337	56.5902875	68.1904874	9.848E-11	2.10243	up	PREDICTED: 40S ribosomal protein S19-1 [Sesamum indicum]
c53882.graph_c0	67.0435898	82.1837206	55.7464905	32.8320253	38.3492953	32.3188969	2.476E-12	-2.07409	down	-
c53904.graph_c0	0.28357327	0	0	5.5646843	6.51309579	5.5497896	3.82E-09	4.89853	up	PREDICTED: alpha-galactosidase-like [Sesamum indicum]
c53924.graph_c0	0	0.25308776	0	6.43155522	5.47525756	5.847879	2.258E-10	5.05081	up	unnamed protein product [Vitis vinifera]
c53936.graph_c0	9.60426716	11.1219447	11.8578689	2.05303706	4.07813995	4.24399574	1.206E-17	-2.74284	down	-
c53939.graph_c0	0.24936784	0	0	10.3306292	7.20024458	6.3592542	1.427E-11	5.52201	up	-
c53960.graph_c0	3.04283179	3.65505447	4.30117525	32.288084	38.138661	54.8591173	2.444E-06	2.41726	up	-
c53979.graph_c0	0	0.258687	0	6.19819672	8.30979196	8.27620021	1.082E-12	5.37941	up	-
c53980.graph_c0	0.99722046	0.95068479	1.34249005	0.0690261	0	0.05632474	7.949E-23	-5.8051	down	hypothetical protein VITISV_003451 [Vitis vinifera]
c53984.graph_c0	2.03709422	1.03247294	1.25916931	0	0	0	1.128E-17	-Inf	down	Animal heme peroxidase homologue [Chondrus crispus]
c54024.graph_c0	4.07715466	5.37981283	3.81040376	0.40407989	0	0	2.17E-26	-6.13046	down	PREDICTED: 17.3 kDa class I heat shock protein-like [Sesamum indicum]

#ID	0 h-1 FPKM	0 h-2 FPKM	0 h-3 FPKM	28 h-1 FPKM	28 h-2 FPKM	28 h-3 FPKM	FDR	log2FC	regulated	nr_annotation
c54082.graph_c0	1.37939147	0.69038636	0.29542878	5.17975783	4.073373	4.09029686	0.004443	1.42111	up	-
c54121.graph_c0	12.6478425	9.64610089	12.7701973	0.43773234	0.19761563	0.53577902	1.429E-32	-5.99224	down	-
c54141.graph_c0	13.2980161	12.9655831	15.0911226	2.5103692	5.32657584	3.48234931	1.099E-25	-2.96286	down	-
c54179.graph_c0	0.35852187	1.4355235	0.4607148	7.03543399	7.0581525	6.37872964	0.0001165	2.09402	up	-
c54197.graph_c0	1.99875535	0.82639955	1.3400764	0	0	0	4.57E-12	-Inf	down	Subunit of cleavage factor I [Komagataella pastoris GS115]
c54205.graph_c0	6.46064145	7.52887526	6.07174361	1.82215644	1.51867977	1.65842609	3.896E-25	-3.09379	down	-
c54229.graph_c0	42.4606142	39.4887255	34.8278108	28.8903342	32.2113475	31.610962	1.194E-13	-1.421	down	-
c54344.graph_c0	0	0.65673867	0.28103032	6.51676417	8.32374557	10.1164599	8.413E-10	3.63905	up	-
c54436.graph_c0	3.74390787	5.30918051	5.0783515	0.90701689	1.16018088	0.37005919	4.137E-20	-3.63315	down	PREDICTED: uncharacterized protein LOC104248461 [Nicotiana sylvestris]
c54544.graph_c0	0	0	0	10.5055761	7.90462516	10.5369873	3.284E-14	Inf	up	-
c54570.graph_c0	1.20042506	1.10919425	1.00861898	0	0	0.02738161	9.468E-29	-7.99519	down	-
c54603.graph_c0	1.87925616	1.20930297	1.29370638	0	0	0	1.398E-25	-Inf	down	hypothetical protein EMIHUDRAFT_349549 [Emiliania huxleyi CCMP1516]
c54893.graph_c0	14.1229337	15.7078618	13.6113967	3.42148893	1.28720155	0.69797676	2.662E-23	-4.09939	down	-
c55206.graph_c0	1.36300467	0.73675954	0.87575748	0	0	0	7.402E-22	-Inf	down	hypothetical protein GUITHDRAFT_143910 [Guillardia theta CCMP2712]
c55419.graph_c0	1.08967852	0.65094319	0.49687356	0	0	0	4.649E-14	-Inf	down	PREDICTED: peroxisomal acyl-coenzyme A oxidase 1-like [Cicer arietinum]
c55533.graph_c0	65.4266014	66.595916	48.6505878	12.2886356	9.64824849	10.8993764	6.8E-36	-3.54659	down	Ubiquitin [Medicago truncatula]
c55599.graph_c0	0.80689701	0.63211695	0.90164748	0.07649323	0.04604414	0.02080595	4.424E-22	-5.1257	down	Disease resistance family protein / LRR family protein, putative [Theobroma cacao]
c56027.graph_c0	5.6848328	10.74877	6.89938882	0	0	0	6.266E-15	-Inf	down	-
c57000.graph_c0	1.01810985	0.84927444	0.65415573	13.4425407	13.3622245	15.296225	9.734E-12	2.9768	up	-
c57052.graph_c0	0.31077248	0	0.39935494	5.42082347	12.4401757	6.26640922	0.0007813	3.98466	up	-
c57433.graph_c0	1.21464826	0.63556556	0.92233293	0	0	0	7.587E-19	-Inf	down	ATP-binding cassette transporter, subfamily F, member 4, SmABCF4 [Selaginella moellendorffii]
c60866.graph_c0	7.69544718	11.5547295	8.72554792	0.82250098	0.59411301	0	4.02E-25	-5.40125	down	hypothetical protein MIMGU_mgv1a015592mg [Erythranthe guttata]
c62784.graph_c0	0.57135043	0.50329185	0.41115644	0	0	0	4.076E-29	-Inf	down	predicted protein [Hordeum vulgare subsp. vulgare]

Transcriptomic analysis of seed germination improvement of *Andrographis paniculata* responding to air plasma treatment

Jia-Yun Tong 1*, Rui He 2*, Xiao-Ting Tang 2, Ming-Zhi Li 3 and Jing-Lin Wan 4
