# Supplementary material for: Transcriptomic analysis of seed germination improvement of Andrographis paniculata responding to air plasma treatment
Source: PLoS One. 2020 Oct 22;15(10):e0240939. doi: 10.1371/journal.pone.0240939 (PMC7580921; doi:10.1371/journal.pone.0240939)
Supplement: S5 Table — (PDF) [file pone.0240939.s007.pdf]

S5 Table. Differentially expressed genes of plasma-treated groups (48 HAS vs. 28 HAS).

#ID	28 h-1 FPKM	28 h-2 FPKM	28 h-3 FPKM	48 h-1 FPKM	48 h-2 FPKM	48 h-3 FPKM	FDR	log2FC	regulated	nr_annotation
c13810.graph_c0	23.024867	23.342368	24.556381	7.4244362	9.8994166	9.2259261	4.154E-06	-1.380393	down	PREDICTED: SNF2 domain-containing protein CLASSY 3-like [Sesamum indicum]
c14030.graph_c0	1.0971573	0.9456015	0.8545772	23.705585	26.184689	32.230237	3.606E-63	4.863565	up	RecName: Full=Galactinol synthase 1; Short=ArGolS1; Short=GolS-1 [Ajuga reptans]
c14063.graph_c0	6.4282357	5.5539176	5.4262632	10.185328	11.75751	12.750483	0.0005064	1.033639	up	PREDICTED: zinc finger protein ZAT5 [Sesamum indicum]
c14105.graph_c0	79.580786	90.90919	68.371208	27.228952	36.605454	28.337026	6.872E-12	-1.334881	down	PREDICTED: protein EXORDIUM-like 2 [Sesamum indicum]
c14260.graph_c0	22.709144	25.785619	23.092904	1.8536125	1.8481673	2.6195293	7.048E-25	-3.46221	down	-
c14261.graph_c0	24.235438	29.233423	21.475413	7.9200523	6.3563145	7.4743189	3.46E-09	-1.739177	down	PREDICTED: probable mitochondrial chaperone BCS1-B [Sesamum indicum]
c14379.graph_c0	0.6776029	0.6118119	0.7372246	7.4439008	28.510599	29.041667	4.067E-05	5.025464	up	-
c14708.graph_c0	0.1447751	0.2287571	0.2658045	1.1928354	1.6038818	1.0205555	2.277E-08	2.61726	up	PREDICTED: hyoscyamine 6-dioxygenase [Sesamum indicum]
c14733.graph_c0	11.047343	11.6449	10.733594	23.121763	31.118624	32.859815	7.317E-13	1.418014	up	PREDICTED: fasciclin-like arabinogalactan protein 17 [Sesamum indicum]
c14818.graph_c0	3.2524346	2.3772829	3.5386135	8.2454009	7.6951212	9.956961	0.0003156	1.5386	up	enolase [Solanum lycopersicum]
c14888.graph_c0	3.4511127	1.9175576	3.4659443	13.460122	20.676082	16.168593	8.84E-09	2.544561	up	-
c15072.graph_c0	1.4645765	1.7113091	1.7926236	8.026453	13.419785	12.534745	2.117E-13	2.807118	up	hypothetical protein MIMGU_mgv1a012722mg [Erythranthe guttata]
c15079.graph_c0	31.693071	29.963558	29.515158	7.7571683	9.6876258	8.9785778	7.098E-18	-1.74824	down	PREDICTED: plasma membrane ATPase 1-like [Sesamum indicum]
c15094.graph_c0	17.458396	12.913434	14.326394	33.757327	40.530034	38.603193	6.571E-09	1.375534	up	-
c15108.graph_c0	44.686228	41.638593	40.741992	6.7971702	8.9609677	10.483341	2.857E-16	-2.239886	down	PREDICTED: elongation of fatty acids protein 3-like [Sesamum indicum]
c15155.graph_c1	12.761072	15.289926	18.457043	9.2760543	5.8473846	7.0259427	0.0003507	-1.020665	down	-
c15159.graph_c0	1.3133048	2.9644777	2.8051909	14.150039	18.621108	16.293744	1.511E-27	2.830255	up	PREDICTED: probable xyloglucan endotransglucosylase/hydrolase protein 23 [Sesamum indicum]
c15187.graph_c0	16.924761	14.262708	12.515736	6.7935708	7.5757521	5.5676512	1.924E-06	-1.089637	down	PREDICTED: ATP sulfurylase 1, chloroplastic-like [Sesamum indicum]
c15187.graph_c1	14.82529	15.33442	12.939534	5.4711314	6.3000004	4.0215399	4.879E-05	-1.405356	down	hypothetical protein MIMGU_mgv1a005900mg [Erythranthe guttata]
c15194.graph_c0	57.696219	87.283787	70.30768	4.7222419	5.2591884	5.1714737	3.89E-23	-3.788054	down	-
c15238.graph_c0	17.348785	16.853503	17.269408	4.7781195	5.5497262	5.1735631	1.822E-11	-1.691601	down	PREDICTED: uncharacterized protein LOC105180187 [Sesamum indicum]
c15329.graph_c0	79.212808	79.097038	69.139391	153.39057	181.37739	207.84065	8.589E-16	1.292407	up	PREDICTED: dof zinc finger protein DOF3.1-like isoform X1 [Sesamum indicum]
c15339.graph_c0	25.510448	29.231323	30.848277	73.633424	67.687472	92.673544	1.048E-11	1.491726	up	hypothetical protein ZEAMMB73_313798 [Zea mays]
c15361.graph_c0	7.6979917	7.0457779	6.1954504	20.651722	21.169969	19.565149	3.429E-11	1.594262	up	hypothetical protein MIMGU_mgv1a014572mg [Erythranthe guttata]
c15371.graph_c0	12.221407	3.7576486	19.323346	1.1144988	0.9445411	0.9586186	0.0072939	-3.505097	down	PREDICTED: protein notum homolog isoform X1 [Sesamum indicum]
c15378.graph_c0	18.062837	21.293969	21.067057	2.5889443	3.9425918	3.4194465	1.112E-32	-2.566786	down	PREDICTED: uncharacterized protein LOC105156846 [Sesamum indicum]

#ID	28 h-1 FPKM	28 h-2 FPKM	28 h-3 FPKM	48 h-1 FPKM	48 h-2 FPKM	48 h-3 FPKM	FDR	log2FC	regulated	nr_annotation
c15396.graph_c0	7.8045974	9.7967981	9.3197382	1.9303224	4.6011206	4.0792744	0.0034524	-1.314727	down	hypothetical protein M569_04751, partial [Genlisea aurea]
c15426.graph_c0	3.3753825	2.6666972	3.2863623	4.9592237	7.9320511	8.9551598	0.0002638	1.260204	up	hypothetical protein MIMGU_mgv1a021331mg [Erythranthe guttata]
c15435.graph_c0	27.789808	26.47278	30.928472	14.327243	9.3578506	13.323406	5.782E-06	-1.155792	down	PREDICTED: uncharacterized protein LOC105156981 [Sesamum indicum]
c15435.graph_c1	15.133615	13.262346	14.164922	6.3943238	6.5747751	6.6936833	0.0072664	-1.072713	down	SEY1 [Theobroma cacao]
c15497.graph_c0	3.3262365	3.4553864	3.2978769	1.1334005	1.2967564	1.6539284	0.0018881	-1.265767	down	-
c15510.graph_c0	4.5897872	4.4801594	4.6562304	68.085075	111.93981	112.20904	1.254E-22	4.44525	up	-
c15515.graph_c0	0	0	0.1085284	7.9237105	15.359619	13.5009	1.354E-19	8.431133	up	-
c15532.graph_c0	1.2955535	2.6514636	2.0438447	6.5688379	16.817311	14.417451	0.0001273	2.685767	up	PREDICTED: laccase-14-like [Sesamum indicum]
c15566.graph_c0	0.2441808	0.2204723	0.4649155	2.1666198	4.262646	4.773712	1.62E-07	3.61964	up	PREDICTED: polygalacturonase At1g48100 [Sesamum indicum]
c15600.graph_c0	0.1735383	0.5092386	0.2478102	1.539808	1.3982056	1.1743816	1.456E-06	2.190674	up	PREDICTED: transcription factor RAX2-like [Sesamum indicum]
c15849.graph_c0	111.62877	125.85772	105.94461	48.45382	45.357087	52.684464	7.88E-15	-1.186782	down	-
c15959.graph_c0	2.0028488	1.9656355	1.207967	5.6294207	5.2620782	7.1697805	8.011E-06	1.844235	up	V-type proton ATPase catalytic subunit A [Gossypium arboreum]
c15999.graph_c0	2.3629175	1.3207337	0.918153	4.7066988	3.6263103	3.9341746	0.0007084	1.461777	up	hypothetical protein EUTSA_v10020227mg [Eutrema salsugineum]
c16017.graph_c0	2.898794	4.0332768	3.1021537	37.045532	21.50512	12.166791	0.0063725	2.876076	up	PREDICTED: uncharacterized protein LOC105174057 [Sesamum indicum]
c16031.graph_c0	11.351758	12.567316	11.264482	6.5799813	6.0514801	4.1176811	0.0006857	-1.023639	down	PREDICTED: uncharacterized protein LOC105156318 [Sesamum indicum]
c16076.graph_c0	12.843375	13.264905	13.948321	4.5677486	3.4741364	1.8758562	5.858E-08	-1.961232	down	PREDICTED: dof zinc finger protein DOF3.1-like [Solanum lycopersicum]
c16083.graph_c0	39.6964	43.788259	41.71226	70.848849	85.993539	102.42823	2.145E-08	1.08736	up	PREDICTED: phosphoglycerate kinase, chloroplastic-like [Fragaria vesca subsp. vesca]
c16084.graph_c0	5.2124317	4.377988	5.6380976	15.672753	13.347817	12.168151	0.0001031	1.481292	up	acetylajmalan acetylesterase [Striga asiatica]
c16100.graph_c0	39.131245	41.063873	40.592849	14.379226	10.201949	10.91645	4.498E-12	-1.718084	down	hypothetical protein M569_09334 [Genlisea aurea]
c16107.graph_c0	26.319475	23.216144	21.785936	41.918664	48.583699	64.931014	1.082E-05	1.16087	up	Fructose-bisphosphate aldolase, chloroplast precursor, putative, expressed [Oryza sativa Japonica Group]
c16197.graph_c0	5.2668286	6.3009744	5.7481592	0.6676093	0.5304383	0.5940347	2.72E-12	-3.226495	down	-
c16349.graph_c0	0.1066916	0.0688089	0.1243707	0.4636782	0.8186848	0.9454915	2.048E-07	2.923506	up	PREDICTED: uncharacterized protein At4g38062-like [Sesamum indicum]
c17172.graph_c0	1.2227068	1.9319819	2.2448673	9.2992259	8.2095019	10.342994	1.222E-07	2.408982	up	PREDICTED: uncharacterized protein LOC105167003 isoform X1 [Sesamum indicum]
c17811.graph_c0	2.2140403	2.2163611	2.0030126	33.18943	24.431152	17.045903	9.701E-08	3.588605	up	PREDICTED: uncharacterized protein LOC105158387 [Sesamum indicum]
c17816.graph_c0	6.5142907	3.9996198	6.8039783	23.781149	24.143525	21.454216	6.412E-07	2.044287	up	PREDICTED: L-ascorbate oxidase homolog [Cucumis melo]
c17845.graph_c0	4.1497568	3.0655974	4.0018345	85.022706	89.969929	106.79879	6.82E-113	4.69	up	hypothetical protein MIMGU_mgv1a009966mg [Erythranthe guttata]
c18297.graph_c0	2.6547021	3.3557259	6.4986456	14.469631	12.476946	13.773245	8.672E-05	1.745654	up	TPA: putative methionine synthase family protein [Zea mays]
c18381.graph_c0	8.893457	8.3645394	6.3498648	14.717674	15.757459	15.603384	0.0048915	1.006167	up	--

#ID	28 h-1 FPKM	28 h-2 FPKM	28 h-3 FPKM	48 h-1 FPKM	48 h-2 FPKM	48 h-3 FPKM	FDR	log2FC	regulated	nr_annotation
c18653.graph_c1	2.3389538	2.5206023	2.7705008	11.189715	10.739654	20.253477	3.304E-05	2.503633	up	unnamed protein product [Coffea canephora]
c18661.graph_c0	1.8435688	1.5314042	1.2034696	2.804231	5.0502627	3.9091277	0.0010735	1.395107	up	-
c18700.graph_c0	2.1265601	2.1600949	1.7352557	10.19263	7.7637474	5.546539	0.0001083	2.015599	up	PREDICTED: NADPH--cytochrome P450 reductase-like [Sesamum indicum]
c18725.graph_c0	2.3330832	2.0363368	1.7451288	0.5421817	0.7310237	0.6578591	0.0003024	-1.625268	down	PREDICTED: basic 7S globulin-like [Nelumbo nucifera]
c18726.graph_c0	0.8288991	1.6137766	1.0568357	2.5282243	3.2696704	4.4991975	0.0001044	1.59251	up	PREDICTED: sulfite reductase 1 [ferredoxin], chloroplastic-like [Sesamum indicum]
c18736.graph_c0	12.887071	13.1301	12.796001	3.7829142	3.1331668	3.8862165	3.842E-15	-1.801265	down	PREDICTED: cation/calcium exchanger 3-like [Sesamum indicum]
c18743.graph_c0	0.3735395	0.786966	0.4064069	2.1306996	1.7556139	2.1767554	6.904E-06	1.99729	up	hypothetical protein MIMGU_mgv1a000037mg [Erythranthe guttata]
c18755.graph_c0	6.6967985	6.1660788	6.4580843	12.6827	13.613505	15.972142	3.952E-09	1.16873	up	PREDICTED: uncharacterized protein LOC105174054 [Sesamum indicum]
c18762.graph_c0	0.7205654	0.4592492	0.4150415	1.9879223	2.2767164	1.8166479	6.551E-06	1.972441	up	PREDICTED: folate-biopterin transporter 1, chloroplastic-like isoform X1 [Sesamum indicum]
c18797.graph_c0	2.6017156	2.7406228	3.1137021	17.258833	12.693738	11.933314	2.963E-09	2.357234	up	-
c18812.graph_c0	2.6080442	1.6428972	2.0786511	0.0768811	0.5701236	1.2313504	0.0035535	-1.735707	down	-
c18813.graph_c0	0.3545422	0.3658495	0.2066454	1.7976332	3.2646418	5.0270729	7.764E-05	3.473879	up	PREDICTED: glyoxylate/succinic semialdehyde reductase 1 [Sesamum indicum]
c18829.graph_c0	1.0512441	2.0881847	2.9737295	5.3301173	8.4699194	10.829179	8.966E-05	2.041633	up	PREDICTED: uncharacterized protein LOC105159002 [Sesamum indicum]
c18840.graph_c0	8.0496162	8.7702594	7.7663094	14.173225	16.756137	18.268376	7.604E-09	1.039171	up	hypothetical protein MIMGU_mgv1a005412mg [Erythranthe guttata]
c18896.graph_c0	9.3899638	10.100552	8.9625291	59.2747	46.975131	39.367269	5.581E-10	2.404213	up	PREDICTED: L-type lectin-domain containing receptor kinase VIII.1-like [Sesamum indicum]
c18926.graph_c0	1.2811335	0.9075987	1.1579755	1.6489147	2.77904	2.7120865	0.0018093	1.125815	up	PREDICTED: BTB/POZ domain-containing protein At5g66560 [Sesamum indicum]
c18956.graph_c0	1.4440204	2.6459775	1.698151	5.1681944	3.7070822	4.8381402	0.0006883	1.2915	up	unnamed protein product [Coffea canephora]
c18979.graph_c0	0.4786971	0.201702	0.1041634	3.3170849	2.485547	3.1314931	9.303E-16	3.555924	up	PREDICTED: uncharacterized protein LOC105171076 [Sesamum indicum]
c18981.graph_c0	11.796301	12.974799	11.404979	26.642959	29.857041	31.084864	1.404E-14	1.315458	up	PREDICTED: carboxymethylenebutenolidase homolog [Sesamum indicum]
c19000.graph_c0	1.6096494	1.7359607	1.714796	0.3400582	0.3602504	0.1512909	1.01E-07	-2.523908	down	-
c19001.graph_c0	1.1135032	1.279586	0.5782061	3.9777152	3.398312	2.3976222	7.75E-05	1.767303	up	V-type proton ATPase subunit B 1 [Aegilops tauschii]
c19019.graph_c0	1.0263527	0.8340303	1.3818675	3.9029331	7.3735204	5.3828499	5.937E-07	2.393265	up	hypothetical protein CICLE_v10023425mg [Citrus clementina]
c19029.graph_c0	36.632746	53.289006	53.639572	13.41447	13.573331	13.236845	7.753E-10	-1.793447	down	-
c19035.graph_c0	4.991373	4.2828043	5.4136943	1.8076951	2.0399273	3.0071512	0.0023602	-1.063359	down	PREDICTED: putative pectinesterase 11 [Sesamum indicum]
c19039.graph_c0	2.7477878	2.8548432	3.5014747	16.365439	11.473889	9.5097919	4.466E-05	2.087355	up	PREDICTED: probable WRKY transcription factor 65 [Sesamum indicum]
c19070.graph_c0	41.747079	40.157339	40.188119	12.624109	12.824108	11.078981	9.259E-10	-1.697873	down	-
c19087.graph_c0	0.4802827	0.6504754	0.326589	1.8263811	2.0423198	2.3925126	8.2E-07	2.142847	up	unnamed protein product [Coffea canephora]

#ID	28 h-1 FPKM	28 h-2 FPKM	28 h-3 FPKM	48 h-1 FPKM	48 h-2 FPKM	48 h-3 FPKM	FDR	log2FC	regulated	nr_annotation
c19088.graph_c0	53.50469	60.471269	61.427241	5.1625043	5.0758478	5.3441478	8.22E-103	-3.450694	down	PREDICTED: LOW QUALITY PROTEIN: uncharacterized protein LOC105159096 [Sesamum indicum]
c19094.graph_c0	0.409021	0.2769807	0.2336305	30.719006	44.571636	45.60211	3.792E-67	7.073862	up	PREDICTED: endoglucanase 5-like [Sesamum indicum]
c19099.graph_c0	0.8606613	0.3586599	1.1074614	13.511016	22.759009	21.841249	4.645E-23	4.673913	up	RecName: Full=Caffeic acid 3-O-methyltransferase 2; Short=CAOMT-2; Short=COMT-2; AltName: Full=S-adenosysl-L-methionine:caffeic acid 3-O-methyltransferase 2 [Ocimum basilicum]
c19100.graph_c0	15.439164	15.069748	15.856395	36.6774	42.358308	44.254348	1.952E-22	1.449862	up	PREDICTED: protein notum homolog [Sesamum indicum]
c19173.graph_c0	9.7141543	10.023966	8.7654717	17.818606	22.155048	26.144624	1.93E-09	1.250809	up	PREDICTED: protein ECERIFERUM 26-like [Sesamum indicum]
c19181.graph_c0	1.675823	2.5755081	2.502157	0.5875527	0.8618412	0.9249554	0.0006772	-1.473511	down	PREDICTED: myb-related protein 308-like [Sesamum indicum]
c19184.graph_c0	0.4179555	0.3564094	0.606308	1.9719937	1.4654798	1.5713446	7.659E-06	1.90576	up	PREDICTED: F-box protein At5g51370-like [Sesamum indicum]
c19213.graph_c0	0.0367591	0.03319	0.0299951	1.4910349	3.1097797	2.9851062	3.058E-13	6.276099	up	PREDICTED: acyl-CoA--sterol O-acyltransferase 1-like [Sesamum indicum]
c19215.graph_c0	0.1421042	0.0320767	0.0579779	2.0714661	3.5779355	3.4459415	1.05E-19	5.324211	up	hypothetical protein MIMGU_mgv1a019872mg [Erythranthe guttata]
c19216.graph_c0	0.9610552	1.6716808	2.0989235	4.7115994	5.0103468	6.9181791	1.352E-08	1.851825	up	PREDICTED: DNA replication licensing factor MCM4 [Sesamum indicum]
c19232.graph_c0	3.2139634	2.8505464	3.0867392	0.7210505	1.2221849	0.9623796	3.834E-05	-1.621033	down	-
c19235.graph_c0	5.7792185	4.9385518	5.642112	17.136637	17.599862	16.295654	7.631E-07	1.683307	up	hypothetical protein MIMGU_mgv1a003115mg [Erythranthe guttata]
c19248.graph_c0	1.2081862	1.8971806	0.8572783	1.997563	3.1037245	3.4955918	0.0060167	1.151946	up	hypothetical protein MIMGU_mgv1a014302mg [Erythranthe guttata]
c19281.graph_c0	363.86343	377.71238	385.79744	208.58597	162.49735	128.96906	6.589E-13	-1.123529	down	PREDICTED: putative DNA-binding protein ESCAROLA [Sesamum indicum]
c19282.graph_c0	12.331916	11.579946	12.322989	40.882676	62.264837	66.33482	5.402E-12	2.259603	up	PREDICTED: cytosolic sulfotransferase 12-like [Sesamum indicum]
c19300.graph_c0	3.9974662	5.3786201	4.0294056	0.8941911	1.0525411	1.7681019	6.093E-05	-1.81637	down	BnaC04g47180D [Brassica napus]
c19358.graph_c0	1.325731	1.6546914	1.7499475	4.7942585	4.1888128	5.4533185	8.151E-06	1.651914	up	hypothetical protein MIMGU_mgv1a000332mg [Erythranthe guttata]
c19359.graph_c0	35.634886	40.828916	30.080458	79.43662	71.282936	78.443412	7.227E-06	1.148866	up	V-type proton ATPase 16 kDa proteolipid subunit [Morus notabilis]
c19372.graph_c0	1.2552546	1.7667351	1.1749066	0.6551702	0.6940734	0.4164047	0.0069075	-1.203533	down	hypothetical protein MIMGU_mgv1a021776mg [Erythranthe guttata]
c19374.graph_c0	7.8682716	8.2793249	7.9234504	3.5529413	3.1455538	4.0646389	2.079E-06	-1.118957	down	PREDICTED: pentatricopeptide repeat-containing protein At1g77360, mitochondrial-like [Sesamum indicum]
c19386.graph_c0	1.468294	1.5856792	1.1511309	0.2919484	0.4252653	0.2273022	3.662E-06	-2.11477	down	PREDICTED: probable (S)-N-methylcoclaurine 3'-hydroxylase isozyme 2 [Sesamum indicum]
c19389.graph_c0	3.1566752	2.7806651	2.355934	5.1236392	5.5312626	6.1654823	0.0016961	1.060205	up	PREDICTED: probable receptor-like serine/threonine-protein kinase At4g34500 [Sesamum indicum]
c19401.graph_c0	1.9515801	1.8993999	1.7992882	3.0841827	5.5816676	7.6897215	0.0061701	1.56318	up	PREDICTED: glucuronoxylan 4-O-methyltransferase 1-like [Sesamum indicum]
c19406.graph_c0	1.0913711	1.7361912	1.3428927	6.3899594	6.1645622	7.561458	1.67E-19	2.311501	up	hypothetical protein MIMGU_mgv1a001005mg [Erythranthe guttata]
c19414.graph_c0	1.6681147	1.5947483	1.2410649	2.5497879	4.4141415	5.3122793	0.0009615	1.478221	up	-

#ID	28 h-1 FPKM	28 h-2 FPKM	28 h-3 FPKM	48 h-1 FPKM	48 h-2 FPKM	48 h-3 FPKM	FDR	log2FC	regulated	nr_annotation
c19428.graph_c0	0.5013553	0.5718024	0.3229752	1.3713525	2.1614557	3.8987557	0.0037835	2.442859	up	PREDICTED: uncharacterized protein LOC105159979 [Sesamum indicum]
c19433.graph_c0	175.36319	160.88837	135.48992	355.73398	399.13138	358.65299	5.253E-16	1.280119	up	PREDICTED: GDSL esterase/lipase At1g71250 [Sesamum indicum]
c19453.graph_c0	10.614269	10.017123	11.620748	19.809698	24.13745	32.305315	1.798E-05	1.277126	up	PREDICTED: protein ECERIFERUM 1-like [Sesamum indicum]
c19468.graph_c0	5.5488619	5.4056364	4.795921	13.65072	17.157454	18.940037	3.334E-14	1.696483	up	PREDICTED: probable sugar phosphate/phosphate translocator At1g12500 [Sesamum indicum]
c19480.graph_c0	0.0369515	0.0667274	0.1809125	10.960254	16.275332	15.462088	1.991E-57	7.262247	up	PREDICTED: peroxidase 44 [Sesamum indicum]
c19533.graph_c0	76.424183	81.26622	76.148522	36.237113	31.831135	25.047059	3.196E-19	-1.281491	down	PREDICTED: shaggy-related protein kinase alpha [Sesamum indicum]
c19541.graph_c0	7.0463153	6.9434764	8.1722133	13.737626	14.409256	15.329999	7.898E-06	1.012551	up	PREDICTED: origin recognition complex subunit 1-like isoform X1 [Sesamum indicum]
c19577.graph_c0	239.32842	250.90539	287.38666	26.94067	21.924693	19.103841	4.251E-86	-3.468778	down	PREDICTED: reticulon-like protein B13 [Sesamum indicum]
c19592.graph_c0	4.6605795	6.3962608	5.3241927	12.406018	15.020197	24.18023	0.0006429	1.690342	up	PREDICTED: protein WALLS ARE THIN 1-like [Nicotiana sylvestris]
c19595.graph_c0	0.7605263	0.5930452	0.6769999	3.3302617	2.8781121	4.9127513	3.872E-08	2.492972	up	PREDICTED: protein IQ-DOMAIN 1 [Sesamum indicum]
c19602.graph_c0	7.2487448	5.9839423	5.0699285	10.238411	16.964812	17.052195	0.0012798	1.307138	up	-
c19602.graph_c1	75.689533	70.941249	71.751039	189.04439	199.83985	220.09955	2.65E-26	1.519767	up	PREDICTED: NAC domain-containing protein 100-like isoform X2 [Nicotiana tomentosiformis]
c19657.graph_c0	5.7536108	4.8304117	6.7952492	20.024108	27.583826	35.406964	1.002E-08	2.289717	up	PREDICTED: auxin-induced protein 15A [Sesamum indicum]
c19688.graph_c0	1.5181074	2.3416273	1.2645708	2.7661998	3.6949194	3.6741861	0.0063517	1.02174	up	PREDICTED: transcription factor bHLH66-like isoform X2 [Sesamum indicum]
c19697.graph_c0	7.178556	6.851938	8.3680615	104.7725	147.34903	187.83986	7.089E-18	4.32955	up	PREDICTED: non-specific lipid-transfer protein 2-like [Sesamum indicum]
c19697.graph_c1	0.279481	2.0187614	1.5963797	21.255768	17.639028	26.163335	4.55E-17	4.10461	up	-
c19702.graph_c0	0.9463373	1.1582597	0.7035632	3.4387164	6.7210149	6.4041261	2.222E-07	2.591994	up	PREDICTED: putative UPF0481 protein At3g02645 [Sesamum indicum]
c19713.graph_c0	0.0851162	0.1793211	0.1620596	1.3306558	2.6669404	2.976024	1.371E-08	4.061003	up	PREDICTED: WAT1-related protein At3g18200 [Sesamum indicum]
c19722.graph_c0	72.316934	76.50246	72.927913	169.52796	159.36304	169.77073	3.844E-16	1.212031	up	hypothetical protein MIMGU_mgv1a026562mg [Erythranthe guttata]
c19728.graph_c0	2.9071727	2.0155516	1.9062556	5.5275832	6.134652	7.904588	1.355E-05	1.556751	up	PREDICTED: uncharacterized protein LOC105160245 [Sesamum indicum]
c19729.graph_c0	7.7297561	10.392173	8.3174741	1.6688961	1.4258009	1.8681927	2.604E-12	-2.370333	down	hypothetical protein MIMGU_mgv1a012036mg [Erythranthe guttata]
c19739.graph_c0	7.8749836	8.4555773	8.5415822	18.836043	18.187699	17.262131	1.232E-12	1.169311	up	PREDICTED: 4-coumarate--CoA ligase-like 5 [Sesamum indicum]
c19740.graph_c0	3.4634524	3.7074729	3.7293511	0.5883748	1.4384117	1.7316839	0.0001953	-1.50954	down	-
c19746.graph_c0	213.46391	193.08538	247.66903	129.19278	79.069372	72.127185	8.927E-06	-1.16942	down	PREDICTED: 17.3 kDa class I heat shock protein-like [Sesamum indicum]
c19751.graph_c0	1.4421873	2.0462512	2.5637712	4.3090423	5.1643402	8.2686177	0.0013371	1.585885	up	PREDICTED: DNA replication licensing factor MCM7 [Sesamum indicum]
c19821.graph_c0	2.5909867	3.1855906	1.3045212	0.279512	0.4441637	0.2487081	0.0002065	-2.82573	down	PREDICTED: uncharacterized protein LOC105163636 [Sesamum indicum]
c19836.graph_c0	26.9439	20.130075	21.813563	1.0045111	0.8513262	0.5362848	2.662E-46	-4.798635	down	PREDICTED: gibberellin 2-beta-dioxygenase [Sesamum indicum]

#ID	28 h-1 FPKM	28 h-2 FPKM	28 h-3 FPKM	48 h-1 FPKM	48 h-2 FPKM	48 h-3 FPKM	FDR	log2FC	regulated	nr_annotation
c19853.graph_c0	12.597554	12.44795	11.388302	2.4042202	5.7021935	6.7690039	7.757E-07	-1.265137	down	PREDICTED: probable LRR receptor-like serine/threonine-protein kinase At4g36180 [Sesamum indicum]
c19875.graph_c0	17.083538	15.321311	16.434887	39.094624	69.02669	81.383238	3.623E-05	1.987045	up	PREDICTED: vestitone reductase-like [Sesamum indicum]
c19877.graph_c0	3.0211067	2.3105868	2.9292358	6.442529	7.6364512	10.663296	2.458E-06	1.618345	up	PREDICTED: putative phospholipid-transporting ATPase 4 isoform X1 [Sesamum indicum]
c19884.graph_c0	25.930676	26.431047	26.449025	8.7950365	10.269405	9.7108103	2.423E-11	-1.413963	down	PREDICTED: uncharacterized protein LOC105166661 [Sesamum indicum]
c19931.graph_c0	31.853033	35.319669	28.271798	15.178972	16.401886	14.271507	3.835E-09	-1.015533	down	PREDICTED: chaperone protein dnaJ 72 [Sesamum indicum]
c19932.graph_c0	8.7538767	6.9693436	7.0224315	0.9746648	2.9784783	4.3028984	0.0001093	-1.438579	down	PREDICTED: uncharacterized protein LOC105155205 [Sesamum indicum]
c19933.graph_c0	1.5952281	2.382103	1.4518884	0.6221751	0.6591191	0.5536077	0.0008318	-1.521772	down	-
c19942.graph_c0	0.0956864	0.3023856	0.1951984	2.1225682	1.9273745	2.536185	8.042E-13	3.514976	up	PREDICTED: cell division cycle-associated 7-like protein isoform X1 [Sesamum indicum]
c19944.graph_c0	8.0590887	8.4004009	9.2929386	1.0254931	1.1281696	0.9124776	3.754E-21	-3.028418	down	PREDICTED: F-box/kelch-repeat protein At1g23390-like [Sesamum indicum]
c19957.graph_c0	3.8731015	3.384757	3.4938579	11.417829	13.431832	16.191102	2.682E-19	1.969937	up	PREDICTED: probable beta-D-xylosidase 7 [Sesamum indicum]
c19973.graph_c0	1.5405475	1.3909699	1.1852414	3.235998	4.5511613	5.014078	1.624E-05	1.67207	up	unnamed protein product [Coffea canephora]
c19987.graph_c0	33.011363	30.519475	34.557641	14.269991	15.723533	14.829489	2.755E-12	-1.089312	down	PREDICTED: uncharacterized protein LOC105163283 [Sesamum indicum]
c19990.graph_c0	4.9102607	3.5668048	4.8201295	0.7019681	0.9419568	0.2914833	1.764E-11	-2.737363	down	PREDICTED: benzoate carboxyl methyltransferase-like [Sesamum indicum]
c20002.graph_c0	1.0844724	0.7663124	0.615597	1.79302	4.3688206	4.945797	0.0016387	2.198414	up	hypothetical protein MIMGU_mgv1a021791mg [Erythranthe guttata]
c20009.graph_c0	0.0775211	0.2799772	0.316283	2.8496477	4.9967276	4.8088954	1.891E-14	4.263357	up	-
c20017.graph_c0	1.0968194	1.4029605	1.3424935	7.41493	5.0322498	4.5359601	1.58E-05	2.195312	up	hypothetical protein MIMGU_mgv1a022798mg, partial [Erythranthe guttata]
c20019.graph_c0	2.9980402	2.3846933	1.8639058	6.1527643	3.834181	4.9111213	0.0081062	1.089623	up	PREDICTED: uncharacterized protein LOC105167731 [Sesamum indicum]
c20054.graph_c0	0.0785333	0.2363607	0.0854434	3.152313	4.3589177	4.0154489	7.036E-27	4.886117	up	PREDICTED: uncharacterized protein LOC105178927 [Sesamum indicum]
c20067.graph_c0	5.7590722	5.9148888	4.8755818	1.0950072	1.5467032	1.9486617	2.787E-05	-1.815772	down	PREDICTED: G-type lectin S-receptor-like serine/threonine-protein kinase SD1-1 [Nelumbo nucifera]
c20076.graph_c0	9.4695723	11.411673	10.500123	3.9688596	4.8710967	6.244668	0.0002775	-1.020479	down	hypothetical protein MIMGU_mgv1a023657mg [Erythranthe guttata]
c20081.graph_c0	0.6920564	0.803394	1.2100979	6.7672214	8.6294126	10.035721	2.193E-13	3.268235	up	PREDICTED: aquaporin TIP1-3-like [Sesamum indicum]
c20082.graph_c0	0.0452377	0.1225362	0.0738272	4.3006574	3.64482	3.5715858	2.06E-23	5.621604	up	PREDICTED: ras-related protein Rab7-like [Sesamum indicum]
c20085.graph_c0	16.59126	19.510589	18.913478	1.5216978	1.4880501	1.1804081	1.885E-50	-3.670203	down	PREDICTED: UDP-glycosyltransferase 86A1-like [Sesamum indicum]
c20086.graph_c0	8.6650383	6.6993506	8.8911743	3.5515749	3.0657106	3.686865	0.0005689	-1.192152	down	-
c20092.graph_c0	2.398767	1.7417769	1.820495	0.5315759	0.8334474	1.3622198	0.0056589	-1.09764	down	PREDICTED: pentatricopeptide repeat-containing protein At4g20770 [Sesamum indicum]
c20108.graph_c0	0.5758538	0.4963083	0.5980444	2.1234536	2.5307343	3.1293787	1.737E-08	2.257145	up	PREDICTED: F-box protein At1g10780 [Sesamum indicum]

#ID	28 h-1 FPKM	28 h-2 FPKM	28 h-3 FPKM	48 h-1 FPKM	48 h-2 FPKM	48 h-3 FPKM	FDR	log2FC	regulated	nr_annotation
c20125.graph_c0	2.3028186	1.5288451	1.768547	19.402713	20.100069	26.660538	3.068E-32	3.601253	up	PREDICTED: glycine cleavage system H protein 3, mitochondrial-like [Sesamum indicum]
c20131.graph_c0	14.434312	12.954906	12.817023	33.885546	53.947846	59.231567	1.106E-07	1.904087	up	PREDICTED: protein ASPARTIC PROTEASE IN GUARD CELL 2 [Sesamum indicum]
c20135.graph_c0	4.014816	5.2384429	3.6926655	37.712105	40.917466	35.047964	1.237E-85	3.176767	up	PREDICTED: membrane protein of ER body-like protein [Sesamum indicum]
c20167.graph_c0	4.2041971	4.507745	4.7365539	0.9083743	1.5397	1.6165321	4.209E-07	-1.693945	down	PREDICTED: pentatricopeptide repeat-containing protein At3g22690 [Sesamum indicum]
c20172.graph_c0	19.885652	20.096287	21.032815	5.2857726	6.9295485	6.9372918	3.543E-19	-1.634664	down	PREDICTED: ethylene-responsive transcription factor ERF008-like [Nelumbo nucifera]
c20195.graph_c0	30.079384	30.616859	28.512389	72.764709	81.014775	71.443421	9.819E-21	1.377289	up	monodehydroascorbate reductase [Acanthus ebracteatus]
c20200.graph_c0	2.1798841	2.0875177	2.2638861	3.7679504	5.4109529	5.5133283	0.003246	1.204794	up	PREDICTED: BAG family molecular chaperone regulator 1-like [Sesamum indicum]
c20213.graph_c0	3.3995111	3.157138	2.932486	5.2940748	6.7822878	8.2162254	0.0009311	1.132685	up	PREDICTED: cyclin-U2-1-like [Sesamum indicum]
c20225.graph_c0	7.2792925	7.8870224	6.557587	19.118426	16.343682	15.041021	2.813E-06	1.263458	up	-
c20226.graph_c0	0.3013372	0.4988119	0.3073608	2.0689881	2.5627692	2.6906532	3.606E-11	2.76351	up	PREDICTED: probable LRR receptor-like serine/threonine-protein kinase At1g05700 [Sesamum indicum]
c20233.graph_c0	6.6019501	4.8570638	6.08547	1.2397715	2.9551222	2.0683899	0.0011698	-1.456835	down	-
c20244.graph_c0	0.2527847	0.2282409	0.1500148	1.8642809	1.7281071	3.4472476	1.713E-06	3.517303	up	PREDICTED: G2/mitotic-specific cyclin S13-7-like [Sesamum indicum]
c20256.graph_c0	0.4157465	0.6256335	0.6332587	1.5809679	1.8609375	2.5321243	7.268E-06	1.871224	up	PREDICTED: uncharacterized protein LOC101258386 [Solanum lycopersicum]
c20309.graph_c0	19.76643	20.908746	21.631424	6.8377324	9.1581676	10.082344	3.287E-09	-1.220671	down	PREDICTED: probable 3-hydroxyisobutyrate dehydrogenase-like 3, mitochondrial [Sesamum indicum]
c20315.graph_c0	28.352038	27.602355	28.903448	3.1457925	4.2919665	4.6227681	3.188E-42	-2.779049	down	BnaA09g38920D [Brassica napus]
c20322.graph_c0	0.6134338	0.484639	0.6882658	3.304683	4.9424625	6.4863685	1.605E-08	3.076267	up	PREDICTED: inorganic phosphate transporter 1-4-like [Sesamum indicum]
c20327.graph_c0	0.4392035	0.264373	0.3285209	2.6908265	2.0642308	2.146597	5.047E-10	2.788312	up	PREDICTED: putative HVA22-like protein g [Sesamum indicum]
c20329.graph_c0	0.3860197	0.6970792	0.0524982	1.5494767	3.0237839	3.4830697	1.349E-05	2.859268	up	hypothetical protein M569_01834 [Genlisea aurea]
c20335.graph_c0	6.334582	9.2099157	7.5016293	42.659554	43.011514	49.719399	8.972E-59	2.59557	up	PREDICTED: perakine reductase-like [Sesamum indicum]
c20375.graph_c0	5.0368605	6.0400639	4.5435175	15.238225	15.773162	16.643471	5.966E-21	1.650662	up	PREDICTED: exopolygalacturonase [Sesamum indicum]
c20382.graph_c0	25.304696	29.840697	27.404711	54.982472	77.517268	83.838146	4.723E-09	1.425268	up	PREDICTED: fasciclin-like arabinogalactan protein 1 [Sesamum indicum]
c20387.graph_c0	32.752339	29.539101	27.805465	128.1824	90.084888	69.362259	0.0009451	1.726606	up	PREDICTED: uncharacterized protein LOC105158345 [Sesamum indicum]
c20394.graph_c0	16.89697	10.953298	12.020124	22.241929	32.580422	26.143316	0.0031828	1.05853	up	-
c20408.graph_c0	2.1871289	2.1178718	2.4830321	6.7902411	9.6196259	13.263614	4.981E-06	2.161113	up	PREDICTED: aspartic proteinase nepenthesin-1 [Sesamum indicum]
c20419.graph_c0	0.69508	0.7531105	1.361231	5.4626035	6.907024	9.5643903	2.259E-10	2.998778	up	PREDICTED: thymidine kinase [Sesamum indicum]

#ID	28 h-1 FPKM	28 h-2 FPKM	28 h-3 FPKM	48 h-1 FPKM	48 h-2 FPKM	48 h-3 FPKM	FDR	log2FC	regulated	nr_annotation
c20472.graph_c0	68.663915	66.211898	76.049111	53.047585	25.656958	14.325723	0.0053995	-1.118583	down	PREDICTED: uncharacterized protein LOC105175062 [Sesamum indicum]
c20493.graph_c0	0.8230203	1.4862203	0.9737879	5.0597084	4.807555	3.6666572	1.312E-07	2.08964	up	Uncharacterized protein F383_18135 [Gossypium arboreum]
c20508.graph_c0	3.0095776	3.4028281	3.8274936	6.358102	9.9760262	10.397386	3.131E-06	1.417794	up	PREDICTED: cellulose synthase-like protein D3 isoform X1 [Sesamum indicum]
c20517.graph_c0	4.5777038	2.1417684	3.7353689	13.662431	19.335507	16.803537	9.88E-20	2.288011	up	PREDICTED: glyoxylate/hydroxypyruvate reductase HPR3 [Sesamum indicum]
c20528.graph_c0	0.7138039	0.5728871	0.5177406	8.9809686	7.4551937	6.2915884	1.386E-20	3.702466	up	PREDICTED: MLO-like protein 1 isoform X3 [Sesamum indicum]
c20552.graph_c0	20.326213	18.965237	19.420484	9.7349627	6.7052802	4.3463597	5.04E-12	-1.444352	down	hypothetical protein MIMGU_mgv1a010327mg [Erythranthe guttata]
c20602.graph_c0	5.2369056	5.3354635	5.139476	18.344655	18.863748	21.072187	4.322E-20	1.931856	up	PREDICTED: anthocyanidin 3-O-glucosyltransferase 2-like [Sesamum indicum]
c20611.graph_c0	9.67864	9.7263513	9.6824802	0.9703811	2.7902886	4.5638931	1.002E-10	-1.781406	down	-
c20613.graph_c0	0.5762467	0.693729	0.9404252	4.8695674	7.4801385	9.9656972	5.625E-08	3.367348	up	hypothetical protein M569_01187, partial [Genlisea aurea]
c20617.graph_c0	0.0299129	0.0540171	0.0610218	1.4029231	1.365722	0.9952762	5.004E-20	4.743554	up	PREDICTED: putative disease resistance protein At1g50180 [Sesamum indicum]
c20619.graph_c0	0.7128964	0.905918	0.7325333	4.7859911	6.098395	8.6659924	3.13E-10	3.090726	up	PREDICTED: G2/mitotic-specific cyclin-1-like [Sesamum indicum]
c20625.graph_c0	1.7606635	2.5914517	1.8106193	26.750676	20.0155	16.893039	6.383E-12	3.418999	up	PREDICTED: probable anion transporter 3, chloroplastic [Sesamum indicum]
c20683.graph_c0	4.3442502	4.0721625	4.4107958	13.073056	10.77664	11.183491	2.536E-09	1.49511	up	PREDICTED: GDSL esterase/lipase At4g10955-like [Sesamum indicum]
c20685.graph_c0	25.360112	23.36011	26.641224	11.415198	9.6663259	8.764385	4.611E-14	-1.290115	down	PREDICTED: F-box/kelch-repeat protein At5g43190 [Sesamum indicum]
c20712.graph_c0	3.3977331	3.3771959	3.6345672	13.825437	16.678462	17.003522	5.221E-25	2.228524	up	PREDICTED: 8-hydroxygeraniol dehydrogenase [Sesamum indicum]
c20717.graph_c0	5.6226536	3.3399533	4.3465636	9.5653554	19.273204	18.023709	0.0001034	1.846182	up	PREDICTED: zinc finger protein WIP2 [Sesamum indicum]
c20718.graph_c0	1.8342944	2.2931943	2.7056983	0.089427	0	0.1591433	3.238E-14	-4.734295	down	-
c20731.graph_c0	2.0756973	2.3989248	2.540628	6.8934798	7.7487788	9.4113675	1.796E-08	1.815803	up	hypothetical protein MIMGU_mgv1a005057mg [Erythranthe guttata]
c20761.graph_c0	3.3295407	3.5011966	3.975919	5.3270163	8.0424237	10.029453	0.0015345	1.147318	up	PREDICTED: inorganic phosphate transporter 2-1, chloroplastic-like [Sesamum indicum]
c20765.graph_c0	0.9142382	0.6390746	0.6738163	1.3083952	1.7029057	2.3949302	0.0013439	1.313881	up	PREDICTED: uncharacterized protein LOC105172642 [Sesamum indicum]
c20793.graph_c0	188.82402	200.57692	201.60241	39.412819	64.862046	78.504108	5.893E-29	-1.661167	down	PREDICTED: aquaporin AQPcic-like [Sesamum indicum]
c20835.graph_c0	0.524342	0.6886279	0.466755	1.7824493	2.0163085	1.8817099	1.242E-05	1.798745	up	unnamed protein product [Coffea canephora]
c20837.graph_c0	1.7948231	2.3122582	1.857492	0.5271505	0.7348053	1.2837304	0.0043602	-1.196323	down	PREDICTED: pentatricopeptide repeat-containing protein At1g71060, mitochondrial [Sesamum indicum]
c20843.graph_c0	1.8014931	2.6131931	2.1206611	5.5777912	5.9883087	5.4294152	8.965E-06	1.420723	up	PREDICTED: aldo-keto reductase [Sesamum indicum]
c20850.graph_c0	7.9598649	10.697431	8.9543443	4.1408657	4.6956708	4.5407785	1.336E-05	-1.005218	down	PREDICTED: uncharacterized protein LOC105161748 [Sesamum indicum]

#ID	28 h-1 FPKM	28 h-2 FPKM	28 h-3 FPKM	48 h-1 FPKM	48 h-2 FPKM	48 h-3 FPKM	FDR	log2FC	regulated	nr_annotation
c20853.graph_c0	1.3533704	1.3252314	1.1198933	2.6336499	2.9692091	2.5153989	0.001108	1.137164	up	PREDICTED: B3 domain-containing protein Os01g0234100-like [Sesamum indicum]
c20865.graph_c0	0.8749144	0.6869267	1.034671	3.584219	4.5802925	4.9768939	1.444E-18	2.375663	up	PREDICTED: 125 kDa kinesin-related protein [Sesamum indicum]
c20872.graph_c0	18.087464	21.165345	22.197872	36.866927	84.038491	92.862979	0.0024671	1.826359	up	hypothetical protein MIMGU_mgv1a013356mg [Erythranthe guttata]
c20878.graph_c0	0.2556392	0.3173751	0.3520117	2.2075331	1.7593241	1.9462291	7.284E-12	2.721939	up	PREDICTED: protein CHUP1, chloroplastic-like [Sesamum indicum]
c20900.graph_c0	1.261541	1.0315954	1.3595943	86.954621	100.49223	95.922651	1.67E-259	6.31685	up	PREDICTED: raucaffricine-O-beta-D-glucosidase-like [Sesamum indicum]
c20941.graph_c0	0.9336581	1.0838645	0.5986022	3.7195098	2.7313926	3.0839431	1.485E-06	1.914036	up	PREDICTED: protein tesmin/TSO1-like CXC 2 isoform X3 [Sesamum indicum]
c20944.graph_c0	822.87573	952.81593	928.70749	101.06855	145.27583	196.14017	3.746E-57	-2.57837	down	PREDICTED: 18 kDa seed maturation protein-like [Nicotiana sylvestris]
c21008.graph_c0	1.0594945	0.7652993	0.7163322	1.2278741	2.235722	1.8778289	0.0085229	1.104559	up	hypothetical protein MIMGU_mgv1a020944mg, partial [Erythranthe guttata]
c21037.graph_c0	0.3988677	1.0804203	1.3018911	24.1674	37.493099	35.720046	1.58E-35	5.163841	up	PREDICTED: defensin J1-2-like [Sesamum indicum]
c21058.graph_c1	4.4448169	4.5149096	4.1558622	0.3521333	0.248695	0.3133261	1.779E-12	-3.79555	down	-
c21062.graph_c0	7.3763836	5.564758	5.9002713	3.4447772	2.1504943	1.2588976	0.0001021	-1.402273	down	-
c21068.graph_c0	146.82512	140.36375	142.03978	59.94759	67.315731	60.058613	3.179E-14	-1.155428	down	PREDICTED: LOW QUALITY PROTEIN: protein SENSITIVE TO PROTON RHIZOTOXICITY 1 [Sesamum indicum]
c21075.graph_c0	0.512621	0.1322425	0.5577261	1.4233384	2.7862528	2.3126989	1.583E-06	2.469118	up	PREDICTED: uncharacterized protein LOC105162896 [Sesamum indicum]
c21084.graph_c0	0.356812	0.2478213	0.3135522	3.4791188	4.3859884	5.0459893	1.332E-21	3.849998	up	PREDICTED: 65-kDa microtubule-associated protein 3 [Sesamum indicum]
c21092.graph_c0	1.1425767	0.8144523	0.8341929	1.8294317	3.3108542	1.8312944	0.0073224	1.355621	up	PREDICTED: uncharacterized protein LOC105166030 [Sesamum indicum]
c21110.graph_c0	2.9327612	3.741751	4.0058567	0.5657051	0.7705234	0.6471785	1.816E-07	-2.391442	down	PREDICTED: uncharacterized protein At1g08160-like [Sesamum indicum]
c21119.graph_c0	20.026317	18.859602	16.648809	39.919695	43.519019	40.984967	3.702E-12	1.204967	up	Signal peptidase complex catalytic subunit SEC11C [Gossypium arboreum]
c21157.graph_c0	6.870051	6.4169089	7.216798	2.6024792	2.3328556	3.1172485	0.0015358	-1.306955	down	PREDICTED: transcription factor PRE6-like [Solanum lycopersicum]
c21161.graph_c0	0.868767	1.8085128	1.0239765	0.1223585	0.129624	0.0816554	2.203E-05	-3.426085	down	PREDICTED: probable 9-cis-epoxycarotenoid dioxygenase NCED5, chloroplastic [Sesamum indicum]
c21182.graph_c0	5.0881154	5.1391529	4.0111196	0.8198595	1.9107917	2.0912506	0.0001199	-1.534019	down	-
c21195.graph_c0	0.8293658	0.7488395	0.7422482	1.7634411	3.161488	3.3192485	1.061E-05	1.860548	up	PREDICTED: glucan endo-1,3-beta-glucosidase 13 [Sesamum indicum]
c21202.graph_c0	0.7981159	1.325018	1.050413	14.130755	8.5739396	8.9146711	4.673E-08	3.369274	up	hypothetical protein MIMGU_mgv1a027064mg [Erythranthe guttata]
c21212.graph_c0	3.4965387	3.9139993	3.8208811	9.3307796	14.607581	15.24426	8.056E-09	1.836413	up	PREDICTED: protein LUTEIN DEFICIENT 5, chloroplastic [Sesamum indicum]
c21236.graph_c0	27.284699	29.386105	28.856515	4.2639491	6.7177936	5.7072683	1.57E-39	-2.322489	down	PREDICTED: probable purine permease 9 [Sesamum indicum]
c21242.graph_c0	9.2025205	10.783388	10.090463	18.633776	20.308127	24.727236	4.718E-12	1.120843	up	PREDICTED: probable inactive receptor kinase At4g23740 [Sesamum indicum]

#ID	28 h-1 FPKM	28 h-2 FPKM	28 h-3 FPKM	48 h-1 FPKM	48 h-2 FPKM	48 h-3 FPKM	FDR	log2FC	regulated	nr_annotation
c21243.graph_c0	2.2649124	1.4157717	1.9903155	0.2944557	1.1697755	0.4585086	0.0021556	-1.536279	down	PREDICTED: uncharacterized protein DDB_G0284459-like isoform X1 [Sesamum indicum]
c21247.graph_c0	5.8990784	5.891941	5.4951714	12.936765	14.791514	16.810263	8.923E-13	1.404047	up	PREDICTED: uncharacterized protein LOC105161548 [Sesamum indicum]
c21248.graph_c0	2.2314319	2.5184673	2.5795095	6.2168168	7.366291	8.0222559	1.8E-09	1.597758	up	PREDICTED: phosphatidylinositol/phosphatidylcholine transfer protein SFH13-like isoform X1 [Sesamum indicum]
c21257.graph_c0	63.516393	63.821257	66.129972	262.28014	244.97221	269.12461	3.146E-43	2.04726	up	PREDICTED: tubulin beta chain-like [Sesamum indicum]
c21269.graph_c0	4.3666751	4.7312379	6.6987619	9.9631482	12.665697	19.306325	0.002068	1.442082	up	hypothetical protein MIMGU_mgv1a022779mg, partial [Erythranthe guttata]
c21292.graph_c0	31.814834	35.774809	32.789107	173.17927	130.80477	112.48421	1.943E-07	2.102189	up	PREDICTED: cytochrome P450 71D95-like [Sesamum indicum]
c21328.graph_c0	23.048331	27.33926	25.998257	244.03499	214.5276	168.97229	2.583E-17	3.085192	up	F-box/WD repeat-containing protein 4 [Theobroma cacao]
c21337.graph_c0	4.5938492	4.5384165	4.0006876	13.682843	14.467651	14.637776	1.852E-20	1.745188	up	PREDICTED: xylem cysteine proteinase 2-like [Sesamum indicum]
c21343.graph_c0	2.7425774	2.6001047	2.6015828	0.9777428	1.2889955	1.5079821	0.0030836	-1.037803	down	PREDICTED: putative pentatricopeptide repeat-containing protein At5g09950 [Sesamum indicum]
c21351.graph_c0	2.7194043	2.5657207	2.368608	10.108751	7.6727286	5.8586204	0.0002012	1.67736	up	PREDICTED: uncharacterized protein LOC105175340 [Sesamum indicum]
c21408.graph_c0	94.82226	100.11249	109.24951	12.166897	12.733118	13.647362	8.517E-85	-2.939747	down	PREDICTED: zinc finger CCCH domain-containing protein 20-like [Sesamum indicum]
c21437.graph_c0	125.46178	119.18766	129.8477	68.680507	48.873694	37.37888	1.565E-10	-1.221836	down	PREDICTED: 7-deoxyloganetic acid glucosyltransferase-like [Sesamum indicum]
c21505.graph_c0	2.2860927	1.3210415	1.7161983	5.8342189	7.2857963	7.3571398	1.168E-10	1.98114	up	PREDICTED: uncharacterized protein LOC105160466 [Sesamum indicum]
c21528.graph_c0	1389.3835	1553.6872	1408.2496	638.56097	570.21918	588.41526	3.475E-10	-1.231414	down	PREDICTED: thiamine thiazole synthase 2, chloroplastic-like [Populus euphratica]
c21536.graph_c0	4.8397998	5.4963448	4.7215324	18.349876	14.760132	13.901516	3.13E-10	1.689899	up	PREDICTED: protein TRANSPORT INHIBITOR RESPONSE 1-like [Sesamum indicum]
c21546.graph_c0	3.8575881	6.0283391	2.7845572	7.1465972	21.716681	18.742313	0.0090833	1.936437	up	-
c21554.graph_c0	6.8662223	6.1067935	5.993998	13.890781	15.98022	18.559213	1.107E-17	1.390659	up	PREDICTED: LRR receptor-like serine/threonine-protein kinase RCH1 [Sesamum indicum]
c21564.graph_c0	7.03375	5.6017461	6.033815	13.899498	11.576431	13.14067	5.049E-06	1.093119	up	PREDICTED: methylsterol monooxygenase 1-1-like [Sesamum indicum]
c21570.graph_c0	0.8812101	0.5569551	1.4740735	14.744383	15.56072	17.293838	6.26E-40	4.069323	up	PREDICTED: uncharacterized protein LOC105175024 [Sesamum indicum]
c21576.graph_c0	3.1548208	3.029365	3.2689626	7.4266544	9.6160043	10.16645	1.491E-06	1.561933	up	hypothetical protein MIMGU_mgv1a000209mg [Erythranthe guttata]
c21578.graph_c0	36.819134	34.827285	37.01612	9.3592344	11.572996	11.196561	5.132E-29	-1.719658	down	PREDICTED: LOW QUALITY PROTEIN: plasma membrane ATPase 1-like [Sesamum indicum]
c21582.graph_c0	9.4294029	11.222824	8.0440574	21.912848	24.940667	18.853361	0.0003157	1.23754	up	-
c21590.graph_c0	8.1105019	9.558813	9.0779294	27.930639	27.468732	29.871589	8.884E-22	1.71447	up	hypothetical protein MIMGU_mgv1a025384mg [Erythranthe guttata]
c21609.graph_c0	8.1827096	6.1012394	7.1078	1.6060161	2.6229597	1.0122249	8.824E-08	-1.990695	down	PREDICTED: pathogenesis-related genes transcriptional activator PTI6 [Sesamum indicum]

#ID	28 h-1 FPKM	28 h-2 FPKM	28 h-3 FPKM	48 h-1 FPKM	48 h-2 FPKM	48 h-3 FPKM	FDR	log2FC	regulated	nr_annotation
c21611.graph_c0	28.321322	35.119935	29.005668	9.2043758	16.43796	15.665159	1.213E-07	-1.129698	down	PREDICTED: subtilisin-like protease SDD1 [Sesamum indicum]
c21617.graph_c0	4.7530277	4.630344	4.9603101	1.2366721	1.2093269	0.9310936	1.216E-09	-2.041923	down	PREDICTED: probable galacturonosyltransferase-like 9 [Sesamum indicum]
c21618.graph_c0	0.4583266	0.6069447	0.523587	1.0457328	1.1898884	1.6197362	0.0023516	1.316137	up	PREDICTED: protein ODORANT1-like [Sesamum indicum]
c21633.graph_c0	3.4189764	3.4562753	3.3504979	1.4515135	1.4498338	1.8266178	0.0005998	-1.072767	down	PREDICTED: pentatricopeptide repeat-containing protein At5g39710 [Sesamum indicum]
c21648.graph_c0	0.6249787	0.5642972	0.7285393	2.3766196	2.2779555	2.316102	1.709E-05	1.904243	up	PREDICTED: probable WRKY transcription factor 71 [Nicotiana tomentosiformis]
c21653.graph_c0	0.2597932	0.2010591	0.4542624	3.6223772	3.5882831	3.3487475	2.689E-15	3.569947	up	PREDICTED: peroxidase 66-like [Sesamum indicum]
c21657.graph_c0	0.4533451	0.6549249	0.6658666	9.0794423	14.184344	15.850875	6.306E-20	4.495718	up	PREDICTED: uncharacterized protein LOC105157568 [Sesamum indicum]
c21669.graph_c0	3.3136747	3.6568126	3.5852129	8.4628805	7.185501	6.8934736	1.395E-05	1.14044	up	PREDICTED: BTB/POZ domain-containing protein At3g19850-like [Sesamum indicum]
c21672.graph_c0	3.103361	3.428817	2.7988769	6.1594065	8.4443044	9.2571527	7.566E-06	1.390436	up	PREDICTED: wall-associated receptor kinase-like 20 [Sesamum indicum]
c21695.graph_c0	0.6512895	0.49617	0.4151929	1.9349002	2.1044532	2.3185055	1.194E-07	2.064535	up	PREDICTED: uncharacterized protein LOC105168358 isoform X2 [Sesamum indicum]
c21716.graph_c0	4.0507537	3.1733764	3.3539905	1.7367126	0.6399431	0.7390644	9.54E-05	-1.70145	down	unnamed protein product [Vitis vinifera]
c21718.graph_c0	0.8423482	0.9778647	0.8051805	3.2947355	4.0559424	5.4153828	1.07E-10	2.317862	up	PREDICTED: uncharacterized protein LOC105157238 isoform X1 [Sesamum indicum]
c21720.graph_c0	0.5703676	1.0871978	0.6205537	1.847623	4.1699694	6.0756664	0.0049478	2.43585	up	PREDICTED: allene oxide synthase [Sesamum indicum]
c21723.graph_c0	0.8941311	0.9477194	0.6502989	5.592969	5.2986331	8.3747024	3.205E-12	2.990133	up	PREDICTED: scarecrow-like protein 28 [Sesamum indicum]
c21726.graph_c0	2.4494788	2.086462	2.4513029	0.9373267	0.868861	0.7037109	0.0002623	-1.431884	down	PREDICTED: uncharacterized protein LOC105173388 [Sesamum indicum]
c21735.graph_c0	13.05009	16.126379	13.369255	3.0789761	3.837414	3.0619667	7.12E-13	-2.052185	down	PREDICTED: RING-H2 finger protein ATL66 [Sesamum indicum]
c21746.graph_c0	2.7697768	2.3113905	2.2601152	0.6383447	1.2961436	0.80466	0.001494	-1.389725	down	unnamed protein product [Coffea canephora]
c21750.graph_c0	0.3853312	0.5025481	0.2096181	5.4813331	3.8520408	3.0422495	4.673E-08	3.548166	up	PREDICTED: uncharacterized protein LOC105162958 [Sesamum indicum]
c21769.graph_c0	23.895381	27.011582	23.950835	98.141634	100.81092	99.926009	1.933E-44	2.03922	up	p-coumaroyl shikimate 3'-hydroxylase isoform 2 [Ocimum basilicum]
c21813.graph_c0	10.961336	9.4022055	9.6897238	24.315026	33.118484	32.040943	5.031E-10	1.610533	up	PREDICTED: probable receptor-like protein kinase At1g80640 [Nicotiana tomentosiformis]
c21818.graph_c0	2.0882465	2.5925499	1.8743913	0.7279267	0.630941	0.8243516	0.0007341	-1.543162	down	-
c21827.graph_c0	0.7221872	0.8624117	0.7793953	3.0711049	5.2555945	5.9645356	5.203E-08	2.627747	up	PREDICTED: L-ascorbate oxidase-like [Sesamum indicum]
c21859.graph_c0	4.9082616	4.7934715	4.273665	0.8706589	1.9984412	2.7114746	5.792E-06	-1.297442	down	PREDICTED: oligopeptide transporter 4-like [Sesamum indicum]
c21871.graph_c0	0.229852	0.181593	0.3985594	3.0956399	6.0187647	5.8978316	4.258E-12	4.241463	up	hypothetical protein MIMGU_mgv1a006316mg [Erythranthe guttata]
c21881.graph_c0	2.5197226	3.8072654	2.0141124	6.8441441	6.5600131	6.1478771	0.000442	1.273613	up	PREDICTED: LOW QUALITY PROTEIN: alkaline/neutral invertase CINV2 [Sesamum indicum]

#ID	28 h-1 FPKM	28 h-2 FPKM	28 h-3 FPKM	48 h-1 FPKM	48 h-2 FPKM	48 h-3 FPKM	FDR	log2FC	regulated	nr_annotation
c21891.graph_c0	0.5246852	0.7698299	0.8027603	2.6603083	2.8182741	1.7013721	6.457E-05	1.820199	up	hypothetical protein JCGZ_14180 [Jatropha curcas]
c21901.graph_c0	3.3176277	2.7426393	2.724736	47.187163	59.998464	57.926307	6.61E-131	4.270153	up	vacuolar invertase CvINV [Elsholtzia haichowensis]
c21908.graph_c0	4.4679313	3.2370584	4.6307483	1.2331666	1.0644664	0.7924686	4.014E-10	-1.950015	down	PREDICTED: lysine-specific demethylase JMJ18 [Sesamum indicum]
c21909.graph_c0	0.2830911	0.4089676	0.231	1.2918198	1.5585996	0.7024438	0.0011501	1.989367	up	hypothetical protein MIMGU_mgv1a0261841mg, partial [Erythranthe guttata]
c21911.graph_c0	4.8720355	5.8295576	4.7512566	16.870106	20.637711	24.750146	7.523E-17	2.047555	up	PREDICTED: inositol monophosphatase 3-like [Sesamum indicum]
c21920.graph_c0	1.4203061	0.5209763	0.9778709	5.8511133	6.0197409	6.8082194	6.608E-13	2.717619	up	PREDICTED: WW domain-containing protein C11B10.08 [Sesamum indicum]
c21921.graph_c0	0.9379721	0.8872295	0.656038	2.2080477	2.8789647	3.5263983	1.115E-05	1.831574	up	hypothetical protein MIMGU_mgv1a000635mg [Erythranthe guttata]
c21940.graph_c0	1.4807877	1.8067736	1.4042529	7.1021905	6.1266121	6.7257391	8.451E-10	2.133178	up	PREDICTED: uncharacterized protein LOC105161910 [Sesamum indicum]
c21946.graph_c0	0.4839311	0.4733565	0.4387598	1.0905215	1.8231689	2.0468099	4.886E-06	1.86145	up	PREDICTED: receptor protein kinase CLAVATA1 [Sesamum indicum]
c21950.graph_c0	5.4640674	6.1804794	5.4222221	14.892401	17.631193	15.508608	3.34E-18	1.53293	up	PREDICTED: uncharacterized protein LOC105173886 [Sesamum indicum]
c21976.graph_c0	53.660856	85.024137	62.571301	202.26104	188.06162	132.77661	4.514E-05	1.425053	up	PREDICTED: epidermis-specific secreted glycoprotein EP1-like [Sesamum indicum]
c21983.graph_c0	0.8940829	0.4843638	0.4620574	3.0599531	5.3627284	7.5295191	1.043E-05	3.14574	up	PREDICTED: mitotic spindle checkpoint protein BUBR1 isoform X2 [Sesamum indicum]
c22011.graph_c0	192.68092	215.20662	215.42011	45.464077	41.863196	45.863164	7.401E-48	-2.183503	down	PREDICTED: salt stress-induced hydrophobic peptide ESI3 [Solanum lycopersicum]
c22045.graph_c0	0.9575412	0.6611417	0.666442	3.5520154	2.5527412	2.5252731	1.48E-07	1.966203	up	PREDICTED: receptor-like protein kinase HERK 1 [Sesamum indicum]
c22049.graph_c0	403.61613	389.22301	332.38593	926.8037	886.29552	741.86794	3.676E-11	1.227902	up	hypothetical protein MIMGU_mgv1a015271mg [Erythranthe guttata]
c22063.graph_c0	0.197907	0.1786915	0.3027948	5.8952514	8.5706831	6.3337337	6.661E-36	4.97253	up	PREDICTED: reticuline oxidase-like protein [Sesamum indicum]
c22065.graph_c0	1.5876037	2.0174584	1.7272956	0.2236005	0.5527145	0.4642363	9.106E-06	-2.075349	down	-
c22068.graph_c0	29.451223	29.864516	26.853523	58.007553	66.831818	79.77562	1.649E-13	1.285785	up	PREDICTED: glycerol-3-phosphate 2-O-acyltransferase 4 [Sesamum indicum]
c22084.graph_c0	2.3904567	2.539245	2.2055726	3.5846043	4.6996111	5.779975	8.038E-05	1.014654	up	PREDICTED: uncharacterized protein LOC105169955 isoform X1 [Sesamum indicum]
c22085.graph_c0	0.3397627	0.3579028	0.4043136	2.3687083	2.4903489	3.3690664	4.048E-17	2.938736	up	PREDICTED: WD repeat and HMG-box DNA-binding protein 1 [Sesamum indicum]
c22097.graph_c0	12.498843	12.016107	11.120546	4.0801021	5.1565157	5.9021357	7.92E-10	-1.198295	down	PREDICTED: MATE efflux family protein 5-like [Sesamum indicum]
c22099.graph_c0	0.1382222	0.2080029	0.0375961	5.2562062	4.5783908	4.3651446	3.48E-24	5.256874	up	PREDICTED: CBL-interacting serine/threonine-protein kinase 5 [Sesamum indicum]
c22111.graph_c0	3.840598	3.4326726	4.558395	13.86711	14.377957	14.132821	1.691E-13	1.881756	up	PREDICTED: (DL)-glycerol-3-phosphatase 2 [Sesamum indicum]
c22114.graph_c0	0.7633248	0.9981672	1.0739082	3.770188	2.7216141	3.3843782	8.581E-07	1.846923	up	PREDICTED: uncharacterized protein LOC105171186 [Sesamum indicum]
c22147.graph_c0	0.3941622	0.5338373	0.4288442	1.3531637	1.2350264	1.2225605	0.0002087	1.534246	up	PREDICTED: glutathione transferase GST 23-like [Sesamum indicum]

#ID	28 h-1 FPKM	28 h-2 FPKM	28 h-3 FPKM	48 h-1 FPKM	48 h-2 FPKM	48 h-3 FPKM	FDR	log2FC	regulated	nr_annotation
c22176.graph_c0	2.0906329	1.8286566	1.412731	7.2047722	9.9135842	10.610918	9.057E-17	2.414761	up	hypothetical protein MIMGU_mgv1a009135mg [Erythranthe guttata]
c22183.graph_c0	4.8965933	5.1618833	4.5813196	14.330867	20.448976	18.794756	1.548E-19	1.90801	up	PREDICTED: malonyl-coenzyme:anthocyanin 5-O-glucoside-6'''-O-malonyltransferase-like [Sesamum indicum]
c22206.graph_c0	5.2906341	5.2292019	5.6454571	1.8650569	2.648415	2.8070544	1.19E-05	-1.107794	down	hypothetical protein MIMGU_mgv1a010455mg [Erythranthe guttata]
c22209.graph_c0	0.2687127	0.0909834	0.1370421	0.8089563	0.7667815	1.1175911	1.437E-08	2.47818	up	PREDICTED: receptor-like protein kinase HSL1 [Sesamum indicum]
c22267.graph_c0	0.5783682	0.9399821	0.8023043	6.671431	7.9218942	8.6107505	1.427E-16	3.360195	up	PREDICTED: lipoxygenase homology domain-containing protein 1-like [Sesamum indicum]
c22287.graph_c0	0.4834525	1.418665	0.9862333	3.830073	8.2772957	4.6348472	0.0003893	2.567819	up	-
c22291.graph_c0	5.0334542	4.3333541	5.0942729	8.7048698	12.208345	13.598705	9.382E-07	1.289726	up	PREDICTED: ras-related protein Rab7 [Sesamum indicum]
c22340.graph_c0	1.0839533	1.4447598	1.7268752	0.490711	0.3465658	0.6112843	0.0006294	-1.511807	down	PREDICTED: cytochrome P450 71A1-like [Sesamum indicum]
c22346.graph_c0	18.952761	20.587034	19.837839	61.313675	41.194131	35.390766	0.0066018	1.267522	up	PREDICTED: uncharacterized protein LOC105163060 [Sesamum indicum]
c22362.graph_c0	192.76588	195.97409	211.78415	37.431011	31.639995	26.226937	4.012E-70	-2.608635	down	hypothetical protein MIMGU_mgv1a011867mg [Erythranthe guttata]
c22371.graph_c0	3.1248936	3.4663969	2.9141573	0.2263447	1.7983859	0.6042005	5.642E-05	-1.835732	down	-
c22378.graph_c0	2.398669	2.1657732	3.0152912	5.1770498	8.5313769	7.3850363	8.247E-05	1.510107	up	PREDICTED: transcription factor PCL1 [Sesamum indicum]
c22389.graph_c0	5.5899405	6.0707486	5.885093	18.011454	17.79489	19.774079	2.278E-24	1.704947	up	PREDICTED: uncharacterized protein LOC105168835 [Sesamum indicum]
c22433.graph_c0	0.109542	0.1978124	0.2234635	2.7076254	3.9716319	3.15053	2.253E-16	4.24723	up	PREDICTED: dirigent protein 18-like [Sesamum indicum]
c22443.graph_c0	2.0841194	1.2303844	1.3081726	3.0482018	4.1979602	2.8930897	0.0077042	1.171882	up	PREDICTED: uncharacterized protein LOC105175907 [Sesamum indicum]
c22488.graph_c0	1.1678977	1.3708527	1.2388934	2.812753	2.8229407	2.7003578	0.0071732	1.184489	up	PREDICTED: dehydration-responsive element-binding protein 3 [Sesamum indicum]
c22518.graph_c0	1.4769632	1.4817325	2.3166428	18.222354	18.004194	24.117609	6.004E-35	3.554789	up	PREDICTED: DNA (cytosine-5)-methyltransferase CMT3 [Sesamum indicum]
c22524.graph_c0	1.4444384	1.9266479	1.8751248	10.902368	11.549737	17.069073	5.452E-13	2.950839	up	PREDICTED: uncharacterized protein LOC105161197 [Sesamum indicum]
c22526.graph_c0	0.1838963	0.5396337	0.5252026	2.5058479	1.8520758	1.6074498	2.106E-06	2.305784	up	hypothetical protein MIMGU_mgv1a014603mg [Erythranthe guttata]
c22529.graph_c1	10.238927	12.528285	14.145677	1.3426131	3.6032507	4.1016286	3.944E-11	-2.002978	down	PREDICTED: CBS domain-containing protein CBSX5 [Sesamum indicum]
c22532.graph_c0	2.3166696	2.4016222	2.3804829	123.22631	132.09879	128.7107	8.39E-228	5.798538	up	unnamed protein product [Coffea canephora]
c22540.graph_c0	3.9263768	3.3425701	3.2954311	1.8011898	1.506428	1.4761598	0.0060206	-1.096987	down	PREDICTED: probable WRKY transcription factor 71 isoform X1 [Sesamum indicum]
c22552.graph_c0	1.8571866	1.8488515	2.0691709	0.8903406	0.7993287	0.711655	0.0002831	-1.220908	down	PREDICTED: pentatricopeptide repeat-containing protein At3g26540 [Sesamum indicum]
c22564.graph_c0	3.4080893	2.3279574	2.780973	55.145793	86.118179	82.995112	3.669E-34	4.752739	up	PREDICTED: polygalacturonase-like [Sesamum indicum]
c22577.graph_c0	4.9839689	3.9706382	3.2694509	1.6103553	2.3621208	1.1022188	0.0060997	-1.228213	down	PREDICTED: uncharacterized protein LOC105177681 [Sesamum indicum]
c22578.graph_c0	0.6602346	0.59613	0.8260773	2.9570326	4.4920553	5.4112327	2.611E-09	2.659203	up	PREDICTED: cysteine-rich repeat secretory protein 60 [Sesamum indicum]

#ID	28 h-1 FPKM	28 h-2 FPKM	28 h-3 FPKM	48 h-1 FPKM	48 h-2 FPKM	48 h-3 FPKM	FDR	log2FC	regulated	nr_annotation
c22593.graph_c0	1.1487536	0.7053074	0.7873938	5.9992548	5.3682235	8.4476741	6.434E-13	2.947103	up	hypothetical protein MIMGU_mgv1a007264mg [Erythranthe guttata]
c22603.graph_c0	1.3002399	1.2702238	0.9914124	4.3230161	5.0376825	6.6594154	1.408E-12	2.206447	up	PREDICTED: uncharacterized protein LOC105179085 [Sesamum indicum]
c22634.graph_c0	1.1659143	2.1631054	2.5152832	6.1747135	7.2276668	10.77228	3.399E-06	2.083762	up	PREDICTED: DNA replication licensing factor MCM3 homolog 2 [Sesamum indicum]
c22642.graph_c0	2.4371252	3.2273935	2.5189874	0.7208217	1.3090683	1.0078872	0.0021593	-1.39609	down	-
c22666.graph_c0	0.1864232	0.1683227	0.0760599	8.1525263	9.1372863	11.564458	2.813E-27	6.10466	up	hypothetical protein OsI_01464 [Oryza sativa Indica Group]
c22670.graph_c0	1.0754378	0.8091829	0.4753388	3.8623904	7.8224333	6.418602	3.545E-08	2.971342	up	PREDICTED: pleckstrin homology domain-containing family A member 8 [Sesamum indicum]
c22683.graph_c0	1.6851897	1.5714558	1.9837523	5.3577642	5.6759015	6.1382965	2.574E-08	1.752252	up	PREDICTED: glycerate dehydrogenase HPR, peroxisomal [Sesamum indicum]
c22704.graph_c0	5.6066352	5.7583275	4.9752783	1.7767058	1.7880941	3.003714	0.0030078	-1.277311	down	-
c22756.graph_c0	0.9829997	0.8875565	1.1586173	7.4761468	10.046757	8.900438	2.01E-26	3.161856	up	PREDICTED: uncharacterized protein LOC105158433 [Sesamum indicum]
c22766.graph_c0	16.030125	14.298262	16.925311	4.4641028	6.0011608	5.8075307	7.537E-15	-1.501399	down	PREDICTED: uncharacterized protein LOC104211375 [Nicotiana sylvestris]
c22770.graph_c0	1.3872102	2.2545374	1.2225084	6.8225576	7.0786483	7.3849396	7.828E-09	2.17171	up	PREDICTED: uncharacterized protein LOC105160016 [Sesamum indicum]
c22778.graph_c0	3.1343625	3.6651279	4.0670259	35.040869	45.677449	49.78233	8.709E-43	3.622369	up	PREDICTED: LOW QUALITY PROTEIN: 4-coumarate--CoA ligase 1-like [Sesamum indicum]
c22778.graph_c1	3.2164838	3.6600666	3.5594223	38.872364	43.42892	46.117861	7.381E-80	3.66075	up	4-coumarate coenzyme A ligase [Paulownia fortunei]
c22797.graph_c0	2.4658752	3.295152	4.1852387	12.252659	16.556386	19.468469	6.891E-11	2.313907	up	PREDICTED: uncharacterized protein LOC105174672 [Sesamum indicum]
c22799.graph_c0	0.1676132	0.3178119	0.2598649	2.0608822	3.0160428	2.8735218	1.194E-18	3.450956	up	PREDICTED: uncharacterized protein LOC105170951 [Sesamum indicum]
c22827.graph_c0	0.3230577	0.3977602	0.6949783	8.115535	26.186653	36.900761	2.269E-05	5.674214	up	unnamed protein product [Coffea canephora]
c22837.graph_c0	23.515631	23.591566	27.876717	3.5189757	2.8507685	2.725954	2.253E-48	-2.996775	down	PREDICTED: cytochrome P450 86B1-like [Sesamum indicum]
c22838.graph_c0	5.3367878	6.7744114	5.1744977	2.3875664	2.2764034	1.4871096	8.328E-06	-1.443688	down	PREDICTED: uncharacterized protein LOC105171100 [Sesamum indicum]
c22864.graph_c0	17.408692	16.35479	15.183047	69.059256	79.87964	86.647964	3.357E-44	2.305782	up	PREDICTED: NADPH--cytochrome P450 reductase-like [Sesamum indicum]
c22886.graph_c0	1.8002641	1.1307615	1.5648052	8.3837494	12.034785	14.036802	8.569E-15	2.971956	up	PREDICTED: alcohol dehydrogenase-like 4 [Sesamum indicum]
c22901.graph_c0	3.7689376	6.2520183	5.2210658	24.886952	42.842651	44.782813	1.659E-10	2.916445	up	PREDICTED: laccase-14-like [Sesamum indicum]
c22914.graph_c0	2.6458664	1.464207	2.2286507	12.117065	13.180398	19.926877	1.904E-10	2.871154	up	PREDICTED: hexose carrier protein HEX6-like [Sesamum indicum]
c22923.graph_c0	7.8504986	7.6692694	8.2962209	16.721086	19.874202	20.539341	9.382E-07	1.300865	up	PREDICTED: uncharacterized protein LOC102582737 [Solanum tuberosum]
c22943.graph_c0	39.179571	32.664461	35.854441	9.1899743	9.9323435	6.1122384	3.258E-19	-2.049337	down	PREDICTED: dof zinc finger protein DOF3.1-like [Sesamum indicum]
c22951.graph_c0	323.5922	379.26215	388.26932	22.31937	25.628834	28.284943	3.3E-104	-3.800654	down	PREDICTED: 1-Cys peroxiredoxin [Phoenix dactylifera]
c23062.graph_c0	2.8961325	3.7547801	2.9085791	0.0941298	1.3960672	1.0888294	5.107E-05	-1.874641	down	-
c23065.graph_c0	0	0.0457413	0.0620073	1.573277	3.2313498	2.7140771	4.141E-15	6.154607	up	PREDICTED: beta-glucosidase 12-like [Sesamum indicum]
c23084.graph_c1	26.587377	18.24449	20.827279	9.4364084	11.266157	8.9295679	0.0006109	-1.107466	down	PREDICTED: elongation of fatty acids protein 3-like [Populus euphratica]

#ID	28 h-1 FPKM	28 h-2 FPKM	28 h-3 FPKM	48 h-1 FPKM	48 h-2 FPKM	48 h-3 FPKM	FDR	log2FC	regulated	nr_annotation
c23103.graph_c0	8.4065243	6.6608783	6.4863406	10.728378	21.11817	23.929615	0.0037039	1.401519	up	PREDICTED: uncharacterized protein LOC105172657 [Sesamum indicum]
c23106.graph_c0	0.1097449	0.0990894	0.089551	8.0683669	4.5684681	2.5993598	7.744E-05	5.733374	up	-
c23112.graph_c0	200.81928	215.11933	210.58055	35.654924	27.70723	24.972322	1.5E-77	-2.778015	down	hypothetical protein MIMGU_mgv1a018508mg [Erythranthe guttata]
c23113.graph_c0	38.219574	43.394528	41.582481	97.404908	101.28784	96.021571	6.468E-18	1.300239	up	-
c23144.graph_c0	0.881961	1.1944921	1.0795094	7.1868246	10.489806	13.002746	2.11E-12	3.314879	up	PREDICTED: WAT1-related protein At2g39510-like [Sesamum indicum]
c23146.graph_c0	3.3341457	4.6063068	3.441768	10.183796	9.9793595	7.6569508	8.114E-06	1.334572	up	PREDICTED: uncharacterized protein LOC105156632 [Sesamum indicum]
c23151.graph_c0	6.8417661	5.4188362	8.5211543	18.94244	20.228401	27.211476	4.78E-10	1.712997	up	PREDICTED: copper transporter 6-like [Sesamum indicum]
c23152.graph_c0	41.697815	36.766187	39.103446	18.933153	12.058306	18.551322	1.218E-07	-1.199863	down	-
c23190.graph_c0	0.8358614	0.686095	1.3021071	23.887267	31.325962	44.823519	1.06E-16	5.17972	up	hypothetical protein MIMGU_mgv1a015689mg [Erythranthe guttata]
c23196.graph_c0	3.5120923	2.8539811	2.3643169	1.6694384	0.3537135	0.0990304	0.000901	-1.958705	down	BnaC01g07200D [Brassica napus]
c23198.graph_c0	0.2873839	0.0432468	0.1954191	13.964075	23.797828	29.118069	6.098E-20	7.020231	up	PREDICTED: peroxidase 27-like [Sesamum indicum]
c23198.graph_c1	0	0.231023	0.4175692	6.1622513	10.994792	14.4293	2.578E-09	5.634428	up	hypothetical protein MIMGU_mgv1a009974mg [Erythranthe guttata]
c23201.graph_c0	6.212079	4.7735531	5.7161129	23.287728	57.505394	70.81013	0.0001499	3.208369	up	PREDICTED: cytochrome P450 71D11-like [Beta vulgaris subsp. vulgaris]
c23217.graph_c0	0	0	0	2.69428	2.7400924	3.4521899	5.579E-22	Inf	up	hypothetical protein MIMGU_mgv1a016855mg [Erythranthe guttata]
c23230.graph_c0	7.5800477	9.3165383	8.3225709	26.460468	23.555119	23.812932	2.889E-16	1.594219	up	PREDICTED: tetraspanin-8-like [Sesamum indicum]
c23258.graph_c1	2.7719515	2.7530936	2.1864935	0.468487	1.240763	0.7294997	0.0003753	-1.631761	down	-
c23261.graph_c0	12.312239	16.599572	14.694134	50.058129	41.445909	40.195918	1.819E-12	1.641107	up	PREDICTED: uncharacterized protein LOC105161807 [Sesamum indicum]
c23279.graph_c0	0.8132503	1.1014332	0.6636056	5.7341293	7.0984262	9.2871393	1.281E-15	3.137589	up	unnamed protein product [Coffea canephora]
c23283.graph_c0	0.5742678	0.51851	1.0649951	43.013852	55.10218	75.369289	7.297E-28	6.362792	up	unnamed protein product [Coffea canephora]
c23287.graph_c0	1.6138935	1.3697628	1.5539702	3.3549953	6.1115087	6.0433204	8.965E-06	1.804662	up	PREDICTED: GDSL esterase/lipase At5g33370-like isoform X1 [Sesamum indicum]
c23299.graph_c0	1.1637528	1.1720012	1.5339899	11.460722	16.709238	16.003292	2.311E-31	3.547874	up	PREDICTED: B-box zinc finger protein 21 [Vitis vinifera]
c23342.graph_c0	0.8862212	0.8535196	0.915989	39.541942	20.151577	12.727611	0.0002774	4.82967	up	PREDICTED: beta-D-xylosidase 1 [Sesamum indicum]
c23354.graph_c0	1.0064587	0.5111651	0.3593022	2.152846	2.7030273	2.4122237	4.833E-06	1.99241	up	PREDICTED: putative late blight resistance protein homolog R1A-10 [Sesamum indicum]
c23376.graph_c0	1.7187516	1.8670954	1.0080377	11.029386	17.274008	17.295452	1.514E-19	3.346152	up	4-coumarate coenzyme A ligase [Paulownia fortunei]
c23392.graph_c0	1.4881805	3.4935873	3.1572921	9.1300712	8.7129765	8.325301	1.606E-06	1.72848	up	PREDICTED: uncharacterized protein LOC105168788 [Sesamum indicum]
c23400.graph_c0	0.2801649	0.2168251	0.326589	1.1668546	1.0749052	1.5348194	5.334E-07	2.236869	up	hypothetical protein MIMGU_mgv1a026825mg [Erythranthe guttata]
c23414.graph_c0	53.073745	55.907385	62.034051	13.407931	16.446826	13.116349	4.699E-29	-1.952922	down	PREDICTED: mannan synthase 1-like [Sesamum indicum]
c23432.graph_c0	3.5446261	2.1541592	2.7811404	15.034519	14.188068	11.993733	2.088E-12	2.325508	up	PREDICTED: beta-galactosidase-like [Sesamum indicum]

#ID	28 h-1 FPKM	28 h-2 FPKM	28 h-3 FPKM	48 h-1 FPKM	48 h-2 FPKM	48 h-3 FPKM	FDR	log2FC	regulated	nr_annotation
c23435.graph_c0	0	0	0.1444898	4.0401462	6.0633972	5.9914964	3.437E-23	6.829127	up	hypothetical protein MIMGU_mgv1a016138mg [Erythranthe guttata]
c23458.graph_c0	5.1955233	6.4153558	4.0561747	11.533889	10.785693	11.561471	3.545E-07	1.156352	up	PREDICTED: uncharacterized protein LOC105155953 [Sesamum indicum]
c23482.graph_c0	4.5570199	5.0024408	4.4230472	12.221558	10.467311	12.010828	1.908E-10	1.355488	up	PREDICTED: serine/threonine-protein kinase AtPK2/AtPK19-like [Sesamum indicum]
c23494.graph_c0	0.4173862	0.3768606	0.3405837	1.3430181	1.3796508	1.158797	4.067E-05	1.81736	up	PREDICTED: O-acyltransferase WSD1-like isoform X1 [Sesamum indicum]
c23519.graph_c0	0	0.0527979	0.0477155	3.6319773	2.1986512	1.780739	3.275E-08	6.297437	up	-
c23541.graph_c0	10.005652	8.8587448	7.3718573	19.332188	26.350204	34.732024	1.028E-05	1.650446	up	PREDICTED: non-specific lipid transfer protein GPI-anchored 2-like [Nicotiana tomentosiformis]
c23542.graph_c0	6.8037947	8.201987	7.7425673	1.8647159	4.1978107	4.6457963	0.0008225	-1.058878	down	PREDICTED: F-box/kelch-repeat protein At1g57790-like [Nicotiana tomentosiformis]
c23565.graph_c0	4.682088	2.9408601	4.4849882	1.032155	0.9567627	0.4592027	1.129E-06	-2.257594	down	PREDICTED: protein CUP-SHAPED COTYLEDON 3 [Sesamum indicum]
c23586.graph_c0	4.980539	4.2775958	4.1962443	9.7520975	9.2980483	8.0836437	0.0003564	1.056358	up	PREDICTED: glucan endo-1,3-beta-glucosidase 14-like [Sesamum indicum]
c23589.graph_c0	2.490801	2.5517044	3.205054	6.5574147	7.9116171	7.8877151	1.738E-05	1.476832	up	PREDICTED: calcium-dependent protein kinase 8-like [Sesamum indicum]
c23594.graph_c0	1.2972183	1.6644313	1.1142311	3.9809846	3.7589606	4.0812951	0.000214	1.579411	up	PREDICTED: uncharacterized protein LOC105160721 [Sesamum indicum]
c23637.graph_c0	0	0.1411526	0.2126086	5.4824713	6.9976067	6.9941461	2.448E-28	5.818978	up	PREDICTED: uncharacterized acetyltransferase At3g50280-like [Sesamum indicum]
c23640.graph_c0	0.382902	0.3951139	0.3124449	6.6563183	4.3337726	3.3932434	9.079E-07	3.775144	up	-
c23652.graph_c0	19.725808	21.51145	20.172383	10.310056	9.202214	9.4627651	5.205E-05	-1.039378	down	PREDICTED: uncharacterized protein LOC105169025 [Sesamum indicum]
c23673.graph_c0	4.3952692	7.3357417	5.8688243	1.0129686	1.2519703	1.3520001	1.435E-06	-2.244853	down	PREDICTED: EPIDERMAL PATTERNING FACTOR-like protein 4-like [Fragaria vesca subsp. vesca]
c23690.graph_c0	20.353493	19.908737	21.353508	48.834865	51.327899	58.210581	7.528E-17	1.401921	up	PREDICTED: uncharacterized protein LOC105170479 [Sesamum indicum]
c23716.graph_c0	20.905995	14.400364	15.652183	52.726582	62.514004	38.40775	3.366E-06	1.634588	up	hypothetical protein [Arachis diogoi]
c23716.graph_c1	42.678444	41.449018	34.825262	183.66068	158.92785	117.30656	1.118E-07	1.998874	up	PREDICTED: abscisic acid receptor PYL4-like [Sesamum indicum]
c23817.graph_c0	10.587921	11.736137	8.0074871	1.0911358	2.1962594	2.3301265	4.338E-09	-2.402385	down	hypothetical protein MIMGU_mgv1a012373mg [Erythranthe guttata]
c23864.graph_c0	1.9777018	0.9236272	1.9476758	45.815441	34.707751	38.151107	3.619E-37	4.659097	up	PREDICTED: histone H2AX-like [Sesamum indicum]
c23868.graph_c0	0.2580585	0.0776675	0	2.1807206	5.5445018	5.142039	2.915E-08	5.289052	up	PREDICTED: uncharacterized protein LOC105162633 [Sesamum indicum]
c23907.graph_c0	1.72706	1.1340896	2.0498427	3.6486263	3.6544438	3.6006922	0.0047017	1.192059	up	hypothetical protein MIMGU_mgv1a015138mg [Erythranthe guttata]
c23921.graph_c0	14.640517	18.268975	16.778852	84.449026	87.696323	88.686392	3.377E-27	2.433573	up	PREDICTED: pectinesterase-like isoform X2 [Sesamum indicum]
c23936.graph_c0	43.021767	43.591419	45.615584	91.073873	98.128974	100.90093	6.95E-14	1.173903	up	actin [Ipomoea nil]
c23950.graph_c0	5.1684394	6.3733878	5.2912804	0.8189222	1.3816517	2.1860161	1.878E-09	-1.910094	down	PREDICTED: LOW QUALITY PROTEIN: H/ACA ribonucleoprotein complex subunit 4-like [Sesamum indicum]
c23951.graph_c0	5.0910667	5.581775	5.3741732	0.3585168	0.5425786	1.3671689	1.18E-11	-2.79475	down	PREDICTED: uncharacterized protein LOC105166244 [Sesamum indicum]

#ID	28 h-1 FPKM	28 h-2 FPKM	28 h-3 FPKM	48 h-1 FPKM	48 h-2 FPKM	48 h-3 FPKM	FDR	log2FC	regulated	nr_annotation
c23967.graph_c0	0.3000017	0.6771834	0.6527971	9.8237504	8.2585155	12.23756	1.101E-26	4.259126	up	PREDICTED: high mobility group B protein 7-like [Sesamum indicum]
c23979.graph_c0	5.49326	4.0903061	4.7153101	22.92401	24.668408	28.846376	2.035E-36	2.457584	up	PREDICTED: ferredoxin--NADP reductase, leaf isozyme, chloroplastic isoform 1 [Glycine max]
c23987.graph_c0	6.1414857	7.2796988	7.0539642	10.662573	14.344371	15.298173	1.605E-06	1.013225	up	PREDICTED: lysophospholipid acyltransferase 1-like [Sesamum indicum]
c23996.graph_c0	0.6763082	1.1067903	0.7933016	0.2946867	0.4540871	0.3337224	0.0067093	-1.214148	down	Quinohemoprotein ethanol dehydrogenase type-1 [Theobroma cacao]
c24030.graph_c0	0.197622	0.1784341	0.2822014	5.3857596	10.216932	11.033243	2.674E-14	5.36832	up	PREDICTED: uncharacterized protein LOC105166144 [Sesamum indicum]
c24031.graph_c0	2.7173132	4.3865231	3.0235982	0.6262536	0.5528664	0.835855	1.268E-06	-2.288139	down	hypothetical protein MIMGU_mgv1a025777mg, partial [Erythranthe guttata]
c24038.graph_c1	5.7633054	4.9707218	4.6326189	13.520468	22.524538	15.426117	1.701E-06	1.779421	up	PREDICTED: luminal-binding protein 5 [Sesamum indicum]
c24097.graph_c0	0.8804379	1.0599371	1.2315945	3.2949157	2.4490245	3.9484675	1.597E-06	1.654482	up	PREDICTED: protein S-acyltransferase 18 isoform X4 [Sesamum indicum]
c24101.graph_c0	17.479117	22.080651	24.560186	68.256586	66.415346	75.85157	1.972E-24	1.756408	up	PREDICTED: methionine gamma-lyase [Sesamum indicum]
c24101.graph_c1	6.85532	6.1897112	9.3522767	28.784376	26.609943	35.156321	6.319E-13	2.0558	up	PREDICTED: methionine gamma-lyase [Sesamum indicum]
c24115.graph_c0	0.0592032	0	0.0483093	1.3507994	4.014773	3.1383811	2.17E-08	6.32959	up	PREDICTED: geraniol 8-hydroxylase-like isoform X1 [Beta vulgaris subsp. vulgaris]
c24123.graph_c0	2.9047158	3.292308	3.0258184	1.2534268	1.161872	1.2547031	0.0038275	-1.286332	down	PREDICTED: fasciclin-like arabinogalactan protein 1 [Sesamum indicum]
c24123.graph_c1	2.1918647	2.4879465	1.9929487	0.3175257	0.588665	0.9888636	0.0001525	-1.78756	down	PREDICTED: fasciclin-like arabinogalactan protein 1 [Sesamum indicum]
c24158.graph_c0	1.2750037	1.0814385	1.6078794	0.3917966	0.3112958	0.4793501	0.0002072	-1.703477	down	-
c24169.graph_c0	1.166563	1.3166213	1.0708941	7.3935226	6.0702191	7.7299866	6.968E-09	2.620246	up	PREDICTED: 2-hydroxyisoflavanone dehydratase-like [Sesamum indicum]
c24236.graph_c0	2.5868675	3.0184415	3.3773804	24.466721	20.094311	17.191836	4.839E-15	2.82861	up	PREDICTED: uncharacterized protein LOC102578886 [Solanum tuberosum]
c24237.graph_c1	1.9765966	1.626043	1.6128867	5.4563824	4.5417237	5.8954255	7.466E-06	1.65094	up	hypothetical protein MIMGU_mgv1a021532mg [Erythranthe guttata]
c24238.graph_c0	91.559685	81.60585	95.192839	10.754469	11.551293	13.290641	9.327E-45	-2.875059	down	-
c24238.graph_c1	2.439995	2.2030867	2.21224	13.574278	15.108418	13.760101	7.33E-11	2.671051	up	PREDICTED: metallothionein-like protein 1 [Sesamum indicum]
c24247.graph_c0	6.8373566	5.5086543	6.0942327	27.867266	74.511241	94.9134	0.0002536	3.444611	up	PREDICTED: cytochrome P450 71D11-like [Sesamum indicum]
c24250.graph_c0	1.7414153	2.1128248	2.3534992	6.208234	8.6960852	11.416369	6.001E-07	2.117669	up	hypothetical protein MIMGU_mgv1a016982mg [Erythranthe guttata]
c24292.graph_c0	29.193156	33.605859	25.986956	65.721917	57.368421	53.295438	7.013E-08	1.0363	up	hypothetical protein MIMGU_mgv1a016421mg [Erythranthe guttata]
c24294.graph_c0	29.36742	27.088315	28.216104	10.980144	10.59186	8.3403006	9.028E-10	-1.456342	down	ZPT2-14 [Petunia x hybrida]
c24304.graph_c0	3.4439821	3.3583604	3.8219554	0	0.5549663	0.3107517	2.8E-11	-3.60644	down	-
c24314.graph_c0	2.9409414	3.1295719	3.1282901	8.3211156	6.2764317	4.9755409	0.0043217	1.139383	up	hypothetical protein MIMGU_mgv1a013918mg [Erythranthe guttata]
c24321.graph_c0	12.132535	12.127598	10.209936	4.2817646	4.4018089	5.5908421	1.118E-09	-1.232143	down	PREDICTED: uncharacterized protein LOC105168724 isoform X1 [Sesamum indicum]
c24323.graph_c0	182.04986	160.68221	185.64771	43.155551	88.826791	113.14398	9.292E-05	-1.079821	down	PREDICTED: alpha-xylosidase 1 [Sesamum indicum]
c24348.graph_c0	3.6994049	3.0618645	3.4211754	8.2843738	13.826796	19.054337	0.0003331	2.046016	up	hypothetical protein MIMGU_mgv1a011727mg [Erythranthe guttata]

#ID	28 h-1 FPKM	28 h-2 FPKM	28 h-3 FPKM	48 h-1 FPKM	48 h-2 FPKM	48 h-3 FPKM	FDR	log2FC	regulated	nr_annotation
c24355.graph_c0	42.91908	41.994742	39.674072	92.530786	75.357586	81.928253	1.136E-09	1.049337	up	PREDICTED: uncharacterized endoplasmic reticulum membrane protein C16E8.02 [Sesamum indicum]
c24366.graph_c0	8.4439793	7.9921827	8.2207431	46.578095	74.367663	68.636973	2.82E-18	2.976935	up	PREDICTED: chaperone protein dnaJ 20, chloroplastic-like [Sesamum indicum]
c24370.graph_c0	0	0	0	2.0974255	2.4518267	2.1880484	1.939E-22	Inf	up	hypothetical protein SELMODRAFT_117229, partial [Selaginella moellendorffii]
c24417.graph_c0	2.2234231	1.822231	1.7305584	0.2601556	0.2756033	0.2700657	4.76E-09	-2.800295	down	PREDICTED: transcription factor TGA3 [Sesamum indicum]
c24418.graph_c0	0	0	0.026675	2.0304331	2.7655685	3.1708853	9.57E-28	8.252555	up	PREDICTED: patatin-like protein 1 [Sesamum indicum]
c24438.graph_c0	1.2171887	1.2480252	1.2962311	7.6097796	9.7238323	10.377755	7.564E-31	2.918047	up	PREDICTED: ornithine decarboxylase-like [Sesamum indicum]
c24442.graph_c0	0.6609523	0.4475834	0.4044987	3.979577	9.7630888	8.7593248	8.232E-07	3.922724	up	-
c24454.graph_c0	1.3919115	1.6392595	0.9876421	3.375279	3.4131671	4.777982	0.0001552	1.564804	up	hypothetical protein TRIUR3_08605 [Triticum urartu]
c24465.graph_c0	86.453862	118.46891	118.24272	6.9369406	8.3858013	8.1415725	1.75E-33	-3.74502	down	legumin-like protein [Perilla frutescens]
c24475.graph_c0	0.4582489	0.5378825	0.2617491	3.020494	3.8767378	3.7730013	2.078E-12	3.123265	up	PREDICTED: uncharacterized protein LOC105167521 [Sesamum indicum]
c24482.graph_c0	37.133275	40.199658	40.268305	96.888964	118.94527	136.53274	6.101E-17	1.620291	up	PREDICTED: probable methylenetetrahydrofolate reductase [Sesamum indicum]
c24492.graph_c0	79.205229	73.832138	72.50206	214.81905	202.14341	199.62893	4.982E-25	1.494441	up	hypothetical protein MIMGU_mgv1a017195mg [Erythranthe guttata]
c24500.graph_c0	5.5089383	4.3771682	5.8438233	21.088615	29.590504	33.179653	4.137E-15	2.448998	up	-
c24502.graph_c0	9.2738665	7.4072667	9.8958299	0.4521273	0.718461	0.4023001	1.691E-13	-4.041844	down	-
c24523.graph_c0	43.997563	47.814421	43.841624	62.708065	91.810658	112.09577	0.0013256	1.008642	up	PREDICTED: anthocyanidin 3-O-glucoside 6''-O-acyltransferase-like [Sesamum indicum]
c24534.graph_c0	70.815181	61.451552	63.030942	250.89527	213.25118	205.42693	3.408E-18	1.823317	up	conserved hypothetical protein [Ricinus communis]
c24542.graph_c0	2.3811742	1.7916476	2.2128824	0.5030523	0.2664615	1.2682366	0.0007487	-1.6119	down	-
c24548.graph_c0	2.2570568	1.817596	1.8915166	15.464788	9.0106869	7.9122731	0.0004631	2.495316	up	PREDICTED: S-norcoclaurine synthase-like [Sesamum indicum]
c24558.graph_c0	1.3976242	1.3145037	1.9007498	7.8983457	5.0047639	6.3054061	4.427E-07	2.106659	up	PREDICTED: uncharacterized protein LOC105160970 [Sesamum indicum]
c24559.graph_c0	65.271953	71.14201	71.077293	10.595432	10.672547	10.818731	9.011E-61	-2.65113	down	PREDICTED: uncharacterized protein LOC105168445 [Sesamum indicum]
c24566.graph_c0	5.4967586	6.954811	6.2853359	25.807425	24.134812	29.733996	3.469E-29	2.129988	up	PREDICTED: 2-C-methyl-D-erythritol 4-phosphate cytidylyltransferase, chloroplastic isoform X2 [Sesamum indicum]
c24575.graph_c0	8.6169592	9.3950868	9.0545451	20.14504	22.814918	25.901761	2.29E-12	1.38592	up	PREDICTED: magnesium protoporphyrin IX methyltransferase, chloroplastic [Sesamum indicum]
c24582.graph_c0	4.3800076	3.4547121	3.4097263	10.912517	12.371751	22.429282	0.0006755	2.057988	up	PREDICTED: protein TIFY 10B-like [Sesamum indicum]
c24644.graph_c0	0.8454992	0.1090581	0.5913604	2.0669126	1.9463496	2.0434738	5.165E-06	2.01155	up	hypothetical protein MIMGU_mgv1a016118mg [Erythranthe guttata]
c24673.graph_c0	9.5850845	9.8304704	9.7562492	4.0640448	3.4084121	3.4654892	5.332E-07	-1.369666	down	PREDICTED: UDP-glycosyltransferase 89A2-like [Sesamum indicum]

#ID	28 h-1 FPKM	28 h-2 FPKM	28 h-3 FPKM	48 h-1 FPKM	48 h-2 FPKM	48 h-3 FPKM	FDR	log2FC	regulated	nr_annotation
c24676.graph_c0	3.7585635	3.8784348	3.3648901	9.0656986	9.6616902	11.094609	1.115E-11	1.478573	up	PREDICTED: LOW QUALITY PROTEIN: uncharacterized protein LOC105175967 [Sesamum indicum]
c24693.graph_c1	3.8623676	4.6498078	5.0557886	1.1219623	1.728848	1.1798272	9.083E-05	-1.714567	down	PREDICTED: mitoferrin-like [Sesamum indicum]
c24749.graph_c0	1.1705425	1.7072841	2.1307263	35.039301	42.07727	33.513552	1.676E-49	4.505104	up	predicted protein [Hordeum vulgare subsp. vulgare]
c24765.graph_c0	1.3099669	0.6989139	1.1660971	7.3966818	10.714377	8.4619581	1.075E-15	3.101381	up	hypothetical protein CICLE_v10022284mg [Citrus clementina]
c24783.graph_c0	0.3221724	0.0581783	0.315468	3.8387459	4.0666858	4.2151106	2.068E-16	4.161539	up	-
c24796.graph_c0	3.6497711	4.0558783	2.5200014	6.7615939	7.7662963	8.2963342	0.0005718	1.198062	up	hypothetical protein L484_021121 [Morus notabilis]
c24797.graph_c0	1.2433059	1.8134124	3.0435824	64.31218	62.223277	50.806376	4.12E-60	4.904938	up	PREDICTED: probable pectinesterase/pectinesterase inhibitor 17 [Sesamum indicum]
c24866.graph_c0	3.5257166	4.334831	3.917557	9.9841718	8.8645496	7.9108623	2.802E-05	1.229648	up	PREDICTED: acidic mammalian chitinase-like [Sesamum indicum]
c24896.graph_c0	144.6271	165.41431	149.30122	284.07327	313.14785	357.39606	1.869E-14	1.094648	up	PREDICTED: thiamine thiazole synthase 2, chloroplastic-like [Sesamum indicum]
c24896.graph_c1	136.11355	166.137	141.38156	278.69866	288.41652	338.91393	4.683E-13	1.070517	up	PREDICTED: thiamine thiazole synthase 2, chloroplastic [Vitis vinifera]
c24911.graph_c0	2.7536113	4.8689117	3.1831428	1.0907516	0.9244152	1.6175737	0.0014743	-1.532014	down	PREDICTED: auxin-induced protein 10A5-like [Sesamum indicum]
c24918.graph_c0	12.324468	13.470542	11.776884	2.6722133	4.1374488	7.4989611	0.0001727	-1.362617	down	-
c24937.graph_c0	0.5224823	0.497961	0.7105688	5.0039296	4.4435327	5.7947541	8.011E-18	3.17978	up	PREDICTED: putative cyclin-A3-1 [Sesamum indicum]
c24946.graph_c0	13.128572	15.384809	13.020619	2.3457337	6.1891074	6.5767112	4.197E-08	-1.432057	down	hypothetical protein MIMGU_mgv1a010693mg [Erythranthe guttata]
c24966.graph_c0	2.5379537	3.6282626	3.5378721	6.5681301	6.2481234	7.3948066	0.0098487	1.100097	up	hypothetical protein MIMGU_mgv1a019945mg [Erythranthe guttata]
c24971.graph_c0	0.9713719	1.5149179	1.3330621	3.3020836	2.4309228	2.3405794	0.009975	1.128889	up	PREDICTED: zinc transporter 8-like [Sesamum indicum]
c24986.graph_c0	3.9758245	5.2650353	4.5419365	20.382586	21.948805	10.164301	0.0009838	1.975751	up	-
c25059.graph_c0	6.8837073	6.5135833	6.2531447	21.035242	16.092237	13.769536	5.935E-05	1.422162	up	PREDICTED: lysine-specific demethylase JMJ706 [Sesamum indicum]
c25082.graph_c0	11.24852	12.829084	12.446658	22.822152	28.71347	26.316633	1.442E-10	1.130369	up	PREDICTED: delta(7)-sterol-C5(6)-desaturase-like [Solanum tuberosum]
c25102.graph_c0	1.910021	1.9640935	2.2512556	19.906759	19.378895	24.594621	4.058E-35	3.422771	up	PREDICTED: ubiquitin-conjugating enzyme E2 20-like [Sesamum indicum]
c25122.graph_c0	184.72747	180.7942	194.76397	368.89501	516.40205	550.71168	8.303E-10	1.39312	up	PREDICTED: pEARLI1-like lipid transfer protein 1 [Sesamum indicum]
c25141.graph_c0	7.2657929	5.9111305	6.5772932	3.3577861	1.2196002	1.1524132	3.328E-07	-1.721352	down	hypothetical protein MIMGU_mgv1a024254mg, partial [Erythranthe guttata]
c25142.graph_c0	6.2330393	6.2998322	4.8583729	39.975874	44.660704	33.261896	1.067E-29	2.80383	up	uncharacterized protein LOC100499662 [Glycine max]
c25198.graph_c0	88.232142	83.853767	91.695378	680.5245	370.57313	263.95023	0.0098601	2.375684	up	hypothetical protein MIMGU_mgv1a026311mg [Erythranthe guttata]
c25203.graph_c0	80.545827	72.92162	70.824835	129.7923	173.55261	176.2363	7.576E-12	1.133032	up	hypothetical protein MIMGU_mgv1a012619mg [Erythranthe guttata]
c25208.graph_c0	0.0719884	0.2599951	0.0587419	4.3800289	7.1535022	5.5212082	1.926E-22	5.485321	up	unnamed protein product [Coffea canephora]
c25218.graph_c0	26.438344	28.213846	25.408258	12.750096	10.960745	7.4393173	4.629E-16	-1.313213	down	PREDICTED: limonoid UDP-glucosyltransferase-like [Sesamum indicum]
c25222.graph_c0	2.0019618	1.6067411	1.4002152	14.661885	16.55661	21.432809	2.229E-27	3.431742	up	PREDICTED: fructose-1,6-bisphosphatase, chloroplastic [Sesamum indicum]

#ID	28 h-1 FPKM	28 h-2 FPKM	28 h-3 FPKM	48 h-1 FPKM	48 h-2 FPKM	48 h-3 FPKM	FDR	log2FC	regulated	nr_annotation
c25224.graph_c0	23.045302	25.07432	24.291977	91.781613	86.688316	89.784203	4.018E-40	1.932321	up	PREDICTED: alpha carbonic anhydrase 1, chloroplastic [Sesamum indicum]
c25228.graph_c0	13.294721	11.146466	12.742546	4.5473755	4.6757049	4.1652341	0.000161	-1.431336	down	PREDICTED: serine/threonine-protein kinase HT1-like [Fragaria vesca subsp. vesca]
c25231.graph_c0	0.2984476	0.5389403	0.4383554	12.748854	10.019187	7.2035151	4.12E-13	4.604477	up	PREDICTED: ectonucleotide pyrophosphatase/phosphodiesterase family member 3 [Eucalyptus grandis]
c25236.graph_c0	2.3224433	1.4336281	2.8233044	7.3897093	12.283745	10.611389	2.895E-10	2.2355	up	PREDICTED: uncharacterized protein LOC105167293 [Sesamum indicum]
c25241.graph_c0	0.4034257	0.5142432	0.4066491	1.082905	1.7845436	1.980656	5.302E-06	1.90504	up	PREDICTED: organic cation/carnitine transporter 7-like isoform X2 [Sesamum indicum]
c25258.graph_c0	2.2717617	2.2250173	1.9794162	3.6117334	7.1870394	9.5542393	0.0061069	1.680871	up	PREDICTED: uncharacterized protein At4g06744-like [Nicotiana tomentosiformis]
c25264.graph_c0	1.0859052	1.2384892	1.0259987	0.2173366	0.306989	0.8057698	0.0065889	-1.304128	down	PREDICTED: F-box protein At3g07870-like [Sesamum indicum]
c25291.graph_c0	1.8191169	1.4108586	1.2560177	29.444119	41.871824	44.296639	8.204E-42	4.723148	up	PREDICTED: purple acid phosphatase 22-like [Sesamum indicum]
c25305.graph_c1	0.6871875	0.3722795	1.0093309	1.6550155	2.9529065	2.7127263	3.024E-05	1.854091	up	hypothetical protein MIMGU_mgv1a002668mg [Erythranthe guttata]
c25327.graph_c0	8.6538194	9.2910105	9.0023112	19.073418	22.086126	21.727974	2.017E-16	1.262134	up	PREDICTED: LOW QUALITY PROTEIN: microtubule-associated protein TORTIFOLIA1 [Sesamum indicum]
c25329.graph_c0	8.4307892	7.5532028	7.7327209	15.118705	14.129567	17.691002	6.131E-07	1.026291	up	PREDICTED: probable plastid-lipid-associated protein 13, chloroplastic [Sesamum indicum]
c25330.graph_c0	5.8613011	6.2738241	5.7856135	15.068969	14.122996	15.087645	1.682E-12	1.347989	up	PREDICTED: probable aspartyl aminopeptidase [Sesamum indicum]
c25352.graph_c0	4.7763305	5.0182733	4.676936	12.072008	18.736218	20.405776	2.66E-08	1.856991	up	PREDICTED: transcription factor bHLH62 isoform X2 [Sesamum indicum]
c25353.graph_c0	515.55858	510.27845	575.03798	67.604325	69.568895	61.639204	4.921E-69	-2.966999	down	hypothetical protein MIMGU_mgv1a005193mg [Erythranthe guttata]
c25364.graph_c1	4.8987215	2.0272479	4.8300911	7.5031341	11.511854	9.4388272	0.0027492	1.309738	up	leucine-rich repeat protein [Dasypyrum villosum]
c25389.graph_c0	60.23967	63.623415	68.926954	12.338071	10.950179	13.518542	5.347E-56	-2.346566	down	PREDICTED: vacuolar cation/proton exchanger 3-like [Sesamum indicum]
c25406.graph_c0	4.7324223	4.5867649	5.192458	2.1012374	2.5132328	1.9902922	0.0006553	-1.095351	down	PREDICTED: uncharacterized protein LOC105160550 [Sesamum indicum]
c25414.graph_c0	7.3223983	8.7601569	7.5434593	2.3204198	2.9498441	2.6841043	0.0002033	-1.532003	down	PREDICTED: protein SOMBRERO-like [Sesamum indicum]
c25434.graph_c0	0	0.0445428	0.0402551	9.536261	12.421122	17.360105	2.927E-30	8.891506	up	hypothetical protein MIMGU_mgv1a005366mg [Erythranthe guttata]
c25449.graph_c0	2.1741395	2.6425594	2.4223017	5.4057685	5.8390455	7.2150304	1.724E-07	1.389476	up	PREDICTED: F-box/LRR-repeat protein 17-like [Sesamum indicum]
c25464.graph_c0	5.550536	5.0995366	5.7806793	67.394726	60.020168	56.068441	1.341E-53	3.526055	up	PREDICTED: alanine--glyoxylate aminotransferase 2 homolog 3, mitochondrial-like [Sesamum indicum]
c25466.graph_c0	3.551938	2.5863444	3.4593238	4.5749615	6.6929472	8.076931	0.0041601	1.044541	up	PREDICTED: uncharacterized protein LOC105177131 [Sesamum indicum]
c25486.graph_c0	0.1384011	0.0999705	0.135521	1.087688	2.5647378	3.6527333	4.703E-05	4.313972	up	PREDICTED: 1-aminocyclopropane-1-carboxylate synthase 7 [Sesamum indicum]
c25506.graph_c0	4.2419686	4.6885705	4.1775658	2.1322616	2.4552963	1.5260679	0.0038236	-1.057223	down	PREDICTED: serine acetyltransferase 1, chloroplastic-like [Sesamum indicum]

#ID	28 h-1 FPKM	28 h-2 FPKM	28 h-3 FPKM	48 h-1 FPKM	48 h-2 FPKM	48 h-3 FPKM	FDR	log2FC	regulated	nr_annotation
c25519.graph_c0	3.378501	4.495429	4.207792	1.4650654	0.8357242	1.3036064	0.0001706	-1.695794	down	PREDICTED: ethylene-responsive transcription factor CRF4-like [Sesamum indicum]
c25527.graph_c0	0.1489112	0.0896352	0.2025171	10.633247	13.064311	13.044414	3.536E-47	6.417253	up	Early nodulin 20 precursor, putative [Ricinus communis]
c25537.graph_c0	18.840337	18.763713	19.193658	35.942887	36.492712	42.499257	2.331E-11	1.057376	up	hypothetical protein MIMGU_mgv1a020674mg [Erythranthe guttata]
c25559.graph_c0	37.065684	38.856759	38.140909	11.918968	10.950127	9.9453373	2.883E-25	-1.752722	down	PREDICTED: dipeptidyl peptidase 9 [Sesamum indicum]
c25564.graph_c0	41.379252	40.80344	37.889977	219.32886	248.15407	310.61945	5.438E-27	2.734038	up	PREDICTED: low-temperature-induced cysteine proteinase [Sesamum indicum]
c25565.graph_c0	0.9293894	1.6503312	1.2133986	10.288473	13.229031	19.497248	3.178E-10	3.53819	up	PREDICTED: glucan endo-1,3-beta-glucosidase 12 [Sesamum indicum]
c25595.graph_c0	0.8766462	0.653872	1.3062659	3.6718362	3.633953	3.8690263	5.517E-07	2.018561	up	-
c25609.graph_c0	17.285338	15.561405	17.280313	37.926842	40.24676	38.022466	8.991E-13	1.254189	up	PREDICTED: histidine-containing phosphotransfer protein 1 [Sesamum indicum]
c25645.graph_c0	2.2618843	2.4098779	2.1409878	4.7593942	5.46723	3.4185178	0.0083138	1.045231	up	-
c25690.graph_c0	143.81036	168.05798	172.19385	380.11516	387.25734	495.78136	3.281E-14	1.423281	up	lipid transfer protein 2 [Tamarix hispida]
c25692.graph_c0	1.8009833	1.9286529	1.264529	2.9730518	3.2058305	4.0153447	0.0067122	1.068841	up	PREDICTED: uncharacterized protein LOC105177785 [Sesamum indicum]
c25695.graph_c0	10.937804	9.8758115	9.5862816	3.5944928	3.8079293	2.2845415	0.0002839	-1.604979	down	PREDICTED: plasma membrane ATPase 4-like [Cicer arietinum]
c25701.graph_c0	0	0.5633189	0.1018187	10.913498	14.912695	24.34728	3.499E-11	6.272913	up	unnamed protein product [Coffea canephora]
c25703.graph_c0	4.383915	3.3440508	2.8371204	1.1497107	2.3344599	0.5967533	0.0044085	-1.336103	down	hypothetical protein MIMGU_mgv1a015966mg [Erythranthe guttata]
c25711.graph_c0	4.0110107	4.1269015	4.0341036	0.7094304	2.0041481	1.4729095	0.000841	-1.512707	down	-
c25720.graph_c0	2.3157039	1.4188002	1.1135116	3.5643235	5.1086634	4.8505503	4.509E-05	1.516253	up	PREDICTED: transcription factor ICE1-like [Sesamum indicum]
c25720.graph_c1	0.6216522	0.3929056	1.0145263	2.679166	2.5043396	3.5758587	1.258E-06	2.14861	up	PREDICTED: transcription factor ICE1-like, partial [Nicotiana sylvestris]
c25727.graph_c0	0.1653251	0.2239096	0.2698079	1.9908368	4.1070977	3.4496358	3.572E-09	3.887035	up	Homeobox-leucine zipper ATHB-13 -like protein [Gossypium arboreum]
c25784.graph_c0	22.259176	20.457424	21.677831	53.99874	61.77375	81.072931	3.834E-10	1.649219	up	PREDICTED: uncharacterized protein LOC105164223 [Sesamum indicum]
c25787.graph_c0	2.6657032	3.2091734	3.0556269	8.4474377	7.1592288	7.0153807	0.0002165	1.386661	up	PREDICTED: uncharacterized protein LOC105177716 [Sesamum indicum]
c25813.graph_c0	7.4811127	6.9876655	6.4202783	23.380158	18.706236	11.347377	0.0089318	1.406025	up	PREDICTED: root phototropism protein 2-like [Malus domestica]
c25818.graph_c0	90.73385	99.552686	101.57035	525.40226	607.84792	599.48294	1.217E-63	2.609112	up	PREDICTED: kirola-like isoform X2 [Sesamum indicum]
c25846.graph_c0	0.7619225	0.8408211	0.5181021	4.6143436	3.5809914	3.1032324	4.032E-09	2.462848	up	PREDICTED: glucan endo-1,3-beta-glucosidase 5-like [Sesamum indicum]
c25849.graph_c0	4.3446971	3.3732756	3.9220259	24.26371	23.985191	25.472062	4.333E-58	2.704615	up	PREDICTED: cell division cycle 20.2, cofactor of APC complex-like [Sesamum indicum]
c25850.graph_c0	2.2813428	1.9514261	2.841324	0.5073281	0.8061789	0.6771262	4.581E-05	-1.795049	down	PREDICTED: vacuolar iron transporter 1 [Nicotiana sylvestris]
c25870.graph_c0	30.332877	30.964701	23.738165	7.3450628	13.259806	18.206294	0.0004029	-1.101412	down	PREDICTED: transcription factor DIVARICATA-like [Sesamum indicum]
c25886.graph_c0	8.195291	8.5095163	7.7275357	26.085761	25.066875	30.965055	1.915E-19	1.790077	up	hypothetical protein MIMGU_mgv1a010584mg [Erythranthe guttata]

#ID	28 h-1 FPKM	28 h-2 FPKM	28 h-3 FPKM	48 h-1 FPKM	48 h-2 FPKM	48 h-3 FPKM	FDR	log2FC	regulated	nr_annotation
c25922.graph_c0	1.8106213	0.9008199	1.2663876	3.2318697	4.9619914	6.5015787	6.617E-05	1.917339	up	hypothetical protein MIMGU_mgv1a027056mg [Erythranthe guttata]
c25927.graph_c0	11.121176	12.336551	14.778941	26.784128	29.014562	25.265881	0.0007404	1.125481	up	PREDICTED: V-type proton ATPase subunit H [Beta vulgaris subsp. vulgaris]
c25928.graph_c0	5.7889772	4.985662	3.9243529	14.563018	18.178437	25.614822	5.912E-07	2.024714	up	BnaA07g08260D [Brassica napus]
c25931.graph_c0	8.9479054	8.38347	8.5016508	2.5636319	3.8268896	4.8732682	3.623E-05	-1.164484	down	PREDICTED: putative GATA transcription factor 22 [Nicotiana tomentosiformis]
c25945.graph_c0	0	0.0753355	0.0340418	9.2013087	1.9047173	14.163035	4.153E-05	7.900294	up	--
c25953.graph_c1	5.3617837	6.051485	4.7781481	9.2109861	11.17898	13.288466	0.0008499	1.094031	up	PREDICTED: probable pectate lyase 5 [Sesamum indicum]
c25955.graph_c0	41.79396	41.834697	40.458027	101.09267	122.01942	117.44913	2.02E-25	1.495465	up	PREDICTED: probable xyloglucan endotransglucosylase/hydrolase protein 7 [Sesamum indicum]
c25956.graph_c0	0.3861575	0.9007155	0.7877535	5.9961684	5.7904529	6.7145619	8.404E-19	3.198389	up	PREDICTED: protein POLYCHOME-like [Sesamum indicum]
c25958.graph_c0	18.842025	19.795505	22.350606	45.85072	49.978936	67.362111	1.678E-08	1.45762	up	-
c25980.graph_c0	8.1076362	9.3315439	9.9115306	17.805983	18.584119	22.475885	4.192E-10	1.145372	up	PREDICTED: thioredoxin-like protein CITRX1, chloroplastic [Sesamum indicum]
c25987.graph_c0	1.6740424	0.8691144	1.2977047	7.1616556	6.519119	8.1189072	3.519E-13	2.546295	up	hypothetical protein MIMGU_mgv1a011925mg [Erythranthe guttata]
c25990.graph_c0	29.997699	29.288204	35.213788	3.4567538	2.5698327	3.5614512	5.379E-47	-3.256379	down	PREDICTED: espin-like [Sesamum indicum]
c25995.graph_c0	2.9884897	3.9524775	3.400278	1.6006186	1.5826173	1.2343247	0.0035535	-1.182422	down	-
c25997.graph_c0	3.6001687	3.4312045	3.1009143	7.3205809	7.6881227	5.4422732	0.0001274	1.057306	up	PREDICTED: LOW QUALITY PROTEIN: ADP-ribosylation factor-binding protein GGA3 [Sesamum indicum]
c25999.graph_c0	20.039997	29.625561	26.439633	112.63901	169.05857	161.01898	2.303E-19	2.575524	up	PREDICTED: glutamate decarboxylase [Sesamum indicum]
c26000.graph_c0	5.7537205	7.2184132	5.9799333	14.970385	11.14218	13.662111	0.0001746	1.116005	up	PREDICTED: uncharacterized protein LOC105169481 [Sesamum indicum]
c26004.graph_c0	10.144985	10.130992	9.974822	25.400951	28.979168	31.82016	6.953E-19	1.549569	up	unnamed protein product [Vitis vinifera]
c26007.graph_c0	0.1929494	0.1088845	0.1968064	2.0789097	2.5910032	2.3666576	3.654E-17	3.856601	up	PREDICTED: mitotic checkpoint serine/threonine-protein kinase BUB1 [Sesamum indicum]
c26014.graph_c0	1.4079411	1.2931568	1.5252219	3.0770157	2.8359608	3.2307339	0.001414	1.15569	up	PREDICTED: uncharacterized protein LOC105174110 [Sesamum indicum]
c26020.graph_c0	6.0381805	5.3265796	6.0031406	1.5835528	1.7707812	1.8787143	5.437E-05	-1.691403	down	PREDICTED: leucoanthocyanidin dioxygenase [Sesamum indicum]
c26026.graph_c0	0.3656065	0.1650542	0.3729149	4.0550399	4.6640364	5.9792201	1.385E-15	4.060112	up	-
c26027.graph_c0	0.4008512	0.1608582	0.4361217	1.1291298	3.0502491	3.114548	0.0003346	2.8947	up	PREDICTED: probable xyloglucan endotransglucosylase/hydrolase protein 32 [Sesamum indicum]
c26030.graph_c0	0	0	0	6.2035125	8.3517505	8.2797721	4.596E-28	Inf	up	PREDICTED: uncharacterized protein LOC105174742 [Sesamum indicum]
c26030.graph_c1	0.1073859	0.0969594	0.087626	3.5391078	8.6521283	7.1459821	4.787E-10	6.078005	up	-
c26035.graph_c0	40.689349	38.18097	40.733313	306.33888	333.67517	335.88737	9.881E-77	3.068793	up	PREDICTED: polyphenol oxidase I, chloroplastic-like [Sesamum indicum]

#ID	28 h-1 FPKM	28 h-2 FPKM	28 h-3 FPKM	48 h-1 FPKM	48 h-2 FPKM	48 h-3 FPKM	FDR	log2FC	regulated	nr_annotation
c26039.graph_c0	10.636519	9.2771202	8.4431408	126.57108	81.909489	66.593986	1.494E-06	3.331397	up	PREDICTED: uncharacterized protein LOC105177716 [Sesamum indicum]
c26040.graph_c0	5.4856861	5.4131806	5.369084	1.6338851	2.1938192	2.2821611	4.674E-08	-1.376483	down	PREDICTED: pentatricopeptide repeat-containing protein At1g06710, mitochondrial [Sesamum indicum]
c26045.graph_c0	2.5627154	2.3879362	2.3253642	6.9126601	7.4883084	9.5962406	2.677E-13	1.760138	up	PREDICTED: uncharacterized protein LOC105165819 [Sesamum indicum]
c26048.graph_c0	15.397411	13.619977	14.124047	48.947583	45.739827	48.923919	4.915E-31	1.77787	up	PREDICTED: uncharacterized protein LOC105164113 [Sesamum indicum]
c26050.graph_c0	17.183576	18.67169	19.872073	45.703486	39.107812	38.661756	7.718E-12	1.193017	up	PREDICTED: uncharacterized protein LOC105163776 [Sesamum indicum]
c26064.graph_c0	0.3677926	0.5396337	0.4126592	3.3799809	2.654642	2.6445141	8.453E-10	2.764488	up	PREDICTED: basic leucine zipper 9-like [Sesamum indicum]
c26066.graph_c0	0.4822727	1.0341866	0.9592307	1.9485702	2.6714132	4.1815804	0.0013069	1.862797	up	PREDICTED: origin of replication complex subunit 6 [Sesamum indicum]
c26068.graph_c0	8.0994335	11.887846	11.679044	0.8907121	0.7946117	0.5422712	3.084E-22	-3.781776	down	PREDICTED: flavonol synthase/flavanone 3-hydroxylase-like [Sesamum indicum]
c26074.graph_c0	1242.653	1420.56	1543.2281	432.2842	418.35904	447.23427	5.812E-17	-1.654411	down	PREDICTED: embryonic protein DC-8 [Nicotiana sylvestris]
c26079.graph_c0	2.8066639	2.7584158	2.5131561	0.8185751	1.3674778	1.2046006	0.0017668	-1.218554	down	PREDICTED: putative E3 ubiquitin-protein ligase XBAT31 [Sesamum indicum]
c26085.graph_c0	1.6270947	2.203671	1.1380251	5.4507935	4.2137913	4.9811666	0.0002313	1.606369	up	PREDICTED: cycloartenol synthase-like isoform X1 [Sesamum indicum]
c26089.graph_c1	17.378655	19.071437	16.955353	37.788407	51.516137	55.665369	1.855E-11	1.476659	up	Phosphoenolpyruvate carboxylase family protein [Theobroma cacao]
c26096.graph_c0	4.0318445	2.9212907	2.9650187	17.224817	17.111307	23.13016	1.378E-21	2.574134	up	PREDICTED: thylakoid lumenal 16.5 kDa protein, chloroplastic [Sesamum indicum]
c26098.graph_c0	13.512093	14.700185	16.448262	5.0540381	2.379618	2.6232808	2.516E-08	-2.093818	down	PREDICTED: UDP-glycosyltransferase 73C3 isoform X2 [Vitis vinifera]
c26098.graph_c1	15.687204	12.96711	16.090933	4.1309735	3.7086996	4.1741175	2.72E-10	-1.854159	down	PREDICTED: UDP-glycosyltransferase 73C3-like [Sesamum indicum]
c26107.graph_c0	1.269223	0.9256068	1.115343	7.6110208	5.4408549	6.6070639	7.902E-12	2.617091	up	PREDICTED: LOW QUALITY PROTEIN: anthranilate synthase alpha subunit 1, chloroplastic [Sesamum indicum]
c26109.graph_c0	0.9787999	0.8837645	0.8681442	1.6183056	1.942985	2.3999309	0.0066537	1.163292	up	PREDICTED: uncharacterized protein LOC105156347 [Sesamum indicum]
c26120.graph_c0	3.0273167	2.8137767	3.2694693	0.1128631	0.0797099	0.3012748	4.205E-17	-4.171176	down	PREDICTED: cytochrome P450 76C1-like [Sesamum indicum]
c26125.graph_c0	1.9504634	1.9401791	1.6455135	3.3942516	3.4626203	4.3997751	0.0035329	1.063719	up	PREDICTED: cytochrome P450 90B1 [Sesamum indicum]
c26134.graph_c0	10.125871	9.2279987	9.4187795	18.05561	22.17193	23.608589	1.514E-14	1.187381	up	PREDICTED: probable methyltransferase PMT2 [Sesamum indicum]
c26138.graph_c0	1.9131598	2.8988618	2.5121516	4.9616395	5.9059049	6.2006137	7.407E-06	1.259201	up	PREDICTED: glycerol-3-phosphate 2-O-acyltransferase 6-like [Sesamum indicum]
c26146.graph_c0	2.0684128	2.5430916	2.5855782	4.5743187	6.0869684	3.8220183	0.0070879	1.048852	up	PREDICTED: uncharacterized protein LOC100267130 [Vitis vinifera]
c26148.graph_c0	0.3211953	1.0440333	0.3145113	3.9085318	2.6741475	3.477788	5.724E-09	2.631812	up	PREDICTED: protein CHUP1, chloroplastic isoform X1 [Sesamum indicum]
c26158.graph_c0	0.3693567	0.1333978	0.4822273	3.7454945	3.4719101	3.9159442	4.817E-13	3.539472	up	PREDICTED: shikimate O-hydroxycinnamoyltransferase-like [Sesamum indicum]
c26161.graph_c0	6.5910129	7.4388334	8.0673191	0.3481081	0.1843892	0.4646167	8.58E-15	-4.425528	down	hypothetical protein MIMGU_mgv1a020481mg, partial [Erythranthe guttata]

#ID	28 h-1 FPKM	28 h-2 FPKM	28 h-3 FPKM	48 h-1 FPKM	48 h-2 FPKM	48 h-3 FPKM	FDR	log2FC	regulated	nr_annotation
c26161.graph_c1	15.575828	21.977513	20.461905	1.6677746	1.7927874	1.6149149	2.589E-29	-3.473134	down	PREDICTED: beta-galactosidase 15-like [Sesamum indicum]
c26162.graph_c1	3.0594438	2.2444426	2.4964811	7.7561406	8.4734621	10.136383	3.307E-05	1.795827	up	hypothetical protein MIMGU_mgv1a0223741mg, partial [Erythranthe guttata]
c26163.graph_c0	4.4396736	4.6099004	3.47179	10.129704	23.101874	26.038566	0.0004875	2.271188	up	PREDICTED: hyoscyamine 6-dioxygenase-like [Sesamum indicum]
c26177.graph_c0	63.293689	64.527301	59.025295	22.206355	27.027413	27.407758	2.644E-16	-1.247226	down	PREDICTED: growth-regulating factor 4-like isoform X1 [Sesamum indicum]
c26178.graph_c1	0.4322627	0.4203152	0.9767708	4.8048786	4.3757742	2.9252409	1.462E-08	2.772077	up	PREDICTED: U-box domain-containing protein 4-like [Nicotiana sylvestris]
c26182.graph_c0	0	0.0268336	0.0242506	1.7705461	4.0307141	4.6257049	1.449E-11	7.70103	up	PREDICTED: agmatine coumaroyltransferase-2-like [Sesamum indicum]
c26184.graph_c0	8.6751072	9.6311574	11.123855	40.899643	62.7635	74.332057	7.235E-10	2.629345	up	hypothetical protein MIMGU_mgv1a010851mg [Erythranthe guttata]
c26186.graph_c0	5.6015708	5.9412662	6.0852697	11.933811	10.558011	12.064899	3.519E-05	1.014411	up	PREDICTED: rop guanine nucleotide exchange factor 14 [Sesamum indicum]
c26191.graph_c0	1.5588583	0.9080664	1.1899501	2.7408585	2.4984527	2.6656645	0.0071213	1.155229	up	hypothetical protein MIMGU_mgv1a008195mg [Erythranthe guttata]
c26193.graph_c0	2.9671099	5.258821	3.0488397	0.5571897	1.4756872	1.2394601	0.0023504	-1.756474	down	PREDICTED: GATA transcription factor 2-like [Sesamum indicum]
c26203.graph_c0	1.7910369	2.004061	1.7214878	0.4456974	0.6639783	0.5700821	1.232E-06	-1.6794	down	PREDICTED: receptor-like protein kinase isoform X1 [Sesamum indicum]
c26216.graph_c0	0.6888661	0.6219815	0.6870223	4.2689218	13.875561	15.193844	9.516E-05	4.084407	up	PREDICTED: blue copper protein [Sesamum indicum]
c26227.graph_c0	0.7307305	0.560814	0.7751512	2.1303859	2.9928264	4.3269234	2.267E-05	2.225547	up	PREDICTED: probable pectinesterase 53 [Sesamum indicum]
c26233.graph_c0	3.2598036	2.8732186	3.4832982	9.6742235	10.90875	10.358859	8.693E-13	1.725924	up	PREDICTED: uncharacterized protein LOC105176469 [Sesamum indicum]
c26240.graph_c0	7.2938894	9.1122869	8.484681	3.6827107	3.3880452	4.8290477	4.813E-07	-1.024104	down	PREDICTED: uncharacterized protein LOC105167378 [Sesamum indicum]
c26248.graph_c0	9.86302	10.313675	10.518737	36.86667	36.653283	49.309094	1.823E-17	2.040088	up	PREDICTED: photosystem II reaction center W protein, chloroplastic [Sesamum indicum]
c26259.graph_c0	1.5457725	1.9938395	2.2523888	10.170118	13.838174	15.940068	3.507E-18	2.82106	up	putative cyclin-dependent kinase B1-2 [Oryza sativa Japonica Group]
c26269.graph_c0	0.6218667	0.4491899	0.4566943	1.9312415	1.5031221	0.9819469	0.0032333	1.582371	up	PREDICTED: phospholipase A1-Igamma3, chloroplastic [Sesamum indicum]
c26275.graph_c0	2.9175786	3.6612304	3.3491489	8.2740413	8.4333229	5.8562892	0.0004501	1.229247	up	PREDICTED: CASP-like protein 4A3 [Sesamum indicum]
c26282.graph_c0	5.4709327	4.2031115	5.5215556	9.2464135	9.3443475	12.286977	0.0003057	1.061978	up	PREDICTED: inorganic pyrophosphatase 3 [Sesamum indicum]
c26286.graph_c0	1.0655355	1.8259861	2.2357756	3.6660358	4.3801358	6.3768745	0.0002761	1.52724	up	PREDICTED: mini-chromosome maintenance complex-binding protein isoform X1 [Sesamum indicum]
c26289.graph_c0	0.1301537	0.1175166	0.1062044	1.8697669	1.1069128	2.1041004	3.177E-09	3.889872	up	PREDICTED: F-box/kelch-repeat protein SKIP25-like [Sesamum indicum]
c26291.graph_c0	12.368733	12.284587	10.092785	5.1215715	4.8720428	5.7661816	0.002344	-1.098665	down	PREDICTED: uncharacterized protein LOC105169169 isoform X2 [Sesamum indicum]
c26294.graph_c1	5.4957445	4.7440259	5.4208032	14.85115	10.461629	9.8086741	0.0010788	1.215023	up	PREDICTED: probable BOI-related E3 ubiquitin-protein ligase 2 [Sesamum indicum]
c26297.graph_c0	2.866032	3.4284512	3.3358533	7.8928124	8.0684352	9.0740801	7.393E-13	1.419019	up	O-Glycosyl hydrolases family 17 protein [Theobroma cacao]
c26323.graph_c0	0.1760033	0.4540413	0.3487847	1.0836145	1.418066	2.1268978	2.977E-05	2.275754	up	PREDICTED: putative glycosyltransferase 5 [Sesamum indicum]
c26325.graph_c0	291.45287	322.60597	326.45968	48.876407	49.913718	45.517006	8.463E-64	-2.661842	down	PREDICTED: uncharacterized protein LOC105175521 [Sesamum indicum]

#ID	28 h-1 FPKM	28 h-2 FPKM	28 h-3 FPKM	48 h-1 FPKM	48 h-2 FPKM	48 h-3 FPKM	FDR	log2FC	regulated	nr_annotation
c26326.graph_c0	0	0	0.0503568	1.6427262	2.4032289	2.2273339	3.346E-20	6.991393	up	PREDICTED: MLP-like protein 423 [Cucumis melo]
c26328.graph_c0	0.9811329	0.6286826	1.1105047	23.228326	19.380074	26.165726	6.463E-51	4.702053	up	PREDICTED: bifunctional L-3-cyanoalanine synthase/cysteine synthase 1, mitochondrial [Sesamum indicum]
c26333.graph_c0	23.605035	23.163675	25.605129	122.90951	135.83867	151.87693	3.197E-65	2.543369	up	4-coumarate coenzyme A ligase [Paulownia fortunei]
c26336.graph_c0	1.6431444	1.9419549	2.3436615	6.9426884	6.7808927	7.4733438	2.445E-18	1.879431	up	PREDICTED: uncharacterized protein LOC104091376 [Nicotiana tomentosiformis]
c26346.graph_c0	0.3700138	0.5396803	0.2090273	6.5301982	5.3126823	5.7784287	7.639E-27	4.024421	up	PREDICTED: G2/mitotic-specific cyclin-2-like isoform X2 [Sesamum indicum]
c26348.graph_c0	6.6828145	6.1467392	7.1094442	0.8708463	0.7548186	0.8453169	3.746E-17	-2.9687	down	hypothetical protein MIMGU_mgv1a024370mg [Erythranthe guttata]
c26351.graph_c0	0.2117855	0.1274816	0.1267312	2.8098156	3.1283358	3.7104241	4.646E-32	4.410612	up	PREDICTED: kinesin-like protein KIF22 [Sesamum indicum]
c26353.graph_c0	0.4804091	0.8218693	0.412642	1.6345604	1.5958053	2.0818197	5.188E-05	1.672526	up	PREDICTED: cytochrome P450 711A1 [Sesamum indicum]
c26367.graph_c0	0.9332333	0.9830592	0.7615107	2.5137515	2.7674469	1.8420055	0.0007304	1.455294	up	PREDICTED: dehydration-responsive protein RD22 [Sesamum indicum]
c26372.graph_c0	1.0208847	1.1137971	0.8330335	3.5586278	3.8270549	3.4063279	3.281E-06	1.904694	up	PREDICTED: protein trichome birefringence-like 38 [Sesamum indicum]
c26373.graph_c0	0.9778958	0.9329264	1.4302966	2.0113532	2.4528802	3.1215523	0.0005316	1.218598	up	PREDICTED: condensin-2 complex subunit H2 isoform X1 [Sesamum indicum]
c26376.graph_c0	0.4110318	0.5904232	0.3353986	0.7104723	0.9533684	1.2432755	0.0059977	1.156267	up	PREDICTED: putative serine/threonine-protein kinase-like protein CCR3 [Sesamum indicum]
c26388.graph_c0	0.206422	0.2631242	0.3071528	2.2625465	2.5273368	2.9855623	1.179E-21	3.361768	up	PREDICTED: kinesin-like protein KCA1 [Sesamum indicum]
c26407.graph_c0	2.2700838	1.5372546	1.6671328	7.1937459	7.0112294	7.4891136	4.758E-08	2.028508	up	PREDICTED: EPIDERMAL PATTERNING FACTOR-like protein 4 [Sesamum indicum]
c26421.graph_c0	2.4037092	2.4169518	2.1397168	9.6946132	8.656368	4.2514885	0.0053446	1.749343	up	PREDICTED: homeobox-leucine zipper protein HAT22-like [Sesamum indicum]
c26422.graph_c0	37.687836	37.75659	43.393601	16.251961	16.258167	15.441297	1.305E-16	-1.267021	down	hypothetical protein MIMGU_mgv1a006265mg [Erythranthe guttata]
c26423.graph_c0	15.775479	13.837706	12.477449	42.624293	42.22854	34.961367	1.461E-17	1.553321	up	hypothetical protein MIMGU_mgv1a008823mg [Erythranthe guttata]
c26424.graph_c0	4.0247933	5.0596614	4.6231409	11.616228	9.3957861	8.9742057	5.078E-06	1.17635	up	unnamed protein product [Coffea canephora]
c26427.graph_c0	0	0.2102695	0.0316715	1.0823764	2.8666165	2.8017223	5.133E-07	4.832445	up	hypothetical protein MIMGU_mgv1a010556mg [Erythranthe guttata]
c26431.graph_c0	9.1072013	8.1847026	8.2609538	4.1880734	3.6972966	3.8698503	0.0005876	-1.075949	down	hypothetical protein MIMGU_mgv1a020357mg [Erythranthe guttata]
c26438.graph_c0	3.4311096	4.7257173	3.9860953	0.14743	0.3123684	0.1967735	3.333E-15	-4.176973	down	PREDICTED: probable trehalose-phosphate phosphatase J [Nicotiana tomentosiformis]
c26446.graph_c0	2.7008475	2.0416287	3.3826834	10.429803	8.097059	7.7218396	1.819E-06	1.738726	up	PREDICTED: uncharacterized protein LOC105170673 [Sesamum indicum]
c26452.graph_c0	9.1731534	7.8404541	7.947788	23.614576	49.791351	75.38784	0.0022997	2.602716	up	PREDICTED: subtilisin-like protease [Sesamum indicum]
c26474.graph_c0	0.8982812	0.7394993	0.970134	4.018721	4.824994	5.2743548	1.079E-12	2.474064	up	PREDICTED: acetylornithine deacetylase [Sesamum indicum]
c26478.graph_c0	5.1150131	3.6357438	4.3514178	12.829362	20.971295	23.997076	3.021E-07	2.173366	up	PREDICTED: 21 kDa protein-like [Sesamum indicum]

#ID	28 h-1 FPKM	28 h-2 FPKM	28 h-3 FPKM	48 h-1 FPKM	48 h-2 FPKM	48 h-3 FPKM	FDR	log2FC	regulated	nr_annotation
c26489.graph_c0	24.521614	21.356807	24.104633	58.300667	81.468474	90.686734	5.196E-11	1.754354	up	PREDICTED: L-gulonolactone oxidase, partial [Sesamum indicum]
c26496.graph_c0	15.239452	16.564341	17.544025	8.4896932	7.6577649	6.815092	5.221E-09	-1.058578	down	PREDICTED: methionine gamma-lyase [Sesamum indicum]
c26497.graph_c0	0.6277148	0.8001425	0.3916901	1.1233056	1.2395896	1.832436	0.0034774	1.243157	up	PREDICTED: uncharacterized protein LOC105170157 isoform X1 [Sesamum indicum]
c26519.graph_c0	2.3982267	2.5984486	2.1441183	5.2075304	4.6766333	5.4333419	5.051E-05	1.14426	up	PREDICTED: uncharacterized protein LOC105156252 [Sesamum indicum]
c26523.graph_c0	4.5037802	3.6104362	3.1942005	9.7104198	11.643541	11.251345	2.979E-08	1.566864	up	PREDICTED: epidermal growth factor receptor substrate 15-like [Sesamum indicum]
c26525.graph_c0	1.8336725	2.4739363	2.5281664	8.4156229	8.4907924	9.0808927	6.428E-16	1.967936	up	PREDICTED: probable receptor-like protein kinase At5g15080 [Sesamum indicum]
c26527.graph_c0	4.6751961	3.9157775	4.2666889	13.645752	12.266966	10.09513	8.933E-10	1.531534	up	PREDICTED: F-box/kelch-repeat protein SKIP25-like [Sesamum indicum]
c26528.graph_c0	177.1579	175.35939	203.43487	84.570643	76.017744	67.83942	8.221E-17	-1.238196	down	unnamed protein product [Coffea canephora]
c26530.graph_c0	2.0573063	1.7718214	2.8926071	7.8634817	10.455509	11.244984	5.511E-15	2.172328	up	hypothetical protein MIMGU_mgv1a009832mg [Erythranthe guttata]
c26547.graph_c0	2.3585725	2.3851182	3.3872536	10.045272	14.822434	15.109853	3.448E-10	2.331909	up	-
c26569.graph_c0	9.2694895	9.1525892	9.5543088	22.674977	24.530933	25.434061	1.815E-10	1.416785	up	PREDICTED: uncharacterized protein LOC105155725 [Sesamum indicum]
c26580.graph_c0	3.4231284	3.7376681	3.0747347	0.739996	1.2828044	1.316888	2.858E-05	-1.583271	down	PREDICTED: transcription factor TCP5-like [Sesamum indicum]
c26585.graph_c0	1.6209402	2.0071639	2.4374992	7.8664168	5.5349463	5.6152628	5.989E-06	1.697432	up	PREDICTED: probable isoprenylcysteine alpha-carbonyl methylesterase ICMEL2 [Sesamum indicum]
c26591.graph_c0	9887.9202	10697.499	11635.043	3386.9316	2759.4004	3193.9919	1.965E-08	-1.741675	down	-
c26595.graph_c0	8.2329145	8.7167221	8.2375365	2.5468384	3.0270989	1.7687114	3.137E-07	-1.735521	down	PREDICTED: homeobox-leucine zipper protein HAT22-like [Sesamum indicum]
c26598.graph_c0	43.286369	43.908484	41.418304	11.009698	19.050992	23.148576	8.552E-12	-1.242047	down	PREDICTED: uncharacterized protein LOC105161463 [Sesamum indicum]
c26605.graph_c0	2.8066555	2.1821819	2.799143	9.437635	9.7363014	9.8044704	7.979E-11	1.936747	up	hypothetical protein MIMGU_mgv1a010920mg [Erythranthe guttata]
c26610.graph_c0	1.4619843	1.0725282	1.4539288	4.5750202	5.2761317	8.0901215	1.498E-06	2.204673	up	PREDICTED: DNA primase small subunit [Sesamum indicum]
c26612.graph_c0	5.7478571	7.2046303	5.7386031	10.714459	12.349529	15.406397	0.0004624	1.079329	up	PREDICTED: gibberellin-regulated protein 9-like [Solanum tuberosum]
c26624.graph_c0	2.267818	2.2092818	2.8488283	13.162763	17.029739	16.12103	6.131E-31	2.697594	up	PREDICTED: vinorine synthase-like [Sesamum indicum]
c26626.graph_c0	0.1944867	0.4097409	0.1057997	1.8900341	1.8281522	3.2903627	4.034E-07	3.342522	up	-
c26629.graph_c0	0.6760081	0.8392614	0.8619017	2.5171144	3.2339344	4.3364661	2.992E-07	2.120257	up	PREDICTED: lysine-rich arabinogalactan protein 19 [Sesamum indicum]
c26648.graph_c0	7.2341188	3.5426338	6.6033357	9.6360383	11.196106	17.009886	0.00612	1.157425	up	-
c26651.graph_c0	37.733157	37.186687	37.178069	13.436654	18.674626	16.891756	6.496E-11	-1.157078	down	hypothetical protein AMTR_s00062p00211520 [Amborella trichopoda]
c26655.graph_c0	7.7162346	5.2252773	6.6899084	13.6525	18.132928	16.680716	0.0004946	1.340904	up	BnaA05g08010D [Brassica napus]
c26663.graph_c0	1.6916762	1.2924365	1.5290518	21.080356	12.686299	10.978377	6.278E-06	3.363483	up	PREDICTED: uncharacterized calcium-binding protein At1g02270 [Sesamum indicum]

#ID	28 h-1 FPKM	28 h-2 FPKM	28 h-3 FPKM	48 h-1 FPKM	48 h-2 FPKM	48 h-3 FPKM	FDR	log2FC	regulated	nr_annotation
c26665.graph_c0	8.1292051	8.1584873	6.781321	21.949449	23.618007	26.04065	2.73E-21	1.674357	up	PREDICTED: uncharacterized protein LOC105157763 [Sesamum indicum]
c26667.graph_c0	3.2615588	3.0037795	4.5776173	52.587864	35.826384	56.356842	2.081E-17	3.783531	up	hypothetical protein CARUB_v10021660mg, partial [Capsella rubella]
c26682.graph_c0	2.5341116	1.9896219	1.9779094	15.502227	15.387062	21.746963	2.706E-16	3.056359	up	PREDICTED: transcription factor bHLH35-like isoform X1 [Nicotiana tomentosiformis]
c26690.graph_c0	9.6204003	8.5192749	8.1795458	2.7928114	4.3137497	4.0784791	1.103E-08	-1.19986	down	PREDICTED: putative pentatricopeptide repeat-containing protein At5g59900 [Sesamum indicum]
c26692.graph_c1	4.4297177	5.4112503	8.5049729	14.20263	14.346152	17.927496	0.0013523	1.380102	up	PREDICTED: fasciclin-like arabinogalactan protein 17 [Sesamum indicum]
c26694.graph_c0	5.1130151	4.2948609	4.4193111	12.60093	16.052481	17.260406	2.549E-22	1.76839	up	PREDICTED: putative inactive cadmium/zinc-transporting ATPase HMA3 [Sesamum indicum]
c26696.graph_c0	1.5991161	1.1594568	1.4037191	3.5318895	3.6765372	5.4928127	1.674E-06	1.647009	up	PREDICTED: glutamate synthase 1 [NADH], chloroplastic isoform X1 [Sesamum indicum]
c26708.graph_c0	11.155897	8.5194574	9.8688036	46.717901	75.32297	64.264856	5.739E-16	2.69108	up	PREDICTED: intracellular ribonuclease LX-like [Sesamum indicum]
c26715.graph_c1	3.8584223	4.2337769	4.2416498	6.5720588	8.2756014	9.8671817	1.292E-06	1.039114	up	hypothetical protein MIMGU_mgv1a001258mg [Erythranthe guttata]
c26734.graph_c0	1209.2895	1213.802	1308.5701	560.33567	351.89552	250.57226	1.048E-15	-1.627371	down	hypothetical protein MIMGU_mgv1a014589mg [Erythranthe guttata]
c26738.graph_c0	3.3681706	3.1097394	3.5336568	6.6769631	12.038439	11.625176	1.677E-05	1.631352	up	PREDICTED: UPF0496 protein At4g34320-like [Sesamum indicum]
c26739.graph_c0	24.303702	17.89939	22.164754	53.035904	65.399979	67.830385	1.265E-16	1.57051	up	PREDICTED: NAC domain-containing protein 100-like [Sesamum indicum]
c26752.graph_c0	1.520038	1.0979614	1.1726838	6.7962084	9.129591	7.761645	2.367E-19	2.681323	up	PREDICTED: cytochrome P450 84A1-like [Sesamum indicum]
c26753.graph_c0	1.1220695	0.6754157	1.2716661	6.55841	4.6039905	4.2185304	7.259E-07	2.374584	up	hypothetical protein CICLE_v10022498mg [Citrus clementina]
c26754.graph_c0	0.0313464	0.1698172	0.0511568	0.7946777	1.1365173	0.8838743	2.501E-12	3.519328	up	PREDICTED: probable receptor-like protein kinase At5g20050 [Sesamum indicum]
c26760.graph_c0	14.889546	12.607357	12.122748	52.384714	52.074008	50.940623	3.699E-40	2.014227	up	PREDICTED: NAC domain-containing protein 18 [Sesamum indicum]
c26763.graph_c0	2.0220502	1.6109309	1.6014477	2.9400236	3.2743216	5.0307988	0.0051608	1.139556	up	unnamed protein product [Coffea canephora]
c26764.graph_c0	13.396864	12.269789	12.87987	5.7943672	6.0320706	5.5003635	1.262E-12	-1.111543	down	PREDICTED: dentin sialophosphoprotein [Sesamum indicum]
c26765.graph_c0	9.5484417	8.6648898	9.4441824	26.651828	26.744952	30.078427	3.256E-16	1.634583	up	PREDICTED: uncharacterized protein LOC105158032 [Sesamum indicum]
c26773.graph_c0	71.432231	65.716122	70.53591	17.092935	17.669091	14.152656	3.225E-45	-2.042856	down	PREDICTED: flavonoid 3'-monooxygenase-like [Sesamum indicum]
c26775.graph_c0	3.0023425	2.7724437	2.6169247	10.725127	10.628941	18.085815	2.988E-06	2.270104	up	PREDICTED: transcription factor bHLH35-like isoform X2 [Sesamum indicum]
c26777.graph_c0	13.942214	13.828127	14.003618	4.8007559	5.7851188	5.1794165	5.934E-12	-1.36644	down	PREDICTED: pentatricopeptide repeat-containing protein At5g47360 [Sesamum indicum]
c26779.graph_c0	1.8384499	0.9808783	1.2955928	8.0238899	8.2197992	10.414875	2.102E-27	2.734915	up	PREDICTED: probable leucine-rich repeat receptor-like protein kinase At1g35710 [Sesamum indicum]
c26780.graph_c0	7.3479053	8.0166507	8.55655	1.5523352	1.8500748	1.7265731	9.2E-08	-2.182346	down	PREDICTED: desiccation-related protein PCC3-06 isoform X2 [Sesamum indicum]

#ID	28 h-1 FPKM	28 h-2 FPKM	28 h-3 FPKM	48 h-1 FPKM	48 h-2 FPKM	48 h-3 FPKM	FDR	log2FC	regulated	nr_annotation
c26781.graph_c0	0.3708786	0.1785966	0.3833363	1.7864407	1.5936988	2.3982899	4.727E-10	2.670473	up	PREDICTED: rop guanine nucleotide exchange factor 5-like [Sesamum indicum]
c26783.graph_c0	4.6679464	4.5127283	5.3479985	26.357393	43.688206	42.54449	2.143E-14	2.987062	up	-
c26785.graph_c0	0.5099809	0.6139532	0.6319167	7.5657229	11.36299	13.037798	2.365E-20	4.21996	up	1-deoxy-D-xylulose-5-phosphate synthase [Andrographis paniculata]
c26786.graph_c0	1.2838468	1.1229685	1.1130841	1.9325024	2.4782523	3.2580549	0.0048006	1.158771	up	hypothetical protein MIMGU_mgv1a013028mg [Erythranthe guttata]
c26790.graph_c0	0.7391871	1.084552	0.7539633	3.9821398	2.2333734	2.3969274	0.0027121	1.794594	up	-
c26792.graph_c0	1.0804157	0.9755141	0.6741727	2.9806906	4.0111073	3.1539682	8.268E-06	1.93274	up	PREDICTED: uncharacterized protein LOC105170774 [Sesamum indicum]
c26803.graph_c0	14.374274	14.557416	14.457032	36.832037	34.253916	33.439985	6.824E-18	1.312285	up	PREDICTED: probable sodium-coupled neutral amino acid transporter 6 [Sesamum indicum]
c26804.graph_c0	2.4385453	2.3176608	1.780377	0.3524865	0.7181091	0.3618928	1.445E-07	-2.156156	down	PREDICTED: NAC domain-containing protein 45 [Sesamum indicum]
c26806.graph_c0	4.2701054	4.5068915	4.2798897	1.5817858	2.3041015	1.7813209	0.0001002	-1.167052	down	PREDICTED: uncharacterized protein LOC105179467 isoform X2 [Sesamum indicum]
c26809.graph_c0	1.5165981	1.7568966	2.428364	6.4200898	6.8013067	7.0358051	8.796E-10	1.869031	up	PREDICTED: aluminum-activated malate transporter 2-like [Sesamum indicum]
c26818.graph_c0	29.068774	31.757607	31.928837	9.9340726	12.826058	12.281497	4.731E-20	-1.366735	down	PREDICTED: uncharacterized protein LOC105165319 [Sesamum indicum]
c26824.graph_c0	65.344814	57.14294	59.195638	117.42127	131.75962	135.8791	8.285E-15	1.12327	up	PREDICTED: arabinogalactan peptide 22-like [Sesamum indicum]
c26839.graph_c0	4.5419362	4.1952171	5.7083736	42.881502	54.611448	67.537949	3.926E-23	3.549393	up	PREDICTED: 36.4 kDa proline-rich protein-like, partial [Cucumis sativus]
c26851.graph_c0	1.6171796	1.2831723	1.3995815	0.248472	0.263226	0.4421778	7.143E-06	-2.1362	down	-
c26854.graph_c0	50.082148	50.141605	48.461306	176.01031	186.22352	221.2976	7.98E-42	2.012153	up	PREDICTED: lipoxygenase homology domain-containing protein 1-like [Sesamum indicum]
c26856.graph_c0	61.394857	55.085888	50.83135	114.94286	131.42668	133.27681	2.024E-14	1.221699	up	PREDICTED: dof zinc finger protein DOF1.4-like isoform X2 [Sesamum indicum]
c26866.graph_c0	0.9385272	1.5951097	0.6532084	2.0294068	2.5947195	2.7708984	0.0013308	1.25286	up	PREDICTED: methylthioribose kinase [Sesamum indicum]
c26871.graph_c0	0.6735503	0.6797001	0.7920872	1.2053288	1.4897163	1.7651469	0.0074606	1.09238	up	PREDICTED: LOW QUALITY PROTEIN: anthranilate synthase alpha subunit 1, chloroplastic [Sesamum indicum]
c26872.graph_c0	0	0.0652477	0.1769007	1.3740007	2.5715373	2.4044026	2.12E-13	4.741899	up	PREDICTED: WAT1-related protein At5g07050-like [Sesamum indicum]
c26873.graph_c0	12.433454	12.364569	12.274044	3.4165727	3.6194445	2.5987489	3.114E-11	-1.90036	down	hypothetical protein MIMGU_mgv1a015234mg [Erythranthe guttata]
c26875.graph_c0	6.9339381	7.8732995	12.516263	73.64349	61.651255	85.019902	1.01E-26	3.05308	up	PREDICTED: histone H2A [Nicotiana sylvestris]
c26877.graph_c0	0.3488699	0.4199957	0.512415	1.4445797	0.9057215	0.996824	0.001264	1.435898	up	PREDICTED: short-chain dehydrogenase TIC 32, chloroplastic [Sesamum indicum]
c26878.graph_c0	7.7148241	7.0501963	8.8896327	18.076532	24.549537	24.68031	1.183E-12	1.544605	up	PREDICTED: probable receptor-like protein kinase At1g80640 isoform X1 [Sesamum indicum]
c26889.graph_c0	8.8210575	8.7683536	8.3205208	0.4103244	0.9780501	1.0040365	5.643E-14	-3.409161	down	PREDICTED: premnaspirodiene oxygenase-like [Sesamum indicum]

#ID	28 h-1 FPKM	28 h-2 FPKM	28 h-3 FPKM	48 h-1 FPKM	48 h-2 FPKM	48 h-3 FPKM	FDR	log2FC	regulated	nr_annotation
c26894.graph_c0	0.6724567	0.7939854	0.50651	6.5568335	6.251553	8.6346619	6.375E-18	3.482917	up	hypothetical protein MIMGU_mgv1a008083mg [Erythranthe guttata]
c26902.graph_c0	0.3639131	0.219053	0.0494917	3.3058881	4.2352036	2.8047426	9.073E-16	4.073808	up	hypothetical protein MIMGU_mgv1a014026mg [Erythranthe guttata]
c26913.graph_c0	4.4765552	5.1175791	4.0947074	0.5948929	0.7756516	0.57005	9.561E-12	-2.778699	down	-
c26916.graph_c0	7.3384487	6.7278685	7.8766733	2.3612831	2.6530987	1.5280404	3.932E-06	-1.702171	down	hypothetical protein MIMGU_mgv1a026902mg, partial [Erythranthe guttata]
c26922.graph_c0	36.824366	47.323188	42.594534	8.1295466	9.1255829	9.8204798	2.869E-31	-2.187375	down	PREDICTED: uncharacterized protein LOC105157862 isoform X2 [Sesamum indicum]
c26928.graph_c0	2.946645	3.0406219	3.6311925	0.9147158	0.6460203	0.4069543	7.036E-07	-2.23628	down	PREDICTED: probable protein phosphatase 2C 51 [Sesamum indicum]
c26934.graph_c0	20.920833	20.864367	22.296059	61.033182	52.483473	60.313258	5.049E-24	1.483477	up	PREDICTED: BAG family molecular chaperone regulator 7 [Sesamum indicum]
c26940.graph_c0	44.385753	49.01547	44.993132	15.933909	20.166425	20.325834	7.286E-16	-1.256597	down	PREDICTED: DELLA protein GAI-like [Sesamum indicum]
c26940.graph_c1	16.319944	16.785519	16.790925	7.7350384	8.7660331	5.7621689	0.0023798	-1.121384	down	DELLA protein GAIP-B, putative [Ricinus communis]
c26940.graph_c2	47.37827	51.364799	47.402086	19.171267	22.540615	23.649254	5.493E-11	-1.122126	down	PREDICTED: LOW QUALITY PROTEIN: DELLA protein GAI [Sesamum indicum]
c26943.graph_c0	0.0760249	0.0686434	0.0930535	3.2282999	4.6961088	5.3163091	1.748E-27	5.833378	up	PREDICTED: dirigent protein 24-like [Sesamum indicum]
c26946.graph_c1	1.6046035	1.7897022	1.412037	3.6292105	9.2526501	8.9425376	0.0008165	2.210476	up	PREDICTED: protein NRT1/ PTR FAMILY 2.10-like [Sesamum indicum]
c26947.graph_c0	98.983739	116.01015	108.48307	12.669613	14.382646	16.826952	3.016E-79	-2.843574	down	hypothetical protein OsJ_02873 [Oryza sativa Japonica Group]
c26952.graph_c0	4.9996988	5.5744262	4.9142002	10.514466	14.546563	15.379247	6.895E-09	1.4205	up	hypothetical protein MIMGU_mgv1a006305mg [Erythranthe guttata]
c26954.graph_c0	2.597932	2.7478072	1.4839239	1.0820088	0.5482099	0.5860308	0.0016492	-1.566829	down	PREDICTED: protein NRT1/ PTR FAMILY 4.4 [Sesamum indicum]
c26957.graph_c0	0.3316144	0.4790667	0.2705946	4.5397329	1.870282	4.413448	4.745E-05	3.374954	up	PREDICTED: heat shock factor-binding protein 1 [Sesamum indicum]
c26958.graph_c0	15.534437	14.94242	15.542399	8.1138969	6.2895292	6.0751106	0.0003374	-1.120503	down	-
c26965.graph_c0	25.150027	24.998458	24.966825	6.8295435	11.697582	14.083296	8.194E-11	-1.172417	down	PREDICTED: ethylene-responsive transcription factor ERF118-like [Sesamum indicum]
c26968.graph_c0	54.60763	59.748304	55.788822	115.93751	124.09959	125.69977	2.244E-15	1.144857	up	PREDICTED: V-type proton ATPase subunit E [Sesamum indicum]
c26975.graph_c0	6.6360705	7.2633105	6.4770814	2.3260672	2.8080261	4.0191086	2.766E-05	-1.118791	down	PREDICTED: uncharacterized protein LOC105175632 [Sesamum indicum]
c26976.graph_c0	3.0246359	3.4781128	2.7009158	11.86354	14.292248	16.92839	2.341E-22	2.264501	up	PREDICTED: 12-oxophytodienoate reductase 3 [Sesamum indicum]
c26985.graph_c0	56.285353	54.161529	52.205798	174.47635	237.04452	296.24527	9.032E-10	2.156258	up	hypothetical protein MIMGU_mgv1a004588mg [Erythranthe guttata]
c26987.graph_c0	66.893128	64.694969	61.662022	31.401263	27.736633	25.390214	2.853E-15	-1.147452	down	PREDICTED: F-box protein At1g67340 [Sesamum indicum]
c26996.graph_c0	9.8176784	10.773708	8.8492345	24.582932	36.832886	41.32843	1.773E-08	1.837418	up	PREDICTED: uncharacterized protein LOC105176754 [Sesamum indicum]
c27021.graph_c0	0.0992836	0.1255014	0.1458264	0.9816248	1.6265297	1.8364717	1.204E-11	3.616053	up	PREDICTED: uncharacterized protein LOC105169333 [Sesamum indicum]
c27023.graph_c0	12.414545	13.321043	12.736116	92.593597	90.420725	107.90756	2.718E-83	2.959859	up	PREDICTED: UPF0483 protein C25G4.2-like [Sesamum indicum]
c27031.graph_c0	17.157003	13.713491	15.319644	54.993325	101.97644	107.06513	1.865E-07	2.545969	up	PREDICTED: uncharacterized protein LOC104093193 [Nicotiana tomentosiformis]

#ID	28 h-1 FPKM	28 h-2 FPKM	28 h-3 FPKM	48 h-1 FPKM	48 h-2 FPKM	48 h-3 FPKM	FDR	log2FC	regulated	nr_annotation
c27045.graph_c0	2.4528703	1.4925232	1.740454	10.679404	11.814767	13.291429	1.074E-16	2.692958	up	hypothetical protein MIMGU_mgv1a002613mg [Erythranthe guttata]
c27045.graph_c1	2.2948353	2.2662731	1.7847903	10.817371	11.893042	12.375297	2.036E-19	2.507347	up	PREDICTED: cysteine-rich receptor-like protein kinase 2 [Sesamum indicum]
c27050.graph_c0	8.1422081	8.7985179	10.531408	30.358666	29.078956	35.952147	4.822E-23	1.8366	up	hypothetical protein MIMGU_mgv1a016029mg [Erythranthe guttata]
c27052.graph_c0	28.652185	27.173523	45.199272	5.0315656	3.2466577	4.3142547	1.3E-08	-2.956659	down	PREDICTED: L-idonate 5-dehydrogenase-like [Cucumis sativus]
c27054.graph_c0	1.6649895	1.5033294	1.4979632	6.2232597	7.452718	5.8744793	4.05E-09	2.10738	up	PREDICTED: uncharacterized protein LOC105156067 [Sesamum indicum]
c27063.graph_c0	7.6427087	7.4091183	6.4825611	16.239638	14.476146	15.538781	3.278E-11	1.146986	up	PREDICTED: uncharacterized protein LOC105165907 [Sesamum indicum]
c27064.graph_c0	7.5970081	8.7401854	7.698878	2.3297824	3.9489951	5.4589695	0.0078319	-1.003257	down	PREDICTED: gibberellin 3-beta-dioxygenase 1-like [Cicer arietinum]
c27077.graph_c0	9.4000546	10.290174	8.5490734	31.650802	27.324241	30.895462	7.29E-29	1.714416	up	PREDICTED: LOW QUALITY PROTEIN: 4-coumarate--CoA ligase-like 7 [Sesamum indicum]
c27079.graph_c0	213.1842	204.3606	212.11969	968.37448	757.49496	940.22497	1.722E-20	2.127067	up	hypothetical protein MIMGU_mgv1a008423mg [Erythranthe guttata]
c27083.graph_c0	0.4936851	0.5571892	0.3021323	9.152065	11.684324	10.161915	4.496E-27	4.557473	up	MYB117 [Jatropha curcas]
c27086.graph_c0	3.2398153	3.1690203	2.2030524	70.099166	98.914714	96.428194	3.463E-62	4.982441	up	PREDICTED: flavonoid 3',5'-methyltransferase-like [Sesamum indicum]
c27087.graph_c0	6.6361784	6.8412658	6.1412258	23.720758	26.153557	29.279655	4.097E-35	2.051908	up	PREDICTED: 3-ketoacyl-CoA thiolase 2, peroxisomal [Sesamum indicum]
c27089.graph_c0	2.2170664	2.755907	2.5154037	3.5994874	6.1582491	6.6453865	0.0011633	1.163472	up	PREDICTED: high affinity nitrate transporter 2.5 [Sesamum indicum]
c27091.graph_c0	24.226534	23.645703	25.299025	2.8259738	3.792117	2.6821702	5.888E-49	-2.936976	down	PREDICTED: uncharacterized protein LOC105176468 [Sesamum indicum]
c27101.graph_c0	0.0600771	0.0361626	0.0490224	0.6092167	1.7210433	1.0615682	9.21E-06	4.572608	up	PREDICTED: UDP-glucuronate:xylan alpha-glucuronosyltransferase 1-like isoform X1 [Sesamum indicum]
c27104.graph_c0	5.0831368	5.518778	5.9290611	9.6055817	16.541152	18.00847	0.0001584	1.449204	up	PREDICTED: protein GLUTAMINE DUMPER 5-like [Sesamum indicum]
c27109.graph_c0	0.0887445	0.060096	0.1086222	1.293637	3.3367517	2.952746	2.547E-08	4.90683	up	PREDICTED: root phototropism protein 2-like [Sesamum indicum]
c27119.graph_c0	18.625535	16.897577	15.998194	2.9370344	2.7524204	0.9046231	4.534E-16	-2.914787	down	-
c27120.graph_c0	4.9087268	4.9031667	5.7082942	114.7044	62.636479	49.667591	5.99E-05	3.927405	up	PREDICTED: aureusidin synthase-like [Sesamum indicum]
c27121.graph_c0	23.800795	25.383348	26.458603	5.2530007	5.1889094	4.5477617	7.295E-21	-2.291811	down	PREDICTED: uncharacterized protein LOC105159027 [Sesamum indicum]
c27133.graph_c0	41.562378	41.333598	35.300924	100.97979	100.42717	85.841264	6.626E-19	1.324942	up	PREDICTED: chaperone protein dnaJ 11, chloroplastic-like [Sesamum indicum]
c27142.graph_c0	17.172915	15.787451	18.135779	5.9007293	8.042583	8.0677391	1.502E-09	-1.178883	down	PREDICTED: LOW QUALITY PROTEIN: uncharacterized protein LOC105176443 [Sesamum indicum]
c27145.graph_c0	11.061859	9.4129826	9.4809526	22.091776	27.517882	25.401678	7.403E-13	1.362862	up	PREDICTED: transmembrane 9 superfamily member 9-like [Sesamum indicum]
c27158.graph_c0	143.56182	171.26548	175.76402	29.087371	23.300651	27.029235	7.602E-50	-2.581823	down	hypothetical protein MIMGU_mgv1a015139mg [Erythranthe guttata]
c27166.graph_c0	7.0022703	7.0096103	7.850258	2.5855863	3.229703	3.2277624	2.733E-05	-1.235834	down	PREDICTED: probable LRR receptor-like serine/threonine-protein kinase RLK [Sesamum indicum]

#ID	28 h-1 FPKM	28 h-2 FPKM	28 h-3 FPKM	48 h-1 FPKM	48 h-2 FPKM	48 h-3 FPKM	FDR	log2FC	regulated	nr_annotation
c27172.graph_c0	3.6204053	2.7428588	3.905005	0.4747376	1.0058539	0.797902	6.026E-07	-2.141955	down	PREDICTED: uncharacterized protein LOC105161718 isoform X1 [Sesamum indicum]
c27173.graph_c0	15.948683	16.94916	16.454477	6.1810238	6.055711	5.4584857	2.029E-10	-1.436278	down	PREDICTED: uncharacterized protein LOC105179147 [Sesamum indicum]
c27176.graph_c0	1.6453853	3.1198202	1.7454073	0.6256941	0.3682484	0.4330191	0.0008554	-2.137207	down	PREDICTED: phospholipase A1-Ibeta2, chloroplastic [Sesamum indicum]
c27180.graph_c0	7.7972555	8.7675237	9.1298281	2.8658753	1.6737184	1.7654113	2.98E-12	-1.974047	down	PREDICTED: uncharacterized protein LOC105169888 [Sesamum indicum]
c27197.graph_c0	0.5143038	0.3572063	0.408907	2.2064917	1.8416749	1.8145865	6.699E-10	2.240748	up	PREDICTED: uncharacterized protein LOC105158016 [Sesamum indicum]
c27204.graph_c0	16.541039	14.067813	13.954521	29.928681	40.698077	43.180301	2.343E-11	1.388646	up	PREDICTED: serine/threonine-protein kinase-like protein ACR4 [Sesamum indicum]
c27205.graph_c0	7.4010121	7.4935808	7.8544059	2.3860025	2.9872585	2.557311	8.681E-06	-1.481342	down	PREDICTED: dnaJ homolog subfamily B member 6 isoform X1 [Sesamum indicum]
c27206.graph_c0	0.8288636	0.8887085	1.0567905	2.3639527	3.3390949	3.6225754	2.932E-05	1.783859	up	PREDICTED: uncharacterized protein LOC105160269 [Sesamum indicum]
c27216.graph_c0	7.2474511	6.1563088	5.3691632	38.015924	40.849509	41.086366	4.394E-47	2.716658	up	PREDICTED: GDSL esterase/lipase CPRD49-like isoform X1 [Sesamum indicum]
c27229.graph_c0	0.0317044	0.0286261	0	1.4065681	2.5970112	2.8606991	2.447E-15	6.863371	up	PREDICTED: probable protein S-acyltransferase 4 [Sesamum indicum]
c27232.graph_c0	6.1162937	5.1886659	4.8537346	2.9818664	2.5722683	2.0088869	0.0018121	-1.047873	down	1-aminocyclopropane-1-carboxylate oxidase-like protein [Medicago truncatula]
c27235.graph_c1	5.4301679	7.2698654	5.1185374	18.513267	16.972409	22.491972	2.16E-08	1.743951	up	PREDICTED: enolase-like [Sesamum indicum]
c27247.graph_c0	7.5512342	9.1866121	7.2778929	29.214012	32.710012	30.939739	9.196E-40	1.991924	up	PREDICTED: NADP-dependent malic enzyme-like isoform X1 [Sesamum indicum]
c27249.graph_c0	24.145795	25.868258	24.066247	6.6218651	8.8102565	8.5365797	1.65E-23	-1.590806	down	unnamed protein product [Coffea canephora]
c27253.graph_c0	4.2933429	4.1001295	3.9075635	64.782259	91.579177	94.98501	7.106E-42	4.388366	up	hypothetical protein MIMGU_mgv1a016583mg [Erythranthe guttata]
c27254.graph_c1	0.6491484	0.7256726	0.7314902	2.3509813	1.950954	3.2424279	3.685E-06	1.881502	up	PREDICTED: cytochrome P450 81E8-like [Sesamum indicum]
c27260.graph_c0	2.2610911	1.4321344	2.5610144	5.5610886	5.8912991	5.519172	1.047E-05	1.480886	up	PREDICTED: WAT1-related protein At1g43650-like [Sesamum indicum]
c27266.graph_c0	8.8119722	11.837548	11.458028	28.514835	38.481541	39.755368	3.605E-17	1.769586	up	PREDICTED: palmitoyl-protein thioesterase 1 [Sesamum indicum]
c27271.graph_c0	6.5223822	6.29417	5.9980469	18.897371	16.080457	11.754768	0.0001584	1.360765	up	PREDICTED: uncharacterized protein LOC105171966 [Sesamum indicum]
c27273.graph_c0	35.486298	36.349577	32.519135	82.112145	102.32663	92.70284	4.548E-23	1.448023	up	unnamed protein product [Coffea canephora]
c27274.graph_c1	0.3477092	0.2092992	0.094576	11.018677	10.894755	13.07247	4.746E-26	5.788407	up	TPX2 family protein, putative isoform 1 [Theobroma cacao]
c27277.graph_c0	0.7015061	1.1480271	0.7334173	1.8062092	1.6485191	2.8434264	0.0018527	1.32538	up	hypothetical protein MIMGU_mgv1a004926mg [Erythranthe guttata]
c27282.graph_c0	198.99404	247.77785	231.6465	26.52375	19.682405	18.94765	1.216E-68	-3.331512	down	hypothetical protein MIMGU_mgv1a005561mg [Erythranthe guttata]
c27288.graph_c0	33.573284	35.839523	33.825576	10.211604	17.74985	18.062177	3.661E-13	-1.13322	down	PREDICTED: pre-rRNA-processing protein esf1 [Sesamum indicum]
c27289.graph_c0	0.7699364	0.8606996	0.6581791	2.1377977	2.7570717	1.9849039	0.0001414	1.627914	up	hypothetical protein MIMGU_mgv1a007466mg [Erythranthe guttata]
c27290.graph_c0	13.550296	13.120721	15.030034	58.273999	49.671224	59.514689	7.062E-33	2.049091	up	-
c27300.graph_c0	2.0445431	2.4613743	2.0608789	5.7421925	5.1679915	5.6067405	0.000107	1.374489	up	PREDICTED: uncharacterized protein LOC105168317 [Sesamum indicum]

#ID	28 h-1 FPKM	28 h-2 FPKM	28 h-3 FPKM	48 h-1 FPKM	48 h-2 FPKM	48 h-3 FPKM	FDR	log2FC	regulated	nr_annotation
c27319.graph_c0	2.7048926	4.2464597	3.2809288	1.2973273	1.079855	0.7695693	4.129E-06	-1.651981	down	hypothetical protein MIMGU_mgv1a003421mg [Erythranthe guttata]
c27320.graph_c0	59.058994	62.027994	66.201104	187.88134	133.49638	118.29568	0.0012677	1.281401	up	PREDICTED: probable 6-phosphogluconolactonase 4, chloroplastic [Sesamum indicum]
c27324.graph_c0	28.145828	25.845047	26.073135	11.285822	12.458781	10.112445	4.21E-15	-1.199818	down	PREDICTED: lysM domain receptor-like kinase 3 [Sesamum indicum]
c27333.graph_c0	1.0002833	0.8638941	1.1711025	6.284536	7.5921184	8.0445486	2.935E-15	2.889983	up	PREDICTED: transcription factor bHLH118-like [Sesamum indicum]
c27338.graph_c0	14.772522	16.524079	15.931582	6.7387081	6.0648587	5.4123745	1.896E-07	-1.329135	down	PREDICTED: clathrin light chain 2-like [Sesamum indicum]
c27348.graph_c0	2.5337304	2.8436158	3.0336264	29.11532	38.157712	40.622674	9.036E-50	3.71766	up	PREDICTED: endoglucanase 25-like [Sesamum indicum]
c27359.graph_c0	15.990978	16.063863	16.447456	95.308058	96.084873	101.04879	2.158E-70	2.633527	up	PREDICTED: pectinesterase-like isoform X1 [Sesamum indicum]
c27363.graph_c0	11.541505	14.225351	10.538937	1.0449791	0.6150159	0.2066258	4.251E-20	-4.21986	down	-
c27369.graph_c0	2.9017847	3.3012498	3.9306022	1.0299032	0.9351922	1.6364316	0.0010397	-1.454156	down	-
c27389.graph_c0	0.8794804	0.4915785	0.9568651	27.870156	48.421083	62.398246	1.19E-14	5.926398	up	PREDICTED: expansin-A18-like [Sesamum indicum]
c27390.graph_c1	1.3002101	2.0218335	1.0609608	3.6624713	3.200954	3.910614	0.0018074	1.341614	up	unnamed protein product [Coffea canephora]
c27395.graph_c0	4.7569321	4.3854861	4.3310683	22.024499	17.078964	16.095738	8.435E-11	2.08246	up	PREDICTED: uncharacterized protein LOC100247103 isoform X1 [Vitis vinifera]
c27396.graph_c0	1220.0857	1249.2786	1362.2089	369.80582	397.56581	407.87418	4.429E-27	-1.664617	down	PREDICTED: abscisic acid 8'-hydroxylase 2 isoform X2 [Sesamum indicum]
c27410.graph_c0	0.669878	0.3316848	0.4231858	10.381174	9.0244116	13.087931	2.626E-34	4.552506	up	PREDICTED: transcription factor MYC2 [Sesamum indicum]
c27414.graph_c0	9.1774581	9.5667876	7.1612354	16.991794	16.132404	18.197355	1.732E-08	1.028874	up	PREDICTED: LOW QUALITY PROTEIN: BAG family molecular chaperone regulator 2 [Sesamum indicum]
c27415.graph_c0	0.390767	0.291465	0.4713623	3.747249	6.9128508	9.3129879	1.089E-07	4.142651	up	PREDICTED: uncharacterized protein LOC105161921 [Sesamum indicum]
c27432.graph_c0	3.8922947	9.957402	6.9873775	26.313441	29.44392	30.29127	4.315E-11	2.086355	up	hypothetical protein MIMGU_mgv1a022792mg, partial [Erythranthe guttata]
c27437.graph_c0	7.1562333	6.8612994	6.6002654	24.494797	26.950928	29.866691	2.756E-36	2.019075	up	hypothetical protein PHAVU_010G112100g [Phaseolus vulgaris]
c27451.graph_c0	3.0586196	1.9247841	2.2689169	12.101019	10.952636	8.1539642	3.98E-08	2.151838	up	PREDICTED: cyclic dof factor 1 [Sesamum indicum]
c27459.graph_c0	2.1900865	2.1100087	1.9767841	3.5670506	4.0910241	4.7057113	2.79E-05	1.016451	up	PREDICTED: filament-like plant protein 7 isoform X2 [Sesamum indicum]
c27462.graph_c0	35.277661	37.431938	35.316326	93.755713	72.902434	70.818986	4.501E-06	1.184071	up	PREDICTED: oxalate--CoA ligase [Sesamum indicum]
c27464.graph_c0	2.8507493	3.6151114	3.5023506	37.556399	51.743893	53.648568	1.673E-42	3.877948	up	PREDICTED: fasciclin-like arabinogalactan protein 2 [Sesamum indicum]
c27468.graph_c0	8.6899358	7.9359707	8.3241196	2.369388	3.6850101	4.6651019	4.99E-07	-1.18641	down	PREDICTED: probable folate-biopterin transporter 9, chloroplastic [Sesamum indicum]
c27479.graph_c0	1.3833147	1.099123	1.2190754	2.7353871	3.343628	3.1204243	0.0015859	1.352138	up	PREDICTED: putative F-box protein At5g62060 [Sesamum indicum]
c27483.graph_c0	0.6612876	0.6965941	1.0342434	4.6801215	5.1060218	5.6560492	1.038E-10	2.729359	up	PREDICTED: uncharacterized protein LOC105176460 isoform X2 [Sesamum indicum]
c27490.graph_c0	10.558493	11.862081	10.040623	0.8513783	2.0564052	2.5453724	2.203E-21	-2.546646	down	PREDICTED: cytochrome P450 76A1 [Sesamum indicum]

#ID	28 h-1 FPKM	28 h-2 FPKM	28 h-3 FPKM	48 h-1 FPKM	48 h-2 FPKM	48 h-3 FPKM	FDR	log2FC	regulated	nr_annotation
c27492.graph_c0	0.6853397	0.5862292	0.6475314	6.2639184	5.8124339	6.2652111	2.115E-18	3.29943	up	PREDICTED: heat stress transcription factor A-6b-like [Sesamum indicum]
c27503.graph_c0	1.3333014	1.1543731	0.8047946	0.0926059	0.2207356	0.1442003	3.367E-09	-2.816806	down	hypothetical protein MIMGU_mgv1a026576mg, partial [Erythranthe guttata]
c27505.graph_c0	2.6512064	3.6398736	3.3191319	124.34218	133.62745	113.42435	7.83E-189	5.314442	up	plasma membrane intrinsic protein [Olea europaea]
c27518.graph_c0	3.3128889	3.113319	2.2619368	25.110283	27.690773	30.959896	1.277E-34	3.308974	up	hypothetical protein MIMGU_mgv1a013970mg [Erythranthe guttata]
c27527.graph_c0	3.8385677	3.496812	3.7475013	10.252656	11.045538	9.934522	1.017E-08	1.536178	up	hypothetical protein MIMGU_mgv1a009219mg [Erythranthe guttata]
c27530.graph_c0	10.342059	11.611324	10.748309	20.547608	22.522163	22.9302	2.316E-11	1.053428	up	hypothetical protein MIMGU_mgv1a003692mg [Erythranthe guttata]
c27533.graph_c0	0	0	0.0340204	3.4879533	3.583092	1.9749926	4.832E-16	8.095699	up	PREDICTED: probable trehalose-phosphate phosphatase J [Sesamum indicum]
c27535.graph_c0	24.883741	19.937812	22.155781	75.937515	60.648487	40.556296	0.002416	1.453197	up	-
c27536.graph_c0	0.4371752	0.3947282	0.4459143	1.3392001	1.4676415	2.0134138	7.375E-06	1.952853	up	PREDICTED: protein IQ-DOMAIN 1 [Sesamum indicum]
c27542.graph_c0	4.1177365	3.4333945	3.8229019	1.251623	2.0594431	1.9667232	0.0011254	-1.074397	down	PREDICTED: pentatricopeptide repeat-containing protein At5g15010, mitochondrial [Sesamum indicum]
c27548.graph_c0	14.635851	17.619735	15.995591	5.0291618	7.3405067	9.016131	2.87E-08	-1.140082	down	PREDICTED: mitochondrial substrate carrier family protein B-like [Sesamum indicum]
c27550.graph_c0	0.0551023	0.0497522	0.044963	3.3526176	5.6975056	6.5256164	6.852E-18	6.73193	up	PREDICTED: galactose oxidase-like [Sesamum indicum]
c27557.graph_c1	3.4334765	2.8094723	2.8892416	5.5762362	9.6534681	8.8342489	0.0004522	1.430769	up	PREDICTED: uncharacterized protein At5g65660 [Sesamum indicum]
c27558.graph_c0	3.13504	3.4294379	3.4436864	7.7185251	15.098821	17.271742	0.0001615	2.031782	up	PREDICTED: D-amino-acid transaminase, chloroplastic-like [Sesamum indicum]
c27563.graph_c0	0.0735182	0	0.0299951	1.7240091	1.1353164	1.3681737	1.085E-16	5.399641	up	PREDICTED: uncharacterized protein LOC105158131 isoform X1 [Sesamum indicum]
c27571.graph_c0	5.3402092	4.8217082	4.122023	0.3658978	0.7752486	0.8139341	1.51E-08	-2.839672	down	-
c27575.graph_c0	3.6839394	3.0431666	4.7329526	0.1987091	0	0.0884051	1.674E-16	-5.244759	down	-
c27584.graph_c0	3.5340899	3.0705385	3.3734884	7.0154093	9.8496061	8.8745512	0.0001033	1.403592	up	PREDICTED: uncharacterized protein LOC105159891 [Sesamum indicum]
c27587.graph_c0	4.3490324	5.6340591	4.4436834	46.402712	58.654488	45.170348	9.854E-48	3.420352	up	Tubulin beta-7 chain [Gossypium arboreum]
c27592.graph_c0	0.871397	0.7867898	1.1293192	7.7968935	9.223514	11.100206	8.04E-20	3.371354	up	PREDICTED: transcription factor MYB44-like [Sesamum indicum]
c27593.graph_c0	0.5146497	0.8983822	0.67192	6.6975261	5.2292669	5.669187	2.938E-26	3.124088	up	PREDICTED: wall-associated receptor kinase 2-like [Sesamum indicum]
c27597.graph_c0	2.9678952	3.966002	3.5842318	9.6307858	8.7679021	12.452432	6.7E-05	1.593302	up	PREDICTED: uncharacterized protein LOC105165182 [Sesamum indicum]
c27601.graph_c0	44.86984	48.814748	46.676499	114.37973	126.34656	139.28003	1.017E-24	1.476339	up	PREDICTED: protein SPIRAL1-like 1-like [Oryza brachyantha]
c27602.graph_c0	12.618497	13.341946	13.818691	2.1687965	2.1670328	2.4341569	8.375E-34	-2.513721	down	hypothetical protein MIMGU_mgv1a002239mg [Erythranthe guttata]
c27606.graph_c0	0.1133648	0.2456586	0.333017	4.7707802	5.6325403	5.1400441	2.892E-32	4.528248	up	PREDICTED: rop guanine nucleotide exchange factor 3 [Sesamum indicum]
c27608.graph_c0	37.143629	35.364915	39.065244	13.756807	14.40992	12.735855	5.879E-21	-1.405644	down	PREDICTED: selenoprotein O [Sesamum indicum]
c27610.graph_c0	0.5178041	0.4921353	0.3780477	1.2781647	2.8911023	3.2582202	0.0002558	2.448227	up	PREDICTED: lecithin-cholesterol acyltransferase-like 1 [Sesamum indicum]

#ID	28 h-1 FPKM	28 h-2 FPKM	28 h-3 FPKM	48 h-1 FPKM	48 h-2 FPKM	48 h-3 FPKM	FDR	log2FC	regulated	nr_annotation
c27614.graph_c0	19.404046	18.980037	21.663165	7.8632849	9.7514626	10.677434	1.465E-08	-1.048595	down	PREDICTED: uncharacterized protein LOC105163129 [Sesamum indicum]
c27618.graph_c0	11.293354	11.667082	10.544	1.531389	2.3276779	4.0878622	8.956E-10	-2.045301	down	-
c27634.graph_c0	0.3128004	0.3177331	0.6700118	1.5859903	1.522649	1.8522061	1.045E-05	1.971245	up	PREDICTED: uncharacterized protein LOC105170116 [Sesamum indicum]
c27637.graph_c0	9.3315747	9.0082831	8.0753079	1.8748277	2.8889492	2.4568189	1.578E-11	-1.835641	down	-
c27651.graph_c0	0.8253892	1.4331713	0.8289364	2.1327233	2.472509	2.2199326	0.0044753	1.185512	up	PREDICTED: serine/threonine-protein kinase D6PKL1-like [Sesamum indicum]
c27654.graph_c0	1.0241679	0.4498675	0.9260599	2.1753759	3.6798412	4.3707919	1.535E-05	2.121773	up	cytochrome P450 [Verbena x hybrida]
c27656.graph_c0	1.0122672	1.2827822	1.0288793	4.1870335	4.3641115	3.8057176	2.818E-10	1.937179	up	PREDICTED: LOW QUALITY PROTEIN: uncharacterized protein ycf45 [Sesamum indicum]
c27662.graph_c0	1.0529686	1.1831331	1.0883375	3.8855045	3.8020055	3.1933826	5.678E-07	1.754792	up	PREDICTED: MATE efflux family protein 5-like [Sesamum indicum]
c27665.graph_c0	2.7865897	3.1450365	3.2130269	5.3367051	5.9383052	7.3449074	0.000413	1.064056	up	PREDICTED: IAA-amino acid hydrolase ILR1-like 2 [Sesamum indicum]
c27670.graph_c1	45.765674	49.327085	51.856125	18.855914	19.128534	22.311219	7.508E-18	-1.244781	down	hypothetical protein MIMGU_mgv1a001800mg [Erythranthe guttata]
c27688.graph_c0	9.4048425	8.2564641	6.9302315	20.111054	33.3397	35.231098	3.421E-07	1.883487	up	PREDICTED: allene oxide cyclase 4, chloroplastic-like [Pyrus x bretschneideri]
c27699.graph_c0	7.0859456	7.0164462	7.2115563	2.7330205	3.8302456	3.7110666	9.16E-08	-1.016702	down	PREDICTED: uncharacterized protein LOC105161282 isoform X2 [Sesamum indicum]
c27705.graph_c0	3.2558168	3.3698969	5.3782379	13.991495	16.848361	23.734749	5.966E-08	2.219605	up	hypothetical protein MIMGU_mgv1a024655mg [Erythranthe guttata]
c27724.graph_c0	10.753314	10.402751	12.260959	22.697412	27.719616	26.422675	9.289E-10	1.239685	up	PREDICTED: pollen-specific protein SF21-like [Nicotiana sylvestris]
c27729.graph_c0	0.4098086	0.3700187	0.3715561	5.6563702	4.0355891	4.3653608	1.926E-16	3.658245	up	PREDICTED: psbP-like protein 2, chloroplastic [Sesamum indicum]
c27731.graph_c0	1.0296407	1.2595515	1.0298961	0.2526088	0.0892028	0.1123849	1.158E-08	-2.807052	down	hypothetical protein MIMGU_mgv1a025181mg, partial [Erythranthe guttata]
c27738.graph_c0	0.4138195	0.7161437	0.253255	2.5353089	3.936163	5.0563335	1.886E-08	3.093052	up	PREDICTED: protein CHUP1, chloroplastic isoform X1 [Sesamum indicum]
c27745.graph_c0	0.4163635	0.5639058	0.5096238	1.3722056	1.7891513	1.3618626	0.0002422	1.641413	up	PREDICTED: equilibrative nucleotide transporter 1 [Sesamum indicum]
c27754.graph_c0	84.145254	95.214076	90.443058	36.012003	36.619171	37.311498	3.987E-17	-1.253424	down	PREDICTED: topless-related protein 4 [Sesamum indicum]
c27760.graph_c0	8.3832855	8.3803194	9.6258316	1.8975771	5.3070675	2.5664481	5.707E-05	-1.404956	down	PREDICTED: uncharacterized protein LOC105179312 [Sesamum indicum]
c27771.graph_c0	0.5634678	0.5489238	0.5928808	2.199098	2.2500306	2.7093381	1.551E-09	2.109574	up	PREDICTED: uncharacterized protein LOC105176570 isoform X1 [Sesamum indicum]
c27778.graph_c0	1.171358	0.9325308	0.9763738	2.7460494	3.7040365	2.6280952	6.63E-08	1.598258	up	PREDICTED: phosphoenolpyruvate carboxylase 4 [Sesamum indicum]
c27786.graph_c1	1.7645386	1.5078622	1.3370027	0.3195261	0.211562	0.6041638	2.338E-05	-1.98254	down	PREDICTED: pentatricopeptide repeat-containing protein At1g33350 [Sesamum indicum]
c27795.graph_c0	1.6557333	2.0149621	1.9972263	3.9237759	3.866758	6.1707621	0.0012162	1.338635	up	PREDICTED: exonuclease DPD1, chloroplastic/mitochondrial [Sesamum indicum]
c27795.graph_c1	3.7633532	2.8482859	3.4773036	6.5942761	7.6546926	7.240803	0.0015848	1.1303	up	PREDICTED: exonuclease DPD1, chloroplastic/mitochondrial [Sesamum indicum]

#ID	28 h-1 FPKM	28 h-2 FPKM	28 h-3 FPKM	48 h-1 FPKM	48 h-2 FPKM	48 h-3 FPKM	FDR	log2FC	regulated	nr_annotation
c27796.graph_c0	19.543537	15.674888	15.86254	52.510584	48.718645	54.110269	6.342E-24	1.647266	up	unnamed protein product [Coffea canephora]
c27800.graph_c0	0.1848976	0.2225936	0.1005833	2.9687033	2.4001172	4.8659843	1.853E-08	4.372704	up	PREDICTED: transcription factor bHLH130-like [Sesamum indicum]
c27801.graph_c0	6.8039252	7.0406434	6.362906	18.993229	23.679843	24.25791	1.012E-24	1.765714	up	PREDICTED: chorismate mutase 1, chloroplastic-like [Sesamum indicum]
c27807.graph_c0	39.499081	37.534863	39.099814	114.58184	142.94976	178.639	9.152E-12	1.945194	up	hypothetical protein MIMGU_mgv1a0223741mg, partial [Erythranthe guttata]
c27825.graph_c0	59.733574	61.124992	58.979698	141.88062	153.81282	178.93495	8.696E-24	1.439434	up	PREDICTED: 2-Cys peroxiredoxin BAS1, chloroplastic-like [Sesamum indicum]
c27828.graph_c0	81.903989	85.901651	86.202031	27.656791	32.067935	33.911531	3.785E-21	-1.40091	down	hypothetical protein MIMGU_mgv1a024518mg [Erythranthe guttata]
c27837.graph_c0	2.3619897	3.1054453	2.7388861	5.0950457	4.8967764	5.9824046	0.0049954	1.002252	up	PREDICTED: uncharacterized FCP1 homology domain-containing protein C1271.03c-like [Sesamum indicum]
c27846.graph_c0	4.1161336	4.1259257	5.6358375	32.27777	33.210709	22.58302	5.822E-14	2.709962	up	PREDICTED: lignin-forming anionic peroxidase-like [Sesamum indicum]
c27854.graph_c0	4.4535844	4.6319797	4.2781042	0.5002117	1.2112312	0.8265871	6.767E-08	-2.367002	down	PREDICTED: hexose carrier protein HEX6-like [Sesamum indicum]
c27870.graph_c0	77.279063	75.396011	91.310351	608.4725	514.68796	562.50003	6.083E-49	2.832816	up	PREDICTED: asparagine synthetase [glutamine-hydrolyzing] [Solanum lycopersicum]
c27881.graph_c0	20.306272	24.094664	29.693582	94.644632	76.857393	57.460247	5.99E-05	1.676189	up	PREDICTED: serine carboxypeptidase-like 50 [Sesamum indicum]
c27892.graph_c0	2.0913478	2.4032794	1.085969	10.60375	11.658894	10.793243	1.329E-13	2.608544	up	PREDICTED: transcription factor MYB86-like [Sesamum indicum]
c27892.graph_c1	1.3417285	1.0297368	1.1495815	5.697478	8.0176873	8.3988591	2.593E-11	2.686201	up	-
c27901.graph_c0	5.0267185	4.846361	4.4146075	1.3499441	2.2309592	3.0269537	0.0064184	-1.081285	down	PREDICTED: pentatricopeptide repeat-containing protein At5g18950 [Sesamum indicum]
c27911.graph_c0	0	0.0240952	0.0871033	3.0444191	4.9811312	3.2506869	1.573E-22	6.696917	up	PREDICTED: protein NRT1/ PTR FAMILY 6.3-like [Sesamum indicum]
c27912.graph_c0	11.177513	13.730653	12.115344	3.192433	4.3804896	4.4367586	2.491E-13	-1.587902	down	PREDICTED: uncharacterized protein LOC105168751 [Sesamum indicum]
c27916.graph_c0	0.5700225	0.6660524	0.6566593	9.2655832	9.2754447	13.387793	2.27E-24	4.115104	up	PREDICTED: uncharacterized protein LOC105157022 [Sesamum indicum]
c27926.graph_c0	49.098529	49.04501	45.709024	13.194195	18.794563	16.833519	1.497E-23	-1.52249	down	PREDICTED: UPF0392 protein RCOM_0530710 [Sesamum indicum]
c27942.graph_c0	6.2662454	6.4043516	5.7523557	18.202542	16.47853	17.2272	8.527E-11	1.538377	up	PREDICTED: 1-aminocyclopropane-1-carboxylate oxidase [Sesamum indicum]
c27945.graph_c0	25.5965	32.984538	28.21427	5.0333036	8.7775802	7.1657653	6.824E-18	-2.015057	down	PREDICTED: uncharacterized protein LOC105169359 [Sesamum indicum]
c27946.graph_c0	1.9149795	1.4048507	2.1485855	0.7079851	0.8035976	0.224986	0.0002209	-1.60805	down	PREDICTED: putative germin-like protein 2-1 [Sesamum indicum]
c27949.graph_c0	2.8232875	3.0756216	2.5541903	0.3889918	0.2472538	0.2076736	9.317E-13	-3.270723	down	PREDICTED: uncharacterized protein LOC105156723 [Sesamum indicum]
c27963.graph_c0	5.0779562	7.5171336	5.5408214	2.7692708	2.0615236	3.0634515	0.0034439	-1.155295	down	-
c27969.graph_c0	0.4659272	0.2734476	0.2281157	9.2428445	4.3483788	4.9660671	6.362E-06	4.318755	up	PREDICTED: S-type anion channel SLAH2-like [Sesamum indicum]
c27970.graph_c0	15.095783	16.149053	16.933401	38.173148	30.535144	24.957426	0.001844	1.007554	up	Os05g0129800 [Oryza sativa Japonica Group]
c27971.graph_c0	1.3532146	1.3440085	1.5090898	6.4609601	8.2377532	11.90485	3.091E-08	2.695379	up	PREDICTED: EH domain-containing protein 1-like [Sesamum indicum]

#ID	28 h-1 FPKM	28 h-2 FPKM	28 h-3 FPKM	48 h-1 FPKM	48 h-2 FPKM	48 h-3 FPKM	FDR	log2FC	regulated	nr_annotation
c27974.graph_c0	362.87318	377.48491	342.1091	1010.4177	848.97164	653.55039	0.000133	1.263172	up	-
c27976.graph_c0	4.8901448	3.6725744	3.9903182	7.588963	7.7941026	10.309334	0.0004716	1.072339	up	PREDICTED: homeobox-leucine zipper protein ATHB-40-like [Sesamum indicum]
c27977.graph_c0	0.1394354	0.2937599	0.3034084	3.770553	5.1802941	6.1333874	9.217E-21	4.390807	up	PREDICTED: pyruvate kinase, cytosolic isozyme-like [Sesamum indicum]
c27977.graph_c1	0.314052	0.1701357	0.2050111	3.9012241	7.0005822	6.1633032	3.777E-17	4.662415	up	Pyruvate kinase, cytosolic isozyme [Glycine soja]
c27988.graph_c0	134.4951	132.17635	140.0733	302.80185	564.28442	671.79679	0.0001853	1.949796	up	-
c27993.graph_c0	3.6483349	3.3929275	2.7686207	6.4743465	6.4178632	9.5876747	3.512E-05	1.235298	up	PREDICTED: protein ECERIFERUM 1 [Sesamum indicum]
c27998.graph_c0	1.0840066	0.5918062	1.2548134	8.9793109	9.7156047	9.4398314	3.079E-31	3.302809	up	PREDICTED: pectinesterase 2.1-like [Sesamum indicum]
c28000.graph_c0	223.66438	176.70447	184.69047	416.17577	398.93732	350.56811	6.739E-11	1.038571	up	PREDICTED: uncharacterized protein LOC105170296 [Sesamum indicum]
c28001.graph_c0	0.7308863	1.03702	0.7667965	1.3235031	2.2433458	2.060879	0.0072781	1.184505	up	ADP-ribosylation factor [Hyacinthus orientalis]
c28002.graph_c0	4.3738998	5.2423293	6.0010853	1.5700503	2.4949169	2.4884442	0.0007634	-1.219326	down	PREDICTED: DELLA protein RGL2-like [Sesamum indicum]
c28007.graph_c0	0.4423395	0.2795737	0.3970399	3.139908	4.3955363	3.4424485	1.498E-13	3.331494	up	PREDICTED: purple acid phosphatase 17-like [Sesamum indicum]
c28016.graph_c0	2.4741231	2.6175004	3.3647738	1.2988027	1.3423648	0.7610489	0.0006004	-1.268226	down	PREDICTED: uncharacterized protein LOC105177164 [Sesamum indicum]
c28020.graph_c0	12.249237	11.900725	9.586114	2.0430013	1.9719289	1.979437	4.625E-17	-2.449442	down	PREDICTED: very-long-chain 3-oxoacyl-CoA reductase-like protein At1g24470 [Sesamum indicum]
c28026.graph_c0	1.0858134	0.8169898	0.7678796	1.3304702	3.2077636	3.4698888	0.0080527	1.611706	up	PREDICTED: uncharacterized protein LOC105166116 [Sesamum indicum]
c28036.graph_c0	8.1446968	8.7838221	7.1998399	3.2979454	3.9494828	3.0620786	0.0049795	-1.1858	down	PREDICTED: MADS-box protein SVP-like isoform X2 [Nicotiana sylvestris]
c28049.graph_c0	12.245376	12.661392	11.979808	4.5063628	6.807292	6.6828909	0.0020282	-1.000564	down	-
c28052.graph_c0	0.5177232	0.3272188	0.5491953	2.0343821	4.3103622	3.503576	8.768E-07	2.850248	up	PREDICTED: pto-interacting protein 1 [Nicotiana sylvestris]
c28058.graph_c0	15.134019	13.837936	14.829527	23.928659	31.493547	30.782568	5.093E-10	1.013826	up	PREDICTED: coatomer subunit epsilon-1 [Sesamum indicum]
c28061.graph_c0	3.4973651	4.4620985	3.1019797	9.0108955	11.587866	16.764576	3.708E-05	1.791128	up	flavonoid 3-O-glucosyltransferase [Perilla frutescens]
c28062.graph_c0	7.9910251	8.1103259	8.7534756	4.373407	4.0739279	3.4888694	5.704E-06	-1.01342	down	PREDICTED: uncharacterized CRM domain-containing protein At3g25440, chloroplastic isoform X1 [Sesamum indicum]
c28068.graph_c0	44.950504	54.623826	48.171366	110.41048	103.25199	87.200379	2.602E-08	1.071124	up	hypothetical protein MIMGU_mgv1a013790mg [Erythranthe guttata]
c28070.graph_c0	27.981699	37.645884	36.464383	1.5881581	1.5889909	1.4131335	4.351E-44	-4.431995	down	-
c28072.graph_c0	83.734671	93.505546	102.76597	13.517364	14.118317	12.705284	6.277E-58	-2.753159	down	PREDICTED: uncharacterized protein LOC104888288 [Beta vulgaris subsp. vulgaris]
c28074.graph_c0	6.8725573	9.3344306	6.6624371	1.7125117	1.3409293	1.6562854	9.567E-09	-2.234059	down	PREDICTED: dof zinc finger protein DOF5.4-like [Sesamum indicum]
c28080.graph_c0	1.0276349	0.8660008	1.2717882	5.0150213	5.2438095	4.674828	3.5E-15	2.279674	up	PREDICTED: receptor-like protein kinase HSL1 [Sesamum indicum]
c28081.graph_c0	0.1386729	0.0250417	0.2036807	6.011611	3.9477704	3.9414387	1.521E-15	5.290227	up	PREDICTED: ent-kaurenoic acid oxidase 1-like [Sesamum indicum]
c28092.graph_c0	1.5288036	1.6989124	1.1995108	6.186281	14.212658	19.100006	0.0002346	3.184713	up	PREDICTED: ammonium transporter 1 member 2-like, partial [Sesamum indicum]

#ID	28 h-1 FPKM	28 h-2 FPKM	28 h-3 FPKM	48 h-1 FPKM	48 h-2 FPKM	48 h-3 FPKM	FDR	log2FC	regulated	nr_annotation
c28093.graph_c0	571.85092	623.61211	643.24863	35.895649	35.168559	27.610771	3.44E-122	-4.175371	down	hypothetical protein MIMGU_mgv1a010438mg [Erythranthe guttata]
c28104.graph_c0	369.96815	385.37216	415.39164	71.250003	54.158496	88.82859	5.649E-47	-2.40759	down	-
c28141.graph_c0	12.549707	12.633646	12.197328	21.644498	29.394596	31.99011	9.714E-09	1.187169	up	PREDICTED: U-box domain-containing protein 11-like [Sesamum indicum]
c28142.graph_c1	8.4668484	6.9744942	6.8925074	2.7466688	2.7211668	2.3760813	3.795E-09	-1.466068	down	PREDICTED: trihelix transcription factor PTL [Sesamum indicum]
c28151.graph_c0	2.9287195	3.0023291	3.422961	8.6729987	12.622753	14.698707	5.601E-09	1.978001	up	PREDICTED: uncharacterized protein LOC105163554 [Sesamum indicum]
c28155.graph_c0	1.9601369	2.4042835	2.5651648	0.1875183	0.4966323	0.3754185	7.698E-09	-2.681699	down	PREDICTED: uncharacterized protein LOC104750095 [Camelina sativa]
c28160.graph_c0	8.0515825	7.384762	7.7905441	14.118936	16.880382	22.146676	3.6E-06	1.230567	up	unnamed protein product [Coffea canephora]
c28167.graph_c0	0.4259931	0.5384846	0.4866496	1.133953	1.1440816	1.7777347	0.0004501	1.520896	up	PREDICTED: uncharacterized protein LOC105171763 [Sesamum indicum]
c28171.graph_c0	5.2272778	7.0191031	8.5308319	72.715659	95.751825	94.482996	8.972E-59	3.698372	up	Protein of unknown function UPF0057 [Medicago truncatula]
c28172.graph_c0	48.629469	47.200148	43.886833	92.751025	101.69539	109.89408	3.594E-16	1.16301	up	PREDICTED: 1,4-dihydroxy-2-naphthoyl-CoA synthase, peroxisomal [Sesamum indicum]
c28174.graph_c0	0.7062933	0.5904784	0.6830573	2.3874102	3.2317193	3.1864548	1.882E-08	2.189402	up	PREDICTED: cytochrome P450 94A1 [Sesamum indicum]
c28183.graph_c0	0.2140103	0.2810637	0.4127634	1.9975621	2.0116724	2.2601745	8.74E-12	2.828027	up	PREDICTED: LOW QUALITY PROTEIN: protein tesmin/TSO1-like CXC 2 [Sesamum indicum]
c28188.graph_c1	0.0456275	0.123592	0.0744633	1.96643	1.8993829	1.1321702	8.949E-11	4.406101	up	PREDICTED: omega-hydroxypalmitate O-feruloyl transferase [Sesamum indicum]
c28198.graph_c0	4.735642	5.3337807	4.8601847	0.3713057	0.2294561	0.0825964	4.373E-27	-4.389331	down	PREDICTED: flavonoid 3'-monooxygenase-like [Sesamum indicum]
c28206.graph_c0	1.3645587	1.2092525	1.5052448	4.6124764	6.6848112	8.3223118	1.796E-08	2.299318	up	PREDICTED: cytokinin dehydrogenase 3-like [Sesamum indicum]
c28207.graph_c0	6.0253454	8.0284363	6.5395987	1.2976452	3.4171059	4.3546307	0.0016432	-1.157257	down	PREDICTED: piriformospora indica-insensitive protein 2-like [Sesamum indicum]
c28209.graph_c0	4.4003084	3.719466	4.5073687	1.4240975	0.5028862	0.950365	6.213E-06	-2.077	down	PREDICTED: zinc finger CCCH domain-containing protein 23 [Sesamum indicum]
c28209.graph_c1	5.5052287	6.1004111	7.5551	0.5709504	1.0080879	0.5080282	3.612E-14	-3.163091	down	PREDICTED: zinc finger CCCH domain-containing protein 20-like [Sesamum indicum]
c28213.graph_c0	18.212922	17.072516	16.510914	37.825037	37.882163	35.004711	1.097E-14	1.138737	up	PREDICTED: probable mitochondrial chaperone BCS1-A [Sesamum indicum]
c28223.graph_c0	22.22045	24.986865	22.183112	50.967151	47.107703	59.487796	3.581E-11	1.224869	up	PREDICTED: 50S ribosomal protein L29, chloroplastic-like [Solanum lycopersicum]
c28226.graph_c0	4.2315633	2.7104146	3.7480426	6.189021	6.4593837	10.034893	0.0018579	1.12226	up	hypothetical protein MIMGU_mgv1a020800mg [Erythranthe guttata]
c28250.graph_c0	1.5350499	1.902822	1.7833459	0.0659589	0.1048133	0.1760696	8.871E-14	-3.881819	down	PREDICTED: probable glycosyltransferase At5g11130 [Sesamum indicum]
c28251.graph_c0	17.171052	17.673972	18.763635	56.684231	72.897992	77.102583	1.721E-27	1.98374	up	PREDICTED: fasciclin-like arabinogalactan protein 7 [Sesamum indicum]
c28256.graph_c0	2.9719861	3.8015185	3.0987598	8.5111069	10.531551	13.035719	1.117E-10	1.736857	up	PREDICTED: malate dehydrogenase [NADP], chloroplastic [Nicotiana sylvestris]
c28262.graph_c0	34.985397	32.439212	35.159407	10.74827	12.482966	12.184921	8.168E-13	-1.495001	down	hypothetical protein MIMGU_mgv1a015596mg [Erythranthe guttata]

#ID	28 h-1 FPKM	28 h-2 FPKM	28 h-3 FPKM	48 h-1 FPKM	48 h-2 FPKM	48 h-3 FPKM	FDR	log2FC	regulated	nr_annotation
c28266.graph_c0	1.1691501	0.867127	1.022161	7.4099192	6.7845658	7.3468217	2.738E-15	2.8592	up	PREDICTED: protein trichome birefringence-like 41 [Sesamum indicum]
c28274.graph_c0	12.166951	12.908914	11.636832	33.270512	37.282297	35.589834	1.231E-21	1.572138	up	PREDICTED: uncharacterized protein LOC105166742 isoform X3 [Sesamum indicum]
c28276.graph_c0	4.9760153	4.7717434	4.9284708	13.963425	11.566764	11.147279	3.45E-08	1.36767	up	PREDICTED: uncharacterized protein LOC105160853 [Sesamum indicum]
c28280.graph_c0	13.616554	13.484259	17.526702	7.9618642	5.426475	3.6165215	6.901E-06	-1.338759	down	-
c28297.graph_c0	0.0822222	0.1855973	0.6709261	5.159021	3.4779545	6.7697779	4.42E-10	4.077047	up	PREDICTED: CTD small phosphatase-like protein [Sesamum indicum]
c28308.graph_c0	2.7686853	2.3528125	3.6214042	268.58093	265.88474	341.75669	1.16E-111	6.686388	up	PREDICTED: uncharacterized oxidoreductase At4g09670-like [Sesamum indicum]
c28312.graph_c0	10.186393	9.5263045	10.024359	3.1217861	4.3638776	5.6376749	1.473E-09	-1.146214	down	PREDICTED: uncharacterized protein LOC105156537 [Sesamum indicum]
c28313.graph_c0	0.4376176	0.3647332	0.3021551	3.9683277	4.656001	5.3534428	2.568E-19	3.699905	up	malate dehydrogenase, putative [Ricinus communis]
c28318.graph_c0	8.0358907	7.3320312	8.9385278	40.503084	36.603937	34.250549	6.263E-34	2.240221	up	PREDICTED: pectinesterase 1-like [Sesamum indicum]
c28334.graph_c0	10.755455	10.260522	10.185707	24.430532	31.310437	38.761185	4.579E-09	1.63451	up	hypothetical protein MIMGU_mgv1a019816mg [Erythranthe guttata]
c28336.graph_c0	2.4395728	2.252767	2.9407646	3.7951423	5.2862038	6.5661843	0.0056264	1.069487	up	PREDICTED: mavicyanin [Sesamum indicum]
c28346.graph_c1	18.120831	19.75859	16.660153	39.731953	42.091187	46.367991	5.834E-18	1.273388	up	PREDICTED: lysM domain-containing GPI-anchored protein 1 [Sesamum indicum]
c28349.graph_c0	0.2281499	0.2832473	0.2792527	1.7713307	1.531845	1.9621078	6.065E-10	2.778121	up	PREDICTED: L-tryptophan--pyruvate aminotransferase 1-like [Sesamum indicum]
c28364.graph_c0	11.816752	8.7072272	10.861524	20.487975	26.081909	26.502549	8.467E-05	1.256357	up	PREDICTED: uncharacterized protein At3g50808-like [Malus domestica]
c28365.graph_c0	0.2980318	0.4709159	0.9119683	2.4555525	3.3017263	3.7816197	3.056E-08	2.538743	up	-
c28379.graph_c0	0.2343368	0.0906789	0.1639002	1.2305906	1.1238461	1.548064	9.71E-11	3.037023	up	PREDICTED: uncharacterized protein LOC105158982 [Sesamum indicum]
c28382.graph_c0	22.802143	23.949028	24.995972	78.182463	77.085971	85.332985	3.23E-35	1.787094	up	hypothetical protein JCGZ_02248 [Jatropha curcas]
c28389.graph_c0	3.7705965	4.6958554	5.1633252	1.5382327	2.6189535	2.2485954	0.0061729	-1.055907	down	PREDICTED: endo-1,3;1,4-beta-D-glucanase-like [Sesamum indicum]
c28401.graph_c0	9.5994765	8.9982451	10.344468	3.0652323	4.723261	3.4299439	2.524E-06	-1.331181	down	-
c28410.graph_c0	2.3307955	1.625537	2.1642425	0.8150221	0.7123191	0.7070717	0.0001463	-1.409258	down	unnamed protein product [Coffea canephora]
c28421.graph_c0	12.190781	16.998356	8.8143094	0.195604	1.0360934	0.5221417	5.668E-10	-4.415307	down	-
c28432.graph_c0	1.8161249	1.2389528	1.3502148	15.091361	10.784766	14.566185	2.393E-20	3.244502	up	PREDICTED: CASP-like protein 4B1 [Sesamum indicum]
c28441.graph_c0	0.6295947	0.6340571	0.7508569	1.8109771	2.8615074	2.6219334	1.741E-06	1.890497	up	PREDICTED: LOW QUALITY PROTEIN: protein NRT1/ PTR FAMILY 2.7-like [Sesamum indicum]
c28447.graph_c0	17.078535	11.301191	12.472353	0.5931041	0.6806821	2.6387025	7.229E-14	-3.356572	down	PREDICTED: putative expansin-B2 [Sesamum indicum]
c28449.graph_c0	12.968606	14.317213	13.33182	2.83549	5.3613158	7.1857649	1.106E-09	-1.371422	down	PREDICTED: L-type lectin-domain containing receptor kinase S.1 [Sesamum indicum]
c28450.graph_c0	6.6092414	5.9524558	6.5915508	33.383866	27.656426	27.972927	5.495E-24	2.262131	up	PREDICTED: beta-galactosidase-like [Sesamum indicum]

#ID	28 h-1 FPKM	28 h-2 FPKM	28 h-3 FPKM	48 h-1 FPKM	48 h-2 FPKM	48 h-3 FPKM	FDR	log2FC	regulated	nr_annotation
c28599.graph_c0	1.9629675	2.1210397	1.7330577	0.4486929	0.5617604	1.016258	0.0009798	-1.487823	down	PREDICTED: uncharacterized protein LOC105178333 [Sesamum indicum]
c28609.graph_c0	11.143646	11.206746	10.857462	48.93816	45.227439	58.528821	3.129E-30	2.24243	up	PREDICTED: allantoinase [Sesamum indicum]
c28711.graph_c0	86.835038	81.998279	80.93715	22.267893	30.246413	25.954968	1.311E-29	-1.632851	down	hypothetical protein MIMGU_mgv1a018814mg [Erythranthe guttata]
c28850.graph_c0	21.329796	22.931314	20.585638	6.3526415	7.1850125	7.1817324	1.893E-20	-1.606113	down	PREDICTED: cyclic dof factor 2-like [Sesamum indicum]
c28866.graph_c0	4.6660957	2.6758542	3.3444222	5.6748381	6.943209	10.525587	0.0065999	1.149452	up	unnamed protein product [Coffea canephora]
c29044.graph_c0	162.96693	165.24018	157.2878	357.47608	360.02481	398.86623	4.813E-18	1.242471	up	Gip1-like protein [Populus tomentosa]
c29248.graph_c0	3.4589166	4.429544	5.251327	12.664195	13.934321	17.951925	7.075E-13	1.799255	up	hypothetical protein MIMGU_mgv1a000889mg [Erythranthe guttata]
c29266.graph_c0	7.1974292	7.1371949	7.1291279	14.713253	18.184725	20.787281	1.543E-13	1.359669	up	PREDICTED: uncharacterized protein LOC105158344 [Sesamum indicum]
c29273.graph_c1	7.7102308	6.3780883	7.7141215	2.3051999	2.9870066	4.3904845	9.595E-07	-1.137115	down	PREDICTED: uncharacterized mitochondrial protein ymf11 [Sesamum indicum]
c29442.graph_c0	2.3779721	3.3529834	2.5783474	21.512695	22.308919	24.689701	2.506E-49	3.084532	up	PREDICTED: uncharacterized protein LOC105176814 [Sesamum indicum]
c29644.graph_c0	13.989773	14.80746	12.2789	27.642722	27.31079	32.15423	3.727E-08	1.125738	up	PREDICTED: homeobox-leucine zipper protein HOX21-like [Sesamum indicum]
c29668.graph_c0	157.60527	169.74096	158.96904	353.4881	365.64055	386.68338	5.934E-16	1.226244	up	PREDICTED: 20 kDa chaperonin, chloroplastic-like [Sesamum indicum]
c29706.graph_c0	0.3113092	1.0540614	0.9208434	2.7622515	1.9856835	2.1506054	0.0001886	1.641695	up	PREDICTED: uncharacterized protein LOC105173288 [Sesamum indicum]
c29711.graph_c0	0.5834154	0.3160616	0.2856373	7.0994109	14.101809	10.967037	1.063E-14	4.794479	up	hypothetical protein MIMGU_mgv1a021530mg [Erythranthe guttata]
c29801.graph_c0	0.4278409	0.7726004	0.0872787	5.2876185	3.8780243	5.7906352	2.161E-13	3.584929	up	PREDICTED: basic blue protein-like [Cucumis sativus]
c29807.graph_c0	69.973135	74.097242	73.332144	245.03831	321.24874	355.50936	3.083E-20	2.120355	up	PREDICTED: annexin D1-like [Sesamum indicum]
c29866.graph_c0	1.1528418	0.5782823	0.6794013	3.8156479	5.4182899	7.8015995	7.88E-07	2.853909	up	Isoflavone reductase-like protein 4 isoform 2 [Theobroma cacao]
c29900.graph_c0	15.799878	15.596151	17.036612	27.140453	41.044244	32.529223	2.255E-06	1.092194	up	PREDICTED: remorin-like [Sesamum indicum]
c29995.graph_c0	0.4027622	0	0.2957856	1.4294861	2.1092971	1.6353619	3.819E-10	2.923632	up	PREDICTED: transcription factor bHLH126-like [Sesamum indicum]
c30053.graph_c0	10.33574	11.207367	11.350268	3.5508228	3.5995254	3.0233154	5.313E-13	-1.649665	down	PREDICTED: pentatricopeptide repeat-containing protein At1g31790 [Sesamum indicum]
c30289.graph_c0	1.9000031	1.6797845	1.905684	3.2613667	8.7704418	10.893453	0.0083307	2.088449	up	PREDICTED: two-component response regulator ARR8 [Sesamum indicum]
c30990.graph_c0	0.6795975	0.5850727	0.5674424	1.4023439	2.0374126	1.0338891	0.0044505	1.327547	up	PREDICTED: sucrose synthase 5-like [Sesamum indicum]
c31084.graph_c0	4.2153545	3.8582078	3.5339331	6.733987	10.700764	9.639081	0.0001785	1.256448	up	PREDICTED: uncharacterized protein LOC105169539 [Sesamum indicum]
c31286.graph_c0	0.3006333	0.348999	0.2628367	2.123131	2.2780358	3.4150005	3.033E-12	3.136271	up	PREDICTED: kinesin KP1-like isoform X2 [Sesamum indicum]
c31599.graph_c0	36.870338	43.126922	38.487336	85.050502	73.019192	78.767025	6.255E-13	1.043899	up	PREDICTED: uncharacterized protein LOC105172751 isoform X2 [Sesamum indicum]
c31795.graph_c0	3.8752211	3.7176464	3.2362616	1.8420481	1.3416058	1.7073368	0.0036058	-1.100218	down	PREDICTED: uncharacterized protein LOC105176312 isoform X1 [Sesamum indicum]

#ID	28 h-1 FPKM	28 h-2 FPKM	28 h-3 FPKM	48 h-1 FPKM	48 h-2 FPKM	48 h-3 FPKM	FDR	log2FC	regulated	nr_annotation
c32206.graph_c0	29.693066	33.136606	32.805628	63.336923	72.496463	83.563551	1.566E-17	1.236287	up	PREDICTED: magnesium-chelatase subunit ChlI, chloroplastic-like [Sesamum indicum]
c32277.graph_c0	18.871264	15.758008	17.438495	67.194949	50.410038	40.689481	9.864E-05	1.654003	up	hypothetical protein MIMGU_mgv1a005557mg [Erythranthe guttata]
c32358.graph_c0	18.679112	23.601847	19.396893	3.727598	5.2652507	4.3916813	8.576E-28	-2.166851	down	PREDICTED: uncharacterized protein LOC105174506 [Sesamum indicum]
c32363.graph_c0	0.7058411	0.7137853	0.5298837	2.7199049	4.2462877	6.9738679	0.0002128	2.869072	up	PREDICTED: omega-3 fatty acid desaturase, endoplasmic reticulum [Sesamum indicum]
c32363.graph_c1	1.5109566	2.2037919	2.3394023	6.1877267	9.3124695	9.919183	1.142E-10	2.103986	up	PREDICTED: disease resistance response protein 206-like [Sesamum indicum]
c32388.graph_c1	26.67738	24.669723	25.064705	43.807308	54.206068	55.115465	6.772E-13	1.040817	up	hypothetical protein CICLE_v10011936mg [Citrus clementina]
c32414.graph_c0	25.099756	28.035451	26.575601	126.2078	96.54419	102.798	2.74E-14	2.077152	up	hypothetical protein MIMGU_mgv1a007303mg [Erythranthe guttata]
c32432.graph_c0	31.269916	38.88373	35.244619	70.346124	67.2304	76.040645	5.47E-13	1.061266	up	PREDICTED: ferredoxin, root R-B1-like [Sesamum indicum]
c32432.graph_c1	4.8981441	3.1993022	2.7212566	6.0766571	7.2771529	9.4034313	0.0050943	1.109668	up	PREDICTED: glucan endo-1,3-beta-glucosidase 14-like [Sesamum indicum]
c32525.graph_c0	8.4988595	7.3400353	6.532971	1.4051648	2.1502025	2.3616914	2.745E-05	-1.884802	down	hypothetical protein MIMGU_mgv1a012373mg [Erythranthe guttata]
c32527.graph_c0	12.840295	15.138383	14.17111	3.2786226	4.1555591	3.0214879	2.095E-12	-1.971164	down	PREDICTED: uncharacterized protein LOC105173965 [Sesamum indicum]
c32529.graph_c0	80.220517	77.35223	76.309875	150.73205	183.10045	172.66013	1.053E-15	1.153667	up	PREDICTED: FK506-binding protein 2-like [Sesamum indicum]
c32550.graph_c0	44.502667	52.331174	44.034072	14.391317	18.163272	14.623344	9.285E-16	-1.538532	down	hypothetical protein MIMGU_mgv1a022013mg [Erythranthe guttata]
c32583.graph_c0	9.5967749	12.171285	11.363896	24.555557	38.480999	40.225023	1.099E-07	1.673788	up	PREDICTED: caffeoylshikimate esterase [Sesamum indicum]
c32620.graph_c0	6.8888993	7.1296788	7.6209524	16.774083	18.720771	18.21154	6.175E-13	1.35154	up	tyrosine aminotransferase [Perilla frutescens]
c32871.graph_c0	4.4760851	4.290192	3.3152998	1.8330634	1.1096619	1.3203728	0.0009573	-1.451515	down	-
c32901.graph_c0	18.509731	23.346542	18.985425	7.4626106	10.245827	11.846092	9.951E-06	-1.006203	down	-
c32907.graph_c0	0.7397592	0.4624153	0.3250356	2.4524444	2.4452399	2.1179888	4.561E-07	2.243628	up	PREDICTED: LOW QUALITY PROTEIN: anthranilate synthase alpha subunit 1, chloroplastic [Sesamum indicum]
c32968.graph_c0	6.1999516	5.1901755	7.3038805	28.937389	39.091474	48.301245	4.399E-13	2.671909	up	PREDICTED: transcription factor DIVARICATA [Sesamum indicum]
c32997.graph_c0	228.16436	205.24896	211.20325	498.90502	440.50411	510.876	9.74E-17	1.213061	up	-
c32998.graph_c0	41.03611	45.356465	45.273834	21.061101	18.450046	12.896649	6.471E-17	-1.281212	down	PREDICTED: cytochrome P450 CYP82D47-like [Sesamum indicum]
c33108.graph_c0	19.436855	21.3765	19.926716	43.609053	53.690753	53.069458	6.476E-21	1.346169	up	unnamed protein product [Coffea canephora]
c33120.graph_c0	17.566748	22.533334	24.119746	261.61843	160.07675	121.43794	9.775E-05	3.135072	up	PREDICTED: universal stress protein A-like protein [Sesamum indicum]
c33186.graph_c0	19.632877	22.060617	23.083311	42.490926	41.157152	42.512004	2.341E-10	1.004101	up	PREDICTED: uncharacterized protein LOC105156000 [Sesamum indicum]
c33275.graph_c0	0.1344479	0.0242788	0.065825	0.7839442	1.5526624	1.3950977	1.39E-10	4.08492	up	PREDICTED: amino acid permease 6 [Sesamum indicum]
c33310.graph_c0	15.533513	16.261008	15.665548	24.146771	42.405236	49.059526	0.0014593	1.315803	up	PREDICTED: beta-xylosidase/alpha-L-arabinofuranosidase 2-like [Sesamum indicum]
c33366.graph_c0	22.264098	21.729568	22.885312	65.580215	86.313481	95.109267	7.054E-18	1.921047	up	PREDICTED: lamin-like protein-like [Solanum tuberosum]

#ID	28 h-1 FPKM	28 h-2 FPKM	28 h-3 FPKM	48 h-1 FPKM	48 h-2 FPKM	48 h-3 FPKM	FDR	log2FC	regulated	nr_annotation
c33432.graph_c0	2.9863243	2.7947786	2.8637038	10.554872	10.274199	11.210989	3.435E-18	1.931842	up	PREDICTED: WEB family protein At2g38370 [Sesamum indicum]
c33448.graph_c0	12.420326	11.057546	12.190211	3.9634435	5.4817508	5.877747	0.0011543	-1.183509	down	hypothetical protein MIMGU_mgv1a007817mg [Erythranthe guttata]
c33463.graph_c0	0.3365733	0.2194791	0.3356724	4.4796304	2.8624406	3.3743477	7.628E-13	3.636288	up	PREDICTED: 187-kDa microtubule-associated protein AIR9-like [Nicotiana sylvestris]
c33473.graph_c0	27.658587	21.952764	20.139166	9.3077777	10.901288	10.628573	1.66E-07	-1.138042	down	-
c33475.graph_c0	5.8934843	4.5045116	6.5537082	0.1737308	0.2576654	0.0618339	4.548E-31	-5.061279	down	PREDICTED: uncharacterized protein LOC102591997 [Solanum tuberosum]
c33480.graph_c0	1.0586357	0.9446035	0.9654661	4.6887519	5.619418	6.1948224	9.605E-25	2.512688	up	PREDICTED: uncharacterized protein LOC105159750 isoform X2 [Sesamum indicum]
c33514.graph_c0	0.4697843	0.381754	0.3450061	2.6201391	2.8388042	3.867978	2.049E-11	3.000599	up	PREDICTED: alpha-mannosidase-like [Sesamum indicum]
c33519.graph_c0	0.2234944	0.1681621	0.455924	2.6913083	3.4013299	3.7391085	4.984E-15	3.571255	up	PREDICTED: protein IQ-DOMAIN 1 [Sesamum indicum]
c33521.graph_c0	0.1168623	0.1055157	0.3814346	4.5920769	10.984916	8.3038114	2.177E-09	5.332372	up	-
c33538.graph_c0	4.4071731	4.2913632	4.4776431	0.7667631	1.7406267	0.7309941	7.119E-07	-1.9923	down	-
c33557.graph_c0	15.965597	15.879013	14.87672	45.667093	69.654162	89.230338	1.866E-06	2.163101	up	PREDICTED: prolyl endopeptidase-like [Sesamum indicum]
c33563.graph_c0	0.4223571	0.5243547	0.3446399	1.3049612	2.2331855	1.9650174	5.258E-07	2.125446	up	PREDICTED: DNA-damage-repair/toleration protein DRT100 [Sesamum indicum]
c33576.graph_c0	2.0428117	1.5510294	2.386723	0.2354013	0.1870343	0.2094587	2.745E-10	-3.197462	down	PREDICTED: pathogenesis-related protein 5-like [Sesamum indicum]
c33588.graph_c0	4.6637173	5.0024219	5.0073095	10.489766	11.913121	14.71249	1.895E-08	1.376706	up	PREDICTED: light-induced protein, chloroplastic-like [Sesamum indicum]
c33596.graph_c0	143.40524	137.01315	153.84276	55.716997	62.781564	56.844134	6.657E-18	-1.267951	down	PREDICTED: uncharacterized protein LOC105160439 [Sesamum indicum]
c33599.graph_c0	44.664023	41.912878	50.406447	13.754234	9.0690518	6.3985177	3.377E-27	-2.175228	down	hypothetical protein MIMGU_mgv1a014857mg [Erythranthe guttata]
c33599.graph_c1	14.154082	10.917424	12.188036	40.61595	42.120321	45.124647	7.438E-31	1.819584	up	PREDICTED: probable plastid-lipid-associated protein 6, chloroplastic [Nicotiana sylvestris]
c33607.graph_c0	4.3740911	5.2658589	5.1375175	24.782157	18.689068	17.154942	1.326E-08	2.085306	up	PREDICTED: neurofilament medium polypeptide [Sesamum indicum]
c33621.graph_c0	0.7237481	0.5601229	1.2936351	3.6259304	2.5453957	4.9755466	6.21E-05	2.153384	up	PREDICTED: E3 ubiquitin ligase BIG BROTHER [Sesamum indicum]
c33632.graph_c0	0.1690232	0.0872069	0.0591092	4.1931662	3.1127482	1.9608459	7.037E-09	4.929887	up	PREDICTED: probable receptor-like protein kinase At5g39020 [Nicotiana sylvestris]
c33637.graph_c0	14.211379	13.45578	13.85296	44.207254	45.388059	33.270427	1.002E-10	1.609284	up	hypothetical protein MIMGU_mgv1a025918mg [Erythranthe guttata]
c33642.graph_c0	28.408707	32.685319	30.125874	58.269554	59.878446	67.868697	1.234E-11	1.068654	up	PREDICTED: 50S ribosomal protein L18, chloroplastic [Nicotiana tomentosiformis]
c33649.graph_c0	7.862962	9.3280656	7.423126	16.760454	23.437483	25.293044	1.856E-09	1.447532	up	hypothetical protein MIMGU_mgv1a010621mg [Erythranthe guttata]
c33657.graph_c0	4.1157812	4.3918308	4.274383	13.516917	17.987345	20.256457	4.404E-16	2.053655	up	PREDICTED: uncharacterized protein LOC105180227 [Sesamum indicum]
c33657.graph_c1	0.7002528	1.1240224	0.6983784	2.3669918	3.2389069	2.9836914	4.324E-05	1.80534	up	hypothetical protein MIMGU_mgv1a008629mg [Erythranthe guttata]
c33662.graph_c0	230.54735	246.19083	270.52799	43.579015	38.491914	38.429243	1.636E-67	-2.588107	down	hypothetical protein MIMGU_mgv1a015081mg [Erythranthe guttata]

#ID	28 h-1 FPKM	28 h-2 FPKM	28 h-3 FPKM	48 h-1 FPKM	48 h-2 FPKM	48 h-3 FPKM	FDR	log2FC	regulated	nr_annotation
c33669.graph_c0	2.9567702	1.9104177	2.2798911	35.106701	35.661387	43.016896	3.492E-96	4.032777	up	PREDICTED: protein notum homolog [Sesamum indicum]
c33688.graph_c0	3.2366468	3.3168214	3.1433682	8.7591135	10.052487	10.951642	1.032E-14	1.656813	up	PREDICTED: peptidyl-prolyl cis-trans isomerase FKBP62 [Sesamum indicum]
c33689.graph_c0	0.4850995	0.2502853	0.3958372	4.3921378	3.7223499	4.6115554	1.173E-13	3.534694	up	PREDICTED: mitoferrin-like [Sesamum indicum]
c33690.graph_c0	26.499359	23.486844	23.855584	10.315006	12.826578	10.851753	1.199E-11	-1.079932	down	PREDICTED: uncharacterized protein At4g22758 [Sesamum indicum]
c33700.graph_c0	238.26003	280.23026	253.99598	70.73804	72.20253	74.281884	5.263E-30	-1.788687	down	PREDICTED: probable protein phosphatase 2C 55 [Sesamum indicum]
c33715.graph_c1	0.5063712	0.653151	0.8263895	2.4757563	1.7485091	2.3660918	6.663E-05	1.776327	up	unnamed protein product [Coffea canephora]
c33717.graph_c0	0.6956415	0.9295867	0.5676378	3.4212944	5.0069674	5.6177344	6.783E-14	2.714383	up	PREDICTED: uncharacterized protein LOC105167945 [Sesamum indicum]
c33724.graph_c0	193.13932	170.75191	188.49324	53.949621	97.180872	115.487	5.165E-06	-1.02035	down	PREDICTED: alpha-xylosidase 1-like [Sesamum indicum]
c33740.graph_c0	1.3979518	1.4161486	1.0294278	0.3457574	0.3662881	0.4999359	0.0004742	-1.62627	down	-
c33741.graph_c0	12.915104	14.382658	13.877749	6.0220419	7.2105825	6.5741428	2.335E-08	-1.016329	down	PREDICTED: probable protein arginine N-methyltransferase 3 [Sesamum indicum]
c33756.graph_c0	9.557499	8.848549	8.0165753	3.874287	3.2574105	3.3652383	2.176E-07	-1.286276	down	hypothetical protein MIMGU_mgv1a023474mg [Erythranthe guttata]
c33766.graph_c0	10.157691	8.9043133	9.4353158	5.0939959	3.9397549	3.6149626	1.819E-06	-1.12371	down	PREDICTED: NAC domain-containing protein 89-like [Sesamum indicum]
c33771.graph_c0	9.085806	9.8864271	10.17041	27.611097	22.211693	21.546433	1.994E-08	1.338971	up	PREDICTED: uncharacterized protein LOC105166582 [Sesamum indicum]
c33774.graph_c1	557.12096	621.8105	659.39554	72.538844	58.847173	48.9852	8.19E-90	-3.301423	down	hypothetical protein MIMGU_mgv1a012797mg [Erythranthe guttata]
c33780.graph_c0	13.446097	12.030529	13.55743	2.2914029	2.0183404	1.832698	2.706E-34	-2.622481	down	PREDICTED: homocysteine S-methyltransferase 2 [Sesamum indicum]
c33792.graph_c0	6.7173895	8.2225834	8.1300338	15.886648	15.377029	15.610446	1.535E-05	1.065427	up	PREDICTED: uncharacterized protein LOC105175481 [Sesamum indicum]
c33797.graph_c0	0.8859336	0.5090368	0.1971585	2.6543308	2.1630321	2.9976789	1.083E-07	2.3395	up	PREDICTED: probable BOI-related E3 ubiquitin-protein ligase 3 [Sesamum indicum]
c33801.graph_c0	2.2380426	1.7720358	1.7138414	9.5144718	8.8310595	9.0096027	9.989E-15	2.300273	up	PREDICTED: sulfate transporter 3.1-like [Sesamum indicum]
c33804.graph_c0	0.3887157	0.1619879	0.0975966	5.9127098	1.9273228	2.0572225	0.0087259	3.998429	up	PREDICTED: uncharacterized protein LOC104593265 [Nelumbo nucifera]
c33821.graph_c0	3.320957	2.3425881	2.7522155	13.286416	7.8041526	6.8767591	0.0050734	1.78694	up	PREDICTED: ras GTPase-activating protein-binding protein 1-like [Sesamum indicum]
c33845.graph_c0	25.871724	28.360186	27.015631	8.3523023	9.0111028	6.7479178	4.783E-19	-1.709585	down	PREDICTED: transcription factor TGA1 [Sesamum indicum]
c33851.graph_c0	34.078883	31.126446	35.592208	15.677855	15.72534	12.614417	2.145E-08	-1.151617	down	PREDICTED: selenoprotein O-like [Malus domestica]
c33853.graph_c0	8.9382937	10.343805	10.169914	0.6383068	1.014313	1.1359231	2.23E-12	-3.367783	down	-
c33856.graph_c0	1.4697628	1.2533326	1.0660573	0.3105054	0.3289429	0.1227937	8.8E-07	-2.265572	down	-
c33870.graph_c0	0.6347059	0.3125891	0.5179149	7.4968175	10.498896	8.9483968	8.162E-38	4.237166	up	PREDICTED: non-specific phospholipase C3-like [Sesamum indicum]
c33873.graph_c0	4.4415857	4.5270044	4.9806299	18.789817	27.47988	24.955665	2.964E-22	2.387848	up	PREDICTED: RING finger and CHY zinc finger domain-containing protein 1-like [Sesamum indicum]
c33877.graph_c0	50.737753	54.873361	55.940167	151.91658	175.95098	205.01091	4.474E-24	1.759819	up	hypothetical protein MIMGU_mgv1a007222mg [Erythranthe guttata]

#ID	28 h-1 FPKM	28 h-2 FPKM	28 h-3 FPKM	48 h-1 FPKM	48 h-2 FPKM	48 h-3 FPKM	FDR	log2FC	regulated	nr_annotation
c33886.graph_c0	0.984142	0.9289783	0.7665495	20.64	23.607615	22.653959	5.982E-62	4.682114	up	hypothetical protein MIMGU_mgv1a026334mg [Erythranthe guttata]
c33887.graph_c0	73.485134	64.115594	70.526747	466.46304	389.43196	472.24342	1.812E-41	2.717688	up	metallothionein class I type 3 [Avicennia marina]
c33905.graph_c0	92.645909	90.69626	89.267462	38.190411	36.250466	32.010665	4.694E-20	-1.312357	down	hypothetical protein MIMGU_mgv1a015035mg [Erythranthe guttata]
c33918.graph_c0	3.6170685	3.4551997	3.7642298	7.8408448	9.0103585	8.6322333	0.0001452	1.273323	up	PREDICTED: ethylene receptor 2-like [Sesamum indicum]
c33924.graph_c0	3.5870343	3.3164858	2.3650085	8.5844153	10.866735	12.655081	1.006E-13	1.829455	up	PREDICTED: F-box protein At2g32560-like [Sesamum indicum]
c33945.graph_c0	6.9810092	7.2036535	6.5932632	12.305965	15.551089	15.747473	3.7E-10	1.107179	up	PREDICTED: ethylene receptor 2-like [Sesamum indicum]
c33985.graph_c0	0.8659434	0.8048618	1.0183393	5.3268167	7.7635601	4.8833988	1.764E-11	2.778523	up	PREDICTED: PI-PLC X domain-containing protein At5g67130-like isoform X2 [Nicotiana tomentosiformis]
c33996.graph_c0	100.2461	117.33912	118.7093	10.302917	11.408011	11.032045	5.26E-94	-3.320071	down	PREDICTED: glutaredoxin [Sesamum indicum]
c33996.graph_c1	3.9173864	4.5476133	3.4705457	41.285216	43.73668	63.624088	2.583E-18	3.676681	up	hypothetical protein CICLE_v10010813mg, partial [Citrus clementina]
c34006.graph_c0	29.022469	25.385676	27.382426	11.137028	11.79833	9.7817937	2.905E-09	-1.279456	down	PREDICTED: uncharacterized protein LOC105156884 [Sesamum indicum]
c34011.graph_c0	0.2715537	0.4903751	0.3877748	1.9792323	2.0967567	1.837679	1.444E-07	2.40528	up	PREDICTED: uncharacterized protein LOC105163738 [Sesamum indicum]
c34018.graph_c0	0.0582215	0.0525686	0.1425249	6.8634078	6.2545792	3.8086785	3.877E-15	6.108402	up	unnamed protein product [Coffea canephora]
c34036.graph_c0	4.4840569	4.409612	4.5382372	9.8917003	12.882939	14.917592	7.071E-11	1.52458	up	PREDICTED: uncharacterized protein LOC105158907 [Sesamum indicum]
c34050.graph_c0	7.8659284	9.5928289	8.1242806	25.923673	35.768445	40.372852	5.027E-13	2.031737	up	PREDICTED: polygalacturonase-1 non-catalytic subunit beta-like [Sesamum indicum]
c34065.graph_c0	2.7450878	2.9319515	2.8682533	0.0424342	0.3146769	0.0755153	9.225E-17	-4.284937	down	PREDICTED: WUSCHEL-related homeobox 9 [Sesamum indicum]
c34069.graph_c0	83.987543	87.871257	86.359505	24.136365	46.179035	50.416053	7.205E-08	-1.066407	down	PREDICTED: uncharacterized protein LOC105174506 [Sesamum indicum]
c34074.graph_c0	23.343895	22.642961	20.442215	64.199378	59.149463	67.48656	1.022E-27	1.565196	up	Phosphoribulokinase family protein [Populus trichocarpa]
c34106.graph_c2	0.060479	0.1092138	0.0987008	3.1431258	6.4158804	9.2087598	4.491E-08	6.155773	up	hypothetical protein MIMGU_mgv1a008571mg [Erythranthe guttata]
c34112.graph_c0	34.745962	32.927243	31.906806	69.980965	66.518667	75.636405	2.972E-14	1.133117	up	hypothetical protein M569_08891, partial [Genlisea aurea]
c34114.graph_c0	11.986721	11.544411	14.345564	3.3764559	2.8615565	1.8026101	1.545E-06	-2.186975	down	hypothetical protein PRUPE_ppb013116mg [Prunus persica]
c34139.graph_c0	0.8774356	0.5166796	0.6537211	1.2572857	2.4589693	2.3665342	0.0003893	1.601129	up	PREDICTED: hyoscyamine 6-dioxygenase [Sesamum indicum]
c34142.graph_c0	3.7360452	3.4780593	3.995347	9.2361099	10.999816	12.30118	7.336E-13	1.575025	up	PREDICTED: probable BOI-related E3 ubiquitin-protein ligase 2 [Sesamum indicum]
c34147.graph_c0	0.0704325	0.0635939	0.344834	3.0354646	2.2699106	3.7336498	1.836E-14	4.278428	up	PREDICTED: bidirectional sugar transporter SWEET1-like [Sesamum indicum]
c34149.graph_c0	0.1570663	0.0945441	0.0854432	0.5309148	1.3123596	1.2006955	1.136E-05	3.202913	up	PREDICTED: subtilisin-like protease [Sesamum indicum]
c34153.graph_c0	4.1427475	3.0992818	4.2497062	8.40198	11.92082	13.483556	4.624E-05	1.590967	up	-
c34161.graph_c0	6.7612429	7.1969059	6.824693	19.65682	14.46575	11.08899	0.0078777	1.172243	up	PREDICTED: uncharacterized protein LOC105166332 [Sesamum indicum]
c34162.graph_c1	1.2633277	1.2193332	1.1730534	12.093036	11.465647	12.774798	5.475E-26	3.355376	up	PREDICTED: serine/threonine-protein kinase Aurora-2 [Sesamum indicum]

#ID	28 h-1 FPKM	28 h-2 FPKM	28 h-3 FPKM	48 h-1 FPKM	48 h-2 FPKM	48 h-3 FPKM	FDR	log2FC	regulated	nr_annotation
c34168.graph_c0	18.7688	17.295049	15.92101	40.768158	32.461478	28.403898	0.0003976	1.015371	up	PREDICTED: probable protein phosphatase 2C 34 [Sesamum indicum]
c34170.graph_c0	205.21889	221.28378	247.14935	81.382845	98.327067	96.697804	1.079E-17	-1.246758	down	hypothetical protein MIMGU_mgv1a014768mg [Erythranthe guttata]
c34177.graph_c0	15.5552	18.263316	17.940518	2.2991977	3.838106	4.2776074	4.614E-27	-2.280895	down	PREDICTED: cytochrome P450 71D95-like [Sesamum indicum]
c34193.graph_c0	6.9508256	7.9661863	6.6680377	15.062761	15.345113	15.238748	5.543E-12	1.122605	up	hypothetical protein MIMGU_mgv1a013779mg [Erythranthe guttata]
c34193.graph_c1	10.315603	9.9600814	8.6120706	20.104966	19.216939	20.781154	7.811E-06	1.09957	up	PREDICTED: abscisic acid receptor PYL2 [Sesamum indicum]
c34195.graph_c0	0.5212359	0.5752109	0.378066	4.2578758	8.2436988	2.6128498	0.0018933	3.394441	up	ubiquitin-conjugating enzyme e2-16kda, ubiquitin protein ligase [Thalassiosira pseudonana CCMP1335]
c34199.graph_c0	0.5716277	0.6635908	0.6330305	2.1737405	3.1800772	2.9473238	3.365E-07	2.187355	up	PREDICTED: (+)-neomenthol dehydrogenase-like [Sesamum indicum]
c34208.graph_c0	29.074371	26.548617	27.305498	225.01744	235.97231	321.15913	5.571E-25	3.275952	up	hypothetical protein JCGZ_15938 [Jatropha curcas]
c34215.graph_c0	0.141089	0.0955426	0.2014731	1.1624645	1.0420302	1.3526167	1.383E-10	3.062034	up	PREDICTED: vinorine synthase-like [Sesamum indicum]
c34231.graph_c0	33.234704	38.158524	30.408814	13.056326	17.561463	16.534185	3.817E-12	-1.072969	down	PREDICTED: uncharacterized protein LOC105161303 [Sesamum indicum]
c34240.graph_c0	0.2713276	0.4899668	0.3874519	2.6654398	2.0950109	2.0656675	1.773E-08	2.618595	up	PREDICTED: MADS-box transcription factor 23 [Sesamum indicum]
c34259.graph_c0	0.049348	0	0.080535	1.3135954	1.5903944	2.0593659	5.737E-18	5.289345	up	PREDICTED: pathogenesis-related protein 5 [Sesamum indicum]
c34261.graph_c0	13.595289	15.329947	15.541327	33.155715	34.535547	50.771285	3.396E-06	1.451492	up	PREDICTED: kinesin KP1-like isoform X2 [Sesamum indicum]
c34267.graph_c0	9.7563737	10.059982	10.79528	1.0140876	0.7074677	0.9463451	1.184E-41	-3.473802	down	hypothetical protein MIMGU_mgv1a010327mg [Erythranthe guttata]
c34277.graph_c0	16.372458	13.508416	14.511498	47.649092	49.088541	59.537442	5.532E-31	1.855473	up	PREDICTED: long chain acyl-CoA synthetase 6, peroxisomal-like isoform X2 [Sesamum indicum]
c34293.graph_c0	0.0673793	0.3346046	0.3023954	1.9643897	4.2525451	4.9777213	5.929E-07	4.018685	up	PREDICTED: GDSL esterase/lipase At5g45960 [Sesamum indicum]
c34294.graph_c1	10.134098	11.080549	10.833883	20.515203	21.216593	21.075644	3.278E-10	1.012244	up	PREDICTED: ABC transporter B family member 19 [Sesamum indicum]
c34305.graph_c0	4.9345704	4.6444737	4.5146381	13.002676	11.365179	14.032059	4.843E-11	1.48898	up	PREDICTED: monoacylglycerol lipase abhd6-B [Sesamum indicum]
c34311.graph_c1	6.0977062	6.126808	6.7873357	1.5458583	1.1337573	1.0933425	1.039E-11	-2.284431	down	PREDICTED: vesicle transport v-SNARE 13-like isoform X2 [Sesamum indicum]
c34317.graph_c0	1.258546	1.9155597	2.3180027	6.8369987	6.2772416	6.0024067	5.957E-08	1.843223	up	PREDICTED: NAC domain-containing protein 21/22-like [Sesamum indicum]
c34324.graph_c0	104.31714	106.1971	100.20963	44.476316	45.810467	37.531993	6.393E-18	-1.238235	down	PREDICTED: chaperone protein dnaJ 1, mitochondrial-like isoform X1 [Sesamum indicum]
c34331.graph_c0	1.6195835	1.949776	1.8298618	0.6843138	0.5576519	0.6557366	0.0011378	-1.463648	down	PREDICTED: putative SNAP25 homologous protein SNAP30 [Sesamum indicum]
c34335.graph_c0	91.497919	105.37933	104.87268	21.867216	17.641544	17.586422	1.273E-50	-2.355372	down	PREDICTED: uncharacterized protein LOC105176201 [Sesamum indicum]
c34336.graph_c0	68.336125	69.388829	66.173244	22.580574	27.611731	22.389085	5.593E-24	-1.450203	down	PREDICTED: membrane-anchored ubiquitin-fold protein 3 [Sesamum indicum]
c34337.graph_c0	2.0520458	3.208516	2.9405022	9.1356383	11.492745	11.685202	2.002E-10	2.016029	up	PREDICTED: MLO-like protein 4 [Sesamum indicum]

#ID	28 h-1 FPKM	28 h-2 FPKM	28 h-3 FPKM	48 h-1 FPKM	48 h-2 FPKM	48 h-3 FPKM	FDR	log2FC	regulated	nr_annotation
c34354.graph_c0	2.4339862	2.924007	3.113821	19.374366	18.696673	15.726985	4.732E-35	2.710991	up	PREDICTED: protein NRT1/ PTR FAMILY 5.5-like isoform X2 [Sesamum indicum]
c34360.graph_c0	3.3011696	4.4552823	3.1757629	6.6511138	6.999386	9.4062852	0.0001201	1.115708	up	PREDICTED: uncharacterized oxidoreductase At1g06690, chloroplastic [Sesamum indicum]
c34364.graph_c0	1.5047072	1.311761	1.5665402	0.3288495	0.2090257	0.2340867	2.757E-07	-2.455124	down	unnamed protein product [Coffea canephora]
c34383.graph_c0	5.6190887	7.4209553	6.752232	13.926295	18.619841	23.521842	2.24E-06	1.537274	up	PREDICTED: probable inactive patatin-like protein 9 [Sesamum indicum]
c34401.graph_c0	11.622163	8.1249115	8.6665743	33.078605	37.221243	38.596157	3.86E-43	1.977799	up	PREDICTED: ABC transporter G family member 28 [Sesamum indicum]
c34406.graph_c0	0.2871736	0.2074327	0.2530778	0.7280274	0.6941311	0.466412	0.0016119	1.382843	up	hypothetical protein VITISV_004365 [Vitis vinifera]
c34414.graph_c0	4.2138621	3.9471451	4.0268795	18.594859	14.010194	11.106643	2.288E-05	1.892607	up	PREDICTED: ABC transporter G family member 8-like [Sesamum indicum]
c34445.graph_c0	14.565975	16.568272	16.554441	34.615349	53.261378	59.081483	4.708E-07	1.657239	up	PREDICTED: glycerophosphodiester phosphodiesterase GDPDL3-like [Sesamum indicum]
c34445.graph_c1	9.500661	9.2020757	9.5848619	20.582219	35.103481	35.718667	1.866E-06	1.724542	up	hypothetical protein MIMGU_mgv1a001772mg [Erythranthe guttata]
c34453.graph_c0	0.997859	0.8509191	0.7991662	1.7567497	2.2829044	1.8757744	0.0015833	1.198379	up	PREDICTED: uncharacterized protein DDB_G0290685 [Sesamum indicum]
c34456.graph_c1	8.0166608	8.0920404	7.3801874	14.95595	21.474989	18.269127	1.165E-08	1.256303	up	hypothetical protein MIMGU_mgv1a023921mg, partial [Erythranthe guttata]
c34457.graph_c0	2.7897926	2.5189212	4.433083	155.96802	160.89144	151.3656	1.49E-140	5.627911	up	--
c34459.graph_c0	57.090983	57.535893	57.560492	117.39801	118.07889	123.08186	2.314E-14	1.099896	up	PREDICTED: uncharacterized protein LOC105175711 [Sesamum indicum]
c34460.graph_c0	0.4967393	0.3261884	0.9580651	11.848937	19.222929	20.220367	1.373E-22	4.879274	up	PREDICTED: transcription factor bHLH93 [Sesamum indicum]
c34472.graph_c0	0.0264072	0.0238432	0.0430961	0.6359881	1.0992801	2.1742412	0.0001081	5.415111	up	PREDICTED: protein NRT1/ PTR FAMILY 6.4 [Sesamum indicum]
c34487.graph_c0	10.623813	11.146922	11.8077	26.282825	26.416857	21.485646	1.789E-10	1.187248	up	PREDICTED: SNAP25 homologous protein SNAP33 [Sesamum indicum]
c34489.graph_c0	4.0845134	5.5791802	7.3495371	24.161301	41.628608	15.828625	0.0044547	2.299305	up	hypothetical protein PAXINDRAFT_10917 [Paxillus involutus ATCC 200175]
c34498.graph_c0	23.400818	24.174913	24.893627	54.911577	66.124441	61.9352	1.241E-20	1.375217	up	PREDICTED: cytochrome P450 71D95-like [Sesamum indicum]
c34501.graph_c0	5.6364736	6.4887391	5.0592481	22.237709	16.84077	12.396662	0.0006225	1.633755	up	PREDICTED: probable galactinol--sucrose galactosyltransferase 1 [Sesamum indicum]
c34502.graph_c0	0.7827235	0.9187437	0.8941744	5.5560943	4.3619525	4.6347931	9.425E-11	2.533682	up	PREDICTED: NAC domain-containing protein 72-like [Sesamum indicum]
c34503.graph_c0	0.5672195	0.5761643	0.3471349	3.5650046	3.0784785	3.4386843	1.655E-13	2.802788	up	hypothetical protein CISIN_1g004365mg [Citrus sinensis]
c34508.graph_c0	4.2125988	3.9883711	3.6183644	13.100835	17.01635	17.27397	1.733E-32	2.040731	up	PREDICTED: cellulose synthase-like protein G2 [Sesamum indicum]
c34523.graph_c0	2.6415065	2.7738968	2.8583112	5.4591915	6.1689082	5.7642995	0.0003131	1.112115	up	Serine carboxypeptidase-like 40 [Theobroma cacao]
c34529.graph_c0	4.2058511	5.4032846	4.3536612	13.891652	11.779671	12.76731	3.161E-13	1.506057	up	PREDICTED: acetolactate synthase small subunit 1, chloroplastic-like isoform X2 [Sesamum indicum]
c34532.graph_c0	0.4785318	0.8024145	0.5020432	3.2928318	3.1211607	5.4743652	5.406E-07	2.775845	up	hypothetical protein MIMGU_mgv1a017766mg, partial [Erythranthe guttata]
c34533.graph_c0	5.1260801	4.9550782	6.397284	50.376163	55.230032	69.311184	4.217E-39	3.445979	up	PREDICTED: GDSL esterase/lipase At2g40250 [Sesamum indicum]

#ID	28 h-1 FPKM	28 h-2 FPKM	28 h-3 FPKM	48 h-1 FPKM	48 h-2 FPKM	48 h-3 FPKM	FDR	log2FC	regulated	nr_annotation
c34533.graph_c1	1.574233	0.8122199	1.1010525	17.103911	16.307568	24.223507	6.196E-22	4.08573	up	PREDICTED: GDSL esterase/lipase At2g30310-like [Cucumis melo]
c34550.graph_c0	0.3725152	0.3027117	0.3799617	0.8027238	0.9254228	1.2394492	0.0003237	1.528378	up	PREDICTED: cell division control protein 45 homolog [Sesamum indicum]
c34563.graph_c0	12.780832	14.231646	13.247067	26.845181	27.507756	32.858751	1.058E-15	1.155404	up	PREDICTED: rho guanine nucleotide exchange factor 8-like [Sesamum indicum]
c34601.graph_c0	3.2647526	3.1320008	2.9970129	12.285621	11.302609	8.1124509	1.267E-07	1.8014	up	PREDICTED: uncharacterized protein LOC105178335 [Sesamum indicum]
c34613.graph_c0	6137.9993	6406.6292	6827.4128	1309.8043	1381.2781	1122.9639	1.603E-08	-2.301914	down	hypothetical protein MIMGU_mgv1a024110mg [Erythranthe guttata]
c34616.graph_c0	0.3931247	0.2615456	0.236369	1.022856	0.9724543	0.8634578	7.397E-05	1.726208	up	Cysteine-rich RLK (RECEPTOR-like protein kinase) 8 [Theobroma cacao]
c34619.graph_c1	2.5414073	2.9830483	2.2811444	8.7622651	7.7809672	4.8728817	0.000835	1.504924	up	PREDICTED: uncharacterized protein LOC105177084 [Sesamum indicum]
c34624.graph_c0	1.0494794	1.6244254	1.2845499	2.8506267	3.1205564	2.8746665	0.0072607	1.201505	up	PREDICTED: zinc finger Ran-binding domain-containing protein 2-like isoform X2 [Sesamum indicum]
c34632.graph_c0	8.4765794	11.172433	11.210018	3.2933912	4.6228569	4.798595	2.228E-06	-1.243649	down	PREDICTED: exocyst complex component EXO70A1-like [Sesamum indicum]
c34632.graph_c2	5.2683748	4.636422	4.2989508	13.187042	13.790968	13.990241	4.276E-07	1.569516	up	PREDICTED: cyclin-D3-1 [Sesamum indicum]
c34640.graph_c0	25.51132	27.082204	22.907442	50.535765	50.741436	56.162878	4.474E-12	1.101686	up	PREDICTED: 50S ribosomal protein L24, chloroplastic [Sesamum indicum]
c34644.graph_c0	6.1405686	5.3294602	6.8750832	24.376618	26.335439	33.233203	6.107E-23	2.232259	up	PREDICTED: cytokinin riboside 5'-monophosphate phosphoribohydrolase LOG3-like isoform X1 [Nicotiana sylvestris]
c34647.graph_c0	21.672217	18.948509	25.291587	74.330639	98.254243	119.57814	6.034E-12	2.183051	up	PREDICTED: probable pectinesterase 15 [Sesamum indicum]
c34662.graph_c0	3.2279073	3.5719032	2.7923789	8.1524566	9.0602185	9.0332916	1.31E-09	1.492687	up	PREDICTED: probable GABA transporter 2 [Sesamum indicum]
c34678.graph_c0	25.448598	22.075003	25.512328	8.064493	10.496119	12.506285	2.676E-05	-1.197644	down	-
c34685.graph_c0	0.1469088	0.2321286	0.2697219	0.9776433	0.937057	0.911325	1.56E-06	2.165899	up	PREDICTED: uncharacterized protein LOC105169662 [Sesamum indicum]
c34687.graph_c0	66.924947	72.160036	77.466332	19.739739	19.601944	17.917944	2.432E-37	-1.876208	down	PREDICTED: probable fructokinase-7 [Sesamum indicum]
c34693.graph_c0	53.318069	56.223319	53.748746	149.1054	116.04571	115.15892	4.475E-07	1.267158	up	PREDICTED: sterol 14-demethylase-like [Sesamum indicum]
c34705.graph_c0	0.0824481	0.0744429	0.1569795	0.9754172	1.0333362	0.6509403	1.714E-10	3.126244	up	PREDICTED: abscisic acid 8'-hydroxylase 4 [Sesamum indicum]
c34730.graph_c0	13.619301	17.69137	14.625441	31.106814	34.161632	32.207232	2.2E-12	1.126528	up	PREDICTED: methylsterol monooxygenase 2-2 [Sesamum indicum]
c34731.graph_c0	1.9332004	1.4624449	1.8332826	0.2649161	0.561293	0.2946509	2.865E-06	-2.189646	down	hypothetical protein MIMGU_mgv1a016001mg [Erythranthe guttata]
c34735.graph_c0	7.5357785	6.6952358	8.1660496	2.9802721	3.4001013	3.7397578	0.0029274	-1.107599	down	PREDICTED: peroxisomal membrane protein 13 [Glycine max]
c34743.graph_c0	3.5507001	2.9053915	3.7122198	0.351623	0.5215026	0.4380208	8.742E-10	-2.919469	down	PREDICTED: uncharacterized protein LOC105168020 [Sesamum indicum]
c34762.graph_c0	7.2149279	6.3321823	5.7226419	12.343163	12.669574	12.576242	0.0003148	1.005844	up	TPA: hypothetical protein ZEAMMB73_114394 [Zea mays]
c34789.graph_c0	2.9633279	3.4400665	3.1089232	1.1805314	1.3643235	0.8594425	0.0012852	-1.439495	down	-
c34792.graph_c0	2.4006588	2.256648	3.9446705	0.6669615	0.6624045	1.4465547	0.0037419	-1.59781	down	unnamed protein product [Coffea canephora]
c34794.graph_c0	54.44811	57.574208	51.715287	182.19993	207.09438	296.32676	7.76E-09	2.102809	up	hypothetical protein MIMGU_mgv1a016621mg [Erythranthe guttata]

#ID	28 h-1 FPKM	28 h-2 FPKM	28 h-3 FPKM	48 h-1 FPKM	48 h-2 FPKM	48 h-3 FPKM	FDR	log2FC	regulated	nr_annotation
c34806.graph_c0	0.0342557	0.0309297	0.0279524	1.215807	2.5760001	3.2068169	2.611E-09	6.259953	up	PREDICTED: O-acyltransferase WSD1-like [Sesamum indicum]
c34819.graph_c0	0.8837088	1.1018704	0.6867611	1.5468958	1.8082743	2.8952253	0.0047824	1.261755	up	PREDICTED: uncharacterized protein LOC105178720 [Sesamum indicum]
c34821.graph_c0	99.499347	106.24235	107.97082	18.736822	21.904284	18.214247	7.046E-59	-2.373659	down	PREDICTED: protein lin-12 [Sesamum indicum]
c34834.graph_c0	3.962405	4.1739603	3.6524204	29.391746	33.534671	39.422009	6.081E-51	3.15647	up	PREDICTED: allene oxide synthase, chloroplastic [Sesamum indicum]
c34847.graph_c0	57.415185	57.101795	61.260812	19.839372	29.135951	27.141136	5.427E-16	-1.17203	down	PREDICTED: DNA-directed RNA polymerase III subunit RPC5 [Sesamum indicum]
c34849.graph_c0	3.1561673	2.2740215	2.6664565	9.2944964	16.374978	18.356219	2.978E-07	2.474139	up	PREDICTED: uncharacterized protein LOC105171410 [Sesamum indicum]
c34854.graph_c0	60.119651	64.041484	58.620631	132.98925	213.45655	276.61632	0.0001318	1.801219	up	chalcone isomerase [Camellia nitidissima]
c34858.graph_c0	17.68392	17.378362	19.851125	46.627073	45.787808	47.108443	3.913E-17	1.387298	up	PREDICTED: rac-like GTP-binding protein 5 isoform X2 [Sesamum indicum]
c34859.graph_c0	0.0892366	0.0805723	0.0364082	3.6762036	4.3738141	6.2401834	2.872E-21	6.151692	up	hypothetical protein MIMGU_mgv1a004092mg [Erythranthe guttata]
c34859.graph_c1	0	0.0882364	0.2392281	2.6013431	5.3803863	4.7395409	1.997E-11	5.307723	up	PREDICTED: cytochrome P450 78A9-like, partial [Cucumis melo]
c34867.graph_c0	1.4274417	1.398535	1.1399982	0.3079808	0.3262684	0.8563734	0.0036751	-1.379257	down	PREDICTED: pentatricopeptide repeat-containing protein At3g03580 [Sesamum indicum]
c34868.graph_c0	87.823126	77.611727	85.723153	225.81809	230.78901	251.26136	4.185E-26	1.53571	up	hypothetical protein VITISV_041718 [Vitis vinifera]
c34872.graph_c0	10.8707	9.0843022	11.481195	1.6124845	1.811761	1.999995	7.391E-16	-2.496246	down	PREDICTED: polyol transporter 5-like [Sesamum indicum]
c34900.graph_c0	9.9152868	9.7936303	9.0535805	26.501127	34.023238	38.172464	1.952E-17	1.815472	up	PREDICTED: sulfate transporter 3.1 [Sesamum indicum]
c34901.graph_c0	0.4537814	0.4682538	0.4760768	2.382977	2.1762717	2.1203586	3.891E-07	2.300443	up	PREDICTED: stress enhanced protein 2, chloroplastic [Sesamum indicum]
c34902.graph_c0	457.86811	550.11184	547.0116	73.330181	65.273873	64.850896	2.755E-67	-2.889596	down	PREDICTED: desiccation-related protein PCC3-06 isoform X2 [Sesamum indicum]
c34907.graph_c1	0.1999122	0.361004	0.3262535	12.036666	11.543361	9.9209835	2.767E-50	5.283254	up	PREDICTED: ethylene-responsive transcription factor RAP2-12-like [Sesamum indicum]
c34919.graph_c0	2.7159951	2.4120875	2.9791942	0	0.0597893	0.0502183	3.636E-19	-6.192037	down	PREDICTED: 3-oxoacyl-[acyl-carrier-protein] reductase, chloroplastic-like isoform X2 [Sesamum indicum]
c34938.graph_c0	24.705253	25.60798	27.009908	12.85256	12.307456	12.304354	4.001E-12	-1.002473	down	unnamed protein product [Coffea canephora]
c34942.graph_c0	1.7048467	1.6419378	1.391141	0.3601698	0.8394237	0.6409538	0.0037807	-1.334401	down	-
c34943.graph_c0	4.6683693	8.3992062	5.2658679	1.0877761	2.1203551	2.0906567	0.0005618	-1.759249	down	PREDICTED: uncharacterized protein LOC104095569 [Nicotiana tomentosiformis]
c34951.graph_c0	0.423119	0.3502004	0.4603489	2.1006436	3.36174	3.4598975	2.273E-11	2.887468	up	PREDICTED: uncharacterized protein LOC105174266 [Sesamum indicum]
c34953.graph_c0	28.078187	41.643989	27.651897	95.839547	69.617454	54.700671	0.0083732	1.228552	up	-
c34966.graph_c0	17.566347	39.06009	20.538262	551.68088	619.64617	490.88233	2.718E-85	4.471879	up	-
c34990.graph_c0	4.6674789	3.1926483	2.7699088	24.561913	34.567205	30.469439	1.348E-19	3.112282	up	PREDICTED: nuclear valosin-containing protein-like [Sesamum indicum]
c34990.graph_c1	5.3142921	4.3838357	4.235572	25.512733	40.565178	42.634214	1.14E-14	2.997355	up	PREDICTED: nuclear valosin-containing protein-like [Sesamum indicum]

#ID	28 h-1 FPKM	28 h-2 FPKM	28 h-3 FPKM	48 h-1 FPKM	48 h-2 FPKM	48 h-3 FPKM	FDR	log2FC	regulated	nr_annotation
c35007.graph_c0	11.983142	13.975386	11.272029	1.2657929	6.0342935	3.7543164	5.349E-05	-1.729745	down	PREDICTED: LOW QUALITY PROTEIN: piriformospora indica-insensitive protein 2 [Sesamum indicum]
c35007.graph_c1	6.4253013	6.3391395	7.0332838	1.668638	2.693668	2.7927329	5.463E-06	-1.435222	down	PREDICTED: LOW QUALITY PROTEIN: piriformospora indica-insensitive protein 2 [Sesamum indicum]
c35027.graph_c0	1.5205343	1.0751626	1.749	0.208994	0.1968034	0.103312	3.068E-11	-3.045664	down	PREDICTED: uncharacterized protein LOC104748542 [Camelina sativa]
c35028.graph_c0	2.0973201	2.1451882	2.6473158	9.6163207	12.761661	12.899499	8.581E-33	2.392573	up	PREDICTED: subtilisin-like protease [Sesamum indicum]
c35040.graph_c0	3.9223446	4.3587808	5.0717218	8.872859	8.9135259	8.4395005	0.0037818	1.016074	up	PREDICTED: serine/threonine-protein kinase tricorner [Sesamum indicum]
c35065.graph_c0	2.9894418	2.7180611	3.0022894	10.414054	14.31689	15.585409	9.616E-18	2.246069	up	PREDICTED: cytokinin dehydrogenase 7 [Sesamum indicum]
c35067.graph_c0	25.642938	28.338513	25.370868	10.496184	14.060704	13.738017	4.787E-10	-1.014108	down	PREDICTED: nuclear transcription factor Y subunit A-1-like [Sesamum indicum]
c35074.graph_c0	4.4365122	4.6449707	3.5431331	12.683026	12.897416	11.072351	6.235E-14	1.581214	up	PREDICTED: protease Do-like 1, chloroplastic isoform X2 [Cucumis melo]
c35094.graph_c0	18.941739	13.617553	16.272864	31.439574	34.842159	37.487769	5.047E-08	1.126773	up	PREDICTED: zeatin O-glucosyltransferase-like [Sesamum indicum]
c35097.graph_c0	5.2718929	8.0478773	8.2932023	0.6200269	1.0947389	0.7968946	7.704E-13	-3.071306	down	PREDICTED: B3 domain-containing transcription factor FUS3-like isoform X2 [Sesamum indicum]
c35104.graph_c1	0.1247791	0.3379914	0.6109121	4.2704992	3.5187258	6.7553147	6.441E-08	3.797887	up	PREDICTED: uncharacterized protein LOC102597032 [Solanum tuberosum]
c35122.graph_c0	19.251347	18.375428	17.691261	7.8436609	8.5248367	8.4526379	1.426E-10	-1.115522	down	PREDICTED: cytochrome P450 82C2 [Sesamum indicum]
c35128.graph_c0	15.516803	10.804929	12.054202	38.829334	51.514824	61.2215	8.275E-12	2.017051	up	PREDICTED: anthocyanidin 3-O-glucosyltransferase 2-like [Sesamum indicum]
c35138.graph_c0	187.02551	187.82525	182.54046	693.88849	571.69706	680.26781	2.73E-24	1.848107	up	hypothetical protein JCGZ_08206 [Jatropha curcas]
c35141.graph_c1	11.839821	11.03371	12.532396	35.982665	26.626023	30.408256	1.706E-08	1.440337	up	hypothetical protein MIMGU_mgv1a011475mg [Erythranthe guttata]
c35155.graph_c0	2.5525198	2.4733216	2.6416448	0.4734888	0	0.2808717	1.883E-10	-3.276086	down	PREDICTED: SUN domain-containing protein 2-like [Sesamum indicum]
c35160.graph_c0	0.0583397	0.0351168	0.1110776	0.7394982	0.5744997	0.7457345	7.78E-12	3.374902	up	PREDICTED: DNA cross-link repair protein SNM1 [Sesamum indicum]
c35164.graph_c0	0.1446515	0.1959102	0.0885259	1.1001389	1.4082687	1.631495	6.723E-12	3.307822	up	PREDICTED: uncharacterized protein LOC105166066 [Sesamum indicum]
c35180.graph_c0	66.421205	70.309313	69.54694	20.789296	22.633814	26.133178	1.037E-23	-1.529058	down	PREDICTED: protein ABSCISIC ACID-INSENSITIVE 5 [Sesamum indicum]
c35185.graph_c0	2.6937725	3.6704471	2.6377156	0.8070766	1.0523075	1.6019866	0.00258	-1.344538	down	PREDICTED: 40S ribosomal protein S23 [Musa acuminata subsp. malaccensis]
c35192.graph_c0	62.398211	65.160109	58.652434	113.45535	123.53221	134.04687	6.134E-13	1.034587	up	Triosephosphate isomerase, chloroplastic [Gossypium arboreum]
c35195.graph_c0	0.6299815	1.2893122	0.6511424	7.3998776	8.3468519	6.2054102	3.035E-17	3.137577	up	PREDICTED: transcription factor bHLH118-like [Sesamum indicum]
c35198.graph_c0	8.2676529	8.3344084	10.579483	1.4886163	1.7977897	2.56965	1.04E-16	-2.179474	down	PREDICTED: uncharacterized protein LOC105165319 [Sesamum indicum]
c35200.graph_c0	11.037672	12.941608	10.031103	20.09143	25.288437	27.907365	5.093E-08	1.14501	up	PREDICTED: probable flavin-containing monooxygenase 1 [Sesamum indicum]
c35206.graph_c0	16.518114	18.155181	19.279583	2.0319506	2.4333799	2.4761978	1.032E-32	-2.919945	down	hypothetical protein MIMGU_mgv1a006135mg [Erythranthe guttata]

#ID	28 h-1 FPKM	28 h-2 FPKM	28 h-3 FPKM	48 h-1 FPKM	48 h-2 FPKM	48 h-3 FPKM	FDR	log2FC	regulated	nr_annotation
c35212.graph_c0	4.6391277	6.4908822	5.7504777	13.017759	17.785296	16.016664	2.95E-11	1.509019	up	PREDICTED: cytokinin riboside 5'-monophosphate phosphoribohydrolase LOG8-like [Sesamum indicum]
c35224.graph_c0	39.003129	40.298139	38.409213	16.752828	17.327694	14.741982	3.357E-17	-1.22673	down	PREDICTED: ruvB-like 2 [Sesamum indicum]
c35243.graph_c0	3.1911553	3.2040212	2.4789667	12.846997	11.210117	13.360373	4.584E-21	2.119701	up	PREDICTED: lycopene epsilon cyclase, chloroplastic [Sesamum indicum]
c35244.graph_c0	6.5427996	6.9816318	6.8677296	0.942441	2.1166121	2.7169937	1.616E-08	-1.792521	down	PREDICTED: zinc finger CCCH domain-containing protein 39-like isoform X2 [Sesamum indicum]
c35247.graph_c0	0.4388243	0.2311267	0.3282373	3.3838065	3.6829445	3.2171161	1.415E-14	3.406093	up	PREDICTED: protein TRANSPARENT TESTA 12-like [Sesamum indicum]
c35275.graph_c0	2.068668	2.3135414	2.1100203	6.1979015	6.4238738	7.2780184	2.997E-16	1.656471	up	PREDICTED: serine/threonine-protein kinase Nek5 [Sesamum indicum]
c35292.graph_c0	257.82198	252.78009	283.87738	38.634204	34.665662	32.782519	1.555E-80	-2.860207	down	hypothetical protein MIMGU_mgv1a0189502mg, partial [Erythranthe guttata]
c35295.graph_c1	2.3383094	3.6155569	2.5520053	4.9276157	5.7304575	8.6702257	0.0020957	1.220497	up	PREDICTED: ras-related protein RABA6a-like [Sesamum indicum]
c35297.graph_c0	2.8945216	1.611647	2.5193664	5.5646793	8.0976689	8.7602013	6.044E-07	1.708329	up	PREDICTED: MATE efflux family protein 9 [Sesamum indicum]
c35304.graph_c0	1.6162921	1.5242207	1.6119659	0.4552821	0.627011	0.6886823	0.0022535	-1.38875	down	PREDICTED: uncharacterized protein LOC105174699 [Sesamum indicum]
c35308.graph_c0	98.545567	106.67246	98.586742	20.679086	35.456596	48.867868	8.628E-15	-1.502139	down	PREDICTED: GDP-L-galactose phosphorylase 2-like [Sesamum indicum]
c35346.graph_c0	0.5509655	0.5685373	0.5780357	2.1949379	3.1708234	3.7285357	4.831E-08	2.455653	up	hypothetical protein MIMGU_mgv1a024479mg [Erythranthe guttata]
c35347.graph_c0	11.073155	8.9495394	10.186206	3.7324301	4.5109665	5.3792365	0.000174	-1.112061	down	-
c35357.graph_c0	0	0.1096835	0.1982506	9.546927	7.0959807	10.481485	2.836E-33	6.503469	up	--
c35359.graph_c0	3.1838191	4.148287	4.834198	10.06377	12.339018	10.31834	2.335E-07	1.466627	up	PREDICTED: sphinganine C(4)-monooxygenase 1-like [Sesamum indicum]
c35370.graph_c0	0.5996024	0.6590771	0.6381791	8.8230756	11.727483	10.555837	2.391E-43	4.073147	up	PREDICTED: protein STICHEL-like 2 [Sesamum indicum]
c35372.graph_c0	5.0774227	5.7258678	6.4768199	13.187248	14.33207	13.954499	3.12E-12	1.30359	up	PREDICTED: vacuolar amino acid transporter 1 isoform X2 [Sesamum indicum]
c35374.graph_c0	2.8799527	2.266952	2.289761	6.3650654	7.6850538	8.8701922	6.955E-08	1.661293	up	PREDICTED: mitogen-activated protein kinase 19 isoform X2 [Sesamum indicum]
c35376.graph_c0	1.4019808	0.8190841	1.4804769	6.1676306	9.7454134	10.045685	5.627E-12	2.842801	up	PREDICTED: uncharacterized protein LOC105165561 [Sesamum indicum]
c35381.graph_c0	0.1964202	0.3063302	0.2477013	2.0144511	1.5825663	2.3160825	2.852E-13	3.02169	up	PREDICTED: uncharacterized protein LOC105165535 [Sesamum indicum]
c35381.graph_c1	0.3893533	0.2163382	0.3177091	1.746351	1.6489551	2.1281573	2.739E-09	2.620986	up	PREDICTED: uncharacterized protein LOC105165535 [Sesamum indicum]
c35382.graph_c0	0.7501702	0.6956397	0.6948534	4.548881	5.6085395	8.0036635	3.825E-11	3.11998	up	PREDICTED: bidirectional sugar transporter SWEET3b [Sesamum indicum]
c35387.graph_c0	8.5123329	8.6856224	7.9060096	25.220549	17.88952	15.650223	0.0015383	1.277588	up	PREDICTED: transcription factor bHLH121 [Sesamum indicum]
c35410.graph_c0	0.0696211	0.0419075	0.0378735	0.9119152	0.5921035	0.8637661	4.12E-14	4.033644	up	PREDICTED: serine/threonine protein phosphatase 2A 57 kDa regulatory subunit B' beta isoform-like [Nicotiana tomentosiformis]
c35413.graph_c0	0.8275783	0.8621834	0.9869728	2.1787266	2.2226116	2.4412224	0.0016206	1.394749	up	PREDICTED: inositol hexakisphosphate and diphosphoinositol-pentakisphosphate kinase 2-like isoform X2 [Sesamum indicum]

#ID	28 h-1 FPKM	28 h-2 FPKM	28 h-3 FPKM	48 h-1 FPKM	48 h-2 FPKM	48 h-3 FPKM	FDR	log2FC	regulated	nr_annotation
c35415.graph_c0	1.749718	2.1327723	1.8560823	4.71303	4.5229653	4.2429625	0.0010028	1.275685	up	hypothetical protein MIMGU_mgv1a019839mg, partial [Erythranthe guttata]
c35424.graph_c0	4.2977703	4.2801347	4.7536005	1.2669164	1.4763587	1.8680881	3.917E-08	-1.494519	down	PREDICTED: transcription factor bHLH13-like [Sesamum indicum]
c35476.graph_c0	1.9135843	1.3943546	1.7258344	0.5532091	1.0368718	0.6058365	0.0084407	-1.162972	down	hypothetical protein MIMGU_mgv1a012316mg [Erythranthe guttata]
c35493.graph_c0	0	0.0194217	0	1.3360237	1.1553919	1.6497436	4.674E-25	7.78266	up	PREDICTED: probable inactive purple acid phosphatase 27 isoform X1 [Sesamum indicum]
c35495.graph_c0	10.257668	9.4575204	13.130378	1.7593031	2.6209242	2.2258278	1.351E-15	-2.278462	down	PREDICTED: CBL-interacting protein kinase 18-like [Sesamum indicum]
c35503.graph_c0	20.743341	18.783035	18.286337	4.7532393	8.0327906	8.8280411	1.512E-12	-1.387202	down	hypothetical protein MIMGU_mgv1a013110mg [Erythranthe guttata]
c35510.graph_c0	10.985052	14.561161	10.870885	3.2588933	5.6493856	6.5903265	0.0026248	-1.200608	down	PREDICTED: beta-glucosidase 24-like [Sesamum indicum]
c35510.graph_c1	26.090285	26.561004	21.241832	83.455239	90.918811	110.20199	3.642E-27	1.984499	up	PREDICTED: NADPH--cytochrome P450 reductase-like [Sesamum indicum]
c35517.graph_c0	7.0622619	6.8561345	7.2877092	3.092034	3.011471	3.7053438	1.454E-05	-1.07163	down	PREDICTED: putative lysine-specific demethylase JMJ16 isoform X1 [Sesamum indicum]
c35535.graph_c0	0.8198599	0.5978995	0.9005755	3.5573737	3.5145427	5.6549148	2.61E-08	2.49405	up	PREDICTED: peregrin [Sesamum indicum]
c35540.graph_c0	9.6644972	11.496336	12.267346	3.4353147	2.677975	3.6334625	3.848E-08	-1.734004	down	PREDICTED: ras-related protein RABC2a-like [Sesamum indicum]
c35549.graph_c0	7.530832	9.0928455	5.7595227	37.771754	38.428286	38.538855	1.633E-50	2.400388	up	allene oxide cyclase [Salvia miltiorrhiza]
c35552.graph_c0	2.0468251	1.8095892	1.5310098	0.0540521	0.2863083	0.4328572	2.415E-08	-2.781127	down	PREDICTED: probable glycosyltransferase At3g07620 [Sesamum indicum]
c35552.graph_c1	4.5195173	3.5079705	5.3053845	0.6030338	1.81005	0.7154345	3.478E-06	-2.063839	down	hypothetical protein MIMGU_mgv1a009597mg [Erythranthe guttata]
c35583.graph_c0	76.960639	90.055163	109.89869	235.47343	170.46129	153.40065	0.0051843	1.063689	up	PREDICTED: probable xyloglucan endotransglucosylase/hydrolase protein 23 [Nicotiana tomentosiformis]
c35583.graph_c1	46.686866	53.992349	64.39341	140.10366	99.222808	93.56665	0.0042711	1.061459	up	PREDICTED: probable xyloglucan endotransglucosylase/hydrolase protein 25 [Prunus mume]
c35586.graph_c0	6.7188489	5.9966001	5.7604419	25.78539	34.447056	45.590535	7.056E-10	2.552557	up	PREDICTED: probable galactinol--sucrose galactosyltransferase 6 [Sesamum indicum]
c35595.graph_c0	628.63883	666.75996	702.63832	80.17062	77.420462	64.55094	1.634E-74	-3.124754	down	PREDICTED: cysteine proteinase 15A [Nicotiana tomentosiformis]
c35626.graph_c0	0.8768406	1.0701066	1.1636618	0.2687046	0.2587818	0.3586376	8.27E-06	-1.771911	down	PREDICTED: phosphatidylinositol/phosphatidylcholine transfer protein SFH2 [Sesamum indicum]
c35641.graph_c0	0.1868631	0	0.0381197	5.507059	5.2067429	4.2678708	1.436E-27	6.101628	up	-
c35651.graph_c0	1.4278708	1.0385491	1.5211431	6.0833804	10.705499	17.044099	0.0002301	3.113389	up	PREDICTED: uncharacterized protein LOC105171337 [Sesamum indicum]
c35664.graph_c0	5.3044575	5.682372	5.7222836	1.8234008	2.1731311	1.1154351	0.0001041	-1.665259	down	hypothetical protein L484_005397 [Morus notabilis]
c35671.graph_c0	0.7130027	0.5211509	0.7064768	1.8290856	1.8465088	1.5126263	0.0001874	1.461868	up	PREDICTED: E3 ubiquitin-protein ligase ORTHRUS 2-like [Sesamum indicum]
c35683.graph_c0	2.786841	2.3710875	2.1865765	0.679332	0.5757359	0.4231259	5.657E-06	-2.081458	down	-
c35685.graph_c0	30.530956	39.321521	45.322627	234.4436	245.75171	260.15961	2.512E-71	2.724821	up	hypothetical protein POPTR_0014s02030g [Populus trichocarpa]

#ID	28 h-1 FPKM	28 h-2 FPKM	28 h-3 FPKM	48 h-1 FPKM	48 h-2 FPKM	48 h-3 FPKM	FDR	log2FC	regulated	nr_annotation
c35719.graph_c0	4.797952	6.1043239	4.5379448	0.6911088	0.8785752	0.4919556	3.613E-09	-2.862727	down	-
c35732.graph_c0	0.5857235	0.6114868	0.5675604	0.1392089	0.1966333	0.3303128	0.0034759	-1.373635	down	PREDICTED: zinc finger BED domain-containing protein DAYSLEEPER-like isoform X1 [Sesamum indicum]
c35738.graph_c0	19.166604	21.985234	19.762526	8.8420876	9.9361503	9.8529278	3.853E-08	-1.049075	down	PREDICTED: F-box/FBD/LRR-repeat protein At1g13570-like [Sesamum indicum]
c35742.graph_c0	0.7864911	1.1744419	1.0120224	2.8374313	2.3966075	3.6165042	3.424E-05	1.615625	up	PREDICTED: thiol-disulfide oxidoreductase LTO1-like [Sesamum indicum]
c35746.graph_c0	6.2332633	7.0612831	6.2867833	19.335661	26.046645	21.178442	1.837E-18	1.803435	up	PREDICTED: probable pectinesterase/pectinesterase inhibitor 61 [Sesamum indicum]
c35752.graph_c0	8.1051323	7.0714945	8.3724268	29.205445	35.382868	38.346836	4.869E-40	2.165556	up	PREDICTED: probable galactinol--sucrose galactosyltransferase 6 [Sesamum indicum]
c35755.graph_c0	7.4552273	11.276119	7.362082	1.7455506	2.2955576	2.1958757	2.843E-07	-2.026726	down	PREDICTED: dynein light chain 1, cytoplasmic-like [Nicotiana tomentosiformis]
c35761.graph_c0	1.4866011	1.1910207	1.3497366	0.0796215	0.2249316	0.3070027	1.069E-08	-2.694987	down	PREDICTED: probable leucine-rich repeat receptor-like protein kinase At5g63930 [Sesamum indicum]
c35774.graph_c0	1.2489055	1.049876	1.3705093	2.5656809	3.5276531	5.0515841	0.0003282	1.635835	up	-
c35787.graph_c0	0.3657998	0.3853301	0.4726086	1.7774332	1.1256915	1.5299771	1.924E-06	1.905128	up	PREDICTED: probable LRR receptor-like serine/threonine-protein kinase At4g36180 [Sesamum indicum]
c35811.graph_c1	0.4603074	0.3562409	0.2682908	0.791857	1.1037848	1.0383425	0.0006754	1.47224	up	PREDICTED: phosphatidylinositol 4-kinase alpha 1-like [Sesamum indicum]
c35812.graph_c0	926.42889	865.54204	945.94862	285.61313	283.01848	310.15068	3.032E-27	-1.598129	down	-
c35818.graph_c0	3.1208962	3.4692452	4.9396863	54.528715	33.884943	24.498455	6.599E-05	3.346166	up	PREDICTED: probable galactinol--sucrose galactosyltransferase 1 [Sesamum indicum]
c35844.graph_c0	1.9728924	2.6098657	1.8532157	9.3924887	9.9502028	11.59166	3.95E-22	2.305246	up	PREDICTED: uncharacterized protein LOC105173814 [Sesamum indicum]
c35848.graph_c0	0.3882218	0.175264	0.3167858	2.3374738	2.6066001	5.3638756	0.0001211	3.582561	up	-
c35849.graph_c0	48.68415	46.266381	46.230909	14.222969	13.829933	10.966378	5.063E-35	-1.810645	down	PREDICTED: cytochrome P450 78A5 [Sesamum indicum]
c35855.graph_c0	244.86365	221.82958	216.79198	549.36526	577.48789	598.76829	3.207E-16	1.37704	up	PREDICTED: cysteine proteinase 3-like [Sesamum indicum]
c35883.graph_c0	9.7050974	10.319634	9.4870565	15.892589	21.433996	24.698359	2.18E-06	1.10716	up	PREDICTED: uncharacterized protein LOC105165376 isoform X1 [Sesamum indicum]
c35887.graph_c0	3.3575928	2.460939	2.1595821	5.9583946	10.608734	15.548811	0.0020993	2.038922	up	PREDICTED: putative UDP-rhamnose:rhamnosyltransferase 1 [Sesamum indicum]
c35887.graph_c1	2.1649296	1.4216207	1.9271617	4.365785	10.571474	16.537507	0.0088249	2.538119	up	hypothetical protein MIMGU_mgv1a005472mg [Erythranthe guttata]
c35893.graph_c2	1.3018305	0.685668	0.7967123	2.612763	3.2049433	3.7931287	2.429E-05	1.824163	up	hypothetical protein M569_00198, partial [Genlisea aurea]
c35894.graph_c0	4.1311155	4.1615813	3.092365	1.6012417	1.6504749	1.6943273	0.002715	-1.160989	down	PREDICTED: protein trichome birefringence-like 10 [Sesamum indicum]
c35916.graph_c0	9.2582663	8.5078628	9.3187043	2.3679568	4.0389444	4.5055212	1.615E-12	-1.27974	down	PREDICTED: pentatricopeptide repeat-containing protein At4g19440, chloroplastic [Sesamum indicum]

#ID	28 h-1 FPKM	28 h-2 FPKM	28 h-3 FPKM	48 h-1 FPKM	48 h-2 FPKM	48 h-3 FPKM	FDR	log2FC	regulated	nr_annotation
c35927.graph_c0	29.826935	29.9292	29.467531	92.302234	97.783025	106.34093	1.106E-32	1.772497	up	PREDICTED: cytochrome b5-like [Sesamum indicum]
c35936.graph_c0	4.154086	3.2543273	3.3897002	32.987498	46.595005	34.312991	1.011E-27	3.436672	up	-
c35944.graph_c0	0.5177701	0.5843723	0.5633283	0.1914243	0.2317609	0.2676585	0.0072394	-1.232123	down	PREDICTED: DNA-directed RNA polymerase III subunit RPC5 [Sesamum indicum]
c35947.graph_c0	1.428842	0.9460809	1.0104669	4.4071619	4.2851123	4.6735282	4.228E-07	2.023038	up	PREDICTED: B3 domain-containing protein At3g19184 isoform X2 [Sesamum indicum]
c35957.graph_c0	0.9068258	0.8057822	0.7634533	1.3501694	1.7009457	1.996876	0.0031914	1.064257	up	PREDICTED: glutamate receptor 3.2-like [Sesamum indicum]
c35963.graph_c0	311.48976	341.0305	328.15011	528.91683	686.3306	800.64973	7.753E-07	1.075668	up	tubulin alpha chain-like [Sesamum indicum]
c35981.graph_c0	10.865767	10.535913	10.524004	23.234722	22.52088	19.555156	6.02E-07	1.076575	up	PREDICTED: uncharacterized protein LOC105169411 [Sesamum indicum]
c35992.graph_c0	1.0589181	0.6733125	0.6815189	2.9680888	4.065599	4.4072532	1.891E-14	2.280566	up	PREDICTED: putative leucine-rich repeat-containing protein DDB_G0290503 [Sesamum indicum]
c35995.graph_c0	14.792999	16.353385	14.918476	26.828663	34.559379	37.519227	3.738E-11	1.13928	up	PREDICTED: LOW QUALITY PROTEIN: interactor of constitutive active ROPs 3 [Sesamum indicum]
c35996.graph_c0	0.0676657	0.0610958	0.1183171	0.7229288	1.1682541	1.1883914	1.649E-16	3.671874	up	hypothetical protein VITISV_035665 [Vitis vinifera]
c36017.graph_c0	0.1135118	0.1317735	0.1720173	2.2815979	2.4170764	2.5239729	1.214E-22	4.153492	up	PREDICTED: potassium channel AKT2/3 [Sesamum indicum]
c36019.graph_c0	1.7916031	1.9450311	1.6881851	0.7299607	0.8878686	0.8419634	0.007391	-1.101989	down	PREDICTED: F-box protein SKIP14 [Sesamum indicum]
c36031.graph_c0	3.6844439	2.9940366	2.4051809	6.5384255	6.5968277	6.7874937	0.0057201	1.175155	up	VHS domain [Oryza sativa Japonica Group]
c36037.graph_c0	18.340198	18.395873	17.577551	59.183739	44.577324	32.594175	0.0029218	1.378807	up	PREDICTED: ethylene-responsive transcription factor ERF107-like [Sesamum indicum]
c36038.graph_c0	0.0520289	0.0939544	0.0636827	0.7254548	1.5719961	1.7604691	1.449E-07	4.303751	up	PREDICTED: endoglucanase 10-like [Sesamum indicum]
c36039.graph_c0	14.852247	19.581246	19.090599	2.0825572	3.9711904	2.2977782	1.662E-17	-2.646161	down	floricaula, partial [Salvia coccinea]
c36040.graph_c0	7.3995672	7.1739846	7.6217206	1.5376224	0.8144622	1.8470256	4.779E-09	-2.356221	down	CYP72A52v1 [Nicotiana tabacum]
c36060.graph_c0	2.7685808	3.6157371	3.7517849	11.092148	21.908244	24.479152	1.038E-05	2.533217	up	PREDICTED: laccase-14-like [Sesamum indicum]
c36070.graph_c1	14.9763	14.090354	13.401515	5.2643493	4.3939526	5.8907192	7.981E-10	-1.4065	down	PREDICTED: uncharacterized protein LOC105157576 isoform X2 [Sesamum indicum]
c36078.graph_c0	4.1183819	3.5325871	4.45275	1.0440705	1.3825826	0.2322519	2.434E-06	-2.141121	down	PREDICTED: LOB domain-containing protein 18-like [Sesamum indicum]
c36082.graph_c0	31.160519	34.73027	32.972317	10.440935	15.495701	16.104614	9.768E-16	-1.198931	down	PREDICTED: uncharacterized membrane protein At1g75140-like [Sesamum indicum]
c36103.graph_c0	2.3197494	3.621028	4.39537	8.5721687	10.348314	14.811471	1.904E-05	1.741559	up	PREDICTED: proliferating cell nuclear antigen [Sesamum indicum]
c36129.graph_c0	2.4115573	3.2798963	2.4161731	0.154776	0.0819832	0.2065782	3.414E-16	-4.147699	down	PREDICTED: glycerol-3-phosphate dehydrogenase [NAD(+)]-like [Cicer arietinum]
c36136.graph_c0	28.914361	28.507112	27.627675	12.177617	14.779453	14.201962	1.759E-07	-1.008279	down	hypothetical protein MIMGU_mgv1a0053871mg, partial [Erythranthe guttata]

#ID	28 h-1 FPKM	28 h-2 FPKM	28 h-3 FPKM	48 h-1 FPKM	48 h-2 FPKM	48 h-3 FPKM	FDR	log2FC	regulated	nr_annotation
c36142.graph_c0	1.295226	1.9647057	1.4373757	0.3940307	1.0435693	0.3506061	0.0036739	-1.362194	down	PREDICTED: uncharacterized protein LOC105159601 [Sesamum indicum]
c36144.graph_c0	1.9290566	1.2689945	1.5066332	0.3842491	0.8511365	0.7459691	0.0065082	-1.21833	down	PREDICTED: type I inositol 1,4,5-trisphosphate 5-phosphatase CVP2 isoform X1 [Cucumis melo]
c36156.graph_c0	11.336439	10.975118	10.565967	21.635751	22.851727	26.87109	7.311E-13	1.157723	up	PREDICTED: UDP-glycosyltransferase 90A1-like [Solanum tuberosum]
c36162.graph_c0	571.3673	579.80157	619.77934	137.81822	148.24057	152.03363	3.323E-29	-1.974827	down	hypothetical protein MIMGU_mgv1a009201mg [Erythranthe guttata]
c36188.graph_c0	1.4229302	1.6118055	1.5622065	0.9510254	0.6948249	0.1750793	0.0019999	-1.278163	down	PREDICTED: WEB family protein At1g12150-like [Sesamum indicum]
c36193.graph_c0	33.533538	36.002176	34.57165	72.557676	71.741655	75.609683	7.935E-15	1.120803	up	PREDICTED: GDSL esterase/lipase At1g54790 [Sesamum indicum]
c36202.graph_c0	3.8679679	2.8993612	4.3472604	10.73095	8.6241066	8.0666968	0.0002675	1.349441	up	PREDICTED: NAC domain-containing protein 8-like [Sesamum indicum]
c36202.graph_c1	4.1139057	3.2144462	2.7759092	8.0225823	8.711427	6.1569093	0.0018128	1.223319	up	PREDICTED: NAC domain-containing protein 8-like [Sesamum indicum]
c36217.graph_c0	0.5247014	0.7994635	0.5084306	1.78745	1.3651438	1.0726373	0.0052795	1.255749	up	hypothetical protein MIMGU_mgv1a009127mg [Erythranthe guttata]
c36224.graph_c0	1.2392694	1.3675983	0.5617965	5.0616768	5.732042	4.0379341	4.642E-07	2.270455	up	PREDICTED: laccase-1-like [Sesamum indicum]
c36224.graph_c1	0.7812991	0.4907408	0.6652525	3.0141213	4.1510249	4.2144882	8.478E-11	2.589992	up	PREDICTED: laccase-1-like [Sesamum indicum]
c36234.graph_c0	0.4102893	0.493937	0.6695854	3.5249316	2.6935484	2.416619	2.135E-08	2.50402	up	PREDICTED: high mobility group B protein 6 [Sesamum indicum]
c36245.graph_c0	3.2774567	2.75747	3.4037537	5.9011617	7.4018532	9.8295316	2.23E-05	1.328494	up	-
c36254.graph_c1	4.1880282	3.9282472	3.317861	1.9069851	1.7472169	1.6968239	0.0088787	-1.05102	down	PREDICTED: pentatricopeptide repeat-containing protein DOT4, chloroplastic isoform X1 [Sesamum indicum]
c36255.graph_c0	0.5924024	0.133721	0.3021222	2.9661092	2.3069559	1.7205082	2.22E-07	2.815048	up	hypothetical protein MIMGU_mgv1a018451mg [Erythranthe guttata]
c36266.graph_c0	209.00085	187.4808	198.82816	529.84794	530.12577	431.65648	1.02E-14	1.368933	up	PREDICTED: uncharacterized protein LOC105176605 [Sesamum indicum]
c36267.graph_c0	12.571699	12.577502	12.121424	1.9049398	3.0270792	4.3352987	1.625E-15	-1.976489	down	PREDICTED: transcription factor bHLH30-like isoform X2 [Sesamum indicum]
c36310.graph_c1	2.632122	2.0370509	2.9367742	8.1707964	5.337847	7.9973581	1.657E-05	1.545698	up	PREDICTED: histone H3-like centromeric protein HTR12 [Sesamum indicum]
c36353.graph_c0	0.5404572	0.5545252	0.4410086	4.4218108	6.2678221	5.6800889	4.228E-23	3.450541	up	hypothetical protein MIMGU_mgv1a004476mg [Erythranthe guttata]
c36375.graph_c0	1.3384621	1.176703	1.2646227	2.6341937	3.9967194	4.628189	2.639E-06	1.608097	up	PREDICTED: oligopeptide transporter 7 [Sesamum indicum]
c36383.graph_c0	1.2745401	1.130949	1.147602	1.559864	2.685291	3.0733348	0.0045267	1.074073	up	hypothetical protein MIMGU_mgv1a023345mg, partial [Erythranthe guttata]
c36388.graph_c0	0.335148	0.488827	0.3576251	1.699299	2.3887285	2.471581	2.44E-09	2.508961	up	PREDICTED: uncharacterized protein LOC105178674 isoform X1 [Sesamum indicum]
c36389.graph_c0	0.5822554	1.051444	1.0452544	9.1518393	17.201275	13.265619	1.279E-12	3.919025	up	-
c36389.graph_c1	2.5610795	2.6716247	2.7796634	4.4755599	6.4775685	6.3380686	7.834E-05	1.145132	up	PREDICTED: transcription factor MYB3-like [Sesamum indicum]
c36394.graph_c0	6.3881412	5.7159296	5.7762031	17.143228	20.479618	17.331073	3.779E-10	1.659875	up	PREDICTED: auxin-induced protein 15A-like [Sesamum indicum]
c36402.graph_c0	0.5456632	1.6188148	1.0495338	2.6678637	1.7795084	2.7255304	0.006187	1.205035	up	hypothetical protein MIMGU_mgv1a005987mg [Erythranthe guttata]
c36402.graph_c2	0.3223872	0.2381608	0.3826405	1.7088982	1.1806764	1.5205674	3.372E-07	2.271686	up	hypothetical protein MIMGU_mgv1a000547mg [Erythranthe guttata]

#ID	28 h-1 FPKM	28 h-2 FPKM	28 h-3 FPKM	48 h-1 FPKM	48 h-2 FPKM	48 h-3 FPKM	FDR	log2FC	regulated	nr_annotation
c36419.graph_c0	0.4345147	0.7846521	0.7091209	1.897129	1.7504519	1.3068805	0.0020481	1.407754	up	PREDICTED: AP-4 complex subunit epsilon [Sesamum indicum]
c36448.graph_c0	2.1790737	2.2954158	3.4673074	4.7416676	5.9498356	5.3250869	0.0028124	1.049937	up	PREDICTED: LOW QUALITY PROTEIN: protein LOW PSII ACCUMULATION 2, chloroplastic [Sesamum indicum]
c36451.graph_c0	69.043318	64.416844	75.345923	17.066076	17.131584	12.678982	7.798E-48	-2.110966	down	hypothetical protein MIMGU_mgv1a024078mg [Erythranthe guttata]
c36463.graph_c1	3.608212	4.2976251	3.2366105	8.1417893	7.010154	8.3990195	1.19E-05	1.123651	up	PREDICTED: leucine-rich repeat receptor-like serine/threonine/tyrosine-protein kinase SOBIR1 [Sesamum indicum]
c36469.graph_c0	10.371874	10.499335	11.185063	3.9817801	5.0618558	5.0503341	4.234E-12	-1.148029	down	PREDICTED: uncharacterized protein LOC105171114 [Sesamum indicum]
c36491.graph_c0	76.957226	81.410225	78.239703	232.55558	269.49981	287.98285	8.954E-33	1.77825	up	acetylajmalan acetylesterase [Striga asiatica]
c36499.graph_c0	2.0505966	1.8355353	1.6876948	0.739451	0.4510247	0.657959	0.0001016	-1.543733	down	-
c36504.graph_c0	49.522648	58.960823	57.874368	123.66598	134.36563	131.35751	1.237E-17	1.267647	up	hypothetical protein MIMGU_mgv1a014610mg [Erythranthe guttata]
c36557.graph_c0	0.2789198	0.1259192	0.2275963	7.9549066	4.9627191	6.2131113	4.386E-16	4.967793	up	hypothetical protein MIMGU_mgv1a015689mg [Erythranthe guttata]
c36561.graph_c0	206.18175	200.14256	235.28572	108.28789	75.903957	48.045325	2.313E-09	-1.412903	down	hypothetical protein MIMGU_mgv1a011962mg [Erythranthe guttata]
c36600.graph_c0	6.9996305	5.6078962	5.8403544	44.737632	48.665	54.351387	1.177E-89	3.0412	up	hypothetical protein MIMGU_mgv1a021873mg [Erythranthe guttata]
c36606.graph_c0	52.931889	70.313475	78.59344	17.101312	11.322979	10.706873	2.323E-13	-2.315781	down	unnamed protein product [Coffea canephora]
c36617.graph_c0	13.084201	13.126452	10.320715	23.956383	24.012328	25.907437	0.0010408	1.057802	up	predicted Rac-like GTPase ortholog, partial [Nicotiana alata]
c36628.graph_c0	0.1956928	0.2061409	0.5056651	0.6201375	0.7664539	0.7909056	0.0039692	1.298853	up	hypothetical protein MIMGU_mgv1a001243mg [Erythranthe guttata]
c36638.graph_c0	26.968844	28.717801	28.569106	55.717894	74.723754	79.345565	2.66E-12	1.352268	up	PREDICTED: probable methyltransferase PMT20 [Sesamum indicum]
c36639.graph_c0	2.7484208	2.8951607	2.7027277	5.047081	9.0374613	10.253473	0.0003148	1.575197	up	PREDICTED: B-box zinc finger protein 24 [Sesamum indicum]
c36647.graph_c0	0.0491895	0	0.0401382	0.7793917	1.2219931	1.9417946	6.864E-08	5.492702	up	hypothetical protein MIMGU_mgv1a003330mg [Erythranthe guttata]
c36648.graph_c0	45.280017	52.599844	42.130984	139.2594	157.35702	157.04497	5.703E-32	1.736237	up	candidate cinnamic acid 4-hydroxylase [Capsicum annuum]
c36665.graph_c0	7.4316994	7.8800988	7.6502291	13.864641	14.790261	17.494865	3.193E-06	1.046549	up	PREDICTED: LOW QUALITY PROTEIN: single-stranded DNA-binding protein WHY1, chloroplastic, partial [Sesamum indicum]
c36675.graph_c0	2.1677651	2.1260204	1.9823639	5.4008519	5.1192796	6.3232179	1.847E-05	1.465629	up	PREDICTED: nuclear transcription factor Y subunit B-3-like [Sesamum indicum]
c36679.graph_c0	22.070399	23.976515	23.53402	11.257217	7.9110791	5.3554121	2.255E-07	-1.451599	down	hypothetical protein MIMGU_mgv1a014406mg [Erythranthe guttata]
c36704.graph_c0	23.873688	26.662977	20.566769	8.6461881	8.9361834	7.4744104	5.526E-17	-1.461322	down	hypothetical protein MIMGU_mgv1a022795mg [Erythranthe guttata]
c36710.graph_c0	6.5258643	5.8922436	4.5262941	1.7922696	1.6065858	2.0854433	0.0004694	-1.585112	down	hypothetical protein MIMGU_mgv1a009490mg [Erythranthe guttata]
c36710.graph_c1	9.3129359	13.527052	9.2512969	2.951208	4.3498394	6.0511417	0.0020607	-1.231868	down	PREDICTED: zinc finger CCCH domain-containing protein 20-like [Sesamum indicum]
c36729.graph_c0	20.269211	18.692667	19.414025	7.2818934	5.4946073	5.9598116	1.766E-17	-1.592444	down	hypothetical protein MIMGU_mgv1a006282mg [Erythranthe guttata]
c36772.graph_c0	26.826784	27.843077	24.114429	68.996929	75.289833	78.607699	3.279E-26	1.540701	up	PREDICTED: protein CREG1 isoform X1 [Sesamum indicum]

#ID	28 h-1 FPKM	28 h-2 FPKM	28 h-3 FPKM	48 h-1 FPKM	48 h-2 FPKM	48 h-3 FPKM	FDR	log2FC	regulated	nr_annotation
c36786.graph_c0	0.2110116	0.2857855	0.602643	1.5780889	1.5584519	1.8563656	1.871E-07	2.222424	up	PREDICTED: uncharacterized protein LOC105170278 isoform X1 [Sesamum indicum]
c36792.graph_c0	44.737219	47.649834	39.225487	19.732627	17.626783	13.764376	1.531E-16	-1.317372	down	PREDICTED: hexose carrier protein HEX6-like [Sesamum indicum]
c36799.graph_c0	22.640738	23.951245	25.006276	6.6392753	7.6858082	7.0033494	3.968E-26	-1.70717	down	PREDICTED: uncharacterized protein LOC105174622 [Sesamum indicum]
c36800.graph_c0	1.2693451	1.462265	2.0715504	0.5548236	0.5877683	0.7898859	0.0048326	-1.275787	down	hypothetical protein MIMGU_mgv1a002177mg [Erythranthe guttata]
c36802.graph_c0	14.327054	13.367186	13.778395	4.5833887	7.0542817	8.1565434	1.199E-05	-1.033854	down	-
c36814.graph_c0	20.375322	18.898742	19.476285	8.4190035	9.5585167	8.237312	2.808E-10	-1.123386	down	PREDICTED: uncharacterized protein At5g19025-like isoform X2 [Sesamum indicum]
c36825.graph_c0	2.5942875	3.4473032	2.8758123	8.5623804	7.0331595	5.5208367	0.0002909	1.292241	up	PREDICTED: transcription factor bHLH104 [Sesamum indicum]
c36831.graph_c0	3.2360602	3.7740678	3.6308232	9.2293753	25.394091	27.450182	0.0010314	2.570154	up	PREDICTED: 1-aminocyclopropane-1-carboxylate oxidase 5-like [Sesamum indicum]
c36832.graph_c0	0.9581694	1.5226414	1.5324425	12.048339	11.168285	10.547628	5.375E-24	3.116749	up	protein with unknown function [Ricinus communis]
c36852.graph_c0	11.257678	9.6296472	9.6138695	21.000493	31.213789	28.633189	1.365E-10	1.441823	up	PREDICTED: probable polygalacturonase isoform X2 [Sesamum indicum]
c36857.graph_c0	13.395852	13.976063	14.2809	5.0304651	6.2275716	7.5801774	4.839E-11	-1.108293	down	PREDICTED: ABC transporter C family member 5-like [Sesamum indicum]
c36865.graph_c0	10.553375	9.8018242	9.7633205	61.091964	62.05673	78.278854	2.053E-40	2.781323	up	PREDICTED: cinnamoyl-CoA reductase 1-like [Sesamum indicum]
c36871.graph_c0	6.6226581	7.9277571	6.8711937	12.079262	16.096243	18.758397	1.449E-06	1.167378	up	PREDICTED: fatty acyl-CoA reductase 3-like isoform X1 [Sesamum indicum]
c36886.graph_c0	13.476877	13.005213	12.427861	4.4453731	5.8530292	4.6617995	1.783E-10	-1.340306	down	PREDICTED: uncharacterized protein LOC105158662 [Sesamum indicum]
c36900.graph_c0	0.451639	0.7476107	0.429956	2.2899426	2.3248367	2.1224762	1.524E-06	2.091546	up	PREDICTED: probable LRR receptor-like serine/threonine-protein kinase At1g06840 [Sesamum indicum]
c36900.graph_c1	1.2704145	1.5294202	1.0654436	2.7286383	3.2697646	2.8657487	0.0021148	1.237707	up	PREDICTED: probable LRR receptor-like serine/threonine-protein kinase At1g06840 [Sesamum indicum]
c36904.graph_c0	8.3002379	7.8604607	8.2585133	2.7902585	3.2559797	3.6214626	4.264E-15	-1.298549	down	PREDICTED: LOW QUALITY PROTEIN: U-box domain-containing protein 17-like [Sesamum indicum]
c36922.graph_c0	27.406965	29.904815	26.116439	9.3033471	12.475656	11.945558	1.112E-10	-1.26955	down	PREDICTED: probable inactive receptor kinase At1g27190 [Sesamum indicum]
c36925.graph_c0	7.4294565	9.5306377	6.9899525	12.607972	15.700841	21.61278	0.0009619	1.095535	up	PREDICTED: protein ECERIFERUM 1-like [Sesamum indicum]
c36933.graph_c0	0.1719677	0.0776354	0.1262918	0.6321471	0.8313309	0.9310026	2.34E-09	2.706854	up	PREDICTED: uncharacterized protein LOC105176404 [Sesamum indicum]
c36943.graph_c0	17.901168	23.504474	20.735726	0.5616596	1.0710185	1.9240813	1.747E-52	-4.099363	down	PREDICTED: probable protein phosphatase 2C 24 [Sesamum indicum]
c36949.graph_c0	30.595678	26.580604	26.051294	7.697805	8.3878879	7.6322561	7.27E-15	-1.769789	down	PREDICTED: probable receptor-like protein kinase At5g47070 [Sesamum indicum]
c36954.graph_c0	1.2793829	0.7599755	0.7417653	3.3714541	3.2551718	1.7847504	0.0015169	1.64426	up	PREDICTED: heat stress transcription factor B-2b [Sesamum indicum]
c36972.graph_c0	153.74083	162.1802	151.26137	687.71664	814.2887	1136.1599	4.924E-11	2.533714	up	chalcone synthase [Misopates orontium]
c36976.graph_c0	2.3647075	3.1264099	2.3086071	41.107762	59.482519	63.438996	7.733E-34	4.429748	up	hypothetical protein MIMGU_mgv1a008959mg [Erythranthe guttata]

#ID	28 h-1 FPKM	28 h-2 FPKM	28 h-3 FPKM	48 h-1 FPKM	48 h-2 FPKM	48 h-3 FPKM	FDR	log2FC	regulated	nr_annotation
c36995.graph_c0	0.1720834	0.1553752	0.1203588	0.6855463	1.2874489	1.1922629	1.126E-08	2.853366	up	PREDICTED: protein ECERIFERUM 3-like [Sesamum indicum]
c37005.graph_c0	25.530233	26.484595	26.278029	9.9346702	11.69166	8.3846909	5.671E-20	-1.341853	down	PREDICTED: MND1-interacting protein 1-like isoform X2 [Sesamum indicum]
c37020.graph_c0	6.8614847	6.987155	5.5147213	33.743441	37.548294	33.632331	6.761E-35	2.479294	up	PREDICTED: 7-deoxyloganetin glucosyltransferase-like [Sesamum indicum]
c37023.graph_c0	5.2198675	4.7421438	6.2575918	12.743017	9.9949563	11.702078	3.474E-06	1.131461	up	PREDICTED: bifunctional riboflavin kinase/FMN phosphatase-like isoform X2 [Sesamum indicum]
c37037.graph_c0	2.227493	1.781364	2.5446626	0.0806718	0.6836956	0.4306875	3.479E-07	-2.438272	down	-
c37042.graph_c0	0.5423386	0.3390098	0.5787112	1.2162649	1.8486962	1.223385	0.0003795	1.590765	up	-
c37051.graph_c0	10.637935	10.511195	10.591263	23.406824	20.753753	21.506401	1.419E-05	1.093076	up	PREDICTED: transcription repressor OFP1 [Sesamum indicum]
c37077.graph_c0	4.7818336	4.1846998	3.8719223	15.386444	13.929152	13.898201	1.679E-13	1.795322	up	hypothetical protein MIMGU_mgv1a0032972mg, partial [Erythranthe guttata]
c37090.graph_c0	20.530299	23.76392	25.664527	41.985857	52.012292	74.885809	0.0004727	1.306393	up	PREDICTED: protein ASPARTIC PROTEASE IN GUARD CELL 2-like [Sesamum indicum]
c37094.graph_c0	1.7435976	1.7796493	1.5155502	0.0480465	0.2035977	0.1710059	1.915E-11	-3.553096	down	PREDICTED: transmembrane 9 superfamily member 12-like [Sesamum indicum]
c37097.graph_c0	5.3611861	5.2924423	5.3079501	16.490895	14.062473	13.464144	1.256E-10	1.509232	up	PREDICTED: LOW QUALITY PROTEIN: transcription factor bHLH96-like [Sesamum indicum]
c37117.graph_c0	4.1081135	3.7805729	4.3191641	14.72074	13.685266	17.553672	2.605E-15	1.953638	up	PREDICTED: psbP-like protein 1, chloroplastic [Sesamum indicum]
c37119.graph_c0	12.266954	11.631027	13.593171	21.89515	25.829294	27.374104	3.866E-10	1.040468	up	PREDICTED: uncharacterized protein LOC105157824 isoform X3 [Sesamum indicum]
c37123.graph_c0	4.03595	3.5665507	2.8378455	21.98733	18.85342	16.561768	2.115E-18	2.50573	up	PREDICTED: phospholipase D alpha 1-like [Sesamum indicum]
c37135.graph_c0	0.9528678	1.0129931	0.9154817	2.220849	3.2401498	3.9001936	7.286E-07	1.733953	up	hypothetical protein MIMGU_mgv1a001054mg [Erythranthe guttata]
c37138.graph_c0	0.3658564	0.4129176	0.1990239	1.8549999	1.5557417	1.7537282	2.746E-08	2.446708	up	PREDICTED: probable polygalacturonase At1g80170 [Sesamum indicum]
c37145.graph_c0	8.118123	8.9895048	8.5491251	15.998533	21.555867	26.121266	1.579E-06	1.346571	up	PREDICTED: ACT domain-containing protein DS12, chloroplastic-like isoform X1 [Sesamum indicum]
c37146.graph_c0	17.549118	22.791392	19.994296	51.222035	68.197058	83.990244	1.091E-08	1.788695	up	PREDICTED: arogenate dehydratase/prephenate dehydratase 6, chloroplastic-like [Sesamum indicum]
c37149.graph_c0	0.7047944	1.2946702	1.4278506	5.883978	7.5061418	9.7309556	8.815E-13	2.789265	up	PREDICTED: replication protein A 70 kDa DNA-binding subunit B [Sesamum indicum]
c37157.graph_c0	16.43261	16.966722	14.9528	1.8069793	0.9397352	0.7600695	1.469E-53	-3.727102	down	PREDICTED: probable protein phosphatase 2C 8 isoform X1 [Sesamum indicum]
c37161.graph_c0	1.2388739	1.2384356	1.6607823	14.806288	11.169954	8.7331273	6.696E-09	3.117911	up	hypothetical protein MIMGU_mgv1a014011mg [Erythranthe guttata]
c37164.graph_c0	0.621321	0.7479928	1.0139857	5.5129907	9.1776853	7.9421183	1.723E-11	3.280718	up	PREDICTED: flavonoid 3'-monooxygenase-like, partial [Solanum tuberosum]

#ID	28 h-1 FPKM	28 h-2 FPKM	28 h-3 FPKM	48 h-1 FPKM	48 h-2 FPKM	48 h-3 FPKM	FDR	log2FC	regulated	nr_annotation
c37167.graph_c0	3.7373593	3.7442916	4.0940569	0.1297912	0.274996	0.8084119	2.382E-11	-3.230837	down	-
c37173.graph_c0	49.141437	43.748432	63.102168	1230.4472	1210.3904	1453.0253	4.006E-94	4.681861	up	PREDICTED: vignain-like [Sesamum indicum]
c37183.graph_c0	5.1226119	4.6969474	4.277219	0.2852349	0.7287672	0.8360479	2.522E-23	-2.903227	down	hypothetical protein MIMGU_mgv1a024814mg, partial [Erythranthe guttata]
c37184.graph_c0	0	0.2492659	0.1126357	2.7995193	3.5681695	4.8263037	1.587E-18	4.987819	up	unnamed protein product [Coffea canephora]
c37191.graph_c0	1.9635046	1.3939285	1.5410509	5.6997405	6.2797113	8.6048058	1.269E-11	2.108559	up	PREDICTED: kinesin-3-like [Sesamum indicum]
c37196.graph_c0	13.518747	12.635027	11.955422	4.6313535	6.5254555	5.4396547	3.681E-06	-1.162101	down	PREDICTED: protein IWS1 homolog A-like isoform X2 [Sesamum indicum]
c37196.graph_c1	12.04893	12.77304	11.18747	5.5305788	5.9265812	4.7128764	5.656E-10	-1.112197	down	hypothetical protein MIMGU_mgv1a000837mg [Erythranthe guttata]
c37200.graph_c0	21.211572	21.744069	22.96951	46.799952	44.117703	42.541718	1.602E-11	1.061039	up	PREDICTED: LIM domain-containing protein WLIM2b [Sesamum indicum]
c37202.graph_c0	0.2425025	0.218957	0.1978801	1.7828609	1.3676974	1.367568	1.766E-09	2.824391	up	PREDICTED: condensin complex subunit 3 [Sesamum indicum]
c37202.graph_c1	0.3795523	0.4378947	0.5505984	3.6617859	3.1147007	3.4960625	2.689E-15	2.9525	up	PREDICTED: condensin complex subunit 3 [Sesamum indicum]
c37214.graph_c0	211.66986	203.32482	227.79727	105.95446	75.395109	53.710378	1.051E-12	-1.399309	down	PREDICTED: uncharacterized protein LOC105174797 [Sesamum indicum]
c37225.graph_c0	0.3431489	0.1032771	0.2800067	6.1257786	4.4159453	3.580033	1.059E-11	4.330642	up	PREDICTED: cytochrome P450 714A1-like [Sesamum indicum]
c37246.graph_c0	0.2356552	0.3647564	0.4395262	1.2375142	1.2657895	0.7973719	0.0001856	1.711154	up	PREDICTED: ALA-interacting subunit 3-like [Sesamum indicum]
c37260.graph_c0	0.3349025	0.2591876	0.117119	3.3354642	2.9553076	1.5109187	5.165E-06	3.506323	up	PREDICTED: uncharacterized protein LOC105156178 [Sesamum indicum]
c37313.graph_c0	15.918971	17.053113	16.166421	8.4524819	6.4699275	5.1299085	3.256E-08	-1.243284	down	PREDICTED: C2 and GRAM domain-containing protein At1g03370 isoform X2 [Sesamum indicum]
c37325.graph_c0	8.3875027	6.6458067	6.8441337	17.358004	18.235461	19.885499	5.376E-08	1.382912	up	PREDICTED: vacuolar iron transporter 1 [Nicotiana sylvestris]
c37350.graph_c0	2.2769633	3.1841136	2.7643169	5.7020418	5.854183	5.4807867	0.0004029	1.092919	up	PREDICTED: OTU domain-containing protein DDB_G0284757-like [Sesamum indicum]
c37359.graph_c0	0.4676757	0.5730771	0.4906537	1.0585954	1.2111697	1.1114795	0.0049954	1.183179	up	PREDICTED: oxysterol-binding protein-related protein 1C-like isoform X1 [Sesamum indicum]
c37384.graph_c0	2.5977184	1.8894274	1.4425852	6.2197321	3.8759129	4.0693237	0.0045478	1.308292	up	hypothetical protein MIMGU_mgv1a022389mg [Erythranthe guttata]
c37386.graph_c0	1.2133739	0.6573377	0.5940619	71.365091	89.289349	102.64046	6.206E-66	6.776079	up	--
c37387.graph_c0	15.136319	12.103117	11.932431	33.982691	30.876061	29.550376	4.726E-16	1.313615	up	hypothetical protein MIMGU_mgv1a011856mg [Erythranthe guttata]
c37389.graph_c0	0.3287735	0.2585482	0.3894336	2.7827785	2.7486336	2.7871072	1.887E-22	3.131857	up	unnamed protein product [Coffea canephora]
c37391.graph_c1	54.066694	48.057946	52.79749	119.10763	95.524256	107.92543	1.142E-09	1.103346	up	PREDICTED: auxin-induced protein AUX28-like [Sesamum indicum]
c37416.graph_c0	8.4268705	9.6020006	9.4905534	25.936345	23.535717	23.229833	1.109E-17	1.445755	up	PREDICTED: rho GTPase-activating protein 2 [Sesamum indicum]
c37435.graph_c1	12.365898	13.398296	12.631776	5.9215484	6.2731625	5.2689581	0.0019259	-1.094067	down	PREDICTED: auxin efflux carrier component 2 [Sesamum indicum]
c37435.graph_c2	0.7972516	0.9997826	0.5421257	1.6281488	1.7248262	2.1980534	0.00271	1.286641	up	PREDICTED: probable auxin efflux carrier component 1b isoform X2 [Sesamum indicum]
c37458.graph_c0	9.9356571	10.53567	9.4743629	16.822745	20.963235	20.865075	1.663E-11	1.007978	up	PREDICTED: probable long-chain-alcohol O-fatty-acyltransferase 5 [Sesamum indicum]

#ID	28 h-1 FPKM	28 h-2 FPKM	28 h-3 FPKM	48 h-1 FPKM	48 h-2 FPKM	48 h-3 FPKM	FDR	log2FC	regulated	nr_annotation
c37460.graph_c0	14.235067	13.28584	13.279581	37.771539	36.624948	40.440064	6.813E-25	1.534806	up	PREDICTED: acetyl-CoA carboxylase 1-like isoform X1 [Sesamum indicum]
c37475.graph_c0	4.8073298	5.5807304	4.3430356	7.5082407	11.758191	11.037823	0.0032066	1.075492	up	PREDICTED: reticulon-like protein B8 [Sesamum indicum]
c37479.graph_c0	0.9791283	0.8840611	0.9987009	0.0443256	0.0939152	0.3944067	1.019E-06	-2.40405	down	hypothetical protein MIMGU_mgv1a022974mg [Erythranthe guttata]
c37481.graph_c0	3.9833089	5.1138509	4.2491523	20.85399	13.7624	12.799493	0.000113	1.880739	up	PREDICTED: uncharacterized protein LOC105162739 [Sesamum indicum]
c37493.graph_c0	3.8924562	3.8773125	4.1393221	24.765371	21.076404	18.353957	4.54E-17	2.476942	up	PREDICTED: serine/threonine-protein kinase-like protein CCR1 [Sesamum indicum]
c37495.graph_c0	8.8940038	8.4726256	8.9847812	13.576625	19.643748	18.459135	1.066E-07	1.007378	up	PREDICTED: uncharacterized protein LOC105159731 [Sesamum indicum]
c37496.graph_c0	2.550306	2.0698312	1.9641172	4.7219224	6.426037	7.3688516	3.548E-07	1.527203	up	hypothetical protein MIMGU_mgv1a012483mg [Erythranthe guttata]
c37503.graph_c0	306.24502	241.87151	298.98658	432.39273	637.07625	687.62961	3.353E-05	1.086734	up	PREDICTED: WAT1-related protein At2g39510-like [Sesamum indicum]
c37505.graph_c1	257.21468	264.93152	258.89417	121.90591	126.65449	123.28845	2.19E-11	-1.029025	down	PREDICTED: uncharacterized protein LOC101224374, partial [Cucumis sativus]
c37508.graph_c0	1.9261639	1.6380323	1.553458	4.8547191	6.1354923	6.8458401	1.061E-10	1.83819	up	PREDICTED: kinesin-4-like [Sesamum indicum]
c37508.graph_c1	2.1057235	2.7806087	2.1478165	6.4727122	6.7863624	8.8765708	8.649E-10	1.693204	up	PREDICTED: kinesin-4-like [Sesamum indicum]
c37516.graph_c0	4.7379014	4.8825601	5.1874104	9.4392427	12.120482	11.848764	9.989E-15	1.211327	up	hypothetical protein MIMGU_mgv1a001577mg [Erythranthe guttata]
c37532.graph_c0	4.7494245	4.339336	4.5214068	67.082734	76.228938	93.616074	2.849E-50	4.159578	up	hypothetical protein JCGZ_04972 [Jatropha curcas]
c37539.graph_c1	6.6532932	7.0752644	6.6354847	2.4363558	2.7795638	2.6681289	0.0030579	-1.328986	down	-
c37544.graph_c0	163.37073	188.03003	195.86996	22.835944	22.905761	19.959197	2.341E-81	-3.01512	down	PREDICTED: uncharacterized GPI-anchored protein At3g06035 [Sesamum indicum]
c37563.graph_c0	0.2251474	0.1355247	0.5511553	5.9932093	8.566217	8.8878693	7.844E-22	4.717461	up	hypothetical protein MIMGU_mgv1a018370mg [Erythranthe guttata]
c37569.graph_c0	2.458805	2.5752817	2.6082743	1.3090209	0.6603566	0.8874354	0.0019043	-1.366139	down	hypothetical protein MIMGU_mgv1a000964mg [Erythranthe guttata]
c37570.graph_c1	2.3851589	1.8245565	2.3517432	3.821197	4.5819095	5.5671608	0.0010296	1.126964	up	PREDICTED: probable prolyl 4-hydroxylase 9 [Sesamum indicum]
c37590.graph_c0	1.339353	1.2886092	1.7916412	3.2841307	3.7444962	3.7889539	7.637E-05	1.330314	up	PREDICTED: LOW QUALITY PROTEIN: interactor of constitutive active ROPs 3 [Sesamum indicum]
c37600.graph_c0	115.5635	104.75438	112.19733	583.07397	636.96844	702.76597	5.619E-41	2.57121	up	PREDICTED: alpha-L-arabinofuranosidase 1-like [Sesamum indicum]
c37606.graph_c0	1.5468362	2.2040853	2.228582	4.9630949	4.2516755	3.4347693	0.0007608	1.128067	up	PREDICTED: aspartic proteinase-like protein 1 [Sesamum indicum]
c37620.graph_c0	9.6671153	8.6582963	10.193443	27.299978	25.927987	29.026855	1.726E-22	1.570074	up	-
c37623.graph_c0	58.54195	59.715832	59.697574	156.40531	155.39682	166.96987	2.749E-24	1.469462	up	ATP binding protein, putative [Ricinus communis]
c37626.graph_c0	14.362503	14.419781	15.248002	2.6991559	3.9098306	4.8033853	5.592E-22	-1.914197	down	PREDICTED: uncharacterized protein LOC105173972 [Sesamum indicum]
c37627.graph_c0	201.28955	202.25114	186.14801	367.62165	446.64027	499.60127	2.263E-12	1.193454	up	PREDICTED: low-temperature-induced cysteine proteinase [Sesamum indicum]
c37629.graph_c0	0.3266846	0.688253	0.6516204	6.6715598	7.7988509	5.9772539	6.227E-22	3.658206	up	PREDICTED: hippocampus abundant transcript 1 protein-like isoform X1 [Sesamum indicum]

#ID	28 h-1 FPKM	28 h-2 FPKM	28 h-3 FPKM	48 h-1 FPKM	48 h-2 FPKM	48 h-3 FPKM	FDR	log2FC	regulated	nr_annotation
c37639.graph_c0	1.4743723	0.7930672	0.8959075	0.1988165	0.3369952	0.1769058	0.0004649	-2.113681	down	PREDICTED: LOW QUALITY PROTEIN: transcription factor GAMYB [Sesamum indicum]
c37646.graph_c0	5.941652	6.66197	6.2988673	1.8828704	1.7003769	1.950017	4.977E-09	-1.72936	down	PREDICTED: trehalose-phosphate phosphatase A-like isoform X2 [Sesamum indicum]
c37673.graph_c1	7.8776431	8.7823308	7.7715844	21.094755	19.524156	17.370094	3.187E-13	1.291942	up	PREDICTED: ethylene receptor 1 [Sesamum indicum]
c37674.graph_c0	6416.4739	7280.4562	6194.2988	1461.4495	1321.9718	1432.3633	9.196E-19	-2.194569	down	2S albumin precursor isoform 3 [Sesamum indicum]
c37711.graph_c0	3.9954726	4.8623327	4.2879678	0.8257428	0.8747744	1.5184647	1.478E-06	-1.993678	down	PREDICTED: telomere repeat-binding protein 5 [Sesamum indicum]
c37711.graph_c1	5.9787632	5.559062	5.8335863	1.2577123	2.0498364	2.0660397	9.904E-08	-1.659534	down	PREDICTED: telomere repeat-binding protein 5 [Sesamum indicum]
c37725.graph_c0	1.5661522	1.4755707	1.1483185	5.5528428	6.2787985	7.4497299	4.842E-15	2.240914	up	PREDICTED: uncharacterized protein LOC105169345 [Sesamum indicum]
c37726.graph_c0	13.257131	15.037454	14.524058	5.8979197	7.4382509	6.2891902	3.889E-06	-1.086472	down	PREDICTED: WPP domain-interacting protein 1 [Sesamum indicum]
c37733.graph_c0	1.1928966	1.2999167	0.9957708	2.3984786	3.0380293	3.170298	9.048E-06	1.340605	up	PREDICTED: protein argonaute 16 isoform X1 [Sesamum indicum]
c37737.graph_c0	3.6976632	3.2121794	3.2229855	7.5277032	7.1471267	7.8671139	1.18E-05	1.195903	up	PREDICTED: glucose-6-phosphate 1-dehydrogenase, chloroplastic [Sesamum indicum]
c37751.graph_c0	2.0048915	2.2200922	1.9292159	4.8429455	4.495549	4.8425435	1.059E-05	1.247512	up	PREDICTED: proline-rich receptor-like protein kinase PERK10 [Sesamum indicum]
c37759.graph_c0	0	0	0.0510812	1.388631	1.5551482	0.9531737	5.761E-18	6.291857	up	hypothetical protein MIMGU_mgv1a007953mg [Erythranthe guttata]
c37764.graph_c0	62.855129	59.036808	71.066067	857.55925	555.06634	813.01661	4.264E-16	3.574737	up	PREDICTED: acid phosphatase 1-like [Camelina sativa]
c37770.graph_c0	8.856469	9.6196255	8.7115215	2.3341659	3.4147718	4.2774802	4.708E-09	-1.405486	down	PREDICTED: pentatricopeptide repeat-containing protein At3g48250, chloroplastic [Sesamum indicum]
c37795.graph_c0	0.7754174	0.8078414	0.8274217	2.0036011	2.242718	2.3546312	0.0003049	1.492738	up	PREDICTED: putative late blight resistance protein homolog R1B-16 [Sesamum indicum]
c37803.graph_c0	10.005451	10.596522	10.639646	3.8577432	4.3405674	4.9245423	6.682E-14	-1.212599	down	PREDICTED: LOW QUALITY PROTEIN: LRR receptor-like serine/threonine-protein kinase RCH1 [Sesamum indicum]
c37805.graph_c0	64.618662	66.836404	65.265331	216.79655	135.5481	123.2228	0.0092622	1.326444	up	hypothetical protein MIMGU_mgv1a026895mg [Erythranthe guttata]
c37818.graph_c0	0.0545057	0.1230338	0.2001429	0.6563549	1.1344832	1.0143516	7.981E-10	2.924846	up	PREDICTED: phosphoinositide phospholipase C 6-like [Sesamum indicum]
c37828.graph_c0	14.504278	14.535002	14.486738	5.684704	6.1383862	5.5737911	2.577E-16	-1.281607	down	PREDICTED: uncharacterized protein LOC105157890 [Sesamum indicum]
c37839.graph_c0	1.1429957	1.1899799	1.0373632	3.769918	3.9937709	4.5778381	8.645E-14	1.912623	up	PREDICTED: histidine kinase 1 [Sesamum indicum]
c37841.graph_c0	14.150314	15.429065	12.472085	24.017556	31.636068	32.003585	6.553E-12	1.096939	up	PREDICTED: CBL-interacting protein kinase 23 [Sesamum indicum]
c37842.graph_c0	47.724326	48.875903	50.445169	172.80027	233.14308	299.04047	1.299E-09	2.295895	up	-
c37847.graph_c0	0.9200024	0.5895119	0.6538481	2.6709073	2.6700938	3.9162985	3.991E-08	2.135874	up	PREDICTED: probable LRR receptor-like serine/threonine-protein kinase At1g56140 isoform X1 [Sesamum indicum]
c37849.graph_c0	290.77141	293.75127	317.85985	57.360881	47.253875	51.21452	2.412E-58	-2.488587	down	hypothetical protein MIMGU_mgv1a008920mg [Erythranthe guttata]
c37852.graph_c0	6.0297383	5.3136254	5.8452181	39.408017	28.987179	22.877951	1.507E-06	2.459217	up	beta-D-xylosidase family protein [Populus trichocarpa]

#ID	28 h-1 FPKM	28 h-2 FPKM	28 h-3 FPKM	48 h-1 FPKM	48 h-2 FPKM	48 h-3 FPKM	FDR	log2FC	regulated	nr_annotation
c37862.graph_c0	0.4383244	0.5371107	0.5875992	1.8652568	1.0931138	1.2006299	0.0038301	1.46414	up	PREDICTED: flavonol synthase/flavanone 3-hydroxylase [Sesamum indicum]
c37863.graph_c0	0.150338	0.4615197	0.4170935	3.2777084	2.8263189	4.4086405	2.254E-15	3.393779	up	PREDICTED: uncharacterized protein LOC105168997 [Sesamum indicum]
c37867.graph_c0	1.0930575	0.9869284	1.0070132	0.0893891	0.1420454	0.1193068	1.366E-09	-3.102845	down	PREDICTED: uncharacterized protein LOC105158642 [Sesamum indicum]
c37873.graph_c0	6.0705015	5.6321474	4.894974	13.996068	13.960616	14.717911	3.362E-13	1.404372	up	PREDICTED: oxygen-evolving enhancer protein 1, chloroplastic-like [Nicotiana sylvestris]
c37874.graph_c0	18.637213	17.222826	18.481512	114.28303	81.300389	62.773635	2.98E-05	2.300668	up	PREDICTED: aspartic proteinase-like protein 1 isoform X1 [Sesamum indicum]
c37888.graph_c0	5.0693649	5.5831307	4.818411	1.9065541	2.244181	2.8274006	0.0066473	-1.111303	down	PREDICTED: myb-related protein 306-like [Sesamum indicum]
c37891.graph_c0	0.7438123	0.5682708	0.9648866	5.8020732	6.940528	7.0341105	9.241E-28	3.156345	up	PREDICTED: receptor-like serine/threonine-protein kinase NCRK isoform X1 [Sesamum indicum]
c37904.graph_c0	7.6245785	6.8051498	10.226298	21.440114	18.476542	17.792286	2.989E-05	1.27164	up	PREDICTED: uncharacterized protein LOC105164967 [Sesamum indicum]
c37906.graph_c0	35.57082	29.984338	32.449601	84.15371	60.677672	51.089798	0.0082896	1.049732	up	PREDICTED: protein TRANSPORT INHIBITOR RESPONSE 1-like [Sesamum indicum]
c37914.graph_c0	0.2569387	0.2029926	0.4193198	2.1983991	4.5284891	6.2306118	1.041E-05	3.908125	up	PREDICTED: geraniol synthase, chloroplastic-like [Sesamum indicum]
c37922.graph_c0	0.7492812	1.1839287	0.9680616	2.8493074	3.6892725	3.0282709	6.11E-05	1.76041	up	PREDICTED: peptidyl-prolyl cis-trans isomerase FKBP18, chloroplastic [Sesamum indicum]
c37931.graph_c0	0.7467922	0.6009917	0.8610753	2.0166996	1.7440398	2.0691075	0.0001007	1.443042	up	hypothetical protein MIMGU_mgv1a001348mg [Erythranthe guttata]
c37938.graph_c1	2.0186482	2.0830286	1.8354519	4.5083359	4.0273595	4.5752672	4.839E-05	1.186382	up	PREDICTED: chromosome-associated kinesin KIF4A [Sesamum indicum]
c37940.graph_c0	0.2234406	0.2567676	0.248626	0.514959	1.0637965	0.7331319	0.0007428	1.697183	up	PREDICTED: uncharacterized protein At5g41620 [Sesamum indicum]
c37960.graph_c0	81.758534	60.21331	89.672893	12.886309	11.632601	6.0560705	4.201E-17	-2.872831	down	hypothetical protein MIMGU_mgv1a009609mg [Erythranthe guttata]
c37964.graph_c0	367.28086	380.42802	372.93669	57.731919	83.876527	95.083186	4.438E-45	-2.208875	down	uncharacterized LOC102580903 [Solanum tuberosum]
c37965.graph_c0	1.4185078	1.430658	1.2436869	2.1423731	3.161207	3.6423386	0.0001244	1.16233	up	PREDICTED: filament-like plant protein 7 [Sesamum indicum]
c37966.graph_c0	2.2876291	2.2791885	2.2850819	0.2999729	0.6355699	0.9786851	6.71E-05	-1.812723	down	-
c37968.graph_c0	84.792128	90.297508	94.393883	18.084123	19.582619	20.41118	2.751E-49	-2.173981	down	BnaC06g30860D [Brassica napus]
c37976.graph_c0	18.907299	17.856104	16.797424	5.2794433	7.4035908	9.192459	3.498E-10	-1.25751	down	PREDICTED: ninja-family protein AFP1-like [Sesamum indicum]
c37983.graph_c0	5.9014984	5.856946	6.427399	2.2256283	3.209205	2.970526	0.0001835	-1.07767	down	PREDICTED: cytochrome P450 71D11-like [Sesamum indicum]
c37983.graph_c1	1.8298942	1.1522081	1.2574136	0.1526004	0.5496494	0.5702881	0.0003415	-1.713164	down	PREDICTED: F-box/kelch-repeat protein At1g57790-like [Sesamum indicum]
c37994.graph_c0	38.905985	40.584537	34.498251	59.418114	80.321931	91.219681	3.288E-06	1.054557	up	PREDICTED: polygalacturonase At1g48100 [Sesamum indicum]
c38004.graph_c0	0.1039939	0.0704226	0.2121454	2.4716237	2.3390911	2.7563756	1.852E-19	4.330856	up	PREDICTED: uncharacterized protein LOC105177313 isoform X1 [Sesamum indicum]
c38009.graph_c0	3.0067807	3.8007774	2.9442102	0.9909421	1.5342983	1.2886887	0.0028504	-1.318615	down	-
c38024.graph_c0	14.329424	19.734625	16.635295	3.6277505	5.7120965	7.1191786	1.226E-09	-1.590428	down	PREDICTED: lysine histidine transporter 1-like [Solanum tuberosum]

#ID	28 h-1 FPKM	28 h-2 FPKM	28 h-3 FPKM	48 h-1 FPKM	48 h-2 FPKM	48 h-3 FPKM	FDR	log2FC	regulated	nr_annotation
c38036.graph_c0	3.5028239	2.9962625	3.4600179	1.3437106	1.0934119	1.4382163	2.109E-05	-1.31825	down	PREDICTED: pentatricopeptide repeat-containing protein At1g74900, mitochondrial [Sesamum indicum]
c38037.graph_c0	29.147971	30.185689	28.476817	12.748555	14.443433	13.811868	6.272E-12	-1.058449	down	hypothetical protein MIMGU_mgv1a006765mg [Erythranthe guttata]
c38039.graph_c0	0.0486727	0.1098672	0.0595748	0.8637471	1.0457546	0.9606962	1.606E-13	3.758226	up	PREDICTED: DNA-directed primase/polymerase protein isoform X1 [Sesamum indicum]
c38055.graph_c0	5.2619017	5.3262375	5.5259325	3.1106017	2.5982215	2.0492326	0.0005187	-1.006739	down	PREDICTED: pyruvate kinase isozyme G, chloroplastic isoform X1 [Sesamum indicum]
c38063.graph_c1	259.15469	305.14184	323.96566	20.54214	20.452451	17.649793	2.468E-78	-3.877579	down	PREDICTED: peroxygenase-like [Nicotiana tomentosiformis]
c38072.graph_c0	7.4249856	7.6586546	7.8138746	14.325525	15.306705	16.200729	7.78E-12	1.041521	up	Lysosomal alpha-mannosidase [Glycine soja]
c38078.graph_c0	195.8512	224.5153	170.46718	66.268422	96.874967	90.708894	8.095E-10	-1.182701	down	PREDICTED: polyol transporter 5-like [Sesamum indicum]
c38081.graph_c0	9.1808656	8.7548341	7.9120875	19.150707	23.488921	29.902072	1.798E-08	1.525238	up	hypothetical protein VITISV_034764 [Vitis vinifera]
c38083.graph_c0	26.275525	22.696567	22.694542	66.347802	71.459333	83.814252	2.085E-31	1.668069	up	PREDICTED: lysosomal beta glucosidase-like [Sesamum indicum]
c38085.graph_c0	2.9759672	2.9912102	2.5200014	0.9964454	0.7540093	0.7599695	0.0001369	-1.709272	down	PREDICTED: uncharacterized protein LOC105176978 [Sesamum indicum]
c38086.graph_c0	5.331539	5.9084041	5.3304979	11.595508	12.917077	13.216673	3.784E-15	1.227096	up	PREDICTED: calcium-transporting ATPase, endoplasmic reticulum-type [Sesamum indicum]
c38090.graph_c0	1.2721808	1.4619309	1.2457069	4.0900965	3.8670514	3.9915377	1.699E-10	1.629319	up	PREDICTED: DNA (cytosine-5)-methyltransferase CMT2 [Sesamum indicum]
c38104.graph_c0	12.749167	12.355146	12.604726	24.27429	28.344064	25.477412	3.196E-13	1.090305	up	PREDICTED: probable sulfate transporter 3.3 [Sesamum indicum]
c38110.graph_c0	0.4050181	0.2612095	0.4249176	3.3737075	7.0703715	7.9615731	4.323E-08	4.104425	up	PREDICTED: uncharacterized protein LOC105166696 [Sesamum indicum]
c38120.graph_c0	4.6599517	4.8972535	4.9245266	1.5251247	2.2055378	2.5202297	5.266E-05	-1.177754	down	PREDICTED: pentatricopeptide repeat-containing protein At1g73400, mitochondrial [Sesamum indicum]
c38124.graph_c1	0.3168428	0.2622394	0.7540784	2.3427928	1.7373335	1.399662	0.000113	2.087646	up	PREDICTED: LOW QUALITY PROTEIN: probable WRKY transcription factor 70 [Sesamum indicum]
c38128.graph_c0	8.2497368	9.3248551	8.0579166	17.680666	23.067825	26.545103	4.486E-10	1.428777	up	PREDICTED: protein cfxQ homolog [Sesamum indicum]
c38136.graph_c0	0.4741258	0.3389055	0.4513633	4.4322969	3.5282429	3.5650451	2.308E-19	3.234605	up	PREDICTED: glutamate receptor 2.1-like [Sesamum indicum]
c38144.graph_c0	0.1161694	0.2307582	0.2275039	2.3560528	2.6831486	3.4328576	3.041E-19	3.920012	up	PREDICTED: cyclin-A2-1-like isoform X2 [Sesamum indicum]
c38146.graph_c1	54.398018	55.534568	50.856767	125.95643	128.19562	120.24663	4.386E-16	1.261889	up	PREDICTED: uncharacterized protein LOC105174863 [Sesamum indicum]
c38157.graph_c0	0.6924473	0.8857213	0.8632424	2.7307093	2.0921544	2.863656	2.843E-06	1.699098	up	hypothetical protein MIMGU_mgv1a002532mg [Erythranthe guttata]
c38172.graph_c0	9.3505719	9.0188313	8.7714849	4.1063913	4.0536175	3.4877586	1.199E-06	-1.176694	down	PREDICTED: uncharacterized GPI-anchored protein At1g61900-like [Sesamum indicum]
c38180.graph_c0	1.4080522	0.9535043	1.5375776	4.4095303	6.1450664	5.5817504	1.956E-12	2.085016	up	PREDICTED: polyadenylate-binding protein 7-like [Sesamum indicum]
c38190.graph_c0	18.046666	16.545775	24.833448	55.571533	47.782315	46.150735	3.125E-10	1.375825	up	PREDICTED: early nodulin-like protein 2 [Sesamum indicum]

#ID	28 h-1 FPKM	28 h-2 FPKM	28 h-3 FPKM	48 h-1 FPKM	48 h-2 FPKM	48 h-3 FPKM	FDR	log2FC	regulated	nr_annotation
c38203.graph_c0	0.904604	0.6466116	0.9841993	3.3444022	4.8083417	3.9536013	9.421E-09	2.291611	up	PREDICTED: psbP domain-containing protein 3, chloroplastic [Sesamum indicum]
c38204.graph_c0	2.448705	2.6737082	2.0910593	11.477263	10.629364	10.083942	5.196E-11	2.20242	up	PREDICTED: uncharacterized protein LOC105164617 isoform X2 [Sesamum indicum]
c38208.graph_c0	4.8204851	4.3638399	4.9116971	1.2956437	1.5589769	1.7790988	5.463E-10	-1.567701	down	PREDICTED: pentatricopeptide repeat-containing protein At2g26790, mitochondrial isoform X1 [Sesamum indicum]
c38235.graph_c0	0.5355854	0.5158223	0.4078978	1.6293455	1.5822528	1.7719556	2.975E-05	1.813996	up	PREDICTED: uncharacterized protein LOC105167746 [Sesamum indicum]
c38241.graph_c0	2.0586781	1.7426187	1.3648899	3.6968287	5.2409867	6.3853368	1.05E-05	1.603152	up	PREDICTED: lipid phosphate phosphatase epsilon 2, chloroplastic isoform X1 [Sesamum indicum]
c38242.graph_c0	5.0956875	5.2821589	4.8305242	12.624052	10.6771	10.905509	3.301E-13	1.214901	up	PREDICTED: filament-like plant protein 4 [Sesamum indicum]
c38245.graph_c0	198.70345	212.7585	178.27011	1403.4397	1078.5883	917.8842	6.498E-10	2.576676	up	PREDICTED: acyl-[acyl-carrier-protein] desaturase, chloroplastic [Sesamum indicum]
c38249.graph_c0	0.4833989	0.5819518	0.6574159	2.0084376	1.8752576	2.1808626	8.787E-06	1.857565	up	PREDICTED: uncharacterized protein LOC105164781 [Sesamum indicum]
c38253.graph_c0	70.716043	69.799231	68.797669	169.80598	142.50287	114.45755	0.0002565	1.07544	up	PREDICTED: uncharacterized protein LOC105177769 isoform X1 [Sesamum indicum]
c38268.graph_c0	2.3086819	2.2929756	2.7787017	5.7065274	6.742917	5.8588082	0.0004787	1.350427	up	hypothetical protein MIMGU_mgv1a003097mg [Erythranthe guttata]
c38269.graph_c0	6.8569596	6.4013864	6.2859913	13.386293	12.447586	12.507789	8.319E-08	1.015754	up	PREDICTED: uncharacterized protein LOC105168472 [Sesamum indicum]
c38271.graph_c0	0.2756159	0.4977106	0.4819292	2.5453621	2.6436299	2.7533445	1.261E-09	2.702631	up	PREDICTED: agamous-like MADS-box protein AGL12 [Sesamum indicum]
c38277.graph_c0	4.9357774	4.380364	4.2685188	27.271755	28.607871	29.738044	1.33E-39	2.696862	up	PREDICTED: threonine dehydratase biosynthetic, chloroplastic [Phoenix dactylifera]
c38281.graph_c0	0.5166787	0.4382389	0.4343815	2.9967929	5.2351651	5.9334669	3.768E-10	3.381435	up	hypothetical protein CICLE_v10027936mg [Citrus clementina]
c38283.graph_c0	2.6797429	2.1848235	2.3987858	4.4867803	5.934786	7.6688442	2.461E-05	1.351284	up	hypothetical protein SORBIDRAFT_03g008610 [Sorghum bicolor]
c38284.graph_c0	15.997772	14.444488	16.61136	33.254528	38.940221	38.809015	4.853E-15	1.277137	up	PREDICTED: E3 ubiquitin ligase BIG BROTHER-like [Sesamum indicum]
c38291.graph_c0	1.2698552	1.093642	1.370961	1.8572711	2.7545749	3.3051773	0.0012887	1.117287	up	PREDICTED: two-component response regulator ARR9 [Sesamum indicum]
c38292.graph_c0	76.301069	76.477492	74.625312	148.27028	146.73046	149.8782	6.925E-12	1.010362	up	PREDICTED: UDP-glucose 4-epimerase GEPI48 [Sesamum indicum]
c38294.graph_c0	0.7986728	0.9134271	1.0427368	8.2339964	9.6524123	12.070809	5.192E-21	3.47984	up	PREDICTED: uncharacterized protein LOC105163186 [Sesamum indicum]
c38300.graph_c0	76.791634	72.592114	67.247519	25.449114	32.449164	25.220786	1.283E-20	-1.342456	down	PREDICTED: pollen-specific leucine-rich repeat extensin-like protein 1 [Sesamum indicum]
c38303.graph_c0	1.8838412	1.6622744	1.8166898	0.434165	0.2299726	0.6277658	1.803E-05	-2.010089	down	PREDICTED: uncharacterized protein LOC104427989 [Eucalyptus grandis]
c38305.graph_c0	2.7983452	2.7337453	2.3208601	0.8722398	0.5544194	0.620891	2.275E-05	-1.88858	down	PREDICTED: probable xyloglucan endotransglucosylase/hydrolase protein 26 [Sesamum indicum]
c38306.graph_c0	3.6486714	4.2552772	4.4349157	8.9608595	9.0336081	6.301494	0.0009653	1.022289	up	PREDICTED: PGR5-like protein 1B, chloroplastic isoform X1 [Sesamum indicum]
c38322.graph_c0	35.772509	37.975915	34.760061	11.514978	12.577779	12.040448	6.85E-24	-1.545483	down	PREDICTED: protein phosphatase 2C 37-like [Sesamum indicum]

#ID	28 h-1 FPKM	28 h-2 FPKM	28 h-3 FPKM	48 h-1 FPKM	48 h-2 FPKM	48 h-3 FPKM	FDR	log2FC	regulated	nr_annotation
c38327.graph_c0	140.10048	161.33054	153.7128	56.98182	57.5396	59.637188	3.468E-19	-1.344212	down	PREDICTED: ent-kaurenoic acid oxidase 1-like [Sesamum indicum]
c38328.graph_c0	3.8791703	3.9453181	4.3883556	1.0965768	1.7980299	1.8333732	2.835E-13	-1.33617	down	PREDICTED: uncharacterized protein LOC104091985 isoform X3 [Nicotiana tomentosiformis]
c38331.graph_c0	22.73741	21.279793	27.288037	345.52517	391.2841	449.21681	2.002E-96	4.09414	up	PREDICTED: raucaffricine-O-beta-D-glucosidase-like [Sesamum indicum]
c38336.graph_c0	26.4384	24.086696	26.510863	78.926563	59.397738	51.637527	0.0001565	1.351233	up	PREDICTED: ankyrin repeat-containing protein At5g02620 [Sesamum indicum]
c38337.graph_c0	0.5504152	1.0560682	0.8982686	2.3983154	1.4320447	3.1040097	0.0054488	1.513502	up	PREDICTED: nicotianamine aminotransferase A-like [Sesamum indicum]
c38340.graph_c0	1.0823247	0.6701059	0.4289674	9.8386714	11.004235	13.183903	1.021E-40	4.002436	up	PREDICTED: protein NRT1/ PTR FAMILY 4.5-like, partial [Sesamum indicum]
c38344.graph_c0	0.7326921	0.6615523	0.8113961	21.891758	10.541665	8.8541611	0.0002053	4.285497	up	PREDICTED: uncharacterized protein LOC105173288 [Sesamum indicum]
c38345.graph_c0	1.0015991	1.6554204	1.7592657	0.2582238	0.2735569	0.3255019	5.624E-07	-2.325659	down	PREDICTED: uncharacterized protein LOC105160841 isoform X1 [Sesamum indicum]
c38355.graph_c0	14.78354	13.197608	14.285426	6.8334772	8.5826048	4.5759613	0.0001782	-1.038063	down	hypothetical protein MIMGU_mgv1a006265mg [Erythranthe guttata]
c38356.graph_c0	0.4141885	0.3902331	0.5583925	1.7804835	1.450928	1.5639525	5.323E-06	1.860022	up	PREDICTED: 65-kDa microtubule-associated protein 3 [Sesamum indicum]
c38360.graph_c0	5.4898192	5.1846905	5.09753	14.637371	14.489699	15.301708	1.92E-07	1.536026	up	unnamed protein product [Coffea canephora]
c38360.graph_c1	2.8012168	2.3116674	2.7527553	7.0633171	6.2288655	8.9347456	7.905E-08	1.539619	up	PREDICTED: auxin transporter-like protein 3 [Sesamum indicum]
c38361.graph_c0	7.0567775	6.9434195	7.2347531	17.316574	16.340245	16.811247	3.715E-13	1.291908	up	PREDICTED: uncharacterized protein LOC105173063 [Sesamum indicum]
c38376.graph_c0	1.4037023	1.9011174	1.1999529	3.092586	4.2635738	4.2972759	0.0001379	1.408089	up	-
c38376.graph_c1	2.4023554	2.3046705	2.5483931	4.6058081	6.2906616	6.028787	6.989E-05	1.258508	up	PREDICTED: receptor-like serine/threonine-protein kinase ALE2 [Sesamum indicum]
c38383.graph_c0	0.9232406	1.2314541	1.1985222	4.2821444	4.8745305	5.5851765	2.075E-12	2.174822	up	PREDICTED: transcription factor LHW-like [Sesamum indicum]
c38383.graph_c1	1.0193466	0.9482645	1.0586272	5.8731676	4.6042126	5.1213911	9.206E-12	2.411756	up	PREDICTED: transcription factor LHW-like [Sesamum indicum]
c38403.graph_c0	1.8891622	1.4433154	3.6759822	4.9735084	7.4153891	9.3425067	0.0004542	1.664142	up	PREDICTED: 2-hydroxyisoflavanone dehydratase [Sesamum indicum]
c38404.graph_c0	0.6462977	0.8753193	0.3691615	4.3692262	6.219769	6.8034945	4.492E-21	3.237697	up	PREDICTED: phosphoinositide phospholipase C 2-like [Sesamum indicum]
c38406.graph_c0	0.9097066	0.4693599	0.7574624	1.882645	1.4708952	2.5546285	0.0002	1.508343	up	PREDICTED: potassium channel SKOR-like [Sesamum indicum]
c38410.graph_c0	0.4552564	0.6850898	0.6191426	1.1541421	1.5894757	2.4646769	0.0027705	1.5986	up	PREDICTED: MLO-like protein 11 [Sesamum indicum]
c38418.graph_c0	36.756072	48.501215	51.87033	118.6634	106.39152	84.351553	1.497E-05	1.220159	up	hypothetical protein MIMGU_mgv1a006647mg [Erythranthe guttata]
c38429.graph_c0	0.4143375	0.3928134	0.6761921	7.1690259	7.0105049	9.7904125	1.834E-30	4.052846	up	PREDICTED: kinesin-like protein KIN12A isoform X2 [Sesamum indicum]
c38442.graph_c0	1177.8314	1132.3058	1260.0717	271.84924	272.96111	255.04368	1.223E-38	-2.11574	down	--
c38443.graph_c0	5.6098524	5.0651708	5.340526	0.4740601	0.7533137	0.4218158	3.851E-10	-3.242418	down	unnamed protein product [Vitis vinifera]
c38447.graph_c1	1.1427845	1.0963165	1.3113321	5.2510427	5.778643	5.9008511	1.964E-16	2.293358	up	PREDICTED: uncharacterized protein LOC105178397 [Sesamum indicum]

#ID	28 h-1 FPKM	28 h-2 FPKM	28 h-3 FPKM	48 h-1 FPKM	48 h-2 FPKM	48 h-3 FPKM	FDR	log2FC	regulated	nr_annotation
c38449.graph_c0	12.369453	15.319564	10.629304	2.2200627	3.1848473	2.5515564	1.431E-14	-2.230225	down	PREDICTED: probably inactive leucine-rich repeat receptor-like protein kinase At5g48380 [Sesamum indicum]
c38457.graph_c0	15.517231	12.36455	16.156074	2.0959018	1.4233038	1.1476436	7.663E-27	-3.186458	down	-
c38464.graph_c0	0.7040139	1.1078621	0.9191514	6.7311664	9.5618278	10.073	3.588E-26	3.306722	up	PREDICTED: cation/H(+) antiporter 14 isoform X1 [Nicotiana tomentosiformis]
c38473.graph_c0	94.613216	94.307395	93.603926	46.142259	36.320642	34.304688	4.15E-17	-1.227156	down	hypothetical protein CISIN_1g011138mg [Citrus sinensis]
c38488.graph_c0	20.522962	22.806536	23.284173	60.93365	56.602083	57.690509	1.614E-21	1.438725	up	PREDICTED: uncharacterized protein LOC105173740 [Sesamum indicum]
c38491.graph_c1	0.4308951	0.3760892	0.5039698	6.1719609	5.4776347	8.0027617	2.219E-27	3.946431	up	PREDICTED: probably inactive leucine-rich repeat receptor-like protein kinase IMK2 [Sesamum indicum]
c38493.graph_c0	0.7093319	0.6117829	0.7602268	2.8315898	2.6869584	2.8777588	2.469E-12	2.054464	up	PREDICTED: structural maintenance of chromosomes protein 2-1 [Sesamum indicum]
c38494.graph_c0	1.9440221	2.6230434	2.1388397	1.2459393	0.7626214	0.7883586	0.0017155	-1.209142	down	Putative gag-pol polyprotein, identical [Solanum demissum]
c38495.graph_c0	4.0298897	3.5519789	4.3844761	10.094741	9.9210822	12.769931	6.294E-05	1.493945	up	PREDICTED: cytochrome c oxidase subunit 5b-1, mitochondrial-like [Sesamum indicum]
c38496.graph_c0	102.01061	98.380126	104.15435	194.42822	202.07241	228.89075	7.471E-14	1.078315	up	PREDICTED: ADP,ATP carrier protein, mitochondrial [Sesamum indicum]
c38509.graph_c0	6.9177222	7.3346526	6.3544374	1.7036375	4.1404174	3.9011782	4.514E-05	-1.052233	down	PREDICTED: sulfate transporter 3.1-like [Sesamum indicum]
c38512.graph_c0	1.1690418	1.2666421	1.090204	3.5987702	4.7095102	3.9556144	9.995E-07	1.836967	up	PREDICTED: microtubule-associated protein RP/EB family member 1B [Sesamum indicum]
c38514.graph_c0	3.1229374	2.9770994	3.2357375	5.8918001	6.2221426	6.2746032	4.477E-06	1.018831	up	PREDICTED: serine/threonine-protein kinase D6PK [Sesamum indicum]
c38516.graph_c0	5.9337297	5.7520262	6.5944554	11.236015	12.22094	12.235426	4.603E-08	1.005593	up	PREDICTED: uncharacterized protein LOC105155823 [Sesamum indicum]
c38532.graph_c0	8.444834	10.88439	9.6656927	3.5545308	4.6961723	5.2713336	5.57E-08	-1.064281	down	PREDICTED: autophagy-related protein 13 isoform X2 [Sesamum indicum]
c38550.graph_c0	65.279875	64.472642	61.374293	30.249431	33.154325	27.336321	6.751E-13	-1.032728	down	PREDICTED: N-(5'-phosphoribosyl)anthranilate isomerase 1, chloroplastic isoform X2 [Sesamum indicum]
c38571.graph_c0	18.904266	19.151079	18.942188	5.8252679	8.36385	9.2653653	7.417E-15	-1.24631	down	PREDICTED: cation/H(+) antiporter 18-like [Sesamum indicum]
c38572.graph_c0	18.599656	17.666148	17.739546	8.0046148	8.8969639	7.6673456	8.682E-08	-1.094983	down	PREDICTED: cyclin-dependent kinase F-4-like [Sesamum indicum]
c38593.graph_c0	10.864898	9.4435192	10.699944	30.710107	34.3364	38.277064	1.74E-28	1.775317	up	PREDICTED: long chain acyl-CoA synthetase 6, peroxisomal-like isoform X2 [Sesamum indicum]
c38596.graph_c0	3.0643548	4.4829571	3.5133446	8.6044519	7.9173529	7.5249406	0.0001579	1.165067	up	PREDICTED: uncharacterized protein LOC105168232 isoform X2 [Sesamum indicum]
c38601.graph_c0	0.5318934	0.4802499	0.6912181	0.2996507	0.1322682	0.1999705	0.0021722	-1.376255	down	-
c38619.graph_c0	1.8532744	2.7251424	2.7652696	5.3359533	7.0393303	6.1513662	4.096E-06	1.372879	up	PREDICTED: uncharacterized protein LOC105177754 isoform X2 [Sesamum indicum]
c38642.graph_c0	2.567233	2.683966	1.8192042	5.7804103	4.762834	4.5718883	0.0008262	1.143211	up	hypothetical protein MIMGU_mgv1a025521mg [Erythranthe guttata]
c38644.graph_c0	55.519061	60.974493	57.740861	10.961361	11.431498	12.751466	5.531E-49	-2.269207	down	PREDICTED: pheophytinase, chloroplastic [Sesamum indicum]

#ID	28 h-1 FPKM	28 h-2 FPKM	28 h-3 FPKM	48 h-1 FPKM	48 h-2 FPKM	48 h-3 FPKM	FDR	log2FC	regulated	nr_annotation
c38667.graph_c0	3.9592276	6.3424072	3.4391295	13.436931	12.519763	9.5072602	0.0004164	1.414791	up	-
c38681.graph_c1	4.6304095	4.3523467	3.352098	17.126565	19.737852	18.211947	5.017E-32	2.199168	up	PREDICTED: 3-ketoacyl-CoA synthase 12 [Sesamum indicum]
c38703.graph_c0	3.6961025	4.3626411	3.9763883	9.1084278	8.0133345	10.386961	1.518E-08	1.235049	up	PREDICTED: BTB/POZ domain-containing protein NPY2 [Sesamum indicum]
c38732.graph_c2	6.1657113	6.2629416	6.3675746	0.8548196	1.4230506	1.6842152	1.537E-08	-2.214249	down	PREDICTED: probable sucrose-phosphate synthase 1 isoform X1 [Sesamum indicum]
c38737.graph_c0	1.1220343	1.1197331	0.807148	2.8445223	3.0332517	4.2794539	8.918E-09	1.774524	up	PREDICTED: beta-1,3-galactosyltransferase 15 [Sesamum indicum]
c38740.graph_c1	0.1798579	0.3247897	0	10.943192	15.215793	13.794347	3.09E-24	6.346833	up	-
c38744.graph_c2	0.6406718	0.7230832	0.8588577	2.2622749	2.9496688	2.554909	1.832E-06	1.842949	up	PREDICTED: uncharacterized protein LOC105160721 [Sesamum indicum]
c38757.graph_c0	20.003617	21.977506	21.11284	42.820932	53.773547	54.548631	1.347E-19	1.298106	up	PREDICTED: uncharacterized protein At5g02240 [Sesamum indicum]
c38761.graph_c0	2.4710635	2.3905057	2.1603939	13.312083	12.996457	15.72699	3.953E-28	2.62274	up	PREDICTED: putative glucose-6-phosphate 1-epimerase [Sesamum indicum]
c38791.graph_c0	35.487531	37.657031	35.78272	68.292022	80.989673	86.230156	8.164E-15	1.150879	up	PREDICTED: probable methyltransferase PMT26 [Sesamum indicum]
c38792.graph_c0	0.2881985	0	0.1175838	3.1051588	2.9992858	3.4943212	5.046E-17	4.604299	up	PREDICTED: uncharacterized protein LOC105110235 [Populus euphratica]
c38803.graph_c0	0.9404563	1.0614298	1.3429581	0.1862653	0.434116	0.4972131	0.0006932	-1.554537	down	-
c38847.graph_c0	6.6263262	6.6303602	7.1219437	26.921818	26.52829	22.672073	4.127E-31	1.944895	up	hypothetical protein EUGRSUZ_J00099 [Eucalyptus grandis]
c38855.graph_c0	2.9368096	3.1633882	3.4474708	17.306929	15.082797	12.261569	8.472E-13	2.272044	up	PREDICTED: UDP-N-acetylglucosamine--peptide N-acetylglucosaminyltransferase 110 kDa subunit [Sesamum indicum]
c38860.graph_c0	0.2438637	0.1223256	0.1989908	0.8929996	1.2371093	1.0085125	3.555E-08	2.510249	up	PREDICTED: probable indole-3-pyruvate monooxygenase YUCCA4 [Sesamum indicum]
c38873.graph_c1	7.527985	6.3639183	7.2870165	3.5081895	2.8740944	2.8302198	0.000277	-1.154828	down	PREDICTED: raucaffricine-O-beta-D-glucosidase-like [Sesamum indicum]
c38886.graph_c0	0.634076	0.9092824	0.8521896	5.7680022	10.217556	13.083244	6.013E-08	3.631588	up	hypothetical protein MIMGU_mgv1a023829mg, partial [Erythranthe guttata]
c38888.graph_c0	23.995682	22.154135	22.681858	57.933933	47.63849	41.702973	1.347E-05	1.144665	up	PREDICTED: putative zinc transporter At3g08650 [Sesamum indicum]
c38892.graph_c0	0.135513	0.0917667	0	2.0612725	1.9107097	2.6747407	7.614E-20	4.91277	up	PREDICTED: peroxidase 19 [Sesamum indicum]
c38893.graph_c0	1.3449056	1.7381496	1.1404685	0.0668536	0.0354116	0.0594859	2.297E-15	-4.656111	down	-
c38900.graph_c0	1.8346447	1.4140957	1.2049468	4.1406085	2.884256	4.2394553	0.0004566	1.3846	up	hypothetical protein AMTR_s00334p00011530 [Amborella trichopoda]
c38916.graph_c0	31.509662	32.157886	34.1682	7.1525223	8.2149178	8.254649	1.407E-34	-2.010905	down	PREDICTED: uncharacterized acetyltransferase At3g50280-like [Sesamum indicum]
c38937.graph_c0	3.0902335	3.5360838	2.7712692	8.571077	9.3265331	11.042885	2.548E-10	1.662184	up	PREDICTED: calcium sensing receptor, chloroplastic [Sesamum indicum]
c38938.graph_c0	15.980243	12.941867	15.788172	4.6489489	5.4778035	5.5717561	1.198E-09	-1.471431	down	PREDICTED: EPIDERMAL PATTERNING FACTOR-like protein 2 [Sesamum indicum]
c38952.graph_c0	8.5686342	7.3682603	8.0740208	3.0601493	3.4701569	4.1418798	0.0001558	-1.131741	down	hypothetical protein MIMGU_mgv1a006823mg [Erythranthe guttata]
c38963.graph_c0	40.966715	39.7133	39.052625	188.31036	158.196	127.62712	2.065E-09	2.033078	up	PREDICTED: uncharacterized protein LOC105177709 [Sesamum indicum]

#ID	28 h-1 FPKM	28 h-2 FPKM	28 h-3 FPKM	48 h-1 FPKM	48 h-2 FPKM	48 h-3 FPKM	FDR	log2FC	regulated	nr_annotation
c38995.graph_c0	31.255019	28.977945	29.721877	9.4202046	12.836617	11.761897	1.223E-20	-1.365909	down	PREDICTED: SNF2 domain-containing protein CLASSY 3-like [Sesamum indicum]
c39001.graph_c0	0	0.0453616	0	1.6875813	2.0239107	2.5498862	6.721E-27	7.15044	up	PREDICTED: putative UDP-glucuronate:xylan alpha-glucuronosyltransferase 4 [Sesamum indicum]
c39033.graph_c0	26.547831	28.503427	28.172881	12.379505	7.1114297	4.5767419	4.875E-13	-1.732128	down	hypothetical protein MIMGU_mgv1a025187mg [Erythranthe guttata]
c39040.graph_c0	10.862811	9.1431439	9.2645963	18.437011	26.124782	23.050262	1.997E-07	1.244399	up	PREDICTED: uncharacterized protein LOC105178874 isoform X1 [Sesamum indicum]
c39068.graph_c0	12.137957	14.353591	13.242922	3.5402545	5.2101129	6.1469464	1.567E-15	-1.381373	down	PREDICTED: protein NRT1/ PTR FAMILY 5.8 [Nicotiana sylvestris]
c39102.graph_c1	370.04556	377.49273	381.13271	41.494126	32.747232	28.826638	7.48E-109	-3.404828	down	15 kDa oleosin [Sesamum indicum]
c39123.graph_c0	0.3266813	0.3754069	0.3877371	2.6351349	5.0647707	4.8234387	1.168E-10	3.553471	up	PREDICTED: probable 1-deoxy-D-xylulose-5-phosphate synthase 2, chloroplastic [Sesamum indicum]
c39124.graph_c0	0.1331799	0.6012449	0.173878	0.7765507	2.2176087	1.6222777	0.0035125	2.37467	up	PREDICTED: aspartic proteinase-like protein 2 isoform X2 [Sesamum indicum]
c39138.graph_c0	0.2566151	0.270316	0.2617448	2.6022286	5.2263302	5.8368568	2.605E-09	4.144546	up	PREDICTED: COBRA-like protein 4 [Sesamum indicum]
c39138.graph_c1	0.0876207	0.0791133	0.3574889	9.218453	19.884648	24.21227	3.017E-11	6.693597	up	hypothetical protein MIMGU_mgv1a006245mg [Erythranthe guttata]
c39142.graph_c0	11.036172	14.832844	12.373868	5.1257993	5.8826764	6.8413581	7.069E-05	-1.060755	down	PREDICTED: uncharacterized protein LOC105155593 [Sesamum indicum]
c39146.graph_c0	7.9272921	7.2438377	7.2089882	16.676756	18.757161	22.137127	3.007E-16	1.401618	up	PREDICTED: phytoene synthase 2, chloroplastic [Sesamum indicum]
c39147.graph_c0	0.2977188	0.2837462	0.3644042	1.6982124	1.9989446	1.9587805	6.704E-11	2.618788	up	hypothetical protein MIMGU_mgv1a023002mg [Erythranthe guttata]
c39170.graph_c0	5.6073413	4.9645946	5.9970731	2.346237	1.973822	2.3946775	0.0013892	-1.259587	down	PREDICTED: protein REVEILLE 6 isoform X2 [Nicotiana sylvestris]
c39200.graph_c0	0.1479982	0.2895283	0.221403	1.2506572	0.7618288	0.7511584	0.0003212	2.121522	up	hypothetical protein MIMGU_mgv1a005105mg [Erythranthe guttata]
c39211.graph_c0	0.1347397	0.1419335	0.219893	1.024755	1.1760706	0.9371492	5.734E-09	2.700214	up	PREDICTED: uncharacterized protein LOC105159159 [Sesamum indicum]
c39225.graph_c0	29.656143	30.740759	29.678228	55.104875	59.215018	63.887222	9.814E-13	1.024504	up	PREDICTED: serine hydroxymethyltransferase, mitochondrial [Sesamum indicum]
c39231.graph_c0	19.073711	19.943653	22.488071	55.690424	57.273062	79.545059	3.866E-10	1.684569	up	PREDICTED: glucose-1-phosphate adenylyltransferase large subunit 1 [Sesamum indicum]
c39252.graph_c0	8.4735128	9.1256382	9.1010742	28.243132	30.468539	34.399805	1.581E-31	1.841731	up	hypothetical protein MIMGU_mgv1a009282mg [Erythranthe guttata]
c39260.graph_c0	2.0327391	2.0189101	2.5591349	11.521291	12.244406	16.605587	3.915E-18	2.648289	up	PREDICTED: probable galactinol--sucrose galactosyltransferase 2 [Sesamum indicum]
c39271.graph_c0	15.051579	13.747824	14.33371	37.051301	34.983889	42.263669	4.184E-20	1.447448	up	PREDICTED: glycerophosphodiester phosphodiesterase GDPD1, chloroplastic-like [Sesamum indicum]
c39285.graph_c0	30.014473	34.604394	39.372923	14.728922	14.968788	18.658972	8.085E-09	-1.065068	down	hypothetical protein MIMGU_mgv11b009377mg [Erythranthe guttata]
c39313.graph_c0	0.3408185	0.6154542	0	3.5641032	2.9748217	3.2674181	2.704E-12	3.406375	up	PREDICTED: malate dehydrogenase, cytoplasmic-like [Sesamum indicum]
c39334.graph_c0	0.0184957	0	0	1.7114559	1.4902027	2.0235039	7.671E-30	8.184529	up	hypothetical protein MIMGU_mgv1a002051mg [Erythranthe guttata]

#ID	28 h-1 FPKM	28 h-2 FPKM	28 h-3 FPKM	48 h-1 FPKM	48 h-2 FPKM	48 h-3 FPKM	FDR	log2FC	regulated	nr_annotation
c39349.graph_c0	0.0994866	0.1197695	0.1082404	2.900453	1.8257943	1.0846864	9.875E-05	4.20572	up	PREDICTED: glucan endo-1,3-beta-glucosidase 14 isoform X1 [Sesamum indicum]
c39362.graph_c0	9.1140556	7.6339485	8.8402015	23.541426	21.013658	23.274466	1.234E-16	1.4497	up	PREDICTED: U-box domain-containing protein 4 [Sesamum indicum]
c39383.graph_c0	19.009276	21.137715	20.248121	9.3188744	6.9598063	9.2631614	1.08E-14	-1.196228	down	PREDICTED: probable auxin efflux carrier component 1c [Sesamum indicum]
c39384.graph_c0	17.168687	15.986143	17.171377	30.301364	34.582215	36.913023	3.795E-08	1.055246	up	PREDICTED: two-on-two hemoglobin-3 [Sesamum indicum]
c39389.graph_c0	35.816748	34.195437	42.663307	8.0734473	6.5499585	7.6838362	1.162E-32	-2.292101	down	hypothetical protein MIMGU_mgv1a004314mg [Erythranthe guttata]
c39403.graph_c0	0.1421282	0.146661	0.1822471	0.8493159	0.7634219	1.4885318	1.945E-05	2.756273	up	PREDICTED: uncharacterized protein LOC105155966 isoform X1 [Sesamum indicum]
c39418.graph_c0	11.292278	8.5985159	10.473709	1.908506	2.8811087	2.97181	8.882E-11	-1.933891	down	PREDICTED: metal transporter Nramp5-like isoform X1 [Sesamum indicum]
c39418.graph_c2	6.0063936	5.7391254	7.0424552	1.8479481	1.8010628	2.1704669	2.858E-05	-1.650227	down	PREDICTED: metal transporter Nramp5-like isoform X2 [Sesamum indicum]
c39433.graph_c0	5.8459617	7.1383471	6.5420667	2.6112087	3.6634241	3.2025765	0.0072939	-1.005948	down	PREDICTED: peroxisome biogenesis factor 10-like [Sesamum indicum]
c39485.graph_c0	5.5972377	4.7217807	4.767328	10.202171	11.411454	11.474009	1.6E-05	1.172979	up	PREDICTED: monothiol glutaredoxin-S10-like isoform X1 [Sesamum indicum]
c39522.graph_c0	39.313446	38.327354	36.783125	14.919234	14.21813	12.159724	1.083E-21	-1.425765	down	hypothetical protein MIMGU_mgv1a011861mg [Erythranthe guttata]
c39525.graph_c0	20.588444	16.692554	18.217146	106.59089	174.49582	201.72502	6.332E-11	3.153141	up	PREDICTED: WAT1-related protein At2g37460-like [Sesamum indicum]
c39546.graph_c0	0.263589	0.1851081	0.286782	1.1508526	1.2585175	1.0570547	4.912E-07	2.277824	up	PREDICTED: flavin-containing monooxygenase FMO GS-OX-like 9 [Sesamum indicum]
c39590.graph_c0	0.9345474	0.675047	0.991358	0.3257671	0.3137371	0.3952713	0.0048062	-1.289525	down	PREDICTED: pentatricopeptide repeat-containing protein At3g09040, mitochondrial [Sesamum indicum]
c39629.graph_c0	0.4049163	0.4160292	0.3076216	0.7079465	1.4624677	1.4173346	0.0004255	1.699026	up	PREDICTED: FK506-binding protein 5 [Solanum lycopersicum]
c39645.graph_c0	0.1659067	0.2995965	0.1353786	1.6823922	3.490319	3.8672047	1.051E-07	3.941631	up	PREDICTED: transcription factor MYB48 [Sesamum indicum]
c39655.graph_c0	6.4081993	5.4288425	6.3910468	32.290894	36.86421	35.335305	4.472E-25	2.558807	up	PREDICTED: UDP-arabinose 4-epimerase 1-like [Sesamum indicum]
c39655.graph_c1	6.6632778	4.062966	5.5784057	22.267242	32.304755	27.816643	1.872E-17	2.373059	up	PREDICTED: probable UDP-arabinose 4-epimerase 3 [Sesamum indicum]
c39661.graph_c0	0.4181847	0.3985583	0.3981078	0.6773243	1.2479008	1.4673926	0.0014635	1.512073	up	PREDICTED: uncharacterized protein LOC105171946 [Sesamum indicum]
c39674.graph_c0	3.9341716	4.5398697	4.9171663	1.2163054	1.9585627	1.7532632	2.773E-06	-1.408067	down	PREDICTED: auxin response factor 18-like [Sesamum indicum]
c39679.graph_c0	3.5746409	4.7045871	4.0210066	8.0126596	9.4105083	9.8174589	4.907E-08	1.186035	up	PREDICTED: fatty acid amide hydrolase [Sesamum indicum]
c39687.graph_c1	11.160263	11.97926	9.9345641	28.886469	27.143299	25.174863	7.69E-08	1.340213	up	RuBisCO large subunit-binding protein subunit beta, chloroplastic [Gossypium arboreum]
c39714.graph_c0	6.6117982	7.5149673	6.2837901	2.8593799	4.0737064	2.6319921	0.0085792	-1.054656	down	PREDICTED: zinc finger protein MAGPIE-like [Sesamum indicum]
c39715.graph_c2	54.080931	55.707152	55.125621	244.98385	268.86927	285.04425	5.43E-50	2.316156	up	PREDICTED: glutamate decarboxylase [Sesamum indicum]
c39733.graph_c0	1.4358941	1.3962068	1.4781167	0.448025	0.4449639	0.5730593	0.0005113	-1.515923	down	unnamed protein product [Coffea canephora]
c39735.graph_c0	11.066575	11.271066	7.224188	5.1621933	4.874292	2.5962066	0.0017573	-1.177714	down	-
c39758.graph_c0	10.642222	4.9781195	7.0099484	2.1128602	0.5165352	2.7477074	0.0033815	-2.024748	down	hypothetical protein M569_00228 [Genlisea aurea]

#ID	28 h-1 FPKM	28 h-2 FPKM	28 h-3 FPKM	48 h-1 FPKM	48 h-2 FPKM	48 h-3 FPKM	FDR	log2FC	regulated	nr_annotation
c39764.graph_c0	22.640779	23.161197	22.490928	0.8807833	1.4773816	0.718406	6.313E-44	-4.435265	down	PREDICTED: putative cytochrome c oxidase subunit 6b-like isoform X2 [Sesamum indicum]
c39766.graph_c0	0.0178489	0.0644634	0.0728226	4.1403284	5.2490302	4.8919211	1.885E-50	6.562248	up	PREDICTED: beta-amyrin synthase [Sesamum indicum]
c39771.graph_c0	0.1349619	0.121858	0.1321534	0.7869412	1.087394	1.7657609	1.014E-05	3.258495	up	PREDICTED: pachytene checkpoint protein 2 homolog [Sesamum indicum]
c39774.graph_c0	0	0	0.0520425	1.4147632	1.798524	1.4746502	3.332E-21	6.527031	up	PREDICTED: uncharacterized protein LOC105171399 isoform X2 [Sesamum indicum]
c39810.graph_c0	0.3088332	0.1533661	0.1764038	0.5676299	0.933106	0.9230656	4.762E-06	1.957466	up	PREDICTED: protein argonaute MEL1 [Sesamum indicum]
c39816.graph_c0	6.4130566	6.4245742	5.6067876	16.141914	27.55748	30.034813	8.942E-07	2.031435	up	PREDICTED: protein IQ-DOMAIN 1 isoform X1 [Sesamum indicum]
c39829.graph_c0	4.1374537	3.5361134	2.809145	0.7606566	0.890647	0.9261853	8.44E-07	-1.984803	down	-
c39843.graph_c0	1.5239386	2.1168826	1.6739712	5.1264464	3.8566894	3.900397	0.0015504	1.325719	up	PREDICTED: uncharacterized protein LOC105177798 [Sesamum indicum]
c39856.graph_c1	2.8931896	4.7021021	3.0353388	0.9430279	1.221029	2.0511348	0.0057769	-1.30071	down	-
c39870.graph_c0	0.4420102	0.5321251	0.3606768	4.8557647	3.7591456	3.822096	2.295E-11	3.2678	up	-
c39870.graph_c1	0.2931868	0.2117762	0.4784761	2.3784696	1.8897751	2.0502121	5.629E-09	2.728359	up	hypothetical protein PFL1_05374 [Pseudozyma flocculosa PF-1]
c39878.graph_c0	38.561055	41.936028	40.465696	2.0753086	2.7192441	2.3325437	2.869E-82	-4.046867	down	PREDICTED: uncharacterized protein LOC105157942 isoform X1 [Sesamum indicum]
c39883.graph_c0	2.5339367	2.5374971	3.0075224	6.8910957	6.6816124	7.6905509	2.77E-05	1.437317	up	PREDICTED: adenine phosphoribosyltransferase 5-like isoform X1 [Sesamum indicum]
c39891.graph_c0	13.91853	13.959508	15.035658	6.3654284	4.1947131	5.0395508	1.503E-08	-1.411077	down	-
c39893.graph_c0	0.1769928	0.3652752	0.3713778	1.5063596	1.4599921	1.6540486	7.3E-08	2.379847	up	PREDICTED: uncharacterized protein LOC105155471 [Sesamum indicum]
c39894.graph_c0	41.616889	31.691342	52.79192	1.7166906	1.4397452	1.0501569	2.603E-21	-4.858178	down	PREDICTED: beta-amyrin 28-oxidase-like [Sesamum indicum]
c39913.graph_c0	98.431038	103.68633	99.970466	25.730896	38.421877	36.351965	9.909E-27	-1.552476	down	PREDICTED: uncharacterized protein LOC105168623 [Sesamum indicum]
c39933.graph_c0	2.8427797	2.9660379	2.5516536	9.4890394	7.5499689	9.4764355	7.456E-10	1.710021	up	hypothetical protein L484_002978 [Morus notabilis]
c39938.graph_c0	0.7029519	0.8189673	0.6106096	6.754683	5.6332638	6.75197	9.91E-25	3.210533	up	PREDICTED: thioredoxin reductase NTRC [Sesamum indicum]
c39945.graph_c0	23.404464	28.209275	24.097525	63.252031	66.118372	75.456756	9.579E-22	1.476213	up	PREDICTED: protodermal factor 1-like [Sesamum indicum]
c39981.graph_c0	43.474453	45.941186	44.97649	108.48015	120.1188	126.89367	1.073E-23	1.443106	up	PREDICTED: 2-aminoethanethiol dioxygenase-like isoform X1 [Sesamum indicum]
c40001.graph_c0	12.195964	10.681068	11.60458	24.172199	25.636449	25.955752	2.585E-14	1.176457	up	PREDICTED: protein IQ-DOMAIN 1-like [Sesamum indicum]
c40024.graph_c0	7.9903023	7.9978858	9.0061912	3.938782	2.6824256	3.709525	1.226E-07	-1.228101	down	PREDICTED: pheophytinase, chloroplastic [Sesamum indicum]
c40027.graph_c0	30.521977	28.283707	31.003872	9.738613	10.738934	12.328443	4.477E-16	-1.413982	down	hypothetical protein MIMGU_mgv1a025482mg [Erythranthe guttata]
c40069.graph_c0	7.33634	6.6968187	5.9863935	12.773834	14.831432	18.003872	7.468E-05	1.225766	up	PREDICTED: villin-3-like [Sesamum indicum]
c40069.graph_c1	6.3340047	6.2045889	6.0461652	10.149637	14.684125	14.490175	0.0001479	1.116751	up	PREDICTED: villin-3-like [Sesamum indicum]
c40069.graph_c2	10.301513	10.052716	10.222957	17.990287	22.290323	25.495543	1.366E-10	1.142077	up	PREDICTED: villin-2-like [Sesamum indicum]

#ID	28 h-1 FPKM	28 h-2 FPKM	28 h-3 FPKM	48 h-1 FPKM	48 h-2 FPKM	48 h-3 FPKM	FDR	log2FC	regulated	nr_annotation
c40070.graph_c0	3.3800174	3.7284257	3.5386515	0.9700576	1.3273921	1.1328862	2.487E-07	-1.596531	down	PREDICTED: protein TRM32 isoform X2 [Sesamum indicum]
c40072.graph_c0	3.8194776	3.054501	3.2502338	6.5359833	8.0231424	11.015942	3.114E-05	1.372503	up	PREDICTED: putative zinc finger protein At1g68190 isoform X1 [Sesamum indicum]
c40085.graph_c0	2.6985029	2.2980579	2.3020452	5.1696966	5.7237344	6.813482	2.134E-05	1.317325	up	PREDICTED: uncharacterized protein LOC105172351 [Sesamum indicum]
c40091.graph_c0	25.450054	27.181193	25.285789	86.250207	73.097309	67.575393	1.761E-12	1.588513	up	PREDICTED: universal stress protein A-like protein [Sesamum indicum]
c40092.graph_c0	0.0237936	0	0.0776614	0.7841626	1.7253524	2.2542472	1.898E-07	5.576799	up	PREDICTED: uncharacterized protein LOC105168414 [Sesamum indicum]
c40102.graph_c0	19.232515	18.456682	19.303097	9.5076654	9.4450109	8.1499784	1.215E-08	-1.028888	down	PREDICTED: protein Brevis radix-like 2 [Sesamum indicum]
c40130.graph_c0	17.138414	15.409994	15.963239	114.34584	91.354398	83.218475	3.026E-15	2.621795	up	PREDICTED: bifunctional 3-dehydroquinate dehydratase/shikimate dehydrogenase, chloroplastic-like isoform X1 [Sesamum indicum]
c40137.graph_c0	130.17107	132.12511	110.42076	34.938096	41.492471	39.48979	1.021E-29	-1.645413	down	PREDICTED: CBL-interacting serine/threonine-protein kinase 7-like isoform X1 [Sesamum indicum]
c40153.graph_c0	7.1317729	8.4346052	7.4587553	24.446325	21.176943	16.314145	4.603E-06	1.475097	up	Lysine decarboxylase family protein isoform 3 [Theobroma cacao]
c40153.graph_c1	27.096448	30.549917	26.422602	61.185756	57.198741	63.243157	3.225E-15	1.154158	up	PREDICTED: 50S ribosomal protein L15, chloroplastic [Sesamum indicum]
c40169.graph_c0	8.1634756	8.4524456	8.0370388	3.5879895	3.4078288	4.603708	4.253E-10	-1.047222	down	PREDICTED: uncharacterized protein LOC105170357 [Sesamum indicum]
c40171.graph_c0	3.7273008	3.2539659	3.5248558	9.3241008	9.2645379	8.9090243	1.588E-15	1.430568	up	PREDICTED: 125 kDa kinesin-related protein-like [Sesamum indicum]
c40184.graph_c0	5.5698021	4.866783	5.864404	1.3664776	2.1111087	3.2423562	0.0012502	-1.247572	down	PREDICTED: ADP-ribosylation factor-like protein 8B isoform X1 [Nelumbo nucifera]
c40189.graph_c0	0.209514	0.3625787	0.3134298	0.7967234	1.1488211	1.5359926	1.599E-05	2.008581	up	PREDICTED: uncharacterized protein LOC105177814 [Sesamum indicum]
c40192.graph_c0	2.6470166	4.3816809	4.2478897	7.9408493	9.5379654	9.6531668	2.679E-05	1.305204	up	PREDICTED: endo-1,3;1,4-beta-D-glucanase-like [Sesamum indicum]
c40204.graph_c0	1.4269451	1.7996666	1.5340186	2.8136236	3.9843958	4.1640601	0.0001914	1.238925	up	PREDICTED: transcription factor EGL1-like [Sesamum indicum]
c40207.graph_c0	0.2926717	0.2789359	0.3449589	3.4212866	2.6200759	3.9061637	1.102E-20	3.483226	up	PREDICTED: kinesin-4 [Sesamum indicum]
c40208.graph_c0	0.4726099	0.5943633	0.426965	5.5841678	7.3663537	9.4615831	7.307E-19	3.942621	up	PREDICTED: cellulose synthase-like protein D5 [Sesamum indicum]
c40228.graph_c0	2.1452499	2.1604549	3.0297227	6.9550362	6.0384505	5.4905927	9.334E-05	1.378562	up	PREDICTED: membrane protein of ER body-like protein [Sesamum indicum]
c40229.graph_c0	2.4702463	2.2750088	1.8947587	3.9453361	4.8098631	5.3215272	0.0002654	1.121912	up	PREDICTED: transcription factor E2FA-like [Sesamum indicum]
c40230.graph_c0	3.0140526	2.27869	2.4947453	1.1516441	1.471779	1.089788	0.0014985	-1.028905	down	-
c40238.graph_c0	12.378603	16.165634	13.858072	4.044858	4.1124848	3.864795	9.331E-20	-1.775859	down	PREDICTED: glucose-1-phosphate adenylyltransferase large subunit 2, chloroplastic/amyloplastic-like [Sesamum indicum]
c40264.graph_c0	1.4133117	1.6844361	1.2455109	6.8076113	7.8191515	7.906464	1.696E-10	2.41508	up	PREDICTED: very-long-chain enoyl-CoA reductase-like [Sesamum indicum]
c40274.graph_c0	3.9035243	5.0054057	3.399378	1.1642364	2.2905392	1.7018858	0.0010176	-1.222368	down	-
c40283.graph_c0	7.4242157	8.1881564	8.2682203	14.959092	17.698366	18.301951	2.724E-10	1.132124	up	unnamed protein product [Coffea canephora]
c40304.graph_c0	31.997003	29.996303	31.621928	12.703957	13.408456	13.020423	1.303E-12	-1.217114	down	PREDICTED: probable serine/threonine-protein kinase Cx32, chloroplastic [Sesamum indicum]

#ID	28 h-1 FPKM	28 h-2 FPKM	28 h-3 FPKM	48 h-1 FPKM	48 h-2 FPKM	48 h-3 FPKM	FDR	log2FC	regulated	nr_annotation
c40319.graph_c0	0.1999893	0.2608256	0.1813218	0.8731707	0.7459827	0.8270674	8.643E-06	1.974571	up	PREDICTED: monofunctional riboflavin biosynthesis protein RIBA 3, chloroplastic [Sesamum indicum]
c40334.graph_c0	7.6496659	7.4223737	5.9625694	2.3879477	2.9130351	2.1891688	0.000115	-1.448847	down	-
c40354.graph_c1	4.1502073	3.9613766	4.5153804	18.48758	13.906129	16.004334	5.021E-14	1.984844	up	PREDICTED: GATA transcription factor 8 [Sesamum indicum]
c40357.graph_c0	1.3709852	1.3168842	1.4519463	6.6185241	3.290324	4.1454161	0.0039112	1.817759	up	ribulose-1,5-bisphosphate carboxylase/oxygenase large subunit (chloroplast) [Andrographis paniculata]
c40362.graph_c0	0.5188646	0.6090319	0.9314564	5.3931323	5.9920706	6.6129483	3.13E-14	3.166032	up	PREDICTED: uncharacterized protein LOC105160931 [Sesamum indicum]
c40362.graph_c1	3.1519289	3.3692795	2.9562623	0.7347682	0.9729972	1.5118947	0.000303	-1.523986	down	PREDICTED: protein SHI RELATED SEQUENCE 5-like [Sesamum indicum]
c40376.graph_c1	101.06051	94.763587	114.44201	26.821951	24.777535	18.042704	2.903E-37	-2.10941	down	PREDICTED: protein SRG1 [Sesamum indicum]
c40408.graph_c0	20.591957	19.835839	20.716384	10.334831	9.722767	8.393195	2.2E-12	-1.05919	down	PREDICTED: nucleolin-like isoform X1 [Sesamum indicum]
c40410.graph_c0	0.4703383	0.1633352	0.2952248	1.0089343	1.4089302	1.2650025	4.316E-06	2.023331	up	Uncharacterized protein TCM_021521 [Theobroma cacao]
c40418.graph_c0	3.3012639	3.461495	4.5186396	28.752218	37.180597	44.44091	3.551E-23	3.325785	up	PREDICTED: organ-specific protein S2-like [Nicotiana tomentosiformis]
c40434.graph_c0	24.514596	20.053371	21.713429	48.60301	45.130245	40.647139	2.441E-11	1.064079	up	PREDICTED: putative glutamine amidotransferase YLR126C [Sesamum indicum]
c40440.graph_c0	5.6919207	5.4309591	5.8496361	14.039861	15.637865	14.817593	3.174E-18	1.43082	up	PREDICTED: armadillo repeat-containing kinesin-like protein 2 isoform X2 [Sesamum indicum]
c40443.graph_c0	3.2320187	3.0813396	3.2270079	0.6361524	1.4017669	1.7207757	0.0002019	-1.316093	down	PREDICTED: pentatricopeptide repeat-containing protein At2g21090 [Sesamum indicum]
c40462.graph_c0	1.5061942	2.1008216	1.7243278	3.1202795	4.4526919	5.1724822	1.551E-05	1.292194	up	PREDICTED: paramyosin-like [Sesamum indicum]
c40484.graph_c0	0.0231955	0.06283	0.1514185	3.1460099	2.4918251	1.9882883	3.746E-17	5.052224	up	PREDICTED: probable receptor-like protein kinase At5g39020 [Sesamum indicum]
c40493.graph_c0	14.965705	17.155531	19.370589	35.561567	33.137268	36.986765	8.166E-10	1.079683	up	PREDICTED: clp protease-related protein At4g12060, chloroplastic isoform X1 [Sesamum indicum]
c40499.graph_c0	214.99057	237.90505	255.42699	61.027328	70.647282	72.02366	1.342E-27	-1.75895	down	PREDICTED: legumin B-like [Sesamum indicum]
c40504.graph_c0	8.3208668	7.6234473	7.6085245	2.4196773	2.6619453	2.8982886	1.452E-08	-1.52187	down	PREDICTED: isochorismate synthase, chloroplastic-like [Sesamum indicum]
c40517.graph_c0	42.560167	44.550958	39.244758	96.7312	87.611395	102.42342	9.99E-16	1.225282	up	hypothetical protein MIMGU_mgv1a015340mg [Erythranthe guttata]
c40523.graph_c1	47.395834	52.310277	48.465655	22.259377	21.164211	15.453638	1.15E-19	-1.285613	down	PREDICTED: uncharacterized protein LOC105164312 [Sesamum indicum]
c40525.graph_c0	16.408521	16.877567	15.915024	4.9140515	5.4883292	6.6773738	1.542E-12	-1.487967	down	PREDICTED: U-box domain-containing protein 4 [Sesamum indicum]
c40570.graph_c0	23.796578	23.950333	23.246326	54.652661	62.345214	63.778805	1.085E-21	1.387916	up	PREDICTED: transmembrane emp24 domain-containing protein p24delta3-like [Sesamum indicum]
c40591.graph_c0	6.9688088	4.9255534	8.3367561	0.079941	0.1270317	0.142262	6.301E-21	-5.824082	down	PREDICTED: cytokinin hydroxylase [Sesamum indicum]
c40597.graph_c0	15.119822	14.944914	15.476323	45.592594	40.139957	31.799048	5.727E-07	1.414475	up	PREDICTED: uncharacterized protein LOC105174543 [Sesamum indicum]
c40600.graph_c1	136.2735	134.07936	125.86941	41.690272	63.193882	69.585451	1.377E-14	-1.149365	down	PREDICTED: auxin response factor 19-like [Sesamum indicum]

#ID	28 h-1 FPKM	28 h-2 FPKM	28 h-3 FPKM	48 h-1 FPKM	48 h-2 FPKM	48 h-3 FPKM	FDR	log2FC	regulated	nr_annotation
c40615.graph_c0	2.307393	2.935643	2.7386383	13.205873	10.046525	7.8468144	1.262E-05	2.01219	up	PREDICTED: cytochrome P450 CYP72A219-like [Sesamum indicum]
c40623.graph_c1	0.1045614	0.0629394	0.0568808	4.9481315	4.6803083	2.5158958	1.806E-12	5.806451	up	PREDICTED: E3 ubiquitin-protein ligase RMA1H1-like [Sesamum indicum]
c40632.graph_c1	13.689308	12.803102	17.460841	1.2732847	1.0038257	1.2120049	1.035E-29	-3.610405	down	PREDICTED: cytochrome P450 71A1-like [Vitis vinifera]
c40642.graph_c0	0.0280562	0.1519927	0.2060426	0.8179558	1.43165	1.5822004	8.11E-09	3.341754	up	PREDICTED: uncharacterized protein LOC105163904 [Sesamum indicum]
c40646.graph_c0	46.722781	45.203823	49.782264	6.8112404	5.6317528	6.8736082	4.921E-42	-2.831085	down	PREDICTED: transcription factor PIF3 isoform X2 [Sesamum indicum]
c40646.graph_c1	30.178975	30.180616	34.717698	4.9028163	4.5527123	4.6317864	4.902E-37	-2.711481	down	unnamed protein product [Vitis vinifera]
c40673.graph_c0	1.620265	1.6350589	1.9054126	9.9065901	6.9112302	4.8374036	0.0008174	2.12158	up	PREDICTED: GDSL esterase/lipase At4g10955-like [Sesamum indicum]
c40691.graph_c0	104.83913	105.68437	102.07325	576.06955	378.36048	261.1033	0.0034582	2.013509	up	PREDICTED: chaperone protein dnaJ 20, chloroplastic-like [Sesamum indicum]
c40692.graph_c0	0.1345239	0.1214625	0.0878163	5.3201922	4.0103008	4.9462925	2.994E-33	5.422345	up	hypothetical protein MIMGU_mgv1a008779mg [Erythranthe guttata]
c40701.graph_c1	126.81322	140.48111	142.9329	60.311979	59.408283	40.161997	1.644E-20	-1.313864	down	PREDICTED: glutathione S-transferase F9-like [Sesamum indicum]
c40704.graph_c0	0.9562406	1.2334223	0.9660666	2.3087722	2.0484112	2.0029733	0.0077681	1.056246	up	PREDICTED: protein trichome birefringence-like 3 [Sesamum indicum]
c40715.graph_c0	3.9340803	3.4515744	3.6341631	10.914367	11.263419	10.381299	4.196E-09	1.605099	up	PREDICTED: apoptosis-inducing factor homolog B [Sesamum indicum]
c40715.graph_c2	100.62109	96.817542	92.957897	549.08868	573.04442	509.70748	2.246E-63	2.532755	up	PREDICTED: uncharacterized protein LOC105174779 [Sesamum indicum]
c40731.graph_c0	19.546239	20.060472	19.76772	6.8565985	8.6451812	10.049741	8.124E-14	-1.179854	down	PREDICTED: probably inactive leucine-rich repeat receptor-like protein kinase At5g48380 [Sesamum indicum]
c40732.graph_c0	0.8481398	0.4594744	1.6609804	8.7081454	9.4530074	4.4003574	2.113E-05	2.969952	up	hypothetical protein L484_002552 [Morus notabilis]
c40759.graph_c0	79.045828	70.038821	79.786845	33.549929	23.114361	17.296311	2.884E-16	-1.577405	down	hypothetical protein MIMGU_mgv1a008881mg [Erythranthe guttata]
c40770.graph_c0	15.586506	18.676522	16.918328	6.8934237	7.9547774	6.5718503	9.38E-07	-1.215625	down	PREDICTED: O-glucosyltransferase rumi homolog isoform X1 [Sesamum indicum]
c40770.graph_c1	13.137674	15.276398	13.583205	4.9909493	7.2242393	5.0564892	4.136E-07	-1.243978	down	PREDICTED: O-glucosyltransferase rumi homolog isoform X1 [Sesamum indicum]
c40773.graph_c0	1472.244	1358.7118	1292.612	335.70093	366.24336	241.12372	1.44E-24	-2.084707	down	hypothetical protein MIMGU_mgv1a016776mg [Erythranthe guttata]
c40776.graph_c0	5.0211583	4.6676343	5.44951	0.9092411	0.6310822	0.697446	7.701E-15	-2.7096	down	-
c40800.graph_c0	0.9340715	1.4272568	0.9380858	1.9126225	2.9910448	3.9709601	0.0020013	1.460227	up	PREDICTED: LOW QUALITY PROTEIN: probable leucine-rich repeat receptor-like protein kinase At1g35710 [Sesamum indicum]
c40802.graph_c0	0.7532819	0.9824286	0.785414	1.8566338	2.135468	2.3128303	0.0016125	1.36157	up	hypothetical protein MIMGU_mgv1a011194mg [Erythranthe guttata]
c40806.graph_c1	35.187821	40.381924	35.983521	71.801815	99.621028	112.11458	4.431E-08	1.381158	up	PREDICTED: phenylalanine ammonia-lyase [Sesamum indicum]
c40828.graph_c0	1.4755066	2.3138977	2.0594759	3.7898453	3.6498923	4.9049914	0.0031515	1.118012	up	PREDICTED: protein ABIL4-like [Sesamum indicum]
c40830.graph_c0	1.946102	2.2554432	1.951427	4.4918353	4.1344821	4.0732497	7.592E-07	1.089595	up	PREDICTED: auxilin-like protein 1 [Sesamum indicum]
c40897.graph_c0	2.5650368	1.8426472	2.4138814	0.9730386	0.9553909	0.9502726	0.0012932	-1.202526	down	PREDICTED: protein root UVB sensitive 6 [Sesamum indicum]

#ID	28 h-1 FPKM	28 h-2 FPKM	28 h-3 FPKM	48 h-1 FPKM	48 h-2 FPKM	48 h-3 FPKM	FDR	log2FC	regulated	nr_annotation
c40910.graph_c0	234.1593	207.00049	211.36502	27.944737	56.643552	49.776946	9.207E-52	-2.249135	down	PREDICTED: hippocampus abundant transcript-like protein 1 isoform X1 [Sesamum indicum]
c40910.graph_c1	222.93651	207.29531	203.2866	31.384307	54.634582	49.773671	1.422E-48	-2.189053	down	PREDICTED: hippocampus abundant transcript-like protein 1 isoform X1 [Sesamum indicum]
c40912.graph_c1	15.558301	17.902041	15.804535	40.873607	31.599038	26.421308	0.0020013	1.054568	up	hypothetical protein MIMGU_mgv1a013233mg [Erythranthe guttata]
c40916.graph_c0	0.3015454	0.4084008	0.3998452	1.6244817	2.024637	2.4657746	1.95E-08	2.498423	up	PREDICTED: uncharacterized protein LOC105162032 [Sesamum indicum]
c40925.graph_c0	3.6911696	4.1796341	4.6660758	0.9587765	1.3407338	0.8531136	1.237E-07	-1.952806	down	PREDICTED: uncharacterized protein LOC105166274 [Sesamum indicum]
c40929.graph_c0	3669.9995	3972.1388	4094.3939	503.03885	459.38986	381.23699	2.553E-37	-3.081252	down	--
c40932.graph_c0	4.4527786	3.994915	4.4177907	8.8157416	10.079512	11.893839	2.458E-13	1.296932	up	PREDICTED: uncharacterized protein LOC105163461 [Sesamum indicum]
c40939.graph_c0	3.8973078	3.4035296	3.2583722	9.2323641	8.3220642	7.9266599	2.255E-06	1.315674	up	PREDICTED: uncharacterized protein LOC105156631 [Sesamum indicum]
c40942.graph_c0	3.0529279	2.7893232	3.143612	4.3765719	9.1264803	11.108853	0.0085792	1.481981	up	PREDICTED: serine carboxypeptidase-like 27 [Sesamum indicum]
c40949.graph_c0	59.684948	78.35717	82.197013	39.276864	30.861344	30.434697	2.579E-06	-1.083747	down	PREDICTED: malate synthase [Tarenaya hassleriana]
c40952.graph_c0	34.772865	38.934204	37.170966	81.655244	100.97998	117.58466	5.425E-13	1.473996	up	putative nitrilase-associated protein [Plantago major]
c40960.graph_c0	4.208119	5.6088402	3.9897378	0.6096115	0.3229047	0.0904048	2.578E-16	-3.689555	down	PREDICTED: transcription factor bHLH113-like [Sesamum indicum]
c40962.graph_c0	1.3451533	1.8555584	1.7684105	2.6049847	3.1108956	4.2986504	0.007391	1.047144	up	PREDICTED: ATP-dependent DNA helicase Q-like 1 isoform X2 [Sesamum indicum]
c40970.graph_c0	0.8100189	1.5313083	1.3219376	9.9467241	13.528595	15.988064	9.603E-21	3.464662	up	PREDICTED: glycerophosphodiester phosphodiesterase GDPDL3-like [Sesamum indicum]
c40977.graph_c0	10.914781	8.5563001	8.6992475	4.2900122	3.7494169	4.7715338	0.0049658	-1.094216	down	class I KNOX homeobox transcription factor STM-like 2 [Prunus persica]
c40980.graph_c0	17.131174	16.654266	16.443994	6.6623875	8.5586395	7.3547872	2.057E-13	-1.115191	down	PREDICTED: putative pentatricopeptide repeat-containing protein At5g65820 isoform X2 [Sesamum indicum]
c40985.graph_c0	11.558468	11.384959	10.460519	2.0169197	4.878085	4.0294815	4.378E-10	-1.583747	down	PREDICTED: zinc transporter 4, chloroplastic-like [Sesamum indicum]
c41001.graph_c0	261.38354	296.77137	258.96303	80.658165	124.45472	134.68887	6.97E-12	-1.231934	down	PREDICTED: uncharacterized protein LOC105155456 [Sesamum indicum]
c41003.graph_c0	118.90689	127.93342	144.2844	19.669734	16.5792	13.334866	1.535E-64	-2.932389	down	hypothetical protein M569_01497, partial [Genlisea aurea]
c41009.graph_c0	5.6535234	4.5374236	4.378296	9.621364	10.860464	13.107235	1.408E-08	1.243367	up	PREDICTED: uncharacterized protein LOC105156446 [Sesamum indicum]
c41020.graph_c0	69.085799	65.033624	64.997597	595.19832	613.76568	766.20865	6.63E-54	3.349881	up	PREDICTED: stachyose synthase [Sesamum indicum]
c41025.graph_c0	5.8813502	5.3210792	5.4805523	11.522807	13.12014	11.961745	1.904E-13	1.173988	up	PREDICTED: uncharacterized protein LOC105177410 [Sesamum indicum]
c41038.graph_c0	5.8560389	5.8067576	6.3144199	14.647092	16.640207	12.67906	1.478E-06	1.331874	up	shikimate kinase, partial [Ipomoea purpurea]
c41040.graph_c0	5.1891421	4.9765577	4.4517345	14.701869	16.271667	16.751453	4.322E-28	1.747175	up	PREDICTED: glucomannan 4-beta-mannosyltransferase 9-like [Nicotiana tomentosiformis]
c41055.graph_c0	1.1931526	2.616312	1.6690333	6.2656936	8.6977317	6.7286613	6.177E-06	2.023953	up	calmodulin, partial [Malus domestica]

#ID	28 h-1 FPKM	28 h-2 FPKM	28 h-3 FPKM	48 h-1 FPKM	48 h-2 FPKM	48 h-3 FPKM	FDR	log2FC	regulated	nr_annotation
c41061.graph_c0	18.275551	17.787416	16.885404	6.6760727	7.5362593	7.0439971	2.55E-16	-1.276261	down	PREDICTED: pentatricopeptide repeat-containing protein At2g17670 [Sesamum indicum]
c41070.graph_c1	21.030291	21.811559	24.011813	9.0534711	9.5697414	9.6131641	1.666E-15	-1.202609	down	probable tocopherol cyclase, chloroplastic [Sesamum indicum]
c41081.graph_c0	0.0457311	0	0.1119487	0.7535788	0.7369157	1.2636909	1.073E-09	4.160533	up	PREDICTED: probable LRR receptor-like serine/threonine-protein kinase At4g31250 [Sesamum indicum]
c41083.graph_c0	3.246595	2.1741	2.2518157	15.500957	14.459537	20.658472	4.373E-19	2.762094	up	hypothetical protein MIMGU_mgv1a013647mg [Erythranthe guttata]
c41101.graph_c0	505.5497	537.54127	474.07211	65.197951	80.310472	86.532433	4.711E-63	-2.671139	down	-
c41104.graph_c0	5.6599779	4.8938858	6.2623671	16.446489	16.263671	13.308543	1.229E-14	1.496003	up	PREDICTED: UPF0496 protein At4g34320 [Sesamum indicum]
c41107.graph_c0	110.41254	122.96525	131.17472	15.395859	14.206714	12.397414	2.497E-92	-3.072861	down	PREDICTED: isopentenyl-diphosphate Delta-isomerase I [Sesamum indicum]
c41123.graph_c1	23.113285	22.283159	20.446628	8.214334	6.4540507	7.4917916	2.341E-10	-1.525279	down	hypothetical protein MIMGU_mgv1a010537mg [Erythranthe guttata]
c41126.graph_c0	57.002776	55.857301	56.973311	118.21077	121.98436	127.75751	2.275E-15	1.156495	up	hypothetical protein MIMGU_mgv1a006861mg [Erythranthe guttata]
c41134.graph_c0	3.8873565	4.9749279	4.1374581	7.6697776	10.031671	12.162137	3.375E-06	1.235795	up	PREDICTED: dynamin-like protein ARC5 [Sesamum indicum]
c41135.graph_c0	5.0178979	4.6976116	4.8272752	11.013804	10.852108	9.5319306	7.026E-07	1.15386	up	hypothetical protein MIMGU_mgv1a008665mg [Erythranthe guttata]
c41136.graph_c1	24.907351	27.80617	33.243451	5.4455412	6.7411755	8.2753067	5.293E-18	-2.034573	down	PREDICTED: protein TRANSPARENT TESTA 12-like [Nicotiana tomentosiformis]
c41136.graph_c2	12.234048	14.713881	14.623364	2.9682432	3.2728405	4.0425383	8.267E-12	-1.976809	down	PREDICTED: protein TRANSPARENT TESTA 12-like [Sesamum indicum]
c41141.graph_c0	0.5149132	0.4349236	0.2710742	2.0212348	1.672854	2.1544322	1.86E-09	2.304372	up	PREDICTED: LOW QUALITY PROTEIN: uncharacterized protein LOC105157816 [Sesamum indicum]
c41142.graph_c0	0	0.0953319	0.0861552	1.4052641	3.969885	3.4534733	1.48E-07	5.630661	up	gibberelin 20-oxidase [Lactuca sativa]
c41146.graph_c2	1.6798507	0.9479673	0.9423868	2.927835	2.6787289	3.5525045	0.0010784	1.400561	up	PREDICTED: protein BRASSINAZOLE-RESISTANT 1 isoform X2 [Sesamum indicum]
c41147.graph_c0	0.0906254	0	0.1478991	7.4093944	10.283263	10.01702	4.933E-43	6.893277	up	hypothetical protein MIMGU_mgv1a010461mg [Erythranthe guttata]
c41154.graph_c1	0.1653251	0	0.1349039	2.724303	3.7740898	4.0090362	2.45E-18	5.16271	up	-
c41154.graph_c2	2.0540376	1.5692798	3.4810849	13.819341	19.307715	24.236316	1.093E-11	3.046199	up	Ubiquitin-conjugating enzyme [Plantago major]
c41157.graph_c1	17.151513	17.342742	15.305014	29.24202	34.951687	40.330029	3.904E-10	1.107406	up	PREDICTED: 4-hydroxy-3-methylbut-2-en-1-yl diphosphate synthase, chloroplastic [Sesamum indicum]
c41173.graph_c0	13.364468	13.863332	13.672464	8.6922893	6.3349941	4.3047003	3.138E-06	-1.029041	down	hypothetical protein MIMGU_mgv1a018356mg [Erythranthe guttata]
c41183.graph_c0	0	0.2039554	0.9830535	2.4815208	4.8532991	2.7175905	5.467E-05	3.112316	up	hypothetical protein VOLCADRAFT_105423 [Volvox carteri f. nagariensis]
c41191.graph_c0	13.885076	13.871651	13.053319	38.412944	31.548373	30.487733	8.726E-10	1.346033	up	PREDICTED: uncharacterized protein DDB_G0287625 [Sesamum indicum]
c41207.graph_c0	15.024631	12.747576	12.143214	2.8113791	4.3233609	3.577483	3.934E-18	-1.862027	down	PREDICTED: pentatricopeptide repeat-containing protein At1g11900 [Sesamum indicum]
c41210.graph_c0	25.744393	23.320243	25.190469	125.2341	111.04501	98.454086	1.984E-21	2.217958	up	PREDICTED: probable beta-1,3-galactosyltransferase 6 [Sesamum indicum]

#ID	28 h-1 FPKM	28 h-2 FPKM	28 h-3 FPKM	48 h-1 FPKM	48 h-2 FPKM	48 h-3 FPKM	FDR	log2FC	regulated	nr_annotation
c41219.graph_c0	29.320652	34.645934	24.89835	66.431803	66.009752	62.961672	1.832E-14	1.179987	up	hypothetical protein MIMGU_mgv1a008823mg [Erythranthe guttata]
c41225.graph_c0	81.752595	77.040012	79.855048	26.750016	27.212693	23.802003	2.079E-27	-1.574823	down	PREDICTED: 1-aminocyclopropane-1-carboxylate oxidase homolog 1-like [Solanum lycopersicum]
c41226.graph_c0	0.3039429	0.2582889	0.3647278	2.0170016	2.1847859	3.6095971	3.754E-08	3.110554	up	PREDICTED: alpha-mannosidase-like [Sesamum indicum]
c41242.graph_c0	11.079988	10.510732	10.122052	3.1803051	4.9595527	6.0990451	3.51E-09	-1.122529	down	PREDICTED: pentatricopeptide repeat-containing protein At3g02490, mitochondrial [Sesamum indicum]
c41258.graph_c0	1.9502384	2.4026994	2.6473402	23.080362	24.769025	24.463217	5.505E-87	3.409424	up	PREDICTED: phosphoenolpyruvate carboxykinase [ATP]-like [Sesamum indicum]
c41261.graph_c1	0.0793039	0.1432079	0.097067	2.1109936	2.9817891	2.5939112	2.812E-18	4.625513	up	hypothetical protein MIMGU_mgv1a019580mg [Erythranthe guttata]
c41287.graph_c1	0.1617404	0.1251741	0.1131247	1.2301071	0.7446568	1.0684814	2.467E-09	2.976415	up	hypothetical protein MIMGU_mgv1a018989mg [Erythranthe guttata]
c41288.graph_c0	8.5182831	7.8336408	6.6505041	3.1326229	4.3777726	3.4397566	0.0033454	-1.032946	down	PREDICTED: transcription initiation factor TFIID subunit 14b [Sesamum indicum]
c41289.graph_c0	88.077064	77.784834	75.201129	240.89555	225.88822	253.0668	9.093E-27	1.620758	up	PREDICTED: uncharacterized protein LOC105166112 [Sesamum indicum]
c41295.graph_c0	7.4954551	6.9536188	5.3096943	2.4013621	2.4886487	2.8334733	0.0001692	-1.314415	down	PREDICTED: NAC transcription factor 29 [Sesamum indicum]
c41298.graph_c0	30.799652	36.423663	30.586452	15.029224	12.215902	11.793189	8.468E-17	-1.278112	down	unnamed protein product [Coffea canephora]
c41313.graph_c0	8.2950544	8.2453392	6.0098741	0.7544102	0.7492558	0.6502925	1.015E-26	-3.344095	down	PREDICTED: uncharacterized protein LOC105168700 [Sesamum indicum]
c41315.graph_c0	2.3066116	2.9969899	2.2953369	0.7487781	1.0576528	1.1104304	0.0010265	-1.34556	down	-
c41321.graph_c0	6.4838391	5.503742	5.8768619	28.273462	26.380969	23.997113	4.839E-37	2.182649	up	PREDICTED: probable amino acid permease 7 [Sesamum indicum]
c41322.graph_c0	0.4619862	0.3932942	0.3877477	0.1003888	0.1595247	0.0744378	4.995E-05	-1.856134	down	-
c41328.graph_c0	0.2611097	0.3143434	0.319595	1.2687402	1.2856383	1.1289174	3.56E-06	2.08388	up	PREDICTED: uncharacterized protein LOC105177311 [Sesamum indicum]
c41351.graph_c0	0.1812197	0.1227183	0.1478738	4.0199136	5.4753571	6.234017	3.273E-25	5.156738	up	-
c41354.graph_c0	10.944043	10.656576	11.395427	4.7847253	5.6760406	5.055686	1.055E-12	-1.048837	down	PREDICTED: uncharacterized protein LOC105169395 [Sesamum indicum]
c41362.graph_c0	22.569382	22.193084	22.119601	77.524393	53.006719	40.124254	0.0086561	1.403982	up	PREDICTED: gibberellin receptor GID1B-like [Sesamum indicum]
c41387.graph_c0	2.2254024	2.4772558	2.3880463	0.4637033	1.2280934	1.1002676	0.0018437	-1.317529	down	PREDICTED: pentatricopeptide repeat-containing protein At5g27110 [Sesamum indicum]
c41394.graph_c1	2.4212661	2.2337019	2.0186841	6.1382663	6.1493386	6.2335675	4.652E-05	1.514793	up	PREDICTED: squamosa promoter-binding-like protein 6, partial [Sesamum indicum]
c41429.graph_c0	1.8411318	1.6623694	1.6756965	15.618308	11.886281	13.817209	6.633E-18	3.042383	up	hypothetical protein PHAVU_003G130600g [Phaseolus vulgaris]
c41431.graph_c0	28.674871	27.39315	28.387606	12.998772	13.770623	13.923122	1.546E-08	-1.012566	down	PREDICTED: uncharacterized protein LOC105163802 [Sesamum indicum]
c41432.graph_c1	4.1411616	3.3620305	4.1742491	0.8822231	1.2149909	1.4129942	1.657E-05	-1.699389	down	-
c41445.graph_c0	0.107276	0.1162322	0.0700291	2.1756844	1.6998444	2.1053031	1.08E-19	4.394633	up	unnamed protein product [Coffea canephora]
c41452.graph_c0	1.1916486	1.4440341	0.9211979	0.3577506	0.3789933	0.6366486	0.0044108	-1.335783	down	PREDICTED: uncharacterized protein LOC105161758 [Sesamum indicum]

#ID	28 h-1 FPKM	28 h-2 FPKM	28 h-3 FPKM	48 h-1 FPKM	48 h-2 FPKM	48 h-3 FPKM	FDR	log2FC	regulated	nr_annotation
c41454.graph_c0	8.9605614	10.089755	11.009963	78.805538	92.404836	105.98097	6.016E-55	3.242737	up	PREDICTED: BURP domain-containing protein 3-like [Sesamum indicum]
c41473.graph_c0	15.207933	13.941941	14.998044	39.894687	42.764732	41.513779	1.947E-26	1.532856	up	PREDICTED: probable polygalacturonase [Sesamum indicum]
c41480.graph_c0	1621.4001	2138.5385	1834.7846	335.10848	446.22771	339.96932	6.221E-27	-2.279924	down	PREDICTED: pathogenesis-related protein STH-2-like [Sesamum indicum]
c41483.graph_c0	1.7212561	2.9446728	1.848067	4.1913888	4.1969659	4.7001572	0.0070026	1.048607	up	PREDICTED: uncharacterized protein LOC105112702 isoform X1 [Populus euphratica]
c41484.graph_c0	0.7963108	1.0545245	1.1696096	1.7495965	2.5663644	3.5925703	0.0024304	1.421043	up	hypothetical protein MIMGU_mgv1a017010mg [Erythranthe guttata]
c41486.graph_c0	58.428829	55.068439	61.399918	9.5539206	10.12122	9.116694	2.309E-66	-2.561078	down	glucose and ribitol dehydrogenase homolog 1-like precursor [Sesamum indicum]
c41489.graph_c0	124.68696	129.56924	133.01179	51.713788	57.711667	62.086089	2.152E-14	-1.135673	down	PREDICTED: protein DJ-1 homolog D [Sesamum indicum]
c41508.graph_c0	4.5079213	4.4312991	4.8056868	1.6589387	2.0503518	2.0091547	2.113E-05	-1.227076	down	hypothetical protein MIMGU_mgv1a001446mg [Erythranthe guttata]
c41513.graph_c0	0.7386018	0.9447582	1.4565081	3.2768125	2.1489529	3.6793206	0.0003026	1.58023	up	hypothetical protein MIMGU_mgv1a001330mg [Erythranthe guttata]
c41529.graph_c0	53.303505	61.293293	58.857111	16.985111	15.316922	17.153331	3.44E-31	-1.767119	down	PREDICTED: LOB domain-containing protein 36 [Sesamum indicum]
c41543.graph_c0	14.465702	13.998898	18.159838	7.6166346	3.28733	4.7691659	1.097E-06	-1.517247	down	hypothetical protein MIMGU_mgv1a008932mg [Erythranthe guttata]
c41543.graph_c1	12.37382	10.354907	11.88237	4.3037365	3.2421596	2.6380579	1.173E-06	-1.715304	down	PREDICTED: gibberellin 2-beta-dioxygenase 8 [Sesamum indicum]
c41549.graph_c0	0.7028568	1.413458	1.1470508	4.3736209	4.8049254	5.0807281	2.358E-09	2.167654	up	PREDICTED: interferon-activable protein 203 isoform X1 [Sesamum indicum]
c41555.graph_c0	0.3522654	0.4505888	0.2155843	2.418663	3.0747361	4.8008647	5.89E-08	3.372562	up	PREDICTED: protein IQ-DOMAIN 1-like [Sesamum indicum]
c41558.graph_c0	1.5153513	1.6513002	1.5349832	0.4305285	0.5613449	0.8545668	0.0032759	-1.314497	down	PREDICTED: NAC domain-containing protein 8-like [Sesamum indicum]
c41561.graph_c0	7.5760629	10.585539	9.048522	25.644347	21.710927	25.014078	3.169E-13	1.455666	up	PREDICTED: probable transcription factor GLK1 [Sesamum indicum]
c41565.graph_c0	6.9740144	5.9513206	6.2112332	2.317825	2.112833	3.0216364	0.0002228	-1.320014	down	PREDICTED: uncharacterized protein LOC105165462 [Sesamum indicum]
c41567.graph_c0	31.402432	31.753774	30.139081	61.102719	61.307651	72.600687	1.14E-14	1.104346	up	chloroplast omega-3 desaturase [Portulaca oleracea]
c41569.graph_c0	1.672773	1.8707833	2.1870527	0.722851	0.5105153	0.8575846	0.0004	-1.410967	down	PREDICTED: uncharacterized protein LOC105179857 isoform X2 [Sesamum indicum]
c41581.graph_c0	3.1425287	3.2243281	2.7640916	9.7256711	11.399251	14.03948	5.526E-17	1.982903	up	PREDICTED: trehalose-phosphate phosphatase A-like isoform X2 [Sesamum indicum]
c41597.graph_c0	29.787001	30.916187	32.091189	13.233154	13.497671	13.410807	5.411E-15	-1.167201	down	PREDICTED: ninja-family protein AFP1-like [Sesamum indicum]
c41598.graph_c0	24.496894	24.040561	22.753999	65.181792	46.504546	43.355047	0.000988	1.170739	up	hypothetical protein MIMGU_mgv1a006127mg [Erythranthe guttata]
c41613.graph_c0	0.7907493	0.5949771	0.9678675	2.8399435	1.5485316	3.0843833	0.0009573	1.713061	up	-
c41640.graph_c0	447.99514	605.11963	547.61033	79.524951	77.389352	71.110724	6.412E-31	-2.767902	down	PREDICTED: ubiquitin carboxyl-terminal hydrolase isozyme L3 [Sesamum indicum]
c41645.graph_c0	5.4457935	5.9522075	5.2233236	2.1496011	2.4696847	2.5592493	8.833E-05	-1.171887	down	PREDICTED: MATE efflux family protein 5-like isoform X3 [Sesamum indicum]
c41664.graph_c0	23.367543	21.430489	27.122782	8.7229977	12.403519	10.983141	9.569E-11	-1.127321	down	PREDICTED: probable inactive purple acid phosphatase 27 [Sesamum indicum]

#ID	28 h-1 FPKM	28 h-2 FPKM	28 h-3 FPKM	48 h-1 FPKM	48 h-2 FPKM	48 h-3 FPKM	FDR	log2FC	regulated	nr_annotation
c41664.graph_c1	0.8183132	1.0159327	1.1128949	2.3338627	2.4266585	3.3841817	0.0002074	1.504766	up	PREDICTED: uncharacterized protein At5g39865-like [Sesamum indicum]
c41681.graph_c0	5.449369	5.1998301	5.3309158	2.295951	2.5985916	2.8810459	6.953E-05	-1.000402	down	PREDICTED: uncharacterized protein LOC105169002 isoform X1 [Sesamum indicum]
c41721.graph_c1	0.1319578	0.1787183	0.215353	2.4253605	4.695755	6.2509649	1.489E-07	4.696545	up	PREDICTED: geraniol synthase, chloroplastic-like [Sesamum indicum]
c41730.graph_c0	1.6677881	2.0078084	2.3150968	6.9739162	11.403806	11.480976	1.588E-10	2.35032	up	PREDICTED: vacuolar amino acid transporter 1-like isoform X1 [Sesamum indicum]
c41730.graph_c1	18.300409	18.318752	19.241917	3.2519321	4.6507875	4.8225827	1.216E-30	-2.098972	down	PREDICTED: glucan endo-1,3-beta-glucosidase 5 [Sesamum indicum]
c41743.graph_c0	19.259886	19.334643	20.477688	4.6350395	5.2129497	3.9264932	3.718E-28	-2.058508	down	1-O-acylglucose:anthocyanin-O-acyltransferase- like protein [Gentiana triflora]
c41744.graph_c0	6.5187258	4.6846149	7.1284879	2.3046365	1.310064	1.3004133	4.954E-07	-1.845564	down	hypothetical protein MIMGU_mgv1a021012mg [Erythranthe guttata]
c41758.graph_c0	0.5263829	0.2970465	0.4295242	1.2232534	3.5931457	6.0111766	0.0097957	3.13408	up	PREDICTED: tetratricopeptide repeat protein 28-like [Sesamum indicum]
c41762.graph_c1	32.412414	34.629583	30.746358	90.011509	115.21052	141.91857	6.883E-11	1.863812	up	PREDICTED: lanC-like protein GCR2 [Sesamum indicum]
c41774.graph_c0	21.395456	20.749877	26.914385	5.2879979	2.1294124	1.2382181	2.581E-22	-2.930346	down	hypothetical protein POPTR_0018s00310g [Populus trichocarpa]
c41777.graph_c0	0.1786047	0.2418949	0.2428999	2.1381709	2.7847809	2.8985628	3.333E-27	3.596308	up	PREDICTED: serine/threonine-protein kinase ULK4 [Sesamum indicum]
c41781.graph_c0	0.9658365	0.7751643	0.8464936	5.1691354	6.2446437	7.0605831	1.668E-15	2.87332	up	PREDICTED: actin-11 [Sesamum indicum]
c41790.graph_c0	221.20904	205.15184	211.41512	41.040593	69.48727	87.303841	1.454E-26	-1.657577	down	PREDICTED: 5'-adenylylsulfate reductase 1, chloroplastic-like [Nicotiana sylvestris]
c41799.graph_c0	0.4933933	1.1582685	1.2883357	4.3778864	5.9629369	5.2309886	6.654E-08	2.442053	up	NOX2 [Striga asiatica]
c41799.graph_c1	0.7247753	1.1417264	0.9059909	2.9906816	4.0379842	4.4525436	3.646E-12	2.086186	up	PREDICTED: respiratory burst oxidase homolog protein A [Sesamum indicum]
c41829.graph_c0	7.093289	5.9663672	6.4277995	1.8928982	2.6068849	2.3580052	1.352E-05	-1.469932	down	PREDICTED: uncharacterized protein LOC105164835 isoform X1 [Sesamum indicum]
c41848.graph_c0	17.014214	16.140075	17.425296	7.097924	8.9654263	8.2589793	2.36E-10	-1.018062	down	PREDICTED: LOW QUALITY PROTEIN: F-box protein SKIP14-like [Sesamum indicum]
c41859.graph_c0	1.8296377	2.4320983	1.7210619	5.2826234	4.8114521	5.6176068	2.98E-06	1.436113	up	PREDICTED: probable microtubule-binding protein TANGLED [Sesamum indicum]
c41861.graph_c0	0.5943664	0.7805922	0.5290889	4.2806999	2.6846505	3.0471529	3.562E-07	2.445939	up	PREDICTED: calcium-transporting ATPase 2, plasma membrane-type-like [Sesamum indicum]
c41869.graph_c0	209.90314	200.4605	205.99769	805.9563	647.75196	492.26897	2.185E-05	1.707623	up	PREDICTED: probable 2-aminoethanethiol dioxygenase [Sesamum indicum]
c41876.graph_c1	58.579208	59.342334	57.934062	151.12472	133.75255	130.66115	5.258E-18	1.285442	up	PREDICTED: phospho-2-dehydro-3-deoxyheptonate aldolase 1, chloroplastic [Sesamum indicum]
c41892.graph_c0	4.7248924	6.9206188	7.4253566	14.329594	14.334497	16.697875	1.019E-08	1.290717	up	PREDICTED: tetraspanin-10 [Nicotiana sylvestris]
c41894.graph_c0	0.860422	1.7609288	1.1701622	6.2530497	5.7000203	5.8227133	1.661E-08	2.273591	up	PREDICTED: pleckstrin homology domain-containing family A member 8-like [Sesamum indicum]
c41898.graph_c0	0.2735775	0.2352522	0.350801	2.0311378	2.5016213	2.8064487	3.443E-18	3.130413	up	PREDICTED: kinesin-3-like [Sesamum indicum]

#ID	28 h-1 FPKM	28 h-2 FPKM	28 h-3 FPKM	48 h-1 FPKM	48 h-2 FPKM	48 h-3 FPKM	FDR	log2FC	regulated	nr_annotation
c41899.graph_c0	18.952038	17.136821	18.27852	245.54619	240.23352	190.17139	4.386E-39	3.680535	up	hypothetical protein AALP_AA7G017200 [Arabis alpina]
c41905.graph_c0	7.1745481	7.4033649	7.7843856	3.1979844	4.0231036	2.6677006	0.0037226	-1.136549	down	-
c41909.graph_c0	496.13506	487.68197	469.37529	1537.8587	1369.5052	1605.16	2.206E-20	1.677777	up	hypothetical protein MIMGU_mgv1a015091mg [Erythranthe guttata]
c41920.graph_c0	5.2261479	5.4177914	6.4757131	1.1040879	0.9097256	0.9824108	9.853E-10	-2.469403	down	-
c41941.graph_c0	3.8467653	4.9872571	7.0022263	25.130454	42.384349	21.137195	6.015E-05	2.52104	up	60S ribosomal protein L35 [Galdieria sulphuraria]
c41953.graph_c0	17.690321	16.386628	16.701654	5.7768593	7.0614023	7.3909596	3.825E-11	-1.289854	down	PREDICTED: potassium transporter 6-like isoform X2 [Sesamum indicum]
c41953.graph_c1	24.821505	24.362673	25.519733	8.0274619	10.23719	9.5462845	1.93E-15	-1.387541	down	PREDICTED: potassium transporter 6-like isoform X2 [Sesamum indicum]
c41962.graph_c0	14.221737	12.618435	11.83379	4.7193176	5.275255	5.9437496	7.277E-17	-1.239759	down	PREDICTED: thyroid adenoma-associated protein homolog [Sesamum indicum]
c41964.graph_c0	0.7493132	0.4566777	0.5044326	1.3297336	1.6099332	1.5423713	0.000385	1.428543	up	hypothetical protein M569_02915, partial [Genlisea aurea]
c41968.graph_c0	1.1711773	1.22015	1.3232369	0.0570982	0.1209773	0	1.873E-13	-4.344309	down	-
c41980.graph_c0	2.2258684	3.2990244	2.0904475	0.2129398	0.1127919	0.4736813	1.527E-09	-3.213262	down	PREDICTED: probable serine/threonine-protein kinase At5g41260, partial [Sesamum indicum]
c41985.graph_c0	1.9389224	1.9279476	1.1816018	4.6043491	4.9766229	5.8685642	5.93E-08	1.653625	up	PREDICTED: probable GABA transporter 2 [Sesamum indicum]
c41997.graph_c0	12.387378	12.573422	12.842314	25.902937	42.415556	47.769413	1.233E-05	1.651082	up	unnamed protein product [Coffea canephora]
c42000.graph_c0	24.859175	24.153181	24.226391	56.847134	67.860105	66.112949	1.573E-22	1.420487	up	hypothetical protein MIMGU_mgv1a002552mg [Erythranthe guttata]
c42048.graph_c0	2.5097143	2.6800241	2.4811173	6.5766178	8.8142284	7.4304695	1.265E-09	1.610941	up	PREDICTED: probable glucan 1,3-beta-glucosidase A [Beta vulgaris subsp. vulgaris]
c42052.graph_c0	1.9739257	1.9172903	3.0993912	0.5686582	0.6827477	0.4047909	1.041E-05	-2.036008	down	PREDICTED: homeobox-leucine zipper protein HOX11-like [Sesamum indicum]
c42077.graph_c0	0.3171219	0.442512	0.4469644	1.8637039	3.0583351	3.8043639	1.302E-07	2.886155	up	PREDICTED: high-affinity nitrate transporter 3.1-like [Sesamum indicum]
c42078.graph_c0	13.623833	16.64485	15.667933	4.6950671	4.5164883	2.2088684	3.127E-14	-1.959651	down	PREDICTED: uncharacterized protein LOC105156356 isoform X1 [Sesamum indicum]
c42081.graph_c0	6.925054	7.4332494	7.2314274	2.4144659	3.6199853	3.6231108	5.029E-07	-1.125862	down	PREDICTED: PHD finger protein MALE STERILITY 1 [Sesamum indicum]
c42089.graph_c0	3.3730158	3.3334017	3.9299771	8.9127052	8.089864	8.2143785	8.806E-10	1.289043	up	PREDICTED: U-box domain-containing protein 9-like [Sesamum indicum]
c42100.graph_c0	20.960362	24.926609	22.186599	50.198578	54.092612	57.567693	3.278E-19	1.289914	up	PREDICTED: 4-hydroxy-3-methylbut-2-en-1-yl diphosphate synthase, chloroplastic [Sesamum indicum]
c42120.graph_c0	107.83328	112.0976	101.08003	358.46448	304.67128	257.05002	1.594E-08	1.566417	up	PREDICTED: chaperone protein dnaJ 11, chloroplastic-like [Sesamum indicum]
c42129.graph_c1	1.4429727	1.578476	2.1058311	6.4017595	5.2913631	5.3832665	2.68E-08	1.781049	up	PREDICTED: cytochrome P450 90A1 [Sesamum indicum]
c42160.graph_c0	26.941863	28.333272	23.973642	8.8744527	10.576582	9.6590317	1.828E-09	-1.405103	down	-
c42202.graph_c0	3.1850737	3.0539713	3.3119925	1.8221513	1.0597991	1.3670115	0.0031222	-1.117801	down	hypothetical protein MIMGU_mgv1a025142mg [Erythranthe guttata]

#ID	28 h-1 FPKM	28 h-2 FPKM	28 h-3 FPKM	48 h-1 FPKM	48 h-2 FPKM	48 h-3 FPKM	FDR	log2FC	regulated	nr_annotation
c42204.graph_c0	0.1962429	0.0354378	0.1601326	9.9003417	17.919557	17.707064	2.786E-25	6.890701	up	PREDICTED: probable 2-oxoglutarate-dependent dioxygenase AOP1 [Sesamum indicum]
c42207.graph_c0	0.0939329	0.1696252	0.3065939	19.526965	27.750119	28.393254	3.041E-53	7.086408	up	-
c42219.graph_c0	41.079276	48.543931	45.981008	106.21373	108.31128	132.20161	1.341E-19	1.39447	up	PREDICTED: uncharacterized protein LOC105161471 [Sesamum indicum]
c42248.graph_c0	0.4055235	0.5315077	0.5443903	1.2767865	1.2120706	1.4459162	0.0001957	1.450798	up	PREDICTED: 65-kDa microtubule-associated protein 3 [Sesamum indicum]
c42251.graph_c0	28.170222	30.028407	28.903072	7.7442302	11.172862	12.226424	6.088E-20	-1.448992	down	PREDICTED: ATPase family AAA domain-containing protein At1g05910 isoform X1 [Sesamum indicum]
c42274.graph_c0	24.733384	24.247763	23.156047	7.5144776	9.4832118	8.9881907	3.596E-21	-1.434797	down	PREDICTED: lysine-specific histone demethylase 1 homolog 1 [Sesamum indicum]
c42279.graph_c0	62.263278	62.281351	64.837261	445.0601	284.96309	220.30786	0.0004077	2.380909	up	PREDICTED: transcription factor bHLH130-like [Sesamum indicum]
c42293.graph_c1	4.1151481	3.7930011	5.8064159	1.7387511	2.0722455	1.8372172	0.0043407	-1.240875	down	hypothetical protein CISIN_1g015935mg [Citrus sinensis]
c42301.graph_c0	11.476845	11.35305	10.769764	22.142526	22.482766	23.014555	3.035E-12	1.051158	up	hypothetical protein JCGZ_08911 [Jatropha curcas]
c42329.graph_c1	109.22825	97.748263	105.81121	45.106877	50.797518	50.227286	4.683E-13	-1.058102	down	PREDICTED: probable N-acetyltransferase HLS1 isoform X2 [Sesamum indicum]
c42335.graph_c0	17.653703	15.229578	17.569571	0.9624675	1.4565966	1.1928398	1.292E-53	-3.768417	down	PREDICTED: vetispiradiene synthase 3-like [Sesamum indicum]
c42358.graph_c0	23.8791	21.727079	31.447077	62.173171	53.855728	63.744296	1.685E-11	1.265068	up	hypothetical protein MIMGU_mgv1a016283mg [Erythranthe guttata]
c42360.graph_c0	46.067126	45.484516	41.975054	77.560324	93.56117	105.37603	7.075E-13	1.087872	up	PREDICTED: UDP-glycosyltransferase 73D1 [Sesamum indicum]
c42372.graph_c1	57.225312	51.268555	59.063028	11.058658	15.984444	15.343615	1.29E-24	-1.947566	down	-
c42373.graph_c0	56.991472	55.666864	54.496121	19.771789	27.566615	26.313397	3.329E-15	-1.146054	down	PREDICTED: ethylene-responsive transcription factor ERF118-like [Sesamum indicum]
c42388.graph_c0	1.7089936	2.0002642	1.3428757	2.8081346	4.3348223	5.1401031	0.0002841	1.315401	up	PREDICTED: ATP-citrate synthase beta chain protein 2-like [Citrus sinensis]
c42389.graph_c0	13.048047	12.843686	12.113846	4.4421869	6.0083454	6.3446294	4.663E-12	-1.141983	down	PREDICTED: protein FAR1-RELATED SEQUENCE 3-like [Sesamum indicum]
c42395.graph_c0	2.3223401	2.2149881	1.8683203	26.825323	25.563182	32.796833	1.299E-57	3.774294	up	PREDICTED: tetraketide alpha-pyrone reductase 1-like [Sesamum indicum]
c42399.graph_c0	11.76225	9.4803906	10.186527	19.227436	22.817738	25.606184	9.073E-14	1.143847	up	PREDICTED: scarecrow-like protein 14 [Sesamum indicum]
c42403.graph_c0	11.628072	10.974937	12.176347	21.587222	29.327227	33.735064	1.162E-07	1.318454	up	PREDICTED: LOW QUALITY PROTEIN: protein YIF1B-like [Sesamum indicum]
c42413.graph_c0	428.57195	499.41029	475.07777	106.85838	100.20372	84.04512	1.402E-48	-2.22395	down	PREDICTED: extra-large guanine nucleotide-binding protein 3-like [Sesamum indicum]
c42434.graph_c1	5.6825936	5.0697677	5.0785643	10.461654	11.446226	15.336492	4.824E-05	1.27215	up	PREDICTED: uncharacterized protein LOC105170092 [Sesamum indicum]
c42438.graph_c0	0.6668897	0.8901184	0.9937138	5.9540766	9.2278176	8.8298359	4.276E-21	3.268986	up	PREDICTED: uncharacterized protein LOC105168396 [Sesamum indicum]
c42443.graph_c0	0.8811301	1.1933668	0.8660621	0.1269202	0.1344565	0.1129328	1.93E-09	-2.930813	down	putative retroelement pol polyprotein [Arabidopsis thaliana]
c42453.graph_c0	1.2222145	1.5633555	1.3713109	4.7122925	4.2398679	5.8299508	6.506E-09	1.871736	up	hypothetical protein MIMGU_mgv1a006178mg [Erythranthe guttata]

#ID	28 h-1 FPKM	28 h-2 FPKM	28 h-3 FPKM	48 h-1 FPKM	48 h-2 FPKM	48 h-3 FPKM	FDR	log2FC	regulated	nr_annotation
c42464.graph_c0	6.5773876	7.1265172	8.2096669	2.8408657	3.3039655	3.2696352	9.343E-06	-1.179822	down	-
c42466.graph_c0	0.6010114	0.5024601	0.8173673	1.6647314	1.4945602	1.9582864	0.0004059	1.454662	up	PREDICTED: TSL-kinase interacting protein 1 isoform X2 [Sesamum indicum]
c42486.graph_c0	0.2174632	0.441785	0.2883533	5.1684493	9.7461147	9.2590283	8.846E-16	4.705152	up	hypothetical protein MIMGU_mgv1a023633mg [Erythranthe guttata]
c42491.graph_c0	43.48765	46.485717	48.674369	14.791553	12.555351	13.243183	1.173E-31	-1.727348	down	PREDICTED: bidirectional sugar transporter SWEET13b [Sesamum indicum]
c42500.graph_c0	0.914178	1.1136579	1.1367037	2.7038386	3.8191856	4.1243303	1.495E-09	1.785537	up	PREDICTED: LOW QUALITY PROTEIN: intracellular protein transport protein USO1-like [Sesamum indicum]
c42500.graph_c1	1.4284215	2.0518443	1.2185612	2.8805469	4.5337911	5.7852677	0.0007106	1.523214	up	PREDICTED: paramyosin-like [Sesamum indicum]
c42509.graph_c0	0.3587528	0.5232556	0.4278498	1.3292563	1.3340708	1.3383911	0.0001618	1.653581	up	PREDICTED: putative late blight resistance protein homolog R1B-14 [Sesamum indicum]
c42522.graph_c0	0.5911654	0.7199647	0.5048227	2.1434771	2.0861399	2.4499673	2.228E-08	1.920857	up	hypothetical protein MIMGU_mgv1a005054mg [Erythranthe guttata]
c42530.graph_c1	0.5096056	0.4001096	0.3615948	0.7302186	0.8033311	1.2245178	0.0064529	1.154133	up	PREDICTED: inactive protein kinase SELMODRAFT_444075 [Sesamum indicum]
c42531.graph_c0	1.8350609	1.4531722	1.0555406	2.4976706	3.7366881	4.1224899	4.199E-05	1.288131	up	PREDICTED: probable LRR receptor-like serine/threonine-protein kinase MRH1 isoform X1 [Sesamum indicum]
c42544.graph_c0	5.4761715	4.7473477	5.1810977	10.193094	13.705594	14.363863	1.613E-12	1.348683	up	PREDICTED: non-lysosomal glucosylceramidase isoform X3 [Sesamum indicum]
c42556.graph_c0	636.66845	652.30468	682.79034	140.44969	133.73377	122.79444	2.123E-33	-2.268581	down	PREDICTED: LOW QUALITY PROTEIN: low-temperature-induced 65 kDa protein [Sesamum indicum]
c42575.graph_c0	1.7656293	1.8780959	1.8551983	2.7286	4.9367907	4.9376228	0.0007393	1.228275	up	PREDICTED: nucleobase-ascorbate transporter 2 [Sesamum indicum]
c42622.graph_c1	2.1706869	2.0895087	2.5617437	1.0005475	0.8672389	1.0926177	0.0016253	-1.161902	down	PREDICTED: glycosyltransferase family protein 64 protein C5-like [Sesamum indicum]
c42629.graph_c0	0.8615546	1.113469	1.406043	2.3019415	2.1890938	3.2676791	8.567E-05	1.237263	up	PREDICTED: DNA polymerase alpha catalytic subunit [Sesamum indicum]
c42672.graph_c0	1.2754914	1.7134292	1.5738784	5.5207097	5.973848	5.8245776	3.676E-09	1.965311	up	PREDICTED: uncharacterized protein LOC105171447 isoform X1 [Sesamum indicum]
c42679.graph_c0	1.7451472	1.9046975	1.9404308	0.534794	0.6180539	0.7137848	0.0001313	-1.544363	down	PREDICTED: probable methyltransferase PMT10 [Sesamum indicum]
c42680.graph_c0	0.0647041	0.7594829	0.2111922	2.952621	4.691916	4.4516859	8.364E-14	3.582923	up	PREDICTED: uncharacterized protein LOC104243668 [Nicotiana sylvestris]
c42692.graph_c0	63.887801	63.236817	60.758675	120.03132	135.12766	155.37116	3.341E-12	1.1664	up	PREDICTED: multiple C2 and transmembrane domain-containing protein 1 [Sesamum indicum]
c42697.graph_c0	0.566938	0.7678378	0.5204439	12.576125	11.467193	12.389106	7.092E-42	4.339501	up	PREDICTED: micronuclear linker histone polyprotein-like [Sesamum indicum]
c42712.graph_c0	6.7085176	7.5548124	7.0982777	15.208254	17.49722	19.144652	7.29E-16	1.31812	up	PREDICTED: phosphoglucomutase, chloroplastic [Sesamum indicum]
c42724.graph_c0	1.8465657	2.1833373	2.242236	4.5141263	5.018326	6.1489341	2.662E-06	1.360317	up	PREDICTED: U-box domain-containing protein 11-like [Sesamum indicum]
c42742.graph_c0	0.2631767	0.4118815	0.3722335	1.1787055	1.5314191	1.3258479	2.445E-06	1.98467	up	PREDICTED: uncharacterized protein LOC105163391 [Sesamum indicum]
c42749.graph_c0	2.0118747	1.5239381	1.4984401	0.8557716	0.6890056	0.7005436	0.0026265	-1.118387	down	-

#ID	28 h-1 FPKM	28 h-2 FPKM	28 h-3 FPKM	48 h-1 FPKM	48 h-2 FPKM	48 h-3 FPKM	FDR	log2FC	regulated	nr_annotation
c42770.graph_c0	26.000943	24.872878	25.62179	9.1365355	11.481628	8.6562503	2.069E-15	-1.345891	down	PREDICTED: E3 ubiquitin-protein ligase Topors [Sesamum indicum]
c42772.graph_c0	0.2853819	0.1220557	0.3186632	0.6854069	0.7261055	1.2366827	0.0003506	1.901674	up	PREDICTED: testis-expressed sequence 11 protein [Sesamum indicum]
c42782.graph_c2	6.622163	7.1002908	5.8680054	13.968273	17.785018	17.213714	1.386E-07	1.359791	up	hypothetical protein MIMGU_mgv1a014334mg [Erythranthe guttata]
c42789.graph_c1	0.2666096	0.3691094	0.3190752	1.2841999	1.3365864	1.002345	4.897E-06	1.967881	up	PREDICTED: ABC transporter G family member 15-like isoform X1 [Sesamum indicum]
c42796.graph_c0	117.35345	109.23029	108.71852	53.246657	53.892018	52.961828	1.524E-11	-1.024414	down	PREDICTED: putative oxidoreductase TDA3 [Sesamum indicum]
c42810.graph_c0	8.7744221	9.6089491	8.8434865	18.665518	20.240493	20.380891	1.451E-12	1.163321	up	hypothetical protein MIMGU_mgv1a004563mg [Erythranthe guttata]
c42825.graph_c0	9.3190224	7.987396	9.9461336	2.0971551	4.0352992	3.008507	6.835E-08	-1.543879	down	PREDICTED: protein SULFUR DEFICIENCY-INDUCED 1 [Sesamum indicum]
c42828.graph_c0	5.237542	4.267192	4.9916549	20.461514	21.649019	18.783418	3.223E-35	2.112577	up	hypothetical protein ARALYDRAFT_319569 [Arabidopsis lyrata subsp. lyrata]
c42843.graph_c0	0.76716	0.9445548	0.8346617	2.0037988	2.3725209	1.8354083	0.0010098	1.327608	up	Receptor-like protein kinase [Medicago truncatula]
c42849.graph_c0	2.1929859	1.3860424	2.2964718	5.2352356	4.5644872	4.369715	0.0003075	1.314551	up	PREDICTED: monothiol glutaredoxin-S10-like [Sesamum indicum]
c42856.graph_c1	0.2579144	0	0.2104561	19.288607	11.082815	12.0722	4.862E-13	6.552599	up	PREDICTED: uncharacterized protein LOC105158077 [Sesamum indicum]
c42860.graph_c1	1.1568571	1.3140906	0.7917302	5.7236967	4.5100879	6.1451652	7.093E-11	2.372472	up	hypothetical protein MIMGU_mgv1a007855mg [Erythranthe guttata]
c42889.graph_c0	107.0236	101.10945	101.59671	38.127932	38.459384	29.90824	6.882E-24	-1.496181	down	hypothetical protein MIMGU_mgv1a002860mg [Erythranthe guttata]
c42906.graph_c0	0.7351761	0.9719857	1.0069711	2.6625425	2.6090928	2.6060257	6.144E-05	1.579731	up	PREDICTED: ABC transporter G family member 24-like [Sesamum indicum]
c42906.graph_c1	1.9986598	2.1268528	1.572644	4.4335348	4.249479	4.4548219	8.118E-05	1.248223	up	PREDICTED: ABC transporter G family member 24-like [Sesamum indicum]
c42921.graph_c0	1.9620868	2.4307731	2.0850842	5.0898576	6.3724664	9.1607795	2.541E-05	1.705835	up	PREDICTED: protein notum homolog isoform X1 [Sesamum indicum]
c42922.graph_c0	2.3279386	2.4337909	2.2994903	16.307239	17.522336	21.955137	1.229E-37	3.02076	up	PREDICTED: root phototropism protein 3-like [Sesamum indicum]
c42923.graph_c1	0.0776514	0.0467413	0.0633629	1.7389091	2.0159524	2.9193791	3.402E-18	5.187441	up	PREDICTED: phosphate transporter PHO1 homolog 1 [Sesamum indicum]
c42926.graph_c0	12.206135	12.639899	13.533365	29.370908	26.94774	25.900718	2.093E-14	1.14324	up	PREDICTED: neutral ceramidase isoform X1 [Sesamum indicum]
c42945.graph_c0	68.381942	75.767394	73.771666	35.533067	34.017191	35.141527	2.056E-12	-1.0149	down	PREDICTED: cell division cycle protein 48 homolog [Solanum lycopersicum]
c42957.graph_c0	1.5107724	1.4651292	1.346924	3.8300305	6.8751285	5.5536769	3.186E-07	1.944437	up	PREDICTED: putative pectate lyase 2 [Sesamum indicum]
c42982.graph_c0	3.3920247	3.8668477	3.6647144	7.0379601	8.7536439	9.0194716	3.136E-08	1.221374	up	PREDICTED: uncharacterized protein LOC105171946 [Sesamum indicum]
c42991.graph_c0	31.82317	30.383864	29.111128	66.698823	79.237678	94.53148	2.076E-13	1.434343	up	PREDICTED: proline transporter 3 [Sesamum indicum]
c43005.graph_c0	3.3711347	3.5210274	3.1820907	0.8147977	1.4578141	2.1750064	0.0001844	-1.150066	down	PREDICTED: myosin heavy chain, striated muscle [Sesamum indicum]
c43044.graph_c1	7.2301238	7.5958709	8.5747512	38.252721	40.04169	53.993283	3.033E-19	2.537433	up	PREDICTED: uncharacterized protein LOC100243914 [Vitis vinifera]
c43055.graph_c0	0.1565787	0.1237039	0.1117961	0.8435189	1.445539	1.6114922	3.502E-10	3.346502	up	PREDICTED: uncharacterized protein LOC105175800 [Sesamum indicum]
c43058.graph_c0	2.3456988	2.4365751	1.863255	3.8942939	4.1255321	4.9869622	0.0006211	1.008619	up	unnamed protein product [Coffea canephora]
c43066.graph_c0	4.7091826	3.6399272	4.483097	1.3566445	1.7246404	1.3680853	8.174E-05	-1.489093	down	PREDICTED: transcription factor TCP5-like [Sesamum indicum]

#ID	28 h-1 FPKM	28 h-2 FPKM	28 h-3 FPKM	48 h-1 FPKM	48 h-2 FPKM	48 h-3 FPKM	FDR	log2FC	regulated	nr_annotation
c43068.graph_c1	1.3533299	1.3177676	1.5590204	0.0336362	0.1425338	0.1496464	2.533E-12	-3.677246	down	hypothetical protein MIMGU_mgv1a002927mg [Erythranthe guttata]
c43079.graph_c0	4.2233444	4.6174459	4.7121709	9.0680042	10.069412	10.54758	1.066E-10	1.170801	up	PREDICTED: ATP-dependent DNA helicase DDM1 [Sesamum indicum]
c43082.graph_c1	8.1270652	7.0473649	9.9145984	0.713976	1.08053	3.7209934	8.828E-07	-2.160367	down	PREDICTED: auxin-responsive protein IAA28-like [Sesamum indicum]
c43111.graph_c0	6.7276351	5.9602905	6.521162	23.660564	30.082371	30.066702	1.712E-47	2.162787	up	PREDICTED: alpha-mannosidase [Sesamum indicum]
c43121.graph_c0	37.097398	36.220825	37.686684	14.440344	14.600458	12.995358	6.844E-18	-1.358081	down	PREDICTED: transcription factor bHLH47 isoform X3 [Sesamum indicum]
c43126.graph_c0	12.153829	11.893725	11.936542	5.627295	5.9858689	5.5201592	2.816E-08	-1.028953	down	PREDICTED: uncharacterized protein LOC105159445 [Sesamum indicum]
c43143.graph_c0	3.9224773	3.5416292	4.0809045	8.9807272	6.7486791	8.1569001	1.399E-05	1.094834	up	PREDICTED: vacuolar amino acid transporter 1-like isoform X1 [Eucalyptus grandis]
c43145.graph_c0	1.1224805	1.1923467	0.8081779	16.153268	12.501826	15.527396	4.621E-38	3.86836	up	PREDICTED: G2/mitotic-specific cyclin-2-like [Sesamum indicum]
c43147.graph_c0	37.846126	38.29565	44.193393	17.920856	7.886066	6.1330268	2.018E-08	-1.851824	down	hypothetical protein [Asterochloris sp. DA2]
c43150.graph_c0	36.539449	39.987767	39.991567	15.465594	15.468976	15.584104	5.231E-19	-1.282618	down	PREDICTED: patellin-3 [Sesamum indicum]
c43172.graph_c0	23.126242	19.834032	22.007663	10.867143	10.283336	9.2909963	5.035E-09	-1.049721	down	PREDICTED: RING-H2 finger protein ATL47 [Sesamum indicum]
c43183.graph_c3	0.1451691	0.1310741	0.1184568	1.1654128	1.8844102	3.0017764	1.096E-05	3.96893	up	PREDICTED: uncharacterized protein LOC105179145 [Sesamum indicum]
c43185.graph_c0	317.30619	365.41056	361.75137	36.489148	33.930727	30.237788	1.012E-80	-3.330641	down	PREDICTED: poly [ADP-ribose] polymerase 3 [Sesamum indicum]
c43205.graph_c0	12.488042	11.996342	12.035843	17.395599	27.336501	30.29241	0.0004157	1.071902	up	PREDICTED: protein TRANSPARENT TESTA 12-like [Sesamum indicum]
c43218.graph_c1	10.545784	11.006914	11.184224	3.6791443	3.2046993	3.0554351	1.735E-10	-1.674381	down	hypothetical protein CICLE_v10026263mg [Citrus clementina]
c43229.graph_c0	12.703475	14.215015	14.667842	2.3702767	2.8512236	2.2995536	8.201E-47	-2.426923	down	unnamed protein product [Vitis vinifera]
c43238.graph_c0	0.2203867	0.1989885	0.1634852	3.8855934	2.8249221	3.4573777	9.32E-25	4.171764	up	PREDICTED: kinesin-like protein KIF22 [Sesamum indicum]
c43247.graph_c0	1.7353528	1.7031097	1.4160338	3.729905	6.2816843	7.829075	9.152E-05	1.909457	up	-
c43253.graph_c0	17.705898	17.390482	13.884049	1.3137676	3.0155178	3.6043627	6.057E-13	-2.598352	down	PREDICTED: ethylene-responsive transcription factor CRF4-like [Sesamum indicum]
c43253.graph_c1	3.7159116	4.2619089	3.1960537	0.7213807	0.7642154	0.7551532	2.221E-08	-2.276488	down	PREDICTED: ethylene-responsive transcription factor CRF4-like [Sesamum indicum]
c43254.graph_c0	2.1010994	2.3426259	1.7404569	5.266067	5.1726429	4.5420913	3.966E-07	1.320551	up	PREDICTED: LOW QUALITY PROTEIN: linoleate 13S-lipoxygenase 3-1, chloroplastic [Sesamum indicum]
c43258.graph_c0	4.1179862	3.4414555	3.8916304	0.9225783	1.9547197	1.2745624	6.962E-06	-1.432226	down	PREDICTED: zinc finger BED domain-containing protein DAYSLEEPER-like isoform X1 [Sesamum indicum]
c43260.graph_c0	10.584786	11.423433	12.214079	23.987963	28.235931	23.997314	4.296E-11	1.195404	up	PREDICTED: UPF0057 membrane protein At2g24040-like [Sesamum indicum]
c43264.graph_c0	13.678072	9.6583461	11.399682	91.505918	65.149572	59.467301	5.748E-09	2.687161	up	PREDICTED: chlorophyll a-b binding protein CP26, chloroplastic [Sesamum indicum]
c43269.graph_c0	29.083759	27.217186	31.419976	12.095161	10.613083	10.300188	1.494E-18	-1.365279	down	PREDICTED: AP2-like ethylene-responsive transcription factor At2g41710 isoform X1 [Sesamum indicum]

#ID	28 h-1 FPKM	28 h-2 FPKM	28 h-3 FPKM	48 h-1 FPKM	48 h-2 FPKM	48 h-3 FPKM	FDR	log2FC	regulated	nr_annotation
c43274.graph_c0	1.2078993	1.2786577	1.3594979	2.560635	2.6567507	4.0401161	0.0001164	1.304775	up	PREDICTED: uncharacterized protein LOC105179654 [Sesamum indicum]
c43280.graph_c1	7.9897837	7.5919031	6.2712326	3.617014	3.5252448	2.9180136	0.0007093	-1.074592	down	hypothetical protein MIMGU_mgv1a000521mg [Erythranthe guttata]
c43284.graph_c0	0.4859381	0.3133975	0.4248445	1.9798789	1.3982944	1.0178621	0.0012521	1.896164	up	-
c43292.graph_c0	0.3774052	0.3097832	0.2239706	1.739596	1.6586019	1.9735497	5.66E-09	2.601648	up	PREDICTED: protein NSP-INTERACTING KINASE 2 [Sesamum indicum]
c43307.graph_c1	1.1093976	0.7929983	0.8298211	10.31245	19.428747	14.858793	5.454E-14	4.061519	up	PREDICTED: uncharacterized protein LOC105160017 [Sesamum indicum]
c43310.graph_c0	13.580142	13.701344	12.782799	5.4195802	5.2943127	5.632616	2.669E-12	-1.251157	down	PREDICTED: mitochondrial aspartate-glutamate transporter AGC1 [Sesamum indicum]
c43313.graph_c0	0.2093104	0.1889877	0.1423297	0.9286074	1.030592	2.0460001	0.0002771	2.923666	up	PREDICTED: 9-cis-epoxycarotenoid dioxygenase NCED1, chloroplastic [Sesamum indicum]
c43320.graph_c0	0.0553434	0.0999399	0.1806391	2.5254675	5.6481227	1.3732557	0.0019899	4.861862	up	hypothetical protein GUITHDRAFT_114321 [Guillardia theta CCMP2712]
c43324.graph_c0	30.854744	31.749854	30.156401	14.42069	13.147455	11.548299	5.314E-13	-1.20053	down	hypothetical protein MIMGU_mgv1a008176mg [Erythranthe guttata]
c43328.graph_c0	0.7066562	0.5742399	0.7908009	0.2175366	0.2168975	0.3985115	0.0035382	-1.278509	down	PREDICTED: leucine-rich repeat receptor protein kinase EXS-like [Nicotiana tomentosiformis]
c43329.graph_c0	102.77636	95.613079	88.263849	43.012386	46.169701	42.296105	8.805E-14	-1.08286	down	PREDICTED: root phototropism protein 2-like [Sesamum indicum]
c43332.graph_c0	0.221587	0.1667269	0.0602711	2.4810873	2.1820772	1.9577332	2.399E-15	3.930541	up	hypothetical protein MIMGU_mgv1a020708mg [Erythranthe guttata]
c43338.graph_c1	11.832087	14.122399	16.267823	23.832506	31.668388	34.094162	2.596E-06	1.121373	up	PREDICTED: calcium-dependent protein kinase 26-like [Sesamum indicum]
c43359.graph_c0	1.0073279	1.1116388	2.5572442	19.011061	19.238127	9.7203468	5.461E-07	3.404095	up	-
c43367.graph_c0	0.7006984	0.8102552	0.9930641	1.8387026	1.6507479	2.1906654	0.0003223	1.222922	up	PREDICTED: centromere-associated protein E isoform X1 [Sesamum indicum]
c43373.graph_c0	2.1205378	2.6638564	2.2569672	8.531281	6.8093449	4.1594973	0.0059783	1.5207	up	Activator of Hsp90 ATPase homolog 1-like protein
c43373.graph_c1	6.3571875	6.833267	6.5813098	24.942058	19.338215	17.900964	4.807E-08	1.701167	up	PREDICTED: probable galactinol--sucrose galactosyltransferase 2 [Sesamum indicum]
c43374.graph_c0	20.851662	22.836019	25.280644	12.631858	9.8929061	6.046467	2.46E-07	-1.220279	down	UPA25 [Capsicum annuum]
c43389.graph_c0	1.3793597	1.5793528	1.2968251	2.3059137	4.5769618	4.9039991	0.001419	1.50004	up	PREDICTED: ABC transporter C family member 8-like isoform X3 [Sesamum indicum]
c43393.graph_c1	1.0531014	0.9437026	1.0273099	0.5219094	0.3296133	0.5179755	0.0031317	-1.096099	down	PREDICTED: piezo-type mechanosensitive ion channel homolog [Sesamum indicum]
c43401.graph_c0	2.476435	3.0998933	2.4187774	0.4993965	0.8313644	0.90988	1.217E-06	-1.802378	down	PREDICTED: ABC transporter B family member 29, chloroplastic [Sesamum indicum]
c43407.graph_c0	58.22134	64.223539	61.459187	27.029371	26.793562	30.320899	3.203E-13	-1.086737	down	PREDICTED: uncharacterized protein LOC105176746 isoform X1 [Sesamum indicum]
c43415.graph_c0	299.6263	302.1091	301.09159	136.21419	142.77725	147.81185	7.24E-10	-1.03995	down	hypothetical protein MIMGU_mgv1a002107mg [Erythranthe guttata]
c43433.graph_c0	15.255601	17.163558	16.972598	39.308141	30.659394	31.830042	1.488E-06	1.09024	up	PREDICTED: probable 1-acylglycerol-3-phosphate O-acyltransferase [Beta vulgaris subsp. vulgaris]

#ID	28 h-1 FPKM	28 h-2 FPKM	28 h-3 FPKM	48 h-1 FPKM	48 h-2 FPKM	48 h-3 FPKM	FDR	log2FC	regulated	nr_annotation
c43442.graph_c0	17.183363	18.686914	17.199687	66.376123	43.482645	34.224989	0.0078153	1.494261	up	PREDICTED: uncharacterized protein LOC105168876 [Sesamum indicum]
c43456.graph_c1	0.2139194	0.1931491	0.5934919	1.3557928	1.1490384	1.5924166	4.443E-06	2.074116	up	PREDICTED: putative Myb family transcription factor At1g14600 [Sesamum indicum]
c43457.graph_c0	1.5158468	1.5102538	3.1989274	6.0293695	10.037321	5.4828065	0.0009591	1.82668	up	ADP ribosylation factor [Karlodinium veneficum]
c43467.graph_c0	5.7212666	5.6722151	5.3092823	14.575353	16.495314	16.385302	1.803E-08	1.546417	up	hypothetical protein VITISV_020777 [Vitis vinifera]
c43471.graph_c0	7.6839637	6.4523538	6.640599	16.798832	17.972844	20.824064	4.325E-23	1.459527	up	PREDICTED: pentatricopeptide repeat-containing protein MRL1, chloroplastic [Sesamum indicum]
c43484.graph_c0	25.241809	24.400345	25.027559	13.399675	11.04695	8.2733832	1.461E-14	-1.141801	down	PREDICTED: histone-lysine N-methyltransferase ASHR2 [Sesamum indicum]
c43489.graph_c0	1.2005745	1.2558609	1.4455944	0.2227049	0.4325361	0.726592	0.0004812	-1.470349	down	PREDICTED: pentatricopeptide repeat-containing protein At1g43980, mitochondrial [Sesamum indicum]
c43491.graph_c0	12.170168	12.082286	11.930702	22.461393	25.913607	30.916359	3.993E-12	1.169787	up	PREDICTED: cytochrome b561 and DOMON domain-containing protein At4g17280 [Sesamum indicum]
c43492.graph_c0	0.3346101	0.6042432	0.307169	1.3254469	1.0671542	0.9906747	0.0008039	1.489489	up	PREDICTED: actin-related protein 2/3 complex subunit 2B [Sesamum indicum]
c43512.graph_c0	15.35754	13.914317	15.107213	27.939416	30.951902	33.027435	1.467E-12	1.089964	up	PREDICTED: malonyl-CoA-acyl carrier protein transacylase, mitochondrial [Sesamum indicum]
c43515.graph_c1	3.7560203	3.1401242	4.0108331	0.6465599	1.1208303	1.7259155	0.0001846	-1.61343	down	-
c43516.graph_c0	31.150448	26.53818	27.967492	67.950481	59.929078	56.01475	5.541E-11	1.147459	up	PREDICTED: solute carrier family 25 member 44-like [Nicotiana tomentosiformis]
c43520.graph_c0	2.2669991	2.3880355	2.7747791	5.2682452	5.0736973	6.0878609	0.0020648	1.185886	up	PREDICTED: uncharacterized protein LOC105165005 [Sesamum indicum]
c43561.graph_c0	3.2723148	3.7389988	3.5444896	0.917677	1.3999212	2.384307	0.0034266	-1.136052	down	PREDICTED: uncharacterized protein LOC105156369 [Sesamum indicum]
c43566.graph_c1	0.1104661	0.797924	1.2619517	2.9405013	4.1534728	3.9869568	2.504E-07	2.386888	up	PREDICTED: protein ECERIFERUM 1-like [Sesamum indicum]
c43570.graph_c0	14.544101	14.007424	16.845779	78.966333	85.988054	76.187432	9.006E-59	2.450353	up	PREDICTED: ferritin-3, chloroplastic [Sesamum indicum]
c43571.graph_c0	0	0.0763483	0.2069968	2.6795985	6.2451614	5.3408111	4.358E-10	5.680253	up	-
c43574.graph_c0	42.5068	43.529081	46.329449	25.57901	20.71039	16.190702	8.956E-11	-1.034546	down	PREDICTED: uncharacterized Rho GTPase-activating protein At5g61530 [Sesamum indicum]
c43576.graph_c1	16.348355	18.285292	17.356009	4.9834973	9.6092878	10.336033	1.189E-06	-1.029996	down	PREDICTED: uncharacterized protein C683.02c [Sesamum indicum]
c43590.graph_c0	2.7702748	3.0640906	2.3452912	6.3207493	5.4870553	6.5615255	0.0001556	1.210945	up	PREDICTED: uncharacterized protein LOC101267356 [Solanum lycopersicum]
c43599.graph_c0	0.1833333	0.3724487	0.5983938	1.626719	1.907952	2.1194727	3.177E-07	2.32959	up	PREDICTED: uncharacterized protein At3g27210-like [Sesamum indicum]
c43607.graph_c0	11.541847	10.806581	13.669963	5.2975087	6.4956703	5.1549731	7.903E-09	-1.048011	down	-
c43632.graph_c0	58.051968	59.890007	57.290484	117.09522	127.39707	119.77819	4.282E-14	1.096819	up	PREDICTED: uncharacterized protein LOC105174142 [Sesamum indicum]
c43633.graph_c0	3.0175863	5.5925949	5.5726321	25.466533	50.864987	18.181714	0.0039891	2.771464	up	hypothetical protein SELMODRAFT_227399 [Selaginella moellendorffii]

#ID	28 h-1 FPKM	28 h-2 FPKM	28 h-3 FPKM	48 h-1 FPKM	48 h-2 FPKM	48 h-3 FPKM	FDR	log2FC	regulated	nr_annotation
c43644.graph_c0	0.5240283	0.5534944	0.71805	2.656978	3.4387699	3.7358237	3.233E-19	2.489131	up	PREDICTED: kinesin-like protein KIN12B [Sesamum indicum]
c43646.graph_c0	16.223519	14.801435	14.391418	4.9440798	7.1353534	5.1642929	1.532E-06	-1.359521	down	--
c43651.graph_c0	94.249744	90.779736	95.785322	161.34687	182.68651	205.1667	5.846E-11	1.006399	up	PREDICTED: UDP-glucose 4-epimerase GEPI48 [Sesamum indicum]
c43672.graph_c0	6.1169661	6.3233251	6.0711654	14.969398	16.193532	16.00896	1.559E-19	1.390279	up	unnamed protein product [Coffea canephora]
c43680.graph_c0	1.6695517	2.2269127	2.3840959	7.1183954	8.7639541	8.9445016	1.54E-10	2.020791	up	PREDICTED: probable receptor protein kinase TMK1 [Sesamum indicum]
c43685.graph_c0	5.1591878	6.0745113	5.7210852	22.948664	22.915583	23.936526	1.025E-38	2.083429	up	PREDICTED: uncharacterized protein LOC105159738 isoform X2 [Sesamum indicum]
c43691.graph_c0	0.7263296	0.6810309	0.524293	1.0269082	2.4383623	2.3631123	0.004017	1.621736	up	PREDICTED: dirigent protein 21-like [Sesamum indicum]
c43693.graph_c0	1.894339	2.5972901	2.1182708	0.3557354	0.9421464	0.3165313	1.175E-05	-2.002684	down	PREDICTED: uncharacterized protein LOC105171344 [Sesamum indicum]
c43695.graph_c0	0	0	0	1.1355793	1.7095385	1.2231541	6.616E-21	Inf	up	PREDICTED: nudix hydrolase 3-like [Cicer arietinum]
c43696.graph_c0	6.7570392	7.3316412	7.5014564	3.6024652	3.1932934	3.0746171	0.0001395	-1.084337	down	PREDICTED: F-box/kelch-repeat protein At1g30090-like isoform X2 [Sesamum indicum]
c43713.graph_c0	35.931112	36.325846	35.287179	181.50249	263.58705	348.12311	3.176E-09	2.916044	up	PREDICTED: fatty-acid-binding protein 1-like [Nicotiana sylvestris]
c43736.graph_c0	0.1831985	0.3308221	0.1494885	3.5993725	7.3801911	6.7153388	1.356E-11	4.768803	up	unnamed protein product [Coffea canephora]
c43736.graph_c1	0.3043056	0.2442306	0.275901	6.3431117	9.1715612	12.088972	7.527E-17	5.098434	up	hypothetical protein MIMGU_mgv1a003774mg [Erythranthe guttata]
c43738.graph_c0	1.1624229	1.1485739	1.1453918	4.0867571	4.6239423	5.1453426	1.221E-10	2.042048	up	Wee1-like protein kinase [Morus notabilis]
c43741.graph_c0	1.3408991	1.2819242	0.7938043	4.0659212	3.9189829	4.5964262	2.012E-08	1.922824	up	unnamed protein product [Coffea canephora]
c43745.graph_c0	18.017736	18.535475	19.298533	40.215226	46.658429	44.049775	1.173E-16	1.268713	up	hypothetical protein L484_019510 [Morus notabilis]
c43764.graph_c1	23.759807	23.674783	26.958286	10.863742	10.331348	9.7622003	3.312E-13	-1.221476	down	PREDICTED: uncharacterized protein LOC105178804 isoform X1 [Sesamum indicum]
c43765.graph_c0	0.2012593	0.4240093	0.3831938	3.5715533	8.19786	2.4212927	0.0034354	3.848177	up	hypothetical protein EMIHUDRAFT_444197 [Emiliania huxleyi CCMP1516]
c43782.graph_c0	16.459056	18.09803	16.289413	34.496356	55.965919	55.50769	4.262E-07	1.555099	up	PREDICTED: pentatricopeptide repeat-containing protein At1g06140, mitochondrial-like [Sesamum indicum]
c43783.graph_c0	51.013848	49.094406	50.276625	115.95097	109.23697	102.10499	1.079E-15	1.165809	up	PREDICTED: uncharacterized protein LOC105163767 [Sesamum indicum]
c43815.graph_c0	103.78905	102.49832	98.737669	41.251548	47.397056	47.881157	2.207E-12	-1.120269	down	PREDICTED: aldehyde dehydrogenase family 2 member B7, mitochondrial [Sesamum indicum]
c43816.graph_c0	4.7684535	5.4715302	6.0797173	10.829489	22.945061	22.745476	0.000422	1.821406	up	hypothetical protein VITISV_012452 [Vitis vinifera]
c43818.graph_c0	20.28739	20.684881	20.043842	8.9375982	9.3657568	9.1871435	1.672E-10	-1.108819	down	PREDICTED: pentatricopeptide repeat-containing protein At3g61360 [Sesamum indicum]
c43837.graph_c0	2.1779041	1.9664432	1.9040914	1.0352452	0.7833691	0.7018324	0.0057611	-1.213927	down	PREDICTED: PHD finger protein ING2 isoform X2 [Sesamum indicum]
c43839.graph_c0	53.592588	48.618233	64.302341	19.382492	20.987805	26.954994	1.593E-09	-1.268465	down	PREDICTED: flavanone 3-dioxygenase-like [Sesamum indicum]
c43841.graph_c0	1.8189915	1.7727263	1.6020824	0.219591	0.4652601	0.3256513	3.272E-07	-2.330279	down	-

#ID	28 h-1 FPKM	28 h-2 FPKM	28 h-3 FPKM	48 h-1 FPKM	48 h-2 FPKM	48 h-3 FPKM	FDR	log2FC	regulated	nr_annotation
c43843.graph_c0	2.6191382	2.5963586	2.7649031	5.362997	7.157145	7.0236677	5.889E-08	1.328936	up	PREDICTED: interactor of constitutive active ROPs 2, chloroplastic-like isoform X2 [Sesamum indicum]
c43849.graph_c1	2.3483875	2.5915678	2.1611211	4.0682306	5.2383305	6.0331451	4.767E-07	1.147726	up	PREDICTED: alpha-1,4 glucan phosphorylase L-2 isozyme, chloroplastic/amyloplastic-like [Sesamum indicum]
c43854.graph_c0	3.5320249	4.2157113	3.7112017	1.0732778	2.2090435	2.2101339	0.0029857	-1.030922	down	PREDICTED: uncharacterized protein LOC105167611 [Sesamum indicum]
c43865.graph_c0	35.993596	30.444482	33.650989	14.420397	16.167801	13.829163	1.223E-12	-1.13106	down	PREDICTED: chavicol O-methyltransferase-like [Sesamum indicum]
c43873.graph_c0	3.4977948	4.2810894	3.4884324	0.5911617	1.0437735	1.8410423	0.0003164	-1.667904	down	hypothetical protein MIMGU_mgv1a024870mg, partial [Erythranthe guttata]
c43877.graph_c0	0.3113209	0.1923272	0.2941462	1.0384796	0.6160802	0.7207456	0.0007557	1.624934	up	PREDICTED: probable cyclic nucleotide-gated ion channel 20, chloroplastic isoform X1 [Sesamum indicum]
c43880.graph_c0	0.3281939	0.1693305	0.3443188	1.4263197	1.1332596	2.7497832	0.0012968	2.693429	up	hypothetical protein MIMGU_mgv1a002490mg [Erythranthe guttata]
c43889.graph_c0	4.5662597	5.0670809	5.3188376	1.1045936	0.8425317	0.9042319	6.419E-10	-2.343712	down	PREDICTED: uncharacterized protein LOC102584927 isoform X1 [Solanum tuberosum]
c43902.graph_c1	8.5115157	8.8254704	9.4546068	2.018582	2.2121823	3.2825681	4.409E-07	-1.797469	down	PREDICTED: uncharacterized protein LOC105173250 [Sesamum indicum]
c43903.graph_c0	3.3875502	4.6971975	3.3565448	18.326048	17.46468	21.901074	1.317E-17	2.375713	up	PREDICTED: uncharacterized protein LOC105156092 [Sesamum indicum]
c43909.graph_c2	1.3049623	0.9257746	1.2930182	2.7175088	2.5033661	3.3642059	0.002416	1.325242	up	unnamed protein product [Coffea canephora]
c43909.graph_c3	1.4648321	1.5728288	1.4214271	4.014665	4.3062139	4.0187528	9.429E-05	1.50999	up	PREDICTED: AP2-like ethylene-responsive transcription factor ANT [Sesamum indicum]
c43921.graph_c0	10.014391	10.11234	9.9060548	23.833592	34.9641	44.857387	7.364E-06	1.820489	up	PREDICTED: hexose carrier protein HEX6-like [Sesamum indicum]
c43921.graph_c1	0.3940568	0.5929939	1.1790062	4.3289657	5.8207104	10.222317	7.986E-05	3.264177	up	PREDICTED: hexose carrier protein HEX6-like [Sesamum indicum]
c43942.graph_c0	0.106672	0.1733667	0.1566783	1.5955331	1.4897329	1.8528233	2.137E-14	3.540829	up	lupeol synthase [Olea europaea]
c43952.graph_c0	0.5994547	0.7134677	0.7781934	1.2779345	1.5733545	1.7517463	0.0061823	1.175456	up	hypothetical protein MIMGU_mgv1a021758mg [Erythranthe guttata]
c43962.graph_c0	5.6605411	4.9553876	5.0206036	14.22552	11.236563	9.5766116	0.0001384	1.212438	up	PREDICTED: uncharacterized protein LOC105172439 [Sesamum indicum]
c43967.graph_c1	55.747206	63.747686	60.990249	22.373554	25.022711	21.308993	6.03E-18	-1.352069	down	phytosulfokine precursor [Avicennia marina]
c43975.graph_c0	622.96461	774.25772	672.94699	116.25821	110.7692	108.66788	9.296E-50	-2.581026	down	PREDICTED: WD repeat-containing protein 82-B isoform X1 [Sesamum indicum]
c43993.graph_c0	85.682307	82.216125	90.813484	58.913171	18.256385	24.248978	0.0006862	-1.28698	down	hypothetical protein M569_00228 [Genlisea aurea]
c44014.graph_c1	71.090609	73.452996	71.84737	296.19546	268.32051	242.90115	2.682E-24	1.94471	up	PREDICTED: uncharacterized protein LOC105173450 [Sesamum indicum]
c44019.graph_c0	0.3376254	0.6774313	0.7040545	3.4237206	2.6698874	2.5809828	3.593E-08	2.382529	up	hypothetical protein MIMGU_mgv1a010335mg [Erythranthe guttata]
c44025.graph_c0	0.0771765	0.5574653	0.4093402	1.1739231	1.7099901	1.1751184	1.247E-05	1.997319	up	PREDICTED: WAT1-related protein At1g25270-like [Sesamum indicum]
c44028.graph_c0	0.1653025	0.7462635	0.269771	56.993016	73.917662	88.00031	1.594E-52	7.571559	up	-
c44030.graph_c0	17.007413	16.942284	19.591122	13.328616	5.6753722	3.7043685	0.0046748	-1.173465	down	PREDICTED: RING-H2 finger protein ATL3 [Sesamum indicum]

#ID	28 h-1 FPKM	28 h-2 FPKM	28 h-3 FPKM	48 h-1 FPKM	48 h-2 FPKM	48 h-3 FPKM	FDR	log2FC	regulated	nr_annotation
c44040.graph_c0	0.5432439	0.5937612	0.9448919	1.3409612	1.6701482	1.7252727	0.0013785	1.222718	up	PREDICTED: LOW QUALITY PROTEIN: ATP-dependent DNA helicase Q-like 5 [Sesamum indicum]
c44041.graph_c0	1.777924	1.1358245	1.3823388	3.7844305	4.1217616	3.5565412	9.596E-07	1.45729	up	PREDICTED: kinesin-like protein NACK1 [Sesamum indicum]
c44044.graph_c0	2.2259841	2.6961469	2.3258587	4.404447	6.0876425	5.9091932	7.696E-05	1.214723	up	PREDICTED: scarecrow-like protein 3 isoform X1 [Sesamum indicum]
c44052.graph_c0	2.4358389	1.9286469	1.9570458	0.6650227	1.0567663	0.5071999	0.0007592	-1.465773	down	PREDICTED: ferredoxin--nitrite reductase, chloroplastic [Sesamum indicum]
c44058.graph_c0	76.513388	84.213655	76.375723	33.05879	38.593927	37.457608	8.319E-14	-1.080165	down	PREDICTED: AP2-like ethylene-responsive transcription factor AIL1, partial [Sesamum indicum]
c44077.graph_c1	2.5165926	2.9308695	2.5594584	6.056313	6.7587652	7.5279647	1.336E-05	1.384548	up	PREDICTED: uncharacterized protein LOC105169472 [Sesamum indicum]
c44088.graph_c0	2.4542899	2.9880173	2.7908322	0.7426242	0.6804067	0.6786415	2.535E-08	-1.925887	down	hypothetical protein MIMGU_mgv1a001438mg [Erythranthe guttata]
c44089.graph_c0	4.9748162	3.392737	3.9298538	9.7272571	8.6702871	7.7001922	0.0006575	1.130896	up	PREDICTED: aquaporin AQPcic-like [Sesamum indicum]
c44094.graph_c0	55.30178	57.231399	55.170414	102.08227	110.77542	113.90287	6.63E-12	1.002695	up	PREDICTED: selenoprotein K [Sesamum indicum]
c44100.graph_c0	4.84769	5.2210609	5.0127016	9.5134432	11.746107	10.995469	3.092E-11	1.135447	up	PREDICTED: probable LRR receptor-like serine/threonine-protein kinase At4g36180 [Sesamum indicum]
c44106.graph_c1	3.9976264	3.2084283	2.7183588	8.8677503	11.48193	12.524557	5.323E-06	1.764783	up	phospholipase D [Plantago major]
c44106.graph_c2	6.4857211	5.1739074	5.1569811	27.162424	25.855703	27.119944	4.179E-49	2.295335	up	PREDICTED: phospholipase D alpha 1 [Sesamum indicum]
c44107.graph_c0	0.1184966	1.176905	0.7735384	3.6048774	4.614541	5.8805968	1.718E-09	2.805103	up	-
c44108.graph_c0	1.2987818	1.156391	1.2511472	4.2758109	4.117912	4.5573709	6.531E-10	1.846882	up	PREDICTED: homeobox-leucine zipper protein GLABRA 2 [Sesamum indicum]
c44112.graph_c1	7.1095737	7.8925555	7.3864235	38.460658	40.274878	51.048275	4.128E-31	2.57454	up	PREDICTED: 2-hydroxyisoflavanone dehydratase-like [Sesamum indicum]
c44131.graph_c0	8.9284555	10.30088	11.932107	4.2754905	4.6892237	4.6099234	1.433E-08	-1.15865	down	PREDICTED: glutamyl-tRNA(Gln) amidotransferase subunit A, mitochondrial isoform X4 [Sesamum indicum]
c44133.graph_c0	38.712873	40.201524	36.284897	18.659255	19.253784	17.788814	4.686E-12	-1.005973	down	PREDICTED: polygalacturonase At1g48100 [Sesamum indicum]
c44140.graph_c0	0.2244199	0.221051	0.2330679	1.2413179	1.5889895	1.5187112	6.259E-10	2.717911	up	PREDICTED: receptor-like serine/threonine-protein kinase At1g78530 [Sesamum indicum]
c44159.graph_c0	82.916308	78.887133	85.27079	21.828689	44.631573	52.058554	1.907E-05	-1.030906	down	PREDICTED: proton-coupled amino acid transporter 3-like [Sesamum indicum]
c44162.graph_c0	0.9179077	0.5697894	0.7490052	1.9270728	2.3881697	2.6529286	3.258E-05	1.676276	up	PREDICTED: probable leucine-rich repeat receptor-like protein kinase At5g49770 isoform X3 [Sesamum indicum]
c44162.graph_c1	1.284154	1.6368997	1.6437006	3.8938566	4.0912569	5.2254947	1.764E-06	1.572079	up	hypothetical protein MIMGU_mgv1a0019072mg, partial [Erythranthe guttata]
c44170.graph_c0	39.574386	41.217624	38.766697	120.05469	116.08185	137.48811	1.923E-28	1.685259	up	PREDICTED: pyruvate, phosphate dikinase, chloroplastic [Populus euphratica]
c44199.graph_c0	122.95975	126.56337	128.4747	65.233485	61.587282	52.818146	3.671E-12	-1.028699	down	PREDICTED: F-box/LRR-repeat protein 14 [Sesamum indicum]
c44225.graph_c0	13.28907	12.886366	12.9234	23.859727	33.441234	35.602746	6.063E-09	1.284017	up	unnamed protein product [Coffea canephora]

#ID	28 h-1 FPKM	28 h-2 FPKM	28 h-3 FPKM	48 h-1 FPKM	48 h-2 FPKM	48 h-3 FPKM	FDR	log2FC	regulated	nr_annotation
c44237.graph_c0	22.96182	22.939125	22.256065	8.2006495	7.8602039	7.181065	4.966E-21	-1.508314	down	PREDICTED: serine/threonine-protein kinase UCNL-like [Sesamum indicum]
c44239.graph_c0	1.7416772	2.4051089	2.5915897	10.77879	23.525524	15.946321	4.127E-06	2.929455	up	ribosomal protein L41 [Cryptococcus gattii R265]
c44265.graph_c0	1.3251867	1.7947791	1.8923477	12.283331	8.8975727	10.742803	4.471E-12	2.717869	up	Cold shock, CspA, partial [Metarhizium brunneum ARSEF 3297]
c44271.graph_c2	0.2358553	0.1597164	0.192456	1.6443017	2.0586543	2.4606509	2.625E-12	3.426034	up	-
c44291.graph_c0	0.937131	0.9589603	1.6313413	3.5372503	5.0895998	5.7311303	1.344E-09	2.058728	up	PREDICTED: ABC transporter G family member 21 [Sesamum indicum]
c44295.graph_c2	14.539249	25.279458	15.992227	3.7357233	8.3768054	7.9222576	0.0025876	-1.448319	down	PREDICTED: spermidine hydroxycinnamoyl transferase-like [Sesamum indicum]
c44303.graph_c0	2581.7455	2630.9395	3104.1934	189.6364	165.78839	151.90483	8.128E-45	-3.989709	down	hypothetical protein MIMGU_mgv1a024110mg [Erythranthe guttata]
c44308.graph_c0	0.6333094	0.6019148	0.4351792	2.1547939	1.7008673	1.7669444	2.903E-05	1.798183	up	PREDICTED: probable leucine-rich repeat receptor-like serine/threonine-protein kinase At3g14840 [Sesamum indicum]
c44316.graph_c0	1.6970796	1.7876877	1.552658	3.4549149	3.6945923	3.3931793	0.0017094	1.107253	up	PREDICTED: uncharacterized protein LOC105169064 [Sesamum indicum]
c44340.graph_c1	1.9518341	2.2719106	3.1661724	0.8048256	0.6315667	0.8222221	2.269E-05	-1.666261	down	PREDICTED: mediator of RNA polymerase II transcription subunit 25-like [Sesamum indicum]
c44359.graph_c0	3.5157066	3.7949037	4.4865254	14.575499	17.819239	21.794205	1.542E-16	2.235931	up	PREDICTED: glutamate dehydrogenase A [Sesamum indicum]
c44360.graph_c0	2.7400208	2.9935181	2.0793262	5.2792308	5.5191167	5.5627435	0.0002837	1.108142	up	PREDICTED: probable WRKY transcription factor 30 [Sesamum indicum]
c44362.graph_c0	0.3804249	0.515232	0.9002285	32.790727	50.318723	50.416125	3.302E-45	6.249458	up	hypothetical protein MIMGU_mgv1a010045mg [Erythranthe guttata]
c44380.graph_c0	22.236963	21.673167	20.7956	9.1619025	10.352986	9.6417446	8.991E-13	-1.109645	down	PREDICTED: peptidyl-prolyl cis-trans isomerase FKBP43-like isoform X3 [Sesamum indicum]
c44382.graph_c0	8.1453199	8.0832806	10.119468	15.905762	15.963372	19.780886	0.0004286	1.010832	up	-
c44385.graph_c1	0.5302095	0.2593118	0.3965927	1.4561723	1.6316364	1.2956934	5.926E-06	1.927083	up	hypothetical protein MIMGU_mgv1a001279mg [Erythranthe guttata]
c44391.graph_c0	89.693835	83.798248	91.097096	566.82457	583.01378	618.60246	2.489E-53	2.781595	up	PREDICTED: raucaffricine-O-beta-D-glucosidase-like [Sesamum indicum]
c44399.graph_c0	66.962792	66.755182	59.76908	108.14202	147.78708	145.98905	9.095E-11	1.091249	up	PREDICTED: leucine-rich repeat extensin-like protein 4 [Sesamum indicum]
c44427.graph_c1	1.242577	0.9284942	1.0488959	2.7156171	2.3014936	2.126379	0.0057554	1.195635	up	hypothetical protein MIMGU_mgv1a004986mg [Erythranthe guttata]
c44457.graph_c0	12.921526	13.659545	11.953188	3.9325784	6.4424071	7.9362949	1.329E-05	-1.041323	down	hypothetical protein MIMGU_mgv1a011349mg [Erythranthe guttata]
c44466.graph_c0	314.81826	329.81211	318.67024	1905.3032	2162.7558	2154.6335	3.818E-43	2.731255	up	PREDICTED: aquaporin TIP2-1-like [Solanum tuberosum]
c44467.graph_c0	8.786939	8.3584613	8.3563595	16.964588	18.230307	18.494978	3.091E-11	1.114675	up	PREDICTED: probable LRR receptor-like serine/threonine-protein kinase At1g51880 isoform X1 [Sesamum indicum]
c44514.graph_c0	265.1639	262.84738	289.97041	13.371558	13.369088	10.846709	7.79E-147	-4.400036	down	PREDICTED: alcohol dehydrogenase 1 [Sesamum indicum]
c44534.graph_c0	110.61833	98.749814	101.7338	32.022704	38.040165	32.765449	1.918E-26	-1.557185	down	PREDICTED: hippocampus abundant transcript-like protein 1 [Solanum lycopersicum]
c44546.graph_c0	14.646446	17.97598	15.779961	455.1348	577.34908	513.31789	1.58E-176	5.035865	up	--
c44560.graph_c1	5.7083725	5.7726202	6.1174875	11.504894	11.753666	12.12557	2.476E-08	1.049001	up	PREDICTED: uncharacterized protein LOC102583109 [Solanum tuberosum]

#ID	28 h-1 FPKM	28 h-2 FPKM	28 h-3 FPKM	48 h-1 FPKM	48 h-2 FPKM	48 h-3 FPKM	FDR	log2FC	regulated	nr_annotation
c44561.graph_c0	92.635826	57.116278	97.637183	28.812059	14.109635	16.92996	6.283E-06	-1.992688	down	BnaCnng13060D [Brassica napus]
c44568.graph_c1	5.5935629	6.5286471	5.7888704	2.248129	2.5648216	2.9236201	0.009929	-1.172384	down	-
c44573.graph_c0	0.4428834	0.1999411	0.4743234	1.1578614	0.8920827	1.9044165	0.002381	1.861927	up	PREDICTED: cell division control protein 6 homolog [Sesamum indicum]
c44575.graph_c0	2.6696717	2.8345263	2.6423553	0.9086682	0.9626237	1.3940129	0.001121	-1.281126	down	unknown protein [Arabidopsis thaliana]
c44590.graph_c0	0.9667671	0.9352501	0.9579185	2.0132369	4.9146678	4.361587	0.000922	2.008993	up	hypothetical protein MIMGU_mgv1a013990mg [Erythranthe guttata]
c44618.graph_c0	14.883763	13.58186	13.913932	4.18878	5.5735057	5.098743	2.592E-12	-1.474426	down	PREDICTED: transcription initiation factor TFIID subunit 8-like [Sesamum indicum]
c44624.graph_c0	0.425232	1.24782	1.6481826	4.1773496	6.1384516	2.6378571	0.0045665	2.003018	up	hypothetical protein SELMODRAFT_29765, partial [Selaginella moellendorffii]
c44646.graph_c1	3.1873755	3.2164778	3.6526957	1.1585963	1.6679946	1.8239212	0.0025135	-1.07814	down	PREDICTED: non-specific lipid-transfer protein-like protein At2g13820 [Sesamum indicum]
c44650.graph_c1	2.085165	3.3309458	2.4540542	0.4574596	0.430776	0.3165904	6.284E-09	-2.661146	down	hypothetical protein MIMGU_mgv1a004154mg [Erythranthe guttata]
c44658.graph_c1	3.5529208	4.3479712	3.8096326	12.840811	10.803766	11.326323	2.709E-19	1.623767	up	PREDICTED: protein LONGIFOLIA 1 [Sesamum indicum]
c44661.graph_c0	1.4487401	0.9883244	0.7618365	0.1224257	0.4323171	0.2904896	0.0012875	-1.895092	down	hypothetical protein MIMGU_mgv1a0244072mg, partial [Erythranthe guttata]
c44667.graph_c0	1.7667302	1.7059689	1.5417511	0.5598652	0.8896639	0.5258405	0.0020414	-1.306789	down	PREDICTED: endoglucanase 24-like [Sesamum indicum]
c44677.graph_c0	78.055598	88.448078	88.391439	12.207788	11.454652	9.2071914	3.041E-53	-2.909822	down	PREDICTED: protein ABSCISIC ACID-INSENSITIVE 5 isoform X2 [Nicotiana tomentosiformis]
c44680.graph_c0	3.3921471	2.7271425	2.730047	0.6479145	0.6863869	1.0482008	3.423E-05	-1.855825	down	PREDICTED: uncharacterized protein LOC102579233 [Solanum tuberosum]
c44683.graph_c0	54.766007	52.753344	58.462176	663.57375	498.87265	389.50108	1.35E-09	3.27505	up	PREDICTED: protein FAR1-RELATED SEQUENCE 5 [Sesamum indicum]
c44686.graph_c0	11.024846	11.221745	10.537197	5.5963914	5.4146491	4.0297628	1.165E-06	-1.078754	down	PREDICTED: cytochrome P450 734A1-like [Sesamum indicum]
c44694.graph_c0	10.655592	12.516641	13.422187	33.439034	29.451042	28.120307	5.664E-07	1.359376	up	PREDICTED: crocetin glucosyltransferase, chloroplastic-like [Sesamum indicum]
c44701.graph_c0	0.1578145	0.0610679	0.1287754	5.2868125	4.4503152	4.653318	2.485E-37	5.415617	up	hypothetical protein MIMGU_mgv1a021943mg [Erythranthe guttata]
c44705.graph_c0	8.0469883	8.6441441	8.1754038	23.020893	26.39525	25.470289	5.809E-20	1.630375	up	PREDICTED: pyridoxal kinase isoform X1 [Sesamum indicum]
c44713.graph_c0	2.2634449	1.9147979	1.8136741	0.9822086	0.9857662	0.8049662	0.0067718	-1.067737	down	PREDICTED: protein ROOT PRIMORDIUM DEFECTIVE 1 [Sesamum indicum]
c44718.graph_c0	73.816799	78.024035	74.072724	25.788369	30.666759	28.018084	2.203E-21	-1.379594	down	PREDICTED: ninja-family protein AFP3-like [Sesamum indicum]
c44743.graph_c0	11.363311	11.138905	11.738869	18.144415	23.58352	25.609776	3.83E-09	1.01216	up	PREDICTED: COBRA-like protein 7 [Sesamum indicum]
c44744.graph_c0	0.6501106	0.7174309	0.6778419	1.465001	1.6489904	1.9145877	0.0020282	1.336467	up	PREDICTED: serine/threonine-protein kinase Nek2 [Sesamum indicum]
c44747.graph_c0	131.0041	136.26108	115.96475	230.22213	303.21931	285.83473	4.026E-11	1.133809	up	PREDICTED: endoplasmin homolog [Sesamum indicum]
c44756.graph_c0	0.0979508	0	0	8.6911927	13.021703	12.704824	1.867E-33	8.492155	up	-

#ID	28 h-1 FPKM	28 h-2 FPKM	28 h-3 FPKM	48 h-1 FPKM	48 h-2 FPKM	48 h-3 FPKM	FDR	log2FC	regulated	nr_annotation
c44762.graph_c5	22.742472	20.53432	20.651954	66.330954	64.046818	73.092621	8.718E-25	1.711941	up	unnamed protein product [Coffea canephora]
c44785.graph_c0	26.703306	27.672004	29.487918	10.193245	8.8801133	7.9652874	1.161E-28	-1.587238	down	PREDICTED: phytosulfokine receptor 2-like [Sesamum indicum]
c44790.graph_c1	0.5640367	0.5092722	0.4602493	6.792101	6.86404	7.753265	4.528E-25	3.844334	up	hypothetical protein SORBIDRAFT_07g024570 [Sorghum bicolor]
c44797.graph_c0	0.8584215	1.0334321	0.9598964	4.0300461	3.116622	4.661684	2.664E-08	2.093336	up	hypothetical protein MIMGU_mgv1a000411mg [Erythranthe guttata]
c44799.graph_c0	7.4387081	7.4039672	6.5000772	1.9118078	2.5562399	2.9728225	2.936E-11	-1.484443	down	PREDICTED: uncharacterized protein LOC105170906 isoform X2 [Sesamum indicum]
c44802.graph_c0	5.6756021	5.4226081	6.299322	2.3176087	2.8644296	2.2054022	4.099E-08	-1.195928	down	PREDICTED: adagio protein 3 isoform X1 [Sesamum indicum]
c44808.graph_c0	128.78315	134.94249	133.98702	27.084358	31.707394	35.625346	3.873E-44	-2.036442	down	hypothetical protein MIMGU_mgv1a003414mg [Erythranthe guttata]
c44811.graph_c0	5.2393231	5.0848928	4.4070806	16.471415	19.681016	20.851846	7.652E-29	1.990693	up	PREDICTED: glucose-1-phosphate adenylyltransferase large subunit 3, chloroplastic/amyloplastic [Sesamum indicum]
c44821.graph_c0	0.1552329	0.0262801	0.1820864	1.5618549	1.4721995	2.2760918	3.861E-17	3.905465	up	PREDICTED: abnormal spindle-like microcephaly-associated protein homolog isoform X2 [Sesamum indicum]
c44832.graph_c0	1.1621462	0.6332037	0.9973519	0.2285854	0.215252	0.1807946	6.942E-06	-2.116352	down	Ty3/gypsy retrotransposon protein [Beta vulgaris subsp. vulgaris]
c44848.graph_c0	70.945833	67.602796	61.888545	120.20342	146.30771	169.41014	1.03E-10	1.15828	up	PREDICTED: L-type lectin-domain containing receptor kinase S.4-like [Sesamum indicum]
c44849.graph_c0	7.1355929	7.5797311	8.1820647	16.493622	19.758886	24.117964	9.199E-11	1.435571	up	hypothetical protein MIMGU_mgv1a011926mg [Erythranthe guttata]
c44854.graph_c0	1.6200281	1.5475296	1.4943553	4.1962928	6.5578466	7.17638	1.537E-08	1.9769	up	PREDICTED: GDSL esterase/lipase At4g18970-like [Sesamum indicum]
c44868.graph_c2	1.9630934	2.6180805	2.2631899	4.8169276	4.4257819	4.8751544	8.969E-05	1.087725	up	PREDICTED: uncharacterized protein LOC105178110 [Sesamum indicum]
c44873.graph_c0	0.1038664	0.1641179	0.1483198	1.1190958	1.0809393	1.2300623	7.781E-11	3.084357	up	PREDICTED: monogalactosyldiacylglycerol synthase 2, chloroplastic [Sesamum indicum]
c44878.graph_c1	35.047293	32.967312	36.739428	5.5420735	7.2764845	4.6690003	2.593E-57	-2.54266	down	Taxane 13-alpha-hydroxylase [Medicago truncatula]
c44880.graph_c0	0.1815323	0.3278133	0.9258054	1.7257916	2.0110936	1.1261059	0.0026706	1.801828	up	hypothetical protein GUITHDRAFT_152339 [Guillardia theta CCMP2712]
c44882.graph_c0	10.687879	10.989821	10.014015	21.038741	25.800043	30.689693	1.651E-10	1.327632	up	PREDICTED: uncharacterized protein LOC105178220 [Sesamum indicum]
c44883.graph_c0	2.8218379	2.9067079	3.0160773	7.2797214	7.8987776	9.4584252	3.429E-11	1.533294	up	PREDICTED: probable protein phosphatase 2C 34 [Sesamum indicum]
c44896.graph_c0	3.6729439	1.938774	2.8587649	0.7878923	1.2140746	0.7647947	0.0009309	-1.577523	down	-
c44910.graph_c0	27.794157	24.107505	27.348427	62.258856	58.440704	57.654362	1.059E-16	1.213589	up	PREDICTED: protein TIFY 6B [Sesamum indicum]
c44910.graph_c1	16.023572	12.933322	17.103293	4.2058252	7.9330734	7.5759119	0.000235	-1.193323	down	-
c44911.graph_c0	27.695884	31.336144	26.845816	9.6488561	14.20656	11.083533	1.531E-16	-1.260482	down	PREDICTED: amino-acid permease BAT1 homolog [Sesamum indicum]
c44914.graph_c0	6.0074174	4.7927539	5.5504167	1.7324777	2.6036362	2.5094972	0.0002936	-1.221369	down	PREDICTED: DNA-directed RNA polymerase III subunit RPC4 [Sesamum indicum]
c44927.graph_c0	128.07641	130.3618	125.80482	267.74059	379.05649	460.17703	1.4E-06	1.560848	up	PREDICTED: S-adenosylmethionine synthase 1 [Sesamum indicum]
c44935.graph_c0	19.672322	19.701159	19.334223	67.795789	81.303115	78.756135	6.222E-43	1.99544	up	PREDICTED: ABC transporter B family member 19 [Sesamum indicum]

#ID	28 h-1 FPKM	28 h-2 FPKM	28 h-3 FPKM	48 h-1 FPKM	48 h-2 FPKM	48 h-3 FPKM	FDR	log2FC	regulated	nr_annotation
c44948.graph_c0	2.160422	2.2596737	2.0596102	0.9218692	0.5457519	0.9167765	0.0004831	-1.394696	down	PREDICTED: protein LURP-one-related 15-like isoform X1 [Sesamum indicum]
c44954.graph_c0	0.675967	0.6634074	0.8633482	1.713676	2.8810115	4.6407522	0.0041643	2.097081	up	PREDICTED: exonuclease 1 [Sesamum indicum]
c44955.graph_c0	0.9287662	0.698824	0.8525988	2.0357126	2.3644548	1.8768358	0.0003144	1.380389	up	PREDICTED: LOW QUALITY PROTEIN: serine carboxypeptidase 24-like [Sesamum indicum]
c44970.graph_c0	1.978412	1.3524998	1.0608704	4.6931365	4.9338564	6.0566846	6.257E-09	1.876156	up	PREDICTED: uncharacterized protein LOC105168255 [Sesamum indicum]
c44970.graph_c1	2.7395407	1.0011982	2.0225435	8.5987438	7.6203019	9.2696147	4.051E-09	2.186831	up	unnamed protein product [Coffea canephora]
c44971.graph_c0	0.034069	0.2768497	0.1668	0.8205135	1.0522313	1.3833246	3.096E-09	2.80517	up	PREDICTED: probable transcription factor GLK1 [Sesamum indicum]
c44979.graph_c0	0.8773089	0.4916655	0.7652477	2.9143476	1.7874409	1.9789974	0.0003862	1.697084	up	hypothetical protein MIMGU_mgv1a001060mg [Erythranthe guttata]
c44994.graph_c0	1.2542832	1.7132694	0.9972416	0.0407666	0.5830277	0.253917	0.0001031	-2.159566	down	PREDICTED: G-type lectin S-receptor-like serine/threonine-protein kinase RLK1 isoform X1 [Vitis vinifera]
c45019.graph_c0	17.2963	19.223579	15.47844	3.1926221	3.6992768	3.3290343	6.009E-36	-2.30644	down	BnaAnng27240D [Brassica napus]
c45027.graph_c0	11.999501	12.516104	8.3962693	0.3096234	0.5740149	0.4821269	3.655E-27	-4.557812	down	PREDICTED: uncharacterized protein LOC101264598 [Solanum lycopersicum]
c45028.graph_c0	3.4595879	2.1800708	2.8229955	7.1260841	8.6622483	9.7143492	2.745E-08	1.628624	up	hypothetical protein MIMGU_mgv1a010001mg [Erythranthe guttata]
c45035.graph_c0	0.9515117	0.3995934	0.5597488	1.150013	2.4365986	2.7204131	0.0026507	1.751235	up	PREDICTED: protein NRT1/ PTR FAMILY 4.5-like [Sesamum indicum]
c45078.graph_c0	28.174122	31.27613	37.213546	3.5854071	2.7504961	3.1745581	9.558E-39	-3.299532	down	PREDICTED: uncharacterized protein LOC105174597 [Sesamum indicum]
c45088.graph_c0	2.0363264	3.0643531	2.252426	4.2446297	5.651221	5.3079998	0.0037667	1.085763	up	PREDICTED: uncharacterized protein LOC104801838 isoform X2 [Tarenaya hassleriana]
c45134.graph_c0	6.9393839	6.980907	6.5053065	9.4698761	14.233584	16.286958	0.0005251	1.003119	up	PREDICTED: filament-like plant protein [Sesamum indicum]
c45137.graph_c0	1.9783724	2.9090923	2.4906894	3.8690733	5.7687012	8.5429421	0.0090749	1.333279	up	PREDICTED: receptor-like protein kinase HAIKU2 [Sesamum indicum]
c45137.graph_c2	6.0220605	6.3765356	6.7306254	10.224137	14.090405	16.342344	1.005E-05	1.122669	up	PREDICTED: receptor-like protein kinase HAIKU2 [Sesamum indicum]
c45143.graph_c0	1.4087933	2.464516	2.1554322	8.4823091	9.1633327	6.6537238	1.61E-09	2.054345	up	PREDICTED: serine/threonine-protein kinase Nek6-like [Sesamum indicum]
c45149.graph_c1	0.9780677	1.0793487	1.3301588	0.1836701	0.0972881	0.4085713	2.458E-06	-2.259098	down	PREDICTED: protein FAR1-RELATED SEQUENCE 5-like [Nicotiana sylvestris]
c45149.graph_c2	358.145	370.43944	358.82891	142.02087	178.24212	181.22241	1.099E-10	-1.078872	down	PREDICTED: carbonic anhydrase 2-like [Sesamum indicum]
c45158.graph_c0	0.755418	0.8261713	0.5990511	4.9360923	5.3149157	5.7841386	2.831E-34	2.918921	up	PREDICTED: uncharacterized protein LOC105177021 [Sesamum indicum]
c45180.graph_c0	45.549878	36.802451	37.776689	89.325335	81.230392	77.269497	1.485E-12	1.089215	up	PREDICTED: pentatricopeptide repeat-containing protein At5g24830 isoform X1 [Sesamum indicum]
c45189.graph_c1	3.0315148	3.3454345	1.9239822	5.5505209	6.5585774	7.978094	0.0022012	1.313129	up	hypothetical protein JCGZ_03401 [Jatropha curcas]
c45190.graph_c0	51.384333	45.658134	50.95073	236.05324	323.07988	343.68064	4.674E-25	2.644548	up	PREDICTED: beta-glucosidase 24-like [Eucalyptus grandis]
c45193.graph_c0	0.1434635	0	0.0668943	0.9871885	1.1558914	2.1959859	1.319E-06	4.398679	up	PREDICTED: cellulose synthase-like protein G2 [Sesamum indicum]

#ID	28 h-1 FPKM	28 h-2 FPKM	28 h-3 FPKM	48 h-1 FPKM	48 h-2 FPKM	48 h-3 FPKM	FDR	log2FC	regulated	nr_annotation
c45210.graph_c1	5.0150308	9.3415899	7.4107085	32.398929	31.57805	29.612656	4.659E-43	2.147774	up	PREDICTED: pleiotropic drug resistance protein 1-like [Sesamum indicum]
c45215.graph_c1	26.918262	27.526309	25.905797	71.775859	52.593568	42.920314	0.005831	1.10849	up	PREDICTED: U-box domain-containing protein 13 [Sesamum indicum]
c45234.graph_c0	8.7676519	9.0246591	9.205241	3.9515181	3.3750869	3.4720866	6.356E-09	-1.276939	down	-
c45240.graph_c0	69.09979	72.146644	69.165753	33.736907	34.395953	29.221935	8.842E-14	-1.068709	down	PREDICTED: crt homolog 1 [Sesamum indicum]
c45242.graph_c0	1.4364414	1.5748945	1.4023632	6.5353545	7.7500922	8.4767657	5.514E-17	2.404916	up	PREDICTED: serine/threonine-protein kinase TIO isoform X2 [Sesamum indicum]
c45246.graph_c0	7.4880083	5.9603283	6.2709472	19.774171	24.167765	28.077817	5.564E-19	1.905842	up	PREDICTED: WAT1-related protein At5g40240-like [Sesamum indicum]
c45258.graph_c0	411.87866	440.61131	405.96126	1129.5795	1120.5769	1065.0884	1.649E-15	1.440336	up	ascorbate peroxidase [Acanthus ebracteatus]
c45271.graph_c0	15.046638	18.495389	15.681718	4.6265514	8.2521387	10.459722	7.781E-05	-1.045947	down	PREDICTED: dof zinc finger protein DOF5.4-like [Sesamum indicum]
c45279.graph_c0	25.71237	25.097708	25.909643	76.10267	99.041991	97.003997	8.346E-35	1.864004	up	PREDICTED: peroxidase 7 [Sesamum indicum]
c45285.graph_c0	3.7732624	4.8230242	3.7652236	0.6915019	1.0072733	0.8716668	1.977E-10	-2.229143	down	PREDICTED: U-box domain-containing protein 19-like [Sesamum indicum]
c45292.graph_c0	47.321518	41.405444	40.977585	94.149375	134.33762	123.01231	6.327E-14	1.474512	up	PREDICTED: pentatricopeptide repeat-containing protein At5g59600 [Sesamum indicum]
c45303.graph_c0	39.774392	38.807822	36.128846	284.52368	369.48589	346.64468	2.604E-79	3.162683	up	PREDICTED: S-type anion channel SLAH2 [Sesamum indicum]
c45320.graph_c1	1.0321709	1.208088	1.0606024	4.021973	4.1067882	4.6135402	2.597E-07	1.989638	up	PREDICTED: laccase-17-like [Sesamum indicum]
c45321.graph_c1	66.549042	66.185585	68.192665	141.90872	155.13812	152.96624	1.649E-15	1.203765	up	PREDICTED: UDP-glucuronate 4-epimerase 1 [Sesamum indicum]
c45334.graph_c0	347.16208	344.07339	373.83317	113.94111	96.596545	83.583105	8.517E-31	-1.809831	down	hypothetical protein MIMGU_mgv1a010052mg [Erythranthe guttata]
c45340.graph_c0	12.647523	11.753042	12.852119	4.5885028	3.9086105	4.1828097	6.076E-18	-1.510183	down	PREDICTED: B3 domain-containing transcription repressor VAL1 [Populus euphratica]
c45351.graph_c2	2.3656805	1.5738857	1.8287773	0.4997791	0.5015892	0.4681057	3.39E-06	-1.930375	down	PREDICTED: potassium channel KAT3-like [Sesamum indicum]
c45353.graph_c0	11.908365	12.527719	13.193899	7.5473649	4.3278492	3.3886054	1.794E-05	-1.245689	down	PREDICTED: protein ZINC INDUCED FACILITATOR-LIKE 1-like isoform X2 [Pyrus x bretschneideri]
c45354.graph_c0	5.2655832	4.2078535	5.2844138	1.380934	1.8693022	1.5700654	0.000157	-1.576901	down	PREDICTED: protein Brevis radix-like 4 [Sesamum indicum]
c45357.graph_c0	0.9325644	1.4434598	1.0327381	3.5462821	2.5045706	2.7046804	0.0014615	1.410104	up	PREDICTED: two-component response regulator-like APRR9 [Sesamum indicum]
c45364.graph_c0	1.1722043	1.1568455	1.7573075	2.7298271	2.8187076	4.3352748	0.0004178	1.311119	up	PREDICTED: maf-like protein DDB_G0281937 [Sesamum indicum]
c45402.graph_c0	1.2938733	0.8836735	0.9745755	1.9554797	2.0938688	3.049632	0.0003315	1.208678	up	PREDICTED: cellulose synthase-like protein E1 [Sesamum indicum]
c45406.graph_c0	296.21273	323.01789	336.27776	28.258609	30.399166	29.39057	1.651E-95	-3.398986	down	15.5 kDa oleosin [Sesamum indicum]
c45434.graph_c1	3.1818256	3.7450176	3.7090625	0.7922354	1.0681711	2.0506946	0.0016123	-1.41189	down	-
c45435.graph_c0	4.3427113	4.8748327	5.1717653	0.5951034	0.5516349	0.7280892	1.901E-10	-2.899211	down	-
c45449.graph_c3	33.38744	37.135019	32.922345	9.6139129	19.057079	20.504108	2.865E-06	-1.042723	down	hypothetical protein MIMGU_mgv1a015476mg [Erythranthe guttata]
c45455.graph_c1	1.5546667	1.5160158	1.1163639	0.2364785	0.5845475	0.7364602	0.0026903	-1.399941	down	-

#ID	28 h-1 FPKM	28 h-2 FPKM	28 h-3 FPKM	48 h-1 FPKM	48 h-2 FPKM	48 h-3 FPKM	FDR	log2FC	regulated	nr_annotation
c45458.graph_c0	53.156982	54.380344	57.241955	19.491371	20.287015	22.103219	2.678E-21	-1.372427	down	PREDICTED: DUF21 domain-containing protein At2g14520-like [Sesamum indicum]
c45467.graph_c1	6.5177134	5.8216058	7.1483883	30.470504	30.303489	34.305576	2.208E-22	2.327387	up	hypothetical protein MIMGU_mgv1a008026mg [Erythranthe guttata]
c45474.graph_c0	0.0498338	0.0899905	0	1.6423692	2.1414043	3.9344582	1.454E-07	5.822061	up	-
c45482.graph_c0	0.2222579	0.3511865	0.3400511	2.2538256	2.3503478	2.7574806	2.51E-12	3.0505	up	PREDICTED: myosin-12 [Sesamum indicum]
c45492.graph_c0	45.942803	42.216048	45.858851	145.85491	137.64329	173.13098	2.775E-28	1.809663	up	PREDICTED: VIN3-like protein 2 [Sesamum indicum]
c45515.graph_c0	6.0068274	6.3533623	6.6584313	14.812878	15.147717	14.324237	1.798E-14	1.261486	up	PREDICTED: V-type proton ATPase subunit a3-like [Sesamum indicum]
c45516.graph_c0	8.5884851	8.7319589	8.9257823	2.7261479	4.0547079	4.4016194	1.106E-09	-1.196639	down	unnamed protein product [Coffea canephora]
c45518.graph_c0	77.797476	81.61141	82.092186	33.568392	30.703493	29.085505	1.833E-20	-1.326801	down	PREDICTED: Fanconi anemia group I protein homolog [Sesamum indicum]
c45537.graph_c2	86.905378	90.77511	90.389742	169.9167	187.81147	206.42252	1.721E-13	1.112875	up	PREDICTED: UDP-glucose 6-dehydrogenase 4 [Sesamum indicum]
c45541.graph_c0	49.117374	41.493775	48.717173	205.09982	255.33622	243.43491	1.077E-54	2.374918	up	PREDICTED: WAT1-related protein At1g09380 [Sesamum indicum]
c45569.graph_c2	5.5429668	7.9145348	5.1541341	2.2875735	1.7310048	2.1808598	0.0006211	-1.539695	down	PREDICTED: heat stress transcription factor C-1-like [Sesamum indicum]
c45569.graph_c3	67.124129	65.036294	65.511248	336.39086	328.655	318.59686	1.579E-53	2.357893	up	PREDICTED: putative glycerol-3-phosphate transporter 1 [Eucalyptus grandis]
c45591.graph_c0	0.3533374	0.7018671	0.8072971	2.8664392	4.1753865	1.9926096	0.0001936	2.31764	up	hypothetical protein H632_c597p1 [Helicosporidium sp. ATCC 50920]
c45616.graph_c0	0.3025919	0.3512728	0.5996448	1.0958788	1.3350933	1.0238616	0.0009133	1.501974	up	PREDICTED: la-related protein 6C-like isoform X1 [Sesamum indicum]
c45622.graph_c0	1.2569193	1.1084877	1.5503799	2.1860705	2.4336331	3.2309311	0.0079922	1.040571	up	PREDICTED: LOW QUALITY PROTEIN: beta-adaptin-like protein A [Sesamum indicum]
c45633.graph_c0	0.2270084	0.1024836	0.5094017	2.5897485	2.2862704	2.0483046	1.659E-10	3.088289	up	-
c45635.graph_c0	1.6373724	1.6590863	1.3212371	2.9287471	4.2264478	4.0013103	1.717E-05	1.30877	up	PREDICTED: laccase-17-like [Sesamum indicum]
c45661.graph_c0	3.5369463	3.3870782	3.4733383	10.305859	9.9308877	12.019555	1.8E-19	1.674479	up	PREDICTED: chaperonin 60 subunit beta 4, chloroplastic isoform X1 [Sesamum indicum]
c45669.graph_c0	8.0659359	7.9330324	7.5454923	3.1402801	3.9843586	3.1516003	2.938E-05	-1.156619	down	PREDICTED: uncharacterized protein LOC105168837 isoform X1 [Sesamum indicum]
c45673.graph_c0	3.7021026	3.4237839	7.2882529	46.243476	26.763151	18.749325	0.0028001	2.725857	up	PREDICTED: probable galactinol--sucrose galactosyltransferase 2-like isoform X1 [Citrus sinensis]
c45673.graph_c1	1.3409717	2.3114733	2.3873934	12.825616	11.459072	15.26204	1.816E-11	2.75255	up	hypothetical protein MIMGU_mgv1a001872mg [Erythranthe guttata]
c45676.graph_c0	23.73304	24.640672	24.980826	6.8461074	8.7868289	7.4973849	8.563E-22	-1.626522	down	PREDICTED: GABA transporter 1 [Sesamum indicum]
c45690.graph_c0	3.8700075	4.238931	4.7238996	10.534804	8.9666153	6.3326855	0.0047361	1.0576	up	PREDICTED: U-box domain-containing protein 35 [Sesamum indicum]
c45696.graph_c0	414.33563	517.71227	357.60014	1868.6563	1392.4908	1050.7956	0.0002468	1.792691	up	PREDICTED: nitrate reductase [NADH] 2 [Sesamum indicum]
c45700.graph_c0	6.6479986	7.1013413	7.0254474	18.212079	15.781147	16.450839	1.328E-15	1.324515	up	hypothetical protein MIMGU_mgv1a010620mg [Erythranthe guttata]
c45745.graph_c0	376.01194	340.07362	393.40551	90.080241	94.78204	86.26224	2.513E-30	-1.991179	down	PREDICTED: alpha-galactosidase [Sesamum indicum]

#ID	28 h-1 FPKM	28 h-2 FPKM	28 h-3 FPKM	48 h-1 FPKM	48 h-2 FPKM	48 h-3 FPKM	FDR	log2FC	regulated	nr_annotation
c45751.graph_c0	5.0342242	5.2021089	4.3211621	1.6133183	2.0600941	2.3327215	9.665E-08	-1.240401	down	hypothetical protein MIMGU_mgv1a003838mg [Erythranthe guttata]
c45753.graph_c0	2.6205732	2.3661319	2.7756278	7.0835086	7.8536892	7.0466795	3.017E-11	1.542639	up	PREDICTED: phosphatidylinositol/phosphatidylcholine transfer protein SFH9 isoform X1 [Sesamum indicum]
c45758.graph_c0	1.5777153	1.6436873	1.6552321	0.5494149	0.6984462	0.9581775	0.0067785	-1.109378	down	PREDICTED: pentatricopeptide repeat-containing protein At1g74630 [Sesamum indicum]
c45770.graph_c0	40.849433	44.440318	44.301029	13.016173	16.573708	17.471196	1.212E-21	-1.424205	down	unnamed protein product [Coffea canephora]
c45783.graph_c0	6.1823084	5.7220334	6.6103138	2.2354995	2.9147577	2.273296	8.943E-07	-1.279773	down	PREDICTED: uncharacterized protein LOC105166430 [Sesamum indicum]
c45800.graph_c0	306.41452	325.10515	317.75506	121.4477	113.58558	99.664594	1.934E-19	-1.459323	down	PREDICTED: uncharacterized protein LOC105158790 isoform X1 [Sesamum indicum]
c45801.graph_c0	60.617379	58.773623	62.783009	27.865156	28.773145	25.887639	2.98E-14	-1.100112	down	PREDICTED: protein CPR-5 [Sesamum indicum]
c45804.graph_c1	0.4761743	0.7255249	0.6556854	3.8101362	3.6767002	2.5175022	9.256E-10	2.475356	up	hypothetical protein CICLE_v10021726mg [Citrus clementina]
c45809.graph_c0	0.3753529	0.3389085	0.8422835	2.4384106	2.4571906	4.0218516	7.173E-07	2.554084	up	PREDICTED: uncharacterized protein LOC105176285 [Sesamum indicum]
c45815.graph_c1	466.37755	414.50447	389.03515	72.9643	104.07088	75.39222	7.106E-42	-2.292902	down	hypothetical protein MIMGU_mgv1a010438mg [Erythranthe guttata]
c45827.graph_c1	17.747762	17.930399	16.48561	38.222371	36.037851	40.520528	7.88E-13	1.180161	up	unnamed protein product [Coffea canephora]
c45833.graph_c0	2.255838	6.5992651	5.1540871	25.391835	42.772739	14.044609	0.0054871	2.592	up	60S ribosomal protein L35a-4 [Auxenochlorella protothecoides]
c45850.graph_c0	74.088984	88.513312	86.143478	18.096987	21.314968	22.503619	2.896E-39	-1.967712	down	PREDICTED: uncharacterized protein LOC105163078 [Sesamum indicum]
c45851.graph_c0	2.0492797	1.9329105	2.4635023	0.2783157	0.6142536	0.5159242	5.069E-08	-2.164846	down	-
c45858.graph_c0	0.0966789	0.0698336	0.0315557	1.1029289	0.4673678	1.0904216	8.882E-06	3.799012	up	PREDICTED: putative ABC transporter B family member 8 [Sesamum indicum]
c45862.graph_c0	24.846578	25.173383	28.254923	9.2651053	10.348133	8.0996561	6.927E-24	-1.45627	down	PREDICTED: uncharacterized protein LOC105160721 [Sesamum indicum]
c45865.graph_c0	1.3607858	1.2100459	1.0262696	2.3521312	2.8240375	3.6742236	8.701E-05	1.335666	up	PREDICTED: type I inositol 1,4,5-trisphosphate 5-phosphatase CVP2-like [Sesamum indicum]
c45870.graph_c0	1.7476133	1.8887356	1.3612183	2.3494849	4.017948	5.1965287	0.0078817	1.241775	up	PREDICTED: kinesin-4-like isoform X2 [Sesamum indicum]
c45871.graph_c0	2.1621735	1.342165	1.3967501	0.799375	0.6048863	0.3048339	0.0021215	-1.466469	down	PREDICTED: pentatricopeptide repeat-containing protein At2g03880, mitochondrial [Sesamum indicum]
c45873.graph_c0	13.053669	12.096403	13.05766	4.6083417	6.9577814	6.5381585	5.592E-10	-1.042624	down	hypothetical protein PHAVU_004G031700g [Phaseolus vulgaris]
c45903.graph_c0	21.131917	24.347525	25.170566	40.401553	50.9034	51.141984	6.458E-13	1.049306	up	PREDICTED: probable xyloglucan glycosyltransferase 12 [Sesamum indicum]
c45908.graph_c0	3.2049961	2.543046	2.5360006	5.8476366	9.3248974	11.501793	5.827E-05	1.719343	up	PREDICTED: chlorophyll a-b binding protein CP24 10A, chloroplastic [Cucumis melo]
c45914.graph_c0	0.3337468	0.3390098	0.2382928	1.9566001	1.4565485	1.7880243	1.74E-08	2.559991	up	PREDICTED: separase [Sesamum indicum]
c45916.graph_c0	324.80061	373.7312	344.78003	52.361465	59.254601	61.224795	5.473E-59	-2.554049	down	PREDICTED: translocator protein homolog [Sesamum indicum]
c45929.graph_c0	0.2632634	0.2925565	0.1487221	0.7187508	0.843011	0.6852216	9.541E-05	1.714954	up	hypothetical protein JCGZ_06305 [Jatropha curcas]

#ID	28 h-1 FPKM	28 h-2 FPKM	28 h-3 FPKM	48 h-1 FPKM	48 h-2 FPKM	48 h-3 FPKM	FDR	log2FC	regulated	nr_annotation
c45931.graph_c0	16.8045	19.613734	19.397937	109.72583	81.897216	65.181377	2.608E-06	2.252148	up	PREDICTED: uncharacterized protein LOC105168927 [Sesamum indicum]
c45932.graph_c0	31.707067	31.758624	34.31561	13.791376	12.569147	12.752718	1.561E-16	-1.278165	down	PREDICTED: uncharacterized protein LOC105174716 [Sesamum indicum]
c45960.graph_c0	14.22521	14.310244	14.390312	5.7634935	5.7277492	4.4811765	1.126E-19	-1.381976	down	PREDICTED: F-box/LRR-repeat protein 3 isoform X1 [Sesamum indicum]
c45964.graph_c2	499.13478	516.30857	514.44255	1844.5511	1529.6716	1630.78	3.418E-21	1.755294	up	hypothetical protein MIMGU_mgv1a027154mg [Erythranthe guttata]
c45974.graph_c2	47.214864	48.768508	44.466233	99.990906	95.67474	93.137964	1.015E-12	1.083446	up	PREDICTED: EIN3-binding F-box protein 1-like [Sesamum indicum]
c45984.graph_c0	4.7045694	4.6429278	4.5382229	1.6642014	2.4486382	2.5913936	0.0004395	-1.015785	down	PREDICTED: uncharacterized protein LOC105157708 [Sesamum indicum]
c45985.graph_c0	10.450097	9.7990261	10.655081	4.2776939	5.1625309	4.9849112	2.632E-11	-1.060534	down	PREDICTED: uncharacterized protein LOC105175876 isoform X1 [Sesamum indicum]
c45987.graph_c0	12.121522	12.3834	11.235196	40.261997	48.278822	53.616158	8.992E-35	2.02985	up	hypothetical protein MIMGU_mgv1a004935mg [Erythranthe guttata]
c45989.graph_c0	2.464604	2.8919593	2.4862926	1.0545374	1.4104076	1.3253785	0.0004127	-1.011664	down	PREDICTED: pentatricopeptide repeat-containing protein At4g21300 [Sesamum indicum]
c45999.graph_c0	1.1869022	0.968617	0.9498775	5.1789036	9.0112157	8.0063515	1.586E-12	2.870587	up	PREDICTED: heparanase-like protein 1 isoform X1 [Sesamum indicum]
c46015.graph_c0	10.221182	11.441344	10.18213	1.9209361	3.0741472	3.091174	1.544E-12	-1.943711	down	PREDICTED: cytochrome P450 724B1 isoform X1 [Vitis vinifera]
c46019.graph_c2	85.551844	80.430893	84.144798	163.65186	178.72788	171.52333	1.131E-12	1.079528	up	PREDICTED: protein ACCUMULATION AND REPLICATION OF CHLOROPLASTS 3 [Sesamum indicum]
c46047.graph_c0	16.66006	15.016174	17.111924	7.9376544	7.9787553	7.3913831	1.983E-07	-1.023238	down	PREDICTED: histone-lysine N-methyltransferase ATXR2 [Sesamum indicum]
c46056.graph_c2	47.494417	56.116665	46.374376	191.29587	139.91671	119.95214	6.924E-05	1.639166	up	PREDICTED: cytochrome P450 93A3-like [Sesamum indicum]
c46064.graph_c0	33.29855	36.563381	38.033798	9.1223006	8.1117272	9.3786731	3.608E-30	-1.976373	down	hypothetical protein M569_12324 [Genlisea aurea]
c46073.graph_c0	146.12736	146.06343	162.39793	75.679475	70.373145	54.145119	1.279E-13	-1.137512	down	PREDICTED: RING finger and CHY zinc finger domain-containing protein 1-like isoform X1 [Sesamum indicum]
c46079.graph_c0	10.692174	11.979597	10.914739	54.596569	58.303477	76.409275	4.886E-22	2.533439	up	PREDICTED: probable beta-D-xylosidase 7 [Sesamum indicum]
c46085.graph_c0	0.1813873	0.4585721	0.6216444	7.0356069	6.5277902	8.5515707	2.385E-27	4.172073	up	PREDICTED: thylakoid lumenal 29 kDa protein, chloroplastic [Sesamum indicum]
c46087.graph_c1	108.8986	110.28176	123.25679	58.136254	49.662364	42.429093	2.313E-14	-1.142159	down	PREDICTED: SUMO-activating enzyme subunit 2 [Sesamum indicum]
c46088.graph_c0	0.249008	0.3747182	0.2031885	3.7876322	2.1177279	4.2127654	6.536E-08	3.660631	up	PREDICTED: protein TIC 62, chloroplastic [Beta vulgaris subsp. vulgaris]
c46092.graph_c0	1.3642021	1.1723853	1.1534131	0.354179	0.4634943	0.4634504	0.0004875	-1.489091	down	hypothetical protein M569_17037, partial [Genlisea aurea]
c46111.graph_c1	0.1419126	0.1281337	0.1157995	9.3539999	7.6226374	11.524338	2.637E-23	6.249218	up	-
c46114.graph_c0	3.2968914	4.048426	5.165254	18.889318	29.485149	17.476931	1.882E-07	2.432901	up	40S ribosomal protein S17 [Auxenochlorella protothecoides]
c46130.graph_c0	16.437594	16.302477	16.206508	37.213263	32.873442	25.57463	0.0002006	1.013541	up	PREDICTED: GATA transcription factor 5-like [Sesamum indicum]
c46137.graph_c0	3.5692166	3.7166536	3.3588858	0.6934974	1.3294144	1.2929082	9.227E-06	-1.651524	down	PREDICTED: uncharacterized protein LOC103331091 [Prunus mume]
c46144.graph_c2	1.1182533	1.475683	1.5442063	106.85531	88.018966	121.56556	2.322E-64	6.29961	up	major latex-like protein 1 [Plantago major]

#ID	28 h-1 FPKM	28 h-2 FPKM	28 h-3 FPKM	48 h-1 FPKM	48 h-2 FPKM	48 h-3 FPKM	FDR	log2FC	regulated	nr_annotation
c46145.graph_c0	48.715007	45.398941	45.960952	19.054923	22.373238	22.465317	5.218E-14	-1.093448	down	PREDICTED: serine/threonine-protein kinase GRIK2 [Sesamum indicum]
c46147.graph_c1	2.3072411	2.5181809	2.1309567	15.844251	12.427075	9.9230169	1.207E-08	2.506356	up	PREDICTED: polyamine oxidase 1 [Sesamum indicum]
c46154.graph_c1	0.5562353	0.3476965	0.4887976	1.8982632	3.0451979	2.1716527	7.548E-08	2.388255	up	hypothetical protein MIMGU_mgv1a012700mg [Erythranthe guttata]
c46159.graph_c0	37.840445	30.274002	43.124264	12.952723	6.2177083	10.444762	2.171E-09	-1.857926	down	dehydration responsive protein, partial [Corchorus olitorius]
c46164.graph_c0	25.40581	21.576887	22.577513	10.326613	10.696688	12.523665	1.243E-08	-1.012059	down	PREDICTED: protein Brevis radix-like 1 [Sesamum indicum]
c46173.graph_c0	28.279539	27.637982	28.817413	9.2030374	13.179883	10.817313	9.863E-12	-1.314959	down	PREDICTED: 1-aminocyclopropane-1-carboxylate oxidase homolog 1-like [Sesamum indicum]
c46178.graph_c0	16100.365	15688.519	18260.994	2624.4807	2587.16	2099.8277	1.105E-10	-2.731226	down	PREDICTED: vicilin-like antimicrobial peptides 2-2 [Sesamum indicum]
c46180.graph_c1	0.5568135	0.2822458	0.3826149	0.074295	0.104942	0.1542501	0.0014569	-1.840666	down	PREDICTED: replication protein A 70 kDa DNA-binding subunit A isoform X1 [Sesamum indicum]
c46183.graph_c0	0.3113759	0.4016333	0.4476652	2.2741774	3.7830652	4.9668939	1.407E-07	3.278598	up	PREDICTED: uncharacterized protein LOC105179348 isoform X2 [Sesamum indicum]
c46204.graph_c0	32.775657	31.72984	31.493478	53.62417	69.904703	72.383167	1.623E-12	1.066009	up	PREDICTED: probable cinnamyl alcohol dehydrogenase 6 [Sesamum indicum]
c46205.graph_c0	109.53947	101.95362	113.56659	25.458782	22.239683	20.522945	1.088E-51	-2.206996	down	PREDICTED: uncharacterized protein LOC104222596 [Nicotiana sylvestris]
c46205.graph_c1	183.05057	179.20703	202.34616	50.638219	51.377132	44.031881	8.804E-39	-1.907868	down	PREDICTED: uncharacterized protein LOC102603928 [Solanum tuberosum]
c46208.graph_c0	5.3625753	5.6305459	4.5912935	1.8796004	2.1548698	2.2910372	1.369E-05	-1.261512	down	PREDICTED: uncharacterized protein LOC105158293 isoform X2 [Sesamum indicum]
c46216.graph_c0	163.78689	157.2194	146.32452	64.392143	69.074807	65.320032	1.161E-15	-1.191861	down	PREDICTED: uncharacterized protein LOC105174095 isoform X1 [Sesamum indicum]
c46216.graph_c1	8.4694836	7.2262056	9.0033595	3.1517635	3.5475932	2.5415058	0.0003538	-1.376286	down	-
c46231.graph_c0	0.7981737	0.8708169	1.5197076	31.532687	32.601194	23.256296	7.548E-36	4.819558	up	PREDICTED: vestitone reductase-like [Sesamum indicum]
c46236.graph_c0	109.52874	102.49613	116.01386	19.746808	16.376086	9.9671098	6.611E-79	-2.781485	down	PREDICTED: cytochrome P450 71B36-like [Sesamum indicum]
c46238.graph_c0	1.1844702	1.2205857	1.2711813	0.652784	0.6051023	0.348506	0.0030006	-1.146451	down	PREDICTED: uncharacterized protein LOC104808395 [Tarenaya hassleriana]
c46239.graph_c0	4.1580851	4.1748494	3.7594036	9.2553126	11.368302	12.512436	1.221E-15	1.491944	up	PREDICTED: subtilisin-like protease SBT3.5 [Sesamum indicum]
c46243.graph_c1	11.985002	13.460033	13.079698	5.1263217	5.1928746	5.7266837	6.94E-08	-1.222512	down	hypothetical protein MIMGU_mgv1a008659mg [Erythranthe guttata]
c46259.graph_c0	26.692417	26.572185	25.477676	299.72578	211.2089	184.55256	1.433E-09	3.193543	up	PREDICTED: phosphoglycerate kinase, cytosolic-like [Sesamum indicum]
c46264.graph_c0	26.652345	26.006683	23.981942	8.2290718	10.975904	10.34368	6.173E-18	-1.337708	down	hypothetical protein L484_013876 [Morus notabilis]
c46269.graph_c0	1.9138183	2.172341	1.7847544	9.6343111	8.8112671	7.4624374	8.956E-10	2.187438	up	hypothetical protein MIMGU_mgv1a009279mg [Erythranthe guttata]
c46279.graph_c0	1.7618473	1.1029428	1.6293401	3.1563566	2.7444208	3.0469653	0.0038399	1.036926	up	BnaA04g06750D [Brassica napus]
c46291.graph_c0	39.411001	32.050318	35.09436	13.960459	16.470512	15.281657	4.502E-16	-1.181507	down	hypothetical protein MIMGU_mgv11b005497mg [Erythranthe guttata]
c46294.graph_c0	193.30575	133.7676	170.08392	51.047765	23.598073	14.86538	4.222E-12	-2.411188	down	-
c46295.graph_c0	0.6243704	0.6910459	0.8874833	2.3743065	2.8939357	3.1803238	5.732E-08	1.97644	up	hypothetical protein MIMGU_mgv1a004572mg [Erythranthe guttata]

#ID	28 h-1 FPKM	28 h-2 FPKM	28 h-3 FPKM	48 h-1 FPKM	48 h-2 FPKM	48 h-3 FPKM	FDR	log2FC	regulated	nr_annotation
c46306.graph_c0	0.7017	0.2851062	0.3435489	7.293543	10.97746	12.504642	5.838E-22	4.56553	up	unnamed protein product [Coffea canephora]
c46315.graph_c0	6.7462567	5.9301709	5.3063968	21.008432	19.599117	15.949559	8.106E-13	1.698573	up	hypothetical protein MIMGU_mgv1a001332mg [Erythranthe guttata]
c46327.graph_c0	5.2152651	5.7002418	5.3568471	1.246761	2.9794612	3.1216886	0.0001959	-1.119334	down	PREDICTED: high affinity nitrate transporter 2.4-like [Sesamum indicum]
c46354.graph_c0	7.130237	7.8095494	8.5765629	3.1225684	3.6204031	3.1952089	9.649E-10	-1.202511	down	PREDICTED: uncharacterized protein LOC105172385 [Sesamum indicum]
c46354.graph_c1	2.4559208	3.7318334	3.1282128	0.8731755	1.5282998	1.5538926	0.0022038	-1.203492	down	PREDICTED: pentatricopeptide repeat-containing protein At4g22760 [Sesamum indicum]
c46391.graph_c0	49.047805	52.830403	52.381392	20.113667	19.496174	16.246636	2.326E-23	-1.421254	down	hypothetical protein MIMGU_mgv1a003239mg [Erythranthe guttata]
c46391.graph_c1	26.579645	24.608424	25.865871	15.651469	10.386627	8.24809	2.424E-07	-1.115492	down	unnamed protein product [Coffea canephora]
c46400.graph_c0	2.3686961	2.6733881	2.1798103	6.2718822	6.3262206	6.8422737	4.392E-12	1.470233	up	PREDICTED: flavin-containing monooxygenase FMO GS-OX-like 2 isoform X1 [Sesamum indicum]
c46405.graph_c0	4.774811	5.54298	6.0214105	2.2008817	2.0817565	2.4479141	0.0023117	-1.237928	down	PREDICTED: uncharacterized protein LOC101504607, partial [Cicer arietinum]
c46408.graph_c1	6.3515939	7.4679661	5.9802101	14.111566	12.371995	13.461706	5.644E-06	1.056924	up	PREDICTED: phosphoglycerate mutase-like protein 4 [Sesamum indicum]
c46427.graph_c0	6.12915	5.0424126	5.4228613	1.9113169	2.4935141	2.4092942	1.841E-07	-1.246804	down	PREDICTED: uncharacterized protein LOC105155870 [Sesamum indicum]
c46430.graph_c0	25.967082	22.54552	21.799168	73.203379	91.219007	109.84073	5.89E-15	2.000272	up	PREDICTED: baicalein 7-O-glucuronosyltransferase-like [Sesamum indicum]
c46432.graph_c0	0.2914201	0.52625	0.7728383	1.9393311	2.3479842	2.1364635	5.9E-06	2.052297	up	--
c46442.graph_c0	14.74257	14.815898	13.26418	4.5824492	6.6448795	6.1306126	5.078E-10	-1.266741	down	PREDICTED: heat stress transcription factor C-1-like [Sesamum indicum]
c46445.graph_c1	43.812963	45.524244	45.429647	20.226132	17.328481	15.164618	4.517E-17	-1.307607	down	PREDICTED: plant intracellular Ras-group-related LRR protein 3-like [Sesamum indicum]
c46486.graph_c0	11.724845	10.422123	10.87202	4.0533011	5.9871362	5.8643951	2.856E-09	-1.018771	down	PREDICTED: uncharacterized protein LOC105176969 [Sesamum indicum]
c46492.graph_c1	13.20491	13.099941	12.705194	4.9105004	7.2779096	6.3439365	1.302E-08	-1.037887	down	PREDICTED: LOW QUALITY PROTEIN: uncharacterized protein LOC105176443 [Sesamum indicum]
c46530.graph_c0	2.1783199	3.5195701	3.2743259	17.584362	18.166636	17.844723	1.36E-12	2.62032	up	PREDICTED: diacylglycerol kinase 3-like [Sesamum indicum]
c46530.graph_c1	2.2759516	3.642903	3.9675646	20.981355	18.337441	15.985403	9.741E-13	2.529721	up	PREDICTED: diacylglycerol kinase 3-like [Sesamum indicum]
c46530.graph_c2	2.4926536	3.232452	2.8666903	8.9699337	9.2554457	9.2644539	1.504E-16	1.719572	up	PREDICTED: glutamate receptor 2.8-like [Sesamum indicum]
c46533.graph_c0	23.612969	25.210863	26.159461	12.016166	3.4717285	1.9439841	2.623E-08	-2.031772	down	Cell wall-associated hydrolase, partial [Medicago truncatula]
c46533.graph_c1	8.4161903	10.638643	12.361577	3.4138051	1.8082563	0.3796979	1.86E-07	-2.422322	down	-
c46544.graph_c0	0.2424045	0.4085545	0.4747202	1.2700308	1.9096619	1.4034688	5.22E-07	2.061907	up	PREDICTED: phosphatidylinositol 4-phosphate 5-kinase 6 [Sesamum indicum]
c46546.graph_c0	4.8850759	4.3569757	4.083406	1.9633784	1.6799688	2.2173491	0.0075754	-1.142287	down	PREDICTED: vacuolar cation/proton exchanger 3-like [Sesamum indicum]
c46550.graph_c1	4.920315	5.928771	5.3869477	13.326244	13.048029	12.35667	6.027E-13	1.297467	up	PREDICTED: G-type lectin S-receptor-like serine/threonine-protein kinase At4g27290 [Sesamum indicum]

#ID	28 h-1 FPKM	28 h-2 FPKM	28 h-3 FPKM	48 h-1 FPKM	48 h-2 FPKM	48 h-3 FPKM	FDR	log2FC	regulated	nr_annotation
c46550.graph_c2	0.6080546	0.6519569	0.9923352	2.4326924	4.0826021	3.4719247	1.416E-09	2.181773	up	PREDICTED: uncharacterized protein LOC105175473 [Sesamum indicum]
c46559.graph_c0	25.396671	19.632546	17.032994	6.6148257	2.3358686	1.1771664	2.784E-09	-2.545244	down	-
c46566.graph_c0	0	0	0.0609157	3.5012137	5.7140364	8.251491	4.528E-12	8.189238	up	-
c46577.graph_c0	0.5372904	0.3880983	0.6576369	2.383693	4.4011216	2.3633952	5.47E-05	2.564779	up	-
c46579.graph_c0	4.1175438	7.435512	7.97972	36.861236	52.700234	29.170708	1.271E-07	2.642899	up	60S ribosomal protein L11 [Candida albicans WO-1]
c46589.graph_c0	1.9214695	2.1810257	1.6350993	5.8106554	3.9440616	5.0464471	1.149E-05	1.415347	up	PREDICTED: uncharacterized protein LOC105173906 [Sesamum indicum]
c46590.graph_c0	3.3478047	3.5162646	2.8432826	1.5588694	1.3761943	1.4256024	0.0055851	-1.109846	down	PREDICTED: uncharacterized protein LOC104210866 [Nicotiana sylvestris]
c46592.graph_c0	6.1642483	4.7517765	5.8451108	47.005368	55.652974	83.347904	8.472E-12	3.507365	up	hypothetical protein MIMGU_mgv1a019962mg [Erythranthe guttata]
c46603.graph_c1	41.435102	37.984196	42.203715	17.177798	20.619802	16.549263	3.944E-11	-1.12199	down	PREDICTED: DEAD-box ATP-dependent RNA helicase 53-like [Sesamum indicum]
c46629.graph_c0	4.2897545	4.9612366	4.7786423	1.3441208	1.8770026	1.6852595	4.779E-06	-1.478992	down	Uncharacterized protein TCM_005954 [Theobroma cacao]
c46642.graph_c0	0.0525316	0.0711467	0.1393125	1.1486379	0.9346762	1.4516088	2.562E-16	3.789169	up	PREDICTED: condensin-2 complex subunit D3 [Sesamum indicum]
c46665.graph_c0	93.841384	89.876026	93.127664	36.779032	39.792492	43.60438	2.523E-15	-1.164155	down	PREDICTED: uncharacterized protein LOC105173922 isoform X1 [Sesamum indicum]
c46666.graph_c0	0.0834899	0.3517898	0.3179263	0.9701043	0.6353103	0.9259707	5.382E-05	1.79591	up	PREDICTED: Fanconi anemia group J protein [Sesamum indicum]
c46679.graph_c0	5.2198711	5.2367269	5.2058993	27.201459	45.526054	21.587079	5.732E-05	2.627367	up	RecName: Full=Tubulin beta-1 chain; AltName: Full=Beta-1-tubulin [Cyanophora paradoxa]
c46688.graph_c0	4.3838432	3.5688683	4.8086685	1.1842455	0.8685446	1.0537345	7.769E-06	-1.992273	down	-
c46692.graph_c1	38.793443	34.943296	36.657286	74.742618	80.516469	75.089416	2.722E-14	1.102355	up	PREDICTED: transcription factor SPATULA-like [Sesamum indicum]
c46698.graph_c0	623.12125	651.43983	662.57237	2675.8193	2917.8153	3528.6468	5.787E-24	2.274266	up	hypothetical protein MIMGU_mgv1a006007mg [Erythranthe guttata]
c46710.graph_c0	108.93387	87.382549	89.390225	183.99363	227.56249	237.2305	4.737E-12	1.220969	up	PREDICTED: uncharacterized protein LOC105177873 [Sesamum indicum]
c46714.graph_c0	2.1441363	3.0611239	2.6318735	6.5507171	7.2842141	8.6191662	2.783E-11	1.55748	up	PREDICTED: boron transporter 4-like [Sesamum indicum]
c46719.graph_c1	0.4070218	0	0.0369029	1.9490694	2.6720976	2.2953577	1.813E-15	3.998921	up	PREDICTED: G2/mitotic-specific cyclin-2-like [Sesamum indicum]
c46730.graph_c0	16.192597	17.82523	17.832799	8.0130511	8.3509859	7.3119359	1.669E-12	-1.088517	down	PREDICTED: protein-lysine N-methyltransferase EFM1 isoform X1 [Sesamum indicum]
c46737.graph_c0	3.485966	4.753368	5.6890396	23.265906	35.155991	20.862343	2.915E-08	2.546541	up	60S ribosomal protein L12 [Triticum urartu]
c46738.graph_c0	40.128174	33.943643	39.465453	20.480023	5.9557923	6.4316474	2.334E-06	-1.720924	down	cell wall-associated hydrolase [Phaseolus vulgaris]
c46739.graph_c0	0.4085861	0.3689149	0.5417797	2.8485211	3.9778281	2.7074104	2.604E-10	2.891194	up	predicted protein [Trichoderma reesei QM6a]
c46745.graph_c1	1.6654854	1.694733	2.211108	9.6341039	8.6086782	9.958855	5.52E-34	2.382262	up	PREDICTED: chloride channel protein CLC-b-like [Nicotiana sylvestris]
c46748.graph_c0	151.43461	144.16504	157.04656	67.605068	77.091077	73.941976	9.351E-12	-1.010039	down	hypothetical protein MIMGU_mgv1a023425mg [Erythranthe guttata]
c46752.graph_c0	1.5021164	1.5500232	1.751021	8.2508537	9.9574442	9.6273937	9.248E-23	2.57347	up	PREDICTED: pollen-specific protein SF21-like [Sesamum indicum]

#ID	28 h-1 FPKM	28 h-2 FPKM	28 h-3 FPKM	48 h-1 FPKM	48 h-2 FPKM	48 h-3 FPKM	FDR	log2FC	regulated	nr_annotation
c46766.graph_c0	0.1011859	0.0228403	0.0619251	1.218475	1.5286103	1.6833503	5.388E-18	4.6106	up	PREDICTED: protein ESKIMO 1-like [Sesamum indicum]
c46784.graph_c0	25.071309	27.123662	23.775494	17.464536	9.7057357	8.1520469	0.0046566	-1.048403	down	-
c46784.graph_c1	26.646809	29.35437	26.22245	60.655509	79.439486	83.972382	2.017E-16	1.483129	up	PREDICTED: soluble inorganic pyrophosphatase [Sesamum indicum]
c46787.graph_c1	6.4107899	6.7646891	6.6177227	1.6643918	1.7632213	3.2232786	0.0002827	-1.537797	down	hypothetical protein MIMGU_mgv1a019568mg [Erythranthe guttata]
c46819.graph_c0	27.119839	26.222582	29.12168	69.479049	51.736958	50.118216	0.0001982	1.10331	up	PREDICTED: putative ABC transporter C family member 15 [Sesamum indicum]
c46841.graph_c0	194.61888	198.162	211.29099	68.107003	77.674079	79.650113	1.586E-21	-1.382752	down	PREDICTED: uncharacterized protein LOC104591752 [Nelumbo nucifera]
c46855.graph_c1	2.6513575	3.4838454	2.9725947	9.5359028	6.5707281	5.6161388	0.0041909	1.305635	up	PREDICTED: peptide-N4-(N-acetyl-beta-glucosaminyl)asparagine amidase A-like [Sesamum indicum]
c46861.graph_c0	4.6126381	4.9277928	4.5396355	1.2497102	1.4657646	2.5218223	2.077E-06	-1.392176	down	PREDICTED: pentatricopeptide repeat-containing protein At3g54980, mitochondrial-like [Sesamum indicum]
c46867.graph_c1	21.090427	22.105486	24.430434	42.998209	56.048013	59.718164	1.396E-12	1.267705	up	-
c46868.graph_c0	21.484877	22.301453	22.177177	7.4966453	8.1395076	10.919089	1.526E-17	-1.274678	down	PREDICTED: putative glycerol-3-phosphate transporter 4 isoform X1 [Sesamum indicum]
c46886.graph_c0	0.1571336	0.0945846	0	9.3613838	3.9387664	11.933333	1.464E-08	6.696091	up	-
c46896.graph_c0	3.488459	1.7998579	1.9428861	0.2807542	0.5948501	0.4371735	0.0005003	-2.430603	down	-
c46898.graph_c0	0.0756853	0	0.0308793	1.2951461	2.2359337	0.9390033	3.908E-07	5.426442	up	hypothetical protein HMPREF1544_02206 [Mucor circinelloides f. circinelloides 1006PhL]
c46903.graph_c1	12.388058	12.000261	13.765921	26.118688	28.923492	33.069965	1.746E-14	1.246293	up	hypothetical protein MIMGU_mgv1a010174mg [Erythranthe guttata]
c46917.graph_c0	0	0.0803383	0	1.6635863	0.9558606	3.060854	4.681E-06	6.188464	up	hypothetical protein CICLE_v10005479mg [Citrus clementina]
c46943.graph_c0	5.9450981	6.4414395	6.3697729	1.1139952	1.5966638	2.0990673	8.903E-07	-1.929881	down	PREDICTED: molybdenum cofactor sulfurase-like [Sesamum indicum]
c46943.graph_c1	7.4340906	7.3698676	7.7929376	14.927415	19.061425	21.12463	6.599E-13	1.322898	up	PREDICTED: SPX domain-containing membrane protein At4g22990-like [Sesamum indicum]
c46948.graph_c0	1.0573752	1.3127271	1.186363	9.7171814	7.6318887	6.1120323	4.218E-09	2.770986	up	Long-chain-alcohol oxidase FAO4A [Glycine soja]
c46948.graph_c1	3.0374994	3.1257313	3.0435439	20.298766	15.415757	13.62816	6.743E-10	2.471044	up	PREDICTED: long-chain-alcohol oxidase FAO4A-like isoform X1 [Sesamum indicum]
c46954.graph_c0	5.83541	5.5514473	5.6738398	2.4089235	2.8521932	2.5469177	0.0001322	-1.087889	down	hypothetical protein MIMGU_mgv1a007683mg [Erythranthe guttata]
c46957.graph_c6	30.947495	30.977087	28.409516	57.172079	58.537708	63.306293	2.279E-12	1.027892	up	PREDICTED: alpha-glucan phosphorylase, H isozyme [Sesamum indicum]
c46968.graph_c1	42.80561	36.502261	47.345616	5.8047636	8.4366044	8.6084062	2.94E-30	-2.435959	down	PREDICTED: lys-63-specific deubiquitinase BRCC36 [Sesamum indicum]
c46979.graph_c0	3.6497813	4.1417024	3.5691947	21.907736	24.448161	25.932431	7.986E-68	2.709482	up	PREDICTED: probable boron transporter 2 [Sesamum indicum]
c46984.graph_c0	70.761857	72.508419	78.104074	13.234427	14.928627	13.915481	2.47E-57	-2.355088	down	PREDICTED: uncharacterized protein LOC105171157 [Sesamum indicum]
c46988.graph_c0	136.20794	160.17861	160.06177	22.691068	21.268678	20.638576	1.475E-73	-2.777202	down	PREDICTED: aspartic proteinase-like [Sesamum indicum]

#ID	28 h-1 FPKM	28 h-2 FPKM	28 h-3 FPKM	48 h-1 FPKM	48 h-2 FPKM	48 h-3 FPKM	FDR	log2FC	regulated	nr_annotation
c47000.graph_c0	11.667974	12.050748	11.631753	21.545519	25.867359	27.406462	4.793E-15	1.119917	up	PREDICTED: peroxidase 7-like [Sesamum indicum]
c47011.graph_c0	0.2100855	0.2608203	0.4285701	3.029149	3.0326965	3.4950284	3.001E-16	3.449263	up	PREDICTED: condensin complex subunit 1 [Sesamum indicum]
c47025.graph_c0	168.34434	164.57779	157.95685	4.8160677	7.9801135	3.6260299	5.41E-148	-4.863993	down	-
c47037.graph_c1	8.4460014	10.379762	8.0405129	1.1895477	1.5752269	2.3815178	9.462E-07	-2.350155	down	glyceraldehyde-3-phosphate dehydrogenase [Leucophyllum frutescens]
c47037.graph_c4	21.318716	23.314555	26.58185	3.8349532	3.4013033	4.2852369	1.559E-19	-2.585718	down	PREDICTED: glyceraldehyde-3-phosphate dehydrogenase, cytosolic [Sesamum indicum]
c47052.graph_c0	0.4045429	0.1369741	0.1237889	2.1793488	1.9692329	1.3688269	7.146E-10	3.099366	up	PREDICTED: putative germin-like protein 2-1 [Sesamum indicum]
c47053.graph_c0	53.012876	38.486575	31.858987	11.350967	3.3669925	4.8480107	1.208E-07	-2.591909	down	-
c47064.graph_c1	3.7168507	3.0832953	2.6727606	6.0659044	7.2683447	9.0393438	3.137E-07	1.277061	up	lipoxygenase [Actinidia arguta]
c47065.graph_c0	1.9345398	3.0082195	3.5517796	17.301434	31.894948	9.2731925	0.0082396	2.820061	up	predicted protein [Physcomitrella patens]
c47073.graph_c0	3.5650255	4.6739956	6.8143072	21.294695	36.133983	21.151135	3.281E-06	2.419185	up	component of cytosolic 80S ribosome and 40S small subunit [Volvox carteri f. nagariensis]
c47105.graph_c0	12.796323	12.178414	14.674815	1.6282909	3.051882	2.7862374	1.214E-14	-2.377417	down	R3-MYB anthocyanin repressor [Petunia x hybrida]
c47114.graph_c0	0.2031919	0.1375974	0.1036268	3.7024352	4.2633491	4.2684025	3.899E-28	4.822724	up	PREDICTED: subtilisin-like protease SBT5.3 [Sesamum indicum]
c47127.graph_c0	0.3288344	0.5938133	0	24.592487	30.026908	33.750585	7.809E-27	6.622253	up	-
c47131.graph_c1	1.2095458	0.8918869	0.8882815	0.1022126	0.4331272	0.5229517	0.0014723	-1.476974	down	hypothetical protein MIMGU_mgv1a003156mg [Erythranthe guttata]
c47142.graph_c0	114.74918	95.782637	104.82124	355.91975	523.13505	527.00801	4.267E-16	2.191497	up	hypothetical protein MIMGU_mgv1a004716mg [Erythranthe guttata]
c47144.graph_c1	0.302339	0.6142134	0.5550888	5.077889	6.9018815	5.8822826	8.127E-15	3.639087	up	Peptide methionine sulfoxide reductase [Ectocarpus siliculosus]
c47150.graph_c1	3.6457171	3.0193208	2.9338426	7.4576848	7.1914924	7.6283377	1.781E-06	1.257063	up	hypothetical protein MIMGU_mgv1a011470mg [Erythranthe guttata]
c47165.graph_c0	3.2340076	4.1941899	8.7324387	25.415925	43.11173	23.211813	2.205E-05	2.539898	up	40S ribosomal protein S8-B [Rozella allomycis CSF55]
c47173.graph_c0	0.8547669	0.7717744	1.243339	2.3082836	2.5950616	3.1437196	0.0002468	1.52489	up	PREDICTED: alpha-N-acetylglucosaminidase-like [Sesamum indicum]
c47176.graph_c1	25.47884	31.087842	26.034982	1.4547885	2.4658753	3.6244939	1.016E-14	-3.42209	down	-
c47178.graph_c2	10.202816	10.528213	10.246664	42.635672	39.094829	36.630247	9.579E-33	1.978386	up	PREDICTED: uncharacterized protein LOC105158099 [Sesamum indicum]
c47182.graph_c0	41.483398	37.982236	40.66178	165.51252	110.08952	90.821871	0.0017429	1.66128	up	PREDICTED: fructose-1,6-bisphosphatase, cytosolic [Sesamum indicum]
c47192.graph_c0	320.4012	300.60714	313.92934	135.91838	152.98836	153.44795	1.119E-10	-1.039907	down	PREDICTED: protein NRT1/ PTR FAMILY 3.1-like [Sesamum indicum]
c47192.graph_c2	8.1109412	7.3234194	7.2487915	1.0403611	1.0373049	1.4157872	3.789E-12	-2.659482	down	-
c47193.graph_c0	207.64791	197.57164	193.71266	59.702849	67.743774	59.454317	3.662E-24	-1.63934	down	PREDICTED: phytosulfokine receptor 1 [Sesamum indicum]
c47199.graph_c0	4.427264	5.5708506	9.4927126	32.41758	63.562078	29.429305	0.0004383	2.71916	up	RecName: Full=60S ribosomal protein L13a [Cyanophora paradoxa]
c47202.graph_c1	0.0285693	0.0515908	0.1631863	1.1588391	1.9565664	0.8055688	1.658E-05	4.045401	up	-
c47211.graph_c0	0.7082971	0.5668525	0.5254224	2.6526465	1.4266952	1.7611535	0.0014782	1.750847	up	PREDICTED: uncharacterized protein LOC104880154 [Vitis vinifera]

#ID	28 h-1 FPKM	28 h-2 FPKM	28 h-3 FPKM	48 h-1 FPKM	48 h-2 FPKM	48 h-3 FPKM	FDR	log2FC	regulated	nr_annotation
c47234.graph_c0	120.17679	135.47106	134.58112	17.336206	19.561003	23.275392	5.982E-62	-2.6588	down	PREDICTED: uncharacterized protein LOC105178088 [Sesamum indicum]
c47250.graph_c2	1.8779243	1.9378166	2.7363765	0.5100873	0.8405843	0.8573149	0.000577	-1.536822	down	-
c47283.graph_c0	40.506442	38.599753	39.55364	10.773895	13.447749	15.376442	1.693E-23	-1.546499	down	PREDICTED: probable trehalose-phosphate phosphatase J [Sesamum indicum]
c47301.graph_c0	12.422161	11.084094	12.121921	5.7148734	5.4654748	5.7279039	3.549E-12	-1.032813	down	PREDICTED: midasin [Sesamum indicum]
c47303.graph_c0	0.7184678	0.9081927	1.0943592	2.3678482	2.7014056	2.3770121	0.0006977	1.492604	up	hypothetical protein M569_00480, partial [Genlisea aurea]
c47312.graph_c1	60.062707	56.176499	68.790773	113.04861	136.48899	135.48698	5.241E-14	1.095442	up	hypothetical protein MIMGU_mgv1a004264mg [Erythranthe guttata]
c47328.graph_c0	127.2492	112.13844	121.471	60.580447	55.345042	44.823655	2.376E-13	-1.120976	down	PREDICTED: DNA repair protein RAD16 [Sesamum indicum]
c47334.graph_c0	304.889	407.31858	341.93891	60.092032	58.54587	51.773878	7.366E-29	-2.584985	down	PREDICTED: em-like protein GEA6 [Sesamum indicum]
c47345.graph_c0	3.1841922	4.6982148	5.0698045	20.377878	32.433981	15.329082	0.0001201	2.433282	up	PREDICTED: 40S ribosomal protein S24-1-like [Musa acuminata subsp. malaccensis]
c47351.graph_c0	0.2980656	0.3289309	0.6756086	1.4693005	2.1346913	2.2038603	6.638E-07	2.190242	up	hypothetical protein MIMGU_mgv1a003544mg [Erythranthe guttata]
c47352.graph_c0	22.604236	21.65453	22.44327	37.922471	43.77837	50.462033	1.303E-12	1.024657	up	RUBISCO SUBUNIT BINDING-protein ALPHA SUBUNIT [Populus trichocarpa]
c47361.graph_c0	0.0925983	0.4737765	0.1763055	1.8388782	3.1500681	1.1488372	0.0009455	3.086703	up	-
c47364.graph_c0	2.4830531	1.9295593	2.8731486	0.5417746	0.5466139	1.239603	4.066E-05	-1.612352	down	PREDICTED: pleiotropic drug resistance protein 1-like [Sesamum indicum]
c47388.graph_c0	1.6093998	1.9002562	1.7678461	0.2353884	0.6649746	0.9774202	0.0017655	-1.466563	down	-
c47422.graph_c1	133.20759	127.57202	128.46847	381.02004	282.13224	225.97291	0.0025949	1.24204	up	PREDICTED: aspartate aminotransferase, cytoplasmic [Sesamum indicum]
c47433.graph_c1	1.8864499	1.654622	1.3634045	0.4782428	0.4342631	0.729493	0.0010406	-1.539254	down	hypothetical protein VITISV_042890 [Vitis vinifera]
c47434.graph_c0	13.625395	13.592922	13.170804	30.460102	34.418331	25.792116	5.561E-11	1.20874	up	PREDICTED: type I inositol 1,4,5-trisphosphate 5-phosphatase 2 isoform X1 [Sesamum indicum]
c47450.graph_c0	0.156775	0.377475	0.5543508	4.1732006	6.3858888	4.538464	1.328E-16	3.829464	up	-
c47459.graph_c0	0.6967511	0.6862919	1.9640584	7.3866246	14.799897	5.0008716	0.0037912	3.055279	up	-
c47464.graph_c1	1.1461723	0.7625477	0.6891443	1.6822564	2.9972467	2.44941	0.0005833	1.489725	up	PREDICTED: glutamate synthase 1 [NADH], chloroplastic isoform X1 [Sesamum indicum]
c47466.graph_c0	4.0170471	4.8683488	4.6801782	51.082407	27.750119	17.92915	0.0056867	2.893506	up	hypothetical protein MIMGU_mgv1a004441mg [Erythranthe guttata]
c47467.graph_c0	0.1333591	0	0.1088199	7.3251733	12.356519	8.3729884	3.75E-25	6.889079	up	Protein bli-3 OS=Neurospora crassa (strain ATCC 24698 / 74-OR23-1A / CBS 708,71 / DSM 1257 / FGSC 987) GN=bli-3 PE=2 SV=1 [Rhizoctonia solani AG-1 IB]
c47468.graph_c1	13005.458	11549.554	11593.664	42781.52	38273.95	43600.174	2.516E-06	1.829121	up	PREDICTED: non-specific lipid-transfer protein 1-like [Sesamum indicum]
c47469.graph_c0	168.99069	176.80353	174.22974	69.408706	84.445918	79.892533	2.417E-13	-1.114781	down	hypothetical protein MIMGU_mgv1a011464mg [Erythranthe guttata]
c47487.graph_c0	2.9999024	3.3242285	3.1155038	27.525619	40.100798	46.369716	6.242E-19	3.628365	up	PREDICTED: vinorine synthase-like [Sesamum indicum]

#ID	28 h-1 FPKM	28 h-2 FPKM	28 h-3 FPKM	48 h-1 FPKM	48 h-2 FPKM	48 h-3 FPKM	FDR	log2FC	regulated	nr_annotation
c47495.graph_c3	44.121634	44.240813	39.413694	168.54759	168.38997	188.89322	5.418E-47	2.082324	up	hypothetical protein M569_11087, partial [Genlisea aurea]
c47497.graph_c0	3.30912	5.2286939	6.6986099	30.329847	42.641667	21.460658	3.056E-06	2.67143	up	alpha-tubulin, partial [Syntrichia caninervis]
c47499.graph_c0	14.016201	13.4966	14.217637	62.377018	42.236715	37.562279	0.0001406	1.819786	up	replication factor C / DNA polymerase III gamma-tau subunit, putative [Ricinus communis]
c47502.graph_c1	7.9349348	7.3237133	8.1375175	2.6821638	2.6572608	2.894825	1.293E-09	-1.46516	down	PREDICTED: uncharacterized protein LOC105160536 [Sesamum indicum]
c47507.graph_c3	0.4975364	0.8086118	0.6495771	1.6397266	2.8728821	3.2547344	2.11E-05	2.021493	up	PREDICTED: uncharacterized protein LOC105173228 [Sesamum indicum]
c47509.graph_c0	0.2738356	0.061812	0.5027571	5.1198251	8.7332918	3.8606691	5.001E-07	4.435625	up	Peptidyl-prolyl cis-trans isomerase,FKBP-type,N-terminal [Ostreococcus tauri]
c47526.graph_c0	11.204607	10.595922	8.7819653	2.7657821	5.5036689	6.1856972	0.0001211	-1.051174	down	-
c47528.graph_c0	0.5885636	0.8695926	1.2661481	5.6292688	4.813932	5.9141105	2.189E-10	2.628539	up	expressed unknown protein [Ectocarpus siliculosus]
c47533.graph_c2	3.6200299	2.7439659	2.7715744	0.963051	0.6601525	0.9073238	3.789E-05	-1.805335	down	hypothetical protein MIMGU_mgv1a0267602mg, partial [Erythranthe guttata]
c47540.graph_c0	4.3904349	3.4872607	4.9293857	1.380839	1.4628315	1.4148232	2.952E-05	-1.54779	down	hypothetical protein MIMGU_mgv1a008287mg [Erythranthe guttata]
c47540.graph_c1	4.8004182	5.3674634	4.8726707	1.9489758	1.6565644	1.5527022	2.321E-13	-1.49773	down	PREDICTED: xylem serine proteinase 1 [Sesamum indicum]
c47545.graph_c0	0.2124094	0.2876787	0.238321	2.0529902	6.8455684	2.2759362	0.0078702	3.947184	up	PREDICTED: KDEL-tailed cysteine endopeptidase CEP1-like [Glycine max]
c47550.graph_c1	1.0447541	0.6603205	0.9093449	5.2530678	6.4067514	9.5054106	1.93E-09	3.051795	up	-
c47554.graph_c5	280.34158	315.70888	304.51554	141.01254	139.06681	157.4735	7.078E-11	-1.00001	down	hypothetical protein POPTR_0003s102801g, partial [Populus trichocarpa]
c47555.graph_c0	0.4244934	0.4216055	0.5195747	1.0761533	0.8360396	1.1969435	0.0028132	1.230243	up	PREDICTED: kinesin-4 [Sesamum indicum]
c47564.graph_c1	65.775273	62.085811	64.279489	154.11178	164.61855	157.81551	4.363E-19	1.351605	up	PREDICTED: pentatricopeptide repeat-containing protein At5g62370 [Sesamum indicum]
c47564.graph_c4	0	0.03319	0.1499755	3.6343975	4.9855199	2.8607268	1.55E-18	6.006508	up	PREDICTED: probable pectinesterase/pectinesterase inhibitor 59 [Sesamum indicum]
c47580.graph_c1	208.64535	200.33243	213.11291	82.753573	85.937635	66.608696	2.74E-17	-1.359258	down	PREDICTED: probable copper-transporting ATPase HMA5 [Nicotiana tomentosiformis]
c47586.graph_c0	6.0553837	7.874751	10.914765	35.456897	72.21182	32.823539	0.00127	2.53268	up	60S ribosomal protein L8 [Rozella allomycis CSF55]
c47596.graph_c5	4.3343017	4.836246	4.0190395	6.4929642	13.492458	15.443436	0.0054652	1.454772	up	PREDICTED: probably inactive leucine-rich repeat receptor-like protein kinase At3g28040 [Sesamum indicum]
c47600.graph_c1	7.5746485	7.5251346	7.2565747	22.318356	23.673597	29.46049	2.685E-22	1.794031	up	PREDICTED: protein TIC 62, chloroplastic [Sesamum indicum]
c47604.graph_c0	2.1881772	5.0804199	5.152543	21.000688	27.284893	22.846629	1.335E-21	2.556104	up	large subunit ribosomal protein L19e, cytoplasmic [Guillardia theta CCMP2712]
c47606.graph_c1	34.880742	29.442957	29.319461	84.278894	116.40938	120.6135	2.622E-16	1.814572	up	PREDICTED: serine carboxypeptidase-like 27 [Sesamum indicum]
c47609.graph_c2	2.0919789	1.7839241	1.8967086	3.3883311	3.9016586	5.1122499	0.0086423	1.140373	up	-
c47615.graph_c0	2.6862624	2.2388705	2.6416027	12.965327	9.6192591	10.60423	2.491E-13	2.180043	up	PREDICTED: vinorine synthase-like [Sesamum indicum]

#ID	28 h-1 FPKM	28 h-2 FPKM	28 h-3 FPKM	48 h-1 FPKM	48 h-2 FPKM	48 h-3 FPKM	FDR	log2FC	regulated	nr_annotation
c47622.graph_c1	710.6689	800.90527	884.3312	94.861854	96.29669	89.438419	8.495E-62	-3.051722	down	PREDICTED: probable glycosyltransferase At5g03795 [Sesamum indicum]
c47627.graph_c0	5.3180031	5.1007778	4.951238	2.121745	2.3648009	2.163237	1.477E-05	-1.167925	down	PREDICTED: pentatricopeptide repeat-containing protein At1g63330-like [Sesamum indicum]
c47639.graph_c0	0.53164	0.3600158	1.247215	4.7172502	12.939579	5.6214923	0.0018829	3.471239	up	PREDICTED: cytochrome b5 [Phoenix dactylifera]
c47660.graph_c0	21.035065	17.363522	18.856347	94.114713	117.3228	139.42241	5.615E-23	2.651889	up	PREDICTED: beta-galactosidase-like [Sesamum indicum]
c47664.graph_c0	1.3488041	1.3669674	1.0556902	3.5240932	5.6554699	6.147247	3.43E-08	2.056323	up	PREDICTED: cytochrome P450 704C1-like [Sesamum indicum]
c47672.graph_c1	1.8248119	1.9100891	1.7657543	3.234223	3.4696375	4.5352547	0.0001692	1.069421	up	PREDICTED: uncharacterized protein LOC105177120 [Sesamum indicum]
c47683.graph_c0	12.19978	12.736391	12.920658	27.609897	24.675929	29.841741	1.425E-13	1.159913	up	alpha-expansin 3 [Striga asiatica]
c47685.graph_c0	0	0.2034627	0.2298466	2.142282	4.0850784	2.8592845	3.226E-11	4.422718	up	-
c47689.graph_c0	47.769476	45.826008	46.300603	10.992029	13.560304	12.5116	8.603E-38	-1.877518	down	PREDICTED: major facilitator superfamily domain-containing protein 12-like [Sesamum indicum]
c47697.graph_c0	1.1120712	1.6734934	2.319016	25.843257	19.911125	8.7799757	0.0004535	3.47044	up	predicted protein, partial [Micromonas pusilla CCMP1545]
c47703.graph_c0	1.3233036	1.4744576	1.8379659	2.9265012	2.9868485	3.4931481	0.0048559	1.06081	up	PREDICTED: PI-PLC X domain-containing protein At5g67130-like [Nicotiana sylvestris]
c47705.graph_c0	36.332974	41.122097	38.625146	72.001041	90.707038	101.0051	6.692E-07	1.220858	up	-
c47708.graph_c0	0.3952947	0.2974284	1.4515077	7.0984233	17.428518	6.1675195	0.0016528	3.868338	up	-
c47709.graph_c0	29.312946	33.378283	34.613477	3.0411908	2.9368542	2.9637508	4.59E-98	-3.401199	down	hypothetical protein MIMGU_mgv1a025551mg [Erythranthe guttata]
c47713.graph_c3	18.321207	18.944583	15.426518	4.8804951	5.431712	5.5624765	1.084E-20	-1.690656	down	hypothetical protein MIMGU_mgv1a016005mg [Erythranthe guttata]
c47721.graph_c0	13.228016	12.126639	14.822894	74.986621	68.801154	56.104373	3.56E-18	2.360042	up	PREDICTED: 17.4 kDa class III heat shock protein [Sesamum indicum]
c47726.graph_c0	23.71689	23.434329	30.550629	2.7415857	1.4021133	1.5141392	8.728E-33	-3.724494	down	PREDICTED: E3 ubiquitin-protein ligase At4g11680-like [Sesamum indicum]
c47730.graph_c0	3.8147618	3.5413968	3.8142938	20.023021	27.561122	32.784534	7.06E-16	2.881706	up	hypothetical protein MIMGU_mgv1a012535mg [Erythranthe guttata]
c47753.graph_c0	46.40827	44.692852	46.615414	18.934975	21.475649	20.223406	5.619E-15	-1.143379	down	PREDICTED: transcription factor PIF1 [Sesamum indicum]
c47756.graph_c0	64.204493	66.362045	64.334582	22.08852	21.892101	14.282075	2.362E-30	-1.696326	down	ent-copalyl diphosphate synthase [Andrographis paniculata]
c47763.graph_c3	1.4653137	1.3002299	1.4224517	4.4833626	5.1906113	6.4113253	2.758E-10	1.978836	up	PREDICTED: U-box domain-containing protein 5 [Sesamum indicum]
c47765.graph_c0	0.8111985	1.0653617	1.2335993	2.710851	2.0300782	2.6200385	0.0023987	1.288141	up	-
c47771.graph_c0	58.180172	60.544189	60.263924	13.373552	14.244376	13.467718	2.047E-46	-2.081781	down	PREDICTED: floral homeotic protein AGAMOUS-like isoform X1 [Sesamum indicum]
c47778.graph_c0	5.5785699	4.9169987	5.1868796	8.8029824	10.293934	12.498303	4.45E-08	1.048011	up	PREDICTED: CBS domain-containing protein CBSCBSPB1-like isoform X3 [Sesamum indicum]
c47795.graph_c0	6.0629141	8.0530186	10.139897	48.143588	60.01853	49.337025	5.092E-51	2.737891	up	unnamed protein product [Vitis vinifera]
c47805.graph_c0	0.616219	0.715356	1.4725726	4.5750316	5.9105991	3.5743923	5.417E-08	2.36537	up	-

#ID	28 h-1 FPKM	28 h-2 FPKM	28 h-3 FPKM	48 h-1 FPKM	48 h-2 FPKM	48 h-3 FPKM	FDR	log2FC	regulated	nr_annotation
c47806.graph_c0	29.045571	27.050223	27.704021	80.625176	94.090154	112.32239	7.651E-20	1.813897	up	hypothetical protein MIMGU_mgv1a0009861mg, partial [Erythranthe guttata]
c47832.graph_c0	3.2362643	2.6532153	2.6090761	7.8762305	12.81138	11.826376	3.447E-10	1.969727	up	hypothetical protein MIMGU_mgv1a000209mg [Erythranthe guttata]
c47836.graph_c1	28.384539	29.348195	28.246996	13.045196	14.407449	13.803098	3.309E-12	-1.018777	down	PREDICTED: uncharacterized protein LOC105173629 [Sesamum indicum]
c47847.graph_c0	0.0960444	0.0867191	0.0783714	1.4609199	2.5794458	1.5707339	1.447E-10	4.460558	up	triosephosphate isomerase [Genlisea aurea]
c47848.graph_c2	5.1059837	5.516508	5.2703698	0.7744525	1.8459867	1.7719796	3.004E-09	-1.827176	down	PREDICTED: auxin response factor 18-like isoform X1 [Sesamum indicum]
c47848.graph_c3	3.3258682	2.3552527	4.3102821	0.5786369	0.9632788	0.8826302	3.479E-05	-2.010376	down	-
c47853.graph_c3	99.028605	100.6816	102.71457	49.509253	42.439343	44.053035	7.207E-14	-1.108075	down	PREDICTED: probable indole-3-acetic acid-amido synthetase GH3.1 [Sesamum indicum]
c47865.graph_c0	20.551422	18.772161	20.301066	8.5667667	8.0863006	8.8480984	1.31E-13	-1.182982	down	PREDICTED: transcription factor GTE4-like isoform X3 [Sesamum indicum]
c47865.graph_c1	16.61539	12.432712	10.786493	5.3525778	5.0540585	6.2122063	0.0007641	-1.219548	down	PREDICTED: transcription factor GTE4-like isoform X3 [Sesamum indicum]
c47865.graph_c4	83.939776	79.862901	81.091076	198.78262	222.66228	200.16824	4.394E-17	1.384642	up	PREDICTED: patellin-3-like [Sesamum indicum]
c47865.graph_c6	90.345113	95.95397	102.19468	38.709866	31.137143	18.475801	6.527E-20	-1.656887	down	hypothetical protein VITISV_027452 [Vitis vinifera]
c47884.graph_c0	0.1022698	0.1385101	0.2920797	6.6761777	6.8666027	4.6139206	1.052E-23	5.133821	up	cysteine peroxiredoxin [Lichtheimia corymbifera JMRC:FSU:9682]
c47891.graph_c1	12.623677	11.925682	12.621681	4.8399419	5.0750118	7.6023811	8.01E-05	-1.047885	down	hypothetical protein MIMGU_mgv1a011954mg [Erythranthe guttata]
c47898.graph_c0	1.7542646	1.5839364	1.5268966	3.1131234	5.3395814	5.2762638	0.0001577	1.52937	up	PREDICTED: chlorophyll a-b binding protein 13, chloroplastic [Sesamum indicum]
c47903.graph_c0	0.3753623	0.4929702	0.4733613	1.0381078	1.0997494	0.8467269	0.0086423	1.196631	up	unknown [Chondrus crispus]
c47912.graph_c0	0.2055644	0.3248094	0.3774124	2.2799699	4.8307034	3.1299998	3.841E-07	3.527103	up	-
c47914.graph_c0	3.6066016	4.5500706	5.5230846	22.858166	44.649319	20.673399	0.0003495	2.722544	up	hypothetical protein JCGZ_08960 [Jatropha curcas]
c47917.graph_c0	67.298281	61.371172	57.29251	28.127385	33.635117	27.555616	1.021E-10	-1.017609	down	PREDICTED: uncharacterized protein LOC105177054 [Sesamum indicum]
c47923.graph_c0	0.9361289	0.9508913	0.98667	6.5758103	9.8994416	2.7275881	0.0060747	2.781834	up	--
c47924.graph_c0	15.570227	17.312083	15.645609	9.599516	7.8665014	5.933472	5.813E-09	-1.003514	down	PREDICTED: cysteine-rich receptor-like protein kinase 3 [Nicotiana sylvestris]
c47927.graph_c0	0.056808	0.0256462	0	1.0801254	1.2968302	0.6727624	2.682E-11	5.253922	up	hypothetical protein MVLG_06307 [Microbotryum violaceum p1A1 Lamole]
c47948.graph_c0	332.61653	275.5077	325.30287	951.60968	1086.049	1074.57	1.927E-17	1.77676	up	PREDICTED: alpha-L-arabinofuranosidase 1-like isoform X2 [Sesamum indicum]
c47954.graph_c0	1.9910543	3.5954707	4.7537051	17.853604	28.71718	11.477864	0.0011192	2.527094	up	40S ribosomal protein S11 [Auxenochlorella protothecoides]
c47961.graph_c0	2.6052594	1.6516184	2.6460302	5.2345891	6.773591	6.7521105	7.229E-07	1.479012	up	PREDICTED: probable polygalacturonase At3g15720-like [Glycine max]
c47968.graph_c0	69.437595	67.51082	76.687071	26.177549	27.14734	25.56358	2.482E-22	-1.39555	down	PREDICTED: alpha-galactosidase-like [Sesamum indicum]
c47983.graph_c0	2.7541966	4.1085952	5.4230787	16.848579	35.537252	13.033381	0.0098601	2.446214	up	PREDICTED: 40S ribosomal protein S19-3 [Prunus mume]

#ID	28 h-1 FPKM	28 h-2 FPKM	28 h-3 FPKM	48 h-1 FPKM	48 h-2 FPKM	48 h-3 FPKM	FDR	log2FC	regulated	nr_annotation
c48011.graph_c0	0.4735725	0.6468694	0.7035032	1.9547843	2.5111178	3.2458835	2.632E-09	2.115186	up	PREDICTED: NADP-dependent malic enzyme-like isoform X1 [Sesamum indicum]
c48037.graph_c1	0.814341	0.2321917	0.769416	4.4005904	13.295025	5.0758022	0.003943	3.675288	up	--
c48042.graph_c0	95.88233	96.966053	96.405556	43.331955	41.106377	39.519534	2.15E-16	-1.178941	down	PREDICTED: LOW QUALITY PROTEIN: cell division protein FtsZ homolog 2-2, chloroplastic-like [Sesamum indicum]
c48045.graph_c1	0.7980271	0.9112757	0.459659	1.8743576	3.1203144	2.2501957	4.84E-06	1.776052	up	PREDICTED: putative nuclease HARBI1 [Brassica rapa]
c48046.graph_c0	28.006456	26.872372	27.355429	13.24636	11.89991	11.833678	2.866E-09	-1.108676	down	PREDICTED: uncharacterized protein LOC105172478 [Sesamum indicum]
c48062.graph_c1	14.711835	15.162835	14.077498	28.576225	29.278157	29.008215	1.265E-09	1.024668	up	PREDICTED: uncharacterized protein LOC102581927 [Solanum tuberosum]
c48068.graph_c1	122.60825	119.42017	122.97285	244.677	293.06764	307.8008	2.524E-12	1.250225	up	truncated cellulose synthase catalytic subunit [Gossypium hirsutum subsp. latifolium]
c48068.graph_c2	0.2689834	0.1734763	0.3449101	1.5586573	1.7028087	1.6469247	6.419E-09	2.679751	up	hypothetical protein MIMGU_mgv1a000605mg [Erythranthe guttata]
c48071.graph_c1	67.715065	65.730295	62.895261	147.57251	160.62353	189.15964	2.703E-20	1.380094	up	hypothetical protein POPTR_0003s13500g [Populus trichocarpa]
c48076.graph_c1	29.034295	24.491341	25.178078	9.3746388	14.646447	12.549374	2.271E-11	-1.070714	down	PREDICTED: aspartokinase 2, chloroplastic isoform X1 [Sesamum indicum]
c48091.graph_c0	0.5341487	0.4913859	0.435861	1.3669206	1.5292877	1.7846318	5.369E-07	1.718295	up	PREDICTED: myosin-2-like [Sesamum indicum]
c48097.graph_c0	0.2489861	0.4721033	0.2641218	0.9783854	0.8693064	0.6178178	0.0024813	1.371787	up	PREDICTED: lisH domain and HEAT repeat-containing protein KIAA1468 homolog isoform X1 [Sesamum indicum]
c48112.graph_c1	2.0009031	2.5014847	2.6374717	7.316227	4.6503932	5.9023408	0.0009295	1.372519	up	PREDICTED: cytochrome P450 90A1 [Sesamum indicum]
c48133.graph_c0	1.7919511	1.3207868	1.3130116	2.7813429	3.8795524	4.2071977	1.282E-05	1.331507	up	PREDICTED: probable LRR receptor-like serine/threonine-protein kinase MRH1 isoform X3 [Nelumbo nucifera]
c48135.graph_c0	1.4355027	1.6664456	2.8781951	0.571876	0.3855303	0.370074	0.0010522	-2.12181	down	PREDICTED: formin-like protein 3 [Nicotiana tomentosiformis]
c48142.graph_c0	113.51757	108.76494	116.89586	36.035243	37.735167	37.235546	5.065E-26	-1.570175	down	PREDICTED: pentatricopeptide repeat-containing protein At5g39350 [Vitis vinifera]
c48143.graph_c3	397.40878	369.06091	357.62412	1194.1574	992.7959	849.46841	1.683E-07	1.48096	up	PREDICTED: haloacid dehalogenase-like hydrolase domain-containing protein 3 [Sesamum indicum]
c48148.graph_c0	2.4095602	1.9391279	2.4790984	0.7303749	1.055105	0.8271241	0.0026163	-1.349289	down	PREDICTED: SNF2 domain-containing protein CLASSY 3-like [Sesamum indicum]
c48149.graph_c0	0.4516758	0.8156418	1.0406506	2.9300184	4.7451942	1.7980088	0.0080932	2.075507	up	citrate synthase, mitochondrial, putative (ISS), partial [Ostreococcus tauri]
c48159.graph_c0	0.0451526	0.0203843	0.0368441	0.6868107	1.8189817	0.53473	0.0017465	4.922524	up	atp-dependent rrna helicase rrp3 [Lichtheimia corymbifera JMRC:FSU:9682]
c48160.graph_c0	0.1850801	0.233954	0.1812287	1.5014574	1.2923723	0.876742	2.46E-07	2.661203	up	hypothetical protein VOLCADRAFT_45407 [Volvox carteri f. nagariensis]
c48181.graph_c0	36.20092	33.772525	35.495304	10.277964	13.017583	11.948467	1.113E-26	-1.543128	down	PREDICTED: probable leucine-rich repeat receptor-like protein kinase At1g35710 [Sesamum indicum]
c48194.graph_c2	0.4417289	0.4985497	0.4730869	1.4698003	1.9278074	2.8336089	9.714E-06	2.174451	up	PREDICTED: 125 kDa kinesin-related protein [Sesamum indicum]
c48197.graph_c0	0.5799103	1.1053875	1.4721842	3.5937196	4.7588877	2.034881	0.0075611	1.759624	up	Heat Shock Protein 70 [Ostreococcus lucimarinus CCE9901]

#ID	28 h-1 FPKM	28 h-2 FPKM	28 h-3 FPKM	48 h-1 FPKM	48 h-2 FPKM	48 h-3 FPKM	FDR	log2FC	regulated	nr_annotation
c48200.graph_c0	0.0871323	0.0786723	0.1421985	1.5462529	4.5046856	2.3094567	5.266E-05	4.789392	up	hypothetical protein EMIHUDRAFT_225441 [Emiliania huxleyi CCMP1516]
c48208.graph_c0	3.5315375	5.247982	5.4632339	28.071285	45.348166	22.156263	1.19E-05	2.783997	up	PREDICTED: 40S ribosomal protein S13-1-like [Camelina sativa]
c48239.graph_c3	230.6108	273.69789	262.57924	70.842242	93.746036	78.84828	3.03E-29	-1.617273	down	PREDICTED: beta-hexosaminidase 2 [Sesamum indicum]
c48244.graph_c1	21.880295	20.500839	37.428865	1735.4834	1713.0052	2250.8296	1.582E-87	6.196914	up	PREDICTED: putative mannan endo-1,4-beta-mannosidase 9 [Sesamum indicum]
c48244.graph_c3	7.8517109	7.5825316	7.9111682	12.981681	24.021062	18.558641	0.0004178	1.283754	up	RecName: Full=Luminal-binding protein 8; Short=BiP 8; AltName: Full=78 kDa glucose-regulated protein homolog 8; Short=GRP-78-8, partial [Nicotiana tabacum]
c48244.graph_c4	6.3510894	6.8902165	5.4636546	12.16932	20.693184	14.604172	7.444E-05	1.378677	up	hypothetical protein JCGZ_10646 [Jatropha curcas]
c48250.graph_c2	31.655001	36.855089	32.627649	14.186086	16.487509	18.545964	1.702E-09	-1.000541	down	hypothetical protein MIMGU_mgv1a010855mg [Erythranthe guttata]
c48250.graph_c3	3.8968002	3.4561718	3.2641754	6.9939557	8.8447898	11.279513	1.077E-06	1.388732	up	PREDICTED: probable pectin methyltransferase QUA2 [Sesamum indicum]
c48251.graph_c1	13.209316	14.967594	14.809254	2.8459684	5.9294183	7.8924147	1.282E-07	-1.338753	down	hypothetical protein Csa_6G486800 [Cucumis sativus]
c48259.graph_c1	90.953397	77.102284	80.110225	514.171	675.19654	671.43406	1.399E-53	2.94356	up	PREDICTED: dehydrodolichyl diphosphate synthase 6 [Sesamum indicum]
c48264.graph_c0	36.00751	38.221381	36.696905	15.139674	17.129714	16.754985	3.205E-15	-1.138029	down	PREDICTED: phytosulfokine receptor 1 [Sesamum indicum]
c48288.graph_c0	18.451318	17.644923	16.957823	31.752158	38.710603	38.315089	1.998E-13	1.074361	up	GIGANTEA family protein [Populus trichocarpa]
c48291.graph_c0	1.1877372	0.9008289	1.1630206	3.0713105	2.9984904	3.7509482	8.379E-05	1.634901	up	PREDICTED: homeobox-leucine zipper protein ATHB-12-like [Sesamum indicum]
c48295.graph_c0	0.3681873	0.2949807	0.3681421	2.2217765	2.36763	3.0823628	5.599E-27	2.933337	up	PREDICTED: phragmoplast orienting kinesin 2 [Sesamum indicum]
c48296.graph_c0	0.1706357	0.2054241	0.1856498	2.0187381	1.6803351	2.8226959	1.089E-11	3.578204	up	hypothetical protein CHLNCDRAFT_48764 [Chlorella variabilis]
c48300.graph_c0	6.3648316	6.3322634	6.1988733	2.6573365	2.6409943	3.2542098	2.407E-08	-1.10322	down	hypothetical protein MIMGU_mgv1a0002422mg, partial [Erythranthe guttata]
c48303.graph_c3	29.368274	29.537699	31.244557	14.607848	9.2352259	13.941376	3.627E-06	-1.207306	down	hexose transport protein [Actinidia deliciosa]
c48309.graph_c0	11.337606	15.022042	12.20746	32.229017	34.052649	38.816349	7.062E-13	1.486571	up	PREDICTED: hydroquinone glucosyltransferase [Sesamum indicum]
c48309.graph_c2	3.5096077	5.0034421	2.9391747	0.9365655	1.7363108	1.7708695	0.0037525	-1.333669	down	-
c48309.graph_c4	5.8665912	7.4227442	6.2988033	16.536064	16.014935	14.496032	1.914E-08	1.308025	up	PREDICTED: hydroquinone glucosyltransferase [Sesamum indicum]
c48322.graph_c0	0.5536435	0.4998882	0.7471557	0.9986925	1.8014485	2.3536701	0.0048119	1.546472	up	PREDICTED: uncharacterized acetyltransferase At3g50280-like [Sesamum indicum]
c48322.graph_c3	0.2060106	0.345444	0.168103	1.5294972	7.3111857	7.6677206	0.0004532	4.541805	up	PREDICTED: omega-hydroxypalmitate O-feruloyl transferase isoform X1 [Sesamum indicum]
c48327.graph_c0	0.358406	0.4179924	0.3533848	1.1736238	1.143045	1.7348578	2.221E-06	1.881496	up	hypothetical protein MIMGU_mgv1a001429mg [Erythranthe guttata]
c48332.graph_c0	20.093594	17.533222	17.968133	48.052776	61.498936	65.610535	7.815E-22	1.692616	up	PREDICTED: probable copper-transporting ATPase HMA5 [Sesamum indicum]
c48342.graph_c0	0.8489263	0.958126	1.1335367	4.6466942	4.4562566	5.462025	1.454E-14	2.349131	up	hypothetical protein MIMGU_mgv11b010420mg [Erythranthe guttata]

#ID	28 h-1 FPKM	28 h-2 FPKM	28 h-3 FPKM	48 h-1 FPKM	48 h-2 FPKM	48 h-3 FPKM	FDR	log2FC	regulated	nr_annotation
c48359.graph_c0	3.8566173	4.7879752	4.032053	0.1527671	0	0	9.958E-17	-6.274163	down	-
c48364.graph_c0	2.7268429	3.8945684	4.9760914	19.544791	35.352197	13.53242	0.0022242	2.59698	up	predicted protein [Physcomitrella patens]
c48372.graph_c1	312.96565	269.95302	305.25374	585.28217	563.97673	805.83817	1.677E-06	1.177721	up	PREDICTED: uncharacterized protein LOC105170680 isoform X1 [Sesamum indicum]
c48374.graph_c0	4.7780894	4.5128455	5.9252746	27.135073	31.236824	23.683715	2.989E-29	2.472083	up	PREDICTED: GDSL esterase/lipase At1g29670-like [Sesamum indicum]
c48392.graph_c0	0.1295155	0.3274329	0.295914	2.1013812	3.2348867	2.2495987	2.868E-14	3.369611	up	hypothetical protein SELMODRAFT_168478 [Selaginella moellendorffii]
c48397.graph_c1	74.452828	80.953886	74.927883	297.26497	218.05047	191.64875	2.258E-05	1.667765	up	unnamed protein product [Vitis vinifera]
c48414.graph_c0	3.8158496	4.0717825	2.5475744	20.116984	12.344369	10.466103	0.001106	2.095152	up	PREDICTED: uncharacterized protein LOC105161268 [Sesamum indicum]
c48428.graph_c0	3.769453	6.7575998	6.1516858	25.205994	44.308867	29.02103	6.175E-08	2.597407	up	hypothetical protein MGL_1430 [Malassezia globosa CBS 7966]
c48429.graph_c6	18.845409	19.31051	19.72797	2.2002054	4.9946803	4.6146481	4.427E-15	-2.264366	down	PREDICTED: protein GLUTAMINE DUMPER 2-like [Sesamum indicum]
c48432.graph_c1	0.764346	0.9457375	0.8547001	2.6912912	2.318892	2.0434729	0.0002517	1.50597	up	PREDICTED: uncharacterized protein LOC105156326 [Sesamum indicum]
c48435.graph_c0	2.5690861	4.327028	5.644028	28.870177	55.529687	28.418959	9.354E-06	3.203123	up	60s ribosomal protein l10 [Lichtheimia corymbifera JMRC:FSU:9682]
c48436.graph_c0	0.0755814	0.2388501	0.1850213	1.101757	2.3343558	1.1934534	8.95E-05	3.245602	up	-
c48465.graph_c0	19.763928	16.945382	13.787764	38.841273	41.147619	46.096263	1.429E-21	1.36051	up	PREDICTED: elongation factor G-1, chloroplastic [Sesamum indicum]
c48472.graph_c0	0	0	0.0302167	1.548987	1.8895948	1.1694491	1.417E-20	7.289309	up	PREDICTED: hydroxyacylglutathione hydrolase cytoplasmic [Sesamum indicum]
c48480.graph_c0	3.6836367	5.7880666	6.7923667	38.870247	43.298258	30.917428	8.833E-22	2.839949	up	hypothetical protein SELMODRAFT_270900 [Selaginella moellendorffii]
c48481.graph_c0	241.38523	257.64002	253.07291	102.73851	109.1157	102.51387	1.54E-13	-1.21696	down	PREDICTED: uncharacterized protein LOC105166397 [Sesamum indicum]
c48482.graph_c0	0.2281373	0.2059866	0.1489266	1.5615768	1.3479492	1.2865571	7.976E-10	2.893407	up	hypothetical protein PHACADRAFT_263295 [Phanerochaete carnosa HHB-10118-sp]
c48498.graph_c0	1741.8381	1873.0071	1923.6713	321.74047	315.58888	298.97274	2.239E-38	-2.521519	down	hypothetical protein MIMGU_mgv1a004937mg [Erythranthe guttata]
c48500.graph_c0	2.7157054	2.5666081	2.3713201	1.1260045	1.2780699	1.1450423	0.0006449	-1.067974	down	PREDICTED: phosphatidylinositol/phosphatidylcholine transfer protein SFH11 [Sesamum indicum]
c48503.graph_c0	72.424973	73.436601	72.767532	145.24856	146.75032	169.89286	7.32E-14	1.119733	up	PREDICTED: potassium transporter 4-like [Sesamum indicum]
c48512.graph_c2	32.397202	34.080235	38.522566	11.544183	14.173282	15.667631	2.308E-19	-1.305999	down	hypothetical protein MIMGU_mgv1a005052mg [Erythranthe guttata]
c48527.graph_c0	0.369283	0.4816181	0.6361451	4.5249029	7.3832243	4.8129662	9.507E-13	3.526558	up	conserved unknown protein [Ectocarpus siliculosus]
c48530.graph_c1	0.0579952	0.0523642	0	1.7643157	10.357811	12.035728	1.662E-05	7.79568	up	PREDICTED: non-specific lipid-transfer protein-like protein At2g13820 [Sesamum indicum]
c48536.graph_c0	1.3590657	1.7359102	1.677004	3.0672746	5.2969761	5.8323539	8.038E-05	1.604735	up	PREDICTED: glycerophosphodiester phosphodiesterase GDPD6 [Solanum lycopersicum]
c48537.graph_c0	72.063438	74.682554	95.750305	809.17753	1101.2907	1026.7032	1.859E-61	3.634833	up	hypothetical protein CARUB_v10002736mg [Capsella rubella]

#ID	28 h-1 FPKM	28 h-2 FPKM	28 h-3 FPKM	48 h-1 FPKM	48 h-2 FPKM	48 h-3 FPKM	FDR	log2FC	regulated	nr_annotation
c48542.graph_c0	0.068365	0.4320903	0.0557853	2.9463606	3.4885249	4.1638033	2.449E-16	4.29274	up	PREDICTED: probable protein phosphatase 2C 60 isoform X3 [Nicotiana sylvestris]
c48543.graph_c0	0.0523352	0.1417612	0	1.3267732	3.4436105	2.1840264	5.06E-07	5.195416	up	-
c48549.graph_c0	27.187796	27.00028	27.425394	13.017586	12.118971	9.5727021	2.132E-13	-1.187939	down	dimethylaniline monooxygenase, putative [Ricinus communis]
c48550.graph_c0	95.588062	89.345183	91.200274	39.239551	40.485123	42.444637	5.274E-15	-1.135389	down	PREDICTED: uncharacterized protein LOC105175870 [Sesamum indicum]
c48550.graph_c1	51.922451	43.580675	45.961128	16.427557	20.52662	16.959632	2.08E-11	-1.35243	down	PREDICTED: putative E3 ubiquitin-protein ligase RF4 [Sesamum indicum]
c48551.graph_c0	4.9572053	4.6223753	4.4274791	10.85353	9.9245888	8.9864704	1.138E-08	1.132331	up	PREDICTED: probable galactinol--sucrose galactosyltransferase 1 [Sesamum indicum]
c48556.graph_c0	0.2931814	0.6764947	0.6911194	5.2440984	6.824062	5.4009967	1.372E-19	3.433618	up	glyceraldehyde 3-phosphate dehydrogenase [Daucus carota]
c48565.graph_c0	9.3111237	6.813796	7.5978009	11.897717	15.225859	22.316377	0.0051388	1.093689	up	PREDICTED: shikimate O-hydroxycinnamoyltransferase-like [Sesamum indicum]
c48566.graph_c0	3.5282175	4.0544632	5.3654029	22.361378	41.92265	20.017608	0.0001413	2.737383	up	predicted protein [Hordeum vulgare subsp. vulgare]
c48579.graph_c0	0	0	0.0617772	1.5674382	2.0332785	1.2239179	4.955E-20	6.322802	up	PREDICTED: thiol protease aleurain-like [Eucalyptus grandis]
c48591.graph_c0	0.0923138	0.062513	0.1883183	1.4334284	2.3552922	0.5986834	0.0024416	3.714671	up	-
c48596.graph_c0	13.574151	14.793431	11.970281	4.9505663	6.3318042	3.1157205	1.396E-07	-1.443664	down	PREDICTED: early nodulin-like protein 1 [Nelumbo nucifera]
c48605.graph_c0	3.8611409	3.6130208	3.4084406	8.2535365	7.3059907	6.1364518	4.586E-06	1.041633	up	PREDICTED: abietadienol/abietadienal oxidase [Sesamum indicum]
c48610.graph_c0	3.727312	4.6991716	4.429033	1.1321724	1.4992492	1.5692195	1.089E-07	-1.577108	down	PREDICTED: putative pentatricopeptide repeat-containing protein At2g01510 isoform X1 [Sesamum indicum]
c48612.graph_c1	2.9392961	3.1132392	3.9205296	0.429897	1.1385594	0.5100264	5.952E-07	-2.233223	down	hypothetical protein CICLE_v10018809mg [Citrus clementina]
c48628.graph_c2	212.7091	221.35786	220.5054	23.517419	45.161567	55.848411	6.339E-57	-2.364531	down	PREDICTED: non-specific lipid-transfer protein-like protein At2g13820 [Sesamum indicum]
c48632.graph_c0	0.0345976	0.0624768	0.1693882	1.0963755	1.4866904	1.0535922	3.019E-13	3.807356	up	26S proteasome complex ubiquitin receptor, subunit Rpn13 domain-containing protein [Rozella allomycis CSF55]
c48643.graph_c1	1.897822	1.8920506	1.8712337	14.684361	22.989344	33.802289	2.174E-07	3.689613	up	PREDICTED: fatty acid desaturase 4, chloroplastic-like [Sesamum indicum]
c48653.graph_c0	14.503735	16.523039	15.445224	39.755766	42.397658	46.919856	1.393E-24	1.513879	up	PREDICTED: serine carboxypeptidase-like 7 [Sesamum indicum]
c48654.graph_c1	79.144896	89.350512	78.626956	28.857186	33.072829	27.96582	1.154E-22	-1.417858	down	PREDICTED: pleiotropic drug resistance protein 3-like [Sesamum indicum]
c48657.graph_c0	0.8152808	0.7361221	0.9503749	5.3147747	5.4739605	6.6338127	1.059E-11	2.839372	up	predicted protein [Physcomitrella patens]
c48659.graph_c1	0.254498	0.8272362	0.8722068	5.4841141	7.2451049	4.9371404	3.283E-14	3.215931	up	-
c48662.graph_c0	63.856872	63.446569	59.979616	121.84768	122.90377	134.83776	2.831E-13	1.060477	up	PREDICTED: chorismate synthase 1, chloroplastic [Sesamum indicum]
c48671.graph_c0	29.93525	31.569707	31.564076	13.553813	14.476075	15.488229	2.959E-12	-1.05641	down	PREDICTED: protein DAMAGED DNA-BINDING 2 [Sesamum indicum]
c48683.graph_c0	5.0068981	4.6547079	5.0388918	9.8725077	11.330285	11.77541	2.824E-10	1.20469	up	PREDICTED: probable ATP-dependent RNA helicase DHX35 isoform X3 [Sesamum indicum]

#ID	28 h-1 FPKM	28 h-2 FPKM	28 h-3 FPKM	48 h-1 FPKM	48 h-2 FPKM	48 h-3 FPKM	FDR	log2FC	regulated	nr_annotation
c48684.graph_c1	143.64341	133.91589	136.71196	481.35095	509.29579	549.64224	9.928E-25	1.934914	up	Beta-galactosidase 8 [Morus notabilis]
c48690.graph_c1	0.1645475	0.1188568	0.1879772	3.0452103	7.7336361	2.7467277	0.0002376	4.87303	up	PREDICTED: fruit bromelain-like [Setaria italica]
c48700.graph_c0	0	0	0	2.2282547	6.9872743	2.9343777	4.53E-06	Inf	up	-
c48706.graph_c0	6.531187	4.7879714	5.2071614	24.722354	22.650014	18.889049	2.044E-16	2.048776	up	PREDICTED: uncharacterized protein LOC105173691 [Sesamum indicum]
c48706.graph_c2	200.34245	207.71061	194.89753	403.64311	485.31871	475.06788	6.687E-11	1.216651	up	PREDICTED: pyruvate kinase, cytosolic isozyme-like [Sesamum indicum]
c48706.graph_c3	12.613392	8.0620079	9.750718	88.06244	108.4909	132.67976	3.181E-28	3.472044	up	-
c48712.graph_c0	13.210242	12.449987	11.461361	23.182188	27.58	32.771782	1.006E-10	1.207652	up	PREDICTED: cystinosin homolog isoform X1 [Sesamum indicum]
c48716.graph_c0	2.2960767	3.691204	6.3518927	19.024381	35.344745	18.06472	0.000136	2.587207	up	unknown [Lotus japonicus]
c48725.graph_c1	1.2078199	1.3714469	1.4932897	2.1805162	2.8260547	3.3437665	0.0014497	1.071668	up	PREDICTED: DNA polymerase epsilon catalytic subunit A [Sesamum indicum]
c48729.graph_c0	0.2858001	0.1843219	0.3664737	1.1385711	1.7544408	1.3814915	1.666E-07	2.38792	up	AaceriAFL219Wp [Saccharomycetaceae sp. 'Ashbya aceri']
c48734.graph_c1	11.38168	12.027953	10.739325	35.762996	27.058752	25.728567	3.257E-06	1.42319	up	PREDICTED: CTP synthase isoform X2 [Elaeis guineensis]
c48739.graph_c2	473.57694	463.12952	448.46291	2796.2865	2121.3092	1830.7133	1.103E-08	2.333693	up	PREDICTED: calreticulin [Sesamum indicum]
c48744.graph_c0	0.0595442	0.0537629	0.0809793	1.9623942	0.7729313	0.850677	0.0009413	4.266529	up	-
c48765.graph_c0	23.248747	22.03114	21.397145	57.165285	55.568505	58.214002	2.899E-22	1.400645	up	PREDICTED: probable galacturonosyltransferase 4 [Sesamum indicum]
c48777.graph_c0	16.03564	14.559793	14.587705	4.2702426	6.7555477	7.0926542	8.445E-07	-1.284816	down	PREDICTED: protein notum homolog isoform X2 [Sesamum indicum]
c48794.graph_c0	0.5693763	0.8354019	0.4936444	2.0749636	1.481377	2.5286147	4.523E-05	1.72479	up	hypothetical protein MIMGU_mgv1a027092mg [Erythranthe guttata]
c48810.graph_c0	80.692829	83.652647	77.789792	34.803321	35.840372	38.858355	3.238E-14	-1.103869	down	hypothetical protein MIMGU_mgv1a006553mg [Erythranthe guttata]
c48810.graph_c3	1.8504065	1.0740495	0.6471071	16.418686	12.069033	10.882399	1.773E-12	3.512629	up	unnamed protein product [Coffea canephora]
c48810.graph_c4	48.961052	56.682388	52.391362	102.91905	103.77548	104.92211	8.562E-12	1.021536	up	PREDICTED: trifunctional UDP-glucose 4,6-dehydratase/UDP-4-keto-6-deoxy-D-glucose 3,5-epimerase/UDP-4-keto-L-rhamnose-reductase RHM1 [Sesamum indicum]
c48815.graph_c0	0.187241	0.2958568	0.6493452	1.7207274	1.7600433	1.214315	6.147E-06	2.094719	up	--
c48816.graph_c4	16.741913	15.267541	18.647625	60.826432	73.487121	78.694313	4.186E-45	2.109715	up	PREDICTED: uncharacterized protein LOC105168334 [Sesamum indicum]
c48822.graph_c0	0.123453	0.148622	0.2350522	1.4605344	3.5918514	1.206748	0.0037282	3.656432	up	hypothetical protein PNEG_01290 [Pneumocystis murina B123]
c48824.graph_c0	4.4469118	3.8074645	4.3793976	14.091971	11.942987	16.776251	2.777E-08	1.80268	up	hypothetical protein CICLE_v100149971mg, partial [Citrus clementina]
c48824.graph_c4	5.9170867	5.1409679	6.4377259	15.283718	15.641543	15.656054	3.196E-10	1.454056	up	PREDICTED: UDP-N-acetylglucosamine diphosphorylase 1 [Vitis vinifera]
c48830.graph_c0	0.0649516	0.0879679	0.212	0.9468064	2.4421517	0.8424627	0.005441	3.564652	up	-
c48831.graph_c0	7.0728035	8.4296235	7.2719009	23.190241	30.265837	39.514075	1.128E-08	2.064633	up	PREDICTED: non-specific lipid-transfer protein 1-like [Sesamum indicum]
c48832.graph_c0	281.4677	254.57729	245.71925	681.57779	709.90704	812.67494	7.88E-13	1.535665	up	PREDICTED: aconitate hydratase, cytoplasmic [Sesamum indicum]

#ID	28 h-1 FPKM	28 h-2 FPKM	28 h-3 FPKM	48 h-1 FPKM	48 h-2 FPKM	48 h-3 FPKM	FDR	log2FC	regulated	nr_annotation
c48832.graph_c2	55.651137	59.563489	62.701026	23.54742	20.303763	21.369019	2.524E-22	-1.403237	down	PREDICTED: (+)-neomenthol dehydrogenase-like isoform X1 [Sesamum indicum]
c48837.graph_c0	31.234746	29.947888	32.769383	13.008912	9.5870364	7.9964299	4.874E-16	-1.568802	down	-
c48842.graph_c0	14.031385	12.424044	14.517454	37.708873	26.597286	22.230314	0.0084611	1.129425	up	PREDICTED: internal alternative NAD(P)H-ubiquinone oxidoreductase A2, mitochondrial-like [Sesamum indicum]
c48848.graph_c0	6.5584999	6.1908791	5.9355022	24.486688	29.943867	30.059519	2.382E-37	2.21526	up	PREDICTED: uncharacterized protein LOC105173962 [Sesamum indicum]
c48848.graph_c1	1.4345719	1.3201932	1.1705989	13.428373	12.336378	12.757498	1.889E-38	3.33854	up	PREDICTED: O-acyltransferase WSD1-like [Nicotiana tomentosiformis]
c48850.graph_c0	2.0797413	3.487364	6.642746	22.97282	31.19913	20.709157	3.312E-12	2.654432	up	-
c48853.graph_c0	0	0	0.1279307	1.2420584	2.7895179	1.2820043	4.279E-06	5.403279	up	-
c48877.graph_c2	17.446747	19.451255	17.764555	41.249351	45.837782	39.098986	4.259E-10	1.248444	up	hypothetical protein L484_013174 [Morus notabilis]
c48880.graph_c0	0.1609717	0.5813695	0.7224339	8.161741	10.807969	6.6268196	8.221E-17	4.166835	up	predicted protein [Thalassiosira pseudonana CCMP1335]
c48882.graph_c1	10.330131	11.769009	12.951461	29.560302	22.325729	20.201981	0.0008626	1.088971	up	PREDICTED: double-stranded RNA-binding protein 2-like [Sesamum indicum]
c48887.graph_c1	0.1149595	0.5189884	0.3752241	13.11477	7.8729883	7.3906476	5.723E-09	4.867626	up	PREDICTED: zinc-finger homeodomain protein 10-like [Sesamum indicum]
c48893.graph_c3	4.5544568	6.2506162	2.6758077	17.088404	15.412092	21.780363	1.642E-07	2.051786	up	PREDICTED: UDP-glucuronate 4-epimerase 6 [Sesamum indicum]
c48903.graph_c0	12.263015	13.321425	14.196751	2.9631274	3.087614	3.0255755	1.006E-14	-2.090526	down	PREDICTED: phosphoenolpyruvate carboxylase kinase 2 [Sesamum indicum]
c48913.graph_c0	0.0440523	0	0	3.2945295	5.6788954	3.6270514	4.045E-20	8.195546	up	-
c48922.graph_c1	1.7294036	2.802673	2.7499902	13.827385	32.333746	13.253796	0.0018933	3.060346	up	-
c48944.graph_c0	0	0.0343525	0	2.652477	6.181951	6.5226186	1.879E-13	8.836943	up	hypothetical protein M378DRAFT_109196 [Amanita muscaria Koide BX008]
c48950.graph_c2	3.9381572	3.9137519	4.248727	10.050814	14.054857	12.699279	1.902E-13	1.641309	up	hypothetical protein MIMGU_mgv1a004080mg [Erythranthe guttata]
c48953.graph_c1	1.6082892	1.136453	1.8544088	7.3124592	9.6246429	12.106186	5.359E-13	2.693187	up	PREDICTED: protein ECERIFERUM 1-like [Sesamum indicum]
c48957.graph_c1	0.9465699	1.5668837	2.1884477	7.7990058	20.549328	8.7188697	0.0044961	3.008059	up	PREDICTED: adenosylhomocysteinase-like [Phoenix dactylifera]
c48957.graph_c2	616.14003	617.11856	611.33848	1167.7898	1252.9252	1515.8613	5.419E-07	1.132777	up	PREDICTED: uncharacterized protein LOC105169334 [Sesamum indicum]
c48972.graph_c0	2.1827798	1.9189812	1.945182	5.6064798	4.3581206	4.7942505	4.919E-05	1.333682	up	PREDICTED: subtilisin-like protease SBT3.5 isoform X2 [Sesamum indicum]
c48982.graph_c0	0.1073369	0.0969152	0.2627581	1.496632	5.1889094	1.755415	0.007858	4.200716	up	-
c48988.graph_c0	3.0118486	5.3551594	6.9570226	35.475614	60.542044	28.900648	1.209E-05	3.063317	up	-
c49003.graph_c0	5.0809553	4.358245	5.5971244	0.9660728	2.2174467	1.0028734	5.932E-05	-1.813181	down	-
c49008.graph_c0	1.9558511	1.9761824	1.6339576	0.6493105	0.500266	0.6302755	0.0004796	-1.599354	down	PREDICTED: uncharacterized protein LOC105161665 [Sesamum indicum]
c49011.graph_c0	22.084289	23.08847	23.026321	7.5304051	9.0181012	15.148977	2.961E-05	-1.071059	down	PREDICTED: uncharacterized protein LOC105176842 [Sesamum indicum]
c49016.graph_c0	0.1207309	0.2616208	0.2955462	0.6427481	0.9403094	0.5991474	0.0001542	1.724412	up	unknown [Picea sitchensis]

#ID	28 h-1 FPKM	28 h-2 FPKM	28 h-3 FPKM	48 h-1 FPKM	48 h-2 FPKM	48 h-3 FPKM	FDR	log2FC	regulated	nr_annotation
c49018.graph_c2	84.506501	76.125662	74.179246	155.84196	166.37015	160.4335	4.981E-11	1.080685	up	1-deoxy-D-xylulose-5-phosphate synthase [Andrographis paniculata]
c49019.graph_c0	0.3226902	0.1165436	0.5002939	1.3498108	1.3866287	1.2010535	3.27E-06	2.10805	up	hypothetical protein HMPREF1120_05211 [Exophiala dermatitidis NIH/UT8656]
c49022.graph_c0	72.755323	65.307735	65.035412	197.5568	166.8462	139.60214	1.094E-06	1.358427	up	PREDICTED: uncharacterized protein LOC105176567 [Sesamum indicum]
c49029.graph_c0	0.2298704	0.6226543	0.6002316	1.748266	2.7781137	1.3481837	0.0010339	2.053716	up	ras-related c3 botulinum toxin substrate 1 (rhosmall gtp binding protein rac1) [Lichtheimia corymbifera JMRC:FSU:9682]
c49030.graph_c2	589.91819	636.89353	681.80081	60.992358	56.654904	48.513615	2.405E-70	-3.477046	down	PREDICTED: probable xyloglucan endotransglucosylase/hydrolase protein 28 [Sesamum indicum]
c49038.graph_c1	18.909995	21.463143	16.303072	1.6637144	2.4152828	2.302787	6.673E-28	-3.114657	down	PREDICTED: E3 ubiquitin-protein ligase RING1-like [Sesamum indicum]
c49050.graph_c0	0.0599908	0.0812492	0.0734281	1.7109633	4.2695815	1.1164303	0.0014635	5.07942	up	PREDICTED: CBS domain-containing protein CBSX3, mitochondrial-like [Pyrus x bretschneideri]
c49054.graph_c0	0	0.0660967	0.0597342	1.9486306	3.8337702	5.0365079	3.914E-10	6.454858	up	short chain dehydrogenase, putative [Ricinus communis]
c49059.graph_c4	2.3446039	2.0581531	1.9663214	0.1651088	0.8745637	0.734564	9.806E-05	-1.823483	down	-
c49060.graph_c0	10.774822	10.70625	10.345402	3.1433047	4.6900707	5.8301444	1.896E-09	-1.186599	down	PREDICTED: uncharacterized protein LOC105172576 [Sesamum indicum]
c49060.graph_c4	7.5726277	6.8681718	6.3462079	2.8969543	3.1605829	3.6164641	0.0007755	-1.064053	down	PREDICTED: F-box protein SKIP28-like isoform X1 [Sesamum indicum]
c49060.graph_c5	5.1119694	5.0517515	5.5508187	2.1939472	2.3782727	2.7239457	0.0035843	-1.067639	down	PREDICTED: uncharacterized protein At1g15400-like [Sesamum indicum]
c49078.graph_c4	7.9052787	7.6257751	7.4798053	17.700069	19.991068	21.617342	4.633E-17	1.405099	up	PREDICTED: reticulon-like protein B2 [Sesamum indicum]
c49081.graph_c0	0.0783064	0.1237308	0.2236407	1.4640712	2.8391295	1.1040015	0.000187	3.701158	up	S-methyl-5'-thioadenosine phosphorylase [Galdieria sulphuraria]
c49084.graph_c0	1.7318556	2.1615898	1.7872571	6.1337995	6.566417	5.4003672	6.083E-06	1.714602	up	PREDICTED: uncharacterized protein LOC105173906 [Sesamum indicum]
c49085.graph_c0	56.000826	53.774614	51.941857	20.22081	20.383298	20.579302	1.142E-20	-1.360296	down	-
c49091.graph_c0	0.4618867	0.6950673	0.544405	2.4069545	3.5146945	2.1995773	3.157E-07	2.293662	up	-
c49105.graph_c0	1.6073543	2.9025804	3.1852851	13.971017	17.678492	32.373246	9.076E-05	3.089027	up	predicted protein [Micromonas sp. RCC299]
c49119.graph_c1	112.17069	120.81623	128.44748	42.636681	40.942112	38.603439	3.624E-26	-1.521358	down	PREDICTED: cylicin-2-like isoform X3 [Nicotiana sylvestris]
c49121.graph_c0	13.725263	16.523501	15.70533	89.188631	127.53294	125.97877	5.52E-28	2.934362	up	PREDICTED: uncharacterized protein LOC105160017 [Sesamum indicum]
c49130.graph_c0	12.755172	11.142805	12.908767	31.04139	28.139124	30.858916	3.407E-17	1.333625	up	PREDICTED: uncharacterized protein LOC104213443 [Nicotiana sylvestris]
c49138.graph_c0	0.1307294	0.2754183	0.2844643	2.0989826	3.74504	2.2608541	7.753E-09	3.587523	up	-
c49138.graph_c1	167.42188	174.37579	175.50365	67.872665	75.704688	74.888962	2.032E-15	-1.203499	down	PREDICTED: uncharacterized protein LOC105179211 isoform X1 [Sesamum indicum]
c49142.graph_c0	664.08624	672.32928	650.4535	1589.6451	1313.0092	1084.5725	0.0002662	1.052674	up	PREDICTED: 4-coumarate--CoA ligase-like 7 [Sesamum indicum]
c49145.graph_c0	0.1106727	0.239825	0.2348008	1.0100577	1.4861578	0.7988829	3.648E-06	2.532015	up	hypothetical protein SELMODRAFT_166421 [Selaginella moellendorffii]
c49148.graph_c0	0	0.0493357	0	1.5930151	1.3207354	1.6023408	2.521E-19	6.565202	up	--
c49150.graph_c0	0	0	0	1.3468224	1.5694745	1.3781536	1.077E-20	Inf	up	predicted protein [Physcomitrella patens]

#ID	28 h-1 FPKM	28 h-2 FPKM	28 h-3 FPKM	48 h-1 FPKM	48 h-2 FPKM	48 h-3 FPKM	FDR	log2FC	regulated	nr_annotation
c49157.graph_c4	11.520129	11.592619	11.337804	3.1769041	4.0740804	4.9096882	5.376E-07	-1.466293	down	MYB-related transcription factor [Salvia miltiorrhiza]
c49158.graph_c0	3.5954668	5.8807184	5.0020106	22.899665	42.938775	22.835728	3.334E-05	2.649484	up	S-adenosyl-methionine synthetase [Undaria pinnatifida]
c49160.graph_c3	3.1114263	1.6053293	1.4961366	5.9863708	6.5656634	8.6479515	4.813E-07	1.808879	up	PREDICTED: dof zinc finger protein DOF1.4-like [Sesamum indicum]
c49168.graph_c0	17.452827	17.911471	17.758463	82.982682	99.699435	146.61902	5.68E-09	2.667441	up	PREDICTED: kinesin-like protein NACK1 [Sesamum indicum]
c49172.graph_c1	1.9891968	2.1809279	2.2801651	9.0652257	11.765887	8.0661833	2.546E-11	2.203324	up	predicted protein [Hordeum vulgare subsp. vulgare]
c49172.graph_c2	0.7424256	0.7541333	1.3630798	5.5288402	6.7294752	4.0821622	1.927E-08	2.555426	up	heat shock protein S [Schizosaccharomyces japonicus yFS275]
c49177.graph_c0	4.4303876	5.8336609	6.577583	20.903414	33.960612	26.511559	1.781E-11	2.307286	up	40S ribosomal protein S12 [Blumeria graminis f. sp. hordei DH14]
c49183.graph_c0	1.3816656	1.2812311	1.9806165	0.4733413	0.5515925	0.7581173	0.0030078	-1.345112	down	-
c49185.graph_c0	5.239263	7.3284955	8.4803064	30.429539	50.171152	26.688529	1.946E-05	2.386319	up	hypothetical protein CHLNCDRAFT_59606 [Chlorella variabilis]
c49217.graph_c0	0.1028234	0.0464199	0.2097575	2.4112192	4.5564872	1.3916681	0.0002943	4.575769	up	conserved unknown protein [Ectocarpus siliculosus]
c49225.graph_c0	0.2638197	0.3970075	0.5023078	1.2261735	1.9484733	1.041449	0.000423	1.894929	up	PREDICTED: V-type proton ATPase subunit E3 [Tarenaya hassleriana]
c49233.graph_c4	141.8109	138.75231	141.96422	44.160044	56.396961	56.770441	3.218E-19	-1.387851	down	-
c49236.graph_c1	11.747795	12.062291	13.081399	31.395155	33.430213	37.215071	6.32E-18	1.507632	up	PREDICTED: kinesin KP1-like isoform X2 [Sesamum indicum]
c49245.graph_c1	1.1667739	2.3703466	1.1900974	6.7478088	4.1617893	4.8115526	0.0002102	1.785638	up	PREDICTED: protein PLANT CADMIUM RESISTANCE 6 [Sesamum indicum]
c49246.graph_c0	9.6756844	8.7690789	8.9143444	21.901148	24.553002	22.961073	2.302E-21	1.383686	up	PREDICTED: uncharacterized protein LOC105164785 isoform X2 [Sesamum indicum]
c49248.graph_c0	0.0347238	0	0	2.7289287	5.595424	4.5038903	6.019E-19	8.560613	up	-
c49251.graph_c0	0	0.0284767	0.0772066	1.1993379	5.0398605	2.9524861	2.688E-05	6.463153	up	-
c49260.graph_c1	1.0618808	0.7190841	1.5163506	15.815643	14.972335	9.8808065	3.561E-12	3.669956	up	predicted protein [Physcomitrella patens]
c49268.graph_c1	25.652429	21.376026	21.691616	172.24076	184.57721	188.75238	1.424E-90	3.029563	up	PREDICTED: auxin-responsive protein IAA14 [Sesamum indicum]
c49270.graph_c0	27.165794	25.109257	24.461164	61.226689	50.534336	47.258552	8.238E-07	1.097934	up	PREDICTED: ultraviolet-B receptor UVR8 isoform X2 [Sesamum indicum]
c49287.graph_c0	52.127831	77.731111	69.926416	7.7589084	5.5681315	6.4584217	2.89E-20	-3.288253	down	PREDICTED: peroxygenase-like [Solanum lycopersicum]
c49287.graph_c1	59.258925	66.413318	67.905485	19.129358	21.031276	22.187986	2.799E-28	-1.594687	down	PREDICTED: probable helicase DDB_G0274399 isoform X1 [Sesamum indicum]
c49292.graph_c0	0.1253075	0.4148499	0.2726663	1.3501096	1.7387687	0.6124374	0.00659	2.231596	up	phosphoenolpyruvate carboxykinase [Neocallimastix frontalis]
c49295.graph_c0	0.1042338	0.3137111	0.4819722	2.3782319	4.0124547	2.7823275	9.642E-12	3.383514	up	unknown [Picea sitchensis]
c49297.graph_c0	3.2125378	2.4655273	1.9005183	0.7126224	0.9706334	0.2717516	5.878E-05	-1.910752	down	-
c49307.graph_c0	322.88959	308.70899	295.22631	1648.3562	1087.8131	791.56508	0.0021175	1.982148	up	PREDICTED: pentatricopeptide repeat-containing protein At3g21470 [Sesamum indicum]
c49309.graph_c0	0	0.0595667	0.0538328	1.2543698	1.3288527	0.930109	6.298E-17	4.997242	up	hypothetical protein EMIHUDRAFT_452099 [Emiliania huxleyi CCMP1516]

#ID	28 h-1 FPKM	28 h-2 FPKM	28 h-3 FPKM	48 h-1 FPKM	48 h-2 FPKM	48 h-3 FPKM	FDR	log2FC	regulated	nr_annotation
c49311.graph_c0	0.0591066	0.160103	0.1929219	2.0978135	2.3811204	1.9999519	3.89E-16	4.015139	up	glucokinase [Galdieria sulphuraria]
c49315.graph_c0	0.805624	0.8313176	1.5025887	3.355331	4.8682104	2.7908433	6.805E-05	1.848541	up	PREDICTED: T-complex protein 1 subunit beta [Phoenix dactylifera]
c49316.graph_c1	0.0539336	0	0.0880187	3.0764144	3.7660573	3.7106639	7.322E-23	6.251459	up	hypothetical protein MG5_02709 [Candida albicans P57072]
c49327.graph_c0	90.606697	98.602149	86.079387	26.787054	28.674956	25.499799	7.701E-33	-1.72363	down	PREDICTED: chromodomain-helicase-DNA-binding protein 4-like isoform X4 [Sesamum indicum]
c49340.graph_c0	27.493075	26.282059	28.070698	131.25192	91.374071	93.879364	1.352E-07	2.000531	up	PREDICTED: uncharacterized protein LOC104715674 [Camelina sativa]
c49353.graph_c2	12.899002	12.894438	13.576985	30.63989	34.788163	37.685553	1.152E-20	1.427986	up	PREDICTED: chloroplast stem-loop binding protein of 41 kDa b, chloroplastic [Nicotiana sylvestris]
c49355.graph_c1	191.72673	188.89941	177.55179	547.27569	929.49289	1206.2138	2.783E-05	2.296126	up	PREDICTED: (RS)-norcoclaurine 6-O-methyltransferase-like [Sesamum indicum]
c49355.graph_c3	564.60556	571.8276	523.22707	1079.8654	1906.0428	2365.7357	0.0004882	1.719961	up	PREDICTED: (RS)-norcoclaurine 6-O-methyltransferase-like [Sesamum indicum]
c49370.graph_c2	0.1900827	0.3432537	0.1163295	1.9877867	2.0420063	1.7151231	3.318E-11	3.189291	up	hypothetical protein M569_09445 [Genlisea aurea]
c49372.graph_c0	3.4253245	5.5397553	8.0165341	28.198175	49.484305	25.897201	2.414E-05	2.643738	up	small subunit ribosomal protein S6e, cytoplasmic [Guillardia theta CCMP2712]
c49380.graph_c0	11.525844	9.7831305	12.680891	2.3528982	3.0722871	3.943748	1.036E-09	-1.824408	down	PREDICTED: uncharacterized protein LOC105167081 [Sesamum indicum]
c49380.graph_c1	56.531022	52.902865	53.059542	15.531617	13.910995	14.856435	6.823E-35	-1.831273	down	PREDICTED: sedoheptulose-1,7-bisphosphatase, chloroplastic [Sesamum indicum]
c49380.graph_c2	8.3972857	6.3261114	7.7753313	185.12186	210.0805	234.24939	1.78E-150	4.844674	up	PREDICTED: gamma-cadinene synthase-like [Sesamum indicum]
c49382.graph_c1	0.2752739	0.7456396	0.8610481	5.4084141	9.2412241	5.1745961	3.284E-08	3.432526	up	-
c49384.graph_c0	0.034373	0.2172494	0.1402406	2.1349474	2.2617179	1.395671	3.372E-13	3.931282	up	-
c49388.graph_c1	55.228102	57.819905	56.91689	193.5146	143.98965	151.76831	1.96E-08	1.573145	up	PREDICTED: zinc finger BED domain-containing protein DAYSLEEPER [Sesamum indicum]
c49395.graph_c0	0.7619708	1.0438441	1.3721642	3.5636685	4.4103675	1.8966315	0.0069885	1.678125	up	-
c49402.graph_c0	3.6814651	2.465002	3.7466185	6.5016796	7.4987505	9.144278	5.684E-05	1.263304	up	Pollen-specific SF21 [Gossypium arboreum]
c49402.graph_c1	0.6253034	0.4362743	0.5102425	1.9815452	2.2900439	2.2760883	4.987E-07	2.09775	up	PREDICTED: uncharacterized protein LOC105173599 [Sesamum indicum]
c49405.graph_c0	135.30151	137.53852	132.68974	32.005402	53.276617	52.871343	3.468E-24	-1.520418	down	hypothetical protein MIMGU_mgv1a009036mg [Erythranthe guttata]
c49416.graph_c0	0.115355	0.5207737	0.2196337	8.2371133	20.343975	5.3777361	0.0006075	5.344466	up	--
c49428.graph_c3	268.21796	244.75478	266.13085	575.84028	739.46722	697.1664	3.407E-14	1.406812	up	PREDICTED: serine carboxypeptidase-like [Sesamum indicum]
c49429.graph_c1	593.31503	558.2572	585.76326	276.80039	272.61826	249.34194	2.073E-11	-1.077908	down	hypothetical protein VITISV_037044 [Vitis vinifera]
c49431.graph_c0	21.638391	19.826388	20.529236	40.845944	37.453332	42.619381	6.604E-12	1.006487	up	PREDICTED: biotin synthase [Sesamum indicum]
c49433.graph_c0	0.1790654	0.3233585	0.2557028	1.418616	1.3826235	1.0603118	1.901E-07	2.393915	up	serine/threonine-protein phosphatase pp2a catalytic subunit [Grosmannia clavigera kw1407]

#ID	28 h-1 FPKM	28 h-2 FPKM	28 h-3 FPKM	48 h-1 FPKM	48 h-2 FPKM	48 h-3 FPKM	FDR	log2FC	regulated	nr_annotation
c49437.graph_c1	0.3443468	0.5652961	0.9195846	3.4521991	5.0023583	3.0011311	9.507E-09	2.684352	up	hypothetical protein MIMGU_mgv1a008893mg [Erythranthe guttata]
c49438.graph_c0	5.6056923	4.6075645	4.5296163	2.0915201	1.892893	2.3293587	1.605E-07	-1.181175	down	PREDICTED: ABC transporter B family member 4-like [Sesamum indicum]
c49441.graph_c0	0.1040478	0.1878907	0.0849021	1.3628446	2.0026468	3.0511862	1.081E-07	4.12309	up	unnamed protein product [Chondrus crispus]
c49449.graph_c0	2.1476985	1.9737984	2.472283	14.486876	18.128105	14.707094	7.609E-28	2.882405	up	PREDICTED: protein DA1-related 2 isoform X2 [Sesamum indicum]
c49452.graph_c0	5.8053613	5.0812368	5.1238317	16.857483	18.653936	20.879297	1.707E-21	1.855676	up	Rop guanine nucleotide exchange factor 1 [Morus notabilis]
c49454.graph_c0	0.3200258	0.2311626	0.3655938	2.1094126	1.890872	1.5159922	1.551E-08	2.634568	up	NADH-malate dehydrogenase [Pyropia haitanensis]
c49460.graph_c0	0.1590147	0.0574301	0.5449696	1.2093769	0.939538	1.0043565	1.611E-05	2.093514	up	acetyl-CoA C-acyltransferase-like protein [Zea mays]
c49463.graph_c0	0.6239366	1.0915027	1.1455361	4.5970225	6.126759	4.2223515	5.191E-10	2.424406	up	Molecular chaperones GRP78/BiP/KAR2, HSP70 superfamily [Ectocarpus siliculosus]
c49470.graph_c0	0	0.3548282	0.3206722	1.7790603	1.8846986	1.4563574	9.032E-10	2.965299	up	hypothetical protein CHLNCDRAFT_144079 [Chlorella variabilis]
c49474.graph_c0	215.38356	220.83253	218.13818	1556.7774	1059.6723	812.50519	6.974E-05	2.442212	up	PREDICTED: pyruvate decarboxylase 1 [Sesamum indicum]
c49474.graph_c6	5.5302634	5.3973921	5.3212749	14.870959	13.993997	11.573572	3.305E-09	1.360389	up	PREDICTED: uncharacterized protein LOC105170638 isoform X1 [Sesamum indicum]
c49477.graph_c0	0.6435137	1.7154295	2.0003884	8.856183	8.6413637	9.0552927	1.277E-18	2.648475	up	hypothetical protein SELMODRAFT_144212 [Selaginella moellendorffii]
c49479.graph_c0	4.2910658	7.5789291	9.613698	33.112578	56.510166	21.609304	0.0030884	2.409505	up	hypothetical protein PISMIDRAFT_677145 [Pisolithus microcarpus 441]
c49480.graph_c0	0.5404276	0.2439777	0.5354812	1.8104412	2.6954874	1.4367662	3.233E-05	2.208442	up	hypothetical protein JCGZ_17423 [Jatropha curcas]
c49485.graph_c0	0.0246992	0.1338061	0.0201543	3.0368752	4.1127106	5.7943939	5.118E-18	6.215811	up	predicted protein [Physcomitrella patens]
c49492.graph_c1	0.211931	0.2296246	0.3804547	1.0745526	1.8213731	1.1473565	2.535E-06	2.332836	up	TPR1 [Medicago sativa]
c49499.graph_c0	0	0.0318807	0	0.5594594	0.6400938	0.6969251	2.832E-19	5.93814	up	PREDICTED: uncharacterized protein LOC102585254 [Solanum tuberosum]
c49500.graph_c8	9.3871897	14.750859	10.081886	3.64004	6.2541423	5.7156807	0.0023453	-1.098655	down	PREDICTED: tyrosine/DOPA decarboxylase 1-like [Sesamum indicum]
c49500.graph_c9	16.665023	15.868549	20.323533	4.0534628	4.363976	2.8736682	4.824E-23	-2.18341	down	hypothetical protein MIMGU_mgv1a019874mg [Erythranthe guttata]
c49511.graph_c1	10.021379	9.6046173	10.121163	28.269015	30.830028	34.835188	4.219E-21	1.698232	up	hypothetical protein MIMGU_mgv1a004264mg [Erythranthe guttata]
c49513.graph_c1	0.0421641	0.0951755	0.0688111	6.8945453	9.7951993	11.342108	5.145E-40	7.122345	up	-
c49522.graph_c0	0.3841664	0.2312442	0.2438152	1.406771	2.1208162	1.2517364	1.593E-07	2.513678	up	predicted protein [Hordeum vulgare subsp. vulgare]
c49528.graph_c1	0.6678207	1.3329021	1.5487662	9.0888653	8.2125874	11.258676	3.927E-22	3.049131	up	PREDICTED: WAT1-related protein At1g21890-like [Sesamum indicum]
c49533.graph_c0	1.3415854	2.0401277	2.7079983	7.6972637	11.852206	10.830944	2.917E-10	2.352971	up	Rotamase cyclophilin 2 [Theobroma cacao]
c49540.graph_c0	0.2637458	0.4365859	1.1836795	4.0118073	8.9722715	3.2722085	0.0037987	3.140141	up	--
c49541.graph_c1	5.6032091	5.3925288	4.5367296	10.240785	10.469743	9.7490627	1.465E-06	1.014209	up	PREDICTED: CBL-interacting serine/threonine-protein kinase 1-like [Sesamum indicum]
c49544.graph_c0	0.2079066	0.1564334	0.3675751	4.260513	3.8155335	5.7450917	2.974E-22	4.278023	up	phenylpropenal double-bond reductase [Pinus pinaster]
c49549.graph_c0	16.11083	17.089443	15.775567	6.1109101	7.4646512	6.0616483	5.21E-17	-1.278445	down	PREDICTED: uncharacterized protein LOC105168432 [Sesamum indicum]

#ID	28 h-1 FPKM	28 h-2 FPKM	28 h-3 FPKM	48 h-1 FPKM	48 h-2 FPKM	48 h-3 FPKM	FDR	log2FC	regulated	nr_annotation
c49552.graph_c0	3.8556682	5.7517244	6.9079472	22.152415	50.143425	19.002054	0.0097711	2.499141	up	predicted protein [Physcomitrella patens]
c49554.graph_c0	4.3867773	3.3435735	3.7887704	7.1854033	9.9071581	12.240876	1.228E-05	1.382908	up	PREDICTED: uncharacterized protein At4g17910 isoform X1 [Sesamum indicum]
c49555.graph_c0	0.0218706	0.1777237	0.3212317	1.386128	1.6446467	0.5673495	0.0021717	2.832471	up	acid protease, partial [Dacryopinax sp. DJM-731 SS1]
c49557.graph_c0	0.4321843	0.5017139	0.6549379	1.0173901	2.0174233	1.2534475	0.0042355	1.464808	up	heat shock protein SSA1 [Spathaspora passalidarum NRRL Y-27907]
c49562.graph_c0	0.0232452	0.0209883	0.0758717	0.7660911	1.3734441	0.7865345	1.55E-09	4.639254	up	predicted protein, partial [Ostreococcus lucimarinus CCE9901]
c49563.graph_c0	28.500618	32.13535	28.701642	11.983468	14.001873	15.994227	3.922E-06	-1.051342	down	-
c49567.graph_c0	8.6262233	13.753229	19.075756	28.169222	31.521288	23.220208	0.001194	1.041634	up	predicted protein [Hordeum vulgare subsp. vulgare]
c49575.graph_c0	0.0784426	0.0141653	0.0128017	1.034091	2.1067194	0.9378228	3.223E-07	5.308255	up	--
c49579.graph_c0	0.0246234	0.2445583	0.2009245	4.9939056	7.3735472	3.5270656	3.575E-11	5.119237	up	hypothetical protein GUITHDRAFT_78647, partial [Guillardia theta CCMP2712]
c49585.graph_c0	3.278834	5.0183742	7.2108036	25.899995	40.002425	21.647587	2.737E-06	2.534338	up	60S ribosomal protein L5 [Rozella allomycis CSF55]
c49603.graph_c0	156.77878	163.04316	164.72789	74.71413	80.009374	78.742867	2.129E-10	-1.012516	down	PREDICTED: GRIP and coiled-coil domain-containing protein 2-like [Sesamum indicum]
c49614.graph_c1	0.5037904	0.4548755	0.462475	3.1131333	4.904699	1.9177251	0.0004408	2.844774	up	-
c49615.graph_c0	0.0179018	0	0.0146077	0.7034477	1.4663958	0.6663057	1.022E-07	6.478286	up	predicted protein [Physcomitrella patens]
c49616.graph_c0	519.61001	479.72672	491.80504	1413.0669	1836.1998	1982.2143	8.431E-21	1.847382	up	hypothetical protein MIMGU_mgv1a012029mg [Erythranthe guttata]
c49618.graph_c2	0.1935768	0.3745323	0.5190019	1.1217046	1.2254447	0.998086	0.0002536	1.662923	up	hypothetical protein NDAI_0G05590 [Naumovozyma dairenensis CBS 421]
c49623.graph_c1	0.3942613	0.5177905	1.0528822	1.9535887	2.8878004	1.6574407	0.0004696	1.762787	up	predicted protein [Physcomitrella patens]
c49627.graph_c0	0.115403	0.1041981	0.131835	0.7021524	1.6426585	0.8069959	0.0003879	3.195728	up	predicted protein [Physcomitrella patens]
c49629.graph_c0	0.494653	0.5582816	0.8745378	2.9260428	4.4282679	1.7667118	0.0020933	2.281561	up	hypothetical protein THAOC_26120, partial [Thalassiosira oceanica]
c49632.graph_c0	0.4055554	0.3661785	0.7091355	1.835969	2.9563795	2.0910517	7.98E-07	2.251224	up	hypothetical protein THAOC_09683, partial [Thalassiosira oceanica]
c49642.graph_c0	0.312397	0.0940217	0.5438152	2.2703206	4.2509264	1.5033434	0.0016472	3.112081	up	-
c49651.graph_c0	0.3740798	0.4101359	0.4578691	6.7400404	13.562897	3.3451983	0.0016749	4.2869	up	Os02g0639600 [Oryza sativa Japonica Group]
c49656.graph_c0	0.6511487	0.4409447	0.7969981	4.6427566	7.9787989	5.1409206	6.551E-10	3.267264	up	PREDICTED: obg-like ATPase 1 [Pyrus x bretschneideri]
c49667.graph_c0	0.471432	0.6191402	0.6994266	2.607599	3.6832466	2.3685639	1.38E-07	2.312754	up	PREDICTED: eukaryotic translation initiation factor 3 subunit D-like [Pyrus x bretschneideri]
c49672.graph_c0	0.2569665	0.3796637	0.4003031	7.0474829	7.9051274	3.9785378	2.291E-10	4.235048	up	nitric oxide dioxygenase [Cladophialophora psammophila CBS 110553]
c49674.graph_c1	0.1048127	0.3312263	0.2138158	4.3843094	9.570782	3.1327274	0.0002075	4.751395	up	pyrophosphate--fructose-6-phosphate 1-phosphotransferase [Galdieria sulphuraria]
c49674.graph_c2	47.124338	38.382173	51.618924	92.183542	89.515409	84.835757	7.374E-12	1.001542	up	PREDICTED: NAC domain-containing protein 100-like [Sesamum indicum]
c49676.graph_c0	0.0604152	0	0.0492983	3.0632281	3.3262467	3.2026293	7.059E-22	6.488768	up	--

#ID	28 h-1 FPKM	28 h-2 FPKM	28 h-3 FPKM	48 h-1 FPKM	48 h-2 FPKM	48 h-3 FPKM	FDR	log2FC	regulated	nr_annotation
c49686.graph_c0	6.8052797	7.5124722	7.6810473	2.3454431	2.0344626	1.6247483	4.744E-19	-1.82635	down	PREDICTED: DNA repair protein REV1 [Sesamum indicum]
c49697.graph_c0	0.1479232	0.2671216	0.3621125	1.1250216	1.2414833	1.3764265	4.086E-07	2.30656	up	26S proteasome regulatory subunit N5 [Mucor circinelloides f. circinelloides 1006PhL]
c49708.graph_c0	266.10321	225.32372	238.68037	1086.0036	1275.7148	1272.0811	4.727E-53	2.354294	up	-
c49708.graph_c1	41.16762	37.573694	39.59605	17.54707	18.988756	17.340091	7.345E-14	-1.093922	down	PREDICTED: uncharacterized protein LOC105169648 [Sesamum indicum]
c49708.graph_c2	6.4126069	6.3163449	5.8215894	1.724217	1.9757089	3.4128076	1.847E-05	-1.348055	down	PREDICTED: LOW QUALITY PROTEIN: multiple C2 and transmembrane domain-containing protein 1 [Sesamum indicum]
c49716.graph_c0	8.2621484	8.1593152	6.9525283	15.491137	14.099576	17.81232	5.353E-06	1.062197	up	-
c49716.graph_c2	33.327555	29.436352	28.284709	92.622818	84.478256	86.802846	4.169E-28	1.579128	up	NADH dehydrogenase subunit 5 [Beta vulgaris subsp. maritima]
c49717.graph_c0	0.4295044	0.6980439	0.9462746	9.9085976	13.553764	10.608998	1.661E-31	4.075559	up	--
c49722.graph_c0	3.2817933	2.7779547	2.7708998	1.169976	1.132335	1.3366409	1.855E-05	-1.237671	down	hypothetical protein VITISV_035665 [Vitis vinifera]
c49723.graph_c0	0.0964731	0.1306592	0.1968031	1.0700088	2.2670891	1.0880999	8.803E-05	3.415965	up	delta-9 fatty acid desaturase [Cryptococcus curvatus]
c49724.graph_c0	0.1884578	0.4821191	0.89705	3.9415876	7.1281028	3.5780535	7.798E-06	3.258094	up	-
c49733.graph_c1	0.1209831	0.4642546	0.6663681	3.7571964	4.9144447	2.2514962	4.94E-06	3.166775	up	-
c49740.graph_c0	231.02103	252.9481	247.74128	696.8045	628.92566	720.6913	7.598E-15	1.526731	up	putative galactinol synthase family protein [Populus trichocarpa]
c49741.graph_c0	0.0790759	0.0713981	0.1290506	2.0046909	1.8051679	1.6945738	6.876E-16	4.3433	up	PREDICTED: serine-threonine kinase receptor-associated protein-like [Setaria italica]
c49743.graph_c0	0.6029889	1.0548571	1.9373838	7.834411	15.435247	6.1208884	0.0009096	3.064882	up	-
c49744.graph_c0	0.4667717	0.379306	0.9141166	4.6741736	6.3306802	3.0534809	1.984E-06	3.037263	up	S-Adenosylmethionine decarboxylase [Ectocarpus siliculosus]
c49750.graph_c2	31.922568	28.308388	27.824593	74.556614	88.378228	99.889961	1.314E-23	1.615542	up	hypothetical protein MIMGU_mgv1a015998mg [Erythranthe guttata]
c49752.graph_c0	9.3389903	11.149285	9.9490381	2.2360399	2.9261809	2.4577595	4.001E-09	-1.959441	down	PREDICTED: probable protein phosphatase 2C 75 [Populus euphratica]
c49755.graph_c0	0.1172358	0.4234117	0.334822	3.4922172	3.0698648	1.5867339	2.109E-05	3.268765	up	-
c49756.graph_c0	0.1349986	0.2285457	0.2478549	0.4491917	0.8384273	0.4948519	0.0028881	1.577638	up	predicted protein [Physcomitrella patens]
c49766.graph_c0	0.3941313	0.2264586	0.7601641	3.3608808	3.9934729	2.1822506	1.579E-07	2.829197	up	predicted protein [Hordeum vulgare subsp. vulgare]
c49767.graph_c0	0.0277164	0.0250253	0.0452328	3.3727315	9.1185938	4.1264247	1.073E-07	7.434414	up	-
c49768.graph_c0	0.4837816	0.4138194	1.1427316	4.0344	7.3170154	2.6995182	0.0020884	2.818751	up	PREDICTED: thiol protease aleurain-like [Eucalyptus grandis]
c49770.graph_c0	12.62167	17.373368	25.663875	122.7997	244.65206	98.981849	0.0005849	3.10091	up	Elongation factor 1-alpha [Auxenochlorella protothecoides]
c49771.graph_c1	55.921668	56.057241	54.990325	29.27368	23.913749	21.955616	9.721E-15	-1.1048	down	PREDICTED: LOW QUALITY PROTEIN: DUF21 domain-containing protein At4g14240 [Sesamum indicum]
c49777.graph_c0	69.591415	62.890252	75.294998	30.316847	31.536845	31.361482	1.179E-14	-1.115279	down	PREDICTED: 1-acyl-sn-glycerol-3-phosphate acyltransferase 2-like [Sesamum indicum]
c49779.graph_c0	0.0289673	0.1046188	0.0709111	1.5788793	3.1507704	1.0128185	0.0001363	4.847775	up	PREDICTED: V-type proton ATPase subunit a1 [Brachypodium distachyon]

#ID	28 h-1 FPKM	28 h-2 FPKM	28 h-3 FPKM	48 h-1 FPKM	48 h-2 FPKM	48 h-3 FPKM	FDR	log2FC	regulated	nr_annotation
c49781.graph_c0	0.1154742	0.2085248	0.2120085	1.2807556	1.2017418	1.4652111	4.087E-10	2.922191	up	predicted protein [Physcomitrella patens]
c49799.graph_c0	0.185814	1.0066358	1.5162271	6.5949223	13.05814	6.077698	9.636E-05	3.281004	up	-
c49804.graph_c0	757.42485	678.76684	777.54111	337.27915	317.68547	283.02668	2.412E-09	-1.194716	down	PREDICTED: histidinol-phosphate aminotransferase, chloroplastic-like [Sesamum indicum]
c49805.graph_c0	0.2199861	0.4125326	0.4832877	5.4053722	9.2030406	3.340047	3.397E-05	4.045364	up	PREDICTED: trifunctional UDP-glucose 4,6-dehydratase/UDP-4-keto-6-deoxy-D-glucose 3,5-epimerase/UDP-4-keto-L-rhamnose-reductase RHM1 [Tarenaya hassleriana]
c49808.graph_c0	0.2844683	0.3210603	0.058031	3.6058457	4.2019519	4.6522667	8.278E-17	4.271336	up	-
c49811.graph_c0	0.3812145	0.5736683	0.8295145	1.5301887	2.5595517	1.7198561	0.0001264	1.737585	up	DEAD-box ATP-dependent RNA helicase 15 [Arabidopsis thaliana]
c49812.graph_c0	1.1625527	1.3901116	1.4101309	4.9386224	14.682994	10.418878	0.000423	2.948772	up	Short-chain dehydrogenase/reductase SDR [Macrophomina phaseolina MS6]
c49814.graph_c0	0.1975512	0.3057776	0.5526864	1.5382375	1.7811646	1.3368839	1.309E-06	2.180823	up	PREDICTED: serine--tRNA ligase-like [Nelumbo nucifera]
c49823.graph_c1	1.5619261	1.1445692	1.4961744	0.3730164	0.3647683	0.6382841	0.0004179	-1.57381	down	-
c49826.graph_c0	0.1218614	0.2672144	0.3125192	0.9047432	1.7299137	1.040655	4.165E-05	2.422409	up	Methylmalonyl-CoA mutase, alpha chain, catalytic domain-containing protein [Rozella allomycis CSF55]
c49840.graph_c0	3.8415819	4.9947671	7.0468043	23.529366	57.405257	22.426786	0.0071973	2.732871	up	predicted protein [Hordeum vulgare subsp. vulgare]
c49846.graph_c0	0.2413242	0.1961038	0.4726045	2.5083486	2.7545091	1.3064859	1.459E-05	2.895835	up	predicted protein [Micromonas sp. RCC299]
c49852.graph_c0	0.1684451	0.1330789	0.2405372	0.8807559	1.4985414	0.9736771	4.796E-08	2.66317	up	hypothetical protein M422DRAFT_230321 [Sphaerobolus stellatus SS14]
c49855.graph_c0	0.1316077	0.3327224	0.3651288	1.3012153	1.5552081	0.8312508	3.233E-05	2.195321	up	enolase [Rhizoctonia solani AG-1 IB]
c49858.graph_c0	2.0397289	2.9639601	3.4718388	35.389122	75.879712	30.554422	5.969E-05	4.097765	up	protein of unknown function [Taphrina deformans PYCC 5710]
c49861.graph_c1	19.793273	25.383814	19.15269	9.935859	10.349427	9.7298934	5.246E-07	-1.057466	down	hypothetical protein POPTR_0001s24240g [Populus trichocarpa]
c49863.graph_c0	0.1609289	0.4116939	0.5033806	2.1758855	4.105936	2.0570955	4.455E-05	2.988415	up	-
c49864.graph_c0	0.2507713	0.3113316	0.306941	5.7216796	6.8611972	3.853692	2.7E-14	4.284109	up	hypothetical protein EUTSA_v10013921mg [Eutrema salsugineum]
c49865.graph_c0	1.9281791	1.7780067	1.6068545	12.220545	28.481608	35.95284	9.255E-06	3.877905	up	PREDICTED: monosaccharide-sensing protein 2-like [Sesamum indicum]
c49870.graph_c0	0.2905987	0.5009136	0.6251507	3.5830914	5.9953159	2.5326927	8.101E-05	3.132986	up	--
c49873.graph_c0	0.2889983	0.0948867	0.3644495	1.0323751	1.5875945	0.8000719	0.0002323	2.228972	up	hypothetical protein MIMGU_mgv1a007681mg [Erythranthe guttata]
c49874.graph_c0	3.5094396	3.1957778	2.6433915	0.4182325	0.6847394	0.4398027	1.226E-09	-2.562906	down	hypothetical protein MIMGU_mgv11b019086mg [Erythranthe guttata]
c49876.graph_c7	4.6939813	3.8377628	3.136583	2.2019563	1.0919048	1.4173569	0.0013537	-1.253815	down	PREDICTED: squalene monooxygenase-like [Sesamum indicum]
c49876.graph_c9	40.579933	43.40019	44.259347	12.678394	16.808213	17.727535	1.45E-14	-1.405153	down	PREDICTED: uncharacterized protein LOC105160027 [Sesamum indicum]
c49882.graph_c0	0.1197477	0.1621814	0.1954263	2.2009406	5.7888736	1.6207313	0.0042414	4.362292	up	-
c49883.graph_c0	0.2737711	0.6709434	0.89358	1.9830004	2.5734169	1.1910117	0.006825	1.686125	up	unnamed protein product [Vitis vinifera]
c49885.graph_c0	0.2992027	0.641611	0.3967388	1.2325997	2.762248	1.8982381	0.0004115	2.170782	up	hypothetical protein E5Q_00045 [Mixia osmundae IAM 14324]

#ID	28 h-1 FPKM	28 h-2 FPKM	28 h-3 FPKM	48 h-1 FPKM	48 h-2 FPKM	48 h-3 FPKM	FDR	log2FC	regulated	nr_annotation
c49905.graph_c0	0.6010852	0.6784045	1.4407869	8.6192177	11.552501	11.821782	2.491E-28	3.59097	up	PREDICTED: 2-methylene-furan-3-one reductase-like [Solanum lycopersicum]
c49908.graph_c0	0.0359911	0.259973	0.0881054	1.505507	1.4982414	1.3801848	1.133E-12	3.557821	up	PREDICTED: probable disease resistance protein At5g63020 [Vitis vinifera]
c49910.graph_c0	0.0477467	0.215554	0.0779218	2.6629881	1.1540917	2.2618058	1.893E-06	4.209029	up	-
c49914.graph_c0	0.4139433	0.3971115	0.3799962	0.8854374	2.0497333	1.2839149	0.0026426	1.85521	up	hypothetical protein GUITHDRAFT_71484 [Guillardia theta CCMP2712]
c49918.graph_c0	18.410415	19.912086	20.536416	54.76643	53.573646	54.452185	1.481E-25	1.510071	up	PREDICTED: uncharacterized protein At1g04910-like [Sesamum indicum]
c49918.graph_c1	0.4773558	0.5746767	0.5842776	2.8237236	6.5169628	6.2813307	2.638E-06	3.28346	up	unnamed protein product [Coffea canephora]
c49922.graph_c0	0.1010075	0.0608002	0.0824213	0.8535632	1.9441304	1.6139277	7.5E-09	4.203973	up	PREDICTED: neutral ceramidase-like [Cicer arietinum]
c49923.graph_c0	0.4092293	0.7105687	0.8733496	5.1074789	7.0170723	3.3374435	2.634E-06	2.996112	up	-
c49925.graph_c0	0.5078722	0.3821341	0.7943042	2.4677674	2.7279655	1.3843116	0.0003413	2.009788	up	-
c49926.graph_c0	7.845358	10.273957	20.720162	42.131571	71.734947	39.71746	0.0003704	2.018121	up	cysteine protease 1 [Brachiaria hybrid cultivar]
c49927.graph_c1	0.0551489	0.3983543	0.2250053	3.495265	3.0363038	3.9808842	1.059E-15	3.997123	up	-
c49928.graph_c0	0.2873559	0.6745842	0.1875841	1.4569792	2.5467632	1.5556936	3.308E-05	2.311089	up	hypothetical protein M422DRAFT_237710 [Sphaerobolus stellatus SS14]
c49929.graph_c0	0.0712657	0	0.0290761	5.2845719	3.0384279	6.3700477	1.35E-16	7.238162	up	-
c49931.graph_c4	4.1771929	4.1793555	4.2745126	11.620158	10.21803	9.9066101	2.945E-10	1.374518	up	PREDICTED: uncharacterized protein LOC105177468 [Sesamum indicum]
c49934.graph_c0	1.029528	0.9295673	1.3879689	4.5958787	6.3714849	2.978688	0.0003451	2.099088	up	ATP-dependent RNA helicase ded1 [Exophiala dermatitidis NIH/UT8656]
c49935.graph_c0	0.4352916	0.7336513	0.9708646	3.5312903	7.5988533	2.8475471	0.0051375	2.740618	up	-
c49936.graph_c1	7.3183245	7.9426621	6.6754279	13.618049	19.224533	20.538197	1.453E-08	1.318541	up	PREDICTED: heat shock protein 83-like [Sesamum indicum]
c49936.graph_c4	35.846275	43.291308	34.51187	53.775948	85.366403	98.19043	0.001234	1.095514	up	PREDICTED: 4-coumarate--CoA ligase 2 [Sesamum indicum]
c49938.graph_c0	0.2671457	0.160805	0.1271601	6.2928146	17.12955	10.646219	5.414E-09	5.968053	up	-
c49940.graph_c0	0.1537376	0.104108	0.3449837	2.0218147	4.5159857	1.430529	0.0022254	3.756013	up	PREDICTED: DEAD-box ATP-dependent RNA helicase 7-like [Musa acuminata subsp. malaccensis]
c49947.graph_c0	0.0334959	0.0907309	0.0273324	0.6368773	0.7646535	0.4155723	2.739E-09	3.628661	up	hypothetical protein SELMODRAFT_141243 [Selaginella moellendorffii]
c49948.graph_c1	0.0653509	0.236023	0.2133033	3.8105069	7.1082255	5.454389	1.668E-15	5.024408	up	hypothetical protein SELMODRAFT_424962 [Selaginella moellendorffii]
c49949.graph_c0	2.1697509	3.7418459	3.1160783	13.724092	21.007269	8.7855104	0.0009152	2.308935	up	ATP synthase subunit alpha [Taphrina deformans PYCC 5710]
c49952.graph_c0	0.025263	0.0684303	0.0206144	1.7292248	1.8658281	3.1058013	5.141E-14	5.911499	up	-
c49954.graph_c0	0.0589593	0.0798521	0.2164964	4.3720129	4.2357528	3.3914484	1.758E-26	5.120354	up	-
c49959.graph_c0	0.5559545	0.2316807	0.139586	2.11414	3.5605089	1.6882107	2.27E-05	3.027001	up	predicted protein [Physcomitrella patens]
c49964.graph_c0	0.0954188	0.2584628	0.2595366	3.5075616	4.2710761	3.44387	2.216E-20	4.233288	up	hypothetical protein GUITHDRAFT_68249 [Guillardia theta CCMP2712]
c49972.graph_c3	0.2955726	0.1642304	0.2411848	1.8156601	2.7172854	1.1026861	8.97E-05	3.046697	up	ABC-2 type transporter domain-containing protein [Rozella allomycis CSF55]

#ID	28 h-1 FPKM	28 h-2 FPKM	28 h-3 FPKM	48 h-1 FPKM	48 h-2 FPKM	48 h-3 FPKM	FDR	log2FC	regulated	nr_annotation
c49978.graph_c0	0.024163	0.1527183	0.0788672	6.2788118	11.356458	10.192593	2.219E-27	6.799339	up	glutathione-independent formaldehyde dehydrogenase [Bathycoccus prasinos]
c49979.graph_c0	0	0.2603516	0.3697413	2.4018758	2.876387	1.5331905	2.035E-08	3.476395	up	-
c49982.graph_c0	0.045492	0.041075	0.1299238	1.6146046	2.474084	1.8471413	1.433E-20	4.81124	up	PREDICTED: glucose-6-phosphate isomerase, cytosolic 2-like [Cucumis sativus]
c49983.graph_c0	25.106364	23.298379	22.117928	10.84379	11.612547	11.641413	1.379E-06	-1.007532	down	PREDICTED: uncharacterized protein LOC104590989 [Nelumbo nucifera]
c49987.graph_c0	0.3086752	0.1045143	0.1889073	1.5161654	2.0725076	1.2620378	1.365E-10	3.0492	up	-
c49989.graph_c0	0.2926539	0.264239	0.5799505	1.8018072	2.9754766	1.6975453	4.817E-06	2.54488	up	threonyl-tRNA synthetase [Mucor circinelloides f. circinelloides 1006PhL]
c49993.graph_c0	0.0540618	0.1098286	0.3198261	0.5824817	0.3992797	0.4725573	0.0037829	1.634116	up	secreted protein [Achlya hypogyna]
c49995.graph_c0	0.3681654	0.4273956	0.8583426	1.2666939	1.6244157	1.2457384	0.0029252	1.36068	up	hypothetical protein GUITHDRAFT_99801 [Guillardia theta CCMP2712]
c49998.graph_c0	0.1633811	0.1475178	0.1333176	5.8815707	11.847317	4.9754024	9.978E-08	5.710121	up	predicted protein [Physcomitrella patens]
c50003.graph_c0	0.0191335	0.0691031	0.0936768	1.9402525	5.8580673	2.1148706	0.0001619	5.795651	up	-
c50012.graph_c0	0.1684845	0.3803142	1.9934883	5.2323787	10.18115	5.6058899	3.994E-05	3.078013	up	-
c50013.graph_c0	0.1160777	0.1048073	0.2367962	2.6484677	3.1174783	2.4220515	2.415E-15	4.20056	up	predicted protein [Micromonas pusilla CCMP1545]
c50016.graph_c0	0.1528512	0.5980447	0.4573263	2.2604188	4.720861	1.7814434	0.0035435	2.893121	up	-
c50016.graph_c1	0.6468668	0.4283107	1.055676	9.8394152	13.898223	10.749259	1.534E-31	4.052584	up	-
c50017.graph_c1	9.8616381	11.136735	9.3051042	18.510847	22.071013	18.439466	3.602E-08	1.002208	up	PREDICTED: RNA-binding protein 38 [Sesamum indicum]
c50018.graph_c0	58.202001	55.327226	60.037508	27.746945	24.085814	19.487021	8.898E-13	-1.236401	down	PREDICTED: uncharacterized protein LOC103433973 [Malus domestica]
c50019.graph_c0	0.2438241	0.3459504	0.2842264	2.0751505	1.730632	0.9428714	0.0003031	2.492851	up	protein of unknown function [Taphrina deformans PYCC 5710]
c50024.graph_c1	1.8037966	2.510227	2.6196822	5.8944134	7.3777445	6.9993064	2.4E-11	1.585913	up	hypothetical protein CICLE_v10007524mg [Citrus clementina]
c50027.graph_c0	0.2734313	0.2160225	0.7251325	4.2024559	8.6286039	4.3175654	4.624E-06	3.850733	up	-
c50029.graph_c0	0.6007573	0.9944504	1.5795754	7.7842643	13.938351	5.0065765	0.0009808	3.109827	up	-
c50037.graph_c1	0.2594386	0.4684975	0.352833	3.2885758	3.9483603	2.6335395	3.657E-11	3.233172	up	-
c50037.graph_c2	0.112502	0.0507894	0.7344054	3.9929263	4.1544852	5.9637659	7.937E-17	4.008694	up	-
c50041.graph_c0	0	0.0469703	0.1697955	2.7035695	5.2741429	3.3443984	1.47E-13	5.736921	up	phosphoric diester hydrolase, putative [Ricinus communis]
c50042.graph_c2	0.0869148	0.0261586	0.0354609	0.4039601	0.6419202	0.5718384	1.308E-12	3.479444	up	PREDICTED: uncharacterized protein LOC105170683 [Sesamum indicum]
c50051.graph_c0	2.287655	3.0693508	3.7334155	13.604534	20.125618	10.803076	5.034E-06	2.330933	up	hypothetical protein GUITHDRAFT_64866 [Guillardia theta CCMP2712]
c50053.graph_c0	0.1196366	0.1440276	0.0325408	2.2747239	4.6053844	0.9895285	0.0020099	4.770023	up	--
c50054.graph_c0	0.6251739	0.4939142	0.7970887	4.7547236	14.533997	7.2274876	0.0005839	3.817118	up	PREDICTED: nucleolar and coiled-body phosphoprotein 1-like [Tarenaya hassleriana]
c50055.graph_c0	0.1025806	0.0926207	0	5.4611955	7.3007177	10.065773	3.386E-18	6.906357	up	-

#ID	28 h-1 FPKM	28 h-2 FPKM	28 h-3 FPKM	48 h-1 FPKM	48 h-2 FPKM	48 h-3 FPKM	FDR	log2FC	regulated	nr_annotation
c50055.graph_c1	0	0.0347403	0.1098867	3.2676773	5.0633936	5.4679479	5.497E-31	6.606951	up	-
c50058.graph_c0	0.0894259	0.2220438	0.3466114	8.5865598	19.363664	11.019134	1.41E-10	5.918128	up	-
c50059.graph_c0	42.810414	35.059946	45.6871	20.405567	6.4139015	6.1852667	7.009E-07	-1.837619	down	hypothetical protein AMTR_s00028p00129850 [Amborella trichopoda]
c50063.graph_c0	12.118374	15.1203	15.338845	45.840889	43.950033	35.623065	5.784E-13	1.603236	up	-
c50064.graph_c0	0.2121932	0.3065449	0.4848142	2.7972933	5.043467	2.4411491	5.879E-06	3.3917	up	hypothetical protein VOLCADRAFT_93958 [Volvox carteri f. nagariensis]
c50070.graph_c1	0.1039988	0.1252015	0.4667419	4.5480015	9.7059392	3.6166914	3.763E-05	4.713766	up	probable SEC14-phosphatidylinositol(PI)/phosphatidylcholine(PC) transfer protein [Piriformospora indica DSM 11827]
c50072.graph_c0	0.1591382	0.2873738	0.3153634	2.3629991	3.8770793	1.6923224	1.332E-05	3.417833	up	hypothetical protein GUITHDRAFT_109518 [Guillardia theta CCMP2712]
c50076.graph_c0	0.583145	0.8424404	0.7137623	20.253511	31.63606	28.808016	4.268E-38	5.272215	up	PREDICTED: peroxidase 73 [Sesamum indicum]
c50077.graph_c0	0.2993941	0.7155656	0.6897971	3.8620119	8.1590173	3.8932567	0.0001012	3.25572	up	hypothetical protein VOLCADRAFT_120457 [Volvox carteri f. nagariensis]
c50078.graph_c0	0.1235882	0.2710008	0.4610146	0.8056652	1.0905893	0.6571364	0.0008387	1.61611	up	predicted protein [Hordeum vulgare subsp. vulgare]
c50079.graph_c2	3.1211377	3.5512576	3.0644698	0.6432963	1.2607646	2.1751243	0.0014616	-1.227653	down	PREDICTED: uncharacterized protein LOC105174831 [Sesamum indicum]
c50080.graph_c0	2.2467483	3.003893	5.182678	23.495331	36.958554	28.508188	1.109E-17	3.1265	up	carbonyl reductase (NADPH) [Galdieria sulphuraria]
c50082.graph_c0	0.3442842	0.2719993	0.4565162	8.128053	6.8769913	7.3293704	1.476E-26	4.423888	up	YALI0E30965p [Yarrowia lipolytica]
c50083.graph_c0	0.3003839	0.9221427	1.1275095	4.7975591	10.084191	3.659005	0.003371	3.013587	up	-
c50084.graph_c0	0.0228515	0	0.0186466	0.9848432	3.4981971	2.3711852	2.332E-07	7.39024	up	hypothetical protein PLEOSDRAFT_1111887 [Pleurotus ostreatus PC15]
c50087.graph_c0	0.4289496	0.9784452	1.1790125	3.519905	4.2139743	2.4190169	1.194E-05	2.015059	up	PREDICTED: mitochondrial-processing peptidase subunit alpha-like [Musa acuminata subsp. malaccensis]
c50088.graph_c0	0.2258927	0.3739266	0.445456	1.5032623	1.3397425	0.8704975	8.076E-05	1.876506	up	unknown [Picea sitchensis]
c50091.graph_c0	0.5281059	1.0573189	0.7869158	9.4882031	7.1532864	7.4325482	6.591E-21	3.391183	up	-
c50099.graph_c0	42.579653	42.880048	41.347556	100.41025	87.361306	83.986492	4.809E-12	1.145055	up	hypothetical protein, partial (mitochondrion) [Nicotiana tabacum]
c50101.graph_c0	0.4120535	0.4340533	0.5183581	1.3928218	2.1671785	1.2780533	1.748E-05	1.863172	up	predicted protein [Micromonas sp. RCC299]
c50102.graph_c0	0.9131871	1.0027974	1.5910028	2.7843334	4.374782	3.256916	9.92E-06	1.60548	up	protein disulfide isomerase [Rhizoctonia solani 123E]
c50106.graph_c0	0.1853374	0.2510135	0.1323296	1.9675291	2.5510059	1.0974509	5.749E-06	3.347574	up	saccharopepsin [Mucor circinelloides f. circinelloides 1006PhL]
c50108.graph_c1	24.125978	21.344004	24.176527	105.47919	105.51871	94.570833	2.716E-49	2.176241	up	PREDICTED: cationic amino acid transporter 7, chloroplastic [Sesamum indicum]
c50108.graph_c2	3.5794578	3.8475176	4.0334972	7.562044	10.528833	9.6123733	0.0024223	1.309935	up	-
c50109.graph_c0	89.262643	91.063343	85.762269	239.41682	207.44227	174.28884	3.861E-07	1.269703	up	PREDICTED: pentatricopeptide repeat-containing protein At5g66520-like [Sesamum indicum]
c50110.graph_c2	1.9406985	2.7314778	2.0726452	14.796046	14.179971	11.716913	1.115E-27	2.637993	up	PREDICTED: uncharacterized protein LOC105163215 [Sesamum indicum]
c50110.graph_c4	1.0145023	1.4588155	1.195748	3.6673569	4.5410493	4.1107745	3.919E-06	1.786585	up	PREDICTED: uncharacterized protein LOC105159914 [Sesamum indicum]

#ID	28 h-1 FPKM	28 h-2 FPKM	28 h-3 FPKM	48 h-1 FPKM	48 h-2 FPKM	48 h-3 FPKM	FDR	log2FC	regulated	nr_annotation
c50112.graph_c0	0.1692594	0.0382064	0.0863215	1.8773021	2.6138172	1.6465491	9.957E-19	4.423275	up	-
c50114.graph_c0	0.1563399	0.1693923	0.4847739	2.3384336	5.8783078	2.3981227	0.0009151	3.739922	up	-
c50115.graph_c0	0.1189688	0.3759617	0.3276367	2.0169723	4.4132618	1.274733	0.0077576	3.262005	up	-
c50116.graph_c0	0.8152906	0.557675	0.7862288	16.503738	23.090439	16.802677	4.191E-40	4.744647	up	hypothetical protein O988_02039 [Pseudogymnoascus pannorum VKM F-3808]
c50124.graph_c0	0.0768371	0.41626	0.5486112	5.7707514	13.310212	5.1781114	6.952E-05	4.573089	up	Formate/nitrite transporter [Trametes versicolor FP-101664 SS1]
c50125.graph_c0	0.4096507	0.2641973	0.6685433	1.8174186	2.750478	1.4851176	3.957E-05	2.209746	up	PREDICTED: 26S proteasome non-ATPase regulatory subunit 1 homolog A-like [Brachypodium distachyon]
c50130.graph_c0	0.2352844	0.1062199	0.447977	2.7338691	4.6339246	3.8036711	1.123E-16	3.854553	up	--
c50131.graph_c1	0.4936243	0.6077679	1.2449978	2.2184092	3.6758531	2.2269948	0.0003298	1.825949	up	hypothetical protein GUITHDRAFT_147173 [Guillardia theta CCMP2712]
c50134.graph_c0	0.1307311	0.0737737	0.3066921	1.6363989	2.6990968	2.1011468	6.815E-17	3.686785	up	-
c50135.graph_c0	0.2631179	0.2850849	0.6011658	2.4902925	2.8972682	1.187061	0.0005588	2.559847	up	hypothetical protein ZEAMMB73_452342 [Zea mays]
c50137.graph_c0	0.3048628	0.3670167	0.3938788	1.2881202	3.0021357	1.461356	0.0026319	2.463307	up	Karyopherin beta [Blumeria graminis f. sp. tritici 96224]
c50137.graph_c1	0.1226193	0.4059503	0.4669295	1.0880028	2.0856699	1.1985979	0.0005864	2.168313	up	-
c50139.graph_c0	1.7133638	1.2155054	1.4313787	10.910784	12.325577	11.778851	3.388E-23	3.045436	up	PREDICTED: putative glucose-6-phosphate 1-epimerase [Sesamum indicum]
c50143.graph_c0	0.2004302	0.0723879	0.0981297	3.7600892	12.703684	6.194075	1.58E-05	5.958514	up	hypothetical protein CHLNCDRAFT_33886 [Chlorella variabilis]
c50144.graph_c0	3.2430838	4.0478067	5.0202434	13.11844	24.720714	12.748535	0.0008688	2.073046	up	molecular chaperone DnaK [Galdieria sulphuraria]
c50145.graph_c2	2.9148281	3.6081355	3.0690012	12.5741	14.01518	15.218289	1.694E-14	2.163479	up	PREDICTED: subtilisin-like protease [Sesamum indicum]
c50147.graph_c0	0.0600788	0.0904091	0.0490238	2.436936	1.9093366	0.4743312	0.0014342	4.651962	up	predicted protein [Thalassiosira pseudonana CCMP1335]
c50150.graph_c0	0.3698816	0.2652102	0.5326242	1.1583406	1.0956441	0.7852835	0.0004791	1.424891	up	Eukaryotic translation initiation factor 3 subunit, putative [Ricinus communis]
c50152.graph_c0	0.0429149	0.2324888	0.1284001	0.9247627	1.4599062	0.6615055	5.465E-05	2.954942	up	predicted protein [Hordeum vulgare subsp. vulgare]
c50153.graph_c1	0.5455261	0.9577534	0.9521153	4.2257995	6.6336883	3.6404517	1.143E-06	2.599606	up	hypothetical protein EMIHUDRAFT_422478 [Emiliania huxleyi CCMP1516]
c50156.graph_c1	57.78576	61.37613	51.163623	6.3445887	8.5727046	8.1131164	5.878E-73	-2.849467	down	hypothetical protein VITISV_035070 [Vitis vinifera]
c50158.graph_c0	0.0263972	0.0476683	0.0323098	3.2456526	3.7751235	5.6419467	3.729E-24	6.931781	up	-
c50162.graph_c0	10.105065	9.5001704	8.7071153	2.9239789	3.5872219	3.3151188	2.122E-21	-1.487702	down	PREDICTED: uncharacterized protein LOC105111104, partial [Populus euphratica]
c50164.graph_c1	0.103267	0.1864808	0.6741202	3.4033616	10.885682	3.901836	0.0035379	4.263218	up	-
c50165.graph_c0	0.0326444	0.2652732	0.1997815	1.0344784	1.3370034	0.5154647	0.0013419	2.580006	up	predicted protein [Hordeum vulgare subsp. vulgare]
c50166.graph_c1	1.1228444	1.1739005	1.0126773	1.6480176	2.241862	2.6828384	0.0021221	1.025185	up	PREDICTED: putative late blight resistance protein homolog R1A-10 [Sesamum indicum]
c50167.graph_c0	0.0137034	0.0371186	0.0782729	2.7965777	5.0051969	2.0710724	2.885E-09	6.291814	up	ATP-dependent Clp protease ATP-binding subunit ClpB [Galdieria sulphuraria]

#ID	28 h-1 FPKM	28 h-2 FPKM	28 h-3 FPKM	48 h-1 FPKM	48 h-2 FPKM	48 h-3 FPKM	FDR	log2FC	regulated	nr_annotation
c50170.graph_c0	0.114655	0.4658522	0.3976196	2.1436716	2.9253046	1.5841335	1.857E-07	2.80656	up	hypothetical protein CHLNCDRAFT_33886 [Chlorella variabilis]
c50175.graph_c0	3.3428033	3.7061155	3.8441543	1.1036557	1.2944596	1.4730393	1.556E-06	-1.454635	down	Putative gag-pol polyprotein, identical [Solanum demissum]
c50176.graph_c1	0.189579	0.085586	0.1325957	0.8753982	1.6183659	0.4734636	0.0069075	2.900106	up	predicted protein [Hordeum vulgare subsp. vulgare]
c50179.graph_c0	2.5770212	3.9906018	5.1214044	16.894811	31.833143	14.407832	0.0009046	2.468005	up	EF2, translation elongation factor 2 [Ectocarpus siliculosus]
c50185.graph_c0	278.57514	215.26942	252.26124	82.875119	40.691523	40.474528	3.912E-17	-2.128203	down	BnaCnng12640D [Brassica napus]
c50187.graph_c1	0.0767911	0.1040028	0.1566523	5.9863108	8.8166077	3.4644445	6.356E-09	5.798177	up	hypothetical protein GUITHDRAFT_163621 [Guillardia theta CCMP2712]
c50199.graph_c0	40.940108	79.290653	69.841394	3.9195319	1.8269983	1.6740364	4.337E-13	-4.619398	down	defensin-like protein 4 [Arabidopsis thaliana]
c50200.graph_c0	136.19936	128.7141	142.0611	48.218809	55.445768	48.456558	6.899E-22	-1.379366	down	PREDICTED: uncharacterized protein LOC105159431 [Sesamum indicum]
c50201.graph_c0	2.1129748	2.4801635	2.4138382	0.4463919	1.0403757	1.1915904	0.0033258	-1.35996	down	-
c50205.graph_c0	0.5667617	0.5629059	0.4624729	1.652348	1.5221412	1.4702493	0.0003068	1.589049	up	PREDICTED: protein FLX-like 4 [Sesamum indicum]
c50207.graph_c0	2.6795275	3.0058741	2.8159116	6.2783759	10.303874	11.905573	1.184E-05	1.776961	up	hypothetical protein MIMGU_mgv1a010254mg [Erythranthe guttata]
c50212.graph_c0	3900.9833	4367.392	4538.9984	1696.6379	1475.104	1677.2971	1.183E-07	-1.357517	down	hypothetical protein MIMGU_mgv1a016953mg [Erythranthe guttata]
c50216.graph_c0	12.219704	15.576348	18.476007	45.89866	39.936887	34.962584	2.022E-08	1.430164	up	-
c50226.graph_c0	7.0901977	8.9129807	8.9819763	39.425161	31.193128	35.168635	4.184E-20	2.127754	up	histone H4 [Zea mays]
c50229.graph_c0	6.7923066	6.2404093	4.5701041	8.7607971	11.20121	15.859413	0.0040054	1.060232	up	PREDICTED: homeobox-leucine zipper protein ATHB-13 [Sesamum indicum]
c50230.graph_c0	28.24324	26.212181	21.148687	104.07311	69.103193	59.733356	0.0009191	1.675644	up	PREDICTED: zinc finger protein CONSTANS-LIKE 4-like [Sesamum indicum]
c50232.graph_c0	0.8230203	0.5944881	0.6044201	2.3994494	3.0392589	2.7383896	1.362E-06	2.054325	up	PREDICTED: putative late blight resistance protein homolog R1A-4 [Sesamum indicum]
c50235.graph_c0	0.4894218	0	0.2995231	4.4977414	6.4078501	6.900109	8.747E-21	4.529588	up	PREDICTED: dirigent protein 22-like [Sesamum indicum]
c50239.graph_c0	3283.0768	4019.6129	3655.1871	691.99444	602.4768	613.48698	6.225E-32	-2.476948	down	hypothetical protein MIMGU_mgv1a015957mg [Erythranthe guttata]
c50240.graph_c0	320.32101	343.09762	310.24407	1298.9207	1529.586	1927.0961	1.079E-17	2.325391	up	hypothetical protein MIMGU_mgv1a027056mg [Erythranthe guttata]
c50246.graph_c0	3.2772781	3.6988436	1.6045393	11.216317	14.962931	26.798715	0.0004295	2.6596	up	-
c50255.graph_c0	1042.1833	1311.2389	1255.4256	187.37416	145.37308	119.03521	3.157E-49	-2.948638	down	oleosin, partial [Genlisea aurea]
c50256.graph_c0	3.8000062	5.1962993	4.2242428	10.82032	12.01747	11.336024	1.122E-09	1.411031	up	PREDICTED: probable indole-3-pyruvate monooxygenase YUCCA5 [Sesamum indicum]
c50257.graph_c0	44.359523	44.408581	43.453434	121.05259	134.29888	143.15502	4.284E-29	1.631276	up	PREDICTED: 5-methyltetrahydropteroyltriglutamate--homocysteine methyltransferase [Sesamum indicum]
c50260.graph_c0	21.08055	21.468311	26.535735	190.3623	132.32259	117.40709	1.05E-07	2.721802	up	hypothetical protein POPTR_0001s05450g [Populus trichocarpa]
c50265.graph_c0	5.0773708	1.4586695	2.6365138	11.116626	40.28877	35.401183	0.0008322	3.265448	up	-
c50266.graph_c0	2.8497496	4.4575489	3.8319517	1.2719252	1.0779604	0.6337808	4.4E-06	-1.850424	down	-

#ID	28 h-1 FPKM	28 h-2 FPKM	28 h-3 FPKM	48 h-1 FPKM	48 h-2 FPKM	48 h-3 FPKM	FDR	log2FC	regulated	nr_annotation
c50270.graph_c0	6.6901338	5.3222263	5.577129	1.0084607	1.1654638	0.9381096	3.009E-11	-2.457786	down	hypothetical protein MIMGU_mgv1a025449mg [Erythranthe guttata]
c50274.graph_c0	10.178188	10.940417	10.606359	4.2446834	2.7808709	3.0811502	8.801E-08	-1.600321	down	PREDICTED: zinc finger protein MAGPIE-like [Sesamum indicum]
c50277.graph_c0	34.319019	38.899809	49.307541	176.66806	113.72901	96.353968	0.0024764	1.710775	up	PREDICTED: serine carboxypeptidase-like 50 [Sesamum indicum]
c50278.graph_c0	1.0785469	1.2029624	1.0612795	5.0664511	3.4929987	4.1503122	8.499E-08	1.975148	up	PREDICTED: probable carboxylesterase 6 [Sesamum indicum]
c50293.graph_c0	1.4169042	0.767599	1.1561823	2.9454899	2.1309976	2.9404986	0.0023802	1.307933	up	PREDICTED: protein PROTON GRADIENT REGULATION 5, chloroplastic [Sesamum indicum]
c50295.graph_c0	32.001808	32.99935	32.042724	142.15777	121.42128	96.251147	5.686E-09	1.938017	up	PREDICTED: transcription factor MYB1R1-like [Sesamum indicum]
c50297.graph_c0	358.93888	423.16367	384.38676	73.023882	86.402824	84.328613	3.206E-48	-2.219412	down	PREDICTED: uncharacterized protein LOC100246645 [Vitis vinifera]
c50303.graph_c0	10.12994	10.581835	9.2305862	15.036497	22.443408	25.379451	0.0001142	1.104088	up	PREDICTED: probable inactive receptor kinase At1g48480 [Sesamum indicum]
c50305.graph_c0	1.0571827	1.1335126	1.9050242	2.484689	3.9039767	4.8440221	0.0009127	1.486982	up	PREDICTED: DELLA protein GAI1 [Sesamum indicum]
c50306.graph_c0	0.2601444	0.352329	0.594372	4.4516546	4.9255877	6.8951706	3.453E-19	3.78932	up	PREDICTED: microtubule-associated protein RP/EB family member 1C [Sesamum indicum]
c50309.graph_c0	6.8625853	6.8958501	6.141731	10.87354	14.045989	18.538953	0.0001663	1.16217	up	PREDICTED: uncharacterized protein LOC105167450 [Sesamum indicum]
c50312.graph_c0	1.1933794	0.6687991	0.9066301	9.8586071	9.5598508	12.392373	6.391E-27	3.562198	up	-
c50314.graph_c0	0.3170754	0.2862893	0.8336885	6.8325785	8.6102516	9.2981923	1.187E-29	4.140911	up	PREDICTED: syntaxin-112-like [Sesamum indicum]
c50329.graph_c0	1.6444382	1.8106994	1.9800442	0.3558811	0.7270963	0.7011776	0.000125	-1.577129	down	PREDICTED: pentatricopeptide repeat-containing protein At2g17525, mitochondrial [Sesamum indicum]
c50349.graph_c0	6.3020433	8.3094316	8.4074418	23.711315	28.611781	33.057585	7.763E-12	1.928416	up	PREDICTED: RNA-binding protein 38-like isoform X1 [Nelumbo nucifera]
c50353.graph_c0	0.1325451	0.0897569	0.1622336	8.9465689	19.311584	18.499993	2.813E-17	6.954415	up	hypothetical protein MIMGU_mgv1a009974mg [Erythranthe guttata]
c50372.graph_c0	1.7045459	1.3496242	1.0913181	4.321275	4.68351	4.1999717	3.268E-07	1.713262	up	PREDICTED: cytochrome P450 CYP82D47-like [Sesamum indicum]
c50375.graph_c0	4.178805	5.609958	5.0699394	0.6969662	1.3290321	3.1007817	0.0005352	-1.509248	down	-
c50378.graph_c0	183.74221	191.36264	199.14526	59.784921	62.795854	62.892319	2.841E-27	-1.589379	down	hypothetical protein MIMGU_mgv1a003144mg [Erythranthe guttata]
c50379.graph_c0	7.5022577	7.724549	8.0818249	2.0437436	4.2418257	2.3380853	2.017E-06	-1.401858	down	PREDICTED: uncharacterized protein LOC105165869 [Sesamum indicum]
c50389.graph_c0	3.2530014	4.0385885	3.1521268	1.6107105	1.5698443	0.9172483	0.0018263	-1.302449	down	PREDICTED: uncharacterized protein At1g08160-like [Sesamum indicum]
c50398.graph_c0	1.9478475	2.7553338	1.8013513	0.8230134	0.8718829	0.8055436	0.0033354	-1.336881	down	-
c50401.graph_c0	0.4694809	0.6023803	0.2217905	1.2215245	1.1613332	0.6967341	0.0082918	1.299975	up	PREDICTED: cytochrome P450 71A21-like [Sesamum indicum]
c50423.graph_c0	2.6609918	2.7843517	3.1251171	0.8511316	1.0018564	1.9354035	0.0036267	-1.144628	down	PREDICTED: pentatricopeptide repeat-containing protein At3g20730 [Sesamum indicum]
c50427.graph_c0	4.5755351	3.2214141	3.6224802	10.529449	10.935238	11.365721	2.632E-11	1.564474	up	PREDICTED: geraniol 8-hydroxylase-like [Sesamum indicum]
c50430.graph_c0	0.7253521	0.9005218	0.8878221	1.953804	2.5568346	2.2498016	0.0008117	1.465331	up	PREDICTED: uncharacterized protein LOC104084947 [Nicotiana tomentosiformis]

#ID	28 h-1 FPKM	28 h-2 FPKM	28 h-3 FPKM	48 h-1 FPKM	48 h-2 FPKM	48 h-3 FPKM	FDR	log2FC	regulated	nr_annotation
c50436.graph_c0	1.4255184	2.6546634	1.9629193	7.4536672	8.6140981	9.8478541	1.07E-07	2.139224	up	-
c50439.graph_c0	9.5245554	8.4399333	9.9099706	2.9172859	2.7576865	2.3162375	1.765E-09	-1.757961	down	PREDICTED: putative clathrin assembly protein At4g40080 [Sesamum indicum]
c50443.graph_c0	28.605785	27.345032	28.179826	54.483848	54.667219	63.078943	2.534E-12	1.0744	up	hypothetical protein MIMGU_mgv1a017588mg [Erythranthe guttata]
c50446.graph_c0	1.0725259	0.9996287	0.7904783	4.0346617	12.54395	13.072347	0.0002317	3.397608	up	PREDICTED: short-chain dehydrogenase reductase 2a [Sesamum indicum]
c50451.graph_c0	0.6141723	0.6992026	0.6754759	1.69241	2.8686451	3.1925001	2.658E-06	1.994997	up	PREDICTED: premnaspirodiene oxygenase-like [Sesamum indicum]
c50454.graph_c0	21.488192	23.055597	23.267997	4.4137272	4.9706796	4.3872629	1.645E-30	-2.259016	down	PREDICTED: LOW QUALITY PROTEIN: putative ETHYLENE INSENSITIVE 3-like 4 protein [Sesamum indicum]
c50456.graph_c0	0.8604348	0.7360029	0.5542956	2.9275732	3.1014088	6.0271092	0.0002809	2.522301	up	PREDICTED: haloacid dehalogenase-like hydrolase domain-containing protein Sgpp [Sesamum indicum]
c50460.graph_c0	2.7152389	3.5769335	2.1429697	1.0438141	1.0759081	0.5522472	4.657E-05	-1.611166	down	unnamed protein product [Coffea canephora]
c50463.graph_c0	1.7235822	1.8526585	1.5180504	7.733383	5.9148234	4.7211256	1.03E-05	1.900353	up	PREDICTED: putative E3 ubiquitin-protein ligase XBAT31 [Sesamum indicum]
c50472.graph_c0	0.3433444	0.1377812	0.2801662	35.445785	41.39265	38.20877	1.3E-164	7.278487	up	PREDICTED: 1-aminocyclopropane-1-carboxylate oxidase homolog 1-like [Sesamum indicum]
c50477.graph_c0	4.3868254	6.0880378	2.6515657	0.205949	0	0.1832522	1.323E-07	-5.013317	down	--
c50482.graph_c0	287.13434	236.65841	283.59505	1003.0008	905.32091	867.86486	7.732E-29	1.826069	up	PREDICTED: putative lipid-binding protein AIR1B [Beta vulgaris subsp. vulgaris]
c50491.graph_c0	16.63514	19.00254	16.110719	29.765712	35.425449	38.56722	4.328E-10	1.042031	up	PREDICTED: adenine phosphoribosyltransferase 1-like [Sesamum indicum]
c50495.graph_c0	1.3402667	1.9286529	1.4695877	11.255125	14.735572	17.573038	1.334E-21	3.23678	up	PREDICTED: glycerophosphodiester phosphodiesterase GDPDL3-like [Sesamum indicum]
c50500.graph_c0	0.0592923	0	0.0645093	2.5553489	2.335522	2.095402	7.578E-28	5.860804	up	PREDICTED: uncharacterized protein LOC105175608 [Sesamum indicum]
c50510.graph_c0	1.1769548	1.1192053	1.5529637	1.9680078	2.5724551	3.219804	0.0004917	1.046345	up	PREDICTED: probable RNA-dependent RNA polymerase 5 [Sesamum indicum]
c50511.graph_c0	0.6559054	0.3230297	0.5352134	2.4186425	4.2437406	3.2953933	1.199E-09	2.750392	up	hypothetical protein MIMGU_mgv1a014011mg [Erythranthe guttata]
c50514.graph_c0	0.8992081	0.5535686	0.4669295	4.5592498	5.0495165	6.9610881	1.045E-15	3.147295	up	PREDICTED: auxin-induced protein 6B-like [Sesamum indicum]
c50516.graph_c0	22.30658	25.191922	26.8696	49.459216	48.497244	54.191972	8.258E-13	1.074139	up	ferritin [Avicennia marina]
c50517.graph_c0	1.7680457	1.8829092	1.4427109	0.1149296	0.6696472	0.9715053	0.0014093	-1.517137	down	-
c50523.graph_c0	0.732406	0.9919409	1.0085129	3.3653722	3.6881418	4.2852155	4.515E-07	2.091795	up	PREDICTED: RAN GTPase-activating protein 2-like isoform X2 [Sesamum indicum]
c50524.graph_c0	0.8676912	1.333972	1.0142037	0.2378081	0.4093844	0.2909503	0.000107	-1.742319	down	PREDICTED: pentatricopeptide repeat-containing protein At5g46460, mitochondrial [Sesamum indicum]
c50528.graph_c0	4.5605744	4.5512207	4.7659926	44.624213	55.439442	74.990868	5.862E-17	3.692444	up	hypothetical protein CICLE_v10032302mg [Citrus clementina]
c50533.graph_c0	0.8741685	0.7498276	0.9808072	2.4377605	2.4651248	2.9085721	1.287E-05	1.624439	up	hypothetical protein MIMGU_mgv1a002430mg [Erythranthe guttata]

#ID	28 h-1 FPKM	28 h-2 FPKM	28 h-3 FPKM	48 h-1 FPKM	48 h-2 FPKM	48 h-3 FPKM	FDR	log2FC	regulated	nr_annotation
c50536.graph_c0	1.4288758	1.3175907	2.4559393	0.1541451	0.2449471	0.2057361	8.69E-06	-3.070803	down	PREDICTED: mitogen-activated protein kinase kinase kinase 2-like [Sesamum indicum]
c50538.graph_c0	1.543287	2.1015869	2.4360416	68.500119	117.9223	135.65591	1.126E-19	5.758238	up	PREDICTED: beta-glucosidase 12-like [Sesamum indicum]
c50540.graph_c0	3.6335696	3.9939841	3.1798155	0.6675097	2.1214368	1.4254707	0.001957	-1.33236	down	-
c50542.graph_c0	1.4037581	1.0139696	1.099637	4.341655	4.599457	4.1165017	3.227E-06	1.93417	up	hypothetical protein L484_017688 [Morus notabilis]
c50555.graph_c0	0.0984059	0.3554052	0.3211936	3.9915748	2.7750119	4.3286091	5.752E-14	3.884702	up	hypothetical protein MIMGU_mgv1a016013mg [Erythranthe guttata]
c50565.graph_c0	0	0.1067748	0.0964966	6.0709193	18.738408	17.606087	4.428E-10	7.730125	up	PREDICTED: pathogenesis-related protein PR-1-like [Sesamum indicum]
c50569.graph_c0	0	0.165236	0.0497768	4.3301405	10.157502	10.664368	9.342E-12	6.900409	up	unnamed protein product [Coffea canephora]
c50572.graph_c0	0.4185125	0.6870501	0.6519598	2.1219816	2.4523442	2.7892777	1.015E-06	2.105205	up	PREDICTED: glyoxylate/succinic semialdehyde reductase 1 [Sesamum indicum]
c50577.graph_c0	0.5950799	0.9919409	1.2699793	2.3209463	4.179894	4.8531356	0.000107	2.020922	up	PREDICTED: uncharacterized protein LOC105168308 [Sesamum indicum]
c50578.graph_c0	8.8249637	8.4219947	7.7935937	3.5399656	3.6001578	3.1498401	0.0003868	-1.239964	down	PREDICTED: stachyose synthase [Sesamum indicum]
c50581.graph_c0	7.6150427	5.5701627	7.9442415	31.157215	33.654493	41.204735	1.872E-19	2.365312	up	-
c50587.graph_c0	1.1046606	0.2992215	0.5408364	8.8215039	10.087005	12.833017	1.5E-18	4.06578	up	PREDICTED: 7-deoxyloganetin glucosyltransferase-like [Eucalyptus grandis]
c50590.graph_c0	114.47406	120.87986	103.22909	259.17377	303.59343	287.63247	3.427E-18	1.368423	up	PREDICTED: uncharacterized protein At1g01500 [Sesamum indicum]
c50592.graph_c0	14.278536	16.006582	15.103973	29.105991	31.938377	31.982748	4.339E-13	1.075717	up	PREDICTED: dihydrolipoyl dehydrogenase 2, chloroplastic-like isoform X1 [Sesamum indicum]
c50596.graph_c0	2.0829777	2.790766	1.8641792	0.4258593	0.9925218	0.5304979	0.0001205	-1.757986	down	-
c50601.graph_c0	1.7614694	2.3588574	2.147942	12.317989	10.046189	12.634679	5.715E-29	2.52538	up	PREDICTED: protein CYPRO4 [Sesamum indicum]
c50603.graph_c0	0.7697799	0.843976	0.4486672	1.6727189	1.6982077	1.5503908	0.0031023	1.298296	up	PREDICTED: L-type lectin-domain containing receptor kinase IX.1-like [Nicotiana sylvestris]
c50608.graph_c0	10.062266	14.78161	12.707077	5.1626016	4.9329592	6.9355136	0.0014745	-1.100549	down	PREDICTED: palmitoyl-monogalactosyldiacylglycerol delta-7 desaturase, chloroplastic [Sesamum indicum]
c50620.graph_c0	0.3727936	0.5289392	0.9125897	2.9702727	4.4339071	4.5049956	4.593E-10	2.748126	up	PREDICTED: zinc finger protein ZAT5 [Sesamum indicum]
c50627.graph_c0	0.0469941	0.0424312	0.0766935	6.4929591	8.5192467	8.5335849	1.423E-38	7.183068	up	hypothetical protein MIMGU_mgv1a009322mg [Erythranthe guttata]
c50628.graph_c0	1.8805164	1.8919791	1.7536983	0.4767386	1.2265421	0.484799	0.0034952	-1.305931	down	-
c50630.graph_c0	211.5804	212.36205	202.32798	1587.4866	1130.546	944.98423	4.206E-07	2.599082	up	hypothetical protein AMTR_s00033p00173430 [Amborella trichopoda]
c50634.graph_c0	3.3007868	3.4609947	3.6925852	1.3496743	1.3583255	1.291002	0.0002308	-1.344064	down	PREDICTED: anthocyanidin 3-O-glucosyltransferase-like [Sesamum indicum]
c50639.graph_c0	17.05187	16.911931	20.258479	6.3835572	6.7626048	5.0821507	1.1E-06	-1.529725	down	-
c50650.graph_c0	0.7860249	0.7514542	1.0564069	2.2271228	2.607721	2.1902784	0.0001778	1.477274	up	PREDICTED: asparagine--tRNA ligase, cytoplasmic 2 [Sesamum indicum]
c50653.graph_c0	0.903917	0.6872862	0.8928708	2.4724756	2.8748284	2.0926771	0.0002123	1.623855	up	unnamed protein product [Coffea canephora]

#ID	28 h-1 FPKM	28 h-2 FPKM	28 h-3 FPKM	48 h-1 FPKM	48 h-2 FPKM	48 h-3 FPKM	FDR	log2FC	regulated	nr_annotation
c50654.graph_c0	7.804128	8.1367473	7.7353381	1.4924564	2.574317	3.0475409	9.798E-15	-1.703198	down	PREDICTED: pentatricopeptide repeat-containing protein At1g15510, chloroplastic [Sesamum indicum]
c50684.graph_c0	11.511938	12.150218	11.846255	2.9881698	5.5134263	6.3147819	5.644E-10	-1.230343	down	PREDICTED: leucine-rich repeat receptor-like protein CLAVATA2 [Sesamum indicum]
c50686.graph_c0	0.2091227	0.314697	0.3697253	8.7034098	11.466755	11.911194	1.508E-46	5.202353	up	PREDICTED: PI-PLC X-box domain-containing protein DDB_G0293730 [Sesamum indicum]
c50691.graph_c0	0.5880363	0.4344068	0.3053481	3.1848023	1.8664193	1.688232	0.0005673	2.398524	up	Leucine-rich repeat transmembrane protein kinase [Theobroma cacao]
c50695.graph_c0	0.8799273	1.0262187	1.166772	0.1858955	0.344634	0.5375781	0.0012541	-1.496222	down	-
c50696.graph_c0	3.5917603	2.6592787	2.989464	15.843816	18.713867	26.007886	2.915E-12	2.748506	up	PREDICTED: (RS)-norcoclaurine 6-O-methyltransferase-like [Sesamum indicum]
c50700.graph_c0	29.308894	38.576612	27.705296	113.28494	142.73904	108.2497	3.346E-20	1.97043	up	-
c50706.graph_c0	1.027825	0.9280296	1.2056267	5.9442254	4.6581924	4.9993203	2.932E-08	2.349049	up	PREDICTED: uncharacterized protein LOC105155422 [Sesamum indicum]
c50708.graph_c0	3.0111048	4.1788123	4.5273181	6.1139435	10.632733	13.081517	0.0033007	1.378842	up	PREDICTED: UPF0496 protein 1-like [Sesamum indicum]
c50709.graph_c0	0.3824305	0.1726494	0.6241203	13.815625	8.2166905	4.9603577	0.0001395	4.572261	up	-
c50710.graph_c0	3.7279642	3.5726865	3.682407	0.6632238	1.8882513	1.5490978	0.0003372	-1.394943	down	PREDICTED: multiple C2 and transmembrane domain-containing protein 1-like [Sesamum indicum]
c50711.graph_c0	1.94236	1.7147963	1.1622966	3.994048	3.4197447	2.7749473	0.0057842	1.127761	up	-
c50713.graph_c0	1.4528208	2.01449	1.8205738	0.1315398	0.1393505	0.4096517	7.325E-09	-2.926658	down	-
c50732.graph_c0	6.828738	8.6457723	7.5333578	14.700571	19.261926	17.813548	3.002E-08	1.208014	up	PREDICTED: heat stress transcription factor C-1-like [Sesamum indicum]
c50735.graph_c0	7.2772061	6.0074375	7.0409383	27.541407	23.034301	13.71877	0.0015781	1.71119	up	PREDICTED: uncharacterized protein LOC104450130 [Eucalyptus grandis]
c50746.graph_c0	4.4087976	4.3426155	3.4885264	7.3496923	8.1807953	9.2520349	2.773E-05	1.057119	up	hypothetical protein MIMGU_mgv1a005428mg [Erythranthe guttata]
c50748.graph_c0	5.4377053	4.3865692	3.8188354	9.8305606	11.31207	11.009374	7.501E-06	1.276632	up	hypothetical protein MIMGU_mgv1a006779mg [Erythranthe guttata]
c50751.graph_c0	5.5693423	4.7076198	5.2213839	0.6008129	0.7956105	1.2028493	1.371E-07	-2.542472	down	-
c50753.graph_c0	1.6984559	1.4289864	1.4174244	3.6697464	2.9027797	3.918382	0.0013522	1.250832	up	PREDICTED: probable WRKY transcription factor 14 [Sesamum indicum]
c50754.graph_c0	0.6792515	0.7199614	0.45787	9.6956236	11.064491	14.756018	5.472E-28	4.295241	up	PREDICTED: chaperonin CPN60, mitochondrial [Sesamum indicum]
c50763.graph_c0	26.024935	29.031686	28.058846	55.822462	56.286085	65.001194	2.912E-15	1.132212	up	PREDICTED: dihydrolipoyllysine-residue acetyltransferase component 5 of pyruvate dehydrogenase complex, chloroplastic [Sesamum indicum]
c50764.graph_c0	0.4500795	0.8127592	1.0099684	6.9887346	8.612487	6.7261695	1.393E-12	3.335776	up	PREDICTED: uncharacterized protein LOC100267736 [Vitis vinifera]
c50767.graph_c0	3.9443212	3.6281276	3.7817773	1.0936882	1.6551858	1.5570515	0.0001048	-1.363911	down	PREDICTED: uncharacterized protein LOC102659506 [Glycine max]
c50769.graph_c0	2.0388226	1.8950088	2.1529751	0.3040421	0.4831436	0.6087034	8.892E-06	-2.092543	down	-
c50770.graph_c0	8.7868418	10.767157	12.97427	21.215179	23.130428	24.304317	1.087E-05	1.117233	up	cyclin D [Scutellaria baicalensis]
c50772.graph_c0	2.2687535	2.2848338	1.9936908	32.297582	48.862364	69.581861	6.821E-11	4.557298	up	PREDICTED: uncharacterized protein LOC105158508 [Sesamum indicum]

#ID	28 h-1 FPKM	28 h-2 FPKM	28 h-3 FPKM	48 h-1 FPKM	48 h-2 FPKM	48 h-3 FPKM	FDR	log2FC	regulated	nr_annotation
c50775.graph_c0	1.0122472	1.0752521	0.6316359	3.8492936	5.0373562	5.1040382	1.144E-08	2.401056	up	kunitz trypsin inhibitor [Populus tremula]
c50777.graph_c0	4.0922528	4.452853	3.8957857	1.2635565	1.9726514	1.4201747	0.0008929	-1.381413	down	hypothetical protein CICLE_v10023373mg [Citrus clementina]
c50781.graph_c0	0.4723926	0.4653013	0.1752129	2.9939618	3.1140715	4.7467801	1.931E-12	3.324862	up	PREDICTED: glutaminyl-peptide cyclotransferase [Sesamum indicum]
c50792.graph_c0	0.4475532	0.5317086	0.6535153	11.137125	8.8567327	12.300838	3.787E-37	4.349057	up	PREDICTED: cell division cycle 20.2, cofactor of APC complex-like [Sesamum indicum]
c50794.graph_c0	3.8818556	2.9207931	5.9493321	0.1261676	0.2339038	0.280658	1.603E-08	-4.28583	down	PREDICTED: cytochrome P450 86B1-like [Sesamum indicum]
c50795.graph_c0	8.8879849	8.3897906	8.6011328	52.699631	54.004074	66.650998	5.223E-48	2.783647	up	PREDICTED: caffeoylshikimate esterase [Sesamum indicum]
c50796.graph_c0	2.5357558	2.4474497	4.0669618	5.4309864	10.919854	10.552513	0.0009153	1.601198	up	PREDICTED: glucomannan 4-beta-mannosyltransferase 2-like [Sesamum indicum]
c50807.graph_c0	0.3860197	0.1161799	0.8924687	4.2406732	3.8877222	4.498965	4.932E-12	3.218301	up	PREDICTED: uncharacterized protein LOC105159678 [Sesamum indicum]
c50809.graph_c0	0.0899949	0.4062847	0.3304579	7.5289584	4.3505555	3.5526177	5.125E-06	4.278326	up	-
c50814.graph_c0	9.315657	10.490329	12.341761	3.6486263	3.5841661	2.3020819	6.245E-08	-1.707721	down	-
c50815.graph_c0	0.0574447	0	0.0468744	5.9708608	1.6970593	2.5268366	0.0003631	6.673278	up	PREDICTED: acid phosphatase 1-like [Nicotiana sylvestris]
c50825.graph_c0	1.4262356	1.4048258	1.375396	3.533542	3.6563044	3.9484353	0.0007022	1.445695	up	hypothetical protein MIMGU_mgv1a000817mg [Erythranthe guttata]
c50826.graph_c0	1.547371	1.9051785	0.9948089	8.499429	12.278338	11.370554	1.311E-18	2.890642	up	PREDICTED: adenosine kinase 2-like [Sesamum indicum]
c50827.graph_c0	1.0475701	1.6552508	0.3205532	4.4815689	7.5611182	6.4984302	4.064E-09	2.65246	up	-
c50830.graph_c0	4.1862454	3.7219332	4.54878	10.450309	15.717144	19.65719	3.472E-06	1.911913	up	PREDICTED: delta(8)-fatty-acid desaturase 2-like [Sesamum indicum]
c50834.graph_c0	0.9639295	1.3055069	0.9438703	5.4983321	5.3070547	6.0882986	3.213E-08	2.436511	up	PREDICTED: receptor-like protein kinase HSL1 [Sesamum indicum]
c50851.graph_c0	14.46353	14.109953	14.605692	2.8799892	5.655511	6.8127581	7.277E-07	-1.462908	down	PREDICTED: fibroin heavy chain [Sesamum indicum]
c50852.graph_c0	4.6325527	4.4302614	3.9814337	1.1813696	2.0981328	1.4530956	3.711E-05	-1.42836	down	PREDICTED: F-box/kelch-repeat protein At3g23880-like [Sesamum indicum]
c50857.graph_c0	2.0248846	1.747024	1.5054191	4.049667	4.4109799	3.4003626	0.0024854	1.211148	up	PREDICTED: uncharacterized protein LOC105165400 [Sesamum indicum]
c50869.graph_c0	5.5325302	6.4153558	6.6227937	10.501535	14.632339	12.385028	1.58E-06	1.051672	up	PREDICTED: cationic amino acid transporter 8, vacuolar [Sesamum indicum]
c50874.graph_c0	474.82047	449.09472	446.16687	142.04915	152.56188	96.141254	4.852E-35	-1.765631	down	hypothetical protein JCGZ_21814 [Jatropha curcas]
c50881.graph_c0	1.9278589	3.3943179	2.8316108	4.2764805	6.4720179	7.5016537	0.0044266	1.196176	up	PREDICTED: cationic amino acid transporter 8, vacuolar [Sesamum indicum]
c50885.graph_c0	4.7050677	3.7607326	4.2484022	1.075479	0.9321869	1.3049383	2.19E-06	-1.898783	down	hypothetical protein MIMGU_mgv1a010676mg [Erythranthe guttata]
c50891.graph_c0	0.0387779	0.1400514	0.0949274	8.356162	12.497422	11.852684	4.294E-51	6.93637	up	PREDICTED: annexin D4 [Sesamum indicum]
c50893.graph_c0	2.6444279	3.9157796	3.1072775	1.2067226	0.8522508	0.2386077	8.365E-06	-2.014519	down	-
c50894.graph_c0	33.53445	35.08207	29.356523	13.984875	12.355826	5.5086546	1.327E-11	-1.570104	down	PREDICTED: auxin-induced protein X15-like [Sesamum indicum]
c50899.graph_c0	0.4903737	0.4743873	0.6859559	6.3934437	11.946958	8.8493187	1.147E-13	4.074191	up	PREDICTED: annexin D1-like [Sesamum indicum]
c50901.graph_c0	1.9527958	1.2734161	1.5049396	3.025382	4.0791232	5.8733819	0.0007182	1.489313	up	hypothetical protein MIMGU_mgv1a012192mg [Erythranthe guttata]

#ID	28 h-1 FPKM	28 h-2 FPKM	28 h-3 FPKM	48 h-1 FPKM	48 h-2 FPKM	48 h-3 FPKM	FDR	log2FC	regulated	nr_annotation
c50914.graph_c0	0.2247464	0.3478713	0.6549687	20.145272	16.512695	9.5600755	8.007E-11	5.283904	up	PREDICTED: probable trehalose-phosphate phosphatase H [Sesamum indicum]
c50920.graph_c0	3.1994789	3.8744301	4.3307713	6.6320582	13.748448	15.198685	0.0017809	1.670346	up	PREDICTED: DNA-damage-repair/toleration protein DRT100-like [Sesamum indicum]
c50925.graph_c0	0.2641625	0.5366564	0.538886	2.5950518	7.0058797	4.2456936	0.0001439	3.397816	up	PREDICTED: transcription factor RAX2-like [Sesamum indicum]
c50929.graph_c0	0.2063416	0.0931536	0.0841865	1.3077659	1.0737	0.7854585	6.942E-09	3.0942	up	PREDICTED: protein HOTHEAD [Sesamum indicum]
c50930.graph_c0	3.6796648	3.4995866	4.1235383	8.2090732	11.134178	12.671999	3.718E-07	1.537265	up	PREDICTED: NADPH:quinone oxidoreductase [Sesamum indicum]
c50940.graph_c0	1.8298158	1.9394829	1.8177049	0.3630406	0.726462	0.7716861	6.254E-05	-1.555863	down	LINE-1 reverse transcriptase like [Glycine soja]
c50945.graph_c0	606.19219	678.56701	653.64873	123.7218	145.19061	99.291464	1.382E-57	-2.354328	down	hypothetical protein POPTR_0011s11140g [Populus trichocarpa]
c50961.graph_c0	8.6803417	8.279086	9.9761802	0.3099429	0.9850407	0.827356	2.57E-13	-3.641002	down	-
c50962.graph_c0	11.365747	11.914593	13.38756	22.587123	25.22174	30.671909	7.504E-11	1.135955	up	PREDICTED: adenylyl-sulfate kinase 3-like isoform X2 [Sesamum indicum]
c50966.graph_c0	3.0824134	3.2801178	3.5332911	10.465677	11.629152	14.320242	3.771E-13	1.917832	up	hypothetical protein MIMGU_mgv1a009312mg [Erythranthe guttata]
c50969.graph_c0	3.3771174	2.1211968	3.4745786	4.6529838	9.4642024	7.2867467	0.0045433	1.284913	up	-
c50975.graph_c0	0.6470364	0.8178984	0.7655658	3.936798	3.2582501	2.7366712	5.164E-08	2.202313	up	PREDICTED: putative cyclin-A3-1 [Sesamum indicum]
c50982.graph_c0	2.279552	2.1628771	2.3960556	11.998771	20.960581	21.17856	2.515E-11	3.016908	up	PREDICTED: hevamine-A [Sesamum indicum]
c50986.graph_c0	1.4693191	1.6706056	1.1989524	3.104117	4.2384281	4.2964828	0.0003823	1.460343	up	PREDICTED: polyadenylate-binding protein-interacting protein 8-like [Sesamum indicum]
c50987.graph_c0	55.139709	54.300741	53.377444	25.2659	20.787463	14.524096	2.56E-15	-1.377187	down	unnamed protein product [Coffea canephora]
c51000.graph_c0	1.6070881	0.9942379	1.2628015	3.3197227	3.037274	3.2224028	0.0003445	1.352882	up	PREDICTED: polygalacturonase-like [Sesamum indicum]
c51005.graph_c0	0.1524457	0.2477595	0.1492733	1.4299472	2.2108704	2.3727765	1.135E-13	3.486674	up	PREDICTED: protein BRANCHLESS TRICHOME [Sesamum indicum]
c51013.graph_c0	0.0336362	0.0303703	0.0274468	4.2636311	7.0462083	10.053446	1.744E-14	7.898787	up	PREDICTED: putative pectate lyase 2 [Sesamum indicum]
c51016.graph_c0	0.8713447	1.4790761	1.6495436	3.1809417	3.2294127	3.8524654	0.0003836	1.399378	up	PREDICTED: cell wall / vacuolar inhibitor of fructosidase 1-like [Malus domestica]
c51021.graph_c0	3.1682628	3.3007434	4.3750834	7.4141657	10.254368	11.636516	7.046E-05	1.468879	up	-
c51022.graph_c0	33.535799	42.954897	33.81979	16.735308	13.39028	11.686589	4.949E-10	-1.351249	down	PREDICTED: uncharacterized protein LOC105178343 [Sesamum indicum]
c51025.graph_c0	7.9010703	8.0337	6.6214577	0.9624205	2.0709972	1.1507284	7.779E-17	-2.398103	down	hypothetical protein MIMGU_mgv1a027162mg [Erythranthe guttata]
c51026.graph_c0	2.4873088	2.0505191	2.6473352	27.895843	40.37115	51.655665	3.453E-15	4.094042	up	PREDICTED: uncharacterized protein LOC105161197 [Sesamum indicum]
c51029.graph_c0	0.6899377	0.6832345	0.5448228	0.141056	0.2988634	0.1506129	0.000263	-1.666365	down	PREDICTED: uncharacterized protein LOC104606632 [Nelumbo nucifera]
c51031.graph_c0	0.9470763	1.2521415	0.9660081	3.3870937	5.0416693	5.6461356	2.341E-09	2.187211	up	PREDICTED: uncharacterized protein C594.04c [Sesamum indicum]
c51035.graph_c0	1.2121587	1.5687341	1.3188155	16.696638	10.41751	9.6613267	6.216E-07	3.218193	up	PREDICTED: uncharacterized protein LOC105158187 [Sesamum indicum]
c51046.graph_c1	8.0593899	11.804706	14.760356	28.604437	33.669925	26.462058	4.715E-05	1.398115	up	-

#ID	28 h-1 FPKM	28 h-2 FPKM	28 h-3 FPKM	48 h-1 FPKM	48 h-2 FPKM	48 h-3 FPKM	FDR	log2FC	regulated	nr_annotation
c51047.graph_c0	0.6458686	0.9476331	0.9552302	4.5539413	4.1196685	6.9203885	1.443E-08	2.652215	up	PREDICTED: uncharacterized protein LOC105160227 isoform X1 [Sesamum indicum]
c51050.graph_c0	23.897905	23.252639	20.357003	66.865964	74.114325	80.861865	1.222E-34	1.756052	up	PREDICTED: protein TRANSPARENT TESTA 12-like [Sesamum indicum]
c51055.graph_c0	0.8108071	1.1544382	0.788845	1.38352	2.2194457	2.4620956	0.0046015	1.172489	up	PREDICTED: uncharacterized protein PB18E9.04c [Sesamum indicum]
c51057.graph_c0	7.2264468	8.4160515	9.5714901	1.8585616	1.5470091	0.9449925	1.07E-08	-2.485252	down	PREDICTED: uncharacterized protein LOC105177738 [Sesamum indicum]
c51058.graph_c0	75.864935	81.796288	60.521591	25.299955	31.876444	29.560329	5.906E-11	-1.291995	down	PREDICTED: heme-binding-like protein At3g10130, chloroplastic [Sesamum indicum]
c51060.graph_c0	2.3959336	2.1196003	1.7378331	4.2947789	7.9946442	9.1715243	0.0002126	1.809792	up	PREDICTED: PTI1-like tyrosine-protein kinase At3g15890 [Sesamum indicum]
c51066.graph_c0	2.2624904	2.6855007	2.2610442	10.311454	10.753052	12.501033	4.621E-21	2.259396	up	PREDICTED: guanine nucleotide-binding protein subunit beta-like protein [Sesamum indicum]
c51068.graph_c0	0.5453271	0.4923792	0.7787193	1.9873215	3.4783649	1.9220725	0.0002336	2.058849	up	PREDICTED: probable ADP-ribosylation factor GTPase-activating protein AGD11 [Sesamum indicum]
c51073.graph_c0	2.1695824	2.0118735	1.8182091	0.4459631	0.4724438	0.8597664	0.0002587	-1.71871	down	-
c51078.graph_c0	14.387684	14.434144	12.001126	3.2422131	2.2898211	3.8465357	1.032E-07	-2.077772	down	Dof zinc finger DOF5.2 -like protein [Gossypium arboreum]
c51087.graph_c0	6.0378529	6.0653731	5.9383089	15.712326	15.088163	20.159315	1.328E-11	1.538362	up	PREDICTED: protein CURVATURE THYLAKOID 1D, chloroplastic [Sesamum indicum]
c51088.graph_c0	33.877467	34.502932	37.417986	96.969227	73.616182	76.668048	6.245E-07	1.271941	up	PREDICTED: indole-3-acetic acid-induced protein ARG7-like [Nicotiana sylvestris]
c51089.graph_c0	1.9338634	1.7165024	1.6850009	7.8525152	9.1550679	12.088828	1.101E-14	2.484093	up	PREDICTED: chloroplast stem-loop binding protein of 41 kDa a, chloroplastic [Sesamum indicum]
c51094.graph_c0	0.1872066	0.236642	0.0916554	7.4986226	12.167211	10.895159	2.387E-34	5.925123	up	PREDICTED: peroxidase 16 [Sesamum indicum]
c51097.graph_c0	26.585956	27.872037	27.559316	59.16162	56.327771	65.635479	9.185E-15	1.184736	up	PREDICTED: 30S ribosomal protein S20, chloroplastic isoform X1 [Sesamum indicum]
c51100.graph_c0	1.0594358	1.7498253	1.454875	7.7954454	12.040505	13.202362	6.523E-14	2.987698	up	hypothetical protein MIMGU_mgv1a004964mg [Erythranthe guttata]
c51101.graph_c0	1.2255809	1.5215539	1.375088	9.7094628	12.137478	17.62442	4.408E-10	3.294342	up	PREDICTED: chlorophyll a-b binding protein 151, chloroplastic-like [Pyrus x bretschneideri]
c51104.graph_c0	33.977064	38.603279	35.753706	6.101361	6.7488133	3.8322031	7.188E-29	-2.6546	down	-
c51106.graph_c0	2.9300084	2.5915326	2.0005179	8.0343704	5.8616529	7.2838201	2.976E-05	1.540394	up	PREDICTED: transcription factor HEC2-like [Sesamum indicum]
c51113.graph_c0	1.9719058	2.2326228	1.7878434	3.0153131	4.7074758	4.1657221	0.0042382	1.023601	up	PREDICTED: metacaspase-1-like [Sesamum indicum]
c51120.graph_c0	10.425116	11.718103	11.588358	4.6520794	5.7854121	5.4591985	0.0006126	-1.046926	down	--
c51125.graph_c0	0.3971893	0.5881445	0.803776	9.9887795	8.3204461	7.2035449	2.119E-24	3.880388	up	PREDICTED: phosphate transporter PHO1 homolog 9-like [Sesamum indicum]
c51131.graph_c0	0.3145184	0.2839806	0.4106311	3.5083416	11.572336	14.757133	6.486E-05	4.908547	up	hypothetical protein CISIN_1g026722mg [Citrus sinensis]

#ID	28 h-1 FPKM	28 h-2 FPKM	28 h-3 FPKM	48 h-1 FPKM	48 h-2 FPKM	48 h-3 FPKM	FDR	log2FC	regulated	nr_annotation
c51132.graph_c0	43.673348	48.567572	48.236068	83.840274	98.889188	109.72819	2.344E-14	1.096018	up	PREDICTED: 24-methylenesterol C-methyltransferase 2-like [Sesamum indicum]
c51133.graph_c0	13.867017	13.54503	15.224319	1.4381562	1.0157014	0.9952932	2.09E-15	-3.578433	down	-
c51134.graph_c0	205.22334	253.35421	239.69087	23.484473	21.689343	22.325153	1.326E-70	-3.327295	down	PREDICTED: uncharacterized protein LOC105179155 [Sesamum indicum]
c51135.graph_c0	0.1189208	0.1073743	0.2425959	3.1655572	13.014867	10.998518	6.383E-06	5.878054	up	PREDICTED: non-specific lipid transfer protein GPI-anchored 2-like [Eucalyptus grandis]
c51137.graph_c0	0.1802111	0.4067844	0.4411525	2.7792426	3.1862654	0.8130258	0.0098222	2.768314	up	hypothetical protein MIMGU_mgv1a008689mg [Erythranthe guttata]
c51138.graph_c0	1.8915876	3.0624885	2.3418925	0.4134011	0.4379484	0.2942735	4.538E-08	-2.626396	down	hypothetical protein MIMGU_mgv1a008111mg [Erythranthe guttata]
c51144.graph_c0	2.8824603	3.4701219	3.1840868	0.5716722	1.1585723	2.0567979	0.0002512	-1.305558	down	PREDICTED: UPF0392 protein RCOM_0530710-like [Sesamum indicum]
c51145.graph_c0	1.606592	0.8199054	1.5959585	19.567856	24.763165	25.368589	7.858E-39	4.151567	up	PREDICTED: proline-rich protein 3 [Sesamum indicum]
c51148.graph_c0	2.4776305	2.2630804	2.4918951	13.730904	17.370362	17.676637	2.141E-35	2.791035	up	PREDICTED: GDSL esterase/lipase 7 [Sesamum indicum]
c51154.graph_c0	13.18559	15.335707	22.430475	7.0820789	3.901354	5.2933366	0.000351	-1.595571	down	PREDICTED: heat stress transcription factor A-2-like [Nicotiana sylvestris]
c51156.graph_c0	1.1824466	1.2344569	1.3266918	0.4683869	0.6946788	0.4584446	0.0092793	-1.169584	down	PREDICTED: uncharacterized protein At4g26485-like [Sesamum indicum]
c51158.graph_c0	7.48531	8.3542978	8.1722133	17.65856	18.893244	25.757525	2.829E-08	1.413835	up	hypothetical protein MIMGU_mgv1a009002mg [Erythranthe guttata]
c51160.graph_c0	0.6246562	0.4834337	0.218449	1.8663803	2.3966105	2.9691195	1.254E-08	2.483552	up	PREDICTED: expansin-B3-like [Sesamum indicum]
c51161.graph_c0	11.067827	10.329116	8.8288754	43.109598	45.669392	56.681304	8.515E-31	2.306171	up	PREDICTED: glyceraldehyde-3-phosphate dehydrogenase A, chloroplastic [Sesamum indicum]
c51162.graph_c0	0.1915553	0.2306087	0.3647177	2.0234206	3.3439673	3.8889234	2.828E-10	3.587441	up	PREDICTED: dirigent protein 23-like [Sesamum indicum]
c51165.graph_c0	2.3163572	1.9813769	1.6911677	0.3605802	0.9276925	0.8250226	0.0012133	-1.475112	down	-
c51169.graph_c0	3.9009827	4.3957325	3.1067739	1.9383549	1.4248442	1.1615572	0.0005336	-1.282639	down	PREDICTED: probable WRKY transcription factor 71 isoform X1 [Sesamum indicum]
c51176.graph_c0	8.6833811	8.7363109	9.2112385	2.6730861	1.6657709	1.6789383	1.431E-06	-2.093912	down	-
c51177.graph_c0	26.754868	25.57444	24.991239	73.531413	132.84284	182.74935	0.0002518	2.361076	up	PREDICTED: trans-resveratrol di-O-methyltransferase [Vitis vinifera]
c51183.graph_c0	0.5254091	1.3045867	0.7502767	3.9959684	5.8207104	9.7778687	0.0001883	2.957201	up	PREDICTED: LOW QUALITY PROTEIN: ethylene-responsive transcription factor 2-like [Sesamum indicum]
c51184.graph_c0	44.612573	76.451639	70.578206	3.0775359	4.4828797	2.738373	5.712E-15	-4.179339	down	hypothetical protein CARUB_v10018292mg, partial [Capsella rubella]
c51186.graph_c0	5.0856825	5.4582897	5.1677694	10.581953	9.9217564	10.822711	0.0058987	1.03827	up	PREDICTED: nucleobase-ascorbate transporter 12 [Sesamum indicum]
c51193.graph_c0	5.0693225	6.0632018	4.6200158	12.93493	10.697173	14.850869	1.665E-05	1.331879	up	PREDICTED: cyclin-D3-1 [Sesamum indicum]
c51195.graph_c0	0.3993565	0.1802907	0.1629358	2.5310688	2.7707391	2.1019878	6.179E-12	3.360436	up	hypothetical protein MIMGU_mgv1a015662mg [Erythranthe guttata]
c51201.graph_c0	71.657079	78.314636	72.884757	40.453976	32.916975	24.676301	1.411E-12	-1.135588	down	PREDICTED: uncharacterized protein LOC105164312 [Sesamum indicum]
c51202.graph_c0	3.4060681	2.8208476	2.8559948	13.220306	8.8637215	6.7824712	0.0027571	1.721093	up	PREDICTED: U-box domain-containing protein 9-like [Sesamum indicum]
c51218.graph_c0	2.0537567	1.8068023	2.0196126	0.2002529	0.4950019	0.9503138	0.0001314	-1.813204	down	-

#ID	28 h-1 FPKM	28 h-2 FPKM	28 h-3 FPKM	48 h-1 FPKM	48 h-2 FPKM	48 h-3 FPKM	FDR	log2FC	regulated	nr_annotation
c51220.graph_c0	0.5205669	0.4700231	0.4247784	22.600099	42.466581	39.631728	2.436E-21	6.24065	up	-
c51235.graph_c0	0	0.0834878	0.1006016	4.2585199	8.8986044	10.081372	4.12E-14	7.008238	up	PREDICTED: probable pectate lyase 16 [Sesamum indicum]
c51236.graph_c0	29.54449	25.159214	23.745232	41.144699	54.998938	62.744603	3.532E-06	1.053995	up	hypothetical protein MIMGU_mgv1a014485mg [Erythranthe guttata]
c51237.graph_c0	0.0544192	0.1474064	0.0444056	3.8629012	4.8230389	3.1302946	4.941E-22	5.627672	up	-
c51246.graph_c0	1.1072454	2.0470841	1.3767668	8.2874223	6.6554419	5.1142941	9.219E-07	2.195814	up	PREDICTED: uncharacterized protein LOC105170022 [Sesamum indicum]
c51248.graph_c0	1.0147003	1.8323584	1.4075781	4.8875878	5.4503227	7.210095	1.465E-06	2.082278	up	PREDICTED: mitogen-activated protein kinase 19 isoform X2 [Sesamum indicum]
c51249.graph_c0	15.253516	17.412368	17.899604	30.613679	35.650244	34.817876	2.18E-10	1.038631	up	PREDICTED: NADP-dependent D-sorbitol-6-phosphate dehydrogenase-like [Sesamum indicum]
c51257.graph_c0	3.8465756	2.9994929	2.1686077	11.52466	18.407386	24.374332	1.794E-06	2.623028	up	PREDICTED: leucine-rich repeat extensin-like protein 2 [Sesamum indicum]
c51262.graph_c0	2.9688734	3.0366334	3.1985579	14.582654	13.424046	13.577247	2.32E-26	2.219288	up	hypothetical protein MIMGU_mgv1a003907mg [Erythranthe guttata]
c51264.graph_c0	0.1626309	0.0978937	0.1769407	7.4899969	6.0420524	7.2759812	2.775E-28	5.615678	up	PREDICTED: protein HEADING DATE 3A [Populus euphratica]
c51270.graph_c0	1.0460551	0.8995139	1.1787428	66.297591	87.023592	132.98302	3.408E-18	6.551086	up	PREDICTED: auxin-binding protein ABP19a [Beta vulgaris subsp. vulgaris]
c51271.graph_c0	56.815943	53.978876	58.317427	9.4039251	10.626473	8.5070152	4.014E-60	-2.525655	down	PREDICTED: alcohol dehydrogenase-like 7 [Sesamum indicum]
c51273.graph_c0	1.5610294	2.0358912	1.9578581	6.046088	5.6675405	6.4557273	2.363E-08	1.752	up	PREDICTED: mitogen-activated protein kinase homolog NTF6 [Sesamum indicum]
c51287.graph_c0	1.1411568	0.9273219	0.6052635	28.930007	40.378521	30.568323	3.298E-51	5.261755	up	hypothetical protein MIMGU_mgv1a013386mg [Erythranthe guttata]
c51310.graph_c0	0.9800087	1.954057	1.2328386	0	0.3289989	0.2763329	0.0001351	-2.770517	down	-
c51315.graph_c0	1.743842	1.8369468	3.438822	5.1577125	8.5862401	4.9171088	0.0048368	1.445715	up	unnamed protein product [Pneumocystis jirovecii]
c51323.graph_c0	1.1452754	1.0340763	1.0226991	4.4921174	6.5579325	5.2887915	6.511E-15	2.387916	up	PREDICTED: uncharacterized protein LOC105175605 isoform X2 [Sesamum indicum]
c51328.graph_c0	25.749343	24.223033	29.701772	3.7594877	6.426664	4.5615948	6.132E-20	-2.39929	down	-
c51331.graph_c0	26.613609	30.254127	26.55691	6.6046872	10.333832	14.556409	1.469E-07	-1.373533	down	--
c51335.graph_c0	41.102345	49.343867	42.095728	71.213377	93.839903	111.62295	8.321E-06	1.097622	up	PREDICTED: uncharacterized protein LOC105168965 [Sesamum indicum]
c51337.graph_c0	1.1856629	0.9607432	1.3892182	6.3199505	11.145094	16.836072	4.05E-05	3.307061	up	PREDICTED: cell division control protein 2 homolog D [Sesamum indicum]
c51351.graph_c0	77.494522	72.57263	76.691781	30.775512	29.322126	26.393568	7.937E-21	-1.346605	down	PREDICTED: putative E3 ubiquitin-protein ligase RF4 [Sesamum indicum]
c51353.graph_c0	0.7354443	0.4426915	0.5751117	5.049578	4.1972338	4.9423926	6.931E-16	3.060706	up	PREDICTED: uncharacterized protein LOC105163262 isoform X2 [Sesamum indicum]
c51360.graph_c0	2.4189419	0.9100324	1.096576	8.5171861	6.6769652	5.7596911	4.323E-08	2.291666	up	PREDICTED: uncharacterized protein LOC105165055 [Sesamum indicum]
c51361.graph_c0	0.3178684	0.1594474	0.259378	2.6861441	7.8729484	6.8914979	1.099E-06	4.59133	up	hypothetical protein MIMGU_mgv1a009735mg [Erythranthe guttata]
c51365.graph_c0	3.1060575	3.8480057	3.0060557	0.4578089	1.4549791	2.0367781	0.0049359	-1.310829	down	-

#ID	28 h-1 FPKM	28 h-2 FPKM	28 h-3 FPKM	48 h-1 FPKM	48 h-2 FPKM	48 h-3 FPKM	FDR	log2FC	regulated	nr_annotation
c51372.graph_c0	5.1395549	6.1255077	5.7755096	14.89089	18.890673	26.102154	1.413E-06	1.848318	up	PREDICTED: 2-alkenal reductase (NADP(+)-dependent) isoform X1 [Sesamum indicum]
c51382.graph_c0	9.1416023	9.3914881	8.5138152	54.867338	31.708648	24.301014	0.0066057	2.091779	up	PREDICTED: abscisic acid receptor PYL4-like [Sesamum indicum]
c51391.graph_c0	4.0181352	3.4586927	4.4591211	0.6451491	0.5395715	0.4531972	4.509E-13	-2.818473	down	hypothetical protein MIMGU_mgv1a026169mg, partial [Erythranthe guttata]
c51401.graph_c0	1.8280938	1.3435094	2.2896008	0.4850045	0.5708927	0.6233559	0.0002378	-1.663968	down	PREDICTED: uncharacterized protein LOC105157495 [Sesamum indicum]
c51406.graph_c0	0.5369927	0.5887513	0.8450646	13.856654	26.577522	29.417916	8.899E-14	5.177002	up	PREDICTED: pectinesterase-like [Sesamum indicum]
c51410.graph_c0	5.7622737	8.5054354	5.9285687	1.1432514	1.345707	0.8477152	6.891E-11	-2.554586	down	PREDICTED: cyclic dof factor 2-like [Sesamum indicum]
c51414.graph_c0	25.089855	26.332295	24.966011	9.4591626	13.62292	14.10367	1.14E-10	-1.003362	down	PREDICTED: methyltransferase-like protein 13 [Sesamum indicum]
c51416.graph_c0	0.7561427	1.0620182	1.3025688	3.7806182	4.1179264	6.0172444	2.018E-08	2.193491	up	PREDICTED: uncharacterized protein LOC105175985 [Sesamum indicum]
c51426.graph_c0	6.9477847	7.8587318	6.9776438	2.7097935	3.6908969	3.5090959	4.692E-06	-1.099423	down	hypothetical protein MIMGU_mgv1a026964mg, partial [Erythranthe guttata]
c51429.graph_c0	2.978299	3.1527669	3.771105	0.1301798	0.1379097	0.1158332	3.981E-14	-4.646749	down	-
c51433.graph_c0	12.56792	13.673922	16.254682	66.431287	102.41352	110.32952	9.164E-14	2.749166	up	PREDICTED: leucine-rich repeat extensin-like protein 1 [Sesamum indicum]
c51437.graph_c0	0.0543309	0.0981115	0.1773344	7.8854396	6.8580324	3.6767249	2.241E-11	5.852267	up	hypothetical protein MIMGU_mgv1a027064mg [Erythranthe guttata]
c51441.graph_c0	5.4189909	9.5759882	6.2221779	14.473901	16.061027	18.510532	2.547E-08	1.248558	up	PREDICTED: phospholipid hydroperoxide glutathione peroxidase 1, chloroplastic-like [Sesamum indicum]
c51448.graph_c0	1.2596456	1.2711468	1.6627156	5.3536198	16.815533	24.194169	0.002447	3.489506	up	PREDICTED: uncharacterized protein LOC105162545 [Sesamum indicum]
c51455.graph_c0	1.6488784	1.8544485	1.6995427	45.17495	26.880932	28.385444	1.64E-10	4.323557	up	PREDICTED: uncharacterized protein LOC105171871 [Sesamum indicum]
c51460.graph_c0	20.140229	26.607144	24.73789	91.636477	129.43698	146.65607	5.306E-14	2.397843	up	PREDICTED: MLP-like protein 423 [Sesamum indicum]
c51462.graph_c0	11.709765	12.224823	12.093139	2.5511389	4.17678	6.1908765	0.0010544	-1.449029	down	-
c51463.graph_c0	12.155406	16.204548	12.602599	1.6314218	1.6322773	1.8548597	6.705E-16	-2.959074	down	PREDICTED: uncharacterized protein LOC105175709 [Sesamum indicum]
c51468.graph_c0	4.6072578	5.4598974	5.1105496	1.368759	1.83671	0.5683597	3.862E-06	-1.964256	down	PREDICTED: oleosin 1-like [Solanum lycopersicum]
c51470.graph_c0	6.3231443	8.1130828	8.0381668	13.920878	16.26692	17.491541	9.47E-05	1.123624	up	PREDICTED: probable stress-associated endoplasmic reticulum protein [Musa acuminata subsp. malaccensis]
c51473.graph_c0	1.7943588	1.8469571	1.5227494	4.6399432	5.0600295	5.9905084	2.39E-06	1.642645	up	PREDICTED: DNA replication complex GINS protein PSF2 [Sesamum indicum]
c51474.graph_c0	0.1761193	0.7950961	0.5748476	5.5811045	7.5680044	6.5551638	3.668E-13	3.709911	up	PREDICTED: subtilisin-like protease [Sesamum indicum]
c51476.graph_c0	83.214611	77.913685	72.904942	23.281904	19.449418	14.324962	2.652E-43	-1.987698	down	PREDICTED: uncharacterized protein LOC104224082 [Nicotiana sylvestris]
c51478.graph_c0	1.2423626	1.1217369	1.2840931	3.6745008	2.6692718	4.0168742	0.0003723	1.550165	up	PREDICTED: GDSL esterase/lipase 5 isoform X1 [Sesamum indicum]
c51483.graph_c0	4.9091437	5.0216258	5.1467196	9.9248147	10.514138	9.1113909	7.962E-05	1.012923	up	PREDICTED: UPF0553 protein-like isoform X1 [Sesamum indicum]
c51485.graph_c0	1.2431441	0.827063	1.1211739	3.7321029	10.279647	7.3795615	0.0003229	2.771775	up	PREDICTED: glutathione transferase GST 23-like [Sesamum indicum]
c51489.graph_c0	3.4089425	3.8069449	2.7084674	62.428316	60.533614	64.604035	7.29E-124	4.283017	up	PREDICTED: chlorophyll a-b binding protein 6, chloroplastic-like [Malus domestica]

#ID	28 h-1 FPKM	28 h-2 FPKM	28 h-3 FPKM	48 h-1 FPKM	48 h-2 FPKM	48 h-3 FPKM	FDR	log2FC	regulated	nr_annotation
c51493.graph_c0	26.750153	31.125887	30.41294	12.059255	13.37651	16.158499	0.0002182	-1.047299	down	Endosomal P24A protein precursor, putative [Ricinus communis]
c51506.graph_c0	0.6630656	0.5559228	0.6569967	5.042907	6.9323331	6.9978146	9.966E-17	3.374109	up	PREDICTED: uncharacterized protein LOC103322890 [Prunus mume]
c51513.graph_c0	0.6844498	0.617994	0.8144871	26.244614	36.725983	40.78611	6.33E-45	5.649739	up	PREDICTED: ectonucleotide pyrophosphatase/phosphodiesterase family member 1-like [Sesamum indicum]
c51514.graph_c0	7.1312613	7.0030903	7.1088406	11.415736	14.611028	19.859248	0.0004132	1.146062	up	PREDICTED: cyclin-D3-3-like [Sesamum indicum]
c51517.graph_c0	0.3465478	0.1564501	0.2356501	8.7122847	4.3433455	8.2732907	2.023E-10	4.900817	up	PREDICTED: uncharacterized protein LOC105157094 [Sesamum indicum]
c51520.graph_c0	0	0.1940491	0.1315274	2.247482	2.4530842	3.6359926	9.075E-16	4.716146	up	PREDICTED: miraculin-like [Nicotiana sylvestris]
c51523.graph_c0	5.0848728	4.9659522	5.3347046	0.9207787	1.3238296	0.9948683	9.205E-08	-2.210474	down	-
c51528.graph_c0	21.443565	19.978015	21.206257	71.780262	50.628135	42.643938	0.0010721	1.448923	up	PREDICTED: phytochrome A-associated F-box protein [Sesamum indicum]
c51530.graph_c0	1.7590478	0.6176548	1.355626	6.0698007	7.2175914	12.565294	2.697E-05	2.825385	up	PREDICTED: ethylene-responsive transcription factor 2 [Nicotiana tomentosiformis]
c51532.graph_c0	0.3234964	0.1947246	0.2419729	2.6311892	2.2444209	3.1317569	3.034E-15	3.438765	up	PREDICTED: E2F transcription factor-like E2FE [Sesamum indicum]
c51537.graph_c0	124.48646	127.74764	132.03571	12.470416	11.876185	7.9617387	2.47E-114	-3.525836	down	PREDICTED: anthocyanidin 3-O-glucosyltransferase 5-like [Sesamum indicum]
c51541.graph_c0	0	0.1852383	0.0558024	3.1206328	4.7752351	5.4763094	7.767E-20	5.829724	up	RecName: Full=Casparian strip membrane protein 3; Short=MgCASP3 [Erythranthe guttata]
c51543.graph_c0	4.8121919	5.9399427	6.4119683	0.3860638	0.3271902	0.6870345	4.12E-14	-3.577796	down	-
c51545.graph_c0	16.056734	18.829363	18.854334	3.3011725	5.0222832	4.0637208	9.403E-32	-2.081287	down	PREDICTED: galactose oxidase [Sesamum indicum]
c51549.graph_c0	0.6545695	0.3283416	0.8308585	7.1908633	7.3248536	6.5624482	1.598E-15	3.580024	up	PREDICTED: cyclin-U4-1 [Sesamum indicum]
c51554.graph_c0	2.6754345	3.7123111	3.8846921	9.8746977	13.86881	14.28907	6.986E-15	1.923946	up	PREDICTED: 3-ketoacyl-CoA synthase 1 [Sesamum indicum]
c51556.graph_c0	0.4096821	0.5044153	0.7901571	39.042086	68.267092	61.917648	1.619E-33	6.66563	up	hypothetical protein MIMGU_mgv1a009759mg [Erythranthe guttata]
c51560.graph_c0	0.1020428	0.2456935	0.2497982	3.147437	3.5170307	3.8747619	3.411E-19	4.179658	up	PREDICTED: homeobox-leucine zipper protein HOX21-like [Sesamum indicum]
c51570.graph_c0	1.4960387	1.1461186	1.4797035	0.4022538	0.4261391	0.3067911	7.003E-05	-1.817138	down	PREDICTED: uncharacterized protein LOC105175073 [Sesamum indicum]
c51590.graph_c0	1.7017942	1.6463149	2.2813538	129.12081	132.21736	127.91554	7.26E-153	6.152947	up	PREDICTED: raucaffricine-O-beta-D-glucosidase-like [Sesamum indicum]
c51599.graph_c0	0.1085813	0	0.1181353	6.3312032	10.643943	10.205555	3.832E-31	6.936159	up	PREDICTED: uncharacterized protein LOC105165189 [Sesamum indicum]
c51601.graph_c0	4.1493945	3.7465142	3.6728103	0.8246082	1.5110442	2.1218719	2.154E-05	-1.346465	down	PREDICTED: uncharacterized protein LOC105171071 [Sesamum indicum]
c51606.graph_c0	2.3446039	1.5877181	2.3383282	4.6230459	5.8595766	5.3623173	0.0007194	1.375084	up	PREDICTED: probable phospholipase A2 homolog 1 [Sesamum indicum]
c51612.graph_c0	0.4189245	0.4728119	0.7691376	2.655088	2.8127438	3.1893498	1.033E-07	2.420953	up	PREDICTED: uncharacterized protein LOC105169203 [Sesamum indicum]
c51614.graph_c0	0.5543903	0.6674166	1.8095119	11.126567	20.348513	18.550134	2.098E-14	4.074784	up	-
c51619.graph_c0	2.6028682	2.9067595	3.4653419	10.418928	12.41729	12.206421	5.61E-09	2.003711	up	hypothetical protein MIMGU_mgv1a023502mg, partial [Erythranthe guttata]
c51624.graph_c0	0.3197263	0.3849105	0.0869647	3.2422131	4.1503008	5.7698035	1.692E-12	4.092256	up	hypothetical protein MIMGU_mgv1a024616mg [Erythranthe guttata]

#ID	28 h-1 FPKM	28 h-2 FPKM	28 h-3 FPKM	48 h-1 FPKM	48 h-2 FPKM	48 h-3 FPKM	FDR	log2FC	regulated	nr_annotation
c51629.graph_c0	25.690256	27.744106	35.020606	9.4500296	11.769878	11.476575	2.989E-07	-1.39808	down	-
c51630.graph_c0	1.1433435	1.327284	1.4993983	9.4720284	12.666457	17.316863	1.785E-11	3.347024	up	hypothetical protein MIMGU_mgv1a010366mg [Erythranthe guttata]
c51636.graph_c0	16.563972	18.9019	16.992482	4.4692267	7.0525868	8.4090445	1.028E-09	-1.363336	down	unnamed protein product [Coffea canephora]
c51638.graph_c0	2.6493893	1.8505313	2.4882016	0.3801845	0.4698859	0.3946668	2.005E-07	-2.45006	down	-
c51639.graph_c0	1.3109112	1.0356762	0.9359813	6.4389966	5.7211207	6.2838367	2.529E-08	2.534098	up	hypothetical protein ARALYDRAFT_486411 [Arabidopsis lyrata subsp. lyrata]
c51648.graph_c0	2.2247683	2.791832	2.1846251	6.8828525	10.633507	12.121052	3.299E-08	2.074822	up	hypothetical protein MIMGU_mgv1a011849mg [Erythranthe guttata]
c51653.graph_c0	2.691293	4.0220445	2.4232531	0.8234443	1.2461991	0.628025	0.0001609	-1.720308	down	-
c51659.graph_c0	0.0543494	0.0490724	0.0443487	1.9978646	2.4084256	3.1875783	4.677E-20	5.719849	up	hypothetical protein MIMGU_mgv1a012062mg [Erythranthe guttata]
c51660.graph_c0	11.241836	10.614339	10.326455	26.871156	22.859648	25.286417	6.188E-08	1.265466	up	PREDICTED: nudix hydrolase 1 [Sesamum indicum]
c51665.graph_c0	19.868045	19.463209	19.54996	6.7487098	7.4981929	7.3963549	2.305E-07	-1.403686	down	PREDICTED: uncharacterized protein LOC104227211 [Nicotiana sylvestris]
c51676.graph_c0	12.072427	14.858789	15.294977	5.0337798	4.0528363	3.0099061	3.22E-14	-1.754456	down	hypothetical protein MIMGU_mgv1a009467mg [Erythranthe guttata]
c51682.graph_c0	0.2068453	0.2334524	0.5274503	4.5228069	6.0412869	7.5238154	4.218E-21	4.257692	up	PREDICTED: serine carboxypeptidase-like 34 [Sesamum indicum]
c51683.graph_c0	2.5202353	2.6601338	2.1723501	10.258664	9.3901694	12.451045	6.548E-15	2.167829	up	hypothetical protein MIMGU_mgv1a008015mg [Erythranthe guttata]
c51688.graph_c0	5.9847352	6.5412663	6.2714357	22.838132	19.96447	21.813351	2.215E-12	1.825454	up	PREDICTED: uncharacterized protein LOC105159746 [Sesamum indicum]
c51690.graph_c0	1.0624356	1.5987996	2.6730613	19.190695	14.147927	17.175623	2.895E-19	3.288743	up	histone H4 [Zea mays]
c51696.graph_c0	0.3186831	0.2517733	0.1300214	1.8682885	4.0119427	4.2233739	5.639E-08	3.880281	up	PREDICTED: carboxylesterase 1-like [Sesamum indicum]
c51710.graph_c0	11.181964	8.9979265	10.040648	4.6851174	4.2722194	5.224179	0.0006542	-1.04954	down	PREDICTED: pentatricopeptide repeat-containing protein At2g13600-like [Sesamum indicum]
c51715.graph_c0	4.9922605	3.977244	5.2717744	0.3722387	1.7745379	1.9872945	0.000164	-1.764353	down	BnaCnng51820D [Brassica napus]
c51716.graph_c0	11.755996	18.096534	14.810342	2.0353392	1.8481673	1.6169934	2.46E-16	-2.975869	down	PREDICTED: SNF1-related protein kinase regulatory subunit gamma-1-like [Sesamum indicum]
c51740.graph_c0	1.6775321	2.3837181	1.7727762	5.1591235	4.4683875	5.9553172	4.129E-06	1.460187	up	hypothetical protein MIMGU_mgv1a007527mg [Erythranthe guttata]
c51746.graph_c0	10.981641	12.076651	11.734195	67.16824	53.342917	52.387899	9.697E-17	2.36014	up	PREDICTED: scarecrow-like protein 8 [Sesamum indicum]
c51753.graph_c0	9.7471124	8.0463805	11.362234	25.239874	22.998923	23.165017	1.212E-07	1.335597	up	Putative non-specific lipid-transfer protein AKCS9 [Aegilops tauschii]
c51754.graph_c0	39.315847	43.469257	40.893896	16.458097	15.724411	17.655296	1.084E-16	-1.269182	down	PREDICTED: uncharacterized protein LOC105174255 [Sesamum indicum]
c51762.graph_c0	3.1594008	2.2475367	2.4218001	14.441455	11.57065	8.6386016	1.527E-06	2.195192	up	PREDICTED: cyclic dof factor 1 [Sesamum indicum]
c51764.graph_c0	75.676176	76.472816	75.632971	24.716904	40.434665	44.023308	2.706E-11	-1.028272	down	PREDICTED: ribosomal RNA-processing protein 14-C-like [Sesamum indicum]
c51766.graph_c0	15.882699	19.323335	20.648379	36.767389	42.294376	40.078264	6.255E-09	1.132641	up	PREDICTED: microsomal glutathione S-transferase 3 [Sesamum indicum]
c51780.graph_c0	2.6593682	4.6689225	3.4358693	11.142846	13.590048	19.746357	1.858E-06	2.082696	up	hypothetical protein M569_05207, partial [Genlisea aurea]

#ID	28 h-1 FPKM	28 h-2 FPKM	28 h-3 FPKM	48 h-1 FPKM	48 h-2 FPKM	48 h-3 FPKM	FDR	log2FC	regulated	nr_annotation
c51782.graph_c0	7.7238432	8.7173828	10.462304	25.161688	32.567735	30.403358	1.798E-12	1.749323	up	PREDICTED: uncharacterized protein LOC105172990 [Sesamum indicum]
c51784.graph_c0	2.2007135	2.7095972	3.5099029	9.8902812	7.5223462	6.5438223	0.0002127	1.557016	up	-
c51786.graph_c0	0.2352312	0.2973485	0.537451	3.5184405	4.2959416	5.9429995	2.593E-14	3.718974	up	PREDICTED: uncharacterized protein LOC100256155 [Vitis vinifera]
c51795.graph_c0	8.8329689	8.5450101	8.3584287	1.9287983	2.442026	1.8836705	1.898E-10	-2.001216	down	PREDICTED: E3 ubiquitin-protein ligase ATL4-like [Sesamum indicum]
c51805.graph_c0	9.9099769	7.2860494	8.2597415	0.6280813	1.4258056	2.554802	3.454E-09	-2.44029	down	-
c51807.graph_c0	0.6071147	0.4604608	0.5152167	1.7546027	2.3479438	2.3007673	4.427E-07	2.053031	up	PREDICTED: uncharacterized protein LOC105172555 [Sesamum indicum]
c51809.graph_c0	0.8501328	0.7310383	0.7267348	2.0525827	2.0657393	3.1048387	5.758E-05	1.684376	up	PREDICTED: LOW QUALITY PROTEIN: myb-like protein AA [Sesamum indicum]
c51820.graph_c0	16.861309	16.757552	16.183783	4.0746993	5.2940045	4.5149516	5.313E-10	-1.804391	down	PREDICTED: uncharacterized protein LOC105171189 [Sesamum indicum]
c51825.graph_c0	12.698039	12.09051	10.110308	32.776697	27.695675	18.054219	0.0050911	1.219445	up	hypothetical protein MIMGU_mgv1a016748mg [Erythranthe guttata]
c51827.graph_c0	0.3158644	0.4277939	0.3221785	7.6072468	7.4227222	6.0563685	6.291E-19	4.351062	up	PREDICTED: photosystem I reaction center subunit psaK, chloroplastic [Malus domestica]
c51832.graph_c0	0.2571473	0.5804498	0.9092638	1.9013935	2.5898089	3.1903429	7.568E-07	2.170372	up	PREDICTED: RAN GTPase-activating protein 2-like isoform X1 [Sesamum indicum]
c51835.graph_c0	9.6240902	10.734275	12.749867	48.03153	54.735327	50.442956	2.424E-45	2.250192	up	hypothetical protein CICLE_v10001854mg [Citrus clementina]
c51842.graph_c0	5.4943979	5.3090613	4.7980072	2.5658883	1.812165	2.1743924	0.0065701	-1.203882	down	-
c51846.graph_c0	2.2229654	3.7826668	2.58135	0.4335034	0.8036776	0.6750253	1.198E-05	-2.133884	down	PREDICTED: uncharacterized protein LOC105176481 [Sesamum indicum]
c51856.graph_c0	5.712081	6.7203444	8.968916	0.3291129	1.0459656	0.7809138	2.284E-12	-3.286322	down	-
c51859.graph_c0	47.258046	46.117632	47.598973	19.362603	24.550695	22.612705	1.195E-10	-1.045229	down	-
c51860.graph_c0	5.5400394	3.050083	4.6308861	17.127824	22.681066	17.678673	3.875E-09	2.158535	up	-
c51862.graph_c0	0.2405746	0.0434433	0.0785227	3.9642976	3.8120286	4.5584967	2.853E-22	5.12946	up	PREDICTED: 65-kDa microtubule-associated protein 3 [Sesamum indicum]
c51863.graph_c0	55.104394	58.248002	53.52573	105.33922	125.85386	143.86832	3.814E-14	1.206118	up	-
c51875.graph_c0	3.4709742	2.8056442	3.2368989	1.0894533	0.7546324	0.7456839	6.803E-06	-1.827486	down	hypothetical protein MIMGU_mgv1a017259mg [Erythranthe guttata]
c51877.graph_c0	3.2176153	3.043548	2.3338202	0.6473912	0.7544157	0.4032313	1.104E-06	-2.207364	down	PREDICTED: myb-related protein 305 [Sesamum indicum]
c51906.graph_c0	0.1235731	0.1859582	0.0672231	4.1770128	9.2925811	9.8956629	8.47E-12	5.984591	up	PREDICTED: uncharacterized protein At1g28695-like [Sesamum indicum]
c51907.graph_c0	3.6418916	4.93243	3.3432231	0.8655686	1.3754475	1.0910851	3.134E-05	-1.80267	down	PREDICTED: dof zinc finger protein DOF5.6 isoform X2 [Sesamum indicum]
c51920.graph_c0	5.1441702	4.4948743	3.655975	16.07016	18.628778	17.742902	7.592E-21	2.019862	up	PREDICTED: protein TIFY 6B [Sesamum indicum]
c51928.graph_c0	6.1528352	10.317233	5.4988217	25.62585	19.278646	27.428165	1.327E-05	1.764672	up	Tubulin beta-8 chain -like protein [Gossypium arboreum]
c51933.graph_c0	4.227814	3.6608721	3.110531	6.0618938	6.7941222	8.7161274	0.0005572	1.009861	up	hypothetical protein MIMGU_mgv1a008682mg [Erythranthe guttata]
c51936.graph_c0	0.4607703	0.3235808	0.3759848	1.752181	2.5437134	2.2520046	2.295E-08	2.532416	up	hypothetical protein MIMGU_mgv1a013390mg [Erythranthe guttata]
c51940.graph_c0	5.186387	4.3561127	4.1336297	36.234144	57.011646	52.034402	1.775E-23	3.443742	up	PREDICTED: kirola-like isoform X2 [Sesamum indicum]

#ID	28 h-1 FPKM	28 h-2 FPKM	28 h-3 FPKM	48 h-1 FPKM	48 h-2 FPKM	48 h-3 FPKM	FDR	log2FC	regulated	nr_annotation
c51945.graph_c0	6.4266915	7.5842305	8.9702185	116.83516	65.179378	41.710857	0.0017089	3.341335	up	PREDICTED: chaperone protein dnaJ 11, chloroplastic-like [Sesamum indicum]
c51947.graph_c0	4.6005533	3.6791405	4.8265894	33.023179	44.021307	40.82899	2.829E-61	3.205785	up	PREDICTED: aldehyde dehydrogenase family 2 member C4-like [Sesamum indicum]
c51952.graph_c0	271.85374	301.27874	351.76553	45.536477	49.810992	64.075108	7.871E-31	-2.498505	down	-
c51961.graph_c0	53.379754	69.21547	65.059948	8.05352	8.7415245	7.0484987	1.439E-40	-2.93406	down	PREDICTED: protein ABSCISIC ACID-INSENSITIVE 5 [Sesamum indicum]
c51962.graph_c0	1.0051831	1.2279105	1.0614627	14.390327	26.916612	25.942302	5.999E-15	4.383031	up	-
c51963.graph_c0	6.8298934	6.8349774	7.2122953	34.09351	38.077168	55.210931	2.771E-11	2.645858	up	PREDICTED: D-3-phosphoglycerate dehydrogenase 2, chloroplastic-like [Sesamum indicum]
c51965.graph_c0	10.934732	9.2894095	8.3512503	26.42386	56.419749	90.280419	0.0041296	2.62587	up	PREDICTED: subtilisin-like protease [Sesamum indicum]
c51971.graph_c0	15.672133	14.592669	15.905489	25.328619	30.252441	39.882099	3.428E-05	1.084299	up	PREDICTED: UDP-glucose 6-dehydrogenase 3 [Nicotiana sylvestris]
c51975.graph_c0	1.2320412	1.5674976	1.6450945	8.0924606	20.154025	28.739328	0.000293	3.70594	up	PREDICTED: vacuolar iron transporter homolog 2-like [Sesamum indicum]
c51977.graph_c0	5.1759146	7.1116432	6.2434407	25.387648	21.757862	16.371243	8.905E-07	1.825117	up	unnamed protein product [Coffea canephora]
c51985.graph_c0	8.3947753	7.1300521	8.475508	1.7133807	1.8151191	1.6850354	6.938E-08	-2.16136	down	PREDICTED: zinc finger protein 3 [Solanum lycopersicum]
c51987.graph_c0	3.8181629	4.5476909	4.64024	15.137255	17.563333	16.309448	1.61E-09	1.953699	up	PREDICTED: RPM1-interacting protein 4 [Sesamum indicum]
c51988.graph_c0	16.101922	17.642256	15.451901	33.940844	30.530385	35.777903	3.679E-08	1.069996	up	PREDICTED: thioredoxin F1, chloroplastic-like [Sesamum indicum]
c51992.graph_c0	1.3060614	1.7688765	1.4653863	2.6212544	3.9461227	4.2964828	0.00186	1.293029	up	PREDICTED: cucumber peeling cupredoxin-like [Sesamum indicum]
c52006.graph_c0	0	0.0590759	0	3.6491673	3.6022695	3.6897808	1.549E-23	7.579981	up	PREDICTED: probable leucine-rich repeat receptor-like protein kinase At5g49770 [Sesamum indicum]
c52017.graph_c0	8.0674152	8.2499696	8.1104791	2.0904066	2.7532023	2.2622001	2.099E-07	-1.743033	down	hypothetical protein MIMGU_mgv1a014249mg [Erythranthe guttata]
c52024.graph_c0	3.4607753	1.3887803	2.0918255	18.684466	19.966047	25.010272	5.067E-18	3.23559	up	Chlorophyll a-b binding protein 151 [Morus notabilis]
c52037.graph_c0	0.2954854	0.6669889	0.4822273	2.4345714	7.0430177	6.582119	6.74E-05	3.500945	up	hypothetical protein MIMGU_mgv1a017917mg [Erythranthe guttata]
c52039.graph_c0	7.4325088	9.5364833	5.5541409	0.8925397	2.2062544	1.5001111	6.039E-07	-2.262042	down	hypothetical protein MIMGU_mgv1a019582mg [Erythranthe guttata]
c52046.graph_c0	3.9641887	3.7849971	3.6251459	83.57508	95.818923	121.23435	5.649E-47	4.761735	up	PREDICTED: serine carboxypeptidase 1 [Sesamum indicum]
c52057.graph_c0	0.1416476	0.2131575	0.0963194	3.6807503	5.1039731	4.3401858	5.532E-31	4.90113	up	hypothetical protein MIMGU_mgv1a003539mg [Erythranthe guttata]
c52059.graph_c0	0.7261046	1.0086221	0.5924954	4.6727547	4.1251808	3.9058018	6.625E-09	2.494594	up	PREDICTED: shikimate O-hydroxycinnamoyltransferase-like [Sesamum indicum]
c52060.graph_c0	4.0307227	2.5020632	5.6872928	272.06692	258.56527	300.99428	1.03E-245	6.129092	up	--
c52063.graph_c0	28.403122	29.775507	31.900536	15.574353	15.570615	12.358186	1.858E-07	-1.006142	down	PREDICTED: uncharacterized protein LOC105166059 [Sesamum indicum]
c52068.graph_c0	14.96007	17.646021	19.68751	64.716249	61.805699	76.539503	4.121E-33	1.9981	up	PREDICTED: outer envelope pore protein 16, chloroplastic [Nicotiana tomentosiformis]
c52071.graph_c0	7.1097987	7.5963866	6.0916145	0.3004064	1.4320989	1.3364993	9.006E-09	-2.739294	down	hypothetical protein MIMGU_mgv1a026849mg [Erythranthe guttata]

#ID	28 h-1 FPKM	28 h-2 FPKM	28 h-3 FPKM	48 h-1 FPKM	48 h-2 FPKM	48 h-3 FPKM	FDR	log2FC	regulated	nr_annotation
c52075.graph_c0	4.4925381	5.5166233	4.7656367	1.0250321	2.0511392	2.6348614	0.0029426	-1.3423	down	-
c52084.graph_c0	14.465154	17.991084	15.580551	4.0338426	3.1079034	2.2433056	5.601E-21	-2.305115	down	PREDICTED: transcription factor TGA5-like [Sesamum indicum]
c52087.graph_c0	0.1824791	0.7139665	0.1985353	2.85277	4.3290456	3.4988448	2.055E-12	3.324224	up	hypothetical protein MIMGU_mgv1a014873mg [Erythranthe guttata]
c52090.graph_c0	0.8086569	0.2433804	0.7331749	21.411782	14.116664	15.809169	2.164E-18	4.894243	up	hypothetical protein glysoja_014339 [Glycine soja]
c52096.graph_c0	9.4586978	9.8022111	10.487819	1.7082802	3.1502459	3.7437466	2.275E-12	-1.759795	down	PREDICTED: pentatricopeptide repeat-containing protein At1g77405 [Sesamum indicum]
c52105.graph_c0	12.364434	13.835047	13.060352	4.9999213	5.1949486	7.357796	0.0008882	-1.122991	down	PREDICTED: ABC transporter C family member 5-like [Sesamum indicum]
c52110.graph_c0	43.474757	48.173123	50.105396	4.0268712	4.382858	2.8959186	3.441E-80	-3.604667	down	PREDICTED: oleosin 1 [Sesamum indicum]
c52113.graph_c0	30.668226	28.159863	28.842398	68.787629	78.456225	68.828357	2.688E-13	1.341963	up	-
c52118.graph_c0	3.230003	5.2533645	3.3163884	16.892239	11.97806	10.350133	0.0001229	1.784255	up	PREDICTED: MLO-like protein 6 [Sesamum indicum]
c52129.graph_c0	0.0734544	0.0994837	0.0599382	11.173067	19.826153	21.54041	8.196E-26	7.850026	up	PREDICTED: peroxidase 60 [Sesamum indicum]
c52131.graph_c0	8.4172742	9.3397706	8.4407175	15.554353	19.133489	20.931827	2.079E-06	1.123797	up	PREDICTED: uncharacterized protein LOC105174462 isoform X1 [Sesamum indicum]
c52133.graph_c0	4.9133774	4.7051869	5.3457011	0.7549173	0.5998075	0.6717209	9.942E-09	-2.838958	down	-
c52161.graph_c0	0.3408294	0.4308318	0.1668684	5.3571275	6.5905862	7.0732269	1.058E-19	4.380094	up	PREDICTED: non-specific lipid-transfer protein-like protein At2g13820 [Sesamum indicum]
c52165.graph_c0	1.5452132	0.8526116	1.5410772	2.8291921	2.1902513	3.5824512	0.0073525	1.168375	up	hypothetical protein MIMGU_mgv1a011603mg [Erythranthe guttata]
c52171.graph_c0	6.2462583	6.2765355	8.1385916	17.495325	17.04603	27.04381	1.536E-05	1.613542	up	enolase [Prunus armeniaca]
c52178.graph_c0	0.7384988	0.6667951	0.7633047	6.9895584	6.2145663	6.6079713	8.865E-16	3.235275	up	PREDICTED: protein MIZU-KUSSEI 1 [Sesamum indicum]
c52190.graph_c0	111.62668	134.44521	110.15871	38.438634	35.715492	23.975815	3.518E-22	-1.812838	down	PREDICTED: probable E3 ubiquitin-protein ligase RHA2B [Sesamum indicum]
c52192.graph_c0	37.321234	33.249602	34.187452	9.9226775	9.2534099	10.259211	4.826E-19	-1.788872	down	hypothetical protein MIMGU_mgv1a015849mg [Erythranthe guttata]
c52196.graph_c0	19.317999	16.86732	18.881351	63.773801	59.578765	63.689092	5.799E-11	1.806838	up	PREDICTED: aldo-keto reductase family 4 member C9-like [Sesamum indicum]
c52203.graph_c0	4.4198441	3.3963445	4.3739092	0.8344116	1.1365173	2.2273633	0.0012415	-1.507119	down	-
c52205.graph_c0	1.3200253	0.4469471	1.750336	66.301708	82.757491	78.907953	2.3E-134	6.054682	up	-
c52226.graph_c0	2.9453509	3.6489109	4.024268	15.454743	13.153129	10.970328	4.797E-09	1.944926	up	hypothetical protein MIMGU_mgv1a016982mg [Erythranthe guttata]
c52228.graph_c0	0.1768915	0.0532388	0.192456	2.4664525	4.0381295	3.1921958	2.81E-17	4.553368	up	NAC domain-containing protein 1 [Salvia miltiorrhiza]
c52231.graph_c0	19.830278	20.574908	26.401141	38.586483	60.498996	50.618156	9.644E-06	1.198754	up	PREDICTED: L-ascorbate oxidase homolog [Nicotiana tomentosiformis]
c52233.graph_c0	58.375412	67.718081	55.264526	137.09454	145.86926	189.21132	3.074E-11	1.419474	up	PREDICTED: probable calcium-binding protein CML18 [Sesamum indicum]
c52235.graph_c0	11.958267	17.687509	6.2912444	64.122264	84.093455	55.014735	1.869E-12	2.541176	up	PREDICTED: pathogenesis-related protein STH-2-like [Sesamum indicum]
c52242.graph_c0	8.052864	6.3493075	6.3859706	3.3066867	2.2845872	3.1981195	0.0066857	-1.195566	down	-

#ID	28 h-1 FPKM	28 h-2 FPKM	28 h-3 FPKM	48 h-1 FPKM	48 h-2 FPKM	48 h-3 FPKM	FDR	log2FC	regulated	nr_annotation
c52256.graph_c0	14.129736	13.148372	13.804325	8.5288004	6.0665115	4.3907064	5.807E-05	-1.061573	down	PREDICTED: uncharacterized protein LOC105163700 [Sesamum indicum]
c52272.graph_c0	0.3720278	0.1679531	0.3794646	22.046021	34.845288	29.267274	4.294E-42	6.583974	up	-
c52290.graph_c0	4.2329298	2.7207022	3.1613212	10.185451	14.258546	19.744291	4.85E-06	2.160438	up	hypothetical protein MIMGU_mgv1a011727mg [Erythranthe guttata]
c52292.graph_c0	20.41134	23.405498	23.317681	56.661599	49.336515	63.539422	4.611E-07	1.379001	up	hypothetical protein OsJ_01227 [Oryza sativa Japonica Group]
c52303.graph_c0	5.7307813	6.3717304	5.7970299	2.1012112	1.4627859	0.373929	1.895E-08	-2.125438	down	PREDICTED: MATE efflux family protein DTX1 [Sesamum indicum]
c52308.graph_c0	19.144409	19.56316	16.236104	7.4924503	6.7745472	6.8790537	7.349E-16	-1.333229	down	PREDICTED: cytochrome P450 703A2 [Sesamum indicum]
c52313.graph_c0	28.360641	29.632381	24.690858	53.79791	54.68066	59.021079	1.271E-13	1.059941	up	PREDICTED: LRR receptor-like serine/threonine-protein kinase GSO1 [Sesamum indicum]
c52322.graph_c0	17.733277	15.210912	17.94306	35.245873	43.577725	40.722033	5.092E-16	1.27055	up	hypothetical protein MIMGU_mgv1a018712mg, partial [Erythranthe guttata]
c52323.graph_c0	10.806844	7.7076577	12.375256	2.5324903	5.3657328	4.0970812	0.0004751	-1.334569	down	PREDICTED: putative defensin-like protein 120 [Solanum lycopersicum]
c52324.graph_c0	14.71209	13.057854	13.059204	6.0225129	6.1002922	5.1237608	3.01E-06	-1.199934	down	PREDICTED: uncharacterized protein LOC105157576 isoform X2 [Sesamum indicum]
c52330.graph_c0	2.5949753	2.4808441	1.4946908	7.3526029	4.0995743	5.8536385	0.0018916	1.448599	up	hypothetical protein MIMGU_mgv1a020446mg, partial [Erythranthe guttata]
c52335.graph_c0	3.9928674	4.0293242	4.5518232	15.481481	13.79871	12.38455	4.717E-09	1.773578	up	PREDICTED: uncharacterized protein LOC105167003 isoform X1 [Sesamum indicum]
c52336.graph_c0	0.3785462	0.7405487	0.87519	64.057997	126.91236	105.10196	1.694E-25	7.24494	up	hypothetical protein MIMGU_mgv1a020596mg [Erythranthe guttata]
c52337.graph_c0	0.7587686	1.21795	1.3070922	3.0991174	3.8491975	3.6133755	0.0001078	1.72371	up	PREDICTED: uncharacterized protein LOC105167330 [Sesamum indicum]
c52347.graph_c0	11.546894	14.866365	13.042727	41.537953	45.799059	54.686657	1.558E-26	1.88676	up	PREDICTED: LIM domain-containing protein WLIM1 [Sesamum indicum]
c52360.graph_c0	1.2431716	0.8017624	0.869501	6.753478	5.0081437	6.4098177	5.194E-09	2.686155	up	hypothetical protein MIMGU_mgv1a0032971mg, partial [Erythranthe guttata]
c52371.graph_c0	44.481635	37.830716	49.878624	2.910129	2.9865873	2.9940114	2.101E-46	-3.853021	down	hypothetical protein MIMGU_mgv1a015982mg [Erythranthe guttata]
c52372.graph_c0	6.5903287	7.5862297	5.4419291	14.876956	22.776675	27.777865	8.034E-06	1.771291	up	PREDICTED: ATP synthase gamma chain, chloroplastic-like [Sesamum indicum]
c52377.graph_c0	8.8334236	7.682527	8.4800018	17.536501	17.792819	18.49755	5.221E-09	1.148004	up	PREDICTED: uncharacterized protein LOC105168503 isoform X2 [Sesamum indicum]
c52393.graph_c0	2.3419079	3.2339767	3.3723136	152.61792	157.05546	146.05331	1.29E-146	5.712501	up	-
c52394.graph_c0	15.814945	18.642567	19.402108	68.353364	94.533925	93.95577	4.472E-25	2.289662	up	PREDICTED: protein RALF-like 34 [Phoenix dactylifera]
c52396.graph_c0	4.3194397	6.8000855	4.0668779	1.4038995	1.3385351	1.3740994	7.452E-05	-1.839549	down	protein with unknown function [Ricinus communis]
c52400.graph_c0	2.6445549	3.7653535	2.3239302	10.443293	12.29267	10.324866	4.99E-07	1.961607	up	hypothetical protein MIMGU_mgv1a005057mg [Erythranthe guttata]
c52403.graph_c0	8.8778617	12.120006	11.359004	28.448452	39.865673	42.215472	6.255E-13	1.807823	up	PREDICTED: dirigent protein 22-like [Sesamum indicum]
c52406.graph_c0	0.1612382	0.6551236	0.3947073	24.525762	31.070226	39.735818	1.955E-35	6.335265	up	hypothetical protein MIMGU_mgv1a016694mg [Erythranthe guttata]
c52420.graph_c0	2.8524712	3.0437893	2.063094	8.0531886	9.1407609	12.35714	2.595E-08	1.930247	up	flavonoid glycosyltransferase [Veronica persica]

#ID	28 h-1 FPKM	28 h-2 FPKM	28 h-3 FPKM	48 h-1 FPKM	48 h-2 FPKM	48 h-3 FPKM	FDR	log2FC	regulated	nr_annotation
c52432.graph_c0	0.9367638	0.7612289	0.6879524	10.805495	10.566565	10.354254	2.923E-26	3.775995	up	hypothetical protein MIMGU_mgv1a020209mg [Erythranthe guttata]
c52443.graph_c0	2.9832242	2.8949603	2.6390397	23.218849	31.785882	35.502483	3.137E-27	3.445186	up	PREDICTED: cinnamoyl-CoA reductase 1-like [Sesamum indicum]
c52447.graph_c0	2.397686	1.1891626	1.7360424	4.2806249	2.947622	3.8088743	0.003067	1.098908	up	ATPase subunit 1 [Silene latifolia]
c52460.graph_c0	4.3812683	4.7329213	3.830442	10.809647	13.027407	19.501444	2.69E-05	1.778865	up	hypothetical protein MIMGU_mgv1a007773mg [Erythranthe guttata]
c52462.graph_c0	0.5262763	0.8145911	0.8588743	4.0978627	3.5335256	3.0526774	3.021E-07	2.326335	up	endo-beta-1,4-D-glucanase [Solanum lycopersicum]
c52465.graph_c0	7.3947248	7.112183	9.1822273	1.0188433	1.0793409	0.1813121	3.04E-11	-3.327134	down	-
c52471.graph_c0	13.016275	10.716817	13.429071	86.983692	125.36506	134.03954	1.314E-23	3.255034	up	PREDICTED: bidirectional sugar transporter SWEET14-like [Solanum tuberosum]
c52484.graph_c0	7.2979916	5.7906867	12.992949	51.019243	33.260739	50.634687	9.395E-09	2.416138	up	histone H4 [Zea mays]
c52487.graph_c0	2.6563055	4.0136403	3.5830492	8.3832993	9.4634556	9.2936903	1.721E-05	1.444509	up	PREDICTED: long chain acyl-CoA synthetase 4 [Sesamum indicum]
c52489.graph_c0	8.8279772	10.656661	8.612949	4.6220025	4.1233273	3.2468132	0.0024919	-1.18151	down	hypothetical protein MIMGU_mgv1a012466mg [Erythranthe guttata]
c52491.graph_c0	6.9695028	5.5936067	7.4563641	3.1411051	1.2478575	1.9215188	0.0003398	-1.60895	down	PREDICTED: cation/calcium exchanger 3-like [Sesamum indicum]
c52492.graph_c0	1.1582337	0.7239991	0.5816057	8.9218138	4.9052503	3.1151375	0.0067306	2.840848	up	PREDICTED: uncharacterized protein LOC105168627 [Sesamum indicum]
c52493.graph_c0	7.6018132	10.189991	6.2984485	4.7438071	2.5127442	2.7040852	0.0024921	-1.218475	down	PREDICTED: E3 ubiquitin-protein ligase RHA2A-like [Sesamum indicum]
c52501.graph_c0	53.611931	78.040042	64.358417	15.46459	24.920403	27.908212	2.273E-07	-1.488034	down	-
c52505.graph_c0	10.992536	8.7973625	9.1736793	2.5334271	1.6774118	2.5360068	1.11E-05	-2.055632	down	PREDICTED: UDP-glycosyltransferase 73C3-like [Sesamum indicum]
c52506.graph_c0	1.078598	1.2984972	0.749738	9.0640459	6.0081164	3.829812	0.0008882	2.651305	up	PREDICTED: uncharacterized protein LOC105159772 [Sesamum indicum]
c52508.graph_c0	7.6353761	6.2916379	6.6538301	14.752515	26.777495	30.266657	7.839E-05	1.833456	up	hypothetical protein MIMGU_mgv1a018889mg [Erythranthe guttata]
c52517.graph_c0	39.893974	42.257286	35.888993	16.081871	15.901006	12.878597	1.065E-06	-1.351333	down	PREDICTED: uncharacterized protein LOC105169022 [Sesamum indicum]
c52518.graph_c0	25.964535	23.309578	23.72927	37.613669	49.211195	58.737071	2.495E-05	1.031289	up	PREDICTED: protein YLS9 [Sesamum indicum]
c52533.graph_c0	12.687838	21.064126	14.138203	267.00855	331.59479	267.74205	1.593E-88	4.217453	up	-
c52540.graph_c0	26.36447	21.996697	23.903547	42.161406	56.565578	50.940054	4.683E-13	1.087681	up	PREDICTED: flavonol synthase/flavanone 3-hydroxylase-like [Sesamum indicum]
c52543.graph_c0	8.4221218	9.4845918	9.2890446	2.1703264	3.6041477	5.636851	0.0002004	-1.222868	down	-
c52548.graph_c0	14.646935	11.172684	11.95178	5.921936	4.7475688	6.1237846	0.0048901	-1.125318	down	-
c52556.graph_c0	10.953361	10.277696	8.0615932	5.1725422	3.3166492	3.9969052	0.0036352	-1.180291	down	-
c52585.graph_c0	1.5074498	2.0416287	1.8963528	2.7069718	4.3859069	4.888137	0.0051562	1.170449	up	hypothetical protein MIMGU_mgv1a017183mg [Erythranthe guttata]
c52587.graph_c0	0.1971621	0.1780189	0.2011034	1.3745464	1.3237866	1.1674694	2.462E-09	2.789687	up	-
c52603.graph_c0	1.6613751	1.6200712	1.5183487	6.9074021	12.493387	10.418499	4.816E-10	2.668121	up	-
c52609.graph_c0	0.7424256	1.3406815	2.1203464	4.1172215	5.3586562	9.001691	0.0010538	2.168135	up	-

#ID	28 h-1 FPKM	28 h-2 FPKM	28 h-3 FPKM	48 h-1 FPKM	48 h-2 FPKM	48 h-3 FPKM	FDR	log2FC	regulated	nr_annotation
c52612.graph_c0	0.1488902	0.1344339	0.1214932	5.2844211	5.5982034	3.5265346	6.654E-16	5.198324	up	-
c52616.graph_c0	0.3392559	0.8423698	0.5536601	14.29856	17.653207	13.009752	9.998E-29	4.73659	up	PREDICTED: reticuline oxidase-like protein [Sesamum indicum]
c52620.graph_c0	0.162164	0.2928379	0.1984868	10.174966	12.738987	18.838853	1.124E-17	6.032934	up	PREDICTED: probable pectate lyase 8 [Sesamum indicum]
c52626.graph_c0	0.5761727	0.6358365	0.9403044	29.173077	48.571671	45.598039	1.285E-33	5.873314	up	PREDICTED: probable pectinesterase/pectinesterase inhibitor 46 [Sesamum indicum]
c52638.graph_c0	23.10821	21.875081	20.682629	57.748205	54.988774	62.893429	2.347E-18	1.461334	up	hypothetical protein MIMGU_mgv1a016092mg [Erythranthe guttata]
c52646.graph_c0	4.713662	6.9841967	5.9173996	29.108554	44.794777	39.53252	3.604E-19	2.722371	up	-
c52659.graph_c0	0.581544	0.6300956	0.9965238	2.5800245	3.514144	2.2956903	1.245E-05	1.964234	up	PREDICTED: LOW QUALITY PROTEIN: phototropin-1 [Sesamum indicum]
c52660.graph_c0	2.1055535	1.442227	1.2441518	10.491711	9.7497332	8.5165614	7.78E-12	2.630331	up	PREDICTED: protein RADIALIS-like 3 [Sesamum indicum]
c52674.graph_c0	122.50936	129.24427	147.31925	29.423299	24.763165	28.071501	2.196E-38	-2.234208	down	hypothetical protein PHAVU_005G056100g [Phaseolus vulgaris]
c52675.graph_c0	29.590502	27.875205	31.550261	6.8764925	5.827848	6.1186617	2.692E-14	-2.196732	down	PREDICTED: uncharacterized protein LOC102593371 [Solanum tuberosum]
c52679.graph_c0	17.566575	29.332496	25.913222	2.0821024	1.8993829	2.5731141	3.285E-14	-3.43229	down	PREDICTED: transcription factor TGA5-like [Sesamum indicum]
c52690.graph_c0	0.1927172	0.406013	1.048371	21.252641	38.732007	45.86333	8.67E-16	6.034414	up	PREDICTED: 21 kDa protein-like [Sesamum indicum]
c52694.graph_c0	18.422984	20.305746	18.283384	3.0505504	6.0176265	13.946202	0.003322	-1.283088	down	PREDICTED: expansin-A10-like isoform X2 [Camelina sativa]
c52697.graph_c0	0.1073859	0.0969594	0.175252	3.9474664	5.4796812	6.9037454	5.391E-18	5.460232	up	MYB-related transcription factor [Salvia miltiorrhiza]
c52698.graph_c0	4.7834846	6.838477	5.8549266	2.5264246	2.6764405	1.5735982	0.0029228	-1.321553	down	-
c52705.graph_c0	5.2464377	3.6091744	2.4463145	19.950739	11.741882	17.18849	3.588E-06	2.16249	up	PREDICTED: succinate dehydrogenase [ubiquinone] iron-sulfur subunit 2, mitochondrial-like [Sesamum indicum]
c52711.graph_c0	37.561981	34.208583	31.579066	7.4201419	11.136049	10.637196	3.003E-08	-1.788656	down	PREDICTED: 7-deoxyloganetic acid glucosyltransferase-like [Sesamum indicum]
c52717.graph_c0	3.6687492	7.6602409	6.9228605	24.995953	28.635545	23.017106	3.64E-11	2.111569	up	PREDICTED: pleiotropic drug resistance protein 1-like [Sesamum indicum]
c52718.graph_c0	5.1341685	7.262554	6.2841593	34.925927	32.403538	25.093133	1.409E-11	2.352717	up	hypothetical protein M569_04419, partial [Genlisea aurea]
c52721.graph_c0	0.2904212	0.1966673	0.177736	2.3928464	4.3873799	4.0126107	6.285E-13	4.053206	up	PREDICTED: transcription repressor OFP17 [Nicotiana tomentosiformis]
c52739.graph_c0	7.8088477	6.385501	8.4157909	2.054362	1.5827981	3.1573836	0.0001959	-1.694712	down	-
c52742.graph_c0	1.6885399	2.2673438	1.3425057	10.591982	14.011616	12.794128	2.555E-21	2.857743	up	PREDICTED: palmitoyl-protein thioesterase 1-like [Sesamum indicum]
c52750.graph_c0	7.6329325	8.452235	5.6799971	17.646781	36.422286	31.024986	3.31E-05	1.998339	up	-
c52758.graph_c0	18.387957	24.545572	21.697043	3.1021558	2.2205121	2.6856774	4.347E-23	-2.965293	down	PREDICTED: auxin-induced protein X15-like [Sesamum indicum]
c52764.graph_c0	2.1602721	3.0341471	2.4809276	6.7950506	5.801802	8.3924798	0.0001723	1.493432	up	-
c52769.graph_c0	10.898518	12.115563	11.103526	26.032276	37.052704	42.845115	2.816E-08	1.6692	up	PREDICTED: uncharacterized protein LOC105168965 [Sesamum indicum]
c52771.graph_c0	2.6809729	1.8577213	2.0859007	10.274023	9.4607782	7.8759809	5.758E-09	2.104568	up	PREDICTED: CASP-like protein 2C1 isoform X2 [Sesamum indicum]

#ID	28 h-1 FPKM	28 h-2 FPKM	28 h-3 FPKM	48 h-1 FPKM	48 h-2 FPKM	48 h-3 FPKM	FDR	log2FC	regulated	nr_annotation
c52778.graph_c0	41.434853	51.929794	38.150357	10.347564	23.584887	10.183722	1.984E-06	-1.543148	down	PREDICTED: uncharacterized protein LOC104588233 isoform X2 [Nelumbo nucifera]
c52789.graph_c0	155.60666	167.92425	146.78322	57.191435	81.101232	66.115104	6.471E-16	-1.164855	down	hypothetical protein MIMGU_mgv1a008833mg [Erythranthe guttata]
c52791.graph_c0	23.78866	24.44154	30.694798	3.4164492	4.5634825	2.2469092	1.259E-17	-2.907173	down	hypothetical protein MIMGU_mgv1a018283mg [Erythranthe guttata]
c52792.graph_c0	39.700647	41.879441	46.027134	15.694951	15.307303	15.295299	3.987E-06	-1.420232	down	-
c52795.graph_c0	37.322778	40.653863	42.118731	11.072406	13.329399	12.718243	4.258E-14	-1.655024	down	PREDICTED: protein ELF4-LIKE 3 [Sesamum indicum]
c52802.graph_c0	0.1447145	0.2613273	0.3542577	1.7426453	4.0808998	2.7747494	4.909E-06	3.528623	up	PREDICTED: adenine phosphoribosyltransferase 3 [Nicotiana tomentosiformis]
c52812.graph_c0	14.951907	12.400156	12.923634	3.5097487	3.1232486	0.999345	2.162E-09	-2.347435	down	PREDICTED: peamaclein [Vitis vinifera]
c52825.graph_c0	0	0	0.3276424	4.4110262	6.1107774	11.774714	1.554E-06	6.114385	up	hypothetical protein MIMGU_mgv1a016882mg [Erythranthe guttata]
c52837.graph_c0	3.2137282	3.5713172	3.4796911	7.4422217	7.8011406	8.2252759	0.0006587	1.233837	up	hypothetical protein CISIN_1g041913mg [Citrus sinensis]
c52841.graph_c0	6.6772697	5.3438407	6.5630834	23.981138	33.013055	32.520897	1.393E-26	2.304032	up	PREDICTED: probable calcium-binding protein CML49 [Sesamum indicum]
c52842.graph_c0	0.2429314	0.2924591	0.506588	5.8507365	13.3387	32.849241	0.0019733	5.664884	up	PREDICTED: endoglucanase 1-like [Sesamum indicum]
c52850.graph_c0	1.3020602	0.8708432	1.4166274	61.922711	97.201387	93.335588	3.146E-45	6.169604	up	PREDICTED: aquaporin TIP4-1 [Sesamum indicum]
c52853.graph_c0	0.6683558	0.1508657	0.5453729	30.49885	21.988636	16.772602	2.566E-13	5.715478	up	-
c52857.graph_c0	0.229622	0.1382181	0.1249131	2.6195656	1.8500748	1.8129018	1.419E-11	3.722159	up	PREDICTED: basic leucine zipper 43 [Sesamum indicum]
c52876.graph_c0	11.574791	14.84338	12.73013	30.194345	24.778854	24.217913	0.0017865	1.063011	up	PREDICTED: muscle M-line assembly protein unc-89-like [Populus euphratica]
c52882.graph_c0	32.420423	36.946003	35.701139	10.373564	7.6081386	7.1002578	3.367E-07	-2.017363	down	-
c52891.graph_c0	1.5768851	0.776607	1.9885756	5.0879004	9.8175247	9.7011069	1.545E-06	2.532036	up	PREDICTED: anthocyanidin 3-O-glucosyltransferase 2-like [Sesamum indicum]
c52908.graph_c0	23.609768	23.962632	23.437199	8.9562912	10.413773	9.7186025	8.221E-08	-1.247732	down	PREDICTED: transcription factor TCP9 [Sesamum indicum]
c52909.graph_c0	1.8537224	1.2553031	0.945389	6.2659457	4.9785073	5.3140539	7.341E-07	2.076679	up	PREDICTED: glucan endo-1,3-beta-glucosidase 4-like isoform X1 [Sesamum indicum]
c52916.graph_c0	0.0450932	0	0.0367957	2.8579494	2.9065448	2.5938459	3.095E-23	6.714055	up	PREDICTED: pectinesterase/pectinesterase inhibitor PPE8B-like [Sesamum indicum]
c52919.graph_c0	0.9258821	2.0899618	1.7628619	7.2373465	7.4598724	9.9206919	6.441E-08	2.404291	up	V-type proton ATPase subunit [Morus notabilis]
c52926.graph_c0	5.3383757	10.088482	8.7121419	16.366176	17.754757	24.574261	3.233E-05	1.319819	up	-
c52935.graph_c0	1.237877	1.8235944	1.5948909	6.9783672	6.999038	8.7444709	1.121E-13	2.327109	up	PREDICTED: GDSL esterase/lipase At4g10955-like [Sesamum indicum]
c52942.graph_c0	0	0.129777	0.0586423	3.1883532	9.457486	8.8351584	9.553E-09	6.860157	up	-
c52943.graph_c0	18.166092	12.900668	21.152468	1.5523726	0.5481835	0.6906459	6.804E-15	-4.164387	down	-
c52953.graph_c0	11.820439	16.332538	12.27594	37.231172	44.492401	37.168082	1.775E-07	1.596781	up	-

#ID	28 h-1 FPKM	28 h-2 FPKM	28 h-3 FPKM	48 h-1 FPKM	48 h-2 FPKM	48 h-3 FPKM	FDR	log2FC	regulated	nr_annotation
c52961.graph_c0	9.6255779	13.238608	11.324939	2.9793454	5.4107233	6.3119135	0.002963	-1.186575	down	-
c52970.graph_c0	25.191927	23.491717	26.903057	12.73815	12.939956	9.9369463	1.18E-05	-1.04188	down	hypothetical protein MIMGU_mgv1a014883mg [Erythranthe guttata]
c52974.graph_c0	29.858446	27.556263	31.556646	308.0893	425.80735	545.66238	1.433E-16	3.880177	up	-
c52975.graph_c0	10.610114	7.0744157	7.4589985	1.0345462	2.4111478	2.0251726	2.879E-06	-2.171382	down	-
c52984.graph_c0	5.8120718	6.1223819	6.5094553	1.3000992	1.300781	0.8354815	5.386E-09	-2.378671	down	-
c52986.graph_c0	0	0.0852407	0.0770354	2.8720285	3.2327263	5.2175071	1.956E-14	6.160328	up	PREDICTED: thioredoxin H2 [Sesamum indicum]
c52989.graph_c0	6.0654077	5.0988052	5.2906558	0.7953467	1.1234311	1.1794911	7.106E-07	-2.37364	down	-
c52991.graph_c0	8.4075419	8.5190381	9.5284522	4.4996976	2.759775	2.844808	0.0007167	-1.336674	down	hypothetical protein M569_07000 [Genlisea aurea]
c53005.graph_c0	5.0727419	5.009605	4.2686696	1.9089282	1.1707925	2.1455404	0.0014781	-1.41163	down	-
c53014.graph_c0	1.9971903	1.5027297	1.2222682	12.235998	18.102881	24.403057	9.397E-10	3.56824	up	unknown [Picea sitchensis]
c53027.graph_c0	2.3663954	1.8398785	2.9500767	0.9998601	0.2648076	0.0741391	1.92E-05	-2.34111	down	-
c53033.graph_c0	3.5112571	5.9255089	5.4233332	22.518812	23.855951	23.997379	7.163E-26	2.284637	up	PREDICTED: pleiotropic drug resistance protein 1-like [Sesamum indicum]
c53039.graph_c0	6.5275401	9.4545681	7.3238267	1.379023	1.0956807	0.766904	2.963E-09	-2.795558	down	PREDICTED: premnaspirodiene oxygenase-like [Sesamum indicum]
c53045.graph_c0	5.6515236	8.4660024	5.9391781	3.4190493	2.9896436	1.0140832	0.0008005	-1.381022	down	PREDICTED: uncharacterized protein At1g76070 [Sesamum indicum]
c53051.graph_c0	6.7428509	5.3671956	9.2667138	104.3641	71.840895	87.15871	1.36E-19	3.669968	up	PREDICTED: histone H2A.1 [Sesamum indicum]
c53052.graph_c0	0.1927172	0.1740056	0.2620928	24.102612	36.575437	37.531129	3.926E-47	7.320702	up	unnamed protein product [Coffea canephora]
c53054.graph_c0	180.1795	208.61068	212.44761	63.107981	66.855254	52.992971	5.424E-23	-1.673597	down	PREDICTED: LOW QUALITY PROTEIN: 36.4 kDa proline-rich protein [Sesamum indicum]
c53073.graph_c0	4.8473404	3.6472449	2.8017347	0.6400376	1.220476	1.7085048	0.0007154	-1.632932	down	-
c53082.graph_c0	4.1493208	4.3458793	4.6047042	21.985001	26.187819	35.661093	6.952E-13	2.714099	up	hypothetical protein SORBIDRAFT_07g000600 [Sorghum bicolor]
c53101.graph_c0	0.863915	0.3600158	1.0845348	9.6029737	14.278156	16.639617	1.894E-18	4.16588	up	-
c53108.graph_c0	17.837362	22.660016	21.240353	0.5258179	2.2281613	1.9884459	2.072E-21	-3.680339	down	-
c53119.graph_c0	33.921463	38.817179	34.192493	7.5878741	7.5512548	5.7286682	1.114E-11	-2.312618	down	-
c53137.graph_c0	0.980642	4.7222816	2.1338557	16.159468	14.924254	28.019831	6.093E-06	2.954074	up	-
c53141.graph_c0	6.2030957	8.0011626	6.7303597	2.3329426	3.2952931	2.6909015	0.0009393	-1.293946	down	PREDICTED: squamosa promoter-binding-like protein 9 [Sesamum indicum]
c53173.graph_c0	14.431817	15.20234	14.516628	28.763193	33.091631	38.850653	2.3E-12	1.227811	up	PREDICTED: 30S ribosomal protein 2, chloroplastic [Sesamum indicum]
c53202.graph_c0	1.0097751	0.663078	0.749062	2.2108473	2.3421247	3.8308606	8.1E-05	1.827933	up	PREDICTED: glutathione S-transferase U17-like [Sesamum indicum]
c53219.graph_c0	0	0.1791172	0	14.081726	7.7253316	9.1736295	9.561E-12	7.492181	up	PREDICTED: CBL-interacting serine/threonine-protein kinase 5 [Sesamum indicum]
c53222.graph_c0	9.6372751	11.948405	7.5118212	24.431912	22.405875	26.444157	2.515E-05	1.375675	up	PREDICTED: zeatin O-glucosyltransferase-like [Solanum lycopersicum]

#ID	28 h-1 FPKM	28 h-2 FPKM	28 h-3 FPKM	48 h-1 FPKM	48 h-2 FPKM	48 h-3 FPKM	FDR	log2FC	regulated	nr_annotation
c53235.graph_c0	4.0305525	4.0347774	3.2888979	18.881169	22.237861	17.294478	7.997E-14	2.404339	up	PREDICTED: elicitor-responsive protein 3-like [Sesamum indicum]
c53256.graph_c0	0.1664325	0.3005459	0.2716152	11.814067	20.114312	18.583866	3.561E-24	6.128998	up	hypothetical protein MIMGU_mgv1a010045mg [Erythranthe guttata]
c53267.graph_c0	6.649608	6.2110061	6.4550997	17.729688	16.781045	19.008509	9.304E-06	1.51234	up	hypothetical protein JCGZ_13755 [Jatropha curcas]
c53275.graph_c0	10.267603	12.301483	9.1838842	3.0034484	3.9772368	2.4497461	0.0001096	-1.710797	down	ATP binding protein, putative [Ricinus communis]
c53282.graph_c0	16.628756	15.218482	14.676597	2.4376118	5.1647083	6.1241572	4.373E-06	-1.732452	down	-
c53299.graph_c0	9.3210463	11.221374	8.5652	29.058791	29.769399	33.14915	7.657E-12	1.70106	up	PREDICTED: probable polygalacturonase [Sesamum indicum]
c53313.graph_c0	5.9663498	6.9433147	6.7077011	0.6722478	2.4925776	1.6449457	1.056E-05	-2.003892	down	PREDICTED: uncharacterized protein LOC100815796 [Glycine max]
c53316.graph_c0	4.8657474	5.0618614	5.6966754	1.3408029	0.8522508	2.6246843	0.0003173	-1.65896	down	-
c53342.graph_c0	29.329529	29.81519	36.484748	10.919218	12.118425	17.118411	0.0005145	-1.215065	down	hypothetical protein MIMGU_mgv1a006294mg [Erythranthe guttata]
c53357.graph_c0	12.563498	13.234271	14.134936	1.9303224	1.0224712	0.4293973	7.718E-13	-3.499205	down	-
c53377.graph_c0	18.12242	20.752878	21.279928	53.973692	52.628038	60.987358	3.438E-10	1.51943	up	-
c53394.graph_c0	3.4626808	2.4425595	2.1191394	6.8581296	8.1371995	10.129855	2.829E-05	1.683988	up	uncharacterized protein [Arabidopsis thaliana]
c53452.graph_c0	2.8721826	3.8035236	4.2967421	1.0922109	1.2856278	1.4037728	0.0009006	-1.498782	down	PREDICTED: trihelix transcription factor GT-3b [Sesamum indicum]
c53474.graph_c0	5.0555212	4.8413078	4.37528	0.5825678	0	0.3455769	5.088E-12	-3.873079	down	-
c53487.graph_c0	6.3186279	6.3237569	6.2119864	29.721355	36.393108	37.264653	7.743E-25	2.493095	up	PREDICTED: rapid alkalinization factor-like [Sesamum indicum]
c53497.graph_c0	12.141353	11.25099	9.6465276	28.75511	24.884902	23.063548	0.0019933	1.261135	up	PREDICTED: UDP-arabinopyranose mutase 3-like [Sesamum indicum]
c53512.graph_c0	4.5079379	3.0877723	2.4100128	19.703943	15.446714	12.09739	5.303E-07	2.289137	up	PREDICTED: uncharacterized protein LOC105165055 [Sesamum indicum]
c53532.graph_c0	5.3338708	6.5497399	5.5710656	22.446766	23.779627	25.267045	8.994E-08	2.074957	up	hypothetical protein MIMGU_mgv1a011661mg [Erythranthe guttata]
c53541.graph_c0	0.5205314	0.3655486	0.8494987	1.6128734	2.2523032	1.8917558	8.387E-05	1.765646	up	-
c53554.graph_c0	42.895024	43.448583	41.575967	19.87222	20.175032	15.471909	0.0002087	-1.160093	down	-
c53594.graph_c0	2.8153338	1.694655	1.9144078	11.003316	15.437223	15.612172	7.263E-10	2.746223	up	PREDICTED: adenosine kinase 2 [Sesamum indicum]
c53622.graph_c0	15.515892	15.780699	16.881122	0.6781909	2.155383	2.2126507	2.797E-12	-3.230829	down	-
c53626.graph_c0	14.205555	22.21137	16.82198	45.455652	60.484229	60.766944	1.792E-10	1.68397	up	-
c53764.graph_c0	16.709857	16.110311	15.021728	45.951944	31.946587	47.595912	2.811E-05	1.436923	up	-
c53783.graph_c0	8.28472	13.984956	11.463056	28.764958	33.375175	30.063836	0.0002574	1.491109	up	-
c53824.graph_c0	20.123768	15.276583	18.826429	11.048206	6.1963594	8.5295148	0.0038941	-1.022458	down	-
c53904.graph_c0	5.5646843	6.5130958	5.5497896	11.494832	16.882278	13.714862	0.0020884	1.292465	up	PREDICTED: alpha-galactosidase-like [Sesamum indicum]
c53936.graph_c0	2.0530371	4.07814	4.2439957	6.2457017	7.1679439	8.7991973	0.0092366	1.135873	up	-
c53939.graph_c0	10.330629	7.2002446	6.3592542	17.115177	29.813466	36.181607	0.0001765	1.829897	up	-
c53976.graph_c0	0.0640676	0	0.0522786	1.0557351	1.7206513	1.951034	1.499E-15	5.374948	up	PREDICTED: uncharacterized protein LOC105155633 [Sesamum indicum]

#ID	28 h-1 FPKM	28 h-2 FPKM	28 h-3 FPKM	48 h-1 FPKM	48 h-2 FPKM	48 h-3 FPKM	FDR	log2FC	regulated	nr_annotation
c53979.graph_c0	6.1981967	8.309792	8.2762002	13.332532	23.204045	22.667199	0.0002338	1.410087	up	-
c53999.graph_c0	3.8149489	4.8223576	4.5656851	21.599588	17.417752	12.621553	1.457E-05	2.016998	up	-
c54007.graph_c0	2.7145251	1.5479758	2.5647719	25.534807	39.137662	48.825371	1.36E-13	4.087299	up	PREDICTED: probable aquaporin PIP2-5 [Sesamum indicum]
c54095.graph_c0	3.5794578	2.4624113	3.0598945	16.636497	13.504373	20.570479	5.221E-10	2.519991	up	-
c54198.graph_c0	6.1894155	7.1125878	5.9687875	17.830773	17.000587	11.106	0.0032392	1.300073	up	-
c54753.graph_c0	4.1691461	3.764348	3.8272378	18.166155	15.045965	22.042003	8.121E-08	2.274299	up	Aldo-keto reductase family 4 member C9 [Gossypium arboreum]
c55343.graph_c0	1.3420048	1.6660937	2.1901299	5.1032696	5.1810331	6.2436806	0.000118	1.70847	up	-
c55911.graph_c0	0.7905649	1.9034825	1.1826732	4.3424298	4.6002778	6.3902431	4.618E-06	2.022695	up	-
c58690.graph_c0	0	0	0.3177979	6.9114058	8.3677673	11.640555	1.178E-18	6.435017	up	hypothetical protein MIMGU_mgv1a015676mg [Erythranthe guttata]
c64432.graph_c0	5.8691018	4.2898679	7.52579	19.130191	16.888431	18.598034	8.906E-05	1.669685	up	-
