# Supplementary material for: Transcriptomic analysis of seed germination improvement of Andrographis paniculata responding to air plasma treatment
Source: PLoS One. 2020 Oct 22;15(10):e0240939. doi: 10.1371/journal.pone.0240939 (PMC7580921; doi:10.1371/journal.pone.0240939)
Supplement: S6 Table — (PDF) [file pone.0240939.s008.pdf]

S6 Table. Differentially expressed genes of plasma-treated groups (48 HAS vs. 0 HAS).

#ID	0 h-1 FPKM	0 h-2 FPKM	0 h-3 FPKM	48 h-1 FPKM	48 h-2 FPKM	48 h-3 FPKM	FDR	log2FC	regulated	nr_annotation
c13791.graph_c0	0	0	0	40.7062495	45.402211	45.9377843	2.754E-37	Inf	up	--
c13810.graph_c0	2.08421284	1.25177992	1.60697761	7.42443619	9.89941658	9.22592609	0.004572	1.404197	up	PREDICTED: SNF2 domain-containing protein CLASSY 3-like [Sesamum indicum]
c13899.graph_c0	1.02487953	0.92331521	0.92190754	0.04720929	0.10002504	0.08401309	2.542E-13	-4.661973	down	-
c13905.graph_c0	33.5266557	27.6762916	27.9760259	28.6807319	17.2103802	8.9230569	3.567E-09	-1.702674	down	PREDICTED: monothiol glutaredoxin-S4-like [Sesamum indicum]
c13919.graph_c0	18.3674562	18.7499246	16.8258357	17.0888363	15.4412631	19.9759104	0.0009064	-1.055537	down	-
c13925.graph_c0	0	0	0	7.1480873	9.73611224	8.17756084	1.095E-12	Inf	up	PREDICTED: homeobox-leucine zipper protein HOX21-like [Sesamum indicum]
c13954.graph_c0	30.8512077	29.2567268	34.4285729	3.3657803	2.77327253	1.33104492	5.178E-23	-4.67144	down	flavin-containing monooxygenase family protein FMO1 [Gossypium hirsutum]
c14030.graph_c0	0.41170484	0.13737237	0.08817617	23.7055848	26.1846888	32.2302366	1.821E-48	6.000527	up	RecName: Full=Galactinol synthase 1; Short=ArGolS1; Short=GolS-1 [Ajuga reptans]
c14063.graph_c0	0.68622231	0.68690946	1.17576379	10.1853277	11.7575097	12.750483	1.215E-10	2.73971	up	PREDICTED: zinc finger protein ZAT5 [Sesamum indicum]
c14105.graph_c0	45.1982713	46.7141615	44.7550132	27.2289516	36.605454	28.3370262	6.444E-22	-1.589295	down	PREDICTED: protein EXORDIUM-like 2 [Sesamum indicum]
c14220.graph_c0	75.8344644	85.6198711	65.9489167	25.833305	36.8339144	39.9007809	1.14E-29	-2.171114	down	PREDICTED: transcription factor GTE7-like [Sesamum indicum]
c14260.graph_c0	44.0279329	43.5981275	43.8020415	1.85361253	1.84816726	2.61952933	2.59E-101	-5.398096	down	-
c14261.graph_c0	0.13025651	0	0	7.92005234	6.35631447	7.47431888	1.372E-12	6.39044	up	PREDICTED: probable mitochondrial chaperone BCS1-B [Sesamum indicum]
c14340.graph_c0	3.26653414	3.38255698	4.92135426	26.4614758	27.3731315	23.6375964	7.855E-08	1.721691	up	-
c14379.graph_c0	0	0	0	7.44390076	28.5105986	29.0416672	5.968E-06	Inf	up	-
c14382.graph_c0	61.137143	69.9492103	62.8952639	70.5766074	29.3007213	19.5656426	3.598E-07	-1.694932	down	PREDICTED: protein SHORT INTERNODES [Sesamum indicum]
c14444.graph_c0	1.00984902	0.44927122	1.00931909	5.2719198	6.89906796	7.45028874	0.0001437	1.965207	up	hypothetical protein PHAVU_001G014100g [Phaseolus vulgaris]
c14524.graph_c0	2.37243356	1.65684362	1.91428182	0	0	0.0452272	8.392E-22	-8.077752	down	hypothetical protein MIMGU_mgv1a007850mg [Erythranthe guttata]
c14644.graph_c0	0.63711619	0.3826525	0.81871952	32.1650237	53.3509135	58.9117445	1.077E-19	5.26396	up	-
c14708.graph_c0	0	0	0	1.19283538	1.6038818	1.02055553	2.134E-11	Inf	up	PREDICTED: hyoscyamine 6-dioxygenase [Sesamum indicum]
c14733.graph_c0	3.18141577	3.89229069	3.90654543	23.1217631	31.1186243	32.8598153	1.195E-14	1.963118	up	PREDICTED: fasciclin-like arabinogalactan protein 17 [Sesamum indicum]
c14818.graph_c0	0	0.21331062	0.54767676	8.24540089	7.69512119	9.95696103	4.197E-09	4.050388	up	enolase [Solanum lycopersicum]
c14888.graph_c0	0	0	0	13.4601215	20.6760816	16.1685925	9.465E-13	Inf	up	-
c14914.graph_c0	38.4457824	40.0916187	43.1551146	36.0950156	36.4472716	32.4179909	1.412E-16	-1.231224	down	PREDICTED: uncharacterized protein LOC105172581 [Sesamum indicum]

#ID	0 h-1 FPKM	0 h-2 FPKM	0 h-3 FPKM	48 h-1 FPKM	48 h-2 FPKM	48 h-3 FPKM	FDR	log2FC	regulated	nr_annotation
c14919.graph_c0	65.4938286	66.8890088	73.0018135	1.97672531	1.19662901	0.83756109	6.42E-189	-6.683442	down	hypothetical protein MIMGU_mgv1a025595mg [Erythranthe guttata]
c14921.graph_c0	2.11867605	1.749658	1.70161412	0	0.05169404	0	1.312E-21	-7.802609	down	-
c14972.graph_c0	0	0	0	2.30379017	2.44058634	2.04989864	1.659E-09	Inf	up	AT3G62290 [Arabidopsis thaliana]
c14990.graph_c0	27.6138836	31.9065371	27.3760719	14.7570197	15.7385475	15.9160089	2.071E-30	-1.922882	down	PREDICTED: F-box protein SKIP1 [Sesamum indicum]
c15003.graph_c0	9.23689238	13.4993669	10.6827952	7.31827986	7.75283012	4.84596198	1.227E-05	-1.761886	down	-
c15064.graph_c0	1.35092647	2.52015672	1.14417786	10.5504879	7.97070257	8.38103032	0.0001043	1.415206	up	cytochrome P450 CYP81B61 [Salvia miltiorrhiza]
c15079.graph_c0	16.6348809	19.5981067	20.0569447	7.75716825	9.68762584	8.97857777	4.931E-22	-2.113489	down	PREDICTED: plasma membrane ATPase 1-like [Sesamum indicum]
c15088.graph_c0	35.864785	35.6411751	38.2027904	17.5156998	8.01271459	9.13869094	7.155E-45	-2.665054	down	PREDICTED: uncharacterized protein LOC105156392 [Sesamum indicum]
c15089.graph_c0	17.7664581	18.5101362	11.6482663	3.8413802	5.13107911	3.12081639	7.944E-15	-3.001948	down	hypothetical protein MIMGU_mgv1a022353mg [Erythranthe guttata]
c15108.graph_c0	8.36182602	9.02668533	13.0628864	6.79717018	8.96096769	10.4833407	0.0027357	-1.243038	down	PREDICTED: elongation of fatty acids protein 3-like [Sesamum indicum]
c15121.graph_c0	4.43412995	1.47952335	1.50364747	0	0	0	5.045E-06	-Inf	down	PREDICTED: oxygen-evolving enhancer protein 1, chloroplastic [Elaeis guineensis]
c15133.graph_c0	0.68325502	0.78916061	1.41832344	4.64831327	5.74504499	5.98864299	0.000377	1.473046	up	PREDICTED: uncharacterized protein LOC105171932 [Sesamum indicum]
c15145.graph_c0	1.4974279	1.32691929	1.45107689	0.04523043	0.02395808	0	1.414E-26	-6.941185	down	PREDICTED: cucumisin-like [Sesamum indicum]
c15147.graph_c0	2.4447761	1.34094475	1.63537116	0	0	0	6.726E-20	-Inf	down	UPF0012 hydrolase C26A3.11 [Taphrina deformans PYCC 5710]
c15152.graph_c0	0.14374472	0	0	8.47532901	10.6620678	11.5476107	2.422E-14	6.736946	up	cytochrome P450 98A3 [Sesamum indicum]
c15155.graph_c1	13.4805697	13.4940685	9.19619544	9.27605428	5.84738463	7.02594268	4.654E-09	-1.713328	down	-
c15159.graph_c0	0	0	0	14.1500394	18.6211077	16.2937435	7.012E-46	Inf	up	PREDICTED: probable xyloglucan endotransglucosylase/hydrolase protein 23 [Sesamum indicum]
c15161.graph_c0	2.07852285	2.68465055	2.0678587	10.2538244	11.1898729	10.4978563	0.0010528	1.207728	up	PREDICTED: transmembrane protein 184A-like [Sesamum indicum]
c15169.graph_c0	6.98435129	7.34091231	7.2549274	5.3620333	6.02124955	5.53448178	3.797E-06	-1.3704	down	PREDICTED: uncharacterized protein LOC105156896 isoform X1 [Sesamum indicum]
c15170.graph_c0	1.59297725	1.01472787	0.18609444	4.93631983	12.1548967	10.2091476	0.0023952	2.271582	up	hypothetical protein MIMGU_mgv1a006023mg [Erythranthe guttata]
c15173.graph_c0	1.22328875	1.34696506	0.94318469	87.9039241	39.756592	34.8965402	0.0001733	4.53536	up	BnaC09g03800D [Brassica napus]
c15194.graph_c0	48.6295964	41.1792034	40.8724636	4.72224186	5.25918842	5.17147373	5.711E-69	-4.126446	down	-
c15220.graph_c0	2.54410705	1.99737615	2.05131187	0	0	0	9.782E-26	-Inf	down	malate dehydrogenase [Symphyocladia latiuscula]
c15232.graph_c0	5.36172628	5.07698198	5.58651126	2.40303501	3.96001544	3.44488761	6.394E-06	-1.734443	down	unnamed protein product [Coffea canephora]

#ID	0 h-1 FPKM	0 h-2 FPKM	0 h-3 FPKM	48 h-1 FPKM	48 h-2 FPKM	48 h-3 FPKM	FDR	log2FC	regulated	nr_annotation
c15238.graph_c0	25.807402	24.5196894	24.7321265	4.77811949	5.54972624	5.17356314	4.981E-59	-3.295108	down	PREDICTED: uncharacterized protein LOC105180187 [Sesamum indicum]
c15272.graph_c0	2.5234047	2.8146094	2.87208352	12.0221493	12.4545506	10.3426316	0.0027357	1.066713	up	hypothetical protein MIMGU_mgv1a014946mg [Erythranthe guttata]
c15278.graph_c0	29.8150822	27.094252	25.9543478	8.73393682	11.2639703	13.6281151	9.061E-35	-2.323459	down	hypothetical protein MIMGU_mgv1a025332mg [Erythranthe guttata]
c15296.graph_c0	7.67964238	3.52336068	6.44203183	0	0	0	1.422E-15	-Inf	down	unnamed protein product [Chondrus crispus]
c15303.graph_c0	0.7314863	1.00680081	0.70499164	17.9423982	18.8293183	17.2392429	4.763E-16	3.450347	up	hypothetical protein MIMGU_mgv1a012143mg [Erythranthe guttata]
c15307.graph_c0	130.942733	103.676379	112.1313	60.5162168	60.5655816	55.3130961	3.667E-30	-1.990962	down	PREDICTED: zinc finger A20 and AN1 domain-containing stress-associated protein 8-like isoform X2 [Sesamum indicum]
c15329.graph_c0	0.5199872	0.20820316	0	153.390571	181.377386	207.840653	3.78E-108	8.538375	up	PREDICTED: dof zinc finger protein DOF3.1-like isoform X1 [Sesamum indicum]
c15339.graph_c0	252.184041	235.786193	251.24662	73.633424	67.687472	92.673544	3.89E-74	-2.678161	down	hypothetical protein ZEAMMB73_313798 [Zea mays]
c15356.graph_c0	0.08034583	0.08042628	0.10324757	10.5848335	7.37101171	5.46657803	5.125E-09	5.460695	up	PREDICTED: putative late blight resistance protein homolog R1B-19 isoform X1 [Sesamum indicum]
c15361.graph_c0	1.23328096	1.23451591	1.30514166	20.6517217	21.1699693	19.5651485	1.784E-18	3.006252	up	hypothetical protein MIMGU_mgv1a014572mg [Erythranthe guttata]
c15363.graph_c0	0.38282205	0	0.24597075	22.3952209	29.5161678	26.2033952	5.561E-20	5.935175	up	PREDICTED: GTP-binding protein SAR1A-like [Elaeis guineensis]
c15363.graph_c1	1.43603069	0.47915622	0.92267815	36.6020354	40.1769395	46.6942681	5.988E-19	4.424925	up	PREDICTED: GTP-binding protein SAR1A-like [Sesamum indicum]
c15364.graph_c0	8.7109368	6.78195738	7.01032038	37.5314495	36.4538411	41.1850807	1.79E-14	1.339222	up	hypothetical protein MIMGU_mgv1a008393mg [Erythranthe guttata]
c15371.graph_c0	33.9535964	34.6334408	21.0903899	1.11449885	0.94454111	0.95861856	9.306E-30	-5.90269	down	PREDICTED: protein notum homolog isoform X1 [Sesamum indicum]
c15378.graph_c0	15.3244002	15.0760247	16.5326391	2.58894432	3.94259183	3.41944645	2.335E-52	-3.262723	down	PREDICTED: uncharacterized protein LOC105156846 [Sesamum indicum]
c15380.graph_c0	22.647299	23.528688	24.8034169	18.3351788	22.7728467	17.6506357	1.57E-10	-1.293233	down	PREDICTED: uncharacterized protein LOC105168435 [Sesamum indicum]
c15395.graph_c0	4.04650337	1.53641754	2.779266	0	0	0	8.566E-11	-Inf	down	--
c15396.graph_c0	5.76206104	4.45696022	8.41417792	1.93032235	4.60112055	4.0792744	0.0013027	-1.848811	down	hypothetical protein M569_04751, partial [Genlisea aurea]
c15399.graph_c0	3.69184075	4.83262605	4.92662928	0	0	0	1.116E-21	-Inf	down	-
c15416.graph_c0	3.14448248	1.41643404	1.91937263	0	0	0	2.26E-11	-Inf	down	predicted protein [Physcomitrella patens]
c15446.graph_c0	4.90674413	3.91113467	2.80238243	21.4301048	25.1856941	30.3902281	1.416E-08	1.711446	up	PREDICTED: UPF0329 protein ECU05_1680/ECU11_0050-like isoform X2 [Sesamum indicum]
c15446.graph_c1	0.46345301	1.15979273	2.08444416	9.17970764	16.5095224	14.0566419	7.065E-06	2.38337	up	-

#ID	0 h-1 FPKM	0 h-2 FPKM	0 h-3 FPKM	48 h-1 FPKM	48 h-2 FPKM	48 h-3 FPKM	FDR	log2FC	regulated	nr_annotation
c15451.graph_c0	2.29466803	1.62468312	2.22953457	0.05156092	0	0.0917572	7.856E-20	-6.435344	down	-
c15452.graph_c0	0.58381897	0.19480119	0	11.4741784	13.4850086	10.6882325	1.346E-10	4.516759	up	-
c15476.graph_c0	1.60801872	1.463299	1.7845903	0.13467379	0	0.1797479	4.094E-14	-4.956344	down	-
c15485.graph_c0	1.7698855	0.61622879	0.69220046	6.23856474	7.96084437	8.20039614	8.355E-05	1.848262	up	hypothetical protein MIMGU_mgv1a013787mg [Erythranthe guttata]
c15495.graph_c0	15.5429054	10.3723128	16.3114865	70.6410819	68.5150812	65.1918752	1.065E-05	1.256349	up	hypothetical protein M569_09676, partial [Genlisea aurea]
c15497.graph_c0	3.29711056	4.28560976	3.66777942	1.13340048	1.29675641	1.65392841	5.345E-10	-2.483746	down	-
c15510.graph_c0	0.17067871	0.85424809	0.4386578	68.0850753	111.939807	112.209044	9.79E-29	6.614711	up	-
c15511.graph_c0	14.0303924	9.36296113	11.5745642	7.18095144	7.26924299	6.5315578	7.952E-06	-1.752988	down	unnamed protein product [Coffea canephora]
c15515.graph_c0	0	0	0	7.92371054	15.3596192	13.5009	3.904E-13	Inf	up	-
c15525.graph_c0	13.7003403	7.67642305	9.63318703	0	0	0	1.395E-26	-Inf	down	--
c15532.graph_c0	1.66372158	1.30851879	0.45813174	6.56883788	16.8173108	14.4174507	0.0008743	2.440656	up	PREDICTED: laccase-14-like [Sesamum indicum]
c15553.graph_c0	8.10423045	4.5333696	5.20712849	0	0	0	4.263E-16	-Inf	down	hypothetical protein OIDMADRAFT_111959 [Oidiodendron maius Zn]
c15566.graph_c0	0	0	0	2.16661976	4.26264599	4.77371201	3.28E-08	Inf	up	PREDICTED: polygalacturonase At1g48100 [Sesamum indicum]
c15569.graph_c0	0	0	0	20.1125354	19.7749316	18.8317523	1.368E-23	Inf	up	PREDICTED: CDGSH iron-sulfur domain-containing protein NEET [Sesamum indicum]
c15575.graph_c0	0	0	0.09336734	5.15411214	6.73655715	8.10012113	9.493E-17	6.686148	up	PREDICTED: probable LRR receptor-like serine/threonine-protein kinase At4g30520 [Sesamum indicum]
c15576.graph_c0	13.9419651	13.4223169	13.0681245	9.67096291	13.6469433	13.2438862	2.97E-10	-1.168116	down	PREDICTED: uncharacterized protein LOC105180173 [Sesamum indicum]
c15577.graph_c0	0.19460632	0.58440357	1.00030732	29.0440141	28.8693141	32.5432751	9.946E-19	4.637335	up	PREDICTED: uncharacterized protein LOC103494735 isoform X1 [Cucumis melo]
c15590.graph_c1	4.87900989	7.32584322	5.82188367	0	0	0	9.987E-23	-Inf	down	PREDICTED: cirhin-like [Phoenix dactylifera]
c15600.graph_c0	0	0	0.23012385	1.53980802	1.39820562	1.17438164	2.482E-07	3.11292	up	PREDICTED: transcription factor RAX2-like [Sesamum indicum]
c15640.graph_c0	2.48649798	1.42227876	1.27809934	0	0	0	1.521E-12	-Inf	down	-
c15678.graph_c0	3.42647893	3.0215874	4.08864957	16.3847548	22.1349988	18.4578878	4.875E-06	1.410671	up	PREDICTED: uncharacterized protein LOC105165199 [Sesamum indicum]
c15749.graph_c0	26.6632874	22.8771314	15.9895773	16.6099155	10.1611809	13.1141181	8.231E-07	-1.721371	down	hypothetical protein MIMGU_mgv1a004686mg [Erythranthe guttata]
c15817.graph_c0	14.6969744	6.85593374	8.61797199	0	0	0	1.458E-14	-Inf	down	hypothetical protein MIMGU_mgv1a016137mg [Erythranthe guttata]
c15849.graph_c0	86.3734634	79.198225	88.2702782	48.45382	45.3570868	52.6844641	8.809E-31	-1.810998	down	-
c15949.graph_c0	28.760182	28.9074541	23.7261058	18.3180532	28.3000611	23.9638424	8.689E-09	-1.227799	down	hypothetical protein MIMGU_mgv1a025131mg [Erythranthe guttata]

#ID	0 h-1 FPKM	0 h-2 FPKM	0 h-3 FPKM	48 h-1 FPKM	48 h-2 FPKM	48 h-3 FPKM	FDR	log2FC	regulated	nr_annotation
c15957.graph_c0	0.10804568	0	0.13884298	5.17601404	5.58880847	8.59118958	1.174E-11	5.261343	up	-
c15959.graph_c0	0	0	0	5.62942075	5.26207825	7.16978049	2.742E-13	Inf	up	V-type proton ATPase catalytic subunit A [Gossypium arboreum]
c15999.graph_c0	12.694969	11.1579639	11.3398982	4.70669883	3.62631033	3.9341746	2.206E-10	-2.532602	down	hypothetical protein EUTSA_v10020227mg [Eutrema salsugineum]
c16031.graph_c0	8.24115128	9.03506103	9.88419196	6.57998128	6.05148013	4.11768107	6.303E-08	-1.711956	down	PREDICTED: uncharacterized protein LOC105156318 [Sesamum indicum]
c16059.graph_c0	0.3694757	0.18492283	0.23739545	9.70097562	3.96656425	4.69452567	0.0031131	3.537958	up	hypothetical protein L484_026688 [Morus notabilis]
c16072.graph_c0	10.3328465	9.14398248	9.04451438	6.13925365	6.64994751	6.62885916	1.172E-07	-1.572567	down	hypothetical protein MIMGU_mgv1a016741mg [Erythranthe guttata]
c16076.graph_c0	16.9084791	16.2891168	15.0299398	4.56774857	3.47413643	1.87585625	1.036E-21	-3.287924	down	PREDICTED: dof zinc finger protein DOF3.1-like [Solanum lycopersicum]
c16083.graph_c0	9.01720259	10.7025322	5.62819599	70.8488485	85.9935388	102.428229	7.339E-17	2.337389	up	PREDICTED: phosphoglycerate kinase, chloroplastic-like [Fragaria vesca subsp. vesca]
c16084.graph_c0	0	0	0	15.6727532	13.3478166	12.1681512	6.29E-17	Inf	up	acetylajmalan acetylesterase [Striga asiatica]
c16100.graph_c0	44.676479	46.7279369	38.8260588	14.3792264	10.2019487	10.9164502	1.94E-39	-2.884661	down	hypothetical protein M569_09334 [Genlisea aurea]
c16107.graph_c0	3.23517637	3.65627602	5.90072605	41.9186642	48.5836992	64.9310144	7.609E-13	2.574159	up	Fructose-bisphosphate aldolase, chloroplast precursor, putative, expressed [Oryza sativa Japonica Group]
c16116.graph_c0	1.48219569	1.48367989	1.90468014	34.9776885	35.6636805	37.0110601	1.372E-45	3.445236	up	PREDICTED: transcription repressor OFP6-like [Sesamum indicum]
c16134.graph_c0	0	0	0	3.22666945	4.10191803	6.07026455	1.418E-10	Inf	up	PREDICTED: uncharacterized protein LOC104223573 [Nicotiana sylvestris]
c16163.graph_c0	14.1685582	17.6940082	16.9697786	6.84332039	10.3374898	10.9942902	2.674E-15	-1.819653	down	PREDICTED: xyloglucan galactosyltransferase KATAMARI1 homolog [Sesamum indicum]
c16187.graph_c0	3.87316065	3.55395245	2.40562964	13.7970718	16.6323693	21.0606358	4.731E-06	1.370522	up	PREDICTED: uncharacterized protein LOC105171815 [Sesamum indicum]
c16197.graph_c0	2.44574674	3.26426105	2.91007537	0.66760931	0.53043833	0.59403475	5.215E-10	-3.280514	down	-
c16231.graph_c0	27.5459711	15.1503045	15.9483978	0	0	0	8.104E-18	-Inf	down	-
c16250.graph_c0	138.859848	149.508724	147.58971	81.5687845	77.16494	65.0223383	4.531E-44	-1.977642	down	hypothetical protein MIMGU_mgv1a014113mg [Erythranthe guttata]
c16273.graph_c0	5.76379997	7.89881818	7.23036089	64.8577164	68.6419184	67.5533306	2.692E-35	2.24699	up	40S ribosomal protein S9-2 [Morus notabilis]
c16349.graph_c0	0.02097108	0.10496039	0.02694867	0.46367823	0.81868476	0.94549147	3.532E-05	2.842253	up	PREDICTED: uncharacterized protein At4g38062-like [Sesamum indicum]
c16382.graph_c0	11.9521371	9.62192599	9.91425535	4.4277392	5.24612483	4.87288048	1.108E-14	-2.132953	down	PREDICTED: peroxidase 9 [Sesamum indicum]
c16384.graph_c0	1.79710656	1.19927073	1.15467654	0	0	0	4.146E-25	-Inf	down	PREDICTED: L-lactate dehydrogenase A-like [Solanum tuberosum]

#ID	0 h-1 FPKM	0 h-2 FPKM	0 h-3 FPKM	48 h-1 FPKM	48 h-2 FPKM	48 h-3 FPKM	FDR	log2FC	regulated	nr_annotation
c16509.graph_c0	11.8766476	13.4251543	14.3275565	73.3157773	68.2857934	64.7723322	4.733E-15	1.363447	up	Peptidylprolyl cis/trans isomerase [Theobroma cacao]
c16681.graph_c0	0.78693784	1.05030112	1.10755545	0	0	0	4.44E-20	-Inf	down	--
c16725.graph_c0	31.6667139	33.0968832	32.7139652	2.57412726	0.75749325	0.89072782	1.557E-61	-5.52244	down	-
c16744.graph_c0	1.77680778	1.7172564	2.83440209	10.9503716	10.5242474	12.1041316	3.833E-05	1.38427	up	PREDICTED: uncharacterized protein LOC105171253 isoform X1 [Sesamum indicum]
c16837.graph_c0	0.26345674	0.5274411	0.50782851	5.09698412	12.4705894	10.798252	1.23E-05	3.413947	up	PREDICTED: uncharacterized protein LOC105156659 [Sesamum indicum]
c16854.graph_c0	1.70989262	1.53605561	1.29583163	0	0	0.03594003	1.537E-21	-8.019862	down	-
c16937.graph_c0	0.68840394	0.92762556	0.61243361	0.02439241	0.12920399	0.17363375	1.138E-11	-3.804688	down	PREDICTED: subtilisin-like protease [Sesamum indicum]
c16945.graph_c0	1.10561168	0.40437802	0.87431064	0	0	0	1.9E-10	-Inf	down	polyunsaturated fatty acids delta-5-elongase [Thraustochytrium sp. FJN-10]
c17004.graph_c0	1.26627964	0.89954993	1.20729186	0	0	0	1.539E-20	-Inf	down	PREDICTED: prostaglandin reductase 1-like [Pyrus x bretschneideri]
c17007.graph_c0	18.7777388	13.4261013	10.7245057	6.86479276	4.36344932	5.86392126	1.45E-07	-2.33484	down	-
c17172.graph_c0	2.10291095	1.26301002	0	9.2992259	8.20950188	10.3429941	0.0004368	2.049466	up	PREDICTED: uncharacterized protein LOC105167003 isoform X1 [Sesamum indicum]
c17223.graph_c0	2.33728246	2.67385474	3.8616435	17.2260564	22.485274	22.1703385	0.0003783	1.77462	up	-
c17322.graph_c0	0.068869	0.03446898	0.06637455	0.41240301	0.52090848	0.84681628	0.0003866	2.363692	up	PREDICTED: uncharacterized protein LOC105167219 [Sesamum indicum]
c17325.graph_c0	1.40953713	0.68826759	1.19281457	0	0	0	5.796E-15	-Inf	down	unnamed protein product [Chondrus crispus]
c17409.graph_c0	0.3694757	0.18492283	0.71218634	10.3817458	9.01491874	8.9347424	2.461E-08	3.458311	up	-
c17428.graph_c0	0.60200763	1.08469881	0.81228382	0	0.05875407	0.04934875	1.965E-14	-5.578713	down	gag-pol polymerase [Arabidopsis lyrata subsp. lyrata]
c17559.graph_c0	0	0	0	50.125956	129.832864	158.152163	1.176E-11	Inf	up	PREDICTED: 3'-hydroxy-N-methyl-(S)-coclaurine 4'-O-methyltransferase-like [Sesamum indicum]
c17663.graph_c0	1.29924554	0.43351551	0.55652732	21.9440785	24.5151134	29.8210829	9.348E-10	4.043589	up	-
c17703.graph_c0	8.90752367	6.17292222	4.40250792	1.44284691	1.71958652	1.76527519	3.175E-07	-2.998165	down	-
c17748.graph_c0	11.2922392	11.5971453	13.9456118	6.48507884	7.44266756	6.97254752	8.037E-07	-1.839377	down	PREDICTED: ABC transporter B family member 20 [Nelumbo nucifera]
c17782.graph_c0	1.51149176	1.56181191	1.56639255	0	0	0	7.646E-22	-Inf	down	--
c17811.graph_c0	0.19867237	0.19887131	0	33.1894296	24.4311519	17.0459026	2.486E-13	6.561382	up	PREDICTED: uncharacterized protein LOC105158387 [Sesamum indicum]
c17816.graph_c0	0	0	0	23.7811487	24.1435249	21.4542165	7.682E-15	Inf	up	PREDICTED: L-ascorbate oxidase homolog [Cucumis melo]
c17821.graph_c0	1.76219832	0.5039894	0.97049769	20.4091113	20.392514	21.6680679	4.556E-09	3.256193	up	-
c17845.graph_c0	0.10381222	0.20783235	0	85.0227055	89.9699295	106.798795	6.99E-117	8.815572	up	hypothetical protein MIMGU_mgv1a009966mg [Erythranthe guttata]

#ID	0 h-1 FPKM	0 h-2 FPKM	0 h-3 FPKM	48 h-1 FPKM	48 h-2 FPKM	48 h-3 FPKM	FDR	log2FC	regulated	nr_annotation
c17960.graph_c0	76.7099437	74.2008113	73.5080526	40.896434	45.1771801	41.9212814	3.508E-37	-1.828539	down	PREDICTED: probable ADP-ribosylation factor GTPase-activating protein AGD6 [Sesamum indicum]
c18032.graph_c0	1.21310507	1.05241051	0.88336989	0	0	0	2.075E-20	-Inf	down	hypothetical protein GUITHDRAFT_137181 [Guillardia theta CCMP2712]
c18118.graph_c0	63.8274276	66.1772567	60.4518029	34.2923945	36.5515089	32.6659359	2.387E-30	-1.896779	down	PREDICTED: E3 ubiquitin-protein ligase ATL42 [Sesamum indicum]
c18221.graph_c0	1.93596322	0.81448046	1.04559264	0	0	0	9.471E-11	-Inf	down	hypothetical protein PHAVU_001G265900g [Phaseolus vulgaris]
c18282.graph_c0	5.85528607	5.86114926	6.84024948	0.32692554	0.69267594	0.43634457	2.217E-15	-4.701623	down	-
c18297.graph_c0	0.36526185	0.7312552	0	14.4696307	12.4769458	13.7732455	1.014E-08	4.214313	up	TPA: putative methionine synthase family protein [Zea mays]
c18320.graph_c0	2.09189538	0.87249587	1.45609121	0	0	0	1.091E-11	-Inf	down	predicted protein [Physcomitrella patens]
c18333.graph_c0	7.39263791	6.66003646	6.3332216	0.45403868	2.40499464	1.01000222	2.15E-10	-3.434765	down	-
c18333.graph_c1	3.97975493	5.80962091	3.83560699	0.45830123	0.16183819	0.67965613	1.395E-13	-4.401403	down	-
c18358.graph_c0	14.726961	13.777297	12.5575297	61.6240597	58.9698212	56.0737875	1.731E-06	1.090476	up	PREDICTED: protein P21-like [Sesamum indicum]
c18369.graph_c0	19.0341702	19.3463566	25.9648722	18.8844051	18.7196543	15.3629533	8.497E-06	-1.299973	down	hypothetical protein MIMGU_mgv1a008825mg [Erythranthe guttata]
c18381.graph_c0	1.52956927	2.04146786	1.31037138	14.7176737	15.7574587	15.6033844	5.853E-06	2.222772	up	--
c18400.graph_c0	3.1384297	0.39269655	2.01650321	13.7337992	11.8691617	9.9691529	0.0026383	1.668547	up	-
c18422.graph_c1	0.83602348	0.74387611	0.67642576	0	0	0	7.243E-20	-Inf	down	--
c18557.graph_c0	1.32208413	1.10284	0.28315507	10.7589128	12.9031285	13.0051235	2.773E-06	2.749947	up	mitochondrial ATP synthase, beta chain [Plantago major]
c18610.graph_c0	1.10290418	1.38001071	3.18886995	89.667936	97.4142056	98.545869	7.618E-36	4.623657	up	betaine aldehyde dehydrogenase 1 [Jatropha curcas]
c18661.graph_c0	0.10146304	0	0	2.80423104	5.05026268	3.90912773	1.008E-10	5.852244	up	-
c18663.graph_c0	13.4998478	10.5211206	12.3913135	0.44417644	0.09411022	0.15809021	4.505E-45	-6.698909	down	-
c18669.graph_c0	311.128593	341.87719	284.800189	29.6535669	22.2340459	19.7251315	1.2E-175	-4.720281	down	PREDICTED: uncharacterized protein LOC105174162 [Sesamum indicum]
c18679.graph_c0	1.28277377	1.5106568	1.93931187	7.71629473	11.7830322	14.8452165	4.113E-05	1.828939	up	PREDICTED: uncharacterized protein LOC105160400 [Sesamum indicum]
c18688.graph_c0	13.7462915	14.5845603	13.1484309	4.01335194	4.87690382	5.56664678	8.112E-56	-2.541933	down	PREDICTED: topless-related protein 4-like isoform X5 [Sesamum indicum]
c18696.graph_c0	10.3862138	11.0746541	12.2828289	9.15243773	12.26678	10.4882067	3.977E-05	-1.104135	down	BnaC04g40780D [Brassica napus]
c18699.graph_c0	18.5435267	18.4034449	16.2934468	7.30064373	7.27009798	7.66536129	1.118E-11	-2.27601	down	-
c18700.graph_c0	1.55440838	1.3729102	0.93998885	10.19263	7.76374735	5.54653903	0.002216	1.598885	up	PREDICTED: NADPH--cytochrome P450 reductase-like [Sesamum indicum]
c18707.graph_c0	0.15319321	0.30669323	0.19685934	3.01080704	2.89056139	4.60452781	1.104E-07	2.979713	up	PREDICTED: protein kinase PVPK-1 [Sesamum indicum]
c18710.graph_c0	88.4938063	51.544474	57.3873292	0	0	0	3.556E-48	-Inf	down	hypothetical protein CHLNCDRAFT_59390 [Chlorella variabilis]

#ID	0 h-1 FPKM	0 h-2 FPKM	0 h-3 FPKM	48 h-1 FPKM	48 h-2 FPKM	48 h-3 FPKM	FDR	log2FC	regulated	nr_annotation
c18715.graph_c0	0.19385313	0.33958268	0.24910895	5.491647	9.64892484	14.8976661	3.263E-06	4.232747	up	PREDICTED: homeobox-leucine zipper protein MERISTEM L1-like [Sesamum indicum]
c18726.graph_c0	0.21384123	0.07135179	0.1831963	2.52822426	3.26967041	4.49919748	9.371E-10	3.432586	up	PREDICTED: sulfite reductase 1 [ferredoxin], chloroplastic-like [Sesamum indicum]
c18731.graph_c0	1.7378418	2.46087208	2.2876637	11.1289943	10.6728897	12.5779278	1.485E-06	1.386664	up	PREDICTED: uncharacterized protein LOC104214549 [Nicotiana sylvestris]
c18732.graph_c0	8.95554731	8.0307113	10.9088457	7.21913002	5.37166406	5.50589013	1.609E-06	-1.638622	down	PREDICTED: uncharacterized protein LOC105163289 [Sesamum indicum]
c18736.graph_c0	34.5668746	32.7331572	33.0030498	3.7829142	3.13316677	3.88621652	5.29E-132	-4.230194	down	PREDICTED: cation/calcium exchanger 3-like [Sesamum indicum]
c18743.graph_c0	0.08565916	0	0	2.13069964	1.75561393	2.17675539	4.519E-10	5.151173	up	hypothetical protein MIMGU_mgv1a000037mg [Erythranthe guttata]
c18762.graph_c0	0	0	0	1.98792233	2.27671639	1.81664786	1.098E-11	Inf	up	PREDICTED: folate-biopterin transporter 1, chloroplastic-like isoform X1 [Sesamum indicum]
c18784.graph_c0	16.284431	14.8157841	16.9835328	6.33636672	7.04166183	7.29631424	7.771E-36	-2.238228	down	PREDICTED: probable carboxylesterase 16 [Sesamum indicum]
c18793.graph_c0	13.2742561	8.70948543	8.02726808	0	0	0	2.774E-27	-Inf	down	Ubiquitin-conjugating enzyme [Pestalotiopsis fici W106-1]
c18797.graph_c0	0	0	0	17.258833	12.6937379	11.9333141	8.386E-20	Inf	up	-
c18808.graph_c0	0.32186867	0.83769654	1.24084197	9.42954005	12.8166583	15.4086895	1.191E-12	2.937199	up	PREDICTED: uncharacterized protein LOC105161274 [Sesamum indicum]
c18813.graph_c0	0	0	0	1.79763317	3.26464178	5.02707287	1.243E-06	Inf	up	PREDICTED: glyoxylate/succinic semialdehyde reductase 1 [Sesamum indicum]
c18817.graph_c0	0.34194817	0.59900852	0.43941694	4.80413066	4.25506703	4.76522437	2.772E-05	2.30798	up	hypothetical protein MIMGU_mgv1a015124mg [Erythranthe guttata]
c18818.graph_c0	2.9100942	2.31636798	3.37914422	2.55176529	2.22422258	2.64417946	0.0007164	-1.232576	down	hypothetical protein MIMGU_mgv1a001568mg [Erythranthe guttata]
c18827.graph_c0	3.78286749	4.13089687	2.82829643	0.12672816	0.53701246	0.56380975	2.912E-16	-4.163639	down	-
c18829.graph_c0	0	0	0	5.33011728	8.46991942	10.8291794	5.965E-13	Inf	up	PREDICTED: uncharacterized protein LOC105159002 [Sesamum indicum]
c18846.graph_c0	5.94156695	5.54720292	6.75417006	31.421482	34.5696735	34.8897673	5.188E-13	1.446226	up	PREDICTED: 50S ribosomal protein L17, chloroplastic-like [Sesamum indicum]
c18851.graph_c0	19.4620607	19.3269336	18.6578948	16.2221274	15.1879537	17.219928	2.642E-14	-1.257099	down	PREDICTED: transmembrane protein 136-like [Sesamum indicum]
c18858.graph_c0	1.9472711	1.79533513	1.18530969	1.46348809	1.150288	0.84013091	0.0007874	-1.518092	down	PREDICTED: cytochrome P450 71A8-like [Sesamum indicum]
c18864.graph_c0	13.9296556	17.5087301	13.3234138	0.36457123	0.15448761	0.38927195	8.285E-65	-6.631518	down	hypothetical protein MIMGU_mgv1a017080mg [Erythranthe guttata]
c18867.graph_c0	31.4213272	21.8243855	32.4669263	0.23630511	0	0	4.086E-45	-9.464763	down	PREDICTED: uncharacterized protein LOC105160499 [Sesamum indicum]

#ID	0 h-1 FPKM	0 h-2 FPKM	0 h-3 FPKM	48 h-1 FPKM	48 h-2 FPKM	48 h-3 FPKM	FDR	log2FC	regulated	nr_annotation
c18874.graph_c0	0.22724936	0.22747691	0.33374219	2.36274248	2.50303916	2.1555782	2.253E-05	2.132781	up	hypothetical protein MIMGU_mgv1a002578mg [Erythranthe guttata]
c18876.graph_c0	18.7801891	15.5112781	18.2893517	12.5688282	12.8219958	13.8760323	9.569E-10	-1.440057	down	hypothetical protein MIMGU_mgv1a016811mg [Erythranthe guttata]
c18878.graph_c0	8.90314232	9.10579785	8.45631027	93.9681718	98.3200636	101.540571	7.144E-41	2.454916	up	40S ribosomal protein S17-like protein [Solanum tuberosum]
c18889.graph_c0	10.7276735	10.4096887	12.5194713	6.65591989	4.48708912	4.75586465	1.733E-09	-2.093716	down	histone h4, partial [Cysticapnos vesicaria]
c18892.graph_c0	1.76071547	0.88123928	0.67877673	5.43398788	6.4440126	7.8661066	0.0016932	1.557394	up	hypothetical protein CICLE_v10006109mg [Citrus clementina]
c18896.graph_c0	3.65449095	3.76574302	4.39229602	59.2746999	46.9751305	39.3672685	5.486E-11	2.610559	up	PREDICTED: L-type lectin-domain containing receptor kinase VIII.1-like [Sesamum indicum]
c18903.graph_c0	31.8662863	27.7924872	35.6787195	11.5782096	13.6513751	15.173146	1.038E-22	-2.261682	down	PREDICTED: transmembrane ascorbate ferrireductase 1 [Sesamum indicum]
c18926.graph_c0	0.05423749	0	0	1.64891467	2.77904002	2.71208655	3.293E-15	6.035368	up	PREDICTED: BTB/POZ domain-containing protein At5g66560 [Sesamum indicum]
c18936.graph_c0	2.13649196	1.9247682	1.83031783	0.06560919	0	0	2.217E-19	-7.447672	down	-
c18949.graph_c0	135.04453	137.152955	138.637056	7.49468851	10.0467916	11.0547004	2.88E-202	-4.868332	down	hypothetical protein MIMGU_mgv1a005690mg [Erythranthe guttata]
c18950.graph_c0	0	0	0	4.66949173	5.2294323	3.56133057	2.06E-11	Inf	up	-
c18956.graph_c0	0.05843633	0.05849484	0.075093	5.1681944	3.70708223	4.83814019	2.469E-13	5.14218	up	unnamed protein product [Coffea canephora]
c18978.graph_c0	5.92669222	7.06941769	6.88635424	5.78700487	6.37308425	6.86565618	6.391E-06	-1.084269	down	hypothetical protein MIMGU_mgv1a018497mg [Erythranthe guttata]
c18979.graph_c0	0	0	0	3.31708491	2.48554702	3.13149307	2.87E-15	Inf	up	PREDICTED: uncharacterized protein LOC105171076 [Sesamum indicum]
c18981.graph_c0	4.91836149	4.4801907	4.48740536	26.6429586	29.8570409	31.0848637	2.339E-15	1.637827	up	PREDICTED: carboxymethylenebutenolidase homolog [Sesamum indicum]
c18990.graph_c0	2.005296	3.19343818	1.75696688	118.989964	209.321417	287.219109	5.78E-11	5.441706	up	-
c19001.graph_c0	0	0.139419	0	3.97771518	3.39831202	2.39762219	4.492E-09	5.131502	up	V-type proton ATPase subunit B 1 [Aegilops tauschii]
c19012.graph_c0	11.1347011	6.74617284	5.91706198	0	0	0	2.907E-23	-Inf	down	uncharacterized protein LOC100191867 precursor [Zea mays]
c19017.graph_c0	4.36811215	4.83623469	3.06174385	17.5601011	17.3755299	19.2598253	9.899E-05	1.129414	up	PREDICTED: ceramide-1-phosphate transfer protein [Sesamum indicum]
c19019.graph_c0	0	0	0	3.90293305	7.37352036	5.38284986	5.083E-14	Inf	up	hypothetical protein CICLE_v10023425mg [Citrus clementina]
c19020.graph_c0	51.9644699	49.6064734	45.3770139	49.3517636	52.0863958	42.2682245	2.204E-07	-1.047828	down	-
c19029.graph_c0	14.7475992	15.8835591	15.1130212	13.4144699	13.573331	13.2368451	2.59E-06	-1.20293	down	-
c19035.graph_c0	8.95781246	8.45439479	8.16743051	1.8076951	2.03992727	3.00715121	1.473E-26	-2.921711	down	PREDICTED: putative pectinesterase 11 [Sesamum indicum]
c19039.graph_c0	0.1035807	0.10368442	0	16.3654386	11.4738889	9.50979188	1.559E-14	6.501351	up	PREDICTED: probable WRKY transcription factor 65 [Sesamum indicum]

#ID	0 h-1 FPKM	0 h-2 FPKM	0 h-3 FPKM	48 h-1 FPKM	48 h-2 FPKM	48 h-3 FPKM	FDR	log2FC	regulated	nr_annotation
c19040.graph_c0	3.43721516	3.56808875	4.49875308	19.967089	22.3019758	24.3671078	5.537E-16	1.511094	up	PREDICTED: UDP-sulfoquinovose synthase, chloroplastic isoform X2 [Sesamum indicum]
c19056.graph_c0	0	0	0	0.68407943	1.18240396	1.31348843	8.631E-10	Inf	up	PREDICTED: cytochrome b561 and DOMON domain-containing protein At2g04850 [Sesamum indicum]
c19060.graph_c0	2.08669996	1.56659211	2.4580344	14.6583314	13.7467399	11.83126	8.232E-06	1.700972	up	PREDICTED: uncharacterized protein LOC105179584 [Sesamum indicum]
c19065.graph_c0	1.63163204	0.94226877	1.33060554	21.7959049	20.3952505	20.6799305	1.036E-39	2.993434	up	PREDICTED: protein SCARECROW [Sesamum indicum]
c19070.graph_c0	37.3547876	42.2776049	44.1428931	12.6241089	12.824108	11.0789815	6.387E-27	-2.778635	down	-
c19074.graph_c0	3.32657844	2.61635747	3.47326282	16.9650879	22.1177129	23.9092184	4.48E-15	1.717716	up	PREDICTED: probable galacturonosyltransferase 9 [Sesamum indicum]
c19087.graph_c0	0.33041174	0.44099013	0.28306144	1.82638107	2.0423198	2.3925126	0.0046096	1.551903	up	unnamed protein product [Coffea canephora]
c19088.graph_c0	17.2140149	13.3450548	15.5786141	5.16250429	5.07584783	5.34414783	3.856E-35	-2.583324	down	PREDICTED: LOW QUALITY PROTEIN: uncharacterized protein LOC105159096 [Sesamum indicum]
c19094.graph_c0	0	0	0	30.7190056	44.5716362	45.6021101	4.063E-68	Inf	up	PREDICTED: endoglucanase 5-like [Sesamum indicum]
c19095.graph_c0	0	0.12329361	0.07913934	3.85806474	10.0976691	8.78414129	2.438E-08	5.773803	up	PREDICTED: probable glutathione S-transferase [Sesamum indicum]
c19099.graph_c0	0	0	0.05852804	13.5110159	22.759009	21.8412487	7.976E-31	8.896707	up	RecName: Full=Caffeic acid 3-O-methyltransferase 2; Short=CAOMT-2; Short=COMT-2; AltName: Full=S-adenosysl-L-methionine:caffeic acid 3-O-methyltransferase 2 [Ocimum basilicum]
c19100.graph_c0	0.95226465	0.32996015	0.32945709	36.6773997	42.3583076	44.2543478	1.06E-109	5.245088	up	PREDICTED: protein notum homolog [Sesamum indicum]
c19110.graph_c0	0.0576275	0.11537041	0.14810723	1.96433778	2.41843353	1.93681334	3.083E-07	3.271758	up	-
c19118.graph_c0	172.324738	179.907123	170.886095	60.3828882	43.404046	31.8213146	5.627E-85	-2.955599	down	hypothetical protein M569_08491 [Genlisea aurea]
c19122.graph_c0	320.795999	336.387866	305.974354	121.457336	176.719401	180.972651	1.321E-44	-2.030955	down	-
c19130.graph_c0	13.0659878	12.9776832	13.1459029	6.99839546	9.48985777	8.05375505	1.513E-08	-1.697278	down	hypothetical protein MIMGU_mgv1a000569mg [Erythranthe guttata]
c19155.graph_c0	0.44680628	0.48452483	0.33492886	5.5569657	8.86673609	12.2392989	5.524E-07	3.371821	up	PREDICTED: transcription factor bHLH143-like [Sesamum indicum]
c19159.graph_c0	7.48541817	7.56461621	7.04170326	4.45438989	5.41798018	4.22772196	2.762E-12	-1.66653	down	hypothetical protein MIMGU_mgv1a026890mg [Erythranthe guttata]
c19173.graph_c0	0.495028	1.02644193	0.63613057	17.8186062	22.1550475	26.1446244	2.075E-31	3.915627	up	PREDICTED: protein ECERIFERUM 26-like [Sesamum indicum]
c19192.graph_c0	2.62112622	2.68338158	1.68412548	17.1776707	19.360449	24.8557679	7.112E-13	2.117948	up	PREDICTED: probable carbohydrate esterase At4g34215 [Sesamum indicum]
c19205.graph_c0	6.71660629	6.22324941	5.42118992	27.0523422	28.9295522	32.8075599	2.044E-09	1.256079	up	PREDICTED: protein MEI2-like 4 [Sesamum indicum]

#ID	0 h-1 FPKM	0 h-2 FPKM	0 h-3 FPKM	48 h-1 FPKM	48 h-2 FPKM	48 h-3 FPKM	FDR	log2FC	regulated	nr_annotation
c19206.graph_c0	0.27214243	0.61293361	0.21857112	16.9859653	19.8869885	21.973874	6.351E-57	4.720698	up	PREDICTED: lysine histidine transporter-like 8 [Sesamum indicum]
c19211.graph_c0	1.13429498	1.33119474	1.18116994	8.34187518	9.65793921	10.5166156	1.133E-16	1.946548	up	hypothetical protein MIMGU_mgv1a001561mg [Erythranthe guttata]
c19213.graph_c0	0.20230804	0.15188296	0.19498038	1.49103487	3.10977971	2.98510619	1.492E-05	2.757861	up	PREDICTED: acyl-CoA--sterol O-acyltransferase 1-like [Sesamum indicum]
c19214.graph_c0	0	0	0	8.41697134	37.8070646	50.6281445	1.696E-06	Inf	up	hypothetical protein MIMGU_mgv1a015553mg [Erythranthe guttata]
c19215.graph_c0	0	0	0.12562666	2.07146605	3.5779355	3.44594155	2.643E-12	5.118735	up	hypothetical protein MIMGU_mgv1a019872mg [Erythranthe guttata]
c19216.graph_c0	0.54448505	0.583961	0.59972987	4.71159936	5.0103468	6.91817911	6.757E-12	2.245429	up	PREDICTED: DNA replication licensing factor MCM4 [Sesamum indicum]
c19228.graph_c0	9.13013374	9.48199904	10.4126677	0.84112747	0.44553628	0.70165328	2.196E-44	-4.87783	down	PREDICTED: zinc finger CCCH domain-containing protein 48-like [Sesamum indicum]
c19232.graph_c0	3.24809785	3.40804192	2.96701357	0.72105051	1.22218493	0.96237961	3.035E-12	-2.751908	down	-
c19235.graph_c0	1.135951	2.84272121	2.91948402	17.1366365	17.5998621	16.2956539	9.854E-06	1.864189	up	hypothetical protein MIMGU_mgv1a003115mg [Erythranthe guttata]
c19248.graph_c0	0.07227607	0	0	1.99756302	3.10372451	3.4955918	6.954E-11	5.889468	up	hypothetical protein MIMGU_mgv1a014302mg [Erythranthe guttata]
c19281.graph_c0	561.128627	554.807207	558.743233	208.585967	162.49735	128.969063	6.04E-70	-2.754235	down	PREDICTED: putative DNA-binding protein ESCAROLA [Sesamum indicum]
c19282.graph_c0	0	0	0	40.8826763	62.2648367	66.3348204	1.14E-47	Inf	up	PREDICTED: cytosolic sulfotransferase 12-like [Sesamum indicum]
c19289.graph_c0	5.41414149	6.02623043	6.59394062	3.16394122	5.12630082	5.92859766	8.95E-07	-1.371482	down	PREDICTED: pentatricopeptide repeat-containing protein At4g37170 [Sesamum indicum]
c19303.graph_c0	0.15964309	0.15980295	0.30772154	3.64007226	3.62250517	4.61299931	4.518E-10	3.216012	up	PREDICTED: aspartic proteinase PCS1 [Sesamum indicum]
c19305.graph_c0	21.3995982	22.6834076	17.2693773	11.8723918	11.0965389	9.43328131	1.266E-24	-1.9334	down	PREDICTED: uncharacterized protein LOC105174914 [Sesamum indicum]
c19324.graph_c0	0.90723732	0.45407289	0.71245526	3.43609213	4.57474913	4.25558991	0.0010113	1.542707	up	PREDICTED: scarecrow-like protein 8 [Sesamum indicum]
c19327.graph_c0	0	0	0.14040454	3.42237997	4.26540776	4.03042913	1.853E-10	5.32945	up	predicted protein [Hordeum vulgare subsp. vulgare]
c19336.graph_c0	101.882514	120.656094	89.2795173	44.9727091	34.1624877	31.8873495	2.818E-26	-2.499106	down	PREDICTED: probable WRKY transcription factor 7 [Sesamum indicum]
c19349.graph_c0	1.00335107	1.86523214	1.84192263	18.4430321	24.5742442	29.4918993	1.787E-15	2.916577	up	PREDICTED: LRR receptor-like serine/threonine-protein kinase HSL2 [Sesamum indicum]
c19358.graph_c0	0.16094826	0.53703142	0.13788326	4.79425849	4.18881282	5.4533185	9.371E-09	3.099447	up	hypothetical protein MIMGU_mgv1a000332mg [Erythranthe guttata]

#ID	0 h-1 FPKM	0 h-2 FPKM	0 h-3 FPKM	48 h-1 FPKM	48 h-2 FPKM	48 h-3 FPKM	FDR	log2FC	regulated	nr_annotation
c19359.graph_c0	9.12976629	9.13890837	8.6904527	79.4366197	71.2829362	78.4434121	1.557E-09	2.071996	up	V-type proton ATPase 16 kDa proteolipid subunit [Morus notabilis]
c19362.graph_c0	0	0.03629732	0	2.2382025	3.92824718	4.31004737	9.443E-14	7.157083	up	PREDICTED: lactosylceramide 4-alpha-galactosyltransferase-like [Sesamum indicum]
c19380.graph_c0	0	0	0	2.82269119	6.32374717	9.01710474	5.215E-08	Inf	up	PREDICTED: uncharacterized protein LOC105170375 [Sesamum indicum]
c19383.graph_c0	5.93652485	2.97123469	4.0773923	0.18859013	0.19978838	0.67122547	2.996E-10	-4.641658	down	-
c19389.graph_c0	0.0529669	0	0	5.12363923	5.53126258	6.16548228	2.073E-18	7.312507	up	PREDICTED: probable receptor-like serine/threonine-protein kinase At4g34500 [Sesamum indicum]
c19395.graph_c0	1.06117983	0.69045759	0.81819486	7.72326179	7.97472369	8.43790955	6.99E-08	2.214388	up	PREDICTED: uncharacterized protein LOC105159543 [Sesamum indicum]
c19405.graph_c0	3.36463884	2.92387509	2.94581358	2.82722149	2.95901282	3.54614973	0.0034868	-1.003532	down	hypothetical protein MIMGU_mgv1a004222mg [Erythranthe guttata]
c19406.graph_c0	0.42903521	0.3340282	0.49006865	6.38995942	6.16456222	7.56145798	1.734E-18	2.983875	up	hypothetical protein MIMGU_mgv1a001005mg [Erythranthe guttata]
c19428.graph_c0	0	0.07268507	0.09330976	1.37135253	2.16145573	3.89875566	8.551E-05	4.442088	up	PREDICTED: uncharacterized protein LOC105159979 [Sesamum indicum]
c19429.graph_c0	1.14471221	1.32815413	1.2369777	10.5458355	9.59779285	9.8528021	4.688E-14	1.999348	up	PREDICTED: uncharacterized protein LOC105163794 isoform X2 [Sesamum indicum]
c19430.graph_c0	8.9739926	6.41641335	7.00153244	0	0	0	3.116E-42	-Inf	down	40S ribosomal protein S6-B [Mucor circinelloides f. circinelloides 1006PhL]
c19433.graph_c0	0.23146885	0.1853605	0.41642529	355.733977	399.131383	358.652991	0	9.357341	up	PREDICTED: GDSL esterase/lipase At1g71250 [Sesamum indicum]
c19445.graph_c0	0	0	0	4.69465207	5.37940764	7.24629021	7.118E-14	Inf	up	PREDICTED: T-complex protein 1 subunit epsilon [Nicotiana tomentosiformis]
c19453.graph_c0	41.5376555	46.7950207	35.8365055	19.8096977	24.1374498	32.305315	4.513E-18	-1.724674	down	PREDICTED: protein ECERIFERUM 1-like [Sesamum indicum]
c19467.graph_c0	0.84134946	0.90381575	0.76472814	3.57304963	3.68507492	3.39795857	0.0023046	1.069873	up	hypothetical protein MIMGU_mgv1a023991mg [Erythranthe guttata]
c19468.graph_c0	0.65296846	0.20111456	0.12909082	13.6507197	17.1574536	18.9400374	2.717E-33	4.650869	up	PREDICTED: probable sugar phosphate/phosphate translocator At1g12500 [Sesamum indicum]
c19480.graph_c0	0.1016834	0	0	10.9602539	16.2753319	15.4620877	4.604E-39	7.711004	up	PREDICTED: peroxidase 44 [Sesamum indicum]
c19492.graph_c0	135.002675	136.635693	133.050524	40.1685838	27.3820563	33.5611419	1.946E-93	-3.012144	down	l-asparaginase, putative [Ricinus communis]
c19498.graph_c0	0	0	0	1.08612261	1.90294053	1.44963805	1.457E-11	Inf	up	predicted protein [Physcomitrella patens]
c19507.graph_c0	347.873114	190.615216	181.239426	0	0	0	7.683E-29	-Inf	down	-
c19511.graph_c0	4.36867519	4.5533817	4.2249093	12.9869461	19.9997861	22.2991909	0.0005461	1.046914	up	PREDICTED: histone-lysine N-methyltransferase family member SUVH9 [Sesamum indicum]

#ID	0 h-1 FPKM	0 h-2 FPKM	0 h-3 FPKM	48 h-1 FPKM	48 h-2 FPKM	48 h-3 FPKM	FDR	log2FC	regulated	nr_annotation
c19514.graph_c0	3.74253578	2.59822878	2.28829913	18.5461936	20.8257007	23.8503883	1.391E-20	1.856515	up	PREDICTED: omega-6 fatty acid desaturase, chloroplastic [Sesamum indicum]
c19516.graph_c0	5.54320052	5.25975374	4.37781975	20.6930085	24.0068339	21.7258204	4.916E-06	1.112278	up	PREDICTED: myosin heavy chain IB isoform X1 [Sesamum indicum]
c19520.graph_c0	16.5809509	17.5435288	20.0927796	22.6361642	14.9247841	14.6483802	1.079E-05	-1.06508	down	PREDICTED: calcium-binding protein PBP1-like [Sesamum indicum]
c19522.graph_c0	0.54875865	0.5053635	0.42310596	11.1828161	13.9034315	13.9449583	6.337E-46	3.704611	up	PREDICTED: putative leucine-rich repeat receptor-like serine/threonine-protein kinase At2g14440 [Sesamum indicum]
c19529.graph_c0	1.00161765	0.91543621	1.06327166	0.0401198	0.38251861	0.28558689	1.167E-08	-3.116793	down	-
c19533.graph_c0	47.965989	46.5902911	55.0205973	36.2371134	31.8311352	25.0470591	1.157E-19	-1.69842	down	PREDICTED: shaggy-related protein kinase alpha [Sesamum indicum]
c19541.graph_c0	0.73820271	0.7389419	0.63241314	13.7376257	14.4092561	15.3299986	1.158E-21	3.348079	up	PREDICTED: origin recognition complex subunit 1-like isoform X1 [Sesamum indicum]
c19554.graph_c0	2.92788845	1.85756215	1.5367764	24.6561664	27.0056497	29.037785	1.684E-32	2.659615	up	PREDICTED: uncharacterized protein LOC105168795 [Sesamum indicum]
c19560.graph_c0	26.3138584	25.35245	26.1457384	8.05182133	9.21782582	6.85631329	7.068E-54	-2.707428	down	PREDICTED: glutathione S-transferase omega-like 2 [Sesamum indicum]
c19565.graph_c0	27.6032281	28.6247847	23.9877457	2.37833542	0.38762431	1.46508131	2.465E-39	-5.239286	down	-
c19567.graph_c0	2.93934189	1.60488283	2.91872281	1.35395673	1.69514443	2.84757332	0.0097677	-1.367189	down	-
c19570.graph_c0	0.85576057	0.32946826	0.84591247	2.66837139	5.0111737	4.42483334	0.0016711	1.544722	up	PREDICTED: probable glucan 1,3-alpha-glucosidase [Sesamum indicum]
c19577.graph_c0	2406.73122	2236.25457	2518.98785	26.9406699	21.9246927	19.1038406	1.07E-241	-7.732284	down	PREDICTED: reticulon-like protein B13 [Sesamum indicum]
c19580.graph_c0	1.13834323	1.82317297	1.1702529	7.27109783	10.2951506	12.75293	8.691E-06	1.85195	up	expansin 5 [Cucumis sativus]
c19592.graph_c0	0	0.12837874	0	12.4060183	15.0201967	24.1802297	1.508E-12	7.638183	up	PREDICTED: protein WALLS ARE THIN 1-like [Nicotiana sylvestris]
c19593.graph_c0	1.57099471	1.50704416	0.7570464	6.81438398	12.3937047	13.682889	6.859E-05	2.078903	up	PREDICTED: transcription factor bHLH95-like isoform X1 [Sesamum indicum]
c19595.graph_c0	0.14269256	0.09522363	0.18336559	3.33026167	2.87811214	4.91275129	1.232E-09	3.698594	up	PREDICTED: protein IQ-DOMAIN 1 [Sesamum indicum]
c19602.graph_c0	0	0	0	10.2384113	16.9648122	17.0521951	2.025E-13	Inf	up	-
c19602.graph_c1	0.11008628	0.66117911	0.4243957	189.044392	199.839847	220.099551	8.8E-195	7.96998	up	PREDICTED: NAC domain-containing protein 100-like isoform X2 [Nicotiana tomentosiformis]
c19608.graph_c0	129.339462	132.063344	124.510637	19.6526959	29.7215073	33.2985591	1.27E-106	-3.247834	down	hypothetical protein MIMGU_mgv1a012664mg [Erythranthe guttata]
c19622.graph_c0	5.11909416	5.32438501	4.00861419	17.2798779	18.9694767	24.1943331	2.838E-06	1.045943	up	PREDICTED: cyclin-D3-3-like [Sesamum indicum]
c19632.graph_c0	0	0	0	5.72467442	5.61201642	4.78967455	4.364E-14	Inf	up	NADH dehydrogenase subunit 1 (mitochondrion) [Cyanoptyche gloeocystis]

#ID	0 h-1 FPKM	0 h-2 FPKM	0 h-3 FPKM	48 h-1 FPKM	48 h-2 FPKM	48 h-3 FPKM	FDR	log2FC	regulated	nr_annotation
c19652.graph_c0	0	0.1268796	0.16288221	3.09448007	3.27822641	3.48077611	1.281E-09	4.055441	up	PREDICTED: probable nucleoredoxin 2 [Sesamum indicum]
c19656.graph_c0	0.03091457	0	0.03972644	2.67716832	2.89647852	4.99233266	6.164E-11	6.192294	up	hypothetical protein M569_04675 [Genlisea aurea]
c19660.graph_c0	18.4637336	19.2435637	16.7838935	14.6662889	15.2043985	13.3724627	8.134E-19	-1.349525	down	PREDICTED: uncharacterized protein LOC105172629 [Sesamum indicum]
c19668.graph_c0	2.41526061	1.53316237	1.89250336	21.0570023	23.9171488	27.525115	2.515E-21	2.614001	up	PREDICTED: uncharacterized protein LOC105169149 [Sesamum indicum]
c19685.graph_c0	0.73197928	0.87925469	0.56437341	5.5746023	7.33439304	6.2803135	9.894E-07	2.122653	up	hypothetical protein MIMGU_mgv1a026794mg [Erythranthe guttata]
c19686.graph_c0	5.6757177	4.99963295	6.12655845	1.88238289	1.88336995	0.83746649	6.454E-10	-2.881404	down	-
c19688.graph_c0	0.43516081	0.13067897	0.55919888	2.76619983	3.69491938	3.67418611	1.114E-05	2.141881	up	PREDICTED: transcription factor bHLH66-like isoform X2 [Sesamum indicum]
c19697.graph_c0	0	0	0	104.772498	147.349032	187.839856	1.769E-29	Inf	up	PREDICTED: non-specific lipid-transfer protein 2-like [Sesamum indicum]
c19697.graph_c1	0	0	0	21.2557679	17.6390285	26.1633353	5.573E-14	Inf	up	-
c19702.graph_c0	0.26041409	0.20274711	0.22309494	3.43871638	6.7210149	6.40412614	2.014E-09	3.565406	up	PREDICTED: putative UPF0481 protein At3g02645 [Sesamum indicum]
c19707.graph_c0	0.54371499	0.44821365	0.32879778	2.85810906	2.49717112	2.80530643	0.0007078	1.616124	up	PREDICTED: histone-lysine N-methyltransferase ASHR3 [Sesamum indicum]
c19713.graph_c0	0.15614904	0.19538175	0.25082213	1.33065582	2.66694038	2.97602399	8.014E-05	2.499185	up	PREDICTED: WAT1-related protein At3g18200 [Sesamum indicum]
c19719.graph_c0	36.3377323	36.5027631	33.0294781	3.16711383	2.53989737	1.76459142	4.82E-173	-4.833818	down	SPla/RYanodine receptor domain-containing protein [Theobroma cacao]
c19722.graph_c0	25.864239	24.2148938	27.8111896	169.527959	159.363043	169.770732	6.22E-33	1.660766	up	hypothetical protein MIMGU_mgv1a026562mg [Erythranthe guttata]
c19727.graph_c0	17.4585684	9.5244475	11.8905243	0	0	0	4.934E-30	-Inf	down	--
c19728.graph_c0	0.14285693	0.07149999	0.27536522	5.52758322	6.13465198	7.904588	8.949E-13	4.288438	up	PREDICTED: uncharacterized protein LOC105160245 [Sesamum indicum]
c19729.graph_c0	15.076573	11.8159586	15.3940644	1.66889611	1.42580086	1.86819275	1.957E-42	-4.10873	down	hypothetical protein MIMGU_mgv1a012036mg [Erythranthe guttata]
c19742.graph_c0	2.04525583	1.78594591	1.73351652	0.16035941	0.21235169	0.60641907	6.406E-12	-3.53537	down	-
c19746.graph_c0	2976.86331	2819.2395	3061.80676	129.192783	79.0693723	72.1271845	8.06E-181	-5.988457	down	PREDICTED: 17.3 kDa class I heat shock protein-like [Sesamum indicum]
c19751.graph_c0	0.5433238	0.78033214	0.66783639	4.30904233	5.16434019	8.26861767	6.628E-05	2.130272	up	PREDICTED: DNA replication licensing factor MCM7 [Sesamum indicum]
c19755.graph_c0	0.06001965	0.06007975	0	32.5313143	50.2983819	49.823489	7.936E-57	9.100557	up	PREDICTED: probable beta-D-xylosidase 2 [Sesamum indicum]
c19764.graph_c0	4.19359951	3.53085878	4.02911507	14.7856956	18.0351935	19.1319433	2.961E-11	1.122353	up	PREDICTED: probable galacturonosyltransferase 4 [Sesamum indicum]

#ID	0 h-1 FPKM	0 h-2 FPKM	0 h-3 FPKM	48 h-1 FPKM	48 h-2 FPKM	48 h-3 FPKM	FDR	log2FC	regulated	nr_annotation
c19769.graph_c0	3.95417972	2.63875949	2.29155651	17.2856722	20.2038168	14.7451216	3.477E-06	1.541831	up	-
c19778.graph_c0	0.7245456	0.72527112	1.70696126	4.33874131	7.07133894	4.94946913	0.0066501	1.339307	up	PREDICTED: uncharacterized protein LOC102622885 [Citrus sinensis]
c19787.graph_c0	493.291072	478.612443	506.13531	55.5794549	44.6724039	38.4439247	8.14E-167	-4.42589	down	hypothetical protein MIMGU_mgv1a013874mg [Erythranthe guttata]
c19791.graph_c0	3.93770048	3.26670454	3.43116439	13.7652552	15.8987395	16.2720317	3.689E-08	1.091247	up	PREDICTED: cytosolic enolase 3 [Sesamum indicum]
c19792.graph_c0	12.6417582	11.3223731	12.458694	0.35026804	0.37106651	0.35062469	5.214E-80	-6.104877	down	hypothetical protein MIMGU_mgv1a004859mg [Erythranthe guttata]
c19801.graph_c0	11.7672543	5.68643188	7.99521649	212.827837	210.416667	192.36653	3.833E-87	3.58192	up	unnamed protein product [Coffea canephora]
c19806.graph_c0	3.35690653	3.2861444	2.66437981	19.4424564	25.1258395	23.9161881	6.342E-25	1.859836	up	PREDICTED: probable dolichyl-diphosphooligosaccharide--protein glycosyltransferase subunit 3B [Sesamum indicum]
c19810.graph_c0	11.2872278	10.9715105	11.0620691	2.24223015	3.76233253	5.18196352	1.515E-54	-2.60287	down	PREDICTED: probable receptor protein kinase TMK1 [Sesamum indicum]
c19815.graph_c0	0.18894528	0.41609586	0.63128564	3.2028644	4.3519509	4.77046718	3.519E-07	2.284281	up	PREDICTED: radical S-adenosyl methionine domain-containing protein 1, mitochondrial [Sesamum indicum]
c19818.graph_c0	4.86687728	5.16075288	5.03510465	19.7966083	21.4551488	20.2013523	6.521E-10	1.009853	up	PREDICTED: nuclear pore complex protein NUP93A-like [Sesamum indicum]
c19819.graph_c0	15.0248126	8.39114011	10.1835179	0	0	0	4.333E-30	-Inf	down	unnamed protein product [Chondrus crispus]
c19832.graph_c0	0	0	0	13.2696991	13.0911746	11.6597072	4.953E-24	Inf	up	ATPase subunit 6 [Cycas taitungensis]
c19836.graph_c0	5.16103918	3.92922799	3.45618546	1.00451111	0.85132617	0.53628477	1.38E-13	-3.398235	down	PREDICTED: gibberellin 2-beta-dioxygenase [Sesamum indicum]
c19839.graph_c1	1.43923688	1.42038682	1.27639919	4.59402884	8.21027987	9.0229366	0.0009223	1.373089	up	PREDICTED: receptor-like protein kinase HSL1 [Sesamum indicum]
c19846.graph_c0	4.48013357	4.09465282	4.6307503	3.40958409	4.37247057	3.5128528	0.0040831	-1.246792	down	hypothetical protein MIMGU_mgv1a0026501mg, partial [Erythranthe guttata]
c19853.graph_c0	9.30920639	10.020342	8.95951079	2.40422018	5.70219349	6.76900388	1.609E-17	-1.958679	down	PREDICTED: probable LRR receptor-like serine/threonine-protein kinase At4g36180 [Sesamum indicum]
c19859.graph_c0	15.988221	19.2431822	17.1212475	16.570566	8.54505025	9.04946688	9.667E-11	-1.61987	down	PREDICTED: uncharacterized protein DDB_G0287625-like [Sesamum indicum]
c19875.graph_c0	0.15775389	0.42109829	0.06757333	39.0946241	69.0266896	81.3832377	6.458E-20	7.176717	up	PREDICTED: vestitone reductase-like [Sesamum indicum]
c19877.graph_c0	1.80941168	1.22379968	1.94811176	6.44252899	7.6364512	10.6632956	0.0002585	1.287015	up	PREDICTED: putative phospholipid-transporting ATPase 4 isoform X1 [Sesamum indicum]
c19884.graph_c0	65.2938074	65.2196819	53.1011106	8.79503655	10.269405	9.71081027	5.723E-93	-3.691552	down	PREDICTED: uncharacterized protein LOC105166661 [Sesamum indicum]
c19885.graph_c0	16.9539225	15.4690498	16.0024453	10.0901887	11.128619	13.897745	4.022E-06	-1.484301	down	atypical dual-speci###city phosphatase 1 [Arabidopsis thaliana]
c19888.graph_c0	6.66948108	5.00711967	5.53514509	0	0	0	7.941E-25	-Inf	down	60S ribosomal protein L26B [Populus trichocarpa]

#ID	0 h-1 FPKM	0 h-2 FPKM	0 h-3 FPKM	48 h-1 FPKM	48 h-2 FPKM	48 h-3 FPKM	FDR	log2FC	regulated	nr_annotation
c19890.graph_c0	0.13293002	0.26612626	0.17082034	4.40869706	5.83809995	8.71740475	2.404E-07	4.031669	up	PREDICTED: uncharacterized protein LOC105158935 [Sesamum indicum]
c19916.graph_c0	1344.43037	1348.4148	1364.68462	32.1143083	29.0223779	22.9905375	8.09E-225	-6.605647	down	hypothetical protein MIMGU_mgv1a010720mg [Erythranthe guttata]
c19917.graph_c0	0.95666917	0	0.52686761	7.93211432	9.33679256	5.71357665	5.126E-07	2.938114	up	-
c19931.graph_c0	22.6273472	21.8253689	22.7958324	15.1789719	16.4018858	14.2715069	1.051E-18	-1.570613	down	PREDICTED: chaperone protein dnaJ 72 [Sesamum indicum]
c19932.graph_c0	0.12207268	0.16292656	0	0.97466476	2.97847825	4.30289841	0.0028928	3.836836	up	PREDICTED: uncharacterized protein LOC105155205 [Sesamum indicum]
c19933.graph_c0	3.79883537	2.53509288	3.3629171	0.6221751	0.65911907	0.55360766	3.587E-11	-3.419103	down	-
c19942.graph_c0	0	0	0.08459125	2.12256815	1.9273745	2.53618496	2.716E-10	5.232507	up	PREDICTED: cell division cycle-associated 7-like protein isoform X1 [Sesamum indicum]
c19944.graph_c0	174.505401	168.680333	188.870882	1.02549305	1.1281696	0.91247755	0	-8.45775	down	PREDICTED: F-box/kelch-repeat protein At1g23390-like [Sesamum indicum]
c19947.graph_c0	0	0	0	2.45453041	3.7247216	4.72221924	8.221E-12	Inf	up	PREDICTED: uncharacterized protein At1g08160-like [Sesamum indicum]
c19957.graph_c0	0.12222524	0.04893905	0.09423857	11.4178293	13.4318316	16.1911022	1.513E-52	6.25113	up	PREDICTED: probable beta-D-xylosidase 7 [Sesamum indicum]
c19964.graph_c0	8.62530099	9.09812814	8.34268525	3.50315897	2.53445903	2.05271766	4.831E-13	-2.696419	down	PREDICTED: probable E3 ubiquitin-protein ligase XERICO [Sesamum indicum]
c19973.graph_c0	0.78729759	0.60621996	0.23347121	3.23599799	4.55116131	5.01407804	0.0001738	1.961657	up	unnamed protein product [Coffea canephora]
c19983.graph_c0	1.65724115	1.45153805	0.8518482	11.4125208	12.3732297	13.7208566	6.705E-13	2.229678	up	PREDICTED: glucuronokinase 1-like [Sesamum indicum]
c19987.graph_c0	39.3644392	33.1474258	39.2107517	14.2699906	15.7235332	14.8294886	9.987E-45	-2.33705	down	PREDICTED: uncharacterized protein LOC105163283 [Sesamum indicum]
c19990.graph_c0	26.4654423	24.9156474	31.5939046	0.70196809	0.94195676	0.29148326	4.725E-77	-6.441017	down	PREDICTED: benzoate carboxyl methyltransferase-like [Sesamum indicum]
c20002.graph_c0	0.12975054	0	0.08336729	1.79301996	4.36882064	4.94579698	8.278E-06	4.671606	up	hypothetical protein MIMGU_mgv1a021791mg [Erythranthe guttata]
c20007.graph_c0	45.7618919	52.6208884	53.2230097	47.7622297	43.5314905	38.9372142	1.563E-11	-1.235043	down	unnamed protein product [Coffea canephora]
c20009.graph_c0	0	0	0	2.84964773	4.99672756	4.80889544	3.721E-12	Inf	up	-
c20017.graph_c0	0.25151993	0.62942947	0.64642613	7.41493002	5.03224983	4.5359601	2.783E-05	2.460796	up	hypothetical protein MIMGU_mgv1a022798mg, partial [Erythranthe guttata]
c20019.graph_c0	0.58928846	0.19662618	0.50483933	6.15276426	3.83418098	4.91112134	7.942E-06	2.517386	up	PREDICTED: uncharacterized protein LOC105167731 [Sesamum indicum]
c20028.graph_c0	5.3707566	6.68656741	6.08206572	0.95865394	1.9328735	3.27443666	4.956E-20	-2.588917	down	PREDICTED: probable L-type lectin-domain containing receptor kinase S.7 [Sesamum indicum]
c20029.graph_c0	8.20482025	6.85923897	6.4883193	0	0.08799614	0	3.655E-36	-8.987078	down	hypothetical protein MIMGU_mgv1a021012mg [Erythranthe guttata]

#ID	0 h-1 FPKM	0 h-2 FPKM	0 h-3 FPKM	48 h-1 FPKM	48 h-2 FPKM	48 h-3 FPKM	FDR	log2FC	regulated	nr_annotation
c20032.graph_c0	32.646766	36.0153002	28.9914869	12.9325503	13.9019464	17.4785334	1.698E-44	-2.158881	down	PREDICTED: DNA topoisomerase 6 subunit A [Sesamum indicum]
c20045.graph_c0	15.3763512	15.2984649	12.4542963	4.29263565	3.18326887	3.66677893	3.071E-22	-2.962999	down	PREDICTED: uncharacterized protein LOC105176125 [Sesamum indicum]
c20049.graph_c0	21.4425848	18.1618938	22.1042158	145.443804	178.341937	198.661717	4.793E-25	2.058778	up	V-type proton ATPase 16 kDa proteolipid subunit [Morus notabilis]
c20054.graph_c0	0	0	0	3.15231304	4.35891774	4.01544894	8.307E-20	Inf	up	PREDICTED: uncharacterized protein LOC105178927 [Sesamum indicum]
c20061.graph_c0	2.35129873	2.22756464	2.05031169	10.2506247	13.5433836	12.8553686	1.799E-12	1.445945	up	PREDICTED: polygalacturonate 4-alpha-galacturonosyltransferase-like [Sesamum indicum]
c20067.graph_c0	2.87242741	2.3795617	2.41836124	1.09500724	1.54670319	1.94866171	0.000382	-1.764689	down	PREDICTED: G-type lectin S-receptor-like serine/threonine-protein kinase SD1-1 [Nelumbo nucifera]
c20070.graph_c0	0.92940246	1.05168092	1.03853823	0.63286282	0.63100369	0.79498946	0.0016302	-1.572455	down	-
c20071.graph_c0	1.76559928	1.3633976	1.23166956	6.55281473	9.50205063	8.55989798	8.8E-05	1.476338	up	PREDICTED: phosphoserine phosphatase, chloroplastic [Sesamum indicum]
c20073.graph_c0	27.71263	30.3129516	26.4203944	18.5703623	27.3790793	33.6060012	1.896E-10	-1.110913	down	PREDICTED: ocs element-binding factor 1 [Sesamum indicum]
c20074.graph_c0	9.16627718	7.23697924	9.12459999	2.25981515	3.5280002	2.22242993	2.284E-10	-2.695826	down	-
c20076.graph_c0	4.78088835	6.62632018	5.87358111	3.96885964	4.87109666	6.24466803	0.0001033	-1.219947	down	hypothetical protein MIMGU_mgv1a023657mg [Erythranthe guttata]
c20081.graph_c0	0.27205828	0.2723307	0.34960566	6.76722139	8.62941263	10.0357215	1.401E-09	3.804442	up	PREDICTED: aquaporin TIP1-3-like [Sesamum indicum]
c20082.graph_c0	0	0.0623051	0	4.30065738	3.64482001	3.5715858	2.999E-13	6.527946	up	PREDICTED: ras-related protein Rab7-like [Sesamum indicum]
c20085.graph_c0	26.8514898	27.2175369	26.994626	1.52169781	1.48805013	1.18040813	2.89E-148	-5.289223	down	PREDICTED: UDP-glycosyltransferase 86A1-like [Sesamum indicum]
c20086.graph_c0	14.8492964	12.2915216	12.2931689	3.55157491	3.06571056	3.68686499	1.8E-25	-2.950916	down	-
c20090.graph_c0	1.11087883	0.9772044	1.03819875	5.08605765	6.50509848	6.92628916	1.315E-05	1.544217	up	PREDICTED: cyclin-D4-1-like [Sesamum indicum]
c20097.graph_c0	2.14236831	1.60838517	2.06477107	0.38377294	0.52272115	0.73174041	3.035E-08	-2.853742	down	hypothetical protein MIMGU_mgv1a023283mg [Erythranthe guttata]
c20099.graph_c0	0	0	0	1.75987471	1.3982804	1.17444445	2.389E-11	Inf	up	predicted protein [Physcomitrella patens]
c20108.graph_c0	0.39616017	0.21630374	0.09256027	2.12345358	2.53073428	3.12937875	5.445E-06	2.453624	up	PREDICTED: F-box protein At1g10780 [Sesamum indicum]
c20112.graph_c0	0.60693055	0.36002269	0.54883948	11.5970609	12.7025153	13.2304258	1.978E-42	3.609803	up	PREDICTED: pentatricopeptide repeat-containing protein At2g15820 isoform X1 [Sesamum indicum]
c20115.graph_c0	4.93631992	4.41559663	3.91399211	0	0	0	3.402E-25	-Inf	down	-
c20120.graph_c0	0.56860528	0.15177991	0.09742404	3.77162117	3.55162249	3.32489208	1.916E-07	2.699995	up	PREDICTED: probable protein phosphatase 2C 38 [Sesamum indicum]
c20121.graph_c0	27.8863766	24.0969603	29.0968744	317.840677	318.569501	363.309332	8.353E-69	2.604569	up	hypothetical protein CICLE_v10026787mg [Citrus clementina]

#ID	0 h-1 FPKM	0 h-2 FPKM	0 h-3 FPKM	48 h-1 FPKM	48 h-2 FPKM	48 h-3 FPKM	FDR	log2FC	regulated	nr_annotation
c20123.graph_c0	26.214793	24.0365111	24.1013018	113.881714	118.771807	121.946935	1.395E-16	1.235905	up	PREDICTED: NADH dehydrogenase [ubiquinone] 1 beta subcomplex subunit 10-B-like [Sesamum indicum]
c20125.graph_c0	2.14337016	1.49253317	1.19752849	19.4027131	20.1000692	26.6605385	4.322E-14	2.759431	up	PREDICTED: glycine cleavage system H protein 3, mitochondrial-like [Sesamum indicum]
c20126.graph_c0	11.6395699	16.8083249	8.21425942	93.5197656	84.7333601	74.6886517	1.638E-11	1.776686	up	PREDICTED: plastid division protein PDV2-like [Sesamum indicum]
c20131.graph_c0	0.83122105	1.01035055	0.68666912	33.8855459	53.9478464	59.2315668	1.514E-21	4.838661	up	PREDICTED: protein ASPARTIC PROTEASE IN GUARD CELL 2 [Sesamum indicum]
c20135.graph_c0	0.09579202	0.03196265	0	37.7121051	40.9174661	35.047964	4.23E-150	8.798398	up	PREDICTED: membrane protein of ER body-like protein [Sesamum indicum]
c20139.graph_c0	0.63491358	0.34221888	0.56484629	9.71873399	12.5361144	13.4519328	1.382E-19	3.51001	up	PREDICTED: protein IQ-DOMAIN 14-like [Sesamum indicum]
c20147.graph_c0	36.6726467	45.3606213	29.1159419	25.6079163	29.636157	30.6363293	5.944E-07	-1.389372	down	PREDICTED: uncharacterized protein LOC105166783 [Sesamum indicum]
c20167.graph_c0	3.91116275	3.68478041	4.77258727	0.90837434	1.53969995	1.61653206	4.413E-15	-2.634909	down	PREDICTED: pentatricopeptide repeat-containing protein At3g22690 [Sesamum indicum]
c20172.graph_c0	30.4805458	30.6905444	29.3995715	5.2857726	6.92954848	6.93729179	2.046E-87	-3.263512	down	PREDICTED: ethylene-responsive transcription factor ERF008-like [Nelumbo nucifera]
c20173.graph_c0	7.63749349	7.40472804	7.90096625	7.65569301	7.594596	6.57573563	2.216E-05	-1.088676	down	PREDICTED: uncharacterized protein At3g17611 [Sesamum indicum]
c20191.graph_c0	6.54293365	6.09637635	7.13880746	4.4355342	4.0964862	4.958689	7.485E-09	-1.570628	down	hypothetical protein MIMGU_mgv1a014744mg [Erythranthe guttata]
c20195.graph_c0	7.69703814	6.34159827	4.71724556	72.7647085	81.0147755	71.4434208	1.243E-48	2.571757	up	monodehydroascorbate reductase [Acanthus ebracteatus]
c20199.graph_c0	81.4503773	87.8036251	75.2413583	39.7175983	39.5281259	42.1884624	5.547E-43	-2.02633	down	PREDICTED: NAC domain-containing protein 18 [Sesamum indicum]
c20225.graph_c0	0.64099947	2.08533434	2.26520167	19.1184257	16.343682	15.0410208	8.468E-11	2.317914	up	-
c20226.graph_c0	0	0.06917109	0	2.06898814	2.56276918	2.69065315	2.838E-13	5.715948	up	PREDICTED: probable LRR receptor-like serine/threonine-protein kinase At1g05700 [Sesamum indicum]
c20239.graph_c0	1.84894046	1.2125878	1.26991056	35.1832885	39.2324972	35.1733827	7.734E-76	3.645088	up	PREDICTED: trihelix transcription factor GTL1 isoform X1 [Sesamum indicum]
c20254.graph_c0	1.37071973	3.56743997	1.58528621	64.528897	36.3876074	21.4613074	0.0028313	3.231131	up	unnamed protein product [Coffea canephora]
c20255.graph_c0	10.6277847	6.17538818	7.43658472	0	0	0	1.621E-38	-Inf	down	--
c20256.graph_c0	0.34321666	0.41990708	0.39204178	1.58096788	1.86093751	2.53212428	0.0077327	1.347224	up	PREDICTED: uncharacterized protein LOC101258386 [Solanum lycopersicum]
c20259.graph_c0	4.28935444	1.66619238	2.7971294	0	0	0	3.469E-11	-Inf	down	uncharacterized protein LOC100273024 [Zea mays]
c20263.graph_c0	6.41187082	4.81371851	5.29682648	0	0	0	1.017E-19	-Inf	down	putative 40s ribosomal protein s21 protein [Neofusicoccum parvum UCRNP2]

#ID	0 h-1 FPKM	0 h-2 FPKM	0 h-3 FPKM	48 h-1 FPKM	48 h-2 FPKM	48 h-3 FPKM	FDR	log2FC	regulated	nr_annotation
c20279.graph_c0	0.60035519	0.41604671	0.29672315	8.76429158	8.56356213	9.4640946	3.947E-15	3.338924	up	PREDICTED: haloacid dehalogenase-like hydrolase domain-containing protein At2g33255 [Sesamum indicum]
c20280.graph_c0	5.58849168	7.14800096	6.71596981	74.0332794	74.6921499	83.6897139	6.924E-54	2.559232	up	PREDICTED: 50S ribosomal protein L3, chloroplastic-like [Sesamum indicum]
c20281.graph_c0	0.08475428	0.04241957	0.10891261	2.30339458	2.64696109	2.01480856	2.849E-09	3.859163	up	PREDICTED: BTB/POZ domain-containing protein At5g60050 [Sesamum indicum]
c20285.graph_c0	7.66766925	8.67029968	6.75130679	8.89535331	6.23617161	5.58708133	0.0031665	-1.163724	down	PREDICTED: dentin sialophosphoprotein [Sesamum indicum]
c20293.graph_c0	24.3837383	23.9031587	22.906285	181.608933	188.822978	207.016076	1.167E-45	2.001607	up	PREDICTED: 40S ribosomal protein S16-like [Sesamum indicum]
c20297.graph_c0	35.682738	39.3392451	36.4945448	19.9040869	20.3703794	19.3533694	5.834E-35	-1.920528	down	PREDICTED: uncharacterized protein At4g18255 [Sesamum indicum]
c20298.graph_c0	1.12751561	1.03459093	0.80494567	5.53995161	5.65493641	7.34277536	3.579E-06	1.626665	up	PREDICTED: uncharacterized protein LOC105161749 [Sesamum indicum]
c20309.graph_c0	13.3067841	15.1244264	13.5571679	6.83773244	9.15816762	10.0823442	1.187E-15	-1.710266	down	PREDICTED: probable 3-hydroxyisobutyrate dehydrogenase-like 3, mitochondrial [Sesamum indicum]
c20315.graph_c0	6.05323205	5.95571578	5.85060316	3.14579252	4.29196646	4.62276806	4.264E-07	-1.589692	down	BnaA09g38920D [Brassica napus]
c20322.graph_c0	0	0	0	3.304683	4.94246249	6.48636853	6.202E-11	Inf	up	PREDICTED: inorganic phosphate transporter 1-4-like [Sesamum indicum]
c20327.graph_c0	0	0.20163582	0	2.69082653	2.06423079	2.146597	6.521E-09	4.096397	up	PREDICTED: putative HVA22-like protein g [Sesamum indicum]
c20329.graph_c0	0	0	0	1.54947673	3.02378394	3.48306967	9.104E-08	Inf	up	hypothetical protein M569_01834 [Genlisea aurea]
c20335.graph_c0	0.89392663	0.85008067	0.516929	42.6595541	43.0115136	49.719399	5.553E-96	4.890595	up	PREDICTED: perakine reductase-like [Sesamum indicum]
c20336.graph_c0	5.72828534	3.71649532	2.86263967	0	0	0	1.299E-15	-Inf	down	probable 40S ribosomal protein S27 [Piriformospora indica DSM 11827]
c20338.graph_c0	3.08081037	2.63695398	2.98356654	11.7643135	12.5500165	12.8102636	9.853E-05	1.07403	up	PREDICTED: BAG family molecular chaperone regulator 8, chloroplastic [Sesamum indicum]
c20366.graph_c0	2.27842677	2.91423834	3.09052797	0.58306537	0.9882993	0.93385452	2.691E-07	-2.753921	down	-
c20368.graph_c0	0.25634208	0.1140439	0	7.60956912	13.398629	16.203581	1.852E-13	5.640661	up	PREDICTED: protein NRT1/ PTR FAMILY 2.13-like [Sesamum indicum]
c20375.graph_c0	1.35356305	1.16522986	1.11320455	15.2382248	15.7731615	16.6434707	7.041E-31	2.696863	up	PREDICTED: exopolygalacturonase [Sesamum indicum]
c20376.graph_c0	20.9526758	23.1441091	19.731745	16.1027958	16.0872467	15.7065771	1.02E-22	-1.43046	down	PREDICTED: pentatricopeptide repeat-containing protein At5g02860 [Sesamum indicum]
c20380.graph_c0	0	0	0	1.25841891	2.25608687	1.46426699	6.459E-10	Inf	up	chlorophyllase [Picrorhiza kurrooa]
c20382.graph_c0	1.76004365	1.66570756	1.80938062	54.9824723	77.5172683	83.8381459	1.186E-33	4.344224	up	PREDICTED: fasciclin-like arabinogalactan protein 1 [Sesamum indicum]
c20385.graph_c0	40.9855089	41.8933078	38.5207617	17.7016823	16.9418946	13.4862432	2.398E-54	-2.348833	down	hypothetical protein MIMGU_mgv1a003020mg [Erythranthe guttata]

#ID	0 h-1 FPKM	0 h-2 FPKM	0 h-3 FPKM	48 h-1 FPKM	48 h-2 FPKM	48 h-3 FPKM	FDR	log2FC	regulated	nr_annotation
c20387.graph_c0	94.224968	102.419745	95.4753915	128.182404	90.0848885	69.362259	2.465E-06	-1.03022	down	PREDICTED: uncharacterized protein LOC105158345 [Sesamum indicum]
c20394.graph_c0	2.08641542	1.79014684	0.76603612	22.2419285	32.5804216	26.1433156	9.089E-09	3.10991	up	-
c20398.graph_c0	15.505617	8.50788608	9.88886834	0	0	0	4.473E-22	-Inf	down	--
c20399.graph_c0	0.83630069	1.17199337	1.1463248	7.85863841	6.74728128	6.3070232	3.688E-05	1.713041	up	myb family transcription factor family protein [Populus trichocarpa]
c20408.graph_c0	0.21806392	0.13096937	0.11208831	6.79024106	9.61962587	13.2636137	1.101E-12	4.987383	up	PREDICTED: aspartic proteinase nepenthesin-1 [Sesamum indicum]
c20415.graph_c0	27.789405	27.1489501	26.9169126	152.532139	132.350112	122.861409	2.66E-11	1.302779	up	PREDICTED: probable E3 ubiquitin-protein ligase RHA2B [Sesamum indicum]
c20419.graph_c0	0	0	0	5.46260354	6.90702402	9.56439027	2.171E-15	Inf	up	PREDICTED: thymidine kinase [Sesamum indicum]
c20444.graph_c0	0.41049386	0.24654294	0.66816775	13.4377727	12.633174	11.6652189	3.058E-38	3.808287	up	PREDICTED: transcription factor DIVARICATA-like [Sesamum indicum]
c20447.graph_c0	1.1749342	0.48428088	0.97695333	32.0270758	35.2778611	37.4490003	1.898E-46	4.292125	up	PREDICTED: uncharacterized protein At5g01610-like [Sesamum indicum]
c20460.graph_c0	4.7822646	5.08013821	4.13873834	1.3486941	1.85741123	1.08005391	1.503E-13	-2.726133	down	PREDICTED: lysM domain receptor-like kinase 3 [Sesamum indicum]
c20472.graph_c0	118.075129	112.735985	114.014348	53.0475847	25.6569575	14.3257234	1.408E-72	-2.887464	down	PREDICTED: uncharacterized protein LOC105175062 [Sesamum indicum]
c20473.graph_c0	0.43637762	0.39313313	0.47664814	3.07544936	3.55618933	4.34623184	1.029E-07	2.048155	up	PREDICTED: uncharacterized protein LOC105174198 [Sesamum indicum]
c20480.graph_c0	1.1853104	1.08479754	0.82686415	25.3964571	38.9354568	42.585677	6.026E-20	4.087316	up	PREDICTED: protein notum homolog [Sesamum indicum]
c20483.graph_c0	2.52553295	2.85426342	3.35010046	0	0.15902202	0.06678293	1.808E-19	-6.324719	down	-
c20493.graph_c0	0	0	0.07275881	5.05970842	4.80755502	3.66665723	2.61E-15	6.492442	up	Uncharacterized protein F383_18135 [Gossypium arboreum]
c20501.graph_c0	9.26691936	8.61986396	6.51589415	26.4172764	32.977289	40.9562229	0.0001664	1.020252	up	PREDICTED: geranylgeranyl pyrophosphate synthase, chloroplastic-like [Sesamum indicum]
c20506.graph_c0	18.1048829	18.0305478	19.7044467	17.7431362	16.6330522	17.7186101	1.739E-10	-1.11777	down	PREDICTED: probable 2-oxoglutarate-dependent dioxygenase At3g49630 isoform X1 [Sesamum indicum]
c20508.graph_c0	11.7511843	12.3604346	10.4986077	6.35810201	9.9760262	10.3973855	4.393E-13	-1.396947	down	PREDICTED: cellulose synthase-like protein D3 isoform X1 [Sesamum indicum]
c20512.graph_c0	10.7333417	15.471489	7.17223993	5.9329527	5.23770332	5.45507582	0.0002604	-2.015563	down	-
c20514.graph_c0	0	0.06217232	0	2.68933507	3.21272965	3.41123254	6E-12	6.217384	up	PREDICTED: uncharacterized protein LOC105156631 [Sesamum indicum]
c20517.graph_c0	0.68710763	0.91706089	0.44148026	13.6624306	19.3355068	16.8035367	8.948E-23	3.587944	up	PREDICTED: glyoxylate/hydroxypyruvate reductase HPR3 [Sesamum indicum]
c20522.graph_c0	44.3085878	46.2779976	42.5554404	28.8787051	25.5633917	23.0791949	3.554E-35	-1.793879	down	PREDICTED: zinc finger protein GIS2-like isoform X2 [Sesamum indicum]

#ID	0 h-1 FPKM	0 h-2 FPKM	0 h-3 FPKM	48 h-1 FPKM	48 h-2 FPKM	48 h-3 FPKM	FDR	log2FC	regulated	nr_annotation
c20528.graph_c0	0	0	0	8.9809686	7.45519373	6.29158844	2.99E-29	Inf	up	PREDICTED: MLO-like protein 1 isoform X3 [Sesamum indicum]
c20541.graph_c0	1.47445508	0.32798478	0.63157772	6.48996223	9.59348097	12.3552349	1.257E-05	2.526273	up	Os06g0708300 [Oryza sativa Japonica Group]
c20547.graph_c0	1.02782737	1.1079994	1.01599913	7.97582516	8.64232876	7.93938863	3.526E-08	1.943998	up	hypothetical protein MIMGU_mgv1a006570mg [Erythranthe guttata]
c20552.graph_c0	165.075907	173.46402	178.445608	9.73496267	6.70528023	4.34635971	2.03E-252	-5.643387	down	hypothetical protein MIMGU_mgv1a010327mg [Erythranthe guttata]
c20557.graph_c0	0.28981944	0.72527412	0.74485891	3.738008	4.45496199	4.1575713	0.0004628	1.785144	up	PREDICTED: UDP-galactose transporter 2-like [Sesamum indicum]
c20560.graph_c0	0.22459248	0.67445212	0.28861023	5.7934587	5.04149075	7.36426388	2.675E-06	2.922355	up	unnamed protein product [Vitis vinifera]
c20560.graph_c1	0.71046499	0.38791441	0.82997786	7.19979361	5.92534695	8.1005699	1.19E-07	2.440088	up	poly(A)-binding protein [Nicotiana tabacum]
c20573.graph_c0	2.41971637	3.41583755	3.26888936	0.5144309	0.30276509	0.35601807	1.048E-14	-3.965887	down	-
c20574.graph_c0	35.9055724	37.7164166	28.2536799	31.6900727	26.5632457	26.2354295	7.088E-12	-1.281101	down	PREDICTED: LOW QUALITY PROTEIN: serrate RNA effector molecule [Pyrus x bretschneideri]
c20588.graph_c0	54.9067961	56.770743	52.3745457	8.28892922	7.28007005	6.02012359	4.18E-139	-3.938479	down	PREDICTED: protein BPS1, chloroplastic-like [Sesamum indicum]
c20601.graph_c0	2182.4846	2192.99073	2245.65722	334.586698	120.404852	68.0260256	1.29E-124	-4.653919	down	PREDICTED: chaperone protein dnaJ 20, chloroplastic-like [Nelumbo nucifera]
c20602.graph_c0	0	0.04873447	0	18.3446547	18.8637483	21.0721875	4.164E-57	9.216737	up	PREDICTED: anthocyanidin 3-O-glucosyltransferase 2-like [Sesamum indicum]
c20607.graph_c0	0	0	0	2.06046511	8.00364745	9.24333855	1.493E-05	Inf	up	unnamed protein product [Coffea canephora]
c20610.graph_c0	0	0	0	1.64170037	2.01379023	1.84518962	8.473E-10	Inf	up	PREDICTED: CEN-like protein 1 [Sesamum indicum]
c20611.graph_c0	4.8903839	3.27607259	3.57723794	0.97038106	2.79028861	4.56389307	0.0005098	-1.529582	down	-
c20613.graph_c0	0	0	0	4.86956743	7.48013854	9.96569719	1.492E-08	Inf	up	hypothetical protein M569_01187, partial [Genlisea aurea]
c20617.graph_c0	0.04115735	0.02059928	0	1.40292305	1.36572198	0.99527618	2.629E-11	4.933042	up	PREDICTED: putative disease resistance protein At1g50180 [Sesamum indicum]
c20625.graph_c0	3.98220196	2.75711443	2.04691487	26.7506764	20.0155	16.8930389	2.796E-05	1.852533	up	PREDICTED: probable anion transporter 3, chloroplastic [Sesamum indicum]
c20635.graph_c0	1.31827676	0.92371777	1.41169819	26.9615123	35.9818289	44.1621242	3.668E-20	3.847328	up	PREDICTED: uncharacterized protein LOC105169209 [Sesamum indicum]
c20639.graph_c0	11.4129153	12.8556998	11.1951967	66.6720229	68.4083628	65.83637	5.179E-25	1.485235	up	PREDICTED: T-complex protein 1 subunit alpha [Sesamum indicum]
c20647.graph_c0	13.8998822	15.255741	15.4138214	8.12530336	6.81735675	6.36226454	4.494E-17	-2.078976	down	PREDICTED: uncharacterized protein LOC105174183 [Sesamum indicum]
c20653.graph_c0	0	0	0	4.50758064	5.60171812	3.77942479	4.446E-13	Inf	up	predicted protein [Hordeum vulgare subsp. vulgare]
c20673.graph_c0	20.6115627	11.1616831	11.7236099	0	0	0	2.927E-18	-Inf	down	-

#ID	0 h-1 FPKM	0 h-2 FPKM	0 h-3 FPKM	48 h-1 FPKM	48 h-2 FPKM	48 h-3 FPKM	FDR	log2FC	regulated	nr_annotation
c20674.graph_c0	0.04193356	0.1679022	0.10777257	1.77706877	1.92351472	2.37183765	1.574E-07	3.234234	up	PREDICTED: probable transcription factor GLK1 [Sesamum indicum]
c20676.graph_c0	3.89879309	2.6017981	3.24183167	0	0	0	3.789E-25	-Inf	down	-
c20678.graph_c0	8.43904527	6.99935358	7.74607663	0.2221314	0.11766064	0.29647677	4.024E-26	-6.201524	down	PREDICTED: homeobox-leucine zipper protein HAT22-like [Sesamum indicum]
c20681.graph_c0	0.63210801	0.2711747	0.46416218	3.9931826	5.19973485	4.73747982	2.76E-05	2.327947	up	PREDICTED: ras-related protein RABC2a-like [Sesamum indicum]
c20682.graph_c0	15.0215957	11.5907415	10.2549022	6.19866221	4.42872555	3.71977764	6.372E-10	-2.36752	down	PREDICTED: protein MKS1 [Sesamum indicum]
c20683.graph_c0	1.18632972	0.86780136	0.70360646	13.0730563	10.7766397	11.1834911	2.594E-14	2.657695	up	PREDICTED: GDSL esterase/lipase At4g10955-like [Sesamum indicum]
c20685.graph_c0	164.27113	165.182303	166.309129	11.4151984	9.66632586	8.76438503	6.83E-218	-5.067463	down	PREDICTED: F-box/kelch-repeat protein At5g43190 [Sesamum indicum]
c20709.graph_c0	23.8910808	23.6049948	22.6277628	20.3388907	21.6329475	22.1956366	1.157E-12	-1.146312	down	PREDICTED: ABSCISIC ACID-INSENSITIVE 5-like protein 2 [Sesamum indicum]
c20712.graph_c0	1.25713176	1.80893648	1.96884686	13.8254369	16.6784625	17.0035221	3.848E-17	2.213684	up	PREDICTED: 8-hydroxygeraniol dehydrogenase [Sesamum indicum]
c20713.graph_c0	234.818836	248.452814	226.559352	89.1981326	44.1057771	26.3340703	1.07E-102	-3.15057	down	PREDICTED: uncharacterized protein At1g76070 [Sesamum indicum]
c20731.graph_c0	1.31374285	1.08635257	1.68821158	6.89347976	7.74877881	9.41136751	6.579E-05	1.531934	up	hypothetical protein MIMGU_mgv1a005057mg [Erythranthe guttata]
c20743.graph_c0	13.3143983	12.5536453	13.7826761	57.0249385	58.639039	60.056184	1.195E-14	1.129114	up	PREDICTED: COP9 signalosome complex subunit 1-like [Sesamum indicum]
c20753.graph_c0	2.63028647	3.92706758	2.17696349	1.64284167	2.74111668	2.08305166	0.0006487	-1.455111	down	-
c20754.graph_c0	0.06319426	0.06325754	0	1.86299745	1.91194427	2.84914425	1.983E-09	4.70641	up	PREDICTED: ATP-dependent 6-phosphofructokinase 6-like [Sesamum indicum]
c20761.graph_c0	0.33520481	0.16777024	0.28716764	5.32701628	8.0424237	10.0294532	5.189E-12	3.861536	up	PREDICTED: inorganic phosphate transporter 2-1, chloroplastic-like [Sesamum indicum]
c20762.graph_c0	21.3045264	10.9775737	12.7460876	0	0	0	4.655E-22	-Inf	down	cytochrome c oxidase subunit I [Lingulodinium polyedrum]
c20763.graph_c0	0.68363213	1.12710982	0.98184662	14.8930714	14.9924579	15.7241097	5.505E-24	3.010098	up	PREDICTED: uncharacterized protein LOC105157658 [Sesamum indicum]
c20780.graph_c0	18.9011012	16.6289306	16.6035784	147.19365	135.469311	149.431576	8.514E-41	2.035413	up	PREDICTED: NADH dehydrogenase [ubiquinone] 1 beta subcomplex subunit 3-B [Sesamum indicum]
c20797.graph_c0	106.189933	109.450259	111.044639	558.811636	548.810644	540.256513	2.651E-20	1.317328	up	PREDICTED: 40S ribosomal protein S3-3-like [Sesamum indicum]
c20802.graph_c0	0.31497287	0.39411034	0.2023763	1.70476962	1.26804024	2.06555746	0.0074232	1.455724	up	PREDICTED: pentatricopeptide repeat-containing protein At1g09220, mitochondrial [Sesamum indicum]

#ID	0 h-1 FPKM	0 h-2 FPKM	0 h-3 FPKM	48 h-1 FPKM	48 h-2 FPKM	48 h-3 FPKM	FDR	log2FC	regulated	nr_annotation
c20804.graph_c0	20.7643723	21.2505042	15.9301735	9.32681804	11.191329	8.4259713	4.266E-22	-2.017518	down	PREDICTED: LOW QUALITY PROTEIN: probable LRR receptor-like serine/threonine-protein kinase At1g56140 [Sesamum indicum]
c20809.graph_c0	2.52116588	2.00154759	1.89919191	0.16018362	0.67878048	0.35632605	2.783E-10	-3.459919	down	-
c20810.graph_c0	27.3130531	30.9896517	30.8597821	238.457961	236.74753	246.948594	8.632E-46	1.999369	up	PREDICTED: adenylate kinase [Sesamum indicum]
c20811.graph_c0	14.046012	12.8267369	11.3997989	11.5779864	12.5059721	9.29202044	0.0026523	-1.212392	down	-
c20815.graph_c0	39.8855724	40.3745011	40.1700804	367.547268	433.046863	421.083767	6.307E-56	2.322256	up	PREDICTED: tubulin alpha-3 chain [Sesamum indicum]
c20826.graph_c0	3.86244748	2.28653046	2.56175377	0	0	0	1.891E-24	-Inf	down	Fructose-bisphosphate aldolase A [Rozella allomycis CSF55]
c20831.graph_c0	6.59956081	4.39115957	5.9863743	28.1823373	28.9085683	28.6406432	4.343E-13	1.318072	up	PREDICTED: isocitrate dehydrogenase [NADP] isoform X1 [Sesamum indicum]
c20835.graph_c0	0.3279295	0.29543209	0.25284144	1.78244929	2.01630847	1.88170993	0.0012401	1.679793	up	unnamed protein product [Coffea canephora]
c20837.graph_c0	2.10811534	1.65803495	1.74150751	0.52715048	0.73480528	1.28373037	4.118E-07	-2.139368	down	PREDICTED: pentatricopeptide repeat-containing protein At1g71060, mitochondrial [Sesamum indicum]
c20841.graph_c0	0.13293002	0.13306313	0.17082034	5.26594372	4.41100885	3.59592946	2.913E-08	3.910238	up	Chain O, Localization Of The Small Subunit Ribosomal Proteins Into A 5.5 A Cryo-em Map Of Triticum Aestivum Translating 80s Ribosome
c20843.graph_c0	1.0564882	1.09822096	1.25319615	5.57779116	5.98830873	5.42941517	0.0006865	1.298393	up	PREDICTED: aldo-keto reductase [Sesamum indicum]
c20853.graph_c0	0.02622689	0.05250631	0.10110779	2.6336499	2.96920913	2.51539893	4.818E-14	4.465953	up	PREDICTED: B3 domain-containing protein Os01g0234100-like [Sesamum indicum]
c20868.graph_c0	2.49157738	2.36280535	4.04434809	0.36243215	0.38395291	0.2149933	7.809E-09	-4.230931	down	-
c20871.graph_c0	196.431259	208.112584	192.338467	95.7817533	98.3687907	97.9429347	4.749E-42	-2.048459	down	PREDICTED: branchpoint-bridging protein [Sesamum indicum]
c20872.graph_c0	1.89138441	1.19575475	2.04674008	36.8669267	84.0384908	92.8629789	5.213E-08	4.346	up	hypothetical protein MIMGU_mgv1a013356mg [Erythranthe guttata]
c20878.graph_c0	0.02198346	0.11002737	0.02824962	2.20753314	1.75932405	1.94622907	5.317E-12	4.196804	up	PREDICTED: protein CHUP1, chloroplastic-like [Sesamum indicum]
c20880.graph_c0	0	0	0	31.6439177	30.9076326	23.0755165	1.142E-58	Inf	up	cytochrome c oxidase subunit I [Lingulodinium polyedrum]
c20899.graph_c0	0.44279184	0.18995796	0.08128644	6.4685841	8.27260529	7.72612953	4.221E-13	3.966695	up	PREDICTED: polyadenylate-binding protein 8-like [Sesamum indicum]
c20900.graph_c0	0	0	0	86.9546207	100.492229	95.9226506	2.24E-268	Inf	up	PREDICTED: raucaffricine-O-beta-D-glucosidase-like [Sesamum indicum]
c20914.graph_c0	59.4792406	60.1244276	57.9388557	10.5639802	11.8383696	10.8065338	7.46E-113	-3.437122	down	PREDICTED: uncharacterized protein LOC105168301 [Sesamum indicum]
c20941.graph_c0	0.2752768	0.18370163	0.35374158	3.71950981	2.73139259	3.08394309	1.588E-06	2.535656	up	PREDICTED: protein tesmin/TSO1-like CXC 2 isoform X3 [Sesamum indicum]
c20944.graph_c0	1673.56929	1658.2327	1660.80325	101.068551	145.275835	196.140168	3.62E-142	-4.521977	down	PREDICTED: 18 kDa seed maturation protein-like [Nicotiana sylvestris]

#ID	0 h-1 FPKM	0 h-2 FPKM	0 h-3 FPKM	48 h-1 FPKM	48 h-2 FPKM	48 h-3 FPKM	FDR	log2FC	regulated	nr_annotation
c20982.graph_c0	7.41297614	7.94902913	6.05740963	32.9933501	32.7953662	34.0069909	1.611E-15	1.204847	up	PREDICTED: phosphatidylinositol 4-phosphate 5-kinase 1-like [Sesamum indicum]
c20998.graph_c0	0.29132832	0.19441336	0.43676317	1.78927103	3.22237669	3.02495695	6.482E-05	2.089322	up	hypothetical protein MIMGU_mgv1a024355mg [Erythranthe guttata]
c21005.graph_c0	0.70997679	0.95435208	0.86021409	3.68151869	5.97886822	5.63702483	7.149E-07	1.57271	up	PREDICTED: probable LRR receptor-like serine/threonine-protein kinase At2g24230 [Sesamum indicum]
c21008.graph_c0	0.1666016	0.12507632	0.05352241	1.22787409	2.23572201	1.87782888	2.068E-06	2.934224	up	hypothetical protein MIMGU_mgv1a020944mg, partial [Erythranthe guttata]
c21009.graph_c0	480.2178	457.952132	475.338859	60.3378977	37.4630804	39.9160828	4.48E-158	-4.367761	down	PREDICTED: histone H1-like [Sesamum indicum]
c21011.graph_c0	0.56152507	0.71822272	0.52114251	2.75900543	3.01417019	2.86410418	0.0056345	1.244887	up	PREDICTED: la-related protein 1B-like [Sesamum indicum]
c21017.graph_c0	1.98691854	1.98890814	2.13640875	0.33621042	0.39574908	0.69803536	7.788E-11	-3.121266	down	PREDICTED: uncharacterized protein LOC105175392 [Sesamum indicum]
c21037.graph_c0	0.65856495	0.3296122	0.56418802	24.1674005	37.4930988	35.7200461	5.374E-27	4.945644	up	PREDICTED: defensin J1-2-like [Sesamum indicum]
c21056.graph_c0	320.714039	178.609573	216.018971	0	0	0	3.647E-54	-Inf	down	--
c21058.graph_c1	5.73341899	4.97393879	7.04021838	0.3521333	0.24869502	0.3133261	3.834E-19	-5.294432	down	-
c21062.graph_c0	28.2441686	28.6066406	25.2262251	3.44477716	2.15049433	1.25889761	1.38E-94	-4.584128	down	-
c21075.graph_c0	0	0.0336202	0	1.42333842	2.78625282	2.31269889	9.341E-12	6.583675	up	PREDICTED: uncharacterized protein LOC105162896 [Sesamum indicum]
c21084.graph_c0	0.03776457	0	0	3.4791188	4.38598835	5.04598934	2.244E-19	7.415967	up	PREDICTED: 65-kDa microtubule-associated protein 3 [Sesamum indicum]
c21092.graph_c0	0	0	0	1.82943165	3.31085422	1.83129445	1.007E-07	Inf	up	PREDICTED: uncharacterized protein LOC105166030 [Sesamum indicum]
c21098.graph_c0	46.04187	48.0986108	38.6839276	55.1778541	31.9767128	24.2704736	9.107E-06	-1.255196	down	PREDICTED: receptor-like protein kinase HAIKU2 [Sesamum indicum]
c21098.graph_c1	67.1025111	66.9482656	50.6952969	72.4847221	42.8203565	29.8001496	1.186E-05	-1.34903	down	unnamed protein product [Coffea canephora]
c21099.graph_c0	12.0112033	7.30823829	6.80950228	0	0	0	8.763E-21	-Inf	down	--
c21103.graph_c0	4.69730471	5.11689144	5.5627933	28.5103476	27.1919197	27.6333659	7.54E-13	1.420017	up	hypothetical protein MIMGU_mgv1a010545mg [Erythranthe guttata]
c21104.graph_c0	3.28913351	4.11553386	2.4201076	72.3573917	101.920411	112.088906	5.17E-27	3.844915	up	PREDICTED: DELLA protein GAI-like [Sesamum indicum]
c21106.graph_c0	2.05893627	2.18223315	1.75090673	53.3621893	59.6927096	65.848424	2.24E-109	3.88163	up	PREDICTED: GDP-mannose 4,6 dehydratase 1 [Sesamum indicum]
c21108.graph_c0	0.43017333	0.28706939	0.3685265	29.0622732	39.6045051	35.7330388	1.438E-29	5.565148	up	-
c21116.graph_c0	6.39291694	5.11945478	7.11979786	0	0.20797625	0.34936705	1.474E-17	-6.11047	down	-
c21117.graph_c0	1.00561267	1.16555958	0.47609287	7.46024761	8.16150316	5.9439048	3.06E-06	2.016531	up	PREDICTED: BURP domain-containing protein 17-like [Sesamum indicum]

#ID	0 h-1 FPKM	0 h-2 FPKM	0 h-3 FPKM	48 h-1 FPKM	48 h-2 FPKM	48 h-3 FPKM	FDR	log2FC	regulated	nr_annotation
c21119.graph_c0	1.40734402	0.37072454	0.95183831	39.919695	43.5190188	40.9849668	1.818E-53	4.492692	up	Signal peptidase complex catalytic subunit SEC11C [Gossypium arboreum]
c21133.graph_c0	0	0	0	1.37634852	2.13850906	1.46959996	1.409E-11	Inf	up	--
c21136.graph_c0	15.9021166	18.8751465	20.4348495	14.7079725	15.3359384	12.417255	1.371E-11	-1.397579	down	Arabinoxylan arabinofuranohydrolase [Gossypium arboreum]
c21139.graph_c0	5.78164929	4.70010176	5.40338054	4.90677412	5.9162952	5.11283699	0.0001021	-1.015338	down	PREDICTED: zinc finger CCCH domain-containing protein 11-like [Sesamum indicum]
c21144.graph_c0	30.1080582	33.1599796	26.6440692	13.1003001	14.5371992	10.4192789	1.33E-46	-2.255794	down	hypothetical protein MIMGU_mgv1a000612mg [Erythranthe guttata]
c21148.graph_c0	158.91922	149.175399	177.046655	107.761088	83.7739867	75.332667	3.051E-24	-1.875196	down	PREDICTED: CBS domain-containing protein CBSX3, mitochondrial-like [Sesamum indicum]
c21151.graph_c0	1.3294581	1.00956433	1.47276404	0.2956374	0.26845027	1.27770242	6.834E-05	-2.080929	down	-
c21154.graph_c0	1.35307603	0.84651933	1.04325376	5.85874689	7.7252759	9.12288607	8.487E-08	1.785467	up	PREDICTED: uncharacterized protein At5g39865-like [Sesamum indicum]
c21157.graph_c0	0	0	0.13961939	2.60247917	2.33285556	3.11724851	6.158E-10	4.799717	up	PREDICTED: transcription factor PRE6-like [Solanum lycopersicum]
c21162.graph_c0	0	0.1213478	0.05192691	0.78177172	1.77469788	0.96061226	0.0005653	3.313074	up	PREDICTED: vegetative incompatibility protein HET-E-1-like [Sesamum indicum]
c21165.graph_c0	15.341046	14.6294772	13.1522885	6.27211782	6.15728143	6.23199687	3.931E-42	-2.22407	down	PREDICTED: protein PHOTOPERIOD-INDEPENDENT EARLY FLOWERING 1 isoform X2 [Sesamum indicum]
c21171.graph_c0	2.97257529	2.04569191	3.46176347	0.77021163	1.72255231	2.96976194	0.0010093	-1.670625	down	-
c21182.graph_c0	4.92426671	4.39470631	4.42189043	0.81985946	1.91079171	2.09125058	2.206E-10	-2.541572	down	-
c21184.graph_c0	62.6680933	76.622035	75.1528136	25.8229991	19.2945476	20.5846367	2.507E-39	-2.719828	down	PREDICTED: uncharacterized protein LOC105158345 [Sesamum indicum]
c21193.graph_c0	1.41290041	1.64522383	1.18571949	1.03601193	1.06938725	0.82728978	0.0002133	-1.546907	down	hypothetical protein MIMGU_mgv1a002822mg [Erythranthe guttata]
c21195.graph_c0	0.36810603	0.22108478	0.09460616	1.76344108	3.161488	3.31924846	5.092E-06	2.574621	up	PREDICTED: glucan endo-1,3-beta-glucosidase 13 [Sesamum indicum]
c21196.graph_c0	0	0	0	1.2648423	2.21382556	1.12544907	5.374E-07	Inf	up	predicted protein [Hordeum vulgare subsp. vulgare]
c21198.graph_c0	7.15436127	6.59478587	6.48184513	23.3295224	31.8981668	29.6609827	1.756E-07	1.047088	up	RecName: Full=Ras-related protein Rab11C [Lotus japonicus]
c21208.graph_c0	3.53909575	3.5848139	2.05737345	0.38814831	0.69903333	0.48352085	1.578E-12	-3.56769	down	-
c21212.graph_c0	0.33760743	0.50691824	0.54229864	9.33077958	14.6075815	15.2442603	2.295E-18	3.791287	up	PREDICTED: protein LUTEIN DEFICIENT 5, chloroplastic [Sesamum indicum]
c21224.graph_c0	9.21333427	6.31781671	9.78856241	80.067193	93.3885937	95.8038125	1.113E-44	2.388083	up	PREDICTED: elongation factor 1-gamma 2-like [Sesamum indicum]
c21231.graph_c0	0	0.07137492	0.18325568	1.77361516	3.89704041	4.61755615	8.095E-06	4.285122	up	PREDICTED: probable xyloglucan glycosyltransferase 5 [Sesamum indicum]

#ID	0 h-1 FPKM	0 h-2 FPKM	0 h-3 FPKM	48 h-1 FPKM	48 h-2 FPKM	48 h-3 FPKM	FDR	log2FC	regulated	nr_annotation
c21236.graph_c0	7.55570237	7.80085784	8.38768143	4.26394913	6.71779359	5.70726827	2.608E-10	-1.534289	down	PREDICTED: probable purine permease 9 [Sesamum indicum]
c21237.graph_c0	3.12424179	2.38667729	2.64129894	15.3001993	15.0853302	17.0849278	1.224E-09	1.524524	up	unnamed protein product [Coffea canephora]
c21242.graph_c0	0.02327536	0	0	18.6337761	20.308127	24.7272355	1.365E-90	10.41832	up	PREDICTED: probable inactive receptor kinase At4g23740 [Sesamum indicum]
c21243.graph_c0	8.39005168	8.15849724	6.1608849	0.29445573	1.16977552	0.45850864	2.838E-26	-4.594366	down	PREDICTED: uncharacterized protein DDB_G0284459-like isoform X1 [Sesamum indicum]
c21247.graph_c0	0.43096847	0.21570001	0.46150962	12.936765	14.7915143	16.8102629	5.307E-39	4.304732	up	PREDICTED: uncharacterized protein LOC105161548 [Sesamum indicum]
c21248.graph_c0	0.19188993	0.12805472	0.08219539	6.21681683	7.36629104	8.02225586	2.456E-24	4.73344	up	PREDICTED: phosphatidylinositol/phosphatidylcholine transfer protein SFH13-like isoform X1 [Sesamum indicum]
c21250.graph_c0	0.06446166	0	0.08283578	2.34575815	4.37242327	7.29213484	4.739E-06	5.531706	up	hypothetical protein MIMGU_mgv1a007352mg [Erythranthe guttata]
c21251.graph_c0	8.07500501	8.16473829	8.28039788	4.05775708	4.37830718	4.34605389	1.4E-08	-1.958888	down	PREDICTED: metal tolerance protein 1-like isoform X1 [Sesamum indicum]
c21257.graph_c0	8.55112898	10.3286946	9.66991458	262.280145	244.972208	269.124615	4.03E-132	3.747807	up	PREDICTED: tubulin beta chain-like [Sesamum indicum]
c21269.graph_c0	0	0.4811318	0.61765492	9.96314822	12.6656969	19.3063253	4.524E-08	4.216031	up	hypothetical protein MIMGU_mgv1a022779mg, partial [Erythranthe guttata]
c21270.graph_c0	1.10116656	1.03987661	1.2281502	0.80391681	1.2369237	0.86860328	0.0027703	-1.236248	down	PREDICTED: pentatricopeptide repeat-containing protein At3g24000, mitochondrial [Sesamum indicum]
c21292.graph_c0	0.28082885	0.52708136	0.45109524	173.179265	130.804774	112.484208	5.288E-33	7.357139	up	PREDICTED: cytochrome P450 71D95-like [Sesamum indicum]
c21295.graph_c0	11.4378769	11.8433355	10.9789635	7.63635612	7.57005803	7.00356203	1.733E-22	-1.641394	down	PREDICTED: uncharacterized protein LOC105161140 [Sesamum indicum]
c21296.graph_c0	1.60040871	1.28160902	1.79217003	1.70609246	1.53963541	1.23694659	0.0046984	-1.076179	down	unnamed protein product [Coffea canephora]
c21299.graph_c0	0.57942761	1.09557032	0.66185548	7.35466738	3.95852284	5.43585724	0.001086	1.835159	up	PREDICTED: ethylene-responsive transcription factor-like protein At4g13040 [Sesamum indicum]
c21305.graph_c0	5.58324641	2.55142567	3.04144581	0	0	0	7.982E-13	-Inf	down	hypothetical protein M569_16047 [Genlisea aurea]
c21328.graph_c0	34.8213697	28.3206934	23.9715207	244.03499	214.5276	168.972294	1.656E-08	1.838952	up	F-box/WD repeat-containing protein 4 [Theobroma cacao]
c21337.graph_c0	1.9840766	2.15629735	2.29465528	13.6828435	14.4676507	14.6377757	1.454E-14	1.713352	up	PREDICTED: xylem cysteine proteinase 2-like [Sesamum indicum]
c21343.graph_c0	2.92448309	2.92741152	2.69732914	0.97774279	1.28899546	1.50798207	1.084E-11	-2.202126	down	PREDICTED: putative pentatricopeptide repeat-containing protein At5g09950 [Sesamum indicum]
c21346.graph_c0	0	0	0	2.20364496	4.16874041	4.20169293	3.09E-12	Inf	up	PREDICTED: transcription factor MYB28-like [Sesamum indicum]
c21350.graph_c0	108.562925	118.51188	111.813428	28.2606354	35.6706153	36.3451654	1.152E-77	-2.778679	down	PREDICTED: ethylene-responsive transcription factor 3-like [Sesamum indicum]
c21351.graph_c0	0	0	0.05402426	10.1087506	7.67272857	5.85862043	1.81E-16	7.731333	up	PREDICTED: uncharacterized protein LOC105175340 [Sesamum indicum]

#ID	0 h-1 FPKM	0 h-2 FPKM	0 h-3 FPKM	48 h-1 FPKM	48 h-2 FPKM	48 h-3 FPKM	FDR	log2FC	regulated	nr_annotation
c21361.graph_c0	2.59261096	2.68318018	2.31518503	20.2008928	23.1587457	23.0176161	4.183E-19	2.109709	up	PREDICTED: cyclin-D3-3-like [Sesamum indicum]
c21372.graph_c0	2.71048774	2.65783042	2.41683384	0.35672544	0.72882132	1.22430402	3.163E-13	-2.783624	down	PREDICTED: galactose oxidase-like [Sesamum indicum]
c21379.graph_c0	0.26965436	0	0.11550551	1.90457772	2.71946712	2.13677271	9.376E-07	3.116217	up	PREDICTED: 60S ribosomal protein L9-like [Musa acuminata subsp. malaccensis]
c21382.graph_c0	1.58291541	2.21830065	1.52558173	2.18742739	0.61795042	1.29757316	0.004671	-1.373983	down	-
c21391.graph_c0	19.9278375	9.84208249	10.7170296	0	0	0	3.869E-17	-Inf	down	-
c21397.graph_c0	0.34141048	0.57835013	0.33748152	9.29072052	9.99617921	10.7210416	4.107E-29	3.559963	up	hypothetical protein MIMGU_mgv1a003249mg [Erythranthe guttata]
c21401.graph_c0	11.997393	4.52710613	3.14799933	4318.80181	6786.24014	6748.86841	2.874E-67	8.81078	up	hypothetical protein MIMGU_mgv1a011768mg [Erythranthe guttata]
c21405.graph_c0	0.36351466	0.7641452	0.14013922	4.85595456	2.87370964	2.68187535	0.0048519	2.041328	up	PREDICTED: uncharacterized protein LOC105157791 isoform X1 [Sesamum indicum]
c21408.graph_c0	384.755952	388.145761	389.462542	12.1668974	12.7331179	13.6473618	3.57E-247	-5.933206	down	PREDICTED: zinc finger CCCH domain-containing protein 20-like [Sesamum indicum]
c21417.graph_c0	10.1092089	11.3741289	9.45725416	14.1561592	5.5251126	3.34790158	0.0005301	-1.418464	down	PREDICTED: uncharacterized protein LOC105168262 [Sesamum indicum]
c21418.graph_c0	222.645538	238.626209	226.084445	1071.1658	1001.28271	1013.98902	2.963E-14	1.150727	up	PREDICTED: heat shock cognate 70 kDa protein 2 [Sesamum indicum]
c21437.graph_c0	287.072556	269.564162	293.561768	68.6805073	48.8736937	37.3788801	3.61E-111	-3.464918	down	PREDICTED: 7-deoxyloganetic acid glucosyltransferase-like [Sesamum indicum]
c21448.graph_c0	6.03100089	4.78406945	5.26420183	0.36691566	0.16658685	0.13991972	2.425E-31	-5.576175	down	PREDICTED: ethylene-responsive transcription factor ERF053-like [Nelumbo nucifera]
c21454.graph_c1	2.29956846	2.79014682	1.52229154	16.4987182	17.0023262	16.2227654	1.542E-08	1.897866	up	PREDICTED: calcium-dependent protein kinase 10-like [Sesamum indicum]
c21469.graph_c0	30.2881285	28.2812489	32.7678562	316.091474	336.262657	363.591963	7.657E-67	2.455134	up	PREDICTED: 40S ribosomal protein S3a [Sesamum indicum]
c21484.graph_c0	0.11535308	0.28867146	0.29646654	2.2848217	2.64565355	2.45853655	2.737E-05	2.373687	up	PREDICTED: uncharacterized protein LOC105160233 [Sesamum indicum]
c21504.graph_c0	4.3992891	2.83094635	4.71105056	2.02645603	2.45346794	2.66176096	0.0002054	-1.765585	down	PREDICTED: EPIDERMAL PATTERNING FACTOR-like protein 6 [Vitis vinifera]
c21505.graph_c0	0	0.08396257	0.05389365	5.83421885	7.28579632	7.3571398	3.38E-23	6.187363	up	PREDICTED: uncharacterized protein LOC105160466 [Sesamum indicum]
c21513.graph_c0	3.98588123	5.98480872	5.44502515	2.61344367	4.41578431	4.1504447	3.141E-06	-1.49139	down	PREDICTED: cyclic nucleotide-gated ion channel 2-like isoform X1 [Sesamum indicum]
c21528.graph_c0	603.345527	616.451107	586.232373	638.560972	570.219182	588.415261	2.324E-10	-1.022116	down	PREDICTED: thiamine thiazole synthase 2, chloroplastic-like [Populus euphratica]
c21536.graph_c0	0.38474834	0.26663096	0.22819239	18.3498765	14.7601318	13.9015162	3.391E-30	4.731798	up	PREDICTED: protein TRANSPORT INHIBITOR RESPONSE 1-like [Sesamum indicum]

#ID	0 h-1 FPKM	0 h-2 FPKM	0 h-3 FPKM	48 h-1 FPKM	48 h-2 FPKM	48 h-3 FPKM	FDR	log2FC	regulated	nr_annotation
c21544.graph_c0	0.50208441	0.40206974	0.32259917	8.32595237	9.8003744	11.3595196	1.435E-16	3.569398	up	hypothetical protein JCGZ_21387 [Jatropha curcas]
c21546.graph_c0	0	0	0	7.14659716	21.7166813	18.7423134	1.139E-07	Inf	up	-
c21554.graph_c0	0	0.04716574	0.03027461	13.890781	15.9802199	18.5592125	3.023E-86	8.261719	up	PREDICTED: LRR receptor-like serine/threonine-protein kinase RCH1 [Sesamum indicum]
c21556.graph_c0	3.77911651	3.58024533	4.33599494	0.55953781	0.69155621	0.66383118	3.363E-22	-3.633429	down	PREDICTED: ribulose bisphosphate carboxylase/oxygenase activase, chloroplastic isoform X2 [Sesamum indicum]
c21558.graph_c0	0.05364942	0.05370314	0	1.68046174	2.56564785	2.55074575	4.14E-10	4.975966	up	hypothetical protein CICLE_v10024014mg, partial [Citrus clementina]
c21564.graph_c0	0.54592569	0.34775513	0.5739841	13.8994977	11.5764308	13.1406701	5.313E-22	3.700571	up	PREDICTED: methylsterol monooxygenase 1-1-like [Sesamum indicum]
c21570.graph_c0	0.66685389	0.54615407	0.46741835	14.7443831	15.5607198	17.2938381	1.686E-22	3.807828	up	PREDICTED: uncharacterized protein LOC105175024 [Sesamum indicum]
c21575.graph_c0	0.07506663	0.0751418	0.03215453	26.8095821	19.4878745	17.3323122	4.438E-26	7.444542	up	PREDICTED: uncharacterized protein LOC105169481 [Sesamum indicum]
c21576.graph_c0	0	0.06896954	0.08853993	7.42665444	9.61600426	10.1664499	2.024E-20	6.396654	up	hypothetical protein MIMGU_mgv1a000209mg [Erythranthe guttata]
c21578.graph_c0	22.3908134	20.8827404	20.8703081	9.35923435	11.5729963	11.1965611	3.283E-35	-2.018098	down	PREDICTED: LOW QUALITY PROTEIN: plasma membrane ATPase 1-like [Sesamum indicum]
c21581.graph_c0	5.12324476	4.68457324	5.15924179	2.20109193	3.8943297	4.94677319	1.368E-09	-1.467786	down	PREDICTED: uncharacterized protein LOC105164695 [Sesamum indicum]
c21582.graph_c0	5.70068152	3.73866924	4.54692504	21.9128482	24.9406671	18.8533612	0.0037469	1.215165	up	-
c21587.graph_c0	2.75427886	3.44629607	2.79064716	11.07682	13.8023094	12.6348968	0.0005479	1.040682	up	hypothetical protein MIMGU_mgv1a003864mg [Erythranthe guttata]
c21589.graph_c0	39.7640546	37.7191384	37.7791953	276.377945	262.441731	256.281876	2.464E-36	1.770362	up	PREDICTED: 40S ribosomal protein S9-2-like [Sesamum indicum]
c21590.graph_c0	3.70330159	3.1633151	2.79188164	27.9306387	27.4687316	29.871589	8.364E-22	2.126875	up	hypothetical protein MIMGU_mgv1a025384mg [Erythranthe guttata]
c21609.graph_c0	21.2824483	22.9760682	21.7483542	1.60601607	2.62295974	1.01222494	1.158E-73	-4.676139	down	PREDICTED: pathogenesis-related genes transcriptional activator PTI6 [Sesamum indicum]
c21617.graph_c0	22.5448093	19.8799707	18.2229628	1.23667206	1.20932686	0.93109362	5.03E-139	-5.1798	down	PREDICTED: probable galacturonosyltransferase-like 9 [Sesamum indicum]
c21625.graph_c0	0.22637184	0.3776642	0	1.32080721	3.38762177	2.53605783	0.002962	2.568115	up	PREDICTED: transcription factor bHLH68-like [Sesamum indicum]
c21625.graph_c1	0.37881644	0.37919577	0.24339706	2.35568529	2.1258499	3.02766332	0.0008317	1.892645	up	PREDICTED: transcription factor bHLH68-like [Sesamum indicum]
c21628.graph_c0	0	0.04455761	0	1.92739068	3.86645677	4.30569608	8.74E-10	6.806443	up	PREDICTED: F-box/kelch-repeat protein At5g26960 [Sesamum indicum]

#ID	0 h-1 FPKM	0 h-2 FPKM	0 h-3 FPKM	48 h-1 FPKM	48 h-2 FPKM	48 h-3 FPKM	FDR	log2FC	regulated	nr_annotation
c21629.graph_c0	0	0	0	88.6056768	85.679014	64.665782	4.444E-75	Inf	up	-
c21636.graph_c0	33.8016237	34.1979938	33.5080991	17.2383585	11.3106274	14.1510841	2.961E-25	-2.260035	down	PREDICTED: probable histone H2A.3-like [Cicer arietinum]
c21641.graph_c0	1.32509301	1.06113591	1.36223758	6.61924343	4.71317569	4.44146062	0.0026208	1.062023	up	PREDICTED: uncharacterized protein LOC105157824 isoform X1 [Sesamum indicum]
c21652.graph_c0	7.82113138	7.85633706	7.52027687	40.8890242	43.1034475	44.4080976	2.207E-21	1.450383	up	PREDICTED: probable phenylalanine--tRNA ligase beta subunit [Sesamum indicum]
c21653.graph_c0	0.10212881	0.10223108	0.19685934	3.62237722	3.5882831	3.3487475	9.238E-10	3.693576	up	PREDICTED: peroxidase 66-like [Sesamum indicum]
c21657.graph_c0	2.12078345	1.74827643	1.68326779	9.07944233	14.1843443	15.8508752	1.844E-06	1.79232	up	PREDICTED: uncharacterized protein LOC105157568 [Sesamum indicum]
c21659.graph_c0	5.7155553	6.348268	4.82940154	51.4292012	68.313891	57.3782415	1.775E-31	2.370848	up	PREDICTED: probable polygalacturonase [Sesamum indicum]
c21669.graph_c0	0.74299742	1.08180571	0.78118454	8.46288053	7.185501	6.89347364	1.681E-09	2.09992	up	PREDICTED: BTB/POZ domain-containing protein At3g19850-like [Sesamum indicum]
c21672.graph_c0	0.1123666	0	0.14439552	6.15940647	8.44430437	9.2571527	7.777E-20	5.505116	up	PREDICTED: wall-associated receptor kinase-like 20 [Sesamum indicum]
c21676.graph_c1	7.24258934	8.09530428	7.29908682	3.26808769	4.53375821	5.43504987	3.335E-21	-1.798343	down	PREDICTED: exocyst complex component SEC15B [Sesamum indicum]
c21695.graph_c0	0.22402816	0.22425249	0.17992816	1.93490015	2.10445321	2.31850553	6.876E-06	2.321781	up	PREDICTED: uncharacterized protein LOC105168358 isoform X2 [Sesamum indicum]
c21696.graph_c0	2.5407762	2.42771493	2.91871034	1.41862349	1.91614595	2.01965224	6.127E-05	-1.583805	down	PREDICTED: uncharacterized protein LOC105164695 [Sesamum indicum]
c21704.graph_c0	15.3339836	16.2165325	15.2517193	8.61966053	8.76509839	9.91856037	6.321E-27	-1.795932	down	PREDICTED: protein DGCR14 [Sesamum indicum]
c21705.graph_c0	104.474391	102.906771	98.8325417	72.6304185	60.5762472	56.9362873	2.646E-33	-1.700193	down	PREDICTED: tetraspanin-3-like [Sesamum indicum]
c21709.graph_c0	1.89049311	2.75256168	2.87106004	0.89721135	2.34826109	3.24029384	0.0065724	-1.248998	down	PREDICTED: uncharacterized protein LOC105173813 [Sesamum indicum]
c21716.graph_c0	7.29466678	5.414945	6.74080763	1.73671264	0.63994312	0.7390644	2.191E-17	-3.640153	down	unnamed protein product [Vitis vinifera]
c21718.graph_c0	0	0.0165736	0	3.29473549	4.0559424	5.41538285	1.387E-26	8.577529	up	PREDICTED: uncharacterized protein LOC105157238 isoform X1 [Sesamum indicum]
c21719.graph_c0	0.24338589	0.16241973	0.3127605	2.61593445	2.77126535	2.39414682	3.473E-05	2.414908	up	hypothetical protein MIMGU_mgv1a015222mg [Erythranthe guttata]
c21720.graph_c0	0	0.43642088	0.11205142	1.84762302	4.16996936	6.07566642	0.0015892	3.432872	up	PREDICTED: allene oxide synthase [Sesamum indicum]
c21723.graph_c0	0.16046604	0.05354224	0	5.59296901	5.2986331	8.37470243	3.175E-18	5.490921	up	PREDICTED: scarecrow-like protein 28 [Sesamum indicum]
c21726.graph_c0	0.98563935	1.17758625	1.06229759	0.93732672	0.868861	0.70371085	0.0029298	-1.376288	down	PREDICTED: uncharacterized protein LOC105173388 [Sesamum indicum]
c21733.graph_c0	14.7732486	15.9710852	14.4279992	8.01050437	5.76729188	4.49806224	9.926E-18	-2.313619	down	-
c21738.graph_c0	0	0	0	1.69670937	2.33669519	1.91231427	1.535E-11	Inf	up	PREDICTED: uncharacterized protein LOC105171956 [Sesamum indicum]

#ID	0 h-1 FPKM	0 h-2 FPKM	0 h-3 FPKM	48 h-1 FPKM	48 h-2 FPKM	48 h-3 FPKM	FDR	log2FC	regulated	nr_annotation
c21746.graph_c0	3.86869203	3.4679695	3.33901506	0.63834473	1.29614361	0.80466002	1.139E-11	-2.988587	down	unnamed protein product [Coffea canephora]
c21750.graph_c0	0	0	0	5.48133308	3.85204079	3.04224955	4.534E-11	Inf	up	PREDICTED: uncharacterized protein LOC105162958 [Sesamum indicum]
c21766.graph_c0	11.0619096	4.23887762	4.66429418	0	0	0	5.765E-09	-Inf	down	60S ribosomal protein L7a [Zea mays]
c21769.graph_c0	6.536724	7.35308013	5.23957224	98.1416338	100.810924	99.9260093	4.039E-84	2.950557	up	p-coumaroyl shikimate 3'-hydroxylase isoform 2 [Ocimum basilicum]
c21771.graph_c0	1.00339128	0.89279646	0.5253101	8.52492529	8.92231591	10.2966844	7.623E-13	2.504754	up	PREDICTED: replication factor C subunit 3 [Sesamum indicum]
c21772.graph_c0	29.819347	26.1671498	25.5250194	13.1031956	12.4452559	11.8199605	4.23E-37	-2.139978	down	PREDICTED: activating signal cointegrator 1 [Sesamum indicum]
c21774.graph_c0	39.6863671	37.3484846	44.1309174	17.7337775	15.3095411	12.8182343	1.927E-41	-2.416131	down	PREDICTED: F-box protein At5g39250 [Sesamum indicum]
c21785.graph_c0	0	0.20678885	0	3.6794582	4.16676324	5.19317758	1.314E-11	4.968842	up	PREDICTED: protein TORNADO 2 [Sesamum indicum]
c21789.graph_c0	3.56497991	4.84683616	3.41876144	0.04901928	0.15578996	0	3.52E-31	-6.880534	down	PREDICTED: COBRA-like protein 10 [Sesamum indicum]
c21813.graph_c0	0.37704398	0.12580718	0	24.3150255	33.1184844	32.0409431	2.483E-31	6.471225	up	PREDICTED: probable receptor-like protein kinase At1g80640 [Nicotiana tomentosiformis]
c21818.graph_c0	6.46476606	7.33407148	4.9844882	0.72792673	0.63094099	0.8243516	8.826E-22	-4.118687	down	-
c21827.graph_c0	0	0	0	3.07110489	5.25559446	5.96453558	3.759E-17	Inf	up	PREDICTED: L-ascorbate oxidase-like [Sesamum indicum]
c21836.graph_c0	0.26628636	0.2132424	0.34218851	1.52098623	1.40339074	1.48433515	0.00866	1.403578	up	PREDICTED: NAC domain-containing protein 21/22-like [Sesamum indicum]
c21839.graph_c0	0.15460317	0.15475798	0.06622373	1.42430521	1.10651102	2.02774113	2.009E-05	2.591049	up	PREDICTED: uncharacterized protein LOC105177291 [Sesamum indicum]
c21856.graph_c0	0	0	0	1.43200292	1.65188087	2.29353766	1.776E-13	Inf	up	PREDICTED: cytochrome P450 71A8-like [Sesamum indicum]
c21857.graph_c0	15.1495907	11.0685323	15.7756504	198.362899	203.768366	205.764495	3.195E-75	2.835949	up	PREDICTED: DNA-binding protein S1FA-like [Nicotiana sylvestris]
c21859.graph_c0	0.09844465	0.03941729	0.20240843	0.87065886	1.99844123	2.71147456	0.0007053	2.991441	up	PREDICTED: oligopeptide transporter 4-like [Sesamum indicum]
c21871.graph_c0	0	0	0.05079996	3.0956399	6.01876467	5.89783155	2.502E-14	7.146061	up	hypothetical protein MIMGU_mgv1a006316mg [Erythranthe guttata]
c21881.graph_c0	0.35376525	0.56659119	0.27276141	6.84414414	6.56001312	6.14787714	1.172E-08	3.022743	up	PREDICTED: LOW QUALITY PROTEIN: alkaline/neutral invertase CINV2 [Sesamum indicum]
c21889.graph_c0	11.0064193	11.5284536	9.86646644	6.09518621	7.57315454	9.59148951	8.895E-20	-1.500015	down	hypothetical protein MIMGU_mgv1a009265mg [Erythranthe guttata]
c21894.graph_c0	0.04729404	0.0946828	0	3.74705049	2.35403288	2.17104381	1.112E-07	4.872288	up	PREDICTED: probable membrane-associated kinase regulator 2 [Sesamum indicum]
c21898.graph_c0	0	0	0	4.57298088	4.28805375	3.71159777	4.504E-22	Inf	up	PREDICTED: uncharacterized protein LOC105164396 [Sesamum indicum]
c21901.graph_c0	0.35569413	0.32637944	0.30472069	47.1871626	59.998464	57.9263072	1.75E-150	6.366184	up	vacuolar invertase CvINV [Elsholtzia haichowensis]
c21905.graph_c0	27.7995146	28.5442154	30.7874461	14.3348092	19.8879311	25.8836322	1.008E-18	-1.561831	down	PREDICTED: auxin-induced protein 22D [Sesamum indicum]

#ID	0 h-1 FPKM	0 h-2 FPKM	0 h-3 FPKM	48 h-1 FPKM	48 h-2 FPKM	48 h-3 FPKM	FDR	log2FC	regulated	nr_annotation
c21908.graph_c0	5.94914524	5.18590168	5.25585993	1.23316664	1.06446636	0.79246863	9.96E-37	-3.41852	down	PREDICTED: lysine-specific demethylase JMJ18 [Sesamum indicum]
c21909.graph_c0	0	0	0	1.2918198	1.55859955	0.7024438	2.519E-07	Inf	up	hypothetical protein MIMGU_mgv1a0261841mg, partial [Erythranthe guttata]
c21911.graph_c0	0.70849569	0.70920514	1.05051348	16.8701063	20.6377107	24.7501464	2.618E-27	3.629701	up	PREDICTED: inositol monophosphatase 3-like [Sesamum indicum]
c21921.graph_c0	0	0	0	2.20804774	2.87896473	3.52639826	6.636E-13	Inf	up	hypothetical protein MIMGU_mgv1a000635mg [Erythranthe guttata]
c21925.graph_c0	0.24587332	0.28127946	0.31595695	9.25473693	12.7524092	13.1008329	2.922E-32	4.354242	up	PREDICTED: inactive rhomboid protein 1-like [Sesamum indicum]
c21938.graph_c0	23.5215631	23.6241269	27.0818099	26.9779225	24.3429345	20.8343489	2.166E-08	-1.056566	down	hypothetical protein MIMGU_mgv1a0149401mg [Erythranthe guttata]
c21940.graph_c0	0.16519651	0	0	7.10219048	6.12661212	6.72573907	3.176E-18	5.922429	up	PREDICTED: uncharacterized protein LOC105161910 [Sesamum indicum]
c21944.graph_c0	0.43855056	0.36582475	1.03318386	2.82814965	3.85210495	2.87596635	0.0063598	1.346339	up	PREDICTED: transcription repressor KAN1-like isoform X4 [Sesamum indicum]
c21946.graph_c0	0.2219478	0.20365588	0.1663735	1.09052154	1.82316889	2.0468099	2.226E-05	2.0425	up	PREDICTED: receptor protein kinase CLAVATA1 [Sesamum indicum]
c21947.graph_c0	9.43796014	9.51871204	9.65675534	53.7112949	59.7160891	62.2726382	7.503E-26	1.598388	up	PREDICTED: diaminopimelate decarboxylase 1, chloroplastic-like [Sesamum indicum]
c21948.graph_c0	1.88287826	2.20421515	1.35332055	1.20542103	1.77533787	2.01435077	0.0031895	-1.144505	down	PREDICTED: U-box domain-containing protein 38-like [Sesamum indicum]
c21950.graph_c0	1.04646697	0.68915451	0.81392853	14.8924005	17.6311931	15.5086077	2.555E-38	3.217341	up	PREDICTED: uncharacterized protein LOC105173886 [Sesamum indicum]
c21965.graph_c0	23.9577838	26.1619352	25.3364236	8.13159536	11.6350868	9.86651544	1.853E-21	-2.3716	down	PREDICTED: probable methyltransferase PMT16 [Sesamum indicum]
c21976.graph_c0	1.50828221	4.75032283	4.11277085	202.261044	188.061623	132.776612	9.688E-24	4.638439	up	PREDICTED: epidermis-specific secreted glycoprotein EP1-like [Sesamum indicum]
c21977.graph_c0	0.05250836	0.21024378	0.13495066	26.1704047	41.7146719	43.7316987	6.805E-32	7.104503	up	gibberellin 3-oxidase [Torenia fournieri]
c21982.graph_c0	4.62797861	5.25374528	3.85400976	0.47637941	0.95886583	0.97492294	1.032E-31	-3.537618	down	PREDICTED: exocyst complex component EXO70B1 [Sesamum indicum]
c21983.graph_c0	0.12301734	0.08209368	0	3.05995311	5.36272844	7.52951906	5.377E-08	5.269633	up	PREDICTED: mitotic spindle checkpoint protein BUBR1 isoform X2 [Sesamum indicum]
c21997.graph_c0	0	0	0	4.05994002	1.16380383	1.48750421	0.0013063	Inf	up	PREDICTED: uncharacterized protein LOC105158717 [Sesamum indicum]
c22004.graph_c0	0.35812537	0.35848398	0.69030792	22.7650632	24.1168241	27.8889935	1.594E-17	4.704505	up	H(+)-transporting atpase plant/fungi plasma membrane type [Theobroma cacao]

#ID	0 h-1 FPKM	0 h-2 FPKM	0 h-3 FPKM	48 h-1 FPKM	48 h-2 FPKM	48 h-3 FPKM	FDR	log2FC	regulated	nr_annotation
c22004.graph_c2	28.6772665	30.1456004	25.060446	21.8880625	21.5876251	20.9377353	4.915E-22	-1.396311	down	PREDICTED: uncharacterized protein LOC105170398 [Sesamum indicum]
c22008.graph_c0	4.48035217	3.91675901	3.68475515	2.44903831	2.06973705	2.2525937	3.668E-10	-1.848491	down	hypothetical protein MIMGU_mgv1a027091mg [Erythranthe guttata]
c22011.graph_c0	107.880538	99.3724548	98.8112878	45.4640774	41.8631962	45.8631641	1.715E-43	-2.216235	down	PREDICTED: salt stress-induced hydrophobic peptide ESI3 [Solanum lycopersicum]
c22023.graph_c0	1.63980724	1.69274455	1.11945915	0.14162788	0.35008765	0.29404582	5.696E-11	-3.529579	down	CLAVATA3/ESR (CLE)-related protein 26 [Arabidopsis thaliana]
c22045.graph_c0	0.07749917	0.21333614	0.14938426	3.55201542	2.55274121	2.52527312	1.505E-11	3.280694	up	PREDICTED: receptor-like protein kinase HERK 1 [Sesamum indicum]
c22047.graph_c0	56.7538854	51.8116829	58.802476	32.3127934	38.6143259	41.6360583	2.66E-28	-1.594337	down	PREDICTED: probable serine incorporator [Sesamum indicum]
c22049.graph_c0	70.8123996	78.4844891	59.6063898	926.803703	886.295521	741.86794	8.642E-30	2.59941	up	hypothetical protein MIMGU_mgv1a015271mg [Erythranthe guttata]
c22059.graph_c0	3.31047319	1.88079867	2.06955687	0	0	0.22003094	1.144E-13	-6.081926	down	-
c22063.graph_c0	0	0	0	5.89525137	8.57068314	6.33373374	8.559E-32	Inf	up	PREDICTED: reticuline oxidase-like protein [Sesamum indicum]
c22065.graph_c0	2.26529571	1.53870418	2.183246	0.22360052	0.55271448	0.46423626	1.928E-09	-3.303138	down	-
c22068.graph_c0	3.93736921	4.33544308	3.66826461	58.0075533	66.8318182	79.7756196	1.653E-36	3.078632	up	PREDICTED: glycerol-3-phosphate 2-O-acyltransferase 4 [Sesamum indicum]
c22075.graph_c0	0	0	0	1.18620829	1.98968609	3.56224838	1.727E-05	Inf	up	PREDICTED: pyruvate dehydrogenase E1 component subunit beta-3, chloroplastic-like [Beta vulgaris subsp. vulgaris]
c22080.graph_c0	6.17516755	6.87150189	5.89371953	1.65698072	2.3697497	1.05663289	1.404E-28	-2.916258	down	PREDICTED: vacuolar protein sorting-associated protein 4-like [Sesamum indicum]
c22084.graph_c0	0.30095808	0.25822238	0.19337152	3.5846043	4.69961109	5.77997496	5.996E-15	3.204293	up	PREDICTED: uncharacterized protein LOC105169955 isoform X1 [Sesamum indicum]
c22085.graph_c0	0.05843516	0.01949789	0.0250305	2.36870827	2.4903489	3.36906638	5.051E-19	5.305843	up	PREDICTED: WD repeat and HMG-box DNA-binding protein 1 [Sesamum indicum]
c22088.graph_c0	9.17962269	9.04744831	11.2517396	0.19515847	0.13783116	0.05788361	2.805E-51	-7.243364	down	PREDICTED: nuclear transcription factor Y subunit C-2 [Sesamum indicum]
c22094.graph_c0	244.890801	271.317847	244.597046	54.1633103	35.1656268	24.0338469	1.35E-138	-3.751586	down	PREDICTED: ethylene-responsive transcription factor ERF008-like [Sesamum indicum]
c22097.graph_c0	0.15539659	0.28517903	0.26625437	4.08010215	5.15651574	5.90213566	6.972E-16	3.395028	up	PREDICTED: MATE efflux family protein 5-like [Sesamum indicum]
c22099.graph_c0	0	0	0.08146325	5.25620623	4.57839081	4.36514465	9.366E-15	6.39878	up	PREDICTED: CBL-interacting serine/threonine-protein kinase 5 [Sesamum indicum]
c22111.graph_c0	0.10675351	0.21372082	0.41154747	13.8671102	14.3779575	14.1328206	3.498E-29	4.825396	up	PREDICTED: (DL)-glycerol-3-phosphatase 2 [Sesamum indicum]
c22114.graph_c0	0.25351165	0.25376551	0.18615572	3.77018801	2.72161409	3.38437815	5.137E-08	2.822515	up	PREDICTED: uncharacterized protein LOC105171186 [Sesamum indicum]

#ID	0 h-1 FPKM	0 h-2 FPKM	0 h-3 FPKM	48 h-1 FPKM	48 h-2 FPKM	48 h-3 FPKM	FDR	log2FC	regulated	nr_annotation
c22122.graph_c0	1.39248111	0.85181279	2.28644707	14.0400195	16.7612234	15.3463931	1.675E-10	2.320463	up	PREDICTED: plastidial pyruvate kinase 2-like [Sesamum indicum]
c22136.graph_c0	3.47190995	3.96411277	3.69471426	0.69968279	0.47650441	1.51196431	2.208E-12	-3.071559	down	hypothetical protein MIMGU_mgv1a003925mg [Erythranthe guttata]
c22145.graph_c0	0.1851713	0.10591813	0.10197962	1.31599448	1.49740596	1.86486856	4.659E-06	2.556215	up	PREDICTED: putative pentatricopeptide repeat-containing protein At3g25060, mitochondrial [Sesamum indicum]
c22152.graph_c0	0.18850776	0.02695665	0.03460571	1.56299083	2.15516729	1.85431979	8.674E-09	3.46828	up	PREDICTED: pyruvate kinase isozyme A, chloroplastic-like [Sesamum indicum]
c22156.graph_c0	31.3519044	30.5419233	26.2468915	3.09741762	3.11727147	3.10057152	1.073E-74	-4.257843	down	PREDICTED: serine/threonine-protein kinase OXI1 [Sesamum indicum]
c22160.graph_c0	1.44161863	1.50719829	1.35852715	0.3541635	0.4689916	0.44643778	4.471E-09	-2.783896	down	-
c22176.graph_c0	0.26967307	0.22495259	0.4620541	7.20477222	9.91358422	10.6109181	4.927E-19	3.826408	up	hypothetical protein MIMGU_mgv1a009135mg [Erythranthe guttata]
c22178.graph_c0	8.85643996	11.2758151	10.4181433	3.37704179	5.06604277	5.417542	4.008E-24	-2.166071	down	PREDICTED: uncharacterized protein LOC105169534 [Sesamum indicum]
c22183.graph_c0	1.26984785	0.88272182	1.17852567	14.3308667	20.4489761	18.7947559	3.779E-28	2.983672	up	PREDICTED: malonyl-coenzyme:anthocyanin 5-O-glucoside-6'''-O-malonyltransferase-like [Sesamum indicum]
c22209.graph_c0	1.75618537	1.1796729	1.30654974	0.80895626	0.76678148	1.11759106	3.485E-05	-1.673135	down	PREDICTED: receptor-like protein kinase HSL1 [Sesamum indicum]
c22236.graph_c0	3.72716238	4.94560443	4.23262666	0	0.08459521	0.28421304	2.477E-22	-6.172748	down	-
c22263.graph_c0	0.96528316	0.66257125	0.77969702	4.82752981	3.49917748	4.58941011	0.0001647	1.4112	up	PREDICTED: probable receptor-like serine/threonine-protein kinase At4g34500 [Sesamum indicum]
c22267.graph_c0	0.87535782	1.2745227	1.32939117	6.67143104	7.92189415	8.61075052	0.0001257	1.713333	up	PREDICTED: lipoxygenase homology domain-containing protein 1-like [Sesamum indicum]
c22278.graph_c0	0.34187654	0.34221888	0.4393249	22.7670343	27.0741939	29.3860674	4.521E-58	5.115926	up	PREDICTED: probable serine/threonine-protein kinase At4g35230 [Sesamum indicum]
c22287.graph_c0	0	0	0	3.83007296	8.27729567	4.6348472	6.504E-07	Inf	up	-
c22291.graph_c0	1.02004313	0.69862311	1.0348388	8.70486982	12.2083449	13.5987052	1.534E-12	2.621941	up	PREDICTED: ras-related protein Rab7 [Sesamum indicum]
c22292.graph_c0	0.85849438	1.0868301	1.00585815	0.18609436	0.24643051	0.55885153	1.357E-07	-2.60286	down	-
c22320.graph_c0	0	0	0	1.68321703	1.30308163	1.61292534	5.771E-11	Inf	up	RecName: Full=60S ribosomal protein L5 [Helianthus annuus]
c22326.graph_c0	70.5696857	74.2135978	70.6502731	13.6322947	16.7048025	16.8318464	7.24E-105	-3.212422	down	PREDICTED: EIN3-binding F-box protein 2, partial [Sesamum indicum]
c22337.graph_c0	0.31795031	0.59981407	0.17286024	4.94578357	6.77907092	8.54082155	5.234E-12	3.198482	up	PREDICTED: myosin-9-like [Sesamum indicum]
c22345.graph_c0	31.6982939	29.4759887	33.8333417	203.619868	235.827767	251.322985	2.244E-30	1.840429	up	ribosomal protein L32 [Medicago sativa]
c22345.graph_c1	59.1561086	55.9854166	59.8929237	432.684788	377.548745	410.275153	1.113E-26	1.786013	up	PREDICTED: 60S ribosomal protein L32-1 [Sesamum indicum]

#ID	0 h-1 FPKM	0 h-2 FPKM	0 h-3 FPKM	48 h-1 FPKM	48 h-2 FPKM	48 h-3 FPKM	FDR	log2FC	regulated	nr_annotation
c22346.graph_c0	2.67204732	3.04621228	2.25733101	61.3136751	41.1941314	35.3907663	1.601E-07	3.106154	up	PREDICTED: uncharacterized protein LOC105163060 [Sesamum indicum]
c22348.graph_c0	0.07990306	0.07998307	0.0684524	1.74214862	1.66363513	1.76848483	7.898E-09	3.486756	up	PREDICTED: LOW QUALITY PROTEIN: recQ-mediated genome instability protein 1 [Sesamum indicum]
c22359.graph_c0	0	0	0	5.23192351	5.10957382	3.70971934	1.753E-13	Inf	up	calmodulin [Chara corallina]
c22362.graph_c0	6685.5471	6693.30449	6723.05173	37.4310113	31.6399947	26.2269368	5.13E-225	-8.73329	down	hypothetical protein MIMGU_mgv1a011867mg [Erythranthe guttata]
c22366.graph_c0	0.08612068	0.02873564	0.03688949	6.77034561	3.89436747	2.84738184	1.958E-06	5.479411	up	PREDICTED: probable LRR receptor-like serine/threonine-protein kinase At4g37250 [Sesamum indicum]
c22379.graph_c0	0.4735244	0.35549892	0.3042488	2.1812078	3.23501525	2.42603198	0.0014703	1.77162	up	hypothetical protein MIMGU_mgv1a016703mg [Erythranthe guttata]
c22389.graph_c0	2.33963942	2.53041757	2.00435286	18.0114537	17.7948899	19.774079	5.494E-23	1.999177	up	PREDICTED: uncharacterized protein LOC105168835 [Sesamum indicum]
c22396.graph_c0	288.044775	309.121092	258.335753	144.519226	166.185021	140.605783	5.552E-41	-1.940115	down	PREDICTED: ethylene-responsive transcription factor 4-like [Sesamum indicum]
c22400.graph_c0	4.6239143	7.98423918	5.12489991	1.49094801	1.29742887	1.27925669	6.643E-09	-3.137441	down	PREDICTED: uncharacterized protein LOC105158187 [Sesamum indicum]
c22403.graph_c0	786.901076	826.627547	722.444604	119.122352	140.830145	160.683875	2.11E-120	-3.493797	down	-
c22409.graph_c0	0.81724096	0.53173855	0.68262154	30.831078	31.8243069	34.7689542	5.82E-75	4.565418	up	GDP-mannose 3,5-epimerase 1 [Theobroma cacao]
c22443.graph_c0	0	0.22080171	0	3.04820179	4.19796024	2.89308973	6.345E-09	4.511728	up	PREDICTED: uncharacterized protein LOC105175907 [Sesamum indicum]
c22444.graph_c0	0.55421266	0.63798277	0.56974817	8.45004695	10.1147228	12.4707605	8.287E-22	3.116634	up	asparagine synthetase [Striga hermonthica]
c22447.graph_c0	0.4365081	0.71783854	0.40066442	28.7242667	31.9513715	34.8875979	1.389E-97	4.924228	up	hypothetical protein SBB1_14t00005 [Solanum bulbocastanum]
c22453.graph_c0	3.71997801	3.79892932	4.29745851	33.6650657	32.4735431	34.5444753	1.658E-41	2.072235	up	PREDICTED: probable receptor protein kinase TMK1 [Sesamum indicum]
c22457.graph_c0	0.11409707	0.14276415	0.10996444	0.97230139	1.08571302	1.05220695	0.0002794	2.065844	up	PREDICTED: putative pentatricopeptide repeat-containing protein At1g68930 [Sesamum indicum]
c22462.graph_c0	0	0.09557537	0	2.19905463	3.26148443	2.81765605	5.479E-10	5.424578	up	unknown [Lotus japonicus]
c22469.graph_c0	4.00702817	4.0110406	2.7355071	0	0	0	2.783E-21	-Inf	down	-
c22473.graph_c0	12.5012327	13.0765385	12.7921647	52.3137693	54.032172	54.5189213	1.453E-12	1.049621	up	hypothetical protein MIMGU_mgv1a005340mg [Erythranthe guttata]
c22477.graph_c0	0.34554891	0.17294746	0.0555055	1.87026038	1.64406914	1.80577593	0.0001692	2.20981	up	PREDICTED: kinetochore protein Spc25 [Sesamum indicum]
c22480.graph_c0	0.20337371	0.67859118	0.37749582	5.47510835	5.73405128	6.26099182	2.176E-15	2.774895	up	PREDICTED: protein LONGIFOLIA 1-like [Sesamum indicum]
c22492.graph_c0	0.38817115	0.98906142	0.68020268	3.12094763	3.13406424	3.0951984	0.0089392	1.1655	up	PREDICTED: uncharacterized protein LOC105155609 [Sesamum indicum]
c22502.graph_c0	4.65501698	4.96690981	5.45600146	19.0862048	25.9109399	24.9919579	4.668E-08	1.190397	up	PREDICTED: NAP1-related protein 2-like [Sesamum indicum]

#ID	0 h-1 FPKM	0 h-2 FPKM	0 h-3 FPKM	48 h-1 FPKM	48 h-2 FPKM	48 h-3 FPKM	FDR	log2FC	regulated	nr_annotation
c22513.graph_c0	2.51744689	1.91997541	2.77287336	0.88351858	1.4039712	1.47403035	3.278E-05	-1.966795	down	PREDICTED: protein IQ-DOMAIN 14-like [Sesamum indicum]
c22518.graph_c0	0.09031828	0.22602181	0.11606257	18.2223538	18.0041938	24.117609	1.072E-52	6.106835	up	PREDICTED: DNA (cytosine-5)-methyltransferase CMT3 [Sesamum indicum]
c22524.graph_c0	0.49685258	0.94948655	0.58043203	10.9023678	11.5497367	17.0690728	1.344E-12	3.264995	up	PREDICTED: uncharacterized protein LOC105161197 [Sesamum indicum]
c22529.graph_c1	4.51780942	3.69567024	2.80914386	1.34261313	3.60325073	4.10162855	0.0006176	-1.313985	down	PREDICTED: CBS domain-containing protein CBSX5 [Sesamum indicum]
c22532.graph_c0	0.35416865	0.23634886	0.60682765	123.226311	132.098793	128.710697	9.81E-206	7.29866	up	unnamed protein product [Coffea canephora]
c22540.graph_c0	1.74932343	3.09013255	1.85125283	1.80118977	1.50642801	1.47615984	0.0011757	-1.495858	down	PREDICTED: probable WRKY transcription factor 71 isoform X1 [Sesamum indicum]
c22552.graph_c0	5.78221411	5.13213963	5.26230457	0.89034055	0.79932869	0.71165496	5.378E-61	-3.765786	down	PREDICTED: pentatricopeptide repeat-containing protein At3g26540 [Sesamum indicum]
c22556.graph_c0	15.6975032	15.9025378	14.6226117	5.45938552	7.59860944	8.37182981	4.895E-34	-2.132246	down	PREDICTED: E3 ubiquitin-protein ligase RING1-like [Sesamum indicum]
c22557.graph_c0	62.6485339	60.626933	64.6838882	51.7941961	54.0744577	53.5816595	4.947E-16	-1.255969	down	PREDICTED: uncharacterized protein LOC105167256 isoform X2 [Sesamum indicum]
c22559.graph_c0	0.64735733	0.43200371	0.55458653	5.0096575	5.98104526	7.18162947	1.208E-08	2.453566	up	PREDICTED: formin-like protein 6 [Sesamum indicum]
c22561.graph_c0	2.83945304	3.35907748	3.0268534	2.1402731	2.55077981	2.56565288	4.387E-05	-1.367063	down	PREDICTED: uncharacterized protein LOC105156990 [Sesamum indicum]
c22564.graph_c0	0	0.0816331	0	55.1457928	86.1181795	82.995112	2.576E-58	10.41027	up	PREDICTED: polygalacturonase-like [Sesamum indicum]
c22572.graph_c0	0.55124348	0.42917425	0.7083697	4.40129256	4.48330364	3.96645191	0.0001198	1.907089	up	PREDICTED: uncharacterized protein LOC105156684 [Sesamum indicum]
c22577.graph_c0	5.37840698	4.1724393	4.14687955	1.61035528	2.36212083	1.1022188	4.17E-07	-2.450878	down	PREDICTED: uncharacterized protein LOC105177681 [Sesamum indicum]
c22578.graph_c0	0.12112271	0.060622	0.23347121	2.95703264	4.49205532	5.41123274	1.611E-10	3.916336	up	PREDICTED: cysteine-rich repeat secretory protein 60 [Sesamum indicum]
c22583.graph_c0	0.19431654	0.25934816	0.16646963	1.25311952	1.32752818	1.53978705	0.0016951	1.715174	up	PREDICTED: uncharacterized protein LOC105155367 [Sesamum indicum]
c22593.graph_c0	0.37933827	0.18985906	0.32497646	5.99925479	5.36822351	8.44767414	5.741E-11	3.450252	up	hypothetical protein MIMGU_mgv1a007264mg [Erythranthe guttata]
c22594.graph_c0	0.26892655	0.40379376	0.11519376	2.39494258	5.5554864	5.73166999	4.752E-05	3.095299	up	hypothetical protein MIMGU_mgv1a000346mg [Erythranthe guttata]
c22603.graph_c0	6.39350022	6.92833465	5.95464378	4.32301609	5.03768249	6.65941537	9.005E-09	-1.288263	down	PREDICTED: uncharacterized protein LOC105179085 [Sesamum indicum]
c22607.graph_c0	10.8315808	11.722795	9.48753044	6.48192874	8.06399296	9.39129179	1.519E-17	-1.441565	down	PREDICTED: uncharacterized protein LOC105179586 [Sesamum indicum]
c22621.graph_c0	6.01081943	6.20845741	5.70700859	3.63292082	5.53008335	5.77465438	1.105E-06	-1.287647	down	PREDICTED: MFP1 attachment factor 1-like [Sesamum indicum]

#ID	0 h-1 FPKM	0 h-2 FPKM	0 h-3 FPKM	48 h-1 FPKM	48 h-2 FPKM	48 h-3 FPKM	FDR	log2FC	regulated	nr_annotation
c22632.graph_c0	0.41524888	0.51958086	1.46743095	7.93800288	7.09222417	7.829075	5.727E-06	2.220512	up	PREDICTED: zinc finger HIT domain-containing protein 3 [Sesamum indicum]
c22634.graph_c0	0.06592552	0.04399435	0.05647793	6.17471347	7.22766684	10.7722797	7.436E-19	6.159667	up	PREDICTED: DNA replication licensing factor MCM3 homolog 2 [Sesamum indicum]
c22635.graph_c0	3.45994573	3.70364689	3.34105053	14.1135619	15.2053553	14.9517548	4.009E-09	1.057249	up	PREDICTED: conserved oligomeric Golgi complex subunit 7 [Sesamum indicum]
c22637.graph_c0	0.19764727	0.16487099	0.29631533	14.7489301	7.10506042	4.4285093	0.0012166	4.314323	up	sucrose transporter 1 [Verbascum phoeniceum]
c22646.graph_c1	0.07766931	0.23324125	0.09980814	2.00351291	2.95631003	3.31075409	3.526E-07	3.309273	up	hypothetical protein MIMGU_mgv1a025026mg, partial [Erythranthe guttata]
c22658.graph_c0	0	0	0.11456155	0.65704789	1.2761147	0.99875532	6.217E-07	3.617682	up	-
c22663.graph_c0	1.68171604	1.37165928	1.62747375	0.45905378	0.66867875	0.47654051	1.895E-10	-2.567482	down	PREDICTED: putative calcium-transporting ATPase 13, plasma membrane-type [Sesamum indicum]
c22666.graph_c0	0	0	0	8.15252632	9.13728631	11.5644577	4.273E-16	Inf	up	hypothetical protein OsI_01464 [Oryza sativa Indica Group]
c22670.graph_c0	0	0.06171593	0.07922807	3.86239043	7.82243325	6.41860199	3.463E-12	5.963338	up	PREDICTED: pleckstrin homology domain-containing family A member 8 [Sesamum indicum]
c22680.graph_c0	0	0	0	1.50602085	2.60309688	2.11586573	1.03E-11	Inf	up	-
c22686.graph_c0	0	0	0.07880629	3.38984849	3.65098566	3.82060461	5.752E-13	6.05508	up	PREDICTED: uncharacterized protein LOC105161798 [Sesamum indicum]
c22691.graph_c0	0.86634041	0.74106859	0.78941796	0	0	0	8.707E-28	-Inf	down	PREDICTED: geraniol 8-hydroxylase [Vitis vinifera]
c22704.graph_c0	4.33923679	5.40534634	3.59348091	1.77670576	1.7880941	3.00371399	4.892E-06	-2.040973	down	-
c22722.graph_c0	4.87144316	2.73875573	4.54492957	0.61477961	0.58615601	0.32821632	4.073E-12	-4.004329	down	PREDICTED: protein UPSTREAM OF FLC [Sesamum indicum]
c22728.graph_c0	2.53850363	2.79515011	1.9572466	0.87698817	1.42456269	3.27742296	0.0020373	-1.413992	down	hypothetical protein MIMGU_mgv1a020957mg, partial [Erythranthe guttata]
c22730.graph_c0	16.6542668	17.4440888	15.7563873	7.42689994	8.71593704	9.14097556	1.061E-24	-1.999518	down	PREDICTED: ABC transporter B family member 20 isoform X2 [Nicotiana tomentosiformis]
c22740.graph_c0	0.16513605	0.08265071	0.15915478	12.0566277	10.153561	8.08823863	3.769E-21	5.20422	up	PREDICTED: probable WRKY transcription factor 29 [Sesamum indicum]
c22752.graph_c0	4.12406373	4.12819335	3.31224073	25.5269265	28.5520424	31.3766074	4.454E-08	1.867534	up	PREDICTED: transcription termination factor 4, mitochondrial [Sesamum indicum]
c22754.graph_c0	23.8640314	24.1237746	21.9291624	13.1786579	17.3118757	19.9760809	8.911E-22	-1.492785	down	PREDICTED: protein AUXIN RESPONSE 4 [Sesamum indicum]
c22756.graph_c0	0.03756984	0	0	7.47614679	10.0467567	8.90043797	2.88E-34	8.457255	up	PREDICTED: uncharacterized protein LOC105158433 [Sesamum indicum]
c22766.graph_c0	88.5579153	79.2467087	81.9792512	4.46410284	6.00116084	5.80753073	2.31E-210	-4.962361	down	PREDICTED: uncharacterized protein LOC104211375 [Nicotiana sylvestris]
c22770.graph_c0	0.91616151	0.61138594	0.3924346	6.82255756	7.07864829	7.38493961	9.643E-07	2.459416	up	PREDICTED: uncharacterized protein LOC105160016 [Sesamum indicum]

#ID	0 h-1 FPKM	0 h-2 FPKM	0 h-3 FPKM	48 h-1 FPKM	48 h-2 FPKM	48 h-3 FPKM	FDR	log2FC	regulated	nr_annotation
c22771.graph_c0	1.47319632	2.06454011	2.27173872	13.0291636	16.6784062	14.4915893	0.0003403	1.903148	up	PREDICTED: CBL-interacting serine/threonine-protein kinase 23-like [Tarenaya hassleriana]
c22778.graph_c0	0.14139626	0	0	35.0408691	45.6774486	49.7823305	2.73E-65	8.848503	up	PREDICTED: LOW QUALITY PROTEIN: 4-coumarate--CoA ligase 1-like [Sesamum indicum]
c22778.graph_c1	0	0	0	38.872364	43.4289198	46.117861	3.37E-102	Inf	up	4-coumarate coenzyme A ligase [Paulownia fortunei]
c22780.graph_c0	1.29267178	2.10868565	2.70703387	35.6826996	55.8845318	66.6794529	6.296E-13	3.662752	up	PREDICTED: uncharacterized protein At4g06744-like [Sesamum indicum]
c22797.graph_c0	3.25709815	2.44526973	3.3135209	12.2526593	16.5563863	19.4684689	0.0005956	1.395534	up	PREDICTED: uncharacterized protein LOC105174672 [Sesamum indicum]
c22799.graph_c0	0.11530995	0.13851049	0.20744898	2.06088224	3.01604276	2.8735218	2.768E-10	3.077396	up	PREDICTED: uncharacterized protein LOC105170951 [Sesamum indicum]
c22800.graph_c0	1.43368979	1.15372827	1.11985868	0	0.02743601	0.02304407	2.977E-23	-7.243938	down	-
c22803.graph_c0	3.70717423	2.83199225	3.25948703	0.80888464	0.76170239	0.15994237	2.684E-11	-3.508675	down	-
c22827.graph_c0	0	0	0.10385382	8.115535	26.1866531	36.9007606	1.167E-07	8.353001	up	unnamed protein product [Coffea canephora]
c22837.graph_c0	106.098355	108.183843	122.423567	3.51897568	2.8507685	2.72595402	2.4E-169	-6.224503	down	PREDICTED: cytochrome P450 86B1-like [Sesamum indicum]
c22838.graph_c0	8.5955291	7.56645145	6.82717693	2.38756645	2.27640343	1.48710959	1.035E-24	-2.913419	down	PREDICTED: uncharacterized protein LOC105171100 [Sesamum indicum]
c22841.graph_c0	1.60960748	1.99484289	2.06840934	0.14122623	0.2244181	0.06283112	3.695E-14	-4.74859	down	-
c22844.graph_c0	0.54395153	0.27224811	0.34949962	4.63539353	5.4415171	5.46223566	6.504E-06	2.718931	up	PREDICTED: solute carrier family 35 member F1-like isoform X1 [Sesamum indicum]
c22846.graph_c0	0.51988252	0.59474641	0.76350814	3.69475474	4.05911296	4.3834274	0.0010527	1.668502	up	PREDICTED: protein MIZU-KUSSEI 1-like [Sesamum indicum]
c22852.graph_c0	0.11113576	0.11124705	0.14281386	6.04074538	5.31478687	4.64620279	7.075E-10	4.436768	up	-
c22852.graph_c1	5.48943407	4.93612437	4.06509769	61.2867063	64.3810017	67.72713	1.015E-35	2.722388	up	PREDICTED: HMG-Y-related protein A-like [Sesamum indicum]
c22864.graph_c0	0.09697454	0.48535822	0	69.0592555	79.8796401	86.6479641	6.14E-106	7.65273	up	PREDICTED: NADPH--cytochrome P450 reductase-like [Sesamum indicum]
c22867.graph_c0	19.4633759	15.8027687	11.9497997	16.735536	10.3420748	14.1820755	0.000712	-1.200795	down	-
c22874.graph_c0	20.5198645	13.9956729	16.1573764	14.1781782	17.3761485	13.3577552	3.011E-06	-1.192264	down	PREDICTED: E3 ubiquitin-protein ligase SDIR1-like isoform X2 [Phoenix dactylifera]
c22886.graph_c0	0	0.10780318	0.13839277	8.38374937	12.0347845	14.0368018	2.719E-23	6.090547	up	PREDICTED: alcohol dehydrogenase-like 4 [Sesamum indicum]
c22889.graph_c0	29.676431	30.4608071	27.3905449	11.996707	12.9766155	13.0904278	7.262E-34	-2.219345	down	PREDICTED: zinc finger protein 598 [Sesamum indicum]
c22901.graph_c0	3.97972406	4.58730144	2.01464663	24.8869524	42.8426511	44.7828127	2.755E-07	2.390082	up	PREDICTED: laccase-14-like [Sesamum indicum]
c22914.graph_c0	0.11743424	0	0.15090765	12.1170653	13.1803979	19.9268768	1.695E-16	6.36546	up	PREDICTED: hexose carrier protein HEX6-like [Sesamum indicum]
c22923.graph_c0	0.44268615	0.88625887	1.13773846	16.7210857	19.8742021	20.5393414	6.182E-18	3.50569	up	PREDICTED: uncharacterized protein LOC102582737 [Solanum tuberosum]

#ID	0 h-1 FPKM	0 h-2 FPKM	0 h-3 FPKM	48 h-1 FPKM	48 h-2 FPKM	48 h-3 FPKM	FDR	log2FC	regulated	nr_annotation
c22943.graph_c0	41.2114345	46.4975438	41.5638688	9.1899743	9.9323435	6.11223845	6.303E-65	-3.372163	down	PREDICTED: dof zinc finger protein DOF3.1-like [Sesamum indicum]
c22951.graph_c0	441.109685	421.822618	432.806823	22.3193697	25.6288336	28.2849432	4.74E-204	-5.107932	down	PREDICTED: 1-Cys peroxiredoxin [Phoenix dactylifera]
c22986.graph_c0	34.8029518	32.7689609	32.1551537	26.1806118	25.7540991	24.5660065	2.51E-21	-1.398606	down	PREDICTED: ninja-family protein mc410 [Sesamum indicum]
c22991.graph_c0	5.41089771	4.92392355	5.82946542	66.4649587	65.0240129	78.7190847	7.175E-53	2.681205	up	PREDICTED: uncharacterized protein LOC105174944 [Sesamum indicum]
c22996.graph_c0	26.1039194	27.9591626	25.1584274	26.0206211	23.6422948	20.0716327	1.206E-13	-1.197594	down	PREDICTED: putative F-box/LRR-repeat protein 23 [Sesamum indicum]
c22998.graph_c0	0.19127284	0.09573219	0.49158635	4.40532543	4.57356996	5.0957817	1.284E-07	3.143106	up	PREDICTED: probable ADP-ribosylation factor GTPase-activating protein AGD11 [Sesamum indicum]
c23011.graph_c0	0.33222277	0.16627772	0.42691942	3.82581611	5.02570544	6.39987375	1.167E-06	3.013148	up	-
c23025.graph_c0	16.4244684	11.6121148	16.2354555	8.57087144	7.0620659	6.7789421	1.312E-10	-1.996465	down	PREDICTED: MATH domain-containing protein At5g43560-like [Sesamum indicum]
c23026.graph_c0	8.11233381	6.11315309	6.44221188	0.16794632	0	0.22415643	1.076E-30	-6.724315	down	-
c23036.graph_c0	1.90976021	1.99856675	1.8963635	10.3964289	12.8776222	8.75256641	0.000465	1.445309	up	PREDICTED: anthocyanidin 3-O-glucoside 2'''-O-xylosyltransferase-like isoform X3 [Sesamum indicum]
c23056.graph_c0	7.20146288	9.84410328	8.75662917	43.8730242	44.5887936	36.8797573	9.676E-10	1.263201	up	PREDICTED: uncharacterized protein At1g66480-like [Sesamum indicum]
c23065.graph_c0	0.03485174	0	0	1.573277	3.2313498	2.71407713	3.158E-11	6.745724	up	PREDICTED: beta-glucosidase 12-like [Sesamum indicum]
c23070.graph_c0	0.27430736	0	0.11749861	249.171896	254.954324	183.185694	4.805E-67	9.766117	up	PREDICTED: EG45-like domain containing protein [Sesamum indicum]
c23071.graph_c0	1.41954971	1.06572838	1.29212579	0.05449091	0.17317953	0.38788546	2.47E-10	-3.654307	down	-
c23072.graph_c0	2.74060658	2.46198156	2.4381609	18.1269526	18.9289753	20.1039623	9.965E-10	1.885381	up	PREDICTED: protein RDM1 [Sesamum indicum]
c23075.graph_c1	23.6754363	25.3668612	23.1559478	9.97667586	9.88444169	9.77568681	2.495E-41	-2.301212	down	PREDICTED: E3 ubiquitin-protein ligase CIP8-like [Sesamum indicum]
c23078.graph_c0	0.09888119	0.29694062	0.12706625	3.09725736	4.43922812	2.83697747	4.918E-07	3.291544	up	PREDICTED: probable sugar phosphate/phosphate translocator At3g17430 [Nicotiana tomentosiformis]
c23084.graph_c1	0.81292692	0.48824456	1.25357193	9.43640835	11.2661565	8.92956794	3.217E-06	2.509619	up	PREDICTED: elongation of fatty acids protein 3-like [Populus euphratica]
c23085.graph_c0	291.313747	326.521369	313.623422	107.451783	26.7746008	20.4686092	1.154E-78	-3.576851	down	PREDICTED: zinc finger protein ZAT10-like [Sesamum indicum]
c23086.graph_c0	0	0.14711949	0	5.68682032	3.01224828	1.92765658	0.0005512	5.185773	up	myb family transcription factor family protein [Populus trichocarpa]
c23103.graph_c0	0.55078956	0.39381507	0.20222468	10.7283777	21.1181703	23.9296152	1.658E-09	4.582844	up	PREDICTED: uncharacterized protein LOC105172657 [Sesamum indicum]
c23106.graph_c0	0	0	0	8.06836688	4.56846806	2.59935984	0.0001632	Inf	up	-

#ID	0 h-1 FPKM	0 h-2 FPKM	0 h-3 FPKM	48 h-1 FPKM	48 h-2 FPKM	48 h-3 FPKM	FDR	log2FC	regulated	nr_annotation
c23112.graph_c0	1160.41187	1150.0643	1254.61612	35.654924	27.7072295	24.9723216	1.51E-234	-6.347187	down	hypothetical protein MIMGU_mgv1a018508mg [Erythranthe guttata]
c23113.graph_c0	8.81079134	7.42704338	9.53449743	97.404908	101.287845	96.0215708	1.393E-43	2.496112	up	-
c23115.graph_c0	2.11809863	2.43825252	1.53103534	20.1718802	20.336062	26.5000077	1.85E-21	2.444518	up	PREDICTED: probable glucan endo-1,3-beta-glucosidase A6 [Sesamum indicum]
c23120.graph_c0	0.54008683	0.84955773	0.39659026	21.4662988	20.4066057	20.9979926	4.097E-25	4.125407	up	hypothetical protein MIMGU_mgv1a016344mg [Erythranthe guttata]
c23128.graph_c0	52.9299247	51.6512187	50.8601451	23.6808905	18.2240362	22.7848459	3.518E-52	-2.278568	down	PREDICTED: omega-6 fatty acid desaturase, endoplasmic reticulum [Sesamum indicum]
c23144.graph_c0	0	0	0	7.1868246	10.4898065	13.0027464	3.278E-18	Inf	up	PREDICTED: WAT1-related protein At2g39510-like [Sesamum indicum]
c23146.graph_c0	0.44216564	0.4426084	0.56820036	10.1837957	9.97935947	7.65695077	1.416E-13	3.241164	up	PREDICTED: uncharacterized protein LOC105156632 [Sesamum indicum]
c23151.graph_c0	0.41287815	0.4959499	0.74279068	18.94244	20.2284008	27.2114756	1.621E-25	4.301898	up	PREDICTED: copper transporter 6-like [Sesamum indicum]
c23152.graph_c0	132.359843	104.083664	138.333754	18.9331529	12.0583057	18.5513216	4.662E-38	-3.933084	down	-
c23170.graph_c0	1.27508361	1.81635905	1.32343044	67.9964899	58.9195338	61.025484	3.699E-75	4.397972	up	PREDICTED: BES1/BZR1 homolog protein 2 [Sesamum indicum]
c23186.graph_c0	1.62755949	1.3033514	1.43415661	16.322192	24.0990145	25.1586254	3.706E-17	2.885053	up	PREDICTED: probable aquaporin SIP2-1 [Sesamum indicum]
c23190.graph_c0	0.20910292	0.20931231	0.4030583	23.8872673	31.3259622	44.8235185	2.419E-16	5.895477	up	hypothetical protein MIMGU_mgv1a015689mg [Erythranthe guttata]
c23198.graph_c0	0	0	0	13.9640752	23.7978281	29.1180693	2.507E-19	Inf	up	PREDICTED: peroxidase 27-like [Sesamum indicum]
c23198.graph_c1	0	0	0	6.16225127	10.9947924	14.4293003	3.035E-07	Inf	up	hypothetical protein MIMGU_mgv1a009974mg [Erythranthe guttata]
c23201.graph_c0	0	0	0.23369216	23.2877278	57.5053938	70.8101302	5.234E-10	8.276369	up	PREDICTED: cytochrome P450 71D11-like [Beta vulgaris subsp. vulgaris]
c23208.graph_c0	8.84916111	8.11985369	8.78708547	12.6609621	4.31825256	4.06662415	0.0017055	-1.285877	down	PREDICTED: uncharacterized protein LOC105168502 [Sesamum indicum]
c23217.graph_c0	0	0	0	2.69427995	2.7400924	3.4521899	1.313E-11	Inf	up	hypothetical protein MIMGU_mgv1a016855mg [Erythranthe guttata]
c23225.graph_c0	3.03923356	3.61629141	3.53708872	13.9468719	14.8869516	14.7601918	0.0001783	1.077062	up	PREDICTED: GATA transcription factor 15-like [Sesamum indicum]
c23230.graph_c0	6.11568512	6.50442212	5.54332949	26.4604677	23.5551187	23.812932	4.402E-06	1.009218	up	PREDICTED: tetraspanin-8-like [Sesamum indicum]
c23258.graph_c1	10.1705138	12.3440963	12.4160372	0.46848703	1.24076301	0.72949965	1.046E-26	-4.872693	down	-
c23259.graph_c0	12.7371725	14.3436677	13.412479	6.41041233	4.086143	5.02720695	1.039E-22	-2.393419	down	PREDICTED: uncharacterized protein LOC105165738 [Sesamum indicum]
c23261.graph_c0	4.78251496	5.07569573	5.99763141	50.0581293	41.4459087	40.1959178	3.691E-16	2.03848	up	PREDICTED: uncharacterized protein LOC105161807 [Sesamum indicum]

#ID	0 h-1 FPKM	0 h-2 FPKM	0 h-3 FPKM	48 h-1 FPKM	48 h-2 FPKM	48 h-3 FPKM	FDR	log2FC	regulated	nr_annotation
c23266.graph_c0	1078.60554	1065.59275	1153.72731	726.334465	367.843646	249.882451	1.689E-26	-2.295704	down	PREDICTED: stem-specific protein TSJT1-like [Sesamum indicum]
c23267.graph_c0	18.0768003	22.4951505	18.9639552	6.69447012	9.76241602	9.08212788	2.452E-32	-2.244482	down	PREDICTED: U-box domain-containing protein 38-like [Sesamum indicum]
c23279.graph_c0	0.13986943	0.35002371	0.3594755	5.73412934	7.09842622	9.28713929	7.948E-12	3.67389	up	unnamed protein product [Coffea canephora]
c23283.graph_c0	0.14366147	0.35951331	0.09230534	43.0138522	55.1021802	75.369289	1.236E-26	7.169407	up	unnamed protein product [Coffea canephora]
c23284.graph_c0	28.1138564	24.8633276	29.4631374	18.859455	28.5038095	23.0459474	0.0001229	-1.252177	down	Zinc finger A20 and AN1 domain-containing stress-associated protein 8 [Morus notabilis]
c23287.graph_c0	0.04441129	0.08891152	0.11414053	3.35499531	6.11150868	6.0433204	5.093E-13	4.935001	up	PREDICTED: GDSL esterase/lipase At5g33370-like isoform X1 [Sesamum indicum]
c23298.graph_c0	2.52925199	2.95374876	2.76867947	1.98491014	1.78278463	2.8412157	0.0009903	-1.339991	down	hypothetical protein MIMGU_mgv1a008021mg [Erythranthe guttata]
c23300.graph_c0	11.854125	11.8242134	13.4093485	4.84514062	6.15125941	7.97225585	6.107E-21	-1.992378	down	PREDICTED: uncharacterized protein LOC105160924 [Sesamum indicum]
c23305.graph_c0	0.20244915	0.33775313	0.26015518	1.55424471	4.21512656	6.36160496	0.0082541	2.88711	up	PREDICTED: uncharacterized protein LOC105163284 [Sesamum indicum]
c23307.graph_c0	0.59926318	0.59986325	1.30320708	8.57847896	9.89766741	8.84227962	4.768E-11	2.420007	up	PREDICTED: thermospermine synthase ACAULIS5-like [Sesamum indicum]
c23310.graph_c0	8.59442473	6.22346905	4.22968444	24.5955472	28.1975889	27.8808521	0.0060889	1.068651	up	-
c23312.graph_c0	0.20642916	0.16071679	0.29474405	5.62075765	7.5214878	8.19764636	6.174E-28	3.982298	up	PREDICTED: probable inactive receptor kinase At5g67200 [Sesamum indicum]
c23318.graph_c0	0.8798262	0.73392268	0.94217625	12.2933914	15.1700649	15.3861426	3.433E-09	3.044455	up	-
c23327.graph_c0	8.5912281	5.29989579	5.26328835	0	0	0	4.555E-23	-Inf	down	--
c23334.graph_c0	2.84465406	3.25428863	2.93744981	16.2388618	16.7073383	12.8669039	1.671E-06	1.326075	up	hypothetical protein MIMGU_mgv1a011374mg [Erythranthe guttata]
c23342.graph_c0	0.3251617	0.56960277	0.31338424	39.5419416	20.151577	12.7276112	0.0002089	4.908667	up	PREDICTED: beta-D-xylosidase 1 [Sesamum indicum]
c23343.graph_c0	0.16433457	0.5263972	0.33788228	8.1148108	10.1042809	10.5344006	1.043E-24	3.781734	up	PREDICTED: probable dolichyl-diphosphooligosaccharide--protein glycosyltransferase subunit 3B [Sesamum indicum]
c23352.graph_c0	2.81004587	1.92458822	1.8055101	16.282174	18.9089336	19.7009568	5.831E-10	2.052129	up	PREDICTED: phosphatidyl-N-methylethanolamine N-methyltransferase [Nicotiana sylvestris]
c23354.graph_c0	0	0	0	2.15284598	2.70302728	2.4122237	1.988E-11	Inf	up	PREDICTED: putative late blight resistance protein homolog R1A-10 [Sesamum indicum]
c23358.graph_c1	3.30421376	3.09181445	2.03071741	28.0582667	32.8089317	35.9181768	2.23E-21	2.505389	up	PREDICTED: polyadenylate-binding protein-interacting protein 12 [Sesamum indicum]
c23362.graph_c0	25.4398285	23.3779827	23.8484887	5.95525991	5.69833939	5.64082248	3.167E-18	-3.087064	down	-
c23376.graph_c0	1.81058011	1.44251698	1.56693908	11.0293864	17.2740078	17.295452	5.621E-11	2.217231	up	4-coumarate coenzyme A ligase [Paulownia fortunei]

#ID	0 h-1 FPKM	0 h-2 FPKM	0 h-3 FPKM	48 h-1 FPKM	48 h-2 FPKM	48 h-3 FPKM	FDR	log2FC	regulated	nr_annotation
c23377.graph_c0	8.47663396	7.43386797	8.86848267	37.8713008	40.9253806	42.1835617	6.092E-10	1.267448	up	PREDICTED: trafficking protein particle complex subunit 5 [Sesamum indicum]
c23392.graph_c0	0.32761525	0.24595748	0.31574892	9.13007116	8.71297654	8.32530099	2.421E-12	3.862201	up	PREDICTED: uncharacterized protein LOC105168788 [Sesamum indicum]
c23395.graph_c0	0.97939151	1.30716296	0.83903791	17.1433074	18.2674617	19.5358398	2.76E-22	3.119382	up	PREDICTED: uncharacterized protein LOC105176156 [Sesamum indicum]
c23400.graph_c0	0.22027449	0.11024753	0.21229608	1.16685458	1.07490516	1.5348194	0.0010097	1.777829	up	hypothetical protein MIMGU_mgv1a026825mg [Erythranthe guttata]
c23407.graph_c0	0.50227045	0.33518227	0.43029161	7.44214914	10.6618562	13.5527561	5.484E-12	3.617022	up	PREDICTED: uncharacterized protein LOC105166016 [Sesamum indicum]
c23414.graph_c0	18.4688862	18.2317941	20.8896348	13.4079308	16.4468259	13.116349	1.72E-10	-1.443613	down	PREDICTED: mannan synthase 1-like [Sesamum indicum]
c23417.graph_c0	3.62999824	3.04211146	4.23076667	0	0	0	4.461E-25	-Inf	down	-
c23429.graph_c0	13.566255	13.698961	13.9159553	92.3654846	93.5527596	93.3557791	2.387E-29	1.743739	up	hypothetical protein MIMGU_mgv1a012296mg [Erythranthe guttata]
c23432.graph_c0	0	0	0	15.0345191	14.1880675	11.9937325	9.677E-25	Inf	up	PREDICTED: beta-galactosidase-like [Sesamum indicum]
c23435.graph_c0	0.12181756	0	0	4.04014624	6.06339716	5.99149639	2.632E-11	6.042412	up	hypothetical protein MIMGU_mgv1a016138mg [Erythranthe guttata]
c23443.graph_c0	11.1443802	12.7330907	14.1763136	3.00748453	7.91023081	8.21267956	1.659E-09	-2.02708	down	PREDICTED: LOW QUALITY PROTEIN: expansin-A11-like [Sesamum indicum]
c23452.graph_c0	1.46810616	1.53637516	1.62931437	0.12295592	0.13025689	0.2735136	3.849E-12	-4.162608	down	PREDICTED: uncharacterized protein LOC105168585 [Sesamum indicum]
c23454.graph_c0	27.7416549	27.769434	28.5408402	24.3220179	16.4413079	18.3438199	1.144E-15	-1.518675	down	hypothetical protein CICLE_v100253982mg, partial [Citrus clementina]
c23455.graph_c0	10.5904054	11.5802199	9.23669179	4.74114555	3.90190801	6.20593734	4.455E-20	-2.09669	down	anthocyanidin synthase [Ipomoea nil]
c23458.graph_c0	1.12058316	1.19906424	0.94344417	11.5338894	10.7856932	11.5614713	7.419E-14	2.361568	up	PREDICTED: uncharacterized protein LOC105155953 [Sesamum indicum]
c23463.graph_c0	1.2423378	0.96277301	1.80244722	5.50109255	6.64909991	5.32190557	0.0020952	1.0994	up	PREDICTED: uncharacterized protein LOC101217532 [Cucumis sativus]
c23479.graph_c0	5.85683121	3.07093597	3.22553999	14.2172279	20.686782	17.6038753	0.0015256	1.092392	up	PREDICTED: heterogeneous nuclear ribonucleoprotein 1-like [Sesamum indicum]
c23482.graph_c0	2.57401129	2.60962196	2.62920254	12.2215581	10.4673113	12.0108283	5.299E-06	1.135319	up	PREDICTED: serine/threonine-protein kinase AtPK2/AtPK19-like [Sesamum indicum]
c23483.graph_c0	7.19505335	6.69860369	5.6898021	6.4894919	6.53108829	6.68170258	0.0002257	-1.00676	down	PREDICTED: uncharacterized protein LOC105168747 [Sesamum indicum]
c23501.graph_c0	7.08762116	5.76445865	5.76357639	49.6350021	47.9347072	49.8387351	7.252E-27	1.969693	up	PREDICTED: protein transport protein Sec24-like At4g32640 [Sesamum indicum]

#ID	0 h-1 FPKM	0 h-2 FPKM	0 h-3 FPKM	48 h-1 FPKM	48 h-2 FPKM	48 h-3 FPKM	FDR	log2FC	regulated	nr_annotation
c23505.graph_c0	26.2081291	13.6700179	19.6135229	0	0	0	1.132E-26	-Inf	down	cytochrome c oxidase subunit 2 (mitochondrion) [Gelidium vagum]
c23513.graph_c0	532.834615	566.225916	524.298132	54.2965876	18.5724985	10.1976813	7.35E-215	-5.278443	down	PREDICTED: zinc finger protein ZAT10-like [Sesamum indicum]
c23519.graph_c0	0	0	0.10339008	3.63197729	2.19865117	1.78073902	1.197E-05	5.164331	up	-
c23524.graph_c0	2.69133504	2.2001245	1.67159484	15.5377825	16.8543954	21.9147701	4.835E-13	2.031929	up	PREDICTED: uncharacterized protein LOC105161815 [Sesamum indicum]
c23535.graph_c0	1.10634827	0.9756161	1.08320102	6.6735965	7.81541581	7.12575124	6.314E-09	1.751675	up	PREDICTED: HIPL1 protein-like [Sesamum indicum]
c23541.graph_c0	0	0	0	19.3321883	26.3502044	34.732024	1.869E-21	Inf	up	PREDICTED: non-specific lipid transfer protein GPI-anchored 2-like [Nicotiana tomentosiformis]
c23552.graph_c0	0.57952362	0.29005196	0.49647386	5.78386205	5.18463889	6.72996741	1.381E-06	2.677593	up	PREDICTED: phosphoglycolate phosphatase 2 [Sesamum indicum]
c23561.graph_c0	3.04000074	4.23852675	3.69724339	17.679084	22.1461317	24.2747386	2.369E-14	1.523057	up	PREDICTED: leukotriene A-4 hydrolase homolog [Sesamum indicum]
c23565.graph_c0	6.30206352	7.00930453	8.818259	1.03215502	0.9567627	0.45920266	3.438E-16	-4.19049	down	PREDICTED: protein CUP-SHAPED COTYLEDON 3 [Sesamum indicum]
c23576.graph_c0	3.77063068	3.71970486	3.65161312	20.5404403	18.720091	20.6509472	2.674E-08	1.410732	up	PREDICTED: protein EARLY RESPONSIVE TO DEHYDRATION 15-like [Sesamum indicum]
c23578.graph_c0	0.45666827	0.71834016	0.8383383	3.00508998	7.00376259	7.75434188	0.0016325	2.104957	up	-
c23583.graph_c0	0.68228463	0.63743665	0.64295936	17.1388752	20.8201141	23.9750459	1.623E-38	3.958159	up	PREDICTED: glyoxylate/succinic semialdehyde reductase 2, chloroplastic [Sesamum indicum]
c23586.graph_c0	1.16998165	0.557692	0.7159394	9.75209748	9.2980483	8.08364375	2.573E-09	2.460783	up	PREDICTED: glucan endo-1,3-beta-glucosidase 14-like [Sesamum indicum]
c23589.graph_c0	0.06590589	0	0	6.55741472	7.91161713	7.88771514	2.958E-19	7.406475	up	PREDICTED: calcium-dependent protein kinase 8-like [Sesamum indicum]
c23594.graph_c0	0.65757611	0.37613404	0.24143181	3.98098458	3.75896062	4.08129512	0.0001346	2.203852	up	PREDICTED: uncharacterized protein LOC105160721 [Sesamum indicum]
c23596.graph_c0	17.9611653	18.232775	16.6775121	10.4780357	9.80884743	9.64637757	2.756E-30	-1.83587	down	PREDICTED: probable 1-acyl-sn-glycerol-3-phosphate acyltransferase 4 [Sesamum indicum]
c23601.graph_c0	6.45407707	5.56097101	6.61399883	28.6006123	25.7540486	25.1138021	2.606E-05	1.076846	up	PREDICTED: uncharacterized protein LOC105163419 [Sesamum indicum]
c23610.graph_c1	0	0	0	5.99339004	6.5208719	5.76527829	1.077E-11	Inf	up	hypothetical protein MIMGU_mgv1a005845mg [Erythranthe guttata]
c23617.graph_c0	2.62195492	1.84692696	1.80944742	13.2853374	9.90406952	8.67685019	0.0005407	1.336029	up	PREDICTED: deSI-like protein At4g17486 [Sesamum indicum]
c23621.graph_c0	2.01378169	1.95901514	1.89528223	8.30932677	9.79925896	10.5091265	1.085E-06	1.265475	up	PREDICTED: probable polygalacturonase [Sesamum indicum]
c23632.graph_c0	6.01974268	5.40455708	5.10390609	28.4721468	36.8252501	39.9855324	7.775E-15	1.649913	up	-
c23633.graph_c0	132.335231	131.755553	135.313385	62.0815716	30.0568219	28.3281072	2.675E-75	-2.73117	down	conserved hypothetical protein [Ricinus communis]

#ID	0 h-1 FPKM	0 h-2 FPKM	0 h-3 FPKM	48 h-1 FPKM	48 h-2 FPKM	48 h-3 FPKM	FDR	log2FC	regulated	nr_annotation
c23637.graph_c0	0.21509725	0.35885439	0.64495311	5.48247128	6.99760668	6.99414612	7.382E-09	2.965806	up	PREDICTED: uncharacterized acetyltransferase At3g50280-like [Sesamum indicum]
c23640.graph_c0	0	0.15067557	0	6.65631835	4.33377258	3.39324335	1.68E-07	5.581971	up	-
c23650.graph_c0	0.46338178	0.49476884	0.27788317	33.5256855	41.9081444	53.4323528	1.222E-33	5.685945	up	PREDICTED: glucan endo-1,3-beta-glucosidase 3 [Sesamum indicum]
c23652.graph_c0	25.0259724	23.9043299	26.384268	10.3100559	9.20221405	9.46276513	1.112E-27	-2.394282	down	PREDICTED: uncharacterized protein LOC105169025 [Sesamum indicum]
c23690.graph_c0	3.50055966	3.17034447	4.391254	48.8348645	51.3278987	58.2105808	6.3E-36	2.817296	up	PREDICTED: uncharacterized protein LOC105170479 [Sesamum indicum]
c23691.graph_c0	1.30835682	2.73839451	1.68129031	7.23206183	9.983157	13.3575937	0.0024061	1.391665	up	-
c23713.graph_c1	10.2787059	11.1869475	9.94243137	6.85241256	7.80648365	6.71002298	1.243E-14	-1.573753	down	PREDICTED: uncharacterized protein LOC105162910 isoform X1 [Sesamum indicum]
c23716.graph_c0	0.29654297	0.29683991	0	52.7265817	62.5140036	38.4077495	1.632E-21	7.015474	up	hypothetical protein [Arachis diogoi]
c23716.graph_c1	0.12336449	0.24697603	0	183.660679	158.927853	117.306556	7.567E-37	9.28094	up	PREDICTED: abscisic acid receptor PYL4-like [Sesamum indicum]
c23720.graph_c0	1.72191326	0.86181875	0.66381801	5.86945358	8.57072141	9.73945163	0.0006736	1.878927	up	unnamed protein product [Coffea canephora]
c23732.graph_c0	0.53606001	0.1341492	1.37771668	5.18546123	5.88575078	6.59141745	7.507E-05	2.07213	up	PREDICTED: methylsterol monooxygenase 1-1-like [Sesamum indicum]
c23737.graph_c0	9.56463597	6.58227178	7.16971261	0	0	0	3.512E-24	-Inf	down	hypothetical protein BOTBODRAFT_366584 [Botryobasidium botryosum FD-172 SS1]
c23761.graph_c0	2.8079309	3.64355525	2.53917575	2.49103987	2.02996515	2.64276466	0.0045081	-1.341304	down	-
c23783.graph_c0	0.05184724	0	0	0.6925934	0.63251615	0.68001704	1.005E-08	4.278368	up	PREDICTED: putative late blight resistance protein homolog R1A-10 [Sesamum indicum]
c23800.graph_c0	2.46207885	2.83422588	3.63844912	1.47434796	2.76334895	1.91734254	0.0020692	-1.56792	down	-
c23802.graph_c0	0.32865828	0.14099459	0	67.4770084	80.5566406	104.416825	2.815E-50	8.065965	up	PREDICTED: nicotianamine synthase-like [Sesamum indicum]
c23803.graph_c1	5.49089093	4.08877735	5.85132722	39.6048772	44.8320929	47.3162562	5.697E-24	2.071947	up	hypothetical protein MIMGU_mgv1a015927mg [Erythranthe guttata]
c23816.graph_c0	5.29489973	4.18436982	3.10365001	0.51347442	0.99726713	1.4468071	1.089E-10	-3.114836	down	PREDICTED: elongation of fatty acids protein 3-like [Sesamum indicum]
c23825.graph_c0	92.7354826	104.654642	96.7769624	18.3889301	21.0077185	25.1184328	1.77E-102	-3.210171	down	PREDICTED: zinc finger A20 and AN1 domain-containing stress-associated protein 5-like [Sesamum indicum]
c23833.graph_c0	79.4240499	80.931372	75.1502342	36.6654331	42.6793039	46.2278054	6.9E-42	-1.927434	down	PREDICTED: U3 small nucleolar RNA-associated protein 15 homolog [Sesamum indicum]
c23834.graph_c0	0.14383872	0	0	2.91529954	3.43936163	3.59624945	1.575E-10	5.112761	up	hypothetical protein MIMGU_mgv1a026155mg [Erythranthe guttata]
c23837.graph_c0	0.46977167	0.70536311	1.60980051	5.77045736	7.03006485	4.62106643	0.0004254	1.61437	up	-

#ID	0 h-1 FPKM	0 h-2 FPKM	0 h-3 FPKM	48 h-1 FPKM	48 h-2 FPKM	48 h-3 FPKM	FDR	log2FC	regulated	nr_annotation
c23837.graph_c1	8.75419606	8.05879547	8.78866291	41.9448701	41.2697126	40.9103592	1.202E-14	1.260094	up	PREDICTED: protein FAR1-RELATED SEQUENCE 7-like [Sesamum indicum]
c23840.graph_c0	0	0.04599782	0.05904989	6.09606715	11.480967	13.0332527	1.665E-14	7.144673	up	Integrase, catalytic region, putative [Theobroma cacao]
c23846.graph_c0	18.973588	16.6566005	13.038402	4.01939782	2.47561896	2.07932326	7.914E-29	-3.507347	down	PREDICTED: galactinol--sucrose galactosyltransferase [Sesamum indicum]
c23859.graph_c0	3.88167234	4.27411517	2.32778123	0	0	0	5.72E-19	-Inf	down	putative histone h2a protein [Eutypa lata UCREL1]
c23864.graph_c0	0.84448869	0.46963017	0.7234676	45.8154412	34.7077506	38.1511072	9.089E-32	4.851159	up	PREDICTED: histone H2AX-like [Sesamum indicum]
c23865.graph_c0	0	0	0	2.94417297	3.7970365	3.53090998	1.272E-11	Inf	up	predicted protein [Physcomitrella patens]
c23868.graph_c0	0	0	0	2.18072062	5.54450177	5.14203905	5.132E-07	Inf	up	PREDICTED: uncharacterized protein LOC105162633 [Sesamum indicum]
c23876.graph_c0	5.41358174	7.90271215	5.79722239	1.87025422	3.96261514	3.88299558	4.821E-05	-2.005132	down	hypothetical protein, partial [Oryza eichingeri]
c23891.graph_c0	56.3928194	51.347141	61.1788759	21.0808247	21.697528	21.5134457	1.783E-42	-2.412806	down	-
c23894.graph_c0	0.90172026	0.77367702	0.6621407	4.27229237	7.16612823	6.23017066	0.00055	1.895113	up	PREDICTED: uncharacterized protein LOC105169824 isoform X1 [Sesamum indicum]
c23905.graph_c0	3.30542899	6.47961363	4.24760712	38.6991216	41.9379422	44.0306748	1.772E-12	2.132343	up	PREDICTED: macrophage migration inhibitory factor homolog [Sesamum indicum]
c23907.graph_c0	0.21602463	0.07208032	0.2776002	3.64862628	3.65444383	3.60069217	9.425E-08	3.244783	up	hypothetical protein MIMGU_mgv1a015138mg [Erythranthe guttata]
c23921.graph_c0	5.20574368	3.39844986	4.07192236	84.4490262	87.696323	88.6863918	1.801E-27	3.345981	up	PREDICTED: pectinesterase-like isoform X2 [Sesamum indicum]
c23936.graph_c0	0.36167337	0.24135702	0	91.0738733	98.1289745	100.900929	2.9E-106	7.908522	up	actin [Ipomoea nil]
c23939.graph_c0	3.83367221	2.16902798	3.85545957	27.4868422	32.5351691	23.774453	8.772E-08	2.066974	up	hypothetical protein PHAVU_002G125300g [Phaseolus vulgaris]
c23950.graph_c0	1.51443986	2.01028993	1.39612611	0.8189222	1.38165174	2.18601614	0.0044824	-1.193044	down	PREDICTED: LOW QUALITY PROTEIN: H/ACA ribonucleoprotein complex subunit 4-like [Sesamum indicum]
c23951.graph_c0	14.6767768	12.9106888	14.7167019	0.35851678	0.54257865	1.36716888	2.622E-68	-5.252318	down	PREDICTED: uncharacterized protein LOC105166244 [Sesamum indicum]
c23961.graph_c0	0.26908484	0.20201572	0.08644617	1.67331492	1.96963807	2.26093066	6.456E-05	2.392383	up	PREDICTED: N-alpha-acetyltransferase 10 [Sesamum indicum]
c23962.graph_c0	37.7891168	37.0900681	40.0466553	21.4760449	19.3984474	15.4885546	5.03E-13	-2.042254	down	PREDICTED: NAC transcription factor 25-like [Sesamum indicum]
c23967.graph_c0	0.13759129	0.27545813	0	9.82375041	8.25851549	12.2375597	4.779E-19	5.195228	up	PREDICTED: high mobility group B protein 7-like [Sesamum indicum]
c23973.graph_c0	0.15981051	0.07998527	0	13.6185774	11.229845	14.4102715	2.199E-23	6.357397	up	PREDICTED: transcription factor MYB1R1-like [Malus domestica]
c23976.graph_c0	1.9675891	1.0743051	1.89632242	0	0	0	1.091E-17	-Inf	down	predicted protein [Physcomitrella patens]
c23978.graph_c0	28.5261757	31.8144139	33.811724	26.1601729	24.0268687	32.7802271	2.644E-08	-1.202262	down	PREDICTED: ran-binding protein 6 isoform X2 [Elaeis guineensis]

#ID	0 h-1 FPKM	0 h-2 FPKM	0 h-3 FPKM	48 h-1 FPKM	48 h-2 FPKM	48 h-3 FPKM	FDR	log2FC	regulated	nr_annotation
c23979.graph_c0	0.04907925	0.1965136	0.06306879	22.9240102	24.6684077	28.8463758	4.739E-67	6.936201	up	PREDICTED: ferredoxin--NADP reductase, leaf isozyme, chloroplastic isoform 1 [Glycine max]
c23985.graph_c0	0.35236662	1.32269797	0.45280505	4.46356777	6.53407763	8.73764266	0.0002055	2.190917	up	hypothetical protein MIMGU_mgv1a016798mg [Erythranthe guttata]
c23986.graph_c0	0.78002256	0.39040182	1.33647983	5.98839715	7.10525832	9.2714852	3.647E-05	2.125626	up	PREDICTED: uncharacterized protein LOC105178874 isoform X1 [Sesamum indicum]
c23987.graph_c0	0.16019151	0.04008798	0.20585244	10.6625734	14.3443711	15.2981733	3.921E-40	5.602462	up	PREDICTED: lysophospholipid acyltransferase 1-like [Sesamum indicum]
c23990.graph_c0	0.50516519	1.01134208	0.32457864	5.35199793	6.65584377	7.45384003	5.66E-05	2.386165	up	PREDICTED: phosphoribosylformylglycinamidine cyclo-ligase, chloroplastic [Sesamum indicum]
c23994.graph_c0	31.4707959	29.7994816	31.2872942	18.5613648	25.4224869	28.3252349	1.679E-17	-1.380206	down	PREDICTED: transmembrane and coiled-coil domain-containing protein 1 [Sesamum indicum]
c24000.graph_c1	8.15317622	5.38480191	6.91276156	25.7860017	29.1218782	30.523404	3.169E-05	1.042953	up	NADP-malic enzyme, partial [Flaveria trinervia]
c24007.graph_c0	13.3183623	14.3071888	12.9701411	6.07074446	8.4919247	8.52101034	4.808E-29	-1.83745	down	PREDICTED: uncharacterized protein LOC105164548 [Sesamum indicum]
c24030.graph_c0	0	0.06804536	0.08735351	5.38575964	10.2169317	11.0332427	7.875E-13	6.379249	up	PREDICTED: uncharacterized protein LOC105166144 [Sesamum indicum]
c24031.graph_c0	8.38389819	9.41297773	12.0839488	0.62625356	0.55286642	0.83585497	5.801E-25	-4.912606	down	hypothetical protein MIMGU_mgv1a025777mg, partial [Erythranthe guttata]
c24033.graph_c0	2.20737218	1.38741228	2.17689537	0	0	0	2.06E-23	-Inf	down	CK1/CK1/CK1-D protein kinase [Coniosporium apollinis CBS 100218]
c24064.graph_c0	30.9563711	29.2147867	30.2565219	11.1175873	11.5857073	13.9245752	1.556E-37	-2.323439	down	PREDICTED: 21 kDa protein [Sesamum indicum]
c24065.graph_c0	3.69052017	3.36584095	3.26703113	26.9351428	26.0532553	21.781823	1.922E-18	1.842128	up	PREDICTED: zinc finger CCCH domain-containing protein 53 [Sesamum indicum]
c24089.graph_c0	5.80369921	6.52878349	7.60004008	1.83317014	0.9170657	1.04211963	4.212E-21	-3.400669	down	PREDICTED: ubiquitin-conjugating enzyme E2 5B-like isoform X1 [Citrus sinensis]
c24097.graph_c0	0.46148536	0.92389494	0.55596278	3.29491573	2.44902452	3.94846747	0.0019152	1.305043	up	PREDICTED: protein S-acyltransferase 18 isoform X4 [Sesamum indicum]
c24100.graph_c0	2.07410805	1.64364642	2.55425634	0.15923363	0.08434436	0	4.873E-16	-5.681464	down	hypothetical protein MIMGU_mgv1a023310mg [Erythranthe guttata]
c24101.graph_c0	0.970612	1.7272603	0.41575828	68.2565856	66.4153456	75.8515705	4.698E-68	5.069993	up	PREDICTED: methionine gamma-lyase [Sesamum indicum]
c24101.graph_c1	0.44213769	1.32774128	0.75755258	28.7843763	26.6099431	35.1563208	3.405E-20	4.144755	up	PREDICTED: methionine gamma-lyase [Sesamum indicum]
c24103.graph_c0	4.20368666	2.08311684	2.78117819	0	0	0	8.667E-18	-Inf	down	isocitrate dehydrogenase [Lichtheimia corymbifera JMRC:FSU:9682]
c24109.graph_c0	3.2107792	2.99972803	3.64461425	51.4688386	52.4359133	54.6357699	7.277E-77	2.98805	up	PREDICTED: dihydroxy-acid dehydratase, chloroplastic [Sesamum indicum]

#ID	0 h-1 FPKM	0 h-2 FPKM	0 h-3 FPKM	48 h-1 FPKM	48 h-2 FPKM	48 h-3 FPKM	FDR	log2FC	regulated	nr_annotation
c24115.graph_c0	0	0	0	1.35079942	4.01477299	3.13838114	1.467E-07	Inf	up	PREDICTED: geraniol 8-hydroxylase-like isoform X1 [Beta vulgaris subsp. vulgaris]
c24143.graph_c0	10.6053634	12.1088557	10.3809407	9.38869809	10.2292088	10.5953261	9.808E-09	-1.150004	down	PREDICTED: phosphoglycerate mutase-like protein 1 isoform X1 [Sesamum indicum]
c24146.graph_c0	0.07800556	0.23425102	0.10024023	5.96469632	3.7304187	2.49381549	7.317E-05	3.883251	up	PREDICTED: glutamate decarboxylase [Sesamum indicum]
c24157.graph_c0	19.1514645	21.5201521	21.0696413	19.8686121	18.0936435	18.8780185	5.891E-13	-1.135811	down	PREDICTED: plant intracellular Ras-group-related LRR protein 1-like [Sesamum indicum]
c24169.graph_c0	0	0	0	7.39352263	6.07021906	7.72998658	8.675E-12	Inf	up	PREDICTED: 2-hydroxyisoflavanone dehydratase-like [Sesamum indicum]
c24197.graph_c0	0.61769397	0.53587083	0.3704219	31.336236	37.2963226	39.2924608	1.461E-89	5.129675	up	PREDICTED: very-long-chain enoyl-CoA reductase [Sesamum indicum]
c24236.graph_c0	2.24508714	3.06953109	2.53319344	24.4667206	20.0943112	17.1918362	4.438E-09	1.963956	up	PREDICTED: uncharacterized protein LOC102578886 [Solanum tuberosum]
c24237.graph_c1	0.18130731	0.36297771	0	5.45638245	4.54172367	5.8954255	8.423E-11	3.866776	up	hypothetical protein MIMGU_mgv1a021532mg [Erythranthe guttata]
c24238.graph_c0	1080.13099	1042.58625	1033.19528	10.754469	11.5512928	13.2906415	0	-7.489689	down	-
c24238.graph_c1	0.55953365	0	0.4793486	13.5742776	15.1084177	13.7601007	1.26E-11	4.3299	up	PREDICTED: metallothionein-like protein 1 [Sesamum indicum]
c24240.graph_c0	10.0380567	4.77285144	8.70702768	39.0715856	34.0439846	36.8228016	0.0011449	1.208769	up	PREDICTED: nudix hydrolase 26, chloroplastic isoform X2 [Vitis vinifera]
c24241.graph_c0	5.79789672	6.33942882	7.22127985	100.171806	108.644473	115.528291	8.68E-63	3.044966	up	PREDICTED: 14 kDa zinc-binding protein-like [Sesamum indicum]
c24245.graph_c0	1.09180025	0.81967014	0.35075164	7.0408632	5.59420561	5.81742361	0.0004447	2.024238	up	-
c24247.graph_c0	0.14473161	0.36219135	0.27897878	27.867266	74.5112408	94.9133998	7.806E-09	6.935714	up	PREDICTED: cytochrome P450 71D11-like [Sesamum indicum]
c24250.graph_c0	0.5241299	0.07495068	0.19243648	6.20823402	8.6960852	11.4163686	6.361E-11	4.037241	up	hypothetical protein MIMGU_mgv1a016982mg [Erythranthe guttata]
c24255.graph_c0	50.4433572	51.7964418	48.0342978	24.0468185	16.4601848	13.8670789	4.737E-67	-2.474185	down	PREDICTED: uncharacterized protein LOC105164653 isoform X2 [Sesamum indicum]
c24261.graph_c0	0.4936893	0.14119533	0.54378026	2.14414572	3.3727775	2.77505159	0.000852	1.785316	up	-
c24269.graph_c0	6.36450519	7.77156782	5.16240469	26.4476644	28.5026832	28.3801731	2.503E-08	1.094437	up	unknown [Glycine max]
c24270.graph_c0	7.26712524	6.96703306	9.60160122	194.530724	190.698008	232.16011	3.947E-75	3.673008	up	PREDICTED: peptidyl-prolyl cis-trans isomerase FKBP12 [Sesamum indicum]
c24272.graph_c0	2.34945165	1.50515473	2.17377964	0	0	0	4.107E-25	-Inf	down	PREDICTED: acyl-[acyl-carrier-protein] desaturase 6, chloroplastic [Sesamum indicum]
c24284.graph_c0	0	0.07344123	0	2.36569086	2.07653474	2.34554636	4.272E-10	5.526041	up	-
c24286.graph_c0	1.26591511	0	0.29577291	4.02884141	3.93111614	4.05652818	0.0007887	1.937628	up	PREDICTED: topless-related protein 3-like [Sesamum indicum]

#ID	0 h-1 FPKM	0 h-2 FPKM	0 h-3 FPKM	48 h-1 FPKM	48 h-2 FPKM	48 h-3 FPKM	FDR	log2FC	regulated	nr_annotation
c24289.graph_c0	6.27219362	6.27847428	5.09053714	34.6701232	53.1600931	36.5320269	2.506E-05	1.796764	up	hypothetical protein MIMGU_mgv1a018579mg [Erythranthe guttata]
c24294.graph_c0	14.4379166	16.877269	13.5725443	10.9801444	10.5918596	8.34030062	2.499E-09	-1.599224	down	ZPT2-14 [Petunia x hybrida]
c24300.graph_c0	5.5979338	2.66168116	2.69758746	0	0	0	1.112E-09	-Inf	down	--
c24309.graph_c0	1.10159605	1.55898842	0.97627209	14.0330936	11.9375767	9.99547754	2.704E-11	2.295701	up	PREDICTED: chitotriosidase-1 [Vitis vinifera]
c24323.graph_c0	4.45600816	3.95866728	3.57884181	43.1555508	88.8267915	113.143976	1.057E-05	3.324486	up	PREDICTED: alpha-xylosidase 1 [Sesamum indicum]
c24336.graph_c0	4.4781711	1.73193501	3.13888754	72.5727609	54.2346175	40.5469714	7.23E-08	3.154074	up	PREDICTED: hydroxymethylglutaryl-CoA synthase isoform X4 [Sesamum indicum]
c24341.graph_c0	0.78328947	0.52271588	0.33551933	4.93105187	5.47867356	4.3876203	0.0002057	2.161494	up	PREDICTED: methylsterol monooxygenase 1-1-like [Sesamum indicum]
c24348.graph_c0	0.42416922	0.08491879	0.2180297	8.28437383	13.8267958	19.0543375	9.841E-09	4.798724	up	hypothetical protein MIMGU_mgv1a011727mg [Erythranthe guttata]
c24351.graph_c0	35.2569645	30.0166206	30.5936288	177.138733	189.253335	186.667368	5.09E-19	1.510704	up	40S ribosomal protein S13, putative [Ricinus communis]
c24361.graph_c0	1.44073375	1.44217643	1.38854993	7.30013846	5.62444474	10.2355213	0.0036743	1.421408	up	unnamed protein product [Coffea canephora]
c24366.graph_c0	1.04162414	0.80205167	0.72074599	46.578095	74.367663	68.6369726	8.703E-31	5.185805	up	PREDICTED: chaperone protein dnaJ 20, chloroplastic-like [Sesamum indicum]
c24370.graph_c0	0	0.0785848	0	2.09742547	2.45182672	2.1880484	5.858E-10	5.413905	up	hypothetical protein SELMODRAFT_117229, partial [Selaginella moellendorffii]
c24372.graph_c0	0	0	0	4.17022757	4.25113911	8.26143251	1.271E-08	Inf	up	early dehydration-responsive 1 [Lindernia subracemosa]
c24386.graph_c0	7.79322947	7.19157749	6.49384765	5.44081435	4.69429652	5.34029707	9.6E-07	-1.486846	down	PREDICTED: uncharacterized protein LOC105125740 [Populus euphratica]
c24393.graph_c0	1.01263412	0.25341203	1.4639343	5.71404067	4.32380945	4.8767958	0.004884	1.428274	up	PREDICTED: serine/threonine-protein kinase STE20 [Sesamum indicum]
c24395.graph_c0	0.68860663	0.34464808	0.70790943	5.01167583	7.39264446	8.80582286	7.319E-08	2.578446	up	PREDICTED: uncharacterized protein LOC104250007 [Nicotiana sylvestris]
c24399.graph_c0	8.34825316	7.66842104	8.96089816	33.5687579	37.1915555	41.4627457	1.347E-06	1.146361	up	putative DNA binding protein [Atriplex canescens]
c24403.graph_c0	4.7488648	6.90041623	7.67731265	2.11691806	3.88720453	1.6324715	5.617E-07	-2.367304	down	-
c24417.graph_c0	10.0718919	11.2126664	12.0355756	0.26015562	0.27560333	0.27006572	1.273E-76	-6.389688	down	PREDICTED: transcription factor TGA3 [Sesamum indicum]
c24418.graph_c0	0	0	0	2.03043312	2.76556846	3.17088526	3.002E-14	Inf	up	PREDICTED: patatin-like protein 1 [Sesamum indicum]
c24438.graph_c0	0.02838536	0.22731027	0.10942894	7.60977958	9.72383228	10.377755	1.01E-36	5.222599	up	PREDICTED: ornithine decarboxylase-like [Sesamum indicum]
c24441.graph_c0	0.61013615	0.34899835	0.50403157	12.4464384	16.03526	20.1489292	8.14E-20	4.031307	up	PREDICTED: cysteine-rich repeat secretory protein 3-like [Sesamum indicum]
c24442.graph_c0	0.22735192	0.91031832	0.29215622	3.97957697	9.76308879	8.75932483	0.0003553	2.948819	up	-
c24465.graph_c0	2528.85708	2899.14667	2538.28124	6.93694057	8.38580132	8.14157253	8.1E-292	-9.427562	down	legumin-like protein [Perilla frutescens]
c24469.graph_c0	19.9370719	21.8370465	21.7212422	19.8313936	20.5859578	22.6198542	0.0001186	-1.029883	down	PREDICTED: uncharacterized protein LOC105159964 [Sesamum indicum]

#ID	0 h-1 FPKM	0 h-2 FPKM	0 h-3 FPKM	48 h-1 FPKM	48 h-2 FPKM	48 h-3 FPKM	FDR	log2FC	regulated	nr_annotation
c24473.graph_c0	31.584226	33.5155178	27.8708881	12.9252617	13.0312621	14.6677126	2.809E-35	-2.211386	down	PREDICTED: uncharacterized protein LOC105159991 [Sesamum indicum]
c24475.graph_c0	0.18915212	0.31556921	0.16204531	3.02049396	3.87673783	3.7730013	4.309E-07	2.981946	up	PREDICTED: uncharacterized protein LOC105167521 [Sesamum indicum]
c24482.graph_c0	5.9714425	5.51365649	4.5975089	96.8889636	118.945273	136.532743	7.926E-40	3.43375	up	PREDICTED: probable methylenetetrahydrofolate reductase [Sesamum indicum]
c24492.graph_c0	1.5829336	1.70640472	2.03413235	214.819055	202.143412	199.628933	5.47E-156	5.837174	up	hypothetical protein MIMGU_mgv1a017195mg [Erythranthe guttata]
c24494.graph_c0	23.9298511	24.1255251	20.2801015	15.3292957	12.4726287	11.038488	5.416E-18	-1.825572	down	-
c24500.graph_c0	4.54786624	5.31115695	5.45457563	21.0886148	29.5905036	33.1796526	3.651E-05	1.427459	up	-
c24502.graph_c0	23.3114681	20.8785151	23.0189436	0.45212726	0.71846102	0.40230012	4.344E-34	-6.440043	down	-
c24503.graph_c0	57.9925772	59.6072111	49.0745289	53.5950827	30.9330201	24.6690937	1.619E-11	-1.612286	down	PREDICTED: uncharacterized protein LOC105178379 [Sesamum indicum]
c24520.graph_c0	2.36183844	1.35097341	1.30073824	94.6507636	118.217706	127.366709	4.173E-67	5.067077	up	-
c24534.graph_c0	39.4289409	38.2034094	44.0906863	250.895268	213.25118	205.42693	8.895E-13	1.444394	up	conserved hypothetical protein [Ricinus communis]
c24539.graph_c0	0.13595458	0.20413607	0.08735351	1.25250224	1.65859282	3.06478965	0.0026193	2.784063	up	PREDICTED: BTB/POZ domain-containing protein At1g30440-like [Camelina sativa]
c24542.graph_c0	21.4777708	20.8615871	22.1032183	0.50305229	0.26646145	1.26823655	1.64E-72	-6.005503	down	-
c24548.graph_c0	0.58752631	0.3360655	0.43142547	15.4647875	9.01068694	7.91227308	1.573E-05	3.575606	up	PREDICTED: S-norcoclaurine synthase-like [Sesamum indicum]
c24559.graph_c0	153.220404	156.896774	146.418925	10.595432	10.6725472	10.8187315	1.09E-203	-4.847758	down	PREDICTED: uncharacterized protein LOC105168445 [Sesamum indicum]
c24566.graph_c0	0.54732374	0.44825875	0.51151467	25.807425	24.1348122	29.7339957	2.313E-53	4.706491	up	PREDICTED: 2-C-methyl-D-erythritol 4-phosphate cytidylyltransferase, chloroplastic isoform X2 [Sesamum indicum]
c24575.graph_c0	2.40477893	3.35886551	3.16210209	20.1450402	22.8149182	25.9017612	2.858E-14	1.925402	up	PREDICTED: magnesium protoporphyrin IX methyltransferase, chloroplastic [Sesamum indicum]
c24580.graph_c0	19.0399117	18.7405695	18.4524695	103.277266	120.452718	121.769906	8.234E-29	1.599079	up	PREDICTED: NAC transcription factor 25 [Sesamum indicum]
c24593.graph_c0	198.631914	203.43666	202.034914	150.711359	137.207085	111.123084	4.728E-30	-1.612275	down	hypothetical protein MIMGU_mgv1a023553mg [Erythranthe guttata]
c24594.graph_c0	0.85792605	0.99090592	0.63603977	11.977258	14.5240917	16.7974072	9.135E-28	3.104571	up	PREDICTED: uncharacterized protein LOC105165456 [Sesamum indicum]
c24616.graph_c0	1.40760658	1.6103041	1.35662264	0.37050828	0.63782647	0.32967604	7.535E-08	-2.731812	down	PREDICTED: uncharacterized protein LOC105170552 [Sesamum indicum]
c24628.graph_c0	0.15081109	0	0	2.63980322	2.6493645	4.45051142	1.824E-07	5.012584	up	hypothetical protein MIMGU_mgv1a009447mg [Erythranthe guttata]
c24644.graph_c0	0.2492843	0.16635595	0	2.0669126	1.94634958	2.04347378	5.125E-06	2.865957	up	hypothetical protein MIMGU_mgv1a016118mg [Erythranthe guttata]

#ID	0 h-1 FPKM	0 h-2 FPKM	0 h-3 FPKM	48 h-1 FPKM	48 h-2 FPKM	48 h-3 FPKM	FDR	log2FC	regulated	nr_annotation
c24648.graph_c0	1.54106536	1.69686936	1.2872145	68.7149285	77.1568265	98.4716745	9.41E-40	4.735582	up	PREDICTED: protein YLS3-like [Sesamum indicum]
c24651.graph_c0	12.8744382	8.25928335	8.22637932	0	0	0	3.13E-32	-Inf	down	uncharacterized protein LOC100274375 precursor [Zea mays]
c24658.graph_c0	0	0.10074915	0	7.32518301	4.32210506	2.47515334	0.000124	6.1407	up	PREDICTED: protein LURP-one-related 11-like [Sesamum indicum]
c24673.graph_c0	6.38730085	6.34769895	6.43643783	4.06404477	3.40841209	3.46548916	4.45E-10	-1.823775	down	PREDICTED: UDP-glycosyltransferase 89A2-like [Sesamum indicum]
c24676.graph_c0	0.59102025	0.50287026	0.49366487	9.06569863	9.66169016	11.0946087	2.812E-23	3.213166	up	PREDICTED: LOW QUALITY PROTEIN: uncharacterized protein LOC105175967 [Sesamum indicum]
c24677.graph_c0	19.7965123	19.5670735	17.1728622	22.8259738	14.4602052	10.6144879	3.871E-07	-1.243088	down	PREDICTED: probable inactive receptor kinase At1g27190 [Sesamum indicum]
c24685.graph_c0	11.3973257	11.1434189	9.48017232	6.91857816	3.66469732	2.06410722	4.562E-24	-2.337904	down	PREDICTED: LOW QUALITY PROTEIN: U-box domain-containing protein 28-like [Sesamum indicum]
c24693.graph_c1	4.53925679	5.76287104	7.96719469	1.12196228	1.72884799	1.17982717	2.51E-08	-3.208725	down	PREDICTED: mitoferrin-like [Sesamum indicum]
c24702.graph_c0	1.24265803	1.1978319	0.65057453	21.2851278	14.9578107	16.0720624	1.164E-12	3.076291	up	PREDICTED: mitogen-activated protein kinase kinase kinase 2 [Sesamum indicum]
c24737.graph_c0	3.63095419	3.98074147	3.61053325	13.4599319	17.1700605	20.5869382	1.676E-06	1.167489	up	hypothetical protein MIMGU_mgv1a010118mg [Erythranthe guttata]
c24739.graph_c0	0.9279034	0.82562893	1.14822991	27.7666363	44.0056832	47.0749204	1.502E-20	4.327143	up	PREDICTED: homeobox-leucine zipper protein ATHB-6-like [Sesamum indicum]
c24749.graph_c0	0.24777779	0	0.15920213	35.0393006	42.0772698	33.513552	2.156E-40	7.067302	up	predicted protein [Hordeum vulgare subsp. vulgare]
c24752.graph_c0	5.66846869	6.35504219	5.19606744	81.2220432	111.603895	134.535986	1.652E-16	3.225665	up	hypothetical protein MIMGU_mgv1a006173mg [Erythranthe guttata]
c24765.graph_c0	0	0	0	7.39668177	10.7143767	8.46195809	5.852E-20	Inf	up	hypothetical protein CICLE_v10022284mg [Citrus clementina]
c24769.graph_c0	4.51302474	4.35903354	4.78197416	35.1580335	46.2093623	46.4707868	6.412E-22	2.20364	up	PREDICTED: inorganic phosphate transporter 1-4-like [Sesamum indicum]
c24783.graph_c0	0	0	0	3.83874592	4.06668583	4.21511055	9.474E-13	Inf	up	-
c24790.graph_c0	89.7149166	82.5351042	77.5364442	71.533812	66.2421097	70.7226175	8.549E-20	-1.27583	down	PREDICTED: cell number regulator 8-like [Sesamum indicum]
c24791.graph_c0	12.9341724	13.8588934	9.71504693	87.7741566	95.2973931	78.6982468	2.089E-24	1.82759	up	unnamed protein product [Coffea canephora]
c24796.graph_c0	0.15451505	0.30933955	0.39711592	6.76159385	7.76629628	8.29633418	1.64E-11	3.700808	up	hypothetical protein L484_021121 [Morus notabilis]
c24797.graph_c0	0.92113059	0.72447018	0.84549234	64.3121799	62.2232769	50.8063758	2.17E-54	5.138456	up	PREDICTED: probable pectinesterase/pectinesterase inhibitor 17 [Sesamum indicum]
c24800.graph_c0	0	0	0	2.69112637	3.4062965	2.51894016	4.138E-16	Inf	up	PREDICTED: uncharacterized protein LOC105163689 [Sesamum indicum]
c24813.graph_c0	4.26672518	4.14538009	3.22524164	14.1623891	17.7590483	17.1279007	0.0002918	1.0569	up	PREDICTED: polyadenylate-binding protein-interacting protein 6-like [Sesamum indicum]
c24821.graph_c0	0.32283847	0.21544116	0.41486022	6.24581696	4.83123085	5.64570451	1.888E-07	3.11488	up	PREDICTED: protein RADIALIS-like 4 [Sesamum indicum]

#ID	0 h-1 FPKM	0 h-2 FPKM	0 h-3 FPKM	48 h-1 FPKM	48 h-2 FPKM	48 h-3 FPKM	FDR	log2FC	regulated	nr_annotation
c24843.graph_c0	630.067796	663.918723	606.604714	155.016444	71.4585767	64.9933542	4.41E-104	-3.704736	down	PREDICTED: zinc finger CCCH domain-containing protein 29 [Sesamum indicum]
c24859.graph_c0	4.36843102	2.55553559	4.15552712	40.662964	39.0908749	45.761895	4.99E-34	2.482707	up	PREDICTED: O-acetyl-ADP-ribose deacetylase MACROD2 isoform X1 [Sesamum indicum]
c24860.graph_c0	13.7655464	8.03794282	6.63347706	0	0	0	3.444E-15	-Inf	down	40S ribosomal protein S26E [Uncinocarpus reesii 1704]
c24866.graph_c0	1.34178033	0.92985502	0.53053564	9.9841718	8.86454958	7.91086228	2.218E-08	2.249091	up	PREDICTED: acidic mammalian chitinase-like [Sesamum indicum]
c24873.graph_c0	3.37351285	3.14400189	3.88663921	18.0579711	14.0780333	16.4969865	1.198E-05	1.20909	up	PREDICTED: plastid division protein PDV2-like [Sesamum indicum]
c24876.graph_c0	2.36562097	2.03564034	3.25324409	0.87939263	0.04050478	0	2.218E-12	-4.025357	down	retrotransposon protein, putative, unclassified [Oryza sativa Japonica Group]
c24909.graph_c0	5.19017097	5.70534292	5.68755184	23.144923	28.5902412	31.0870825	4.987E-14	1.297736	up	PREDICTED: DEAD-box ATP-dependent RNA helicase 31-like [Sesamum indicum]
c24920.graph_c0	873.207101	776.991163	836.622975	29.5383047	20.6869282	25.1832986	8.7E-269	-6.055379	down	-
c24925.graph_c0	6.92784282	7.23305011	6.22221398	1.50981048	2.32648892	2.99216337	5.669E-12	-2.603106	down	PREDICTED: protein MKS1 [Sesamum indicum]
c24930.graph_c0	1.87441894	1.96782251	1.76246528	8.42359505	8.87915865	9.93122509	0.0001519	1.262258	up	PREDICTED: potassium transporter 8-like [Sesamum indicum]
c24937.graph_c0	0	0	0	5.00392955	4.44353274	5.7947541	2.131E-22	Inf	up	PREDICTED: putative cyclin-A3-1 [Sesamum indicum]
c24966.graph_c0	14.1134348	12.9624071	11.779261	6.56813008	6.24812337	7.39480661	5.162E-08	-1.959146	down	hypothetical protein MIMGU_mgv1a019945mg [Erythranthe guttata]
c24971.graph_c0	5.89281907	7.23657382	7.3383046	3.30208359	2.43092277	2.34057938	4.342E-12	-2.354505	down	PREDICTED: zinc transporter 8-like [Sesamum indicum]
c24979.graph_c0	27.8598697	28.1644315	29.6210949	136.988939	128.129193	134.851448	1.532E-17	1.206294	up	PREDICTED: adrenodoxin-like protein, mitochondrial [Sesamum indicum]
c24986.graph_c0	0.12156351	0	0	20.3825861	21.948805	10.1643014	2.27E-11	7.761724	up	-
c24998.graph_c0	20.2942048	20.4249314	17.4331488	5.28374972	3.33696647	3.43567407	1.235E-28	-3.276631	down	PREDICTED: uncharacterized protein LOC105177785 [Sesamum indicum]
c24998.graph_c1	14.3980554	14.0898056	16.2928687	3.46458933	2.51678557	2.7304541	1.184E-23	-3.374961	down	PREDICTED: uncharacterized protein LOC105177785 [Sesamum indicum]
c24999.graph_c0	22.369013	21.9608081	22.9407748	2.67505015	2.51901446	1.67498619	8.241E-47	-4.305379	down	hypothetical protein MIMGU_mgv1a011964mg [Erythranthe guttata]
c25013.graph_c0	12.5422114	15.5491554	10.7448254	6.77344669	5.48725828	5.49518047	1.082E-14	-2.139896	down	PREDICTED: probable LRR receptor-like serine/threonine-protein kinase RLK [Sesamum indicum]
c25017.graph_c1	5.1605565	5.32226111	6.93295212	7.20339583	4.42605217	4.23029527	0.0020989	-1.145221	down	PREDICTED: ethylene-responsive transcription factor ERF014 [Sesamum indicum]
c25019.graph_c0	1.00467236	1.08017308	1.91265742	17.825787	19.2111028	25.2255326	1.396E-20	2.933017	up	PREDICTED: 4-diphosphocytidyl-2-C-methyl-D-erythritol kinase, chloroplastic [Sesamum indicum]
c25041.graph_c0	3.58343813	4.52481108	3.03980946	2.91290906	2.94872399	3.18706347	2.863E-07	-1.316683	down	PREDICTED: TATA box-binding protein-associated factor RNA polymerase I subunit B isoform X1 [Sesamum indicum]

#ID	0 h-1 FPKM	0 h-2 FPKM	0 h-3 FPKM	48 h-1 FPKM	48 h-2 FPKM	48 h-3 FPKM	FDR	log2FC	regulated	nr_annotation
c25044.graph_c0	28.3636885	27.3958768	27.3541094	20.0690235	20.5592019	17.9479426	2.652E-25	-1.521372	down	PREDICTED: zinc finger Ran-binding domain-containing protein 2-like isoform X1 [Sesamum indicum]
c25045.graph_c0	1.86562029	1.7806285	2.28588914	0.11991147	0.3387511	0.10669651	4.256E-16	-4.42425	down	-
c25047.graph_c0	148.446321	148.429078	136.401223	23.0920603	26.848909	30.7358589	1.47E-117	-3.445402	down	hypothetical protein MIMGU_mgv1a010693mg [Erythranthe guttata]
c25052.graph_c0	0.73141491	1.46429462	1.35762916	0.26204388	0.07931535	0.19985577	6.936E-08	-3.721293	down	-
c25062.graph_c0	1.14013102	1.14127269	0.45784797	9.97844906	10.6405026	12.0330617	3.159E-09	2.563927	up	hypothetical protein MIMGU_mgv1a015838mg [Erythranthe guttata]
c25075.graph_c0	4.25003957	4.2004435	2.41963821	0.59474638	1.89018501	1.85220613	3.887E-07	-2.354773	down	-
c25081.graph_c0	0.32712571	0.53211157	0.63055423	3.08903779	2.87338037	2.54748965	0.001725	1.493916	up	PREDICTED: putative RING-H2 finger protein ATL21A [Sesamum indicum]
c25082.graph_c0	2.10830795	1.48688618	1.72407403	22.8221519	28.7134696	26.3166326	8.772E-31	2.851341	up	PREDICTED: delta(7)-sterol-C5(6)-desaturase-like [Solanum tuberosum]
c25091.graph_c0	62.6735645	34.4773403	37.7854548	0	0	0	6.918E-31	-Inf	down	-
c25102.graph_c0	0	0.07307332	0	19.906759	19.3788948	24.5946211	3.612E-42	8.76467	up	PREDICTED: ubiquitin-conjugating enzyme E2 20-like [Sesamum indicum]
c25105.graph_c0	0	0	0	6.02430569	14.5589863	19.933954	4.551E-08	Inf	up	PREDICTED: protein IWS1 homolog [Sesamum indicum]
c25108.graph_c0	28.4828901	28.7615115	25.0432284	10.4500829	12.7287465	16.6716181	2.063E-34	-2.067726	down	PREDICTED: LOW QUALITY PROTEIN: probable carboxylesterase 13 [Sesamum indicum]
c25122.graph_c0	7.11268892	10.0122345	8.56883143	368.89501	516.402047	550.711684	1.237E-42	4.779994	up	PREDICTED: pEARLI1-like lipid transfer protein 1 [Sesamum indicum]
c25137.graph_c0	6.01441083	9.73893631	7.04680698	4.4001007	4.1434428	3.91518314	2.683E-05	-1.887258	down	PREDICTED: PHD finger protein ALFIN-LIKE 4-like [Sesamum indicum]
c25141.graph_c0	29.3142446	27.9363566	29.4401147	3.35778613	1.2196002	1.15241319	4.26E-126	-4.915138	down	hypothetical protein MIMGU_mgv1a024254mg, partial [Erythranthe guttata]
c25142.graph_c0	2.17601813	1.98600322	2.38505336	39.9758737	44.6607043	33.2618955	1.472E-30	3.15169	up	uncharacterized protein LOC100499662 [Glycine max]
c25145.graph_c0	14.5828492	14.7371403	12.9562839	92.1142814	98.7756203	104.583991	2.192E-35	1.787231	up	PREDICTED: protein STRICTOSIDINE SYNTHASE-LIKE 11 [Sesamum indicum]
c25153.graph_c0	0.3743785	0.67455608	0.38487295	3.31105815	2.92305385	1.90272809	0.0074292	1.495088	up	hypothetical protein PRUPE_ppa015674mg [Prunus persica]
c25166.graph_c0	0	0	0	16.0258569	51.0272026	56.2422126	2.592E-10	Inf	up	hypothetical protein MIMGU_mgv1a015417mg [Erythranthe guttata]
c25175.graph_c0	27.3722234	29.8866101	26.4220775	28.8477053	25.2093973	24.158602	2.404E-15	-1.111114	down	PREDICTED: WD repeat-containing protein 70 [Nicotiana sylvestris]
c25182.graph_c0	63.8314566	57.7060787	53.7491973	40.0414889	30.7050405	24.5972031	5.797E-22	-1.887266	down	-
c25187.graph_c0	2.79521788	3.37691691	3.03459166	3.32991857	2.96322176	1.97529435	0.0020709	-1.167251	down	hypothetical protein MIMGU_mgv1a002261mg [Erythranthe guttata]

#ID	0 h-1 FPKM	0 h-2 FPKM	0 h-3 FPKM	48 h-1 FPKM	48 h-2 FPKM	48 h-3 FPKM	FDR	log2FC	regulated	nr_annotation
c25187.graph_c1	3.43660287	4.49851922	2.24205592	3.55568705	2.734814	1.56024442	0.0032095	-1.377238	down	hypothetical protein MIMGU_mgv1a002261mg [Erythranthe guttata]
c25189.graph_c0	16.8727294	17.1730079	14.6972796	10.901846	14.9752576	17.1265382	7.335E-10	-1.203308	down	hypothetical protein MIMGU_mgv1a017391mg [Erythranthe guttata]
c25203.graph_c0	2.31816883	2.17078108	0.67266358	129.792305	173.552611	176.236298	1.537E-75	5.525102	up	hypothetical protein MIMGU_mgv1a012619mg [Erythranthe guttata]
c25206.graph_c0	1.41362179	1.50347715	1.19211748	0.52906774	0.17245634	0.18106204	5.812E-10	-3.211337	down	-
c25208.graph_c0	0	0	0	4.38002895	7.15350224	5.52120818	5.255E-14	Inf	up	unnamed protein product [Coffea canephora]
c25210.graph_c0	1.86423018	1.73280429	4.19231679	19.4439925	19.1040201	22.1039898	5.967E-12	1.932776	up	PREDICTED: 60S acidic ribosomal protein P1-like [Tarenaya hassleriana]
c25211.graph_c0	9.3878586	7.5745149	9.87981162	39.4783682	41.3091418	41.6290428	6.095E-13	1.169492	up	PREDICTED: 2-methyl-6-phytyl-1,4-hydroquinone methyltransferase, chloroplastic isoform X1 [Sesamum indicum]
c25217.graph_c0	1.51844679	1.29478695	1.27675315	7.01173139	8.34286727	12.262855	2.376E-05	1.733177	up	PREDICTED: importin subunit beta-1-like [Sesamum indicum]
c25218.graph_c0	14.7094792	16.2761174	11.7592486	12.7500961	10.9607451	7.43931729	1.102E-09	-1.465268	down	PREDICTED: limonoid UDP-glucosyltransferase-like [Sesamum indicum]
c25220.graph_c0	3.4938305	3.49732904	2.87927088	12.2802102	13.5653506	15.0361026	3.142E-05	1.032233	up	PREDICTED: probable RNA 3'-terminal phosphate cyclase-like protein [Sesamum indicum]
c25222.graph_c0	0	0	0	14.6618853	16.5566098	21.4328085	2.338E-45	Inf	up	PREDICTED: fructose-1,6-bisphosphatase, chloroplastic [Sesamum indicum]
c25224.graph_c0	0.30007715	0.15018882	0.38561102	91.781613	86.6883163	89.7842033	4.16E-185	7.304082	up	PREDICTED: alpha carbonic anhydrase 1, chloroplastic [Sesamum indicum]
c25226.graph_c0	1.53320517	1.98947836	1.82428643	14.8049732	18.6767947	23.041771	4.331E-13	2.378056	up	PREDICTED: uncharacterized protein LOC105161424 [Sesamum indicum]
c25229.graph_c0	5.18272245	3.2709317	3.78604852	0	0	0	8.227E-46	-Inf	down	--
c25231.graph_c0	0	0	0	12.7488536	10.0191873	7.20351512	1.218E-18	Inf	up	PREDICTED: ectonucleotide pyrophosphatase/phosphodiesterase family member 3 [Eucalyptus grandis]
c25236.graph_c0	2.34768629	2.64379179	2.01124656	7.38970928	12.2837454	10.6113886	0.000239	1.089119	up	PREDICTED: uncharacterized protein LOC105167293 [Sesamum indicum]
c25237.graph_c0	0	0	0	2.44300934	2.77978121	2.09326483	1.728E-11	Inf	up	PREDICTED: uncharacterized protein LOC105159159 [Sesamum indicum]
c25241.graph_c0	0	0	0	1.08290503	1.78454356	1.98065601	5.352E-13	Inf	up	PREDICTED: organic cation/carnitine transporter 7-like isoform X2 [Sesamum indicum]
c25243.graph_c0	3.0344506	2.87180792	2.22147019	0	0.03589732	0.01507545	2.274E-58	-8.363178	down	hypothetical protein MIMGU_mgv1a019304mg, partial [Erythranthe guttata]
c25252.graph_c0	103.496319	104.193991	106.68728	20.0098662	19.1129751	21.1369513	2.91E-114	-3.400867	down	hypothetical protein MIMGU_mgv1a004537mg [Erythranthe guttata]

#ID	0 h-1 FPKM	0 h-2 FPKM	0 h-3 FPKM	48 h-1 FPKM	48 h-2 FPKM	48 h-3 FPKM	FDR	log2FC	regulated	nr_annotation
c25257.graph_c0	22.0544113	11.7446956	14.5104853	0	0	0	7.086E-29	-Inf	down	secreted protein [Achlya hypogyna]
c25258.graph_c0	0	0.15909452	0.13615882	3.61173342	7.18703936	9.55423932	1.968E-07	5.067917	up	PREDICTED: uncharacterized protein At4g06744-like [Nicotiana tomentosiformis]
c25262.graph_c0	4.35303401	2.31486498	2.85517949	0	0	0	9.453E-20	-Inf	down	--
c25282.graph_c0	0.8141608	0.67914672	0.87185739	5.62543526	8.21082056	13.6260247	0.000585	2.508454	up	unnamed protein product [Coffea canephora]
c25287.graph_c0	0	0	0	3.88581469	12.6891577	23.4544748	7.558E-06	Inf	up	PREDICTED: GDSL esterase/lipase At3g48460 [Sesamum indicum]
c25288.graph_c0	12.0281435	10.3785908	10.2387938	6.82276434	8.00052819	7.95491303	1.083E-18	-1.538402	down	PREDICTED: uncharacterized aarF domain-containing protein kinase At5g05200, chloroplastic [Sesamum indicum]
c25291.graph_c0	0.09626664	0.03212101	0.08247096	29.4441187	41.8718243	44.2966389	2.149E-57	8.073235	up	PREDICTED: purple acid phosphatase 22-like [Sesamum indicum]
c25305.graph_c1	0	0	0	1.65501548	2.95290648	2.71272625	1.485E-11	Inf	up	hypothetical protein MIMGU_mgv1a002668mg [Erythranthe guttata]
c25311.graph_c0	23.2037019	12.4851816	14.5117501	0	0	0	1.016E-26	-Inf	down	putative Trypsin [Glarea lozoyensis 74030]
c25312.graph_c0	0.40427861	0.32374675	0.41561122	23.5759473	23.9105365	18.9561849	2.348E-49	4.843293	up	PREDICTED: uncharacterized protein LOC105158282 [Sesamum indicum]
c25316.graph_c0	5.95631368	4.25877002	4.81114788	1.56781276	2.15917977	2.79006073	7.974E-06	-2.228977	down	-
c25324.graph_c0	0.85452749	0.85538317	1.2478428	4.75926272	5.11767923	6.78198942	9.759E-05	1.468374	up	PREDICTED: protein BASIC PENTACYSTEINE4 isoform X2 [Sesamum indicum]
c25327.graph_c0	2.36744097	3.29914949	2.74399505	19.0734177	22.0861255	21.7279742	1.753E-25	1.882245	up	PREDICTED: LOW QUALITY PROTEIN: microtubule-associated protein TORTIFOLIA1 [Sesamum indicum]
c25329.graph_c0	0.94418337	1.03514109	0.80887534	15.118705	14.1295671	17.691002	1.602E-22	3.057409	up	PREDICTED: probable plastid-lipid-associated protein 13, chloroplastic [Sesamum indicum]
c25330.graph_c0	2.95918604	2.21347414	2.50725575	15.068969	14.1229955	15.0876449	5.59E-11	1.511602	up	PREDICTED: probable aspartyl aminopeptidase [Sesamum indicum]
c25337.graph_c0	4.66134813	3.70602209	3.98379115	27.5330758	30.6916544	30.276086	2.523E-29	1.822559	up	PREDICTED: peroxisomal fatty acid beta-oxidation multifunctional protein AIM1 [Sesamum indicum]
c25340.graph_c0	1.87493837	2.27899065	1.37678288	13.6951444	12.7438165	13.5032471	3.286E-07	1.839717	up	PREDICTED: thylakoid lumenal 15 kDa protein 1, chloroplastic [Sesamum indicum]
c25345.graph_c0	1.99657925	2.15231533	1.9736026	10.1671171	11.5631152	11.0610007	1.127E-10	1.402	up	hypothetical protein MIMGU_mgv1a003320mg [Erythranthe guttata]
c25352.graph_c0	0.67709229	0.79737682	0.61418146	12.0720083	18.7362175	20.4057758	1.001E-15	3.591783	up	PREDICTED: transcription factor bHLH62 isoform X2 [Sesamum indicum]
c25353.graph_c0	3544.28683	3972.98757	3769.0428	67.6043251	69.5688948	61.6392039	9.92E-163	-6.844294	down	hypothetical protein MIMGU_mgv1a005193mg [Erythranthe guttata]
c25360.graph_c0	0.20923553	0.38398259	0.26887594	3.14843651	3.57362863	5.86019668	2.983E-06	2.843869	up	PREDICTED: subtilisin-like protease [Sesamum indicum]
c25364.graph_c0	4.27758307	4.0677731	3.02327498	16.7483512	17.9515867	22.0908866	0.0044417	1.30366	up	-

#ID	0 h-1 FPKM	0 h-2 FPKM	0 h-3 FPKM	48 h-1 FPKM	48 h-2 FPKM	48 h-3 FPKM	FDR	log2FC	regulated	nr_annotation
c25364.graph_c1	1.68504415	1.40560956	0.36089141	7.50313414	11.5118536	9.43882722	0.0005657	2.031061	up	leucine-rich repeat protein [Dasypyrum villosum]
c25365.graph_c0	11.9350735	14.7216069	10.518026	5.61785203	6.26969147	4.89180623	1.185E-17	-2.163273	down	PREDICTED: uncharacterized protein LOC105168447 isoform X2 [Sesamum indicum]
c25367.graph_c0	0.49928036	0.58307703	0.85546004	5.59629721	6.33466669	6.82130357	7.247E-06	2.248733	up	hypothetical protein MIMGU_mgv1a014003mg [Erythranthe guttata]
c25379.graph_c0	3.26135044	4.64579996	4.02977349	23.4587483	28.4631531	29.6649814	4.932E-13	1.750813	up	PREDICTED: CBL-interacting protein kinase 23-like [Malus domestica]
c25389.graph_c0	105.964818	101.507201	100.604851	12.3380705	10.9501788	13.5185422	6.01E-157	-4.081688	down	PREDICTED: vacuolar cation/proton exchanger 3-like [Sesamum indicum]
c25391.graph_c0	6.69734689	3.86335273	4.23024274	0	0	0	2.409E-19	-Inf	down	PREDICTED: 60S ribosomal protein L9-like [Phoenix dactylifera]
c25397.graph_c0	26.4134521	24.0362738	18.5139991	15.9275748	15.467223	10.0386844	2.385E-12	-1.745403	down	PREDICTED: U3 small nucleolar ribonucleoprotein protein MPP10 [Sesamum indicum]
c25411.graph_c0	114.718066	124.119637	134.439611	64.6540789	23.3544959	12.5721498	1.27E-28	-2.884587	down	PREDICTED: basic 7S globulin [Sesamum indicum]
c25413.graph_c0	21.1148988	19.5792799	22.2140963	17.6339236	15.4702063	14.9550642	3.987E-14	-1.403841	down	PREDICTED: protein LTV1 homolog isoform X2 [Sesamum indicum]
c25414.graph_c0	8.31181414	8.19407448	6.79700652	2.32041979	2.94984409	2.68410425	1.535E-09	-2.569863	down	PREDICTED: protein SOMBRERO-like [Sesamum indicum]
c25418.graph_c0	0.37335694	0.2802981	0.9595569	4.47148997	8.65413722	9.33465376	7.17E-06	2.757827	up	-
c25429.graph_c0	2.31751076	2.3198314	3.37517216	19.2153787	16.8882425	18.6175265	2.828E-05	1.751777	up	PREDICTED: alpha-soluble NSF attachment protein-like [Sesamum indicum]
c25434.graph_c0	0	0	0	9.53626104	12.4211216	17.360105	2.803E-24	Inf	up	hypothetical protein MIMGU_mgv1a005366mg [Erythranthe guttata]
c25448.graph_c0	3.72238792	2.78248872	3.35460332	34.0703832	39.0186597	41.9663318	5.365E-54	2.524269	up	PREDICTED: putative invertase inhibitor [Sesamum indicum]
c25449.graph_c0	0.02876356	0.37430067	0.18481155	5.40576845	5.83904553	7.21503039	6.416E-20	3.950358	up	PREDICTED: F-box/LRR-repeat protein 17-like [Sesamum indicum]
c25457.graph_c0	3.1410753	3.98267945	3.5366606	57.4894338	66.2751683	70.5755032	4.016E-86	3.167387	up	PREDICTED: biotin carboxylase 2, chloroplastic [Sesamum indicum]
c25460.graph_c0	0.2311598	0.23139128	0.198033	3.9752438	3.083265	4.80041518	2.026E-07	3.15133	up	-
c25462.graph_c0	13.5460463	13.3702305	11.8155056	3.72990683	3.36052285	2.76053768	1.438E-45	-2.987585	down	PREDICTED: uncharacterized protein LOC105160924 [Sesamum indicum]
c25464.graph_c0	0.13398266	0.10058762	0.30130279	67.394726	60.0201684	56.0684412	1.08E-100	7.394041	up	PREDICTED: alanine--glyoxylate aminotransferase 2 homolog 3, mitochondrial-like [Sesamum indicum]
c25466.graph_c0	0.07882468	0.23671083	0	4.57496147	6.6929472	8.07693096	5.983E-13	4.926617	up	PREDICTED: uncharacterized protein LOC105177131 [Sesamum indicum]
c25476.graph_c0	30.0340494	26.6689042	32.5491046	106.242145	127.340298	151.083384	5.309E-08	1.084235	up	PREDICTED: 29 kDa ribonucleoprotein A, chloroplastic [Sesamum indicum]
c25483.graph_c0	1.05602619	1.31334634	0.74020116	0.02948122	0	0.02623221	1.973E-19	-6.795955	down	hypothetical protein VITISV_009673 [Vitis vinifera]

#ID	0 h-1 FPKM	0 h-2 FPKM	0 h-3 FPKM	48 h-1 FPKM	48 h-2 FPKM	48 h-3 FPKM	FDR	log2FC	regulated	nr_annotation
c25484.graph_c0	23.2951542	26.6633448	22.5740818	9.85095793	14.6288891	11.4262248	6.641E-20	-2.036628	down	hypothetical protein MIMGU_mgv1a012871mg [Erythranthe guttata]
c25486.graph_c0	0	0	0	1.08768797	2.56473782	3.65273325	2.134E-06	Inf	up	PREDICTED: 1-aminocyclopropane-1-carboxylate synthase 7 [Sesamum indicum]
c25495.graph_c0	29.027103	33.7022065	30.579227	29.6601667	24.9860851	23.9455496	1.611E-17	-1.261241	down	PREDICTED: uncharacterized protein LOC105164353 [Sesamum indicum]
c25502.graph_c0	7.84809105	6.90371339	8.12920804	3.81227323	5.01348566	3.23617744	2.312E-12	-1.944355	down	PREDICTED: chaperone protein dnaJ 10 [Sesamum indicum]
c25506.graph_c0	13.3334905	15.1599978	12.672741	2.13226163	2.45529635	1.52606787	2.921E-51	-3.767268	down	PREDICTED: serine acetyltransferase 1, chloroplastic-like [Sesamum indicum]
c25512.graph_c0	1.26174657	0.84200668	0.54046477	18.2109841	13.9561532	14.8249582	8.987E-07	3.146039	up	-
c25523.graph_c0	3.1296977	4.20084239	4.47880652	2.2935195	2.01318481	2.91537122	5.577E-05	-1.730194	down	PREDICTED: uncharacterized protein LOC105168847 isoform X1 [Sesamum indicum]
c25527.graph_c0	0	0	0	10.6332468	13.0643112	13.0444141	8.027E-29	Inf	up	Early nodulin 20 precursor, putative [Ricinus communis]
c25529.graph_c0	4.67367251	4.8516248	5.00487851	0	0	0	2.002E-29	-Inf	down	-
c25539.graph_c0	1.18988269	1.48884272	0.99388013	10.3590668	15.9096507	20.3856506	2.671E-07	2.643235	up	PREDICTED: E3 ubiquitin-protein ligase ATL6-like [Sesamum indicum]
c25559.graph_c0	27.4320229	25.4712313	23.5928034	11.9189681	10.9501269	9.94533734	7.091E-37	-2.234691	down	PREDICTED: dipeptidyl peptidase 9 [Sesamum indicum]
c25564.graph_c0	0	0	0	219.328856	248.154066	310.619451	1.59E-115	Inf	up	PREDICTED: low-temperature-induced cysteine proteinase [Sesamum indicum]
c25565.graph_c0	0.08525011	0.38400965	0.21909954	10.2884733	13.2290305	19.4972477	3.153E-13	4.939399	up	PREDICTED: glucan endo-1,3-beta-glucosidase 12 [Sesamum indicum]
c25568.graph_c0	4.28856398	4.68760393	6.01773079	35.9214454	32.3775243	32.8920689	2.323E-19	1.735671	up	PREDICTED: uncharacterized membrane protein At4g09580 [Sesamum indicum]
c25571.graph_c0	50.8473951	48.2284602	45.1278551	14.9635979	16.6864395	15.220598	9.42E-72	-2.639088	down	PREDICTED: leucine-rich repeat extensin-like protein 5 [Nicotiana tomentosiformis]
c25584.graph_c0	5.09613289	5.14637958	4.34650389	3.15762515	3.38913571	3.32720037	6.869E-07	-1.580088	down	PREDICTED: uncharacterized protein At5g19025-like [Sesamum indicum]
c25595.graph_c0	0	0	0	3.67183619	3.63395303	3.8690263	1.289E-15	Inf	up	-
c25603.graph_c0	9.74293119	6.38969165	6.23604138	0	0	0	6.356E-37	-Inf	down	-
c25604.graph_c0	2.5255257	3.63002717	3.32861592	16.5850452	17.8226479	14.2264363	7.605E-06	1.339782	up	PREDICTED: anthocyanidin 3-O-glucoside 2'''-O-xylosyltransferase-like isoform X1 [Sesamum indicum]
c25609.graph_c0	2.64255414	2.36675813	2.94897266	37.926842	40.24676	38.0224656	5.997E-32	2.848164	up	PREDICTED: histidine-containing phosphotransfer protein 1 [Sesamum indicum]
c25616.graph_c0	3.780455	3.37513347	3.15115742	1.88259553	2.19381986	2.17765884	0.0002206	-1.739429	down	PREDICTED: mediator-associated protein 2-like isoform X2 [Sesamum indicum]

#ID	0 h-1 FPKM	0 h-2 FPKM	0 h-3 FPKM	48 h-1 FPKM	48 h-2 FPKM	48 h-3 FPKM	FDR	log2FC	regulated	nr_annotation
c25625.graph_c0	10.4362608	9.55972623	10.9438889	1.76871699	2.64245552	2.2598064	6.038E-32	-3.238081	down	PREDICTED: LOW QUALITY PROTEIN: benzyl alcohol O-benzoyltransferase-like [Sesamum indicum]
c25626.graph_c0	0.30293634	0.30323968	0.06488086	16.0938852	7.98272316	9.56062415	4.081E-07	4.654641	up	PREDICTED: serine/threonine-protein kinase WAG1 [Sesamum indicum]
c25628.graph_c0	0.51863669	0.43718402	0.66646868	6.31201519	7.67252048	6.84707692	9.743E-15	2.658806	up	PREDICTED: uncharacterized protein At5g08430 [Sesamum indicum]
c25645.graph_c0	0	0.0623051	0	4.75939417	5.46723001	3.41851784	1.676E-14	6.769578	up	-
c25649.graph_c0	10.8224193	6.1270056	12.0833373	94.8814738	104.757669	103.69509	1.94E-42	2.362622	up	PREDICTED: V-type proton ATPase subunit F [Sesamum indicum]
c25651.graph_c0	7.02898925	5.73159561	6.54604664	32.8871308	40.42819	41.9195672	8.71E-20	1.555917	up	PREDICTED: probable prolyl 4-hydroxylase 10 [Nicotiana tomentosiformis]
c25652.graph_c0	5.05442305	4.17957398	5.50674382	2.63191454	3.86057675	4.68372347	0.0015361	-1.426625	down	-
c25675.graph_c0	11.2946547	13.5940764	12.267852	9.78600516	13.1228935	14.6595101	0.002374	-1.007786	down	PREDICTED: chaperone protein dnaJ 6-like [Sesamum indicum]
c25683.graph_c0	0.15225949	0.30482391	0.13043965	9.02412505	29.1752338	38.3590801	5.291E-06	5.994378	up	PREDICTED: DNA-damage-repair/toleration protein DRT100-like [Sesamum indicum]
c25690.graph_c0	16.7306371	16.3517826	17.7752028	380.115159	387.25734	495.781361	2.469E-44	3.613917	up	lipid transfer protein 2 [Tamarix hispida]
c25692.graph_c0	0.17288249	0.46148163	0.22216085	2.97305177	3.20583049	4.01534473	4.625E-06	2.554557	up	PREDICTED: uncharacterized protein LOC105177785 [Sesamum indicum]
c25695.graph_c0	16.1641509	19.5279928	12.8927011	3.5944928	3.80792926	2.28454148	2.268E-13	-3.338409	down	PREDICTED: plasma membrane ATPase 4-like [Cicer arietinum]
c25697.graph_c0	0.62249915	1.63569652	0.19998401	7.0252135	7.44236185	8.73863269	5.65E-06	2.229562	up	hypothetical protein F383_00208 [Gossypium arboreum]
c25701.graph_c0	0	0	0	10.9134979	14.9126952	24.34728	3.57E-10	Inf	up	unnamed protein product [Coffea canephora]
c25703.graph_c0	5.61586181	4.26816472	5.34563316	1.14971071	2.33445992	0.59675331	3.736E-10	-2.924781	down	hypothetical protein MIMGU_mgv1a015966mg [Erythranthe guttata]
c25707.graph_c0	16.1342975	18.9433891	17.7713238	3.98607642	3.63078828	4.44177042	2.045E-58	-3.149471	down	PREDICTED: heterogeneous nuclear ribonucleoprotein 1-like [Sesamum indicum]
c25716.graph_c0	1.2244194	1.13809937	1.01148905	15.3087752	16.8152886	19.4287832	3.498E-13	2.914992	up	hypothetical protein MIMGU_mgv1a000915mg [Erythranthe guttata]
c25720.graph_c0	0.28448117	0.39867245	0.14622783	3.56432354	5.10866339	4.85055029	5.286E-08	3.010751	up	PREDICTED: transcription factor ICE1-like [Sesamum indicum]
c25720.graph_c1	0.17106692	0.34247643	0.32974164	2.67916602	2.50433959	3.57585872	4.321E-05	2.353834	up	PREDICTED: transcription factor ICE1-like, partial [Nicotiana sylvestris]
c25721.graph_c0	16.3504774	15.2447728	17.2359829	137.241817	138.977862	140.146145	2.193E-48	2.073395	up	PREDICTED: phospho-2-dehydro-3-deoxyheptonate aldolase 2, chloroplastic [Sesamum indicum]
c25727.graph_c0	0	0	0	1.99083678	4.1070977	3.4496358	9.572E-09	Inf	up	Homeobox-leucine zipper ATHB-13 -like protein [Gossypium arboreum]
c25731.graph_c0	1.6763807	1.38622293	2.4352002	156.722137	201.097447	229.429989	8.423E-59	5.710896	up	PREDICTED: major pollen allergen Lol p 11-like [Sesamum indicum]

#ID	0 h-1 FPKM	0 h-2 FPKM	0 h-3 FPKM	48 h-1 FPKM	48 h-2 FPKM	48 h-3 FPKM	FDR	log2FC	regulated	nr_annotation
c25741.graph_c0	0.66570715	0.49978031	0.32079752	4.67635794	4.14189745	3.81993	0.0001874	2.079969	up	PREDICTED: uncharacterized protein LOC105170714 [Sesamum indicum]
c25751.graph_c0	0.29458342	0.0737196	0.18927568	10.4484944	13.6564509	15.8773569	2.975E-22	5.142234	up	-
c25755.graph_c0	75.0770244	66.9483368	74.5678409	18.3523734	12.2024081	10.396169	3.08E-106	-3.411057	down	PREDICTED: zinc finger protein CONSTANS-LIKE 1-like [Sesamum indicum]
c25776.graph_c0	0	0	0	16.3861891	16.459741	11.0296816	4.379E-24	Inf	up	--
c25778.graph_c0	9.34441613	9.18370455	9.16970322	7.30433932	9.5343976	10.9560593	4.158E-08	-1.018106	down	PREDICTED: uncharacterized protein LOC105170433 [Sesamum indicum]
c25782.graph_c0	5.23884172	5.57479585	3.63898326	60.8295623	58.3612851	54.5513567	5.848E-48	2.575375	up	PREDICTED: uncharacterized protein LOC105165646 [Sesamum indicum]
c25784.graph_c0	0	0.04984908	0	53.9987401	61.7737504	81.0729311	3.24E-57	10.9369	up	PREDICTED: uncharacterized protein LOC105164223 [Sesamum indicum]
c25786.graph_c1	1.69570615	2.73470667	2.54222446	7.89776414	10.9410999	13.7458701	0.0006495	1.197301	up	PREDICTED: pentatricopeptide repeat-containing protein At1g59720, mitochondrial [Sesamum indicum]
c25787.graph_c0	0.2619825	0.61190461	0.44887717	8.44743773	7.15922878	7.01538068	9.532E-09	3.080595	up	PREDICTED: uncharacterized protein LOC105177716 [Sesamum indicum]
c25799.graph_c0	19.0708916	18.6908944	22.2867179	300.331803	304.286747	315.875799	1.277E-89	2.918423	up	PREDICTED: 60S ribosomal protein L11 [Sesamum indicum]
c25801.graph_c0	2.22893739	1.673377	2.27839881	9.47383701	11.4948705	10.5683484	1.172E-08	1.329431	up	hypothetical protein MIMGU_mgv1a003796mg [Erythranthe guttata]
c25802.graph_c0	45.4154571	42.6499905	42.1889659	31.1402208	30.9591779	25.1222656	6.881E-29	-1.593744	down	hypothetical protein MIMGU_mgv1a008591mg [Erythranthe guttata]
c25803.graph_c0	17.365853	16.4683348	11.6864367	7.24145189	7.58223741	7.41740272	1.88E-13	-2.047558	down	PREDICTED: uncharacterized protein LOC105171564 [Sesamum indicum]
c25804.graph_c0	8.46040903	7.84616902	8.63361574	172.964257	145.713445	169.30301	3.825E-51	3.274475	up	PREDICTED: photosystem II 10 kDa polypeptide, chloroplastic [Sesamum indicum]
c25813.graph_c0	0	0	0	23.3801582	18.7062364	11.3473768	3.395E-11	Inf	up	PREDICTED: root phototropism protein 2-like [Malus domestica]
c25815.graph_c0	3.17671004	2.62686651	3.99345473	61.7129286	62.8161896	64.5921191	1.222E-57	3.249467	up	hypothetical protein MIMGU_mgv1a012869mg [Erythranthe guttata]
c25818.graph_c0	0.43468377	0.34809523	0.44686869	525.402264	607.847917	599.482943	0	9.43937	up	PREDICTED: kirola-like isoform X2 [Sesamum indicum]
c25826.graph_c0	20.352421	20.2879142	17.4357778	21.3280996	18.7046337	16.1970119	3.678E-07	-1.058641	down	PREDICTED: uncharacterized protein LOC105155579 [Sesamum indicum]
c25832.graph_c0	0.25351165	0.03625222	0	1.5681313	3.07507047	2.34531468	3.869E-07	3.584567	up	unnamed protein product [Coffea canephora]
c25837.graph_c0	1.12303395	1.51329029	1.38763759	0.11937832	0.12646686	0.31866634	6.646E-12	-3.860666	down	PREDICTED: uncharacterized protein LOC104248035 [Nicotiana sylvestris]
c25838.graph_c0	24.6119354	26.6700761	21.2856826	12.3088081	8.92189294	7.10938995	9.982E-39	-2.363641	down	PREDICTED: myb-related protein 306-like [Sesamum indicum]
c25840.graph_c0	0.40941551	0.51228184	0.7891727	4.33757309	4.8948153	4.69857829	0.0001943	1.999513	up	-

#ID	0 h-1 FPKM	0 h-2 FPKM	0 h-3 FPKM	48 h-1 FPKM	48 h-2 FPKM	48 h-3 FPKM	FDR	log2FC	regulated	nr_annotation
c25843.graph_c0	0.27103202	0.13565171	0.08707172	1.24846189	1.68630733	1.49967974	0.000176	2.153751	up	PREDICTED: serine/threonine-protein kinase D6PKL1-like [Sesamum indicum]
c25846.graph_c0	0.17472218	0.1165981	0.07484164	4.61434356	3.58099141	3.10323238	8.425E-10	3.94351	up	PREDICTED: glucan endo-1,3-beta-glucosidase 5-like [Sesamum indicum]
c25849.graph_c0	0.43318055	0.28907621	0.33399249	24.2637098	23.9851909	25.472062	7.591E-88	5.108881	up	PREDICTED: cell division cycle 20.2, cofactor of APC complex-like [Sesamum indicum]
c25850.graph_c0	30.1776056	31.3102992	29.579921	0.50732808	0.80617887	0.67712621	1.5E-164	-6.539944	down	PREDICTED: vacuolar iron transporter 1 [Nicotiana sylvestris]
c25855.graph_c0	9.58700699	12.079826	6.77416562	166.740059	253.057471	300.054302	2.203E-14	3.639988	up	PREDICTED: luminal-binding protein 5 [Sesamum indicum]
c25856.graph_c0	0.63795113	0.77741385	0.89107877	3.16858923	3.65451105	4.27456152	0.0011196	1.242353	up	PREDICTED: bifunctional epoxide hydrolase 2-like [Sesamum indicum]
c25862.graph_c0	1.24426506	1.09723589	1.25630204	16.0754667	18.273285	20.5208086	3.87E-36	2.909529	up	PREDICTED: cell division protein FtsZ homolog 2-2, chloroplastic [Sesamum indicum]
c25863.graph_c0	2.09072745	2.65755047	2.64402203	2.32356669	3.01214416	1.985884	0.0042997	-1.035053	down	PREDICTED: protein MID1-COMPLEMENTING ACTIVITY 1 [Sesamum indicum]
c25866.graph_c0	2.3414135	2.61161614	2.40704724	10.2922713	16.0613155	14.1482891	2.01E-05	1.435707	up	PREDICTED: protein DA1-related 1-like [Sesamum indicum]
c25870.graph_c0	0.73219586	0.97723873	0.41817808	7.34506285	13.2598059	18.2062941	3.123E-05	3.166058	up	PREDICTED: transcription factor DIVARICATA-like [Sesamum indicum]
c25871.graph_c0	33.5445046	30.3021827	29.8062333	15.3386316	17.5669409	16.9345263	2.331E-35	-1.928834	down	hypothetical protein MIMGU_mgv1a005352mg [Erythranthe guttata]
c25878.graph_c0	1.558299	1.49746502	1.52188169	8.35523086	9.21635826	11.215521	6.068E-09	1.632076	up	hypothetical protein MIMGU_mgv1a004635mg [Erythranthe guttata]
c25880.graph_c0	7.72696086	9.0117668	9.06333032	2.01803991	3.68841063	4.22271332	5.97E-33	-2.407362	down	PREDICTED: uncharacterized protein LOC105170968 [Sesamum indicum]
c25882.graph_c0	37.6074378	40.7464205	36.596318	33.4741293	31.8305595	29.6830364	4.682E-20	-1.29047	down	PREDICTED: putative nuclear matrix constituent protein 1-like protein [Sesamum indicum]
c25883.graph_c0	11.3283925	12.1217869	12.5495111	8.63706542	8.5780533	9.28629406	0.0002746	-1.460677	down	hypothetical protein CICLE_v10005060mg [Citrus clementina]
c25884.graph_c0	0.77774645	1.02876551	1.0708232	0.02558967	0.02710915	0.04553907	2.358E-18	-5.897324	down	-
c25886.graph_c0	0	0	0	26.085761	25.0668745	30.9650549	4.318E-64	Inf	up	hypothetical protein MIMGU_mgv1a010584mg [Erythranthe guttata]
c25892.graph_c0	1.11969429	0.74721033	0.83933004	8.9399736	10.1993425	9.48449143	5.989E-07	2.385258	up	PREDICTED: 3-phosphoinositide-dependent protein kinase 2 [Nicotiana sylvestris]
c25894.graph_c0	75.0972479	76.0151868	76.0195032	35.471267	46.7253832	41.8221096	4.177E-39	-1.894841	down	PREDICTED: uncharacterized protein LOC105164821 isoform X1 [Sesamum indicum]
c25905.graph_c0	1.26373636	1.58125225	1.50795474	13.4718779	15.9897255	16.6118858	3.397E-16	2.38197	up	PREDICTED: uncharacterized protein LOC102616798 [Citrus sinensis]
c25908.graph_c0	0	0.018544	0.04761188	3.05496718	2.33235408	2.12603798	3.448E-19	5.796058	up	PREDICTED: putative receptor-like protein kinase At3g47110 [Sesamum indicum]

#ID	0 h-1 FPKM	0 h-2 FPKM	0 h-3 FPKM	48 h-1 FPKM	48 h-2 FPKM	48 h-3 FPKM	FDR	log2FC	regulated	nr_annotation
c25914.graph_c0	5.57398691	5.69828264	3.04799739	22.0700915	20.255458	20.5127878	0.0019669	1.123656	up	PREDICTED: uncharacterized protein LOC105157918 [Sesamum indicum]
c25915.graph_c0	339.022346	362.994381	337.218742	49.78607	54.9892654	43.921892	1.61E-126	-3.822465	down	MYB-related transcription factor [Salvia miltiorrhiza]
c25916.graph_c0	0.35866772	0.37897281	0.20484543	1.3400695	1.98360817	1.68240734	0.0036243	1.391246	up	PREDICTED: putative pentatricopeptide repeat-containing protein At3g25970 [Sesamum indicum]
c25918.graph_c0	173.437929	173.357225	168.502999	76.6721086	96.9737332	107.228918	3.829E-40	-1.897648	down	PREDICTED: probable calcium-binding protein CML49 [Sesamum indicum]
c25922.graph_c0	0	0	0	3.23186973	4.96199144	6.50157865	1.293E-13	Inf	up	hypothetical protein MIMGU_mgv1a027056mg [Erythranthe guttata]
c25924.graph_c0	27.2664905	32.9951641	30.9896242	282.1218	245.059989	218.447007	4.836E-16	2.01624	up	hypothetical protein MIMGU_mgv1a012865mg [Erythranthe guttata]
c25927.graph_c0	2.18595484	3.71984436	2.52813466	26.7841275	29.0145625	25.2658807	4.281E-07	2.247643	up	PREDICTED: V-type proton ATPase subunit H [Beta vulgaris subsp. vulgaris]
c25928.graph_c0	0	0.12266246	0	14.5630184	18.1784367	25.6148216	2.857E-18	7.881743	up	BnaA07g08260D [Brassica napus]
c25929.graph_c0	0.18163122	0.44443201	0.18153591	4.98273124	6.16493158	8.3874529	2.219E-13	3.576487	up	PREDICTED: leucine-rich repeat receptor-like protein kinase TDR [Sesamum indicum]
c25930.graph_c0	38.1017241	34.8856215	29.3272024	26.5958981	28.8097041	27.1826212	1.328E-16	-1.32507	down	-
c25931.graph_c0	0.37946311	0.25322873	0.43344456	2.56363192	3.82688964	4.87326821	1.949E-06	2.37206	up	PREDICTED: putative GATA transcription factor 22 [Nicotiana tomentosiformis]
c25936.graph_c0	90.9913325	99.3036651	89.1537202	2.58848683	2.95312546	2.48039079	5.68E-131	-6.140603	down	PREDICTED: heat shock 70 kDa protein-like [Sesamum indicum]
c25937.graph_c0	25.4693758	31.0309105	25.43523	9.92194123	9.8008846	12.168991	6.396E-19	-2.379421	down	PREDICTED: DEAD-box ATP-dependent RNA helicase 37-like [Vitis vinifera]
c25938.graph_c0	1.93217941	1.02078249	2.20704626	13.4492644	14.3002467	15.3548126	4.417E-11	2.039454	up	PREDICTED: ataxin-3 homolog [Sesamum indicum]
c25940.graph_c0	17.1041175	15.133554	17.1660226	10.6019639	14.3146507	13.687916	5.86E-14	-1.378998	down	PREDICTED: clavaminate synthase-like protein At3g21360 [Sesamum indicum]
c25945.graph_c0	0	0	0	9.2013087	1.90471729	14.1630346	4.74E-05	Inf	up	--
c25949.graph_c0	3.9174485	4.09643244	4.26997553	0.06444659	0.40964009	0.86016283	7.635E-27	-4.243954	down	PREDICTED: abscisic acid 8'-hydroxylase 4-like [Sesamum indicum]
c25951.graph_c0	11.5817472	13.5495928	12.6360138	9.24847627	10.4200302	11.315203	2.049E-16	-1.306229	down	PREDICTED: LOW QUALITY PROTEIN: pentatricopeptide repeat-containing protein At1g74750-like [Sesamum indicum]
c25952.graph_c0	0.25653754	0.237041	0.40573648	2.45429077	4.33337246	4.61027225	9.435E-08	2.630507	up	PREDICTED: probably inactive leucine-rich repeat receptor-like protein kinase At2g25790 isoform X1 [Sesamum indicum]
c25953.graph_c0	0.20442775	0.13642164	0.26269776	22.0976426	30.991347	37.202059	3.215E-27	6.195733	up	PREDICTED: probable pectate lyase 5 [Sesamum indicum]
c25953.graph_c1	0	0.09716702	0	9.21098613	11.1789797	13.2884657	1.383E-19	7.426555	up	PREDICTED: probable pectate lyase 5 [Sesamum indicum]
c25955.graph_c0	1.07686384	1.02404504	0.48433443	101.092667	122.019417	117.449133	8.43E-178	6.028086	up	PREDICTED: probable xyloglucan endotransglucosylase/hydrolase protein 7 [Sesamum indicum]

#ID	0 h-1 FPKM	0 h-2 FPKM	0 h-3 FPKM	48 h-1 FPKM	48 h-2 FPKM	48 h-3 FPKM	FDR	log2FC	regulated	nr_annotation
c25956.graph_c0	0	0	0.05689686	5.99616837	5.79045295	6.71456185	1.696E-22	7.294693	up	PREDICTED: protein POLYCHOME-like [Sesamum indicum]
c25958.graph_c0	2.64049313	2.40285198	2.36491451	45.8507205	49.9789362	67.3621111	1.555E-21	3.440765	up	-
c25959.graph_c0	8.09861645	6.99532001	7.72135432	41.276032	44.4918538	46.8994119	2.407E-15	1.52047	up	hypothetical protein MIMGU_mgv1a015224mg [Erythranthe guttata]
c25961.graph_c0	3.85559542	5.89074896	3.2596005	2.3368592	2.47561896	3.16057136	0.0001384	-1.723069	down	--
c25963.graph_c0	22.6491364	21.6619356	22.7524899	126.403439	117.859715	114.457837	1.244E-22	1.403232	up	PREDICTED: uncharacterized protein LOC105175919 isoform X1 [Sesamum indicum]
c25966.graph_c0	0.47030611	0.21969596	0.40290798	1.96418756	1.62181446	1.87623127	0.0082936	1.305816	up	PREDICTED: protein NRT1/ PTR FAMILY 6.2-like [Sesamum indicum]
c25968.graph_c0	0.52086309	0.78207698	0.7529959	16.0150955	13.2169991	14.0900217	2.015E-17	3.380888	up	PREDICTED: probable lipid phosphate phosphatase beta [Sesamum indicum]
c25969.graph_c0	59.5538359	65.0205875	55.0106683	274.946441	279.674238	293.464128	4.792E-17	1.222609	up	unnamed protein product [Coffea canephora]
c25975.graph_c0	0.77442366	0.65593772	1.76067664	4.00629381	4.94185634	5.22508284	0.0075713	1.118813	up	hypothetical protein MIMGU_mgv1a014840mg [Erythranthe guttata]
c25976.graph_c0	19.3563291	10.6376456	13.8186941	0	0	0	4.579E-30	-Inf	down	cytochrome c oxidase subunit I [Lingulodinium polyedrum]
c25977.graph_c0	0	0	0	2.07516035	1.51444008	1.55923728	1.156E-11	Inf	up	ATP synthase F1, gamma subunit [Mucor circinelloides f. circinelloides 1006PhL]
c25980.graph_c0	0.85810301	1.18618599	1.10269634	17.8059828	18.5841191	22.4758847	4.803E-32	3.204333	up	PREDICTED: thioredoxin-like protein CITRX1, chloroplastic [Sesamum indicum]
c25983.graph_c0	12.9533551	7.0480859	8.0810464	0	0	0	3.143E-24	-Inf	down	ats1, partial [Pyramimonas parkeae]
c25985.graph_c1	3.75394778	4.0395348	3.85917722	2.42086708	2.47302173	2.61566004	0.0002595	-1.652132	down	PREDICTED: plasma membrane ATPase 4 [Sesamum indicum]
c25987.graph_c0	0	0	0	7.16165563	6.51911901	8.11890722	2.903E-22	Inf	up	hypothetical protein MIMGU_mgv1a011925mg [Erythranthe guttata]
c25990.graph_c0	109.405698	118.806256	113.26768	3.45675385	2.56983266	3.56145123	6.76E-261	-6.16891	down	PREDICTED: espin-like [Sesamum indicum]
c25992.graph_c0	26.401189	29.9315132	31.1819514	17.4356778	20.960806	22.0310817	8.224E-20	-1.556866	down	hypothetical protein MIMGU_mgv1a027010mg [Erythranthe guttata]
c25994.graph_c0	1.67740909	1.4815489	1.33136114	0.63631592	0.28889981	0.16176859	2.835E-09	-3.040708	down	PREDICTED: pathogenesis-related genes transcriptional activator PTI6-like [Nicotiana tomentosiformis]
c25995.graph_c0	2.08489621	1.62320971	1.48843009	1.60061864	1.58261728	1.23432473	0.0069035	-1.24691	down	-
c25999.graph_c0	5.2469955	5.07599959	4.57048519	112.639008	169.058565	161.018983	1.651E-31	3.870561	up	PREDICTED: glutamate decarboxylase [Sesamum indicum]
c26000.graph_c0	2.08330899	1.25123707	0.96376843	14.970385	11.1421803	13.6621113	7.415E-08	2.203017	up	PREDICTED: uncharacterized protein LOC105169481 [Sesamum indicum]
c26003.graph_c0	0.27318352	0.41018561	0.35105164	4.0267917	8.59844972	7.55792235	5.521E-07	3.255882	up	PREDICTED: uncharacterized protein LOC105170399 [Sesamum indicum]
c26004.graph_c0	3.00873073	1.28369396	2.47192109	25.4009508	28.9791679	31.8201603	2.106E-30	2.650702	up	unnamed protein product [Vitis vinifera]
c26005.graph_c0	5.20278063	2.25036624	3.30161931	0	0	0	3.129E-13	-Inf	down	uncharacterized protein LOC100273290 [Zea mays]

#ID	0 h-1 FPKM	0 h-2 FPKM	0 h-3 FPKM	48 h-1 FPKM	48 h-2 FPKM	48 h-3 FPKM	FDR	log2FC	regulated	nr_annotation
c26007.graph_c0	0	0	0	2.07890971	2.59100324	2.36665762	1.493E-15	Inf	up	PREDICTED: mitotic checkpoint serine/threonine-protein kinase BUB1 [Sesamum indicum]
c26009.graph_c0	177.484823	173.577147	170.383878	737.147414	807.881738	836.743352	1.893E-16	1.17238	up	PREDICTED: 40S ribosomal protein S20-2-like [Glycine max]
c26010.graph_c0	24.0990123	22.6988992	22.8547491	16.5487807	18.5728974	22.0146123	3.446E-10	-1.306302	down	PREDICTED: uncharacterized protein LOC105158897 [Sesamum indicum]
c26011.graph_c0	38.663364	40.9215536	34.1911078	8.26646604	10.921289	10.9621791	2.963E-81	-2.937029	down	PREDICTED: LOW QUALITY PROTEIN: benzyl alcohol O-benzoyltransferase-like [Sesamum indicum]
c26012.graph_c0	5.10174751	7.76242133	4.72028223	28.5763804	32.2648686	28.7727614	0.0006222	1.332997	up	Os03g0113500 [Oryza sativa Japonica Group]
c26013.graph_c0	11.1937394	11.6105573	12.8873795	45.1692578	56.1066985	54.9724445	6.08E-12	1.107437	up	PREDICTED: COBW domain-containing protein 1 [Sesamum indicum]
c26016.graph_c0	0.94593453	0.69623657	1.21556332	6.38213795	6.51672412	6.43139842	5.36E-08	1.736095	up	PREDICTED: probable aminotransferase ACS10 [Sesamum indicum]
c26017.graph_c0	17.4103225	14.6648193	16.6432948	148.659009	147.746916	151.072993	6.18E-27	2.181672	up	PREDICTED: 60S ribosomal protein L34-like [Sesamum indicum]
c26026.graph_c0	0	0	0	4.05503985	4.66403643	5.97922013	3.906E-12	Inf	up	-
c26027.graph_c0	0.12256295	0.06134284	0.07874912	1.1291298	3.05024907	3.114548	0.0001541	3.765179	up	PREDICTED: probable xyloglucan endotransglucosylase/hydrolase protein 32 [Sesamum indicum]
c26029.graph_c0	42.8225006	37.366942	36.8778111	30.4659942	27.1329038	26.0057415	1.963E-22	-1.499341	down	PREDICTED: abscisic acid receptor PYL9-like [Sesamum indicum]
c26030.graph_c0	0	0	0	6.20351248	8.35175051	8.27977215	1.087E-13	Inf	up	PREDICTED: uncharacterized protein LOC105174742 [Sesamum indicum]
c26030.graph_c1	0	0	0	3.5391078	8.65212825	7.14598206	5.282E-08	Inf	up	-
c26034.graph_c0	1.68880236	1.37352592	1.28854312	0.14585952	0.2060273	0.12978492	4.54E-13	-4.194012	down	-
c26035.graph_c0	0.09157801	0	0.11768136	306.338876	333.675169	335.887365	0	11.15897	up	PREDICTED: polyphenol oxidase I, chloroplastic-like [Sesamum indicum]
c26038.graph_c0	0.58814789	0.5352153	0.27483394	2.39723239	2.66133758	3.63786095	2.879E-05	1.621978	up	PREDICTED: DNA mismatch repair protein MSH2-like isoform X1 [Sesamum indicum]
c26039.graph_c0	0.398227	0	0.12793436	126.571076	81.9094889	66.5939862	3.008E-16	8.031451	up	PREDICTED: uncharacterized protein LOC105177716 [Sesamum indicum]
c26040.graph_c0	2.16534794	1.89915706	2.43805067	1.63388514	2.19381915	2.28216114	0.000522	-1.114814	down	PREDICTED: pentatricopeptide repeat-containing protein At1g06710, mitochondrial [Sesamum indicum]
c26045.graph_c0	0.11283371	0.02823667	0.07249789	6.9126601	7.48830845	9.59624063	6.343E-35	5.792748	up	PREDICTED: uncharacterized protein LOC105165819 [Sesamum indicum]
c26048.graph_c0	1.43467578	2.20203899	1.78216134	48.9475826	45.7398272	48.9239193	1.371E-71	3.710948	up	PREDICTED: uncharacterized protein LOC105164113 [Sesamum indicum]
c26049.graph_c0	1.23404064	1.28278697	0.97587143	22.4314918	24.8751858	25.8733047	7.964E-36	3.371947	up	PREDICTED: LOW QUALITY PROTEIN: plasma membrane ATPase 3 [Sesamum indicum]
c26053.graph_c0	7.23232055	8.49084506	7.74484854	1.97418827	2.30926862	2.41535367	7.94E-23	-2.829509	down	-

#ID	0 h-1 FPKM	0 h-2 FPKM	0 h-3 FPKM	48 h-1 FPKM	48 h-2 FPKM	48 h-3 FPKM	FDR	log2FC	regulated	nr_annotation
c26054.graph_c0	1.75640471	2.05119073	2.91535572	0.13484288	0.42854905	0.23996477	3.246E-11	-4.103434	down	PREDICTED: solute carrier family 40 member 2 isoform X2 [Sesamum indicum]
c26056.graph_c0	10.7537969	12.8011046	9.33719918	6.96174534	9.07707696	7.38577282	0.000777	-1.508572	down	PREDICTED: ubiquitin-activating enzyme E1 1-like isoform X1 [Sesamum indicum]
c26057.graph_c0	120.065047	116.530154	120.726648	43.6175209	45.2410436	46.0654011	4.703E-63	-2.42351	down	hypothetical protein MIMGU_mgv1a015990mg [Erythranthe guttata]
c26058.graph_c0	6.13329594	5.28936155	7.33588386	26.6003082	32.6001621	35.7739129	1.34E-12	1.31549	up	hypothetical protein MIMGU_mgv1a007272mg [Erythranthe guttata]
c26061.graph_c0	1.34106015	1.94416989	1.78274005	5.92174548	9.70341603	10.7657117	0.0003237	1.352365	up	PREDICTED: uncharacterized protein LOC105157642 [Sesamum indicum]
c26065.graph_c0	143.336503	154.010305	146.326241	98.4862748	118.803114	136.176636	1.549E-18	-1.349777	down	PREDICTED: DEAD-box ATP-dependent RNA helicase 3, chloroplastic [Sesamum indicum]
c26067.graph_c0	1.53308309	0.86739292	1.3704852	6.69343475	6.18012694	8.08674442	0.0006516	1.455908	up	PREDICTED: probable glutathione peroxidase 2 [Sesamum indicum]
c26068.graph_c0	3.91822867	2.85247432	2.68101608	0.89071206	0.79461172	0.54227121	1.869E-12	-3.094381	down	PREDICTED: flavonol synthase/flavanone 3-hydroxylase-like [Sesamum indicum]
c26072.graph_c0	0	0	0	3.66255461	7.76006501	6.60836367	1.563E-10	Inf	up	PREDICTED: uncharacterized protein LOC105161043 [Sesamum indicum]
c26073.graph_c0	7.93912324	11.6185279	9.54581304	70.6595967	111.693857	134.041701	1.167E-07	2.415489	up	-
c26074.graph_c0	2292.59361	2294.71831	2444.78119	432.284196	418.359036	447.234273	1.272E-62	-3.455813	down	PREDICTED: embryonic protein DC-8 [Nicotiana sylvestris]
c26076.graph_c0	8.36624157	9.43246572	8.31784154	10.3848791	8.12221603	7.50782739	6.213E-06	-1.0169	down	-
c26077.graph_c0	0.24586172	0.04101798	0.10531401	0.83051411	1.27975127	1.78028557	0.000984	2.288854	up	PREDICTED: probable magnesium transporter NIPA1 [Sesamum indicum]
c26079.graph_c0	0	0	0	0.81857508	1.36747779	1.20460065	1.28E-11	Inf	up	PREDICTED: putative E3 ubiquitin-protein ligase XBAT31 [Sesamum indicum]
c26080.graph_c0	6.52652376	6.48201956	6.22460887	29.5935579	33.4408386	29.048981	3.041E-10	1.241097	up	hypothetical protein MIMGU_mgv1a007824mg [Erythranthe guttata]
c26082.graph_c0	1.18204939	1.38723873	1.414223	15.6963468	19.4130324	22.5535828	1.03E-24	2.831779	up	PREDICTED: uncharacterized protein LOC105156132 [Sesamum indicum]
c26084.graph_c0	7.69898482	3.51334588	4.75275995	0	0	0	2.939E-17	-Inf	down	NADH dehydrogenase subunit 4 (mitochondrion) [Saccharina latissima]
c26085.graph_c0	0.15990915	0	0	5.45079348	4.2137913	4.98116659	4.499E-10	5.524671	up	PREDICTED: cycloartenol synthase-like isoform X1 [Sesamum indicum]
c26086.graph_c0	0.39647589	0.46690929	0.29969836	14.5888921	15.136499	15.7914515	1.451E-58	4.275363	up	PREDICTED: protein FAR1-RELATED SEQUENCE 6 isoform X1 [Sesamum indicum]
c26089.graph_c1	0	0.04730302	0	37.788407	51.5161368	55.6653692	1.235E-66	10.56962	up	Phosphoenolpyruvate carboxylase family protein [Theobroma cacao]

#ID	0 h-1 FPKM	0 h-2 FPKM	0 h-3 FPKM	48 h-1 FPKM	48 h-2 FPKM	48 h-3 FPKM	FDR	log2FC	regulated	nr_annotation
c26091.graph_c0	1.50137122	1.42773089	1.35052502	0.41494833	0.14652915	0.12307284	1.39E-10	-3.637283	down	PREDICTED: uncharacterized protein LOC105162488 [Sesamum indicum]
c26092.graph_c0	1.28159516	0.85525232	0.90030562	5.35245703	10.5567257	13.9235546	0.0007735	2.268336	up	PREDICTED: receptor-like protein kinase THESEUS 1 [Sesamum indicum]
c26096.graph_c0	0.13697373	0	0	17.2248167	17.111307	23.1301605	3.365E-39	7.714241	up	PREDICTED: thylakoid lumenal 16.5 kDa protein, chloroplastic [Sesamum indicum]
c26098.graph_c0	12.8006086	18.9150582	14.2952153	5.05403811	2.37961802	2.62328075	5.46E-17	-3.195876	down	PREDICTED: UDP-glycosyltransferase 73C3 isoform X2 [Vitis vinifera]
c26098.graph_c1	13.0720641	12.9330008	13.6728883	4.13097352	3.70869961	4.17411748	1.321E-21	-2.740454	down	PREDICTED: UDP-glycosyltransferase 73C3-like [Sesamum indicum]
c26103.graph_c0	2.67710326	1.67486499	2.04260906	10.4818691	11.2267429	11.2811972	4.725E-06	1.349762	up	hypothetical protein MIMGU_mgv1a009994mg [Erythranthe guttata]
c26107.graph_c0	0	0	0	7.61102078	5.44085489	6.60706392	8.773E-19	Inf	up	PREDICTED: LOW QUALITY PROTEIN: anthranilate synthase alpha subunit 1, chloroplastic [Sesamum indicum]
c26115.graph_c0	1.88605357	1.61823615	1.21182676	25.5048335	32.0155441	36.2221294	1.814E-30	3.294555	up	PREDICTED: macrophage migration inhibitory factor homolog isoform X2 [Sesamum indicum]
c26117.graph_c0	12.9357711	14.4314026	15.1637089	12.9634491	14.5523759	14.4488536	1.168E-07	-1.040205	down	PREDICTED: uncharacterized protein LOC105176303 [Sesamum indicum]
c26118.graph_c0	3.40203987	2.12840406	1.40520726	0	0	0	1.336E-11	-Inf	down	hypothetical protein AALP_AAs67754U000100, partial [Arabis alpina]
c26120.graph_c0	5.39038939	6.62210229	6.8219113	0.11286314	0.07970988	0.30127484	9.276E-51	-6.278856	down	PREDICTED: cytochrome P450 76C1-like [Sesamum indicum]
c26125.graph_c0	0	0	0.05845085	3.39425156	3.46262025	4.39977514	1.09E-15	6.5372	up	PREDICTED: cytochrome P450 90B1 [Sesamum indicum]
c26126.graph_c0	47.4308217	49.2774948	47.6055357	51.2857356	46.7295483	43.3827475	5.134E-13	-1.044327	down	PREDICTED: transcription factor bHLH104 [Sesamum indicum]
c26131.graph_c0	0.73867046	1.30484139	1.11673033	0.52039107	0.76332633	0.49865937	0.0002216	-1.849966	down	hypothetical protein L484_013769 [Morus notabilis]
c26134.graph_c0	0.12996492	0.28620913	0.46762818	18.0556096	22.1719299	23.6085892	3.796E-86	5.142374	up	PREDICTED: probable methyltransferase PMT2 [Sesamum indicum]
c26136.graph_c0	1.19146389	0.62225581	0.73225483	71.3475263	71.640537	71.1706952	6.49E-129	5.380192	up	PREDICTED: uncharacterized protein LOC105162013 [Sesamum indicum]
c26138.graph_c0	0	0.09086078	0.07776193	4.96163946	5.90590492	6.2006137	1.567E-24	5.631502	up	PREDICTED: glycerol-3-phosphate 2-O-acyltransferase 6-like [Sesamum indicum]
c26141.graph_c0	2.42333751	2.89027214	2.84905617	10.5451728	11.3222958	13.4405584	0.0002862	1.091746	up	PREDICTED: short-chain dehydrogenase/reductase 2b-like [Sesamum indicum]
c26142.graph_c0	0.07748707	0.19391165	0	9.81559459	8.81030807	8.54330016	1.576E-28	5.644814	up	PREDICTED: uncharacterized protein LOC105156886 [Sesamum indicum]
c26146.graph_c0	0.90827874	1.03041335	1.01155093	4.57431874	6.08696843	3.8220183	0.0061416	1.274965	up	PREDICTED: uncharacterized protein LOC100267130 [Vitis vinifera]

#ID	0 h-1 FPKM	0 h-2 FPKM	0 h-3 FPKM	48 h-1 FPKM	48 h-2 FPKM	48 h-3 FPKM	FDR	log2FC	regulated	nr_annotation
c26148.graph_c0	0.97225551	0.6193276	0	3.90853175	2.67414748	3.47778805	0.0074292	1.665581	up	PREDICTED: protein CHUP1, chloroplastic isoform X1 [Sesamum indicum]
c26155.graph_c0	60.6819133	63.1452779	53.3829576	15.6486224	8.46910264	10.5439365	1.508E-97	-3.359324	down	hypothetical protein MIMGU_mgv1a001787mg [Erythranthe guttata]
c26159.graph_c0	6.53295605	2.61579913	4.4773905	0	0	0	8.174E-11	-Inf	down	metallothionein type 2a-FL [Elaeis guineensis]
c26161.graph_c0	9.63539728	10.0232828	12.381867	0.3481081	0.18438916	0.46461674	1.72E-23	-6.023067	down	hypothetical protein MIMGU_mgv1a020481mg, partial [Erythranthe guttata]
c26161.graph_c1	22.6554834	22.6515206	24.1526609	1.66777464	1.79278743	1.61491489	1.03E-153	-4.793299	down	PREDICTED: beta-galactosidase 15-like [Sesamum indicum]
c26162.graph_c1	0	0	0.33808626	7.75614063	8.47346209	10.1363832	2.713E-10	5.232243	up	hypothetical protein MIMGU_mgv1a0223741mg, partial [Erythranthe guttata]
c26163.graph_c0	0.1018095	0.10191145	0.13082924	10.1297039	23.1018738	26.0385656	5.958E-11	6.434725	up	PREDICTED: hyoscyamine 6-dioxygenase-like [Sesamum indicum]
c26166.graph_c0	5.00892175	4.23445976	4.38125785	38.0215276	43.2780574	42.4573661	2.642E-45	2.164335	up	PREDICTED: kinesin-13A [Sesamum indicum]
c26171.graph_c0	1.54884731	2.08707456	1.07171622	1.15249548	1.16278973	1.31847885	0.0030669	-1.387452	down	PREDICTED: peroxidase 11 [Sesamum indicum]
c26174.graph_c0	0.70239315	0.31248733	0.75216917	5.53621772	6.93130616	6.36553606	3.068E-09	2.389332	up	hypothetical protein L484_002404 [Morus notabilis]
c26175.graph_c0	4.80588221	2.61151991	2.8231995	0	0	0	6.846E-14	-Inf	down	PREDICTED: protein translation factor SUI1 homolog 2-like [Tarenaya hassleriana]
c26177.graph_c0	27.6929072	30.0556372	25.9020399	22.2063553	27.0274133	27.4077581	4.246E-12	-1.146414	down	PREDICTED: growth-regulating factor 4-like isoform X1 [Sesamum indicum]
c26178.graph_c1	0.09150038	0.045796	0.17637241	4.80487862	4.37577417	2.92524091	1.281E-11	4.247222	up	PREDICTED: U-box domain-containing protein 4-like [Nicotiana sylvestris]
c26182.graph_c0	0.16356285	0.16372664	0.10509237	1.77054605	4.03071413	4.62570489	2.442E-05	3.564888	up	PREDICTED: agmatine coumaroyltransferase-2-like [Sesamum indicum]
c26184.graph_c0	8.58670255	8.47338168	6.96488294	40.8996426	62.7635005	74.3320566	2.281E-06	1.865289	up	hypothetical protein MIMGU_mgv1a010851mg [Erythranthe guttata]
c26185.graph_c0	0	0	0	4.91024136	6.2021525	5.88148512	8.52E-14	Inf	up	PREDICTED: probable receptor-like serine/threonine-protein kinase At4g34500 [Sesamum indicum]
c26186.graph_c0	2.92503333	1.44074336	1.55124404	11.9338109	10.5580113	12.0648986	1.056E-06	1.534612	up	PREDICTED: rop guanine nucleotide exchange factor 14 [Sesamum indicum]
c26189.graph_c0	0.22273528	0.22295831	0.28622365	6.89466172	8.73878376	6.53651374	2.026E-16	3.897998	up	PREDICTED: calcium-dependent protein kinase 13 [Sesamum indicum]
c26189.graph_c1	0.74588258	0.44797768	0.28754659	8.17715259	10.1914137	10.577687	2.45E-11	3.274381	up	PREDICTED: calcium-dependent protein kinase 13 [Sesamum indicum]
c26190.graph_c0	1.44332529	1.6098872	1.11283809	9.23181577	8.41159582	9.29614771	7.053E-07	1.679638	up	PREDICTED: RING-H2 finger protein ATL65 [Sesamum indicum]
c26199.graph_c0	3.26428577	2.33396747	3.14605234	36.0872756	33.4513311	41.1253183	2.12E-43	2.64326	up	hypothetical protein MIMGU_mgv1a007643mg [Erythranthe guttata]

#ID	0 h-1 FPKM	0 h-2 FPKM	0 h-3 FPKM	48 h-1 FPKM	48 h-2 FPKM	48 h-3 FPKM	FDR	log2FC	regulated	nr_annotation
c26203.graph_c0	2.11657411	1.78575599	1.94277252	0.4456974	0.66397831	0.57008212	1.715E-17	-2.822349	down	PREDICTED: receptor-like protein kinase isoform X1 [Sesamum indicum]
c26206.graph_c0	1.59242128	1.34232914	0.96931155	13.8982959	12.0242401	11.1299581	1.114E-07	2.237107	up	hypothetical protein MIMGU_mgv1a006425mg [Erythranthe guttata]
c26208.graph_c0	2.09529116	2.85244942	2.69253209	0.6949148	0.24539265	0.20611034	1.134E-11	-3.731882	down	-
c26211.graph_c0	4.0661284	4.92708423	4.05635557	2.51375155	1.93199124	1.88586281	3.816E-08	-2.054472	down	hypothetical protein MIMGU_mgv1a013710mg [Erythranthe guttata]
c26213.graph_c0	17.5979997	17.4408633	14.8517377	10.0362632	11.10929	9.1591863	1.213E-24	-1.735432	down	PREDICTED: myb-related protein 3R-1-like isoform X1 [Sesamum indicum]
c26214.graph_c0	0	0	0	7.57575648	6.75839611	11.086945	1.053E-15	Inf	up	hypothetical protein MIMGU_mgv1a024317mg, partial [Erythranthe guttata]
c26216.graph_c0	0	0	0.13533102	4.26892176	13.8755607	15.1938436	1.516E-06	6.876897	up	PREDICTED: blue copper protein [Sesamum indicum]
c26221.graph_c0	29.8270016	31.4896663	27.8483253	23.6144041	24.3343269	23.8772213	3.965E-06	-1.328876	down	putative phosphoprotein phosphatase [Arabidopsis thaliana]
c26223.graph_c0	8.71151389	6.51901224	7.93406542	51.2704324	56.2133482	57.9611282	1.861E-19	1.816956	up	PREDICTED: UPF0587 protein C1orf123 homolog [Sesamum indicum]
c26227.graph_c0	0	0.05032113	0.06459996	2.13038588	2.99282645	4.32692342	3.152E-09	5.322306	up	PREDICTED: probable pectinesterase 53 [Sesamum indicum]
c26228.graph_c0	8.39223796	7.03591636	6.50176098	69.1367094	71.526969	69.6882938	3.491E-50	2.24607	up	hypothetical protein MIMGU_mgv1a005139mg [Erythranthe guttata]
c26229.graph_c0	4.18597011	3.60672148	4.0173304	16.027607	22.0989549	23.1799905	5.516E-10	1.352324	up	PREDICTED: uncharacterized protein LOC105163825 [Sesamum indicum]
c26231.graph_c0	0.67543264	0.72118291	0.6365023	3.56758157	5.31755617	4.79853072	0.0001241	1.72759	up	PREDICTED: alpha-ketoglutarate-dependent dioxygenase alkB isoform X1 [Sesamum indicum]
c26233.graph_c0	0	0.07126464	0	9.67422346	10.9087503	10.3588589	1.709E-40	7.754527	up	PREDICTED: uncharacterized protein LOC105176469 [Sesamum indicum]
c26234.graph_c0	0	0	0.04671307	1.97587117	2.44797488	2.29449335	3.049E-13	6.114727	up	PREDICTED: glucan endo-1,3-beta-glucosidase 12 [Sesamum indicum]
c26239.graph_c0	2.37674512	2.73599383	3.35963274	0	0	0	2.516E-19	-Inf	down	-
c26246.graph_c0	2.71421837	3.23444791	2.6574309	0.35721643	0.50456999	0.31784903	1.59E-11	-3.887102	down	hypothetical protein MIMGU_mgv1a027137mg [Erythranthe guttata]
c26248.graph_c0	4.22897175	4.17002424	3.56885716	36.8666705	36.653283	49.3090944	6.77E-18	2.341455	up	PREDICTED: photosystem II reaction center W protein, chloroplastic [Sesamum indicum]
c26252.graph_c0	2.32820571	2.19543346	2.55823702	1.11907563	1.38311242	1.49362015	3.193E-06	-1.848839	down	-
c26257.graph_c0	4.5352181	4.580293	4.63112677	19.0627579	21.6569177	19.9161574	7.806E-08	1.121664	up	PREDICTED: mitochondrial outer membrane import complex protein METAXIN [Sesamum indicum]
c26259.graph_c0	0.35447275	0.86172441	0.52058459	10.1701178	13.8381736	15.9400684	7.825E-19	3.499914	up	putative cyclin-dependent kinase B1-2 [Oryza sativa Japonica Group]
c26261.graph_c0	91.2087954	97.6281411	86.1395797	66.6605856	85.2094702	103.198751	3.349E-15	-1.130846	down	PREDICTED: beta-amylase 1, chloroplastic [Sesamum indicum]

#ID	0 h-1 FPKM	0 h-2 FPKM	0 h-3 FPKM	48 h-1 FPKM	48 h-2 FPKM	48 h-3 FPKM	FDR	log2FC	regulated	nr_annotation
c26269.graph_c0	0.25668889	0.29977025	0.16492769	1.93124149	1.50312209	0.98194689	0.006519	1.609825	up	PREDICTED: phospholipase A1-Igamma3, chloroplastic [Sesamum indicum]
c26272.graph_c0	0.20855303	0.13917457	0.08933297	1.8572823	1.22124748	1.25369551	6.278E-05	2.307252	up	PREDICTED: zinc finger protein WIP2-like [Sesamum indicum]
c26274.graph_c0	14.6624301	13.7049104	13.2858868	13.4791237	11.8333306	11.6340908	1.446E-12	-1.18687	down	PREDICTED: protein FIZZY-RELATED 2-like [Sesamum indicum]
c26278.graph_c0	119.257931	119.064571	113.767239	694.808321	721.223913	727.60799	3.303E-29	1.588352	up	PREDICTED: 60S acidic ribosomal protein P2A-like [Nicotiana tomentosiformis]
c26279.graph_c0	93.3152559	90.7774661	100.535586	24.0889117	12.2870612	12.3048006	1.43E-116	-3.551969	down	hypothetical protein MIMGU_mgv1a015010mg [Erythranthe guttata]
c26282.graph_c0	0.33015285	0.39658014	0.67881517	9.24641353	9.34434752	12.2869771	4.808E-14	3.429978	up	PREDICTED: inorganic pyrophosphatase 3 [Sesamum indicum]
c26284.graph_c0	288.192432	294.360382	271.715883	73.9508716	61.5271172	66.1220216	2.819E-76	-3.096838	down	PREDICTED: 18.2 kDa class I heat shock protein-like [Sesamum indicum]
c26286.graph_c0	0.1795195	0.14974938	0.07689656	3.66603578	4.38013576	6.37687454	4.586E-12	4.134085	up	PREDICTED: mini-chromosome maintenance complex-binding protein isoform X1 [Sesamum indicum]
c26288.graph_c0	1.54517531	1.66883225	1.30632332	0.07492191	0.23811204	0.13333013	6.493E-15	-4.3714	down	hypothetical protein MIMGU_mgv1a008148mg [Erythranthe guttata]
c26289.graph_c0	0	0	0	1.86976688	1.10691278	2.10410044	1.082E-08	Inf	up	PREDICTED: F-box/kelch-repeat protein SKIP25-like [Sesamum indicum]
c26290.graph_c0	0.2538172	0	0	1.11070656	1.7959531	1.76853688	9.026E-07	3.198638	up	PREDICTED: exopolygalacturonase-like [Sesamum indicum]
c26291.graph_c0	16.7912953	15.6724262	15.5999343	5.12157153	4.87204279	5.76618163	4.314E-17	-2.625969	down	PREDICTED: uncharacterized protein LOC105169169 isoform X2 [Sesamum indicum]
c26292.graph_c0	0.41129652	0.09149075	0.11745162	2.14717469	2.18546843	2.77216061	1.243E-05	2.507811	up	PREDICTED: putative kinase-like protein TMKL1 [Sesamum indicum]
c26293.graph_c0	0.73942009	0.41120028	0.52788005	4.54134677	5.45247373	5.65720827	3.155E-05	2.201758	up	-
c26293.graph_c1	3.13299168	2.75099026	4.16728215	32.3571246	43.4515564	34.5133646	5.475E-25	2.431073	up	-
c26294.graph_c1	1.82808486	1.74673743	2.02882123	14.8511498	10.461629	9.80867415	5.715E-05	1.636846	up	PREDICTED: probable BOI-related E3 ubiquitin-protein ligase 2 [Sesamum indicum]
c26296.graph_c0	3.88331017	2.8120161	2.65436559	0.07611827	0	0.33864789	2.916E-18	-5.517334	down	-
c26297.graph_c0	0.0200172	0	0	7.8928124	8.06843525	9.07408011	1.492E-59	9.291058	up	O-Glycosyl hydrolases family 17 protein [Theobroma cacao]
c26298.graph_c0	2.37951036	2.54068595	1.90260934	14.8579743	16.7723679	17.4251002	3.153E-10	1.828549	up	hypothetical protein MIMGU_mgv1a013638mg [Erythranthe guttata]
c26299.graph_c0	0.80123465	0.26734566	0.51480913	5.2900723	6.64683018	6.02067672	1.652E-05	2.484507	up	PREDICTED: cytochrome b561 and DOMON domain-containing protein At3g25290-like [Sesamum indicum]
c26299.graph_c1	0.53625235	0.53678933	0	3.85343902	3.03552027	4.13210367	0.0001028	2.361564	up	PREDICTED: cytochrome b561 and DOMON domain-containing protein At3g25290-like [Sesamum indicum]
c26306.graph_c0	96.3485699	94.7159539	90.9473592	18.6984932	9.92780774	7.78790956	1.47E-148	-3.954728	down	PREDICTED: uncharacterized protein LOC105172717 [Sesamum indicum]

#ID	0 h-1 FPKM	0 h-2 FPKM	0 h-3 FPKM	48 h-1 FPKM	48 h-2 FPKM	48 h-3 FPKM	FDR	log2FC	regulated	nr_annotation
c26309.graph_c0	0.03435869	0.03439309	0.04415228	1.36109902	0.67066017	0.76045671	0.0003631	3.62157	up	PREDICTED: ankyrin-3-like [Sesamum indicum]
c26310.graph_c0	0.35862941	0.40386208	0.34563974	13.5461048	5.99394026	5.40191056	0.0020867	3.493885	up	PREDICTED: uncharacterized protein LOC105176355 [Sesamum indicum]
c26314.graph_c0	2.42770198	2.36618209	2.70920722	35.1963508	36.2886401	38.9635803	3.542E-33	2.859867	up	PREDICTED: galactokinase [Sesamum indicum]
c26315.graph_c0	2.53734641	2.96320171	2.53601491	1.03891956	3.02667558	2.1955079	0.0055544	-1.392426	down	PREDICTED: cyclin-dependent protein kinase inhibitor SMR3-like [Sesamum indicum]
c26316.graph_c0	0.47401167	0.91247369	0.98396915	91.2011244	112.381219	144.815019	9.088E-40	6.171402	up	PREDICTED: uncharacterized protein LOC105167296 [Sesamum indicum]
c26318.graph_c0	1.0357385	0.69118376	0.51759749	3.7637489	4.21186857	4.43383587	0.0034159	1.452821	up	PREDICTED: alpha-ketoglutarate-dependent dioxygenase alkB homolog 2 [Sesamum indicum]
c26323.graph_c0	0.31135348	0.13851789	0.3111901	1.08361452	1.418066	2.12689783	0.0026413	1.576642	up	PREDICTED: putative glycosyltransferase 5 [Sesamum indicum]
c26325.graph_c0	272.386606	270.484086	285.607093	48.8764066	49.9137181	45.5170058	1.19E-111	-3.538993	down	PREDICTED: uncharacterized protein LOC105175521 [Sesamum indicum]
c26326.graph_c0	0	0	0	1.6427262	2.40322892	2.22733393	1.191E-11	Inf	up	PREDICTED: MLP-like protein 423 [Cucumis melo]
c26328.graph_c0	0.30482649	0.39231222	0.55959158	23.2283256	19.3800744	26.1657256	4.087E-41	4.751839	up	PREDICTED: bifunctional L-3-cyanoalanine synthase/cysteine synthase 1, mitochondrial [Sesamum indicum]
c26329.graph_c0	11.5352725	9.4579508	11.9182166	7.20930219	8.08996707	7.74526947	8.345E-10	-1.534572	down	PREDICTED: septum-promoting GTP-binding protein 1 [Sesamum indicum]
c26332.graph_c0	21.4378022	22.5129576	20.8510232	87.1360933	101.305601	123.750014	9.48E-10	1.247081	up	PREDICTED: plastidic ATP/ADP-transporter-like [Sesamum indicum]
c26333.graph_c0	0.06482693	0.03244592	0	122.909514	135.838673	151.876935	7.75E-269	11.04143	up	4-coumarate coenzyme A ligase [Paulownia fortunei]
c26334.graph_c0	5.24746078	4.54205384	4.20458032	47.6889786	47.4177498	49.4676394	2.233E-47	2.353145	up	PREDICTED: aspartate aminotransferase, chloroplastic [Nicotiana sylvestris]
c26336.graph_c0	26.376128	26.8809132	27.3989322	6.94268842	6.78089271	7.47334379	5.959E-86	-2.945999	down	PREDICTED: uncharacterized protein LOC104091376 [Nicotiana tomentosiformis]
c26336.graph_c1	13.9967733	13.7413507	15.2192682	11.9028374	7.09290726	14.3420782	0.0011272	-1.38083	down	-
c26338.graph_c0	58.6710932	52.3582094	59.3021816	6.94767515	6.75249484	6.6924399	1.54E-120	-4.079568	down	PREDICTED: uncharacterized protein LOC105158260 [Sesamum indicum]
c26341.graph_c0	0.05422336	0	0	1.89825566	2.96353724	1.24456793	3.878E-06	5.815542	up	PREDICTED: transcription factor JUNGBRUNNEN 1-like [Sesamum indicum]
c26342.graph_c0	1.49965636	1.47482194	1.58902696	0.65443323	0.87303519	0.88424966	4.261E-06	-1.943595	down	PREDICTED: uncharacterized protein LOC105158927 [Sesamum indicum]
c26343.graph_c0	0.12657926	0	0	2.7987138	3.58258497	2.3865171	5.397E-10	5.115968	up	40S ribosomal protein S16 [Arabidopsis lyrata subsp. lyrata]
c26346.graph_c0	0.03916184	0	0	6.53019822	5.31268228	5.77842873	8.242E-24	7.820953	up	PREDICTED: G2/mitotic-specific cyclin-2-like isoform X2 [Sesamum indicum]
c26349.graph_c0	2.01499525	1.85472457	1.36908091	9.11102688	11.9576649	11.7711965	2.823E-12	1.629497	up	PREDICTED: FACT complex subunit SPT16-like [Sesamum indicum]

#ID	0 h-1 FPKM	0 h-2 FPKM	0 h-3 FPKM	48 h-1 FPKM	48 h-2 FPKM	48 h-3 FPKM	FDR	log2FC	regulated	nr_annotation
c26351.graph_c0	0.05827936	0	0	2.80981557	3.12833577	3.71042408	1.505E-23	6.37175	up	PREDICTED: kinesin-like protein KIF22 [Sesamum indicum]
c26353.graph_c0	0.03478933	0.31341753	0.08941134	1.63456039	1.59580532	2.08181974	7.5E-06	2.585766	up	PREDICTED: cytochrome P450 711A1 [Sesamum indicum]
c26358.graph_c0	60.6539279	63.2800719	57.594479	9.30368981	13.786673	15.1561648	4.71E-107	-3.271165	down	PREDICTED: amidophosphoribosyltransferase, chloroplastic [Sesamum indicum]
c26361.graph_c0	2.01996468	0.86656602	1.3596705	18.1661552	18.1183116	15.7698885	8.847E-11	2.601402	up	-
c26363.graph_c0	37.6848377	41.6316483	42.4045592	18.3482306	19.8188574	13.2850005	3.706E-19	-2.259292	down	PREDICTED: protein phosphatase 2C 37-like [Sesamum indicum]
c26363.graph_c1	45.0172776	42.4791632	40.0152845	21.7138119	19.2532454	16.077178	1.995E-43	-2.173033	down	PREDICTED: protein phosphatase 2C 37-like [Sesamum indicum]
c26364.graph_c0	0.77478315	1.24089436	0.73012648	167.548139	264.55664	399.700825	1.053E-14	7.217362	up	PREDICTED: protein YLS3-like [Sesamum indicum]
c26365.graph_c0	1.43056541	1.56931277	1.43541085	6.31884326	6.29240534	7.24495257	7.25E-06	1.144365	up	PREDICTED: mechanosensitive ion channel protein 10-like [Sesamum indicum]
c26367.graph_c0	11.021348	12.1570448	10.3815202	2.51375155	2.76744692	1.84200554	7.447E-33	-3.251583	down	PREDICTED: dehydration-responsive protein RD22 [Sesamum indicum]
c26371.graph_c0	22.0280349	21.665498	20.2975812	7.82643188	5.99963016	4.54928926	1.004E-54	-2.809088	down	PREDICTED: uncharacterized protein LOC105176235 [Sesamum indicum]
c26372.graph_c0	0	0	0	3.55862784	3.82705491	3.4063279	1.72E-14	Inf	up	PREDICTED: protein trichome birefringence-like 38 [Sesamum indicum]
c26376.graph_c0	0.07711919	0.15439283	0.13213496	0.71047231	0.9533684	1.24327551	0.00035	1.971876	up	PREDICTED: putative serine/threonine-protein kinase-like protein CCR3 [Sesamum indicum]
c26381.graph_c0	77.2222938	80.5114303	71.957321	36.4948472	38.8726388	37.6321407	6.136E-47	-2.040841	down	PREDICTED: protein SDE2 homolog [Sesamum indicum]
c26382.graph_c0	5.93040678	6.67838834	5.71560563	0	0.18087162	0.1519178	7.204E-20	-6.829487	down	PREDICTED: myb-related protein Myb4-like [Sesamum indicum]
c26384.graph_c0	17.7179454	18.4199344	15.7761563	12.1666261	15.3708105	14.3895603	1.223E-17	-1.328183	down	PREDICTED: uncharacterized protein LOC105161020 isoform X2 [Sesamum indicum]
c26387.graph_c0	3.18311774	3.71735601	3.18144736	29.7865533	34.9495154	34.9116953	6.502E-39	2.285265	up	PREDICTED: serine carboxypeptidase-like 45 [Sesamum indicum]
c26390.graph_c0	5.64961927	8.58041953	7.059709	3.48183023	3.91673633	3.64107961	6.536E-10	-1.96716	down	PREDICTED: uncharacterized protein LOC105171007 isoform X3 [Sesamum indicum]
c26391.graph_c0	4.59645726	4.53339727	6.51466496	5.10637664	5.14570458	4.32198307	0.0025598	-1.122572	down	PREDICTED: E3 ubiquitin-protein ligase RNF5-like [Sesamum indicum]
c26393.graph_c0	34.7435714	37.8765216	33.7678471	13.8375387	16.4952955	18.6553921	1.924E-47	-2.139788	down	PREDICTED: uncharacterized protein LOC105156254 [Sesamum indicum]
c26394.graph_c0	7.63524136	8.10409559	7.52785588	61.305666	59.8067765	62.9665331	8.783E-28	1.967002	up	hypothetical protein M569_01696, partial [Genlisea aurea]
c26398.graph_c0	0.07263453	0	0.15556375	8.49828991	13.5161764	11.9082319	1.492E-34	6.175337	up	PREDICTED: glycerophosphodiester phosphodiesterase GDPDL3-like [Sesamum indicum]

#ID	0 h-1 FPKM	0 h-2 FPKM	0 h-3 FPKM	48 h-1 FPKM	48 h-2 FPKM	48 h-3 FPKM	FDR	log2FC	regulated	nr_annotation
c26401.graph_c0	8.38353329	9.46781635	8.19497714	4.4888472	5.10505029	4.34657418	8.262E-10	-1.919799	down	PREDICTED: LOW QUALITY PROTEIN: uncharacterized protein LOC105173575 [Sesamum indicum]
c26404.graph_c0	0	0	0	2.81089225	3.46255756	2.09395677	4.89E-12	Inf	up	--
c26407.graph_c0	0	0	0	7.19374588	7.01122936	7.48911364	3.707E-18	Inf	up	PREDICTED: EPIDERMAL PATTERNING FACTOR-like protein 4 [Sesamum indicum]
c26409.graph_c0	0.36403001	0.36439453	0.24948959	2.05692491	2.74751362	2.62599568	3.626E-05	1.907066	up	PREDICTED: zinc finger CCCH domain-containing protein 31 isoform X1 [Sesamum indicum]
c26413.graph_c0	0.1375434	0.51630423	0.22093589	19.9891258	19.8001147	23.3109202	1.745E-65	5.155956	up	PREDICTED: protein WVD2-like 1 isoform X2 [Sesamum indicum]
c26418.graph_c0	31.8411698	33.9911999	33.8818287	27.941718	29.9942291	32.4890363	2.594E-15	-1.160876	down	PREDICTED: GATA transcription factor 15-like [Sesamum indicum]
c26420.graph_c0	0.05548801	0.44434855	0.07130428	1.84029013	5.90284731	9.14259161	0.0040237	3.856848	up	PREDICTED: 3-ketoacyl-CoA synthase 11 [Sesamum indicum]
c26422.graph_c0	285.842613	280.100125	294.371219	16.251961	16.258167	15.4412969	1.42E-212	-5.182645	down	hypothetical protein MIMGU_mgv1a006265mg [Erythranthe guttata]
c26423.graph_c0	0.23799961	0.19059035	0.24467114	42.6242935	42.22854	34.9613669	2.301E-77	6.458462	up	hypothetical protein MIMGU_mgv1a008823mg [Erythranthe guttata]
c26427.graph_c0	0	0	0	1.08237644	2.86661647	2.80172231	6.164E-07	Inf	up	hypothetical protein MIMGU_mgv1a010556mg [Erythranthe guttata]
c26429.graph_c0	7.54124191	7.95409766	6.79656388	4.21978051	4.81611837	4.89567699	4.845E-17	-1.69689	down	PREDICTED: mitogen-activated protein kinase homolog NTF6 [Sesamum indicum]
c26430.graph_c0	3.44928972	3.08090973	2.96634789	2.71327604	2.95207309	2.32725646	1.628E-05	-1.264853	down	PREDICTED: uncharacterized protein LOC105156229 [Sesamum indicum]
c26431.graph_c0	4.48771975	4.43387309	4.34389686	4.18807339	3.69729661	3.86985031	0.0009868	-1.189395	down	hypothetical protein MIMGU_mgv1a020357mg [Erythranthe guttata]
c26438.graph_c0	7.36137478	9.13083753	8.2257896	0.14742997	0.31236835	0.19677345	1.654E-36	-6.262755	down	PREDICTED: probable trehalose-phosphate phosphatase J [Nicotiana tomentosiformis]
c26442.graph_c0	90.8368424	90.4089556	79.0126698	35.6346933	30.3522706	29.848635	3.84E-63	-2.453927	down	PREDICTED: uncharacterized protein At4g13200, chloroplastic-like [Sesamum indicum]
c26443.graph_c0	0.80862639	1.4839662	1.21230308	6.33215468	6.83968242	6.62860371	0.0044585	1.477899	up	PREDICTED: myosin-6-like [Sesamum indicum]
c26444.graph_c0	34.0354039	39.1989703	32.0796162	17.8309252	4.9336255	4.48444418	2.065E-31	-2.938986	down	PREDICTED: receptor-like protein kinase FERONIA [Sesamum indicum]
c26444.graph_c1	19.6387413	20.0094495	17.0604297	8.86162749	2.10248048	2.5462053	3.983E-45	-3.057269	down	PREDICTED: receptor-like protein kinase FERONIA [Sesamum indicum]
c26446.graph_c0	2.16052922	1.90316954	1.4437101	10.4298031	8.09705895	7.72183963	0.004961	1.244612	up	PREDICTED: uncharacterized protein LOC105170673 [Sesamum indicum]
c26447.graph_c0	122.9693	111.146755	119.45988	67.6377997	36.4600099	30.5526241	6.564E-44	-2.395851	down	PREDICTED: uncharacterized protein LOC105156412 [Sesamum indicum]

#ID	0 h-1 FPKM	0 h-2 FPKM	0 h-3 FPKM	48 h-1 FPKM	48 h-2 FPKM	48 h-3 FPKM	FDR	log2FC	regulated	nr_annotation
c26450.graph_c0	1.74992952	2.00192207	2.64136389	30.3749383	34.9708067	37.273728	5.167E-62	2.979964	up	PREDICTED: coatomer subunit alpha-1-like [Sesamum indicum]
c26452.graph_c0	0	0.07097769	0	23.6145764	49.7913512	75.3878402	4.437E-12	10.01362	up	PREDICTED: subtilisin-like protease [Sesamum indicum]
c26453.graph_c0	6.83268368	5.57294677	5.04052482	32.930679	41.9250189	46.9342934	9.702E-15	1.783265	up	PREDICTED: DAG protein, chloroplastic [Sesamum indicum]
c26454.graph_c0	9.03314413	4.63702024	5.45672762	0	0	0	7.053E-18	-Inf	down	hypothetical protein SNOG_05978 [Phaeosphaeria nodorum SN15]
c26459.graph_c0	0.70506285	0.84692263	0.96643592	11.302168	11.6521541	12.0987461	3.347E-15	2.777912	up	hypothetical protein MIMGU_mgv1a021565mg [Erythranthe guttata]
c26464.graph_c0	19.0647235	22.1372242	18.1292041	203.211625	215.557175	240.906315	1.323E-58	2.456616	up	PREDICTED: 60S ribosomal protein L12 [Sesamum indicum]
c26468.graph_c0	2.01543682	1.21420901	1.82253356	0	0	0	2.664E-30	-Inf	down	PREDICTED: uncharacterized protein LOC104812256 [Tarenaya hassleriana]
c26470.graph_c0	0	0	0	7.67220422	9.85001131	9.44061487	1.83E-43	Inf	up	PREDICTED: tyrosine decarboxylase 1-like [Setaria italica]
c26474.graph_c0	0.18175733	0.07277573	0.04671307	4.01872102	4.82499397	5.27435485	8.415E-16	4.539499	up	PREDICTED: acetylornithine deacetylase [Sesamum indicum]
c26475.graph_c0	6.21392652	3.52784561	4.05216141	0	0	0	3.286E-20	-Inf	down	predicted protein [Hordeum vulgare subsp. vulgare]
c26478.graph_c0	2.32096779	1.42395309	2.02042728	12.8293615	20.971295	23.9970757	4.431E-07	2.298567	up	PREDICTED: 21 kDa protein-like [Sesamum indicum]
c26481.graph_c0	1.11494777	0.98476255	0.80065555	18.8212443	20.3335487	18.9960961	1.139E-83	3.310136	up	PREDICTED: LOW QUALITY PROTEIN: UDP-glucose:glycoprotein glucosyltransferase [Sesamum indicum]
c26482.graph_c0	2.62904921	2.6316818	1.68921616	0.30275645	0.72165094	1.07756348	7.378E-08	-2.753854	down	-
c26483.graph_c0	4.86044051	6.25539537	5.71049919	5.62917749	5.15023585	3.98427932	0.0006174	-1.20305	down	-
c26487.graph_c0	189.426176	201.646716	189.169362	63.7665889	72.3420144	69.9227347	2.874E-58	-2.513097	down	hypothetical protein H632_c16p1 [Helicosporidium sp. ATCC 50920]
c26488.graph_c0	11.5626991	11.3112256	9.79311945	7.98923441	11.0924784	8.9936746	8.15E-07	-1.238957	down	PREDICTED: mechanosensitive ion channel protein 2, chloroplastic-like [Sesamum indicum]
c26489.graph_c0	5.62323625	6.12467401	4.90474942	58.3006665	81.4684737	90.6867342	7.57E-18	2.76822	up	PREDICTED: L-gulonolactone oxidase, partial [Sesamum indicum]
c26490.graph_c0	2.19642595	2.5983754	3.25014496	59.3557316	60.9963927	66.2362075	1.326E-64	3.513343	up	PREDICTED: 50S ribosomal protein L21, chloroplastic [Sesamum indicum]
c26491.graph_c0	3.08618573	2.87172143	1.95500676	35.9604175	37.6714736	41.9742694	5.937E-46	2.854102	up	hypothetical protein MIMGU_mgv1a006583mg [Erythranthe guttata]
c26496.graph_c0	317.258495	348.859207	361.694843	8.48969317	7.65776491	6.81509205	3.71E-265	-6.499663	down	PREDICTED: methionine gamma-lyase [Sesamum indicum]
c26497.graph_c0	0	0	0	1.12330557	1.23958963	1.832436	1.404E-11	Inf	up	PREDICTED: uncharacterized protein LOC105170157 isoform X1 [Sesamum indicum]
c26498.graph_c0	1.85233419	1.66237636	0.82080095	6.76711976	9.84950407	8.37751996	0.0003354	1.511394	up	PREDICTED: uncharacterized protein LOC105161253 [Sesamum indicum]
c26502.graph_c0	34.216199	34.344556	31.5676095	10.680597	10.1527371	9.16962311	1.407E-72	-2.753419	down	PREDICTED: uncharacterized protein LOC105166690 [Sesamum indicum]

#ID	0 h-1 FPKM	0 h-2 FPKM	0 h-3 FPKM	48 h-1 FPKM	48 h-2 FPKM	48 h-3 FPKM	FDR	log2FC	regulated	nr_annotation
c26507.graph_c0	62.246062	63.0164419	60.9004526	19.1421962	15.0149677	13.0462553	2.784E-71	-2.990683	down	hypothetical protein MIMGU_mgv1a002702mg [Erythranthe guttata]
c26512.graph_c0	78.9197461	79.4784248	69.0262374	57.9176048	63.0870126	63.5935506	3.965E-21	-1.318652	down	PREDICTED: PRA1 family protein F2-like [Nicotiana tomentosiformis]
c26519.graph_c0	0.28693327	0.31594265	0.25810443	5.20753042	4.67663327	5.43334191	1.88E-13	3.138002	up	PREDICTED: uncharacterized protein LOC105156252 [Sesamum indicum]
c26520.graph_c0	5.38868501	4.34157736	3.37788955	3.63249374	1.92409332	3.8786053	0.0012869	-1.481942	down	-
c26523.graph_c0	1.0424481	0.52174598	0.37210752	9.71041977	11.6435414	11.2513447	3.077E-13	3.061389	up	PREDICTED: epidermal growth factor receptor substrate 15-like [Sesamum indicum]
c26524.graph_c0	0	0	0	2.71268744	3.61881318	2.86072056	2.118E-11	Inf	up	60S ribosomal protein L17 [Drechslerella stenobrocha 248]
c26525.graph_c0	0.34799442	0.5515429	0.52171771	8.41562294	8.49079243	9.0808927	7.696E-21	3.17212	up	PREDICTED: probable receptor-like protein kinase At5g15080 [Sesamum indicum]
c26527.graph_c0	17.0549249	18.6817699	18.8164362	13.6457524	12.2669656	10.0951296	3.55E-20	-1.613847	down	PREDICTED: F-box/kelch-repeat protein SKIP25-like [Sesamum indicum]
c26528.graph_c0	112.661632	130.003875	122.209472	84.570643	76.0177444	67.8394205	2.858E-27	-1.690372	down	unnamed protein product [Coffea canephora]
c26529.graph_c0	0	0	0.03575775	6.1012008	6.11043508	8.23445821	2.985E-50	8.107062	up	PREDICTED: protein STICHEL [Sesamum indicum]
c26530.graph_c0	0	0.1307766	0.05596167	7.86348171	10.4555088	11.2449837	5.601E-30	6.281857	up	hypothetical protein MIMGU_mgv1a009832mg [Erythranthe guttata]
c26531.graph_c0	6.7972667	6.29055818	9.06437029	28.2384607	35.1722939	38.2678417	2.37E-05	1.172776	up	hypothetical protein MIMGU_mgv1a013949mg [Erythranthe guttata]
c26533.graph_c0	2.65958304	3.03496068	3.34931611	12.0058887	12.5111297	15.3483028	9.515E-05	1.118297	up	PREDICTED: 54S ribosomal protein L12, mitochondrial [Sesamum indicum]
c26539.graph_c1	24.134704	23.6555614	25.1989157	27.5249005	17.3302804	16.0528816	4.922E-10	-1.268822	down	PREDICTED: grpE protein homolog, mitochondrial isoform X2 [Sesamum indicum]
c26540.graph_c0	0.73732778	0.54552712	0.6385295	10.336785	10.3717544	10.8400393	5.645E-40	3.020057	up	PREDICTED: chromosome-associated kinesin KIF4A [Sesamum indicum]
c26542.graph_c0	2.87400532	2.40612049	2.75312064	15.2123616	16.1156525	19.2757612	2.454E-09	1.635096	up	PREDICTED: 50S ribosomal protein L21, mitochondrial [Sesamum indicum]
c26546.graph_c0	11.765041	6.03440461	5.49765217	0	0	0	2.885E-13	-Inf	down	heat shock protein 70-1 [Nicotiana tabacum]
c26547.graph_c0	0	0	0.33361398	10.0452722	14.8224344	15.1098528	5.587E-17	5.847882	up	-
c26548.graph_c0	0.12173276	0.12185466	0.26071903	6.50459317	9.3065777	10.7439228	1.713E-20	4.684701	up	PREDICTED: amino acid permease 6 [Sesamum indicum]
c26549.graph_c0	2.76018101	2.32669045	2.24017382	0.06691734	0.77979884	0.65496908	2.296E-10	-3.328354	down	PREDICTED: uncharacterized protein LOC105160363 [Sesamum indicum]
c26550.graph_c0	3.8962461	4.0290781	4.01374015	14.3875693	18.8873668	19.3217401	2.834E-08	1.116493	up	PREDICTED: glycoprotein 3-alpha-L-fucosyltransferase A [Sesamum indicum]
c26551.graph_c0	1.50967329	0.86353428	1.10856568	6.15929922	9.89278844	11.6681771	0.0003425	1.967889	up	S-adenosylmethionine synthetase [Gentiana triflora]

#ID	0 h-1 FPKM	0 h-2 FPKM	0 h-3 FPKM	48 h-1 FPKM	48 h-2 FPKM	48 h-3 FPKM	FDR	log2FC	regulated	nr_annotation
c26554.graph_c0	16.216429	14.0597906	16.4084672	77.756668	84.4922998	86.2487602	4.068E-13	1.392381	up	hypothetical protein MIMGU_mgv1a016711mg [Erythranthe guttata]
c26555.graph_c0	3.04088308	1.88933466	2.56018786	33.617518	32.2365243	38.0784792	2.911E-19	2.776825	up	PREDICTED: cytochrome c oxidase subunit 6b-2-like [Sesamum indicum]
c26558.graph_c0	1.42630536	1.00781195	1.83285886	0.15458889	0	0.06877613	9.192E-13	-5.244669	down	-
c26561.graph_c0	2.72465804	2.35547005	2.54639524	14.7185054	16.1968311	14.4162336	6.402E-07	1.553224	up	PREDICTED: vesicle-associated membrane protein 724 [Sesamum indicum]
c26569.graph_c0	0.3729222	0.07465913	0.57506375	22.6749768	24.530933	25.434061	9.727E-37	5.121348	up	PREDICTED: uncharacterized protein LOC105155725 [Sesamum indicum]
c26574.graph_c0	6.91418328	7.11335976	5.75879582	0.53081638	0.81226253	0.57727661	2.913E-26	-4.386133	down	PREDICTED: zinc finger CCCH domain-containing protein 48-like [Sesamum indicum]
c26576.graph_c0	1.62758065	2.50415677	2.40135826	8.49678959	15.737599	18.6786162	0.0008737	1.684379	up	PREDICTED: ATP-dependent zinc metalloprotease FTSH 7, chloroplastic [Sesamum indicum]
c26578.graph_c0	0.63278934	0.63342298	0.5227453	14.5325298	11.3370529	13.2273692	6.076E-22	3.438102	up	PREDICTED: transcription repressor KAN1 [Sesamum indicum]
c26579.graph_c0	44.8314832	44.2409039	41.6829249	13.0338431	13.6307535	13.4559985	1.084E-75	-2.721896	down	PREDICTED: uncharacterized protein LOC105161451 [Sesamum indicum]
c26580.graph_c0	2.70180332	2.66796135	1.9236337	0.739996	1.28280441	1.31688797	8.369E-08	-2.150574	down	PREDICTED: transcription factor TCP5-like [Sesamum indicum]
c26585.graph_c0	0.03186086	0	0	7.86641678	5.53494635	5.61526278	2.957E-22	8.232175	up	PREDICTED: probable isoprenylcysteine alpha-carbonyl methylesterase ICMEL2 [Sesamum indicum]
c26586.graph_c0	1.90805198	1.90996261	2.29114055	28.3845018	24.117006	24.4102069	9.187E-32	2.637831	up	hypothetical protein NitaMp096 [Nicotiana tabacum]
c26587.graph_c0	0.13548058	0.3390406	0.43524476	6.92715166	9.18962464	15.7147552	1.276E-06	4.094288	up	Selenoprotein H [Gossypium arboreum]
c26588.graph_c0	18.3667864	17.6398329	17.861009	124.96152	136.620695	157.476792	2.675E-30	1.939619	up	-
c26591.graph_c0	20784.3207	19032.3289	20528.9155	3386.93157	2759.40044	3193.99188	4.937E-34	-3.706491	down	-
c26599.graph_c1	6.26271054	5.1676741	7.12341797	0.89662974	2.31272813	1.66500678	8.099E-20	-2.961934	down	PREDICTED: uncharacterized protein LOC103430095 [Malus domestica]
c26601.graph_c0	0	0	0	2.21144954	1.87421011	3.34514865	1.504E-09	Inf	up	PREDICTED: uncharacterized protein LOC105161958 [Sesamum indicum]
c26604.graph_c0	0.93702604	0.39081847	0.50171482	11.5099934	13.2603686	12.353837	3.05E-13	3.327533	up	-
c26605.graph_c0	0.96542299	0.37581822	0.55138078	9.437635	9.73630137	9.80447038	2.171E-12	2.922002	up	hypothetical protein MIMGU_mgv1a010920mg [Erythranthe guttata]
c26607.graph_c0	14.4314151	14.445866	13.5996225	1.92041213	2.81692217	1.97165904	5.692E-19	-3.684131	down	PREDICTED: heat stress transcription factor A-8 [Sesamum indicum]
c26607.graph_c1	41.5186745	43.9383561	42.3772219	5.62802287	6.18303031	4.72957086	2.02E-112	-3.96751	down	PREDICTED: heat stress transcription factor A-8 [Sesamum indicum]
c26609.graph_c1	20.3570047	22.8790233	21.357922	19.04479	17.9496942	17.2045044	7.99E-18	-1.269105	down	PREDICTED: homeobox-leucine zipper protein HDG5 isoform X2 [Sesamum indicum]

#ID	0 h-1 FPKM	0 h-2 FPKM	0 h-3 FPKM	48 h-1 FPKM	48 h-2 FPKM	48 h-3 FPKM	FDR	log2FC	regulated	nr_annotation
c26610.graph_c0	0	0.06292394	0	4.57502021	5.27613174	8.0901215	1.479E-13	7.1438	up	PREDICTED: DNA primase small subunit [Sesamum indicum]
c26612.graph_c0	0	0	0	10.7144591	12.3495292	15.4063967	1.108E-23	Inf	up	PREDICTED: gibberellin-regulated protein 9-like [Solanum tuberosum]
c26616.graph_c0	62.770186	64.0623829	57.3402847	68.9326397	38.7991038	27.2562341	3.039E-08	-1.449686	down	PREDICTED: uncharacterized protein LOC105165909 [Sesamum indicum]
c26617.graph_c0	28.5772278	29.1004691	26.5279516	29.0332687	18.7811992	17.0123891	1.042E-14	-1.384061	down	PREDICTED: probable F-box protein At5g04010 [Sesamum indicum]
c26624.graph_c0	0.04105661	0.08219545	0	13.1627627	17.0297391	16.1210301	4.1E-51	7.546743	up	PREDICTED: vinorine synthase-like [Sesamum indicum]
c26625.graph_c0	0.22899539	0.45844938	0.58853628	5.06317167	5.5873088	4.88061135	9.467E-06	2.580032	up	PREDICTED: V-type proton ATPase subunit c''2 [Sesamum indicum]
c26626.graph_c0	0	0	0.11462349	1.89003407	1.82815218	3.29036272	7.461E-07	4.879947	up	-
c26627.graph_c0	70.4537045	71.0029247	72.3740179	68.5287837	65.9085554	63.241341	1.862E-15	-1.129904	down	PREDICTED: protein MODIFIER OF SNC1 11-like [Sesamum indicum]
c26630.graph_c0	32.3789992	30.748145	30.4127211	11.4602107	9.81775312	9.68184329	1.032E-56	-2.608925	down	PREDICTED: annexin D5 [Sesamum indicum]
c26632.graph_c0	0.98645955	0.70531953	0.09054568	4.67378167	4.12608747	3.81214426	0.0009457	1.823381	up	PREDICTED: ethylene-responsive transcription factor-like protein At4g13040 isoform X2 [Sesamum indicum]
c26634.graph_c0	1.45007138	1.6203052	2.16674318	13.0793963	14.5801028	13.0477933	2.316E-14	1.935654	up	PREDICTED: pentatricopeptide repeat-containing protein At3g59040 [Sesamum indicum]
c26636.graph_c0	56.2039248	60.248626	50.3582056	76.5236817	47.5791559	38.6178123	5.809E-05	-1.040206	down	PREDICTED: ethylene-responsive transcription factor ERF008-like [Sesamum indicum]
c26638.graph_c0	0.47739881	0.47787686	0.24539055	13.8100631	17.984757	20.8193475	1.103E-19	4.436602	up	-
c26639.graph_c0	3.9333911	3.82795953	4.49294515	0.60394941	0.63981116	0.62695564	3.174E-12	-3.731592	down	PREDICTED: UDP-glucuronate 4-epimerase 3-like [Sesamum indicum]
c26640.graph_c0	1.22171486	1.22293822	1.50956919	0.3463149	0.18343932	0.46222337	1.842E-08	-3.011799	down	-
c26644.graph_c0	0.04765713	0	0	4.69781991	5.72096054	6.71940007	4.321E-20	7.48936	up	PREDICTED: protein polybromo-1-like isoform X1 [Cucumis melo]
c26648.graph_c0	0.16870275	0	0	9.63603833	11.1961059	17.0098862	1.548E-12	6.807266	up	-
c26649.graph_c0	26.3751529	21.7601928	23.4466074	21.0809001	21.8624962	18.8070204	1.612E-12	-1.229555	down	PREDICTED: ganglioside-induced differentiation-associated protein 2 [Sesamum indicum]
c26650.graph_c0	4.71048956	5.52987557	6.05316558	3.86248031	5.00647378	5.05413493	3.812E-08	-1.25122	down	PREDICTED: uncharacterized protein LOC105161159 [Sesamum indicum]
c26653.graph_c0	0	0.04438812	0	3.47244758	3.67863715	5.92507488	1.507E-13	7.191904	up	PREDICTED: basic leucine zipper 61-like [Sesamum indicum]
c26655.graph_c0	0	0.44280995	0	13.6525002	18.1329277	16.6807158	1.578E-13	5.763735	up	BnaA05g08010D [Brassica napus]
c26655.graph_c1	0.9964087	0.66493764	0.42680821	28.8937796	30.0074545	30.2991064	7.648E-58	4.405647	up	PREDICTED: uncharacterized protein LOC105174078 [Sesamum indicum]
c26658.graph_c0	1.16106525	1.93704647	1.65779424	0.07923326	0	0.07050128	3.874E-19	-5.988474	down	-

#ID	0 h-1 FPKM	0 h-2 FPKM	0 h-3 FPKM	48 h-1 FPKM	48 h-2 FPKM	48 h-3 FPKM	FDR	log2FC	regulated	nr_annotation
c26663.graph_c0	0.35809017	0.39429362	0.27609603	21.0803558	12.6862995	10.9783772	7.373E-08	4.440517	up	PREDICTED: uncharacterized calcium-binding protein At1g02270 [Sesamum indicum]
c26665.graph_c0	3.49271871	3.45459452	2.08384513	21.9494491	23.6180067	26.0406504	3.907E-19	1.972657	up	PREDICTED: uncharacterized protein LOC105157763 [Sesamum indicum]
c26666.graph_c0	22.505543	21.3951387	20.0821362	9.54466275	15.2096042	19.9473361	9.959E-20	-1.54361	down	PREDICTED: potassium/sodium hyperpolarization-activated cyclic nucleotide-gated channel 4 [Sesamum indicum]
c26667.graph_c0	70.096248	73.1312179	67.1248351	52.5878642	35.8263843	56.3568425	3.159E-24	-1.552383	down	hypothetical protein CARUB_v10021660mg, partial [Capsella rubella]
c26670.graph_c0	1.28484957	1.45762098	1.68777329	6.31299856	8.66634736	10.3685408	1.614E-06	1.489482	up	hypothetical protein MIMGU_mgv1a011972mg [Erythranthe guttata]
c26671.graph_c0	18.012511	10.3855956	10.9252848	0	0	0	3.463E-22	-Inf	down	predicted protein [Hordeum vulgare subsp. vulgare]
c26672.graph_c0	1.08462259	0.82513859	0.50176187	11.9507319	13.6765668	13.4788237	5.247E-19	3.0062	up	PREDICTED: alpha/beta hydrolase domain-containing protein 17B-like [Sesamum indicum]
c26673.graph_c0	25.9940895	25.4979758	26.2535353	24.5881852	9.92716447	5.34489072	2.414E-10	-1.95752	down	PREDICTED: uncharacterized protein LOC105177028 [Sesamum indicum]
c26674.graph_c0	19.1689178	19.787741	19.1674117	84.7663552	84.2456585	89.1247633	9.521E-14	1.133316	up	PREDICTED: 50S ribosomal protein L5, chloroplastic [Sesamum indicum]
c26676.graph_c0	1.04607464	0.4430132	0.93063276	10.0819121	11.622966	12.1369982	7.138E-17	2.784138	up	PREDICTED: probable receptor-like protein kinase At2g42960 [Sesamum indicum]
c26682.graph_c0	1.21276433	0.60698937	1.36364391	15.5022268	15.3870619	21.7469633	3.957E-10	3.02355	up	PREDICTED: transcription factor bHLH35-like isoform X1 [Nicotiana tomentosiformis]
c26684.graph_c0	3.21498733	1.68572729	2.16405933	0	0	0	7.019E-16	-Inf	down	predicted protein [Nectria haematococca mpVI 77-13-4]
c26685.graph_c0	0.08453855	0	0.07242359	6.8276928	7.94817319	8.47761627	1.942E-35	6.186397	up	PREDICTED: kinesin-like protein NACK1 [Sesamum indicum]
c26690.graph_c0	4.65137867	5.16565224	4.69850004	2.79281143	4.31374968	4.07847913	1.618E-09	-1.400579	down	PREDICTED: putative pentatricopeptide repeat-containing protein At5g59900 [Sesamum indicum]
c26691.graph_c0	3.25755322	4.6441913	4.18608486	16.3694755	20.3280616	26.9461026	3.52E-05	1.37181	up	Ferredoxin [Medicago truncatula]
c26692.graph_c1	1.43408748	2.15328525	2.30357399	14.2026305	14.3461525	17.9274959	0.0003078	1.957119	up	PREDICTED: fasciclin-like arabinogalactan protein 17 [Sesamum indicum]
c26693.graph_c0	1.77994775	1.83829296	1.23441767	12.2855154	15.0555033	13.062309	5.513E-17	2.0412	up	PREDICTED: ATP-dependent 6-phosphofructokinase 4, chloroplastic [Sesamum indicum]
c26694.graph_c0	0.09804912	0.07361048	0.0629985	12.6009296	16.0524808	17.2604063	1.778E-70	6.593504	up	PREDICTED: putative inactive cadmium/zinc-transporting ATPase HMA3 [Sesamum indicum]
c26695.graph_c0	4.80948768	5.11519763	4.63528656	0.4615431	0.39115916	0.53388176	5.667E-25	-4.408619	down	-
c26696.graph_c0	0.23335796	0.13348094	0.12851752	3.53188949	3.67653719	5.49281269	1.712E-12	3.663547	up	PREDICTED: glutamate synthase 1 [NADH], chloroplastic isoform X1 [Sesamum indicum]
c26697.graph_c0	2.15414048	1.64463371	1.73596212	0	0	0	8.456E-28	-Inf	down	hypothetical protein VHEM06393 [Torrubiella hemipterigena]
c26698.graph_c0	1.19765436	1.04416284	1.16668651	22.8857663	23.3209074	23.1030463	8.219E-70	3.327915	up	PREDICTED: auxin response factor 9 [Sesamum indicum]

#ID	0 h-1 FPKM	0 h-2 FPKM	0 h-3 FPKM	48 h-1 FPKM	48 h-2 FPKM	48 h-3 FPKM	FDR	log2FC	regulated	nr_annotation
c26701.graph_c0	1.0291294	1.07698537	1.38258438	10.989354	12.7832496	12.577528	7.606E-13	2.357882	up	PREDICTED: 3-deoxy-manno-octulosonate cytidylyltransferase, mitochondrial [Sesamum indicum]
c26703.graph_c0	1.41739228	1.0286384	1.13837825	11.4909891	12.8303916	11.4736424	3.589E-15	2.302532	up	PREDICTED: probable arabinosyltransferase ARAD1 [Sesamum indicum]
c26708.graph_c0	0	0	0	46.7179013	75.3229702	64.2648561	1.216E-48	Inf	up	PREDICTED: intracellular ribonuclease LX-like [Sesamum indicum]
c26709.graph_c0	0	0	0	1.9558971	2.98616926	2.35458506	4.674E-12	Inf	up	RecName: Full=60S ribosomal protein L5 [Helianthus annuus]
c26715.graph_c1	0.51613562	0.53510432	0.54481636	6.57205883	8.27560138	9.86718168	2.186E-20	2.929407	up	hypothetical protein MIMGU_mgv1a001258mg [Erythranthe guttata]
c26721.graph_c0	0	0	0	30.4092298	54.1609199	41.745143	4.582E-33	Inf	up	hypothetical protein MIMGU_mgv1a020088mg [Erythranthe guttata]
c26721.graph_c1	0	0	0	40.8621787	60.7143226	53.5780819	1.029E-59	Inf	up	hypothetical protein MIMGU_mgv1a026045mg [Erythranthe guttata]
c26722.graph_c0	4.91935208	4.67389105	4.98224817	41.9404626	38.4497607	33.1832771	1.977E-18	1.947124	up	PREDICTED: aldose 1-epimerase-like [Sesamum indicum]
c26726.graph_c0	104.171036	104.337232	103.595568	79.8224139	92.5869765	98.1890216	1.918E-17	-1.226451	down	PREDICTED: receptor homology region, transmembrane domain- and RING domain-containing protein 2-like [Sesamum indicum]
c26727.graph_c0	1.23801183	1.40448505	0.58332788	8.93420844	15.2240876	14.9181948	4.784E-09	2.579206	up	PREDICTED: uncharacterized protein LOC105174183 [Sesamum indicum]
c26734.graph_c0	1130.10945	1108.20134	1247.469	560.33567	351.895523	250.572263	1.775E-44	-2.590055	down	hypothetical protein MIMGU_mgv1a014589mg [Erythranthe guttata]
c26737.graph_c0	15.3864814	8.15842617	10.8160014	0	0	0	3.614E-29	-Inf	down	cytochrome c oxidase subunit III (mitochondrion) [Chlorella sorokiniana]
c26738.graph_c0	0.20906542	0.13951651	0.13432867	6.67696312	12.0384385	11.6251755	3.2E-16	4.949333	up	PREDICTED: UPF0496 protein At4g34320-like [Sesamum indicum]
c26739.graph_c0	0.13113565	0.13126696	0	53.0359041	65.3999793	67.8303849	1.55E-65	8.466271	up	PREDICTED: NAC domain-containing protein 100-like [Sesamum indicum]
c26741.graph_c0	2.21161621	1.64360166	1.55018967	12.8423304	14.2916772	14.7507553	3.571E-14	1.937057	up	PREDICTED: calcium uptake protein 1, mitochondrial [Sesamum indicum]
c26743.graph_c0	26.9953053	28.7808405	24.7194726	9.79148641	12.258873	12.0725622	2.083E-49	-2.258485	down	PREDICTED: endo-1,3(4)-beta-glucanase 2 [Sesamum indicum]
c26744.graph_c0	1.74652054	1.20193522	1.4728534	0	0.21306869	0	2.574E-16	-5.42726	down	-
c26746.graph_c0	0	0.0352468	0	6.55271254	10.1377836	11.3147583	1.256E-25	8.619815	up	PREDICTED: uncharacterized protein LOC105157258 [Sesamum indicum]
c26750.graph_c1	3.65393183	4.07165756	4.16388702	0.76216762	1.21113627	1.52588737	5.749E-10	-2.792598	down	hypothetical protein CICLE_v10032119mg [Citrus clementina]
c26753.graph_c0	0.3430803	0.08585596	0.22043589	6.55841001	4.60399046	4.21853045	1.915E-08	3.55808	up	hypothetical protein CICLE_v10022498mg [Citrus clementina]
c26754.graph_c0	0.08625939	0.08634576	0.11084673	0.79467772	1.13651732	0.88387432	5.307E-05	2.286755	up	PREDICTED: probable receptor-like protein kinase At5g20050 [Sesamum indicum]
c26755.graph_c0	4.74696891	2.40113626	2.95268038	0	0	0	4.132E-20	-Inf	down	NADH dehydrogenase subunit 5 [Pneumocystis carinii]

#ID	0 h-1 FPKM	0 h-2 FPKM	0 h-3 FPKM	48 h-1 FPKM	48 h-2 FPKM	48 h-3 FPKM	FDR	log2FC	regulated	nr_annotation
c26756.graph_c0	3.06699925	1.94767906	2.7122343	2.00520757	2.05990233	2.05455825	0.0002122	-1.354534	down	PREDICTED: uncharacterized protein LOC105171097 [Sesamum indicum]
c26757.graph_c0	56.2531835	55.7077798	53.1382741	35.6472871	32.8543523	28.0878146	8.903E-37	-1.7874	down	PREDICTED: protein SPA1-RELATED 4-like [Solanum lycopersicum]
c26758.graph_c0	0.75856059	0.89737839	0.443082	2.92240299	3.56705163	4.80496981	0.0071142	1.410233	up	hypothetical protein MIMGU_mgv1a001946mg [Erythranthe guttata]
c26760.graph_c0	3.27785664	2.91656793	2.63260972	52.3847138	52.0740078	50.9406233	1.18E-61	3.122702	up	PREDICTED: NAC domain-containing protein 18 [Sesamum indicum]
c26762.graph_c0	1.09647608	1.20733144	0.98631062	8.01381925	8.86421402	9.54248506	7.234E-17	1.987052	up	PREDICTED: uncharacterized protein LOC105177385 [Sesamum indicum]
c26763.graph_c0	0	0	0	2.94002364	3.27432155	5.03079876	8.474E-12	Inf	up	unnamed protein product [Coffea canephora]
c26765.graph_c0	0.46446595	0.19925616	0.42632652	26.6518282	26.744952	30.0784265	9.139E-47	5.238789	up	PREDICTED: uncharacterized protein LOC105158032 [Sesamum indicum]
c26766.graph_c0	1.81079752	2.24763734	1.58232362	7.24009557	10.6743825	10.5687598	9.852E-06	1.314021	up	hypothetical protein MIMGU_mgv1a004061mg [Erythranthe guttata]
c26767.graph_c0	54.6529913	54.2397096	49.0132595	15.4608879	14.9619899	14.9672988	6.348E-84	-2.814249	down	unnamed protein product [Coffea canephora]
c26773.graph_c0	31.1427437	34.0242877	31.8154361	17.0929354	17.6690905	14.1526561	5.62E-42	-2.003764	down	PREDICTED: flavonoid 3'-monooxygenase-like [Sesamum indicum]
c26775.graph_c0	0.28165492	0.56387391	0.12064591	10.7251272	10.6289413	18.0858154	4.843E-10	4.338442	up	PREDICTED: transcription factor bHLH35-like isoform X2 [Sesamum indicum]
c26777.graph_c0	14.1023944	13.2036696	14.6483772	4.80075588	5.7851188	5.1794165	8.133E-39	-2.432904	down	PREDICTED: pentatricopeptide repeat-containing protein At5g47360 [Sesamum indicum]
c26779.graph_c0	0.05748938	0.28773475	0.18469033	8.0238899	8.21979918	10.4148751	6.569E-32	4.630126	up	PREDICTED: probable leucine-rich repeat receptor-like protein kinase At1g35710 [Sesamum indicum]
c26780.graph_c0	31.5938168	31.4146168	29.3668309	1.55233519	1.85007476	1.72657314	4.536E-79	-5.190229	down	PREDICTED: desiccation-related protein PCC3-06 isoform X2 [Sesamum indicum]
c26783.graph_c0	1.42725599	2.07808751	0.66693834	26.3573935	43.6882064	42.5444902	2.461E-16	3.73575	up	-
c26785.graph_c0	1.50732417	1.19666108	0.80150534	7.56572291	11.3629898	13.0377977	4.933E-08	2.168884	up	1-deoxy-D-xylulose-5-phosphate synthase [Andrographis paniculata]
c26786.graph_c0	9.49467719	8.73058825	10.2148209	1.93250235	2.47825232	3.2580549	5.6E-23	-2.915933	down	hypothetical protein MIMGU_mgv1a013028mg [Erythranthe guttata]
c26787.graph_c0	15.3789918	18.2412996	17.7998881	12.5184165	12.6449191	10.1889906	5.641E-16	-1.55743	down	PREDICTED: CASP-like protein 4A3 [Sesamum indicum]
c26788.graph_c0	2.5283713	2.60426259	1.93059922	11.9840826	9.36977119	8.59076698	0.0003523	1.075063	up	PREDICTED: uncharacterized protein LOC105161938 [Sesamum indicum]
c26789.graph_c0	1.84425579	2.50318987	1.56657164	11.0582154	13.4232523	15.7330042	2.643E-12	1.746163	up	PREDICTED: uncharacterized protein LOC105174843 [Sesamum indicum]
c26790.graph_c0	4.95812546	3.69050302	3.1040093	3.98213978	2.23337342	2.39692743	0.0015825	-1.450648	down	-

#ID	0 h-1 FPKM	0 h-2 FPKM	0 h-3 FPKM	48 h-1 FPKM	48 h-2 FPKM	48 h-3 FPKM	FDR	log2FC	regulated	nr_annotation
c26792.graph_c0	0	0	0	2.98069059	4.01110734	3.15396823	5.027E-12	Inf	up	PREDICTED: uncharacterized protein LOC105170774 [Sesamum indicum]
c26796.graph_c0	5.56169541	4.86335758	4.92896468	75.2668048	77.6771409	76.8238491	4.001E-57	2.886372	up	PREDICTED: acylpyruvase FAHD1, mitochondrial [Sesamum indicum]
c26803.graph_c0	5.51934091	4.32072987	3.77361181	36.8320374	34.2539159	33.439985	3.345E-27	1.928118	up	PREDICTED: probable sodium-coupled neutral amino acid transporter 6 [Sesamum indicum]
c26806.graph_c0	0	0	0	1.58178576	2.30410154	1.78132093	1.698E-15	Inf	up	PREDICTED: uncharacterized protein LOC105179467 isoform X2 [Sesamum indicum]
c26809.graph_c0	0.82680378	1.18233101	1.11306967	6.42008985	6.80130672	7.03580508	5.104E-06	1.677339	up	PREDICTED: aluminum-activated malate transporter 2-like [Sesamum indicum]
c26810.graph_c0	18.5002897	11.3270422	13.3870784	0	0	0	4.155E-30	-Inf	down	-
c26813.graph_c0	2.84650355	2.21616414	1.4225049	196.970231	230.736526	281.042918	6.791E-57	5.756223	up	PREDICTED: non-specific lipid-transfer protein 2-like [Sesamum indicum]
c26813.graph_c1	2.35516353	3.17358713	2.79367235	1.50212095	1.591315	0.81679778	1.477E-05	-2.104465	down	PREDICTED: PRA1 family protein F2-like [Sesamum indicum]
c26814.graph_c0	6.87094134	6.14418725	9.18260892	58.657623	63.4818039	66.536943	4.631E-25	2.064357	up	hypothetical protein MIMGU_mgv1a016555mg [Erythranthe guttata]
c26815.graph_c0	27.5765226	24.5707147	28.2976164	119.767802	118.30253	119.900104	4.881E-13	1.13576	up	PREDICTED: tubulin-folding cofactor A [Sesamum indicum]
c26817.graph_c0	0.3319957	0.66465629	1.27988287	27.8329048	19.1170294	25.854147	8.468E-11	3.974596	up	unknown [Medicago truncatula]
c26817.graph_c1	1.3492705	1.75080577	1.92651779	46.9130991	56.6243741	63.6181245	2.562E-57	4.030857	up	PREDICTED: protein BRASSINAZOLE-RESISTANT 1 [Sesamum indicum]
c26818.graph_c0	64.4910871	62.5058671	62.554912	9.93407262	12.8260584	12.2814969	5.32E-119	-3.457101	down	PREDICTED: uncharacterized protein LOC105165319 [Sesamum indicum]
c26819.graph_c0	1.19267902	1.42029756	1.13626741	14.2651138	15.9952061	15.1877878	4.009E-35	2.581532	up	PREDICTED: BTB/POZ domain-containing protein At1g63850-like [Sesamum indicum]
c26821.graph_c0	4.8033793	6.01023645	4.56230668	61.8083088	82.2429194	74.3845896	1.564E-46	2.807864	up	hypothetical protein MIMGU_mgv1a008879mg [Erythranthe guttata]
c26822.graph_c0	1.39291113	1.81865989	1.08953231	0.33475841	0.23642396	0.19857735	1.719E-10	-3.487614	down	-
c26824.graph_c0	0	0.2833105	0.36370102	117.421271	131.759615	135.879099	4.31E-163	8.183969	up	PREDICTED: arabinogalactan peptide 22-like [Sesamum indicum]
c26826.graph_c0	10.2826405	10.1519378	12.1275508	8.30513016	9.21069766	7.62078784	0.0001707	-1.393347	down	hypothetical protein CICLE_v10002915mg [Citrus clementina]
c26829.graph_c0	78.0159633	93.9857191	79.7650926	7.78366887	11.1053025	11.0031836	3.1E-97	-4.097555	down	-
c26830.graph_c0	5.89609683	5.41016747	6.3981173	3.31947548	4.699432	4.80639299	6.074E-09	-1.490249	down	PREDICTED: F-box/kelch-repeat protein At3g06240-like [Sesamum indicum]
c26830.graph_c1	8.35110496	8.1365482	7.44050702	1.43613542	2.82547819	2.09934908	2.379E-12	-2.937754	down	PREDICTED: type 1 phosphatases regulator ypi1 [Sesamum indicum]
c26833.graph_c0	1.62011843	0.94601542	0.86746513	33.7069521	26.8142628	33.8657803	2.385E-33	3.770801	up	unnamed protein product [Coffea canephora]

#ID	0 h-1 FPKM	0 h-2 FPKM	0 h-3 FPKM	48 h-1 FPKM	48 h-2 FPKM	48 h-3 FPKM	FDR	log2FC	regulated	nr_annotation
c26839.graph_c0	0	0	0	42.8815019	54.6114483	67.5379488	1.191E-47	Inf	up	PREDICTED: 36.4 kDa proline-rich protein-like, partial [Cucumis sativus]
c26840.graph_c0	0.08246542	0.082548	0	27.4260823	34.6079826	36.2335452	4.94E-112	8.212418	up	PREDICTED: uncharacterized protein LOC105157872 [Sesamum indicum]
c26843.graph_c0	5.75859373	7.30152277	4.44001256	0.47157264	1.37382857	0.20980125	1.682E-15	-4.116347	down	-
c26844.graph_c0	17.7153225	15.8396879	17.9036381	159.719997	176.903245	182.204366	7.382E-59	2.313644	up	PREDICTED: alcohol dehydrogenase class-3 [Sesamum indicum]
c26847.graph_c0	24.4006139	27.7004369	23.9072614	3.70350879	4.01466078	2.14581572	2.508E-54	-3.960037	down	PREDICTED: WRKY transcription factor 22 [Sesamum indicum]
c26848.graph_c0	56.9290091	50.0111641	52.961918	443.065489	475.595829	505.799762	6.14E-50	2.136227	up	TPA: hypothetical protein ZEAMMB73_262778, partial [Zea mays]
c26851.graph_c0	1.21368323	1.95733655	1.73292334	0.24847203	0.26322599	0.44217784	1.132E-09	-3.386466	down	-
c26853.graph_c0	49.7629291	54.8733634	47.231831	41.8419279	14.1087193	7.36997529	5.442E-13	-2.250841	down	PREDICTED: scarecrow-like protein 21 [Sesamum indicum]
c26854.graph_c0	1.58550047	2.01438107	1.56725452	176.010307	186.223517	221.297595	1.17E-126	5.800551	up	PREDICTED: lipoxygenase homology domain-containing protein 1-like [Sesamum indicum]
c26856.graph_c0	0.17672281	0.35379955	0.22709581	114.94286	131.426675	133.27681	4.561E-97	7.949454	up	PREDICTED: dof zinc finger protein DOF1.4-like isoform X2 [Sesamum indicum]
c26859.graph_c0	2.232391	2.00737626	1.7017776	9.72539238	11.5584213	11.7242264	3.978E-07	1.45563	up	hypothetical protein MIMGU_mgv1a011616mg [Erythranthe guttata]
c26860.graph_c0	2.32599749	2.12586344	2.46917297	22.2671166	22.7997097	25.4089096	1.216E-20	2.328336	up	hypothetical protein MIMGU_mgv1a011282mg [Erythranthe guttata]
c26862.graph_c0	73.3254192	76.640103	74.0922918	10.3926398	13.0995538	11.6447588	5.92E-125	-3.693689	down	PREDICTED: probable serine/threonine-protein kinase WNK11 [Sesamum indicum]
c26866.graph_c0	0.03798013	0.03801816	0	2.02940685	2.59471954	2.77089839	1.262E-12	5.598507	up	PREDICTED: methylthioribose kinase [Sesamum indicum]
c26869.graph_c1	2.51480591	1.67821607	2.89499754	9.50854659	10.3799487	9.36254402	0.0015143	1.023564	up	PREDICTED: GDSL esterase/lipase 5 isoform X2 [Sesamum indicum]
c26871.graph_c0	1.74445425	1.30965079	2.03153408	1.20532878	1.4897163	1.76514687	0.0028494	-1.21474	down	PREDICTED: LOW QUALITY PROTEIN: anthranilate synthase alpha subunit 1, chloroplastic [Sesamum indicum]
c26872.graph_c0	0	0.04976405	0.0638848	1.37400074	2.57153735	2.4044026	8.486E-10	4.762997	up	PREDICTED: WAT1-related protein At5g07050-like [Sesamum indicum]
c26873.graph_c0	4.24690599	3.05364915	2.69028848	3.4165727	3.6194445	2.59874887	0.0054033	-1.064807	down	hypothetical protein MIMGU_mgv1a015234mg [Erythranthe guttata]
c26875.graph_c0	4.33656446	4.34090687	7.0586989	73.6434899	61.651255	85.0199023	4.245E-22	2.785035	up	PREDICTED: histone H2A [Nicotiana sylvestris]
c26878.graph_c0	0	0.06439695	0	18.0765325	24.5495366	24.6803097	5.584E-50	9.018161	up	PREDICTED: probable receptor-like protein kinase At1g80640 isoform X1 [Sesamum indicum]
c26885.graph_c0	3.42620939	4.15236134	4.20038645	56.2597474	61.2146642	60.5621977	4.284E-88	2.897504	up	PREDICTED: LOW QUALITY PROTEIN: SNF2 domain-containing protein CLASSY 1-like [Sesamum indicum]
c26891.graph_c0	14.3484255	4.78759775	4.43884875	0	0	0	5.773E-07	-Inf	down	PREDICTED: chlorophyll a-b binding protein of LHCII type 1 [Elaeis guineensis]

#ID	0 h-1 FPKM	0 h-2 FPKM	0 h-3 FPKM	48 h-1 FPKM	48 h-2 FPKM	48 h-3 FPKM	FDR	log2FC	regulated	nr_annotation
c26893.graph_c0	1.40606647	1.11770029	1.85999377	0.72387773	0.72649959	0.50850162	8.691E-06	-2.1812	down	hypothetical protein MIMGU_mgv1a018417mg [Erythranthe guttata]
c26894.graph_c0	0.07117205	0.14248664	0.36583561	6.55683349	6.25155302	8.63466189	3.093E-13	4.175234	up	hypothetical protein MIMGU_mgv1a008083mg [Erythranthe guttata]
c26895.graph_c0	3.57527569	2.72674727	2.84413431	17.2923301	17.2390877	17.0613445	1.546E-09	1.4802	up	hypothetical protein MIMGU_mgv1a000048mg [Erythranthe guttata]
c26897.graph_c0	1.82912893	1.09857631	1.5983421	27.1640195	27.7058812	26.809331	4.931E-25	3.155894	up	PREDICTED: hypoxanthine-guanine phosphoribosyltransferase [Nicotiana sylvestris]
c26898.graph_c0	0.6932653	0.56778504	0.48593092	7.34483391	9.04190716	8.05945211	2.154E-15	2.78921	up	PREDICTED: probable receptor-like protein kinase At2g42960 [Nelumbo nucifera]
c26899.graph_c0	10.3937641	12.5877635	8.90426558	55.4428259	56.4807313	60.9032607	9.429E-10	1.422317	up	hypothetical protein MIMGU_mgv1a011204mg [Erythranthe guttata]
c26901.graph_c0	3.23386552	3.39895893	3.15829037	35.4530494	35.8223234	37.7490555	1.401E-43	2.459462	up	PREDICTED: LL-diaminopimelate aminotransferase, chloroplastic [Sesamum indicum]
c26902.graph_c0	0	0	0	3.3058881	4.23520359	2.80474264	2.998E-12	Inf	up	hypothetical protein MIMGU_mgv1a014026mg [Erythranthe guttata]
c26916.graph_c0	14.7571685	17.4153464	13.4740984	2.36128308	2.65309874	1.52804035	1.063E-38	-3.817017	down	hypothetical protein MIMGU_mgv1a026902mg, partial [Erythranthe guttata]
c26918.graph_c0	0	0.09688252	0	2.82356507	4.12474159	3.88759483	3.981E-17	5.793153	up	PREDICTED: probable beta-1,4-xylosyltransferase IRX14 [Sesamum indicum]
c26924.graph_c0	1.51205362	1.18247477	1.21440548	9.38100065	9.13099987	8.69576317	7.41E-12	1.784181	up	PREDICTED: cell wall protein RBR3 isoform X2 [Sesamum indicum]
c26928.graph_c0	21.2644127	16.8132229	15.7361502	0.91471583	0.64602034	0.40695432	7.891E-62	-5.777669	down	PREDICTED: probable protein phosphatase 2C 51 [Sesamum indicum]
c26932.graph_c0	5.84509046	5.65176238	4.76240309	20.5541961	23.5467537	23.1008217	1.715E-09	1.029431	up	PREDICTED: uncharacterized protein LOC105170351 [Sesamum indicum]
c26933.graph_c0	2.64060791	2.10481184	2.10509392	15.6999266	15.9401557	18.0183078	3.55E-20	1.840737	up	PREDICTED: ABC transporter G family member 7 isoform X2 [Sesamum indicum]
c26934.graph_c0	0.39252387	0.7421764	0.78463577	61.0331816	52.4834732	60.3132575	2.47E-99	5.480753	up	PREDICTED: BAG family molecular chaperone regulator 7 [Sesamum indicum]
c26939.graph_c0	0.37174691	0.42936826	0.33072196	18.5464739	24.2247706	29.7233266	1.085E-28	4.979164	up	PREDICTED: protein NRT1/ PTR FAMILY 5.10-like [Sesamum indicum]
c26940.graph_c0	3.92851751	3.26853097	2.88474316	15.9339094	20.1664254	20.3258343	1.27E-08	1.465395	up	PREDICTED: DELLA protein GAI-like [Sesamum indicum]
c26940.graph_c1	0.97629113	0.97726874	0	7.73503839	8.76603306	5.76216891	4.625E-05	2.510008	up	DELLA protein GAIP-B, putative [Ricinus communis]
c26940.graph_c2	4.88713012	3.94518052	4.05171335	19.1712671	22.5406153	23.6492539	4.03E-06	1.323312	up	PREDICTED: LOW QUALITY PROTEIN: DELLA protein GAI [Sesamum indicum]
c26943.graph_c0	0	0	0.06720954	3.22829989	4.6961088	5.31630906	6.955E-16	6.564692	up	PREDICTED: dirigent protein 24-like [Sesamum indicum]

#ID	0 h-1 FPKM	0 h-2 FPKM	0 h-3 FPKM	48 h-1 FPKM	48 h-2 FPKM	48 h-3 FPKM	FDR	log2FC	regulated	nr_annotation
c26946.graph_c1	0	0	0	3.62921052	9.25265009	8.94253763	2.924E-11	Inf	up	PREDICTED: protein NRT1/ PTR FAMILY 2.10-like [Sesamum indicum]
c26947.graph_c0	90.3026384	84.5098337	90.8546653	12.6696131	14.3826456	16.8269516	1.38E-127	-3.61932	down	hypothetical protein OsJ_02873 [Oryza sativa Japonica Group]
c26950.graph_c0	0.1146561	0.09564243	0.1718939	39.1002154	58.9717087	69.7859132	1.112E-35	7.747415	up	PREDICTED: alpha-xylosidase 1 [Sesamum indicum]
c26952.graph_c0	0.46903035	0.26083334	0.73666091	10.5144661	14.5465632	15.3792472	1.5E-22	3.75399	up	hypothetical protein MIMGU_mgv1a006305mg [Erythranthe guttata]
c26957.graph_c0	0	0	0	4.53973286	1.87028197	4.41344802	1.026E-06	Inf	up	PREDICTED: heat shock factor-binding protein 1 [Sesamum indicum]
c26958.graph_c0	12.9961981	12.9016977	14.9063467	8.11389689	6.28952919	6.0751106	1.382E-10	-2.008162	down	-
c26959.graph_c0	0.30637837	0.30668517	0	7.19752142	7.92391693	6.27873654	5.558E-09	4.124333	up	-
c26965.graph_c0	1.62333324	1.57079347	1.59930307	6.82954346	11.6975818	14.0832958	0.0001915	1.738395	up	PREDICTED: ethylene-responsive transcription factor ERF118-like [Sesamum indicum]
c26968.graph_c0	4.9854943	4.18394324	4.14161296	115.937509	124.099589	125.699766	4.9E-114	3.762673	up	PREDICTED: V-type proton ATPase subunit E [Sesamum indicum]
c26969.graph_c0	0.10425848	0.27830102	0.13397628	7.94010983	7.93673612	8.29005257	4.142E-24	4.532313	up	PREDICTED: protein trichome birefringence-like 5 [Sesamum indicum]
c26972.graph_c1	7.72154434	8.80066114	8.84182294	7.81790616	8.72980596	8.14704834	0.0005299	-1.058546	down	PREDICTED: telomere repeat-binding factor 4-like isoform X2 [Sesamum indicum]
c26974.graph_c0	299.484742	322.388712	290.318029	54.935071	64.6054948	75.487507	1.35E-99	-3.246638	down	PREDICTED: WW domain-containing protein C11B10.08 [Sesamum indicum]
c26975.graph_c0	5.10844096	4.73150898	4.22545675	2.32606721	2.80802609	4.01910864	1.23E-09	-1.641628	down	PREDICTED: uncharacterized protein LOC105175632 [Sesamum indicum]
c26976.graph_c0	0.15704198	0.39299808	0.45406161	11.8635397	14.2922475	16.9283899	9.019E-36	4.394105	up	PREDICTED: 12-oxophytodienoate reductase 3 [Sesamum indicum]
c26982.graph_c0	0.14325226	0.14339571	0.18408483	0.95020795	0.64312479	0.98640421	0.0072198	1.437817	up	PREDICTED: pentatricopeptide repeat-containing protein At2g22410, mitochondrial [Sesamum indicum]
c26985.graph_c0	2.9476693	2.74944225	2.84090382	174.476351	237.044523	296.24527	4.28E-28	5.348843	up	hypothetical protein MIMGU_mgv1a004588mg [Erythranthe guttata]
c26992.graph_c0	2.53283036	2.30487874	2.36711803	11.3488506	11.4609212	14.8168956	0.0012166	1.36544	up	hypothetical protein L484_015058 [Morus notabilis]
c26994.graph_c0	1.21261836	0.58957584	0.97947935	2.87265375	4.12526506	5.14054084	0.0056929	1.10031	up	PREDICTED: plastidal glycolate/glycerate translocator 1, chloroplastic [Sesamum indicum]
c26995.graph_c0	36.6078112	32.13438	37.9441597	156.767999	169.453186	156.045501	8.06E-16	1.157259	up	hypothetical protein PHAVU_003G132300g [Phaseolus vulgaris]
c26996.graph_c0	4.03167672	4.16052972	3.73876798	24.5829316	36.8328859	41.3284304	3.231E-09	2.08181	up	PREDICTED: uncharacterized protein LOC105176754 [Sesamum indicum]
c27002.graph_c0	4.82077705	4.53136017	3.24853955	21.1228483	26.3361213	25.5900928	7.593E-10	1.517859	up	PREDICTED: ER membrane protein complex subunit 4-like [Fragaria vesca subsp. vesca]

#ID	0 h-1 FPKM	0 h-2 FPKM	0 h-3 FPKM	48 h-1 FPKM	48 h-2 FPKM	48 h-3 FPKM	FDR	log2FC	regulated	nr_annotation
c27003.graph_c0	0.96064464	1.44240987	1.32264256	5.94219991	6.56291433	5.68107137	0.0045506	1.267309	up	PREDICTED: bet1-like SNARE 1-2 isoform X1 [Sesamum indicum]
c27004.graph_c1	4.7158969	4.50105548	3.94612089	32.4328831	29.6491023	23.5543586	5.309E-08	1.689587	up	PREDICTED: membrin-11 [Sesamum indicum]
c27013.graph_c0	3.30644709	2.71748552	2.59407468	13.8838703	17.2219318	19.2296781	1.258E-11	1.525855	up	PREDICTED: probable magnesium transporter NIPA4 [Sesamum indicum]
c27014.graph_c0	1.38727706	1.41589495	1.36324572	34.6075922	35.9723041	35.453923	1.855E-82	3.651793	up	PREDICTED: lysosomal beta glucosidase-like [Sesamum indicum]
c27015.graph_c0	3.40258416	5.03494371	2.70902125	2.45322975	2.85157023	2.39509246	0.0003447	-1.548982	down	PREDICTED: uncharacterized protein LOC105163552 [Sesamum indicum]
c27017.graph_c0	26.4437438	25.7548118	24.9051826	24.7102792	25.0286868	24.0203438	3.33E-12	-1.080772	down	PREDICTED: monoglyceride lipase [Sesamum indicum]
c27018.graph_c0	3.2162122	3.01172741	3.11083011	2.29392345	2.59889303	3.20342333	0.0003302	-1.227216	down	PREDICTED: vesicle-associated protein 2-2-like [Sesamum indicum]
c27021.graph_c0	0.02732098	0	0.14043415	0.98162479	1.62652971	1.83647165	1.152E-08	3.676828	up	PREDICTED: uncharacterized protein LOC105169333 [Sesamum indicum]
c27022.graph_c1	11.7363788	12.7271419	10.6444051	8.27473289	8.29855207	8.11545011	1.123E-21	-1.523892	down	PREDICTED: uncharacterized protein LOC105173472 isoform X1 [Sesamum indicum]
c27023.graph_c0	0	0.06195051	0.07952923	92.5935971	90.4207252	107.907564	6.25E-169	9.974167	up	PREDICTED: UPF0483 protein C25G4.2-like [Sesamum indicum]
c27028.graph_c0	3.09944386	4.48145748	4.42545342	1.42770492	0.84026681	0.42345442	5.221E-09	-3.160829	down	PREDICTED: protein UPSTREAM OF FLC-like [Sesamum indicum]
c27031.graph_c0	17.3178179	21.69316	18.0270494	54.9933252	101.976435	107.065131	0.0032572	1.182957	up	PREDICTED: uncharacterized protein LOC104093193 [Nicotiana tomentosiformis]
c27034.graph_c0	56.9919777	54.9058802	55.4190141	52.2855661	56.086741	58.9203315	5.242E-13	-1.019178	down	PREDICTED: capsanthin/capsorubin synthase, chromoplast-like [Sesamum indicum]
c27037.graph_c0	1.9051786	2.20979847	1.90417884	12.3140835	13.0157641	11.4279996	1.297E-10	1.593495	up	PREDICTED: metal transporter Nramp5-like isoform X2 [Sesamum indicum]
c27038.graph_c0	0.51375486	0.42855776	0.22006511	3.03703712	3.46807692	3.47443376	4.471E-05	2.089403	up	PREDICTED: uncharacterized protein LOC105163339 [Sesamum indicum]
c27043.graph_c0	1.68280096	2.52672905	2.04232887	0.86128111	1.36863436	0.38318138	6.353E-06	-2.278776	down	PREDICTED: lysine histidine transporter 1-like [Musa acuminata subsp. malaccensis]
c27044.graph_c0	3.78439433	3.76223737	4.23022782	4.44161744	2.55505842	2.27353398	4.826E-07	-1.351458	down	PREDICTED: BTB/POZ domain-containing protein At1g67900 [Sesamum indicum]
c27045.graph_c0	0	0.36720614	0.75424383	10.6794045	11.8147666	13.2914294	4.742E-17	3.957588	up	hypothetical protein MIMGU_mgv1a002613mg [Erythranthe guttata]
c27045.graph_c1	0.49335561	0.44446467	0.69737965	10.8173706	11.8930422	12.3752974	1.184E-18	3.399391	up	PREDICTED: cysteine-rich receptor-like protein kinase 2 [Sesamum indicum]
c27052.graph_c0	282.016234	255.957385	280.989177	5.03156557	3.24665775	4.31425473	0	-7.034643	down	PREDICTED: L-idonate 5-dehydrogenase-like [Cucumis sativus]

#ID	0 h-1 FPKM	0 h-2 FPKM	0 h-3 FPKM	48 h-1 FPKM	48 h-2 FPKM	48 h-3 FPKM	FDR	log2FC	regulated	nr_annotation
c27054.graph_c0	0.29370099	0.17639705	0.07548348	6.22325968	7.45271802	5.87447933	3.193E-13	4.154721	up	PREDICTED: uncharacterized protein LOC105156067 [Sesamum indicum]
c27056.graph_c0	0	0	0	1.39055528	2.65740142	3.34800913	4.571E-10	Inf	up	PREDICTED: probable polygalacturonase non-catalytic subunit JP650 [Sesamum indicum]
c27063.graph_c0	0.33207294	0.16620273	0.24892401	16.2396381	14.4761461	15.5387812	5.98E-58	4.937292	up	PREDICTED: uncharacterized protein LOC105165907 [Sesamum indicum]
c27064.graph_c0	7.41809007	6.41294751	4.98291834	2.32978242	3.94899515	5.45896948	5.759E-06	-1.705502	down	PREDICTED: gibberellin 3-beta-dioxygenase 1-like [Cicer arietinum]
c27067.graph_c0	28.0643843	29.9903109	25.566529	14.7063666	16.6304365	15.4648432	1.28E-37	-1.85529	down	PREDICTED: probable beta-1,3-galactosyltransferase 19 [Sesamum indicum]
c27067.graph_c1	119.804016	125.846153	145.313571	92.4096183	113.906715	126.975606	8.993E-12	-1.254296	down	PREDICTED: non-specific lipid-transfer protein 2-like [Sesamum indicum]
c27068.graph_c0	0	0	0	11.9224902	18.1219251	12.9147688	3.496E-19	Inf	up	--
c27069.graph_c0	2.4004483	2.24266185	1.64515792	0.9582948	1.01519714	1.4430053	0.0001063	-1.897797	down	hypothetical protein MIMGU_mgv1a001232mg [Erythranthe guttata]
c27071.graph_c0	2.01053102	3.45007588	2.46058282	19.7277442	20.8368603	20.7975177	8.675E-21	1.935361	up	PREDICTED: uncharacterized protein LOC105167501 [Sesamum indicum]
c27072.graph_c0	110.208019	110.008119	100.540574	23.4960827	27.7001591	27.2585269	1.256E-96	-3.05097	down	PREDICTED: growth-regulating factor 8 isoform X2 [Sesamum indicum]
c27073.graph_c0	2.75900836	1.87405895	2.91232159	12.0280588	15.8677322	16.6393497	2.842E-08	1.5364	up	PREDICTED: uncharacterized protein LOC105170966 [Sesamum indicum]
c27075.graph_c0	11.2147964	11.8962368	10.1095298	10.9486259	8.82157808	7.40942479	0.0005362	-1.29988	down	PREDICTED: putative RNA-binding protein Luc7-like 2 isoform X2 [Nicotiana sylvestris]
c27077.graph_c0	0.89516456	0.83426362	0.79332544	31.6508022	27.3242405	30.8954616	1.148E-75	4.140282	up	PREDICTED: LOW QUALITY PROTEIN: 4-coumarate--CoA ligase-like 7 [Sesamum indicum]
c27079.graph_c0	6.95490107	6.42113795	7.07176547	968.37448	757.494963	940.224969	1.865E-74	6.011738	up	hypothetical protein MIMGU_mgv1a008423mg [Erythranthe guttata]
c27081.graph_c0	19.2862261	17.4931817	19.1187495	86.8805709	80.8226047	84.7266511	7.759E-13	1.158357	up	PREDICTED: uncharacterized protein LOC105166843 [Sesamum indicum]
c27082.graph_c0	32.4560732	31.4931015	34.4463121	19.1564063	14.2939571	9.22667072	6.537E-42	-2.213957	down	PREDICTED: probable BOI-related E3 ubiquitin-protein ligase 2 [Sesamum indicum]
c27083.graph_c0	0	0	0	9.15206503	11.6843243	10.161915	1.222E-21	Inf	up	MYB117 [Jatropha curcas]
c27085.graph_c0	31.8839211	24.8918969	25.3109504	0.68046829	0.24029121	0.60547659	4.761E-76	-6.753704	down	-
c27086.graph_c0	0	0	0	70.0991655	98.9147139	96.4281939	9.534E-87	Inf	up	PREDICTED: flavonoid 3',5'-methyltransferase-like [Sesamum indicum]
c27087.graph_c0	0.41980472	0.14007503	0.26973284	23.7207579	26.1535575	29.279655	2.917E-84	5.557962	up	PREDICTED: 3-ketoacyl-CoA thiolase 2, peroxisomal [Sesamum indicum]

#ID	0 h-1 FPKM	0 h-2 FPKM	0 h-3 FPKM	48 h-1 FPKM	48 h-2 FPKM	48 h-3 FPKM	FDR	log2FC	regulated	nr_annotation
c27089.graph_c0	1.00289533	0.79475383	1.18136352	3.5994874	6.15824905	6.64538647	0.0002347	1.431069	up	PREDICTED: high affinity nitrate transporter 2.5 [Sesamum indicum]
c27090.graph_c0	0.21709742	0.21731481	0.09299293	2.00004301	2.4719369	3.85585687	1.632E-05	2.964102	up	PREDICTED: uncharacterized protein LOC103992241 isoform X1 [Musa acuminata subsp. malaccensis]
c27091.graph_c0	11.2883711	10.6036802	9.2502053	2.82597384	3.79211705	2.68217016	2.47E-31	-2.762325	down	PREDICTED: uncharacterized protein LOC105176468 [Sesamum indicum]
c27092.graph_c0	3.78141317	3.53900783	4.30617849	2.94555434	3.00044005	3.20056673	4.863E-06	-1.366123	down	PREDICTED: F-box/FBD/LRR-repeat protein At5g53840-like isoform X1 [Sesamum indicum]
c27097.graph_c0	9.88172009	5.50240971	5.83881486	0	0	0	7.836E-25	-Inf	down	--
c27099.graph_c0	0.32293863	0.25860961	0.74698007	6.78327663	7.94248634	7.25345148	3.553E-10	3.017493	up	PREDICTED: GDSL esterase/lipase CPRD49-like [Sesamum indicum]
c27099.graph_c1	0.5003733	0	0	7.06830665	6.99966509	6.56278618	3.893E-09	4.370228	up	PREDICTED: GDSL esterase/lipase CPRD49-like [Sesamum indicum]
c27101.graph_c0	0	0	0.03540727	0.60921673	1.72104335	1.06156821	1.999E-05	5.517401	up	PREDICTED: UDP-glucuronate:xylan alpha-glucuronosyltransferase 1-like isoform X1 [Sesamum indicum]
c27102.graph_c0	11.3355401	8.54902738	10.5757636	58.0327776	58.397179	64.7904927	1.158E-21	1.554142	up	PREDICTED: ATP-dependent Clp protease proteolytic subunit 3, chloroplastic [Sesamum indicum]
c27104.graph_c0	0.30035216	0.60130584	0.77192884	9.60558175	16.5411519	18.0084704	8.828E-12	3.687074	up	PREDICTED: protein GLUTAMINE DUMPER 5-like [Sesamum indicum]
c27105.graph_c0	0.04911234	0.14748455	0.0631113	2.6242383	3.21145125	3.05969639	6.56E-10	4.079877	up	PREDICTED: squamosa promoter-binding-like protein 9 [Sesamum indicum]
c27109.graph_c0	0	0	0	1.29363704	3.33675169	2.95274597	2.941E-09	Inf	up	PREDICTED: root phototropism protein 2-like [Sesamum indicum]
c27114.graph_c0	5.58817179	5.91524839	4.95242896	41.9547763	77.7334806	84.8242798	6.071E-07	2.608569	up	PREDICTED: 14-3-3-like protein 16R [Sesamum indicum]
c27115.graph_c0	10.8440727	11.3338255	13.7301354	7.63961603	3.2684266	1.8301458	4.302E-10	-2.492606	down	-
c27116.graph_c0	2.22823056	1.21661552	2.16921552	12.5033653	17.265669	21.3098827	3.063E-08	2.159155	up	PREDICTED: growth-regulating factor 6-like [Sesamum indicum]
c27117.graph_c0	5.15371226	5.6610641	6.0366425	66.1349654	71.3528262	79.371733	5.477E-59	2.664775	up	PREDICTED: very-long-chain 3-oxoacyl-CoA reductase 1-like [Sesamum indicum]
c27118.graph_c0	26.4244873	27.8541252	23.3635861	13.2802766	13.5583622	12.6913869	4.389E-43	-1.989696	down	PREDICTED: LOW QUALITY PROTEIN: putative SWI/SNF-related matrix-associated actin-dependent regulator of chromatin subfamily A member 3-like 1 [Sesamum indicum]
c27119.graph_c0	4.41421521	2.70027718	3.62405973	2.93703437	2.75242042	0.90462306	0.0003132	-1.712285	down	-
c27120.graph_c0	0.65255488	0.6205479	0.37735148	114.704403	62.6364792	49.6675907	2.875E-08	6.106186	up	PREDICTED: aureusidin synthase-like [Sesamum indicum]
c27121.graph_c0	172.753427	189.200933	170.704446	5.25300073	5.18890939	4.54776175	9.58E-277	-6.166855	down	PREDICTED: uncharacterized protein LOC105159027 [Sesamum indicum]
c27128.graph_c0	45.9495475	46.4489654	43.2666318	44.5108997	41.5052566	39.8943212	1.237E-14	-1.122687	down	PREDICTED: uncharacterized protein LOC105174416 isoform X1 [Sesamum indicum]

#ID	0 h-1 FPKM	0 h-2 FPKM	0 h-3 FPKM	48 h-1 FPKM	48 h-2 FPKM	48 h-3 FPKM	FDR	log2FC	regulated	nr_annotation
c27129.graph_c0	0	0	0	4.38422532	6.3033248	6.52830002	3.218E-21	Inf	up	PREDICTED: kinesin-like protein NACK1 [Sesamum indicum]
c27130.graph_c0	0.02561602	0	0	0.84956935	1.62502837	0.90293037	1.124E-07	6.038259	up	gelsolin with villin headpeace [Emiliania huxleyi CCMP1516]
c27132.graph_c0	1.13231781	1.29537332	1.03933801	24.4398662	23.9966059	24.5310519	2.61E-22	3.37968	up	hypothetical protein MIMGU_mgv1a012883mg [Erythranthe guttata]
c27142.graph_c0	9.37321123	9.69535032	9.38502162	5.90072926	8.04258298	8.06773915	1.022E-10	-1.39338	down	PREDICTED: LOW QUALITY PROTEIN: uncharacterized protein LOC105176443 [Sesamum indicum]
c27143.graph_c0	138.692907	145.008625	139.333365	136.841583	145.755708	135.550743	1.611E-13	-1.034725	down	hypothetical protein MIMGU_mgv1a010222mg [Erythranthe guttata]
c27145.graph_c0	2.29943494	2.41134402	3.16592671	22.0917756	27.5178816	25.4016783	2.377E-19	2.227034	up	PREDICTED: transmembrane 9 superfamily member 9-like [Sesamum indicum]
c27150.graph_c0	1.68049299	1.17361099	1.65729072	0	0	0	9.271E-24	-Inf	down	mRNA cleavage factor complex subunit [Schizosaccharomyces japonicus yFS275]
c27151.graph_c0	138.37461	137.403401	140.775385	38.8120519	46.2562456	40.4987874	2.059E-79	-2.750059	down	PREDICTED: probable protein phosphatase 2C 63 [Sesamum indicum]
c27152.graph_c0	1.44769156	1.16736375	1.55028414	0	0	0	1.547E-22	-Inf	down	hypothetical protein KNAG_0E02970 [Kazachstania naganishii CBS 8797]
c27153.graph_c0	2.7411428	3.36347517	2.91645947	11.7305165	14.9872643	17.7344987	1.955E-07	1.277923	up	PREDICTED: ERAD-associated E3 ubiquitin-protein ligase HRD1B-like [Sesamum indicum]
c27155.graph_c1	3.96400649	4.21597433	3.37471225	20.2224036	24.1071275	18.1562451	7.669E-13	1.418161	up	PREDICTED: plant intracellular Ras-group-related LRR protein 5-like [Sesamum indicum]
c27156.graph_c0	10.0181041	10.138335	11.2703474	7.87704803	7.80755983	7.85122722	1.684E-12	-1.435683	down	PREDICTED: polynucleotide 5'-hydroxyl-kinase NOL9 [Sesamum indicum]
c27157.graph_c0	1.54452424	1.11660672	0.90049968	14.5453684	14.7949251	14.5015631	3.876E-44	2.608648	up	PREDICTED: serine/threonine-protein kinase D6PKL1-like [Sesamum indicum]
c27158.graph_c0	562.953248	538.606693	581.511915	29.0873706	23.3006513	27.029235	6.21E-225	-5.420299	down	hypothetical protein MIMGU_mgv1a015139mg [Erythranthe guttata]
c27158.graph_c1	1.85968772	3.38018273	2.95577105	43.8235312	24.7890405	19.3364885	0.0023405	2.418076	up	PREDICTED: uncharacterized protein LOC104093419 [Nicotiana tomentosiformis]
c27160.graph_c0	0.41872408	0.7335009	0.58291677	13.3407261	10.8976437	10.0684705	5.652E-20	3.291667	up	PREDICTED: NAC domain-containing protein 8 [Sesamum indicum]
c27163.graph_c0	57.2848429	51.7780018	62.1050138	325.182167	318.119274	328.329521	8.792E-25	1.486464	up	hypothetical protein MIMGU_mgv1a017510mg [Erythranthe guttata]
c27165.graph_c0	13.2215243	13.8147732	12.6805875	4.14105686	3.95853477	3.6846875	8.387E-65	-2.76824	down	PREDICTED: uncharacterized protein LOC105161407 [Sesamum indicum]
c27166.graph_c0	19.0175871	20.5880738	16.9022966	2.58558633	3.22970299	3.22776241	2.777E-75	-3.663581	down	PREDICTED: probable LRR receptor-like serine/threonine-protein kinase RLK [Sesamum indicum]

#ID	0 h-1 FPKM	0 h-2 FPKM	0 h-3 FPKM	48 h-1 FPKM	48 h-2 FPKM	48 h-3 FPKM	FDR	log2FC	regulated	nr_annotation
c27171.graph_c0	0	0.10623295	0.06818849	3.07978075	4.91987547	4.4802881	2.737E-13	5.129976	up	PREDICTED: short-chain dehydrogenase TIC 32, chloroplastic [Sesamum indicum]
c27173.graph_c0	14.1247942	12.4725632	12.900118	6.18102383	6.05571103	5.45848567	2.021E-22	-2.174087	down	PREDICTED: uncharacterized protein LOC105179147 [Sesamum indicum]
c27176.graph_c0	3.62223083	3.77693536	2.61827439	0.62569412	0.36824836	0.43301912	7.82E-14	-3.816024	down	PREDICTED: phospholipase A1-Ibeta2, chloroplastic [Sesamum indicum]
c27178.graph_c0	83.9686391	74.4664161	82.4040443	54.0654732	56.4907007	58.1556202	3.958E-27	-1.532236	down	PREDICTED: multiprotein-bridging factor 1b-like [Sesamum indicum]
c27180.graph_c0	0.1196463	0	0	2.86587531	1.67371843	1.76541135	3.767E-07	4.734172	up	PREDICTED: uncharacterized protein LOC105169888 [Sesamum indicum]
c27182.graph_c0	0	0	0	2.38399381	8.61278114	6.20061596	1.048E-06	Inf	up	PREDICTED: heme oxygenase 1, chloroplastic-like [Sesamum indicum]
c27183.graph_c0	1.22659341	1.49640765	1.42845037	6.81542021	9.24024607	11.9086904	3.908E-06	1.726363	up	PREDICTED: putative MO25-like protein At5g47540 [Sesamum indicum]
c27185.graph_c0	49.7589501	46.0797769	48.726024	16.6695238	14.283289	14.832442	5.405E-40	-2.673485	down	hypothetical protein MIMGU_mgv1a015987mg [Erythranthe guttata]
c27187.graph_c0	5.13882144	4.53879458	5.16078805	26.2967879	28.8276075	28.6731713	2.867E-14	1.477923	up	biotin carboxyl carrier protein subunit [Camellia chekiangoleosa]
c27188.graph_c0	8.74792019	7.05399214	7.26008619	39.7361098	40.5540614	42.2789463	3.446E-13	1.393137	up	hypothetical protein M569_02688 [Genlisea aurea]
c27197.graph_c0	0.03628889	0.01816261	0.09326532	2.20649172	1.84167488	1.81458647	5.152E-14	4.282972	up	PREDICTED: uncharacterized protein LOC105158016 [Sesamum indicum]
c27200.graph_c0	0.43515993	0.47519528	0.47447081	2.87917446	4.04110875	3.52392537	1.302E-09	1.891586	up	hypothetical protein MIMGU_mgv1a000304mg [Erythranthe guttata]
c27204.graph_c0	0	0.11023399	0.09434223	29.9286807	40.6980769	43.1803014	2.09E-67	8.087471	up	PREDICTED: serine/threonine-protein kinase-like protein ACR4 [Sesamum indicum]
c27205.graph_c0	3.06081358	2.65143334	2.87431023	2.38600248	2.98725855	2.55731102	0.0068737	-1.135066	down	PREDICTED: dnaJ homolog subfamily B member 6 isoform X1 [Sesamum indicum]
c27206.graph_c0	0.14255472	0.2140462	0	2.36395267	3.33909495	3.62257539	4.571E-08	3.700123	up	PREDICTED: uncharacterized protein LOC105160269 [Sesamum indicum]
c27209.graph_c0	14.0279383	12.5993155	13.9519831	84.1352115	80.4101834	71.8700603	2.013E-23	1.526505	up	PREDICTED: heme oxygenase 1, chloroplastic-like [Sesamum indicum]
c27212.graph_c0	48.8195597	54.169429	46.8386391	9.14761275	10.2157041	9.83521807	1.11E-104	-3.377727	down	PREDICTED: uncharacterized protein LOC105171942 [Sesamum indicum]
c27216.graph_c0	0.52483189	0.85370582	0.33721488	38.0159241	40.8495089	41.0863661	1.8E-64	5.114465	up	PREDICTED: GDSL esterase/lipase CPRD49-like isoform X1 [Sesamum indicum]
c27217.graph_c0	0.4430605	0.49894218	0.35584393	2.50007942	2.70258265	2.76934477	0.0027484	1.602443	up	-
c27218.graph_c0	0.89082739	1.48619903	1.14474846	50.5179239	36.0648186	29.5613391	2.794E-11	4.034182	up	PREDICTED: glucan endo-1,3-beta-glucosidase 13-like [Sesamum indicum]

#ID	0 h-1 FPKM	0 h-2 FPKM	0 h-3 FPKM	48 h-1 FPKM	48 h-2 FPKM	48 h-3 FPKM	FDR	log2FC	regulated	nr_annotation
c27220.graph_c0	1.11348969	0.97950108	0.82383904	0.21759804	0	0	4.851E-15	-4.702285	down	-
c27222.graph_c0	1.88874381	1.96477765	1.80843533	18.1509428	20.3130495	19.3078599	1.083E-22	2.332428	up	hypothetical protein MIMGU_mgv1a006093mg [Erythranthe guttata]
c27224.graph_c0	19.5930506	17.7634754	15.1426758	7.71009777	11.3367364	10.8527376	8.571E-26	-1.833907	down	PREDICTED: uncharacterized protein LOC105158173 [Sesamum indicum]
c27229.graph_c0	0.0872445	0	0.05605632	1.40656815	2.5970112	2.86069915	1.58E-08	4.553591	up	PREDICTED: probable protein S-acyltransferase 4 [Sesamum indicum]
c27230.graph_c0	5.36102275	5.9030301	4.97001268	105.234356	103.709499	114.040512	9.15E-99	3.297664	up	PREDICTED: BAG family molecular chaperone regulator 7 [Sesamum indicum]
c27233.graph_c1	15.7967045	15.1800216	15.3695387	3.20165	2.42268568	2.77481388	9.712E-56	-3.476989	down	hypothetical protein MIMGU_mgv1a022117mg [Erythranthe guttata]
c27235.graph_c1	0.38645154	0.25789234	0.33107035	18.513267	16.972409	22.491972	9.045E-19	4.875582	up	PREDICTED: enolase-like [Sesamum indicum]
c27237.graph_c0	6.85949209	6.67294221	6.60483163	4.04086314	5.31800385	5.17947217	1.399E-15	-1.492012	down	PREDICTED: U-box domain-containing protein 45 [Sesamum indicum]
c27242.graph_c0	11.0970101	9.87815704	9.47383632	3.53747043	7.15776507	8.62447857	3.004E-15	-1.685184	down	PREDICTED: RING-H2 finger protein ATL46-like [Sesamum indicum]
c27244.graph_c0	18.9355193	17.0427624	17.9624782	222.400384	223.470992	232.432264	4.969E-75	2.634853	up	PREDICTED: ATP synthase subunit gamma, mitochondrial [Sesamum indicum]
c27247.graph_c0	5.15621971	6.24527329	5.69831396	29.2140123	32.710012	30.9397386	9.343E-19	1.421675	up	PREDICTED: NADP-dependent malic enzyme-like isoform X1 [Sesamum indicum]
c27249.graph_c0	8.14994023	8.21475464	8.10929888	6.62186512	8.81025648	8.53657969	5.658E-08	-1.052484	down	unnamed protein product [Coffea canephora]
c27253.graph_c0	1.13600635	1.13714389	1.31383182	64.7822586	91.5791774	94.9850103	3.514E-41	5.105191	up	hypothetical protein MIMGU_mgv1a016583mg [Erythranthe guttata]
c27254.graph_c1	0.29772224	0.17029735	0.21861992	2.35098126	1.950954	3.24242794	7.817E-06	2.441079	up	PREDICTED: cytochrome P450 81E8-like [Sesamum indicum]
c27255.graph_c0	0.4255344	0.73021801	0.54682855	3.08023603	4.09375316	5.97987382	0.0002128	1.92418	up	PREDICTED: uncharacterized protein LOC105159742 [Sesamum indicum]
c27256.graph_c0	2.70946149	1.59539683	1.63847778	6.75424129	13.5328493	11.7584673	0.0046209	1.406031	up	hypothetical protein M569_10374, partial [Genlisea aurea]
c27258.graph_c0	3.07647529	2.5662966	1.67719684	0.81592037	0.31845162	0.57315871	4.365E-10	-3.098448	down	-
c27260.graph_c0	0	0	0.11933787	5.5610886	5.89129906	5.51917203	2.849E-19	6.101457	up	PREDICTED: WAT1-related protein At1g43650-like [Sesamum indicum]
c27262.graph_c0	7.37727859	7.68005245	7.58407605	34.9256555	50.6032499	50.6418295	4.203E-11	1.564099	up	PREDICTED: ER membrane protein complex subunit 1 [Sesamum indicum]
c27264.graph_c0	0.75378519	0.51739885	1.27307472	0	0.0630575	0.10592658	4.92E-06	-4.965352	down	PREDICTED: probable leucine-rich repeat receptor-like protein kinase At5g63930 [Sesamum indicum]
c27265.graph_c0	5.21926027	5.5443531	3.55879303	14.1305818	21.5188547	24.0988379	0.0024851	1.039055	up	PREDICTED: probable methyltransferase PMT11 [Sesamum indicum]

#ID	0 h-1 FPKM	0 h-2 FPKM	0 h-3 FPKM	48 h-1 FPKM	48 h-2 FPKM	48 h-3 FPKM	FDR	log2FC	regulated	nr_annotation
c27266.graph_c0	0.04928634	0.09867138	0	28.5148347	38.4815411	39.7553682	3.08E-70	8.486611	up	PREDICTED: palmitoyl-protein thioesterase 1 [Sesamum indicum]
c27269.graph_c0	9.3056852	10.6632276	10.4634022	7.55777999	7.11029571	6.9256095	9.139E-09	-1.511732	down	hypothetical protein glysoja_049431 [Glycine soja]
c27273.graph_c0	2.51463262	2.30738809	2.87235776	82.112145	102.32663	92.7028398	1.211E-97	4.147982	up	unnamed protein product [Coffea canephora]
c27274.graph_c1	0.15947166	0	0	11.018677	10.8947553	13.07247	1.381E-14	6.77998	up	TPX2 family protein, putative isoform 1 [Theobroma cacao]
c27276.graph_c0	0.29370078	0.04199927	0.10783346	8.29123131	6.36756494	5.45143035	3.083E-16	4.499095	up	hypothetical protein PHAVU_002G220200g [Phaseolus vulgaris]
c27279.graph_c0	2.68775679	2.12993814	2.15867118	0	0	0	6.78E-19	-Inf	down	PREDICTED: inorganic phosphate transporter 1-11-like [Sesamum indicum]
c27280.graph_c0	4.0199322	2.12720223	2.00259004	0	0	0	4.344E-17	-Inf	down	predicted protein [Hordeum vulgare subsp. vulgare]
c27282.graph_c0	806.354253	753.647759	837.92116	26.5237501	19.6824049	18.9476496	1.84E-199	-6.213533	down	hypothetical protein MIMGU_mgv1a005561mg [Erythranthe guttata]
c27283.graph_c0	45.0235688	47.2002786	43.4709866	264.846774	267.984369	245.783679	7.732E-27	1.504122	up	PREDICTED: blue copper protein-like [Nicotiana tomentosiformis]
c27284.graph_c0	4.86722414	5.10410259	6.15529674	0	0.15080187	0.18999239	2.249E-28	-6.61298	down	-
c27285.graph_c0	0.16678156	0.38954664	0.2857612	8.55318594	6.99926453	8.74988082	8.091E-15	3.834055	up	PREDICTED: protein XRI1-like [Sesamum indicum]
c27287.graph_c0	0.04954524	0	0.12733518	1.27804053	1.69241121	1.21842083	9.165E-08	3.527596	up	PREDICTED: calmodulin-like protein 3 [Sesamum indicum]
c27288.graph_c0	19.2092436	20.790456	18.9110606	10.2116036	17.7498497	18.0621772	2.58E-19	-1.382817	down	PREDICTED: pre-rRNA-processing protein esf1 [Sesamum indicum]
c27289.graph_c0	0	0.15148862	0.06482471	2.13779771	2.75707169	1.98490388	7.485E-09	3.969453	up	hypothetical protein MIMGU_mgv1a007466mg [Erythranthe guttata]
c27290.graph_c0	1.76571973	1.61153302	1.93534039	58.2739987	49.671224	59.5146891	1.542E-62	3.961078	up	-
c27304.graph_c0	8.09818085	6.74261501	6.74314164	5.22937318	6.94332478	6.61769217	4.499E-10	-1.220963	down	PREDICTED: F-box/kelch-repeat protein At1g55270 [Sesamum indicum]
c27312.graph_c0	2.18560687	2.22907458	1.90772243	7.40824722	10.826408	10.918748	0.0001122	1.182312	up	PREDICTED: uncharacterized protein LOC105166961 [Sesamum indicum]
c27313.graph_c0	10.1805561	7.69113235	11.3545511	44.4174918	43.3055485	47.5528684	6.217E-05	1.190595	up	unnamed protein product [Coffea canephora]
c27314.graph_c0	6.78563859	7.7220821	6.64091813	35.1898472	40.3494424	44.2661689	2.919E-25	1.482112	up	PREDICTED: carbamoyl-phosphate synthase large chain, chloroplastic [Sesamum indicum]
c27317.graph_c0	0.08865708	0.17749172	0	7.7592853	9.69097321	8.50302476	1.832E-15	5.601745	up	PREDICTED: transcription factor bHLH79-like [Sesamum indicum]
c27318.graph_c0	28.8449762	32.9883852	29.8388838	2.4580833	2.25214374	1.71428228	1.24E-102	-4.84826	down	hypothetical protein MIMGU_mgv1a022206mg [Erythranthe guttata]
c27323.graph_c0	3.3804496	4.59854447	4.12124175	20.1231089	31.1310386	38.0845468	1.327E-05	1.856988	up	1-deoxy-D-xylulose 5-phosphate reductoisomerase [Lavandula angustifolia]
c27324.graph_c0	42.3037815	43.5208323	39.1715604	11.2858222	12.4587809	10.112445	3.56E-85	-2.901237	down	PREDICTED: lysM domain receptor-like kinase 3 [Sesamum indicum]

#ID	0 h-1 FPKM	0 h-2 FPKM	0 h-3 FPKM	48 h-1 FPKM	48 h-2 FPKM	48 h-3 FPKM	FDR	log2FC	regulated	nr_annotation
c27330.graph_c0	8.78339159	8.31693348	7.62635536	1.96828376	1.62178954	2.33515607	1.727E-09	-3.076623	down	PREDICTED: T-complex protein 1 subunit alpha [Sesamum indicum]
c27332.graph_c0	0	0	0	3.27084031	3.86268839	3.19664034	3.225E-14	Inf	up	predicted protein [Hordeum vulgare subsp. vulgare]
c27333.graph_c0	0	0	0	6.28453598	7.59211841	8.04454861	1.018E-21	Inf	up	PREDICTED: transcription factor bHLH118-like [Sesamum indicum]
c27335.graph_c0	8.7574214	8.31014604	8.97688662	29.4735129	37.6461917	43.4876132	4.331E-07	1.062967	up	PREDICTED: L-ascorbate oxidase homolog [Sesamum indicum]
c27338.graph_c0	6.27892446	8.48827581	7.07048098	6.73870812	6.06485874	5.41237452	2.715E-05	-1.276058	down	PREDICTED: clathrin light chain 2-like [Sesamum indicum]
c27340.graph_c0	100.811686	100.912633	98.9307675	409.459278	412.174857	421.465819	1.517E-13	1.030255	up	PREDICTED: 60S ribosomal protein L22-2-like [Sesamum indicum]
c27341.graph_c0	1.52639033	0.9822335	0.84063086	8.23637481	8.7254404	7.68617321	0.000204	1.866802	up	hypothetical protein MIMGU_mgv1a004267mg [Erythranthe guttata]
c27342.graph_c0	4.64636634	3.89387635	4.95249476	30.4946693	38.3868353	45.7645906	3.523E-14	2.062609	up	PREDICTED: pyruvate dehydrogenase E1 component subunit beta-like [Sesamum indicum]
c27348.graph_c0	1.4661475	0.97841041	0.92109482	29.1153196	38.1577122	40.622674	1.434E-45	3.983401	up	PREDICTED: endoglucanase 25-like [Sesamum indicum]
c27349.graph_c0	4.4570799	2.43563826	3.0683165	0	0	0	2.473E-30	-Inf	down	alpha-2 macroglobulin family-like protein [Micromonas sp. RCC299]
c27350.graph_c0	0.07439785	0	0	6.44277453	11.3997673	9.75785878	9.762E-19	7.530007	up	-
c27353.graph_c0	64.6405875	67.1728908	61.3753555	51.665648	63.2876854	66.0949626	2.052E-15	-1.114447	down	PREDICTED: xyloglucan 6-xylosyltransferase 2-like [Sesamum indicum]
c27356.graph_c0	6.4844187	5.75435449	5.20057205	34.2332377	37.6573566	40.6768699	1.045E-19	1.672489	up	PREDICTED: protein TRIGALACTOSYLDIACYLGLYCEROL 1, chloroplastic-like [Sesamum indicum]
c27359.graph_c0	8.01399895	8.65406199	8.98761245	95.3080576	96.0848726	101.048786	4.363E-58	2.491882	up	PREDICTED: pectinesterase-like isoform X1 [Sesamum indicum]
c27361.graph_c0	11.4866023	11.9153744	10.7729585	1.96282877	1.80815572	1.67057788	1.896E-44	-3.665187	down	unnamed protein product [Coffea canephora]
c27363.graph_c0	43.6070316	38.3520579	51.9878631	1.04497912	0.61501593	0.20662581	5.537E-60	-7.166625	down	-
c27366.graph_c0	4.64074435	4.25213071	4.66986219	19.5218654	20.6570851	18.4573489	3.636E-10	1.094074	up	PREDICTED: uncharacterized protein LOC105171129 [Sesamum indicum]
c27369.graph_c0	4.71124535	3.75678404	5.13062441	1.02990324	0.93519223	1.63643162	1.636E-10	-2.93903	down	-
c27373.graph_c0	0.15111292	0.09075854	0.34953496	2.97920274	4.18847454	4.48419327	5.71E-12	3.264065	up	PREDICTED: probable glycerol-3-phosphate dehydrogenase [NAD(+)] 1, cytosolic [Sesamum indicum]
c27376.graph_c0	15.7918563	20.2768535	14.5558272	7.99785992	9.76385046	8.33641012	1.099E-14	-1.974139	down	-
c27382.graph_c0	0.38009145	0.35120497	0.41328909	12.7944917	16.9498994	16.9448725	4.095E-49	4.326348	up	PREDICTED: protein NRT1/ PTR FAMILY 5.2-like [Sesamum indicum]
c27384.graph_c0	54.5700025	54.3273114	49.4034993	9.30410581	10.1050569	10.2962448	3.91E-108	-3.431952	down	unnamed protein product [Coffea canephora]
c27386.graph_c0	5.34526878	6.85548349	7.72749227	26.3148173	25.4319758	33.6844004	0.0014233	1.077851	up	hypothetical protein MIMGU_mgv1a013949mg [Erythranthe guttata]
c27388.graph_c0	0.44618129	0.31902005	0.81908659	3.93434818	3.85692268	4.44126186	0.0001029	1.92203	up	ribosomal protein S4 (chloroplast) [Andrographis paniculata]

#ID	0 h-1 FPKM	0 h-2 FPKM	0 h-3 FPKM	48 h-1 FPKM	48 h-2 FPKM	48 h-3 FPKM	FDR	log2FC	regulated	nr_annotation
c27389.graph_c0	2.8235241	2.42258694	2.07333729	27.870156	48.4210834	62.3982458	4.903E-07	3.218278	up	PREDICTED: expansin-A18-like [Sesamum indicum]
c27390.graph_c0	0	0	0	2.70991751	3.3241178	5.83780748	2.584E-06	Inf	up	hypothetical protein M569_07000 [Genlisea aurea]
c27390.graph_c1	0	0	0	3.66247135	3.20095404	3.91061398	7.365E-12	Inf	up	unnamed protein product [Coffea canephora]
c27391.graph_c0	4.0257316	3.45408237	4.2230404	1.28671492	0.96220125	0.74082489	3.831E-10	-2.979283	down	unnamed protein product [Coffea canephora]
c27391.graph_c1	4.07624457	3.62695672	3.37568742	2.25318142	1.32609584	1.78210425	7.75E-06	-2.054885	down	serine glyoxylate aminotransferase 2 [Glycine max]
c27395.graph_c0	0	0.1379293	0	22.0244985	17.0789641	16.0957375	1.177E-29	7.644474	up	PREDICTED: uncharacterized protein LOC100247103 isoform X1 [Vitis vinifera]
c27396.graph_c0	1665.78492	1427.69534	1516.48269	369.805818	397.565812	407.874184	2.032E-80	-2.990326	down	PREDICTED: abscisic acid 8'-hydroxylase 2 isoform X2 [Sesamum indicum]
c27400.graph_c0	9.40437917	11.4977664	8.25654551	52.1489071	52.6881086	47.6375956	3.09E-18	1.371851	up	PREDICTED: ferredoxin--NADP reductase, root-type isozyme, chloroplastic-like [Sesamum indicum]
c27405.graph_c0	259.280677	273.745016	369.65664	345.132815	254.830499	244.115609	0.0008522	-1.111564	down	ATP synthase subunit alpha [Medicago truncatula]
c27410.graph_c0	0.14865947	0.11904666	0.11461998	10.3811738	9.02441157	13.0879312	2.195E-31	5.393329	up	PREDICTED: transcription factor MYC2 [Sesamum indicum]
c27411.graph_c0	18.4209297	19.7246235	18.1830397	11.9735401	11.6359956	10.4177061	3.634E-29	-1.742772	down	PREDICTED: ankyrin repeat and zinc finger domain-containing protein 1 [Sesamum indicum]
c27414.graph_c0	18.8857477	16.6935877	16.0846733	16.9917935	16.1324041	18.1973553	3.883E-10	-1.025686	down	PREDICTED: LOW QUALITY PROTEIN: BAG family molecular chaperone regulator 2 [Sesamum indicum]
c27415.graph_c0	0.11688227	0.14039917	0.12015867	3.74724899	6.91285085	9.31298791	3.154E-08	4.696619	up	PREDICTED: uncharacterized protein LOC105161921 [Sesamum indicum]
c27419.graph_c1	59.3911928	52.2657175	56.158485	27.1527046	35.2123253	35.0428682	1.338E-32	-1.806825	down	PREDICTED: uncharacterized protein LOC105161274 [Sesamum indicum]
c27427.graph_c0	1.58099053	1.0550491	1.40279588	0	0	0	5.103E-23	-Inf	down	DEAD-domain-containing protein [Punctularia strigosozonata HHB-11173 SS5]
c27428.graph_c0	0	0.14587534	0.2809021	8.4245288	5.4757533	5.19649618	1.159E-10	4.458087	up	PREDICTED: U-box domain-containing protein 26-like [Sesamum indicum]
c27432.graph_c0	1.24960002	1.25085131	1.14698965	26.3134411	29.4439195	30.2912695	1.936E-14	3.54136	up	hypothetical protein MIMGU_mgv1a022792mg, partial [Erythranthe guttata]
c27434.graph_c0	1.41243565	1.18580967	0.93679264	5.33083668	4.71355714	6.01321869	0.004855	1.17007	up	PREDICTED: putative thiol protease ulp-4 [Sesamum indicum]
c27437.graph_c0	0.22450844	0.0642095	0.08242921	24.4947972	26.9509279	29.8666914	7.36E-105	6.762876	up	hypothetical protein PHAVU_010G112100g [Phaseolus vulgaris]
c27438.graph_c0	0.13861709	0.13875589	0	5.61893654	5.81729598	10.2266508	4.45E-08	5.281049	up	unknown [Lotus japonicus]
c27438.graph_c1	0	0	0	3.24983446	5.37413549	12.4130789	7.414E-05	Inf	up	PREDICTED: auxin-induced protein IAA6 [Sesamum indicum]
c27438.graph_c2	0.250355	0.50121139	0.32171609	10.6095944	11.321025	13.7500798	2.717E-15	4.035422	up	PREDICTED: PGR5-like protein 1A, chloroplastic [Sesamum indicum]
c27439.graph_c1	0.40540756	0.20290676	0.39072358	14.6905236	14.4088052	13.1546214	7.222E-40	4.38419	up	PREDICTED: uncharacterized protein LOC105174869 [Sesamum indicum]

#ID	0 h-1 FPKM	0 h-2 FPKM	0 h-3 FPKM	48 h-1 FPKM	48 h-2 FPKM	48 h-3 FPKM	FDR	log2FC	regulated	nr_annotation
c27440.graph_c0	0.04680039	0.09369451	0.12028072	1.85396971	2.146759	1.34273927	2.256E-07	3.332666	up	hypothetical protein MIMGU_mgv1a018556mg, partial [Erythranthe guttata]
c27456.graph_c0	716.622161	716.657582	798.109808	103.541435	59.407438	50.8807311	1.39E-117	-4.389134	down	PREDICTED: CBL-interacting protein kinase 2 [Sesamum indicum]
c27457.graph_c0	28.7382681	31.5315435	26.1368711	24.5299346	24.3851273	23.224875	3.803E-20	-1.275903	down	PREDICTED: protein OBERON 3 [Sesamum indicum]
c27459.graph_c0	0.01683437	0	0	3.56705058	4.09102406	4.70571128	1.042E-35	8.52076	up	PREDICTED: filament-like plant protein 7 isoform X2 [Sesamum indicum]
c27464.graph_c0	0.26442882	0.1764624	0.11326717	37.5563994	51.7438928	53.6485683	7.916E-64	6.994586	up	PREDICTED: fasciclin-like arabinogalactan protein 2 [Sesamum indicum]
c27465.graph_c0	12.7363586	12.353177	11.9954879	6.26761818	6.89713735	7.69524214	2.411E-26	-1.849626	down	PREDICTED: uncharacterized protein LOC105174651 [Sesamum indicum]
c27466.graph_c0	29.8422622	31.4036274	28.7930768	17.2473112	19.8770091	18.6774842	1.076E-33	-1.709312	down	PREDICTED: flowering time control protein FPA [Sesamum indicum]
c27470.graph_c1	7.12226207	7.45102073	6.19334577	6.11748161	4.49469983	5.75058132	2.129E-06	-1.355631	down	PREDICTED: RING-H2 finger protein ATL43-like [Sesamum indicum]
c27470.graph_c2	0.07236581	0.07243827	0	2.00004301	2.96632428	2.90672287	1.696E-09	4.756847	up	-
c27474.graph_c0	19.1257073	23.0283355	19.6793256	0.37622453	0.79712855	0.27896861	2.86E-99	-6.437514	down	PREDICTED: uncharacterized protein LOC105176742 [Sesamum indicum]
c27475.graph_c0	38.3030693	36.6442182	41.4961418	175.158419	190.673827	213.281787	3.559E-20	1.293269	up	PREDICTED: 60S ribosomal protein L22-2 [Sesamum indicum]
c27476.graph_c0	0	0.08282376	0.31897602	3.12527908	2.98784405	3.18780885	5.183E-08	3.492328	up	hypothetical protein PRUPE_ppa003008mg [Prunus persica]
c27477.graph_c0	3.89801961	4.43806496	3.8619852	5.78415413	3.31065181	2.70750861	0.0002455	-1.051056	down	PREDICTED: LOW QUALITY PROTEIN: receptor-like protein kinase HSL1 [Sesamum indicum]
c27478.graph_c0	0.07219573	0.07226802	0.04638719	0.73162557	1.3739852	2.17491855	0.000437	3.461152	up	PREDICTED: probably inactive leucine-rich repeat receptor-like protein kinase At3g28040 [Sesamum indicum]
c27479.graph_c0	0.0761324	0	0	2.73538706	3.34362797	3.1204243	6.664E-11	5.917497	up	PREDICTED: putative F-box protein At5g62060 [Sesamum indicum]
c27481.graph_c0	1.04726577	0.58239692	0.29906177	5.57445511	4.88336054	5.15089035	0.0004104	2.011345	up	PREDICTED: 60S ribosomal protein L32-1-like [Brachypodium distachyon]
c27482.graph_c0	4.83637779	8.06870114	5.14951917	3.35229854	5.52932303	5.58813358	0.0011907	-1.344102	down	PREDICTED: uncharacterized protein LOC105173594 [Sesamum indicum]
c27483.graph_c0	0	0.15179669	0	4.68012147	5.10602183	5.65604923	5.704E-13	5.660437	up	PREDICTED: uncharacterized protein LOC105176460 isoform X2 [Sesamum indicum]
c27486.graph_c0	28.0810388	28.8087069	25.2270163	12.1741574	14.447169	15.4154695	1.687E-28	-1.985883	down	PREDICTED: probable membrane-associated kinase regulator 1 [Solanum lycopersicum]
c27490.graph_c0	0	0.03700263	0	0.85137832	2.05640517	2.54537239	6.053E-06	6.182571	up	PREDICTED: cytochrome P450 76A1 [Sesamum indicum]
c27498.graph_c0	1.8214495	2.31415471	1.98053707	14.3924036	15.5888675	15.3330726	4.928E-08	1.870539	up	unnamed protein product [Coffea canephora]
c27501.graph_c0	37.1437689	32.9831121	34.2936872	17.9626041	21.0490603	23.6620086	8.379E-28	-1.7573	down	unknown [Picea sitchensis]

#ID	0 h-1 FPKM	0 h-2 FPKM	0 h-3 FPKM	48 h-1 FPKM	48 h-2 FPKM	48 h-3 FPKM	FDR	log2FC	regulated	nr_annotation
c27502.graph_c0	31.3811051	35.0796924	31.9688173	37.4395195	27.0792387	27.0744607	1.212E-12	-1.114453	down	PREDICTED: calcium-dependent protein kinase 32 isoform X1 [Sesamum indicum]
c27503.graph_c0	3.44282208	3.37080379	4.32728321	0.09260591	0.22073564	0.14420031	5.689E-45	-5.63837	down	hypothetical protein MIMGU_mgv1a026576mg, partial [Erythranthe guttata]
c27505.graph_c0	0	0	0	124.342175	133.627454	113.42435	3.48E-224	Inf	up	plasma membrane intrinsic protein [Olea europaea]
c27507.graph_c0	1.36373328	0.85950668	0.71396162	29.9199818	28.7881962	27.0780613	1.037E-45	3.857316	up	PREDICTED: uncharacterized protein LOC105172180 [Sesamum indicum]
c27508.graph_c0	0.25709585	0.20016367	0.25696094	2.0790471	2.34189693	2.55242624	5.523E-06	2.266179	up	-
c27510.graph_c0	0.47363158	0.60956467	0.78253115	4.73740905	3.8300685	3.38334741	0.0009539	1.662592	up	PREDICTED: uncharacterized protein LOC105170878 [Sesamum indicum]
c27511.graph_c0	227.333194	220.181959	209.626456	93.8597073	88.9523253	75.9438031	9.562E-58	-2.358997	down	PREDICTED: glutathione S-transferase-like [Sesamum indicum]
c27512.graph_c0	28.5727871	29.0564207	28.5392492	19.44321	22.1187861	23.2624531	1.166E-16	-1.430774	down	PREDICTED: multiple myeloma tumor-associated protein 2 homolog [Sesamum indicum]
c27515.graph_c0	128.590267	134.819816	148.612866	37.4321242	88.26864	141.430877	6.174E-08	-1.659802	down	hypothetical protein MIMGU_mgv1a022248mg, partial [Erythranthe guttata]
c27518.graph_c0	0	0	0	25.1102833	27.6907734	30.9598959	6.522E-45	Inf	up	hypothetical protein MIMGU_mgv1a013970mg [Erythranthe guttata]
c27519.graph_c0	15.6308104	18.75908	15.4481865	7.80113183	8.75208556	7.3510566	1.011E-36	-2.077494	down	PREDICTED: F-box protein At5g51370-like [Sesamum indicum]
c27527.graph_c0	10.8459486	13.2641886	12.7255192	10.252656	11.0455382	9.93452197	1.262E-09	-1.257017	down	hypothetical protein MIMGU_mgv1a009219mg [Erythranthe guttata]
c27529.graph_c0	0.84356256	0.50664436	0.57813935	4.6628691	3.84202343	5.39369076	0.0001406	1.835408	up	PREDICTED: alpha/beta hydrolase domain-containing protein 11 isoform X1 [Sesamum indicum]
c27531.graph_c0	1.7063004	1.39171104	0.81209762	232.008947	292.968732	301.034832	1.77E-147	6.707445	up	PREDICTED: RING-H2 finger protein ATL79-like [Sesamum indicum]
c27533.graph_c0	0	0	0	3.4879533	3.58309196	1.97499264	5.604E-11	Inf	up	PREDICTED: probable trehalose-phosphate phosphatase J [Sesamum indicum]
c27536.graph_c0	0.05012597	0	0.12882772	1.33920014	1.46764155	2.01341377	3.075E-08	3.712265	up	PREDICTED: protein IQ-DOMAIN 1 [Sesamum indicum]
c27537.graph_c0	2.72745383	3.08020867	2.6061974	2.57713678	0.54603279	1.71984061	8.196E-05	-1.794667	down	BnaC03g60650D [Brassica napus]
c27538.graph_c0	38.5841192	40.574808	31.1454477	15.5274046	16.3134569	12.4459905	1.035E-25	-2.33053	down	-
c27545.graph_c0	0.16552945	0.2711376	0.25138678	5.5591914	9.10563418	10.4728386	5.811E-14	4.161958	up	PREDICTED: probably inactive leucine-rich repeat receptor-like protein kinase At3g28040 [Sesamum indicum]
c27546.graph_c0	0	0.06114516	0.07849535	1.29431488	2.50387506	2.90422038	5.254E-08	4.542256	up	PREDICTED: peptide-N4-(N-acetyl-beta-glucosaminyl)asparagine amidase A-like [Sesamum indicum]
c27548.graph_c0	16.9430342	16.5147495	18.9145083	5.02916183	7.34050674	9.016131	2.622E-35	-2.319032	down	PREDICTED: mitochondrial substrate carrier family protein B-like [Sesamum indicum]
c27550.graph_c0	0	0	0	3.35261756	5.69750557	6.52561645	4.892E-13	Inf	up	PREDICTED: galactose oxidase-like [Sesamum indicum]

#ID	0 h-1 FPKM	0 h-2 FPKM	0 h-3 FPKM	48 h-1 FPKM	48 h-2 FPKM	48 h-3 FPKM	FDR	log2FC	regulated	nr_annotation
c27555.graph_c0	4.9016927	5.15679596	4.47879934	21.9007917	24.1227775	21.1718332	3.458E-15	1.191447	up	PREDICTED: primary amine oxidase-like [Sesamum indicum]
c27557.graph_c0	5.87585541	6.26120624	5.84570852	1.22234028	2.58984263	3.41826897	2.698E-06	-2.345309	down	PREDICTED: uncharacterized protein At5g65660-like [Sesamum indicum]
c27558.graph_c0	1.78347379	2.20043633	1.97204357	7.71852513	15.0988211	17.2717416	0.0009526	1.72114	up	PREDICTED: D-amino-acid transaminase, chloroplastic-like [Sesamum indicum]
c27561.graph_c0	59.9359416	60.3875769	56.9606564	21.5893749	20.122184	19.6270096	5.499E-68	-2.546451	down	PREDICTED: cytosolic Fe-S cluster assembly factor NBP35-like [Sesamum indicum]
c27563.graph_c0	0	0	0.06499346	1.72400907	1.1353164	1.36817367	6.953E-10	4.98033	up	PREDICTED: uncharacterized protein LOC105158131 isoform X1 [Sesamum indicum]
c27568.graph_c0	10.6424111	9.11429141	10.6368264	70.9190957	86.9017318	76.7709383	1.89E-21	1.92736	up	PREDICTED: probable stress-associated endoplasmic reticulum protein [Sesamum indicum]
c27569.graph_c0	6.23966465	6.47512054	5.48034037	7.04045449	3.60354873	3.87135364	6.303E-08	-1.328573	down	hypothetical protein MIMGU_mgv1a002973mg [Erythranthe guttata]
c27572.graph_c0	2.77776659	1.60092163	1.83885396	0.07754727	0	0	2.452E-16	-7.28272	down	PREDICTED: protein IAL1-like [Sesamum indicum]
c27575.graph_c0	11.1081151	10.6874231	15.7988147	0.19870913	0	0.08840509	3.674E-29	-8.021633	down	-
c27576.graph_c0	8.84781085	8.11241256	8.50345433	41.5093728	47.3846833	43.2124856	1.251E-11	1.356028	up	PREDICTED: 60S ribosomal protein L14-2 [Sesamum indicum]
c27577.graph_c0	0.62653174	0.49276788	0.69010122	9.31768794	9.34683874	10.5652962	6.108E-15	2.993402	up	PREDICTED: protein INVOLVED IN DE NOVO 2-like [Sesamum indicum]
c27582.graph_c0	1.73758231	2.48014467	2.06746487	26.8870771	19.3135752	19.5453737	7.507E-10	2.375724	up	PREDICTED: glycerol-3-phosphate acyltransferase 1 [Sesamum indicum]
c27584.graph_c0	0.45873328	0.09183853	0.35369426	7.01540933	9.84960612	8.87455116	2.131E-11	3.807229	up	PREDICTED: uncharacterized protein LOC105159891 [Sesamum indicum]
c27587.graph_c0	0.72846948	0.36459947	0.73551667	46.4027119	58.6544875	45.1703475	1.853E-66	5.337762	up	Tubulin beta-7 chain [Gossypium arboreum]
c27588.graph_c0	31.0915017	31.8990834	32.1457894	27.5716204	31.4167964	25.433844	4.682E-13	-1.191054	down	PREDICTED: E3 ubiquitin-protein ligase CIP8-like [Sesamum indicum]
c27592.graph_c0	0.14105409	0.14119533	0.6344103	7.79689352	9.22351397	11.1002063	8.943E-15	3.899627	up	PREDICTED: transcription factor MYB44-like [Sesamum indicum]
c27593.graph_c0	0	0.04725455	0	6.69752607	5.22926689	5.66918701	4.867E-35	7.539126	up	PREDICTED: wall-associated receptor kinase 2-like [Sesamum indicum]
c27596.graph_c0	0	0.31733097	0	2.26341496	2.70720901	1.55920492	4.96E-06	3.357403	up	PREDICTED: myb-related protein Myb4-like [Sesamum indicum]
c27600.graph_c0	3.29282473	2.19741466	3.12318368	23.257331	22.8784419	25.9641933	3.372E-12	2.0464	up	PREDICTED: 50S ribosomal protein 5, chloroplastic [Sesamum indicum]
c27601.graph_c0	0.83325537	1.13739511	0.68139664	114.37973	126.346563	139.280031	4.27E-160	6.145949	up	PREDICTED: protein SPIRAL1-like 1-like [Oryza brachyantha]
c27602.graph_c0	7.75799421	7.28374981	8.21610276	2.1687965	2.16703277	2.43415695	3.583E-35	-2.799694	down	hypothetical protein MIMGU_mgv1a002239mg [Erythranthe guttata]

#ID	0 h-1 FPKM	0 h-2 FPKM	0 h-3 FPKM	48 h-1 FPKM	48 h-2 FPKM	48 h-3 FPKM	FDR	log2FC	regulated	nr_annotation
c27604.graph_c0	1.83039173	1.32327332	1.43741024	10.4611582	8.86586292	10.4752834	7.035E-09	1.684485	up	PREDICTED: deoxyribodipyrimidine photo-lyase [Sesamum indicum]
c27606.graph_c0	0.09358751	0	0	4.77078022	5.63254026	5.1400441	1.422E-23	6.377169	up	PREDICTED: rop guanine nucleotide exchange factor 3 [Sesamum indicum]
c27608.graph_c0	33.9924446	32.8172496	35.4886351	13.7568069	14.4099198	12.7358545	1.81E-53	-2.340523	down	PREDICTED: selenoprotein O [Sesamum indicum]
c27610.graph_c0	0	0	0	1.27816473	2.89110225	3.25822024	1.143E-08	Inf	up	PREDICTED: lecithin-cholesterol acyltransferase-like 1 [Sesamum indicum]
c27612.graph_c0	0.07281783	0.07289074	0	2.41504313	2.48737743	2.92487922	1.811E-09	4.744593	up	PREDICTED: uncharacterized protein LOC105160557 [Sesamum indicum]
c27614.graph_c0	30.9455574	27.0083076	33.1645464	7.86328486	9.75146264	10.6774339	1.014E-40	-2.710709	down	PREDICTED: uncharacterized protein LOC105163129 [Sesamum indicum]
c27616.graph_c0	0	0	0.14474571	2.23106561	2.69334024	2.07752312	5.605E-10	4.544519	up	PREDICTED: myb-related protein Zm38-like [Sesamum indicum]
c27618.graph_c0	3.83044196	4.55772616	3.34342838	1.53138902	2.32767793	4.08786224	0.0001547	-1.58829	down	-
c27622.graph_c0	23.4439774	24.3941047	21.7100697	11.8118056	14.5008283	14.4750653	1.02E-33	-1.790143	down	PREDICTED: 1-phosphatidylinositol-3-phosphate 5-kinase FAB1A isoform X2 [Sesamum indicum]
c27630.graph_c0	0.72589485	0.7556866	0.52237003	0.10699867	0.31171832	0.14281016	3.689E-08	-2.863615	down	-
c27631.graph_c0	3.67344539	4.33245278	3.78576578	47.4460592	51.5057315	47.1664118	2.895E-52	2.613295	up	PREDICTED: uncharacterized protein LOC100264843 [Vitis vinifera]
c27632.graph_c0	54.1287191	50.2338521	56.3110863	26.184034	24.3853112	25.3500458	4.256E-46	-2.098652	down	PREDICTED: monothiol glutaredoxin-S16, chloroplastic [Sesamum indicum]
c27634.graph_c0	0	0.05385184	0.20739756	1.58599034	1.52264904	1.85220613	2.683E-07	3.205424	up	PREDICTED: uncharacterized protein LOC105170116 [Sesamum indicum]
c27641.graph_c0	1.37360702	1.85093796	1.15412961	0.43804259	0.82498313	0.90945789	3.284E-05	-2.036964	down	PREDICTED: heat shock factor protein HSF30-like [Sesamum indicum]
c27652.graph_c0	3.15962805	4.99388201	3.84654992	0.76601459	0	0.54527614	1.709E-12	-4.187372	down	-
c27654.graph_c0	0.1523414	0.26686441	0.04894118	2.17537593	3.67984121	4.37079192	1.293E-07	3.43168	up	cytochrome P450 [Verbena x hybrida]
c27656.graph_c0	0.17104361	0.26905196	0.21979781	4.1870335	4.36411148	3.8057176	2.349E-13	3.209047	up	PREDICTED: LOW QUALITY PROTEIN: uncharacterized protein ycf45 [Sesamum indicum]
c27659.graph_c0	3.23727108	2.27404401	1.60562234	12.0865087	10.919591	13.687549	4.894E-05	1.354838	up	PREDICTED: methyltransferase-like protein 13 [Sesamum indicum]
c27662.graph_c0	0.19317134	0.12890985	0.12411641	3.88550448	3.80200552	3.1933826	1.016E-11	3.595707	up	PREDICTED: MATE efflux family protein 5-like [Sesamum indicum]
c27665.graph_c0	1.04187048	1.0846303	1.07107585	5.33670506	5.93830516	7.34490745	7.244E-05	1.520594	up	PREDICTED: IAA-amino acid hydrolase ILR1-like 2 [Sesamum indicum]
c27667.graph_c0	75.2251956	73.1638146	71.5073582	66.2508831	58.9782008	63.8369666	2.15E-17	-1.233262	down	PREDICTED: uncharacterized protein LOC105180257 [Sesamum indicum]

#ID	0 h-1 FPKM	0 h-2 FPKM	0 h-3 FPKM	48 h-1 FPKM	48 h-2 FPKM	48 h-3 FPKM	FDR	log2FC	regulated	nr_annotation
c27670.graph_c1	53.4949168	53.789802	60.9363003	18.8559137	19.1285343	22.3112191	9.28E-55	-2.500568	down	hypothetical protein MIMGU_mgv1a001800mg [Erythranthe guttata]
c27672.graph_c0	1.19885073	1.16368601	1.21378294	9.50504527	11.1685723	11.2866362	2.346E-12	2.138947	up	PREDICTED: uncharacterized protein LOC105164990 isoform X1 [Sesamum indicum]
c27675.graph_c0	41.2079142	43.5771737	47.2568315	16.537856	19.2931176	20.7920421	1.656E-40	-2.243695	down	PREDICTED: DNL-type zinc finger protein-like [Sesamum indicum]
c27682.graph_c0	1.21841638	1.55226456	1.20986936	0.3571542	0.43241322	0.13619728	4.225E-09	-3.117902	down	PREDICTED: ammonium transporter 3 member 1-like [Solanum tuberosum]
c27685.graph_c0	20.9534597	22.9118396	18.4913668	23.8001365	18.1503331	16.6934425	4.469E-08	-1.098141	down	PREDICTED: probable WRKY transcription factor 65 [Sesamum indicum]
c27686.graph_c0	1.20948591	1.41247987	1.46789076	0.06190318	0	0	3.531E-17	-7.007732	down	-
c27688.graph_c0	0.10753606	0.03588125	0.18425079	20.1110536	33.3396998	35.2310981	1.396E-28	7.043447	up	PREDICTED: allene oxide cyclase 4, chloroplastic-like [Pyrus x bretschneideri]
c27692.graph_c0	1.12071689	0.85105037	0.74491323	13.7070289	12.0693486	10.0422569	4.995E-16	2.710738	up	PREDICTED: uncharacterized protein LOC105172843 [Sesamum indicum]
c27693.graph_c0	3.25245818	3.47769559	3.46711636	19.7828956	23.8433043	26.6918762	2.987E-18	1.763555	up	PREDICTED: uncharacterized protein LOC105170243 [Sesamum indicum]
c27694.graph_c0	1.73179554	0.95344132	1.66906938	0	0	0	1.389E-19	-Inf	down	hypothetical protein V501_06377 [Pseudogymnoascus pannorum VKM F-4519 (FW-2642)]
c27696.graph_c0	10.579298	10.1759544	9.96362789	3.9683792	4.1367523	4.57622777	3.274E-27	-2.294906	down	hypothetical protein MIMGU_mgv1a008679mg [Erythranthe guttata]
c27698.graph_c0	0.03000741	0.09011236	0	3.67675568	3.86579052	3.71432195	7.06E-18	5.5462	up	hypothetical protein MIMGU_mgv1a004055mg [Erythranthe guttata]
c27705.graph_c0	0	0	0.14040454	13.9914946	16.8483606	23.7347493	5.664E-20	7.546655	up	hypothetical protein MIMGU_mgv1a024655mg [Erythranthe guttata]
c27710.graph_c0	0.28420376	0.22759068	0.29217047	8.90211935	16.64244	26.0928781	1.729E-06	4.973537	up	PREDICTED: glucan endo-1,3-beta-glucosidase 4-like [Nicotiana sylvestris]
c27714.graph_c0	1.04243035	0.72049408	0.82925407	6.76821499	9.6893285	9.46073447	1.338E-15	2.299201	up	PREDICTED: pentatricopeptide repeat-containing protein At3g62470, mitochondrial-like [Sesamum indicum]
c27717.graph_c0	14.520095	12.5935271	10.8792917	10.980353	10.1090683	10.9333773	2.621E-10	-1.260506	down	PREDICTED: uncharacterized protein LOC105170997 [Sesamum indicum]
c27723.graph_c0	101.139013	90.7827489	99.2717622	392.784874	409.604538	422.776392	9E-14	1.054259	up	PREDICTED: 40S ribosomal protein S5 [Sesamum indicum]
c27725.graph_c0	5.76024151	5.59388984	7.18117905	42.0576584	46.0653279	49.5444897	1.299E-17	1.870525	up	PREDICTED: 40S ribosomal protein S13-like [Sesamum indicum]
c27727.graph_c0	27.3597056	31.0483826	25.2925727	12.8279711	14.4379304	15.525757	5.89E-44	-1.986784	down	PREDICTED: autophagy-related protein 9 [Sesamum indicum]
c27733.graph_c0	0.79409193	0.67259677	0.74570584	0.33764736	0.20865625	0.25036383	1.446E-06	-2.482712	down	-
c27737.graph_c0	5.23289862	6.26859207	4.96068337	4.50476376	5.77693538	5.55537937	0.0043657	-1.076344	down	PREDICTED: uncharacterized protein LOC105168421 isoform X1 [Sesamum indicum]

#ID	0 h-1 FPKM	0 h-2 FPKM	0 h-3 FPKM	48 h-1 FPKM	48 h-2 FPKM	48 h-3 FPKM	FDR	log2FC	regulated	nr_annotation
c27738.graph_c0	0.09489607	0.04749555	0.0609726	2.53530895	3.93616301	5.05633354	3.688E-10	4.800887	up	PREDICTED: protein CHUP1, chloroplastic isoform X1 [Sesamum indicum]
c27740.graph_c0	3.33067998	3.6515404	3.26099477	0.43834852	0.15479237	0	7.957E-15	-5.093463	down	PREDICTED: uncharacterized protein LOC103423918 [Malus domestica]
c27741.graph_c0	0.78114804	0.54735117	0.90342554	7.05251636	11.2069289	18.5056922	0.0002618	3.010307	up	PREDICTED: transcription factor TCP4-like [Sesamum indicum]
c27744.graph_c0	1.59169862	1.49672928	1.30161547	0.13330696	0.32951931	0.39538578	2.553E-10	-3.385502	down	-
c27745.graph_c1	6.9930733	7.36635884	6.47855018	41.8760891	45.9498529	45.6931641	5.482E-23	1.661631	up	PREDICTED: equilibrative nucleotide transporter 1 [Sesamum indicum]
c27749.graph_c0	1.38113785	1.6671575	1.40941336	6.324548	7.73087506	8.92415386	9.061E-05	1.344324	up	PREDICTED: tRNA (adenine(58)-N(1))-methyltransferase catalytic subunit trmt61a [Sesamum indicum]
c27751.graph_c0	26.0567852	30.0224784	17.9627712	20.0043418	29.2716909	22.9171691	0.0005147	-1.055113	down	PREDICTED: probable glutathione S-transferase [Sesamum indicum]
c27752.graph_c0	0.72781003	1.11304542	0.88330555	3.96716091	6.49153444	5.43580067	1.976E-06	1.519285	up	PREDICTED: CSC1-like protein At1g32090 [Sesamum indicum]
c27754.graph_c0	104.926041	103.51742	102.236858	36.0120026	36.6191707	37.3114982	4.785E-63	-2.51633	down	PREDICTED: topless-related protein 4 [Sesamum indicum]
c27755.graph_c0	0.13297161	0.15211973	0.29292649	1.03251788	1.61293207	1.52602311	0.0001317	1.817659	up	PREDICTED: glutamate receptor 3.2-like [Sesamum indicum]
c27760.graph_c0	11.6993865	11.7935742	9.31695343	1.8975771	5.30706751	2.5664481	5.133E-18	-2.776385	down	PREDICTED: uncharacterized protein LOC105179312 [Sesamum indicum]
c27765.graph_c0	40.4608418	41.8292706	40.9131412	14.2073381	8.73926243	5.8450427	6.381E-36	-3.101389	down	PREDICTED: polyubiquitin 4-like [Cicer arietinum]
c27772.graph_c0	1.51363599	1.59705176	1.73480302	5.05020722	7.74564137	7.8471097	0.0020267	1.064489	up	PREDICTED: uncharacterized protein At2g39795, mitochondrial isoform X1 [Sesamum indicum]
c27774.graph_c0	0.39431181	0	0	5.93334068	2.95040949	2.58585348	0.0021482	3.882021	up	PREDICTED: transmembrane protein 56 isoform X2 [Sesamum indicum]
c27778.graph_c0	0.29460773	0.15612498	0.26723477	2.74604936	3.70403654	2.62809517	7.714E-11	2.638616	up	PREDICTED: phosphoenolpyruvate carboxylase 4 [Sesamum indicum]
c27779.graph_c0	1.39791127	2.13579058	1.41818789	19.3177057	22.9779837	21.1693375	6.316E-16	2.661403	up	PREDICTED: transcription factor BEE 1-like [Sesamum indicum]
c27785.graph_c0	5.04004086	4.71426228	6.05195356	51.303713	51.2858161	53.8450142	1.189E-26	2.286866	up	hypothetical protein MIMGU_mgv1a013628mg [Erythranthe guttata]
c27785.graph_c1	12.4363384	14.1367632	15.1685845	159.429379	147.089778	147.734109	4.189E-32	2.426398	up	PREDICTED: polyadenylate-binding protein 2 isoform X1 [Sesamum indicum]
c27788.graph_c0	2.1744026	2.53934326	2.9494264	0.11128897	0	0.19804852	2.652E-15	-5.644838	down	-
c27790.graph_c0	3.45263063	4.63262849	3.87037126	39.5229895	46.7450674	51.6068353	6.677E-30	2.506312	up	PREDICTED: nucleoside diphosphate kinase 2, chloroplastic [Sesamum indicum]
c27793.graph_c0	1.47155985	1.09113585	0.98052509	0.55232097	0.53192463	0.49145187	1.408E-05	-2.181835	down	-
c27795.graph_c0	0.39619688	0.0991484	0.25456434	3.92377593	3.86675797	6.17076208	2.033E-07	3.199131	up	PREDICTED: exonuclease DPD1, chloroplastic/mitochondrial [Sesamum indicum]

#ID	0 h-1 FPKM	0 h-2 FPKM	0 h-3 FPKM	48 h-1 FPKM	48 h-2 FPKM	48 h-3 FPKM	FDR	log2FC	regulated	nr_annotation
c27795.graph_c1	0.60917852	0.99090635	0.39140926	6.59427614	7.65469261	7.240803	1.27E-06	2.417718	up	PREDICTED: exonuclease DPD1, chloroplastic/mitochondrial [Sesamum indicum]
c27796.graph_c0	2.21699923	3.00668411	2.29752564	52.5105841	48.7186454	54.1102693	5.616E-49	3.352387	up	unnamed protein product [Coffea canephora]
c27798.graph_c0	23.9436946	22.5806526	21.6163628	6.51031916	7.43782658	9.54115291	5.513E-58	-2.55743	down	PREDICTED: uncharacterized protein LOC105164093 [Sesamum indicum]
c27800.graph_c0	0	0	0	2.96870328	2.40011719	4.86598428	3.318E-08	Inf	up	PREDICTED: transcription factor bHLH130-like [Sesamum indicum]
c27801.graph_c0	0.77136419	0.66684524	0.49561676	18.9932287	23.6798431	24.2579102	9.918E-55	4.0955	up	PREDICTED: chorismate mutase 1, chloroplastic-like [Sesamum indicum]
c27803.graph_c0	2.39313581	1.75998282	2.44766727	2.33969522	2.33562588	0.96085234	0.0037922	-1.241013	down	hypothetical protein MIMGU_mgv1a020303mg [Erythranthe guttata]
c27807.graph_c0	0.53456058	1.60528759	0.68693151	114.581836	142.949758	178.638999	6.842E-38	6.249119	up	hypothetical protein MIMGU_mgv1a0223741mg, partial [Erythranthe guttata]
c27818.graph_c0	0	0	0	20.1353695	27.3291497	24.1823233	6.157E-89	Inf	up	hypothetical protein MIMGU_mgv1a023677mg [Erythranthe guttata]
c27824.graph_c0	3.13417841	3.64009194	2.94320475	18.9162345	24.059113	24.7203123	9.942E-26	1.77959	up	PREDICTED: LOW QUALITY PROTEIN: uncharacterized protein LOC105170443 [Sesamum indicum]
c27825.graph_c0	1.17831881	0.8256491	1.58989564	141.880619	153.81282	178.934948	1.69E-139	6.020116	up	PREDICTED: 2-Cys peroxiredoxin BAS1, chloroplastic-like [Sesamum indicum]
c27827.graph_c0	0	0	0	2.71497051	1.30735548	1.18592072	5.669E-05	Inf	up	PREDICTED: cyclin-D5-3-like [Sesamum indicum]
c27828.graph_c0	55.422358	49.2035739	44.5972383	27.656791	32.0679346	33.9115311	2.595E-28	-1.691055	down	hypothetical protein MIMGU_mgv1a024518mg [Erythranthe guttata]
c27833.graph_c0	1.49385644	1.07665366	1.07501222	10.8998049	11.6636582	11.9517865	3.383E-09	2.226577	up	PREDICTED: uncharacterized protein LOC105175923 [Sesamum indicum]
c27839.graph_c0	14.6785035	14.7674099	13.4323566	8.19563323	9.47815527	9.05476125	6.44E-12	-1.700728	down	-
c27841.graph_c0	1.95382384	2.1335785	2.47269945	13.609015	14.5037771	13.6865729	4.107E-13	1.651213	up	PREDICTED: uncharacterized protein LOC105159050 [Sesamum indicum]
c27846.graph_c0	0.047995	0.14412917	0.18502642	32.2777698	33.2107092	22.58302	1.678E-34	6.843248	up	PREDICTED: lignin-forming anionic peroxidase-like [Sesamum indicum]
c27850.graph_c0	0.04795551	0.33602472	0.18487419	3.31348089	3.65064047	3.1841806	3.521E-08	3.136763	up	PREDICTED: protein COBRA-like [Sesamum indicum]
c27852.graph_c0	2.67230775	2.07447712	2.10246199	14.4197246	13.0936712	13.9482309	3.014E-07	1.58344	up	PREDICTED: uncharacterized protein LOC105168786 [Sesamum indicum]
c27858.graph_c0	0.29546901	0.39435318	0.50625251	10.6462528	19.352733	18.8382961	6.609E-15	4.319213	up	hypothetical protein MIMGU_mgv1a026536mg, partial [Erythranthe guttata]
c27862.graph_c0	2.91723347	2.27123139	3.60991753	0	0	0.17713793	8.63E-18	-6.678613	down	PREDICTED: ethylene-responsive transcription factor ERF109-like [Sesamum indicum]

#ID	0 h-1 FPKM	0 h-2 FPKM	0 h-3 FPKM	48 h-1 FPKM	48 h-2 FPKM	48 h-3 FPKM	FDR	log2FC	regulated	nr_annotation
c27868.graph_c0	1.43253145	1.28141635	1.33168569	5.08240842	5.97913417	9.21948834	0.0016505	1.302355	up	PREDICTED: F-box/kelch-repeat protein At3g27150-like [Sesamum indicum]
c27869.graph_c0	34.8237735	33.6503089	36.3358131	33.4636114	32.1661887	33.0441527	5.89E-14	-1.104493	down	hypothetical protein MIMGU_mgv1a009154mg [Erythranthe guttata]
c27870.graph_c0	8.45255533	10.0579666	9.53110955	608.472499	514.687963	562.500031	5.557E-97	4.894168	up	PREDICTED: asparagine synthetase [glutamine-hydrolyzing] [Solanum lycopersicum]
c27877.graph_c0	0.7519986	0.37637581	0.48317399	4.75056109	3.93175268	4.79942974	2.86E-05	2.051868	up	PREDICTED: RING-H2 finger protein ATL3 [Sesamum indicum]
c27879.graph_c0	3.57069196	3.06365783	3.47413527	26.4574156	27.431015	23.45802	5.719E-17	1.918547	up	PREDICTED: hsp70 nucleotide exchange factor FES1 [Sesamum indicum]
c27879.graph_c1	2.20511295	2.0601663	2.83365745	22.7527315	21.0907901	15.5454549	8.969E-07	2.047766	up	-
c27884.graph_c0	29.0378283	28.6356455	30.2282765	6.9855657	1.93418495	0.91823053	5.843E-72	-4.145432	down	hypothetical protein CICLE_v10017323mg [Citrus clementina]
c27885.graph_c0	0.58582963	0.39094416	0.15056285	2.69852232	3.23992481	6.21092017	0.0030347	2.414855	up	PREDICTED: cytochrome P450 81E8-like isoform X1 [Sesamum indicum]
c27887.graph_c0	46.0270315	43.2362351	38.1046808	25.3354917	33.9596209	38.2893085	6.644E-21	-1.406098	down	PREDICTED: E3 ubiquitin-protein ligase RMA1H1-like [Sesamum indicum]
c27888.graph_c1	7.2733907	7.48535148	8.07035312	31.2969462	30.874644	30.3859862	3.416E-10	1.001416	up	PREDICTED: aspartokinase 2, chloroplastic [Sesamum indicum]
c27892.graph_c0	0.43598431	0.08728418	0.22410284	10.6037495	11.6588939	10.7932427	1.825E-15	4.452145	up	PREDICTED: transcription factor MYB86-like [Sesamum indicum]
c27892.graph_c1	0.18460913	0	0	5.69747795	8.01768726	8.3988591	2.548E-14	5.901714	up	-
c27896.graph_c0	8.03810346	9.25392581	7.59955572	3.85134334	5.60589662	5.976183	7.295E-24	-1.713061	down	PREDICTED: U-box domain-containing protein 44 [Sesamum indicum]
c27898.graph_c0	16.7651894	16.5227961	19.9634893	9.66069551	12.1927577	11.4083098	1.045E-15	-1.70228	down	PREDICTED: uncharacterized protein At2g34460, chloroplastic [Sesamum indicum]
c27905.graph_c0	13.3845651	12.7640854	13.0939622	8.72432604	8.98953489	8.98980831	7.687E-20	-1.573317	down	PREDICTED: transcription factor RF2b-like [Sesamum indicum]
c27907.graph_c0	0.49605815	0.16551829	0.84993914	6.55034938	9.03722908	7.04837203	9.78E-07	2.872588	up	PREDICTED: protein RER1A-like [Sesamum indicum]
c27908.graph_c0	8.67355296	8.84814085	7.09927321	52.2191046	60.3880961	65.1676891	1.667E-26	1.833555	up	PREDICTED: vacuolar protein sorting-associated protein 29 [Sesamum indicum]
c27911.graph_c0	0.03671785	0	0	3.04441907	4.98113118	3.25068694	2.975E-16	7.260042	up	PREDICTED: protein NRT1/ PTR FAMILY 6.3-like [Sesamum indicum]
c27912.graph_c0	5.54443239	5.97944737	5.04674405	3.19243297	4.38048958	4.43675863	9.532E-09	-1.486432	down	PREDICTED: uncharacterized protein LOC105168751 [Sesamum indicum]
c27913.graph_c0	1.4030446	1.40444954	1.54540102	10.7099171	13.8888957	14.4587335	5.458E-07	2.141985	up	hypothetical protein OsI_11409 [Oryza sativa Indica Group]
c27915.graph_c0	6.10573662	7.95961938	7.4811778	47.2238609	50.9980256	53.0772726	2.115E-11	1.791598	up	PREDICTED: mitochondrial import inner membrane translocase subunit TIM8 [Sesamum indicum]
c27916.graph_c0	0.41521602	0.23090655	0.35571268	9.26558319	9.27544471	13.3877932	7.704E-19	3.973514	up	PREDICTED: uncharacterized protein LOC105157022 [Sesamum indicum]

#ID	0 h-1 FPKM	0 h-2 FPKM	0 h-3 FPKM	48 h-1 FPKM	48 h-2 FPKM	48 h-3 FPKM	FDR	log2FC	regulated	nr_annotation
c27918.graph_c0	2.73976431	2.87520976	2.10106625	16.976269	12.5501939	11.2294207	0.0002117	1.393643	up	PREDICTED: G-box-binding factor 4-like [Sesamum indicum]
c27920.graph_c0	4.39591329	4.25943876	3.99999041	23.4751237	25.5638938	26.2355752	6.265E-28	1.554079	up	PREDICTED: LOW QUALITY PROTEIN: uncharacterized protein LOC105171130 [Sesamum indicum]
c27923.graph_c0	0.97241116	1.36994909	1.57355379	71.3692705	100.633702	107.138924	4.759E-44	5.127584	up	PREDICTED: protein disulfide isomerase-like 2-3 [Sesamum indicum]
c27926.graph_c0	27.8504317	31.1866645	26.7850525	13.1941948	18.7945626	16.833519	1.227E-30	-1.836118	down	PREDICTED: UPF0392 protein RCOM_0530710 [Sesamum indicum]
c27934.graph_c0	1.70236905	1.57784603	1.45840775	0	0.55381969	0.51684949	3.846E-09	-3.191594	down	PREDICTED: ADP,ATP carrier protein 1, mitochondrial-like [Malus domestica]
c27935.graph_c0	28.4094746	31.453245	28.1395948	8.29216372	8.46766472	8.01412432	2.746E-83	-2.845614	down	PREDICTED: DNA ligase 1-like [Sesamum indicum]
c27941.graph_c0	9.59838261	9.97753216	9.07282767	3.31599604	3.37778435	4.16103676	5.463E-21	-2.418807	down	hypothetical protein JCGZ_04255 [Jatropha curcas]
c27942.graph_c0	0.23949333	0.29966643	0	18.2025424	16.4785303	17.2272	4.036E-34	5.589247	up	PREDICTED: 1-aminocyclopropane-1-carboxylate oxidase [Sesamum indicum]
c27943.graph_c0	8.94174521	7.78022299	10.4298354	5.89313737	9.06251294	9.30330003	4.717E-05	-1.190096	down	PREDICTED: LOW QUALITY PROTEIN: auxilin-related protein 2-like [Sesamum indicum]
c27945.graph_c0	10.1704569	7.15169003	10.0451066	5.03330363	8.77758018	7.16576528	1.118E-05	-1.410842	down	PREDICTED: uncharacterized protein LOC105169359 [Sesamum indicum]
c27946.graph_c0	1.59187668	1.53852343	2.11616333	0.70798513	0.80359757	0.22498604	2.161E-07	-2.611394	down	PREDICTED: putative germin-like protein 2-1 [Sesamum indicum]
c27947.graph_c0	1.77093816	1.64300089	1.22112108	0	0	0	2.237E-23	-Inf	down	-
c27949.graph_c0	5.61574579	6.50895371	6.4025697	0.38899183	0.2472538	0.20767355	7.981E-44	-5.463819	down	PREDICTED: uncharacterized protein LOC105156723 [Sesamum indicum]
c27952.graph_c0	0	0.06881418	0	1.66776085	1.67733265	2.29169058	9.582E-14	5.347665	up	PREDICTED: uncharacterized protein LOC105176065 [Sesamum indicum]
c27954.graph_c0	2.38106559	3.85018825	2.11829785	0.50621375	1.78757363	3.00283969	0.0037303	-1.69051	down	PREDICTED: probable polyamine transporter At3g13620 [Sesamum indicum]
c27954.graph_c1	3.6205255	2.83342707	4.22956237	0.90967206	2.12011195	3.50748984	7.905E-05	-1.74457	down	PREDICTED: probable polyamine transporter At3g13620 [Sesamum indicum]
c27955.graph_c0	0.0774356	0.07751314	0	7.24088055	8.80445587	12.3462314	3.25E-20	6.510481	up	hypothetical protein MIMGU_mgv1a005882mg [Erythranthe guttata]
c27956.graph_c0	0.80611286	1.07589342	1.43051054	5.905786	7.2679883	8.24426594	1.671E-06	1.665417	up	PREDICTED: surfeit locus protein 1 [Sesamum indicum]
c27958.graph_c0	110.868602	106.039489	112.652421	117.613844	104.486226	99.3999684	1.244E-13	-1.050744	down	PREDICTED: peptide methionine sulfoxide reductase B5-like [Sesamum indicum]
c27959.graph_c0	0.20719884	0.24197403	0.1775058	2.60876278	2.29182211	2.66095823	1.369E-06	2.578974	up	PREDICTED: uncharacterized protein LOC105161116 [Sesamum indicum]
c27961.graph_c0	1.65015259	1.82184372	1.30972764	8.92016253	12.8129282	13.0494791	6.318E-12	1.841415	up	hypothetical protein MIMGU_mgv1a003065mg [Erythranthe guttata]

#ID	0 h-1 FPKM	0 h-2 FPKM	0 h-3 FPKM	48 h-1 FPKM	48 h-2 FPKM	48 h-3 FPKM	FDR	log2FC	regulated	nr_annotation
c27962.graph_c0	4.91038074	4.05827147	4.62735802	25.9594401	31.850884	36.5778705	1.052E-16	1.773155	up	PREDICTED: ent-kaurene oxidase, chloroplastic-like [Sesamum indicum]
c27967.graph_c0	1.50655594	1.37692848	0.9048621	6.68322127	7.94309501	10.0946071	1.853E-08	1.688496	up	PREDICTED: LOW QUALITY PROTEIN: alpha,alpha-trehalose-phosphate synthase [UDP-forming] 1-like [Sesamum indicum]
c27969.graph_c0	0.16026793	0.06417137	0.24714077	9.24284452	4.34837875	4.96606707	9.065E-06	4.28704	up	PREDICTED: S-type anion channel SLAH2-like [Sesamum indicum]
c27970.graph_c0	2.57657718	2.26334206	2.36500263	38.1731475	30.5351441	24.9574255	1.449E-10	2.68963	up	Os05g0129800 [Oryza sativa Japonica Group]
c27971.graph_c0	1.05507385	0.49700252	0.55827539	6.4609601	8.23775323	11.9048503	8.373E-07	2.636184	up	PREDICTED: EH domain-containing protein 1-like [Sesamum indicum]
c27974.graph_c0	105.560633	101.232028	88.4429794	1010.41766	848.971641	653.550391	3.406E-08	2.079295	up	-
c27976.graph_c0	0	0	0	7.58896304	7.79410259	10.3093339	1.692E-23	Inf	up	PREDICTED: homeobox-leucine zipper protein ATHB-40-like [Sesamum indicum]
c27977.graph_c0	0	0.06401401	0	3.77055297	5.18029413	6.13338745	3.276E-15	6.867791	up	PREDICTED: pyruvate kinase, cytosolic isozyme-like [Sesamum indicum]
c27977.graph_c1	0	0	0	3.90122406	7.00058222	6.16330319	5.561E-15	Inf	up	Pyruvate kinase, cytosolic isozyme [Glycine soja]
c27983.graph_c0	8.96596812	8.00468172	4.67092865	4.91137194	5.91250346	3.17826125	0.0003354	-1.639184	down	-
c27986.graph_c0	3.8487797	3.92601717	3.29722287	2.87036654	2.53996678	4.2967881	0.000169	-1.207735	down	-
c27987.graph_c0	0.75989639	0.82404542	1.22062118	9.91760332	12.8550095	11.004825	8.893E-11	2.563894	up	PREDICTED: uncharacterized protein LOC105171687 [Sesamum indicum]
c27988.graph_c0	6.72300873	9.51680518	6.02135288	302.801847	564.28442	671.796794	1.159E-12	5.084731	up	-
c27993.graph_c0	10.5415026	10.5018104	10.9660104	6.47434651	6.41786318	9.58767472	1.018E-10	-1.530861	down	PREDICTED: protein ECERIFERUM 1 [Sesamum indicum]
c27997.graph_c0	2.78044175	2.27718486	2.31431512	16.533475	18.2244547	16.9129145	2.424E-16	1.791785	up	PREDICTED: probable lysine--tRNA ligase, cytoplasmic [Sesamum indicum]
c27998.graph_c0	1.24868964	1.11105779	0.93602578	8.97931086	9.71560475	9.43983139	6.595E-11	2.077582	up	PREDICTED: pectinesterase 2.1-like [Sesamum indicum]
c28000.graph_c0	435.520406	440.309382	438.873921	416.175769	398.937317	350.568105	3.56E-17	-1.189194	down	PREDICTED: uncharacterized protein LOC105170296 [Sesamum indicum]
c28000.graph_c1	26.4632623	32.5866111	19.4321942	10.6419253	11.0688507	10.8464478	1.306E-09	-2.283551	down	PREDICTED: probable membrane-associated kinase regulator 1 [Sesamum indicum]
c28001.graph_c0	0	0	0	1.32350315	2.24334576	2.060879	1.596E-11	Inf	up	ADP-ribosylation factor [Hyacinthus orientalis]
c28005.graph_c0	24.0958689	25.097835	24.5481514	25.9651298	22.8106055	23.548487	8.522E-12	-1.043162	down	PREDICTED: transcription initiation factor IIA large subunit-like [Sesamum indicum]
c28007.graph_c0	0	0	0.23462916	3.13990798	4.39553627	3.44244849	3.034E-11	4.493587	up	PREDICTED: purple acid phosphatase 17-like [Sesamum indicum]
c28009.graph_c0	2.28776084	1.55723514	2.23429607	11.7184478	14.7363691	18.0784753	1.235E-06	1.848681	up	hypothetical protein MIMGU_mgv1a006302mg [Erythranthe guttata]

#ID	0 h-1 FPKM	0 h-2 FPKM	0 h-3 FPKM	48 h-1 FPKM	48 h-2 FPKM	48 h-3 FPKM	FDR	log2FC	regulated	nr_annotation
c28010.graph_c0	26.4577206	25.4793929	27.9794833	16.6680718	14.9986341	15.4385023	1.569E-33	-1.778945	down	PREDICTED: uncharacterized protein LOC105174992 [Sesamum indicum]
c28013.graph_c0	171.941286	160.79534	163.834681	636.667238	694.35391	717.676596	4.686E-13	1.025589	up	PREDICTED: 60S ribosomal protein L21-1 [Sesamum indicum]
c28016.graph_c0	2.44136654	3.3043081	1.8558396	1.29880267	1.34236482	0.76104887	6.866E-07	-2.170143	down	PREDICTED: uncharacterized protein LOC105177164 [Sesamum indicum]
c28020.graph_c0	6.06145057	6.31416736	7.66255025	2.04300133	1.97192887	1.97943699	2.218E-17	-2.760296	down	PREDICTED: very-long-chain 3-oxoacyl-CoA reductase-like protein At1g24470 [Sesamum indicum]
c28022.graph_c0	3.58059824	3.80362348	3.94389471	23.6293246	22.251026	23.6608724	6.187E-09	1.600406	up	PREDICTED: uncharacterized protein LOC105178683 [Sesamum indicum]
c28025.graph_c0	81.2593957	89.3854557	79.5592691	24.2225711	14.4281912	12.5251966	3.854E-90	-3.294423	down	PREDICTED: probable mitochondrial chaperone BCS1-B [Sesamum indicum]
c28026.graph_c0	0.24899607	0.54833988	0.12798792	1.33047023	3.20776359	3.46988879	0.0069182	2.089619	up	PREDICTED: uncharacterized protein LOC105166116 [Sesamum indicum]
c28033.graph_c0	1.44047719	1.04609854	1.81477472	12.7501991	13.9759102	17.5269454	2.027E-19	2.3381	up	PREDICTED: ATP sulfurylase 2 [Sesamum indicum]
c28034.graph_c0	0.45793189	0.36115611	0.39230708	6.03411566	6.41950036	5.71037744	1.465E-25	2.889447	up	PREDICTED: phytosulfokine receptor 2-like [Sesamum indicum]
c28035.graph_c0	0.03140662	0.22006647	0.12107625	2.66191195	2.78932106	3.34686872	1.466E-10	3.53996	up	PREDICTED: ADP-ribosylation factor GTPase-activating protein AGD3-like [Sesamum indicum]
c28036.graph_c0	0.15564349	0	0	3.29794543	3.94948284	3.06207862	1.183E-09	5.051804	up	PREDICTED: MADS-box protein SVP-like isoform X2 [Nicotiana sylvestris]
c28037.graph_c0	1.40623608	1.54840864	0.72282762	9.71637302	9.05812076	7.60810185	0.0005332	1.834676	up	hypothetical protein CICLE_v10003220mg [Citrus clementina]
c28038.graph_c0	17.1969734	17.4843714	16.6979659	103.938432	99.0088328	93.590057	5.255E-26	1.513317	up	PREDICTED: aspartic proteinase PCS1-like [Sesamum indicum]
c28041.graph_c0	0.25444783	0.08490087	0.16348777	1.87531153	2.60749804	1.73816762	6.56E-06	2.609971	up	PREDICTED: uncharacterized protein LOC105158497 [Sesamum indicum]
c28042.graph_c0	0.59308971	0.25973657	0.47633985	1.84407611	3.18360376	2.58281604	0.0021613	1.491583	up	PREDICTED: regulatory protein NPR5-like [Sesamum indicum]
c28043.graph_c1	1.64646237	2.07343003	2.25227058	8.26916196	12.0258016	11.7116083	2.356E-05	1.395518	up	PREDICTED: uncharacterized protein LOC105176055 isoform X2 [Sesamum indicum]
c28045.graph_c0	2.96450156	3.37835053	2.52013201	1.13445368	1.64693319	1.98147383	1.714E-06	-1.919109	down	PREDICTED: cysteine-rich receptor-like protein kinase 10 [Sesamum indicum]
c28045.graph_c1	1.41740089	1.48976122	1.91248708	0.91406151	1.452506	1.04570541	0.0027624	-1.525224	down	hypothetical protein MIMGU_mgv1a002249mg [Erythranthe guttata]
c28048.graph_c0	57.3049691	57.8002319	64.9261244	60.1142524	54.4336059	55.0431794	2.533E-13	-1.103093	down	PREDICTED: alanine aminotransferase 2 [Sesamum indicum]
c28049.graph_c0	0.27174964	0.18134784	0	4.50636285	6.80729196	6.68289094	1.519E-10	4.304493	up	-
c28052.graph_c0	0	0	0	2.03438205	4.3103622	3.50357597	6.326E-10	Inf	up	PREDICTED: pto-interacting protein 1 [Nicotiana sylvestris]
c28056.graph_c0	12.4253844	12.8860365	12.5147626	104.364319	112.746348	112.040919	8.828E-32	2.102512	up	hypothetical protein MIMGU_mgv1a017013mg [Erythranthe guttata]
c28058.graph_c0	3.6539261	3.78978682	3.22457907	23.9286588	31.4935469	30.7825677	5.946E-22	1.993569	up	PREDICTED: coatomer subunit epsilon-1 [Sesamum indicum]

#ID	0 h-1 FPKM	0 h-2 FPKM	0 h-3 FPKM	48 h-1 FPKM	48 h-2 FPKM	48 h-3 FPKM	FDR	log2FC	regulated	nr_annotation
c28061.graph_c0	3.13828889	2.93200265	2.82297783	9.01089552	11.5878659	16.7645759	0.0062649	1.047058	up	flavonoid 3-O-glucosyltransferase [Perilla frutescens]
c28063.graph_c0	0.67928371	0.42497744	0.32733992	3.91125285	4.80645783	5.15070971	4.563E-05	2.260353	up	hypothetical protein MIMGU_mgv1a014814mg [Erythranthe guttata]
c28067.graph_c1	0.16786466	0	0	1.77845056	2.37554474	2.95850211	6.647E-09	4.402068	up	PREDICTED: ATP-dependent 6-phosphofructokinase 6-like [Sesamum indicum]
c28070.graph_c0	17.4304236	10.9288903	7.99956585	1.58815811	1.58899089	1.41313353	7.049E-08	-3.995747	down	-
c28072.graph_c0	198.493523	183.280961	193.992839	13.5173636	14.1183174	12.705284	3.21E-197	-4.852685	down	PREDICTED: uncharacterized protein LOC104888288 [Beta vulgaris subsp. vulgaris]
c28074.graph_c0	3.15199587	3.47875746	3.42729495	1.7125117	1.34092932	1.65628545	5.759E-06	-2.109538	down	PREDICTED: dof zinc finger protein DOF5.4-like [Sesamum indicum]
c28078.graph_c0	4.19414264	2.87664204	2.69481967	41.8591091	48.4380028	49.1519646	4.38E-33	2.819071	up	hypothetical protein MIMGU_mgv1a007042mg [Erythranthe guttata]
c28080.graph_c0	0.8954879	1.43893317	1.12045484	5.01502132	5.24380949	4.67482799	0.0005813	1.09414	up	PREDICTED: receptor-like protein kinase HSL1 [Sesamum indicum]
c28081.graph_c0	0.80136317	0.87856234	0.83363418	6.01161102	3.94777035	3.94143867	0.0006868	1.458516	up	PREDICTED: ent-kaurenoic acid oxidase 1-like [Sesamum indicum]
c28092.graph_c0	0	0.08098443	0	6.18628098	14.2126581	19.100006	2.358E-08	7.909449	up	PREDICTED: ammonium transporter 1 member 2-like, partial [Sesamum indicum]
c28093.graph_c0	4386.13677	4135.01396	4879.89869	35.8956489	35.1685592	27.6107711	9.41E-198	-8.102075	down	hypothetical protein MIMGU_mgv1a010438mg [Erythranthe guttata]
c28095.graph_c0	2.71875862	3.06166618	1.52849921	10.4101501	11.9404061	13.0237612	0.0017248	1.260222	up	PREDICTED: transcription factor TCP2-like [Sesamum indicum]
c28098.graph_c0	25.4807645	15.972379	16.2924204	0	0	0	2.888E-37	-Inf	down	hypothetical protein GYMLUDRAFT_153379 [Gymnopus luxurians FD-317 M1]
c28103.graph_c0	6.44589236	5.37695579	5.13560067	3.84015012	5.03279185	4.57940745	1.828E-06	-1.354142	down	hypothetical protein MIMGU_mgv1a006429mg [Erythranthe guttata]
c28104.graph_c0	232.45483	235.749278	272.885036	71.2500031	54.1584962	88.82859	1.428E-43	-2.808713	down	-
c28105.graph_c0	0.58816115	0.58875011	1.07073135	14.6977468	12.8199348	12.0936036	9.825E-32	3.118175	up	PREDICTED: probable receptor-like serine/threonine-protein kinase At5g57670 [Sesamum indicum]
c28106.graph_c0	1.21782295	0.87074458	0.96877901	10.7919744	13.357119	16.0677736	1.294E-13	2.696093	up	PREDICTED: protein GLUTAMINE DUMPER 5-like [Sesamum indicum]
c28107.graph_c1	5.37034887	6.11550534	7.59755331	45.7985624	42.6516223	42.2456339	2.848E-23	1.756513	up	PREDICTED: isoaspartyl peptidase/L-asparaginase 1 [Sesamum indicum]
c28115.graph_c0	2.26394451	1.51080767	1.31609305	13.5073474	14.9406937	12.7699288	8.823E-10	2.003474	up	PREDICTED: CASP-like protein 5A1 [Sesamum indicum]
c28116.graph_c0	4.94836881	5.70811606	4.5420356	36.0359132	41.8092694	39.9840937	4.694E-25	1.936248	up	PREDICTED: coatomer subunit alpha-1-like [Sesamum indicum]
c28117.graph_c0	0.70203915	0.95572931	0.59541775	2.89750388	4.79617816	4.16652551	3.249E-05	1.373438	up	PREDICTED: protein STICHEL [Sesamum indicum]
c28118.graph_c0	0.08732436	0.20978832	0.22443052	8.8332887	11.3861735	15.3330664	2.805E-20	5.060952	up	PREDICTED: phosphoenolpyruvate carboxylase [Sesamum indicum]

#ID	0 h-1 FPKM	0 h-2 FPKM	0 h-3 FPKM	48 h-1 FPKM	48 h-2 FPKM	48 h-3 FPKM	FDR	log2FC	regulated	nr_annotation
c28123.graph_c0	7.96623088	8.27856694	7.34557767	4.81797701	5.57885893	5.13443858	3.793E-08	-1.621606	down	PREDICTED: uncharacterized protein LOC105171936 [Sesamum indicum]
c28130.graph_c0	62.9381511	58.4921438	65.9244265	40.922205	47.2599103	40.6176999	2.442E-20	-1.560519	down	unnamed protein product [Coffea canephora]
c28139.graph_c0	20.4293854	21.2997059	20.1837934	83.6429343	82.4467553	88.822799	5.693E-12	1.024261	up	PREDICTED: adenylate kinase 4 [Sesamum indicum]
c28141.graph_c0	3.37405204	2.80624752	2.93303472	21.6444984	29.3945955	31.9901102	1.74E-16	2.164841	up	PREDICTED: U-box domain-containing protein 11-like [Sesamum indicum]
c28142.graph_c1	19.6552174	17.9340021	19.6882606	2.74666878	2.7211668	2.37608133	3.13E-104	-3.884891	down	PREDICTED: trihelix transcription factor PTL [Sesamum indicum]
c28145.graph_c0	0.04488097	0.02246296	0.02883691	0.41347276	1.029357	1.67397038	0.0023233	3.987107	up	PREDICTED: G-type lectin S-receptor-like serine/threonine-protein kinase At2g19130 isoform X2 [Vitis vinifera]
c28146.graph_c0	0.88948183	1.26136106	1.04776776	6.21415249	8.02998525	7.47369021	0.0001257	1.741403	up	PREDICTED: T-complex protein 1 subunit gamma-like [Malus domestica]
c28148.graph_c0	0.219217	0.13166191	0.16902152	5.25088203	7.44542361	8.37402584	1.254E-17	4.317519	up	PREDICTED: uncharacterized protein LOC105177785 [Sesamum indicum]
c28150.graph_c0	9.49960364	7.88414053	11.5892723	0.44312175	0.41075454	0.39428706	8.635E-31	-5.555441	down	-
c28151.graph_c0	0.45751377	0.68695785	0.54269843	8.67299866	12.6227532	14.6987075	1.707E-15	3.389861	up	PREDICTED: uncharacterized protein LOC105163554 [Sesamum indicum]
c28152.graph_c0	9.27489285	8.2624762	8.2688883	48.7739284	55.5680561	51.2927368	9.589E-22	1.573876	up	PREDICTED: probable prolyl 4-hydroxylase 4 [Sesamum indicum]
c28153.graph_c0	6.55382365	7.17194775	6.56624495	4.09343039	5.09537944	5.14476252	4.934E-07	-1.522748	down	PREDICTED: probable alpha,alpha-trehalose-phosphate synthase [UDP-forming] 7 [Sesamum indicum]
c28156.graph_c0	46.5717805	42.5833036	46.7310325	204.410932	217.050835	224.137988	3.072E-18	1.229052	up	PREDICTED: 60S ribosomal protein L13a-4 [Sesamum indicum]
c28158.graph_c0	46.0224572	49.6505449	47.9001184	43.3146321	49.1850031	45.7115362	6.84E-11	-1.074348	down	hypothetical protein MIMGU_mgv1a015858mg [Erythranthe guttata]
c28161.graph_c0	1.86998308	1.3370397	1.65921587	5.86555488	7.16982018	7.3359876	7.125E-05	1.044473	up	PREDICTED: uncharacterized protein LOC105175750 [Sesamum indicum]
c28167.graph_c0	0	0.17601392	0.15063906	1.13395301	1.14408164	1.77773472	6.405E-06	2.604121	up	PREDICTED: uncharacterized protein LOC105171763 [Sesamum indicum]
c28171.graph_c0	5.16365868	2.58441465	6.16154114	72.7156588	95.7518252	94.4829965	2.907E-32	3.213721	up	Protein of unknown function UPF0057 [Medicago truncatula]
c28172.graph_c0	0.77184763	1.01406443	0.92986384	92.7510252	101.695392	109.894076	1.53E-173	5.787555	up	PREDICTED: 1,4-dihydroxy-2-naphthoyl-CoA synthase, peroxisomal [Sesamum indicum]
c28174.graph_c0	0.28793858	0.03602836	0.18500623	2.38741017	3.23171925	3.18645482	1.367E-08	3.09074	up	PREDICTED: cytochrome P450 94A1 [Sesamum indicum]
c28176.graph_c0	1.23246868	1.96270901	1.43979186	23.7408203	28.485698	31.1355863	4.777E-32	3.147655	up	PREDICTED: PRA1 family protein B1 [Sesamum indicum]
c28177.graph_c0	0.63623994	0.38212622	1.14463091	6.21314474	4.84341255	5.21548946	0.0003553	1.889191	up	-
c28178.graph_c0	10.5705561	5.18882872	5.74690054	0	0	0	1.813E-14	-Inf	down	60S ribosomal protein L13a [Rozella allomycis CSF55]

#ID	0 h-1 FPKM	0 h-2 FPKM	0 h-3 FPKM	48 h-1 FPKM	48 h-2 FPKM	48 h-3 FPKM	FDR	log2FC	regulated	nr_annotation
c28179.graph_c0	7.23674932	6.44892312	6.12406716	17.8869806	34.8835466	33.7124052	0.0059577	1.099003	up	PREDICTED: heavy metal-associated isoprenylated plant protein 26-like [Sesamum indicum]
c28183.graph_c0	0.18738233	0	0.0343991	1.9975621	2.01167237	2.26017445	3.045E-10	3.815491	up	PREDICTED: LOW QUALITY PROTEIN: protein tesmin/TSO1-like CXC 2 [Sesamum indicum]
c28185.graph_c0	0	0.03425619	0.13192957	1.19804367	3.10615595	3.19803617	4.404E-06	4.440424	up	PREDICTED: lysosomal Pro-X carboxypeptidase [Sesamum indicum]
c28188.graph_c1	0	0	0	1.96643	1.8993829	1.13217021	2.694E-09	Inf	up	PREDICTED: omega-hydroxypalmitate O-feruloyl transferase [Sesamum indicum]
c28192.graph_c0	12.8697776	11.857519	11.668844	7.64223948	8.16266001	7.72347806	1.002E-18	-1.646892	down	PREDICTED: probable WRKY transcription factor 21 isoform X1 [Sesamum indicum]
c28196.graph_c0	0.75146804	0.81008364	0.96566618	21.5678505	24.5974216	26.5830109	7.393E-35	3.824307	up	PREDICTED: uncharacterized protein LOC105157188 [Sesamum indicum]
c28198.graph_c0	10.2774906	9.98520013	10.9626585	0.37130567	0.22945611	0.08259639	4.34E-102	-6.515188	down	PREDICTED: flavonoid 3'-monooxygenase-like [Sesamum indicum]
c28199.graph_c0	2.54924495	2.87895117	2.14192199	8.91237298	12.0890481	12.0292195	1.988E-05	1.10471	up	PREDICTED: aspartate carbamoyltransferase 1, chloroplastic [Sesamum indicum]
c28203.graph_c0	8.12679349	7.49468202	7.90496112	4.17673766	3.5985495	4.00943308	2.941E-28	-2.01274	down	PREDICTED: disease resistance protein RGA2-like [Sesamum indicum]
c28206.graph_c0	0	0	0	4.6124764	6.68481119	8.32231179	1.666E-21	Inf	up	PREDICTED: cytokinin dehydrogenase 3-like [Sesamum indicum]
c28208.graph_c0	4.26456225	3.59480637	3.51882117	25.6405431	28.6088214	31.646337	3.959E-20	1.897528	up	PREDICTED: biotin carboxyl carrier protein of acetyl-CoA carboxylase 2, chloroplastic-like isoform X2 [Sesamum indicum]
c28209.graph_c0	11.0782775	9.92885514	13.5738844	1.42409746	0.50288619	0.95036502	2.009E-24	-4.592395	down	PREDICTED: zinc finger CCCH domain-containing protein 23 [Sesamum indicum]
c28209.graph_c1	14.116436	14.4752195	15.6624961	0.57095041	1.00808788	0.50802824	1.811E-61	-5.431358	down	PREDICTED: zinc finger CCCH domain-containing protein 20-like [Sesamum indicum]
c28210.graph_c0	0.03074336	0	0	6.51424106	9.78148615	13.4827735	2.448E-17	8.914363	up	PREDICTED: LRR receptor-like serine/threonine-protein kinase GSO1 [Sesamum indicum]
c28213.graph_c0	3.08007553	3.11309335	2.03665055	37.8250366	37.8821629	35.0047108	1.016E-52	2.737178	up	PREDICTED: probable mitochondrial chaperone BCS1-A [Sesamum indicum]
c28214.graph_c0	1.66114017	1.24710266	2.25322149	16.238721	16.7976506	18.2694324	6.048E-17	2.289864	up	PREDICTED: myb family transcription factor APL-like [Sesamum indicum]
c28215.graph_c0	8.34731783	6.18320054	6.65051302	39.9884869	45.7845762	41.9451324	8.851E-13	1.574201	up	Uncharacterized protein isoform 1 [Theobroma cacao]
c28216.graph_c0	0.34826645	0.34861519	0.22376809	2.56676618	2.12435745	2.49800809	0.0006534	1.954496	up	-
c28219.graph_c0	67.100205	65.0535516	76.6607825	34.9696966	31.5845507	33.3229289	2.554E-33	-2.081457	down	PREDICTED: light-induced protein, chloroplastic-like [Sesamum indicum]
c28222.graph_c0	236.478816	231.936076	229.20001	119.761352	75.0612955	54.7124051	8.175E-65	-2.488062	down	PREDICTED: uncharacterized protein LOC105155796 [Sesamum indicum]

#ID	0 h-1 FPKM	0 h-2 FPKM	0 h-3 FPKM	48 h-1 FPKM	48 h-2 FPKM	48 h-3 FPKM	FDR	log2FC	regulated	nr_annotation
c28223.graph_c0	4.92755412	4.70828431	5.46863366	50.9671505	47.1077032	59.4877958	2.508E-21	2.363717	up	PREDICTED: 50S ribosomal protein L29, chloroplastic-like [Solanum lycopersicum]
c28226.graph_c0	0.04976265	0.04981248	0.06394698	6.18902095	6.45938365	10.0348933	1.73E-16	6.091378	up	hypothetical protein MIMGU_mgv1a020800mg [Erythranthe guttata]
c28230.graph_c0	20.5152463	17.7396215	19.1248744	2.23593549	2.36870253	3.30335721	6.068E-76	-3.880074	down	PREDICTED: inorganic pyrophosphatase 2 [Sesamum indicum]
c28233.graph_c0	0.36569308	0.41597644	0.7262556	3.8743537	3.63394223	3.28386469	3.376E-08	1.816549	up	hypothetical protein MIMGU_mgv1a025694mg [Erythranthe guttata]
c28237.graph_c0	1.43895253	1.46362558	0.83508237	14.689134	11.5294474	10.0452985	6.56E-09	2.27231	up	hypothetical protein MIMGU_mgv1a003416mg [Erythranthe guttata]
c28238.graph_c0	23.6006966	18.8208611	23.712887	18.2058281	17.9670154	19.5609088	6.586E-12	-1.265578	down	PREDICTED: dnaJ homolog subfamily C member 2-like [Sesamum indicum]
c28239.graph_c0	25.9110514	21.2928728	25.197523	213.790169	242.802599	253.375831	3.172E-54	2.273103	up	PREDICTED: cytochrome c oxidase subunit 6a, mitochondrial [Sesamum indicum]
c28240.graph_c0	1.77790439	1.73574186	1.55132425	75.303946	77.8045788	77.9265971	4.66E-161	4.494729	up	PREDICTED: LOW QUALITY PROTEIN: pyrophosphate--fructose 6-phosphate 1-phosphotransferase subunit beta [Sesamum indicum]
c28244.graph_c0	66.8375991	60.0360013	52.9049874	56.8926345	62.7511294	51.4560199	1.21E-07	-1.087151	down	-
c28248.graph_c0	1.8767961	2.75247795	2.74828156	0.56293902	1.32052389	1.75314913	2.612E-06	-2.055425	down	-
c28251.graph_c0	5.24663146	6.01579574	7.10988165	56.6842315	72.8979919	77.1025834	1.182E-31	2.466529	up	PREDICTED: fasciclin-like arabinogalactan protein 7 [Sesamum indicum]
c28252.graph_c0	14.8777449	17.8869307	17.0953705	17.2598469	17.0554904	14.3252571	2.104E-06	-1.052342	down	PREDICTED: uncharacterized protein LOC105177997 [Sesamum indicum]
c28254.graph_c0	2.06750906	2.00194605	1.73649085	9.47382429	8.49332594	8.10851298	1.862E-09	1.153957	up	PREDICTED: protein SCAR1-like isoform X2 [Sesamum indicum]
c28257.graph_c0	40.8207442	39.6693712	39.0987071	14.4641277	11.5891116	12.1304333	1.066E-68	-2.660061	down	PREDICTED: lanC-like protein GCL1 [Sesamum indicum]
c28260.graph_c0	10.598748	9.68277929	11.1218542	52.3603003	59.3992237	63.58684	2.517E-20	1.46002	up	hypothetical protein M569_06331, partial [Genlisea aurea]
c28262.graph_c0	22.037398	23.6166039	16.4360843	10.7482704	12.4829659	12.1849213	5.265E-14	-1.826768	down	hypothetical protein MIMGU_mgv1a015596mg [Erythranthe guttata]
c28264.graph_c0	0.63848138	0.69998937	0.66419312	8.76714037	8.90197011	8.02525721	7.824E-16	2.664472	up	hypothetical protein MIMGU_mgv1a005920mg [Erythranthe guttata]
c28266.graph_c0	1.03412553	0.74761632	0.51679143	7.40991916	6.78456581	7.34682168	6.857E-07	2.217931	up	PREDICTED: protein trichome birefringence-like 41 [Sesamum indicum]
c28269.graph_c1	4.0257316	4.60544316	2.74497626	14.9107552	21.088244	20.1369675	6.646E-05	1.283768	up	PREDICTED: vesicle-fusing ATPase [Sesamum indicum]
c28271.graph_c0	0	0	0	6.59589085	6.07612745	7.91036976	1.26E-12	Inf	up	PREDICTED: transcription factor bHLH79-like [Sesamum indicum]
c28274.graph_c0	2.88116668	2.88405173	4.5322645	33.2705116	37.2822971	35.5898344	5.35E-31	2.341151	up	PREDICTED: uncharacterized protein LOC105166742 isoform X3 [Sesamum indicum]

#ID	0 h-1 FPKM	0 h-2 FPKM	0 h-3 FPKM	48 h-1 FPKM	48 h-2 FPKM	48 h-3 FPKM	FDR	log2FC	regulated	nr_annotation
c28275.graph_c0	0.03595314	0.05398371	0.04620121	1.57331285	2.05271474	2.47565563	1.406E-14	4.461524	up	PREDICTED: protein argonaute 7 isoform X1 [Sesamum indicum]
c28276.graph_c0	0.28330451	0.28358819	0.242705	13.9634246	11.5667643	11.1472788	2.343E-26	4.489845	up	PREDICTED: uncharacterized protein LOC105160853 [Sesamum indicum]
c28278.graph_c0	1.39430743	1.18097998	0.82695704	5.9285859	5.65255609	6.24234377	0.0082674	1.376685	up	PREDICTED: RNA-binding protein 42, partial [Sesamum indicum]
c28279.graph_c0	2.2700451	2.18262144	2.07267477	1.98124447	1.2243515	1.88532299	0.0002344	-1.369084	down	PREDICTED: QWRF motif-containing protein 2-like [Sesamum indicum]
c28280.graph_c0	26.4104309	27.949284	31.2202668	7.96186418	5.42647504	3.61652152	6.844E-58	-3.339259	down	-
c28281.graph_c0	1.96001472	1.80501919	1.41047005	0	0	0	2.3E-19	-Inf	down	PREDICTED: extracellular ribonuclease LE-like isoform X1 [Phoenix dactylifera]
c28283.graph_c0	0	0	0	0.9447499	1.36479268	1.03168568	1.107E-11	Inf	up	-
c28292.graph_c0	9.82851532	10.3144066	9.4928945	8.49970577	9.7160962	9.66814844	3.442E-09	-1.10726	down	PREDICTED: uncharacterized protein LOC105175287 [Sesamum indicum]
c28293.graph_c0	0.15330805	0.46038468	0	28.106237	17.7304285	10.3679535	2.92E-06	5.527435	up	PREDICTED: nudix hydrolase 17, mitochondrial [Sesamum indicum]
c28296.graph_c1	0.25151993	0.37765768	0.32321307	3.24403188	4.29582302	2.98961006	3.814E-05	2.445466	up	-
c28297.graph_c0	0.28282476	0.50959434	0.58150562	5.15902097	3.47795455	6.76977789	1.21E-05	2.465952	up	PREDICTED: CTD small phosphatase-like protein [Sesamum indicum]
c28302.graph_c0	3.36936384	2.56730783	3.48966209	87.5164603	97.7683699	99.5965241	5.51E-109	3.896452	up	PREDICTED: reticulon-like protein B2 isoform X1 [Nicotiana tomentosiformis]
c28305.graph_c0	0.74081264	1.01121062	0.77888747	4.7153504	6.37563385	8.2257615	3.838E-05	1.908209	up	PREDICTED: uncharacterized protein LOC105178720 [Sesamum indicum]
c28308.graph_c0	0	0	0	268.580932	265.88474	341.756686	6.98E-139	Inf	up	PREDICTED: uncharacterized oxidoreductase At4g09670-like [Sesamum indicum]
c28309.graph_c0	0.13270456	0.13283744	0.0852653	1.28368897	2.39603686	2.12126335	1.811E-06	3.024486	up	PREDICTED: uncharacterized protein LOC105156370 [Sesamum indicum]
c28310.graph_c1	38.6845136	31.5784137	29.1019608	202.872675	202.534711	193.228219	7.877E-27	1.576183	up	PREDICTED: 14-3-3-like protein A [Populus euphratica]
c28311.graph_c1	0.0633247	0.25355244	0.32549898	1.86684298	1.97769377	3.21839221	3.621E-05	2.426448	up	PREDICTED: uncharacterized protein LOC105155826 [Sesamum indicum]
c28312.graph_c0	0.40101606	0.6221973	0.77298226	3.12178614	4.3638776	5.63767492	9.931E-06	1.838566	up	PREDICTED: uncharacterized protein LOC105156537 [Sesamum indicum]
c28313.graph_c0	0	0.04636332	0	3.96832773	4.65600098	5.35344275	8.825E-18	7.226184	up	malate dehydrogenase, putative [Ricinus communis]
c28316.graph_c0	9.75125918	8.86960591	10.1275958	7.46573639	6.86609178	7.6284625	1.139E-09	-1.406007	down	hypothetical protein MIMGU_mgv1a003597mg [Erythranthe guttata]
c28317.graph_c0	0.4857728	0.91173606	1.24847457	7.83169782	8.59304613	10.2040179	1.789E-08	2.30306	up	PREDICTED: cyclin-P3-1-like [Sesamum indicum]

#ID	0 h-1 FPKM	0 h-2 FPKM	0 h-3 FPKM	48 h-1 FPKM	48 h-2 FPKM	48 h-3 FPKM	FDR	log2FC	regulated	nr_annotation
c28321.graph_c0	151.958408	144.991047	155.320504	159.141158	111.271565	94.0178658	3.902E-13	-1.320655	down	PREDICTED: AP2/ERF and B3 domain-containing transcription factor RAV1-like [Sesamum indicum]
c28325.graph_c0	7.84559573	8.28293755	7.48266099	32.8077742	33.0210567	39.2913412	1.034E-08	1.136295	up	PREDICTED: upstream activation factor subunit UAF30 [Sesamum indicum]
c28326.graph_c0	2.18156737	1.84120256	1.81396499	9.61552401	9.81075086	11.7467413	5.436E-06	1.399226	up	PREDICTED: WEB family protein At2g38370-like [Sesamum indicum]
c28328.graph_c0	3.09425412	2.49684542	2.2721366	15.707546	20.5537784	25.5458996	5.398E-09	1.953254	up	PREDICTED: protein ASPARTIC PROTEASE IN GUARD CELL 2 [Sesamum indicum]
c28329.graph_c0	2.4695985	2.89829064	3.28296398	0.5491754	0.41556053	0.97730593	1.723E-09	-3.177918	down	PREDICTED: uncharacterized protein LOC105163289 [Sesamum indicum]
c28334.graph_c0	3.56459905	3.18972635	3.43548818	24.4305319	31.310437	38.7611851	7.199E-12	2.189707	up	hypothetical protein MIMGU_mgv1a019816mg [Erythranthe guttata]
c28336.graph_c0	6.02666034	5.57451576	6.37205461	3.79514228	5.28620384	6.5661843	0.0008482	-1.225722	down	PREDICTED: mavicyanin [Sesamum indicum]
c28344.graph_c0	2.67553266	2.00865885	2.66457818	7.64111312	11.2935974	12.7755713	0.0021892	1.08314	up	PREDICTED: CASP-like protein 2D1 isoform X2 [Sesamum indicum]
c28346.graph_c1	0.26645469	0.2667215	0.24457487	39.7319531	42.0911866	46.3679911	5.27E-127	6.346436	up	PREDICTED: lysM domain-containing GPI-anchored protein 1 [Sesamum indicum]
c28347.graph_c0	5.34714701	5.40347757	5.36615467	3.04951561	2.28626512	1.96202632	2.527E-10	-2.153335	down	PREDICTED: 60S ribosomal protein L3-like [Nicotiana tomentosiformis]
c28348.graph_c0	22.4992334	25.0919129	22.0808716	21.9256348	16.3524533	16.0418778	6.468E-22	-1.369787	down	PREDICTED: scarecrow-like protein 4 [Sesamum indicum]
c28349.graph_c0	0.03923909	0.11783514	0.40339024	1.77133068	1.53184496	1.96210781	3.035E-05	2.195604	up	PREDICTED: L-tryptophan--pyruvate aminotransferase 1-like [Sesamum indicum]
c28350.graph_c0	9.52936894	9.28248882	8.42709084	90.3867256	93.4536851	99.030539	6.207E-51	2.359119	up	PREDICTED: gamma carbonic anhydrase 1, mitochondrial [Sesamum indicum]
c28351.graph_c0	3.35829492	2.49723147	1.84951797	0	0	0	4.731E-19	-Inf	down	-
c28352.graph_c0	5.9863192	6.70326606	7.04073708	0.56084621	0.29707428	0.08317293	9.614E-24	-5.387228	down	-
c28353.graph_c0	15.5665914	14.0127509	12.9520271	0.30951711	0.43719446	0.18360431	7.422E-45	-6.532129	down	hypothetical protein MIMGU_mgv1a016475mg [Erythranthe guttata]
c28357.graph_c0	2.24564729	1.70530039	2.18918638	12.4843597	15.2661966	15.5519115	6.112E-07	1.796072	up	hypothetical protein MIMGU_mgv1a013733mg [Erythranthe guttata]
c28360.graph_c0	0.15079841	0.03773735	0.04844548	1.45871596	1.58212632	1.54518653	3.835E-07	3.264242	up	-
c28362.graph_c0	8.88730854	6.44208154	7.87623679	2.25863953	3.58913207	1.50729291	2.099E-07	-2.678822	down	MAP kinase 20 [Theobroma cacao]
c28364.graph_c0	0.37376394	0.93534551	0.72045218	20.4879751	26.0819086	26.502549	6.985E-15	4.145698	up	PREDICTED: uncharacterized protein At3g50808-like [Malus domestica]
c28364.graph_c1	0.74604487	0.18669798	0.7190229	20.103677	20.5692915	22.7806111	4.458E-14	4.241564	up	hypothetical protein glysoja_041042 [Glycine soja]
c28365.graph_c0	0.10251589	0	0	2.45555248	3.30172628	3.7816197	3.722E-10	5.537447	up	-

#ID	0 h-1 FPKM	0 h-2 FPKM	0 h-3 FPKM	48 h-1 FPKM	48 h-2 FPKM	48 h-3 FPKM	FDR	log2FC	regulated	nr_annotation
c28372.graph_c0	0.11173369	0.33553672	0.23930369	8.98977558	12.2861415	12.5481253	5.197E-31	4.596915	up	PREDICTED: endoplasmic reticulum-Golgi intermediate compartment protein 3 [Sesamum indicum]
c28382.graph_c0	2.3141341	1.55695911	1.75500233	78.1824634	77.0859707	85.3329853	4.12E-132	4.402114	up	hypothetical protein JCGZ_02248 [Jatropha curcas]
c28384.graph_c0	30.2889641	30.1068996	27.9478676	10.5556928	11.1824766	12.479706	5.618E-44	-2.386896	down	PREDICTED: uncharacterized protein LOC102593344 [Solanum tuberosum]
c28385.graph_c0	7.20413563	6.50780319	9.0318044	85.9562031	108.551959	131.937315	7.091E-18	2.817189	up	hypothetical protein M569_02037, partial [Genlisea aurea]
c28386.graph_c0	25.0636073	26.0149755	27.0096451	11.3259039	12.0242249	11.8331952	2.944E-46	-2.169332	down	hypothetical protein MIMGU_mgv1a002759mg [Erythranthe guttata]
c28387.graph_c0	0.20487204	0.30761578	0.13163434	2.45363932	1.19969231	2.12725317	0.0008885	2.162858	up	PREDICTED: transcription factor HBP-1b(c38)-like [Sesamum indicum]
c28389.graph_c0	7.81179128	6.50639606	7.81620634	1.53823272	2.61895347	2.24859537	1.087E-16	-2.81545	down	PREDICTED: endo-1,3;1,4-beta-D-glucanase-like [Sesamum indicum]
c28395.graph_c0	1.08222216	1.44440779	0.92713222	14.2905289	13.3787241	10.3499287	3.951E-05	2.449017	up	PREDICTED: protein transport protein SEC31 homolog B [Sesamum indicum]
c28395.graph_c1	7.1100379	8.56694887	6.26031791	46.3368169	46.3896708	48.9473959	4.573E-18	1.675305	up	PREDICTED: protein transport protein SEC31 homolog B [Sesamum indicum]
c28396.graph_c0	0	0	0	1.90654754	3.42480355	3.24535303	1.429E-11	Inf	up	PREDICTED: leucine-rich repeat extensin-like protein 3 [Vitis vinifera]
c28399.graph_c0	3.89165192	3.72617714	3.7688144	0.25980026	0.27522687	0.18493497	1.247E-22	-4.998835	down	hypothetical protein MIMGU_mgv1a013527mg [Erythranthe guttata]
c28400.graph_c0	9.01366711	10.3792517	12.595412	0.52262799	0.18455366	0.12917535	7.163E-45	-6.253012	down	-
c28401.graph_c0	13.2583821	12.8174952	10.6889547	3.06523232	4.72326105	3.42994393	1.248E-29	-2.733646	down	-
c28409.graph_c0	37.3504938	36.3941964	35.9577829	3.77249324	4.60202932	4.22135711	7.83E-105	-4.142805	down	PREDICTED: uncharacterized protein LOC105172478 [Sesamum indicum]
c28414.graph_c0	0.1132684	0.22676365	0.65499471	7.20016651	11.4968282	15.3667107	3.568E-09	4.053486	up	PREDICTED: vacuole membrane protein KMS1-like [Sesamum indicum]
c28414.graph_c1	0.14955928	0.07485452	0.28828439	5.78691805	9.12282436	9.8079331	7.832E-15	4.554816	up	PREDICTED: vacuole membrane protein KMS1-like [Sesamum indicum]
c28415.graph_c0	19.8742326	20.4280158	18.4329362	5.58593067	8.60246061	9.71054728	2.287E-46	-2.322229	down	PREDICTED: DEAD-box ATP-dependent RNA helicase 18 [Sesamum indicum]
c28416.graph_c0	0.53584134	1.0727558	0.64554125	12.742392	17.0617143	17.2405064	1.047E-32	3.361703	up	PREDICTED: serine carboxypeptidase-like 51 [Sesamum indicum]
c28424.graph_c0	8.26895633	8.27723644	7.08395751	3.87948709	7.32299861	4.83272199	1.037E-06	-1.584062	down	autophagy protein 8 [Trichophyton rubrum CBS 118892]
c28425.graph_c0	2.75118341	1.74815214	1.47563671	0	0	0	9.421E-21	-Inf	down	ATP dependent RNA helicase dbp2 [Aspergillus fumigatus var. RP-2014]
c28426.graph_c0	22.1569478	22.3221233	21.170854	17.6488521	16.3113092	16.8748001	1.61E-22	-1.384591	down	PREDICTED: methyltransferase-like protein 1 [Sesamum indicum]

#ID	0 h-1 FPKM	0 h-2 FPKM	0 h-3 FPKM	48 h-1 FPKM	48 h-2 FPKM	48 h-3 FPKM	FDR	log2FC	regulated	nr_annotation
c28428.graph_c0	64.5639069	70.791766	63.864618	35.381172	37.814055	34.6887076	3.617E-41	-1.902457	down	PREDICTED: uncharacterized protein LOC105164194 [Sesamum indicum]
c28432.graph_c0	0.94399704	0.6670181	0.99900177	15.091361	10.7847659	14.5661855	1.87E-15	2.938411	up	PREDICTED: CASP-like protein 4B1 [Sesamum indicum]
c28433.graph_c0	99.3998819	100.29014	110.983717	90.7246273	98.6173285	96.3210672	1.548E-15	-1.141111	down	PREDICTED: iron-sulfur cluster assembly protein 1-like [Sesamum indicum]
c28434.graph_c0	131.03845	116.822983	145.114592	108.823168	80.2383257	71.7834552	1.836E-13	-1.603142	down	Uncharacterized protein TCM_029816 [Theobroma cacao]
c28436.graph_c0	0.30932448	0	0	8.89106036	6.76235923	4.31059631	4.418E-08	5.025068	up	-
c28439.graph_c0	33.684611	38.1317364	28.555201	13.3228365	10.3272657	6.21642601	2.074E-24	-2.754954	down	PREDICTED: probable protein phosphatase 2C 23 [Sesamum indicum]
c28441.graph_c0	0.0333178	0	0	1.81097712	2.86150743	2.62193337	1.592E-14	6.77061	up	PREDICTED: LOW QUALITY PROTEIN: protein NRT1/ PTR FAMILY 2.7-like [Sesamum indicum]
c28442.graph_c0	0.6238483	0.07805912	0.10020872	5.24440888	5.02306588	4.53859572	8.505E-08	3.204001	up	--
c28444.graph_c0	1.27017838	0.9535877	1.22417213	70.5028129	70.0404771	76.0084226	8.615E-26	4.954271	up	enolase [Solanum lycopersicum]
c28444.graph_c1	3.93914738	4.02698741	2.69253209	168.014955	163.758698	176.499151	4.33E-134	4.561932	up	PREDICTED: enolase [Sesamum indicum]
c28449.graph_c0	7.05171772	7.1367765	7.8101154	2.83548996	5.3613158	7.18576494	1.243E-09	-1.546987	down	PREDICTED: L-type lectin-domain containing receptor kinase S.1 [Sesamum indicum]
c28450.graph_c0	0.29853022	0.22986858	0.32460421	33.3838663	27.6564255	27.9729269	4.983E-63	5.689631	up	PREDICTED: beta-galactosidase-like [Sesamum indicum]
c28452.graph_c0	6.89150819	9.14312938	6.81760718	5.24036756	6.67251683	7.84613769	7.351E-06	-1.231597	down	hypothetical protein MIMGU_mgv1a0119852mg, partial [Erythranthe guttata]
c28463.graph_c0	31.6080866	30.7130504	32.8000211	26.9263358	26.4600158	24.500948	1.196E-09	-1.305379	down	Vacuolar protein sorting-associated protein 32-2-like protein [Morus notabilis]
c28547.graph_c1	0.09233161	0.09242407	0.05932489	6.71989044	8.33543153	7.45522574	1.715E-21	5.510862	up	PREDICTED: uncharacterized protein LOC105173291 [Sesamum indicum]
c28558.graph_c0	0.45161367	0.7749701	0.33162368	7.01351487	6.67437854	6.39924225	6.538E-08	2.677151	up	PREDICTED: thylakoid lumenal 17.9 kDa protein, chloroplastic [Nicotiana tomentosiformis]
c28609.graph_c0	0.62932319	0.22907395	0.51463059	48.9381598	45.2274393	58.5288213	1.249E-78	5.77846	up	PREDICTED: allantoinase [Sesamum indicum]
c28614.graph_c0	38.2836168	42.6919992	25.8926123	9.90017259	6.88277126	12.6631014	1.024E-12	-2.871417	down	-
c28655.graph_c0	0	0	0	4.72895958	4.23902707	2.34665758	2.303E-08	Inf	up	hypothetical protein PGUG_03256 [Meyerozyma guilliermondii ATCC 6260]
c28687.graph_c0	3.66268429	3.86453309	3.68903068	1.611154	3.60686977	3.94914888	2.181E-05	-1.321929	down	PREDICTED: pentatricopeptide repeat-containing protein At5g50390, chloroplastic [Sesamum indicum]
c28687.graph_c1	5.44770495	5.97561844	5.32374611	3.45604688	4.52086339	5.32138067	1.133E-07	-1.355547	down	PREDICTED: SWI/SNF complex component SNF12 homolog [Sesamum indicum]
c28690.graph_c0	12.6881673	14.885969	11.8341271	61.6509309	71.76962	84.1023118	4.169E-14	1.444511	up	PREDICTED: protein TIC 20-I, chloroplastic-like [Sesamum indicum]

#ID	0 h-1 FPKM	0 h-2 FPKM	0 h-3 FPKM	48 h-1 FPKM	48 h-2 FPKM	48 h-3 FPKM	FDR	log2FC	regulated	nr_annotation
c28741.graph_c0	1.65159489	1.53923156	1.2807374	10.4147949	14.8406433	14.8225665	3.242E-16	2.142543	up	PREDICTED: LOW QUALITY PROTEIN: replication factor C subunit 2 [Sesamum indicum]
c28782.graph_c0	0.12874747	0	0.0827228	1.30471623	2.32458999	1.6886235	9.155E-08	3.626567	up	PREDICTED: uncharacterized protein LOC104211388 [Nicotiana sylvestris]
c28785.graph_c0	1.07642633	0.30785834	0.98803604	3.68336111	4.05215425	5.16825904	0.0074577	1.419777	up	-
c28816.graph_c0	6.05164464	5.1012248	6.54871828	120.232937	99.4731173	99.606689	5.492E-32	3.15783	up	hypothetical protein MIMGU_mgv1a005703mg [Erythranthe guttata]
c28850.graph_c0	31.6129873	30.3441781	27.6105811	6.35264151	7.18501255	7.18173238	1.862E-84	-3.130447	down	PREDICTED: cyclic dof factor 2-like [Sesamum indicum]
c28909.graph_c0	1.53850122	1.89543606	0.45623884	14.1191626	17.3830879	17.3167688	1.41E-14	2.638486	up	PREDICTED: uncharacterized protein LOC105163227 [Sesamum indicum]
c28984.graph_c0	0.26828908	0.98471168	0.68952418	18.7845704	21.1219029	19.426822	2.818E-20	3.91098	up	PREDICTED: cyclin-dependent kinase inhibitor 7-like isoform X1 [Sesamum indicum]
c29044.graph_c0	0.15069022	0	0	357.476083	360.024807	398.866231	2.46E-246	11.85789	up	Gip1-like protein [Populus tomentosa]
c29248.graph_c0	0.30337157	0.49347244	0.31674864	12.6641947	13.9343207	17.9519248	5.63E-35	4.302563	up	hypothetical protein MIMGU_mgv1a000889mg [Erythranthe guttata]
c29266.graph_c0	4.15009301	3.81044881	4.33998615	14.7132531	18.1847255	20.7872814	6.022E-09	1.102663	up	PREDICTED: uncharacterized protein LOC105158344 [Sesamum indicum]
c29280.graph_c1	0.10651454	0.1599318	0.0684377	2.7966521	3.1186461	5.06420228	3.891E-08	4.017346	up	PREDICTED: proteasome subunit beta type-3-A [Sesamum indicum]
c29287.graph_c0	9.62217067	10.6168769	9.97620215	69.2040958	77.368526	74.3051746	2.202E-20	1.850655	up	hypothetical protein MIMGU_mgv1a016558mg [Erythranthe guttata]
c29351.graph_c0	2.9997934	3.3781469	2.08804553	14.3937778	18.1761671	24.5903942	1.242E-05	1.73543	up	Rho GDP-dissociation inhibitor 1 [Arabidopsis thaliana]
c29353.graph_c0	37.7367091	30.4723635	33.7108621	226.298966	249.594118	265.757687	1.358E-33	1.844018	up	hypothetical protein PHAVU_003G132300g [Phaseolus vulgaris]
c29363.graph_c0	47.3257733	50.7745232	42.7181486	11.4656359	16.1020386	13.9942647	2.56E-75	-2.781879	down	PREDICTED: cell division cycle protein 48 homolog [Nicotiana sylvestris]
c29442.graph_c0	0.04482006	0.40378445	0.05759555	21.5126949	22.3089189	24.6897012	4.292E-61	6.069042	up	PREDICTED: uncharacterized protein LOC105176814 [Sesamum indicum]
c29564.graph_c0	4.52365339	4.8111946	3.84078084	20.0560195	25.8983801	26.9506875	9.515E-13	1.44768	up	hypothetical protein CICLE_v10001386mg [Citrus clementina]
c29644.graph_c0	0.16175295	0.08095746	0	27.6427224	27.3107896	32.1542295	6.312E-49	7.487028	up	PREDICTED: homeobox-leucine zipper protein HOX21-like [Sesamum indicum]
c29668.graph_c0	13.3825095	12.329569	15.8281348	353.488103	365.640549	386.683377	1.28E-133	3.713732	up	PREDICTED: 20 kDa chaperonin, chloroplastic-like [Sesamum indicum]
c29678.graph_c1	25.479594	24.2227282	25.5170338	8.85172521	6.32101472	5.48417715	8.052E-44	-2.874051	down	PREDICTED: myelin transcription factor 1 [Sesamum indicum]
c29682.graph_c0	0.66166739	0.27597081	0.14171146	6.7052827	7.42631732	8.18109061	7.209E-12	3.36123	up	PREDICTED: topless-related protein 3-like [Sesamum indicum]
c29711.graph_c0	0.32108971	0.16070562	0.2063065	7.09941086	14.1018093	10.9670366	7.333E-12	4.521777	up	hypothetical protein MIMGU_mgv1a021530mg [Erythranthe guttata]

#ID	0 h-1 FPKM	0 h-2 FPKM	0 h-3 FPKM	48 h-1 FPKM	48 h-2 FPKM	48 h-3 FPKM	FDR	log2FC	regulated	nr_annotation
c29739.graph_c0	84.5375655	82.962958	77.4497364	41.4146432	42.0618957	44.785859	5.606E-41	-1.950718	down	PREDICTED: mitochondrial fission 1 protein A [Sesamum indicum]
c29798.graph_c0	2.82753416	2.66054358	2.10742614	11.6699918	12.3629413	10.3375319	0.0004925	1.163593	up	PREDICTED: chorismate mutase 1, chloroplastic-like isoform X1 [Sesamum indicum]
c29807.graph_c0	1.59721223	0.9326401	2.22352103	245.038312	321.248738	355.509358	1.171E-77	6.570312	up	PREDICTED: annexin D1-like [Sesamum indicum]
c29866.graph_c0	0	0	0	3.81564786	5.41828985	7.8015995	6.395E-11	Inf	up	isoflavone reductase-like protein 4 isoform 2 [Theobroma cacao]
c29876.graph_c0	138.1294	140.868528	135.528206	48.0682347	42.0731351	38.337265	3.921E-78	-2.703676	down	PREDICTED: heat stress transcription factor B-2a-like [Sesamum indicum]
c29887.graph_c0	0.30527121	1.22230759	0.72853039	12.8564916	13.5773318	16.9092015	2.618E-23	3.242299	up	PREDICTED: ribokinase [Sesamum indicum]
c29995.graph_c0	0.38791383	0.22188701	0.21363627	1.42948607	2.10929712	1.63536186	0.0037199	1.633251	up	PREDICTED: transcription factor bHLH126-like [Sesamum indicum]
c30053.graph_c0	5.74820753	5.75396349	5.76416777	3.55082283	3.59952544	3.02331542	1.256E-11	-1.779523	down	PREDICTED: pentatricopeptide repeat-containing protein At1g31790 [Sesamum indicum]
c30136.graph_c0	71.4869259	70.6695836	61.4800954	4.09058502	4.55015276	5.64165583	2.88E-131	-4.853192	down	PREDICTED: peroxisomal membrane protein 13 [Glycine max]
c30145.graph_c0	0.39657595	0.5954596	0.54146679	9.36213563	13.9094568	17.5547351	3.023E-12	3.707008	up	PREDICTED: oligopeptide transporter 3 [Sesamum indicum]
c30224.graph_c0	0.47901618	0.2996849	0.23083304	3.58557025	4.49973418	5.15375352	6.575E-07	2.696453	up	PREDICTED: glucosidase 2 subunit beta [Sesamum indicum]
c30289.graph_c0	0.59909305	0.49065787	0.48990982	3.26136667	8.7704418	10.893453	0.001578	2.82707	up	PREDICTED: two-component response regulator ARR8 [Sesamum indicum]
c30362.graph_c0	1.10057778	1.00725014	1.2930615	7.24231451	6.07650441	6.39261456	4.015E-06	1.518527	up	PREDICTED: putative E3 ubiquitin-protein ligase XBAT34 isoform X2 [Sesamum indicum]
c30461.graph_c0	1.40598168	2.5802142	2.10786582	25.7977005	28.9304288	29.0054431	1.97E-14	2.759184	up	PREDICTED: trafficking protein particle complex subunit 2-like protein [Vitis vinifera]
c30494.graph_c0	1.7030138	1.42059926	0.36474003	10.7210038	8.03342693	10.2374958	0.0004866	2.052928	up	-
c30514.graph_c1	1.92862843	2.46454425	2.3729016	11.6057647	11.5740268	13.152297	4.083E-07	1.405248	up	PREDICTED: uncharacterized protein LOC105170005 [Sesamum indicum]
c30648.graph_c0	1.00648748	1.00749532	1.50893897	0.5151344	1.09144486	0.91672698	0.0038249	-1.515268	down	PREDICTED: WEB family protein At1g75720 [Sesamum indicum]
c30774.graph_c0	0.36960455	0.09249366	0.23747824	8.17209167	7.93589519	9.84679316	3.414E-19	4.196358	up	PREDICTED: probable 6-phosphogluconolactonase 4, chloroplastic [Sesamum indicum]
c30802.graph_c0	1.93148443	1.41288276	1.43194267	9.30768014	10.150369	11.1440512	4.043E-05	1.662055	up	-
c30813.graph_c0	0.36799448	0.49115063	0.55170206	2.54265388	2.09504826	2.66464936	0.0095241	1.352886	up	--
c30953.graph_c0	4.2618401	2.80807088	2.07973415	18.0907312	20.1653037	21.624848	2.258E-10	1.696291	up	PREDICTED: protein sym-1-like [Sesamum indicum]
c30987.graph_c0	0.55284466	0.69174781	0.17760682	18.8447127	19.1543494	22.8292857	1.758E-28	4.409255	up	-
c30990.graph_c0	0	0	0	1.40234393	2.0374126	1.03388911	2.379E-11	Inf	up	PREDICTED: sucrose synthase 5-like [Sesamum indicum]
c31084.graph_c0	0.71505966	0.63624506	0.61258665	6.73398704	10.7007641	9.63908102	8.854E-09	2.761307	up	PREDICTED: uncharacterized protein LOC105169539 [Sesamum indicum]

#ID	0 h-1 FPKM	0 h-2 FPKM	0 h-3 FPKM	48 h-1 FPKM	48 h-2 FPKM	48 h-3 FPKM	FDR	log2FC	regulated	nr_annotation
c31162.graph_c0	0.12962359	0.19463008	0.33314288	2.86602483	4.80732583	4.9409576	2.077E-08	3.226531	up	-
c31209.graph_c0	3.6357687	3.2438214	2.84389105	1.82038438	1.85133746	1.49018571	1.988E-05	-1.9275	down	hypothetical protein MIMGU_mgv1a021709mg [Erythranthe guttata]
c31272.graph_c0	7.02627349	9.43944135	8.3162207	8.34684237	8.21086257	5.38076409	0.0003689	-1.190452	down	PREDICTED: choline-phosphate cytidylyltransferase 2-like isoform X1 [Sesamum indicum]
c31286.graph_c0	0	0	0	2.12313101	2.27803575	3.41500048	5.627E-17	Inf	up	PREDICTED: kinesin KP1-like isoform X2 [Sesamum indicum]
c31392.graph_c0	0.25057653	0.18812058	0.16100038	1.38508465	1.58960668	1.28379166	0.0012359	1.813185	up	PREDICTED: transcription repressor MYB5-like [Sesamum indicum]
c31417.graph_c0	1.83118465	0.6110061	1.08606682	5.14753339	6.00308872	7.62091062	0.0004621	1.392339	up	PREDICTED: probable polygalacturonase [Sesamum indicum]
c31482.graph_c0	1.98169641	1.7569744	2.54655834	34.7398537	38.0736254	36.8986393	2.658E-41	3.10332	up	hypothetical protein MIMGU_mgv1a010578mg [Erythranthe guttata]
c31565.graph_c0	0.74818645	0.27234023	0.87404474	3.00775924	3.71741557	4.68350065	0.0015383	1.562076	up	PREDICTED: dual specificity tyrosine-phosphorylation-regulated kinase 3-like isoform X3 [Cucumis melo]
c31628.graph_c0	38.3451462	42.4442705	36.9873029	29.5421877	33.7472859	35.1541827	1.9E-13	-1.278136	down	hypothetical protein MIMGU_mgv1a014722mg [Erythranthe guttata]
c31685.graph_c0	0.6702548	0.31309878	0.8038837	11.5263389	12.9085161	11.0252721	9.7E-20	3.287176	up	PREDICTED: uncharacterized protein At1g04910 [Sesamum indicum]
c31685.graph_c1	0.23303277	0.93306445	0.49909388	12.451729	11.3716354	11.9709255	1.965E-13	3.409578	up	PREDICTED: uncharacterized protein At1g04910 [Sesamum indicum]
c31695.graph_c0	1.26413638	0.67488118	0.32489306	3.72673564	5.75753575	5.25037602	0.00168	1.688284	up	hypothetical protein MIMGU_mgv1a022283mg [Erythranthe guttata]
c31852.graph_c1	1.81190232	1.40150833	1.37585276	5.34916377	6.18926043	8.20280753	0.0026449	1.08468	up	PREDICTED: uracil-DNA glycosylase [Sesamum indicum]
c31982.graph_c0	9.82888783	10.3481788	10.4232364	48.9380938	54.3275201	49.2551904	5.978E-18	1.298252	up	PREDICTED: LOW QUALITY PROTEIN: probable phenylalanine--tRNA ligase alpha subunit [Sesamum indicum]
c32009.graph_c0	3.39750251	2.93411377	4.02349973	21.9100064	21.2604913	23.9733251	3.797E-10	1.676618	up	hypothetical protein MIMGU_mgv1a016742mg [Erythranthe guttata]
c32020.graph_c0	2.30050561	1.66953668	1.77374447	18.8094604	19.6456898	18.8109334	5.865E-15	2.301412	up	PREDICTED: uncharacterized protein LOC105165412 [Sesamum indicum]
c32191.graph_c0	0.73024081	0.73097204	1.02369639	5.8100745	5.8311184	6.25814185	7.19E-05	1.826738	up	PREDICTED: structure-specific endonuclease subunit SLX1 homolog [Sesamum indicum]
c32206.graph_c0	1.65948116	1.42383676	2.38636793	63.3369234	72.4964631	83.5635512	1.024E-74	4.300066	up	PREDICTED: magnesium-chelatase subunit ChlI, chloroplastic-like [Sesamum indicum]
c32223.graph_c0	3.31717611	3.73555998	3.06381752	13.4654956	14.5685717	15.125603	0.0001245	1.075066	up	PREDICTED: cell division topological specificity factor homolog, chloroplastic-like [Sesamum indicum]
c32244.graph_c0	5.81760121	5.5128439	5.88100033	37.8027187	36.7921262	34.8447527	6.728E-13	1.651905	up	PREDICTED: calcineurin subunit B-like isoform X1 [Sesamum indicum]

#ID	0 h-1 FPKM	0 h-2 FPKM	0 h-3 FPKM	48 h-1 FPKM	48 h-2 FPKM	48 h-3 FPKM	FDR	log2FC	regulated	nr_annotation
c32266.graph_c0	19.0759164	20.8309287	17.363593	17.307706	21.2267699	19.2503659	3.008E-07	-1.00633	down	PREDICTED: uncharacterized protein LOC105169439 [Sesamum indicum]
c32277.graph_c0	9.54324801	11.5998336	10.3920807	67.1949493	50.4100382	40.689481	0.000888	1.317623	up	hypothetical protein MIMGU_mgv1a005557mg [Erythranthe guttata]
c32295.graph_c0	1.02712074	0.69914149	0.52795633	0	0	0	7.353E-17	-Inf	down	--
c32333.graph_c0	1.64870335	1.2519929	0.94973924	8.27534842	10.8196962	8.24882203	4.372E-06	1.81224	up	Alpha-soluble NSF attachment protein 2 [Theobroma cacao]
c32363.graph_c0	0.31077455	0.27220002	0.1497591	2.71990494	4.24628766	6.97386788	0.000171	3.228028	up	PREDICTED: omega-3 fatty acid desaturase, endoplasmic reticulum [Sesamum indicum]
c32363.graph_c1	0.15991793	0.37351548	0.41100174	6.1877267	9.31246952	9.91918299	2.393E-15	3.719115	up	PREDICTED: disease resistance response protein 206-like [Sesamum indicum]
c32364.graph_c0	1.01538057	1.01639732	0.86986949	8.93859928	5.94587852	9.15578695	6.148E-05	2.038877	up	CTP synthase 1a [Malus domestica]
c32388.graph_c1	8.14392261	7.45663962	7.34056361	43.8073082	54.2060685	55.1154652	1.274E-26	1.718027	up	hypothetical protein CICLE_v10011936mg [Citrus clementina]
c32392.graph_c0	1.47489552	1.29182586	1.73735755	0.79261581	0.65974884	0.906769	0.0001397	-1.951931	down	hypothetical protein MIMGU_mgv1a018953mg, partial [Erythranthe guttata]
c32414.graph_c0	1.24662515	1.31532607	0.82262948	126.207804	96.54419	102.798002	4.979E-46	5.579545	up	hypothetical protein MIMGU_mgv1a007303mg [Erythranthe guttata]
c32432.graph_c0	9.63233761	8.06420392	8.2519568	70.3461242	67.2303995	76.0406453	1.551E-33	2.024821	up	PREDICTED: ferredoxin, root R-B1-like [Sesamum indicum]
c32432.graph_c1	0.86034665	1.00474285	0.73705301	6.07665713	7.27715289	9.40343126	0.0001041	2.108086	up	PREDICTED: glucan endo-1,3-beta-glucosidase 14-like [Sesamum indicum]
c32453.graph_c1	6.1624421	6.42563839	5.54328746	30.6098051	33.27941	34.3086286	5.278E-13	1.419252	up	PREDICTED: mitochondrial import inner membrane translocase subunit TIM17-2-like [Sesamum indicum]
c32471.graph_c0	16.6823588	17.4124218	15.5265716	10.0569157	11.8238188	10.8338929	2.105E-22	-1.619945	down	PREDICTED: double-stranded RNA-binding protein 3-like isoform X1 [Sesamum indicum]
c32488.graph_c0	2.38068354	3.40438205	2.07593498	33.1336044	33.1924693	37.0791197	9.512E-22	2.701921	up	PREDICTED: 30S ribosomal protein S31, chloroplastic [Sesamum indicum]
c32509.graph_c0	167.441543	163.402152	166.561554	813.417055	817.416754	886.801818	2.099E-21	1.321391	up	-
c32527.graph_c0	14.108203	13.2317329	10.8614267	3.27862263	4.15555909	3.02148794	5.418E-27	-2.886589	down	PREDICTED: uncharacterized protein LOC105173965 [Sesamum indicum]
c32529.graph_c0	2.62442529	3.15246391	3.56520414	150.732046	183.100448	172.660126	2.02E-148	4.737352	up	PREDICTED: FK506-binding protein 2-like [Sesamum indicum]
c32549.graph_c0	14.6502265	10.7778155	9.52647094	81.630911	90.2679268	106.781681	4.989E-18	1.977982	up	vacuolar ATPase subunit c [Cucumis sativus]
c32550.graph_c0	3.6642444	3.95748571	3.46956778	14.3913167	18.1632716	14.6233444	0.0029514	1.069244	up	hypothetical protein MIMGU_mgv1a022013mg [Erythranthe guttata]
c32563.graph_c0	22.282251	22.0951308	17.7447506	2.31299865	2.34824404	2.05809229	3.882E-44	-4.223068	down	-
c32583.graph_c0	4.48330449	3.93450419	4.02496455	24.5555566	38.4809988	40.2250228	3.516E-09	2.027937	up	PREDICTED: caffeoylshikimate esterase [Sesamum indicum]
c32594.graph_c0	24.8678592	17.1878585	15.5976611	0	0	0	2.665E-29	-Inf	down	-
c32600.graph_c0	11.1649506	9.61147237	8.60844217	12.6859196	7.55500613	5.67443521	5.53E-05	-1.183286	down	hypothetical protein JCGZ_03792 [Jatropha curcas]

#ID	0 h-1 FPKM	0 h-2 FPKM	0 h-3 FPKM	48 h-1 FPKM	48 h-2 FPKM	48 h-3 FPKM	FDR	log2FC	regulated	nr_annotation
c32609.graph_c0	7.67890211	8.43650272	8.18296511	64.1863814	72.5674252	64.6355598	1.474E-13	2.031819	up	hypothetical protein MIMGU_mgv1a016321mg [Erythranthe guttata]
c32611.graph_c0	39.6636704	37.8349929	36.1782706	17.7688403	14.5733665	12.1129652	5.372E-24	-2.365649	down	-
c32612.graph_c0	0.10284691	0.82359916	0.5286494	4.4532162	3.31238734	3.11937159	0.0007044	1.888415	up	-
c32620.graph_c0	2.02307321	1.53757518	2.02201158	16.774083	18.7207711	18.2115402	3.626E-20	2.245759	up	tyrosine aminotransferase [Perilla frutescens]
c32655.graph_c0	3.88549272	3.05049683	2.44755489	0.98262687	1.56146103	1.93602857	5.183E-06	-2.089514	down	hypothetical protein AMTR_s00033p00078670, partial [Amborella trichopoda]
c32658.graph_c0	1.90706085	0.82998717	1.1187744	0	0	0	6.711E-11	-Inf	down	Glucose-6-phosphate isomerase 2 [Neocallimastix frontalis]
c32693.graph_c0	1.19626477	0.61491325	0.66475571	5.71888672	5.995358	5.98974276	9.543E-07	1.824026	up	PREDICTED: BTB/POZ domain-containing protein At3g05675 isoform X2 [Sesamum indicum]
c32721.graph_c0	1.13821912	1.22481079	1.42609022	4.56141737	5.80427665	6.60125433	0.000374	1.137787	up	PREDICTED: histone-lysine N-methyltransferase setd3 [Sesamum indicum]
c32725.graph_c0	0.3001718	0.26291333	0.48216581	24.7501367	30.5775236	31.5881717	2.542E-84	5.349992	up	PREDICTED: putative E3 ubiquitin-protein ligase XBAT31 [Sesamum indicum]
c32747.graph_c1	6.03968112	4.45474765	4.79970763	3.22134895	2.32679223	0.91201629	2.978E-08	-2.246198	down	PREDICTED: protein ODORANT1-like [Sesamum indicum]
c32759.graph_c0	16.6886803	16.2864475	15.1262174	12.9648731	14.7558791	13.5087763	6.282E-11	-1.24067	down	hypothetical protein MIMGU_mgv1a024738mg [Erythranthe guttata]
c32765.graph_c0	2.98241767	1.83717176	2.21107199	8.50698515	11.4750587	10.766505	0.0013277	1.107983	up	hypothetical protein MIMGU_mgv1a014004mg [Erythranthe guttata]
c32792.graph_c1	9.29295874	9.2530459	8.52987136	6.56817757	4.65478763	2.98262098	3.828E-14	-1.936513	down	PREDICTED: alpha/beta hydrolase domain-containing protein 17C-like [Sesamum indicum]
c32815.graph_c0	1.06229048	0.54686788	0.62403901	0	0	0	8.366E-13	-Inf	down	PREDICTED: aldehyde dehydrogenase family 2 member B7, mitochondrial-like [Solanum tuberosum]
c32819.graph_c0	5.50723908	2.78958623	2.72848974	0	0	0	7.751E-13	-Inf	down	--
c32832.graph_c0	4.05438797	3.70296334	3.72689641	66.115176	77.4639152	82.0208097	2.5E-95	3.275801	up	Cytochrome P450 77A3 [Morus notabilis]
c32838.graph_c0	10.3007404	10.2225481	9.48736261	7.20892429	8.37047653	8.51637011	1.316E-09	-1.3362	down	PREDICTED: F-box protein SKIP19-like [Sesamum indicum]
c32844.graph_c0	36.8034373	33.7666071	31.1722436	31.2789424	33.3931168	31.7872407	1.359E-13	-1.093761	down	PREDICTED: uncharacterized protein LOC105156000 [Sesamum indicum]
c32856.graph_c0	1.478013	0.87029	1.00551453	7.12859625	6.87306237	5.91536531	0.0013199	1.55709	up	-
c32883.graph_c0	1.50947781	0.94436833	0.60616836	15.8184096	15.6527845	19.4886356	7.506E-13	3.046323	up	PREDICTED: uncharacterized protein LOC105167888 isoform X3 [Sesamum indicum]
c32889.graph_c0	1.38257699	0.5766506	0.61689797	0	0	0	5.046E-09	-Inf	down	phosphoenolpyruvate carboxylase [Dendrobium catenatum]
c32893.graph_c0	0.67508453	0.59129046	0.54219396	3.80933287	3.62373788	5.11886918	0.0008486	1.777534	up	-
c32893.graph_c1	0.59268189	1.18655074	0.30464788	4.58654459	5.09026346	5.63577686	0.000159	1.865087	up	PREDICTED: RNA polymerase sigma factor sigE, chloroplastic/mitochondrial-like [Sesamum indicum]
c32901.graph_c0	13.7382669	11.4167744	10.0762276	7.46261061	10.2458274	11.8460919	1.553E-07	-1.275005	down	-

#ID	0 h-1 FPKM	0 h-2 FPKM	0 h-3 FPKM	48 h-1 FPKM	48 h-2 FPKM	48 h-3 FPKM	FDR	log2FC	regulated	nr_annotation
c32907.graph_c0	0	0	0	2.45244438	2.44523995	2.11798878	2.065E-11	Inf	up	PREDICTED: LOW QUALITY PROTEIN: anthranilate synthase alpha subunit 1, chloroplastic [Sesamum indicum]
c32928.graph_c0	1.4789883	1.0908721	1.00029396	8.46209356	7.67305734	6.18952065	0.0003906	1.633902	up	-
c32947.graph_c0	110.849502	116.44729	113.390789	44.6811018	69.6400378	66.6561202	2.978E-39	-1.937772	down	PREDICTED: uncharacterized protein LOC105174506 [Sesamum indicum]
c32955.graph_c0	2.34736609	4.06681723	2.55238785	0	0.02202842	0	1.75E-23	-9.720335	down	Uncharacterized protein TCM_033752 [Theobroma cacao]
c32968.graph_c0	7.2876849	5.37227387	6.24330909	28.9373893	39.0914738	48.3012455	1.155E-06	1.597794	up	PREDICTED: transcription factor DIVARICATA [Sesamum indicum]
c32986.graph_c0	0.68964474	0.86291914	0.92314666	0.02647274	0.02804466	0.07066585	6.037E-16	-5.334103	down	PREDICTED: nucleoprotein TPR-like [Phoenix dactylifera]
c32989.graph_c1	3.35152678	1.54143265	2.59962693	0	0	0	2.267E-17	-Inf	down	hypothetical protein CHLNCDRAFT_59390 [Chlorella variabilis]
c32997.graph_c0	7.35476974	6.58717291	5.47173018	498.905019	440.504108	510.876	3.77E-108	5.209643	up	-
c32998.graph_c0	203.362964	201.806297	195.028611	21.0611015	18.4500458	12.8966494	4.13E-175	-4.529216	down	PREDICTED: cytochrome P450 CYP82D47-like [Sesamum indicum]
c33046.graph_c0	1.31151944	1.14159368	1.90518324	7.51220813	10.6852345	10.8912308	2.502E-06	1.709765	up	PREDICTED: E3 ubiquitin-protein ligase RING1-like [Sesamum indicum]
c33052.graph_c0	0.68591139	0.21126099	0.40681075	2.74636202	4.53151335	4.00074192	5.085E-07	2.089822	up	PREDICTED: uncharacterized protein LOC105165576 [Sesamum indicum]
c33085.graph_c0	12.9225782	14.7560727	11.9317362	8.4658645	10.9304287	10.6715734	4.068E-07	-1.418288	down	PREDICTED: uncharacterized protein LOC105171255 [Sesamum indicum]
c33101.graph_c1	3.10871924	4.38485439	2.90532894	0.78107862	0.96536776	1.96916399	1.186E-06	-2.509308	down	PREDICTED: E3 ubiquitin-protein ligase RHA1B-like [Sesamum indicum]
c33104.graph_c0	1.56109463	1.11618416	1.0030342	0	0.38089491	0.4113276	2.651E-09	-3.25887	down	hypothetical protein MIMGU_mgv1a001430mg [Erythranthe guttata]
c33108.graph_c0	1.11620127	1.02610926	1.02454487	43.6090526	53.6907535	53.0694576	1.15E-139	4.548707	up	unnamed protein product [Coffea canephora]
c33114.graph_c0	3.43614575	4.28702089	5.24750482	2.66094046	3.69378838	4.4496221	0.0003611	-1.292077	down	PREDICTED: uncharacterized protein LOC105172002 isoform X2 [Sesamum indicum]
c33115.graph_c0	2.63172072	1.39465905	1.88986591	0	0	0	1.606E-21	-Inf	down	--
c33120.graph_c0	9.81077428	10.177711	5.95978305	261.618431	160.076751	121.437936	2.971E-05	3.387708	up	PREDICTED: universal stress protein A-like protein [Sesamum indicum]
c33125.graph_c0	9.02732222	8.25736501	8.60034582	1.00372252	1.0633223	0.76551965	6.651E-17	-4.207934	down	-
c33132.graph_c0	0	0	0.10843565	1.7102632	1.4823953	1.66012572	2.144E-08	4.435937	up	-
c33148.graph_c0	1.38654401	0.76701529	0.70332787	0	0	0	4.34E-12	-Inf	down	PREDICTED: serpin-ZX-like isoform X2 [Pyrus x bretschneideri]
c33167.graph_c0	6.35116868	6.61182955	9.14088359	2.34044434	3.47118378	3.33202128	7.123E-06	-2.301729	down	hypothetical protein EUGRSUZ_A02667 [Eucalyptus grandis]
c33253.graph_c0	3.90726761	3.64601539	4.93589198	1.09819213	1.61583528	3.31150279	1.185E-06	-2.083473	down	PREDICTED: uncharacterized protein LOC105179909 [Sesamum indicum]

#ID	0 h-1 FPKM	0 h-2 FPKM	0 h-3 FPKM	48 h-1 FPKM	48 h-2 FPKM	48 h-3 FPKM	FDR	log2FC	regulated	nr_annotation
c33275.graph_c0	0	0	0	0.7839442	1.55266235	1.39509766	7.877E-10	Inf	up	PREDICTED: amino acid permease 6 [Sesamum indicum]
c33309.graph_c0	6.61061767	6.61723721	6.04445194	3.22074298	3.90827562	2.65737608	4.564E-09	-1.99524	down	PREDICTED: transcription factor DIVARICATA-like [Sesamum indicum]
c33310.graph_c0	0.06813807	0	0.02918671	24.1467712	42.4052358	49.0595255	1.353E-26	9.191185	up	PREDICTED: beta-xylosidase/alpha-L-arabinofuranosidase 2-like [Sesamum indicum]
c33328.graph_c0	0.30731526	0.30762299	0.23694737	2.20832714	3.17925913	4.88719877	0.0002059	2.568205	up	PREDICTED: uncharacterized protein LOC105177453 isoform X1 [Sesamum indicum]
c33329.graph_c0	3.67809447	3.02931062	2.45299212	2.65932953	2.63547994	3.47305231	0.0035472	-1.0791	down	PREDICTED: uncharacterized protein LOC105171097 [Sesamum indicum]
c33366.graph_c0	2.11798459	2.48207465	1.92510067	65.5802149	86.3134811	95.1092673	1.13E-41	4.220851	up	PREDICTED: lamin-like protein-like [Solanum tuberosum]
c33370.graph_c0	7.72416574	8.75524007	8.32020516	36.36488	32.9257028	32.2641371	1.156E-07	1.018272	up	PREDICTED: probable 6-phosphogluconolactonase 1 [Solanum lycopersicum]
c33380.graph_c0	0	0	0	1.2761206	0.91451723	1.83681306	2.246E-08	Inf	up	PREDICTED: protein PHYTOCHROME KINASE SUBSTRATE 4-like [Sesamum indicum]
c33390.graph_c0	30.1209212	23.5398819	32.0197259	24.061735	19.0446194	22.2302932	1.317E-08	-1.406778	down	Uncharacterized protein isoform 2, partial [Theobroma cacao]
c33412.graph_c0	10.3104198	12.384893	8.89595703	38.0623724	64.7603278	93.8756274	0.0069069	1.611816	up	PREDICTED: plastidic ATP/ADP-transporter-like [Nicotiana sylvestris]
c33412.graph_c1	6.9414468	8.6468948	7.58202887	25.3309631	45.8417001	59.9046939	0.0054325	1.470949	up	PREDICTED: plastidic ATP/ADP-transporter [Sesamum indicum]
c33413.graph_c0	7.32342005	7.59492464	7.12175059	3.0391046	5.40886553	5.1379363	8.938E-08	-1.724712	down	PREDICTED: pentatricopeptide repeat-containing protein At1g12775, mitochondrial-like [Sesamum indicum]
c33416.graph_c0	0	0	0	2.36530848	3.47151824	4.69158723	3.071E-15	Inf	up	PREDICTED: uncharacterized protein LOC105176193 [Sesamum indicum]
c33420.graph_c0	2.97469568	2.90999996	2.60631935	11.5432494	13.482332	12.4694279	2.961E-08	1.124227	up	PREDICTED: ceramide kinase isoform X1 [Sesamum indicum]
c33421.graph_c0	16.2218277	17.6521978	15.7125177	12.2967627	10.1743168	10.9815183	4.668E-17	-1.581443	down	PREDICTED: C-terminal binding protein AN-like isoform X2 [Sesamum indicum]
c33422.graph_c0	2.06308793	1.00128669	2.89216327	13.7077266	13.24035	15.0669474	2.476E-08	1.793955	up	hypothetical protein MIMGU_mgv1a008498mg [Erythranthe guttata]
c33425.graph_c0	3.10626215	2.37570041	1.92855959	10.1927656	10.8320623	12.3596448	1.77E-05	1.156004	up	PREDICTED: uncharacterized protein LOC105176055 isoform X2 [Sesamum indicum]
c33426.graph_c1	26.7865892	29.9615453	23.1336979	10.7901852	12.2776256	9.24544774	8.958E-24	-2.321686	down	PREDICTED: dnaJ homolog subfamily C member 21 [Sesamum indicum]
c33429.graph_c0	268.414368	245.710036	279.17083	113.440201	118.759832	125.302852	7.667E-48	-2.169387	down	hypothetical protein MIMGU_mgv1a013716mg [Erythranthe guttata]
c33432.graph_c0	0.44987949	0.39028598	0.30832691	10.554872	10.2741992	11.2109888	2.06E-29	3.787921	up	PREDICTED: WEB family protein At2g38370 [Sesamum indicum]

#ID	0 h-1 FPKM	0 h-2 FPKM	0 h-3 FPKM	48 h-1 FPKM	48 h-2 FPKM	48 h-3 FPKM	FDR	log2FC	regulated	nr_annotation
c33433.graph_c0	81.8200111	78.4391756	87.4572317	54.593575	51.2297214	54.1251732	4.859E-29	-1.648599	down	PREDICTED: uncharacterized protein LOC105158873 [Sesamum indicum]
c33444.graph_c0	1.09699236	0.93541071	1.30525797	6.81233354	10.9838739	7.46039805	5.853E-07	1.893349	up	PREDICTED: uncharacterized protein LOC105178046 isoform X1 [Sesamum indicum]
c33454.graph_c0	2.74468706	3.32584291	3.52703154	0.13308341	0.56394293	0.11841682	1.945E-13	-4.594543	down	-
c33454.graph_c1	3.1991485	2.73141785	3.14373072	1.04021092	1.28564022	1.07983565	9.855E-07	-2.434445	down	-
c33455.graph_c0	1.48042556	1.55083393	1.37150161	14.2412837	14.5828961	13.7724779	1.711E-17	2.25782	up	hypothetical protein MIMGU_mgv1a006888mg [Erythranthe guttata]
c33456.graph_c0	3.85122789	3.85508431	2.41603763	61.7128909	139.159883	202.793179	2.268E-05	4.289879	up	PREDICTED: hexose carrier protein HEX6-like [Sesamum indicum]
c33458.graph_c0	1.94462046	2.50890948	2.08242856	23.0308718	20.982216	21.5554485	2.796E-37	2.311048	up	PREDICTED: protein argonaute 10 [Sesamum indicum]
c33459.graph_c0	0.57983108	0.90286263	0.49673726	8.07202629	11.066431	9.08367469	2.975E-10	2.814989	up	-
c33463.graph_c0	0.54027516	0.59232246	0.95876067	4.47963042	2.86244063	3.37434769	0.0009383	1.34129	up	PREDICTED: 187-kDa microtubule-associated protein AIR9-like [Nicotiana sylvestris]
c33470.graph_c0	13.7988598	14.1859928	15.7715316	14.7426471	14.1621273	13.7849315	8.345E-10	-1.053397	down	PREDICTED: protein AIR2 [Sesamum indicum]
c33471.graph_c0	0.62858041	0.96628653	1.06738487	3.08158219	4.42578295	5.37353157	0.0004405	1.245368	up	hypothetical protein MIMGU_mgv1a002735mg [Erythranthe guttata]
c33473.graph_c0	26.1000759	28.8792958	20.9171892	9.30777767	10.9012883	10.6285726	5.848E-24	-2.316892	down	-
c33474.graph_c0	0	0	0	2.58956466	3.70349517	1.61292534	1.71E-07	Inf	up	60s ribosomal protein l6 [Lichtheimia corymbifera JMRC:FSU:9682]
c33475.graph_c0	6.1476594	6.90888471	5.96133892	0.17373082	0.25766544	0.06183386	1.869E-56	-6.28624	down	PREDICTED: uncharacterized protein LOC102591997 [Solanum tuberosum]
c33480.graph_c0	0.13709011	0.20584107	0.15414544	4.68875192	5.619418	6.19482242	9.358E-29	4.032087	up	PREDICTED: uncharacterized protein LOC105159750 isoform X2 [Sesamum indicum]
c33489.graph_c0	7.22543633	6.93543844	5.34203854	4.46809066	2.60820015	1.78499927	6.957E-08	-2.138208	down	hypothetical protein 2_207_02, partial [Pinus radiata]
c33494.graph_c0	0	0	0	2.7858723	4.50460648	4.56630709	3.419E-13	Inf	up	putative receptor protein kinase [Arabidopsis thaliana]
c33494.graph_c1	0	0.04276892	0	2.12555342	3.00235481	3.16968649	3.911E-20	6.587631	up	PREDICTED: probable LRR receptor-like serine/threonine-protein kinase At4g36180 [Sesamum indicum]
c33496.graph_c0	4.2681701	5.40194072	4.16085815	40.7222659	51.7587874	58.4335437	6.593E-22	2.426258	up	PREDICTED: myosin heavy chain, muscle [Sesamum indicum]
c33503.graph_c0	80.1748543	86.0690748	84.2955235	16.0000022	16.8944882	17.0840469	1.16E-106	-3.344467	down	hypothetical protein MIMGU_mgv1a015119mg [Erythranthe guttata]
c33510.graph_c0	8.33610726	9.37372655	6.84261252	1.86073619	2.61635234	3.28124062	2.417E-26	-2.684658	down	PREDICTED: non-structural maintenance of chromosomes element 4 homolog A-like [Sesamum indicum]
c33512.graph_c0	0.53952087	0.74258403	0.38998444	9.25740377	11.0905759	10.6419971	5.892E-21	3.195908	up	PREDICTED: probable Histone-lysine N-methyltransferase ATXR5 [Sesamum indicum]
c33514.graph_c0	0.19391359	0.12940518	0	2.62013908	2.83880418	3.86797799	1.378E-08	3.846471	up	PREDICTED: alpha-mannosidase-like [Sesamum indicum]

#ID	0 h-1 FPKM	0 h-2 FPKM	0 h-3 FPKM	48 h-1 FPKM	48 h-2 FPKM	48 h-3 FPKM	FDR	log2FC	regulated	nr_annotation
c33518.graph_c0	1.44939576	1.16585928	1.79601197	5.79415236	6.744444	8.14712403	1.415E-05	1.204375	up	unnamed protein product [Coffea canephora]
c33518.graph_c1	1.24683606	0.91770925	1.03673945	5.13521585	5.97699429	7.66557544	2.687E-05	1.531135	up	PREDICTED: protein STICHEL isoform X2 [Sesamum indicum]
c33520.graph_c0	16.1483398	13.5875591	14.4356563	6.42509763	8.0857067	9.05513506	1.617E-21	-1.9283	down	-
c33521.graph_c0	0.16079139	0.1609524	0.2066233	4.59207691	10.9849164	8.30381138	1.456E-06	4.464887	up	-
c33525.graph_c0	19.6987782	18.7226195	21.9896874	2.47469991	2.13615476	3.42529148	6.878E-40	-3.930366	down	PREDICTED: mitogen-activated protein kinase 9-like [Sesamum indicum]
c33528.graph_c0	32.9971994	34.6324543	32.3821673	20.8332636	22.4708669	21.632472	3.033E-27	-1.641256	down	-
c33540.graph_c0	0.92892036	1.77516919	1.62777196	9.29690469	11.0027943	13.0488216	5.749E-10	1.919724	up	PREDICTED: uncharacterized protein LOC105174130 [Sesamum indicum]
c33557.graph_c0	0.0239816	0.02400562	0.03081731	45.6670931	69.6541622	89.2303385	7.425E-33	10.31256	up	PREDICTED: prolyl endopeptidase-like [Sesamum indicum]
c33558.graph_c0	71.811315	73.8799793	77.1104292	20.847372	18.9894962	12.4648668	1.52E-99	-3.103951	down	PREDICTED: uncharacterized protein LOC102628603 [Citrus sinensis]
c33563.graph_c0	0.07264042	0.14542631	0	1.30496124	2.23318555	1.96501743	3.123E-08	3.646185	up	PREDICTED: DNA-damage-repair/toleration protein DRT100 [Sesamum indicum]
c33565.graph_c0	3.91661588	3.25977298	3.95854441	22.3792089	23.9228051	20.8508112	5.526E-13	1.573858	up	PREDICTED: protein FLX-like 3 [Nicotiana sylvestris]
c33566.graph_c0	18.9745102	18.0292589	18.6830755	15.7263093	12.92143	12.7811681	8.579E-22	-1.440124	down	PREDICTED: exocyst complex component EXO70A1 [Sesamum indicum]
c33567.graph_c0	11.5390552	11.8696875	11.5921669	5.66765844	7.21748018	7.47311835	9.683E-22	-1.803863	down	PREDICTED: WPP domain-interacting protein 1 [Sesamum indicum]
c33571.graph_c0	0.04007596	0	0.0514992	0.81225302	1.95564451	2.62813765	7.654E-05	4.838909	up	PREDICTED: transcription factor bHLH130-like [Sesamum indicum]
c33572.graph_c0	2.34996888	2.17411581	1.83018367	17.3523591	15.498475	16.5199509	1.161E-15	1.944512	up	hypothetical protein MIMGU_mgv1a022739mg, partial [Erythranthe guttata]
c33583.graph_c0	56.9118346	53.0603592	51.4674546	29.703573	24.4810084	22.016968	1.346E-43	-2.094953	down	PREDICTED: nifU-like protein 2, chloroplastic [Sesamum indicum]
c33589.graph_c0	4.73223879	4.73697741	4.67207555	57.3399918	63.617261	58.968384	2.082E-64	2.650715	up	PREDICTED: protein TRANSPARENT TESTA 12 [Sesamum indicum]
c33596.graph_c0	206.761828	196.890444	210.455799	55.7169966	62.7815638	56.8441345	6.369E-77	-2.827671	down	PREDICTED: uncharacterized protein LOC105160439 [Sesamum indicum]
c33599.graph_c0	157.350601	150.934988	142.178082	13.754234	9.06905176	6.39851774	5.66E-212	-4.951353	down	hypothetical protein MIMGU_mgv1a014857mg [Erythranthe guttata]
c33599.graph_c1	0.04893134	0	0.18863615	40.6159504	42.1203206	45.124647	1.93E-116	8.031851	up	PREDICTED: probable plastid-lipid-associated protein 6, chloroplastic [Nicotiana sylvestris]
c33602.graph_c0	1.13821794	1.82987751	1.37400935	11.05797	10.7383635	11.1964622	2.775E-11	1.908586	up	PREDICTED: protein ABIL2-like [Sesamum indicum]
c33604.graph_c0	7.98694398	6.14104217	7.58609422	4.15892255	5.98747	6.64207435	0.000485	-1.396225	down	-

#ID	0 h-1 FPKM	0 h-2 FPKM	0 h-3 FPKM	48 h-1 FPKM	48 h-2 FPKM	48 h-3 FPKM	FDR	log2FC	regulated	nr_annotation
c33606.graph_c0	2.4955156	1.9775948	2.93959998	12.5009851	11.619581	12.1461337	2.485E-05	1.271459	up	PREDICTED: uncharacterized protein LOC105160133 isoform X1 [Sesamum indicum]
c33607.graph_c0	0.18237379	0.45639101	0.64448322	24.7821575	18.6890681	17.1549419	1.136E-19	4.541786	up	PREDICTED: neurofilament medium polypeptide [Sesamum indicum]
c33613.graph_c0	0.28145908	0.22539273	0.21701163	2.07438767	3.68091668	3.0916776	5.791E-06	2.586953	up	PREDICTED: uncharacterized protein LOC105174187 [Sesamum indicum]
c33615.graph_c0	0.2635421	0.35174134	0.11288735	3.23722901	4.88696854	4.53673538	5.304E-07	3.103811	up	PREDICTED: putative axial regulator YABBY 2 [Sesamum indicum]
c33617.graph_c0	17.430542	14.2707375	15.6930821	167.718479	180.93271	201.537667	5.85E-67	2.51788	up	PREDICTED: 20 kDa chaperonin, chloroplastic-like [Sesamum indicum]
c33621.graph_c0	0	0	0	3.62593036	2.54539572	4.97554665	4.452E-12	Inf	up	PREDICTED: E3 ubiquitin ligase BIG BROTHER [Sesamum indicum]
c33624.graph_c0	0.97174121	0.94133638	0.80562963	5.02484711	5.445589	5.26765024	4.552E-05	1.516435	up	PREDICTED: uncharacterized protein LOC105177218 isoform X2 [Sesamum indicum]
c33625.graph_c0	0	0	0	3.30929094	3.8814131	6.30982966	4.075E-11	Inf	up	PREDICTED: growth-regulating factor 1-like [Sesamum indicum]
c33625.graph_c1	0	0	0	8.64839215	11.1600017	13.5486176	7.317E-33	Inf	up	PREDICTED: growth-regulating factor 1-like [Sesamum indicum]
c33632.graph_c0	0.09966854	0	0.04269267	4.19316615	3.11274821	1.96084591	1.342E-07	5.021653	up	PREDICTED: probable receptor-like protein kinase At5g39020 [Nicotiana sylvestris]
c33637.graph_c0	0.6341679	0.95220439	1.08657448	44.2072538	45.3880595	33.270427	1.905E-30	4.50267	up	hypothetical protein MIMGU_mgv1a025918mg [Erythranthe guttata]
c33642.graph_c0	2.72129482	1.65092108	1.58953254	58.2695544	59.8784463	67.8686971	5.682E-61	3.948776	up	PREDICTED: 50S ribosomal protein L18, chloroplastic [Nicotiana tomentosiformis]
c33647.graph_c0	0.21202422	0.09095851	0.15569115	2.90205368	4.02033521	5.88450471	3.033E-08	3.778352	up	PREDICTED: U-box domain-containing protein 45-like [Sesamum indicum]
c33648.graph_c0	0	0	0	10.9150102	5.13916878	2.87766234	0.0006069	Inf	up	PREDICTED: protein IQ-DOMAIN 1 [Sesamum indicum]
c33648.graph_c1	0	0.06394386	0.08208819	5.29652914	2.18206737	1.4662106	0.0046591	4.927968	up	PREDICTED: protein IQ-DOMAIN 1 [Sesamum indicum]
c33649.graph_c0	22.3165817	25.4955161	19.2015844	16.7604543	23.4374832	25.2930441	3.804E-08	-1.055104	down	hypothetical protein MIMGU_mgv1a010621mg [Erythranthe guttata]
c33657.graph_c0	0.82369816	0.41226149	0.92617416	13.5169172	17.9873454	20.2564575	2.017E-24	3.554089	up	PREDICTED: uncharacterized protein LOC105180227 [Sesamum indicum]
c33657.graph_c1	0.74937445	0.21432138	0.13756798	2.36699184	3.23890692	2.9836914	0.0008318	1.953406	up	hypothetical protein MIMGU_mgv1a008629mg [Erythranthe guttata]
c33659.graph_c0	11.8054162	9.39318881	14.7814419	6.13513402	6.49943057	7.19596161	7.572E-05	-1.882266	down	-
c33661.graph_c0	14.9879852	15.3551764	13.0209495	11.0636146	13.5290036	12.8822388	2.114E-15	-1.229837	down	PREDICTED: xylosyltransferase 1-like [Sesamum indicum]
c33662.graph_c0	205.606645	213.199739	223.014468	43.5790153	38.4919143	38.4292429	5.43E-116	-3.428833	down	hypothetical protein MIMGU_mgv1a015081mg [Erythranthe guttata]

#ID	0 h-1 FPKM	0 h-2 FPKM	0 h-3 FPKM	48 h-1 FPKM	48 h-2 FPKM	48 h-3 FPKM	FDR	log2FC	regulated	nr_annotation
c33663.graph_c0	0.29355603	0.58769997	1.50892449	6.76107409	6.01653207	11.5506491	0.0007934	2.311345	up	hypothetical protein MIMGU_mgv1a005080mg [Erythranthe guttata]
c33667.graph_c0	8.30689252	7.30347396	8.52351335	5.4122826	4.9630043	5.46310368	6.587E-14	-1.624911	down	PREDICTED: rop guanine nucleotide exchange factor 9-like [Sesamum indicum]
c33669.graph_c0	0.8957583	0.93401591	0.76738992	35.106701	35.6613866	43.0168956	1.608E-82	4.435819	up	PREDICTED: protein notum homolog [Sesamum indicum]
c33672.graph_c0	12.9979819	13.7580403	13.8258631	50.2187894	58.4509885	61.9667542	3.985E-13	1.050862	up	PREDICTED: carbamoyl-phosphate synthase small chain, chloroplastic [Sesamum indicum]
c33678.graph_c0	8.20972946	8.17834568	7.37216854	8.01896105	8.90056564	6.8271114	1.646E-09	-1.017227	down	PREDICTED: U-box domain-containing protein 5 [Sesamum indicum]
c33687.graph_c0	97.6515586	102.595896	97.1648796	25.7492253	20.865201	18.2725058	2.48E-105	-3.208329	down	PREDICTED: probable WRKY transcription factor 4 isoform X1 [Sesamum indicum]
c33688.graph_c0	30.5721737	27.6491669	36.2671194	8.75911351	10.0524869	10.9516419	4.953E-23	-2.689592	down	PREDICTED: peptidyl-prolyl cis-trans isomerase FKBP62 [Sesamum indicum]
c33689.graph_c0	0	0	0	4.39213778	3.72234992	4.61155538	1.176E-12	Inf	up	PREDICTED: mitoferrin-like [Sesamum indicum]
c33690.graph_c0	14.1312766	14.8159777	14.0600965	10.3150056	12.8265784	10.8517533	1.222E-15	-1.359457	down	PREDICTED: uncharacterized protein At4g22758 [Sesamum indicum]
c33691.graph_c0	1.31387749	1.57823177	1.01303076	1.24501335	1.06247999	0.52313032	0.0009271	-1.470182	down	PREDICTED: probable LRR receptor-like serine/threonine-protein kinase At2g16250 [Nicotiana tomentosiformis]
c33693.graph_c0	7.48936881	7.16681748	7.80826083	1.30182925	1.01136218	1.15835984	2.894E-30	-3.708158	down	PREDICTED: probable calcium-binding protein CML45 [Sesamum indicum]
c33693.graph_c1	4.8921337	5.67024808	6.61745842	5.4557848	5.40280272	2.63832854	0.0018514	-1.363276	down	PREDICTED: acyl-CoA-binding domain-containing protein 3-like [Sesamum indicum]
c33699.graph_c0	44.1213133	44.5058104	43.4455332	23.6646006	27.7610889	25.608565	6.932E-35	-1.797419	down	PREDICTED: trihelix transcription factor ASIL1-like [Sesamum indicum]
c33700.graph_c0	133.914216	125.508259	127.21374	70.7380399	72.2025304	74.2818842	9.105E-37	-1.849518	down	PREDICTED: probable protein phosphatase 2C 55 [Sesamum indicum]
c33701.graph_c0	93.8671965	91.4860221	88.1758263	76.488407	60.2389404	49.5434669	3.806E-29	-1.564724	down	PREDICTED: protein slowmo homolog 1-like [Sesamum indicum]
c33707.graph_c0	8.68004208	6.1489501	4.63328214	3.81377306	3.90990011	3.61240603	3.532E-05	-1.79174	down	40S ribosomal protein S12 [Pseudozyma brasiliensis GHG001]
c33709.graph_c0	23.1156598	21.4283404	24.8859374	19.4590674	19.8270013	19.0265627	3.823E-14	-1.27033	down	PREDICTED: E3 ubiquitin-protein ligase RLIM [Sesamum indicum]
c33713.graph_c0	38.0141503	36.917967	35.9327034	3.56945977	4.42353601	3.92516022	3.56E-139	-4.237683	down	PREDICTED: uncharacterized protein LOC105174809 [Sesamum indicum]
c33715.graph_c1	0	0	0	2.47575633	1.74850909	2.36609185	4.124E-11	Inf	up	unnamed protein product [Coffea canephora]
c33717.graph_c0	0.03828547	0	0.14759505	3.42129436	5.00696736	5.61773443	1.349E-17	5.193097	up	PREDICTED: uncharacterized protein LOC105167945 [Sesamum indicum]
c33724.graph_c0	3.97449361	3.72452835	3.74903546	53.9496214	97.1808725	115.486997	7.872E-09	3.513495	up	PREDICTED: alpha-xylosidase 1-like [Sesamum indicum]
c33729.graph_c0	29.0432488	26.2009899	31.6389929	116.385423	125.045953	122.370154	7.643E-11	1.046013	up	hypothetical protein F383_12160 [Gossypium arboreum]

#ID	0 h-1 FPKM	0 h-2 FPKM	0 h-3 FPKM	48 h-1 FPKM	48 h-2 FPKM	48 h-3 FPKM	FDR	log2FC	regulated	nr_annotation
c33735.graph_c0	10.6203626	13.2123256	9.98497532	119.410646	168.822672	198.817081	6.611E-14	2.82496	up	unnamed protein product [Coffea canephora]
c33740.graph_c0	4.5505993	3.94467121	2.89370735	0.34575743	0.36628808	0.49993594	1.532E-21	-4.249013	down	-
c33743.graph_c0	30.3353515	29.7161935	23.0348936	8.07022909	10.7659478	8.17818038	1.74E-34	-2.639086	down	PREDICTED: uncharacterized protein LOC105160397 [Sesamum indicum]
c33751.graph_c0	2.46029839	1.96015752	2.25827203	10.6853404	11.8833634	12.6564226	5.584E-10	1.379173	up	PREDICTED: two-component response regulator-like APRR1 isoform X2 [Sesamum indicum]
c33753.graph_c0	3.70779552	4.2293932	3.98902106	17.4765087	20.0500824	25.2342081	2.433E-09	1.373734	up	hypothetical protein MIMGU_mgv1a003089mg [Erythranthe guttata]
c33759.graph_c0	18.7282955	18.166645	19.6705347	13.6747808	12.3929808	10.5517101	2.454E-17	-1.642182	down	PREDICTED: uncharacterized protein LOC105159346 [Sesamum indicum]
c33766.graph_c0	4.81697791	4.65201964	4.8386677	5.09399592	3.9397549	3.61496259	1.268E-05	-1.189498	down	PREDICTED: NAC domain-containing protein 89-like [Sesamum indicum]
c33768.graph_c0	43.7196028	42.9529483	36.5526032	33.8628088	24.4424706	24.2905684	4.136E-25	-1.586067	down	PREDICTED: pollen-specific leucine-rich repeat extensin-like protein 1 [Sesamum indicum]
c33769.graph_c0	31.0238986	31.1485034	32.7821508	254.906773	250.890844	222.065273	1.396E-39	1.921595	up	hypothetical protein MIMGU_mgv1a013324mg [Erythranthe guttata]
c33771.graph_c0	4.80815698	4.33167446	3.91315898	27.6110974	22.2116929	21.5464326	3.791E-07	1.439946	up	PREDICTED: uncharacterized protein LOC105166582 [Sesamum indicum]
c33774.graph_c1	4021.99952	4070.62006	4025.80509	72.5388442	58.8471729	48.9852001	8.15E-191	-7.081786	down	hypothetical protein MIMGU_mgv1a012797mg [Erythranthe guttata]
c33780.graph_c0	14.7557296	14.0431698	15.5141156	2.29140287	2.01834043	1.83269797	3.721E-89	-3.865721	down	PREDICTED: homocysteine S-methyltransferase 2 [Sesamum indicum]
c33788.graph_c0	0	0.10286187	0	1.84603122	1.10318506	1.43199934	1.219E-07	4.414931	up	PREDICTED: WUSCHEL-related homeobox 1 [Sesamum indicum]
c33796.graph_c0	3.38657107	3.36194599	2.84130871	18.8227191	20.0223353	21.061614	2.841E-17	1.625992	up	hypothetical protein MIMGU_mgv1a003584mg [Erythranthe guttata]
c33797.graph_c0	0.11081461	0.22185115	0.14240116	2.65433085	2.16303206	2.99767891	1.051E-06	3.024351	up	PREDICTED: probable BOI-related E3 ubiquitin-protein ligase 3 [Sesamum indicum]
c33799.graph_c0	0.49523629	0.2478661	0.15909956	3.72599242	3.82640338	3.75515906	3.001E-07	2.638963	up	PREDICTED: beta-galactosidase 8-like isoform X1 [Sesamum indicum]
c33801.graph_c0	0	0	0	9.51447176	8.83105948	9.00960268	1.603E-30	Inf	up	PREDICTED: sulfate transporter 3.1-like [Sesamum indicum]
c33802.graph_c0	0.04103234	0.08214685	0.05272818	0.98284328	2.80323961	4.23809744	0.0009031	4.477119	up	PREDICTED: dof zinc finger protein DOF1.1-like [Sesamum indicum]
c33805.graph_c0	0.20154904	0.10087543	0.12949925	3.89928252	4.81928652	4.21303488	5.104E-11	3.886273	up	PREDICTED: 7-methylguanosine phosphate-specific 5'-nucleotidase A [Sesamum indicum]
c33806.graph_c0	14.5045286	15.1770158	14.3592684	15.0176937	13.9848982	12.9675213	2.292E-09	-1.084611	down	PREDICTED: protein Mpv17-like [Sesamum indicum]

#ID	0 h-1 FPKM	0 h-2 FPKM	0 h-3 FPKM	48 h-1 FPKM	48 h-2 FPKM	48 h-3 FPKM	FDR	log2FC	regulated	nr_annotation
c33810.graph_c0	1.9885819	1.97086452	1.84697695	14.0212354	14.4694831	15.6555377	5.035E-23	1.908703	up	PREDICTED: conserved oligomeric Golgi complex subunit 3 [Sesamum indicum]
c33812.graph_c0	32.5236056	27.8026402	35.6917535	16.6856033	15.5207181	13.669873	9.31E-21	-2.082211	down	-
c33813.graph_c0	42.3387302	44.4961151	45.4261438	27.9998939	25.3796727	28.1782889	9.207E-25	-1.71441	down	PREDICTED: uncharacterized protein C9orf85 homolog [Sesamum indicum]
c33814.graph_c1	2.0155078	2.01752603	3.40790437	2.0522717	1.55295215	1.39131354	0.0030592	-1.591535	down	PREDICTED: probable VAMP-like protein At1g33475 [Sesamum indicum]
c33815.graph_c0	57.4628091	58.5116176	62.0451516	4.31496756	5.72051141	4.19046599	1.33E-190	-4.66688	down	PREDICTED: heat shock 70 kDa protein 8 [Sesamum indicum]
c33816.graph_c0	0.0842782	0	0	1.55285142	3.12560979	2.34892035	7.273E-08	5.375084	up	PREDICTED: dehydrodolichyl diphosphate synthase 2 [Vitis vinifera]
c33818.graph_c0	0.29695994	0.77286899	0.45792634	16.0864375	9.62214483	7.35155076	7.249E-05	3.431289	up	unnamed protein product [Coffea canephora]
c33821.graph_c0	0.10709349	0.10720072	0.04587312	13.286416	7.8041526	6.87675906	2.89E-09	5.75062	up	PREDICTED: ras GTPase-activating protein-binding protein 1-like [Sesamum indicum]
c33827.graph_c0	0.55581659	0.8559587	0.4395362	4.80543449	6.63469877	5.88805121	3.613E-07	2.20789	up	hypothetical protein MIMGU_mgv1a007488mg [Erythranthe guttata]
c33832.graph_c0	1.4508947	1.633891	1.16528571	10.1363295	13.9242749	12.8350795	8.642E-09	2.097889	up	PREDICTED: G-type lectin S-receptor-like serine/threonine-protein kinase At1g11330 [Sesamum indicum]
c33838.graph_c0	3.03543869	2.18390622	2.37696401	0	0	0	9.79E-29	-Inf	down	-
c33842.graph_c0	2.36571081	2.63119967	3.04003205	0.12108039	0.12827	0.21547321	2.619E-14	-5.136948	down	PREDICTED: ethylene-responsive transcription factor ERF109-like [Solanum tuberosum]
c33845.graph_c0	48.5565885	51.0549577	48.8170053	8.35230231	9.01110278	6.74791778	1.93E-106	-3.638666	down	PREDICTED: transcription factor TGA1 [Sesamum indicum]
c33850.graph_c1	13.5062008	13.0465348	14.694749	14.7032339	12.9619367	10.6102091	8.913E-13	-1.122305	down	PREDICTED: uncharacterized protein LOC105173341 [Sesamum indicum]
c33851.graph_c0	34.3975658	34.5226203	33.0353538	15.6778549	15.7253402	12.6144169	1.853E-31	-2.227167	down	PREDICTED: selenoprotein O-like [Malus domestica]
c33853.graph_c0	14.3768218	13.5242772	13.5778542	0.63830679	1.01431298	1.1359231	1.24E-24	-4.920709	down	-
c33856.graph_c0	1.53541534	1.19957294	1.01059657	0.31050543	0.32894286	0.12279372	1.217E-10	-3.305848	down	-
c33857.graph_c0	0.30573958	0.68860289	0.98221865	2.95750601	6.78842449	5.51376793	0.0013854	1.909582	up	PREDICTED: vesicle transport protein SFT2B isoform X1 [Nicotiana sylvestris]
c33860.graph_c0	55.1157944	52.7660955	47.3989237	50.5158737	52.9637372	48.887526	2.06E-09	-1.0436	down	hypothetical protein MIMGU_mgv1a005676mg [Erythranthe guttata]
c33869.graph_c0	0.66265423	0.47379841	0.30412033	3.44485309	5.54339869	6.40201998	1.189E-06	2.397275	up	hypothetical protein MIMGU_mgv1a014159mg [Erythranthe guttata]
c33870.graph_c0	0.03969524	0	0	7.49681746	10.4988957	8.94839683	3.492E-33	8.405526	up	PREDICTED: non-specific phospholipase C3-like [Sesamum indicum]
c33873.graph_c0	45.96523	36.703923	45.5289133	18.7898168	27.4798803	24.9556647	1.157E-19	-1.872346	down	PREDICTED: RING finger and CHY zinc finger domain-containing protein 1-like [Sesamum indicum]

#ID	0 h-1 FPKM	0 h-2 FPKM	0 h-3 FPKM	48 h-1 FPKM	48 h-2 FPKM	48 h-3 FPKM	FDR	log2FC	regulated	nr_annotation
c33874.graph_c0	0.15929443	0.21260525	0.27293286	2.1034513	2.28017372	2.82922071	1.342E-05	2.458147	up	-
c33875.graph_c0	0.4266468	0.65317203	0.32250472	4.18487821	4.26191468	4.25857265	5.703E-08	2.16611	up	PREDICTED: probable serine/threonine-protein kinase At1g54610 isoform X2 [Sesamum indicum]
c33877.graph_c0	6.20736275	7.65440828	7.91890467	151.916581	175.950978	205.010911	4.709E-54	3.589569	up	hypothetical protein MIMGU_mgv1a007222mg [Erythranthe guttata]
c33886.graph_c0	0.67704309	0.67772104	0.71184041	20.6399996	23.6076145	22.6539589	6.929E-32	3.996492	up	hypothetical protein MIMGU_mgv1a026334mg [Erythranthe guttata]
c33887.graph_c0	5.58803901	4.1078254	5.49783933	466.463044	389.431958	472.243416	6.778E-91	5.433358	up	metallothionein class I type 3 [Avicennia marina]
c33905.graph_c0	57.8702117	61.2893211	54.9218081	38.1904109	36.2504661	32.0106653	7.375E-34	-1.724207	down	hypothetical protein MIMGU_mgv1a015035mg [Erythranthe guttata]
c33911.graph_c0	6.59394844	6.1964359	5.53370437	5.51688786	4.26843587	3.36452261	1.033E-05	-1.487354	down	PREDICTED: uncharacterized protein LOC105171510 [Sesamum indicum]
c33912.graph_c0	3.19434873	2.41126524	1.68232155	20.2621936	18.6544002	13.2213972	2.277E-06	1.831118	up	-
c33919.graph_c0	20.5139896	18.4276646	15.8091544	302.408515	300.604528	319.286454	1.496E-92	3.058888	up	PREDICTED: malate dehydrogenase, mitochondrial [Sesamum indicum]
c33924.graph_c0	0.59225031	0.71141203	0.50737673	8.58441526	10.866735	12.6550813	1.573E-18	3.12741	up	PREDICTED: F-box protein At2g32560-like [Sesamum indicum]
c33931.graph_c0	180.579699	179.955865	188.371675	9.09832405	12.2723667	12.5199922	4.44E-216	-5.041127	down	hypothetical protein MIMGU_mgv1a010609mg [Erythranthe guttata]
c33945.graph_c0	2.74434491	4.26079724	2.91483613	12.305965	15.5510893	15.7474732	9.709E-08	1.115429	up	PREDICTED: ethylene receptor 2-like [Sesamum indicum]
c33952.graph_c0	2.49342949	2.53752506	2.45651931	19.525434	23.442807	19.6560297	2.469E-18	2.044752	up	PREDICTED: Golgi to ER traffic protein 4 homolog [Sesamum indicum]
c33967.graph_c0	3.20188913	3.70112199	2.98795014	90.9869474	103.681174	107.800368	5.86E-124	3.915652	up	PREDICTED: auxin-responsive protein IAA9-like [Sesamum indicum]
c33968.graph_c0	11.7109947	10.6646136	11.6174699	72.1141451	79.1382022	82.9333357	3.99E-35	1.764533	up	PREDICTED: glucose-6-phosphate isomerase, cytosolic [Sesamum indicum]
c33974.graph_c0	5.30592887	4.33070498	4.93017611	4.02333808	5.6165951	4.91823811	0.0004724	-1.023467	down	hypothetical protein MIMGU_mgv1a020463mg [Erythranthe guttata]
c33982.graph_c1	21.7316012	21.0058239	26.0066574	16.511809	19.2414854	15.1206282	7.043E-13	-1.455122	down	hypothetical protein MIMGU_mgv1a015522mg [Erythranthe guttata]
c33984.graph_c0	0	0	0	1.90333269	1.62608886	2.07599391	1.79E-11	Inf	up	-
c33985.graph_c0	0.03504283	0.07015583	0.09006284	5.3268167	7.76356011	4.88339884	1.19E-18	5.495632	up	PREDICTED: PI-PLC X domain-containing protein At5g67130-like isoform X2 [Nicotiana tomentosiformis]
c33987.graph_c0	2.23637455	2.09572369	1.89550479	6.77266892	8.80018559	11.4284794	0.0004266	1.093748	up	PREDICTED: cellulose synthase-like protein E6 [Sesamum indicum]
c33996.graph_c0	611.235202	582.437609	654.740645	10.3029174	11.4080111	11.032045	0	-6.839024	down	PREDICTED: glutaredoxin [Sesamum indicum]
c33996.graph_c1	1.53998641	1.23322278	0.19789435	41.2852155	43.7366797	63.624088	5.225E-19	4.636921	up	hypothetical protein CICLE_v10010813mg, partial [Citrus clementina]

#ID	0 h-1 FPKM	0 h-2 FPKM	0 h-3 FPKM	48 h-1 FPKM	48 h-2 FPKM	48 h-3 FPKM	FDR	log2FC	regulated	nr_annotation
c34003.graph_c1	5.44525343	5.97607528	5.47986513	52.7641476	48.1497213	46.3038711	1.047E-26	2.107754	up	PREDICTED: 3-oxoacyl-[acyl-carrier-protein] reductase, chloroplastic-like isoform X2 [Sesamum indicum]
c34006.graph_c0	50.929112	57.2257588	61.0360927	11.1370277	11.7983304	9.78179368	1.311E-64	-3.389214	down	PREDICTED: uncharacterized protein LOC105156884 [Sesamum indicum]
c34007.graph_c0	249.198178	261.628215	242.405968	47.6306395	51.7176228	74.7421349	3.399E-96	-3.134637	down	-
c34008.graph_c0	0.19626193	0.19645846	0.08406812	3.97780447	4.2140015	6.05992718	3.502E-10	3.887988	up	PREDICTED: acyl-protein thioesterase 1 homolog 1 [Sesamum indicum]
c34010.graph_c0	0.51714305	0.29118425	0.5399463	7.59303787	10.7567486	11.1809139	2.994E-23	3.426526	up	PREDICTED: retinol dehydrogenase 11-like [Sesamum indicum]
c34011.graph_c0	10.1814765	11.3136906	10.3228406	1.97923234	2.09675667	1.83767899	8.814E-21	-3.445	down	PREDICTED: uncharacterized protein LOC105163738 [Sesamum indicum]
c34014.graph_c0	3.4001042	3.64151651	2.9026609	17.6115231	19.6551125	19.3933759	4.104E-16	1.492909	up	PREDICTED: NEDD8-activating enzyme E1 regulatory subunit [Sesamum indicum]
c34018.graph_c0	0	0	0	6.86340778	6.25457919	3.8086785	2.277E-12	Inf	up	unnamed protein product [Coffea canephora]
c34025.graph_c0	3.65065378	2.79447186	2.20763983	23.9777829	23.7668001	25.8417503	1.209E-20	2.074625	up	hypothetical protein MIMGU_mgv1a009463mg [Erythranthe guttata]
c34026.graph_c0	34.4049956	31.0973565	30.4824298	23.1129791	31.6541277	27.577447	8.863E-18	-1.242501	down	PREDICTED: probable receptor-like protein kinase At5g24010 [Sesamum indicum]
c34030.graph_c0	18.9270343	17.9279935	17.2068992	11.0346433	11.965571	9.40173365	2.835E-19	-1.754948	down	PREDICTED: dnaJ homolog subfamily B member 4 [Sesamum indicum]
c34031.graph_c0	0.30020852	0.50084855	0.77155966	2.02819223	3.80892389	3.69137709	0.0029298	1.562716	up	PREDICTED: maltose excess protein 1-like, chloroplastic [Sesamum indicum]
c34032.graph_c0	2.40924644	2.94104748	3.77558178	0.37894841	0.86024976	1.01155805	1.84E-10	-3.055274	down	PREDICTED: uncharacterized protein LOC105176742 [Sesamum indicum]
c34034.graph_c0	25.6378604	25.5681293	21.9229522	13.960832	19.5337095	17.8911858	3.333E-13	-1.530412	down	hypothetical protein MIMGU_mgv1a010704mg [Erythranthe guttata]
c34036.graph_c0	0.26304655	0.23937269	0.3687548	9.89170029	12.8829387	14.9175922	7.342E-32	4.40757	up	PREDICTED: uncharacterized protein LOC105158907 [Sesamum indicum]
c34037.graph_c0	19.9600514	18.1636713	18.3901762	12.9949614	17.1414043	15.4358282	1.64E-16	-1.331757	down	PREDICTED: cyclin-C1-2-like [Sesamum indicum]
c34044.graph_c0	0	0	0	2.89854951	3.98356117	5.99469257	1.534E-09	Inf	up	PREDICTED: receptor-like serine/threonine-protein kinase At3g01300 [Sesamum indicum]
c34044.graph_c1	0	0.0749921	0.09627142	5.17638761	5.9224554	7.43088196	1.164E-14	5.722546	up	PREDICTED: receptor-like serine/threonine-protein kinase At3g01300 [Sesamum indicum]
c34050.graph_c0	1.24536008	1.4840561	1.21930439	25.9236734	35.7684446	40.3728519	2.007E-24	3.668785	up	PREDICTED: polygalacturonase-1 non-catalytic subunit beta-like [Sesamum indicum]
c34051.graph_c0	155.160122	128.920345	163.32293	3.43689589	5.79245935	5.00421157	7.935E-93	-6.00127	down	PREDICTED: HVA22-like protein e [Sesamum indicum]
c34059.graph_c0	1.96676463	1.76149888	2.60718211	12.2447096	12.4666533	12.6093222	9.667E-15	1.53692	up	PREDICTED: vacuolar protein sorting-associated protein 35B-like isoform X1 [Sesamum indicum]

#ID	0 h-1 FPKM	0 h-2 FPKM	0 h-3 FPKM	48 h-1 FPKM	48 h-2 FPKM	48 h-3 FPKM	FDR	log2FC	regulated	nr_annotation
c34063.graph_c0	2.0533487	1.6747743	1.73465779	10.2465933	13.1744725	14.6812827	5.865E-15	1.780461	up	PREDICTED: homeobox-leucine zipper protein MERISTEM L1-like [Sesamum indicum]
c34069.graph_c0	94.6321948	97.3658158	93.841366	24.1363646	46.1790347	50.4160533	1.239E-54	-2.272105	down	PREDICTED: uncharacterized protein LOC105174506 [Sesamum indicum]
c34071.graph_c0	0.82150735	1.19611631	1.03647548	7.56826154	7.90103496	7.5790288	5.273E-17	1.897034	up	PREDICTED: conserved oligomeric Golgi complex subunit 4 [Sesamum indicum]
c34074.graph_c0	3.09795119	1.31883877	2.47095926	64.1993782	59.149463	67.4865601	6.03E-102	3.775239	up	Phosphoribulokinase family protein [Populus trichocarpa]
c34076.graph_c0	0.80230215	0.49421879	0.71376233	4.77593678	7.1676617	7.13325685	1.783E-06	2.221324	up	PREDICTED: uncharacterized protein ycf49 [Sesamum indicum]
c34081.graph_c0	1.00355862	1.00456353	0.62827278	5.54726193	6.203132	6.43357134	9.511E-08	1.770899	up	PREDICTED: probable glycosyltransferase At3g07620 [Sesamum indicum]
c34084.graph_c0	0.61081395	0.44738458	0.61262058	6.56051098	7.09546475	6.48475776	7.025E-23	2.57206	up	PREDICTED: conserved oligomeric Golgi complex subunit 1 [Sesamum indicum]
c34085.graph_c1	13.5662347	16.4503502	12.0472979	6.50307658	6.45864478	9.22207251	2.533E-10	-1.941292	down	-
c34103.graph_c0	5.10096457	3.07716947	3.16894519	0	0	0	4.038E-26	-Inf	down	carboxylesterase [Cryptococcus neoformans var. grubii H99]
c34106.graph_c2	0	0.08329672	0	3.14312583	6.41588036	9.20875982	8.988E-07	6.796382	up	hypothetical protein MIMGU_mgv1a008571mg [Erythranthe guttata]
c34109.graph_c0	1.90663785	1.8351414	1.88469625	0.20267529	0.21470989	0.06011308	6.946E-18	-4.57004	down	PREDICTED: ATP-dependent 6-phosphofructokinase 6 [Sesamum indicum]
c34111.graph_c0	0	0.2155088	0.41499046	6.04943571	7.24912145	8.29473185	1.131E-10	4.058663	up	PREDICTED: uncharacterized protein At5g01610 [Sesamum indicum]
c34112.graph_c0	5.0873499	6.41787477	7.6120947	69.9809646	66.5186668	75.6364049	7.757E-42	2.451057	up	hypothetical protein M569_08891, partial [Genlisea aurea]
c34113.graph_c0	1.85048995	3.41221068	3.50435158	0.0897259	0.09505371	0	3.281E-13	-6.579473	down	-
c34114.graph_c0	28.9536618	34.1188211	27.3162392	3.37645592	2.8615565	1.8026101	3.208E-23	-4.500139	down	hypothetical protein PRUPE_ppb013116mg [Prunus persica]
c34116.graph_c0	7.11396317	6.33711388	6.0385702	4.02851139	5.00023832	4.89531174	2.906E-10	-1.505088	down	PREDICTED: GATA transcription factor 26 [Sesamum indicum]
c34122.graph_c0	39.3733105	21.1584167	23.9666508	0	0	0	2.639E-28	-Inf	down	-
c34134.graph_c0	5.07503065	4.72802552	3.61594512	30.737607	40.9977012	41.8078161	2.919E-21	2.06131	up	PREDICTED: F-box only protein 6-like [Sesamum indicum]
c34139.graph_c0	8.29340796	8.1440851	7.62203422	1.25728568	2.45896933	2.36653415	3.785E-22	-3.011563	down	PREDICTED: hyoscyamine 6-dioxygenase [Sesamum indicum]
c34142.graph_c0	0.47892337	0.38352235	0.45131936	9.23610992	10.9998156	12.3011799	5.629E-27	3.608644	up	PREDICTED: probable BOI-related E3 ubiquitin-protein ligase 2 [Sesamum indicum]
c34147.graph_c0	0	0.09700547	0	3.03546463	2.26991062	3.73364983	3.508E-10	5.537397	up	PREDICTED: bidirectional sugar transporter SWEET1-like [Sesamum indicum]
c34149.graph_c0	0.04802406	0.0961443	0	0.53091479	1.31235963	1.20069549	0.0001255	3.383444	up	PREDICTED: subtilisin-like protease [Sesamum indicum]
c34151.graph_c0	4.45434735	5.80039586	4.05239837	3.70416639	4.38577574	3.36057135	4.104E-06	-1.3381	down	PREDICTED: uncharacterized protein ycf36 isoform X1 [Brassica rapa]
c34152.graph_c1	1.15109959	2.17647646	1.97227806	0.53023401	0.81137137	0.73390961	6.839E-06	-2.379756	down	PREDICTED: UDP-glucuronic acid decarboxylase 1-like [Nicotiana tomentosiformis]

#ID	0 h-1 FPKM	0 h-2 FPKM	0 h-3 FPKM	48 h-1 FPKM	48 h-2 FPKM	48 h-3 FPKM	FDR	log2FC	regulated	nr_annotation
c34153.graph_c0	0.32571588	0	0	8.40198003	11.9208202	13.4835565	4.079E-13	5.694277	up	-
c34155.graph_c0	24.3538805	25.5281855	26.8178567	10.3362583	17.1911456	17.5467629	8.155E-29	-1.794278	down	PREDICTED: acyl-CoA-binding domain-containing protein 1 [Sesamum indicum]
c34158.graph_c0	0.37637809	0.4362426	0.63639571	4.47117318	6.14799891	7.46969218	2.191E-11	2.612033	up	hypothetical protein MIMGU_mgv1a022010mg [Erythranthe guttata]
c34160.graph_c0	2.80760472	2.41826502	3.60788322	17.2035558	26.2543129	30.8293335	3.135E-07	2.04257	up	PREDICTED: uncharacterized protein LOC105157271 [Sesamum indicum]
c34162.graph_c1	0.17981579	0.05999861	0.38511734	12.0930362	11.4656469	12.7747983	1.729E-23	4.83126	up	PREDICTED: serine/threonine-protein kinase Aurora-2 [Sesamum indicum]
c34163.graph_c0	1.04513087	0.7692481	0.82952134	13.9895385	12.9001912	11.2383037	9.285E-24	2.836834	up	hypothetical protein MIMGU_mgv1a004090mg [Erythranthe guttata]
c34168.graph_c0	6.86463158	5.31723638	4.56818531	40.7681578	32.461478	28.4038983	1.932E-06	1.592826	up	PREDICTED: probable protein phosphatase 2C 34 [Sesamum indicum]
c34174.graph_c0	24.4183235	25.3425088	22.7542342	13.5250535	14.0942252	15.3255817	3.706E-31	-1.773747	down	PREDICTED: mitochondrial import inner membrane translocase subunit TIM17-2-like [Sesamum indicum]
c34177.graph_c0	0.03781357	0.07570287	0	2.29919766	3.83810603	4.27760744	5.501E-13	5.507465	up	PREDICTED: cytochrome P450 71D95-like [Sesamum indicum]
c34185.graph_c0	2.9523746	3.94044127	5.05855767	0	0	0	7.736E-19	-Inf	down	hypothetical protein SORBIDRAFT_07g005790 [Sorghum bicolor]
c34191.graph_c0	23.0175205	23.3794009	25.0111683	7.0164401	5.946454	8.87919816	1.084E-17	-2.727521	down	Mitogen-activated protein kinase 10 [Morus notabilis]
c34193.graph_c0	2.88926084	3.04909258	1.89957955	15.0627608	15.3451128	15.2387477	9.796E-15	1.528629	up	hypothetical protein MIMGU_mgv1a013779mg [Erythranthe guttata]
c34193.graph_c1	3.03555952	2.21735615	1.37055633	20.104966	19.2169386	20.7811536	2.077E-10	2.171441	up	PREDICTED: abscisic acid receptor PYL2 [Sesamum indicum]
c34195.graph_c0	0	0	0	4.25787579	8.24369878	2.61284984	9.766E-06	Inf	up	ubiquitin-conjugating enzyme e2-16kda, ubiquitin protein ligase [Thalassiosira pseudonana CCMP1335]
c34199.graph_c0	0	0.05623522	0	2.17374055	3.18007721	2.94732384	7.495E-12	6.193554	up	PREDICTED: (+)-neomenthol dehydrogenase-like [Sesamum indicum]
c34201.graph_c0	45.2777152	46.2227672	43.2840205	12.6069904	15.8994972	15.6567827	1.317E-73	-2.630801	down	PREDICTED: uncharacterized protein LOC105177120 [Sesamum indicum]
c34203.graph_c0	0.61121199	0.35689735	0.42544212	7.22630046	8.12763713	9.85364164	2.175E-22	3.158174	up	PREDICTED: zinc finger CCCH domain-containing protein 46-like [Sesamum indicum]
c34208.graph_c0	11.6236815	9.11685315	8.47070439	225.017436	235.972312	321.159125	4.066E-27	3.724622	up	hypothetical protein JCGZ_15938 [Jatropha curcas]
c34210.graph_c0	1.23512998	1.64204963	0.94239461	6.72061734	7.72240331	7.4512261	6.084E-09	1.503037	up	PREDICTED: protein DENND6A [Sesamum indicum]
c34213.graph_c0	34.3865302	34.8636764	36.586571	239.274083	233.680292	245.080523	8.301E-36	1.744254	up	PREDICTED: probable ATP synthase 24 kDa subunit, mitochondrial [Sesamum indicum]
c34214.graph_c0	17.5602657	17.5225734	17.1725914	16.9407594	16.3837563	18.1519163	2.482E-08	-1.039018	down	PREDICTED: uncharacterized protein LOC105176117, partial [Sesamum indicum]
c34215.graph_c0	1.84418838	2.23467402	2.30749048	1.16246453	1.04203018	1.35261671	4.577E-05	-1.863428	down	PREDICTED: vinorine synthase-like [Sesamum indicum]

#ID	0 h-1 FPKM	0 h-2 FPKM	0 h-3 FPKM	48 h-1 FPKM	48 h-2 FPKM	48 h-3 FPKM	FDR	log2FC	regulated	nr_annotation
c34218.graph_c0	12.5829769	13.7842005	11.0504733	9.29850913	9.47680448	9.37273858	2.649E-20	-1.426662	down	PREDICTED: uncharacterized protein LOC105168895 [Sesamum indicum]
c34223.graph_c0	0.20827172	0.1563602	0.40145611	2.25451355	1.67695028	2.21946245	0.0002295	1.982185	up	PREDICTED: putative receptor-like protein kinase At1g80870 [Sesamum indicum]
c34225.graph_c0	2.1745378	2.94259657	2.38038684	14.7281564	18.8254188	19.4429715	1.445E-13	1.799512	up	PREDICTED: CAP-Gly domain-containing linker protein 1 isoform X1 [Sesamum indicum]
c34228.graph_c0	0	0	0	1.30889747	2.03659542	2.40208877	1.864E-12	Inf	up	PREDICTED: transcription factor SPEECHLESS-like [Sesamum indicum]
c34229.graph_c0	0.93262725	1.72349748	1.50576113	6.34483978	6.5348776	7.39024759	5.089E-06	1.26279	up	PREDICTED: gamma-tubulin complex component 4 isoform X1 [Sesamum indicum]
c34231.graph_c0	16.0034043	17.8524983	16.3019468	13.0563265	17.561463	16.5341854	8.822E-13	-1.1114	down	PREDICTED: uncharacterized protein LOC105161303 [Sesamum indicum]
c34232.graph_c0	9.51475593	9.09963393	9.81262164	46.0613462	53.6464786	51.0699296	3.99E-15	1.386629	up	PREDICTED: probable signal peptidase complex subunit 2 [Sesamum indicum]
c34234.graph_c1	4.93255127	4.62695648	4.34527723	22.6066206	22.0415329	24.6926589	1.124E-12	1.301423	up	PREDICTED: xylosyltransferase 1 [Sesamum indicum]
c34240.graph_c0	0	0.09342372	0	2.66543985	2.09501088	2.06566753	6.615E-10	5.190369	up	PREDICTED: MADS-box transcription factor 23 [Sesamum indicum]
c34242.graph_c0	1.74887411	1.86236739	1.67357525	26.498718	31.5948193	31.3870291	1.481E-47	3.061688	up	hypothetical protein MIMGU_mgv1a006918mg [Erythranthe guttata]
c34252.graph_c0	11.348652	11.5246538	11.73018	50.4194297	47.9956975	44.8291992	5.827E-11	1.033271	up	PREDICTED: uncharacterized protein At1g03900 [Sesamum indicum]
c34255.graph_c0	0.04321715	0.02163021	0	3.58330135	5.48321696	6.97905331	3.859E-17	6.9425	up	PREDICTED: subtilisin-like protease [Sesamum indicum]
c34257.graph_c0	2.4359044	2.77700242	2.05783875	9.82838823	11.402555	14.143994	7.322E-08	1.262991	up	PREDICTED: ABC transporter B family member 25, mitochondrial [Sesamum indicum]
c34259.graph_c0	0.0678981	0	0	1.31359541	1.59039435	2.05936591	4.165E-10	5.190237	up	PREDICTED: pathogenesis-related protein 5 [Sesamum indicum]
c34261.graph_c0	39.3081028	38.5708692	40.9306076	33.155715	34.5355466	50.7712852	1.753E-10	-1.026028	down	PREDICTED: kinesin KP1-like isoform X2 [Sesamum indicum]
c34266.graph_c0	9.29976489	10.4463853	9.13866818	5.38864004	6.77640841	6.89896332	8.028E-13	-1.620622	down	PREDICTED: aspartic proteinase nepenthesin-2-like [Sesamum indicum]
c34267.graph_c0	52.2993027	51.1691912	54.7865499	1.01408762	0.70746774	0.94634512	0	-6.903817	down	hypothetical protein MIMGU_mgv1a010327mg [Erythranthe guttata]
c34268.graph_c0	0.06412093	0	0	2.89454654	4.13028146	4.99341347	2.301E-13	6.54643	up	PREDICTED: auxin transporter-like protein 2 [Sesamum indicum]
c34277.graph_c0	7.89737937	7.64609765	5.98924632	47.6490918	49.0885407	59.537442	1.305E-22	1.84275	up	PREDICTED: long chain acyl-CoA synthetase 6, peroxisomal-like isoform X2 [Sesamum indicum]
c34279.graph_c0	0.15336048	0.15351405	0.49268573	6.92299833	5.91216433	5.53146654	5.262E-10	3.494154	up	-
c34284.graph_c0	1.6180142	1.19090764	2.20151891	12.451063	16.6273245	12.990378	5.211E-12	2.043751	up	PREDICTED: putative lipoyltransferase-like protein, chloroplastic isoform X1 [Sesamum indicum]

#ID	0 h-1 FPKM	0 h-2 FPKM	0 h-3 FPKM	48 h-1 FPKM	48 h-2 FPKM	48 h-3 FPKM	FDR	log2FC	regulated	nr_annotation
c34292.graph_c0	0.54698231	0.37642689	0.57110131	4.9761683	8.97514571	10.2567206	1.716E-07	2.98758	up	PREDICTED: probable fructokinase-6, chloroplastic [Sesamum indicum]
c34293.graph_c0	0.23176889	0.23200097	0.05956644	1.96438971	4.25254508	4.97772134	3.65E-05	3.398883	up	PREDICTED: GDSL esterase/lipase At5g45960 [Sesamum indicum]
c34294.graph_c0	0.57971382	0.23211773	0.07449552	15.4346283	17.0300563	17.8204665	2.176E-30	4.820718	up	PREDICTED: ABC transporter B family member 19 [Sesamum indicum]
c34294.graph_c1	0.38241826	0.44169369	0.34021565	20.5152035	21.2165929	21.0756437	1.015E-70	4.737138	up	PREDICTED: ABC transporter B family member 19 [Sesamum indicum]
c34294.graph_c2	0.33295022	0.49992543	0.07130904	20.4490308	21.7174267	21.1976697	3.396E-41	5.124205	up	PREDICTED: ABC transporter B family member 19 [Sesamum indicum]
c34297.graph_c1	0.03189573	0.03192767	0	0.61707292	0.68484317	1.04584338	2.307E-08	4.194862	up	PREDICTED: zinc finger protein 91-like [Sesamum indicum]
c34299.graph_c0	1.8366291	1.92601432	2.0229781	0.161145	0.25607039	0.50185049	1.625E-10	-3.684813	down	PREDICTED: receptor-like cytosolic serine/threonine-protein kinase RBK2 [Sesamum indicum]
c34311.graph_c1	14.0691597	16.7965341	18.1900048	1.54585834	1.13375732	1.09334254	5.369E-54	-4.71358	down	PREDICTED: vesicle transport v-SNARE 13-like isoform X2 [Sesamum indicum]
c34317.graph_c0	0.29685257	0.4952497	0.25431153	6.83699866	6.27724158	6.00240669	5.183E-11	3.179474	up	PREDICTED: NAC domain-containing protein 21/22-like [Sesamum indicum]
c34330.graph_c0	31.704338	31.6636834	31.4157477	139.576978	136.99385	139.6104	1.572E-15	1.117396	up	PREDICTED: NADH dehydrogenase [ubiquinone] iron-sulfur protein 1, mitochondrial [Sesamum indicum]
c34335.graph_c0	275.155832	250.574913	259.617161	21.8672163	17.6415442	17.5864217	3.8E-202	-4.794696	down	PREDICTED: uncharacterized protein LOC105176201 [Sesamum indicum]
c34337.graph_c0	0	0.06893287	0	9.13563827	11.4927449	11.6852017	1.562E-24	7.862622	up	PREDICTED: MLO-like protein 4 [Sesamum indicum]
c34342.graph_c0	0.28931237	0.28960207	0.18588892	2.59870087	3.45890806	3.37952767	1.02E-05	2.607102	up	-
c34344.graph_c0	1.63603508	1.12051333	2.2130209	10.1539182	12.3535643	16.3051627	6.482E-07	1.937232	up	hypothetical protein MIMGU_mgv1a014272mg [Erythranthe guttata]
c34351.graph_c0	26.5837143	36.219621	31.3143649	16.327101	19.3685703	19.7486687	1.902E-14	-1.784652	down	hypothetical protein MIMGU_mgv11b020149mg [Erythranthe guttata]
c34354.graph_c0	1.30551677	1.27841483	1.20352488	19.3743664	18.6966728	15.7269847	8.284E-33	2.813655	up	PREDICTED: protein NRT1/ PTR FAMILY 5.5-like isoform X2 [Sesamum indicum]
c34359.graph_c0	0.88264154	0.48826402	0.56711466	0.06419581	0.15868458	0.1142421	4.764E-09	-3.550987	down	-
c34360.graph_c0	0	0.09571884	0.12287945	6.65111382	6.99938603	9.40628516	3.717E-22	5.686763	up	PREDICTED: uncharacterized oxidoreductase At1g06690, chloroplastic [Sesamum indicum]
c34362.graph_c0	1.15759876	1.18702031	1.66897002	0.67628884	0.90933534	1.18037096	0.0005605	-1.5658	down	PREDICTED: uncharacterized protein LOC105166158 [Sesamum indicum]
c34370.graph_c1	4.15431181	4.15847172	3.99262612	58.9842604	60.476468	65.247086	3.521E-69	2.889829	up	PREDICTED: uridine 5'-monophosphate synthase [Sesamum indicum]

#ID	0 h-1 FPKM	0 h-2 FPKM	0 h-3 FPKM	48 h-1 FPKM	48 h-2 FPKM	48 h-3 FPKM	FDR	log2FC	regulated	nr_annotation
c34376.graph_c0	8.16160689	7.1708545	8.56442687	0.36117489	0.10435118	0.0876467	1.158E-73	-6.423932	down	hypothetical protein MIMGU_mgv1a019938mg [Erythranthe guttata]
c34383.graph_c0	2.34632512	2.04065171	2.57026681	13.9262948	18.6198409	23.521842	3.132E-08	1.984684	up	PREDICTED: probable inactive patatin-like protein 9 [Sesamum indicum]
c34387.graph_c0	2.1132288	2.60892536	3.80181575	12.7193822	12.7184131	16.4567576	6.294E-05	1.271866	up	PREDICTED: protein LURP-one-related 5-like [Sesamum indicum]
c34389.graph_c0	15.4784927	14.8601469	13.6520994	1.750066	4.25729366	5.19066069	1.201E-28	-3.005224	down	PREDICTED: serine/threonine-protein kinase OXI1 [Sesamum indicum]
c34389.graph_c1	11.4592633	12.4145329	13.4208064	1.53678151	3.89312398	4.28061583	1.534E-23	-2.97532	down	PREDICTED: serine/threonine-protein kinase OXI1 [Sesamum indicum]
c34390.graph_c0	6.49634188	6.29307772	5.95434855	4.22586455	4.6585897	4.90537187	1.087E-12	-1.461643	down	PREDICTED: ubiquitin carboxyl-terminal hydrolase 20-like [Sesamum indicum]
c34400.graph_c0	2.25400177	1.62450634	2.20132595	0.08306134	0	0.07390748	1.135E-16	-6.274112	down	PREDICTED: serine/threonine-protein kinase CDL1-like [Sesamum indicum]
c34401.graph_c0	0.01743838	0.01745584	0.02240901	33.0786049	37.2212433	38.5961574	2.63E-220	9.869284	up	PREDICTED: ABC transporter G family member 28 [Sesamum indicum]
c34402.graph_c1	0.96339031	0.91613725	1.08324536	6.52340424	7.68645287	8.03546172	5.498E-11	1.886144	up	PREDICTED: uncharacterized protein LOC105169037 [Sesamum indicum]
c34406.graph_c0	0	0	0	0.72802739	0.69413107	0.46641197	4.282E-12	Inf	up	hypothetical protein VITISV_004365 [Vitis vinifera]
c34409.graph_c0	10.9000268	12.3341078	11.3425975	12.3885443	11.7365841	10.4405252	8.929E-06	-1.015843	down	hypothetical protein MIMGU_mgv1a015267mg [Erythranthe guttata]
c34410.graph_c0	7.08433936	7.47381446	6.47073628	1.43968136	1.6607383	1.48028956	3.276E-31	-3.216864	down	PREDICTED: pentatricopeptide repeat-containing protein At5g18390, mitochondrial [Sesamum indicum]
c34411.graph_c0	82.9953131	73.9079305	79.0164569	47.9220451	43.9480681	43.3408195	1.561E-34	-1.818579	down	PREDICTED: polyadenylate-binding protein 1 isoform X1 [Sesamum indicum]
c34413.graph_c0	46.5133807	29.6182932	34.2203449	0	0	0	1.51E-47	-Inf	down	-
c34414.graph_c0	0.12401871	0.2482858	0.15936896	18.5948595	14.0101943	11.1066431	4.512E-16	5.352095	up	PREDICTED: ABC transporter G family member 8-like [Sesamum indicum]
c34416.graph_c1	2.81888424	2.01550495	2.24242439	12.6137087	15.9828311	15.1298558	1.369E-06	1.607232	up	PREDICTED: outer envelope pore protein 21B, chloroplastic [Sesamum indicum]
c34420.graph_c0	0.85186696	1.05737277	0.87574622	16.8887972	22.2481455	24.8876342	1.505E-28	3.500204	up	PREDICTED: dnaJ protein P58IPK homolog [Sesamum indicum]
c34428.graph_c0	22.0660752	21.6567614	21.9314238	14.3730316	16.9510307	14.484821	2.809E-20	-1.538704	down	hypothetical protein PGTG_21261 [Puccinia graminis f. sp. tritici CRL 75-36-700-3]
c34430.graph_c0	159.04022	165.53313	147.142251	66.9034705	51.194945	43.3343148	1.43E-69	-2.556628	down	hypothetical protein PRUPE_ppa024708mg, partial [Prunus persica]
c34441.graph_c0	3.13104731	3.89245256	2.5958192	19.0750847	23.1649694	18.7529798	8.034E-11	1.647753	up	PREDICTED: 1-aminocyclopropane-1-carboxylate oxidase 5-like [Sesamum indicum]

#ID	0 h-1 FPKM	0 h-2 FPKM	0 h-3 FPKM	48 h-1 FPKM	48 h-2 FPKM	48 h-3 FPKM	FDR	log2FC	regulated	nr_annotation
c34451.graph_c0	2.95018253	2.44397519	3.07209937	1.31208993	1.092143	1.20918579	2.746E-07	-2.244155	down	PREDICTED: putative nuclease HARBI1 isoform X2 [Sesamum indicum]
c34453.graph_c0	0.2034014	0.05090127	0.03267236	1.75674974	2.28290435	1.87577443	7.351E-09	3.357877	up	PREDICTED: uncharacterized protein DDB_G0290685 [Sesamum indicum]
c34455.graph_c0	9.49446596	8.52340456	8.1338431	62.2003294	58.9072089	53.3688548	5.865E-20	1.723752	up	hypothetical protein MIMGU_mgv1a015463mg [Erythranthe guttata]
c34456.graph_c1	0.33938971	0.28310796	0.58150562	14.9559497	21.4749892	18.2691266	2.198E-32	4.475512	up	hypothetical protein MIMGU_mgv1a023921mg, partial [Erythranthe guttata]
c34457.graph_c0	0	0.80891242	0.51922232	155.968015	160.891441	151.365601	3.19E-101	7.438388	up	--
c34459.graph_c0	10.0183833	7.21790395	8.7740131	117.39801	118.078889	123.081864	1.676E-66	2.76752	up	PREDICTED: uncharacterized protein LOC105175711 [Sesamum indicum]
c34460.graph_c0	0	0	0	11.8489373	19.2229295	20.2203668	3.87E-27	Inf	up	PREDICTED: transcription factor bHLH93 [Sesamum indicum]
c34467.graph_c0	0.74174273	0.78156365	0.40133422	13.4510434	14.6688598	13.632749	5.789E-26	3.426727	up	hypothetical protein MIMGU_mgv1a014915mg [Erythranthe guttata]
c34469.graph_c0	0.08690824	0.26098579	0.11168053	3.60294916	3.30796938	3.34836616	1.416E-07	3.467301	up	-
c34472.graph_c0	0	0	0	0.63598812	1.09928006	2.17424124	0.0001624	Inf	up	PREDICTED: protein NRT1/ PTR FAMILY 6.4 [Sesamum indicum]
c34475.graph_c0	9.18529466	7.71680607	8.74721237	6.34656941	6.12311531	6.79001273	1.014E-09	-1.430986	down	PREDICTED: uncharacterized protein LOC105155514 [Sesamum indicum]
c34477.graph_c0	7.66906458	7.29290679	6.1902043	36.3197623	34.2194225	31.4134058	9.57E-16	1.255533	up	hypothetical protein MIMGU_mgv1a003188mg [Erythranthe guttata]
c34486.graph_c0	0.52757718	0.28165625	0.40677454	9.04031632	9.98903804	9.19728348	3.755E-21	3.519018	up	hypothetical protein MIMGU_mgv1a001741mg [Erythranthe guttata]
c34489.graph_c0	0	0	0	24.1613008	41.6286082	15.828625	8.496E-10	Inf	up	hypothetical protein PAXINDRAFT_10917 [Paxillus involutus ATCC 200175]
c34493.graph_c0	59.8991401	62.6598233	54.425757	30.0447923	36.1889258	35.9680313	2.292E-33	-1.811876	down	hypothetical protein MIMGU_mgv1a000078mg [Erythranthe guttata]
c34495.graph_c0	9.53498196	11.2984391	11.3626665	45.4603568	51.5002954	50.137059	7.746E-14	1.171004	up	PREDICTED: uncharacterized protein LOC105179402 [Sesamum indicum]
c34498.graph_c0	1.67900073	1.4829547	1.77683258	54.911577	66.1244408	61.9351999	1.204E-94	4.189753	up	PREDICTED: cytochrome P450 71D95-like [Sesamum indicum]
c34501.graph_c0	0	0	0	22.2377088	16.8407702	12.3966619	4.186E-16	Inf	up	PREDICTED: probable galactinol--sucrose galactosyltransferase 1 [Sesamum indicum]
c34502.graph_c0	0.70001969	0.91632701	0.89955302	5.55609425	4.36195247	4.63479305	0.0008068	1.517475	up	PREDICTED: NAC domain-containing protein 72-like [Sesamum indicum]
c34503.graph_c0	0	0	0	3.56500459	3.07847847	3.43868432	1.841E-19	Inf	up	hypothetical protein CISIN_1g004365mg [Citrus sinensis]
c34508.graph_c0	0.35199211	0.16442747	0.21108444	13.1008349	17.0163503	17.2739697	3.928E-69	5.006514	up	PREDICTED: cellulose synthase-like protein G2 [Sesamum indicum]

#ID	0 h-1 FPKM	0 h-2 FPKM	0 h-3 FPKM	48 h-1 FPKM	48 h-2 FPKM	48 h-3 FPKM	FDR	log2FC	regulated	nr_annotation
c34509.graph_c0	0.42647199	0.25613942	0.21921336	5.73624968	6.40983966	8.80978273	8.805E-10	3.521753	up	hypothetical protein CARUB_v10021088mg [Capsella rubella]
c34512.graph_c0	3.3126713	2.90876179	2.76325982	15.1655473	17.6684102	17.7580505	2.205E-19	1.474513	up	PREDICTED: uncharacterized protein LOC105177031 isoform X1 [Sesamum indicum]
c34518.graph_c0	0.04734052	0	0	3.40182746	4.4816782	4.15232104	6.869E-16	6.989344	up	PREDICTED: serine/threonine-protein kinase At5g01020 [Sesamum indicum]
c34519.graph_c0	24.7758305	22.3902104	23.5174291	103.674316	116.149661	114.488504	2.564E-17	1.222471	up	PREDICTED: proteasome subunit alpha type-3 [Sesamum indicum]
c34521.graph_c0	0.1861241	0.18631048	0.47835369	1.37175732	2.42201757	2.03430235	0.0010295	1.738546	up	PREDICTED: LOW QUALITY PROTEIN: uncharacterized protein LOC103453887 [Malus domestica]
c34523.graph_c0	0.03950503	0	0	5.45919147	6.16890816	5.76429948	2.363E-23	7.784263	up	Serine carboxypeptidase-like 40 [Theobroma cacao]
c34526.graph_c0	5.58722477	5.59281953	5.23273927	23.3374068	30.2685327	31.2452613	1.416E-11	1.348382	up	PREDICTED: uncharacterized protein LOC105164370 [Sesamum indicum]
c34527.graph_c1	18.7416056	17.4139342	15.9789814	21.0936969	15.4906523	15.853227	7.466E-11	-1.001563	down	PREDICTED: uncharacterized protein LOC105168318 [Sesamum indicum]
c34529.graph_c0	0.2314741	0.23170589	0.08498668	13.8916518	11.7796711	12.7673105	3.534E-42	5.125779	up	PREDICTED: acetolactate synthase small subunit 1, chloroplastic-like isoform X2 [Sesamum indicum]
c34532.graph_c0	0	0	0	3.29283183	3.12116068	5.47436525	6.257E-10	Inf	up	hypothetical protein MIMGU_mgv1a017766mg, partial [Erythranthe guttata]
c34533.graph_c0	0	0	0	50.3761628	55.2300324	69.3111843	1.548E-69	Inf	up	PREDICTED: GDSL esterase/lipase At2g40250 [Sesamum indicum]
c34533.graph_c1	0	0	0	17.1039113	16.3075681	24.2235072	4.979E-22	Inf	up	PREDICTED: GDSL esterase/lipase At2g30310-like [Cucumis melo]
c34535.graph_c0	1.24343366	1.75177012	1.1836011	30.0808268	35.7323778	36.4678472	4.682E-54	3.594391	up	PREDICTED: poly(U)-specific endoribonuclease-B [Sesamum indicum]
c34537.graph_c0	4.89573012	5.17074605	4.8546322	17.3662994	21.5201706	22.4676419	4.637E-07	1.01839	up	PREDICTED: protein disulfide isomerase-like 5-2 [Sesamum indicum]
c34541.graph_c0	18.9688625	20.3064581	18.6203564	14.4733738	13.952428	14.321875	1.329E-24	-1.454049	down	PREDICTED: lysine-specific demethylase REF6 isoform X1 [Sesamum indicum]
c34545.graph_c0	0.40782283	0.57152368	0.52406848	29.6061837	29.8516767	35.6705739	1.881E-79	4.963305	up	PREDICTED: LOW QUALITY PROTEIN: signal peptide peptidase-like [Sesamum indicum]
c34552.graph_c0	576.771256	604.116531	584.298523	136.923428	63.1655267	51.4655806	8.72E-133	-3.810213	down	PREDICTED: zinc finger CCCH domain-containing protein 29 [Sesamum indicum]
c34555.graph_c0	30.1506338	27.1497523	28.6277625	16.6580679	12.3277112	9.57417974	4.33E-41	-2.16509	down	PREDICTED: RNA-directed DNA methylation 4 [Sesamum indicum]
c34558.graph_c0	0.26038098	0.14034554	0.18016916	4.68688503	5.49298178	5.87791196	9.75E-23	3.770075	up	PREDICTED: histidine kinase 5-like [Sesamum indicum]
c34574.graph_c0	131.093961	130.672372	136.877674	75.4508907	81.2016504	79.1011761	8.09E-35	-1.776935	down	PREDICTED: heat shock 70 kDa protein 14-like [Sesamum indicum]

#ID	0 h-1 FPKM	0 h-2 FPKM	0 h-3 FPKM	48 h-1 FPKM	48 h-2 FPKM	48 h-3 FPKM	FDR	log2FC	regulated	nr_annotation
c34580.graph_c0	109.402773	104.024982	126.257267	17.7405854	12.8494251	9.83230985	7.992E-66	-4.081443	down	hypothetical protein MIMGU_mgv1a010728mg [Erythranthe guttata]
c34593.graph_c0	0.53802107	0.17951994	0.23045946	85.9474622	109.429127	133.457306	3.666E-55	7.421221	up	PREDICTED: uncharacterized protein LOC105177617 [Sesamum indicum]
c34594.graph_c0	5.32388129	5.42471795	4.708059	3.34012284	4.73035582	5.38092262	1.264E-09	-1.223662	down	PREDICTED: uncharacterized protein LOC103331091 [Prunus mume]
c34605.graph_c0	9.96441786	9.18989268	10.6465808	52.2943251	60.7536884	60.5731285	1.278E-12	1.521234	up	40S ribosomal protein s13, partial [Camellia sinensis]
c34610.graph_c0	1.8238076	1.4605071	1.61452476	9.74522102	11.2336472	11.0965282	3.016E-08	1.691475	up	PREDICTED: F-actin-capping protein subunit alpha [Sesamum indicum]
c34613.graph_c0	8338.39758	8509.41383	8986.97563	1309.80425	1381.27807	1122.96387	2.092E-29	-3.777595	down	hypothetical protein MIMGU_mgv1a024110mg [Erythranthe guttata]
c34616.graph_c0	0	0.02849705	0.0365832	1.02285603	0.9724543	0.86345775	3.567E-09	4.428254	up	Cysteine-rich RLK (RECEPTOR-like protein kinase) 8 [Theobroma cacao]
c34619.graph_c1	0.34967357	0.70004743	0.53921325	8.76226507	7.7809672	4.87288173	1.48E-06	2.739625	up	PREDICTED: uncharacterized protein LOC105177084 [Sesamum indicum]
c34623.graph_c0	1.28444308	1.19389145	1.41476589	44.3742279	44.0990258	39.5967323	6.745E-75	4.02203	up	hypothetical protein MIMGU_mgv1a008681mg [Erythranthe guttata]
c34624.graph_c0	0.20628346	0.10324501	0.53016482	2.85062666	3.1205564	2.87466653	3.039E-05	2.364512	up	PREDICTED: zinc finger Ran-binding domain-containing protein 2-like isoform X2 [Sesamum indicum]
c34627.graph_c0	65.4916	61.7546709	67.7846923	47.0011577	49.2288753	47.2608818	2.767E-24	-1.461351	down	PREDICTED: quinone oxidoreductase-like protein 2 homolog [Sesamum indicum]
c34632.graph_c0	8.53497042	11.4957269	6.43136539	3.29339118	4.62285691	4.79859496	1.336E-06	-2.077333	down	PREDICTED: exocyst complex component EXO70A1-like [Sesamum indicum]
c34632.graph_c1	2.7183966	2.21213244	2.03563329	10.7021034	11.6238834	13.2419581	2.298E-11	1.334528	up	PREDICTED: chloroplastic group IIA intron splicing facilitator CRS1, chloroplastic [Sesamum indicum]
c34639.graph_c0	90.6784481	92.1497697	91.8246703	445.329046	480.01636	472.349973	1.75E-21	1.328563	up	40S ribosomal protein S15D [Hevea brasiliensis]
c34641.graph_c0	32.55315	34.5534854	33.0412858	6.95422262	7.52063804	7.5843833	1.418E-96	-3.201581	down	PREDICTED: BTB/POZ domain-containing protein POB1-like [Sesamum indicum]
c34644.graph_c0	0.45846422	0.4589233	0.58914467	24.3766178	26.3354385	33.2332029	1.328E-42	4.776618	up	PREDICTED: cytokinin riboside 5'-monophosphate phosphoribohydrolase LOG3-like isoform X1 [Nicotiana sylvestris]
c34645.graph_c0	18.9693013	18.9540213	20.2402518	11.5769008	12.5650826	12.7991332	3.538E-24	-1.674727	down	PREDICTED: stress response protein NST1-like [Sesamum indicum]
c34647.graph_c0	0.05552871	0	0.14271318	74.3306393	98.2542426	119.57814	1.511E-52	9.483823	up	PREDICTED: probable pectinesterase 15 [Sesamum indicum]
c34648.graph_c0	97.3794263	110.931612	93.1389953	31.7061312	39.5868017	44.3330248	7.001E-63	-2.403814	down	PREDICTED: uncharacterized protein LOC105163464 [Sesamum indicum]
c34652.graph_c0	3.1243141	2.16515259	2.05890632	1.1070469	1.01641103	0.78803494	1.523E-06	-2.346207	down	PREDICTED: protein YLS9-like [Sesamum indicum]

#ID	0 h-1 FPKM	0 h-2 FPKM	0 h-3 FPKM	48 h-1 FPKM	48 h-2 FPKM	48 h-3 FPKM	FDR	log2FC	regulated	nr_annotation
c34659.graph_c0	7.03077974	5.0428474	4.97982774	21.6757124	29.1222194	22.0097776	3.91E-06	1.075519	up	hypothetical protein MIMGU_mgv1a010012mg [Erythranthe guttata]
c34662.graph_c0	0	0	0	8.1524566	9.06021851	9.03329156	1.36E-39	Inf	up	PREDICTED: probable GABA transporter 2 [Sesamum indicum]
c34664.graph_c0	24.9426898	24.1067121	24.3155712	13.4703502	13.7105869	14.5710171	6.974E-12	-1.831329	down	PREDICTED: cyclin-dependent kinase F-4-like isoform X1 [Sesamum indicum]
c34673.graph_c0	1.0042957	0.50265068	0.43018658	7.09337409	6.20768804	9.46746241	5.108E-07	2.543283	up	hypothetical protein MIMGU_mgv1a009336mg [Erythranthe guttata]
c34676.graph_c0	0.9102361	0.79725412	0.77979295	4.22778469	6.55166998	6.49775369	6.538E-06	1.772227	up	PREDICTED: uncharacterized protein LOC105168235 isoform X2 [Sesamum indicum]
c34682.graph_c0	27.0744031	25.633923	25.7902224	13.4168347	15.9305777	13.834446	2.171E-34	-1.881592	down	PREDICTED: E3 ubiquitin-protein ligase At1g63170-like [Sesamum indicum]
c34684.graph_c0	1.13317547	1.18586972	1.25760597	22.8721083	28.7042711	34.1161479	1.551E-26	3.558663	up	hypothetical protein CISIN_1g0023491mg, partial [Citrus sinensis]
c34685.graph_c0	3.08252285	1.92218298	2.27279896	0.97764333	0.93705699	0.91132502	1.401E-07	-2.379242	down	PREDICTED: uncharacterized protein LOC105169662 [Sesamum indicum]
c34687.graph_c0	70.415942	62.1374789	71.1455417	19.7397387	19.6019444	17.9179444	8.612E-81	-2.848028	down	PREDICTED: probable fructokinase-7 [Sesamum indicum]
c34693.graph_c0	9.34410696	11.6851294	8.29174476	149.105395	116.045707	115.15892	5.346E-17	2.686971	up	PREDICTED: sterol 14-demethylase-like [Sesamum indicum]
c34696.graph_c1	0.46945007	0.36549345	0.13405821	2.06633168	2.23993548	2.69377671	0.0010604	1.842561	up	PREDICTED: 2,3-bisphosphoglycerate-independent phosphoglycerate mutase [Sesamum indicum]
c34700.graph_c0	0.53247906	0.79951838	0.25659625	5.18148126	6.07379443	6.30185888	1.715E-09	2.453231	up	PREDICTED: tRNA (guanine(37)-N1)-methyltransferase 2 [Sesamum indicum]
c34704.graph_c0	0.44075183	0.66178977	0.64729549	3.97348281	3.4412803	3.81945989	6.933E-05	1.664769	up	PREDICTED: putative cyclin-A3-1 [Sesamum indicum]
c34705.graph_c0	0	0	0	0.97541719	1.03333624	0.65094026	1.517E-11	Inf	up	PREDICTED: abscisic acid 8'-hydroxylase 4 [Sesamum indicum]
c34722.graph_c0	4.77646478	3.92307503	5.19364691	1.83349621	1.94236691	1.95772068	1.074E-14	-2.29715	down	PREDICTED: probable protein phosphatase 2C 50 [Sesamum indicum]
c34723.graph_c0	1.16332272	1.00386863	0.67013465	8.20426853	8.61312654	10.0623121	3.757E-10	2.229413	up	hypothetical protein MIMGU_mgv1a001992mg [Erythranthe guttata]
c34724.graph_c0	4.19810803	4.65161557	4.81793946	37.4594921	35.2258055	29.6518092	3.75E-18	1.888513	up	PREDICTED: protein TRANSPORT INHIBITOR RESPONSE 1-like [Sesamum indicum]
c34729.graph_c0	9.91080928	10.7115166	7.56763876	6.48396686	6.58861027	6.77020809	1.029E-07	-1.521441	down	PREDICTED: DNA-directed RNA polymerase I subunit RPA12-like [Sesamum indicum]
c34730.graph_c0	0.9281043	1.15023215	1.07906498	31.1068136	34.1616316	32.2072316	6.512E-60	3.928712	up	PREDICTED: methylsterol monooxygenase 2-2 [Sesamum indicum]
c34731.graph_c0	64.4846162	61.3109342	67.6225067	0.2649161	0.56129296	0.29465094	4.23E-255	-8.458997	down	hypothetical protein MIMGU_mgv1a016001mg [Erythranthe guttata]
c34735.graph_c0	93.5654991	90.0888493	86.5524122	2.98027215	3.40010135	3.73975777	9.82E-210	-5.758676	down	PREDICTED: peroxisomal membrane protein 13 [Glycine max]

#ID	0 h-1 FPKM	0 h-2 FPKM	0 h-3 FPKM	48 h-1 FPKM	48 h-2 FPKM	48 h-3 FPKM	FDR	log2FC	regulated	nr_annotation
c34743.graph_c0	1.67936548	2.1395145	1.96186466	0.35162296	0.52150264	0.43802079	4.411E-09	-3.164588	down	PREDICTED: uncharacterized protein LOC105168020 [Sesamum indicum]
c34752.graph_c0	14.5666705	11.9343408	12.8765912	8.80405096	12.6319613	14.0704024	3.328E-09	-1.173317	down	-
c34754.graph_c0	1.77904446	1.97162868	2.12284667	11.8240108	11.7819806	10.4167661	2.043E-05	1.516258	up	PREDICTED: uncharacterized protein LOC105178220 [Sesamum indicum]
c34755.graph_c0	0.26437485	0.26463958	0.39635417	2.76033937	4.90241048	3.21464514	1.034E-06	2.525208	up	PREDICTED: beta-galactosidase 8-like isoform X1 [Sesamum indicum]
c34762.graph_c0	1.18013965	1.45927935	0.89207422	12.3431631	12.6695742	12.5762424	1.407E-09	2.397698	up	TPA: hypothetical protein ZEAMMB73_114394 [Zea mays]
c34763.graph_c0	0.11420885	0.17148482	0.25683507	5.4186571	5.71254408	5.50024174	1.24E-18	3.912551	up	PREDICTED: transmembrane 9 superfamily member 5 isoform X1 [Sesamum indicum]
c34764.graph_c0	758.64986	773.011633	758.835563	9.54472895	14.1020682	16.1540415	1.89E-244	-6.871949	down	PREDICTED: proline dehydrogenase 2, mitochondrial [Sesamum indicum]
c34771.graph_c0	2.16343673	2.27571849	2.00261574	1.53705094	1.53885101	1.54800928	9.543E-07	-1.494804	down	PREDICTED: pentatricopeptide repeat-containing protein At5g13270, chloroplastic [Sesamum indicum]
c34773.graph_c0	24.0805876	22.7954997	24.2217399	19.5621463	20.9865228	22.8299762	6.488E-14	-1.185485	down	Glutamate-rich WD repeat-containing protein 1 [Glycine soja]
c34777.graph_c0	23.4607797	23.8180587	22.0371392	15.9084876	15.3886362	13.1009526	1.673E-29	-1.657491	down	PREDICTED: zinc finger CCCH domain-containing protein 1 [Sesamum indicum]
c34783.graph_c1	10.5904577	10.8242427	8.8817659	8.78095836	8.48636408	7.76755246	3.477E-13	-1.28933	down	hypothetical protein MIMGU_mgv1a012299mg [Erythranthe guttata]
c34786.graph_c0	0.93215901	0.69981932	0.74866327	8.42663826	7.96038347	7.97554989	3.153E-13	2.340321	up	PREDICTED: uncharacterized protein LOC103931436 [Pyrus x bretschneideri]
c34789.graph_c0	3.96076528	5.83048733	4.19155075	1.18053142	1.36432352	0.85944253	5.334E-10	-3.054312	down	-
c34792.graph_c0	3.93654626	4.30283185	3.0816867	0.6669615	0.66240454	1.44655469	3.877E-14	-3.049025	down	unnamed protein product [Coffea canephora]
c34794.graph_c0	0.93477376	1.06938263	0	182.199932	207.094376	296.326761	8.018E-34	7.411876	up	hypothetical protein MIMGU_mgv1a016621mg [Erythranthe guttata]
c34805.graph_c0	0.13341277	0.22257728	0.34288139	9.87365346	12.4130392	11.118601	2.795E-26	4.549645	up	PREDICTED: 1-aminocyclopropane-1-carboxylate oxidase homolog 11-like [Sesamum indicum]
c34806.graph_c0	0.14139797	0.04717985	0.06056733	1.21580698	2.57600015	3.20681688	4.259E-05	3.787374	up	PREDICTED: O-acyltransferase WSD1-like [Sesamum indicum]
c34821.graph_c0	133.452253	123.586002	147.946617	18.7368223	21.9042842	18.2142473	2.191E-76	-3.803167	down	PREDICTED: protein lin-12 [Sesamum indicum]
c34824.graph_c0	5.58922581	3.53249747	3.69604044	0	0	0	1.235E-36	-Inf	down	VIT-domain-containing protein [Auricularia delicata TFB-10046 SS5]
c34825.graph_c0	33.3319134	33.7597567	32.9158681	164.640781	164.064744	171.370073	8.848E-21	1.304537	up	PREDICTED: 28 kDa ribonucleoprotein, chloroplastic-like [Sesamum indicum]
c34827.graph_c0	1.60551838	1.60712607	1.26684937	6.30567203	7.77969901	7.52717774	2.874E-05	1.251831	up	quercetin 3-O-glucoside-6''-O-malonyltransferase [Glandularia x hybrida]
c34832.graph_c0	173.156058	179.205022	177.717436	18.2090889	20.4594321	20.6211572	6.8E-163	-4.180191	down	-

#ID	0 h-1 FPKM	0 h-2 FPKM	0 h-3 FPKM	48 h-1 FPKM	48 h-2 FPKM	48 h-3 FPKM	FDR	log2FC	regulated	nr_annotation
c34834.graph_c0	0.87499531	1.28011986	0.77843327	29.3917465	33.5346712	39.4220088	8.386E-56	4.106275	up	PREDICTED: allene oxide synthase, chloroplastic [Sesamum indicum]
c34836.graph_c0	2.67308576	2.67576246	2.95940274	19.7388541	25.0048075	27.811706	1.568E-20	2.102791	up	hypothetical protein MIMGU_mgv1a008311mg [Erythranthe guttata]
c34840.graph_c0	42.3422532	42.3145953	40.4713426	21.2773339	19.9793058	19.8503804	1.775E-44	-2.049638	down	PREDICTED: tubby-like F-box protein 6 [Sesamum indicum]
c34841.graph_c0	43.8769888	45.7336544	40.4333438	29.7172004	26.1427567	27.648321	2.239E-30	-1.653317	down	PREDICTED: uncharacterized protein LOC105169164 isoform X2 [Sesamum indicum]
c34847.graph_c0	56.8883055	56.8357077	57.4562224	19.8393719	29.1359506	27.1411361	1.132E-52	-2.193276	down	PREDICTED: DNA-directed RNA polymerase III subunit RPC5 [Sesamum indicum]
c34848.graph_c0	6.71469516	7.63279775	5.65493389	71.6109889	81.453856	69.1300135	5.729E-55	2.457151	up	hypothetical protein MIMGU_mgv1a0036351mg, partial [Erythranthe guttata]
c34849.graph_c0	0.02193226	0.02195422	0	9.29449638	16.3749778	18.3562194	1.504E-23	8.958983	up	PREDICTED: uncharacterized protein LOC105171410 [Sesamum indicum]
c34854.graph_c0	2.32926988	1.97289425	2.07221817	132.989249	213.456546	276.616318	5.709E-16	5.584292	up	chalcone isomerase [Camellia nitidissima]
c34856.graph_c0	86.1756905	90.9279293	88.2098911	45.8055947	50.3587585	46.042336	5.388E-40	-1.918323	down	PREDICTED: DEAD-box ATP-dependent RNA helicase 21 [Sesamum indicum]
c34858.graph_c0	2.21805564	2.15299557	2.85028931	46.6270726	45.7878078	47.1084435	5.705E-46	3.251839	up	PREDICTED: rac-like GTP-binding protein 5 isoform X2 [Sesamum indicum]
c34859.graph_c0	0.2455622	0.36871214	0.6311143	3.67620364	4.37381408	6.24018342	8.065E-07	2.488076	up	hypothetical protein MIMGU_mgv1a004092mg [Erythranthe guttata]
c34859.graph_c1	0.26892035	0.94216371	0.3455733	2.60134314	5.38038633	4.73954085	0.0014724	2.005142	up	PREDICTED: cytochrome P450 78A9-like, partial [Cucumis melo]
c34868.graph_c0	13.103545	11.7644326	11.891158	225.818085	230.789007	251.261363	9.32E-102	3.249468	up	hypothetical protein VITISV_041718 [Vitis vinifera]
c34871.graph_c0	15.4722335	13.294186	15.6158162	63.990198	78.9369446	87.9111098	8.216E-14	1.356055	up	PREDICTED: uncharacterized protein LOC105170170 isoform X1 [Sesamum indicum]
c34872.graph_c0	3.18235179	4.93758456	5.24812498	1.61248452	1.81176102	1.99999501	1.145E-07	-2.325195	down	PREDICTED: polyol transporter 5-like [Sesamum indicum]
c34875.graph_c0	15.0346524	13.2284052	15.2592059	59.5500584	59.8149367	63.74174	1.451E-08	1.05421	up	hypothetical protein PRUPE_ppa013245mg [Prunus persica]
c34877.graph_c0	83.1645899	91.8660379	79.3284569	13.8382486	11.6206892	10.2109384	2.428E-82	-3.846077	down	hypothetical protein MIMGU_mgv1a003677mg [Erythranthe guttata]
c34877.graph_c1	109.843143	110.3038	98.8369625	14.8457096	14.3976325	12.9224574	1.98E-149	-3.934171	down	PREDICTED: uncharacterized protein LOC105162623 [Sesamum indicum]
c34882.graph_c0	85.8428415	82.1328394	91.1382003	409.85197	380.743784	378.376346	2.019E-14	1.156828	up	PREDICTED: probable galactinol--sucrose galactosyltransferase 2 [Sesamum indicum]
c34882.graph_c1	3.1976442	1.82905495	2.935071	18.0961102	15.6040048	17.2251921	0.0022024	1.660994	up	-
c34898.graph_c0	6.39533017	6.66847304	5.33208047	2.24449344	3.15797398	4.30632615	1.825E-12	-1.945617	down	PREDICTED: probable trehalose-phosphate phosphatase F [Sesamum indicum]
c34900.graph_c0	0.2563308	0.59870412	0.47579311	26.501127	34.0232379	38.1724639	2.858E-52	5.187358	up	PREDICTED: sulfate transporter 3.1 [Sesamum indicum]

#ID	0 h-1 FPKM	0 h-2 FPKM	0 h-3 FPKM	48 h-1 FPKM	48 h-2 FPKM	48 h-3 FPKM	FDR	log2FC	regulated	nr_annotation
c34901.graph_c0	0	0	0	2.38297703	2.17627174	2.12035864	1.612E-11	Inf	up	PREDICTED: stress enhanced protein 2, chloroplastic [Sesamum indicum]
c34902.graph_c0	1487.49793	1517.3602	1443.9736	73.3301809	65.2738727	64.8508957	5.93E-202	-5.465308	down	PREDICTED: desiccation-related protein PCC3-06 isoform X2 [Sesamum indicum]
c34906.graph_c0	0.08028496	0.24109606	0.05158468	4.14196947	3.95695853	4.54106192	1.236E-12	4.072736	up	PREDICTED: manganese-dependent ADP-ribose/CDP-alcohol diphosphatase-like [Sesamum indicum]
c34907.graph_c1	0.2750603	0.32122501	0.05891056	12.0366655	11.5433614	9.92098348	1.947E-25	4.672392	up	PREDICTED: ethylene-responsive transcription factor RAP2-12-like [Sesamum indicum]
c34910.graph_c0	1.54666663	2.22135251	1.98752786	7.4961614	8.53194733	10.4736155	0.0022302	1.180515	up	PREDICTED: uncharacterized protein LOC105173417 [Sesamum indicum]
c34912.graph_c0	13.7709284	13.806392	13.2164927	68.7195027	71.0248866	74.2274048	1.29E-22	1.373065	up	PREDICTED: probable cytosolic oligopeptidase A [Sesamum indicum]
c34914.graph_c0	5.08203703	3.8463635	6.84918556	19.4127726	26.2512287	27.5357813	0.0001834	1.186112	up	PREDICTED: uncharacterized protein LOC105169952 [Sesamum indicum]
c34917.graph_c0	0.6557594	0.82052005	0.80054295	6.56989559	7.48800832	6.84032064	2.612E-17	2.177794	up	PREDICTED: 125 kDa kinesin-related protein-like [Sesamum indicum]
c34918.graph_c0	22.0498627	19.7591648	21.4287068	11.7532985	14.1178899	13.0931045	6.595E-22	-1.71907	down	PREDICTED: putative quinone-oxidoreductase homolog, chloroplastic [Sesamum indicum]
c34919.graph_c0	1.53153767	2.08497692	2.67659769	0	0.05978932	0.05021828	2.512E-16	-6.890368	down	PREDICTED: 3-oxoacyl-[acyl-carrier-protein] reductase, chloroplastic-like isoform X2 [Sesamum indicum]
c34925.graph_c0	23.363432	23.2810042	20.812279	15.4855334	12.4637071	9.06463237	4.687E-38	-1.875209	down	PREDICTED: probable serine/threonine-protein kinase At1g54610 [Sesamum indicum]
c34927.graph_c0	0.94727966	1.65939938	1.21729187	6.98156647	6.93386543	8.05638964	0.0026747	1.503887	up	-
c34929.graph_c0	0.59966604	0.60026651	0.32446088	8.43215397	8.90204177	7.73572789	3.472E-17	3.028167	up	hypothetical protein CISIN_1g018827mg [Citrus sinensis]
c34938.graph_c0	81.6703747	76.036959	88.8084703	12.8525601	12.3074561	12.3043542	8.614E-97	-3.735869	down	unnamed protein product [Coffea canephora]
c34943.graph_c0	2.12533473	2.3638477	1.5172996	1.0877761	2.12035505	2.09065672	0.0047084	-1.205741	down	PREDICTED: uncharacterized protein LOC104095569 [Nicotiana tomentosiformis]
c34944.graph_c0	0.09559642	0	0	1.27701009	1.44613647	1.9982783	3.056E-09	4.625199	up	PREDICTED: AP2-like ethylene-responsive transcription factor ANT isoform X2 [Sesamum indicum]
c34945.graph_c0	33.1832292	35.9700245	33.9796575	19.4519141	20.2683325	19.2584155	7.32E-37	-1.823999	down	chloroplast glyceraldehyde-3-phosphate dehydrogenase, partial [Chlorokybus atmophyticus]
c34946.graph_c0	55.2857926	61.0010436	56.0877097	58.7616758	52.1500293	57.4628646	7.61E-14	-1.049502	down	PREDICTED: zinc finger CCCH domain-containing protein 49-like [Sesamum indicum]
c34949.graph_c0	33.3360881	36.594438	36.7430945	8.69812203	8.90890777	8.80326447	1.025E-75	-3.032508	down	PREDICTED: fructose-1,6-bisphosphatase, chloroplastic [Sesamum indicum]
c34951.graph_c0	0	0	0	2.10064361	3.36174001	3.45989755	1.726E-14	Inf	up	PREDICTED: uncharacterized protein LOC105174266 [Sesamum indicum]

#ID	0 h-1 FPKM	0 h-2 FPKM	0 h-3 FPKM	48 h-1 FPKM	48 h-2 FPKM	48 h-3 FPKM	FDR	log2FC	regulated	nr_annotation
c34953.graph_c0	0	0.84859333	0.15562645	95.8395472	69.6174543	54.7006711	1.048E-17	6.772158	up	-
c34956.graph_c0	19.5573359	19.1571416	15.1869489	5.05754203	4.76253557	4.06265391	1.41E-48	-2.969462	down	PREDICTED: receptor-like serine/threonine-protein kinase At4g25390 [Sesamum indicum]
c34956.graph_c1	15.6560784	16.5783861	20.9500311	3.6952514	2.77815346	3.50014231	1.467E-23	-3.431638	down	PREDICTED: receptor-like serine/threonine-protein kinase At4g25390 [Sesamum indicum]
c34957.graph_c0	2.76577343	2.16173901	3.01857477	15.7767249	21.0028393	20.8396493	2.92E-16	1.833669	up	PREDICTED: uncharacterized protein LOC105178545 [Sesamum indicum]
c34960.graph_c0	2.27331914	2.45064134	2.24715777	1.66472374	1.99113075	1.57682641	0.001348	-1.432623	down	PREDICTED: plasma membrane ATPase 4-like [Sesamum indicum]
c34964.graph_c0	21.4608283	23.542867	16.2091185	8.23123965	13.3792162	11.2015762	4.142E-13	-1.921417	down	PREDICTED: uncharacterized protein LOC105159829 [Sesamum indicum]
c34966.graph_c0	0	0.36110195	0	551.680883	619.64617	490.882332	3.16E-127	11.16211	up	-
c34970.graph_c0	4.15548272	4.51282111	4.886567	4.11723016	3.74953683	3.08504138	1.554E-05	-1.323154	down	PREDICTED: uncharacterized protein LOC105172443 [Sesamum indicum]
c34973.graph_c0	2.30438953	1.99214743	2.82663033	0.09649791	0.10222783	0.17172651	7.23E-15	-5.290763	down	-
c34974.graph_c0	3.94865975	3.88987384	4.10767286	12.1258764	19.5950885	21.6475688	0.0005744	1.132458	up	PREDICTED: protein HOTHEAD isoform X3 [Sesamum indicum]
c34977.graph_c0	5.2267147	4.60520464	3.46321353	16.050635	18.4118206	19.4477383	2.439E-10	1.003057	up	PREDICTED: coiled-coil domain-containing protein 150 [Sesamum indicum]
c34978.graph_c0	20.9348545	20.5897771	21.8506277	10.1064909	10.4389366	12.4398945	7.897E-17	-1.962047	down	PREDICTED: uncharacterized protein LOC104117218 isoform X2 [Nicotiana tomentosiformis]
c34985.graph_c0	16.7360592	19.8950562	16.2691648	15.1786859	15.8599354	15.0413114	2.408E-17	-1.216012	down	PREDICTED: uncharacterized protein LOC105167921 [Sesamum indicum]
c34990.graph_c0	0	0	0	24.5619131	34.5672051	30.469439	1.483E-25	Inf	up	PREDICTED: nuclear valosin-containing protein-like [Sesamum indicum]
c34990.graph_c1	0.4372602	0.21884903	0.09364945	25.5127326	40.5651784	42.6342139	4.071E-31	6.165614	up	PREDICTED: nuclear valosin-containing protein-like [Sesamum indicum]
c34991.graph_c0	0	0	0	0.93655735	1.19060274	0.81250952	5.481E-12	Inf	up	--
c34993.graph_c0	4.21012724	3.77837652	3.17148493	0.40123902	0	0	6.423E-17	-5.755472	down	-
c34999.graph_c1	0	0	0	69.7075237	186.40565	274.754008	1.236E-12	Inf	up	PREDICTED: probable chalcone--flavonone isomerase 3 isoform X2 [Pyrus x bretschneideri]
c35003.graph_c0	0.92955856	1.24065249	0.72998417	4.4721543	6.70334885	7.28126503	0.0001228	1.648238	up	PREDICTED: uncharacterized protein LOC105158948 [Sesamum indicum]
c35005.graph_c0	11.5890668	10.9424774	11.3013322	90.448254	103.078577	112.551161	1.778E-48	2.156918	up	PREDICTED: GDSL esterase/lipase At4g01130-like [Sesamum indicum]
c35010.graph_c0	2.94554705	2.39217646	3.49947328	1.84322042	2.11539091	2.09566601	0.0002745	-1.568374	down	PREDICTED: uncharacterized aarF domain-containing protein kinase 1-like [Sesamum indicum]

#ID	0 h-1 FPKM	0 h-2 FPKM	0 h-3 FPKM	48 h-1 FPKM	48 h-2 FPKM	48 h-3 FPKM	FDR	log2FC	regulated	nr_annotation
c35017.graph_c0	27.1286185	25.4474251	21.7483644	19.3259045	19.5365318	25.9689858	1.301E-15	-1.214946	down	PREDICTED: beta-carotene hydroxylase 2, chloroplastic isoform X1 [Sesamum indicum]
c35018.graph_c0	1.29171394	1.12343266	1.22451902	9.09090473	12.0487224	13.5048652	2.085E-16	2.227441	up	PREDICTED: uncharacterized protein At5g41620 [Sesamum indicum]
c35023.graph_c0	5.10816099	6.3243151	4.70721456	3.83908167	4.65741851	5.31682593	1.274E-06	-1.24484	down	PREDICTED: uncharacterized protein LOC105166163 [Sesamum indicum]
c35025.graph_c0	62.1968559	59.6482696	53.3695561	53.0485249	57.5691743	57.0703264	1.331E-08	-1.080775	down	-
c35026.graph_c0	1.62000661	1.49189849	1.08252177	29.1924345	32.4436774	35.5366954	1.427E-36	3.517754	up	hypothetical protein MIMGU_mgv1a011760mg [Erythranthe guttata]
c35027.graph_c0	9.7547834	10.7485759	11.401601	0.20899402	0.19680339	0.10331201	1.25E-140	-6.982746	down	PREDICTED: uncharacterized protein LOC104748542 [Camelina sativa]
c35028.graph_c0	0.63124987	0.47391148	0.69529806	9.61632071	12.7616608	12.8994988	1.348E-35	3.267423	up	PREDICTED: subtilisin-like protease [Sesamum indicum]
c35029.graph_c0	2.46838058	2.05496626	1.94714792	8.21804074	10.8050028	10.1772069	1.287E-06	1.153854	up	PREDICTED: beta-galactosidase 16 [Sesamum indicum]
c35030.graph_c0	6.9167932	8.48713982	6.88130563	0.10277744	0	0	1.072E-31	-8.719894	down	-
c35031.graph_c0	2.23440318	1.84611605	1.64074089	7.64577525	9.24204728	9.33254664	9.476E-05	1.177856	up	PREDICTED: F-box/LRR-repeat protein At1g67190-like [Sesamum indicum]
c35033.graph_c0	9.23747792	6.88479194	7.74164664	31.7743742	32.4851631	32.7172554	7.42E-08	1.00582	up	hypothetical protein MIMGU_mgv1a011835mg [Erythranthe guttata]
c35039.graph_c0	4.87328522	4.1584358	2.87452794	41.1422919	44.5209103	43.6154511	2.923E-24	2.426735	up	PREDICTED: acyl carrier protein 1, chloroplastic-like [Sesamum indicum]
c35040.graph_c0	16.60546	16.7051983	14.7236847	8.87285905	8.91352595	8.43950053	5.542E-14	-1.888848	down	PREDICTED: serine/threonine-protein kinase tricorner [Sesamum indicum]
c35042.graph_c0	0.82717687	0.50748703	0.61719969	0	0	0	7.881E-20	-Inf	down	-
c35055.graph_c0	13.9821822	13.3631901	13.2274465	102.079434	114.998156	122.882114	2.517E-46	2.047042	up	hypothetical protein MIMGU_mgv1a005945mg [Erythranthe guttata]
c35065.graph_c0	0.57527114	0.69101663	0.59139696	10.4140539	14.3168905	15.5854088	1.137E-24	3.416433	up	PREDICTED: cytokinin dehydrogenase 7 [Sesamum indicum]
c35069.graph_c0	0.85332483	0.64063448	0.65108024	2.6532174	3.09704345	4.24072664	0.0026844	1.198887	up	PREDICTED: probable leucine-rich repeat receptor-like protein kinase At1g68400 [Sesamum indicum]
c35070.graph_c0	6.84740432	7.77862694	5.79895441	3.93263993	2.92481096	4.57043508	7.016E-18	-1.851194	down	PREDICTED: LOW QUALITY PROTEIN: uncharacterized protein LOC105172331 [Sesamum indicum]
c35074.graph_c0	0.64938514	0.61753363	1.33517699	12.6830261	12.8974156	11.0723506	1.901E-22	2.790822	up	PREDICTED: protease Do-like 1, chloroplastic isoform X2 [Cucumis melo]
c35077.graph_c0	72.9451706	30.0316848	42.7110074	0	0	0	7.132E-15	-Inf	down	hypothetical protein ASPNIDRAFT_138440 [Aspergillus niger ATCC 1015]
c35089.graph_c0	10.1193128	10.5030728	12.2576026	1.68111935	2.26665359	3.8076179	1.361E-36	-3.112397	down	PREDICTED: uncharacterized protein LOC105179874 [Sesamum indicum]

#ID	0 h-1 FPKM	0 h-2 FPKM	0 h-3 FPKM	48 h-1 FPKM	48 h-2 FPKM	48 h-3 FPKM	FDR	log2FC	regulated	nr_annotation
c35092.graph_c0	2.72584703	1.50269433	1.3199037	0	0	0	3.335E-13	-Inf	down	Chain G, Crystal Structure Of The Fab Complexed With Antigen Asf1p, Northeast Structural Genomics Consortium (nesg) Target Pdr16
c35093.graph_c0	18.5941435	19.473137	15.7944782	7.89200495	10.4298064	11.6724498	3.947E-31	-1.865994	down	PREDICTED: U-box domain-containing protein 17 isoform X1 [Sesamum indicum]
c35093.graph_c1	6.2623213	6.52620544	7.60638117	3.63542338	4.18618506	5.76634315	2.047E-05	-1.61038	down	PREDICTED: U-box domain-containing protein 17 isoform X1 [Sesamum indicum]
c35096.graph_c0	3.29030414	2.84447176	3.55550751	0	0.07298259	0.18389875	5.833E-21	-6.283051	down	--
c35103.graph_c0	1.17678102	1.09381943	1.26017517	8.64720691	9.98102545	8.49810777	1.297E-11	1.921585	up	hypothetical protein MIMGU_mgv1a002945mg [Erythranthe guttata]
c35104.graph_c1	0.51505245	0	0	4.2704992	3.51872583	6.75531467	1.713E-05	3.821334	up	PREDICTED: uncharacterized protein LOC102597032 [Solanum tuberosum]
c35106.graph_c0	6.50055684	6.26783579	6.69506285	30.5601724	35.4536683	34.3625504	4.617E-13	1.346112	up	hypothetical protein MIMGU_mgv1a011325mg [Erythranthe guttata]
c35108.graph_c0	7.24279257	7.67296443	7.91118392	1.97395766	2.12062187	3.36440182	6.34E-28	-2.636877	down	PREDICTED: ethylene-responsive transcription factor RAP2-11-like [Sesamum indicum]
c35113.graph_c0	249.308429	270.460022	251.555118	22.6409572	21.2095962	19.3885769	6.92E-167	-4.62365	down	hypothetical protein MIMGU_mgv1a002687mg [Erythranthe guttata]
c35115.graph_c0	71.904909	78.0509118	149.322577	37.1746179	25.7611579	31.834227	0.0011051	-2.679113	down	hypothetical protein EUGRSUZ_J02069 [Eucalyptus grandis]
c35119.graph_c0	0.03961242	0	0	1.20428695	0.96651211	0.68190645	1.387E-07	5.181284	up	hypothetical protein EUTSA_v10024694mg [Eutrema salsugineum]
c35120.graph_c0	9.50994623	9.60761223	10.9759608	50.0117515	43.3562198	44.8971564	2.34E-13	1.18348	up	PREDICTED: uncharacterized protein LOC105156523 [Sesamum indicum]
c35121.graph_c0	1.86752454	1.38172643	1.90422323	8.19100664	11.4905923	13.1122992	4.672E-08	1.64404	up	PREDICTED: LETM1 and EF-hand domain-containing protein 1, mitochondrial [Sesamum indicum]
c35127.graph_c0	0.67464013	0.8441446	1.1378578	3.84567042	4.1974767	4.42421581	0.0037495	1.206032	up	PREDICTED: uncharacterized protein LOC105162824 isoform X1 [Sesamum indicum]
c35128.graph_c0	0	0	0.1012367	38.8293343	51.514824	61.2215001	7.115E-45	9.491773	up	PREDICTED: anthocyanidin 3-O-glucosyltransferase 2-like [Sesamum indicum]
c35134.graph_c0	1.17905054	0.56651097	1.09089117	5.95247607	7.54870048	7.77074423	1.663E-06	1.883263	up	PREDICTED: protein-ribulosamine 3-kinase, chloroplastic [Sesamum indicum]
c35135.graph_c0	2.91442428	2.56852993	2.40178136	15.9346405	14.6547773	14.3862916	4.221E-11	1.498015	up	PREDICTED: cytochrome b561 and DOMON domain-containing protein At3g61750 [Sesamum indicum]
c35136.graph_c0	62.3449402	64.945192	60.1238094	48.8367166	50.2744587	46.9498794	1.736E-17	-1.376563	down	hypothetical protein MIMGU_mgv1a007434mg [Erythranthe guttata]
c35139.graph_c0	0.81062676	0.44260281	0.37879545	4.41292236	6.83262821	6.40336505	3.211E-06	2.416061	up	PREDICTED: thaumatin-like protein 1 [Sesamum indicum]

#ID	0 h-1 FPKM	0 h-2 FPKM	0 h-3 FPKM	48 h-1 FPKM	48 h-2 FPKM	48 h-3 FPKM	FDR	log2FC	regulated	nr_annotation
c35141.graph_c1	0.19627074	0	0	35.9826649	26.626023	30.4082564	1.649E-41	7.897298	up	hypothetical protein MIMGU_mgv1a011475mg [Erythranthe guttata]
c35143.graph_c0	15.1794938	14.7319112	16.3377225	129.905288	107.613469	119.252419	1.486E-23	1.932028	up	PREDICTED: CBL-interacting serine/threonine-protein kinase 6-like [Sesamum indicum]
c35152.graph_c0	1.1013109	0.91867808	0.7665819	6.63717132	8.66593844	9.64850912	1.731E-14	2.142676	up	PREDICTED: AP-3 complex subunit mu [Sesamum indicum]
c35154.graph_c0	0.39700398	0.50578376	0.41740841	11.8368694	12.0818164	11.8933001	2.505E-27	3.744382	up	PREDICTED: transcription factor bHLH66-like [Sesamum indicum]
c35156.graph_c0	245.809332	239.388072	248.401916	46.0222516	22.544863	18.1807816	4.99E-158	-4.080896	down	hypothetical protein MIMGU_mgv1a014857mg [Erythranthe guttata]
c35156.graph_c1	276.095132	240.603564	258.441988	57.9228456	32.2056432	23.3687961	1.45E-128	-3.774075	down	Lactoylglutathione lyase / glyoxalase I family protein [Theobroma cacao]
c35160.graph_c0	0	0.02678341	0.03438331	0.7394982	0.5744997	0.74573453	1.089E-08	4.045602	up	PREDICTED: DNA cross-link repair protein SNM1 [Sesamum indicum]
c35164.graph_c0	0	0	0.06393933	1.1001389	1.40826872	1.63149496	5.749E-10	4.961306	up	PREDICTED: uncharacterized protein LOC105166066 [Sesamum indicum]
c35178.graph_c0	3.25823607	2.85381136	2.70407985	9.63045284	11.9910114	14.6899183	0.0027577	1.020794	up	PREDICTED: NEDD8-specific protease 1 [Sesamum indicum]
c35179.graph_c0	8.00535723	7.89943917	8.82456578	34.9528779	37.5467326	29.8257253	6.978E-10	1.030598	up	hypothetical protein JCGZ_23362 [Jatropha curcas]
c35180.graph_c0	25.9415548	26.092676	27.5523428	20.7892961	22.6338142	26.1331779	2.883E-12	-1.215117	down	PREDICTED: protein ABSCISIC ACID-INSENSITIVE 5 [Sesamum indicum]
c35183.graph_c0	16.2801119	17.9519227	19.0609805	18.0455748	16.1915266	15.7602795	1.556E-09	-1.108496	down	PREDICTED: NADPH-dependent pterin aldehyde reductase isoform X2 [Sesamum indicum]
c35189.graph_c0	0.19264081	0.23140045	0.14853064	9.1576212	9.40057097	12.6963384	9.766E-29	4.753459	up	PREDICTED: BTB/POZ and TAZ domain-containing protein 3 [Sesamum indicum]
c35192.graph_c0	8.13406141	6.40454043	6.88341356	113.455354	123.532207	134.046874	5.843E-87	3.095705	up	Triosephosphate isomerase, chloroplastic [Gossypium arboreum]
c35195.graph_c0	0	0	0	7.39987759	8.34685186	6.20541023	3.054E-22	Inf	up	PREDICTED: transcription factor bHLH118-like [Sesamum indicum]
c35198.graph_c0	18.1620311	16.7568554	17.6495459	1.48861634	1.7977897	2.56965002	2.82E-100	-4.189756	down	PREDICTED: uncharacterized protein LOC105165319 [Sesamum indicum]
c35200.graph_c0	0.07197531	0.14409477	0.09249112	20.0914303	25.2884367	27.9073651	4.761E-45	6.870021	up	PREDICTED: probable flavin-containing monooxygenase 1 [Sesamum indicum]
c35204.graph_c0	7.45130019	7.97179805	7.9540152	4.46745355	4.34795091	4.13670328	8.387E-14	-1.868853	down	hypothetical protein MIMGU_mgv1a009915mg [Erythranthe guttata]
c35207.graph_c0	0.65666315	1.18317726	1.18137341	12.17988	11.7067915	10.6222677	6.182E-14	2.494223	up	PREDICTED: nitrilase-like protein 2 [Sesamum indicum]
c35208.graph_c0	1.61861697	1.41117483	0.40257811	6.59003379	8.00051569	6.71979761	0.0001394	1.625365	up	PREDICTED: uncharacterized protein LOC105165447 [Sesamum indicum]
c35209.graph_c0	7.45048521	7.14934109	6.40478856	36.7808915	35.5046917	35.5073511	1.023E-12	1.344226	up	PREDICTED: protein bem46-like isoform X2 [Sesamum indicum]

#ID	0 h-1 FPKM	0 h-2 FPKM	0 h-3 FPKM	48 h-1 FPKM	48 h-2 FPKM	48 h-3 FPKM	FDR	log2FC	regulated	nr_annotation
c35210.graph_c0	1.1140593	0.84391611	0.96730426	4.88206918	4.73116824	4.73894814	0.0005021	1.278379	up	PREDICTED: uncharacterized protein LOC105169721 [Sesamum indicum]
c35212.graph_c0	1.70538173	1.60954144	1.37750362	13.0177593	17.7852959	16.0166645	2.049E-14	2.298295	up	PREDICTED: cytokinin riboside 5'-monophosphate phosphoribohydrolase LOG8-like [Sesamum indicum]
c35214.graph_c0	70.4413124	66.5048305	74.8131162	54.1538684	21.3736787	15.7380354	4.338E-16	-2.210415	down	hypothetical protein MIMGU_mgv1a007518mg [Erythranthe guttata]
c35218.graph_c0	0.08753076	0.05257105	0.1124805	4.01582459	4.20302284	4.11857284	5.21E-25	4.587162	up	PREDICTED: probable disease resistance protein At5g63020 [Vitis vinifera]
c35223.graph_c0	5.76352858	4.9039049	5.18445369	25.7522315	19.6875842	21.2605801	0.0004576	1.06093	up	PREDICTED: uncharacterized protein LOC105165107 isoform X2 [Sesamum indicum]
c35224.graph_c0	18.8442804	19.8967474	19.2766866	16.7528278	17.327694	14.7419819	2.022E-16	-1.265847	down	PREDICTED: ruvB-like 2 [Sesamum indicum]
c35227.graph_c0	9.04413256	9.11649791	8.12732057	71.7837708	77.2189913	78.8559087	4.524E-35	2.097872	up	PREDICTED: V-type proton ATPase subunit C-like [Sesamum indicum]
c35231.graph_c0	0.09021589	0.04515311	0	1.20513503	1.58486206	1.62697117	2.806E-08	4.023531	up	PREDICTED: transcription factor TGA4 isoform X1 [Sesamum indicum]
c35234.graph_c0	84.8779191	86.3763098	78.860794	32.9918502	33.5039093	33.0019328	4.455E-57	-2.346594	down	PREDICTED: uncharacterized protein LOC105155881 [Sesamum indicum]
c35235.graph_c0	10.3705674	12.5182067	9.82509356	3.05354711	3.25470868	3.23376321	6.107E-42	-2.794814	down	PREDICTED: uncharacterized protein LOC105164201 isoform X1 [Sesamum indicum]
c35239.graph_c0	3.06959217	2.9702437	2.80501153	39.8735101	44.1717926	49.4863679	3.322E-60	2.896834	up	PREDICTED: ran-binding protein 1 homolog c-like [Sesamum indicum]
c35240.graph_c0	17.5115622	19.7327554	17.616674	17.239042	16.7000929	13.6166124	3.761E-07	-1.220998	down	PREDICTED: disease resistance protein RPM1-like [Sesamum indicum]
c35243.graph_c0	0	0	0	12.8469974	11.2101168	13.3603728	8.984E-53	Inf	up	PREDICTED: lycopene epsilon cyclase, chloroplastic [Sesamum indicum]
c35244.graph_c0	6.79262366	6.71750466	6.62546736	0.94244096	2.11661207	2.71699368	2.611E-21	-2.833103	down	PREDICTED: zinc finger CCCH domain-containing protein 39-like isoform X2 [Sesamum indicum]
c35245.graph_c0	6.36424758	5.67467028	7.42233187	46.1662261	47.7592097	49.2327399	2.316E-25	1.858438	up	PREDICTED: alpha-soluble NSF attachment protein 2-like isoform X1 [Sesamum indicum]
c35247.graph_c0	0	0	0	3.38380649	3.68294452	3.21711606	2.959E-15	Inf	up	PREDICTED: protein TRANSPARENT TESTA 12-like [Sesamum indicum]
c35253.graph_c0	3.74979124	4.56200217	4.78156382	35.5287414	35.52704	38.8013167	1.868E-35	2.048753	up	PREDICTED: argininosuccinate synthase, chloroplastic-like isoform X2 [Sesamum indicum]
c35256.graph_c0	3.88442926	3.78322923	2.02364002	0.87046774	1.53692504	1.3769546	2.156E-06	-2.377168	down	PREDICTED: uncharacterized protein LOC105170847 [Sesamum indicum]
c35258.graph_c0	143.807394	142.294968	135.535553	45.2625985	28.0703058	25.4177573	6.52E-99	-3.100234	down	PREDICTED: zinc finger protein 622 [Sesamum indicum]

#ID	0 h-1 FPKM	0 h-2 FPKM	0 h-3 FPKM	48 h-1 FPKM	48 h-2 FPKM	48 h-3 FPKM	FDR	log2FC	regulated	nr_annotation
c35265.graph_c0	0.14203193	0.3317397	0.06083889	2.0063526	3.09581845	3.18238519	6.42E-07	2.937673	up	PREDICTED: auxin response factor 4 isoform X2 [Sesamum indicum]
c35265.graph_c1	0.23165513	0.40580243	0.14884301	3.52136102	5.31307229	5.26961712	1.574E-08	3.146176	up	PREDICTED: auxin response factor 4 isoform X2 [Sesamum indicum]
c35272.graph_c0	0.23412227	0.14061403	0.15042819	4.70201384	5.23255915	5.04745696	4.726E-19	3.818288	up	PREDICTED: interactor of constitutive active ROPs 2, chloroplastic-like [Sesamum indicum]
c35275.graph_c0	0.14554898	0.43708418	0.35329061	6.19790149	6.42387383	7.27801841	7.727E-30	3.387496	up	PREDICTED: serine/threonine-protein kinase Nek5 [Sesamum indicum]
c35277.graph_c0	3.37189249	3.28644606	3.99093519	2.04368901	1.5588293	2.69132404	0.0001552	-1.777859	down	-
c35281.graph_c0	7.87877539	7.18666496	6.47011915	4.85327764	4.45896459	6.38208569	4.384E-08	-1.47351	down	PREDICTED: uncharacterized protein LOC105158079 isoform X1 [Sesamum indicum]
c35283.graph_c0	67.2061862	68.4781565	65.4847995	43.8981271	42.6874632	39.9543293	8.344E-33	-1.684816	down	PREDICTED: DNA-damage-repair/toleration protein DRT111, chloroplastic-like [Sesamum indicum]
c35285.graph_c0	8.86805911	8.59751974	9.93339114	6.34991977	6.64314486	6.16056745	4.33E-18	-1.535612	down	-
c35288.graph_c0	4.17401583	4.35156458	4.13967743	47.58054	47.7350822	44.8640294	5.469E-61	2.45158	up	PREDICTED: valine--tRNA ligase [Sesamum indicum]
c35288.graph_c1	148.275141	158.617944	134.860455	62.6212955	60.7150516	62.4828227	1.818E-46	-2.265111	down	PREDICTED: protein TIME FOR COFFEE [Sesamum indicum]
c35290.graph_c0	14.9940452	14.9224687	12.8946995	10.014782	10.1591789	10.0929354	6.05E-20	-1.516299	down	PREDICTED: protein FRIGIDA [Sesamum indicum]
c35292.graph_c0	586.521041	592.817883	629.515466	38.6342039	34.6656621	32.7825187	1.01E-198	-5.107383	down	hypothetical protein MIMGU_mgv1a0189502mg, partial [Erythranthe guttata]
c35295.graph_c1	0.04021615	0.12076927	0.25839673	4.92761567	5.73045752	8.67022567	2.152E-12	4.48817	up	PREDICTED: ras-related protein RABA6a-like [Sesamum indicum]
c35297.graph_c0	0.53101166	1.06308677	0.25588913	5.56467926	8.09766886	8.76020133	8.644E-08	2.582918	up	PREDICTED: MATE efflux family protein 9 [Sesamum indicum]
c35299.graph_c0	3.58774883	4.33805597	3.97133404	34.1980723	39.1408752	37.1265836	4.327E-35	2.194991	up	PREDICTED: ammonium transporter 2 [Sesamum indicum]
c35304.graph_c0	1.38373812	1.58299854	1.27011305	0.45528207	0.627011	0.68868233	4.729E-06	-2.280903	down	PREDICTED: uncharacterized protein LOC105174699 [Sesamum indicum]
c35306.graph_c0	7.0319596	7.12717225	7.20643917	5.27460739	5.48751298	4.70534808	1.111E-18	-1.483304	down	PREDICTED: uncharacterized protein LOC105157223 [Sesamum indicum]
c35309.graph_c0	2.80381689	2.71094411	2.57943171	20.6057661	21.3006806	21.8347085	6.256E-22	1.959798	up	PREDICTED: WD-40 repeat-containing protein MSI4-like [Sesamum indicum]
c35310.graph_c0	1.25281019	1.07491259	1.30326104	7.63950927	11.1207809	10.7587746	2.365E-07	1.99818	up	PREDICTED: uncharacterized protein LOC105164773 [Sesamum indicum]
c35323.graph_c0	0.7032665	0.4399817	0.53658689	6.31697596	6.8636616	6.80982414	5.984E-16	2.555406	up	PREDICTED: probable thimet oligopeptidase isoform X2 [Sesamum indicum]
c35324.graph_c0	5.71594504	2.83358831	3.56767619	5294.48772	6083.47243	6133.63085	2.67E-294	9.479979	up	PREDICTED: vignain-like [Sesamum indicum]
c35328.graph_c0	1.31134471	0.99761995	1.01107792	11.6702101	8.11413185	8.12667346	5.719E-07	2.063401	up	PREDICTED: uncharacterized protein LOC105175059 [Sesamum indicum]

#ID	0 h-1 FPKM	0 h-2 FPKM	0 h-3 FPKM	48 h-1 FPKM	48 h-2 FPKM	48 h-3 FPKM	FDR	log2FC	regulated	nr_annotation
c35329.graph_c0	16.4931436	17.5116904	20.0304914	4.87455528	5.44313539	6.83816437	1.609E-36	-2.678369	down	PREDICTED: chromo domain protein LHP1 isoform X3 [Sesamum indicum]
c35338.graph_c0	0	0	0	4.77653716	8.71750669	11.1513418	7.6E-13	Inf	up	PREDICTED: dehydrodolichyl diphosphate synthase 2-like [Nicotiana sylvestris]
c35341.graph_c0	0.26674821	0.13350766	0.25708651	4.30055241	4.71862502	5.63057692	2.126E-13	3.455682	up	PREDICTED: transcription factor bHLH79-like [Sesamum indicum]
c35344.graph_c0	30.7258265	29.9497374	29.1998387	28.3773578	28.320438	27.9401895	3.875E-15	-1.103435	down	PREDICTED: microfibrillar-associated protein 1-like [Sesamum indicum]
c35346.graph_c0	0	0	0	2.19493787	3.17082343	3.72853571	1.552E-11	Inf	up	hypothetical protein MIMGU_mgv1a024479mg [Erythranthe guttata]
c35347.graph_c0	6.50509292	6.28312936	6.5261217	3.73243007	4.51096653	5.3792365	1.487E-06	-1.526069	down	-
c35350.graph_c0	0	0	0	19.0565744	48.5317293	8.19747712	0.0001511	Inf	up	hypothetical protein EMIHUDRAFT_425447 [Emiliania huxleyi CCMP1516]
c35357.graph_c0	0	0	0	9.54692705	7.09598071	10.481485	4.376E-20	Inf	up	--
c35358.graph_c0	11.7751118	7.24052602	6.26875802	1.54971444	1.97008144	1.24103393	3.337E-08	-3.422889	down	ubiquitin-like protein [Vanilla planifolia]
c35359.graph_c0	1.1090211	0.44405265	0.78382481	10.0637704	12.3390179	10.3183396	1.597E-12	2.78878	up	PREDICTED: sphinganine C(4)-monooxygenase 1-like [Sesamum indicum]
c35363.graph_c0	4.96527998	4.18921236	4.14737788	42.083926	42.6444333	42.7373103	5.76E-39	2.244059	up	PREDICTED: F-box protein At4g00755-like [Sesamum indicum]
c35370.graph_c0	0.10760819	0.14362125	0.09218719	8.82307563	11.7274827	10.5558365	4.954E-34	5.481825	up	PREDICTED: protein STICHEL-like 2 [Sesamum indicum]
c35372.graph_c0	0.912464	1.3986096	0.4763494	13.1872475	14.3320701	13.9544987	3.089E-27	2.883568	up	PREDICTED: vacuolar amino acid transporter 1 isoform X2 [Sesamum indicum]
c35373.graph_c0	40.5765862	44.2626839	41.4219381	21.1774666	20.9224889	22.2100202	7.715E-44	-1.990826	down	PREDICTED: E3 ubiquitin-protein ligase KEG [Sesamum indicum]
c35374.graph_c0	0.05080182	0	0	6.36506538	7.68505382	8.8701922	8.172E-24	7.81686	up	PREDICTED: mitogen-activated protein kinase 19 isoform X2 [Sesamum indicum]
c35375.graph_c0	0.5971671	0.52304444	0.38369169	24.7796408	28.8008467	28.3309426	5.16E-99	4.751228	up	PREDICTED: uncharacterized protein At1g04910 [Sesamum indicum]
c35376.graph_c0	0	0	0	6.16763055	9.74541339	10.0456851	3.297E-16	Inf	up	PREDICTED: uncharacterized protein LOC105165561 [Sesamum indicum]
c35379.graph_c0	1.04975661	0.52540389	0.86720066	4.76644243	4.09811879	4.54848165	0.0038719	1.441942	up	PREDICTED: uncharacterized protein LOC105136996 isoform X1 [Populus euphratica]
c35381.graph_c0	0.04913738	0.09837318	0.03157174	2.01445109	1.58256629	2.31608252	5.197E-11	4.035603	up	PREDICTED: uncharacterized protein LOC105165535 [Sesamum indicum]
c35381.graph_c1	0	0.08249995	0.05295483	1.74635102	1.64895507	2.1281573	1.463E-09	4.325566	up	PREDICTED: uncharacterized protein LOC105165535 [Sesamum indicum]
c35382.graph_c0	0.0278963	0.02792424	0	4.54888102	5.60853954	8.00366351	3.723E-19	7.338434	up	PREDICTED: bidirectional sugar transporter SWEET3b [Sesamum indicum]

#ID	0 h-1 FPKM	0 h-2 FPKM	0 h-3 FPKM	48 h-1 FPKM	48 h-2 FPKM	48 h-3 FPKM	FDR	log2FC	regulated	nr_annotation
c35387.graph_c0	0.90459799	0.9531619	0.79535658	25.2205493	17.8895197	15.6502232	3.607E-10	3.461359	up	PREDICTED: transcription factor bHLH121 [Sesamum indicum]
c35389.graph_c0	9.09735913	9.86534115	7.79364444	5.41278122	8.13884376	6.52525573	1.548E-05	-1.435871	down	PREDICTED: protein ETHYLENE INSENSITIVE 3 [Sesamum indicum]
c35396.graph_c0	0.52541626	0.5976618	0.55242057	3.08030373	3.75268954	3.44562206	5.046E-05	1.596428	up	PREDICTED: ycf20-like protein [Sesamum indicum]
c35397.graph_c0	0.27248524	0.27275809	0.30013228	4.73373076	4.82485868	4.78641496	1.819E-10	3.066022	up	PREDICTED: 2-amino-3-ketobutyrate coenzyme A ligase, mitochondrial [Sesamum indicum]
c35400.graph_c0	0.35725835	0.22351005	0.05738639	2.01591936	2.22279037	2.26964656	5.475E-05	2.343911	up	PREDICTED: transcription factor bHLH68-like [Sesamum indicum]
c35407.graph_c0	0	0.09979194	0.25621664	21.4912342	23.837617	21.492687	2E-57	6.513419	up	PREDICTED: rac-like GTP-binding protein ARAC8 [Sesamum indicum]
c35410.graph_c0	0.03193067	0	0.12309654	0.91191518	0.59210347	0.8637661	1.041E-06	2.899188	up	PREDICTED: serine/threonine protein phosphatase 2A 57 kDa regulatory subunit B' beta isoform-like [Nicotiana tomentosiformis]
c35413.graph_c0	0	0.08767766	0	2.17872659	2.22261157	2.44122243	6.121E-10	5.279346	up	PREDICTED: inositol hexakisphosphate and diphosphoinositol-pentakisphosphate kinase 2-like isoform X2 [Sesamum indicum]
c35415.graph_c0	8.12513095	6.38612078	7.81150082	4.71303	4.52296533	4.24296253	1.502E-08	-1.744362	down	hypothetical protein MIMGU_mgv1a019839mg, partial [Erythranthe guttata]
c35424.graph_c0	1.96455968	1.71087839	2.29732864	1.26691636	1.47635869	1.86808813	3.304E-05	-1.397569	down	PREDICTED: transcription factor bHLH13-like [Sesamum indicum]
c35426.graph_c0	22.5116206	22.8495262	18.4758505	101.505852	122.767236	146.830486	2.461E-11	1.519227	up	PREDICTED: aspartic proteinase nepenthesin-1 [Sesamum indicum]
c35429.graph_c0	6.25550346	5.86732536	4.17752436	26.8636446	28.6029908	30.121148	3.038E-11	1.3774	up	hypothetical protein MIMGU_mgv1a011469mg [Erythranthe guttata]
c35430.graph_c0	4.42442609	4.19369597	4.07549185	0.39678557	0.15285316	0.19257677	7.604E-32	-5.095069	down	unnamed protein product [Coffea canephora]
c35431.graph_c0	1.88937126	2.30641851	2.25026441	0.76417114	0.58467259	0.56662907	4.442E-09	-2.763549	down	PREDICTED: cysteine-rich repeat secretory protein 3-like isoform X1 [Sesamum indicum]
c35434.graph_c1	1.22029238	4.6417544	5.01799686	15.2892959	20.0082504	16.4052137	0.0073722	1.219683	up	PREDICTED: coatomer subunit beta'-2-like isoform X1 [Solanum tuberosum]
c35440.graph_c0	1.77447552	1.42567626	1.23014636	0	0	0	3.419E-32	-Inf	down	predicted protein [Thalassiosira pseudonana CCMP1335]
c35441.graph_c0	5.69298877	4.73280987	4.3398315	0.26668233	0.65920773	0.23729234	1.904E-18	-4.6925	down	-
c35445.graph_c0	48.3048656	53.0593711	48.4506276	214.524854	217.816268	204.258332	7.389E-14	1.070324	up	PREDICTED: uncharacterized protein LOC105177185 [Sesamum indicum]
c35457.graph_c0	3.68143262	4.25605295	4.99730987	1.57636624	3.59296337	6.248129	0.0063567	-1.216028	down	PREDICTED: U6 snRNA-associated Sm-like protein LSm1 [Vitis vinifera]
c35463.graph_c0	131.951969	132.147419	134.691624	20.1050587	20.8049849	22.1931775	1.18E-130	-3.678639	down	PREDICTED: BTB/POZ and TAZ domain-containing protein 1 [Sesamum indicum]

#ID	0 h-1 FPKM	0 h-2 FPKM	0 h-3 FPKM	48 h-1 FPKM	48 h-2 FPKM	48 h-3 FPKM	FDR	log2FC	regulated	nr_annotation
c35467.graph_c0	18.3740197	20.9125335	19.6382803	13.1024164	13.8804214	13.9032463	4.853E-19	-1.546003	down	PREDICTED: common plant regulatory factor 1-like isoform X2 [Sesamum indicum]
c35468.graph_c0	1.35796447	1.50829131	1.60161032	14.7383436	16.1944549	16.9263248	4.882E-36	2.400198	up	PREDICTED: alpha,alpha-trehalose-phosphate synthase [UDP-forming] 5 isoform X1 [Sesamum indicum]
c35469.graph_c0	11.2656893	9.65232197	13.6180602	60.6889373	59.0746224	73.3313194	6.504E-15	1.461712	up	hypothetical protein MIMGU_mgv1a016480mg [Erythranthe guttata]
c35476.graph_c0	2.30957079	2.03445746	2.07752293	0.55320909	1.03687175	0.60583645	2.587E-08	-2.573101	down	hypothetical protein MIMGU_mgv1a012316mg [Erythranthe guttata]
c35479.graph_c0	1.70482551	1.80691691	1.54642467	11.8718615	12.185302	13.1119066	7.98E-09	1.860193	up	PREDICTED: glycine-rich RNA-binding protein 8-like [Sesamum indicum]
c35484.graph_c0	15.6273844	16.5143954	14.3821965	135.148175	152.397028	167.553727	3.549E-56	2.270836	up	PREDICTED: 60S ribosomal protein L4-1 [Sesamum indicum]
c35493.graph_c0	0	0	0	1.3360237	1.15539192	1.64974363	6.625E-13	Inf	up	PREDICTED: probable inactive purple acid phosphatase 27 isoform X1 [Sesamum indicum]
c35495.graph_c0	32.7029237	34.5576378	37.8834462	1.75930314	2.62092425	2.22582781	3.05E-137	-5.017471	down	PREDICTED: CBL-interacting protein kinase 18-like [Sesamum indicum]
c35500.graph_c0	7.25863693	5.95967518	6.39309872	132.014461	143.674008	147.350225	3.2E-87	3.412479	up	PREDICTED: nascent polypeptide-associated complex subunit alpha-like protein 2 [Vitis vinifera]
c35503.graph_c0	6.8383437	9.01761724	6.68274182	4.75323928	8.03279057	8.82804111	5.603E-05	-1.086216	down	hypothetical protein MIMGU_mgv1a013110mg [Erythranthe guttata]
c35504.graph_c0	58.6007934	60.1087073	63.167052	421.034097	426.992819	445.276541	4.278E-38	1.811291	up	PREDICTED: 40S ribosomal protein S8 [Sesamum indicum]
c35508.graph_c0	3.01749558	2.41641372	2.4898282	33.8564232	37.943762	35.5670529	4.14E-51	2.742415	up	PREDICTED: acyl-CoA-binding domain-containing protein 4 [Sesamum indicum]
c35509.graph_c0	1.3811396	0.81694517	0.72606235	6.13063472	5.51433745	5.50646419	0.0009583	1.542038	up	PREDICTED: uncharacterized protein LOC103335354 [Prunus mume]
c35510.graph_c0	0	0.32190479	0.2066233	3.25889329	5.64938555	6.59032649	5.16E-07	3.840454	up	PREDICTED: beta-glucosidase 24-like [Sesamum indicum]
c35510.graph_c1	0.24092456	0.48233162	0.4127968	83.4552386	90.9188109	110.201987	6.292E-89	6.945634	up	PREDICTED: NADPH--cytochrome P450 reductase-like [Sesamum indicum]
c35515.graph_c0	2.33709346	2.8898887	1.94328435	11.5253263	12.2096857	11.6074956	0.0004061	1.285863	up	PREDICTED: putative 1-phosphatidylinositol-3-phosphate 5-kinase FAB1D [Sesamum indicum]
c35517.graph_c0	10.8808782	10.0247669	10.6432884	3.09203399	3.01147097	3.70534382	1.153E-42	-2.704088	down	PREDICTED: putative lysine-specific demethylase JMJ16 isoform X1 [Sesamum indicum]
c35522.graph_c0	2.78382228	1.42728798	1.48327979	0	0	0	6.843E-12	-Inf	down	hypothetical protein SNOG_05978 [Phaeosphaeria nodorum SN15]
c35526.graph_c1	0	0.0675518	0	1.58535714	2.43691236	2.79362287	6.591E-13	5.642529	up	PREDICTED: L-ascorbate oxidase homolog [Sesamum indicum]
c35528.graph_c0	16.2079293	11.9416399	13.6385288	8.48914611	9.71589049	11.869929	9.815E-09	-1.495163	down	PREDICTED: protein N-terminal glutamine amidohydrolase [Sesamum indicum]

#ID	0 h-1 FPKM	0 h-2 FPKM	0 h-3 FPKM	48 h-1 FPKM	48 h-2 FPKM	48 h-3 FPKM	FDR	log2FC	regulated	nr_annotation
c35529.graph_c0	97.179447	102.059079	100.1361	85.2933853	83.3992324	80.0175453	1.459E-19	-1.284275	down	PREDICTED: uncharacterized protein LOC105166406 [Sesamum indicum]
c35533.graph_c0	55.4728921	48.070331	53.5441039	21.6591648	20.1343882	18.8102778	4.782E-61	-2.389674	down	hypothetical protein MIMGU_mgv1a026579mg [Erythranthe guttata]
c35537.graph_c0	2.37791456	1.45139981	3.57742169	16.1363019	17.6604968	14.0251834	7.42E-09	1.666512	up	hypothetical protein MIMGU_mgv1a013138mg [Erythranthe guttata]
c35540.graph_c0	41.3697896	37.3264354	44.5727484	3.43531475	2.67797505	3.63346248	3.44E-101	-4.677153	down	PREDICTED: ras-related protein RABC2a-like [Sesamum indicum]
c35549.graph_c0	3.1694646	3.29466289	3.13299038	37.7717535	38.4282859	38.5388549	1.088E-41	2.562117	up	allene oxide cyclase [Salvia miltiorrhiza]
c35557.graph_c0	1.41596184	1.0838786	0.21406671	6.98278546	10.8928961	10.7195468	5.755E-07	2.385237	up	--
c35559.graph_c0	4.54759744	3.37962739	2.74483523	26.7876865	28.3783056	27.2809414	1.845E-13	1.935672	up	PREDICTED: AP-2 complex subunit sigma-like [Camelina sativa]
c35583.graph_c0	628.040481	704.20716	655.342701	235.473429	170.46129	153.400652	2.927E-85	-2.839196	down	PREDICTED: probable xyloglucan endotransglucosylase/hydrolase protein 23 [Nicotiana tomentosiformis]
c35583.graph_c1	355.409371	376.017552	381.101463	140.103661	99.222808	93.5666502	3.615E-80	-2.751142	down	PREDICTED: probable xyloglucan endotransglucosylase/hydrolase protein 25 [Prunus mume]
c35586.graph_c0	1.21413932	1.51386337	1.39598372	25.7853904	34.4470559	45.5905346	3.039E-14	3.656222	up	PREDICTED: probable galactinol--sucrose galactosyltransferase 6 [Sesamum indicum]
c35595.graph_c0	1584.50283	1516.13061	1578.39165	80.1706195	77.4204623	64.5509401	2.03E-156	-5.412127	down	PREDICTED: cysteine proteinase 15A [Nicotiana tomentosiformis]
c35599.graph_c0	3.14639204	2.04720274	3.23459067	1.73919694	3.22431947	1.54752698	0.0060394	-1.398908	down	-
c35599.graph_c1	18.4584802	18.9017213	20.7208128	14.3175594	13.2005738	12.9135988	1.029E-16	-1.539408	down	PREDICTED: uncharacterized protein LOC105161992 [Sesamum indicum]
c35601.graph_c0	7.37851039	7.21108468	7.91074908	33.2236019	34.5359129	31.4411121	6.736E-14	1.122082	up	PREDICTED: protein transport protein SEC24 [Sesamum indicum]
c35616.graph_c0	32.1422369	29.4444715	29.6638156	153.979931	160.936684	177.57061	5.073E-21	1.413705	up	PREDICTED: uncharacterized protein LOC105173072 [Sesamum indicum]
c35618.graph_c1	9.14005645	8.25857789	10.2901602	53.4286583	60.3114439	64.9123409	5.011E-19	1.667791	up	PREDICTED: uncharacterized protein LOC105168327 [Sesamum indicum]
c35626.graph_c0	1.88259169	1.51288984	1.9592152	0.26870463	0.2587818	0.35863765	5.496E-25	-3.615884	down	PREDICTED: phosphatidylinositol/phosphatidylcholine transfer protein SFH2 [Sesamum indicum]
c35628.graph_c0	2.23381846	1.63404041	1.43527258	23.5079986	26.664754	23.170989	8.885E-31	2.774573	up	PREDICTED: uncharacterized protein At4g15545 [Sesamum indicum]
c35629.graph_c0	7.59210431	6.84393472	6.73767584	33.7351397	31.7673688	28.882649	2.588E-10	1.141708	up	PREDICTED: KH domain-containing protein At1g09660/At1g09670 [Sesamum indicum]
c35636.graph_c0	5.90986294	5.98620673	5.67320349	25.3754976	27.8092374	26.9765286	1.067E-14	1.171403	up	PREDICTED: extra-large guanine nucleotide-binding protein 3 [Sesamum indicum]

#ID	0 h-1 FPKM	0 h-2 FPKM	0 h-3 FPKM	48 h-1 FPKM	48 h-2 FPKM	48 h-3 FPKM	FDR	log2FC	regulated	nr_annotation
c35641.graph_c0	0	0	0	5.50705902	5.20674285	4.26787084	2.296E-16	Inf	up	-
c35645.graph_c0	0.03630618	0	0	1.27100966	1.38191432	2.02378156	3.023E-11	6.008444	up	hypothetical protein MIMGU_mgv1a021537mg [Erythranthe guttata]
c35649.graph_c0	0.21863511	0.25532972	0.18730318	1.34280618	1.7426119	2.50912382	0.000148	2.058132	up	PREDICTED: pto-interacting protein 1-like [Sesamum indicum]
c35653.graph_c0	16.875585	14.2025975	14.0888604	140.714205	155.259017	160.451313	1.623E-44	2.318834	up	PREDICTED: macrophage migration inhibitory factor homolog isoform X1 [Eucalyptus grandis]
c35660.graph_c0	1.6693277	1.0152907	1.00469153	0	0	0	2.115E-21	-Inf	down	hypothetical protein THAOC_20916 [Thalassiosira oceanica]
c35664.graph_c0	2.10293773	4.33391309	2.38443374	1.82340085	2.17313111	1.11543514	0.0034151	-1.801789	down	hypothetical protein L484_005397 [Morus notabilis]
c35667.graph_c0	81.2537371	79.4280037	80.1965109	89.3720917	81.4620422	69.7723489	3.792E-13	-1.015945	down	PREDICTED: putative glutathione-specific gamma-glutamylcyclotransferase 2 isoform X1 [Sesamum indicum]
c35671.graph_c0	0.23357718	0.18704886	0.06003119	1.82908557	1.84650876	1.51262633	6.577E-06	2.426802	up	PREDICTED: E3 ubiquitin-protein ligase ORTHRUS 2-like [Sesamum indicum]
c35679.graph_c0	1.44421421	1.22325109	1.4989739	15.5054928	13.2819024	13.7966943	1.909E-12	2.337721	up	PREDICTED: serine/threonine-protein kinase At5g01020 [Sesamum indicum]
c35683.graph_c0	3.46573409	2.36201157	2.17942398	0.67933198	0.57573589	0.42312586	1.226E-10	-3.26243	down	-
c35685.graph_c0	6.61998551	6.77278975	5.75469706	234.443603	245.751706	260.159609	4.83E-160	4.255811	up	hypothetical protein POPTR_0014s02030g [Populus trichocarpa]
c35685.graph_c2	0.38816723	0.51807457	0.49881026	4.41046134	4.29350989	5.09111609	7.396E-05	2.275687	up	-
c35687.graph_c0	246.167261	242.351856	259.752647	49.1790052	33.2814957	32.3132023	2.79E-116	-3.714603	down	PREDICTED: ubiquitin carboxyl-terminal hydrolase 19-like [Sesamum indicum]
c35689.graph_c0	33.5426055	31.0305894	30.5282525	22.064124	19.6356384	20.1866687	1.197E-28	-1.63445	down	hypothetical protein MIMGU_mgv1a010259mg [Erythranthe guttata]
c35690.graph_c0	4.75461583	4.66124538	4.93408712	1.70092657	4.01845322	2.98676773	2.011E-17	-1.751632	down	PREDICTED: UDP-glycosyltransferase 73C3-like [Sesamum indicum]
c35693.graph_c0	130.313951	138.415702	144.453854	33.9232775	49.1920363	55.1320393	5.567E-68	-2.605287	down	hypothetical protein MIMGU_mgv1a013946mg [Erythranthe guttata]
c35699.graph_c0	11.9143396	14.201954	12.1184776	7.91222055	7.88897719	9.317973	3.781E-16	-1.62426	down	PREDICTED: trihelix transcription factor GT-4-like isoform X2 [Sesamum indicum]
c35713.graph_c0	28.3624123	28.9570848	28.05381	22.5995976	16.9397641	13.4059907	8.351E-32	-1.698739	down	PREDICTED: uncharacterized protein LOC105178130 [Sesamum indicum]
c35716.graph_c0	4.26491438	3.57420144	3.44129683	1.55337196	1.64560923	1.62609533	2.108E-06	-2.241946	down	PREDICTED: wound-induced protein 1 [Sesamum indicum]
c35719.graph_c1	9.51224092	8.90185935	8.84938156	5.75091351	4.83523455	2.98499148	8.047E-27	-2.01622	down	PREDICTED: solute carrier family 25 member 44-like [Sesamum indicum]
c35725.graph_c0	1.12722524	1.16154087	0.85207592	3.48194028	6.50374909	6.79431912	0.0014996	1.392183	up	PREDICTED: LOW QUALITY PROTEIN: ankyrin repeat-containing protein At3g12360-like [Sesamum indicum]
c35732.graph_c0	1.71253766	1.61341412	1.52105733	0.13920895	0.19663333	0.33031275	1.794E-16	-3.889244	down	PREDICTED: zinc finger BED domain-containing protein DAYSLEEPER-like isoform X1 [Sesamum indicum]

#ID	0 h-1 FPKM	0 h-2 FPKM	0 h-3 FPKM	48 h-1 FPKM	48 h-2 FPKM	48 h-3 FPKM	FDR	log2FC	regulated	nr_annotation
c35735.graph_c0	14.4521098	15.1856804	16.0736077	9.42117756	11.2591013	9.21426983	2.427E-18	-1.633049	down	PREDICTED: uncharacterized protein LOC105166332 [Sesamum indicum]
c35736.graph_c1	0.60692411	0.70878716	0.12998692	9.45875246	7.55232216	6.26043103	9.951E-10	3.009049	up	PREDICTED: RING-H2 finger protein ATL57 [Solanum lycopersicum]
c35738.graph_c0	9.73231362	11.3133589	10.4316125	8.8420876	9.93615028	9.85292779	6.176E-08	-1.15644	down	PREDICTED: F-box/FBD/LRR-repeat protein At1g13570-like [Sesamum indicum]
c35739.graph_c0	26.0704352	25.0224454	23.6451428	22.8095736	25.2499989	24.9218353	8.429E-12	-1.052669	down	PREDICTED: 5'-adenylylsulfate reductase-like 4 [Sesamum indicum]
c35742.graph_c0	0.04162066	0	0	2.83743133	2.39660746	3.61650419	2.344E-14	6.735906	up	PREDICTED: thiol-disulfide oxidoreductase LTO1-like [Sesamum indicum]
c35746.graph_c0	0.53269439	0.5332278	0.47917337	19.335661	26.0466445	21.178442	1.244E-39	4.408458	up	PREDICTED: probable pectinesterase/pectinesterase inhibitor 61 [Sesamum indicum]
c35749.graph_c0	1.55297444	1.34011164	1.10103925	0.09866906	0.1045279	0.30728297	5.693E-12	-3.992223	down	PREDICTED: oligopeptide transporter 7 isoform X1 [Malus domestica]
c35752.graph_c0	0.08353479	0	0	29.2054448	35.3828678	38.3468362	9.63E-110	9.266794	up	PREDICTED: probable galactinol--sucrose galactosyltransferase 6 [Sesamum indicum]
c35760.graph_c0	32.9544458	37.5759238	31.8404267	6.84807178	6.52923151	6.49959797	1.793E-44	-3.380202	down	Ubiquitin-like protein [Medicago truncatula]
c35761.graph_c0	1.03711553	1.1535045	1.55485694	0.07962154	0.22493164	0.30700266	3.232E-12	-3.653229	down	PREDICTED: probable leucine-rich repeat receptor-like protein kinase At5g63930 [Sesamum indicum]
c35764.graph_c0	1.83027332	3.09167898	1.76398026	272.210611	278.214613	313.758896	1.88E-193	5.997814	up	PREDICTED: pistil-specific extensin-like protein [Sesamum indicum]
c35766.graph_c0	19.9220317	20.9637428	20.8287563	158.793228	165.869052	168.6315	6.756E-43	1.9801	up	PREDICTED: staphylococcal nuclease domain-containing protein 1 [Sesamum indicum]
c35772.graph_c0	176.962155	175.50605	158.591457	10.2491039	6.94891699	6.93088916	5.74E-237	-5.412967	down	-
c35780.graph_c0	0.85803083	0.73619144	0.68256412	0.26349132	0.79753459	0.4354127	0.0010122	-1.636194	down	unnamed protein product [Coffea canephora]
c35783.graph_c0	14.938512	8.19708525	8.41843347	0	0	0	1.536E-22	-Inf	down	uncharacterized protein LOC100273336 [Zea mays]
c35784.graph_c0	2.61913173	1.74783626	2.46817118	0	0	0	5.427E-24	-Inf	down	NAD(P)-binding protein [Auricularia delicata TFB-10046 SS5]
c35787.graph_c0	4.00547614	3.35873258	3.66501873	1.7774332	1.12569154	1.52997711	2.996E-17	-2.325607	down	PREDICTED: probable LRR receptor-like serine/threonine-protein kinase At4g36180 [Sesamum indicum]
c35789.graph_c0	2.0794413	2.04974456	1.87663454	9.68095133	9.06289959	7.41693323	1.31E-07	1.109446	up	PREDICTED: pentatricopeptide repeat-containing protein At4g01570 [Sesamum indicum]
c35796.graph_c0	13.9498832	11.5140533	12.8942498	11.6114812	13.1369437	12.4884695	2.113E-06	-1.062033	down	PREDICTED: cytochrome c oxidase-assembly factor COX23, mitochondrial-like [Citrus sinensis]
c35799.graph_c0	66.5904956	72.6654824	69.2457515	18.0273396	18.7037409	21.8876113	9.863E-85	-2.850056	down	PREDICTED: GTP cyclohydrolase 1 [Sesamum indicum]
c35804.graph_c0	2.2056856	2.48929255	3.33458038	24.1053524	24.882452	27.192112	2.031E-39	2.22311	up	hypothetical protein MIMGU_mgv1a001397mg [Erythranthe guttata]
c35805.graph_c0	27.6421366	29.4276632	32.0109056	18.9944455	22.2805408	24.1521071	3.063E-17	-1.467607	down	PREDICTED: DAG protein, chloroplastic [Sesamum indicum]

#ID	0 h-1 FPKM	0 h-2 FPKM	0 h-3 FPKM	48 h-1 FPKM	48 h-2 FPKM	48 h-3 FPKM	FDR	log2FC	regulated	nr_annotation
c35811.graph_c1	0.04523853	0	0.1743999	0.79185696	1.10378478	1.03834252	1.915E-06	2.69504	up	PREDICTED: phosphatidylinositol 4-kinase alpha 1-like [Sesamum indicum]
c35812.graph_c0	1294.50295	1071.59934	1135.28011	285.613133	283.018478	310.150684	4.679E-80	-3.011558	down	-
c35813.graph_c0	6.53720099	6.71371446	7.74597284	779.052868	881.40725	962.388293	3.83E-244	5.942619	up	PREDICTED: cysteine proteinase COT44 [Sesamum indicum]
c35814.graph_c0	300.204405	299.751082	328.509247	204.635945	210.783827	202.61264	4.015E-27	-1.605855	down	PREDICTED: thioredoxin H-type-like [Nelumbo nucifera]
c35828.graph_c0	0.04272613	0.06415337	0.08235716	1.08245776	1.16758243	1.47101473	3.867E-08	3.271261	up	PREDICTED: K(+) efflux antiporter 3, chloroplastic [Sesamum indicum]
c35844.graph_c0	0.31564105	0.34755283	0.24336675	9.39248866	9.95020283	11.5916601	2.001E-29	4.07599	up	PREDICTED: uncharacterized protein LOC105173814 [Sesamum indicum]
c35848.graph_c0	0	0	0	2.33747381	2.60660007	5.36387562	2.926E-05	Inf	up	-
c35849.graph_c0	0.25361014	0.2855971	0.04073738	14.2229689	13.829933	10.9663781	4.641E-39	5.069745	up	PREDICTED: cytochrome P450 78A5 [Sesamum indicum]
c35855.graph_c0	4.04441503	4.18969042	2.77991556	549.365265	577.487886	598.768288	2.26E-267	6.276905	up	PREDICTED: cysteine proteinase 3-like [Sesamum indicum]
c35856.graph_c0	12.7984981	13.56216	13.6753587	2.02991988	2.15045397	2.42109067	1.084E-49	-3.620369	down	PREDICTED: probable methyltransferase PMT19 [Sesamum indicum]
c35864.graph_c0	0.28652612	0.38241737	0.42956364	3.69553168	2.98283254	3.17082394	2.684E-05	2.14756	up	PREDICTED: ras-related protein RABA5a [Beta vulgaris subsp. vulgaris]
c35874.graph_c0	11.9576837	12.2941362	10.3906289	6.48694803	7.31152967	7.38113472	1.008E-27	-1.728026	down	hypothetical protein JCGZ_25649 [Jatropha curcas]
c35883.graph_c0	0.03389161	0	0	15.8925894	21.4339958	24.6983595	7.996E-47	9.835158	up	PREDICTED: uncharacterized protein LOC105165376 isoform X1 [Sesamum indicum]
c35884.graph_c0	8.8338195	8.07630092	6.50836981	1.08510543	0.63224569	0.57931217	3.67E-32	-4.352603	down	-
c35887.graph_c0	0	0.10880836	0.20952478	5.95839458	10.6087337	15.5488113	1.954E-08	5.609635	up	PREDICTED: putative UDP-rhamnose:rhamnosyltransferase 1 [Sesamum indicum]
c35887.graph_c1	0	0	0	4.36578495	10.5714738	16.5375066	5.732E-06	Inf	up	hypothetical protein MIMGU_mgv1a005472mg [Erythranthe guttata]
c35890.graph_c1	134.996533	137.792093	139.724935	102.835524	100.778764	92.8480544	4.687E-26	-1.493277	down	PREDICTED: probable prefoldin subunit 5 [Sesamum indicum]
c35891.graph_c0	34.6483865	33.3618214	36.8917687	401.892452	371.973185	386.274526	1.123E-44	2.450141	up	40S ribosomal protein S6 [Morus notabilis]
c35893.graph_c2	0	0.44824727	0	2.61276298	3.20494328	3.79312865	1.21E-07	3.411555	up	hypothetical protein M569_00198, partial [Genlisea aurea]
c35894.graph_c0	2.5366716	2.3040995	2.23351421	1.60124169	1.65047489	1.6943273	0.0002932	-1.533486	down	PREDICTED: protein trichome birefringence-like 10 [Sesamum indicum]
c35901.graph_c0	8.9690827	10.2983674	9.15270329	3.28085894	3.34694362	4.54111553	4.767E-09	-2.367342	down	PREDICTED: uncharacterized protein LOC104435912 [Eucalyptus grandis]
c35910.graph_c0	2.5865758	2.07133269	2.06078843	12.4388546	17.2165258	16.2839805	2.156E-09	1.752146	up	PREDICTED: exosome complex component RRP42 isoform X1 [Sesamum indicum]
c35914.graph_c0	0.94110453	0.82429104	0.30233915	6.17742402	6.5442317	6.55738882	7.862E-05	2.211878	up	PREDICTED: GTP-binding protein OBGC, chloroplastic [Sesamum indicum]

#ID	0 h-1 FPKM	0 h-2 FPKM	0 h-3 FPKM	48 h-1 FPKM	48 h-2 FPKM	48 h-3 FPKM	FDR	log2FC	regulated	nr_annotation
c35914.graph_c1	3.48304068	3.5666785	3.54980825	10.732897	19.0089475	20.6425852	0.0014428	1.221096	up	PREDICTED: uncharacterized protein LOC105163283 [Sesamum indicum]
c35916.graph_c0	4.00907018	4.53090204	3.57303223	2.36795683	4.03894442	4.50552123	3.356E-08	-1.176259	down	PREDICTED: pentatricopeptide repeat-containing protein At4g19440, chloroplastic [Sesamum indicum]
c35922.graph_c0	38.4544225	39.4585438	39.6112487	30.4580941	27.4737322	28.0000175	7.485E-23	-1.467453	down	PREDICTED: protein DEHYDRATION-INDUCED 19 homolog 7-like [Sesamum indicum]
c35923.graph_c0	14.1166392	15.9854391	18.1404403	84.3710327	75.8618055	75.2898111	6.421E-14	1.26995	up	PREDICTED: uncharacterized protein At5g64816 [Sesamum indicum]
c35927.graph_c0	9.13791663	7.92201327	9.43600552	92.3022343	97.783025	106.340926	5.935E-46	2.463763	up	PREDICTED: cytochrome b5-like [Sesamum indicum]
c35929.graph_c1	9.63998418	8.10569522	6.11129559	8.28891672	6.52310448	6.32179564	0.0015153	-1.183087	down	-
c35931.graph_c0	40.3877794	40.9242735	45.3195407	2.39682512	1.85397932	1.15097047	1.01E-187	-5.560849	down	PREDICTED: 22.0 kDa heat shock protein-like [Sesamum indicum]
c35933.graph_c0	4.42916949	3.81782621	3.95254039	0.62340053	0.5403414	0.55469804	4.007E-27	-3.841754	down	hypothetical protein VITISV_009861 [Vitis vinifera]
c35935.graph_c0	28.8407443	28.9535473	35.2838392	158.415751	168.804175	177.210439	5.067E-25	1.416885	up	hypothetical protein MIMGU_mgv1a007974mg [Erythranthe guttata]
c35936.graph_c0	3.78240134	4.96411426	2.91632128	32.9874976	46.5950051	34.3129915	5.635E-14	2.269476	up	-
c35937.graph_c0	5.09852834	3.60774109	3.5018299	19.4362629	27.5396115	23.8516804	4.609E-07	1.516471	up	hypothetical protein MIMGU_mgv1a016751mg [Erythranthe guttata]
c35939.graph_c0	1.89737421	1.89927414	2.14266147	17.1620334	22.6141377	24.3670941	8.83E-18	2.408572	up	PREDICTED: uncharacterized protein LOC105156528 [Sesamum indicum]
c35942.graph_c0	11.5174834	12.7009219	11.1409771	5.33447862	5.87769433	5.92242639	1.975E-44	-2.063763	down	PREDICTED: putative uncharacterized protein At4g01020, chloroplastic [Sesamum indicum]
c35944.graph_c0	0.68271918	0.77254232	0.83917678	0.19142426	0.23176091	0.26765854	1.744E-07	-2.755519	down	PREDICTED: DNA-directed RNA polymerase III subunit RPC5 [Sesamum indicum]
c35947.graph_c0	0.06553171	0	0	4.40716191	4.28511225	4.67352817	5.543E-14	6.675886	up	PREDICTED: B3 domain-containing protein At3g19184 isoform X2 [Sesamum indicum]
c35957.graph_c0	0.01980486	0	0	1.35016936	1.70094573	1.99687605	6.044E-16	6.992184	up	PREDICTED: glutamate receptor 3.2-like [Sesamum indicum]
c35958.graph_c0	14.1848823	14.036346	15.1988177	12.468931	11.54328	13.0272039	1.077E-11	-1.246997	down	PREDICTED: uncharacterized protein LOC105160232 [Sesamum indicum]
c35959.graph_c0	0	0.04814732	0	1.41798666	1.36135509	1.65600226	4.067E-10	5.518924	up	PREDICTED: membrane steroid-binding protein 2-like [Sesamum indicum]
c35961.graph_c0	1.61313137	0	1.81382047	7.05907872	10.8237654	12.231666	6.674E-05	2.102273	up	-
c35962.graph_c0	10.1818575	13.019906	10.1344975	783.598403	663.205925	639.1538	3.999E-62	4.954722	up	PREDICTED: (-)-isopiperitenol/(-)-carveol dehydrogenase, mitochondrial-like [Sesamum indicum]
c35963.graph_c0	1.65706473	1.90576803	2.03878128	528.91683	686.330599	800.649729	7.063E-74	7.466355	up	tubulin alpha chain-like [Sesamum indicum]
c35970.graph_c0	0	0.28523274	0.13731326	1.05004855	1.87717351	3.53292355	0.0085039	2.900696	up	PREDICTED: cytochrome P450 81D1-like [Sesamum indicum]

#ID	0 h-1 FPKM	0 h-2 FPKM	0 h-3 FPKM	48 h-1 FPKM	48 h-2 FPKM	48 h-3 FPKM	FDR	log2FC	regulated	nr_annotation
c35979.graph_c0	1.77228675	2.17956118	1.43154543	25.9373388	24.2651718	23.493976	2.647E-26	2.762118	up	PREDICTED: uncharacterized protein LOC105171592 [Sesamum indicum]
c35981.graph_c0	3.44506121	2.73278224	3.92586571	23.2347218	22.5208801	19.5551559	4.888E-10	1.674334	up	PREDICTED: uncharacterized protein LOC105169411 [Sesamum indicum]
c35982.graph_c0	8.87077623	10.191001	7.59953244	4.13789248	5.29684437	7.17965011	2.4E-14	-1.704301	down	PREDICTED: uncharacterized protein LOC105177291 [Sesamum indicum]
c35983.graph_c0	0.16718133	0.35860443	0.49105057	1.25414935	1.95796509	1.85989092	0.0045379	1.285177	up	PREDICTED: uncharacterized mitochondrial protein ymf11 [Sesamum indicum]
c35991.graph_c2	4.9122176	4.19074129	4.23217332	53.7912068	56.222552	62.734512	5.335E-47	2.677298	up	PREDICTED: ribonuclease 2 [Sesamum indicum]
c35992.graph_c0	0.06156216	0.04108253	0.07910981	2.96808884	4.06559896	4.4072532	3.644E-22	4.947892	up	PREDICTED: putative leucine-rich repeat-containing protein DDB_G0290503 [Sesamum indicum]
c35995.graph_c0	0	0.0522414	0	26.8286626	34.5593792	37.5192273	1.718E-89	9.875692	up	PREDICTED: LOW QUALITY PROTEIN: interactor of constitutive active ROPs 3 [Sesamum indicum]
c35996.graph_c0	0	0.05325416	0.03418262	0.72292885	1.16825408	1.18839142	3.502E-11	4.106621	up	hypothetical protein VITISV_035665 [Vitis vinifera]
c36002.graph_c0	17.2559374	14.8549662	16.4978746	12.018355	9.02691344	11.2030915	8.218E-13	-1.605645	down	PREDICTED: uncharacterized protein LOC105166164 [Sesamum indicum]
c36012.graph_c0	4.00026863	3.66105078	4.40614591	0	0	0.09369044	2.099E-21	-8.051321	down	-
c36015.graph_c0	21.7776219	20.9819504	22.3880851	12.1004762	13.6160228	10.7111438	1.076E-20	-1.857108	down	PREDICTED: uncharacterized protein LOC105158384 [Sesamum indicum]
c36017.graph_c0	0.02231164	0.02233398	0	2.28159795	2.41707637	2.52397292	1.376E-17	6.335505	up	PREDICTED: potassium channel AKT2/3 [Sesamum indicum]
c36019.graph_c0	2.69984528	2.73192429	3.58254094	0.72996072	0.88786856	0.84196345	9.703E-15	-2.897567	down	PREDICTED: F-box protein SKIP14 [Sesamum indicum]
c36022.graph_c0	34.8079776	35.6322684	37.4512756	14.9933988	17.5280345	16.9260722	1.475E-45	-2.146443	down	PREDICTED: protein trichome birefringence-like 39 [Sesamum indicum]
c36025.graph_c0	9.97672382	10.7549228	7.53091433	5.01338657	7.01334285	6.40536792	9.808E-09	-1.636482	down	-
c36034.graph_c0	13.969574	15.3585152	13.9330335	4.90017207	6.61727498	5.89338097	6.897E-40	-2.334879	down	hypothetical protein MIMGU_mgv1a022687mg [Erythranthe guttata]
c36037.graph_c0	74.9731039	74.7315191	79.5513232	59.1837389	44.5773244	32.5941747	1.705E-27	-1.759412	down	PREDICTED: ethylene-responsive transcription factor ERF107-like [Sesamum indicum]
c36038.graph_c0	0.07158681	0.10748774	0.04599594	0.72545482	1.57199607	1.76046909	0.000112	3.14719	up	PREDICTED: endoglucanase 10-like [Sesamum indicum]
c36039.graph_c0	2.89349168	4.16355929	4.76400956	2.08255724	3.97119037	2.29777816	0.0007187	-1.531119	down	floricaula, partial [Salvia coccinea]
c36040.graph_c0	7.34374668	8.10291741	7.7211861	1.53762237	0.81446223	1.84702564	7.624E-18	-3.477242	down	CYP72A52v1 [Nicotiana tabacum]
c36046.graph_c0	7.99675015	5.56852709	9.82935183	2.80271167	4.41128357	6.91148832	0.0003821	-1.759854	down	PREDICTED: uncharacterized protein LOC105174651 [Sesamum indicum]
c36056.graph_c0	3.89290927	3.89680743	5.40504595	28.0728703	30.0018267	28.4636699	7.487E-18	1.691326	up	PREDICTED: uncharacterized protein At2g34160-like [Sesamum indicum]
c36060.graph_c0	1.97267571	1.97465104	2.18531585	11.0921477	21.9082441	24.4791517	7.484E-05	2.197876	up	PREDICTED: laccase-14-like [Sesamum indicum]

#ID	0 h-1 FPKM	0 h-2 FPKM	0 h-3 FPKM	48 h-1 FPKM	48 h-2 FPKM	48 h-3 FPKM	FDR	log2FC	regulated	nr_annotation
c36076.graph_c0	0.08161367	0.49017238	0.83901441	18.1202693	17.5235277	20.338113	2.281E-23	4.278246	up	PREDICTED: uncharacterized protein LOC105165595 [Sesamum indicum]
c36082.graph_c0	17.7215771	17.257712	17.7237269	10.4409346	15.4957007	16.1046142	9.714E-18	-1.350952	down	PREDICTED: uncharacterized membrane protein At1g75140-like [Sesamum indicum]
c36085.graph_c0	0.53327678	0.86744252	0.770942	11.1768329	8.84784552	8.08720683	1.194E-09	2.680542	up	PREDICTED: putative receptor-like protein kinase At1g72540 [Sesamum indicum]
c36087.graph_c0	13.8644332	14.5545902	13.5887381	13.6658351	13.0438997	11.317021	1.681E-12	-1.15864	down	PREDICTED: uncharacterized protein LOC105173071 [Sesamum indicum]
c36088.graph_c0	34.1609475	35.2392814	31.5664703	17.4413425	17.0008646	16.4597594	1.37E-35	-2.003781	down	PREDICTED: uncharacterized protein LOC105178899 isoform X2 [Sesamum indicum]
c36103.graph_c0	0.75736568	0.86642751	0.55613993	8.57216873	10.3483138	14.8114712	2.352E-09	2.931802	up	PREDICTED: proliferating cell nuclear antigen [Sesamum indicum]
c36106.graph_c0	7.29621738	4.97967507	4.04869627	0	0	0	8.188E-19	-Inf	down	-
c36109.graph_c0	13.2016679	12.6791487	13.5258823	56.4968804	59.633952	58.0213946	3.148E-13	1.125165	up	hypothetical protein MIMGU_mgv1a016596mg [Erythranthe guttata]
c36119.graph_c0	0.36667573	0.3670429	0.34554141	5.35984888	8.89888664	11.2415945	1.067E-08	3.53538	up	PREDICTED: uncharacterized protein LOC105159055 [Sesamum indicum]
c36121.graph_c0	18.155595	17.5135423	15.7024699	16.5342317	18.1597356	15.764954	7.824E-11	-1.042625	down	PREDICTED: ATPase family AAA domain-containing protein 1 [Sesamum indicum]
c36125.graph_c0	5.31800136	4.47159429	5.74042753	26.7501219	29.2727471	28.5537984	8.094E-12	1.424721	up	PREDICTED: protein OS-9 homolog [Sesamum indicum]
c36141.graph_c0	9.28059381	7.1264886	7.02486316	0.81985231	1.73706821	0.83371389	4.519E-16	-3.813751	down	ubiquitin extension protein [Capsicum annuum]
c36144.graph_c0	11.3751387	11.1967537	11.9863529	0.38424906	0.8511365	0.74596909	7.222E-81	-5.15506	down	PREDICTED: type I inositol 1,4,5-trisphosphate 5-phosphatase CVP2 isoform X1 [Cucumis melo]
c36150.graph_c0	1.48202465	0.52493384	0.70318536	0	0	0	5.79E-08	-Inf	down	hypothetical protein EMIHUDRAFT_239943 [Emiliania huxleyi CCMP1516]
c36151.graph_c0	87.0632639	84.1047475	82.7228639	80.8090191	88.873842	80.9536559	1.271E-13	-1.036738	down	hypothetical protein MIMGU_mgv1a022801mg, partial [Erythranthe guttata]
c36157.graph_c0	1.30711437	1.49099394	1.87500695	7.5052404	11.6593306	14.7516631	4.436E-05	1.8293	up	PREDICTED: katanin p60 ATPase-containing subunit A-like 2 isoform X1 [Sesamum indicum]
c36159.graph_c0	6.2124685	6.70452445	4.36584968	4.56077954	4.35790732	5.64958723	0.0010815	-1.261338	down	PREDICTED: uncharacterized protein LOC105167009 [Sesamum indicum]
c36162.graph_c0	560.709512	561.688486	606.77246	137.818225	148.240571	152.033634	9.183E-70	-3.000641	down	hypothetical protein MIMGU_mgv1a009201mg [Erythranthe guttata]
c36172.graph_c0	17.8985072	18.2125692	20.3390998	284.678428	242.103148	211.229094	1.611E-18	2.694151	up	-
c36188.graph_c0	1.74423702	2.13793912	1.23506465	0.95102536	0.69482486	0.17507931	2.864E-08	-2.489179	down	PREDICTED: WEB family protein At1g12150-like [Sesamum indicum]
c36190.graph_c0	0.0931565	0.09324978	0.17956468	71.4466825	115.147437	131.498391	1.107E-34	8.729144	up	unnamed protein product [Coffea canephora]

#ID	0 h-1 FPKM	0 h-2 FPKM	0 h-3 FPKM	48 h-1 FPKM	48 h-2 FPKM	48 h-3 FPKM	FDR	log2FC	regulated	nr_annotation
c36193.graph_c0	1.73641286	2.15199724	0.95629683	72.5576756	71.7416553	75.6096831	4.23E-123	4.49304	up	PREDICTED: GDSL esterase/lipase At1g54790 [Sesamum indicum]
c36202.graph_c0	0	0.20102967	0.12903634	10.7309502	8.62410661	8.06669677	9.237E-15	5.358229	up	PREDICTED: NAC domain-containing protein 8-like [Sesamum indicum]
c36202.graph_c1	0.21770556	0	0	8.02258234	8.71142703	6.15690926	3.61E-13	5.720993	up	PREDICTED: NAC domain-containing protein 8-like [Sesamum indicum]
c36206.graph_c0	80.255123	77.7708505	80.5293913	44.5276146	43.7249722	38.7689623	2.45E-39	-1.925548	down	PREDICTED: early nodulin-93-like [Solanum tuberosum]
c36209.graph_c0	19.0339883	17.5515849	17.9457763	16.9635465	16.1535466	17.3836104	4.264E-10	-1.127492	down	PREDICTED: uncharacterized protein LOC101497815 [Cicer arietinum]
c36211.graph_c0	210.783866	241.942607	201.227282	76.7981351	41.723304	33.6482177	5.208E-65	-3.105308	down	PREDICTED: probable WRKY transcription factor 17 [Sesamum indicum]
c36215.graph_c0	14.2416674	14.7663707	11.6546688	2.01335339	2.09735539	1.64218059	1.113E-69	-3.835531	down	PREDICTED: phosphoenolpyruvate carboxylase kinase 2 [Sesamum indicum]
c36217.graph_c0	0.09024243	0.0451664	0	1.78745	1.36514378	1.07263734	1.904E-07	3.971727	up	hypothetical protein MIMGU_mgv1a009127mg [Erythranthe guttata]
c36224.graph_c0	0.37891494	0	0.24346035	5.06167682	5.732042	4.03793412	1.278E-07	3.557412	up	PREDICTED: laccase-1-like [Sesamum indicum]
c36224.graph_c1	0.1869554	0	0.12012254	3.01412132	4.15102488	4.21448819	1.292E-11	4.188381	up	PREDICTED: laccase-1-like [Sesamum indicum]
c36234.graph_c0	12.293973	11.5528376	12.574111	3.52493161	2.69354845	2.416619	3.027E-28	-3.088048	down	PREDICTED: high mobility group B protein 6 [Sesamum indicum]
c36238.graph_c0	3.63785479	3.9289842	2.89098696	31.7944788	33.682395	32.0181379	5.113E-26	2.205271	up	PREDICTED: probable UDP-arabinopyranose mutase 5 isoform X1 [Sesamum indicum]
c36244.graph_c0	0.11539944	0.13861799	0.17795141	4.93294579	5.36100942	5.01364637	1.631E-21	4.124546	up	PREDICTED: uncharacterized protein LOC105158603 isoform X1 [Sesamum indicum]
c36245.graph_c0	0	0	0	5.90116167	7.40185324	9.82953161	1.209E-22	Inf	up	-
c36254.graph_c1	0.55944952	0.33600583	0	1.90698512	1.74721687	1.6968239	0.0082841	1.581357	up	PREDICTED: pentatricopeptide repeat-containing protein DOT4, chloroplastic isoform X1 [Sesamum indicum]
c36255.graph_c0	0.06113172	0.06119294	0	2.96610923	2.30695593	1.72050819	4.981E-11	4.843739	up	hypothetical protein MIMGU_mgv1a018451mg [Erythranthe guttata]
c36264.graph_c0	4.88198056	6.14799665	7.08302712	27.5659289	26.66673	22.8497663	2.089E-05	1.070625	up	-
c36267.graph_c0	0.03976429	0.19902054	0.15329606	1.90493983	3.02707915	4.33529871	4.582E-06	3.530384	up	PREDICTED: transcription factor bHLH30-like isoform X2 [Sesamum indicum]
c36268.graph_c0	0	0	0	10.702059	10.1370891	7.28200945	2.727E-15	Inf	up	-
c36275.graph_c0	17.0330058	17.3390459	17.3744418	139.89742	140.643865	146.173146	5.44E-45	2.025773	up	PREDICTED: gamma carbonic anhydrase 2, mitochondrial [Sesamum indicum]
c36277.graph_c0	17.281138	20.282027	14.3020885	12.6487733	8.72691763	7.14796922	3.892E-12	-1.8682	down	PREDICTED: ethylene-responsive transcription factor ABR1-like [Sesamum indicum]
c36280.graph_c0	7.91456347	7.64929945	10.0536248	42.3237139	49.2761343	43.3269373	1.095E-10	1.374731	up	-

#ID	0 h-1 FPKM	0 h-2 FPKM	0 h-3 FPKM	48 h-1 FPKM	48 h-2 FPKM	48 h-3 FPKM	FDR	log2FC	regulated	nr_annotation
c36282.graph_c0	32.0829521	32.858483	32.4478557	190.204177	200.33856	220.258808	6.221E-25	1.629699	up	hypothetical protein M569_11398, partial [Genlisea aurea]
c36287.graph_c0	2.86635903	2.15192194	3.03879249	1.1883046	1.85332849	1.67413162	3.459E-06	-1.799255	down	PREDICTED: glutamate decarboxylase-like [Sesamum indicum]
c36289.graph_c0	5.40734069	4.31506368	4.61623394	1.81148907	2.02976761	3.13071269	6.151E-11	-2.062271	down	PREDICTED: transcription factor GAMYB-like [Sesamum indicum]
c36291.graph_c0	9.35718559	8.34845155	7.49343797	42.8523804	53.2056972	56.9723544	3.449E-17	1.582405	up	PREDICTED: 50S ribosomal protein L27, chloroplastic-like [Sesamum indicum]
c36295.graph_c0	0.44881411	0.39934536	0.83307452	4.68607075	5.9863904	5.43688037	6.851E-07	2.231164	up	PREDICTED: uncharacterized protein LOC105171863 [Sesamum indicum]
c36303.graph_c0	4.8731903	3.67994759	3.07618929	17.0916082	17.9396063	17.450666	0.0004945	1.159792	up	hypothetical protein MIMGU_mgv1a014569mg [Erythranthe guttata]
c36304.graph_c0	0.34367535	0.11467316	0.36803036	2.53292922	2.5715259	3.61544708	1.474E-05	2.374787	up	PREDICTED: E3 ubiquitin-protein ligase RNF5-like [Sesamum indicum]
c36310.graph_c1	0.07390919	0.14796641	0.18995248	8.17079641	5.33784695	7.99735806	4.727E-14	4.686791	up	PREDICTED: histone H3-like centromeric protein HTR12 [Sesamum indicum]
c36317.graph_c0	22.8430936	25.3102606	21.2565474	17.1259079	12.4539715	12.1391678	5.497E-18	-1.744006	down	PREDICTED: zinc finger CCCH domain-containing protein 44-like isoform X3 [Sesamum indicum]
c36318.graph_c0	8.95597731	10.0274574	9.420154	48.4354306	56.8149036	60.4997222	6.538E-24	1.523889	up	PREDICTED: 3-oxoacyl-[acyl-carrier-protein] synthase I, chloroplastic [Sesamum indicum]
c36321.graph_c0	0.42772549	0.58384608	0.34977358	2.05979961	2.61852951	2.47029202	0.0009899	1.374363	up	PREDICTED: putative receptor protein kinase ZmPK1 [Sesamum indicum]
c36336.graph_c0	6.33676463	3.41552074	4.38468877	0	0	0	3.394E-20	-Inf	down	hypothetical protein EMIHUDRAFT_109093 [Emiliania huxleyi CCMP1516]
c36337.graph_c0	0.64222967	0.61492178	0.68176176	0.4887658	0.19076404	0.20600567	2.535E-05	-2.12981	down	-
c36340.graph_c0	2.91235034	3.44531509	3.74248549	1.21956686	2.32556977	1.88094965	4.815E-05	-1.925878	down	PREDICTED: putative DNA-binding protein ESCAROLA [Sesamum indicum]
c36343.graph_c0	1.93851606	2.04826037	1.52232049	0.39686388	0.63064373	0.44140886	5.064E-09	-2.928044	down	PREDICTED: brefeldin A-inhibited guanine nucleotide-exchange protein 1-like [Sesamum indicum]
c36346.graph_c0	3.5450544	3.9775564	2.50304135	25.8403675	25.5877742	26.7927701	2.778E-21	1.95019	up	PREDICTED: xylosyltransferase 1 [Sesamum indicum]
c36353.graph_c0	0.06760162	0.03383466	0.04343538	4.42181076	6.26782212	5.68008892	3.926E-22	5.799716	up	hypothetical protein MIMGU_mgv1a004476mg [Erythranthe guttata]
c36357.graph_c0	0	0	0	7.63511541	5.69870109	4.78645621	9.872E-11	Inf	up	-
c36361.graph_c0	17.2751067	19.5704465	15.6856595	72.903836	98.5348857	93.700237	9.114E-14	1.314378	up	PREDICTED: protein NIM1-INTERACTING 1 [Sesamum indicum]
c36365.graph_c0	42.4170837	38.7494995	35.1036205	11.6346904	16.7466627	17.3291473	2.133E-27	-2.369424	down	unnamed protein product [Vitis vinifera]
c36372.graph_c0	0.55630159	0.87506358	0.20424845	14.0571787	17.9167887	18.3059691	4.16E-19	3.929369	up	PREDICTED: uncharacterized protein LOC105168457 [Sesamum indicum]
c36375.graph_c0	0.82387237	0.55788351	0.52935431	2.63419371	3.99671941	4.62818902	6.462E-05	1.536258	up	PREDICTED: oligopeptide transporter 7 [Sesamum indicum]

#ID	0 h-1 FPKM	0 h-2 FPKM	0 h-3 FPKM	48 h-1 FPKM	48 h-2 FPKM	48 h-3 FPKM	FDR	log2FC	regulated	nr_annotation
c36376.graph_c0	0.23981899	0.28807096	0.18490614	3.93267678	2.66823673	3.61722515	2.937E-07	2.832437	up	hypothetical protein CICLE_v10011295mg [Citrus clementina]
c36380.graph_c0	0.35686282	0.17861008	0.34393714	10.7670637	11.203231	13.9197138	3.223E-38	4.329139	up	PREDICTED: uncharacterized protein LOC105162690 isoform X1 [Sesamum indicum]
c36386.graph_c0	44.502157	45.4816503	45.0436141	28.3951117	28.0435936	26.3466895	1.289E-29	-1.722541	down	PREDICTED: uncharacterized protein LOC105179031 [Sesamum indicum]
c36388.graph_c0	0.14188673	0.0355072	0.22791257	1.69929898	2.38872854	2.47158098	1.126E-07	2.982701	up	PREDICTED: uncharacterized protein LOC105178674 isoform X1 [Sesamum indicum]
c36389.graph_c0	0.32045128	0	0.20589629	9.15183926	17.2012748	13.2656194	3.232E-12	5.207111	up	-
c36389.graph_c1	0.30790509	0.13698374	0.30774351	4.47555993	6.47756855	6.33806864	7.218E-15	3.495332	up	PREDICTED: transcription factor MYB3-like [Sesamum indicum]
c36392.graph_c0	20.5208776	19.7855017	22.4040711	265.307606	274.267831	281.689583	6.214E-77	2.691689	up	PREDICTED: aspartic proteinase-like isoform X1 [Sesamum indicum]
c36394.graph_c0	0.15836895	0.07926377	0.10175519	17.1432278	20.4796185	17.3310734	1.313E-30	6.322287	up	PREDICTED: auxin-induced protein 15A-like [Sesamum indicum]
c36395.graph_c0	5.75674353	5.06954821	5.80574542	5.77341691	3.6981887	3.67366077	4.879E-07	-1.348209	down	PREDICTED: uncharacterized protein LOC105174263 [Sesamum indicum]
c36397.graph_c0	0.3102294	0.31054005	0.33221422	3.42964274	4.54161327	4.83181952	1.761E-07	2.724134	up	PREDICTED: RING-H2 finger protein ATL38-like [Sesamum indicum]
c36398.graph_c0	3.81032755	4.10753863	4.1431261	2.6327417	0.78666094	0.54059948	3.519E-09	-2.596503	down	hypothetical protein MIMGU_mgv1a020815mg [Erythranthe guttata]
c36402.graph_c2	0	0.08073063	0.10363828	1.70889823	1.18067639	1.52056737	6.489E-08	3.554638	up	hypothetical protein MIMGU_mgv1a000547mg [Erythranthe guttata]
c36403.graph_c0	0.43806562	0.24556239	0.29272447	1.32372476	1.98377806	1.85294708	0.001848	1.380326	up	hypothetical protein MIMGU_mgv1a000577mg [Erythranthe guttata]
c36405.graph_c0	14.2342176	13.5487693	14.8618707	212.040445	256.198575	250.555998	7.219E-94	3.053656	up	cyclophilin [Ricinus communis]
c36419.graph_c0	0.19928369	0.26597765	0.17072495	1.89712902	1.75045194	1.30688051	0.0006772	1.950746	up	PREDICTED: AP-4 complex subunit epsilon [Sesamum indicum]
c36420.graph_c0	0.35493163	0.25839058	0.49756494	2.11054709	1.7949932	2.64500297	0.0011901	1.537058	up	PREDICTED: DNA polymerase epsilon subunit 2 isoform X1 [Sesamum indicum]
c36426.graph_c0	20.3023279	21.0407728	25.167412	135.619281	156.134975	151.782448	8.356E-32	1.715251	up	-
c36427.graph_c1	14.2841077	14.5932236	14.3817416	14.6065391	13.6054966	12.8358595	1.229E-08	-1.091244	down	PREDICTED: uncharacterized protein LOC105169363 [Sesamum indicum]
c36428.graph_c0	10.021112	6.88412024	10.9837719	6.69779949	6.99962119	7.16770125	3.262E-05	-1.439487	down	-
c36433.graph_c0	36.2507744	34.6861738	32.761706	8.89805015	8.6612749	6.60642825	3.279E-90	-3.115299	down	PREDICTED: uncharacterized protein At5g19025-like [Sesamum indicum]
c36438.graph_c0	3.44900567	3.10431217	3.87343966	2.96383897	2.63066671	2.63720609	8.267E-06	-1.357823	down	PREDICTED: uncharacterized protein LOC105171186 [Sesamum indicum]
c36443.graph_c0	0.58104785	1.2974816	0.68923338	14.2471936	13.7408954	15.2051528	6.228E-21	3.05657	up	PREDICTED: (S)-ureidoglycine aminohydrolase [Sesamum indicum]

#ID	0 h-1 FPKM	0 h-2 FPKM	0 h-3 FPKM	48 h-1 FPKM	48 h-2 FPKM	48 h-3 FPKM	FDR	log2FC	regulated	nr_annotation
c36444.graph_c0	2.20677469	2.65078133	3.48397407	0.11617284	0.30767761	0.51684949	1.8E-14	-4.187443	down	M protein repeat-containing [Populus trichocarpa]
c36448.graph_c0	6.74594766	6.15246247	4.2380848	4.74166756	5.94983555	5.32508686	0.0001033	-1.114003	down	PREDICTED: LOW QUALITY PROTEIN: protein LOW PSII ACCUMULATION 2, chloroplastic [Sesamum indicum]
c36451.graph_c0	780.803198	806.213247	747.287511	17.0660764	17.1315845	12.6789818	1.28E-246	-6.652667	down	hypothetical protein MIMGU_mgv1a024078mg [Erythranthe guttata]
c36459.graph_c0	28.1867476	34.7984659	27.3304562	241.930265	262.547914	289.171323	4.812E-50	2.116909	up	PREDICTED: shikimate O-hydroxycinnamoyltransferase [Sesamum indicum]
c36463.graph_c0	0.7417568	0.49499971	0.95318686	4.78348322	5.91210719	6.68849438	0.0002311	1.962089	up	PREDICTED: leucine-rich repeat receptor-like serine/threonine/tyrosine-protein kinase SOBIR1 [Sesamum indicum]
c36463.graph_c1	0.56335408	0.77538751	0.40721191	8.14178927	7.01015399	8.39901953	2.827E-13	2.742063	up	PREDICTED: leucine-rich repeat receptor-like serine/threonine/tyrosine-protein kinase SOBIR1 [Sesamum indicum]
c36466.graph_c0	36.5167621	37.0914858	32.2048062	152.435032	144.91803	142.059914	5.443E-13	1.039193	up	hypothetical protein MIMGU_mgv1a002222mg [Erythranthe guttata]
c36477.graph_c0	18.8265878	18.5641646	16.9034423	9.30313561	8.86141967	7.39969965	1.584E-45	-2.100643	down	PREDICTED: uncharacterized protein LOC105159352 [Sesamum indicum]
c36479.graph_c0	6.2517644	7.10045098	4.37737183	7.90089802	5.04549346	3.67934212	0.0026449	-1.094262	down	PREDICTED: UDP-glycosyltransferase 74B1 [Vitis vinifera]
c36480.graph_c0	2.50489207	2.10127212	1.81345661	20.9802952	20.0396248	21.1986785	9.508E-39	2.262621	up	PREDICTED: methionine S-methyltransferase-like isoform X1 [Sesamum indicum]
c36483.graph_c0	2.3160295	1.86963601	2.35214985	10.8748721	13.0518285	14.5145937	5.166E-09	1.533198	up	PREDICTED: triose phosphate/phosphate translocator, chloroplastic isoform X2 [Sesamum indicum]
c36491.graph_c0	0.25380118	0.10162213	0.26091565	232.555579	269.499807	287.982846	1.77E-296	9.300048	up	acetylajmalan acetylesterase [Striga asiatica]
c36492.graph_c0	3.93749026	5.30577528	5.64365752	2.92988192	3.99067024	2.60699132	0.0005198	-1.667072	down	-
c36493.graph_c0	4.94490717	5.2052086	3.60587068	2.45856213	3.31313886	3.16882294	2.691E-11	-1.640823	down	PREDICTED: plastidial pyruvate kinase 4, chloroplastic [Sesamum indicum]
c36499.graph_c0	1.48368126	0.90083896	0.9376668	0.73945099	0.45102468	0.65795904	2.457E-05	-1.85343	down	-
c36504.graph_c0	4.08400932	4.84116968	5.66243844	123.66598	134.365629	131.357513	1.752E-81	3.716621	up	hypothetical protein MIMGU_mgv1a014610mg [Erythranthe guttata]
c36508.graph_c0	0.18668804	0.22424997	0.16793106	10.6117334	12.5536971	11.6765669	9.962E-64	4.893192	up	PREDICTED: uncharacterized protein LOC105176006 [Sesamum indicum]
c36516.graph_c0	0.27344945	0.18248218	0.58565563	3.52687262	2.31294384	2.68987719	0.0002427	2.012727	up	-
c36519.graph_c1	5.93133978	6.01540121	5.44070855	2.67824416	2.513559	2.55901804	1.732E-25	-2.180957	down	PREDICTED: uncharacterized protein LOC105168551 [Sesamum indicum]
c36532.graph_c0	1.25095327	1.14608677	1.30781653	7.69608712	10.9052499	14.4432272	1.612E-06	2.130508	up	PREDICTED: uncharacterized protein LOC105171185 [Sesamum indicum]

#ID	0 h-1 FPKM	0 h-2 FPKM	0 h-3 FPKM	48 h-1 FPKM	48 h-2 FPKM	48 h-3 FPKM	FDR	log2FC	regulated	nr_annotation
c36534.graph_c0	56.5780661	60.2174521	57.9930426	26.1251128	25.9522129	21.252382	7.713E-57	-2.268949	down	hypothetical protein MIMGU_mgv1a004539mg [Erythranthe guttata]
c36536.graph_c0	7.92304631	7.7668908	9.54947748	34.2811974	45.9692471	44.8813652	6.665E-13	1.284856	up	unnamed protein product [Coffea canephora]
c36540.graph_c0	130.661301	119.746792	122.726095	505.54155	555.241486	582.886043	2.913E-15	1.119943	up	hypothetical protein POPTR_0009s16030g [Populus trichocarpa]
c36541.graph_c0	0.49983457	0.44474229	0.2141024	1.73959188	3.08954529	3.59654098	0.0006277	1.841684	up	PREDICTED: organic cation/carnitine transporter 7-like [Sesamum indicum]
c36549.graph_c0	2.89047464	2.36193389	2.50151742	12.8797107	14.4504954	12.862603	1.856E-05	1.356021	up	-
c36557.graph_c0	0	0	0	7.95490657	4.96271915	6.21311127	3.762E-14	Inf	up	hypothetical protein MIMGU_mgv1a015689mg [Erythranthe guttata]
c36558.graph_c0	1.01066323	1.46692912	1.10393092	12.15071	10.0610329	8.20192519	3.337E-08	2.074703	up	PREDICTED: uncharacterized protein LOC105159405 [Sesamum indicum]
c36561.graph_c0	1032.83313	1046.67282	1077.48139	108.287888	75.9039569	48.0453247	6.72E-153	-4.771835	down	hypothetical protein MIMGU_mgv1a011962mg [Erythranthe guttata]
c36567.graph_c0	0.15150885	0.25276761	0.19469487	24.7521578	19.2556881	16.3526444	4.849E-25	5.643571	up	PREDICTED: uncharacterized protein LOC105164077 [Sesamum indicum]
c36568.graph_c0	1.49904116	0.69255795	0.59271607	12.6415942	10.4662014	12.9498553	5.59E-08	2.686725	up	hypothetical protein MIMGU_mgv1a007969mg [Erythranthe guttata]
c36568.graph_c1	0.74314382	0.83687396	0.71622693	8.72906515	9.79134996	8.68084258	2.268E-07	2.548448	up	PREDICTED: probable purine permease 9 [Sesamum indicum]
c36569.graph_c0	0.21343015	0	0.45711032	10.6833338	14.511648	14.1714711	4.979E-22	4.837072	up	PREDICTED: uncharacterized protein LOC105167095 [Sesamum indicum]
c36570.graph_c0	6.17352697	5.38430076	4.79135466	35.7577661	36.1510181	33.2200016	2.036E-23	1.670097	up	PREDICTED: protein trichome birefringence-like 25 [Sesamum indicum]
c36574.graph_c0	29.5570487	29.8924507	25.6157524	41.8650626	22.5481759	17.0604606	0.0008352	-1.062794	down	PREDICTED: uncharacterized protein LOC105176146 [Sesamum indicum]
c36575.graph_c0	32.6154049	31.9629003	31.2691433	17.7825343	17.9031972	17.4219041	1.381E-35	-1.868774	down	PREDICTED: PCI domain-containing protein 2 [Sesamum indicum]
c36583.graph_c1	3.72816883	2.44008978	3.31673853	2.64202484	2.09917872	2.46840071	0.0049726	-1.410113	down	PREDICTED: WD repeat-containing protein 48-like [Nicotiana tomentosiformis]
c36586.graph_c0	1.40038005	1.86904309	1.39964519	50.7447768	57.2506853	56.1216237	3.407E-60	4.117059	up	PREDICTED: GDP-mannose 3,5-epimerase 2 [Sesamum indicum]
c36586.graph_c1	1.44949874	1.10548586	1.41917201	33.7022654	36.5118439	38.3055198	9.321E-42	3.751099	up	GDP-mannose 3,5-epimerase 1 [Gossypium arboreum]
c36599.graph_c0	3.99975509	4.05860627	4.92861719	35.2835597	33.6354403	34.7636721	1.029E-21	1.978004	up	PREDICTED: trafficking protein particle complex subunit 3-like isoform X1 [Sesamum indicum]
c36600.graph_c0	0.08138728	0.10862504	0.06972392	44.7376323	48.6649999	54.3513872	4.16E-194	8.134772	up	hypothetical protein MIMGU_mgv1a021873mg [Erythranthe guttata]
c36602.graph_c0	19.1787674	17.6649216	19.5016829	14.0451985	17.86861	18.6033361	4.903E-13	-1.180191	down	PREDICTED: TOM1-like protein 2 [Sesamum indicum]
c36604.graph_c0	0	0	0	2.69885069	1.75445083	2.23768786	1.224E-11	Inf	up	Peptidase S1A, chymotrypsin-type [Penicillium italicum]

#ID	0 h-1 FPKM	0 h-2 FPKM	0 h-3 FPKM	48 h-1 FPKM	48 h-2 FPKM	48 h-3 FPKM	FDR	log2FC	regulated	nr_annotation
c36605.graph_c0	9.60312159	10.0071064	9.80902909	3.99250222	3.9892554	3.71398194	1.759E-19	-2.347697	down	PREDICTED: protein FAR-RED IMPAIRED RESPONSE 1 isoform X1 [Sesamum indicum]
c36606.graph_c0	170.246824	155.806892	158.850346	17.1013123	11.3229792	10.7068731	3.08E-190	-4.639364	down	unnamed protein product [Coffea canephora]
c36607.graph_c0	54.7426035	48.1577325	54.3772636	40.0146574	37.8669254	35.5720464	3.979E-25	-1.487464	down	PREDICTED: nuclear pore complex protein NUP50A-like [Sesamum indicum]
c36608.graph_c0	1.95914512	2.54943898	4.02812648	26.3514272	27.9161418	23.9291361	2.05E-07	2.170838	up	-
c36608.graph_c1	34.2109807	35.5447223	36.2101551	159.064312	143.6913	166.949326	3.058E-15	1.131022	up	PREDICTED: gamma carbonic anhydrase-like 2, mitochondrial [Sesamum indicum]
c36617.graph_c0	2.60037857	1.60183535	1.54227204	23.9563834	24.0123281	25.9074374	9.569E-09	2.670826	up	predicted Rac-like GTPase ortholog, partial [Nicotiana alata]
c36625.graph_c0	3.05897799	3.33422252	4.28032185	42.2093256	45.8435054	44.3001413	8.516E-33	2.610093	up	PREDICTED: uncharacterized protein LOC105164615 [Sesamum indicum]
c36630.graph_c0	33.0186308	34.6436334	35.6180521	129.489699	147.895715	161.77862	1.071E-13	1.06696	up	hypothetical protein M569_05689 [Genlisea aurea]
c36632.graph_c0	0.67359575	0.41205405	0.76941974	13.4454741	18.1152547	17.9148768	2.582E-35	3.711016	up	PREDICTED: protein trichome birefringence-like 23 [Sesamum indicum]
c36638.graph_c0	7.5533667	7.27012524	7.83976463	55.7178945	74.7237544	79.3455655	6.098E-21	2.186728	up	PREDICTED: probable methyltransferase PMT20 [Sesamum indicum]
c36639.graph_c0	0.09696325	0.19412068	0.31150402	5.04708096	9.03746129	10.2534732	1.208E-10	4.297969	up	PREDICTED: B-box zinc finger protein 24 [Sesamum indicum]
c36654.graph_c0	20.0131601	18.9028003	23.2991103	106.289752	107.88643	107.741535	4.412E-20	1.351725	up	PREDICTED: uncharacterized protein LOC105177676 [Sesamum indicum]
c36664.graph_c0	5.84940272	3.00895306	3.34076181	0	0	0	6.084E-15	-Inf	down	predicted protein [Hordeum vulgare subsp. vulgare]
c36665.graph_c0	0.57681259	0.73486023	0.33692137	13.8646408	14.7902605	17.4948653	1.493E-24	3.792968	up	PREDICTED: LOW QUALITY PROTEIN: single-stranded DNA-binding protein WHY1, chloroplastic, partial [Sesamum indicum]
c36666.graph_c0	12.7048608	11.8970936	13.16633	3.39809414	2.47263709	3.08468663	2.576E-46	-3.089988	down	PREDICTED: uncharacterized protein LOC105167713 [Sesamum indicum]
c36669.graph_c0	1.30437649	1.45490349	1.67617543	44.9770421	46.5201808	52.2096912	3.024E-92	3.99659	up	PREDICTED: enoyl-[acyl-carrier-protein] reductase [NADH], chloroplastic-like [Sesamum indicum]
c36674.graph_c0	9.79846317	10.4599875	10.4579843	96.2978193	99.124725	101.642792	1.515E-54	2.255079	up	PREDICTED: peroxidase 17 [Sesamum indicum]
c36676.graph_c1	0.14926629	0.74707878	0.19181309	4.53795675	6.70124504	2.81425674	0.0016939	2.676153	up	PREDICTED: uncharacterized protein At1g04910 [Sesamum indicum]
c36679.graph_c0	23.9546694	28.701725	18.656181	11.2572171	7.9110791	5.3554121	7.884E-14	-2.543845	down	hypothetical protein MIMGU_mgv1a014406mg [Erythranthe guttata]
c36684.graph_c0	18.2798318	18.9581893	22.1251394	7.79593676	9.47443796	7.98780457	6.417E-29	-2.254721	down	PREDICTED: uncharacterized protein LOC105176815 isoform X1 [Sesamum indicum]
c36688.graph_c0	31.037148	25.0472528	25.9709533	103.401877	139.363406	151.667518	2.623E-08	1.242825	up	hypothetical protein PHAVU_002G171200g, partial [Phaseolus vulgaris]

#ID	0 h-1 FPKM	0 h-2 FPKM	0 h-3 FPKM	48 h-1 FPKM	48 h-2 FPKM	48 h-3 FPKM	FDR	log2FC	regulated	nr_annotation
c36691.graph_c0	0.63963765	0.57436716	0.54394392	2.07113155	2.65313214	3.06889706	5.911E-05	1.126655	up	PREDICTED: uncharacterized protein LOC105169716 isoform X1 [Sesamum indicum]
c36695.graph_c0	7.15790988	6.79397782	6.55967079	2.8111302	4.03567737	4.62862333	3.012E-16	-1.861986	down	PREDICTED: two-pore potassium channel 3-like [Sesamum indicum]
c36697.graph_c0	0.15812729	0.11871422	0.30479974	1.89380323	2.00625489	2.46283076	7.522E-06	2.422333	up	PREDICTED: rho GTPase-activating protein REN1 [Sesamum indicum]
c36704.graph_c0	12.5134983	12.5642178	10.9816962	8.64618815	8.93618336	7.47441044	9.59E-16	-1.540494	down	hypothetical protein MIMGU_mgv1a022795mg [Erythranthe guttata]
c36707.graph_c0	0.13521344	0	0.08687731	1.68166044	3.29910245	3.21434141	8.54E-08	4.176882	up	PREDICTED: uncharacterized protein LOC105163665 [Sesamum indicum]
c36710.graph_c0	6.73422813	6.74097144	6.34608316	1.79226961	1.60658582	2.08544325	2.31E-09	-2.870501	down	hypothetical protein MIMGU_mgv1a009490mg [Erythranthe guttata]
c36710.graph_c1	11.2816424	9.61991121	10.3808236	2.95120803	4.34983939	6.05114172	1.49E-08	-2.254455	down	PREDICTED: zinc finger CCCH domain-containing protein 20-like [Sesamum indicum]
c36719.graph_c0	0.27511764	0.55078625	0.61868984	8.55415048	13.8280707	12.9112433	1.876E-15	3.579568	up	PREDICTED: uncharacterized protein LOC105157890 [Sesamum indicum]
c36719.graph_c1	0.30257204	0.70670837	0.64802841	9.15226196	18.3086537	16.039226	3.981E-10	3.68162	up	PREDICTED: uncharacterized protein LOC105157890 [Sesamum indicum]
c36723.graph_c0	0.91393027	0.77562982	0.56168706	19.2006263	28.9889413	30.1627098	6.859E-25	4.100547	up	PREDICTED: dolichyl-diphosphooligosaccharide--protein glycosyltransferase subunit STT3A [Sesamum indicum]
c36725.graph_c0	1.43323798	2.08679731	1.50690072	22.2065979	23.2708714	22.7498937	2.874E-11	2.745489	up	PREDICTED: uncharacterized protein LOC104897046 [Beta vulgaris subsp. vulgaris]
c36729.graph_c1	0.17854026	0.40211785	0.34414696	33.4722772	44.956429	52.5783665	9.887E-40	6.11934	up	PREDICTED: probable inactive receptor kinase At5g58300-like isoform X1 [Glycine max]
c36731.graph_c0	6.63703525	8.00648764	7.21672182	199.112221	225.883422	219.717283	5.655E-87	3.862525	up	hypothetical protein 0_2321_02, partial [Pinus lambertiana]
c36731.graph_c2	5.3185548	5.89943518	6.46511816	119.846449	114.336648	112.17679	2.023E-52	3.273742	up	PREDICTED: tubulin beta-1 chain [Sesamum indicum]
c36731.graph_c3	2.76625666	2.42289832	1.333031	63.0742876	52.9831862	56.6900221	2.195E-15	3.719259	up	PREDICTED: tubulin beta-6 chain-like [Citrus sinensis]
c36732.graph_c0	1.26794341	1.60766988	1.62935748	11.1360027	19.6070755	20.6259025	5.992E-08	2.482046	up	PREDICTED: LOW QUALITY PROTEIN: probable galacturonosyltransferase-like 7 [Sesamum indicum]
c36736.graph_c0	86.5260092	84.9418416	88.079676	28.1253887	26.8526796	24.7168412	2.174E-76	-2.719568	down	PREDICTED: uncharacterized protein LOC105162600 [Sesamum indicum]
c36738.graph_c0	3.09743751	3.62989947	2.62119282	15.9381848	17.5481601	17.8355017	8.49E-06	1.439726	up	PREDICTED: uncharacterized protein LOC105156818 [Sesamum indicum]
c36747.graph_c0	2.03885284	2.13366237	1.54818573	1.02454048	2.1255288	1.89922908	0.0052946	-1.20621	down	-
c36759.graph_c0	1418.62285	1555.23514	1461.46938	31.039205	25.6951484	23.9097282	5.11E-224	-6.794334	down	PREDICTED: GEM-like protein 5 [Sesamum indicum]
c36760.graph_c0	16.3360711	16.2620843	16.701214	3.46451525	4.02257626	4.73503463	3.976E-63	-3.033769	down	PREDICTED: sodium/hydrogen exchanger 1-like [Sesamum indicum]

#ID	0 h-1 FPKM	0 h-2 FPKM	0 h-3 FPKM	48 h-1 FPKM	48 h-2 FPKM	48 h-3 FPKM	FDR	log2FC	regulated	nr_annotation
c36762.graph_c0	0	0.42407859	0.09073544	22.3120376	24.8773186	31.6029207	4.932E-42	6.240376	up	PREDICTED: glucomannan 4-beta-mannosyltransferase 2-like [Sesamum indicum]
c36762.graph_c1	0.10188959	0	0.06546608	16.2390487	19.6893295	24.5974025	2.269E-39	7.475906	up	PREDICTED: glucomannan 4-beta-mannosyltransferase 2-like [Sesamum indicum]
c36764.graph_c1	0.32280271	0	0	22.6014054	23.3133577	23.2859516	2.266E-14	6.747294	up	PREDICTED: probable small nuclear ribonucleoprotein F isoform X2 [Nicotiana tomentosiformis]
c36764.graph_c2	2.20539106	0.36793324	0	10.4973755	8.60957143	12.6548731	2.321E-05	2.628598	up	hypothetical protein MIMGU_mgv1a017211mg [Erythranthe guttata]
c36767.graph_c0	77.8982265	71.4447157	75.248726	17.1089066	24.7869051	23.5071274	3.073E-82	-2.803631	down	PREDICTED: uncharacterized protein LOC105169073 [Sesamum indicum]
c36770.graph_c0	99.3520527	106.805938	103.460261	6.02505713	6.08404727	7.39141834	1.19E-213	-5.008212	down	PREDICTED: probable protein phosphatase 2C 49 [Sesamum indicum]
c36772.graph_c0	5.19648461	5.89882156	4.95663473	68.9969289	75.2898333	78.6076995	3.354E-58	2.777136	up	PREDICTED: protein CREG1 isoform X1 [Sesamum indicum]
c36783.graph_c0	0.60219266	0.42698026	0.4191641	61.83476	84.8277663	82.0469415	2.792E-90	6.28272	up	PREDICTED: probable methyltransferase PMT3 [Sesamum indicum]
c36786.graph_c0	0	0	0.0373088	1.57808888	1.55845186	1.85636562	8.667E-13	6.013215	up	PREDICTED: uncharacterized protein LOC105170278 isoform X1 [Sesamum indicum]
c36791.graph_c0	64.7031365	64.4122763	59.0494516	261.056501	266.540293	282.154644	2.209E-14	1.088192	up	PREDICTED: FRIGIDA-like protein 4a [Sesamum indicum]
c36799.graph_c0	105.804709	112.891859	112.811027	6.63927533	7.68580822	7.00334941	5.77E-212	-4.978163	down	PREDICTED: uncharacterized protein LOC105174622 [Sesamum indicum]
c36800.graph_c0	8.61204457	9.34408094	7.35208204	0.55482358	0.58776831	0.78988589	1.45E-36	-4.729956	down	hypothetical protein MIMGU_mgv1a002177mg [Erythranthe guttata]
c36801.graph_c0	0.0635073	0.0211903	0.02720313	2.78883705	2.89245354	3.01943591	1.491E-18	5.268647	up	PREDICTED: BTB/POZ domain-containing protein At3g08570 [Sesamum indicum]
c36802.graph_c0	6.89943425	6.76539721	6.51382984	4.58338869	7.05428171	8.1565434	9.895E-05	-1.053204	down	-
c36803.graph_c0	0	0	0	2.90866382	2.92335731	1.85813139	2.006E-11	Inf	up	--
c36807.graph_c0	117.928822	125.60817	114.891674	89.4465993	88.4660589	87.4358535	5.435E-25	-1.450378	down	PREDICTED: uncharacterized protein LOC105162095 [Sesamum indicum]
c36813.graph_c0	0.90059375	0.95300959	0.82664187	6.6374856	5.22348257	4.40840324	9.424E-06	1.591653	up	PREDICTED: WPP domain-interacting tail-anchored protein 2-like [Sesamum indicum]
c36814.graph_c0	32.9132465	32.54531	36.2593907	8.41900352	9.55851672	8.23731204	1.038E-77	-2.976011	down	PREDICTED: uncharacterized protein At5g19025-like isoform X2 [Sesamum indicum]
c36825.graph_c0	0	0.13483276	0.1730921	8.56238041	7.03315947	5.52083667	4.714E-15	5.074822	up	PREDICTED: transcription factor bHLH104 [Sesamum indicum]
c36829.graph_c0	77.2286372	76.2254546	76.291389	41.0434794	44.6298563	40.9444994	7.529E-34	-1.877858	down	PREDICTED: transcription factor ILR3-like [Sesamum indicum]
c36831.graph_c0	0.41742301	0.64997489	0.65560623	9.22937534	25.3940911	27.4501815	6.529E-06	4.134592	up	PREDICTED: 1-aminocyclopropane-1-carboxylate oxidase 5-like [Sesamum indicum]
c36832.graph_c0	0.68554216	0.21114727	0.33882647	12.0483389	11.1682848	10.5476276	9.916E-19	3.761581	up	protein with unknown function [Ricinus communis]

#ID	0 h-1 FPKM	0 h-2 FPKM	0 h-3 FPKM	48 h-1 FPKM	48 h-2 FPKM	48 h-3 FPKM	FDR	log2FC	regulated	nr_annotation
c36835.graph_c0	52.0723284	52.790362	50.4355811	40.8225252	41.5152121	40.837584	1.478E-21	-1.351542	down	PREDICTED: NAC transcription factor 29-like isoform X1 [Sesamum indicum]
c36843.graph_c0	2.24591195	1.54040654	1.33615082	0.30653046	1.09596999	0.88643411	1.859E-06	-2.193408	down	--
c36851.graph_c0	1.51207359	2.09949261	1.5669955	12.9865489	11.8058931	16.0335522	1.684E-10	1.961697	up	PREDICTED: serine/threonine-protein kinase D6PKL2 [Sesamum indicum]
c36851.graph_c1	0	1.18320614	1.32907752	9.25612809	9.94994749	11.748479	7.193E-07	2.59119	up	PREDICTED: serine/threonine-protein kinase D6PKL2 [Sesamum indicum]
c36852.graph_c0	1.28556562	0.7218931	0.68497674	21.0004925	31.2137894	28.6331895	1.982E-31	3.889065	up	PREDICTED: probable polygalacturonase isoform X2 [Sesamum indicum]
c36853.graph_c0	171.511192	167.237817	150.169434	20.024545	35.1278593	48.1525473	1.07E-104	-3.270259	down	PREDICTED: uncharacterized protein LOC105157533 [Sesamum indicum]
c36854.graph_c0	0.294792	0.20656103	0.35987842	9.07082613	9.47997188	9.41233344	3.859E-54	3.999105	up	PREDICTED: aldehyde dehydrogenase 22A1 [Sesamum indicum]
c36857.graph_c0	11.778537	11.5599697	10.1085138	5.0304651	6.22757157	7.58017739	2.729E-28	-1.849448	down	PREDICTED: ABC transporter C family member 5-like [Sesamum indicum]
c36865.graph_c0	0	0.09257952	0	61.0919642	62.0567304	78.2788541	4.84E-102	10.07941	up	PREDICTED: cinnamoyl-CoA reductase 1-like [Sesamum indicum]
c36866.graph_c0	0.87074821	0.87162013	1.35045198	18.2290772	13.3627354	10.2883331	6.011E-07	2.745231	up	PREDICTED: adenine/guanine permease AZG1 [Sesamum indicum]
c36868.graph_c0	5.85884804	1.90060202	2.43990564	0	0	0	1.916E-07	-Inf	down	PREDICTED: ribulose bisphosphate carboxylase/oxygenase activase 2, chloroplastic-like isoform X2 [Phoenix dactylifera]
c36871.graph_c0	2.92743281	3.3018188	3.0730745	12.0792615	16.0962427	18.7583966	3.976E-07	1.311192	up	PREDICTED: fatty acyl-CoA reductase 3-like isoform X1 [Sesamum indicum]
c36878.graph_c0	6.97047104	8.00166389	6.20439	24.655611	31.6743425	33.4449424	1.774E-09	1.063168	up	PREDICTED: transcription factor bHLH143-like [Sesamum indicum]
c36881.graph_c0	3.71753715	3.63794792	2.95900105	60.8803239	62.7624493	62.448958	8.619E-86	3.157631	up	PREDICTED: 65-kDa microtubule-associated protein 1 [Sesamum indicum]
c36885.graph_c0	20.9165267	22.1464593	16.2259053	26.2239454	16.0471293	8.66302309	0.0005128	-1.218776	down	PREDICTED: uncharacterized protein LOC105173633 [Sesamum indicum]
c36890.graph_c0	25.8125898	27.6692356	24.5228884	8.36123459	10.844806	9.33508048	9.987E-54	-2.470952	down	PREDICTED: uncharacterized protein LOC105180144 isoform X2 [Sesamum indicum]
c36892.graph_c0	2.12702127	2.23053931	2.21267672	30.3263803	27.9753099	26.9012024	6.263E-16	2.681126	up	PREDICTED: uncharacterized protein At1g76660-like [Sesamum indicum]
c36892.graph_c1	4.15314489	3.34391814	2.78449858	48.9913294	46.9658723	39.0035386	5.741E-26	2.702694	up	PREDICTED: uncharacterized protein At1g76660-like [Sesamum indicum]
c36897.graph_c0	0.54051123	0.59023906	0.50514788	2.17289107	3.45287191	4.51132594	0.0008195	1.605324	up	PREDICTED: probable LRR receptor-like serine/threonine-protein kinase At2g24230 [Sesamum indicum]

#ID	0 h-1 FPKM	0 h-2 FPKM	0 h-3 FPKM	48 h-1 FPKM	48 h-2 FPKM	48 h-3 FPKM	FDR	log2FC	regulated	nr_annotation
c36899.graph_c0	2.8531731	3.56236015	2.77939836	14.1460091	15.5399087	15.303117	5.259E-14	1.272729	up	PREDICTED: starch synthase 3, chloroplastic/amyloplastic [Sesamum indicum]
c36900.graph_c0	0	0.15550867	0	2.28994264	2.3248367	2.12247624	2.289E-09	4.431773	up	PREDICTED: probable LRR receptor-like serine/threonine-protein kinase At1g06840 [Sesamum indicum]
c36900.graph_c1	0.09710944	0	0	2.72863832	3.26976458	2.86574869	3.837E-12	5.513626	up	PREDICTED: probable LRR receptor-like serine/threonine-protein kinase At1g06840 [Sesamum indicum]
c36904.graph_c0	15.496594	15.6374849	15.348648	2.79025854	3.25597966	3.62146257	6.23E-102	-3.286413	down	PREDICTED: LOW QUALITY PROTEIN: U-box domain-containing protein 17-like [Sesamum indicum]
c36905.graph_c0	3.06531597	3.42937193	3.01221652	17.8297651	16.2487805	15.6184696	2.175E-06	1.371473	up	PREDICTED: outer envelope pore protein 24A, chloroplastic-like [Sesamum indicum]
c36918.graph_c0	9.2002345	9.91016595	9.76654983	55.0009358	50.6540717	52.8743363	8.711E-12	1.440259	up	PREDICTED: probable small nuclear ribonucleoprotein F isoform X1 [Sesamum indicum]
c36920.graph_c0	0.25863176	0.17259383	0.08308801	2.1841274	1.87208923	2.06709583	4.124E-07	2.567677	up	hypothetical protein MIMGU_mgv1a002741mg [Erythranthe guttata]
c36923.graph_c0	10.4806406	10.4436642	8.71461288	47.2723827	47.3948817	48.0882971	1.015E-13	1.252346	up	PREDICTED: uncharacterized protein LOC105173973 [Sesamum indicum]
c36925.graph_c0	25.5276409	29.5790905	24.1902797	12.6079722	15.7008405	21.6127796	6.468E-21	-1.690245	down	PREDICTED: protein ECERIFERUM 1-like [Sesamum indicum]
c36926.graph_c0	4.22277186	4.93941611	5.36545791	191.148884	199.674358	212.671974	2.09E-162	4.355728	up	hypothetical protein CICLE_v10008112mg [Citrus clementina]
c36926.graph_c1	3.65784063	3.50230761	3.47426046	137.284407	138.606736	150.705438	5.252E-78	4.308202	up	PREDICTED: serine carboxypeptidase II-2 [Sesamum indicum]
c36930.graph_c0	19.5663794	22.4875976	16.451967	215.864817	227.503822	218.213286	1.5E-38	2.483483	up	PREDICTED: protein transport protein Sec61 subunit beta-like [Vitis vinifera]
c36933.graph_c0	0	0.02368482	0	0.63214713	0.83133091	0.93100256	2.44E-10	5.648064	up	PREDICTED: uncharacterized protein LOC105176404 [Sesamum indicum]
c36938.graph_c0	71.2579824	67.6811949	61.30252	41.8941306	39.5507234	34.5125725	3.551E-36	-1.801951	down	PREDICTED: uncharacterized protein LOC105174627 [Sesamum indicum]
c36942.graph_c0	2.08721367	1.8716679	1.89985781	0.56083923	0.63657982	1.03370785	2.369E-07	-2.415744	down	-
c36943.graph_c0	23.1671247	22.1223476	23.6598502	0.56165964	1.07101851	1.92408128	1.92E-160	-5.308714	down	PREDICTED: probable protein phosphatase 2C 24 [Sesamum indicum]
c36948.graph_c0	17.9822936	18.579709	17.9012302	17.5263211	18.152738	15.5631827	1.984E-11	-1.104721	down	PREDICTED: mitochondrial ribosome-associated GTPase 1 [Sesamum indicum]
c36949.graph_c0	64.2194328	65.8768922	56.4479945	7.69780505	8.38788793	7.63225614	6.05E-116	-3.992363	down	PREDICTED: probable receptor-like protein kinase At5g47070 [Sesamum indicum]
c36951.graph_c0	13.2609844	13.3270437	13.9918242	127.269326	131.558679	126.383183	7.859E-56	2.228493	up	PREDICTED: mechanosensitive ion channel protein 8-like [Sesamum indicum]
c36954.graph_c0	34.8819259	33.943078	34.1691267	3.37145408	3.25517175	1.78475037	6.54E-138	-4.626503	down	PREDICTED: heat stress transcription factor B-2b [Sesamum indicum]
c36959.graph_c0	3.43120575	3.00193083	2.96842214	16.3652813	18.0094179	18.5039156	1.986E-20	1.473353	up	PREDICTED: protein PIR [Sesamum indicum]

#ID	0 h-1 FPKM	0 h-2 FPKM	0 h-3 FPKM	48 h-1 FPKM	48 h-2 FPKM	48 h-3 FPKM	FDR	log2FC	regulated	nr_annotation
c36972.graph_c0	0.03841162	0	0	687.716644	814.288698	1136.15986	8.22E-70	15.06588	up	chalcone synthase [Misopates orontium]
c36976.graph_c0	12.0267499	10.9337829	13.7376442	41.1077618	59.4825193	63.4389962	6.56E-06	1.133785	up	hypothetical protein MIMGU_mgv1a008959mg [Erythranthe guttata]
c36981.graph_c0	25.9928419	26.9650105	24.3714545	14.6895888	21.3774735	24.3896713	8.007E-22	-1.379311	down	PREDICTED: uncharacterized protein LOC105173636 [Sesamum indicum]
c36990.graph_c1	0	0	0	1.80130504	1.4311982	1.53600746	8.473E-10	Inf	up	NADH dehydrogenase subunit 2 [Amorphotheca resinae]
c36991.graph_c0	12.4553961	12.6987547	11.6189359	45.6438992	53.3516753	50.7481761	4.385E-10	1.006589	up	hypothetical protein CISIN_1g024570mg [Citrus sinensis]
c36993.graph_c0	3.63666985	1.96694246	2.78888379	0	0	0	1.891E-24	-Inf	down	--
c36995.graph_c0	0	0	0	0.68554632	1.28744885	1.19226294	1.469E-10	Inf	up	PREDICTED: protein ECERIFERUM 3-like [Sesamum indicum]
c37003.graph_c0	49.5921483	54.221855	49.1235695	38.3448842	37.3086334	30.610351	2.014E-23	-1.540219	down	PREDICTED: BTB/POZ and MATH domain-containing protein 2-like [Sesamum indicum]
c37003.graph_c1	29.1925526	28.1356651	28.483767	22.6576704	21.2800188	21.0077454	2.212E-18	-1.417906	down	PREDICTED: BTB/POZ and MATH domain-containing protein 2-like [Sesamum indicum]
c37005.graph_c0	12.9191153	11.6709096	12.1836349	9.93467022	11.6916604	8.3846909	1.522E-16	-1.311176	down	PREDICTED: MND1-interacting protein 1-like isoform X2 [Sesamum indicum]
c37013.graph_c0	3.75058623	1.62688148	2.4098259	0	0	0	1.883E-12	-Inf	down	-
c37014.graph_c0	4.76361864	5.0929387	5.19200548	4.04390519	4.77084847	5.70403218	3.752E-06	-1.074215	down	PREDICTED: putative pentatricopeptide repeat-containing protein At1g26500 [Sesamum indicum]
c37020.graph_c0	0.28393242	0.21316255	0.2736483	33.7434409	37.5482937	33.6323311	1.116E-59	6.069592	up	PREDICTED: 7-deoxyloganetin glucosyltransferase-like [Sesamum indicum]
c37021.graph_c0	1.83691161	1.99198025	1.34885951	10.3954514	12.3786391	12.655748	1.866E-15	1.757153	up	PREDICTED: putative late blight resistance protein homolog R1B-16 [Sesamum indicum]
c37023.graph_c0	0.26600159	0.17751197	0.11394086	12.7430169	9.9949563	11.7020784	1.709E-28	4.942278	up	PREDICTED: bifunctional riboflavin kinase/FMN phosphatase-like isoform X2 [Sesamum indicum]
c37025.graph_c0	9.5134281	7.9357953	8.61316101	8.69799358	6.18987265	7.50310415	3.051E-05	-1.230892	down	PREDICTED: syntaxin-51-like [Sesamum indicum]
c37038.graph_c0	0.25122182	0.12573669	0.08070749	1.73581442	1.53240408	2.36825976	1.72E-05	2.612715	up	-
c37038.graph_c1	0.72828745	0.24300557	0.10398647	2.46013496	2.96160759	3.98002417	7.599E-05	2.11902	up	unnamed protein product [Coffea canephora]
c37039.graph_c0	2.75929989	1.49611741	1.9945179	0	0	0	1.443E-18	-Inf	down	hypothetical protein POPTR_0005s04730g [Populus trichocarpa]
c37041.graph_c0	67.1090667	63.5866948	62.1199475	57.7493308	49.3608894	51.8812964	5.952E-20	-1.292351	down	hypothetical protein JCGZ_06000 [Jatropha curcas]
c37042.graph_c0	0	0	0.14752383	1.21626494	1.8486962	1.22338505	1.717E-08	3.806358	up	-
c37048.graph_c0	1.00423355	1.03990256	1.06798334	10.432038	11.2880553	10.7584594	6.851E-15	2.364411	up	PREDICTED: mannose-6-phosphate isomerase 2-like [Sesamum indicum]
c37049.graph_c0	30.3509647	31.4121526	31.4106933	16.4272569	18.7250794	17.0604248	3.185E-29	-1.854997	down	PREDICTED: uncharacterized protein LOC105155538 isoform X3 [Sesamum indicum]
c37051.graph_c0	1.56493984	1.38221196	0.94635748	23.4068238	20.7537527	21.5064005	4.563E-16	3.065366	up	PREDICTED: transcription repressor OFP1 [Sesamum indicum]

#ID	0 h-1 FPKM	0 h-2 FPKM	0 h-3 FPKM	48 h-1 FPKM	48 h-2 FPKM	48 h-3 FPKM	FDR	log2FC	regulated	nr_annotation
c37056.graph_c0	0.45666316	0.26121168	0	1.50252817	2.16477512	2.03214961	0.0009214	1.983172	up	PREDICTED: AP2-like ethylene-responsive transcription factor At1g16060 isoform X1 [Sesamum indicum]
c37057.graph_c0	7.94047466	7.75917761	7.08020463	35.2825563	42.464949	42.0655798	5.15E-19	1.375271	up	hypothetical protein MIMGU_mgv1a003064mg [Erythranthe guttata]
c37063.graph_c0	0.17172866	0.34380124	0.1765425	30.0278108	51.8224812	71.2025839	3.741E-14	6.762867	up	PREDICTED: fasciclin-like arabinogalactan protein 8 [Sesamum indicum]
c37064.graph_c0	1.53315439	0.98902219	0.83184706	11.7703485	17.8559761	19.0193046	1.498E-13	2.838048	up	hypothetical protein MIMGU_mgv1a007836mg [Erythranthe guttata]
c37065.graph_c0	1.91116503	1.70810605	1.66651899	0.18864534	0.13323125	0.16785549	1.594E-13	-4.443465	down	--
c37067.graph_c0	8.36272045	6.4243283	6.78940923	30.7872726	40.8404058	46.5517616	1.247E-08	1.431291	up	PREDICTED: polyadenylate-binding protein-interacting protein 8-like [Sesamum indicum]
c37072.graph_c0	0	0	0	2.18259673	2.60122116	2.34665758	1.122E-11	Inf	up	-
c37074.graph_c0	10.3307979	11.0275997	11.0013115	7.7035954	6.71449429	6.58260441	5.09E-22	-1.638719	down	PREDICTED: uncharacterized protein LOC105165632 [Sesamum indicum]
c37077.graph_c0	0.05061038	0.10132213	0.06503635	15.3864437	13.929152	13.8982009	1.039E-37	6.622967	up	hypothetical protein MIMGU_mgv1a0032972mg, partial [Erythranthe guttata]
c37078.graph_c0	45.1292829	50.4367324	43.3387915	885.067935	943.910509	626.800433	6.459E-17	3.129077	up	3-hydroxy-3-methylglutaryl-coenzyme A reductase [Andrographis paniculata]
c37079.graph_c0	19.780804	20.3681449	23.3953013	104.813377	124.563174	120.889853	7.751E-23	1.440407	up	PREDICTED: translocon-associated protein subunit beta [Sesamum indicum]
c37080.graph_c1	5.78507286	5.85056538	5.518056	25.1094949	27.240739	25.0311732	3.554E-07	1.15567	up	PREDICTED: GDSL esterase/lipase CPRD49-like [Sesamum indicum]
c37096.graph_c0	9.22197731	9.32445629	8.25951324	9.61150853	6.90936889	5.03972611	4.389E-05	-1.321062	down	-
c37104.graph_c0	13.75557	13.6207541	12.6532876	77.7977756	98.063721	102.308194	3.169E-28	1.775673	up	PREDICTED: calcium-transporting ATPase 4, plasma membrane-type-like [Sesamum indicum]
c37111.graph_c0	0.57547862	1.06482871	0.82915073	4.75545026	4.57829178	4.60304553	5.69E-07	1.478856	up	PREDICTED: probable LRR receptor-like serine/threonine-protein kinase At1g67720 [Sesamum indicum]
c37113.graph_c0	0.10534813	0.08436289	0	9.68594573	7.11490064	5.75142136	7.136E-16	5.900958	up	PREDICTED: LOW QUALITY PROTEIN: respiratory burst oxidase homolog protein C-like [Sesamum indicum]
c37117.graph_c0	0.38044831	0.598446	0.41904955	14.7207395	13.6852665	17.5536723	1.515E-25	4.021839	up	PREDICTED: psbP-like protein 1, chloroplastic [Sesamum indicum]
c37119.graph_c0	2.57804928	3.10482143	1.91526765	21.8951503	25.8292944	27.3741042	2.785E-25	2.287794	up	PREDICTED: uncharacterized protein LOC105157824 isoform X3 [Sesamum indicum]
c37122.graph_c0	15.3210229	14.9801652	16.1823653	0.30961334	0.19293987	0.12964333	5.7E-218	-7.205295	down	PREDICTED: uncharacterized protein LOC105166759 [Sesamum indicum]
c37123.graph_c0	0.41352759	0.11826905	0	21.9873298	18.8534196	16.5617681	2.342E-34	5.759947	up	PREDICTED: phospholipase D alpha 1-like [Sesamum indicum]

#ID	0 h-1 FPKM	0 h-2 FPKM	0 h-3 FPKM	48 h-1 FPKM	48 h-2 FPKM	48 h-3 FPKM	FDR	log2FC	regulated	nr_annotation
c37127.graph_c0	0.46082408	0.49011587	0.55516611	3.84740461	3.87909308	4.69831793	6.238E-07	2.023404	up	PREDICTED: uncharacterized protein LOC105170631 [Sesamum indicum]
c37135.graph_c0	0.14802243	0.06350171	0	2.22084901	3.24014979	3.9001936	3.683E-14	4.460797	up	hypothetical protein MIMGU_mgv1a001054mg [Erythranthe guttata]
c37136.graph_c0	361.428755	351.449907	357.726262	51.2585309	31.8826423	27.1874412	5.77E-155	-4.284786	down	PREDICTED: uncharacterized protein LOC105171465 [Sesamum indicum]
c37138.graph_c0	0	0	0	1.85499988	1.55574173	1.75372822	2.736E-12	Inf	up	PREDICTED: probable polygalacturonase At1g80170 [Sesamum indicum]
c37142.graph_c0	0.88301914	0.49105742	0.50431758	0	0	0	7.571E-15	-Inf	down	predicted protein [Hordeum vulgare subsp. vulgare]
c37145.graph_c0	0.84300135	0.25315365	0.37915154	15.9985334	21.5558671	26.1212657	4.042E-22	4.413142	up	PREDICTED: ACT domain-containing protein DS12, chloroplastic-like isoform X1 [Sesamum indicum]
c37146.graph_c0	4.97232749	4.26087604	3.43685057	51.2220351	68.1970581	83.9902438	4.99E-15	2.984121	up	PREDICTED: arogenate dehydratase/prephenate dehydratase 6, chloroplastic-like [Sesamum indicum]
c37147.graph_c0	7.1172503	7.96253917	8.37738132	4.73858208	3.73577907	4.65760901	1.266E-09	-1.852893	down	conserved hypothetical protein [Ricinus communis]
c37149.graph_c0	0.46814569	0.2677797	0.77346739	5.88397801	7.50614179	9.73095564	8.312E-12	2.905259	up	PREDICTED: replication protein A 70 kDa DNA-binding subunit B [Sesamum indicum]
c37153.graph_c0	12.85877	12.7120803	13.3148164	14.4407835	12.0571025	8.71140497	5.424E-12	-1.154984	down	PREDICTED: F-box/LRR-repeat protein 10 [Sesamum indicum]
c37155.graph_c1	29.5413518	30.7747055	30.8397686	177.59868	170.335113	168.055567	3.643E-26	1.484133	up	PREDICTED: cytochrome c oxidase subunit 6b-1 [Sesamum indicum]
c37156.graph_c0	1.58653351	0.68062379	1.45625598	33.6172175	41.7332608	45.5807969	1.195E-45	3.997664	up	hypothetical protein MIMGU_mgv1a016103mg [Erythranthe guttata]
c37157.graph_c0	28.7792422	27.8442218	28.0461821	1.80697934	0.93973525	0.76006951	1.96E-177	-5.595378	down	PREDICTED: probable protein phosphatase 2C 8 isoform X1 [Sesamum indicum]
c37160.graph_c0	0.23686964	0.23710683	0.25365572	3.12781656	2.96677632	3.26853894	3.652E-07	2.667794	up	hypothetical protein MIMGU_mgv1a007966mg [Erythranthe guttata]
c37161.graph_c0	0.06087761	0	0	14.8062883	11.1699539	8.7331273	5.716E-17	8.166803	up	hypothetical protein MIMGU_mgv1a014011mg [Erythranthe guttata]
c37164.graph_c0	0	0	0	5.51299072	9.1776853	7.94211828	1.771E-11	Inf	up	PREDICTED: flavonoid 3'-monooxygenase-like, partial [Solanum tuberosum]
c37173.graph_c0	1.89470668	1.97788697	1.80868859	1230.44718	1210.39044	1453.02534	6.42E-278	8.402872	up	PREDICTED: vignain-like [Sesamum indicum]
c37180.graph_c1	3.05762296	1.70038039	2.61944437	20.3441456	21.5521555	18.3805923	0.0001285	2.012861	up	PREDICTED: LOW QUALITY PROTEIN: protein transport protein SEC31 homolog B-like [Sesamum indicum]
c37182.graph_c0	326.859368	311.578992	348.117387	160.035555	153.013886	140.359927	3.454E-49	-2.138337	down	hypothetical protein CISIN_1g033114mg [Citrus sinensis]
c37184.graph_c0	0.04748089	0	0	2.79951932	3.56816949	4.8263037	3.509E-15	6.878426	up	unnamed protein product [Coffea canephora]
c37190.graph_c0	25.8032818	26.6355598	32.1821264	16.6465281	14.4224916	12.3024348	2.212E-17	-1.979922	down	PREDICTED: zinc finger CCCH domain-containing protein 30-like [Sesamum indicum]
c37191.graph_c0	13.4873583	11.6429468	12.9590903	5.69974047	6.27971126	8.60480584	4.477E-31	-1.909647	down	PREDICTED: kinesin-3-like [Sesamum indicum]

#ID	0 h-1 FPKM	0 h-2 FPKM	0 h-3 FPKM	48 h-1 FPKM	48 h-2 FPKM	48 h-3 FPKM	FDR	log2FC	regulated	nr_annotation
c37195.graph_c0	10.7141459	10.3123793	10.061305	4.39294599	4.71124785	6.32166688	4.319E-14	-2.031511	down	PREDICTED: ethylene-responsive transcription factor ERF069-like [Sesamum indicum]
c37198.graph_c0	31.3791121	31.8243249	34.2386725	29.1508313	30.0748839	26.2462894	5.574E-16	-1.207003	down	PREDICTED: uncharacterized protein LOC105173158 [Sesamum indicum]
c37199.graph_c0	4.71465978	4.7193808	4.66670129	31.049813	24.3747763	22.0919042	1.896E-06	1.447121	up	hypothetical protein M569_02394, partial [Genlisea aurea]
c37202.graph_c1	0.4932154	0.49370928	0.70836617	3.66178591	3.11470071	3.49606246	0.0001205	1.579857	up	PREDICTED: condensin complex subunit 3 [Sesamum indicum]
c37204.graph_c0	3.61841721	3.09455888	3.47607109	16.6676097	14.4001375	13.1892785	1.209E-06	1.104948	up	PREDICTED: rab3 GTPase-activating protein non-catalytic subunit [Sesamum indicum]
c37208.graph_c0	0.28928245	0.19304808	0	4.08642325	2.16453496	2.92466889	6.565E-05	3.259979	up	hypothetical protein MIMGU_mgv1a022771mg, partial [Erythranthe guttata]
c37212.graph_c1	5.43275581	6.3861383	4.67555377	56.9836347	60.3186073	55.2388366	3.106E-41	2.371214	up	Pollen Ole e 1 allergen and extensin family protein [Theobroma cacao]
c37215.graph_c0	29.1000146	26.8138398	27.3933034	22.4318794	22.0250375	19.4216836	3.043E-21	-1.39885	down	PREDICTED: elongator complex protein 3 [Sesamum indicum]
c37217.graph_c0	12.9364216	13.1049235	11.8812845	7.22946194	8.68243105	8.15237293	4.294E-18	-1.675621	down	PREDICTED: IAA-amino acid hydrolase ILR1-like 4 isoform X2 [Sesamum indicum]
c37225.graph_c0	0.07869007	0.15753772	0.15167977	6.12577857	4.41594529	3.58003304	1.016E-09	4.173252	up	PREDICTED: cytochrome P450 714A1-like [Sesamum indicum]
c37229.graph_c1	27.085447	30.0051519	25.7718636	28.4891962	23.8668638	21.0008539	7.74E-15	-1.187699	down	PREDICTED: NAC domain-containing protein 100-like [Sesamum indicum]
c37234.graph_c0	22.8842419	19.0063006	21.309534	116.029895	126.965674	132.740618	7.197E-24	1.552357	up	PREDICTED: 60S ribosomal protein L21-1-like [Sesamum indicum]
c37236.graph_c0	0	0	0	23.0252565	24.597446	18.077413	1.629E-19	Inf	up	-
c37239.graph_c0	4.89723339	4.87428418	4.7198541	27.4031519	28.1884646	24.7024902	2.193E-17	1.453511	up	BnaC09g00400D [Brassica napus]
c37240.graph_c0	1.34908544	1.10035554	1.6052113	10.4953087	11.1185069	7.70030071	2.006E-07	1.835225	up	PREDICTED: uncharacterized protein LOC105173599 [Sesamum indicum]
c37244.graph_c0	0.32655398	0.14009185	0.05994783	1.50421412	1.86670946	2.25622858	4.952E-05	2.407393	up	PREDICTED: F-box/kelch-repeat protein At1g57790-like [Sesamum indicum]
c37246.graph_c0	0.27791934	0.09273254	0.11904578	1.2375142	1.26578947	0.79737195	0.002249	1.744437	up	PREDICTED: ALA-interacting subunit 3-like [Sesamum indicum]
c37251.graph_c0	19.3012341	22.3646369	19.4594707	10.1772248	7.38959186	10.1748691	1.301E-26	-2.153122	down	PREDICTED: uncharacterized protein LOC105176792 [Sesamum indicum]
c37253.graph_c0	19.8458937	19.8657664	24.4075487	84.013592	91.2599466	89.8255141	4.585E-09	1.026495	up	PREDICTED: uncharacterized protein LOC105167377 [Sesamum indicum]
c37256.graph_c0	6.79662951	3.25381689	5.69604653	0	0	0	6.423E-15	-Inf	down	predicted protein [Hordeum vulgare subsp. vulgare]
c37266.graph_c0	2.61480383	2.7076781	2.08559573	28.4087352	19.9757413	16.0389153	7.852E-05	2.114505	up	PREDICTED: protein TRANSPORT INHIBITOR RESPONSE 1-like [Sesamum indicum]
c37269.graph_c0	1.99189318	1.46489713	1.72385373	12.1338788	14.9174209	13.9290145	2.865E-12	1.96343	up	PREDICTED: GPN-loop GTPase 2 isoform X1 [Sesamum indicum]

#ID	0 h-1 FPKM	0 h-2 FPKM	0 h-3 FPKM	48 h-1 FPKM	48 h-2 FPKM	48 h-3 FPKM	FDR	log2FC	regulated	nr_annotation
c37274.graph_c0	0.08933512	0.17884915	0	4.44427233	2.96440184	3.80802432	3.109E-09	4.389672	up	cytochrome P450 CYP72A219-like [Sesamum indicum]
c37276.graph_c1	0.06086077	0.09138256	0.03910425	0.58872353	1.06916775	1.4966933	0.0004129	3.019295	up	PREDICTED: AT-rich interactive domain-containing protein 2 isoform X1 [Nicotiana tomentosiformis]
c37277.graph_c0	7.38132822	5.73881126	6.53840999	111.377394	104.667041	106.710422	4.928E-88	3.021292	up	hypothetical protein MIMGU_mgv1a004994mg [Erythranthe guttata]
c37279.graph_c0	7.88963908	7.81352299	9.49136038	4.948732	5.89790418	4.40335198	3.297E-07	-1.745176	down	-
c37282.graph_c0	33.0854992	31.7422707	30.20305	18.2487268	18.200052	15.9910494	1.836E-33	-1.873281	down	hypothetical protein MIMGU_mgv1a011134mg [Erythranthe guttata]
c37283.graph_c0	3.72411766	3.7278468	3.50779795	2.8560896	2.83538624	3.37245859	9.574E-08	-1.292017	down	PREDICTED: pentatricopeptide repeat-containing protein At3g06920 isoform X2 [Sesamum indicum]
c37288.graph_c0	6.72958949	5.9675081	7.00282908	177.568548	220.273488	251.599845	2.656E-48	4.01966	up	monodehydroascorbate reductase [Acanthus ebracteatus]
c37289.graph_c0	6.93863421	6.50274307	7.77942656	5.62009554	5.24935135	5.00072722	4.11E-13	-1.436389	down	PREDICTED: uncharacterized protein LOC105168518 isoform X1 [Sesamum indicum]
c37290.graph_c0	85.4188277	79.5799022	85.7165954	46.5676273	36.9475271	36.6276671	4.743E-47	-2.074308	down	hypothetical protein MIMGU_mgv1a012378mg [Erythranthe guttata]
c37293.graph_c0	3.2043211	1.98112131	2.66438084	18.9277192	22.5350813	23.2540045	2.248E-09	2.022928	up	PREDICTED: cytochrome P450 734A1 [Sesamum indicum]
c37293.graph_c1	4.99997804	5.30329511	4.04232533	18.1201702	33.1892567	38.3537167	0.0007424	1.617764	up	PREDICTED: BTB/POZ domain-containing protein At3g50780-like [Sesamum indicum]
c37295.graph_c0	2.73084264	1.98259443	1.85102817	18.5784396	23.6354976	20.8359275	2.096E-27	2.245762	up	PREDICTED: ATP-dependent 6-phosphofructokinase 5, chloroplastic-like [Sesamum indicum]
c37299.graph_c0	1.65082269	2.06559468	1.25956493	5.75069719	11.0766661	9.98013921	0.0011673	1.405072	up	PREDICTED: peptide methionine sulfoxide reductase A5 isoform X1 [Sesamum indicum]
c37303.graph_c0	21.0586751	20.390881	23.9659924	11.5389338	15.5152054	17.1497323	1.314E-15	-1.59056	down	PREDICTED: uncharacterized protein LOC105163935 [Sesamum indicum]
c37304.graph_c0	0	0.22251673	0.19043784	30.991834	12.8001761	8.92890549	0.0003304	5.991646	up	PREDICTED: uncharacterized protein LOC105169888 [Sesamum indicum]
c37307.graph_c0	0	0.03710522	0.04763398	0.71714071	0.79590094	0.69887963	1.083E-07	3.674386	up	PREDICTED: ferric reduction oxidase 7, chloroplastic [Sesamum indicum]
c37313.graph_c0	8.64452375	9.18404987	7.36026975	8.45248189	6.46992749	5.12990851	1.73E-07	-1.337214	down	PREDICTED: C2 and GRAM domain-containing protein At1g03370 isoform X2 [Sesamum indicum]
c37319.graph_c0	0.31052036	0.36411666	0.39903095	5.03486025	5.50700003	6.50180131	1.46E-38	2.966106	up	PREDICTED: sporulation-specific protein 15 [Sesamum indicum]
c37321.graph_c1	1.60097217	1.9910784	3.05479752	1.2961418	1.32575662	1.86914005	0.0052075	-1.592715	down	PREDICTED: uncharacterized protein LOC105164751 [Sesamum indicum]
c37329.graph_c0	0.47381605	0.62022604	0.23418168	3.89501452	3.66386591	3.67490122	1.258E-05	2.071253	up	PREDICTED: E3 ubiquitin-protein ligase RGLG2-like [Sesamum indicum]
c37333.graph_c0	0.39916062	0.44656741	0.39224609	2.70391761	2.97905176	2.598404	5.906E-05	1.72424	up	PREDICTED: uncharacterized protein LOC105172792, partial [Sesamum indicum]

#ID	0 h-1 FPKM	0 h-2 FPKM	0 h-3 FPKM	48 h-1 FPKM	48 h-2 FPKM	48 h-3 FPKM	FDR	log2FC	regulated	nr_annotation
c37339.graph_c0	0.44937542	0.2249127	0.3849768	2.20796772	2.11978587	2.08742617	0.0039528	1.581456	up	PREDICTED: uncharacterized protein LOC105170136 [Sesamum indicum]
c37340.graph_c0	2.01715435	1.72126327	1.97596277	15.4607309	29.7399729	32.9622626	1.106E-06	2.744395	up	PREDICTED: probable glucan 1,3-alpha-glucosidase [Sesamum indicum]
c37343.graph_c0	10.4264017	11.1453484	9.58523468	9.17912836	11.0260437	11.2247793	5.732E-09	-1.007247	down	PREDICTED: uncharacterized protein LOC105164939 [Sesamum indicum]
c37349.graph_c0	0.17787149	0.1780496	0	7.18282867	6.53882106	6.97446858	3.111E-25	4.862056	up	PREDICTED: uncharacterized protein LOC105169807 [Sesamum indicum]
c37350.graph_c0	0.07641185	0.03824418	0.14728837	5.70204183	5.85418296	5.48078668	3.133E-19	4.995312	up	PREDICTED: OTU domain-containing protein DDB_G0284757-like [Sesamum indicum]
c37352.graph_c0	14.5202732	14.5348131	14.3065191	5.01318636	5.66311419	4.64277259	1.58E-46	-2.519257	down	hypothetical protein MIMGU_mgv1a003689mg [Erythranthe guttata]
c37358.graph_c0	1381.26568	1564.69499	1419.82621	126.540303	68.7441512	47.8211626	2.46E-117	-5.168044	down	hypothetical protein MIMGU_mgv1a011603mg [Erythranthe guttata]
c37361.graph_c0	19.3089128	21.3015505	17.2130108	10.260283	10.4584242	10.5935791	4.121E-39	-1.901218	down	PREDICTED: uncharacterized protein LOC105159352 [Sesamum indicum]
c37364.graph_c0	8.08760645	7.47295843	7.46156527	2.61097482	2.46242462	2.71987866	1.377E-24	-2.579211	down	PREDICTED: uncharacterized protein LOC105156132 [Sesamum indicum]
c37366.graph_c0	0.23917368	0.50542781	0.71714451	6.61027178	7.49556974	7.90772655	8.56E-18	2.883858	up	hypothetical protein MIMGU_mgv1a005273mg [Erythranthe guttata]
c37371.graph_c0	0	0	0	1.73577701	1.87289796	1.00105441	3.29E-10	Inf	up	-
c37372.graph_c0	6.05579039	6.27681372	6.07100954	33.1178289	42.084422	46.1912485	1.941E-17	1.699233	up	PREDICTED: interactor of constitutive active ROPs 1-like [Sesamum indicum]
c37374.graph_c0	1.58615318	2.64623578	1.92503243	0.64945234	0.43001	0.57787879	4.237E-08	-2.90505	down	-
c37384.graph_c0	9.63052192	9.59047385	8.10155385	6.21973212	3.87591294	4.06932371	2.862E-14	-1.954075	down	hypothetical protein MIMGU_mgv1a022389mg [Erythranthe guttata]
c37384.graph_c1	14.7307393	15.3613949	11.3019379	9.56966442	7.523946	6.64587696	7.321E-16	-1.810787	down	PREDICTED: ankyrin repeat-containing protein At5g02620-like [Sesamum indicum]
c37386.graph_c0	0	0.66846369	2.1453575	71.365091	89.289349	102.640456	3.014E-32	5.503369	up	--
c37387.graph_c0	0.04412318	0	0	33.9826905	30.8760612	29.5503756	2E-101	10.06931	up	hypothetical protein MIMGU_mgv1a011856mg [Erythranthe guttata]
c37388.graph_c0	4.53429769	4.52103874	4.93560174	4.02985699	3.31466123	3.65862346	5.535E-11	-1.36189	down	PREDICTED: uncharacterized protein LOC105155378 [Sesamum indicum]
c37389.graph_c0	0.3210305	0.49663485	0.22502013	2.78277846	2.7486336	2.78710721	1.234E-07	1.98373	up	unnamed protein product [Coffea canephora]
c37391.graph_c1	17.8745803	18.8189504	18.7324431	119.107629	95.5242563	107.925427	2.388E-14	1.526556	up	PREDICTED: auxin-induced protein AUX28-like [Sesamum indicum]

#ID	0 h-1 FPKM	0 h-2 FPKM	0 h-3 FPKM	48 h-1 FPKM	48 h-2 FPKM	48 h-3 FPKM	FDR	log2FC	regulated	nr_annotation
c37394.graph_c0	11.0471451	9.18226129	10.6470141	41.9815713	44.3781194	40.1848701	4.745E-06	1.017045	up	PREDICTED: 20 kDa chaperonin, chloroplastic [Solanum lycopersicum]
c37395.graph_c0	3.85326308	2.62985559	3.03847912	11.5371011	14.102492	13.855029	0.0050577	1.032638	up	hypothetical protein MIMGU_mgv1a013530mg [Erythranthe guttata]
c37400.graph_c0	1.36499	1.58373179	1.47501021	7.88806635	7.05453916	6.89160267	2.888E-05	1.287995	up	PREDICTED: aspartic proteinase-like protein 2 [Sesamum indicum]
c37417.graph_c0	1.66005351	1.89091798	2.05967467	10.3362936	13.0730186	13.4672875	3.707E-07	1.692621	up	PREDICTED: uncharacterized protein LOC105162643 [Sesamum indicum]
c37423.graph_c0	0.86393071	0.95679536	1.0865642	9.85572813	10.7997444	9.99007446	3.486E-25	2.3772	up	PREDICTED: vacuolar protein sorting-associated protein 18 homolog [Sesamum indicum]
c37424.graph_c0	20.6869037	17.4916626	16.1112063	98.3225996	88.1008257	74.9612064	5.049E-08	1.255531	up	hypothetical protein MIMGU_mgv1a016992mg [Erythranthe guttata]
c37427.graph_c0	21.3741468	19.7314514	25.0251471	196.584394	211.502295	212.687452	6.953E-46	2.209719	up	hypothetical protein MIMGU_mgv1a021808mg [Erythranthe guttata]
c37428.graph_c0	0.9061252	0.73696395	0.83691736	0	0.16581561	0.16248393	3.753E-12	-3.964016	down	-
c37432.graph_c0	2.55203528	2.69919023	2.75351421	18.8753514	17.881395	17.130683	4.924E-20	1.734395	up	PREDICTED: uncharacterized protein LOC105168937 [Sesamum indicum]
c37434.graph_c0	8.20562597	7.76743817	5.38689184	3.28676428	3.2207836	5.70285904	7.496E-07	-1.824541	down	PREDICTED: uncharacterized protein LOC105168847 isoform X2 [Sesamum indicum]
c37435.graph_c2	0	0	0	1.62814881	1.72482624	2.19805341	1.55E-11	Inf	up	PREDICTED: probable auxin efflux carrier component 1b isoform X2 [Sesamum indicum]
c37435.graph_c3	1.33209216	1.03232985	0.66262884	8.03624735	9.60381962	8.27779346	1.795E-08	2.08433	up	PREDICTED: auxin efflux carrier component 2 [Sesamum indicum]
c37436.graph_c0	3.64172577	1.6004074	1.99745931	0	0	0	5.796E-12	-Inf	down	--
c37437.graph_c0	9.39612095	11.2866357	10.4884977	8.60463996	9.77255088	8.09320609	2.365E-12	-1.254785	down	PREDICTED: heterogeneous nuclear ribonucleoprotein 1 [Sesamum indicum]
c37439.graph_c1	0.83341551	1.48973224	1.22396792	11.4621083	11.1550291	9.75972734	1.178E-08	2.172931	up	hypothetical protein MIMGU_mgv1a015229mg [Erythranthe guttata]
c37444.graph_c1	11.7362554	16.7731668	12.2919015	9.37474335	8.40858858	8.67525387	8.746E-09	-1.639413	down	PREDICTED: E3 ubiquitin-protein ligase RNF170-like [Sesamum indicum]
c37447.graph_c0	18.5128669	21.2036997	16.6371415	11.7988325	13.6744471	14.8753016	1.579E-21	-1.501161	down	PREDICTED: uncharacterized protein LOC105157015 isoform X1 [Sesamum indicum]
c37456.graph_c0	8.64740309	3.82864287	6.62461375	0	0	0	1.792E-13	-Inf	down	hypothetical protein TSTA_040370 [Talaromyces stipitatus ATCC 10500]
c37458.graph_c0	4.01372903	4.11719738	3.14064103	16.8227453	20.9632352	20.8650752	1.802E-15	1.360629	up	PREDICTED: probable long-chain-alcohol O-fatty-acyltransferase 5 [Sesamum indicum]

#ID	0 h-1 FPKM	0 h-2 FPKM	0 h-3 FPKM	48 h-1 FPKM	48 h-2 FPKM	48 h-3 FPKM	FDR	log2FC	regulated	nr_annotation
c37460.graph_c0	1.10707358	1.07023071	0.69182856	37.7715389	36.6249481	40.4400641	1.5E-161	4.309822	up	PREDICTED: acetyl-CoA carboxylase 1-like isoform X1 [Sesamum indicum]
c37473.graph_c0	1.60810108	2.53734163	2.06647356	14.9655358	15.2423485	12.3108227	2.497E-15	1.758122	up	PREDICTED: uncharacterized protein LOC105162786 [Sesamum indicum]
c37475.graph_c0	0.35434448	0.1182331	0.75891114	7.50824067	11.7581913	11.0378227	1.393E-10	3.583051	up	PREDICTED: reticulon-like protein B8 [Sesamum indicum]
c37477.graph_c0	0.66450709	0.95618546	0.96065766	3.06093639	6.24218007	6.6387818	0.0018122	1.59452	up	hypothetical protein MIMGU_mgv1a010755mg [Erythranthe guttata]
c37479.graph_c0	1.39530165	1.54118492	1.73118966	0.04432562	0.09391523	0.39440667	6.515E-13	-4.169534	down	hypothetical protein MIMGU_mgv1a022974mg [Erythranthe guttata]
c37480.graph_c0	6.91765299	6.92457997	5.15808055	26.0033856	29.9229313	31.0135602	5.692E-10	1.176706	up	PREDICTED: 60S ribosomal protein L11-like [Sesamum indicum]
c37481.graph_c0	0.97053277	0.85720995	0.917039	20.8539897	13.7623998	12.7994928	4.373E-08	3.102502	up	PREDICTED: uncharacterized protein LOC105162739 [Sesamum indicum]
c37483.graph_c0	59.6199521	57.8878846	61.5742993	407.693959	406.43701	396.455278	2.384E-35	1.739381	up	hypothetical protein MIMGU_mgv1a016379mg [Erythranthe guttata]
c37485.graph_c0	44.8184972	50.0323316	45.5885568	16.0147474	17.0810958	16.6409532	2E-63	-2.515766	down	PREDICTED: replication protein A 70 kDa DNA-binding subunit A [Sesamum indicum]
c37486.graph_c0	0.08022014	0.08030046	0.03436202	3.27641163	3.28827869	2.7180533	7.921E-15	4.565559	up	PREDICTED: nucleobase-ascorbate transporter 1 [Sesamum indicum]
c37489.graph_c0	0.14161268	0.14175448	0.36395579	5.21851337	4.07718183	4.58535929	6.369E-09	3.397783	up	PREDICTED: CASP-like protein 3A1 [Sesamum indicum]
c37490.graph_c0	0.76319402	0.51949161	0.54921134	5.7935779	6.37594642	7.33224077	6.294E-11	2.394181	up	PREDICTED: beta-hexosaminidase 3 [Sesamum indicum]
c37493.graph_c0	0	0.06917437	0.08880288	24.7653709	21.0764039	18.3539571	6.121E-46	7.640254	up	PREDICTED: serine/threonine-protein kinase-like protein CCR1 [Sesamum indicum]
c37494.graph_c0	14.1052788	11.8313975	13.1789169	8.09051862	7.66099664	7.91385355	7.697E-23	-1.741473	down	hypothetical protein MIMGU_mgv1a003260mg [Erythranthe guttata]
c37495.graph_c0	0.73902028	0.69624499	0.81001292	13.5766246	19.6437482	18.4591347	3.701E-32	3.49964	up	PREDICTED: uncharacterized protein LOC105159731 [Sesamum indicum]
c37496.graph_c0	1.18280206	0.94718917	0.70930882	4.72192244	6.42603701	7.3688516	1.62E-05	1.685767	up	hypothetical protein MIMGU_mgv1a012483mg [Erythranthe guttata]
c37503.graph_c0	0.0873203	0	0.05610502	432.392731	637.076251	687.629609	8.538E-85	12.55577	up	PREDICTED: WAT1-related protein At2g39510-like [Sesamum indicum]
c37506.graph_c0	1.62265211	0.84744884	1.6318739	34.7075148	36.9061128	35.7404653	6.29E-40	3.689288	up	PREDICTED: cold-regulated 413 plasma membrane protein 2-like [Sesamum indicum]
c37508.graph_c0	0.1540825	0.27762622	0	4.85471907	6.1354923	6.84584009	1.102E-19	4.361182	up	PREDICTED: kinesin-4-like [Sesamum indicum]
c37508.graph_c1	0.2172957	0.29001772	0.23269465	6.47271217	6.78636241	8.87657076	7.575E-19	3.882709	up	PREDICTED: kinesin-4-like [Sesamum indicum]
c37510.graph_c0	1.72032766	2.30754741	1.5474824	9.54096583	12.6259756	14.4970097	1.995E-09	1.695958	up	PREDICTED: laccase-4-like [Sesamum indicum]
c37512.graph_c1	6.17331981	6.14860395	6.3067035	52.0100372	56.9058181	53.3882125	8.218E-42	2.10419	up	PREDICTED: dolichyl-diphosphooligosaccharide--protein glycosyltransferase 48 kDa subunit-like [Sesamum indicum]

#ID	0 h-1 FPKM	0 h-2 FPKM	0 h-3 FPKM	48 h-1 FPKM	48 h-2 FPKM	48 h-3 FPKM	FDR	log2FC	regulated	nr_annotation
c37514.graph_c0	1.46084451	2.05513462	1.57282525	12.4034165	10.8279071	11.8456119	1.453E-09	1.770287	up	PREDICTED: uncharacterized protein LOC105161074 [Sesamum indicum]
c37515.graph_c0	4.88266602	4.18374731	4.54267946	19.7562279	23.5407286	27.7132987	6.443E-12	1.361787	up	PREDICTED: uncharacterized protein LOC105164119 [Sesamum indicum]
c37516.graph_c0	2.25548555	2.36787794	1.6789444	9.4392427	12.1204818	11.8487644	2.617E-14	1.387571	up	hypothetical protein MIMGU_mgv1a001577mg [Erythranthe guttata]
c37517.graph_c0	0	0	0	0.66123508	0.53884491	0.56573365	1.128E-10	Inf	up	hypothetical protein [Tuber melanosporum Mel28]
c37518.graph_c0	36.3781275	36.9126261	38.0088507	17.7756116	16.9965562	15.6353528	1.803E-40	-2.159048	down	hypothetical protein MIMGU_mgv1a009711mg [Erythranthe guttata]
c37520.graph_c0	102.078254	107.261005	101.367731	52.1916707	54.4574503	49.6671634	1.847E-40	-2.008361	down	PREDICTED: probable E3 ubiquitin ligase SUD1 [Sesamum indicum]
c37521.graph_c0	1.79678597	2.13581991	2.54945581	10.1732989	13.5173876	14.9437027	2.572E-09	1.548841	up	PREDICTED: inactive beta-amylase 9 [Sesamum indicum]
c37522.graph_c0	3.41678101	2.16012783	3.00416239	16.0701509	16.8488673	20.0482559	0.0003006	1.606065	up	PREDICTED: elongation factor Tu, mitochondrial [Sesamum indicum]
c37522.graph_c1	1.66852606	1.27720935	1.51349792	8.22830049	10.6326859	9.17197936	0.0003148	1.631177	up	hypothetical protein MIMGU_mgv1a006302mg [Erythranthe guttata]
c37524.graph_c0	24.907545	20.8541537	25.9619803	6.13324305	8.79304833	7.16166019	1.735E-36	-2.723043	down	PREDICTED: uncharacterized protein LOC105159531 [Sesamum indicum]
c37525.graph_c0	11.7932581	12.5444242	11.1050857	7.55313418	9.1790468	12.5332595	1.024E-14	-1.298738	down	PREDICTED: probable protein phosphatase 2C 33 [Sesamum indicum]
c37526.graph_c0	12.0341671	11.0155251	12.3218866	11.1459466	8.03933882	8.28224793	2.655E-08	-1.376515	down	-
c37527.graph_c0	9.40616473	10.6507462	9.92977707	5.85159087	7.08745001	7.44526419	1.1E-20	-1.57831	down	PREDICTED: uncharacterized protein LOC105178831 [Sesamum indicum]
c37528.graph_c0	0	0	0	2.31613051	1.54230021	1.2365274	7.603E-07	Inf	up	--
c37531.graph_c0	59.2718691	60.1059929	60.9333588	26.8334399	25.325673	22.2399601	4.887E-56	-2.292651	down	PREDICTED: F-box/LRR-repeat protein 14 [Sesamum indicum]
c37536.graph_c0	3.09622239	2.86293377	2.89978033	20.1121336	18.4894193	18.4333855	1.982E-18	1.671325	up	PREDICTED: lysM domain receptor-like kinase 3 [Sesamum indicum]
c37539.graph_c0	0.58002341	0.77413895	0.49690203	4.51233445	7.73649155	9.66781309	1.803E-05	2.540228	up	PREDICTED: growth-regulating factor 3 [Sesamum indicum]
c37539.graph_c1	15.4605972	13.0324873	14.116367	2.43635575	2.77956376	2.66812886	5.433E-15	-3.453414	down	-
c37540.graph_c0	1.722227	1.72395155	2.28690087	11.0799511	12.6903406	12.4706519	7.402E-12	1.637154	up	hypothetical protein MIMGU_mgv1a025916mg, partial [Erythranthe guttata]
c37541.graph_c0	549.678837	496.755883	524.75179	420.005128	412.839974	356.213281	1.45E-20	-1.417862	down	PREDICTED: protein translation factor SUI1 homolog [Nicotiana tomentosiformis]
c37544.graph_c0	379.406992	354.534787	356.753969	22.8359436	22.9057609	19.9591967	2.46E-210	-5.069208	down	PREDICTED: uncharacterized GPI-anchored protein At3g06035 [Sesamum indicum]
c37546.graph_c0	7.00358441	7.70395323	7.34331133	29.4424058	30.6836464	30.2984557	8.343E-12	1.01763	up	PREDICTED: calcium-transporting ATPase 4, endoplasmic reticulum-type-like [Sesamum indicum]

#ID	0 h-1 FPKM	0 h-2 FPKM	0 h-3 FPKM	48 h-1 FPKM	48 h-2 FPKM	48 h-3 FPKM	FDR	log2FC	regulated	nr_annotation
c37546.graph_c1	6.74675816	6.66580604	5.29198719	27.283859	31.640408	29.4484485	3.344E-06	1.223007	up	PREDICTED: calcium-transporting ATPase 4, endoplasmic reticulum-type-like [Sesamum indicum]
c37547.graph_c0	105.359496	113.329673	98.6421047	88.3005439	57.7303418	44.170551	1.228E-20	-1.743914	down	PREDICTED: F-box protein SKIP2 [Sesamum indicum]
c37548.graph_c0	1.61628554	1.9985873	1.5271992	7.79549775	8.72233881	9.19655808	0.0002128	1.303646	up	PREDICTED: protein-S-isoprenylcysteine O-methyltransferase A isoform X2 [Sesamum indicum]
c37552.graph_c0	1.18961132	1.16833456	1.70175776	11.0215102	10.4053996	9.7700747	3.495E-16	1.921977	up	PREDICTED: uncharacterized protein LOC105164201 isoform X1 [Sesamum indicum]
c37555.graph_c0	24.6451084	25.2972646	26.4976071	20.5466728	23.602067	21.5276857	3.406E-16	-1.239035	down	hypothetical protein M569_08441 [Genlisea aurea]
c37557.graph_c0	0.37618014	0.25103788	0.32227092	3.52337709	3.42664073	3.1864745	1.547E-05	2.400284	up	PREDICTED: cancer-related nucleoside-triphosphatase homolog isoform X1 [Sesamum indicum]
c37559.graph_c0	4.8194351	4.69503976	3.76013584	125.587948	131.449332	140.707191	3.99E-128	3.888796	up	PREDICTED: betaine aldehyde dehydrogenase 1, chloroplastic [Sesamum indicum]
c37563.graph_c0	0	0	0	5.99320925	8.56621701	8.88786925	3.811E-16	Inf	up	hypothetical protein MIMGU_mgv1a018370mg [Erythranthe guttata]
c37565.graph_c0	0.19413641	0.16194234	0.2910518	5.00783139	8.30514884	9.17711094	2.074E-13	4.086726	up	PREDICTED: uncharacterized protein LOC105172319 [Sesamum indicum]
c37573.graph_c0	0.61506506	0.41045397	0.50057587	16.6591587	16.1276384	18.2685253	9.218E-69	4.04726	up	PREDICTED: putative potassium transporter 12 [Sesamum indicum]
c37575.graph_c0	23.6741513	22.1975614	23.4185448	13.2116296	13.8298949	12.7329374	2.359E-30	-1.818614	down	hypothetical protein MIMGU_mgv1a005560mg [Erythranthe guttata]
c37577.graph_c0	0.9835976	1.8707068	1.39035805	0.09061543	0.09599606	0.16125812	3.187E-11	-4.632432	down	PREDICTED: glycerol-3-phosphate acyltransferase 5-like [Sesamum indicum]
c37578.graph_c0	18.8518484	20.0154901	16.5788799	15.6719525	14.3005014	13.0366387	5.469E-22	-1.379733	down	PREDICTED: lysine-specific demethylase JMJ25-like [Sesamum indicum]
c37579.graph_c0	0	0	0	1.49849569	1.34935331	2.0666963	8.908E-17	Inf	up	PREDICTED: protein STICHEL-like 2 [Sesamum indicum]
c37580.graph_c0	50.9575476	48.1747642	44.4817762	2.2524316	2.26058984	1.95145799	1.71E-168	-5.48864	down	PREDICTED: protein PLANT CADMIUM RESISTANCE 2-like [Sesamum indicum]
c37581.graph_c0	47.0961353	44.1759596	46.0903576	41.2538976	46.2134359	48.1815717	2.676E-13	-1.038	down	PREDICTED: uncharacterized protein LOC105174416 isoform X1 [Sesamum indicum]
c37584.graph_c0	32.6052918	35.8628143	28.2658262	6.03832766	8.34846525	8.24143256	1.1E-63	-3.117292	down	PREDICTED: LOW QUALITY PROTEIN: ethylene-responsive transcription factor ERF061 [Sesamum indicum]
c37587.graph_c0	0.38234673	0.51941873	0.42114059	4.88107804	5.83726474	4.25360432	3.176E-10	2.482055	up	PREDICTED: G-type lectin S-receptor-like serine/threonine-protein kinase At1g11300 [Sesamum indicum]
c37588.graph_c0	12.5258256	13.3281868	11.0265183	10.540114	7.02686214	8.0040903	1.919E-07	-1.537083	down	PREDICTED: CBL-interacting serine/threonine-protein kinase 1-like [Sesamum indicum]
c37590.graph_c0	0.03021021	0.03024046	0.03882132	3.28413069	3.74449625	3.7889539	4.861E-18	5.744948	up	PREDICTED: LOW QUALITY PROTEIN: interactor of constitutive active ROPs 3 [Sesamum indicum]

#ID	0 h-1 FPKM	0 h-2 FPKM	0 h-3 FPKM	48 h-1 FPKM	48 h-2 FPKM	48 h-3 FPKM	FDR	log2FC	regulated	nr_annotation
c37594.graph_c0	0.44419793	0.63917392	0.60648768	0.15345904	0.3522377	0.52342991	0.0007805	-1.749898	down	PREDICTED: beta-xylosidase/alpha-L-arabinofuranosidase 2-like [Sesamum indicum]
c37595.graph_c0	1488.66418	1562.82425	1488.39846	67.2423407	88.3162126	98.6260173	1.07E-173	-5.181712	down	hypothetical protein CISIN_1g036208mg, partial [Citrus sinensis]
c37599.graph_c0	2.03544939	2.95635453	2.7182069	0.03676841	0	0	9.789E-31	-8.673981	down	-
c37600.graph_c0	7.88081605	7.91111859	7.13504499	583.073966	636.968444	702.765967	3.34E-201	5.371263	up	PREDICTED: alpha-L-arabinofuranosidase 1-like [Sesamum indicum]
c37601.graph_c0	10.6611181	13.0718757	11.7192419	5.75891861	9.0677403	8.73930504	1.09E-14	-1.614643	down	PREDICTED: UDP-glycosyltransferase 74F2-like [Sesamum indicum]
c37602.graph_c0	1.18989297	0.9966217	1.09218541	5.83898023	9.64588682	11.1474104	5.348E-06	1.995191	up	PREDICTED: probable serine/threonine-protein kinase At1g54610 isoform X1 [Sesamum indicum]
c37605.graph_c0	0.27667303	0.43520726	0.43172204	1.31085895	1.96731949	1.92779077	0.007969	1.159157	up	PREDICTED: uncharacterized protein LOC105155780 [Sesamum indicum]
c37606.graph_c0	0.13301875	0.16643994	0.21366796	4.96309489	4.25167554	3.43476932	7.967E-13	3.606592	up	PREDICTED: aspartic proteinase-like protein 1 [Sesamum indicum]
c37607.graph_c0	4.21568496	3.49731963	4.41548854	23.8346062	24.4326359	24.3085963	1.637E-18	1.562006	up	PREDICTED: protein IQ-DOMAIN 1 [Sesamum indicum]
c37608.graph_c0	2.74216312	3.81237359	4.60050123	2.73678115	4.53478345	2.93469375	0.0079047	-1.156615	down	PREDICTED: glyoxylate/hydroxypyruvate reductase HPR3-like [Nicotiana tomentosiformis]
c37609.graph_c0	3.39339231	3.28046185	3.07634437	1.9913557	2.54059343	3.14368704	1.081E-06	-1.367634	down	PREDICTED: uncharacterized protein LOC105159694 [Sesamum indicum]
c37612.graph_c0	25.1190175	27.3367703	21.4284893	18.7148164	23.0327314	22.7630125	7.734E-16	-1.214961	down	hypothetical protein MIMGU_mgv1a010621mg [Erythranthe guttata]
c37614.graph_c0	0.61995837	0.84221458	0.62595582	7.38410295	6.61244167	6.93333208	1.755E-08	2.310802	up	PREDICTED: 3-phosphoinositide-dependent protein kinase 2-like [Sesamum indicum]
c37617.graph_c0	7.18120744	6.93349059	6.47932945	0.32844381	0.7455994	1.1689893	1.176E-30	-4.230441	down	PREDICTED: uncharacterized protein LOC104880778 [Vitis vinifera]
c37620.graph_c0	0.67753259	0.64251572	0.59571214	27.2999778	25.9279868	29.0268551	3.129E-70	4.40798	up	-
c37621.graph_c0	2.97544777	2.52227171	2.41125922	0.56799154	0.5755565	0.54934289	2.849E-17	-3.239643	down	PREDICTED: polyadenylate-binding protein-interacting protein 4-like [Sesamum indicum]
c37623.graph_c0	14.4927563	11.0531571	11.9090746	156.405305	155.396816	166.969873	1.19E-58	2.659448	up	ATP binding protein, putative [Ricinus communis]
c37627.graph_c0	19.1109789	16.4950192	14.4977823	367.621652	446.640272	499.601271	3.748E-58	3.693305	up	PREDICTED: low-temperature-induced cysteine proteinase [Sesamum indicum]
c37629.graph_c0	0	0	0	6.67155981	7.79885093	5.9772539	1.851E-23	Inf	up	PREDICTED: hippocampus abundant transcript 1 protein-like isoform X1 [Sesamum indicum]
c37630.graph_c0	0.03845902	0.11549258	0.09884272	5.52722893	7.46942525	6.49440758	1.637E-21	5.242369	up	hypothetical protein MIMGU_mgv1a004581mg [Erythranthe guttata]
c37632.graph_c0	17.6676907	18.5985369	16.1246383	9.98208443	11.2834292	11.7968814	7.737E-30	-1.68302	down	PREDICTED: uncharacterized protein LOC105164604 [Sesamum indicum]

#ID	0 h-1 FPKM	0 h-2 FPKM	0 h-3 FPKM	48 h-1 FPKM	48 h-2 FPKM	48 h-3 FPKM	FDR	log2FC	regulated	nr_annotation
c37634.graph_c0	0.88851321	0.64683849	0.72658387	9.07853372	11.7855086	14.0372233	5.333E-17	2.92531	up	PREDICTED: ureidoglycolate hydrolase [Sesamum indicum]
c37636.graph_c0	2.26648961	2.11229301	1.22527077	15.5666838	23.1423386	31.0849857	8.988E-07	2.617936	up	PREDICTED: LRR receptor-like serine/threonine-protein kinase RCH1 [Sesamum indicum]
c37639.graph_c0	1.59698029	1.51216972	2.1631117	0.19881654	0.33699523	0.17690576	2.963E-13	-3.914207	down	PREDICTED: LOW QUALITY PROTEIN: transcription factor GAMYB [Sesamum indicum]
c37640.graph_c0	3.72685691	4.61882422	3.02173107	45.3293672	31.9129427	31.0959401	1.149E-07	2.24547	up	hypothetical protein MIMGU_mgv1a011727mg [Erythranthe guttata]
c37641.graph_c0	64.0167555	57.8258215	59.7119228	34.4794592	39.705871	38.9080736	7.279E-30	-1.702449	down	PREDICTED: probable prefoldin subunit 2 [Sesamum indicum]
c37642.graph_c0	5.1036899	5.35054182	5.21365232	21.3140737	29.5405651	38.6297488	5.912E-05	1.488628	up	PREDICTED: LOW QUALITY PROTEIN: uncharacterized protein LOC105173535 [Sesamum indicum]
c37644.graph_c0	28.9225562	32.2920775	24.5054702	20.2036134	20.1625089	16.4138317	1.155E-10	-1.607938	down	PREDICTED: uncharacterized protein LOC102585852 isoform X1 [Solanum tuberosum]
c37647.graph_c0	3.54267988	3.2608987	2.61637006	15.6434807	20.5863025	23.065605	5.379E-11	1.633757	up	PREDICTED: acetyl-coenzyme A carboxylase carboxyl transferase subunit alpha, chloroplastic-like [Sesamum indicum]
c37648.graph_c0	0.12406632	0.28386413	0.09110294	2.31861636	4.2725658	5.76793722	4.56E-06	3.60672	up	PREDICTED: probable LRR receptor-like serine/threonine-protein kinase At2g24230 [Sesamum indicum]
c37651.graph_c0	3.97891729	3.7173748	3.54505927	32.11099	39.2989871	38.13971	1.069E-27	2.264702	up	PREDICTED: ras-related protein RABA1d [Sesamum indicum]
c37654.graph_c0	0.37448132	0.56228446	0.42107041	7.84867307	8.31471741	9.01741754	7.983E-14	3.194287	up	Alpha/beta-Hydrolases superfamily protein isoform 1 [Theobroma cacao]
c37664.graph_c0	17.9047204	9.02770482	10.0554682	0	0	0	1.484E-16	-Inf	down	metallothionein-like protein type 2 [Narcissus pseudonarcissus]
c37666.graph_c0	0.56297104	0.81968693	0.52613823	8.67552704	7.89198746	8.76826763	1.104E-10	2.716223	up	PREDICTED: beta-galactosidase 9-like [Sesamum indicum]
c37666.graph_c1	0.33227813	0.20468361	0.3612997	4.61529174	6.06178621	5.53141803	1.667E-15	3.148906	up	PREDICTED: beta-galactosidase 9-like [Sesamum indicum]
c37668.graph_c0	4.75903982	5.31047146	5.11300466	5.10308181	5.63452301	4.63662161	0.0003265	-1.000153	down	PREDICTED: uncharacterized protein LOC105157340 isoform X2 [Sesamum indicum]
c37669.graph_c0	1.57992616	1.42721474	1.93123022	11.7152498	11.4330592	7.99185508	5.101E-07	1.639662	up	PREDICTED: alkylated DNA repair protein alkB homolog 8 [Sesamum indicum]
c37670.graph_c0	22.5102761	22.7994773	20.0261049	21.6670221	14.7638335	10.981025	1.398E-13	-1.468445	down	PREDICTED: cationic amino acid transporter 1-like isoform X1 [Sesamum indicum]
c37671.graph_c0	81.6068808	78.2675227	84.3015049	408.315085	444.438036	468.819482	2.022E-24	1.416306	up	BnaC02g12080D [Brassica napus]
c37672.graph_c0	2.52881597	3.25459054	1.85693066	1.76392345	2.04495197	1.59914222	3.68E-05	-1.51306	down	PREDICTED: probable serine/threonine-protein kinase At1g18390 isoform X1 [Nicotiana sylvestris]
c37673.graph_c0	106.354669	106.088201	105.920549	150.194091	91.5159084	60.7123357	0.0002237	-1.077887	down	PREDICTED: ethylene receptor 1 [Sesamum indicum]
c37673.graph_c1	0.2788139	0.31397973	0.31350104	21.0947549	19.5241557	17.3700937	3.747E-58	4.983878	up	PREDICTED: ethylene receptor 1 [Sesamum indicum]
c37674.graph_c0	6820.08107	6352.57917	6186.29338	1461.44949	1321.97182	1432.3633	2.839E-49	-3.214433	down	2S albumin precursor isoform 3 [Sesamum indicum]
c37675.graph_c0	1.92646068	1.24412241	2.23600615	11.5074525	9.03692454	9.52607959	5.802E-05	1.459196	up	hypothetical protein EUGRSUZ_B02864 [Eucalyptus grandis]

#ID	0 h-1 FPKM	0 h-2 FPKM	0 h-3 FPKM	48 h-1 FPKM	48 h-2 FPKM	48 h-3 FPKM	FDR	log2FC	regulated	nr_annotation
c37676.graph_c0	4.50914426	6.53847869	4.16982266	17.0823536	22.6619816	23.2832924	8.452E-06	1.029738	up	PREDICTED: cytochrome b561 and DOMON domain-containing protein At5g47530-like [Nicotiana sylvestris]
c37679.graph_c0	0.69448552	0.91637488	0.6896138	0.75613361	0.43132486	0.72455723	0.0088555	-1.276753	down	PREDICTED: uncharacterized protein LOC105042593 [Elaeis guineensis]
c37684.graph_c0	0.70571057	0.57797774	0.61831775	2.09820043	5.60393266	5.2590595	0.0071842	1.736711	up	PREDICTED: uncharacterized protein LOC105162014 [Sesamum indicum]
c37685.graph_c0	240.100628	228.699912	242.721351	148.699308	141.449685	119.124269	9.466E-35	-1.813266	down	PREDICTED: protein DEHYDRATION-INDUCED 19 homolog 3-like isoform X2 [Sesamum indicum]
c37686.graph_c1	72.3527657	65.9933744	62.5288385	338.768309	384.817132	397.614166	2.719E-25	1.461836	up	PREDICTED: calmodulin-7 isoform X1 [Vitis vinifera]
c37688.graph_c0	0	0	0	2.67835114	2.93196788	2.4626201	2.159E-11	Inf	up	-
c37693.graph_c0	1.55631132	1.3052422	1.24319454	8.99015986	7.67665973	8.5855524	3.458E-06	1.607618	up	PREDICTED: uncharacterized protein LOC105162119 isoform X1 [Sesamum indicum]
c37694.graph_c0	8.1426415	8.06705408	8.09855266	60.0893302	82.8715941	98.2935596	1.1E-11	2.286645	up	PREDICTED: transcription factor DIVARICATA [Sesamum indicum]
c37697.graph_c0	0.90584938	0.90675645	0.6008012	140.68519	147.727038	147.721052	8.34E-257	6.483356	up	PREDICTED: granule-bound starch synthase 1, chloroplastic/amyloplastic [Sesamum indicum]
c37698.graph_c0	67.4279551	57.5471615	61.5964174	318.553712	333.365384	330.573482	4.532E-23	1.378901	up	PREDICTED: 40S ribosomal protein S8 [Sesamum indicum]
c37704.graph_c1	0	0	0	1.80407712	1.88700854	1.19886281	6.027E-15	Inf	up	--
c37707.graph_c0	1.07914519	1.02878647	0.79242531	27.1268824	32.5743435	35.8900715	1.005E-68	4.024076	up	Dihydrolipoyllysine-residue acetyltransferase component of pyruvate dehydrogenase, putative [Ricinus communis]
c37710.graph_c0	502.178741	501.604659	521.849556	831.081948	422.748819	263.728642	0.0091863	-1.007506	down	PREDICTED: uncharacterized protein LOC105175543 [Sesamum indicum]
c37711.graph_c0	2.09139902	4.42624284	2.76431713	0.82574279	0.87477436	1.51846468	0.0001238	-2.55024	down	PREDICTED: telomere repeat-binding protein 5 [Sesamum indicum]
c37711.graph_c1	4.51566514	4.34498585	3.37372587	1.25771229	2.04983641	2.06603969	1.192E-11	-2.210172	down	PREDICTED: telomere repeat-binding protein 5 [Sesamum indicum]
c37713.graph_c1	3.91029662	3.71348336	2.89897268	2.17068915	2.2017274	3.49307709	0.0001345	-1.438437	down	PREDICTED: phosphoenolpyruvate/phosphate translocator 2, chloroplastic [Sesamum indicum]
c37714.graph_c0	10.2166985	9.04119804	11.4164017	4.77434949	6.21392308	5.09782496	1.065E-06	-1.953956	down	hypothetical protein glysoja_022734 [Glycine soja]
c37717.graph_c0	18.8792366	18.2802185	17.5839529	4.54961112	5.02058471	4.5542437	2.973E-46	-2.972429	down	PREDICTED: uncharacterized protein LOC105174387 [Sesamum indicum]
c37721.graph_c0	47.8418295	55.7522298	57.6247598	30.9182933	32.7541827	31.1400104	1.35E-21	-1.785434	down	-
c37725.graph_c0	0.40599132	0.40639786	0.64211067	5.55284282	6.27879851	7.44972989	8.023E-13	2.702277	up	PREDICTED: uncharacterized protein LOC105169345 [Sesamum indicum]
c37726.graph_c0	7.57059307	8.03591592	8.61854739	5.89791973	7.43825095	6.28919016	5.633E-07	-1.325598	down	PREDICTED: WPP domain-interacting protein 1 [Sesamum indicum]

#ID	0 h-1 FPKM	0 h-2 FPKM	0 h-3 FPKM	48 h-1 FPKM	48 h-2 FPKM	48 h-3 FPKM	FDR	log2FC	regulated	nr_annotation
c37727.graph_c0	0.60325684	0.84540528	0.15504178	4.05704471	5.00445875	5.34072425	3.244E-05	2.155986	up	PREDICTED: mitochondrial inner membrane protease subunit 2 [Sesamum indicum]
c37733.graph_c0	0.2075223	0.15107644	0.24243124	2.39847859	3.03802931	3.17029801	7.175E-11	2.814869	up	PREDICTED: protein argonaute 16 isoform X1 [Sesamum indicum]
c37734.graph_c0	3.19433115	2.1604931	2.36675574	1.45815713	1.54474067	1.13232824	6.681E-08	-1.915284	down	Adenosylhomocysteinase [Rozella allomycis CSF55]
c37737.graph_c0	0.19271342	0.11574383	0.34670212	7.52770321	7.14712672	7.86711395	1.33E-19	4.078399	up	PREDICTED: glucose-6-phosphate 1-dehydrogenase, chloroplastic [Sesamum indicum]
c37741.graph_c0	15.8906927	16.2166751	16.7182672	18.3493682	14.6321033	11.1471573	9.784E-12	-1.157089	down	PREDICTED: sphingoid long-chain bases kinase 1-like [Sesamum indicum]
c37742.graph_c0	1.47448983	0.95223633	0.97794984	0	0	0	8.341E-20	-Inf	down	hypothetical protein WALSEDRAFT_61591 [Wallemia sebi CBS 633.66]
c37744.graph_c0	10.9755882	12.8017524	10.3020999	2.02228555	1.63006133	1.76029975	3.189E-43	-3.666983	down	PREDICTED: uncharacterized protein LOC105167616 isoform X1 [Sesamum indicum]
c37745.graph_c0	37.2538958	40.8591036	34.67465	14.180708	13.322053	13.7000621	4.318E-64	-2.468086	down	PREDICTED: U-box domain-containing protein 14 [Sesamum indicum]
c37751.graph_c0	0.15614381	0.10420011	0.10032549	4.84294548	4.49554896	4.84254354	4.962E-19	4.283987	up	PREDICTED: proline-rich receptor-like protein kinase PERK10 [Sesamum indicum]
c37753.graph_c0	0.32946337	0.17357541	0.1559797	0.94251924	1.21848998	1.59200967	0.0024828	1.491525	up	PREDICTED: BTB/POZ domain-containing protein At5g67385 [Sesamum indicum]
c37754.graph_c0	38.7499354	40.5537953	37.4283009	36.1491195	35.0818463	35.6482436	2.988E-16	-1.143624	down	PREDICTED: nucleolar GTP-binding protein 1-like [Sesamum indicum]
c37755.graph_c0	0.59490187	0.92302125	0.80269609	8.87861103	9.11550897	9.1192913	7.069E-16	2.52721	up	PREDICTED: uncharacterized protein LOC105157935 [Sesamum indicum]
c37758.graph_c0	0	0	0	22.241942	26.9088144	26.4657317	2.697E-52	Inf	up	PREDICTED: non-specific phospholipase C6-like [Eucalyptus grandis]
c37758.graph_c1	0.11457534	0	0	16.8359071	20.3515962	18.3616651	4.441E-45	7.922365	up	PREDICTED: non-specific phospholipase C6 [Sesamum indicum]
c37760.graph_c0	0.15844967	0.23791251	0.35632468	1.82467719	2.28096859	1.81841721	0.0002133	1.94901	up	-
c37761.graph_c0	10.7545561	8.95094452	11.1026087	64.5676759	71.9396384	74.8856188	1.383E-26	1.757723	up	unnamed protein product [Triticum aestivum]
c37763.graph_c0	0	0	0.03116116	1.16167625	1.08865646	0.97401874	2.623E-11	5.645417	up	PREDICTED: probable membrane-associated kinase regulator 4 [Sesamum indicum]
c37764.graph_c0	1.16043173	2.01750489	1.49120071	857.559248	555.066342	813.016613	4.024E-42	7.884455	up	PREDICTED: acid phosphatase 1-like [Camelina sativa]
c37767.graph_c0	47.2597556	45.3227218	47.7897285	18.5551131	17.7995483	16.2827249	1.664E-59	-2.43129	down	PREDICTED: U-box domain-containing protein 4-like [Sesamum indicum]
c37770.graph_c0	5.7610432	6.8537504	5.69772662	2.33416595	3.4147718	4.27748024	2.647E-14	-1.89393	down	PREDICTED: pentatricopeptide repeat-containing protein At3g48250, chloroplastic [Sesamum indicum]
c37772.graph_c0	21.0443411	18.507643	18.8649166	24.9615803	14.9628436	11.1646132	8.488E-06	-1.197004	down	PREDICTED: histone-lysine N-methyltransferase setd3 [Sesamum indicum]

#ID	0 h-1 FPKM	0 h-2 FPKM	0 h-3 FPKM	48 h-1 FPKM	48 h-2 FPKM	48 h-3 FPKM	FDR	log2FC	regulated	nr_annotation
c37774.graph_c0	33.5319755	30.3475503	33.2784971	163.015145	148.72755	162.764796	5.352E-20	1.27138	up	PREDICTED: cysteine proteinase inhibitor A-like [Nicotiana sylvestris]
c37775.graph_c0	191.417992	192.68639	195.677962	92.2448986	82.5458907	82.2514714	1.658E-45	-2.188914	down	PREDICTED: acetate/butyrate--CoA ligase AAE7, peroxisomal [Sesamum indicum]
c37776.graph_c0	1.28799006	1.38845516	0.9548759	6.06981541	9.62117632	8.36528091	1.031E-05	1.706371	up	PREDICTED: GDSL esterase/lipase At2g04570-like [Sesamum indicum]
c37780.graph_c0	12.3177879	12.3113263	11.1236244	7.40384517	8.08171262	8.66585505	1.33E-23	-1.584465	down	PREDICTED: AP-3 complex subunit delta isoform X1 [Sesamum indicum]
c37786.graph_c0	3.80051131	3.62940583	3.36814311	11.0672609	15.8173097	19.1222142	0.0005742	1.067087	up	PREDICTED: THO complex subunit 7A [Sesamum indicum]
c37787.graph_c0	0	0	0	1.30094266	1.89884094	1.36336135	5.213E-13	Inf	up	--
c37795.graph_c0	0	0	0	2.00360107	2.24271799	2.35463118	1.262E-13	Inf	up	PREDICTED: putative late blight resistance protein homolog R1B-16 [Sesamum indicum]
c37803.graph_c0	0.46527115	0.36985001	0.29894593	3.85774319	4.34056738	4.92454232	3.704E-16	2.515744	up	PREDICTED: LOW QUALITY PROTEIN: LRR receptor-like serine/threonine-protein kinase RCH1 [Sesamum indicum]
c37804.graph_c0	147.625928	129.726671	142.60595	20.9475321	17.4715639	17.9087292	3.66E-146	-3.912438	down	PREDICTED: SPX domain-containing protein 1-like [Sesamum indicum]
c37807.graph_c0	28.2497598	28.5007095	29.5847495	12.6029099	15.4679173	14.7240646	3.009E-32	-2.033894	down	PREDICTED: pollen-specific leucine-rich repeat extensin-like protein 1 [Nicotiana sylvestris]
c37809.graph_c1	2.43424903	2.7248968	3.12810638	21.7989295	18.4184574	19.3751	5.667E-21	1.829341	up	PREDICTED: CBL-interacting serine/threonine-protein kinase 3 isoform X1 [Sesamum indicum]
c37812.graph_c0	0.22038068	0.11030068	0.2831979	3.29922215	3.65643932	3.16144557	1.327E-07	3.018933	up	PREDICTED: AMSH-like ubiquitin thioesterase 2 isoform X2 [Sesamum indicum]
c37818.graph_c0	0	0	0	0.65635486	1.13448316	1.01435159	1.252E-11	Inf	up	PREDICTED: phosphoinositide phospholipase C 6-like [Sesamum indicum]
c37826.graph_c0	1.23976729	2.1510818	1.27452004	20.1019191	26.9017595	28.8285384	1.709E-25	3.001543	up	PREDICTED: UDP-N-acetylglucosamine--dolichyl-phosphate N-acetylglucosaminephosphotransferase-like [Sesamum indicum]
c37828.graph_c0	47.1714843	44.257978	50.2413035	5.68470402	6.13838623	5.57379113	6.49E-150	-4.044768	down	PREDICTED: uncharacterized protein LOC105157890 [Sesamum indicum]
c37829.graph_c0	0.31868284	0.27912671	0.23035504	9.08298451	10.7109065	11.4453969	1.017E-46	4.219246	up	PREDICTED: uncharacterized aarF domain-containing protein kinase At1g79600, chloroplastic [Sesamum indicum]
c37835.graph_c0	105.53838	105.748638	106.434692	71.3768632	81.4269743	75.0549901	4.619E-24	-1.49904	down	PREDICTED: DEAD-box ATP-dependent RNA helicase 38 [Sesamum indicum]
c37837.graph_c1	0.89538416	0.30729626	0.49311604	10.8649309	15.9043795	15.5184102	2.702E-26	3.619796	up	PREDICTED: serine/threonine protein phosphatase 2A 57 kDa regulatory subunit B' iota isoform [Sesamum indicum]
c37842.graph_c0	38.6688887	41.8115218	38.4402485	172.800272	233.143084	299.040473	7.062E-06	1.543371	up	-
c37845.graph_c0	22.0777098	21.0817161	18.3654852	15.5413249	13.9483177	12.6929666	7.469E-28	-1.557256	down	PREDICTED: retinoblastoma-related protein [Sesamum indicum]

#ID	0 h-1 FPKM	0 h-2 FPKM	0 h-3 FPKM	48 h-1 FPKM	48 h-2 FPKM	48 h-3 FPKM	FDR	log2FC	regulated	nr_annotation
c37849.graph_c0	905.230238	914.777562	981.434342	57.3608814	47.2538751	51.2145196	4.53E-167	-5.183217	down	hypothetical protein MIMGU_mgv1a008920mg [Erythranthe guttata]
c37852.graph_c0	4.87825295	4.15232805	5.03205636	39.4080165	28.9871788	22.8779508	0.0001883	1.688131	up	beta-D-xylosidase family protein [Populus trichocarpa]
c37854.graph_c0	0.50872867	0.44932773	0.36532333	8.17422659	8.17770162	7.7026664	1.489E-34	3.170085	up	PREDICTED: uncharacterized protein LOC105166467 isoform X1 [Sesamum indicum]
c37856.graph_c0	40.3831501	40.113489	40.3208857	33.6565097	33.2362393	30.4915276	3.869E-21	-1.327585	down	PREDICTED: VHS domain-containing protein At3g16270 [Sesamum indicum]
c37858.graph_c0	8.86561839	9.212572	8.46312675	7.23414753	5.76837794	5.39869114	1.706E-06	-1.540107	down	PREDICTED: kxDL motif-containing protein 1 [Sesamum indicum]
c37863.graph_c0	0.04137016	0.08282317	0.05316229	3.27770845	2.82631891	4.40864052	2.492E-13	4.871463	up	PREDICTED: uncharacterized protein LOC105168997 [Sesamum indicum]
c37867.graph_c0	5.67617404	5.09910322	3.86525401	0.08938909	0.14204535	0.11930681	7.133E-37	-6.404476	down	PREDICTED: uncharacterized protein LOC105158642 [Sesamum indicum]
c37868.graph_c0	0.06329101	0	0	4.8103938	4.72540866	6.07018617	2.015E-24	6.948087	up	Nucleobase-ascorbate transporter 7 [Theobroma cacao]
c37870.graph_c0	4.93389022	4.29678277	4.69178076	1.13635537	2.8169639	2.95247675	7.906E-15	-2.043608	down	PREDICTED: putative pentatricopeptide repeat-containing protein At5g43820 [Sesamum indicum]
c37873.graph_c0	1.54552928	1.38249424	1.43672882	13.9960676	13.9606156	14.7179108	1.537E-18	2.27201	up	PREDICTED: oxygen-evolving enhancer protein 1, chloroplastic-like [Nicotiana sylvestris]
c37874.graph_c0	4.31565832	3.51626263	3.67569922	114.28303	81.300389	62.7736354	2.086E-08	3.481379	up	PREDICTED: aspartic proteinase-like protein 1 isoform X1 [Sesamum indicum]
c37877.graph_c0	43.0877936	24.3011224	26.8181863	0	0	0	2.411E-31	-Inf	down	hypothetical protein SELMODRAFT_140819 [Selaginella moellendorffii]
c37878.graph_c0	21.499304	24.4615671	21.5642882	19.2734653	20.3744571	21.4914991	1.006E-12	-1.161731	down	hypothetical protein MIMGU_mgv1a013275mg [Erythranthe guttata]
c37880.graph_c0	64.6268924	64.6916065	71.5000047	347.484116	348.709954	360.337689	3.153E-23	1.376516	up	elongation factor-like protein [Solanum tuberosum]
c37881.graph_c1	157.250435	162.222933	179.707888	59.9459409	119.755584	188.910863	8.27E-07	-1.470112	down	heat shock protein 70 [Saussurea medusa]
c37881.graph_c2	21.9345614	24.5703976	30.8712351	6.73584955	14.7813328	26.114616	0.000136	-1.73622	down	70 kD heatshockprotein [Medicago sativa]
c37883.graph_c0	10.1072245	10.9949946	8.70051823	40.297196	43.973545	40.7674643	2.4E-12	1.05222	up	PREDICTED: uncharacterized protein LOC105172480 [Sesamum indicum]
c37887.graph_c0	18.7813448	21.029819	18.0197621	13.3617112	13.586932	14.0263161	2.242E-23	-1.514197	down	PREDICTED: zinc finger Ran-binding domain-containing protein 2 isoform X1 [Sesamum indicum]
c37888.graph_c0	17.7056823	19.0277322	12.410456	1.90655408	2.24418096	2.82740059	5.54E-24	-3.83443	down	PREDICTED: myb-related protein 306-like [Sesamum indicum]
c37890.graph_c0	39.8098354	39.8496989	36.5630472	25.4891365	24.8801704	28.2263009	1.448E-23	-1.581403	down	PREDICTED: probable acetyltransferase NATA1-like [Sesamum indicum]
c37891.graph_c0	0.02624142	0.07880308	0.10116378	5.80207324	6.940528	7.03411047	1.031E-30	5.553757	up	PREDICTED: receptor-like serine/threonine-protein kinase NCRK isoform X1 [Sesamum indicum]

#ID	0 h-1 FPKM	0 h-2 FPKM	0 h-3 FPKM	48 h-1 FPKM	48 h-2 FPKM	48 h-3 FPKM	FDR	log2FC	regulated	nr_annotation
c37892.graph_c0	2.56655709	2.47031452	2.9175743	1.45506449	1.83048896	0.80919227	6.605E-05	-1.976519	down	PREDICTED: serine/threonine-protein kinase BLUS1-like isoform X2 [Sesamum indicum]
c37893.graph_c0	0.28549307	0.50805146	0.7337398	5.90322191	5.51072827	6.29278034	5.351E-11	2.510199	up	PREDICTED: riboflavin biosynthesis protein PYRD, chloroplastic [Sesamum indicum]
c37895.graph_c0	11.3090435	12.4943319	8.82718985	4.63050156	7.93048547	12.4292472	9.375E-06	-1.41128	down	-
c37899.graph_c0	0.11160091	0.13405519	0.02868232	0.49350738	1.39416327	1.82966625	0.0078864	2.736804	up	PREDICTED: G-type lectin S-receptor-like serine/threonine-protein kinase At1g34300 [Sesamum indicum]
c37902.graph_c0	18.9312674	21.0384032	18.831976	13.0685272	11.961258	13.039083	2.779E-19	-1.643152	down	PREDICTED: small nuclear ribonucleoprotein-associated protein N-like [Sesamum indicum]
c37905.graph_c0	2.95536001	2.23872816	3.38718656	11.8472956	15.5910225	13.6845052	0.000355	1.236415	up	PREDICTED: uncharacterized protein LOC104093968 [Nicotiana tomentosiformis]
c37906.graph_c0	7.41778373	6.69800008	6.43665513	84.1537096	60.6776721	51.089798	2.043E-06	2.244728	up	PREDICTED: protein TRANSPORT INHIBITOR RESPONSE 1-like [Sesamum indicum]
c37907.graph_c0	20.9241907	21.2702341	21.4630352	94.289279	98.5296528	95.2695258	8.154E-16	1.159889	up	PREDICTED: coatomer subunit delta-2-like [Sesamum indicum]
c37908.graph_c0	0.64828552	0.58404121	0.54149716	2.12021232	2.799726	2.69697247	0.003096	1.081596	up	PREDICTED: pentatricopeptide repeat-containing protein ELI1, chloroplastic [Sesamum indicum]
c37909.graph_c0	9.41483184	8.64700086	7.82656275	50.9906802	55.2262203	53.1087705	1.148E-27	1.604617	up	PREDICTED: UDP-sugar pyrophosphorylase [Sesamum indicum]
c37914.graph_c0	0	0	0	2.1983991	4.52848915	6.23061177	1.432E-08	Inf	up	PREDICTED: geraniol synthase, chloroplastic-like [Sesamum indicum]
c37916.graph_c0	92.6675273	94.2689492	97.8823277	31.0714262	38.7602636	35.6608402	1.019E-64	-2.454415	down	PREDICTED: protein DJ-1 homolog B [Sesamum indicum]
c37918.graph_c0	32.7384782	34.5541533	33.4600101	14.4162476	15.6033723	18.4590716	5.42E-40	-2.075633	down	hypothetical protein MIMGU_mgv1a008971mg [Erythranthe guttata]
c37919.graph_c0	0.03302333	0.09916919	0.29705399	5.47618388	6.92939362	7.30901323	5.747E-22	4.48089	up	PREDICTED: uncharacterized protein LOC104231484 isoform X1 [Nicotiana sylvestris]
c37920.graph_c0	0.46892763	0.19558216	0.35151116	2.80804366	2.89850531	2.11418338	0.0001609	1.929277	up	PREDICTED: squamosa promoter-binding-like protein 16 isoform X1 [Nicotiana tomentosiformis]
c37921.graph_c0	13.9914072	15.0682232	14.1592449	7.08612783	8.56614807	6.13112416	8.23E-35	-2.007036	down	PREDICTED: uncharacterized protein LOC105178567 [Sesamum indicum]
c37922.graph_c0	0.25773499	0.42998845	0.22079978	2.84930738	3.68927248	3.0282709	5.399E-05	2.379074	up	PREDICTED: peptidyl-prolyl cis-trans isomerase FKBP18, chloroplastic [Sesamum indicum]
c37924.graph_c0	61.5983715	65.247547	58.8795286	46.9349704	55.405802	58.8795523	4.046E-18	-1.224407	down	PREDICTED: la-related protein 1B [Sesamum indicum]
c37926.graph_c0	1.5109383	1.47727799	1.62553858	10.5531121	11.5226795	11.6656097	5.518E-11	1.850421	up	PREDICTED: uncharacterized protein C630.12 isoform X1 [Sesamum indicum]
c37927.graph_c0	7.06482747	7.18460145	7.19776103	37.4956434	43.1009374	40.4929208	1.744E-21	1.477466	up	PREDICTED: sphingosine-1-phosphate lyase [Sesamum indicum]
c37930.graph_c0	1.81476984	1.68835738	1.28948707	0.94412292	1.37525267	1.12009987	3.736E-05	-1.498327	down	PREDICTED: U-box domain-containing protein 52-like [Sesamum indicum]

#ID	0 h-1 FPKM	0 h-2 FPKM	0 h-3 FPKM	48 h-1 FPKM	48 h-2 FPKM	48 h-3 FPKM	FDR	log2FC	regulated	nr_annotation
c37932.graph_c0	43.620691	45.3524261	41.5206447	26.9496287	36.9107386	40.4022458	1.451E-21	-1.346845	down	PREDICTED: serine/threonine-protein kinase WNK8-like isoform X1 [Sesamum indicum]
c37933.graph_c0	119.012437	121.993927	117.457654	43.1936871	43.6894789	40.0904471	2.184E-68	-2.514042	down	PREDICTED: protein PPLZ12 [Sesamum indicum]
c37934.graph_c1	37.8179274	33.1704678	34.7581486	32.0928008	33.392038	30.4234948	4.276E-15	-1.157927	down	hypothetical protein MIMGU_mgv1a007540mg [Erythranthe guttata]
c37935.graph_c0	1.1858099	1.02659227	1.11197141	0.91529211	0.8132473	0.97205137	0.0005331	-1.31622	down	PREDICTED: uncharacterized protein LOC105178399 [Sesamum indicum]
c37936.graph_c0	0.11044925	0.38695946	0.21289748	1.83155491	1.67082279	2.30875094	0.0003564	2.013749	up	-
c37937.graph_c0	1.72103201	0.77934171	1.05314013	0	0	0	1.269E-11	-Inf	down	Mitochondrial uncoupling protein 3 [Aegilops tauschii]
c37938.graph_c1	0	0	0	4.50833594	4.02735946	4.57526721	4.039E-27	Inf	up	PREDICTED: chromosome-associated kinesin KIF4A [Sesamum indicum]
c37943.graph_c1	4.81558472	5.28390745	3.86763564	1.66366153	2.25954828	2.54310701	6.953E-10	-2.132214	down	hypothetical protein MIMGU_mgv1a008064mg [Erythranthe guttata]
c37947.graph_c0	0.46530971	0.98330414	0.5979414	4.15972213	4.37308211	4.97273321	3.54E-08	1.703736	up	PREDICTED: pentatricopeptide repeat-containing protein At5g57250, mitochondrial [Sesamum indicum]
c37953.graph_c0	127.815646	127.108393	128.669186	47.5900217	60.2251101	61.9945363	7.587E-52	-2.197748	down	PREDICTED: uncharacterized protein LOC105167724 [Sesamum indicum]
c37954.graph_c0	1.97482734	2.56012428	1.66408611	14.3161161	14.2498985	12.738394	4.206E-12	1.722457	up	PREDICTED: O-acyltransferase WSD1-like isoform X1 [Sesamum indicum]
c37960.graph_c0	1043.70309	1022.36532	1039.80801	12.8863089	11.6326006	6.05607051	0	-7.67734	down	hypothetical protein MIMGU_mgv1a009609mg [Erythranthe guttata]
c37964.graph_c0	346.864251	338.928495	364.522104	57.7319189	83.8765271	95.0831859	3.539E-91	-3.175346	down	uncharacterized LOC102580903 [Solanum tuberosum]
c37966.graph_c0	4.23292668	2.93342214	3.48684511	0.29997293	0.6355699	0.97868508	1.574E-14	-3.50744	down	-
c37968.graph_c0	54.1024366	50.5752096	49.4556955	18.0841229	19.5826187	20.41118	5.642E-58	-2.426644	down	BnaC06g30860D [Brassica napus]
c37969.graph_c0	0.21115021	0.04696926	0.09044546	2.35591812	1.4196348	2.44245674	1.345E-07	3.15035	up	PREDICTED: uncharacterized protein At5g41620 [Sesamum indicum]
c37971.graph_c0	46.3680145	48.7252638	44.4978457	26.5539596	22.9165431	23.7897564	6.347E-35	-1.944272	down	PREDICTED: protein vip1-like [Sesamum indicum]
c37977.graph_c0	11.6354543	10.7115949	11.6493645	74.1743846	98.0068305	81.6284576	1.463E-26	1.87858	up	PREDICTED: glutathione S-transferase L3-like [Malus domestica]
c37980.graph_c0	0.95162421	1.25339095	1.22287479	17.0264037	15.7399623	14.287797	2.807E-18	2.762255	up	PREDICTED: methionine adenosyltransferase 2 subunit beta [Sesamum indicum]
c37983.graph_c1	0.82821085	0.72955536	0.72371294	0.15260037	0.5496494	0.57028815	0.0002115	-1.877336	down	PREDICTED: F-box/kelch-repeat protein At1g57790-like [Sesamum indicum]
c37985.graph_c0	4.30828312	3.27757388	4.5397778	2.14329857	1.85008995	2.401526	1.787E-05	-1.941431	down	PREDICTED: uncharacterized protein LOC100267301 [Vitis vinifera]
c37985.graph_c1	2.69168717	2.31716894	2.69796117	2.18218685	2.57446264	2.11821462	0.0053875	-1.184588	down	hypothetical protein MIMGU_mgv1a007684mg [Erythranthe guttata]

#ID	0 h-1 FPKM	0 h-2 FPKM	0 h-3 FPKM	48 h-1 FPKM	48 h-2 FPKM	48 h-3 FPKM	FDR	log2FC	regulated	nr_annotation
c37986.graph_c0	1.58161942	1.93861613	1.74209449	1.87340268	1.26009078	1.21713257	0.0015503	-1.283691	down	PREDICTED: tonoplast dicarboxylate transporter [Sesamum indicum]
c37987.graph_c0	5.0978445	5.50581363	4.82700253	0.3707737	0.65464963	0.76979515	5.272E-14	-4.130836	down	-
c37991.graph_c0	107.092761	106.998747	119.877889	65.7533112	56.6418157	46.4759044	1.049E-38	-1.997931	down	hypothetical protein EUTSA_v10023737mg [Eutrema salsugineum]
c37994.graph_c0	16.568386	17.2483758	17.768334	59.4181136	80.3219313	91.2196812	1.252E-06	1.138211	up	PREDICTED: polygalacturonase At1g48100 [Sesamum indicum]
c37997.graph_c0	4.02702729	4.03105975	4.15268911	19.6490485	24.4549075	28.4872728	5.715E-12	1.548737	up	PREDICTED: mitochondrial inner membrane protease subunit 2-like isoform X1 [Sesamum indicum]
c38000.graph_c0	0.06897801	0.20714124	0.17727894	1.17562023	1.64934938	1.92248831	2.476E-05	2.359446	up	hypothetical protein MIMGU_mgv1a004734mg [Erythranthe guttata]
c38001.graph_c0	26.4993893	25.1191064	25.4266978	19.5235582	20.1775026	18.0692478	6.388E-24	-1.432352	down	PREDICTED: KH domain-containing protein At4g18375 isoform X2 [Sesamum indicum]
c38004.graph_c0	0.07154288	0.07161452	0.09193543	2.47162367	2.33909111	2.75637563	1.753E-10	3.98792	up	PREDICTED: uncharacterized protein LOC105177313 isoform X1 [Sesamum indicum]
c38007.graph_c0	1.53917311	1.40064942	1.43847155	15.9845769	16.3874719	15.8288008	1.236E-07	2.443373	up	-
c38008.graph_c0	1.04353948	0.81750086	1.04947008	6.35344558	6.55358138	6.99218713	8.417E-06	1.753883	up	PREDICTED: uncharacterized protein LOC105167885 [Sesamum indicum]
c38010.graph_c0	17.3164442	19.0637367	14.7322648	9.26915636	10.6378428	10.5900814	7.417E-27	-1.762938	down	PREDICTED: tyrosine-sulfated glycopeptide receptor 1 [Sesamum indicum]
c38012.graph_c0	0.07426168	0.07433604	0.06361946	2.71378314	3.67217205	3.18579112	1.02E-15	4.474752	up	PREDICTED: uncharacterized protein LOC105173003 [Sesamum indicum]
c38014.graph_c0	0.12532598	0.06272574	0.08052442	4.38742168	6.54381205	7.19139737	1.372E-14	5.053883	up	acyl-CoA thioesterase, putative [Ricinus communis]
c38015.graph_c0	13.0393057	9.841621	13.9782875	59.8063393	64.1742034	58.6454559	4.134E-14	1.288849	up	PREDICTED: protein yippee-like [Sesamum indicum]
c38018.graph_c0	5.59850988	6.80499792	6.42348825	1.1052241	3.31741093	3.60587978	5.834E-06	-2.264678	down	-
c38021.graph_c0	36.3865117	37.6805789	37.6610791	26.5991669	25.0188168	27.6685246	2.92E-26	-1.512117	down	hypothetical protein MIMGU_mgv1a011020mg [Erythranthe guttata]
c38028.graph_c0	0.23139901	0.32428301	0.05947138	3.49614976	4.47159668	4.81802848	1.279E-09	3.365922	up	hypothetical protein MIMGU_mgv1a013394mg [Erythranthe guttata]
c38030.graph_c0	7.49861061	7.22017193	8.25943845	186.850611	264.438547	250.324492	9.406E-46	3.907716	up	PREDICTED: serine/threonine-protein kinase Nek6-like [Sesamum indicum]
c38031.graph_c0	20.1300671	17.3326082	18.304851	8.32959263	9.53179379	10.4182226	1.113E-28	-1.999796	down	PREDICTED: uncharacterized protein LOC105175857 [Sesamum indicum]
c38035.graph_c0	14.3065347	16.2534334	14.5040058	7.84421946	3.38197648	1.78551471	2.043E-37	-2.786577	down	hypothetical protein MIMGU_mgv1a008930mg [Erythranthe guttata]
c38036.graph_c0	7.77892878	6.96149534	6.68226342	1.34371061	1.09341186	1.43821632	2.745E-55	-3.481237	down	PREDICTED: pentatricopeptide repeat-containing protein At1g74900, mitochondrial [Sesamum indicum]

#ID	0 h-1 FPKM	0 h-2 FPKM	0 h-3 FPKM	48 h-1 FPKM	48 h-2 FPKM	48 h-3 FPKM	FDR	log2FC	regulated	nr_annotation
c38039.graph_c0	0.06696901	0.03351803	0	0.86374706	1.0457546	0.96069618	6.771E-08	3.833744	up	PREDICTED: DNA-directed primase/polymerase protein isoform X1 [Sesamum indicum]
c38041.graph_c0	85.7662091	92.3659067	84.0673572	49.1985469	46.3617261	49.1464025	6.191E-40	-1.873748	down	hypothetical protein MIMGU_mgv1a027042mg [Erythranthe guttata]
c38045.graph_c0	20.2820253	18.6030203	21.1835305	6.37928263	6.80167691	5.01707021	2.203E-48	-2.740128	down	PREDICTED: cyclin-dependent kinase inhibitor 3 isoform X2 [Sesamum indicum]
c38046.graph_c0	5.40152958	5.46137066	5.35728608	22.3930861	28.0568952	27.9933928	2.624E-16	1.25239	up	PREDICTED: alpha-L-fucosidase 2 isoform X1 [Sesamum indicum]
c38047.graph_c0	297.951936	281.049148	303.908687	31.1252784	32.4049504	32.6292675	6.43E-165	-4.217452	down	PREDICTED: uncharacterized protein C6C3.02c-like [Malus domestica]
c38051.graph_c0	4.08551253	3.13167839	3.216244	19.0226539	23.6366211	24.5294753	5.633E-16	1.666947	up	PREDICTED: cysteine desulfurase 2, chloroplastic isoform X1 [Sesamum indicum]
c38063.graph_c1	798.182712	762.336116	876.599721	20.5421399	20.4524507	17.6497933	6.81E-224	-6.394478	down	PREDICTED: peroxygenase-like [Nicotiana tomentosiformis]
c38065.graph_c1	0.45008328	0.09010679	0.23134994	3.81474391	2.89916379	3.76328578	5.159E-06	2.752986	up	PREDICTED: nudix hydrolase 16, mitochondrial-like [Sesamum indicum]
c38066.graph_c0	103.788976	96.7965648	98.691183	471.382928	473.943143	469.2886	8.94E-18	1.223701	up	RecName: Full=40S ribosomal protein S7 [Avicennia marina]
c38067.graph_c0	0.56454144	0.79533542	0.69858929	5.50912689	4.122108	4.01071744	4.951E-07	1.716261	up	PREDICTED: LOW QUALITY PROTEIN: auxin response factor 19-like [Sesamum indicum]
c38068.graph_c0	4.17200056	3.57420656	2.60814449	2.21608305	3.19136564	4.62154071	0.0015167	-1.068196	down	hypothetical protein MIMGU_mgv1a007990mg [Erythranthe guttata]
c38070.graph_c0	27.6106125	30.4999876	29.4867104	124.341784	140.022105	139.19245	7.192E-16	1.183864	up	hypothetical protein MIMGU_mgv1a013689mg [Erythranthe guttata]
c38071.graph_c0	1.1213697	1.06013189	1.32092076	12.0525873	11.126622	11.0562629	2.732E-16	2.271204	up	PREDICTED: calcium-dependent protein kinase 26-like isoform X1 [Sesamum indicum]
c38072.graph_c0	0.08360128	0.20084399	0.21486191	14.3255254	15.3067047	16.2007288	2.01E-99	5.495324	up	Lysosomal alpha-mannosidase [Glycine soja]
c38073.graph_c1	5.58353899	4.12312873	6.35170012	33.9836046	27.7828017	37.516634	3.116E-10	1.609535	up	hypothetical protein MIMGU_mgv1a015825mg [Erythranthe guttata]
c38074.graph_c0	54.3237903	53.6378902	55.8984148	235.181945	235.760939	256.164514	2.955E-16	1.131226	up	PREDICTED: 60S ribosomal protein L13a-4-like [Sesamum indicum]
c38075.graph_c0	18.112022	18.0124302	16.9270294	5.11414331	6.3360884	5.68816746	4.245E-60	-2.650137	down	PREDICTED: MAR-binding filament-like protein 1 [Sesamum indicum]
c38080.graph_c0	56.9674212	56.2433084	63.8935513	311.29796	334.460979	358.507391	2.474E-26	1.482826	up	--
c38081.graph_c0	0.17729124	0.22183597	0.39869597	19.1507073	23.4889206	29.9020716	2.787E-31	5.475207	up	hypothetical protein VITISV_034764 [Vitis vinifera]
c38083.graph_c0	2.24485664	2.56065399	2.91827302	66.3478018	71.459333	83.8142525	1.591E-83	3.820207	up	PREDICTED: lysosomal beta glucosidase-like [Sesamum indicum]
c38086.graph_c0	0.32431482	0.23188541	0.31752944	11.5955084	12.9170774	13.2166731	3.382E-74	4.411736	up	PREDICTED: calcium-transporting ATPase, endoplasmic reticulum-type [Sesamum indicum]

#ID	0 h-1 FPKM	0 h-2 FPKM	0 h-3 FPKM	48 h-1 FPKM	48 h-2 FPKM	48 h-3 FPKM	FDR	log2FC	regulated	nr_annotation
c38087.graph_c0	0.34216005	0.30444682	0.26869896	2.27659003	3.72896914	3.75532896	2.557E-09	2.390676	up	hypothetical protein MIMGU_mgv1a023205mg [Erythranthe guttata]
c38089.graph_c0	46.9909455	41.5770028	43.1828002	14.4866883	16.1075227	15.2953307	8.214E-61	-2.540163	down	RING/U-box superfamily protein isoform 1 [Theobroma cacao]
c38090.graph_c0	0.14321456	0.19114396	0.10224243	4.09009645	3.86705143	3.99153774	2.929E-21	3.762186	up	PREDICTED: DNA (cytosine-5)-methyltransferase CMT2 [Sesamum indicum]
c38091.graph_c0	2.70131736	1.73057428	2.29105784	14.1354169	18.19117	20.1950342	7.988E-12	1.943258	up	unnamed protein product [Coffea canephora]
c38093.graph_c0	10.6724823	10.4568309	11.15761	6.16594766	5.47281867	4.22602865	1.145E-18	-2.038739	down	PREDICTED: transcription intermediary factor 1-alpha isoform X1 [Sesamum indicum]
c38094.graph_c0	23.2188981	23.9118373	22.9594751	12.8707136	11.33046	10.0973655	2.121E-35	-2.044796	down	PREDICTED: beta-D-glucosyl crocetin beta-1,6-glucosyltransferase-like [Sesamum indicum]
c38097.graph_c0	0.99420141	0.89224554	0.68284885	17.1972473	17.9841856	18.8148648	6.61E-65	3.378311	up	PREDICTED: uncharacterized protein LOC105172430 [Sesamum indicum]
c38098.graph_c0	0.07345591	0.02450982	0.15732291	1.06020049	1.52940101	1.72614813	8.75E-08	3.041638	up	PREDICTED: pentatricopeptide repeat-containing protein At2g37310 [Sesamum indicum]
c38100.graph_c0	0.48094641	0.44439509	0.42787051	3.95362985	3.89953684	3.07817655	3.881E-08	1.999605	up	PREDICTED: DNA-binding protein SMUBP-2 [Sesamum indicum]
c38102.graph_c0	0.38793186	0.20135127	0.24002227	6.72419148	7.86666173	7.04315117	7.175E-39	3.688883	up	PREDICTED: histidine kinase 1 [Sesamum indicum]
c38104.graph_c0	2.40196804	1.72434849	2.21363946	24.2742899	28.3440636	25.4774117	2.132E-46	2.602835	up	PREDICTED: probable sulfate transporter 3.3 [Sesamum indicum]
c38107.graph_c0	21.9477474	24.4785408	22.3944194	18.8170495	21.5574798	21.3899465	6.861E-16	-1.175786	down	PREDICTED: uncharacterized aarF domain-containing protein kinase At1g79600, chloroplastic-like [Citrus sinensis]
c38108.graph_c0	2.51842523	1.86736819	2.63696574	1.80455146	1.82066996	1.52921813	0.0032691	-1.464135	down	PREDICTED: plasma membrane ATPase 4 isoform X1 [Eucalyptus grandis]
c38110.graph_c0	0.39804751	0.55782454	0.61380818	3.37370746	7.07037154	7.96157309	0.0001368	2.517998	up	PREDICTED: uncharacterized protein LOC105166696 [Sesamum indicum]
c38111.graph_c0	0	0	0	2.4579197	1.82270752	1.92459711	4.731E-12	Inf	up	-
c38114.graph_c0	33.3256603	33.6683882	35.7424856	10.3446465	11.914032	12.2024341	2.683E-54	-2.596903	down	PREDICTED: iron-sulfur assembly protein IscA-like 2, mitochondrial isoform X1 [Sesamum indicum]
c38115.graph_c0	25.058219	22.2678373	22.3434147	10.8359411	8.98385136	9.6417552	7.328E-10	-2.255159	down	PREDICTED: pyrophosphate--fructose 6-phosphate 1-phosphotransferase subunit alpha 2 [Tarenaya hassleriana]
c38123.graph_c0	1.30693137	1.04659205	1.59548563	3.85289736	7.525593	7.44580626	0.0045821	1.221156	up	-
c38124.graph_c1	0.3632881	0.18182594	0.32678771	2.34279285	1.73733346	1.39966201	0.0017496	1.641263	up	PREDICTED: LOW QUALITY PROTEIN: probable WRKY transcription factor 70 [Sesamum indicum]
c38128.graph_c0	3.84966689	4.53355499	4.29222776	17.6806658	23.0678252	26.5451028	7.211E-09	1.384627	up	PREDICTED: protein cfxQ homolog [Sesamum indicum]
c38130.graph_c0	41.029151	41.5377929	39.5430626	29.6227753	36.6333019	36.0223181	1.283E-19	-1.276502	down	hypothetical protein MIMGU_mgv1a023381mg, partial [Erythranthe guttata]
c38136.graph_c0	0.02718134	0.08162568	0	4.43229689	3.52824287	3.56504508	7.92E-20	5.728085	up	PREDICTED: glutamate receptor 2.1-like [Sesamum indicum]

#ID	0 h-1 FPKM	0 h-2 FPKM	0 h-3 FPKM	48 h-1 FPKM	48 h-2 FPKM	48 h-3 FPKM	FDR	log2FC	regulated	nr_annotation
c38138.graph_c0	6.80330923	5.78860345	6.19261954	21.5189392	28.3852199	26.4438921	4.057E-06	1.001419	up	PREDICTED: xanthoxin dehydrogenase-like [Malus domestica]
c38141.graph_c0	17.6129728	17.878928	15.7112913	13.6143531	13.1430393	13.1452547	2.935E-21	-1.375198	down	PREDICTED: RNA polymerase II-associated factor 1 homolog [Sesamum indicum]
c38142.graph_c0	2.88268274	1.79546535	1.72870201	29.2718849	30.8849723	30.6144904	3.082E-26	2.810107	up	PREDICTED: uncharacterized protein LOC105179724 [Sesamum indicum]
c38144.graph_c0	0	0.06399927	0	2.35605278	2.68314865	3.43285761	2.521E-15	6.03848	up	PREDICTED: cyclin-A2-1-like isoform X2 [Sesamum indicum]
c38146.graph_c1	22.8463035	20.7081652	19.1541275	125.956433	128.195615	120.246631	4.847E-28	1.562107	up	PREDICTED: uncharacterized protein LOC105174863 [Sesamum indicum]
c38148.graph_c0	11.0669437	7.9761784	10.4527737	55.6679234	61.5656528	70.8521247	1.01E-23	1.651875	up	PREDICTED: eukaryotic translation initiation factor 3 subunit K [Sesamum indicum]
c38149.graph_c0	43.1585196	42.2047732	39.7288489	17.5863499	12.3529013	11.5660819	4.69E-65	-2.600465	down	PREDICTED: homeobox-leucine zipper protein HAT4-like [Sesamum indicum]
c38151.graph_c0	0	0	0	2.90599499	5.64400709	3.55538819	1.906E-08	Inf	up	--
c38152.graph_c0	0.23631917	0.2183592	0.30367948	1.55748265	1.57899782	1.71367151	0.0002711	1.655575	up	PREDICTED: serine/threonine-protein kinase-like protein ACR4 [Sesamum indicum]
c38155.graph_c0	1.89042535	2.28191328	2.2863731	14.3936724	19.6437826	22.2420382	2.337E-12	2.098053	up	PREDICTED: glucuronoxylan 4-O-methyltransferase 1-like [Sesamum indicum]
c38157.graph_c0	0.42344085	0.15894932	0.40810369	2.73070932	2.09215436	2.863656	2.03E-05	1.938594	up	hypothetical protein MIMGU_mgv1a002532mg [Erythranthe guttata]
c38161.graph_c0	7.51549101	7.21734102	7.12882402	2.54756877	2.6988402	2.32243864	2.475E-42	-2.547255	down	PREDICTED: uncharacterized protein LOC105168894 [Sesamum indicum]
c38166.graph_c0	21.7951588	22.3415785	20.6392949	6.60310586	7.95796883	7.88441296	4.665E-67	-2.549206	down	hypothetical protein MIMGU_mgv1a001457mg [Erythranthe guttata]
c38167.graph_c0	3.88114464	3.30227637	4.05228243	0	0	0	1.775E-36	-Inf	down	-
c38168.graph_c0	17.9023545	16.7508193	18.3688627	8.10395598	13.4478463	12.4781528	7.594E-23	-1.666245	down	hypothetical protein MIMGU_mgv1a012288mg [Erythranthe guttata]
c38169.graph_c0	0.25023633	0.15655431	0.04019545	6.19560192	3.81598196	2.74358418	4.409E-05	3.843085	up	PREDICTED: nudix hydrolase 8-like isoform X3 [Sesamum indicum]
c38175.graph_c0	3.72770066	3.01951518	3.27753464	15.2053767	16.8263028	16.8668331	2.079E-11	1.26751	up	PREDICTED: protein CASP isoform X1 [Sesamum indicum]
c38176.graph_c0	44.8262197	46.266063	38.3824138	35.3911756	31.2816624	28.406503	1.065E-23	-1.457901	down	PREDICTED: probable protein phosphatase 2C 9 [Sesamum indicum]
c38178.graph_c0	133.34149	131.759037	145.747944	125.318516	120.324848	114.280162	4.478E-17	-1.208166	down	PREDICTED: zinc finger CCCH domain-containing protein 14-like [Sesamum indicum]
c38182.graph_c0	4.25202691	4.93729022	3.9340965	30.8678869	58.2223336	66.0858503	4.705E-06	2.536194	up	PREDICTED: homeobox-leucine zipper protein ATHB-6 [Sesamum indicum]
c38184.graph_c0	1.26098834	1.61287632	1.44037329	15.7475528	12.7170812	13.1793928	4.872E-17	2.257077	up	hypothetical protein CICLE_v10008173mg [Citrus clementina]
c38185.graph_c0	3.73059573	4.04552563	4.26130063	1.1456227	1.41592306	1.35915759	6.814E-08	-2.640577	down	-

#ID	0 h-1 FPKM	0 h-2 FPKM	0 h-3 FPKM	48 h-1 FPKM	48 h-2 FPKM	48 h-3 FPKM	FDR	log2FC	regulated	nr_annotation
c38190.graph_c0	311.498325	330.084368	288.486018	55.5715333	47.7823153	46.1507351	1.01E-126	-3.650234	down	PREDICTED: early nodulin-like protein 2 [Sesamum indicum]
c38193.graph_c0	7.60540689	7.02989742	7.07000989	29.4277069	36.0708709	36.9555655	9.586E-14	1.217964	up	PREDICTED: dolichyl-diphosphooligosaccharide--protein glycosyltransferase subunit 1B [Sesamum indicum]
c38200.graph_c0	6.98927563	6.19585312	6.85029349	2.04628493	1.76313649	1.69938723	3.955E-29	-2.87713	down	hypothetical protein MIMGU_mgv1a005237mg [Erythranthe guttata]
c38202.graph_c0	0	0	0	4.28978702	3.8575485	2.75180994	4.051E-15	Inf	up	--
c38203.graph_c0	0	0.05191232	0.13328531	3.34440219	4.80834166	3.95360133	3.15E-13	4.987537	up	PREDICTED: psbP domain-containing protein 3, chloroplastic [Sesamum indicum]
c38204.graph_c0	0	0.15686325	0.30206075	11.4772632	10.6293638	10.0839419	1.383E-18	5.099252	up	PREDICTED: uncharacterized protein LOC105164617 isoform X2 [Sesamum indicum]
c38206.graph_c0	0.21455029	0	0.0689264	1.53184732	1.77985228	3.25368102	0.000119	3.516774	up	PREDICTED: putative UDP-glucuronate:xylan alpha-glucuronosyltransferase 4 [Sesamum indicum]
c38209.graph_c0	0.14603641	0.19491019	0.18766257	3.72222535	4.79840829	5.18749139	5.193E-11	3.672527	up	PREDICTED: T-complex protein 1 subunit epsilon [Sesamum indicum]
c38212.graph_c0	39.3199978	41.4080447	39.609296	13.2555237	13.9820915	11.9980419	9.548E-56	-2.634038	down	hypothetical protein MIMGU_mgv1a000768mg [Erythranthe guttata]
c38212.graph_c1	17.5635205	20.3617931	19.6910158	3.21962126	1.92404008	2.93825604	3.308E-41	-3.845372	down	PREDICTED: uncharacterized protein At4g14450, chloroplastic-like [Sesamum indicum]
c38217.graph_c0	112.276987	111.548597	112.617847	28.728885	16.4641517	13.7711995	1.08E-123	-3.516812	down	PREDICTED: dnaJ protein homolog [Sesamum indicum]
c38218.graph_c0	3.28902837	2.64198666	2.33068002	9.40193773	13.6193363	15.1560556	4.492E-05	1.186301	up	PREDICTED: histidine kinase 4 [Sesamum indicum]
c38219.graph_c0	62.5610414	67.4230299	65.1892216	322.871735	316.156227	336.861317	6.165E-21	1.304303	up	PREDICTED: 40S ribosomal protein S10 [Solanum lycopersicum]
c38224.graph_c0	3.86148587	2.64132425	2.81189193	16.5421548	15.8913083	12.6615999	5.407E-05	1.262651	up	PREDICTED: transcription factor MYB1R1-like [Sesamum indicum]
c38225.graph_c0	10.3170679	11.0158921	8.41767662	123.742869	93.94664	73.3232283	1.58E-06	2.282636	up	PREDICTED: LOW QUALITY PROTEIN: transcription factor BIM2 [Sesamum indicum]
c38238.graph_c0	3.18338658	2.37298084	3.04632389	12.9165505	13.1546874	14.1858895	2.883E-07	1.207455	up	PREDICTED: probable glucuronosyltransferase Os03g0107900 [Sesamum indicum]
c38241.graph_c0	0	0	0	3.69682868	5.24098672	6.38533681	1.941E-17	Inf	up	PREDICTED: lipid phosphate phosphatase epsilon 2, chloroplastic isoform X1 [Sesamum indicum]
c38242.graph_c0	1.86858413	1.83848164	1.76498799	12.6240516	10.6770997	10.905509	1.595E-17	1.63042	up	PREDICTED: filament-like plant protein 4 [Sesamum indicum]
c38243.graph_c0	5.53297976	6.31585637	4.98954249	0.62598935	0.47368558	0.39785826	8.709E-19	-4.499824	down	-
c38245.graph_c0	55.8168583	70.1049219	50.509392	1403.43966	1078.58828	917.884202	5.258E-13	3.259485	up	PREDICTED: acyl-[acyl-carrier-protein] desaturase, chloroplastic [Sesamum indicum]
c38246.graph_c0	1.68565245	1.30854968	1.19358191	31.6925099	34.6487512	37.0263436	1.085E-68	3.608645	up	PREDICTED: uncharacterized GPI-anchored protein At4g28100 [Nicotiana sylvestris]
c38248.graph_c0	8.74166794	8.40860806	9.30265059	4.02669734	5.53220622	6.49406258	1.087E-08	-1.745714	down	hypothetical protein MIMGU_mgv1a027006mg [Erythranthe guttata]

#ID	0 h-1 FPKM	0 h-2 FPKM	0 h-3 FPKM	48 h-1 FPKM	48 h-2 FPKM	48 h-3 FPKM	FDR	log2FC	regulated	nr_annotation
c38249.graph_c0	0.07390123	0	0.094966	2.00843765	1.87525763	2.1808626	5.945E-10	4.139973	up	PREDICTED: uncharacterized protein LOC105164781 [Sesamum indicum]
c38255.graph_c0	0.5989865	0.666207	0.14254099	18.7825027	21.7598184	23.7685672	1.21E-84	4.50401	up	PREDICTED: probable protein S-acyltransferase 22 isoform X1 [Sesamum indicum]
c38256.graph_c0	4.09997867	4.97050195	5.09301375	30.595059	26.9875657	26.3644025	8.547E-15	1.550977	up	PREDICTED: cyclin-dependent kinase inhibitor 4-like [Musa acuminata subsp. malaccensis]
c38257.graph_c2	31.1715947	31.2581652	32.2396298	157.410237	145.581845	152.030494	2.165E-18	1.248378	up	PREDICTED: chaperonin CPN60-2, mitochondrial [Sesamum indicum]
c38258.graph_c0	2.7594256	2.89372154	2.47655127	2.3000521	2.50074805	3.4289066	0.0004436	-1.002491	down	PREDICTED: probable rhamnogalacturonate lyase B [Sesamum indicum]
c38259.graph_c0	6.63049026	6.39016673	7.68821323	61.3685145	49.8128181	35.2153851	4.213E-05	1.809239	up	PREDICTED: lysine histidine transporter 1-like [Sesamum indicum]
c38268.graph_c0	0	0	0.10204909	5.70652745	6.74291702	5.85880824	5.503E-15	6.43507	up	hypothetical protein MIMGU_mgv1a003097mg [Erythranthe guttata]
c38269.graph_c0	0.23295128	0.26233261	0.22451371	13.3862931	12.4475864	12.5077887	2.069E-44	4.720012	up	PREDICTED: uncharacterized protein LOC105168472 [Sesamum indicum]
c38271.graph_c0	0.05417448	0.05422872	0	2.5453621	2.64362991	2.7533445	7.898E-11	5.193119	up	PREDICTED: agamous-like MADS-box protein AGL12 [Sesamum indicum]
c38277.graph_c0	1.21892652	1.27824933	1.71554682	27.2717549	28.6078712	29.7380435	1.684E-34	3.322756	up	PREDICTED: threonine dehydratase biosynthetic, chloroplastic [Phoenix dactylifera]
c38278.graph_c0	44.7620074	45.1987262	44.0490864	26.089145	25.7702602	27.6713308	5.78E-33	-1.770279	down	PREDICTED: probable BOI-related E3 ubiquitin-protein ligase 2 [Sesamum indicum]
c38278.graph_c1	3.11786497	3.64115155	3.56140438	0.1595767	0.50715649	0.56796155	9.607E-12	-4.099149	down	zinc ion binding protein, partial [Genlisea aurea]
c38281.graph_c0	0.32313676	0.10782011	0.55365804	2.9967929	5.23516514	5.93346695	2.302E-07	2.808684	up	hypothetical protein CICLE_v10027936mg [Citrus clementina]
c38284.graph_c0	3.53468035	2.68046955	2.89049358	33.2545278	38.9402214	38.809015	1.565E-33	2.588561	up	PREDICTED: E3 ubiquitin ligase BIG BROTHER-like [Sesamum indicum]
c38288.graph_c0	4.53247844	4.92357902	4.20507037	2.73381056	1.76545569	1.69943751	2.186E-19	-2.14702	down	PREDICTED: G-type lectin S-receptor-like serine/threonine-protein kinase At4g27290 [Sesamum indicum]
c38291.graph_c0	0.26880006	0.18834845	0.06908374	1.85727108	2.7545749	3.30517728	2.246E-08	2.896239	up	PREDICTED: two-component response regulator ARR9 [Sesamum indicum]
c38292.graph_c0	21.6333976	19.7340468	17.8232412	148.270277	146.730458	149.878202	4.36E-40	1.894332	up	PREDICTED: UDP-glucose 4-epimerase GEPI48 [Sesamum indicum]
c38294.graph_c0	0.07325987	0.14666646	0	8.2339964	9.65241232	12.0708087	4.768E-20	6.082572	up	PREDICTED: uncharacterized protein LOC105163186 [Sesamum indicum]
c38296.graph_c2	5.19854445	6.95127799	6.68033544	41.6735207	48.9187566	47.7344365	3.556E-29	1.855201	up	PREDICTED: LOW QUALITY PROTEIN: succinate dehydrogenase [ubiquinone] flavoprotein subunit 1, mitochondrial-like [Sesamum indicum]

#ID	0 h-1 FPKM	0 h-2 FPKM	0 h-3 FPKM	48 h-1 FPKM	48 h-2 FPKM	48 h-3 FPKM	FDR	log2FC	regulated	nr_annotation
c38298.graph_c0	1309.473	1284.57546	1277.70993	184.065184	189.123727	192.558827	2.187E-92	-3.79264	down	hypothetical protein ZEAMMB73_433257, partial [Zea mays]
c38300.graph_c0	31.2218391	32.5363431	29.4931406	25.4491142	32.4491639	25.2207856	2.091E-15	-1.185467	down	PREDICTED: pollen-specific leucine-rich repeat extensin-like protein 1 [Sesamum indicum]
c38303.graph_c0	2.5330782	2.06387242	2.34670416	0.434165	0.22997258	0.62776578	3.201E-11	-3.441487	down	PREDICTED: uncharacterized protein LOC104427989 [Eucalyptus grandis]
c38304.graph_c1	4.56562355	4.32783648	4.26691419	36.8356791	39.1579629	51.5459077	4.715E-17	2.256467	up	PREDICTED: delta(8)-fatty-acid desaturase [Sesamum indicum]
c38304.graph_c2	0.7076121	0.81456877	1.18210244	12.809804	19.4751268	19.8958967	2.218E-18	3.23986	up	PREDICTED: peroxidase 31 [Sesamum indicum]
c38306.graph_c0	1.51652664	1.20396689	2.15039644	8.96085949	9.03360806	6.30149396	0.0003159	1.29848	up	PREDICTED: PGR5-like protein 1B, chloroplastic isoform X1 [Sesamum indicum]
c38308.graph_c0	55.8993162	52.9116976	55.2271269	299.022941	306.392366	302.592763	2.475E-25	1.450807	up	PREDICTED: 60S ribosomal protein L7a [Sesamum indicum]
c38310.graph_c0	31.655055	33.9297018	29.7362978	23.4577714	23.2204608	21.8076757	4.139E-23	-1.492781	down	PREDICTED: histone acetyltransferase HAC1-like isoform X1 [Sesamum indicum]
c38315.graph_c0	10.4488424	10.2221926	8.99654625	93.0851627	80.2976025	74.649674	3.683E-19	2.0512	up	PREDICTED: transcription factor PIF3 isoform X2 [Sesamum indicum]
c38316.graph_c1	13.0848142	12.2247222	17.3749878	11.6527648	11.4933333	10.0110235	0.0042271	-1.384015	down	-
c38316.graph_c2	64.2956726	51.4410659	51.2621257	57.9362863	48.0935027	44.8189055	2.482E-08	-1.158188	down	PREDICTED: uncharacterized protein LOC105160920 [Sesamum indicum]
c38321.graph_c0	2.12384068	2.25481389	1.53001691	33.1736123	31.7201554	32.0500287	3.572E-54	3.023181	up	hypothetical protein MIMGU_mgv1a017270mg [Erythranthe guttata]
c38322.graph_c0	107.160284	109.211482	110.300283	11.5149776	12.5777789	12.0404477	7.05E-164	-4.195468	down	PREDICTED: protein phosphatase 2C 37-like [Sesamum indicum]
c38324.graph_c0	37.469595	33.3396579	38.4989501	11.0552439	11.5523476	12.3128413	9.606E-46	-2.665757	down	Mitogen-activated protein kinase 10 [Morus notabilis]
c38330.graph_c0	25.0624638	21.9308472	21.9685073	11.9268593	11.7441274	12.8573885	7.497E-20	-1.93367	down	PREDICTED: U-box domain-containing protein 4 [Sesamum indicum]
c38331.graph_c0	0.09796823	0.09806633	0.04196435	345.525174	391.284105	449.216809	3.56E-230	11.26984	up	PREDICTED: raucaffricine-O-beta-D-glucosidase-like [Sesamum indicum]
c38333.graph_c0	5.46421833	5.69895836	4.87737413	22.6680463	27.558673	28.9673971	6.347E-13	1.283612	up	PREDICTED: uncharacterized protein At2g39795, mitochondrial-like [Sesamum indicum]
c38336.graph_c0	11.7291383	14.8197861	10.3820426	78.9265629	59.397738	51.6375267	0.0002563	1.354484	up	PREDICTED: ankyrin repeat-containing protein At5g02620 [Sesamum indicum]
c38337.graph_c0	0.47332445	0.09475968	0.1824722	2.39831544	1.43204466	3.10400969	0.0016906	2.199666	up	PREDICTED: nicotianamine aminotransferase A-like [Sesamum indicum]
c38338.graph_c0	0.17699739	0.35434925	0.34117298	2.28286157	2.01534585	1.3783662	0.0007531	1.685894	up	PREDICTED: uncharacterized protein LOC105156552 [Sesamum indicum]
c38340.graph_c0	0.04254789	0.0425905	0	9.83867139	11.0042352	13.183903	2.302E-37	7.638233	up	PREDICTED: protein NRT1/ PTR FAMILY 4.5-like, partial [Sesamum indicum]

#ID	0 h-1 FPKM	0 h-2 FPKM	0 h-3 FPKM	48 h-1 FPKM	48 h-2 FPKM	48 h-3 FPKM	FDR	log2FC	regulated	nr_annotation
c38344.graph_c0	0.14401642	0.64872284	0.462667	21.8917577	10.5416649	8.85416108	0.0003355	4.035286	up	PREDICTED: uncharacterized protein LOC105173288 [Sesamum indicum]
c38347.graph_c0	1.40535855	1.44814126	1.75282545	38.0034865	40.4214488	39.0671449	4.978E-68	3.652096	up	cyclin dependent kinase A [Camellia sinensis]
c38350.graph_c0	0	0	0	1.82542809	2.43433776	2.90455	2.479E-19	Inf	up	PREDICTED: uncharacterized protein LOC105176095 isoform X2 [Sesamum indicum]
c38351.graph_c0	2.71687605	2.126864	2.95417124	23.4572598	27.3657299	26.2686161	3.216E-30	2.2835	up	PREDICTED: uncharacterized protein LOC105170284 [Sesamum indicum]
c38352.graph_c0	12.5143996	13.8736829	13.4469872	7.02368939	7.89928069	6.79232211	1.884E-31	-1.894273	down	PREDICTED: U-box domain-containing protein 33-like [Sesamum indicum]
c38354.graph_c0	1.36312996	1.15457263	1.61693166	30.9121041	29.0635181	30.9435799	2.651E-22	3.439443	up	PREDICTED: auxin-responsive protein IAA26 [Sesamum indicum]
c38355.graph_c0	110.727094	100.198137	112.907262	6.83347724	8.58260475	4.57596132	7.74E-196	-5.03619	down	hypothetical protein MIMGU_mgv1a006265mg [Erythranthe guttata]
c38360.graph_c0	1.47596559	1.21671821	2.67765772	14.6373709	14.4896987	15.3017082	5.621E-08	2.022883	up	unnamed protein product [Coffea canephora]
c38360.graph_c1	0.58020349	0.7467229	0.53256032	7.06331714	6.2288655	8.93474563	1.612E-10	2.563154	up	PREDICTED: auxin transporter-like protein 3 [Sesamum indicum]
c38361.graph_c0	1.40040248	1.15259504	1.95953464	17.3165741	16.3402451	16.8112465	3.526E-26	2.462161	up	PREDICTED: uncharacterized protein LOC105173063 [Sesamum indicum]
c38362.graph_c0	1.17260636	1.45833341	0.95890175	11.7521529	12.9701752	15.6709284	2.711E-18	2.474275	up	PREDICTED: phospholipase D delta [Sesamum indicum]
c38362.graph_c1	0.74452019	0	0	12.6891604	9.08285593	12.2062039	5.452E-09	4.520081	up	-
c38369.graph_c0	5.18625011	3.20323101	4.46665759	0.8132616	1.40002208	1.26636174	2.639E-12	-2.911769	down	-
c38371.graph_c0	7.67124357	3.02830852	2.77685964	0	0	0	3.687E-07	-Inf	down	PREDICTED: plastocyanin, chloroplastic-like [Solanum tuberosum]
c38372.graph_c0	45.264079	48.5567486	50.3537522	13.6490568	15.1077061	16.24897	9.65E-76	-2.700421	down	PREDICTED: myosin-14 [Sesamum indicum]
c38374.graph_c0	0	0	0	1.50010609	2.11890734	1.4619078	1.692E-12	Inf	up	Fructose-bisphosphate aldolase A [Rozella allomycis CSF55]
c38375.graph_c0	22.603314	26.5740998	21.2485283	28.4403504	14.5093467	9.32655506	2.058E-05	-1.428365	down	PREDICTED: WRKY transcription factor 22 [Sesamum indicum]
c38376.graph_c0	0.13795438	0.36824673	0.05909228	3.092586	4.2635738	4.29727595	3.314E-09	3.35162	up	-
c38376.graph_c1	0.247906	0.16543616	0.26547419	4.60580812	6.2906616	6.02878698	2.04E-13	3.614892	up	PREDICTED: receptor-like serine/threonine-protein kinase ALE2 [Sesamum indicum]
c38378.graph_c0	27.9984278	26.1916121	26.4647209	113.937199	122.842392	127.720154	1.035E-16	1.1573	up	PREDICTED: acyl-coenzyme A oxidase 4, peroxisomal [Sesamum indicum]
c38383.graph_c0	0.02887026	0.08669752	0.1112983	4.28214442	4.8745305	5.58517652	4.687E-21	4.992134	up	PREDICTED: transcription factor LHW-like [Sesamum indicum]
c38383.graph_c1	0.21250368	0.04254329	0.05461513	5.87316765	4.60421263	5.12139107	1.292E-15	4.651505	up	PREDICTED: transcription factor LHW-like [Sesamum indicum]
c38384.graph_c0	0.10878371	0.21778527	0.06989568	2.4052475	4.03444112	2.45228358	1.091E-07	3.469721	up	-
c38388.graph_c0	10.8320539	14.4572008	10.0530575	4.6199975	6.46051183	7.39952278	2.304E-06	-1.957922	down	-
c38389.graph_c0	10.4665468	9.61721066	9.5253283	6.50646617	6.70651974	8.05823907	1.95E-14	-1.49587	down	PREDICTED: glucan endo-1,3-beta-glucosidase 4-like [Sesamum indicum]

#ID	0 h-1 FPKM	0 h-2 FPKM	0 h-3 FPKM	48 h-1 FPKM	48 h-2 FPKM	48 h-3 FPKM	FDR	log2FC	regulated	nr_annotation
c38390.graph_c0	23.5147851	21.6576917	21.5338128	12.7858454	10.5580434	10.0602428	1.311E-37	-2.010337	down	PREDICTED: F-box protein At3g07870-like [Sesamum indicum]
c38392.graph_c0	19.2928959	16.704361	23.2539369	15.3737524	18.4169447	15.8728056	8.373E-07	-1.277147	down	PREDICTED: lactoylglutathione lyase [Nicotiana sylvestris]
c38393.graph_c1	46.040942	48.2769969	46.2825319	22.7722344	17.4001769	19.1116175	2.122E-54	-2.258576	down	PREDICTED: light-inducible protein CPRF2 [Sesamum indicum]
c38399.graph_c0	0.86999904	0.54429388	0.65215687	3.873916	3.92705011	4.1007261	0.0004568	1.50955	up	PREDICTED: probable flavin-containing monooxygenase 1 [Sesamum indicum]
c38402.graph_c0	0.22160395	0.35492136	0.39867778	1.22493605	2.20604106	2.54319504	0.0022265	1.582279	up	hypothetical protein MIMGU_mgv1a004574mg [Erythranthe guttata]
c38403.graph_c0	0.19994678	0	0.25693953	4.97350845	7.41538914	9.34250669	3.004E-09	4.536598	up	PREDICTED: 2-hydroxyisoflavanone dehydratase [Sesamum indicum]
c38404.graph_c0	0.02964148	0.02967116	0	4.36922616	6.21976902	6.80349454	3.703E-28	7.187994	up	PREDICTED: phosphoinositide phospholipase C 2-like [Sesamum indicum]
c38405.graph_c0	1.13613035	1.63766593	1.10957908	0.08373419	0.04435311	0.26077163	2.092E-13	-4.342757	down	PREDICTED: BEACH domain-containing protein lvsA-like [Sesamum indicum]
c38406.graph_c0	0	0	0	1.88264503	1.47089517	2.5546285	8.79E-15	Inf	up	PREDICTED: potassium channel SKOR-like [Sesamum indicum]
c38416.graph_c0	3.67987069	2.9835133	3.14538729	24.4979576	26.0501207	29.2916709	3.279E-37	2.006431	up	PREDICTED: probable sucrose-phosphate synthase 2 [Sesamum indicum]
c38416.graph_c1	0.86826032	0.65184731	0.83681166	5.59928625	6.14361358	9.25267801	0.0001639	2.1321	up	-
c38419.graph_c0	2.61087281	1.65220455	2.19815254	1.54824315	1.64017589	2.70603427	0.0022566	-1.154625	down	hypothetical protein MIMGU_mgv1a007101mg [Erythranthe guttata]
c38423.graph_c0	15.3066269	14.6835394	15.0254054	6.56109741	7.62500753	7.79855582	6.499E-22	-2.054301	down	PREDICTED: coiled-coil domain-containing protein 97 [Sesamum indicum]
c38428.graph_c0	1.66741002	1.65238888	1.60701592	9.57007037	8.7876368	8.41971252	1.178E-11	1.427262	up	PREDICTED: structural maintenance of chromosomes protein 5 [Sesamum indicum]
c38429.graph_c0	0.17102676	0.28533003	0.1831468	7.16902586	7.01050487	9.79041247	5.256E-25	4.20986	up	PREDICTED: kinesin-like protein KIN12A isoform X2 [Sesamum indicum]
c38433.graph_c0	7.73187122	8.5326067	8.10334576	38.3888601	34.1119252	30.2877827	4.468E-08	1.062384	up	PREDICTED: uncharacterized protein LOC105163069 [Sesamum indicum]
c38435.graph_c0	0.09024382	0.31616966	0.23193377	2.16160091	2.5982171	2.10831983	1.711E-05	2.404896	up	PREDICTED: BEL1-like homeodomain protein 4 [Sesamum indicum]
c38436.graph_c0	3.04965361	2.93252205	2.25261079	13.0964376	13.3116246	12.7948233	3.66E-09	1.236742	up	PREDICTED: putative tRNA pseudouridine synthase Pus10 [Sesamum indicum]
c38437.graph_c0	1.39223764	1.27066425	1.15764032	0.67903418	0.83924678	1.07413428	0.000927	-1.580968	down	PREDICTED: probably inactive leucine-rich repeat receptor-like protein kinase At2g25790 [Sesamum indicum]
c38439.graph_c0	2.77462121	2.01992696	2.64711223	0.6971351	0.94367736	1.44738211	1.258E-07	-2.295363	down	PREDICTED: telomere repeat-binding protein 5-like isoform X2 [Nicotiana sylvestris]
c38441.graph_c0	56.0023774	55.3104843	52.6599815	22.6444184	24.3728397	22.7279223	5.405E-54	-2.250979	down	PREDICTED: UBA and UBX domain-containing protein At4g15410-like [Sesamum indicum]

#ID	0 h-1 FPKM	0 h-2 FPKM	0 h-3 FPKM	48 h-1 FPKM	48 h-2 FPKM	48 h-3 FPKM	FDR	log2FC	regulated	nr_annotation
c38442.graph_c0	1438.04255	1446.16627	1541.26606	271.849243	272.961106	255.043684	8.053E-97	-3.48574	down	--
c38443.graph_c1	5.15573105	5.25919648	5.33180396	28.385684	30.6941791	29.362998	6.913E-20	1.47082	up	PREDICTED: pantothenate kinase 2 [Sesamum indicum]
c38446.graph_c0	28.3744938	23.159293	28.6089191	117.833035	123.977732	119.995687	1.931E-11	1.155575	up	PREDICTED: flavonol synthase/flavanone 3-hydroxylase-like [Sesamum indicum]
c38447.graph_c1	0.51593167	0.22133498	0.31571077	5.25104275	5.77864301	5.90085109	2.648E-15	2.990665	up	PREDICTED: uncharacterized protein LOC105178397 [Sesamum indicum]
c38448.graph_c0	817.785776	823.214906	818.062214	129.202261	58.0292291	36.5387535	1.32E-106	-4.454807	down	PREDICTED: probable alpha,alpha-trehalose-phosphate synthase [UDP-forming] 11 [Sesamum indicum]
c38449.graph_c0	5.42204461	5.42747397	5.16114222	2.22006273	3.18484734	2.55155636	1.576E-09	-2.030923	down	PREDICTED: probably inactive leucine-rich repeat receptor-like protein kinase At5g48380 [Sesamum indicum]
c38450.graph_c1	225.332758	232.468593	226.179785	195.441906	118.392226	83.6732724	4.4E-19	-1.787052	down	PREDICTED: uncharacterized protein LOC104242030 [Nicotiana sylvestris]
c38451.graph_c0	5.35131419	5.02775422	4.48389791	50.323272	55.2050209	56.7059987	1.58E-62	2.430564	up	PREDICTED: delta(24)-sterol reductase [Sesamum indicum]
c38454.graph_c0	357.327064	350.468193	373.693893	339.336876	354.81679	353.624288	1.632E-12	-1.064504	down	PREDICTED: endo-1,3;1,4-beta-D-glucanase-like isoform X1 [Nicotiana tomentosiformis]
c38457.graph_c0	6.7667424	6.94869546	7.04637929	2.09590184	1.42330376	1.1476436	2.996E-19	-3.161433	down	-
c38459.graph_c0	1.47218704	0.85963571	0.86708354	0	0	0	5.585E-17	-Inf	down	PREDICTED: metalloendoproteinase 1-like [Glycine max]
c38460.graph_c0	29.9799067	24.2994157	27.9872092	11.1682198	7.88758311	5.09048945	2.195E-41	-2.77536	down	PREDICTED: uncharacterized protein LOC105164190 [Sesamum indicum]
c38462.graph_c0	35.8331971	38.8422571	36.5925441	17.1711976	21.9672547	23.5883666	2.596E-37	-1.849268	down	PREDICTED: NPL4-like protein 2 [Sesamum indicum]
c38464.graph_c0	0	0.05540722	0.07112925	6.73116641	9.56182778	10.0730001	4.465E-38	6.665576	up	PREDICTED: cation/H(+) antiporter 14 isoform X1 [Nicotiana tomentosiformis]
c38465.graph_c0	0.97865154	0.76193562	2.23574395	9.91757394	11.8861041	10.3399326	7.606E-06	1.9842	up	PREDICTED: F-box protein PP2-A15-like [Nicotiana tomentosiformis]
c38467.graph_c0	1.28403239	1.15349065	1.48079836	12.6482985	13.6564002	16.3552859	1.702E-20	2.422277	up	PREDICTED: uncharacterized protein LOC105163544 [Sesamum indicum]
c38473.graph_c0	57.8817688	54.1850575	52.8317964	46.1422589	36.3206416	34.3046879	3.025E-23	-1.509321	down	hypothetical protein CISIN_1g011138mg [Citrus sinensis]
c38474.graph_c0	50.1529913	51.4234289	49.1756088	50.2149887	48.1404566	47.8571999	1.006E-13	-1.060342	down	PREDICTED: transcription elongation factor S-II [Sesamum indicum]
c38475.graph_c0	4.32248864	3.4504996	4.00772702	19.4067216	19.5978652	17.6267995	1.045E-06	1.248389	up	hypothetical protein MIMGU_mgv1a011554mg [Erythranthe guttata]
c38476.graph_c1	17.9179692	15.5727339	16.413305	351.391485	354.946528	374.494609	1.57E-116	3.419259	up	hypothetical protein CISIN_1g024108mg [Citrus sinensis]
c38478.graph_c0	0.47106089	0.38312023	0.49183216	11.879984	17.4701477	19.4279551	1.196E-21	4.153162	up	hypothetical protein MIMGU_mgv1a002464mg [Erythranthe guttata]
c38479.graph_c1	545.50221	518.186758	554.788777	104.394227	97.407124	93.5138914	1.52E-101	-3.470425	down	PREDICTED: putative G3BP-like protein isoform X1 [Sesamum indicum]

#ID	0 h-1 FPKM	0 h-2 FPKM	0 h-3 FPKM	48 h-1 FPKM	48 h-2 FPKM	48 h-3 FPKM	FDR	log2FC	regulated	nr_annotation
c38482.graph_c0	3.85380163	5.65437928	3.52765697	42.0694933	49.1530876	50.329238	1.34E-36	2.422683	up	PREDICTED: 40S ribosomal protein S7 [Sesamum indicum]
c38484.graph_c0	567.450345	568.018561	576.68671	221.443895	193.830647	176.328114	7.622E-63	-2.547298	down	-
c38486.graph_c0	9.02993316	8.7180649	6.79750497	29.5840059	35.9817975	34.6456297	1.638E-07	1.01153	up	PREDICTED: 40S ribosomal protein S8 [Sesamum indicum]
c38489.graph_c0	4.28451968	4.72644365	3.90460746	2.53747221	2.96549232	3.45841833	1.486E-10	-1.547435	down	PREDICTED: uncharacterized protein LOC105171501 [Sesamum indicum]
c38491.graph_c1	0.31619775	0.27695008	0.25395414	6.1719609	5.47763465	8.00276166	4.64E-20	3.519545	up	PREDICTED: probably inactive leucine-rich repeat receptor-like protein kinase IMK2 [Sesamum indicum]
c38492.graph_c1	7.48986328	6.56548758	6.19900495	3.43058334	3.7168845	4.23189222	1.471E-11	-1.850187	down	PREDICTED: uncharacterized protein LOC105168847 isoform X2 [Sesamum indicum]
c38493.graph_c0	4.26806306	4.21401146	3.40683503	2.83158981	2.68695841	2.87775883	1.057E-13	-1.516405	down	PREDICTED: structural maintenance of chromosomes protein 2-1 [Sesamum indicum]
c38494.graph_c0	0.12021529	0.06016783	0	1.24593934	0.7626214	0.78835864	0.0001012	2.965194	up	Putative gag-pol polyprotein, identical [Solanum demissum]
c38495.graph_c0	0.26403554	0.13214997	0.8482403	10.0947406	9.92108224	12.7699308	8.171E-11	3.683851	up	PREDICTED: cytochrome c oxidase subunit 5b-1, mitochondrial-like [Sesamum indicum]
c38495.graph_c1	0	0	0	4.54276825	6.54501648	5.65897902	1.953E-11	Inf	up	PREDICTED: cytochrome c oxidase subunit 5b-2, mitochondrial [Malus domestica]
c38496.graph_c0	43.5725591	47.4600634	41.9943477	194.428224	202.072411	228.890751	1.001E-17	1.214646	up	PREDICTED: ADP,ATP carrier protein, mitochondrial [Sesamum indicum]
c38500.graph_c0	19.7577445	20.0498889	19.1496955	13.6515922	15.463122	17.0025151	1.364E-20	-1.37445	down	PREDICTED: uncharacterized protein LOC105159907 [Sesamum indicum]
c38502.graph_c0	2.29561308	2.81494195	2.87620486	1.05743425	0.72814519	0.84680877	2.459E-08	-2.614059	down	PREDICTED: protein ROOT HAIR DEFECTIVE 3 homolog 1-like [Sesamum indicum]
c38503.graph_c0	11.351094	10.2980033	10.8366718	7.47280337	8.7130091	7.50068456	1.421E-17	-1.474872	down	PREDICTED: pentatricopeptide repeat-containing protein At5g42310, mitochondrial [Sesamum indicum]
c38504.graph_c0	3.85063158	3.78244091	4.48576412	122.834703	155.697577	164.728121	3.84E-72	4.168836	up	PREDICTED: probable pectinesterase/pectinesterase inhibitor 51 [Sesamum indicum]
c38505.graph_c0	2.48048904	2.01271286	2.41479289	13.330265	15.0571394	13.309179	3.419E-17	1.574264	up	PREDICTED: nuclear pore complex protein NUP96 [Sesamum indicum]
c38506.graph_c0	0.84158946	0.58970253	0.64888546	4.41936447	4.02468874	2.75952556	0.0082621	1.418069	up	-
c38507.graph_c0	0	0.31884833	0.51165356	3.30131285	3.73049671	5.28747899	4.845E-07	2.85275	up	hypothetical protein EUGRSUZ_J00099 [Eucalyptus grandis]
c38509.graph_c0	0.02719466	0.08166567	0.10483864	1.70363749	4.14041745	3.90117823	1.812E-07	4.470659	up	PREDICTED: sulfate transporter 3.1-like [Sesamum indicum]
c38512.graph_c0	0.09191375	0.09200579	0	3.59877017	4.70951018	3.95561442	1.044E-13	5.054429	up	PREDICTED: microtubule-associated protein RP/EB family member 1B [Sesamum indicum]
c38513.graph_c0	178.006279	170.99115	188.079318	791.104706	781.199487	832.868665	5.188E-15	1.144499	up	PREDICTED: 60S ribosomal protein L18-2-like [Sesamum indicum]
c38514.graph_c1	5.15052449	6.70238655	7.05986975	2.68883283	3.35116746	3.23692161	1.991E-05	-2.051285	down	-

#ID	0 h-1 FPKM	0 h-2 FPKM	0 h-3 FPKM	48 h-1 FPKM	48 h-2 FPKM	48 h-3 FPKM	FDR	log2FC	regulated	nr_annotation
c38515.graph_c0	9.20949729	7.23236535	7.7807385	46.5938302	57.5541598	61.1456357	4.845E-25	1.749803	up	hypothetical protein MIMGU_mgv1a012548mg [Erythranthe guttata]
c38516.graph_c0	0.02504371	0.02506879	0	11.2360153	12.2209402	12.2354257	8.118E-66	8.47369	up	PREDICTED: uncharacterized protein LOC105155823 [Sesamum indicum]
c38524.graph_c0	222.006809	224.180776	212.295353	26.58374	28.5428201	34.2024806	3.72E-140	-3.901591	down	Histone H3.3 [Arabidopsis thaliana]
c38525.graph_c1	29.1873033	30.4911892	27.5164994	20.8383951	24.6267287	24.7499743	2.417E-21	-1.33226	down	PREDICTED: pectinesterase 3-like [Sesamum indicum]
c38527.graph_c0	0.15792058	0.07903936	0.1014671	9.40814237	14.1024885	15.1244517	5.566E-30	5.812559	up	PREDICTED: auxin transporter-like protein 2 [Sesamum indicum]
c38531.graph_c0	35.6877425	37.389506	38.7857693	26.5971697	22.4599628	20.5700059	2.113E-31	-1.698071	down	PREDICTED: deSI-like protein At4g17486 [Sesamum indicum]
c38532.graph_c0	15.411083	15.9814255	14.5893124	3.55453081	4.6961723	5.27133356	1.466E-62	-2.788351	down	PREDICTED: autophagy-related protein 13 isoform X2 [Sesamum indicum]
c38536.graph_c0	0.22331707	0.29805426	0.0318857	1.60015531	1.57408691	2.21707335	2.212E-05	2.277684	up	PREDICTED: uncharacterized protein LOC105175444 [Sesamum indicum]
c38540.graph_c0	3.11451672	1.8339032	2.04037666	0	0	0	2.825E-20	-Inf	down	hypothetical protein PAXRUDRAFT_143168 [Paxillus rubicundulus Ve08.2h10]
c38540.graph_c1	1.45384243	0.81398037	0.82329444	0.70373913	0.93792015	0.80797787	0.0035033	-1.353025	down	PREDICTED: heat shock protein 83-like [Nelumbo nucifera]
c38541.graph_c0	6.75266628	6.81244317	4.83214502	4.53766812	6.46116822	5.31833066	1.478E-07	-1.192289	down	PREDICTED: probable glutathione S-transferase [Sesamum indicum]
c38542.graph_c0	1.36876631	0.77070202	0.76952702	13.2404665	13.0247638	14.7266093	1.356E-18	2.803038	up	PREDICTED: homoserine dehydrogenase [Sesamum indicum]
c38545.graph_c1	32.4407005	33.8787945	33.3593212	14.0642302	15.9886856	16.1741004	4.314E-44	-2.128618	down	PREDICTED: protein SSUH2 homolog [Sesamum indicum]
c38547.graph_c0	35.5152993	35.436551	38.8881669	209.149171	256.675821	269.507527	1.318E-31	1.720389	up	-
c38552.graph_c0	4.53231428	6.00035358	6.26258418	3.14282201	2.23548036	1.67787848	1.207E-11	-2.261571	down	PREDICTED: uncharacterized protein LOC105175383 [Sesamum indicum]
c38553.graph_c0	1.59876792	2.29242023	1.88790061	8.04117934	8.56082603	9.91781211	0.0002711	1.178166	up	PREDICTED: uncharacterized protein LOC105174166 [Sesamum indicum]
c38556.graph_c0	2.95573304	3.12571573	2.84867548	45.6104855	55.1815809	78.5886325	2.564E-12	3.305245	up	PREDICTED: thaumatin-like protein [Sesamum indicum]
c38557.graph_c0	8.38302345	9.1863942	8.24003057	2.98080238	6.34430462	8.24624192	9.417E-13	-1.585272	down	PREDICTED: MACPF domain-containing protein CAD1-like [Sesamum indicum]
c38559.graph_c0	13.8974043	14.3373014	12.7964019	7.89698396	7.61830616	7.25592833	1.699E-37	-1.864833	down	PREDICTED: filaggrin-like [Sesamum indicum]
c38563.graph_c0	6.21138793	5.34405951	6.06886699	24.733732	27.7053951	24.9535375	7.626E-08	1.1155	up	PREDICTED: uncharacterized protein LOC105157934 [Sesamum indicum]
c38568.graph_c1	7.18425653	5.62240674	5.03566586	4.633024	4.97186905	5.62150485	0.0001682	-1.245501	down	PREDICTED: floral homeotic protein GLOBOSA [Sesamum indicum]
c38569.graph_c1	6.64626937	6.61772394	7.23023854	3.88760435	3.67228049	3.08442388	1.404E-14	-1.961039	down	PREDICTED: transcription factor HBP-1b(c38)-like isoform X1 [Sesamum indicum]
c38571.graph_c0	12.6230727	12.3111257	10.5958326	5.82526793	8.36385004	9.26536531	2.263E-22	-1.622032	down	PREDICTED: cation/H(+) antiporter 18-like [Sesamum indicum]

#ID	0 h-1 FPKM	0 h-2 FPKM	0 h-3 FPKM	48 h-1 FPKM	48 h-2 FPKM	48 h-3 FPKM	FDR	log2FC	regulated	nr_annotation
c38572.graph_c0	22.5052068	22.4802155	21.9036062	8.00461475	8.8969639	7.66734557	2.492E-42	-2.463007	down	PREDICTED: cyclin-dependent kinase F-4-like [Sesamum indicum]
c38576.graph_c0	108.547577	111.653137	111.229456	30.7301428	33.0202989	35.7919633	4.758E-77	-2.754881	down	PREDICTED: HIPL1 protein-like [Solanum tuberosum]
c38580.graph_c0	3.26708642	3.66769111	2.98199523	37.9185232	44.9380392	48.6321858	5.938E-57	2.709159	up	PREDICTED: NADP-dependent glyceraldehyde-3-phosphate dehydrogenase [Sesamum indicum]
c38581.graph_c1	13.6162419	11.0460136	9.53650303	67.5363852	68.649477	66.3355728	1.005E-19	1.551632	up	-
c38583.graph_c0	11.0792944	11.435305	10.626908	8.20464463	9.23506525	10.4074774	2.25E-13	-1.271086	down	PREDICTED: internal alternative NAD(P)H-ubiquinone oxidoreductase A1, mitochondrial-like [Sesamum indicum]
c38584.graph_c0	24.3240699	23.9261997	23.8495714	188.662379	225.045286	237.024368	2.219E-50	2.153045	up	hypothetical protein M569_17345, partial [Genlisea aurea]
c38588.graph_c0	1.06743738	0.94639126	1.29331648	7.7828527	9.10818248	11.0502163	2.298E-12	2.054275	up	hypothetical protein MIMGU_mgv1a003578mg [Erythranthe guttata]
c38590.graph_c0	21.3098578	21.779186	22.2080347	17.5387934	18.513027	17.4402426	1.025E-16	-1.306068	down	PREDICTED: peroxisomal membrane protein 2 [Sesamum indicum]
c38591.graph_c0	66.1167518	62.4407873	63.2072657	21.008905	15.6368062	13.9654744	5.317E-83	-2.932018	down	PREDICTED: E3 ubiquitin-protein ligase RNF170-like [Sesamum indicum]
c38593.graph_c0	5.2407659	4.25701115	4.02971469	30.710107	34.3364004	38.2770641	4.334E-24	1.914946	up	PREDICTED: long chain acyl-CoA synthetase 6, peroxisomal-like isoform X2 [Sesamum indicum]
c38596.graph_c0	0	0	0	8.60445187	7.9173529	7.52494057	3.231E-25	Inf	up	PREDICTED: uncharacterized protein LOC105168232 isoform X2 [Sesamum indicum]
c38600.graph_c0	0.23745343	0.29051147	0.54246589	2.60078468	4.06844981	4.39042058	1.877E-08	2.33446	up	PREDICTED: phosphatidylinositol 4-kinase gamma 8 [Sesamum indicum]
c38601.graph_c0	1.32814492	1.35660699	1.35840884	0.29965071	0.13226816	0.19997055	1.248E-13	-3.682449	down	-
c38602.graph_c0	0.81465389	1.25456868	1.12739038	5.59997637	6.4829337	8.47593995	0.0001118	1.66136	up	hypothetical protein MIMGU_mgv1a015466mg [Erythranthe guttata]
c38603.graph_c0	19.6823514	18.282092	18.7529824	15.3229195	15.921272	14.971502	1.197E-19	-1.312848	down	PREDICTED: uncharacterized protein LOC105171881 isoform X1 [Sesamum indicum]
c38604.graph_c1	3.29310271	4.14891756	3.35622928	19.1444567	17.2335046	15.7314273	4.238E-06	1.256867	up	PREDICTED: pentatricopeptide repeat-containing protein At4g21190 isoform X1 [Sesamum indicum]
c38607.graph_c0	75.9802896	71.987783	71.4380957	63.8980867	77.8013291	73.7839991	2.739E-13	-1.0463	down	hypothetical protein M569_16671 [Genlisea aurea]
c38608.graph_c0	16.7925579	16.8093731	15.6409274	8.61073128	12.8321033	11.0781289	7.085E-28	-1.621501	down	PREDICTED: DNA ligase 1-like [Sesamum indicum]
c38610.graph_c0	1.12794784	0.74281402	1.37317039	9.48898093	10.9504799	11.3387579	3.872E-16	2.267987	up	hypothetical protein MIMGU_mgv1a005256mg [Erythranthe guttata]
c38612.graph_c0	12.8415438	14.0229847	13.9631395	16.2565518	12.9273395	11.3364236	5.152E-08	-1.023194	down	PREDICTED: uncharacterized protein LOC105173848 isoform X3 [Sesamum indicum]
c38614.graph_c0	13.8997873	13.7090926	12.7834287	13.9980497	10.573312	7.87537506	1.114E-08	-1.324512	down	PREDICTED: sphingoid long-chain bases kinase 1-like [Sesamum indicum]

#ID	0 h-1 FPKM	0 h-2 FPKM	0 h-3 FPKM	48 h-1 FPKM	48 h-2 FPKM	48 h-3 FPKM	FDR	log2FC	regulated	nr_annotation
c38617.graph_c0	0.25804548	0.54008991	0.45217999	6.18093648	3.59450552	3.23062832	0.0006135	2.37112	up	PREDICTED: plant intracellular Ras-group-related LRR protein 1-like [Sesamum indicum]
c38620.graph_c0	7.86075107	8.68261785	8.57746181	6.89847366	7.83718931	5.69382434	3.889E-10	-1.316432	down	PREDICTED: protein LTV1 homolog isoform X1 [Sesamum indicum]
c38629.graph_c0	21.7974091	18.6656745	18.7101612	93.1108533	106.617255	108.674504	2.206E-17	1.362772	up	hypothetical protein M569_15947, partial [Genlisea aurea]
c38630.graph_c0	43.1488629	43.0498036	45.5491782	21.6826619	17.1166465	13.2814834	4E-59	-2.350174	down	PREDICTED: WPP domain-interacting protein 1-like [Sesamum indicum]
c38632.graph_c1	1.04988829	1.00715045	0.89943202	13.2993517	12.4239809	11.0089113	2.549E-15	2.621469	up	PREDICTED: protein PHR1-LIKE 1-like isoform X1 [Sesamum indicum]
c38638.graph_c0	0.16199196	0	0.20816608	3.28322623	4.58487375	4.02798396	9E-11	3.974339	up	PREDICTED: ABC transporter I family member 17-like [Sesamum indicum]
c38642.graph_c0	0	0.09304754	0.29862539	5.78041025	4.76283403	4.57188825	4.164E-14	4.234924	up	hypothetical protein MIMGU_mgv1a025521mg [Erythranthe guttata]
c38644.graph_c0	241.157655	235.6063	248.322257	10.9613614	11.4314981	12.751466	2.41E-231	-5.38627	down	PREDICTED: pheophytinase, chloroplastic [Sesamum indicum]
c38645.graph_c0	43.2521494	42.3623681	35.2170969	14.8852619	14.0102656	15.1805974	7.81E-51	-2.469708	down	PREDICTED: cell number regulator 6-like [Sesamum indicum]
c38646.graph_c0	2.07624441	2.80573667	2.80145909	0.09563859	0	0.17019728	2.398E-16	-5.866523	down	PREDICTED: uncharacterized protein LOC105168686 [Sesamum indicum]
c38650.graph_c0	31.4532265	33.7951118	29.1363155	15.96849	15.3265994	15.8038952	3.883E-37	-2.018618	down	PREDICTED: probable histone-lysine N-methyltransferase ATXR3 isoform X1 [Sesamum indicum]
c38664.graph_c0	0.71242512	1.1410216	1.92253841	9.97625377	9.17802182	8.7016104	3.832E-06	1.858549	up	PREDICTED: uncharacterized protein LOC105179255 [Sesamum indicum]
c38667.graph_c0	2.1087182	0.8795124	1.12907766	13.4369305	12.5197626	9.50726023	6.392E-05	2.096978	up	-
c38669.graph_c0	217.033901	218.697641	226.070098	33.6127237	36.3137259	36.719223	3.56E-130	-3.652969	down	PREDICTED: BTB/POZ and TAZ domain-containing protein 1 [Sesamum indicum]
c38680.graph_c0	7.40715314	6.63919038	6.9677799	4.81690772	4.96118161	5.7147407	1.658E-07	-1.458588	down	PREDICTED: zinc finger CCCH domain-containing protein 39-like [Sesamum indicum]
c38681.graph_c1	0.39206183	0.32704535	0.25190749	17.1265648	19.7378525	18.2119468	1.166E-56	4.810226	up	PREDICTED: 3-ketoacyl-CoA synthase 12 [Sesamum indicum]
c38697.graph_c0	1.61620645	1.01114052	2.07688928	12.6561158	10.055715	12.254983	0.0002417	1.8747	up	hypothetical protein AMTR_s00007p00249170 [Amborella trichopoda]
c38697.graph_c1	1.9088292	2.66707543	2.50402362	21.5420901	22.3943045	18.679042	2.371E-20	2.126378	up	PREDICTED: uncharacterized membrane protein At1g16860-like [Sesamum indicum]
c38703.graph_c0	0.08523162	0.08531697	0.10952601	9.1084278	8.01333453	10.386961	1.276E-38	5.59799	up	PREDICTED: BTB/POZ domain-containing protein NPY2 [Sesamum indicum]
c38706.graph_c0	1.33702047	1.59739658	1.27473734	0.51654052	0.50511881	0.91922929	1.233E-05	-2.137653	down	-
c38710.graph_c0	6.98902706	6.70229925	7.438608	85.2273917	104.294717	107.476397	4.821E-73	2.790834	up	PREDICTED: protein NRT1/ PTR FAMILY 8.3-like [Sesamum indicum]
c38711.graph_c0	22.1611926	21.4027367	21.4628989	175.424823	197.51191	201.374601	1.024E-48	2.123131	up	unnamed protein product [Coffea canephora]

#ID	0 h-1 FPKM	0 h-2 FPKM	0 h-3 FPKM	48 h-1 FPKM	48 h-2 FPKM	48 h-3 FPKM	FDR	log2FC	regulated	nr_annotation
c38714.graph_c1	32.2512875	31.2928692	29.1644239	19.8079931	21.7169473	18.7161066	2.183E-28	-1.639083	down	PREDICTED: putative E3 ubiquitin-protein ligase XBAT34 isoform X2 [Sesamum indicum]
c38715.graph_c0	0.45691732	0.3201624	0.41100979	4.71455083	2.58643488	2.65931703	0.002027	2.062274	up	PREDICTED: F-box/kelch-repeat protein At3g24760 [Sesamum indicum]
c38732.graph_c2	3.51267211	2.58738474	2.12920884	0.85481957	1.42305065	1.6842152	5.39E-06	-2.077624	down	PREDICTED: probable sucrose-phosphate synthase 1 isoform X1 [Sesamum indicum]
c38737.graph_c0	0.38595323	0.20333669	0.41765489	2.84452235	3.03325169	4.2794539	4.269E-09	2.309951	up	PREDICTED: beta-1,3-galactosyltransferase 15 [Sesamum indicum]
c38740.graph_c1	0	0	0	10.943192	15.2157928	13.7943474	1.255E-13	Inf	up	-
c38744.graph_c2	0.15741137	0.34665179	0.08091194	2.26227488	2.94966883	2.55490898	3.938E-07	2.717575	up	PREDICTED: uncharacterized protein LOC105160721 [Sesamum indicum]
c38756.graph_c0	1.41345916	1.67212444	1.0732983	7.99059335	7.02286853	5.10865367	0.0041561	1.265517	up	PREDICTED: ras-related protein Rab11D-like [Sesamum indicum]
c38759.graph_c0	1.188999	1.56603895	1.36707807	4.554489	6.29073	7.79732472	0.0067632	1.152055	up	-
c38760.graph_c0	12.4644723	9.45761962	8.60718292	77.5619043	75.4139451	78.3953816	1.261E-34	1.907771	up	PREDICTED: NADPH-dependent 1-acyldihydroxyacetone phosphate reductase-like [Sesamum indicum]
c38761.graph_c0	0.16190234	0.08103223	0.15603819	13.3120835	12.9964568	15.7269905	1.185E-41	5.698808	up	PREDICTED: putative glucose-6-phosphate 1-epimerase [Sesamum indicum]
c38762.graph_c0	6.79808054	6.42102233	7.12303908	2.76207091	2.04145061	1.97185479	4.556E-22	-2.597836	down	PREDICTED: exocyst complex component EXO70A1-like [Sesamum indicum]
c38763.graph_c0	2.79422498	2.89237603	3.2234601	17.1127278	23.0252018	24.4669986	1.42E-15	1.833413	up	PREDICTED: 54S ribosomal protein L10, mitochondrial [Sesamum indicum]
c38770.graph_c0	3.89767001	1.91177074	2.85493636	0	0	0	1.21E-18	-Inf	down	cytochrome P450 [Aureobasidium pullulans var. namibiae CBS 147.97]
c38773.graph_c0	8.43545388	7.81749858	6.85132653	5.30384799	6.15623271	4.86296389	3.345E-15	-1.517921	down	PREDICTED: uncharacterized protein LOC105166108 isoform X1 [Sesamum indicum]
c38775.graph_c0	16.4387003	15.5180791	15.3500625	86.4162398	86.2118233	85.1804811	4.542E-23	1.429433	up	PREDICTED: uncharacterized protein LOC105168156 [Sesamum indicum]
c38777.graph_c0	62.5234583	59.4662169	67.6015581	417.822512	423.643898	415.673703	1.026E-34	1.71058	up	hypothetical protein [Solanum tuberosum]
c38791.graph_c0	0.26355243	0.14070205	0.22578354	68.2920221	80.9896733	86.2301559	2.99E-218	7.524765	up	PREDICTED: probable methyltransferase PMT26 [Sesamum indicum]
c38792.graph_c0	0.29740035	0.09923272	0	3.10515879	2.99928576	3.49432117	1.738E-07	3.597896	up	PREDICTED: uncharacterized protein LOC105110235 [Populus euphratica]
c38793.graph_c0	44.4013752	45.1631675	41.1079392	55.2976097	34.2422547	26.9984714	1.092E-06	-1.169755	down	PREDICTED: uncharacterized protein LOC105159283 [Sesamum indicum]
c38793.graph_c1	0.92475413	0.92568013	0.7130074	7.09953183	8.24311805	10.6127628	6.039E-08	2.320486	up	PREDICTED: uncharacterized protein LOC105166230 isoform X2 [Sesamum indicum]

#ID	0 h-1 FPKM	0 h-2 FPKM	0 h-3 FPKM	48 h-1 FPKM	48 h-2 FPKM	48 h-3 FPKM	FDR	log2FC	regulated	nr_annotation
c38793.graph_c2	1.48580459	2.08220936	1.81385184	17.1786923	23.7091174	21.8320746	2.91E-27	2.519248	up	PREDICTED: uncharacterized protein LOC105166230 isoform X2 [Sesamum indicum]
c38806.graph_c0	1.45459062	1.30392284	1.6739163	9.72046248	7.9669072	8.8805665	8.807E-11	1.567124	up	PREDICTED: uncharacterized protein LOC105171651 isoform X2 [Sesamum indicum]
c38807.graph_c0	0	0	0	1.82142171	2.70140562	2.10689711	4.124E-11	Inf	up	--
c38814.graph_c0	0.99765733	0.99865633	0.82416161	5.84289452	5.98119202	4.76085675	6.487E-05	1.5417	up	PREDICTED: probable amino acid permease 7 [Sesamum indicum]
c38825.graph_c0	0.52418989	0.38161075	0.48989437	2.85360585	2.97654093	3.55476986	0.0005338	1.729364	up	PREDICTED: tRNA (guanine(9)-N1)-methyltransferase [Sesamum indicum]
c38829.graph_c0	1.75342253	1.48515088	1.64658193	11.7735417	14.6446563	15.6449369	5.878E-16	2.08421	up	PREDICTED: heparan-alpha-glucosaminide N-acetyltransferase isoform X1 [Sesamum indicum]
c38844.graph_c0	0.89775221	1.02702991	1.81287406	8.27067888	9.63795954	9.98748616	0.0001019	1.87169	up	PREDICTED: 14 kDa zinc-binding protein [Sesamum indicum]
c38844.graph_c2	4.12499604	4.7026164	3.53385548	24.8069617	32.318769	29.211615	2.286E-08	1.784602	up	PREDICTED: 14 kDa zinc-binding protein isoform X2 [Tarenaya hassleriana]
c38846.graph_c0	3.9000548	2.92797009	4.87251025	0.49902568	0.42292575	1.24328413	1.276E-10	-3.461872	down	-
c38847.graph_c0	85.8989643	87.0406362	89.5746276	26.9218182	26.52829	22.6720732	8.586E-83	-2.802349	down	hypothetical protein EUGRSUZ_J00099 [Eucalyptus grandis]
c38848.graph_c0	5.98185102	4.25802023	4.66908935	2.57173995	3.50285997	2.54257639	1.514E-08	-1.810464	down	predicted protein [Hordeum vulgare subsp. vulgare]
c38855.graph_c0	0	0.07096172	0.36438958	17.3069288	15.0827968	12.2615692	3.353E-26	5.641999	up	PREDICTED: UDP-N-acetylglucosamine--peptide N-acetylglucosaminyltransferase 110 kDa subunit [Sesamum indicum]
c38857.graph_c0	2.01215942	1.63651662	1.29285226	0.07723885	0.2454756	0.24054334	1.844E-14	-4.163896	down	-
c38860.graph_c0	0.11184446	0	0	0.89299956	1.2371093	1.00851254	7.646E-08	3.809765	up	PREDICTED: probable indole-3-pyruvate monooxygenase YUCCA4 [Sesamum indicum]
c38868.graph_c0	0.94802055	0.72568282	1.14658253	0.0770628	0.13606448	0.25142338	3.843E-11	-3.635806	down	-
c38873.graph_c1	0.5585085	0.35577039	0.78295149	3.50818949	2.8740944	2.83021976	0.0032494	1.421392	up	PREDICTED: raucaffricine-O-beta-D-glucosidase-like [Sesamum indicum]
c38886.graph_c0	0	0	0	5.7680022	10.2175556	13.083244	4.489E-14	Inf	up	hypothetical protein MIMGU_mgv1a023829mg, partial [Erythranthe guttata]
c38888.graph_c0	11.9477598	10.8956913	9.28850018	57.9339335	47.6384898	41.7029725	1.649E-05	1.186356	up	PREDICTED: putative zinc transporter At3g08650 [Sesamum indicum]
c38892.graph_c0	0	0	0	2.06127251	1.91070975	2.67474066	5.33E-13	Inf	up	PREDICTED: peroxidase 19 [Sesamum indicum]
c38898.graph_c0	6.23957423	5.81594781	6.07038334	25.7859087	32.1493004	31.5585553	9.225E-16	1.282363	up	PREDICTED: CDPK-related kinase 1 [Sesamum indicum]
c38902.graph_c0	1.79614463	0.8989716	1.61568188	11.7485517	13.8485508	13.9874664	1.706E-05	2.177273	up	PREDICTED: copper transporter 6-like [Nicotiana sylvestris]
c38904.graph_c0	28.1343657	29.5194352	27.1953166	15.1386553	17.3065355	15.7153318	1.942E-35	-1.835761	down	PREDICTED: 20S-pre-rRNA D-site endonuclease nob1 [Sesamum indicum]
c38906.graph_c0	0.77160191	0.80595605	0.47421431	0.61813044	0.42564226	0.19250306	0.0002944	-1.729935	down	gag-pol polyprotein [Oryza sativa Japonica Group]

#ID	0 h-1 FPKM	0 h-2 FPKM	0 h-3 FPKM	48 h-1 FPKM	48 h-2 FPKM	48 h-3 FPKM	FDR	log2FC	regulated	nr_annotation
c38908.graph_c0	23.2929401	22.8641486	20.1483687	21.3400894	15.4913101	20.4692636	1.649E-11	-1.222595	down	PREDICTED: uncharacterized protein LOC105158092 [Sesamum indicum]
c38916.graph_c0	12.4528336	12.8500348	12.0017882	7.15252234	8.21491782	8.25464901	6.252E-18	-1.678772	down	PREDICTED: uncharacterized acetyltransferase At3g50280-like [Sesamum indicum]
c38923.graph_c0	57.3795797	56.9894027	51.0003629	32.5719393	28.5868057	26.874511	1.838E-42	-1.922662	down	PREDICTED: large subunit GTPase 1 homolog isoform X1 [Cicer arietinum]
c38930.graph_c0	1.30892097	1.94936905	2.01021337	10.7351252	12.8992911	11.5671398	3.524E-11	1.717113	up	PREDICTED: uncharacterized protein LOC105159306 [Sesamum indicum]
c38931.graph_c0	1.76168665	2.29248592	1.47149411	35.056382	40.4047531	42.2043608	1.97E-58	3.394824	up	PREDICTED: uncharacterized protein LOC105171035 [Sesamum indicum]
c38934.graph_c0	27.9185733	28.9288329	26.3556508	16.7702502	18.0054812	14.2383073	1.196E-27	-1.779775	down	PREDICTED: formin-like protein 14 isoform X2 [Sesamum indicum]
c38934.graph_c1	23.6241239	23.3396655	18.4916409	14.1785562	13.293108	13.3098737	7.266E-17	-1.696233	down	PREDICTED: formin-like protein 5 [Sesamum indicum]
c38944.graph_c0	4.21522924	4.81165368	3.80121857	1.43070573	2.88696997	2.00048098	9.231E-07	-2.047731	down	hypothetical protein MIMGU_mgv1a003797mg [Erythranthe guttata]
c38944.graph_c1	14.2897216	15.1454442	14.6238145	3.21669536	3.66012081	4.92933788	1.143E-29	-2.92203	down	PREDICTED: probable protein S-acyltransferase 22 isoform X2 [Sesamum indicum]
c38952.graph_c0	7.6726155	7.21198758	7.75542863	3.06014929	3.4701569	4.14187976	9.144E-14	-2.106498	down	hypothetical protein MIMGU_mgv1a006823mg [Erythranthe guttata]
c38956.graph_c0	42.9873913	36.8091687	36.3833011	153.75902	222.998336	271.929471	2.754E-05	1.457002	up	PREDICTED: REF/SRPP-like protein At1g67360 [Sesamum indicum]
c38961.graph_c0	28.1427639	25.1174506	26.8073005	26.3348912	27.562496	21.9403607	6.235E-12	-1.094824	down	PREDICTED: protein KTI12 homolog [Sesamum indicum]
c38963.graph_c0	19.6139553	18.4517676	19.4785907	188.310365	158.196002	127.627124	2.242E-09	2.030165	up	PREDICTED: uncharacterized protein LOC105177709 [Sesamum indicum]
c38964.graph_c0	1.83947561	1.39121772	1.83851033	12.0508083	12.3674211	12.6327242	6.602E-19	1.850578	up	PREDICTED: DNA gyrase subunit B, chloroplastic/mitochondrial-like isoform X1 [Sesamum indicum]
c38967.graph_c1	0.90296948	1.02439015	0.69621092	10.5093463	10.3989864	8.78366893	2.971E-16	2.487666	up	PREDICTED: phosphomevalonate kinase [Sesamum indicum]
c38969.graph_c0	14.3940413	13.4971508	12.5842245	7.23106457	7.15614407	7.42247668	1.937E-30	-1.908366	down	PREDICTED: chaperone protein ClpB4, mitochondrial [Sesamum indicum]
c38969.graph_c1	19.2277736	19.1335871	18.2522148	10.1619896	10.5073212	10.7142422	7.076E-28	-1.868801	down	PREDICTED: chaperone protein ClpB4, mitochondrial [Sesamum indicum]
c38972.graph_c0	103.586863	96.6875213	110.202666	48.8589957	45.4828828	40.0519846	2.044E-47	-2.223989	down	PREDICTED: zinc finger A20 and AN1 domain-containing stress-associated protein 8-like isoform X1 [Sesamum indicum]
c38985.graph_c0	10.0946014	7.90803362	8.8628315	36.5060678	40.264761	37.9823544	2.037E-08	1.076407	up	PREDICTED: uncharacterized protein LOC105174450 [Sesamum indicum]
c38986.graph_c0	17.807236	15.1012368	14.6039841	11.6863919	11.2086765	11.3497618	3.833E-17	-1.487569	down	PREDICTED: ABC transporter I family member 1 [Sesamum indicum]

#ID	0 h-1 FPKM	0 h-2 FPKM	0 h-3 FPKM	48 h-1 FPKM	48 h-2 FPKM	48 h-3 FPKM	FDR	log2FC	regulated	nr_annotation
c38987.graph_c0	1.40863495	1.16121393	1.17127462	36.4890128	39.9495986	42.0449976	2.649E-40	3.966856	up	PREDICTED: NADPH:quinone oxidoreductase [Sesamum indicum]
c38988.graph_c1	0.75812376	0.67456258	0.61339718	30.9121297	35.3510231	33.1676322	1.13E-100	4.585098	up	PREDICTED: probable protein S-acyltransferase 7 [Sesamum indicum]
c38992.graph_c0	2.13092138	1.59979138	2.00107878	8.7586414	8.27885661	9.80891742	0.0001096	1.209635	up	PREDICTED: traB domain-containing protein [Sesamum indicum]
c38995.graph_c0	2.20585906	2.2080679	2.6226823	9.42020465	12.8366173	11.7618969	5.483E-10	1.248905	up	PREDICTED: SNF2 domain-containing protein CLASSY 3-like [Sesamum indicum]
c38996.graph_c0	11.6308942	13.628801	10.5133091	11.1651179	12.6325178	12.1868435	2.827E-09	-1.009321	down	PREDICTED: uncharacterized protein LOC105173283 [Sesamum indicum]
c39001.graph_c0	0	0	0	1.68758128	2.02391065	2.54988625	2.051E-14	Inf	up	PREDICTED: putative UDP-glucuronate:xylan alpha-glucuronosyltransferase 4 [Sesamum indicum]
c39015.graph_c0	0.6633793	0.88539143	0.87141241	0.02716215	0.0143875	0	2.099E-25	-6.857571	down	PREDICTED: uncharacterized protein LOC103452707 [Malus domestica]
c39021.graph_c0	9.53150499	9.37424778	10.363998	45.9009123	49.1468676	48.5190794	5.542E-17	1.274617	up	PREDICTED: uncharacterized protein LOC105158512 [Sesamum indicum]
c39024.graph_c0	71.5366187	73.8872967	66.4862285	32.0097425	41.6071125	37.7056168	3.763E-43	-1.949419	down	PREDICTED: uncharacterized protein LOC105169073 [Sesamum indicum]
c39028.graph_c0	8.6009005	10.4413243	10.5821786	1.01153752	1.78600224	0.90006	9.135E-16	-4.027637	down	-
c39028.graph_c1	9.99733907	6.60485093	8.22206497	0.18420402	0.19514182	0.32780725	1.446E-20	-6.155522	down	-
c39029.graph_c0	19.4573194	18.1234299	18.2804503	10.8851834	10.4988574	10.119253	2.037E-20	-1.842351	down	PREDICTED: CBL-interacting serine/threonine-protein kinase 1-like [Sesamum indicum]
c39033.graph_c0	15.8978921	18.5661133	17.9973468	12.3795052	7.11142969	4.57674187	4.089E-16	-2.127178	down	hypothetical protein MIMGU_mgv1a025187mg [Erythranthe guttata]
c39040.graph_c0	0.5066508	0.84526356	0.65106634	18.4370106	26.1247821	23.0502617	1.705E-24	4.05368	up	PREDICTED: uncharacterized protein LOC105178874 isoform X1 [Sesamum indicum]
c39047.graph_c0	2.45067276	2.1720393	2.29630007	17.7795418	20.3550456	19.8797922	6.402E-26	2.048226	up	PREDICTED: transcription factor LHW-like [Nicotiana sylvestris]
c39052.graph_c0	0.62843976	0.41937936	0.71783997	8.42707999	12.8119408	9.67346337	6.584E-12	3.103555	up	unnamed protein product [Vitis vinifera]
c39052.graph_c1	1.18180146	1.26457002	0.68077937	13.2902902	12.6078699	12.1262717	3.254E-16	2.593505	up	hypothetical protein MIMGU_mgv1a002912mg [Erythranthe guttata]
c39055.graph_c0	7.39969894	6.56058192	7.33544186	31.1151251	32.4984387	31.4988407	3.648E-09	1.140694	up	PREDICTED: ubiquitin thioesterase OTU1 isoform X1 [Sesamum indicum]
c39060.graph_c0	16.2703123	17.1321845	15.1972337	13.3989889	13.29848	10.718063	2.013E-16	-1.392033	down	hypothetical protein MIMGU_mgv1a009934mg [Erythranthe guttata]
c39068.graph_c0	1.41249524	0.99805387	1.17448444	3.54025454	5.21011294	6.14694642	0.0015367	1.0304	up	PREDICTED: protein NRT1/ PTR FAMILY 5.8 [Nicotiana sylvestris]
c39070.graph_c0	14.0818408	17.0703147	11.4550907	4.28469696	6.80867533	9.21353585	3.025E-10	-2.093727	down	-

#ID	0 h-1 FPKM	0 h-2 FPKM	0 h-3 FPKM	48 h-1 FPKM	48 h-2 FPKM	48 h-3 FPKM	FDR	log2FC	regulated	nr_annotation
c39073.graph_c0	1.75695129	2.08439776	2.92671551	10.8507363	17.2743133	21.1234712	4.269E-05	1.831312	up	LEC14B protein [Morus notabilis]
c39074.graph_c0	0.34321666	0.34356034	0.441047	2.25852554	2.82330806	2.04981489	0.0017531	1.638355	up	-
c39078.graph_c0	24.7075079	24.249197	26.4170969	101.985182	103.519604	104.2746	1.499E-10	1.020533	up	PREDICTED: uncharacterized protein LOC100800807 isoform X2 [Glycine max]
c39087.graph_c0	81.4900172	86.8394515	79.3069015	10.2840985	11.6729522	13.9875429	3.894E-86	-3.805003	down	ubiquitin [Galdieria sulphuraria]
c39094.graph_c0	3.98382019	3.29427732	5.56452899	18.0316311	23.8356466	23.8536762	0.0006096	1.328188	up	-
c39102.graph_c1	12419.2522	12680.8182	12985.2777	41.4941264	32.7472315	28.8266381	5.71E-221	-9.541503	down	15 kDa oleosin [Sesamum indicum]
c39103.graph_c0	1.88858879	1.73719777	2.23013477	18.8566584	21.0224858	20.7116657	3.446E-18	2.349542	up	hypothetical protein MIMGU_mgv1a011278mg [Erythranthe guttata]
c39104.graph_c0	0	0.06766919	0.02895686	1.24557809	0.90168495	0.84970272	9.429E-09	3.942928	up	PREDICTED: probable RNA helicase SDE3 [Sesamum indicum]
c39110.graph_c0	1.54254487	1.22955274	2.16577115	16.7636334	16.2814405	18.0071312	5.112E-27	2.347492	up	PREDICTED: tubulin gamma-1 chain [Sesamum indicum]
c39112.graph_c0	10.6251316	9.24459426	6.3371672	101.809329	124.831353	144.869425	4.918E-27	2.807859	up	PREDICTED: probable voltage-gated potassium channel subunit beta [Sesamum indicum]
c39115.graph_c0	3.65323793	2.58133842	3.3138038	0.09898827	0	0.26423749	4.922E-17	-5.734947	down	PREDICTED: stem-specific protein TSJT1-like [Sesamum indicum]
c39123.graph_c0	0.49034458	0.65444744	0.57760284	2.63513487	5.06477065	4.82343874	7.474E-05	1.833281	up	PREDICTED: probable 1-deoxy-D-xylulose-5-phosphate synthase 2, chloroplastic [Sesamum indicum]
c39130.graph_c0	1.13022838	0.95730473	1.00549953	9.5313517	6.70325714	6.41415587	7.186E-06	1.863142	up	PREDICTED: xylosyltransferase 1 [Sesamum indicum]
c39132.graph_c0	84.8566816	87.7806117	84.7716573	49.1732318	39.8586771	33.8501015	1.271E-47	-2.078519	down	PREDICTED: histidine kinase 3 isoform X2 [Sesamum indicum]
c39133.graph_c0	0.95594446	0.8657682	0.93594398	0.46130773	0	0	2.204E-10	-3.540131	down	-
c39134.graph_c0	150.985195	143.330085	159.722013	69.9931054	85.9189237	88.1797847	1.363E-40	-1.917647	down	PREDICTED: high mobility group B protein 1 [Sesamum indicum]
c39136.graph_c0	9.42813239	10.8532092	7.55200958	39.0282419	42.3271899	43.9498694	3.035E-13	1.154122	up	PREDICTED: patatin-like protein 6 [Sesamum indicum]
c39138.graph_c0	0	0	0	2.60222856	5.22633025	5.8368568	1.753E-13	Inf	up	PREDICTED: COBRA-like protein 4 [Sesamum indicum]
c39138.graph_c1	0	0	0	9.21845303	19.8846483	24.2122699	8.347E-11	Inf	up	hypothetical protein MIMGU_mgv1a006245mg [Erythranthe guttata]
c39142.graph_c0	7.70827725	8.41217601	9.08619441	5.1257993	5.88267639	6.84135809	8.487E-08	-1.520498	down	PREDICTED: uncharacterized protein LOC105155593 [Sesamum indicum]
c39144.graph_c0	27.0993377	27.5085366	24.4721188	17.5259426	19.7625596	19.8924285	1.432E-25	-1.486374	down	PREDICTED: ankyrin repeat domain-containing protein 50 [Sesamum indicum]
c39146.graph_c0	1.80691612	1.28255079	1.09765323	16.6767562	18.7571608	22.1371274	1.809E-31	2.764528	up	PREDICTED: phytoene synthase 2, chloroplastic [Sesamum indicum]
c39147.graph_c0	0	0	0	1.69821245	1.99894463	1.95878048	5.541E-17	Inf	up	hypothetical protein MIMGU_mgv1a023002mg [Erythranthe guttata]
c39157.graph_c0	0.5576278	0.41863964	0.3582869	6.38943109	7.2110122	6.91375438	4.071E-13	2.926474	up	PREDICTED: probable beta-1,3-galactosyltransferase 11 [Sesamum indicum]

#ID	0 h-1 FPKM	0 h-2 FPKM	0 h-3 FPKM	48 h-1 FPKM	48 h-2 FPKM	48 h-3 FPKM	FDR	log2FC	regulated	nr_annotation
c39161.graph_c0	48.4175286	47.8879764	50.8022134	50.7846636	51.8926923	44.6052886	1.661E-12	-1.015559	down	PREDICTED: phytanoyl-CoA dioxygenase [Sesamum indicum]
c39166.graph_c1	18.5246289	18.0239695	16.1397097	80.927429	82.0084294	103.928261	2.375E-13	1.322362	up	hypothetical protein MIMGU_mgv1a005459mg [Erythranthe guttata]
c39167.graph_c0	14.0149597	14.9351726	13.2703628	6.67196872	10.6349356	9.75703965	5.079E-21	-1.665942	down	PREDICTED: uncharacterized protein LOC105176476 isoform X2 [Sesamum indicum]
c39168.graph_c0	34.043788	32.5673988	31.9239356	24.7177168	23.4196108	21.450622	1.472E-24	-1.51694	down	PREDICTED: uncharacterized protein LOC105169489 [Sesamum indicum]
c39170.graph_c0	65.9160176	62.6829214	66.9937112	2.34623703	1.973822	2.39467754	7.31E-194	-5.880396	down	PREDICTED: protein REVEILLE 6 isoform X2 [Nicotiana sylvestris]
c39171.graph_c0	0	0	0	5.464825	6.4982157	4.56486015	1.093E-12	Inf	up	-
c39174.graph_c0	16.9039931	18.7466838	16.8672197	15.3030079	16.1481061	12.3883673	1.609E-15	-1.276686	down	PREDICTED: B3 domain-containing protein Os01g0234100-like [Sesamum indicum]
c39178.graph_c0	15.6781098	18.6506137	17.2271379	2.51194737	2.88286229	1.67633675	2.241E-18	-3.882929	down	hypothetical protein SERLADRAFT_461711 [Serpula lacrymans var. lacrymans S7.9]
c39191.graph_c1	3.09783151	3.52480213	2.89255	16.4254644	18.1403214	17.1181461	8.695E-15	1.423758	up	PREDICTED: protein FAR1-RELATED SEQUENCE 5-like [Prunus mume]
c39191.graph_c2	1.24203038	0.88805292	0.22800832	6.04812253	7.79260432	9.59958058	7.968E-05	2.302154	up	-
c39195.graph_c0	1.89823259	1.9544229	2.26506803	12.6661324	12.3595938	10.9145798	3.685E-11	1.536696	up	PREDICTED: armadillo repeat-containing protein LFR [Sesamum indicum]
c39211.graph_c0	0	0	0.03970538	1.02475501	1.17607064	0.9371492	2.982E-10	5.253477	up	PREDICTED: uncharacterized protein LOC105159159 [Sesamum indicum]
c39213.graph_c0	2.3393633	1.36599506	1.25257269	0	0	0	1.001E-13	-Inf	down	PREDICTED: uncharacterized protein LOC104436401 [Eucalyptus grandis]
c39216.graph_c0	0.41945304	0.74285234	0.82925195	25.1177877	29.2859206	32.2945924	7.261E-81	4.418161	up	PREDICTED: cell division protein FtsZ homolog 2-2, chloroplastic [Sesamum indicum]
c39217.graph_c0	15.3370613	12.7351668	14.7508174	13.0719918	12.7590068	10.376759	1.108E-13	-1.257727	down	PREDICTED: uncharacterized protein LOC105168440 [Sesamum indicum]
c39218.graph_c0	28.9893274	25.5058176	27.2603777	23.7312669	29.9271941	25.038404	1.876E-12	-1.075367	down	PREDICTED: F-box protein At4g00755-like [Sesamum indicum]
c39224.graph_c0	0.91197553	1.46406684	1.37093073	0.07926145	0.11755505	0	5.593E-21	-5.267938	down	hypothetical protein VITISV_033646 [Vitis vinifera]
c39225.graph_c0	4.94504722	4.80179538	5.32719404	55.1048748	59.2150177	63.8872217	4.405E-60	2.543107	up	PREDICTED: serine hydroxymethyltransferase, mitochondrial [Sesamum indicum]
c39227.graph_c1	1.81839612	2.18426037	1.76551499	0.03722722	0	0.03312456	2.324E-24	-7.354052	down	PREDICTED: phospholipase D delta-like isoform X2 [Sesamum indicum]
c39231.graph_c0	3.20044132	2.51165853	3.15854885	55.6904237	57.273062	79.5450595	1.104E-22	3.4184	up	PREDICTED: glucose-1-phosphate adenylyltransferase large subunit 1 [Sesamum indicum]
c39235.graph_c0	1.58915372	1.08840449	2.14960573	8.86124668	8.97926784	8.63887118	0.0005885	1.433241	up	-

#ID	0 h-1 FPKM	0 h-2 FPKM	0 h-3 FPKM	48 h-1 FPKM	48 h-2 FPKM	48 h-3 FPKM	FDR	log2FC	regulated	nr_annotation
c39252.graph_c0	1.05350156	0.35151883	0.67689561	28.2431317	30.4685386	34.3998049	4.075E-80	4.465192	up	hypothetical protein MIMGU_mgv1a009282mg [Erythranthe guttata]
c39255.graph_c0	14.9621014	14.1074465	16.1257809	184.706453	176.832098	192.362316	4.41E-61	2.597079	up	hypothetical protein MIMGU_mgv1a016658mg [Erythranthe guttata]
c39260.graph_c0	0.11986528	0.0399951	0	11.5212911	12.2444058	16.6055872	1.7E-35	6.978293	up	PREDICTED: probable galactinol--sucrose galactosyltransferase 2 [Sesamum indicum]
c39272.graph_c0	5.02700417	4.80839182	4.47407846	19.553973	24.1130606	27.6247401	2.651E-11	1.295563	up	PREDICTED: ATP-dependent zinc metalloprotease FtsH [Sesamum indicum]
c39274.graph_c0	2.43911463	2.40052246	2.39686266	10.0079655	10.4221881	12.04699	2.183E-08	1.146868	up	PREDICTED: uncharacterized protein LOC105158090 isoform X1 [Sesamum indicum]
c39285.graph_c0	128.443647	127.704265	124.870853	14.7289225	14.9687884	18.6589722	1.39E-150	-3.997271	down	hypothetical protein MIMGU_mgv11b009377mg [Erythranthe guttata]
c39287.graph_c0	6.82663674	6.19645395	6.46785905	35.9227322	40.6530576	47.5189299	4.731E-18	1.649867	up	-
c39293.graph_c0	11.0793087	10.564216	10.6520322	10.4304924	10.18164	10.7062842	1.121E-07	-1.061417	down	hypothetical protein MIMGU_mgv1a0098172mg, partial [Erythranthe guttata]
c39296.graph_c0	1.54794011	1.0151832	0.96028623	0	0	0	2.277E-19	-Inf	down	glycosyltransferase family 35 protein [Micromonas sp. RCC299]
c39313.graph_c0	0	0	0	3.5641032	2.97482165	3.26741813	2.005E-11	Inf	up	PREDICTED: malate dehydrogenase, cytoplasmic-like [Sesamum indicum]
c39314.graph_c1	8.04413931	10.3372765	7.40355388	80.8176237	82.3276119	75.5645083	1.516E-27	2.195385	up	PREDICTED: mitochondrial import inner membrane translocase subunit Tim9 [Elaeis guineensis]
c39316.graph_c0	21.5655409	23.4283912	23.3926727	10.6934343	9.47128276	8.16310224	2.107E-31	-2.285885	down	PREDICTED: splicing factor 3B subunit 2 [Solanum lycopersicum]
c39319.graph_c0	0	0	0	1.08095846	1.17882513	1.27301074	1.068E-11	Inf	up	PREDICTED: auxilin-like protein 1 [Sesamum indicum]
c39321.graph_c0	0.87327499	0.34965978	0.33665788	3.05716794	4.2614457	3.65086137	0.0013132	1.799129	up	-
c39321.graph_c1	7.32547495	7.75790076	5.32068823	34.7215999	27.9069732	29.2125223	6.486E-07	1.160326	up	PREDICTED: uncharacterized protein LOC105156443 [Sesamum indicum]
c39327.graph_c0	0.95615242	0.90925437	1.16725903	11.8917446	11.2914166	11.6785128	4.07E-13	2.503852	up	unknown [Glycine max]
c39328.graph_c0	11.1172394	9.09180693	9.99092767	46.9254261	47.7970792	46.8764167	1.665E-10	1.212012	up	PREDICTED: DNA-directed RNA polymerases II, IV and V subunit 9A [Sesamum indicum]
c39334.graph_c0	0	0	0	1.71145586	1.49020271	2.02350389	2.39E-15	Inf	up	hypothetical protein MIMGU_mgv1a002051mg [Erythranthe guttata]
c39339.graph_c0	1.88498907	1.95807949	2.23947158	7.86373532	10.1356534	9.73763695	4.207E-05	1.165442	up	PREDICTED: WD repeat domain-containing protein 83 [Sesamum indicum]
c39340.graph_c0	23.7726243	25.4663538	24.3235768	18.6497475	18.4734012	19.1191013	2.827E-20	-1.404793	down	PREDICTED: protein BASIC PENTACYSTEINE1-like [Sesamum indicum]
c39349.graph_c0	0	0	0	2.90045299	1.82579432	1.08468643	1.008E-05	Inf	up	PREDICTED: glucan endo-1,3-beta-glucosidase 14 isoform X1 [Sesamum indicum]

#ID	0 h-1 FPKM	0 h-2 FPKM	0 h-3 FPKM	48 h-1 FPKM	48 h-2 FPKM	48 h-3 FPKM	FDR	log2FC	regulated	nr_annotation
c39351.graph_c0	3.29674166	3.50956938	3.63123733	23.6224601	15.4236529	15.1423478	0.0012256	1.366877	up	PREDICTED: uncharacterized protein LOC105175733 [Sesamum indicum]
c39353.graph_c0	11.1604376	10.550968	8.32167159	6.79101464	10.2871145	10.6169117	1.522E-05	-1.139118	down	hypothetical protein AALP_AA4G241200 [Arabis alpina]
c39362.graph_c0	0.82811828	0.4736843	0.60809416	23.5414258	21.0136578	23.2744657	2.092E-50	4.135944	up	PREDICTED: U-box domain-containing protein 4 [Sesamum indicum]
c39383.graph_c0	0.06582625	0.08785622	0.02819645	9.31887445	6.95980627	9.26316139	1.012E-36	6.12782	up	PREDICTED: probable auxin efflux carrier component 1c [Sesamum indicum]
c39388.graph_c0	16.8600469	13.855047	16.3957615	15.5850498	13.7865208	11.7663497	1.397E-09	-1.20974	down	-
c39389.graph_c0	53.9672251	50.7178065	61.9374802	8.07344727	6.54995854	7.68383618	1.137E-61	-3.917418	down	hypothetical protein MIMGU_mgv1a004314mg [Erythranthe guttata]
c39394.graph_c0	6.55395423	4.50621371	5.27444062	19.3945634	25.1335447	26.8083018	7.413E-06	1.105163	up	hypothetical protein MIMGU_mgv1a006129mg [Erythranthe guttata]
c39396.graph_c0	6.74864984	3.8413102	3.91102925	1.46289327	1.29146503	0.75930962	2.449E-07	-3.052074	down	hypothetical protein AALP_AA2G024800 [Arabis alpina]
c39400.graph_c0	16.1498689	16.8577729	13.7477755	53.8543043	73.0636637	76.7043465	4.868E-08	1.101219	up	PREDICTED: uncharacterized protein At4g22758-like [Sesamum indicum]
c39403.graph_c0	0.05587282	0.05592877	0.10769819	0.84931588	0.76342186	1.48853184	0.0001121	2.791597	up	PREDICTED: uncharacterized protein LOC105155966 isoform X1 [Sesamum indicum]
c39411.graph_c0	4.92007806	4.22908019	2.74891091	25.5703223	23.6438557	22.3577701	6.002E-11	1.578555	up	PREDICTED: protein DEHYDRATION-INDUCED 19-like [Nelumbo nucifera]
c39416.graph_c1	18.7431915	14.07147	15.3272911	3.00872244	3.88028456	2.67714314	4.699E-20	-3.349878	down	MYB-related transcription factor [Salvia miltiorrhiza]
c39426.graph_c0	0.03494053	0.27980412	0.17959984	9.91436364	13.6062447	14.7219966	7.925E-36	5.245971	up	PREDICTED: serine/threonine-protein kinase HT1-like [Sesamum indicum]
c39438.graph_c0	9.51397561	6.44341056	9.77157843	5.01782086	6.52390294	7.88592791	2.428E-05	-1.430571	down	PREDICTED: probable polyamine transporter At1g31830 isoform X1 [Sesamum indicum]
c39439.graph_c0	169.34639	172.639868	155.49463	658.932261	702.13903	777.069935	2.727E-13	1.085227	up	PREDICTED: 60S ribosomal protein L8-1 [Sesamum indicum]
c39444.graph_c0	5.22940674	5.48793238	6.71999469	23.3113092	24.5308688	25.3747015	7.262E-05	1.048716	up	PREDICTED: uncharacterized protein LOC104232221 [Nicotiana sylvestris]
c39445.graph_c0	0.12673338	0.25372058	0	2.21834615	3.21588356	2.38942286	3.953E-07	3.354553	up	-
c39446.graph_c0	2.88144344	1.71500629	1.90142187	0.50221663	0.07600538	0.12767695	1.674E-12	-4.183928	down	PREDICTED: polyubiquitin [Sesamum indicum]
c39449.graph_c0	56.9708962	62.4224792	53.9887364	30.2955721	27.8545782	30.382768	4.884E-41	-1.985212	down	-
c39464.graph_c0	2.32825901	1.84360137	2.27742023	18.3120598	16.0418172	15.2114948	3.199E-16	1.927145	up	PREDICTED: ribulose-1,5 bisphosphate carboxylase/oxygenase large subunit N-methyltransferase, chloroplastic isoform X1 [Sesamum indicum]
c39481.graph_c0	10.0800191	5.35552135	6.37697162	0	0	0	5.432E-24	-Inf	down	-
c39483.graph_c0	0.25629874	0.15393323	0.19761242	1.79450532	1.45080955	0.92442844	0.0023898	1.769693	up	PREDICTED: protein DYAD-like [Sesamum indicum]

#ID	0 h-1 FPKM	0 h-2 FPKM	0 h-3 FPKM	48 h-1 FPKM	48 h-2 FPKM	48 h-3 FPKM	FDR	log2FC	regulated	nr_annotation
c39485.graph_c0	2.24854561	1.91317761	2.1671026	10.2021714	11.4114538	11.4740094	4.906E-05	1.3665	up	PREDICTED: monothiol glutaredoxin-S10-like isoform X1 [Sesamum indicum]
c39498.graph_c0	29.2454485	28.4553388	31.8917302	27.6283825	28.215821	30.4702047	1.421E-12	-1.073266	down	hypothetical protein M569_06909, partial [Genlisea aurea]
c39502.graph_c1	1.86295396	1.6735559	1.35044439	12.0359264	14.6853399	12.8435625	4.635E-19	1.999972	up	PREDICTED: guanylate-binding protein 4 isoform X1 [Sesamum indicum]
c39522.graph_c0	62.5869409	58.4308949	58.7635145	14.9192344	14.2181303	12.1597236	9.1E-100	-3.13697	down	hypothetical protein MIMGU_mgv1a011861mg [Erythranthe guttata]
c39525.graph_c0	2.92912773	2.70058233	2.57539928	106.590889	174.495818	201.725019	9.78E-18	4.852939	up	PREDICTED: WAT1-related protein At2g37460-like [Sesamum indicum]
c39536.graph_c0	96.1937466	99.5586813	85.9855126	51.9292216	57.1024652	60.5991575	2.659E-35	-1.750298	down	PREDICTED: ubiquitin-activating enzyme E1 1-like isoform X1 [Sesamum indicum]
c39539.graph_c0	0.36494821	0.18265683	0.30148258	5.1873032	6.38576392	5.64132702	7.101E-17	3.321046	up	PREDICTED: probable inactive leucine-rich repeat receptor-like protein kinase At5g20690 [Sesamum indicum]
c39541.graph_c0	3.516826	2.57138431	2.98664182	22.3414269	22.7428007	22.1855631	4.177E-22	1.873147	up	PREDICTED: UPF0160 protein-like [Sesamum indicum]
c39546.graph_c0	0	0	0	1.15085263	1.25851748	1.05705471	2.163E-11	Inf	up	PREDICTED: flavin-containing monooxygenase FMO GS-OX-like 9 [Sesamum indicum]
c39547.graph_c1	0	0	0	3.43443797	3.63837058	5.04230472	5.771E-11	Inf	up	PREDICTED: homeobox-leucine zipper protein ATHB-14-like [Sesamum indicum]
c39547.graph_c2	0.19264372	0.16528853	0.14145989	6.64267166	8.11147236	10.3774056	7.621E-24	4.632983	up	PREDICTED: homeobox-leucine zipper protein ATHB-14-like [Sesamum indicum]
c39550.graph_c0	26.0202306	29.5692131	22.0555771	8.11344214	9.71844557	8.87371312	2.032E-32	-2.557721	down	PREDICTED: uncharacterized protein LOC105155212 [Sesamum indicum]
c39552.graph_c0	0.77605837	0.83982214	0.37734379	4.03853292	6.46868017	6.70550383	1.633E-07	2.091271	up	PREDICTED: anaphase-promoting complex subunit 2 [Sesamum indicum]
c39554.graph_c0	1.60274	1.79089664	1.5566626	12.9112159	13.1140194	11.5799844	3.763E-21	1.909315	up	PREDICTED: cleavage and polyadenylation specificity factor subunit 6 [Nicotiana sylvestris]
c39579.graph_c0	2.31561616	2.14749851	2.01294542	18.8859497	19.940904	17.4187262	1.191E-20	2.102321	up	PREDICTED: parafibromin [Sesamum indicum]
c39586.graph_c0	1.5916474	1.41621439	1.5150592	11.5134012	11.44912	10.9694058	1.705E-07	1.890446	up	PREDICTED: uncharacterized protein LOC105155506 isoform X4 [Sesamum indicum]
c39594.graph_c0	1.82864045	1.54446037	1.3952385	7.63362855	7.80804507	7.96345278	0.0011715	1.279552	up	unnamed protein product [Coffea canephora]
c39612.graph_c0	2.11962849	2.81456762	3.05733386	12.7924397	14.3964025	12.5882734	1.284E-06	1.293859	up	PREDICTED: MOB kinase activator-like 1 [Sesamum indicum]
c39629.graph_c0	0.09605628	0.30768788	0.22218501	0.70794646	1.46246769	1.41733463	0.0068142	1.486926	up	PREDICTED: FK506-binding protein 5 [Solanum lycopersicum]
c39645.graph_c0	0	0.07616685	0	1.6823922	3.49031896	3.86720468	2.578E-07	5.872615	up	PREDICTED: transcription factor MYB48 [Sesamum indicum]
c39649.graph_c0	4.9320028	9.35420486	7.00495769	2.63055312	2.53341097	2.34065035	1.632E-06	-2.521242	down	-
c39654.graph_c0	4.34557966	4.26070175	4.15238501	20.5920088	24.7291678	28.9545297	5.385E-14	1.519844	up	PREDICTED: glucan endo-1,3-beta-glucosidase 3 [Sesamum indicum]

#ID	0 h-1 FPKM	0 h-2 FPKM	0 h-3 FPKM	48 h-1 FPKM	48 h-2 FPKM	48 h-3 FPKM	FDR	log2FC	regulated	nr_annotation
c39655.graph_c0	0.76196945	0.65377067	1.95832182	32.2908939	36.8642095	35.3353053	9.263E-29	3.921165	up	PREDICTED: UDP-arabinose 4-epimerase 1-like [Sesamum indicum]
c39655.graph_c1	0.11906544	0.357554	0.45901141	22.2672422	32.3047546	27.8166434	4.462E-28	5.428193	up	PREDICTED: probable UDP-arabinose 4-epimerase 3 [Sesamum indicum]
c39661.graph_c0	0.03196569	0.06399541	0.08215437	0.67732431	1.24790083	1.46739259	8.804E-06	3.21563	up	PREDICTED: uncharacterized protein LOC105171946 [Sesamum indicum]
c39662.graph_c0	0.46078635	0.6259791	0.33835926	9.58171677	10.2470342	11.3316983	1.625E-23	3.435261	up	PREDICTED: folic acid synthesis protein fol1 isoform X2 [Sesamum indicum]
c39666.graph_c0	22.2231736	21.7295908	20.3628517	15.6666301	15.9053576	16.3690254	1.655E-14	-1.440978	down	hypothetical protein AMTR_s00071p00136090 [Amborella trichopoda]
c39675.graph_c0	49.7075032	52.2378459	50.2017518	23.098336	24.7544198	20.7917461	1.56E-47	-2.165638	down	LIM domain and RING finger protein [Medicago truncatula]
c39679.graph_c0	1.75060525	1.55765174	1.60685503	8.01265957	9.41050833	9.81745887	6.603E-08	1.450213	up	PREDICTED: fatty acid amide hydrolase [Sesamum indicum]
c39682.graph_c0	25.042137	26.16026	23.8544624	9.38913661	11.8649346	8.83179143	1.082E-31	-2.338064	down	PREDICTED: uncharacterized protein LOC105170284 [Sesamum indicum]
c39687.graph_c1	3.2214262	3.54711717	2.75977347	28.8864692	27.1432989	25.1748626	2.615E-10	2.077656	up	RuBisCO large subunit-binding protein subunit beta, chloroplastic [Gossypium arboreum]
c39688.graph_c1	65.0624152	69.5398775	66.6738114	47.4611072	49.6967295	51.7908345	2.015E-24	-1.452979	down	PREDICTED: delta(3,5)-Delta(2,4)-dienoyl-CoA isomerase, mitochondrial [Sesamum indicum]
c39689.graph_c0	0.45674427	0.3657613	0.23477379	3.53457394	3.59586312	3.46953117	5.579E-07	2.314054	up	PREDICTED: uncharacterized protein LOC105163339 [Sesamum indicum]
c39695.graph_c0	28.1681222	25.4109025	25.5952134	238.589952	464.682418	847.302768	0.0021381	3.260571	up	hypothetical protein MIMGU_mgv1a004702mg [Erythranthe guttata]
c39709.graph_c0	31.446007	35.061271	31.2079908	10.3346892	14.2095607	14.6251465	4.101E-42	-2.341778	down	PREDICTED: uncharacterized protein LOC105156369 [Sesamum indicum]
c39709.graph_c1	15.2156393	16.5279087	14.5099104	8.56065081	8.41860701	9.80223363	7.303E-24	-1.805802	down	PREDICTED: F-box/kelch-repeat protein At5g15710-like [Sesamum indicum]
c39711.graph_c0	0.097301	0.06493229	0	0.71712019	1.99421745	2.04720295	0.0001908	3.858446	up	PREDICTED: LOW QUALITY PROTEIN: BTB/POZ domain-containing protein At3g22104-like [Sesamum indicum]
c39713.graph_c0	27.0728429	27.4641989	25.5778927	20.1139264	22.0185416	21.5960159	7.818E-19	-1.348464	down	PREDICTED: eukaryotic translation initiation factor 4G-like [Sesamum indicum]
c39715.graph_c0	19.5371557	20.5964435	19.5763385	4.6933846	6.75815596	7.78465938	1.2E-42	-2.658886	down	PREDICTED: serine carboxypeptidase-like 50 [Sesamum indicum]
c39715.graph_c1	26.3388863	24.9054392	21.9775168	16.8145743	17.3385643	17.0264125	2.24E-21	-1.532848	down	PREDICTED: zinc finger CCHC domain-containing protein 10 [Sesamum indicum]
c39715.graph_c2	24.8501448	24.5241818	24.4567659	244.983848	268.86927	285.044249	1.872E-63	2.416206	up	PREDICTED: glutamate decarboxylase [Sesamum indicum]
c39718.graph_c0	0.35238792	0.61729637	0.56604053	47.5195804	56.2738467	62.1782244	3.03E-103	5.732004	up	PREDICTED: GDSL esterase/lipase At1g71691 [Sesamum indicum]

#ID	0 h-1 FPKM	0 h-2 FPKM	0 h-3 FPKM	48 h-1 FPKM	48 h-2 FPKM	48 h-3 FPKM	FDR	log2FC	regulated	nr_annotation
c39719.graph_c0	28.3655906	30.0774328	26.7018501	24.3074518	26.3708595	28.337787	5.478E-14	-1.126413	down	PREDICTED: dynamin-related protein 3A-like [Sesamum indicum]
c39721.graph_c0	76.9641607	73.1921743	69.1001439	10.0738499	15.5389337	17.9765529	8.39E-103	-3.355636	down	PREDICTED: UDP-glucuronic acid decarboxylase 2-like [Sesamum indicum]
c39727.graph_c0	4.19624405	3.85483964	3.78828931	2.12866629	1.76257862	1.54573893	1.712E-13	-2.134494	down	-
c39733.graph_c0	0.759867	0.76062789	0.70305061	0.44802499	0.44496389	0.57305929	0.001092	-1.619415	down	unnamed protein product [Coffea canephora]
c39733.graph_c1	16.846834	19.4661269	17.9738898	15.9994271	14.9195783	18.8821375	3.979E-10	-1.142547	down	PREDICTED: uncharacterized protein LOC105173212 [Sesamum indicum]
c39737.graph_c0	0.33116918	0.57259229	0.38687774	18.2502073	20.9793185	20.5331085	5.128E-63	4.513949	up	PREDICTED: LRR receptor-like serine/threonine-protein kinase FEI 1 [Sesamum indicum]
c39750.graph_c0	1.53840651	1.11770346	0.98845664	11.5439776	12.5684786	13.2821	8.089E-22	2.343164	up	PREDICTED: uncharacterized membrane protein At3g27390 [Sesamum indicum]
c39753.graph_c0	30.1535127	28.5156599	27.756132	17.8226671	17.2546214	16.756153	2.275E-35	-1.753434	down	PREDICTED: RNA polymerase-associated protein RTF1 homolog [Sesamum indicum]
c39760.graph_c0	75.0796781	73.5094568	75.9283297	108.581947	67.2486279	40.787573	0.0006736	-1.055408	down	PREDICTED: uncharacterized protein LOC105175444 [Sesamum indicum]
c39762.graph_c0	2.35388898	2.7282849	2.8656384	10.7666906	16.1215593	13.1684563	6.735E-07	1.30836	up	PREDICTED: flavonol synthase/flavanone 3-hydroxylase-like [Sesamum indicum]
c39764.graph_c0	59.6739735	53.9118827	65.3191202	0.88078328	1.47738156	0.71840603	4.63E-134	-6.886105	down	PREDICTED: putative cytochrome c oxidase subunit 6b-like isoform X2 [Sesamum indicum]
c39766.graph_c0	0	0	0	4.14032844	5.24903023	4.8919211	1.274E-30	Inf	up	PREDICTED: beta-amyrin synthase [Sesamum indicum]
c39771.graph_c0	0.07427792	0.0743523	0	0.78694117	1.08739402	1.76576089	0.0001001	3.604081	up	PREDICTED: pachytene checkpoint protein 2 homolog [Sesamum indicum]
c39774.graph_c0	0	0	0	1.41476316	1.79852403	1.47465023	9.14E-12	Inf	up	PREDICTED: uncharacterized protein LOC105171399 isoform X2 [Sesamum indicum]
c39775.graph_c0	1.27748844	1.0893206	1.45922065	9.02294354	9.53562591	10.5302139	3.616E-14	1.905177	up	PREDICTED: LOW QUALITY PROTEIN: ARF guanine-nucleotide exchange factor GNOM [Sesamum indicum]
c39778.graph_c0	1.32659878	0.90687709	1.24736485	11.1483866	8.43146289	8.59359522	5.438E-10	2.004226	up	PREDICTED: uncharacterized protein LOC105176769 [Sesamum indicum]
c39779.graph_c0	12.5355525	14.2915345	10.7783864	8.76171108	14.4485613	14.4275144	1.51E-06	-1.022906	down	PREDICTED: pentatricopeptide repeat-containing protein At1g10270, partial [Sesamum indicum]
c39798.graph_c1	44.6331686	44.1613549	43.8572172	41.1868953	43.9922289	40.5754863	1.526E-14	-1.094962	down	PREDICTED: LOW QUALITY PROTEIN: mitogen-activated protein kinase homolog MMK1-like [Sesamum indicum]
c39800.graph_c0	5.72728228	6.36588284	4.97024407	4.04291447	4.57334902	4.38999984	1.044E-07	-1.409268	down	PREDICTED: cyclin-dependent kinase inhibitor 3 isoform X2 [Sesamum indicum]
c39801.graph_c0	19.2034721	23.0502305	19.6544245	6.10591283	5.63918269	6.68677479	1.482E-29	-2.764704	down	hypothetical protein VITISV_001313 [Vitis vinifera]
c39812.graph_c0	105.887973	109.314788	96.136581	31.1239788	30.5272579	32.5778285	1.484E-77	-2.74064	down	hypothetical protein M569_12244, partial [Genlisea aurea]

#ID	0 h-1 FPKM	0 h-2 FPKM	0 h-3 FPKM	48 h-1 FPKM	48 h-2 FPKM	48 h-3 FPKM	FDR	log2FC	regulated	nr_annotation
c39816.graph_c0	2.0588769	2.35535834	2.59174367	16.1419142	27.5574803	30.0348128	1.127E-07	2.366434	up	PREDICTED: protein IQ-DOMAIN 1 isoform X1 [Sesamum indicum]
c39822.graph_c0	3.20402339	2.15168711	2.60588412	10.3871896	11.2414636	12.1681293	0.0001158	1.067156	up	PREDICTED: uric acid degradation bifunctional protein TTL isoform X1 [Sesamum indicum]
c39823.graph_c0	18.4329858	19.8707854	15.8110033	10.8295009	13.4813541	12.1481402	3.476E-19	-1.588648	down	hypothetical protein MIMGU_mgv1a001998mg [Erythranthe guttata]
c39824.graph_c0	0.41940471	0.64881996	0.19598242	8.32479687	10.0470906	11.1266586	4.497E-20	3.5306	up	PREDICTED: apurinic endonuclease-redox protein [Sesamum indicum]
c39829.graph_c0	2.21625918	1.56598478	2.17786767	0.76065662	0.89064698	0.92618526	1.316E-06	-2.231107	down	-
c39835.graph_c0	1.29639201	0.97326761	0.83295753	4.52846667	6.06259948	3.85228582	0.0091542	1.202502	up	PREDICTED: methionine aminopeptidase 2B [Sesamum indicum]
c39843.graph_c0	0	0	0.5181663	5.12644643	3.85668943	3.90039697	3.69E-09	3.591772	up	PREDICTED: uncharacterized protein LOC105177798 [Sesamum indicum]
c39865.graph_c0	0.7211291	0.56988253	0.56088493	14.5632768	15.6132363	13.2694402	1.617E-54	3.53647	up	PREDICTED: uncharacterized protein LOC105169651 [Sesamum indicum]
c39870.graph_c0	0	0	0	4.85576471	3.75914556	3.82209598	3.094E-10	Inf	up	-
c39870.graph_c1	0	0.08076024	0	2.37846963	1.88977513	2.0502121	6.849E-10	5.28797	up	hypothetical protein PFL1_05374 [Pseudozyma flocculosa PF-1]
c39874.graph_c0	0.59010848	0.72968747	0.75831276	4.06135855	5.72539654	4.63814885	1.099E-05	1.770965	up	PREDICTED: uncharacterized protein LOC105156620 [Sesamum indicum]
c39877.graph_c0	12.6592941	14.1862311	14.3749121	14.8165951	13.6372066	11.7804998	6.504E-09	-1.050503	down	hypothetical protein MIMGU_mgv1a019094mg, partial [Erythranthe guttata]
c39878.graph_c0	71.4927016	67.0544349	76.025879	2.07530855	2.71924408	2.33254367	3.11E-224	-5.934359	down	PREDICTED: uncharacterized protein LOC105157942 isoform X1 [Sesamum indicum]
c39879.graph_c0	2.91136735	2.2481609	2.24473338	12.5677487	14.4099758	13.807916	9.626E-08	1.443843	up	PREDICTED: 3-ketoacyl-CoA thiolase 2, peroxisomal [Sesamum indicum]
c39881.graph_c0	652.275533	554.684526	688.800879	30.9852551	35.8750137	33.9529544	9.032E-95	-5.254046	down	-
c39883.graph_c0	0	0.1269072	0	6.89109566	6.68161244	7.69055094	4.535E-18	6.382194	up	PREDICTED: adenine phosphoribosyltransferase 5-like isoform X1 [Sesamum indicum]
c39885.graph_c0	0.05817756	0.01941194	0.04984031	0.66102921	0.83276576	0.47690256	2.535E-06	2.931252	up	--
c39886.graph_c0	85.567761	80.6418064	81.518036	37.7904684	42.7552047	39.1289719	1.638E-46	-2.068457	down	PREDICTED: membrane-anchored ubiquitin-fold protein 3 [Sesamum indicum]
c39891.graph_c0	9.19445396	8.33230831	9.50812	6.36542839	4.1947131	5.03955078	2.645E-11	-1.804647	down	-
c39893.graph_c0	0.208736	0.17412085	0.17882269	1.50635958	1.4599921	1.65404856	0.0002502	2.02342	up	PREDICTED: uncharacterized protein LOC105155471 [Sesamum indicum]
c39894.graph_c0	392.557405	381.642285	443.490563	1.7166906	1.43974521	1.05015692	1.48E-274	-9.190192	down	PREDICTED: beta-amyrin 28-oxidase-like [Sesamum indicum]
c39900.graph_c0	18.3967698	19.5719245	16.9879527	14.989455	15.0524522	13.122813	6.819E-22	-1.363809	down	PREDICTED: septin and tuftelin-interacting protein 1 homolog 1 [Sesamum indicum]

#ID	0 h-1 FPKM	0 h-2 FPKM	0 h-3 FPKM	48 h-1 FPKM	48 h-2 FPKM	48 h-3 FPKM	FDR	log2FC	regulated	nr_annotation
c39908.graph_c0	5.05182786	6.08067333	5.45629715	0.54249934	1.72413682	0.6097423	2.697E-32	-3.560346	down	-
c39913.graph_c0	0.4939992	1.29329165	0.1953257	25.7308961	38.4218774	36.3519653	9.317E-35	4.649082	up	PREDICTED: uncharacterized protein LOC105168623 [Sesamum indicum]
c39917.graph_c0	11.3335328	10.501396	12.9397962	6.94785483	6.00346268	9.60134261	3.393E-14	-1.645225	down	PREDICTED: protein OPI10 homolog [Sesamum indicum]
c39921.graph_c0	17.4650339	19.7850986	18.2283063	4.55227242	6.40239154	6.32030877	2.2E-46	-2.706758	down	PREDICTED: autophagy-related protein 18a-like [Sesamum indicum]
c39926.graph_c0	1055.18742	1151.14368	1166.76017	69.102389	33.5930216	28.0523743	1.44E-228	-5.690681	down	-
c39931.graph_c0	0	0	0.11764625	2.53027368	4.19947844	3.37713346	1.535E-10	5.367155	up	PREDICTED: uncharacterized protein LOC105157879 [Sesamum indicum]
c39933.graph_c0	2.17299753	1.60962836	1.61958496	9.48903945	7.54996891	9.47643546	0.0001125	1.282507	up	hypothetical protein L484_002978 [Morus notabilis]
c39935.graph_c0	5.86637835	6.32396438	4.73574222	3.04868688	1.61485704	3.20592202	4.473E-08	-2.11393	down	MYB5b [Vitis vinifera]
c39936.graph_c0	4.58045339	5.42316561	5.94935496	31.4444087	32.6385778	36.2960493	1.991E-17	1.632408	up	PREDICTED: ABC transporter I family member 19-like [Nicotiana sylvestris]
c39937.graph_c0	0	0.089801	0.1152824	0.95044946	0.87555301	0.88247396	2.701E-06	2.694059	up	hypothetical protein MIMGU_mgv1a019040mg [Erythranthe guttata]
c39938.graph_c0	1.43519285	1.40539889	1.08251196	6.75468299	5.63326384	6.75196998	0.0001354	1.274015	up	PREDICTED: thioredoxin reductase NTRC [Sesamum indicum]
c39942.graph_c0	0.84248656	1.05416273	0.99240953	3.84842811	5.17326373	4.92064455	0.0013745	1.24742	up	PREDICTED: uncharacterized protein LOC105158797 [Sesamum indicum]
c39944.graph_c1	1.46626252	1.01222811	1.23447939	9.96809544	11.6949405	10.6103045	2.919E-09	2.100573	up	Reticulon-4-interacting 1, mitochondrial [Gossypium arboreum]
c39945.graph_c0	0.07594888	0.15204986	0.19519463	63.2520307	66.118372	75.4567558	1.95E-118	7.892454	up	PREDICTED: protodermal factor 1-like [Sesamum indicum]
c39951.graph_c0	10.0060133	10.246817	9.48064385	37.0426889	41.7623809	48.1553911	1.54E-10	1.074139	up	PREDICTED: peptide chain release factor PrfB2, chloroplastic isoform X1 [Sesamum indicum]
c39953.graph_c0	0.30958405	0.2213529	0.28416269	9.24892118	9.88443747	8.77344289	2.61E-20	4.079446	up	hypothetical protein MIMGU_mgv1a010093mg [Erythranthe guttata]
c39981.graph_c0	4.54478588	3.90599624	4.01147097	108.480151	120.118798	126.893666	4.98E-119	3.815224	up	PREDICTED: 2-aminoethanethiol dioxygenase-like isoform X1 [Sesamum indicum]
c39982.graph_c0	11.1973922	13.5376654	10.4647944	6.7878647	7.3801543	6.11927226	2.953E-16	-1.811202	down	hypothetical protein MIMGU_mgv1a011446mg [Erythranthe guttata]
c39988.graph_c0	86.2966659	90.8810335	99.1667717	27.8463108	22.0635547	20.6364606	9.514E-74	-2.983339	down	hypothetical protein JCGZ_17893 [Jatropha curcas]
c39996.graph_c0	1.90481188	1.82381842	1.27709148	7.43898269	7.59780321	7.26410606	0.0012805	1.141901	up	PREDICTED: exosome complex component MTR3 [Sesamum indicum]
c39999.graph_c1	8.01069946	7.40970418	7.55766868	6.72605666	5.54201002	6.56666079	0.0003782	-1.301543	down	-
c40001.graph_c0	2.34215324	1.8399862	2.43828629	24.1721991	25.636449	25.9557517	4.836E-38	2.496528	up	PREDICTED: protein IQ-DOMAIN 1-like [Sesamum indicum]
c40010.graph_c0	4.97607701	4.71889876	3.77684241	23.2967552	21.4992245	17.31816	1.145E-06	1.193511	up	PREDICTED: protein RRP45A-like isoform X1 [Sesamum indicum]

#ID	0 h-1 FPKM	0 h-2 FPKM	0 h-3 FPKM	48 h-1 FPKM	48 h-2 FPKM	48 h-3 FPKM	FDR	log2FC	regulated	nr_annotation
c40012.graph_c0	75.9246889	72.7705332	74.4852062	75.3316116	71.842064	60.2418059	2.736E-15	-1.120774	down	PREDICTED: uncharacterized protein At3g28850-like [Sesamum indicum]
c40013.graph_c0	2.67608149	2.47270263	2.99798981	0	0.06696851	0	1.033E-22	-7.980961	down	PREDICTED: transcription repressor OFP7 [Sesamum indicum]
c40024.graph_c0	16.1854622	15.7848169	16.3394915	3.93878205	2.68242556	3.70952498	1.962E-72	-3.238231	down	PREDICTED: pheophytinase, chloroplastic [Sesamum indicum]
c40027.graph_c0	74.765173	72.4832512	77.1820432	9.73861303	10.7389338	12.328443	1.59E-131	-3.795029	down	hypothetical protein MIMGU_mgv1a025482mg [Erythranthe guttata]
c40032.graph_c0	92.7411866	103.667521	88.1293072	55.5797551	63.8441339	61.7081372	1.068E-31	-1.670398	down	PREDICTED: ubiquitin-activating enzyme E1 1-like isoform X2 [Sesamum indicum]
c40037.graph_c0	61.9454695	58.230392	60.2068769	53.5919709	30.3818664	34.2822335	5.57E-11	-1.615866	down	elicitor and UV light related transcription factor [Oryza sativa]
c40039.graph_c0	1.7391013	1.56380789	1.32573714	0.10862194	0.2013756	0.07248836	1.937E-18	-4.61832	down	hypothetical protein [Vitis hybrid cultivar]
c40040.graph_c0	0.86367174	1.40487193	1.47980317	9.2496723	13.4866666	11.6817201	9.963E-14	2.171764	up	PREDICTED: G-type lectin S-receptor-like serine/threonine-protein kinase At1g11330 [Sesamum indicum]
c40041.graph_c0	0	0	0	2.8271641	2.39603004	3.01871234	7.768E-12	Inf	up	PREDICTED: transmembrane protein 184 homolog DDB_G0279555 isoform X1 [Sesamum indicum]
c40045.graph_c0	7.77920686	8.26375145	8.10947765	6.06976693	5.73293341	4.75013824	5.895E-11	-1.560154	down	unnamed protein product [Coffea canephora]
c40053.graph_c0	18.8819513	18.0194715	17.0979869	90.6717968	89.4674557	87.2554581	6.004E-14	1.291894	up	PREDICTED: serine/threonine-protein phosphatase PP2A-2 catalytic subunit [Sesamum indicum]
c40054.graph_c0	32.9003982	33.5709682	29.754438	29.6057915	23.2508248	21.4608439	6.976E-22	-1.383455	down	PREDICTED: receptor-like cytosolic serine/threonine-protein kinase RBK2 [Sesamum indicum]
c40056.graph_c0	0.69253372	0.9358567	1.02342324	5.29546004	6.04922716	5.81887376	6.507E-06	1.671618	up	PREDICTED: uncharacterized protein LOC105176075 isoform X2 [Sesamum indicum]
c40061.graph_c0	1.74351121	0.96958726	1.12024047	10.8420947	12.0530888	13.9745893	1.489E-11	2.248207	up	PREDICTED: uncharacterized protein LOC105157824 isoform X1 [Sesamum indicum]
c40063.graph_c0	2.69619634	2.6624246	2.43466743	10.4727097	12.4813869	13.0818164	5.13E-06	1.189131	up	PREDICTED: zinc finger protein VAR3, chloroplastic [Sesamum indicum]
c40069.graph_c1	2.79537423	2.22207886	2.85260285	10.1496372	14.6841247	14.4901755	0.0003516	1.295849	up	PREDICTED: villin-3-like [Sesamum indicum]
c40069.graph_c2	5.72526765	5.26632491	4.69269773	17.9902875	22.2903229	25.4955433	6.085E-08	1.047702	up	PREDICTED: villin-2-like [Sesamum indicum]
c40070.graph_c0	6.73457168	7.90512548	5.29963503	0.97005759	1.32739209	1.13288623	6.895E-26	-3.558313	down	PREDICTED: protein TRM32 isoform X2 [Sesamum indicum]
c40072.graph_c0	0.90089815	0.75150022	0.72355612	6.53598333	8.02314241	11.0159416	8.97E-09	2.406339	up	PREDICTED: putative zinc finger protein At1g68190 isoform X1 [Sesamum indicum]
c40085.graph_c0	0.29534307	0.25340469	0.16265466	5.16969664	5.7237344	6.81348196	2.124E-13	3.622272	up	PREDICTED: uncharacterized protein LOC105172351 [Sesamum indicum]
c40091.graph_c0	2.26961146	2.59643901	1.24994596	86.2502071	73.0973088	67.5753932	2.876E-36	4.206096	up	PREDICTED: universal stress protein A-like protein [Sesamum indicum]
c40095.graph_c0	0.03654176	0.18289173	0.23478802	3.33280025	6.02715069	9.31588129	6.875E-06	4.321143	up	PREDICTED: alcohol-forming fatty acyl-CoA reductase-like isoform X1 [Sesamum indicum]

#ID	0 h-1 FPKM	0 h-2 FPKM	0 h-3 FPKM	48 h-1 FPKM	48 h-2 FPKM	48 h-3 FPKM	FDR	log2FC	regulated	nr_annotation
c40096.graph_c0	51.9092037	47.4031844	47.1640833	34.1609359	36.0049008	36.0444225	1.461E-25	-1.481475	down	PREDICTED: uncharacterized protein LOC105168199 [Sesamum indicum]
c40099.graph_c0	0	0.09194924	0	4.69668208	6.3203125	6.7015869	4.916E-20	6.578643	up	PREDICTED: glucan 1,3-beta-glucosidase A-like [Sesamum indicum]
c40100.graph_c0	0.40807616	0.22280988	0.19068874	2.25567825	2.35341126	2.09832026	0.0001656	2.01798	up	PREDICTED: uncharacterized protein LOC105169180 [Sesamum indicum]
c40100.graph_c1	0.36630627	0.22917067	0.11767952	0.97021254	1.65345365	2.0268531	0.0042005	1.686452	up	-
c40102.graph_c0	1.28530288	1.85420312	1.06872447	9.5076654	9.44501088	8.14997839	2.828E-07	1.674247	up	PREDICTED: protein Brevis radix-like 2 [Sesamum indicum]
c40108.graph_c0	15.1528986	15.7391843	13.5420409	12.1201699	12.3812843	11.9261759	5.36E-19	-1.302832	down	PREDICTED: serine/threonine-protein phosphatase 7 long form homolog [Sesamum indicum]
c40109.graph_c0	0.43065006	0.64662194	0.33204143	2.89622335	5.33782297	6.8485777	0.0002602	2.394691	up	PREDICTED: uncharacterized protein LOC101260668 [Solanum lycopersicum]
c40115.graph_c0	31.1628105	34.853573	29.5746882	18.2943561	18.6170121	17.7047629	2.228E-38	-1.823923	down	PREDICTED: LOW QUALITY PROTEIN: DEAD-box ATP-dependent RNA helicase 42 [Sesamum indicum]
c40116.graph_c0	34.6005774	36.3686526	35.5191365	13.9286277	13.2931235	13.4309597	5.261E-57	-2.405945	down	PREDICTED: LOW QUALITY PROTEIN: uncharacterized protein LOC105178147 [Sesamum indicum]
c40121.graph_c0	2.04138988	2.64889595	2.67184583	11.5974126	12.5443178	12.9223767	5.134E-07	1.310841	up	hypothetical protein MIMGU_mgv1a007621mg [Erythranthe guttata]
c40126.graph_c0	19.5486758	20.5900916	19.5129103	7.26493499	6.62530909	5.62749346	3.206E-60	-2.625873	down	PREDICTED: cryptochrome-1 isoform X1 [Sesamum indicum]
c40127.graph_c0	312.704077	328.723827	320.467489	218.714143	220.827382	198.644303	2.331E-26	-1.608772	down	PREDICTED: protein YLS9-like [Sesamum indicum]
c40129.graph_c0	0.02007546	0.04019112	0	0.99872028	1.21476719	1.38235268	3.235E-11	4.891713	up	PREDICTED: probable linoleate 9S-lipoxygenase 5 isoform X1 [Sesamum indicum]
c40130.graph_c0	0	0.03273848	0	114.345836	91.3543977	83.2184751	2.683E-64	12.10695	up	PREDICTED: bifunctional 3-dehydroquinate dehydratase/shikimate dehydrogenase, chloroplastic-like isoform X1 [Sesamum indicum]
c40136.graph_c1	1.12724178	1.60347392	1.44855031	0	0	0	1.892E-19	-Inf	down	-
c40137.graph_c0	61.2310522	67.0468893	57.6674793	34.9380955	41.4924708	39.4897904	7.712E-33	-1.701058	down	PREDICTED: CBL-interacting serine/threonine-protein kinase 7-like isoform X1 [Sesamum indicum]
c40141.graph_c0	14.8600693	12.4502782	12.8004661	13.5897716	11.5811214	12.7390911	1.702E-11	-1.095471	down	PREDICTED: activating signal cointegrator 1 complex subunit 2 [Sesamum indicum]
c40144.graph_c0	10.7704373	10.9415379	9.98158009	9.47979311	11.1759133	9.74791316	5.907E-08	-1.078636	down	PREDICTED: coenzyme Q-binding protein COQ10 homolog, mitochondrial-like [Sesamum indicum]
c40148.graph_c0	3.6894133	3.13750742	4.61517378	53.9015866	55.6682672	55.5890826	5.902E-67	2.830195	up	PREDICTED: signal peptide peptidase-like 2 [Sesamum indicum]
c40150.graph_c0	16.3633943	17.2885654	16.1665373	15.0949403	16.6663516	15.0615819	1.204E-13	-1.107692	down	PREDICTED: translocase of chloroplast 90, chloroplastic [Sesamum indicum]
c40152.graph_c0	43.6727573	45.0671868	44.0680585	34.806973	38.3512876	46.0249008	2.137E-16	-1.176785	down	PREDICTED: peptide methionine sulfoxide reductase A1-like [Nicotiana tomentosiformis]

#ID	0 h-1 FPKM	0 h-2 FPKM	0 h-3 FPKM	48 h-1 FPKM	48 h-2 FPKM	48 h-3 FPKM	FDR	log2FC	regulated	nr_annotation
c40153.graph_c0	0.13820625	0.27668928	0.88800251	24.4463254	21.176943	16.314145	2.959E-17	4.540964	up	Lysine decarboxylase family protein isoform 3 [Theobroma cacao]
c40153.graph_c1	8.05177203	8.54830949	7.33685432	61.1857559	57.1987406	63.2431569	1.461E-30	1.907984	up	PREDICTED: 50S ribosomal protein L15, chloroplastic [Sesamum indicum]
c40154.graph_c0	8.15228533	6.81220058	6.14896205	31.9682532	34.385382	35.8833216	1.464E-13	1.258925	up	PREDICTED: serine/threonine-protein kinase At5g01020 [Sesamum indicum]
c40158.graph_c0	4.88525344	5.8344492	4.45936304	4.25230437	4.11021976	3.25893163	1.312E-07	-1.398641	down	PREDICTED: uncharacterized protein LOC105158947 [Sesamum indicum]
c40159.graph_c0	51.0736622	52.3218119	48.485756	31.8611062	33.1080173	31.9354668	5.808E-32	-1.665533	down	PREDICTED: pentatricopeptide repeat-containing protein At5g01110 [Sesamum indicum]
c40161.graph_c0	73.0220927	73.9855503	65.692891	28.4109009	30.0836738	30.9812614	2.473E-53	-2.266806	down	PREDICTED: DNA-directed RNA polymerase II subunit RPB2 [Sesamum indicum]
c40166.graph_c0	0	0	0	1.00474873	0.72227784	0.76630233	1.128E-10	Inf	up	--
c40167.graph_c0	5.18621546	4.80968744	5.09637638	18.8655995	20.7673833	21.8193201	4.727E-07	1.005946	up	PREDICTED: probable methyltransferase PMT23 [Sesamum indicum]
c40169.graph_c0	4.83471616	5.54838146	5.19302363	3.58798945	3.40782875	4.60370803	2.119E-16	-1.444402	down	PREDICTED: uncharacterized protein LOC105170357 [Sesamum indicum]
c40171.graph_c0	0.033963	0.0169985	0.0218219	9.32410081	9.26453792	8.90902425	3.337E-72	7.547256	up	PREDICTED: 125 kDa kinesin-related protein-like [Sesamum indicum]
c40175.graph_c0	379.855921	371.686769	378.37508	241.522835	269.144793	271.984295	2.141E-25	-1.549371	down	hypothetical protein EUTSA_v10026962mg [Eutrema salsugineum]
c40179.graph_c0	0	0	0	1.94867105	2.94284055	1.99215856	2.832E-13	Inf	up	PREDICTED: RING-H2 finger protein ATL46-like [Sesamum indicum]
c40181.graph_c0	4.19194185	4.52250585	5.98534583	83.4168295	88.6882208	87.6634925	1.381E-80	3.121121	up	PREDICTED: histone deacetylase HDT1 [Sesamum indicum]
c40182.graph_c0	10.017294	9.83705296	7.98737481	6.514295	6.56718119	6.21708556	1.15E-20	-1.543588	down	PREDICTED: WPP domain-associated protein [Sesamum indicum]
c40184.graph_c0	54.8806914	54.255137	58.6110469	1.36647764	2.11110865	3.24235622	9.42E-189	-5.670287	down	PREDICTED: ADP-ribosylation factor-like protein 8B isoform X1 [Nelumbo nucifera]
c40189.graph_c0	0.16815834	0.07214003	0	0.79672337	1.14882106	1.5359926	2.17E-05	2.849325	up	PREDICTED: uncharacterized protein LOC105177814 [Sesamum indicum]
c40190.graph_c0	50.8635568	53.8755901	42.9760993	13.626153	17.2421123	19.6012249	6.449E-52	-2.570027	down	PREDICTED: SNF1-related protein kinase regulatory subunit beta-3-like [Sesamum indicum]
c40192.graph_c0	0.3642044	0.18228455	0.07800284	7.94084935	9.53796541	9.65316676	6.999E-17	4.43191	up	PREDICTED: endo-1,3;1,4-beta-D-glucanase-like [Sesamum indicum]
c40195.graph_c0	1.9488645	1.43744336	2.10894151	15.2138313	20.2215921	19.9273952	2.03E-09	2.308058	up	PREDICTED: growth-regulating factor 6-like [Sesamum indicum]
c40198.graph_c0	40.2043098	38.1351979	37.0556518	11.5458982	16.3447198	11.9554106	5.965E-55	-2.555255	down	hypothetical protein MIMGU_mgv1a012947mg [Erythranthe guttata]

#ID	0 h-1 FPKM	0 h-2 FPKM	0 h-3 FPKM	48 h-1 FPKM	48 h-2 FPKM	48 h-3 FPKM	FDR	log2FC	regulated	nr_annotation
c40199.graph_c0	2.57280587	2.41834665	1.93531165	15.9846442	15.402708	17.0522165	2.407E-15	1.791249	up	hypothetical protein MIMGU_mgv1a006303mg [Erythranthe guttata]
c40200.graph_c0	0.88965153	1.00186018	0.85742808	12.1916247	14.6520923	10.9695809	2.175E-19	2.763872	up	PREDICTED: patatin-like protein 2 [Sesamum indicum]
c40204.graph_c0	0.59211879	0.37434424	0.28033014	2.8136236	3.9843958	4.16406014	2.773E-06	2.11838	up	PREDICTED: transcription factor EGL1-like [Sesamum indicum]
c40207.graph_c0	0.44743164	0.20154585	0.31623205	3.42128664	2.62007591	3.90616374	1.881E-08	2.351179	up	PREDICTED: kinesin-4 [Sesamum indicum]
c40208.graph_c0	0.58059499	0.30221171	0.17906101	5.58416781	7.36635372	9.46158312	5.553E-13	3.384746	up	PREDICTED: cellulose synthase-like protein D5 [Sesamum indicum]
c40209.graph_c0	0	0	0.04902479	3.79583779	6.73928339	7.31794622	2.593E-16	7.448211	up	PREDICTED: leucine-rich repeat receptor-like tyrosine-protein kinase At2g41820 [Sesamum indicum]
c40211.graph_c1	0.93400196	1.32180779	0.53803391	8.39693716	8.54977382	8.23717946	7.532E-11	2.160979	up	PREDICTED: histone deacetylase 15 isoform X8 [Sesamum indicum]
c40212.graph_c0	5.23069748	5.28227094	4.40179069	32.4953193	30.946224	27.5101723	1.163E-15	1.59468	up	PREDICTED: protein TIFY 7 [Sesamum indicum]
c40215.graph_c1	0.02279387	0	0.05858205	19.3822618	46.2719013	59.4929194	9.006E-14	9.536697	up	PREDICTED: subtilisin-like protease [Sesamum indicum]
c40216.graph_c0	7.74045047	8.61715852	9.8538247	75.1088397	86.4171241	82.9610648	2.277E-37	2.199458	up	PREDICTED: ubiquitin-conjugating enzyme E2 36-like [Brassica rapa]
c40220.graph_c0	205.697226	220.488227	195.631504	125.824926	184.357724	215.968083	4.723E-18	-1.265516	down	PREDICTED: uncharacterized protein At5g48480 [Sesamum indicum]
c40223.graph_c1	0.33240682	0.76054783	0.42715592	4.37478349	2.50265842	2.60807955	0.0080183	1.63656	up	PREDICTED: cyclin-D4-1-like [Sesamum indicum]
c40227.graph_c0	3.90026395	3.96817225	2.95789786	27.8029476	20.9047464	17.6631521	6.87E-05	1.608691	up	PREDICTED: potassium/sodium hyperpolarization-activated cyclic nucleotide-gated channel 4 [Sesamum indicum]
c40228.graph_c0	0.22705082	0.51137589	0.58353858	6.95503623	6.03845047	5.49059271	7.02E-09	2.78536	up	PREDICTED: membrane protein of ER body-like protein [Sesamum indicum]
c40229.graph_c0	1.05363569	1.05469074	0.69881991	3.94533605	4.80986307	5.32152724	0.001014	1.30845	up	PREDICTED: transcription factor E2FA-like [Sesamum indicum]
c40236.graph_c0	2.29268373	2.24201844	1.69972463	1.20231204	1.41140152	1.04089623	6.375E-09	-1.785896	down	PREDICTED: uveal autoantigen with coiled-coil domains and ankyrin repeats [Sesamum indicum]
c40238.graph_c0	6.12908535	5.60428997	6.28574749	4.04485801	4.11248479	3.86479499	2.969E-11	-1.601721	down	PREDICTED: glucose-1-phosphate adenylyltransferase large subunit 2, chloroplastic/amyloplastic-like [Sesamum indicum]
c40242.graph_c0	49.9966987	46.8033141	44.3689949	42.6578622	46.5849815	46.0107288	1.559E-14	-1.079718	down	hypothetical protein MIMGU_mgv1a007434mg [Erythranthe guttata]
c40244.graph_c0	0.21722552	0.54360759	0.1860956	3.36872567	3.03874305	3.2645733	3.233E-06	2.341622	up	PREDICTED: sulfhydryl oxidase 2-like [Sesamum indicum]
c40250.graph_c0	1.19326248	0.75648966	1.58450252	8.79448983	9.31669559	7.85788951	2.458E-08	1.854979	up	PREDICTED: Werner Syndrome-like exonuclease isoform X2 [Sesamum indicum]
c40251.graph_c0	1.16045182	0.68181682	0.97253904	10.179538	11.5472364	11.4772121	7.157E-22	2.540693	up	PREDICTED: APO protein 2, chloroplastic [Sesamum indicum]
c40255.graph_c0	43.0542947	46.2197702	43.8659895	19.6703166	21.9102451	20.2395505	1.968E-45	-2.125614	down	PREDICTED: DEAD-box ATP-dependent RNA helicase 37-like [Sesamum indicum]
c40260.graph_c0	0.21187402	0.26510773	0.38571088	14.2978264	14.7677122	14.5312951	3.651E-79	4.63544	up	PREDICTED: proteoglycan 4-like [Sesamum indicum]

#ID	0 h-1 FPKM	0 h-2 FPKM	0 h-3 FPKM	48 h-1 FPKM	48 h-2 FPKM	48 h-3 FPKM	FDR	log2FC	regulated	nr_annotation
c40264.graph_c0	1.16674978	1.0900569	1.59927431	6.80761135	7.81915149	7.90646403	0.0004798	1.522822	up	PREDICTED: very-long-chain enoyl-CoA reductase-like [Sesamum indicum]
c40265.graph_c0	2.71320613	2.74987204	2.87642627	10.3107776	15.2591261	13.6504963	4.634E-07	1.209163	up	PREDICTED: putative GPI-anchor transamidase [Sesamum indicum]
c40269.graph_c0	37.3000991	33.4931819	31.6462927	25.6066172	31.6717161	31.835497	8.338E-16	-1.221098	down	PREDICTED: COP9 signalosome complex subunit 5b-like [Sesamum indicum]
c40273.graph_c0	121.146377	122.925756	121.547661	100.820755	104.967781	89.335131	3.624E-21	-1.326002	down	hypothetical protein 29 [Hevea brasiliensis]
c40274.graph_c0	4.19741006	4.15643449	5.16184507	1.16423636	2.29053919	1.70188585	2.648E-11	-2.420005	down	-
c40281.graph_c1	0.32566811	0.08149855	0.15693616	3.60032047	3.09895867	2.83647081	3.055E-08	3.070376	up	hypothetical protein MIMGU_mgv1a006975mg [Erythranthe guttata]
c40283.graph_c0	0.96493648	1.13243768	1.15446561	14.9590919	17.6983657	18.3019513	1.312E-30	2.947887	up	unnamed protein product [Coffea canephora]
c40284.graph_c0	3.3152135	3.97218367	3.09831317	13.7901347	16.6189354	15.6879677	2.873E-05	1.130977	up	hypothetical protein CICLE_v10022933mg [Citrus clementina]
c40289.graph_c0	4.1767579	3.66213748	4.03526879	16.2482569	17.6296229	17.2066698	9.29E-12	1.086151	up	PREDICTED: RNA polymerase-associated protein CTR9 homolog [Sesamum indicum]
c40295.graph_c0	5.10845251	5.73971902	6.49757838	78.1331828	92.2352696	96.784461	3.329E-70	2.921923	up	hypothetical protein MIMGU_mgv1a011840mg [Erythranthe guttata]
c40300.graph_c0	4.67852065	4.52263843	3.53854102	41.7469626	46.1566486	46.0656664	3.465E-49	2.377979	up	PREDICTED: protein INVOLVED IN DE NOVO 2 [Sesamum indicum]
c40302.graph_c0	10.5792869	10.4204424	11.7459094	1.8712952	2.02371057	1.90788952	2.433E-43	-3.516161	down	hypothetical protein MIMGU_mgv1a018285mg [Erythranthe guttata]
c40303.graph_c0	51.5860484	51.1423423	42.0865146	53.3268341	25.8025165	18.3653226	4.022E-07	-1.568015	down	PREDICTED: leucine-rich repeat receptor-like serine/threonine/tyrosine-protein kinase SOBIR1 [Sesamum indicum]
c40304.graph_c0	66.2415585	60.8887404	65.8932286	12.7039568	13.4084562	13.0204232	5.5E-102	-3.32082	down	PREDICTED: probable serine/threonine-protein kinase Cx32, chloroplastic [Sesamum indicum]
c40310.graph_c0	77.7729053	81.0945657	76.5792865	83.7934516	74.9926528	76.7200371	5.188E-13	-1.014716	down	hypothetical protein MIMGU_mgv1a008821mg [Erythranthe guttata]
c40312.graph_c0	2.45464406	2.11819139	2.0122353	16.3365081	14.2913272	12.1423493	7.93E-10	1.687478	up	hypothetical protein MIMGU_mgv1a010279mg [Erythranthe guttata]
c40316.graph_c0	63.1802962	64.4881269	57.2234473	43.6769645	48.122543	48.8408793	5.994E-24	-1.412905	down	PREDICTED: glucose-6-phosphate/phosphate translocator 2, chloroplastic-like [Solanum tuberosum]
c40319.graph_c0	0	0	0	0.87317073	0.74598267	0.82706736	1.965E-11	Inf	up	PREDICTED: monofunctional riboflavin biosynthesis protein RIBA 3, chloroplastic [Sesamum indicum]
c40320.graph_c0	5.78919409	4.83416355	4.58695279	35.1782218	26.4645896	22.2281529	0.0001711	1.454776	up	PREDICTED: sugar transport protein 14-like [Sesamum indicum]
c40322.graph_c0	305.692846	299.893572	311.850951	1577.63904	1642.49197	1783.29615	8.639E-16	1.428117	up	RecName: Full=Catalase [Avicennia marina]
c40323.graph_c0	14.7054921	13.3299746	13.7529182	7.827536	7.97338967	8.17035696	8.445E-12	-1.81939	down	PREDICTED: RPA-interacting protein B [Sesamum indicum]

#ID	0 h-1 FPKM	0 h-2 FPKM	0 h-3 FPKM	48 h-1 FPKM	48 h-2 FPKM	48 h-3 FPKM	FDR	log2FC	regulated	nr_annotation
c40323.graph_c1	19.3780981	16.6860235	19.5464355	12.2855005	7.32093643	10.7607588	2.018E-07	-1.884059	down	PREDICTED: RPA-interacting protein B [Sesamum indicum]
c40324.graph_c0	28.2732633	26.7412673	29.7684188	15.6857453	18.7235438	20.2672555	1.913E-29	-1.654951	down	PREDICTED: uncharacterized protein LOC105177636 [Sesamum indicum]
c40325.graph_c0	45.9245234	45.4296803	43.9486916	48.2318797	40.5497336	38.4874957	6.627E-14	-1.101402	down	PREDICTED: uncharacterized protein LOC105172600 [Sesamum indicum]
c40326.graph_c1	1.70774865	1.157446	1.32585863	8.24338117	7.29185362	6.31414803	4.508E-08	1.369826	up	PREDICTED: autophagy-related protein 18g-like [Sesamum indicum]
c40329.graph_c0	222.876342	228.095854	236.780308	56.487043	64.080136	67.0301313	1.941E-69	-2.894963	down	PREDICTED: transmembrane 9 superfamily member 8 [Sesamum indicum]
c40330.graph_c0	16.5960412	17.0397781	14.2273329	15.0410846	16.0218764	15.2059745	8.308E-13	-1.065377	down	PREDICTED: uncharacterized protein LOC105164225 [Sesamum indicum]
c40336.graph_c0	25.1821953	26.2332945	24.8031524	11.4648597	16.8371754	19.5626142	2.689E-30	-1.696154	down	YSL transporter [Medicago truncatula]
c40338.graph_c0	38.2156083	37.0261328	39.8177592	36.6934809	36.6672203	31.3796365	2.942E-13	-1.152364	down	PREDICTED: vesicle transport v-SNARE 12 [Sesamum indicum]
c40343.graph_c0	1.77867698	2.07030007	2.28567034	30.4099845	34.0727361	34.3488514	2.25E-46	2.988127	up	hypothetical protein MIMGU_mgv1a009694mg [Erythranthe guttata]
c40344.graph_c0	2.74609602	2.22879391	1.52598578	8.82040208	10.5755456	10.3427746	0.0034968	1.178204	up	unnamed protein product [Coffea canephora]
c40349.graph_c0	2.3494512	1.6518622	2.65971664	10.8481059	10.0181871	10.1569897	5.545E-07	1.200828	up	hypothetical protein MIMGU_mgv1a009342mg [Erythranthe guttata]
c40351.graph_c0	13.0413442	9.573229	12.1881045	0.50970763	0.77139061	1.03665093	2.743E-39	-4.935439	down	PREDICTED: calcium-dependent protein kinase 11 [Sesamum indicum]
c40354.graph_c0	4.79289792	5.48308261	5.5724861	0.94618504	0.55687129	1.12254634	1.071E-12	-3.608531	down	PREDICTED: UDP-glucuronic acid decarboxylase 2-like [Sesamum indicum]
c40354.graph_c1	0.87013974	1.46983115	0.76873776	18.4875799	13.9061287	16.0043343	5.444E-18	2.950962	up	PREDICTED: GATA transcription factor 8 [Sesamum indicum]
c40357.graph_c0	0.68229528	0.64280329	0.61890102	6.61852409	3.29032399	4.14541615	0.0036681	1.849643	up	ribulose-1,5-bisphosphate carboxylase/oxygenase large subunit (chloroplast) [Andrographis paniculata]
c40359.graph_c0	6.08160605	7.57249973	5.33714457	33.616633	52.4781324	63.7147318	7.097E-06	1.955406	up	chloroplast 1-deoxy-D-xylulose 5-phosphate reductoisomerase [Mitragyna speciosa]
c40360.graph_c0	5.91991072	4.12484845	5.70548974	5.0527926	3.51190378	3.3541173	2.947E-06	-1.412616	down	PREDICTED: type I inositol 1,4,5-trisphosphate 5-phosphatase CVP2-like isoform X1 [Sesamum indicum]
c40365.graph_c1	2.85125968	2.9001489	2.48205204	0.4660392	0.26929747	0.41467888	7.352E-16	-3.84872	down	-
c40366.graph_c0	21.9938837	22.3515156	22.6621097	183.595302	176.696431	167.044786	4.493E-44	1.959838	up	PREDICTED: F-box/kelch-repeat protein At1g67480-like [Sesamum indicum]
c40367.graph_c0	21.1762944	22.6695478	23.0549303	14.6994773	15.7517183	13.9233456	1.615E-23	-1.610786	down	PREDICTED: probable protein phosphatase 2C 55 [Sesamum indicum]
c40370.graph_c0	2.85247401	2.52119593	2.41769794	12.5523448	11.2245512	11.1192504	2.101E-06	1.149324	up	PREDICTED: carboxyl-terminal-processing peptidase 2, chloroplastic [Sesamum indicum]
c40376.graph_c1	316.99601	287.237436	317.935424	26.8219508	24.7775352	18.042704	7.72E-192	-4.740865	down	PREDICTED: protein SRG1 [Sesamum indicum]

#ID	0 h-1 FPKM	0 h-2 FPKM	0 h-3 FPKM	48 h-1 FPKM	48 h-2 FPKM	48 h-3 FPKM	FDR	log2FC	regulated	nr_annotation
c40377.graph_c0	9.57542268	4.56429096	7.03131271	0	0	0	8.944E-16	-Inf	down	predicted protein [Hordeum vulgare subsp. vulgare]
c40378.graph_c0	24.386558	24.6129595	24.4478452	24.6706468	22.4220026	20.7466893	1.902E-14	-1.129492	down	PREDICTED: plectin-like [Solanum tuberosum]
c40382.graph_c0	0.11301803	0.1319864	0.14523265	1.68326661	2.1876578	1.80657711	1.331E-08	2.839667	up	PREDICTED: uncharacterized protein LOC105160401 [Sesamum indicum]
c40383.graph_c0	7.55999292	8.86647319	8.2649066	7.77904627	7.61691949	6.53635056	2.282E-12	-1.187106	down	PREDICTED: uncharacterized protein LOC105169210 [Sesamum indicum]
c40384.graph_c0	18.2566152	16.8821998	16.2217399	9.31620267	10.1674558	11.794458	1.119E-24	-1.734731	down	PREDICTED: uncharacterized protein LOC105179476 isoform X3 [Sesamum indicum]
c40386.graph_c0	12.9190282	12.8332474	11.2788564	8.08599067	6.44865713	4.76963019	1.843E-10	-1.94886	down	PREDICTED: uncharacterized protein LOC105178241 [Sesamum indicum]
c40386.graph_c1	25.1870859	26.5214887	24.2341406	17.0240302	14.6996753	11.4819545	9.755E-30	-1.825631	down	PREDICTED: uncharacterized protein LOC105178241 [Sesamum indicum]
c40388.graph_c0	14.1305795	13.4018987	14.9001811	8.28936835	8.23839008	6.26058432	1.55E-27	-1.912938	down	PREDICTED: uncharacterized protein LOC105162418 [Sesamum indicum]
c40392.graph_c0	1.58224697	1.64359857	1.07416981	1.1826372	1.07804294	0.95441478	0.0005251	-1.430061	down	hypothetical protein [Lotus japonicus]
c40394.graph_c0	32.8013802	30.6669502	31.9958634	22.8386091	22.3457942	22.5845489	6.783E-21	-1.511391	down	-
c40395.graph_c0	49.7588082	51.0113994	46.6849543	499.042444	601.933414	676.786176	5.343E-37	2.570727	up	PREDICTED: bidirectional sugar transporter SWEET7-like [Sesamum indicum]
c40408.graph_c0	31.5894447	32.1750848	29.2963874	10.3348311	9.72276695	8.39319496	1.678E-76	-2.7239	down	PREDICTED: nucleolin-like isoform X1 [Sesamum indicum]
c40413.graph_c1	3.09140736	4.54505118	4.22086715	4.36100933	1.97998311	3.2468652	0.0035072	-1.314272	down	PREDICTED: uncharacterized protein LOC100784245 [Glycine max]
c40414.graph_c0	1.70245234	1.54923371	1.29274284	0.10693705	0.22657369	0.12686926	4.199E-15	-4.328169	down	hypothetical protein MIMGU_mgv1a005040mg [Erythranthe guttata]
c40418.graph_c0	0	0.2933403	0.75315363	28.7522184	37.1805969	44.4409102	7.437E-30	5.677089	up	PREDICTED: organ-specific protein S2-like [Nicotiana tomentosiformis]
c40426.graph_c0	0.80322063	1.05141723	0.63518173	4.78140431	4.94471528	6.68558755	0.0003032	1.703525	up	hypothetical protein JCGZ_05663 [Jatropha curcas]
c40427.graph_c0	3.83103857	4.44845474	3.93842899	1.41176105	0.59823586	0.87932354	5.919E-09	-3.083254	down	-
c40428.graph_c0	0.50569709	0.78231445	0.53168791	21.8964138	22.7030508	24.1939592	7.968E-45	4.2232	up	PREDICTED: cytokinin hydroxylase [Sesamum indicum]
c40431.graph_c0	19.2174528	18.3661613	19.0801671	18.6423509	17.1202092	14.5882679	6.908E-16	-1.184993	down	PREDICTED: DNA repair helicase UVH6 isoform X1 [Sesamum indicum]
c40435.graph_c0	139.760249	132.304054	124.560655	82.1827046	66.4958003	60.3040691	4.612E-40	-1.935718	down	PREDICTED: probable polyamine oxidase 4 [Sesamum indicum]
c40439.graph_c0	15.9636002	16.5513596	15.1825731	288.927299	320.841872	284.662363	7.44E-102	3.211116	up	PREDICTED: transcription factor GAMYB-like isoform X1 [Sesamum indicum]
c40440.graph_c0	3.34428078	4.08919308	3.12795138	14.0398614	15.637865	14.817593	1.023E-08	1.057267	up	PREDICTED: armadillo repeat-containing kinesin-like protein 2 isoform X2 [Sesamum indicum]

#ID	0 h-1 FPKM	0 h-2 FPKM	0 h-3 FPKM	48 h-1 FPKM	48 h-2 FPKM	48 h-3 FPKM	FDR	log2FC	regulated	nr_annotation
c40441.graph_c0	3.36191983	1.762769	2.88013339	78.5366456	81.871977	91.3379883	6.32E-80	3.955545	up	PREDICTED: 60S acidic ribosomal protein P2B-like [Sesamum indicum]
c40442.graph_c0	142.320902	142.543857	140.857834	79.1430001	93.1364493	88.8002448	5.214E-33	-1.725454	down	PREDICTED: la-related protein 6A [Sesamum indicum]
c40444.graph_c0	90.0435533	88.2219808	89.9605393	9.86448751	8.3724052	6.95516085	2.71E-179	-4.425142	down	PREDICTED: UDP-glycosyltransferase 76C4-like [Sesamum indicum]
c40447.graph_c0	54.1137869	55.1197409	54.7435283	53.1665374	53.9430652	47.6291987	6.948E-15	-1.10047	down	PREDICTED: E3 ubiquitin-protein ligase MBR2 [Sesamum indicum]
c40451.graph_c0	0.18339036	0.30595666	0.23566387	4.33641309	5.22034528	5.93810316	2.722E-14	3.395428	up	PREDICTED: rop guanine nucleotide exchange factor 1-like [Sesamum indicum]
c40453.graph_c0	10.3888921	11.1311602	9.72309531	9.74323296	10.023073	9.408187	5.126E-13	-1.115338	down	PREDICTED: protein NLP9 [Sesamum indicum]
c40458.graph_c0	8.07987442	8.35982957	6.54388143	2.87739947	3.77718658	3.89609766	7.148E-11	-2.143582	down	PREDICTED: protein tesmin/TSO1-like CXC 5 [Sesamum indicum]
c40461.graph_c0	3.034377	4.13847858	3.65558867	2.97018264	3.0354937	3.82436035	0.000478	-1.159922	down	PREDICTED: protein phosphatase 2C 16-like [Sesamum indicum]
c40462.graph_c0	0.74234526	0.86693671	0.5962144	3.12027947	4.45269195	5.17248225	4.411E-06	1.508808	up	PREDICTED: paramyosin-like [Sesamum indicum]
c40463.graph_c0	68.4468411	69.4612898	63.7515502	27.6548471	26.9913205	25.5366615	1.273E-60	-2.346245	down	PREDICTED: lipase 3-like [Sesamum indicum]
c40465.graph_c0	1.99538377	2.09725094	1.88037458	11.1828559	12.0091659	10.5501904	2.938E-08	1.481022	up	PREDICTED: uncharacterized protein LOC105160301 [Sesamum indicum]
c40469.graph_c0	1.74211814	1.22070383	1.56708355	11.8766512	14.4521501	15.4803591	1.725E-16	2.184615	up	PREDICTED: pentatricopeptide repeat-containing protein At3g58590 [Sesamum indicum]
c40470.graph_c0	10.989404	12.3754593	11.5181088	7.11854702	9.78685031	11.0634571	8.227E-13	-1.342781	down	PREDICTED: mechanosensitive ion channel protein 2, chloroplastic-like [Sesamum indicum]
c40473.graph_c0	6.33380191	6.95869492	5.75698538	4.17471388	4.77440112	5.57195296	1.873E-06	-1.41133	down	PREDICTED: uncharacterized protein LOC105164092 [Sesamum indicum]
c40477.graph_c0	8.3372749	8.41403017	8.29874859	33.0528013	36.2493123	38.877462	1.487E-11	1.090927	up	PREDICTED: dolichyl-diphosphooligosaccharide--protein glycosyltransferase 48 kDa subunit-like [Sesamum indicum]
c40478.graph_c0	4.12455477	4.46612548	5.01991629	1.33358677	1.12247756	1.04032218	1.078E-30	-2.976015	down	PREDICTED: disease resistance protein RPM1-like [Solanum tuberosum]
c40482.graph_c0	21.3120606	22.0530265	21.3223995	20.0506698	19.2155825	17.8029453	1.37E-17	-1.196633	down	PREDICTED: FIP1[V]-like protein isoform X1 [Sesamum indicum]
c40483.graph_c0	2.18100831	1.09159613	1.33461049	0	0	0	1.935E-13	-Inf	down	-
c40484.graph_c0	0	0	0	3.14600991	2.49182506	1.98828835	9.188E-16	Inf	up	PREDICTED: probable receptor-like protein kinase At5g39020 [Sesamum indicum]
c40490.graph_c0	1.36277783	0.62006475	0.63680853	6.73393725	5.68284965	5.38247957	0.0013197	1.756581	up	-
c40492.graph_c0	7.27085128	7.62470965	8.2628104	3.32640761	2.89637713	2.83818115	5.692E-16	-2.369528	down	PREDICTED: zinc finger CCCH domain-containing protein 32 [Sesamum indicum]

#ID	0 h-1 FPKM	0 h-2 FPKM	0 h-3 FPKM	48 h-1 FPKM	48 h-2 FPKM	48 h-3 FPKM	FDR	log2FC	regulated	nr_annotation
c40493.graph_c0	1.35554618	1.61536137	1.74193051	35.5615666	33.1372679	36.9867646	6.827E-40	3.468067	up	PREDICTED: clp protease-related protein At4g12060, chloroplastic isoform X1 [Sesamum indicum]
c40496.graph_c0	0.03726599	0.03730331	0.04788828	2.05991183	2.58230172	2.65770088	7.768E-12	4.87286	up	PREDICTED: uncharacterized protein LOC105173890 [Sesamum indicum]
c40497.graph_c0	4.46580523	3.62933385	3.8637038	3.62537628	3.06388659	2.46468522	3.708E-05	-1.397103	down	PREDICTED: probable tyrosine-protein phosphatase At1g05000 [Sesamum indicum]
c40498.graph_c0	19.9657721	20.5087568	21.2447959	19.425198	19.6316601	19.6094089	1.13E-11	-1.091378	down	hypothetical protein M569_11555, partial [Genlisea aurea]
c40499.graph_c0	1114.41988	1191.90246	1168.8041	61.0273284	70.647282	72.0236598	1.7E-126	-5.113137	down	PREDICTED: legumin B-like [Sesamum indicum]
c40500.graph_c0	6.92641418	6.08928995	6.03700768	0.38841262	0.47025837	0.54309705	4.128E-29	-4.785687	down	hypothetical protein M569_14517 [Genlisea aurea]
c40512.graph_c0	1.30129141	1.6673209	1.5384341	0.38362705	1.06681664	0.72536671	2.699E-05	-2.083522	down	hypothetical protein MIMGU_mgv1a023619mg [Erythranthe guttata]
c40522.graph_c0	1.59900543	1.65988832	1.90257897	17.6772972	19.4783429	20.6323762	2.191E-16	2.46343	up	PREDICTED: branched-chain-amino-acid aminotransferase-like protein 1 [Sesamum indicum]
c40522.graph_c1	1.08163354	1.79208271	1.62958709	18.7955117	19.0743334	20.3318988	1.439E-27	2.671659	up	hypothetical protein MIMGU_mgv1a0045111mg, partial [Erythranthe guttata]
c40523.graph_c1	28.6683702	29.1660567	25.1142661	22.2593773	21.1642106	15.4536378	1.108E-26	-1.50711	down	PREDICTED: uncharacterized protein LOC105164312 [Sesamum indicum]
c40525.graph_c0	25.8431431	24.2547942	27.5240022	4.9140515	5.48832924	6.67737376	1.856E-73	-3.206122	down	PREDICTED: U-box domain-containing protein 4 [Sesamum indicum]
c40526.graph_c0	0.25772169	0	0.11039419	1.50372354	1.34148443	1.58447862	1.676E-05	2.577394	up	PREDICTED: SNARE-interacting protein KEULE-like isoform X1 [Sesamum indicum]
c40527.graph_c0	19.0173576	20.4612809	18.5114627	10.4910361	14.0036156	12.3453534	1.272E-26	-1.67588	down	PREDICTED: serine/threonine-protein kinase PEPKR2-like [Sesamum indicum]
c40528.graph_c0	1.38491192	1.27450042	1.34910214	15.2075615	16.1977694	19.3360841	1.613E-37	2.642004	up	PREDICTED: magnesium-chelatase subunit ChlH, chloroplastic [Sesamum indicum]
c40528.graph_c1	0.85256645	0.85342017	0.76214375	9.56187614	9.73169786	10.7566691	1.092E-14	2.588555	up	PREDICTED: magnesium-chelatase subunit ChlH, chloroplastic [Sesamum indicum]
c40531.graph_c2	0.7105644	0	0	2.53664579	2.77395472	3.13080527	3.393E-05	2.571716	up	PREDICTED: AP-4 complex subunit epsilon [Sesamum indicum]
c40537.graph_c2	2.44661951	2.72737278	2.88200269	13.950804	21.2552542	22.5628056	8.109E-09	1.815469	up	PREDICTED: transcription factor bHLH143-like [Sesamum indicum]
c40538.graph_c0	2.03557613	2.51147827	2.61579591	13.9121501	15.2002498	13.9311666	2.024E-08	1.566841	up	PREDICTED: DNA-repair protein XRCC1 [Sesamum indicum]
c40553.graph_c0	0.47623041	0.39725607	0.4419819	3.09551576	3.5117166	3.49176205	5.629E-06	1.920949	up	PREDICTED: uncharacterized protein LOC105177782 [Sesamum indicum]
c40558.graph_c0	74.463538	64.9781049	76.5125234	30.932165	27.3316716	29.3173628	4.844E-48	-2.318404	down	unnamed protein product [Coffea canephora]
c40559.graph_c0	1.08706613	0.92074625	0.6447337	4.31405141	6.16162829	5.6551054	0.0001519	1.585751	up	PREDICTED: chromatin assembly factor 1 subunit FAS2 isoform X2 [Sesamum indicum]

#ID	0 h-1 FPKM	0 h-2 FPKM	0 h-3 FPKM	48 h-1 FPKM	48 h-2 FPKM	48 h-3 FPKM	FDR	log2FC	regulated	nr_annotation
c40564.graph_c0	2.72879673	2.91671763	3.80378233	2.04524382	2.39238448	1.78193221	4.231E-05	-1.625268	down	hypothetical protein MIMGU_mgv1a001712mg [Erythranthe guttata]
c40566.graph_c0	37.5048816	34.4490528	32.6115227	8.1527057	4.79822396	5.02806315	1.219E-95	-3.545716	down	hypothetical protein F775_27779 [Aegilops tauschii]
c40570.graph_c0	0.92824794	0.25341203	0.27109894	54.6526611	62.3452144	63.7788047	8.49E-142	5.948648	up	PREDICTED: transmembrane emp24 domain-containing protein p24delta3-like [Sesamum indicum]
c40576.graph_c0	67.6700635	69.7781208	69.1478905	450.383946	453.753219	481.742482	2.628E-33	1.727776	up	PREDICTED: 40S ribosomal protein SA-like [Sesamum indicum]
c40579.graph_c0	68.5207675	69.2775685	58.8975914	19.4233232	21.4712929	19.1613166	2.2E-34	-2.728442	down	-
c40586.graph_c0	0.49447727	0.44547517	0.38125372	4.41878369	6.03242978	5.22889817	1.252E-07	2.54917	up	PREDICTED: uncharacterized protein LOC105171062 [Sesamum indicum]
c40588.graph_c0	0.20644942	0.28046191	0.32214475	7.49910404	7.46945497	7.7364147	1.21E-41	3.789393	up	PREDICTED: protein phosphatase 2C and cyclic nucleotide-binding/kinase domain-containing protein isoform X1 [Sesamum indicum]
c40590.graph_c0	4.55473685	3.98938551	4.04833688	29.652586	32.2653316	30.9584544	1.625E-23	1.864823	up	PREDICTED: urease accessory protein G isoform X1 [Sesamum indicum]
c40591.graph_c0	50.1982016	51.0302069	60.5481856	0.07994098	0.12703166	0.14226201	9.97E-147	-9.883933	down	PREDICTED: cytokinin hydroxylase [Sesamum indicum]
c40592.graph_c0	2.85022471	4.10564995	3.9306505	0	0.27138833	0	9.566E-24	-6.380724	down	-
c40593.graph_c0	0.24220263	0.09697806	0.18674398	2.23132863	1.5601226	1.6280467	4.92E-05	2.354961	up	Phototropic-responsive NPH3 family protein [Theobroma cacao]
c40597.graph_c0	3.99741375	4.39592242	4.26863748	45.5925945	40.1399573	31.7990481	1.029E-11	2.201071	up	PREDICTED: uncharacterized protein LOC105174543 [Sesamum indicum]
c40600.graph_c1	62.2609693	67.7213966	57.4738119	41.690272	63.1938817	69.5854507	1.505E-12	-1.127864	down	PREDICTED: auxin response factor 19-like [Sesamum indicum]
c40608.graph_c0	88.1698792	88.6187733	90.1553533	46.8781733	48.9585652	43.1884447	4.291E-39	-1.958734	down	cellular nucleic acid binding protein, putative [Ricinus communis]
c40610.graph_c0	1.36968901	0.82263633	1.12646695	9.4386145	8.55535193	8.03913162	6.41E-07	1.957121	up	PREDICTED: vesicle transport protein SFT2B [Sesamum indicum]
c40615.graph_c0	0.33671635	1.15561206	0.49450724	13.2058726	10.0465249	7.84681442	1.214E-07	2.960658	up	PREDICTED: cytochrome P450 CYP72A219-like [Sesamum indicum]
c40618.graph_c0	5.17681386	4.4309835	5.88111676	0.95038776	2.39806359	2.16793733	3.235E-18	-2.523281	down	PREDICTED: uncharacterized protein LOC105170597 [Sesamum indicum]
c40622.graph_c0	12.2280945	11.4572871	13.0682008	90.3501282	86.9150111	89.3705566	6.322E-36	1.841041	up	PREDICTED: NADH dehydrogenase [ubiquinone] 1 alpha subcomplex subunit 9, mitochondrial [Sesamum indicum]
c40623.graph_c1	0.19182205	0	0	4.94813146	4.6803083	2.51589578	1.578E-08	4.993688	up	PREDICTED: E3 ubiquitin-protein ligase RMA1H1-like [Sesamum indicum]
c40624.graph_c0	37.5408725	34.771925	36.0290599	5.13241548	6.36113571	7.19344428	9.43E-106	-3.557611	down	hypothetical protein MIMGU_mgv1a008212mg [Erythranthe guttata]
c40632.graph_c1	61.9054208	61.6134941	67.3663266	1.27328473	1.0038257	1.21200489	0	-6.788967	down	PREDICTED: cytochrome P450 71A1-like [Vitis vinifera]
c40642.graph_c0	0.03860267	0.03864132	0	0.81795582	1.43165001	1.58220036	2.38E-08	4.62126	up	PREDICTED: uncharacterized protein LOC105163904 [Sesamum indicum]

#ID	0 h-1 FPKM	0 h-2 FPKM	0 h-3 FPKM	48 h-1 FPKM	48 h-2 FPKM	48 h-3 FPKM	FDR	log2FC	regulated	nr_annotation
c40645.graph_c0	0.52365902	0.10483668	0	3.18403039	3.57752406	3.69165566	2.432E-06	3.055737	up	PREDICTED: auxin response factor 17-like isoform X3 [Zea mays]
c40646.graph_c0	389.052741	403.702315	394.744712	6.81124041	5.63175283	6.87360818	0	-6.957313	down	PREDICTED: transcription factor PIF3 isoform X2 [Sesamum indicum]
c40646.graph_c1	308.731804	313.052762	320.830782	4.90281631	4.5527123	4.63178638	0	-7.080667	down	unnamed protein product [Vitis vinifera]
c40647.graph_c0	35.1366365	36.1097357	32.0168507	14.7529463	16.1693206	14.1744565	5.283E-46	-2.212343	down	PREDICTED: protein FAM63A-like [Sesamum indicum]
c40647.graph_c1	17.5658582	15.8073419	13.680499	7.84621301	8.13894229	6.98151315	1.259E-08	-2.049474	down	-
c40651.graph_c0	203.782242	200.262068	196.890152	74.4718021	81.8647421	75.8299482	5.137E-58	-2.390403	down	PREDICTED: type I inositol 1,4,5-trisphosphate 5-phosphatase CVP2 [Sesamum indicum]
c40652.graph_c0	49.848473	49.6224529	49.3565627	22.6655507	21.7470219	18.0398025	7.397E-57	-2.267899	down	PREDICTED: uncharacterized protein LOC105173727 [Sesamum indicum]
c40655.graph_c0	18.4506715	20.7190961	19.3395968	8.93113258	14.230329	14.6280051	8.671E-29	-1.656576	down	PREDICTED: DEAD-box ATP-dependent RNA helicase 24 isoform X1 [Sesamum indicum]
c40656.graph_c0	11.4675273	10.3231377	10.5404954	7.29986872	6.95610709	7.37666191	1.474E-14	-1.596129	down	PREDICTED: putative GTP diphosphokinase RSH1, chloroplastic [Sesamum indicum]
c40658.graph_c0	17.5678315	16.7950669	14.9149563	6.80118253	6.62708476	6.01928954	4.273E-37	-2.355932	down	PREDICTED: protein DOS2-like [Sesamum indicum]
c40661.graph_c0	0.59885284	1.02763286	0.9344531	2.73880783	4.4878308	5.17199674	0.0018811	1.246149	up	unnamed protein product [Coffea canephora]
c40668.graph_c0	3.17720903	3.92001622	2.94344235	0.06807098	0.2884518	0.06056915	1.832E-20	-5.623284	down	hypothetical protein MIMGU_mgv1a010491mg [Erythranthe guttata]
c40673.graph_c0	0.13113713	0.06563422	0	9.9065901	6.91123015	4.83740358	1.042E-08	5.792976	up	PREDICTED: GDSL esterase/lipase At4g10955-like [Sesamum indicum]
c40682.graph_c0	149.821317	165.358277	144.258667	41.464196	41.1125518	39.9075386	2.106E-74	-2.923501	down	PREDICTED: splicing factor 3B subunit 1-like [Sesamum indicum]
c40684.graph_c0	5.27320862	6.64698608	5.64690155	5.21787734	5.81362429	6.08371598	0.006746	-1.057192	down	unknown protein [Ipomoea batatas]
c40685.graph_c0	8.03374693	8.59009548	5.27915602	46.4257823	53.1028301	62.1137446	6E-18	1.866911	up	PREDICTED: uncharacterized protein LOC105159330 [Sesamum indicum]
c40689.graph_c0	97.5574953	99.0684287	91.0304068	6.2757349	6.37279963	7.20437142	1.55E-206	-4.874793	down	PREDICTED: zinc finger AN1 domain-containing stress-associated protein 12 [Sesamum indicum]
c40691.graph_c0	57.0112977	60.2426038	46.2979736	576.069551	378.360484	261.103301	0.0043302	1.890403	up	PREDICTED: chaperone protein dnaJ 20, chloroplastic-like [Sesamum indicum]
c40692.graph_c0	0	0	0	5.32019215	4.01030084	4.94629245	1.417E-22	Inf	up	hypothetical protein MIMGU_mgv1a008779mg [Erythranthe guttata]
c40701.graph_c1	161.828015	154.559325	174.613496	60.3119791	59.4082828	40.1619965	2.167E-71	-2.634027	down	PREDICTED: glutathione S-transferase F9-like [Sesamum indicum]
c40704.graph_c0	0.5325438	0.31357474	0.32204229	2.30877218	2.04841124	2.00297331	0.004006	1.433688	up	PREDICTED: protein trichome birefringence-like 3 [Sesamum indicum]

#ID	0 h-1 FPKM	0 h-2 FPKM	0 h-3 FPKM	48 h-1 FPKM	48 h-2 FPKM	48 h-3 FPKM	FDR	log2FC	regulated	nr_annotation
c40707.graph_c0	57.3609618	56.7819892	55.1017757	9.17622779	8.41116512	7.87541499	3.04E-106	-3.747426	down	PREDICTED: uncharacterized protein LOC104230730, partial [Nicotiana sylvestris]
c40708.graph_c0	5.82118911	5.82701815	6.10247928	2.82255742	3.43868093	4.01839139	0.0001081	-1.810826	down	PREDICTED: nuclear transcription factor Y subunit B-3-like [Sesamum indicum]
c40708.graph_c1	5.65590466	4.83185561	5.45104606	1.93151117	2.4268906	3.83697941	4.373E-09	-1.984799	down	PREDICTED: nuclear transcription factor Y subunit B-3-like [Solanum tuberosum]
c40711.graph_c1	5.62647142	6.16011537	7.23023854	3.07768678	3.08883406	4.61222263	0.0001369	-1.842293	down	-
c40711.graph_c2	24.2935632	23.7818238	23.1477565	16.796824	17.2952956	15.304889	7.01E-26	-1.544541	down	PREDICTED: DEAD-box ATP-dependent RNA helicase 32 [Sesamum indicum]
c40713.graph_c0	8.09539644	8.96080028	7.02261593	3.83232613	3.98027926	4.21232916	5.234E-28	-2.018434	down	PREDICTED: protein tipD-like [Sesamum indicum]
c40715.graph_c1	84.0139855	94.3883788	88.1125125	68.9862107	61.8782357	59.330269	1.857E-26	-1.501718	down	PREDICTED: nematode resistance protein-like HSPRO2 [Sesamum indicum]
c40718.graph_c0	6.9313805	6.05257809	7.51733833	35.2342395	40.1090927	42.0702672	7.126E-17	1.496154	up	PREDICTED: uncharacterized protein DDB_G0290685 [Sesamum indicum]
c40728.graph_c0	0.73221957	0.99681578	0.63983318	35.8060923	29.8140906	30.6355827	8.56E-51	4.333192	up	PREDICTED: shaggy-related protein kinase kappa isoform X1 [Sesamum indicum]
c40732.graph_c0	0.350088	0	0	8.7081454	9.45300745	4.40035739	2.085E-07	5.017184	up	hypothetical protein L484_002552 [Morus notabilis]
c40734.graph_c0	15.0200536	13.4674538	12.3492136	48.9229383	56.2742841	63.0212375	6.26E-09	1.023205	up	unnamed protein product [Coffea canephora]
c40735.graph_c0	3.24786543	2.75388792	2.74968938	14.3270966	14.5438585	12.5289085	1.132E-06	1.226658	up	-
c40736.graph_c0	131.097979	129.870194	126.182798	38.6645099	36.2836299	33.7160341	6.935E-85	-2.84823	down	hypothetical protein MIMGU_mgv1a008470mg [Erythranthe guttata]
c40739.graph_c0	0	0	0	1.05879991	0.85994709	0.72228724	2.117E-11	Inf	up	PREDICTED: filament-like plant protein 4 isoform X1 [Sesamum indicum]
c40740.graph_c0	31.4146665	26.4433311	26.2136949	1.12758077	1.29407969	1.25414324	3.033E-79	-5.533947	down	-
c40743.graph_c0	0.3403906	0.2433796	0.06248791	3.85267738	4.12890318	4.02601169	1.228E-08	3.209547	up	PREDICTED: protein N-terminal asparagine amidohydrolase [Sesamum indicum]
c40748.graph_c0	83.5682079	89.8793576	79.3639643	37.7231066	35.2823014	38.3873991	3.796E-52	-2.198357	down	PREDICTED: uncharacterized protein LOC105170953 [Sesamum indicum]
c40751.graph_c0	0.62681183	0.75292738	0.72493022	8.37316915	6.9127613	6.26860868	2.951E-07	2.342714	up	PREDICTED: 15-cis-zeta-carotene isomerase, chloroplastic [Sesamum indicum]
c40757.graph_c0	2.94296752	2.94591446	2.73385286	2.90724788	2.0071106	1.80207645	0.0001019	-1.368577	down	PREDICTED: uncharacterized protein LOC105167000 [Sesamum indicum]
c40759.graph_c0	328.124177	320.817172	313.354095	33.549929	23.1143605	17.296311	2.08E-191	-4.70864	down	hypothetical protein MIMGU_mgv1a008881mg [Erythranthe guttata]
c40760.graph_c0	1.23405132	1.38969791	0.5451204	4.47649872	6.96291193	9.51533058	0.0023557	1.70577	up	PREDICTED: UDP-glycosyltransferase 86A1-like [Sesamum indicum]

#ID	0 h-1 FPKM	0 h-2 FPKM	0 h-3 FPKM	48 h-1 FPKM	48 h-2 FPKM	48 h-3 FPKM	FDR	log2FC	regulated	nr_annotation
c40763.graph_c0	2.80398929	2.90615271	2.4234162	14.5163126	17.2672577	16.8016564	3.44E-15	1.559751	up	PREDICTED: probable ADP-ribosylation factor GTPase-activating protein AGD14 [Sesamum indicum]
c40764.graph_c0	12.2247323	12.6052158	14.2183394	11.0275911	13.6432706	12.0391857	5.73E-11	-1.111192	down	hypothetical protein MIMGU_mgv1a002739mg [Erythranthe guttata]
c40767.graph_c0	0.25073821	0.37648394	0.08055213	1.67472388	1.89652321	1.23323523	0.0027518	1.756123	up	conserved hypothetical protein [Ricinus communis]
c40770.graph_c0	34.2059952	37.4502706	32.7094886	6.89342367	7.95477744	6.57185028	3.411E-71	-3.302578	down	PREDICTED: O-glucosyltransferase rumi homolog isoform X1 [Sesamum indicum]
c40770.graph_c1	21.0263151	24.5373673	18.8172544	4.99094925	7.22423927	5.05648924	6.469E-42	-2.91849	down	PREDICTED: O-glucosyltransferase rumi homolog isoform X1 [Sesamum indicum]
c40771.graph_c0	16.7109041	19.1074742	15.1520072	14.4456369	14.6998485	13.4281682	2.138E-19	-1.275213	down	PREDICTED: uncharacterized protein LOC105178452 [Sesamum indicum]
c40773.graph_c0	416.987627	388.928516	443.193209	335.700926	366.243363	241.123722	1.051E-18	-1.422626	down	hypothetical protein MIMGU_mgv1a016776mg [Erythranthe guttata]
c40774.graph_c0	27.3662502	23.9728404	28.9977274	6.37160708	7.38523406	7.35912646	4.521E-53	-2.949066	down	PREDICTED: uncharacterized protein LOC105165423 [Sesamum indicum]
c40776.graph_c0	3.50536808	3.30447751	2.66773537	0.90924114	0.63108221	0.69744595	9.913E-16	-3.090687	down	-
c40777.graph_c0	16.3683458	19.1213732	17.0703973	1.6145169	2.29191574	1.83883191	3.205E-97	-4.215801	down	PREDICTED: formin-like protein 6 [Sesamum indicum]
c40778.graph_c0	53.9724016	58.0442363	53.7002087	41.566873	47.4522936	47.8600574	3.439E-20	-1.295465	down	hypothetical protein MIMGU_mgv1a004204mg [Erythranthe guttata]
c40780.graph_c0	0.32711161	0.28066214	0.18015059	4.17594082	3.6029725	3.14113007	2.73E-07	2.784838	up	hypothetical protein MIMGU_mgv1a009114mg [Erythranthe guttata]
c40783.graph_c0	42.3671141	41.4588598	37.8335624	33.9096848	35.4484433	34.748848	5.439E-19	-1.241765	down	PREDICTED: uncharacterized protein LOC105128229 [Populus euphratica]
c40785.graph_c0	3.5182278	3.71923212	3.78867814	25.4249043	30.8924282	31.6523319	1.884E-42	1.974249	up	PREDICTED: alpha-mannosidase-like [Sesamum indicum]
c40788.graph_c0	2.49911446	2.15192855	2.4862924	23.4195752	26.2526536	26.720104	2.854E-38	2.399746	up	PREDICTED: phosphoribosylaminoimidazole carboxylase, chloroplastic-like isoform X1 [Sesamum indicum]
c40790.graph_c0	15.459566	15.6310448	13.6179284	15.390938	13.9016949	12.187318	4.354E-12	-1.121105	down	PREDICTED: 5'-3' exoribonuclease 3 isoform X4 [Sesamum indicum]
c40796.graph_c0	126.990996	124.539503	135.663433	104.071366	101.730752	102.5349	4.988E-21	-1.346389	down	PREDICTED: 2-alkenal reductase (NADP(+)-dependent)-like [Sesamum indicum]
c40799.graph_c0	0.15345267	0.05120211	0.19719276	3.44001817	4.74255893	4.10916347	5.968E-11	3.905987	up	hypothetical protein MIMGU_mgv1a026254mg [Erythranthe guttata]
c40800.graph_c0	0	0	0	1.91262255	2.99104485	3.97096009	1.553E-08	Inf	up	PREDICTED: LOW QUALITY PROTEIN: probable leucine-rich repeat receptor-like protein kinase At1g35710 [Sesamum indicum]
c40802.graph_c0	0.1151605	0.40346535	0.2959716	1.85663383	2.13546799	2.31283026	0.0004958	1.928521	up	hypothetical protein MIMGU_mgv1a011194mg [Erythranthe guttata]
c40806.graph_c1	5.57899117	5.58457769	5.29230309	71.8018153	99.621028	112.114581	1.917E-20	3.083273	up	PREDICTED: phenylalanine ammonia-lyase [Sesamum indicum]

#ID	0 h-1 FPKM	0 h-2 FPKM	0 h-3 FPKM	48 h-1 FPKM	48 h-2 FPKM	48 h-3 FPKM	FDR	log2FC	regulated	nr_annotation
c40807.graph_c0	9.53061026	10.0157626	9.44579657	4.96944992	8.01954252	7.03361919	1.102E-16	-1.558898	down	PREDICTED: probable protein phosphatase 2C 38 isoform X2 [Sesamum indicum]
c40809.graph_c0	3.27471402	2.99295026	3.75073095	1.70519313	2.91810403	2.68440181	0.0008049	-1.483406	down	PREDICTED: protein tesmin/TSO1-like CXC 2 isoform X4 [Sesamum indicum]
c40809.graph_c1	0.23852944	0.28652195	0.2758678	3.34018298	2.88668643	2.97207473	2.02E-08	2.506146	up	PREDICTED: LOW QUALITY PROTEIN: protein tesmin/TSO1-like CXC 2 [Sesamum indicum]
c40812.graph_c0	8.49866481	8.50717493	8.41936622	0.58635204	1.46157386	3.09970499	5.354E-35	-3.338998	down	hypothetical protein MIMGU_mgv1a008008mg [Erythranthe guttata]
c40815.graph_c0	0.24014702	0.32051666	0.30859843	7.04277809	7.03128006	6.75876465	2.162E-15	3.564341	up	PREDICTED: myosin-11 [Sesamum indicum]
c40816.graph_c0	4.2552552	3.62058876	2.05056427	1.56808745	1.76502343	2.00570751	8.562E-05	-1.908811	down	PREDICTED: uncharacterized protein LOC105168692 [Sesamum indicum]
c40827.graph_c0	16.3515963	18.5999658	17.1920034	10.5563222	14.2660639	11.5254059	4.515E-14	-1.542253	down	PREDICTED: random slug protein 5 [Sesamum indicum]
c40830.graph_c0	0.30639798	0.40004973	0.23966389	4.49183532	4.13448209	4.07324974	7.184E-18	2.734431	up	PREDICTED: auxilin-like protein 1 [Sesamum indicum]
c40838.graph_c0	0.15097515	0.22668949	0.19400904	4.31173281	8.32510747	13.0257194	1.482E-05	4.456559	up	PREDICTED: indole-3-acetic acid-amido synthetase GH3.6 [Sesamum indicum]
c40843.graph_c0	2.92246105	3.05257821	3.18399235	0	0	0	7.732E-27	-Inf	down	-
c40844.graph_c0	30.0473897	30.9553371	28.7096559	24.4101517	25.6912208	23.1577928	7.694E-21	-1.309317	down	PREDICTED: uncharacterized protein LOC105156064 [Sesamum indicum]
c40848.graph_c0	313.954084	307.037415	302.055882	113.985391	128.403935	129.141743	5.843E-54	-2.332307	down	Histone [Morus notabilis]
c40850.graph_c0	22.2386991	21.9184914	21.103465	5.75232704	9.6834464	9.25516374	1.814E-25	-2.428024	down	unnamed protein product [Coffea canephora]
c40853.graph_c0	20.3112139	24.2025065	22.896178	17.3801991	18.0947615	17.2423594	4.364E-18	-1.373095	down	PREDICTED: protein ROOT INITIATION DEFECTIVE 3 [Sesamum indicum]
c40855.graph_c0	0.6828788	0.54685008	0.96527892	8.55592988	9.73043864	10.0200712	1.145E-09	2.66383	up	PREDICTED: transcription factor BEE 1-like [Sesamum indicum]
c40857.graph_c0	35.6586579	31.5272806	30.4781365	25.1807829	24.4841716	24.8303751	6.489E-19	-1.406408	down	PREDICTED: vacuolar protein sorting-associated protein 32 homolog 2 [Sesamum indicum]
c40858.graph_c0	0.66286219	0.60320541	0.77436742	8.32734393	10.2333009	10.8674435	2.354E-11	2.826422	up	PREDICTED: GATA transcription factor 5-like [Sesamum indicum]
c40868.graph_c0	13.1143528	12.1037819	12.5233896	13.4119033	12.916623	10.3558037	1.791E-06	-1.055792	down	PREDICTED: FH protein interacting protein FIP2-like [Sesamum indicum]
c40879.graph_c0	221.066044	222.780212	215.274793	93.9589712	70.1254664	71.1416114	3.757E-56	-2.497805	down	PREDICTED: triacylglycerol lipase SDP1-like [Sesamum indicum]
c40880.graph_c0	1.14507785	0.86741311	0.87492833	8.23978367	9.45395562	8.84117966	1.69E-22	2.182448	up	PREDICTED: chlorophyllide a oxygenase, chloroplastic-like [Sesamum indicum]
c40884.graph_c0	3.15467743	2.7187789	2.68813791	34.7512019	35.7115613	35.3328282	9.9E-63	2.610592	up	PREDICTED: bifunctional aspartokinase/homoserine dehydrogenase 1, chloroplastic-like [Sesamum indicum]
c40885.graph_c0	1.10372681	0.80841367	0.72646306	6.84496914	6.80476969	7.083647	2.348E-17	1.959885	up	PREDICTED: putative ATP-dependent RNA helicase C550.03c isoform X1 [Sesamum indicum]

#ID	0 h-1 FPKM	0 h-2 FPKM	0 h-3 FPKM	48 h-1 FPKM	48 h-2 FPKM	48 h-3 FPKM	FDR	log2FC	regulated	nr_annotation
c40889.graph_c0	5.84827626	6.45382403	4.61913943	25.9929151	29.9864915	32.1084026	8.147E-16	1.362292	up	PREDICTED: putative E3 ubiquitin-protein ligase RF298 [Sesamum indicum]
c40890.graph_c0	23.7750733	24.3378795	25.5752491	13.2411018	12.1678095	10.9839463	2.12E-43	-2.033432	down	PREDICTED: low molecular weight phosphotyrosine protein phosphatase [Sesamum indicum]
c40893.graph_c0	60.2639037	49.9360852	59.1924473	338.984826	351.650301	359.086464	1.114E-27	1.612781	up	PREDICTED: 40S ribosomal protein S25-2 [Eucalyptus grandis]
c40896.graph_c0	7.67777897	3.53935985	5.58222037	0	0	0	1.423E-15	-Inf	down	-
c40897.graph_c0	10.1755677	10.8046385	10.990467	0.97303864	0.95539086	0.95027261	2.789E-91	-4.490881	down	PREDICTED: protein root UVB sensitive 6 [Sesamum indicum]
c40898.graph_c0	2.06255478	2.47754414	1.32523228	0	0.20129891	0.22543346	1.23E-13	-4.822335	down	PREDICTED: probable 2-oxoglutarate-dependent dioxygenase AOP1 [Sesamum indicum]
c40899.graph_c1	11.314966	9.25124955	11.3768578	60.5949654	54.9623742	53.3152042	8.515E-19	1.386297	up	hypothetical protein ARALYDRAFT_496862 [Arabidopsis lyrata subsp. lyrata]
c40901.graph_c0	4.90022557	5.26847555	5.13087575	19.8968327	24.2665931	24.944407	1.518E-06	1.153815	up	hypothetical protein MIMGU_mgv1a003594mg [Erythranthe guttata]
c40903.graph_c0	23.852298	23.4216042	20.8138797	7.61031715	6.90141908	5.26484159	7.59E-75	-2.795619	down	PREDICTED: serine/threonine-protein kinase/endoribonuclease IRE1a-like isoform X1 [Sesamum indicum]
c40904.graph_c0	4.47052244	6.29094061	4.49592913	22.3833525	21.0566537	22.5721155	1.892E-05	1.09778	up	hypothetical protein MIMGU_mgv1a017323mg [Erythranthe guttata]
c40909.graph_c0	0.10059509	0.06713055	0.17235824	1.05031396	1.63629454	1.15446024	1.769E-05	2.466115	up	PREDICTED: pentatricopeptide repeat-containing protein At2g17140-like [Sesamum indicum]
c40910.graph_c0	9.82537833	10.9788468	11.4514885	27.9447375	56.6435516	49.7769456	0.0085603	1.028596	up	PREDICTED: hippocampus abundant transcript-like protein 1 isoform X1 [Sesamum indicum]
c40912.graph_c1	6.21329628	6.55964784	6.86153506	40.8736072	31.5990379	26.4213082	0.0001571	1.320865	up	hypothetical protein MIMGU_mgv1a013233mg [Erythranthe guttata]
c40916.graph_c0	0.05186224	0	0.06664503	1.62448173	2.02463696	2.4657746	7.93E-10	4.657208	up	PREDICTED: uncharacterized protein LOC105162032 [Sesamum indicum]
c40921.graph_c0	50.1843338	46.869116	45.6822535	35.2555716	38.5071117	33.8830536	1.912E-22	-1.424463	down	PREDICTED: prostaglandin E synthase 2-like [Sesamum indicum]
c40925.graph_c0	3.74658171	3.79200372	2.62123095	0.95877651	1.34073384	0.85311357	3.144E-12	-2.705429	down	PREDICTED: uncharacterized protein LOC105166274 [Sesamum indicum]
c40929.graph_c0	20132.0579	19213.0311	21254.6743	503.03885	459.389858	381.236989	7.827E-81	-6.510022	down	--
c40931.graph_c0	4.36942192	5.84498358	4.3898154	3.98880196	4.14811711	4.36325145	1.867E-05	-1.241863	down	PREDICTED: cyclin-dependent kinase inhibitor 1 [Sesamum indicum]
c40935.graph_c0	117.450405	118.36284	110.369065	53.1261363	48.9186803	45.5084735	8.313E-55	-2.244812	down	hypothetical protein M569_02358 [Genlisea aurea]
c40938.graph_c0	1.73324258	1.80437728	1.3363712	20.055508	20.9080607	18.9250798	9.536E-40	2.604479	up	PREDICTED: endoglucanase 10-like [Sesamum indicum]
c40939.graph_c0	0	0	0.05648198	9.23236409	8.32206418	7.92665987	3.726E-29	7.770015	up	PREDICTED: uncharacterized protein LOC105156631 [Sesamum indicum]

#ID	0 h-1 FPKM	0 h-2 FPKM	0 h-3 FPKM	48 h-1 FPKM	48 h-2 FPKM	48 h-3 FPKM	FDR	log2FC	regulated	nr_annotation
c40941.graph_c0	2.07162152	1.67980956	1.6061927	9.09475686	7.11596602	6.01479439	0.0015682	1.043459	up	PREDICTED: protein PHYLLO, chloroplastic [Sesamum indicum]
c40942.graph_c0	0.95012202	0.95107342	0.70686255	4.37657188	9.12648032	11.1088529	0.0008333	2.210792	up	PREDICTED: serine carboxypeptidase-like 27 [Sesamum indicum]
c40943.graph_c0	7.04410164	7.0123059	4.8127445	23.4193568	32.8968822	31.7507267	6.968E-08	1.203048	up	PREDICTED: uncharacterized protein LOC105157708 [Sesamum indicum]
c40944.graph_c1	6.26101104	6.24544328	6.2514958	5.30580369	6.74928501	6.97431124	2.45E-07	-1.001378	down	unnamed protein product [Vitis vinifera]
c40949.graph_c0	65.7595936	65.0380562	77.2258523	39.2768643	30.8613435	30.4346973	1.791E-25	-2.062805	down	PREDICTED: malate synthase [Tarenaya hassleriana]
c40949.graph_c1	121.071073	130.770536	139.118586	70.7770867	51.517218	45.7012902	9.714E-41	-2.229776	down	PREDICTED: malate synthase [Tarenaya hassleriana]
c40952.graph_c0	8.95382337	6.24679252	8.13556794	81.6552442	100.979981	117.584657	7.955E-24	2.662721	up	putative nitrilase-associated protein [Plantago major]
c40953.graph_c0	3.67825963	3.13462702	2.29948018	0.59530385	1.21279278	1.01864959	2.391E-10	-2.712983	down	-
c40954.graph_c0	28.0425268	30.1028683	29.7580348	148.461304	137.338516	148.846507	1.958E-19	1.288943	up	PREDICTED: putative 4-hydroxy-4-methyl-2-oxoglutarate aldolase 3 [Sesamum indicum]
c40955.graph_c0	5.9564231	5.48003711	5.72777923	74.9498732	80.2624973	79.5275966	4.247E-81	2.75504	up	PREDICTED: probable alanine--tRNA ligase, chloroplastic isoform X2 [Sesamum indicum]
c40956.graph_c0	1.21421697	0.83161193	1.02652426	12.805208	15.1248255	18.2042022	6.998E-25	2.887073	up	PREDICTED: uncharacterized protein LOC105174334 [Sesamum indicum]
c40959.graph_c0	52.1536428	52.0088636	50.2266864	43.9134534	38.0311885	37.1379766	1.448E-22	-1.388577	down	hypothetical protein MIMGU_mgv1a008968mg [Erythranthe guttata]
c40960.graph_c0	13.5099371	12.088322	12.0462722	0.60961152	0.32290474	0.09040478	1.498E-73	-6.196804	down	PREDICTED: transcription factor bHLH113-like [Sesamum indicum]
c40970.graph_c0	0.13931366	0.13945316	0.04475589	9.94672412	13.5285953	15.9880638	3.124E-29	5.915963	up	PREDICTED: glycerophosphodiester phosphodiesterase GDPDL3-like [Sesamum indicum]
c40971.graph_c0	1.43337841	1.34322987	2.11628069	33.8559284	29.050175	27.6748176	1.653E-33	3.191689	up	PREDICTED: uncharacterized protein LOC105164832 [Sesamum indicum]
c40977.graph_c0	0.11641632	0.11653289	0.14959958	4.29001217	3.74941686	4.77153379	1.09E-08	4.045938	up	class I KNOX homeobox transcription factor STM-like 2 [Prunus persica]
c40979.graph_c0	0.88026104	0.68088284	1.07975346	16.8825855	23.8988034	25.5833581	4.93E-25	3.623789	up	PREDICTED: uncharacterized protein LOC105165458 [Sesamum indicum]
c40980.graph_c0	12.0642313	12.279275	11.8769596	6.66238754	8.55863952	7.35478717	8.851E-28	-1.702855	down	PREDICTED: putative pentatricopeptide repeat-containing protein At5g65820 isoform X2 [Sesamum indicum]
c40984.graph_c1	21.3471099	20.4042601	20.5239986	8.50204653	9.43145445	9.09336682	1.668E-42	-2.223135	down	PREDICTED: uncharacterized protein LOC105159405 [Sesamum indicum]
c40986.graph_c0	6.38586465	6.72996338	7.18419858	27.1953469	27.3755434	29.0379073	5.57E-11	1.023079	up	PREDICTED: glutamate--tRNA ligase, chloroplastic/mitochondrial [Sesamum indicum]
c40989.graph_c0	2.48932088	1.86886017	2.13258393	17.9643773	22.844044	25.3939664	3.526E-22	2.328502	up	PREDICTED: GDSL esterase/lipase At4g10955-like [Sesamum indicum]

#ID	0 h-1 FPKM	0 h-2 FPKM	0 h-3 FPKM	48 h-1 FPKM	48 h-2 FPKM	48 h-3 FPKM	FDR	log2FC	regulated	nr_annotation
c40992.graph_c0	0	0	0	2.23100536	4.5754542	1.98513515	4.42E-08	Inf	up	PREDICTED: hippocampus abundant transcript 1 protein-like [Sesamum indicum]
c40996.graph_c0	69.4210047	75.6683288	65.3658601	21.4028868	23.5561602	20.9453536	8.263E-77	-2.692482	down	PREDICTED: probable methionine--tRNA ligase [Sesamum indicum]
c40998.graph_c0	15.0762415	15.0650006	14.3695743	12.5802855	14.0206142	13.3291079	1.393E-13	-1.175276	down	PREDICTED: protein FAM63A-like [Sesamum indicum]
c40999.graph_c0	0.15939825	0.10637191	0.17069422	1.41953127	3.50027362	2.28662916	9.267E-05	3.012729	up	PREDICTED: tyrosine-sulfated glycopeptide receptor 1 [Sesamum indicum]
c41000.graph_c0	3.27286716	3.53209323	4.20576388	25.9588041	29.6463193	29.8471276	1.627E-30	1.933724	up	PREDICTED: ATP-dependent zinc metalloprotease FTSH 2, chloroplastic [Sesamum indicum]
c41003.graph_c0	2001.21231	1906.87187	2046.74872	19.6697342	16.5792004	13.3348657	8.64E-248	-7.920755	down	hypothetical protein M569_01497, partial [Genlisea aurea]
c41009.graph_c0	2.08872836	2.34316024	2.68409863	9.621364	10.860464	13.1072349	3.335E-06	1.215407	up	PREDICTED: uncharacterized protein LOC105156446 [Sesamum indicum]
c41011.graph_c0	15.9990072	14.413525	13.3372244	5.93352475	8.48789936	10.8282542	1.234E-21	-1.816919	down	PREDICTED: xyloglucan galactosyltransferase KATAMARI1 homolog [Sesamum indicum]
c41012.graph_c0	3.32648265	3.48468867	2.38585781	17.8885528	16.8367244	14.0146813	3.584E-05	1.394612	up	PREDICTED: UMP-CMP kinase isoform X2 [Sesamum indicum]
c41013.graph_c0	27.2127615	30.0006168	27.7641192	9.27848988	10.424257	10.3542783	1.015E-67	-2.518789	down	PREDICTED: uncharacterized protein LOC105172362 [Sesamum indicum]
c41014.graph_c0	16.5728759	15.0507375	16.9139816	237.871236	243.97875	222.760973	2.209E-85	2.84184	up	PREDICTED: short-chain dehydrogenase TIC 32, chloroplastic-like [Sesamum indicum]
c41017.graph_c0	0.10299957	0.13747028	0.22059754	4.39656631	4.1885152	4.55935395	9.689E-14	3.808706	up	PREDICTED: protein PNS1 isoform X1 [Sesamum indicum]
c41019.graph_c0	0.75708509	0.80836607	0.64858954	4.6498411	4.28557025	4.09602664	0.0009902	1.543672	up	PREDICTED: carbon catabolite repressor protein 4 homolog 4-like isoform X1 [Sesamum indicum]
c41020.graph_c0	33.6641047	35.5006696	33.4591264	595.198323	613.76568	766.208655	8.623E-48	3.247054	up	PREDICTED: stachyose synthase [Sesamum indicum]
c41021.graph_c0	165.918449	174.937366	170.78556	47.236062	66.7211733	74.3648343	8.41E-59	-2.466436	down	PREDICTED: glycosyltransferase 6 [Sesamum indicum]
c41024.graph_c0	6.13656721	5.25154139	5.43420897	56.1239681	59.5806652	58.9569395	5.754E-51	2.358388	up	PREDICTED: glycerol-3-phosphate acyltransferase, chloroplastic [Sesamum indicum]
c41025.graph_c0	2.77399294	2.61246472	2.53114137	11.5228072	13.1201404	11.9617454	5.298E-11	1.190444	up	PREDICTED: uncharacterized protein LOC105177410 [Sesamum indicum]
c41027.graph_c0	7.8006417	9.98193974	7.49226467	4.81566903	5.53329272	5.53747817	8.977E-13	-1.688552	down	PREDICTED: E3 ubiquitin-protein ligase At3g02290 [Sesamum indicum]
c41028.graph_c0	1.64702555	1.56057767	1.26020196	9.1388326	8.52805079	9.1004714	3.723E-16	1.568869	up	PREDICTED: DNA-directed RNA polymerase V subunit 1 [Sesamum indicum]
c41038.graph_c0	2.44598264	1.65629218	1.66403887	14.6470919	16.6402073	12.67906	8.467E-08	1.915579	up	shikimate kinase, partial [Ipomoea purpurea]
c41039.graph_c0	58.8712785	61.5639824	63.5645213	33.8748139	34.6665102	37.4748049	3.414E-33	-1.814552	down	hypothetical protein MIMGU_mgv1a0073611mg, partial [Erythranthe guttata]
c41040.graph_c0	0.01929667	0.01931599	0.07439095	14.7018692	16.2716667	16.7514525	3.62E-109	7.686768	up	PREDICTED: glucomannan 4-beta-mannosyltransferase 9-like [Nicotiana tomentosiformis]

#ID	0 h-1 FPKM	0 h-2 FPKM	0 h-3 FPKM	48 h-1 FPKM	48 h-2 FPKM	48 h-3 FPKM	FDR	log2FC	regulated	nr_annotation
c41042.graph_c0	7.67215682	7.38925081	5.54237096	26.1328348	31.5701233	36.5790278	1.684E-08	1.175292	up	PREDICTED: MLO-like protein 1 [Sesamum indicum]
c41044.graph_c0	2.03609404	2.44575945	1.15124304	12.1550715	16.2947468	16.6906011	1.783E-07	1.985464	up	PREDICTED: phytochrome B [Sesamum indicum]
c41044.graph_c1	1.18362858	1.51586472	0.87234373	8.17826888	10.345697	12.2424394	9.027E-13	2.087604	up	PREDICTED: phytochrome B [Sesamum indicum]
c41045.graph_c0	25.6669068	28.094442	26.3998969	16.3368161	17.3923418	17.8026044	4.899E-31	-1.656182	down	PREDICTED: probable pre-mRNA-splicing factor ATP-dependent RNA helicase [Sesamum indicum]
c41055.graph_c0	0	0.2347584	0	6.26569362	8.69773173	6.7286613	4.704E-10	5.519709	up	calmodulin, partial [Malus domestica]
c41055.graph_c2	3.07661226	3.8151421	2.83240733	2.79207177	3.04749365	3.38777546	0.0026568	-1.093836	down	hypothetical protein VITISV_041204 [Vitis vinifera]
c41058.graph_c0	0.55793016	0.40617371	0.0651784	4.67274233	6.83128139	7.27611155	1.934E-10	3.178875	up	PREDICTED: axial regulator YABBY 5 [Beta vulgaris subsp. vulgaris]
c41061.graph_c0	12.5529139	12.7240388	13.7902166	6.6760727	7.53625931	7.04399708	6.819E-32	-1.898283	down	PREDICTED: pentatricopeptide repeat-containing protein At2g17670 [Sesamum indicum]
c41070.graph_c1	41.580037	39.7417026	43.6099361	9.05347109	9.56974138	9.61316414	2.27E-100	-3.164731	down	probable tocopherol cyclase, chloroplastic [Sesamum indicum]
c41073.graph_c0	32.1156884	28.8266129	28.3149991	20.3386593	25.9469452	28.5580356	1.056E-16	-1.27572	down	PREDICTED: uncharacterized protein LOC105174078 [Sesamum indicum]
c41074.graph_c0	37.9708001	35.4263411	35.6179063	32.0559224	26.5993488	24.4561755	3.124E-18	-1.40397	down	PREDICTED: uncharacterized protein LOC105177909 [Sesamum indicum]
c41079.graph_c0	0.33164247	0.96272622	0.59664319	9.92974695	11.9435227	10.4393993	2.341E-21	3.074201	up	PREDICTED: HIPL1 protein-like [Sesamum indicum]
c41083.graph_c0	0.03722506	0	0	15.5009567	14.4595373	20.6584723	7.373E-43	9.411427	up	hypothetical protein MIMGU_mgv1a013647mg [Erythranthe guttata]
c41088.graph_c0	6.69602699	7.99171898	6.50865291	75.6075496	94.4659021	84.6217149	8.991E-60	2.567055	up	PREDICTED: transcription factor ILR3-like [Sesamum indicum]
c41089.graph_c0	8.20251258	7.60456516	6.54363215	7.37918346	8.11285132	6.92696768	1.451E-07	-1.011562	down	PREDICTED: uncharacterized protein LOC105176739 isoform X2 [Sesamum indicum]
c41090.graph_c0	24.2619082	21.2504275	22.7576597	17.5225917	22.4729972	19.028992	5.308E-15	-1.230669	down	PREDICTED: developmentally-regulated G-protein 3 [Sesamum indicum]
c41093.graph_c0	2.29088888	3.16374301	2.53501028	17.0600064	17.8452841	17.8750608	1.119E-20	1.705775	up	PREDICTED: uncharacterized protein LOC105174509 [Sesamum indicum]
c41098.graph_c0	6.36257991	5.58061185	7.16413337	0.45823768	0.5865821	0.98536473	1.188E-48	-4.261985	down	-
c41099.graph_c0	4.14188601	0.99504804	1.9160955	0	0	0	4.977E-06	-Inf	down	PREDICTED: LOW QUALITY PROTEIN: oryzain gamma chain-like [Phoenix dactylifera]
c41100.graph_c0	501.726751	516.389476	501.810512	238.389226	171.083109	147.481895	6.614E-55	-2.457675	down	PREDICTED: SNF1-related protein kinase catalytic subunit alpha KIN10-like isoform X2 [Sesamum indicum]
c41100.graph_c1	708.821262	714.438608	702.827095	287.734323	202.323271	187.026322	9.271E-60	-2.660237	down	PREDICTED: uncharacterized protein LOC105162997 [Sesamum indicum]
c41103.graph_c1	4.4612299	4.46569715	3.25679817	20.0061476	22.4647163	25.7842094	1.663E-18	1.468585	up	PREDICTED: translation initiation factor IF-2, chloroplastic isoform X2 [Sesamum indicum]
c41104.graph_c0	16.8621193	18.4314762	18.4457799	16.4464891	16.2636706	13.3085428	1.497E-14	-1.240074	down	PREDICTED: UPF0496 protein At4g34320 [Sesamum indicum]

#ID	0 h-1 FPKM	0 h-2 FPKM	0 h-3 FPKM	48 h-1 FPKM	48 h-2 FPKM	48 h-3 FPKM	FDR	log2FC	regulated	nr_annotation
c41105.graph_c0	41.6796703	39.1225199	34.6740104	18.9213878	18.7327206	18.2803204	2.629E-41	-2.061083	down	PREDICTED: probable BOI-related E3 ubiquitin-protein ligase 2 [Sesamum indicum]
c41105.graph_c1	0.28385933	0.5276952	0.10422014	4.52038246	3.99726038	4.4543473	2.129E-08	2.817942	up	PREDICTED: B3 domain-containing protein At3g19184-like isoform X1 [Sesamum indicum]
c41107.graph_c0	129.647597	121.413229	135.118396	15.3958593	14.2067143	12.3974145	9.58E-165	-4.216288	down	PREDICTED: isopentenyl-diphosphate Delta-isomerase I [Sesamum indicum]
c41111.graph_c0	0	0	0	0.78719681	1.02852546	0.79383538	1.947E-11	Inf	up	-
c41118.graph_c0	25.5906067	24.277726	25.4784241	20.772135	21.5780474	19.2010711	1.457E-19	-1.309188	down	hypothetical protein MIMGU_mgv1a002787mg [Erythranthe guttata]
c41123.graph_c0	31.3594113	29.6833629	31.0244672	139.012073	139.262824	129.123803	4.821E-15	1.128719	up	PREDICTED: NADH dehydrogenase [ubiquinone] iron-sulfur protein 8, mitochondrial [Sesamum indicum]
c41123.graph_c1	18.6500566	19.6356382	14.1313827	8.21433399	6.4540507	7.49179161	1.437E-20	-2.252902	down	hypothetical protein MIMGU_mgv1a010537mg [Erythranthe guttata]
c41124.graph_c0	1.83300727	1.49080974	1.71754254	11.4338105	16.7342104	14.7753948	2.084E-14	2.066963	up	hypothetical protein MIMGU_mgv1a016809mg [Erythranthe guttata]
c41125.graph_c0	15.7388567	14.1114973	14.43465	245.219222	260.56534	266.371532	1.979E-98	3.105794	up	PREDICTED: isocitrate dehydrogenase [NADP] [Sesamum indicum]
c41126.graph_c0	30.0419517	30.7452886	26.6010247	118.210775	121.984359	127.757507	6.318E-14	1.057018	up	hypothetical protein MIMGU_mgv1a006861mg [Erythranthe guttata]
c41129.graph_c0	1.86144977	1.77664797	1.39071898	11.246481	10.9425506	10.4328831	1.052E-07	1.683919	up	hypothetical protein MIMGU_mgv1a010701mg [Erythranthe guttata]
c41131.graph_c0	13.9506951	15.3581019	13.2995681	11.5977283	9.68734349	9.82345484	2.442E-18	-1.466885	down	PREDICTED: lysM domain receptor-like kinase 4 [Sesamum indicum]
c41133.graph_c0	5.58721534	6.03580494	5.11826821	21.9142782	25.0511663	28.1151154	2.391E-07	1.14546	up	PREDICTED: dolichol-phosphate mannosyltransferase subunit 1 isoform X1 [Sesamum indicum]
c41136.graph_c1	146.777213	135.821772	148.843321	5.44554121	6.74117554	8.27530666	2.31E-232	-5.421455	down	PREDICTED: protein TRANSPARENT TESTA 12-like [Nicotiana tomentosiformis]
c41136.graph_c2	69.5671798	64.7003918	77.7361568	2.96824325	3.27284049	4.04253825	1.09E-109	-5.388084	down	PREDICTED: protein TRANSPARENT TESTA 12-like [Sesamum indicum]
c41137.graph_c0	2.12897569	2.04042211	2.34775876	12.2688782	13.7194659	12.3772948	4.176E-18	1.537441	up	PREDICTED: ABC transporter G family member 29-like [Vitis vinifera]
c41141.graph_c0	0.06856178	0	0	2.02123478	1.67285403	2.15443222	4.043E-14	5.419966	up	PREDICTED: LOW QUALITY PROTEIN: uncharacterized protein LOC105157816 [Sesamum indicum]
c41142.graph_c0	0	0	0.09334058	1.40526406	3.96988501	3.45347332	6.708E-06	5.498064	up	gibberelin 20-oxidase [Lactuca sativa]
c41143.graph_c0	17.8342171	19.3024231	15.9629684	10.4233612	11.1878542	11.5452727	1.975E-29	-1.696939	down	PREDICTED: formin-like protein 5 [Sesamum indicum]
c41144.graph_c0	48.3231579	52.4413695	46.3143668	282.717486	294.958175	317.245128	5.03E-29	1.587154	up	hypothetical protein MIMGU_mgv1a008949mg [Erythranthe guttata]

#ID	0 h-1 FPKM	0 h-2 FPKM	0 h-3 FPKM	48 h-1 FPKM	48 h-2 FPKM	48 h-3 FPKM	FDR	log2FC	regulated	nr_annotation
c41146.graph_c0	0	0	0	3.51981351	3.41808098	5.35034437	1.207E-11	Inf	up	Brassinosteroid signaling positive regulator family protein [Theobroma cacao]
c41146.graph_c2	0	0	0	2.92783496	2.67872893	3.55250447	1.128E-10	Inf	up	PREDICTED: protein BRASSINAZOLE-RESISTANT 1 isoform X2 [Sesamum indicum]
c41147.graph_c0	0	0	0	7.40939437	10.2832634	10.0170199	6.547E-25	Inf	up	hypothetical protein MIMGU_mgv1a010461mg [Erythranthe guttata]
c41152.graph_c0	16.428353	15.0418203	14.7629958	14.4363378	16.3475037	14.3144848	3.861E-12	-1.053854	down	PREDICTED: AP2-associated protein kinase 1 isoform X4 [Sesamum indicum]
c41154.graph_c1	0	0.11384974	0.43846521	2.72430297	3.77408978	4.0090362	2.661E-07	3.202566	up	-
c41156.graph_c0	2.22115306	2.91509456	3.04455426	10.5951369	12.7657924	12.4620886	0.0001462	1.107514	up	PREDICTED: uncharacterized protein LOC105177301 [Sesamum indicum]
c41157.graph_c1	6.0032179	4.55872561	4.87690227	29.2420196	34.9516872	40.3300285	1.059E-14	1.738699	up	PREDICTED: 4-hydroxy-3-methylbut-2-en-1-yl diphosphate synthase, chloroplastic [Sesamum indicum]
c41163.graph_c0	0.33364585	0.61229656	0.28583208	41.9056379	59.9155403	57.8000045	3.142E-56	5.997997	up	-
c41164.graph_c1	47.8637264	48.9719402	46.4491061	1109.08848	959.791277	1144.23693	2.363E-69	3.471384	up	PREDICTED: CASP-like protein 2A1 [Sesamum indicum]
c41165.graph_c0	1.03587911	0.95050668	0.49917962	0.15905323	0.75823926	0.45995498	0.0006573	-1.88293	down	-
c41167.graph_c0	2.66078802	2.43351406	2.58286248	14.1257875	13.7875713	13.0711748	6.067E-14	1.399896	up	PREDICTED: uncharacterized protein LOC105165931 isoform X2 [Sesamum indicum]
c41168.graph_c0	6.02115991	5.01326952	5.53190008	24.5212273	26.471549	26.1549315	1.382E-12	1.200839	up	hypothetical protein MIMGU_mgv1a003607mg [Erythranthe guttata]
c41176.graph_c0	11.4634383	10.9321847	12.0933318	52.8400141	53.4451783	62.8582843	4.059E-17	1.274344	up	PREDICTED: uroporphyrinogen decarboxylase, chloroplastic-like [Fragaria vesca subsp. vesca]
c41178.graph_c0	15.3907975	16.7594571	13.7642686	8.23915242	7.61196186	8.3540992	1.138E-11	-1.938742	down	PREDICTED: probable E3 ubiquitin-protein ligase ARI2 isoform X1 [Sesamum indicum]
c41183.graph_c0	0	0	0	2.48152076	4.85329911	2.71759047	1.433E-07	Inf	up	hypothetical protein VOLCADRAFT_105423 [Volvox carteri f. nagariensis]
c41190.graph_c0	0.39633158	0.34005296	0.36378698	9.25856491	8.26109784	9.56097109	1.873E-25	3.605448	up	PREDICTED: pectinesterase-like [Sesamum indicum]
c41191.graph_c0	8.93482682	7.78035598	6.34755465	38.4129445	31.5483734	30.4877331	3.339E-06	1.112732	up	PREDICTED: uncharacterized protein DDB_G0287625 [Sesamum indicum]
c41192.graph_c1	66.3198153	65.3430125	71.1008223	28.1924273	31.3459112	36.1137316	4.724E-39	-2.105257	down	hypothetical protein MIMGU_mgv1a014126mg [Erythranthe guttata]
c41194.graph_c0	12.9402526	14.1307749	11.2117999	2.98035119	2.43974781	2.20991547	8.413E-42	-3.337692	down	unnamed protein product [Coffea canephora]
c41196.graph_c0	362.369009	351.300466	367.666676	47.8527482	57.0459379	57.577395	1.99E-136	-3.755583	down	-
c41198.graph_c0	67.9110422	67.3090005	62.6361745	14.5758613	13.837832	12.520569	1.655E-97	-3.287849	down	-
c41199.graph_c0	0.02624224	0.05253704	0.13488929	2.94948018	2.35626826	2.53838285	3.919E-13	4.169446	up	PREDICTED: U-box domain-containing protein 13-like [Sesamum indicum]

#ID	0 h-1 FPKM	0 h-2 FPKM	0 h-3 FPKM	48 h-1 FPKM	48 h-2 FPKM	48 h-3 FPKM	FDR	log2FC	regulated	nr_annotation
c41200.graph_c0	0.14849897	0.2147133	0.10601503	1.04885318	1.48151036	1.8394754	8.591E-06	2.198737	up	PREDICTED: uncharacterized protein LOC105159953 isoform X1 [Sesamum indicum]
c41201.graph_c0	1.7541774	1.47271879	1.81789307	23.8238911	31.7552543	27.7851525	2.148E-30	3.02365	up	cytochrome P450 98A3 [Sesamum indicum]
c41202.graph_c0	162.028601	148.498406	157.668906	43.0361708	42.5185793	32.2425574	7.339E-94	-3.005966	down	PREDICTED: GATA transcription factor 15-like [Sesamum indicum]
c41210.graph_c0	1.22673056	0.95934292	0.83746108	125.234104	111.045007	98.4540858	1.681E-65	5.779754	up	PREDICTED: probable beta-1,3-galactosyltransferase 6 [Sesamum indicum]
c41219.graph_c0	5.21990891	4.70262227	3.73719707	66.4318025	66.0097525	62.9616722	5.295E-45	2.824651	up	hypothetical protein MIMGU_mgv1a008823mg [Erythranthe guttata]
c41223.graph_c0	15.4207032	16.2877941	15.7163078	134.292793	142.61291	136.405585	1.217E-49	2.105287	up	PREDICTED: 6-phosphogluconate dehydrogenase, decarboxylating 3 [Sesamum indicum]
c41223.graph_c1	1.3510688	1.35242169	0.43404422	21.1597759	15.1639114	19.3816014	2.34E-07	3.146816	up	-
c41225.graph_c0	41.6736771	44.4763545	40.9214517	26.7500156	27.212693	23.8020031	2.848E-30	-1.724741	down	PREDICTED: 1-aminocyclopropane-1-carboxylate oxidase homolog 1-like [Solanum lycopersicum]
c41226.graph_c0	0.0245998	0.12312216	0.09483515	2.0170016	2.1847859	3.60959714	8.563E-09	3.981502	up	PREDICTED: alpha-mannosidase-like [Sesamum indicum]
c41230.graph_c0	2.33233694	1.92045634	1.98198318	0.24259526	0.44057189	0.61674231	2.68E-12	-3.290338	down	hypothetical protein MIMGU_mgv1a002463mg [Erythranthe guttata]
c41232.graph_c0	55.8667625	56.227385	51.5942266	296.649007	280.236652	227.460325	1.421E-09	1.283045	up	PREDICTED: uncharacterized protein LOC105163662 isoform X2 [Sesamum indicum]
c41239.graph_c0	0	0	0	9.0744898	7.30125691	7.05234716	1.223E-14	Inf	up	--
c41241.graph_c0	18.8661384	18.7323209	16.6634835	8.62008619	11.1171396	10.0044794	1.889E-33	-1.887586	down	PREDICTED: uncharacterized protein LOC105172605 [Sesamum indicum]
c41241.graph_c1	10.7234493	9.94875889	9.18670461	4.25686561	4.76489518	4.5024001	4.867E-11	-2.160386	down	PREDICTED: uncharacterized protein LOC105172605 [Sesamum indicum]
c41246.graph_c0	0.85830699	0.91644421	0.77207092	8.9879459	9.91255656	10.5772332	2.458E-17	2.514466	up	PREDICTED: phosphoglycolate phosphatase 2 [Sesamum indicum]
c41253.graph_c0	0.14028516	0.28085128	0.18027199	65.9123201	79.2731729	64.973212	1.861E-66	7.429928	up	-
c41257.graph_c0	5.532751	3.81951118	3.67748487	0	0	0	1.571E-19	-Inf	down	metallothionein-like protein [Oryza officinalis]
c41258.graph_c0	3.28521468	3.76546297	3.89936903	23.0803625	24.7690246	24.4632168	7.648E-23	1.703307	up	PREDICTED: phosphoenolpyruvate carboxykinase [ATP]-like [Sesamum indicum]
c41275.graph_c0	0.08226679	0.74114247	0.31714823	3.9410555	3.29188245	2.56260855	0.0002466	2.090528	up	-
c41277.graph_c0	1.36006256	1.32976342	1.30063943	16.2887184	18.398085	20.7940432	3.748E-32	2.777125	up	PREDICTED: preprotein translocase subunit SCY1, chloroplastic [Sesamum indicum]
c41278.graph_c2	0.62091596	0.62153771	0.3989508	20.7360252	27.5727544	29.5212648	6.851E-19	4.54865	up	PREDICTED: uncharacterized protein LOC101261197 [Solanum lycopersicum]
c41279.graph_c1	53.6534929	55.5784361	57.1888097	44.5345289	43.6901248	42.7988312	7.671E-22	-1.362452	down	PREDICTED: nucleolar GTP-binding protein 2 [Sesamum indicum]

#ID	0 h-1 FPKM	0 h-2 FPKM	0 h-3 FPKM	48 h-1 FPKM	48 h-2 FPKM	48 h-3 FPKM	FDR	log2FC	regulated	nr_annotation
c41280.graph_c0	3.53934305	3.71159608	3.97064726	1.19040467	1.48040931	2.0263242	4.293E-08	-2.281232	down	hypothetical protein MIMGU_mgv1a019699mg [Erythranthe guttata]
c41283.graph_c0	1.33806722	1.42490116	1.2804559	8.47163575	11.4197824	12.5789092	1.021E-13	1.982647	up	PREDICTED: armadillo repeat-containing protein 6 [Sesamum indicum]
c41284.graph_c0	0.06186708	0.08257205	0.0530011	0.91193589	0.9660855	0.6254812	1.021E-05	2.65165	up	PREDICTED: putative late blight resistance protein homolog R1A-3 [Sesamum indicum]
c41289.graph_c0	7.09874359	6.98872248	7.31778197	240.895554	225.888216	253.066795	2.44E-152	4.054186	up	PREDICTED: uncharacterized protein LOC105166112 [Sesamum indicum]
c41291.graph_c0	5.90919927	5.50634823	5.49452354	1.46056921	2.86015317	3.34746923	8.37E-20	-2.169967	down	hypothetical protein MIMGU_mgv11b004345mg [Erythranthe guttata]
c41293.graph_c0	10.3931163	10.5630867	10.3239344	7.84195105	9.30326321	10.2183104	9.131E-11	-1.214163	down	PREDICTED: cell wall / vacuolar inhibitor of fructosidase 1-like [Sesamum indicum]
c41296.graph_c0	8.3318276	7.75145274	7.93557512	94.09656	99.3012318	108.153494	8.263E-46	2.631321	up	-
c41298.graph_c0	35.1650395	38.2370615	34.4981956	15.0292243	12.2159022	11.7931892	1.341E-66	-2.479175	down	unnamed protein product [Coffea canephora]
c41305.graph_c0	0.44239496	0.55354744	0.52112046	2.92086885	4.13773527	3.95889851	5.132E-05	1.836118	up	PREDICTED: dehydrogenase/reductase SDR family member on chromosome X-like isoform X1 [Sesamum indicum]
c41313.graph_c0	0	0	0	0.75441022	0.74925576	0.6502925	2.198E-11	Inf	up	PREDICTED: uncharacterized protein LOC105168700 [Sesamum indicum]
c41315.graph_c0	11.3013936	10.6928357	9.94708098	0.74877808	1.05765278	1.11043039	3.187E-63	-4.47578	down	-
c41316.graph_c0	6.97131525	5.88440092	6.68249417	21.627968	30.8928669	33.9171599	1.059E-05	1.120719	up	PREDICTED: zinc finger protein-like 1 homolog [Sesamum indicum]
c41319.graph_c0	6.81877715	7.48402362	7.635402	3.11065257	3.12417172	2.8397319	1.838E-29	-2.291185	down	hypothetical protein MIMGU_mgv1a0011691mg, partial [Erythranthe guttata]
c41326.graph_c0	6.11389799	6.25939947	5.87211791	92.6508989	123.799261	134.674829	1.529E-31	3.243595	up	PREDICTED: ABC transporter C family member 4 [Sesamum indicum]
c41330.graph_c0	24.176733	21.7297193	25.543633	8.50929715	9.05611032	9.0369772	3.337E-45	-2.445	down	PREDICTED: E3 ubiquitin-protein ligase RNF5-like [Sesamum indicum]
c41334.graph_c1	49.1611456	54.8161931	49.1688643	44.7343637	49.714346	46.8141149	6.232E-16	-1.135088	down	PREDICTED: dnaJ homolog subfamily B member 4-like [Malus domestica]
c41336.graph_c0	0	0.06135122	0	1.24221256	3.94792073	2.36135106	8.424E-05	5.92175	up	-
c41339.graph_c0	4.67610746	4.08896587	4.83481408	0.24758248	0.2098269	0.13217844	1.143E-28	-5.539265	down	PREDICTED: C2 and GRAM domain-containing protein At1g03370-like [Sesamum indicum]
c41341.graph_c0	17.8974575	18.1076042	19.0012714	14.1530517	11.582434	11.3969368	1.764E-20	-1.580805	down	PREDICTED: E3 ubiquitin-protein ligase PRT1 [Nicotiana tomentosiformis]
c41344.graph_c0	0.16754379	0.06708463	0.25836049	3.61184313	6.44259007	4.94430199	8.878E-13	3.892651	up	PREDICTED: O-glucosyltransferase rumi homolog isoform X2 [Sesamum indicum]

#ID	0 h-1 FPKM	0 h-2 FPKM	0 h-3 FPKM	48 h-1 FPKM	48 h-2 FPKM	48 h-3 FPKM	FDR	log2FC	regulated	nr_annotation
c41345.graph_c0	43.3034388	43.3468007	40.9385707	15.2578575	15.7005549	13.9870993	1.679E-67	-2.52174	down	PREDICTED: F-box/kelch-repeat protein At1g51550 [Sesamum indicum]
c41349.graph_c0	5.42297203	4.36154868	5.23662074	27.896715	32.5207429	33.019351	2.466E-21	1.616159	up	PREDICTED: peptidyl-prolyl cis-trans isomerase CYP23 isoform X2 [Sesamum indicum]
c41351.graph_c0	0	0	0	4.01991364	5.47535707	6.23401696	3.816E-17	Inf	up	-
c41356.graph_c0	0.99640199	0.96300664	0.83889325	0	0	0.08449519	2.588E-17	-6.088717	down	hypothetical protein MIMGU_mgv1a004102mg [Erythranthe guttata]
c41357.graph_c0	0	0	0	2.15143113	2.03926668	2.77074149	5.033E-12	Inf	up	PREDICTED: probable plastid-lipid-associated protein 4, chloroplastic isoform X1 [Sesamum indicum]
c41358.graph_c0	12.2850868	11.2995097	12.1697943	72.6609658	73.9422481	75.2766192	1.928E-29	1.615481	up	PREDICTED: glucose-6-phosphate isomerase 1, chloroplastic [Sesamum indicum]
c41361.graph_c0	0.39625075	0.57694187	0.55548862	6.83642909	6.60953942	7.44135631	2.642E-12	2.752521	up	hypothetical protein MIMGU_mgv1a007271mg [Erythranthe guttata]
c41362.graph_c0	9.21961252	8.3479094	6.08534128	77.524393	53.0067189	40.1242544	0.0012717	1.847593	up	PREDICTED: gibberellin receptor GID1B-like [Sesamum indicum]
c41366.graph_c0	11.2083167	10.1431267	11.3736899	7.73484863	7.44065062	7.11974669	1.064E-24	-1.570519	down	PREDICTED: LRR receptor-like serine/threonine-protein kinase GSO2 [Sesamum indicum]
c41376.graph_c0	6.53293407	4.12399375	3.93282977	49.612495	58.8194807	59.5353074	4.847E-40	2.508372	up	hypothetical protein MIMGU_mgv1a014628mg [Erythranthe guttata]
c41377.graph_c0	1.03828247	0.62359329	1.51213196	19.6408219	19.7261825	19.9728974	1.271E-20	3.200627	up	hypothetical protein MIMGU_mgv1a013935mg [Erythranthe guttata]
c41379.graph_c0	20.4963394	20.7757512	21.1456645	10.9900101	11.5479279	10.4148673	3.537E-33	-1.939443	down	PREDICTED: uncharacterized protein LOC105169116 [Sesamum indicum]
c41381.graph_c0	10.1966006	9.42319765	8.23874046	6.88328983	7.68852612	6.7745465	4.552E-22	-1.40076	down	PREDICTED: uncharacterized protein LOC105157976 isoform X1 [Sesamum indicum]
c41384.graph_c0	14.7876585	14.0111393	13.3258115	4.54111852	4.71999529	4.61245145	7.873E-34	-2.619385	down	hypothetical protein MIMGU_mgv1a000172mg [Erythranthe guttata]
c41385.graph_c0	8.48109528	9.81132005	9.07124256	5.75473409	5.35297432	4.87074594	4.466E-12	-1.791523	down	PREDICTED: uncharacterized protein LOC105163010 [Sesamum indicum]
c41389.graph_c0	45.3886524	47.1378811	46.7824852	286.258433	293.012207	301.839549	2.998E-31	1.642759	up	unnamed protein product [Vitis vinifera]
c41390.graph_c0	9.56401049	7.9779895	8.1043625	46.2896228	46.6708791	51.755139	7.678E-15	1.479156	up	PREDICTED: uncharacterized protein LOC105157940 [Sesamum indicum]
c41394.graph_c1	0.21726735	0.36247486	0.09306572	6.1382663	6.14933858	6.23356746	1.583E-10	3.773876	up	PREDICTED: squamosa promoter-binding-like protein 6, partial [Sesamum indicum]
c41406.graph_c0	2.63190909	2.53321591	2.73170212	15.9096725	15.3526901	14.5712293	1.748E-16	1.520191	up	PREDICTED: uncharacterized protein LOC105161164 [Sesamum indicum]

#ID	0 h-1 FPKM	0 h-2 FPKM	0 h-3 FPKM	48 h-1 FPKM	48 h-2 FPKM	48 h-3 FPKM	FDR	log2FC	regulated	nr_annotation
c41408.graph_c0	3.45172411	2.4149111	2.52781628	0.03419305	0	0	1.035E-34	-8.897762	down	PREDICTED: probable glycosyltransferase At3g07620 isoform X1 [Sesamum indicum]
c41411.graph_c0	3.00497114	3.18492017	2.61219583	10.503542	13.0680249	13.5118361	0.0001567	1.054834	up	PREDICTED: ABC transporter I family member 20-like [Sesamum indicum]
c41415.graph_c0	33.5689255	33.128597	32.946264	20.784462	23.8669797	23.888749	1.146E-27	-1.559718	down	PREDICTED: nuclear pore complex protein NUP50A-like [Sesamum indicum]
c41420.graph_c0	16.1887771	15.569498	14.6846256	11.0540226	13.3833103	12.5939909	2.127E-10	-1.346387	down	PREDICTED: 60S ribosomal protein L18a-like protein [Nicotiana sylvestris]
c41421.graph_c0	28.9822446	28.9454807	30.8249798	10.1715558	12.1866114	14.3839722	4.876E-36	-2.295047	down	hypothetical protein MIMGU_mgv1a015977mg [Erythranthe guttata]
c41425.graph_c0	0.37406076	0.65526182	0.90128041	4.09223391	4.60902934	4.67612532	6.915E-05	1.76565	up	PREDICTED: uncharacterized protein LOC105166018 [Sesamum indicum]
c41426.graph_c0	7.68078262	9.09622248	7.61110577	1.54476884	2.00602647	1.79575175	4.929E-45	-3.210255	down	PREDICTED: uncharacterized protein LOC105177245 [Sesamum indicum]
c41428.graph_c0	1.54016858	1.71201609	1.85260229	7.25950756	7.54200021	8.04497151	1.753E-11	1.141788	up	PREDICTED: uncharacterized protein LOC105169752 [Sesamum indicum]
c41429.graph_c0	0.38972661	0.39011686	0.37561059	15.6183083	11.8862815	13.8172086	1.177E-15	4.147526	up	hypothetical protein PHAVU_003G130600g [Phaseolus vulgaris]
c41431.graph_c0	61.0710242	61.4519623	64.2471529	12.9987724	13.7706231	13.9231215	7.843E-95	-3.217568	down	PREDICTED: uncharacterized protein LOC105163802 [Sesamum indicum]
c41432.graph_c1	0.33516741	0.047929	0.06152906	0.88222309	1.2149909	1.41299424	0.0010969	1.969349	up	-
c41433.graph_c0	2.81908084	2.55568639	2.39230316	16.5627726	19.5708164	21.6265619	3.774E-12	1.874761	up	PREDICTED: palmitoyl-monogalactosyldiacylglycerol delta-7 desaturase, chloroplastic [Sesamum indicum]
c41436.graph_c0	2.70928434	1.94556327	2.49762483	16.4408422	17.5895251	20.0846483	5.449E-11	1.899517	up	PREDICTED: protein N-lysine methyltransferase METTL21A [Sesamum indicum]
c41438.graph_c0	1.53207402	1.60663712	1.82814893	7.29247368	8.36631579	8.55205796	4.912E-05	1.263292	up	-
c41439.graph_c0	23.4714234	24.4408261	20.499516	13.1144554	11.9693523	12.3521553	5.43E-41	-1.884101	down	PREDICTED: mediator of RNA polymerase II transcription subunit 14 [Sesamum indicum]
c41443.graph_c0	9.54768272	8.99367119	7.53633311	7.58569477	8.33375801	7.19199399	8.113E-12	-1.190072	down	PREDICTED: uncharacterized protein LOC105168944 isoform X1 [Sesamum indicum]
c41444.graph_c0	0.10022092	0.20064255	0.25757569	3.32388166	4.49937447	4.27204544	1.303E-09	3.40713	up	PREDICTED: squamosa promoter-binding-like protein 6 [Sesamum indicum]
c41446.graph_c0	3.13854744	2.29188877	3.10751497	26.8050209	28.8988209	24.4414208	8.716E-36	2.21198	up	PREDICTED: ADP-ribosylation factor GTPase-activating protein AGD3-like [Sesamum indicum]
c41448.graph_c0	0.72569242	0.90075967	0.59682783	11.980509	12.7485568	13.0158953	3.459E-27	3.06958	up	PREDICTED: phosphoacetylglucosamine mutase [Sesamum indicum]
c41449.graph_c0	15.8612738	17.3102489	13.7496237	8.78134752	11.1412656	11.1181521	3.491E-20	-1.616066	down	PREDICTED: sodium-coupled neutral amino acid transporter 2-like [Sesamum indicum]

#ID	0 h-1 FPKM	0 h-2 FPKM	0 h-3 FPKM	48 h-1 FPKM	48 h-2 FPKM	48 h-3 FPKM	FDR	log2FC	regulated	nr_annotation
c41451.graph_c0	1.86128018	0.95429325	1.34175197	0	0	0	6.605E-16	-Inf	down	hypothetical protein GUITHDRAFT_154386 [Guillardia theta CCMP2712]
c41454.graph_c0	1.52325973	1.28653738	1.16223538	78.805538	92.4048357	105.980971	2.099E-80	5.105734	up	PREDICTED: BURP domain-containing protein 3-like [Sesamum indicum]
c41459.graph_c0	1.96138289	2.39964623	1.40025259	0.90347692	2.12694278	2.14375556	0.0080032	-1.182295	down	PREDICTED: heptahelical transmembrane protein 4-like [Sesamum indicum]
c41461.graph_c1	24.512277	22.5589085	19.764233	14.9072754	16.0009319	13.8335039	5.27E-20	-1.59471	down	-
c41464.graph_c0	0.42753476	0.53495359	0.85843608	16.0257038	20.524005	20.4146295	3.517E-59	3.938286	up	PREDICTED: nucleobase-ascorbate transporter 12 [Sesamum indicum]
c41469.graph_c0	5.97028658	5.43647325	5.49418106	5.50021741	4.99978183	5.93602162	7.017E-05	-1.056654	down	hypothetical protein MIMGU_mgv1a025668mg [Erythranthe guttata]
c41473.graph_c0	6.54698428	5.5897842	6.48856087	39.8946866	42.7647322	41.5137795	7.548E-31	1.717995	up	PREDICTED: probable polygalacturonase [Sesamum indicum]
c41478.graph_c0	8.23187447	7.13506858	7.68852142	4.74611523	5.44027735	5.16152364	8.965E-23	-1.606472	down	PREDICTED: LOW QUALITY PROTEIN: putative pentatricopeptide repeat-containing protein At5g13230, mitochondrial [Sesamum indicum]
c41480.graph_c0	1375.94165	1236.64402	1542.88942	335.10848	446.227708	339.969319	2.965E-38	-2.912522	down	PREDICTED: pathogenesis-related protein STH-2-like [Sesamum indicum]
c41482.graph_c0	22.3336105	23.8194916	20.8776458	7.71053793	7.65876626	6.92193814	5.242E-71	-2.603884	down	PREDICTED: autophagy-related protein 18g-like [Sesamum indicum]
c41483.graph_c0	0	0	0	4.19138882	4.19696585	4.70015721	1.671E-15	Inf	up	PREDICTED: uncharacterized protein LOC105112702 isoform X1 [Populus euphratica]
c41484.graph_c0	0.21912965	0.14623271	0.28159027	1.74959653	2.56636436	3.59257031	4.061E-05	2.580731	up	hypothetical protein MIMGU_mgv1a017010mg [Erythranthe guttata]
c41486.graph_c0	1250.05621	1227.48123	1331.97371	9.55392064	10.1212204	9.11669397	7.85E-258	-8.066325	down	glucose and ribitol dehydrogenase homolog 1-like precursor [Sesamum indicum]
c41487.graph_c0	6.01185273	7.28036346	6.15876532	1.2006562	0.53339824	0.41354968	7.281E-35	-4.176688	down	hypothetical protein JCGZ_16285 [Jatropha curcas]
c41489.graph_c0	397.669757	375.472013	386.714498	51.7137875	57.7116674	62.0860892	1.98E-114	-3.777527	down	PREDICTED: protein DJ-1 homolog D [Sesamum indicum]
c41491.graph_c0	33.9097872	36.7992544	33.6127894	21.363519	25.1018262	21.9887366	1.877E-30	-1.626952	down	PREDICTED: CTD small phosphatase-like protein 2 isoform X2 [Sesamum indicum]
c41493.graph_c0	28.24164	27.478362	26.7106364	12.6274225	16.7582793	16.9771773	3.655E-32	-1.852429	down	PREDICTED: ethylene-responsive transcription factor 3-like [Sesamum indicum]
c41493.graph_c1	10.7867313	10.9230853	9.50954919	4.62206532	7.34477663	9.87044623	1.988E-05	-1.541653	down	PREDICTED: ethylene-responsive transcription factor 3-like [Sesamum indicum]
c41495.graph_c0	3.38945407	3.21659625	2.09294232	0.56774294	0.77329903	1.11860013	2.088E-11	-2.843306	down	ATP binding protein, putative [Ricinus communis]
c41499.graph_c0	2.76138325	2.33225017	1.7742434	0.07949895	0	0	2.712E-19	-7.389139	down	-
c41499.graph_c1	99.2115304	106.476872	99.9499896	13.8622164	15.7311774	15.3601945	3.07E-127	-3.784763	down	PREDICTED: UV-stimulated scaffold protein A homolog [Sesamum indicum]

#ID	0 h-1 FPKM	0 h-2 FPKM	0 h-3 FPKM	48 h-1 FPKM	48 h-2 FPKM	48 h-3 FPKM	FDR	log2FC	regulated	nr_annotation
c41504.graph_c0	0.48058256	1.30574458	0.44111973	6.26166516	7.63854693	8.27296994	1.195E-06	2.298556	up	PREDICTED: uncharacterized protein LOC105130684 isoform X1 [Populus euphratica]
c41505.graph_c0	2.29642184	3.0944326	4.08599028	2.68520185	2.58604161	2.60648341	0.0074928	-1.288572	down	-
c41507.graph_c0	0.722804	0.28311956	0.60575984	2.40300946	3.74186809	5.25529406	0.0015554	1.794244	up	PREDICTED: endoglucanase 6 [Sesamum indicum]
c41508.graph_c0	2.1508574	2.05287111	2.66752051	1.65893871	2.05035181	2.00915475	0.0002089	-1.288815	down	hypothetical protein MIMGU_mgv1a001446mg [Erythranthe guttata]
c41510.graph_c0	1.12753426	1.12866332	0.94250537	7.1603714	7.58554526	8.6595232	4.077E-21	1.853646	up	PREDICTED: 1-phosphatidylinositol-3-phosphate 5-kinase FAB1B-like [Sesamum indicum]
c41513.graph_c0	0	0.25431592	0	3.2768125	2.14895293	3.67932064	2.964E-08	4.159553	up	hypothetical protein MIMGU_mgv1a001330mg [Erythranthe guttata]
c41514.graph_c0	0.25111178	0.4608326	0.26890715	2.58330285	2.49161886	3.01906636	0.0001019	2.028369	up	PREDICTED: shugoshin-1-like isoform X2 [Sesamum indicum]
c41516.graph_c0	16.7274208	17.5464099	13.9429609	2.12034865	3.52982503	4.26186063	1.108E-67	-3.307635	down	PREDICTED: LOW QUALITY PROTEIN: ubiquitin carboxyl-terminal hydrolase 22 [Sesamum indicum]
c41519.graph_c0	0	0	0.06244676	1.52214834	1.70738639	3.94368957	8.086E-05	5.785219	up	PREDICTED: solute carrier family 35 member F1-like isoform X1 [Sesamum indicum]
c41521.graph_c0	77.8841589	79.4215844	77.1180402	52.9474057	51.5930314	43.2569409	1.27E-32	-1.680883	down	PREDICTED: uncharacterized protein LOC105162845 [Sesamum indicum]
c41523.graph_c0	4.16878059	4.62005731	3.63513978	0.41148778	0.29061427	0.61023225	3.482E-13	-4.25881	down	-
c41525.graph_c0	0.30398167	0.30428606	0	5.88100262	5.78519423	5.85584276	4.084E-08	3.847621	up	PREDICTED: LOW QUALITY PROTEIN: LOB domain-containing protein 6 [Sesamum indicum]
c41525.graph_c1	18.0441911	18.0622596	18.057522	6.64939429	6.07798345	5.62861539	2.765E-50	-2.57612	down	PREDICTED: LOB domain-containing protein 36 [Sesamum indicum]
c41527.graph_c0	54.6246814	55.2877734	60.5296586	30.4462607	32.6248585	29.7377117	2.583E-39	-1.896094	down	PREDICTED: phosphoribosylaminoimidazole-succinocarboxamide synthase, chloroplastic [Sesamum indicum]
c41529.graph_c0	28.9502255	26.9455857	27.0858021	16.9851114	15.3169223	17.1533305	2.775E-26	-1.762524	down	PREDICTED: LOB domain-containing protein 36 [Sesamum indicum]
c41530.graph_c0	1.55962074	1.40506422	1.5031308	1.14945948	0.45664235	0.44746718	3.039E-05	-2.119229	down	retrotransposon protein, putative, unclassified [Oryza sativa Japonica Group]
c41532.graph_c0	4.69804527	4.11896004	4.37174682	2.59688744	3.32027784	3.4793221	1.653E-07	-1.511112	down	PREDICTED: uncharacterized protein LOC105160266 isoform X1 [Sesamum indicum]
c41535.graph_c0	260.403141	293.444909	249.876169	57.5357101	65.4373755	71.079119	1.138E-84	-3.069811	down	PREDICTED: adagio protein 1-like [Sesamum indicum]
c41538.graph_c0	3.03051511	3.981534	2.79905105	20.8811466	22.2134706	19.2527291	1.588E-16	1.652193	up	PREDICTED: allantoate deiminase [Sesamum indicum]
c41539.graph_c0	0.80211589	0.94890438	1.26501219	16.5762495	16.9200208	16.3334809	1.585E-52	3.024282	up	PREDICTED: poly [ADP-ribose] polymerase 1 [Sesamum indicum]
c41543.graph_c0	155.144725	159.08041	169.199722	7.61663464	3.28732997	4.76916587	2.93E-245	-5.951387	down	hypothetical protein MIMGU_mgv1a008932mg [Erythranthe guttata]

#ID	0 h-1 FPKM	0 h-2 FPKM	0 h-3 FPKM	48 h-1 FPKM	48 h-2 FPKM	48 h-3 FPKM	FDR	log2FC	regulated	nr_annotation
c41543.graph_c1	99.0368586	94.355885	93.6487752	4.3037365	3.24215962	2.63805788	9.77E-199	-5.826448	down	PREDICTED: gibberellin 2-beta-dioxygenase 8 [Sesamum indicum]
c41545.graph_c0	2.96274525	2.04775352	2.49283895	57.6114012	73.9754086	84.5697485	2.331E-35	3.826062	up	PREDICTED: 60S ribosomal protein L5-like [Nicotiana sylvestris]
c41551.graph_c0	34.8039768	34.1373748	29.7162706	17.0002133	13.2222854	9.76532997	1.198E-14	-2.311177	down	-
c41551.graph_c1	41.1516742	39.9078984	38.7656497	15.5092935	15.1415715	12.7477827	4.687E-67	-2.480315	down	PREDICTED: uncharacterized protein LOC105172898 isoform X1 [Sesamum indicum]
c41555.graph_c0	0	0	0	2.41866295	3.07473615	4.80086468	1.013E-11	Inf	up	PREDICTED: protein IQ-DOMAIN 1-like [Sesamum indicum]
c41556.graph_c0	4.00111236	4.14564936	4.41996144	3.427413	3.01435572	2.41673664	0.0001653	-1.518567	down	transcription factor, putative [Ricinus communis]
c41564.graph_c0	0.70928964	0.75018856	0.46433136	0	0	0.01097039	7.028E-26	-8.490664	down	Strong similarity to gi|3600044 T12H20.12 protease homolog from Arabidopsis thaliana BAC gb|AF080119 and is a member of the reverse transcriptase family PF|00078 [Arabidopsis thaliana]
c41565.graph_c0	3.51057913	4.56832278	3.30823766	2.31782504	2.11283301	3.02163645	3.369E-05	-1.628635	down	PREDICTED: uncharacterized protein LOC105165462 [Sesamum indicum]
c41567.graph_c0	3.7865713	6.06458077	4.91711398	61.1027187	61.3076515	72.6006874	5.55E-58	2.703446	up	chloroplast omega-3 desaturase [Portulaca oleracea]
c41569.graph_c0	1.17694282	0.78541424	0.94106017	0.722851	0.51051532	0.85758463	0.0012927	-1.487867	down	PREDICTED: uncharacterized protein LOC105179857 isoform X2 [Sesamum indicum]
c41570.graph_c0	31.9958752	32.5131857	28.8746219	22.2191695	23.5385159	22.313822	3.187E-25	-1.473082	down	PREDICTED: RNA polymerase-associated protein LEO1 [Sesamum indicum]
c41572.graph_c0	4.56175876	4.76272782	4.22319055	39.0434141	46.8192178	50.98504	1.536E-35	2.31601	up	PREDICTED: ER lumen protein-retaining receptor-like [Sesamum indicum]
c41575.graph_c0	3.22592004	2.95432901	2.55782158	18.2320403	20.8106829	20.2362518	1.096E-21	1.744559	up	PREDICTED: glycosylphosphatidylinositol anchor attachment 1 protein [Sesamum indicum]
c41577.graph_c0	7.76203014	5.06346689	6.94853927	51.9042015	52.0921969	59.6960971	3.472E-22	2.030506	up	PREDICTED: ergosterol biosynthetic protein 28 [Sesamum indicum]
c41581.graph_c0	15.1052992	15.7949419	14.0350242	9.7256711	11.3992511	14.0394804	4.201E-17	-1.374712	down	PREDICTED: trehalose-phosphate phosphatase A-like isoform X2 [Sesamum indicum]
c41582.graph_c0	5.75070916	7.40431141	5.35907977	40.6646334	30.8688199	26.7730093	0.0001233	1.399738	up	PREDICTED: lysine histidine transporter-like 8 [Sesamum indicum]
c41586.graph_c0	0.03510107	0.21081731	0.09021253	8.21369835	8.3930995	10.7613338	1.963E-30	5.328229	up	PREDICTED: protein Brevis radix-like 4 [Sesamum indicum]
c41597.graph_c0	31.5110882	27.7158806	34.7495647	13.233154	13.4976706	13.4108072	1.522E-27	-2.246548	down	PREDICTED: ninja-family protein AFP1-like [Sesamum indicum]
c41598.graph_c0	4.86810013	4.00575047	3.55196605	65.1817922	46.5045464	43.3550474	4.561E-09	2.633961	up	hypothetical protein MIMGU_mgv1a006127mg [Erythranthe guttata]
c41604.graph_c0	13.4411416	13.8476272	13.1519144	8.37982977	8.41757629	7.35976545	5.269E-33	-1.75949	down	Calcium-transporting ATPase 12, plasma membrane-type [Morus notabilis]
c41605.graph_c1	25.130711	24.4762786	23.2095517	17.2051899	19.5204639	20.5652195	3.134E-22	-1.365394	down	PREDICTED: WD repeat-containing protein LWD1 [Sesamum indicum]

#ID	0 h-1 FPKM	0 h-2 FPKM	0 h-3 FPKM	48 h-1 FPKM	48 h-2 FPKM	48 h-3 FPKM	FDR	log2FC	regulated	nr_annotation
c41610.graph_c0	1.06087295	1.00293885	0.83310562	11.3480656	10.6989153	7.53684555	7.247E-09	2.340913	up	PREDICTED: uncharacterized protein LOC105179150 [Sesamum indicum]
c41615.graph_c0	2.68720295	2.07855428	1.80506205	11.0559235	11.7422129	11.4144971	8.108E-08	1.365353	up	hypothetical protein MIMGU_mgv1a003382mg [Erythranthe guttata]
c41630.graph_c0	70.7283071	67.3570377	70.6141754	451.582251	458.721178	465.928734	1.426E-33	1.703217	up	unknown [Lotus japonicus]
c41631.graph_c0	8.71157875	6.31074493	7.43859924	5.49125102	8.11067901	9.06744088	0.0003333	-1.011202	down	hypothetical protein VITISV_026847 [Vitis vinifera]
c41639.graph_c0	6.0705277	5.55016774	5.52239696	41.3628401	41.6631638	43.9115491	1.333E-38	1.871151	up	PREDICTED: alpha-1,4 glucan phosphorylase L-1 isozyme, chloroplastic/amyloplastic [Sesamum indicum]
c41640.graph_c0	1186.59981	1029.49954	1151.16288	79.5249507	77.3893523	71.1107243	1.93E-130	-4.900738	down	PREDICTED: ubiquitin carboxyl-terminal hydrolase isozyme L3 [Sesamum indicum]
c41642.graph_c0	0.19054544	0.25431499	0.24485844	3.62788637	4.02927211	5.2065669	2.708E-08	3.198285	up	PREDICTED: protein polybromo-1-like [Sesamum indicum]
c41645.graph_c0	0	0.06579297	0	2.1496011	2.46968468	2.55924934	1.608E-13	5.761004	up	PREDICTED: MATE efflux family protein 5-like isoform X3 [Sesamum indicum]
c41648.graph_c0	3.74827025	2.98237771	3.21111711	1.06250801	0.65659907	0.70905993	2.238E-09	-3.040974	down	hypothetical protein VITISV_011460 [Vitis vinifera]
c41653.graph_c0	18.8351338	19.0782242	18.9025445	3.66304139	4.20848183	4.08566684	1.225E-93	-3.268238	down	hypothetical protein MIMGU_mgv1a026893mg [Erythranthe guttata]
c41653.graph_c1	9.52065653	9.3408485	10.0468089	4.47265452	2.70756293	2.84267221	1.895E-17	-2.536365	down	PREDICTED: uncharacterized protein LOC105160086 [Sesamum indicum]
c41654.graph_c0	7.4687153	7.35998382	6.98685015	53.9936332	59.6357464	60.3514079	6.158E-43	1.976767	up	PREDICTED: coatomer subunit beta-1 [Sesamum indicum]
c41654.graph_c1	0.19815456	0.06611766	0	2.31233683	1.41821292	1.89506893	6.226E-07	3.420658	up	PREDICTED: coatomer subunit beta-1 [Sesamum indicum]
c41657.graph_c0	16.1589179	15.4092761	14.6575406	4.01533721	4.50265327	4.10494546	1.036E-65	-2.8907	down	PREDICTED: cysteine synthase 2 [Sesamum indicum]
c41660.graph_c0	27.2929906	29.6002339	27.929079	15.7906264	16.276139	15.2845616	1.426E-32	-1.858491	down	hypothetical protein MIMGU_mgv1a007698mg [Erythranthe guttata]
c41662.graph_c0	287.467525	307.739525	280.128695	64.2303498	64.2428227	54.3596515	1.244E-81	-3.274926	down	PREDICTED: uncharacterized protein LOC105179373 [Sesamum indicum]
c41664.graph_c0	21.2811381	21.1184095	21.972188	8.72299769	12.4035193	10.9831411	1.634E-40	-2.026869	down	PREDICTED: probable inactive purple acid phosphatase 27 [Sesamum indicum]
c41665.graph_c0	1.03419893	1.03523452	1.52833455	33.3470027	39.1626202	42.0070212	1.948E-61	3.966864	up	NAD(P)-binding Rossmann-fold superfamily protein [Theobroma cacao]
c41667.graph_c0	2.87274291	2.95549785	2.51233098	1.58058334	1.28503258	1.36278445	2.988E-16	-1.992278	down	PREDICTED: phospholipase A I-like [Sesamum indicum]
c41668.graph_c0	51.5986078	57.7922768	57.4070139	37.3874961	39.2573196	37.7969164	7.619E-28	-1.562265	down	PREDICTED: NASP-related protein sim3 isoform X1 [Sesamum indicum]
c41676.graph_c0	5.00707781	6.00433313	5.61775855	3.13767235	3.07592493	3.12524103	8.264E-15	-1.850059	down	PREDICTED: uncharacterized protein LOC105161819 isoform X2 [Sesamum indicum]
c41679.graph_c0	2.24829945	1.45623874	1.78447735	0.18276001	0.38722414	0.32523751	4.861E-11	-3.644284	down	hypothetical protein MIMGU_mgv1a009883mg [Erythranthe guttata]

#ID	0 h-1 FPKM	0 h-2 FPKM	0 h-3 FPKM	48 h-1 FPKM	48 h-2 FPKM	48 h-3 FPKM	FDR	log2FC	regulated	nr_annotation
c41681.graph_c0	4.66483403	5.37312921	4.92698335	2.29595103	2.59859158	2.88104585	3.1E-17	-1.965115	down	PREDICTED: uncharacterized protein LOC105169002 isoform X1 [Sesamum indicum]
c41686.graph_c0	2.0055034	2.14134572	1.34011866	29.7586861	36.5103324	46.0534677	2.108E-20	3.336864	up	PREDICTED: BTB/POZ domain-containing protein NPY4-like [Sesamum indicum]
c41688.graph_c0	19.395995	17.6853305	17.8503065	146.017448	160.123094	160.15043	1.381E-44	2.066905	up	PREDICTED: 60S ribosomal protein L18a-2-like [Sesamum indicum]
c41690.graph_c0	14.8190637	15.8464558	13.8436068	87.2721347	70.7845096	63.5578859	1.575E-06	1.304535	up	PREDICTED: uncharacterized protein LOC105167598 [Sesamum indicum]
c41690.graph_c1	35.2247556	35.4659258	32.7759736	185.896449	153.924278	147.62085	1.209E-08	1.223567	up	PREDICTED: uncharacterized protein LOC105167598 [Sesamum indicum]
c41692.graph_c0	34.778187	33.7123665	31.5037365	14.0315403	14.6659894	13.6201965	1.231E-47	-2.257345	down	PREDICTED: protein kinase 2B, chloroplastic-like isoform X2 [Sesamum indicum]
c41697.graph_c0	1.40906561	1.07859974	1.27814478	0.11454028	0.08089436	0.2038345	2.855E-13	-4.257088	down	-
c41699.graph_c0	12.2198912	11.5591627	10.5703338	7.03154642	8.91572724	7.26157828	2.738E-15	-1.584741	down	PREDICTED: box C/D snoRNA protein 1 [Sesamum indicum]
c41702.graph_c0	7.15259152	7.41677056	6.92887386	29.0610928	33.471591	34.2773949	1.699E-11	1.151161	up	hypothetical protein MIMGU_mgv1a006372mg [Erythranthe guttata]
c41702.graph_c1	1.8885449	1.30876338	1.96015209	1.80676808	0.85068964	1.19085287	0.0044447	-1.429262	down	-
c41704.graph_c0	1.63627444	1.55801473	0.9744115	5.95623718	7.01101229	7.099147	0.0007309	1.25153	up	PREDICTED: GTP-binding protein At3g49725, chloroplastic [Sesamum indicum]
c41705.graph_c0	2.503111	2.21329545	1.60829837	21.0232988	34.3439211	51.7589465	3.178E-05	3.057802	up	PREDICTED: putative phospholipid:diacylglycerol acyltransferase 2 [Sesamum indicum]
c41709.graph_c0	33.9643868	32.2742311	32.3710495	13.5376021	14.8142429	19.7231413	1.922E-35	-2.057455	down	PREDICTED: B-box zinc finger protein 20 [Sesamum indicum]
c41713.graph_c0	3.26564156	3.6899078	2.35257139	14.9286509	13.3522867	11.742141	5.028E-06	1.093835	up	PREDICTED: poly(A)-specific ribonuclease PARN [Sesamum indicum]
c41721.graph_c1	0	0	0	2.42536053	4.69575503	6.25096491	1.123E-07	Inf	up	PREDICTED: geraniol synthase, chloroplastic-like [Sesamum indicum]
c41722.graph_c0	1.39038517	1.06281186	1.03953454	9.82810003	10.4856969	10.7343646	4.816E-16	2.13545	up	PREDICTED: uncharacterized protein LOC105159385 [Sesamum indicum]
c41723.graph_c0	0.62676782	0.84882912	0.94755467	6.31764753	7.79025374	8.17521502	9.373E-17	2.176054	up	PREDICTED: katanin p80 WD40 repeat-containing subunit B1 homolog [Sesamum indicum]
c41725.graph_c0	0	0	0	1.30368377	1.7820579	1.42194771	1.429E-11	Inf	up	similar to AHNAK nucleoprotein [Ectocarpus siliculosus]
c41728.graph_c0	18.5340737	20.789308	16.0190606	73.1962768	78.3689661	84.5836548	2.978E-14	1.075902	up	PREDICTED: transketolase, chloroplastic [Sesamum indicum]
c41730.graph_c0	2.08370984	1.92738144	2.03365795	6.97391622	11.4038057	11.4809762	3.985E-05	1.278231	up	PREDICTED: vacuolar amino acid transporter 1-like isoform X1 [Sesamum indicum]
c41730.graph_c1	26.2679998	26.5593074	28.1988165	3.25193211	4.65078746	4.82258272	1.57E-108	-3.695569	down	PREDICTED: glucan endo-1,3-beta-glucosidase 5 [Sesamum indicum]
c41733.graph_c0	230.359054	237.078221	228.966949	35.0219516	39.7250382	35.5381746	6.48E-110	-3.677521	down	PREDICTED: protein NLP5-like [Sesamum indicum]

#ID	0 h-1 FPKM	0 h-2 FPKM	0 h-3 FPKM	48 h-1 FPKM	48 h-2 FPKM	48 h-3 FPKM	FDR	log2FC	regulated	nr_annotation
c41734.graph_c0	13.216879	12.8711572	14.4168318	9.67492815	8.349522	7.18090968	1.702E-15	-1.698715	down	hypothetical protein M569_06103, partial [Genlisea aurea]
c41738.graph_c0	8.03273279	7.20404582	7.15230875	1.99093757	1.79079363	1.88851173	4.909E-48	-2.996216	down	hypothetical protein MIMGU_mgv1a0006002mg, partial [Erythranthe guttata]
c41741.graph_c0	0.62758698	0.80770553	0.63365824	4.04721843	6.343805	7.45960914	2.123E-06	2.083536	up	hypothetical protein MIMGU_mgv1a006910mg [Erythranthe guttata]
c41743.graph_c0	9.7521942	9.21004664	9.96356898	4.6350395	5.21294973	3.92649321	2E-22	-2.088499	down	1-O-acylglucose:anthocyanin-O-acyltransferase- like protein [Gentiana triflora]
c41744.graph_c0	38.6832684	41.8368653	33.7146664	2.30463651	1.31006401	1.30041328	1.21E-153	-5.542522	down	hypothetical protein MIMGU_mgv1a021012mg [Erythranthe guttata]
c41745.graph_c1	1.69524092	1.08604061	1.13279488	10.4326003	10.1917338	11.1727896	5.244E-07	2.007364	up	hypothetical protein MIMGU_mgv1a015492mg [Erythranthe guttata]
c41751.graph_c0	0.69692502	0.41857373	0.53734575	2.6966217	3.99037217	3.19925037	0.0014577	1.558497	up	PREDICTED: protein DA1-related 2 isoform X2 [Sesamum indicum]
c41751.graph_c1	1.30680877	0.96387594	0.26515279	3.61175243	5.43725112	6.03276435	0.0023636	1.558817	up	hypothetical protein MIMGU_mgv1a005034mg [Erythranthe guttata]
c41753.graph_c2	5.67388346	3.27142944	4.60801621	34.3737484	32.2062932	35.4598949	5.208E-23	1.89601	up	PREDICTED: omega-amidase,chloroplastic-like isoform X2 [Nicotiana sylvestris]
c41755.graph_c0	84.0368231	91.9837531	102.920662	16.9511583	15.8223762	10.7904001	5.578E-58	-3.693865	down	PREDICTED: protein PHLOEM PROTEIN 2-LIKE A10 [Sesamum indicum]
c41756.graph_c0	90.7807759	91.4953694	98.8821281	65.2648706	61.0525748	59.3491647	1.9E-29	-1.615464	down	PREDICTED: protein DEHYDRATION-INDUCED 19 homolog 4-like [Sesamum indicum]
c41757.graph_c0	36.5353333	34.9573465	36.8026987	28.7688854	26.5050492	24.1685618	3.568E-25	-1.462312	down	PREDICTED: uncharacterized protein LOC105168956 [Sesamum indicum]
c41762.graph_c1	1.66857317	1.33282097	1.12623732	90.0115085	115.210519	141.918567	1.128E-37	5.373985	up	PREDICTED: lanC-like protein GCR2 [Sesamum indicum]
c41766.graph_c0	0.13032058	0.04348369	0.19537829	0.60029909	0.67834037	0.78340903	0.004884	1.452371	up	PREDICTED: uncharacterized protein LOC102666339 [Glycine max]
c41767.graph_c0	12.6033443	12.4176004	11.3064994	9.38375114	11.991025	12.5731411	3.144E-09	-1.118917	down	PREDICTED: uncharacterized protein LOC105162543 [Sesamum indicum]
c41768.graph_c0	29.5767065	30.7752522	24.1738145	25.1415605	27.8220184	27.9212799	7.823E-15	-1.080631	down	PREDICTED: ATP-dependent helicase BRM isoform X1 [Sesamum indicum]
c41770.graph_c0	17.0092243	17.6703419	15.4584482	15.2318653	15.4537291	14.2182702	3.989E-14	-1.174958	down	PREDICTED: nuclear pore complex protein NUP1 [Sesamum indicum]
c41771.graph_c0	0.44417966	0.36378363	0.41511887	16.591495	14.5815483	15.0609208	2.078E-35	4.225247	up	PREDICTED: uncharacterized protein LOC105163485 [Sesamum indicum]
c41771.graph_c1	0.22919949	0.34414349	0.34361882	9.53710054	8.87309808	8.64262399	3.417E-21	3.864356	up	PREDICTED: uncharacterized protein LOC105163485 [Sesamum indicum]
c41774.graph_c0	13.1246353	12.1633644	12.8541469	5.28799791	2.12941238	1.23821809	1.26E-53	-3.133702	down	hypothetical protein POPTR_0018s00310g [Populus trichocarpa]

#ID	0 h-1 FPKM	0 h-2 FPKM	0 h-3 FPKM	48 h-1 FPKM	48 h-2 FPKM	48 h-3 FPKM	FDR	log2FC	regulated	nr_annotation
c41776.graph_c0	0.22895205	0.08333866	0.24071927	1.03545275	1.82822772	1.211391	0.0002133	1.852453	up	unnamed protein product [Coffea canephora]
c41777.graph_c0	0.01365239	0.01366606	0.03508773	2.13817086	2.78478086	2.89856281	1.087E-25	5.935822	up	PREDICTED: serine/threonine-protein kinase ULK4 [Sesamum indicum]
c41779.graph_c0	2.00547986	2.0271693	2.22339853	13.6575927	12.856681	12.4103207	1.211E-16	1.620307	up	gtpase-activating protein [Gossypium arboreum]
c41780.graph_c0	45.7214835	52.4199517	48.87585	10.2168886	9.72947686	8.72991451	1.56E-104	-3.373843	down	PREDICTED: E3 ubiquitin-protein ligase PUB23-like [Sesamum indicum]
c41780.graph_c1	10.6371096	11.942759	12.3760833	3.44310241	10.5217774	17.7927471	0.0066859	-1.175468	down	PREDICTED: E3 ubiquitin-protein ligase PUB23-like [Sesamum indicum]
c41781.graph_c0	0.83671451	0.3448745	0.8854681	5.16913539	6.24464368	7.06058313	5.8E-07	2.134496	up	PREDICTED: actin-11 [Sesamum indicum]
c41782.graph_c0	61.3542228	65.5060849	60.933056	30.9533517	28.5912592	28.8690467	1.202E-48	-2.102406	down	PREDICTED: protein NLP7 isoform X1 [Sesamum indicum]
c41785.graph_c0	4.02981264	2.72792591	3.53924126	15.4644079	17.090034	16.7308058	6.299E-10	1.239859	up	PREDICTED: heterogeneous nuclear ribonucleoprotein 1 [Sesamum indicum]
c41786.graph_c0	19.7581168	21.6576195	16.524459	7.10798773	11.3946805	9.13559795	1.59E-26	-2.09024	down	PREDICTED: transmembrane protein 45B-like [Sesamum indicum]
c41791.graph_c0	3.84125074	4.14471513	4.64768244	3.17114208	3.74894086	3.88489855	1.438E-06	-1.248211	down	PREDICTED: probable beta-1,3-galactosyltransferase 16 [Sesamum indicum]
c41795.graph_c0	0.2326994	0.31057656	0.24918996	1.78648223	1.28694169	1.27168092	0.0080098	1.444777	up	PREDICTED: myosin-11-like isoform X1 [Sesamum indicum]
c41797.graph_c0	0.90058274	0.73967962	0.77152298	27.6133044	30.4924794	32.9368759	1.02E-98	4.219466	up	PREDICTED: probable acyl-activating enzyme 17, peroxisomal isoform X1 [Sesamum indicum]
c41799.graph_c0	0	0	0	4.37788642	5.96293691	5.23098856	5.261E-12	Inf	up	NOX2 [Striga asiatica]
c41799.graph_c1	0	0	0	2.99068164	4.03798424	4.45254365	5.975E-29	Inf	up	PREDICTED: respiratory burst oxidase homolog protein A [Sesamum indicum]
c41800.graph_c0	0.42404402	0.12127675	0.31137906	84.8283799	73.1929892	76.5722529	4.04E-109	7.082491	up	PREDICTED: acyl-CoA-binding domain-containing protein 3-like [Sesamum indicum]
c41802.graph_c0	156.84102	162.890867	151.74885	118.919612	101.553322	89.898719	3.303E-29	-1.615987	down	PREDICTED: uncharacterized protein LOC105170559 [Sesamum indicum]
c41805.graph_c0	2.68317814	2.24735637	3.02578726	14.9828542	16.9948165	16.0249169	2.834E-08	1.571505	up	hypothetical protein MIMGU_mgv1a014951mg [Erythranthe guttata]
c41811.graph_c0	3.47575943	3.17669728	3.49551272	16.2193138	17.3298847	16.7855399	4.107E-05	1.29151	up	PREDICTED: zinc finger SWIM domain-containing protein 7 isoform X2 [Vitis vinifera]
c41815.graph_c0	2.04901019	2.58824486	2.38229168	10.6968637	14.0460037	16.2365968	1.406E-07	1.521361	up	PREDICTED: calcineurin B-like protein 10 isoform X3 [Sesamum indicum]
c41816.graph_c0	0	0.02992607	0.03841771	1.12923288	1.45888453	1.22534711	4.145E-10	4.767849	up	--
c41819.graph_c0	13.6183594	13.1737778	13.4314731	13.3895577	13.6633934	11.0696524	9.901E-10	-1.093621	down	PREDICTED: deoxyhypusine synthase [Sesamum indicum]
c41820.graph_c0	1.08798645	1.01128477	0.99864691	6.12133638	6.29519921	6.97563449	1.305E-05	1.628008	up	PREDICTED: uncharacterized protein LOC105162931 [Sesamum indicum]

#ID	0 h-1 FPKM	0 h-2 FPKM	0 h-3 FPKM	48 h-1 FPKM	48 h-2 FPKM	48 h-3 FPKM	FDR	log2FC	regulated	nr_annotation
c41824.graph_c0	1.03872036	0.86646707	0.62290517	4.78468475	6.96114282	9.47977441	5.164E-05	2.048121	up	PREDICTED: probable protein phosphatase 2C 53 [Sesamum indicum]
c41826.graph_c0	0.53388599	0.41566046	0.22868821	11.0939634	10.3053311	8.65565942	3.151E-15	3.665638	up	hypothetical protein MIMGU_mgv1a008654mg [Erythranthe guttata]
c41827.graph_c0	0.43734405	1.24038228	0.65567182	3.82764369	4.69517558	4.54108578	0.0040209	1.465012	up	PREDICTED: agamous-like MADS-box protein AGL15 [Sesamum indicum]
c41836.graph_c0	6.96168487	5.72978378	6.16282803	5.04533244	4.52959147	4.43857983	8.573E-10	-1.442569	down	PREDICTED: putative protease Do-like 14 isoform X1 [Sesamum indicum]
c41839.graph_c0	13.4470995	13.961423	12.7390175	8.29731027	7.84384893	7.17781632	2.557E-24	-1.798697	down	PREDICTED: putative F-box protein At5g62060 [Sesamum indicum]
c41840.graph_c0	0.98608859	0.82914385	0.55755167	3.37943077	5.27390651	5.78766123	0.000196	1.584776	up	PREDICTED: probable glycosyltransferase At3g07620 [Sesamum indicum]
c41841.graph_c0	4.396981	3.37852708	4.57594286	16.3172494	17.1350445	17.4887839	1.66E-07	1.024204	up	hypothetical protein MIMGU_mgv1a007444mg [Erythranthe guttata]
c41843.graph_c0	13.0686798	12.9281641	11.3711247	9.64826895	9.3725268	8.37532647	6.566E-24	-1.462365	down	PREDICTED: uncharacterized protein LOC105155405 [Sesamum indicum]
c41844.graph_c0	25.3759477	27.855275	25.542374	105.963891	123.701339	110.688327	1.703E-13	1.091989	up	PREDICTED: ADP-ribosylation factor-like protein 8A [Nicotiana tomentosiformis]
c41846.graph_c2	3.29210692	3.12196118	2.95020855	14.2067591	17.0795977	15.9433957	2.015E-07	1.315045	up	PREDICTED: BRCA1-A complex subunit BRE [Sesamum indicum]
c41847.graph_c0	19.6602845	17.4562457	20.8870115	271.139289	242.323068	314.342714	2.687E-36	2.816529	up	hypothetical protein MIMGU_mgv1a012525mg [Erythranthe guttata]
c41848.graph_c0	35.7417674	36.5305289	33.8320612	7.09792398	8.96542629	8.25897933	6.25E-98	-3.145666	down	PREDICTED: LOW QUALITY PROTEIN: F-box protein SKIP14-like [Sesamum indicum]
c41849.graph_c0	3.63086695	2.95606221	3.48380315	4.10318952	1.60643783	1.9048661	0.0002022	-1.403003	down	PREDICTED: uncharacterized protein LOC105177931 [Sesamum indicum]
c41851.graph_c0	1.36077476	1.2054313	0.71183959	6.54552507	7.14574169	6.46581457	1.282E-13	1.60797	up	hypothetical protein MIMGU_mgv1a000152mg [Erythranthe guttata]
c41852.graph_c0	67.6675866	76.6758049	67.6320772	62.8976449	67.1741551	65.3970148	7.502E-16	-1.134934	down	PREDICTED: PHD finger protein ALFIN-LIKE 4-like [Sesamum indicum]
c41853.graph_c0	1.08130329	1.04075582	1.22918859	8.92718996	9.90375609	12.3411858	2.504E-11	2.194837	up	PREDICTED: homogentisate solanesyltransferase, chloroplastic [Sesamum indicum]
c41856.graph_c0	40.1971167	40.5042826	44.1165282	31.6892507	29.8625032	32.4592275	4.612E-19	-1.426851	down	PREDICTED: uncharacterized protein LOC105160829 [Sesamum indicum]
c41857.graph_c0	21.0082901	17.8420694	19.5724513	4.71758799	6.47139687	10.0905803	5.883E-47	-2.483086	down	PREDICTED: monoglyceride lipase-like [Sesamum indicum]
c41859.graph_c0	10.1745242	11.2696818	10.8281602	5.28262336	4.8114521	5.61760679	2.685E-23	-2.055594	down	PREDICTED: probable microtubule-binding protein TANGLED [Sesamum indicum]

#ID	0 h-1 FPKM	0 h-2 FPKM	0 h-3 FPKM	48 h-1 FPKM	48 h-2 FPKM	48 h-3 FPKM	FDR	log2FC	regulated	nr_annotation
c41862.graph_c0	11.1015496	11.4808569	10.6349936	10.2120688	10.1494344	8.20949594	7.841E-16	-1.2325	down	PREDICTED: G patch domain-containing protein 1 [Sesamum indicum]
c41876.graph_c1	30.6039797	26.5602583	29.5078302	151.124719	133.752548	130.661146	1.829E-15	1.246405	up	PREDICTED: phospho-2-dehydro-3-deoxyheptonate aldolase 1, chloroplastic [Sesamum indicum]
c41879.graph_c0	3.7606396	1.5724731	1.65163811	0.08771004	0.78980427	1.24870182	0.0035405	-2.751519	down	PREDICTED: heat shock cognate 70 kDa protein 2-like [Elaeis guineensis]
c41882.graph_c0	0.5052771	0.39338682	0.64930108	3.87911852	4.8765541	4.92430597	5.374E-08	2.117987	up	hypothetical protein MIMGU_mgv1a004014mg [Erythranthe guttata]
c41887.graph_c0	0.88975544	0.68822676	0.46774267	0.42847994	0.25656491	0.14918825	2.95E-06	-2.290959	down	hypothetical protein VITISV_000584 [Vitis vinifera]
c41892.graph_c0	0.09631117	0.04820381	0	14.3295939	14.3344969	16.6978745	1.016E-42	7.29358	up	PREDICTED: tetraspanin-10 [Nicotiana sylvestris]
c41894.graph_c0	0.07892394	0.39501485	0.20284077	6.25304969	5.70002031	5.82271327	6.264E-10	3.698754	up	PREDICTED: pleckstrin homology domain-containing family A member 8-like [Sesamum indicum]
c41896.graph_c0	1.39901912	1.56517532	1.00465012	5.79990449	6.42540709	5.66667309	0.0029284	1.157812	up	PREDICTED: eukaryotic translation initiation factor 1A-like [Cucumis sativus]
c41898.graph_c0	0	0.10765532	0.02303383	2.03113779	2.50162131	2.80644871	3.62E-17	4.793112	up	PREDICTED: kinesin-3-like [Sesamum indicum]
c41899.graph_c0	40.9931655	47.5692925	42.5324788	245.546192	240.233525	190.171394	2.207E-10	1.351273	up	hypothetical protein AALP_AA7G017200 [Arabis alpina]
c41900.graph_c2	14.246858	15.5889754	14.6769047	4.91275996	6.18840378	6.56789039	2.655E-55	-2.355083	down	PREDICTED: LRR receptor-like serine/threonine-protein kinase RPK2 [Sesamum indicum]
c41900.graph_c3	53.1642155	52.1606176	52.4294853	56.3920992	44.4905974	34.1567554	3.632E-15	-1.234738	down	PREDICTED: heat stress transcription factor A-4b-like [Sesamum indicum]
c41909.graph_c0	1.24568401	0.73348904	0.28248587	1537.85868	1369.50518	1605.15998	1.91E-284	9.957254	up	hypothetical protein MIMGU_mgv1a015091mg [Erythranthe guttata]
c41910.graph_c0	101.022111	101.512477	95.7505618	64.2163778	75.2739827	75.0171242	5.662E-26	-1.495455	down	hypothetical protein MIMGU_mgv1a007193mg [Erythranthe guttata]
c41912.graph_c0	1.33830157	0.79386173	0.89173285	0.913282	0.72563365	0.85326447	0.0087936	-1.290478	down	hypothetical protein MIMGU_mgv1a009757mg [Erythranthe guttata]
c41915.graph_c0	13.7134237	13.37176	13.0598866	8.76999587	10.5251821	10.5683624	1.197E-19	-1.447276	down	PREDICTED: zinc finger BED domain-containing protein RICESLEEPER 1-like [Sesamum indicum]
c41918.graph_c0	18.5711946	15.2824048	17.4227099	15.324622	15.6786001	16.3442287	2.04E-05	-1.133195	down	PREDICTED: uncharacterized protein At4g28440 [Vitis vinifera]
c41920.graph_c0	3.86166378	4.26541313	4.27792314	1.10408789	0.90972564	0.98241077	6E-12	-3.064632	down	-
c41924.graph_c0	8.26105437	8.10459895	7.44373554	3.82047166	5.30007038	5.28800644	4.918E-15	-1.74694	down	PREDICTED: ABC transporter C family member 5-like [Sesamum indicum]
c41925.graph_c0	68.3496947	72.5522755	68.8982057	61.4599766	63.2574246	63.1047511	5.507E-17	-1.177453	down	hypothetical protein MIMGU_mgv1a014620mg [Erythranthe guttata]
c41925.graph_c1	2.53734641	2.53988718	2.71715883	0.38959484	0.68788081	0.92442438	4.747E-08	-2.990703	down	-
c41927.graph_c0	1.11476237	1.56920433	1.611578	7.70243853	6.18784807	5.85410256	0.0003592	1.185408	up	hypothetical protein MIMGU_mgv1a006603mg [Erythranthe guttata]

#ID	0 h-1 FPKM	0 h-2 FPKM	0 h-3 FPKM	48 h-1 FPKM	48 h-2 FPKM	48 h-3 FPKM	FDR	log2FC	regulated	nr_annotation
c41932.graph_c0	3.35247379	3.29919229	3.07199325	24.7732987	24.90517	25.1391429	6.743E-36	1.9271	up	PREDICTED: kinesin-like calmodulin-binding protein homolog [Sesamum indicum]
c41936.graph_c0	2.1474924	3.15512087	1.86941509	1.18066407	2.73817309	2.41342061	0.003392	-1.207348	down	PREDICTED: cyclic nucleotide-gated ion channel 4 [Sesamum indicum]
c41938.graph_c0	0	0.06870519	0	1.16979913	1.27275386	1.18153972	2.287E-09	4.71427	up	PREDICTED: uncharacterized protein LOC104906987 [Beta vulgaris subsp. vulgaris]
c41939.graph_c0	15.1215216	14.5021926	13.7884003	11.4700788	11.8860415	10.4657964	3.156E-13	-1.37645	down	PREDICTED: phosphatidylinositol/phosphatidylcholine transfer protein SFH8-like isoform X2 [Sesamum indicum]
c41941.graph_c0	0	0	0	25.1304543	42.3843489	21.1371948	1.997E-14	Inf	up	60S ribosomal protein L35 [Galdieria sulphuraria]
c41943.graph_c0	19.9312612	20.1256182	20.1496624	16.3716936	18.5340866	16.938213	9.456E-15	-1.234951	down	PREDICTED: uncharacterized protein LOC105176972 [Sesamum indicum]
c41944.graph_c0	24.0428783	9.7337457	15.2420516	0	0	0	3.545E-15	-Inf	down	--
c41954.graph_c0	49.0201818	50.2668909	48.1735337	19.8706591	20.7262002	22.421098	8.677E-54	-2.245039	down	PREDICTED: pheophorbide a oxygenase, chloroplastic-like [Citrus sinensis]
c41957.graph_c0	0.18666452	0.18685144	0.3198284	1.31841775	1.76106123	1.63216721	0.0013176	1.736051	up	PREDICTED: RING-H2 finger protein ATL39-like [Sesamum indicum]
c41961.graph_c0	141.998008	118.799433	141.841033	123.435631	106.10572	95.2747706	9.461E-15	-1.324057	down	PREDICTED: transcription factor HY5 [Sesamum indicum]
c41963.graph_c0	6.52687248	5.02569858	4.70978567	4.44035494	4.01801506	4.77412635	2.184E-05	-1.311746	down	PREDICTED: lachrymatory-factor synthase-like [Sesamum indicum]
c41966.graph_c0	0.34811046	0.34845904	0.33550179	2.16473891	2.71795957	1.71215259	0.0030432	1.657455	up	PREDICTED: uncharacterized protein LOC105157153 [Sesamum indicum]
c41968.graph_c0	5.08219914	5.45952879	6.05296171	0.05709821	0.12097726	0	1.048E-36	-7.566229	down	-
c41974.graph_c0	0.41854669	0.37706922	0.32270944	1.92796253	2.45093103	2.98495168	0.0009983	1.698252	up	unnamed protein product [Coffea canephora]
c41976.graph_c0	13.7249927	12.3799601	14.0192803	64.7039153	69.0856844	65.4034335	8.758E-17	1.292726	up	hypothetical protein JCGZ_26392 [Jatropha curcas]
c41981.graph_c1	63.2914967	67.4521272	57.6906595	38.8320542	39.6504304	39.5676422	4.862E-33	-1.69123	down	PREDICTED: homeobox-leucine zipper protein ANTHOCYANINLESS 2-like [Sesamum indicum]
c41985.graph_c0	0.06753857	0.1014093	0.08678973	4.60434912	4.97662289	5.86856417	5.055E-19	4.895666	up	PREDICTED: probable GABA transporter 2 [Sesamum indicum]
c41988.graph_c0	13.3213072	16.58585	17.6023197	6.41810474	7.62613393	8.68194579	1.366E-16	-2.087609	down	hypothetical protein MIMGU_mgv1a002241mg [Erythranthe guttata]
c41989.graph_c0	0.38546974	0.16077322	0.04127866	2.16031253	3.66801279	4.00245796	1.517E-07	3.052262	up	PREDICTED: transcription factor bHLH18-like [Sesamum indicum]
c41994.graph_c0	66.2339926	66.5577929	61.4385831	46.9787629	48.2620742	47.8897739	2.666E-25	-1.457482	down	PREDICTED: neurofilament medium polypeptide [Sesamum indicum]
c41997.graph_c0	2.00293685	2.0806007	2.71954295	25.9029365	42.4155557	47.7694131	7.644E-11	3.06277	up	unnamed protein product [Coffea canephora]
c41999.graph_c0	4.73631583	6.77294076	5.73856174	38.0862754	39.1591486	43.3734045	1.484E-17	1.786693	up	-

#ID	0 h-1 FPKM	0 h-2 FPKM	0 h-3 FPKM	48 h-1 FPKM	48 h-2 FPKM	48 h-3 FPKM	FDR	log2FC	regulated	nr_annotation
c41999.graph_c1	5.80139966	7.43322737	6.73082551	46.5746134	47.0753614	46.6594028	1.408E-29	1.794907	up	PREDICTED: uncharacterized protein LOC105156173 isoform X1 [Sesamum indicum]
c42000.graph_c0	12.0752577	11.8249882	12.5340356	56.8471339	67.8601046	66.1129493	8.552E-23	1.367789	up	hypothetical protein MIMGU_mgv1a002552mg [Erythranthe guttata]
c42001.graph_c0	47.2488427	49.0984337	45.555003	34.4937427	35.5701485	31.7768988	1.179E-25	-1.495201	down	PREDICTED: ubiquitin-activating enzyme E1 1 [Sesamum indicum]
c42002.graph_c0	3.24942901	2.11139061	1.75816629	0	0	0	4.048E-18	-Inf	down	predicted protein [Hordeum vulgare subsp. vulgare]
c42004.graph_c0	95.13054	94.1304626	93.8600093	60.2016233	57.5355184	55.4170815	6.288E-34	-1.725488	down	PREDICTED: uncharacterized protein At1g04910-like [Sesamum indicum]
c42005.graph_c0	4.10026854	3.22753073	3.28115461	2.14626978	3.52880194	3.62086315	3.357E-07	-1.215365	down	PREDICTED: chromatin assembly factor 1 subunit FAS1-like [Sesamum indicum]
c42006.graph_c0	116.269162	112.846983	116.559007	71.7275538	76.4073914	82.4818878	2.845E-29	-1.603271	down	PREDICTED: 50S ribosomal protein L1, chloroplastic [Sesamum indicum]
c42007.graph_c0	9.85294818	8.82462342	9.66266912	7.64393981	4.85869641	5.35620619	4.808E-11	-1.675424	down	PREDICTED: phytochromobilin:ferredoxin oxidoreductase, chloroplastic isoform X1 [Sesamum indicum]
c42013.graph_c0	44.9178524	45.2853762	40.2475068	28.1713137	35.6619591	38.8282385	6.24E-22	-1.36679	down	PREDICTED: CBL-interacting protein kinase 18 [Sesamum indicum]
c42014.graph_c0	0	0	0	1.24249158	2.02141337	1.77679533	9.713E-12	Inf	up	hypothetical protein MIMGU_mgv1a013619mg [Erythranthe guttata]
c42015.graph_c1	3.90010917	4.25646029	2.47109321	2.66982458	3.88568453	3.5966935	0.00081	-1.084959	down	PREDICTED: exocyst complex component EXO70A1-like isoform X1 [Sesamum indicum]
c42016.graph_c1	2.02107492	1.9545191	2.2890235	0.53649293	0.90267226	0.8985754	1.482E-08	-2.450157	down	PREDICTED: uncharacterized protein LOC105163887 isoform X1 [Sesamum indicum]
c42018.graph_c0	1.83423134	1.29937123	1.23292364	9.59294152	11.5946804	11.7973666	5.471E-13	1.899251	up	PREDICTED: GPI transamidase component PIG-S [Sesamum indicum]
c42019.graph_c0	2.11082769	2.65241576	2.53935904	19.322196	19.5052207	18.9967265	3.421E-15	1.966736	up	PREDICTED: uncharacterized protein LOC105165798 [Sesamum indicum]
c42024.graph_c0	13.8284968	14.5289681	16.0072525	6.09180729	8.89034167	10.4360598	1.346E-25	-1.830236	down	PREDICTED: transmembrane protein 64-like [Sesamum indicum]
c42029.graph_c1	6.9755975	5.4582159	6.56512374	34.2589167	36.724659	35.5974983	3.033E-15	1.469224	up	PREDICTED: vesicle-associated membrane protein 724 [Nicotiana sylvestris]
c42030.graph_c0	2.16296909	0.88372856	1.07776566	0	0	0	6.398E-09	-Inf	down	--
c42033.graph_c0	1.86908674	1.48456477	1.7491737	7.33689618	8.44670176	7.94390701	5.245E-07	1.19744	up	PREDICTED: protein ROOT PRIMORDIUM DEFECTIVE 1 [Sesamum indicum]
c42041.graph_c0	5.26157556	6.01925055	4.24998068	1.59268645	2.34748963	1.54039438	5.224E-10	-2.522037	down	PREDICTED: probable LRR receptor-like serine/threonine-protein kinase RLK [Sesamum indicum]
c42043.graph_c0	39.3487022	40.7362088	39.7293602	35.0638157	29.6595414	29.8795905	1.44E-21	-1.354958	down	PREDICTED: probable zinc metalloprotease EGY1, chloroplastic [Sesamum indicum]

#ID	0 h-1 FPKM	0 h-2 FPKM	0 h-3 FPKM	48 h-1 FPKM	48 h-2 FPKM	48 h-3 FPKM	FDR	log2FC	regulated	nr_annotation
c42048.graph_c0	0.49804745	0.565019	0.34133904	6.57661779	8.81422844	7.43046951	3.646E-15	3.004856	up	PREDICTED: probable glucan 1,3-beta-glucosidase A [Beta vulgaris subsp. vulgaris]
c42052.graph_c0	14.4849852	12.5634783	13.8545745	0.56865823	0.6827477	0.40479094	5.672E-97	-5.64382	down	PREDICTED: homeobox-leucine zipper protein HOX11-like [Sesamum indicum]
c42061.graph_c0	0.56910493	0.55068564	0.51192566	4.73614578	4.99885788	5.53598921	6.864E-12	2.208115	up	PREDICTED: putative ion channel POLLUX-like 2 isoform X4 [Sesamum indicum]
c42066.graph_c1	4.25717172	3.92998972	3.55591224	19.8931757	20.5435079	22.1405758	1.731E-15	1.39685	up	PREDICTED: uncharacterized protein LOC105165741 [Sesamum indicum]
c42067.graph_c0	0	0	0	2.8276737	2.13146854	2.27411945	2.956E-12	Inf	up	-
c42073.graph_c0	0.33216219	0.53199168	0.2987891	2.63168149	2.30167753	2.15105459	0.0013986	1.595603	up	PREDICTED: protein OBERON 2 isoform X2 [Sesamum indicum]
c42075.graph_c0	16.8105268	15.0266462	17.1382708	138.753962	154.621136	151.073687	2.308E-48	2.161936	up	PREDICTED: ATP synthase subunit d, mitochondrial [Sesamum indicum]
c42077.graph_c0	0.15866533	0.03970605	0	1.86370385	3.05833513	3.80436393	9.8E-09	4.451081	up	PREDICTED: high-affinity nitrate transporter 3.1-like [Sesamum indicum]
c42079.graph_c0	0.82334184	1.06766997	0.72138195	5.24065743	6.97632663	7.13271067	1.727E-11	1.868404	up	PREDICTED: uncharacterized protein LOC105156622 [Sesamum indicum]
c42083.graph_c0	3.31687134	2.95128239	2.55010059	24.7330355	25.4546145	28.002961	7.337E-31	2.132091	up	PREDICTED: imidazole glycerol phosphate synthase hisHF, chloroplastic [Sesamum indicum]
c42091.graph_c0	15.2817122	14.8003582	13.3892763	254.083585	310.653649	318.570156	4.58E-99	3.324722	up	PREDICTED: calnexin homolog 1 [Sesamum indicum]
c42092.graph_c0	1.93549567	2.2572044	2.36645126	15.4074109	16.6157179	17.1598827	2.132E-25	1.885894	up	PREDICTED: protein PAT1 homolog 1-like [Sesamum indicum]
c42095.graph_c0	0	0	0	1.66884173	3.97785478	2.82135814	4.408E-07	Inf	up	-
c42096.graph_c0	27.0197639	26.7402737	26.9114068	11.2634171	14.1393414	12.4745473	4.004E-46	-2.111535	down	PREDICTED: uncharacterized protein LOC105170420 [Sesamum indicum]
c42097.graph_c0	4.29819141	2.34921528	2.91415808	0	0	0	1.632E-24	-Inf	down	predicted protein [Hordeum vulgare subsp. vulgare]
c42099.graph_c0	11.5852891	12.1767345	11.3517608	136.08233	107.698038	106.958628	1.797E-15	2.308041	up	--
c42100.graph_c0	1.99919642	2.08635569	2.15909231	50.198578	54.0926123	57.5676929	6.85E-113	3.675981	up	PREDICTED: 4-hydroxy-3-methylbut-2-en-1-yl diphosphate synthase, chloroplastic [Sesamum indicum]
c42109.graph_c0	3.86010652	3.42488412	3.45724131	14.170846	16.667641	16.2528376	4.954E-08	1.112628	up	PREDICTED: probable beta-1,3-galactosyltransferase 20 [Sesamum indicum]
c42109.graph_c1	2.43700469	1.14186787	1.46587756	10.4135709	15.02972	14.4939591	1.04E-09	1.964363	up	hypothetical protein MIMGU_mgv1a002479mg [Erythranthe guttata]
c42114.graph_c0	30.5710525	32.5541932	30.1252146	21.9522056	25.8644749	26.0077304	8.672E-22	-1.357156	down	PREDICTED: cryptochrome-1 [Sesamum indicum]
c42117.graph_c0	28.7682554	30.5436298	27.6168865	129.359891	161.878394	174.774754	2.898E-17	1.401009	up	PREDICTED: monosaccharide-sensing protein 2-like [Sesamum indicum]
c42120.graph_c0	9.24910311	8.30060283	7.45915307	358.464483	304.67128	257.050017	3.425E-28	4.190508	up	PREDICTED: chaperone protein dnaJ 11, chloroplastic-like [Sesamum indicum]

#ID	0 h-1 FPKM	0 h-2 FPKM	0 h-3 FPKM	48 h-1 FPKM	48 h-2 FPKM	48 h-3 FPKM	FDR	log2FC	regulated	nr_annotation
c42122.graph_c0	69.1541318	62.415697	65.9593961	51.4422893	42.9686843	40.9023188	7.965E-26	-1.559048	down	PREDICTED: cyclic dof factor 1 [Sesamum indicum]
c42123.graph_c1	42.254896	39.1095922	41.3933141	220.309722	250.425127	264.251811	3.788E-29	1.561595	up	PREDICTED: elongation factor 1-delta-like [Sesamum indicum]
c42128.graph_c0	156.365536	164.873214	150.780296	152.369268	149.846181	147.495259	2.241E-14	-1.086215	down	PREDICTED: glycine-rich RNA-binding protein 2, mitochondrial-like [Sesamum indicum]
c42129.graph_c1	0.19090324	0.34396992	0.34344551	6.40175953	5.29136314	5.38326652	1.025E-12	3.263694	up	PREDICTED: cytochrome P450 90A1 [Sesamum indicum]
c42134.graph_c1	47.2791889	46.4541533	48.4365157	13.8498175	15.948046	17.1457174	1.915E-24	-2.619564	down	70 kDa heat shock protein-like protein, partial [Arachis diogoi]
c42138.graph_c0	0.49482988	0.2251479	0.28903454	6.23712444	7.79287161	8.03884897	2.168E-24	3.432793	up	PREDICTED: LOW QUALITY PROTEIN: putative kinase-like protein TMKL1 [Sesamum indicum]
c42144.graph_c1	81.6563307	82.959765	87.2424157	37.1036874	27.5341907	28.9430653	6.738E-64	-2.440816	down	hypothetical protein MIMGU_mgv1a005576mg [Erythranthe guttata]
c42147.graph_c0	38.616984	36.8917054	38.0431809	21.1046942	21.7190683	21.0900706	2.268E-32	-1.846907	down	PREDICTED: F-box protein CPR30-like [Sesamum indicum]
c42150.graph_c0	79.1508207	80.669379	73.952322	61.7855201	64.1846076	58.9333369	3.462E-22	-1.355036	down	PREDICTED: LOW QUALITY PROTEIN: cleavage and polyadenylation specificity factor subunit 6 [Sesamum indicum]
c42152.graph_c2	53.1071726	51.3777763	50.1919797	32.3012304	28.580401	21.799377	1.487E-40	-1.916034	down	PREDICTED: shaggy-related protein kinase alpha [Sesamum indicum]
c42157.graph_c0	6.90854159	6.42950824	6.28640637	6.48501046	6.05004589	6.90295971	8.575E-09	-1.030149	down	PREDICTED: uncharacterized protein LOC105167143 isoform X1 [Sesamum indicum]
c42159.graph_c0	13.3463733	13.6369107	11.6353779	117.77708	128.364748	145.926377	1.653E-59	2.325245	up	PREDICTED: serine/threonine-protein kinase SRK2E isoform X1 [Sesamum indicum]
c42160.graph_c0	11.4390648	13.8611549	12.2681299	8.87445271	10.5765824	9.65903168	4.151E-07	-1.388059	down	-
c42163.graph_c0	109.164041	99.6404399	111.103397	11.0819873	8.95176664	8.75135855	1.99E-128	-4.487758	down	-
c42163.graph_c1	117.671901	119.655907	113.345388	15.106806	12.3256875	8.72576144	2.47E-162	-4.287894	down	PREDICTED: BAG family molecular chaperone regulator 6 [Sesamum indicum]
c42164.graph_c0	1.44810974	2.07079972	1.68365184	0.69881069	0.94220661	1.13054101	0.000119	-1.932739	down	-
c42171.graph_c0	131.356436	174.77254	108.555958	1509.4964	1078.22573	725.600417	0.0011864	1.993823	up	PREDICTED: glyceraldehyde-3-phosphate dehydrogenase, cytosolic [Sesamum indicum]
c42173.graph_c0	1.67635	2.30728934	1.11555549	0.63429193	1.02254081	0.66254378	5.112E-05	-2.155033	down	PREDICTED: rop guanine nucleotide exchange factor 12-like [Sesamum indicum]
c42176.graph_c0	3.35393568	3.28430949	3.65407968	13.0311669	14.8011741	16.3167511	0.0007013	1.07923	up	ribosomal protein L7Ae/L30e/S12e/Gadd45 family protein [Medicago truncatula]
c42178.graph_c0	9.02368383	8.27999304	11.0160014	7.75899237	9.54073667	7.39704081	0.0021576	-1.219688	down	-
c42181.graph_c0	0.42933367	0.64464537	0.27585538	4.74636216	5.02819518	4.92716526	3.465E-05	2.432359	up	-
c42181.graph_c1	7.0167447	6.37741163	5.4211404	27.3641423	28.9469777	27.2420331	3.352E-09	1.135134	up	PREDICTED: ethanolamine-phosphate cytidylyltransferase [Sesamum indicum]
c42184.graph_c0	52.8714781	48.6642508	47.0475165	41.8723518	41.5363385	40.8359438	3.173E-18	-1.274257	down	hypothetical protein MIMGU_mgv1a003601mg [Erythranthe guttata]

#ID	0 h-1 FPKM	0 h-2 FPKM	0 h-3 FPKM	48 h-1 FPKM	48 h-2 FPKM	48 h-3 FPKM	FDR	log2FC	regulated	nr_annotation
c42189.graph_c0	0.02244671	0.06740755	0.11537963	9.49183098	12.5528745	13.8002845	1.978E-50	6.412904	up	PREDICTED: uncharacterized protein At1g04910 [Sesamum indicum]
c42192.graph_c2	3.42697869	3.04310591	3.12527976	44.9640163	50.1693641	56.8187135	1.956E-50	2.965271	up	PREDICTED: rac-like GTP-binding protein ARAC7 isoform X3 [Sesamum indicum]
c42204.graph_c0	0	0	0	9.9003417	17.9195574	17.7070643	7.499E-23	Inf	up	PREDICTED: probable 2-oxoglutarate-dependent dioxygenase AOP1 [Sesamum indicum]
c42207.graph_c0	0	0	0	19.5269654	27.7501187	28.3932541	8.885E-31	Inf	up	-
c42210.graph_c0	10.3724751	12.7522064	11.3335597	40.9871622	55.2509611	59.0986662	3.315E-08	1.14902	up	PREDICTED: cellulose synthase-like protein D3 isoform X1 [Sesamum indicum]
c42211.graph_c0	1.28695203	1.53130501	1.84100518	4.87671821	6.77779542	7.42453855	0.000224	1.006512	up	PREDICTED: uncharacterized protein LOC105176468 [Sesamum indicum]
c42219.graph_c0	191.547767	182.752306	196.438422	106.213725	108.311278	132.201614	3.441E-27	-1.738863	down	PREDICTED: uncharacterized protein LOC105161471 [Sesamum indicum]
c42226.graph_c0	2.82484639	3.33261702	2.56695647	22.8456417	22.2920065	21.7834529	4.47E-29	1.924223	up	PREDICTED: filament-like plant protein 3 [Sesamum indicum]
c42227.graph_c0	5.48431191	5.06281889	4.62006576	19.1714527	23.0158244	22.6532789	4.188E-09	1.076749	up	PREDICTED: serine/threonine-protein kinase CDL1 [Sesamum indicum]
c42229.graph_c0	58.4229465	54.4847262	54.6657454	38.3793358	34.755786	27.9165994	2.75E-35	-1.742993	down	PREDICTED: zinc finger CCCH domain-containing protein 17 isoform X1 [Sesamum indicum]
c42233.graph_c2	2.87116539	2.10762965	1.47582448	13.2255242	13.6372168	9.57130005	0.0039941	1.487132	up	PREDICTED: probable serine/threonine protein kinase IREH1 [Sesamum indicum]
c42237.graph_c1	132.721667	131.735007	135.459963	66.2019674	57.4890128	50.3490638	1.631E-52	-2.214083	down	PREDICTED: E3 ubiquitin-protein ligase RNF5-like [Sesamum indicum]
c42239.graph_c0	0	0	0	15.5301222	22.4670939	14.4129582	1.59E-18	Inf	up	--
c42243.graph_c0	87.3297975	79.3778156	83.5142306	488.931822	521.748026	523.738272	3.466E-30	1.597853	up	unnamed protein product [Coffea canephora]
c42246.graph_c0	1.17864354	1.02758845	1.12373817	0.63048966	0.37107075	0.40517094	5.819E-06	-2.250429	down	hypothetical protein VITISV_034155 [Vitis vinifera]
c42248.graph_c0	1.76387973	1.40531007	1.54965209	1.27678655	1.2120706	1.44591617	0.0002109	-1.279388	down	PREDICTED: 65-kDa microtubule-associated protein 3 [Sesamum indicum]
c42253.graph_c0	14.2631371	14.9428925	12.8533863	4.00885391	3.33263258	3.24502838	9.793E-58	-3.002421	down	PREDICTED: lysM domain receptor-like kinase 3 [Sesamum indicum]
c42256.graph_c0	77.1738581	79.1782509	78.9217926	103.000638	71.0743043	56.0923626	1.474E-06	-1.039658	down	PREDICTED: mpv17-like protein isoform X2 [Sesamum indicum]
c42268.graph_c0	64.7965441	62.6401462	56.4612762	24.2867896	29.6272282	34.5618306	1.692E-38	-2.076433	down	-
c42274.graph_c0	16.6476766	18.6051743	18.0708632	7.51447758	9.48321183	8.98819068	1.274E-39	-2.058883	down	PREDICTED: lysine-specific histone demethylase 1 homolog 1 [Sesamum indicum]
c42284.graph_c0	26.2859333	27.5477867	23.6155529	3.45345728	3.48005488	3.18528743	9.956E-97	-3.951781	down	PREDICTED: DNA repair protein RAD51 homolog [Sesamum indicum]
c42286.graph_c0	123.154121	105.34313	125.982343	514.839934	589.125031	586.301262	1.146E-18	1.232694	up	PREDICTED: 60S ribosomal protein L24-like [Sesamum indicum]

#ID	0 h-1 FPKM	0 h-2 FPKM	0 h-3 FPKM	48 h-1 FPKM	48 h-2 FPKM	48 h-3 FPKM	FDR	log2FC	regulated	nr_annotation
c42290.graph_c1	2.90700641	2.50854942	2.5762885	0.1846982	0.19566535	0.16434335	7.231E-15	-4.891447	down	-
c42293.graph_c1	10.0265538	7.91119751	11.2171103	1.73875112	2.07224547	1.83721722	6.511E-16	-3.389937	down	hypothetical protein CISIN_1g015935mg [Citrus sinensis]
c42295.graph_c0	79.9278393	68.8629501	77.469028	300.126189	323.689616	358.911688	1.704E-15	1.098952	up	PREDICTED: 40S ribosomal protein S25-2 [Solanum lycopersicum]
c42299.graph_c0	6.71034372	7.32770522	5.48740013	4.87067165	3.96914308	2.75035602	6.217E-06	-1.760484	down	hypothetical protein MIMGU_mgv1a012462mg [Erythranthe guttata]
c42300.graph_c0	0.3715127	0	0.11935213	4.02157538	4.89489489	4.03518724	2.381E-08	3.709927	up	PREDICTED: putative B3 domain-containing protein At5g66980 [Sesamum indicum]
c42303.graph_c0	29.961936	31.3316359	29.4037539	10.5797819	7.62649359	7.99821337	5.149E-64	-2.802065	down	PREDICTED: high mobility group B protein 10 [Sesamum indicum]
c42308.graph_c0	0.43596474	0.5727767	0.28011598	30.8509075	33.8794806	33.5710178	5.74E-115	5.239124	up	PREDICTED: zinc finger CCCH domain-containing protein 53 [Sesamum indicum]
c42309.graph_c1	1.84959068	1.51757603	1.0909889	8.21254573	13.9972547	17.4733132	7.209E-05	2.13075	up	PREDICTED: serine/threonine-protein kinase-like protein At3g51990 [Sesamum indicum]
c42316.graph_c0	0	0.12859377	0	7.21937815	7.52267779	7.37152703	9.229E-13	6.419609	up	-
c42318.graph_c0	4.9334718	4.74474872	4.47508701	18.2247032	20.3453742	23.9476864	6.355E-07	1.1233	up	PREDICTED: triphosphate tunel metalloenzyme 3 [Sesamum indicum]
c42329.graph_c0	8.13081282	9.18914229	10.6506438	6.23414147	5.17082181	4.81608821	9.323E-12	-1.801651	down	PREDICTED: AP2-like ethylene-responsive transcription factor BBM2 [Sesamum indicum]
c42333.graph_c0	2.00739047	1.50705043	2.07799197	0.05137053	0.16326254	0.13712756	4.798E-16	-5.026564	down	PREDICTED: uncharacterized protein LOC105169088 [Sesamum indicum]
c42334.graph_c1	689.972084	727.482483	684.610474	265.533856	387.272079	352.007466	1.06E-31	-2.088251	down	PREDICTED: protein EXORDIUM-like 2 [Sesamum indicum]
c42335.graph_c0	9.47713519	10.7191394	13.2333241	0.96246749	1.45659657	1.19283977	6.928E-28	-4.237234	down	PREDICTED: vetispiradiene synthase 3-like [Sesamum indicum]
c42337.graph_c0	50.0922986	47.3485971	48.2307477	15.0218783	14.9819659	15.7747332	8.104E-58	-2.687542	down	unnamed protein product [Coffea canephora]
c42338.graph_c0	6.79704067	7.46103663	6.99749862	34.120115	43.0813559	45.8722171	7.86E-19	1.511476	up	PREDICTED: kinetochore protein NDC80 homolog [Sesamum indicum]
c42339.graph_c0	40.4468872	38.1878028	40.6385207	15.3101941	14.8597357	15.0653453	7.939E-62	-2.415963	down	PREDICTED: uncharacterized protein LOC105156535 [Sesamum indicum]
c42340.graph_c0	189.989555	195.368149	196.193148	127.572861	145.691574	153.250638	1.464E-20	-1.467853	down	phosphoglycerate kinase [Gossypium hirsutum]
c42341.graph_c0	0	0	0	2.95964576	6.82817353	2.16530166	5.63E-05	Inf	up	--
c42352.graph_c0	2.5301706	2.58386992	2.52884286	11.1132552	16.3127917	18.0381463	2.01E-07	1.547655	up	PREDICTED: putative ABC transporter C family member 15 [Sesamum indicum]
c42353.graph_c0	2.75948121	1.18381903	1.74999505	0	0	0	6.434E-13	-Inf	down	predicted protein [Physcomitrella patens]
c42358.graph_c0	248.889005	279.994799	241.911836	62.1731711	53.8557277	63.7442958	2.43E-99	-3.115405	down	hypothetical protein MIMGU_mgv1a016283mg [Erythranthe guttata]

#ID	0 h-1 FPKM	0 h-2 FPKM	0 h-3 FPKM	48 h-1 FPKM	48 h-2 FPKM	48 h-3 FPKM	FDR	log2FC	regulated	nr_annotation
c42358.graph_c1	21.538499	22.9809305	22.1263979	19.2741122	20.2378893	23.6760912	1.139E-09	-1.096469	down	PREDICTED: GATA transcription factor 12-like [Sesamum indicum]
c42361.graph_c1	2.58740504	2.73388459	2.77076493	1.7215512	1.96406512	2.00315696	0.0026216	-1.528978	down	copalyl diphosphate synthase [Isodon eriocalyx]
c42363.graph_c0	4.31296147	3.2294616	2.9239044	1.62689633	1.52463408	1.97653428	1.745E-09	-2.044423	down	PREDICTED: uncharacterized protein LOC105168320 [Sesamum indicum]
c42368.graph_c0	13.5472044	15.0006903	12.7757351	11.8766698	12.6352047	13.687377	3.864E-14	-1.131612	down	PREDICTED: pentatricopeptide repeat-containing protein At4g19191, mitochondrial-like [Sesamum indicum]
c42372.graph_c1	0.50863441	0.71280122	1.04578456	11.0586577	15.9844442	15.3436152	5.38E-12	3.194069	up	-
c42379.graph_c0	2.1364789	2.24045723	2.18983117	0.51549764	0.74469169	0.43783731	5.042E-14	-2.972474	down	-
c42385.graph_c0	69.4182426	60.1528676	70.25138	25.4670789	30.4619776	33.5031492	1.003E-45	-2.181992	down	PREDICTED: uncharacterized protein LOC105170258 [Sesamum indicum]
c42387.graph_c0	88.5108245	85.3409809	88.8007943	57.1793251	40.725954	33.5966421	2.694E-45	-2.007142	down	PREDICTED: homogentisate 1,2-dioxygenase isoform X1 [Sesamum indicum]
c42388.graph_c0	0	0	0	2.80813456	4.33482232	5.14010313	1.594E-16	Inf	up	PREDICTED: ATP-citrate synthase beta chain protein 2-like [Citrus sinensis]
c42390.graph_c0	0.95799343	1.66774386	1.76630275	14.9268777	15.6912642	13.7257124	2.291E-16	2.314238	up	hypothetical protein JCGZ_15647 [Jatropha curcas]
c42391.graph_c0	14.5398584	12.8320016	12.3824905	1.26817192	3.52662025	3.24418379	1.117E-25	-3.337783	down	-
c42395.graph_c0	0	0.18019828	0.28916286	26.8253227	25.5631821	32.7968325	4.327E-70	6.469585	up	PREDICTED: tetraketide alpha-pyrone reductase 1-like [Sesamum indicum]
c42396.graph_c0	19.2956679	21.0623114	19.8244327	10.1703768	12.7081263	14.7345076	1.499E-21	-1.700661	down	BnaA08g23740D [Brassica napus]
c42397.graph_c0	1.3424782	1.91974641	2.30018352	10.0709105	9.17151785	7.65094268	0.0008127	1.254883	up	hypothetical protein MIMGU_mgv1a015275mg [Erythranthe guttata]
c42401.graph_c0	2.28542801	2.94688908	3.03642024	1.74862343	1.43659736	1.49240789	2.883E-06	-1.837748	down	PREDICTED: serine/threonine-protein kinase BLUS1-like isoform X2 [Sesamum indicum]
c42402.graph_c0	0.28747763	0.95921833	0.61570023	15.3609025	19.3125132	21.4054054	4.376E-33	3.888253	up	PREDICTED: transcription factor bHLH71-like [Nicotiana sylvestris]
c42403.graph_c0	0.90646604	0.63516161	1.04836027	21.5872223	29.3272272	33.7350637	1.969E-25	4.003507	up	PREDICTED: LOW QUALITY PROTEIN: protein YIF1B-like [Sesamum indicum]
c42407.graph_c0	10.3476127	11.0485059	11.0316615	7.20263235	8.45319279	9.42479258	7.686E-07	-1.392321	down	PREDICTED: myb family transcription factor APL-like [Sesamum indicum]
c42409.graph_c0	51.6321754	46.8900103	54.6180482	301.21125	318.463861	333.077508	5.294E-31	1.617423	up	PREDICTED: 60S ribosomal protein L5-like [Sesamum indicum]
c42413.graph_c0	560.082674	502.626025	566.234461	106.85838	100.203715	84.0451197	2.34E-109	-3.499641	down	PREDICTED: extra-large guanine nucleotide-binding protein 3-like [Sesamum indicum]
c42425.graph_c0	1.02563823	1.13142701	0.96831605	3.95308961	5.51566418	5.52494725	4.884E-05	1.239741	up	PREDICTED: anaphase-promoting complex subunit 4 [Sesamum indicum]
c42426.graph_c0	14.681265	14.9537901	14.6873518	10.0253609	12.254601	8.78854568	2.474E-19	-1.53151	down	PREDICTED: uncharacterized membrane protein At3g27390 [Sesamum indicum]

#ID	0 h-1 FPKM	0 h-2 FPKM	0 h-3 FPKM	48 h-1 FPKM	48 h-2 FPKM	48 h-3 FPKM	FDR	log2FC	regulated	nr_annotation
c42427.graph_c0	6.4723034	3.23939222	3.366472	0	0	0	2.037E-13	-Inf	down	PREDICTED: serpin-ZX-like [Malus domestica]
c42430.graph_c0	36.654546	32.323244	31.650915	31.8925275	30.6631655	24.085342	8.084E-12	-1.229612	down	PREDICTED: bet1-like SNARE 1-1 [Sesamum indicum]
c42432.graph_c0	0.06562578	0.11496011	0.08433172	1.01268398	0.76858451	0.95487594	1.849E-05	2.354572	up	PREDICTED: uncharacterized protein LOC103330039 [Prunus mume]
c42434.graph_c1	1.21003851	1.58394254	1.07650204	10.4616539	11.446226	15.3364923	2.054E-07	2.247305	up	PREDICTED: uncharacterized protein LOC105170092 [Sesamum indicum]
c42437.graph_c0	2.68665745	3.41619847	3.07922215	1.03687631	1.70081763	1.27974422	4.061E-09	-2.217633	down	PREDICTED: post-GPI attachment to proteins factor 3 [Sesamum indicum]
c42438.graph_c0	0.7181038	0.31947683	0.4101297	5.95407656	9.22781758	8.82983586	6.087E-14	3.030685	up	PREDICTED: uncharacterized protein LOC105168396 [Sesamum indicum]
c42439.graph_c0	4.87239703	5.51279984	4.45874978	117.292919	83.7654616	68.5287389	1.148E-08	3.175338	up	PREDICTED: probable serine/threonine-protein kinase At1g54610 [Nicotiana tomentosiformis]
c42441.graph_c0	26.6614838	29.1544158	22.6522535	19.7930508	20.3099458	18.4292108	3.783E-21	-1.438009	down	PREDICTED: uncharacterized protein LOC105162071 [Sesamum indicum]
c42442.graph_c0	1.80249619	2.49418096	1.97564971	47.3264462	32.2861278	18.1654376	0.0005376	2.957667	up	PREDICTED: uncharacterized protein LOC101292603 isoform 2 [Fragaria vesca subsp. vesca]
c42445.graph_c0	2.81482136	2.96593682	2.63065956	37.2229239	49.3309012	56.1838049	8.792E-24	3.062318	up	PREDICTED: ABC transporter B family member 1-like [Sesamum indicum]
c42449.graph_c0	2.75377124	2.65808127	3.12796251	26.0037752	32.1071593	39.2418915	3.779E-17	2.4866	up	PREDICTED: heme-binding protein 2-like [Sesamum indicum]
c42450.graph_c0	2.47121235	3.21579296	2.06414371	12.0662026	10.4914437	14.585345	0.0045132	1.24594	up	PREDICTED: protein PLANT CADMIUM RESISTANCE 2-like [Nicotiana tomentosiformis]
c42453.graph_c0	0.0700688	0.03506948	0	4.71229248	4.23986787	5.82995076	1.336E-20	6.135456	up	hypothetical protein MIMGU_mgv1a006178mg [Erythranthe guttata]
c42460.graph_c0	3.42680096	2.52017073	3.14541065	0	0	0	2.348E-25	-Inf	down	hypothetical protein [Dendrobium catenatum]
c42462.graph_c0	0	0	0	1.30477099	1.62413982	1.42219729	2.937E-12	Inf	up	PREDICTED: 65-kDa microtubule-associated protein 8 isoform X1 [Sesamum indicum]
c42463.graph_c0	19.3639746	20.1828797	19.4261326	21.2851878	18.6514697	16.7037488	8.675E-14	-1.072006	down	PREDICTED: TBC1 domain family member 5 homolog A-like [Sesamum indicum]
c42465.graph_c0	12.1460642	14.2561167	11.1705554	3.44468668	5.13681118	5.09542071	3.763E-37	-2.481	down	PREDICTED: uncharacterized protein LOC105168867 isoform X1 [Sesamum indicum]
c42465.graph_c1	1.43261352	2.02070409	2.00832555	24.2966314	23.0065166	21.4588558	2.186E-35	2.636205	up	PREDICTED: transcription factor bHLH93-like [Nicotiana sylvestris]
c42470.graph_c0	0	0	0	1.68351501	1.41867729	1.12348615	1.341E-11	Inf	up	DEAD-domain-containing protein [Punctularia strigosozonata HHB-11173 SS5]
c42472.graph_c0	48.0496938	44.8838994	40.2627875	4.58984571	4.86238498	5.17308834	9.551E-83	-4.204367	down	-
c42474.graph_c0	192.617103	181.893038	175.72238	1284.66804	834.48457	680.063528	0.0095642	1.340883	up	PREDICTED: protein EARLY RESPONSIVE TO DEHYDRATION 15 [Sesamum indicum]

#ID	0 h-1 FPKM	0 h-2 FPKM	0 h-3 FPKM	48 h-1 FPKM	48 h-2 FPKM	48 h-3 FPKM	FDR	log2FC	regulated	nr_annotation
c42475.graph_c0	0	0	0	3.79330322	5.06686084	3.3752584	1.093E-12	Inf	up	--
c42478.graph_c0	0.35313514	0.45448553	0.25931008	2.13789023	5.6128531	5.83090746	0.0008403	2.641354	up	PREDICTED: pentatricopeptide repeat-containing protein At4g21170 [Sesamum indicum]
c42478.graph_c1	0.65361224	0.55612672	0.79792172	3.13118117	2.90246843	3.18794841	0.005671	1.17989	up	PREDICTED: pentatricopeptide repeat-containing protein At3g16610 [Sesamum indicum]
c42479.graph_c0	0.70890333	0.90066289	1.0511179	2.96401471	4.94951387	4.60420904	0.0008271	1.204699	up	PREDICTED: uncharacterized protein LOC105164847 [Sesamum indicum]
c42481.graph_c0	0.14125679	0.25451683	0.2541288	1.66572763	1.76463645	1.22740914	0.0004764	1.821718	up	hypothetical protein PGTG_21261 [Puccinia graminis f. sp. tritici CRL 75-36-700-3]
c42486.graph_c0	0	0.03743855	0	5.16844933	9.74611474	9.25902834	8.598E-19	8.318379	up	hypothetical protein MIMGU_mgv1a023633mg [Erythranthe guttata]
c42487.graph_c1	0.18445075	0.2051505	0.1316814	3.00206238	2.78028069	3.2256206	1.377E-11	3.097719	up	PREDICTED: bifunctional dethiobiotin synthetase/7,8-diamino-pelargonic acid aminotransferase, mitochondrial [Sesamum indicum]
c42492.graph_c0	13.8314218	14.6004686	11.5415095	5.95748943	7.53842277	8.48150124	5.217E-24	-1.883524	down	PREDICTED: uncharacterized protein At5g01610 [Sesamum indicum]
c42493.graph_c0	0.25370258	0.15872289	0.2445134	3.24296723	6.12824368	10.346468	0.0002078	3.875386	up	PREDICTED: MATE efflux family protein 1 [Sesamum indicum]
c42496.graph_c0	31.472329	30.1165185	30.3509435	18.088226	20.7403524	17.1362226	2.02E-25	-1.734615	down	hypothetical protein MIMGU_mgv1a013377mg [Erythranthe guttata]
c42498.graph_c0	15.6880956	12.6855248	13.9827192	55.1142505	58.6427612	63.7989959	6.967E-12	1.048871	up	hypothetical protein L484_022782 [Morus notabilis]
c42499.graph_c0	0.07723091	0.27057886	0	1.9566292	2.07281139	2.08919628	4.294E-07	3.132081	up	PREDICTED: protein PHR1-LIKE 1-like isoform X1 [Sesamum indicum]
c42500.graph_c0	0	0	0	2.70383863	3.81918556	4.12433032	1.291E-28	Inf	up	PREDICTED: LOW QUALITY PROTEIN: intracellular protein transport protein USO1-like [Sesamum indicum]
c42500.graph_c1	0	0	0	2.88054688	4.53379105	5.78526771	7.355E-11	Inf	up	PREDICTED: paramyosin-like [Sesamum indicum]
c42503.graph_c0	14.305093	13.9216558	13.0720789	10.5430278	14.8145165	12.3127236	2.68E-06	-1.15428	down	-
c42504.graph_c0	2.0602098	2.64228701	2.19242049	1.00831231	0.78542987	1.3193974	6.025E-08	-2.164048	down	PREDICTED: crossover junction endonuclease EME1B-like isoform X1 [Sesamum indicum]
c42506.graph_c0	92.2368761	103.075513	94.2908575	4.53681935	2.83566389	2.26062736	9.2E-258	-5.91552	down	PREDICTED: mitogen-activated protein kinase kinase kinase 2-like [Sesamum indicum]
c42517.graph_c1	6.91811984	5.26037246	4.9579169	29.9673458	30.1237237	28.3006392	5.812E-09	1.352437	up	hypothetical protein MIMGU_mgv1a017465mg [Erythranthe guttata]
c42520.graph_c1	37.4656734	39.2756967	33.5426202	24.7161922	22.0933656	20.551227	1.558E-34	-1.724501	down	PREDICTED: uncharacterized protein LOC105178356 [Sesamum indicum]
c42522.graph_c0	0.11349594	0.03786986	0.04861559	2.14347709	2.08613992	2.4499673	1.531E-13	4.049888	up	hypothetical protein MIMGU_mgv1a005054mg [Erythranthe guttata]
c42524.graph_c0	8.12616914	8.90322079	7.58506251	64.7700244	96.393822	107.04502	8.295E-12	2.421645	up	T1N15.23 [Arabidopsis thaliana]

#ID	0 h-1 FPKM	0 h-2 FPKM	0 h-3 FPKM	48 h-1 FPKM	48 h-2 FPKM	48 h-3 FPKM	FDR	log2FC	regulated	nr_annotation
c42526.graph_c0	32.915255	30.4137366	34.3666422	19.4382563	21.0558062	25.2089835	8.92E-23	-1.593441	down	PREDICTED: BI1-like protein [Sesamum indicum]
c42531.graph_c0	0.02069566	0.04143276	0.07978421	2.49767058	3.73668808	4.12248993	1.939E-20	5.152267	up	PREDICTED: probable LRR receptor-like serine/threonine-protein kinase MRH1 isoform X1 [Sesamum indicum]
c42532.graph_c0	12.3444632	12.5976981	13.1728865	11.516621	11.7542482	9.83318366	3.866E-17	-1.220469	down	PREDICTED: histidine kinase 2 isoform X2 [Sesamum indicum]
c42536.graph_c0	10.8401826	12.1836209	12.3008165	45.6116935	62.2406901	71.7123107	4.966E-07	1.320797	up	PREDICTED: signal peptidase complex subunit 3B-like [Sesamum indicum]
c42537.graph_c0	18.8283772	17.2151643	16.7613779	18.5591332	14.764989	13.9821167	1.579E-16	-1.170082	down	PREDICTED: uncharacterized protein LOC105173836 [Sesamum indicum]
c42538.graph_c0	8.43460466	7.9057656	8.57250619	53.7931074	55.1538035	57.8274469	4.243E-28	1.724243	up	PREDICTED: triose phosphate/phosphate translocator, non-green plastid, chloroplastic-like [Sesamum indicum]
c42540.graph_c0	89.8721145	87.2081657	91.3307649	361.387878	372.553857	375.404804	1.231E-13	1.028841	up	PREDICTED: 60S ribosomal protein L36-2-like [Sesamum indicum]
c42542.graph_c0	18.1468028	17.8972797	17.6981669	7.90146342	9.54514232	7.23423537	2.319E-45	-2.141557	down	PREDICTED: mitogen-activated protein kinase kinase kinase 2-like [Sesamum indicum]
c42544.graph_c0	1.22657794	1.07746256	0.93285378	10.1930937	13.705594	14.363863	2.122E-23	2.542523	up	PREDICTED: non-lysosomal glucosylceramidase isoform X3 [Sesamum indicum]
c42546.graph_c0	89.9819463	86.4951812	91.7320303	355.156308	376.482822	382.521093	7.172E-14	1.035506	up	PREDICTED: 40S ribosomal protein S9-2-like [Sesamum indicum]
c42549.graph_c0	21.9241144	23.6924395	20.9245077	205.622369	211.077927	213.328016	4.01E-54	2.225901	up	hypothetical protein M569_16894, partial [Genlisea aurea]
c42549.graph_c1	21.9392806	21.7048925	23.3660538	204.321037	218.036356	223.650656	1.959E-52	2.249507	up	hypothetical protein M569_06257, partial [Genlisea aurea]
c42552.graph_c0	1.78712238	1.78891191	1.86592495	1.92081599	1.0901098	1.2513277	0.0013493	-1.359968	down	PREDICTED: 3-ketoacyl-CoA synthase 11-like [Sesamum indicum]
c42554.graph_c0	26.900506	26.2749199	28.9836893	16.8553034	15.7778809	15.0689958	9.405E-29	-1.801039	down	PREDICTED: protein REVEILLE 6 isoform X2 [Sesamum indicum]
c42556.graph_c0	1263.48934	1254.2425	1261.84737	140.449692	133.733767	122.794436	6.441E-93	-4.266898	down	PREDICTED: LOW QUALITY PROTEIN: low-temperature-induced 65 kDa protein [Sesamum indicum]
c42557.graph_c0	0	0	0	0.95759049	1.76194123	2.01803273	2.074E-08	Inf	up	PREDICTED: MLO-like protein 11 [Sesamum indicum]
c42557.graph_c1	0.03129634	0.09398302	0	2.04708801	2.01592029	2.20630771	2.831E-11	4.641346	up	PREDICTED: MLO-like protein 11 [Sesamum indicum]
c42559.graph_c0	0.25652133	0.19971637	0.36626673	2.38950001	4.97931876	6.37848821	7.374E-05	3.026999	up	PREDICTED: caffeoylshikimate esterase [Sesamum indicum]
c42560.graph_c0	7.36087219	7.70535215	7.32609768	4.83113332	4.85975224	4.45646432	1.983E-17	-1.67911	down	hypothetical protein PRUPE_ppa004658mg [Prunus persica]
c42560.graph_c1	3.32325057	3.85883083	3.75804753	2.32681234	2.07576887	3.81386458	0.0038683	-1.43434	down	PREDICTED: probable folate-biopterin transporter 2 [Sesamum indicum]
c42561.graph_c0	19.6541767	20.2197469	20.9956049	182.936045	147.962208	124.50896	2.162E-08	1.890995	up	PREDICTED: probable WRKY transcription factor 4 [Sesamum indicum]
c42562.graph_c0	9.62075456	9.87668979	8.82171635	5.25902265	5.28312628	6.57542946	1.948E-21	-1.744523	down	PREDICTED: NADPH-dependent diflavin oxidoreductase 1 isoform X1 [Sesamum indicum]

#ID	0 h-1 FPKM	0 h-2 FPKM	0 h-3 FPKM	48 h-1 FPKM	48 h-2 FPKM	48 h-3 FPKM	FDR	log2FC	regulated	nr_annotation
c42564.graph_c0	16.0732871	16.3873335	16.9573442	3.38201936	1.83983646	1.21998668	1.77E-34	-3.941331	down	PREDICTED: uncharacterized protein LOC103940260 [Pyrus x bretschneideri]
c42566.graph_c0	9.43290269	9.38854576	8.61638897	5.84298699	7.40956348	7.63335783	7.006E-20	-1.414716	down	PREDICTED: protein CHROMATIN REMODELING 8 [Sesamum indicum]
c42567.graph_c0	20.6577659	22.2729048	20.5957974	20.2825909	21.968986	22.0657172	9.815E-13	-1.000799	down	PREDICTED: RNA-binding protein pno1 [Sesamum indicum]
c42569.graph_c0	4.37158891	4.37596639	3.32174964	29.3474365	37.2858003	39.699501	1.38E-25	2.119735	up	PREDICTED: probable leucine-rich repeat receptor-like protein kinase At1g35710 [Sesamum indicum]
c42571.graph_c0	14.1956888	12.949547	13.2294317	70.1435388	72.7905973	70.9536402	9.465E-23	1.387823	up	hypothetical protein MIMGU_mgv1a020880mg [Erythranthe guttata]
c42576.graph_c0	33.7830607	34.7894989	35.0085884	28.9197495	33.846555	31.1854838	3.058E-15	-1.160853	down	PREDICTED: rRNA-processing protein EFG1-like isoform X2 [Sesamum indicum]
c42579.graph_c0	1.52251327	1.41708781	1.37297532	0.7628385	1.1991679	1.22616355	0.0004422	-1.46057	down	PREDICTED: arginine decarboxylase [Sesamum indicum]
c42588.graph_c0	2.71037449	1.92177104	2.08008829	24.5535754	29.8324394	29.4387472	3.42E-36	2.623249	up	PREDICTED: alpha-galactosidase-like [Sesamum indicum]
c42589.graph_c0	35.5341043	35.5248034	32.0935534	18.9808779	20.326741	20.527848	7.438E-37	-1.803414	down	hypothetical protein M569_03173, partial [Genlisea aurea]
c42590.graph_c0	16.8800917	14.6160503	16.836986	13.4794107	13.7238253	13.198204	5.288E-15	-1.276585	down	PREDICTED: LRR receptor-like serine/threonine-protein kinase GSO1 [Sesamum indicum]
c42592.graph_c1	233.05321	226.491291	245.03122	97.6935156	78.1150554	66.597739	3.606E-67	-2.551611	down	hypothetical protein MIMGU_mgv1a008823mg [Erythranthe guttata]
c42595.graph_c0	1.59394858	1.50690331	1.650009	0.97896626	0.47533569	0.36294929	7.409E-07	-2.386979	down	-
c42595.graph_c1	61.3563007	62.5279665	62.7683648	53.9505152	54.8159097	51.0596978	2.212E-18	-1.241209	down	PREDICTED: ABSCISIC ACID-INSENSITIVE 5-like protein 6 isoform X2 [Sesamum indicum]
c42597.graph_c0	5.93219562	5.26589403	6.9998334	141.955777	188.727628	177.447632	2.815E-72	3.778743	up	hypothetical protein MIMGU_mgv1a007920mg [Erythranthe guttata]
c42598.graph_c1	29.7761856	29.6310156	27.2562469	19.7820062	21.9234288	19.7991635	3.706E-25	-1.512432	down	PREDICTED: mechanosensitive ion channel protein 1, mitochondrial-like [Sesamum indicum]
c42599.graph_c0	27.4138918	25.7845069	22.9092366	14.4226179	25.0050837	22.1329586	3.251E-17	-1.330702	down	-
c42602.graph_c0	0.1963911	0.27522286	0.35331847	1.66454028	3.18174832	2.73681139	2.675E-05	2.167592	up	PREDICTED: uncharacterized protein LOC105171438 isoform X1 [Sesamum indicum]
c42604.graph_c0	3.37282137	3.14600337	3.4279653	2.25982766	2.27431272	2.38780252	3.531E-11	-1.541419	down	PREDICTED: uncharacterized protein LOC105176546 [Sesamum indicum]
c42608.graph_c0	0	0	0	1.46470398	1.12849183	0.86885653	3.104E-09	Inf	up	PREDICTED: uncharacterized protein LOC105177151 [Sesamum indicum]
c42613.graph_c0	157.675664	174.582388	159.039181	120.865139	101.1852	75.4374139	8.961E-33	-1.735016	down	hypothetical protein MIMGU_mgv1a008930mg [Erythranthe guttata]
c42619.graph_c0	1.46118793	1.15651482	1.00434279	16.6232838	17.444531	21.9501748	2.409E-30	2.934032	up	PREDICTED: uncharacterized protein LOC103434215 [Malus domestica]

#ID	0 h-1 FPKM	0 h-2 FPKM	0 h-3 FPKM	48 h-1 FPKM	48 h-2 FPKM	48 h-3 FPKM	FDR	log2FC	regulated	nr_annotation
c42622.graph_c1	1.23415681	1.33422404	1.17359587	1.00054746	0.86723895	1.09261773	0.0012338	-1.353729	down	PREDICTED: glycosyltransferase family protein 64 protein C5-like [Sesamum indicum]
c42624.graph_c0	0.1111824	0	0.22859806	2.76556896	3.88467785	5.03094155	1.231E-12	4.063078	up	PREDICTED: uncharacterized protein LOC105179248 isoform X1 [Sesamum indicum]
c42629.graph_c0	0.18594783	0.17450065	0.13440956	2.30194145	2.18909383	3.2676791	4.211E-13	2.954075	up	PREDICTED: DNA polymerase alpha catalytic subunit [Sesamum indicum]
c42630.graph_c0	1.25021141	1.0092446	1.14014728	14.4157808	15.2717717	15.6701896	7.689E-21	2.719429	up	PREDICTED: uncharacterized protein LOC101231346, partial [Cucumis sativus]
c42633.graph_c0	35.0491982	33.1463051	32.5143071	8.57981	7.19974302	6.51238235	1.279E-90	-3.188103	down	PREDICTED: probable leucine-rich repeat receptor-like serine/threonine-protein kinase At5g15730 [Sesamum indicum]
c42636.graph_c0	21.2249058	19.1262543	22.9205454	1000.23374	802.137619	737.849054	5.64E-30	4.313691	up	hypothetical protein MIMGU_mgv1a009141mg [Erythranthe guttata]
c42642.graph_c0	585.482624	572.994012	617.057386	181.307504	172.025619	175.383311	1.773E-60	-2.764892	down	PREDICTED: leucine-rich repeat receptor-like protein kinase PXL1 [Sesamum indicum]
c42644.graph_c0	0.61175993	0.30618626	0.78613572	17.1895909	21.7926388	23.3189033	9.128E-11	4.164464	up	-
c42644.graph_c1	55.9377296	58.82255	50.1199421	14.2042518	18.3938652	17.4934057	3.808E-80	-2.739289	down	PREDICTED: scarecrow-like protein 14 [Sesamum indicum]
c42653.graph_c0	0.13414578	0.1342801	0	2.52111029	3.06357693	4.04668093	1.017E-13	4.158555	up	PREDICTED: protein trichome birefringence-like 14 [Sesamum indicum]
c42659.graph_c0	0.27310139	0.18224991	0.29245509	5.49324084	4.35346117	5.22365934	7.722E-11	3.316285	up	hypothetical protein MIMGU_mgv1a021608mg, partial [Erythranthe guttata]
c42665.graph_c0	2.34147002	2.27487892	2.25666125	17.1934746	16.265259	16.5970598	1.851E-15	1.848326	up	PREDICTED: uncharacterized protein LOC105168054 [Sesamum indicum]
c42669.graph_c0	19.0756285	15.4865284	16.1506647	188.050486	163.134153	149.911089	8.78E-22	2.291822	up	PREDICTED: zeaxanthin epoxidase, chloroplastic-like [Sesamum indicum]
c42672.graph_c0	0.77046818	1.67101931	0.82506842	5.52070973	5.97384802	5.82457755	0.0005833	1.390453	up	PREDICTED: uncharacterized protein LOC105171447 isoform X1 [Sesamum indicum]
c42676.graph_c0	18.9072705	21.1725374	17.2809236	8.67073452	11.5391955	9.65082333	2.508E-41	-1.961961	down	PREDICTED: uncharacterized protein At1g04910 [Sesamum indicum]
c42679.graph_c0	1.10822638	0.63390634	1.18676231	0.53479403	0.61805392	0.71378477	0.0005111	-1.673891	down	PREDICTED: probable methyltransferase PMT10 [Sesamum indicum]
c42680.graph_c0	0	0	0	2.95262104	4.69191596	4.45168587	1.019E-12	Inf	up	PREDICTED: uncharacterized protein LOC104243668 [Nicotiana sylvestris]
c42680.graph_c1	99.6258533	92.3567258	86.7151985	37.0744355	29.9358757	25.1437574	1.262E-40	-2.607071	down	PREDICTED: alpha,alpha-trehalose-phosphate synthase [UDP-forming] 6 [Solanum lycopersicum]
c42680.graph_c2	39.1565275	40.7807795	40.8986589	19.0896063	15.593902	14.2958085	5.529E-54	-2.315772	down	PREDICTED: nuclear pore complex protein GP210 [Sesamum indicum]

#ID	0 h-1 FPKM	0 h-2 FPKM	0 h-3 FPKM	48 h-1 FPKM	48 h-2 FPKM	48 h-3 FPKM	FDR	log2FC	regulated	nr_annotation
c42680.graph_c3	154.441695	154.307919	167.731442	66.946755	52.7555965	47.4755465	3.071E-68	-2.523701	down	PREDICTED: alpha,alpha-trehalose-phosphate synthase [UDP-forming] 6 [Sesamum indicum]
c42683.graph_c1	52.0569167	53.0425934	51.3153725	50.4576786	51.3437741	48.9974335	4.921E-14	-1.069917	down	hypothetical protein PRUPE_ppa012854mg [Prunus persica]
c42686.graph_c0	46.7055174	44.6532037	44.0951797	26.4608604	26.6676801	25.7845877	1.909E-30	-1.796315	down	unnamed protein product [Coffea canephora]
c42690.graph_c0	30.0734074	28.976493	29.7361089	14.0848094	19.3743585	21.7619972	2.515E-30	-1.709142	down	PREDICTED: probable polyamine oxidase 2 isoform X1 [Sesamum indicum]
c42691.graph_c0	0.69246996	0.83179604	0.62289592	4.92815211	9.27575402	11.3812368	3.217E-05	2.547353	up	PREDICTED: pentatricopeptide repeat-containing protein At5g46100 [Sesamum indicum]
c42694.graph_c0	0.85502949	0.76579245	0.4915446	81.9217297	135.273942	135.789896	5.338E-34	6.363493	up	hypothetical protein MIMGU_mgv1a002094mg [Erythranthe guttata]
c42697.graph_c0	0	0	0.06264994	12.5761249	11.4671926	12.389106	2.302E-36	8.135032	up	PREDICTED: micronuclear linker histone polyprotein-like [Sesamum indicum]
c42698.graph_c0	3.78177587	4.48087018	2.90922715	0.26070887	0.15064879	0.50613205	2.097E-28	-4.622194	down	hypothetical protein MIMGU_mgv1a010940mg [Erythranthe guttata]
c42706.graph_c0	1.52113436	1.38423413	2.19165337	13.4616277	14.4409113	14.6230763	2.043E-12	2.037131	up	PREDICTED: uncharacterized protein LOC105176645 [Sesamum indicum]
c42707.graph_c0	0.03246437	0.06499377	0	1.97394775	2.56642153	2.44832468	1.531E-12	5.157023	up	PREDICTED: uncharacterized protein LOC105170754 [Sesamum indicum]
c42707.graph_c1	40.6117638	39.1322234	42.732695	3.90786675	5.51988129	5.68800635	5.5E-145	-4.042986	down	PREDICTED: uncharacterized protein LOC105175855 [Sesamum indicum]
c42708.graph_c0	11.8608327	5.03462997	4.05157507	0	0	0	1.533E-08	-Inf	down	PREDICTED: ribulose bisphosphate carboxylase small chain, chloroplastic-like [Phoenix dactylifera]
c42710.graph_c0	53.80395	55.4573044	45.4090252	19.3782228	22.855313	19.669048	8.852E-61	-2.338705	down	PREDICTED: cysteine-rich receptor-like protein kinase 10 [Sesamum indicum]
c42712.graph_c0	2.33292904	2.005282	2.11808589	15.2082544	17.4972199	19.144652	7.326E-23	1.985342	up	PREDICTED: phosphoglucomutase, chloroplastic [Sesamum indicum]
c42713.graph_c1	100.954797	95.7371566	93.8120854	82.4576334	87.1347491	82.5109215	4.106E-18	-1.222013	down	PREDICTED: probable CCR4-associated factor 1 homolog 7 [Sesamum indicum]
c42720.graph_c0	74.3202553	73.2753577	69.5371078	3.86308974	4.83301828	5.69618666	8.86E-201	-4.937285	down	PREDICTED: mitochondrial carrier protein MTM1-like [Sesamum indicum]
c42722.graph_c0	19.5932882	20.6451663	16.6970897	11.4003885	11.9263626	11.8346633	1.089E-20	-1.711676	down	PREDICTED: DNA-directed RNA polymerase II subunit 1 [Sesamum indicum]
c42722.graph_c1	28.2533191	29.3702851	24.8801804	18.706824	19.374369	19.4863694	4.419E-28	-1.535839	down	PREDICTED: DNA-directed RNA polymerase II subunit 1 [Sesamum indicum]
c42723.graph_c0	19.3115776	17.3099559	16.6944954	88.7128186	90.1253044	81.1667798	4.821E-14	1.270143	up	PREDICTED: uncharacterized protein LOC105052633 [Elaeis guineensis]

#ID	0 h-1 FPKM	0 h-2 FPKM	0 h-3 FPKM	48 h-1 FPKM	48 h-2 FPKM	48 h-3 FPKM	FDR	log2FC	regulated	nr_annotation
c42724.graph_c0	0.45369634	0.51470406	0.4664142	4.51412631	5.01832597	6.14893411	1.118E-09	2.429353	up	PREDICTED: U-box domain-containing protein 11-like [Sesamum indicum]
c42727.graph_c0	0.19755014	0.13183197	0.08461992	1.8806261	2.82777399	2.59102419	9.717E-07	3.12296	up	-
c42728.graph_c0	9.35726117	8.38223989	7.95247666	3.07483491	3.7324544	3.89224249	3.002E-49	-2.283763	down	PREDICTED: G-type lectin S-receptor-like serine/threonine-protein kinase SD2-2 [Sesamum indicum]
c42730.graph_c0	2.06079656	2.29206681	2.64820516	0.31642337	0.39108089	0.18770107	1.036E-13	-3.986629	down	PREDICTED: uncharacterized protein LOC104646173 [Solanum lycopersicum]
c42731.graph_c0	33.0929906	33.2099919	29.4450814	21.8428868	23.7531379	23.9340309	1.594E-22	-1.479417	down	PREDICTED: lipase-like [Sesamum indicum]
c42733.graph_c0	20.9317812	20.3779777	24.0809031	16.9754461	17.168314	13.863758	1.331E-16	-1.463547	down	PREDICTED: uncharacterized protein LOC105161097 [Sesamum indicum]
c42736.graph_c0	0.30251359	0.20187767	0.21596774	10.1104442	9.84146066	10.566761	2.738E-56	4.389693	up	PREDICTED: probable polyribonucleotide nucleotidyltransferase 1, chloroplastic-like [Solanum tuberosum]
c42737.graph_c0	0	0	0	2.84662216	4.6072446	3.65864385	1.805E-12	Inf	up	hypothetical protein MIMGU_mgv1a011219mg [Erythranthe guttata]
c42739.graph_c0	2.71986182	3.27718607	2.86945868	16.9677061	18.5815019	19.1440183	8.057E-22	1.605731	up	PREDICTED: nardilysin isoform X1 [Sesamum indicum]
c42741.graph_c0	20.6137178	20.8071593	20.3172726	12.7041243	13.2701795	12.4777438	2.414E-33	-1.700699	down	PREDICTED: zinc finger CCCH domain-containing protein 41 [Sesamum indicum]
c42742.graph_c0	0.19312334	0.2416459	0.06204278	1.17870552	1.5314191	1.32584794	0.0002796	2.010648	up	PREDICTED: uncharacterized protein LOC105163391 [Sesamum indicum]
c42744.graph_c0	21.3280843	21.6784565	20.508644	11.0188173	14.5067238	14.5645138	5.342E-31	-1.685891	down	PREDICTED: MAR-binding filament-like protein 1-1 [Sesamum indicum]
c42747.graph_c0	239.098083	246.669119	227.588765	46.4202767	17.4400569	9.29531711	2.24E-165	-4.276915	down	hypothetical protein MIMGU_mgv1a015419mg [Erythranthe guttata]
c42749.graph_c0	0.05573454	0	0	0.85577161	0.68900555	0.7005436	4.876E-09	4.340297	up	-
c42749.graph_c1	0.11477048	0	0	3.91216006	7.61684396	7.52652216	2.407E-10	6.367957	up	-
c42754.graph_c0	0.20685667	0.37271485	0.0531638	2.17249635	2.22074219	1.66176553	0.0001018	2.253787	up	-
c42755.graph_c0	4.00330412	3.96950799	2.81486273	1.80925899	2.65387918	3.00302258	3.301E-06	-1.552473	down	PREDICTED: uncharacterized protein LOC105156766 [Sesamum indicum]
c42757.graph_c1	2.69903379	2.66368383	2.00285882	16.5301445	15.6937335	13.7424038	1.127E-10	1.629385	up	hypothetical protein MIMGU_mgv1a010783mg [Erythranthe guttata]
c42758.graph_c0	0.24055833	0.36119882	0.92738093	4.32155143	5.04771457	4.43687363	8.315E-05	2.141326	up	PREDICTED: uncharacterized protein LOC100262706 [Vitis vinifera]
c42760.graph_c0	16.6812682	16.9842973	15.1205468	6.43222715	7.5120773	6.90639732	5.12E-53	-2.245007	down	PREDICTED: protein PHOTOPERIOD-INDEPENDENT EARLY FLOWERING 1 isoform X1 [Sesamum indicum]
c42764.graph_c0	2.26045893	2.0437493	1.40553833	10.8154721	10.6748583	14.6445205	1.58E-05	1.646621	up	PREDICTED: LOW QUALITY PROTEIN: protein IQ-DOMAIN 14 [Sesamum indicum]

#ID	0 h-1 FPKM	0 h-2 FPKM	0 h-3 FPKM	48 h-1 FPKM	48 h-2 FPKM	48 h-3 FPKM	FDR	log2FC	regulated	nr_annotation
c42766.graph_c0	57.2857277	58.8910446	57.496092	36.2494148	46.3505413	46.9873579	2.598E-24	-1.444453	down	PREDICTED: LOW QUALITY PROTEIN: neurofilament heavy polypeptide [Sesamum indicum]
c42770.graph_c0	13.0491816	12.7005245	12.2282629	9.13653546	11.4816278	8.6562503	2.312E-13	-1.394752	down	PREDICTED: E3 ubiquitin-protein ligase Topors [Sesamum indicum]
c42771.graph_c0	1.0375451	0.99863851	1.07688528	36.7636239	26.8342086	24.4686145	1.889E-14	3.81138	up	PREDICTED: uncharacterized protein LOC105176977 [Sesamum indicum]
c42773.graph_c0	0.08966356	0.26926003	0.23044244	2.18900382	1.92519436	2.7562676	6.769E-06	2.520898	up	PREDICTED: probable beta-1,3-galactosyltransferase 20 [Sesamum indicum]
c42773.graph_c1	0	0	0	1.50511155	1.93615815	1.65810568	1.568E-12	Inf	up	PREDICTED: probable beta-1,3-galactosyltransferase 20 [Sesamum indicum]
c42774.graph_c0	12.4726889	11.6455319	11.5757049	9.9619271	9.61022776	7.84761043	2.287E-19	-1.39508	down	PREDICTED: uncharacterized protein LOC105163756 [Sesamum indicum]
c42776.graph_c0	1.71231077	0.99984815	0.97794984	0	0	0	4.795E-15	-Inf	down	unnamed protein product [Coffea canephora]
c42779.graph_c0	27.2465449	29.8257653	27.8465081	15.5598667	21.0712624	24.5554174	1.019E-15	-1.496746	down	PREDICTED: serine-threonine kinase receptor-associated protein [Solanum lycopersicum]
c42780.graph_c0	24.2585071	26.1674334	29.8703839	17.2784072	18.0216843	19.4700583	4.177E-17	-1.572778	down	hypothetical protein MIMGU_mgv1a013973mg [Erythranthe guttata]
c42782.graph_c2	2.56260058	2.63642127	2.65272925	13.9682726	17.7850183	17.2137139	5.304E-07	1.618771	up	hypothetical protein MIMGU_mgv1a014334mg [Erythranthe guttata]
c42783.graph_c0	7.20083563	4.83577782	5.1537695	0	0	0	5.973E-32	-Inf	down	PREDICTED: glycine-rich RNA-binding protein-like isoform X1 [Eucalyptus grandis]
c42786.graph_c0	6.00304211	4.81427073	4.73675679	29.6248003	32.6173114	33.0650705	1.767E-19	1.597722	up	PREDICTED: protein TIC 40, chloroplastic-like [Sesamum indicum]
c42789.graph_c1	0	0.02447979	0.09427811	1.28419986	1.33658641	1.00234501	2.622E-09	3.891589	up	PREDICTED: ABC transporter G family member 15-like isoform X1 [Sesamum indicum]
c42792.graph_c0	2.89511177	1.97912932	2.26849588	0.52042259	0.41349348	0.4630689	1.342E-10	-3.366565	down	PREDICTED: external alternative NAD(P)H-ubiquinone oxidoreductase B1, mitochondrial-like isoform X2 [Sesamum indicum]
c42794.graph_c0	5.53287244	5.83162286	5.31155846	210.666054	122.006221	114.00103	6.314E-07	3.738523	up	PREDICTED: LOW QUALITY PROTEIN: cytochrome P450 78A7-like [Sesamum indicum]
c42796.graph_c0	57.9906899	56.3711733	58.0093436	53.2466573	53.8920181	52.9618276	2.597E-15	-1.124167	down	PREDICTED: putative oxidoreductase TDA3 [Sesamum indicum]
c42797.graph_c0	68.4494852	70.999687	61.7742379	58.464317	23.8124034	13.6063066	1.418E-12	-2.062302	down	PREDICTED: LOW QUALITY PROTEIN: probable WRKY transcription factor 33 [Sesamum indicum]
c42798.graph_c0	3.75476919	4.34472162	3.80690208	19.8979789	20.4743429	23.3244246	1.425E-12	1.400934	up	PREDICTED: uncharacterized protein LOC105176928 [Sesamum indicum]
c42799.graph_c0	38.4660335	44.0537368	36.9758597	17.3365413	13.9492988	13.1383343	3.087E-59	-2.439145	down	PREDICTED: uncharacterized protein LOC105164926 [Sesamum indicum]

#ID	0 h-1 FPKM	0 h-2 FPKM	0 h-3 FPKM	48 h-1 FPKM	48 h-2 FPKM	48 h-3 FPKM	FDR	log2FC	regulated	nr_annotation
c42801.graph_c0	21.5200614	19.3198301	23.5618156	4.40076848	4.42662196	3.67845749	1.61E-56	-3.382075	down	vacuolar H+-ATPase A subunit [Citrus unshiu]
c42802.graph_c1	98.4030965	98.6034954	97.247202	79.5304612	89.649037	78.6625694	4.539E-19	-1.266262	down	PREDICTED: cAMP-regulated phosphoprotein 21-like [Sesamum indicum]
c42803.graph_c0	30.9510395	31.7744116	29.6011088	298.438027	266.972334	256.701797	3.616E-34	2.140204	up	PREDICTED: 2,3-bisphosphoglycerate-independent phosphoglycerate mutase [Sesamum indicum]
c42808.graph_c0	6.0338462	5.73329487	6.45488287	5.95381236	4.4838926	5.3729887	2.433E-07	-1.219049	down	PREDICTED: receptor-like cytosolic serine/threonine-protein kinase RBK1 [Sesamum indicum]
c42810.graph_c0	0.80684377	0.56834749	0.7680194	18.6655185	20.2404931	20.3808905	1.157E-52	3.77015	up	hypothetical protein MIMGU_mgv1a004563mg [Erythranthe guttata]
c42811.graph_c1	2.18045566	1.69154526	2.17152818	45.6494794	32.8252743	25.2023197	1.979E-07	3.091139	up	hypothetical protein MIMGU_mgv1a016107mg [Erythranthe guttata]
c42812.graph_c0	0.68415176	0.59144999	0.31969531	3.38062073	3.33855383	3.415928	0.0002342	1.656946	up	PREDICTED: protein TPX2-like isoform X2 [Sesamum indicum]
c42815.graph_c0	12.4726274	13.7336286	12.6219083	11.7060466	11.9446534	8.94623181	5.379E-07	-1.267814	down	hypothetical protein MIMGU_mgv1a002605mg [Erythranthe guttata]
c42816.graph_c0	11.8001791	11.7601882	11.0402358	57.8122908	64.5283551	68.6658185	7.152E-24	1.445492	up	hypothetical protein MIMGU_mgv1a003367mg [Erythranthe guttata]
c42817.graph_c0	1.84452032	1.93777165	2.06519588	10.8350923	12.3874755	12.3656304	6.257E-16	1.584518	up	PREDICTED: probable glutamate carboxypeptidase 2 isoform X2 [Sesamum indicum]
c42818.graph_c0	21.1990576	20.7275065	19.808499	141.556739	140.293031	140.811818	6.431E-36	1.758723	up	V-type proton ATPase subunit B2 -like protein [Gossypium arboreum]
c42819.graph_c0	14.4972572	13.2313234	15.7213166	12.7817	12.5163087	11.4537399	2.534E-13	-1.259123	down	PREDICTED: uncharacterized protein At2g02148 isoform X1 [Sesamum indicum]
c42820.graph_c0	4.37690861	4.43107883	5.04926781	52.6260149	51.9403076	50.3326789	7.333E-58	2.464155	up	PREDICTED: glucosidase 2 subunit beta [Sesamum indicum]
c42821.graph_c0	10.60605	9.49283359	9.56192334	8.46158498	8.96402327	8.6941637	5.604E-16	-1.200991	down	PREDICTED: disease resistance protein RPS2 isoform X1 [Vitis vinifera]
c42822.graph_c0	16.7051912	17.6386214	17.1653496	2.82196869	6.93448526	8.10241083	1.088E-47	-2.56069	down	PREDICTED: uncharacterized protein LOC105177905 [Sesamum indicum]
c42825.graph_c0	4.59922855	4.9293576	4.95500843	2.09715512	4.03529916	3.00850705	3.556E-07	-1.691716	down	PREDICTED: protein SULFUR DEFICIENCY-INDUCED 1 [Sesamum indicum]
c42826.graph_c0	2.21721753	1.65104515	2.60598687	9.29154556	10.0543815	10.5505609	7.051E-07	1.18492	up	PREDICTED: B3 domain-containing protein Os01g0723500-like [Sesamum indicum]
c42831.graph_c0	35.1806492	32.2477965	31.8210362	15.4301552	13.7653713	14.7807337	1.925E-32	-2.189317	down	PREDICTED: uncharacterized protein LOC105163175 [Sesamum indicum]
c42834.graph_c0	15.5343019	15.1394041	12.7271646	11.6959652	7.71132787	6.52861462	1.921E-19	-1.7482	down	PREDICTED: calmodulin-binding transcription activator 1 [Sesamum indicum]
c42838.graph_c0	0.80017006	0.84004308	0.62698183	18.2853835	20.4187504	21.1976865	6.26E-72	3.706541	up	PREDICTED: WD repeat-containing protein 44 [Sesamum indicum]

#ID	0 h-1 FPKM	0 h-2 FPKM	0 h-3 FPKM	48 h-1 FPKM	48 h-2 FPKM	48 h-3 FPKM	FDR	log2FC	regulated	nr_annotation
c42839.graph_c0	11.3705617	11.4394322	12.8405163	7.72421341	9.13566714	7.62615959	6.2E-11	-1.562628	down	PREDICTED: probable complex I intermediate-associated protein 30-like [Citrus sinensis]
c42843.graph_c0	0.03198604	0.16009036	0.28772332	2.00379877	2.37252086	1.83540833	6.27E-07	2.661333	up	Receptor-like protein kinase [Medicago truncatula]
c42844.graph_c0	12.37926	13.5028642	13.6599923	8.46046417	11.1296753	9.70652267	5.708E-16	-1.454977	down	PREDICTED: 5'-3' exoribonuclease 3 isoform X2 [Sesamum indicum]
c42847.graph_c0	71.487786	70.375624	70.0994816	57.8464577	58.7496377	56.5372697	1.36E-20	-1.309194	down	PREDICTED: uncharacterized protein LOC105164616 [Sesamum indicum]
c42849.graph_c0	0.30173407	0.20135748	0.4523636	5.23523558	4.56448715	4.36971505	1.373E-08	2.869729	up	PREDICTED: monothiol glutaredoxin-S10-like [Sesamum indicum]
c42851.graph_c0	10.3016933	9.7616489	8.99892214	7.6511232	8.67027611	8.20746167	6.175E-12	-1.262807	down	hypothetical protein MIMGU_mgv1a008129mg [Erythranthe guttata]
c42852.graph_c0	18.0885264	19.9161652	16.9767927	12.7679564	13.9699442	13.5976058	1.782E-25	-1.464779	down	PREDICTED: E3 ubiquitin-protein ligase UPL4 isoform X1 [Sesamum indicum]
c42854.graph_c0	9.42569303	10.6269375	13.6423747	4.11326678	1.93666995	1.30131913	8.43E-15	-3.19845	down	-
c42856.graph_c1	0	0.17761058	0	19.288607	11.082815	12.0721998	6.751E-10	6.905639	up	PREDICTED: uncharacterized protein LOC105158077 [Sesamum indicum]
c42859.graph_c0	44.975602	45.2581446	45.2945422	40.8029834	39.5722029	45.5773093	2.115E-15	-1.123722	down	PREDICTED: soluble starch synthase 1, chloroplastic/amyloplastic-like, partial [Sesamum indicum]
c42860.graph_c1	0.61615193	0.46257668	0.79177962	5.72369671	4.51008791	6.14516523	2.597E-06	2.110884	up	hypothetical protein MIMGU_mgv1a007855mg [Erythranthe guttata]
c42863.graph_c1	31.0968348	31.1681906	31.5451752	16.8041193	20.7427738	18.6737882	4.55E-30	-1.75943	down	PREDICTED: uncharacterized protein LOC105166783 [Sesamum indicum]
c42865.graph_c0	11.4783505	11.0424521	10.9385955	7.74847171	10.9051001	13.3227894	1.564E-11	-1.089969	down	PREDICTED: putative amidase C869.01 isoform X1 [Sesamum indicum]
c42871.graph_c1	7.4223364	7.90910867	7.24750961	6.51932671	5.81594622	5.95351185	3.005E-11	-1.318866	down	PREDICTED: uncharacterized protein LOC105178500 [Sesamum indicum]
c42875.graph_c0	1.02767578	0.85725404	0.88040271	0.35503573	0.62686218	0.70201925	0.0005664	-1.74249	down	--
c42877.graph_c1	2.38608402	3.00172998	2.48611819	11.2584318	11.7695958	13.2952388	1.165E-06	1.18702	up	PREDICTED: protein DEK-like [Sesamum indicum]
c42878.graph_c0	13.7380393	14.3018677	12.2871314	10.6651077	10.1542478	9.9552269	1.603E-20	-1.404962	down	PREDICTED: long chain acyl-CoA synthetase 4 [Sesamum indicum]
c42882.graph_c0	0.3361044	0.37849608	0.10797687	3.52216975	5.20743511	5.51033928	9.456E-10	3.09856	up	PREDICTED: transcription factor bHLH110 [Sesamum indicum]
c42883.graph_c0	0.49300285	0.41757398	0.29239762	3.03955503	3.51613562	3.60610372	2.313E-05	2.062871	up	PREDICTED: uncharacterized protein LOC101301730 isoform 2 [Fragaria vesca subsp. vesca]
c42883.graph_c1	1.48822958	1.42891493	1.24893645	12.9270821	17.8742199	21.4861594	3.214E-12	2.626874	up	PREDICTED: uncharacterized protein At1g01500-like [Sesamum indicum]
c42886.graph_c0	3.60303267	3.66928692	3.03307807	16.1849137	15.6276859	15.9183159	1.258E-15	1.196538	up	PREDICTED: LOW QUALITY PROTEIN: translational activator GCN1 [Sesamum indicum]

#ID	0 h-1 FPKM	0 h-2 FPKM	0 h-3 FPKM	48 h-1 FPKM	48 h-2 FPKM	48 h-3 FPKM	FDR	log2FC	regulated	nr_annotation
c42887.graph_c0	10.0495079	10.9984642	9.03981086	49.9328938	54.7286742	57.0615059	6.793E-15	1.408267	up	-
c42891.graph_c0	0.08844409	0	0	4.39994492	3.8843401	5.19589243	5.197E-22	6.255713	up	PREDICTED: uncharacterized protein LOC104093672 [Nicotiana tomentosiformis]
c42892.graph_c0	11.0482363	11.210108	10.7771332	5.64436097	6.61667758	7.53347768	8.518E-14	-1.759946	down	PREDICTED: UDP-galactose/UDP-glucose transporter 5B-like [Sesamum indicum]
c42901.graph_c0	28.4815192	25.9411133	25.7039791	20.5137318	18.9324594	16.1286353	1.688E-26	-1.541668	down	PREDICTED: MACPF domain-containing protein At4g24290-like [Sesamum indicum]
c42906.graph_c0	0.28900933	0.18081171	0.32496488	2.66254247	2.60909282	2.6060257	7.451E-06	2.289053	up	PREDICTED: ABC transporter G family member 24-like [Sesamum indicum]
c42906.graph_c1	0.8511801	0.8848029	0.715177	4.43353484	4.24947899	4.45482189	0.0004521	1.40721	up	PREDICTED: ABC transporter G family member 24-like [Sesamum indicum]
c42911.graph_c1	49.251218	48.519865	47.5584319	9.34030873	7.08098329	6.3155475	2.34E-132	-3.686761	down	hypothetical protein MIMGU_mgv1a023461mg [Erythranthe guttata]
c42916.graph_c0	40.1535778	39.1804968	38.6630692	27.614547	25.8513291	23.4187599	8.575E-26	-1.63301	down	hypothetical protein MIMGU_mgv1a013052mg [Erythranthe guttata]
c42917.graph_c0	3.8081908	4.12967113	3.4663538	2.83591928	3.87153686	4.08423996	0.0001514	-1.101805	down	PREDICTED: uncharacterized protein LOC105168516 isoform X1 [Sesamum indicum]
c42919.graph_c0	18.8064427	18.8478197	16.6709048	10.0426937	10.4192019	10.0621509	9.512E-34	-1.848235	down	PREDICTED: pentatricopeptide repeat-containing protein At4g02820, mitochondrial [Sesamum indicum]
c42920.graph_c0	1.18665484	0.67006534	0.54739944	14.8286003	13.6006987	13.6433581	4.186E-29	3.119795	up	PREDICTED: uncharacterized protein LOC105169238 [Sesamum indicum]
c42921.graph_c0	0.31391227	0.25138128	0.32271175	5.08985764	6.37246645	9.16077955	1.408E-09	3.512207	up	PREDICTED: protein notum homolog isoform X1 [Sesamum indicum]
c42922.graph_c0	2.42333975	2.10936205	2.03092665	16.3072388	17.5223356	21.9551375	2.894E-20	2.068174	up	PREDICTED: root phototropism protein 3-like [Sesamum indicum]
c42923.graph_c1	0.07122728	0.3208437	0.09152987	1.73890915	2.01595238	2.91937906	1.021E-06	2.768365	up	PREDICTED: phosphate transporter PHO1 homolog 1 [Sesamum indicum]
c42925.graph_c0	11.7413575	12.8380176	13.7948428	5.77694015	3.82812788	4.33645578	4.91E-43	-2.472315	down	PREDICTED: formin-like protein 5 [Sesamum indicum]
c42926.graph_c0	6.04886106	5.17636134	5.94407867	29.3709082	26.9477396	25.9007177	7.04E-15	1.243852	up	PREDICTED: neutral ceramidase isoform X1 [Sesamum indicum]
c42928.graph_c0	15.591991	15.8634664	15.4583538	1.56006359	1.90216209	2.26554141	3.01E-128	-4.056183	down	PREDICTED: probably inactive leucine-rich repeat receptor-like protein kinase At5g06940 [Sesamum indicum]
c42929.graph_c0	2.4391503	1.05432414	2.49327647	0	0.21641224	0.13632681	7.055E-08	-5.136255	down	PREDICTED: mitochondrial phosphate carrier protein 3, mitochondrial-like [Sesamum indicum]
c42932.graph_c0	18.6742762	21.0004891	22.1219796	8.70914008	12.4668274	11.7713695	1.285E-30	-1.932334	down	PREDICTED: sulfate transporter 4.1, chloroplastic [Sesamum indicum]
c42935.graph_c0	79.7319639	76.3232277	78.9194378	501.53302	479.256402	485.072434	1.165E-28	1.624495	up	glutamine synthetase [Hevea brasiliensis]

#ID	0 h-1 FPKM	0 h-2 FPKM	0 h-3 FPKM	48 h-1 FPKM	48 h-2 FPKM	48 h-3 FPKM	FDR	log2FC	regulated	nr_annotation
c42937.graph_c0	23.8166415	23.4105015	23.4599954	20.5343771	17.4914514	15.3956793	2.559E-22	-1.417119	down	hypothetical protein MIMGU_mgv1a002036mg [Erythranthe guttata]
c42939.graph_c0	32.9914529	34.189712	30.9396865	21.2173434	21.5136884	21.1861031	2.922E-31	-1.635093	down	PREDICTED: protein transport protein SEC16B homolog [Sesamum indicum]
c42942.graph_c1	14.6034759	14.7327509	13.0258428	5.2759508	3.9124611	4.83534458	9.322E-28	-2.606935	down	PREDICTED: uncharacterized protein LOC105166066 [Sesamum indicum]
c42944.graph_c0	32.155664	32.6903622	32.2542682	30.1891385	29.6137225	26.2219954	7.759E-17	-1.190798	down	PREDICTED: uncharacterized protein LOC105162695 [Sesamum indicum]
c42945.graph_c0	111.577083	104.961575	115.648847	35.5330668	34.0171912	35.1415272	6.782E-76	-2.683304	down	PREDICTED: cell division cycle protein 48 homolog [Solanum lycopersicum]
c42945.graph_c1	100.088888	104.116071	109.249175	36.5695044	33.0482015	33.1740324	8.334E-73	-2.624788	down	PREDICTED: cell division cycle protein 48 homolog [Sesamum indicum]
c42947.graph_c0	2.72746655	2.1234871	2.53131848	11.2141767	11.682881	11.8000726	7.836E-05	1.214319	up	PREDICTED: 39S ribosomal protein L45, mitochondrial [Sesamum indicum]
c42950.graph_c0	0.05386135	0.16174586	0	7.56714004	9.85631227	9.49270215	1.637E-40	5.955955	up	hypothetical protein CISIN_1g0086311mg, partial [Citrus sinensis]
c42952.graph_c0	14.3514206	14.0426346	13.1998984	71.1363472	75.847269	79.9689133	1.512E-23	1.429721	up	hypothetical protein MIMGU_mgv1a007743mg [Erythranthe guttata]
c42953.graph_c0	0.78871099	0.48056568	0.44066298	9.38277248	11.1447473	9.72613517	9.259E-20	3.129962	up	hypothetical protein CICLE_v10018965mg [Citrus clementina]
c42957.graph_c0	0.1154822	0.23119568	0.2967984	3.83003046	6.87512846	5.55367686	5.123E-12	3.625612	up	PREDICTED: putative pectate lyase 2 [Sesamum indicum]
c42958.graph_c0	1.40473928	1.32802669	1.50428799	7.5491351	7.95931044	6.94108085	4.624E-05	1.387149	up	hypothetical protein MIMGU_mgv1a008941mg [Erythranthe guttata]
c42960.graph_c0	0.39332024	0.31497127	0.40434566	3.1162294	5.45092954	4.7717991	4.401E-06	2.556001	up	PREDICTED: SEC14 cytosolic factor [Sesamum indicum]
c42961.graph_c0	49.3736662	49.5166223	50.1214207	44.5395082	39.4797337	37.0272116	1.104E-19	-1.31453	down	PREDICTED: LOW QUALITY PROTEIN: serine/arginine-rich SC35-like splicing factor SCL28 [Sesamum indicum]
c42963.graph_c0	20.3407552	20.31743	19.8283732	16.7084769	17.6580046	17.2465543	2.047E-17	-1.246905	down	PREDICTED: ubiquitin carboxyl-terminal hydrolase 25 isoform X1 [Sesamum indicum]
c42964.graph_c0	0.77642246	0.24543156	0.57763524	5.4964467	5.224584	6.49860831	1.695E-08	2.410364	up	PREDICTED: uncharacterized protein LOC105168903 [Sesamum indicum]
c42966.graph_c0	2.16349917	4.84089956	3.10726234	15.8280079	16.5194425	16.5874292	0.0016738	1.256597	up	NAD(P)H-quinone oxidoreductase subunit H [Medicago truncatula]
c42978.graph_c0	24.9943599	26.39646	23.8953906	20.3350331	19.1287762	18.3727491	2.527E-23	-1.395604	down	PREDICTED: uncharacterized protein LOC105176008 [Sesamum indicum]
c42979.graph_c0	103.278708	97.5754529	92.6361014	81.1825091	67.6246078	60.5269763	3.643E-26	-1.499403	down	PREDICTED: heterogeneous nuclear ribonucleoprotein A2 homolog 1 [Sesamum indicum]
c42981.graph_c0	1.03973855	0.59473125	0.71577064	16.8312316	16.9971024	16.9853483	1.115E-32	3.419056	up	PREDICTED: petal death protein [Sesamum indicum]

#ID	0 h-1 FPKM	0 h-2 FPKM	0 h-3 FPKM	48 h-1 FPKM	48 h-2 FPKM	48 h-3 FPKM	FDR	log2FC	regulated	nr_annotation
c42985.graph_c1	2.03857477	1.90636504	1.65451534	23.3522935	32.0951535	37.5776192	6.603E-17	3.031964	up	PREDICTED: protein NSP-INTERACTING KINASE 3 [Sesamum indicum]
c42986.graph_c0	72.7450531	62.785977	70.1657158	47.0395905	39.6300996	47.3088053	1.975E-26	-1.6342	down	thioredoxin peroxidase, partial [Fargesia spathacea]
c42986.graph_c1	0.1851051	0.92645228	0.23786738	62.1755634	31.1452328	15.0826939	0.000533	5.335608	up	PREDICTED: cytochrome P450 CYP82D47-like [Sesamum indicum]
c42991.graph_c0	13.3872175	13.211119	10.9474301	66.6988227	79.2376779	94.5314805	4.099E-14	1.659264	up	PREDICTED: proline transporter 3 [Sesamum indicum]
c42996.graph_c0	0.12918691	0.25863253	0.16601029	4.76061613	8.25839644	6.40690101	1.471E-12	4.107238	up	-
c42998.graph_c0	3.10135118	3.79433598	2.9386991	22.5687332	29.6261666	27.040721	2.571E-26	1.98976	up	PREDICTED: exocyst complex component EXO70B1 [Sesamum indicum]
c43001.graph_c0	11.0073657	12.6099328	11.0539779	53.520378	47.1093666	42.5089997	1.185E-07	1.032034	up	aldose 1-epimerase, putative [Ricinus communis]
c43008.graph_c0	6.22339052	5.33592121	6.20887676	34.5938919	38.3651094	38.5306099	2.179E-25	1.629567	up	PREDICTED: uncharacterized protein LOC105169979 [Sesamum indicum]
c43010.graph_c0	8.68574599	9.40816642	7.37160584	60.9993736	92.3615292	95.1107271	1.225E-12	2.26345	up	hypothetical protein MIMGU_mgv1a004602mg [Erythranthe guttata]
c43011.graph_c0	11.4300259	12.139122	9.7323164	7.02005692	7.93571548	6.24640443	2.591E-14	-1.667525	down	PREDICTED: uncharacterized protein LOC105158337 [Sesamum indicum]
c43012.graph_c0	0.67954997	0.71511405	0.49260178	6.40492965	8.0266573	8.09867541	2.778E-21	2.559283	up	PREDICTED: heat shock 70 kDa protein 16 [Sesamum indicum]
c43015.graph_c0	3.93982718	3.38437199	3.91382196	14.2095988	17.2622619	19.4235253	1.536E-09	1.156746	up	PREDICTED: protein PHLOEM PROTEIN 2-LIKE A9 [Sesamum indicum]
c43017.graph_c0	1.24395948	0.96849287	0.8436724	12.6380165	14.4676155	17.136939	2.568E-24	2.837679	up	hypothetical protein MIMGU_mgv1a007209mg [Erythranthe guttata]
c43022.graph_c1	16.1807069	13.1064646	14.0052983	62.4711614	70.1739657	73.4851221	5.316E-17	1.232224	up	PREDICTED: eukaryotic translation initiation factor 3 subunit M [Sesamum indicum]
c43024.graph_c0	2.52998112	2.85193076	2.31386438	9.55411305	13.5916269	14.3118974	2.269E-07	1.260378	up	PREDICTED: uncharacterized protein LOC105166682 isoform X3 [Sesamum indicum]
c43027.graph_c0	0	0.22369319	0.04102386	7.99969884	7.94504148	7.37977818	5.047E-29	5.449306	up	PREDICTED: phototropin-2 isoform X1 [Sesamum indicum]
c43030.graph_c0	6.81193458	6.97372742	6.56520422	100.552202	104.860807	103.938355	5.167E-38	2.908419	up	Succinyl-CoA ligase [ADP-forming] subunit alpha-1 [Morus notabilis]
c43030.graph_c1	5.46072553	6.41901636	4.18459773	80.8616559	77.2043753	73.5107792	2.355E-62	2.836738	up	hypothetical protein M569_06331, partial [Genlisea aurea]
c43031.graph_c0	2.40877652	2.15051951	2.09146838	13.1947797	12.8981302	15.636392	1.133E-06	1.632053	up	PREDICTED: serine protease SPPA, chloroplastic-like [Sesamum indicum]
c43032.graph_c0	0	0	0.03968561	1.36565952	1.23576619	1.9746279	6.096E-12	5.797392	up	Protein kinase G11A [Glycine soja]
c43035.graph_c0	21.437088	22.8353196	22.6108961	21.7591225	18.9315685	18.5447029	9.349E-13	-1.190005	down	PREDICTED: uncharacterized protein LOC105155972 [Sesamum indicum]
c43040.graph_c0	11.0554949	12.5801538	11.8177214	6.75958011	6.48356781	6.8680615	3.206E-26	-1.835187	down	PREDICTED: mitogen-activated protein kinase kinase kinase YODA-like isoform X5 [Sesamum indicum]

#ID	0 h-1 FPKM	0 h-2 FPKM	0 h-3 FPKM	48 h-1 FPKM	48 h-2 FPKM	48 h-3 FPKM	FDR	log2FC	regulated	nr_annotation
c43041.graph_c0	5.45498038	4.247011	4.15399452	19.1712898	23.0208975	23.1449043	1.241E-07	1.218567	up	PREDICTED: uncharacterized protein LOC105157371 [Sesamum indicum]
c43042.graph_c0	0.40868792	0.88113234	0.82816871	5.82142335	7.5324434	7.74403075	1.079E-19	2.290063	up	hypothetical protein MIMGU_mgv1a000774mg [Erythranthe guttata]
c43044.graph_c1	61.4178725	66.468645	55.7393634	38.2527212	40.0416898	53.9932828	9.533E-26	-1.492415	down	PREDICTED: uncharacterized protein LOC100243914 [Vitis vinifera]
c43045.graph_c0	19.9331374	18.370342	20.5518534	14.5190292	17.2784968	18.2735076	2.942E-16	-1.25494	down	PREDICTED: putative MO25-like protein At5g47540 [Sesamum indicum]
c43047.graph_c0	16.9779486	17.8855145	16.767909	64.1812591	76.3616531	77.5284471	4.153E-14	1.058025	up	PREDICTED: putative serine/threonine-protein kinase {ECO:0000250|UniProtKB:Q9FE20, ECO:0000303|Ref.2} isoform X1 [Nicotiana tomentosiformis]
c43050.graph_c0	11.7228524	13.0100901	11.2436662	6.57382555	7.25434485	8.04285686	6.023E-17	-1.737251	down	PREDICTED: putative vesicle-associated membrane protein 726 [Pyrus x bretschneideri]
c43052.graph_c0	56.9464359	55.611655	55.3110811	52.9083148	52.9229472	51.0914767	2.357E-14	-1.114203	down	PLAC8 family protein isoform 1 [Theobroma cacao]
c43053.graph_c0	19.8850525	21.5596558	20.7423533	12.4070314	15.848415	16.1410805	9.017E-18	-1.508393	down	PREDICTED: F-box protein PP2-A15 [Sesamum indicum]
c43055.graph_c0	0	0.13478323	0.06921141	0.84351886	1.44553904	1.6114922	4.993E-07	3.226223	up	PREDICTED: uncharacterized protein LOC105175800 [Sesamum indicum]
c43056.graph_c0	0.06354077	0	0	1.87321272	2.26350386	3.35959016	6.004E-12	5.879798	up	PREDICTED: flap endonuclease GEN-like 2 isoform X2 [Sesamum indicum]
c43058.graph_c0	0.48554697	0.22872149	0.33032492	3.89429387	4.12553214	4.98696219	8.828E-10	2.62027	up	unnamed protein product [Coffea canephora]
c43063.graph_c0	2.19149854	2.48342603	1.85972988	23.1227075	22.4375813	24.2351383	2.983E-25	2.401695	up	PREDICTED: uncharacterized protein LOC105177601 [Sesamum indicum]
c43066.graph_c1	1.61440524	2.50098616	1.87699612	14.6250715	13.6552792	14.3366872	8.218E-12	1.814052	up	PREDICTED: zinc finger CCCH domain-containing protein 67 isoform X1 [Sesamum indicum]
c43072.graph_c0	6.42113669	8.38467807	6.82328537	50.2444241	47.3627657	44.3529031	6.749E-30	1.699148	up	PREDICTED: phosphatidylinositol 4-phosphate 5-kinase 9-like [Sesamum indicum]
c43077.graph_c0	1.35488899	0.96874693	0.64668908	4.70754282	6.57386605	8.37748658	6.18E-05	1.707712	up	PREDICTED: microtubule-associated protein TORTIFOLIA1-like [Sesamum indicum]
c43079.graph_c0	0.39530079	0.49462079	0.38098288	9.0680042	10.0694124	10.5475799	5.687E-37	3.527332	up	PREDICTED: ATP-dependent DNA helicase DDM1 [Sesamum indicum]
c43088.graph_c0	1.5536107	1.43553822	1.68930491	0	0	0	4.063E-20	-Inf	down	PREDICTED: putative clathrin assembly protein At5g57200 [Sesamum indicum]
c43090.graph_c0	0.19142737	0.22994287	0.04919835	2.32789101	1.64407883	1.4122796	4.358E-05	2.518133	up	hypothetical protein MIMGU_mgv1a018481mg, partial [Erythranthe guttata]
c43092.graph_c0	1.5705004	2.37935376	1.36361837	7.97721272	11.9720936	11.1342367	1.46E-06	1.527193	up	PREDICTED: uncharacterized protein LOC105160850 [Sesamum indicum]

#ID	0 h-1 FPKM	0 h-2 FPKM	0 h-3 FPKM	48 h-1 FPKM	48 h-2 FPKM	48 h-3 FPKM	FDR	log2FC	regulated	nr_annotation
c43096.graph_c0	0	0.08865349	0.11380928	7.13927182	7.99537526	9.07497305	1.099E-24	5.867991	up	PREDICTED: uncharacterized protein LOC104227860 [Nicotiana sylvestris]
c43097.graph_c0	30.9847032	34.7956099	31.574075	28.354511	25.8984526	26.1201324	1.379E-16	-1.291976	down	PREDICTED: uncharacterized protein LOC105164803 [Sesamum indicum]
c43099.graph_c0	5.5004032	5.26800128	6.37013851	0.07819954	0	0.02783259	2.03E-113	-8.319476	down	PREDICTED: putative RING-H2 finger protein ATL21A [Sesamum indicum]
c43104.graph_c0	1.4032262	1.66001883	0.88520788	5.31200058	6.15032241	6.6715973	0.000169	1.183623	up	hypothetical protein MIMGU_mgv1a007414mg [Erythranthe guttata]
c43107.graph_c0	5.90546584	6.1268983	6.8773114	5.89387534	5.79356888	4.21059576	4.67E-08	-1.266555	down	PREDICTED: secretory carrier-associated membrane protein 2 [Sesamum indicum]
c43108.graph_c0	26.367832	27.9986795	23.2660393	23.8966096	17.6922496	17.0807693	1.129E-19	-1.413521	down	PREDICTED: U-box domain-containing protein 32 isoform X3 [Sesamum indicum]
c43111.graph_c0	5.14040189	5.74521849	5.56262732	23.6605642	30.0823707	30.0667018	4.372E-18	1.326892	up	PREDICTED: alpha-mannosidase [Sesamum indicum]
c43115.graph_c0	0	0.06908632	0	3.56065677	4.88350173	4.97868011	3.727E-20	6.590441	up	PREDICTED: MLO-like protein 4 [Sesamum indicum]
c43116.graph_c0	1.82084575	1.57249879	2.20222012	11.4792387	12.8229141	12.5847786	1.598E-10	1.698619	up	PREDICTED: tRNA (adenine(58)-N(1))-methyltransferase non-catalytic subunit trm6 [Sesamum indicum]
c43119.graph_c0	1.34189086	1.46534679	1.61987482	1.64831651	0.63497872	1.36666243	0.005954	-1.286185	down	hypothetical protein CICLE_v10024008mg, partial [Citrus clementina]
c43121.graph_c0	32.1974152	30.7545123	30.9881758	14.440344	14.6004581	12.9953578	4.867E-42	-2.176614	down	PREDICTED: transcription factor bHLH47 isoform X3 [Sesamum indicum]
c43123.graph_c0	14.8565587	14.3694459	13.6941571	52.7260616	70.7234836	80.2134	2.077E-07	1.223556	up	PREDICTED: GDSL esterase/lipase At4g01130-like [Sesamum indicum]
c43123.graph_c2	11.8304575	14.1036484	8.40345069	40.035985	52.1607812	56.6764085	5.746E-06	1.097403	up	hypothetical protein MIMGU_mgv1a003725mg [Erythranthe guttata]
c43124.graph_c0	157.487903	165.811311	167.884458	87.8848413	84.63054	85.050031	9.331E-38	-1.948537	down	PREDICTED: putative DNA-binding protein ESCAROLA [Sesamum indicum]
c43126.graph_c0	6.43366295	7.19186856	7.84931064	5.62729505	5.98586888	5.52015925	2.657E-11	-1.345592	down	PREDICTED: uncharacterized protein LOC105159445 [Sesamum indicum]
c43128.graph_c0	8.88543989	7.97590031	9.21518529	4.35985318	6.22115432	6.33366717	2.504E-18	-1.649286	down	PREDICTED: transmembrane protein 53-B [Sesamum indicum]
c43139.graph_c0	76.7318865	76.2418678	74.1527519	54.7263832	24.1520473	18.6610802	2.278E-25	-2.217078	down	PREDICTED: transcription factor bHLH112 isoform X1 [Sesamum indicum]
c43140.graph_c0	0.67926502	0.60439573	0.43644122	20.3379552	16.6840267	20.3857316	1.714E-43	4.049596	up	PREDICTED: uncharacterized protein LOC102583254 [Solanum tuberosum]
c43141.graph_c0	15.0176196	13.9518782	15.2304704	13.9482435	15.2554507	15.5289153	2.072E-10	-1.002415	down	hypothetical protein MIMGU_mgv1a005112mg [Erythranthe guttata]
c43142.graph_c0	1.80162025	1.3917731	1.81185914	0.59535231	1.12762156	1.13974559	7.396E-07	-1.835454	down	PREDICTED: protein WEAK CHLOROPLAST MOVEMENT UNDER BLUE LIGHT 1 isoform X1 [Sesamum indicum]

#ID	0 h-1 FPKM	0 h-2 FPKM	0 h-3 FPKM	48 h-1 FPKM	48 h-2 FPKM	48 h-3 FPKM	FDR	log2FC	regulated	nr_annotation
c43143.graph_c0	12.2106154	11.1086055	13.263768	8.98072716	6.74867906	8.15690008	4.564E-18	-1.629636	down	PREDICTED: vacuolar amino acid transporter 1-like isoform X1 [Eucalyptus grandis]
c43145.graph_c0	0.45424319	0.86392628	0.58372048	16.1532682	12.5018264	15.5273957	3.881E-25	3.524044	up	PREDICTED: G2/mitotic-specific cyclin-2-like [Sesamum indicum]
c43146.graph_c0	81.3470457	80.5463857	78.0199031	54.3100024	67.707263	59.6835122	8.522E-24	-1.421214	down	hypothetical protein MIMGU_mgv1a000959mg [Erythranthe guttata]
c43150.graph_c0	42.3389847	41.5084378	46.338634	15.4655944	15.4689757	15.5841042	1.942E-66	-2.50325	down	PREDICTED: patellin-3 [Sesamum indicum]
c43151.graph_c0	7.89256139	6.96094989	6.46911328	10.0616889	4.74664798	3.42725533	0.0006201	-1.222829	down	PREDICTED: F-box protein At1g61340-like [Sesamum indicum]
c43153.graph_c0	0.28949557	0.28978546	0.12400442	2.35586945	3.2491944	3.75740858	1.329E-06	2.718202	up	PREDICTED: uncharacterized protein LOC105157169 [Sesamum indicum]
c43154.graph_c0	69.1316805	72.8231992	59.0470216	46.0127658	49.2599869	44.5571277	1.799E-28	-1.539658	down	PREDICTED: homeobox protein knotted-1-like LET12 isoform X1 [Sesamum indicum]
c43159.graph_c1	0.5095856	0.53328205	0.68460302	8.77043162	10.9188659	11.1646712	3.45E-29	3.133904	up	PREDICTED: endonuclease 4-like [Sesamum indicum]
c43163.graph_c0	0.99496473	0.89433236	1.04372985	20.6521541	23.68184	23.4029751	1.035E-71	3.508613	up	hypothetical protein SELMODRAFT_91337 [Selaginella moellendorffii]
c43164.graph_c0	59.0424375	54.8653309	56.4947116	30.4710955	33.3631286	31.626217	6.842E-38	-1.854521	down	PREDICTED: F-box protein SKP2A-like [Sesamum indicum]
c43165.graph_c0	3.44385889	2.99680699	3.09281754	14.2591567	14.914876	14.0992399	1.265E-10	1.165205	up	PREDICTED: uncharacterized protein LOC105161979 [Sesamum indicum]
c43166.graph_c1	14.8243822	14.4805746	14.8485767	14.2760936	10.8401733	11.3811055	5.666E-12	-1.287133	down	PREDICTED: syntaxin-81 [Sesamum indicum]
c43167.graph_c0	9.03897159	7.95870306	8.59028586	6.03575088	8.45327801	7.70085816	4.668E-12	-1.228456	down	hypothetical protein MIMGU_mgv1a016655mg [Erythranthe guttata]
c43168.graph_c0	1.51507641	2.21655977	2.54598953	12.9915578	15.4691348	12.7062375	9.615E-08	1.68976	up	PREDICTED: annexin D5 [Sesamum indicum]
c43170.graph_c0	40.7768342	46.7402294	36.9881269	2.79905611	2.65312786	2.35949996	3.038E-68	-5.008324	down	PREDICTED: hyoscyamine 6-dioxygenase [Sesamum indicum]
c43170.graph_c1	36.9351945	38.3454319	29.1559579	1.21525394	1.02993135	1.55710893	2.533E-94	-5.794815	down	hypothetical protein MIMGU_mgv1a019348mg [Erythranthe guttata]
c43172.graph_c0	102.133094	104.588501	106.484902	10.8671428	10.2833361	9.29099628	5.65E-175	-4.378908	down	PREDICTED: RING-H2 finger protein ATL47 [Sesamum indicum]
c43177.graph_c0	1.50481999	1.25527237	0.87479314	23.7988079	28.6038504	30.5158136	8.226E-54	3.495276	up	PREDICTED: formin-like protein 1 [Sesamum indicum]
c43181.graph_c0	38.8984659	43.7248041	39.8015535	40.32577	32.8959365	25.4270431	1.081E-16	-1.322586	down	hypothetical protein MIMGU_mgv1a021597mg [Erythranthe guttata]
c43181.graph_c1	3.18198331	2.81429368	3.24877429	0.72282343	0.97845038	1.46498453	3.286E-16	-2.572317	down	PREDICTED: nodulation-signaling pathway 2 protein-like [Sesamum indicum]
c43182.graph_c0	0.29576804	0.70315248	0.28505523	4.73435401	6.56702351	8.12213473	4.122E-10	2.8983	up	PREDICTED: rho GTPase-activating protein 5-like [Sesamum indicum]
c43183.graph_c1	18.9229477	20.0018218	19.400718	6.16765077	6.36719756	5.87993418	1.052E-53	-2.680744	down	PREDICTED: pentatricopeptide repeat-containing protein At5g42310, mitochondrial-like [Sesamum indicum]

#ID	0 h-1 FPKM	0 h-2 FPKM	0 h-3 FPKM	48 h-1 FPKM	48 h-2 FPKM	48 h-3 FPKM	FDR	log2FC	regulated	nr_annotation
c43183.graph_c2	3.6544276	2.48749913	3.6159848	18.6851687	18.8316866	19.1722676	1.126E-12	1.51946	up	PREDICTED: O-glucosyltransferase rumi homolog [Beta vulgaris subsp. vulgaris]
c43185.graph_c0	198.038236	182.61301	204.596862	36.489148	33.9307266	30.2377877	3.36E-110	-3.555348	down	PREDICTED: poly [ADP-ribose] polymerase 3 [Sesamum indicum]
c43191.graph_c0	1.5130821	1.80309193	1.6666032	8.47433047	8.2274451	9.41074455	5.65E-08	1.371469	up	hypothetical protein MIMGU_mgv1a002186mg [Erythranthe guttata]
c43192.graph_c0	1.89631175	2.24333982	2.52544788	12.29257	16.6566698	18.0319157	9.731E-13	1.791381	up	PREDICTED: heparan-alpha-glucosaminide N-acetyltransferase-like [Sesamum indicum]
c43193.graph_c0	3.62297285	3.98331553	2.97657177	3.4471523	3.45862083	3.26200176	5.285E-06	-1.072353	down	PREDICTED: rho GTPase-activating protein 7-like [Sesamum indicum]
c43196.graph_c0	0.71781438	0.42458778	0.3773536	4.62908201	3.37544685	3.95845157	7.122E-06	1.969318	up	PREDICTED: basic leucine zipper 43-like [Sesamum indicum]
c43198.graph_c0	18.0700348	16.1288185	19.0650414	8.57191939	7.53051006	8.09231808	1.17E-38	-2.154951	down	hypothetical protein MIMGU_mgv1a005420mg [Erythranthe guttata]
c43199.graph_c1	3.12593216	4.14499163	3.3387608	14.9600654	16.720036	16.4728281	1.014E-06	1.163279	up	PREDICTED: single-stranded DNA-binding protein, mitochondrial [Sesamum indicum]
c43200.graph_c0	3.30326925	3.26472157	2.79406648	21.9571727	22.4855966	23.6162126	6.391E-17	1.845372	up	PREDICTED: uncharacterized aarF domain-containing protein kinase At1g79600, chloroplastic [Sesamum indicum]
c43201.graph_c0	1.6494096	1.81616735	1.37771181	13.7138819	14.9708824	16.3263762	4.224E-15	2.198723	up	PREDICTED: ATP-dependent RNA helicase-like protein DB10 [Sesamum indicum]
c43201.graph_c1	0.5038334	0.60520549	0.77693504	7.98362403	8.26099133	7.26898486	4.219E-10	2.618878	up	PREDICTED: ATP-dependent RNA helicase-like protein DB10 [Sesamum indicum]
c43202.graph_c0	20.1522391	25.2777837	23.8982442	16.846524	15.1151888	13.4603505	5.014E-18	-1.625311	down	PREDICTED: probable phytol kinase 1, chloroplastic [Nicotiana sylvestris]
c43205.graph_c0	28.2188275	26.440737	26.9530797	17.3955993	27.3365009	30.29241	1.863E-14	-1.147246	down	PREDICTED: protein TRANSPARENT TESTA 12-like [Sesamum indicum]
c43206.graph_c0	0.12642643	0.21092172	0.16246297	1.5141396	1.68630614	1.58909041	9.309E-05	2.241001	up	hypothetical protein MIMGU_mgv1a010976mg [Erythranthe guttata]
c43207.graph_c1	8.04206005	8.29405577	7.08530366	2.55381513	3.22573774	2.75930563	2.863E-45	-2.474974	down	PREDICTED: BRCT domain-containing protein At4g02110 [Sesamum indicum]
c43211.graph_c0	2.81169693	5.62902484	4.30135937	1.69604194	0.45735474	1.48168923	2.859E-06	-2.814997	down	-
c43214.graph_c0	0.42180977	0.14074405	0.54204225	5.37561494	5.21452683	6.56968244	2.681E-08	2.932219	up	-
c43218.graph_c0	6.65294303	6.16169055	5.75279676	55.9067855	60.6221657	64.3735592	3.152E-35	2.266233	up	-
c43222.graph_c0	40.6669995	40.9720572	41.3997528	12.8938305	13.0151363	8.92934302	8.591E-62	-2.835421	down	--
c43223.graph_c0	1.64077962	2.02144322	1.73002402	0.0387588	0	0.03448735	8.773E-23	-7.199419	down	PREDICTED: uncharacterized protein LOC105164927 [Sesamum indicum]
c43225.graph_c0	0	0	0	22.7429854	50.2819518	4.04731432	0.0011486	Inf	up	hypothetical protein Csa_2G382530 [Cucumis sativus]
c43229.graph_c0	2.85503296	2.67511969	3.30621343	2.37027675	2.85122362	2.29955356	6.251E-07	-1.253859	down	unnamed protein product [Vitis vinifera]

#ID	0 h-1 FPKM	0 h-2 FPKM	0 h-3 FPKM	48 h-1 FPKM	48 h-2 FPKM	48 h-3 FPKM	FDR	log2FC	regulated	nr_annotation
c43231.graph_c0	69.6869052	72.5409009	71.2906911	17.8813356	11.448482	9.31841907	3.57E-121	-3.47246	down	hypothetical protein MIMGU_mgv1a010924mg [Erythranthe guttata]
c43238.graph_c0	0.02756648	0	0	3.88559341	2.8249221	3.45737765	1.625E-20	7.5354	up	PREDICTED: kinesin-like protein KIF22 [Sesamum indicum]
c43245.graph_c0	16.4294765	16.9589967	16.1993257	5.53877145	6.53013411	7.12001195	1.014E-57	-2.390577	down	PREDICTED: uncharacterized protein LOC105163648 [Sesamum indicum]
c43246.graph_c0	20.8235224	19.9166689	19.5017282	86.6080974	100.060922	102.395037	6.254E-19	1.24278	up	unnamed protein product [Coffea canephora]
c43247.graph_c0	0	0	0	3.72990497	6.28168427	7.829075	9.42E-11	Inf	up	-
c43248.graph_c0	23.7665796	26.5266518	26.1501178	3.00798372	2.92722008	2.9877051	4.49E-111	-4.116452	down	PREDICTED: cell cycle checkpoint protein RAD17 [Sesamum indicum]
c43250.graph_c0	0.64251763	0.71462335	0.45870033	7.76084887	10.1029098	12.113985	5.773E-11	3.025433	up	PREDICTED: uncharacterized protein LOC105177156 [Sesamum indicum]
c43250.graph_c1	0.83391374	0.34781199	0.44650507	5.82593929	6.71445877	7.0637564	2.772E-07	2.573645	up	PREDICTED: uncharacterized protein LOC102585981 isoform X1 [Solanum tuberosum]
c43253.graph_c0	3.56511768	5.35303141	5.0394491	1.31376758	3.0155178	3.60436268	9.481E-05	-1.848664	down	PREDICTED: ethylene-responsive transcription factor CRF4-like [Sesamum indicum]
c43253.graph_c1	1.38182222	1.38320591	2.13083552	0.7213807	0.76421538	0.75515319	1.378E-05	-2.151021	down	PREDICTED: ethylene-responsive transcription factor CRF4-like [Sesamum indicum]
c43254.graph_c0	0.43801723	0.526147	0.50658253	5.26606703	5.17264289	4.54209134	3.099E-11	2.331592	up	PREDICTED: LOW QUALITY PROTEIN: linoleate 13S-lipoxygenase 3-1, chloroplastic [Sesamum indicum]
c43254.graph_c1	0.17018039	0.5110524	0.65606562	4.70343828	2.98963352	3.62707895	0.0001738	2.062648	up	PREDICTED: LOW QUALITY PROTEIN: linoleate 13S-lipoxygenase 3-1, chloroplastic [Sesamum indicum]
c43257.graph_c0	4.51445744	3.49193754	4.74648263	27.1596129	29.5066449	28.8763968	1.77E-12	1.725451	up	PREDICTED: transcriptional regulator ATRX homolog [Solanum tuberosum]
c43258.graph_c0	4.87535993	5.64525276	6.29889557	0.92257829	1.95471966	1.27456243	3.726E-29	-3.048657	down	PREDICTED: zinc finger BED domain-containing protein DAYSLEEPER-like isoform X1 [Sesamum indicum]
c43259.graph_c0	0.03502619	0	0.09002008	15.4243009	18.8014583	20.6440997	6.302E-71	7.738044	up	PREDICTED: 1-acyl-sn-glycerol-3-phosphate acyltransferase-like [Sesamum indicum]
c43260.graph_c0	1.47107704	1.3743801	1.44930072	23.9879626	28.2359307	23.9973143	2.992E-33	3.130083	up	PREDICTED: UPF0057 membrane protein At2g24040-like [Sesamum indicum]
c43263.graph_c0	0.64995008	1.1325275	0.86614534	13.3504961	15.3884	16.7926772	1.742E-39	3.082257	up	PREDICTED: oxysterol-binding protein-related protein 2A-like isoform X3 [Sesamum indicum]
c43264.graph_c0	1.89001568	2.01266834	1.96366615	91.5059176	65.1495725	59.4673009	4.391E-15	4.193353	up	PREDICTED: chlorophyll a-b binding protein CP26, chloroplastic [Sesamum indicum]
c43269.graph_c0	66.6720576	61.9267217	58.9636536	12.095161	10.6130831	10.3001878	8.66E-122	-3.519993	down	PREDICTED: AP2-like ethylene-responsive transcription factor At2g41710 isoform X1 [Sesamum indicum]
c43272.graph_c0	0.08719686	0.61098923	0	4.73955471	3.8295637	4.07427043	4.87E-07	3.17784	up	PREDICTED: histone-lysine N-methyltransferase CLF isoform X3 [Sesamum indicum]

#ID	0 h-1 FPKM	0 h-2 FPKM	0 h-3 FPKM	48 h-1 FPKM	48 h-2 FPKM	48 h-3 FPKM	FDR	log2FC	regulated	nr_annotation
c43272.graph_c1	0.23600488	0.33073769	0.24262049	6.06610422	5.09036815	6.8098539	8.324E-20	3.456979	up	PREDICTED: histone-lysine N-methyltransferase CLF isoform X2 [Sesamum indicum]
c43274.graph_c0	0.08596319	0.05736618	0.14728813	2.56063496	2.65675065	4.04011613	1.811E-12	3.963882	up	PREDICTED: uncharacterized protein LOC105179654 [Sesamum indicum]
c43275.graph_c0	40.6047863	41.362044	38.6741006	36.1151252	38.6788026	37.3695791	1.491E-15	-1.122991	down	PREDICTED: ABC transporter A family member 2 [Sesamum indicum]
c43278.graph_c1	9.20153643	8.7773033	8.06837223	38.5456702	37.1366467	42.7723003	1.405E-10	1.168542	up	hypothetical protein MIMGU_mgv1a009792mg [Erythranthe guttata]
c43279.graph_c0	1.63864136	1.52517469	1.6993522	6.8330318	6.03230721	6.85766383	0.0007736	1.003204	up	PREDICTED: magnesium transporter MRS2-I-like [Sesamum indicum]
c43280.graph_c1	2.98386457	3.19645614	4.50708308	3.61701403	3.52524476	2.91801359	0.0046279	-1.107157	down	hypothetical protein MIMGU_mgv1a000521mg [Erythranthe guttata]
c43281.graph_c0	0.0623444	0.16641822	0.16023006	6.50938298	7.30154326	8.53469684	5.747E-38	4.818211	up	PREDICTED: probable beta-D-xylosidase 6 [Sesamum indicum]
c43283.graph_c0	13.2937308	6.34000454	6.70797269	0	0	0	1.128E-14	-Inf	down	glyceraldehyde 3-phosphate dehydrogenase [Cymbidium faberi]
c43284.graph_c0	0.04775748	0	0	1.97987888	1.3982944	1.01786205	1.393E-06	5.537909	up	-
c43285.graph_c0	17.4310852	17.0272917	16.7356863	6.36632734	7.26314815	7.77083355	1.001E-46	-2.2784	down	PREDICTED: pentatricopeptide repeat-containing protein At2g15980 [Sesamum indicum]
c43287.graph_c0	35.2367142	39.1911094	38.5723399	258.195989	276.287028	277.436839	2.014E-36	1.825708	up	PREDICTED: 60S ribosomal protein L31 [Eucalyptus grandis]
c43288.graph_c0	10.2166752	5.68019914	6.86688603	0	0	0	1.854E-35	-Inf	down	alpha-2 macroglobulin family-like protein [Micromonas sp. RCC299]
c43296.graph_c0	0.58775737	0.05883459	0.1510583	5.08991596	4.30224637	5.87803348	4.068E-09	3.252274	up	hypothetical protein VITISV_034944 [Vitis vinifera]
c43299.graph_c1	63.0447828	61.1304937	63.4629916	44.6136695	51.22888	51.9576955	6.766E-21	-1.364634	down	unnamed protein product [Coffea canephora]
c43303.graph_c1	5.45970807	6.23672928	4.95242896	26.8849836	27.4365753	28.0992493	4.539E-16	1.291138	up	PREDICTED: protein MEI2-like 4 [Sesamum indicum]
c43304.graph_c0	0	0	0	4.7068035	7.65337163	4.48027746	2.981E-11	Inf	up	metallothionein-like type 1 protein [Ipomoea batatas]
c43305.graph_c2	2.61758962	2.27084931	2.82551329	28.2305598	34.6756018	32.0429479	6.032E-42	2.599482	up	PREDICTED: probable methyltransferase PMT26 [Sesamum indicum]
c43305.graph_c3	1.65111495	1.71179574	1.81864122	26.6195088	31.1928099	24.6526495	2.841E-28	2.972605	up	PREDICTED: probable methyltransferase PMT26 [Sesamum indicum]
c43307.graph_c1	2.98925096	2.80124993	2.69708707	10.3124502	19.4287469	14.8587927	0.0004515	1.36753	up	PREDICTED: uncharacterized protein LOC105160017 [Sesamum indicum]
c43310.graph_c0	5.76220946	6.10585271	5.05004506	5.41958017	5.29431273	5.632616	2.919E-07	-1.065417	down	PREDICTED: mitochondrial aspartate-glutamate transporter AGC1 [Sesamum indicum]
c43312.graph_c0	7.62454427	7.78636453	6.87828249	54.2958595	58.2903188	58.3344102	1.575E-40	1.921312	up	PREDICTED: IAA-amino acid hydrolase ILR1-like 4 [Sesamum indicum]
c43314.graph_c0	0.44278704	0.32659084	0.44920964	8.22289511	9.32526297	9.60911581	4.868E-29	3.456752	up	PREDICTED: LOW QUALITY PROTEIN: probable serine/threonine-protein kinase WNK3 [Sesamum indicum]

#ID	0 h-1 FPKM	0 h-2 FPKM	0 h-3 FPKM	48 h-1 FPKM	48 h-2 FPKM	48 h-3 FPKM	FDR	log2FC	regulated	nr_annotation
c43319.graph_c0	3.70325272	1.9247682	2.47092907	0	0	0	2.008E-16	-Inf	down	predicted protein [Hordeum vulgare subsp. vulgare]
c43320.graph_c0	0	0	0	2.52546746	5.6481227	1.37325574	0.0005184	Inf	up	hypothetical protein GUITHDRAFT_114321 [Guillardia theta CCMP2712]
c43322.graph_c0	2.64912464	3.5085054	2.35677465	16.0325126	21.3599019	22.4174237	1.585E-14	1.791687	up	hypothetical protein MIMGU_mgv1a008032mg [Erythranthe guttata]
c43323.graph_c0	22.9477132	24.0933197	20.7307919	24.0418473	20.7123234	20.1193615	1.107E-11	-1.076002	down	Ribosome production factor 1 [Gossypium arboreum]
c43324.graph_c0	24.7604159	25.8297971	24.6864684	14.4206903	13.1474554	11.5482992	3.424E-31	-1.9588	down	hypothetical protein MIMGU_mgv1a008176mg [Erythranthe guttata]
c43328.graph_c0	0.87506275	0.82032382	0.66041493	0.21753656	0.21689751	0.39851148	9.038E-09	-2.519658	down	PREDICTED: leucine-rich repeat receptor protein kinase EXS-like [Nicotiana tomentosiformis]
c43332.graph_c0	0.30488267	0.30518797	0.06529772	2.48108727	2.18207715	1.95773315	9.97E-05	2.290968	up	hypothetical protein MIMGU_mgv1a020708mg [Erythranthe guttata]
c43336.graph_c0	20.0470329	20.8037649	18.3183192	12.6304868	12.4095207	11.4954757	1.481E-33	-1.710918	down	PREDICTED: uncharacterized protein LOC105160884 [Sesamum indicum]
c43338.graph_c1	34.1207419	32.3690311	34.6759935	23.8325055	31.6683883	34.0941616	6.929E-10	-1.198758	down	PREDICTED: calcium-dependent protein kinase 26-like [Sesamum indicum]
c43339.graph_c0	68.7471161	67.0296694	70.3734715	62.418001	60.1285764	65.0834566	4.949E-16	-1.15348	down	unnamed protein product [Coffea canephora]
c43342.graph_c0	1.80407069	1.54603875	1.70120022	10.8211149	12.4643542	12.8522697	1.202E-24	1.818536	up	PREDICTED: tRNA(adenine(34)) deaminase, chloroplastic [Sesamum indicum]
c43349.graph_c0	0.84691833	0.93700496	1.37472453	9.56816228	8.96171414	8.40407489	5.052E-09	2.071286	up	Transcription factor SPEECHLESS [Glycine soja]
c43349.graph_c1	19.9871317	19.500208	17.6579257	9.36224369	11.4338944	12.8416519	2.196E-27	-1.785293	down	PREDICTED: protein DEFECTIVE IN MERISTEM SILENCING 3-like [Sesamum indicum]
c43350.graph_c0	3.31847026	3.0993517	2.96982609	2.78422775	2.94955169	3.50963457	2.611E-06	-1.041259	down	PREDICTED: pentatricopeptide repeat-containing protein At2g32630 [Sesamum indicum]
c43353.graph_c0	49.9218738	52.2618993	52.3922104	198.944304	211.235817	225.510583	3.105E-13	1.020598	up	PREDICTED: uncharacterized protein LOC105157922 [Sesamum indicum]
c43359.graph_c0	0	0	0	19.0110614	19.2381272	9.72034677	5.463E-12	Inf	up	-
c43360.graph_c0	3.59412775	3.66968126	4.71096839	26.389825	28.7636036	26.5161315	6.205E-21	1.748352	up	PREDICTED: palmitoyl-protein thioesterase 1-like [Sesamum indicum]
c43361.graph_c0	20.3230543	22.0975153	20.5686972	7.32379329	8.73893312	8.65385379	2.63E-54	-2.370097	down	hypothetical protein MIMGU_mgv1a001131mg [Erythranthe guttata]
c43362.graph_c0	59.988471	58.8307072	59.168611	19.6144368	15.6642577	14.3458245	3.496E-80	-2.854749	down	PREDICTED: protein SRG1-like [Citrus sinensis]
c43363.graph_c0	7.46029793	8.54862949	8.45150184	4.97381866	4.02372047	4.10380674	2.462E-07	-1.914886	down	-
c43363.graph_c2	12.8422355	12.2228772	12.038914	6.59438636	5.80450485	4.78903406	9.526E-20	-2.123033	down	hypothetical protein MIMGU_mgv1a021390mg, partial [Erythranthe guttata]
c43369.graph_c0	2.37230986	2.10388792	2.13930673	12.2504516	13.5262287	12.998573	2.07E-14	1.532883	up	PREDICTED: B2 protein [Sesamum indicum]

#ID	0 h-1 FPKM	0 h-2 FPKM	0 h-3 FPKM	48 h-1 FPKM	48 h-2 FPKM	48 h-3 FPKM	FDR	log2FC	regulated	nr_annotation
c43372.graph_c0	13.468978	13.423071	11.8946014	6.38190528	6.18176659	6.71215563	2.139E-40	-2.024899	down	PREDICTED: nuclear pore complex protein NUP214 [Sesamum indicum]
c43374.graph_c0	17.6483677	17.7113374	20.469117	12.6318582	9.8929061	6.04646697	1.318E-19	-1.976815	down	UPA25 [Capsicum annuum]
c43375.graph_c0	16.5546918	17.9359616	16.3680307	132.847463	103.746066	81.9018449	3.435E-05	1.636629	up	hypothetical protein MIMGU_mgv1a007068mg [Erythranthe guttata]
c43376.graph_c0	1.45034717	1.01625963	2.23650424	5.56731003	8.63451083	7.64860388	0.0007566	1.184879	up	PREDICTED: transmembrane protein 64-like [Tarenaya hassleriana]
c43379.graph_c0	0	0	0	23.060192	33.487598	7.60810185	1.443E-06	Inf	up	unknown [Medicago truncatula]
c43386.graph_c0	18.1090614	17.5927321	15.8379077	12.1742624	12.7234558	11.0149523	6.645E-18	-1.537058	down	PREDICTED: vacuolar protein sorting-associated protein VTA1 homolog [Sesamum indicum]
c43387.graph_c0	11.575851	11.5874424	14.8754268	0.15020322	0	0	5.308E-35	-8.947594	down	PREDICTED: uncharacterized protein LOC104587006 isoform X2 [Nelumbo nucifera]
c43389.graph_c0	0.02750534	0.01376644	0.03534545	2.30591368	4.57696184	4.90399909	7.752E-14	6.230538	up	PREDICTED: ABC transporter C family member 8-like isoform X3 [Sesamum indicum]
c43390.graph_c0	19.6959177	18.6887839	17.0616747	5.94038109	6.00706284	5.45389722	2.165E-57	-2.687444	down	hypothetical protein MIMGU_mgv1a008049mg [Erythranthe guttata]
c43392.graph_c0	0.8192594	0.72895979	0.70185384	3.85763766	3.48702063	4.0294598	0.0002711	1.322328	up	PREDICTED: probable receptor-like protein kinase At5g18500 [Sesamum indicum]
c43393.graph_c0	2.1698033	1.3637989	1.49140726	0.55785051	0.73871871	0.99274406	8.725E-06	-2.156264	down	hypothetical protein MIMGU_mgv1a0000292mg, partial [Erythranthe guttata]
c43400.graph_c1	21.7139436	20.1424951	20.0143925	12.5681473	11.2802794	12.3945603	5.74E-27	-1.787014	down	PREDICTED: putative G3BP-like protein isoform X2 [Sesamum indicum]
c43401.graph_c0	1.65204358	1.55034173	1.09464166	0.4993965	0.83136435	0.90988001	2.681E-06	-1.962328	down	PREDICTED: ABC transporter B family member 29, chloroplastic [Sesamum indicum]
c43403.graph_c0	0.4404849	0.94064209	0.67924864	2.81357037	2.78001687	3.89967958	0.0043864	1.183068	up	-
c43405.graph_c1	35.5250553	36.8875174	34.3518869	28.5759386	33.334515	30.9684147	2.715E-16	-1.220305	down	hypothetical protein CISIN_1g022174mg [Citrus sinensis]
c43406.graph_c0	19.2957298	20.0925319	15.7346523	12.6413677	15.2945217	16.666331	2.17E-17	-1.324846	down	PREDICTED: uncharacterized protein LOC105165773 [Sesamum indicum]
c43406.graph_c1	17.927768	17.6133918	9.81243534	4.28198535	6.8043664	3.26578699	4.71E-08	-2.676063	down	-
c43407.graph_c0	213.601327	203.116589	209.315054	27.029371	26.7935621	30.3208995	5.38E-144	-3.913507	down	PREDICTED: uncharacterized protein LOC105176746 isoform X1 [Sesamum indicum]
c43408.graph_c0	14.5380062	12.990172	13.753556	64.6660978	83.5214751	93.1087161	9.19E-13	1.524519	up	PREDICTED: porphobilinogen deaminase, chloroplastic [Sesamum indicum]
c43409.graph_c2	9.46113231	11.5927822	10.0435071	4.51843776	7.9676008	10.3235519	4.403E-10	-1.475635	down	PREDICTED: uncharacterized membrane protein At4g09580 isoform X3 [Sesamum indicum]
c43412.graph_c0	0.23895416	0.31892458	0.25588796	54.4479983	72.6066073	79.5596773	2.392E-66	6.965246	up	PREDICTED: uncharacterized protein LOC105177232 [Sesamum indicum]

#ID	0 h-1 FPKM	0 h-2 FPKM	0 h-3 FPKM	48 h-1 FPKM	48 h-2 FPKM	48 h-3 FPKM	FDR	log2FC	regulated	nr_annotation
c43415.graph_c0	253.243437	257.284246	266.521775	136.214191	142.777249	147.811847	3.897E-33	-1.883519	down	hypothetical protein MIMGU_mgv1a002107mg [Erythranthe guttata]
c43418.graph_c0	24.5804177	24.3306257	22.4278093	22.5328408	18.2524021	15.8049665	1.664E-19	-1.3452	down	PREDICTED: UBX domain-containing protein 4 [Sesamum indicum]
c43421.graph_c0	1.19591357	0.88928253	1.09771163	6.64200018	7.0363938	8.06674599	2.307E-07	1.752591	up	PREDICTED: GDT1-like protein 2, chloroplastic [Sesamum indicum]
c43426.graph_c0	133.800454	122.405109	138.516376	31.9193301	32.1040718	30.223232	1.96E-94	-3.084115	down	PREDICTED: F-box protein SKIP23-like [Sesamum indicum]
c43433.graph_c0	73.5132952	69.2945357	66.3677534	39.3081411	30.6593944	31.8300421	4.49E-47	-2.050869	down	PREDICTED: probable 1-acylglycerol-3-phosphate O-acyltransferase [Beta vulgaris subsp. vulgaris]
c43435.graph_c0	0	0	0	1.41257207	1.78608408	1.45962363	1.742E-11	Inf	up	predicted protein [Hordeum vulgare subsp. vulgare]
c43443.graph_c0	0.21466815	0.10744156	0.13792854	2.04358514	1.85066653	2.87419738	4.036E-07	2.862482	up	PREDICTED: CDT1-like protein a, chloroplastic [Sesamum indicum]
c43444.graph_c0	82.7826441	75.1905826	76.1535934	12.4348735	12.9393961	11.2936216	1.14E-131	-3.69132	down	PREDICTED: probable polygalacturonase At1g80170 [Sesamum indicum]
c43453.graph_c0	8.57484837	10.4126914	8.87714932	3.84810211	3.62581996	2.86432285	7.596E-42	-2.444485	down	PREDICTED: uncharacterized protein LOC105177800 [Sesamum indicum]
c43454.graph_c0	2.54483334	2.21511444	2.65408478	20.7943982	21.1652555	22.5842151	3.147E-22	2.102688	up	hypothetical protein MIMGU_mgv1a003626mg [Erythranthe guttata]
c43456.graph_c1	2.94332776	2.59272205	4.91698052	1.35579276	1.14903842	1.59241664	0.0003445	-2.374758	down	PREDICTED: putative Myb family transcription factor At1g14600 [Sesamum indicum]
c43457.graph_c0	0	0	0	6.0293695	10.0373207	5.48280654	5.286E-13	Inf	up	ADP ribosylation factor [Karlodinium veneficum]
c43463.graph_c0	1.62124122	2.20245917	1.87502324	17.2830066	14.6021918	14.7138202	2.696E-20	2.017192	up	F3H9.11 protein isoform 5 [Theobroma cacao]
c43466.graph_c0	32.9210148	31.8206423	27.6436176	24.1107416	24.479911	27.093639	8.576E-18	-1.304435	down	PREDICTED: 50S ribosomal protein L3-2, chloroplastic-like [Sesamum indicum]
c43467.graph_c0	0.07717569	0.38626486	0.29752146	14.5753529	16.495314	16.3853019	9.643E-24	4.938146	up	hypothetical protein VITISV_020777 [Vitis vinifera]
c43471.graph_c0	0.01644231	0	0	16.7988318	17.9728444	20.8240638	1.56E-149	10.72481	up	PREDICTED: pentatricopeptide repeat-containing protein MRL1, chloroplastic [Sesamum indicum]
c43477.graph_c0	25.4891655	22.8653083	26.026218	9.64734829	10.9502097	11.5084242	9.959E-34	-2.232917	down	PREDICTED: high mobility group B protein 14-like isoform X2 [Nelumbo nucifera]
c43479.graph_c0	0.38280194	0.07663705	0.29514939	2.68023207	2.91410144	3.07520696	1.807E-05	2.50186	up	PREDICTED: diacylglycerol kinase 4-like [Sesamum indicum]
c43481.graph_c0	2.48324815	1.42041986	1.08268521	0.3268186	0.2596685	0	1.413E-06	-4.081608	down	-
c43482.graph_c0	19.4626231	20.0520674	19.4893635	15.3074637	18.3886922	18.9244179	2.387E-11	-1.18615	down	PREDICTED: ribosome biogenesis protein TSR3 homolog isoform X2 [Sesamum indicum]
c43483.graph_c0	0.52873211	0.45365276	0.42707764	0.12525493	0.26538482	0.21051872	4.229E-06	-2.256485	down	hypothetical protein MIMGU_mgv1a000607mg [Erythranthe guttata]
c43484.graph_c0	16.7331936	17.6941404	15.2240909	13.3996752	11.0469503	8.27338323	7.528E-28	-1.611707	down	PREDICTED: histone-lysine N-methyltransferase ASHR2 [Sesamum indicum]

#ID	0 h-1 FPKM	0 h-2 FPKM	0 h-3 FPKM	48 h-1 FPKM	48 h-2 FPKM	48 h-3 FPKM	FDR	log2FC	regulated	nr_annotation
c43488.graph_c1	56.2874843	53.5079984	55.470229	37.1575294	35.5075446	31.3819177	3.57E-32	-1.683155	down	hypothetical protein CISIN_1g018310mg [Citrus sinensis]
c43491.graph_c0	1.70550907	1.62961611	1.89278576	22.4613926	25.9136066	30.916359	1.624E-31	2.900091	up	PREDICTED: cytochrome b561 and DOMON domain-containing protein At4g17280 [Sesamum indicum]
c43492.graph_c0	0	0	0.07395272	1.32544688	1.06715419	0.99067472	1.554E-08	4.470791	up	PREDICTED: actin-related protein 2/3 complex subunit 2B [Sesamum indicum]
c43493.graph_c0	89.8759357	92.2204127	87.608149	37.7126404	20.8225507	14.196013	3.47E-89	-2.892215	down	PREDICTED: E3 ubiquitin-protein ligase RNF170-like [Sesamum indicum]
c43494.graph_c0	0.83879053	1.02621499	1.25752554	7.04059784	6.50358742	6.72307296	1.522E-05	1.678545	up	PREDICTED: uncharacterized protein LOC105160516 isoform X2 [Sesamum indicum]
c43495.graph_c0	39.2122608	34.8156935	40.388011	26.896406	23.9868588	20.6049435	3.478E-30	-1.693243	down	PREDICTED: jmjC domain-containing protein 7 [Sesamum indicum]
c43496.graph_c0	0.89059982	0.89149162	1.14445602	6.68104036	6.58106778	5.57972105	0.0001729	1.66796	up	-
c43498.graph_c0	2.73053086	2.57658109	2.45842311	2.40338515	2.29148563	3.15074935	0.000137	-1.003414	down	PREDICTED: chromosome transmission fidelity protein 18 homolog [Sesamum indicum]
c43500.graph_c0	6.88810466	5.97006276	4.4257436	22.9066866	27.7101271	26.1663789	1.172E-08	1.13483	up	unnamed protein product [Vitis vinifera]
c43504.graph_c0	85.2011869	75.5729352	75.8802576	345.736456	373.58714	367.096253	3.792E-17	1.180779	up	PREDICTED: 40S ribosomal protein S12-like [Sesamum indicum]
c43506.graph_c0	1.21393017	1.65971125	1.82628098	0.16366122	0.34675843	0.31552033	4.676E-13	-3.541181	down	PREDICTED: uncharacterized protein LOC103328554 [Prunus mume]
c43510.graph_c1	39.609164	38.2971621	31.6192894	38.2874283	34.118003	34.8058568	2.154E-07	-1.043858	down	PREDICTED: dynamin-related protein 3A [Sesamum indicum]
c43510.graph_c2	33.25454	31.5453352	29.0803104	27.4288459	27.4171915	29.671031	3.93E-13	-1.168358	down	PREDICTED: dynamin-related protein 3A-like [Nicotiana sylvestris]
c43511.graph_c0	22.2370477	23.6881221	21.2868068	10.1738992	13.3808659	12.1933981	3.743E-32	-1.932003	down	PREDICTED: uncharacterized protein LOC105169670 [Sesamum indicum]
c43512.graph_c0	3.3210317	3.14170022	3.6579919	27.9394164	30.9519017	33.027435	1.664E-30	2.1619	up	PREDICTED: malonyl-CoA-acyl carrier protein transacylase, mitochondrial [Sesamum indicum]
c43516.graph_c0	11.8872059	13.5087231	12.3401565	67.9504806	59.929078	56.0147497	1.769E-12	1.270585	up	PREDICTED: solute carrier family 25 member 44-like [Nicotiana tomentosiformis]
c43522.graph_c0	63.0121231	63.8412191	60.1075259	30.4684493	32.9544542	32.1093516	2.332E-44	-1.986838	down	PREDICTED: TOM1-like protein 2 isoform X2 [Sesamum indicum]
c43530.graph_c0	6.447602	6.97384823	5.8387215	77.2991005	70.6973832	61.0118992	6.125E-24	2.426725	up	hypothetical protein MIMGU_mgv1a026892mg [Erythranthe guttata]
c43538.graph_c0	21.4193634	20.1342287	18.5056376	19.9103334	21.7190964	18.7685362	1.862E-12	-1.008563	down	PREDICTED: cyclin-T1-4-like isoform X2 [Sesamum indicum]
c43539.graph_c0	30.3404579	29.7981048	28.6339145	12.4896398	15.1391661	14.1618468	2.892E-49	-2.106936	down	PREDICTED: autophagy-related protein 18f [Sesamum indicum]
c43541.graph_c0	0.94771995	0.94866895	0.635404	7.0986943	6.87676577	8.57946191	2.451E-08	2.140668	up	PREDICTED: DNA-3-methyladenine glycosylase isoform X1 [Sesamum indicum]
c43545.graph_c0	14.1602913	13.4182404	13.6660707	10.3684926	14.916528	16.5143808	5.357E-11	-1.005195	down	PREDICTED: probable beta-1,4-xylosyltransferase IRX10L [Sesamum indicum]

#ID	0 h-1 FPKM	0 h-2 FPKM	0 h-3 FPKM	48 h-1 FPKM	48 h-2 FPKM	48 h-3 FPKM	FDR	log2FC	regulated	nr_annotation
c43547.graph_c0	2.7057567	2.22613652	2.02428343	19.5319943	21.7589256	19.3847724	2.484E-33	2.108524	up	PREDICTED: protein LHY-like [Sesamum indicum]
c43548.graph_c0	29.7826751	31.9806796	28.1588526	13.3364059	14.9034239	13.9677322	1.301E-42	-2.109358	down	unnamed protein product [Coffea canephora]
c43550.graph_c0	2.08605274	1.87932745	1.80944574	0.57654235	0.30538836	0.76950588	1.586E-08	-2.819304	down	PREDICTED: subtilisin-like protease SDD1, partial [Sesamum indicum]
c43552.graph_c1	2.18504381	2.08430324	1.81685564	0.59206026	0.60212741	0.56895659	2.44E-12	-2.802908	down	hypothetical protein MIMGU_mgv1a005717mg [Erythranthe guttata]
c43556.graph_c0	15.5227679	14.1914389	13.9167707	62.4025582	74.1152043	70.2407337	3.078E-17	1.225096	up	PREDICTED: peroxisomal nicotinamide adenine dinucleotide carrier isoform X1 [Sesamum indicum]
c43560.graph_c0	11.7519198	10.9725245	12.0214053	8.48703144	8.71277877	7.40301203	3.684E-24	-1.515241	down	PREDICTED: protein HASTY 1 [Sesamum indicum]
c43561.graph_c0	10.9970005	12.3241878	11.9811231	0.91767701	1.39992122	2.38430697	7.333E-57	-3.937221	down	PREDICTED: uncharacterized protein LOC105156369 [Sesamum indicum]
c43566.graph_c1	0	0	0	2.94050131	4.15347278	3.98695678	1.044E-11	Inf	up	PREDICTED: protein ECERIFERUM 1-like [Sesamum indicum]
c43567.graph_c0	0.53306695	0.50551648	0.43263924	9.14989463	8.10505183	8.48650093	1.25E-38	3.11563	up	PREDICTED: uncharacterized protein LOC105158074 [Sesamum indicum]
c43568.graph_c0	89.4368897	94.3236194	84.0180281	41.4555704	43.0363774	45.9300792	3.634E-43	-2.055613	down	PREDICTED: nucleolin [Sesamum indicum]
c43570.graph_c0	5.66986986	4.89655068	5.50022116	78.9663331	85.9880536	76.1874323	2.474E-63	2.889365	up	PREDICTED: ferritin-3, chloroplastic [Sesamum indicum]
c43571.graph_c0	0	0	0	2.67959855	6.24516143	5.34081111	2.753E-08	Inf	up	-
c43573.graph_c0	1.22333915	1.36062682	1.68848659	0	0	0.03714131	3.704E-20	-7.889975	down	PREDICTED: phospholipase D delta-like [Vitis vinifera]
c43574.graph_c0	76.5668623	74.5711822	80.2836467	25.5790097	20.7103903	16.1907024	2.423E-84	-2.900889	down	PREDICTED: uncharacterized Rho GTPase-activating protein At5g61530 [Sesamum indicum]
c43575.graph_c1	23.4937614	25.1418363	23.0400524	14.3818587	15.688385	15.1408764	1.983E-17	-1.683145	down	PREDICTED: ELMO domain-containing protein A [Sesamum indicum]
c43575.graph_c2	23.3680183	20.2602832	25.2998793	15.0866512	14.5458503	13.0469325	9.99E-15	-1.708856	down	PREDICTED: ELMO domain-containing protein A [Sesamum indicum]
c43576.graph_c1	9.14539795	10.440089	10.6519926	4.98349726	9.60928777	10.3360333	4.716E-09	-1.308599	down	PREDICTED: uncharacterized protein C683.02c [Sesamum indicum]
c43581.graph_c0	49.7945921	44.593777	46.234669	46.8949614	46.8674767	47.3590716	5.419E-11	-1.011979	down	PREDICTED: enhancer of rudimentary homolog isoform X1 [Sesamum indicum]
c43584.graph_c0	2.92859049	3.715535	3.06319571	16.6272622	19.3427899	22.9459561	3.241E-14	1.580477	up	PREDICTED: nodulation-signaling pathway 1 protein [Sesamum indicum]
c43587.graph_c0	0	0.07643826	0	0.80901887	1.2669544	1.50231678	9.061E-09	4.534103	up	PREDICTED: vacuolar cation/proton exchanger 3-like [Sesamum indicum]
c43592.graph_c0	0	0	0	4.88113413	6.96720134	4.70894728	7.68E-19	Inf	up	PREDICTED: suppressor of disruption of TFIIS-like [Sesamum indicum]
c43599.graph_c0	0.18918691	0	0.08103755	1.62671896	1.90795201	2.11947267	3.075E-07	3.370874	up	PREDICTED: uncharacterized protein At3g27210-like [Sesamum indicum]

#ID	0 h-1 FPKM	0 h-2 FPKM	0 h-3 FPKM	48 h-1 FPKM	48 h-2 FPKM	48 h-3 FPKM	FDR	log2FC	regulated	nr_annotation
c43600.graph_c0	2.97471956	3.43580572	2.74445404	2.90446757	3.10174516	3.14710519	0.0003634	-1.017634	down	PREDICTED: poly(ADP-ribose) glycohydrolase 1-like [Sesamum indicum]
c43601.graph_c0	17.1452169	19.1723943	17.625826	14.8276526	16.944246	14.7889381	7.237E-15	-1.231168	down	hypothetical protein MIMGU_mgv1a008619mg [Erythranthe guttata]
c43602.graph_c0	6.79684083	6.32526542	6.96007325	2.20137648	2.43573996	3.39520403	1.406E-23	-2.344467	down	PREDICTED: DDT domain-containing protein DDB_G0282237 [Sesamum indicum]
c43605.graph_c0	154.036641	143.834088	152.571266	53.2157253	53.7945563	43.9280662	1.932E-71	-2.593655	down	PREDICTED: probable proteasome inhibitor [Sesamum indicum]
c43609.graph_c0	4.54861651	4.852722	4.92223352	22.8050453	23.4932841	24.9722491	3.957E-19	1.295848	up	PREDICTED: 116 kDa U5 small nuclear ribonucleoprotein component-like [Sesamum indicum]
c43611.graph_c0	0	0	0	0.74986514	0.73481185	0.7172674	8.089E-12	Inf	up	--
c43613.graph_c0	10.485471	8.67777099	10.2913528	0	0	0	3.921E-51	-Inf	down	PREDICTED: benzoate carboxyl methyltransferase-like [Sesamum indicum]
c43614.graph_c0	0.05233699	0.23575227	0.10088265	1.73578481	2.04317059	2.617054	2.845E-08	3.018525	up	PREDICTED: taxadiene 5-alpha hydroxylase [Sesamum indicum]
c43618.graph_c0	8.06847105	7.20810412	7.02369031	30.7185723	35.8730684	39.2336925	1.076E-14	1.226647	up	PREDICTED: 50S ribosomal protein L10, chloroplastic [Sesamum indicum]
c43620.graph_c0	0.53285022	0.33336487	0.42795852	2.08630961	2.86024854	2.51158071	0.0054611	1.505273	up	PREDICTED: uncharacterized protein LOC105161412 [Sesamum indicum]
c43623.graph_c0	17.8156993	16.3125549	16.5968419	14.3482378	9.97282605	9.6530792	2.886E-20	-1.587538	down	PREDICTED: uncharacterized protein LOC105169540 isoform X1 [Sesamum indicum]
c43628.graph_c0	35.8837256	37.9151942	36.6382622	29.4545747	26.9084734	23.3101513	3.631E-24	-1.485574	down	PREDICTED: uncharacterized protein LOC105165517 [Sesamum indicum]
c43630.graph_c1	0.13951178	0.46550492	0.05975939	1.32811594	2.40547817	2.24913629	0.0004871	2.15222	up	-
c43633.graph_c0	0	0	0	25.4665333	50.8649875	18.1817136	3.258E-09	Inf	up	hypothetical protein SELMODRAFT_227399 [Selaginella moellendorffii]
c43638.graph_c0	0.41890706	0.35169322	0.45148762	3.34882882	2.87506996	2.90222732	1.618E-08	1.884676	up	PREDICTED: protein ROS1-like isoform X1 [Sesamum indicum]
c43640.graph_c0	18.2771894	18.2954912	17.668696	6.90545053	7.596284	7.22435819	6.183E-56	-2.33846	down	PREDICTED: LOW QUALITY PROTEIN: ubiquitin carboxyl-terminal hydrolase 2-like [Sesamum indicum]
c43644.graph_c0	0.51695295	0.46300001	0.31467079	2.65697801	3.43876991	3.7358237	9.572E-09	1.90678	up	PREDICTED: kinesin-like protein KIN12B [Sesamum indicum]
c43650.graph_c0	0.63211513	0.80531576	0.29537926	6.1411	7.45917981	6.07669386	1.655E-07	2.493201	up	PREDICTED: solute carrier family 35 member F1-like isoform X2 [Sesamum indicum]
c43651.graph_c0	28.5596623	25.6400961	29.6278437	161.346871	182.686511	205.166697	1.437E-33	1.690379	up	PREDICTED: UDP-glucose 4-epimerase GEPI48 [Sesamum indicum]
c43652.graph_c0	116.078884	121.311759	105.998491	53.3042252	49.9824789	46.1178631	1.17E-48	-2.214998	down	PREDICTED: protein SMG7-like [Sesamum indicum]
c43654.graph_c0	3.93957905	3.959111	3.28163031	2.38134361	2.99385979	3.47194109	2.092E-10	-1.358438	down	PREDICTED: uncharacterized protein LOC105159805 [Sesamum indicum]
c43656.graph_c0	0.22641141	0.05665953	0.14547381	3.23306687	3.09358661	3.4799558	1.482E-08	3.500315	up	-

#ID	0 h-1 FPKM	0 h-2 FPKM	0 h-3 FPKM	48 h-1 FPKM	48 h-2 FPKM	48 h-3 FPKM	FDR	log2FC	regulated	nr_annotation
c43658.graph_c0	1.05304781	1.51240762	1.23553786	5.98953735	6.56861068	7.80652463	0.0002324	1.400966	up	PREDICTED: potassium transporter 8-like [Sesamum indicum]
c43663.graph_c0	43.7727795	43.181588	39.4020021	35.0385518	35.2616679	32.9352925	1.339E-20	-1.307494	down	PREDICTED: VIN3-like protein 2 isoform X2 [Sesamum indicum]
c43666.graph_c0	3.08869046	2.41309917	2.75900126	20.751352	21.7997323	21.6447561	3.467E-19	1.940176	up	PREDICTED: uncharacterized protein LOC105173066 [Sesamum indicum]
c43670.graph_c0	1.26944227	1.31378845	1.02300834	8.44413601	12.8723355	13.6160892	6.771E-11	2.253082	up	PREDICTED: ABC transporter B family member 15-like [Sesamum indicum]
c43672.graph_c0	1.68327315	1.58179796	2.14100047	14.969398	16.1935318	16.0089604	3.055E-31	2.103448	up	unnamed protein product [Coffea canephora]
c43680.graph_c1	98.750186	95.0656514	101.959183	74.3818304	74.8685349	72.8935217	5.813E-21	-1.430841	down	PREDICTED: probable receptor protein kinase TMK1 [Sesamum indicum]
c43682.graph_c0	24.6962476	24.2806544	20.7937186	21.9991902	21.4349035	20.5534845	1.563E-14	-1.139642	down	-
c43684.graph_c0	1.42779806	1.60246751	1.64944602	12.0908731	14.3289809	15.4637023	9.899E-33	2.139614	up	PREDICTED: villin-4-like [Sesamum indicum]
c43685.graph_c1	0.34445747	0.30423741	0.15622641	45.9391528	51.4750516	52.0712951	6.19E-203	6.524383	up	PREDICTED: LOW QUALITY PROTEIN: LRR receptor-like serine/threonine-protein kinase ERECTA [Sesamum indicum]
c43688.graph_c0	10.8972209	9.32603718	11.0102756	9.50278319	10.6353475	11.0467269	0.0004007	-1.022792	down	hypothetical protein MIMGU_mgv1a020146mg, partial [Erythranthe guttata]
c43691.graph_c0	0.07687391	0	0.04939301	1.02690821	2.43836232	2.36311228	2.025E-06	4.497318	up	PREDICTED: dirigent protein 21-like [Sesamum indicum]
c43694.graph_c0	0.17641527	0.13244394	0	12.4738129	11.0407728	11.4787354	2.352E-61	5.825841	up	PREDICTED: probable LRR receptor-like serine/threonine-protein kinase At1g67720 [Sesamum indicum]
c43695.graph_c0	0	0	0	1.13557931	1.70953851	1.22315409	2.211E-10	Inf	up	PREDICTED: nudix hydrolase 3-like [Cicer arietinum]
c43696.graph_c0	3.75073942	3.55478803	3.2303241	3.60246519	3.19329341	3.0746171	0.0008559	-1.107449	down	PREDICTED: F-box/kelch-repeat protein At1g30090-like isoform X2 [Sesamum indicum]
c43698.graph_c0	1.74698648	1.35385999	0.86901166	8.30677622	8.52502147	9.14675792	2.004E-05	1.697815	up	PREDICTED: coiled-coil domain-containing protein 93 [Sesamum indicum]
c43699.graph_c0	8.02988129	6.23996577	5.97399311	6.71628786	6.80574075	6.92882615	0.0072875	-1.000828	down	-
c43702.graph_c0	1.07028636	1.93535655	1.46409327	5.0573161	6.70544039	7.55655248	0.0011096	1.087729	up	PREDICTED: probable beta-1,3-galactosyltransferase 11 [Sesamum indicum]
c43708.graph_c0	43.6127045	46.8695278	41.6580484	26.1016004	19.2369383	14.7874832	1.571E-51	-2.144305	down	PREDICTED: uncharacterized protein LOC105161926 [Sesamum indicum]
c43713.graph_c0	1.47369333	1.19857482	1.42031571	181.502494	263.587046	348.12311	4.773E-24	6.572253	up	PREDICTED: fatty-acid-binding protein 1-like [Nicotiana sylvestris]
c43714.graph_c0	1.75249546	1.65679196	2.00180093	1.16603725	1.71038088	1.8356352	0.0087374	-1.226055	down	PREDICTED: uncharacterized protein LOC105158948 [Sesamum indicum]
c43717.graph_c0	2.026549	1.50843	1.20193648	0	0	0	4.67E-28	-Inf	down	glutathione peroxidase [Micromonas pusilla CCMP1545]
c43730.graph_c0	0.62776199	0.86403708	0.40334951	3.54230367	3.6760567	4.24544395	0.0033998	1.582798	up	PREDICTED: basic leucine zipper 43-like [Sesamum indicum]

#ID	0 h-1 FPKM	0 h-2 FPKM	0 h-3 FPKM	48 h-1 FPKM	48 h-2 FPKM	48 h-3 FPKM	FDR	log2FC	regulated	nr_annotation
c43731.graph_c2	20.2268938	20.1556699	22.3910084	2.10478169	3.50815728	3.62078937	4.37E-99	-3.79289	down	hypothetical protein MIMGU_mgv1a004451mg [Erythranthe guttata]
c43734.graph_c0	14.8540763	16.4922579	15.5681649	11.864123	16.7315327	16.3097068	4.65E-14	-1.086756	down	PREDICTED: uncharacterized protein LOC105168011 [Sesamum indicum]
c43736.graph_c0	0	0	0	3.59937253	7.38019113	6.71533878	2.493E-10	Inf	up	unnamed protein product [Coffea canephora]
c43736.graph_c1	0	0	0	6.34311168	9.17156117	12.088972	5.318E-19	Inf	up	hypothetical protein MIMGU_mgv1a003774mg [Erythranthe guttata]
c43741.graph_c0	0.07235098	0.10863515	0.04648694	4.06592123	3.91898285	4.59642623	1.73E-15	4.777055	up	unnamed protein product [Coffea canephora]
c43743.graph_c0	0.12629723	0.06321185	0.10819796	4.01418689	3.53351504	3.08865741	1.306E-17	4.144679	up	hypothetical protein MIMGU_mgv1a003562mg [Erythranthe guttata]
c43744.graph_c0	5.9561807	4.83484861	4.82390421	28.5888885	33.0952858	37.193141	1.704E-24	1.64322	up	hypothetical protein MIMGU_mgv1a003216mg [Erythranthe guttata]
c43744.graph_c1	7.61706708	9.36267624	6.69342388	65.2715725	73.2469597	78.9221474	2.045E-38	2.181278	up	PREDICTED: D-3-phosphoglycerate dehydrogenase 3, chloroplastic [Sesamum indicum]
c43745.graph_c0	8.0768186	8.36530769	7.61928915	40.2152261	46.6584294	44.049775	1.431E-16	1.424877	up	hypothetical protein L484_019510 [Morus notabilis]
c43748.graph_c0	15.0342719	16.2010606	15.2782825	9.15125748	9.99409623	12.0097465	5.799E-19	-1.598563	down	PREDICTED: phosphoglucan phosphatase LSF1, chloroplastic [Sesamum indicum]
c43749.graph_c0	0.134143	0.13427732	0.08618953	0.83417363	1.44011317	1.70440911	3.174E-05	2.464574	up	PREDICTED: cytochrome c oxidase copper chaperone 1 [Sesamum indicum]
c43754.graph_c1	7.96811394	7.65598205	6.78056754	4.5910353	6.47619068	6.94681306	2.22E-11	-1.33723	down	PREDICTED: folylpolyglutamate synthase [Sesamum indicum]
c43759.graph_c0	0.84949411	1.20838465	1.35017836	4.92220114	7.54898928	7.03691242	1.458E-07	1.488806	up	PREDICTED: BTB/POZ domain-containing protein At5g66560 [Sesamum indicum]
c43761.graph_c0	0.04954292	0.17357386	0.03183231	1.30080187	1.3296895	1.48234271	5.489E-07	3.001131	up	PREDICTED: probable inactive leucine-rich repeat receptor-like protein kinase At5g20690 [Sesamum indicum]
c43763.graph_c0	0.69913324	0.75815275	0.82354606	7.29965423	8.01740362	9.93381761	8.144E-09	2.446375	up	PREDICTED: squamosa promoter-binding-like protein 16-like isoform X1 [Solanum tuberosum]
c43763.graph_c1	0.43552266	0.43595877	0.47971188	2.69398546	3.15756274	2.6010999	0.002251	1.625026	up	-
c43764.graph_c1	19.8093363	19.4396012	20.6046797	10.8637422	10.3313478	9.76220031	1.581E-30	-1.967652	down	PREDICTED: uncharacterized protein LOC105178804 isoform X1 [Sesamum indicum]
c43765.graph_c0	0	0	0	3.57155334	8.19786	2.42129271	0.0001165	Inf	up	hypothetical protein EMIHUDRAFT_444197 [Emiliania huxleyi CCMP1516]
c43768.graph_c0	8.77071002	7.53841246	8.99886501	0.71917567	1.14281912	1.11044626	1.649E-73	-4.116214	down	PREDICTED: QWRF motif-containing protein 2 [Sesamum indicum]
c43769.graph_c0	1.70178386	1.3448589	0.95914896	12.7623956	15.5307592	17.9883376	7.166E-22	2.513188	up	PREDICTED: telomere repeat-binding protein 4 [Sesamum indicum]
c43777.graph_c0	4.08575449	3.60584034	3.47952606	40.268798	43.6509021	41.0034309	2.871E-53	2.465978	up	PREDICTED: probable sugar phosphate/phosphate translocator At3g14410 [Sesamum indicum]

#ID	0 h-1 FPKM	0 h-2 FPKM	0 h-3 FPKM	48 h-1 FPKM	48 h-2 FPKM	48 h-3 FPKM	FDR	log2FC	regulated	nr_annotation
c43779.graph_c0	1.9627234	0.71135283	0.65228723	0	0	0	5.05E-06	-Inf	down	PREDICTED: 2,3-bisphosphoglycerate-independent phosphoglycerate mutase [Elaeis guineensis]
c43780.graph_c0	19.9620858	23.3258495	23.9970983	3.48527277	3.18159718	3.89296406	3.327E-67	-3.690391	down	PREDICTED: mitogen-activated protein kinase 9-like [Sesamum indicum]
c43781.graph_c0	12.1876941	12.3957682	10.8841419	5.8973271	7.72071328	9.44074826	1.516E-20	-1.643678	down	PREDICTED: probable serine/threonine-protein kinase abkC isoform X1 [Sesamum indicum]
c43782.graph_c0	3.64355587	3.47887183	4.0338209	34.4963557	55.9659195	55.5076902	1.01E-12	2.682501	up	PREDICTED: pentatricopeptide repeat-containing protein At1g06140, mitochondrial-like [Sesamum indicum]
c43784.graph_c0	8.94943769	9.95694687	10.2917429	4.70006441	4.67316715	5.60727023	1.097E-23	-1.982657	down	PREDICTED: histone-lysine N-methyltransferase ATXR4 [Sesamum indicum]
c43789.graph_c2	5.64326574	5.16472377	5.38706622	1.18833017	0.78680733	0.66085565	1.323E-11	-3.626429	down	PREDICTED: coiled-coil domain-containing protein 94-like [Sesamum indicum]
c43789.graph_c3	15.9609367	15.9198587	14.7601884	16.1484239	16.1615278	14.8126837	1.94E-10	-1.001107	down	PREDICTED: lysophospholipid acyltransferase LPEAT1-like isoform X1 [Sesamum indicum]
c43789.graph_c4	3.07860923	3.03637299	3.49070713	2.83621563	1.76742738	1.48449859	1.508E-05	-1.665501	down	PREDICTED: uncharacterized protein LOC105175802 [Sesamum indicum]
c43791.graph_c1	2.29487247	1.08477493	1.8840846	11.2169323	11.6341215	11.7051833	2.633E-06	1.696506	up	PREDICTED: uncharacterized protein LOC105165414 [Sesamum indicum]
c43796.graph_c0	0	0	0.25520617	5.85476305	5.76630447	5.94212353	3.965E-17	5.054964	up	geranyl diphosphate synthase large subunit [Antirrhinum majus]
c43798.graph_c0	4.99631328	4.80441411	4.19604891	41.8250461	45.8059946	45.8584828	1.786E-37	2.236117	up	PREDICTED: methyltransferase-like protein 13 isoform X1 [Sesamum indicum]
c43799.graph_c0	6.27907187	2.86538444	4.47940165	0	0	0	2.277E-20	-Inf	down	hypothetical protein THAPSDRAFT_261727 [Thalassiosira pseudonana CCMP1335]
c43802.graph_c0	23.0745188	20.4920997	24.2275842	209.661058	189.772282	198.319368	8.413E-49	2.123503	up	PREDICTED: ethylene-responsive transcription factor RAP2-3 [Sesamum indicum]
c43808.graph_c0	38.483976	32.7868766	38.1609167	20.0237406	19.8146748	19.375145	4.28E-39	-1.903577	down	PREDICTED: (S)-coclaurine N-methyltransferase isoform X1 [Sesamum indicum]
c43809.graph_c0	47.8107847	49.4611671	44.7585473	48.5507072	44.5082109	40.4336167	7.278E-15	-1.103544	down	hypothetical protein [Nicotiana tabacum]
c43813.graph_c0	2.30854631	2.00274357	1.87882982	0.49624947	0.52571614	0.69387967	3.861E-08	-2.870044	down	-
c43813.graph_c1	74.0821308	75.3966033	70.979846	49.7751211	53.1125812	50.8114279	8.506E-28	-1.538197	down	PREDICTED: FACT complex subunit SSRP1 [Sesamum indicum]
c43816.graph_c0	0	0.27364625	0.87823623	10.8294891	22.9450611	22.7454755	1.795E-09	4.564764	up	hypothetical protein VITISV_012452 [Vitis vinifera]
c43818.graph_c0	9.5613458	10.2720863	8.9112387	8.93759818	9.36575675	9.18714353	3.29E-08	-1.081501	down	PREDICTED: pentatricopeptide repeat-containing protein At3g61360 [Sesamum indicum]
c43820.graph_c0	14.2792034	14.7357513	13.1715571	4.3307083	4.3291464	3.80997838	1.106E-66	-2.77379	down	PREDICTED: metal tolerance protein 9-like [Sesamum indicum]
c43826.graph_c0	24.632141	24.5708941	23.4918044	9.56732029	8.16696724	8.61847643	2.786E-47	-2.478116	down	PREDICTED: protein REVEILLE 6-like isoform X1 [Sesamum indicum]
c43829.graph_c0	6.39743516	3.75497963	3.84890281	0	0	0	7.178E-26	-Inf	down	predicted protein, partial [Micromonas sp. RCC299]

#ID	0 h-1 FPKM	0 h-2 FPKM	0 h-3 FPKM	48 h-1 FPKM	48 h-2 FPKM	48 h-3 FPKM	FDR	log2FC	regulated	nr_annotation
c43831.graph_c1	1.34132746	1.50298947	1.56930137	15.1052734	19.714561	22.0563388	6.948E-23	2.663423	up	ABC transporter-like [Arabidopsis thaliana]
c43836.graph_c0	9.87198853	10.3000378	8.81514384	7.41351319	8.43309095	7.65986959	5.426E-15	-1.320162	down	hypothetical protein MIMGU_mgv1a020553mg, partial [Erythranthe guttata]
c43838.graph_c0	19.6826702	19.8437841	18.7882291	14.5974663	17.5322724	16.4819941	7.742E-18	-1.282448	down	PREDICTED: uncharacterized protein LOC105159284 [Sesamum indicum]
c43840.graph_c0	10.5318327	10.1948277	10.9063766	5.65097135	8.6220933	8.00084705	2.366E-13	-1.531136	down	hypothetical protein M569_07122 [Genlisea aurea]
c43843.graph_c0	0.27720661	0.27748419	0.25907016	5.36299699	7.15714502	7.02366771	8.788E-21	3.56409	up	PREDICTED: interactor of constitutive active ROPs 2, chloroplastic-like isoform X2 [Sesamum indicum]
c43844.graph_c0	51.616544	52.0817138	46.4681109	11.4005558	11.9431253	11.0188846	3.58E-102	-3.144198	down	PREDICTED: uncharacterized protein LOC105180214 [Sesamum indicum]
c43848.graph_c0	7.4494775	7.30976063	7.02220656	31.808845	30.9711927	30.0107584	6.389E-12	1.074803	up	PREDICTED: dihydrolipoyllysine-residue acetyltransferase component 1 of pyruvate dehydrogenase complex, mitochondrial [Sesamum indicum]
c43849.graph_c0	20.6584543	16.4357332	16.4192005	94.554517	95.7986683	93.9717034	8.324E-20	1.39373	up	PREDICTED: probable ubiquitin-conjugating enzyme E2 33 [Sesamum indicum]
c43851.graph_c0	0.41669978	0.48663655	0.3792953	8.14168859	6.26974219	5.76422858	2.054E-13	2.965588	up	PREDICTED: serine/threonine-protein kinase D6PKL1-like [Sesamum indicum]
c43851.graph_c1	0.68565278	0.11438989	0	3.89528639	3.68046646	2.90394796	1.781E-05	2.716807	up	PREDICTED: serine/threonine-protein kinase D6PKL1-like [Sesamum indicum]
c43852.graph_c0	4.85052311	5.35687484	4.76994188	18.0632879	24.6699282	27.1236876	1.747E-07	1.198441	up	PREDICTED: putative 3,4-dihydroxy-2-butanone kinase [Sesamum indicum]
c43853.graph_c0	88.2481205	80.8890157	85.1622751	22.6625521	9.56971245	6.62765819	3.01E-134	-3.706307	down	PREDICTED: BTB/POZ and TAZ domain-containing protein 1 [Sesamum indicum]
c43855.graph_c1	4.79136477	4.8345319	5.22121523	36.2663914	36.6428796	34.9090014	1.965E-34	1.842189	up	hypothetical protein MIMGU_mgv1a001401mg [Erythranthe guttata]
c43856.graph_c0	50.840675	47.2664303	54.5330632	7.5282587	6.38928461	6.20381642	1.32E-116	-3.938309	down	PREDICTED: uncharacterized protein LOC105161639 [Sesamum indicum]
c43857.graph_c0	297.189927	299.829413	286.296771	210.669847	216.068217	202.356929	1.543E-20	-1.506618	down	PREDICTED: uncharacterized protein LOC105170581 [Sesamum indicum]
c43858.graph_c0	34.501068	33.7625643	33.4649118	23.9584332	24.2767687	21.4652941	1.322E-28	-1.561975	down	PREDICTED: ubiquitin carboxyl-terminal hydrolase 23 [Sesamum indicum]
c43860.graph_c0	0.35614146	0.89124521	0.53393186	3.71847508	4.4027174	4.7197301	0.0002442	1.828862	up	hypothetical protein SORBIDRAFT_02g006760 [Sorghum bicolor]
c43865.graph_c0	31.1317159	31.8157435	31.7920704	14.420397	16.1678006	13.8291634	2.5E-40	-2.111466	down	PREDICTED: chavicol O-methyltransferase-like [Sesamum indicum]
c43867.graph_c0	0.40732267	0.32618444	0.23554159	2.06388718	2.54421901	2.55431331	2.289E-05	1.869297	up	unnamed protein product [Coffea canephora]

#ID	0 h-1 FPKM	0 h-2 FPKM	0 h-3 FPKM	48 h-1 FPKM	48 h-2 FPKM	48 h-3 FPKM	FDR	log2FC	regulated	nr_annotation
c43870.graph_c0	1.36595579	1.8406279	1.8903309	22.2641339	21.9966487	24.5477841	3.574E-24	2.734554	up	PREDICTED: nitrogen regulatory protein P-II homolog [Musa acuminata subsp. malaccensis]
c43874.graph_c0	8.93365234	9.16656293	7.27004351	44.7732209	35.2190073	31.2711464	0.000188	1.123427	up	PREDICTED: putative lysine-specific demethylase JMJ16 [Sesamum indicum]
c43875.graph_c0	38.9510379	41.6269995	36.0781266	25.6005699	26.2503562	22.518755	2.516E-31	-1.665155	down	PREDICTED: uncharacterized protein LOC105157181 [Sesamum indicum]
c43877.graph_c0	0.02254464	0	0.02897075	1.03847956	0.61608018	0.72074564	1.071E-07	4.509688	up	PREDICTED: probable cyclic nucleotide-gated ion channel 20, chloroplastic isoform X1 [Sesamum indicum]
c43878.graph_c0	8.27592199	8.39041688	7.60121761	5.47387015	6.93797165	7.06674304	4.075E-12	-1.338123	down	PREDICTED: putative GTP-binding protein 6 [Sesamum indicum]
c43879.graph_c0	66.2147348	66.3561874	64.1058337	37.5552421	39.7852252	42.3397843	2.721E-34	-1.735277	down	PREDICTED: probable 3-hydroxyacyl-CoA dehydrogenase B0272.3 [Sesamum indicum]
c43881.graph_c0	0	0	0	7.25371459	6.98584641	6.87342277	2.366E-15	Inf	up	hypothetical protein MIMGU_mgv1a004314mg [Erythranthe guttata]
c43887.graph_c0	10.8990558	7.88874721	9.39460482	8.43437371	8.67335897	8.19211279	1.073E-08	-1.17268	down	PREDICTED: uncharacterized protein LOC105168322 isoform X1 [Sesamum indicum]
c43888.graph_c1	5.04964576	5.52710429	4.36642635	4.52163136	4.28347283	4.83572163	0.000187	-1.147023	down	PREDICTED: probable tetraacyldisaccharide 4'-kinase, mitochondrial isoform X1 [Sesamum indicum]
c43889.graph_c0	18.800262	14.1623236	18.3042016	1.1045936	0.84253174	0.90423188	1.043E-53	-5.181616	down	PREDICTED: uncharacterized protein LOC102584927 isoform X1 [Solanum tuberosum]
c43890.graph_c0	28.9198883	32.6976188	27.3153987	130.051792	141.496814	144.116154	7.819E-18	1.206554	up	PREDICTED: plasma membrane ATPase 3 [Sesamum indicum]
c43900.graph_c0	0.33211551	0.49867211	0.42678158	3.41662556	4.15972445	3.81145973	2.97E-05	2.157351	up	PREDICTED: 5'-nucleotidase domain-containing protein 4 [Sesamum indicum]
c43900.graph_c1	0.85613143	0.73456176	1.02157973	4.45065753	6.38604711	7.82007918	4.461E-05	1.808443	up	PREDICTED: 5'-nucleotidase domain-containing protein 4 [Sesamum indicum]
c43902.graph_c1	0	0	0	2.01858205	2.21218232	3.2825681	1.042E-11	Inf	up	PREDICTED: uncharacterized protein LOC105173250 [Sesamum indicum]
c43902.graph_c4	0.79336444	0.73533229	0.75518868	5.46820788	6.56720214	6.52758302	1.2E-08	2.002305	up	PREDICTED: la-related protein 1A-like [Sesamum indicum]
c43903.graph_c0	0.66584973	0.08331456	0.32086623	18.3260481	17.4646801	21.9010744	4.203E-25	4.738547	up	PREDICTED: uncharacterized protein LOC105156092 [Sesamum indicum]
c43907.graph_c0	0.5841318	0.45345378	0.45957091	10.5321747	11.9254443	11.5604212	1.032E-63	3.488167	up	PREDICTED: uncharacterized protein LOC105157864 isoform X2 [Sesamum indicum]
c43908.graph_c0	0.17455214	0.17472692	0.22430641	69.2817969	89.2389047	105.717234	6.557E-64	7.821501	up	hypothetical protein MIMGU_mgv1a002930mg [Erythranthe guttata]
c43909.graph_c0	23.8778326	27.2802181	23.3307773	7.59469713	7.93207491	9.22229456	1.435E-68	-2.608299	down	PREDICTED: heterogeneous nuclear ribonucleoprotein 1-like [Sesamum indicum]

#ID	0 h-1 FPKM	0 h-2 FPKM	0 h-3 FPKM	48 h-1 FPKM	48 h-2 FPKM	48 h-3 FPKM	FDR	log2FC	regulated	nr_annotation
c43910.graph_c0	0.80762656	0.73914082	0.74130865	7.37660795	9.03072993	11.0277078	3.558E-17	2.561788	up	PREDICTED: tetratricopeptide repeat protein 7A [Sesamum indicum]
c43915.graph_c0	58.4444727	59.7196961	56.5336858	54.3716463	60.4669939	55.022067	3.704E-14	-1.058823	down	PREDICTED: uncharacterized protein LOC105160776 [Sesamum indicum]
c43917.graph_c1	17.828969	18.6893759	15.585333	10.6799107	12.583636	12.4196582	4.497E-20	-1.56495	down	PREDICTED: nicotinate phosphoribosyltransferase-like [Sesamum indicum]
c43920.graph_c0	73.1107296	68.1635414	66.642173	48.1579002	47.7374017	45.2665197	2.105E-28	-1.574743	down	PREDICTED: serine/arginine-rich splicing factor RS31-like [Eucalyptus grandis]
c43921.graph_c0	3.99305541	4.50372264	3.83035799	23.8335918	34.9640999	44.8573871	2.765E-06	2.046885	up	PREDICTED: hexose carrier protein HEX6-like [Sesamum indicum]
c43921.graph_c1	0	0	0	4.32896573	5.82071043	10.2223173	1.361E-06	Inf	up	PREDICTED: hexose carrier protein HEX6-like [Sesamum indicum]
c43922.graph_c0	3.4797001	3.39537312	3.30669421	19.2074213	21.4894729	21.3333474	9.211E-17	1.587946	up	PREDICTED: zinc finger CCCH domain-containing protein 33 isoform X4 [Sesamum indicum]
c43924.graph_c0	11.7778451	10.3944153	10.5676312	7.47979572	8.33203633	9.85467662	1.121E-12	-1.371174	down	PREDICTED: uncharacterized vacuolar membrane protein YML018C isoform X2 [Sesamum indicum]
c43925.graph_c0	82.8736261	86.0661668	79.0938349	44.3345686	58.3748634	53.864216	1.807E-32	-1.685015	down	-
c43928.graph_c0	33.1265789	29.5127387	33.0611762	20.9394198	24.7265471	24.1684609	4.881E-24	-1.475393	down	hypothetical protein MIMGU_mgv1a006580mg [Erythranthe guttata]
c43929.graph_c0	185.05218	189.018643	198.966735	205.680556	137.786794	114.08497	4.347E-11	-1.333012	down	PREDICTED: sucrose synthase 2, partial [Sesamum indicum]
c43936.graph_c0	16.1762239	17.4801845	16.3480193	15.7805067	17.6010748	17.0684055	3.181E-11	-1.006281	down	PREDICTED: LMBR1 domain-containing protein 2 homolog A [Sesamum indicum]
c43937.graph_c1	87.1924424	88.2269745	83.0064956	80.3688779	77.0271711	72.6383498	1.914E-16	-1.183448	down	PREDICTED: protein decapping 5-like isoform X2 [Sesamum indicum]
c43942.graph_c0	0	0	0	1.59553313	1.48973291	1.85282331	6.365E-14	Inf	up	lupeol synthase [Olea europaea]
c43945.graph_c0	20.3014171	19.1729229	18.2557083	15.288158	15.4694023	15.5611708	6.576E-21	-1.33448	down	PREDICTED: putative nuclear matrix constituent protein 1-like protein [Sesamum indicum]
c43947.graph_c0	10.0423446	8.52222537	8.10302049	6.12213631	7.54243014	7.98841852	1.751E-14	-1.320517	down	PREDICTED: squamosa promoter-binding-like protein 7 [Sesamum indicum]
c43949.graph_c0	0.35461754	0.26622948	0.45569758	2.45022609	2.68224135	2.90692858	0.0006663	1.876903	up	PREDICTED: probable nucleoside diphosphate kinase 5 [Sesamum indicum]
c43951.graph_c0	8.55668398	8.33718336	8.36061883	4.36129112	4.76850272	4.56547131	3.562E-22	-1.901509	down	PREDICTED: switch 2 isoform X1 [Sesamum indicum]
c43953.graph_c0	1.48204522	1.83675052	1.99517663	0	0.03443881	0.14462931	1.347E-21	-5.936762	down	PREDICTED: G-type lectin S-receptor-like serine/threonine-protein kinase At5g24080 isoform X1 [Sesamum indicum]
c43955.graph_c0	1.02749211	1.10470773	1.36927056	7.43250603	7.24244616	7.54925754	1.748E-06	1.64592	up	PREDICTED: alkylated DNA repair protein alkB homolog 8 isoform X2 [Sesamum indicum]

#ID	0 h-1 FPKM	0 h-2 FPKM	0 h-3 FPKM	48 h-1 FPKM	48 h-2 FPKM	48 h-3 FPKM	FDR	log2FC	regulated	nr_annotation
c43957.graph_c0	78.5587312	79.7624499	80.8803915	75.3369173	86.4296688	76.8829825	4.103E-13	-1.022782	down	PREDICTED: beta-1,3-galactosyltransferase 7-like [Sesamum indicum]
c43962.graph_c0	1.55767363	1.5592334	1.87112843	14.2255202	11.2365628	9.57661161	4.572E-07	1.798992	up	PREDICTED: uncharacterized protein LOC105172439 [Sesamum indicum]
c43963.graph_c0	1.88259878	1.72295673	1.58976918	131.614118	135.596996	138.141532	1.98E-172	5.269179	up	PREDICTED: uncharacterized protein LOC105158952 [Sesamum indicum]
c43967.graph_c1	67.5156205	71.504195	74.5881856	22.3735543	25.022711	21.3089934	5.455E-66	-2.655742	down	phytosulfokine precursor [Avicennia marina]
c43968.graph_c0	0.9393793	1.27334993	0.70416476	7.95102314	10.6196566	9.4330301	2.274E-19	2.245115	up	PREDICTED: probable serine/threonine-protein kinase At1g54610 isoform X2 [Sesamum indicum]
c43969.graph_c0	5.62735155	4.78093812	5.10449615	24.2659311	23.0069041	23.1810132	1.894E-12	1.167259	up	PREDICTED: external alternative NAD(P)H-ubiquinone oxidoreductase B2, mitochondrial [Sesamum indicum]
c43970.graph_c0	9.91376341	9.11756442	8.35031194	31.518513	42.1711903	40.0414981	2.215E-11	1.033443	up	PREDICTED: putative UDP-glucuronate:xylan alpha-glucuronosyltransferase 3 [Sesamum indicum]
c43972.graph_c0	105.992448	103.077721	106.598932	462.739024	471.610095	477.329152	4.142E-16	1.142882	up	hypothetical protein JCGZ_19923 [Jatropha curcas]
c43975.graph_c0	522.283969	501.898149	520.240243	116.258207	110.769196	108.66788	7.823E-87	-3.21827	down	PREDICTED: WD repeat-containing protein 82-B isoform X1 [Sesamum indicum]
c43977.graph_c0	68.4922666	72.6386535	66.0114557	55.5317223	56.3824637	49.7053643	2.651E-22	-1.374153	down	PREDICTED: LOW QUALITY PROTEIN: protein EARLY FLOWERING 3 [Sesamum indicum]
c43978.graph_c0	3.57675935	3.61883922	3.06418492	13.8892092	15.4271083	12.9890723	3.027E-05	1.02734	up	PREDICTED: tryptophan--tRNA ligase, mitochondrial isoform X2 [Sesamum indicum]
c43983.graph_c0	6.17394724	5.53711026	5.96178996	3.84669144	4.77170197	4.82697333	6.409E-08	-1.415972	down	PREDICTED: cryptochrome DASH, chloroplastic/mitochondrial [Sesamum indicum]
c43985.graph_c0	21.9929503	21.524073	21.4993371	6.6553116	5.61462446	6.49394157	1.245E-75	-2.807958	down	PREDICTED: uncharacterized protein LOC105162976 [Sesamum indicum]
c43986.graph_c0	0	0.33387802	0	9.37211653	10.5796782	14.901288	3.357E-13	5.69519	up	hypothetical protein MIMGU_mgv1a005877mg [Erythranthe guttata]
c43987.graph_c0	2.58477753	2.82856091	2.61782505	1.89694044	2.5867977	2.01109844	6.678E-06	-1.327636	down	unnamed protein product [Coffea canephora]
c43988.graph_c0	12.5089188	13.2398881	5.66558692	3.68391184	2.80190823	5.71535223	0.0001764	-2.375737	down	uncharacterized protein UHOR_12387 [Ustilago hordei]
c43990.graph_c0	29.2198758	32.0074033	27.9955415	24.6980203	27.6772837	27.0113705	1.544E-16	-1.187106	down	PREDICTED: ethanolamine-phosphate cytidylyltransferase-like [Sesamum indicum]
c43994.graph_c0	3.11305743	3.16643557	2.70994978	12.3044373	13.3290832	14.735084	6.199E-05	1.148767	up	hypothetical protein MIMGU_mgv1a014320mg [Erythranthe guttata]
c43994.graph_c1	8.25180505	7.66620689	8.17816831	80.5922084	89.6939297	84.9738032	3.48E-49	2.385848	up	PREDICTED: heat stress transcription factor A-6b-like [Sesamum indicum]
c43998.graph_c0	7.78864019	4.847786	5.26098912	0	0	0	6.453E-32	-Inf	down	--
c43999.graph_c0	3.89604689	4.39877877	4.51173865	75.0829911	77.7948643	85.3765968	6.22E-100	3.197611	up	hypothetical protein MIMGU_mgv1a001702mg [Erythranthe guttata]

#ID	0 h-1 FPKM	0 h-2 FPKM	0 h-3 FPKM	48 h-1 FPKM	48 h-2 FPKM	48 h-3 FPKM	FDR	log2FC	regulated	nr_annotation
c44006.graph_c0	0.29301634	0.37711254	0.26895543	3.81780445	3.84023316	4.32353019	1.178E-07	2.656841	up	PREDICTED: uncharacterized protein LOC105178295 isoform X2 [Sesamum indicum]
c44010.graph_c0	76.7650805	69.464068	78.9000209	29.2220502	32.2738641	33.5539378	1.565E-55	-2.264283	down	photosystem II protein I (chloroplast) [Salvia miltiorrhiza]
c44013.graph_c0	2.99082791	1.78941131	2.95980761	0.34837705	1.00653611	0.70450864	3.545E-09	-2.944462	down	PREDICTED: peroxidase 46 [Sesamum indicum]
c44014.graph_c0	1.54717674	1.8472756	1.07793074	12.1069746	12.6985225	15.2651638	5.832E-26	2.147893	up	PREDICTED: uncharacterized protein At1g04910-like isoform X1 [Sesamum indicum]
c44014.graph_c1	16.4009547	23.3566818	16.0785034	296.195463	268.320509	242.901147	2.78E-35	2.841011	up	PREDICTED: uncharacterized protein LOC105173450 [Sesamum indicum]
c44019.graph_c0	0.5677718	0.31000382	0.53062491	3.42372056	2.66988735	2.58098283	0.0016301	1.609346	up	hypothetical protein MIMGU_mgv1a010335mg [Erythranthe guttata]
c44021.graph_c0	56.9874514	57.1641059	55.5758271	48.7308632	52.3823375	51.0482747	5.743E-17	-1.175938	down	PREDICTED: protein disulfide isomerase-like 1-4 [Sesamum indicum]
c44025.graph_c0	7.9640684	9.30071708	6.54985165	1.17392308	1.7099901	1.17511841	5.413E-28	-3.572078	down	PREDICTED: WAT1-related protein At1g25270-like [Sesamum indicum]
c44028.graph_c0	0.6823219	0.45533676	0.58454044	56.993016	73.9176623	88.0003097	1.182E-32	5.966851	up	-
c44029.graph_c0	4.84019714	3.88921889	4.06193957	2.00204728	2.37800832	2.26724862	1.286E-10	-1.963519	down	PREDICTED: vesicle-associated protein 1-4-like [Sesamum indicum]
c44030.graph_c0	120.681183	113.648583	119.742543	13.3286161	5.67537224	3.70436853	2.15E-214	-4.958828	down	PREDICTED: RING-H2 finger protein ATL3 [Sesamum indicum]
c44032.graph_c1	50.561844	56.6022477	49.2521569	14.5706105	8.55373169	6.3274262	9.02E-119	-3.412065	down	PREDICTED: glycosyltransferase family protein 64 protein C5-like [Sesamum indicum]
c44033.graph_c0	1.44739227	1.7317107	1.29311253	5.72105898	6.37692378	6.03409791	6.103E-09	1.002276	up	hypothetical protein MIMGU_mgv1a000017mg [Erythranthe guttata]
c44036.graph_c0	23.1416118	24.3781781	20.4270964	18.4008126	17.8532929	17.5472669	6.129E-20	-1.351931	down	PREDICTED: glucose-6-phosphate 1-dehydrogenase, chloroplastic-like [Sesamum indicum]
c44040.graph_c0	0.07867918	0.11813695	0.17693525	1.34096123	1.6701482	1.72527268	2.749E-07	2.634524	up	PREDICTED: LOW QUALITY PROTEIN: ATP-dependent DNA helicase Q-like 5 [Sesamum indicum]
c44043.graph_c0	2.31655134	2.95656055	2.38148815	0.05335395	0	0	2.417E-24	-8.124238	down	PREDICTED: uncharacterized protein LOC105165513 [Sesamum indicum]
c44044.graph_c0	0.11205158	0.44865514	0.14399072	4.40444695	6.08764249	5.9091932	2.644E-13	3.521598	up	PREDICTED: scarecrow-like protein 3 isoform X1 [Sesamum indicum]
c44050.graph_c2	4.75962045	4.35172309	4.67150527	26.3092368	31.6022698	30.5371941	2.176E-19	1.661211	up	PREDICTED: elongation factor Ts, mitochondrial [Sesamum indicum]
c44052.graph_c0	13.0450051	13.3677452	8.21602826	0.66502266	1.05676631	0.50719986	3.254E-24	-4.974731	down	PREDICTED: ferredoxin--nitrite reductase, chloroplastic [Sesamum indicum]
c44052.graph_c1	5.90335295	9.88273507	7.19366077	0.18753634	0	0.41717176	2.342E-21	-6.263229	down	PREDICTED: ferredoxin--nitrite reductase, chloroplastic [Sesamum indicum]

#ID	0 h-1 FPKM	0 h-2 FPKM	0 h-3 FPKM	48 h-1 FPKM	48 h-2 FPKM	48 h-3 FPKM	FDR	log2FC	regulated	nr_annotation
c44054.graph_c0	195.776595	182.911959	221.898007	111.231006	64.7670198	34.38968	1.163E-22	-2.516283	down	PREDICTED: uncharacterized protein LOC105157696 [Sesamum indicum]
c44056.graph_c0	71.3816532	66.5944965	68.839105	331.55578	349.852428	361.013561	2.469E-20	1.314973	up	hypothetical protein Csa_5G600925 [Cucumis sativus]
c44062.graph_c0	47.6143051	50.2536293	46.7344556	11.4042595	10.0678585	11.3744198	6.5E-93	-3.153838	down	PREDICTED: transcription factor E2FB [Sesamum indicum]
c44066.graph_c0	6.03388104	4.96350113	5.22036406	27.4087602	34.2837744	36.582225	8.519E-14	1.578086	up	PREDICTED: 6,7-dimethyl-8-ribityllumazine synthase, chloroplastic [Sesamum indicum]
c44077.graph_c0	8.9808397	9.55761155	9.71851333	1.82893096	1.6607405	2.78978018	9.431E-30	-3.190558	down	PREDICTED: PXMP2/4 family protein 4-like [Sesamum indicum]
c44077.graph_c1	10.5885087	10.8000425	9.41502994	6.05631304	6.75876522	7.52796473	1.207E-11	-1.61754	down	PREDICTED: uncharacterized protein LOC105169472 [Sesamum indicum]
c44080.graph_c0	0.33077737	0.35870098	0.24795284	13.3320989	12.4019907	12.1565675	2.764E-42	4.324399	up	PREDICTED: 6-phosphogluconate dehydrogenase, decarboxylating 3-like [Citrus sinensis]
c44081.graph_c0	1.10936792	1.53169487	1.27810773	17.4096176	17.0246574	16.8080193	6.943E-24	2.69168	up	PREDICTED: pheophytinase, chloroplastic isoform X1 [Nicotiana sylvestris]
c44084.graph_c1	131.224501	128.075532	145.127529	7.85606923	7.49717562	9.04115136	2.99E-173	-5.070508	down	PREDICTED: chaperone protein dnaJ 6 [Sesamum indicum]
c44085.graph_c1	4.71437121	4.63329027	4.95667118	2.36901069	4.43376723	3.23216158	0.0002083	-1.538674	down	-
c44085.graph_c2	8.98922812	9.03988794	8.98451092	5.25259476	5.28017037	5.56071196	9.746E-14	-1.765103	down	PREDICTED: CTD small phosphatase-like protein 2 isoform X3 [Sesamum indicum]
c44086.graph_c0	3.57736257	3.20958752	3.74574651	20.5798139	24.491484	23.0689512	8.85E-21	1.672429	up	PREDICTED: probable apyrase 7 [Sesamum indicum]
c44089.graph_c1	3.62370135	3.75241028	3.85373768	1.78431192	2.3170955	3.12412486	5.815E-05	-1.661024	down	PREDICTED: COBRA-like protein 1 [Sesamum indicum]
c44090.graph_c0	7.59320991	6.98348841	8.32118101	5.93007695	7.03455645	6.79315907	2.138E-07	-1.234531	down	PREDICTED: tobamovirus multiplication protein 1-like isoform X1 [Sesamum indicum]
c44091.graph_c0	25.2333758	24.6210464	21.218493	22.9983667	20.7775263	15.7545217	1.12E-17	-1.267203	down	PREDICTED: methyl-CpG-binding domain-containing protein 9-like [Sesamum indicum]
c44095.graph_c0	2.92365619	2.1356152	1.69234863	16.7674124	16.0150132	13.8183898	1.386E-15	1.776301	up	hypothetical protein MIMGU_mgv1a003860mg [Erythranthe guttata]
c44100.graph_c0	0.49611288	0.49660966	0.56668861	9.5134432	11.7461074	10.9954685	8.46E-41	3.348065	up	PREDICTED: probable LRR receptor-like serine/threonine-protein kinase At4g36180 [Sesamum indicum]
c44101.graph_c0	27.3294547	27.4973527	23.3778749	14.0007712	15.4486971	14.0689727	3.353E-40	-1.862558	down	PREDICTED: uncharacterized protein LOC105175040 isoform X2 [Sesamum indicum]
c44102.graph_c0	1.44589862	1.8790112	1.56466271	9.53471747	9.28389432	8.48393427	1.506E-08	1.465293	up	hypothetical protein MIMGU_mgv1a001743mg [Erythranthe guttata]
c44103.graph_c0	1.15224998	0.57670189	0.74034342	5.30763919	8.4342002	9.28798735	0.000108	2.197885	up	hypothetical protein M569_03256, partial [Genlisea aurea]
c44103.graph_c1	0.35422066	0.38412331	0.34139068	3.48086589	3.25541994	3.38762105	1.021E-06	2.213308	up	ABC transporter family protein [Hevea brasiliensis]
c44105.graph_c0	0.19193416	0.48031589	0.49328599	3.27121198	4.68304383	5.27073562	1.164E-05	2.473933	up	PREDICTED: UDP-galactose transporter 2-like [Sesamum indicum]

#ID	0 h-1 FPKM	0 h-2 FPKM	0 h-3 FPKM	48 h-1 FPKM	48 h-2 FPKM	48 h-3 FPKM	FDR	log2FC	regulated	nr_annotation
c44106.graph_c1	0.91672586	0.30588127	0.39267634	8.86775035	11.4819298	12.524557	1.07E-08	3.330557	up	phospholipase D [Plantago major]
c44106.graph_c2	0.35492139	0.3806537	0.32577717	27.1624241	25.8557033	27.1199441	1.76E-105	5.222864	up	PREDICTED: phospholipase D alpha 1 [Sesamum indicum]
c44107.graph_c0	0	0	0	3.60487739	4.614541	5.88059675	1.55E-11	Inf	up	-
c44108.graph_c0	0.32265289	0.29813167	0.22325786	4.27581093	4.11791201	4.55737086	4.362E-12	2.925649	up	PREDICTED: homeobox-leucine zipper protein GLABRA 2 [Sesamum indicum]
c44110.graph_c0	0.98344868	0.80309045	1.06422778	4.816196	4.57175147	4.07327263	0.0004341	1.222226	up	PREDICTED: glucan endo-1,3-beta-glucosidase 9 [Sesamum indicum]
c44111.graph_c0	24.1230649	27.3692634	23.9813077	21.390767	24.3551985	23.2136032	4.016E-14	-1.149192	down	PREDICTED: serine/threonine-protein kinase HT1-like [Sesamum indicum]
c44112.graph_c1	0.21381639	0.21403049	0.27476252	38.4606581	40.2748778	51.0482747	5.847E-66	6.50617	up	PREDICTED: 2-hydroxyisoflavanone dehydratase-like [Sesamum indicum]
c44115.graph_c0	11.5105652	11.0495765	10.3587385	8.2291916	9.78098074	10.4625335	5.284E-15	-1.229499	down	PREDICTED: F-box protein At-B isoform X1 [Sesamum indicum]
c44123.graph_c0	3.78015682	4.14188254	4.39814363	17.8832469	19.4436877	19.4298847	1.067E-06	1.183458	up	PREDICTED: ribonuclease H2 subunit B [Sesamum indicum]
c44127.graph_c0	32.4685377	33.6063247	33.2099509	13.1690372	16.8926491	17.0995686	6.079E-45	-2.096202	down	PREDICTED: ran-binding protein 9 [Sesamum indicum]
c44131.graph_c0	8.65389477	7.7881063	7.50728027	4.27549052	4.68922367	4.60992344	4.181E-19	-1.836972	down	PREDICTED: glutamyl-tRNA(Gln) amidotransferase subunit A, mitochondrial isoform X4 [Sesamum indicum]
c44133.graph_c1	119.040684	124.472746	112.049548	30.246027	17.5379989	15.4349392	7.07E-119	-3.495844	down	PREDICTED: syntaxin-121 [Sesamum indicum]
c44135.graph_c0	6.83304309	7.05034336	5.40352671	5.13288283	5.84805693	3.79164461	1.308E-06	-1.399127	down	hypothetical protein MIMGU_mgv1a025425mg [Erythranthe guttata]
c44140.graph_c0	0.36492235	0.19669341	0.14428913	1.24131793	1.58898954	1.51871119	0.0028428	1.608941	up	PREDICTED: receptor-like serine/threonine-protein kinase At1g78530 [Sesamum indicum]
c44142.graph_c0	1.29787847	1.85960787	0.75215647	9.51863261	8.76747409	7.05106467	4.264E-07	1.688297	up	PREDICTED: probable LRR receptor-like serine/threonine-protein kinase At1g56130 [Sesamum indicum]
c44144.graph_c0	0.98932602	1.01637765	0.86985265	11.1770304	12.2472563	12.5916311	1.029E-23	2.628827	up	hypothetical protein MIMGU_mgv1a003454mg [Erythranthe guttata]
c44149.graph_c0	22.5975799	25.3908039	22.4124849	15.6137734	16.6889166	17.6848785	1.661E-21	-1.512606	down	PREDICTED: 1-phosphatidylinositol-3-phosphate 5-kinase FAB1A isoform X1 [Sesamum indicum]
c44152.graph_c0	78.1455376	76.3379313	70.460519	19.9598227	20.7236439	21.7175505	8.614E-86	-2.867395	down	PREDICTED: WD repeat-containing protein 55 homolog [Sesamum indicum]
c44156.graph_c0	0	0	0	17.9725041	40.926434	10.4619423	1.933E-05	Inf	up	60S ribosomal protein L29 [Zea mays]
c44159.graph_c0	40.6097206	40.9865123	36.08458	21.828689	44.6315732	52.058554	3.531E-05	-1.018592	down	PREDICTED: proton-coupled amino acid transporter 3-like [Sesamum indicum]
c44162.graph_c0	0	0	0	1.92707275	2.38816971	2.65292859	3.192E-14	Inf	up	PREDICTED: probable leucine-rich repeat receptor-like protein kinase At5g49770 isoform X3 [Sesamum indicum]
c44162.graph_c1	0	0	0	3.89385659	4.09125686	5.2254947	2.293E-22	Inf	up	hypothetical protein MIMGU_mgv1a0019072mg, partial [Erythranthe guttata]

#ID	0 h-1 FPKM	0 h-2 FPKM	0 h-3 FPKM	48 h-1 FPKM	48 h-2 FPKM	48 h-3 FPKM	FDR	log2FC	regulated	nr_annotation
c44163.graph_c0	0.24736507	0.30951596	0.27813967	8.46037293	9.05327196	9.27691568	6.798E-26	3.98453	up	PREDICTED: probable LRR receptor-like serine/threonine-protein kinase At4g30520 [Sesamum indicum]
c44166.graph_c0	90.6443452	96.4799792	80.9080568	32.3233275	30.288856	26.6855589	5.39E-72	-2.599416	down	PREDICTED: probable ADP-ribosylation factor GTPase-activating protein AGD14 [Sesamum indicum]
c44169.graph_c0	2.06064815	0.68757053	1.0788207	0	0	0	8.469E-08	-Inf	down	PREDICTED: vacuolar-processing enzyme-like [Musa acuminata subsp. malaccensis]
c44170.graph_c0	0.87792774	0.74507538	0.68671334	120.05469	116.08185	137.488108	8.15E-204	6.321648	up	PREDICTED: pyruvate, phosphate dikinase, chloroplastic [Populus euphratica]
c44173.graph_c0	2.84483524	3.09411809	3.26906259	0.85681562	0.88099542	0.91935191	2.11E-15	-2.812479	down	hypothetical protein MIMGU_mgv1a025700mg, partial [Erythranthe guttata]
c44178.graph_c0	3.00183179	2.81703532	2.7859525	16.2856235	21.6064944	24.6754566	2.813E-12	1.839252	up	PREDICTED: LOW QUALITY PROTEIN: homeobox-leucine zipper protein ATHB-15 [Sesamum indicum]
c44178.graph_c1	1.11294206	0.92838042	1.43017461	7.00631794	9.50422136	10.4156415	2.204E-05	1.928153	up	PREDICTED: homeobox-leucine zipper protein ATHB-15-like [Sesamum indicum]
c44188.graph_c0	54.9997636	56.3748336	57.1204704	30.9280567	29.278938	25.9206697	3.365E-44	-1.983868	down	PREDICTED: uncharacterized protein LOC105157865 [Sesamum indicum]
c44190.graph_c0	0.14990815	0.18006991	0.07705516	2.43065089	2.7505467	2.50685696	5.892E-09	3.22705	up	PREDICTED: DNA replication complex GINS protein PSF1 isoform X2 [Sesamum indicum]
c44197.graph_c0	1.67694022	2.01434331	1.58028536	23.01911	25.6679915	25.3622685	5.763E-45	2.794129	up	PREDICTED: 5'-methylthioadenosine/S-adenosylhomocysteine nucleosidase 1-like isoform X3 [Populus euphratica]
c44204.graph_c0	14.0562408	14.1952004	16.0855343	8.35206102	7.38685897	6.51118445	3.448E-25	-2.010139	down	PREDICTED: uncharacterized protein At2g39910 [Sesamum indicum]
c44207.graph_c0	35.3215556	37.9991985	35.5103109	14.1956313	18.5969221	18.5951634	4.344E-49	-2.104773	down	PREDICTED: stress response protein NST1-like [Sesamum indicum]
c44208.graph_c1	11.0236324	11.9504113	12.1085297	1.22205389	1.38390191	1.38734205	1.624E-53	-4.155603	down	PREDICTED: uncharacterized protein LOC105160931 [Sesamum indicum]
c44209.graph_c0	43.2573315	43.8281678	43.0590596	6.99929464	8.61500403	7.77591295	2.04E-103	-3.49664	down	PREDICTED: probable RNA methyltransferase At5g51130 [Sesamum indicum]
c44211.graph_c1	0.99313384	0.76021577	0.90085857	6.37766772	8.09623559	10.128459	7.98E-11	2.189146	up	PREDICTED: glucose-6-phosphate 1-dehydrogenase 4, chloroplastic [Sesamum indicum]
c44213.graph_c0	4.06784255	1.9434144	1.90085045	0	0	0	1.181E-09	-Inf	down	PREDICTED: small heat shock protein, chloroplastic-like isoform X2 [Phoenix dactylifera]
c44215.graph_c0	157.672713	160.102614	147.915422	18.1851503	16.0692659	13.4511546	1.14E-154	-4.299954	down	PREDICTED: PAX3- and PAX7-binding protein 1 [Sesamum indicum]
c44217.graph_c0	7.82077464	9.10812006	8.04000404	6.05840433	7.2224647	6.47990772	2.382E-16	-1.35715	down	hypothetical protein MIMGU_mgv1a015770mg [Erythranthe guttata]

#ID	0 h-1 FPKM	0 h-2 FPKM	0 h-3 FPKM	48 h-1 FPKM	48 h-2 FPKM	48 h-3 FPKM	FDR	log2FC	regulated	nr_annotation
c44221.graph_c0	4.67099423	4.72173726	3.81434096	28.5115124	26.9033303	27.4253289	4.35E-23	1.634763	up	PREDICTED: kinesin-like protein KCA2 [Sesamum indicum]
c44223.graph_c0	11.8722385	12.1755631	9.43645589	185.102701	176.487637	180.958802	7.393E-93	3.004011	up	PREDICTED: 14-3-3 protein 7 isoform X1 [Sesamum indicum]
c44226.graph_c1	5.4613548	5.94219947	4.71260841	22.0212984	27.1397967	28.3102586	1.731E-13	1.24541	up	PREDICTED: uncharacterized protein LOC105161158 [Sesamum indicum]
c44227.graph_c0	2.21307737	3.40814374	3.28141389	17.5130374	16.7253366	14.3270525	4.208E-07	1.429934	up	PREDICTED: E3 SUMO-protein ligase MMS21 [Sesamum indicum]
c44229.graph_c0	1.1655477	1.27783052	1.2838071	7.41418535	8.8294631	9.46342268	5.283E-06	1.76412	up	PREDICTED: uncharacterized protein LOC105172855 [Sesamum indicum]
c44232.graph_c0	68.9403254	69.4893841	64.7633131	20.8692368	21.2838172	18.4195688	4.73E-81	-2.762119	down	PREDICTED: WEB family protein At5g55860-like [Sesamum indicum]
c44236.graph_c0	3.04913507	2.93010079	3.13460743	35.5439992	42.0985092	39.158623	1.219E-44	2.659294	up	PREDICTED: actin-depolymerizing factor 5-like [Sesamum indicum]
c44237.graph_c0	14.864105	15.6144525	13.8354887	8.20064954	7.86020392	7.18106502	7.2E-31	-1.945958	down	PREDICTED: serine/threonine-protein kinase UCNL-like [Sesamum indicum]
c44238.graph_c0	3.02738353	2.37612085	1.85673776	80.7548982	70.4075343	62.7746177	2.07E-36	3.871502	up	hypothetical protein MIMGU_mgv1a008109mg [Erythranthe guttata]
c44239.graph_c0	0	0	0	10.7787905	23.5255238	15.9463205	2.848E-12	Inf	up	ribosomal protein L41 [Cryptococcus gattii R265]
c44241.graph_c0	4.34738849	1.90076076	2.37589603	0	0	0	5.177E-12	-Inf	down	DNAJ [Theobroma cacao]
c44242.graph_c0	5.65740275	7.69823276	6.9292059	31.9233065	36.1482335	37.2455495	2.914E-14	1.355388	up	PREDICTED: 50S ribosomal protein L4, chloroplastic [Sesamum indicum]
c44253.graph_c0	7.45631982	8.03393653	7.41913735	4.34098321	6.82231452	6.81257375	3.647E-12	-1.374989	down	PREDICTED: probable fructokinase-4 [Solanum lycopersicum]
c44256.graph_c0	68.1445947	61.3118152	62.0497487	31.4275787	26.5920766	21.3445562	5.184E-58	-2.282538	down	hypothetical protein MIMGU_mgv1a026885mg [Erythranthe guttata]
c44260.graph_c0	11.134091	11.3516334	10.3612637	45.4986928	47.6284257	47.4942978	1.179E-14	1.080559	up	PREDICTED: tripeptidyl-peptidase 2 isoform X4 [Sesamum indicum]
c44261.graph_c0	29.4386291	31.2518912	29.3799264	20.5211753	22.4179755	19.4574494	5.129E-28	-1.547409	down	PREDICTED: phosphatidylinositol:ceramide inositolphosphotransferase 1 [Sesamum indicum]
c44263.graph_c0	18.5141564	19.5845513	17.1255386	16.7721744	17.629717	17.2686523	1.357E-15	-1.113126	down	PREDICTED: calpain-type cysteine protease DEK1 [Sesamum indicum]
c44264.graph_c0	1.39285728	1.32940308	1.1238761	0.71619961	0.34774971	0.31863505	1.405E-07	-2.475438	down	-
c44265.graph_c0	0	0	0	12.2833313	8.8975727	10.742803	2.81E-18	Inf	up	Cold shock, CspA, partial [Metarhizium brunneum ARSEF 3297]
c44266.graph_c0	81.9136492	82.7533336	85.7732698	28.641271	25.7728768	27.5300516	6.07E-73	-2.628095	down	PREDICTED: interferon-related developmental regulator 1 [Sesamum indicum]
c44271.graph_c0	7.30424283	7.47915709	6.13198567	49.7455412	51.6576373	56.1526073	3.078E-38	1.896378	up	PREDICTED: DNA-directed RNA polymerases IV and V subunit 2-like [Sesamum indicum]
c44280.graph_c0	28.2803148	27.3034849	26.2999512	24.3717035	26.7454215	21.25178	1.235E-17	-1.195109	down	PREDICTED: uncharacterized protein LOC105178865 isoform X2 [Sesamum indicum]

#ID	0 h-1 FPKM	0 h-2 FPKM	0 h-3 FPKM	48 h-1 FPKM	48 h-2 FPKM	48 h-3 FPKM	FDR	log2FC	regulated	nr_annotation
c44287.graph_c0	0.93233776	0.93327136	0.98025622	0.42946518	0.20680286	0.31265635	7.353E-07	-2.591445	down	-
c44291.graph_c0	0.11461364	0.34418522	0.1104623	3.53725028	5.08959979	5.73113032	4.981E-15	3.636797	up	PREDICTED: ABC transporter G family member 21 [Sesamum indicum]
c44292.graph_c0	10.4866312	13.452207	13.4191156	10.3916302	12.169743	8.95745973	4.746E-09	-1.265175	down	hypothetical protein MIMGU_mgv1a008797mg [Erythranthe guttata]
c44293.graph_c0	1.66438254	1.41251995	0.92991194	0.06666686	0.24718909	0.17795931	6.611E-14	-4.056595	down	-
c44295.graph_c0	0	0.11657946	0	3.86255385	9.88877632	7.92391129	1.082E-07	6.518729	up	hypothetical protein MIMGU_mgv1a006801mg [Erythranthe guttata]
c44295.graph_c1	25.5056696	26.2643476	22.3403783	21.118144	20.9425047	21.3397734	3.837E-19	-1.241061	down	PREDICTED: E3 ubiquitin-protein ligase KEG [Sesamum indicum]
c44295.graph_c2	0.0675832	0.06765088	0	3.73572332	8.37680545	7.92225755	7.654E-10	6.196966	up	PREDICTED: spermidine hydroxycinnamoyl transferase-like [Sesamum indicum]
c44297.graph_c0	19.1119704	17.7689248	18.3031484	8.85931583	7.64405992	6.12293267	1.428E-43	-2.298782	down	PREDICTED: BTB/POZ domain-containing protein At5g48130 [Sesamum indicum]
c44298.graph_c0	3.24298538	3.29541809	2.74667213	33.6563261	20.8605736	17.9240114	0.001037	1.959408	up	PREDICTED: uncharacterized protein LOC105160865 [Sesamum indicum]
c44299.graph_c0	4.37925802	4.64591243	5.14653533	1.94826397	2.00915432	2.31651848	1.976E-21	-2.196247	down	PREDICTED: DNA polymerase alpha subunit B isoform X1 [Sesamum indicum]
c44300.graph_c0	69.6511896	71.7715504	68.7737061	73.0531204	67.5192067	67.2062856	1.761E-13	-1.032266	down	PREDICTED: peptidyl-prolyl cis-trans isomerase FKBP17-2, chloroplastic-like isoform X1 [Nicotiana sylvestris]
c44301.graph_c1	16.115923	14.9000178	15.4209903	16.7959348	13.1384804	13.0531595	6.801E-11	-1.123591	down	PREDICTED: E3 ubiquitin-protein ligase At1g63170-like [Sesamum indicum]
c44303.graph_c0	5251.31481	5319.33153	4740.25765	189.636404	165.788394	151.904828	1.806E-84	-5.92839	down	hypothetical protein MIMGU_mgv1a024110mg [Erythranthe guttata]
c44308.graph_c0	0.04586178	0	0	2.15479393	1.70086725	1.76694444	5.837E-11	5.946115	up	PREDICTED: probable leucine-rich repeat receptor-like serine/threonine-protein kinase At3g14840 [Sesamum indicum]
c44309.graph_c0	3.24948701	1.87032601	1.77468077	0	0	0	3.104E-14	-Inf	down	PREDICTED: probable rRNA-processing protein EBP2 homolog [Phoenix dactylifera]
c44310.graph_c0	10.6305019	9.8791881	9.30945673	36.3689918	44.6510902	50.9081872	6.623E-10	1.124423	up	PREDICTED: glutamyl-tRNA(Gln) amidotransferase subunit B, chloroplastic/mitochondrial [Sesamum indicum]
c44315.graph_c0	46.691315	50.6506636	45.0560884	34.8505971	33.3507355	29.5029792	1.073E-28	-1.558339	down	PREDICTED: exocyst complex component EXO70B1 [Sesamum indicum]
c44315.graph_c1	32.2840125	30.3414526	27.2733577	16.7437222	21.5305957	22.2496787	1.615E-27	-1.59152	down	PREDICTED: translation initiation factor eIF-2B subunit beta [Sesamum indicum]
c44315.graph_c2	8.2984365	11.2543012	6.36389225	7.3984461	7.18461016	5.15675607	0.0001998	-1.403383	down	PREDICTED: exocyst complex component EXO70B1 [Sesamum indicum]

#ID	0 h-1 FPKM	0 h-2 FPKM	0 h-3 FPKM	48 h-1 FPKM	48 h-2 FPKM	48 h-3 FPKM	FDR	log2FC	regulated	nr_annotation
c44316.graph_c0	0.38917012	0.35414529	0.18185419	3.45491485	3.69459226	3.39317932	4.507E-07	2.499234	up	PREDICTED: uncharacterized protein LOC105169064 [Sesamum indicum]
c44319.graph_c0	53.2209163	51.8539499	50.493235	20.8073188	18.8494467	15.5946759	1.008E-67	-2.506973	down	PREDICTED: F-box-like/WD repeat-containing protein TBL1XR1 [Sesamum indicum]
c44321.graph_c0	8.45361575	4.47281412	5.27642645	0	0	0	2.321E-22	-Inf	down	--
c44329.graph_c0	0.67141641	1.23805819	1.40772088	0.0651108	0.0344885	0.0289676	1.956E-10	-5.697279	down	hypothetical protein MIMGU_mgv1a000906mg [Erythranthe guttata]
c44339.graph_c0	38.1048276	36.0641561	34.8730585	33.9868069	32.6116495	28.3667193	3.431E-18	-1.214191	down	PREDICTED: angio-associated migratory cell protein [Sesamum indicum]
c44340.graph_c1	1.77957386	1.00403692	1.33051512	0.80482558	0.63156675	0.82222214	3.567E-05	-1.878679	down	PREDICTED: mediator of RNA polymerase II transcription subunit 25-like [Sesamum indicum]
c44347.graph_c0	16.3022131	17.386762	16.2575705	10.8791514	9.09951737	9.31187231	1.004E-34	-1.783717	down	hypothetical protein MIMGU_mgv1a000491mg [Erythranthe guttata]
c44349.graph_c0	13.9799296	14.1407179	13.7562347	87.4526263	99.1334975	100.413976	6.132E-32	1.757156	up	PREDICTED: proteasome subunit alpha type-6-like [Sesamum indicum]
c44353.graph_c0	19.6075407	18.6032991	18.2902729	6.32470662	8.52760332	7.73096247	6.254E-55	-2.344746	down	PREDICTED: uncharacterized TPR repeat-containing protein At1g05150-like [Sesamum indicum]
c44355.graph_c0	0.31372567	0.45361306	0.5375332	3.72520525	5.51135628	5.91496296	3.325E-09	2.508197	up	PREDICTED: potassium transporter 8-like [Sesamum indicum]
c44355.graph_c1	0.09680832	0.13566737	0.14928304	7.72351996	7.70972335	8.66581645	2.304E-44	4.958549	up	PREDICTED: potassium transporter 6-like [Sesamum indicum]
c44358.graph_c0	16.7876969	15.2704858	16.1125059	23.0063602	13.3589574	9.9927787	0.0003111	-1.058916	down	PREDICTED: protein HEADING DATE 3A-like [Sesamum indicum]
c44359.graph_c0	2.43682481	3.09458982	2.29014032	14.5754993	17.8192391	21.7942047	9.078E-11	1.771451	up	PREDICTED: glutamate dehydrogenase A [Sesamum indicum]
c44360.graph_c0	0.45240108	0.37737841	0.43601497	5.27923081	5.51911668	5.56274348	8.934E-10	2.673559	up	PREDICTED: probable WRKY transcription factor 30 [Sesamum indicum]
c44361.graph_c0	8.25534365	7.78316767	7.27788561	5.26184861	7.44800169	7.86884245	2.813E-06	-1.203143	down	PREDICTED: pentatricopeptide repeat-containing protein At1g71490-like [Sesamum indicum]
c44362.graph_c0	0	0	0	32.7907267	50.3187233	50.4161249	9.937E-50	Inf	up	hypothetical protein MIMGU_mgv1a010045mg [Erythranthe guttata]
c44363.graph_c0	93.8099031	89.480965	90.4238108	30.2096433	34.2130466	36.5189732	4.006E-65	-2.459187	down	PREDICTED: pentatricopeptide repeat-containing protein At2g17210 isoform X1 [Sesamum indicum]
c44363.graph_c1	19.1002982	20.8752897	19.4639706	8.28520448	9.13043021	8.39920138	1.081E-43	-2.221737	down	PREDICTED: uncharacterized protein LOC105157994 [Sesamum indicum]
c44364.graph_c0	52.6839602	53.3589774	51.5531653	23.6645752	28.774957	31.4120016	9.119E-42	-1.931738	down	PREDICTED: probable pre-mRNA-splicing factor ATP-dependent RNA helicase [Sesamum indicum]
c44365.graph_c0	15.7639615	15.139469	15.1134416	10.2077722	12.6464195	13.3789686	4.744E-19	-1.366356	down	PREDICTED: U-box domain-containing protein 62 [Sesamum indicum]

#ID	0 h-1 FPKM	0 h-2 FPKM	0 h-3 FPKM	48 h-1 FPKM	48 h-2 FPKM	48 h-3 FPKM	FDR	log2FC	regulated	nr_annotation
c44366.graph_c0	53.6055241	58.5797295	50.5884348	40.9523838	53.723476	51.0287536	6.672E-17	-1.18095	down	PREDICTED: rac-like GTP-binding protein RAC1 [Sesamum indicum]
c44373.graph_c0	0.43515432	0.91473912	1.00654295	5.97329885	12.5285626	12.6275967	6.246E-06	2.688231	up	PREDICTED: inositol polyphosphate multikinase alpha [Nicotiana sylvestris]
c44376.graph_c0	8.47384316	6.79550175	6.99137674	2.3065123	2.77239877	2.28912702	1.633E-18	-2.61341	down	hypothetical protein F383_25407 [Gossypium arboreum]
c44382.graph_c0	3.93766077	4.3458708	3.37336666	15.905762	15.9633722	19.7808859	0.0014429	1.130381	up	-
c44385.graph_c1	3.22203769	2.89056686	3.47641221	1.45617233	1.63163636	1.29569338	4.472E-10	-2.148531	down	hypothetical protein MIMGU_mgv1a001279mg [Erythranthe guttata]
c44391.graph_c0	0.34419685	0.68908303	0.32167752	566.824573	583.013784	618.602456	0	9.335113	up	PREDICTED: raucaffricine-O-beta-D-glucosidase-like [Sesamum indicum]
c44394.graph_c0	0.63478391	0.37248732	0.50631049	12.9665239	14.0141799	15.8977543	1.99E-52	3.805119	up	PREDICTED: protein ENHANCED DISEASE RESISTANCE 2 [Sesamum indicum]
c44395.graph_c0	4.97552992	5.53050737	5.9230472	2.27783066	3.51535896	3.60320005	4.44E-12	-1.832891	down	unnamed protein product [Coffea canephora]
c44396.graph_c0	46.7776308	48.8594674	50.3395987	6.32102224	7.05883744	6.24934175	3.91E-146	-3.913941	down	PREDICTED: uncharacterized protein LOC105156238 [Sesamum indicum]
c44399.graph_c0	18.5649475	18.9835745	17.413985	108.142016	147.78708	145.989048	2.972E-20	1.848095	up	PREDICTED: leucine-rich repeat extensin-like protein 4 [Sesamum indicum]
c44400.graph_c0	16.26139	16.1547302	15.8775749	15.229983	16.6137948	15.5852862	1.017E-11	-1.044436	down	PREDICTED: RING finger and transmembrane domain-containing protein 2 [Sesamum indicum]
c44401.graph_c0	0	0	0	4.47994003	7.49361039	2.41271435	2.561E-05	Inf	up	--
c44403.graph_c0	6.2723033	6.94405611	5.27438915	5.72519727	5.5573721	5.49704695	1.2E-07	-1.154647	down	PREDICTED: WD repeat-containing protein 82-B [Sesamum indicum]
c44407.graph_c0	8.46238671	9.03558454	10.0380018	9.43528314	8.55550321	6.93692521	2.964E-07	-1.159052	down	PREDICTED: shikimate kinase, chloroplastic-like [Sesamum indicum]
c44409.graph_c0	31.0580677	30.7784702	30.3880838	32.0798429	30.5389008	29.7689208	5.17E-13	-1.013498	down	PREDICTED: nuclear poly(A) polymerase 1 isoform X1 [Sesamum indicum]
c44411.graph_c0	0.59465044	0.51021077	0.21832839	4.85221541	5.05742525	4.24783545	2.12E-05	2.411156	up	PREDICTED: uncharacterized protein LOC105170709 [Sesamum indicum]
c44414.graph_c2	10.117702	9.80374264	9.41319286	45.5241888	47.7928772	50.4265906	1.735E-15	1.274725	up	PREDICTED: actin-depolymerizing factor 2-like [Tarenaya hassleriana]
c44416.graph_c1	17.2405838	15.1573859	16.2517359	109.405647	69.3806966	63.8297387	0.0055542	1.311027	up	PREDICTED: ATP-dependent 6-phosphofructokinase 6-like [Sesamum indicum]
c44418.graph_c0	0	0	0	1.46877067	1.55598443	0.77347339	1.309E-08	Inf	up	hypothetical protein AN2366.2 [Aspergillus nidulans FGSC A4]
c44423.graph_c0	8.665834	7.59822953	6.57845331	48.9952286	43.4155957	47.3316789	8.786E-29	1.599689	up	PREDICTED: cytochrome P450 734A1 [Sesamum indicum]
c44427.graph_c1	5.54168326	6.25538975	4.24246611	2.71561706	2.30149359	2.12637896	1.156E-09	-2.177227	down	hypothetical protein MIMGU_mgv1a004986mg [Erythranthe guttata]

#ID	0 h-1 FPKM	0 h-2 FPKM	0 h-3 FPKM	48 h-1 FPKM	48 h-2 FPKM	48 h-3 FPKM	FDR	log2FC	regulated	nr_annotation
c44434.graph_c0	20.6673665	19.0927137	19.6435147	15.1247436	17.8291636	16.6889271	6.063E-19	-1.278761	down	PREDICTED: COP9 signalosome complex subunit 6a [Sesamum indicum]
c44436.graph_c0	23.0952057	24.0459195	22.7624878	17.1068178	16.0181515	14.4327112	3.526E-28	-1.570681	down	PREDICTED: LOW QUALITY PROTEIN: U-box domain-containing protein 4-like [Sesamum indicum]
c44439.graph_c0	7.75157382	7.08877596	8.11643495	35.6621952	29.8875819	32.006573	4.022E-09	1.071981	up	PREDICTED: methionine aminopeptidase 1D, chloroplastic/mitochondrial isoform X2 [Sesamum indicum]
c44446.graph_c0	1751.22491	1783.3599	1734.69118	314.834882	319.290486	324.265912	2.306E-68	-3.476549	down	PREDICTED: REF/SRPP-like protein At3g05500 [Sesamum indicum]
c44448.graph_c1	1.35834615	1.54512083	1.42815975	6.31386705	5.96566557	8.19930299	0.0031559	1.222162	up	-
c44449.graph_c0	1.22877053	1.05428654	0.95868998	5.33668251	8.09488148	6.1515281	6.103E-05	1.573558	up	PREDICTED: protein REVEILLE 8 [Sesamum indicum]
c44457.graph_c0	0.35205536	0.48456085	0.39585444	3.93257837	6.44240711	7.93629493	3.823E-07	2.865868	up	hypothetical protein MIMGU_mgv1a011349mg [Erythranthe guttata]
c44460.graph_c0	8.76199046	11.8956773	8.32663966	76.1292482	67.9249757	66.449074	4.684E-24	1.847268	up	PREDICTED: ammonium transporter 1 member 1-like [Sesamum indicum]
c44463.graph_c1	9.39043954	8.11804592	8.36468502	1.86784798	1.35388736	1.22463151	2.159E-29	-3.550057	down	PREDICTED: metalloendoproteinase 1-like [Sesamum indicum]
c44465.graph_c0	4.59300877	4.27684462	3.84329179	34.2446507	34.8185931	29.9453277	3.595E-20	1.946272	up	PREDICTED: tubulin-folding cofactor B isoform X2 [Sesamum indicum]
c44466.graph_c0	124.332409	125.271582	135.07417	1905.30318	2162.75583	2154.63349	4.031E-74	2.995134	up	PREDICTED: aquaporin TIP2-1-like [Solanum tuberosum]
c44467.graph_c0	0.91189746	0.58891006	0.79381649	16.9645883	18.2303065	18.4949781	5.339E-45	3.529467	up	PREDICTED: probable LRR receptor-like serine/threonine-protein kinase At1g51880 isoform X1 [Sesamum indicum]
c44471.graph_c0	7.38942699	6.86303479	6.460996	4.14071861	3.34570368	3.2160323	4.373E-14	-1.964267	down	PREDICTED: probable WRKY transcription factor 31 [Sesamum indicum]
c44480.graph_c0	0.55805642	0.71096484	0.45635201	10.1888835	17.5772006	19.795502	7.415E-11	3.760207	up	hypothetical protein M569_00618, partial [Genlisea aurea]
c44482.graph_c0	0.77400233	0.85633288	1.04697224	3.64036623	4.77096075	5.81048063	0.0006421	1.38251	up	PREDICTED: outer envelope protein 80, chloroplastic [Sesamum indicum]
c44483.graph_c0	48.5004093	49.8281828	47.936251	15.9553618	17.3779017	17.7587022	7.186E-67	-2.536392	down	PREDICTED: uncharacterized protein LOC105160446 [Sesamum indicum]
c44492.graph_c0	2.40922853	1.99752083	2.18905842	9.19202798	9.38157665	9.11660519	1.015E-05	1.052533	up	PREDICTED: methionine--tRNA ligase, mitochondrial [Sesamum indicum]
c44496.graph_c0	2.49586733	2.58921626	3.20728834	19.5236209	18.9999314	19.1203603	2.865E-13	1.777698	up	PREDICTED: uncharacterized protein LOC105165753 [Sesamum indicum]
c44497.graph_c0	14.3968365	14.5867619	14.3948448	5.4560821	6.61664466	6.18027421	7.934E-46	-2.269354	down	PREDICTED: uncharacterized protein LOC105166424 [Sesamum indicum]
c44501.graph_c0	3.76538453	3.50231216	2.6548445	13.8142745	17.5126767	17.1539713	1.295E-13	1.270313	up	PREDICTED: calcium-transporting ATPase 3, endoplasmic reticulum-type [Sesamum indicum]
c44503.graph_c0	1.78622287	2.56666167	2.33238916	7.32550749	9.64437324	11.6430033	0.0001301	1.072516	up	unnamed protein product [Coffea canephora]
c44505.graph_c0	4.36260543	3.87721983	2.56728892	22.1239226	23.6763666	24.598811	4.629E-15	1.689506	up	hypothetical protein CICLE_v10012334mg [Citrus clementina]

#ID	0 h-1 FPKM	0 h-2 FPKM	0 h-3 FPKM	48 h-1 FPKM	48 h-2 FPKM	48 h-3 FPKM	FDR	log2FC	regulated	nr_annotation
c44506.graph_c0	1.47243791	1.2452018	1.4027942	7.15673773	9.34084747	10.1139176	3.508E-10	1.668216	up	PREDICTED: uncharacterized protein LOC105166535 [Sesamum indicum]
c44511.graph_c0	0.36177906	0.18107066	0.17433766	1.4581633	2.5598668	2.96563414	8.479E-05	2.261222	up	PREDICTED: uncharacterized protein LOC105162704 [Sesamum indicum]
c44513.graph_c0	4.57034652	6.6336384	4.84528789	34.5261108	26.2612856	26.927816	9.508E-07	1.439139	up	PREDICTED: uncharacterized protein LOC105176019 [Sesamum indicum]
c44514.graph_c0	609.893772	607.120252	654.001086	13.3715576	13.3690876	10.8467087	3.99E-246	-6.654069	down	PREDICTED: alcohol dehydrogenase 1 [Sesamum indicum]
c44519.graph_c0	37.7609227	39.1440583	33.0639288	9.3384127	8.84989283	8.37563138	4.32E-98	-3.063935	down	PREDICTED: calmodulin-binding transcription activator 4 [Sesamum indicum]
c44521.graph_c0	6.52005997	7.2927536	6.84854182	36.8759425	33.8365553	32.254281	3.37E-16	1.301747	up	PREDICTED: AP3-complex subunit beta-A isoform X2 [Sesamum indicum]
c44533.graph_c0	4.79128194	3.6333937	5.3640417	25.1466603	25.470802	27.0765504	1.421E-15	1.473839	up	unnamed protein product [Coffea canephora]
c44536.graph_c1	5.3411715	3.82698265	3.46793272	30.1453673	31.3866438	28.758859	3.351E-16	1.822964	up	uncharacterized protein LOC100499956 [Glycine max]
c44543.graph_c0	10.074636	9.17229679	8.75416891	9.10462301	7.07005756	8.33719721	2.43E-07	-1.204621	down	PREDICTED: probable inactive purple acid phosphatase 16 [Nelumbo nucifera]
c44545.graph_c1	15.7193474	17.1230344	14.7578022	14.2734184	15.5181018	14.5659278	4.374E-15	-1.119332	down	hypothetical protein MIMGU_mgv1a002529mg [Erythranthe guttata]
c44547.graph_c0	0	0	0	7.00242733	6.61625265	6.06231995	8.983E-15	Inf	up	-
c44548.graph_c0	127.798123	118.148303	121.648628	9.83216139	10.0334749	10.1078435	1.21E-189	-4.633978	down	PREDICTED: uncharacterized protein LOC102619543 isoform X1 [Citrus sinensis]
c44550.graph_c0	0.04855391	0.12960675	0.0831916	1.46121367	1.5163873	1.76452882	1.516E-09	3.161423	up	hypothetical protein MIMGU_mgv1a020534mg, partial [Erythranthe guttata]
c44552.graph_c0	31.4538671	33.2299891	29.0893669	28.1273012	28.1965266	27.8680999	4.823E-17	-1.171817	down	PREDICTED: serrate RNA effector molecule-like [Sesamum indicum]
c44554.graph_c0	5.75338883	5.9495351	5.49917289	24.9250077	26.6370534	24.9844924	3.033E-08	1.136157	up	unnamed protein product [Coffea canephora]
c44555.graph_c0	7.06763217	7.67468468	6.57896949	3.65821567	4.24104308	4.68810284	9.977E-18	-1.780175	down	PREDICTED: protein tesmin/TSO1-like CXC 5 [Sesamum indicum]
c44559.graph_c0	0.15133577	0.23805149	0.08334534	0.77677071	1.49808976	1.63044265	0.0006558	2.024207	up	PREDICTED: probable receptor-like protein kinase At5g24010 [Sesamum indicum]
c44560.graph_c0	15.4539397	13.6193828	13.4414094	59.8830891	61.4815926	58.1832552	2.322E-12	1.06188	up	PREDICTED: serine/threonine-protein phosphatase PP2A-2 catalytic subunit [Sesamum indicum]
c44560.graph_c1	0.60215378	0.5241363	0.40371728	11.5048944	11.753666	12.1255702	3.274E-33	3.517089	up	PREDICTED: uncharacterized protein LOC102583109 [Solanum tuberosum]
c44563.graph_c0	71.2716066	72.9695942	61.399815	12.3703539	14.2456554	12.1288058	6.5E-119	-3.425341	down	PREDICTED: uncharacterized protein LOC105165633 [Sesamum indicum]
c44566.graph_c0	82.9759821	81.9871424	73.9550815	38.8979291	40.013215	39.0503988	9.787E-47	-2.034784	down	hypothetical protein MIMGU_mgv1a005930mg [Erythranthe guttata]

#ID	0 h-1 FPKM	0 h-2 FPKM	0 h-3 FPKM	48 h-1 FPKM	48 h-2 FPKM	48 h-3 FPKM	FDR	log2FC	regulated	nr_annotation
c44568.graph_c0	103.587954	104.441589	109.834879	60.8606317	75.2091247	74.5865718	3.681E-29	-1.615449	down	PREDICTED: probable cyclic nucleotide-gated ion channel 17 [Sesamum indicum]
c44568.graph_c1	15.5801375	10.1466252	10.1311558	2.24812899	2.56482161	2.92362011	1.358E-12	-3.230184	down	-
c44572.graph_c0	5.6724729	3.15452946	3.41326812	0	0	0	2.841E-24	-Inf	down	--
c44573.graph_c0	0.1523414	0.03812349	0	1.15786138	0.89208272	1.90441648	0.0001785	3.375762	up	PREDICTED: cell division control protein 6 homolog [Sesamum indicum]
c44575.graph_c0	7.21038567	7.62614951	6.55587103	0.90866816	0.96262373	1.39401287	4.818E-39	-3.732036	down	unknown protein [Arabidopsis thaliana]
c44576.graph_c0	1.81151411	2.00565074	2.0104309	8.44556932	9.85783483	11.5872175	9.034E-08	1.336472	up	PREDICTED: phosphoinositide phosphatase SAC6 [Sesamum indicum]
c44583.graph_c0	2.41395468	1.89519364	2.00719416	0	0	0.03880012	1.883E-24	-8.386615	down	actin [Dendrobium catenatum]
c44585.graph_c0	0.95387859	0.65102302	1.50435625	8.34836417	7.78617529	7.00181758	2.159E-07	1.873383	up	PREDICTED: methyltransferase-like protein 6 isoform X3 [Sesamum indicum]
c44591.graph_c0	56.0964009	60.1213003	52.3803175	28.1943958	25.2315641	23.7979257	4.871E-51	-2.140218	down	PREDICTED: uncharacterized protein LOC105165989 [Sesamum indicum]
c44592.graph_c0	38.7344985	39.1203607	40.2913114	29.2961729	30.4556355	31.2648288	4.321E-20	-1.395127	down	PREDICTED: E3 ubiquitin-protein ligase RNF185-like [Sesamum indicum]
c44594.graph_c0	154.948309	150.189297	159.134055	118.495379	97.173109	79.4546345	1.312E-29	-1.665888	down	PREDICTED: uncharacterized protein LOC105169586 [Sesamum indicum]
c44595.graph_c0	0.15148391	0.1010904	0.12977522	7.67562746	7.34289751	8.52681087	2.278E-19	4.926971	up	PREDICTED: putative 4-hydroxy-tetrahydrodipicolinate reductase 3, chloroplastic isoform X2 [Sesamum indicum]
c44602.graph_c0	30.5111821	30.7493927	28.2782103	15.3187576	17.7857909	15.4095722	2.912E-41	-1.902494	down	PREDICTED: uncharacterized protein At1g51745 [Sesamum indicum]
c44606.graph_c0	0	0	0.06420405	5.3853812	6.97297134	5.28335466	8.587E-20	7.049254	up	--
c44609.graph_c0	21.8897191	20.2400288	20.8213751	12.2244818	15.4170907	13.9110669	4.047E-21	-1.620088	down	PREDICTED: extra-large guanine nucleotide-binding protein 3-like [Sesamum indicum]
c44609.graph_c1	38.9641467	34.7739047	39.2118148	29.4090278	27.3067032	27.1685164	4.873E-16	-1.445632	down	Extra-large GTP-binding protein 3 isoform 2 [Theobroma cacao]
c44609.graph_c2	47.058659	50.3437491	47.0909062	32.0456707	31.4834519	33.8899415	1.645E-27	-1.586122	down	PREDICTED: extra-large guanine nucleotide-binding protein 3 [Sesamum indicum]
c44615.graph_c0	10.7782511	12.0866952	10.2569701	6.39795614	5.47650986	5.85985355	6.76E-32	-1.91516	down	PREDICTED: pentatricopeptide repeat-containing protein At3g22470, mitochondrial-like [Sesamum indicum]
c44618.graph_c0	6.91108868	8.01032632	8.22663144	4.18878005	5.57350571	5.09874297	3.458E-12	-1.662768	down	PREDICTED: transcription initiation factor TFIID subunit 8-like [Sesamum indicum]
c44618.graph_c1	0.89725626	0.73485387	0.8385526	3.40663924	5.65220746	6.79792771	0.0002091	1.654769	up	hypothetical protein MIMGU_mgv1a005181mg [Erythranthe guttata]
c44622.graph_c0	446.771074	481.905289	406.651674	32.3842536	22.1126911	15.1929085	5.46E-207	-5.265372	down	PREDICTED: telomere repeat-binding protein 2-like [Sesamum indicum]

#ID	0 h-1 FPKM	0 h-2 FPKM	0 h-3 FPKM	48 h-1 FPKM	48 h-2 FPKM	48 h-3 FPKM	FDR	log2FC	regulated	nr_annotation
c44624.graph_c0	0	0	0	4.17734956	6.13845159	2.63785715	1.037E-06	Inf	up	hypothetical protein SELMODRAFT_29765, partial [Selaginella moellendorffii]
c44627.graph_c0	7.23527912	6.6917173	7.34181007	3.7426651	4.47907995	4.38588419	1.533E-27	-1.775549	down	PREDICTED: uncharacterized protein LOC105176363 [Sesamum indicum]
c44631.graph_c0	4.01527079	5.80103925	5.37255645	2.78388085	4.03997836	3.83438479	5.285E-07	-1.536444	down	PREDICTED: WD repeat-containing protein 48-like [Sesamum indicum]
c44635.graph_c0	17.0762981	18.2978102	15.9671777	1.59876012	2.16792638	2.97790721	5.34E-110	-3.952473	down	PREDICTED: uncharacterized protein LOC105175356 [Sesamum indicum]
c44641.graph_c0	20.7809077	21.1100979	22.0616297	9.90778357	9.1840834	10.2163265	1.285E-41	-2.143093	down	PREDICTED: chloroplast envelope membrane protein [Sesamum indicum]
c44642.graph_c0	23.2092946	10.7227086	13.4375754	0	0	0	8.828E-14	-Inf	down	-
c44646.graph_c0	1.84479263	1.49821729	2.72846275	8.40151593	9.61377722	10.813967	5.987E-06	1.221152	up	PREDICTED: GDP-mannose transporter GONST4 [Sesamum indicum]
c44646.graph_c1	3.70834971	3.87345711	3.35648237	1.15859627	1.66799462	1.82392123	1.533E-11	-2.257283	down	PREDICTED: non-specific lipid-transfer protein-like protein At2g13820 [Sesamum indicum]
c44646.graph_c3	82.7252259	84.897333	78.015323	45.3242421	50.6184012	47.6754057	3.468E-34	-1.792647	down	PREDICTED: heat shock 70 kDa protein, mitochondrial [Sesamum indicum]
c44650.graph_c1	1.93105057	1.76729987	2.05608048	0.45745965	0.43077602	0.31659043	3.111E-10	-3.270814	down	hypothetical protein MIMGU_mgv1a004154mg [Erythranthe guttata]
c44656.graph_c0	73.4399385	72.7223295	71.4604	30.6687549	29.7129187	23.5311465	9.692E-63	-2.389643	down	PREDICTED: MLO-like protein 10 isoform X1 [Sesamum indicum]
c44658.graph_c0	62.4680854	67.0787825	61.4205088	44.9453372	39.4885177	34.0411345	1.03E-33	-1.701949	down	PREDICTED: serine/threonine-protein kinase CDL1 [Sesamum indicum]
c44658.graph_c1	0.04040068	0.08088227	0.05191647	12.840811	10.8037661	11.3263227	8.785E-71	6.643745	up	PREDICTED: protein LONGIFOLIA 1 [Sesamum indicum]
c44661.graph_c0	4.29673155	2.88213624	2.56150677	0.12242565	0.43231711	0.29048962	8.187E-16	-4.557356	down	hypothetical protein MIMGU_mgv1a0244072mg, partial [Erythranthe guttata]
c44662.graph_c0	0.32044688	0.48115163	0.51473365	1.77129938	3.9093269	3.80888788	0.0018088	1.816055	up	PREDICTED: transcription factor JUNGBRUNNEN 1 [Sesamum indicum]
c44663.graph_c0	46.4128499	47.6857362	43.0184339	26.6908928	26.5524903	23.3358492	4.455E-38	-1.855793	down	hypothetical protein MIMGU_mgv1a008429mg [Erythranthe guttata]
c44666.graph_c0	14.6297915	14.0954958	12.7302567	76.3042475	84.5643548	82.2078381	1.02E-27	1.533959	up	PREDICTED: coatomer subunit beta'-2-like isoform X1 [Sesamum indicum]
c44667.graph_c0	0.74276073	0.94627844	0.73755036	0.55986515	0.88966386	0.52584046	0.0062711	-1.318615	down	PREDICTED: endoglucanase 24-like [Sesamum indicum]
c44669.graph_c0	33.7555244	34.0504796	30.1731907	24.2570164	24.1304549	22.0772781	1.672E-26	-1.49097	down	unnamed protein product [Coffea canephora]
c44676.graph_c1	2.62481372	2.35183626	2.57102001	14.4919875	16.7139857	15.7988652	1.475E-19	1.618804	up	PREDICTED: zinc-metallopeptidase, peroxisomal isoform X1 [Sesamum indicum]

#ID	0 h-1 FPKM	0 h-2 FPKM	0 h-3 FPKM	48 h-1 FPKM	48 h-2 FPKM	48 h-3 FPKM	FDR	log2FC	regulated	nr_annotation
c44677.graph_c0	50.9852301	53.3101779	51.7333033	12.2077885	11.454652	9.20719136	8.723E-60	-3.261459	down	PREDICTED: protein ABSCISIC ACID-INSENSITIVE 5 isoform X2 [Nicotiana tomentosiformis]
c44677.graph_c1	0.06156367	0	0	1.13432942	1.56218982	1.11025145	5.936E-09	4.950454	up	-
c44677.graph_c2	116.144763	116.617148	120.451878	58.6616342	60.799573	69.1825895	5.041E-41	-1.92437	down	PREDICTED: putative peptidyl-tRNA hydrolase PTRHD1 [Sesamum indicum]
c44677.graph_c3	42.1865402	43.3901115	40.5513753	33.9484173	33.1335168	33.6902458	3.057E-21	-1.340327	down	PREDICTED: DEAD-box ATP-dependent RNA helicase 37-like [Sesamum indicum]
c44677.graph_c4	14.6817979	11.8388469	14.3746222	9.92967671	9.78009519	11.7008909	2.793E-10	-1.399803	down	PREDICTED: purple acid phosphatase 17-like [Sesamum indicum]
c44681.graph_c0	66.765262	68.2191021	64.4953827	28.3667244	23.3897766	22.9688921	3.978E-61	-2.429525	down	PREDICTED: tubby-like F-box protein 8 [Sesamum indicum]
c44681.graph_c1	39.3070642	51.1637803	41.8914863	17.5499551	14.009502	15.2859323	2.321E-27	-2.511764	down	unknown [Lotus japonicus]
c44683.graph_c0	640.030326	738.274837	640.229928	663.573751	498.872648	389.501078	1.426E-13	-1.388282	down	PREDICTED: protein FAR1-RELATED SEQUENCE 5 [Sesamum indicum]
c44684.graph_c0	0	0	0	5.19362618	4.15246579	3.74932199	2.986E-12	Inf	up	-
c44688.graph_c0	1.99229932	2.58719261	2.07582446	13.5921482	18.0778691	18.362015	1.642E-19	1.888128	up	hypothetical protein MIMGU_mgv1a002066mg [Erythranthe guttata]
c44689.graph_c0	3.59108013	3.85770113	4.61468014	19.6886411	30.431767	36.4018405	6.899E-06	1.813629	up	PREDICTED: uncharacterized protein LOC105180344 [Sesamum indicum]
c44691.graph_c0	1.36869887	1.41900047	1.75883224	0.04503338	0	0.08014086	4.472E-18	-6.19683	down	PREDICTED: uncharacterized protein LOC103492548 [Cucumis melo]
c44691.graph_c1	24.9304715	24.5824096	20.4000147	94.5824708	94.415869	100.318647	5.736E-13	1.033234	up	hypothetical protein MIMGU_mgv1a007945mg [Erythranthe guttata]
c44694.graph_c0	3.84319686	2.42221367	1.64622004	33.4390342	29.4510417	28.1203067	2.323E-11	2.516491	up	PREDICTED: crocetin glucosyltransferase, chloroplastic-like [Sesamum indicum]
c44701.graph_c0	0	0	0	5.28681251	4.45031518	4.65331805	8.538E-26	Inf	up	hypothetical protein MIMGU_mgv1a021943mg [Erythranthe guttata]
c44702.graph_c0	8.86931675	9.59035295	9.99560107	81.6662358	66.1480004	54.0427579	2.758E-07	1.814009	up	PREDICTED: hydroxymethylglutaryl-CoA synthase isoform X1 [Sesamum indicum]
c44705.graph_c0	2.01307151	1.88366856	1.68709343	23.0208926	26.39525	25.4702885	1.992E-30	2.727271	up	PREDICTED: pyridoxal kinase isoform X1 [Sesamum indicum]
c44710.graph_c0	14.711464	14.7817658	15.908579	1.32974402	3.35921376	3.27654894	6.294E-46	-3.543836	down	PREDICTED: uncharacterized protein LOC100244117 [Vitis vinifera]
c44710.graph_c1	13.4005448	13.3491617	12.7279965	10.3773398	7.32902211	4.88218331	6.112E-14	-1.811976	down	PREDICTED: dehydration-responsive element-binding protein 1D-like [Sesamum indicum]
c44711.graph_c0	32.1643932	32.7140464	31.3315253	10.1330642	13.2292515	11.2527684	1.053E-61	-2.495731	down	PREDICTED: gamma-glutamyltranspeptidase 3 [Sesamum indicum]
c44713.graph_c0	1.23449162	1.06721944	1.00950857	0.98220863	0.98576615	0.80496622	0.0038861	-1.270276	down	PREDICTED: protein ROOT PRIMORDIUM DEFECTIVE 1 [Sesamum indicum]

#ID	0 h-1 FPKM	0 h-2 FPKM	0 h-3 FPKM	48 h-1 FPKM	48 h-2 FPKM	48 h-3 FPKM	FDR	log2FC	regulated	nr_annotation
c44714.graph_c0	4.70995938	5.04360655	4.081908	16.8519381	21.7010465	21.1004012	0.0015771	1.088084	up	hypothetical protein MIMGU_mgv1a014633mg [Erythranthe guttata]
c44715.graph_c0	6.48985561	6.67075299	7.16432442	0.3611403	0.9352062	0.85690791	3.458E-35	-4.271806	down	PREDICTED: uncharacterized membrane protein At1g06890 isoform X2 [Sesamum indicum]
c44718.graph_c0	50.0988726	48.8268858	54.9693331	25.788369	30.6667594	28.0180837	1.36E-39	-1.886563	down	PREDICTED: ninja-family protein AFP3-like [Sesamum indicum]
c44720.graph_c0	23.5586769	20.9489141	23.7145021	19.8227946	18.2024224	14.6126722	4.517E-18	-1.388696	down	hypothetical protein MIMGU_mgv1a011515mg [Erythranthe guttata]
c44722.graph_c0	7.45885883	8.15711004	7.33964044	3.12467846	4.71455282	5.10327153	9.16E-28	-1.851491	down	PREDICTED: protein NRT1/ PTR FAMILY 5.5-like isoform X2 [Sesamum indicum]
c44724.graph_c0	73.364664	73.0149469	72.4625209	49.3430641	44.8144909	43.2120626	7.161E-33	-1.686936	down	PREDICTED: cytochrome P450 78A3-like [Sesamum indicum]
c44725.graph_c0	1.78859779	1.15096423	1.64172784	14.4180133	18.7030247	19.3221376	2.624E-14	2.493906	up	hypothetical protein MIMGU_mgv1a006677mg [Erythranthe guttata]
c44728.graph_c0	67.008013	63.5359012	65.2806575	481.166817	505.597054	542.866308	1.071E-37	1.94672	up	PREDICTED: ruBisCO large subunit-binding protein subunit beta, chloroplastic [Sesamum indicum]
c44735.graph_c0	2.99180356	1.68754569	2.13588113	0	0	0	2.046E-33	-Inf	down	hypothetical protein SELMODRAFT_444263 [Selaginella moellendorffii]
c44740.graph_c0	5.70418914	5.00137315	6.74155999	35.9416176	37.7100631	33.9943572	8.401E-20	1.605251	up	PREDICTED: transcription factor TGA1-like [Sesamum indicum]
c44741.graph_c0	6.68674714	6.65646256	5.83926178	39.4801681	34.0725053	39.6717873	9.614E-20	1.546971	up	PREDICTED: aspartate-semialdehyde dehydrogenase [Sesamum indicum]
c44742.graph_c0	10.7137706	11.3754077	11.579125	7.81630478	7.31336995	9.36627177	1.727E-15	-1.477632	down	PREDICTED: probable zinc metallopeptidase EGY3, chloroplastic [Sesamum indicum]
c44743.graph_c0	28.4500503	27.1378741	26.1604722	18.1444146	23.58352	25.6097759	2.78E-20	-1.30187	down	PREDICTED: COBRA-like protein 7 [Sesamum indicum]
c44744.graph_c0	3.37918669	3.28308307	4.34238868	1.46500103	1.64899038	1.91458775	8.305E-08	-2.153638	down	PREDICTED: serine/threonine-protein kinase Nek2 [Sesamum indicum]
c44747.graph_c0	2.83974747	2.43650661	2.22243776	230.222132	303.219309	285.834735	2.25E-137	5.751528	up	PREDICTED: endoplasmin homolog [Sesamum indicum]
c44750.graph_c0	10.0240111	10.452134	10.9295465	8.01046587	8.52317455	9.61767811	2.956E-10	-1.28446	down	PREDICTED: transcription factor HBP-1b(c38)-like isoform X1 [Sesamum indicum]
c44753.graph_c0	0	0	0	14.1902212	14.7459328	11.6625282	2.678E-35	Inf	up	apocytochrome b [Chara vulgaris]
c44753.graph_c1	0	0	0	1.2233363	0.46459534	0.55452767	0.0003025	Inf	up	Citrate synthase, mitochondrial [Rozella allomycis CSF55]
c44756.graph_c0	0	0	0	8.6911927	13.0217031	12.7048238	3.064E-17	Inf	up	-
c44762.graph_c1	2.42039588	2.4810604	3.05049099	31.9787145	33.3892246	36.2286521	4.737E-72	2.654019	up	PREDICTED: LOW QUALITY PROTEIN: cullin-associated NEDD8-dissociated protein 1 [Sesamum indicum]
c44762.graph_c4	165.397321	146.537528	173.860517	255.301118	132.797807	93.375867	0.0069917	-1.014704	down	PREDICTED: uncharacterized protein LOC105162036 [Sesamum indicum]
c44762.graph_c5	0	0	0	66.3309543	64.0468181	73.092621	3.01E-100	Inf	up	unnamed protein product [Coffea canephora]

#ID	0 h-1 FPKM	0 h-2 FPKM	0 h-3 FPKM	48 h-1 FPKM	48 h-2 FPKM	48 h-3 FPKM	FDR	log2FC	regulated	nr_annotation
c44762.graph_c6	5.31181574	4.9787716	5.58482198	24.9128275	21.7263702	22.879804	1.313E-07	1.114672	up	PREDICTED: UPF0496 protein At1g20180-like isoform X2 [Nicotiana sylvestris]
c44764.graph_c0	2.26997505	1.97370454	2.4485832	8.68555274	9.63790652	9.77928624	6.356E-08	1.04925	up	PREDICTED: LOW QUALITY PROTEIN: protein DA1-related 1 [Sesamum indicum]
c44767.graph_c0	0.9973398	0.51996796	0.77431277	30.1868839	30.1542697	28.1716032	2.5E-104	4.25534	up	PREDICTED: putative E3 ubiquitin-protein ligase XBAT31 [Sesamum indicum]
c44770.graph_c0	2.96117197	3.02892701	2.76554474	34.5847947	40.3022361	40.3502188	5.747E-69	2.698759	up	PREDICTED: SNF2 domain-containing protein CLASSY 3-like [Sesamum indicum]
c44773.graph_c0	12.3859457	12.6237729	13.0225341	8.52693559	8.98929687	9.06404638	6.308E-21	-1.535835	down	PREDICTED: zinc finger CCCH domain-containing protein 5 [Sesamum indicum]
c44775.graph_c1	15.1884356	15.5365711	14.768899	12.5967243	14.6070387	16.3280429	3.146E-10	-1.084239	down	PREDICTED: uncharacterized protein LOC105172565 isoform X4 [Sesamum indicum]
c44777.graph_c0	2.03512254	1.46984992	1.72140605	7.54701601	8.44770391	10.8542729	1.752E-07	1.340234	up	PREDICTED: gibberellin 2-beta-dioxygenase 8-like [Sesamum indicum]
c44781.graph_c0	1.06605236	1.3042576	1.48407945	12.6856775	13.6990456	15.292931	5.127E-22	2.412144	up	PREDICTED: protein S-acyltransferase 21 [Sesamum indicum]
c44781.graph_c1	1.50655171	0.94253768	1.20998662	12.664895	17.4603761	18.6789978	1.434E-07	2.71459	up	-
c44785.graph_c0	59.8466496	56.3426245	61.1143862	10.1932453	8.88011331	7.9652874	2.17E-130	-3.727396	down	PREDICTED: phytosulfokine receptor 2-like [Sesamum indicum]
c44790.graph_c1	0	0	0	6.79210101	6.86403998	7.75326496	7.688E-25	Inf	up	hypothetical protein SORBIDRAFT_07g024570 [Sorghum bicolor]
c44796.graph_c0	23.0028605	22.5559782	20.4730299	8.62265767	8.61922488	9.28383114	1.17E-48	-2.332582	down	PREDICTED: AMP deaminase-like [Sesamum indicum]
c44797.graph_c0	0	0	0.05621366	4.03004607	3.11662196	4.66168399	1.369E-16	6.666283	up	hypothetical protein MIMGU_mgv1a000411mg [Erythranthe guttata]
c44797.graph_c1	84.3931498	82.4960087	75.5298408	56.3188572	52.8620364	53.6661207	3.232E-28	-1.589171	down	PREDICTED: uncharacterized protein LOC105172555 [Sesamum indicum]
c44799.graph_c0	3.94892059	4.2755585	3.59876624	1.91180782	2.55623993	2.9728225	7.107E-12	-1.690562	down	PREDICTED: uncharacterized protein LOC105170906 isoform X2 [Sesamum indicum]
c44802.graph_c0	77.6366872	74.9863793	74.1287236	2.31760872	2.86442964	2.20540224	3.46E-272	-5.959172	down	PREDICTED: adagio protein 3 isoform X1 [Sesamum indicum]
c44803.graph_c0	15.1091255	16.79711	15.3847965	7.59705001	10.2406735	9.2526295	8.845E-36	-1.825816	down	PREDICTED: ribosome biogenesis protein BMS1 homolog [Sesamum indicum]
c44808.graph_c0	48.4658882	59.1769292	38.5318345	27.0843576	31.7073942	35.6253457	9.448E-09	-1.648723	down	hypothetical protein MIMGU_mgv1a003414mg [Erythranthe guttata]
c44811.graph_c0	2.15946794	2.41593977	2.6117664	16.4714155	19.6810156	20.851846	1.866E-20	1.964752	up	PREDICTED: glucose-1-phosphate adenylyltransferase large subunit 3, chloroplastic/amyloplastic [Sesamum indicum]
c44812.graph_c0	5.76585752	5.14920034	4.06788128	41.0021898	42.5173553	43.6813477	5.444E-43	2.070637	up	PREDICTED: pyrophosphate-energized membrane proton pump 3 [Sesamum indicum]
c44815.graph_c0	16.7146686	17.0005437	16.929837	5.14664205	4.5338172	3.66111201	6.839E-82	-2.937789	down	PREDICTED: LOW QUALITY PROTEIN: leucine-rich repeat receptor-like protein kinase PXL2 [Sesamum indicum]

#ID	0 h-1 FPKM	0 h-2 FPKM	0 h-3 FPKM	48 h-1 FPKM	48 h-2 FPKM	48 h-3 FPKM	FDR	log2FC	regulated	nr_annotation
c44817.graph_c0	9.44345537	8.9553899	8.8352874	6.0288968	6.91238833	7.57138086	3.316E-10	-1.42905	down	PREDICTED: nuclear transcription factor Y subunit A-1-like isoform X2 [Sesamum indicum]
c44820.graph_c0	71.7394847	67.3604614	60.3653355	35.0273308	39.6587071	40.6336745	5.068E-37	-1.808834	down	PREDICTED: protein TIME FOR COFFEE [Sesamum indicum]
c44821.graph_c0	0.01334911	0.01336248	0.01715413	1.56185492	1.47219951	2.27609179	1.874E-18	5.896717	up	PREDICTED: abnormal spindle-like microcephaly-associated protein homolog isoform X2 [Sesamum indicum]
c44826.graph_c0	2.38916166	1.73931203	2.58173162	0.50023899	0.4769483	0.66766447	1.199E-09	-3.049757	down	PREDICTED: uncharacterized protein LOC105177426 isoform X1 [Sesamum indicum]
c44832.graph_c0	1.17168327	1.33843628	1.09824567	0.2285854	0.21525201	0.18079459	5.257E-18	-3.54395	down	Ty3/gypsy retrotransposon protein [Beta vulgaris subsp. vulgaris]
c44838.graph_c0	34.1279597	34.5649438	31.9376451	27.9102004	32.7654729	35.2644322	8.762E-15	-1.089068	down	PREDICTED: uncharacterized protein LOC105170843 [Sesamum indicum]
c44839.graph_c0	6.42331474	6.9332811	5.37021135	55.7892001	34.856899	29.1501484	0.0039911	1.673392	up	PREDICTED: crocetin glucosyltransferase, chloroplastic-like, partial [Sesamum indicum]
c44846.graph_c0	1.96276064	2.10848649	2.24539554	7.80623153	10.3255142	10.3404216	8.305E-07	1.148538	up	PREDICTED: uncharacterized protein LOC105173505 [Sesamum indicum]
c44848.graph_c0	2.93735929	3.09781672	3.16798715	120.203417	146.307706	169.410144	3.538E-57	4.543034	up	PREDICTED: L-type lectin-domain containing receptor kinase S.4-like [Sesamum indicum]
c44849.graph_c0	111.012853	111.252483	134.409905	16.4936222	19.7588861	24.1179642	3.294E-46	-3.58709	down	hypothetical protein MIMGU_mgv1a011926mg [Erythranthe guttata]
c44852.graph_c0	6.84805344	3.05580358	4.02892472	0	0	0	4.712E-13	-Inf	down	Ribosomal protein L7 eukaryotic [Macrophomina phaseolina MS6]
c44853.graph_c0	3.41032651	4.42283482	3.22478828	2.92445694	2.86784277	3.1472121	1.155E-07	-1.322971	down	hypothetical protein MIMGU_mgv1a001275mg [Erythranthe guttata]
c44854.graph_c0	0.12921769	0.19402063	0.16604985	4.19629276	6.55784665	7.17637995	9.213E-16	4.168754	up	PREDICTED: GDSL esterase/lipase At4g18970-like [Sesamum indicum]
c44856.graph_c0	3.37680543	3.86307061	2.58293373	16.1101909	22.9911291	23.4299033	2.768E-10	1.649602	up	PREDICTED: UDP-galactose transporter 2 [Sesamum indicum]
c44858.graph_c0	60.6791425	64.8343482	61.9891189	25.5060709	25.7668867	24.8288804	2.056E-58	-2.318205	down	PREDICTED: F-box protein SKIP22-like [Sesamum indicum]
c44859.graph_c0	0.43534477	0.34862456	0.11188705	3.89838096	5.14958091	4.86767808	2.141E-16	2.945866	up	PREDICTED: DNA topoisomerase 2-binding protein 1-A isoform X1 [Sesamum indicum]
c44861.graph_c0	80.2069051	78.9718656	71.2455868	112.863139	52.8275215	33.247119	0.0012152	-1.208453	down	PREDICTED: scopoletin glucosyltransferase-like [Sesamum indicum]
c44864.graph_c0	38.759963	38.7213326	37.5797543	26.3713298	27.1066601	23.4333412	1.416E-27	-1.597657	down	PREDICTED: UPF0183 protein At3g51130 isoform X1 [Sesamum indicum]
c44866.graph_c0	8.02596963	7.93892942	7.75052348	3.10637466	3.38352664	3.46477437	5.495E-16	-2.271267	down	PREDICTED: transcription repressor OFP13-like [Sesamum indicum]
c44868.graph_c2	0.14868058	0.22324419	0.12737362	4.81692763	4.42578192	4.8751544	2.149E-17	3.808076	up	PREDICTED: uncharacterized protein LOC105178110 [Sesamum indicum]

#ID	0 h-1 FPKM	0 h-2 FPKM	0 h-3 FPKM	48 h-1 FPKM	48 h-2 FPKM	48 h-3 FPKM	FDR	log2FC	regulated	nr_annotation
c44869.graph_c0	3.84943796	3.42350226	3.08216227	20.2278538	23.1629266	22.7197718	6.226E-26	1.656647	up	PREDICTED: kinesin-like protein KIF3A [Sesamum indicum]
c44871.graph_c0	0.91417554	0.84972731	0.75519757	19.5209497	24.3604323	25.5994531	1.352E-68	3.765517	up	PREDICTED: synaptotagmin-2-like [Sesamum indicum]
c44873.graph_c0	0	0	0	1.11909583	1.0809393	1.23006232	1.447E-11	Inf	up	PREDICTED: monogalactosyldiacylglycerol synthase 2, chloroplastic [Sesamum indicum]
c44877.graph_c0	13.1432057	11.7605445	14.2207637	10.851075	12.3640681	11.9170237	9.031E-12	-1.176221	down	PREDICTED: vinorine synthase-like [Sesamum indicum]
c44878.graph_c1	6.7836841	6.72641583	8.01827366	5.54207352	7.27648451	4.6690003	3.498E-09	-1.321539	down	Taxane 13-alpha-hydroxylase [Medicago truncatula]
c44880.graph_c0	0	0	0	1.72579156	2.01109358	1.1261059	5.253E-10	Inf	up	hypothetical protein GUITHDRAFT_152339 [Guillardia theta CCMP2712]
c44881.graph_c0	5.48615953	4.24949346	3.44103834	0	0.19122573	0.32122885	1.524E-25	-5.725727	down	PREDICTED: xylosyltransferase 2-like [Sesamum indicum]
c44881.graph_c1	32.9856868	30.6118085	26.6338841	21.0286382	19.6992081	16.6290539	1.358E-29	-1.666479	down	PREDICTED: protein SPA1-RELATED 3-like isoform X1 [Solanum tuberosum]
c44882.graph_c0	0.51901797	0.17317923	0.62249473	21.0387409	25.8000426	30.6896926	1.271E-41	4.853829	up	PREDICTED: uncharacterized protein LOC105178220 [Sesamum indicum]
c44883.graph_c0	1.25773768	1.86112617	0.84325707	7.27972144	7.89877757	9.45842525	2.99E-08	1.621338	up	PREDICTED: probable protein phosphatase 2C 34 [Sesamum indicum]
c44892.graph_c0	0.04949118	0	0.25439251	1.77818565	2.92226182	3.57013495	7.92E-09	3.71526	up	PREDICTED: uncharacterized protein LOC105176998 [Sesamum indicum]
c44894.graph_c0	6.37475982	4.44746343	4.22002784	0	0	0	2.089E-28	-Inf	down	unknown [Lotus japonicus]
c44896.graph_c0	3.18766978	2.49042868	1.59854902	0.78789226	1.21407463	0.76479468	2.062E-06	-2.411625	down	-
c44899.graph_c0	2.51658082	2.04327064	1.97627588	11.4891503	14.5183057	17.0994186	3.829E-11	1.70037	up	PREDICTED: uncharacterized protein LOC105166230 isoform X2 [Sesamum indicum]
c44900.graph_c1	31.8178694	33.2519196	30.4886242	23.8050437	25.6335771	24.7494412	9.043E-23	-1.383136	down	PREDICTED: cell division cycle and apoptosis regulator protein 1 isoform X2 [Sesamum indicum]
c44901.graph_c0	1.75540613	3.5593833	2.42928687	32.6341582	31.8483505	34.793558	8.544E-37	2.662496	up	unnamed protein product [Coffea canephora]
c44902.graph_c0	7.4690821	7.51510023	7.37171064	7.90960953	7.0641398	7.10104338	7.525E-06	-1.033313	down	PREDICTED: uncharacterized aarF domain-containing protein kinase At1g71810, chloroplastic [Sesamum indicum]
c44904.graph_c0	0	0.08000995	0	2.94546578	3.66642751	2.78466135	4.073E-14	5.867806	up	PREDICTED: uncharacterized protein LOC105161004 [Sesamum indicum]
c44906.graph_c0	16.1707019	17.6508254	15.5178752	9.58521658	10.8884259	9.79620991	1.454E-21	-1.723156	down	PREDICTED: pentatricopeptide repeat-containing protein At1g66345, mitochondrial [Sesamum indicum]
c44908.graph_c0	38.1384558	37.1558797	36.3311852	29.8511963	26.7221702	22.7893178	1.516E-26	-1.505675	down	hypothetical protein MIMGU_mgv1a001607mg [Erythranthe guttata]
c44910.graph_c1	9.90997789	9.02822472	8.01285372	4.20582515	7.93307339	7.5759119	3.939E-05	-1.477169	down	-
c44912.graph_c0	1.84846455	2.20614541	1.73583278	11.1345169	10.8242623	11.1312847	8.7E-05	1.498927	up	PREDICTED: uncharacterized protein LOC105178220 [Sesamum indicum]

#ID	0 h-1 FPKM	0 h-2 FPKM	0 h-3 FPKM	48 h-1 FPKM	48 h-2 FPKM	48 h-3 FPKM	FDR	log2FC	regulated	nr_annotation
c44917.graph_c0	10.4436366	10.5841608	9.05830657	4.62363918	4.5177435	5.56533362	3.776E-36	-2.04971	down	PREDICTED: serine/threonine-protein kinase CTR1-like [Sesamum indicum]
c44919.graph_c0	84.2917399	83.6764122	80.546239	109.085576	58.0846857	42.2285945	2.57E-05	-1.248171	down	PREDICTED: tubby-like F-box protein 6 [Sesamum indicum]
c44920.graph_c0	5.49603322	5.87362127	5.2201976	50.8776469	62.7868002	69.7341732	1.85E-31	2.445337	up	PREDICTED: molybdate-anion transporter-like [Sesamum indicum]
c44922.graph_c0	24.7942644	24.8613734	24.5339836	16.6937958	12.3671696	11.0453112	4.05E-31	-1.897648	down	PREDICTED: transcription factor IIIA-like isoform X2 [Sesamum indicum]
c44927.graph_c0	19.9533199	19.9733001	18.8580677	267.74059	379.056493	460.177031	1.419E-14	3.210659	up	PREDICTED: S-adenosylmethionine synthase 1 [Sesamum indicum]
c44928.graph_c0	3.06764635	3.20821298	2.05927829	12.3589845	14.7908937	13.8494013	5.829E-06	1.282156	up	hypothetical protein MIMGU_mgv1a008719mg [Erythranthe guttata]
c44930.graph_c0	0.3678848	0.08183404	0.21010957	1.7322551	1.67553908	1.40731972	0.0008585	1.85477	up	PREDICTED: FHA domain-containing protein At4g14490 [Sesamum indicum]
c44935.graph_c0	2.50390849	3.20821219	3.31415019	67.7957886	81.3031152	78.7561353	1.71E-117	3.635201	up	PREDICTED: ABC transporter B family member 19 [Sesamum indicum]
c44938.graph_c0	10.8244615	8.93291954	7.22038616	35.8542456	38.2251571	35.3573454	8.853E-06	1.005759	up	PREDICTED: signal recognition particle 14 kDa protein-like [Sesamum indicum]
c44940.graph_c0	0.4173852	0.35352574	0.41258193	2.10008427	2.00543968	1.81600455	0.0081483	1.306684	up	PREDICTED: trimethylguanosine synthase [Sesamum indicum]
c44943.graph_c0	0.81198698	0.78377149	0.63351436	5.10280629	7.24547621	6.7512396	3.185E-09	2.078018	up	PREDICTED: GDSL esterase/lipase At4g10955-like [Sesamum indicum]
c44944.graph_c0	10.7029136	9.47341988	11.0106071	9.04885101	10.4165725	10.3643106	2.298E-12	-1.085018	down	PREDICTED: uncharacterized protein LOC105173941 [Sesamum indicum]
c44946.graph_c0	1.19838373	1.55945884	1.65546832	1.13162999	1.22806438	1.47353789	0.0047042	-1.226114	down	PREDICTED: protein TONSOKU [Sesamum indicum]
c44948.graph_c0	2.11903549	1.85601271	2.26920375	0.92186925	0.54575191	0.9167765	4.964E-09	-2.402048	down	PREDICTED: protein LURP-one-related 15-like isoform X1 [Sesamum indicum]
c44950.graph_c0	1.01188815	1.01290141	1.23187864	0	0	0	1.64E-18	-Inf	down	hypothetical protein MIMGU_mgv1a010141mg [Erythranthe guttata]
c44950.graph_c1	7.54125966	10.7320447	10.5081151	36.327893	41.2339261	42.2301871	1.759E-06	1.03524	up	PREDICTED: U6 snRNA-associated Sm-like protein LSm7 [Sesamum indicum]
c44953.graph_c0	2.8128905	2.66142186	3.0204824	8.16474226	12.2221964	14.845744	0.0020717	1.025379	up	PREDICTED: sucrose nonfermenting 4-like protein [Sesamum indicum]
c44954.graph_c0	0.08087535	0	0.10392803	1.71367599	2.88101153	4.64075224	1.164E-05	4.604702	up	PREDICTED: exonuclease 1 [Sesamum indicum]
c44955.graph_c0	9.85043514	10.0468451	9.47654369	2.03571259	2.3644548	1.87683584	3.296E-54	-3.244542	down	PREDICTED: LOW QUALITY PROTEIN: serine carboxypeptidase 24-like [Sesamum indicum]
c44957.graph_c1	1.39953572	1.24527746	1.59863005	6.13154507	9.10602129	8.08174011	1.773E-06	1.432426	up	PREDICTED: digalactosyldiacylglycerol synthase 2, chloroplastic isoform X1 [Sesamum indicum]

#ID	0 h-1 FPKM	0 h-2 FPKM	0 h-3 FPKM	48 h-1 FPKM	48 h-2 FPKM	48 h-3 FPKM	FDR	log2FC	regulated	nr_annotation
c44963.graph_c1	9.59943051	9.40603494	8.51333472	5.57397271	6.13586797	6.45590595	5.391E-15	-1.61761	down	PREDICTED: glutathione gamma-glutamylcysteinyltransferase 1 isoform X2 [Sesamum indicum]
c44965.graph_c0	14.4222345	13.6446391	15.2054958	5.26829375	8.88064754	12.3236287	3.697E-18	-1.738178	down	-
c44970.graph_c0	1.2055047	1.40134278	1.04940473	4.69313654	4.93385635	6.05668464	0.0054097	1.082876	up	PREDICTED: uncharacterized protein LOC105168255 [Sesamum indicum]
c44970.graph_c1	1.70518142	1.7068889	1.03794898	8.59874378	7.62030188	9.26961465	0.0010679	1.506217	up	unnamed protein product [Coffea canephora]
c44971.graph_c0	0	0.04692261	0	0.82051352	1.05223133	1.38332462	2.854E-10	5.10446	up	PREDICTED: probable transcription factor GLK1 [Sesamum indicum]
c44973.graph_c0	0	0	0	3.04803484	2.12435745	2.78349473	1.734E-11	Inf	up	--
c44975.graph_c0	9.42345285	9.75538094	6.83100564	0.1484017	0.55024762	0.72625817	1.586E-32	-5.223532	down	-
c44976.graph_c1	12.0529966	12.841891	12.7772976	12.7796616	12.2130541	10.5363129	9.905E-12	-1.100276	down	PREDICTED: N-alpha-acetyltransferase 35, NatC auxiliary subunit isoform X1 [Sesamum indicum]
c44978.graph_c0	2.5745445	2.65703329	2.51335176	18.4415026	22.8672912	22.4296394	3.313E-31	2.019922	up	PREDICTED: uncharacterized protein LOC105173611 isoform X1 [Sesamum indicum]
c44979.graph_c0	0.04162393	0	0.0534884	2.91434758	1.78744087	1.97899736	1.996E-09	5.116791	up	hypothetical protein MIMGU_mgv1a001060mg [Erythranthe guttata]
c44980.graph_c0	8.39767196	7.47569918	9.01024584	5.99389666	6.9545502	6.80367569	6.314E-16	-1.354496	down	PREDICTED: probable inactive leucine-rich repeat receptor-like protein kinase At3g03770 [Sesamum indicum]
c44981.graph_c2	2.13231431	2.08152926	2.19661575	37.7786915	41.6902403	41.9504484	2.369E-90	3.223666	up	hypothetical protein MIMGU_mgv1a006718mg [Erythranthe guttata]
c44983.graph_c0	2.69227651	2.51997421	3.14516537	2.8346294	2.11571205	1.57639762	0.0001606	-1.367813	down	PREDICTED: uncharacterized protein LOC105163946 [Sesamum indicum]
c44987.graph_c0	2.65136654	2.48814514	3.46518673	2.80345217	1.67609213	1.40778424	1.476E-05	-1.556088	down	unnamed protein product [Coffea canephora]
c44990.graph_c0	17.7974046	18.1043169	17.2107649	8.68030411	8.2092146	7.69408975	2.67E-34	-2.126736	down	PREDICTED: protein WEAK CHLOROPLAST MOVEMENT UNDER BLUE LIGHT 1-like isoform X2 [Sesamum indicum]
c44994.graph_c0	0.7522608	0.7308666	0.59707009	0.04076657	0.58302773	0.25391695	2.614E-06	-2.287481	down	PREDICTED: G-type lectin S-receptor-like serine/threonine-protein kinase RLK1 isoform X1 [Vitis vinifera]
c44996.graph_c0	69.7346194	65.9890045	71.4421605	14.6982161	13.4275325	10.2067936	2.87E-117	-3.448158	down	PREDICTED: protein FAR1-RELATED SEQUENCE 5-like [Sesamum indicum]
c45006.graph_c0	49.9460539	43.7946321	47.8284221	22.9625375	21.4668961	18.3060625	1.427E-47	-2.189023	down	PREDICTED: uncharacterized protein LOC103488200 [Cucumis melo]
c45009.graph_c0	0	0	0	0.87305274	0.60696137	0.50979933	5.268E-10	Inf	up	--
c45012.graph_c0	16.6244461	17.0790165	16.7472278	5.13345822	4.65174893	5.53033899	5.047E-62	-2.736771	down	PREDICTED: peptide-N(4)-(N-acetyl-beta-glucosaminyl)asparagine amidase isoform X2 [Sesamum indicum]
c45014.graph_c1	32.0951012	33.760828	29.0601729	27.1816518	26.3686371	23.5702705	1.238E-20	-1.31424	down	PREDICTED: TBC1 domain family member 15 [Sesamum indicum]

#ID	0 h-1 FPKM	0 h-2 FPKM	0 h-3 FPKM	48 h-1 FPKM	48 h-2 FPKM	48 h-3 FPKM	FDR	log2FC	regulated	nr_annotation
c45015.graph_c0	13.7244357	16.8696752	14.1350905	5.02035035	6.79580035	6.94878731	1.585E-14	-2.275782	down	PREDICTED: protein HEADING DATE 3A-like [Sesamum indicum]
c45017.graph_c0	0.24815557	0.34155558	0.2590979	2.01468814	1.92239972	2.51734868	3.449E-06	1.90891	up	PREDICTED: calcium-transporting ATPase 12, plasma membrane-type-like [Sesamum indicum]
c45018.graph_c0	0	0	0	15.063166	37.7080222	15.7899705	3.889E-08	Inf	up	hypothetical protein DI09_20p90 [Microsporidia sp. UGP3]
c45019.graph_c0	55.6912742	51.0043415	45.9243801	3.19262207	3.69927682	3.32903428	8.32E-203	-4.91806	down	BnaAnng27240D [Brassica napus]
c45024.graph_c0	0.05279945	0	0.06784939	0.94041782	1.21955801	1.24073998	8.393E-10	3.785448	up	PREDICTED: uncharacterized protein LOC105156561 isoform X1 [Sesamum indicum]
c45030.graph_c0	0.96081742	1.37633968	1.04309884	6.92872629	5.56169604	5.96144497	1.61E-08	1.435585	up	PREDICTED: protein FAM91A1 isoform X1 [Sesamum indicum]
c45031.graph_c0	28.4434448	30.0712008	28.2764391	16.7928334	18.6404873	16.7479215	1.209E-33	-1.752365	down	PREDICTED: uncharacterized protein LOC105156468 [Sesamum indicum]
c45033.graph_c1	1.22881852	1.02156611	0.66910188	3.95264339	3.92309638	4.23410439	0.0024287	1.039995	up	hypothetical protein MIMGU_mgv1a021825mg [Erythranthe guttata]
c45035.graph_c0	4.04935496	4.48008449	4.30371398	1.15001298	2.43659861	2.72041305	8.393E-12	-2.055498	down	PREDICTED: protein NRT1/ PTR FAMILY 4.5-like [Sesamum indicum]
c45041.graph_c0	97.5218459	95.8197111	88.4167488	43.1136746	37.6165516	32.0308501	6.718E-59	-2.333627	down	PREDICTED: NF-kappa-B-activating protein [Sesamum indicum]
c45043.graph_c0	1.46163766	0.88250552	1.40124327	8.8060484	9.12515295	8.2349547	5.922E-12	1.785912	up	PREDICTED: protein downstream neighbor of Son [Sesamum indicum]
c45045.graph_c0	0.43575289	0.43618923	0	24.4880832	10.8446713	20.3605394	4.486E-07	5.00645	up	-
c45047.graph_c0	0.44647374	0.37988269	0.45898914	3.53735825	4.0088488	4.08079641	2.25E-08	2.156408	up	PREDICTED: subtilisin-like protease SBT3.5 [Sesamum indicum]
c45048.graph_c2	3.06581004	3.23599721	3.41309541	12.1785815	14.8125492	17.4925586	7.996E-09	1.171765	up	PREDICTED: metalloendoproteinase 1-like [Sesamum indicum]
c45055.graph_c0	56.1000721	60.1566661	52.4367033	24.139055	24.9858788	20.93689	9.363E-58	-2.283483	down	PREDICTED: transcription factor GTE9-like isoform X2 [Sesamum indicum]
c45056.graph_c0	2.14655714	1.32928459	2.22075454	9.73691226	8.84149532	8.23139927	1.625E-08	1.21755	up	PREDICTED: uncharacterized protein LOC105167041 isoform X1 [Sesamum indicum]
c45058.graph_c0	0.60482662	0.53816201	1.29537693	10.091615	11.0187823	10.7973855	8.424E-11	2.681403	up	-
c45060.graph_c0	16.8203486	15.4547261	14.5952971	12.229688	14.525061	13.669968	2.025E-16	-1.23245	down	PREDICTED: chitin elicitor receptor kinase 1-like [Sesamum indicum]
c45063.graph_c0	89.4575033	81.6406427	82.1117382	81.4329668	77.2604135	83.298249	1.022E-13	-1.081776	down	PREDICTED: serine/arginine-rich splicing factor RSZ22 isoform X2 [Vitis vinifera]
c45063.graph_c2	0	0	0	2.84546801	2.28365782	2.07153845	2.095E-11	Inf	up	PREDICTED: KH domain-containing protein At4g18375 [Sesamum indicum]
c45070.graph_c0	20.2945197	21.5979651	17.9122309	15.2882471	14.2782886	14.5471964	2.429E-25	-1.453694	down	PREDICTED: CCR4-NOT transcription complex subunit 1 isoform X1 [Sesamum indicum]
c45072.graph_c0	51.0523768	51.2982707	46.9138125	16.2900575	21.8387254	23.5764722	1.583E-55	-2.296793	down	PREDICTED: extensin [Sesamum indicum]

#ID	0 h-1 FPKM	0 h-2 FPKM	0 h-3 FPKM	48 h-1 FPKM	48 h-2 FPKM	48 h-3 FPKM	FDR	log2FC	regulated	nr_annotation
c45073.graph_c0	46.9195677	48.8014571	49.6028436	22.4408174	25.5967493	21.3547519	1.157E-46	-2.085517	down	hypothetical protein MIMGU_mgv1a005118mg [Erythranthe guttata]
c45073.graph_c2	2.90514396	2.90805303	2.28141529	1.58601962	1.68019547	1.7640384	5.848E-05	-1.70269	down	PREDICTED: phosphatidylcholine:diacylglycerol cholinephosphotransferase 1-like [Sesamum indicum]
c45075.graph_c0	152.483753	147.835513	148.12963	42.2957559	57.0420505	54.0114859	2.699E-67	-2.570485	down	PREDICTED: uncharacterized protein LOC105179151 [Sesamum indicum]
c45078.graph_c0	273.655042	248.88511	259.444151	3.58540714	2.75049608	3.17455808	0	-7.374601	down	PREDICTED: uncharacterized protein LOC105174597 [Sesamum indicum]
c45079.graph_c0	1.24903221	1.25620844	1.19428326	6.99139577	7.80517179	8.55980576	1.262E-24	1.638716	up	PREDICTED: probable LRR receptor-like serine/threonine-protein kinase At4g20940 [Sesamum indicum]
c45081.graph_c0	4.7870349	5.10893468	3.79947402	18.5484828	23.6691597	22.2173653	8.947E-10	1.215269	up	PREDICTED: uncharacterized protein LOC105177718 [Sesamum indicum]
c45085.graph_c0	39.9913624	40.7488164	36.8022175	33.039539	35.4210662	35.8608575	2.95E-17	-1.190021	down	PREDICTED: protein DEK [Sesamum indicum]
c45088.graph_c0	0.56035845	0.56091956	0.72008279	4.24462973	5.65122105	5.3079998	3.053E-05	2.020472	up	PREDICTED: uncharacterized protein LOC104801838 isoform X2 [Tarenaya hassleriana]
c45092.graph_c0	1.01112717	0.82470639	0.67373105	0	0	0	1.804E-19	-Inf	down	-
c45094.graph_c0	27.2853971	27.3875487	26.8975024	22.065542	17.4077959	18.1876026	6.466E-27	-1.513315	down	PREDICTED: pentatricopeptide repeat-containing protein At1g63330-like [Sesamum indicum]
c45095.graph_c1	0.3721209	0.10642672	0.06831287	1.86103394	2.02342243	1.91740052	6.381E-05	2.401269	up	PREDICTED: putative phospholipid-transporting ATPase 5 [Sesamum indicum]
c45095.graph_c2	0.54124028	0.61917972	0.39743725	5.55611663	5.20687433	5.54592855	4.087E-08	2.37499	up	PREDICTED: putative phospholipid-transporting ATPase 5 [Sesamum indicum]
c45104.graph_c0	64.4749031	62.2293275	58.7939802	40.7683569	40.9930733	35.9680199	1.001E-32	-1.671598	down	PREDICTED: mRNA-decapping enzyme-like protein [Sesamum indicum]
c45105.graph_c0	0.63454337	0.40116554	0.47208141	6.09196747	6.4862953	5.94075423	5.863E-11	2.602264	up	hypothetical protein VITISV_036763 [Vitis vinifera]
c45113.graph_c0	5.26336289	2.4085181	3.60726917	0	0	0	3.828E-16	-Inf	down	--
c45114.graph_c0	161.019614	176.007028	192.62196	33.8581599	41.5068253	39.8499809	1.061E-60	-3.223282	down	PREDICTED: serine/arginine repetitive matrix protein 1-like [Sesamum indicum]
c45124.graph_c0	24.886053	22.1069053	21.1803644	14.7349963	14.4987257	14.2074074	2.658E-31	-1.665778	down	PREDICTED: elongation factor Tu GTP-binding domain-containing protein 1 isoform X1 [Sesamum indicum]
c45132.graph_c0	2.04244581	2.68173496	2.82913987	3.05460114	1.70859718	1.86996042	0.0003357	-1.196055	down	ribosomal protein S3 (mitochondrion) [Rhazya stricta]
c45134.graph_c0	1.35216416	1.43638661	1.89715857	9.4698761	14.2335839	16.2869582	1.701E-08	2.064148	up	PREDICTED: filament-like plant protein [Sesamum indicum]
c45138.graph_c0	34.3803299	30.8617326	31.9815022	30.9509599	32.2651351	30.1789439	5.266E-13	-1.075285	down	PREDICTED: actin-related protein 7 isoform X1 [Sesamum indicum]
c45139.graph_c0	25.9409548	26.0996407	25.1575352	18.0358352	16.2170417	15.0700721	1.017E-24	-1.660678	down	unnamed protein product [Coffea canephora]
c45141.graph_c0	2.39613337	2.47121555	1.95944398	18.7523831	16.5824554	17.6182555	6.471E-23	1.942178	up	PREDICTED: uncharacterized protein LOC105159160 [Sesamum indicum]

#ID	0 h-1 FPKM	0 h-2 FPKM	0 h-3 FPKM	48 h-1 FPKM	48 h-2 FPKM	48 h-3 FPKM	FDR	log2FC	regulated	nr_annotation
c45143.graph_c0	0.72688729	0.90951894	0.54487938	8.48230906	9.16333275	6.6537238	3.294E-08	2.464744	up	PREDICTED: serine/threonine-protein kinase Nek6-like [Sesamum indicum]
c45144.graph_c0	15.3843443	14.0689068	13.6677966	70.4653616	75.6822592	70.0616932	4.255E-20	1.308702	up	PREDICTED: protein COBRA-like [Sesamum indicum]
c45147.graph_c0	108.327671	112.504683	107.060343	26.5004433	26.6609424	22.4645713	6.832E-93	-3.132241	down	PREDICTED: phytochrome C [Sesamum indicum]
c45149.graph_c0	1.07932017	0.92034154	1.07875378	4.52977173	5.46198491	5.57069772	0.0008443	1.316146	up	PREDICTED: pheophytinase, chloroplastic [Sesamum indicum]
c45149.graph_c2	208.329895	197.995608	222.777664	142.02087	178.242117	181.222405	7.53E-19	-1.349796	down	PREDICTED: carbonic anhydrase 2-like [Sesamum indicum]
c45150.graph_c0	5.02032182	3.96531437	3.85566712	15.8805356	18.8522745	20.0462061	2.049E-10	1.073767	up	hypothetical protein MIMGU_mgv1a000966mg [Erythranthe guttata]
c45151.graph_c0	0	0	0	4.98844812	5.08746725	4.43869114	2.009E-21	Inf	up	mitochondrial carrier, adenine nucleotidetranslocator [Galdieria sulphuraria]
c45155.graph_c0	0.67468297	0.95675797	0.57799622	4.26460567	5.77989919	5.37699258	6.749E-09	1.783583	up	PREDICTED: proline-rich receptor-like protein kinase PERK8 [Sesamum indicum]
c45156.graph_c1	58.1948419	55.7806183	55.4940473	36.9838879	37.5904946	32.9302913	1.571E-32	-1.673003	down	PREDICTED: casein kinase I isoform alpha-like isoform X2 [Sesamum indicum]
c45158.graph_c0	0.01463921	0	0.03762392	4.93609232	5.31491572	5.78413862	9.126E-50	7.224178	up	PREDICTED: uncharacterized protein LOC105177021 [Sesamum indicum]
c45162.graph_c0	0	0	0	1.15971879	1.10572329	1.05254902	1.462E-12	Inf	up	-
c45163.graph_c0	1.31455666	0.83547492	0.64352661	0	0	0	2.185E-15	-Inf	down	-
c45169.graph_c0	21.094801	20.0008919	22.0081947	22.7690984	21.5391221	17.862872	3.311E-11	-1.037049	down	hypothetical protein MIMGU_mgv1a012191mg [Erythranthe guttata]
c45170.graph_c0	0.48349414	0.31393186	0.50376415	5.03211378	6.7975535	7.41258577	8.635E-22	2.860835	up	PREDICTED: probable LRR receptor-like serine/threonine-protein kinase At4g26540 [Sesamum indicum]
c45175.graph_c0	0	0	0	67.7934434	143.749559	39.964629	1.133E-08	Inf	up	predicted protein [Hordeum vulgare subsp. vulgare]
c45180.graph_c0	6.30938785	6.25944111	5.86868437	89.3253349	81.2303921	77.2694965	2.056E-51	2.734218	up	PREDICTED: pentatricopeptide repeat-containing protein At5g24830 isoform X1 [Sesamum indicum]
c45181.graph_c0	10.0014826	9.9856281	10.1954947	6.78551724	8.55045156	9.57560007	3.785E-13	-1.299623	down	hypothetical protein MIMGU_mgv1a002860mg [Erythranthe guttata]
c45183.graph_c0	2.35360189	2.35595866	1.84098272	11.6899198	15.0139992	15.9100053	1.324E-11	1.681759	up	hypothetical protein OsJ_01978 [Oryza sativa Japonica Group]
c45186.graph_c2	8.98318035	8.35648199	7.95301533	6.55263402	7.15583272	7.29758302	2.257E-17	-1.286111	down	PREDICTED: E3 ubiquitin-protein ligase listerin isoform X2 [Sesamum indicum]
c45187.graph_c0	1.02135072	0.4868445	0.62498863	4.97351306	4.84163126	5.74106207	3.765E-05	1.851323	up	PREDICTED: dicer-like protein 4 isoform X1 [Sesamum indicum]
c45187.graph_c1	0.39843388	0.15953314	0.20480132	2.128967	2.25538239	2.48224129	0.0002001	2.155707	up	PREDICTED: dicer-like protein 4 isoform X1 [Sesamum indicum]
c45187.graph_c2	0.19175547	0.28792123	0.12320669	2.22588635	2.82592508	2.57789815	6.939E-09	2.646035	up	PREDICTED: dicer-like protein 4 isoform X2 [Sesamum indicum]
c45189.graph_c1	0	0	0	5.5505209	6.55857738	7.97809404	1.644E-11	Inf	up	hypothetical protein JCGZ_03401 [Jatropha curcas]
c45190.graph_c0	14.0886466	12.5286427	14.7640086	236.053237	323.079885	343.680642	8.516E-33	3.422834	up	PREDICTED: beta-glucosidase 24-like [Eucalyptus grandis]

#ID	0 h-1 FPKM	0 h-2 FPKM	0 h-3 FPKM	48 h-1 FPKM	48 h-2 FPKM	48 h-3 FPKM	FDR	log2FC	regulated	nr_annotation
c45193.graph_c0	0	0	0.03623667	0.98718845	1.15589135	2.19598587	1.608E-06	5.84608	up	PREDICTED: cellulose synthase-like protein G2 [Sesamum indicum]
c45194.graph_c0	1.76913513	1.46947573	2.0799271	17.5814944	20.8296581	22.7098941	2.018E-26	2.498769	up	hypothetical protein OsI_30754 [Oryza sativa Indica Group]
c45195.graph_c0	0.05059501	0.05064567	0.06501659	1.39834266	1.28385789	2.19815184	9.166E-08	3.852244	up	PREDICTED: glutamine-dependent NAD(+) synthetase-like [Sesamum indicum]
c45195.graph_c1	0.19245222	0.04816123	0.24730873	1.55137097	2.01914416	1.53816063	3.628E-05	2.360557	up	PREDICTED: glutamine-dependent NAD(+) synthetase-like [Sesamum indicum]
c45196.graph_c0	13.9914558	14.8162022	14.362844	5.57751469	6.95606213	7.26997825	9.174E-41	-2.146188	down	PREDICTED: eukaryotic translation initiation factor 2D [Sesamum indicum]
c45198.graph_c0	36.1787492	37.5813431	35.6287127	7.36056857	4.75435427	3.38761534	2.78E-143	-3.824198	down	hypothetical protein MIMGU_mgv1a000338mg [Erythranthe guttata]
c45198.graph_c1	34.7965878	35.8175002	35.6305148	11.3974109	12.5831564	15.6276505	7.559E-60	-2.444614	down	cryptochrome 1 family protein [Populus trichocarpa]
c45201.graph_c0	22.0402413	20.0152927	25.4027288	18.4209474	14.8578287	8.94046266	6.217E-07	-1.687339	down	-
c45201.graph_c2	2.78827122	2.20688722	2.83310054	11.8878773	11.549558	12.331863	1.315E-06	1.173534	up	PREDICTED: ras-related protein RABH1e-like [Sesamum indicum]
c45202.graph_c0	31.0236304	31.3234782	26.2503734	23.0436625	26.2463915	26.0915253	3.298E-19	-1.251087	down	PREDICTED: OTU domain-containing protein 5 isoform X2 [Sesamum indicum]
c45206.graph_c0	15.4898248	17.1338545	14.781552	11.948157	14.4690502	13.2422893	6.238E-17	-1.276882	down	PREDICTED: uncharacterized protein LOC105171856 [Sesamum indicum]
c45208.graph_c0	18.2590242	18.9000901	15.6076185	12.2360139	14.388192	13.282345	3.764E-20	-1.421123	down	PREDICTED: uncharacterized protein At2g33490 [Sesamum indicum]
c45210.graph_c1	4.20390776	4.49833232	2.42167044	32.398929	31.5780496	29.6126561	4.24E-31	2.061844	up	PREDICTED: pleiotropic drug resistance protein 1-like [Sesamum indicum]
c45213.graph_c1	0.04501424	0.3604745	0.17353523	80.2860196	104.822923	96.0501856	1.98E-152	7.90084	up	PREDICTED: nudix hydrolase 18, mitochondrial-like [Sesamum indicum]
c45215.graph_c1	2.21491176	3.56569307	2.64084987	71.7758586	52.5935676	42.9203144	8.728E-10	3.30293	up	PREDICTED: U-box domain-containing protein 13 [Sesamum indicum]
c45216.graph_c1	2.836692	2.14696361	2.93399099	0.19122042	0.1350499	0.17014678	7.668E-17	-5.009346	down	-
c45216.graph_c2	31.437625	31.3199624	27.139813	31.6390098	30.5003748	25.8010949	6.153E-13	-1.045616	down	PREDICTED: calpain-15 [Sesamum indicum]
c45217.graph_c0	4.34449378	3.34526472	3.81136637	22.0903406	22.6274053	24.1417697	1.093E-13	1.564009	up	PREDICTED: pyridoxal reductase, chloroplastic isoform X2 [Sesamum indicum]
c45218.graph_c0	98.9272002	98.191523	96.7822241	39.7870356	38.3800688	34.6827068	9.528E-63	-2.396642	down	PREDICTED: 4-coumarate--CoA ligase 1-like [Sesamum indicum]
c45223.graph_c0	3.90105376	3.37465685	2.78500474	20.232355	16.545333	17.0551211	3.623E-08	1.409133	up	PREDICTED: uncharacterized protein LOC105166056 isoform X1 [Sesamum indicum]
c45225.graph_c0	5.29028877	3.32024849	3.56097789	23.2470572	24.6684157	25.3659111	5.139E-14	1.57395	up	hypothetical protein MIMGU_mgv1a011604mg [Erythranthe guttata]

#ID	0 h-1 FPKM	0 h-2 FPKM	0 h-3 FPKM	48 h-1 FPKM	48 h-2 FPKM	48 h-3 FPKM	FDR	log2FC	regulated	nr_annotation
c45227.graph_c0	123.706499	131.599807	115.83258	45.5939698	43.0689689	38.2734869	7.376E-71	-2.562287	down	PREDICTED: uncharacterized protein LOC105178162 [Sesamum indicum]
c45227.graph_c1	6.32629514	6.54094017	6.73896056	5.36807501	5.36186315	5.39059983	9.034E-07	-1.300405	down	PREDICTED: DNA-(apurinic or apyrimidinic site) lyase 2 isoform X1 [Sesamum indicum]
c45234.graph_c0	9.91884346	10.1702864	9.95574268	3.95151808	3.37508687	3.47208656	6.515E-37	-2.490724	down	-
c45235.graph_c0	48.9696439	50.1154831	42.9989478	16.8239355	17.2745221	16.2825761	7.965E-68	-2.511795	down	PREDICTED: outer envelope protein 61 [Sesamum indicum]
c45238.graph_c0	403.374093	396.509747	391.610168	289.31224	385.159853	427.133978	5.817E-13	-1.136285	down	PREDICTED: 8-hydroxygeraniol dehydrogenase [Sesamum indicum]
c45240.graph_c0	71.0528903	74.2869097	66.4231309	33.7369074	34.3959529	29.2219354	6.171E-51	-2.136802	down	PREDICTED: crt homolog 1 [Sesamum indicum]
c45246.graph_c0	0.13556302	0.09046585	0.05806795	19.7741709	24.1677649	28.0778175	5.768E-57	6.970744	up	PREDICTED: WAT1-related protein At5g40240-like [Sesamum indicum]
c45248.graph_c0	194.914687	191.294407	202.196762	167.340918	165.341161	154.541097	3.456E-17	-1.289365	down	PREDICTED: aldehyde dehydrogenase [Nicotiana sylvestris]
c45251.graph_c0	0.60149642	0.88730339	0.44749547	4.52059733	4.85081859	5.39780172	1.845E-06	1.915978	up	PREDICTED: uncharacterized protein LOC105179935 isoform X1 [Sesamum indicum]
c45253.graph_c0	0	0	0	13.6669756	23.4211085	25.3945965	2.7E-12	Inf	up	-
c45255.graph_c1	78.5206363	81.9231123	67.3684844	84.1427958	71.7154093	68.2089715	4.05E-14	-1.035959	down	PREDICTED: anamorsin homolog isoform X1 [Sesamum indicum]
c45257.graph_c0	2.92607756	3.2014734	2.66706577	18.462366	19.492225	16.9576281	5.811E-14	1.626335	up	PREDICTED: acyl-coenzyme A thioesterase 8-like [Sesamum indicum]
c45258.graph_c0	29.6324645	25.4331977	23.9331176	1129.5795	1120.57687	1065.08841	2.07E-157	4.376023	up	ascorbate peroxidase [Acanthus ebracteatus]
c45259.graph_c0	0.08349071	0.16714864	0.21457775	7.48659694	8.74598498	10.5626298	1.942E-34	4.819063	up	PREDICTED: LRR receptor-like serine/threonine-protein kinase FEI 2 isoform X1 [Sesamum indicum]
c45266.graph_c0	2.253657	1.73531823	2.3231957	78.0520258	97.7689353	98.5011642	7.02E-116	4.418608	up	PREDICTED: phosphomethylpyrimidine synthase, chloroplastic isoform X1 [Sesamum indicum]
c45267.graph_c1	28.5193895	29.3636029	27.7206285	124.178851	127.157588	133.168864	2.626E-16	1.149452	up	PREDICTED: uncharacterized protein LOC105158383 [Sesamum indicum]
c45267.graph_c2	0.54284464	1.19545407	0.13951537	34.6071889	41.0064756	45.299924	2.033E-39	4.998587	up	-
c45279.graph_c0	8.66155866	7.87936615	7.5957772	76.1026704	99.0419908	97.0039974	1.304E-44	2.474012	up	PREDICTED: peroxidase 7 [Sesamum indicum]
c45282.graph_c0	144.030981	136.330166	148.096257	40.2264538	37.1868303	42.4841384	1.714E-82	-2.854838	down	branched-chain amino acid aminotransferase, putative [Ricinus communis]
c45285.graph_c0	11.6343034	11.0198269	11.7353705	0.69150188	1.00727329	0.87166681	4.175E-88	-4.765533	down	PREDICTED: U-box domain-containing protein 19-like [Sesamum indicum]
c45290.graph_c0	0.80401608	0.98367033	0.688795	6.50180581	8.02132263	7.61604863	1.72E-05	2.141623	up	-
c45290.graph_c1	18.9139961	18.949558	17.1139575	12.2080737	14.4402	14.864707	5.267E-23	-1.424983	down	PREDICTED: WD repeat-containing protein 26-like [Sesamum indicum]

#ID	0 h-1 FPKM	0 h-2 FPKM	0 h-3 FPKM	48 h-1 FPKM	48 h-2 FPKM	48 h-3 FPKM	FDR	log2FC	regulated	nr_annotation
c45292.graph_c0	0.62928407	0.71390276	0.43128264	94.1493748	134.337617	123.012307	5.91E-80	6.61088	up	PREDICTED: pentatricopeptide repeat-containing protein At5g59600 [Sesamum indicum]
c45297.graph_c0	5.17558453	2.10743068	3.15632659	0	0	0	5.366E-11	-Inf	down	hypothetical protein SORBIDRAFT_05g002010 [Sorghum bicolor]
c45300.graph_c0	68.3283747	74.0017582	60.5873056	13.9055004	12.5927927	12.9717256	5.005E-93	-3.376188	down	PREDICTED: uncharacterized protein LOC105166619 [Sesamum indicum]
c45303.graph_c0	2.46053958	3.39724612	3.05286006	284.523681	369.485888	346.644677	5.08E-143	5.788411	up	PREDICTED: S-type anion channel SLAH2 [Sesamum indicum]
c45304.graph_c0	62.763009	60.6965632	58.2143091	59.5441747	58.4714691	59.8610922	1.043E-13	-1.047029	down	PREDICTED: cytochrome b-c1 complex subunit Rieske-4, mitochondrial-like [Sesamum indicum]
c45320.graph_c1	7.15344582	10.0037919	7.29989557	4.02197303	4.10678818	4.61354017	1.33E-11	-1.958196	down	PREDICTED: laccase-17-like [Sesamum indicum]
c45322.graph_c0	7.08618777	7.57888727	6.54565614	4.74056904	7.05117244	7.5841122	2.2E-11	-1.154444	down	PREDICTED: tetratricopeptide repeat protein 7A [Sesamum indicum]
c45326.graph_c0	1.7671061	1.96541733	2.46003475	0.04522148	0	0	2.414E-23	-8.061023	down	conserved hypothetical protein [Ricinus communis]
c45328.graph_c0	5.61503585	5.2279487	7.02648984	40.8413562	46.4731606	52.6815102	4.496E-31	1.946185	up	PREDICTED: RNA polymerase sigma factor sigA [Sesamum indicum]
c45331.graph_c0	138.570724	153.051674	120.498966	85.5626318	86.7977629	69.6943091	1.534E-28	-1.781954	down	PREDICTED: ferredoxin--nitrite reductase, chloroplastic [Sesamum indicum]
c45334.graph_c0	549.462852	508.971473	599.222047	113.941114	96.5965453	83.583105	5.553E-81	-3.508891	down	hypothetical protein MIMGU_mgv1a010052mg [Erythranthe guttata]
c45339.graph_c0	0.7027507	0.4689696	0.66893517	0	0	0	2.047E-19	-Inf	down	-
c45340.graph_c0	12.746407	11.6195955	10.9720061	4.58850282	3.90861055	4.1828097	1.301E-48	-2.492197	down	PREDICTED: B3 domain-containing transcription repressor VAL1 [Populus euphratica]
c45342.graph_c0	62.5889661	61.4687891	59.2969945	28.8486628	23.2768367	21.2458406	1.62E-59	-2.333189	down	PREDICTED: uncharacterized protein At4g26450 [Sesamum indicum]
c45348.graph_c0	22.3975059	18.5857152	20.6281244	13.630321	12.3564785	15.7234873	6.609E-24	-1.580087	down	PREDICTED: uncharacterized protein LOC105163463 [Sesamum indicum]
c45349.graph_c0	7.64421738	6.17467656	6.56139036	49.0789372	61.7004973	61.2833643	1.555E-41	2.056959	up	PREDICTED: uncharacterized protein LOC105168326 [Sesamum indicum]
c45351.graph_c1	45.7970811	52.5144262	43.708727	54.5098738	28.1392338	19.9525	1.267E-06	-1.468701	down	PREDICTED: cytochrome P450 710A1-like [Sesamum indicum]
c45352.graph_c0	1.72126275	1.66357301	1.37289773	23.403328	25.6039738	23.3541763	4.501E-24	2.910677	up	PREDICTED: uncharacterized protein LOC105164615 [Sesamum indicum]
c45356.graph_c1	19.9560017	19.3257717	18.904714	7.3915864	9.27449283	8.41105207	3.889E-54	-2.234833	down	PREDICTED: uncharacterized protein LOC105174921 [Sesamum indicum]
c45357.graph_c0	4.949178	4.6789042	4.71102939	3.54628212	2.5045706	2.70468044	4.876E-05	-1.722398	down	PREDICTED: two-component response regulator-like APRR9 [Sesamum indicum]
c45364.graph_c0	0.41258749	0.18772756	0.28919523	2.72982712	2.81870764	4.3352748	8.734E-07	2.454075	up	PREDICTED: maf-like protein DDB_G0281937 [Sesamum indicum]

#ID	0 h-1 FPKM	0 h-2 FPKM	0 h-3 FPKM	48 h-1 FPKM	48 h-2 FPKM	48 h-3 FPKM	FDR	log2FC	regulated	nr_annotation
c45371.graph_c0	0.07905292	0.07913208	0.05079307	2.18486122	2.70036146	3.43453493	5.03E-11	4.296478	up	-
c45372.graph_c0	72.9417579	74.0937109	66.8371382	30.5511638	29.0871402	25.396616	1.258E-60	-2.345028	down	PREDICTED: uncharacterized RING finger protein C4G3.12c [Sesamum indicum]
c45377.graph_c0	15.2341062	17.0401115	15.4097033	73.649735	101.157045	105.471615	1.802E-12	1.532108	up	PREDICTED: transmembrane 9 superfamily member 1-like [Sesamum indicum]
c45381.graph_c0	31.5292555	32.5896406	28.2842264	29.3090146	28.4104612	28.0491667	5.507E-16	-1.122911	down	PREDICTED: LOW QUALITY PROTEIN: uncharacterized protein LOC105165795 [Sesamum indicum]
c45382.graph_c0	1684.32576	1714.14098	1965.92845	168.938782	164.343385	155.032413	1.9E-99	-4.475611	down	Polyubiquitin [Triticum urartu]
c45383.graph_c1	3.59887452	3.34515837	2.1471804	20.3272826	20.5725655	21.9152361	3.513E-14	1.775348	up	PREDICTED: GTP-binding protein OBGC, chloroplastic [Sesamum indicum]
c45387.graph_c0	0.67738532	1.0431748	0.80350796	11.9529872	11.2896723	11.1909734	1.953E-13	2.753819	up	PREDICTED: nudix hydrolase 9 isoform X1 [Sesamum indicum]
c45390.graph_c0	16.8134457	17.7510875	17.9940076	13.7476466	13.16742	12.8190665	2.943E-14	-1.420435	down	General transcription factor IIE subunit 1 isoform 2 [Theobroma cacao]
c45393.graph_c1	13.3086455	12.0405483	13.4706482	91.2772593	96.5793224	102.462927	4.362E-41	1.883309	up	PREDICTED: aldehyde dehydrogenase family 7 member B4 isoform X1 [Sesamum indicum]
c45395.graph_c0	45.9040321	48.8606238	45.3026363	24.6320582	20.1167473	17.672184	6.375E-53	-2.17793	down	PREDICTED: probable protein phosphatase 2C 4 [Sesamum indicum]
c45397.graph_c0	0.47487361	0.50036749	0.32117441	7.75959419	12.7573965	13.2352178	7.121E-15	3.680114	up	PREDICTED: choline transporter-like protein 2 [Sesamum indicum]
c45399.graph_c1	0.04074551	0.04078631	0.05235959	0.71321096	0.71579419	0.93521595	9.53E-07	3.119751	up	PREDICTED: pentatricopeptide repeat-containing protein At5g04780-like [Sesamum indicum]
c45401.graph_c1	5.49494379	5.15231664	4.06690537	29.9252455	30.2765896	33.497946	2.71E-20	1.655322	up	PREDICTED: mitogen-activated protein kinase kinase 2 [Sesamum indicum]
c45405.graph_c0	0.82191259	0.68931902	0.91346181	10.9077655	10.9267341	10.3976252	2.771E-26	2.713685	up	PREDICTED: beta-amylase 7 [Sesamum indicum]
c45406.graph_c0	931.010988	935.738944	932.214349	28.2586091	30.3991658	29.3905701	5.14E-194	-6.009011	down	15.5 kDa oleosin [Sesamum indicum]
c45407.graph_c0	0.29075334	0.40746228	0.44835547	3.53576446	3.46194752	3.47977944	3.814E-05	2.171533	up	PREDICTED: root phototropism protein 3 [Populus euphratica]
c45407.graph_c1	0.48980468	0.57681782	0.62941841	9.8211063	9.75751926	10.6990781	1.05E-23	3.137897	up	hypothetical protein MIMGU_mgv1a005061mg [Erythranthe guttata]
c45418.graph_c0	18.0738016	17.6396023	16.9913238	19.5865576	16.9895913	16.2583367	1.196E-11	-1.010108	down	PREDICTED: probable LRR receptor-like serine/threonine-protein kinase At4g08850 [Sesamum indicum]
c45419.graph_c0	15.9825759	14.1368123	14.0972554	9.72900217	13.4756232	13.2048618	8.461E-15	-1.302605	down	PREDICTED: probable folate-biopterin transporter 7 [Sesamum indicum]
c45423.graph_c0	19.8316666	20.0199366	20.7010702	7.03297093	7.32742983	7.53360943	3.515E-58	-2.486505	down	PREDICTED: aspartic proteinase nepenthesin-1 [Sesamum indicum]
c45424.graph_c0	139.42402	138.631966	132.480324	19.6070983	25.343461	23.1938859	5.53E-125	-3.611895	down	hypothetical protein F383_22559 [Gossypium arboreum]
c45426.graph_c0	0.33770523	0.28170283	0.28930973	1.97040187	2.85644465	3.27581246	0.0001056	2.132516	up	hypothetical protein MIMGU_mgv1a010549mg [Erythranthe guttata]

#ID	0 h-1 FPKM	0 h-2 FPKM	0 h-3 FPKM	48 h-1 FPKM	48 h-2 FPKM	48 h-3 FPKM	FDR	log2FC	regulated	nr_annotation
c45427.graph_c0	1.10820511	0.82828839	1.13926995	6.37073317	6.90765009	7.52184931	5.051E-14	1.736587	up	PREDICTED: alpha-N-acetylglucosaminidase [Sesamum indicum]
c45430.graph_c0	0	0	0	24.3944783	56.2599644	19.4970363	5.13E-08	Inf	up	predicted protein [Bathycoccus prasinos]
c45432.graph_c1	0.48075456	0.54139046	0.23167074	3.0449544	3.40171051	3.64535048	0.000249	1.995877	up	PREDICTED: charged multivesicular body protein 5-like [Nelumbo nucifera]
c45434.graph_c1	6.8795423	6.88643112	5.82668279	0.79223539	1.06817114	2.05069464	6.108E-16	-3.350303	down	-
c45443.graph_c0	27.7890135	28.1340218	22.0693393	23.966321	26.9356602	27.3252045	2.397E-12	-1.012945	down	PREDICTED: uncharacterized protein LOC105158089 [Sesamum indicum]
c45443.graph_c2	1.59609211	1.71751713	1.48700512	0.73521208	1.36301908	1.24295584	0.0005959	-1.549369	down	PREDICTED: protein IQ-DOMAIN 1-like isoform X1 [Sesamum indicum]
c45444.graph_c0	2.29272264	0.8980507	1.47311968	0	0	0	3.292E-10	-Inf	down	PREDICTED: malate dehydrogenase-like [Musa acuminata subsp. malaccensis]
c45449.graph_c0	447.950348	445.741815	402.582816	107.570628	79.2555097	62.6971976	2.018E-93	-3.385283	down	PREDICTED: putative DNA-binding protein ESCAROLA [Sesamum indicum]
c45449.graph_c1	13.869955	16.3972981	14.7504341	1.10154372	0.2333904	0.39205879	6.607E-40	-5.694687	down	-
c45449.graph_c2	1.92429955	1.85353865	1.58632524	7.89393398	8.46897094	6.72634893	0.0007226	1.090604	up	PREDICTED: protein IQ-DOMAIN 1-like [Sesamum indicum]
c45449.graph_c4	23.8629523	24.3710403	19.890706	0.89124763	0.62944586	0.13217113	1.075E-48	-6.364416	down	-
c45455.graph_c1	1.45457039	1.19908099	1.15449386	0.23647855	0.58454748	0.73646017	3.586E-06	-2.32084	down	-
c45458.graph_c0	23.5045425	20.2817604	19.5275949	19.4913714	20.2870147	22.1032194	3.922E-12	-1.05039	down	PREDICTED: DUF21 domain-containing protein At2g14520-like [Sesamum indicum]
c45458.graph_c1	6.16025705	5.6766734	5.97285346	34.09236	32.2749789	31.7206778	7.405E-21	1.445232	up	PREDICTED: protein IQ-DOMAIN 32 [Sesamum indicum]
c45461.graph_c0	0.16278119	0.10184012	0.20918028	2.38068884	2.80007254	3.2525426	1.153E-11	3.127477	up	PREDICTED: putative pentatricopeptide repeat-containing protein At4g17915 [Sesamum indicum]
c45462.graph_c0	2.43419505	0.82470639	0.86622563	0	0	0	3.637E-06	-Inf	down	PREDICTED: serine hydroxymethyltransferase, mitochondrial [Musa acuminata subsp. malaccensis]
c45467.graph_c1	0.19285497	0	0.24782627	30.4705038	30.3034895	34.3055756	1.057E-43	6.725805	up	hypothetical protein MIMGU_mgv1a008026mg [Erythranthe guttata]
c45473.graph_c0	5.55256967	4.16859729	4.21179242	142.129672	137.886873	138.509015	1.27E-133	3.893884	up	PREDICTED: peroxisomal (S)-2-hydroxy-acid oxidase GLO5 [Musa acuminata subsp. malaccensis]
c45474.graph_c0	0	0	0	1.64236916	2.14140429	3.93445825	2.745E-06	Inf	up	-
c45476.graph_c1	2.13410973	2.00391285	1.71097604	8.43848958	9.69527157	8.87862817	2.766E-12	1.190055	up	PREDICTED: nuclear pore complex protein NUP205 isoform X2 [Sesamum indicum]
c45478.graph_c0	73.9427566	77.6110509	66.1571566	72.9573367	72.8666969	69.0986448	1.501E-13	-1.034208	down	PREDICTED: ankyrin repeat-containing protein At5g02620 [Sesamum indicum]
c45479.graph_c0	3.84863039	3.91778055	4.06548572	16.6463354	20.9134412	23.1000056	6.309E-12	1.33517	up	PREDICTED: reticulon-like protein B17 [Sesamum indicum]
c45482.graph_c0	0	0	0	2.25382557	2.35034779	2.7574806	7.702E-15	Inf	up	PREDICTED: myosin-12 [Sesamum indicum]
c45487.graph_c2	2.27426744	2.07858436	2.28719246	0.18219161	0	0.162113	5.521E-15	-5.267551	down	unnamed protein product [Coffea canephora]

#ID	0 h-1 FPKM	0 h-2 FPKM	0 h-3 FPKM	48 h-1 FPKM	48 h-2 FPKM	48 h-3 FPKM	FDR	log2FC	regulated	nr_annotation
c45492.graph_c0	20.0347034	18.8857271	20.0805564	145.854907	137.643289	173.130984	3.379E-28	1.933783	up	PREDICTED: VIN3-like protein 2 [Sesamum indicum]
c45497.graph_c0	4.36330373	3.47796177	3.11501002	24.0813399	25.7478401	20.5001238	4.331E-15	1.668111	up	PREDICTED: serine carboxypeptidase-like 31 [Sesamum indicum]
c45501.graph_c0	12.3552923	12.8230627	12.4923995	11.9339789	9.81496024	8.04750625	8.888E-17	-1.350183	down	PREDICTED: probable serine/threonine-protein kinase At1g18390 [Sesamum indicum]
c45502.graph_c0	14.1513631	13.5775303	13.4272092	7.23193334	8.75583595	7.99624053	2.411E-31	-1.798997	down	PREDICTED: uncharacterized protein LOC105163310 [Sesamum indicum]
c45503.graph_c0	0	0	0.11497538	28.1902635	63.8324717	9.3879867	8.568E-05	8.726844	up	40s ribosomal protein s22 [Lichtheimia corymbifera JMRC:FSU:9682]
c45509.graph_c0	27.0874232	26.084288	24.1073798	108.783533	116.612728	117.312084	2.504E-15	1.131153	up	hypothetical protein glysoja_038462 [Glycine soja]
c45510.graph_c0	28.3430575	30.8218336	33.9205475	4.78212204	5.78569186	6.55189223	1.314E-64	-3.466427	down	PREDICTED: 26.5 kDa heat shock protein, mitochondrial [Sesamum indicum]
c45512.graph_c0	3.51025659	2.83804627	2.77588843	2.23883815	2.70119109	2.54546714	0.0023233	-1.304344	down	hypothetical protein MIMGU_mgv1a000874mg [Erythranthe guttata]
c45515.graph_c0	20.157587	20.672009	19.7516091	14.812878	15.1477169	14.3242371	2.096E-23	-1.46916	down	PREDICTED: V-type proton ATPase subunit a3-like [Sesamum indicum]
c45519.graph_c0	1.17599104	1.37336338	0.37779876	3.38562389	5.92994121	5.34218047	0.0073797	1.307241	up	predicted protein [Arabidopsis lyrata subsp. lyrata]
c45522.graph_c1	41.8510652	41.5023623	49.743621	4.92509329	7.04558205	7.82099888	9.671E-56	-3.776068	down	PREDICTED: G-type lectin S-receptor-like serine/threonine-protein kinase At4g27290 [Sesamum indicum]
c45525.graph_c0	31.5596968	24.7218889	26.0970026	11.6519947	12.6164532	12.8535483	6.82E-30	-2.167862	down	PREDICTED: MATE efflux family protein 5 [Sesamum indicum]
c45528.graph_c0	0.47557095	0.38348244	0.54322444	14.190446	12.1063812	10.9805708	1.87E-31	3.71665	up	PREDICTED: uncharacterized protein LOC105168379 [Sesamum indicum]
c45530.graph_c0	1.90536193	1.21371718	2.00328997	71.490361	37.6986323	47.8507604	6.564E-08	3.929542	up	hypothetical protein AMTR_s00028p00129850 [Amborella trichopoda]
c45532.graph_c0	9.32956503	8.22229871	8.81789372	2.3978815	2.9361501	2.77093577	9.766E-31	-2.722668	down	PREDICTED: uncharacterized protein LOC105175116 [Sesamum indicum]
c45533.graph_c0	39.4281412	40.4786158	33.0831234	13.0264574	15.0226126	12.8168201	6.818E-66	-2.484274	down	PREDICTED: uncharacterized protein LOC105157181 [Sesamum indicum]
c45535.graph_c0	8.54727045	9.18625877	6.62868743	7.65326996	8.48821784	7.91612425	6.605E-08	-1.034142	down	PREDICTED: polypyrimidine tract-binding protein homolog 1 isoform X1 [Sesamum indicum]
c45541.graph_c0	0.11651871	0.19439231	0.14973116	205.099821	255.336224	243.43491	0	9.555873	up	PREDICTED: WAT1-related protein At1g09380 [Sesamum indicum]
c45543.graph_c0	1.50325583	0.77832471	0.46628286	17.8603999	18.9715188	17.8467169	2.18E-24	3.305812	up	PREDICTED: protein SCARECROW [Sesamum indicum]
c45543.graph_c1	69.7189926	72.7736629	75.1767135	14.1279141	25.9147576	22.930313	3.9E-54	-2.817232	down	-
c45543.graph_c2	146.990011	142.961081	135.276899	130.715073	113.453705	100.538145	2.115E-20	-1.315704	down	PREDICTED: E3 ubiquitin-protein ligase UPL5 [Sesamum indicum]

#ID	0 h-1 FPKM	0 h-2 FPKM	0 h-3 FPKM	48 h-1 FPKM	48 h-2 FPKM	48 h-3 FPKM	FDR	log2FC	regulated	nr_annotation
c45543.graph_c5	31.7486814	31.3963133	32.055817	203.328457	205.459174	214.267456	1.641E-33	1.692223	up	PREDICTED: ATP synthase subunit O, mitochondrial-like [Sesamum indicum]
c45544.graph_c0	25.022116	23.3852742	25.6524122	18.9732935	17.4379204	17.0767446	1.113E-20	-1.4856	down	PREDICTED: F-box protein FBW2 [Sesamum indicum]
c45547.graph_c1	0	0	0.29692782	6.17329538	6.99088258	5.4929611	8.004E-10	4.922946	up	-
c45550.graph_c0	55.5144229	56.6075405	62.3876542	39.5703313	42.8910868	44.5895647	1.44E-24	-1.478365	down	PREDICTED: uncharacterized protein LOC104114664 [Nicotiana tomentosiformis]
c45551.graph_c0	0.20950179	0.29958796	0.23075837	2.01278429	1.54810887	1.32482221	0.001141	1.712916	up	hypothetical protein CICLE_v10013877mg [Citrus clementina]
c45552.graph_c0	2.02343353	1.56060009	1.87554847	10.3901712	10.8128831	10.6862616	2.041E-08	1.528019	up	PREDICTED: probable anion transporter 6, chloroplastic isoform X2 [Sesamum indicum]
c45554.graph_c0	48.8193415	51.1306446	46.7025634	28.5678105	32.9111397	24.8265921	3.904E-27	-1.782382	down	PREDICTED: protein polybromo-1-like [Sesamum indicum]
c45554.graph_c1	58.8663967	59.2507585	57.8107582	40.6621541	37.2191774	34.0490321	2.952E-32	-1.667127	down	PREDICTED: vacuolar protein 8 [Sesamum indicum]
c45555.graph_c0	17.85357	16.570461	14.635761	10.9967581	9.88057481	9.44840742	5.381E-24	-1.707092	down	PREDICTED: abscisic acid 8'-hydroxylase 3-like [Solanum lycopersicum]
c45559.graph_c0	6.60479391	6.89475365	6.09275627	4.04202376	4.71944613	5.72357127	1.038E-12	-1.45645	down	PREDICTED: uncharacterized protein LOC105170176 [Sesamum indicum]
c45560.graph_c0	7.42109233	6.20120216	7.46326757	2.85361849	2.64517992	3.22681431	2.96E-11	-2.291242	down	PREDICTED: uncharacterized protein LOC105171076 [Sesamum indicum]
c45565.graph_c0	48.0017798	43.8730288	46.695901	30.3896372	33.8936269	32.8579981	1.007E-26	-1.531771	down	hypothetical protein MIMGU_mgv1a003857mg [Erythranthe guttata]
c45566.graph_c0	74.0480001	73.2147613	76.4440263	38.7107143	25.5271422	25.4193118	2.076E-57	-2.328364	down	hypothetical protein MIMGU_mgv1a020815mg [Erythranthe guttata]
c45569.graph_c0	20.3805894	19.7634663	29.4635973	2.05361716	0.6215881	0.26104231	3.735E-20	-5.558075	down	-
c45569.graph_c1	0	0.0523539	0.06720954	1.83097606	1.63342915	1.80068533	3.231E-09	4.431365	up	-
c45569.graph_c2	4.25670538	4.26096783	3.87460809	2.28757354	1.73100485	2.18085984	6.26E-05	-2.012098	down	PREDICTED: heat stress transcription factor C-1-like [Sesamum indicum]
c45569.graph_c3	74.1422938	70.1946862	68.5655201	336.390861	328.654995	318.596859	3.222E-17	1.191867	up	PREDICTED: putative glycerol-3-phosphate transporter 1 [Eucalyptus grandis]
c45573.graph_c0	18.3785319	19.2096512	16.0293046	16.1835243	17.6007073	16.0336163	5.766E-16	-1.12276	down	PREDICTED: sister chromatid cohesion 1 protein 4-like isoform X3 [Sesamum indicum]
c45574.graph_c0	1.04175747	0.82704878	0.73859288	4.07059987	5.64456441	4.65264435	0.0003681	1.442823	up	PREDICTED: probable lipid-A-disaccharide synthase, mitochondrial isoform X1 [Sesamum indicum]
c45580.graph_c1	1.45148261	0.72646802	1.17802902	31.8816739	32.2090499	32.3759799	1.747E-65	3.82773	up	PREDICTED: ABC transporter G family member 15-like isoform X2 [Sesamum indicum]
c45582.graph_c0	10.7400739	11.6225173	10.8173294	10.863846	10.1986804	9.10146139	1.048E-09	-1.152535	down	PREDICTED: aspartic proteinase-like protein 2 [Sesamum indicum]
c45584.graph_c1	13.0360801	10.9090758	13.5355183	11.865571	11.7558741	12.4814203	4.448E-07	-1.072836	down	PREDICTED: uncharacterized protein At5g43822 [Sesamum indicum]

#ID	0 h-1 FPKM	0 h-2 FPKM	0 h-3 FPKM	48 h-1 FPKM	48 h-2 FPKM	48 h-3 FPKM	FDR	log2FC	regulated	nr_annotation
c45588.graph_c0	9.91802783	11.5826191	10.0898379	52.049032	48.8593658	49.4101221	1.521E-12	1.234632	up	PREDICTED: probable receptor-like protein kinase At5g56460 [Sesamum indicum]
c45590.graph_c0	1.87274859	1.79311848	2.5635054	21.341188	21.1779925	20.1239442	1.023E-22	2.309346	up	hypothetical protein MIMGU_mgv1a014871mg [Erythranthe guttata]
c45591.graph_c0	0	0	0	2.86643917	4.17538651	1.99260964	8.523E-08	Inf	up	hypothetical protein H632_c597p1 [Helicosporidium sp. ATCC 50920]
c45594.graph_c0	0.13529975	0.47402332	0.43466384	7.85275819	9.57350429	4.10367412	1.138E-05	3.344402	up	hypothetical protein MIMGU_mgv1a011617mg [Erythranthe guttata]
c45596.graph_c0	46.0805474	47.9573992	43.8239507	36.6709498	29.8187909	26.4417268	1.73E-29	-1.580574	down	PREDICTED: scarecrow-like protein 13 [Sesamum indicum]
c45602.graph_c0	1.74774985	0.78899018	2.28996556	6.53527883	7.09056581	9.12991226	0.0003196	1.21021	up	PREDICTED: probable alpha-mannosidase I MNS5 isoform X1 [Sesamum indicum]
c45604.graph_c0	6.03702796	6.08670543	5.09719577	453.630241	461.635418	429.685503	1.3E-205	5.271864	up	PREDICTED: glucose-6-phosphate 1-dehydrogenase, chloroplastic-like [Sesamum indicum]
c45606.graph_c0	1.02930215	1.03033284	1.20767714	0.12368612	0.48044498	0.14674022	5.104E-09	-3.158081	down	hypothetical protein VITISV_002159 [Vitis vinifera]
c45611.graph_c0	12.4630261	12.1824571	14.2419554	62.841404	62.0013216	54.1674601	4.834E-15	1.18496	up	PREDICTED: AP-4 complex subunit mu [Sesamum indicum]
c45616.graph_c0	2.02221232	2.14331003	3.21006357	1.09587876	1.33509326	1.02386164	8.807E-06	-2.118572	down	PREDICTED: la-related protein 6C-like isoform X1 [Sesamum indicum]
c45617.graph_c0	20.7012453	19.3619831	17.5508348	17.8855548	15.8768827	12.3388348	5.643E-17	-1.333188	down	PREDICTED: uncharacterized protein LOC105158248 [Sesamum indicum]
c45622.graph_c0	0.24131189	0.56362491	0.10336511	2.18607052	2.43363308	3.2309311	9.444E-05	2.099088	up	PREDICTED: LOW QUALITY PROTEIN: beta-adaptin-like protein A [Sesamum indicum]
c45623.graph_c0	2.73168274	2.11783363	1.27335155	0	0	0	1.449E-18	-Inf	down	--
c45625.graph_c0	19.6003543	20.1151225	20.7377519	13.8039219	12.6925445	12.2319618	6.012E-29	-1.658412	down	PREDICTED: protein VACUOLELESS1 [Sesamum indicum]
c45627.graph_c0	2.71028197	1.84096151	1.74140982	0.35669836	0.66128766	0.39673507	3.596E-09	-3.175322	down	-
c45628.graph_c0	26.5138553	27.8998425	21.3986359	13.6760292	15.9970588	16.2493949	9.339E-24	-1.741291	down	PREDICTED: E3 ubiquitin-protein ligase UPL2-like [Sesamum indicum]
c45632.graph_c0	0	0.04848218	0.1867176	2.0079121	2.74164633	2.66009074	1.598E-09	3.93107	up	PREDICTED: probable protein phosphatase 2C 14 [Sesamum indicum]
c45632.graph_c1	0.34123575	0.38176303	0.23214783	4.19774582	3.93765437	4.57429894	1.29E-12	2.720921	up	PREDICTED: protein-tyrosine-phosphatase MKP1-like isoform X1 [Sesamum indicum]
c45633.graph_c0	0	0	0	2.58974852	2.28627044	2.04830458	2.144E-11	Inf	up	-
c45637.graph_c0	56.9376088	54.8948213	57.735979	58.8635705	55.1515151	53.6924423	3.914E-13	-1.032138	down	PREDICTED: activator of 90 kDa heat shock protein ATPase homolog [Sesamum indicum]
c45639.graph_c0	15.0454639	15.0247564	14.6956897	60.0856873	64.874252	60.9634745	1.983E-12	1.036063	up	PREDICTED: dolichyl-diphosphooligosaccharide--protein glycosyltransferase subunit 1A [Sesamum indicum]
c45641.graph_c0	26.0770213	29.771682	27.8948148	16.232333	14.4447984	12.8257679	6.076E-15	-1.959223	down	PREDICTED: F-box protein PP2-A15-like [Nicotiana sylvestris]

#ID	0 h-1 FPKM	0 h-2 FPKM	0 h-3 FPKM	48 h-1 FPKM	48 h-2 FPKM	48 h-3 FPKM	FDR	log2FC	regulated	nr_annotation
c45642.graph_c0	95.6197768	98.4392499	91.0526769	45.729423	49.464104	49.5216527	8.117E-44	-1.996596	down	PREDICTED: uncharacterized protein LOC105169167 [Sesamum indicum]
c45643.graph_c0	0	0	0	11.5908989	34.7473873	5.48591071	0.0008552	Inf	up	hypothetical protein SELMODRAFT_171412 [Selaginella moellendorffii]
c45653.graph_c0	3.32709187	2.65350812	2.50269939	1.66962586	1.66316817	2.01778653	2.738E-07	-1.681553	down	PREDICTED: integrator complex subunit 9 homolog isoform X2 [Sesamum indicum]
c45655.graph_c0	205.455409	191.938992	199.265751	120.464539	135.091124	136.809113	2.553E-27	-1.624325	down	hypothetical protein MIMGU_mgv1a018120mg, partial [Erythranthe guttata]
c45660.graph_c0	30.8114009	29.6885536	28.3871262	23.8905158	25.6090644	22.5803348	1.505E-18	-1.319452	down	PREDICTED: uncharacterized protein LOC105168018 [Sesamum indicum]
c45661.graph_c0	0.12640267	0.06326462	0.08121622	10.3058592	9.93088766	12.019555	1.718E-60	5.880279	up	PREDICTED: chaperonin 60 subunit beta 4, chloroplastic isoform X1 [Sesamum indicum]
c45662.graph_c1	399.967544	413.856287	388.466041	227.451003	226.672128	209.564263	8.91E-29	-1.873506	down	PREDICTED: glucan endo-1,3-beta-glucosidase-like [Sesamum indicum]
c45663.graph_c0	31.8507435	34.345873	32.695842	20.3604368	17.4458495	16.6132214	4.861E-39	-1.875667	down	PREDICTED: uncharacterized protein LOC105167456 isoform X2 [Sesamum indicum]
c45665.graph_c0	3.44833649	3.29488996	2.25590854	3.1768325	2.41701852	2.41556624	0.0001732	-1.175834	down	hypothetical protein MIMGU_mgv1a015231mg [Erythranthe guttata]
c45667.graph_c0	21.9553657	21.9371727	19.3935674	14.8095052	17.8042289	16.63216	9.194E-22	-1.380963	down	hypothetical protein MIMGU_mgv1a002103mg [Erythranthe guttata]
c45668.graph_c0	128.196915	135.435528	121.037185	925.912411	863.372926	823.073666	2.205E-30	1.748829	up	triosephosphate isomerase, putative [Ricinus communis]
c45672.graph_c0	1.36223194	1.16473826	1.45876831	8.88941981	9.55575154	8.77051945	3.241E-10	1.751981	up	PREDICTED: alpha-1,3-mannosyl-glycoprotein 2-beta-N-acetylglucosaminyltransferase isoform X1 [Sesamum indicum]
c45673.graph_c0	66.7131895	59.4534997	68.8247622	46.2434764	26.7631506	18.749325	2.505E-20	-2.091419	down	PREDICTED: probable galactinol--sucrose galactosyltransferase 2-like isoform X1 [Citrus sinensis]
c45673.graph_c1	0	0.16789978	0	12.8256161	11.4590718	15.2620401	3.122E-15	6.873867	up	hypothetical protein MIMGU_mgv1a001872mg [Erythranthe guttata]
c45681.graph_c1	2.10616574	2.55506145	2.04332269	25.429983	28.4237926	30.6539579	5.722E-63	2.636866	up	PREDICTED: uncharacterized protein LOC105164103 [Sesamum indicum]
c45682.graph_c0	21.3627598	23.4535854	21.6121612	10.6382528	13.2149122	14.8552828	7.6E-35	-1.801194	down	PREDICTED: GTPase-activating protein gyp7 isoform X3 [Sesamum indicum]
c45686.graph_c0	6.80864701	7.10318894	6.02529929	5.04784634	6.53982871	6.98030011	1.783E-10	-1.123833	down	PREDICTED: F-box/WD-40 repeat-containing protein At5g21040-like [Sesamum indicum]
c45690.graph_c0	0.63286126	0.72087359	0.4767338	10.5348044	8.96661534	6.33268546	7.183E-09	2.810835	up	PREDICTED: U-box domain-containing protein 35 [Sesamum indicum]
c45693.graph_c1	20.7942628	21.3227701	17.4785544	9.85461472	11.3847485	12.2457712	1.252E-25	-1.850432	down	PREDICTED: abl interactor 2-like isoform X1 [Sesamum indicum]

#ID	0 h-1 FPKM	0 h-2 FPKM	0 h-3 FPKM	48 h-1 FPKM	48 h-2 FPKM	48 h-3 FPKM	FDR	log2FC	regulated	nr_annotation
c45697.graph_c0	13.8478987	13.023352	12.0547438	5.812933	5.12266531	6.11065277	2.481E-35	-2.206421	down	PREDICTED: uncharacterized protein LOC105177954 [Sesamum indicum]
c45699.graph_c0	0	0	0	23.9949124	50.2382588	12.9112995	4.248E-07	Inf	up	60s ribosomal protein l9 [Pfiesteria piscicida]
c45700.graph_c0	4.27360591	4.97835772	2.8903962	18.2120789	15.7811473	16.4508389	1.561E-06	1.044135	up	hypothetical protein MIMGU_mgv1a010620mg [Erythranthe guttata]
c45702.graph_c0	43.5171089	44.6480028	47.3716316	23.2664232	21.0037674	20.9805805	2.446E-37	-2.070841	down	PREDICTED: uncharacterized protein LOC105178309 [Sesamum indicum]
c45702.graph_c1	12.4147356	12.8182317	14.0568655	1.03975129	1.31331549	1.45891316	7.174E-67	-4.389284	down	PREDICTED: uncharacterized protein LOC105162982 [Sesamum indicum]
c45711.graph_c0	0.19728714	0.19748469	0.38028268	4.36209077	3.56210272	4.20480898	1.037E-06	2.945489	up	hypothetical protein VITISV_029424 [Vitis vinifera]
c45712.graph_c0	98.326615	99.7086369	95.5472928	56.343768	58.5467492	65.3376511	4.649E-31	-1.72258	down	PREDICTED: plastidial pyruvate kinase 2-like [Sesamum indicum]
c45713.graph_c0	1.42197674	1.49457067	2.13184582	18.2747473	17.0237405	18.2812131	3.425E-33	2.386752	up	PREDICTED: probable sodium/metabolite cotransporter BASS2, chloroplastic isoform X2 [Sesamum indicum]
c45717.graph_c0	10.7116447	10.1326404	10.8742611	45.2953398	51.2257318	52.2453049	6.434E-13	1.209373	up	chloroplast monodehydroascorbate reductase [Avicennia marina]
c45718.graph_c1	10.5754309	9.94873506	9.97649331	6.37027708	6.29978437	6.33508533	2.221E-25	-1.699287	down	PREDICTED: DNA mismatch repair protein MSH3 isoform X1 [Sesamum indicum]
c45720.graph_c0	1.18509867	1.54217098	0.91373923	7.8062999	10.2649965	7.99032719	1.372E-09	1.82223	up	PREDICTED: serine/threonine-protein kinase BLUS1-like isoform X4 [Sesamum indicum]
c45721.graph_c0	9.089999	7.71155503	9.00910634	4.71514837	6.34797512	6.70844675	1.313E-15	-1.562161	down	hypothetical protein MIMGU_mgv1a008794mg [Erythranthe guttata]
c45722.graph_c0	1.97190877	2.20163911	1.31572082	18.5508013	19.6893347	15.8846818	8.268E-20	2.289018	up	hypothetical protein MIMGU_mgv1a007079mg [Erythranthe guttata]
c45729.graph_c0	9.08714367	3.88254277	4.12979076	0	0	0	1.102E-09	-Inf	down	chaperone [Agave tequilana]
c45742.graph_c0	0.60578347	0.92081455	0.86495083	11.2650944	13.0945559	18.5206946	9.555E-13	3.139427	up	PREDICTED: DNA replication licensing factor MCM5 [Sesamum indicum]
c45746.graph_c0	1.89722416	1.76347223	1.69789854	24.4074299	30.6180508	30.326857	2.111E-47	2.972858	up	PREDICTED: LOW QUALITY PROTEIN: uncharacterized protein LOC105161466 [Sesamum indicum]
c45747.graph_c0	7.71565169	6.6859091	6.75792911	29.1399261	34.3918209	30.8373306	8.338E-11	1.138149	up	hypothetical protein CISIN_1g034493mg [Citrus sinensis]
c45752.graph_c0	0	0	0	23.2272088	52.4981472	16.0496397	4.27E-08	Inf	up	PREDICTED: 60S ribosomal protein L6-3-like isoform X2 [Cicer arietinum]
c45754.graph_c1	5.05019401	4.27204311	3.19914752	2.75222339	3.60984862	3.73167516	0.0006341	-1.329452	down	PREDICTED: uncharacterized protein LOC105170987 isoform X2 [Sesamum indicum]
c45758.graph_c0	1.19273995	0.93126875	1.103557	0.54941492	0.69844618	0.95817754	0.000433	-1.573129	down	PREDICTED: pentatricopeptide repeat-containing protein At1g74630 [Sesamum indicum]
c45764.graph_c0	1.22255414	0.99834549	1.36431593	4.38662891	5.52628277	6.69872831	0.0005339	1.186977	up	PREDICTED: tRNA pseudouridine(38/39) synthase isoform X3 [Sesamum indicum]

#ID	0 h-1 FPKM	0 h-2 FPKM	0 h-3 FPKM	48 h-1 FPKM	48 h-2 FPKM	48 h-3 FPKM	FDR	log2FC	regulated	nr_annotation
c45766.graph_c0	60.1959704	53.9878771	53.1341736	30.3723773	35.1717402	35.4799569	5.153E-35	-1.747194	down	PREDICTED: uncharacterized protein DDB_G0286299-like [Sesamum indicum]
c45770.graph_c0	30.594743	27.7693044	28.625186	13.0161727	16.5737081	17.4711961	1.975E-35	-1.908205	down	unnamed protein product [Coffea canephora]
c45772.graph_c0	11.7065894	10.960068	11.5922908	6.02684118	7.1911968	8.29799068	3.015E-10	-1.692982	down	PREDICTED: diphthamide biosynthesis protein 3-like [Sesamum indicum]
c45779.graph_c0	119.360801	115.23231	119.872952	25.1568349	26.2441797	32.0404162	8.956E-91	-3.106786	down	hypothetical protein MIMGU_mgv1a025324mg, partial [Erythranthe guttata]
c45783.graph_c0	9.67954925	10.0095474	9.73158	2.23549951	2.91475774	2.27329603	2.074E-48	-3.007308	down	PREDICTED: uncharacterized protein LOC105166430 [Sesamum indicum]
c45785.graph_c0	0.30562976	0.24474864	0.47129561	19.062023	22.1029267	19.5417922	2.407E-68	4.867501	up	hypothetical protein EUTSA_v10017458mg [Eutrema salsugineum]
c45789.graph_c0	0.04423468	0	0	1.01879691	1.77003853	0.90651936	2.097E-06	5.381877	up	--
c45791.graph_c0	169.044855	171.633505	169.544844	1178.49199	882.843715	715.49988	0.0003214	1.434435	up	PREDICTED: telomere repeat-binding protein 3-like [Sesamum indicum]
c45791.graph_c2	31.9947119	34.0797465	30.2208936	27.0193142	25.7546462	23.7614179	6.237E-16	-1.346329	down	PREDICTED: uncharacterized protein LOC105178732 [Sesamum indicum]
c45793.graph_c0	23.6009353	21.3390718	23.6816938	18.5446307	23.6022309	23.2234902	1.085E-13	-1.09224	down	PREDICTED: probable inactive receptor kinase At2g26730 [Sesamum indicum]
c45800.graph_c0	332.182346	330.019441	328.140203	121.447703	113.585584	99.6645938	3.008E-59	-2.580039	down	PREDICTED: uncharacterized protein LOC105158790 isoform X1 [Sesamum indicum]
c45801.graph_c0	60.3363111	58.3350719	63.5450575	27.8651558	28.7731451	25.887639	1.048E-50	-2.160679	down	PREDICTED: protein CPR-5 [Sesamum indicum]
c45803.graph_c0	0.03215615	0	0	1.51084141	2.5734386	2.60959652	3.998E-14	6.6961	up	unnamed protein product [Vitis vinifera]
c45804.graph_c1	0.61422249	0.65582671	0.47358013	3.81013625	3.67670016	2.51750222	0.001682	1.509443	up	hypothetical protein CICLE_v10021726mg [Citrus clementina]
c45804.graph_c2	38.3731817	40.2275865	35.2861276	18.1037983	18.6546913	19.4636284	1.296E-46	-2.035632	down	PREDICTED: putative SWI/SNF-related matrix-associated actin-dependent regulator of chromatin subfamily A member 3-like 2 isoform X2 [Sesamum indicum]
c45806.graph_c0	0.59181134	0.52658129	0.63375084	8.87489206	8.69592939	9.8912457	5.86E-18	2.950981	up	unnamed protein product [Coffea canephora]
c45809.graph_c0	0.19366876	0.12924179	0	2.43841064	2.45719062	4.02185164	9.671E-08	3.78325	up	PREDICTED: uncharacterized protein LOC105176285 [Sesamum indicum]
c45813.graph_c1	5.60474374	6.2478965	4.71970055	4.43978939	2.56926826	3.11515033	2.891E-14	-1.716708	down	hypothetical protein MIMGU_mgv1a004624mg [Erythranthe guttata]
c45817.graph_c0	2.58666228	2.58925243	2.86862599	11.7517907	11.9654463	12.6351462	4.134E-06	1.156638	up	PREDICTED: LOW QUALITY PROTEIN: actin-related protein 2 [Tarenaya hassleriana]
c45818.graph_c0	105.119902	102.011447	102.794686	22.8116648	23.4344525	23.3549418	1.498E-96	-3.172522	down	PREDICTED: uncharacterized protein LOC101291143 [Fragaria vesca subsp. vesca]
c45819.graph_c1	20.2784516	20.6903733	17.5958481	18.2012707	18.9638538	16.5695319	1.284E-09	-1.139795	down	-

#ID	0 h-1 FPKM	0 h-2 FPKM	0 h-3 FPKM	48 h-1 FPKM	48 h-2 FPKM	48 h-3 FPKM	FDR	log2FC	regulated	nr_annotation
c45819.graph_c2	1.674902	1.67657916	1.78682786	18.3416651	16.1614659	14.7141782	3.138E-21	2.244936	up	PREDICTED: uncharacterized protein LOC105166519 [Sesamum indicum]
c45820.graph_c2	44.53843	46.8490689	50.6713957	22.3103843	21.3743937	20.3695148	7.913E-45	-2.166546	down	PREDICTED: uncharacterized protein LOC105161926 [Sesamum indicum]
c45822.graph_c0	40.7677868	36.5746038	39.4935479	19.8982486	18.049563	17.8528865	9.323E-42	-2.081594	down	hypothetical protein EUGRSUZ_K00825 [Eucalyptus grandis]
c45823.graph_c0	4.58625593	5.09395504	5.00545572	21.0389722	21.1232425	21.4498656	2.467E-09	1.096748	up	PREDICTED: cysteine--tRNA ligase-like [Sesamum indicum]
c45829.graph_c0	48.0823459	50.3227045	47.5484003	29.4206469	37.292665	39.7095827	3.649E-25	-1.477657	down	PREDICTED: nucleobase-ascorbate transporter 3 [Sesamum indicum]
c45829.graph_c3	0.39198713	0.50448811	0.21587956	3.04376034	4.09893433	4.49856484	9.707E-06	2.370441	up	PREDICTED: uncharacterized protein LOC105167094 [Sesamum indicum]
c45831.graph_c0	128.125325	127.479651	134.797021	55.560982	36.0958449	29.446142	2.247E-75	-2.696124	down	PREDICTED: uncharacterized protein LOC105166052 [Sesamum indicum]
c45832.graph_c1	52.6036305	56.1343713	57.7678654	31.1912968	25.5251697	23.0729917	7.172E-48	-2.074646	down	PREDICTED: probable mitochondrial chaperone BCS1-B [Sesamum indicum]
c45833.graph_c0	0	0	0	25.3918345	42.7727393	14.0446091	6.046E-09	Inf	up	60S ribosomal protein L35a-4 [Auxenochlorella protothecoides]
c45835.graph_c1	21.184085	19.9350968	19.7939826	88.3098159	94.2243615	90.3952358	2.773E-16	1.145863	up	PREDICTED: putative aconitate hydratase, cytoplasmic [Sesamum indicum]
c45837.graph_c0	1.50607729	0.89512883	1.02816475	0	0	0	3.592E-19	-Inf	down	hypothetical protein EMIHUDRAFT_61288, partial [Emiliania huxleyi CCMP1516]
c45838.graph_c0	33.8427139	36.1916321	35.2916565	19.1045856	18.7530436	17.0623392	6.756E-43	-1.955975	down	PREDICTED: uncharacterized protein LOC105173340 isoform X4 [Sesamum indicum]
c45839.graph_c0	2.71880769	2.93212476	2.16281342	1.4312817	2.06907597	2.33483406	3.037E-08	-1.443436	down	-
c45840.graph_c0	1.72307764	1.14986869	1.19937078	8.56540792	8.33828046	8.03341865	8.37E-07	1.600354	up	PREDICTED: uncharacterized protein LOC101257656 isoform X1 [Solanum lycopersicum]
c45844.graph_c0	113.01763	106.896536	109.187362	32.0101329	30.2775485	26.3136138	1.22E-84	-2.908203	down	PREDICTED: LOW QUALITY PROTEIN: uncharacterized protein LOC105169583 [Sesamum indicum]
c45845.graph_c0	60.6352427	58.4281516	57.5669965	23.2344539	22.4238501	22.4784616	3.841E-62	-2.390544	down	hypothetical protein MIMGU_mgv1a000962mg [Erythranthe guttata]
c45846.graph_c0	0	0	0	6.18919501	7.3280783	3.56342315	3.493E-09	Inf	up	--
c45850.graph_c0	126.036413	122.193321	116.101354	18.0969867	21.3149676	22.5036195	8.18E-125	-3.576995	down	PREDICTED: uncharacterized protein LOC105163078 [Sesamum indicum]
c45850.graph_c1	2.63649726	2.32601932	2.87118929	2.09956907	2.74758916	2.71069826	0.0077932	-1.075461	down	hypothetical protein MIMGU_mgv1a017816mg, partial [Erythranthe guttata]
c45851.graph_c0	2.19023645	2.01602725	2.26457286	0.27831568	0.61425357	0.5159242	2.182E-15	-3.230214	down	-
c45857.graph_c0	0.51056181	0.70763963	0.45421764	2.2794507	2.75977328	3.28381961	0.0076216	1.295407	up	PREDICTED: prolyl 3-hydroxylase 1 isoform X1 [Sesamum indicum]

#ID	0 h-1 FPKM	0 h-2 FPKM	0 h-3 FPKM	48 h-1 FPKM	48 h-2 FPKM	48 h-3 FPKM	FDR	log2FC	regulated	nr_annotation
c45860.graph_c0	12.606571	12.7884175	11.7930619	12.8376923	12.2564797	11.3041191	5.908E-11	-1.04607	down	PREDICTED: serine/threonine-protein kinase svkA-like isoform X2 [Sesamum indicum]
c45862.graph_c0	10.0745487	9.97885396	9.59647946	9.2651053	10.3481329	8.09965609	1.453E-12	-1.114777	down	PREDICTED: uncharacterized protein LOC105160721 [Sesamum indicum]
c45863.graph_c0	39.592594	38.1032184	37.1378365	29.3828644	33.930257	32.2551423	2.03E-19	-1.284169	down	hypothetical protein MIMGU_mgv1a004300mg [Erythranthe guttata]
c45865.graph_c0	0.05673674	0.02839677	0	2.35213118	2.82403748	3.67422361	3.003E-16	5.6952	up	PREDICTED: type I inositol 1,4,5-trisphosphate 5-phosphatase CVP2-like [Sesamum indicum]
c45867.graph_c0	50.7797155	52.3265368	48.8047738	29.0626488	31.8347124	28.7094049	5.948E-36	-1.779405	down	PREDICTED: uncharacterized protein LOC105177822 isoform X2 [Sesamum indicum]
c45870.graph_c0	0.07286516	0.40115969	0.09363462	2.34948487	4.01794799	5.19652866	3.769E-06	3.325787	up	PREDICTED: kinesin-4-like isoform X2 [Sesamum indicum]
c45871.graph_c1	1.94439037	1.9014219	1.94123445	20.3656943	16.8892125	14.9702798	5.578E-13	2.161087	up	PREDICTED: pentatricopeptide repeat-containing protein At2g03880, mitochondrial [Sesamum indicum]
c45873.graph_c0	18.2166288	20.3047742	18.0692862	4.60834167	6.95778139	6.53815849	5.392E-66	-2.668164	down	hypothetical protein PHAVU_004G031700g [Phaseolus vulgaris]
c45879.graph_c0	26.6741422	25.1693099	26.1531567	16.1166878	20.9617414	18.6947528	6.461E-24	-1.505376	down	PREDICTED: oral cancer-overexpressed protein 1 homolog [Sesamum indicum]
c45882.graph_c0	7.03864343	6.50716737	5.87632751	5.08018002	6.7382321	6.90909726	5.095E-05	-1.073688	down	PREDICTED: auxin-binding protein T92-like [Sesamum indicum]
c45884.graph_c1	0.28737606	0.14383191	0.46161201	7.08205095	5.81975384	7.83278807	6.675E-11	3.513146	up	hypothetical protein MIMGU_mgv1a007716mg [Erythranthe guttata]
c45885.graph_c0	2.70944439	2.32967374	2.36579995	1.31206145	2.84774343	2.64815092	0.0019624	-1.150253	down	PREDICTED: ADP,ATP carrier protein ER-ANT1 [Sesamum indicum]
c45895.graph_c1	17.2213579	15.8637446	15.8848149	7.10538029	12.476739	13.0776852	4.508E-09	-1.611258	down	hypothetical protein MIMGU_mgv1a014929mg [Erythranthe guttata]
c45898.graph_c0	0.90639556	1.20973757	1.39770502	6.17922725	10.8512624	10.1544058	1.434E-06	1.921258	up	PREDICTED: uncharacterized protein LOC105158788 [Sesamum indicum]
c45900.graph_c0	2.3085226	2.05407488	1.75795137	20.2436196	21.3622142	19.4144137	2.963E-11	2.302286	up	-
c45902.graph_c1	1.16714335	0.89870159	1.09602627	0	0	0	1.861E-19	-Inf	down	--
c45904.graph_c0	16.3871601	15.1170149	19.4065299	3.25619858	5.01752373	4.87280904	9.908E-16	-2.980497	down	-
c45905.graph_c0	0.79129968	0.77277273	1.24006271	4.96073487	5.08577137	3.60716598	3.293E-05	1.262918	up	PREDICTED: uncharacterized protein LOC105156411 [Sesamum indicum]
c45908.graph_c0	0	0	0.08585955	5.84763662	9.32489745	11.5017926	4.554E-15	7.219894	up	PREDICTED: chlorophyll a-b binding protein CP24 10A, chloroplastic [Cucumis melo]
c45909.graph_c0	2.62817017	3.34266593	2.86077407	11.1946826	11.6481725	14.3965038	4.722E-06	1.056315	up	hypothetical protein MIMGU_mgv1a014735mg [Erythranthe guttata]
c45916.graph_c0	749.525642	742.087625	782.110347	52.3614647	59.2546007	61.224795	1.49E-153	-4.738052	down	PREDICTED: translocator protein homolog [Sesamum indicum]
c45917.graph_c1	104.635673	113.471287	106.383371	98.3583818	91.5310238	88.2215608	7.509E-18	-1.238083	down	PREDICTED: signal recognition particle receptor subunit alpha homolog [Sesamum indicum]

#ID	0 h-1 FPKM	0 h-2 FPKM	0 h-3 FPKM	48 h-1 FPKM	48 h-2 FPKM	48 h-3 FPKM	FDR	log2FC	regulated	nr_annotation
c45918.graph_c0	40.7104172	37.6025027	37.5451745	31.9322697	34.3597034	35.5536169	9.701E-15	-1.204467	down	PREDICTED: E3 ubiquitin-protein ligase At3g02290-like [Sesamum indicum]
c45918.graph_c1	15.5233174	16.0421446	17.4444726	9.26386559	9.62993088	8.44900519	1.946E-17	-1.860263	down	-
c45918.graph_c4	33.7718362	36.3636482	33.8041943	23.2192101	21.7615949	18.6887528	3.133E-28	-1.72175	down	PREDICTED: branchpoint-bridging protein [Sesamum indicum]
c45921.graph_c0	1.02637137	0.68493275	1.09910654	0.55157682	0.22260143	0.32719316	2.218E-06	-2.356059	down	PREDICTED: serine/threonine-protein kinase CTR1-like [Sesamum indicum]
c45923.graph_c0	4.52820123	2.98442488	3.6008155	20.8377198	22.5782345	22.8228612	9.664E-20	1.55746	up	PREDICTED: alpha-glucosidase 2 [Sesamum indicum]
c45925.graph_c2	0.91159577	1.3687629	1.03627089	7.97830412	7.04907464	8.90973514	3.595E-08	1.834868	up	PREDICTED: protein LATERAL ROOT PRIMORDIUM 1-like [Sesamum indicum]
c45928.graph_c0	0.56570624	0.52432659	0.64618215	3.26210992	5.11214484	5.34148166	1.902E-08	1.954339	up	hypothetical protein MIMGU_mgv1a002000mg [Erythranthe guttata]
c45931.graph_c1	18.3572667	18.1581854	20.3328382	10.2739028	13.2515677	10.8037764	4.094E-25	-1.749666	down	PREDICTED: uncharacterized protein LOC105168924 isoform X1 [Sesamum indicum]
c45932.graph_c0	23.3356479	22.4040867	24.0935145	13.7913757	12.5691467	12.7527178	2.426E-30	-1.852473	down	PREDICTED: uncharacterized protein LOC105174716 [Sesamum indicum]
c45933.graph_c0	26.5326757	25.6508661	27.2097947	20.1839106	17.2071432	14.0275559	1.305E-24	-1.639748	down	PREDICTED: N-acylphosphatidylethanolamine synthase isoform X3 [Sesamum indicum]
c45935.graph_c0	7.12576487	6.12941238	6.16262177	48.4990485	52.9654445	56.5024124	9.3E-40	2.005618	up	PREDICTED: 4-hydroxy-tetrahydrodipicolinate reductase 2, chloroplastic-like [Sesamum indicum]
c45939.graph_c0	66.0537789	60.5230487	66.2147865	26.0329851	21.7206385	22.242826	4.913E-66	-2.476055	down	PREDICTED: pentatricopeptide repeat-containing protein At5g65560-like isoform X1 [Sesamum indicum]
c45940.graph_c0	51.6757703	50.202837	47.9302893	59.2093932	44.6327673	46.8371336	7.748E-13	-1.003069	down	hypothetical protein MIMGU_mgv1a013847mg [Erythranthe guttata]
c45941.graph_c0	6.12962639	4.95454763	5.77071296	4.80146978	5.05458399	3.68118047	1.336E-07	-1.332488	down	PREDICTED: exonuclease V, chloroplastic-like [Sesamum indicum]
c45944.graph_c0	33.8870908	35.6901473	32.6350039	15.9280604	14.1365354	14.7081052	6.996E-47	-2.205653	down	PREDICTED: probable E3 ubiquitin-protein ligase LUL4 isoform X2 [Sesamum indicum]
c45947.graph_c0	28.3022102	36.5447339	26.6852847	195.507531	229.737203	200.621233	6.102E-35	1.755615	up	hypothetical protein JCGZ_17777 [Jatropha curcas]
c45950.graph_c0	2.59269447	3.89293599	2.57450708	1.737134	1.55273864	1.88381138	1.842E-05	-1.823432	down	Gag-pol polyprotein, putative [Solanum demissum]
c45951.graph_c0	0.80840209	0.35964959	0.46170164	15.515682	15.0343611	19.4016955	9.176E-32	3.922695	up	PREDICTED: probable ribose-5-phosphate isomerase 4, chloroplastic [Sesamum indicum]
c45956.graph_c0	1.51541035	1.3555525	1.61589637	7.69337378	7.77258136	6.63407316	3.416E-05	1.282698	up	PREDICTED: rop guanine nucleotide exchange factor 12-like [Sesamum indicum]
c45957.graph_c1	53.6807718	50.5769009	52.5207238	14.8842823	20.5868641	25.5210144	1.323E-60	-2.386777	down	hypothetical protein MIMGU_mgv1a008606mg [Erythranthe guttata]
c45958.graph_c0	2.32126634	1.93632561	1.41497447	8.22500751	9.29429134	12.0024421	1.104E-06	1.362497	up	unnamed protein product [Vitis vinifera]

#ID	0 h-1 FPKM	0 h-2 FPKM	0 h-3 FPKM	48 h-1 FPKM	48 h-2 FPKM	48 h-3 FPKM	FDR	log2FC	regulated	nr_annotation
c45960.graph_c0	24.532705	23.6030113	24.6538193	5.76349348	5.72774919	4.48117647	9.53E-102	-3.203819	down	PREDICTED: F-box/LRR-repeat protein 3 isoform X1 [Sesamum indicum]
c45961.graph_c1	28.8907065	29.7745868	28.4169092	13.9922176	16.2183824	13.6982543	1.293E-44	-2.006599	down	PREDICTED: digalactosyldiacylglycerol synthase 1, chloroplastic [Sesamum indicum]
c45963.graph_c0	65.9154636	64.7431545	62.5543002	26.8013312	27.3665163	23.7800584	5.863E-59	-2.325874	down	PREDICTED: protein MEI2-like 2 [Sesamum indicum]
c45964.graph_c0	5.35484837	5.60760477	4.97563126	27.8791058	33.0722437	34.9364223	1.328E-21	1.56881	up	PREDICTED: probable inactive receptor kinase At4g23740 [Sesamum indicum]
c45964.graph_c5	179.862678	185.174559	169.028982	123.326369	83.2390912	70.8840304	9.48E-41	-1.952009	down	PREDICTED: uncharacterized protein LOC105172390 isoform X1 [Sesamum indicum]
c45964.graph_c6	32.6480749	30.9616051	31.9690862	8.92494815	6.41175687	5.72707748	2.48E-103	-3.19163	down	hypothetical protein MIMGU_mgv1a000896mg [Erythranthe guttata]
c45965.graph_c0	1.27235411	1.62741379	1.36252117	7.27725683	6.82977851	7.17058923	5.415E-08	1.30335	up	PREDICTED: LOW QUALITY PROTEIN: WD repeat-containing protein 76 [Sesamum indicum]
c45969.graph_c0	14.497262	7.60140798	9.31477785	0	0	0	1.376E-21	-Inf	down	--
c45970.graph_c0	5.58367768	3.9088351	5.01798311	20.7106772	20.3020409	20.283026	7.388E-08	1.061606	up	PREDICTED: magnesium/proton exchanger-like isoform X3 [Sesamum indicum]
c45973.graph_c0	9.00342193	10.1389922	9.50711314	4.89513809	6.51826908	6.45787747	4.761E-24	-1.703614	down	PREDICTED: F-box/kelch-repeat protein At1g55270-like [Sesamum indicum]
c45974.graph_c1	5.67069907	2.73307061	4.04837379	3.4828119	3.89459562	2.41032174	0.0058107	-1.362154	down	hypothetical protein POPTR_0012s15190g, partial [Populus trichocarpa]
c45974.graph_c3	3.96752017	2.69761791	3.55927469	2.5861828	2.55709728	2.57731029	3.659E-05	-1.422627	down	hypothetical protein MIMGU_mgv1a004105mg [Erythranthe guttata]
c45979.graph_c0	1.15627696	1.3268155	0.83353216	4.7805792	5.2295889	5.45587188	0.0006337	1.205666	up	PREDICTED: uncharacterized protein LOC105161942 [Sesamum indicum]
c45981.graph_c1	44.5813044	46.6543979	41.9212747	33.0252458	32.4235568	31.3743447	1.001E-25	-1.475667	down	PREDICTED: DEAD-box ATP-dependent RNA helicase 51-like [Sesamum indicum]
c45983.graph_c1	4.4683854	4.10152806	4.07360681	17.1963806	19.2542473	19.2958677	3.502E-12	1.121588	up	PREDICTED: epidermal growth factor receptor substrate 15-like [Sesamum indicum]
c45984.graph_c0	2.60928811	2.68724421	3.09511249	1.66420135	2.44863825	2.59139362	2.17E-05	-1.35041	down	PREDICTED: uncharacterized protein LOC105157708 [Sesamum indicum]
c45985.graph_c0	12.0303205	11.738667	10.8826308	4.27769386	5.1625309	4.98491123	1.538E-49	-2.284051	down	PREDICTED: uncharacterized protein LOC105175876 isoform X1 [Sesamum indicum]
c45987.graph_c0	2.70987778	2.53997187	2.05772287	40.2619971	48.2788215	53.6161579	1.022E-53	3.263031	up	hypothetical protein MIMGU_mgv1a004935mg [Erythranthe guttata]
c45991.graph_c0	0.54265933	0.50698921	0.46489248	6.13251355	5.36680154	5.9015112	1.522E-09	2.508045	up	PREDICTED: uncharacterized protein LOC105179859 isoform X3 [Sesamum indicum]

#ID	0 h-1 FPKM	0 h-2 FPKM	0 h-3 FPKM	48 h-1 FPKM	48 h-2 FPKM	48 h-3 FPKM	FDR	log2FC	regulated	nr_annotation
c45995.graph_c0	0	0	0	2.52062402	4.41179257	2.73040916	3.075E-09	Inf	up	PREDICTED: auxin-repressed 12.5 kDa protein-like [Nicotiana tomentosiformis]
c45999.graph_c0	0.12562038	0.25149235	0	5.17890364	9.01121567	8.00635153	1.345E-17	4.867642	up	PREDICTED: heparanase-like protein 1 isoform X1 [Sesamum indicum]
c46005.graph_c0	0.31399005	0.12572179	0.48418758	2.11165726	2.26768927	1.90467884	0.0003061	1.739976	up	MYB-related transcription factor [Salvia miltiorrhiza]
c46005.graph_c1	28.772816	26.4903859	28.928894	27.4891502	25.9579336	24.5324772	5.015E-13	-1.126811	down	PREDICTED: CD2 antigen cytoplasmic tail-binding protein 2 [Sesamum indicum]
c46007.graph_c1	39.7646948	34.6807407	37.4546317	475.401896	551.398887	572.451114	1.566E-70	2.816773	up	PREDICTED: glyoxysomal fatty acid beta-oxidation multifunctional protein MFP-a [Sesamum indicum]
c46010.graph_c0	59.179845	60.9146952	53.910594	43.3720978	45.4709861	41.6510235	1.373E-24	-1.431704	down	PREDICTED: phosphoinositide phosphatase SAC1 isoform X4 [Sesamum indicum]
c46014.graph_c0	29.2703722	31.4810163	31.9457863	24.8136533	32.7354676	33.5830051	2.064E-13	-1.047934	down	PREDICTED: RNA polymerase I termination factor [Sesamum indicum]
c46015.graph_c0	0.08872793	0	0	1.92093607	3.07414719	3.09117396	3.794E-12	5.505458	up	PREDICTED: cytochrome P450 724B1 isoform X1 [Vitis vinifera]
c46016.graph_c0	6.24084055	5.66746292	6.8622408	29.3104392	26.3100279	30.3522454	2.368E-12	1.17749	up	PREDICTED: uncharacterized protein LOC105163003 isoform X1 [Sesamum indicum]
c46019.graph_c0	0.06797729	0.34022679	0.08735351	1.31512735	2.18934252	2.45183172	3.219E-05	2.565402	up	Malate dehydrogenase [Morus notabilis]
c46020.graph_c0	99.4260407	99.697493	90.9464743	53.7427729	48.6979213	43.054084	3.423E-44	-2.008959	down	PREDICTED: flowering time control protein FCA isoform X4 [Sesamum indicum]
c46021.graph_c1	1.42281992	1.24621407	0.45709498	7.0455205	10.4147088	8.89332019	1.959E-05	2.061529	up	PREDICTED: probable cyclic nucleotide-gated ion channel 5 [Sesamum indicum]
c46028.graph_c0	126.903244	126.947635	116.654494	548.233727	590.756714	604.594567	7.222E-15	1.21638	up	PREDICTED: mannose-6-phosphate isomerase 2-like [Sesamum indicum]
c46030.graph_c0	0.67837597	1.01858288	0.87173997	5.11875429	6.13206424	6.60687138	1.416E-11	1.775169	up	PREDICTED: uncharacterized protein LOC105166222 isoform X2 [Sesamum indicum]
c46032.graph_c1	5.85874743	5.36901289	5.00147522	73.169796	60.5485765	61.0474858	1.959E-26	2.57275	up	PREDICTED: CAP-Gly domain-containing linker protein 1 [Sesamum indicum]
c46041.graph_c1	19.4887847	20.9781032	18.6971283	15.0336851	18.6296147	17.8358435	9.284E-15	-1.220564	down	PREDICTED: pentatricopeptide repeat-containing protein At2g03880, mitochondrial-like isoform X1 [Sesamum indicum]
c46042.graph_c0	3.70115574	3.16002925	3.82355682	22.9322138	22.6648628	20.7618838	1.363E-15	1.617262	up	PREDICTED: methylmalonate-semialdehyde dehydrogenase [acylating], mitochondrial-like [Sesamum indicum]
c46051.graph_c0	194.468858	201.839392	190.278287	150.796006	155.86322	141.229037	1.02E-21	-1.406136	down	PREDICTED: uncharacterized protein LOC105174291 isoform X1 [Sesamum indicum]
c46053.graph_c0	3.00966655	2.68402425	3.51594607	14.8213479	16.5025138	19.064208	1.276E-07	1.42911	up	PREDICTED: (RS)-norcoclaurine 6-O-methyltransferase-like [Cucumis melo]

#ID	0 h-1 FPKM	0 h-2 FPKM	0 h-3 FPKM	48 h-1 FPKM	48 h-2 FPKM	48 h-3 FPKM	FDR	log2FC	regulated	nr_annotation
c46054.graph_c0	6.91518015	6.48947312	6.14898858	0.39816969	0.81349554	0.43020806	2.227E-51	-4.599724	down	PREDICTED: exocyst complex component EXO70A1-like [Sesamum indicum]
c46055.graph_c0	1.55264924	1.46169184	1.97146318	12.2095007	10.4810824	11.0457635	1.16E-17	1.740618	up	PREDICTED: importin-13 [Sesamum indicum]
c46056.graph_c2	8.33841775	8.82307751	8.35668891	191.295868	139.916714	119.952144	2.059E-10	3.13448	up	PREDICTED: cytochrome P450 93A3-like [Sesamum indicum]
c46061.graph_c0	13.950679	13.4544018	11.3940987	11.6164797	14.0605472	13.1182709	8.163E-13	-1.01865	down	PREDICTED: probable methyltransferase PMT18 [Sesamum indicum]
c46064.graph_c0	59.719391	52.4351838	58.8749237	9.12230056	8.11172721	9.37867309	1.14E-116	-3.700705	down	hypothetical protein M569_12324 [Genlisea aurea]
c46067.graph_c1	2.32595713	1.57501715	2.32962046	14.4640581	14.8555501	15.9543533	2.193E-14	1.841335	up	PREDICTED: diacylglycerol kinase 5 [Sesamum indicum]
c46068.graph_c0	23.8187642	22.487921	22.8864775	10.8387093	10.425645	9.03775674	2.187E-51	-2.206548	down	PREDICTED: cation/H(+) antiporter 18-like [Sesamum indicum]
c46070.graph_c0	66.8278468	60.9005303	73.2896106	14.6224784	15.5341951	17.8102798	2.18E-56	-3.088236	down	PREDICTED: glucan endo-1,3-beta-glucosidase 14-like [Sesamum indicum]
c46073.graph_c0	200.953026	197.225619	206.108563	75.6794746	70.3731448	54.145119	7.827E-62	-2.608148	down	PREDICTED: RING finger and CHY zinc finger domain-containing protein 1-like isoform X1 [Sesamum indicum]
c46074.graph_c0	0.26143258	0.57572761	0.47033171	3.9017497	5.46021092	5.1433384	6.275E-07	2.446097	up	PREDICTED: violaxanthin de-epoxidase, chloroplastic [Sesamum indicum]
c46077.graph_c0	9.29998946	11.3156171	10.3024609	50.2247818	52.1898723	64.8000777	4.644E-14	1.41502	up	PREDICTED: 40S ribosomal protein S20-2-like [Sesamum indicum]
c46079.graph_c0	0.38714259	0.44715029	0.30614988	54.5965688	58.3034772	76.4092748	2.662E-58	6.357168	up	PREDICTED: probable beta-D-xylosidase 7 [Sesamum indicum]
c46082.graph_c0	22.2352414	23.1487972	22.31186	5.8331991	5.50367698	5.43364293	3.459E-80	-3.029194	down	PREDICTED: uncharacterized protein LOC105165640 isoform X3 [Sesamum indicum]
c46085.graph_c0	0.04991435	0.19985734	0.12828384	7.03560692	6.52779023	8.55157068	1.68E-18	4.849895	up	PREDICTED: thylakoid lumenal 29 kDa protein, chloroplastic [Sesamum indicum]
c46087.graph_c1	1107.63223	1134.59727	1185.00262	58.1362544	49.6623638	42.429093	7.45E-138	-5.525486	down	PREDICTED: SUMO-activating enzyme subunit 2 [Sesamum indicum]
c46087.graph_c2	102.103117	108.075582	96.7042365	54.5518933	53.305189	41.9917311	6.134E-47	-2.04855	down	PREDICTED: zinc finger protein JACKDAW-like [Sesamum indicum]
c46088.graph_c0	0	0	0.29351285	3.78763216	2.11772786	4.21276539	2.415E-06	4.062309	up	PREDICTED: protein TIC 62, chloroplastic [Beta vulgaris subsp. vulgaris]
c46089.graph_c0	2.08686183	1.88005636	1.60902005	6.98527676	9.53860135	9.4087197	6.905E-05	1.196821	up	PREDICTED: 1-(5-phosphoribosyl)-5-[(5-phosphoribosylamino)methylideneamino] imidazole-4-carboxamide isomerase, chloroplastic [Sesamum indicum]
c46091.graph_c0	0.55278043	0.59022289	0.49724132	2.58023745	2.64353263	1.8427456	0.0063378	1.093167	up	PREDICTED: aspartic proteinase-like protein 1 isoform X2 [Sesamum indicum]
c46093.graph_c0	0.53558359	0.31427718	0.71197874	6.49946047	5.0649075	6.01026909	1.639E-16	2.472913	up	PREDICTED: uncharacterized protein LOC105169441 [Sesamum indicum]
c46094.graph_c0	11.6622083	13.5975969	11.8149497	6.17802544	5.50835115	5.13235017	4.31E-49	-2.154474	down	PREDICTED: probable protein phosphatase 2C 23 [Sesamum indicum]

#ID	0 h-1 FPKM	0 h-2 FPKM	0 h-3 FPKM	48 h-1 FPKM	48 h-2 FPKM	48 h-3 FPKM	FDR	log2FC	regulated	nr_annotation
c46097.graph_c0	5.39513255	5.26881459	6.73004305	4.6551552	4.59766392	4.96192549	1.139E-08	-1.311575	down	PREDICTED: uncharacterized protein LOC105179152 isoform X1 [Sesamum indicum]
c46100.graph_c0	1.43916961	1.31349801	1.19666407	0.31196633	0.70229232	0.76336086	9.226E-06	-2.181078	down	hypothetical protein M569_02521, partial [Genlisea aurea]
c46102.graph_c0	0	0	0	1.49049485	1.13211218	0.87581448	2.782E-10	Inf	up	predicted protein [Hordeum vulgare subsp. vulgare]
c46103.graph_c1	11.3382725	11.0237995	10.0852249	7.89664055	7.02422403	6.67061703	9.662E-24	-1.601165	down	PREDICTED: uncharacterized protein LOC105171484 [Sesamum indicum]
c46111.graph_c1	0	0	0	9.35399991	7.62263744	11.5243378	3.984E-13	Inf	up	-
c46114.graph_c0	0	0	0	18.889318	29.485149	17.4769308	2.816E-21	Inf	up	40S ribosomal protein S17 [Auxenochlorella protothecoides]
c46120.graph_c0	10.1528231	10.2850676	9.83405916	8.8759398	8.77810024	8.92246695	7.046E-11	-1.20476	down	PREDICTED: PHD finger protein ALFIN-LIKE 3-like isoform X2 [Elaeis guineensis]
c46122.graph_c0	27.8332174	27.5182783	26.0849493	26.9898328	28.8355366	25.4700958	2.477E-12	-1.019612	down	PREDICTED: dipeptidyl peptidase 9 [Sesamum indicum]
c46130.graph_c0	110.017843	121.043923	115.133348	37.2132632	32.873442	25.5746304	1.468E-85	-2.868694	down	PREDICTED: GATA transcription factor 5-like [Sesamum indicum]
c46131.graph_c0	59.2527075	60.6103451	54.0363113	46.4433622	48.2683882	44.8363924	5.807E-21	-1.334017	down	PREDICTED: protein MODIFIER OF SNC1 1, partial [Sesamum indicum]
c46142.graph_c0	29.4889497	27.600508	26.8262817	17.7527871	19.7152143	18.4290437	9.607E-30	-1.604313	down	PREDICTED: homeobox protein LUMINIDEPENDENS [Sesamum indicum]
c46143.graph_c0	0.32799317	0.2872814	0.52685526	2.30403633	2.92101376	3.26002318	0.0001351	1.864502	up	hypothetical protein MIMGU_mgv1a018015mg [Erythranthe guttata]
c46144.graph_c1	0.7761794	1.06831536	0.78962516	50.5909966	56.120116	64.0504146	7.33E-115	4.99926	up	hypothetical protein MIMGU_mgv1a007954mg [Erythranthe guttata]
c46144.graph_c2	0	0	0	106.85531	88.0189656	121.565565	4.636E-65	Inf	up	major latex-like protein 1 [Plantago major]
c46146.graph_c0	11.1367911	11.2785829	9.72715566	40.2782738	47.3827472	46.2523954	2.627E-10	1.039927	up	PREDICTED: phosphatidylinositol 3,4,5-trisphosphate 3-phosphatase and dual-specificity protein phosphatase PTEN-like [Sesamum indicum]
c46147.graph_c1	1.81402578	1.78092221	0.76208873	15.8442508	12.4270747	9.92301689	4.039E-06	2.129096	up	PREDICTED: polyamine oxidase 1 [Sesamum indicum]
c46148.graph_c0	2.97624609	2.78492898	2.41115713	18.8953503	20.7750863	19.3587814	3.513E-10	1.835897	up	PREDICTED: proline synthase co-transcribed bacterial homolog protein-like [Sesamum indicum]
c46149.graph_c1	2.07611406	0.95103746	2.21570423	0	0.03434275	0	7.282E-12	-8.310345	down	PREDICTED: geraniol 8-hydroxylase-like [Vitis vinifera]
c46151.graph_c0	1.46209687	0.75395564	0.91095873	9.79621096	12.5832009	11.9853382	5.242E-13	2.439483	up	PREDICTED: uncharacterized protein LOC105155656 [Sesamum indicum]
c46155.graph_c0	41.1855307	44.2292818	39.9976894	38.0278231	37.0431816	35.4635219	9.39E-18	-1.198153	down	PREDICTED: uncharacterized protein LOC105178641 [Sesamum indicum]
c46157.graph_c0	4.12663321	4.38111483	5.30288706	28.1481157	27.256583	25.604121	7.267E-26	1.533424	up	PREDICTED: probable receptor-like protein kinase At1g49730 isoform X1 [Sesamum indicum]
c46164.graph_c0	31.6762686	30.7937336	30.6756541	10.3266135	10.6966882	12.523665	1.311E-53	-2.492389	down	PREDICTED: protein Brevis radix-like 1 [Sesamum indicum]

#ID	0 h-1 FPKM	0 h-2 FPKM	0 h-3 FPKM	48 h-1 FPKM	48 h-2 FPKM	48 h-3 FPKM	FDR	log2FC	regulated	nr_annotation
c46166.graph_c0	0	0	0	19.9405137	45.9416164	14.3530361	1.672E-07	Inf	up	ribosomal protein S18 [Micromonas pusilla CCMP1545]
c46170.graph_c0	3.99701356	3.4453193	3.40043452	18.5820253	17.3737223	14.7594052	4.758E-10	1.212067	up	PREDICTED: E3 ubiquitin-protein ligase UPL7 [Sesamum indicum]
c46178.graph_c0	10317.9225	9801.13597	10053.0653	2624.4807	2587.15999	2099.82766	3.969E-25	-3.060461	down	PREDICTED: vicilin-like antimicrobial peptides 2-2 [Sesamum indicum]
c46179.graph_c0	82.1261691	76.7874023	83.9799927	11.7909236	12.2139148	12.4201984	4.33E-134	-3.75605	down	PREDICTED: peptidyl-prolyl cis-trans isomerase FKBP53 [Sesamum indicum]
c46180.graph_c1	4.35480189	3.86135696	3.93799126	0.07429495	0.10494199	0.15425014	2.517E-95	-6.213255	down	PREDICTED: replication protein A 70 kDa DNA-binding subunit A isoform X1 [Sesamum indicum]
c46182.graph_c0	25.0297438	22.9480968	19.0280772	12.672081	12.4708778	14.8492103	2.281E-18	-1.760499	down	PREDICTED: uncharacterized protein LOC105163032 [Sesamum indicum]
c46183.graph_c0	0	0.02042156	0	2.2741774	3.78306519	4.96689393	1.397E-13	8.060132	up	PREDICTED: uncharacterized protein LOC105179348 isoform X2 [Sesamum indicum]
c46184.graph_c0	3.41155127	3.45765452	4.05518007	21.6470237	20.9346562	19.820706	9.263E-12	1.495833	up	PREDICTED: DNA repair protein RAD50 [Sesamum indicum]
c46185.graph_c0	0	0	0	1.85317845	1.4724134	1.29560125	1.128E-10	Inf	up	--
c46186.graph_c0	0	0	0	6.97249021	5.35085613	4.83625123	1.467E-18	Inf	up	ATP synthase F0 subunit 9 (mitochondrion) [Hildenbrandia rubra]
c46190.graph_c1	38.0099736	40.559052	37.6405934	17.2761244	16.4359354	15.7608849	7.77E-56	-2.247766	down	PREDICTED: protein ASPARTIC PROTEASE IN GUARD CELL 1-like [Sesamum indicum]
c46190.graph_c3	1.82599866	1.82782712	4.35774963	42.8968411	43.1527075	48.9679633	2.431E-23	3.046065	up	-
c46199.graph_c0	1.2621641	1.34117738	1.47221414	7.03039449	10.0252237	10.4737573	2.842E-11	1.730298	up	PREDICTED: IAA-amino acid hydrolase ILR1-like 3 [Sesamum indicum]
c46204.graph_c0	6.73143247	5.54908363	5.99291939	53.6241702	69.9047032	72.3831668	3.43E-31	2.400403	up	PREDICTED: probable cinnamyl alcohol dehydrogenase 6 [Sesamum indicum]
c46206.graph_c0	3.60387609	3.48948299	4.32815277	1.56697522	2.67904257	2.76096066	4.248E-13	-1.73404	down	hypothetical protein MIMGU_mgv1a005772mg [Erythranthe guttata]
c46208.graph_c0	17.8031739	17.5971895	17.8496991	1.87960045	2.15486976	2.29103723	4.68E-107	-4.094055	down	PREDICTED: uncharacterized protein LOC105158293 isoform X2 [Sesamum indicum]
c46211.graph_c0	128.458572	128.041881	123.665751	115.558955	124.467692	118.543068	5.923E-14	-1.102452	down	PREDICTED: interferon-induced guanylate-binding protein 1 [Sesamum indicum]
c46212.graph_c0	9.1786692	8.27444724	8.09322333	4.96150607	4.66240018	4.86939024	1.829E-26	-1.833294	down	PREDICTED: protein root UVB sensitive 1, chloroplastic [Sesamum indicum]
c46212.graph_c1	3.87999963	3.157076	3.32396876	36.664844	48.8514001	48.062043	3.229E-38	2.666796	up	PREDICTED: heterogeneous nuclear ribonucleoprotein 1-like [Sesamum indicum]
c46212.graph_c2	130.254003	133.060098	121.799553	31.9796291	41.528532	39.4998395	5.108E-81	-2.789976	down	PREDICTED: ubiquinol oxidase 2, mitochondrial-like [Sesamum indicum]
c46213.graph_c1	22.4747594	20.6553832	21.9562821	30.149698	19.8822977	14.0060747	8.633E-05	-1.029481	down	GAL83 [Nicotiana attenuata]

#ID	0 h-1 FPKM	0 h-2 FPKM	0 h-3 FPKM	48 h-1 FPKM	48 h-2 FPKM	48 h-3 FPKM	FDR	log2FC	regulated	nr_annotation
c46213.graph_c2	64.445769	67.0081753	59.7274448	40.3607249	41.4654689	45.6424299	1.432E-29	-1.602516	down	PREDICTED: protein ETHYLENE INSENSITIVE 3-like [Sesamum indicum]
c46214.graph_c0	5.22047418	6.81613264	8.26411392	1.25463687	1.10761302	0.86828649	1.13E-14	-3.668728	down	-
c46216.graph_c0	81.620852	78.0368933	81.3115571	64.3921434	69.0748074	65.3200323	4.893E-20	-1.296103	down	PREDICTED: uncharacterized protein LOC105174095 isoform X1 [Sesamum indicum]
c46218.graph_c0	0.96392878	0.69126735	0.7025387	0.1325424	0.11233008	0.07076126	1.308E-15	-3.909423	down	hypothetical protein MIMGU_mgv1a020311mg [Erythranthe guttata]
c46221.graph_c0	1.26585852	1.50620648	1.65737036	0.24203973	0.13986095	0.03915736	7.8E-19	-4.395149	down	hypothetical protein MIMGU_mgv1a023003mg, partial [Erythranthe guttata]
c46227.graph_c0	1.44304694	1.06212642	0.54540366	5.59142181	5.05355858	5.39271394	0.001099	1.386578	up	-
c46231.graph_c0	0.04575881	0.04580463	0	31.5326874	32.6011937	23.2562958	8.443E-47	8.899337	up	PREDICTED: vestitone reductase-like [Sesamum indicum]
c46232.graph_c0	0.68352591	0.45614024	0.29278595	2.87866812	3.11313315	3.2818214	0.00034	1.682451	up	PREDICTED: uncharacterized protein LOC103980779 [Musa acuminata subsp. malaccensis]
c46234.graph_c2	64.9349124	75.9587399	51.0114136	36.1089418	45.7568411	33.3626352	6.939E-11	-1.75263	down	PREDICTED: uncharacterized protein LOC105177376 [Sesamum indicum]
c46234.graph_c3	0	0	0	5.91067337	6.11602216	5.13697275	5.406E-12	Inf	up	-
c46236.graph_c0	101.023277	94.8997898	107.810409	19.7468076	16.3760862	9.96710976	3.45E-122	-3.731186	down	PREDICTED: cytochrome P450 71B36-like [Sesamum indicum]
c46237.graph_c0	81.7192519	88.312154	77.621328	587.651447	601.980709	671.75981	1.169E-34	1.892109	up	PREDICTED: ATP synthase subunit beta, mitochondrial-like [Sesamum indicum]
c46239.graph_c0	0.22884524	0.13744464	0.02940752	9.25531257	11.3683021	12.512436	4.353E-53	5.379831	up	PREDICTED: subtilisin-like protease SBT3.5 [Sesamum indicum]
c46242.graph_c0	0	0	0	3.75593609	4.73685579	6.5248754	1.922E-13	Inf	up	unknown [Picea sitchensis]
c46243.graph_c0	2.37126989	2.27334952	1.9742265	8.49212538	8.60523029	10.0476118	0.0002975	1.018935	up	PREDICTED: serine/threonine-protein kinase TOUSLED isoform X2 [Sesamum indicum]
c46243.graph_c2	170.124301	154.721201	168.292911	54.1411687	64.4020037	63.1116344	5.159E-59	-2.461575	down	PREDICTED: probable phospholipid hydroperoxide glutathione peroxidase [Sesamum indicum]
c46246.graph_c0	24.3230999	23.9463445	23.0172939	109.160681	138.755363	147.124649	3.526E-18	1.448559	up	PREDICTED: serine carboxypeptidase-like 25 [Sesamum indicum]
c46247.graph_c0	37.8996991	40.8348492	34.403446	37.8703553	32.5036828	29.4807049	5.479E-18	-1.19245	down	PREDICTED: UDP-glycosyltransferase 91C1-like [Sesamum indicum]
c46248.graph_c0	32.373241	28.5816922	28.8877631	137.886818	132.403792	144.571267	3.119E-17	1.190612	up	PREDICTED: nijmegen breakage syndrome 1 protein [Sesamum indicum]
c46249.graph_c2	6.22398768	3.36768652	3.45862512	0	0.32834691	0.55157063	2.151E-09	-4.931474	down	-
c46249.graph_c6	11.7563845	14.7516331	11.9157112	0.76272875	1.45443352	0.40720287	2.057E-24	-4.894664	down	-
c46250.graph_c0	0.86089941	1.16337798	1.2169188	11.4605361	12.1410483	10.9737976	2.208E-13	2.39486	up	PREDICTED: uncharacterized protein LOC105168273 [Sesamum indicum]

#ID	0 h-1 FPKM	0 h-2 FPKM	0 h-3 FPKM	48 h-1 FPKM	48 h-2 FPKM	48 h-3 FPKM	FDR	log2FC	regulated	nr_annotation
c46259.graph_c0	1.42853113	2.33993715	1.60208209	299.725785	211.2089	184.552557	8.722E-21	6.008591	up	PREDICTED: phosphoglycerate kinase, cytosolic-like [Sesamum indicum]
c46264.graph_c0	11.326877	11.796057	12.3082014	8.22907178	10.9759041	10.34368	9.544E-14	-1.285014	down	hypothetical protein L484_013876 [Morus notabilis]
c46268.graph_c0	1.60683248	1.68503393	1.44211281	6.17974391	7.26856763	6.12592699	0.0004044	1.029871	up	PREDICTED: trimethylguanosine synthase isoform X2 [Sesamum indicum]
c46269.graph_c0	0	0.07531056	0.2900407	9.63431111	8.8112671	7.46243743	9.802E-17	5.11017	up	hypothetical protein MIMGU_mgv1a009279mg [Erythranthe guttata]
c46271.graph_c0	3.34180549	3.24300977	3.73707	15.9104777	14.8136736	14.7425678	5.492E-09	1.121764	up	PREDICTED: serine/threonine-protein kinase Nek2 [Sesamum indicum]
c46272.graph_c0	6.12378538	4.98055791	5.016683	0	0	0	2.244E-34	-Inf	down	hypothetical protein S7711_01318 [Stachybotrys chartarum IBT 7711]
c46273.graph_c1	36.0555233	38.9338431	34.2862534	20.1376242	22.4770157	17.8075019	6.407E-40	-1.871853	down	hypothetical protein MIMGU_mgv1a002387mg [Erythranthe guttata]
c46275.graph_c0	35.1155901	33.8258052	33.4657133	15.7584946	13.1831295	12.4081923	7.19E-59	-2.321339	down	PREDICTED: DIS3-like exonuclease 2 [Sesamum indicum]
c46279.graph_c0	0.64643598	0.48531246	0.62302187	3.15635663	2.74442084	3.0469653	0.0025563	1.334088	up	BnaA04g06750D [Brassica napus]
c46280.graph_c0	27.4148293	30.5477586	26.8083187	8.57431951	10.4119839	10.380106	6.426E-60	-2.549773	down	PREDICTED: uncharacterized protein LOC105162504 [Sesamum indicum]
c46282.graph_c0	20.1072363	19.2435909	18.0796966	16.5824783	17.8450859	17.5098767	2.693E-14	-1.162739	down	PREDICTED: uncharacterized PKHD-type hydroxylase At1g22950 [Sesamum indicum]
c46288.graph_c0	24.90261	25.2628943	22.6732407	23.9706937	23.6139173	20.7187079	3.153E-13	-1.107812	down	PREDICTED: magnesium transporter MRS2-1-like [Sesamum indicum]
c46290.graph_c0	14.0633634	15.0561789	13.171813	12.1153681	14.1071068	13.1713249	1.728E-14	-1.121235	down	PREDICTED: translation initiation factor IF-2, chloroplastic isoform X2 [Sesamum indicum]
c46293.graph_c0	38.7463702	39.5814826	39.3273735	206.241727	228.809654	229.045573	2.796E-26	1.477348	up	hypothetical protein POPTR_0016s08070g [Populus trichocarpa]
c46294.graph_c0	34.9254048	38.3219521	47.4697891	51.0477649	23.5980729	14.8653799	0.00095	-1.431998	down	-
c46295.graph_c0	0.19398458	0.16643899	0.17805562	2.37430654	2.89393567	3.18032382	5.205E-09	2.950329	up	hypothetical protein MIMGU_mgv1a004572mg [Erythranthe guttata]
c46297.graph_c0	0	0	0	7.91479969	23.9843437	5.15907085	0.0003292	Inf	up	hypothetical protein CHLNCDRAFT_30572 [Chlorella variabilis]
c46302.graph_c0	0	0	0	2.83323012	2.13437423	1.23248455	2.335E-06	Inf	up	-
c46303.graph_c0	3.51617589	3.21789653	2.74728305	1.63409438	1.88016209	1.57597706	5.352E-34	-1.913903	down	PREDICTED: sacsin [Sesamum indicum]
c46306.graph_c0	0	0	0	7.29354298	10.9774603	12.5046416	1.339E-25	Inf	up	unnamed protein product [Coffea canephora]
c46307.graph_c0	27.9436035	28.797924	26.6618534	18.1890102	22.5991724	21.2484634	7.441E-25	-1.447411	down	PREDICTED: LOW QUALITY PROTEIN: translocase of chloroplast 120, chloroplastic-like [Sesamum indicum]
c46308.graph_c0	3.0007704	2.83454844	2.87850372	19.974525	21.614323	21.8267519	6.955E-16	1.8447	up	PREDICTED: glycine--tRNA ligase 2, chloroplastic/mitochondrial isoform X1 [Cucumis melo]

#ID	0 h-1 FPKM	0 h-2 FPKM	0 h-3 FPKM	48 h-1 FPKM	48 h-2 FPKM	48 h-3 FPKM	FDR	log2FC	regulated	nr_annotation
c46309.graph_c0	4.48997595	4.11196373	5.15598869	25.9188742	31.4204203	33.1253647	2.239E-17	1.693786	up	PREDICTED: persulfide dioxygenase ETHE1 homolog, mitochondrial [Sesamum indicum]
c46314.graph_c0	30.6954798	32.321003	29.0377299	35.17711	25.6559275	21.3095083	2.06E-11	-1.173014	down	PREDICTED: uncharacterized protein LOC105166052 [Sesamum indicum]
c46315.graph_c0	0.53551212	0.75940183	0.83151974	21.0084317	19.5991168	15.9495594	1.485E-31	3.715401	up	hypothetical protein MIMGU_mgv1a001332mg [Erythranthe guttata]
c46317.graph_c0	19.9311319	20.9376823	18.9136937	14.3061075	14.3646741	14.2376366	4.632E-24	-1.495133	down	PREDICTED: LRR receptor-like serine/threonine-protein kinase RPK2 [Sesamum indicum]
c46320.graph_c0	1.05642002	2.04673135	1.22616656	5.02318981	6.05316133	6.564731	0.0040616	1.006613	up	PREDICTED: MORC family CW-type zinc finger protein 3-like isoform X1 [Sesamum indicum]
c46324.graph_c1	5.10169226	4.81288424	4.90511626	1.79209078	2.84775432	2.96353344	7.237E-20	-1.98847	down	PREDICTED: kinesin-4-like [Sesamum indicum]
c46327.graph_c0	14.571728	20.0680031	15.2066804	1.24676097	2.97946119	3.12168861	4.977E-29	-3.792878	down	PREDICTED: high affinity nitrate transporter 2.4-like [Sesamum indicum]
c46328.graph_c0	1.56935355	1.90415154	1.99631092	20.5185469	22.0618058	24.7935157	3.885E-54	2.601086	up	PREDICTED: kinesin-13A [Sesamum indicum]
c46330.graph_c0	21.6713697	19.2910694	15.4178923	15.1292469	18.29635	14.9371911	8.214E-10	-1.237755	down	PREDICTED: ATP-dependent helicase BRM isoform X1 [Sesamum indicum]
c46332.graph_c0	0.93237986	1.28059293	0.89164281	10.6072701	9.94622359	11.7489764	5.299E-22	2.363374	up	PREDICTED: uncharacterized protein LOC105162624 [Sesamum indicum]
c46334.graph_c0	25.8960103	25.3758781	25.0302118	16.8211727	19.4172153	20.5176715	1.612E-21	-1.447187	down	PREDICTED: glycine-rich RNA-binding protein RZ1A [Populus euphratica]
c46335.graph_c1	19.7252948	21.1200054	17.1379759	17.2893669	16.913061	18.5393261	2.522E-16	-1.152663	down	PREDICTED: phospholipid-transporting ATPase 1-like [Sesamum indicum]
c46342.graph_c0	2.41318285	2.37663801	2.70090189	1.25502178	1.63343903	1.91436143	3.917E-05	-1.665913	down	PREDICTED: protein GAMETE EXPRESSED 3-like [Solanum tuberosum]
c46342.graph_c1	18.536616	15.897535	18.1133421	14.5422518	15.6413144	12.5834722	4.043E-13	-1.314688	down	PREDICTED: zinc finger CCHC domain-containing protein 8 [Sesamum indicum]
c46345.graph_c0	1.21612964	1.58255164	1.40649726	0.28009449	0.29672615	0.04984528	3.178E-11	-3.756675	down	-
c46346.graph_c0	39.7848791	38.4177762	39.9056616	39.5316061	35.9723177	37.2624412	2.061E-14	-1.082786	down	PREDICTED: acyl-coenzyme A thioesterase 9, mitochondrial-like isoform X2 [Sesamum indicum]
c46348.graph_c0	8.25514268	6.34725615	7.84083252	56.1019496	52.5440978	43.6425028	1.016E-12	1.747375	up	PREDICTED: transmembrane protein 33 homolog [Sesamum indicum]
c46350.graph_c0	4.14487504	4.35910274	4.51726596	38.8619937	41.7328353	43.912103	1.15E-30	2.237376	up	PREDICTED: uncharacterized protein LOC105177167 [Sesamum indicum]
c46351.graph_c0	21.1344624	22.2418556	19.7547426	19.3752353	18.5336593	16.456333	7.416E-17	-1.230369	down	PREDICTED: chromodomain-helicase-DNA-binding protein 1-like isoform X2 [Sesamum indicum]
c46352.graph_c0	17.3037824	15.1401669	18.1377962	7.33070437	10.1081171	11.4666975	6.35E-25	-1.832427	down	PREDICTED: CDP-diacylglycerol--serine O-phosphatidyltransferase 1-like [Sesamum indicum]

#ID	0 h-1 FPKM	0 h-2 FPKM	0 h-3 FPKM	48 h-1 FPKM	48 h-2 FPKM	48 h-3 FPKM	FDR	log2FC	regulated	nr_annotation
c46354.graph_c0	83.8131888	79.9199658	97.8790633	3.12256835	3.62040311	3.19520892	6.129E-97	-5.739575	down	PREDICTED: uncharacterized protein LOC105172385 [Sesamum indicum]
c46354.graph_c1	6.14009796	5.77499656	7.20185625	0.87317551	1.52829982	1.55389263	6.859E-25	-3.30225	down	PREDICTED: pentatricopeptide repeat-containing protein At4g22760 [Sesamum indicum]
c46355.graph_c0	64.4795694	65.6111541	65.9915949	48.484996	46.8966547	43.5014573	3.033E-26	-1.513953	down	PREDICTED: fimbrin-2 [Sesamum indicum]
c46360.graph_c0	14.0195403	14.0785582	12.1836705	6.12668531	6.57818938	7.36687534	1.562E-22	-2.023088	down	unnamed protein product [Coffea canephora]
c46360.graph_c1	19.5923747	19.1431809	19.0582696	10.2114257	12.265215	10.3657926	1.633E-16	-1.834851	down	PREDICTED: mitochondrial ubiquitin ligase activator of NFKB 1 [Sesamum indicum]
c46363.graph_c0	1.71102938	1.80012755	1.43591216	8.01023787	8.55403538	7.55682664	1.82E-08	1.269921	up	PREDICTED: transcription termination factor 2 [Sesamum indicum]
c46364.graph_c0	0	0.05099425	0.03273204	3.02712891	3.03285949	3.09024126	4.257E-18	5.748671	up	PREDICTED: uncharacterized protein LOC105172001 isoform X1 [Sesamum indicum]
c46365.graph_c0	14.5925212	14.5475124	14.5423421	9.90435349	11.4516062	11.7667204	2.894E-17	-1.419564	down	PREDICTED: CDK5RAP3-like protein [Sesamum indicum]
c46368.graph_c2	1.65880447	1.71490701	1.15317657	0	0	0	1.049E-29	-Inf	down	PREDICTED: uncharacterized protein LOC104097920 [Nicotiana tomentosiformis]
c46368.graph_c3	63.8863421	64.8428042	59.6003692	29.0174341	31.8281662	30.2328944	1.421E-46	-2.066074	down	PREDICTED: LOW QUALITY PROTEIN: nuclear pore complex protein NUP1-like [Sesamum indicum]
c46369.graph_c0	20.9958161	20.5922576	19.2587759	15.695604	15.592677	14.7771484	9.876E-13	-1.41751	down	PREDICTED: retrovirus-related Pol polyprotein from transposon TNT 1-94 [Sesamum indicum]
c46371.graph_c0	7.04244905	6.74370073	7.41755133	4.53268677	6.52798078	7.77634929	5.36E-12	-1.196633	down	PREDICTED: uncharacterized protein LOC105180200 [Sesamum indicum]
c46372.graph_c1	69.8299143	77.8195245	81.8511504	69.3495464	70.4459262	63.774669	1.057E-16	-1.191248	down	hypothetical protein MIMGU_mgv1a026333mg, partial [Erythranthe guttata]
c46373.graph_c0	0	0	0.0963358	4.21294429	3.7314473	3.62574586	4.246E-12	5.860907	up	PREDICTED: uncharacterized protein LOC104110773 [Nicotiana tomentosiformis]
c46375.graph_c0	17.5890931	17.022043	15.740387	7.8868736	7.98058725	6.97665357	3.889E-47	-2.155769	down	PREDICTED: ubiquitin carboxyl-terminal hydrolase 8-like [Sesamum indicum]
c46380.graph_c0	5.10847992	4.67528712	3.65741942	3.56331486	2.70653209	2.75185551	5.061E-05	-1.584033	down	-
c46382.graph_c0	6.84045399	3.22801459	4.77185228	0	0	0	1.6E-15	-Inf	down	-
c46385.graph_c0	0.6459963	0.43109545	0.79938524	9.34580689	13.1932153	12.9052466	1.183E-28	3.211888	up	PREDICTED: uncharacterized protein LOC105177282 [Sesamum indicum]
c46386.graph_c0	2.77812002	1.80979329	1.41666437	0	0	0	7.205E-17	-Inf	down	--
c46387.graph_c0	0	0	0	1.39182676	4.34262208	0.5428774	0.0025989	Inf	up	-
c46388.graph_c0	13.0316093	12.525181	13.7451675	90.0152028	99.9185728	101.176346	5.774E-40	1.86865	up	hypothetical protein MIMGU_mgv1a008721mg [Erythranthe guttata]
c46390.graph_c0	2.3626663	1.91215365	2.77772657	10.1885384	11.3331971	11.4969562	8.966E-05	1.204846	up	hypothetical protein PHAVU_011G040900g [Phaseolus vulgaris]

#ID	0 h-1 FPKM	0 h-2 FPKM	0 h-3 FPKM	48 h-1 FPKM	48 h-2 FPKM	48 h-3 FPKM	FDR	log2FC	regulated	nr_annotation
c46391.graph_c0	70.1259689	67.290978	59.8748322	20.113667	19.496174	16.246636	5.732E-86	-2.834414	down	hypothetical protein MIMGU_mgv1a003239mg [Erythranthe guttata]
c46392.graph_c0	89.0341303	90.9149978	91.0031045	51.1719418	53.6469491	49.2479749	1.837E-37	-1.832324	down	PREDICTED: tobamovirus multiplication protein 1 [Sesamum indicum]
c46392.graph_c1	18.1681775	16.1954412	16.8588669	16.3148627	14.8571928	15.3320687	6.605E-12	-1.154857	down	PREDICTED: TBC1 domain family member 13-like [Sesamum indicum]
c46394.graph_c0	2.28902889	3.27331572	3.99202588	1.28034451	2.63295305	1.13924284	6.361E-05	-1.949931	down	-
c46398.graph_c0	0.1280409	0.08544608	0.10969175	3.89266774	5.33179405	4.54825662	6.44E-14	4.391485	up	PREDICTED: squalene synthase-like [Sesamum indicum]
c46400.graph_c0	0.32591014	0.23561524	0.37227328	6.27188221	6.32622057	6.84227366	2.764E-26	3.358854	up	PREDICTED: flavin-containing monooxygenase FMO GS-OX-like 2 isoform X1 [Sesamum indicum]
c46401.graph_c0	7.2657167	4.94563471	5.60204018	3.48069911	5.67289023	4.76477713	0.0055993	-1.379509	down	-
c46401.graph_c4	40.3328396	41.6413163	39.1579569	36.1163351	36.7571751	33.3710221	2.425E-17	-1.205772	down	PREDICTED: transcription factor HBP-1b(c1)-like isoform X1 [Sesamum indicum]
c46401.graph_c5	29.3382453	29.3676231	27.239682	14.5124954	16.1996911	16.8564027	3.944E-29	-1.872411	down	-
c46403.graph_c0	18.3709167	18.5904106	15.4609532	9.06889265	10.4788109	9.56988738	2.989E-34	-1.865926	down	PREDICTED: LOW QUALITY PROTEIN: uncharacterized protein LOC105173575 [Sesamum indicum]
c46404.graph_c0	23.3969713	24.9736908	23.9803169	18.3058128	18.1733641	15.9414889	6.366E-25	-1.481163	down	PREDICTED: kanadaptin [Sesamum indicum]
c46405.graph_c0	3.15686282	2.56218157	2.30244778	2.20088168	2.08175649	2.4479141	0.0062799	-1.268163	down	PREDICTED: uncharacterized protein LOC101504607, partial [Cicer arietinum]
c46405.graph_c1	60.5520054	59.3129529	55.7675971	28.3090714	27.8396551	27.2856572	7.436E-48	-2.089967	down	PREDICTED: protein RTF2 homolog [Sesamum indicum]
c46407.graph_c0	142.081968	140.840865	136.576304	106.15908	102.892748	111.506695	2.088E-23	-1.40513	down	PREDICTED: ras-related protein Rab11D [Sesamum indicum]
c46408.graph_c1	1.63259619	1.34583728	1.91283744	14.1115664	12.3719946	13.4617063	4.476E-11	2.011669	up	PREDICTED: phosphoglycerate mutase-like protein 4 [Sesamum indicum]
c46411.graph_c0	15.918535	17.1278057	15.2293179	6.39583293	8.0417587	6.55324242	1.358E-35	-2.221106	down	PREDICTED: pentatricopeptide repeat-containing protein At1g80150, mitochondrial [Sesamum indicum]
c46412.graph_c0	1.83850493	1.86955776	1.65003606	10.135619	10.1393515	9.35352157	5.169E-08	1.45161	up	PREDICTED: uncharacterized protein LOC105174617 [Sesamum indicum]
c46413.graph_c0	11.2996115	10.4929875	8.79027131	8.8828613	9.09132073	8.7268425	3.431E-13	-1.210993	down	PREDICTED: serine racemase isoform X1 [Sesamum indicum]
c46416.graph_c0	616.78973	611.26002	629.19279	249.384401	279.430503	310.439193	7.242E-37	-2.166597	down	PREDICTED: xylose isomerase-like [Sesamum indicum]
c46430.graph_c0	0.62881709	0.68666918	0.36729766	73.2033789	91.2190067	109.840728	1.985E-51	6.331246	up	PREDICTED: baicalein 7-O-glucuronosyltransferase-like [Sesamum indicum]
c46432.graph_c0	0	0	0	1.93933111	2.34798417	2.13646353	1.128E-10	Inf	up	--
c46434.graph_c0	0.30644173	0.10224953	0.42004241	1.59977995	2.47237455	2.12683849	1.978E-05	1.87138	up	PREDICTED: putative ion channel POLLUX-like 2 isoform X4 [Sesamum indicum]
c46435.graph_c0	5.03191463	4.57255338	4.08150814	2.23567022	0.80108375	0.87762614	1.086E-17	-2.800639	down	PREDICTED: putative beta-glucosidase 41 isoform X1 [Sesamum indicum]

#ID	0 h-1 FPKM	0 h-2 FPKM	0 h-3 FPKM	48 h-1 FPKM	48 h-2 FPKM	48 h-3 FPKM	FDR	log2FC	regulated	nr_annotation
c46436.graph_c0	10.6985563	10.2197027	10.9984547	53.1108801	60.8289458	60.6633047	1.883E-22	1.431245	up	PREDICTED: cyprosin-like [Sesamum indicum]
c46439.graph_c0	219.066072	233.960244	228.421032	54.0530916	71.5705261	69.6800714	9.893E-75	-2.825497	down	PREDICTED: uncharacterized protein LOC105159071 [Sesamum indicum]
c46440.graph_c0	24.9885251	27.6370659	24.3469561	11.943672	12.535716	13.6286662	2.592E-44	-2.03256	down	PREDICTED: uncharacterized protein DDB_G0271670 isoform X1 [Sesamum indicum]
c46442.graph_c0	0	0	0.04533257	4.58244922	6.64487953	6.13061263	1.873E-25	7.524748	up	PREDICTED: heat stress transcription factor C-1-like [Sesamum indicum]
c46445.graph_c0	53.0146281	54.763369	53.855738	17.3398343	17.1448218	16.2517539	1.909E-64	-2.68846	down	PREDICTED: DNA-directed RNA polymerase III subunit RPC9-like [Sesamum indicum]
c46448.graph_c1	80.2274773	88.015929	81.8090286	41.0798782	43.3443741	41.5437506	6.217E-45	-2.007146	down	hypothetical protein MIMGU_mgv1a021536mg, partial [Erythranthe guttata]
c46448.graph_c2	194.303433	192.913263	192.615114	172.858455	143.832752	130.031492	3.291E-20	-1.389107	down	PREDICTED: pentatricopeptide repeat-containing protein At4g21065-like [Sesamum indicum]
c46450.graph_c0	3.20957617	3.23125439	3.31850887	24.1987597	29.6142183	26.9148885	1.241E-36	2.027328	up	PREDICTED: probable metal-nicotianamine transporter YSL7 [Sesamum indicum]
c46452.graph_c0	27.9603367	30.4089475	29.3915016	18.6577876	20.7490299	17.4688343	4.175E-24	-1.644187	down	PREDICTED: TATA-box-binding protein-like isoform X4 [Sesamum indicum]
c46453.graph_c0	1.32540352	1.71369384	1.987062	0.05087701	0.37728614	0.36216044	3.356E-11	-3.71356	down	acidic class III chitinase [Rehmannia glutinosa]
c46454.graph_c0	18.3829866	20.823987	18.5939083	6.30934353	6.18142921	4.55874978	5.029E-44	-2.775752	down	PREDICTED: kinesin-like protein KIF3A isoform X1 [Sesamum indicum]
c46456.graph_c0	3.4420495	3.90489568	3.70703761	17.4105224	24.2191906	31.9912608	1.717E-05	1.709975	up	PREDICTED: purple acid phosphatase 15 isoform X2 [Sesamum indicum]
c46459.graph_c0	20.2472482	15.3482211	21.7241974	10.1414835	7.67405143	8.2181318	1.929E-09	-2.152883	down	-
c46459.graph_c1	12.7457258	13.2310253	14.8116585	5.50868358	4.95273628	6.03024992	1.525E-30	-2.324916	down	PREDICTED: putative lipid phosphate phosphatase 3, chloroplastic [Sesamum indicum]
c46462.graph_c0	4.48746923	5.98928366	4.08465773	1.37804978	2.18981497	2.06917944	5.995E-08	-2.392349	down	-
c46462.graph_c2	3.59510361	2.29704483	2.01504116	0.88086275	1.38108756	1.72433003	2.946E-05	-2.010226	down	PREDICTED: restin homolog [Sesamum indicum]
c46463.graph_c0	1.00365875	1.64764857	0.82543439	5.54781544	5.13278763	4.77186572	0.0053254	1.140565	up	PREDICTED: uncharacterized protein LOC105170218 [Sesamum indicum]
c46468.graph_c0	4.39359714	3.17471282	3.62686615	1.09903575	2.44218	2.31360473	2.113E-10	-1.964516	down	hypothetical protein MIMGU_mgv1a003714mg [Erythranthe guttata]
c46470.graph_c0	1.60120634	1.14486408	1.69018255	13.4342717	13.0599363	14.1569715	9.52E-11	2.176523	up	hypothetical protein MIMGU_mgv1a002711mg [Erythranthe guttata]
c46471.graph_c0	52.0607448	49.9500884	48.1918836	3.03315439	3.66512387	3.41577321	4.25E-167	-4.912277	down	hypothetical protein AMTR_s00109p00039350 [Amborella trichopoda]
c46472.graph_c1	12.7187652	12.7719186	14.1389582	13.0006855	13.0239222	11.898917	3.899E-12	-1.081405	down	PREDICTED: uncharacterized protein LOC105165093 [Sesamum indicum]

#ID	0 h-1 FPKM	0 h-2 FPKM	0 h-3 FPKM	48 h-1 FPKM	48 h-2 FPKM	48 h-3 FPKM	FDR	log2FC	regulated	nr_annotation
c46473.graph_c1	1.0615993	0.62972583	0.55578402	3.83961255	4.64320825	3.59373353	3.985E-05	1.411955	up	PREDICTED: sulfate transporter 1.3-like [Sesamum indicum]
c46474.graph_c0	0.56489301	0	1.08886483	5.46437105	4.27271416	5.32522778	7.209E-05	2.158386	up	PREDICTED: non-specific lipid-transfer protein 1-like [Pyrus x bretschneideri]
c46477.graph_c0	1.17490262	1.17607911	0.75489819	9.38077339	11.0080157	9.59899912	1.888E-11	2.256268	up	PREDICTED: UPF0554 protein isoform X2 [Sesamum indicum]
c46478.graph_c0	5.64172623	4.89947988	5.58433743	21.5908047	24.9562472	24.7985391	4.185E-14	1.124946	up	PREDICTED: ultraviolet-B receptor UVR8 isoform X1 [Sesamum indicum]
c46479.graph_c0	4.27347372	3.97219917	3.35596734	19.8099978	25.3556761	23.2151296	3.896E-15	1.540144	up	hypothetical protein MIMGU_mgv1a004800mg [Erythranthe guttata]
c46484.graph_c0	1.407838	2.18748903	1.21508568	42.2393577	49.4847222	52.1392101	1.27E-115	3.884821	up	PREDICTED: formin-like protein 1 [Nelumbo nucifera]
c46485.graph_c0	2.54937237	1.6769794	1.26361714	0.06710423	0.31989959	0.41796259	1.94E-08	-3.802642	down	PREDICTED: heat shock protein 81-1 [Musa acuminata subsp. malaccensis]
c46486.graph_c0	9.37174301	10.3299819	8.50368798	4.05330113	5.98713619	5.86439507	2.757E-28	-1.84943	down	PREDICTED: uncharacterized protein LOC105176969 [Sesamum indicum]
c46488.graph_c0	32.5373723	33.8236084	31.8336271	19.1298927	20.6813851	19.4239972	5.99E-34	-1.747244	down	PREDICTED: pentatricopeptide repeat-containing protein At5g67570, chloroplastic [Sesamum indicum]
c46488.graph_c1	19.0863946	21.3799718	18.6870649	3.55859211	7.53979367	9.49924157	3.4E-11	-2.552254	down	-
c46488.graph_c2	34.2171521	37.567284	34.876408	10.1609023	17.7812084	23.0810584	1.951E-42	-2.092212	down	PREDICTED: F-box/kelch-repeat protein SKIP11-like isoform X1 [Sesamum indicum]
c46489.graph_c0	12.7370054	11.5805676	10.8997918	5.66211657	6.75829484	5.19769565	3.234E-29	-2.016873	down	PREDICTED: dr1-associated corepressor [Sesamum indicum]
c46492.graph_c0	10.7156424	13.1484566	9.92034038	8.2796989	9.67096133	9.3507103	1.546E-08	-1.325883	down	PREDICTED: LOW QUALITY PROTEIN: uncharacterized protein LOC105176443 [Sesamum indicum]
c46492.graph_c1	7.17522905	7.84935239	5.59813242	4.9105004	7.27790965	6.34393647	4.09E-08	-1.174987	down	PREDICTED: LOW QUALITY PROTEIN: uncharacterized protein LOC105176443 [Sesamum indicum]
c46493.graph_c0	21.072462	22.2695106	19.7666885	88.2731393	99.4265355	102.409319	8.67E-17	1.181692	up	PREDICTED: probable receptor-like protein kinase At1g67000 [Sesamum indicum]
c46493.graph_c1	0.70953726	1.04993146	1.38749658	6.53672001	8.97221281	8.6233476	5.219E-10	1.910174	up	PREDICTED: uncharacterized protein LOC105170658 [Sesamum indicum]
c46496.graph_c0	0.17937402	0.29925607	0.30733696	1.79021971	1.77981174	1.5439136	0.0009705	1.682115	up	PREDICTED: pentatricopeptide repeat-containing protein At3g12770 [Sesamum indicum]
c46499.graph_c1	0.24637613	0.57545329	0.36937026	6.58235502	9.57814064	9.7952271	8.704E-17	3.421053	up	PREDICTED: probable serine incorporator [Sesamum indicum]
c46501.graph_c0	70.039327	65.1664263	65.9015167	49.5830906	48.1942144	46.012722	3.607E-26	-1.500108	down	MADS box protein [Paulownia kawakamii]
c46502.graph_c0	1.48035784	1.39111528	1.68879289	10.4929271	10.039768	9.31177808	7.855E-18	1.692585	up	PREDICTED: uncharacterized protein ycf39 isoform X2 [Nicotiana sylvestris]
c46504.graph_c0	19.0700761	17.1340034	17.1648575	14.4341124	14.1433276	13.5320399	6.015E-20	-1.357781	down	PREDICTED: uncharacterized protein LOC105157171 isoform X1 [Sesamum indicum]
c46506.graph_c0	2.8602521	2.12265512	1.90113992	0	0	0	1.108E-26	-Inf	down	putative Trypsin [Glarea lozoyensis 74030]

#ID	0 h-1 FPKM	0 h-2 FPKM	0 h-3 FPKM	48 h-1 FPKM	48 h-2 FPKM	48 h-3 FPKM	FDR	log2FC	regulated	nr_annotation
c46507.graph_c0	50.8021785	56.2191993	54.3379608	35.4017249	31.416185	29.5699794	2.078E-35	-1.758245	down	PREDICTED: mitogen-activated protein kinase kinase kinase YODA-like [Sesamum indicum]
c46508.graph_c0	154.12016	149.047117	154.001047	101.770699	96.1829019	98.5676971	2.216E-29	-1.641215	down	hypothetical protein VITISV_004365 [Vitis vinifera]
c46509.graph_c0	0.11284093	0.22590785	0.09667004	1.10886856	0.86879726	0.89416478	0.0005844	1.712983	up	PREDICTED: probable LRR receptor-like serine/threonine-protein kinase At3g47570 [Solanum lycopersicum]
c46518.graph_c0	68.9973951	67.0993531	70.4304216	25.5029396	30.6417244	34.3493108	1.927E-50	-2.212452	down	PREDICTED: uncharacterized protein LOC105175391 [Sesamum indicum]
c46523.graph_c1	1.05373545	0.86048707	0.78394787	18.2402541	18.0242896	18.2986066	1.162E-44	3.32302	up	PREDICTED: glutathione synthetase, chloroplastic isoform X2 [Sesamum indicum]
c46525.graph_c1	31.4372141	33.0560381	29.5160067	7.51536351	10.2591569	8.91169999	2.173E-84	-2.838068	down	PREDICTED: lon protease homolog 2, peroxisomal [Sesamum indicum]
c46529.graph_c0	0.63351753	0.34146641	1.12720861	2.82839792	3.66219901	3.63522101	0.0054862	1.236628	up	PREDICTED: uncharacterized protein LOC105164965 [Sesamum indicum]
c46536.graph_c0	1.26075889	1.5324545	1.08008339	7.30081517	9.02338314	9.00612518	1.679E-08	1.690218	up	zeaxanthin epoxidase [Scutellaria baicalensis]
c46537.graph_c0	0.23128401	0.08681835	0.03715114	1.01210111	0.84647242	0.94795945	0.0007721	1.980293	up	hypothetical protein MIMGU_mgv1a001125mg [Erythranthe guttata]
c46540.graph_c0	11.6547932	11.5366921	12.3446731	12.3853508	11.9693881	11.5623981	1.005E-11	-1.001767	down	PREDICTED: L-ascorbate oxidase homolog [Sesamum indicum]
c46540.graph_c1	2.02336532	0.31159868	1.80007238	8.02978017	8.50657843	9.56899568	0.0011276	1.635901	up	PREDICTED: L-ascorbate oxidase homolog [Tarenaya hassleriana]
c46542.graph_c0	17.0487352	15.439336	15.7689895	14.2318366	16.6863708	16.579714	1.101E-11	-1.042767	down	hypothetical protein MIMGU_mgv1a012248mg [Erythranthe guttata]
c46544.graph_c0	0	0	0.11429162	1.27003079	1.90966191	1.40346876	1.765E-11	4.270144	up	PREDICTED: phosphatidylinositol 4-phosphate 5-kinase 6 [Sesamum indicum]
c46547.graph_c0	2.21648948	1.69943665	1.81804898	0.78203285	0.78244293	0.50255698	1.369E-07	-2.485021	down	PREDICTED: NAC domain-containing protein 7 [Sesamum indicum]
c46550.graph_c2	0	0	0	2.43269236	4.08260211	3.47192471	2.672E-22	Inf	up	PREDICTED: uncharacterized protein LOC105175473 [Sesamum indicum]
c46552.graph_c0	15.7998193	16.7733349	15.8422671	14.5054579	17.3943342	14.789121	7.255E-12	-1.071899	down	PREDICTED: serine/threonine protein phosphatase 2A 57 kDa regulatory subunit B' theta isoform-like [Sesamum indicum]
c46552.graph_c1	141.748757	144.262824	143.286029	71.9297962	56.0551328	52.0916545	1.028E-56	-2.265197	down	PREDICTED: uncharacterized protein LOC105165908 isoform X2 [Sesamum indicum]
c46558.graph_c0	31.4553008	30.3819986	32.3167843	20.8079958	16.1191044	15.9298621	5.214E-33	-1.845539	down	-
c46560.graph_c0	1.82739429	1.52935134	2.27128143	13.1369353	12.8059698	12.1803075	5.583E-13	1.740242	up	hypothetical protein L484_026123 [Morus notabilis]
c46564.graph_c0	4.22969034	3.38197726	2.75080197	13.1156096	15.3794877	15.200846	1.869E-07	1.059527	up	PREDICTED: probable methyltransferase PMT28 isoform X2 [Sesamum indicum]
c46566.graph_c0	0	0	0	3.50121367	5.71403638	8.25149105	1.183E-08	Inf	up	-

#ID	0 h-1 FPKM	0 h-2 FPKM	0 h-3 FPKM	48 h-1 FPKM	48 h-2 FPKM	48 h-3 FPKM	FDR	log2FC	regulated	nr_annotation
c46568.graph_c0	31.5271648	32.5024765	31.6694659	16.5896297	17.9795633	17.8991063	8.25E-40	-1.88591	down	PREDICTED: uncharacterized protein LOC105175670 isoform X1 [Sesamum indicum]
c46569.graph_c0	3.14319745	2.81762229	3.37599191	0.51863615	0.73257615	0.26919627	1.232E-15	-3.638204	down	PREDICTED: U-box domain-containing protein 33 [Sesamum indicum]
c46569.graph_c1	1.85324107	2.66670167	1.04190107	0.26676977	0.11304409	0.28484417	2.439E-06	-4.068364	down	PREDICTED: U-box domain-containing protein 33 [Sesamum indicum]
c46571.graph_c1	0.99602202	1.20153618	1.41120237	5.10562303	5.58326556	6.25964446	7.715E-05	1.208493	up	PREDICTED: uncharacterized protein LOC105165701 isoform X1 [Sesamum indicum]
c46572.graph_c0	722.003728	756.032668	673.132973	317.89303	139.090893	105.05462	1.873E-73	-2.933072	down	PREDICTED: short-chain type dehydrogenase/reductase [Sesamum indicum]
c46573.graph_c0	20.5928927	19.2796978	17.5120693	10.6296288	16.0784166	15.8629314	2.26E-20	-1.454065	down	PREDICTED: uncharacterized protein LOC105161259 isoform X2 [Sesamum indicum]
c46574.graph_c0	196.454845	189.888292	187.093228	81.307996	93.3254077	92.0015413	5.154E-48	-2.124289	down	hypothetical protein MIMGU_mgv1a002799mg [Erythranthe guttata]
c46576.graph_c0	120.374546	128.847253	117.145289	53.5791787	46.4350152	46.8428016	1.291E-54	-2.332969	down	PREDICTED: EIN3-binding F-box protein 1-like [Sesamum indicum]
c46577.graph_c0	0	0	0	2.383693	4.40112159	2.36339521	2.193E-08	Inf	up	-
c46579.graph_c0	0	0	0	36.8612359	52.7002345	29.1707085	2.676E-24	Inf	up	60S ribosomal protein L11 [Candida albicans WO-1]
c46581.graph_c0	5.91682064	2.01763856	1.5039591	0	0	0	2.443E-05	-Inf	down	PREDICTED: photosystem II 22 kDa protein, chloroplastic [Phoenix dactylifera]
c46582.graph_c0	0.66518186	0.52566942	0.53986422	7.12793525	7.44867834	8.06431082	7.901E-13	2.692057	up	PREDICTED: uncharacterized protein LOC105162786 [Sesamum indicum]
c46584.graph_c0	86.9018845	85.9442331	86.5525282	35.425948	26.2471425	21.6506696	2.097E-75	-2.648107	down	PREDICTED: phosphoglucan phosphatase DSP4, amyloplastic [Sesamum indicum]
c46585.graph_c0	2.69711072	1.52171192	1.63842264	23.8988188	29.1945546	28.6213572	2.608E-30	2.78487	up	PREDICTED: uncharacterized protein LOC105160704 isoform X2 [Sesamum indicum]
c46587.graph_c0	32.6414798	31.2751334	31.748875	8.68084972	8.0522694	9.86771686	8.533E-66	-2.864015	down	PREDICTED: inositol-tetrakisphosphate 1-kinase 2-like isoform X1 [Sesamum indicum]
c46589.graph_c0	0.22660799	0.34025236	0.33973362	5.81065544	3.94406156	5.04644708	1.042E-10	3.014663	up	PREDICTED: uncharacterized protein LOC105173906 [Sesamum indicum]
c46589.graph_c1	70.948567	73.5744107	66.2065761	20.6176848	37.1591763	43.9511148	3.271E-46	-2.078269	down	PREDICTED: transcription factor GTE7-like [Sesamum indicum]
c46590.graph_c0	8.74249731	7.52795836	7.85203856	1.55886944	1.37619429	1.42560245	5.874E-30	-3.482375	down	PREDICTED: uncharacterized protein LOC104210866 [Nicotiana sylvestris]
c46592.graph_c0	0.16761703	0.06711395	0.0430789	47.0053681	55.6529736	83.3479039	1.106E-28	8.373965	up	hypothetical protein MIMGU_mgv1a019962mg [Erythranthe guttata]
c46594.graph_c0	54.9240364	57.5870143	58.5448412	55.1730306	53.4666992	51.4880972	3.751E-15	-1.112263	down	unnamed protein product [Coffea canephora]

#ID	0 h-1 FPKM	0 h-2 FPKM	0 h-3 FPKM	48 h-1 FPKM	48 h-2 FPKM	48 h-3 FPKM	FDR	log2FC	regulated	nr_annotation
c46598.graph_c0	3.84894542	3.57157331	3.57415094	19.5412714	27.995154	27.2676074	4.6E-14	1.743058	up	PREDICTED: uncharacterized protein LOC105157371 [Sesamum indicum]
c46603.graph_c0	14.8610357	10.3405763	11.6445239	8.84903213	10.7894919	10.6965042	1.093E-07	-1.300164	down	flowering locus T [Camellia sinensis]
c46603.graph_c1	31.3894613	32.2936956	29.4768196	17.1777977	20.6198022	16.5492627	2.322E-25	-1.796085	down	PREDICTED: DEAD-box ATP-dependent RNA helicase 53-like [Sesamum indicum]
c46604.graph_c0	0.25677747	0.42060206	0.44995802	8.92476943	9.4319286	11.5769391	2.23E-34	3.706783	up	PREDICTED: carboxy-terminal kinesin 2-like [Sesamum indicum]
c46610.graph_c0	0	0	0	3.70032765	2.74403392	2.38708374	4.794E-11	Inf	up	-
c46615.graph_c0	0.74333743	0.59526541	0.68229859	4.12843155	4.2906613	4.45689154	1.493E-06	1.653301	up	hypothetical protein MIMGU_mgv1a001336mg [Erythranthe guttata]
c46617.graph_c0	150.514395	151.506556	164.671464	9.48660905	7.3836088	5.74226373	1.71E-225	-5.378578	down	BnaC09g43060D [Brassica napus]
c46618.graph_c0	12.0894006	5.22443884	4.92751455	0	0	0	6.569E-11	-Inf	down	PREDICTED: mitochondrial uncoupling protein 5-like [Musa acuminata subsp. malaccensis]
c46620.graph_c0	8.96307387	5.17731354	5.13585263	0	0	0	3.929E-25	-Inf	down	PREDICTED: alpha carbonic anhydrase 1, chloroplastic-like [Camelina sativa]
c46621.graph_c0	6.54938935	6.19573068	5.17921515	4.60817433	5.51397772	5.75225563	1.786E-06	-1.194027	down	PREDICTED: uncharacterized protein At1g04910-like [Sesamum indicum]
c46622.graph_c0	2.18972226	2.23036958	2.12275142	12.8471111	14.8097322	14.7378515	1.405E-10	1.676231	up	PREDICTED: uncharacterized protein LOC105163866 [Sesamum indicum]
c46624.graph_c0	1.4546899	1.12733926	1.20602201	0.30261569	0.59537146	1.11552902	0.0001185	-1.942557	down	PREDICTED: folylpolyglutamate synthase-like [Solanum tuberosum]
c46629.graph_c0	6.26704428	6.63843363	6.73247198	1.34412084	1.87700265	1.68525954	1.114E-27	-3.024306	down	Uncharacterized protein TCM_005954 [Theobroma cacao]
c46634.graph_c0	21.6318072	20.8683716	19.474666	97.8717246	94.4482815	86.9819739	1.844E-16	1.156991	up	PREDICTED: L-arabinokinase-like [Sesamum indicum]
c46636.graph_c0	78.3586473	83.2555768	63.7277783	47.1346871	38.6808691	29.9960758	1.461E-22	-1.969383	down	PREDICTED: ABC transporter B family member 4-like [Sesamum indicum]
c46638.graph_c0	16.0591194	14.1359697	14.2818404	128.83138	123.89291	158.054492	7.553E-28	2.189389	up	unknown [Populus trichocarpa x Populus deltoides]
c46642.graph_c0	0	0	0.04644039	1.1486379	0.93467618	1.45160881	9.894E-13	5.200429	up	PREDICTED: condensin-2 complex subunit D3 [Sesamum indicum]
c46643.graph_c0	0.73854566	0.42564906	0.4313911	0	0	0	7.214E-15	-Inf	down	alpha-2 macroglobulin family-like protein [Micromonas sp. RCC299]
c46644.graph_c0	4.35236168	2.59328566	3.72863805	0	0.10113736	0.1698947	3.273E-19	-6.344845	down	PREDICTED: protein ROOT HAIR DEFECTIVE 3-like [Sesamum indicum]
c46648.graph_c0	10.2794665	11.6319024	11.1993769	8.69809477	7.41528066	5.90767516	3.782E-11	-1.601289	down	PREDICTED: ABC transporter G family member 12-like [Sesamum indicum]
c46653.graph_c0	0.42861882	0.58227945	0.29506723	2.00256319	2.91329021	3.32531831	9.927E-06	1.637434	up	PREDICTED: DNA mismatch repair protein MSH1, mitochondrial-like [Sesamum indicum]
c46657.graph_c2	106.950267	103.157921	108.30996	36.1601316	33.1228316	36.4812046	2.198E-71	-2.606832	down	PREDICTED: putative clathrin assembly protein At5g35200 [Sesamum indicum]

#ID	0 h-1 FPKM	0 h-2 FPKM	0 h-3 FPKM	48 h-1 FPKM	48 h-2 FPKM	48 h-3 FPKM	FDR	log2FC	regulated	nr_annotation
c46658.graph_c0	2.64291225	3.21246416	2.72912695	10.5001748	11.123662	13.7533491	8.477E-07	1.023283	up	PREDICTED: QWRF motif-containing protein 8 [Sesamum indicum]
c46659.graph_c0	2.77244759	3.11784399	2.90294474	0.66218951	0.70150949	1.06619359	4.13E-13	-2.877388	down	PREDICTED: probable serine/threonine-protein kinase At5g41260 [Sesamum indicum]
c46660.graph_c0	9.9894918	9.84014027	9.6148836	6.26975344	6.99162476	8.13981188	1.401E-18	-1.48058	down	PREDICTED: GDP-mannose transporter GONST1-like isoform X2 [Sesamum indicum]
c46665.graph_c0	72.1946641	67.4912439	70.6456014	36.779032	39.7924924	43.60438	1.871E-37	-1.82715	down	PREDICTED: uncharacterized protein LOC105173922 isoform X1 [Sesamum indicum]
c46666.graph_c0	0.03829138	0.03832973	0	0.97010433	0.63531034	0.92597067	9.021E-09	4.050135	up	PREDICTED: Fanconi anemia group J protein [Sesamum indicum]
c46670.graph_c1	48.443091	45.170257	45.6225207	37.7511883	40.3975919	42.3624619	1.706E-17	-1.227045	down	PREDICTED: UDP-galactose transporter 1 [Sesamum indicum]
c46679.graph_c0	0	0	0	27.2014587	45.5260544	21.5870788	3.086E-16	Inf	up	RecName: Full=Tubulin beta-1 chain; AltName: Full=Beta-1-tubulin [Cyanophora paradoxa]
c46684.graph_c0	0.18360544	0.22054716	0	4.80384041	5.30411824	5.59889905	1.086E-15	4.276697	up	hypothetical protein MIMGU_mgv1a002647mg [Erythranthe guttata]
c46687.graph_c0	112.375138	124.833601	107.757127	22.561813	18.5852571	17.6612393	1.7E-120	-3.56462	down	PREDICTED: uncharacterized protein LOC105168969 [Sesamum indicum]
c46688.graph_c0	3.65860414	4.15716872	3.30372245	1.18424546	0.86854463	1.05373449	7.266E-09	-2.851041	down	-
c46690.graph_c0	4.89292819	5.03238342	4.31841102	49.8322055	55.1001374	60.4501532	8.623E-59	2.518823	up	tyrosine aminotransferase 2 [Salvia miltiorrhiza]
c46691.graph_c0	20.2031272	23.0500745	19.7270852	7.45545145	8.10161193	7.93882882	1.698E-59	-2.440571	down	PREDICTED: fimbrin-1 [Sesamum indicum]
c46692.graph_c0	38.2432148	41.4168223	37.1535737	47.9245944	36.5759905	31.8341336	5.088E-10	-1.016106	down	PREDICTED: E3 ubiquitin-protein ligase MBR2-like isoform X2 [Sesamum indicum]
c46698.graph_c0	20.651992	23.8739517	19.7820739	2675.81929	2917.81528	3528.64679	1.43E-115	6.128759	up	hypothetical protein MIMGU_mgv1a006007mg [Erythranthe guttata]
c46699.graph_c0	0.03100056	0.0310316	0	5.05507298	12.5863194	13.5193329	2.268E-11	7.956731	up	PREDICTED: gamma-cadinene synthase-like [Sesamum indicum]
c46701.graph_c0	18.6410966	18.8880168	16.848764	15.7554074	14.521121	15.8415454	2.219E-11	-1.253076	down	PREDICTED: transcription initiation factor TFIID subunit 9-like [Sesamum indicum]
c46707.graph_c0	32.8465576	33.3292358	34.529491	34.0688127	33.9867878	32.7084222	1.68E-11	-1.016722	down	PREDICTED: F-box/kelch-repeat protein At1g30090-like [Citrus sinensis]
c46710.graph_c0	5.29739268	5.23557447	4.8254699	183.993625	227.562491	237.230504	1.23E-111	4.379926	up	PREDICTED: uncharacterized protein LOC105177873 [Sesamum indicum]
c46712.graph_c0	2.11783371	3.34300502	2.61682709	21.1617849	19.4769311	19.0967159	7.366E-09	1.870692	up	PREDICTED: sucrose transport protein SUC3 isoform X2 [Nicotiana tomentosiformis]
c46713.graph_c0	2.71437042	2.71708845	3.1821021	12.2839197	13.1527519	12.5306453	6.425E-05	1.119935	up	PREDICTED: probable plastid-lipid-associated protein 10, chloroplastic [Sesamum indicum]
c46714.graph_c0	0.45386591	0.63100054	0.61563764	6.55071709	7.28421408	8.61916623	4.981E-17	2.700335	up	PREDICTED: boron transporter 4-like [Sesamum indicum]

#ID	0 h-1 FPKM	0 h-2 FPKM	0 h-3 FPKM	48 h-1 FPKM	48 h-2 FPKM	48 h-3 FPKM	FDR	log2FC	regulated	nr_annotation
c46718.graph_c0	0.18582979	0.14881269	0.04775973	5.99194819	7.76238231	8.07356491	4.742E-22	4.823217	up	PREDICTED: probable receptor-like protein kinase At1g80640 isoform X1 [Sesamum indicum]
c46718.graph_c1	0.44147464	0.47137783	0.60513322	2.11491923	3.50437254	2.67800651	0.0011827	1.422319	up	PREDICTED: uncharacterized protein LOC105166978 [Sesamum indicum]
c46719.graph_c1	0.49779863	0.18686141	0.47976821	1.9490694	2.6720976	2.29535766	0.0037276	1.545587	up	PREDICTED: G2/mitotic-specific cyclin-2-like [Sesamum indicum]
c46719.graph_c2	4.02228851	4.22541978	4.31679999	21.1951863	20.6634709	21.8848165	3.199E-15	1.324969	up	PREDICTED: 1,4-alpha-glucan-branching enzyme-like isoform X1 [Sesamum indicum]
c46722.graph_c0	31.590426	31.940224	27.8423708	32.6820852	27.7119521	25.519467	1.367E-15	-1.10095	down	PREDICTED: E3 ubiquitin-protein ligase MBR2-like isoform X1 [Sesamum indicum]
c46725.graph_c0	1.28400595	1.28529169	1.59677273	0.80132615	0.88933208	0.64510898	0.0001218	-1.85442	down	PREDICTED: GDSL esterase/lipase At1g33811 [Sesamum indicum]
c46728.graph_c0	2.05177401	2.02966586	2.66762978	18.6354247	17.715947	16.1265828	1.131E-23	1.940233	up	hypothetical protein MIMGU_mgv1a007859mg [Erythranthe guttata]
c46730.graph_c0	17.9809927	14.5244439	16.493377	8.01305106	8.35098591	7.3119359	2.948E-36	-2.066372	down	PREDICTED: protein-lysine N-methyltransferase EFM1 isoform X1 [Sesamum indicum]
c46732.graph_c0	2.16230317	1.39487963	2.03767313	7.61407579	7.83170778	7.60213406	0.003634	1.023865	up	hypothetical protein MIMGU_mgv1a009658mg [Erythranthe guttata]
c46734.graph_c0	49.2876437	48.4121098	49.0515233	37.8258599	48.2441022	47.2905281	2.562E-16	-1.15986	down	PREDICTED: protein RCC2 homolog isoform X1 [Sesamum indicum]
c46737.graph_c0	0	0	0	23.2659064	35.155991	20.8623432	6.604E-23	Inf	up	60S ribosomal protein L12 [Triticum urartu]
c46739.graph_c0	0	0	0	2.84852112	3.97782814	2.70741039	1.177E-12	Inf	up	predicted protein [Trichoderma reesei QM6a]
c46740.graph_c0	267.531722	273.896711	257.507924	11702.299	12755.1806	14170.128	3.811E-74	4.576057	up	hypothetical protein MIMGU_mgv1a004735mg [Erythranthe guttata]
c46742.graph_c0	0	0	0	1.97722944	1.14252809	1.33282338	1.304E-07	Inf	up	PREDICTED: homeobox-leucine zipper protein REVOLUTA-like isoform X3 [Sesamum indicum]
c46745.graph_c0	6.56906508	7.54414856	6.34749104	27.069133	32.8512006	35.1479573	6.301E-10	1.195496	up	PREDICTED: uncharacterized protein LOC105166530 [Sesamum indicum]
c46745.graph_c1	0.61835478	0.80102513	0.63101412	9.63410393	8.60867823	9.95885504	2.465E-28	2.766229	up	PREDICTED: chloride channel protein CLC-b-like [Nicotiana sylvestris]
c46745.graph_c2	3.83353249	3.98785634	4.53986918	34.9707566	34.4796432	41.6533382	1.177E-19	2.147622	up	PREDICTED: BTB/POZ domain-containing protein At5g48800-like isoform X2 [Sesamum indicum]
c46746.graph_c0	0.87470229	0.78243156	0.90878785	7.78400197	7.81028343	7.85676529	3.997E-17	2.173724	up	PREDICTED: putative leucine--tRNA ligase, mitochondrial isoform X3 [Sesamum indicum]
c46749.graph_c1	0.10474247	0	0.04486607	9.23141185	11.6536935	15.9988035	7.744E-23	6.926641	up	PREDICTED: BTB/POZ domain-containing protein NPY1 [Sesamum indicum]
c46751.graph_c1	2.20664946	2.04879684	1.19178733	8.13163766	10.2687147	9.96189591	2.286E-06	1.364607	up	transposase [Pisum sativum]

#ID	0 h-1 FPKM	0 h-2 FPKM	0 h-3 FPKM	48 h-1 FPKM	48 h-2 FPKM	48 h-3 FPKM	FDR	log2FC	regulated	nr_annotation
c46752.graph_c0	0.85295262	0.91948416	0.67450947	8.25085368	9.95744421	9.62739368	2.271E-14	2.489882	up	PREDICTED: pollen-specific protein SF21-like [Sesamum indicum]
c46753.graph_c0	1.32854418	1.14431063	0.43673374	12.9225457	12.4535604	11.7010185	1.794E-20	2.664782	up	PREDICTED: uncharacterized protein LOC105158178 [Sesamum indicum]
c46756.graph_c1	100.530254	95.6048031	97.4615591	35.8924276	32.8963774	35.4684688	2.388E-61	-2.509856	down	PREDICTED: uncharacterized protein LOC105173509 [Sesamum indicum]
c46756.graph_c2	144.928747	140.16661	140.734433	100.807046	106.653486	113.263094	1.598E-23	-1.427709	down	PREDICTED: trihelix transcription factor ASIL2 [Sesamum indicum]
c46759.graph_c0	12.3478613	10.4228863	10.2964594	55.1309184	54.2111841	44.5494464	8.412E-11	1.204193	up	farnesyl diphosphate synthase [Bacopa monnieri]
c46761.graph_c1	41.007158	37.9467994	39.0534589	20.3164209	20.3666018	17.7412786	3.076E-40	-2.030414	down	PREDICTED: uncharacterized protein LOC105178437 [Sesamum indicum]
c46762.graph_c0	1.07059126	0.98477167	0.96674476	0.29322388	0.05647912	0.09487594	2.558E-12	-3.752961	down	PREDICTED: phospholipase D beta 1-like [Sesamum indicum]
c46762.graph_c2	4.18122222	3.10530351	3.23537657	0.20709756	0.0877579	0.25798383	5.65E-26	-5.26066	down	hypothetical protein JCGZ_20700 [Jatropha curcas]
c46766.graph_c0	0	0	0.04472649	1.21847499	1.52861034	1.6833503	3.771E-11	5.575435	up	PREDICTED: protein ESKIMO 1-like [Sesamum indicum]
c46767.graph_c0	15.7976799	15.0567473	14.8737392	10.1348614	10.2532528	9.14615587	2.086E-24	-1.646994	down	PREDICTED: MYST-like histone acetyltransferase 2 isoform X2 [Sesamum indicum]
c46769.graph_c0	1.39768571	1.25917675	1.05199057	1.2324547	0.87691991	1.03116028	0.0010679	-1.249316	down	PREDICTED: endoglucanase 11-like [Sesamum indicum]
c46770.graph_c0	29.2311509	27.517102	27.1630984	6.29197262	5.93236802	6.13042254	1.557E-84	-3.208666	down	PREDICTED: diphthamide biosynthesis protein 2 [Sesamum indicum]
c46771.graph_c0	5.29875796	5.3757404	4.69276759	0.46176933	0.83860904	0.52827373	1.127E-31	-4.095137	down	PREDICTED: LOW QUALITY PROTEIN: uncharacterized protein LOC105163603 [Sesamum indicum]
c46775.graph_c0	12.6482602	13.7921422	11.7851948	80.2854521	84.2042954	82.8390108	7.155E-29	1.676701	up	PREDICTED: gamma-tubulin complex component 3 [Sesamum indicum]
c46778.graph_c0	0.22975133	0.17248604	0.07380989	1.53454882	1.56961087	1.36543232	0.0001775	2.223533	up	PREDICTED: probable ubiquitin-like-specific protease 2B isoform X1 [Sesamum indicum]
c46782.graph_c0	0	0	0	0.79332118	0.80040723	0.73950634	1.659E-09	Inf	up	UTP-glucose-1-phosphate uridylyltransferase [Mucor circinelloides f. circinelloides 1006PhL]
c46784.graph_c0	20.8217559	17.1096017	18.769682	17.4645364	9.70573568	8.15204694	2.493E-05	-1.686039	down	-
c46784.graph_c1	7.93945177	9.04582334	8.46061793	60.6555093	79.4394855	83.9723824	4.233E-23	2.115488	up	PREDICTED: soluble inorganic pyrophosphatase [Sesamum indicum]
c46792.graph_c1	24.4781464	26.6396106	22.8953966	18.6765525	19.8952013	18.9866856	7.628E-23	-1.379991	down	PREDICTED: LOW QUALITY PROTEIN: protein ALWAYS EARLY 3-like [Sesamum indicum]
c46797.graph_c0	0	0	0	0.68318037	1.09809853	0.79654523	1.315E-11	Inf	up	hypothetical protein GUITHDRAFT_89280 [Guillardia theta CCMP2712]
c46802.graph_c0	0.11272624	0.1253768	0.0482859	2.71165893	2.40815636	2.35121395	3.337E-17	3.698302	up	PREDICTED: callose synthase 7 [Sesamum indicum]
c46804.graph_c0	1.04418354	1.47773774	0.97166073	0.82928447	0.59739791	0.61982956	0.0001448	-1.780499	down	hypothetical protein MIMGU_mgv1a013877mg [Erythranthe guttata]

#ID	0 h-1 FPKM	0 h-2 FPKM	0 h-3 FPKM	48 h-1 FPKM	48 h-2 FPKM	48 h-3 FPKM	FDR	log2FC	regulated	nr_annotation
c46804.graph_c1	6.44340444	6.29443831	5.48676674	4.8394584	5.3288615	5.93947722	1.608E-08	-1.196569	down	PREDICTED: probable serine/threonine-protein kinase abkC isoform X1 [Sesamum indicum]
c46807.graph_c1	63.8047329	61.1200834	62.5552312	21.9517971	30.70422	32.0837838	1.829E-49	-2.169393	down	hypothetical protein MIMGU_mgv1a012384mg [Erythranthe guttata]
c46809.graph_c1	515.97447	510.28739	517.220676	378.818383	370.806109	361.399211	6.689E-20	-1.491166	down	PREDICTED: pentatricopeptide repeat-containing protein At5g14080-like [Sesamum indicum]
c46810.graph_c0	18.9105109	20.2215166	17.5210217	96.8289772	70.3874585	61.9346164	0.0045476	1.00729	up	PREDICTED: putative 1-phosphatidylinositol-3-phosphate 5-kinase FAB1C isoform X1 [Sesamum indicum]
c46818.graph_c0	11.6693665	12.8491568	10.6079971	7.644858	8.26752506	8.60922254	6.083E-14	-1.536342	down	PREDICTED: geranylgeranyl transferase type-1 subunit beta [Sesamum indicum]
c46821.graph_c0	7.15802122	7.05567727	6.90879735	27.803179	37.6298585	38.562763	8.694E-12	1.276869	up	PREDICTED: nodal modulator 1 [Sesamum indicum]
c46822.graph_c0	5.57503254	5.70744725	7.13157328	4.78589609	4.82275578	5.11015387	2.589E-09	-1.343847	down	PREDICTED: uncharacterized protein LOC105160395 [Sesamum indicum]
c46832.graph_c0	1.56198078	1.53911448	1.16041663	8.38666358	10.3376393	11.4036719	1.245E-12	1.802683	up	PREDICTED: cytochrome c-type biogenesis ccda-like chloroplastic protein [Sesamum indicum]
c46833.graph_c0	61.0235244	57.9088477	62.939766	24.921492	24.4146936	23.1991631	6.737E-57	-2.34333	down	PREDICTED: F-box protein SKIP17-like [Sesamum indicum]
c46836.graph_c0	2.22752375	2.90837515	2.57206211	39.317816	51.6717338	54.9382406	9.13E-37	3.219451	up	PREDICTED: probable pectate lyase 12 [Sesamum indicum]
c46837.graph_c0	4.60673214	4.98025269	4.69943681	2.53335474	3.76282861	3.79954417	1.106E-15	-1.525413	down	PREDICTED: protein ROOT PRIMORDIUM DEFECTIVE 1-like [Sesamum indicum]
c46838.graph_c0	69.644263	73.0703166	71.0439443	25.2540453	27.6278994	26.4783126	1.295E-64	-2.44834	down	PREDICTED: regulator of nonsense transcripts UPF3-like [Sesamum indicum]
c46841.graph_c0	241.047589	221.643557	249.413677	68.1070027	77.6740788	79.6501134	4.732E-75	-2.680196	down	PREDICTED: uncharacterized protein LOC104591752 [Nelumbo nucifera]
c46842.graph_c0	12.5275414	13.1638074	11.1255866	7.88547155	8.22567488	8.81760159	3.131E-18	-1.57987	down	PREDICTED: uncharacterized protein LOC105174093 isoform X2 [Sesamum indicum]
c46843.graph_c1	4.82520577	5.5687491	4.44982832	2.09190319	2.4931325	2.72224321	2.364E-14	-2.042385	down	hypothetical protein F383_09329 [Gossypium arboreum]
c46844.graph_c1	22.3812256	23.4670243	23.1140959	10.7210136	13.6904018	13.1613343	2.221E-36	-1.89798	down	PREDICTED: uncharacterized protein LOC105173956 [Sesamum indicum]
c46848.graph_c0	28.8429776	30.2958117	27.6301838	22.0680427	24.1443975	22.7858156	6.545E-22	-1.348673	down	PREDICTED: serine/threonine-protein phosphatase 4 regulatory subunit 3A-like isoform X1 [Beta vulgaris subsp. vulgaris]
c46849.graph_c0	3.4500809	3.13766347	3.67655314	13.0044692	15.7066222	14.38221	7.135E-09	1.0484	up	PREDICTED: patellin-3 [Sesamum indicum]
c46852.graph_c0	476.026893	447.305302	455.919056	452.214102	476.913781	464.619756	1.579E-10	-1.002921	down	PREDICTED: uncharacterized protein LOC105178178 [Sesamum indicum]
c46855.graph_c0	2.49235888	3.11856826	2.0017374	15.4988341	13.5305838	9.44923087	0.0052494	1.328683	up	-
c46855.graph_c1	0.50419743	0.6234558	0.26678798	9.53590276	6.57072814	5.61613881	5.784E-07	2.959572	up	PREDICTED: peptide-N4-(N-acetyl-beta-glucosaminyl)asparagine amidase A-like [Sesamum indicum]
c46858.graph_c0	0	0	0	3.03674433	2.80956782	2.39584147	4.417E-22	Inf	up	predicted protein [Hordeum vulgare subsp. vulgare]

#ID	0 h-1 FPKM	0 h-2 FPKM	0 h-3 FPKM	48 h-1 FPKM	48 h-2 FPKM	48 h-3 FPKM	FDR	log2FC	regulated	nr_annotation
c46859.graph_c0	33.7597557	31.6079078	33.3463443	147.152767	149.989295	152.276368	1.67E-15	1.168716	up	PREDICTED: NADH dehydrogenase [ubiquinone] 1 alpha subcomplex subunit 8-B-like [Sesamum indicum]
c46861.graph_c0	6.85524604	5.64972351	5.35403944	1.24971021	1.46576462	2.52182228	4.563E-30	-2.792313	down	PREDICTED: pentatricopeptide repeat-containing protein At3g54980, mitochondrial-like [Sesamum indicum]
c46863.graph_c0	2.52902963	3.68674088	2.90282616	14.0699242	15.7680693	15.800124	2.679E-11	1.303892	up	PREDICTED: myosin-2 heavy chain [Sesamum indicum]
c46867.graph_c0	6.22695421	6.55615275	6.34346141	4.96385986	6.61262037	5.84500358	6.516E-07	-1.156614	down	PREDICTED: U11/U12 small nuclear ribonucleoprotein 59 kDa protein isoform X1 [Sesamum indicum]
c46867.graph_c1	5.9863192	7.61734779	6.519201	42.9982092	56.0480133	59.7181641	6.169E-18	1.957308	up	-
c46868.graph_c0	18.2499191	18.7244759	19.8288694	7.49664534	8.13950755	10.9190888	3.647E-45	-2.119056	down	PREDICTED: putative glycerol-3-phosphate transporter 4 isoform X1 [Sesamum indicum]
c46875.graph_c0	39.2994449	39.2313143	34.1229017	34.444412	29.2273727	21.0278865	1.19E-19	-1.421551	down	PREDICTED: uncharacterized protein LOC105163168 [Sesamum indicum]
c46878.graph_c0	3.52640345	2.99599492	2.43714584	10.8928773	12.4073279	14.8665639	2.742E-06	1.072893	up	PREDICTED: LOW QUALITY PROTEIN: ATP-dependent Clp protease ATP-binding subunit clpX-like, mitochondrial [Sesamum indicum]
c46883.graph_c0	26.405815	23.1759245	22.8285177	122.265963	158.149662	183.103918	1.779E-11	1.65628	up	PREDICTED: serine hydroxymethyltransferase 4 [Sesamum indicum]
c46886.graph_c0	0	0	0	9.36138381	3.93876636	11.9333334	3.594E-08	Inf	up	-
c46888.graph_c1	6.11006754	4.64954945	4.80715023	45.3478549	49.6226364	53.6384875	3.796E-44	2.236914	up	PREDICTED: probable sphingolipid transporter spinster homolog 2 [Sesamum indicum]
c46890.graph_c0	6.58533004	7.02472736	6.58187431	27.1475571	32.2652231	29.390384	5.293E-11	1.117074	up	PREDICTED: cleavage and polyadenylation specificity factor subunit 2 [Sesamum indicum]
c46891.graph_c0	125.717258	134.820094	117.264677	83.133956	85.2606654	77.7411566	3.992E-28	-1.634367	down	PREDICTED: primary amine oxidase-like [Sesamum indicum]
c46895.graph_c0	0	0	0	1.67162478	1.70763791	1.16867274	1.517E-11	Inf	up	trypsin-like protease [Conidiobolus coronatus]
c46898.graph_c0	0	0	0	1.29514608	2.2359337	0.93900334	5.573E-06	Inf	up	hypothetical protein HMPREF1544_02206 [Mucor circinelloides f. circinelloides 1006PhL]
c46899.graph_c0	19.4485729	20.029369	16.8368412	10.0943656	9.83373856	10.4922669	1.099E-41	-1.904253	down	PREDICTED: nuclear-pore anchor [Sesamum indicum]
c46900.graph_c2	87.7322834	89.5017231	85.6229526	29.3735727	35.9359691	36.478585	9.204E-58	-2.389663	down	PREDICTED: 1,4-alpha-glucan-branching enzyme 3, chloroplastic/amyloplastic [Sesamum indicum]
c46903.graph_c1	3.51174154	2.65787802	3.90736111	26.1186882	28.9234917	33.0699654	1.467E-25	2.105639	up	hypothetical protein MIMGU_mgv1a010174mg [Erythranthe guttata]
c46907.graph_c0	6.59517583	5.79668479	4.7543031	4.89036348	3.29683908	3.56024947	0.0006188	-1.552614	down	-
c46907.graph_c1	12.9990798	11.7261951	12.6559882	13.0181819	11.9404201	10.3048019	1.521E-10	-1.098797	down	PREDICTED: endophilin-A2 isoform X1 [Sesamum indicum]
c46910.graph_c0	1.78511556	1.1562314	1.07950314	21.0891262	24.8265934	23.865092	1.249E-51	3.100984	up	PREDICTED: cytosolic endo-beta-N-acetylglucosaminidase [Sesamum indicum]

#ID	0 h-1 FPKM	0 h-2 FPKM	0 h-3 FPKM	48 h-1 FPKM	48 h-2 FPKM	48 h-3 FPKM	FDR	log2FC	regulated	nr_annotation
c46912.graph_c0	48.2427553	44.1536762	49.1718293	26.4762328	28.8677485	25.7024712	9.742E-37	-1.823451	down	PREDICTED: uncharacterized protein LOC105158577 [Sesamum indicum]
c46913.graph_c2	220.391984	217.392697	199.780805	99.9972111	96.3437485	79.4212123	7.532E-49	-2.223359	down	PREDICTED: serine/threonine-protein kinase HT1-like [Sesamum indicum]
c46915.graph_c1	158.037291	149.981082	155.610949	70.597821	93.6037837	89.8231779	6.532E-37	-1.890381	down	PREDICTED: uncharacterized protein At1g04910-like [Sesamum indicum]
c46916.graph_c0	5.17659731	3.10478607	3.10616104	14.0508703	18.663903	22.5674094	0.0001239	1.259277	up	plastidic aldolase [Solanum tuberosum]
c46917.graph_c0	0	0	0	1.66358627	0.95586057	3.06085398	9.389E-06	Inf	up	hypothetical protein CICLE_v10005479mg [Citrus clementina]
c46919.graph_c0	50.1402062	46.5304008	50.9920916	31.9351836	33.8867801	32.8302802	2.668E-29	-1.600672	down	PREDICTED: protein REVEILLE 7-like isoform X2 [Sesamum indicum]
c46925.graph_c0	148.998171	142.405006	153.303666	1000.49512	993.187301	901.251725	2.207E-25	1.685586	up	PREDICTED: aquaporin PIP2-7 [Sesamum indicum]
c46929.graph_c1	0.12891214	0.30109621	0.27609535	2.3356581	2.18078	1.90213126	7.295E-05	2.165309	up	-
c46933.graph_c1	0.16645887	0.45822027	0.40107419	7.22674912	7.06695176	7.64133269	3.437E-26	3.396699	up	PREDICTED: probable LRR receptor-like serine/threonine-protein kinase At1g07650 [Sesamum indicum]
c46938.graph_c0	22.2719481	21.0287552	23.0898356	19.7253908	21.5267097	19.734451	2.593E-14	-1.141558	down	PREDICTED: EH domain-containing protein 1-like [Sesamum indicum]
c46941.graph_c0	19.2166944	19.9168552	18.0508142	562.569439	552.356528	586.945453	9.15E-132	3.878631	up	Expansin A1, ALPHA 1.2,EXPA1 [Theobroma cacao]
c46943.graph_c0	4.83680553	4.69924742	4.57021074	1.11399518	1.59666381	2.09906728	1.089E-09	-2.577514	down	PREDICTED: molybdenum cofactor sulfurase-like [Sesamum indicum]
c46943.graph_c1	2.24991479	1.77902325	2.13805213	14.9274151	19.0614249	21.1246299	1.464E-20	2.136811	up	PREDICTED: SPX domain-containing membrane protein At4g22990-like [Sesamum indicum]
c46945.graph_c0	1.96555781	2.17739546	1.48181417	23.5162567	23.019877	22.5358628	3.879E-40	2.604666	up	PREDICTED: mitogen-activated protein kinase 9-like [Sesamum indicum]
c46945.graph_c1	2.59052261	2.24736774	2.21928274	22.9109606	22.2487692	24.0668468	1.383E-07	2.277855	up	PREDICTED: mitogen-activated protein kinase 9-like [Sesamum indicum]
c46948.graph_c0	0	0	0	9.7171814	7.63188868	6.11203229	2.019E-12	Inf	up	Long-chain-alcohol oxidase FAO4A [Glycine soja]
c46948.graph_c1	0.06146046	0.15380501	0.11846869	20.2987664	15.4157572	13.6281599	7.841E-25	6.194791	up	PREDICTED: long-chain-alcohol oxidase FAO4A-like isoform X1 [Sesamum indicum]
c46954.graph_c0	5.29112283	4.8345239	5.49478501	2.40892346	2.85219316	2.54691772	1.507E-13	-2.020981	down	hypothetical protein MIMGU_mgv1a007683mg [Erythranthe guttata]
c46956.graph_c0	0.73203614	0.51724882	0.40117893	6.40679472	6.71367639	6.19490812	2.039E-28	2.536161	up	PREDICTED: uncharacterized protein LOC105155208 [Sesamum indicum]
c46957.graph_c0	4.26585206	2.92474224	3.90483585	0.10767072	0.28516017	0.14370713	2.642E-23	-5.401074	down	BRI1 kinase inhibitor 1 [Glycine soja]
c46957.graph_c2	16.1265408	15.694281	10.2177103	2.88882635	4.59054184	4.03929478	2.355E-14	-2.888161	down	PREDICTED: CBL-interacting serine/threonine-protein kinase 6-like [Sesamum indicum]
c46957.graph_c3	19.2861535	18.8324374	17.1928213	17.8220811	22.6275758	15.3737675	3.31E-11	-1.004961	down	PREDICTED: RNA-binding protein 42, partial [Sesamum indicum]

#ID	0 h-1 FPKM	0 h-2 FPKM	0 h-3 FPKM	48 h-1 FPKM	48 h-2 FPKM	48 h-3 FPKM	FDR	log2FC	regulated	nr_annotation
c46957.graph_c6	3.64265611	3.87940072	3.82598795	57.172079	58.5377079	63.3062934	1.942E-88	2.960808	up	PREDICTED: alpha-glucan phosphorylase, H isozyme [Sesamum indicum]
c46959.graph_c0	54.6267508	55.5915447	55.4959728	277.663991	291.267493	277.372223	1.023E-21	1.334406	up	PREDICTED: 60S ribosomal protein L27a-3-like [Sesamum indicum]
c46964.graph_c0	5.20673258	4.92830299	4.18747544	28.7154276	27.6550124	25.3766047	7.544E-16	1.498981	up	hypothetical protein MIMGU_mgv1a000577mg [Erythranthe guttata]
c46965.graph_c0	0.38176897	0.70970946	0.49058824	3.81857084	3.72594598	4.11305534	0.0001981	1.863819	up	-
c46968.graph_c1	30.9294866	29.1015025	31.2794296	5.80476357	8.43660444	8.60840619	2.038E-88	-3.023268	down	PREDICTED: lys-63-specific deubiquitinase BRCC36 [Sesamum indicum]
c46969.graph_c0	23.9850753	23.5650673	23.4522329	15.9083958	14.9048695	16.336913	2.171E-25	-1.607309	down	PREDICTED: serine/threonine-protein kinase HT1-like [Sesamum indicum]
c46970.graph_c2	38.3747714	43.0643976	38.3924617	31.6546656	32.9773642	29.1350727	1.131E-22	-1.370685	down	PREDICTED: preprotein translocase subunit SCY2, chloroplastic [Sesamum indicum]
c46971.graph_c1	45.4522506	51.2434819	48.1615199	28.7611661	35.5386798	34.1716953	9.003E-29	-1.578103	down	PREDICTED: uncharacterized protein LOC105159071 [Sesamum indicum]
c46972.graph_c0	127.101446	120.24303	134.709411	94.3182375	96.0784944	104.067947	1.697E-22	-1.394937	down	unnamed protein product [Coffea canephora]
c46974.graph_c0	1.37497985	1.26810391	1.44925796	0.93936622	1.17607999	1.43106353	0.00042	-1.23041	down	PREDICTED: LOW QUALITY PROTEIN: U3 small nucleolar RNA-associated protein 21 homolog [Sesamum indicum]
c46976.graph_c0	23.5194074	26.5507881	22.4805265	21.1893304	20.7739419	23.0195881	4.815E-17	-1.17582	down	PREDICTED: serine/threonine-protein kinase EDR1 [Sesamum indicum]
c46979.graph_c0	3.98613871	4.69427085	4.59504573	21.9077359	24.4481611	25.9324311	3.077E-19	1.424179	up	PREDICTED: probable boron transporter 2 [Sesamum indicum]
c46981.graph_c0	5.13779598	4.68579042	3.81464561	77.2750606	88.5864713	89.9418618	5.433E-88	3.211992	up	PREDICTED: heterodimeric geranylgeranyl pyrophosphate synthase small subunit, chloroplastic [Sesamum indicum]
c46981.graph_c1	0.68697652	0.9578183	0.4729243	6.55490707	7.7582685	7.58227196	9.763E-14	2.354431	up	PREDICTED: putative membrane-bound O-acyltransferase C24H6.01c [Sesamum indicum]
c46982.graph_c0	0.22131996	0.16615619	0.28440492	3.36425738	3.67202345	4.03668398	8.043E-08	3.018861	up	PREDICTED: LOW QUALITY PROTEIN: ABC transporter B family member 13-like [Sesamum indicum]
c46982.graph_c1	0.16741026	0.12568342	0.18823771	1.60012847	1.87895268	2.84756939	3.955E-07	2.690356	up	PREDICTED: LOW QUALITY PROTEIN: ABC transporter B family member 13-like [Sesamum indicum]
c46984.graph_c0	34.1939153	35.3015827	33.1504036	13.2344273	14.9286266	13.9154813	1.317E-57	-2.305258	down	PREDICTED: uncharacterized protein LOC105171157 [Sesamum indicum]
c46985.graph_c0	3.9280582	3.84194595	3.9688104	3.25966751	2.95572432	3.19539179	3.727E-06	-1.335226	down	PREDICTED: sucrose nonfermenting 4-like protein [Sesamum indicum]
c46988.graph_c0	41.5278838	37.9989241	45.6027967	22.6910675	21.2686784	20.6385762	2.803E-31	-1.971156	down	PREDICTED: aspartic proteinase-like [Sesamum indicum]
c46991.graph_c0	0	0	0	3.47036779	7.47964133	1.59719607	0.0002557	Inf	up	NADH dehydrogenase subunit 10 (mitochondrion) [Nephroselmis olivacea]

#ID	0 h-1 FPKM	0 h-2 FPKM	0 h-3 FPKM	48 h-1 FPKM	48 h-2 FPKM	48 h-3 FPKM	FDR	log2FC	regulated	nr_annotation
c46992.graph_c0	6.99707137	7.04527834	6.82297219	4.39855523	3.73582276	3.913808	1.197E-11	-1.806549	down	PREDICTED: pentatricopeptide repeat-containing protein At4g16835, mitochondrial [Sesamum indicum]
c46994.graph_c0	29.029699	28.0913366	26.4028057	21.0061175	22.7425208	20.9211404	3.014E-20	-1.386517	down	RNA-binding (RRM/RBD/RNP motif) family protein [Medicago truncatula]
c47000.graph_c0	5.5406586	4.91452031	5.6116358	21.5455194	25.8673594	27.4064619	8.739E-17	1.197468	up	PREDICTED: peroxidase 7-like [Sesamum indicum]
c47001.graph_c0	2.41325432	3.07210312	3.10112734	1.5466068	1.40803642	1.78470936	1.903E-08	-1.876673	down	PREDICTED: ATP-dependent zinc metalloprotease FTSH 10, mitochondrial-like [Sesamum indicum]
c47003.graph_c0	0.19581067	0.50090613	0.25162447	1.52332342	1.46513915	1.7299746	0.0073848	1.298646	up	PREDICTED: transcription factor HBP-1b(c38)-like [Sesamum indicum]
c47004.graph_c1	42.0591468	41.5198265	42.2067169	456.628528	516.519445	456.668738	1.324E-59	2.487824	up	PREDICTED: glucan endo-1,3-beta-glucosidase 5 [Sesamum indicum]
c47005.graph_c0	0.36377804	0.23172692	0.29748039	5.78871369	6.55202825	6.47911721	2.314E-15	3.378502	up	PREDICTED: (6-4)DNA photolyase [Sesamum indicum]
c47005.graph_c1	4.93305483	4.47793294	4.21298927	55.7778391	62.1998496	60.2300731	4.953E-64	2.69143	up	PREDICTED: 3-oxoacyl-[acyl-carrier-protein] reductase 4-like [Sesamum indicum]
c47006.graph_c0	0.18585978	0.08586733	0.16534881	1.75177523	2.10695345	2.01578706	9.511E-10	2.726814	up	PREDICTED: putative ATP-dependent helicase hrq1 isoform X1 [Sesamum indicum]
c47007.graph_c1	38.7305071	40.2961273	37.347542	31.3704772	32.4486553	30.261896	3.223E-21	-1.323788	down	PREDICTED: uncharacterized protein LOC105162827 [Sesamum indicum]
c47008.graph_c0	2.69800569	2.99006883	2.16690258	0.31069735	0.18808353	0.03949381	5.945E-20	-4.862399	down	PREDICTED: pyruvate decarboxylase 1-like [Sesamum indicum]
c47010.graph_c0	39.3244757	37.4175294	37.7316861	34.4168584	34.9466317	36.0681649	6.943E-16	-1.1364	down	PREDICTED: probable transcriptional regulator SLK2 [Sesamum indicum]
c47011.graph_c0	0	0	0	3.02914896	3.03269645	3.49502842	1.232E-17	Inf	up	PREDICTED: condensin complex subunit 1 [Sesamum indicum]
c47018.graph_c0	66.0199894	64.3955238	65.0755799	24.2344706	24.5246696	22.4866456	3.445E-66	-2.47315	down	PREDICTED: 65-kDa microtubule-associated protein 6-like [Sesamum indicum]
c47019.graph_c1	2.66330801	2.78854847	3.61914913	2.03057632	1.88225572	2.03264393	2.404E-06	-1.629312	down	PREDICTED: G-type lectin S-receptor-like serine/threonine-protein kinase At5g35370 [Sesamum indicum]
c47021.graph_c0	7.36544331	8.18995728	6.79469165	58.6939521	58.3042467	59.0388732	2.329E-42	1.961412	up	PREDICTED: uncharacterized protein LOC105163284 [Sesamum indicum]
c47022.graph_c0	1.89150628	1.24792295	1.10484566	8.237646	7.55202728	10.9594724	1.869E-06	1.641334	up	PREDICTED: dihydroorotase, mitochondrial isoform X3 [Vitis vinifera]
c47023.graph_c0	113.86264	113.315573	111.71551	64.6034814	65.1582064	56.7209479	9.737E-39	-1.878136	down	unnamed protein product [Coffea canephora]
c47025.graph_c0	83.9107555	74.0656841	75.6178503	4.8160677	7.98011346	3.62602991	5.58E-128	-4.852076	down	-
c47026.graph_c0	14.5558059	16.1095062	15.7629542	13.7560192	12.3385554	12.632152	5.13E-14	-1.277598	down	PREDICTED: uncharacterized protein LOC105170563 isoform X2 [Sesamum indicum]
c47027.graph_c0	0	0	0	0.69955772	0.78010166	0.7862681	1.659E-09	Inf	up	hypothetical protein JAAARDRAFT_31728 [Jaapia argillacea MUCL 33604]

#ID	0 h-1 FPKM	0 h-2 FPKM	0 h-3 FPKM	48 h-1 FPKM	48 h-2 FPKM	48 h-3 FPKM	FDR	log2FC	regulated	nr_annotation
c47028.graph_c0	2.05268382	2.44186406	1.72029129	1.31552433	1.16136542	1.75581845	3.618E-05	-1.570214	down	PREDICTED: probable LRR receptor-like serine/threonine-protein kinase At4g36180 [Sesamum indicum]
c47029.graph_c0	0.07593404	0	0.19515649	1.53901853	1.77862202	1.86737651	1.73E-07	3.219418	up	PREDICTED: uncharacterized protein LOC105170663 [Sesamum indicum]
c47033.graph_c0	27.1938824	27.5824552	24.7994467	9.31165208	10.1355708	9.18835385	1.872E-67	-2.491795	down	PREDICTED: protein CHROMATIN REMODELING 5 isoform X2 [Sesamum indicum]
c47033.graph_c2	1.61314425	1.67686571	2.07295423	0.45727191	1.15050733	2.13610842	0.0022823	-1.555586	down	PREDICTED: probable receptor-like protein kinase At5g24010 [Sesamum indicum]
c47033.graph_c3	2.85110597	1.70444889	1.22126135	24.0044321	31.0336131	32.6552482	1.252E-30	2.908559	up	PREDICTED: polyadenylate-binding protein-interacting protein 9 [Sesamum indicum]
c47033.graph_c4	1.32063384	1.68549423	1.44250676	0.36499621	1.03111794	1.24495716	7.722E-05	-1.786259	down	PREDICTED: probable receptor-like protein kinase At5g24010 [Sesamum indicum]
c47034.graph_c0	0.91002078	0.82812003	1.11625761	12.2706955	12.9589435	11.5287359	8.94E-17	2.666515	up	PREDICTED: primase homolog protein isoform X2 [Sesamum indicum]
c47036.graph_c0	4.378654	3.05247328	2.81337187	0	0	0	8.808E-26	-Inf	down	hypothetical protein SNOG_05978 [Phaeosphaeria nodorum SN15]
c47037.graph_c0	32.9645728	33.5582204	33.9811606	20.3443583	20.1077465	21.4144816	2.047E-29	-1.717966	down	PREDICTED: L-2-hydroxyglutarate dehydrogenase, mitochondrial isoform X1 [Sesamum indicum]
c47037.graph_c1	27.1154278	23.9113203	17.0073849	1.18954765	1.57522687	2.38151778	3.17E-22	-4.745769	down	glyceraldehyde-3-phosphate dehydrogenase [Leucophyllum frutescens]
c47037.graph_c3	252.046144	242.631421	248.036854	1590.49944	1695.76144	1781.43725	2.466E-25	1.751463	up	glyceraldehyde 3-phosphate dehydrogenase [Salvia miltiorrhiza]
c47037.graph_c4	67.8617337	63.2782961	76.63094	3.83495318	3.40130334	4.28523691	3.318E-94	-5.191244	down	PREDICTED: glyceraldehyde-3-phosphate dehydrogenase, cytosolic [Sesamum indicum]
c47049.graph_c0	16.1878922	15.1297968	14.2511503	9.92849573	10.9981137	9.12757109	8.021E-18	-1.617395	down	PREDICTED: lycopene beta cyclase, chloroplastic/chromoplastic [Sesamum indicum]
c47049.graph_c1	9.83851594	9.92459348	9.08095586	4.74240297	4.44430849	4.18230814	6.518E-39	-2.124145	down	PREDICTED: uncharacterized protein At5g05190 [Sesamum indicum]
c47051.graph_c0	46.7080568	51.5133968	42.9557578	45.9501876	44.1863709	46.1438814	3.441E-12	-1.066655	down	PREDICTED: nuclear transcription factor Y subunit C-9-like isoform X1 [Sesamum indicum]
c47051.graph_c1	36.2044343	35.7173563	35.2710022	34.7386144	30.4232995	28.1245414	3.14E-14	-1.214387	down	PREDICTED: probable ribonuclease P/MRP protein subunit POP5 [Sesamum indicum]
c47052.graph_c0	0	0	0	2.17934882	1.9692329	1.36882692	2.615E-11	Inf	up	PREDICTED: putative germin-like protein 2-1 [Sesamum indicum]
c47053.graph_c0	6.40695285	11.8400648	12.033121	11.350967	3.3669925	4.84801066	0.0057985	-1.629612	down	-
c47064.graph_c0	122.479737	121.544928	116.027181	71.0450917	77.1437353	73.9127186	1.618E-32	-1.715102	down	PREDICTED: pentatricopeptide repeat-containing protein At3g46790, chloroplastic-like [Nicotiana tomentosiformis]
c47064.graph_c1	0.89495602	0.73587858	0.28751348	6.0659044	7.26834473	9.03934376	3.658E-12	2.530917	up	lipoxygenase [Actinidia arguta]

#ID	0 h-1 FPKM	0 h-2 FPKM	0 h-3 FPKM	48 h-1 FPKM	48 h-2 FPKM	48 h-3 FPKM	FDR	log2FC	regulated	nr_annotation
c47064.graph_c2	61.5252139	62.2719659	59.6143693	28.9360563	22.3412279	21.757259	3.75E-59	-2.340026	down	PREDICTED: RNA pseudouridine synthase 6, chloroplastic [Sesamum indicum]
c47065.graph_c0	0	0	0	17.3014338	31.8949483	9.27319252	5.419E-08	Inf	up	predicted protein [Physcomitrella patens]
c47067.graph_c0	1.64347234	2.58518548	2.21249498	0.54074068	0.45827935	0.41699477	6.39E-12	-3.200027	down	-
c47068.graph_c0	1.61141186	1.55098601	1.39375926	6.33783408	8.68002632	8.86549081	4.895E-06	1.368214	up	hypothetical protein MIMGU_mgv1a009241mg [Erythranthe guttata]
c47069.graph_c0	28.0906474	24.8591972	26.316961	120.900898	169.930743	193.381122	2.626E-08	1.586659	up	PREDICTED: 60S ribosomal protein L27a-3-like [Sesamum indicum]
c47072.graph_c0	0	0	0	4.45124666	5.92467304	2.33579302	1.381E-06	Inf	up	hypothetical protein GUITHDRAFT_106940 [Guillardia theta CCMP2712]
c47073.graph_c0	0	0	0	21.2946952	36.1339833	21.151135	1.575E-21	Inf	up	component of cytosolic 80S ribosome and 40S small subunit [Volvox carteri f. nagariensis]
c47075.graph_c0	36.5411009	37.9470607	31.3528357	29.2465328	31.2997312	31.3413628	1.709E-18	-1.220918	down	PREDICTED: zinc finger CCCH domain-containing protein 19 [Sesamum indicum]
c47078.graph_c0	40.1341192	38.9268861	37.4440877	23.8221697	24.3066446	20.5658761	1.388E-36	-1.778043	down	PREDICTED: signal peptide peptidase-like 4 isoform X1 [Sesamum indicum]
c47081.graph_c0	1.71550716	1.52642221	1.98676676	7.59000035	9.96300819	12.2744227	7.895E-07	1.486132	up	hypothetical protein MIMGU_mgv1a0002972mg, partial [Erythranthe guttata]
c47082.graph_c2	6.89630178	7.22771713	7.08204228	29.2144255	32.1859565	32.6867952	2.882E-12	1.130039	up	PREDICTED: uncharacterized protein LOC103719963 isoform X1 [Phoenix dactylifera]
c47083.graph_c0	1.55411614	1.69179368	1.57271686	0.23265989	0.34127299	0.46181244	3.904E-15	-3.244001	down	PREDICTED: pleiotropic drug resistance protein 2-like [Sesamum indicum]
c47085.graph_c0	227.289717	228.624879	221.957023	183.105843	198.297911	198.060931	5.129E-16	-1.244942	down	hypothetical protein MIMGU_mgv1a006811mg [Erythranthe guttata]
c47086.graph_c0	56.3256488	52.4709027	46.2040406	32.2346193	36.9765404	35.3758765	1.567E-28	-1.585415	down	hypothetical protein MIMGU_mgv1a001579mg [Erythranthe guttata]
c47088.graph_c0	7.75824143	8.73952708	7.78257375	37.6103823	39.5416303	40.9485717	1.518E-17	1.26395	up	PREDICTED: serine/threonine-protein kinase D6PKL2-like [Sesamum indicum]
c47102.graph_c0	4.81657012	3.97340946	4.66543967	49.769546	63.68551	68.9467444	1.673E-33	2.737964	up	PREDICTED: dolichyl-diphosphooligosaccharide--protein glycosyltransferase subunit 2 [Sesamum indicum]
c47103.graph_c0	3.49395298	4.18709	3.85749215	12.6034134	18.4907859	18.5966097	1.832E-05	1.081808	up	PREDICTED: uncharacterized protein LOC105164063 [Sesamum indicum]
c47103.graph_c2	398.471809	399.301431	397.543187	174.490548	166.738283	164.177297	2.871E-50	-2.258247	down	PREDICTED: two-pore potassium channel 1 [Sesamum indicum]
c47105.graph_c0	48.8088317	44.366608	52.1512602	1.62829089	3.05188196	2.7862374	5.51E-136	-5.311572	down	R3-MYB anthocyanin repressor [Petunia x hybrida]
c47107.graph_c0	45.672473	42.5626968	43.9318457	8.9826153	7.67957226	7.37169214	7.56E-118	-3.473026	down	hypothetical protein MIMGU_mgv1a008454mg [Erythranthe guttata]

#ID	0 h-1 FPKM	0 h-2 FPKM	0 h-3 FPKM	48 h-1 FPKM	48 h-2 FPKM	48 h-3 FPKM	FDR	log2FC	regulated	nr_annotation
c47111.graph_c0	6.25376666	5.92164892	5.90779424	4.57795962	5.37766175	6.26256906	1.771E-06	-1.178185	down	PREDICTED: putative white-brown complex homolog protein 30 [Sesamum indicum]
c47114.graph_c0	0	0.0349816	0	3.70243524	4.26334912	4.2684025	1.073E-19	7.441706	up	PREDICTED: subtilisin-like protease SBT5.3 [Sesamum indicum]
c47116.graph_c0	19.7034079	19.1324082	18.4944944	15.3338345	15.339263	14.9668841	1.154E-21	-1.345655	down	hypothetical protein JCGZ_07037 [Jatropha curcas]
c47120.graph_c0	12.9238548	12.9367961	12.3946912	9.84895974	9.22809751	9.81517057	5.433E-12	-1.421158	down	hypothetical protein MIMGU_mgv1a010617mg [Erythranthe guttata]
c47126.graph_c0	30.6717857	33.7837532	30.2318789	20.7622013	21.9950347	23.6107778	2.829E-21	-1.531212	down	PREDICTED: INO80 complex subunit D-like [Sesamum indicum]
c47127.graph_c0	0	0.45289803	0	24.5924873	30.0269079	33.7505848	1.183E-13	6.597939	up	-
c47130.graph_c0	36.5491793	36.8770078	31.2646948	633.157988	352.058951	282.226695	0.0013913	2.596117	up	PREDICTED: uncharacterized protein LOC105169217 [Sesamum indicum]
c47131.graph_c0	38.0367345	31.9165308	37.9323113	152.375133	160.152839	136.794767	1.265E-13	1.04041	up	PREDICTED: remorin-like [Sesamum indicum]
c47134.graph_c0	33.974959	34.7371813	31.2031666	10.5963367	12.3960596	13.5384403	3.761E-65	-2.471606	down	PREDICTED: dynamin-related protein 4C-like [Sesamum indicum]
c47142.graph_c0	1.01793628	0.81100547	0.85426183	355.919746	523.135049	527.008007	3.26E-79	8.010174	up	hypothetical protein MIMGU_mgv1a004716mg [Erythranthe guttata]
c47143.graph_c0	3.14062802	4.16549907	3.32956086	18.2281137	17.6246409	18.0214066	2.577E-05	1.324287	up	PREDICTED: 15-cis-phytoene desaturase, chloroplastic/chromoplastic [Sesamum indicum]
c47144.graph_c1	0	0	0	5.07788897	6.9018815	5.88228264	5.269E-14	Inf	up	Peptide methionine sulfoxide reductase [Ectocarpus siliculosus]
c47145.graph_c0	88.0486774	91.6675493	90.4272971	81.6377031	71.3556414	75.2465229	4.384E-19	-1.258498	down	PREDICTED: uncharacterized protein LOC105156360 [Sesamum indicum]
c47147.graph_c0	11.76611	11.9960011	10.0488338	6.13357961	9.00238418	11.014474	1.499E-16	-1.394524	down	PREDICTED: CDT1-like protein a, chloroplastic [Sesamum indicum]
c47150.graph_c1	0.20756529	0.10388657	0.40009443	7.45768478	7.19149236	7.62833772	1.805E-20	3.940779	up	hypothetical protein MIMGU_mgv1a011470mg [Erythranthe guttata]
c47165.graph_c0	0	0	0	25.4159255	43.1117296	23.2118128	1.022E-18	Inf	up	40S ribosomal protein S8-B [Rozella allomycis CSF55]
c47166.graph_c0	154.787709	159.036635	155.603271	105.479796	113.148243	113.491861	3.436E-23	-1.517933	down	PREDICTED: splicing factor U2af large subunit B isoform X2 [Sesamum indicum]
c47166.graph_c1	2.54027767	2.27037623	2.68143642	0.16716211	0.17708798	0.07436992	1.183E-15	-5.17578	down	-
c47173.graph_c0	0.25566919	0.25592521	0.39425405	2.30828355	2.59506163	3.14371963	6.509E-05	2.125035	up	PREDICTED: alpha-N-acetylglucosaminidase-like [Sesamum indicum]
c47175.graph_c0	5.75987232	4.30643479	5.57409262	3.27553708	4.68454638	4.05122894	4.083E-07	-1.404602	down	PREDICTED: O-acyltransferase WSD1-like isoform X1 [Sesamum indicum]
c47176.graph_c0	463.042836	472.071847	416.757961	101.983206	119.644961	120.872392	5.922E-75	-3.000082	down	PREDICTED: very-long-chain (3R)-3-hydroxyacyl-CoA dehydratase PASTICCINO 2 [Sesamum indicum]
c47176.graph_c1	11.3696656	12.6456118	14.204624	1.45478855	2.4658753	3.62449389	6.673E-11	-3.371578	down	-

#ID	0 h-1 FPKM	0 h-2 FPKM	0 h-3 FPKM	48 h-1 FPKM	48 h-2 FPKM	48 h-3 FPKM	FDR	log2FC	regulated	nr_annotation
c47178.graph_c2	0.87416788	0.87504323	1.05726149	42.6356718	39.0948286	36.6302467	4.402E-70	4.380965	up	PREDICTED: uncharacterized protein LOC105158099 [Sesamum indicum]
c47182.graph_c0	15.0968758	16.4675539	13.083777	165.512518	110.089519	90.8218714	0.0002438	2.031258	up	PREDICTED: fructose-1,6-bisphosphatase, cytosolic [Sesamum indicum]
c47184.graph_c2	14.1375266	14.4634706	13.0528692	8.14623542	9.72769162	10.9507237	1.529E-24	-1.551803	down	PREDICTED: myosin-6-like [Sesamum indicum]
c47184.graph_c4	12.7942346	15.6758244	10.1277164	6.97783498	5.69396882	7.04786744	1.45E-10	-1.980967	down	PREDICTED: calcium-dependent protein kinase 10-like [Sesamum indicum]
c47185.graph_c0	1.15154259	1.56171674	1.33657352	9.03455813	10.8399039	10.9925806	4.178E-10	1.908876	up	PREDICTED: protein LOW PSII ACCUMULATION 1, chloroplastic [Sesamum indicum]
c47185.graph_c1	0.33072419	0.60693484	0.92081965	2.69138151	4.572667	6.50655873	0.003306	1.852061	up	PREDICTED: rapid alkalinization factor-like [Sesamum indicum]
c47186.graph_c0	39.490125	41.8058489	38.6685423	14.0705319	13.6484423	11.8053409	2.64E-66	-2.617053	down	PREDICTED: BTB/POZ domain-containing protein At1g04390 [Sesamum indicum]
c47186.graph_c1	42.6558914	41.117175	37.8349306	13.8268592	13.9470252	12.067591	2.462E-52	-2.625233	down	PREDICTED: BTB/POZ domain-containing protein At1g04390 [Sesamum indicum]
c47188.graph_c1	51.8692558	52.1398106	54.6563188	208.671042	222.100765	233.318019	1.952E-13	1.04585	up	PREDICTED: 60S acidic ribosomal protein P1-like [Sesamum indicum]
c47189.graph_c1	42.9880869	50.8915353	46.4177896	33.4122392	36.3586509	36.3167186	3.452E-23	-1.4224	down	PREDICTED: inactive rhomboid protein 1-like [Sesamum indicum]
c47189.graph_c3	9.8133779	9.61270728	8.42206535	6.50609231	6.63587436	6.82335946	4.391E-20	-1.49654	down	PREDICTED: probable apyrase 7 [Sesamum indicum]
c47197.graph_c0	0.40936998	0.29026076	0.24110929	1.74426476	1.98101445	2.64265639	4.189E-05	1.741352	up	PREDICTED: acyltransferase-like protein At1g54570, chloroplastic isoform X1 [Nelumbo nucifera]
c47199.graph_c0	0	0	0	32.4175805	63.5620776	29.4293045	8.806E-15	Inf	up	RecName: Full=60S ribosomal protein L13a [Cyanophora paradoxa]
c47200.graph_c0	0	0	0	15.2795572	27.1021006	6.19878665	7.232E-07	Inf	up	--
c47201.graph_c0	8.70754544	7.86230636	8.61897049	100.437092	88.7727056	86.2816654	3.511E-37	2.436136	up	PREDICTED: probable serine/threonine-protein kinase At1g01540 [Sesamum indicum]
c47202.graph_c1	0	0	0.05051321	1.15883909	1.95656639	0.80556879	1.594E-05	5.223546	up	-
c47205.graph_c0	0.77524342	0.51734647	0.79697479	6.9039798	7.01128409	5.42289188	4.845E-07	2.192573	up	unnamed protein product [Vitis vinifera]
c47206.graph_c0	15.7871858	9.25087252	10.2685883	0	0	0	1.187E-40	-Inf	down	alpha-2 macroglobulin family-like protein [Micromonas sp. RCC299]
c47207.graph_c0	39.2797961	37.869003	34.4131004	182.651484	226.056319	188.478241	2.326E-23	1.401438	up	hypothetical protein MIMGU_mgv1a001548mg [Erythranthe guttata]
c47210.graph_c0	0	0	0	2.02643002	2.36581372	2.06069171	5.637E-13	Inf	up	hypothetical protein V492_07146 [Pseudogymnoascus pannorum VKM F-4246]
c47211.graph_c0	0.17719087	0.1773683	0.19923512	2.65264647	1.42669524	1.76115355	0.0001373	2.390798	up	PREDICTED: uncharacterized protein LOC104880154 [Vitis vinifera]
c47214.graph_c1	3.53836707	3.52044408	3.47221008	2.84489414	2.51151698	2.60168552	5.329E-08	-1.419204	down	PREDICTED: protein LONGIFOLIA 2-like [Sesamum indicum]

#ID	0 h-1 FPKM	0 h-2 FPKM	0 h-3 FPKM	48 h-1 FPKM	48 h-2 FPKM	48 h-3 FPKM	FDR	log2FC	regulated	nr_annotation
c47218.graph_c1	24.5605814	24.6833196	26.049113	22.5139014	21.506342	20.012641	1.053E-17	-1.250288	down	PREDICTED: KH domain-containing protein At4g18375-like [Sesamum indicum]
c47221.graph_c0	6.51291471	6.30913201	5.77755354	92.5954286	109.699188	102.161905	2.45E-82	3.013952	up	hypothetical protein MIMGU_mgv1a010747mg [Erythranthe guttata]
c47222.graph_c0	0.54154259	0.49691113	0.57991973	2.97263847	3.0610619	4.16176967	2.02E-05	1.633778	up	hypothetical protein MIMGU_mgv1a000984mg [Erythranthe guttata]
c47224.graph_c0	0.10302809	0.06875417	0	1.01243944	0.90496981	0.78825471	3.762E-06	2.980681	up	-
c47226.graph_c1	1.28941407	1.46512485	1.79129527	7.54472163	8.57091432	8.17016557	5.574E-06	1.394838	up	hypothetical protein MIMGU_mgv1a008054mg [Erythranthe guttata]
c47233.graph_c0	9.60038565	8.52548499	9.66839785	8.34543867	6.96117056	5.99485129	5.66E-13	-1.396948	down	PREDICTED: glycerophosphodiester phosphodiesterase GDPD4 isoform X3 [Sesamum indicum]
c47234.graph_c0	179.270387	158.829879	176.282782	17.3362061	19.5610029	23.275392	7.31E-149	-4.116945	down	PREDICTED: uncharacterized protein LOC105178088 [Sesamum indicum]
c47234.graph_c3	71.2458435	69.2673502	78.725243	452.536866	505.139577	509.538211	4.417E-35	1.721712	up	hypothetical protein OsJ_19397 [Oryza sativa Japonica Group]
c47234.graph_c6	65.6647471	70.6830291	59.3397382	39.0378072	43.1624715	41.7158351	5.809E-33	-1.676909	down	PREDICTED: uncharacterized protein LOC105169758 [Sesamum indicum]
c47235.graph_c0	25.0054393	25.482642	26.6185456	9.00658378	7.7464697	7.35278263	1.221E-62	-2.692081	down	PREDICTED: aspartic proteinase-like protein 1 isoform X1 [Sesamum indicum]
c47238.graph_c0	3.17265212	3.22322949	3.04252507	13.5672779	12.7322509	14.2264249	3.891E-08	1.085673	up	PREDICTED: WUSCHEL-related homeobox 8 [Sesamum indicum]
c47239.graph_c0	108.309949	111.207238	107.326854	37.7133961	34.7130626	30.7232157	1.785E-71	-2.678484	down	PREDICTED: transcription factor GTE8 [Sesamum indicum]
c47240.graph_c0	1.36206804	0.43824598	0.71887788	0	0	0	1.167E-07	-Inf	down	sucrose synthase 1 [Oncidium hybrid cultivar]
c47246.graph_c0	0	0	0	9.85104757	9.14759653	7.79146924	2.355E-23	Inf	up	allergen Ale o 13 [Metarhizium acridum CQMa 102]
c47250.graph_c1	2.91526028	1.93114818	2.75457712	1.50084795	1.96653741	2.10859813	0.0003413	-1.470654	down	PREDICTED: uncharacterized protein LOC105163644 [Sesamum indicum]
c47250.graph_c3	74.8056247	74.3808517	79.9999135	311.927852	337.409503	328.350237	7.478E-15	1.073372	up	PREDICTED: 60S ribosomal protein L6-like [Sesamum indicum]
c47253.graph_c0	1171.19964	1109.53842	1166.40438	674.59415	595.219902	495.510692	1.055E-28	-1.979117	down	PREDICTED: ABC transporter G family member 3 [Sesamum indicum]
c47259.graph_c0	31.5861763	33.5153306	31.0217555	17.632081	20.7966054	20.1260007	9.375E-34	-1.734971	down	PREDICTED: uncharacterized protein LOC105174955 isoform X1 [Sesamum indicum]
c47266.graph_c0	0	0	0	1.58430503	2.21240878	1.6019376	8.39E-21	Inf	up	--
c47269.graph_c0	30.0996563	31.892566	29.9059188	15.4997166	25.3043595	27.4720661	1.965E-23	-1.454899	down	PREDICTED: uncharacterized protein LOC105174622 [Sesamum indicum]
c47269.graph_c1	59.7904738	62.7437367	62.0786895	44.8503879	45.7373402	44.4490427	3.072E-25	-1.468945	down	PREDICTED: uncharacterized protein LOC105168306 isoform X2 [Sesamum indicum]
c47270.graph_c0	5.09966745	3.14139938	3.94113016	0	0	0	2.702E-35	-Inf	down	-

#ID	0 h-1 FPKM	0 h-2 FPKM	0 h-3 FPKM	48 h-1 FPKM	48 h-2 FPKM	48 h-3 FPKM	FDR	log2FC	regulated	nr_annotation
c47271.graph_c0	0	0	0	1.02429284	0.76596287	0.77737879	2.134E-11	Inf	up	-
c47274.graph_c0	0	0	0	2.34708602	5.75567833	0.81216451	0.0018545	Inf	up	-
c47277.graph_c0	65.7468331	64.0880763	63.1333548	69.2231669	67.475546	58.2230688	1.566E-12	-1.000912	down	PREDICTED: uncharacterized protein LOC105171105 isoform X1 [Sesamum indicum]
c47277.graph_c1	62.921997	67.0361067	56.6805659	36.1633261	36.3827829	33.3429894	7.24E-36	-1.833206	down	PREDICTED: zinc finger CCCH domain-containing protein 44-like isoform X4 [Sesamum indicum]
c47287.graph_c0	36.5231047	35.0221205	31.4718537	28.7733006	26.5258487	28.4353523	9.63E-19	-1.313933	down	PREDICTED: GATA transcription factor 15-like [Sesamum indicum]
c47290.graph_c0	0	0	0	5.98378327	5.68707183	4.07691889	4.257E-24	Inf	up	--
c47293.graph_c0	1.03268034	0.91028582	0.75264678	5.46684189	6.30290935	5.92567861	6.788E-11	1.697903	up	PREDICTED: uncharacterized protein LOC105174459 isoform X2 [Sesamum indicum]
c47294.graph_c0	3.57713678	2.97327538	2.38046624	23.7451948	22.7549726	19.1909133	2.026E-17	1.86716	up	PREDICTED: uncharacterized protein LOC105159979 [Sesamum indicum]
c47295.graph_c0	0	0	0	2.33984459	2.77387468	1.6854121	5.28E-16	Inf	up	--
c47296.graph_c0	166.821295	170.652449	175.434841	127.957725	133.901769	125.413872	1.217E-23	-1.42361	down	PREDICTED: NEDD8-activating enzyme E1 catalytic subunit [Sesamum indicum]
c47296.graph_c2	24.5949374	23.1021809	20.8430515	22.6933734	22.7278756	21.4971686	6.61E-14	-1.05002	down	PREDICTED: uncharacterized protein At1g10890 isoform X2 [Sesamum indicum]
c47297.graph_c0	318.434749	304.932656	307.966573	279.229494	259.106715	238.905926	5.611E-17	-1.276038	down	PREDICTED: pentatricopeptide repeat-containing protein At5g15340, mitochondrial [Sesamum indicum]
c47300.graph_c0	161.61969	170.012016	149.513931	28.4569723	29.3101636	33.9866606	4.82E-111	-3.408748	down	PREDICTED: chaperone protein ClpD, chloroplastic [Sesamum indicum]
c47301.graph_c0	8.68661381	9.53062569	7.88103244	5.7148734	5.46547484	5.72790387	3.551E-28	-1.641754	down	PREDICTED: midasin [Sesamum indicum]
c47302.graph_c1	2.07434105	2.44186779	1.91923947	12.4751273	12.9729456	11.6580286	8.213E-16	1.511631	up	PREDICTED: paladin [Sesamum indicum]
c47303.graph_c0	3.95417446	2.17697368	2.37125967	2.36784822	2.70140562	2.37701213	0.0052279	-1.206686	down	hypothetical protein M569_00480, partial [Genlisea aurea]
c47315.graph_c0	9.77123146	10.2157277	9.46560838	3.29300118	3.58634486	3.40931341	4.748E-50	-2.535496	down	PREDICTED: uncharacterized protein LOC105167449 isoform X2 [Sesamum indicum]
c47316.graph_c0	4.10239494	4.36862007	5.1034929	1.60825268	1.7037487	1.53833958	2.919E-12	-2.504696	down	PREDICTED: putative pentatricopeptide repeat-containing protein At1g53330 [Sesamum indicum]
c47317.graph_c0	15.8130088	15.1183215	16.2157346	18.9637981	14.3169007	13.7706324	3.811E-09	-1.014791	down	PREDICTED: histone-lysine N-methyltransferase ASHH3-like [Sesamum indicum]
c47323.graph_c0	4.13565675	2.51987703	2.77277356	27.9126605	55.2793766	20.193489	0.0055528	2.432698	up	component of cytosolic 80S ribosome and 40S small subunit [Volvox carteri f. nagariensis]
c47327.graph_c0	5.97779165	3.08805661	3.81341483	0	0	0	6.006E-27	-Inf	down	alpha-2 macroglobulin family-like protein [Micromonas sp. RCC299]
c47329.graph_c0	0	0	0	9.27296399	37.7593892	6.3601671	0.0015236	Inf	up	-

#ID	0 h-1 FPKM	0 h-2 FPKM	0 h-3 FPKM	48 h-1 FPKM	48 h-2 FPKM	48 h-3 FPKM	FDR	log2FC	regulated	nr_annotation
c47331.graph_c0	15.4930184	17.4470989	15.879911	11.2712803	11.6405409	14.5145017	1.647E-17	-1.402991	down	PREDICTED: uncharacterized protein LOC104812881 [Tarenaya hassleriana]
c47334.graph_c0	477.83099	438.829954	522.768726	60.0920324	58.54587	51.7738781	5.612E-91	-4.0957	down	PREDICTED: em-like protein GEA6 [Sesamum indicum]
c47334.graph_c2	67.917916	64.7581071	65.3499988	51.4921707	53.3367669	55.0690191	5.062E-21	-1.326637	down	PREDICTED: probable E3 ubiquitin-protein ligase ARI8 [Sesamum indicum]
c47345.graph_c0	0	0	0.27463156	20.3778783	32.4339814	15.3290815	2.882E-12	6.900575	up	PREDICTED: 40S ribosomal protein S24-1-like [Musa acuminata subsp. malaccensis]
c47346.graph_c0	0	2.0899367	1.60977889	70.7833262	86.8048521	102.346689	1.12E-24	5.104519	up	PREDICTED: probable aquaporin PIP-type pTOM75 [Sesamum indicum]
c47346.graph_c1	45.0719357	49.3725165	46.1958131	301.539957	310.450408	303.271462	4.69E-28	1.684471	up	PREDICTED: BTB/POZ domain-containing protein At5g41330-like [Sesamum indicum]
c47348.graph_c0	23.7092651	25.8805406	19.1569613	24.9339224	20.2403541	19.5255315	1.616E-08	-1.097929	down	hypothetical protein MIMGU_mgv1a010183mg [Erythranthe guttata]
c47349.graph_c0	1.34228618	2.39516702	0.82494782	7.90349707	11.5624337	11.1945666	6.048E-06	1.73084	up	Ubiquitin carboxyl-terminal hydrolase [Medicago truncatula]
c47350.graph_c3	11.9110197	11.0872262	9.63189605	10.2895192	10.8642837	10.8589109	1.262E-11	-1.044017	down	PREDICTED: uncharacterized protein LOC105166536 [Sesamum indicum]
c47351.graph_c0	1.9138469	2.37189745	2.75215106	1.46930049	2.13469126	2.20386028	0.0025149	-1.304661	down	hypothetical protein MIMGU_mgv1a003544mg [Erythranthe guttata]
c47352.graph_c0	3.93001002	4.30699187	4.09241842	37.9224707	43.7783699	50.4620327	5.923E-35	2.400889	up	RUBISCO SUBUNIT BINDING-protein ALPHA SUBUNIT [Populus trichocarpa]
c47357.graph_c0	33.1769776	31.6526021	33.1595676	23.0729223	25.1118907	21.2742135	8.65E-27	-1.514471	down	hypothetical protein MIMGU_mgv1a004228mg [Erythranthe guttata]
c47358.graph_c0	7.38837045	6.65939354	6.74057646	0.91344637	0.43701933	0.76034173	3.096E-48	-4.307167	down	hypothetical protein MIMGU_mgv1a009769mg [Erythranthe guttata]
c47359.graph_c0	58.4066837	63.282334	52.6404133	30.5363387	27.817645	24.2919988	1.185E-48	-2.089897	down	hypothetical protein VITISV_031499 [Vitis vinifera]
c47360.graph_c0	58.5019996	55.7408457	61.6685393	51.3195381	55.721516	55.8373599	4.107E-15	-1.130831	down	PREDICTED: mitogen-activated protein kinase kinase kinase 1-like [Sesamum indicum]
c47360.graph_c1	1.3065725	0.85012254	1.17529817	0.30092495	0.35067285	0.26776121	1.397E-08	-2.876453	down	-
c47361.graph_c0	0	0	0	1.83887816	3.15006811	1.14883724	2.597E-06	Inf	up	-
c47362.graph_c2	2.3749482	1.65092108	1.86505151	0.79009565	0.57946884	1.08157286	4.972E-06	-2.280815	down	hypothetical protein MIMGU_mgv1a003741mg [Erythranthe guttata]
c47364.graph_c0	11.817542	11.5490585	13.0268611	0.54177465	0.54661385	1.23960297	1.55E-105	-4.99218	down	PREDICTED: pleiotropic drug resistance protein 1-like [Sesamum indicum]
c47367.graph_c4	37.5855224	36.9899498	36.8056015	18.1306138	19.961755	16.6510562	7.546E-47	-2.042532	down	PREDICTED: ubiquitin carboxyl-terminal hydrolase 5 [Sesamum indicum]
c47370.graph_c0	39.1110723	38.2916783	35.4586122	18.4222076	22.7448609	18.7021123	5.779E-42	-1.933736	down	PREDICTED: hexokinase-3-like [Sesamum indicum]

#ID	0 h-1 FPKM	0 h-2 FPKM	0 h-3 FPKM	48 h-1 FPKM	48 h-2 FPKM	48 h-3 FPKM	FDR	log2FC	regulated	nr_annotation
c47371.graph_c0	0.18052678	0.18070755	0.27838087	4.59023995	4.40471253	4.67630528	4.582E-12	3.395706	up	PREDICTED: DNA repair protein RAD51 homolog 2 isoform X3 [Sesamum indicum]
c47373.graph_c1	3.24609284	3.05359974	3.11595403	31.2382445	33.2076414	35.1051723	3.917E-35	2.38351	up	PREDICTED: acyl-protein thioesterase 2-like [Sesamum indicum]
c47377.graph_c0	2.7913822	3.03270468	2.62466096	10.1923304	11.9935731	13.4501347	2.331E-05	1.056195	up	PREDICTED: proline-rich receptor-like protein kinase PERK8 [Sesamum indicum]
c47378.graph_c0	29.9811228	31.1516573	30.7756101	16.4241517	15.6769004	14.2112161	1.189E-43	-2.004676	down	PREDICTED: E3 ubiquitin protein ligase RIE1-like isoform X2 [Sesamum indicum]
c47382.graph_c0	0	0	0	29.4187655	68.7904244	25.4984893	3.899E-08	Inf	up	ubiquitin/40s ribosomal protein S27a fusion [Wallemia sebi CBS 633.66]
c47383.graph_c0	6.23280714	3.44126882	4.59732603	0	0	0	5.918E-29	-Inf	down	--
c47385.graph_c0	36.6675556	38.3904731	35.6666626	29.9008339	31.3513247	31.6312595	1.334E-19	-1.2714	down	PREDICTED: pentatricopeptide repeat-containing protein At2g26790, mitochondrial isoform X1 [Sesamum indicum]
c47389.graph_c0	4.23416533	2.78842448	4.24785297	2.73739599	1.92120967	2.13125368	1.131E-06	-1.743774	down	PREDICTED: uncharacterized protein LOC104811210 [Tarenaya hassleriana]
c47391.graph_c0	4.97260435	7.27492997	6.38999346	4.05250045	3.54650104	2.19488989	5.006E-05	-1.940518	down	-
c47395.graph_c0	4.86723221	4.33076089	3.99598555	19.6175515	25.5986598	23.8282512	2.116E-07	1.367473	up	hypothetical protein MIMGU_mgv1a009112mg [Erythranthe guttata]
c47396.graph_c0	8.15458655	8.95142384	9.31463695	7.21009994	6.77956691	6.72766733	2.666E-18	-1.368062	down	hypothetical protein MIMGU_mgv1a001351mg [Erythranthe guttata]
c47400.graph_c0	11.8269707	7.08214741	6.78487747	0	0	0	6.221E-25	-Inf	down	glyceraldehyde-3-phosphate dehydrogenase 3 [Pilobolus crystallinus]
c47411.graph_c0	23.8290284	24.1512061	22.0204888	10.9821475	14.9669825	15.4517359	3.712E-36	-1.780109	down	PREDICTED: mediator of RNA polymerase II transcription subunit 13 [Sesamum indicum]
c47412.graph_c0	260.870499	263.526052	267.529967	45.724851	46.4848294	42.1562649	7.76E-111	-3.576429	down	PREDICTED: L-type lectin-domain containing receptor kinase IV.1 [Sesamum indicum]
c47421.graph_c2	12.0693212	14.9352824	12.1308335	6.27452721	7.69827017	6.43996778	1.042E-26	-1.958243	down	PREDICTED: transcription factor bHLH48-like isoform X2 [Sesamum indicum]
c47422.graph_c1	62.361885	68.6985011	55.5352501	381.020037	282.132244	225.97291	0.0023858	1.244577	up	PREDICTED: aspartate aminotransferase, cytoplasmic [Sesamum indicum]
c47423.graph_c0	19.045105	20.6490619	17.1888961	12.5074174	12.370897	12.8073295	1.24E-30	-1.610057	down	PREDICTED: mediator of RNA polymerase II transcription subunit 12 isoform X1 [Sesamum indicum]
c47425.graph_c0	15.6229509	17.2343699	17.6178112	80.0909995	83.6020076	86.8154817	7.117E-18	1.291762	up	hypothetical protein MIMGU_mgv1a005589mg [Erythranthe guttata]
c47426.graph_c0	18.8616889	18.1192624	17.8713489	6.8665668	9.05575489	10.0108582	6.984E-42	-2.103515	down	PREDICTED: sucrose transport protein SUC3 isoform X2 [Sesamum indicum]
c47429.graph_c0	31.8594916	29.723375	31.1251361	20.1816971	17.9108434	17.2392506	2.239E-35	-1.759338	down	PREDICTED: pentatricopeptide repeat-containing protein At1g03560, mitochondrial [Sesamum indicum]

#ID	0 h-1 FPKM	0 h-2 FPKM	0 h-3 FPKM	48 h-1 FPKM	48 h-2 FPKM	48 h-3 FPKM	FDR	log2FC	regulated	nr_annotation
c47433.graph_c1	3.41132882	2.59817536	2.28714396	0.47824283	0.43426311	0.72949302	6.395E-11	-3.353812	down	hypothetical protein VITISV_042890 [Vitis vinifera]
c47435.graph_c0	21.3837584	24.1377461	23.0355673	2.59873831	1.95377609	2.23775006	7.912E-56	-4.348954	down	PREDICTED: protein TIC 62, chloroplastic [Sesamum indicum]
c47437.graph_c1	13.0304915	11.5467399	13.7937876	2.80433895	4.27386456	4.50902184	2.055E-28	-2.753875	down	hypothetical protein MIMGU_mgv1a006304mg [Erythranthe guttata]
c47438.graph_c0	6.17812351	6.47089994	5.38311966	32.9007466	34.2366796	33.5611252	1.332E-23	1.465006	up	PREDICTED: clustered mitochondria protein [Sesamum indicum]
c47439.graph_c1	50.7544499	55.6217636	50.9926915	38.0795829	36.3801831	34.8571791	6.485E-28	-1.541508	down	PREDICTED: long chain base biosynthesis protein 2a [Sesamum indicum]
c47443.graph_c0	25.9543674	27.4170585	28.53653	24.8658212	27.7430958	25.4589836	1.653E-12	-1.088979	down	hypothetical protein MIMGU_mgv1a014801mg [Erythranthe guttata]
c47445.graph_c0	14.4111638	13.7780615	14.5472782	9.81666698	10.5749405	10.1194188	5.464E-23	-1.504928	down	PREDICTED: LOW QUALITY PROTEIN: nuclear pore complex protein NUP88 [Sesamum indicum]
c47448.graph_c0	20.6730004	23.2276239	21.2645471	18.7430098	17.6598968	16.7926991	6.149E-15	-1.308485	down	PREDICTED: ADP-ribosylation factor-related protein 1 [Nicotiana tomentosiformis]
c47449.graph_c0	1.86459419	2.5451745	2.55944627	0.03904059	0.08271753	0	2.118E-25	-6.863524	down	PREDICTED: E3 ubiquitin-protein ligase CHIP-like [Glycine max]
c47449.graph_c1	9.40506853	8.95524305	8.35203464	5.49460066	5.59698379	3.88617798	4.068E-09	-1.848741	down	hypothetical protein MIMGU_mgv1a013929mg [Erythranthe guttata]
c47450.graph_c0	0	0	0	4.17320055	6.38588883	4.53846403	1.951E-15	Inf	up	-
c47451.graph_c0	0.57854054	0.71073802	0.60827523	3.14948213	3.07984101	2.414367	0.0053265	1.17265	up	PREDICTED: uncharacterized protein LOC105174442 [Sesamum indicum]
c47452.graph_c0	15.2717543	9.33425657	10.1289336	0	0	0	6.67E-38	-Inf	down	lectin [Bryopsis maxima]
c47453.graph_c0	45.5899734	45.7866362	44.5881263	9.28401897	9.15801174	8.53292865	4.37E-108	-3.349884	down	PREDICTED: probable plastidic glucose transporter 2-like isoform X2 [Glycine max]
c47456.graph_c0	0.48088662	0.30632519	0.33706819	1.75195902	2.49598405	2.81315595	0.0001989	1.628669	up	PREDICTED: SWI/SNF-related matrix-associated actin-dependent regulator of chromatin subfamily A-like protein 1 isoform X3 [Sesamum indicum]
c47458.graph_c0	0	0	0	13.6696092	19.6249222	15.9516538	1.178E-31	Inf	up	--
c47458.graph_c1	0	0	0	9.97705526	11.9245418	9.52247208	9.378E-35	Inf	up	--
c47459.graph_c0	0	0	0	7.38662458	14.7998973	5.00087162	8.761E-07	Inf	up	-
c47462.graph_c0	0.51065417	0.96852413	0.69074824	2.59984381	3.54113906	3.67928895	0.0039761	1.154519	up	PREDICTED: leucine-rich repeat receptor-like serine/threonine-protein kinase BAM3 [Sesamum indicum]
c47464.graph_c1	0.33200516	0	0.10665994	1.68225644	2.99724675	2.44941	2.125E-06	3.004156	up	PREDICTED: glutamate synthase 1 [NADH], chloroplastic isoform X1 [Sesamum indicum]
c47466.graph_c0	0.29556557	0.32544769	0.34183211	51.0824069	27.7501187	17.9291501	5.077E-06	5.648667	up	hypothetical protein MIMGU_mgv1a004441mg [Erythranthe guttata]

#ID	0 h-1 FPKM	0 h-2 FPKM	0 h-3 FPKM	48 h-1 FPKM	48 h-2 FPKM	48 h-3 FPKM	FDR	log2FC	regulated	nr_annotation
c47467.graph_c0	0	0	0	7.32517328	12.3565193	8.37298837	3.379E-20	Inf	up	Protein bli-3 OS=Neurospora crassa (strain ATCC 24698 / 74-OR23-1A / CBS 708,71 / DSM 1257 / FGSC 987) GN=bli-3 PE=2 SV=1 [Rhizoctonia solani AG-1 IB]
c47468.graph_c0	80.0914389	73.4736252	75.0244425	28.09306	35.6436371	35.8416461	1.039E-52	-2.220346	down	hypothetical protein MIMGU_mgv1a012537mg [Erythranthe guttata]
c47468.graph_c1	17.1266498	2.60112821	2.42851565	42781.5201	38273.9501	43600.1741	3.64E-229	11.45583	up	PREDICTED: non-specific lipid-transfer protein 1-like [Sesamum indicum]
c47468.graph_c2	16.1569824	19.8251653	18.52985	15.5250753	9.49513762	5.83895738	3.087E-08	-1.824868	down	PREDICTED: zinc finger protein ZAT11-like [Nicotiana tomentosiformis]
c47468.graph_c3	63.8762735	63.5020891	82.5335188	2.06999461	2.19290829	2.72692257	2.318E-53	-5.931535	down	PREDICTED: LOW QUALITY PROTEIN: small heat shock protein, chloroplastic-like [Sesamum indicum]
c47469.graph_c0	89.663122	81.4182633	95.4183581	69.4087065	84.4459182	79.8925334	4.992E-17	-1.210962	down	hypothetical protein MIMGU_mgv1a011464mg [Erythranthe guttata]
c47471.graph_c2	12.6595489	13.0119973	11.0661319	38.4513439	59.1018862	85.750849	0.0096681	1.293051	up	PREDICTED: probable E3 ubiquitin-protein ligase ARI2 isoform X1 [Sesamum indicum]
c47474.graph_c0	95.7833967	93.7327576	89.6997632	45.3873442	40.2475343	34.1878518	1.284E-54	-2.233594	down	PREDICTED: ankyrin repeat domain-containing protein 6 [Sesamum indicum]
c47475.graph_c0	462.409017	271.598512	313.741882	0	0	0	1.09E-60	-Inf	down	--
c47476.graph_c0	30.2165419	29.5019636	26.8563452	6.85504639	9.40914325	7.34601477	1.412E-74	-2.895043	down	PREDICTED: uncharacterized protein LOC105163261 [Sesamum indicum]
c47476.graph_c1	27.4282689	24.2658563	22.7419792	17.4574285	22.8259633	24.303187	4.643E-13	-1.225992	down	PREDICTED: protein YIPF1 homolog [Sesamum indicum]
c47482.graph_c3	41.7415311	41.750762	41.2643853	10.3405941	11.1451205	12.4546849	1.556E-81	-2.897609	down	PREDICTED: YTH domain-containing family protein 2-like isoform X2 [Sesamum indicum]
c47487.graph_c0	0.28142601	0.42256172	0.18082178	27.5256187	40.1007983	46.3697156	3.929E-29	5.989864	up	PREDICTED: vinorine synthase-like [Sesamum indicum]
c47489.graph_c0	10.3287823	9.24098151	7.92649796	7.26537832	7.63618274	5.41269315	2.887E-16	-1.450265	down	PREDICTED: uncharacterized protein LOC105164519 [Sesamum indicum]
c47492.graph_c0	13.5790167	12.7012951	16.5198836	139.915784	159.466874	156.794987	7.274E-60	2.391642	up	PREDICTED: uncharacterized protein LOC105161817 [Sesamum indicum]
c47495.graph_c2	72.8984159	76.5802252	74.4589671	54.6333543	59.2937858	58.0919792	2.831E-21	-1.399394	down	PREDICTED: uncharacterized protein LOC105175572 isoform X2 [Sesamum indicum]
c47495.graph_c3	0	0	0.16423398	168.547586	168.389965	188.893223	7.85E-258	10.5939	up	hypothetical protein M569_11087, partial [Genlisea aurea]
c47497.graph_c0	0	0	0	30.3298467	42.6416666	21.4606576	2.988E-19	Inf	up	alpha-tubulin, partial [Syntrichia caninervis]
c47498.graph_c0	93.9221566	89.3994331	82.4562682	38.8089938	37.8981957	33.3472582	7.744E-56	-2.286756	down	hypothetical protein MIMGU_mgv1a005594mg [Erythranthe guttata]
c47500.graph_c0	4.81071851	3.5400695	3.27476998	59.0047952	60.7065182	58.3001527	1.602E-76	2.922006	up	hypothetical protein MIMGU_mgv1a001160mg [Erythranthe guttata]

#ID	0 h-1 FPKM	0 h-2 FPKM	0 h-3 FPKM	48 h-1 FPKM	48 h-2 FPKM	48 h-3 FPKM	FDR	log2FC	regulated	nr_annotation
c47502.graph_c0	898.215909	944.828922	878.431335	342.399151	291.316442	245.708644	5.42E-46	-2.642153	down	PREDICTED: probable inactive receptor kinase At1g27190 [Sesamum indicum]
c47503.graph_c0	1.68796457	1.72037581	1.18314617	12.2708612	14.2575047	15.5727543	4.996E-18	2.179626	up	PREDICTED: pentatricopeptide repeat-containing protein At1g80270, mitochondrial [Sesamum indicum]
c47506.graph_c0	9.98219732	9.61448122	8.90432574	207.595102	226.885416	236.484838	5.63E-123	3.538822	up	-
c47507.graph_c0	43.7112974	41.0298385	40.2718994	39.517254	40.210446	43.5436175	1.986E-13	-1.03796	down	Fas-associated factor 1-like protein [Capsicum annuum]
c47508.graph_c0	5.72549101	6.22959155	6.20587665	25.9606765	31.8753285	31.0172031	1.136E-10	1.268971	up	PREDICTED: probable serine/threonine-protein kinase At5g41260 [Sesamum indicum]
c47509.graph_c0	0	0	0	5.11982505	8.73329176	3.8606691	2.429E-08	Inf	up	Peptidyl-prolyl cis-trans isomerase,FKBP-type,N-terminal [Ostreococcus tauri]
c47511.graph_c1	63.0128752	64.7325457	65.0141737	47.3066576	50.9616963	47.2869972	1.542E-22	-1.423759	down	hypothetical protein MIMGU_mgv1a002091mg [Erythranthe guttata]
c47512.graph_c1	3.81854803	2.31229895	2.48378273	19.2398483	22.3607024	30.8769335	4.039E-08	2.053055	up	PREDICTED: glycosyltransferase family protein 64 protein C5 [Sesamum indicum]
c47517.graph_c0	113.42782	115.788633	121.05734	29.3946585	32.454697	30.4568795	5.946E-87	-2.943459	down	PREDICTED: calcium and calcium/calmodulin-dependent serine/threonine-protein kinase-like [Sesamum indicum]
c47522.graph_c1	8.09712044	8.69990557	8.79345768	1.17569711	0.88044569	0.93790806	1.747E-77	-4.108185	down	PREDICTED: ABC transporter C family member 4-like [Sesamum indicum]
c47524.graph_c0	63.113418	67.9381945	60.760191	61.0540365	54.0944422	49.537133	3.392E-18	-1.233594	down	PREDICTED: histone-lysine N-methyltransferase ASHH2 [Sesamum indicum]
c47525.graph_c0	0	0	0	1.47786726	1.74528261	1.37966941	4.052E-14	Inf	up	glycoside hydrolase family 13 protein [Serpula lacrymans var. lacrymans S7.9]
c47528.graph_c0	0	0	0	5.62926879	4.813932	5.91411051	5.464E-16	Inf	up	expressed unknown protein [Ectocarpus siliculosus]
c47533.graph_c1	1.72960211	2.17909285	1.83939905	13.2693614	13.358781	13.4448217	1.187E-13	1.784164	up	PREDICTED: riboflavin biosynthesis protein PYRR, chloroplastic [Sesamum indicum]
c47533.graph_c2	23.9202255	23.2670932	24.17985	0.96305096	0.66015252	0.90732383	1.57E-113	-5.830704	down	hypothetical protein MIMGU_mgv1a0267602mg, partial [Erythranthe guttata]
c47533.graph_c4	17.9144635	18.9130803	18.2547709	88.8377879	104.289074	103.59904	9.882E-20	1.409084	up	Pectinesterase 1 [Morus notabilis]
c47536.graph_c0	83.8487191	81.7977941	83.7426868	72.4439925	75.2037749	67.7301852	2.514E-18	-1.228962	down	hypothetical protein MIMGU_mgv1a006250mg [Erythranthe guttata]
c47545.graph_c0	0	0	0	2.05299016	6.84556843	2.27593624	8.748E-05	Inf	up	PREDICTED: KDEL-tailed cysteine endopeptidase CEP1-like [Glycine max]
c47547.graph_c0	4.81950626	3.17027549	5.83935599	0	0	0	4.69E-17	-Inf	down	PREDICTED: uncharacterized protein LOC101507298 [Cicer arietinum]
c47550.graph_c0	15.6573649	18.6497067	20.7442202	1.1740942	0.71074882	0.99495424	1.033E-51	-5.269496	down	PREDICTED: uncharacterized protein LOC105155796 [Sesamum indicum]
c47550.graph_c1	0.04791609	0.14389221	0.12314814	5.25306784	6.40675136	9.50541057	7.484E-13	5.042111	up	-

#ID	0 h-1 FPKM	0 h-2 FPKM	0 h-3 FPKM	48 h-1 FPKM	48 h-2 FPKM	48 h-3 FPKM	FDR	log2FC	regulated	nr_annotation
c47550.graph_c2	5.16204908	4.92827159	6.32668889	1.12705358	2.67916701	3.49772941	7.569E-13	-2.203404	down	PREDICTED: chloride channel protein CLC-a-like [Sesamum indicum]
c47550.graph_c6	8.5943049	8.39807959	10.6934182	1.00541387	2.39650669	3.74618447	6.208E-19	-2.989305	down	PREDICTED: chloride channel protein CLC-b-like [Sesamum indicum]
c47550.graph_c7	0.92628527	0.52983589	0.680179	10.2402529	10.7191598	12.148954	3.81E-08	2.936365	up	hypothetical protein MIMGU_mgv1a002946mg [Erythranthe guttata]
c47553.graph_c1	20.9018075	18.0081273	16.1692421	17.9148869	16.9488482	15.855315	1.281E-10	-1.132575	down	PREDICTED: mediator of RNA polymerase II transcription subunit 8 [Sesamum indicum]
c47554.graph_c4	17.1231238	17.733467	15.9117397	6.75247822	8.64499855	7.36338119	1.072E-37	-2.177398	down	PREDICTED: zinc finger protein JACKDAW [Sesamum indicum]
c47554.graph_c5	394.290269	392.242733	369.627339	141.012543	139.066807	157.473502	2.041E-56	-2.419268	down	hypothetical protein POPTR_0003s102801g, partial [Populus trichocarpa]
c47555.graph_c0	0	0	0	1.07615334	0.83603958	1.19694346	1.281E-13	Inf	up	PREDICTED: kinesin-4 [Sesamum indicum]
c47556.graph_c0	55.1822147	28.1452627	38.8353215	0	0	0	5.904E-29	-Inf	down	mannose-binding lectin precursor [Dendrobium catenatum]
c47557.graph_c0	37.320859	36.4957381	31.3503644	169.371923	184.186238	186.505884	7.484E-21	1.343109	up	PREDICTED: pentatricopeptide repeat-containing protein At5g16860 [Sesamum indicum]
c47558.graph_c0	104.06505	91.3569944	109.651955	527.122423	501.032157	501.495261	3.622E-21	1.308457	up	PREDICTED: NADH dehydrogenase [ubiquinone] 1 beta subcomplex subunit 2-like [Fragaria vesca subsp. vesca]
c47560.graph_c0	35.6117574	33.2993837	33.3399583	7.46526467	8.18603543	10.6630369	1.16E-78	-2.978935	down	PREDICTED: CBL-interacting serine/threonine-protein kinase 24 [Sesamum indicum]
c47563.graph_c2	1.28905493	1.34195955	1.25892983	0.52252705	0.90581572	0.67627844	0.0001215	-1.911275	down	-
c47564.graph_c1	22.487663	22.9157699	20.2319953	154.111775	164.618554	157.815513	7.605E-39	1.842907	up	PREDICTED: pentatricopeptide repeat-containing protein At5g62370 [Sesamum indicum]
c47564.graph_c2	17.6401378	18.6787521	17.5746156	13.4964351	13.800127	14.6312754	8.888E-20	-1.380236	down	PREDICTED: uncharacterized protein LOC105170859 [Sesamum indicum]
c47564.graph_c3	63.1922398	59.5292584	52.3862557	60.3079342	58.3064145	48.8239029	8.765E-13	-1.078233	down	PREDICTED: uncharacterized protein LOC105179697 [Sesamum indicum]
c47564.graph_c4	0	0	0	3.6343975	4.98551986	2.86072676	6.457E-15	Inf	up	PREDICTED: probable pectinesterase/pectinesterase inhibitor 59 [Sesamum indicum]
c47564.graph_c8	0.92208555	0.57688055	1.03680189	12.1051663	7.53688635	9.1648869	7.718E-07	2.493336	up	PREDICTED: probable transcription factor KAN2 isoform X2 [Sesamum indicum]
c47564.graph_c9	3.18029712	1.91008903	1.86825496	0.75340212	1.33023032	1.86214633	0.0001869	-1.843541	down	-
c47565.graph_c0	0.31190328	0.43229853	0.83244753	2.27666168	2.20110308	3.30415203	0.0017932	1.273207	up	PREDICTED: uncharacterized protein LOC105160289 [Sesamum indicum]
c47568.graph_c0	1.42892391	1.04296702	0.68858393	9.73600747	9.76209607	10.3224403	5.258E-14	2.226465	up	PREDICTED: D-2-hydroxyglutarate dehydrogenase, mitochondrial isoform X1 [Sesamum indicum]
c47569.graph_c0	2.02096065	1.99233305	1.84938907	8.85784848	11.2067871	9.83952498	6.311E-07	1.330691	up	hypothetical protein JCGZ_11229 [Jatropha curcas]

#ID	0 h-1 FPKM	0 h-2 FPKM	0 h-3 FPKM	48 h-1 FPKM	48 h-2 FPKM	48 h-3 FPKM	FDR	log2FC	regulated	nr_annotation
c47574.graph_c1	947.355017	933.596707	956.402819	322.955621	133.832145	103.255117	2.856E-83	-3.337734	down	PREDICTED: NAC domain-containing protein 2 [Sesamum indicum]
c47575.graph_c0	0.01750384	0.07008547	0.11246563	6.04712949	7.73869013	5.36635092	8.332E-35	5.548842	up	PREDICTED: uncharacterized protein At5g05190 [Sesamum indicum]
c47575.graph_c1	109.836365	114.841146	108.062994	87.2091083	99.813571	95.6810053	2.114E-17	-1.254427	down	PREDICTED: protein STICHEL-like 4 [Sesamum indicum]
c47580.graph_c0	40.5618678	39.8265258	38.6409666	13.2200523	15.0606989	14.0684587	4.806E-68	-2.509853	down	PREDICTED: two-component response regulator ARR2-like isoform X1 [Sesamum indicum]
c47580.graph_c1	388.161765	366.175821	396.904401	82.7535734	85.9376349	66.6086963	7.937E-82	-3.307379	down	PREDICTED: probable copper-transporting ATPase HMA5 [Nicotiana tomentosiformis]
c47585.graph_c1	251.066046	270.32407	300.990548	317.293165	245.167892	254.149801	2.723E-08	-1.024412	down	Mitochondrial protein, putative [Medicago truncatula]
c47585.graph_c2	217.199107	229.446516	276.854933	242.283423	153.933454	168.69785	2.048E-07	-1.368409	down	unnamed protein product [Triticum aestivum]
c47586.graph_c0	0	0	0	35.4568966	72.2118195	32.8235391	2.276E-14	Inf	up	60S ribosomal protein L8 [Rozella allomycis CSF55]
c47588.graph_c0	2.91550826	3.14292213	4.13080441	14.5317127	14.2272254	14.0332834	0.0010344	1.049761	up	PREDICTED: uncharacterized protein LOC101244714 [Solanum lycopersicum]
c47591.graph_c0	3.65889165	2.95934483	3.27246716	13.564138	14.0553146	14.6367116	3.568E-07	1.077063	up	PREDICTED: uncharacterized protein LOC100250241 isoform X4 [Vitis vinifera]
c47593.graph_c0	1.28051448	1.6022459	1.09700787	11.7477807	11.4038974	12.7274203	7.426E-10	2.157129	up	PREDICTED: beta-galactosidase-like [Sesamum indicum]
c47593.graph_c2	4.35404072	4.03264388	4.29727882	24.9667823	34.7290525	47.0982603	3.57E-06	2.048152	up	PREDICTED: pentatricopeptide repeat-containing protein At3g09060 [Sesamum indicum]
c47596.graph_c5	0.42355031	0.35613852	0.37010967	6.49296417	13.4924584	15.4434359	6.979E-08	3.91606	up	PREDICTED: probably inactive leucine-rich repeat receptor-like protein kinase At3g28040 [Sesamum indicum]
c47598.graph_c0	211.556645	225.315966	237.99075	24.0617012	24.0228226	20.1123839	7.95E-172	-4.32391	down	PREDICTED: B2 protein [Sesamum indicum]
c47598.graph_c1	162.165233	168.562163	161.354457	68.7184844	69.4701249	70.2513348	9.475E-45	-2.256772	down	transferase, transferring glycosyl groups, putative [Ricinus communis]
c47600.graph_c1	4.05812334	4.49302495	3.79261757	22.3183563	23.6735969	29.4604901	4.159E-16	1.592653	up	PREDICTED: protein TIC 62, chloroplastic [Sesamum indicum]
c47601.graph_c0	12.819353	12.8195347	11.5346125	8.1994645	8.84673954	8.4358083	1.386E-26	-1.562179	down	PREDICTED: LOW QUALITY PROTEIN: mediator of RNA polymerase II transcription subunit 16 [Sesamum indicum]
c47604.graph_c0	0	0	0.11054	21.0006878	27.2848933	22.8466286	7.855E-40	8.276252	up	large subunit ribosomal protein L19e, cytoplasmic [Guillardia theta CCMP2712]
c47605.graph_c0	8.30276232	7.17540126	8.18427466	34.2544243	38.8552728	39.1243646	6.73E-16	1.225591	up	PREDICTED: arginine--tRNA ligase, cytoplasmic-like isoform X1 [Sesamum indicum]
c47606.graph_c0	118.684614	114.426685	118.706984	104.902231	99.3779757	84.5934678	7.687E-20	-1.299646	down	-
c47606.graph_c1	0.03360826	0.13456765	0.04318794	84.2788939	116.409376	120.613503	3.402E-93	9.550514	up	PREDICTED: serine carboxypeptidase-like 27 [Sesamum indicum]
c47608.graph_c1	46.6197267	47.5679916	40.9005878	38.0990948	46.5022424	47.8140456	2.006E-13	-1.048705	down	PREDICTED: transcription elongation factor SPT6 [Sesamum indicum]

#ID	0 h-1 FPKM	0 h-2 FPKM	0 h-3 FPKM	48 h-1 FPKM	48 h-2 FPKM	48 h-3 FPKM	FDR	log2FC	regulated	nr_annotation
c47609.graph_c2	0.47972746	0	0	3.38833108	3.90165861	5.11224992	8.303E-08	3.691446	up	-
c47610.graph_c0	0.08159668	0.5717487	0.10485496	68.1341873	35.3981187	28.2266332	5.839E-08	6.445875	up	PREDICTED: uncharacterized protein LOC105174809 [Sesamum indicum]
c47615.graph_c0	0.42646644	0.18973044	0.24356723	12.9653272	9.61925908	10.6042302	1.29E-22	4.262142	up	PREDICTED: vinorine synthase-like [Sesamum indicum]
c47616.graph_c0	1.35021542	1.19440845	1.0087676	7.46343908	7.5082137	7.46460361	3.488E-07	1.643722	up	PREDICTED: UPF0505 protein isoform X2 [Sesamum indicum]
c47617.graph_c1	0.2719415	0	0.06989112	15.3324513	16.986011	15.782758	3.739E-37	6.127962	up	PREDICTED: transcription factor UNE12-like [Sesamum indicum]
c47617.graph_c3	5.23907807	3.74594586	4.56842972	0	0.18261345	0.61352318	8.473E-15	-5.132765	down	-
c47618.graph_c0	4.49150342	1.38847089	1.78245521	0.24340388	0.64464223	0.70388275	0.007366	-3.293167	down	HSP70 [Dendrobium catenatum]
c47622.graph_c0	11.8354658	14.8091466	11.5588721	3.1620449	4.27388678	3.88078419	6.144E-15	-2.776437	down	-
c47622.graph_c1	2339.59651	2200.16903	2471.13004	94.8618542	96.2966901	89.4384185	5.95E-154	-5.661017	down	PREDICTED: probable glycosyltransferase At5g03795 [Sesamum indicum]
c47626.graph_c0	1.64031481	2.12846322	2.10786948	8.58192467	9.38797082	10.5744008	6.042E-09	1.258469	up	PREDICTED: serine/threonine-protein kinase HT1-like [Sesamum indicum]
c47627.graph_c0	3.14274081	2.78567163	2.15800187	2.12174496	2.36480086	2.16323698	1.372E-05	-1.297545	down	PREDICTED: pentatricopeptide repeat-containing protein At1g63330-like [Sesamum indicum]
c47628.graph_c0	2.56348723	1.65271286	2.40084493	10.4072784	12.8486562	10.6493838	4.939E-06	1.336358	up	-
c47631.graph_c0	2.54274883	1.05536622	0.9563511	0.51421743	0.54475102	0.5083862	0.0080555	-2.549318	down	hypothetical protein EUGRSUZ_H03284 [Eucalyptus grandis]
c47633.graph_c0	205.02863	213.141217	183.744597	177.19282	188.320675	198.118174	1.475E-15	-1.112457	down	-
c47633.graph_c1	23.3385939	25.169735	24.3007573	17.8055446	20.2864242	18.9606748	6.094E-22	-1.371371	down	PREDICTED: adenine nucleotide transporter BT1, chloroplastic/mitochondrial-like [Sesamum indicum]
c47636.graph_c0	2.8274006	3.29420424	3.45463376	13.7925546	18.1400223	17.933855	6.972E-08	1.356557	up	UbiA prenyltransferase family protein isoform 1 [Theobroma cacao]
c47636.graph_c1	2.8935163	2.70954831	3.718283	21.4115577	22.0452759	21.8063633	2.942E-08	1.786637	up	PREDICTED: chlorophyll synthase, chloroplastic [Sesamum indicum]
c47637.graph_c0	3.68997875	3.56175678	2.54023327	0.72845338	0.90032603	0.32408672	4.373E-10	-3.337971	down	hypothetical protein VITISV_006175 [Vitis vinifera]
c47639.graph_c0	0	0	0	4.71725023	12.9395789	5.62149226	3.363E-06	Inf	up	PREDICTED: cytochrome b5 [Phoenix dactylifera]
c47647.graph_c0	18.3206339	19.3931524	17.84955	10.6387627	10.986945	12.5622063	2.857E-31	-1.719224	down	zeaxanthin epoxidase [Scutellaria baicalensis]
c47647.graph_c1	12.3028679	15.8636311	17.149475	4.41841864	5.90185152	7.52109664	7.161E-09	-2.371767	down	zeaxanthin epoxidase [Scutellaria baicalensis]
c47648.graph_c1	6.96330141	6.86739183	6.04245607	207.506664	218.599358	199.171646	6.76E-146	3.959346	up	PREDICTED: ATP-citrate synthase beta chain protein 2 [Sesamum indicum]
c47650.graph_c0	8.6783183	8.56881773	9.7485018	7.45115307	8.03763685	7.76725246	2.186E-10	-1.235079	down	PREDICTED: uncharacterized protein LOC105179099 isoform X1 [Sesamum indicum]
c47652.graph_c0	18.1838584	17.5773468	16.1126431	10.9503175	11.7389648	10.8597302	5.705E-25	-1.645735	down	PREDICTED: E3 ubiquitin-protein ligase SDIR1 [Sesamum indicum]

#ID	0 h-1 FPKM	0 h-2 FPKM	0 h-3 FPKM	48 h-1 FPKM	48 h-2 FPKM	48 h-3 FPKM	FDR	log2FC	regulated	nr_annotation
c47652.graph_c1	2.82717454	2.83000552	2.69113423	43.650791	47.8267249	49.7841383	6.388E-52	3.061821	up	PREDICTED: auxin-responsive protein IAA9-like [Sesamum indicum]
c47652.graph_c5	0.34374291	2.06452271	0.44172325	38.001407	36.2320945	30.9956382	8.681E-13	4.197073	up	AUX/IAA8A [Malus domestica]
c47657.graph_c0	3.12529107	3.24547033	3.29212785	26.1480364	22.5755305	20.4517453	5.854E-15	1.825612	up	PREDICTED: uncharacterized protein LOC105164082 isoform X2 [Sesamum indicum]
c47658.graph_c0	0.66328963	0.86515194	1.08481346	3.98118543	4.47259988	3.80605791	0.0001411	1.207155	up	PREDICTED: dihydrolipoyl dehydrogenase 2, chloroplastic [Sesamum indicum]
c47660.graph_c0	0.19599722	0.13079566	0.16790946	94.1147132	117.322801	139.422412	9.273E-83	8.447705	up	PREDICTED: beta-galactosidase-like [Sesamum indicum]
c47661.graph_c1	6.8209654	6.23407421	4.14442023	28.9606666	34.8047909	31.5123597	1.193E-15	1.454309	up	hypothetical protein MIMGU_mgv1a014073mg [Erythranthe guttata]
c47663.graph_c2	43.9531541	43.9558547	45.1322003	19.6189074	11.1169449	8.32242117	8.161E-74	-2.771611	down	PREDICTED: zinc finger protein ZAT10-like [Sesamum indicum]
c47664.graph_c0	5.37810993	5.99008632	6.18104009	3.52409318	5.65546989	6.14724703	5.458E-06	-1.222728	down	PREDICTED: cytochrome P450 704C1-like [Sesamum indicum]
c47671.graph_c2	16.5305629	14.6853915	14.8928859	9.96088705	11.2431992	9.8871199	6.965E-28	-1.586638	down	PREDICTED: pentatricopeptide repeat-containing protein At1g77360, mitochondrial-like [Sesamum indicum]
c47676.graph_c1	83.2216796	79.775591	89.024426	23.5541223	20.7507164	21.3504678	5.86E-86	-2.956621	down	PREDICTED: BI1-like protein-like [Solanum tuberosum]
c47677.graph_c0	17.8458459	20.1039538	19.4964608	17.8599106	12.0840997	10.8882758	1.518E-16	-1.501512	down	UDP-galactose/UDP-glucose transporter 4 [Morus notabilis]
c47678.graph_c2	38.7139371	41.7304083	38.3629327	12.9195558	15.9470825	14.770288	2.262E-65	-2.465438	down	hypothetical protein MIMGU_mgv1a024577mg, partial [Erythranthe guttata]
c47679.graph_c0	0.32395783	0.59867487	0.28820675	9.13719459	9.04740427	8.33449771	4.107E-26	3.439977	up	PREDICTED: outer envelope protein 80, chloroplastic isoform X1 [Sesamum indicum]
c47680.graph_c0	2.0719942	1.58024304	1.85958986	7.26173338	8.15300362	7.40505157	1.418E-08	1.030983	up	PREDICTED: pentatricopeptide repeat-containing protein At5g21222-like [Sesamum indicum]
c47681.graph_c0	0	0	0	1.54531963	6.10183906	1.75002051	0.0029812	Inf	up	-
c47682.graph_c0	9.26123854	8.96854118	10.5830257	67.283746	60.7093089	64.6660796	3.677E-32	1.72374	up	PREDICTED: probable tRNA N6-adenosine threonylcarbamoyltransferase, mitochondrial isoform X1 [Sesamum indicum]
c47685.graph_c0	0	0	0	2.14228201	4.08507844	2.85928447	2.156E-10	Inf	up	-
c47687.graph_c0	7.05108117	6.92240827	6.85377166	28.6486547	27.6147669	29.7152664	4.25E-08	1.028462	up	DUF250 domain-containing protein [Rozella allomycis CSF55]
c47689.graph_c0	0.98137459	0.80139674	0.49780469	10.992029	13.5603039	12.5115996	1.184E-28	3.007801	up	PREDICTED: major facilitator superfamily domain-containing protein 12-like [Sesamum indicum]
c47694.graph_c0	28.3421579	28.2006548	28.8603904	26.6665597	23.1705101	21.2081208	1.265E-19	-1.279682	down	PREDICTED: truncated FRIGIDA-like protein 1 [Sesamum indicum]
c47695.graph_c0	183.845786	162.403378	185.7542	68.0988538	51.1488789	44.146837	2.271E-64	-2.714057	down	hypothetical protein VITISV_032357 [Vitis vinifera]
c47697.graph_c0	0	0	0	25.8432567	19.911125	8.77997568	1.233E-07	Inf	up	predicted protein, partial [Micromonas pusilla CCMP1545]
c47702.graph_c1	109.090209	108.07668	101.749072	99.1997844	108.049858	102.195364	4.227E-14	-1.061414	down	-
c47705.graph_c0	0.70409477	0	7.23831726	72.0010412	90.7070376	101.005102	1.743E-24	4.003041	up	-

#ID	0 h-1 FPKM	0 h-2 FPKM	0 h-3 FPKM	48 h-1 FPKM	48 h-2 FPKM	48 h-3 FPKM	FDR	log2FC	regulated	nr_annotation
c47705.graph_c1	0.38095805	0.10895415	0.27974067	3.48456518	3.05409743	2.96670866	2.108E-08	2.612094	up	hypothetical protein MIMGU_mgv1a003510mg [Erythranthe guttata]
c47708.graph_c0	0	0	0	7.09842332	17.4285177	6.16751946	3.084E-06	Inf	up	-
c47709.graph_c0	18.6837904	15.6453601	18.641922	3.04119084	2.93685424	2.96375078	5.39E-72	-3.584015	down	hypothetical protein MIMGU_mgv1a025551mg [Erythranthe guttata]
c47713.graph_c1	12.6775252	12.8424263	12.5786023	10.3836016	10.6106163	10.2675826	4.179E-17	-1.302781	down	PREDICTED: pumilio homolog 23 isoform X1 [Sesamum indicum]
c47713.graph_c3	10.5059155	11.3208088	8.26093978	4.88049506	5.43171202	5.56247649	5.318E-20	-1.939308	down	hypothetical protein MIMGU_mgv1a016005mg [Erythranthe guttata]
c47714.graph_c2	2.32424863	2.57147875	2.82955406	0.22539468	0	0.10027741	2.442E-15	-5.554536	down	hypothetical protein MIMGU_mgv1a022738mg [Erythranthe guttata]
c47718.graph_c0	0.48949962	0.3392237	0.43547981	4.85648134	5.36534681	6.11150745	8.859E-10	2.671242	up	hypothetical protein MIMGU_mgv1a004721mg [Erythranthe guttata]
c47719.graph_c0	22.4539097	22.0858774	22.5875735	17.471289	17.3876131	15.6169146	1.267E-22	-1.427939	down	PREDICTED: arginine/serine-rich coiled-coil protein 2 [Sesamum indicum]
c47719.graph_c1	168.941026	182.266193	153.5034	156.3102	160.019625	157.882815	5.736E-13	-1.106375	down	PREDICTED: eukaryotic translation initiation factor isoform 4G-1-like [Sesamum indicum]
c47722.graph_c0	19.8341018	17.4742069	17.8936961	157.38102	171.830644	179.681265	2.062E-49	2.185797	up	Heterogeneous nuclear ribonucleoprotein F [Gossypium arboreum]
c47725.graph_c0	3.28782342	2.10837098	3.03670672	13.5829808	10.9300195	10.3594754	0.0006031	1.034723	up	PREDICTED: RING finger and CHY zinc finger domain-containing protein 1-like [Sesamum indicum]
c47726.graph_c0	84.864226	74.369074	77.2739779	2.74158567	1.40211332	1.51413917	1.21E-197	-6.389109	down	PREDICTED: E3 ubiquitin-protein ligase At4g11680-like [Sesamum indicum]
c47726.graph_c1	37.2192574	37.5429686	37.2868896	9.89471996	9.43961597	9.35159657	1.479E-91	-2.982121	down	PREDICTED: LOW QUALITY PROTEIN: uncharacterized protein LOC105174988 [Sesamum indicum]
c47730.graph_c0	1.108891	0.59200074	0.56998753	20.0230212	27.5611221	32.7845335	6.475E-20	4.126048	up	hypothetical protein MIMGU_mgv1a012535mg [Erythranthe guttata]
c47747.graph_c3	9.61239059	7.95131221	7.46698671	55.0126823	61.9895923	70.1311969	4.241E-34	1.883737	up	PREDICTED: ultraviolet-B receptor UVR8 [Sesamum indicum]
c47748.graph_c0	3.92149342	3.6712563	4.20543754	13.788174	19.0743241	18.9290805	2.956E-09	1.109814	up	PREDICTED: pentatricopeptide repeat-containing protein At1g11710, mitochondrial [Sesamum indicum]
c47749.graph_c0	1.80626656	1.90045867	1.98227382	9.25552216	10.5771588	10.3754431	1.727E-14	1.388061	up	PREDICTED: importin-11 [Sesamum indicum]
c47753.graph_c0	173.504354	165.705319	160.335146	18.9349754	21.4756493	20.2234058	1.39E-150	-4.061105	down	PREDICTED: transcription factor PIF1 [Sesamum indicum]
c47755.graph_c0	2.67213774	1.83415781	1.76595572	0.07033564	0.07451209	0.06258423	1.924E-17	-5.932334	down	--
c47756.graph_c0	42.2515463	40.6938351	50.1183783	22.0885197	21.8921013	14.2820753	5.616E-22	-2.207727	down	ent-copalyl diphosphate synthase [Andrographis paniculata]
c47757.graph_c0	35.521868	32.9087715	36.1915554	10.9862707	10.0467129	9.59724071	7.376E-71	-2.78795	down	PREDICTED: uncharacterized protein LOC105172510 isoform X1 [Sesamum indicum]

#ID	0 h-1 FPKM	0 h-2 FPKM	0 h-3 FPKM	48 h-1 FPKM	48 h-2 FPKM	48 h-3 FPKM	FDR	log2FC	regulated	nr_annotation
c47763.graph_c2	212.122713	218.481498	219.793375	107.537105	72.1282786	53.0838635	1.334E-60	-2.489511	down	PREDICTED: uncharacterized protein LOC105161925 [Sesamum indicum]
c47763.graph_c3	0.03476091	0.10438716	0	4.48336257	5.19061134	6.41132526	2.493E-21	5.845321	up	PREDICTED: U-box domain-containing protein 5 [Sesamum indicum]
c47765.graph_c0	0	0.15235226	0.39116568	2.71085105	2.03007822	2.62003852	1.1E-06	2.725398	up	-
c47770.graph_c0	11.1935456	11.3458721	8.65947527	6.44189329	7.15461795	7.41918746	1.655E-18	-1.586987	down	PREDICTED: probable serine/threonine-protein kinase At1g18390 isoform X1 [Sesamum indicum]
c47771.graph_c0	42.2917684	41.0488807	46.8714143	13.3735516	14.2443765	13.4677176	2.742E-69	-2.683629	down	PREDICTED: floral homeotic protein AGAMOUS-like isoform X1 [Sesamum indicum]
c47775.graph_c0	0	0	0	1.25410228	1.43485485	1.24979979	1.204E-11	Inf	up	catalase [Ipomoea batatas]
c47776.graph_c0	9.66083816	10.066574	9.4486238	6.31822663	6.53249607	6.18951776	4.939E-16	-1.632981	down	GTPase-activating protein gyp7 [Glycine soja]
c47778.graph_c0	11.617805	10.8454314	11.5744317	8.80298243	10.2939338	12.4983034	5.197E-11	-1.12944	down	PREDICTED: CBS domain-containing protein CBSCBSPB1-like isoform X3 [Sesamum indicum]
c47779.graph_c1	25.7539634	19.0502967	35.1275605	23.4529922	19.8282372	18.6396746	0.0036747	-1.386723	down	hypothetical protein VITISV_019132 [Vitis vinifera]
c47782.graph_c0	0	0	0	1.41060268	2.85686948	2.99020025	4.98E-10	Inf	up	squalene epoxidase [Chlorophytum borivilianum]
c47795.graph_c0	0	0	0	48.1435881	60.0185305	49.3370251	1.01E-108	Inf	up	unnamed protein product [Vitis vinifera]
c47797.graph_c0	48.0699332	50.945166	43.7173886	40.8247608	45.2637137	42.5659721	1.673E-16	-1.167512	down	PREDICTED: chromatin structure-remodeling complex protein SYD [Sesamum indicum]
c47800.graph_c0	51.2482869	53.4222098	49.3283923	40.3120218	47.3476228	46.6886956	6.719E-18	-1.216719	down	PREDICTED: probable alpha,alpha-trehalose-phosphate synthase [UDP-forming] 7 [Sesamum indicum]
c47804.graph_c0	9.08082723	8.67799038	8.24954099	6.29334361	6.10475443	5.46484486	6.982E-16	-1.557103	down	PREDICTED: MORC family CW-type zinc finger protein 3 isoform X1 [Sesamum indicum]
c47805.graph_c0	0	0	0	4.57503164	5.91059911	3.57439227	2.782E-16	Inf	up	-
c47806.graph_c0	10.2411996	10.9970149	9.98989971	80.6251761	94.0901543	112.322389	1.084E-22	2.179239	up	hypothetical protein MIMGU_mgv1a0009861mg, partial [Erythranthe guttata]
c47809.graph_c1	2.74325731	2.70501913	2.36766778	15.5785299	21.5385489	17.9228531	1.269E-13	1.795311	up	PREDICTED: probable E3 ubiquitin-protein ligase LUL4 [Sesamum indicum]
c47810.graph_c1	3.04350875	3.04655637	2.3806265	0	0	0.32541841	9.167E-16	-5.740425	down	-
c47810.graph_c2	325.27905	329.012967	313.966124	199.097363	194.898057	180.445064	2.655E-29	-1.769209	down	PREDICTED: E3 ubiquitin-protein ligase RKP [Sesamum indicum]
c47810.graph_c3	23.8975471	23.0594417	26.8360499	3.27264555	1.47083624	0.79417645	1.067E-56	-4.733366	down	-
c47816.graph_c0	2.89179242	3.09998514	2.29289287	20.0280444	18.5150832	17.8376456	1.954E-22	1.75378	up	PREDICTED: malonate--CoA ligase isoform X2 [Sesamum indicum]
c47820.graph_c0	0	0	0	6.12857911	5.91537653	3.97475709	1.591E-23	Inf	up	predicted protein [Hordeum vulgare subsp. vulgare]
c47823.graph_c0	21.4256518	24.1513067	20.0510687	20.9401945	19.3197309	22.0687733	2.959E-11	-1.090172	down	hypothetical protein MIMGU_mgv1a017876mg [Erythranthe guttata]

#ID	0 h-1 FPKM	0 h-2 FPKM	0 h-3 FPKM	48 h-1 FPKM	48 h-2 FPKM	48 h-3 FPKM	FDR	log2FC	regulated	nr_annotation
c47826.graph_c1	22.0862609	22.6116289	20.8518757	20.82644	21.4878233	22.5813973	2.932E-13	-1.032157	down	hypothetical protein MIMGU_mgv1a000321mg [Erythranthe guttata]
c47828.graph_c0	12.2428195	12.4663733	11.4603181	3.88927029	3.86269713	3.24435873	1.033E-27	-2.732935	down	-
c47832.graph_c0	0.17811172	0.05348702	0.04577612	7.87623048	12.8113802	11.8263755	1.936E-30	5.857965	up	hypothetical protein MIMGU_mgv1a000209mg [Erythranthe guttata]
c47833.graph_c0	12.0750729	11.6972557	10.8739449	9.45383692	9.51705597	9.73321865	2.604E-18	-1.28815	down	PREDICTED: uncharacterized protein LOC105171928 isoform X1 [Sesamum indicum]
c47836.graph_c1	50.9098757	50.9400709	53.6283132	13.0451965	14.4074495	13.8030979	3.217E-89	-2.933613	down	PREDICTED: uncharacterized protein LOC105173629 [Sesamum indicum]
c47837.graph_c0	16.7129929	16.6597295	15.0068419	13.8831584	13.6837974	13.786231	8.109E-14	-1.242446	down	hypothetical protein MIMGU_mgv1a003968mg [Erythranthe guttata]
c47838.graph_c0	0.73643065	0.56257563	0.34865251	3.24940858	2.32642631	2.52591962	0.001373	1.292953	up	putative terpenesynthase-1 [Marrubium vulgare]
c47840.graph_c3	8.34547202	12.8401442	4.76633865	140.669119	83.2591852	59.4160988	0.0033272	2.451472	up	-
c47843.graph_c1	14.1898011	14.8365773	14.1741522	75.8950032	71.7110087	63.1512973	2.72E-15	1.271334	up	hypothetical protein MIMGU_mgv1a027115mg, partial [Erythranthe guttata]
c47843.graph_c3	0.18382992	0.061338	0.11811436	0.95968466	1.91373087	2.46130354	0.0002673	2.847985	up	PREDICTED: serine/threonine-protein kinase STN7, chloroplastic [Sesamum indicum]
c47846.graph_c0	5.50867929	4.54466653	5.60086495	112.485534	119.578345	144.898234	4.484E-58	3.568811	up	hypothetical protein MIMGU_mgv1a012852mg [Erythranthe guttata]
c47847.graph_c0	0	0	0	1.46091991	2.57944583	1.57073395	5.015E-09	Inf	up	triosephosphate isomerase [Genlisea aurea]
c47848.graph_c2	3.48265198	3.60635103	2.9707036	0.77445252	1.84598671	1.77197965	3.08E-11	-2.224976	down	PREDICTED: auxin response factor 18-like isoform X1 [Sesamum indicum]
c47848.graph_c3	4.12744426	3.68249279	3.68968994	0.57863687	0.9632788	0.88263019	5.269E-11	-3.270902	down	-
c47849.graph_c0	0	0	0	13.417687	30.4137203	10.2180436	7.405E-08	Inf	up	Eukaryotic translation initiation factor 5A [Rozella allomycis CSF55]
c47850.graph_c0	22.9760556	23.569759	21.7592231	10.7148297	12.9090519	13.9738398	8.887E-25	-1.882137	down	unnamed protein product [Coffea canephora]
c47851.graph_c0	122.008247	117.662985	119.289821	88.9517993	77.6819276	66.5081842	7.49E-30	-1.636001	down	hypothetical protein MIMGU_mgv1a004485mg [Erythranthe guttata]
c47851.graph_c1	170.694755	170.459897	163.089613	70.7849232	71.9142636	81.6695928	1.498E-48	-2.186481	down	PREDICTED: NADH dehydrogenase [ubiquinone] iron-sulfur protein 7, mitochondrial-like [Sesamum indicum]
c47853.graph_c0	26.7383802	23.8044074	25.5418556	22.3442198	22.8049796	22.8397692	5.327E-12	-1.180058	down	PREDICTED: mediator of RNA polymerase II transcription subunit 6 [Sesamum indicum]
c47853.graph_c1	16.1809588	14.9424519	14.203787	6.87609529	11.0655989	10.5552493	1.226E-15	-1.694035	down	hypothetical protein MIMGU_mgv1a026972mg [Erythranthe guttata]
c47853.graph_c3	96.7143345	106.93826	94.7916698	49.5092527	42.4393435	44.0530353	4.215E-49	-2.147894	down	PREDICTED: probable indole-3-acetic acid-amido synthetase GH3.1 [Sesamum indicum]

#ID	0 h-1 FPKM	0 h-2 FPKM	0 h-3 FPKM	48 h-1 FPKM	48 h-2 FPKM	48 h-3 FPKM	FDR	log2FC	regulated	nr_annotation
c47861.graph_c0	0.77935794	0.78013835	0.70694527	4.70216572	5.10068877	4.89798768	1.833E-09	1.679779	up	PREDICTED: uncharacterized protein LOC105167319 isoform X5 [Sesamum indicum]
c47863.graph_c0	1.06299099	1.50219588	0.7231688	11.9243767	15.3175856	13.0962124	1.954E-21	2.600628	up	PREDICTED: LOW QUALITY PROTEIN: uncharacterized protein LOC105171608 [Sesamum indicum]
c47863.graph_c1	2.01928437	1.69671703	1.74253396	19.3090626	23.5627918	22.9443641	1.183E-53	2.571871	up	PREDICTED: LOW QUALITY PROTEIN: uncharacterized protein LOC105171608 [Sesamum indicum]
c47865.graph_c0	49.8391422	45.7295142	42.6208742	8.56676672	8.08630064	8.84809837	2.56E-117	-3.453658	down	PREDICTED: transcription factor GTE4-like isoform X3 [Sesamum indicum]
c47865.graph_c1	28.0397058	34.1364932	26.7806387	5.35257779	5.05405853	6.21220635	5.783E-39	-3.437013	down	PREDICTED: transcription factor GTE4-like isoform X3 [Sesamum indicum]
c47865.graph_c3	0.60747661	0.55280446	0.3548325	10.3789095	12.1809525	13.128316	1.432E-17	3.54203	up	hypothetical protein MIMGU_mgv1a012348mg [Erythranthe guttata]
c47865.graph_c4	235.782617	232.609558	223.254425	198.782616	222.662276	200.168241	2.066E-13	-1.172285	down	PREDICTED: patellin-3-like [Sesamum indicum]
c47865.graph_c6	375.978214	345.168882	378.285883	38.7098664	31.1371431	18.475801	3.63E-184	-4.647334	down	hypothetical protein VITISV_027452 [Vitis vinifera]
c47874.graph_c1	22.4028717	23.0570035	22.1996422	24.2483869	23.0706442	20.2181344	3.298E-12	-1.017817	down	PREDICTED: dymeclin-like [Sesamum indicum]
c47881.graph_c1	23.9163023	23.692392	21.4310217	11.4594409	12.0972422	11.3069378	7.914E-45	-2.002512	down	PREDICTED: uncharacterized protein LOC105166430 [Sesamum indicum]
c47882.graph_c0	40.0267518	37.9725079	35.6569771	32.9254629	33.1063816	33.3736998	2.533E-17	-1.20981	down	PREDICTED: survival of motor neuron-related-splicing factor 30 isoform X2 [Sesamum indicum]
c47884.graph_c0	0	0	0	6.67617772	6.86660268	4.61392061	2.53E-17	Inf	up	cysteine peroxiredoxin [Lichtheimia corymbifera JMRC:FSU:9682]
c47886.graph_c0	1.35183649	1.46144536	1.70242014	0.64760797	1.37212424	1.21896432	0.0003462	-1.511183	down	PREDICTED: ferric reduction oxidase 2-like isoform X2 [Sesamum indicum]
c47889.graph_c0	36.6568199	34.6722726	34.8966332	19.7949452	22.6378655	19.4596495	5.722E-36	-1.797865	down	PREDICTED: serine/threonine-protein kinase EDR1 [Sesamum indicum]
c47891.graph_c1	54.680133	51.1395561	58.0040062	4.83994186	5.07501181	7.60238113	9.75E-138	-4.247766	down	hypothetical protein MIMGU_mgv1a011954mg [Erythranthe guttata]
c47891.graph_c2	786.407989	791.290727	812.491392	444.150629	502.163682	521.486047	2.361E-20	-1.723827	down	PREDICTED: fumarate hydratase 1, mitochondrial [Sesamum indicum]
c47894.graph_c0	2.97689395	2.88957562	3.12989448	22.937347	26.0601594	21.5187261	6.507E-10	1.951378	up	PREDICTED: uncharacterized protein LOC105169824 isoform X1 [Sesamum indicum]
c47896.graph_c0	26.4078868	26.2650151	24.5887414	23.8417048	23.4415042	21.5955067	6.864E-17	-1.181395	down	PREDICTED: transducin beta-like protein 3 [Sesamum indicum]
c47896.graph_c1	886.559198	830.280553	912.005776	159.816314	160.787636	151.781365	1.74E-74	-3.49405	down	PREDICTED: uncharacterized protein LOC105160195 [Sesamum indicum]
c47896.graph_c2	22.0835764	20.8888628	22.7807214	13.7187622	12.5560365	13.7015911	2.715E-13	-1.73486	down	PREDICTED: cytochrome P450 CYP72A219-like [Sesamum indicum]

#ID	0 h-1 FPKM	0 h-2 FPKM	0 h-3 FPKM	48 h-1 FPKM	48 h-2 FPKM	48 h-3 FPKM	FDR	log2FC	regulated	nr_annotation
c47898.graph_c0	0.5631971	1.36913398	0.41356032	3.11312339	5.33958141	5.27626376	0.0038905	1.528344	up	PREDICTED: chlorophyll a-b binding protein 13, chloroplastic [Sesamum indicum]
c47900.graph_c0	109.067233	113.480735	98.9436018	56.6566049	58.2721998	60.5466517	7.279E-41	-1.89057	down	PREDICTED: glycine-rich RNA-binding protein RZ1C-like isoform X1 [Sesamum indicum]
c47902.graph_c0	5.50678276	5.2285758	5.09919499	78.0378677	91.4051365	92.7383964	3.032E-80	3.029554	up	PREDICTED: probable starch synthase 4, chloroplastic/amyloplastic isoform X3 [Sesamum indicum]
c47903.graph_c0	0	0	0	1.03810782	1.09974936	0.84672692	1.581E-10	Inf	up	unknown [Chondrus crispus]
c47904.graph_c0	153.121301	143.562816	152.233291	96.0444802	95.7376707	91.7988521	1.258E-30	-1.6799	down	PREDICTED: ATP-dependent RNA helicase DHX36 isoform X1 [Sesamum indicum]
c47909.graph_c0	9.7236531	9.84678858	10.0447487	8.38405163	9.84009785	8.49706062	6.516E-13	-1.168076	down	PREDICTED: transcription initiation factor TFIID subunit 8-like [Sesamum indicum]
c47910.graph_c0	23.4819139	21.0411488	19.9951185	19.306934	20.7305847	16.4030254	1.34E-15	-1.208738	down	PREDICTED: cytochrome P450 CYP72A219-like [Sesamum indicum]
c47911.graph_c2	120.574232	119.426922	129.895818	18.8848049	18.4607411	16.7799853	2.15E-135	-3.78984	down	hypothetical protein MIMGU_mgv1a008076mg [Erythranthe guttata]
c47912.graph_c0	0	0	0	2.27996994	4.83070338	3.12999975	4.443E-09	Inf	up	-
c47913.graph_c1	29.2355588	30.0596812	25.5097134	30.6578701	24.3763349	22.6636208	2.745E-11	-1.136923	down	PREDICTED: lysophospholipid acyltransferase LPEAT2 [Sesamum indicum]
c47914.graph_c0	0	0	0	22.8581659	44.6493186	20.6733993	1.48E-13	Inf	up	hypothetical protein JCGZ_08960 [Jatropha curcas]
c47915.graph_c0	4.24654579	1.92614287	2.13163261	0	0	0	9.561E-11	-Inf	down	PREDICTED: stem-specific protein TSJT1-like isoform X1 [Elaeis guineensis]
c47920.graph_c0	6.58369259	6.11647381	6.58023772	35.9162503	34.6051916	35.3443484	6.531E-16	1.439897	up	indole-3-glycerol phosphate lyase IGL1 [Aphelandra squarrosa]
c47923.graph_c0	0	0	0	6.57581033	9.89944156	2.72758814	6.013E-07	Inf	up	--
c47924.graph_c0	22.1390296	22.7450864	20.6812748	9.59951596	7.86650137	5.93347201	3.362E-57	-2.496813	down	PREDICTED: cysteine-rich receptor-like protein kinase 3 [Nicotiana sylvestris]
c47927.graph_c0	0	0	0	1.08012541	1.29683015	0.67276237	1.349E-08	Inf	up	hypothetical protein MVLG_06307 [Microbotryum violaceum p1A1 Lamole]
c47934.graph_c0	14.2326956	13.5532759	11.6450493	7.75921285	7.59564553	9.22003352	7.238E-15	-1.698759	down	hypothetical protein MIMGU_mgv1a010597mg [Erythranthe guttata]
c47937.graph_c1	39.199188	36.420621	38.9785371	7.20886035	7.50378181	7.58342764	4.61E-114	-3.380211	down	PREDICTED: ATP-dependent DNA helicase 2 subunit KU80 isoform X2 [Sesamum indicum]
c47938.graph_c0	197.426065	198.959785	181.989296	147.15375	163.636844	153.261967	1.345E-19	-1.335773	down	hypothetical protein MIMGU_mgv1a002698mg [Erythranthe guttata]
c47942.graph_c0	0	0	0	1.04333436	0.7368575	0.61890176	2.164E-08	Inf	up	-
c47943.graph_c0	0	0	0	2.00613797	1.63948623	1.22403395	1.609E-10	Inf	up	--
c47944.graph_c1	4.77697983	4.49263338	4.65392157	30.9084035	43.651046	43.8394586	1.192E-17	2.064381	up	PREDICTED: MATE efflux family protein DTX1 [Sesamum indicum]

#ID	0 h-1 FPKM	0 h-2 FPKM	0 h-3 FPKM	48 h-1 FPKM	48 h-2 FPKM	48 h-3 FPKM	FDR	log2FC	regulated	nr_annotation
c47946.graph_c0	42.6491057	44.1070106	41.161116	34.4068805	28.0560882	23.5648853	2.44E-29	-1.583732	down	PREDICTED: scarecrow-like protein 1 [Sesamum indicum]
c47948.graph_c0	1.8445318	2.20539692	1.48143524	951.60968	1086.04896	1074.57003	0	8.119168	up	PREDICTED: alpha-L-arabinofuranosidase 1-like isoform X2 [Sesamum indicum]
c47952.graph_c0	1.9012037	1.93770942	2.13217922	12.9611951	11.9427724	11.7594823	9.637E-10	1.601397	up	PREDICTED: ABC transporter I family member 11, chloroplastic isoform X2 [Sesamum indicum]
c47953.graph_c0	1.75841377	1.40813964	1.53152789	8.15382612	7.77991159	7.68765611	4.062E-08	1.314264	up	PREDICTED: serine carboxypeptidase-like 45 [Sesamum indicum]
c47954.graph_c0	0	0	0	17.8536043	28.71718	11.4778644	7.925E-11	Inf	up	40S ribosomal protein S11 [Auxenochlorella protothecoides]
c47956.graph_c0	20.4064264	16.8019499	19.0541701	11.4060718	9.83861701	9.98859475	9.407E-24	-1.863655	down	hypothetical protein MIMGU_mgv1a0158002mg, partial [Erythranthe guttata]
c47957.graph_c0	56.5884466	58.3645245	53.1592309	39.6052312	45.402904	41.5767456	2.042E-23	-1.427905	down	PREDICTED: nucleolar complex protein 4 homolog isoform X1 [Sesamum indicum]
c47958.graph_c0	3.44671142	3.58159755	3.24805113	17.8423607	19.894964	20.3159306	4.28E-13	1.479192	up	PREDICTED: 3-oxoacyl-[acyl-carrier-protein] synthase II, chloroplastic [Vitis vinifera]
c47958.graph_c1	11.1702903	10.6537363	11.009171	8.74262652	9.64765906	9.04864836	7.291E-12	-1.277779	down	hypothetical protein MIMGU_mgv1a024121mg, partial [Erythranthe guttata]
c47961.graph_c0	0.3050715	0.45806547	0.29402171	5.23458907	6.77359097	6.75211053	1.236E-12	3.129278	up	PREDICTED: probable polygalacturonase At3g15720-like [Glycine max]
c47963.graph_c0	2.90894205	3.95713616	2.87546592	27.2800805	30.0646704	22.8673695	2.841E-13	2.025619	up	PREDICTED: protein ENHANCED DISEASE RESISTANCE 2-like [Sesamum indicum]
c47963.graph_c2	7.13039425	4.48170756	8.94974964	2.44427322	2.91308748	2.31083084	3.339E-05	-2.446553	down	-
c47967.graph_c0	0	0	0	3.3104713	8.86780853	3.11395959	1.191E-05	Inf	up	hypothetical protein FOMPIDRAFT_1023471 [Fomitopsis pinicola FP-58527 SS1]
c47968.graph_c0	179.286408	160.616204	172.227328	26.1775491	27.1473395	25.56358	1.96E-130	-3.716258	down	PREDICTED: alpha-galactosidase-like [Sesamum indicum]
c47971.graph_c0	221.818253	214.295197	224.086907	61.3226875	65.2462502	68.0204573	3.058E-74	-2.781636	down	PREDICTED: LOW QUALITY PROTEIN: manganese-dependent ADP-ribose/CDP-alcohol diphosphatase-like [Sesamum indicum]
c47972.graph_c0	3.26540758	2.21116412	2.83859101	13.8028178	12.4665434	13.6200508	0.0011806	1.246259	up	-
c47972.graph_c1	0.15624427	0.25024115	0.40156013	7.02438953	8.81389471	9.09361535	4.078E-24	3.916976	up	PREDICTED: NF-X1-type zinc finger protein NFXL1 [Prunus mume]
c47972.graph_c3	170.258586	168.897847	173.211786	105.104174	118.462606	117.229395	1.613E-27	-1.608105	down	PREDICTED: putative lactoylglutathione lyase [Sesamum indicum]
c47972.graph_c5	0.19527785	0.0651578	0.50187954	13.1928642	14.2938817	16.3277861	1.742E-25	4.809819	up	hypothetical protein MIMGU_mgv1a026403mg, partial [Erythranthe guttata]
c47975.graph_c0	57.0319485	50.9962193	49.8231944	41.7106171	40.1185252	43.2653242	5.444E-18	-1.351754	down	hypothetical protein PRUPE_ppa009199mg [Prunus persica]
c47977.graph_c0	0	0	0	9.82939458	25.6982652	7.78325881	9.827E-06	Inf	up	hypothetical protein CY34DRAFT_809502 [Suillus luteus UH-Slu-Lm8-n1]

#ID	0 h-1 FPKM	0 h-2 FPKM	0 h-3 FPKM	48 h-1 FPKM	48 h-2 FPKM	48 h-3 FPKM	FDR	log2FC	regulated	nr_annotation
c47978.graph_c0	5.30333039	5.22482023	3.71237587	25.0460197	27.7863299	28.738154	1.925E-23	1.502007	up	PREDICTED: ABC transporter D family member 1 [Sesamum indicum]
c47978.graph_c1	9.75787293	9.262421	8.51804431	6.63369671	8.27548839	6.84042343	1.462E-11	-1.359459	down	hypothetical protein MIMGU_mgv1a010030mg [Erythranthe guttata]
c47979.graph_c0	8.03468871	7.19364792	6.75205619	54.6364622	61.9739787	64.1442448	3.814E-43	2.021154	up	hypothetical protein MIMGU_mgv1a024130mg [Erythranthe guttata]
c47980.graph_c1	306.487179	306.85427	302.475695	273.354048	282.748563	269.846185	1.54E-13	-1.166571	down	PREDICTED: protein CLP1 homolog [Sesamum indicum]
c47981.graph_c1	0.82393781	0.48348167	0.40161102	4.24029553	4.10387459	5.14710291	8.95E-07	1.967947	up	PREDICTED: uncharacterized protein LOC105174256 [Sesamum indicum]
c47982.graph_c3	98.4547239	96.138865	89.1826638	34.8064855	31.7799057	30.5032563	2.879E-68	-2.561682	down	PREDICTED: uncharacterized protein LOC105178089 [Sesamum indicum]
c47983.graph_c0	0	0	0	16.8485794	35.5372518	13.033381	7.229E-09	Inf	up	PREDICTED: 40S ribosomal protein S19-3 [Prunus mume]
c47984.graph_c0	0.78341904	0.54291013	0.54208241	0.19431373	0.11762962	0.22229896	1.055E-07	-2.817131	down	hypothetical protein [Beta vulgaris subsp. vulgaris]
c47984.graph_c1	3.17213568	3.21475696	3.443351	18.0788123	18.3446819	15.8603041	9.169E-16	1.393607	up	PREDICTED: putative nuclease HARBI1 [Sesamum indicum]
c47990.graph_c0	0.48098542	0.64195608	0.41205687	4.67732652	5.37232853	5.1694645	2.492E-06	2.292509	up	PREDICTED: kinetochore protein Spc25 [Sesamum indicum]
c47991.graph_c0	0	0	0	0.50955216	0.46619845	0.53583217	4.331E-10	Inf	up	P-ATPase family transporter: sodium/potassium ion [Ostreococcus lucimarinus CCE9901]
c47997.graph_c0	54.2459656	56.1560151	47.3352951	45.7917807	56.849459	56.536789	3.794E-13	-1.006721	down	PREDICTED: importin subunit beta-1-like [Sesamum indicum]
c47999.graph_c0	30.6202899	15.7295958	16.8407466	0	0	0	8.862E-22	-Inf	down	--
c48000.graph_c0	7.69961942	8.11796583	7.7248654	3.51762453	3.80349039	3.4274368	4.941E-35	-2.149098	down	PREDICTED: uncharacterized protein LOC105159735 isoform X3 [Sesamum indicum]
c48004.graph_c0	2.402911	2.18355032	2.62794533	8.3524692	11.476345	14.0676705	6.718E-05	1.20623	up	PREDICTED: inactive protein kinase SELMODRAFT_444075 [Sesamum indicum]
c48005.graph_c0	6.15667496	6.36164123	5.44452203	112.035875	112.227433	121.667065	1.908E-81	3.250747	up	PREDICTED: uncharacterized protein LOC105173858 [Sesamum indicum]
c48007.graph_c0	2.01089875	2.09918003	1.40278873	0.95275085	0.756993	0.30613266	1.676E-09	-2.454181	down	PREDICTED: uncharacterized protein LOC104226160 [Nicotiana sylvestris]
c48013.graph_c0	47.3825275	51.1030022	46.3069789	45.268611	40.0546638	36.69968	1.937E-19	-1.260581	down	PREDICTED: OBERON-like protein [Sesamum indicum]
c48014.graph_c0	2.84142316	3.35513216	3.03096774	18.3620523	17.9715648	16.7681052	3.6E-21	1.50826	up	PREDICTED: uncharacterized protein LOC105158471, partial [Sesamum indicum]
c48016.graph_c0	65.9167395	62.5008061	61.0645323	36.7636592	40.7195128	39.5238	4.175E-33	-1.713982	down	PREDICTED: probable F-box protein At1g60180 [Sesamum indicum]
c48017.graph_c1	4.90076062	4.83729631	4.65193848	20.0889468	24.4149113	23.3201175	5.788E-15	1.21649	up	PREDICTED: bifunctional purple acid phosphatase 26 isoform X1 [Sesamum indicum]
c48024.graph_c0	4.30159372	3.12296125	3.94837017	3.17903665	3.32166956	3.83616551	0.0012266	-1.157369	down	hypothetical protein MIMGU_mgv1a020521mg, partial [Erythranthe guttata]

#ID	0 h-1 FPKM	0 h-2 FPKM	0 h-3 FPKM	48 h-1 FPKM	48 h-2 FPKM	48 h-3 FPKM	FDR	log2FC	regulated	nr_annotation
c48025.graph_c0	16.2406698	15.389896	14.3306672	14.496481	15.9560562	14.6739571	4.65E-10	-1.043931	down	PREDICTED: fructose-bisphosphate aldolase cytoplasmic isozyme [Elaeis guineensis]
c48031.graph_c1	1.06918099	1.19723062	0.9547718	3.99008177	7.30441099	6.64019579	0.000129	1.451376	up	PREDICTED: cytochrome P450 CYP749A22-like [Sesamum indicum]
c48033.graph_c0	8.15502801	9.31484797	9.34896175	36.0995387	41.7437462	44.5202501	8.263E-14	1.168268	up	PREDICTED: protease Do-like 2, chloroplastic [Sesamum indicum]
c48037.graph_c0	0	0	0	3.56164418	8.65217772	2.51344759	6.308E-05	Inf	up	PREDICTED: thiol protease aleurain-like [Eucalyptus grandis]
c48037.graph_c1	0	0	0	4.40059038	13.2950251	5.07580223	7.008E-06	Inf	up	--
c48045.graph_c1	0.4198272	0.38792086	0.45649541	1.87435759	3.12031435	2.25019571	0.0017298	1.492794	up	PREDICTED: putative nuclease HARBI1 [Brassica rapa]
c48046.graph_c0	31.8626314	36.6153891	35.1060521	13.2463602	11.8999098	11.8336778	3.63E-49	-2.501734	down	PREDICTED: uncharacterized protein LOC105172478 [Sesamum indicum]
c48046.graph_c1	33.6910287	32.8451899	32.0027229	25.3291871	23.9219467	24.9450715	9.782E-24	-1.425518	down	PREDICTED: probable NOT transcription complex subunit VIP2 isoform X2 [Sesamum indicum]
c48050.graph_c1	0	0	0	21.3826713	54.0399138	19.2748805	2.78E-08	Inf	up	unknown [Lotus japonicus]
c48052.graph_c1	0	0	0	1.79404181	1.69759629	1.67381924	5.739E-18	Inf	up	--
c48054.graph_c0	0.53730539	0.53784342	0.39188196	0.28094638	0.46770212	0.27379244	0.0007062	-1.541267	down	PREDICTED: protein LONGIFOLIA 2-like [Sesamum indicum]
c48055.graph_c0	0.36873413	0.30199366	0.17230471	2.90290742	3.50058287	3.57221961	2.807E-07	2.550422	up	farnesyl diphosphate synthase [Leibnitzia anandria]
c48059.graph_c1	3.91055298	3.66788809	3.75901939	33.2749294	37.0388346	35.6025978	3.97E-49	2.204777	up	PREDICTED: protein transport protein Sec24-like At4g32640 [Sesamum indicum]
c48062.graph_c3	76.9083771	74.9822743	73.076858	46.3777686	46.7996556	48.8564931	5.216E-30	-1.680946	down	PREDICTED: probable protein phosphatase 2C 6 [Sesamum indicum]
c48066.graph_c0	0	0	0	3.58420529	3.48387371	3.09056913	3.628E-17	Inf	up	predicted protein [Hordeum vulgare subsp. vulgare]
c48067.graph_c0	0	0	0	10.0023618	13.0716521	11.8180877	1.902E-39	Inf	up	--
c48068.graph_c2	0.10574163	0.15877127	0	1.55865734	1.70280872	1.64692466	1.17E-06	3.210511	up	hypothetical protein MIMGU_mgv1a000605mg [Erythranthe guttata]
c48071.graph_c1	28.575571	28.4845024	26.0425333	147.572509	160.62353	189.159636	9.763E-25	1.56202	up	hypothetical protein POPTR_0003s13500g [Populus trichocarpa]
c48076.graph_c2	37.8500862	36.1069281	35.3082089	31.7803499	36.5511017	36.5695336	5.717E-14	-1.078296	down	PREDICTED: eukaryotic translation initiation factor 4G-like [Sesamum indicum]
c48076.graph_c3	0	0	0	2.038228	1.38192347	1.41461035	1.243E-11	Inf	up	-
c48078.graph_c0	27.8483402	29.5806582	26.6249475	8.47371365	9.45833066	9.69224148	8.774E-73	-2.624348	down	PREDICTED: ankyrin repeat protein SKIP35-like [Sesamum indicum]
c48078.graph_c1	38.3818327	36.728808	38.3099909	26.797872	13.0754757	14.3463273	2.827E-24	-2.06824	down	-
c48090.graph_c0	0.32247233	0.96838571	0.20719485	10.397889	13.8478089	17.0500757	1.493E-10	3.768301	up	hypothetical protein [Arabidopsis thaliana]
c48090.graph_c2	51.6566556	53.9833048	47.0427886	28.9724304	37.8264497	41.0860692	4.594E-24	-1.522779	down	hypothetical protein MIMGU_mgv1a003910mg [Erythranthe guttata]

#ID	0 h-1 FPKM	0 h-2 FPKM	0 h-3 FPKM	48 h-1 FPKM	48 h-2 FPKM	48 h-3 FPKM	FDR	log2FC	regulated	nr_annotation
c48090.graph_c3	0.99459919	0.9244812	1.46068496	4.45054972	5.82418666	5.4742177	0.0083352	1.193523	up	hypothetical protein MIMGU_mgv1a003194mg [Erythranthe guttata]
c48091.graph_c0	0.26346833	0.36089663	0.37420589	1.36692056	1.5292877	1.78463176	0.0029078	1.205625	up	PREDICTED: myosin-2-like [Sesamum indicum]
c48091.graph_c1	21.4541658	22.0810455	17.3024902	330.960726	332.501183	350.665334	2.192E-91	3.04357	up	PREDICTED: pentatricopeptide repeat-containing protein At2g13420, mitochondrial-like [Sesamum indicum]
c48095.graph_c0	5.04741889	5.35952949	5.08856851	25.4594552	24.9299944	29.1000223	2.437E-15	1.340813	up	PREDICTED: maltose excess protein 1-like, chloroplastic [Sesamum indicum]
c48096.graph_c0	8.72749305	8.21178235	6.18340563	3.37871933	3.64662433	4.13657623	9.191E-18	-2.067206	down	PREDICTED: putative SWI/SNF-related matrix-associated actin-dependent regulator of chromatin subfamily A member 3-like 2 isoform X3 [Sesamum indicum]
c48097.graph_c0	0	0.06858486	0	0.97838538	0.86930637	0.61781779	1.471E-08	4.167074	up	PREDICTED: lisH domain and HEAT repeat-containing protein KIAA1468 homolog isoform X1 [Sesamum indicum]
c48100.graph_c0	51.3881423	53.4628498	47.4214159	36.536371	34.1328851	31.078908	5.565E-30	-1.59598	down	PREDICTED: uncharacterized protein LOC105166426 [Sesamum indicum]
c48105.graph_c0	27.7789966	15.5684852	17.6882389	3.33306685	5.92555293	2.14760688	1.443E-09	-3.43968	down	hypothetical protein AOL_s00169g48 [Arthrobotrys oligospora ATCC 24927]
c48109.graph_c0	116.935893	113.453806	107.41553	69.386252	80.2141216	73.9465133	1.821E-29	-1.614392	down	PREDICTED: cleft lip and palate transmembrane protein 1 homolog [Sesamum indicum]
c48111.graph_c5	18.4257432	17.7992919	16.5579088	12.503727	14.6714048	16.7238197	1.447E-13	-1.286245	down	PREDICTED: ribonuclease II, chloroplastic/mitochondrial [Sesamum indicum]
c48112.graph_c1	0.52943327	0.52996342	1.08854835	7.31622696	4.6503932	5.90234084	7.884E-05	2.03754	up	PREDICTED: cytochrome P450 90A1 [Sesamum indicum]
c48112.graph_c3	7.9967825	6.88784262	7.24908971	0.74242674	1.21001709	1.06713414	3.488E-25	-3.89865	down	PREDICTED: uncharacterized protein LOC105163706 [Sesamum indicum]
c48114.graph_c0	159.373403	156.0553	157.89243	65.816627	60.459942	74.9689682	1.835E-54	-2.251384	down	ribosomal protein L20 (chloroplast) [Andrographis paniculata]
c48118.graph_c0	2.55953286	2.19608216	3.21078914	0.50528756	0.41633737	0.39964607	2.593E-12	-3.608418	down	PREDICTED: sodium/hydrogen exchanger 4 isoform X3 [Nelumbo nucifera]
c48119.graph_c1	12.7431571	13.399073	11.1463225	9.3326696	9.94953898	9.41020331	1.132E-18	-1.394681	down	PREDICTED: uncharacterized protein LOC105173907 [Sesamum indicum]
c48122.graph_c1	181.404108	201.097442	159.934232	68.424096	64.9249116	59.3528477	1.702E-52	-2.507044	down	PREDICTED: NF-X1-type zinc finger protein NFXL1 [Sesamum indicum]
c48125.graph_c1	11.4530462	9.20635274	13.1566584	55.6873809	57.2439656	62.637265	1.213E-16	1.355185	up	PREDICTED: sister chromatid cohesion 1 protein 3-like [Sesamum indicum]
c48126.graph_c0	0.08555543	0.0856411	0.05497106	2.26605318	2.40060857	2.61245003	4.511E-13	3.993576	up	PREDICTED: cysteine-rich receptor-like protein kinase 2 [Sesamum indicum]
c48133.graph_c0	0.32706341	0.27702308	0.45261938	2.78134286	3.87955242	4.20719766	1.999E-08	2.333998	up	PREDICTED: probable LRR receptor-like serine/threonine-protein kinase MRH1 isoform X3 [Nelumbo nucifera]

#ID	0 h-1 FPKM	0 h-2 FPKM	0 h-3 FPKM	48 h-1 FPKM	48 h-2 FPKM	48 h-3 FPKM	FDR	log2FC	regulated	nr_annotation
c48134.graph_c1	295.021142	296.319902	290.191209	120.044349	118.730501	116.522405	1.303E-51	-2.327844	down	PREDICTED: protein ALUMINUM SENSITIVE 3 [Sesamum indicum]
c48135.graph_c0	3.10375323	2.9938844	2.75565345	0.57187598	0.38553026	0.37007404	2.133E-14	-3.745374	down	PREDICTED: formin-like protein 3 [Nicotiana tomentosiformis]
c48139.graph_c0	0	0	0.18038052	1.487153	1.71245464	2.53145352	4.068E-08	3.934216	up	hypothetical protein VITISV_002420 [Vitis vinifera]
c48141.graph_c0	13.2559406	10.3778681	10.7045019	8.26820724	9.26256317	7.54726835	3.058E-16	-1.470063	down	PREDICTED: peptidyl-prolyl cis-trans isomerase CYP40-like isoform X1 [Sesamum indicum]
c48143.graph_c2	3.07489539	2.25351699	2.75184161	26.0515894	24.5974981	24.6208923	9.709E-19	2.203432	up	PREDICTED: protein transport protein SEC13 homolog B-like [Sesamum indicum]
c48143.graph_c4	1.16297857	1.11936838	1.4944735	4.86256084	5.67515396	6.89335042	0.0019052	1.181645	up	PREDICTED: NAC transcription factor 29-like isoform X3 [Sesamum indicum]
c48149.graph_c0	0	0	0	2.93001842	4.74519423	1.79800881	4.195E-08	Inf	up	citrate synthase, mitochondrial, putative (ISS), partial [Ostreococcus tauri]
c48150.graph_c0	4.66837668	5.34250533	4.53298916	1.31682199	1.5101978	1.60168204	2.816E-42	-2.73505	down	PREDICTED: CST complex subunit CTC1 [Sesamum indicum]
c48154.graph_c0	32.8359313	30.4537196	31.3819369	13.9868373	12.864683	12.9277837	1.137E-39	-2.266512	down	hypothetical protein MIMGU_mgv1a024172mg [Erythranthe guttata]
c48157.graph_c1	269.174604	258.621146	281.562204	250.768761	220.654153	208.911846	8.889E-17	-1.265625	down	Metal tolerance protein 3 [Triticum urartu]
c48159.graph_c0	0	0	0	0.68681072	1.8189817	0.53473004	0.0009212	Inf	up	atp-dependent rrna helicase rrp3 [Lichtheimia corymbifera JMRC:FSU:9682]
c48160.graph_c0	0	0	0	1.50145741	1.2923723	0.87674197	1.538E-09	Inf	up	hypothetical protein VOLCADRAFT_45407 [Volvox carteri f. nagariensis]
c48161.graph_c2	1.02361066	0.81970852	0.78922814	23.3239516	18.7814269	20.5856937	4.141E-24	3.561878	up	hypothetical protein MIMGU_mgv1a011390mg [Erythranthe guttata]
c48161.graph_c4	0.32271534	0.43071798	0.59901398	26.5593733	35.450509	38.5936601	2.621E-48	5.187908	up	PREDICTED: WD repeat-containing protein 44 [Sesamum indicum]
c48162.graph_c0	3.25757107	2.73334534	3.93986616	0.48546995	0.28052539	0.62831744	1.22E-16	-3.849886	down	PREDICTED: aluminum-activated malate transporter 9-like [Sesamum indicum]
c48163.graph_c0	2.00399786	3.26400743	2.66251208	2.31558987	1.88953974	2.00471151	0.0001711	-1.367587	down	PREDICTED: uncharacterized aarF domain-containing protein kinase 1 [Sesamum indicum]
c48166.graph_c0	68.3290786	69.0716222	64.9055587	60.0857072	64.3107853	67.7747306	1.237E-14	-1.092665	down	PREDICTED: uroporphyrinogen decarboxylase 1, chloroplastic isoform X2 [Sesamum indicum]
c48168.graph_c0	21.9666424	21.6329401	21.2955392	106.036472	123.934935	121.916989	3.227E-24	1.418971	up	PREDICTED: dnaJ protein ERDJ3B [Sesamum indicum]
c48169.graph_c0	2.37666436	2.60973943	2.79497138	8.24062569	11.2884779	12.4333004	6.936E-06	1.013053	up	PREDICTED: GPI inositol-deacylase A isoform X1 [Sesamum indicum]
c48175.graph_c2	35.2834311	32.6548165	35.412006	6.99933754	8.33658154	7.07243875	4.109E-84	-3.225346	down	PREDICTED: transcription factor TGA1-like [Sesamum indicum]
c48181.graph_c0	48.6639999	47.3497024	47.9800816	10.2779641	13.0175829	11.9484673	1.469E-95	-3.051744	down	PREDICTED: probable leucine-rich repeat receptor-like protein kinase At1g35710 [Sesamum indicum]

#ID	0 h-1 FPKM	0 h-2 FPKM	0 h-3 FPKM	48 h-1 FPKM	48 h-2 FPKM	48 h-3 FPKM	FDR	log2FC	regulated	nr_annotation
c48187.graph_c1	2.00113514	2.12381	2.10680212	40.6919473	43.8611654	45.9708126	1.47E-87	3.369002	up	PREDICTED: protein IQ-DOMAIN 31-like isoform X1 [Sesamum indicum]
c48188.graph_c0	31.15958	30.3344349	29.3016016	14.6714033	13.1983418	10.7888648	3.209E-49	-2.245017	down	PREDICTED: nitronate monooxygenase [Sesamum indicum]
c48194.graph_c2	0.03798607	0.07604822	0.09762721	1.46980033	1.92780742	2.8336089	7.651E-08	3.847456	up	PREDICTED: 125 kDa kinesin-related protein [Sesamum indicum]
c48195.graph_c0	0.30643743	0.15337214	0.06563071	1.92911937	2.14335918	1.46532094	7.351E-05	2.392584	up	hypothetical protein VITISV_040416 [Vitis vinifera]
c48196.graph_c0	11.050566	8.74808766	10.4878881	11.1785907	9.93912469	9.23616827	0.0001845	-1.011572	down	hypothetical protein MIMGU_mgv1a003019mg [Erythranthe guttata]
c48197.graph_c0	0	0	0	3.59371958	4.75888767	2.03488096	1.484E-07	Inf	up	Heat Shock Protein 70 [Ostreococcus lucimarinus CCE9901]
c48200.graph_c0	0	0	0	1.54625292	4.50468555	2.30945671	1.457E-05	Inf	up	hypothetical protein EMIHUDRAFT_225441 [Emiliania huxleyi CCMP1516]
c48202.graph_c0	3.2066345	3.30026364	2.90186781	50.0959108	40.3759281	35.7266726	2.097E-14	2.734642	up	hypothetical protein MIMGU_mgv1a010335mg [Erythranthe guttata]
c48203.graph_c1	4.53447169	3.53565167	3.98687808	20.9312286	22.5467726	23.8675076	7.503E-11	1.462967	up	Hypothetical protein [Arabidopsis thaliana]
c48205.graph_c0	8.51564998	7.11107824	7.43184127	4.23723204	3.28884835	4.33020373	3.497E-13	-1.973623	down	PREDICTED: uncharacterized protein LOC105167185 isoform X1 [Sesamum indicum]
c48208.graph_c0	0	0	0	28.0712845	45.3481663	22.1562633	4.142E-16	Inf	up	PREDICTED: 40S ribosomal protein S13-1-like [Camelina sativa]
c48210.graph_c1	9.16181657	9.92822852	11.1252256	5.03330363	4.51184028	5.51212714	4.251E-10	-2.023597	down	-
c48211.graph_c0	0.50948854	0.35999909	0.23107516	7.37193396	6.31793539	6.24012598	1.019E-15	3.171111	up	PREDICTED: wall-associated receptor kinase-like 2 [Sesamum indicum]
c48212.graph_c0	53.8124509	59.4094277	57.3868298	51.4680499	54.524158	47.8459804	2.424E-16	-1.167142	down	PREDICTED: putative late blight resistance protein homolog R1A-10 [Sesamum indicum]
c48213.graph_c0	17.6485428	19.7836098	17.3081469	16.165074	14.1775067	13.986653	6.725E-21	-1.318393	down	PREDICTED: probable ATP-dependent DNA helicase CHR12 [Sesamum indicum]
c48215.graph_c0	2.44028257	1.35129531	1.80145133	0	0	0	1.447E-19	-Inf	down	--
c48219.graph_c0	24.9993548	26.5097389	24.4332208	16.3348899	15.4884411	15.5242825	9.779E-31	-1.697527	down	PREDICTED: transmembrane protein 45B-like [Sesamum indicum]
c48220.graph_c0	1.77918022	3.49831782	1.55142941	0.11707835	0	0.10417561	7.966E-08	-5.941561	down	hypothetical protein VITISV_010987 [Vitis vinifera]
c48221.graph_c0	36.4775065	39.4000221	37.0087301	8.69007125	14.3749371	15.4133684	1.384E-65	-2.579275	down	hypothetical protein MIMGU_mgv1a025040mg, partial [Erythranthe guttata]
c48222.graph_c0	15.1128631	14.33699	14.8510681	12.5352097	9.68851716	7.87442902	8.792E-23	-1.568193	down	hypothetical protein MIMGU_mgv1a001261mg [Erythranthe guttata]
c48223.graph_c0	6.10230826	4.32679665	5.88128078	2.8109222	2.48152602	1.66742778	1.32E-06	-2.241426	down	-
c48223.graph_c1	0.0411732	0.20607214	0.15872757	4.02073224	4.82205087	5.973956	2.801E-14	4.162562	up	PREDICTED: uncharacterized protein LOC100255010 isoform X5 [Vitis vinifera]
c48224.graph_c0	573.009602	567.068485	517.664962	468.578701	425.84796	349.954424	7.584E-15	-1.426737	down	-

#ID	0 h-1 FPKM	0 h-2 FPKM	0 h-3 FPKM	48 h-1 FPKM	48 h-2 FPKM	48 h-3 FPKM	FDR	log2FC	regulated	nr_annotation
c48226.graph_c1	7.03031564	8.2179644	5.97329283	7.13724698	6.68080636	6.76773899	9.448E-09	-1.057817	down	PREDICTED: pentatricopeptide repeat-containing protein At1g06270 isoform X2 [Sesamum indicum]
c48231.graph_c0	68.1693603	69.4663903	74.6127635	31.0617637	27.155579	25.0222981	6.958E-60	-2.365425	down	PREDICTED: putative lysine-specific demethylase JMJD5 [Sesamum indicum]
c48235.graph_c0	1.13225851	0.89574553	0.96217545	6.54467651	6.75505747	6.8862991	4.014E-11	1.737826	up	PREDICTED: pentatricopeptide repeat-containing protein At1g19720 isoform X1 [Sesamum indicum]
c48237.graph_c0	2.62342209	2.1224232	2.90939286	9.56814684	12.1705644	9.42691158	0.0001552	1.003223	up	PREDICTED: BTB/POZ domain-containing protein At1g55760 [Sesamum indicum]
c48238.graph_c0	0	0	0	6.40608403	10.5420882	1.21749603	0.000316	Inf	up	-
c48239.graph_c2	0.38343273	0.47977086	0.49272624	2.29608109	2.80663788	2.4752205	0.0042576	1.460388	up	-
c48239.graph_c3	5507.69182	5503.88487	5444.07256	70.8422421	93.7460357	78.8482798	3.98E-217	-7.100241	down	PREDICTED: beta-hexosaminidase 2 [Sesamum indicum]
c48239.graph_c5	125.71397	116.522349	113.908123	405.996364	506.695621	547.887414	2.645E-11	1.014702	up	PREDICTED: beta-hexosaminidase 2 [Sesamum indicum]
c48239.graph_c6	79.6979073	80.0794895	73.5143895	42.6050631	42.1925972	39.6989495	1.343E-37	-1.921895	down	PREDICTED: putative transcription elongation factor SPT5 homolog 1 [Sesamum indicum]
c48244.graph_c0	21.9910376	21.0604066	21.9261139	20.1343258	21.197187	21.3981784	6.191E-12	-1.069402	down	hypothetical protein MIMGU_mgv1a007762mg [Erythranthe guttata]
c48244.graph_c1	0	0	0	1735.48337	1713.00518	2250.82956	1.04E-181	Inf	up	PREDICTED: putative mannan endo-1,4-beta-mannosidase 9 [Sesamum indicum]
c48244.graph_c2	2586.72989	2714.42794	2573.21681	553.813235	547.352286	534.486715	5.803E-46	-3.284055	down	PREDICTED: heat shock cognate 70 kDa protein 2 [Sesamum indicum]
c48245.graph_c0	6.666108	6.59801802	7.10251809	1.2901793	1.93172779	2.05108223	2.12E-46	-2.9753	down	PREDICTED: putative pentatricopeptide repeat-containing protein At3g16890, mitochondrial [Sesamum indicum]
c48249.graph_c0	2.50763188	2.02994164	1.90542282	1.7376677	1.69187785	1.92153611	3.554E-08	-1.283613	down	hypothetical protein MIMGU_mgv1a022404mg [Erythranthe guttata]
c48250.graph_c2	46.6439445	42.8995301	42.008751	14.1860857	16.4875093	18.5459643	4.686E-55	-2.438622	down	hypothetical protein MIMGU_mgv1a010855mg [Erythranthe guttata]
c48250.graph_c3	0.2846882	0.33246882	0.42680821	6.99395572	8.84478983	11.2795133	1.838E-16	3.671703	up	PREDICTED: probable pectin methyltransferase QUA2 [Sesamum indicum]
c48251.graph_c0	1.45375946	1.9029737	1.50888107	0.30906878	0.43656119	0.50418049	1.104E-08	-2.984838	down	PREDICTED: uncharacterized protein LOC104593668 [Nelumbo nucifera]
c48251.graph_c1	9.88542567	9.74070998	8.73342481	2.84596841	5.92941826	7.89241471	4.185E-12	-1.796486	down	hypothetical protein Csa_6G486800 [Cucumis sativus]
c48253.graph_c0	2.99646679	3.15133906	3.16819746	1.08325013	2.7393658	3.35799679	0.0001184	-1.408944	down	PREDICTED: serine/threonine-protein kinase-like protein At3g51990 [Sesamum indicum]
c48253.graph_c1	5.06731958	6.01172591	4.52201825	42.1015587	50.2568196	47.4425297	2.495E-44	2.145166	up	PREDICTED: uncharacterized protein LOC105168293 [Sesamum indicum]
c48253.graph_c2	28.369835	24.9372072	26.8865664	0.32670178	0	0	5.23E-116	-8.899607	down	-

#ID	0 h-1 FPKM	0 h-2 FPKM	0 h-3 FPKM	48 h-1 FPKM	48 h-2 FPKM	48 h-3 FPKM	FDR	log2FC	regulated	nr_annotation
c48256.graph_c0	87.7635569	89.0117409	89.2813527	49.0351494	48.4836753	44.6589378	3.451E-40	-1.920732	down	PREDICTED: uncharacterized protein LOC105170589 [Sesamum indicum]
c48257.graph_c0	2.59611333	2.41130576	2.38980843	9.84280214	10.8170593	11.0294338	3.447E-10	1.080202	up	PREDICTED: protein SHOOT GRAVITROPISM 6 [Sesamum indicum]
c48258.graph_c1	180.188273	192.747522	186.461088	38.5311924	31.6533252	28.7619543	2.86E-113	-3.511953	down	PREDICTED: pentatricopeptide repeat-containing protein At1g79540 [Sesamum indicum]
c48259.graph_c1	82.9022101	79.572592	86.6579304	514.170998	675.196542	671.434063	7.172E-28	1.877875	up	PREDICTED: dehydrodolichyl diphosphate synthase 6 [Sesamum indicum]
c48263.graph_c0	1.49969698	1.02713595	1.82573999	5.23560761	6.00869903	8.54078897	0.0075419	1.158079	up	PREDICTED: hsp70-Hsp90 organizing protein 3-like [Sesamum indicum]
c48264.graph_c0	50.9217011	50.0028489	48.4607675	15.1396744	17.1297138	16.754985	1.004E-73	-2.626599	down	PREDICTED: phytosulfokine receptor 1 [Sesamum indicum]
c48265.graph_c0	0.17151973	0.26534138	0.28052137	3.9647441	4.35234554	3.96239019	5.399E-18	3.075395	up	PREDICTED: osmotic avoidance abnormal protein 3-like [Sesamum indicum]
c48267.graph_c0	0.36827736	0.12288205	0.15775033	1.2440308	2.27637236	1.71071174	0.0005538	1.990866	up	hypothetical protein MIMGU_mgv1a013848mg [Erythranthe guttata]
c48268.graph_c0	1.99837233	0.75014002	0.49384376	0	0	0	9.745E-06	-Inf	down	PREDICTED: BAG family molecular chaperone regulator 6-like [Phoenix dactylifera]
c48270.graph_c0	6.5982191	6.36216899	5.72403673	6.0577728	5.41197263	5.24734273	1.183E-15	-1.174062	down	PREDICTED: calcium-transporting ATPase 1, chloroplastic [Sesamum indicum]
c48272.graph_c0	1.12104021	1.2871867	1.31347096	4.92087084	6.11408971	5.40563121	0.0014262	1.121415	up	PREDICTED: uncharacterized protein LOC105162718 [Sesamum indicum]
c48274.graph_c0	1.73625584	1.74833965	1.59368393	9.11173446	11.0648874	12.0808672	3.403E-22	1.646493	up	PREDICTED: aminomethyltransferase, mitochondrial [Eucalyptus grandis]
c48275.graph_c0	64.1686189	58.9482889	62.3491818	745.988864	842.422438	864.90828	3.896E-73	2.705584	up	PREDICTED: actin-1-like [Tarenaya hassleriana]
c48277.graph_c0	2.61766503	2.47196814	2.22137947	14.4238814	15.8587894	16.0732187	5.197E-09	1.646831	up	PREDICTED: histone deacetylase 5 [Sesamum indicum]
c48278.graph_c0	23.6692639	24.3387699	23.7835043	7.61526796	9.68094361	9.12283619	1.185E-47	-2.463658	down	G patch domain-containing protein 8 [Glycine soja]
c48280.graph_c0	0.89800829	0.91653315	1.24448415	12.052681	15.432008	18.027967	1.545E-21	2.868409	up	PREDICTED: LRR receptor-like serine/threonine-protein kinase ERL2 isoform X2 [Sesamum indicum]
c48283.graph_c1	15.1411206	15.9848716	15.2907611	8.83329984	9.96371146	11.5635438	8.287E-21	-1.633153	down	PREDICTED: probable mitochondrial chaperone BCS1-B [Sesamum indicum]
c48284.graph_c0	0	0	0	0.92045892	0.79782105	0.78179068	3.251E-09	Inf	up	Annexin-like protein RJ4 isoform 2 [Theobroma cacao]
c48286.graph_c0	1.3564722	1.07312411	0.7872157	8.14301533	11.2529336	13.7738254	1.643E-09	2.346292	up	PREDICTED: uncharacterized protein LOC105179489 [Sesamum indicum]
c48287.graph_c0	8.18102663	9.46046924	9.75388585	38.0926118	42.3722284	45.5639956	5.414E-15	1.180428	up	PREDICTED: uncharacterized protein LOC105157447 [Sesamum indicum]
c48288.graph_c0	5.892378	6.06183544	5.24917783	31.7521576	38.7106034	38.3150894	3.888E-31	1.640904	up	GIGANTEA family protein [Populus trichocarpa]

#ID	0 h-1 FPKM	0 h-2 FPKM	0 h-3 FPKM	48 h-1 FPKM	48 h-2 FPKM	48 h-3 FPKM	FDR	log2FC	regulated	nr_annotation
c48291.graph_c1	7.70223242	6.36908503	8.00420561	40.1066894	44.5145333	48.2593868	5.258E-16	1.568456	up	PREDICTED: pyridoxal reductase, chloroplastic [Sesamum indicum]
c48293.graph_c0	25.2260864	22.4793291	24.0760773	325.219307	367.735602	354.393838	9.392E-78	2.847584	up	PREDICTED: apyrase 2-like [Sesamum indicum]
c48294.graph_c0	0	0	0	1.65185814	1.37495553	0.97987562	2.241E-10	Inf	up	--
c48295.graph_c0	0.27826821	0.37853803	0.33007908	2.2217765	2.36763004	3.08236277	8.59E-13	1.937373	up	PREDICTED: phragmoplast orienting kinesin 2 [Sesamum indicum]
c48296.graph_c0	0	0	0	2.01873812	1.68033505	2.82269592	2.289E-11	Inf	up	hypothetical protein CHLNCDRAFT_48764 [Chlorella variabilis]
c48297.graph_c0	0	0	0	1.11836737	0.90832721	0.7629227	2.117E-11	Inf	up	hypothetical protein DI09_7p140 [Microsporidia sp. UGP3]
c48300.graph_c0	8.19241125	8.48339453	7.6616274	2.65733651	2.64099432	3.25420983	1.002E-47	-2.52687	down	hypothetical protein MIMGU_mgv1a0002422mg, partial [Erythranthe guttata]
c48302.graph_c0	16.7475409	16.4509594	18.1019886	404.252308	448.649459	439.37828	1.55E-128	3.634622	up	hypothetical protein MIMGU_mgv1a010747mg [Erythranthe guttata]
c48303.graph_c0	2.40777323	2.29894498	2.23726069	9.72594398	12.36415	13.087408	8.968E-07	1.319175	up	PREDICTED: altered inheritance rate of mitochondria protein 25 [Sesamum indicum]
c48303.graph_c3	38.8734828	46.5924893	44.0383634	14.6078477	9.23522585	13.9413764	7.222E-40	-2.789796	down	hexose transport protein [Actinidia deliciosa]
c48305.graph_c0	0	0	0	3.87450972	11.1097116	2.06850927	0.0006534	Inf	up	predicted protein [Physcomitrella patens]
c48309.graph_c0	5.07675099	3.23389474	4.27014066	32.2290171	34.0526492	38.8163488	2.2E-14	2.043358	up	PREDICTED: hydroquinone glucosyltransferase [Sesamum indicum]
c48309.graph_c1	2.60754843	2.28388956	2.42002437	12.1113119	16.0822651	13.9828255	1.574E-09	1.506788	up	unnamed protein product [Coffea canephora]
c48309.graph_c3	2.9383954	2.36081057	2.90649124	1.99466216	2.92438319	2.99503979	9.305E-05	-1.077373	down	PREDICTED: aluminum-activated malate transporter 4-like [Sesamum indicum]
c48309.graph_c4	1.80555066	1.80735864	1.842515	16.5360638	16.0149347	14.496032	4.917E-12	2.092104	up	PREDICTED: hydroquinone glucosyltransferase [Sesamum indicum]
c48309.graph_c5	97.5959066	95.2622035	98.7630002	94.3872182	94.8858478	92.8431905	1.155E-13	-1.065378	down	PREDICTED: transmembrane protein 53-A-like [Sesamum indicum]
c48311.graph_c0	6.94288388	7.82352981	7.03554018	55.0441923	66.3277322	66.9626056	1.215E-39	2.090115	up	cytochrome P450 CYP72A219-like [Sesamum indicum]
c48316.graph_c1	0.08012273	0.18714024	0.13728118	0.63972381	0.91230166	0.83194043	0.0050513	1.534146	up	PREDICTED: probable inactive receptor kinase At5g10020 [Nicotiana sylvestris]
c48322.graph_c0	0.2050895	0.20529486	0	0.99869248	1.80144854	2.35367014	0.000573	2.63806	up	PREDICTED: uncharacterized acetyltransferase At3g50280-like [Sesamum indicum]
c48322.graph_c3	0.04049302	0	0.05203513	1.52949721	7.31118571	7.66772059	3.657E-05	6.431477	up	PREDICTED: omega-hydroxypalmitate O-feruloyl transferase isoform X1 [Sesamum indicum]
c48327.graph_c0	0.20547194	0.08227108	0.07921187	1.17362375	1.14304496	1.73485784	6.352E-06	2.451806	up	hypothetical protein MIMGU_mgv1a001429mg [Erythranthe guttata]
c48331.graph_c0	62.6073514	62.6990035	62.9979121	11.9540536	13.1862373	14.0280391	1.94E-105	-3.285116	down	PREDICTED: uncharacterized protein LOC105164372 [Sesamum indicum]

#ID	0 h-1 FPKM	0 h-2 FPKM	0 h-3 FPKM	48 h-1 FPKM	48 h-2 FPKM	48 h-3 FPKM	FDR	log2FC	regulated	nr_annotation
c48332.graph_c0	5.41716349	5.2289241	5.86735577	48.0527755	61.4989361	65.6105347	3.62E-31	2.383515	up	PREDICTED: probable copper-transporting ATPase HMA5 [Sesamum indicum]
c48334.graph_c1	4.16459769	4.73402457	6.07732348	24.4833421	25.3860112	26.7902137	1.153E-13	1.333551	up	PREDICTED: 3-hydroxyisobutyryl-CoA hydrolase-like protein 1, mitochondrial isoform X2 [Sesamum indicum]
c48336.graph_c0	11.0866325	12.4591662	10.1157304	10.5934215	10.6190883	10.3381527	1.417E-08	-1.109263	down	PREDICTED: pentatricopeptide repeat-containing protein At2g21090-like [Sesamum indicum]
c48336.graph_c2	3.95123763	3.41063847	3.56896513	1.92558015	2.01197459	2.37055252	3.037E-09	-1.81209	down	PREDICTED: ABC transporter G family member 5 [Sesamum indicum]
c48336.graph_c3	2.06430917	1.52259304	1.25655111	1.03429937	1.37847986	0.65312561	8.646E-05	-1.673991	down	PREDICTED: pentatricopeptide repeat-containing protein At2g13600 [Sesamum indicum]
c48336.graph_c4	5.65315089	6.75679005	6.18026559	28.2167557	31.5117368	32.7153115	1.728E-15	1.294002	up	hypothetical protein MIMGU_mgv1a004418mg [Erythranthe guttata]
c48340.graph_c0	81.3504491	87.2309412	81.811064	45.3230107	46.7577869	43.8207354	1.136E-40	-1.898941	down	PREDICTED: serine/threonine-protein kinase PBS1 [Sesamum indicum]
c48340.graph_c3	140.626259	152.314374	135.124054	132.404059	131.857419	119.850529	1.11E-15	-1.172165	down	hypothetical protein MIMGU_mgv1a000810mg [Erythranthe guttata]
c48344.graph_c0	0.96360532	0.88418937	1.13508174	8.43349267	10.2665652	11.9143584	9.344E-08	2.335081	up	PREDICTED: uncharacterized protein LOC105167681 [Sesamum indicum]
c48346.graph_c0	17.9901654	17.0281429	17.4115724	15.3818238	17.421336	15.721738	6.461E-15	-1.130562	down	PREDICTED: sec1 family domain-containing protein MIP3 [Sesamum indicum]
c48347.graph_c1	1.67334669	1.82067641	1.68285659	7.57392876	11.3609333	9.95975625	0.0002757	1.456989	up	PREDICTED: sulfhydryl oxidase 2 isoform X3 [Sesamum indicum]
c48347.graph_c4	3.68510194	3.50435241	3.39377635	13.4666598	14.6858901	16.3627299	0.0003681	1.053337	up	PREDICTED: sulfhydryl oxidase 2 isoform X1 [Sesamum indicum]
c48348.graph_c0	523.793944	520.219895	501.906429	584.593556	349.963704	243.526987	1.509E-08	-1.395367	down	PREDICTED: uncharacterized protein LOC105164444 [Sesamum indicum]
c48351.graph_c0	0.40058979	0.86881367	0.3431825	3.19842854	5.47348396	5.52769598	4.785E-05	2.1161	up	-
c48354.graph_c0	1.17986169	0.81196716	1.08974645	5.80847212	8.6003268	9.24861479	2.449E-08	1.91518	up	PREDICTED: uncharacterized protein LOC105158848 isoform X1 [Sesamum indicum]
c48360.graph_c0	0.14221097	0.14235338	0	3.27535061	3.60863029	3.06982126	2.31E-10	4.126809	up	hypothetical protein MIMGU_mgv1a011575mg [Erythranthe guttata]
c48361.graph_c0	21.6718372	20.3474745	21.9417693	21.3195922	20.3574915	20.0210879	3.511E-12	-1.068726	down	PREDICTED: protein TRIGALACTOSYLDIACYLGLYCEROL 3, chloroplastic isoform X1 [Sesamum indicum]
c48364.graph_c0	0	0	0	19.5447905	35.3521969	13.5324199	5.382E-11	Inf	up	predicted protein [Physcomitrella patens]
c48368.graph_c0	50.6210105	48.2156127	48.397202	36.7596499	38.9888901	38.6058119	9.827E-23	-1.382764	down	PREDICTED: protein strawberry notch isoform X1 [Sesamum indicum]

#ID	0 h-1 FPKM	0 h-2 FPKM	0 h-3 FPKM	48 h-1 FPKM	48 h-2 FPKM	48 h-3 FPKM	FDR	log2FC	regulated	nr_annotation
c48371.graph_c0	76.4087186	75.4317148	70.8348274	69.2291797	70.771997	71.4780923	1.089E-14	-1.091571	down	PREDICTED: phosphatidylinositol N-acetylglucosaminyltransferase subunit A-like [Sesamum indicum]
c48372.graph_c0	10.5213438	9.1822564	10.1789313	49.0857995	47.3244065	44.6433239	4.371E-17	1.222478	up	PREDICTED: acyl-CoA-binding domain-containing protein 4-like [Sesamum indicum]
c48372.graph_c1	80.5995502	82.9509676	79.8214989	585.282165	563.976733	805.838171	8.652E-12	1.986561	up	PREDICTED: uncharacterized protein LOC105170680 isoform X1 [Sesamum indicum]
c48373.graph_c0	91.0589542	96.7124474	92.5211978	47.2841442	42.3360412	34.3834102	1.927E-52	-2.190091	down	PREDICTED: uncharacterized protein LOC105162592 [Sesamum indicum]
c48374.graph_c0	0	0	0	27.1350735	31.2368236	23.6837146	2.061E-77	Inf	up	PREDICTED: GDSL esterase/lipase At1g29670-like [Sesamum indicum]
c48377.graph_c0	0	0	0	2.61713301	2.60551489	2.13231205	1.177E-15	Inf	up	-
c48384.graph_c0	0.90514359	0.78319572	0.94628774	4.70646009	5.39018786	3.73504692	6.329E-07	1.373751	up	PREDICTED: LOW QUALITY PROTEIN: probable serine/threonine-protein kinase GCN2 [Sesamum indicum]
c48386.graph_c0	1102.79499	1085.33769	1175.1755	472.273636	473.192363	475.826646	1.97E-35	-2.260938	down	PREDICTED: uncharacterized protein LOC105166153 [Sesamum indicum]
c48390.graph_c0	0	0.10518253	0.0450095	2.83958612	3.17911747	4.30678406	7.702E-15	5.079709	up	PREDICTED: 7-hydroxymethyl chlorophyll a reductase, chloroplastic isoform X1 [Sesamum indicum]
c48392.graph_c0	0	0	0	2.1013812	3.23488668	2.24959874	1.525E-15	Inf	up	hypothetical protein SELMODRAFT_168478 [Selaginella moellendorffii]
c48395.graph_c0	0.95892001	0.93854955	1.01318348	6.06614834	6.11438994	5.67711085	4.698E-08	1.599434	up	PREDICTED: protein FAM135B-like [Sesamum indicum]
c48398.graph_c0	4.69575535	4.625847	6.13001299	16.5488048	25.5333596	29.1445555	0.0004348	1.175605	up	hypothetical protein MIMGU_mgv1a027078mg [Erythranthe guttata]
c48399.graph_c0	37.3037488	37.7549821	35.5393473	216.611449	202.706133	222.640936	1.582E-27	1.520849	up	PREDICTED: uncharacterized protein LOC105163762 isoform X1 [Sesamum indicum]
c48400.graph_c0	3.17388977	1.96335659	1.87889365	0	0	0	2.893E-26	-Inf	down	lectin [Bryopsis plumosa]
c48404.graph_c0	12.1733976	5.50316851	5.92931452	0	0	0	5.907E-14	-Inf	down	PREDICTED: BTB/POZ and TAZ domain-containing protein 1-like [Phoenix dactylifera]
c48409.graph_c2	2.03328549	1.50666658	1.59485793	0.24327211	0.18040211	0.43292411	4.695E-15	-3.602021	down	PREDICTED: subtilisin-like protease SDD1 [Sesamum indicum]
c48410.graph_c0	28.4445447	30.4858485	29.365708	17.367064	19.8497198	18.8184529	1.528E-31	-1.675654	down	PREDICTED: DNA polymerase eta [Sesamum indicum]
c48412.graph_c0	5.0552222	3.99496124	5.47045109	0.73534743	0.43278413	0.79970944	5.328E-15	-3.898018	down	PREDICTED: auxin-induced protein 15A-like [Sesamum indicum]
c48415.graph_c3	1.20581159	1.11761021	1.31995776	18.5556815	16.8243745	18.8903296	4.066E-24	2.879064	up	hypothetical protein MTR_4g125190 [Medicago truncatula]
c48417.graph_c0	140.228979	140.396089	133.290965	50.5550624	53.9212915	55.7595429	8.776E-58	-2.387491	down	PREDICTED: E3 ubiquitin-protein ligase UPL1-like [Sesamum indicum]
c48419.graph_c0	135.851885	127.64814	133.021202	110.330407	109.047496	102.537275	5.087E-20	-1.317435	down	PREDICTED: asparagine--tRNA ligase, chloroplastic/mitochondrial [Sesamum indicum]
c48420.graph_c1	0.98505564	1.23567292	0.6569526	7.11064505	8.05615095	7.16515905	5.249E-19	1.941942	up	PREDICTED: protein MON2 homolog isoform X2 [Sesamum indicum]

#ID	0 h-1 FPKM	0 h-2 FPKM	0 h-3 FPKM	48 h-1 FPKM	48 h-2 FPKM	48 h-3 FPKM	FDR	log2FC	regulated	nr_annotation
c48421.graph_c2	26.7137185	26.9526942	26.5362326	13.9459046	15.8499721	12.9651269	9.9E-33	-1.925682	down	PREDICTED: peptide chain release factor PrfB1, chloroplastic [Sesamum indicum]
c48422.graph_c0	3.24039605	2.90114458	3.02603947	16.1315519	17.6394276	16.6800565	5.356E-16	1.441895	up	PREDICTED: myosin-1 [Sesamum indicum]
c48428.graph_c0	0	0	0.09659047	25.2059943	44.3088668	29.0210301	9.429E-24	8.937015	up	hypothetical protein MGL_1430 [Malassezia globosa CBS 7966]
c48429.graph_c0	28.010976	29.3651948	25.7533319	18.1723684	19.415612	19.7035459	9.392E-28	-1.554753	down	PREDICTED: uncharacterized protein LOC105159568 [Sesamum indicum]
c48430.graph_c0	43.6533389	61.5389069	39.2730717	585.357052	346.014227	263.722847	0.0051348	2.046555	up	PREDICTED: alanine aminotransferase 2 [Sesamum indicum]
c48431.graph_c0	0.43342642	0.82433483	0.61266713	3.31419574	4.27240772	3.73060122	0.0006757	1.575136	up	PREDICTED: urease accessory protein D isoform X1 [Sesamum indicum]
c48432.graph_c0	6.10234456	5.49143946	6.23775915	3.25103541	4.03062381	3.94121637	4.624E-20	-1.689914	down	PREDICTED: uncharacterized protein LOC105165127 isoform X1 [Sesamum indicum]
c48432.graph_c1	0.4284572	0.38989658	0.55058446	2.69129125	2.31889202	2.04347287	0.0058675	1.348627	up	PREDICTED: uncharacterized protein LOC105156326 [Sesamum indicum]
c48435.graph_c0	0	0	0	28.8701767	55.5296874	28.4189586	1.537E-16	Inf	up	60s ribosomal protein l10 [Lichtheimia corymbifera JMRC:FSU:9682]
c48436.graph_c0	0	0	0	1.10175695	2.33435579	1.19345339	3.133E-06	Inf	up	-
c48437.graph_c0	52.1613896	54.9147417	49.9702234	24.6154328	22.3383345	21.2311667	8.619E-55	-2.218097	down	PREDICTED: uncharacterized protein LOC105158158 [Sesamum indicum]
c48443.graph_c2	0.31996502	0	0.41116772	1.22120098	1.3383252	2.06082589	0.0060113	1.628099	up	hypothetical protein CICLE_v10031670mg [Citrus clementina]
c48450.graph_c1	28.2379813	29.6384057	26.6675958	15.1651581	14.227578	12.0086618	1.856E-47	-2.04407	down	PREDICTED: uncharacterized protein LOC105178025 [Sesamum indicum]
c48453.graph_c0	36.1283643	36.7912194	34.191268	27.4681516	30.9067363	31.5616012	2.901E-19	-1.271272	down	PREDICTED: uncharacterized protein LOC105172605 [Sesamum indicum]
c48454.graph_c0	22.4617397	21.8653997	21.2823135	3.26735048	3.32375399	3.28957021	4.41E-135	-3.748404	down	PREDICTED: DNA repair protein UVH3 [Sesamum indicum]
c48455.graph_c0	15.3308962	14.7329961	15.2076417	14.4266715	15.8520582	14.9925582	2.076E-12	-1.018878	down	PREDICTED: probable inactive leucine-rich repeat receptor-like protein kinase At3g03770 [Sesamum indicum]
c48458.graph_c0	0	0	0	1.44094111	1.34226934	0.97265849	8.741E-13	Inf	up	Inositol-3-phosphate synthase [Ectocarpus siliculosus]
c48459.graph_c1	17.1861742	16.1318925	13.9081467	12.0528215	13.2328121	12.9181816	6.694E-11	-1.322874	down	PREDICTED: carboxyl-terminal-processing peptidase 1, chloroplastic [Sesamum indicum]
c48463.graph_c0	2.34722391	1.9509858	2.20834468	9.13234516	11.2290955	10.685652	6.524E-09	1.233541	up	PREDICTED: uncharacterized protein LOC105169809 [Sesamum indicum]
c48464.graph_c0	0.8999303	0.40946884	0.10513146	5.27592859	6.30781888	5.70045158	2.737E-06	2.605447	up	PREDICTED: auxin response factor 8-like isoform X2 [Nicotiana tomentosiformis]
c48464.graph_c2	289.038472	267.831424	288.709742	97.0513576	98.4683361	100.910048	3.467E-64	-2.530414	down	PREDICTED: probable LRR receptor-like serine/threonine-protein kinase At5g10290 [Sesamum indicum]
c48465.graph_c0	1.93038801	2.35239079	1.79755436	38.8412727	41.1476187	46.0962635	2.187E-79	3.356236	up	PREDICTED: elongation factor G-1, chloroplastic [Sesamum indicum]

#ID	0 h-1 FPKM	0 h-2 FPKM	0 h-3 FPKM	48 h-1 FPKM	48 h-2 FPKM	48 h-3 FPKM	FDR	log2FC	regulated	nr_annotation
c48468.graph_c0	1.48522514	1.37658552	1.48444575	0.05067727	0.10737284	0.22546165	1.369E-13	-4.535991	down	PREDICTED: scarecrow-like protein 21 [Sesamum indicum]
c48469.graph_c2	168.329457	173.348289	155.66403	91.3684494	65.9597354	53.8167419	1.478E-51	-2.244145	down	PREDICTED: rhomboid protein 2-like [Sesamum indicum]
c48469.graph_c3	4.62646793	5.03658871	3.80821212	35.9439325	31.2220744	30.9340821	1.532E-22	1.852171	up	PREDICTED: probable ubiquitin-conjugating enzyme E2 24 [Sesamum indicum]
c48472.graph_c0	0	0	0	1.548987	1.8895948	1.16944912	2.297E-11	Inf	up	PREDICTED: hydroxyacylglutathione hydrolase cytoplasmic [Sesamum indicum]
c48474.graph_c2	3.94079196	4.24639451	3.00865555	49.5458462	53.6868914	53.3207683	1.143E-70	2.789773	up	PREDICTED: LOW QUALITY PROTEIN: probable acyl-activating enzyme 16, chloroplastic [Sesamum indicum]
c48475.graph_c2	0.29560054	0.19726436	0.12661947	29.0481601	27.695703	31.5816542	9.189E-38	6.145187	up	PREDICTED: MADS-box protein SVP-like isoform X1 [Sesamum indicum]
c48479.graph_c0	231.330674	244.350768	246.453848	223.079175	232.510525	216.310516	3.689E-11	-1.122168	down	PREDICTED: notchless protein homolog [Sesamum indicum]
c48479.graph_c1	2.41993983	2.11956765	1.63260221	9.25203661	13.5212235	12.299017	2.39E-05	1.486415	up	PREDICTED: dystrophia myotonica WD repeat-containing protein-like [Sesamum indicum]
c48480.graph_c0	0	0	0	38.8702467	43.2982575	30.9174279	3.528E-55	Inf	up	hypothetical protein SELMODRAFT_270900 [Selaginella moellendorffii]
c48482.graph_c0	0	0	0	1.56157677	1.34794915	1.28655705	4.124E-11	Inf	up	hypothetical protein PHACADRAFT_263295 [Phanerochaete carnosa HHB-10118-sp]
c48484.graph_c0	26.3009618	24.5694197	24.4561556	15.4072223	19.1321129	20.6726842	2.169E-24	-1.469638	down	PREDICTED: uncharacterized protein LOC105160250 [Sesamum indicum]
c48489.graph_c0	13.960049	13.6982247	12.451232	6.11316173	6.27447407	5.79706575	1.543E-38	-2.15738	down	PREDICTED: BTB/POZ domain-containing protein At2g04740 isoform X1 [Sesamum indicum]
c48492.graph_c0	26.7311619	26.7579291	29.2324602	5.1510996	4.11141231	4.14391167	9.384E-62	-3.639289	down	hypothetical protein MIMGU_mgv1a013606mg [Erythranthe guttata]
c48492.graph_c2	10.8446446	11.3489358	10.4518482	0.72660319	0.57731098	0.76775099	4.947E-58	-4.992804	down	hypothetical protein VITISV_043907 [Vitis vinifera]
c48492.graph_c3	1.03781954	1.45440226	1.1669343	0.56171289	0.45579577	0.53171144	4.539E-10	-2.254378	down	OSJNBb0012A12.6 [Oryza sativa Japonica Group]
c48494.graph_c0	40.4070459	40.307389	39.7329529	26.5078412	27.7327179	27.6408489	1.166E-28	-1.57488	down	PREDICTED: chromatin modification-related protein eaf-1 isoform X2 [Sesamum indicum]
c48495.graph_c0	0.92749032	1.04744714	1.1001801	9.11428881	10.212531	9.7863927	3.275E-17	2.222304	up	PREDICTED: uncharacterized protein LOC105160781 [Sesamum indicum]
c48498.graph_c0	3530.09566	3650.77638	3524.72972	321.74047	315.588884	298.972741	9.665E-98	-4.531719	down	hypothetical protein MIMGU_mgv1a004937mg [Erythranthe guttata]
c48500.graph_c0	1.97303984	1.71284533	2.10912234	1.12600451	1.2780699	1.14504233	1.154E-07	-1.727679	down	PREDICTED: phosphatidylinositol/phosphatidylcholine transfer protein SFH11 [Sesamum indicum]
c48503.graph_c0	31.6259776	30.0653977	29.979481	145.248562	146.750324	169.892863	3.716E-21	1.31464	up	PREDICTED: potassium transporter 4-like [Sesamum indicum]
c48504.graph_c0	42.1068286	41.3585222	41.1343932	38.6876278	38.7828438	37.1982847	7.556E-16	-1.136783	down	PREDICTED: uncharacterized protein C594.04c [Sesamum indicum]

#ID	0 h-1 FPKM	0 h-2 FPKM	0 h-3 FPKM	48 h-1 FPKM	48 h-2 FPKM	48 h-3 FPKM	FDR	log2FC	regulated	nr_annotation
c48506.graph_c0	41.3151423	40.6495642	35.4952345	29.7485221	25.4264011	25.2800293	7.28E-27	-1.558603	down	hypothetical protein MIMGU_mgv1a010122mg [Erythranthe guttata]
c48506.graph_c1	0.88428517	0.66387798	0.67470273	4.7097599	4.98941953	5.54986718	1.4E-06	1.760827	up	PREDICTED: uncharacterized protein LOC105174972 isoform X1 [Sesamum indicum]
c48506.graph_c2	0.55393766	0.63370553	0.33049335	5.30371924	6.98952017	6.38959344	1.854E-16	2.604864	up	PREDICTED: probable L-type lectin-domain containing receptor kinase S.7 [Sesamum indicum]
c48506.graph_c5	2.19585028	2.97277131	2.24352616	20.5280746	20.0430805	20.818242	2.138E-32	2.033788	up	PREDICTED: CO(2)-response secreted protease-like [Sesamum indicum]
c48508.graph_c0	387.044072	387.885637	411.473351	360.905286	373.703793	376.527673	4.536E-13	-1.113368	down	PREDICTED: F-box protein CPR30-like [Solanum tuberosum]
c48512.graph_c2	29.3807632	28.4393368	29.8454125	11.5441829	14.1732822	15.6676306	1.41E-48	-2.105157	down	hypothetical protein MIMGU_mgv1a005052mg [Erythranthe guttata]
c48519.graph_c2	33.0446698	32.2610242	34.3379375	15.127493	14.0349688	13.9898485	2.384E-48	-2.223773	down	hypothetical protein MIMGU_mgv1a005028mg [Erythranthe guttata]
c48526.graph_c0	3.15509773	3.20152088	3.6101049	0.15927024	0.1687275	0.10628827	7.243E-27	-5.53718	down	hypothetical protein MIMGU_mgv1a026399mg [Erythranthe guttata]
c48527.graph_c0	0	0	0	4.52490288	7.3832243	4.81296624	2.983E-17	Inf	up	conserved unknown protein [Ectocarpus siliculosus]
c48529.graph_c0	8.18357843	6.66427142	7.02015265	3.52259933	3.32752757	3.2559033	1.965E-41	-2.128795	down	PREDICTED: LOW QUALITY PROTEIN: uncharacterized protein LOC105155975 [Sesamum indicum]
c48530.graph_c1	0	0	0	1.76431574	10.3578105	12.0357278	4.581E-05	Inf	up	PREDICTED: non-specific lipid-transfer protein-like protein At2g13820 [Sesamum indicum]
c48533.graph_c0	0	0	0	2.19900306	1.80012776	1.24514708	1.619E-09	Inf	up	predicted protein [Hordeum vulgare subsp. vulgare]
c48536.graph_c0	0	0	0.29304319	3.06727462	5.29697614	5.83235386	1.457E-11	4.538389	up	PREDICTED: glycerophosphodiester phosphodiesterase GDPD6 [Solanum lycopersicum]
c48537.graph_c0	0.87745541	0.61483384	1.80410391	809.177529	1101.29067	1026.70315	7.69E-163	8.76659	up	hypothetical protein CARUB_v10002736mg [Capsella rubella]
c48542.graph_c0	0	0	0	2.94636059	3.48852493	4.16380331	5.354E-12	Inf	up	PREDICTED: probable protein phosphatase 2C 60 isoform X3 [Nicotiana sylvestris]
c48543.graph_c0	0	0	0	1.32677319	3.44361053	2.1840264	3.445E-06	Inf	up	-
c48546.graph_c0	11.0039397	9.90831546	8.45786808	2.60544432	3.3121828	3.3299343	1.445E-42	-2.686708	down	PREDICTED: cytosolic Fe-S cluster assembly factor narfl [Sesamum indicum]
c48549.graph_c0	1.36240801	1.55859686	1.50064134	13.0175857	12.1189714	9.57270207	4.716E-11	1.957954	up	dimethylaniline monooxygenase, putative [Ricinus communis]
c48550.graph_c1	44.8074016	47.9415839	39.0716577	16.4275565	20.5266203	16.9596315	4.036E-34	-2.308447	down	PREDICTED: putative E3 ubiquitin-protein ligase RF4 [Sesamum indicum]
c48553.graph_c0	2.35059646	2.43903377	2.24703878	1.18839664	0.95121584	0.98693298	7.704E-09	-2.182964	down	PREDICTED: uncharacterized protein LOC103423938 [Malus domestica]
c48554.graph_c1	57.8001558	60.7583452	54.0284198	11.9825478	10.733186	12.204955	6.46E-112	-3.320565	down	unnamed protein product [Coffea canephora]
c48555.graph_c0	42.8824173	43.8463307	41.8432478	36.7052756	36.2885654	33.758642	5.01E-20	-1.284589	down	PREDICTED: cytokinesis protein sepH-like [Sesamum indicum]

#ID	0 h-1 FPKM	0 h-2 FPKM	0 h-3 FPKM	48 h-1 FPKM	48 h-2 FPKM	48 h-3 FPKM	FDR	log2FC	regulated	nr_annotation
c48556.graph_c0	0	0	0	5.24409842	6.82406203	5.40099672	1.229E-22	Inf	up	glyceraldehyde 3-phosphate dehydrogenase [Daucus carota]
c48557.graph_c0	16.8826467	17.7657426	15.874018	16.1960304	16.3390541	15.4678984	4.546E-15	-1.090105	down	PREDICTED: uncharacterized protein LOC105167386 [Sesamum indicum]
c48559.graph_c0	0	0	0	0.97478482	0.95890444	0.92931179	1.664E-11	Inf	up	--
c48560.graph_c0	12.1310594	12.5340227	11.4318539	12.4348203	9.74380162	9.92140888	2.362E-12	-1.181207	down	PREDICTED: putative methyltransferase At1g22800 [Sesamum indicum]
c48560.graph_c1	20.9709972	19.6728273	20.2482362	15.3608647	18.677571	18.0361304	7.823E-12	-1.246373	down	PREDICTED: glucose-induced degradation protein 8 homolog isoform X1 [Sesamum indicum]
c48562.graph_c0	18.0271954	13.9705138	12.8105031	9.33712003	9.16184224	9.12530472	1.415E-11	-1.712074	down	TBC1 domain family member 15 [Morus notabilis]
c48565.graph_c0	0.05165816	0	0.06638278	11.8977168	15.2258594	22.3163775	1.051E-18	7.676397	up	PREDICTED: shikimate O-hydroxycinnamoyltransferase-like [Sesamum indicum]
c48566.graph_c0	0	0	0.09451842	22.3613779	41.9226495	20.0176076	2.506E-13	8.743428	up	predicted protein [Hordeum vulgare subsp. vulgare]
c48568.graph_c1	31.3670313	29.8177411	28.0172029	22.6318531	23.205119	24.3446834	2.805E-22	-1.363219	down	PREDICTED: DEAD-box ATP-dependent RNA helicase ISE2, chloroplastic [Sesamum indicum]
c48569.graph_c0	44.540369	47.4072044	39.8739664	31.5703991	36.0498094	32.5122453	6.74E-22	-1.414458	down	PREDICTED: LOW QUALITY PROTEIN: U5 small nuclear ribonucleoprotein 200 kDa helicase-like [Sesamum indicum]
c48571.graph_c0	4.71332335	3.59082585	4.60973743	1.52940293	2.24337732	2.06507144	7.659E-19	-2.169999	down	hypothetical protein VITISV_032357 [Vitis vinifera]
c48572.graph_c0	44.3257829	42.8917044	43.4242122	32.402484	33.2018227	35.1247132	1.135E-22	-1.393358	down	hypothetical protein MIMGU_mgv1a000350mg [Erythranthe guttata]
c48573.graph_c0	31.5285691	32.7981346	29.2785752	11.7949934	11.764344	11.3518583	2.023E-64	-2.439041	down	PREDICTED: transcription initiation factor TFIID subunit 1 isoform X2 [Sesamum indicum]
c48574.graph_c0	1.14820929	0.53876205	0.6455288	6.90879079	7.87933432	11.0888516	5.214E-09	2.453257	up	PREDICTED: glucan endo-1,3-beta-glucosidase 4-like isoform X1 [Sesamum indicum]
c48574.graph_c1	57.9622402	58.3229425	55.3674373	36.4625783	38.2440594	38.7892637	2.645E-30	-1.614771	down	PREDICTED: thylakoid lumenal 19 kDa protein, chloroplastic [Sesamum indicum]
c48575.graph_c1	34.2174392	37.123103	37.5857879	8.58869357	10.0985343	8.6499263	1.358E-71	-3.014518	down	hypothetical protein MIMGU_mgv1a015601mg [Erythranthe guttata]
c48578.graph_c0	0	0	0	0.86191011	0.94569958	0.87648278	2.033E-11	Inf	up	putative Trypsin [Glarea lozoyensis 74030]
c48579.graph_c0	0	0	0	1.56743825	2.03327849	1.22391789	9.415E-13	Inf	up	PREDICTED: thiol protease aleurain-like [Eucalyptus grandis]
c48580.graph_c1	13.6976803	14.0131142	11.9642374	10.4902856	11.0478139	10.8990431	5.925E-14	-1.307366	down	PREDICTED: uncharacterized protein LOC105168601 [Sesamum indicum]
c48580.graph_c2	0.69241881	1.07320593	0.6601635	14.9803935	13.9297703	15.8928226	1.179E-42	3.192267	up	PREDICTED: cinnamoyl-CoA reductase 2 [Sesamum indicum]
c48580.graph_c3	2.58146671	2.13614937	2.56536855	1.2683816	1.10854962	1.07216841	1.509E-07	-2.093423	down	-
c48580.graph_c4	40.6532393	39.579848	41.740125	9.95673011	9.17585741	8.69137335	1.262E-95	-3.147935	down	PREDICTED: uncharacterized protein LOC105176765 [Sesamum indicum]

#ID	0 h-1 FPKM	0 h-2 FPKM	0 h-3 FPKM	48 h-1 FPKM	48 h-2 FPKM	48 h-3 FPKM	FDR	log2FC	regulated	nr_annotation
c48582.graph_c0	55.5944087	53.9995446	52.3078534	25.0077763	24.4030734	25.4190651	1.634E-50	-2.13014	down	PREDICTED: uncharacterized protein LOC105174456 [Sesamum indicum]
c48587.graph_c1	3.66530488	3.17457423	3.03982718	14.2253223	14.055189	14.7885164	4.419E-08	1.108029	up	PREDICTED: rab3 GTPase-activating protein catalytic subunit isoform X4 [Sesamum indicum]
c48588.graph_c0	446.523713	412.155812	456.124903	202.145027	242.053349	222.808228	4.251E-35	-1.999847	down	vacuolar-processing enzyme precursor [Sesamum indicum]
c48589.graph_c2	1.13414962	1.07336065	1.24543776	5.96515852	5.75585214	5.86558432	1.947E-06	1.330448	up	PREDICTED: GPI ethanolamine phosphate transferase 2 isoform X1 [Sesamum indicum]
c48591.graph_c0	0	0	0	1.43342844	2.35529215	0.59868343	6.933E-05	Inf	up	-
c48596.graph_c1	18.9954413	19.1119724	17.7754101	11.644124	12.4781448	12.2972964	1.202E-26	-1.635582	down	hypothetical protein MIMGU_mgv1a011046mg [Erythranthe guttata]
c48601.graph_c0	0.40448621	0.17352482	0.185636	1.70349295	4.03225192	5.75514282	0.0023633	2.884546	up	PREDICTED: lactosylceramide alpha-2,3-sialyltransferase [Sesamum indicum]
c48601.graph_c2	289.713444	303.527481	314.682415	245.811628	174.127982	156.011039	7.716E-27	-1.666808	down	PREDICTED: B2 protein [Sesamum indicum]
c48601.graph_c3	0.89603625	1.02981253	0.76762804	33.6309535	19.9192446	11.1537221	0.0008268	3.586449	up	PREDICTED: cytochrome P450 CYP736A12-like [Sesamum indicum]
c48601.graph_c5	11.9823753	9.69818577	11.6078741	11.2489613	11.0825877	10.4413965	8.855E-12	-1.039609	down	PREDICTED: SUN domain-containing protein 2 [Sesamum indicum]
c48601.graph_c8	39.9759573	38.244221	29.4577054	28.198032	20.4345725	17.34036	2.473E-13	-1.713249	down	PREDICTED: B2 protein [Sesamum indicum]
c48602.graph_c0	26.7012916	24.2013024	22.655186	21.0017806	21.5944623	19.657185	4.818E-16	-1.256559	down	PREDICTED: mediator of RNA polymerase II transcription subunit 15a isoform X2 [Sesamum indicum]
c48604.graph_c0	0.71166757	0.61523745	0.58196791	6.40733802	5.17766894	4.37534785	1.928E-07	2.053571	up	PREDICTED: probable serine/threonine-protein kinase At5g41260 [Sesamum indicum]
c48605.graph_c0	1.54547315	1.6437095	1.365371	8.25353652	7.30599072	6.13645181	7.687E-06	1.239974	up	PREDICTED: abietadienol/abietadienal oxidase [Sesamum indicum]
c48612.graph_c0	23.4005982	25.7291448	23.5430479	8.29879864	9.022928	8.96655422	8.557E-53	-2.485655	down	PREDICTED: autophagy-related protein 9 [Sesamum indicum]
c48612.graph_c1	37.4865573	37.6019452	35.079399	0.42989703	1.13855945	0.51002639	1.32E-151	-6.757578	down	hypothetical protein CICLE_v10018809mg [Citrus clementina]
c48616.graph_c0	10.0101968	9.12893406	8.8761612	6.23917413	7.82097845	7.25465385	1.844E-14	-1.414517	down	PREDICTED: deSI-like protein At4g17486 [Sesamum indicum]
c48617.graph_c0	0.6290517	0.53708137	0.66570522	0.39203057	0.28891051	0.21232914	2.291E-05	-2.046088	down	hypothetical protein VITISV_012155 [Vitis vinifera]
c48618.graph_c3	2.68109055	3.70992462	3.95197037	1.88882008	2.53970011	3.2966807	0.0014889	-1.448495	down	PREDICTED: dnaJ homolog subfamily C member 21 [Sesamum indicum]
c48620.graph_c1	236.748792	237.107703	249.172695	117.034703	124.459263	110.955056	1.951E-42	-2.054945	down	hypothetical protein VITISV_027576 [Vitis vinifera]
c48620.graph_c2	7.48928205	7.03237904	8.00582523	1.89281477	2.06989185	2.77080564	4.988E-15	-2.764617	down	-
c48620.graph_c4	0.60275155	0.42234858	0.54219172	6.8856461	6.76506708	6.57149406	1.978E-08	2.672677	up	-
c48622.graph_c1	56.6734261	62.1634886	53.7146262	25.3457462	32.2780229	33.4143313	9.638E-43	-1.943696	down	PREDICTED: uncharacterized protein LOC105161451 [Sesamum indicum]
c48623.graph_c1	2.70995311	1.87800004	2.74573613	1.44033782	1.1189664	0.81168249	2.326E-06	-2.132611	down	-

#ID	0 h-1 FPKM	0 h-2 FPKM	0 h-3 FPKM	48 h-1 FPKM	48 h-2 FPKM	48 h-3 FPKM	FDR	log2FC	regulated	nr_annotation
c48623.graph_c2	11.4937931	12.2537886	10.7617692	45.2682404	50.6876306	46.0060891	1.312E-12	1.022529	up	unnamed protein product [Coffea canephora]
c48623.graph_c3	584.225012	601.156751	572.264226	3016.77032	3248.48021	3754.0922	9.544E-14	1.49147	up	PREDICTED: CBL-interacting serine/threonine-protein kinase 3-like [Sesamum indicum]
c48623.graph_c4	3.074017	1.53854758	0.65837226	11.3279399	15.5007475	11.3394744	0.0010015	1.846679	up	-
c48624.graph_c0	1.86339681	2.08048534	1.93405047	15.6482229	16.3675543	17.9186788	9.601E-10	2.067714	up	PREDICTED: pyridoxal biosynthesis protein PDX2 [Sesamum indicum]
c48625.graph_c0	222.081868	232.184438	219.954492	36.1065343	35.3840574	39.369851	3.43E-112	-3.62207	down	hypothetical protein MIMGU_mgv1a004857mg [Erythranthe guttata]
c48627.graph_c1	6.95907194	9.27134874	7.5272943	2.16779925	3.61579815	3.77570885	6.191E-16	-2.339719	down	hypothetical protein MIMGU_mgv1a018596mg [Erythranthe guttata]
c48632.graph_c0	0	0	0	1.09637547	1.48669039	1.05359216	1.931E-11	Inf	up	26S proteasome complex ubiquitin receptor, subunit Rpn13 domain-containing protein [Rozella allomycis CSF55]
c48637.graph_c0	14.8027536	14.0899275	13.5023116	2.67874792	3.22478251	3.08775968	3.55E-33	-3.257081	down	PREDICTED: kinesin-1-like protein PSS1 isoform X2 [Sesamum indicum]
c48639.graph_c1	4.74010102	7.5648984	5.11432411	0.61795617	0.78557955	1.35630574	1.639E-13	-3.682635	down	PREDICTED: multiple C2 and transmembrane domain-containing protein 1 [Sesamum indicum]
c48639.graph_c2	216.740662	209.092159	224.914944	145.525107	166.91001	160.398882	6.446E-23	-1.481041	down	PREDICTED: uncharacterized protein LOC105176680 [Sesamum indicum]
c48642.graph_c0	0.06520378	0.17405086	0.22343851	0.9410963	1.37879855	1.47878067	8.822E-05	2.004165	up	PREDICTED: protein BREAST CANCER SUSCEPTIBILITY 2 homolog B [Sesamum indicum]
c48643.graph_c1	1.68641498	1.74255862	1.81757614	14.6843611	22.9893444	33.8022887	2.88E-05	2.739586	up	PREDICTED: fatty acid desaturase 4, chloroplastic-like [Sesamum indicum]
c48646.graph_c1	3.09969039	2.3270957	3.37042105	0.38441267	0.63994639	0.39091209	2.583E-13	-3.661839	down	BnaC03g12490D [Brassica napus]
c48646.graph_c2	152.514859	159.352951	149.523227	142.196145	149.17031	155.559448	1.056E-12	-1.064225	down	PREDICTED: WD-40 repeat-containing protein MSI1 [Sesamum indicum]
c48649.graph_c1	20.6978063	20.4363731	20.5173327	22.3142097	20.0039111	18.7987544	9.465E-13	-1.027276	down	PREDICTED: uncharacterized protein LOC105175481 [Sesamum indicum]
c48652.graph_c0	8.5244646	7.71251974	8.84768326	43.2686228	58.3172765	69.7437461	9.299E-09	1.747003	up	PREDICTED: uncharacterized protein LOC105158144 [Sesamum indicum]
c48653.graph_c0	1.69299816	2.95669919	3.19392142	39.7557658	42.3976581	46.9198558	5.756E-62	3.016712	up	PREDICTED: serine carboxypeptidase-like 7 [Sesamum indicum]
c48657.graph_c0	0	0	0	5.3147747	5.47396048	6.63381266	8.433E-16	Inf	up	predicted protein [Physcomitrella patens]
c48658.graph_c0	53.7132597	51.0684712	50.0749632	40.8667649	39.1474228	41.9882174	1.594E-21	-1.360588	down	PREDICTED: calmodulin-binding receptor-like cytoplasmic kinase 2 [Sesamum indicum]
c48659.graph_c1	0	0	0	5.4841141	7.24510487	4.93714036	4.414E-17	Inf	up	-
c48666.graph_c0	131.80232	133.054318	126.212006	117.860768	114.679448	102.555482	2.052E-16	-1.238362	down	PREDICTED: sister chromatid cohesion protein PDS5 homolog A [Sesamum indicum]

#ID	0 h-1 FPKM	0 h-2 FPKM	0 h-3 FPKM	48 h-1 FPKM	48 h-2 FPKM	48 h-3 FPKM	FDR	log2FC	regulated	nr_annotation
c48669.graph_c0	2.2211366	2.56304084	2.21997103	1.10838862	1.53549668	1.87132249	1.118E-05	-1.657513	down	PREDICTED: pentatricopeptide repeat-containing protein At4g25270, chloroplastic [Sesamum indicum]
c48669.graph_c1	3.17070809	2.67513002	2.91034673	0.62594209	1.67987803	1.44809473	6.07E-08	-2.253987	down	PREDICTED: probable serine/threonine protein kinase IRE [Sesamum indicum]
c48670.graph_c0	0	0	0	0.83580484	1.5771791	0.55777059	3.019E-05	Inf	up	hypothetical protein AMTR_s00036p00173790 [Amborella trichopoda]
c48671.graph_c0	59.0592952	57.1006374	57.103794	13.5538133	14.4760746	15.4882286	9.023E-93	-3.012233	down	PREDICTED: protein DAMAGED DNA-BINDING 2 [Sesamum indicum]
c48676.graph_c0	2.27398857	2.32368782	2.37425934	14.533692	15.3966843	15.8057676	9.714E-10	1.694594	up	PREDICTED: probable receptor-like protein kinase At1g80640 isoform X2 [Sesamum indicum]
c48680.graph_c0	29.2128647	26.8476754	34.0815793	12.0102141	12.8692757	10.0738516	4.002E-24	-2.385829	down	PREDICTED: zinc finger A20 and AN1 domain-containing stress-associated protein 5 [Sesamum indicum]
c48681.graph_c1	12.0052704	12.2244866	9.73512533	10.1447246	11.7167693	9.73935038	6.925E-09	-1.120665	down	PREDICTED: uncharacterized protein LOC105173885 isoform X2 [Sesamum indicum]
c48681.graph_c2	1.96716664	1.8351816	2.13236728	13.6599231	15.594235	19.0545097	1.402E-18	2.002442	up	PREDICTED: subtilisin-like protease [Sesamum indicum]
c48683.graph_c0	0.51029758	0.178783	0.3278763	9.87250767	11.3302852	11.7754104	4.007E-37	4.000966	up	PREDICTED: probable ATP-dependent RNA helicase DHX35 isoform X3 [Sesamum indicum]
c48684.graph_c0	7.35794011	7.43512132	7.21466513	6.52160528	7.72565986	6.94631081	1.359E-06	-1.073996	down	PREDICTED: ATPase ASNA1 homolog 2-like [Sesamum indicum]
c48684.graph_c1	15.2928437	18.0014422	15.558821	481.350951	509.295791	549.642236	3.65E-127	3.959993	up	Beta-galactosidase 8 [Morus notabilis]
c48685.graph_c0	15.6322026	14.1381853	14.1200485	55.73101	69.3787157	76.9347588	7.424E-12	1.181204	up	PREDICTED: blue-light photoreceptor PHR2 [Sesamum indicum]
c48690.graph_c1	0	0	0	3.04521029	7.73363609	2.74672775	6.512E-06	Inf	up	PREDICTED: fruit bromelain-like [Setaria italica]
c48695.graph_c0	96.0625346	90.8982787	98.029186	428.283453	452.004754	455.661081	1.581E-17	1.209933	up	PREDICTED: pre-mRNA-splicing factor 18 [Sesamum indicum]
c48696.graph_c1	17.9791517	16.820126	16.9136305	110.214116	128.56761	125.025636	1.391E-36	1.794867	up	hypothetical protein MIMGU_mgv1a0087951mg, partial [Erythranthe guttata]
c48697.graph_c0	1.85551436	1.92880977	1.696599	18.1790333	21.5917875	20.6509398	4.728E-26	2.443342	up	PREDICTED: pentatricopeptide repeat-containing protein At1g07740, mitochondrial [Sesamum indicum]
c48700.graph_c0	0	0	0	2.22825467	6.98727427	2.93437765	0.0001646	Inf	up	-
c48702.graph_c0	8.30269898	9.40098178	8.81528793	9.3292573	8.44855637	8.07796874	1.04E-08	-1.051788	down	PREDICTED: phospholipase SGR2-like [Sesamum indicum]
c48702.graph_c1	19.5881959	22.3311175	23.6857832	10.5894483	11.1518563	10.4817536	8.664E-24	-2.045221	down	PREDICTED: phospholipase SGR2-like isoform X2 [Sesamum indicum]
c48703.graph_c0	8.64905604	9.78068421	8.97521018	5.73434655	5.08591661	4.80573666	8.84E-17	-1.824981	down	calcium-dependent protein kinase 34 [Brassica napus]
c48704.graph_c0	1.82642369	2.25484485	1.2517476	0.56087354	0.29708876	0.34934333	1.165E-09	-3.144434	down	PREDICTED: uncharacterized protein LOC104774238, partial [Camelina sativa]
c48706.graph_c0	1.07175988	1.32040996	0.79456955	24.7223537	22.6500141	18.8890494	8.306E-27	3.367508	up	PREDICTED: uncharacterized protein LOC105173691 [Sesamum indicum]

#ID	0 h-1 FPKM	0 h-2 FPKM	0 h-3 FPKM	48 h-1 FPKM	48 h-2 FPKM	48 h-3 FPKM	FDR	log2FC	regulated	nr_annotation
c48706.graph_c2	105.852654	113.658579	96.2411806	403.643112	485.318713	475.067877	4.815E-12	1.091568	up	PREDICTED: pyruvate kinase, cytosolic isozyme-like [Sesamum indicum]
c48706.graph_c3	0.68505914	0.68574513	0.52819687	88.0624402	108.490901	132.679757	5.534E-53	6.417695	up	-
c48708.graph_c1	660.78492	638.12528	697.367549	435.943154	437.662806	409.488871	4.203E-22	-1.655398	down	PREDICTED: 2-alkenal reductase (NADP(+)-dependent)-like [Sesamum indicum]
c48712.graph_c0	3.09567612	2.74463015	3.06879406	23.1821879	27.5800004	32.7717816	3.691E-20	2.206719	up	PREDICTED: cystinosin homolog isoform X1 [Sesamum indicum]
c48714.graph_c0	39.1761932	44.4406617	39.1071889	24.4298009	27.2049997	32.6490252	4.936E-28	-1.56241	down	hypothetical protein MIMGU_mgv1a0225922mg, partial [Erythranthe guttata]
c48714.graph_c2	1.3731092	0.8246905	1.76449969	16.1919713	16.0813408	13.0568138	7.594E-08	2.495497	up	-
c48716.graph_c0	0	0	0	19.024381	35.3447451	18.0647203	6.319E-15	Inf	up	unknown [Lotus japonicus]
c48717.graph_c0	48.3775243	48.5310695	47.8817715	10.9426263	13.872435	14.6749565	1.244E-85	-2.896495	down	hypothetical protein MIMGU_mgv1a002354mg [Erythranthe guttata]
c48720.graph_c1	37.7618974	35.6697266	35.9705143	34.0432503	34.0172272	32.5762382	1.349E-15	-1.137235	down	PREDICTED: polycomb group protein FERTILIZATION-INDEPENDENT ENDOSPERM-like isoform X1 [Sesamum indicum]
c48722.graph_c0	194.910592	182.679612	183.06037	1342.09534	1496.10495	1516.40701	7.669E-37	1.938225	up	PREDICTED: 60S ribosomal protein L7-2-like [Sesamum indicum]
c48723.graph_c0	19.8951487	18.6093997	17.829928	113.326353	129.778041	136.662363	2.172E-33	1.7336	up	PREDICTED: lysosomal beta glucosidase-like [Sesamum indicum]
c48725.graph_c1	0.25179482	0.20163756	0.19413978	2.1805162	2.82605467	3.34376652	2.597E-08	2.667371	up	PREDICTED: DNA polymerase epsilon catalytic subunit A [Sesamum indicum]
c48728.graph_c0	45.9005398	46.6562834	42.187843	28.7644493	33.3789777	31.3488222	3.182E-28	-1.546123	down	PREDICTED: protein cfxQ homolog [Sesamum indicum]
c48729.graph_c0	0	0	0	1.13857113	1.75444081	1.38149147	1.642E-11	Inf	up	AaceriAFL219Wp [Saccharomycetaceae sp. 'Ashbya aceri']
c48730.graph_c1	1.41100183	2.82482947	2.53847051	16.6388086	15.4234501	14.1111222	0.00074	1.750671	up	PREDICTED: 3-oxoacyl-[acyl-carrier-protein] reductase 4 [Sesamum indicum]
c48730.graph_c2	67.0908578	73.4480572	68.4497681	476.061812	516.972603	540.432137	4.124E-35	1.856117	up	PREDICTED: LOW QUALITY PROTEIN: dedicator of cytokinesis protein 7 [Sesamum indicum]
c48734.graph_c1	3.90400008	2.49487998	2.72097338	35.7629963	27.058752	25.7285669	4.116E-10	2.270517	up	PREDICTED: CTP synthase isoform X2 [Elaeis guineensis]
c48735.graph_c0	0	0	0	1.5312819	1.07577971	1.04699322	4.998E-15	Inf	up	predicted protein [Hordeum vulgare subsp. vulgare]
c48736.graph_c1	75.128419	79.189465	73.340915	48.0254856	45.4723159	44.777509	6.347E-34	-1.735087	down	PREDICTED: crossover junction endonuclease MUS81 isoform X1 [Sesamum indicum]
c48736.graph_c4	84.7408446	88.8407055	87.1460658	63.6511639	75.9613511	73.9124324	1.93E-19	-1.308808	down	PREDICTED: vacuolar-sorting receptor 1-like [Sesamum indicum]
c48739.graph_c1	8.28706107	8.59810965	9.45732012	80.0237852	103.765366	113.319245	3.422E-26	2.471383	up	PREDICTED: pentatricopeptide repeat-containing protein At1g74850, chloroplastic [Sesamum indicum]
c48739.graph_c2	162.101675	200.672228	137.504826	2796.28652	2121.3092	1830.71327	2.606E-10	2.74563	up	PREDICTED: calreticulin [Sesamum indicum]

#ID	0 h-1 FPKM	0 h-2 FPKM	0 h-3 FPKM	48 h-1 FPKM	48 h-2 FPKM	48 h-3 FPKM	FDR	log2FC	regulated	nr_annotation
c48739.graph_c3	11.343087	12.3185021	12.5136297	6.40801371	7.41514553	7.01761485	2.149E-08	-1.816125	down	Os01g0916200 [Oryza sativa Japonica Group]
c48743.graph_c0	85.088583	88.4511418	79.1937555	47.2437703	45.2833397	40.7182876	1.828E-40	-1.938371	down	PREDICTED: non-lysosomal glucosylceramidase [Sesamum indicum]
c48744.graph_c0	0	0	0	1.96239415	0.77293129	0.85067702	9.424E-05	Inf	up	-
c48745.graph_c1	21.221314	21.4460368	20.2437043	19.0073904	18.2910496	17.9790758	7.273E-17	-1.202598	down	PREDICTED: phosphoinositide phosphatase SAC2 [Sesamum indicum]
c48750.graph_c0	61.3891537	64.918972	47.9578857	31.7257698	41.3021274	50.1060994	1.266E-12	-1.521915	down	PREDICTED: ABC transporter C family member 3-like [Sesamum indicum]
c48751.graph_c0	680.067546	668.498249	714.345067	252.885961	267.74639	258.902845	2.838E-46	-2.422865	down	PREDICTED: UBP1-associated protein 2C [Sesamum indicum]
c48751.graph_c1	193.601193	208.676203	192.893131	52.5134926	59.4454739	60.3749727	5.036E-83	-2.807645	down	PREDICTED: UBP1-associated protein 2C [Sesamum indicum]
c48751.graph_c2	7.33233933	6.47193596	7.42647987	7.08778931	6.20433327	7.49102391	7.441E-06	-1.04774	down	hypothetical protein MIMGU_mgv1a001442mg [Erythranthe guttata]
c48755.graph_c0	0	0	0	1.22448522	0.95582687	1.0035233	1.921E-11	Inf	up	predicted protein [Physcomitrella patens]
c48756.graph_c1	6.89438335	6.50916845	7.09771213	29.8811822	31.8848768	32.7534518	1.149E-11	1.185493	up	PREDICTED: probable beta-1,3-galactosyltransferase 9 isoform X1 [Sesamum indicum]
c48756.graph_c3	45.550011	34.1967168	32.5925547	37.6719051	15.1552464	7.21321505	4.564E-06	-1.893965	down	-
c48761.graph_c0	20.7644521	21.0356692	20.1195185	16.7287685	15.4025615	15.9618108	3.347E-21	-1.380253	down	PREDICTED: metalloendoproteinase 1-like [Sesamum indicum]
c48762.graph_c1	2.27142245	2.16001209	2.06753134	1.18579676	1.86583817	2.26539359	1.926E-05	-1.315398	down	PREDICTED: serine carboxypeptidase-like 40 [Sesamum indicum]
c48762.graph_c2	4.24403252	4.43709482	2.54508324	47.8743032	61.9465588	62.0032853	9.692E-35	2.918966	up	PREDICTED: uncharacterized GPI-anchored protein At3g06035-like [Sesamum indicum]
c48762.graph_c3	0.10589435	0.31800116	0.13607843	2.82914667	3.82393449	3.81943926	6.993E-07	3.204808	up	-
c48764.graph_c0	12.7365586	11.0150309	12.726532	11.2376525	13.0474379	10.5557727	2.288E-11	-1.085565	down	PREDICTED: elongator complex protein 1 [Sesamum indicum]
c48765.graph_c0	2.08574268	2.48933724	2.88643591	57.1652853	55.568505	58.2140024	1.22E-109	3.497826	up	PREDICTED: probable galacturonosyltransferase 4 [Sesamum indicum]
c48768.graph_c0	4.43713728	4.90911518	4.03770702	3.18807744	3.43954187	4.05496066	3.999E-09	-1.343983	down	PREDICTED: serine/threonine-protein kinase ATR [Sesamum indicum]
c48769.graph_c0	0.06857308	0.16016406	0.08811912	4.10630934	3.12317516	3.07291372	1.1E-15	4.012596	up	PREDICTED: uncharacterized protein At5g41620-like [Sesamum indicum]
c48772.graph_c0	1.18838513	0.97876434	0.96653288	4.75344986	6.04871703	6.09159768	2.28E-08	1.410161	up	hypothetical protein MIMGU_mgv1a002385mg [Erythranthe guttata]
c48773.graph_c0	140.508411	130.60974	137.682426	109.11824	121.040799	110.567833	2.028E-19	-1.281572	down	PREDICTED: uncharacterized protein LOC105180349 [Sesamum indicum]
c48774.graph_c0	996.611934	1006.31246	973.257363	597.412127	631.749332	705.159198	4.919E-24	-1.640672	down	-
c48776.graph_c0	12.2160605	11.8554792	10.3377872	77.5444015	84.547929	78.5240046	1.554E-25	1.789007	up	PREDICTED: uncharacterized protein LOC104109628 isoform X2 [Nicotiana tomentosiformis]

#ID	0 h-1 FPKM	0 h-2 FPKM	0 h-3 FPKM	48 h-1 FPKM	48 h-2 FPKM	48 h-3 FPKM	FDR	log2FC	regulated	nr_annotation
c48777.graph_c0	11.3098722	13.1152669	11.9128144	4.27024261	6.75554768	7.09265423	2.306E-15	-2.02984	down	PREDICTED: protein notum homolog isoform X2 [Sesamum indicum]
c48781.graph_c0	0.46229822	0.42419771	0.29703576	5.46570733	5.11347121	4.10542829	1.23E-08	2.622923	up	PREDICTED: pullulanase 1, chloroplastic isoform X2 [Sesamum indicum]
c48782.graph_c0	0.53919581	0.28407144	0.36467787	2.09154672	2.09110476	2.25651791	0.0001976	1.422717	up	PREDICTED: uncharacterized protein LOC105163648 [Sesamum indicum]
c48784.graph_c0	11.4591164	11.5804625	11.1992165	56.1213736	59.2823919	57.1704994	2.052E-20	1.315608	up	PREDICTED: acetyl-coenzyme A synthetase, chloroplastic/glyoxysomal isoform X1 [Sesamum indicum]
c48784.graph_c1	11.2425973	11.7204328	10.3262616	43.4049504	45.1270514	47.0732523	1.565E-12	1.008866	up	PREDICTED: glutamine synthetase nodule isozyme-like [Sesamum indicum]
c48784.graph_c2	85.6961487	81.1126469	79.9804274	11.5210525	14.9800431	14.8398952	4.19E-126	-3.599304	down	beta-D-glucosidase [Tropaeolum majus]
c48784.graph_c3	77.8386146	76.3447544	78.6625433	61.2853069	70.2759278	67.300897	3.446E-18	-1.247497	down	PREDICTED: ubiquitin domain-containing protein DSK2a-like isoform X2 [Sesamum indicum]
c48784.graph_c4	64.5642357	62.0357526	59.6649709	38.7359434	50.6953861	50.9654358	3.775E-22	-1.429568	down	PREDICTED: probable LRR receptor-like serine/threonine-protein kinase At3g47570 [Sesamum indicum]
c48784.graph_c5	2.73719116	2.83778676	2.00994229	0.81053969	0.95407613	1.20202223	2.376E-06	-2.373186	down	-
c48784.graph_c6	0.05383449	0.0538884	0	1.19030023	1.47114189	2.18443909	4.067E-09	4.483622	up	PREDICTED: auxin response factor 18-like [Sesamum indicum]
c48786.graph_c0	7.04226388	7.17033394	5.55403893	5.68029929	5.01465694	5.52503942	3.391E-09	-1.298505	down	--
c48787.graph_c0	0.04887022	0	0	1.26062809	1.62165745	2.24339859	2.562E-10	5.709598	up	PREDICTED: phosphate transporter PHO1 homolog 3-like isoform X3 [Sesamum indicum]
c48792.graph_c0	0	0	0	1.22996759	1.82420212	1.31330132	1.525E-11	Inf	up	PREDICTED: LOW QUALITY PROTEIN: calreticulin-like [Phoenix dactylifera]
c48795.graph_c1	69.509698	65.2057454	64.9619738	17.9303229	13.708824	14.8887006	1.696E-98	-3.113739	down	hypothetical protein MIMGU_mgv1a013489mg [Erythranthe guttata]
c48796.graph_c1	124.216675	134.935878	129.675488	144.29477	106.089679	85.6848957	1.293E-11	-1.219917	down	PREDICTED: probable methyltransferase PMT15 [Sesamum indicum]
c48796.graph_c2	14.9474235	14.2009462	13.5873603	13.5252646	14.4026181	13.2194623	8.26E-10	-1.071788	down	PREDICTED: Down syndrome critical region protein 3 homolog isoform X1 [Sesamum indicum]
c48796.graph_c3	34.8816774	35.8196218	26.9847658	28.8109374	34.8749492	30.2370961	2.064E-10	-1.073934	down	PREDICTED: UDP-glycosyltransferase 74E2-like [Sesamum indicum]
c48796.graph_c6	4.67929302	4.18509333	3.87825663	27.0385925	32.373249	30.5501074	5.874E-26	1.800864	up	hypothetical protein MIMGU_mgv1a006632mg [Erythranthe guttata]
c48796.graph_c7	2.24395288	3.025124	2.48824038	24.0737596	28.0111645	30.1877404	3.158E-48	2.386104	up	hypothetical protein MIMGU_mgv1a025096mg, partial [Erythranthe guttata]
c48798.graph_c0	3.66660695	1.92845142	2.71523717	1.43132096	1.69826833	1.52829694	6.581E-05	-1.853808	down	PREDICTED: uncharacterized protein LOC105168156 [Sesamum indicum]
c48800.graph_c1	165.431296	162.251253	166.810563	136.17793	147.878686	142.101739	1.614E-16	-1.233424	down	photosystem II protein D1 [Phaseolus vulgaris]

#ID	0 h-1 FPKM	0 h-2 FPKM	0 h-3 FPKM	48 h-1 FPKM	48 h-2 FPKM	48 h-3 FPKM	FDR	log2FC	regulated	nr_annotation
c48801.graph_c1	17.2652332	16.1643563	15.6136608	7.4564356	8.68337041	7.66282778	5.43E-41	-2.061508	down	PREDICTED: molybdenum cofactor sulfurase isoform X3 [Sesamum indicum]
c48802.graph_c0	39.0645936	40.8979432	39.1570034	25.692446	25.1856829	23.4227851	8.913E-33	-1.697269	down	PREDICTED: cyclin-T1-3-like [Sesamum indicum]
c48803.graph_c0	29.4923089	27.5320608	29.1408811	27.4692233	27.9521358	26.2709392	6.773E-13	-1.094316	down	PREDICTED: WAT1-related protein At4g08300 [Tarenaya hassleriana]
c48804.graph_c0	11.634823	14.1539296	14.1888237	11.4778767	11.805573	10.4831294	7.392E-13	-1.262023	down	PREDICTED: uncharacterized protein LOC105155609 [Sesamum indicum]
c48804.graph_c2	7.35712958	6.26232707	6.36711617	24.8060734	31.3589875	32.7392033	7.138E-09	1.132135	up	PREDICTED: putative serine/threonine-protein kinase [Sesamum indicum]
c48808.graph_c0	18.1327747	16.6296111	18.5871908	10.187211	11.2012945	11.9428261	1.402E-18	-1.69891	down	hypothetical protein MIMGU_mgv1a005510mg [Erythranthe guttata]
c48808.graph_c1	23.3944742	22.5298975	21.5618532	19.3809186	19.8143594	18.6786474	3.131E-17	-1.238331	down	PREDICTED: ubiquitin domain-containing protein DSK2a-like [Sesamum indicum]
c48808.graph_c2	487.48894	468.605443	478.682683	113.112366	126.780378	133.806355	4.805E-67	-2.960812	down	PREDICTED: 26S proteasome non-ATPase regulatory subunit 14-like [Cucumis sativus]
c48810.graph_c0	2.20864639	2.83781775	2.28750291	34.8033206	35.8403721	38.8583555	1.841E-54	2.88226	up	hypothetical protein MIMGU_mgv1a006553mg [Erythranthe guttata]
c48810.graph_c2	54.8362296	54.7939873	53.5671838	44.7068243	48.7823031	45.3092644	5.537E-19	-1.251916	down	PREDICTED: uncharacterized protein LOC105170911 [Sesamum indicum]
c48810.graph_c3	0	0	0	16.4186858	12.0690333	10.882399	2.254E-15	Inf	up	unnamed protein product [Coffea canephora]
c48814.graph_c0	2.37542317	2.48788521	2.26112061	1.23603778	1.28796605	1.31617682	7.715E-09	-1.909262	down	PREDICTED: molybdopterin biosynthesis protein CNX1 [Sesamum indicum]
c48815.graph_c0	0	0	0	1.72072739	1.76004333	1.21431504	1.687E-11	Inf	up	--
c48816.graph_c1	20.7865124	20.5770937	21.3913603	18.8850239	15.5449392	13.8578163	9.653E-20	-1.391008	down	PREDICTED: serrate RNA effector molecule-like [Sesamum indicum]
c48816.graph_c4	6.16193879	6.88868253	8.02934001	60.8264317	73.4871209	78.6943127	5.952E-44	2.313012	up	PREDICTED: uncharacterized protein LOC105168334 [Sesamum indicum]
c48822.graph_c0	0	0	0	1.46053439	3.59185145	1.20674795	0.0002159	Inf	up	hypothetical protein PNEG_01290 [Pneumocystis murina B123]
c48827.graph_c0	11.0691104	12.5315046	11.1787519	10.4801393	12.6263007	10.9936381	6.267E-10	-1.048035	down	PREDICTED: callose synthase 5 [Sesamum indicum]
c48828.graph_c1	78.5264157	82.2280246	74.9812531	29.2459708	27.7473005	26.2863066	3.282E-65	-2.516418	down	PREDICTED: LOW QUALITY PROTEIN: B3 domain-containing transcription repressor VAL1 [Sesamum indicum]
c48830.graph_c0	0	0	0	0.94680641	2.4421517	0.84246265	0.0003889	Inf	up	-
c48831.graph_c0	2.91945223	2.14307545	2.25096702	23.1902411	30.265837	39.5140748	1.718E-10	2.645721	up	PREDICTED: non-specific lipid-transfer protein 1-like [Sesamum indicum]
c48831.graph_c1	4.62922438	4.28413457	4.32125206	2.89680686	3.62290732	3.7589436	2.207E-05	-1.386045	down	hypothetical protein MIMGU_mgv1a001779mg [Erythranthe guttata]

#ID	0 h-1 FPKM	0 h-2 FPKM	0 h-3 FPKM	48 h-1 FPKM	48 h-2 FPKM	48 h-3 FPKM	FDR	log2FC	regulated	nr_annotation
c48832.graph_c0	34.3337096	34.6522615	32.0600891	681.577794	709.907039	812.674938	4.017E-86	3.42867	up	PREDICTED: aconitate hydratase, cytoplasmic [Sesamum indicum]
c48832.graph_c2	207.492414	196.034086	211.442555	23.5474198	20.3037634	21.3690186	1.13E-158	-4.252452	down	PREDICTED: (+)-neomenthol dehydrogenase-like isoform X1 [Sesamum indicum]
c48837.graph_c0	39.2241732	34.1421307	41.024974	13.008912	9.58703644	7.99642992	8.2E-50	-2.913479	down	-
c48837.graph_c5	4.68802065	4.3493456	4.16312871	39.6075685	62.6787379	88.1689276	5.235E-06	2.824176	up	PREDICTED: 3-ketoacyl-CoA synthase 1 [Sesamum indicum]
c48837.graph_c8	0.32542795	0.21716921	0.13939594	4.29721156	6.45801976	5.69098866	3.047E-08	3.574979	up	TPA: hypothetical protein ZEAMMB73_870894 [Zea mays]
c48837.graph_c9	32.7406247	31.6624465	28.1080912	23.6019642	18.0530102	12.0167524	6.627E-26	-1.79276	down	PREDICTED: probable WRKY transcription factor 33-like [Cicer arietinum]
c48839.graph_c1	1.27716826	1.30303267	1.19934707	5.20423965	6.42414744	6.66418295	2.793E-05	1.25412	up	hypothetical protein MIMGU_mgv1a005831mg [Erythranthe guttata]
c48845.graph_c0	0	0	0	1.69128533	2.15005407	3.00979103	1.312E-10	Inf	up	-
c48847.graph_c0	15.1463903	15.3393309	14.8993569	4.48766801	5.17507958	3.63954428	5.251E-59	-2.78805	down	PREDICTED: uncharacterized protein LOC105164002 isoform X1 [Sesamum indicum]
c48847.graph_c1	1.34141809	1.31693899	1.65747631	0.09506168	0.07552974	0.08458531	2.27E-20	-5.096325	down	PREDICTED: ABC transporter B family member 19-like [Sesamum indicum]
c48848.graph_c0	1.76375725	1.10858445	1.73940537	24.4866877	29.9438672	30.0595186	4.358E-44	3.172705	up	PREDICTED: uncharacterized protein LOC105173962 [Sesamum indicum]
c48848.graph_c1	1.02487585	1.44386223	1.21945005	13.4283731	12.3363779	12.7574977	6.321E-16	2.368282	up	PREDICTED: O-acyltransferase WSD1-like [Nicotiana tomentosiformis]
c48848.graph_c2	0.40972361	0.31363179	0.46456858	4.75160707	6.65678356	7.50757725	5.287E-16	2.966295	up	PREDICTED: probable inactive leucine-rich repeat receptor-like protein kinase At1g66830 [Sesamum indicum]
c48848.graph_c3	54.7236139	56.2630125	52.9959838	51.7036726	52.059756	52.1535147	2.262E-14	-1.089871	down	PREDICTED: myb-related protein 3R-1-like isoform X1 [Sesamum indicum]
c48848.graph_c4	34.794188	34.006121	33.6338626	38.0316647	29.2091202	26.955712	5.448E-16	-1.132087	down	PREDICTED: protein STRUBBELIG-RECEPTOR FAMILY 3-like [Sesamum indicum]
c48849.graph_c1	244.99828	253.804355	247.589247	91.7683172	99.4243189	102.472618	3.58E-52	-2.364911	down	PREDICTED: probable inactive receptor kinase At5g10020 [Sesamum indicum]
c48850.graph_c0	0.16351582	0	0	22.9728199	31.1991298	20.7091567	5.764E-32	7.839784	up	-
c48853.graph_c0	0	0	0	1.24205845	2.78951787	1.28200432	1.269E-05	Inf	up	-
c48861.graph_c0	12.0432512	11.6298291	10.8514453	10.6642804	10.67525	9.80260291	2.861E-15	-1.164543	down	PREDICTED: phospholipase A I [Sesamum indicum]
c48861.graph_c1	5.48297191	5.3085127	4.62022032	4.8856559	3.68443933	4.34722724	0.0013913	-1.266216	down	PREDICTED: phospholipase A I [Sesamum indicum]
c48862.graph_c0	18.3840319	16.5086773	19.1847602	18.8310402	17.6612668	16.8906014	1.076E-09	-1.035342	down	PREDICTED: uncharacterized protein LOC105177107 isoform X1 [Sesamum indicum]
c48863.graph_c0	60.8142887	33.5626899	40.4483079	0	0	0	1.083E-33	-Inf	down	predicted protein [Hordeum vulgare subsp. vulgare]

#ID	0 h-1 FPKM	0 h-2 FPKM	0 h-3 FPKM	48 h-1 FPKM	48 h-2 FPKM	48 h-3 FPKM	FDR	log2FC	regulated	nr_annotation
c48865.graph_c3	73.1994211	78.8623462	71.667927	59.3840117	62.5885851	61.7811746	3.719E-19	-1.30179	down	PREDICTED: enhancer of mRNA-decapping protein 4-like [Sesamum indicum]
c48866.graph_c0	14.3838429	14.1116041	12.6810808	112.545022	112.735414	117.765122	4.63E-47	2.041998	up	hypothetical protein MIMGU_mgv1a005571mg [Erythranthe guttata]
c48869.graph_c0	101.644942	102.615364	98.1677696	37.2276051	34.5542416	32.2001525	2.408E-69	-2.555235	down	PREDICTED: uncharacterized protein LOC105165944 [Sesamum indicum]
c48870.graph_c0	226.363051	235.333954	204.303126	177.772533	124.769755	107.059079	3.897E-28	-1.708958	down	PREDICTED: DNA repair helicase XPB1-like [Sesamum indicum]
c48871.graph_c0	6.586903	4.34890346	6.24326836	22.1212647	24.7569944	24.1255293	4.71E-07	1.027014	up	hypothetical protein MIMGU_mgv1a012747mg [Erythranthe guttata]
c48872.graph_c0	5.22314178	4.48146169	4.83990518	0.09820202	0.10403313	0.02912652	8.451E-50	-6.985266	down	-
c48872.graph_c1	119.354218	127.462319	112.999087	71.6435313	78.3496672	74.0455481	5.357E-32	-1.701377	down	PREDICTED: FACT complex subunit SPT16-like [Sesamum indicum]
c48872.graph_c2	0.47902338	0.2950788	0.37880862	1.52760316	2.48140915	2.32583138	0.0006306	1.433391	up	PREDICTED: putative late blight resistance protein homolog R1A-10 [Sesamum indicum]
c48879.graph_c1	1.17368585	1.07487294	1.37987255	9.7544895	9.45630931	9.15030728	1.347E-13	1.947766	up	PREDICTED: multisubstrate pseudouridine synthase 7 isoform X2 [Sesamum indicum]
c48880.graph_c0	0	0	0	8.16174103	10.8079687	6.62681963	3.044E-16	Inf	up	predicted protein [Thalassiosira pseudonana CCMP1335]
c48887.graph_c1	0	0	0	13.1147701	7.87298826	7.39064761	3.264E-09	Inf	up	PREDICTED: zinc-finger homeodomain protein 10-like [Sesamum indicum]
c48887.graph_c2	37.0780082	37.0570531	35.0453099	164.538992	169.948551	165.574597	6.851E-17	1.178162	up	hypothetical protein MIMGU_mgv1a000942mg [Erythranthe guttata]
c48889.graph_c0	1.44584185	0.692182	0.76741992	0	0	0	3.492E-11	-Inf	down	PREDICTED: granule-bound starch synthase 1b, chloroplastic/amyloplastic-like [Elaeis guineensis]
c48892.graph_c0	0	0	0	7.14335724	12.4457574	2.83661389	9.029E-06	Inf	up	predicted protein [Micromonas sp. RCC299]
c48893.graph_c0	5.81286319	4.52564303	5.60231951	4.31390202	4.25487982	5.02973776	0.0084521	-1.248272	down	PREDICTED: calcium-dependent protein kinase 20-like [Malus domestica]
c48893.graph_c3	0.50132008	0.50182208	0	17.088404	15.4120918	21.780363	6.644E-12	4.755988	up	PREDICTED: UDP-glucuronate 4-epimerase 6 [Sesamum indicum]
c48895.graph_c0	61.2924419	60.7836143	62.2200115	46.8368367	49.2843832	47.6053791	2.063E-22	-1.377029	down	neutral/alkaline invertase 1 [Orobanche ramosa]
c48896.graph_c0	1.52905525	1.1704484	1.04023934	9.52920733	10.8850863	11.2070676	3.695E-06	2.062913	up	PREDICTED: N-acetyl-D-glucosamine kinase-like isoform X1 [Sesamum indicum]
c48898.graph_c0	0	0	0	2.01837124	1.97374116	1.79593459	9.459E-13	Inf	up	hypothetical protein EUGRSUZ_K01803 [Eucalyptus grandis]
c48899.graph_c0	1.95226345	1.60815884	2.14287851	0.31849366	0.4366423	0.61679814	5.254E-15	-3.082891	down	PREDICTED: phosphate transporter PHO1 homolog 9-like [Sesamum indicum]
c48900.graph_c0	5.32465352	2.43914584	2.9573044	0	0	0	3.814E-16	-Inf	down	--

#ID	0 h-1 FPKM	0 h-2 FPKM	0 h-3 FPKM	48 h-1 FPKM	48 h-2 FPKM	48 h-3 FPKM	FDR	log2FC	regulated	nr_annotation
c48903.graph_c0	54.4673358	53.2023733	52.5114514	2.9631274	3.08761395	3.02557551	4.11E-180	-5.159198	down	PREDICTED: phosphoenolpyruvate carboxylase kinase 2 [Sesamum indicum]
c48903.graph_c1	50.9896189	55.0331229	45.2233981	23.6315764	23.8135804	23.7197463	8.133E-39	-2.10363	down	hypothetical protein MIMGU_mgv1a0000982mg, partial [Erythranthe guttata]
c48903.graph_c2	53.4103412	52.3878221	53.5693674	23.3337345	21.6375506	18.4767207	6.483E-59	-2.343519	down	PREDICTED: suppressor of mec-8 and unc-52 protein homolog 2 [Sesamum indicum]
c48904.graph_c0	0	0	0	2.68726759	2.49497825	2.41798099	2.449E-16	Inf	up	PREDICTED: cytochrome P450 6k1-like [Pyrus x bretschneideri]
c48905.graph_c0	1.29792537	1.72483325	1.61037219	0.90710818	1.70353972	1.39414988	0.0011404	-1.238621	down	PREDICTED: putative serine/threonine-protein kinase [Sesamum indicum]
c48907.graph_c4	25.7544416	22.9393171	27.9787421	21.3266874	21.9522486	19.5666566	5.036E-16	-1.305386	down	PREDICTED: flowering time control protein FCA [Sesamum indicum]
c48909.graph_c1	17.5864922	16.6317828	16.7524168	10.9739097	9.28046331	10.3931359	1.796E-19	-1.748624	down	hypothetical protein MIMGU_mgv1a025365mg [Erythranthe guttata]
c48913.graph_c0	0	0	0	3.29452945	5.67889541	3.62705139	1.44E-13	Inf	up	-
c48914.graph_c1	34.865895	34.5033867	34.6466143	5.88547659	4.33276103	3.88573164	2.37E-146	-3.892887	down	hypothetical protein MIMGU_mgv1a000556mg [Erythranthe guttata]
c48916.graph_c0	54.68476	52.3309797	53.1256937	245.951097	235.150949	225.121598	5.173E-15	1.124733	up	PREDICTED: D-lactate dehydrogenase [cytochrome], mitochondrial [Sesamum indicum]
c48916.graph_c1	18.8779626	18.0631808	19.6211929	98.9787242	99.1660984	113.331254	8.057E-09	1.442101	up	BnaC03g39320D [Brassica napus]
c48920.graph_c0	7.31708244	7.25690332	6.71624331	24.2302277	31.2964202	31.2618708	3.878E-09	1.006054	up	PREDICTED: cell division cycle protein 27 homolog B [Sesamum indicum]
c48922.graph_c1	0	0	0	13.8273848	32.3337464	13.253796	1.949E-09	Inf	up	-
c48923.graph_c1	28.3671846	29.6125439	28.901986	20.4216204	23.8095188	24.8149235	1.881E-18	-1.352141	down	PREDICTED: NAD-dependent protein deacetylase SRT2 [Sesamum indicum]
c48927.graph_c0	39.5444731	38.8384806	37.9816391	33.8421049	31.3247217	27.475923	6.059E-21	-1.343399	down	PREDICTED: coiled-coil domain-containing protein 130-like [Sesamum indicum]
c48938.graph_c0	3.07291205	3.54204806	4.54711875	23.2482443	18.0852785	23.5867886	9.205E-07	1.521467	up	cytochrome b, partial (mitochondrion) [Erythranthe guttata]
c48940.graph_c0	0	0	0	1.30521093	0.91200201	0.79071921	1.542E-09	Inf	up	hypothetical protein AALP_AA3G363600 [Arabis alpina]
c48942.graph_c0	16.3103917	16.7348922	16.567985	86.5616259	124.637543	134.975549	1.992E-10	1.778103	up	PREDICTED: heparanase-like protein 2 isoform X1 [Sesamum indicum]
c48943.graph_c0	389.230273	381.899263	396.053806	370.02678	301.200626	260.337134	1.667E-13	-1.33767	down	PREDICTED: DNA mismatch repair protein msh2 isoform X2 [Sesamum indicum]
c48944.graph_c0	0	0	0	2.65247696	6.18195096	6.5226186	4.383E-10	Inf	up	hypothetical protein M378DRAFT_109196 [Amanita muscaria Koide BX008]
c48949.graph_c1	26.430601	24.8035006	25.3767869	134.199465	154.186391	151.10889	2.565E-24	1.500528	up	PREDICTED: palmitoyl-acyl carrier protein thioesterase, chloroplastic [Sesamum indicum]

#ID	0 h-1 FPKM	0 h-2 FPKM	0 h-3 FPKM	48 h-1 FPKM	48 h-2 FPKM	48 h-3 FPKM	FDR	log2FC	regulated	nr_annotation
c48950.graph_c0	245.361664	237.41112	250.336273	116.316974	113.841489	102.971261	1.012E-43	-2.154515	down	PREDICTED: methyl-CpG-binding domain-containing protein 11 [Sesamum indicum]
c48950.graph_c2	0.5818557	0.47323115	0.37385381	10.0508142	14.0548569	12.6992787	6.504E-27	3.667876	up	hypothetical protein MIMGU_mgv1a004080mg [Erythranthe guttata]
c48950.graph_c3	9.2544081	10.5071213	10.9345132	2.23157591	4.24322784	2.39295417	1.509E-21	-2.818516	down	-
c48952.graph_c2	4.05129797	4.06505654	3.6118725	2.7054855	2.86613383	2.86813277	2.347E-17	-1.491997	down	PREDICTED: protein RST1 [Sesamum indicum]
c48953.graph_c1	0	0	0	7.31245919	9.62464295	12.1061863	1.042E-26	Inf	up	PREDICTED: protein ECERIFERUM 1-like [Sesamum indicum]
c48956.graph_c2	31.8432848	30.8904097	33.3347329	9.94565714	9.8034832	10.4624601	7.028E-71	-2.687232	down	PREDICTED: DEAD-box ATP-dependent RNA helicase 13 [Sesamum indicum]
c48956.graph_c3	64.2968181	58.1402709	58.0125655	11.0907004	11.6729584	11.3422962	2.15E-112	-3.420938	down	PREDICTED: spermatogenesis-associated protein 5 [Sesamum indicum]
c48957.graph_c1	0	0	0	7.79900585	20.5493282	8.71886966	1.345E-05	Inf	up	PREDICTED: adenosylhomocysteinase-like [Phoenix dactylifera]
c48957.graph_c2	74.5098353	81.0202516	69.7018076	1167.78978	1252.92518	1515.86126	7.898E-51	3.108265	up	PREDICTED: uncharacterized protein LOC105169334 [Sesamum indicum]
c48958.graph_c0	1.30096611	1.16020314	1.55020802	0.58837165	0.46170994	0.60108949	1.723E-07	-2.297968	down	PREDICTED: probable plastidic glucose transporter 2 [Sesamum indicum]
c48960.graph_c0	0.49217669	0.4187691	0.98032313	3.49137459	3.67467057	3.04608585	0.0001497	1.406942	up	PREDICTED: uncharacterized protein LOC105166139 [Sesamum indicum]
c48962.graph_c0	0	0	0	3.52403958	4.75146333	2.06669108	9.776E-08	Inf	up	hypothetical protein SS1G_02090 [Sclerotinia sclerotiorum 1980]
c48967.graph_c3	47.4255923	46.8446461	45.8170711	33.6663507	37.0185066	34.3519675	2.418E-24	-1.433784	down	PREDICTED: mitochondrial carnitine/acylcarnitine carrier-like protein [Sesamum indicum]
c48971.graph_c0	14.8837985	14.3502839	12.5161687	63.6244582	68.3529833	70.2855285	4.503E-18	1.25909	up	PREDICTED: LOW QUALITY PROTEIN: T-complex protein 1 subunit beta-like [Sesamum indicum]
c48972.graph_c0	0.23710249	0.75157639	0.7109342	5.60647981	4.35812056	4.79425052	8.841E-07	2.099511	up	PREDICTED: subtilisin-like protease SBT3.5 isoform X2 [Sesamum indicum]
c48978.graph_c0	26.86928	23.5513447	25.3519696	16.151073	16.4846688	14.8589205	1.061E-24	-1.69079	down	PREDICTED: LOW QUALITY PROTEIN: ADP-ribosylation factor-binding protein GGA3 [Sesamum indicum]
c48979.graph_c0	8854.65546	8951.01217	8042.78709	2805.19595	2966.13783	2995.85732	5.82E-35	-2.577379	down	hypothetical protein PHAVU_003G1236000g, partial [Phaseolus vulgaris]
c48981.graph_c0	55.7266216	55.5421113	59.2322916	33.0648585	31.0743553	31.487264	2.045E-38	-1.851288	down	hypothetical protein MIMGU_mgv1a010195mg [Erythranthe guttata]
c48982.graph_c0	0	0	0	1.49663196	5.18890939	1.75541498	0.0011374	Inf	up	-
c48983.graph_c0	70.7943042	70.8280431	67.6041484	49.9538154	40.6589401	37.3751613	3.842E-33	-1.721153	down	PREDICTED: protein SPA1-RELATED 4-like isoform X6 [Sesamum indicum]
c48984.graph_c0	204.912448	194.785218	204.100268	148.179763	155.791315	147.775099	9.183E-22	-1.436686	down	PREDICTED: uncharacterized protein LOC105157959 [Sesamum indicum]
c48986.graph_c0	13.0754007	12.2623098	13.7322002	0.52025533	0.55114744	0.85462167	8.795E-83	-5.365177	down	anion exchange family protein [Populus trichocarpa]

#ID	0 h-1 FPKM	0 h-2 FPKM	0 h-3 FPKM	48 h-1 FPKM	48 h-2 FPKM	48 h-3 FPKM	FDR	log2FC	regulated	nr_annotation
c48988.graph_c0	0	0	0	35.4756139	60.5420436	28.9006479	9.324E-18	Inf	up	-
c48989.graph_c0	0	0	0	3.21816473	12.5460595	4.00890508	0.0001279	Inf	up	hypothetical protein COCSADRAFT_262301 [Bipolaris sorokiniana ND90Pr]
c48990.graph_c0	1.30586357	1.20584009	1.27482532	24.8816169	27.1099137	26.7947855	9.73E-100	3.359991	up	PREDICTED: alpha-glucan water dikinase, chloroplastic-like [Sesamum indicum]
c48992.graph_c0	1.75179028	1.98735035	1.50074657	20.980225	42.9702752	16.6576696	0.001225	2.919697	up	Papain family cysteine protease [Arabidopsis thaliana]
c48992.graph_c1	1.87943482	1.99069568	1.57265462	54.7825241	79.8760487	85.3443384	6.197E-30	4.314472	up	PREDICTED: subtilisin-like protease [Sesamum indicum]
c49000.graph_c0	20.4026732	23.3356155	19.3331043	14.6447486	17.730669	18.0627958	2.072E-17	-1.342504	down	PREDICTED: probable U3 small nucleolar RNA-associated protein 7 [Sesamum indicum]
c49001.graph_c2	8.36795122	8.6936157	9.28681017	2.68650541	3.15537745	3.89745058	4.649E-15	-2.458816	down	PREDICTED: uncharacterized protein LOC105156426 [Sesamum indicum]
c49003.graph_c1	2.80076841	4.15344142	3.15476517	1.52903736	1.14737935	1.4739056	1.276E-10	-2.297828	down	hypothetical protein MIMGU_mgv1a000068mg [Erythranthe guttata]
c49008.graph_c0	44.787024	41.1119165	50.6368468	0.64931045	0.50026596	0.63027549	2.62E-117	-7.277339	down	PREDICTED: uncharacterized protein LOC105161665 [Sesamum indicum]
c49013.graph_c0	64.9923843	66.5123608	57.117181	36.6054933	39.2966471	40.5894057	1.683E-33	-1.712826	down	PREDICTED: probable histone-lysine N-methyltransferase ATXR3 isoform X2 [Sesamum indicum]
c49015.graph_c1	56.4582066	56.6532574	55.6580211	66.7159987	52.7155192	45.0330712	2.152E-13	-1.048492	down	PREDICTED: E3 ubiquitin-protein ligase SINAT5 isoform X1 [Vitis vinifera]
c49015.graph_c3	47.2820439	49.031176	44.4217278	25.7179208	23.8303738	20.9245038	6.494E-46	-2.011927	down	PREDICTED: AP-5 complex subunit mu [Sesamum indicum]
c49016.graph_c0	0	0	0	0.6427481	0.94030936	0.59914736	8.064E-11	Inf	up	unknown [Picea sitchensis]
c49018.graph_c1	4.21720788	4.24814869	3.77291582	39.3680526	43.2165646	45.0066159	2.772E-48	2.36378	up	hypothetical protein MIMGU_mgv1a008196mg [Erythranthe guttata]
c49019.graph_c0	0	0	0	1.3498108	1.38662872	1.20105353	2.089E-11	Inf	up	hypothetical protein HMPREF1120_05211 [Exophiala dermatitidis NIH/UT8656]
c49020.graph_c0	1.54733418	1.67116388	1.72675599	0	0	0	1.888E-24	-Inf	down	-
c49020.graph_c1	37.2319069	35.4916125	34.2295677	23.1059376	19.4927742	17.0429788	1.713E-39	-1.854792	down	PREDICTED: uncharacterized protein LOC105161915 [Sesamum indicum]
c49022.graph_c0	8.69725988	8.56832509	9.67436679	197.556796	166.846199	139.602139	4.153E-19	3.211758	up	PREDICTED: uncharacterized protein LOC105176567 [Sesamum indicum]
c49022.graph_c1	14.803806	17.6722172	15.1673666	14.3754334	13.7158879	13.119153	5.694E-11	-1.22496	down	PREDICTED: ribosomal L1 domain-containing protein 1 [Sesamum indicum]
c49023.graph_c0	17.8032157	16.0876892	14.9505622	3.04100454	4.17220442	3.23816936	5.705E-46	-3.244546	down	PREDICTED: DNA polymerase zeta catalytic subunit [Sesamum indicum]
c49023.graph_c1	36.0681975	35.9791453	30.405413	9.85587426	9.80378957	8.45079955	1.531E-88	-2.880162	down	PREDICTED: DNA polymerase zeta catalytic subunit [Sesamum indicum]

#ID	0 h-1 FPKM	0 h-2 FPKM	0 h-3 FPKM	48 h-1 FPKM	48 h-2 FPKM	48 h-3 FPKM	FDR	log2FC	regulated	nr_annotation
c49028.graph_c0	14.909801	8.67079527	10.043641	0	0	0	2.277E-42	-Inf	down	alpha-2 macroglobulin family-like protein [Micromonas sp. RCC299]
c49029.graph_c0	0	0	0	1.74826597	2.77811372	1.34818368	1.349E-07	Inf	up	ras-related C3 botulinum toxin substrate 1 (rho small gtp binding protein rac1) [Lichtheimia corymbifera [illegible]
c49030.graph_c2	2432.47793	2372.10496	2596.92043	60.9923576	56.6549037	48.5136152	3.47E-134	-6.492745	down	PREDICTED: probable xyloglucan endotransglucosylase/hydrolase protein 28 [Sesamum [illegible]
c49032.graph_c0	17.067669	17.748962	18.142975	84.5963502	83.2875618	78.8088431	4.05E-17	1.202553	up	PREDICTED: tyrosine--tRNA ligase, cytoplasmic-like [Sesamum indicum]
c49034.graph_c1	8.22911111	7.20409471	7.81130146	46.8871748	37.6942024	36.1024263	4.005E-08	1.363666	up	PREDICTED: probable NOT transcription complex subunit VIP2 isoform X1 [Sesamum indicum]
c49035.graph_c1	11.325483	12.1186736	12.2117203	1.67899805	1.14344669	1.70738557	3.572E-21	-3.990603	down	PREDICTED: histone-lysine N-methyltransferase, H3 lysine-9 specific SUVH5-like isoform X1 [Sesamum [illegible]
c49038.graph_c1	28.0918035	25.9105099	26.0429075	1.66371439	2.4152828	2.30278699	5.065E-93	-4.672135	down	PREDICTED: E3 ubiquitin-protein ligase RING1-like [Sesamum indicum]
c49038.graph_c2	1.16361174	0.98829557	1.08748157	45.7709395	55.3026529	58.8499467	2.53E-102	4.604252	up	ATP-citrate synthase [Camellia sinensis]
c49045.graph_c1	8.4857651	8.25924319	8.49089008	40.6640894	51.9170013	49.3524915	3.255E-21	1.469941	up	hypothetical protein MIMGU_mgv1a008892mg [Erythranthe guttata]
c49047.graph_c0	0.25494122	0.55292576	0.49141434	3.75789568	4.72747883	5.22461403	3.094E-07	2.373764	up	PREDICTED: uncharacterized protein LOC105176173 [Sesamum indicum]
c49050.graph_c0	0	0	0	1.71096334	4.26958153	1.11643026	0.0002802	Inf	up	PREDICTED: CBS domain-containing protein CBSX3, mitochondrial-like [Pyrus x bretschneideri]
c49051.graph_c0	59.9717302	58.9440628	56.1870667	260.279799	247.15138	253.440021	3.331E-15	1.103488	up	PREDICTED: ethylene-responsive transcription factor RAP2-12-like [Sesamum indicum]
c49051.graph_c3	6.38798023	6.80741073	5.57727591	3.21001311	3.56468464	3.55778554	1.51E-25	-1.879167	down	alcohol dehydrogenase class III [Solanum lycopersicum]
c49054.graph_c0	0	0	0	1.94863057	3.83377021	5.03650788	1.035E-08	Inf	up	short chain dehydrogenase, putative [Ricinus communis]
c49058.graph_c0	0	0	0	1.8142482	4.04007195	1.48252696	8.769E-06	Inf	up	PREDICTED: proliferation-associated protein 2G4-like isoform X2 [Solanum lycopersicum]
c49059.graph_c1	20.3157567	17.4309427	18.6475369	144.382115	159.092441	175.126747	3.762E-31	2.065594	up	PREDICTED: 60S ribosomal protein L35a-3-like [Sesamum indicum]
c49059.graph_c2	46.0043692	44.8957786	43.7713168	40.6944378	41.3228159	43.1067453	2.838E-13	-1.123676	down	PREDICTED: thiosulfate/3-mercaptopyruvate sulfurtransferase 1, mitochondrial-like [Sesamum indicum]
c49059.graph_c5	23.1102402	21.1041376	19.2773724	90.2051675	107.828123	104.359838	9.147E-09	1.232855	up	unknown [Glycine max]
c49060.graph_c2	1.43217298	0.86016425	1.04289276	8.04840812	8.52631248	9.07896521	2.781E-07	1.926139	up	PREDICTED: probable serine/threonine-protein kinase DDB_G0291350 [Sesamum indicum]
c49060.graph_c3	21.159166	20.9125305	21.3740917	84.8334371	104.928985	119.093361	8.082E-12	1.260903	up	PREDICTED: 1,4-alpha-glucan-branching enzyme 2-2, chloroplastic/amyloplastic-like isoform X1 [Nicotiana [illegible]

#ID	0 h-1 FPKM	0 h-2 FPKM	0 h-3 FPKM	48 h-1 FPKM	48 h-2 FPKM	48 h-3 FPKM	FDR	log2FC	regulated	nr_annotation
c49060.graph_c5	6.59052495	5.15573585	5.26649014	2.19394725	2.37827273	2.72394568	1.306E-10	-2.239825	down	PREDICTED: uncharacterized protein At1g15400-like [Sesamum indicum]
c49064.graph_c0	218.66595	216.272591	220.475587	147.021952	168.202031	176.54617	1.422E-18	-1.434875	down	PREDICTED: phosphoglucan, water dikinase, chloroplastic [Sesamum indicum]
c49072.graph_c0	13.4745374	14.3609506	12.7244061	10.3144506	10.2954551	11.5067596	4.584E-16	-1.354128	down	PREDICTED: magnesium transporter MRS2-I-like [Sesamum indicum]
c49074.graph_c0	4.37574243	5.82016487	4.6216457	0.49699964	0.9360193	0.19654546	1.22E-21	-4.206698	down	PREDICTED: uncharacterized protein LOC105167219 [Sesamum indicum]
c49075.graph_c0	5.39123566	4.22979434	5.49242983	1.29762144	1.75389259	1.23424443	9.312E-15	-2.840118	down	-
c49078.graph_c3	4.64072708	4.54992117	5.39167384	30.8937274	30.4325338	33.0650314	8.901E-23	1.674894	up	hypothetical protein EUGRSUZ_H01869 [Eucalyptus grandis]
c49080.graph_c0	11.8418121	13.0516472	12.2223235	12.3891889	11.4650271	12.8568273	2.725E-09	-1.032606	down	hypothetical protein MIMGU_mgv1a006759mg [Erythranthe guttata]
c49080.graph_c1	211.977807	213.744404	211.812821	120.5539	91.1574238	74.5978333	1.247E-49	-2.164999	down	hypothetical protein MIMGU_mgv1a009220mg [Erythranthe guttata]
c49080.graph_c2	1.42076941	1.19464136	0.87635757	1.09948058	0.94290609	0.65220747	0.0038364	-1.381055	down	PREDICTED: uncharacterized protein LOC105158955 [Sesamum indicum]
c49081.graph_c0	0	0	0	1.46407123	2.83912952	1.1040015	7.209E-07	Inf	up	S-methyl-5'-thioadenosine phosphorylase [Galdieria sulphuraria]
c49082.graph_c0	7.6777854	8.83643818	8.38870264	4.98888115	6.33489769	5.53364324	4.381E-12	-1.584285	down	PREDICTED: chitin elicitor receptor kinase 1-like isoform X3 [Populus euphratica]
c49084.graph_c0	0.14016878	0.28061827	0.36024486	6.13379951	6.566417	5.4003672	3.432E-10	3.510076	up	PREDICTED: uncharacterized protein LOC105173906 [Sesamum indicum]
c49085.graph_c0	101.41198	97.0057601	98.2855975	20.2208103	20.3832981	20.5793015	7.33E-110	-3.295279	down	-
c49085.graph_c1	33.6047021	30.5007184	32.6919676	13.6997919	14.7978422	14.3411676	1.102E-33	-2.194772	down	PREDICTED: cellulose synthase A catalytic subunit 2 [UDP-forming]-like [Nicotiana sylvestris]
c49086.graph_c0	4.32450516	3.39287107	5.05651527	2.97903916	3.57418664	2.84235025	5.981E-06	-1.46516	down	PREDICTED: uncharacterized protein LOC105159651 [Sesamum indicum]
c49091.graph_c0	0	0	0	2.4069545	3.51469454	2.19957725	5.862E-12	Inf	up	-
c49094.graph_c0	88.2778485	84.7222765	79.1623751	44.37875	45.2987392	44.4786038	4.142E-42	-1.92709	down	PREDICTED: uridine-cytidine kinase C-like isoform X1 [Sesamum indicum]
c49097.graph_c1	11.4378988	11.6163219	11.8503857	3.90759634	5.55825987	5.56703586	1.538E-36	-2.239456	down	PREDICTED: probable galacturonosyltransferase 14 [Sesamum indicum]
c49099.graph_c0	131.282506	133.492378	116.369645	36.2099729	40.2087539	36.4595988	5.698E-81	-2.772921	down	PREDICTED: uncharacterized protein LOC105161154 isoform X1 [Sesamum indicum]
c49103.graph_c5	16.4305875	17.1247313	14.1540462	158.70986	178.731164	174.901622	7.019E-63	2.406984	up	PREDICTED: V-type proton ATPase catalytic subunit A [Sesamum indicum]
c49105.graph_c0	0.10531272	0	0	13.9710167	17.6784922	32.3732464	4.844E-10	8.242913	up	predicted protein [Micromonas sp. RCC299]

#ID	0 h-1 FPKM	0 h-2 FPKM	0 h-3 FPKM	48 h-1 FPKM	48 h-2 FPKM	48 h-3 FPKM	FDR	log2FC	regulated	nr_annotation
c49108.graph_c0	0	0	0	1.81929642	3.23398427	0.74080633	9.131E-05	Inf	up	predicted protein [Micromonas pusilla CCMP1545]
c49109.graph_c0	12.8755917	12.205651	12.3270534	55.4599696	57.2135484	62.4538117	9.083E-18	1.208553	up	PREDICTED: transmembrane protein 56-B-like [Sesamum indicum]
c49112.graph_c0	358.668528	352.973443	347.767126	92.3056071	88.0970377	85.8981817	2.265E-76	-3.008158	down	PREDICTED: THO complex subunit 4D [Solanum lycopersicum]
c49114.graph_c0	5.4511327	5.00628997	5.44071561	38.7371896	36.6469523	34.6633437	8.006E-29	1.775003	up	PREDICTED: signal recognition particle subunit SRP68 [Sesamum indicum]
c49115.graph_c1	0.46621198	0.46667882	0.68468669	2.69974498	4.16007629	3.43953777	0.0013504	1.642174	up	ALA-interacting subunit 5 [Arabidopsis thaliana]
c49119.graph_c0	43.201845	43.689934	45.0633665	17.6040043	20.7046917	19.7501816	2.996E-53	-2.205021	down	PREDICTED: pentatricopeptide repeat-containing protein At1g62680, mitochondrial-like [Sesamum indicum]
c49119.graph_c1	45.3333087	46.8687501	47.5605041	42.6366814	40.9421115	38.6034393	3.652E-15	-1.210499	down	PREDICTED: cyclin-2-like isoform X3 [Nicotiana sylvestris]
c49121.graph_c0	4.1242365	3.91108386	1.67362332	89.188631	127.532944	125.97877	2.145E-33	4.125699	up	PREDICTED: uncharacterized protein LOC105160017 [Sesamum indicum]
c49124.graph_c0	0	0	0	8.393718	8.6005813	5.38724445	8.683E-18	Inf	up	-
c49128.graph_c4	12.2497484	12.8202852	11.7228724	4.36730666	4.84046777	5.42081119	5.645E-45	-2.35021	down	PREDICTED: uncharacterized protein LOC105173091 [Sesamum indicum]
c49132.graph_c3	1.79096041	1.51263601	2.01377316	0	0.05462255	0	2.714E-20	-7.659828	down	-
c49134.graph_c0	1.2336301	2.14759198	1.58526351	0.93884825	0.05234715	0.48364193	3.471E-07	-2.741352	down	retrotransposon protein, putative, unclassified [Oryza sativa Japonica Group]
c49138.graph_c0	0	0	0	2.09898257	3.74504003	2.26085408	2.573E-10	Inf	up	-
c49138.graph_c1	109.845277	111.347893	106.94925	67.8726648	75.7046881	74.8889618	6.232E-29	-1.605984	down	PREDICTED: uncharacterized protein LOC105179211 isoform X1 [Sesamum indicum]
c49138.graph_c2	29.5557684	30.2277262	28.6972984	8.88479967	8.37450225	8.40162262	8.775E-81	-2.801666	down	hypothetical protein MIMGU_mgv1a026550mg, partial [Erythranthe guttata]
c49142.graph_c0	268.274287	287.897936	262.634416	1589.64515	1313.00922	1084.57255	1.988E-05	1.272245	up	PREDICTED: 4-coumarate--CoA ligase-like 7 [Sesamum indicum]
c49142.graph_c4	10.6228811	10.7155248	10.3872584	54.1904921	56.448786	59.3887401	5.695E-22	1.403738	up	hypothetical protein MIMGU_mgv1a002611mg [Erythranthe guttata]
c49145.graph_c0	0	0	0	1.01005767	1.48615781	0.79888288	9.173E-10	Inf	up	hypothetical protein SELMODRAFT_166421 [Selaginella moellendorffii]
c49146.graph_c0	18.8429163	20.460241	17.1002073	15.0545546	10.8054815	10.9956154	1.444E-18	-1.623385	down	PREDICTED: beta-galactosidase 3 [Sesamum indicum]
c49148.graph_c0	0	0	0	1.59301513	1.32073542	1.60234076	4.331E-10	Inf	up	--
c49149.graph_c0	0	0	0	1.13602362	1.03499218	0.80866159	3.694E-12	Inf	up	--
c49150.graph_c0	0	0	0	1.34682237	1.56947449	1.37815362	8.473E-10	Inf	up	predicted protein [Physcomitrella patens]
c49152.graph_c0	143.379752	132.474897	143.607577	131.797461	119.270311	111.269763	5.382E-18	-1.226417	down	PREDICTED: xyloglucan galactosyltransferase KATAMARI1 homolog [Sesamum indicum]
c49154.graph_c0	87.9182565	90.2744968	89.7898008	88.1695329	79.1308223	76.8316566	3.079E-16	-1.149645	down	PREDICTED: uncharacterized protein LOC105158432 [Sesamum indicum]

#ID	0 h-1 FPKM	0 h-2 FPKM	0 h-3 FPKM	48 h-1 FPKM	48 h-2 FPKM	48 h-3 FPKM	FDR	log2FC	regulated	nr_annotation
c49154.graph_c1	102.157883	108.387619	101.910049	102.385553	88.6838098	80.2857719	4.217E-16	-1.216969	down	hypothetical protein MIMGU_mgv1a005704mg [Erythranthe guttata]
c49158.graph_c0	0	0	0	22.8996653	42.9387754	22.8357282	3.806E-19	Inf	up	S-adenosyl-methionine synthetase [Undaria pinnatifida]
c49159.graph_c0	19.1985957	19.0516719	18.6276379	111.27008	124.302894	139.871265	1.764E-31	1.702555	up	PREDICTED: DNA ligase 1-like isoform X2 [Sesamum indicum]
c49159.graph_c1	0	0.6754867	0.43357941	7.46015871	7.40918771	4.28704448	3.979E-06	3.091222	up	-
c49160.graph_c0	472.266599	439.129256	451.413504	302.921343	251.269474	213.151817	4.698E-28	-1.84077	down	PREDICTED: G-type lectin S-receptor-like serine/threonine-protein kinase At1g11300 [Sesamum indicum]
c49161.graph_c0	20.2874361	20.4244621	18.4289052	6.55227974	6.25859055	5.30450722	2.52E-39	-2.721126	down	hypothetical protein CICLE_v10004556mg [Citrus clementina]
c49162.graph_c0	123.546295	120.695891	122.63301	491.798989	497.240823	589.076631	1.34E-13	1.085863	up	PREDICTED: uncharacterized protein At1g04910-like [Sesamum indicum]
c49163.graph_c0	0.95841634	1.26463206	0.8117383	3.53182258	6.16503382	7.58865147	0.0026735	1.484419	up	putative retroelement pol polyprotein [Arabidopsis thaliana]
c49165.graph_c0	202.012994	190.75215	186.988449	157.378365	145.986797	136.232837	1.29E-22	-1.414021	down	PREDICTED: UDP-D-apiose/UDP-D-xylose synthase 2 isoform X1 [Phoenix dactylifera]
c49168.graph_c0	19.5656038	20.2420905	18.0526815	82.982682	99.6994349	146.619023	0.0001466	1.486054	up	PREDICTED: kinesin-like protein NACK1 [Sesamum indicum]
c49169.graph_c1	57.2586257	52.1416039	64.3821508	57.1462457	31.8220543	40.7438989	2.983E-07	-1.431337	down	hypothetical protein (mitochondrion) [Vicia faba]
c49169.graph_c2	303.595306	327.392522	335.249452	386.501771	190.717027	252.253479	5.511E-07	-1.226422	down	Cell wall-associated hydrolase, partial [Medicago truncatula]
c49170.graph_c0	20.5873319	20.7043961	18.4487127	6.7462365	8.46333894	7.34548345	4.649E-48	-2.424755	down	PREDICTED: type I inositol 1,4,5-trisphosphate 5-phosphatase 1-like isoform X2 [Sesamum indicum]
c49171.graph_c0	0	0	0	2.70405513	7.16154633	2.1052955	0.0004368	Inf	up	-
c49172.graph_c1	0	0	0	9.06522565	11.7658865	8.06618327	7.758E-25	Inf	up	predicted protein [Hordeum vulgare subsp. vulgare]
c49172.graph_c2	0	0	0	5.52884024	6.7294752	4.08216219	2.276E-12	Inf	up	heat shock protein S [Schizosaccharomyces japonicus yFS275]
c49175.graph_c0	0.06271153	0	0.0805868	24.5539295	57.2874086	19.4832207	8.72E-08	8.429019	up	60S ribosomal protein L7a, putative [Ricinus communis]
c49177.graph_c0	0	0	0	20.9034141	33.9606119	26.5115585	2.04E-33	Inf	up	40S ribosomal protein S12 [Blumeria graminis f. sp. hordei DH14]
c49179.graph_c2	36.4475922	37.3449011	38.1433725	17.5877946	18.3383859	18.7629682	1.7E-46	-2.052143	down	hypothetical protein MIMGU_mgv1a003978mg [Erythranthe guttata]
c49183.graph_c0	1.43862561	1.23434244	1.18844413	0.47334134	0.55159253	0.7581173	2.324E-05	-2.135946	down	-
c49183.graph_c1	7.72243628	8.58048773	8.15722909	3.32954481	4.4015244	4.10207394	1.098E-33	-2.06988	down	PREDICTED: mitochondrial fission protein ELM1 [Sesamum indicum]
c49185.graph_c0	0	0	0	30.4295392	50.1711523	26.6885288	2.187E-21	Inf	up	hypothetical protein CHLNCDRAFT_59606 [Chlorella variabilis]
c49189.graph_c0	20.1221532	16.9169011	18.3370415	11.2680988	10.4931701	11.9668582	9.255E-26	-1.732022	down	PREDICTED: josephin-like protein [Sesamum indicum]
c49191.graph_c0	0	0	0	5.29891978	10.395487	1.65896282	0.0001502	Inf	up	putative salivary secreted peptide [Trichosporon asahii var. asahii CBS 8904]
c49192.graph_c0	1.45165346	1.78401264	1.90237142	8.30221161	6.99967794	6.11481162	3.972E-05	1.044406	up	PREDICTED: 2-oxoisovalerate dehydrogenase subunit alpha 1, mitochondrial isoform X1 [Sesamum indicum]

#ID	0 h-1 FPKM	0 h-2 FPKM	0 h-3 FPKM	48 h-1 FPKM	48 h-2 FPKM	48 h-3 FPKM	FDR	log2FC	regulated	nr_annotation
c49193.graph_c0	1.527031	1.22856231	1.44879746	0.74941712	0.7103464	0.87740374	7.925E-08	-1.865457	down	PREDICTED: probable ubiquitin conjugation factor E4 [Sesamum indicum]
c49194.graph_c1	0.17018834	0.13628701	0.13121925	1.53652828	1.561326	1.08817452	9.419E-05	2.244476	up	-
c49197.graph_c1	49.0470471	51.2891769	48.7045885	243.915051	217.131647	214.452544	5.448E-16	1.165482	up	PREDICTED: LOW QUALITY PROTEIN: uncharacterized protein LOC101209660 [Cucumis sativus]
c49197.graph_c2	2.13997294	1.80132465	1.06248066	0	0	0.03986849	4.678E-16	-8.006223	down	-
c49199.graph_c0	36.1157866	38.600282	29.3574707	19.2010424	23.7186748	27.7710782	2.263E-17	-1.578077	down	PREDICTED: ABC transporter C family member 3-like [Sesamum indicum]
c49213.graph_c0	0	0	0	1.64957926	3.58951945	1.9834942	8.501E-08	Inf	up	predicted protein [Bathycoccus prasinos]
c49217.graph_c0	0	0	0	2.4112192	4.55648724	1.39166811	6.586E-05	Inf	up	conserved unknown protein [Ectocarpus siliculosus]
c49218.graph_c0	16.3324169	14.0230768	15.3261823	15.1416183	14.4786291	13.7973997	1.244E-13	-1.089361	down	PREDICTED: F-box protein 7 [Sesamum indicum]
c49221.graph_c0	76.4187236	74.4084694	75.0094027	61.6771238	69.5830092	81.5875173	1.312E-14	-1.10625	down	PREDICTED: mannosyl-oligosaccharide 1,2-alpha-mannosidase MNS3 [Sesamum indicum]
c49225.graph_c0	0	0	0	1.22617353	1.94847326	1.04144902	6.331E-08	Inf	up	PREDICTED: V-type proton ATPase subunit E3 [Tarenaya hassleriana]
c49230.graph_c0	34.3969378	35.601853	36.1374839	25.3330082	26.7801903	24.8735532	1.321E-25	-1.481573	down	PREDICTED: protein NLP7 isoform X1 [Sesamum indicum]
c49230.graph_c1	39.8531658	41.0831385	41.0500256	12.0479636	18.312642	20.0421235	2.42E-47	-2.30082	down	PREDICTED: uncharacterized protein LOC105156041 [Sesamum indicum]
c49231.graph_c0	1.06036903	1.06143083	0.93679863	11.7225734	13.7769344	16.8955209	3.33E-13	2.772328	up	PREDICTED: methyl-CpG-binding domain-containing protein 13-like isoform X1 [Sesamum indicum]
c49233.graph_c1	9.14926237	9.11034825	9.56619673	45.9939039	47.9046785	48.956576	2.91E-20	1.341186	up	PREDICTED: pentatricopeptide repeat-containing protein At4g02750 [Sesamum indicum]
c49233.graph_c4	71.7541522	73.4450319	74.4457562	44.1600436	56.3969605	56.7704409	4.628E-19	-1.503719	down	-
c49238.graph_c0	19.7238033	20.0019772	18.3458967	17.6000419	15.117657	13.3113497	2.977E-19	-1.347822	down	PREDICTED: guanine nucleotide-binding protein alpha-1 subunit [Sesamum indicum]
c49238.graph_c1	265.148778	266.512525	279.593719	326.16042	258.022124	195.059547	5.425E-09	-1.069107	down	PREDICTED: uncharacterized protein LOC105157579 [Sesamum indicum]
c49239.graph_c1	57.7586141	58.1576098	53.7923488	40.2083834	43.3394274	43.7313138	4.052E-24	-1.433188	down	hypothetical protein MIMGU_mgv1a000365mg [Erythranthe guttata]
c49244.graph_c1	25.2409747	25.8404827	25.8625178	20.1707643	20.7619501	20.8868791	3.013E-16	-1.333936	down	PREDICTED: protein ENHANCED DISEASE RESISTANCE 2 [Sesamum indicum]
c49245.graph_c0	4.99249682	5.64934336	5.29980719	89.58858	97.2385735	101.601522	4.513E-29	3.157878	up	-
c49245.graph_c1	0.2508391	0.70305278	0.58020872	6.7478088	4.16178934	4.81155263	7.836E-06	2.344812	up	PREDICTED: protein PLANT CADMIUM RESISTANCE 6 [Sesamum indicum]
c49245.graph_c3	85.9601126	92.9550067	71.1150471	30.0583011	29.3936541	30.4489457	1.694E-38	-2.490676	down	hypothetical protein JCGZ_25510 [Jatropha curcas]

#ID	0 h-1 FPKM	0 h-2 FPKM	0 h-3 FPKM	48 h-1 FPKM	48 h-2 FPKM	48 h-3 FPKM	FDR	log2FC	regulated	nr_annotation
c49246.graph_c0	0.96759881	0.70137662	1.02902298	21.9011476	24.5530023	22.9610725	1.542E-86	3.664291	up	PREDICTED: uncharacterized protein LOC105164785 isoform X2 [Sesamum indicum]
c49248.graph_c0	0	0	0	2.72892871	5.595424	4.50389034	2.932E-13	Inf	up	-
c49249.graph_c2	5.21867007	5.51411221	5.29044481	26.2825266	27.2772323	26.760848	4.343E-09	1.307688	up	PREDICTED: 15 kDa selenoprotein [Sesamum indicum]
c49250.graph_c0	0.02842689	0	0	0.9689821	1.33170038	0.72237913	3.674E-09	5.73421	up	ADP-glucose pyrophosphorylase beta subunit IbAGPb1A [Ipomoea batatas]
c49251.graph_c0	0	0	0	1.19933789	5.03986052	2.95248609	2.816E-05	Inf	up	-
c49255.graph_c1	21.9081039	21.3548273	23.5683389	17.6464911	20.0262878	19.5879228	6.06E-17	-1.243545	down	PREDICTED: uncharacterized protein LOC105170478 isoform X2 [Sesamum indicum]
c49255.graph_c2	1233.81734	1168.46724	1280.4424	813.11441	526.675448	367.118111	1.923E-31	-2.115529	down	PREDICTED: rop guanine nucleotide exchange factor 7-like isoform X1 [Sesamum indicum]
c49256.graph_c0	2.12491994	0.96683988	1.48942159	17.8854811	17.4392399	18.6855566	9.267E-11	2.543579	up	PREDICTED: uncharacterized protein LOC105160829 [Sesamum indicum]
c49257.graph_c0	18.0550498	19.5696303	18.805	14.2232227	12.6546903	12.9621153	5.251E-22	-1.517662	down	PREDICTED: regulation of nuclear pre-mRNA domain-containing protein 1B-like [Sesamum indicum]
c49257.graph_c1	16.9616975	16.4120265	15.815226	87.518433	82.3407271	82.5825577	7.572E-22	1.343936	up	PREDICTED: uncharacterized protein LOC105163499 [Sesamum indicum]
c49257.graph_c2	2.6646774	2.13387653	2.22574037	11.7424836	14.9536915	14.7806511	3.077E-08	1.54122	up	PREDICTED: uncharacterized protein LOC105174437 [Sesamum indicum]
c49258.graph_c1	42.6192204	38.4618197	42.3361376	32.6779143	20.6268878	17.6115731	1.132E-22	-1.806242	down	PREDICTED: probable serine/threonine-protein kinase NAK [Sesamum indicum]
c49260.graph_c1	0	0	0	15.8156428	14.972335	9.88080655	7.745E-12	Inf	up	predicted protein [Physcomitrella patens]
c49260.graph_c2	37004.0695	37864.8467	39206.8195	9069.95781	3937.25311	4954.4692	7.751E-14	-3.669722	down	hypothetical protein MTR_5g051050, partial [Medicago truncatula]
c49262.graph_c0	38.0841397	39.5639604	36.6981106	27.1624287	29.618679	26.4796066	7.551E-26	-1.475406	down	PREDICTED: probable LRR receptor-like serine/threonine-protein kinase At1g56140 [Nicotiana tomentosiformis]
c49266.graph_c0	35.7557528	36.7780297	36.5911811	14.8467377	14.1781172	12.9349224	8.61E-62	-2.394841	down	PREDICTED: C2 and GRAM domain-containing protein At1g03370 isoform X1 [Sesamum indicum]
c49268.graph_c1	1.92083326	2.16310127	3.18828127	172.240764	184.577211	188.752378	6.55E-165	5.204999	up	PREDICTED: auxin-responsive protein IAA14 [Sesamum indicum]
c49270.graph_c0	10.7192501	10.1701586	10.4208303	61.2266886	50.5343355	47.2585521	1.448E-08	1.33158	up	PREDICTED: ultraviolet-B receptor UVR8 isoform X2 [Sesamum indicum]
c49273.graph_c1	25.5562439	26.4904791	25.124098	23.9300473	23.0339581	21.350051	1.931E-11	-1.191625	down	hypothetical protein MIMGU_mgv1a007843mg [Erythranthe guttata]
c49275.graph_c0	14.2492332	12.783624	11.6413216	59.9857806	64.4072428	71.0119391	2.047E-19	1.319276	up	PREDICTED: cyclic pyranopterin monophosphate synthase, mitochondrial [Sesamum indicum]
c49275.graph_c1	3.37275195	2.5932587	3.70598648	2.83700786	3.91187614	3.04525067	0.0093063	-1.005712	down	hypothetical protein CICLE_v10022328mg [Citrus clementina]

#ID	0 h-1 FPKM	0 h-2 FPKM	0 h-3 FPKM	48 h-1 FPKM	48 h-2 FPKM	48 h-3 FPKM	FDR	log2FC	regulated	nr_annotation
c49275.graph_c2	31.8384719	27.6126244	30.4642262	14.2048298	14.7430555	11.5625899	2.011E-46	-2.166678	down	hypothetical protein MIMGU_mgv1a010148mg [Erythranthe guttata]
c49277.graph_c0	7.4625693	9.28615305	8.05006675	1.73451976	1.83751336	2.83417982	3.27E-32	-2.974626	down	PREDICTED: cyclic pyranopterin monophosphate synthase, mitochondrial [Sesamum indicum]
c49280.graph_c0	49.5763299	46.0856972	48.5182005	44.6154399	41.6025799	37.3334219	1.575E-18	-1.237994	down	PREDICTED: nuclear pore complex protein NUP35 [Sesamum indicum]
c49281.graph_c0	15.6738367	15.3750791	16.9545544	8.92584365	10.811295	10.5308189	2.464E-29	-1.687123	down	PREDICTED: peroxisome biogenesis protein 1 isoform X2 [Sesamum indicum]
c49284.graph_c2	63.9069131	67.2292996	56.1713899	52.8469331	59.2914964	53.1764279	4.783E-18	-1.197454	down	PREDICTED: probable glycerol-3-phosphate dehydrogenase [NAD(+)] 1, cytosolic [Sesamum indicum]
c49285.graph_c0	3.15042979	3.40928051	3.22779992	15.2571131	18.2405756	14.0293737	3.767E-07	1.260778	up	PREDICTED: carotenoid 9,10(9',10')-cleavage dioxygenase 1 [Nelumbo nucifera]
c49287.graph_c0	271.677864	218.103826	265.328776	7.75890842	5.56813148	6.45842166	4.21E-109	-6.266751	down	PREDICTED: peroxygenase-like [Solanum lycopersicum]
c49287.graph_c1	178.244103	178.37497	185.343425	19.1293584	21.0312755	22.1879863	3.35E-145	-4.139813	down	PREDICTED: probable helicase DDB_G0274399 isoform X1 [Sesamum indicum]
c49288.graph_c0	6.55130786	5.84677388	5.17292998	2.61780381	2.77324571	3.42925618	1.842E-07	-2.012363	down	PREDICTED: GPI-anchored protein LORELEI-like [Pyrus x bretschneideri]
c49288.graph_c3	26.2166147	27.550227	23.291934	7.01389653	4.38360912	3.73410872	1.412E-91	-3.353285	down	PREDICTED: probable protein phosphatase 2C 25 [Sesamum indicum]
c49289.graph_c0	146.296335	153.475486	135.25508	100.81988	106.902114	110.040089	4.922E-24	-1.470891	down	PREDICTED: BTB/POZ domain-containing protein At5g17580 [Sesamum indicum]
c49290.graph_c0	189.868841	179.435037	181.311991	95.080608	96.0042312	93.7820466	9.003E-41	-1.96789	down	PREDICTED: probable acyl-activating enzyme 18, peroxisomal isoform X2 [Vitis vinifera]
c49292.graph_c0	0	0	0	1.35010964	1.73876867	0.61243736	2.086E-06	Inf	up	phosphoenolpyruvate carboxykinase [Neocallimastix frontalis]
c49295.graph_c0	0	0	0	2.37823189	4.01245472	2.78232754	9.737E-15	Inf	up	unknown [Picea sitchensis]
c49303.graph_c0	0	0	0	1.17887243	0.90989274	0.92907319	8.932E-14	Inf	up	PREDICTED: delta-1-pyrroline-5-carboxylate synthase [Brachypodium distachyon]
c49306.graph_c0	22.6785808	22.3437894	23.1060099	2.96121088	2.25223662	2.53352657	1.06E-153	-4.149753	down	unnamed protein product [Coffea canephora]
c49307.graph_c0	54.4230837	56.9109965	51.6257427	1648.3562	1087.81311	791.565076	3.736E-06	3.430642	up	PREDICTED: pentatricopeptide repeat-containing protein At3g21470 [Sesamum indicum]
c49309.graph_c0	0	0	0	1.25436984	1.32885275	0.93010895	2.281E-11	Inf	up	hypothetical protein EMIHUDRAFT_452099 [Emiliania huxleyi CCMP1516]
c49310.graph_c0	0	0	0	1.24100868	1.23957261	1.10424206	1.829E-11	Inf	up	hypothetical protein PNEG_02956 [Pneumocystis murina B123]
c49311.graph_c0	0	0	0	2.09781347	2.38112035	1.99995194	1.541E-13	Inf	up	glucokinase [Galdieria sulphuraria]
c49315.graph_c0	0	0	0	3.35533099	4.86821041	2.79084334	5.021E-12	Inf	up	PREDICTED: T-complex protein 1 subunit beta [Phoenix dactylifera]
c49316.graph_c1	0	0	0	3.07641438	3.76605725	3.71066391	6.112E-13	Inf	up	hypothetical protein MG5_02709 [Candida albicans P57072]

#ID	0 h-1 FPKM	0 h-2 FPKM	0 h-3 FPKM	48 h-1 FPKM	48 h-2 FPKM	48 h-3 FPKM	FDR	log2FC	regulated	nr_annotation
c49321.graph_c0	19.0625171	20.768629	18.7013154	13.8059202	17.5375016	16.7463967	1.52E-18	-1.304517	down	PREDICTED: serine hydroxymethyltransferase 6-like [Sesamum indicum]
c49322.graph_c1	2.90130667	4.44900545	3.56964314	19.904373	23.0744037	22.0625859	3.512E-09	1.554143	up	-
c49327.graph_c0	53.1430843	54.9243317	49.7175641	26.7870535	28.6749561	25.4997991	8.65E-44	-1.979661	down	PREDICTED: chromodomain-helicase-DNA-binding protein 4-like isoform X4 [Sesamum indicum]
c49328.graph_c0	115.048104	123.448797	105.787476	33.217712	32.5732271	31.3180487	4.071E-77	-2.841484	down	PREDICTED: uncharacterized protein LOC105166619 [Sesamum indicum]
c49330.graph_c0	186.515212	187.99822	181.881244	135.761987	147.362075	134.706023	8.607E-23	-1.431213	down	PREDICTED: membrin-11 [Sesamum indicum]
c49334.graph_c0	2.90482883	2.38339145	2.08058822	2.14966838	2.09140989	2.06890584	0.0022198	-1.237741	down	PREDICTED: uncharacterized protein LOC104590989 [Nelumbo nucifera]
c49340.graph_c0	12.2940564	10.3184154	10.4512156	131.251919	91.3740715	93.8793636	1.421E-08	2.249761	up	PREDICTED: uncharacterized protein LOC104715674 [Camelina sativa]
c49343.graph_c0	59.796504	54.5843116	59.8105951	39.3811255	25.0556244	22.4102321	6.317E-35	-2.011616	down	PREDICTED: serine/threonine-protein kinase HT1 [Sesamum indicum]
c49344.graph_c0	0	0	0	1.19079783	0.72469488	0.92430116	1.049E-10	Inf	up	lipoyltransferase and lipoate-protein ligase [Lichtheimia corymbifera JMRC:FSU:9682]
c49345.graph_c0	0	0	0	3.04106212	2.84012697	1.56658456	3.727E-10	Inf	up	-
c49348.graph_c1	0.7656602	1.44088255	1.29875284	11.3142329	13.3311266	14.4608123	1.529E-20	2.455953	up	PREDICTED: LRR receptor-like serine/threonine-protein kinase GSO2 isoform X2 [Sesamum indicum]
c49350.graph_c0	104.359473	105.154074	104.206274	50.3604899	56.1543445	57.8321032	2.297E-41	-1.952392	down	PREDICTED: autophagy-related protein 18f [Sesamum indicum]
c49351.graph_c0	0	0	0	19.7105002	77.1456964	1.76116665	0.0050344	Inf	up	-
c49353.graph_c5	101.752668	99.3385947	95.9004453	54.4154318	55.6432319	51.0454864	2.405E-38	-1.89907	down	PREDICTED: nuclear poly(A) polymerase 4-like isoform X2 [Sesamum indicum]
c49354.graph_c0	6.67941997	6.86205862	9.7127032	4.69610534	9.60680806	6.19580519	0.007187	-1.213657	down	-
c49354.graph_c1	22.9590578	23.0316851	23.2585397	14.8927654	16.2852366	15.1416601	7.243E-27	-1.598893	down	fructose-6-phosphate 2-kinase/fructose-2,6-bisphosphatase [Solanum tuberosum]
c49354.graph_c2	1.45919827	1.13606845	1.66678005	7.169646	7.75360746	9.30344902	0.0036088	1.484192	up	PREDICTED: 6-phosphofructo-2-kinase/fructose-2,6-bisphosphatase [Sesamum indicum]
c49355.graph_c0	352.786116	364.423489	360.325599	297.165634	292.807109	269.873326	2.757E-18	-1.342186	down	PREDICTED: protein-tyrosine-phosphatase MKP1-like isoform X1 [Sesamum indicum]
c49355.graph_c1	0	0	0	547.275685	929.492889	1206.21381	6.102E-35	Inf	up	PREDICTED: (RS)-norcoclaurine 6-O-methyltransferase-like [Sesamum indicum]
c49355.graph_c3	2.63241936	2.24260028	2.66302627	1079.86539	1906.04278	2365.73568	1.922E-24	8.442398	up	PREDICTED: (RS)-norcoclaurine 6-O-methyltransferase-like [Sesamum indicum]
c49357.graph_c0	0	0	0	4.04734144	12.0397703	2.73698806	0.0001037	Inf	up	PREDICTED: omega-6 fatty acid desaturase, endoplasmic reticulum isozyme 2-like [Solanum tuberosum]

#ID	0 h-1 FPKM	0 h-2 FPKM	0 h-3 FPKM	48 h-1 FPKM	48 h-2 FPKM	48 h-3 FPKM	FDR	log2FC	regulated	nr_annotation
c49360.graph_c2	2.44384408	2.96763197	3.06321255	1.99306791	2.28736493	2.43024201	1.567E-06	-1.358844	down	PREDICTED: rho GTPase-activating protein 2-like [Sesamum indicum]
c49362.graph_c1	4.52959179	4.64748067	4.87484025	30.1496027	40.7813288	51.2867879	9.759E-09	2.095411	up	-
c49364.graph_c1	7.75482441	8.62128324	9.78888017	5.02626442	6.26240382	5.56932887	3.925E-14	-1.657096	down	Cationic amino acid transporter 2 isoform 1 [Theobroma cacao]
c49366.graph_c0	23.5762998	22.6509606	27.1450093	1.31003648	1.14646397	1.23323723	2.37E-100	-5.330768	down	PREDICTED: MLO-like protein 3 [Sesamum indicum]
c49366.graph_c1	124.967387	116.459377	122.476653	788.627542	796.375153	821.830901	1.495E-32	1.707614	up	PREDICTED: 40S ribosomal protein S26-like [Musa acuminata subsp. malaccensis]
c49369.graph_c4	98.3436651	100.658719	78.001304	41.2802728	42.3330544	37.5851464	4.22E-33	-2.207033	down	hypothetical protein MIMGU_mgv1a000459mg [Erythranthe guttata]
c49370.graph_c2	0	0	0	1.98778673	2.04200631	1.71512308	2.157E-11	Inf	up	hypothetical protein M569_09445 [Genlisea aurea]
c49372.graph_c0	0	0	0	28.1981755	49.4843049	25.8972011	7.274E-20	Inf	up	small subunit ribosomal protein S6e, cytoplasmic [Guillardia theta CCMP2712]
c49375.graph_c0	2.25532291	2.05641067	2.29558751	1.23430727	1.19863231	1.26302106	7.914E-08	-1.855924	down	PREDICTED: protein-L-isoaspartate O-methyltransferase 1 isoform X1 [Sesamum indicum]
c49376.graph_c0	0.71572306	1.09573137	1.1361401	3.91743806	5.99908306	5.55643332	0.0006406	1.364369	up	hypothetical protein MIMGU_mgv1a024598mg, partial [Erythranthe guttata]
c49376.graph_c1	1.55062723	1.02117102	1.31093249	5.88332487	7.28804143	6.08792569	1.53E-06	1.290636	up	PREDICTED: uncharacterized protein LOC105163785 [Sesamum indicum]
c49378.graph_c0	53.6530519	49.6193589	49.9097965	10.3056634	10.7137066	9.77709347	1.83E-112	-3.331394	down	PREDICTED: uncharacterized protein LOC105170029 [Sesamum indicum]
c49378.graph_c2	143.158693	145.333797	137.015717	17.0044986	14.6094579	13.986614	6.92E-166	-4.235572	down	PREDICTED: uncharacterized protein LOC105158337 [Sesamum indicum]
c49380.graph_c0	0.29697507	0.17836347	0.53427462	2.35289815	3.0722871	3.94374802	3.42E-05	2.180969	up	PREDICTED: uncharacterized protein LOC105167081 [Sesamum indicum]
c49380.graph_c1	28.9285242	26.5411712	33.3337753	15.5316173	13.9109952	14.8564352	2.309E-21	-2.021061	down	PREDICTED: sedoheptulose-1,7-bisphosphatase, chloroplastic [Sesamum indicum]
c49380.graph_c2	0.38560504	0.17155163	0.36705018	185.121861	210.080504	234.249386	1.11E-268	8.389007	up	PREDICTED: gamma-cadinene synthase-like [Sesamum indicum]
c49382.graph_c1	0	0	0	5.40841409	9.24122408	5.17459606	2.625E-13	Inf	up	-
c49384.graph_c0	0	0	0	2.13494737	2.26171787	1.39567102	3.488E-12	Inf	up	-
c49386.graph_c0	21.7069217	22.2178101	20.6893399	14.9035976	15.8387566	20.2606477	1.342E-19	-1.36116	down	hypothetical protein MIMGU_mgv1a000652mg [Erythranthe guttata]
c49386.graph_c1	529.836112	515.772176	543.915697	149.274585	186.157265	192.417656	5.139E-60	-2.612554	down	chloroplast chlorophyll a/b binding protein cab-BO3 [Bambusa oldhamii]
c49388.graph_c1	20.0880459	19.5703694	19.4993711	193.514601	143.989655	151.768313	2.24E-11	2.036321	up	PREDICTED: zinc finger BED domain-containing protein DAYSLEEPER [Sesamum indicum]
c49389.graph_c0	0	0	0	0.66276025	2.07123665	0.76663611	0.0006879	Inf	up	putative Sec61/secY [Ectocarpus siliculosus]

#ID	0 h-1 FPKM	0 h-2 FPKM	0 h-3 FPKM	48 h-1 FPKM	48 h-2 FPKM	48 h-3 FPKM	FDR	log2FC	regulated	nr_annotation
c49392.graph_c1	5.53459272	4.68780635	4.10317544	0	0.06925115	0	4.71E-32	-8.742721	down	-
c49392.graph_c2	11.0139353	9.73256395	9.68386617	61.9728873	72.441764	71.1050111	2.77E-33	1.736406	up	PREDICTED: auxin response factor 6-like isoform X2 [Nicotiana tomentosiformis]
c49392.graph_c4	16.2406554	17.255807	14.5864789	6.57314594	6.93910336	6.54405117	3.374E-44	-2.278112	down	PREDICTED: uncharacterized protein LOC105175883 [Sesamum indicum]
c49398.graph_c0	18.6131658	18.2039785	16.6414523	13.938628	13.9528891	13.8915089	2.543E-20	-1.371783	down	PREDICTED: SWI/SNF complex subunit SWI3C [Sesamum indicum]
c49405.graph_c0	8.42025304	9.34609251	8.09686693	32.0054024	53.2766172	52.8713431	1.766E-05	1.391789	up	hypothetical protein MIMGU_mgv1a009036mg [Erythranthe guttata]
c49405.graph_c1	0	0	0	4.80589558	5.42219576	6.71372347	1.536E-33	Inf	up	PREDICTED: subtilisin-like protease SBT5.3 [Sesamum indicum]
c49405.graph_c2	14.4546168	13.4286169	12.8562348	6.13458389	7.1962851	7.40228004	4.174E-28	-1.994124	down	unnamed protein product [Coffea canephora]
c49407.graph_c0	0	0	0	0.81553696	1.0472273	0.52775264	1.349E-08	Inf	up	-
c49409.graph_c0	15.1282886	14.5832847	16.3160918	14.2571522	16.2336784	14.489165	2.824E-12	-1.053271	down	PREDICTED: copper chaperone for superoxide dismutase, chloroplastic/cytosolic [Sesamum indicum]
c49409.graph_c1	21.5742805	20.9018763	18.0283342	15.9681373	13.6524534	15.7796292	4.711E-18	-1.428086	down	PREDICTED: bifunctional riboflavin kinase/FMN phosphatase-like [Sesamum indicum]
c49414.graph_c0	34.4797943	33.5515373	35.6560621	31.9798455	30.9773123	30.3787871	1.781E-16	-1.168851	down	PREDICTED: E3 ubiquitin-protein ligase RGLG2-like isoform X1 [Sesamum indicum]
c49415.graph_c0	237.305843	232.929864	236.419897	44.8121685	41.2647574	40.4980866	1.59E-112	-3.496688	down	-
c49416.graph_c0	0	0	0	8.23711327	20.3439748	5.37773608	6.166E-06	Inf	up	--
c49417.graph_c0	15.59661	15.3923371	12.4205563	13.3567402	12.8634969	17.016797	0.0001859	-1.022384	down	-
c49417.graph_c1	20.8426626	19.5735837	20.0397356	20.1479367	15.8943006	13.992952	3.61E-19	-1.284343	down	PREDICTED: exocyst complex component EXO70A1 [Sesamum indicum]
c49417.graph_c6	143.411344	149.959197	132.326661	88.2585323	91.0934802	84.1851192	2.694E-31	-1.708217	down	PREDICTED: omega-amidase,chloroplastic isoform X2 [Nicotiana sylvestris]
c49418.graph_c0	28.9705904	30.5409174	26.8952884	12.5567201	13.8589049	12.7623434	7.47E-36	-2.1588	down	PREDICTED: putative pentatricopeptide repeat-containing protein At1g13630 [Sesamum indicum]
c49421.graph_c0	4.56497822	6.12769078	4.058241	3.51612033	4.14851957	4.46595558	3.884E-06	-1.300853	down	PREDICTED: pentatricopeptide repeat-containing protein At1g69290 [Sesamum indicum]
c49421.graph_c1	81.3259596	74.4969283	72.9869367	39.0272994	43.5395331	49.08835	9.545E-37	-1.81693	down	hypothetical protein MIMGU_mgv1a006935mg [Erythranthe guttata]
c49428.graph_c1	7.59385121	6.89103892	6.61199963	50.0177808	57.4207379	55.9664194	2.661E-34	1.934642	up	PREDICTED: magnesium-protoporphyrin IX monomethyl ester [oxidative] cyclase, chloroplastic [Sesamum indicum]
c49428.graph_c2	0.06473118	0.58316399	0.33272852	15.5049921	18.3209275	22.286248	5.502E-31	4.81368	up	PREDICTED: GATA transcription factor 12-like [Sesamum indicum]
c49428.graph_c3	113.335929	112.473509	117.17849	575.840278	739.467218	697.166399	4.455E-23	1.53068	up	PREDICTED: serine carboxypeptidase-like [Sesamum indicum]

#ID	0 h-1 FPKM	0 h-2 FPKM	0 h-3 FPKM	48 h-1 FPKM	48 h-2 FPKM	48 h-3 FPKM	FDR	log2FC	regulated	nr_annotation
c49429.graph_c1	283.76474	265.577878	262.929304	276.800387	272.618258	249.341941	7.186E-13	-1.04005	down	hypothetical protein VITISV_037044 [Vitis vinifera]
c49430.graph_c0	0.05039946	0	0	6.59324012	14.4613599	5.74268262	1.243E-07	8.048808	up	hypothetical protein A1O3_04014 [Capronia epimyces CBS 606.96]
c49431.graph_c0	8.63703729	8.81520923	8.79209743	40.8459442	37.4533317	42.6193811	6.074E-14	1.187068	up	PREDICTED: biotin synthase [Sesamum indicum]
c49433.graph_c0	0	0	0	1.41861599	1.3826235	1.06031182	2.211E-10	Inf	up	serine/threonine-protein phosphatase pp2a catalytic subunit [Grosmannia clavigera kw1407]
c49434.graph_c0	216.622941	223.61775	201.209839	138.934876	140.087701	131.376878	1.472E-27	-1.660477	down	PREDICTED: protein misato homolog 1 isoform X1 [Sesamum indicum]
c49437.graph_c0	2.22736594	1.6827142	1.94417213	15.7577713	11.5869755	13.7627948	1.724E-10	1.799602	up	FIZZY-related 3 isoform 1 [Theobroma cacao]
c49437.graph_c1	0	0	0	3.45219907	5.00235829	3.00113112	1.32E-16	Inf	up	hypothetical protein MIMGU_mgv1a008893mg [Erythranthe guttata]
c49439.graph_c0	8.04482393	8.41677542	8.79804339	52.6179564	42.7660208	36.7312828	2.795E-06	1.374065	up	PREDICTED: random slug protein 5 [Sesamum indicum]
c49439.graph_c2	2.77931852	3.55490759	1.78576717	49.2182983	50.1817709	41.2626923	5.5E-20	3.103416	up	hypothetical protein EUGRSUZ_H01271 [Eucalyptus grandis]
c49441.graph_c0	0	0	0	1.36284461	2.00264678	3.05118623	3.269E-08	Inf	up	unnamed protein product [Chondrus crispus]
c49447.graph_c0	0.39736854	0.53638202	0.61121379	0.31616157	0.48771214	0.54783108	0.0007192	-1.222143	down	PREDICTED: uncharacterized protein LOC105155261 [Sesamum indicum]
c49449.graph_c0	0.26384192	0.10564245	0.13561893	14.4868761	18.1281046	14.7070938	2.63E-35	5.534264	up	PREDICTED: protein DA1-related 2 isoform X2 [Sesamum indicum]
c49451.graph_c1	8.09365087	7.24893908	5.83896804	60.1744488	57.649942	54.9396331	1.507E-14	2.014754	up	hypothetical protein M569_10128, partial [Genlisea aurea]
c49452.graph_c0	0.57054479	0.97905618	0.68080317	16.8574828	18.653936	20.8792968	1.471E-35	3.640403	up	Rop guanine nucleotide exchange factor 1 [Morus notabilis]
c49454.graph_c0	0	0	0	2.10941262	1.89087199	1.51599215	2.211E-10	Inf	up	NADH-malate dehydrogenase [Pyropia haitanensis]
c49456.graph_c0	0.68837441	0.76562635	0.88458837	5.7780426	6.79296745	10.4079319	6.819E-06	2.270576	up	PREDICTED: uncharacterized protein LOC105161573 [Sesamum indicum]
c49456.graph_c3	1.68681526	1.83896513	2.38224066	11.2780703	13.675526	11.3494501	9.09E-18	1.596019	up	PREDICTED: pentatricopeptide repeat-containing protein At1g31920 [Sesamum indicum]
c49460.graph_c0	0	0	0	1.20937687	0.93953798	1.00435652	1.221E-11	Inf	up	acetyl-CoA C-acyltransferase-like protein [Zea mays]
c49463.graph_c0	0	0	0	4.59702254	6.12675905	4.2223515	4.054E-18	Inf	up	Molecular chaperones GRP78/BiP/KAR2, HSP70 superfamily [Ectocarpus siliculosus]
c49466.graph_c0	2.40642745	1.83947562	2.02408661	10.762323	10.8035513	12.5890032	1.64E-06	1.427802	up	PREDICTED: growth hormone-regulated TBC protein 1-like [Solanum lycopersicum]
c49470.graph_c0	0	0	0	1.77906026	1.88469862	1.45635735	5.771E-11	Inf	up	hypothetical protein CHLNCDRAFT_144079 [Chlorella variabilis]
c49471.graph_c0	14.0316067	14.6214675	13.4375777	8.2141212	9.66428369	10.7107026	7.048E-24	-1.578632	down	PREDICTED: pleiotropic drug resistance protein 1-like [Sesamum indicum]
c49472.graph_c0	0.19057451	0.06358845	0.2448958	7.08131014	29.7591647	7.02993776	0.0072189	5.41582	up	cathepsin B-like cysteine protease [Guillardia theta CCMP2712]
c49474.graph_c1	10.2082867	9.46929023	10.7936726	5.88023147	5.58007447	5.59009717	8.967E-27	-1.854958	down	PREDICTED: lipoxygenase 6, chloroplastic [Sesamum indicum]

#ID	0 h-1 FPKM	0 h-2 FPKM	0 h-3 FPKM	48 h-1 FPKM	48 h-2 FPKM	48 h-3 FPKM	FDR	log2FC	regulated	nr_annotation
c49477.graph_c0	0	0	0	8.856183	8.64136371	9.0552927	1.039E-32	Inf	up	hypothetical protein SELMODRAFT_144212 [Selaginella moellendorffii]
c49479.graph_c0	0.05179035	0	0	33.1125783	56.5101664	21.6093039	1.089E-12	10.06755	up	hypothetical protein PISMIDRAFT_677145 [Pisolithus microcarpus 441]
c49480.graph_c0	0	0	0	1.81044123	2.6954874	1.43676624	1.137E-09	Inf	up	hypothetical protein JCGZ_17423 [Jatropha curcas]
c49485.graph_c0	0	0	0	3.03687523	4.11271064	5.79439392	1.267E-15	Inf	up	predicted protein [Physcomitrella patens]
c49487.graph_c0	231.275744	219.907485	219.599078	161.974756	164.946291	154.949668	1.486E-23	-1.494115	down	dead box ATP-dependent RNA helicase, putative [Ricinus communis]
c49488.graph_c0	2.3323775	0.94971377	1.26999891	0	0	0	4.822E-10	-Inf	down	Fructose-bisphosphate aldolase A [Rozella allomycis CSF55]
c49492.graph_c1	0	0	0	1.07455261	1.82137311	1.14735654	8.853E-10	Inf	up	TPR1 [Medicago sativa]
c49494.graph_c0	218.726948	233.092716	219.219013	95.0864173	106.56672	105.624608	1.125E-45	-2.146146	down	hypothetical protein MIMGU_mgv1a000743mg [Erythranthe guttata]
c49495.graph_c0	85.7893061	86.2121234	78.7172924	42.3769339	37.3015235	34.6411523	7.792E-52	-2.146343	down	PREDICTED: E3 ubiquitin-protein ligase RHF2A [Sesamum indicum]
c49495.graph_c1	10.9415026	10.8015391	11.2648178	5.49639203	7.3572791	5.61454438	7.944E-28	-1.859223	down	PREDICTED: BI1-like protein [Sesamum indicum]
c49495.graph_c2	3.32389025	2.580292	3.05095041	22.4976821	31.6787817	32.2517091	3.972E-18	2.246982	up	PREDICTED: transcription factor bHLH143 [Sesamum indicum]
c49495.graph_c3	1.54483092	1.34467638	1.81254551	9.96239305	13.4382561	16.848013	3.224E-08	2.070787	up	-
c49495.graph_c4	20.628298	15.5388594	12.9863304	9.5020668	11.0830843	7.51545058	3.45E-08	-1.820527	down	-
c49496.graph_c0	0	0	0	1.80526013	2.98820969	1.15453503	6.525E-06	Inf	up	eukaryotic translation initiation factor 2 gamma [Volvox carteri f. nagariensis]
c49499.graph_c0	0	0	0	0.55945944	0.64009379	0.6969251	1.612E-11	Inf	up	PREDICTED: uncharacterized protein LOC102585254 [Solanum tuberosum]
c49500.graph_c1	117.000092	116.371935	103.367516	58.9203857	25.7594285	17.1319077	4.895E-46	-2.721054	down	PREDICTED: pectinesterase-like isoform X1 [Sesamum indicum]
c49500.graph_c5	3.9267669	3.73047569	3.36854338	14.528567	16.3159633	14.7396883	1.841E-11	1.030215	up	PREDICTED: brefeldin A-inhibited guanine nucleotide-exchange protein 5-like [Sesamum indicum]
c49500.graph_c6	40.8543465	39.5447419	40.5800724	194.946914	203.230764	216.560391	1.387E-21	1.326458	up	ADP/ATP carrier 2 [Theobroma cacao]
c49500.graph_c8	0.03320279	0.03323603	0.04266689	3.64004001	6.25414231	5.71568066	8.542E-20	6.131362	up	PREDICTED: tyrosine/DOPA decarboxylase 1-like [Sesamum indicum]
c49500.graph_c9	64.8536178	62.4836653	70.6064096	4.05346281	4.36397596	2.87366821	7.19E-191	-5.148866	down	hypothetical protein MIMGU_mgv1a019874mg [Erythranthe guttata]
c49506.graph_c0	59.4601009	59.1284323	59.440856	38.6535777	40.2131718	34.6912049	3.989E-32	-1.665786	down	PREDICTED: cullin-4 [Sesamum indicum]
c49508.graph_c0	3.61428907	3.54093146	3.09633636	1.51136174	1.40096644	1.38682561	1.862E-13	-2.267987	down	PREDICTED: uncharacterized protein LOC103341621 [Prunus mume]
c49510.graph_c0	48.9470891	48.6587011	50.5954309	8.89611876	10.0556134	9.70048222	4.17E-108	-3.390709	down	PREDICTED: nuclear valosin-containing protein-like [Sesamum indicum]
c49511.graph_c0	16.1213842	16.5432193	15.4506612	94.6093494	120.026443	128.812096	6.673E-23	1.813758	up	PREDICTED: L-ascorbate oxidase homolog [Sesamum indicum]

#ID	0 h-1 FPKM	0 h-2 FPKM	0 h-3 FPKM	48 h-1 FPKM	48 h-2 FPKM	48 h-3 FPKM	FDR	log2FC	regulated	nr_annotation
c49511.graph_c1	0.56510112	0.50910029	0.36308866	28.2690152	30.8300275	34.8351878	5.578E-58	5.014394	up	hypothetical protein MIMGU_mgv1a004264mg [Erythranthe guttata]
c49513.graph_c1	0	0	0	6.89454531	9.79519931	11.3421078	5.657E-33	Inf	up	-
c49514.graph_c0	39.179932	37.6256715	38.8145107	10.0383595	11.1835575	9.8320288	1.229E-88	-2.915144	down	ankyrin repeat BTB/POZ domain-containing protein [Populus trichocarpa]
c49516.graph_c0	0	0	0	3.90927697	6.17513057	1.21124638	2.508E-05	Inf	up	ATP-grasp fold, succinyl-CoA synthetase-type domain-containing protein [Rozella allomycis CSF55]
c49519.graph_c0	1.73371723	2.23782134	1.87612234	11.6848769	12.1115438	13.3517142	3.804E-08	1.648703	up	PREDICTED: synaptotagmin-5 [Sesamum indicum]
c49521.graph_c0	81.4697907	83.8754225	77.4490231	68.989149	74.7015085	75.3949595	1.105E-15	-1.166587	down	PREDICTED: rubisco accumulation factor 1, chloroplastic [Sesamum indicum]
c49522.graph_c0	0	0	0	1.40677101	2.12081625	1.25173639	1.625E-10	Inf	up	predicted protein [Hordeum vulgare subsp. vulgare]
c49523.graph_c2	75.5408435	80.3946368	71.7848138	29.2063065	29.6000765	29.6605587	4.08E-61	-2.380952	down	PREDICTED: pentatricopeptide repeat-containing protein At2g34400 [Sesamum indicum]
c49527.graph_c0	2.00300178	2.38331079	2.47680691	14.8668178	16.0077808	16.0785533	1.206E-12	1.753804	up	PREDICTED: fe-S cluster assembly factor HCF101, chloroplastic [Sesamum indicum]
c49528.graph_c0	156.84621	160.007235	149.925649	91.6858876	95.038048	92.1746022	1.561E-35	-1.760309	down	hypothetical protein MIMGU_mgv1a021345mg [Erythranthe guttata]
c49528.graph_c1	0.14508285	0.04840938	0	9.08886532	8.21258739	11.2586756	1.895E-26	6.206368	up	PREDICTED: WAT1-related protein At1g21890-like [Sesamum indicum]
c49528.graph_c2	1107.05383	1085.44464	1217.30885	251.000693	262.043186	281.047796	9.575E-61	-3.122228	down	PREDICTED: LOW QUALITY PROTEIN: pre-mRNA-splicing factor 38B-like [Sesamum indicum]
c49533.graph_c0	0	0	0	7.69726373	11.8522058	10.8309435	1.664E-19	Inf	up	Rotamase cyclophilin 2 [Theobroma cacao]
c49537.graph_c0	24.4895838	17.9551482	19.4714571	81.7190579	93.1262413	89.6996646	3.309E-11	1.07693	up	-
c49537.graph_c1	59.0317979	53.5093324	54.5397344	218.299947	237.918649	270.397529	2.85E-15	1.101115	up	PREDICTED: putative disease resistance RPP13-like protein 1 [Sesamum indicum]
c49537.graph_c2	13.7156755	13.4548214	14.2008641	10.3252332	13.9215141	11.6608424	1.877E-11	-1.226586	down	hypothetical protein M569_00556, partial [Genlisea aurea]
c49537.graph_c3	0.61164438	0.97961095	1.25757958	5.63486693	7.99907406	7.52080607	0.0002588	1.863212	up	-
c49537.graph_c4	94.5968071	92.4953245	92.3043667	61.4581764	63.0903329	56.4054293	6.276E-31	-1.643589	down	PREDICTED: uncharacterized protein LOC105163060 [Sesamum indicum]
c49540.graph_c0	0	0	0	4.01180728	8.97227154	3.2722085	2.062E-06	Inf	up	--
c49541.graph_c1	0.23905379	0.65805621	0.49918958	10.2407854	10.4697432	9.7490627	1.678E-25	3.427303	up	PREDICTED: CBL-interacting serine/threonine-protein kinase 1-like [Sesamum indicum]
c49542.graph_c0	8.50682335	8.14656701	7.78984437	7.34336869	7.48522937	7.74206746	4.801E-08	-1.13224	down	PREDICTED: putative Peroxidase 48 [Nicotiana sylvestris]
c49544.graph_c0	0	0	0	4.26051303	3.8155335	5.7450917	1.111E-18	Inf	up	phenylpropenal double-bond reductase [Pinus pinaster]
c49545.graph_c0	0	0	0	2.09392304	2.64078281	0.93158006	5.632E-06	Inf	up	Lysine--tRNA ligase [Rozella allomycis CSF55]

#ID	0 h-1 FPKM	0 h-2 FPKM	0 h-3 FPKM	48 h-1 FPKM	48 h-2 FPKM	48 h-3 FPKM	FDR	log2FC	regulated	nr_annotation
c49548.graph_c1	6.92287001	7.01902714	7.48346679	3.91427174	4.87164318	5.67492756	1.113E-12	-1.590544	down	PREDICTED: 4-hydroxy-3-methylbut-2-enyl diphosphate reductase, chloroplastic-like [Sesamum indicum]
c49548.graph_c2	5.73956642	5.68147691	6.84289003	33.3416525	30.871248	32.2549138	5.716E-17	1.382776	up	hypothetical protein MIMGU_mgv1a010472mg [Erythranthe guttata]
c49549.graph_c0	18.8673114	20.0549468	16.7216065	6.11091011	7.46465117	6.06164825	2.21E-63	-2.520884	down	PREDICTED: uncharacterized protein LOC105168432 [Sesamum indicum]
c49552.graph_c0	0	0	0	22.1524154	50.1434245	19.0020541	5.051E-10	Inf	up	predicted protein [Physcomitrella patens]
c49554.graph_c0	0.70548194	0.82388644	0.65474661	7.18540332	9.90715808	12.2408765	6.388E-11	2.724134	up	PREDICTED: uncharacterized protein At4g17910 isoform X1 [Sesamum indicum]
c49555.graph_c0	0	0	0	1.38612804	1.64464674	0.56734946	4.278E-06	Inf	up	acid protease, partial [Dacryopinax sp. DJM-731 SS1]
c49557.graph_c0	0	0	0	1.01739012	2.01742326	1.25344746	1.088E-09	Inf	up	heat shock protein SSA1 [Spathaspora passalidarum NRRL Y-27907]
c49561.graph_c1	0.37164562	0.31690402	0.35376247	3.2336284	4.1241985	4.33281973	1.901E-14	2.465175	up	PREDICTED: probable inactive leucine-rich repeat receptor-like protein kinase At3g03770 [Sesamum indicum]
c49562.graph_c0	0	0	0	0.76609109	1.37344412	0.78653451	2.253E-08	Inf	up	predicted protein, partial [Ostreococcus lucimarinus CCE9901]
c49563.graph_c0	2.39110668	1.43610061	0.73744002	11.9834683	14.001873	15.994227	2.763E-07	2.189265	up	-
c49564.graph_c2	90.9966295	95.7442115	86.5154596	625.626346	520.133762	533.554845	3.476E-16	1.606339	up	PREDICTED: uncharacterized protein LOC105178313 [Sesamum indicum]
c49567.graph_c0	2.64733068	0.75082811	1.13397491	28.1692222	31.5212881	23.2202085	4.159E-24	3.181951	up	predicted protein [Hordeum vulgare subsp. vulgare]
c49570.graph_c2	4.23444696	5.16589993	2.89076075	1.58480105	1.29146503	1.19320084	1.19E-07	-2.602892	down	-
c49570.graph_c3	14.2008194	12.9441583	12.5383697	3.4223012	4.31390172	3.87388449	2.871E-57	-2.793156	down	PREDICTED: DNA polymerase kappa isoform X1 [Sesamum indicum]
c49571.graph_c2	25.2670932	33.5799508	20.450877	310.519923	198.715655	156.046461	0.0011404	2.065646	up	PREDICTED: cannabidiolic acid synthase-like [Sesamum indicum]
c49571.graph_c4	68.4677408	70.6447687	66.3012319	27.6574445	23.3488816	23.4098545	3.088E-66	-2.478441	down	PREDICTED: scarecrow-like protein 21 [Sesamum indicum]
c49573.graph_c0	0.83758354	0.52401391	0.134541	5.30500156	7.86800824	7.03762538	1.566E-06	2.744975	up	-
c49575.graph_c0	0	0	0	1.03409105	2.10671937	0.93782276	2.409E-07	Inf	up	--
c49576.graph_c0	1.35331597	1.5882351	1.25932265	20.8295396	21.1099343	22.7419245	1.544E-49	2.927722	up	PREDICTED: DNA (cytosine-5)-methyltransferase 1B-like [Sesamum indicum]
c49579.graph_c0	0	0	0	4.99390561	7.3735472	3.52706557	1.433E-13	Inf	up	hypothetical protein GUITHDRAFT_78647, partial [Guillardia theta CCMP2712]
c49581.graph_c0	18.318413	16.9262364	15.4367329	72.729573	75.088438	77.6225511	5.055E-15	1.13648	up	PREDICTED: magnesium-chelatase subunit ChlD, chloroplastic [Sesamum indicum]
c49585.graph_c0	0	0	0	25.8999954	40.0024245	21.6475869	8.253E-23	Inf	up	60S ribosomal protein L5 [Rozella allomycis CSF55]
c49587.graph_c1	15.9137105	15.8693061	15.9570004	80.1065497	85.333821	86.0528285	1.656E-22	1.378517	up	hypothetical protein MIMGU_mgv1a009436mg [Erythranthe guttata]

#ID	0 h-1 FPKM	0 h-2 FPKM	0 h-3 FPKM	48 h-1 FPKM	48 h-2 FPKM	48 h-3 FPKM	FDR	log2FC	regulated	nr_annotation
c49588.graph_c0	7.74047362	7.06770293	5.89071105	6.51366278	6.74877796	6.16203724	1.577E-13	-1.106538	down	PREDICTED: uncharacterized protein LOC105173993 [Sesamum indicum]
c49592.graph_c3	45.4048134	48.163729	45.9809059	38.4168908	34.2604281	31.6647521	6.155E-23	-1.433879	down	PREDICTED: DNA annealing helicase and endonuclease ZRANB3 isoform X1 [Sesamum indicum]
c49593.graph_c0	1.15023478	1.08365794	0.95641585	9.66170893	10.4335138	8.20868203	4.414E-07	2.133813	up	-
c49594.graph_c0	1.11344498	0.86288511	1.06157679	11.8791727	12.2339987	11.30609	5.886E-17	2.525597	up	hypothetical protein MIMGU_mgv1a0042561mg, partial [Erythranthe guttata]
c49595.graph_c0	15.9516749	15.9833334	15.1423354	4.28745564	4.7714353	5.43341684	6.261E-69	-2.719598	down	PREDICTED: uncharacterized protein LOC105169231 [Sesamum indicum]
c49599.graph_c0	13.7381142	5.44885449	8.32736824	0	0	0	2.689E-13	-Inf	down	--
c49600.graph_c2	96.4460323	99.0295844	86.5096975	69.6037853	71.3996305	67.7729904	1.036E-24	-1.449908	down	PREDICTED: transcription initiation factor TFIID subunit 15b [Sesamum indicum]
c49602.graph_c0	0	0	0	4.9131308	7.44585045	4.12880345	4.771E-13	Inf	up	sporamin A [Ipomoea trifida]
c49603.graph_c0	730.120419	773.602999	730.564182	74.71413	80.0093738	78.742867	6.28E-102	-4.276916	down	PREDICTED: GRIP and coiled-coil domain-containing protein 2-like [Sesamum indicum]
c49606.graph_c0	0	0	0	0.55972302	1.18591734	0.4382736	8.376E-05	Inf	up	-
c49607.graph_c1	25.1913399	27.4172837	22.9153595	22.6171588	24.7051664	22.2271832	8.185E-15	-1.135894	down	PREDICTED: uncharacterized protein LOC105164316 isoform X1 [Sesamum indicum]
c49610.graph_c2	112.899898	116.166523	105.436952	89.2068332	83.9453848	73.0737244	5.391E-23	-1.456336	down	PREDICTED: alpha,alpha-trehalose-phosphate synthase [UDP-forming] 1-like [Sesamum indicum]
c49611.graph_c0	38.1760675	36.995986	33.0701077	29.0780877	28.6797903	26.438867	6.557E-23	-1.377524	down	PREDICTED: nipped-B-like protein isoform X2 [Sesamum indicum]
c49614.graph_c1	0	0	0	3.11313331	4.904699	1.91772505	1.753E-06	Inf	up	-
c49615.graph_c0	0	0	0	0.7034477	1.4663958	0.66630569	3.138E-06	Inf	up	predicted protein [Physcomitrella patens]
c49616.graph_c0	23.0603137	19.6015507	20.0315271	1413.06694	1836.19981	1982.21427	1.431E-76	5.360923	up	hypothetical protein MIMGU_mgv1a012029mg [Erythranthe guttata]
c49618.graph_c2	0	0	0	1.12170462	1.22544472	0.99808602	2.155E-11	Inf	up	hypothetical protein NDAI_0G05590 [Naumovozyma dairenensis CBS 421]
c49618.graph_c3	1.60934337	1.93314586	2.06806995	0.29652642	0.12565351	0.21107794	8.262E-13	-4.154189	down	-
c49623.graph_c1	0	0	0	1.95358867	2.88780043	1.65744068	1.185E-11	Inf	up	predicted protein [Physcomitrella patens]
c49624.graph_c0	0	0	0	20.0166367	17.2907922	13.4342193	1.378E-38	Inf	up	-
c49627.graph_c0	0	0	0	0.70215243	1.6426585	0.80699589	9.47E-06	Inf	up	predicted protein [Physcomitrella patens]
c49632.graph_c0	0	0	0	1.83596897	2.95637949	2.09105171	2.148E-11	Inf	up	hypothetical protein THAOC_09683, partial [Thalassiosira oceanica]
c49634.graph_c0	5.48318464	5.36449705	4.78242716	24.2059	25.7400742	26.2770892	2.845E-14	1.268532	up	PREDICTED: uncharacterized protein At1g04910 [Sesamum indicum]

#ID	0 h-1 FPKM	0 h-2 FPKM	0 h-3 FPKM	48 h-1 FPKM	48 h-2 FPKM	48 h-3 FPKM	FDR	log2FC	regulated	nr_annotation
c49635.graph_c0	7.04059481	7.0476449	7.26979297	2.73906376	2.77072615	2.8687904	1.378E-47	-2.368255	down	PREDICTED: uncharacterized protein LOC105155337 [Sesamum indicum]
c49636.graph_c0	19.5286373	16.1401193	16.4273663	1.1244409	0.81503759	1.73774657	1.515E-67	-4.84165	down	-
c49640.graph_c0	0	0	0	1.66195916	4.08602336	1.78572229	1.625E-06	Inf	up	Sterol methyltransferase [Ectocarpus siliculosus]
c49641.graph_c0	26.1821501	28.1659934	21.1416593	9.8373563	9.85541657	9.76813079	3.497E-31	-2.372362	down	PREDICTED: LOW QUALITY PROTEIN: auxin transport protein BIG [Sesamum indicum]
c49642.graph_c0	0	0	0	2.27032064	4.25092643	1.50334337	3.828E-07	Inf	up	-
c49643.graph_c2	9.02041435	7.85418558	9.53086253	127.581265	134.653815	135.258115	4.692E-88	2.892389	up	PREDICTED: phosphoglucomutase, cytoplasmic [Sesamum indicum]
c49643.graph_c4	41.7463142	43.3194826	35.6860304	38.9132966	40.8190189	40.6188004	1.407E-13	-1.021294	down	PREDICTED: protein NYNRIN-like [Prunus mume]
c49647.graph_c0	5.62087615	3.34073711	3.72438433	0.40455607	0.25714686	0.43196587	1.931E-14	-4.546472	down	PREDICTED: uncharacterized protein LOC105167219 [Sesamum indicum]
c49648.graph_c1	157.417783	145.413425	153.844003	800.296952	742.796214	672.105684	4.035E-16	1.262985	up	PREDICTED: uncharacterized protein LOC105179524 [Sesamum indicum]
c49651.graph_c0	0	0	0	6.74004038	13.5628974	3.3451983	1.828E-06	Inf	up	Os02g0639600 [Oryza sativa Japonica Group]
c49652.graph_c0	0	0	0	1.39312546	3.26059332	0.60538344	0.0011684	Inf	up	-
c49656.graph_c0	0	0	0	4.64275663	7.97879891	5.14092062	1.747E-12	Inf	up	PREDICTED: obg-like ATPase 1 [Pyrus x bretschneideri]
c49658.graph_c1	0	0	0	10.2502512	26.5030736	6.44111007	1.42E-05	Inf	up	-
c49659.graph_c0	48.105363	49.030048	48.4551956	12.1004227	10.6824421	10.4079872	1.888E-84	-3.147801	down	hypothetical protein MIMGU_mgv1a000745mg [Erythranthe guttata]
c49661.graph_c0	0	0	0	2.16141167	2.21079656	1.39267011	1.387E-15	Inf	up	transferrin-like protein IDI-100 [Dunaliella salina]
c49662.graph_c0	0	0	0	3.98140655	3.25414394	2.96783376	2.94E-35	Inf	up	predicted protein [Hordeum vulgare subsp. vulgare]
c49663.graph_c0	0.15239218	0.15254478	0.32638331	4.21180854	6.14750727	5.99622705	5.387E-12	3.662152	up	PREDICTED: uncharacterized protein LOC105174845 isoform X2 [Sesamum indicum]
c49665.graph_c1	0	0	0	1.38069452	3.86565014	1.05302887	0.0010538	Inf	up	--
c49667.graph_c0	0	0	0	2.60759897	3.68324657	2.36856393	4.803E-13	Inf	up	PREDICTED: eukaryotic translation initiation factor 3 subunit D-like [Pyrus x bretschneideri]
c49668.graph_c0	4.20392411	4.96343975	5.84084981	3.21088993	2.6300633	4.43281544	1.867E-07	-1.56798	down	NADH-ubiquinone oxidoreductase chain [Medicago truncatula]
c49671.graph_c1	0.14142313	0.18875299	0.24231243	1.95432317	3.45061404	3.51653323	1.958E-07	2.929771	up	PREDICTED: systemin receptor SR160-like [Sesamum indicum]
c49671.graph_c2	37.6918059	37.214366	33.5741401	15.1726407	15.0689747	16.3296784	4.814E-52	-2.236641	down	PREDICTED: uncharacterized protein LOC104231501 [Nicotiana sylvestris]
c49671.graph_c3	35.6883239	40.5239555	29.5534999	324.113329	253.606774	231.423803	5.634E-09	1.926246	up	PREDICTED: receptor-like protein kinase BRI1-like 3 [Sesamum indicum]
c49671.graph_c5	4.50009095	5.35161536	3.3609407	0.53150991	0.1876901	0.31528955	3.126E-25	-4.672378	down	-
c49672.graph_c0	0	0	0	7.04748293	7.90512735	3.9785378	7.68E-16	Inf	up	nitric oxide dioxygenase [Cladophialophora psammophila CBS 110553]

#ID	0 h-1 FPKM	0 h-2 FPKM	0 h-3 FPKM	48 h-1 FPKM	48 h-2 FPKM	48 h-3 FPKM	FDR	log2FC	regulated	nr_annotation
c49674.graph_c1	0	0	0	4.38430944	9.57078204	3.13272735	8.299E-06	Inf	up	pyrophosphate--fructose-6-phosphate 1-phosphotransferase [Galdieria sulphuraria]
c49674.graph_c2	1.3440458	1.02064195	1.78670904	92.1835421	89.5154086	84.8357569	7.53E-146	4.983762	up	PREDICTED: NAC domain-containing protein 100-like [Sesamum indicum]
c49676.graph_c0	0	0	0	3.06322813	3.32624674	3.20262931	4.957E-12	Inf	up	--
c49679.graph_c2	56.1167668	56.1167863	54.5168739	14.7340514	15.2803315	17.2963566	1.219E-73	-2.836215	down	hypothetical protein MIMGU_mgv1a010138mg [Erythranthe guttata]
c49680.graph_c0	70.0135872	73.9470045	61.7497552	32.8014049	40.6644178	41.0838876	7.773E-40	-1.8646	down	OSJNBa0006B20.7 [Oryza sativa Japonica Group]
c49682.graph_c0	52.9688171	51.8598523	52.818245	36.9948372	38.14252	40.4952178	3.35E-24	-1.465665	down	hypothetical protein MIMGU_mgv1a027053mg [Erythranthe guttata]
c49684.graph_c2	4.49946205	3.54858052	2.45296413	0.37683617	1.46377819	0.44707542	8.466E-10	-3.228046	down	-
c49686.graph_c0	7.96232432	7.16642619	7.24576751	2.34544306	2.0344626	1.62474826	7.838E-53	-2.909885	down	PREDICTED: DNA repair protein REV1 [Sesamum indicum]
c49687.graph_c2	60.988951	83.4833389	68.2567925	0.166727	0.50044329	0.54395984	4.208E-97	-8.490798	down	PREDICTED: uncharacterized protein LOC103716664 [Phoenix dactylifera]
c49689.graph_c1	36.0723116	38.3812911	34.6006034	14.2085271	18.2926183	18.5074524	2.517E-49	-2.117724	down	PREDICTED: TBC1 domain family member 5 homolog A-like [Sesamum indicum]
c49689.graph_c2	4.01115458	4.32562253	3.56031953	18.1338198	23.4885703	23.3556002	1.702E-17	1.428722	up	hypothetical protein MIMGU_mgv1a021246mg [Erythranthe guttata]
c49693.graph_c0	3.33164391	3.50325886	3.41717988	17.7542303	19.7481311	17.8772289	1.296E-18	1.414635	up	PREDICTED: beta-galactosidase 8-like [Sesamum indicum]
c49697.graph_c0	0	0	0	1.12502158	1.24148328	1.37642647	1.128E-11	Inf	up	26S proteasome regulatory subunit N5 [Mucor circinelloides f. circinelloides 1006PhL]
c49702.graph_c0	335.328193	327.490437	321.635988	141.10231	157.420189	148.679327	2.93E-42	-2.157119	down	hypothetical protein MIMGU_mgv1a004852mg [Erythranthe guttata]
c49703.graph_c0	2.4340113	2.90390676	2.50951467	0.24770366	0.53863514	0.31320743	6.734E-37	-3.863013	down	PREDICTED: stromal 70 kDa heat shock-related protein, chloroplastic-like [Sesamum indicum]
c49707.graph_c1	18.2925959	17.133783	15.2233931	10.7533931	9.69163009	11.6033543	1.856E-20	-1.675457	down	PREDICTED: non-structural maintenance of chromosomes element 1 homolog [Sesamum indicum]
c49708.graph_c0	0	0	0	1086.00356	1275.71479	1272.0811	0	Inf	up	-
c49708.graph_c1	33.9388347	30.5462504	32.5215737	17.5470698	18.9887561	17.3400913	7.388E-37	-1.866631	down	PREDICTED: uncharacterized protein LOC105169648 [Sesamum indicum]
c49708.graph_c2	5.34735724	6.49972149	4.95734388	1.724217	1.97570894	3.41280761	3.185E-14	-2.262996	down	PREDICTED: LOW QUALITY PROTEIN: multiple C2 and transmembrane domain-containing protein 1 [Sesamum indicum]
c49716.graph_c0	0.35524791	0.35560364	0.15216921	15.4911374	14.0995758	17.8123203	2.62E-28	4.769655	up	-
c49716.graph_c1	1.92697895	2.36729684	3.03902703	2.09803301	1.79518627	1.57961452	0.0038619	-1.439724	down	NADH dehydrogenase subunit 5 [Arabidopsis thaliana]
c49716.graph_c2	5.67202895	7.15711158	9.29063028	92.6228176	84.4782558	86.8028465	4.405E-60	2.556205	up	NADH dehydrogenase subunit 5 [Beta vulgaris subsp. maritima]
c49717.graph_c0	0	0	0	9.90859763	13.5537641	10.6089985	2.228E-30	Inf	up	--

#ID	0 h-1 FPKM	0 h-2 FPKM	0 h-3 FPKM	48 h-1 FPKM	48 h-2 FPKM	48 h-3 FPKM	FDR	log2FC	regulated	nr_annotation
c49722.graph_c0	3.66879225	4.42579235	4.31159088	1.16997602	1.13233496	1.3366409	5.669E-32	-2.788571	down	hypothetical protein VITISV_035665 [Vitis vinifera]
c49723.graph_c0	0	0	0	1.07000878	2.26708911	1.08809986	8.788E-07	Inf	up	delta-9 fatty acid desaturase [Cryptococcus curvatus]
c49724.graph_c0	0	0	0	3.94158764	7.12810277	3.57805353	3.526E-11	Inf	up	-
c49726.graph_c0	0.02036919	0.04077918	0.05235044	0.52543204	0.37771426	0.63449994	4.191E-06	2.737134	up	unnamed protein product [Coffea canephora]
c49728.graph_c0	1.94088206	1.77388421	1.9519124	8.35727037	7.82403615	7.40166172	0.0017496	1.041081	up	PREDICTED: origin recognition complex subunit 3 [Sesamum indicum]
c49733.graph_c1	0	0	0	3.75719643	4.91444467	2.25149625	5.895E-11	Inf	up	-
c49734.graph_c0	0	0	0	1.46357626	2.87126218	1.20581607	1.113E-05	Inf	up	PREDICTED: ABC transporter E family member 2 [Nicotiana sylvestris]
c49737.graph_c0	0	0.083685	0	2.07951175	6.44578715	3.7006741	4.39E-05	6.169399	up	KLTH0G03806p [Lachancea thermotolerans]
c49738.graph_c0	0	0	0	4.96758694	8.18619825	2.16913737	2.818E-06	Inf	up	F-type H+-transporting ATPase subunit gamma [Galdieria sulphuraria]
c49740.graph_c1	0.70167913	1.05357264	1.40888354	9.61568471	9.28782976	7.04609989	1.399E-08	2.015138	up	PREDICTED: RPM1-interacting protein 4-like [Sesamum indicum]
c49740.graph_c2	506.353189	494.623171	508.834806	37.6520508	41.7939241	49.4533487	5.06E-173	-4.570867	down	galactinol synthase [Boea hygrometrica]
c49740.graph_c3	68.3056307	77.5616299	59.8202329	63.6750719	60.774754	57.9463157	1.729E-14	-1.187628	down	hypothetical protein MIMGU_mgv1a010537mg [Erythranthe guttata]
c49741.graph_c0	0	0	0	2.00469093	1.80516792	1.69457384	1.265E-11	Inf	up	PREDICTED: serine-threonine kinase receptor-associated protein-like [Setaria italica]
c49743.graph_c0	0	0	0	7.834411	15.4352475	6.1208884	2.835E-09	Inf	up	-
c49744.graph_c0	0	0	0	4.67417362	6.33068023	3.05348091	4.982E-11	Inf	up	S-Adenosylmethionine decarboxylase [Ectocarpus siliculosus]
c49747.graph_c0	0.75169278	0.44589362	0.60819388	4.05245669	5.32560095	4.38179434	7.58E-07	1.909197	up	--
c49750.graph_c0	9.78968098	8.16623655	8.38675176	5.01049592	3.53867525	4.01247822	2.602E-06	-2.07883	down	-
c49750.graph_c2	13.4761998	12.4190835	13.0109944	74.556614	88.3782281	99.889961	3.579E-23	1.734602	up	hypothetical protein MIMGU_mgv1a015998mg [Erythranthe guttata]
c49751.graph_c1	34.6220885	38.2947594	36.2255521	17.8865017	19.5319719	19.0317215	1.11E-42	-1.970156	down	PREDICTED: polycomb group protein EMBRYONIC FLOWER 2 isoform X5 [Sesamum indicum]
c49752.graph_c0	6.28200992	6.50267428	5.04539466	2.23603986	2.92618089	2.45775948	1.983E-09	-2.245293	down	PREDICTED: probable protein phosphatase 2C 75 [Populus euphratica]
c49755.graph_c0	0	0	0	3.49221718	3.0698648	1.58673388	3.727E-07	Inf	up	-
c49756.graph_c0	0	0	0	0.44919169	0.83842727	0.49485189	8.283E-08	Inf	up	predicted protein [Physcomitrella patens]
c49757.graph_c0	0	0	0	2.50883285	5.77561297	1.63132859	8.434E-05	Inf	up	S-(hydroxymethyl)glutathione dehydrogenase-like protein [Chaetomium thermophilum var. thermophilum DSM 1495]
c49759.graph_c1	96.6580848	98.2601691	96.5452081	92.7117229	79.388396	69.3724468	2.034E-18	-1.284621	down	PREDICTED: 6-phosphofructo-2-kinase/fructose-2,6-bisphosphatase [Sesamum indicum]

#ID	0 h-1 FPKM	0 h-2 FPKM	0 h-3 FPKM	48 h-1 FPKM	48 h-2 FPKM	48 h-3 FPKM	FDR	log2FC	regulated	nr_annotation
c49762.graph_c2	37.5980002	36.279015	36.8541706	19.6146216	15.0191339	15.2310333	2.27E-51	-2.163044	down	PREDICTED: autophagy-related protein 18a-like [Sesamum indicum]
c49766.graph_c0	0	0	0	3.36088084	3.99347291	2.1822506	6.637E-13	Inf	up	predicted protein [Hordeum vulgare subsp. vulgare]
c49767.graph_c0	0	0	0	3.37273147	9.11859382	4.12642475	1.812E-07	Inf	up	-
c49768.graph_c0	0	0	0	4.03439999	7.3170154	2.69951821	2.227E-08	Inf	up	PREDICTED: thiol protease aleurain-like [Eucalyptus grandis]
c49770.graph_c0	0	0	0	122.799697	244.652058	98.9818488	1.594E-17	Inf	up	Elongation factor 1-alpha [Auxenochlorella protothecoides]
c49771.graph_c1	35.6669464	35.0041895	36.7633146	29.2736805	23.9137485	21.9556164	1.15E-26	-1.528814	down	PREDICTED: LOW QUALITY PROTEIN: DUF21 domain-containing protein At4g14240 [Sesamum indicum]
c49772.graph_c0	14.7893504	13.0742354	13.1539538	47.5953464	54.2327084	67.7137709	4.727E-07	1.026599	up	PREDICTED: long-chain-alcohol oxidase FAO1 [Sesamum indicum]
c49772.graph_c1	7.67555309	6.96949548	6.35999985	36.9403631	37.0870868	41.017877	1.587E-16	1.436774	up	PREDICTED: vacuolar protein sorting-associated protein 25 [Nicotiana tomentosiformis]
c49775.graph_c0	451.031763	419.995523	492.212876	239.906549	205.251787	158.112566	1.295E-36	-2.189677	down	-
c49777.graph_c1	0.77990659	0	0.66814057	3.67233754	4.8207085	6.46421242	1.547E-05	2.34047	up	-
c49779.graph_c0	0	0	0	1.57887933	3.15077041	1.01281855	3.043E-05	Inf	up	PREDICTED: V-type proton ATPase subunit a1 [Brachypodium distachyon]
c49781.graph_c0	0	0	0	1.2807556	1.2017418	1.4652111	1.207E-11	Inf	up	predicted protein [Physcomitrella patens]
c49784.graph_c0	8.0961977	7.8419849	7.74534823	33.7994255	34.0699175	32.7540979	5.13E-14	1.070105	up	hypothetical protein MIMGU_mgv1a000301mg [Erythranthe guttata]
c49784.graph_c1	46.7565191	48.4307929	47.2843138	25.617838	23.9107661	22.6720466	1.046E-44	-1.996215	down	PREDICTED: BTB/POZ domain-containing protein POB1-like isoform X1 [Sesamum indicum]
c49785.graph_c0	863.708163	889.458883	721.286403	143.43044	186.045688	193.916706	4.887E-62	-3.261646	down	-
c49787.graph_c1	100.509869	97.815778	93.5030827	64.8110221	57.1881492	52.8933625	2.885E-35	-1.752281	down	PREDICTED: uncharacterized protein LOC105163549 [Sesamum indicum]
c49788.graph_c0	1.68460848	1.54068211	1.48942279	1.07643686	1.36934114	1.21553022	1.002E-13	-1.384906	down	PREDICTED: uncharacterized protein LOC105155957 isoform X2 [Sesamum indicum]
c49790.graph_c0	5.88601469	6.66791612	5.64514914	30.2341936	30.0118581	24.7133104	1.138E-11	1.208032	up	PREDICTED: UDP-glycosyltransferase 76F1-like [Sesamum indicum]
c49795.graph_c1	35.7040434	37.0489456	35.9934222	14.1973601	14.4234493	14.5446091	2.981E-59	-2.350747	down	PREDICTED: putative receptor-like protein kinase At5g39000 [Sesamum indicum]
c49798.graph_c2	16.1605151	17.7541267	14.7744625	10.8290708	11.8644297	10.6110696	4.764E-27	-1.564911	down	PREDICTED: leukocyte receptor cluster member 8 homolog isoform X1 [Sesamum indicum]
c49799.graph_c0	0	0	0	6.59492231	13.0581395	6.077698	4.183E-09	Inf	up	-
c49801.graph_c0	9.99770143	9.03211799	8.92856829	41.3315502	42.8652521	37.7816706	1.35E-12	1.10895	up	PREDICTED: uncharacterized protein LOC105163678 [Sesamum indicum]

#ID	0 h-1 FPKM	0 h-2 FPKM	0 h-3 FPKM	48 h-1 FPKM	48 h-2 FPKM	48 h-3 FPKM	FDR	log2FC	regulated	nr_annotation
c49804.graph_c0	652.740692	620.700423	647.682178	337.27915	317.68547	283.026675	9.359E-29	-2.049751	down	PREDICTED: histidinol-phosphate aminotransferase, chloroplastic-like [Sesamum indicum]
c49805.graph_c0	0	0	0	5.40537216	9.20304059	3.34004697	3.599E-10	Inf	up	PREDICTED: trifunctional UDP-glucose 4,6-dehydratase/UDP-4-keto-6-deoxy-D-glucose 3,5-epimerase/UDP-4-keto-L-rhamnose-reductase RHM1 [Tarenaya hassleriana]
c49806.graph_c0	3.19524847	2.43463955	3.15612046	18.4968003	13.3349193	9.77336203	0.0098317	1.23439	up	PREDICTED: pentatricopeptide repeat-containing protein At5g66520-like [Sesamum indicum]
c49807.graph_c0	0	0	0	2.26039577	4.12405956	1.92748296	3.491E-09	Inf	up	eukaryotic initiation factor 4A [Ectocarpus siliculosus]
c49808.graph_c0	0	0	0	3.60584565	4.20195186	4.65226668	1.917E-12	Inf	up	-
c49809.graph_c0	3.83310858	2.33046664	2.86887083	0	0	0	6.854E-51	-Inf	down	--
c49811.graph_c0	0	0	0	1.53018874	2.55955172	1.71985609	5.771E-11	Inf	up	DEAD-box ATP-dependent RNA helicase 15 [Arabidopsis thaliana]
c49812.graph_c0	0	0	0	4.93862241	14.6829945	10.4188776	2.234E-10	Inf	up	Short-chain dehydrogenase/reductase SDR [Macrophomina phaseolina MS6]
c49813.graph_c0	0	0	0	0.74035706	1.41924305	0.65876526	8.437E-06	Inf	up	predicted protein [Physcomitrella patens]
c49814.graph_c0	0	0	0	1.53823748	1.78116456	1.33688389	3.917E-12	Inf	up	PREDICTED: serine--tRNA ligase-like [Nelumbo nucifera]
c49817.graph_c0	0.57292792	0.55500157	0.48686512	2.22194593	2.47112546	2.08312408	0.0004284	1.052523	up	PREDICTED: pleiotropic drug resistance protein 2-like [Sesamum indicum]
c49818.graph_c0	75.8049888	74.9820914	77.9369225	49.5072102	50.4552322	50.2518451	5.445E-30	-1.624683	down	PREDICTED: uncharacterized protein LOC105158481 isoform X1 [Sesamum indicum]
c49823.graph_c0	10.8938452	11.1220586	9.82721226	6.69074716	6.75096842	5.80778746	4.141E-33	-1.741433	down	PREDICTED: uncharacterized protein LOC105179488 isoform X2 [Sesamum indicum]
c49823.graph_c1	0.96552072	1.30943473	1.1606851	0.37301641	0.36476829	0.63828414	1.587E-06	-2.342422	down	-
c49823.graph_c2	2.6870455	2.21654184	2.68563544	8.48081651	13.1609271	14.05221	5.692E-05	1.207289	up	PREDICTED: transmembrane protein 87B [Sesamum indicum]
c49825.graph_c0	1.51217241	0.86496378	0.83280061	5.83780206	5.20055468	4.89930246	0.0076021	1.301775	up	PREDICTED: protein CHROMATIN REMODELING 4 [Sesamum indicum]
c49826.graph_c0	0	0	0	0.90474317	1.72991366	1.04065504	7.2E-10	Inf	up	Methylmalonyl-CoA mutase, alpha chain, catalytic domain-containing protein [Rozella allomycis CSF55]
c49829.graph_c1	0	0	0	2.96922311	8.58356997	1.83596393	0.0006335	Inf	up	PREDICTED: thiol protease SEN102-like [Oryza brachyantha]
c49830.graph_c0	9.71242979	9.13837694	10.4919905	6.86061041	9.21633017	9.32227907	1.27E-12	-1.232241	down	PREDICTED: uncharacterized protein LOC105178939 [Sesamum indicum]
c49837.graph_c0	20.6940312	22.6322339	19.4247506	4.17868774	4.77553346	4.01920478	3.95E-110	-3.291791	down	PREDICTED: leucine-rich repeat receptor protein kinase EXS [Sesamum indicum]
c49840.graph_c0	0	0	0	23.5293658	57.4052572	22.4267855	1.624E-10	Inf	up	predicted protein [Hordeum vulgare subsp. vulgare]
c49842.graph_c0	2.06303875	1.6826778	1.27644904	8.30635155	8.65042697	9.42658237	1.794E-05	1.378649	up	-
c49846.graph_c0	0	0	0	2.50834859	2.75450915	1.30648595	5.559E-10	Inf	up	predicted protein [Micromonas sp. RCC299]

#ID	0 h-1 FPKM	0 h-2 FPKM	0 h-3 FPKM	48 h-1 FPKM	48 h-2 FPKM	48 h-3 FPKM	FDR	log2FC	regulated	nr_annotation
c49847.graph_c1	13.4277615	12.5970736	12.170338	12.8187373	8.04031249	7.17862368	6.412E-15	-1.452013	down	hypothetical protein JCGZ_09714 [Jatropha curcas]
c49852.graph_c0	0	0	0	0.88075592	1.49854144	0.9736771	1.693E-11	Inf	up	hypothetical protein M422DRAFT_230321 [Sphaerobolus stellatus SS14]
c49855.graph_c0	0	0	0	1.30121533	1.55520805	0.83125077	7.305E-10	Inf	up	enolase [Rhizoctonia solani AG-1 IB]
c49857.graph_c2	1.87316663	3.303646	2.98021073	0.82175394	0.87054866	0.43871503	1.165E-08	-2.95294	down	-
c49858.graph_c0	0.04385115	0	0	35.3891221	75.8797118	30.5544222	4.012E-13	10.65388	up	protein of unknown function [Taphrina deformans PYCC 5710]
c49861.graph_c1	25.3056211	29.4924759	24.1567672	9.93585901	10.3494271	9.72989339	1.613E-39	-2.411976	down	hypothetical protein POPTR_0001s24240g [Populus trichocarpa]
c49862.graph_c0	1.96765452	2.70823414	3.0552877	0.90636584	1.20023088	1.68016432	4.221E-05	-2.058119	down	hypothetical protein PHAVU_001G023000g [Phaseolus vulgaris]
c49863.graph_c0	0	0	0	2.17588553	4.105936	2.05709547	2.182E-09	Inf	up	-
c49864.graph_c0	0	0	0	5.72167958	6.86119718	3.85369204	1.863E-17	Inf	up	hypothetical protein EUTSA_v10013921mg [Eutrema salsugineum]
c49865.graph_c1	4.24873402	4.04552563	3.8618037	174.8984	141.491169	117.057448	1.328E-19	4.145331	up	PREDICTED: uncharacterized protein LOC105161592 [Sesamum indicum]
c49867.graph_c0	0	0	0	3.22549734	9.45376452	2.0090197	0.0026404	Inf	up	GAMM1 protein [Cryptococcus neoformans var. neoformans JEC21]
c49868.graph_c0	5.37990237	4.49091282	5.03235984	2.41686128	3.24684787	3.33484672	5.598E-16	-1.750806	down	PREDICTED: protein FAR1-RELATED SEQUENCE 8 isoform X1 [Sesamum indicum]
c49870.graph_c0	0	0	0	3.58309142	5.99531589	2.53269273	1.212E-09	Inf	up	--
c49872.graph_c2	145.743505	148.195115	144.080977	90.5961038	86.1133427	82.0736627	1.434E-34	-1.775105	down	PREDICTED: putative RNA polymerase II subunit B1 CTD phosphatase RPAP2 homolog isoform X3 [Sesamum indicum]
c49873.graph_c0	0	0	0	1.03237505	1.58759446	0.80007194	3.264E-08	Inf	up	hypothetical protein MIMGU_mgv1a007681mg [Erythranthe guttata]
c49876.graph_c0	12.4322794	11.0334706	11.8172777	49.5626377	51.8801658	50.4831638	3.039E-13	1.088344	up	PREDICTED: serine/threonine-protein kinase HT1 [Sesamum indicum]
c49876.graph_c4	4.31846532	5.22337078	6.01184882	2.81807012	2.98540358	3.39250249	0.0003613	-1.780577	down	-
c49876.graph_c7	0	0	0	2.20195629	1.09190479	1.41735689	9.71E-07	Inf	up	PREDICTED: squalene monooxygenase-like [Sesamum indicum]
c49878.graph_c0	0	0	0	2.34702434	2.30878858	1.62729985	2.988E-22	Inf	up	--
c49879.graph_c0	10.3102536	9.60881382	9.9531297	2.62026983	2.50322924	3.22662994	9.787E-47	-2.85743	down	PREDICTED: protein RMD5 homolog A-like isoform X1 [Sesamum indicum]
c49879.graph_c1	53.227144	45.1155428	49.1187359	30.4968592	26.2645529	22.8898334	1.671E-35	-1.901636	down	-
c49880.graph_c0	0.68619741	0.75335723	0.56889732	0.30588862	0.25924152	0.21774228	1.353E-06	-2.369647	down	PREDICTED: probable sulfate transporter 3.4 [Eucalyptus grandis]
c49880.graph_c1	7.40400713	6.72698909	7.30528419	4.31940114	3.79416862	4.14764452	4.3E-22	-1.821762	down	hypothetical protein MIMGU_mgv1a013752mg [Erythranthe guttata]
c49882.graph_c0	0	0	0	2.20094056	5.7888736	1.62073132	0.0007145	Inf	up	-
c49883.graph_c0	0	0	0	1.98300039	2.57341687	1.19101173	4.439E-08	Inf	up	unnamed protein product [Vitis vinifera]

#ID	0 h-1 FPKM	0 h-2 FPKM	0 h-3 FPKM	48 h-1 FPKM	48 h-2 FPKM	48 h-3 FPKM	FDR	log2FC	regulated	nr_annotation
c49885.graph_c0	0	0	0	1.23259972	2.76224795	1.89823815	4.424E-08	Inf	up	hypothetical protein E5Q_00045 [Mixia osmundae IAM 14324]
c49886.graph_c0	18.3498417	16.2323772	24.67703	0	0	0	5.832E-26	-Inf	down	-
c49891.graph_c1	155.030149	154.995171	162.306206	36.0925733	40.8630573	36.2688696	2.506E-92	-3.080042	down	PREDICTED: zinc finger protein AZF2-like [Sesamum indicum]
c49894.graph_c0	3.85440493	3.59445158	3.64071293	11.5632824	17.5228165	17.0402115	1.24E-05	1.032036	up	PREDICTED: beta-hexosaminidase 1 [Sesamum indicum]
c49895.graph_c0	0	0	0	0.85889289	1.98522082	1.04214236	5.924E-06	Inf	up	Diphosphomevalonate decarboxylase [Ectocarpus siliculosus]
c49898.graph_c0	63.9589222	65.5561734	64.8034546	50.4139124	66.1590733	65.9933793	7.248E-15	-1.112471	down	PREDICTED: LOW QUALITY PROTEIN: uncharacterized protein LOC105160697 [Sesamum indicum]
c49903.graph_c0	0	0	0	1.26885995	4.59859011	1.3072909	0.0008631	Inf	up	-
c49905.graph_c0	0	0	0	8.6192177	11.5525009	11.821782	3.502E-32	Inf	up	PREDICTED: 2-methylene-furan-3-one reductase-like [Solanum lycopersicum]
c49906.graph_c0	0.12087279	0.12099383	0	4.45423594	8.25776502	12.3854761	2.906E-06	5.683556	up	-
c49907.graph_c0	0.20853313	0.24353227	0.17864889	1.92114322	2.44226195	2.64960361	4.842E-06	2.454741	up	hypothetical protein MIMGU_mgv1a022633mg, partial [Erythranthe guttata]
c49908.graph_c0	0	0	0	1.50550704	1.49824142	1.38018478	2.04E-11	Inf	up	PREDICTED: probable disease resistance protein At5g63020 [Vitis vinifera]
c49909.graph_c0	30.2317121	30.0268748	27.2071981	121.88476	129.51509	116.34627	6.35E-14	1.055239	up	PREDICTED: putative late blight resistance protein homolog R1A-10 [Sesamum indicum]
c49910.graph_c0	0	0	0	2.66298809	1.15409167	2.26180576	1.456E-06	Inf	up	-
c49911.graph_c1	6.96848646	6.68482	5.96985399	0.53498535	0.9918162	0.9520535	1.667E-14	-4.011116	down	-
c49912.graph_c2	34.5771473	29.1693685	33.6465087	131.549023	162.26112	152.120074	3.119E-17	1.173697	up	PREDICTED: translocon-associated protein subunit alpha-like [Sesamum indicum]
c49914.graph_c0	0	0	0	0.8854374	2.04973334	1.28391491	1.982E-07	Inf	up	hypothetical protein GUITHDRAFT_71484 [Guillardia theta CCMP2712]
c49917.graph_c0	37.262477	37.772604	37.9023551	35.4406783	33.3961883	30.1151865	2.185E-15	-1.206296	down	PREDICTED: deSI-like protein At4g17486 [Sesamum indicum]
c49918.graph_c1	0.10946606	0	0	2.82372359	6.51696277	6.28133067	1.259E-07	6.147373	up	unnamed protein product [Coffea canephora]
c49920.graph_c0	0.03615507	0.4704866	0.0464607	31.076748	32.8514738	27.2073391	2.37E-100	6.355779	up	hypothetical protein MIMGU_mgv1a020088mg [Erythranthe guttata]
c49921.graph_c0	4.93395916	4.10583234	5.19448957	28.5325022	28.8923411	24.9007543	2.512E-11	1.514154	up	PREDICTED: transcription factor GTE1-like isoform X2 [Sesamum indicum]
c49922.graph_c0	0	0	0	0.8535632	1.94413044	1.61392774	5.116E-10	Inf	up	PREDICTED: neutral ceramidase-like [Cicer arietinum]
c49923.graph_c0	0	0	0	5.10747886	7.01707229	3.33744355	1.061E-12	Inf	up	-
c49925.graph_c0	0	0	0	2.46776739	2.72796555	1.38431159	5.033E-09	Inf	up	-
c49926.graph_c0	0	0	0	42.1315714	71.7349472	39.71746	3.964E-22	Inf	up	cysteine protease 1 [Brachiaria hybrid cultivar]
c49927.graph_c1	0	0	0	3.49526504	3.03630378	3.98088424	7.644E-13	Inf	up	-

#ID	0 h-1 FPKM	0 h-2 FPKM	0 h-3 FPKM	48 h-1 FPKM	48 h-2 FPKM	48 h-3 FPKM	FDR	log2FC	regulated	nr_annotation
c49928.graph_c0	0	0	0	1.45697923	2.54676316	1.55569363	9.027E-09	Inf	up	hypothetical protein M422DRAFT_237710 [Sphaerobolus stellatus SS14]
c49929.graph_c0	0	0	0	5.28457191	3.03842786	6.37004768	9.924E-15	Inf	up	-
c49930.graph_c0	0	0	0	1.34971484	1.42985922	0.95632649	1.014E-12	Inf	up	hypothetical protein CHLNCDRAFT_58466 [Chlorella variabilis]
c49931.graph_c2	0.40644173	0.40684872	0.52229371	2.4338641	3.37173278	3.88704192	0.0005267	1.832279	up	PREDICTED: ethylene-responsive transcription factor CRF2-like [Sesamum indicum]
c49931.graph_c4	0.18640286	0.06219651	0.19961255	11.6201581	10.2180297	9.90661014	3.505E-38	5.12739	up	PREDICTED: uncharacterized protein LOC105177468 [Sesamum indicum]
c49932.graph_c0	3.60354619	1.69085372	2.46005912	94.5111766	45.2807137	58.6175934	5.106E-06	3.674684	up	Cell wall-associated hydrolase, partial [Medicago truncatula]
c49934.graph_c0	0.06158839	0	0	4.59587873	6.37148495	2.97868803	1.168E-09	6.825127	up	ATP-dependent RNA helicase ded1 [Exophiala dermatitidis NIH/UT8656]
c49934.graph_c1	0	0	0	1.14422831	2.25904624	1.2957986	2.837E-07	Inf	up	hypothetical protein THAOC_34093 [Thalassiosira oceanica]
c49935.graph_c0	0	0	0	3.53129033	7.59885325	2.84754706	3.021E-07	Inf	up	-
c49936.graph_c1	138.427737	146.948886	155.535118	13.6180495	19.2245333	20.5381974	2.59E-151	-4.071261	down	PREDICTED: heat shock protein 83-like [Sesamum indicum]
c49936.graph_c4	10.9950024	10.7599429	8.95986217	53.7759483	85.3664027	98.1904297	1.39E-06	1.925542	up	PREDICTED: 4-coumarate--CoA ligase 2 [Sesamum indicum]
c49938.graph_c0	0	0	0	6.2928146	17.1295498	10.646219	5.901E-12	Inf	up	-
c49940.graph_c0	0	0	0	2.0218147	4.51598573	1.43052898	5.803E-06	Inf	up	PREDICTED: DEAD-box ATP-dependent RNA helicase 7-like [Musa acuminata subsp. malaccensis]
c49941.graph_c0	10.7739427	9.93111341	9.94270028	9.61907465	9.05348302	9.65018137	5.277E-12	-1.129993	down	hypothetical protein MIMGU_mgv1a019692mg [Erythranthe guttata]
c49943.graph_c0	0	0	0	0.67065262	1.72009767	0.94222526	1.663E-05	Inf	up	predicted protein [Ostreococcus lucimarinus CCE9901]
c49944.graph_c0	0	0	0	1.24576383	3.60252183	1.52789544	2.858E-05	Inf	up	predicted protein [Hordeum vulgare subsp. vulgare]
c49947.graph_c0	0	0	0	0.63687725	0.76465346	0.41557234	1.768E-09	Inf	up	hypothetical protein SELMODRAFT_141243 [Selaginella moellendorffii]
c49948.graph_c1	0	0	0	3.81050694	7.10822553	5.45438904	1.952E-13	Inf	up	hypothetical protein SELMODRAFT_424962 [Selaginella moellendorffii]
c49949.graph_c0	0	0	0	13.7240922	21.0072692	8.78551038	6.542E-15	Inf	up	ATP synthase subunit alpha [Taphrina deformans PYCC 5710]
c49952.graph_c0	0	0	0	1.7292248	1.86582815	3.10580125	2.601E-11	Inf	up	-
c49954.graph_c0	0	0	0	4.37201289	4.23575278	3.39144837	7.56E-19	Inf	up	-
c49959.graph_c0	0	0	0	2.11413996	3.56050886	1.68821066	6.694E-08	Inf	up	predicted protein [Physcomitrella patens]
c49963.graph_c0	2.83546742	2.2924785	2.24226948	2.0093981	1.1707925	1.60915531	0.0009739	-1.62947	down	-
c49964.graph_c0	0	0	0	3.50756156	4.27107605	3.44387	2.872E-17	Inf	up	hypothetical protein GUITHDRAFT_68249 [Guillardia theta CCMP2712]
c49965.graph_c0	43.7600898	51.4851631	39.4137542	40.420796	38.8402047	33.0004825	5.096E-13	-1.276104	down	PREDICTED: uncharacterized protein LOC105161315 [Sesamum indicum]

#ID	0 h-1 FPKM	0 h-2 FPKM	0 h-3 FPKM	48 h-1 FPKM	48 h-2 FPKM	48 h-3 FPKM	FDR	log2FC	regulated	nr_annotation
c49968.graph_c0	26.0905411	22.190632	21.8037525	89.3899306	102.436716	104.280866	2.993E-11	1.060177	up	PREDICTED: uncharacterized protein LOC105161736 [Sesamum indicum]
c49971.graph_c0	0	0	0	0.61774899	1.37118698	0.91611556	2.078E-07	Inf	up	predicted protein [Physcomitrella patens]
c49972.graph_c3	0	0	0	1.81566006	2.71728538	1.10268613	4.978E-08	Inf	up	ABC-2 type transporter domain-containing protein [Rozella allomycis CSF55]
c49974.graph_c0	18.2763036	11.0260411	12.2568852	0.22676529	0.48046066	0.25221799	1.137E-24	-6.461407	down	predicted protein [Hordeum vulgare subsp. vulgare]
c49975.graph_c1	48.5475955	48.8760332	50.7706082	346.69908	357.55481	370.005586	2.223E-36	1.839043	up	PREDICTED: 60S ribosomal protein L3-like [Sesamum indicum]
c49978.graph_c0	0	0	0	6.27881178	11.3564581	10.1925927	1.117E-24	Inf	up	glutathione-independent formaldehyde dehydrogenase [Bathycoccus prasinos]
c49979.graph_c0	0	0	0	2.4018758	2.87638697	1.5331905	2.131E-10	Inf	up	-
c49982.graph_c0	0	0	0	1.61460462	2.47408399	1.84714134	1.151E-14	Inf	up	PREDICTED: glucose-6-phosphate isomerase, cytosolic 2-like [Cucumis sativus]
c49985.graph_c0	0	0	0	2.82144046	6.54547482	1.84315245	2.493E-05	Inf	up	lipoamide dehydrogenase [Chondrus crispus]
c49987.graph_c0	0	0	0	1.5161654	2.07250761	1.26203777	2.2E-11	Inf	up	-
c49989.graph_c0	0	0	0	1.80180723	2.97547655	1.69754531	8.198E-10	Inf	up	threonyl-tRNA synthetase [Mucor circinelloides f. circinelloides 1006PhL]
c49993.graph_c0	0	0	0	0.58248165	0.39927974	0.47255732	1.961E-11	Inf	up	secreted protein [Achlya hypogyna]
c49995.graph_c0	0	0	0	1.26669391	1.62441568	1.24573837	1.659E-09	Inf	up	hypothetical protein GUITHDRAFT_99801 [Guillardia theta CCMP2712]
c49998.graph_c0	0	0	0	5.88157072	11.8473167	4.97540243	2.352E-09	Inf	up	predicted protein [Physcomitrella patens]
c50003.graph_c0	0	0	0	1.94025247	5.85806733	2.1148706	1.245E-05	Inf	up	-
c50004.graph_c1	19.910867	20.6706954	17.7896681	10.7250202	11.2867139	9.68191254	8.943E-40	-1.896986	down	hypothetical protein MIMGU_mgv1a002674mg [Erythranthe guttata]
c50010.graph_c0	49.3063131	50.1481665	45.8621427	176.912202	204.26107	259.278554	6.392E-07	1.118829	up	PREDICTED: putative GTP diphosphokinase RSH1, chloroplastic [Sesamum indicum]
c50012.graph_c0	0	0	0	5.23237868	10.1811504	5.60588992	2.366E-09	Inf	up	-
c50013.graph_c0	0	0	0	2.64846774	3.11747826	2.42205152	1.22E-11	Inf	up	predicted protein [Micromonas pusilla CCMP1545]
c50014.graph_c2	27.2344229	32.187331	23.2898031	9.96423636	10.936293	13.2991764	1.436E-21	-2.292522	down	PREDICTED: uncharacterized protein LOC104210641 [Nicotiana sylvestris]
c50016.graph_c0	0	0	0	2.2604188	4.72086099	1.78144339	1.738E-05	Inf	up	-
c50016.graph_c1	0	0	0	9.83941516	13.898223	10.7492588	1.453E-30	Inf	up	-
c50017.graph_c0	22.9884978	24.6798523	22.8943756	14.1895974	19.8559101	24.2623004	1.548E-13	-1.299744	down	PREDICTED: bifunctional epoxide hydrolase 2-like [Sesamum indicum]
c50017.graph_c2	218.172584	227.042797	205.305941	58.1509608	32.9746973	23.1874545	2.83E-99	-3.510473	down	hypothetical protein MIMGU_mgv1a011129mg [Erythranthe guttata]

#ID	0 h-1 FPKM	0 h-2 FPKM	0 h-3 FPKM	48 h-1 FPKM	48 h-2 FPKM	48 h-3 FPKM	FDR	log2FC	regulated	nr_annotation
c50017.graph_c3	111.650546	107.283219	107.866323	75.5430725	74.5040598	86.9279138	1.324E-25	-1.481829	down	PREDICTED: bifunctional epoxide hydrolase 2 [Sesamum indicum]
c50018.graph_c0	327.876509	340.304545	331.371981	27.7469452	24.0858139	19.4870212	2.37E-204	-4.82181	down	PREDICTED: uncharacterized protein LOC103433973 [Malus domestica]
c50018.graph_c5	236.112247	241.150458	235.173646	114.730916	119.654387	117.898687	2.146E-35	-2.034008	down	PREDICTED: UBP1-associated protein 2A-like [Sesamum indicum]
c50019.graph_c0	0	0	0	2.07515049	1.73063195	0.94287137	4.488E-07	Inf	up	protein of unknown function [Taphrina deformans PYCC 5710]
c50020.graph_c0	0	0	0	1.8463254	3.13691358	1.11589759	1.987E-06	Inf	up	mitochondrial translation elongation factor Tu [Guillardia theta CCMP2712]
c50021.graph_c0	17.2431989	16.592825	16.5115092	9.75904678	10.5108355	9.64837904	1.609E-31	-1.768774	down	PREDICTED: pentatricopeptide repeat-containing protein At3g53170-like isoform X1 [Sesamum indicum]
c50022.graph_c0	5.43715381	5.11274386	4.41095828	17.481116	27.0148707	22.5552837	3.913E-06	1.141913	up	PREDICTED: xyloglucan galactosyltransferase KATAMARI1 homolog [Sesamum indicum]
c50023.graph_c0	3.18766239	3.56080848	3.14641211	36.9426147	40.3085039	37.4536083	3.534E-37	2.517132	up	hypothetical protein MIMGU_mgv1a000846mg [Erythranthe guttata]
c50024.graph_c0	5.75482065	5.70821429	4.43710343	1.06034323	2.75720337	3.21643305	2.599E-10	-2.207001	down	hypothetical protein MIMGU_mgv1a022598mg, partial [Erythranthe guttata]
c50024.graph_c1	0.22769295	0.15954467	0.14629723	5.8944134	7.37774448	6.9993064	5.94E-28	4.23	up	hypothetical protein CICLE_v10007524mg [Citrus clementina]
c50026.graph_c0	0	0	0	1.73049411	1.31269658	1.02652217	3.634E-12	Inf	up	PREDICTED: uncharacterized protein LOC100820133 [Glycine max]
c50027.graph_c0	0	0	0	4.20245591	8.62860394	4.31756536	4.829E-10	Inf	up	-
c50029.graph_c0	0	0	0	7.78426428	13.9383509	5.0065765	4.371E-09	Inf	up	-
c50030.graph_c0	1.84634767	1.97259435	1.27756992	10.7782992	10.9678066	10.2599065	6.065E-15	1.637472	up	PREDICTED: uncharacterized protein LOC105168379 [Sesamum indicum]
c50031.graph_c0	183.628845	188.776871	193.922113	153.927557	148.683258	143.42258	1.146E-20	-1.361431	down	PREDICTED: putative ATP-dependent RNA helicase DHX33 [Sesamum indicum]
c50037.graph_c1	0	0	0	3.28857584	3.94836032	2.63353951	2.417E-11	Inf	up	-
c50037.graph_c2	0	0	0	3.99292632	4.15448518	5.96376593	1.783E-14	Inf	up	-
c50039.graph_c2	0.18258516	0.18276799	0.11731458	23.5493099	34.9266936	43.4047854	3.632E-21	6.700206	up	hypothetical protein MIMGU_mgv1a001711mg [Erythranthe guttata]
c50041.graph_c0	0	0	0	2.70356951	5.27414287	3.34439839	4.384E-13	Inf	up	phosphoric diester hydrolase, putative [Ricinus communis]
c50042.graph_c1	51.2449039	51.0799607	50.3049475	28.5609453	28.4857252	27.0249361	5.529E-40	-1.877114	down	PREDICTED: uncharacterized calcium-binding protein At1g02270 [Sesamum indicum]
c50044.graph_c0	12.9025087	11.6519628	12.254956	69.5109603	62.414732	65.6442344	2.856E-10	1.408693	up	Gag-protease-integrase-RT-RNaseH polyprotein [Glycine max]
c50046.graph_c0	9.20526874	8.54151832	8.24052086	37.542861	48.3523862	52.3549286	2.738E-13	1.389674	up	PREDICTED: UMP-CMP kinase 3-like [Sesamum indicum]
c50048.graph_c0	0	0	0	1.47174932	4.44813457	0.63551871	0.0019987	Inf	up	--

#ID	0 h-1 FPKM	0 h-2 FPKM	0 h-3 FPKM	48 h-1 FPKM	48 h-2 FPKM	48 h-3 FPKM	FDR	log2FC	regulated	nr_annotation
c50050.graph_c0	3.75610476	4.73464599	4.24574644	33.1939071	37.167555	39.3429727	9.27E-33	2.086229	up	hypothetical protein MIMGU_mgv1a001814mg [Erythranthe guttata]
c50051.graph_c0	0	0	0	13.6045339	20.1256179	10.803076	4.949E-23	Inf	up	hypothetical protein GUITHDRAFT_64866 [Guillardia theta CCMP2712]
c50052.graph_c4	1.27677951	1.38112721	0.89974541	9.78945685	9.2653339	10.5506296	3.556E-17	2.043292	up	hypothetical protein MIMGU_mgv1a002839mg [Erythranthe guttata]
c50053.graph_c0	0	0	0	2.27472392	4.60538442	0.98952855	0.0003978	Inf	up	--
c50054.graph_c0	0	0	0	4.7547236	14.533997	7.22748762	1.78E-07	Inf	up	PREDICTED: nucleolar and coiled-body phosphoprotein 1-like [Tarenaya hassleriana]
c50055.graph_c0	0	0	0	5.46119547	7.30071769	10.0657734	1.983E-12	Inf	up	-
c50055.graph_c1	0	0	0	3.26767727	5.06339355	5.46794787	6.651E-25	Inf	up	-
c50057.graph_c0	4.76717722	4.65190174	5.50955748	2.59643051	2.13610681	2.26113319	4.319E-14	-2.109683	down	PREDICTED: transcription factor bHLH79-like [Sesamum indicum]
c50057.graph_c3	372.787274	393.753475	357.477558	374.636858	340.000167	278.843669	2.222E-11	-1.191393	down	hypothetical protein MIMGU_mgv1a006830mg [Erythranthe guttata]
c50058.graph_c0	0	0	0	8.58655982	19.3636641	11.0191343	2.14E-14	Inf	up	-
c50061.graph_c0	13.5722587	14.8570398	13.3611495	115.603921	136.334139	115.355657	7.312E-40	2.116842	up	-
c50062.graph_c0	0	0	0	0.80675508	1.5872242	1.02549403	3.485E-08	Inf	up	bifunctional aminoacyl-tRNA synthetase [Zea mays]
c50064.graph_c0	0	0	0	2.79729328	5.043467	2.44114907	7.38E-11	Inf	up	hypothetical protein VOLCADRAFT_93958 [Volvox carteri f. nagariensis]
c50066.graph_c0	29.1815625	31.9101297	28.5268193	12.9983497	12.3602598	14.4474483	3.964E-40	-2.187916	down	PREDICTED: flowering time control protein FPA [Vitis vinifera]
c50067.graph_c0	1.6672662	1.68614124	1.63448688	1.61516934	1.3587957	1.80350489	0.0008947	-1.078382	down	PREDICTED: uncharacterized protein LOC105173219 [Sesamum indicum]
c50070.graph_c1	0	0	0	4.54800153	9.7059392	3.61669138	8.601E-09	Inf	up	probable SEC14-phosphatidylinositol(PI)/phosphatidylcholine(PC) transfer protein [Piriformospora indica DSM 11827]
c50071.graph_c0	28.4115149	27.669285	27.0506425	15.5351868	17.9238067	18.702768	2.658E-32	-1.692399	down	PREDICTED: uncharacterized protein LOC105171564 [Sesamum indicum]
c50072.graph_c0	0	0	0	2.36299911	3.87707928	1.69232242	2.618E-09	Inf	up	hypothetical protein GUITHDRAFT_109518 [Guillardia theta CCMP2712]
c50073.graph_c0	0	0	0	3.27833668	9.63477474	2.00742831	0.0002896	Inf	up	-
c50075.graph_c0	20.6042023	21.2753393	23.136862	10.917254	9.81202673	9.27540532	1.834E-36	-2.131628	down	PREDICTED: uncharacterized protein LOC104218703 [Nicotiana sylvestris]
c50076.graph_c0	0.16047045	0	0	20.2535114	31.6360597	28.8080156	9.366E-37	7.970553	up	PREDICTED: peroxidase 73 [Sesamum indicum]
c50077.graph_c0	0	0	0	3.86201191	8.15901733	3.89325674	5.973E-11	Inf	up	hypothetical protein VOLCADRAFT_120457 [Volvox carteri f. nagariensis]

#ID	0 h-1 FPKM	0 h-2 FPKM	0 h-3 FPKM	48 h-1 FPKM	48 h-2 FPKM	48 h-3 FPKM	FDR	log2FC	regulated	nr_annotation
c50078.graph_c0	0	0	0	0.80566524	1.09058925	0.65713641	1.151E-11	Inf	up	predicted protein [Hordeum vulgare subsp. vulgare]
c50080.graph_c0	0	0	0	23.4953314	36.9585538	28.508188	1.459E-41	Inf	up	carbonyl reductase (NADPH) [Galdieria sulphuraria]
c50081.graph_c0	0	0	0	0.99045934	1.18785461	0.4157098	2.528E-06	Inf	up	RHTO0S07e00188g1_1 [Rhodosporidium toruloides]
c50082.graph_c0	0	0	0	8.12805299	6.87699128	7.32937042	3.367E-22	Inf	up	YALI0E30965p [Yarrowia lipolytica]
c50083.graph_c0	0	0	0	4.79755908	10.0841907	3.65900504	2.451E-06	Inf	up	-
c50084.graph_c0	0	0	0	0.9848432	3.49819714	2.37118516	2.436E-06	Inf	up	hypothetical protein PLEOSDRAFT_1111887 [Pleurotus ostreatus PC15]
c50085.graph_c0	5.84936635	5.74262315	6.33134672	37.7217246	66.1342411	91.6386358	0.000151	2.417309	up	PREDICTED: subtilisin-like protease [Sesamum indicum]
c50086.graph_c0	7.44825842	7.39226382	7.45340074	4.58428073	4.54715901	5.04037384	1.365E-13	-1.671735	down	hypothetical protein VITISV_036599 [Vitis vinifera]
c50087.graph_c0	0	0	0	3.51990496	4.21397428	2.41901687	2.745E-17	Inf	up	PREDICTED: mitochondrial-processing peptidase subunit alpha-like [Musa acuminata subsp. malaccensis]
c50088.graph_c0	0	0	0	1.50326232	1.33974254	0.87049754	1.401E-11	Inf	up	unknown [Picea sitchensis]
c50091.graph_c0	0	0	0	9.48820311	7.15328638	7.43254822	2.187E-34	Inf	up	-
c50092.graph_c0	28.3427544	28.0747716	27.4807925	13.8371494	14.2920346	11.5747864	2.851E-49	-2.095546	down	PREDICTED: RNA polymerase II C-terminal domain phosphatase-like 2 [Sesamum indicum]
c50097.graph_c0	0	0	0	0.77834934	2.73873955	0.54416258	0.0051172	Inf	up	NADPH-dependent thioredoxin reductase [Euglena gracilis]
c50099.graph_c0	20.4209386	20.644692	19.7168759	100.410247	87.3613056	83.9864919	1.2E-11	1.146685	up	hypothetical protein, partial (mitochondrion) [Nicotiana tabacum]
c50101.graph_c0	0	0	0	1.39282176	2.16717847	1.27805332	1.191E-13	Inf	up	predicted protein [Micromonas sp. RCC299]
c50102.graph_c0	0	0	0	2.78433342	4.37478203	3.25691603	3.072E-19	Inf	up	protein disulfide isomerase [Rhizoctonia solani 123E]
c50103.graph_c0	0	0.08453615	0	7.00220797	5.52228165	8.03045128	1.539E-15	6.921331	up	hypothetical protein MIMGU_mgv1a018847mg, partial [Erythranthe guttata]
c50104.graph_c0	0	0	0	0.89972352	1.7275807	0.65046197	1.686E-05	Inf	up	unknown [Picea sitchensis]
c50106.graph_c0	0	0	0	1.96752909	2.55100593	1.09745095	8.414E-09	Inf	up	saccharopepsin [Mucor circinelloides f. circinelloides 1006PhL]
c50107.graph_c2	0	0	0	1.23161585	3.13139437	0.75146357	0.0001563	Inf	up	--
c50108.graph_c1	0.46589557	0.34977157	0.07483678	105.479189	105.518711	94.5708325	3.34E-226	7.419014	up	PREDICTED: cationic amino acid transporter 7, chloroplastic [Sesamum indicum]
c50108.graph_c2	0	0	0	7.56204402	10.5288331	9.61237328	4.049E-12	Inf	up	-
c50109.graph_c0	7.02373725	5.91373217	6.41083471	239.416821	207.44227	174.288837	8.027E-29	3.991848	up	PREDICTED: pentatricopeptide repeat-containing protein At5g66520-like [Sesamum indicum]
c50109.graph_c3	57.8991141	60.5651605	53.8489208	287.104921	260.218091	221.350917	3.144E-08	1.144086	up	PREDICTED: DEAD-box ATP-dependent RNA helicase 7-like [Sesamum indicum]
c50110.graph_c2	1.21730491	1.41505996	2.06889306	14.7960457	14.1799708	11.716913	3.68E-14	2.093094	up	PREDICTED: uncharacterized protein LOC105163215 [Sesamum indicum]
c50110.graph_c3	0.24452432	0.12238459	0	4.84334276	5.48890653	5.81291817	1.836E-09	4.457419	up	PREDICTED: uncharacterized protein LOC101248594 [Solanum lycopersicum]

#ID	0 h-1 FPKM	0 h-2 FPKM	0 h-3 FPKM	48 h-1 FPKM	48 h-2 FPKM	48 h-3 FPKM	FDR	log2FC	regulated	nr_annotation
c50110.graph_c4	0	0.1035005	0.06643459	3.66735694	4.54104933	4.11077453	2.051E-13	5.153329	up	PREDICTED: uncharacterized protein LOC105159914 [Sesamum indicum]
c50111.graph_c0	1.153938	1.18076224	1.31809424	1.01583482	1.37647581	0.86184261	0.005272	-1.188292	down	gag-pol polyprotein [Oryza sativa Japonica Group]
c50112.graph_c0	0	0	0	1.87730214	2.61381723	1.64654912	1.853E-15	Inf	up	-
c50114.graph_c0	0	0	0	2.33843359	5.87830776	2.39812275	2.899E-06	Inf	up	-
c50115.graph_c0	0	0	0	2.01697233	4.41326179	1.27473298	5.298E-06	Inf	up	-
c50116.graph_c0	0	0	0	16.503738	23.0904395	16.8026772	1.016E-51	Inf	up	hypothetical protein O988_02039 [Pseudogymnoascus pannorum VKM F-3808]
c50124.graph_c0	0	0	0	5.77075139	13.3102121	5.17811136	5.491E-09	Inf	up	Formate/nitrite transporter [Trametes versicolor FP-101664 SS1]
c50125.graph_c0	0	0	0	1.81741856	2.75047799	1.48511765	7.422E-11	Inf	up	PREDICTED: 26S proteasome non-ATPase regulatory subunit 1 homolog A-like [Brachypodium distachyon]
c50126.graph_c0	8.12288733	3.70678907	3.12123599	0	0	0	1.23E-10	-Inf	down	-
c50128.graph_c0	90.1259294	87.5077218	88.6948628	44.521301	49.2294854	48.2313715	3.442E-38	-1.926617	down	PREDICTED: uncharacterized protein LOC105173692 [Sesamum indicum]
c50129.graph_c0	2.8710987	3.75719485	3.02357017	32.5275824	38.8193747	40.7564886	1.582E-63	2.517343	up	PREDICTED: geranylgeranyl diphosphate reductase, chloroplastic [Sesamum indicum]
c50130.graph_c0	0	0	0	2.73386915	4.63392458	3.80367106	2.437E-15	Inf	up	--
c50131.graph_c0	0	0	0	1.01666657	1.3670059	1.11338313	2.029E-11	Inf	up	hypothetical protein [Tuber melanosporum Mel28]
c50131.graph_c1	0	0	0	2.21840922	3.67585308	2.22699477	2.859E-11	Inf	up	hypothetical protein GUITHDRAFT_147173 [Guillardia theta CCMP2712]
c50133.graph_c0	1.68122665	1.87387867	1.99189762	14.7306059	18.4912804	18.3559508	9.159E-42	2.193936	up	PREDICTED: purple acid phosphatase 2-like [Sesamum indicum]
c50133.graph_c1	1.54310524	2.08527807	2.08209889	7.60560919	8.81023199	11.004972	0.0019344	1.239757	up	PREDICTED: purple acid phosphatase 2-like [Sesamum indicum]
c50134.graph_c0	0	0	0	1.6363989	2.69909676	2.10114684	2.002E-18	Inf	up	-
c50135.graph_c0	0	0	0	2.49029247	2.8972682	1.18706104	1.892E-09	Inf	up	hypothetical protein ZEAMMB73_452342 [Zea mays]
c50137.graph_c1	0	0	0	1.08800279	2.08566986	1.19859795	2.302E-07	Inf	up	-
c50139.graph_c0	0.33677517	0.84278101	0.9376671	10.9107838	12.3255773	11.778851	9.871E-15	3.021823	up	PREDICTED: putative glucose-6-phosphate 1-epimerase [Sesamum indicum]
c50140.graph_c0	34.3826433	38.7797997	32.4725675	22.6717327	21.3540644	20.1370835	2.829E-30	-1.733977	down	PREDICTED: uncharacterized protein LOC105161920 [Sesamum indicum]
c50140.graph_c1	3.71336722	5.03605145	4.00217488	18.1533502	24.3752006	20.9641969	4.317E-07	1.294505	up	PREDICTED: uncharacterized protein LOC105179028 [Sesamum indicum]
c50141.graph_c0	4.06501043	3.69231417	3.43409864	2.11520479	1.57958244	1.45013993	8.175E-10	-2.130492	down	hypothetical protein VITISV_003658 [Vitis vinifera]
c50143.graph_c0	0	0	0	3.76008919	12.7036841	6.19407502	1.597E-06	Inf	up	hypothetical protein CHLNCDRAFT_33886 [Chlorella variabilis]
c50144.graph_c0	0	0	0	13.1184399	24.7207143	12.7485346	4.282E-14	Inf	up	molecular chaperone DnaK [Galdieria sulphuraria]

#ID	0 h-1 FPKM	0 h-2 FPKM	0 h-3 FPKM	48 h-1 FPKM	48 h-2 FPKM	48 h-3 FPKM	FDR	log2FC	regulated	nr_annotation
c50145.graph_c0	3.33884596	3.8085413	2.39472516	17.5973679	17.8086797	20.622774	1.47E-06	1.538809	up	PREDICTED: E3 ubiquitin-protein ligase MARCH11 isoform X1 [Sesamum indicum]
c50145.graph_c2	1.0996607	0.51800557	0.83123973	12.5741002	14.0151803	15.2182889	3.69E-15	3.074291	up	PREDICTED: subtilisin-like protease [Sesamum indicum]
c50147.graph_c0	0	0	0	2.43693604	1.90933662	0.47433116	0.0001319	Inf	up	predicted protein [Thalassiosira pseudonana CCMP1335]
c50150.graph_c0	0	0	0	1.15834063	1.09564415	0.78528345	3.475E-15	Inf	up	Eukaryotic translation initiation factor 3 subunit, putative [Ricinus communis]
c50151.graph_c0	100.433113	107.00824	95.6950621	50.8866165	55.1038742	53.1292007	2.325E-37	-1.947758	down	PREDICTED: ethylene-insensitive protein 2-like [Sesamum indicum]
c50152.graph_c0	0	0	0	0.92476267	1.45990622	0.66150546	2.004E-08	Inf	up	predicted protein [Hordeum vulgare subsp. vulgare]
c50153.graph_c1	0	0	0	4.22579949	6.63368828	3.64045171	3.098E-18	Inf	up	hypothetical protein EMIHUDRAFT_422478 [Emiliania huxleyi CCMP1516]
c50154.graph_c0	54.7983168	55.7814292	51.5380863	48.8819714	38.3505249	35.5132998	3.906E-24	-1.412531	down	PREDICTED: splicing factor U2AF-associated protein 2 isoform X3 [Sesamum indicum]
c50155.graph_c0	13.2604841	13.7299055	12.2970855	13.832685	13.6533964	11.8413113	6.12E-11	-1.013667	down	PREDICTED: nephrocystin-3 [Sesamum indicum]
c50156.graph_c0	100.412428	112.336573	104.531815	94.1946355	95.4134689	102.224848	7.984E-15	-1.138806	down	PREDICTED: acetolactate synthase 1, chloroplastic [Sesamum indicum]
c50156.graph_c1	29.1555834	26.3777558	30.6829699	6.34458868	8.5727046	8.11311644	3.253E-67	-2.927967	down	hypothetical protein VITISV_035070 [Vitis vinifera]
c50157.graph_c0	12.3520956	12.2686158	11.5663224	133.202556	187.183608	158.161264	3.358E-30	2.703225	up	ATP-binding cassette superfamily [Micromonas sp. RCC299]
c50158.graph_c0	0	0	0	3.2456526	3.77512347	5.6419467	5.576E-21	Inf	up	-
c50162.graph_c0	3.22506455	3.38202225	3.4996612	2.92397887	3.58722187	3.31511881	1.005E-08	-1.062008	down	PREDICTED: uncharacterized protein LOC105111104, partial [Populus euphratica]
c50163.graph_c0	0	0	0	4.47319633	10.8056921	3.70135405	4.05E-07	Inf	up	-
c50164.graph_c1	0	0	0	3.40336155	10.8856822	3.90183602	6.513E-05	Inf	up	-
c50165.graph_c0	0	0	0	1.03447844	1.33700345	0.51546471	5.589E-07	Inf	up	predicted protein [Hordeum vulgare subsp. vulgare]
c50166.graph_c1	2.60198304	2.4214534	3.39589577	1.64801759	2.24186196	2.68283839	1.227E-05	-1.384806	down	PREDICTED: putative late blight resistance protein homolog R1A-10 [Sesamum indicum]
c50167.graph_c0	0	0	0	2.79657767	5.00519692	2.07107237	3.126E-10	Inf	up	ATP-dependent Clp protease ATP-binding subunit ClpB [Galdieria sulphuraria]
c50170.graph_c0	0	0	0	2.14367159	2.92530455	1.58413353	1.75E-15	Inf	up	hypothetical protein CHLNCDRAFT_33886 [Chlorella variabilis]
c50173.graph_c0	9.96485562	8.69094441	8.68473791	31.2674712	41.9347182	38.4164643	1.421E-08	1.008207	up	PREDICTED: uncharacterized protein LOC102612426 [Citrus sinensis]
c50176.graph_c1	0	0	0	0.87539817	1.61836589	0.47346362	2.775E-05	Inf	up	predicted protein [Hordeum vulgare subsp. vulgare]
c50178.graph_c0	39.384112	41.0047076	36.5367343	20.0576317	22.8930207	24.1702772	3.666E-37	-1.820114	down	PREDICTED: DDB1- and CUL4-associated factor homolog 1 [Sesamum indicum]
c50179.graph_c0	0	0	0	16.8948107	31.8331432	14.4078324	9.82E-17	Inf	up	EF2, translation elongation factor 2 [Ectocarpus siliculosus]
c50180.graph_c0	0	0	0	5.17444152	4.28183773	3.7841279	1.073E-39	Inf	up	Myosin tail domain-containing protein [Rozella allomycis CSF55]

#ID	0 h-1 FPKM	0 h-2 FPKM	0 h-3 FPKM	48 h-1 FPKM	48 h-2 FPKM	48 h-3 FPKM	FDR	log2FC	regulated	nr_annotation
c50181.graph_c0	0	0	0	2.92756165	2.43896236	2.02324408	8.524E-30	Inf	up	--
c50182.graph_c0	1.29921872	1.3005197	1.14394921	9.64189608	11.4354538	11.8729639	1.484E-17	2.11823	up	PREDICTED: putative late blight resistance protein homolog R1A-10 [Sesamum indicum]
c50183.graph_c0	270.542582	300.15235	362.054404	395.434034	214.224733	265.605554	0.0005548	-1.101384	down	Cell wall-associated hydrolase, partial [Medicago truncatula]
c50187.graph_c1	0	0	0	5.98631083	8.81660769	3.46444447	1.377E-09	Inf	up	hypothetical protein GUITHDRAFT_163621 [Guillardia theta CCMP2712]
c50188.graph_c0	16.4507368	15.6830569	13.5899024	5.41267563	12.0415544	8.50856033	7.64E-07	-1.843795	down	PREDICTED: putative ribonuclease H protein At1g65750 [Malus domestica]
c50188.graph_c1	7.27170714	7.16381479	6.41690396	3.47678491	3.54847964	3.0558952	1.212E-23	-2.063959	down	PREDICTED: uncharacterized protein LOC105167219 [Sesamum indicum]
c50189.graph_c0	13.9401126	11.8138765	13.4077226	50.3853066	53.8779246	56.6803923	3.744E-11	1.01977	up	PREDICTED: probable LRR receptor-like serine/threonine-protein kinase At2g16250 [Sesamum indicum]
c50190.graph_c1	21.3741212	16.7208718	14.3103252	1.15830791	0.35059622	0.14723649	2.127E-34	-5.966924	down	-
c50191.graph_c0	8.19328842	8.51999734	8.89317379	0.76947585	0.4269919	0.32603571	9.351E-58	-5.075587	down	PREDICTED: uncharacterized protein LOC105157045 [Sesamum indicum]
c50194.graph_c0	7.04809722	6.26054694	5.90410594	26.9699116	30.2147319	24.7075385	2.871E-11	1.074983	up	hypothetical protein M569_07983, partial [Genlisea aurea]
c50195.graph_c0	37.0903537	42.949412	34.4193271	18.2835635	16.780017	18.1923987	2.056E-39	-2.118843	down	hypothetical protein VITISV_039063 [Vitis vinifera]
c50196.graph_c1	4.8048227	4.55732533	4.73707099	2.27856729	1.06087093	0.86521985	3.685E-36	-2.745184	down	hypothetical protein VITISV_021503 [Vitis vinifera]
c50197.graph_c0	6.71677483	6.65212592	5.845842	2.87062195	2.85318332	3.03939661	3.047E-45	-2.149028	down	PREDICTED: uncharacterized protein LOC103415359 [Malus domestica]
c50198.graph_c0	7.81267953	8.4845077	6.91406588	2.68210588	7.44509816	9.57629589	5.973E-05	-1.268865	down	PREDICTED: uncharacterized protein LOC105162629 [Sesamum indicum]
c50199.graph_c0	257.142571	245.816206	258.271166	3.9195319	1.82699826	1.67403644	0	-7.681498	down	defensin-like protein 4 [Arabidopsis thaliana]
c50200.graph_c0	82.1260941	77.9300751	81.453973	48.2188086	55.4457682	48.4565585	2.606E-31	-1.686085	down	PREDICTED: uncharacterized protein LOC105159431 [Sesamum indicum]
c50201.graph_c0	2.22889401	3.68620803	2.11702999	0.44639186	1.0403757	1.19159037	2.581E-06	-2.61357	down	-
c50202.graph_c0	44.0154056	40.8946444	41.9431692	19.2183503	18.1813768	16.1856424	4.079E-54	-2.258421	down	PREDICTED: squalene monooxygenase-like [Sesamum indicum]
c50203.graph_c0	12.0397471	13.9189839	11.5491865	7.73301574	8.10944309	8.96587906	6.136E-09	-1.614459	down	-
c50205.graph_c0	0.23394311	0.15611825	0	1.652348	1.52214118	1.47024932	1.471E-05	2.575753	up	PREDICTED: protein FLX-like 4 [Sesamum indicum]
c50207.graph_c0	1.39650603	1.56565294	1.07673932	6.2783759	10.3038741	11.9055729	4.316E-05	1.794419	up	hypothetical protein MIMGU_mgv1a010254mg [Erythranthe guttata]
c50212.graph_c0	10560.0812	9984.95233	10690.9067	1696.63794	1475.10397	1677.2971	5.626E-41	-3.703514	down	hypothetical protein MIMGU_mgv1a016953mg [Erythranthe guttata]
c50213.graph_c0	0.60983977	0.61045044	0.65305688	6.74189013	6.94382023	5.41566756	1.366E-05	2.333067	up	PREDICTED: LOW QUALITY PROTEIN: uncharacterized protein LOC105161466 [Sesamum indicum]
c50216.graph_c0	239.587447	251.5836	226.064149	45.8986605	39.9368873	34.9625843	6E-126	-3.582622	down	-

#ID	0 h-1 FPKM	0 h-2 FPKM	0 h-3 FPKM	48 h-1 FPKM	48 h-2 FPKM	48 h-3 FPKM	FDR	log2FC	regulated	nr_annotation
c50218.graph_c0	2.02802411	1.47640354	1.97431157	0	0	0.10075424	9.872E-19	-6.806669	down	hypothetical protein VITISV_044399 [Vitis vinifera]
c50219.graph_c0	40.1403106	34.9634463	33.7221001	368.113991	345.085184	344.260186	4.799E-56	2.265761	up	60S ribosomal L34-1 -like protein [Gossypium arboreum]
c50224.graph_c0	12.64576	6.25386368	7.83496719	0	0	0	5.166E-25	-Inf	down	predicted protein [Hordeum vulgare subsp. vulgare]
c50226.graph_c0	6.30600278	6.20441443	6.71825584	39.4251611	31.1931276	35.1686347	8.006E-12	1.445302	up	histone H4 [Zea mays]
c50229.graph_c0	0	0	0	8.76079712	11.2012096	15.8594128	5.766E-14	Inf	up	PREDICTED: homeobox-leucine zipper protein ATHB-13 [Sesamum indicum]
c50230.graph_c0	1.34178033	0.61990335	0.99475433	104.073105	69.1031933	59.7333558	1.527E-13	5.293003	up	PREDICTED: zinc finger protein CONSTANS-LIKE 4-like [Sesamum indicum]
c50231.graph_c0	29.052712	14.837655	21.6193672	0	0	0	8.893E-28	-Inf	down	predicted protein [Physcomitrella patens]
c50232.graph_c0	0	0	0	2.39944935	3.03925892	2.73838958	5.257E-13	Inf	up	PREDICTED: putative late blight resistance protein homolog R1A-4 [Sesamum indicum]
c50235.graph_c0	0	0	0	4.49774139	6.40785012	6.90010905	1.497E-15	Inf	up	PREDICTED: dirigent protein 22-like [Sesamum indicum]
c50236.graph_c0	14.0486943	16.3309493	8.73538468	5.74065759	6.85554362	6.593967	2.319E-06	-2.042693	down	-
c50238.graph_c0	30.0943133	26.2713211	20.2355498	2.9014372	3.41524544	5.16336274	2.547E-17	-3.758171	down	-
c50239.graph_c0	7223.6407	7097.40063	7562.77428	691.994445	602.476798	613.486984	4.081E-85	-4.535157	down	hypothetical protein MIMGU_mgv1a015957mg [Erythranthe guttata]
c50240.graph_c0	1.71133495	2.29799201	1.39457225	1298.92067	1529.58602	1927.09611	7.666E-89	8.762411	up	hypothetical protein MIMGU_mgv1a027056mg [Erythranthe guttata]
c50245.graph_c0	0.20082858	0.10051484	0	9.99088466	10.2901272	7.07893798	1.12E-14	5.507287	up	PREDICTED: protein RALF-like 24 [Sesamum indicum]
c50246.graph_c0	2.70553448	1.35412183	0.86917973	11.2163167	14.962931	26.7987152	0.002798	2.408366	up	-
c50247.graph_c0	9.55358467	4.91080734	6.4701709	0	0	0	6.895E-18	-Inf	down	40S ribosomal protein S11 [Lachancea thermotolerans]
c50248.graph_c0	40.850923	46.8697947	42.7797947	12.232687	16.2453145	15.2071523	1.108E-55	-2.601003	down	PREDICTED: uncharacterized protein LOC105176358 [Sesamum indicum]
c50251.graph_c0	25.3623616	20.8886618	18.5648575	0.88729602	3.44660264	6.84242571	3.653E-15	-3.571227	down	PREDICTED: trihelix transcription factor ASIL2 [Sesamum indicum]
c50253.graph_c0	24.9242288	19.3233897	35.7966938	6.07813022	5.00814374	6.81043135	3.532E-07	-3.182644	down	conserved hypothetical protein [Ricinus communis]
c50255.graph_c0	3759.77414	3929.75954	3850.37212	187.374158	145.373082	119.035213	1.6E-150	-5.685606	down	oleosin, partial [Genlisea aurea]
c50256.graph_c0	0.03788731	0.22755147	0.09737338	10.8203202	12.0174697	11.3360238	1.362E-35	5.539246	up	PREDICTED: probable indole-3-pyruvate monooxygenase YUCCA5 [Sesamum indicum]
c50257.graph_c0	1.14820929	0.61059699	0.5533104	121.052593	134.298876	143.155015	3.49E-235	6.415119	up	PREDICTED: 5-methyltetrahydropteroyltriglutamate--homocysteine methyltransferase [Sesamum indicum]
c50258.graph_c0	6.10865909	4.4162271	5.88740159	0	0	0	6.508E-22	-Inf	down	RecName: Full=60S ribosomal protein L31 [Cyanophora paradoxa]
c50261.graph_c0	2.09356461	2.10918139	2.11753701	24.3268631	18.8638458	19.2875456	3.821E-15	2.292707	up	PREDICTED: U-box domain-containing protein 30-like [Sesamum indicum]
c50265.graph_c0	0	0	0.40805738	11.1166261	40.28877	35.4011831	3.156E-06	6.664676	up	-

#ID	0 h-1 FPKM	0 h-2 FPKM	0 h-3 FPKM	48 h-1 FPKM	48 h-2 FPKM	48 h-3 FPKM	FDR	log2FC	regulated	nr_annotation
c50266.graph_c0	1.49107896	1.21616982	0.7806315	1.27192518	1.0779604	0.63378077	0.0082485	-1.229672	down	-
c50267.graph_c0	8.13674358	9.44533612	7.7321959	1.38583341	1.00099258	1.62545822	4.185E-24	-3.671784	down	hypothetical protein MIMGU_mgv1a021404mg, partial [Erythranthe guttata]
c50270.graph_c0	0	0	0	1.00846066	1.16546377	0.9381096	2.211E-10	Inf	up	hypothetical protein MIMGU_mgv1a025449mg [Erythranthe guttata]
c50271.graph_c0	0.06204401	0.12421227	0	18.0622404	25.9772445	18.6655188	2.397E-36	7.387983	up	PREDICTED: uncharacterized protein LOC105180227 [Sesamum indicum]
c50272.graph_c0	77.9722749	90.1034846	87.7539312	7.9125063	9.12195931	9.07672372	1.8E-141	-4.313217	down	hypothetical protein MIMGU_mgv1a027115mg, partial [Erythranthe guttata]
c50273.graph_c0	0.28382527	0.21308211	0.18236335	5.03345822	5.5400921	5.46755378	1.715E-09	3.546186	up	-
c50276.graph_c0	0.73507531	0.55185853	0.59037555	10.8351891	14.1240205	14.4616292	6.418E-40	3.371134	up	PREDICTED: glycerophosphodiester phosphodiesterase GDPDL3-like [Sesamum indicum]
c50282.graph_c0	32.6504941	27.5795075	33.8933357	1.8969221	1.1483194	1.20562134	9.653E-90	-5.477295	down	hypothetical protein MIMGU_mgv1a026849mg [Erythranthe guttata]
c50283.graph_c0	42.5126105	44.5375646	34.2712369	30.5295429	25.3842339	29.1130916	2.918E-15	-1.525333	down	hypothetical protein PHAVU_005G057600g [Phaseolus vulgaris]
c50286.graph_c0	13.480203	7.25231509	8.46380843	0	0	0	3.212E-25	-Inf	down	predicted protein [Physcomitrella patens]
c50290.graph_c0	0.43567418	0.2907403	0.09330976	329.258398	436.188854	389.96485	9.67E-209	9.450791	up	PREDICTED: GDSL esterase/lipase At5g03810-like [Sesamum indicum]
c50293.graph_c0	0.07798104	0	0.10020872	2.94548992	2.13099765	2.94049864	2.608E-09	4.468559	up	PREDICTED: protein PROTON GRADIENT REGULATION 5, chloroplastic [Sesamum indicum]
c50294.graph_c0	7.15865176	7.06536465	7.99552371	5.11576079	5.58276705	7.15701809	4.917E-09	-1.337734	down	PREDICTED: mitoferrin-like [Sesamum indicum]
c50295.graph_c0	15.2189258	16.8118134	18.0379379	142.157767	121.421278	96.2511473	2.353E-08	1.831422	up	PREDICTED: transcription factor MYB1R1-like [Sesamum indicum]
c50297.graph_c0	2217.19253	2063.96425	2415.48696	73.0238815	86.4028241	84.3286129	5.29E-168	-5.801584	down	PREDICTED: uncharacterized protein LOC100246645 [Vitis vinifera]
c50300.graph_c0	196.129956	231.129658	194.181325	111.697856	160.52902	183.612171	3.847E-18	-1.471181	down	putative heat shock protein 90, partial [Liriodendron tulipifera]
c50301.graph_c0	121.356152	122.957718	118.531762	60.144227	78.9228774	78.6889895	8.894E-33	-1.758753	down	-
c50305.graph_c0	0.54546873	0.39434412	0.27259125	2.48468902	3.90397672	4.84402205	1.619E-05	2.190955	up	PREDICTED: DELLA protein GAI1 [Sesamum indicum]
c50306.graph_c0	0	0	0	4.45165457	4.9255877	6.89517062	1.328E-24	Inf	up	PREDICTED: microtubule-associated protein RP/EB family member 1C [Sesamum indicum]
c50309.graph_c0	39.9011934	43.4474324	39.2387785	10.8735404	14.0459894	18.5389525	1.239E-46	-2.519672	down	PREDICTED: uncharacterized protein LOC105167450 [Sesamum indicum]
c50310.graph_c0	18.7132801	16.7008359	14.4863288	194.000737	205.080171	189.624012	8.579E-39	2.544832	up	PREDICTED: 60S ribosomal protein L15 [Solanum lycopersicum]
c50311.graph_c0	174.21718	181.944648	187.984746	18.9979863	22.6707375	26.7165566	3.89E-147	-4.014826	down	PREDICTED: vegetative incompatibility protein HET-E-1-like [Sesamum indicum]

#ID	0 h-1 FPKM	0 h-2 FPKM	0 h-3 FPKM	48 h-1 FPKM	48 h-2 FPKM	48 h-3 FPKM	FDR	log2FC	regulated	nr_annotation
c50312.graph_c0	0.39633928	0.11335319	0.07275881	9.85860712	9.55985078	12.3923732	3.574E-21	4.764913	up	-
c50313.graph_c0	0.70889398	1.48371711	0.41407129	6.64953547	9.18284798	8.55810805	8.963E-07	2.210348	up	unnamed protein product [Coffea canephora]
c50314.graph_c0	0.29084377	0.43670252	0.24916384	6.83257852	8.61025162	9.29819232	2.321E-15	3.643721	up	PREDICTED: syntaxin-112-like [Sesamum indicum]
c50316.graph_c0	13.0823184	12.2296055	11.2885776	2.16642312	3.56130405	5.11829692	2.527E-49	-2.781644	down	PREDICTED: scarecrow-like protein 8 [Sesamum indicum]
c50321.graph_c0	5.17771059	6.27403113	7.28390065	3.91645776	5.5852086	6.79097136	9.793E-05	-1.229687	down	hypothetical protein MIMGU_mgv1a011362mg [Erythranthe guttata]
c50322.graph_c0	4.884407	3.13653079	2.96068697	16.9805069	18.1686759	18.5087123	0.0002101	1.273745	up	PREDICTED: 40S ribosomal protein S17-like [Sesamum indicum]
c50323.graph_c0	16.9367803	13.488742	20.6523819	137.126519	145.389575	146.640172	2.581E-34	2.04896	up	hypothetical protein MIMGU_mgv1a017626mg [Erythranthe guttata]
c50326.graph_c0	0.83072792	0.76996274	0.86982969	3.93998455	4.47421904	5.06950134	0.0001807	1.426773	up	PREDICTED: poly(A) RNA polymerase cid11 isoform X1 [Sesamum indicum]
c50328.graph_c0	1.29036242	0.86893122	0.81400915	5.53316548	5.40377119	5.59649538	4.769E-06	1.461627	up	PREDICTED: uncharacterized protein LOC105167120 isoform X1 [Sesamum indicum]
c50329.graph_c0	0.74499951	0.60764449	1.02826897	0.35588111	0.72709627	0.70117764	0.0042771	-1.450076	down	PREDICTED: pentatricopeptide repeat-containing protein At2g17525, mitochondrial [Sesamum indicum]
c50330.graph_c0	0.2403748	0.7218465	0.51481856	9.89138625	13.3721034	14.1871637	1.203E-14	3.638572	up	hypothetical protein MIMGU_mgv1a027157mg [Erythranthe guttata]
c50331.graph_c0	26.9534761	26.160114	26.5622853	7.38227846	5.08785231	5.61699091	2.672E-92	-3.149861	down	PREDICTED: UPF0392 protein RCOM_0530710-like [Sesamum indicum]
c50341.graph_c0	9.9267909	10.2727558	7.82634084	1.14867337	1.40409249	1.76898922	8.166E-37	-3.717297	down	PREDICTED: U-box domain-containing protein 8 [Sesamum indicum]
c50344.graph_c0	4.62674263	5.47937397	5.86180736	1.80103821	1.27198773	0.96153178	1.379E-13	-2.994628	down	-
c50349.graph_c0	0.8258109	0.68886485	1.0611997	23.7113151	28.6117811	33.057585	5.883E-20	4.025189	up	PREDICTED: RNA-binding protein 38-like isoform X1 [Nelumbo nucifera]
c50350.graph_c0	0	0.19286941	0.49519378	6.2127146	7.89794135	4.42242887	5.953E-08	3.71288	up	-
c50353.graph_c0	0	0	0	8.94656894	19.3115838	18.4999935	1.516E-17	Inf	up	hypothetical protein MIMGU_mgv1a009974mg [Erythranthe guttata]
c50364.graph_c0	0	0	0	6.39852097	9.89288334	8.46311085	1.458E-12	Inf	up	-
c50366.graph_c0	0.04692523	0.25051849	0.10050129	4.38069892	4.64081931	4.68006788	1.278E-26	4.088588	up	PREDICTED: BTB/POZ domain-containing protein At1g67900 [Sesamum indicum]
c50372.graph_c0	0	0	0	4.32127501	4.68350997	4.1999717	1.072E-21	Inf	up	PREDICTED: cytochrome P450 CYP82D47-like [Sesamum indicum]
c50373.graph_c0	0.3941234	0.07890361	0.20258567	9.14992294	13.0012423	13.9569367	2.153E-18	4.719596	up	PREDICTED: auxin-induced in root cultures protein 12-like [Sesamum indicum]
c50374.graph_c0	1.17101687	0.67338544	0.92849549	27.4524526	31.2223975	27.2863782	6.025E-84	3.936097	up	PREDICTED: succinate dehydrogenase [ubiquinone] flavoprotein subunit 1, mitochondrial isoform X1 [Nicotiana sylvestris]

#ID	0 h-1 FPKM	0 h-2 FPKM	0 h-3 FPKM	48 h-1 FPKM	48 h-2 FPKM	48 h-3 FPKM	FDR	log2FC	regulated	nr_annotation
c50376.graph_c0	5.09929737	4.92838963	6.02556231	1.78192616	1.02967347	1.00898457	4.615E-15	-3.078888	down	-
c50378.graph_c0	86.0655746	82.3279082	82.0315344	59.7849206	62.7958539	62.8923193	8.1E-25	-1.451185	down	hypothetical protein MIMGU_mgv1a003144mg [Erythranthe guttata]
c50379.graph_c0	4.57264018	5.07572799	3.95613475	2.04374361	4.24182571	2.33808528	3.226E-07	-1.682939	down	PREDICTED: uncharacterized protein LOC105165869 [Sesamum indicum]
c50380.graph_c0	13.7275447	11.8834122	6.07515101	265.096971	356.336618	300.672366	3.143E-61	3.848497	up	hypothetical protein MIMGU_mgv1a001548mg [Erythranthe guttata]
c50390.graph_c0	2.72507421	1.70487685	2.40750692	0.15690726	0.08311211	0.2094227	8.054E-15	-4.94187	down	-
c50392.graph_c0	0.28398775	0	0	2.7470944	3.04879501	2.79354066	7.956E-08	3.921202	up	60S ribosomal protein L11 [Zea mays]
c50393.graph_c0	73.6508277	72.5687614	72.1225064	14.8924765	4.46740256	3.54943822	1.37E-163	-4.242661	down	hypothetical protein MIMGU_mgv1a014429mg [Erythranthe guttata]
c50401.graph_c0	2.54984714	2.04192034	2.44656871	1.22152446	1.16133325	0.69673405	3.855E-08	-2.205626	down	PREDICTED: cytochrome P450 71A21-like [Sesamum indicum]
c50411.graph_c0	0	0	0	3.31929961	4.10246136	7.38373208	6.381E-08	Inf	up	hypothetical protein MIMGU_mgv1a015512mg [Erythranthe guttata]
c50414.graph_c0	0	0	0	1.85834811	3.12675006	2.77212286	1.561E-12	Inf	up	60S ribosomal protein L13a [Rozella allomycis CSF55]
c50419.graph_c0	15.4534843	17.6788099	14.3779534	7.51127638	6.70860465	6.99195726	1.082E-40	-2.177604	down	PREDICTED: uncharacterized protein At5g05190 [Sesamum indicum]
c50427.graph_c0	0	0.03751075	0.04815458	10.5294491	10.9352384	11.3657213	7.155E-41	7.550391	up	PREDICTED: geraniol 8-hydroxylase-like [Sesamum indicum]
c50429.graph_c0	0.14737923	0.88516085	0	4.20903616	5.32198862	4.95329605	9.305E-06	2.801986	up	hypothetical protein MIMGU_mgv1a000543mg [Erythranthe guttata]
c50432.graph_c0	0.80679455	0.08076024	0.31102888	6.39213714	7.95280367	8.33312016	1.876E-09	3.226603	up	hypothetical protein M569_10692 [Genlisea aurea]
c50437.graph_c0	0.21504522	0.4783568	0.27634163	8.43083798	8.97808997	7.9326165	2.039E-28	3.691137	up	PREDICTED: probable leucine-rich repeat receptor-like protein kinase At1g68400 [Sesamum indicum]
c50439.graph_c0	6.28449911	8.29019112	7.38719349	2.91728591	2.75768645	2.31623755	1.19E-16	-2.474066	down	PREDICTED: putative clathrin assembly protein At4g40080 [Sesamum indicum]
c50443.graph_c0	8.15727464	7.55303469	7.86181565	54.4838475	54.6672192	63.0789433	5.76E-24	1.850682	up	hypothetical protein MIMGU_mgv1a017588mg [Erythranthe guttata]
c50444.graph_c0	3.46002735	3.73899709	2.5768168	2.57181595	2.1105492	2.03054002	8.961E-06	-1.552127	down	PREDICTED: exocyst complex component EXO70A1-like [Sesamum indicum]
c50446.graph_c0	0	0.1429521	0	4.03466173	12.5439502	13.0723472	1.655E-07	6.673461	up	PREDICTED: short-chain dehydrogenase reductase 2a [Sesamum indicum]
c50451.graph_c0	0	0	0	1.69241003	2.86864514	3.19250009	7.233E-14	Inf	up	PREDICTED: premnaspirodiene oxygenase-like [Sesamum indicum]
c50454.graph_c0	87.9634008	84.0334318	84.5277496	4.41372722	4.97067962	4.38726292	3.51E-221	-5.237843	down	PREDICTED: LOW QUALITY PROTEIN: putative ETHYLENE INSENSITIVE 3-like 4 protein [Sesamum indicum]
c50458.graph_c0	0.05331743	0.10674165	0.13703003	1.4490252	1.48303035	2.0323399	9.591E-08	3.035129	up	PREDICTED: uncharacterized protein LOC105166786 isoform X1 [Sesamum indicum]

#ID	0 h-1 FPKM	0 h-2 FPKM	0 h-3 FPKM	48 h-1 FPKM	48 h-2 FPKM	48 h-3 FPKM	FDR	log2FC	regulated	nr_annotation
c50460.graph_c0	0.18373335	0.06130578	0	1.04381405	1.07590808	0.55224725	0.002027	2.45216	up	unnamed protein product [Coffea canephora]
c50463.graph_c0	0.6022821	0.6028852	0.53209499	7.73338298	5.91482345	4.72112558	6.853E-07	2.393585	up	PREDICTED: putative E3 ubiquitin-protein ligase XBAT31 [Sesamum indicum]
c50469.graph_c0	0	0	0	0.37919845	1.00428695	0.84352126	4.601E-07	Inf	up	PREDICTED: uncharacterized protein LOC105170283 [Sesamum indicum]
c50472.graph_c0	0	0	0	35.4457847	41.3926504	38.2087696	1.29E-105	Inf	up	PREDICTED: 1-aminocyclopropane-1-carboxylate oxidase homolog 1-like [Sesamum indicum]
c50479.graph_c0	0.85391267	0.44773548	0.41802346	2.62226684	4.08758994	2.79993272	0.0029591	1.449611	up	PREDICTED: putative receptor-like protein kinase At5g39000 [Sesamum indicum]
c50480.graph_c0	50.399795	48.3273557	55.547837	38.1566437	48.092841	52.8192068	1.34E-14	-1.17333	down	PREDICTED: calcyclin-binding protein-like [Sesamum indicum]
c50481.graph_c0	0.75901224	0.81605171	0.43349368	11.9649715	12.7028725	13.2272954	1.383E-29	3.224568	up	hypothetical protein MIMGU_mgv1a003752mg [Erythranthe guttata]
c50482.graph_c0	0.44260548	0.08860974	0	1003.00075	905.320913	867.864859	9.75E-218	11.35617	up	PREDICTED: putative lipid-binding protein AIR1B [Beta vulgaris subsp. vulgaris]
c50489.graph_c0	36.6009765	38.3560896	32.826525	34.5416265	27.5226881	26.6632099	1.373E-20	-1.291937	down	PREDICTED: transcription factor MYC4-like [Sesamum indicum]
c50491.graph_c0	0	0.05785681	0.07427391	29.765712	35.4254492	38.5672203	4.055E-83	8.582472	up	PREDICTED: adenine phosphoribosyltransferase 1-like [Sesamum indicum]
c50495.graph_c0	0.0576275	0.0576852	0.14810723	11.2551246	14.7355717	17.5730381	1.236E-30	6.334806	up	PREDICTED: glycerophosphodiester phosphodiesterase GDPDL3-like [Sesamum indicum]
c50497.graph_c0	0	0	0	3.02735892	3.42578694	2.93860992	2.401E-12	Inf	up	60S ribosomal protein L7a [Zea mays]
c50500.graph_c0	0	0.05444149	0	2.55534894	2.33552203	2.09540204	5.526E-15	6.000583	up	PREDICTED: uncharacterized protein LOC105175608 [Sesamum indicum]
c50502.graph_c0	41.2494328	42.6694525	35.4893789	7.84109758	11.3743087	12.8538211	8.035E-81	-2.91984	down	PREDICTED: myb-like protein X [Sesamum indicum]
c50507.graph_c0	2.55333697	2.2757958	1.57314888	25.6819663	27.6165693	29.0161595	3.396E-38	2.669837	up	hypothetical protein MIMGU_mgv1a005093mg [Erythranthe guttata]
c50509.graph_c0	19.5535103	18.8324868	18.7434098	95.3279768	91.085583	88.8944221	4.835E-13	1.252727	up	PREDICTED: serine/threonine-protein phosphatase PP2A-2 catalytic subunit [Sesamum indicum]
c50510.graph_c0	0.08613714	0.03448936	0.04427585	1.96800776	2.57245512	3.21980399	1.432E-17	4.537203	up	PREDICTED: probable RNA-dependent RNA polymerase 5 [Sesamum indicum]
c50511.graph_c0	0	0	0	2.41864253	4.24374061	3.29539329	3.349E-12	Inf	up	hypothetical protein MIMGU_mgv1a014011mg [Erythranthe guttata]
c50512.graph_c0	3.64857777	3.68734888	3.47134279	1.77761471	2.08860367	1.5529525	3.106E-09	-2.013732	down	-
c50514.graph_c0	0	0	0	4.55924979	5.04951651	6.96108807	7.518E-19	Inf	up	PREDICTED: auxin-induced protein 6B-like [Sesamum indicum]
c50519.graph_c0	4.96080443	4.28862121	3.76694506	4.59989334	3.26964542	2.82546094	2.454E-06	-1.290212	down	PREDICTED: vinorine synthase-like isoform X1 [Sesamum indicum]
c50520.graph_c0	31.6957508	41.3448845	14.5101675	571.411276	567.543401	394.198025	1.788E-15	3.123342	up	PREDICTED: dirigent protein 22-like [Sesamum indicum]

#ID	0 h-1 FPKM	0 h-2 FPKM	0 h-3 FPKM	48 h-1 FPKM	48 h-2 FPKM	48 h-3 FPKM	FDR	log2FC	regulated	nr_annotation
c50523.graph_c0	0.12596515	0.12609129	0.08093511	3.36537217	3.6881418	4.28521547	6.805E-10	4.073776	up	PREDICTED: RAN GTPase-activating protein 2-like isoform X2 [Sesamum indicum]
c50525.graph_c0	2.29575436	2.01079656	2.67356006	0.06609371	0.28007308	0	5.766E-17	-5.370622	down	PREDICTED: uncharacterized protein LOC105162053 [Sesamum indicum]
c50526.graph_c0	19.7784066	26.8037327	19.1597806	16.8195307	9.50306841	9.47841425	7.797E-07	-1.880809	down	hypothetical protein M569_14808, partial [Genlisea aurea]
c50528.graph_c0	0.44034514	0.22039304	0.14146523	44.6242127	55.4394416	74.9908681	9.677E-27	6.755051	up	hypothetical protein CICLE_v10032302mg [Citrus clementina]
c50530.graph_c0	0.06171555	0.06177735	0.39653463	1.1371278	2.4695305	2.12479991	0.0005416	2.411683	up	-
c50531.graph_c0	0	0	0	2.16147167	2.21085793	2.38750075	1.868E-11	Inf	up	ADP/ATP translocase [Cyanidioschyzon merolae strain 10D]
c50532.graph_c0	0	0	0.14587044	5.22884135	4.87460457	6.79278372	6.02E-12	5.804602	up	PREDICTED: chromosome-associated kinesin KIF4A [Sesamum indicum]
c50533.graph_c0	0	0	0	2.43776052	2.46512478	2.90857214	1.884E-17	Inf	up	hypothetical protein MIMGU_mgv1a002430mg [Erythranthe guttata]
c50535.graph_c0	9.42879944	3.88114582	7.24718091	0	0.344008	0.07223485	2.251E-11	-6.676435	down	60s ribosomal protein l10 [Lichtheimia corymbifera JMRC:FSU:9682]
c50538.graph_c0	0	0	0	68.5001193	117.922302	135.655911	7.256E-36	Inf	up	PREDICTED: beta-glucosidase 12-like [Sesamum indicum]
c50542.graph_c0	0.07725753	0	0.09927898	4.341655	4.59945702	4.11650169	1.2E-11	5.18192	up	hypothetical protein L484_017688 [Morus notabilis]
c50544.graph_c0	0	0	0	2.32720774	3.45155217	4.1414709	1.355E-11	Inf	up	PREDICTED: glutaredoxin-C5-like [Sesamum indicum]
c50545.graph_c0	1.11761958	0.88727553	1.18856723	8.69854804	12.4121174	12.4470482	4.018E-14	2.367989	up	PREDICTED: uncharacterized protein LOC105163839 [Sesamum indicum]
c50546.graph_c0	5.41415791	4.57277009	3.91354159	18.8604039	22.126956	28.5707937	0.0009194	1.303612	up	hypothetical protein ZEAMMB73_107586 [Zea mays]
c50549.graph_c0	1.53756357	2.19871886	1.62300326	1.06237788	1.23264727	0.81025496	0.0001268	-1.803842	down	PREDICTED: probable N-acetyltransferase HLS1 [Sesamum indicum]
c50555.graph_c0	0	0	0	3.99157481	2.77501187	4.3286091	1.642E-11	Inf	up	hypothetical protein MIMGU_mgv1a016013mg [Erythranthe guttata]
c50557.graph_c0	0	0	0	2.22673317	2.90937634	1.84924487	2.407E-11	Inf	up	hypothetical protein SELMODRAFT_103994 [Selaginella moellendorffii]
c50559.graph_c0	713.74997	751.060965	713.806162	188.906157	116.779812	88.6346652	3.03E-82	-3.470929	down	PREDICTED: nematode resistance protein-like HSPRO2 [Sesamum indicum]
c50561.graph_c0	0.31962102	0.55989687	0.10268142	2.42925975	3.50932656	2.29254319	0.0005181	2.055515	up	-
c50562.graph_c0	1.31345813	1.23509013	1.32981782	35.9712872	46.6920233	47.6679696	3.589E-62	4.047832	up	PREDICTED: coatomer subunit gamma-2 isoform X1 [Sesamum indicum]
c50563.graph_c0	0	0	0	0.53666284	1.20812457	1.0346253	1.799E-08	Inf	up	PREDICTED: putative transcription factor bHLH086 [Sesamum indicum]
c50565.graph_c0	0	0	0	6.07091929	18.7384083	17.6060866	2.484E-09	Inf	up	PREDICTED: pathogenesis-related protein PR-1-like [Sesamum indicum]
c50569.graph_c0	0.08393233	0	0	4.3301405	10.1575016	10.6643681	3.208E-09	7.217101	up	unnamed protein product [Coffea canephora]

#ID	0 h-1 FPKM	0 h-2 FPKM	0 h-3 FPKM	48 h-1 FPKM	48 h-2 FPKM	48 h-3 FPKM	FDR	log2FC	regulated	nr_annotation
c50570.graph_c0	0.42947954	0.19107093	0.49057619	11.9138996	13.645942	14.8256356	8.865E-27	4.158851	up	PREDICTED: uncharacterized protein LOC105162704 [Sesamum indicum]
c50572.graph_c0	0	0	0	2.12198157	2.45234418	2.78927769	6.61E-13	Inf	up	PREDICTED: glyoxylate/succinic semialdehyde reductase 1 [Sesamum indicum]
c50575.graph_c0	0.61530483	0.57192661	0.5930183	3.27968388	3.64600405	3.94503219	1.94E-05	1.590405	up	PREDICTED: probable beta-D-xylosidase 7 [Sesamum indicum]
c50578.graph_c0	48.7998054	50.9257011	53.0316523	3.53996565	3.60015779	3.14984015	1.62E-138	-4.909412	down	PREDICTED: stachyose synthase [Sesamum indicum]
c50580.graph_c0	0.61297274	0.68176282	0.35008633	7.94777694	12.097788	13.1574265	3.161E-15	3.315595	up	PREDICTED: centrosome-associated protein CEP250 [Sesamum indicum]
c50581.graph_c0	0.26525512	0	0	31.1572152	33.6544933	41.2047354	2.325E-37	7.643553	up	-
c50587.graph_c0	0	0	0	8.82150393	10.0870053	12.8330171	1.792E-15	Inf	up	PREDICTED: 7-deoxyloganetin glucosyltransferase-like [Eucalyptus grandis]
c50588.graph_c0	20.8989179	22.2468501	26.0542672	9.84224681	10.7310953	9.97212581	6.646E-24	-2.200559	down	PREDICTED: 1,2-dihydroxy-3-keto-5-methylthiopentene dioxygenase 2 [Sesamum indicum]
c50589.graph_c0	286.708522	275.459707	283.258028	246.185405	228.92552	207.049888	1.233E-19	-1.324784	down	PREDICTED: coiled-coil domain-containing protein 9-like [Sesamum indicum]
c50592.graph_c0	0.44148119	0.41430306	0.31911796	29.1059908	31.9383771	31.982748	3.59E-111	5.29099	up	PREDICTED: dihydrolipoyl dehydrogenase 2, chloroplastic-like isoform X1 [Sesamum indicum]
c50601.graph_c0	0.08169489	0.16355339	0.27994977	12.3179886	10.0461894	12.6346787	9.256E-47	5.032417	up	PREDICTED: protein CYPRO4 [Sesamum indicum]
c50602.graph_c0	0.17728643	0.4056319	0.45564016	2.54324753	3.60882849	3.50863591	2.946E-07	2.187725	up	PREDICTED: putative 1-phosphatidylinositol-3-phosphate 5-kinase FAB1C isoform X2 [Populus euphratica]
c50608.graph_c0	0.54939381	0.43995516	0.84719136	5.1626016	4.93295919	6.9355136	4.212E-05	2.187739	up	PREDICTED: palmitoyl-monogalactosyldiacylglycerol delta-7 desaturase, chloroplastic [Sesamum indicum]
c50610.graph_c0	41.4271626	37.0731854	38.8513093	45.2944763	25.7834993	24.9605628	1.308E-08	-1.294303	down	PREDICTED: uncharacterized acetyltransferase At3g50280-like [Sesamum indicum]
c50612.graph_c0	2.89303925	3.20901037	2.11002891	0.28813583	0.30524498	0.12819077	2.385E-15	-4.518041	down	PREDICTED: uncharacterized protein LOC105161205 [Sesamum indicum]
c50616.graph_c0	1.44832106	1.67989377	1.8611499	2.13909636	0.76284999	0.73495889	0.0004034	-1.453693	down	PREDICTED: G-type lectin S-receptor-like serine/threonine-protein kinase At2g19130 [Sesamum indicum]
c50618.graph_c0	14.5959802	8.74138216	10.3400735	0	0	0	2.766E-34	-Inf	down	NADH dehydrogenase subunit 2 [Amorphotheca resinae]
c50620.graph_c0	0.51292871	0.51344233	0.47080978	2.97027269	4.4339071	4.50499564	0.000223	1.967955	up	PREDICTED: zinc finger protein ZAT5 [Sesamum indicum]
c50621.graph_c0	6.86703354	9.87630723	6.69437998	24.7964194	32.5086024	41.1510742	0.0012291	1.048354	up	PREDICTED: plastidic ATP/ADP-transporter [Sesamum indicum]
c50625.graph_c0	2.81922329	3.03108678	1.94558505	0.72145899	0.45857899	0.98432314	5.622E-10	-2.860776	down	PREDICTED: salicylate carboxymethyltransferase [Sesamum indicum]
c50627.graph_c0	0.38795624	0.6472412	0.08308986	6.49295915	8.51924672	8.53358492	3.696E-11	3.385349	up	hypothetical protein MIMGU_mgv1a009322mg [Erythranthe guttata]

#ID	0 h-1 FPKM	0 h-2 FPKM	0 h-3 FPKM	48 h-1 FPKM	48 h-2 FPKM	48 h-3 FPKM	FDR	log2FC	regulated	nr_annotation
c50631.graph_c0	1.58383495	1.05694728	1.91193967	31.7913924	35.5059486	36.1170946	9.122E-52	3.482214	up	PREDICTED: uncharacterized protein LOC105167007 [Sesamum indicum]
c50634.graph_c0	2.78354283	2.67634341	1.74141607	1.34967432	1.35832547	1.291002	1.674E-06	-1.861186	down	PREDICTED: anthocyanidin 3-O-glucosyltransferase-like [Sesamum indicum]
c50640.graph_c0	303.97244	300.207546	317.481325	53.8815791	76.5346137	87.5022715	1.081E-97	-3.105631	down	PREDICTED: uncharacterized protein LOC105162278 [Sesamum indicum]
c50641.graph_c0	0	0.16129723	0	2.00405985	1.96579482	1.78320041	1.192E-08	4.151941	up	PREDICTED: KH domain-containing protein At4g18375-like [Glycine max]
c50642.graph_c0	49.6168481	42.571313	48.8371513	201.319729	231.819394	237.113358	4.406E-16	1.228101	up	PREDICTED: 60S ribosomal protein L6-like [Sesamum indicum]
c50648.graph_c0	4.53925679	2.54896219	3.84132601	0	0	0	4.904E-20	-Inf	down	60S ribosomal protein L30 [Nectria haematococca mpVI 77-13-4]
c50650.graph_c0	0	0	0	2.22712283	2.60772096	2.19027845	6.941E-16	Inf	up	PREDICTED: asparagine--tRNA ligase, cytoplasmic 2 [Sesamum indicum]
c50651.graph_c0	0.07805779	0	0	1.65397439	3.12346076	4.09515537	8.168E-08	5.82032	up	PREDICTED: scarecrow-like protein 32 [Sesamum indicum]
c50652.graph_c0	4.50628147	5.2537481	5.99512951	0.29304568	0.36218742	0.2172919	5.073E-30	-5.195189	down	PREDICTED: uncharacterized protein LOC105162763 [Sesamum indicum]
c50653.graph_c0	0	0	0	2.4724756	2.8748284	2.09267715	6.207E-12	Inf	up	unnamed protein product [Coffea canephora]
c50654.graph_c0	3.82155568	4.36592555	4.13685763	1.49245639	2.57431699	3.04754091	4.834E-13	-1.82089	down	PREDICTED: pentatricopeptide repeat-containing protein At1g15510, chloroplastic [Sesamum indicum]
c50658.graph_c0	0.20065574	0.26780889	0.08595019	6.77809567	7.44168213	9.64977404	1.137E-14	4.415091	up	hypothetical protein MIMGU_mgv1a000413mg [Erythranthe guttata]
c50662.graph_c0	9.02014408	10.3940519	9.70429585	7.05379058	7.21672362	9.11370639	6.235E-08	-1.336331	down	hypothetical protein MIMGU_mgv1a005797mg [Erythranthe guttata]
c50663.graph_c0	1.23502862	0.78671429	1.0099477	0.17239226	0.25568018	0.18407232	7.214E-10	-3.330085	down	PREDICTED: cell division cycle 20.2, cofactor of APC complex-like [Sesamum indicum]
c50664.graph_c0	0	0	0	4.50097689	3.97353271	3.22620342	2.36E-11	Inf	up	-
c50666.graph_c0	0.23901213	0.13956335	0.10238002	17.4503399	11.64398	11.5270331	3.472E-17	5.398066	up	PREDICTED: protein LONGIFOLIA 1 [Sesamum indicum]
c50667.graph_c0	0.79374257	0.39726869	0.50999531	4.29607864	4.2606739	4.55461695	0.0004598	1.931338	up	PREDICTED: BEL1-like homeodomain protein 4 [Sesamum indicum]
c50671.graph_c0	5.04412659	5.86457265	5.91825869	1.03907592	2.87424568	3.90371258	5.65E-15	-2.14146	down	PREDICTED: uncharacterized protein LOC105159806 [Sesamum indicum]
c50675.graph_c0	4.38530982	4.81111235	3.69675466	2.94114434	2.53371593	3.50852232	2.819E-07	-1.536439	down	PREDICTED: BTB/POZ domain-containing protein NPY4-like [Sesamum indicum]
c50677.graph_c0	0.0459518	0.09199564	0	1.52401679	0.94179808	1.73274458	5.115E-07	3.928861	up	hypothetical protein MIMGU_mgv1a012682mg [Erythranthe guttata]
c50679.graph_c0	2.58355395	2.58614099	2.45923638	7.31672179	13.8836388	16.707348	0.0082355	1.284196	up	PREDICTED: uncharacterized protein LOC105164931 [Sesamum indicum]

#ID	0 h-1 FPKM	0 h-2 FPKM	0 h-3 FPKM	48 h-1 FPKM	48 h-2 FPKM	48 h-3 FPKM	FDR	log2FC	regulated	nr_annotation
c50680.graph_c0	345.41294	353.58575	361.081399	121.960805	75.0942403	62.6640729	6.223E-84	-3.035386	down	PREDICTED: transcription factor HBP-1b(c38)-like [Sesamum indicum]
c50683.graph_c0	7.53892895	5.65985854	5.65122961	0.86816913	1.53286655	1.28748613	1.112E-09	-3.377709	down	hypothetical protein MIMGU_mgv1a022729mg [Erythranthe guttata]
c50685.graph_c0	40.9749491	44.192071	38.0988354	16.5642093	18.5133981	17.591392	1.317E-52	-2.244839	down	hypothetical protein MIMGU_mgv1a008021mg [Erythranthe guttata]
c50686.graph_c0	0.14386653	0.1440106	0.06162473	8.7034098	11.4667553	11.9111941	3.837E-27	5.505274	up	PREDICTED: PI-PLC X-box domain-containing protein DDB_G0293730 [Sesamum indicum]
c50688.graph_c0	0	0	0	1.87568236	1.98705802	1.66897088	2.211E-10	Inf	up	hypothetical protein L969DRAFT_613963, partial [Mixia osmundae IAM 14324]
c50690.graph_c0	0	0.03794991	0	2.61952208	3.99609506	3.3564024	3.279E-16	7.025369	up	PREDICTED: uncharacterized protein LOC105174731 [Sesamum indicum]
c50691.graph_c0	0	0	0	3.1848023	1.86641933	1.68823196	1.155E-06	Inf	up	Leucine-rich repeat transmembrane protein kinase [Theobroma cacao]
c50692.graph_c0	0	0	0	2.31375012	2.05579291	1.9923493	2.036E-11	Inf	up	actin [Pseudozyma antarctica]
c50693.graph_c0	273.781085	280.142221	299.616389	170.968345	100.506341	68.7939032	2.79E-34	-2.330949	down	PREDICTED: organic cation/carnitine transporter 3-like [Sesamum indicum]
c50694.graph_c0	0	0	0	1.28533383	0.94999209	1.14368231	9.972E-12	Inf	up	PREDICTED: protein kinase PINOID [Sesamum indicum]
c50696.graph_c0	0	0.0494687	0	15.8438162	18.7138667	26.0078856	1.787E-29	9.246474	up	PREDICTED: (RS)-norcoclaurine 6-O-methyltransferase-like [Sesamum indicum]
c50700.graph_c0	0.99395712	2.27417697	1.64220976	113.284941	142.739039	108.249701	8.719E-59	5.191844	up	-
c50702.graph_c0	0.20345731	0.13577403	0.17430047	1.74942271	1.85330123	1.77900092	5.163E-05	2.371654	up	-
c50703.graph_c0	0.7552396	0.54431701	0.23292307	12.6352917	15.5968303	16.2698765	3.073E-39	3.847892	up	PREDICTED: fructokinase-like 2, chloroplastic [Sesamum indicum]
c50706.graph_c0	0	0	0	5.94422537	4.65819239	4.99932032	3.247E-14	Inf	up	PREDICTED: uncharacterized protein LOC105155422 [Sesamum indicum]
c50709.graph_c0	0	0.79007248	0	13.8156255	8.21669052	4.96035774	0.0015144	4.103527	up	-
c50710.graph_c0	2.69964503	2.83746573	1.90803291	0.66322376	1.88825134	1.54909784	5.495E-06	-1.889729	down	PREDICTED: multiple C2 and transmembrane domain-containing protein 1-like [Sesamum indicum]
c50711.graph_c0	0	0.05944843	0.15263434	3.99404802	3.41974472	2.77494733	6.494E-11	4.55334	up	-
c50715.graph_c0	0.20363145	0.47561584	0.34889931	2.75144581	4.90220266	5.7310603	3.864E-06	2.672666	up	PREDICTED: aspartic proteinase nepenthesin-2-like [Sesamum indicum]
c50718.graph_c0	11.2470347	12.1916272	10.6711715	3.97280385	3.92433207	4.77699592	4.437E-37	-2.446182	down	PREDICTED: formin BNI1-like [Sesamum indicum]
c50719.graph_c0	6.27084837	5.7064797	6.34895451	41.9278464	40.9864747	42.4475873	2.776E-13	1.756367	up	PREDICTED: transmembrane protein 256 homolog [Sesamum indicum]
c50721.graph_c0	2.07970969	0.80958586	1.33625465	10.4313607	8.23168994	5.58799961	0.005925	1.513491	up	PREDICTED: LOW QUALITY PROTEIN: GDSL esterase/lipase At2g27360-like [Sesamum indicum]

#ID	0 h-1 FPKM	0 h-2 FPKM	0 h-3 FPKM	48 h-1 FPKM	48 h-2 FPKM	48 h-3 FPKM	FDR	log2FC	regulated	nr_annotation
c50723.graph_c0	2.82617691	2.73470667	2.23957869	50.4675807	40.224632	38.4189347	1.414E-19	3.038763	up	hypothetical protein MIMGU_mgv1a005711mg [Erythranthe guttata]
c50724.graph_c0	2.2845635	2.68456439	2.72301872	38.860488	56.8725557	64.4330965	2.438E-17	3.353696	up	PREDICTED: protein ASPARTIC PROTEASE IN GUARD CELL 1-like [Sesamum indicum]
c50726.graph_c0	5.91185096	6.075578	7.09049837	4.28448773	4.69275608	7.94770007	0.001162	-1.197986	down	PREDICTED: magnesium-dependent phosphatase 1-like [Sesamum indicum]
c50728.graph_c0	1.95585023	2.84772177	1.82788726	0.32761033	0.0578439	0.24292142	4.953E-15	-4.39682	down	PREDICTED: UDP-glycosyltransferase 76C4-like [Sesamum indicum]
c50732.graph_c0	3.83176444	2.94237913	2.76551684	14.700571	19.2619264	17.813548	1.493E-07	1.420959	up	PREDICTED: heat stress transcription factor C-1-like [Sesamum indicum]
c50734.graph_c0	7.20201781	7.8138746	7.40390242	4.83710611	6.16733209	3.88505176	1.396E-09	-1.609503	down	PREDICTED: putative chromatin-remodeling complex ATPase chain isoform X1 [Sesamum indicum]
c50735.graph_c0	0.28607846	0.28636492	0.18381107	27.541407	23.0343009	13.7187697	3.757E-10	5.404147	up	PREDICTED: uncharacterized protein LOC104450130 [Eucalyptus grandis]
c50737.graph_c0	4.20627716	5.62608458	5.12565168	4.17575094	4.98993561	3.92362933	1.243E-05	-1.212578	down	PREDICTED: uncharacterized protein LOC105171448 [Sesamum indicum]
c50738.graph_c0	0	0	0.24172594	7.84169608	10.3267866	6.59199711	5.09E-22	5.626347	up	elongation factor 1-alpha [Lichtheimia corymbifera JMRC:FSU:9682]
c50739.graph_c0	0.31447309	0.44969712	0.34638032	7.16005287	7.10291136	9.7221769	6.875E-15	3.41345	up	PREDICTED: uncharacterized protein LOC105163344 [Sesamum indicum]
c50740.graph_c0	3.76372764	2.47925572	2.87071994	0	0	0	1.681E-43	-Inf	down	hypothetical protein SELMODRAFT_118280 [Selaginella moellendorffii]
c50741.graph_c0	0	0	0	2.60735781	2.76217944	2.3200113	1.659E-09	Inf	up	hypothetical protein M569_01958 [Genlisea aurea]
c50742.graph_c0	7.37808179	7.71371292	7.37421005	47.2780948	46.5650285	54.0295249	3.017E-09	1.700411	up	hypothetical protein MIMGU_mgv1a016086mg [Erythranthe guttata]
c50746.graph_c0	0.73528336	0.44161178	0.18897365	7.34969227	8.18079526	9.25203493	1.052E-15	3.17171	up	hypothetical protein MIMGU_mgv1a005428mg [Erythranthe guttata]
c50747.graph_c0	10.3226229	11.1191629	11.044537	6.14007053	6.94268446	6.86143903	1.146E-23	-1.72397	down	PREDICTED: exocyst complex component EXO70B1 [Sesamum indicum]
c50748.graph_c0	0.12265192	0	0.07880629	9.83056062	11.3120703	11.0093738	1.202E-24	6.298452	up	hypothetical protein MIMGU_mgv1a006779mg [Erythranthe guttata]
c50749.graph_c0	0.23840847	0.2386472	0.20424288	2.34279924	3.17994938	3.12691301	1.042E-05	2.645019	up	PREDICTED: protein IRX15-LIKE-like [Nicotiana tomentosiformis]
c50754.graph_c0	0	0.0813497	0	9.69562355	11.064491	14.7560179	2.805E-32	7.759731	up	PREDICTED: chaperonin CPN60, mitochondrial [Sesamum indicum]
c50762.graph_c1	2.89160879	3.67107862	2.26575108	2.59896451	2.40912678	3.12193304	0.0078086	-1.133637	down	-

#ID	0 h-1 FPKM	0 h-2 FPKM	0 h-3 FPKM	48 h-1 FPKM	48 h-2 FPKM	48 h-3 FPKM	FDR	log2FC	regulated	nr_annotation
c50763.graph_c0	1.80695601	2.08008021	2.28330984	55.8224615	56.2860847	65.0011937	2.94E-108	3.822421	up	PREDICTED: dihydrolipoyllysine-residue acetyltransferase component 5 of pyruvate dehydrogenase complex, chloroplastic [Sesamum indicum]
c50764.graph_c0	0	0	0	6.98873463	8.61248703	6.72616948	5.615E-13	Inf	up	PREDICTED: uncharacterized protein LOC100267736 [Vitis vinifera]
c50766.graph_c0	1.18041195	0.89903888	1.15414486	22.529593	37.5666924	39.5254381	4.964E-16	3.917814	up	PREDICTED: uncharacterized protein LOC105178721 [Sesamum indicum]
c50767.graph_c0	6.10538876	6.48498308	5.84066267	1.09368823	1.65518581	1.55705154	4.335E-27	-3.121522	down	PREDICTED: uncharacterized protein LOC102659506 [Glycine max]
c50770.graph_c0	0.47975642	0.38418946	0.1233012	21.2151794	23.1304283	24.3043165	4.495E-27	5.11102	up	cyclin D [Scutellaria baicalensis]
c50771.graph_c0	2.89812941	2.53129215	3.10350919	1.33497292	1.27559077	1.74683953	1.446E-08	-1.990002	down	PREDICTED: pentatricopeptide repeat-containing protein At3g05340 [Sesamum indicum]
c50772.graph_c0	0	0	0	32.2975823	48.8623637	69.5818614	4.711E-18	Inf	up	PREDICTED: uncharacterized protein LOC105158508 [Sesamum indicum]
c50773.graph_c0	0.19101167	0	0.08181919	1.52509424	2.60990007	2.9750047	2.707E-07	3.681225	up	PREDICTED: probable leucine-rich repeat receptor-like protein kinase At1g68400 [Sesamum indicum]
c50774.graph_c0	2.83675133	2.97481057	2.43022518	0.37334291	0.39551154	0.11073276	1.387E-12	-4.2376	down	-
c50775.graph_c0	0	0	0	3.84929357	5.03735621	5.10403821	5.425E-14	Inf	up	kunitz trypsin inhibitor [Populus tremula]
c50776.graph_c0	17.667833	19.8000983	19.0021478	3.71534743	8.05934686	7.39890518	2.1E-12	-2.588554	down	PREDICTED: RNA-binding protein cabeza [Nicotiana sylvestris]
c50777.graph_c0	0	0	0	1.26355647	1.97265138	1.42017467	1.128E-10	Inf	up	hypothetical protein CICLE_v10023373mg [Citrus clementina]
c50781.graph_c0	0.17726384	0	0.07593035	2.99396182	3.11407152	4.74678007	1.044E-10	4.405362	up	PREDICTED: glutaminyl-peptide cyclotransferase [Sesamum indicum]
c50786.graph_c0	0.35852555	0.29907046	0.46071952	5.78019956	4.4905084	5.04522135	8.698E-08	2.758661	up	PREDICTED: uncharacterized protein LOC105162253 isoform X1 [Sesamum indicum]
c50789.graph_c0	21.9447532	22.9743756	25.0953031	11.3140662	9.0385334	5.94129132	4.92E-13	-2.423883	down	PREDICTED: uncharacterized protein LOC102593092 [Solanum tuberosum]
c50792.graph_c0	0.03241004	0.03244249	0.08329637	11.1371248	8.85673273	12.3008383	1.054E-39	6.741099	up	PREDICTED: cell division cycle 20.2, cofactor of APC complex-like [Sesamum indicum]
c50794.graph_c0	24.1375075	19.2950702	22.6582854	0.12616764	0.2339038	0.28065802	3.61E-137	-7.717658	down	PREDICTED: cytochrome P450 86B1-like [Sesamum indicum]
c50795.graph_c0	10.965696	9.76266621	8.57169893	52.699631	54.0040737	66.6509982	3.32E-18	1.547403	up	PREDICTED: caffeoylshikimate esterase [Sesamum indicum]
c50796.graph_c0	0.72185373	0.12042943	0.15460177	5.43098638	10.9198541	10.5525126	1.587E-07	3.736879	up	PREDICTED: glucomannan 4-beta-mannosyltransferase 2-like [Sesamum indicum]
c50803.graph_c0	176.448717	201.715778	152.243778	25.0022361	27.9827719	24.6858616	5.888E-58	-3.788265	down	-
c50807.graph_c0	0	0	0	4.24067316	3.88772221	4.49896499	5.679E-13	Inf	up	PREDICTED: uncharacterized protein LOC105159678 [Sesamum indicum]
c50809.graph_c0	0.06191221	0.12394841	0	7.52895838	4.35055553	3.55261767	2.006E-06	5.386181	up	-

#ID	0 h-1 FPKM	0 h-2 FPKM	0 h-3 FPKM	48 h-1 FPKM	48 h-2 FPKM	48 h-3 FPKM	FDR	log2FC	regulated	nr_annotation
c50811.graph_c0	0.10369449	0.18164706	0.1665644	1.86283743	3.82039753	4.97262464	1.911E-05	3.526243	up	PREDICTED: probable inactive leucine-rich repeat receptor-like protein kinase At1g66830 [Sesamum indicum]
c50812.graph_c0	29.4804301	34.1652633	28.9697505	181.618709	179.709479	192.812496	4.657E-27	1.563934	up	PREDICTED: NADH dehydrogenase [ubiquinone] 1 alpha subcomplex subunit 13-B-like [Sesamum indicum]
c50814.graph_c0	25.2028735	24.4352269	29.795755	3.64862628	3.58416607	2.30208188	5.556E-56	-4.07437	down	-
c50815.graph_c0	0	0	0.20313507	5.97086078	1.69705934	2.52683664	0.0044565	4.621734	up	PREDICTED: acid phosphatase 1-like [Nicotiana sylvestris]
c50818.graph_c0	1.40460155	1.15788898	1.27409549	26.5652757	26.691203	22.0121127	8.758E-22	3.278455	up	PREDICTED: uncharacterized protein LOC105164615 [Sesamum indicum]
c50820.graph_c0	0.11291623	0.24220563	0.24874598	3.29910374	4.75445997	3.84791485	2.478E-18	3.27312	up	PREDICTED: probable RNA-dependent RNA polymerase 1 [Sesamum indicum]
c50823.graph_c0	3.65353427	4.65460893	2.1340619	0.30598855	0.38898932	0.16336006	3.632E-10	-4.615758	down	PREDICTED: uncharacterized protein LOC105158347 [Sesamum indicum]
c50824.graph_c0	1.25416558	1.65487372	1.025597	10.8189247	11.8508018	12.1500955	5.984E-09	2.129361	up	PREDICTED: tetraspanin-3-like [Sesamum indicum]
c50826.graph_c0	0	0	0	8.49942903	12.2783379	11.3705544	3.92E-27	Inf	up	PREDICTED: adenosine kinase 2-like [Sesamum indicum]
c50827.graph_c0	0.3603393	0.54105019	0.46305027	4.48156894	7.5611182	6.49843022	6.33E-06	2.73765	up	-
c50830.graph_c0	0.02938713	0.02941655	0.03776362	10.4503087	15.7171438	19.6571902	1.006E-23	7.861402	up	PREDICTED: delta(8)-fatty-acid desaturase 2-like [Sesamum indicum]
c50831.graph_c0	19.8766683	22.3290681	21.1384599	1.4350849	1.70273425	1.37908431	1.483E-84	-4.829205	down	PREDICTED: uncharacterized protein LOC105179506 [Sesamum indicum]
c50833.graph_c0	0	0.03865387	0.04962206	2.20564013	0.97986798	0.75970271	0.0030877	4.470506	up	PREDICTED: U-box domain-containing protein 21 [Sesamum indicum]
c50834.graph_c0	0.66313779	0.92932255	1.19302165	5.49833209	5.30705471	6.08829863	0.0025089	1.577537	up	PREDICTED: receptor-like protein kinase HSL1 [Sesamum indicum]
c50835.graph_c0	46.864079	46.3310625	44.5048653	15.8938159	12.3140443	11.5037476	8.576E-65	-2.805082	down	PREDICTED: RING-H2 finger protein ATL80-like [Sesamum indicum]
c50838.graph_c0	24.7811436	29.5796133	23.7467728	14.2001067	14.2121701	10.8899804	5.08E-23	-2.005164	down	ring finger protein, putative [Ricinus communis]
c50839.graph_c0	5.95162086	6.64189718	5.16761381	0.29637953	0.15698909	0.03296459	1.096E-49	-6.1837	down	PREDICTED: E3 ubiquitin-protein ligase PUB23-like [Sesamum indicum]
c50841.graph_c0	3.75864793	3.69970479	4.66901102	28.7982184	30.8750528	32.3515499	1.286E-16	1.902139	up	hypothetical protein MIMGU_mgv1a012263mg [Erythranthe guttata]
c50849.graph_c0	29.5396833	28.7478944	34.309695	16.5725569	16.1397649	18.4715076	3.318E-25	-1.875052	down	PREDICTED: polyadenylate-binding protein-interacting protein 6-like [Sesamum indicum]
c50852.graph_c0	5.35562966	5.39874598	5.81594041	1.18136959	2.09813284	1.45309563	1.452E-19	-2.834317	down	PREDICTED: F-box/kelch-repeat protein At3g23880-like [Sesamum indicum]
c50857.graph_c0	0.06191221	0	0	4.04966701	4.41097991	3.40036263	1.819E-13	6.585891	up	PREDICTED: uncharacterized protein LOC105165400 [Sesamum indicum]
c50859.graph_c0	0.36126166	0.39778575	0.13927066	3.96053192	6.59324808	8.79533674	9.251E-07	3.409479	up	-

#ID	0 h-1 FPKM	0 h-2 FPKM	0 h-3 FPKM	48 h-1 FPKM	48 h-2 FPKM	48 h-3 FPKM	FDR	log2FC	regulated	nr_annotation
c50868.graph_c0	5.20226469	3.85738813	5.44713163	21.655748	20.4970383	23.6915833	0.003857	1.163181	up	hypothetical protein PHAVU_008G011200g [Phaseolus vulgaris]
c50871.graph_c0	1.21390322	1.57515395	2.13765952	17.9760683	18.1658856	21.7812062	6.31E-22	2.531097	up	PREDICTED: protein arginine N-methyltransferase PRMT10 isoform X1 [Sesamum indicum]
c50873.graph_c0	25.2996934	27.9379269	24.7187628	22.8267746	19.5965963	17.5459167	2.716E-19	-1.391292	down	PREDICTED: trihelix transcription factor ASIL2 [Sesamum indicum]
c50874.graph_c0	7.45218227	11.7222985	14.1365156	142.049148	152.561876	96.141254	2.665E-10	2.531485	up	hypothetical protein JCGZ_21814 [Jatropha curcas]
c50879.graph_c0	5.33924024	4.9882809	4.80279457	0.32792376	0	0	1.726E-18	-6.487391	down	-
c50880.graph_c0	1.48593803	1.40629364	1.21512949	10.5284164	13.0520623	14.9934239	2.488E-19	2.210877	up	PREDICTED: peptide-N4-(N-acetyl-beta-glucosaminyl)asparagine amidase A-like [Sesamum indicum]
c50885.graph_c0	0.05306334	0	0.06818849	1.07547899	0.93218693	1.30493828	3.156E-08	3.745213	up	hypothetical protein MIMGU_mgv1a010676mg [Erythranthe guttata]
c50890.graph_c0	3.04018261	3.62846283	3.75649521	26.284567	25.4487888	28.1804418	8.477E-09	1.919182	up	PREDICTED: ADP-ribosylation factor GTPase-activating protein AGD3-like [Sesamum indicum]
c50891.graph_c0	0	0.0534082	0	8.35616203	12.4974218	11.8526835	2.092E-30	8.245643	up	PREDICTED: annexin D4 [Sesamum indicum]
c50892.graph_c0	0.84437723	1.2960082	0.86804653	32.9827639	42.4129504	41.0685532	9.347E-62	4.254358	up	PREDICTED: endo-1,3;1,4-beta-D-glucanase-like [Sesamum indicum]
c50896.graph_c0	0.27197032	0.11667543	0.17474632	20.8499053	24.0028672	23.3613396	1.34E-124	5.902351	up	PREDICTED: WEB family protein At3g02930, chloroplastic-like [Sesamum indicum]
c50897.graph_c0	1.38358736	1.84663042	1.64626346	0.28325576	0.60015026	0.71411127	2.088E-07	-2.641352	down	PREDICTED: RING-H2 finger protein ATL16-like [Sesamum indicum]
c50899.graph_c0	0	0	0.06193044	6.39344366	11.9469576	8.84931868	1.948E-18	7.719086	up	PREDICTED: annexin D1-like [Sesamum indicum]
c50901.graph_c0	0	0	0.12787867	3.02538198	4.07912318	5.87338187	2.648E-11	5.607325	up	hypothetical protein MIMGU_mgv1a012192mg [Erythranthe guttata]
c50904.graph_c0	3.83982119	1.38371983	1.77635601	18.3949674	12.5917534	15.1086751	0.0001851	1.71344	up	hypothetical protein EMIHUDRAFT_447448 [Emiliania huxleyi CCMP1516]
c50905.graph_c0	0	0.18186599	0.15564747	1.11586138	1.95049771	2.38292818	3.507E-05	2.976011	up	PREDICTED: fasciclin-like arabinogalactan protein 12 [Sesamum indicum]
c50906.graph_c0	0.02424172	0	0	3.21595957	6.50626932	10.0750106	6.759E-09	8.663546	up	PREDICTED: probable LRR receptor-like serine/threonine-protein kinase At4g26540 [Sesamum indicum]
c50907.graph_c0	563.298612	588.693763	568.599103	191.91551	122.431197	101.588082	1.004E-73	-3.054968	down	hypothetical protein MIMGU_mgv1a008386mg [Erythranthe guttata]
c50912.graph_c0	0	0	0	14.9383721	6.36653774	5.5001684	1.39E-05	Inf	up	PREDICTED: uncharacterized protein LOC105157276 [Sesamum indicum]
c50914.graph_c0	0	0	0	20.1452717	16.512695	9.56007554	1.069E-15	Inf	up	PREDICTED: probable trehalose-phosphate phosphatase H [Sesamum indicum]
c50915.graph_c0	2308.10462	2107.65866	1983.73098	450.082801	327.923969	354.106913	1.976E-98	-3.509323	down	hypothetical protein NitaMp027 [Nicotiana tabacum]
c50917.graph_c0	7.37066986	4.01693859	4.84104132	0	0	0	3.859E-24	-Inf	down	--

#ID	0 h-1 FPKM	0 h-2 FPKM	0 h-3 FPKM	48 h-1 FPKM	48 h-2 FPKM	48 h-3 FPKM	FDR	log2FC	regulated	nr_annotation
c50919.graph_c0	0	0	0	1.32187416	3.01078558	1.8231033	1.395E-06	Inf	up	hypothetical protein AOL_s00079g69 [Arthrobotrys oligospora ATCC 24927]
c50920.graph_c0	0	0.05184221	0	6.63205819	13.7484483	15.1986852	1.322E-13	8.404324	up	PREDICTED: DNA-damage-repair/toleration protein DRT100-like [Sesamum indicum]
c50921.graph_c0	0.58956557	0.46591257	0.43861926	10.6342319	10.7811339	13.0996557	3.987E-28	3.513003	up	PREDICTED: glucan endo-1,3-beta-glucosidase 2-like [Sesamum indicum]
c50924.graph_c0	68.1932181	64.159336	65.1342588	46.0665473	47.3985588	44.9973602	2.218E-27	-1.529561	down	PREDICTED: KH domain-containing protein At4g18375-like [Sesamum indicum]
c50925.graph_c0	0	0	0.11676593	2.59505176	7.00587971	4.24569363	4.694E-06	5.825957	up	PREDICTED: transcription factor RAX2-like [Sesamum indicum]
c50926.graph_c0	11.1841858	12.0510833	9.52038623	1.96884104	2.95481016	4.02928471	1.898E-39	-2.895984	down	PREDICTED: putative nuclease HARBI1 [Sesamum indicum]
c50929.graph_c0	0.03548831	0.07104769	0.04560388	1.30776586	1.0737	0.78545849	3.982E-07	3.369147	up	PREDICTED: protein HOTHEAD [Sesamum indicum]
c50930.graph_c0	0.06750493	0.06757253	0	8.20907321	11.1341779	12.6719995	1.33E-23	6.881454	up	PREDICTED: NADPH:quinone oxidoreductase [Sesamum indicum]
c50933.graph_c0	0	0.08524384	0.43772853	1.9613407	2.32713897	1.53576647	2.516E-05	2.434029	up	hypothetical protein MIMGU_mgv1a0012201mg, partial [Erythranthe guttata]
c50941.graph_c0	8.25200183	8.26026496	6.47635949	35.9195084	34.4849546	39.1794187	1.118E-10	1.237934	up	PREDICTED: protein TIC 21, chloroplastic [Sesamum indicum]
c50942.graph_c0	108.411885	114.415537	109.367658	34.7699505	59.0217984	74.4902068	6.209E-44	-2.009016	down	PREDICTED: uncharacterized protein LOC105163758 [Sesamum indicum]
c50943.graph_c0	0.61806021	0.30933955	0.89351082	4.69752836	3.16683926	3.86317851	0.0010039	1.669038	up	hypothetical protein MIMGU_mgv1a007960mg [Erythranthe guttata]
c50945.graph_c0	494.747321	464.483328	481.162691	123.721798	145.190609	99.2914644	5.731E-93	-2.985727	down	hypothetical protein POPTR_0011s11140g [Populus trichocarpa]
c50948.graph_c0	2.92707014	2.74687609	2.40964753	11.5869872	17.051102	13.9091698	6.434E-07	1.374657	up	hypothetical protein MIMGU_mgv1a009797mg [Erythranthe guttata]
c50952.graph_c0	5.13309968	4.71005306	4.39749088	39.6049247	38.6167853	35.4155483	1.803E-07	1.981446	up	-
c50955.graph_c0	7.16955544	6.45906119	6.26495056	7.66187204	5.17797417	3.29120094	0.0017877	-1.30716	down	-
c50957.graph_c0	6.70870104	6.41977142	5.80151742	542.095314	526.18702	454.16458	3.02E-108	5.315264	up	PREDICTED: basic 7S globulin-like [Sesamum indicum]
c50959.graph_c0	0.15850677	0.23799823	0	11.3170748	11.8343708	11.62907	1.314E-19	5.452184	up	hypothetical protein JCGZ_13997 [Jatropha curcas]
c50962.graph_c0	4.24086537	4.51043145	4.76846991	22.5871228	25.2217404	30.671909	5.971E-14	1.515232	up	PREDICTED: adenylyl-sulfate kinase 3-like isoform X2 [Sesamum indicum]
c50965.graph_c0	91.1674743	90.4654259	90.8650201	30.620431	46.5066553	56.8265315	1.161E-44	-2.050643	down	PREDICTED: probable ribose-5-phosphate isomerase 1 [Sesamum indicum]
c50966.graph_c0	0.85831962	1.21295873	0.51904692	10.4656768	11.6291523	14.3202422	2.232E-14	2.798234	up	hypothetical protein MIMGU_mgv1a009312mg [Erythranthe guttata]
c50969.graph_c0	12.3235742	12.9425987	8.56716827	4.65298375	9.4642024	7.28674665	3.271E-05	-1.68488	down	-
c50970.graph_c0	0.18549034	0.18567608	0.11918122	3.84493058	5.06891806	4.40954165	1.633E-08	3.746844	up	PREDICTED: putative B3 domain-containing protein At5g66980 [Sesamum indicum]

#ID	0 h-1 FPKM	0 h-2 FPKM	0 h-3 FPKM	48 h-1 FPKM	48 h-2 FPKM	48 h-3 FPKM	FDR	log2FC	regulated	nr_annotation
c50974.graph_c0	0.29301845	0.58662373	0.37654031	1.77394208	2.65549944	3.53433975	0.0011775	1.638494	up	PREDICTED: serine/threonine-protein kinase CDL1-like [Sesamum indicum]
c50975.graph_c0	0.13353911	0.08911522	0.17160304	3.93679799	3.25825009	2.73667124	7.514E-10	3.638139	up	PREDICTED: putative cyclin-A3-1 [Sesamum indicum]
c50977.graph_c0	15.0390245	16.7979375	14.6656339	170.35341	185.222525	159.360964	1.994E-65	2.451791	up	hypothetical protein MIMGU_mgv1a020088mg [Erythranthe guttata]
c50978.graph_c0	2.86038043	1.82206479	1.44800386	0.87838967	1.35352342	1.49211844	0.0006046	-1.739264	down	-
c50981.graph_c0	21.0499929	11.1748953	12.5615121	0	0	0	3.533E-24	-Inf	down	cytochrome b (mitochondrion) [Gracilaria salicornia]
c50982.graph_c0	0.63792154	0.47892024	0.40987722	11.9987715	20.960581	21.1785603	2.819E-14	4.124647	up	PREDICTED: hevamine-A [Sesamum indicum]
c50984.graph_c0	0.03940448	0.09860984	0.15190895	6.80662068	6.99927278	6.33105104	3.818E-38	5.09052	up	PREDICTED: putative late blight resistance protein homolog R1A-10 [Sesamum indicum]
c50986.graph_c0	0	0	0	3.10411701	4.23842808	4.29648279	2.007E-13	Inf	up	PREDICTED: polyadenylate-binding protein-interacting protein 8-like [Sesamum indicum]
c50990.graph_c0	0	0	0	2.09949412	2.50217938	1.4594666	2.629E-11	Inf	up	actin [Pseudozyma antarctica]
c50996.graph_c0	11.5997631	12.7790032	14.239958	0.89551327	0.63245848	0.69057935	4.63E-54	-5.134631	down	SLF-interacting SKP1-like protein 1, partial [Antirrhinum hispanicum]
c50997.graph_c0	29.8601201	24.7167477	23.6131917	23.9822838	18.8679301	16.6320952	4.463E-07	-1.404215	down	hypothetical protein MIMGU_mgv1a007364mg [Erythranthe guttata]
c50999.graph_c0	1.45655389	1.12154801	1.0798439	15.2250912	13.4500609	13.7768081	2.274E-13	2.523878	up	PREDICTED: probable F-actin-capping protein subunit beta isoform X2 [Sesamum indicum]
c51000.graph_c0	0	0	0	3.31972267	3.03727405	3.22240284	1.987E-16	Inf	up	PREDICTED: polygalacturonase-like [Sesamum indicum]
c51004.graph_c0	6.4841629	6.62875487	6.91412417	61.0073239	75.1322147	82.6699321	1.477E-24	2.427053	up	putative. proteolipid subunit of vacuolar H+ ATPase, partial [Zea mays]
c51005.graph_c0	0.12585047	0.33593732	0.05390761	1.4299472	2.21087043	2.37277651	1.683E-05	2.527364	up	PREDICTED: protein BRANCHLESS TRICHOME [Sesamum indicum]
c51010.graph_c0	0.04377754	0	0	1.04859894	0.85451033	0.86126495	3.121E-09	4.988536	up	PREDICTED: uncharacterized protein LOC105169592 [Sesamum indicum]
c51013.graph_c0	0	0	0	4.26363112	7.04620828	10.0534463	5.518E-12	Inf	up	PREDICTED: putative pectate lyase 2 [Sesamum indicum]
c51016.graph_c0	0	0.24001766	0.36974839	3.18094165	3.22941272	3.85246542	2.495E-08	3.038702	up	PREDICTED: cell wall / vacuolar inhibitor of fructosidase 1-like [Malus domestica]
c51018.graph_c0	8.81333327	7.81710246	7.1680267	3.49443283	1.52432324	0.91450745	5.937E-13	-2.998044	down	PREDICTED: pathogenesis-related genes transcriptional activator PTI6-like [Sesamum indicum]
c51019.graph_c0	2.0326933	2.03472874	1.52132794	14.3844188	17.8328067	16.1866462	2.499E-25	2.09676	up	PREDICTED: two-component response regulator ARR12-like [Sesamum indicum]
c51021.graph_c0	0.67065068	0.44754815	0.71817722	7.41416574	10.2543681	11.6365163	1.543E-08	2.969784	up	-
c51025.graph_c0	1.9914071	1.76465023	1.92976558	0.96242051	2.07099717	1.15072836	0.000272	-1.470298	down	hypothetical protein MIMGU_mgv1a027162mg [Erythranthe guttata]

#ID	0 h-1 FPKM	0 h-2 FPKM	0 h-3 FPKM	48 h-1 FPKM	48 h-2 FPKM	48 h-3 FPKM	FDR	log2FC	regulated	nr_annotation
c51026.graph_c0	0.37198926	0.22341705	0	27.8958429	40.3711505	51.6556649	1.166E-21	6.646023	up	PREDICTED: uncharacterized protein LOC105161197 [Sesamum indicum]
c51029.graph_c0	3.3684447	3.73965234	4.87949559	0.14105595	0.29886336	0.15061289	2.48E-27	-5.374308	down	PREDICTED: uncharacterized protein LOC104606632 [Nelumbo nucifera]
c51031.graph_c0	0	0	0.05980424	3.38709369	5.04166926	5.64613555	8.325E-18	6.820953	up	PREDICTED: uncharacterized protein C594.04c [Sesamum indicum]
c51035.graph_c0	0.77831392	1.16863993	0.5715224	16.6966384	10.41751	9.66132674	8.796E-06	2.865593	up	PREDICTED: uncharacterized protein LOC105158187 [Sesamum indicum]
c51036.graph_c0	0.20898547	0.17432895	0.0895182	2.14993001	3.26341276	4.85386739	6.993E-06	3.420655	up	PREDICTED: beta-amyrin 28-oxidase-like [Sesamum indicum]
c51037.graph_c0	13.7724466	16.1810301	17.6150493	86.4933641	92.2602867	103.357084	7.212E-24	1.546603	up	unnamed protein product [Coffea canephora]
c51043.graph_c0	5.25502725	6.1185471	6.25533463	39.3417202	35.6582579	35.7315396	6.116E-25	1.634267	up	PREDICTED: probable acyl-activating enzyme 1, peroxisomal [Sesamum indicum]
c51044.graph_c0	30.9481898	31.6448481	38.0605403	5.37241319	0	0.08386563	2.483E-63	-5.169123	down	PREDICTED: uncharacterized protein LOC104420277 [Eucalyptus grandis]
c51046.graph_c1	0.24642126	0.49333603	0.94998324	28.6044369	33.669925	26.4620582	6.321E-16	4.683902	up	-
c51047.graph_c0	0	0	0	4.55394131	4.11966849	6.92038852	1.737E-15	Inf	up	PREDICTED: uncharacterized protein LOC105160227 isoform X1 [Sesamum indicum]
c51050.graph_c0	0.30227914	0.33620202	0.30212051	66.8659635	74.1143249	80.8618649	1.13E-199	6.862133	up	PREDICTED: protein TRANSPARENT TESTA 12-like [Sesamum indicum]
c51051.graph_c0	0.63924203	0.2399558	0.30804425	3.31262693	3.74328166	3.34056293	0.0001842	2.116726	up	PREDICTED: uncharacterized protein LOC105159339 isoform X2 [Sesamum indicum]
c51053.graph_c0	7.72952234	7.57935897	5.47314368	0	0.15395454	0	1.635E-24	-8.12598	down	hypothetical protein MIMGU_mgv1a015250mg [Erythranthe guttata]
c51054.graph_c0	34.2469858	20.1978457	17.9680625	0	0	0	3.001E-29	-Inf	down	uncharacterized protein LOC100274422 [Zea mays]
c51055.graph_c0	0.25744471	0.04295042	0.11027555	1.38352	2.21944569	2.46209556	1.646E-06	2.863753	up	PREDICTED: uncharacterized protein PB18E9.04c [Sesamum indicum]
c51058.graph_c0	53.4579408	48.0402232	48.1382925	25.2999552	31.8764442	29.5603293	2.556E-31	-1.807213	down	PREDICTED: heme-binding-like protein At3g10130, chloroplastic [Sesamum indicum]
c51059.graph_c0	7.69323132	9.64732507	6.73558927	2.3365406	2.2277533	2.70275171	1.745E-13	-2.743976	down	PREDICTED: E3 ubiquitin-protein ligase RHA1B-like [Sesamum indicum]
c51060.graph_c0	0.33298763	0.26665685	0.21395114	4.29477894	7.9946442	9.17152433	2.051E-08	3.696341	up	PREDICTED: PTI1-like tyrosine-protein kinase At3g15890 [Sesamum indicum]
c51061.graph_c0	9.63089819	10.5843714	9.52006575	0.99273976	1.24887878	0.3312502	6.068E-37	-4.54514	down	-
c51063.graph_c0	6.07591027	4.18137113	4.3918792	1.04953591	1.55659844	2.36580587	1.253E-09	-2.583844	down	-
c51066.graph_c0	0.48968106	0.14004897	0.26968267	10.3114545	10.7530523	12.5010331	6.929E-30	4.205959	up	PREDICTED: guanine nucleotide-binding protein subunit beta-like protein [Sesamum indicum]

#ID	0 h-1 FPKM	0 h-2 FPKM	0 h-3 FPKM	48 h-1 FPKM	48 h-2 FPKM	48 h-3 FPKM	FDR	log2FC	regulated	nr_annotation
c51067.graph_c0	0.4224885	0.53568798	0.61530305	4.69663587	5.85516548	5.40273467	8.375E-10	2.317774	up	PREDICTED: spindle pole body protein pcp1 [Sesamum indicum]
c51068.graph_c0	0	0	0	1.98732149	3.47836494	1.92207252	8.063E-08	Inf	up	PREDICTED: probable ADP-ribosylation factor GTPase-activating protein AGD11 [Sesamum indicum]
c51070.graph_c0	10.0991523	7.37703126	8.41803926	50.0404206	49.815069	56.8317538	6.333E-06	1.579718	up	PREDICTED: ER membrane protein complex subunit 4 [Sesamum indicum]
c51071.graph_c0	0.59232435	0.75462224	0.4843747	3.52216689	5.36047171	5.29690635	6.169E-05	1.930947	up	PREDICTED: protein disulfide-isomerase SCO2 [Sesamum indicum]
c51073.graph_c0	3.54989601	1.85748559	2.90293622	0.44596306	0.47244378	0.85976637	2.265E-08	-3.247691	down	-
c51074.graph_c0	0	0.27968341	0	5.01939444	7.63529815	6.41304389	6.252E-14	5.078968	up	-
c51078.graph_c0	19.5028141	15.5591607	12.6251617	3.24221313	2.28982111	3.84653566	1.479E-19	-3.357757	down	Dof zinc finger DOF5.2 -like protein [Gossypium arboreum]
c51082.graph_c0	2.21896047	1.91576984	2.49502055	10.4256807	10.2055605	10.3226113	5.939E-07	1.204107	up	hypothetical protein MIMGU_mgv1a003778mg [Erythranthe guttata]
c51083.graph_c0	1.39590849	0.23288438	0.89689884	5.14400781	7.03887619	7.24708643	0.0006024	1.921298	up	-
c51084.graph_c0	20.8119302	20.2289218	15.891454	11.809646	14.4243178	11.6207798	4.651E-08	-1.605768	down	PREDICTED: 5'-adenylylsulfate reductase-like 5 isoform X2 [Sesamum indicum]
c51086.graph_c0	3.83020153	3.91391267	3.07622538	18.6723416	13.7844546	14.7830134	0.0008488	1.116741	up	hypothetical protein MIMGU_mgv1a006570mg [Erythranthe guttata]
c51087.graph_c0	0.33009987	0.49564563	0.70698568	15.712326	15.0881628	20.1593153	2.662E-28	4.029374	up	PREDICTED: protein CURVATURE THYLAKOID 1D, chloroplastic [Sesamum indicum]
c51088.graph_c0	1.01111028	0.70848593	0.5197267	96.9692274	73.6161818	76.6680478	2.366E-37	5.780206	up	PREDICTED: indole-3-acetic acid-induced protein ARG7-like [Nicotiana sylvestris]
c51089.graph_c0	0.8568719	0.31600576	0.75339401	7.85251516	9.15506789	12.088828	9.701E-14	2.893075	up	PREDICTED: chloroplast stem-loop binding protein of 41 kDa a, chloroplastic [Sesamum indicum]
c51090.graph_c0	13.1168316	15.8465108	15.6931894	9.68809926	17.7677634	17.6115953	0.0003519	-1.015771	down	hypothetical protein MIMGU_mgv1a017353mg [Erythranthe guttata]
c51091.graph_c0	27.2776708	26.914914	19.5294656	131.034841	127.40603	117.232885	1.04E-06	1.336484	up	60S ribosomal protein L29-1 [Medicago truncatula]
c51094.graph_c0	0	0	0	7.49862256	12.1672107	10.8951592	2.503E-29	Inf	up	PREDICTED: peroxidase 16 [Sesamum indicum]
c51096.graph_c0	0	0	0	2.85254475	4.68398417	3.0458126	2.951E-11	Inf	up	predicted protein [Hordeum vulgare subsp. vulgare]
c51097.graph_c0	2.50639066	1.69520299	1.82802799	59.1616196	56.3277712	65.6354792	1.699E-69	3.893198	up	PREDICTED: 30S ribosomal protein S20, chloroplastic isoform X1 [Sesamum indicum]
c51098.graph_c0	20.595011	17.8920236	19.687679	14.6543041	15.6470198	17.0540471	9.03E-15	-1.316221	down	PREDICTED: pre-mRNA-splicing factor 38 [Sesamum indicum]
c51099.graph_c0	0.33070543	0.66207316	0.42496957	4.11301008	3.06620272	4.33745971	0.0003444	2.007897	up	-
c51100.graph_c0	0.03555325	0	0	7.7954454	12.0405051	13.2023623	2.844E-28	8.855514	up	hypothetical protein MIMGU_mgv1a004964mg [Erythranthe guttata]

#ID	0 h-1 FPKM	0 h-2 FPKM	0 h-3 FPKM	48 h-1 FPKM	48 h-2 FPKM	48 h-3 FPKM	FDR	log2FC	regulated	nr_annotation
c51101.graph_c0	0	0.42199293	0.27086758	9.70946279	12.1374782	17.6244201	2.531E-10	4.802134	up	PREDICTED: chlorophyll a-b binding protein 151, chloroplastic-like [Pyrus x bretschneideri]
c51103.graph_c0	5.4975839	2.82209687	6.15889336	0	0	0	6.976E-13	-Inf	down	40S ribosomal protein S16 [Arabidopsis lyrata subsp. lyrata]
c51104.graph_c0	118.04178	114.260312	116.895156	6.101361	6.74881328	3.83220306	2.59E-203	-5.403267	down	-
c51106.graph_c0	0.08227375	0	0	8.03437043	5.86165294	7.28382005	4.536E-16	7.016339	up	PREDICTED: transcription factor HEC2-like [Sesamum indicum]
c51110.graph_c0	8.75421562	4.8196399	6.56221631	317.355957	330.931453	367.538378	3.36E-146	4.63899	up	beta-tubulin [Deschampsia antarctica]
c51113.graph_c0	0.68905537	0.60352718	0.71943904	3.01531306	4.70747578	4.16572206	0.0006453	1.537204	up	PREDICTED: metacaspase-1-like [Sesamum indicum]
c51116.graph_c0	6.77944461	3.98322383	5.68164439	0	0	0	3.205E-23	-Inf	down	ribosomal protein L27
c51117.graph_c0	6.73336377	2.77533785	4.07183013	0	0	0	1.058E-10	-Inf	down	hypothetical protein MVLG_03147 [Microbotryum violaceum p1A1 Lamole]
c51118.graph_c0	0.36927205	0.36964182	0.42707629	3.47001451	4.43289541	5.99357267	1.433E-08	2.549394	up	PREDICTED: beta-1,4-mannosyl-glycoprotein 4-beta-N-acetylglucosaminyltransferase [Sesamum indicum]
c51119.graph_c0	4.64115492	3.98211629	2.98203391	0.71262244	0.43139261	0.54350328	1.344E-12	-3.787059	down	-
c51120.graph_c0	10.8311624	12.2338875	8.36989222	4.65207941	5.78541213	5.45919852	3.861E-12	-2.001642	down	--
c51125.graph_c0	0.32789691	0.45951535	0.19663491	9.98877948	8.32044608	7.20354492	5.763E-20	3.689697	up	PREDICTED: phosphate transporter PHO1 homolog 9-like [Sesamum indicum]
c51126.graph_c0	14.5385011	13.2796666	12.610721	5.27381486	6.5624696	7.96998629	1.595E-12	-2.051094	down	PREDICTED: suppressor of glycerol defect protein 1 isoform X2 [Sesamum indicum]
c51129.graph_c0	2.18549819	3.37528796	3.530625	13.5187005	14.6870774	20.1163463	5.166E-06	1.38534	up	PREDICTED: UPF0483 protein AGAP003155 [Sesamum indicum]
c51131.graph_c0	0	0	0	3.5083416	11.5723356	14.7571332	1.401E-06	Inf	up	hypothetical protein CISIN_1g026722mg [Citrus sinensis]
c51132.graph_c0	3.72277151	3.07841247	3.0679415	83.8402739	98.8891883	109.728186	3.043E-77	3.869301	up	PREDICTED: 24-methylenesterol C-methyltransferase 2-like [Sesamum indicum]
c51134.graph_c0	222.233975	203.046088	211.104814	23.4844732	21.6893425	22.3251528	1.16E-137	-4.252825	down	PREDICTED: uncharacterized protein LOC105179155 [Sesamum indicum]
c51135.graph_c0	0	0	0	3.16555715	13.0148668	10.9985183	1.097E-06	Inf	up	PREDICTED: non-specific lipid transfer protein GPI-anchored 2-like [Eucalyptus grandis]
c51137.graph_c0	0.08265115	0	0.053105	2.7792426	3.18626537	0.81302576	0.0004849	4.629166	up	hypothetical protein MIMGU_mgv1a008689mg [Erythranthe guttata]
c51138.graph_c0	19.295474	16.1705264	15.9152178	0.41340114	0.43794838	0.29427348	8.48E-65	-6.501329	down	hypothetical protein MIMGU_mgv1a008111mg [Erythranthe guttata]
c51144.graph_c0	2.050445	1.43134744	2.66957278	0.57167217	1.1585723	2.05679793	0.0026919	-1.736333	down	PREDICTED: UPF0392 protein RCOM_0530710-like [Sesamum indicum]
c51145.graph_c0	2.88328481	2.02032039	1.48205405	19.5678559	24.7631648	25.3685886	4.384E-12	2.431915	up	PREDICTED: proline-rich protein 3 [Sesamum indicum]
c51147.graph_c0	0	0	0	0.90734652	1.5177215	1.10479676	2.951E-11	Inf	up	-
c51148.graph_c0	0.03963936	0.0793581	0.05093814	13.7309039	17.3703623	17.6766372	1.266E-54	7.143727	up	PREDICTED: GDSL esterase/lipase 7 [Sesamum indicum]

#ID	0 h-1 FPKM	0 h-2 FPKM	0 h-3 FPKM	48 h-1 FPKM	48 h-2 FPKM	48 h-3 FPKM	FDR	log2FC	regulated	nr_annotation
c51153.graph_c0	7.0969408	8.28051375	6.33161554	5.74692189	4.19112939	4.18720398	6.953E-10	-1.629239	down	hypothetical protein MIMGU_mgv1a020747mg [Erythranthe guttata]
c51154.graph_c0	55.6563405	54.7886674	54.1343776	7.08207888	3.90135399	5.29333661	3.849E-41	-4.345715	down	PREDICTED: heat stress transcription factor A-2-like [Nicotiana sylvestris]
c51158.graph_c0	2.81317387	1.95687499	1.47052466	17.6585603	18.8932438	25.7575245	7.814E-12	2.304029	up	hypothetical protein MIMGU_mgv1a009002mg [Erythranthe guttata]
c51160.graph_c0	0	0	0	1.86638031	2.39661046	2.96911953	3.659E-12	Inf	up	PREDICTED: expansin-B3-like [Sesamum indicum]
c51161.graph_c0	3.24187499	3.24512123	3.28889819	43.1095984	45.6693922	56.6813037	1.69E-35	2.874896	up	PREDICTED: glyceraldehyde-3-phosphate dehydrogenase A, chloroplastic [Sesamum indicum]
c51162.graph_c0	0	0	0.33868756	2.02342061	3.3439673	3.88892338	2.259E-07	3.712858	up	PREDICTED: dirigent protein 23-like [Sesamum indicum]
c51164.graph_c0	30.5132635	29.6111823	21.2516288	27.5743428	16.4812939	11.7426637	1.505E-06	-1.544702	down	PREDICTED: probable WRKY transcription factor 31 [Sesamum indicum]
c51166.graph_c0	0.50195686	0.25122975	0.80629305	2.89022126	3.5517332	2.98317373	0.0030594	1.567101	up	-
c51170.graph_c0	2.23454477	1.2340868	2.2773795	52.8849398	79.8640836	54.1309974	3.985E-20	3.998516	up	PREDICTED: CASP-like protein 1 [Sesamum indicum]
c51172.graph_c0	75.4740889	80.4397044	66.5649364	30.8911214	38.2266496	38.1259958	2.029E-48	-2.072578	down	PREDICTED: ethylene-responsive transcription factor TINY-like [Sesamum indicum]
c51176.graph_c0	88.2408922	86.9624552	72.5978651	2.6730861	1.66577094	1.67893831	1.72E-148	-6.369546	down	-
c51177.graph_c0	0	0	0	73.5314125	132.842837	182.749351	8.095E-22	Inf	up	PREDICTED: trans-resveratrol di-O-methyltransferase [Vitis vinifera]
c51178.graph_c0	0.90265324	1.39008786	1.2491717	0.12793585	1.15202644	1.36603862	0.0052742	-1.464356	down	-
c51180.graph_c0	1.01104823	1.16776227	0.79953046	5.1587644	6.60364479	7.84165579	0.0003733	1.698331	up	hypothetical protein MIMGU_mgv1a0076692mg, partial [Erythranthe guttata]
c51184.graph_c0	151.995192	113.692556	130.928683	3.07753592	4.48287969	2.73837296	7.565E-76	-6.287999	down	hypothetical protein CARUB_v10018292mg, partial [Capsella rubella]
c51186.graph_c0	0.13202674	0.26431789	0	10.5819531	9.92175639	10.8227106	2.154E-13	5.302513	up	PREDICTED: nucleobase-ascorbate transporter 12 [Sesamum indicum]
c51195.graph_c0	0	0	0	2.53106876	2.77073913	2.10198778	2.367E-11	Inf	up	hypothetical protein MIMGU_mgv1a015662mg [Erythranthe guttata]
c51201.graph_c0	56.3045362	54.2184416	45.9141218	40.4539761	32.9169752	24.6763007	8.226E-23	-1.683006	down	PREDICTED: uncharacterized protein LOC105164312 [Sesamum indicum]
c51202.graph_c0	0.35552205	0.2588204	0.49839262	13.2203057	8.86372152	6.78247123	3.646E-07	3.685013	up	PREDICTED: U-box domain-containing protein 9-like [Sesamum indicum]
c51203.graph_c0	0.03031958	0.09104983	0.03896186	12.9047577	16.0087123	16.0805769	1.085E-64	7.113708	up	PREDICTED: aldehyde dehydrogenase family 3 member F1 [Sesamum indicum]
c51204.graph_c0	34.2745661	21.986681	22.0220872	0	0.15704651	0	2.295E-37	-10.01083	down	histone H3 [Neurospora crassa OR74A]
c51207.graph_c0	0.25671103	0.32121011	0.28864836	22.7925858	21.9224211	21.7537532	4.084E-75	5.244283	up	PREDICTED: argininosuccinate lyase, chloroplastic isoform X1 [Sesamum indicum]

#ID	0 h-1 FPKM	0 h-2 FPKM	0 h-3 FPKM	48 h-1 FPKM	48 h-2 FPKM	48 h-3 FPKM	FDR	log2FC	regulated	nr_annotation
c51211.graph_c0	3.60094432	3.69466387	2.89209751	171.34499	181.255659	202.273782	9.58E-142	4.750315	up	PREDICTED: acyl carrier protein 1, chloroplastic-like [Sesamum indicum]
c51213.graph_c0	10.4737729	8.15442505	6.48036173	3.75243662	4.54314468	4.76985077	1.113E-05	-1.959624	down	-
c51215.graph_c0	24.476775	25.8742017	22.641187	9.62245934	8.95821326	11.8484332	2.772E-21	-2.279596	down	PREDICTED: uncharacterized protein LOC105162031 [Sesamum indicum]
c51220.graph_c0	0	0	0	22.600099	42.4665815	39.6317276	1.01E-19	Inf	up	-
c51221.graph_c0	0.32666738	0.28612018	0.20989026	5.41705136	4.10477107	4.6192239	4.708E-10	3.094639	up	PREDICTED: uncharacterized protein LOC105171540 [Sesamum indicum]
c51222.graph_c0	4.34024535	3.33422134	2.98325357	0.18597754	0.29553098	0.33096339	8.059E-16	-4.736663	down	PREDICTED: transcription factor PAR2-like [Sesamum indicum]
c51225.graph_c0	11.030075	10.4398708	7.64839133	82.9530632	92.675003	105.994404	2.667E-39	2.256812	up	PREDICTED: S-formylglutathione hydrolase [Sesamum indicum]
c51228.graph_c0	39.5638551	37.7930278	40.7697364	172.802043	167.39693	176.491455	4.411E-15	1.111242	up	PREDICTED: nascent polypeptide-associated complex subunit alpha-like protein [Sesamum indicum]
c51231.graph_c0	4.90061722	4.57848948	4.47821308	3.81247094	4.198279	3.12449948	0.0001006	-1.34235	down	PREDICTED: uncharacterized protein LOC105173527 isoform X1 [Sesamum indicum]
c51232.graph_c0	28.1604181	25.2025342	24.6870661	133.233793	144.755194	150.927079	5.376E-19	1.439929	up	hypothetical protein MIMGU_mgv1a017391mg [Erythranthe guttata]
c51233.graph_c0	7.17971124	7.99542695	5.99703858	32.3279694	33.4592565	27.220297	1.403E-05	1.1209	up	hypothetical protein MIMGU_mgv1a002611mg [Erythranthe guttata]
c51234.graph_c0	2.86224339	2.75491297	3.53663084	0.50709333	0.75208545	0.27072516	7.755E-11	-3.60289	down	hypothetical protein MIMGU_mgv1a020559mg [Erythranthe guttata]
c51235.graph_c0	0.55130364	0.21225219	0.16348777	4.25851993	8.89860441	10.0813722	1.435E-06	3.626948	up	PREDICTED: probable pectate lyase 16 [Sesamum indicum]
c51236.graph_c0	0	0.20413607	0.08735351	41.1446987	54.9989377	62.7446026	8.195E-51	8.065043	up	hypothetical protein MIMGU_mgv1a014485mg [Erythranthe guttata]
c51237.graph_c0	0	0	0	3.86290117	4.82303885	3.1302946	1.899E-13	Inf	up	-
c51241.graph_c0	8.55215087	4.65256226	5.2560164	0	0	0	8.415E-16	-Inf	down	predicted protein [Hordeum vulgare subsp. vulgare]
c51242.graph_c0	3.00497207	3.43769267	2.28538233	16.4972626	16.1601005	17.5446186	4.637E-07	1.509634	up	hypothetical protein MIMGU_mgv1a003845mg [Erythranthe guttata]
c51246.graph_c0	0.07254594	0	0.18644881	8.28742231	6.65544185	5.11429415	2.436E-13	5.246569	up	PREDICTED: uncharacterized protein LOC105170022 [Sesamum indicum]
c51248.graph_c0	0	0	0	4.88758775	5.45032271	7.210095	2.033E-12	Inf	up	PREDICTED: mitogen-activated protein kinase 19 isoform X2 [Sesamum indicum]
c51249.graph_c0	4.0475686	3.85154154	3.66016415	30.6136789	35.6502442	34.8178756	1.366E-24	2.109177	up	PREDICTED: NADP-dependent D-sorbitol-6-phosphate dehydrogenase-like [Sesamum indicum]
c51257.graph_c0	2.35757864	2.21545332	2.22581527	11.5246603	18.4073855	24.3743323	0.0001197	1.970768	up	PREDICTED: leucine-rich repeat extensin-like protein 2 [Sesamum indicum]
c51262.graph_c0	0.19147915	0.19167088	0.16403885	14.5826544	13.424046	13.5772474	5.919E-48	5.233615	up	hypothetical protein MIMGU_mgv1a003907mg [Erythranthe guttata]

#ID	0 h-1 FPKM	0 h-2 FPKM	0 h-3 FPKM	48 h-1 FPKM	48 h-2 FPKM	48 h-3 FPKM	FDR	log2FC	regulated	nr_annotation
c51264.graph_c0	0	0	0	7.48999691	6.0420524	7.2759812	3.168E-18	Inf	up	PREDICTED: protein HEADING DATE 3A [Populus euphratica]
c51265.graph_c0	4.01757012	4.09340728	4.24081941	1.38796787	1.33034712	2.99929898	8.88E-07	-2.135394	down	kunitz trypsin inhibitor [Populus tremula]
c51267.graph_c0	20.3306598	21.6700653	20.319991	6.86268594	6.71901446	7.9581299	1.512E-56	-2.550814	down	PREDICTED: exocyst complex component EXO70B1-like [Sesamum indicum]
c51268.graph_c0	0	0	0	2.31080782	2.87376341	2.14554042	4.124E-11	Inf	up	large subunit ribosomal protein L3e, cytoplasmic [Guillardia theta CCMP2712]
c51269.graph_c0	0.07245576	0.07252831	0.09310852	3.07054474	2.89929702	3.32609838	2.476E-09	4.266757	up	PREDICTED: uncharacterized protein LOC102579011 [Solanum tuberosum]
c51270.graph_c0	0	0	0.26421747	66.2975915	87.0235916	132.983015	1.211E-22	9.02379	up	PREDICTED: auxin-binding protein ABP19a [Beta vulgaris subsp. vulgaris]
c51271.graph_c0	91.1880693	91.236806	93.4053626	9.40392506	10.6264729	8.50701523	4.47E-168	-4.291294	down	PREDICTED: alcohol dehydrogenase-like 7 [Sesamum indicum]
c51273.graph_c0	0.39774602	0.27870101	0.71556713	6.04608798	5.66754046	6.45572728	1.624E-10	2.680591	up	PREDICTED: mitogen-activated protein kinase homolog NTF6 [Sesamum indicum]
c51274.graph_c0	0.61682243	0.49395206	0.95116949	6.13717616	3.49159676	5.05631708	0.0006878	1.816846	up	hypothetical protein glysoja_042469, partial [Glycine soja]
c51275.graph_c0	0.0678191	0	0	3.43636604	4.63325308	3.89156452	6.716E-13	6.461958	up	PREDICTED: homeobox-leucine zipper protein ATHB-52 [Sesamum indicum]
c51276.graph_c0	12.4014537	12.0828352	11.6866632	8.98768869	9.52136635	8.81046504	0.0001325	-1.422299	down	PREDICTED: uncharacterized protein LOC103341433 [Prunus mume]
c51279.graph_c0	0	0.04741835	0	1.96385281	8.18315811	12.8532774	8.507E-05	7.895657	up	hypothetical protein MIMGU_mgv1a008158mg [Erythranthe guttata]
c51281.graph_c0	3.86986257	2.55314527	2.42995278	18.1499554	15.6654064	13.6263272	7.594E-07	1.41216	up	PREDICTED: protein TRANSPARENT TESTA GLABRA 1 [Sesamum indicum]
c51282.graph_c0	6.09062293	6.61754724	6.37147759	3.04520876	4.48059643	3.83861196	2.561E-13	-1.771136	down	PREDICTED: scarecrow-like protein 15 [Sesamum indicum]
c51286.graph_c0	10.8798077	11.261593	8.70024062	33.9485085	43.8870002	52.3241349	2.681E-05	1.056415	up	hypothetical protein JCGZ_13359 [Jatropha curcas]
c51287.graph_c0	0	0	0	28.9300065	40.3785213	30.5683232	1.056E-52	Inf	up	hypothetical protein MIMGU_mgv1a013386mg [Erythranthe guttata]
c51295.graph_c0	0	0	0	3.19555126	2.79655141	3.33788357	2.211E-10	Inf	up	hypothetical protein M569_00609, partial [Genlisea aurea]
c51305.graph_c0	0.76571954	0.447117	0.49198996	5.99616167	6.60131234	4.39381649	3.661E-06	2.303729	up	PREDICTED: uncharacterized protein LOC105156993 [Sesamum indicum]
c51308.graph_c0	2.43760221	2.40131225	1.54134723	97.1344913	127.032313	137.938607	8.051E-57	4.808267	up	PREDICTED: adenosine kinase 2-like [Sesamum indicum]
c51311.graph_c0	0.54382398	0.54436854	0.51493131	5.90659852	10.4195634	13.9603358	3.445E-06	3.211457	up	PREDICTED: pentatricopeptide repeat-containing protein At1g02150 [Sesamum indicum]
c51312.graph_c0	9.49344487	5.9551827	6.01839657	0	0	0	2.957E-24	-Inf	down	60S ribosomal protein L21-a, putative [Candida maltosa Xu316]
c51314.graph_c0	14.7694852	11.4822031	12.5244595	74.318354	77.4873887	82.8444024	8.173E-21	1.57945	up	PREDICTED: 30S ribosomal protein S10, chloroplastic [Sesamum indicum]

#ID	0 h-1 FPKM	0 h-2 FPKM	0 h-3 FPKM	48 h-1 FPKM	48 h-2 FPKM	48 h-3 FPKM	FDR	log2FC	regulated	nr_annotation
c51315.graph_c0	0	0	0	5.15771246	8.58624006	4.91710879	3.549E-09	Inf	up	unnamed protein product [Pneumocystis jirovecii]
c51319.graph_c0	11.9139568	10.316258	9.39258052	48.2132179	50.505376	49.1910078	1.68E-10	1.209598	up	hypothetical protein MIMGU_mgv1a015541mg [Erythranthe guttata]
c51323.graph_c0	0.02973189	0.17856999	0.07641332	4.4921174	6.55793252	5.28879155	3.435E-21	4.820378	up	PREDICTED: uncharacterized protein LOC105175605 isoform X2 [Sesamum indicum]
c51327.graph_c0	177.348475	185.347825	159.404353	122.827971	137.543229	140.403377	1.787E-23	-1.39995	down	PREDICTED: uncharacterized protein LOC103445335 [Malus domestica]
c51329.graph_c0	0	0	0	2.52858474	3.10168612	2.72358676	1.185E-09	Inf	up	-
c51331.graph_c0	0.88235673	1.76648056	1.700795	6.60468716	10.3338317	14.5564086	0.0012908	1.824893	up	--
c51334.graph_c0	23.8753218	23.1104759	22.58021	9.25553374	16.918631	15.922002	9.461E-27	-1.751705	down	PREDICTED: GATA transcription factor 8-like [Sesamum indicum]
c51335.graph_c0	0.84250178	0.73792724	0.13533102	71.2133766	93.8399031	111.622954	2.224E-39	6.322388	up	PREDICTED: uncharacterized protein LOC105168965 [Sesamum indicum]
c51336.graph_c0	3.1215495	3.29132461	2.6742098	0.99693533	1.70605955	1.91060599	2.673E-07	-2.003519	down	-
c51337.graph_c0	0.04182974	0.08374325	0	6.31995048	11.1450938	16.8360725	1.101E-09	7.078959	up	PREDICTED: cell division control protein 2 homolog D [Sesamum indicum]
c51341.graph_c0	5.90274641	7.72670547	5.39720625	2.61442067	0.77550527	1.11662198	1.072E-11	-3.071527	down	PREDICTED: putative calcium-binding protein CML23 [Sesamum indicum]
c51343.graph_c0	14.9802184	18.2611564	15.9591604	9.82536133	12.3261856	12.5674119	4.199E-19	-1.524601	down	PREDICTED: F-box/kelch-repeat protein SKIP30 [Sesamum indicum]
c51346.graph_c0	2.38803602	2.34696496	1.78543757	11.040073	10.7633592	10.1437185	3.243E-05	1.279149	up	PREDICTED: serine protease SPPA, chloroplastic-like [Sesamum indicum]
c51348.graph_c0	0.07858398	0.07866267	0.05049176	2.28049557	3.75807993	4.41264222	6.537E-11	4.630071	up	PREDICTED: BTB/POZ domain-containing protein At1g63850-like [Sesamum indicum]
c51349.graph_c0	2.93372511	4.01859119	4.56362721	0	0	0.12657268	2.869E-18	-7.551949	down	-
c51351.graph_c0	60.1670312	60.3324339	58.0468221	30.7755117	29.3221257	26.3935683	8.567E-48	-2.060921	down	PREDICTED: putative E3 ubiquitin-protein ligase RF4 [Sesamum indicum]
c51353.graph_c0	0.54811339	0.59086703	0.59598626	5.04957803	4.1972338	4.94239258	5.662E-06	2.016055	up	PREDICTED: uncharacterized protein LOC105163262 isoform X2 [Sesamum indicum]
c51356.graph_c0	1.5981637	1.59976402	0.55505504	13.6887143	13.27902	13.6318379	1.149E-14	2.427476	up	PREDICTED: A-kinase anchor protein 17B [Sesamum indicum]
c51358.graph_c0	10.4842046	8.3514185	10.1519022	66.5228235	72.4184351	75.1091881	2.308E-26	1.864655	up	PREDICTED: 50S ribosomal protein HLP, mitochondrial [Sesamum indicum]
c51360.graph_c0	0	0	0	8.51718609	6.67696517	5.75969114	3.973E-16	Inf	up	PREDICTED: uncharacterized protein LOC105165055 [Sesamum indicum]
c51361.graph_c0	0.0971904	0.09728772	0.06244676	2.68614412	7.87294837	6.89149793	1.598E-06	5.056736	up	hypothetical protein MIMGU_mgv1a009735mg [Erythranthe guttata]
c51363.graph_c0	6.98448335	7.28898692	10.5030547	0.27381113	0.29006969	0.06090887	1.868E-23	-6.324373	down	-

#ID	0 h-1 FPKM	0 h-2 FPKM	0 h-3 FPKM	48 h-1 FPKM	48 h-2 FPKM	48 h-3 FPKM	FDR	log2FC	regulated	nr_annotation
c51366.graph_c0	0.80679455	0.48456146	0.31102888	81.7598937	113.858952	115.539372	2.328E-64	6.585552	up	PREDICTED: putative invertase inhibitor [Sesamum indicum]
c51369.graph_c0	0.87150579	1.96285155	1.3998993	9.63465568	13.183718	10.7160734	0.0002657	1.962505	up	-
c51370.graph_c0	4.26194463	2.34641678	3.55989893	113.079784	128.737298	166.822768	1.462E-29	4.306935	up	putative plasma membrane intrinsic protein PIP family member 2 aquaporin [Cedrela odorata]
c51372.graph_c0	0.08081761	0	0	14.8908899	18.890673	26.1021539	3.127E-26	8.530464	up	PREDICTED: 2-alkenal reductase (NADP(+)-dependent) isoform X1 [Sesamum indicum]
c51373.graph_c0	6.53177224	6.30480165	8.54347139	2.84756442	4.43959696	4.58942617	5.171E-09	-1.876884	down	PREDICTED: F-box protein At5g52880 [Sesamum indicum]
c51375.graph_c0	71.8959022	72.6455589	71.3362017	324.44019	336.569296	358.941586	6.679E-18	1.221722	up	hypothetical protein MIMGU_mgv1a013134mg [Erythranthe guttata]
c51377.graph_c0	9.39448121	6.12677575	5.85322681	0	0	0	1.927E-24	-Inf	down	unknown [Lotus japonicus]
c51379.graph_c0	90.0258061	92.7453312	87.5782263	46.5506779	45.9587821	49.8377253	2.074E-42	-1.942803	down	PREDICTED: glutelin type-B 5 [Sesamum indicum]
c51380.graph_c0	24.7052913	25.7848653	23.3716134	16.5397439	18.4741277	16.8925159	3.397E-22	-1.527028	down	PREDICTED: protein YLS9 [Sesamum indicum]
c51381.graph_c0	4.40598105	2.3816122	3.96330206	0	0	0	6.574E-19	-Inf	down	--
c51382.graph_c0	4.31117991	5.0718211	4.05507853	54.8673378	31.7086478	24.3010137	0.0061428	2.042082	up	PREDICTED: abscisic acid receptor PYL4-like [Sesamum indicum]
c51384.graph_c0	0.8088742	0.64099996	0.77957653	11.8919532	12.4665098	12.4324407	2.577E-23	3.025841	up	PREDICTED: afadin- and alpha-actinin-binding protein [Sesamum indicum]
c51386.graph_c0	0.20327992	0.5087087	0.13061138	5.8055165	3.47191012	4.08258016	7.857E-06	2.985658	up	-
c51391.graph_c0	5.89714488	4.83312217	5.35200816	0.6451491	0.53957151	0.4531972	5.935E-34	-4.307638	down	hypothetical protein MIMGU_mgv1a026169mg, partial [Erythranthe guttata]
c51392.graph_c0	0	0.07816369	0	3.02137327	3.81045074	4.03259965	1.471E-11	6.108466	up	PREDICTED: 21 kDa protein-like [Sesamum indicum]
c51395.graph_c0	50.7126173	58.1688823	51.3674977	37.2658878	37.6153884	33.8436612	4.993E-20	-1.576018	down	PREDICTED: E3 ubiquitin-protein ligase RING1-like [Sesamum indicum]
c51401.graph_c0	8.24779118	7.43630044	7.66717233	0.48500446	0.57089268	0.62335585	1.989E-35	-4.818322	down	PREDICTED: uncharacterized protein LOC105157495 [Sesamum indicum]
c51405.graph_c0	4.32583663	4.54667672	3.61326637	23.24725	20.1946669	23.2856964	4.103E-07	1.404247	up	PREDICTED: DEAD-box ATP-dependent RNA helicase 31-like [Sesamum indicum]
c51406.graph_c0	0	0	0	13.8566545	26.5775218	29.4179162	7.56E-20	Inf	up	PREDICTED: pectinesterase-like [Sesamum indicum]
c51408.graph_c0	2.3589027	2.95158098	3.03128336	11.7713647	15.5399541	15.4694246	0.0045972	1.334209	up	hypothetical protein POPTR_0013s01180g [Populus trichocarpa]
c51409.graph_c0	5.61440049	4.29766423	5.80007457	0.10141867	0.10744078	0.27072516	1.593E-21	-6.061411	down	-
c51410.graph_c0	3.10239495	3.03649038	3.18936027	1.14325143	1.34570696	0.84771521	7.078E-08	-2.501054	down	PREDICTED: cyclic dof factor 2-like [Sesamum indicum]
c51412.graph_c0	3.93199542	3.18224347	1.93510332	13.0252638	17.1871227	13.7500307	2.053E-06	1.265563	up	PREDICTED: probable peroxidase 61 [Sesamum indicum]
c51413.graph_c0	10.6799548	10.3895041	8.31181809	6.92892321	10.2764956	11.3441781	0.0044515	-1.064027	down	unknown [Lotus japonicus]
c51414.graph_c0	21.4786633	20.972389	18.9711837	9.45916262	13.62292	14.1036695	2.145E-29	-1.747017	down	PREDICTED: methyltransferase-like protein 13 [Sesamum indicum]

#ID	0 h-1 FPKM	0 h-2 FPKM	0 h-3 FPKM	48 h-1 FPKM	48 h-2 FPKM	48 h-3 FPKM	FDR	log2FC	regulated	nr_annotation
c51416.graph_c0	0.11559786	0.11571361	0	3.78061816	4.11792642	6.01724445	8.328E-13	4.905223	up	PREDICTED: uncharacterized protein LOC105175985 [Sesamum indicum]
c51417.graph_c0	3.20253016	3.20573701	3.35492776	22.640957	23.6456128	25.5959934	4.511E-21	1.860849	up	PREDICTED: ubiquitin receptor RAD23c-like [Sesamum indicum]
c51418.graph_c0	4.5246781	4.62820798	4.00335083	15.3070165	21.3799287	21.5651444	2.724E-08	1.124385	up	PREDICTED: LOW QUALITY PROTEIN: BAG family molecular chaperone regulator 2 [Sesamum indicum]
c51420.graph_c0	2.53872621	2.80877029	2.40384693	0.55393583	1.23885878	1.86202425	8.613E-06	-2.116039	down	PREDICTED: embryonic protein DC-8-like isoform X2 [Sesamum indicum]
c51422.graph_c0	2.01278187	1.67899781	1.2932522	12.8256161	12.6049789	12.0996354	0.0001622	1.89958	up	PREDICTED: GTP-binding protein OBGC, chloroplastic [Sesamum indicum]
c51424.graph_c0	0.2234788	0.22370258	0.09572637	1.85294923	1.89027225	3.48067984	0.0002727	2.718892	up	PREDICTED: LOW QUALITY PROTEIN: subtilisin-like protease [Sesamum indicum]
c51433.graph_c0	4.78419389	5.25057341	4.22203709	66.4312873	102.413521	110.329515	2.872E-16	3.267519	up	PREDICTED: leucine-rich repeat extensin-like protein 1 [Sesamum indicum]
c51435.graph_c0	11.2078908	12.4902449	9.18785494	6.1037084	7.67853999	8.16919389	1.033E-15	-1.602805	down	PREDICTED: pentatricopeptide repeat-containing protein At1g09820 [Sesamum indicum]
c51437.graph_c0	0.11213134	0.22448724	0.09606214	7.88543958	6.85803236	3.67672492	8.374E-08	4.405926	up	hypothetical protein MIMGU_mgv1a027064mg [Erythranthe guttata]
c51439.graph_c0	41.2766784	45.8542974	39.308467	32.9820462	38.7367543	39.501119	3.714E-17	-1.204688	down	PREDICTED: uncharacterized protein At3g15000, mitochondrial [Sesamum indicum]
c51440.graph_c0	17.8315849	19.7384743	16.6784376	8.26703079	8.15651469	8.208361	2.187E-34	-2.155149	down	hypothetical protein MIMGU_mgv1a026899mg [Erythranthe guttata]
c51444.graph_c0	1.33021638	1.22502452	1.09400366	0.09803857	0.4154399	0.34893651	7.731E-09	-3.116533	down	-
c51446.graph_c0	1.66639466	1.40117318	1.45614052	10.4393019	14.116711	16.2827692	2.792E-09	2.151299	up	PREDICTED: phospholipase D delta [Sesamum indicum]
c51448.graph_c0	0	0.10205227	0	5.35361982	16.8155327	24.1941688	1.497E-06	7.803602	up	PREDICTED: uncharacterized protein LOC105162545 [Sesamum indicum]
c51450.graph_c0	1.9617302	1.79042741	1.6682433	8.23904241	8.84088919	9.29388001	3.681E-06	1.2649	up	PREDICTED: probable indole-3-acetic acid-amido synthetase GH3.5 isoform X1 [Sesamum indicum]
c51455.graph_c0	0.15920705	0.07968323	0.30688104	45.1749499	26.8809319	28.3854439	3.704E-16	6.506111	up	PREDICTED: uncharacterized protein LOC105171871 [Sesamum indicum]
c51460.graph_c0	0	0	0	91.6364774	129.436982	146.65607	9.788E-54	Inf	up	PREDICTED: MLP-like protein 423 [Sesamum indicum]
c51462.graph_c0	18.6289537	10.3317827	17.4689584	2.55113891	4.17677997	6.19087654	8.681E-08	-2.875248	down	-
c51463.graph_c0	47.6161678	37.0281134	40.8345815	1.6314218	1.63227727	1.85485967	3.14E-91	-5.633209	down	PREDICTED: uncharacterized protein LOC105175709 [Sesamum indicum]
c51466.graph_c0	125.48772	128.249015	126.701684	26.7858385	8.85735716	9.18453874	9.99E-158	-4.079705	down	PREDICTED: zinc-finger homeodomain protein 6 [Sesamum indicum]
c51468.graph_c0	9.40967601	14.971409	11.3281132	1.36875905	1.83671004	0.56835967	3.958E-19	-4.25895	down	PREDICTED: oleosin 1-like [Solanum lycopersicum]

#ID	0 h-1 FPKM	0 h-2 FPKM	0 h-3 FPKM	48 h-1 FPKM	48 h-2 FPKM	48 h-3 FPKM	FDR	log2FC	regulated	nr_annotation
c51470.graph_c0	0.73263557	0.1833423	0.11768321	13.9208782	16.26692	17.4915412	4.534E-19	4.520034	up	PREDICTED: probable stress-associated endoplasmic reticulum protein [Musa acuminata subsp. malaccensis]
c51473.graph_c0	0	0.29656079	0	4.63994324	5.06002951	5.99050835	2.417E-14	4.716756	up	PREDICTED: DNA replication complex GINS protein PSF2 [Sesamum indicum]
c51474.graph_c0	0	0	0	5.58110448	7.56800442	6.55516383	1.889E-11	Inf	up	PREDICTED: subtilisin-like protease [Sesamum indicum]
c51476.graph_c0	63.4480049	63.545178	60.3293845	23.2819043	19.4494178	14.3249617	3.569E-79	-2.72594	down	PREDICTED: uncharacterized protein LOC104224082 [Nicotiana sylvestris]
c51477.graph_c0	5.36844353	3.72503377	4.15487627	0	0	0	4.17E-35	-Inf	down	RecName: Full=60S ribosomal protein L5 [Helianthus annuus]
c51478.graph_c0	0	0	0	3.67450081	2.66927181	4.01687415	1.593E-11	Inf	up	PREDICTED: GDSL esterase/lipase 5 isoform X1 [Sesamum indicum]
c51480.graph_c0	0.92155709	0.46123994	0.78949156	28.0169504	30.4300708	26.1883702	3.219E-19	4.265593	up	PREDICTED: uncharacterized protein LOC105179276 [Sesamum indicum]
c51483.graph_c0	0.29925074	0.25675748	0.32961346	9.92481465	10.5141377	9.11139088	1.075E-21	4.041189	up	PREDICTED: UPF0553 protein-like isoform X1 [Sesamum indicum]
c51489.graph_c0	2.83891531	3.0270901	2.45851469	62.4283165	60.5336135	64.6040355	3.254E-66	3.478319	up	PREDICTED: chlorophyll a-b binding protein 6, chloroplastic-like [Malus domestica]
c51490.graph_c0	7.48351737	9.56683331	7.18349637	0	0	0	4.151E-41	-Inf	down	unnamed protein product [Coffea canephora]
c51492.graph_c0	2.56160418	3.05518038	3.43183781	10.3434654	13.4045006	17.2008156	0.0001962	1.151595	up	hypothetical protein MIMGU_mgv1a000973mg [Erythranthe guttata]
c51500.graph_c0	0.43468377	0.87023808	0.44686869	9.61101703	7.72112792	8.55182516	1.625E-08	2.873912	up	PREDICTED: BRI1 kinase inhibitor 1 [Sesamum indicum]
c51506.graph_c0	0	0	0.16748021	5.04290698	6.93233313	6.99781463	2.524E-16	5.768084	up	PREDICTED: uncharacterized protein LOC103322890 [Prunus mume]
c51508.graph_c0	4.92387019	4.7355144	5.45890585	34.0659003	30.8120153	33.3586251	4.535E-18	1.68257	up	PREDICTED: 30S ribosomal protein 3-1, chloroplastic [Sesamum indicum]
c51511.graph_c0	4.18386607	4.34316875	4.11529481	22.0322283	26.6172148	28.0301156	1.24E-12	1.579576	up	PREDICTED: protein FLUORESCENT IN BLUE LIGHT, chloroplastic [Sesamum indicum]
c51513.graph_c0	0	0	0.05042378	26.2446138	36.7259829	40.7861099	2.984E-52	9.950161	up	PREDICTED: ectonucleotide pyrophosphatase/phosphodiesterase family member 1-like [Sesamum indicum]
c51514.graph_c0	0.40461607	0.25313827	0.25997384	11.4157357	14.6110285	19.8592481	2.581E-16	4.622948	up	PREDICTED: cyclin-D3-3-like [Sesamum indicum]
c51516.graph_c0	12.2523612	6.67875898	8.41799546	0	0	0	6.133E-22	-Inf	down	predicted protein [Physcomitrella patens]
c51517.graph_c0	0	0	0	8.71228466	4.3433455	8.27329067	3.512E-11	Inf	up	PREDICTED: uncharacterized protein LOC105157094 [Sesamum indicum]
c51518.graph_c0	0	0.07172588	0	2.97056065	3.77633857	4.69899911	2.474E-12	6.306415	up	PREDICTED: uncharacterized protein LOC105177191 [Sesamum indicum]
c51527.graph_c0	0	0	0.27893722	6.39918429	6.35546232	8.54093355	2.969E-10	5.202354	up	-

#ID	0 h-1 FPKM	0 h-2 FPKM	0 h-3 FPKM	48 h-1 FPKM	48 h-2 FPKM	48 h-3 FPKM	FDR	log2FC	regulated	nr_annotation
c51528.graph_c0	10.2457763	10.4911313	9.46912115	71.7802621	50.6281346	42.6439382	0.0009223	1.4421	up	PREDICTED: phytochrome A-associated F-box protein [Sesamum indicum]
c51532.graph_c0	0.03709169	0.07425767	0	2.63118922	2.24442088	3.13175693	1.287E-12	5.16587	up	PREDICTED: E2F transcription factor-like E2FE [Sesamum indicum]
c51533.graph_c0	16.9552304	16.2521754	15.7138724	1.04135095	1.50434326	1.01082307	3.658E-79	-4.803028	down	PREDICTED: uncharacterized protein LOC105155796 [Sesamum indicum]
c51534.graph_c0	434.593424	445.102392	438.608057	161.516627	72.0614956	47.5871027	3.265E-95	-3.226212	down	PREDICTED: zinc finger A20 and AN1 domain-containing stress-associated protein 5 [Sesamum indicum]
c51535.graph_c0	32.2664208	19.5524884	19.3814661	0	0	0	2.184E-28	-Inf	down	-
c51537.graph_c0	134.719924	125.495728	131.122502	12.4704162	11.876185	7.9617387	4.9E-187	-4.611938	down	PREDICTED: anthocyanidin 3-O-glucosyltransferase 5-like [Sesamum indicum]
c51540.graph_c0	857.850664	933.26925	780.863924	135.627994	116.886772	105.481694	1.07E-123	-3.85717	down	Polyubiquitin-A [Triticum urartu]
c51541.graph_c0	0	0	0	3.12063275	4.77523511	5.47630939	5.739E-13	Inf	up	RecName: Full=Casparian strip membrane protein 3; Short=MgCASP3 [Erythranthe guttata]
c51543.graph_c0	5.28013372	5.53710769	5.16966162	0.38606378	0.32719021	0.68703445	1.424E-19	-4.533634	down	-
c51544.graph_c0	0.58553734	0.41028657	0.82768253	8.63096304	5.94324821	5.03985498	1.58E-05	2.413244	up	PREDICTED: polyol transporter 5-like [Sesamum indicum]
c51545.graph_c0	16.2191611	15.6690509	13.9179239	3.30117247	5.02228317	4.06372081	1.36E-61	-2.908899	down	PREDICTED: galactose oxidase [Sesamum indicum]
c51547.graph_c0	0.47543626	0	1.22190884	10.9500724	12.0642849	17.9276877	5.96E-08	3.552312	up	-
c51549.graph_c0	0	0	0	7.19086335	7.32485363	6.56244817	1.682E-15	Inf	up	PREDICTED: cyclin-U4-1 [Sesamum indicum]
c51553.graph_c0	3.9010621	3.52144474	4.02831904	15.5950147	15.9431311	16.7886708	6.939E-07	1.058381	up	hypothetical protein MIMGU_mgv1a005667mg [Erythranthe guttata]
c51556.graph_c0	0	0	0	39.0420856	68.2670923	61.9176478	1.713E-39	Inf	up	hypothetical protein MIMGU_mgv1a009759mg [Erythranthe guttata]
c51558.graph_c0	4.28800689	5.74095216	1.99746875	17.1348237	43.7328417	40.1592717	0.0025987	2.046145	up	PREDICTED: blue copper protein-like [Sesamum indicum]
c51560.graph_c0	0.04680039	0	0	3.14743696	3.5170307	3.87476189	8.494E-15	6.816092	up	PREDICTED: homeobox-leucine zipper protein HOX21-like [Sesamum indicum]
c51565.graph_c1	11.2072158	15.3643826	12.2101505	7.29470849	7.01451828	10.584977	4.406E-07	-1.659268	down	PREDICTED: floral homeotic protein APETALA 2-like isoform X2 [Sesamum indicum]
c51566.graph_c0	2.87210383	3.13372799	2.17755261	17.198801	18.948846	17.3281459	3.068E-16	1.692662	up	PREDICTED: ankyrin repeat-containing protein At2g01680 [Sesamum indicum]
c51569.graph_c0	5.84030455	5.84615273	6.54284019	2.75921512	3.87304635	4.84888772	6.19E-06	-1.693312	down	PREDICTED: uncharacterized protein LOC105171557 [Sesamum indicum]
c51570.graph_c0	2.12078345	1.93559176	2.32451266	0.40225377	0.4261391	0.30679113	4.443E-11	-3.508407	down	PREDICTED: uncharacterized protein LOC105175073 [Sesamum indicum]
c51573.graph_c0	34.7559086	35.4565623	35.9011326	27.5765253	13.7954928	6.13435659	2.513E-17	-2.156613	down	-
c51574.graph_c0	3.72890961	4.08477973	4.88220773	1.16671067	1.57931862	1.78789424	1.017E-08	-2.511977	down	hypothetical protein JCGZ_21459 [Jatropha curcas]

#ID	0 h-1 FPKM	0 h-2 FPKM	0 h-3 FPKM	48 h-1 FPKM	48 h-2 FPKM	48 h-3 FPKM	FDR	log2FC	regulated	nr_annotation
c51578.graph_c0	0.40585215	0.10156464	0.13038402	2.80423104	3.16879227	3.41009015	4.54E-06	2.867246	up	hypothetical protein CICLE_v10033008mg [Citrus clementina]
c51579.graph_c0	6.82941825	4.91730758	5.38881665	0.77266559	1.28628579	0.78572937	3.482E-13	-3.613036	down	-
c51582.graph_c0	13.0688342	12.9828152	11.8321148	0.2280277	0.38650832	0.16231814	1.16E-85	-6.629327	down	PREDICTED: LOW QUALITY PROTEIN: EID1-like F-box protein 3 [Sesamum indicum]
c51583.graph_c0	32.5875606	32.1644967	28.2750375	28.3442011	35.2847887	28.8278708	1.638E-12	-1.027563	down	PREDICTED: probable ubiquitin-conjugating enzyme E2 26 [Sesamum indicum]
c51588.graph_c0	2.25541527	2.33552455	3.39800446	1.86288714	1.59398323	0.57377969	3.58E-05	-1.999229	down	-
c51590.graph_c0	0	0	0	129.120805	132.21736	127.915542	7.12E-111	Inf	up	PREDICTED: raucaffricine-O-beta-D-glucosidase-like [Sesamum indicum]
c51592.graph_c0	8.02379469	6.34447021	6.41181635	3.04378242	2.82967941	5.58249528	3.597E-08	-1.879427	down	PREDICTED: glutaredoxin-C9-like [Sesamum indicum]
c51597.graph_c0	11.9019547	10.1736441	11.3419761	2.03281056	2.38191954	2.21986957	2.606E-52	-3.352489	down	PREDICTED: pentatricopeptide repeat-containing protein At3g26630, chloroplastic [Sesamum indicum]
c51599.graph_c0	0.04979921	0	0	6.33120317	10.6439428	10.2055553	3.493E-23	8.087249	up	PREDICTED: uncharacterized protein LOC105165189 [Sesamum indicum]
c51602.graph_c0	8.66082409	10.8368707	9.37221208	8.29387326	7.89659577	6.81934449	0.000116	-1.342568	down	-
c51603.graph_c0	0.32182691	0.08053729	0.20678016	4.74380708	2.82683721	1.91264561	0.0027292	2.958643	up	hypothetical protein MIMGU_mgv1a020884mg [Erythranthe guttata]
c51604.graph_c0	14.084173	13.0977533	14.4789759	60.4395926	54.8054894	63.6854045	4.143E-09	1.08531	up	PREDICTED: 50S ribosomal protein L31, chloroplastic [Sesamum indicum]
c51605.graph_c0	1.08699855	0.63471743	0.23280603	3.9222047	4.77394501	3.93548022	0.0027858	1.684043	up	PREDICTED: 29 kDa ribonucleoprotein A, chloroplastic [Sesamum indicum]
c51606.graph_c0	0.1792196	0.6278967	0.69091283	4.62304591	5.85957661	5.36231727	8.96E-06	2.37396	up	PREDICTED: probable phospholipase A2 homolog 1 [Sesamum indicum]
c51610.graph_c0	30.121116	13.0104829	18.0278371	0	0	0	1.927E-14	-Inf	down	-
c51614.graph_c0	1.01705138	0.76355235	1.14358237	11.126567	20.3485134	18.550134	1.473E-09	3.06678	up	-
c51619.graph_c0	0	0	0	10.418928	12.4172897	12.206421	6.178E-22	Inf	up	hypothetical protein MIMGU_mgv1a023502mg, partial [Erythranthe guttata]
c51620.graph_c0	67.9826049	59.5785063	75.0807732	43.0948127	32.5082969	24.9917203	4.064E-18	-2.021577	down	PREDICTED: ethylene-responsive transcription factor ERF069-like [Sesamum indicum]
c51622.graph_c0	25.9222439	26.1828141	25.1791028	166.261949	166.115303	163.001751	3.891E-32	1.663393	up	PREDICTED: 40S ribosomal protein SA-like [Sesamum indicum]
c51624.graph_c0	0	0	0	3.24221313	4.15030077	5.76980348	5.611E-11	Inf	up	hypothetical protein MIMGU_mgv1a024616mg [Erythranthe guttata]
c51629.graph_c0	51.9814082	43.846862	53.7947909	9.45002964	11.7698779	11.4765748	6.199E-51	-3.216496	down	-
c51632.graph_c0	0.19285497	0.38609617	0.86739194	271.214134	249.768513	228.993669	3.34E-132	7.98951	up	PREDICTED: non-specific lipid-transfer protein 2-like [Sesamum indicum]
c51635.graph_c0	0	0	0	0.94429641	1.15832032	1.10556483	6.063E-10	Inf	up	-

#ID	0 h-1 FPKM	0 h-2 FPKM	0 h-3 FPKM	48 h-1 FPKM	48 h-2 FPKM	48 h-3 FPKM	FDR	log2FC	regulated	nr_annotation
c51636.graph_c0	7.42837473	9.25682858	7.27295667	4.46922665	7.05258679	8.40904452	8.841E-07	-1.290984	down	unnamed protein product [Coffea canephora]
c51639.graph_c0	0.22546121	0	0.57945315	6.43899664	5.72112066	6.28383667	9.61E-08	3.484731	up	hypothetical protein ARALYDRAFT_486411 [Arabidopsis lyrata subsp. lyrata]
c51642.graph_c0	7.14506391	6.94185924	6.95377993	23.4743527	33.7900284	41.6885424	0.0002751	1.208383	up	hypothetical protein MIMGU_mgv1a019984mg [Erythranthe guttata]
c51644.graph_c0	1.59097771	1.20815718	1.90347146	5.35743166	7.71018008	6.97062853	0.0057179	1.064697	up	-
c51645.graph_c0	56.3960131	62.0483583	55.7795129	35.8994096	22.4656118	18.4649865	1.636E-29	-2.186774	down	PREDICTED: uncharacterized protein LOC105177839 [Sesamum indicum]
c51647.graph_c0	1.89602712	1.59015397	1.77796453	31.1581336	29.0572752	21.2133054	1.654E-14	2.937651	up	hypothetical protein MIMGU_mgv1a009839mg [Erythranthe guttata]
c51648.graph_c0	0.67447312	0.311607	0.20001338	6.88285251	10.633507	12.1210515	2.316E-12	3.626744	up	hypothetical protein MIMGU_mgv1a011849mg [Erythranthe guttata]
c51657.graph_c0	3.41874635	2.85180809	4.2467836	0	0.22243932	0	7.549E-19	-6.619193	down	-
c51658.graph_c0	49.7268238	55.1871197	51.3985801	8.79715384	4.04431904	3.39690671	2.816E-63	-4.266456	down	hypothetical protein MIMGU_mgv1a005887mg [Erythranthe guttata]
c51659.graph_c0	0	0	0	1.99786456	2.40842563	3.18757826	9.804E-12	Inf	up	hypothetical protein MIMGU_mgv1a012062mg [Erythranthe guttata]
c51660.graph_c0	0.35354746	0.17695074	0.68148372	26.8711558	22.8596478	25.2864173	1.585E-31	4.926193	up	PREDICTED: nudix hydrolase 1 [Sesamum indicum]
c51668.graph_c0	77.04052	83.0838721	72.3068655	48.8206261	38.9912296	38.0223557	2.925E-41	-1.897004	down	hypothetical protein MIMGU_mgv1a003993mg [Erythranthe guttata]
c51669.graph_c0	2.44309736	2.90408321	4.218672	28.9782857	33.2323909	37.3002034	3.586E-20	2.352515	up	PREDICTED: probable calcium-binding protein CML13 [Sesamum indicum]
c51671.graph_c0	0	0	0	5.40839234	8.51245331	5.22484255	1.021E-11	Inf	up	PREDICTED: 60S ribosomal protein L35-like [Musa acuminata subsp. malaccensis]
c51672.graph_c0	29.5589602	16.8348698	21.7755592	0	0	0	2.107E-31	-Inf	down	--
c51676.graph_c0	158.237019	168.590537	155.25803	5.03377981	4.05283629	3.00990615	0	-6.326874	down	hypothetical protein MIMGU_mgv1a009467mg [Erythranthe guttata]
c51680.graph_c0	6.24373048	5.87684934	5.98764244	84.2215114	88.5858252	90.5236048	1.805E-48	2.843838	up	PREDICTED: cytochrome b-c1 complex subunit 6 [Sesamum indicum]
c51682.graph_c0	0	0	0	4.52280687	6.04128689	7.52381536	5.262E-25	Inf	up	PREDICTED: serine carboxypeptidase-like 34 [Sesamum indicum]
c51683.graph_c0	0.48839506	0.92887982	1.44349608	10.2586637	9.39016935	12.4510449	3.19E-12	2.460905	up	hypothetical protein MIMGU_mgv1a008015mg [Erythranthe guttata]
c51684.graph_c0	1.81063396	0.55767601	0.35795944	18.9903821	28.545821	27.8580864	2.518E-16	3.777033	up	PREDICTED: 60S ribosomal protein L12-1-like [Cucumis sativus]
c51685.graph_c0	2.10277208	2.0692018	1.87776204	8.33985664	10.1220648	10.28388	2.028E-05	1.228404	up	PREDICTED: uncharacterized protein LOC105162245 [Sesamum indicum]

#ID	0 h-1 FPKM	0 h-2 FPKM	0 h-3 FPKM	48 h-1 FPKM	48 h-2 FPKM	48 h-3 FPKM	FDR	log2FC	regulated	nr_annotation
c51687.graph_c0	4.11647333	5.75328408	4.39155798	0.93020242	1.13704232	1.84638209	6.468E-11	-2.889553	down	PREDICTED: LOB domain-containing protein 37-like [Sesamum indicum]
c51688.graph_c0	0.86678195	1.7352998	1.33661895	22.8381315	19.9644704	21.8133505	1.099E-16	3.019263	up	PREDICTED: uncharacterized protein LOC105159746 [Sesamum indicum]
c51689.graph_c0	0	0	0	4.35599833	2.23289615	2.58396075	2.997E-08	Inf	up	PREDICTED: transcription factor PCF7-like [Sesamum indicum]
c51690.graph_c0	0.48727025	0.24387909	0.31308078	19.1906947	14.1479267	17.175623	7.485E-17	4.585933	up	histone H4 [Zea mays]
c51694.graph_c0	2.19521648	1.09870733	0.90673074	6.71717945	8.87591728	8.61188589	0.0008325	1.511295	up	PREDICTED: uncharacterized protein LOC105176347 isoform X3 [Sesamum indicum]
c51696.graph_c0	0	0.10972919	0	1.86828846	4.01194274	4.22337386	5.238E-08	5.506164	up	PREDICTED: carboxylesterase 1-like [Sesamum indicum]
c51700.graph_c0	0	0	0	73.5011737	71.3767826	68.595774	5.496E-90	Inf	up	--
c51703.graph_c0	2.32665346	1.66355946	1.38814084	18.755318	16.9494609	16.415906	6.981E-10	2.265753	up	hypothetical protein MIMGU_mgv1a001920mg [Erythranthe guttata]
c51705.graph_c0	0	0	0	2.94548992	4.41420941	3.38796582	9.463E-13	Inf	up	60S ribosomal protein L8 [Rozella allomycis CSF55]
c51711.graph_c0	0	0	0	2.82228415	6.2944586	2.90776485	3.731E-05	Inf	up	predicted protein [Physcomitrella patens]
c51715.graph_c0	7.67698065	7.88689609	9.60561291	0.3722387	1.77453795	1.98729454	3.904E-12	-3.646966	down	BnaCnng51820D [Brassica napus]
c51716.graph_c0	22.053418	19.903493	19.8224516	2.03533924	1.84816726	1.61699341	4.5E-109	-4.503169	down	PREDICTED: SNF1-related protein kinase regulatory subunit gamma-1-like [Sesamum indicum]
c51717.graph_c0	11.6189151	6.44395319	8.37333493	0	0	0	4.508E-26	-Inf	down	60S ribosomal protein L8 [Rozella allomycis CSF55]
c51719.graph_c0	2.54892225	1.02954239	1.32167927	0	0	0	1.827E-09	-Inf	down	Bifunctional monodehydroascorbate reductase and carbonic anhydrase nectarin-3 [Gossypium arboreum]
c51720.graph_c0	12.6927661	15.6642855	11.8420202	161.064627	177.67079	189.066601	4.239E-65	2.696569	up	hypothetical protein MIMGU_mgv1a021151mg [Erythranthe guttata]
c51724.graph_c0	13.1862118	12.733554	13.456168	176.074517	191.071718	213.259471	2.2E-77	2.861507	up	PREDICTED: 60S acidic ribosomal protein P3-like [Sesamum indicum]
c51728.graph_c0	7.28198627	6.66448281	7.75347369	33.7987708	39.2576666	40.534445	3.189E-11	1.366564	up	PREDICTED: proliferation-associated protein 2G4-like [Sesamum indicum]
c51729.graph_c0	14.4472132	13.7921577	13.3222794	1.41723819	1.76250366	2.08347394	2.886E-42	-4.003194	down	PREDICTED: homeobox-leucine zipper protein HOX3 [Sesamum indicum]
c51730.graph_c0	0.16859296	0.25314266	0.21664862	2.71807948	4.77170247	4.90616244	1.36E-07	3.251029	up	PREDICTED: uncharacterized protein LOC105170277 [Sesamum indicum]
c51731.graph_c0	7.3777705	8.67463029	8.57780079	4.31548152	2.97162411	2.78391989	4.006E-14	-2.300438	down	hypothetical protein MIMGU_mgv1a020203mg [Erythranthe guttata]
c51733.graph_c0	3.61757604	2.80350851	4.04889251	0	0	0	4.14E-21	-Inf	down	-
c51740.graph_c0	0.07567624	0.11362802	0.2431174	5.15912346	4.46838753	5.95531723	9.934E-16	4.142689	up	hypothetical protein MIMGU_mgv1a007527mg [Erythranthe guttata]
c51743.graph_c0	1.02070686	0.40869158	1.04931898	11.2841008	29.0883988	33.6357588	3.468E-05	3.863838	up	-

#ID	0 h-1 FPKM	0 h-2 FPKM	0 h-3 FPKM	48 h-1 FPKM	48 h-2 FPKM	48 h-3 FPKM	FDR	log2FC	regulated	nr_annotation
c51745.graph_c0	8.36342989	3.88525739	5.99713366	0	0	0	2.835E-19	-Inf	down	predicted protein [Physcomitrella patens]
c51746.graph_c0	5.83269513	6.01469841	5.75066147	67.1682399	53.3429174	52.3878992	1.429E-15	2.284077	up	PREDICTED: scarecrow-like protein 8 [Sesamum indicum]
c51751.graph_c0	5.70270474	4.1100589	5.56943304	0	0	0	3.982E-19	-Inf	down	30S ribosomal protein S28e [Mucor circinelloides f. circinelloides 1006PhL]
c51753.graph_c0	0	0	0	25.2398736	22.9989233	23.1650171	8.289E-38	Inf	up	Putative non-specific lipid-transfer protein AKCS9 [Aegilops tauschii]
c51754.graph_c0	47.5834595	48.0071422	40.6035074	16.4580967	15.7244115	17.6552962	1.337E-60	-2.466093	down	PREDICTED: uncharacterized protein LOC105174255 [Sesamum indicum]
c51757.graph_c0	3.0903147	4.29640164	4.41241859	0.31633327	0.16755837	0.84441433	5.3E-12	-4.176272	down	-
c51758.graph_c0	21.0961927	18.8902051	15.3976124	5.5070484	6.4880915	6.2405352	1.299E-30	-2.620024	down	PREDICTED: uncharacterized protein LOC105123074 [Populus euphratica]
c51759.graph_c0	0.12874747	0.0644382	0.41361399	9.72606646	5.96854187	5.01310103	1.175E-06	4.070444	up	PREDICTED: uncharacterized protein LOC105158157 [Sesamum indicum]
c51760.graph_c0	4.71381345	4.58746325	4.54307769	2.01049649	2.30026742	2.64761297	2.941E-09	-2.013181	down	PREDICTED: uncharacterized protein LOC105156249 [Sesamum indicum]
c51762.graph_c0	1.31728368	2.63720549	3.2162472	14.441455	11.5706499	8.63860157	0.0051535	1.255528	up	PREDICTED: cyclic dof factor 1 [Sesamum indicum]
c51765.graph_c0	3.75401154	3.91219954	3.63456082	13.454754	16.461997	17.1148431	9.069E-05	1.036254	up	PREDICTED: arogenate dehydrogenase 2, chloroplastic-like [Sesamum indicum]
c51766.graph_c0	5.09288097	5.74681463	6.54455368	36.7673886	42.2943761	40.0782642	4.046E-13	1.754824	up	PREDICTED: microsomal glutathione S-transferase 3 [Sesamum indicum]
c51767.graph_c0	6.03785111	4.80412334	2.98418374	0	0	0	2.568E-16	-Inf	down	predicted protein [Physcomitrella patens]
c51779.graph_c0	0.05179035	0.15552663	0.06655265	18.1308066	21.0270387	22.1612114	1.602E-51	6.788751	up	PREDICTED: acid phosphatase 1-like [Sesamum indicum]
c51780.graph_c0	0	0.10174174	0	11.1428462	13.5900482	19.7463571	7.018E-17	7.759918	up	hypothetical protein M569_05207, partial [Genlisea aurea]
c51782.graph_c0	44.2094699	43.083568	45.3394835	25.1616883	32.5677352	30.4033576	1.575E-19	-1.611802	down	PREDICTED: uncharacterized protein LOC105172990 [Sesamum indicum]
c51784.graph_c0	0.68817575	1.23995673	0.53059985	9.89028118	7.52234621	6.54382228	3.759E-05	2.278427	up	-
c51786.graph_c0	0.12946236	0.194388	0.41591065	3.51844052	4.29594161	5.94299947	1.141E-08	3.181868	up	PREDICTED: uncharacterized protein LOC100256155 [Vitis vinifera]
c51793.graph_c0	2.95948388	2.73009854	3.20649647	3.47491221	1.86894136	1.76003649	0.0014626	-1.329731	down	PREDICTED: uncharacterized protein LOC105170241 [Sesamum indicum]
c51794.graph_c0	22.7299907	25.0232962	23.0149428	18.8506685	16.8338316	17.1993208	5.312E-18	-1.435386	down	PREDICTED: uncharacterized protein LOC105156609 [Sesamum indicum]
c51795.graph_c0	22.8768532	24.6376892	20.3421322	1.92879826	2.442026	1.88367047	1.026E-96	-4.458112	down	PREDICTED: E3 ubiquitin-protein ligase ATL4-like [Sesamum indicum]
c51799.graph_c0	14.9477272	11.2514754	12.2510403	14.6382746	6.49016627	4.5828875	1.757E-05	-1.576786	down	PREDICTED: uncharacterized protein LOC105166323 [Sesamum indicum]

#ID	0 h-1 FPKM	0 h-2 FPKM	0 h-3 FPKM	48 h-1 FPKM	48 h-2 FPKM	48 h-3 FPKM	FDR	log2FC	regulated	nr_annotation
c51802.graph_c0	1.59199747	1.45699805	1.34436957	16.6360231	11.7640303	8.50126043	0.0004231	2.064063	up	hypothetical protein MIMGU_mgv1a010707mg [Erythranthe guttata]
c51805.graph_c0	1.85048995	2.43729334	1.8773312	0.62808128	1.42580562	2.55480204	0.0044515	-1.452085	down	-
c51806.graph_c0	0.74150419	0.90461316	1.13152391	4.95263467	10.7874288	9.4404819	5.913E-05	2.146752	up	PREDICTED: uncharacterized protein LOC105177377 [Sesamum indicum]
c51807.graph_c0	0.10023988	0.0668935	0.08587481	1.75460273	2.3479438	2.30076731	3.347E-09	3.639244	up	PREDICTED: uncharacterized protein LOC105172555 [Sesamum indicum]
c51809.graph_c0	13.9807256	13.1026311	14.3153673	2.05258268	2.06573934	3.10483873	7.724E-42	-3.540269	down	PREDICTED: LOW QUALITY PROTEIN: myb-like protein AA [Sesamum indicum]
c51812.graph_c0	0	0.27372327	0.35139338	2.01535578	2.57982197	2.68987719	1.412E-05	2.507183	up	PREDICTED: calmodulin-like protein 1 [Sesamum indicum]
c51816.graph_c0	9.10935506	9.20615436	9.45475077	0.40346789	0.34194024	0.28720264	7.21E-35	-5.762258	down	hypothetical protein Csa_5G205920 [Cucumis sativus]
c51818.graph_c0	1.42221111	1.63299336	1.39757462	10.0194337	11.553339	10.869725	4.606E-09	1.846163	up	PREDICTED: FACT complex subunit SPT16-like [Sesamum indicum]
c51820.graph_c0	18.3593667	15.1198768	16.8364752	4.07469928	5.29400448	4.51495156	2.486E-26	-2.878604	down	PREDICTED: uncharacterized protein LOC105171189 [Sesamum indicum]
c51821.graph_c0	1.29892749	1.74348778	1.44155872	10.7427138	12.9652436	15.2456845	7.092E-17	2.096914	up	PREDICTED: bifunctional aspartate aminotransferase and glutamate/aspartate-prephenate aminotransferase [Sesamum indicum]
c51827.graph_c0	0	0	0.13961939	7.60724682	7.42272224	6.05636853	1.396E-13	6.190838	up	PREDICTED: photosystem I reaction center subunit psaK, chloroplastic [Malus domestica]
c51832.graph_c0	0.11793678	0.23610976	0.15155343	1.90139349	2.58980894	3.19034287	1.546E-06	2.901402	up	PREDICTED: RAN GTPase-activating protein 2-like isoform X1 [Sesamum indicum]
c51833.graph_c0	2.89418265	2.4731177	2.99345358	12.9738735	14.0542663	12.3831176	5.853E-07	1.217905	up	PREDICTED: uncharacterized CRM domain-containing protein At3g25440, chloroplastic [Sesamum indicum]
c51834.graph_c0	53.7006178	49.0866426	49.0977161	8.03755093	11.010531	11.0975656	3.594E-52	-3.355551	down	unnamed protein product [Coffea canephora]
c51835.graph_c0	60.1854436	70.9537485	54.5188385	48.03153	54.7353272	50.4429556	2.794E-15	-1.294771	down	hypothetical protein CICLE_v10001854mg [Citrus clementina]
c51837.graph_c0	36.9800656	37.5546882	34.5561356	23.7136192	20.3669255	21.8549387	9.819E-32	-1.7406	down	PREDICTED: protein MOS2 [Sesamum indicum]
c51840.graph_c0	1.04933545	1.47054068	1.48328136	15.3707886	11.7773646	11.4403716	3.147E-07	2.254833	up	PREDICTED: putative DNA-binding protein ESCAROLA [Populus euphratica]
c51841.graph_c0	8.05330231	3.9531701	4.37834367	0	0	0	6.783E-17	-Inf	down	uncharacterized protein LOC100191800 [Zea mays]
c51842.graph_c0	4.90721966	5.70869569	4.77208658	2.56588831	1.81216502	2.17439237	1.944E-06	-2.242821	down	-
c51844.graph_c0	0	0	0	8.01874622	18.0441088	19.0773555	1.75E-16	Inf	up	hypothetical protein MIMGU_mgv1a004963mg [Erythranthe guttata]
c51845.graph_c0	15.8772504	17.3494934	16.988865	11.4220044	10.5568321	14.000369	9.56E-13	-1.4996	down	PREDICTED: replication protein A 32 kDa subunit A-like isoform X2 [Sesamum indicum]
c51847.graph_c0	0.31437334	0.47203221	0.80796439	35.0441688	64.2784913	80.5322589	2.773E-13	5.779703	up	PREDICTED: S-antigen protein-like [Sesamum indicum]

#ID	0 h-1 FPKM	0 h-2 FPKM	0 h-3 FPKM	48 h-1 FPKM	48 h-2 FPKM	48 h-3 FPKM	FDR	log2FC	regulated	nr_annotation
c51849.graph_c0	21.8346699	20.3599879	21.3565645	16.9124135	15.7788691	13.452504	1.445E-20	-1.476619	down	PREDICTED: uncharacterized protein LOC104243797 [Nicotiana sylvestris]
c51856.graph_c0	45.6076529	38.5013656	45.1416546	0.32911288	1.04596557	0.78091377	3.37E-123	-6.940199	down	-
c51857.graph_c0	4.34570843	4.93564499	3.00698859	0.07699135	0	0	3.245E-26	-8.274586	down	-
c51858.graph_c0	1.25155202	0.8816037	1.25089525	13.5799115	18.0054568	19.11293	4.622E-22	2.880833	up	PREDICTED: LRR receptor-like serine/threonine-protein kinase HSL2 [Sesamum indicum]
c51860.graph_c0	0.55774897	0.37220498	0.47781967	17.1278243	22.6810663	17.6786727	2.049E-13	4.331074	up	-
c51862.graph_c0	0.19860467	0	0	3.96429755	3.81202863	4.55849665	1.724E-11	4.959896	up	PREDICTED: 65-kDa microtubule-associated protein 3 [Sesamum indicum]
c51863.graph_c0	1.62342693	1.40545085	1.74787038	105.339223	125.85386	143.868315	3.907E-80	5.271846	up	-
c51865.graph_c0	92.9783406	98.260481	95.5279859	11.1771629	10.5491198	11.7536218	3.27E-125	-4.115747	down	PREDICTED: ethylene-responsive transcription factor 14-like [Sesamum indicum]
c51866.graph_c0	1426.45982	1423.86615	1367.47214	118.50651	106.328042	112.974904	1.74E-135	-4.657531	down	hypothetical protein M569_15403, partial [Genlisea aurea]
c51869.graph_c0	9.83560339	5.21229826	5.20435167	0	0	0	1.828E-15	-Inf	down	predicted protein [Hordeum vulgare subsp. vulgare]
c51870.graph_c0	737.950664	768.931021	704.285105	31.593638	38.693724	36.8131096	1.17E-220	-5.387977	down	PREDICTED: zinc finger A20 and AN1 domain-containing stress-associated protein 1-like [Sesamum indicum]
c51872.graph_c0	0.85324734	0.72756814	0.73097107	9.46191878	13.4472537	13.0302667	4.455E-23	2.935978	up	PREDICTED: amino-acid permease BAT1 homolog isoform X1 [Sesamum indicum]
c51875.graph_c0	20.1945219	17.9838373	19.9306514	1.08945334	0.75463242	0.74568387	5.12E-117	-5.497988	down	hypothetical protein MIMGU_mgv1a017259mg [Erythranthe guttata]
c51887.graph_c0	6.61988652	3.15915266	4.3523246	0	0	0	7.882E-19	-Inf	down	predicted protein [Physcomitrella patens]
c51894.graph_c0	1.73999691	2.40077573	1.81294453	11.0910177	13.3100806	15.4969757	6.759E-09	1.723413	up	PREDICTED: activating signal cointegrator 1 [Sesamum indicum]
c51895.graph_c0	0.27571083	0.18399127	0	5.41872636	5.78533105	7.38299864	2.327E-15	4.334341	up	PREDICTED: cyclin-D1-1-like [Sesamum indicum]
c51898.graph_c0	0.12030896	0.36128828	0	3.99011244	5.16638259	4.14210773	6.43E-08	3.78062	up	PREDICTED: uncharacterized protein LOC105163238 [Sesamum indicum]
c51905.graph_c0	28.3646374	24.1678855	30.4832163	4.58854134	7.16791991	8.16571298	9.44E-38	-3.086578	down	PREDICTED: cysteine proteinase inhibitor isoform X4 [Sesamum indicum]
c51906.graph_c0	0	0	0	4.17701284	9.29258108	9.8956629	8.274E-12	Inf	up	PREDICTED: uncharacterized protein At1g28695-like [Sesamum indicum]
c51908.graph_c0	1.7674532	1.34460951	0.99934883	8.72765141	9.31488755	10.3157798	2.286E-05	1.771562	up	hypothetical protein MIMGU_mgv1a014525mg [Erythranthe guttata]
c51910.graph_c0	3.46672274	4.16423297	4.32759381	20.9876387	26.3906231	26.2257388	2.16E-07	1.59842	up	PREDICTED: uncharacterized protein LOC105168519 isoform X2 [Sesamum indicum]
c51913.graph_c0	2.28435017	1.82155877	1.79114059	13.3046721	13.9813248	17.7417567	3.033E-13	1.914344	up	PREDICTED: uncharacterized protein LOC105168217 [Sesamum indicum]

#ID	0 h-1 FPKM	0 h-2 FPKM	0 h-3 FPKM	48 h-1 FPKM	48 h-2 FPKM	48 h-3 FPKM	FDR	log2FC	regulated	nr_annotation
c51919.graph_c0	0.45510028	0.075926	0	8.94438537	9.84562813	9.9483254	5.313E-16	4.758655	up	PREDICTED: F-box/kelch-repeat protein At5g26960 [Sesamum indicum]
c51920.graph_c0	0.04566381	0.13712861	0.05867981	16.07016	18.6287783	17.7429017	6.532E-50	6.745501	up	PREDICTED: protein TIFY 6B [Sesamum indicum]
c51924.graph_c0	5.64326574	3.71214521	6.21584563	0	0	0	6.278E-21	-Inf	down	-
c51926.graph_c0	0	0.15227936	0.13032617	4.48477896	9.9475727	12.6366763	8.344E-08	5.541344	up	PREDICTED: WD repeat-containing protein RUP2-like [Sesamum indicum]
c51927.graph_c0	0.31568965	0.13543104	0.28976693	56.504871	68.6189944	85.8417695	1.392E-51	7.129186	up	geranyl diphosphate synthase large subunit [Antirrhinum majus]
c51928.graph_c0	2.82190608	2.01766556	3.62626103	25.6258502	19.278646	27.4281649	6.111E-05	2.075776	up	Tubulin beta-8 chain -like protein [Gossypium arboreum]
c51931.graph_c0	10.5402208	9.80476082	7.6615925	7.06206465	5.92277593	7.94200308	2.786E-05	-1.433834	down	PREDICTED: histone H3.2-like [Oryza brachyantha]
c51933.graph_c0	0.14304274	0.09545732	0.3063593	6.06189385	6.79412216	8.71612741	3.502E-17	4.274865	up	hypothetical protein MIMGU_mgv1a008682mg [Erythranthe guttata]
c51936.graph_c0	0	0	0	1.75218096	2.54371337	2.25200457	1.685E-11	Inf	up	hypothetical protein MIMGU_mgv1a013390mg [Erythranthe guttata]
c51938.graph_c0	0	0.21471573	0.2756422	3.26060225	4.50094384	5.71461145	1.533E-08	3.742267	up	PREDICTED: ABC transporter B family member 15-like [Sesamum indicum]
c51939.graph_c0	0.09201314	0.09210528	0.11824053	1.61060167	6.01673107	6.10954725	0.0004301	4.466443	up	PREDICTED: uncharacterized protein LOC105163738 [Sesamum indicum]
c51940.graph_c0	0	0	0	36.2341444	57.0116464	52.0344023	4.865E-46	Inf	up	PREDICTED: kirola-like isoform X2 [Sesamum indicum]
c51942.graph_c0	19.6257528	23.2457581	22.506975	15.2711992	11.9808653	9.29383052	9.113E-19	-1.850385	down	PREDICTED: set1/Ash2 histone methyltransferase complex subunit ASH2 [Sesamum indicum]
c51943.graph_c0	34.3959697	34.9382352	34.4213813	5.42151334	5.05026268	5.57258634	1.64E-60	-3.709823	down	PREDICTED: ethylene-responsive transcription factor ERF008-like [Sesamum indicum]
c51947.graph_c0	0.14468401	0.25345056	0.09296234	33.0231791	44.0213071	40.8289896	1.33E-102	6.890839	up	PREDICTED: aldehyde dehydrogenase family 2 member C4-like [Sesamum indicum]
c51950.graph_c0	16.5715046	15.6070819	13.7387352	6.36114623	3.6085525	3.87078467	1.838E-40	-2.734685	down	PREDICTED: U-box domain-containing protein 26-like [Sesamum indicum]
c51952.graph_c0	201.161257	176.738909	238.706451	45.5364767	49.8109924	64.0751082	1.762E-22	-2.975013	down	-
c51953.graph_c0	3.72458321	3.976867	3.03128336	0.22875527	0.60584617	1.11949783	1.543E-10	-3.48992	down	-
c51958.graph_c0	7.06947151	3.6076532	5.16572495	0	0	0	7.596E-17	-Inf	down	predicted protein [Physcomitrella patens]
c51961.graph_c0	41.4160875	41.0272042	41.4352777	8.05351998	8.74152446	7.04849867	4.13E-78	-3.394738	down	PREDICTED: protein ABSCISIC ACID-INSENSITIVE 5 [Sesamum indicum]
c51962.graph_c0	0.56948567	0.24430968	0.31363356	14.3903272	26.9166119	25.9423019	1.922E-14	4.874541	up	-
c51963.graph_c0	1.22193814	0.78631825	1.53285224	34.0935103	38.077168	55.2109308	3.684E-18	4.142132	up	PREDICTED: D-3-phosphoglycerate dehydrogenase 2, chloroplastic-like [Sesamum indicum]
c51965.graph_c0	0.07411406	0	0	26.4238597	56.4197487	90.2804189	3.387E-10	10.17895	up	PREDICTED: subtilisin-like protease [Sesamum indicum]
c51967.graph_c0	5.16119783	4.25998599	3.60706404	4.46286871	3.53485476	3.30300937	0.0001318	-1.214282	down	-

#ID	0 h-1 FPKM	0 h-2 FPKM	0 h-3 FPKM	48 h-1 FPKM	48 h-2 FPKM	48 h-3 FPKM	FDR	log2FC	regulated	nr_annotation
c51971.graph_c0	1.48248172	1.07924814	0.17318615	25.3286187	30.2524415	39.882099	3.635E-18	4.116653	up	PREDICTED: UDP-glucose 6-dehydrogenase 3 [Nicotiana sylvestris]
c51972.graph_c0	47.7576768	49.4687654	46.5616854	18.8631201	22.755725	26.1870087	7.69E-43	-2.105992	down	PREDICTED: transcription factor GTE7-like [Sesamum indicum]
c51974.graph_c0	14.6783689	12.8137213	15.5723517	6.60409506	6.82966084	5.59646102	6.472E-09	-2.195985	down	hypothetical protein MIMGU_mgv1a0003262mg, partial [Erythranthe guttata]
c51975.graph_c0	0	0	0	8.09246058	20.1540249	28.7393277	9.256E-09	Inf	up	PREDICTED: vacuolar iron transporter homolog 2-like [Sesamum indicum]
c51977.graph_c0	1.39335026	2.78949097	1.79051024	25.3876483	21.757862	16.3712427	4.561E-08	2.398944	up	unnamed protein product [Coffea canephora]
c51983.graph_c0	2.59302187	3.91991348	3.12812745	22.4747931	21.2269626	21.1691815	4.115E-12	1.733817	up	hypothetical protein MIMGU_mgv1a013022mg [Erythranthe guttata]
c51984.graph_c0	4.90800446	3.94113289	4.22775585	21.96193	33.4251384	37.1378696	1.355E-07	1.798085	up	PREDICTED: uncharacterized protein LOC105164204 [Sesamum indicum]
c51985.graph_c0	6.94982616	8.52451164	6.66665485	1.71338071	1.8151191	1.68503541	9.138E-14	-3.103107	down	PREDICTED: zinc finger protein 3 [Solanum lycopersicum]
c51987.graph_c0	0.78242579	1.34264446	0.57454178	15.1372551	17.5633326	16.309448	9.017E-12	3.167503	up	PREDICTED: RPM1-interacting protein 4 [Sesamum indicum]
c51988.graph_c0	4.89561213	4.31909739	4.26512232	33.9408441	30.5303845	35.7779028	2.277E-13	1.879099	up	PREDICTED: thioredoxin F1, chloroplastic-like [Sesamum indicum]
c51992.graph_c0	0.4492542	0.14990135	0	2.62125437	3.9461227	4.29648279	4.757E-07	3.174073	up	PREDICTED: cucumber peeling cupredoxin-like [Sesamum indicum]
c51995.graph_c0	5.28104004	2.67158522	3.64857287	0	0	0	1.048E-18	-Inf	down	--
c52000.graph_c0	1.30455013	1.89942755	0.76199935	34.6347476	24.7695315	23.5265149	3.331E-11	3.382249	up	PREDICTED: probable serine/threonine-protein kinase DDB_G0282963 isoform X2 [Nicotiana tomentosiformis]
c52001.graph_c0	1.90659104	1.93928246	2.7661798	14.2217897	17.2571723	21.7797928	6.169E-11	1.98293	up	PREDICTED: kynurenine--oxoglutarate transaminase 1-like [Sesamum indicum]
c52003.graph_c0	16.1798278	8.14178779	9.32817679	0	0	0	2.851E-18	-Inf	down	-
c52004.graph_c0	0.116696	0	0	4.62284262	2.8472914	2.58281847	2.541E-06	5.443466	up	hypothetical protein MIMGU_mgv1a000070mg [Erythranthe guttata]
c52006.graph_c0	0	0	0	3.64916726	3.60226953	3.68978077	3.131E-12	Inf	up	PREDICTED: probable leucine-rich repeat receptor-like protein kinase At5g49770 [Sesamum indicum]
c52011.graph_c0	3.68857871	3.69227227	3.87815701	0.51487262	2.72722555	1.74089614	5.941E-06	-2.215351	down	-
c52012.graph_c0	18.7140174	20.116707	19.2893641	9.09792792	9.05986237	9.39052661	4.665E-29	-2.094729	down	PREDICTED: RING-H2 finger protein ATL52-like [Sesamum indicum]
c52015.graph_c0	9.05995962	8.54437707	10.3915697	36.8355219	47.4265487	51.4350368	9.506E-11	1.252674	up	PREDICTED: uncharacterized protein LOC105168722 [Sesamum indicum]
c52017.graph_c0	10.854695	11.0497265	8.27466016	2.09040657	2.7532023	2.2622001	3.694E-25	-3.104836	down	hypothetical protein MIMGU_mgv1a014249mg [Erythranthe guttata]
c52018.graph_c0	2.24672554	1.12448765	0.36089141	20.1808436	18.9123309	22.1006686	1.071E-07	3.031035	up	hypothetical protein JCGZ_17967 [Jatropha curcas]

#ID	0 h-1 FPKM	0 h-2 FPKM	0 h-3 FPKM	48 h-1 FPKM	48 h-2 FPKM	48 h-3 FPKM	FDR	log2FC	regulated	nr_annotation
c52023.graph_c0	11.0624211	9.50093058	9.25278932	0.3618257	0.63885076	0.37560872	1.472E-45	-5.460557	down	-
c52024.graph_c0	0.17635922	0	0.67988574	18.6844659	19.9660471	25.0102721	2.1E-17	5.174279	up	Chlorophyll a-b binding protein 151 [Morus notabilis]
c52026.graph_c0	1.28557144	1.96415282	1.56506229	7.10611357	7.19788735	10.4273658	0.0009252	1.339862	up	PREDICTED: uncharacterized protein LOC105168444 [Sesamum indicum]
c52029.graph_c0	0.18194257	0.31871832	0.05845085	34.9063154	11.3201047	12.3417421	0.0005715	5.726406	up	PREDICTED: epidermis-specific secreted glycoprotein EP1-like [Vitis vinifera]
c52032.graph_c0	19.9690778	20.7203814	20.698726	11.3778223	12.596676	10.8653879	3.87E-29	-1.835762	down	PREDICTED: cyclin-dependent kinase F-1 [Sesamum indicum]
c52038.graph_c0	0.54335573	0.54389982	0.34911681	11.2629316	9.54536824	9.13087201	2.844E-07	3.372222	up	PREDICTED: uncharacterized protein LOC104246111 isoform X2 [Nicotiana sylvestris]
c52040.graph_c0	0.47198428	0.52495212	0.4717366	30.0510804	29.8393608	24.1169306	1.15E-49	4.822674	up	PREDICTED: transmembrane protein 87A-like [Sesamum indicum]
c52041.graph_c0	9.28622104	10.302041	7.14469554	3.81436354	3.63677009	3.00611229	3.72E-16	-2.366066	down	PREDICTED: protein MKS1 [Sesamum indicum]
c52044.graph_c0	0.5181383	0.20746285	0	2.10030829	3.4386703	2.37852578	7.692E-05	2.442197	up	PREDICTED: LOW QUALITY PROTEIN: BTB/POZ domain-containing protein At1g63850 [Sesamum indicum]
c52046.graph_c0	1.00309849	1.09823759	0.92648404	83.5750801	95.8189225	121.234347	7.759E-52	5.612995	up	PREDICTED: serine carboxypeptidase 1 [Sesamum indicum]
c52048.graph_c0	4.26825674	1.67550226	3.22639935	0	0	0	2.862E-11	-Inf	down	tamavidin1 [Pleurotus cornucopiae]
c52049.graph_c0	0.84173703	0.96294846	1.5452363	6.64682619	7.04150638	7.93502718	4.424E-05	1.6645	up	PREDICTED: serine/threonine-protein kinase BLUS1 [Sesamum indicum]
c52052.graph_c0	12.045374	14.2846053	11.8309166	8.41108996	8.94796912	6.91811008	1.783E-18	-1.668381	down	PREDICTED: 5'-adenylylsulfate reductase-like 5 isoform X2 [Sesamum indicum]
c52053.graph_c0	33.9792454	28.0625141	30.3159903	3.63194387	1.95433861	1.64148918	3.38E-110	-4.678023	down	PREDICTED: uncharacterized protein LOC105180043 [Sesamum indicum]
c52056.graph_c0	2.08753852	1.23611849	1.54908951	0	0	0	1.193E-25	-Inf	down	putative Bax inhibitor 1 [Auxenochlorella protothecoides]
c52057.graph_c0	0	0	0	3.68075033	5.10397313	4.34018578	3.151E-23	Inf	up	hypothetical protein MIMGU_mgv1a003539mg [Erythranthe guttata]
c52060.graph_c0	0	0	0	272.066919	258.565274	300.994284	8.7E-238	Inf	up	--
c52063.graph_c0	19.3204518	14.8711328	14.1065599	15.5743528	15.5706154	12.3581857	9.97E-08	-1.164069	down	PREDICTED: uncharacterized protein LOC105166059 [Sesamum indicum]
c52067.graph_c0	0.33936995	0	0	5.62769519	5.13382446	5.42478012	4.511E-09	4.581021	up	PREDICTED: protein RADIALIS-like 3 [Sesamum indicum]
c52068.graph_c0	1.05107943	0.61374362	1.57579179	64.716249	61.8056988	76.5395034	2.025E-77	4.94437	up	PREDICTED: outer envelope pore protein 16, chloroplastic [Nicotiana tomentosiformis]
c52074.graph_c0	0.83946836	0.31511586	0.26968752	2.2234474	2.76512038	3.18265977	0.0075911	1.506717	up	-
c52075.graph_c0	3.46153174	4.33124742	2.38296714	1.02503212	2.05113923	2.63486142	0.0001228	-1.859142	down	-
c52081.graph_c0	61.5450933	66.7713567	76.5621209	27.387712	26.9757892	27.189024	1.237E-27	-2.348254	down	PREDICTED: probable cyclic nucleotide-gated ion channel 17 [Sesamum indicum]
c52084.graph_c0	75.9287299	78.5947004	82.9932558	4.03384263	3.10790338	2.24330557	1.72E-233	-5.671912	down	PREDICTED: transcription factor TGA5-like [Sesamum indicum]

#ID	0 h-1 FPKM	0 h-2 FPKM	0 h-3 FPKM	48 h-1 FPKM	48 h-2 FPKM	48 h-3 FPKM	FDR	log2FC	regulated	nr_annotation
c52087.graph_c0	0	0	0.32263993	2.85277001	4.3290456	3.4988448	2.856E-09	3.992838	up	hypothetical protein MIMGU_mgv1a014873mg [Erythranthe guttata]
c52089.graph_c0	17.250026	17.7537021	15.9227505	8.58010631	10.0380604	10.8211122	4.624E-24	-1.810623	down	PREDICTED: uncharacterized protein LOC105176476 isoform X2 [Sesamum indicum]
c52090.graph_c0	0.12362613	0.37124978	0.15886448	21.4117821	14.1166641	15.8091685	3.049E-15	5.287872	up	hypothetical protein glysoja_014339 [Glycine soja]
c52091.graph_c0	6.74689105	4.890572	4.18552792	0	0	0	1.411E-19	-Inf	down	-
c52094.graph_c0	4.14557806	4.8136859	5.11414265	2.90257444	1.78022012	2.17489961	4.015E-05	-2.048129	down	-
c52096.graph_c0	4.60135206	4.33097695	4.94214173	1.70828018	3.15024587	3.74374665	1.027E-08	-1.719498	down	PREDICTED: pentatricopeptide repeat-containing protein At1g77405 [Sesamum indicum]
c52098.graph_c0	47.7270132	45.610789	53.2106072	35.8494829	28.727085	31.2170591	1.621E-15	-1.628466	down	putative lipid transfer protein family protein [Cucumis sativus]
c52102.graph_c0	47.4168469	48.1497331	51.4736859	26.4939591	29.7378001	34.238669	1.694E-16	-1.722203	down	PREDICTED: transcription factor ILR3-like [Sesamum indicum]
c52103.graph_c0	0.64829814	0.64894731	0.29403133	12.928804	13.5104064	12.8794469	1.089E-26	3.616381	up	PREDICTED: brefeldin A-inhibited guanine nucleotide-exchange protein 2 [Sesamum indicum]
c52105.graph_c0	10.541357	9.50716875	7.64480675	4.99992131	5.19494862	7.35779602	1.785E-06	-1.675784	down	PREDICTED: ABC transporter C family member 5-like [Sesamum indicum]
c52106.graph_c0	6.04812392	4.88041057	5.07564323	0.45485112	0.18069735	0.15177142	1.702E-28	-5.340653	down	PREDICTED: ethylene-responsive transcription factor ERF054-like [Sesamum indicum]
c52108.graph_c0	35.6084285	35.644085	33.0370699	10.7534429	19.4672886	30.8851767	6.4E-15	-1.800519	down	-
c52109.graph_c0	0	0	0	3.73897472	2.871718	4.40816532	6.715E-12	Inf	up	PREDICTED: serine/threonine-protein kinase D6PK-like [Sesamum indicum]
c52110.graph_c0	181.367503	177.293589	183.896985	4.0268712	4.38285797	2.89591864	0	-6.601087	down	PREDICTED: oleosin 1 [Sesamum indicum]
c52113.graph_c0	0	0	0	68.7876291	78.456225	68.8283574	4.964E-73	Inf	up	-
c52114.graph_c0	12.5223818	12.214426	10.6516456	87.309837	84.7168966	82.382862	1.554E-35	1.830829	up	PREDICTED: protein rough sheath 2 homolog [Sesamum indicum]
c52117.graph_c0	0.04752541	0.09514599	0.06107203	3.02106182	5.28769781	5.99957806	2.412E-11	5.106669	up	PREDICTED: serine/threonine protein phosphatase 2A 57 kDa regulatory subunit B' iota isoform [Sesamum indicum]
c52118.graph_c0	0	0.32407226	0.03782083	16.8922395	11.9780602	10.3501333	2.187E-16	5.75774	up	PREDICTED: MLO-like protein 6 [Sesamum indicum]
c52120.graph_c0	0.32428432	0.05410151	0.2083591	10.9044433	10.6024613	9.79131724	8.186E-22	4.722071	up	PREDICTED: probable pectinesterase/pectinesterase inhibitor 12 [Sesamum indicum]
c52127.graph_c0	6.36372991	4.39996751	5.31125389	0	0	0	3.505E-38	-Inf	down	NADH dehydrogenase subunit 1 (mitochondrion) [Micromonas pusilla CCMP1545]
c52129.graph_c0	0	0	0	11.1730666	19.8261531	21.5404096	2.641E-22	Inf	up	PREDICTED: peroxidase 60 [Sesamum indicum]
c52131.graph_c0	1.60464789	0.62853445	1.34480613	15.5543532	19.1334888	20.9318273	3.672E-17	2.93566	up	PREDICTED: uncharacterized protein LOC105174462 isoform X1 [Sesamum indicum]
c52132.graph_c0	2.72443107	2.0094857	3.50100178	2.5099236	1.11956198	0.82280024	0.0002901	-1.889124	down	-
c52133.graph_c0	10.6526597	9.22787887	8.95055847	0.7549173	0.59980751	0.67172087	4.286E-18	-4.84303	down	-

#ID	0 h-1 FPKM	0 h-2 FPKM	0 h-3 FPKM	48 h-1 FPKM	48 h-2 FPKM	48 h-3 FPKM	FDR	log2FC	regulated	nr_annotation
c52136.graph_c0	52.1327056	62.5931119	51.5990727	14.8321915	15.0114393	16.4969936	1.248E-56	-2.861202	down	PREDICTED: cell division cycle protein 48 homolog [Nicotiana tomentosiformis]
c52137.graph_c0	6.85728129	4.94857168	4.71333007	0	0	0	6.591E-23	-Inf	down	rpl13 protein isoform 1 [Lichtheimia corymbifera JMRC:FSU:9682]
c52139.graph_c0	32.2669683	40.9124198	30.8678915	6.60587463	4.54878006	6.1717609	1.364E-19	-3.597476	down	PREDICTED: ethylene-responsive transcription factor ERF008-like [Sesamum indicum]
c52140.graph_c0	54.1557491	43.2526419	48.1223224	201.513368	216.852659	206.697074	1.689E-11	1.084701	up	PREDICTED: 60S ribosomal protein L17-2 [Sesamum indicum]
c52153.graph_c0	0.06339357	0.1269141	0	26.8066518	25.3048898	28.4773722	9.373E-58	7.723748	up	PREDICTED: probable 3-beta-hydroxysteroid-Delta(8),Delta(7)-isomerase [Sesamum indicum]
c52155.graph_c0	17.9292118	15.781128	13.5060586	122.742553	92.0171918	103.133975	6.685E-10	1.74112	up	hypothetical protein MIMGU_mgv1a013595mg [Erythranthe guttata]
c52157.graph_c0	0.30978149	0.41345559	0.39808148	11.7961579	10.0779024	12.9508952	5.213E-13	3.938886	up	PREDICTED: choline transporter-like protein 2 [Sesamum indicum]
c52158.graph_c0	6.61206508	6.61868606	7.33405014	67.1984839	76.3511808	84.7254945	3.7E-43	2.450772	up	PREDICTED: 50S ribosomal protein L12, chloroplastic-like [Sesamum indicum]
c52160.graph_c0	6.08778224	3.14850375	2.34691236	0	0	0	3.179E-09	-Inf	down	-
c52161.graph_c0	0	0.09388372	0	5.35712751	6.5905862	7.07322686	6.202E-14	6.652406	up	PREDICTED: non-specific lipid-transfer protein-like protein At2g13820 [Sesamum indicum]
c52162.graph_c0	0	0.38492448	0	25.5069214	24.7696995	26.4785562	8.184E-14	6.633866	up	-
c52163.graph_c0	46.0414579	56.0951462	42.9369556	49.2030171	36.7183357	35.5851751	1.096E-07	-1.26547	down	PREDICTED: trihelix transcription factor ASIL2 [Sesamum indicum]
c52165.graph_c0	23.8591949	21.9913567	20.4906008	2.82919214	2.19025132	3.58245121	2.803E-38	-3.962698	down	hypothetical protein MIMGU_mgv1a011603mg [Erythranthe guttata]
c52171.graph_c0	0.27723418	0.27751178	0.71251379	17.4953251	17.0460301	27.0438099	3.412E-14	4.570807	up	enolase [Prunus armeniaca]
c52173.graph_c0	12.3555667	11.1271425	9.58295692	4.86253568	6.92689304	6.55553789	3.249E-28	-1.871136	down	PREDICTED: pentatricopeptide repeat-containing protein At3g09650, chloroplastic [Sesamum indicum]
c52175.graph_c0	19.5522181	20.6951228	18.3587173	12.2868622	12.8479431	12.1003542	3.177E-22	-1.671312	down	PREDICTED: mitogen-activated protein kinase kinase kinase 1-like [Sesamum indicum]
c52177.graph_c0	9.61483347	8.85450437	9.94613055	3.36596845	6.05253646	8.86683105	3.526E-11	-1.666284	down	PREDICTED: dnaJ protein ERDJ3A [Sesamum indicum]
c52178.graph_c0	0.06774029	0.06780812	0.08704895	6.98955844	6.2145663	6.60797126	1.649E-15	5.457362	up	PREDICTED: protein MIZU-KUSSEI 1 [Sesamum indicum]
c52179.graph_c0	16.4029273	16.0772825	12.7348733	6.61125786	7.00382604	7.84354331	3.479E-06	-2.092058	down	hypothetical protein MIMGU_mgv1a0261451mg, partial [Erythranthe guttata]
c52180.graph_c0	0.69492397	1.39123967	0.66975362	8.80287776	8.98646953	9.68412443	3.731E-05	2.302286	up	-
c52181.graph_c0	1.34331797	0.67233155	1.23301198	24.2206631	39.5183836	48.7659425	2.916E-11	4.085432	up	Alpha-xylosidase, partial [Medicago truncatula]
c52192.graph_c0	17.2179607	12.6796419	19.1041815	9.92267752	9.2534099	10.2592106	7.66E-08	-1.754512	down	hypothetical protein MIMGU_mgv1a015849mg [Erythranthe guttata]

#ID	0 h-1 FPKM	0 h-2 FPKM	0 h-3 FPKM	48 h-1 FPKM	48 h-2 FPKM	48 h-3 FPKM	FDR	log2FC	regulated	nr_annotation
c52194.graph_c0	18.0588731	12.3008874	12.495736	0	0	0	4.988E-42	-Inf	down	--
c52196.graph_c0	1.16834006	1.75426497	1.87670404	63.7738009	59.578765	63.6890922	1.365E-21	4.264354	up	PREDICTED: aldo-keto reductase family 4 member C9-like [Sesamum indicum]
c52197.graph_c0	19.237124	20.7376476	20.2834592	10.9061498	8.66530743	8.62597909	3.686E-11	-2.108637	down	PREDICTED: splicing factor 3B subunit 2 [Sesamum indicum]
c52199.graph_c0	4.31632389	2.19890022	3.16951262	0	0	0	1.253E-20	-Inf	down	--
c52205.graph_c0	0	0.34088406	0.43761132	66.3017083	82.7574913	78.9079534	5.557E-88	7.159419	up	-
c52213.graph_c0	0	0.1486544	0.1908357	4.78845967	6.66709857	5.96503967	9.292E-10	4.645243	up	hypothetical protein MIMGU_mgv1a0183231mg, partial [Erythranthe guttata]
c52214.graph_c0	3.15333585	2.59946518	3.81379897	0	0	0	2.216E-22	-Inf	down	PREDICTED: uncharacterized protein LOC105178332 [Sesamum indicum]
c52215.graph_c0	0	0.71665974	0.46000744	5.27658588	4.54179612	5.67321488	4.196E-06	2.696637	up	-
c52217.graph_c0	0.43598431	0.52370505	0.11205142	3.29358887	6.38260617	6.64749385	7.677E-06	2.910994	up	PREDICTED: zinc finger protein JACKDAW-like [Sesamum indicum]
c52218.graph_c0	158.251382	160.732572	149.09039	35.9134475	21.7405388	23.5862538	2.326E-57	-3.533587	down	-
c52220.graph_c0	11.7609519	14.333564	14.7023535	3.89534672	1.56049696	0.96117522	4.306E-41	-3.664359	down	PREDICTED: transcription factor MYB44-like [Sesamum indicum]
c52228.graph_c0	0	0	0.1042535	2.46645248	4.03812951	3.19219576	7.767E-11	5.482071	up	NAC domain-containing protein 1 [Salvia miltiorrhiza]
c52232.graph_c0	14.1648219	14.5200816	15.3875595	84.3961097	99.2888201	103.944138	4.174E-30	1.684636	up	PREDICTED: cytochrome c1-2, heme protein, mitochondrial-like [Sesamum indicum]
c52233.graph_c0	234.458802	248.35368	215.778928	137.094545	145.869261	189.21132	4.021E-29	-1.584654	down	PREDICTED: probable calcium-binding protein CML18 [Sesamum indicum]
c52235.graph_c0	0	0	0	64.1222638	84.093455	55.0147355	5.871E-43	Inf	up	PREDICTED: pathogenesis-related protein STH-2-like [Sesamum indicum]
c52237.graph_c0	22.6625815	26.6021566	18.2276357	6.00812899	7.00137256	9.08819498	4.665E-17	-2.630228	down	-
c52239.graph_c0	4.56232354	5.04761749	4.01136647	3.2076366	3.2809261	3.54306508	0.0012297	-1.458015	down	-
c52242.graph_c0	5.30590483	2.65560895	4.61237791	3.30668674	2.28458716	3.19811954	0.0020384	-1.530312	down	-
c52248.graph_c0	9.2696467	7.38250543	7.30362403	0	0	0	3.966E-51	-Inf	down	-
c52251.graph_c0	17.6023506	16.5380483	14.2861374	0.99574592	1.50696009	3.54403492	1.728E-23	-4.029812	down	PREDICTED: dynamin-related protein 4C-like [Sesamum indicum]
c52252.graph_c0	6.68791822	3.01914017	4.54989161	0	0	0	3.835E-13	-Inf	down	--
c52253.graph_c0	12.0430756	7.90500647	9.13327607	0.63659071	0	0.16183845	5.72E-32	-6.161473	down	hypothetical protein L484_021523 [Morus notabilis]
c52257.graph_c0	9.27455744	7.17387985	6.22995433	0	0	0	2.328E-23	-Inf	down	60S ribosomal protein l43 [Moniliophthora roreri MCA 2997]
c52260.graph_c0	0.13731092	0.54979367	0.70579988	14.5474742	18.6275531	14.9703124	6.603E-14	4.080744	up	PREDICTED: uncharacterized protein LOC105162704 [Sesamum indicum]
c52262.graph_c0	0.56132979	0.56189188	0.24044367	18.0996766	16.8004378	15.644841	5.111E-12	4.204362	up	hypothetical protein MIMGU_mgv1a010766mg [Erythranthe guttata]

#ID	0 h-1 FPKM	0 h-2 FPKM	0 h-3 FPKM	48 h-1 FPKM	48 h-2 FPKM	48 h-3 FPKM	FDR	log2FC	regulated	nr_annotation
c52267.graph_c0	7.57177698	3.15806624	5.6758527	0	0	0	5.123E-12	-Inf	down	-
c52269.graph_c0	5.31268265	4.78022696	6.13663599	44.9843914	46.8981467	49.1772313	3.993E-28	2.098986	up	PREDICTED: probable protein Pop3 [Sesamum indicum]
c52272.graph_c0	0	0	0	22.0460207	34.8452883	29.267274	2.094E-33	Inf	up	-
c52273.graph_c0	28.8567249	28.4654661	26.2945332	20.8810712	21.5064918	21.7625114	1.064E-11	-1.399464	down	hypothetical protein MIMGU_mgv1a000759mg [Erythranthe guttata]
c52275.graph_c0	0	0.07901046	0	3.49040507	3.00434952	3.62336502	3.51E-11	5.996334	up	-
c52276.graph_c0	8.31860792	11.0629316	9.00992415	2.18961263	4.17533233	4.96817558	1.696E-09	-2.353876	down	-
c52278.graph_c0	3.93935063	2.55154401	3.87111057	18.3594153	23.5204154	22.7945544	0.0003272	1.618744	up	-
c52279.graph_c0	9.20084667	9.09774212	7.06523366	2.27416148	1.09509015	4.50696479	3.537E-11	-2.703267	down	PREDICTED: 8-hydroxygeraniol dehydrogenase-like [Sesamum indicum]
c52290.graph_c0	0.49356867	0.39525032	1.01480845	10.1854514	14.2585456	19.7442914	5.319E-09	3.501597	up	hypothetical protein MIMGU_mgv1a011727mg [Erythranthe guttata]
c52293.graph_c0	6.16745921	6.51661471	6.8246752	2.20961444	1.08680862	1.75544789	1.04E-12	-2.957719	down	PREDICTED: putative calcium-binding protein CML23 [Sesamum indicum]
c52298.graph_c0	1.16670308	1.07803509	0.80729365	11.575232	14.5398863	13.904422	7.333E-10	2.695029	up	PREDICTED: LOW QUALITY PROTEIN: uncharacterized protein LOC105174495 [Sesamum indicum]
c52301.graph_c0	0.28142714	0.28170895	0.09041125	9.52813713	9.54457773	9.86225531	4.1E-16	4.460299	up	hypothetical protein MIMGU_mgv1a015992mg [Erythranthe guttata]
c52303.graph_c0	6.84236587	5.47937397	4.10326515	2.10121124	1.46278589	0.37392903	1.22E-12	-3.057501	down	PREDICTED: MATE efflux family protein DTX1 [Sesamum indicum]
c52307.graph_c0	3.54989601	3.95725191	4.25072804	0.96625329	2.28347828	3.83588071	9.092E-05	-1.765638	down	PREDICTED: probable L-type lectin-domain containing receptor kinase S.7 [Sesamum indicum]
c52309.graph_c0	0.67481098	0.50661503	0.65036912	3.73007935	3.29297232	3.04241866	0.0069066	1.443143	up	PREDICTED: trihelix transcription factor GTL1-like isoform X1 [Glycine max]
c52310.graph_c0	8.72220717	4.91115439	5.90441527	0	0	0	1.053E-23	-Inf	down	unknown [Picea sitchensis]
c52312.graph_c0	5.99810416	4.36662571	3.92397158	1.40657867	0.31930705	0.35759007	1.262E-13	-3.762146	down	-
c52313.graph_c0	6.34669649	6.47464125	5.3851405	53.7979103	54.6806599	59.0210787	1.069E-44	2.185125	up	PREDICTED: LRR receptor-like serine/threonine-protein kinase GSO1 [Sesamum indicum]
c52320.graph_c0	1.44784459	1.44929438	0.37210752	14.6723376	18.539231	18.514871	3.017E-18	2.972949	up	PREDICTED: coatomer subunit alpha-1-like [Nicotiana sylvestris]
c52322.graph_c0	0	0	0	35.2458726	43.5777249	40.7220327	4.37E-116	Inf	up	hypothetical protein MIMGU_mgv1a018712mg, partial [Erythranthe guttata]
c52323.graph_c0	97.8367903	87.5534244	108.864589	2.53249032	5.36573282	4.09708119	7.522E-91	-5.646905	down	PREDICTED: putative defensin-like protein 120 [Solanum lycopersicum]
c52327.graph_c0	42.8914015	45.0568116	39.4175844	27.9986378	31.6122947	32.8255932	4.641E-26	-1.481347	down	PREDICTED: F-box/kelch-repeat protein At5g60570 [Sesamum indicum]

#ID	0 h-1 FPKM	0 h-2 FPKM	0 h-3 FPKM	48 h-1 FPKM	48 h-2 FPKM	48 h-3 FPKM	FDR	log2FC	regulated	nr_annotation
c52330.graph_c0	0.21002589	1.051181	0.53978317	7.35260291	4.09957433	5.8536385	0.0003909	2.254973	up	hypothetical protein MIMGU_mgv1a020446mg, partial [Erythranthe guttata]
c52334.graph_c0	2.39493683	1.71238214	1.86853596	0	0	0	1.516E-19	-Inf	down	Serpin-ZX [Aegilops tauschii]
c52335.graph_c0	3.07006869	2.42616544	3.01077986	15.4814812	13.7987097	12.3845505	0.0003022	1.277174	up	PREDICTED: uncharacterized protein LOC105167003 isoform X1 [Sesamum indicum]
c52336.graph_c0	0	0	0	64.0579967	126.912356	105.101956	1.114E-29	Inf	up	hypothetical protein MIMGU_mgv1a020596mg [Erythranthe guttata]
c52337.graph_c0	0.57999647	0.11611545	0.14906369	3.09911738	3.84919754	3.61337546	1.766E-05	2.63278	up	PREDICTED: uncharacterized protein LOC105167330 [Sesamum indicum]
c52338.graph_c0	9.35784155	10.6363182	10.9390501	6.8968432	5.47977682	5.69135891	1.963E-11	-1.790043	down	unnamed protein product [Coffea canephora]
c52343.graph_c0	4.27145965	3.39110166	5.48899476	46.0005143	49.0194712	45.338004	9.196E-29	2.39394	up	PREDICTED: uncharacterized protein LOC105158873 [Sesamum indicum]
c52347.graph_c0	1.47105848	1.32527837	1.41777629	41.5379534	45.7990589	54.6866568	9.357E-55	4.054149	up	PREDICTED: LIM domain-containing protein WLIM1 [Sesamum indicum]
c52349.graph_c0	7.56385219	8.29251445	9.25699955	4.20311002	5.74162067	5.51143456	1.233E-05	-1.725017	down	-
c52354.graph_c0	3.56132927	3.46004554	2.28822456	1.64046441	1.73787312	2.5758977	0.0004531	-1.662087	down	hypothetical protein MIMGU_mgv1a024937mg [Erythranthe guttata]
c52357.graph_c0	50.7529527	54.7072751	44.6514604	13.0297409	8.35022343	6.53641383	4.529E-91	-3.431399	down	hypothetical protein MIMGU_mgv1a001601mg [Erythranthe guttata]
c52360.graph_c0	0.48871037	0.48919974	0.94201826	6.75347802	5.00814374	6.40981774	7.944E-05	2.221205	up	hypothetical protein MIMGU_mgv1a0032971mg, partial [Erythranthe guttata]
c52362.graph_c0	16.5844453	13.7952405	19.8108471	11.8355916	11.8544628	14.5522564	0.0002778	-1.414084	down	-
c52366.graph_c0	0.27867893	0.092986	0.11937115	2.48179303	4.07972915	5.40648968	4.45E-06	3.585656	up	PREDICTED: myosin-9-like [Sesamum indicum]
c52369.graph_c0	37.9097464	40.601043	38.6896386	35.4317572	34.9486681	39.7674325	1.764E-13	-1.107664	down	-
c52371.graph_c0	1003.62253	984.370931	1005.92888	2.91012897	2.98658725	2.9940114	0	-9.413554	down	hypothetical protein MIMGU_mgv1a015982mg [Erythranthe guttata]
c52372.graph_c0	0.36126166	0.28929873	0.37138843	14.876956	22.7766752	27.7778649	2.569E-17	4.973197	up	PREDICTED: ATP synthase gamma chain, chloroplastic-like [Sesamum indicum]
c52377.graph_c0	0.08936731	0.0447284	0	17.5365014	17.7928195	18.4975496	1.03E-54	7.649017	up	PREDICTED: uncharacterized protein LOC105168503 isoform X2 [Sesamum indicum]
c52380.graph_c0	4.44109999	3.27065249	3.95412831	15.9273563	17.9876563	23.0653623	2.262E-07	1.266771	up	PREDICTED: LOW QUALITY PROTEIN: ATP-dependent Clp protease ATP-binding subunit clpA homolog CD4B, chloroplastic [Sesamum indicum]
c52381.graph_c0	33.0319057	35.7936458	31.3203428	12.8519889	15.8060633	11.0412906	1.262E-20	-2.352986	down	PREDICTED: importin subunit beta-1-like [Nicotiana sylvestris]
c52383.graph_c0	12.6465586	7.46286027	7.80631501	0	0	0	2.066E-27	-Inf	down	-
c52393.graph_c0	0.37908745	0.37946704	0.24357119	152.617915	157.055457	146.05331	3.21E-106	7.81599	up	-

#ID	0 h-1 FPKM	0 h-2 FPKM	0 h-3 FPKM	48 h-1 FPKM	48 h-2 FPKM	48 h-3 FPKM	FDR	log2FC	regulated	nr_annotation
c52394.graph_c0	0.37777545	0.07563075	0	68.3533645	94.5339246	93.9557699	1.128E-77	8.142259	up	PREDICTED: protein RALF-like 34 [Phoenix dactylifera]
c52398.graph_c0	60.1105209	48.6154372	58.0001198	291.739093	304.088779	313.892242	2.445E-24	1.4291	up	PREDICTED: uncharacterized protein LOC105159752 [Sesamum indicum]
c52401.graph_c0	424.357015	410.235511	394.499635	66.4583099	54.631873	60.7367575	2.15E-128	-3.770705	down	unnamed protein product [Coffea canephora]
c52403.graph_c0	0	0.09781862	0.12557506	28.4484525	39.8656731	42.2154717	1.992E-44	7.913417	up	PREDICTED: dirigent protein 22-like [Sesamum indicum]
c52404.graph_c0	8.48318608	10.6789318	8.58884786	1.89460954	1.12899885	1.36972239	1.25E-16	-3.666926	down	PREDICTED: cyclic pyranopterin monophosphate synthase, mitochondrial [Sesamum indicum]
c52406.graph_c0	0	0	0	24.5257615	31.0702259	39.7358185	2.25E-31	Inf	up	hypothetical protein MIMGU_mgv1a016694mg [Erythranthe guttata]
c52410.graph_c0	4.0525019	4.59743452	5.3812129	4.35566726	2.90041796	2.65758619	0.0013712	-1.51212	down	-
c52413.graph_c0	9.06071015	5.62952053	8.02991122	4.60541637	4.26902018	4.35398878	0.0003426	-1.796685	down	-
c52415.graph_c0	0	0	0	16.76397	25.480869	21.6308031	7.737E-49	Inf	up	PREDICTED: GDSL esterase/lipase 1-like [Sesamum indicum]
c52420.graph_c0	0.17839682	0	0.22924698	8.05318864	9.14076092	12.35714	3.029E-16	5.148501	up	flavonoid glycosyltransferase [Veronica persica]
c52421.graph_c0	2.96697983	2.90321035	2.69886801	15.2946171	16.3980066	15.576633	1.324E-10	1.446265	up	PREDICTED: uncharacterized protein LOC105173356 [Sesamum indicum]
c52432.graph_c0	0	0	0	10.8054951	10.5665648	10.3542542	5.968E-27	Inf	up	hypothetical protein MIMGU_mgv1a020209mg [Erythranthe guttata]
c52437.graph_c0	0	0	0	3.73082442	6.37317418	8.46514608	3.539E-12	Inf	up	PREDICTED: probable LRR receptor-like serine/threonine-protein kinase At4g26540 [Sesamum indicum]
c52440.graph_c0	182.010223	202.337482	169.066375	45.4932229	36.427122	24.7092688	7.22E-110	-3.384481	down	hypothetical protein MIMGU_mgv1a009891mg [Erythranthe guttata]
c52441.graph_c0	0	0	0	8.40143232	22.8441003	29.4038062	8.139E-08	Inf	up	-
c52442.graph_c0	4.62906739	4.75564225	3.28734822	0.89781028	1.90224225	2.49645683	2.1E-06	-2.286074	down	PREDICTED: mitoferrin-like [Sesamum indicum]
c52443.graph_c0	0.26852745	0.57599215	0.19718194	23.2188492	31.7858819	35.5024831	4.787E-38	5.42282	up	PREDICTED: cinnamoyl-CoA reductase 1-like [Sesamum indicum]
c52446.graph_c0	0.21005617	0.77097719	0.26993049	16.8360174	18.1090633	18.3096064	3.681E-25	4.39656	up	PREDICTED: vegetative incompatibility protein HET-E-1-like [Sesamum indicum]
c52454.graph_c0	2.04273284	1.61429868	1.42762754	0.03301577	0	0.08813169	1.375E-22	-6.406813	down	PREDICTED: cytochrome P450 86B1-like [Sesamum indicum]
c52455.graph_c0	2.37234374	3.26523901	1.90534727	19.1236307	16.4967509	16.7730045	0.0006954	1.78498	up	-
c52456.graph_c0	0.24203836	0.40380122	0	5.79751973	11.3386508	15.277387	1.118E-06	4.634316	up	PREDICTED: uncharacterized protein LOC105159948 [Sesamum indicum]
c52457.graph_c0	0.61674411	0.61736168	1.0567208	3.59850437	4.61474304	3.87601756	0.0077739	1.372616	up	PREDICTED: methylsterol monooxygenase 1-1-like [Sesamum indicum]
c52460.graph_c0	0	0	0	10.8096474	13.0274067	19.501444	1.886E-16	Inf	up	hypothetical protein MIMGU_mgv1a007773mg [Erythranthe guttata]
c52461.graph_c0	63.6210898	64.2637493	82.8704685	46.8895181	42.0533526	27.7356552	1.678E-10	-1.868141	down	PREDICTED: branchpoint-bridging protein [Sesamum indicum]

#ID	0 h-1 FPKM	0 h-2 FPKM	0 h-3 FPKM	48 h-1 FPKM	48 h-2 FPKM	48 h-3 FPKM	FDR	log2FC	regulated	nr_annotation
c52462.graph_c0	0.31033126	0.20709467	0.13292932	4.09786268	3.53352562	3.0526774	1.97E-06	3.031907	up	endo-beta-1,4-D-glucanase [Solanum lycopersicum]
c52465.graph_c0	12.6074591	9.96322383	11.0849522	1.0188433	1.07934093	0.18131213	3.989E-19	-4.893299	down	-
c52466.graph_c0	3.94576305	4.42846736	2.91935676	0	0	0	4.215E-22	-Inf	down	-
c52471.graph_c0	0.20585242	0.20605855	0.08817617	86.9836923	125.365057	134.039535	4.486E-57	8.419274	up	PREDICTED: bidirectional sugar transporter SWEET14-like [Solanum tuberosum]
c52472.graph_c0	15.2130213	5.55625516	6.60450498	0	0	0	7.712E-08	-Inf	down	RecName: Full=40S ribosomal protein S29 [Griffithsia japonica]
c52474.graph_c0	1.11228281	1.94844404	1.78665932	10.311119	8.8879678	8.60491008	0.0001505	1.503261	up	PREDICTED: probable E3 ubiquitin-protein ligase XERICO [Sesamum indicum]
c52482.graph_c0	50.727242	44.5804408	45.4768609	18.6025534	19.0891555	22.0891449	2.145E-43	-2.254076	down	hypothetical protein MIMGU_mgv1a027140mg [Erythranthe guttata]
c52484.graph_c0	55.6838217	43.5560657	44.5758162	51.019243	33.2607394	50.6346867	1.701E-05	-1.103379	down	histone H4 [Zea mays]
c52487.graph_c0	0.07458826	0.07466295	0	8.38329929	9.46345557	9.29369027	3.601E-19	6.503462	up	PREDICTED: long chain acyl-CoA synthetase 4 [Sesamum indicum]
c52491.graph_c0	18.5394591	14.7184325	16.9779795	3.1411051	1.24785749	1.9215188	2.209E-19	-3.995274	down	PREDICTED: cation/calcium exchanger 3-like [Sesamum indicum]
c52492.graph_c0	0.12258614	0	0	8.92181378	4.90525032	3.11513752	0.0001587	6.130744	up	PREDICTED: uncharacterized protein LOC105168627 [Sesamum indicum]
c52493.graph_c0	9.41343717	8.61749037	8.0644263	4.74380708	2.51274419	2.70408518	2.603E-12	-2.393019	down	PREDICTED: E3 ubiquitin-protein ligase RHA2A-like [Sesamum indicum]
c52496.graph_c0	5.86007934	6.00396962	5.13841377	0.88919556	1.21113627	0.73468651	9.288E-18	-3.602936	down	-
c52500.graph_c0	7.01101327	5.48846228	5.54426438	0	0	0	2.796E-33	-Inf	down	-
c52505.graph_c0	8.59357281	10.6667007	11.0430813	2.53342713	1.67741178	2.53600676	2.54E-09	-3.182043	down	PREDICTED: UDP-glycosyltransferase 73C3-like [Sesamum indicum]
c52506.graph_c0	0.10992953	0	0	9.06404587	6.00811644	3.829812	1.44E-07	6.441751	up	PREDICTED: uncharacterized protein LOC105159772 [Sesamum indicum]
c52508.graph_c0	0.30598701	0	0	14.7525151	26.7774952	30.2666568	3.181E-15	6.867274	up	hypothetical protein MIMGU_mgv1a018889mg [Erythranthe guttata]
c52510.graph_c0	18.9334696	18.3259021	13.6732029	15.2804231	14.9660381	17.3162062	0.0001926	-1.113687	down	PREDICTED: zinc-finger homeodomain protein 1-like [Sesamum indicum]
c52513.graph_c0	27.9032811	33.2602052	29.4875173	12.6840407	6.80818293	5.86881525	3.624E-22	-2.840729	down	PREDICTED: cyclin-dependent protein kinase inhibitor SMR3-like [Sesamum indicum]
c52514.graph_c0	0	0	0	4.44039318	5.0525069	3.36569661	1.485E-11	Inf	up	predicted protein [Hordeum vulgare subsp. vulgare]
c52517.graph_c0	25.6025559	27.7638758	25.9214469	16.0818712	15.9010064	12.8785971	4.082E-10	-1.837049	down	PREDICTED: uncharacterized protein LOC105169022 [Sesamum indicum]
c52518.graph_c0	0	0	0	37.6136692	49.2111953	58.7370713	2.619E-34	Inf	up	PREDICTED: protein YLS9 [Sesamum indicum]
c52520.graph_c0	11.2256034	9.39473856	8.86803986	5.59472157	5.92692911	6.41125329	9.871E-08	-1.734759	down	unnamed protein product [Coffea canephora]
c52522.graph_c0	21.8215658	12.1088506	16.6115858	0	0	0	1.734E-28	-Inf	down	--

#ID	0 h-1 FPKM	0 h-2 FPKM	0 h-3 FPKM	48 h-1 FPKM	48 h-2 FPKM	48 h-3 FPKM	FDR	log2FC	regulated	nr_annotation
c52531.graph_c0	53.8185969	32.4843062	33.0999924	0	0	0	1.148E-38	-Inf	down	uncharacterized protein LOC100191904 [Zea mays]
c52533.graph_c0	0	0	0	267.008551	331.594793	267.742052	6.85E-138	Inf	up	-
c52540.graph_c0	0.71427591	0.35749557	0.65562345	42.1614064	56.5655784	50.940054	8.643E-84	5.413575	up	PREDICTED: flavonol synthase/flavanone 3-hydroxylase-like [Sesamum indicum]
c52543.graph_c0	5.22098575	3.50538729	3.68186345	2.17032642	3.60414771	5.63685101	0.003201	-1.147293	down	-
c52546.graph_c0	17.3418837	13.403977	16.3611445	5.86478173	2.78514918	2.87914346	7.435E-13	-3.033091	down	-
c52553.graph_c0	51.9726757	54.1495753	55.6117917	34.4753072	38.2599787	34.9138571	2.161E-29	-1.606605	down	hypothetical protein MIMGU_mgv1a011657mg [Erythranthe guttata]
c52555.graph_c0	0.47528673	0.88355922	0.43625876	2.75229515	4.57238376	5.89980503	0.0011951	1.856638	up	PREDICTED: regulatory protein NPR5-like [Sesamum indicum]
c52558.graph_c0	2.28589468	2.37971101	2.58496935	91.9021428	101.642623	114.828348	7.246E-94	4.388985	up	eukaryotic translation initiation factor 5A [Quercus suber]
c52562.graph_c0	1.23367469	1.67594933	1.35884642	28.3323583	30.0146982	28.1715908	1.229E-23	3.323415	up	hypothetical protein MIMGU_mgv1a012628mg [Erythranthe guttata]
c52564.graph_c0	19.8700416	10.659967	12.572193	0	0	0	1.54E-27	-Inf	down	--
c52569.graph_c0	0	0	0	3.24101234	3.58952592	5.11224992	9.898E-12	Inf	up	PREDICTED: protein IQ-DOMAIN 14-like [Sesamum indicum]
c52571.graph_c0	40.9594764	46.2112559	39.2558675	46.8211198	30.482739	21.672782	1.774E-08	-1.357993	down	PREDICTED: uncharacterized protein LOC105157856 [Sesamum indicum]
c52573.graph_c0	0.63465124	0.4235245	0.54370132	5.06724504	6.81339791	8.84419279	8.591E-06	2.669235	up	PREDICTED: microtubule-associated protein 70-2 [Sesamum indicum]
c52580.graph_c0	0.08629323	0.25913891	0.11089021	7.39340223	3.87409684	4.739425	2.498E-06	4.129225	up	hypothetical protein POPTR_0001s10630g [Populus trichocarpa]
c52587.graph_c0	0.13563821	0.06788702	0	1.37454642	1.3237866	1.16746936	1.705E-06	3.249873	up	-
c52590.graph_c0	0.31192415	0.23417737	0.40083488	24.8570613	24.0498306	26.9119549	2.443E-37	5.301442	up	PREDICTED: probable serine/threonine-protein kinase At4g35230 [Sesamum indicum]
c52595.graph_c0	35.4807389	34.605594	30.5631144	27.0597093	32.8522944	27.3802416	1.634E-09	-1.223731	down	-
c52596.graph_c0	6.16315085	3.50677268	4.41846653	0	0	0	1.482E-24	-Inf	down	translationally-controlled tumor protein [Tilletiaria anomala UBC 951]
c52599.graph_c0	2.03440068	0.29091969	0.74693869	22.4906712	15.3168036	25.2533188	8.216E-09	3.352061	up	-
c52600.graph_c0	0	0	0	3.10373576	4.72654555	7.53709977	1.437E-08	Inf	up	PREDICTED: LOW QUALITY PROTEIN: protein E6-like [Sesamum indicum]
c52601.graph_c0	21.3518917	28.1342055	21.2784693	3.61299588	2.55168736	2.85761956	3.136E-24	-3.981677	down	-
c52603.graph_c0	0.18287157	0	0	6.90740212	12.4933865	10.418499	5.499E-16	6.34385	up	-
c52604.graph_c0	14.8941634	13.0454429	15.949658	0.32670178	0.69220184	0.58139456	3.229E-51	-5.807879	down	-
c52605.graph_c0	15.6961061	17.2067917	15.804943	13.6239548	13.5183663	15.4111948	4.35E-14	-1.212893	down	PREDICTED: inositol-tetrakisphosphate 1-kinase 1-like [Sesamum indicum]
c52608.graph_c0	1.24514789	0.31159868	1.00004021	7.88639124	7.89896568	5.6138108	0.0001891	2.049079	up	-
c52609.graph_c0	0	0	0	4.11722145	5.35865618	9.00169099	4.771E-08	Inf	up	-

#ID	0 h-1 FPKM	0 h-2 FPKM	0 h-3 FPKM	48 h-1 FPKM	48 h-2 FPKM	48 h-3 FPKM	FDR	log2FC	regulated	nr_annotation
c52610.graph_c0	113.926978	111.547765	102.007316	71.4345869	71.9770233	78.9643263	3.755E-29	-1.575593	down	PREDICTED: cysteine proteinase inhibitor 1-like [Sesamum indicum]
c52611.graph_c0	16.3868986	12.6622023	9.05116543	67.9350591	52.8894678	55.9705621	2.836E-05	1.206518	up	PREDICTED: trihelix transcription factor GT-2-like [Sesamum indicum]
c52612.graph_c0	0	0.41012795	0	5.2844211	5.59820339	3.52653459	1.462E-08	4.131127	up	-
c52616.graph_c0	0	0	0	14.2985597	17.6532067	13.0097523	1.881E-22	Inf	up	PREDICTED: reticuline oxidase-like protein [Sesamum indicum]
c52620.graph_c0	0	0	0	10.1749662	12.7389871	18.8388534	1.331E-15	Inf	up	PREDICTED: probable pectate lyase 8 [Sesamum indicum]
c52621.graph_c0	7.67071966	5.04975904	5.50581139	0	0	0	1.064E-36	-Inf	down	-
c52622.graph_c0	21.3740828	19.0840621	19.097236	0.21818503	0.11557029	0.04853493	5.04E-126	-8.280453	down	PREDICTED: LOW QUALITY PROTEIN: EID1-like F-box protein 3 [Sesamum indicum]
c52626.graph_c0	0.04404217	0	0.16978778	29.1730771	48.5716713	45.5980391	2.672E-38	8.124436	up	PREDICTED: probable pectinesterase/pectinesterase inhibitor 46 [Sesamum indicum]
c52631.graph_c0	28.0337786	25.6098439	22.5007491	6.35216002	11.4221748	10.932378	2.687E-28	-2.432667	down	hypothetical protein MIMGU_mgv1a017584mg [Erythranthe guttata]
c52638.graph_c0	0.7246657	0.45336959	0.34920904	57.7482054	54.988774	62.8934291	1.252E-75	5.833927	up	hypothetical protein MIMGU_mgv1a016092mg [Erythranthe guttata]
c52646.graph_c0	1.66296213	1.16523914	1.28218331	29.1085545	44.7947766	39.5325203	9.233E-20	3.763688	up	-
c52659.graph_c0	0.08001494	0.08009507	0	2.58002446	3.51414395	2.29569025	2.71E-09	4.707484	up	PREDICTED: LOW QUALITY PROTEIN: phototropin-1 [Sesamum indicum]
c52660.graph_c0	0.099898	0	0	10.4917112	9.74973319	8.51656141	3.242E-17	7.175683	up	PREDICTED: protein RADIALIS-like 3 [Sesamum indicum]
c52662.graph_c0	5.31967927	4.56429096	4.29691333	0	0	0	8.313E-22	-Inf	down	hypothetical protein M569_00609, partial [Genlisea aurea]
c52666.graph_c0	3.21245743	4.56418277	4.12813499	0.47734279	0.20227472	0.25484205	6.064E-16	-4.674139	down	-
c52667.graph_c0	1.04980163	1.40113713	0.44967888	44.1663218	60.1083197	69.13169	8.511E-20	4.885881	up	PIP2;1 [Karelinia caspia]
c52673.graph_c0	1.21000952	0.69878144	0.47843389	14.7488637	16.0788371	15.2216763	9.177E-23	3.25869	up	PREDICTED: uncharacterized protein LOC105176754 [Sesamum indicum]
c52674.graph_c0	2819.40896	2596.66673	2878.14897	29.4232988	24.7631648	28.0715008	0	-7.671407	down	hypothetical protein PHAVU_005G056100g [Phaseolus vulgaris]
c52675.graph_c0	98.5272191	84.2259575	90.8602309	6.87649248	5.82784797	6.11866166	8.16E-139	-4.875793	down	PREDICTED: uncharacterized protein LOC102593371 [Solanum tuberosum]
c52676.graph_c0	25.7252892	24.7461302	22.5762054	21.0408108	12.9821972	11.8298269	5.558E-12	-1.677206	down	PREDICTED: zinc finger protein NUTCRACKER-like [Sesamum indicum]
c52679.graph_c0	43.5058974	55.9921654	58.3270085	2.08210236	1.8993829	2.57311411	3.89E-54	-5.610016	down	PREDICTED: transcription factor TGA5-like [Sesamum indicum]
c52682.graph_c0	5.57455068	6.07249741	8.0062852	49.0906735	49.4453395	49.7286672	5.165E-11	1.893973	up	PREDICTED: uncharacterized protein At2g34160-like [Sesamum indicum]
c52686.graph_c0	1.00870062	0.72122191	0.55552296	7.16873761	6.60994792	6.85119589	0.0001174	2.163125	up	-
c52687.graph_c0	7.90859801	8.05788365	6.71474781	4.35853923	2.41204528	2.37322809	9.508E-12	-2.313804	down	-
c52690.graph_c0	0.08838686	0.44237685	0.11358062	21.2526414	38.7320071	45.8633299	1.421E-14	6.336433	up	PREDICTED: 21 kDa protein-like [Sesamum indicum]

#ID	0 h-1 FPKM	0 h-2 FPKM	0 h-3 FPKM	48 h-1 FPKM	48 h-2 FPKM	48 h-3 FPKM	FDR	log2FC	regulated	nr_annotation
c52691.graph_c0	20.2010636	16.9915023	18.387743	0.38771953	0.13691394	0.45998734	2.201E-47	-6.828708	down	RNA recognition motif containing protein [Zea mays]
c52692.graph_c0	49.4771286	46.7136256	53.7311771	36.4024835	33.9928514	33.2284017	1.719E-21	-1.549891	down	PREDICTED: universal stress protein A-like protein [Sesamum indicum]
c52697.graph_c0	0	0	0	3.94746639	5.47968123	6.90374538	5.843E-12	Inf	up	MYB-related transcription factor [Salvia miltiorrhiza]
c52700.graph_c0	10.0736203	6.09223998	8.22546936	0	0	0	9.424E-31	-Inf	down	PREDICTED: 60S ribosomal protein L18-3 [Nelumbo nucifera]
c52705.graph_c0	0.68748583	3.09678407	0.8834465	19.9507387	11.7418825	17.1884902	8.608E-05	2.382881	up	PREDICTED: succinate dehydrogenase [ubiquinone] iron-sulfur subunit 2, mitochondrial-like [Sesamum indicum]
c52710.graph_c0	0.29504194	0.59067476	0.37914057	6.5234851	8.63855182	9.91611961	4.174E-07	3.286404	up	-
c52711.graph_c0	12.9763642	14.1091303	12.3626051	7.42014193	11.1360492	10.6371962	0.0002911	-1.457912	down	PREDICTED: 7-deoxyloganetic acid glucosyltransferase-like [Sesamum indicum]
c52712.graph_c0	10.8234076	11.3670773	11.8564324	7.19236192	7.27309737	4.79979029	4.73E-06	-1.837003	down	PREDICTED: C-terminal binding protein AN-like isoform X2 [Sesamum indicum]
c52717.graph_c0	2.20843495	3.15806624	2.4325083	24.9959531	28.635545	23.0171063	1.806E-08	2.278901	up	PREDICTED: pleiotropic drug resistance protein 1-like [Sesamum indicum]
c52718.graph_c0	0.70641293	0.23570677	0	34.9259269	32.403538	25.0931327	1.508E-18	5.6211	up	hypothetical protein M569_04419, partial [Genlisea aurea]
c52719.graph_c0	9.83682169	8.11685106	8.37019677	5.14347991	6.48677772	5.99321577	4.198E-05	-1.59901	down	-
c52721.graph_c0	0.099898	0.09999803	0.25674577	2.39284642	4.38737994	4.01261066	7.477E-08	3.524251	up	PREDICTED: transcription repressor OFP17 [Nicotiana tomentosiformis]
c52726.graph_c0	9.46555246	8.79824286	6.95063409	8.63130182	2.73371869	1.82105001	1.095E-06	-1.922297	down	-
c52732.graph_c0	10.5895353	9.6783879	10.0580664	4.24163941	4.04415236	4.52902229	1.088E-05	-2.259915	down	-
c52734.graph_c0	5.56920824	5.95925289	4.68884167	0.70768523	0.18742667	0.78711757	2.727E-13	-4.274769	down	-
c52737.graph_c0	0.87150579	0.87237847	1.67987916	4.61660585	8.50562453	6.07244157	0.0057959	1.453047	up	PREDICTED: serine/threonine-protein kinase At5g01020 [Sesamum indicum]
c52742.graph_c0	0	0	0	10.5919823	14.0116162	12.7941281	1.527E-32	Inf	up	PREDICTED: palmitoyl-protein thioesterase 1-like [Sesamum indicum]
c52743.graph_c0	8.30412485	4.64201204	4.85051327	0	0	0	9.968E-18	-Inf	down	-
c52746.graph_c0	4.23704957	2.5447754	3.42243091	0.89221633	0.23629875	0.19847218	3.131E-12	-3.930531	down	-
c52747.graph_c0	25.4571872	29.4920563	25.6362928	11.5667469	8.64515382	7.29281305	1.159E-55	-2.560436	down	hypothetical protein MIMGU_mgv1a022353mg [Erythranthe guttata]
c52748.graph_c0	45.7607402	49.9707956	49.6400466	10.0375663	6.96016434	8.68778345	3.983E-74	-3.513272	down	PREDICTED: pentatricopeptide repeat-containing protein At3g57430, chloroplastic [Sesamum indicum]
c52749.graph_c0	7.20043393	4.67522859	4.00122926	0	0	0	6.829E-17	-Inf	down	-
c52750.graph_c0	2.8402154	2.18188283	1.61269757	17.6467811	36.4222862	31.0249856	1.124E-06	2.657029	up	-
c52753.graph_c0	8.49020982	11.1488473	8.44664962	1.51388421	1.0691845	1.27220923	1.788E-22	-3.875711	down	-
c52754.graph_c0	0.62824408	1.25774634	0.40365926	8.39230164	8.27747935	11.3298779	1.871E-05	2.598929	up	hypothetical protein M569_06445, partial [Genlisea aurea]

#ID	0 h-1 FPKM	0 h-2 FPKM	0 h-3 FPKM	48 h-1 FPKM	48 h-2 FPKM	48 h-3 FPKM	FDR	log2FC	regulated	nr_annotation
c52758.graph_c0	24.5720259	24.5055324	21.0506841	3.10215578	2.22051209	2.6856774	3.14E-54	-4.141149	down	PREDICTED: auxin-induced protein X15-like [Sesamum indicum]
c52760.graph_c0	50.8722032	51.144549	47.4663339	18.7467167	25.472446	22.1200796	8.707E-20	-2.193426	down	hypothetical protein MIMGU_mgv1a001181mg [Erythranthe guttata]
c52761.graph_c0	1.29663561	0.353982	0	30.2980125	30.601508	33.3363847	3.56E-28	4.836162	up	PREDICTED: callose synthase 3-like [Cucumis sativus]
c52765.graph_c0	0	0	0	6.5503719	9.67299815	8.65441197	4.899E-12	Inf	up	PREDICTED: transcription factor HEC2-like [Sesamum indicum]
c52769.graph_c0	0.86678195	1.12794487	1.67077368	26.0322758	37.0527035	42.8451152	5.554E-19	3.821847	up	PREDICTED: uncharacterized protein LOC105168965 [Sesamum indicum]
c52771.graph_c0	0.17157045	0.17174225	0.11023741	10.2740226	9.46077823	7.87598089	3.976E-15	4.917906	up	PREDICTED: CASP-like protein 2C1 isoform X2 [Sesamum indicum]
c52778.graph_c0	0.17018039	0.5110524	0	10.3475642	23.5848867	10.1837217	3.166E-05	5.00326	up	PREDICTED: uncharacterized protein LOC104588233 isoform X2 [Nelumbo nucifera]
c52779.graph_c0	4.50510001	5.26121306	5.54801389	0	0	0	2.023E-20	-Inf	down	-
c52787.graph_c0	3.51001911	4.99291654	4.74790897	0	0	0.15143631	3.707E-18	-7.493875	down	-
c52791.graph_c0	96.25799	99.4209325	98.4175559	3.41644923	4.56348249	2.24690921	3.34E-160	-5.864642	down	hypothetical protein MIMGU_mgv1a018283mg [Erythranthe guttata]
c52793.graph_c0	26.1962352	31.9392131	29.3555862	24.5911911	29.0806159	26.1555402	6.268E-08	-1.152508	down	hypothetical protein PHAVU_002G223700g [Phaseolus vulgaris]
c52795.graph_c0	27.315216	27.4519383	25.8344346	11.0724058	13.3293992	12.718243	1.26E-20	-2.138561	down	PREDICTED: protein ELF4-LIKE 3 [Sesamum indicum]
c52799.graph_c0	28.4638294	29.7728858	26.0971784	3.38833108	4.52592399	2.75274996	4.372E-37	-4.001609	down	PREDICTED: uncharacterized protein LOC105178281 [Sesamum indicum]
c52801.graph_c0	85.7546789	77.8276281	75.5081715	47.6998059	43.1351831	45.3749183	2.001E-34	-1.826552	down	PREDICTED: UPF0369 protein C6orf57 homolog [Nicotiana tomentosiformis]
c52802.graph_c0	0	0	0	1.74264525	4.0808998	2.77474943	6.861E-07	Inf	up	PREDICTED: adenine phosphoribosyltransferase 3 [Nicotiana tomentosiformis]
c52803.graph_c0	203.439453	244.526075	194.231766	244.520402	82.6219584	75.3518349	8.445E-06	-1.666606	down	PREDICTED: probable CCR4-associated factor 1 homolog 11 [Sesamum indicum]
c52805.graph_c0	10.4744979	11.1140857	9.82590545	4.43890369	6.84926466	10.6470438	4.363E-06	-1.545146	down	PREDICTED: dnaJ protein ERDJ3A [Sesamum indicum]
c52810.graph_c0	1.05815532	0.17653582	1.81302864	5.84905018	6.19635943	4.91531359	0.0047289	1.448101	up	PREDICTED: UDP-galactose transporter 2-like [Nelumbo nucifera]
c52812.graph_c0	4.41925774	3.96606058	5.87474603	3.50974869	3.12324865	0.99934505	8.478E-05	-1.913513	down	PREDICTED: peamaclein [Vitis vinifera]
c52817.graph_c0	1.05714778	2.11641271	0.67923838	40.4174097	41.7856015	40.2960799	1.183E-15	3.979373	up	-
c52825.graph_c0	0	0	0	4.41102625	6.11077742	11.7747139	1.113E-05	Inf	up	hypothetical protein MIMGU_mgv1a016882mg [Erythranthe guttata]
c52828.graph_c0	0	0	0	15.8966347	13.7630947	13.2831221	6.945E-27	Inf	up	cytochrome c oxidase subunit 3 (mitochondrion) [Microbotryum lychnidis-dioicae]
c52831.graph_c0	0	0	0	6.93369479	9.63064719	10.0083972	1.488E-13	Inf	up	-

#ID	0 h-1 FPKM	0 h-2 FPKM	0 h-3 FPKM	48 h-1 FPKM	48 h-2 FPKM	48 h-3 FPKM	FDR	log2FC	regulated	nr_annotation
c52832.graph_c0	68.4917306	67.576749	56.2996251	20.6602795	21.7178311	25.1113351	1.493E-63	-2.528609	down	PREDICTED: acidic leucine-rich nuclear phosphoprotein 32-related protein [Sesamum indicum]
c52837.graph_c0	0.08503434	0	0	7.44222171	7.80114063	8.22527586	9.223E-17	7.111055	up	hypothetical protein CISIN_1g041913mg [Citrus sinensis]
c52838.graph_c0	1.62089096	1.62251403	1.92268528	8.84475588	10.8301978	12.3671595	0.0004646	1.608768	up	-
c52840.graph_c0	6.19315683	5.36160722	5.05469231	4.08640029	5.14584666	5.28256961	0.0013176	-1.214702	down	hypothetical protein MIMGU_mgv1a013481mg [Erythranthe guttata]
c52841.graph_c0	2.50562466	1.32373721	1.60991345	23.9811383	33.0130551	32.5208969	1.812E-26	3.020347	up	PREDICTED: probable calcium-binding protein CML49 [Sesamum indicum]
c52842.graph_c0	0	0	0	5.85073655	13.3387	32.8492413	1.55E-05	Inf	up	PREDICTED: endoglucanase 1-like [Sesamum indicum]
c52849.graph_c0	8.79298705	4.28805246	6.08425811	0	0	0	5.335E-14	-Inf	down	unknown [Picea sitchensis]
c52850.graph_c0	0	0.06641872	0	61.922711	97.2013872	93.3355876	2.296E-53	10.87866	up	PREDICTED: aquaporin TIP4-1 [Sesamum indicum]
c52852.graph_c0	1.10882214	1.87540311	1.32661274	0.10567441	0	0	2.357E-18	-6.310202	down	PREDICTED: uncharacterized protein LOC103504028 [Cucumis melo]
c52853.graph_c0	1.60929005	1.8410303	0.88628633	30.4988495	21.9886363	16.7726019	3.874E-06	2.994954	up	-
c52856.graph_c0	3.62546949	4.42296544	2.6206154	29.3295415	30.739373	29.9979062	1.678E-10	2.063396	up	PREDICTED: protein transport protein SEC31 homolog B [Sesamum indicum]
c52857.graph_c0	0	0	0	2.61956562	1.85007476	1.8129018	6.063E-10	Inf	up	PREDICTED: basic leucine zipper 43 [Sesamum indicum]
c52858.graph_c0	0.46345301	1.62370983	2.97777736	17.7189706	13.3433126	10.8274133	0.0001869	2.024475	up	PREDICTED: membrin-11 [Sesamum indicum]
c52861.graph_c0	119.662825	122.156934	119.786297	2.62218909	4.62981898	2.52764209	3.22E-221	-6.232799	down	PREDICTED: heat shock 70 kDa protein-like [Sesamum indicum]
c52868.graph_c0	40.0305047	40.8296532	42.3629981	41.4747627	37.7052151	34.3521225	5.394E-15	-1.133514	down	hypothetical protein MIMGU_mgv1a007230mg [Erythranthe guttata]
c52869.graph_c0	143.492551	139.341466	131.097117	43.918504	49.0852778	44.3540076	3.268E-52	-2.609389	down	-
c52870.graph_c0	4.53542719	3.92088209	4.90098932	3.03878644	2.91742335	2.53490053	0.0002118	-1.67057	down	hypothetical protein M569_00403, partial [Genlisea aurea]
c52871.graph_c0	0.4355304	0	0	7.75728763	4.81739295	5.11728757	2.437E-07	4.357319	up	PREDICTED: uncharacterized protein LOC105159124 [Sesamum indicum]
c52872.graph_c0	79.3171032	94.7481216	82.9877765	40.7305148	39.6409895	33.9827952	7.632E-43	-2.184033	down	-
c52876.graph_c0	1.84647139	0.69312013	1.48299303	30.1943454	24.7788539	24.2179128	7.049E-11	3.285494	up	PREDICTED: muscle M-line assembly protein unc-89-like [Populus euphratica]
c52879.graph_c0	0.07585005	0.075926	0	3.84329059	6.07023689	5.9068182	3.932E-13	5.694794	up	-
c52880.graph_c0	3.23132494	3.00352058	1.48299303	58.687601	77.9407585	100.844902	3.769E-15	3.926132	up	hypothetical protein MIMGU_mgv1a011350mg [Erythranthe guttata]
c52882.graph_c0	9.96088246	10.4043723	10.5740191	10.3735644	7.60813863	7.10025784	0.00752	-1.313813	down	-
c52884.graph_c0	1.26417293	1.01235105	0.64980491	5.82319282	9.37682937	5.38869963	0.0018022	1.797624	up	-
c52890.graph_c0	6.41122314	3.14333535	4.37154214	0	0	0	7.278E-15	-Inf	down	60S ribosomal protein L11 [Zea mays]

#ID	0 h-1 FPKM	0 h-2 FPKM	0 h-3 FPKM	48 h-1 FPKM	48 h-2 FPKM	48 h-3 FPKM	FDR	log2FC	regulated	nr_annotation
c52891.graph_c0	0	0	0	5.08790044	9.81752472	9.70110688	1.977E-10	Inf	up	PREDICTED: anthocyanidin 3-O-glucosyltransferase 2-like [Sesamum indicum]
c52898.graph_c0	0.40444411	0.50606138	0.5197267	20.2135661	22.8940404	16.5768752	1.324E-20	4.363656	up	PREDICTED: vegetative incompatibility protein HET-E-1-like [Sesamum indicum]
c52903.graph_c0	1.4682682	1.11700122	1.43395491	0.64927893	1.14638716	0.91473044	0.0015333	-1.595277	down	-
c52907.graph_c0	0.44285432	0.55412222	0.426814	40.2886457	71.6391388	68.8837553	5.562E-22	5.962701	up	-
c52908.graph_c0	43.1549937	46.3629107	45.2991217	8.95629117	10.4137732	9.71860247	1.133E-71	-3.232494	down	PREDICTED: transcription factor TCP9 [Sesamum indicum]
c52909.graph_c0	0	0	0	6.26594573	4.97850729	5.31405392	1.953E-13	Inf	up	PREDICTED: glucan endo-1,3-beta-glucosidase 4-like isoform X1 [Sesamum indicum]
c52911.graph_c0	20.7480141	23.906521	18.6058698	18.5643685	19.5210182	19.3327202	1.54E-05	-1.156411	down	-
c52916.graph_c0	0	0	0	2.85794942	2.90654483	2.59384593	1.177E-12	Inf	up	PREDICTED: pectinesterase/pectinesterase inhibitor PPE8B-like [Sesamum indicum]
c52917.graph_c0	0.52372489	1.04849865	0.26920289	5.21088694	4.29356889	7.12665031	0.0001023	2.164693	up	PREDICTED: scarecrow-like protein 8 [Sesamum indicum]
c52919.graph_c0	0	0.21253361	0.2728409	7.23734649	7.45987235	9.92069191	9.134E-10	4.630921	up	V-type proton ATPase subunit [Morus notabilis]
c52924.graph_c0	5.7802941	5.40034338	4.20914717	5.14767781	3.00873956	1.26355111	0.0002902	-1.705103	down	PREDICTED: probable galacturonosyltransferase-like 1 [Sesamum indicum]
c52926.graph_c0	3.92877393	3.07777148	3.51209046	16.3661761	17.7547574	24.5742611	3.744E-06	1.45915	up	-
c52927.graph_c0	1.03620904	0.86437221	0.44385655	19.251571	29.3279231	37.6575368	9.278E-11	4.181953	up	unknown [Lotus japonicus]
c52930.graph_c0	2.16052922	1.73015413	3.88691182	23.8054284	27.327574	31.5249416	5.385E-16	2.380773	up	PREDICTED: uncharacterized protein LOC105172794 [Sesamum indicum]
c52932.graph_c0	34.0637966	22.0360619	20.5466638	0	0	0	5.786E-28	-Inf	down	-
c52935.graph_c0	0	0.04486597	0.17279063	6.97836719	6.99903798	8.74447089	3.739E-23	5.663183	up	PREDICTED: GDSL esterase/lipase At4g10955-like [Sesamum indicum]
c52939.graph_c0	7.01848112	14.0510181	9.01902611	4.38756318	6.6051822	5.54782832	0.0005746	-1.886406	down	-
c52942.graph_c0	0	0	0	3.18835316	9.45748598	8.83515841	1.495E-07	Inf	up	-
c52943.graph_c0	46.0578735	46.9473593	46.1941003	1.55237258	0.54818351	0.69064589	2.427E-57	-6.637873	down	-
c52952.graph_c0	6.42665612	7.07640059	4.78993438	32.6820145	41.2711802	35.8235086	1.192E-07	1.567744	up	unnamed protein product [Coffea canephora]
c52953.graph_c0	0	0	0	37.2311719	44.4924009	37.1680818	1.713E-26	Inf	up	-
c52958.graph_c0	3.47695978	1.28226789	1.64611667	9.77819598	12.3234154	10.65071	0.0090617	1.338891	up	PREDICTED: phosphatidylinositol/phosphatidylcholine transfer protein SFH6-like isoform X2 [Populus euphratica]
c52961.graph_c0	0.30799728	0.15415285	0.19789435	2.97934545	5.41072327	6.31191349	4.177E-06	3.451552	up	-
c52974.graph_c0	0	0	0	308.0893	425.807347	545.662377	1.782E-45	Inf	up	-
c52984.graph_c0	15.6017172	16.9514845	19.4443371	1.30009925	1.30078098	0.83548147	4.106E-57	-4.935886	down	-
c52985.graph_c0	8.9894115	6.9987657	9.71813862	6.17836054	8.91264521	7.95378357	0.00339	-1.182877	down	-
c52986.graph_c0	0.06494756	0.19503778	0.16692037	2.87202852	3.23272634	5.21750715	1.496E-07	3.70146	up	PREDICTED: thioredoxin H2 [Sesamum indicum]

#ID	0 h-1 FPKM	0 h-2 FPKM	0 h-3 FPKM	48 h-1 FPKM	48 h-2 FPKM	48 h-3 FPKM	FDR	log2FC	regulated	nr_annotation
c52991.graph_c0	17.3519715	14.9247722	13.5439524	4.49969765	2.75977497	2.84480804	1.069E-19	-3.186542	down	hypothetical protein M569_07000 [Genlisea aurea]
c52992.graph_c0	6.13009916	6.68411587	8.29942476	1.51270907	1.17519001	1.34599943	8.945E-14	-3.404524	down	-
c53000.graph_c0	7.86357134	8.01370055	8.94840406	1.04738817	1.29451089	1.009623	3.357E-35	-3.910036	down	-
c53001.graph_c0	1.25343881	1.25469393	1.38061585	6.76353327	11.8836524	14.5316335	0.0001968	2.06352	up	hypothetical protein MIMGU_mgv1a027115mg, partial [Erythranthe guttata]
c53003.graph_c0	24.2430563	29.8225044	20.2684037	12.1089495	16.8188858	14.6053933	5.209E-07	-1.791811	down	-
c53007.graph_c0	20.8453868	30.102146	21.078411	147.651425	99.7211422	122.695428	6.56E-05	1.351261	up	-
c53009.graph_c0	0	0	0	4.59871156	6.33331046	6.54704943	1.09E-11	Inf	up	-
c53010.graph_c0	4.12118102	3.00022382	1.44433112	17.6028853	25.5954623	20.883922	0.0005049	1.889055	up	-
c53014.graph_c0	0.45799077	0.68767407	0.29426814	12.2359982	18.1028805	24.4030567	5.918E-09	4.227811	up	unknown [Picea sitchensis]
c53027.graph_c0	1.80885321	2.80652997	2.44067038	0.99986013	0.26480764	0.07413913	3.803E-10	-3.381765	down	-
c53031.graph_c0	11.73195	13.0815874	15.2668559	3.42032834	4.63798161	8.03454323	7.968E-10	-2.345737	down	-
c53033.graph_c0	3.85342293	3.10885379	1.84768695	22.5188124	23.8559512	23.9973785	5.302E-14	1.985727	up	PREDICTED: pleiotropic drug resistance protein 1-like [Sesamum indicum]
c53036.graph_c0	11.6687294	7.32680506	6.40686022	0	0	0	5.047E-21	-Inf	down	hypothetical protein GUITHDRAFT_93413, partial [Guillardia theta CCMP2712]
c53038.graph_c0	1.89370724	1.08067115	1.2736016	0	0	0	6.538E-24	-Inf	down	predicted protein [Physcomitrella patens]
c53043.graph_c0	2.51894925	1.14612345	2.64841324	13.2908256	9.16318643	12.3892442	0.0040876	1.448488	up	hypothetical protein M569_02184 [Genlisea aurea]
c53045.graph_c0	12.2530294	12.0883956	9.46251678	3.41904935	2.9896436	1.01408318	2.032E-30	-3.192389	down	PREDICTED: uncharacterized protein At1g76070 [Sesamum indicum]
c53052.graph_c0	0	0	0	24.1026125	36.5754365	37.5311294	3.599E-38	Inf	up	unnamed protein product [Coffea canephora]
c53054.graph_c0	2.07580077	3.56207892	4.19176399	63.1079812	66.8552538	52.9929709	1.655E-15	3.196084	up	PREDICTED: LOW QUALITY PROTEIN: 36.4 kDa proline-rich protein [Sesamum indicum]
c53056.graph_c0	0.62487656	1.25100456	0.80299112	6.90812456	7.62325033	9.22021153	0.0001896	2.128132	up	putative S-adenosine-L-methionine synthetase [Zinnia [illegible]
c53061.graph_c0	0	0	0	2.77300051	6.21427611	11.2930962	6.667E-05	Inf	up	PREDICTED: dirigent protein 21-like [Sesamum indicum]
c53074.graph_c0	5.47554551	7.30803792	7.03629277	6.96611716	4.07158976	2.56485896	0.0014974	-1.547099	down	PREDICTED: probable galacturonosyltransferase-like 1 [Sesamum indicum]
c53076.graph_c0	7.84791442	8.10918495	8.29562771	0.4664523	0.24707483	0.10376161	2.552E-25	-5.889477	down	-
c53082.graph_c0	0.91345156	2.40021138	1.61400445	21.9850014	26.1878189	35.6610933	2.903E-12	3.064435	up	hypothetical protein SORBIDRAFT_07g000600 [Sorghum bicolor]
c53092.graph_c0	6.059923	6.86414782	6.35274922	4.55438648	2.80150852	3.5295669	6.235E-05	-1.834095	down	-
c53100.graph_c0	0.36775843	0	0	9.03474814	6.46057359	8.5415061	7.251E-12	5.03845	up	hypothetical protein MIMGU_mgv1a006779mg [Erythranthe guttata]
c53101.graph_c0	0	0	0	9.60297367	14.2781561	16.6396171	5.364E-22	Inf	up	-
c53102.graph_c0	0.29062867	1.45459844	0.74693869	12.3163199	6.52382377	12.3884206	0.0001373	2.635826	up	-

#ID	0 h-1 FPKM	0 h-2 FPKM	0 h-3 FPKM	48 h-1 FPKM	48 h-2 FPKM	48 h-3 FPKM	FDR	log2FC	regulated	nr_annotation
c53107.graph_c0	57.2474771	57.6249403	58.9755134	25.3388237	23.2538853	21.1044163	1.325E-32	-2.333912	down	PREDICTED: R3H domain-containing protein 1-like, partial [Sesamum indicum]
c53108.graph_c0	39.3821837	38.7074593	42.5397766	0.52581792	2.2281613	1.98844589	1.308E-82	-5.707005	down	-
c53115.graph_c0	1.6316135	2.17766308	1.16482663	10.8560745	12.7392309	11.1457728	0.0005844	1.788994	up	-
c53119.graph_c0	33.9437957	51.7161879	37.2045447	7.58787407	7.55125478	5.72866825	4.005E-17	-3.572183	down	-
c53120.graph_c0	0.29504194	0	0	14.4060296	16.1252967	15.962534	3.22E-12	6.301797	up	-
c53137.graph_c0	0.89951367	0.90041439	0	16.1594679	14.9242536	28.0198308	2.791E-06	4.032363	up	-
c53138.graph_c0	1.16135656	0.98367033	1.49238916	5.76109375	5.49286224	7.61604863	0.003706	1.352352	up	unnamed protein product [Coffea canephora]
c53141.graph_c0	0.0937898	0.09388372	0	2.33294262	3.2952931	2.69090152	6.965E-09	4.464162	up	PREDICTED: squamosa promoter-binding-like protein 9 [Sesamum indicum]
c53164.graph_c0	23.5806769	24.3419234	20.6433079	3.12283328	4.02745085	3.26192667	2.308E-30	-3.738443	down	PREDICTED: uncharacterized protein LOC103930947 [Pyrus x bretschneideri]
c53168.graph_c0	2.12647036	0.38701813	0.74525416	11.7542592	13.3955617	11.8850785	5.981E-06	2.495158	up	hypothetical protein M569_03938, partial [Genlisea aurea]
c53169.graph_c0	1.16791868	1.16908818	0.30016435	9.0380881	8.20693579	7.85055939	0.0001618	2.246051	up	-
c53171.graph_c0	2.59364375	1.46454615	1.965577	21.3211016	25.1184372	23.1690801	1.348E-17	2.511826	up	PREDICTED: uncharacterized protein LOC105166615 isoform X1 [Sesamum indicum]
c53173.graph_c0	1.24885611	0.81256932	0.64193182	28.7631927	33.0916308	38.8506535	1.241E-47	4.203488	up	PREDICTED: 30S ribosomal protein 2, chloroplastic [Sesamum indicum]
c53178.graph_c0	15.4743775	15.4898727	11.2255092	6.89806734	8.52561023	8.18381179	6.566E-06	-1.855432	down	PREDICTED: uncharacterized protein LOC105161374 [Sesamum indicum]
c53184.graph_c0	0	0	0.14672768	2.84016765	3.56600087	2.99515744	7.245E-10	4.948723	up	PREDICTED: MATE efflux family protein LAL5-like [Sesamum indicum]
c53185.graph_c0	6.03893014	4.21316594	5.87898812	81.9345387	100.016125	106.95713	3.961E-34	3.140079	up	PREDICTED: 60S ribosomal protein L38 [Sesamum indicum]
c53194.graph_c0	33.6240074	33.975202	22.4193391	27.7620729	26.1599102	28.9929642	9.605E-06	-1.131541	down	hypothetical protein M569_16135, partial [Genlisea aurea]
c53202.graph_c0	0.37891494	0.25286291	0.3246138	2.21084735	2.34212469	3.83086058	0.0001295	2.109795	up	PREDICTED: glutathione S-transferase U17-like [Sesamum indicum]
c53215.graph_c0	1.81856002	0.72815241	2.10322946	34.5127477	28.9301827	23.7027594	3.842E-13	3.211116	up	-
c53217.graph_c0	16.6896165	10.8591136	7.5063863	10.5066349	10.5875542	7.29657802	0.0097951	-1.312416	down	-
c53219.graph_c0	0	0	0	14.0817264	7.72533156	9.17362955	3.447E-08	Inf	up	PREDICTED: CBL-interacting serine/threonine-protein kinase 5 [Sesamum indicum]
c53222.graph_c0	0.59373033	0.19810829	0.76296698	24.4319121	22.4058747	26.4441572	9.2E-16	4.535045	up	PREDICTED: zeatin O-glucosyltransferase-like [Solanum lycopersicum]
c53226.graph_c0	2.54984714	1.1911202	1.26697308	0	0	0	2.006E-11	-Inf	down	carboxypeptidase, partial [Metarhizium majus ARSEF 297]
c53235.graph_c0	0.24111558	0.36203553	0.9295292	18.8811689	22.2378611	17.2944785	8.446E-18	4.219637	up	PREDICTED: elicitor-responsive protein 3-like [Sesamum indicum]
c53238.graph_c0	1.99943352	1.50107674	1.28467562	16.5780771	12.6840007	10.653553	0.000402	2.053431	up	-

#ID	0 h-1 FPKM	0 h-2 FPKM	0 h-3 FPKM	48 h-1 FPKM	48 h-2 FPKM	48 h-3 FPKM	FDR	log2FC	regulated	nr_annotation
c53256.graph_c0	0	0	0	11.8140672	20.1143117	18.5838663	7.086E-16	Inf	up	hypothetical protein MIMGU_mgv1a010045mg [Erythranthe guttata]
c53264.graph_c0	1.09180025	1.63934028	0.70150327	14.5846452	7.19255007	8.94988248	0.0043724	2.16292	up	PREDICTED: coatomer subunit beta'-2-like [Glycine max]
c53267.graph_c0	0	0.15790331	0	17.7296877	16.7810452	19.0085091	2.721E-19	7.399216	up	hypothetical protein JCGZ_13755 [Jatropha curcas]
c53270.graph_c0	49.0072083	46.0440538	43.6414038	9.50497616	7.97158435	8.80986524	1.071E-19	-3.413244	down	Histone H3.3 [Triticum urartu]
c53273.graph_c0	0.25877816	0.51807457	0.66508035	2.74163813	2.65187376	2.33342821	0.0071244	1.398985	up	-
c53274.graph_c0	0.89951367	1.80082879	2.31182135	30.2472092	24.5811236	26.9137848	1.448E-07	3.006398	up	PREDICTED: 8-hydroxygeraniol dehydrogenase-like [Sesamum indicum]
c53277.graph_c0	3.22802712	5.1700152	2.07407133	16.0588933	24.2584938	20.3752077	0.003458	1.516951	up	prohibitin-like protein [Petunia x hybrida]
c53281.graph_c0	31.4517278	24.711691	32.6269016	27.8422676	28.1236224	24.9899336	4.004E-08	-1.1513	down	-
c53282.graph_c0	5.13623505	5.76457558	6.00024127	2.43761184	5.16470833	6.12415724	0.0047864	-1.332062	down	-
c53293.graph_c0	5.21768387	3.13374515	2.23497678	32.6867208	32.930185	31.6507224	7.946E-06	2.189458	up	-
c53299.graph_c0	1.38647313	1.15655123	1.33625465	29.0587906	29.7693992	33.1491502	2.796E-21	3.548433	up	PREDICTED: probable polygalacturonase [Sesamum indicum]
c53303.graph_c0	13.0587973	5.58277938	10.6629725	0	0	0	5.804E-15	-Inf	down	hypothetical protein CICLE_v10032980mg [Citrus clementina]
c53304.graph_c0	16.9487067	16.7771708	17.4238074	16.4817127	18.9307236	12.6584944	0.0008326	-1.107246	down	PREDICTED: vacuolar-sorting receptor 3-like [Sesamum indicum]
c53313.graph_c0	10.3982339	12.9651557	11.4867538	0.67224781	2.49257763	1.64494566	1.578E-16	-3.892745	down	PREDICTED: uncharacterized protein LOC100815796 [Glycine max]
c53316.graph_c0	3.49294296	2.76801549	3.55345125	1.34080287	0.85225084	2.62468433	8.704E-05	-2.047686	down	-
c53319.graph_c0	3.57671215	2.68522027	0.38301806	19.2214197	27.9260756	27.609296	2.612E-06	2.482037	up	PREDICTED: succinyl-CoA ligase [ADP-forming] subunit beta, mitochondrial [Prunus mume]
c53339.graph_c0	0	0	0	8.43032372	6.3792184	7.50125135	1.108E-11	Inf	up	hypothetical protein MIMGU_mgv1a021216mg, partial [Erythranthe guttata]
c53342.graph_c0	45.9986597	38.7001638	42.7913599	10.9192181	12.1184251	17.1184105	1.926E-18	-2.688597	down	hypothetical protein MIMGU_mgv1a006294mg [Erythranthe guttata]
c53351.graph_c0	87.9821338	90.7781042	81.4433066	54.1157034	68.8954083	63.5688599	4.859E-22	-1.500262	down	PREDICTED: zinc finger A20 and AN1 domain-containing stress-associated protein 8-like [Sesamum indicum]
c53354.graph_c0	0	0	0	4.39544527	8.22637961	7.43097747	8.498E-12	Inf	up	PREDICTED: uncharacterized protein DDB_G0271670-like [Solanum tuberosum]
c53360.graph_c0	16.9475988	15.3741409	14.8024624	9.94123848	12.7929097	12.8072049	4.96E-11	-1.427966	down	PREDICTED: uncharacterized protein LOC105157237 [Sesamum indicum]
c53362.graph_c0	10.8480194	12.4761624	10.6775498	3.40217977	0.90104923	2.45963177	5.666E-12	-3.330521	down	PREDICTED: uncharacterized protein LOC105161926 [Sesamum indicum]
c53364.graph_c0	12.0072761	8.82429586	8.00788393	5.32090713	5.34017929	3.73777198	4.76E-07	-2.013726	down	-
c53370.graph_c0	8.2008455	8.40451115	8.02926564	2.33849998	3.23962091	2.40090371	2.738E-08	-2.647625	down	-
c53377.graph_c0	0	0	0	53.9736924	52.6280378	60.9873576	1.592E-41	Inf	up	-

#ID	0 h-1 FPKM	0 h-2 FPKM	0 h-3 FPKM	48 h-1 FPKM	48 h-2 FPKM	48 h-3 FPKM	FDR	log2FC	regulated	nr_annotation
c53394.graph_c0	0	0	0	6.85812962	8.13719954	10.1298553	6.037E-14	Inf	up	uncharacterized protein [Arabidopsis thaliana]
c53452.graph_c0	7.11333189	5.53813152	5.75538973	1.09221088	1.28562777	1.40377276	1.445E-11	-3.30287	down	PREDICTED: trihelix transcription factor GT-3b [Sesamum indicum]
c53470.graph_c0	0.97742074	0.24459987	0.31400609	12.6064923	14.7859482	15.0230103	4.595E-09	3.775411	up	-
c53487.graph_c0	1.15219476	1.25819838	0.53840578	29.7213551	36.3931077	37.2646534	1.38E-29	4.117565	up	PREDICTED: rapid alkalinization factor-like [Sesamum indicum]
c53493.graph_c0	39.7799322	37.4424663	46.0323412	0.91163851	0.96577046	0.16223409	1.915E-72	-6.928872	down	-
c53497.graph_c0	0	0.8801094	1.12984406	28.7551097	24.8849016	23.0635481	2.583E-10	4.226856	up	PREDICTED: UDP-arabinopyranose mutase 3-like [Sesamum indicum]
c53512.graph_c0	0	0	0	19.7039426	15.4467141	12.0973903	2.125E-14	Inf	up	PREDICTED: uncharacterized protein LOC105165055 [Sesamum indicum]
c53522.graph_c0	118.232222	138.137357	123.920424	6.63750964	14.7844667	16.0522491	1.48E-121	-4.37393	down	PREDICTED: uncharacterized protein LOC103335027 [Prunus mume]
c53531.graph_c0	12.9358859	9.55747655	11.4778724	7.09367964	6.16221261	6.31191349	2.39E-06	-1.810406	down	-
c53532.graph_c0	2.64200429	3.82004979	2.26338674	22.446766	23.7796268	25.2670449	4.783E-05	2.018525	up	hypothetical protein MIMGU_mgv1a011661mg [Erythranthe guttata]
c53537.graph_c0	23.7420201	23.0345389	29.570684	1.68251519	1.78242083	2.69476542	5.386E-21	-4.656066	down	--
c53541.graph_c0	0.31831193	0	0	1.61287344	2.25230324	1.8917558	9.922E-07	3.175664	up	-
c53554.graph_c0	31.4570878	42.5845657	33.8788294	19.8722201	20.1750323	15.4719092	3.462E-10	-1.974206	down	-
c53566.graph_c0	4.81448288	4.81930385	3.68261971	0	0.3356275	0.18793363	2.978E-19	-5.714403	down	-
c53570.graph_c0	39.5187624	31.4438043	24.7405208	14.0027929	14.8342611	13.290241	4.582E-10	-2.197432	down	-
c53579.graph_c0	0	0.37847426	0.16195596	2.55439341	3.81309875	4.44245488	3.853E-07	3.294866	up	-
c53583.graph_c0	17.4130014	13.7392863	9.47706191	2.83093988	1.19961501	2.85481372	1.265E-11	-3.564796	down	-
c53594.graph_c0	0	0	0	11.0033158	15.4372233	15.6121721	1.558E-12	Inf	up	PREDICTED: adenosine kinase 2 [Sesamum indicum]
c53626.graph_c0	6.43571241	4.2947712	7.04493855	45.4556517	60.4842285	60.7669443	8.101E-11	2.204122	up	-
c53646.graph_c0	0.15199084	0.60857213	0.39062848	3.08052518	3.11510458	3.23940238	0.0002466	2.014788	up	-
c53652.graph_c0	1.65933192	0.95203286	1.01414612	0	0	0	5.439E-20	-Inf	down	hypothetical protein VHEM109090 [Torrubiella hemipterigena]
c53655.graph_c0	1.15083192	0.92158744	0.29577291	19.2960299	15.2751941	11.5092195	1.634E-08	3.282602	up	-
c53698.graph_c0	0.970612	0.64772261	1.52444703	4.57030996	8.52558319	8.5752941	0.0002906	1.750573	up	hypothetical protein POPTR_0007s07730g [Populus trichocarpa]
c53715.graph_c0	0	1.38558188	0	17.8529605	12.6987601	19.0625483	6.971E-10	4.15953	up	PREDICTED: purple acid phosphatase 2 [Sesamum indicum]
c53744.graph_c0	0	0.19286941	0	7.10024526	5.64138668	7.58130664	1.871E-10	5.715576	up	-
c53761.graph_c0	4.78310974	5.54388339	6.14648538	1.62345204	0.49138588	0.61908765	2.416E-10	-3.587853	down	-
c53783.graph_c0	0	1.48831356	1.91062865	28.7649584	33.3751746	30.0638355	1.58E-09	3.728906	up	-
c53789.graph_c0	0.99292187	3.57809809	1.78632149	11.7087282	10.8534806	12.8601582	0.0040574	1.461481	up	PREDICTED: PLASMODESMATA CALLOSE-BINDING PROTEIN 3 [Sesamum indicum]
c53797.graph_c0	7.16445354	9.05889809	7.75293175	0	0	0	6.093E-19	-Inf	down	-

#ID	0 h-1 FPKM	0 h-2 FPKM	0 h-3 FPKM	48 h-1 FPKM	48 h-2 FPKM	48 h-3 FPKM	FDR	log2FC	regulated	nr_annotation
c53803.graph_c0	5.85961153	5.44651625	4.3027616	5.0126666	2.04242786	5.6610746	0.0072764	-1.303197	down	-
c53824.graph_c0	1.93995142	0.52960745	1.1331429	11.0482059	6.19635943	8.52951475	0.0015075	1.832967	up	-
c53867.graph_c0	8.42534016	5.95325426	6.36876217	74.8802092	59.4948764	74.7533208	5.369E-08	2.320422	up	PREDICTED: 40S ribosomal protein S19-1 [Sesamum indicum]
c53882.graph_c0	67.0435898	82.1837206	55.7464905	43.5987726	32.5412727	28.8015509	7.346E-12	-1.97365	down	-
c53904.graph_c0	0.28357327	0	0	11.4948325	16.8822785	13.7148622	7.41E-12	6.211863	up	PREDICTED: alpha-galactosidase-like [Sesamum indicum]
c53924.graph_c0	0	0.25308776	0	17.9354339	5.67544936	6.01047266	0.0047269	5.888772	up	unnamed protein product [Vitis vinifera]
c53936.graph_c0	9.60426716	11.1219447	11.8578689	6.24570165	7.16794387	8.79919731	0.0001611	-1.576044	down	-
c53939.graph_c0	0.24936784	0	0	17.115177	29.8134657	36.1816072	7.437E-15	7.373783	up	-
c53960.graph_c0	3.04283179	3.65505447	4.30117525	46.8143603	46.0304876	42.9022961	1.004E-09	2.605828	up	-
c53976.graph_c0	0	0	0	1.05573512	1.7206513	1.95103405	1.563E-11	Inf	up	PREDICTED: uncharacterized protein LOC105155633 [Sesamum indicum]
c53979.graph_c0	0	0.258687	0	13.3325324	23.2040451	22.6671993	6.412E-15	6.823092	up	-
c53980.graph_c0	0.99722046	0.95068479	1.34249005	0.04374783	0.04634553	0	1.198E-16	-6.20145	down	hypothetical protein VITISV_003451 [Vitis vinifera]
c53984.graph_c0	2.03709422	1.03247294	1.25916931	0	0	0	2.671E-16	-Inf	down	Animal heme peroxidase homologue [Chondrus crispus]
c53999.graph_c0	2.79947101	1.40113713	0.44967888	21.599588	17.4177517	12.6215534	5.57E-05	2.475643	up	-
c54007.graph_c0	0	0.19677207	0.25260695	25.5348066	39.1376623	48.8253713	3.46E-17	6.940836	up	PREDICTED: probable aquaporin PIP2-5 [Sesamum indicum]
c54024.graph_c0	4.07715466	5.37981283	3.81040376	0	0	0.1519178	1.974E-18	-7.48753	down	PREDICTED: 17.3 kDa class I heat shock protein-like [Sesamum indicum]
c54095.graph_c0	0	0	0	16.6364968	13.5043729	20.5704788	8.762E-16	Inf	up	-
c54121.graph_c0	12.6478425	9.64610089	12.7701973	1.66457393	1.46951186	2.22169181	2.552E-12	-3.730503	down	-
c54141.graph_c0	13.2980161	12.9655831	15.0911226	0.79551946	0	0.42470906	1.402E-29	-6.072215	down	-
c54197.graph_c0	1.99875535	0.82639955	1.3400764	0	0	0	3.281E-11	-Inf	down	Subunit of cleavage factor I [Komagataella pastoris GS115]
c54198.graph_c0	1.54837258	1.54992304	0.99486003	17.8307726	17.0005874	11.106	3.058E-05	2.479449	up	-
c54205.graph_c0	6.46064145	7.52887526	6.07174361	1.24369403	0.94110216	1.42281189	3.399E-15	-3.489682	down	-
c54319.graph_c0	0.30912434	0.92830165	0.79447403	5.69571011	7.84408848	8.10881376	3.067E-05	2.385607	up	-
c54344.graph_c0	0	0.65673867	0.28103032	8.86490382	10.0316059	8.96356604	1.515E-08	3.872192	up	-
c54436.graph_c0	3.74390787	5.30918051	5.0783515	2.39523065	0.30449477	0.51150284	1.1E-10	-3.120984	down	PREDICTED: uncharacterized protein LOC104248461 [Nicotiana sylvestris]
c54544.graph_c0	0	0	0	10.2648726	7.93536406	5.67765684	2.445E-08	Inf	up	-
c54570.graph_c0	1.20042506	1.10919425	1.00861898	0	0	0.03784738	2.15E-18	-7.49339	down	-
c54603.graph_c0	1.87925616	1.20930297	1.29370638	0	0	0	3.255E-19	-Inf	down	hypothetical protein EMIHUDRAFT_349549 [Emiliania huxleyi CCMP1516]
c54753.graph_c0	0.35852187	0	0	18.1661552	15.0459648	22.0420032	3.869E-12	6.27211	up	Aldo-keto reductase family 4 member C9 [Gossypium arboreum]

#ID	0 h-1 FPKM	0 h-2 FPKM	0 h-3 FPKM	48 h-1 FPKM	48 h-2 FPKM	48 h-3 FPKM	FDR	log2FC	regulated	nr_annotation
c54836.graph_c0	0.20630901	0.2065156	0.5302305	4.94169832	7.0472906	5.41180732	5.497E-07	3.172171	up	PREDICTED: 3'-hydroxy-N-methyl-(S)-coclaurine 4'-O-methyltransferase-like [Sesamum indicum]
c54893.graph_c0	14.1229337	15.7078618	13.6113967	1.80707884	0.38287618	0.32158558	2.861E-17	-5.095862	down	-
c55206.graph_c0	1.36300467	0.73675954	0.87575748	0	0	0	4.092E-20	-Inf	down	hypothetical protein GUITHDRAFT_143910 [Guillardia theta CCMP2712]
c55343.graph_c0	0.23080892	0.23104004	0.59319721	5.10326965	5.18103308	6.24368064	1.125E-06	2.939128	up	-
c55419.graph_c0	1.08967852	0.65094319	0.49687356	0	0	0	5.633E-13	-Inf	down	PREDICTED: peroxisomal acyl-coenzyme A oxidase 1-like [Cicer arietinum]
c55533.graph_c0	65.4266014	66.595916	48.6505878	10.1587505	7.533375	9.94311457	7.355E-32	-3.719595	down	Ubiquitin [Medicago truncatula]
c55599.graph_c0	0.80689701	0.63211695	0.90164748	0.03232027	0.30815464	0.20130866	1.039E-08	-3.156002	down	Disease resistance family protein / LRR family protein, putative [Theobroma cacao]
c55911.graph_c0	0.18129039	0.18147192	0.23296533	4.34242979	4.60027782	6.39024309	6.425E-08	3.661885	up	-
c56027.graph_c0	5.6848328	10.74877	6.89938882	0	0	0	3.711E-14	-Inf	down	-
c56545.graph_c0	0.49493492	0	0.31800548	4.78764648	6.038013	4.26001905	5.298E-07	3.194437	up	-
c57000.graph_c0	1.01810985	0.84927444	0.65415573	20.3222326	28.3188382	24.3419621	1.828E-14	3.836611	up	-
c57433.graph_c0	1.21464826	0.63556556	0.92233293	0	0	0	2.707E-17	-Inf	down	ATP-binding cassette transporter, subfamily F, member 4, SmABCF4 [Selaginella moellendorffii]
c58690.graph_c0	0	0	0.68860503	6.91140582	8.36776734	11.6405553	2.891E-09	4.233007	up	hypothetical protein MIMGU_mgv1a015676mg [Erythranthe guttata]
c60866.graph_c0	7.69544718	11.5547295	8.72554792	0	0	0	7.788E-25	-Inf	down	hypothetical protein MIMGU_mgv1a015592mg [Erythranthe guttata]
c62784.graph_c0	0.57135043	0.50329185	0.41115644	0	0	0	2.922E-19	-Inf	down	predicted protein [Hordeum vulgare subsp. vulgare]
c64432.graph_c0	2.69177596	0.38492448	0.49414838	19.1301911	16.8884315	18.5980335	1.604E-06	2.931964	up	-
