# Supplementary material for: Transcriptomic analysis of seed germination improvement of Andrographis paniculata responding to air plasma treatment
Source: PLoS One. 2020 Oct 22;15(10):e0240939. doi: 10.1371/journal.pone.0240939 (PMC7580921; doi:10.1371/journal.pone.0240939)
Supplement: S7 Table — (PDF) [file pone.0240939.s009.pdf]

S7 Table. Differentially expressed genes at 28 HAS (plasma vs. control).

#ID	ctrl 28h-1_FPKM	ctrl 28h-2_FPKM	ctrl 28h-3_FPKM	plasma 28h-1_FPKM	plasma 28h-2_FPKM	plasma 28h-3_FPKM	FDR	log2FC	regulated	annotation	database
c15435.graph_c0	14.6175374	13.8463951	9.81990012	27.7898082	26.4727798	30.9284723	1.60E-11	1.145803	up	uncharacterized protein LOC105156981 [Sesamum indicum]	nr
c15435.graph_c1	5.37260991	6.93213681	4.95270675	15.1336147	13.2623459	14.1649216	1.09E-05	1.295031	up	SEY1 [Theobroma cacao]	nr
c18820.graph_c0	8.67781393	6.59073165	7.11669063	23.4226483	26.1157082	23.4317495	1.75E-47	1.696298	up	hypothetical protein MIMGU_mgv1a014625mg [Erythranthe guttata]	nr
c20200.graph_c0	0.76885908	1.02878076	0.42404918	2.1798841	2.08751768	2.26388607	0.003297	1.547356	up	BAG family molecular chaperone regulator 1-like [Sesamum indicum]	nr
c20387.graph_c0	10.030714	8.76350242	6.84320239	32.7523391	29.5391014	27.8054652	2.60E-36	1.804428	up	uncharacterized protein LOC105158345 [Sesamum indicum]	nr
c21137.graph_c0	69.9251861	67.4232596	54.2832789	228.51831	215.28593	195.903522	4.25E-48	1.730672	up	Wound-induced protein,hypothetical protein L484_018717 [Morus notabilis]	nr
c21161.graph_c0	6.24188275	7.11206895	5.88682602	0.86876704	1.80851278	1.0239765	5.39E-32	-2.38697	down	9-cis-epoxycarotenoid dioxygenase NCED5, chloroplastic [Sesamum indicum]	nr
c21351.graph_c0	1.14595231	1.1713691	1.37302542	2.71940428	2.56572068	2.36860798	0.001363	1.045399	up	Senescence regulator, uncharacterized protein LOC105175340 [Sesamum indicum]	nr
c21712.graph_c0	6.93433492	5.13889851	5.70396374	1.68517372	0.82993848	1.25008	6.55E-07	-2.24677	down	-	
c22005.graph_c0	3.40998049	2.97024916	3.38527445	0.62966898	0.67513181	0.9954967	1.92E-11	-2.09224	down	uncharacterized protein LOC105178969 [Sesamum indicum]	nr
c23208.graph_c0	21.1720884	19.7295128	17.7032436	9.20876626	9.5464529	8.90581104	7.85E-17	-1.0913	down	uncharacterized protein LOC105168502 [Sesamum indicum]	nr
c24261.graph_c0	8.4851964	11.4833546	11.780045	3.79314001	4.07279434	3.97353069	2.58E-09	-1.42935	down	-	
c24366.graph_c0	4.36809352	3.76732368	3.58878268	8.44397933	7.99218268	8.22074307	8.05E-06	1.064474	up	chaperone protein dnaJ 20, chloroplastic-like [Sesamum indicum]	nr
c25198.graph_c0	42.9737056	37.6538174	30.6293808	88.2321425	83.8537665	91.6953778	4.06E-27	1.237179	up	hypothetical protein MIMGU_mgv1a026311mg [Erythranthe guttata]	nr
c26100.graph_c0	9.550106	11.2714064	11.2366381	3.22731725	5.97609748	3.3029894	1.32E-08	-1.36629	down	Zinc finger CCCH domain-containing protein 30 [Arabidopsis thaliana]	Swissprot
c26100.graph_c1	12.8521207	16.8709201	15.3591805	3.76788424	7.56955278	4.49654805	4.19E-08	-1.51752	down	Zinc finger CCCH domain-containing protein 29-like [Sesamum indicum]	nr
c26161.graph_c0	3.19645213	3.45454008	3.52588128	6.59101291	7.43883336	8.06731906	0.007062	1.11181	up	Ethylene-responsive transcription factor RAP2-10 [Arabidopsis thaliana]	Swissprot
c26293.graph_c1	13.2257589	13.0174159	12.3606033	29.8811604	28.3144527	29.0115999	1.45E-31	1.168167	up	-	
c26653.graph_c0	1.20872176	1.11969975	1.48961087	2.96504573	2.99725331	2.05127546	0.001329	1.061949	up	bZIP transcription factor,basic leucine zipper 61-like [Sesamum indicum]	nr
c26801.graph_c0	3.27078823	4.13167584	3.98506581	10.1164384	9.54938608	9.66202167	1.36E-05	1.358196	up	hypothetical protein MIMGU_mgv1a027149mg, partial [Erythranthe guttata]	nr
c27133.graph_c0	13.9158086	11.4208969	12.4011713	41.5623776	41.3335983	35.3009243	2.60E-37	1.638828	up	chaperone protein dnaJ 11, chloroplastic-like [Sesamum indicum]	nr
c28248.graph_c0	0.4922958	0.79806713	0.93673061	2.28398163	1.97629539	2.07078929	2.59E-05	1.502117	up	-	

#ID	ctrl 28h-1_FPKM	ctrl 28h-2_FPKM	ctrl 28h-3_FPKM	plasma 28h-1_FPKM	plasma 28h-2_FPKM	plasma 28h-3_FPKM	FDR	log2FC	regulated	annotation	database
c34236.graph_c0	1.01207751	1.22214813	1.00474479	2.30581743	2.61188453	2.15520502	2.37E-07	1.119042	up	receptor protein kinase CLAVATA1-like [Sesamum indicum]	nr
c35279.graph_c0	89.2379896	95.7212728	85.9478828	37.7655757	52.3724521	39.3810661	1.55E-16	-1.07276	down	secoisolariciresinol dehydrogenase-like [Sesamum indicum]	nr
c37513.graph_c0	2.2757353	3.05678568	2.48517182	10.5581596	9.29470247	9.90767779	2.25E-12	1.921796	up	-	
c38035.graph_c0	4.88564784	6.12063449	6.37461464	2.19471393	2.2739912	1.70279314	1.67E-12	-1.50096	down	hypothetical protein MIMGU_mgv1a008930mg [Erythranthe guttata]	nr
c40715.graph_c2	39.6985105	36.9751338	26.5244929	100.621086	96.8175419	92.9578975	5.57E-40	1.483893	up	Wound-induced protein, uncharacterized protein LOC105174779 [Sesamum indicum]	nr
c41309.graph_c0	19.5000822	11.3597764	10.9766391	2.02079977	1.82459272	2.02948444	8.21E-05	-2.84153	down	-	
c42341.graph_c0	2.4156905	1.61617126	2.99773538	0.25943228	0.60903187	1.10081205	0.008269	-1.84048	down	Zinc knuckle	Pfam
c42797.graph_c0	69.5098685	69.6027991	62.4328869	27.9946258	37.3394153	28.8321544	2.41E-18	-1.10614	down	WRKY transcription factor 33 [Sesamum indicum]	nr
c43225.graph_c0	11.7027842	11.4016126	13.4207873	2.65231651	4.36697571	5.98356617	3.26E-06	-1.49591	down	60S ribosomal protein L39	Swissprot
c43253.graph_c0	7.0370302	7.45000565	7.54049345	17.7058977	17.3904815	13.8840487	1.89E-08	1.145208	up	ethylene-responsive transcription factor CRF4-like [Sesamum indicum]	nr
c43253.graph_c1	1.55858205	2.36621619	1.50430951	3.71591159	4.26190889	3.1960537	0.000348	1.033479	up	ethylene-responsive transcription factor CRF4-like [Sesamum indicum]	nr
c43379.graph_c0	13.2176573	9.18313549	10.7599519	2.14629299	2.76843048	5.33747064	4.76E-05	-1.69981	down	60S ribosomal protein L37-2	Swissprot
c43483.graph_c0	0.70996003	1.01281438	0.88806843	0.40623828	0.43618874	0.43003734	0.001211	-1.04341	down	NB-ARC domain;Leucine rich repeat	Pfam
c43633.graph_c0	14.7884744	9.70367612	10.7051355	3.01758632	5.5925949	5.57263205	0.004418	-1.3199	down	60S ribosomal protein L26-1	Swissprot
c44035.graph_c0	6.97128182	4.06526883	3.78940707	0.10604233	0.06383086	0.23074581	9.25E-10	-5.2163	down	hypothetical protein EPUS_08767 [Endocarpon pusillum Z07020]	nr
c44054.graph_c0	34.7965078	32.0706837	31.5152111	63.5673822	65.7527442	71.2709812	2.08E-33	1.020281	up	PREDICTED: uncharacterized protein LOC105157696 [Sesamum indicum]	nr
c44265.graph_c0	7.60501761	5.75499711	5.21050653	1.32518674	1.79477907	1.89234773	1.30E-07	-1.89799	down	Cold shock, CspA, partial [Metarhizium brunneum ARSEF 3297]	nr
c45752.graph_c0	13.753782	9.92946525	13.8464446	3.09563284	4.28276275	7.94471239	0.006738	-1.29792	down	60S ribosomal protein L6	nr
c46166.graph_c0	10.5030765	7.04816195	9.44175302	2.68582259	4.09948248	4.95721151	0.002133	-1.20815	down	40S ribosomal protein S18	nr
c46906.graph_c0	3.02772904	2.3239255	1.8034755	0.20067138	0.24158326	0.70956691	6.92E-06	-2.64097	down	Domain of unknown function (DUF1857)	Pfam
c47065.graph_c0	10.0769233	6.75965437	6.89925091	1.93453983	3.00821948	3.55177959	0.001139	-1.49073	down	60S ribosomal protein L30-2	nr
c47311.graph_c0	1.47479581	1.45725602	1.0736811	0.2280747	0.44618182	0.37221419	4.56E-05	-1.9452	down	hypothetical protein SELMODRAFT_271243 [Selaginella moellendorffii]	nr
c47343.graph_c0	1.01211424	1.07151122	1.72248215	0.03726709	0.13459475	0.21286748	1.65E-06	-3.31018	down	udp-d-glucose 6-dehydrogenase [Thalassiosira pseudonana CCMP1335]	nr
c47459.graph_c0	3.358564	2.80767436	2.84629431	0.69675113	0.68629194	1.96405844	0.00279	-1.43402	down	-	
c47708.graph_c0	2.38568211	2.05212178	2.11464047	0.39529472	0.29742839	1.4515077	0.006283	-1.61522	down	-	
c47954.graph_c0	12.3978809	7.95100193	9.94112185	1.99105427	3.59547065	4.75370511	0.000735	-1.55797	down	40S ribosomal protein S11 [Auxenochlorella protothecoides]	nr

#ID	ctrl 28h-1_FPKM	ctrl 28h-2_FPKM	ctrl 28h-3_FPKM	plasma 28h-1_FPKM	plasma 28h-2_FPKM	plasma 28h-3_FPKM	FDR	log2FC	regulated	annotation	database
c47967.graph_c0	3.71530168	2.63712436	3.64048243	0.8208074	1.98752797	1.43087684	0.003295	-1.24556	down	hypothetical protein FOMPIDRAFT_1023471 [Fomitopsis pinicola FP-58527 SS1]	nr
c48037.graph_c1	1.60744268	1.59174844	1.48574149	0.81434104	0.23219167	0.76941601	0.00418	-1.37287	down	Cathepsin propeptide inhibitor domain (I29)	Pfam
c48143.graph_c3	179.044313	154.091627	145.432786	397.408776	369.060911	357.624118	6.48E-41	1.223739	up	haloacid dehalogenase-like hydrolase domain-containing protein 3 [Sesamum indicum]	nr
c48359.graph_c0	0.15586239	0.57753269	0.46419941	3.85661726	4.78797522	4.032053	1.58E-09	3.399291	up	-	
c48392.graph_c0	0.90448258	0.77580342	1.16398256	0.12951548	0.32743293	0.295914	4.74E-05	-1.92431	down	6-phosphogluconate dehydrogenase, decarboxylating 3	Swissprot
c48428.graph_c0	16.3202197	10.2751676	12.2742244	3.76945304	6.7575998	6.15168579	0.009415	-1.2294	down	60S ribosomal protein L10a-2	Swissprot
c48527.graph_c0	2.38788878	2.31033352	1.63307174	0.369283	0.48161811	0.63614515	1.04E-07	-2.09765	down	Intracellular ribonuclease LX (Precursor)	Swissprot
c48617.graph_c0	0.92167869	0.85379731	0.67330257	0.30927637	0.25496517	0.34014681	5.75E-05	-1.44472	down	hypothetical protein VITISV_012155 [Vitis vinifera]	nr
c48651.graph_c0	1.21540834	1.19626223	1.09348346	0.29368885	0.55686434	0.62308403	0.008846	-1.25728	down	pyruvate dehydrogenase E1 component subunit alpha [Galdieria sulphuraria]	nr
c48670.graph_c0	0.87938776	0.56776622	0.60846598	0.18544888	0.26046681	0.26902181	0.002527	-1.53239	down	hypothetical protein AMTR_s00036p00173790 [Amborella trichopoda]	nr
c48880.graph_c0	4.78808914	3.66406553	5.47674164	0.16097173	0.58136952	0.7224339	5.74E-15	-3.25539	down	Glutathione S-transferase, C-terminal domain	Pfam
c48922.graph_c1	7.68460083	5.79052916	5.0100398	1.72940355	2.80267303	2.74999019	0.001185	-1.35278	down	-	
c48989.graph_c0	1.75113074	1.01385029	1.7819045	0.37236297	0.58072438	0.49720115	0.002179	-1.6565	down	Probable cinnamyl alcohol dehydrogenase 2	Swissprot
c49019.graph_c0	1.29369597	1.19841563	0.91145493	0.32269018	0.11654359	0.50029385	9.94E-05	-1.86247	down	Guanine nucleotide-binding protein-like NSN1	Swissprot
c49029.graph_c0	1.96205764	1.43201119	1.23953589	0.2298704	0.62265426	0.60023158	0.00147	-1.68226	down	Rac-like GTP-binding protein ARAC2 (Precursor)	Swissprot
c49091.graph_c0	2.12386891	1.41410222	1.97670095	0.46188671	0.69506732	0.54440502	0.00035	-1.70504	down	-	
c49145.graph_c0	0.80151799	0.6629343	0.73887433	0.11067269	0.23982495	0.23480083	0.000108	-1.92007	down	Pescadillo homolog	Swissprot
c49147.graph_c0	1.12034788	0.66306098	0.95775423	0.2165749	0.19554683	0.29453892	4.15E-05	-1.96288	down	Probable alpha-glucosidase Os06g0675700 (Precursor)	Swissprot
c49158.graph_c0	14.1401541	9.25032017	10.9728696	3.59546684	5.88071844	5.00201057	0.001676	-1.25584	down	S-adenosyl-methionine synthetase [Undaria pinnatifida]	nr
c49175.graph_c0	17.5065309	11.7397094	14.0441958	3.91974055	5.72026733	8.62844764	0.006589	-1.25173	down	60S ribosomal protein L7a, putative [Ricinus communis]	nr
c49225.graph_c0	2.55890015	1.89635031	1.12904905	0.26381975	0.3970075	0.50230777	0.00717	-2.27251	down	V-type proton ATPase subunit E3	nr
c49292.graph_c0	0.97232832	0.90071662	0.77758956	0.12530745	0.4148499	0.27266629	8.60E-05	-1.71449	down	phosphoenolpyruvate carboxykinase [Neocallimastix frontalis]	nr
c49371.graph_c0	1.10321689	0.88866546	0.90475013	0.3708927	0.33488134	0.30264542	0.00711	-1.53072	down	CD36 family	Pfam
c49390.graph_c0	4.43849672	3.06501293	7.13025743	0.06240055	0.1126837	0.05091834	1.19E-07	-6.02465	down	hypothetical protein CHLNCDRAFT_33886 [Chlorella variabilis]	nr
c49437.graph_c1	1.45743989	1.42510541	1.56745379	0.34434681	0.56529615	0.9195846	0.003249	-1.28809	down	Lipase (class 3)	Pfam
c49480.graph_c0	1.64743474	1.0174011	1.93286212	0.54042762	0.24397774	0.53548121	0.003297	-1.80603	down	GDP dissociation inhibitor	Pfam
c49497.graph_c0	5.54590979	2.96391642	4.12929636	0.32987141	1.26583255	1.48044806	0.003297	-2.04669	down	-	
c49567.graph_c0	36.1634352	29.3893424	34.1722263	8.62622332	13.7532292	19.075756	1.28E-05	-1.27297	down	predicted protein [Hordeum vulgare subsp. vulgare]	nr
c49590.graph_c0	1.36555296	1.04174854	1.11642525	0.09316786	0.11216245	0.25341404	1.59E-09	-2.94708	down	lupus La protein [Galdieria sulphuraria]	nr

#ID	ctrl 28h-1_FPKM	ctrl 28h-2_FPKM	ctrl 28h-3_FPKM	plasma 28h-1_FPKM	plasma 28h-2_FPKM	plasma 28h-3_FPKM	FDR	log2FC	regulated	annotation	database
c49618.graph_c2	1.25172275	0.79510884	1.4911845	0.19357683	0.37453227	0.51900193	0.007879	-1.70825	down	KH domain	Pfam
c49630.graph_c1	1.77661589	1.47697185	1.4471745	0.07044889	0.09541311	0.22994288	8.43E-14	-3.57544	down	abhydrolase domain-containing protein 4 [Malus domestica]	nr
c49667.graph_c0	2.43873052	1.79702625	1.76077192	0.47143199	0.61914019	0.69942664	2.38E-05	-1.75237	down	eukaryotic translation initiation factor 3 subunit D-like [Pyrus x bretschneideri]	nr
c49672.graph_c0	1.84288605	1.31535126	1.46962563	0.25696653	0.37966366	0.40030312	6.48E-10	-2.1661	down	nitric oxide dioxygenase [Cladophialophora psammophila CBS 110553]	nr
c49717.graph_c0	2.66620547	2.4698402	2.37117071	0.42950438	0.69804394	0.94627459	5.05E-07	-1.86271	down	L,D-transpeptidase catalytic domain	Pfam
c49737.graph_c0	2.51454357	3.05726945	2.18428451	0.91141395	1.04236703	1.38825183	0.007448	-1.22137	down	Cycloartenol-C-24-methyltransferase	Swissprot
c49744.graph_c0	2.77681632	1.95718828	1.91770274	0.46677173	0.379306	0.91411661	4.06E-05	-1.92445	down	S-Adenosylmethionine decarboxylase [Ectocarpus siliculosus]	nr
c49756.graph_c0	0.63288089	0.50540465	0.47663799	0.13499858	0.22854574	0.24785491	0.007879	-1.40931	down	Glycine dehydrogenase (decarboxylating), mitochondrial (Precursor)	Swissprot
c49770.graph_c0	57.0974024	37.2775798	45.1750063	12.6216705	17.3733678	25.6638752	0.00309	-1.33329	down	Elongation factor 1-alpha [Auxenochlorella protothecoides]	nr
c49789.graph_c0	6.01490475	3.71460583	3.12783715	0.36912498	0.33328525	0.36144356	8.51E-06	-3.60485	down	-	
c49807.graph_c0	2.69056103	2.25503036	1.93970366	0.54488075	0.98395246	1.15196545	9.66E-05	-1.36871	down	eukaryotic initiation factor 4A [Ectocarpus siliculosus]	nr
c49812.graph_c0	0.60951861	0.26349294	0.24204101	1.16255272	1.3901116	1.41013092	4.52E-05	1.818459	up	Short-chain dehydrogenase/reductase SDR [Macrophomina phaseolina MS6]	nr
c49858.graph_c0	6.82142727	4.86813575	5.40123626	2.03972892	2.96396014	3.47183884	0.004836	-1.01964	down	ABC transporter F family member 1	Swissprot
c49863.graph_c0	1.45685702	0.89970661	1.0330727	0.16092893	0.41169391	0.50338065	0.00442	-1.66342	down	-	
c49864.graph_c0	0.89185714	0.82617212	0.96588594	0.2507713	0.31133158	0.30694099	0.002507	-1.63398	down	Fructose-bisphosphate aldolase, cytoplasmic isozyme	Swissprot
c49883.graph_c0	2.27607671	1.68675545	2.10894697	0.27377113	0.67094336	0.89358002	1.51E-05	-1.73042	down	Aconitate hydratase, cytoplasmic	Swissprot
c49887.graph_c0	1.31957399	0.98731318	0.70539183	0.17005826	0.02362256	0.23483502	2.61E-05	-2.81932	down	Geranylgeranyl pyrophosphate synthase 10, mitochondrial (Precursor)	Swissprot
c49903.graph_c0	1.22643396	1.07299037	0.71023644	0.13171247	0.19027842	0.4299052	0.000968	-2.00746	down	-	
c49905.graph_c0	12.8263998	7.24606063	9.50186952	0.60108523	0.6784045	1.44078687	6.61E-09	-3.44907	down	2-methylene-furan-3-one reductase-like [Solanum lycopersicum]	nr
c49926.graph_c0	57.8557927	36.0168066	44.8267022	7.845358	10.2739566	20.7201617	0.00034	-1.84274	down	cysteine protease 1 [Brachiaria hybrid cultivar]	nr
c49934.graph_c0	4.68899892	3.48564947	2.4711867	1.02952803	0.92956728	1.38796894	0.006283	-1.67778	down	ATP-dependent RNA helicase ded1 [Exophiala dermatitidis NIH/UT8656]	nr
c49956.graph_c0	1.8961795	2.16187868	3.18566985	0.62031747	0.35641996	1.01234797	0.001249	-1.86779	down	GTP cyclohydrolase-2 (Precursor)	Swissprot
c49972.graph_c3	2.4993351	1.11677237	1.69307246	0.29557258	0.16423035	0.24118481	0.000691	-2.92963	down	ABC-2 type transporter domain-containing protein [Rozella allomycis CSF55]	nr
c50003.graph_c0	1.08876088	1.37532811	2.16174514	0.01913353	0.06910313	0.09367682	3.61E-08	-4.67217	down	-	
c50016.graph_c1	2.67700751	1.65323112	2.21467668	0.64686676	0.42831069	1.05567599	0.001334	-1.62505	down	-	
c50028.graph_c0	1.07880956	0.77093142	0.9179943	0.02383373	0.21519621	0.27227378	2.13E-07	-2.44337	down	KDEL-tailed cysteine endopeptidase CEP1 (Precursor)	Swissprot
c50051.graph_c0	8.35023051	5.22512795	6.78001202	2.28765497	3.06935079	3.73341555	0.008148	-1.17073	down	ATP synthase subunit beta, mitochondrial (Precursor)	Swissprot

#ID	ctrl 28h-1_FPKM	ctrl 28h-2_FPKM	ctrl 28h-3_FPKM	plasma 28h-1_FPKM	plasma 28h-2_FPKM	plasma 28h-3_FPKM	FDR	log2FC	regulated	annotation	database
c50072.graph_c0	0.79382549	0.62641818	0.70051027	0.15913824	0.28737382	0.31536336	0.005392	-1.48444	down	B-box zinc finger; Kinesin protein 1B; FHA domain	Pfam
c50080.graph_c0	31.2913198	20.0318961	25.5728286	2.2467483	3.003893	5.18267799	1.14E-11	-2.88835	down	carbonyl reductase (NADPH) [Galdieria sulphuraria]	nr
c50091.graph_c0	3.50396655	2.69574781	3.30170263	0.52810588	1.05731891	0.78691581	1.81E-15	-2.01027	down	-	
c50102.graph_c0	4.14940817	2.69066382	3.54897399	0.91318707	1.0027974	1.59100276	9.37E-05	-1.5736	down	protein disulfide isomerase [Rhizoctonia solani 123E]	nr
c50104.graph_c0	1.26218596	1.142653	0.99673728	0.46580612	0.28038617	0.47059256	0.000146	-1.49019	down	NADP-dependent malic enzyme, chloroplastic (Precursor)	Swissprot
c50106.graph_c0	1.10856116	0.5550897	0.86257709	0.18533743	0.25101349	0.1323296	0.0014	-2.16156	down	saccharopepsin [Mucor circinelloides f. circinelloides 1006PhL]	nr
c50116.graph_c0	2.8755822	1.62787557	2.79130914	0.81529061	0.55767499	0.78622875	0.000806	-1.76347	down	Multicopper oxidase	Pfam
c50137.graph_c0	1.74134106	0.97394221	1.07637574	0.30486281	0.36701671	0.3938788	0.005145	-1.8401	down	Karyopherin beta [Blumeria graminis f. sp. tritici 96224]	nr
c50150.graph_c0	1.15367221	0.92534177	0.97770673	0.36988159	0.2652102	0.53262416	1.52E-06	-1.39462	down	Eukaryotic translation initiation factor 3 subunit A	nr
c50153.graph_c1	3.1943712	1.92432704	2.02335974	0.54552606	0.95775339	0.95211529	0.009357	-1.5495	down	Polyadenylate-binding protein 2	Swissprot
c50311.graph_c0	97.0259825	109.383295	111.84233	38.7899494	42.5158011	34.1825902	4.86E-47	-1.46982	down	vegetative incompatibility protein HET-E-1-like [Sesamum indicum]	nr
c50590.graph_c0	57.7019286	39.0672224	33.6747535	114.474056	120.879864	103.229092	3.17E-13	1.366327	up	uncharacterized protein At1g01500 [Sesamum indicum]	nr
c50630.graph_c0	116.194377	87.6542192	70.6331756	211.580395	212.362054	202.327983	6.26E-15	1.180839	up	hypothetical protein AMTR_s00033p00173430 [Amborella trichopoda]	nr
c51165.graph_c0	4.30952406	4.08949784	6.15657895	2.31635723	1.9813769	1.69116768	0.000571	-1.28787	down	-	
c51619.graph_c0	1.32875411	1.4770701	0.7914762	2.60286824	2.9067595	3.4653419	0.003701	1.310857	up	hypothetical protein MIMGU_mgv1a023502mg, partial [Erythranthe guttata]	nr
c51753.graph_c0	6.0326415	3.75336214	3.03916039	9.74711242	8.04638046	11.3622345	0.002221	1.175766	up	Putative non-specific lipid-transfer protein AKCS9 [Aegilops tauschii]	nr
c52090.graph_c0	3.02120284	4.5209642	5.19111965	0.80865685	0.24338044	0.73317488	4.27E-08	-2.83811	down	hypothetical protein glysoja_014339 [Glycine soja]	nr
c52750.graph_c0	3.41461514	3.45068697	1.78738891	7.63293249	8.452235	5.67999707	0.000527	1.320736	up	-	
c53027.graph_c0	0.85009914	1.18123438	0.75954599	2.36639535	1.83987853	2.95007668	0.00717	1.352242	up	-	
c53093.graph_c0	1.00414103	2.44173911	1.12147403	3.78516274	4.29396682	4.35580522	0.001814	1.438563	up	-	
c53169.graph_c0	1.31731641	2.03382739	1.5257342	5.60232135	4.75180298	4.57144847	0.004546	1.606939	up	-	

Transcriptomic analysis of seed germination improvement of *Andrographis paniculata* responding to air plasma treatment

Jia-Yun Tong 1*, Rui He 2*, Xiao-Ting Tang 2, Ming-Zhi Li 3 and Jing-Lin Wan 4
