# Supplementary material for: Transcriptomic analysis of seed germination improvement of Andrographis paniculata responding to air plasma treatment
Source: PLoS One. 2020 Oct 22;15(10):e0240939. doi: 10.1371/journal.pone.0240939 (PMC7580921; doi:10.1371/journal.pone.0240939)
Supplement: S8 Table — (PDF) [file pone.0240939.s010.pdf]

S8 Table. Differentially expressed genes at 48 HAS (plasma vs. control).

#ID	control-1 FPKM	control-2 FPKM	control-3 FPKM	plasma-1 FPKM	plasma-2 FPKM	plasma-3 FPKM	FDR	log2FC	regulated	nr_annotation
c19480.graph_c0	1.25100608	4.72859349	5.95669365	10.9602539	16.2753319	15.4620877	4.79E-05	1.84687	up	PREDICTED: peroxidase 44 [Sesamum indicum]
c25604.graph_c0	37.5406524	34.5961352	27.3256807	16.5850452	17.8226479	14.2264363	0.000503	-1.01703	down	PREDICTED: anthocyanidin 3-O-glucoside 2'''-O-xylosyltransferase-like isoform X1 [Sesamum indicum]
c27182.graph_c0	16.7837227	15.1409018	13.1461702	2.38399381	8.61278114	6.20061596	0.002597	-1.39725	down	PREDICTED: heme oxygenase 1, chloroplastic-like [Sesamum indicum]
c27911.graph_c0	0.39752941	1.74720303	0.83939922	3.04441907	4.98113118	3.25068694	0.009305	1.932924	up	PREDICTED: protein NRT1/ PTR FAMILY 6.3-like [Sesamum indicum]
c44035.graph_c0	14.4322219	10.677167	24.4602305	0.22402745	0.30852889	0.19933827	4.71E-07	-6.07538	down	hypothetical protein EPUS_08767 [Endocarpon pusillum Z07020]
c49500.graph_c8	11.6011811	11.4904741	12.0181658	3.64004001	6.25414231	5.71568066	0.000838	-1.16612	down	PREDICTED: tyrosine/DOPA decarboxylase 1-like [Sesamum indicum]
c49929.graph_c0	0.31365482	0.98089936	0.65380372	5.28457191	3.03842786	6.37004768	0.000824	2.93931	up	-
c50472.graph_c0	7.07776019	16.8595447	15.3995041	35.4457847	41.3926504	38.2087696	3.92E-13	1.562901	up	PREDICTED: 1-aminocyclopropane-1-carboxylate oxidase homolog 1-like [Sesamum indicum]
c51145.graph_c0	4.25674993	10.2899896	10.8026019	19.5678559	24.7631648	25.3685886	0.000381	1.470417	up	PREDICTED: proline-rich protein 3 [Sesamum indicum]

Transcriptomic analysis of seed germination improvement of *Andrographis paniculata* responding to air plasma treatment

Jia-Yun Tong 1*, Rui He 2*, Xiao-Ting Tang 2, Ming-Zhi Li 3 and Jing-Lin Wan 4
